# A survey of TIR domain sequence and structure divergence

**DOI:** 10.1007/s00251-020-01157-7

**Published:** 2020-01-30

**Authors:** Vladimir Y. Toshchakov, Andrew F. Neuwald

**Affiliations:** 1 Department of Microbiology and Immunology, University of Maryland School of Medicine, Baltimore, MD 21201 USA; 2 Department of Biochemistry and Molecular Biology, University of Maryland School of Medicine, Baltimore, MD 21201 USA

**Keywords:** TIR domains, Bayesian partitioning with pattern selection, Protein function, Protein structure

## Abstract

Toll-interleukin-1R resistance (TIR) domains are ubiquitously present in all forms of cellular life. They are most commonly found in signaling proteins, as units responsible for signal-dependent formation of protein complexes that enable amplification and spatial propagation of the signal. A less common function of TIR domains is their ability to catalyze nicotinamide adenine dinucleotide degradation. This survey analyzes 26,414 TIR domains, automatically classified based on group-specific sequence patterns presumably determining biological function, using a statistical approach termed Bayesian partitioning with pattern selection (BPPS). We examine these groups and patterns in the light of available structures and biochemical analyses. Proteins within each of thirteen eukaryotic groups (10 metazoans and 3 plants) typically appear to perform similar functions, whereas proteins within each prokaryotic group typically exhibit diverse domain architectures, suggesting divergent functions. Groups are often uniquely characterized by structural fold variations associated with group-specific sequence patterns and by herein identified sequence motifs defining TIR domain functional divergence. For example, BPPS identifies, in helices C and D of TIRAP and MyD88 orthologs, conserved surface-exposed residues apparently responsible for specificity of TIR domain interactions. In addition, BPPS clarifies the functional significance of the previously described Box 2 and Box 3 motifs, each of which is a part of a larger, group-specific block of conserved, intramolecularly interacting residues.

**Electronic supplementary material:**

The online version of this article (10.1007/s00251-020-01157-7) contains supplementary material, which is available to authorized users.

## Introduction

Toll-interleukin-1R resistance (TIR) domains most commonly function as signaling units responsible for transient, signal-dependent associations among TIR-containing proteins that enable amplification and spatial propagation of the signal (Pawson and Nash 2003). TIR domains were first recognized as a region of homology between *Drosophila* Toll protein and human IL-1 receptor (IL-1R) (Gay and Keith 1991). Other TIR domains were found in resistance proteins (R proteins) (Whitham et al. 1994), plant receptors that sense the presence of phytopathogens on the gene-for-gene basis and that activate programmed cell death at the infection site (Spoel and Dong 2012; Van der Biezen and Jones 1998). Metazoan TIR-containing proteins are generally involved in the innate immune defense mechanisms against single-celled pathogens, viruses, and multicellular parasites (Kawai and Akira 2011). The Toll proteins of insects, in addition to their role in immunity, participate in dorsoventral polarization in early embryogenesis (Anderson et al. 1985; Lemaitre et al. 1996). TIR domains, however, are not restricted to multicellular organisms, as they are widespread among all major bacterial phyla (Ve et al. 2015), and occur in archaea and fungi. Some TIR domains found in pathogenic microbes may function to subvert immune defenses of higher organisms (Rana et al. 2013). The broad phylogenetic distribution of TIR-containing proteins among prokaryotes however suggests that bacterial TIR domains mediate a broad spectrum of functions beyond interference with antimicrobial defenses of multicellular organisms. The signaling functions of TIR domains are due to their ability to mutually interact to form transitory oligomeric complexes that typically serve as platforms for recruitment of other signaling components (Kagan et al. 2014). These complexes can contain either additional interaction domains, such as the death domain within the adapter TIR protein MyD88, or a catalytic domain capable of transducing the signal spatially (Ferrao et al. 2012). TIR domain interactions involve distinct structural surfaces, thereby allowing multiple interactions simultaneously (Toshchakov et al. 2011).

A second recognized function, found in a subset of phylogenetically distant TIR domains, is enzymatic degradation of nicotinamide adenine dinucleotides (NAD) (Essuman et al. 2017, 2018; Horsefield et al. 2019; Wan et al. 2019). Although associated with TIR domains as phylogenetically distant as bacteria, plants, and animals, this function is not as common as their signaling function and often depends on or is regulated by either homo- or heteromeric TIR association (Essuman et al. 2018; Horsefield et al. 2019; Wan et al. 2019).

The consensus TIR domain consists of 5 β-strands, which form a central parallel β-sheet, alternating with 5 α-helices, which surround the β-sheet (Rock et al. 1998; Xu et al. 2000). An early survey of 29 TIR-encoding genes identified three conserved TIR-specific sequence motifs, named Box 1, Box 2, and Box 3 (Slack et al. 2000), corresponding to the first β-strand, the loop that connects the second strand and second helix, and the fifth helix, respectively.^1^ Later analyses, however, found that Boxes 2 and 3 are poorly conserved among diverse TIR domains (Ve et al. 2015).

Here we apply Bayesian partitioning with pattern selection (BPPS) (Neuwald 2014a, b) to classify the TIR domains based on those residue patterns that best distinguish each group from other, closely-related groups and that presumably correspond to group-specific functional determinants. To obtain biological insights, we contrast and compare such features among various subgroups in the light of available structures and of published studies. In particular, we compare TIR domains of Toll proteins of arthropods and Toll-like receptors of other metazoans with IL-1R family and plant TIRs. We also examine residue patterns characteristic of MyD88, TIRAP/Mal, and SARM1 proteins and consider the functional implications. Our survey reveals many group-specific structural variations and conserved surface patches that likely define group-specific interactions.

## Materials and methods

### Database searches

TIR domain sequences in the NCBI non-redundant (nr) and environmental (env_nr) protein sequence databases and from the translated NCBI EST database (Agarwala et al. 2018) were identified and multiply aligned using MAPGAPS (Neuwald 2009). A curated multiple sequence alignment (MSA) of TIR domains (smart00255) from the SMART database (Letunic and Bork 2018) served as the initial MAPGAPS query. The TRIF and TRAM query MSAs were constructed manually. The identified TIR pool was refined by removal of sequence fragments (i.e., sequences that fail to match at least 75% of the columns in the query MSA) and all but one sequence among those sharing ≥ 98% identity. The refined MSA served as input to BPPS.

### BPPS classification

BPPS was performed as previously described (Neuwald 2014a, b). BPPS identifies subgroup-specific sets of co-conserved residues presumably associated with functional specialization. BPPS uses Markov chain Monte Carlo (MCMC) sampling to stochastically move sequences between hierarchically arranged partitions, each defined by an evolving characteristic pattern. Upon convergence, this defines subgroups and corresponding conserved patterns that best distinguish each subgroup from those further up the hierarchy. The benefit of BPPS over construction of a phylogenetic tree is identification of subgroup-specific residue patterns and the ability to analyze very large numbers of sequences; modeling thousands of sequences as a tree introduces more complexity than either is necessary or can be reliably inferred.

### Primary data presentation

The primary data are presented in Supplemental Fig. 1, which presents a series of MSAs for each group of the hierarchy, and in Supplemental Fig. 2, which lists all of the sequences. The first line of each group-specific MSA shows the consensus sequence for the group, followed by representative sequences for each major phylum. The first MSA in each group-specific series shows major phyla represented in the group and the corresponding TIR sequence, for which all residues conserved in the subgroup are highlighted. The subsequent MSAs are “contrast alignments” highlighting those residues specifically conserved in the group (and subgroup) of interest. The three lines immediately below each contrast MSA show the most frequent residues at each position and below this their corresponding frequencies as integer tenths (for example, number 4 indicates the corresponding residue is present in 40–50% of the sequences). The lines that present the group-specific residues, referred to as “foreground residues,” are followed by analogous presentation of “background” residues, i.e., parent group residues. The 25 most characteristic foreground residues (i.e., those most divergent from the background) are denoted by black dots above the alignments. The red bars above the dots are proportional to a measure of selective constraint, as defined by an urn model in which residues correspond to distinctly colored balls in an urn, and where some of the colors are similar (representing biochemically similar residues). The selective constraint is then defined as the expected number of random trials required to draw by chance at least as many of the same- or similarly colored balls from the background urn as are observed in the foreground. (The background for the root node consists of overall residue frequencies in proteins.) Because the associated probabilities vary over a wide range, a column with *p* = 0.01 (100 trials) would disappear relative to a column with *p* = 0.00001 (100,000 trials). To work around this problem, the BPPS program optimally adjusts (so as to span the full range of) bar heights using the formula $h=\frac{t^{1-\sigma }}{1-\sigma }$, where *t* is the number of trials and 0 ≤ *σ* < 1 is a scaling parameter that converges to linear scaling at *σ* = 0 (for further details, see Neuwald et al. 2003). 3D images were generated using DeepView - Swiss-PdbViewer (Guex and Peitsch 1997).

## Results

### Detection and classification of TIR domains (general description of identified groups)

**Fig. 1:**
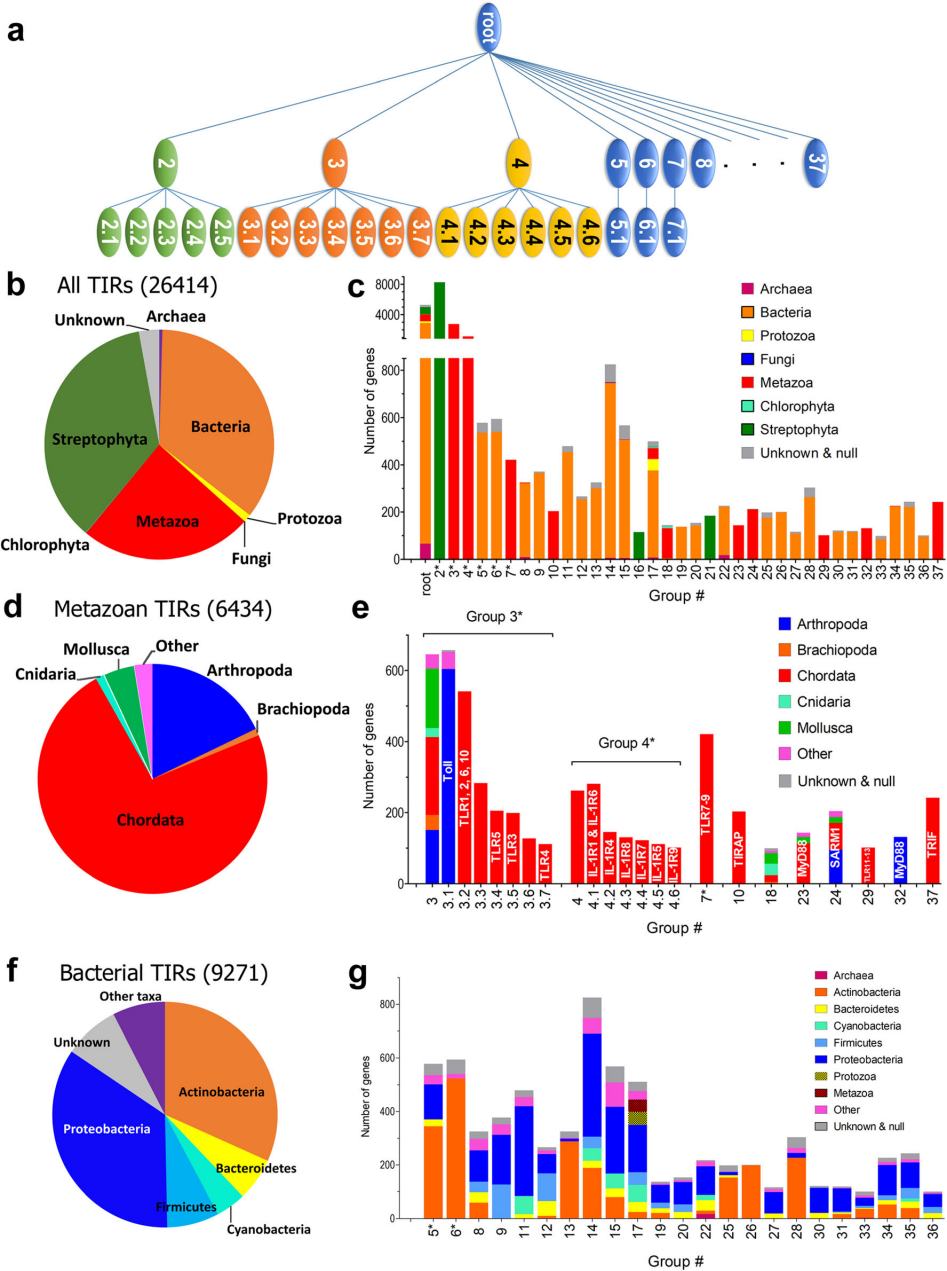
Hierarchical classification and phylogenetic composition of BPPS-defined TIR domain groups. **a** Bayesian classification of TIR domains. **b** Phylogenetic composition of the analyzed pool of TIR domains. Plant, animal, and bacterial TIR domains are nearly equally represented. **c** Phylogenetic composition of individual groups. The partition has differentiated plant (green columns), animal (red), and bacterial (brown) TIR domains into separate groups. Only group 17 combines bacterial TIRs with a significant number of eukaryotic TIRs. **d** Phylogenetic composition of metazoan groups. **e** Phylogenetic composition of individual metazoan groups. Except for group 18, the metazoan groups represent a single protein or a protein family. Characterized protein subgroups are indicated on column. Group 18 combines TIRs from architecturally diverse protozoan and lower metazoan proteins. Taxa accounting for less than 3% of a group are grouped into “Other.” **f** Phylogenetic composition of the analyzed pool of bacterial TIRs. **g** Phylogenetic composition of individual bacterial groups. Bacterial groups typically comprise proteins with diverse domain architecture. Phylogenetic composition of bacterial groups varies; there are, however, some common features. For example, cyanobacterial TIRs typically co-partition with proteobacterial TIRs

BPPS classified TIR domains into 36 groups, 6 of which were subdivided further into subgroups (Fig. 1a). Among these were 9271 bacterial, 6434 metazoan, 9522 higher plant, 118 archaeal, 292 protozoan, 16 chlorophytan, and 758 phylogenetically unclassified TIR domains (Fig. 1b). Twenty-two TIR groups predominantly contained prokaryotic proteins, whereas 13 exclusively comprised eukaryotic proteins. Only group 17 combines prokaryotic TIRs with a significant percentage of eukaryotic TIRs: ~ 10% protozoan, ~ 10% lower metazoan, and ~ 1.5% plant TIR domains (Fig. 1c).

Of the 13 eukaryotic TIR groups, 3 are plant (Streptophyta) (Fig. 1c), 9 are metazoan, and one (group 18) is a combination of protozoan and metazoan TIRs (Fig. 1c). Most group 18 TIRs are from lower metazoans, including 24% from Cnidaria, 22% from mollusks, and 16% from echinoderms. Thirteen percent of group 18 TIRs are from evolutionally early chordates, such as lancelet and *Ascidia*, or from bony fish. Seven TIRs in this group are from green algae (chlorophytes). Group 18 is the only eukaryotic group that contains TIR proteins with diverse domain architectures, whereas all other eukaryotic groups represent either a single TIR-containing protein or a protein family (Fig. 1e).

There are 3 groups of Streptophyta TIRs, i.e., groups 2, 16, and 21 (Fig. 1c). Group 2 is the largest of all groups and contains several subgroups (Fig. 1a, c). Group 2 TIR proteins are large, cytoplasmic receptors that contain the TIR in tandem with a nucleotide-binding (NB) domain followed by multiple leucine-rich repeats (LRRs), whereas group 21 proteins lack a NB domain and LRRs, but contain another interaction domain and may contain a catalytic unit. Group 16 TIRs are often paired with an ATPase.

The phylogenetic composition of individual metazoan groups is shown in Fig. 1e. Three of 10 metazoan groups correspond to Toll-like receptors (TLRs) (Fig. 1e). The largest TLR group, i.e., group 3, combines Toll proteins of arthropods with TLRs of other metazoan lineages, but excludes endosomal TLRs (TLR7–9) and TLR11–13. TLR7–9 and TLR11–13 are associated with groups 7 and 29, respectively (Fig. 1e). TIRs of the IL-1R family are associated with group 4. SARM1 and TIRAP/Mal are associated with groups 24 and 10, respectively (Fig. 1e). MyD88 is associated with groups 23 and 32. Group 32 consists exclusively of arthropod TIRs, whereas group 23 consists of other metazoan TIRs. Interestingly, proteins of groups 23 and 32 have different domain architectures. Group 23 TIR proteins typically contain an N-terminal death domain followed by the TIR, whereas group 32 (arthropod) TIR proteins are typically larger and have a domain architecture similar to that of *Drosophila* MyD88 (Horng and Medzhitov 2001); all group 32 proteins examined here have a C-terminal localization domain, in addition to the death and TIR domain (Marek and Kagan 2012).

Unlike most eukaryotic groups, the majority of prokaryotic TIR groups are polyphyletic at the phylum level (Fig. 1g). However, more than 95% of group 6, 13, and 26 TIRs are from Actinobacteria, and most group 11, 27, 30, and 31 TIRs are from Proteobacteria (Fig. 1g). Group 22 contains the largest percentage of archaeal genes (8%). Group 12 contains the largest portion of TIRs from Firmicutes (41%) (Fig. 1g).

### Sequence features generally conserved in TIR domains

**Fig. 2:**
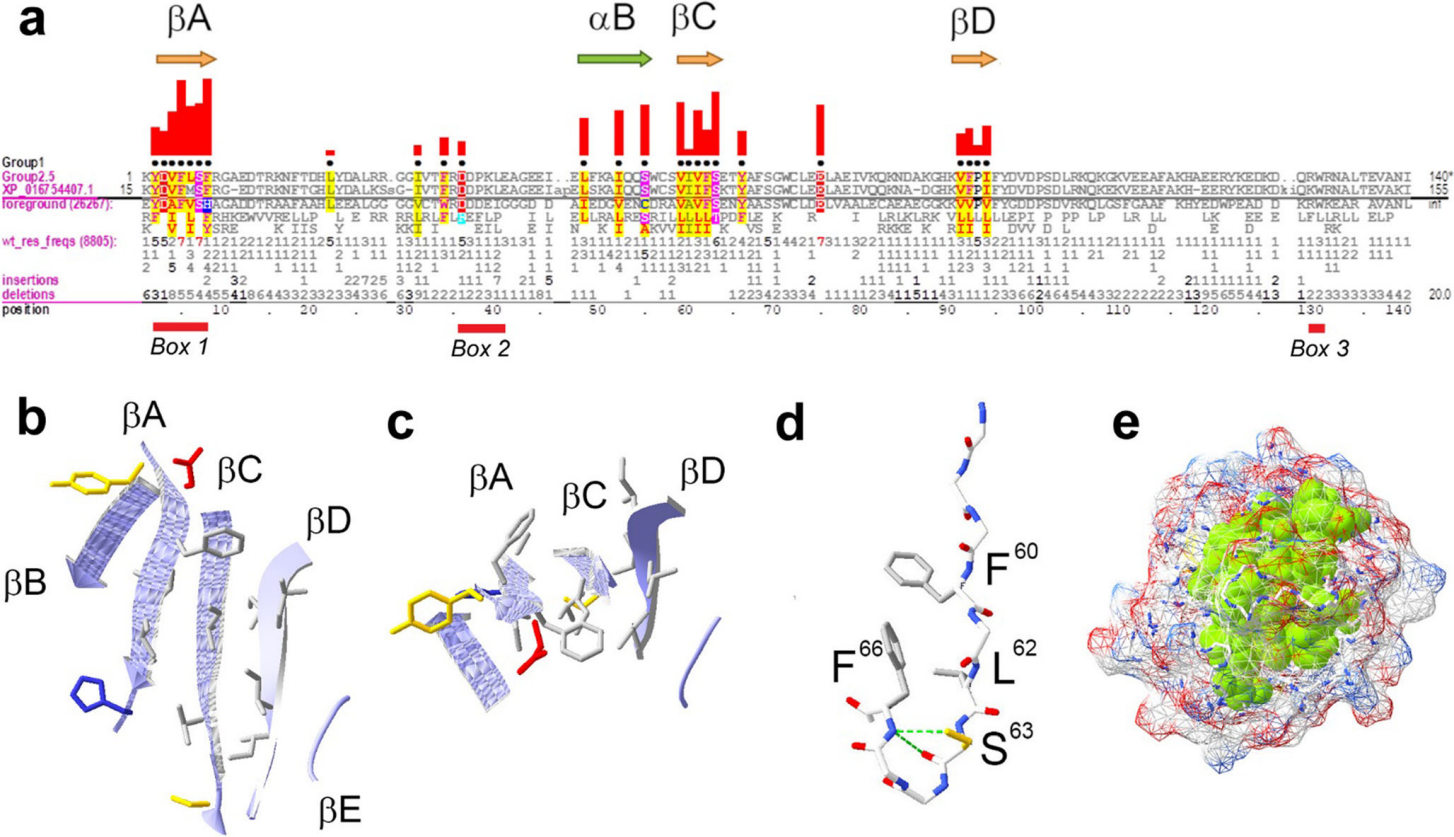
Pattern residues and structural features characteristic of TIR domains. **a** TIR domain residue patterns. Pattern positions are indicated by black dots above the alignment with the heights of red bars indicating the selective constraint imposed at those positions (Neuwald et al. 2003). Arrows indicate selected secondary structural elements. Horizontal bars beneath alignment indicate three motifs, named Boxes 1–3, previously identified in a subset of TIRs (Slack et al. 2000). **b**, **c** The β-sheet of TcpB TIR domain in “front” (**b**) and “top” (**c**) view. Side chains are shown for conserved residues. Images correspond to coordinate file 4lqc (Snyder et al. 2014). **d** Interactions of the conserved tyrosine/phenylalanine at position 66 proposed to stabilize the CC loop conformation. The backbone nitrogen of F66 interacts with S63 through two hydrogen bonds. The F66 side chain interacts with conserved hydrophobic residues of βC. The image shows residues 698–707 of TLR2 (PDB ID: 1FYW) (Xu et al. 2000). **e** Residues generally conserved in TIR domains form the tightly packed core of *Hydra magnipapillata* Toll-related receptor-2 (TRR-2) TIR domain (PDB ID: 4W8H) (Weisse and Scheidig 2015). Conserved residues are shown in green in the space-filling mode

The residue patterns conserved among all TIR domains are highlighted in Fig. 2a and largely occur in regions corresponding to three β-strands (labeled βA, βC, and βD in Fig. 2a–c), which form the TIR domain inner core. The surface-exposed strands, i.e., βB and βE, which form lateral edges of the β-sheet, are not conserved (Fig. 2b, c). Three pattern residue positions prior to βC correspond to α-helix B (Fig. 2a) with the two hydrophobic residues packing against β-strands in TLR2, MyD88, IL1RAPL1, and TcpB (PDB IDs: 1fyw, 4eo7, 1t3g, and 4lqc, respectively).

The conserved aromatic residue at the CC loop (position 66^2^) forms a structural element conserved in most TIR domains (Fig. 2d). The side chain of this residue is involved in intramolecular interactions with three conserved residues of βC (Fig. 2d), one of which is serine/threonine-63 at the end of βC (Fig. 2a, d). This serine/threonine forms two hydrogen bonds with the backbone nitrogen of the conserved tyrosine/phenylalanine-66 (Fig. 2d). The other two interactions are through the aromatic side chain-66 and hydrophobic side chains of two core βC residues at positions 60 and 62 (Fig. 2d). An apparent function of these residues is to stabilize the drastic turn of polypeptide backbone after βC, thereby ensuring correct folding.

The glutamic acid-75 (E75) located between βC and βD is conserved in more than 70% of all TIR domains (Fig. 2a). This glutamic acid was found to be critically important for the NADase activity present in a subclass of bacterial, archaeal, and plant TIR domains, and in metazoan SARM1 (Essuman et al. 2017, 2018; Horsefield et al. 2019; Wan et al. 2019). We confirm the high frequency of E75 (> 90%) in TIR groups, for which the NADase activity has been reported, i.e., groups 2 (plant NB-LRR receptors) and 24 (SARM1 orthologs) (Supplemental Table 1). The E75 frequency, however, does not necessarily correlate with reported enzymatic activity. Thus, E75 is frequent (> 90%) in subgroup 3.2 (TIRs of TLRs that heterodimerize, i.e., TLR1, TLR2, TLR6, and TLR10), but is rare (< 10%) in endosomal TLRs, in TIRAP and in MyD88, i.e., groups 7, 10, and 23, respectively (Supplemental Table 1). The high global conservation of E75 suggests that this residue plays a role beyond catalysis. E75 is the only highly TIR-conserved residue with significant surface exposure; other TIR-conserved residues form a tightly packed core maintaining structural integrity (Fig. 2e). Surface-exposed residues mediating intermolecular TIR-TIR interactions are likely to be subgroup-specific.

### TIR domains of green plants

Plant TIR domains form three separate groups, i.e., 2, 16, and 21 (Fig. 1c), all from higher plants (Embryophyta) and each associated with a characteristic protein domain architecture. Group 2 includes 5 subgroups and consists of 8258 domains (Fig. 1a, c). Nearly all proteins harboring a group 2 domain have the TIR located at their N-terminus, followed by a nucleotide-binding (NB) domain, and C-terminal LRRs. This domain architecture represents NB-LRR proteins, a large, polyphyletic class of intracellular receptors that, upon recognition of pathogen-associated molecules or effectors, activate cell death-inducing proteases (Dodds and Rathjen 2010; Urbach and Ausubel 2017). The large size of group 2 is due to the abundance of genes of this type in plant genomes (Van der Biezen and Jones 1998). Group 16, which consists of 114 TIR domains, occur in proteins lacking LRRs, but typically having a TIR preceded by ~ 150–180 N-terminal residues and followed by a P-loop NTPase domain. Examination of domain architecture of group 21 proteins suggested these usually occur as single-domain adapter proteins, though some have a second interaction domain and a few have an amylase domain. Predominance of single-domain architecture in group 21 proteins suggests this group has evolved as adapters that facilitate recruitment of group 16 proteins to plant TIR-containing NB-LRR proteins. Finally, there are also ~ 1000 unclassified green plant TIR domains assigned to the root (Fig. 1c).

These observations suggest that group 21 corresponds to signaling adapter proteins that act downstream of group 2 TIR-containing NB-LRR receptors and that facilitate recruitment of group 16 TIR-containing NTPases. The relatively large number of group 2 TIR proteins suggests that plant NB-LRR receptors use common downstream group 16 and 21 TIR signaling proteins. This notion is compatible with the general observation that different pathogens elicit significantly overlapping gene expression profiles in plants and animals (Boller and Felix 2009). Because BPPS, based solely on the TIR domain residue patterns, almost perfectly differentiates plant protein families with different domain architectures, the sequence features conserved in these groups are likely to be functionally important.

#### Group 2: TIR domains of phytopathogen-sensing NB-LRR receptors

**Fig. 3:**
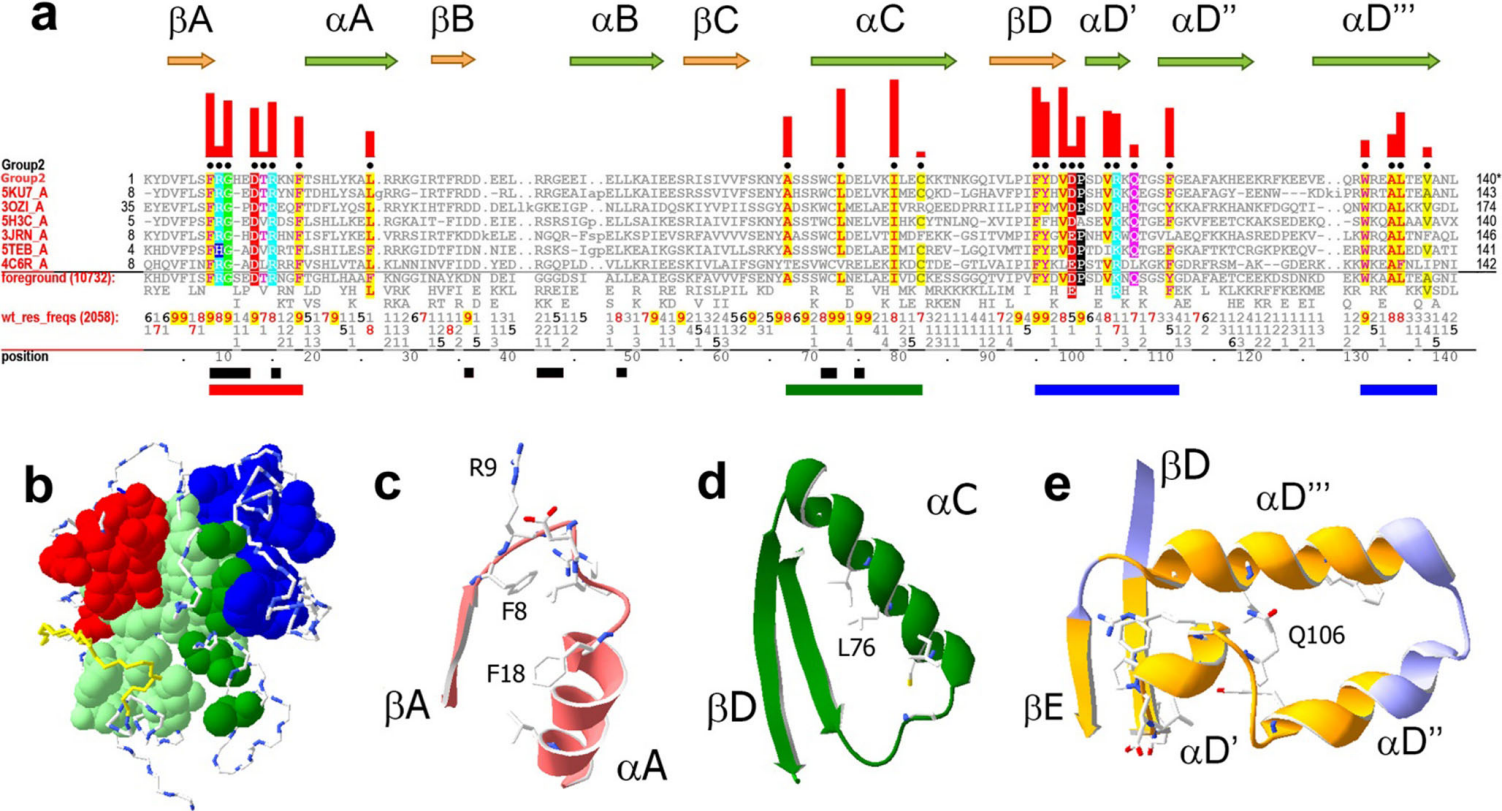
Group 2–specific residues and structural features. **a** Group 2 residue patterns. Formatting is as for Fig. 2a. Arrows above the alignment indicate secondary structure elements. Black bars directly below the alignment indicate residue positions forming the NADP-binding pocket of *Vitis rotundifolia* RUN1 NB-LRR receptor (PDB ID: 6O0W) (Horsefield et al. 2019). The colored horizontal bars beneath the alignment show clusters of pattern residues shown in same color in panel **b**. **b** Group 2–specific residue clusters in relation to residues conserved in all TIR domains for the grape RUN1 protein (PDB ID: 6O0W). Conserved residues and the backbone of non-conserved residues are shown in space-filling and stick modes, respectively. Group 2–specific residues of the AA loop, of αC, and of the αDs are colored red, dark green, and blue, respectively (i.e., as the horizontal colored bars in panel **a**); residues conserved among all TIR domains are colored light green. **c** Group 2–specific residues in the AA loop region of the grape Rpv1 protein (PDB ID: 5ku7) (Williams et al. 2016) and corresponding to the red cluster in panel **b**. **d** Conserved group 2 residues in the αC region, which contact core β-strands and helices αD″ and αD‴ (αDs are shown separately in panel **e**) (PDB ID: 3ozi; Bernoux et al. 2011). **e** The group 2–specific residues between βD and βE. This region comprises three αD helices that together form a hairpin-like structure oriented perpendicularly to β-strands. Sidechains of group 2 residues at the three-helix structural interface are shown. Highlighted in yellow are residues in van der Waals contacts with conserved residues

The pattern residues characteristic of group 2 TIR domains (Fig. 3a) form several clusters, as shown in Fig. 3b–e

##### AA loop conserved residues

Group 2–specific residues in the AA loop form a surface-exposed cluster (Fig. 3a, b), which includes both an -F_9_R_8_G_9_- motif (subscript numbers indicate the frequency of occurrence in tenth parts) and R15_8_ and which forms part of the ligand-binding pocket of the NAD-cleaving NB-LRR protein, RUN1 (black bars in Fig. 3a indicate pocket residues). The lack of conservation among residues corresponding to the ligand-binding pocket of RUN1 (such as those within the BB loop) is consistent with some plant-derived TIRs being incapable of cleaving NAD (Horsefield et al. 2019) and with plant NB-LRR receptors utilizing multiple signaling mechanisms (Wan et al. 2019).

##### Hydrophobic residues in the αC region

The side chains of conserved group 2 residues between β-strands C and D (Fig. 3a) interact with the TIR core and are less than 5% surface exposed (Fig. 3d).

##### Antiparallel, hairpin-like α-helical structure between βD and βE

**Fig. 4:**
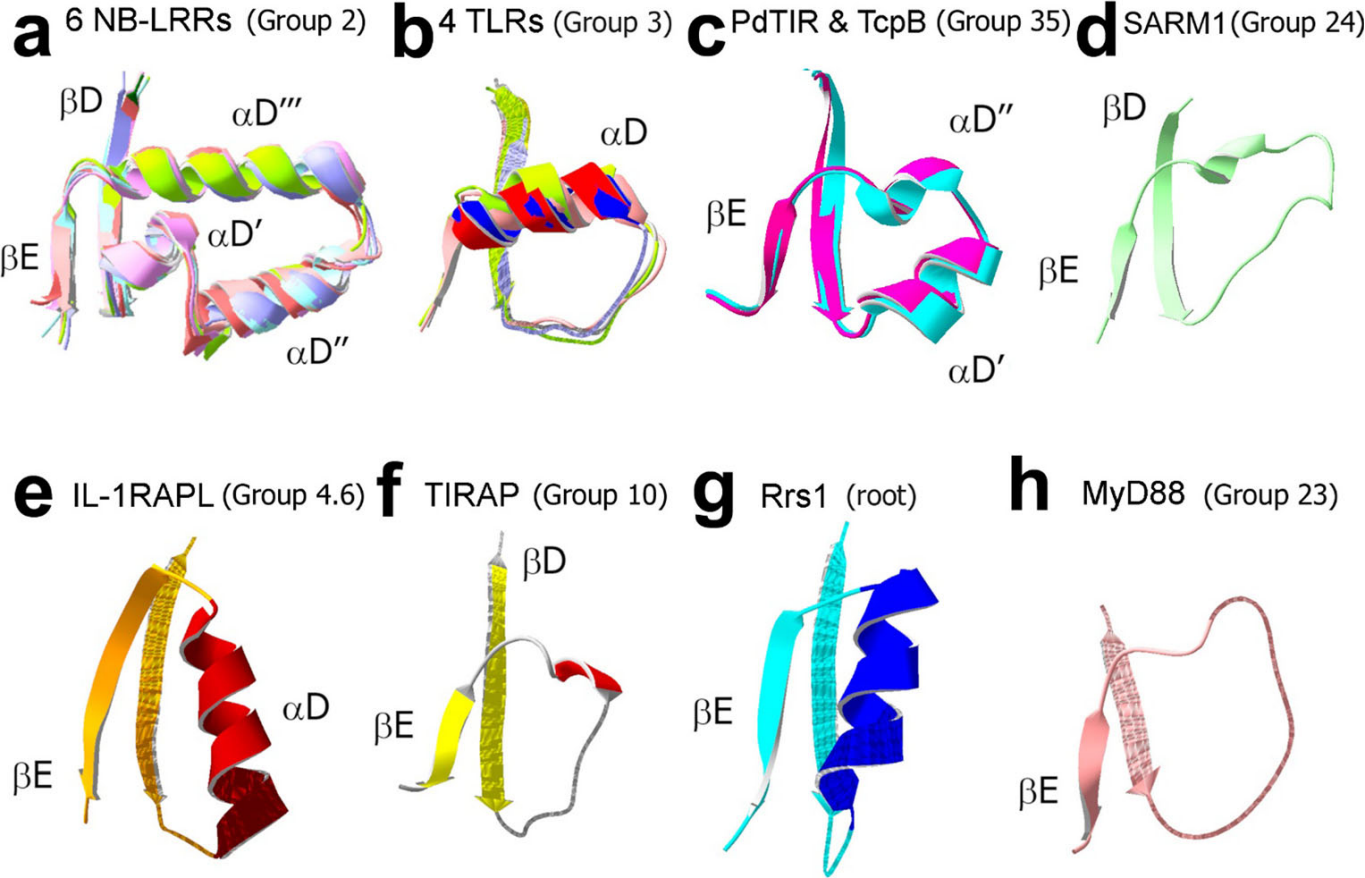
The fourth helical region of TIR domains differ among, but are highly similar within different groups. Shown are the βD- and βE-strands, together with the connecting segment, for TIR domains of different groups. Within each group, the region between βD and βE is nearly identically folded across multiple 3D structures, i.e., for groups 2, 3 (subgroup 3.2), and 35 (panels a-c), whereas across groups, this region differs drastically (panels a–h). **a** Region between βD and βE superimposed across six structures of group 2 TIRs: flax disease resistance protein L6 (*Linum usitatissimum*) (PDB ID: 3ozi) (Bernoux et al. 2011), NP_177436 protein from *Arabidopsis thaliana* (PDB ID 3jrn) (Chan et al. 2010), *Arabidopsis thaliana* suppressor of Npr1-1, constitutive 1 (SNC1) (PDB ID: 5h3c) (Hyun et al. 2016), RPS4, and RPP1 (PDB IDs: 4c6t) (Williams et al. 2014) and 5teb (Zhang et al. 2017), and grape RPV1 protein (PDB ID: 5ku7) (Williams et al. 2016). **b** Superimposed group 3.2 (TLRs which heterodimerize: TLR1, TLR2, TLR6, and TLR10) TIR regions corresponding to the region shown in panel **a** (PDB IDs: 1fyv, 1fyw, 4om7, and 2j67). **c** Superimposed regions for the bacterial group 35 TIRs: PdTIR from *Paracoccus denitrificans* (PDB ID: 3h16) (Chan et al. 2009) and TcpB from *Brucella abortus* (PDB ID: 4lqc) (Snyder et al. 2014). **d** Corresponding region for group 24 TIR (hSARM1, PDB ID: 6O0Q) (Horsefield et al. 2019). **e** Corresponding region for group 4 TIR IL-1R accessory protein-like (IL-1RAPL), also known as IL-1R9 (PDB ID: 1t3g) (Khan et al. 2004). **f** Corresponding region for TIRAP (PDB ID: 4lqd) (Snyder et al. 2014). **g** Corresponding region for Rrs1, a plant TIR assigned to the root (PDB ID: 4c6t) (Williams et al. 2014). **h** Corresponding region for group 23 TIR MyD88 (PDB ID: 2js7)

The largest cluster of group 2 residues occurs near the junction of the βD- and βE-strands with a hairpin-like structure of three α-helices (denoted αD′, αD″, and αD‴) that is oriented perpendicular to the β-strands (Chan et al. 2010) (Fig. 3e). All available group 2 TIR domain structures conserve this structural arrangement (Fig. 4a), which appears to uniquely characterize this group; in other groups, this forth helical region typically consists of one or two helices of varying length and orientation in relation to β-strands (Fig. 4b–h).

#### Groups 16 and 21: plant TIR domains

The structures of group 16 and 21 TIR domains are currently unknown. Group 16 TIRs are characterized by the presence of a conserved G7 in β-strand A, rather than the Ser present at this position in > 70% of all TIRs (Supplemental Fig. 1). Other group 16–specific residues are W21 (αA) and C33 (βB) (Supplemental Fig. 1). Box 2 is not conserved in group 16; although W130 of Box 3 is conserved, it is typically flanked by 3 polar residues, which rarely occurs in other TIRs. Group 21 characteristics include as follows: a C67 at the start of αC, followed by a group-specific highly conserved αC segment; a -Pro-Gly- pair in the C-terminal half of the BB loop; and a conserved αE region distinct from other TIRs (Supplemental Fig. 1).

### Toll and Toll-like receptor TIR domains

Arthropod Toll proteins and Toll-like receptors (TLRs) of other metazoans form groups 3, 7, and 29. With few exceptions, proteins in these groups are single-span transmembrane receptors consisting of N-terminal LRRs, a transmembrane helix, and a cytoplasmic TIR domain (Medzhitov et al. 1997; Rock et al. 1998). Group 3 is the largest group of metazoan TIR domains, represented here by 2763 sequences (Fig. 1c). Like group 2, the largest group of plant TIR domains, group 3 represents a family of (germline) receptors that detect pathogens. Most group 3 proteins are from insects and vertebrates, though they are also present in lower protostomes and deuterostomes, but not in sponges or flatworms (Gauthier et al. 2010; Nie et al. 2018; Wiens et al. 2007) (Supplemental Fig. 2)—suggesting group 3–specific features emerged during early evolution of metazoan body plans.

Group 7 and 29 TIRs are present only among chordates (Fig. 1c). Group 7 includes TIRs of TLR7, TLR8, and TLR9, which are endosomal TLRs that sense viral single-stranded RNA and bacterial CpG motif-enriched DNA. Group 29 TIRs are related to TLR11, TLR12, and TLR13. This group has a patchy distribution among chordates. For example, TLR11–13 are functional in many rodents and ungulates, whereas many distantly related species (such as humans and dogs) have a pseudogene at the syntenic position (Roach et al. 2005).

#### Group 3: Toll proteins and plasma membrane TLRs (TLR1–6 and TLR10s)

##### Group 3 subgroups

Group 3 TIRs are subdivided into 7 subgroups (Fig. 1a, e). Subgroup 3.1 is the largest (652 invertebrate TIRs) (Fig. 1e) and includes 604 arthropod Toll proteins along with representatives from 9 other phyla, i.e., Mollusca, Cnidaria, Echinodermata, Brachiopoda, Annelida, Priapulida, Tardigrada, and Chordata—though all 14 chordate proteins are from lancelets, which are primitive chordates. Subgroup 3.2 includes TLR2 and TLRs, which heterodimerize with TLR2, i.e., TLR1, TLR6, and TLR10 (Fig. 1e). Subgroup 3.3 TIRs are absent in mammals, but present in TLRs of cold-blooded vertebrates and birds. TLR3-, TLR4-, and TLR5-related TIR domains form subgroups 3.5, 3.7, and 3.4, respectively (Fig. 1e). As for group 3.3, group 3.6 TIRs are present in vertebrates, excluding mammals.

##### Group 3 proteins with atypical domain architectures

Only a few group 3 TIR proteins have a domain architecture different from that of Toll proteins and TLRs. Such atypical proteins are well documented for cnidarians; for example, in several actiniarian TIR proteins, Ig-like domains replace LRRs (van der Burg et al. 2016) and in *Hydra magnipapillata*, a membrane-anchored TIR protein lacks a ligand-binding domain (Bosch et al. 2009).

##### Group 3 TIR domain residue patterns and structural features

**Fig. 5:**
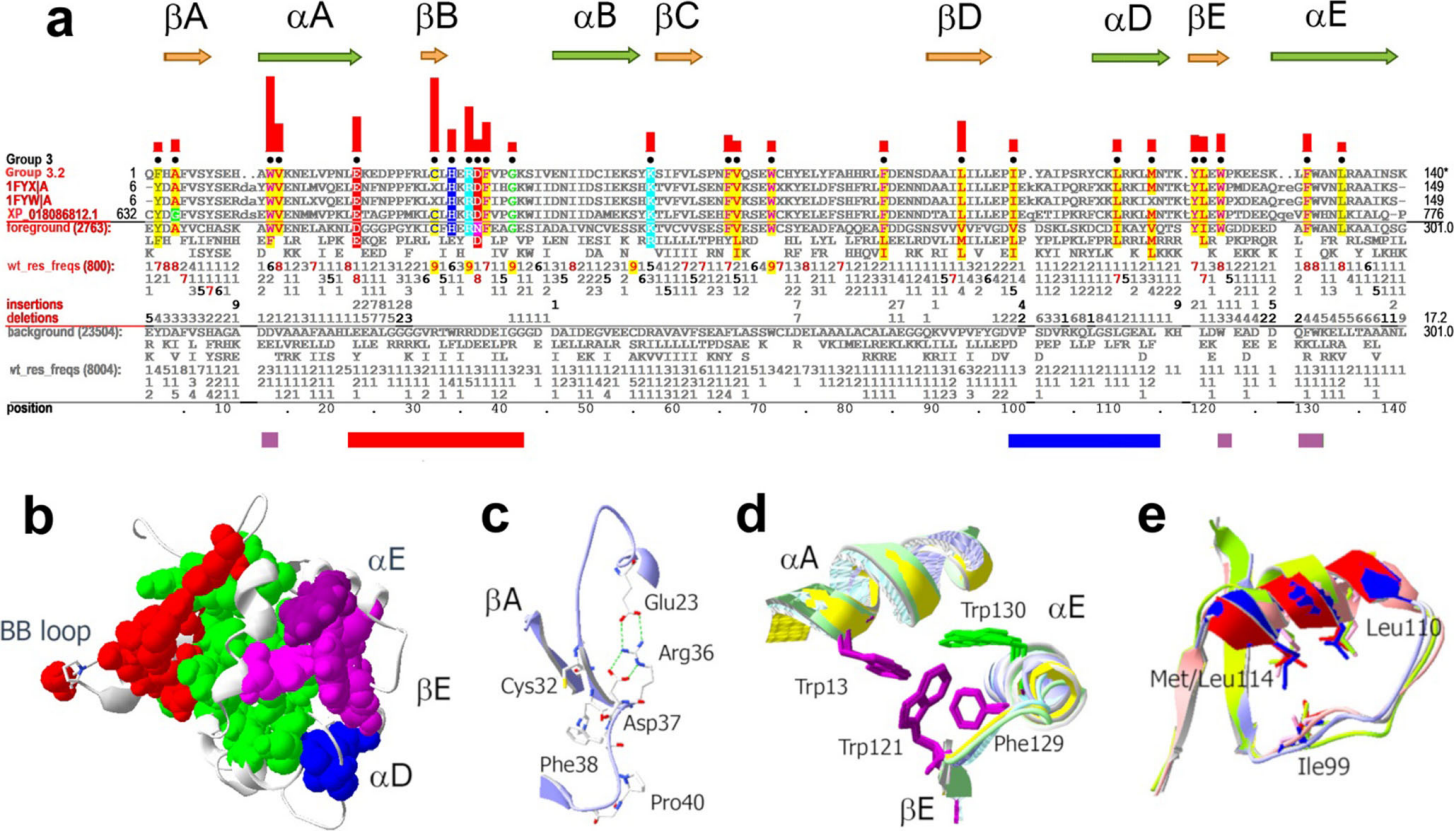
Residue patterns and structural features associated with Toll protein and Toll-like receptor TIR domains. **a** Group 3–specific TIR domain residue patterns. Red, purple, and blue bars correspond to the similarly colored clusters in panel **b**. **b** The βB, W/F13-V14/βE, and DD-loop/αD group 3 residue clusters shown in red, purple, and blue, respectively, for TLR2 TIR domain (PDB ID: 1FYW). Residue positions conserved in all TIR domains are light green. **c** Side chains of group 3 residues clustered near βB. R36 forms a salt bridge with E23 and with D37. The βA-strand is shown to reveal orientation relative to the β-sheet. **d** The W/F13-V14/βE cluster consisting of W121 of βE, F129 and W130 of αE, and W13 of αA. To reveal the similar side-chain orientations of group 3 residues, the backbone atoms of αA, βE, the EE loop, and αE are superimposed for all four currently available structures: TLR1 (PDB ID: 1FYV) (Xu et al. 2000), TLR2 (PDB ID: 1FYW) (Xu et al. 2000), TLR6 (PDB ID: 4OM7) (Jang and Park 2014), and TLR10 (PDB ID: 2J67) (Nyman et al. 2008). **e** Three buried hydrophobic group 3 residues in the αD region

Group 3–specific residue clusters (Fig. 5a) and their structural locations (Fig. 5b–e) differ from those of group 2. Two largest clusters (highlighted by red and purple in Fig. 5b) are located at opposite TIR surfaces near β-strands, which form the lateral edges of the β-sheet, i.e., strands βB and βE (Fig. 5a, b). These clusters correspond to two out of four sites that mediate assembly of TLR complexes and initiation of intracellular signaling (Javmen et al. 2018; Toshchakov and Javmen 2020; Ve et al. 2017). The TIR sites near βB and βE mediate TLR TIR dimerization and lateral extension of the initial complex through recruitment of adapter TIRs. Many group 3 TLR residues are within these interaction sites; these conserved features are discussed below in more detail.

##### Cluster of conserved residues near βB (extended Box 2)

The largest group 3–specific cluster includes 7 conserved residues near βB that form the motif: -E_8_-x-(x-x)-x[5]-C_9_-x-H_6_-x-R_9_-D_8_-F_7_-x-x-G_9_- (Fig. 5a, c). This cluster corresponds to the red bar below the Fig. 5a alignment. The first residue of this motif, E23, is at the C-terminal end of αA, followed by a variable AB loop region. E23 is present in more than 80% of group 3 TIRs, while another ~ 10% have aspartic acid at this position (Fig. 5a). The three charged residues of the motif, i.e., E23, R36, and D37, can form two salt bridges, where R36 interacts with both acidic residues, thereby creating an atomic layer that covers the edge-forming strand of the β-sheet (Fig. 5c), which may prevent non-specific interactions with other β-sheet proteins (Richardson and Richardson 2002).

C32 in the βB is buried in available structures of group 3 TIRs, i.e., of human TLR1, TLR2, TLR6, and TLR10 TIRs. In TLR1 and TLR6 (PDB IDs: 1FYV and 4OM7), this cysteine forms a disulfide bond with C51 situated at the buried side of βB (not shown). The TLR2 and TLR10 TIRs lack C51, which is replaced by a serine or phenylalanine residue that contacts C32 (PDB IDs: 1FYW and 4J67). The group 3 BB loop pattern residues (-H-x-R-D-F-x-x-G-) have a high surface exposure and backbone torsion angles that vary significantly among available TIR structures, indicating that this region is conformationally flexible.

The BB loop region of TLRs has long been defined as Box 2, one of three TIR domain conserved regions (Slack et al. 2000) (Fig. 2a). This survey, which analyzes ~ 500 times more sequences than the initial survey, identifies additional TLR-specific conserved positions near Box 2, including the acidic residue at the C-terminal end of αA, F38 of the BB loop, and C32 of βB.

##### Cluster of aromatic residues near βE (extended Box 3)

The short βE-strand is a conserved feature of group 3 TIR domains that forms one edge of the β-sheet; it contains two group 3 residues and is preceded by the group 3 residue Y118 (Fig. 5a). The most frequent variant of the βE motif is -Y_7_-I/L_8_-x-W_8_-P_5_- (Fig. 5a). These βE motif residues, unlike those of the βB motif, have minimal surface exposure and therefore seem unlikely to mediate intermolecular interactions. The I/L119 and W121 side chains are on the side of β-sheet facing helices A and E (Fig. 5d). W121 contacts group 3 residues W/F13 of αA, and F129 and W130 of αE, whereas W/F13 also contacts W130 (Fig. 5d); these are distantly located in the primary sequence of TIR domains but interact in the 3D structure, suggesting a role in maintaining structural integrity. Two of these 4 residues, F129 and W130, constitute Box 3 (Slack et al. 2000). Hence, our findings clarify the functional significance of Box 3, as it is a part of a larger set of aromatic residues mediating intramolecular contacts involving helices A and E.

##### Conserved residues in the region between β-strands D and E

Two αD (positions 110 and 114) and one DD loop (position 99) hydrophobic residues (Fig. 5a, e) form another group 3–specific cluster. The region containing this cluster (blue bar in Fig. 5a) forms the group 3–specific fold variant of the fourth helical region (Figs. 4b and 5e), homologous to the hairpin structure of group 2 TIRs, shown in Figs. 3e and 4a. In both groups, the conserved residues contact the core residues of the β-sheet and apparently determine the fold of the αD region and its orientation in relation to the β-sheet, distinct from that in other groups (Fig. 4). The side-chain and backbone conformation for residues 99, 110, and 114 are remarkably well conserved for all four group 3 proteins of known 3D structure (Fig. 5e).

#### Group 7: endosomal, nucleic acid–sensing TLRs

The most prominent feature of group 7 TIR domains, which include TLR7, TLR8, and TLR9, is a > 90% conserved pattern in the N-terminal half of the BB loop, -E_9_E_9_R_9_D_9_W_9_xP_9_G_9_-, with the glutamate and tryptophan residues being the most distinguishing feature (Supplemental Fig. 1). Cell-permeable decoy peptides, which included either a large segment of this region or the entire motif, potently inhibited TLR9 signaling in cultured macrophages and in mice (Javmen et al. 2018). Another highly group 7–specific residue is D9 at the C-terminal end of βA. Group 7 TIRs also conserve characteristic αC and αD motifs (Supplemental Fig. 1). However, a lack of structural data hinders functional interpretations.

#### Group 29: TLR11–13

The most prominent features of group 29 are C61 in βC and a highly conserved residue cluster between βD and βE (residues 98–115) (Supplemental Fig. 1), which suggests yet another αD conformation variant among those shown in Fig. 4.

### TIR domains of cytokine receptors

Group 4 TIRs, the third largest eukaryotic group (2203 sequences; Fig. 1c), correspond to the IL-1R family of cytokine receptors (Boraschi and Tagliabue 2013; Garlanda et al. 2013). In humans, all 10 of these TIR proteins contain a single transmembrane helix, a cytosolic TIR, and one or three extracellular Ig-like domains. The IL-1R family members function as heterodimers that recognize cytokines IL-1s, IL-18, IL-33, and IL-36s and activate downstream signaling molecules, many of which can also be activated by TLRs (Boraschi and Tagliabue 2013).

#### Group 4 subgroups

Group 4 includes six offspring subgroups (4.1–4.6), each of which includes characterized members of the IL-1R family (Fig. 1a). Members of subgroup 4.1, which is the largest in our analysis (281 sequences), correspond to TIRs within the main subunits of IL-1 or IL-36 receptors, namely IL-1R1 and IL-1R6, respectively (Garlanda et al. 2013). Subgroup 4.2 includes 145 TIR domains related to the IL-33 receptor IL-1R4 (Fig. 1e), also known as ST2 (Boraschi and Tagliabue 2013; Garlanda et al. 2013). Subgroup 4.3 includes SIGIRR (single Ig and TIR domain containing), an orphan IL-1R family receptor, also known as IL-1R8 (Garlanda et al. 2013). Subgroups 4.4 and 4.5 correspond to TIRs in IL-18R’s main and auxiliary subunits: IL-1R7 and IL-1R5, respectively. Subgroup 4.6 TIRs are related to IL-1R9, an orphan receptor also known as IL-1RAPL (Boraschi and Tagliabue 2013), and includes the only group 4 TIR of known structure, human IL-1R9 (PDB ID: 1T3G) (Khan et al. 2004).

#### Evolutionary distribution of group 4 TIRs

As previously noted for IL-1R family (Buchmann 2014; Venkatesh et al. 2014; Wang and Secombes 2013), we find that all group 4 TIR domains occur in jawed vertebrates, but not in jawless fishes and lancelets. The elephant shark genome, which is reported to be the slowest evolving genome of any vertebrate, encodes (at least) 7 IL-1R family members, all belonging to group 4 (Supplemental Fig. 2; Venkatesh et al. 2014). This phylogenetic distribution pattern suggests that the group 4–specific traits arose simultaneously with IL-1R family and many other novel immune molecules in a series of macroevolutionary events, which followed two rounds of whole genome duplication, leading to the emergence of the adaptive immunity in jawed vertebrates (Flajnik and Kasahara 2010). Cnidarians appear to have multiple proteins with IL-1R-like domain architectures (van der Burg et al. 2016), but our analysis assigned these to group 3 (Supplemental Fig. 2). Thus, the similarity of cnidarian IL-1R-like proteins and IL-1R family members appears due to convergent evolution.

#### Group 4 residue patterns and structural features

In addition to the three TIR domain conserved boxes described by Slack et al. (Slack et al. 2000), group 4 TIRs conserve several group-specific motifs.

##### Conserved αA-αE contacts through four aromatic residues (extended Box 3)

**Fig. 6:**
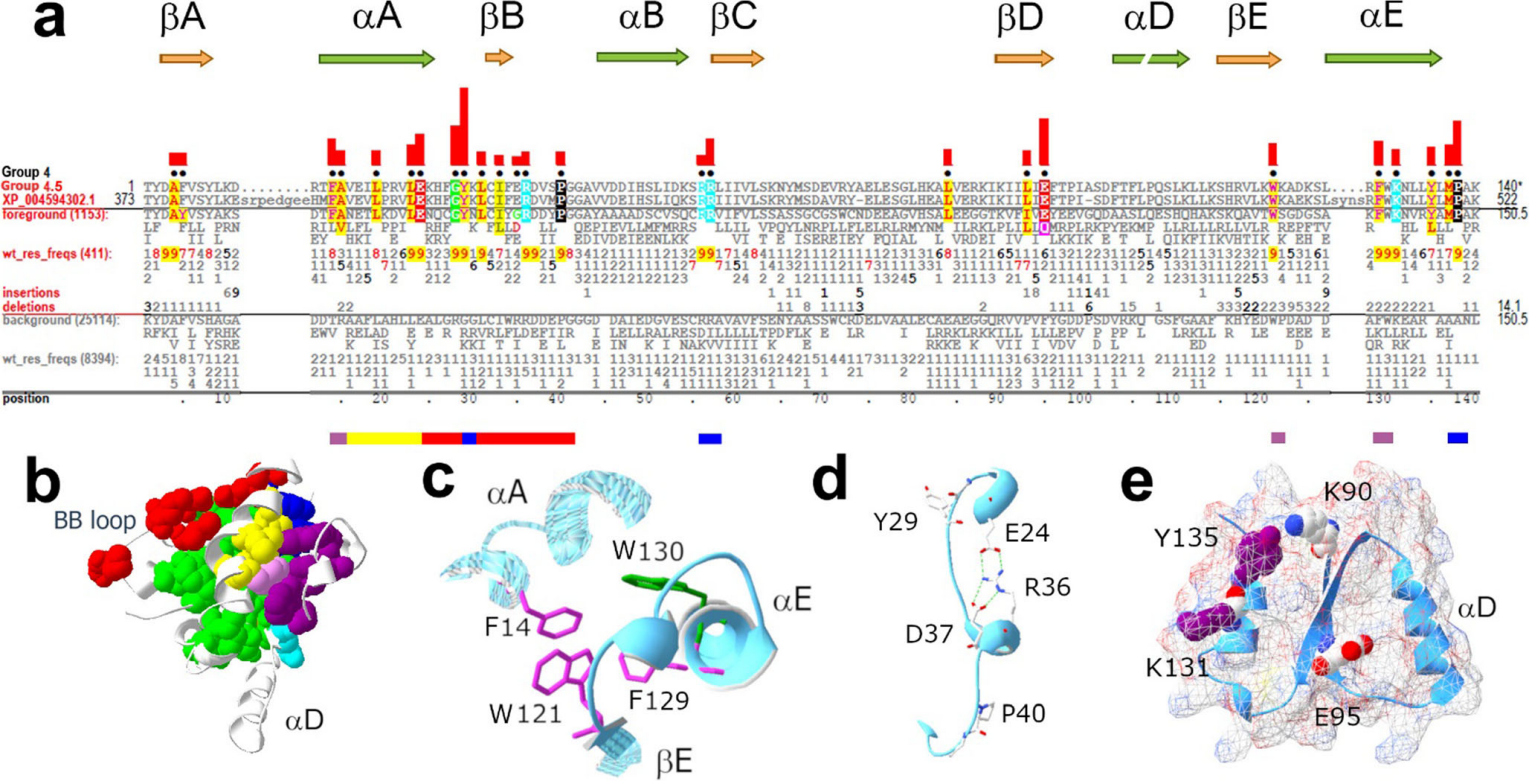
Group 4 (IL-1R family) TIR domain sequence and structural features. **a** IL-1R family TIR domain conserved residue patterns. Bars below alignments correspond to structurally interacting regions colored to correspond to the clusters of mutually interacting residues shown in panel **b**. **b** Four 3D clusters of group 4–specific residues with side chains shown in red (the βB cluster), purple (the βE cluster, including F14), yellow (the hydrophobic side of αA), and blue for the IL-1R9 TIR domain (PDB ID: 1T3G) (Khan et al. 2004). Residues conserved in all TIR domains are colored light green. **c** A conserved cluster of four group 4 aromatic residues in the IL-1R9 TIR. This cluster, which is similar to the analogous group 3 cluster, includes F129 and W130 of Box 3, W121 at the C-terminus of βE, and F14 in αA. **d** Group 4–specific cluster of conserved residues near the βB-strand and homologous to the group 3 cluster that, as shown in Fig. 5c, also conserves both the three charged residues and the proline-glycine pair of the BB loop (see Fig. 5a). **e** Conserved structural features in the vicinity of βE

More than 90% of group 4 TIRs conserve the Box 3 aromatic residues F129 and W130 (Fig. 6a), which contact the conserved W121 at the C-terminal end of βE and F14 in αA (Fig. 6c)—as is seen for the homologous group 3 cluster (Fig. 5d), except that the W121 aromatic ring is turned by ~ 180°.

##### AB loop, β-strand B, and BB loop (extended Box 2)

The cluster of residues conserved near βB in group 3 (-E_8_-x(8)-C_9_-x-H_6_-x-R_9_-D_8_-F_7_-x-x-G_9_-) is partly conserved in group 4 (-E_9_-x(3)-G_9_-Y_9_-x-L_9_-x-I_7_-x-x-R_9_-D_9_-x-x-P_9_-G_8_-), as they share 4 out of 7 residues. In both cases, all 3 charged residues interact with each other in a similar manner, with nearly identical backbone folds for the entire region (compare Fig. 6d with Fig. 5c). The most notable difference is the presence of a conserved -G28-Y29- pair in the AB loop of group 4 TIRs (Fig. 6a). The side chain of Y29 interacts with group 4–specific M138 at the C-terminal end of αE.

##### The βE-strand is atypically long and solvent-exposed: conserved polar or charged residues near βE

In the only known group 4 TIR structure, IL-1RAPL (PDB ID: 1T3G) (Khan et al. 2004), αD is long and parallel to the β-strands and differs from that of other metazoan TIRs of known structure (Fig. 4e). βE is similarly elongated (to 7 residues, compared with 3 residues in group 3 TIRs) (Fig. 4e) and forms a significant surface patch (Fig. 6e). This feature is contrary to the general tendency that, in globular domains, strands which form β-sheet edges are short and buried (Richardson and Richardson 2002), suggesting a possibility of the edge-to-edge type interaction of group 4 TIRs with other β-sheet proteins. However, group 4 fails to conserve residues in the αD region (Fig. 6a), suggesting that other IL-1R family members may not share IL-1RAPL unusually shaped αD and βE regions.

In addition to the extended backbone of βE, the regional surface features include several conserved charged residues. Two of these (most frequently K90 and E95) are located at the ends of βD (Fig. 6e). This pair is conserved in > 90% of subgroup 4.1, 4.2, 4.5, and 4.6 TIRs, whereas subgroup 4.4 (the TIR of the main subunit of IL-18R) has leucine in both positions and subgroup 4.3 (the TIRs of SIGIRRs) lacks this βD feature (Supplemental Fig. 1). Two other surface-exposed conserved residues near βE are K131 and Y135 of αE (Fig. 6e), which are present in > 90% and 70% of group 4 TIRs, respectively (Fig. 6a).

##### Conserved contacts at the αE C-terminus

Group 4 TIRs conserve two buried residues at the αE C-terminus, M137 and P138, which contact Y29 of the AB loop and two positively charged residues (positions 56 and 57) at the βC N-terminus, respectively (Fig. 6a, b).

### TIR domains of SARM1 orthologs (group 24)

**Fig. 7:**
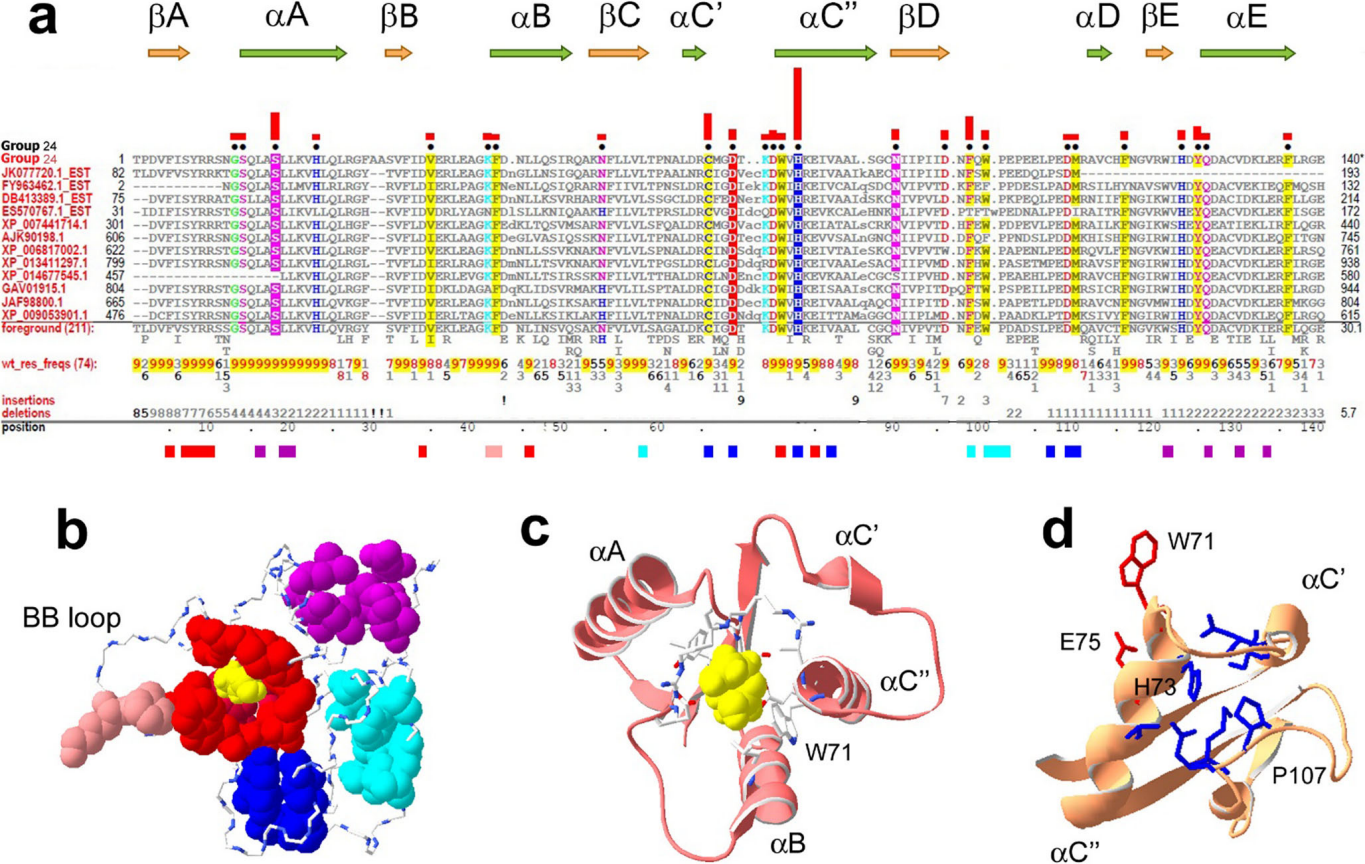
Group 24 (SARM1-related) TIR domain sequence and structural features. **a** Group 24 contrast alignment. Colored horizontal bars indicate the mutually interacting residues shown in panel **b**. **b** Clusters of group 24–specific residues. Color scheme: residues (also shown in panel **c**) contacting ribose, red; ribose, yellow; residues in the αC and αD regions, light and dark blue, respectively; residues mediating αA-αE contacts, purple. **c** Ligand-binding pocket of SARM1 showing side chains for residues contacting ribose in human SARM1 bound to ribose (PDB ID: 6O0Q) (Horsefield et al. 2019). **d** H73 is on the opposite side of αC″ from W71 and E75, which bind ligand, and interacts with the other group 24 residues shown in blue; this cluster likely stabilizes this SARM1-specific structural motif

Group 24 includes SARM1 TIRs (Supplemental Fig. 2). These proteins harbor multiple Sterile α motifs (SAM) or Armadillo (Arm) repeats or both, along with a C-terminal TIR (Mink et al. 2001; O’Neill et al. 2003). Group 24 TIRs occur among diverse metazoans (Fig. 7a), with typically one SARM1-like protein per genome (Fig. 1e).

Among metazoan TIRs, only SARM1 TIRs have known NADase activity (Essuman et al. 2017, 2018; Horsefield et al. 2019), which many bacterial and plant TIRs possess. SARM1’s catalytic activity mediates neuron-specific programmed cell death in response to axonal injury due to mechanical or chemical trauma (Gerdts et al. 2013; Osterloh et al. 2012). It was proposed that metazoans acquired these TIRs from bacteria through multiple lateral gene transfers (Zhang et al. 2011), though our analysis fails to find bacterial SARM1 homologs. A crystal structure of human SARM1 reveals the ligand-binding pocket and catalytic site (Horsefield et al. 2019).

#### Group 24 residue patterns and structural features

##### Conservation of Box 1

Group 24 TIR domains have a moderately conserved Box 1 with a consensus (-P_6_D_9_V_9_F_9_I_6_S_9_Y_9_-) that differs from the global consensus (-Y-D-V-F-I-S-Y-) only by the N-terminal residue (Fig. 7a).

##### The BB loop region

Group 24 conserves the BB loop region (typically as E_8_(R/K)_9_L_9_E_7_A_9_G_9_-) (Fig. 7a), but not the consensus Box 2 motif (Carlsson et al. 2016). The first, second, and sixth residues of -E_8_(R/K)_9_L_9_E_7_A_9_G_9_- are important for catalysis (Summers et al. 2016), and this region is associated with binding of SARM1 to TLR adapters MyD88 and TRIF (Carlsson et al. 2016).

##### Group 24–specific αA-αE contacts are aliphatic

Group 24 TIRs lack the cluster of group 3 and 4 4 aromatic residues mediating helix-helix and helix-strand contacts for αA and αE (Figs. 5d and 6c). Instead, the buried surface of αA (positions 16, 19, 20, and 24) mainly consists of leucines (> 90% for each position) (Fig. 7a). The αA-αE contacts are mostly through αA leucine residues, but also involve W121 of βE (Fig. 7a, b).

##### Conservation of group 24 residues forming the NAD-binding pocket

The residues that bind ribose in the ribose-SARM1-TIR complex (PDB ID: 6O0Q) are shown in red in Fig. 7a, b. As for TIRs of catalytically active plant NB-LRR TIRs, the SARM1 ligand-binding pocket consists of αC″, αB, and βA residues and of the AA and BB loops (Fig. 7c). Each group 24 residue forming the ribose-binding part of the NAD-binding pocket is > 90% conserved, suggesting that NADase activity is a common function of these proteins.

##### Clusters of group 24 residues in the αC and αD regions

The group 24 residues shown in light and dark blue in Fig. 7 a, b, and d are likely determinants of the SARM1-specific fold variant shown in Fig. 4d.

##### The group 24–specific EE loop residues

The side chains of all residues that form the group 24–specific EE loop motif, -H_9_D/E_9_Y_9_Q_9_- (Fig. 7a), correspond to a surface patch, possibly mediating intermolecular interactions.

### Group 10: TIRAP/Mal-related TIR domains

Group 10 TIR domains occur in chordate proteins, in the adapter protein TIRAP/Mal, which is involved in TLR signaling (Fitzgerald et al. 2001; Horng et al. 2001). Based on X-ray crystallography (Lin et al. 2012; Snyder et al. 2013; Valkov et al. 2011; Woo et al. 2012), the TIRAP TIR domain has an abnormal fold, in that βB is shifted C-terminally by ~ 15 residues relative to other TIR domains, resulting in the absence of αB and in an abnormally long, unstructured AB loop. NMR spectroscopy reveals, however, that in solution, the size and backbone conformation of the TIRAP TIR βB region is similar to that of groups 3 and 4 (Hughes et al. 2017). High-resolution cryo-EM of the tertiary structure of self-assembled, oligomeric complexes of TIRAP TIR domains in solution (Ve et al. 2017) revealed an open-ended, multifilamentous organization of TIR oligomers, with monomers of the assembly having a rather typical TIR structure, which resembles the NMR structure.

#### Group 10 sequence patterns and features

**Fig. 8:**
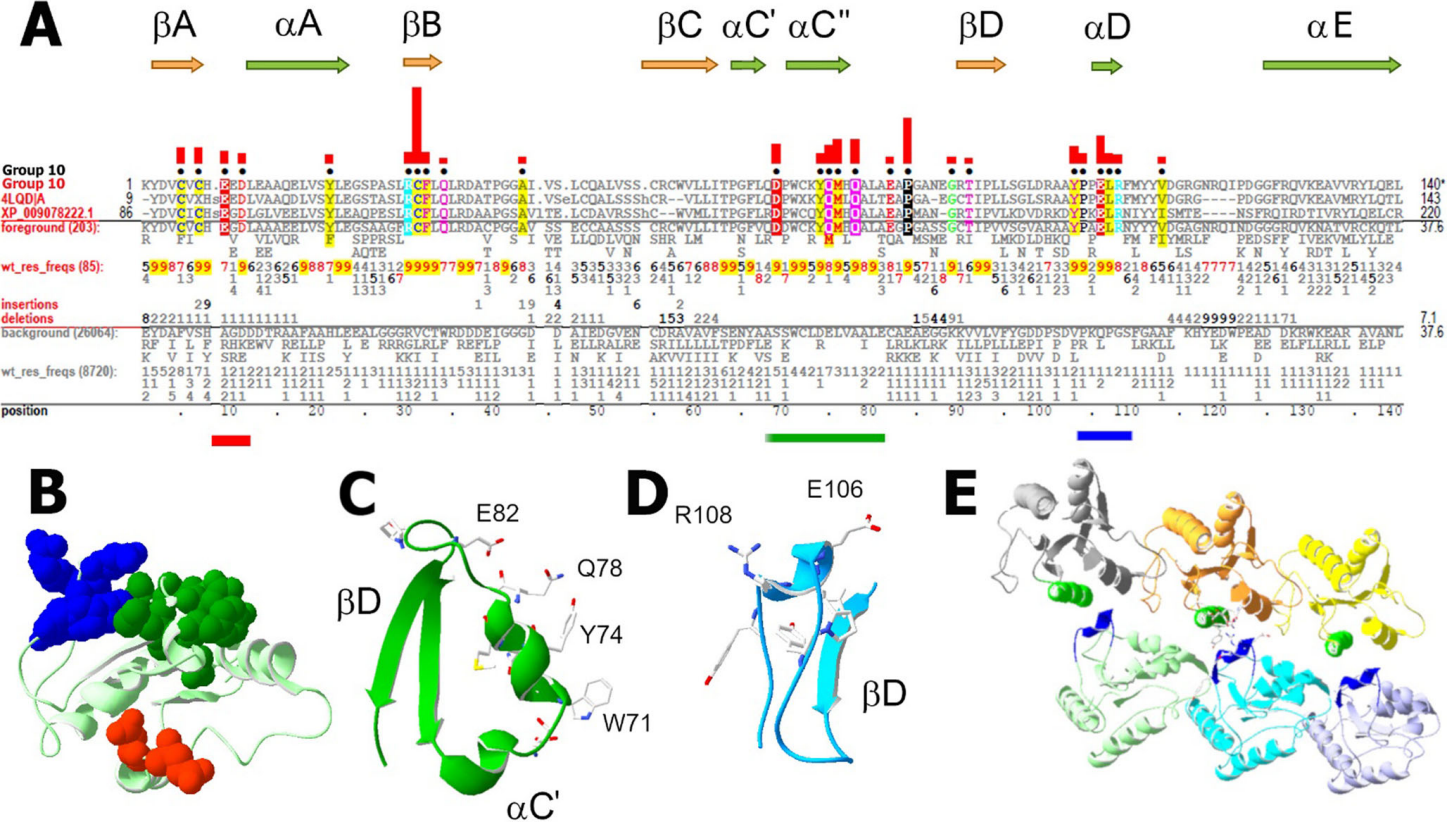
Group 10 (TIRAP-related) TIR domains. **a** Group 10 contrast alignment. Horizontal bars beneath indicate residues shown in panel **b** using the same color scheme. **b** Clusters of surface-exposed group 10 residues in chain A of the oligomeric TIRAP assembly (PDB ID: 5UZB) (Ve et al. 2017). Color scheme: AA loop residues, red; αC region, dark green; αD region, blue. **c** Residues in the αC region. W71, Y74, Q78, and E82 are juxtaposed on the surface to form a large patch. **d** Residues in the αD region. E106-L107-R108 are characteristic of TIRAP-related TIR domains. **e** αC and αD conserved surface patches mutually interact in oligomeric TIRAP TIR protofilaments. The image represents the double-stranded fragment of TIRAP signalosome composed of six TIRAP monomers (PDB ID: 5UZB) (Ve et al. 2017). αC″ helices are shown in green for 3 TIRs forming the “upper” strand of the double-stranded protofilament. αDs are shown in dark blue for 3 TIRs forming the lower strand. For clarity, reciprocal interactions of upper TIR αDs with lower TIR αCs are not shown

The consensus Box 1 of group 10 TIRs (-Y_9_D_9_V_9_C_7_V_6_C_9_H_9_-) differs from the global consensus by two cysteines (Fig. 8a), and Box 2, the BB loop, is highly conserved. However, group 10 lacks both Box 3 and the aromatic residues of αA and βE, with which the conserved aromatic residues of Box 3 interact in groups 3 and 4 (Figs. 5d and 6c).

##### AA loop charged residues

As for group 2 (plant NB-LRR receptor; Fig. 3a, c) and group 24 (SARM1-related; Fig. 7a) TIRs, group 10 TIRs conserve AA loop charged residues (Fig. 8a, b).

##### Conserved β-sheet cysteines

Group 10 TIRs conserve βA cysteines (C5 and C7 in Fig. 8a), the side chains of which are on the convex side of β-sheet facing αB, αC, and αD. In available structures of human TIRAP, C5 forms a disulfide bond with C48 (Lin et al. 2012; Snyder et al. 2013; Valkov et al. 2011; Woo et al. 2012) that, however, appears to be a crystallographic artifact, which accounts for TIRAP’s significant structural deviation from other TIRs. The NMR structure of human TIRAP and the cryo-EM structures of oligomeric TIRAP complexes (Hughes et al. 2017; Ve et al. 2017) lack the C5-C48 disulfide bond, and, unlike the BB loop Box 2 motif (-R_9_D_9_-ϕ-ϕ-P_8_G_9_-), C48 is only ~ 50% conserved in TIRAP-related proteins (Fig. 8a). C7, which was proposed to be involved in the redox regulation of TIRAP through glutathionylation (Hughes et al. 2017), occurs in > 90% of group 10 TIRs and in < 10% of other TIRs (Figs. 2a and 8a). C31 in βB is another group 10–specific cysteine on the same side of the β-sheet and contacts several residues of αA and the BB loop; it is neither surface-exposed in resolved structures nor involved in intermolecular contacts in the oligomeric TIRAP complex (Ve et al. 2017). An additional disulfide bond connecting C55 of βC to C90 of βD occurs in human TIRAP (Snyder et al. 2014; Valkov et al. 2011) but not in other group 10 TIRs, < 10% of which conserve C90 (Fig. 8a).

##### Conformational flexibility of βB and adjacent loops

The significant difference between crystal and solution structures of group 10 TIRs indicates that the βB region is conformationally flexible. This flexibility might be important for function because protein interfaces formed by disordered regions typically have a better fit and bind a larger set of targets (Wright and Dyson 2015). Indeed, this region mediates homomeric interactions in TIRAP filaments and also heteromeric interactions with MyD88 and certain TLR TIRs (Javmen et al. 2018; Ve et al. 2017). The high flexibility of group 10 BB loops might be due to a high number of residues with a small side chain, as the most frequent BB loop motif (-R_9_D_9_A_7_T_3_P_8_G_9_G_6_A_8_-) has two glycines and two alanines (Fig. 8a).

##### Two group 10 residue clusters forming surface patches

Surface patches are formed by clusters associated with the αC- and αD-regions (Fig. 8a–d). The N-terminal cluster (green bar in Fig. 8a) includes nine group 10 residues in the 23-residue long region between βC and βD (Fig. 8a–c). Notably, four residues in the αC region, -W_9_-x-x-Y_9_-x(3)-Q_9_-x(3)-E_8_-, are juxtaposed on the TIR surface (Fig. 8c). The C-terminal cluster (the dark blue bar in Fig. 8a) is a more compact, 6-residue motif (-Y_9_P_9_-x-E_9_L_9_R_8_-x-x-Y_8_-) centered on the short 3_10_-helix D (Fig. 8a, b, d). Cell-permeating decoy peptides based on these segments inhibited TLR signaling (Couture et al. 2012). These two conserved surface patches, located in αC and 3_10_-D helix of TIRAP TIRs, correspond to TIR interfaces that mediate self-assembly of filamentous TIRAP complexes in solution (Ve et al. 2017 and Fig. 8e). Other TIR-derived decoy peptide studies suggest that nearly the same interfaces mediate the heterotypic TIRAP interactions with both MyD88 and TLR TIRs (Javmen et al. 2018; Toshchakov and Javmen 2020; Ve et al. 2017). Notably, the group 10 surface patches are larger (especially the αC site) than the actual TIR-TIR contact areas in TIRAP protofilaments (Fig. 8c–e), which provides an explanation for the multispecificity of TIR-TIR interactions mediated by these regions: different segments of αC″ may be critical for interactions with TIRs in different TIRAP heterocomplexes (for detailed discussion, see references Javmen et al. 2018, Toshchakov and Javmen 2020, and Ve et al. 2017).

### Groups 23 and 32: MyD88-like proteins

**Fig. 9:**
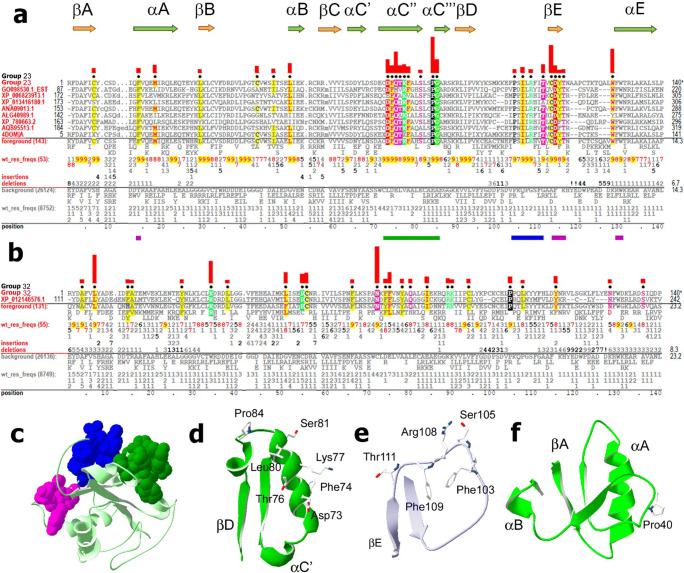
Group 23 and 32 (MyD88-like) TIR domains. **a** Group 23 TIR domain contrast alignment. Horizontal bars indicate three groups of residues shown in the same colors as in panel **c**. **b** Group 32 contrast alignment. **c** Solution NMR structure of human MyD88 (PDB ID: 2JS7), a representative group 23 TIR. Group 23–specific residues in βE are shown in magenta; residues in the αC and αD regions are shown in green and blue, respectively. **d** Group 32 residues in the αC region form the exposed surface of αC″. **e** Segment between the βD and βE strands. **f** Atypical BB loop structure of the human MyD88 TIR domain

MyD88 is an adapter protein utilized by two large metazoan receptor families, i.e., the IL-1R family and TLRs (Medzhitov et al. 1998; Wesche et al. 1997), which harbor group 4 and 3 TIRs, respectively. MyD88-related TIRs belong to two groups: group 23 TIRs from non-arthropod metazoans and group 32 TIRs from arthropods (Fig. 1e). Group 23 is formed mostly by chordate proteins, but also includes representative cnidarian TIRs, several TIRs of lower deuterostomes (both echinoderms and hemichordates), and TIRs of two protostome phyla, i.e., Annelida and Brachiopoda. These organisms typically have one gene encoding a protein with a group 23 TIR; however, some species of protostomes and deuterostomes, including echinoderms, may have multiple genes encoding MyD88-like proteins (Ren et al. 2014; Tassia et al. 2017). A typical MyD88-like protein has two protein interaction domains, an N-terminal death domain, and a C-terminal TIR (Hardiman et al. 1996). Invertebrates may have more complex domain architectures (Lee et al. 2011; Supplemental Fig. 2). For example, Arthropoda MyD88s typically have a phosphoinositide-binding domain, C-terminally to the TIR (Horng and Medzhitov 2001; Marek and Kagan 2012). All 3 “Boxes” of conserved residues are present in MyD88-like TIRs. The typical Box 1 sequence of group 23 is -F_8_D_9_A_9_F_9_I_7_C_9_Y_9_- (Fig. 9a). Box 2 is highly conserved, with a consensus (-R_9_D_9_V_7_L_9_P_9_G_9_-) similar to that of TLRs (group 3), IL-1R family (group 4), and TIRAPs (group 10). E23 at the C-terminal end of αA is conserved in all four groups, along with the Box 2 -RD- motif (Figs. 5c, 6d, 8a, and 9a). Box 3 (residues 129 and 130) has the typical -F_8_W_9_- pattern, which is similar to that of groups 3 and 4, but dissimilar to that of TIRAP (group 10).

#### Group 23 residue patterns and structural features

##### Conserved motifs in the αC and αD regions mark sites mediating TLR signalosome assembly

Two motifs in the αC and αD regions and a third in βE distinguish the MyD88-like TIRs of non-arthropods from other TIRs (Fig. 9a, c). These motifs, together with a group 23 conserved βB region (which is similar to the homologous region in groups 3, 4, and 10), correspond to TIR sites mediating the assembly of TLR signaling complexes (Javmen et al. 2018; Toshchakov and Javmen 2020; Ve et al. 2017). The αC and αD clusters correspond to homologous regions conserved in TIRAP TIRs (group 10), though the group 23 variant of the αC″ surface-forming motif (-73D_9_F_9_-x-x-K_9_-x(3)-S_8_-) differs from the group 10 motif (-71W_9_-x-x-Y_9_-x(3)-Q_9_-x(3)-E_8_-) (Fig. 8a versus Fig. 9a)—though both form the exposed surface of αC″ (Figs. 9d and 8c). For both groups 23 and 10, residues conserved in the region between βD and βE (i.e., -F_7_P_9_-S_7_-I_9_L_9_R_8_-x-x-T_9_- and -Y_9_P_9_-x-E_9_L_9_R_8_-x-x-Y_8_-, respectively) are mostly in the segment oriented orthogonally to the β-sheet (Figs. 9e and 8d). These regions in both adapters play a critical role in signaling, as these can interact in homo- and heterotypic fashion with the αC motif of certain TIRs, leading to signal-dependent adapter oligomerization and initiation of intracellular signaling (Fig. 8e; Javmen et al. 2018; Toshchakov and Javmen 2020; Ve et al. 2017).

##### The MyD88-like-specific sequence of βE

MyD88-like proteins conserve a βE motif (-C_9_D_9_Y_8_T_9_-) that differs from that of other TIRs (Fig. 9a). Motif residues are largely surface exposed and may be involved in signaling interactions. C113 and Y115 of the βE motif also interact intramolecularly with Box 3 aromatic residues (not shown).

##### Conserved contacts between αA and αE

αA residues F14 and M18, which are juxtaposed in 3D, are > 90% and > 80% conserved in MyD88-like TIR domains, respectively (Fig. 9a), and their side chains interact with the Box 3 residues W130 and F129, respectively, through aromatic and sulfur-aromatic interactions. This forms a network of interactions, analogous to those shown in Figs. 5d and 6c for group 3 and 4 TIRs, that presumably stabilize core structural elements on the concave side of the β-sheet.

##### Unusual structure of BB loop

The known structure of the MyD88 BB loop differs from that of other TIRs having the conserved Box 2 motif, i.e., TIRs of TLRs, IL-1Rs, and TIRAPs, inasmuch as residues on the N-terminal side of αB are in an extended conformation (though residues of βB and those near the αB C-terminal end are more typical) (Fig. 9f), despite all residues of the Box 2 motif -RD-ϕ-ϕ-PG- are highly conserved in all four groups, as is the E23 in the C-terminal turn of αA (Fig. 9a). Unlike the TIRAP TIR, which has drastically different BB loop conformations in solution and crystal, the NMR and X-ray structures of the MyD88 BB loop are nearly identical (Ohnishi et al. 2009; Snyder et al. 2013). This apparent discord between conservation of motifs yet dissimilar folds suggests that the BB loop requires conformational flexibility, which manifests in different BB loop conformations in monomeric adapter TIRs, including the differences in solution and crystal structures of the TIRAP BB loop.

### Group 37: TRIF-like TIRs

Group 37 consists of TIR domains related to those of TRIF, also known as TICAM-1, a TLR adapter protein that participates in TLR3 and TLR4 signaling, leading to a robust activation of type I interferons (Oshiumi et al. 2003; Yamamoto et al. 2002). TRIF-like proteins occur in vertebrates (Tassia et al. 2017). Group 37 consists exclusively of chordate TIRs (Supplemental Fig. 3), which include TIRs of elephant shark, many bony fishes, and tetrapods, but not TIRs of lancelet, lamprey, or *Ciona* (Supplemental Fig. 3)—suggesting that they are restricted to gnathostomes.

#### Group 37 residue patterns and structural features

##### BB loop and αB motifs

**Fig. 10:**
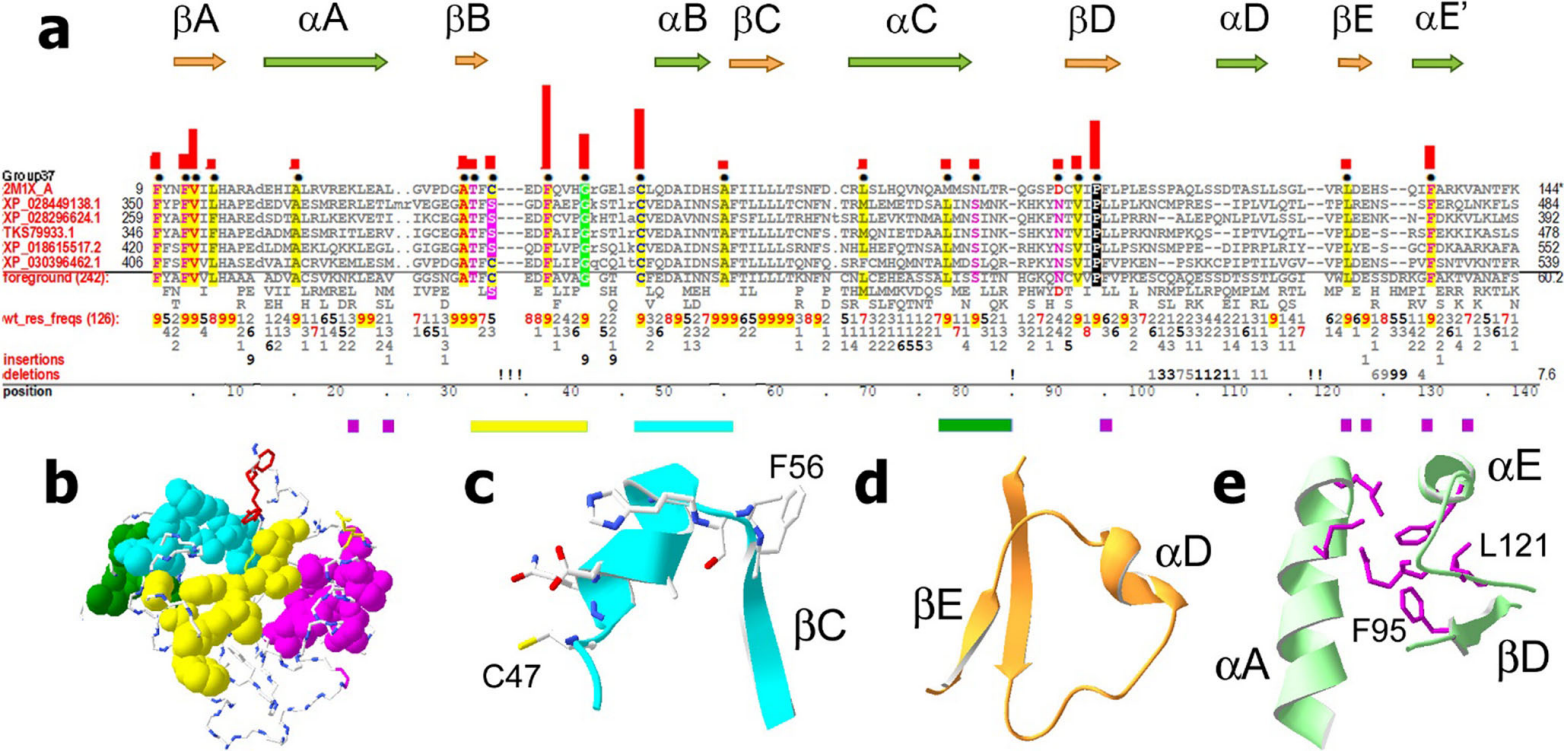
TRIF-like TIR domains. **a** Group 37 TIR domain contrast alignment. Horizontal bars beneath the alignment indicate groups of residues shown in the same colors as in panel **b**. **b** Group 37 conserved residue clusters. The NMR structure of human TRIF (PDB ID: 2M1X) (Enokizono et al. 2013) was used to generate images for panels **b**–**e**. **c** Conserved αB residues. **d** TRIF-specific conformation of the αD region. **e** Residues mediating interaction of βD and βE with αA and αE′

Many TRIF-like TIR residues are within βA, βB, and βD (Fig. 10a) and appear to play a predominantly structural role not directly related to signaling. Among the group 37 surface-exposed residues are those of the BB loop and αB (Fig. 10a–c). The BB loop motif differs in its N-terminal half (-35E_8_D_9_F_9_xxP_6_G_9_-), from the Box 2 motif (-xRDxxPG-) typical of groups 3, 4, 10, 23, and 32. Cell-permeable peptides derived from both conserved regions, but not from other surface-exposed segments of TRIF TIR, potently inhibited the TLR4-mediated signaling (Piao et al. 2013).

##### αE contacts

In group 37, αE contacts the β-sheet and αA through conserved F129 and V133, which interact with conserved L121 and F95 of the β-sheet but not with conserved αA residues (Fig. 10a, e).

### Prokaryotic TIR domains

BPPS partitioned bacterial TIRs into 23 groups, only one of which, group 17, contained a ~ 20% admixture of eukaryotic genes, all of which are from protozoans and lower metazoans (Fig. 1b, c). Unlike most metazoan groups, each bacterial group typically contains proteins with different domain architectures. Here, we review, as an example, the features of two of the largest bacterial groups (groups 14 and 6) and of the only bacterial TIR group with a known structure (group 35).

#### Group 14

**Fig. 11:**
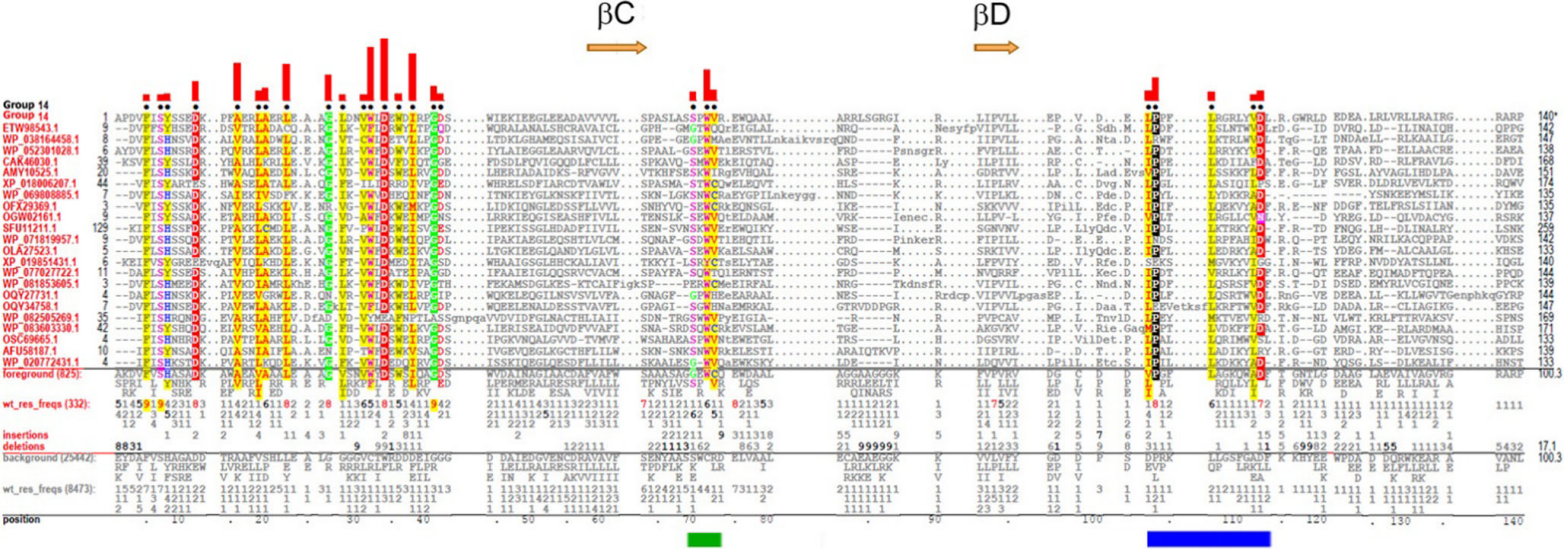
Group 14 TIR domain contrast alignment. Green and blue bars beneath the alignment indicate pattern residues of third and fourth helical regions

Group 14 is the largest prokaryotic group (825 sequences) that consists of ~ 50% Proteobacteria, ~ 25% Actinobacteria, ~ 5% Cyanobacteria, and ~ 5% Firmicutes proteins (Fig. 1g). Box 1 of group 14 TIRs has a consensus of -P_2_D_4_V_5_F_9_I_4_S_9_Y_5_-, which corresponds to the consensus of all TIR domains except for the N-terminal residue. Boxes 2 and 3 do not correspond to typical TIR domains. Most group 14–specific residues are located in the N-terminal region of the TIR and include D12 of the AA loop, a pattern of non-polar residues in αA, and a motif that spans βB and adjacent loops (Fig. 11). Additional conserved residues are located in the third and fourth helical regions (positions 70–73 and 102–106) and near βE (Fig. 11). Because proteins of this group are not well studied and lack structural information, it is difficult to interpret the functional significance of pattern residues.

#### Group 6

Group 6 bacterial TIR domains mainly occur in actinobacterial proteins (Fig. 1g). Proteins of this class can have different domain architectures, but often combine an N-terminal TIR with multiple C-terminal tetratricopeptide repeats and are large (> 700 aa).

##### Residue patterns of Group 6 TIRs

**Fig. 12:**
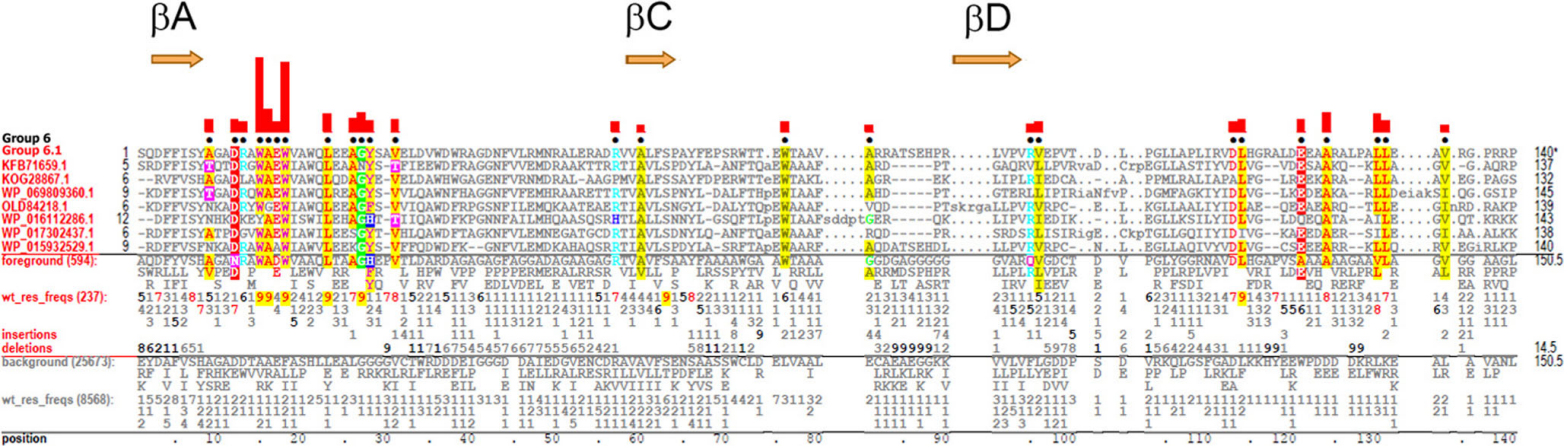
Group 6 TIR domain contrast alignment

Boxes 1, 2, and 3 are not conserved in group 6. Instead, the group 6–specific consensus patterns for βA (Box 1) and the BB loop (Box 2) are -R_3_**D**_**7**_F_3_F_5_V_4_S_8_**Y**_**7**_- and -W_4_**D**_5_A_1_V_1_**P**_**3**_**G**_**6**_-, respectively, and the two conserved aromatic residues of Box 3, i.e., F129 and W130, are replaced by two conserved aliphatic residues (Fig. 12). The two most characteristic residues of group 6 are W15 and W18 (Fig. 12), which likely form the hydrophobic side of αA and thus part of the structural core.

#### Group 35

Group 35 is phylogenetically similar to group 14, with the most from Proteobacteria, followed by Actinobacteria, Firmicutes, Bacteroides, and Cyanobacteria (Fig. 1g). These proteins are relatively small (< 300 aa) and typically have a C-terminal TIR along with an N-terminal domain of different types. Three structures for two representative proteins are available.

##### Group 35 residue patterns and structural features

**Fig. 13:**
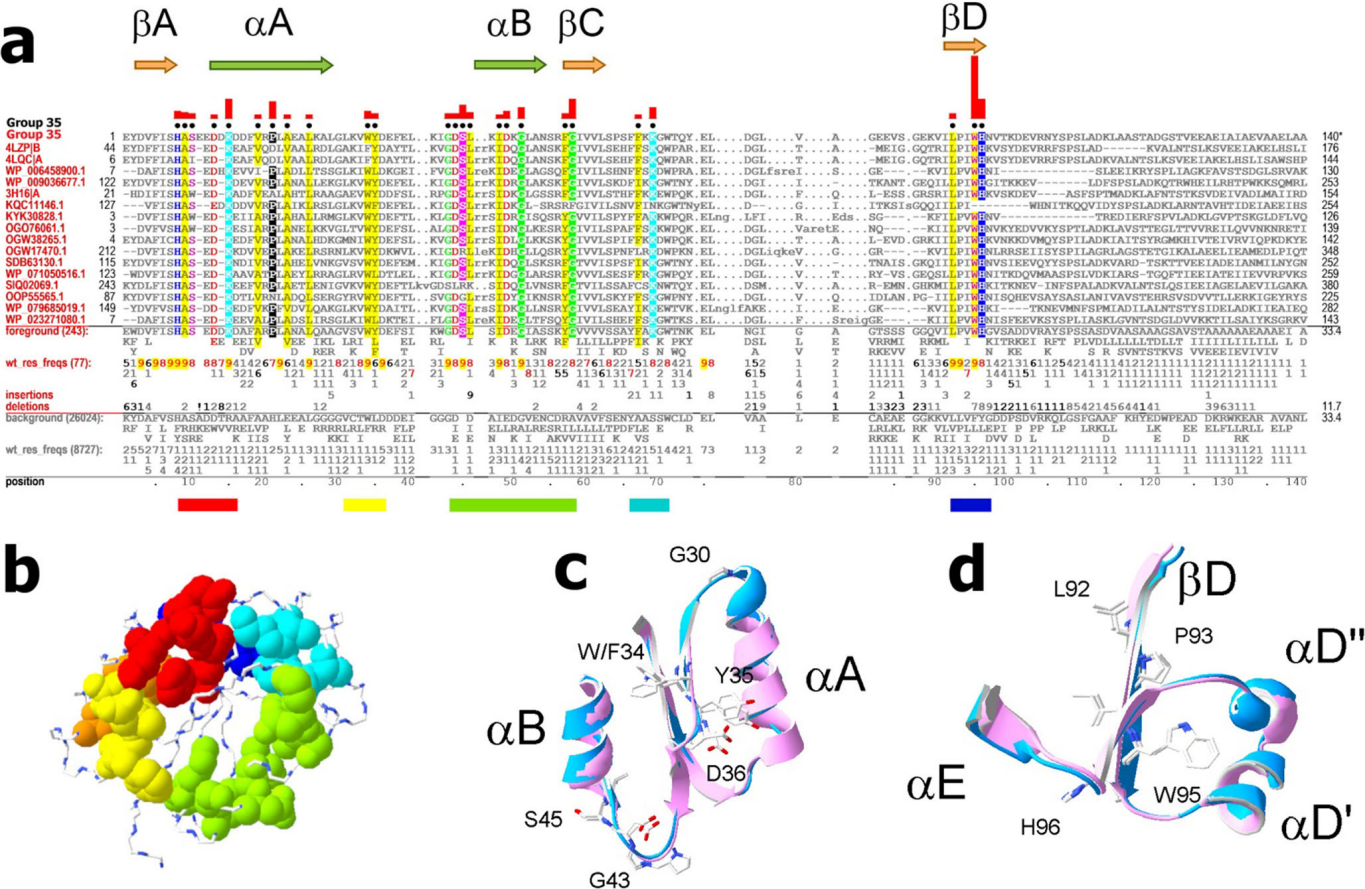
Group 35 bacterial TIR domains. **a** Group 35 contrast alignment. Horizontal bars indicate clusters of conserved residues, shown in the same colors as in panel **b**. **b** Group 35 conserved residue clusters for the TIR domain of *Paracoccus denitrificans* PdTIR protein (PDB ID: 3h16) (Chan et al. 2009). Colors correspond to the color of horizontal bars below the alignment in panel **a**. **c** Conserved residues in the segment between αA and αB. Shown are superimposed regions of TIR domains from *Paracoccus denitrificans* PdTIR protein (PDB ID: 3h16) (Chan et al. 2009) and *Brucella* protein TcpB (PDB ID: 4lqc) (Snyder et al. 2014). **d** Conserved βD residues. Shown are superimposed regions of TIR domains as in panel **c**

Most Group 35 conserved residues are in the N-terminal half of the TIRs (Fig. 13a). Many of these form a surface-exposed patch (Fig. 13b). In addition to the N-terminal residues, there are 5 conserved residues in the C-terminal half (Fig. 13a).

Box 2 of group 35 TIRs differs from the typical regional sequence described above for groups 3 and 4. There are two motifs in the BB loop region of group 35 TIRs: -V_8_W_9_Y_6_D_9_-, which forms a 4-residue “bulge,” immediately after βB′ (Chan et al. 2009) (Fig. 13a, c); and -G_9_D_8_S_9_L_8_- in the C-terminal half of BB loop (Fig. 13a, c). Two conserved glycines, i.e., G30 and G43, are located at the tips of the AB and BB loops, respectively (Fig. 13c).

The group 35 C-terminal conserved segment corresponds to βD (Fig. 13a, d), the residue side chains of which are buried with the aromatic ring of W95 pointing towards αD′ and αD″ (Fig. 13d). The αD region in group 35 has a unique fold (Fig. 4c), which is formed by two antiparallel helices oriented orthogonal to the β-sheet (Figs. 4c and 13d).

## Discussion

TIR domain–containing proteins occur among all major forms of cellular life. The primary and nearly universal function of TIR domains appears to be the mediation of regulated protein interactions in signaling. Prokaryotes use TIR domains as a signaling unit in a variety of functionally diverse signaling cascades (Spear et al. 2009). The multicellular organisms, however, use this type of protein domain in a more specialized fashion, primarily as a unit mediating the assembly of intracellular signaling complexes to signal microbial presence, and initiate and regulate antimicrobial defense mechanisms. A later acquired function of TIR domains, unique to arthropods and mediated by Toll orthologs, is in early embryogenesis, in addition to participating in defense. The functional similarity of TIR domains in plant and animal immune defense is apparently coincidental and likely stems from a strong evolutionary pressure to develop non-self-sensing and antimicrobial defense mechanisms concomitantly with development of multicellularity—together with the efficacy of TIRs as a general signaling unit, as indicated by their universal evolutionary distribution in this role.

The universal evolutionary distribution of TIR domains suggests this protein fold emerged early in the development of cellular life. Therefore, it is not surprising that some groups of TIR domains have acquired additional functions. One such function, found in SARM1 and some plant and bacterial TIRs, is the ability to catalyze NAD degradation (Essuman et al. 2018; Horsefield et al. 2019; Wan et al. 2019). Another function, found in some pathogenic bacteria, is the secretion of cell-permeable TIR proteins to subvert the antibacterial defense mechanisms mediated by host TIRs (Alaidarous et al. 2014; Cirl et al. 2008). Yet another, more recently evolved function is their participation in cytokine signaling, the emergence of which (and of cytokine signaling in general) coincides with the development of the adaptive immunity in jawed vertebrates (Flajnik and Kasahara 2010; Liongue et al. 2016; Rivers-Auty et al. 2018), and reflects the increased complexity of associated regulatory mechanisms.

The emergence of groups 4 and 37 of our hierarchy can be directly associated with the emergence of cytokine signaling in gnathostomes. Group 4 TIRs mediate downstream signaling for the IL-1R family, whereas group 37 mediates upstream signaling that induces type I interferons, cytokines important for antiviral defense. Notably, our statistical analysis has identified IL-1R and TRIF groups but failed to identify a group of TRAM-related TIRs. TRAM is an auxiliary TLR adapter that function in conjunction with TRIF to mediate the TLR4-dependent TRIF signaling (Oshiumi et al. 2003). The failure to identify a TRAM group is likely due to this group’s small size. Thus, Sullivan et al. found that TRAM evolved simultaneously with TRIF in early chordates as a product of the second whole genome duplication (Sullivan et al. 2007). The TRAM gene, however, was lost in the early evolution of rayfin fish (Sullivan et al. 2007).

The long evolutionary history of TIR domains explains their abundance and diversity, but complicates the analysis of TIR functions. Particularly, the molecular mechanisms that underlie selectivity of TIR domain interactions are not well articulated at this time. The literature typically cites the absence of specific binding motifs, which are common for the entire group, yet recognizes that the TIR domains interact specifically with certain members of the family, while also often demonstrating “multispecificity,” being able to interact with a subgroup of TIRs (Gay et al. 2014; Jiang et al. 2006; Toshchakov et al. 2011; Ve et al. 2017). There are two mechanistic explanations for the multispecificity of TIR-TIR interactions. The first is that individual TIRs often can simultaneously interact with several other TIRs through non-overlapping interaction sites. The second is that the individual sites can also be “multispecific” and may interact with different TIRs with a similar affinity (Javmen et al. 2018; Ve et al. 2017). Recent studies have advanced our understanding of the topology of TIR domain interactions for certain TLRs and TLR adapters (Javmen et al. 2018; Nanson et al. 2019; Toshchakov and Javmen 2020; Ve et al. 2017). This new knowledge has led to plausible models for the architecture of TLR signaling complexes (Javmen et al. 2018; Ve et al. 2017). The known examples of TIR-TIR interaction modes in signaling complexes however cover a miniscule portion of the TIR domains existing in nature; and, even for these few examples, the molecular features responsible for the functional specificity of TIR-TIR interactions remain obscure.

The functional diversity of TIR domains is confirmed by our observation that the sequence features common for all TIR domains are restricted to the structural core, which has minimal or no surface exposure (Fig. 2e) and, therefore, cannot be directly associated with a specific binding or catalytic function. This observed diversity and complexity of TIR functions emphasizes the need for automated, computational methods to analyze and classify the large numbers of TIR sequences into smaller, functionally related subgroups. Our study has applied Bayesian partitioning with pattern selection (BPPS) to automatically classify TIR domains based on those sequence motifs that best distinguish each group. The algorithm was applied to phylogenetically diverse TIR domain sequences retrieved from the NCBI non-redundant (nr) and environmental (env_nr) protein sequence databases and from the translated NCBI EST database. This sequence pool reflects the state of global genome sequencing projects at the time of sampling and the abundance of proteins with TIR domains in individual genomes. Metazoan, plant, and bacterial TIRs were nearly equally represented in this set and together accounted for ~ 95% of the sequences (Fig. 1b). The two largest of the minor phylogenetic groups in this set, i.e., archaea and protozoans, together account for ~ 1.5% of the sequences. The TIR domains referred to as of “unknown” phylogenetic origin (Fig. 1b) corresponded to ~ 3% of the total; these sequences were derived from environmental samples and co-partitioned with bacterial groups (Fig. 1c, g).

Results of partitioning have clearly demonstrated the biological significance of identified groups and confirmed BPPS as a valuable method for exploration and analysis of large sets of related, but functionally diverse protein sequences. Many traits indicate the validity of BPPS for functional analysis of TIR domains. First, the analysis has clearly differentiated phylogenetically distant sequences. Thus, representatives of three main taxonomic categories of the analyzed sequences, i.e., prokaryotic, plant, and metazoan TIRs, partitioned separately with only group 17 combining the bacterial TIRs with TIRs of protozoans and lower metazoans (Fig. 1c).

The second indication of the functional relatedness of individual groups is that, in most cases, the algorithm has automatically grouped architecturally similar eukaryotic TIRs to the same groups. For example, the plant TIR domains formed 3 groups, each of which is characterized by similar domain architectures among full-length proteins. The largest group of plant TIR domains (and of the entire partitioning), group 2, is formed by the NB-LRR family of pathogen-sensing receptors. The predominant domain architecture for proteins with other two types of plant TIR domains, i.e., groups 21 and 16, suggests that these TIRs belong to adapter proteins and protein kinases, respectively. Notably, the size of group 2 is larger than the sizes of other two Streptophyta groups by more than an order of magnitude. This significant size difference is however consistent with the relative abundance of corresponding genes in plant genomes (Van der Biezen and Jones 1998). This correlation is another clear indication that the sequence features identified by BPPS for groups of TIRs are functionally important.

Unlike most eukaryotic TIR groups, the prokaryotic groups typically contain proteins with different domain architectures. This likely reflects faster evolution together with sparser sampling of prokaryotic TIRs. It should be noted that the physiological functions of prokaryotic TIR domains are much less studied compared with that of eukaryotes. Moreover, only 3 experimentally determined structures of prokaryotic TIRs are currently available, all of which belong to just one group. The lack of specific biochemical and structural data on the prokaryotic TIRs makes it difficult to assess the functional implications. For this reason, most prokaryotic groups are not reviewed in detail here. Nevertheless, the motifs defining bacterial subgroups are important for understanding the diversity of prokaryotic TIR functions and should be instructive for planning future research. For this reason, motifs for prokaryotic TIR groups are published in Supplemental Fig. 1 as a series of contrast alignments.

The third line of evidence indicating the biological significance of identified groups is the remarkable conservation of regional backbone fold variants observed within individual groups. Thus, each group with multiple structural representatives can be uniquely characterized by a highly conserved backbone configuration of the fourth helical region (Fig. 4a–c). Each fold variant is associated with a group-specific motif presumably responsible for the fold (Figs. 3c, 5e, and 12d).

BPPS identification of four large, conserved surface patches, which are formed by the surface-exposed side of α-helices C″ and short 3_10_-helices D in TIRs of groups 10 and 23 (Figs. 8c, d and 9d, e), is, in our opinion, another significant result. These group-defining surface features are highly conserved in proteins related to TIRAP (group 10) and to MyD88 (group 23) (Figs. 8a and 9a). Current models of adapter recruitment to activated TLRs suggest that the third and fourth helical regions belong to two of the four TIR sites mediating signal-dependent assembly of signaling complexes. The sites, which include αCs and 3_10_-helices D (previously termed “sites 2 and 3”; (Toshchakov and Javmen 2020), were deemed highly multispecific, being able to mutually interact with the corresponding helical region of TIRAP, MyD88, or a TLR TIR (Couture et al. 2012; Javmen et al. 2018; Toshchakov and Javmen 2020; Ve et al. 2017; Fig. 8e). This discovery of conserved surface patches, each of which corresponds to a multispecific interaction site within adapters TIRAP and MyD88, suggests mechanisms of TIR-TIR recognition in signaling.

BPPS confirms the TIR domain Box 2 and 3 motifs identified by Slack et al. (2000) and identifies the groups associated with these motifs: the Box 2 motif (-RDxxPG-) is present in group 3 (the Toll proteins and TLRs), group 4 (the IL-1R family), group 10 (TIRAP orthologs), and both groups of MyD88-like TIRs (group 32 of arthropod TIRs and group 23 of other metazoan TIRs). Box 2 is typically absent in prokaryotic TIRs, except perhaps the TIRs of group 28, in which the BB loop motif (-W_9_**D**_9_**F**_**7**_**V**_**6**_**P**_**7**_**G**_**9**_-) resembles Box 2 for the most part (Supplemental Fig. 1). Box 3 (-FW- in the N-terminal round of αE) features the same groups of eukaryotic TIRs as does Box 2, with the exception of group 10 (TIRAP-related TIRs). The Box 3 motif is absent from prokaryotic TIRs.

BPPS conclusively clarifies the functional significance of Boxes 2 and 3, each of which is in fact part of a larger conserved group of intramolecularly interacting residues. Thus, the presence of Box 3 within a group, in all available cases, signified the presence of conserved aromatic or sulfur-containing residues at group-specific positions in αA and βE. Notably, in all TIR structures with a Box 3, these residues, although distantly located in the sequence (Figs. 5a and 6a), establish mutual contacts (Figs. 5d and 6c). Analogously, the presence of Box 2 in a TIR group in all cases correlated with the presence of an acidic residue in αA, i.e., 12–14 residues away from R36 of Box 2 (Figs. 5a, 6a, 8a, and 9a, b). The charged residues of Box 2 contact the conserved negatively charged residue of αA through salt bridges and hydrogen bonds in groups 3 and 4, and in certain conformations of group 10 TIRs (Figs. 5c, 6d, and not shown). Many lines of evidence indicate that the region near βB is conformationally flexible (see, for example, Hughes et al. 2017); therefore, it is not surprising that these interactions are absent from some conformation variants (e.g., see Fig. 9f).

## Electronic supplementary material

ESM 1 (PDF 3.38 mb)

ESM 2 (PDF 21511 kb)

ESM 3 (PDF 424 kb)

ESM 4 (PDF 87 kb)

## Footnotes

1 Strands and helices of the TIR domains are alphabetized starting from the N-terminus. Loops are indicated by two capital letters, according to the structural elements they connect. Thus, the BB loop is the loop that connects second β-strand with second helix, while the CD loop connects the third helix with the fourth strand.

2 Here and throughout the text, residues are numbered to correspond to multiple sequence alignments, not to individual protein sequences.
