# Supplementary material for: A survey of TIR domain sequence and structure divergence
Source: Immunogenetics. 2020 Jan 30;72(3):181–203. doi: 10.1007/s00251-020-01157-7 (PMC7075850; doi:10.1007/s00251-020-01157-7)
Supplement: Supplementary file 1 — (PDF 3.38 mb) [file 251_2020_1157_MOESM1_ESM.pdf]

Supplemental Figure 1. A BPPS output showing the contrast alignments for each node of the hierarchy presented in Figure 1A, except Group 37. Group 37 contrast alignments and contents are shown in Supplemental Figure 3.

Group35 (bacterial TIRs)

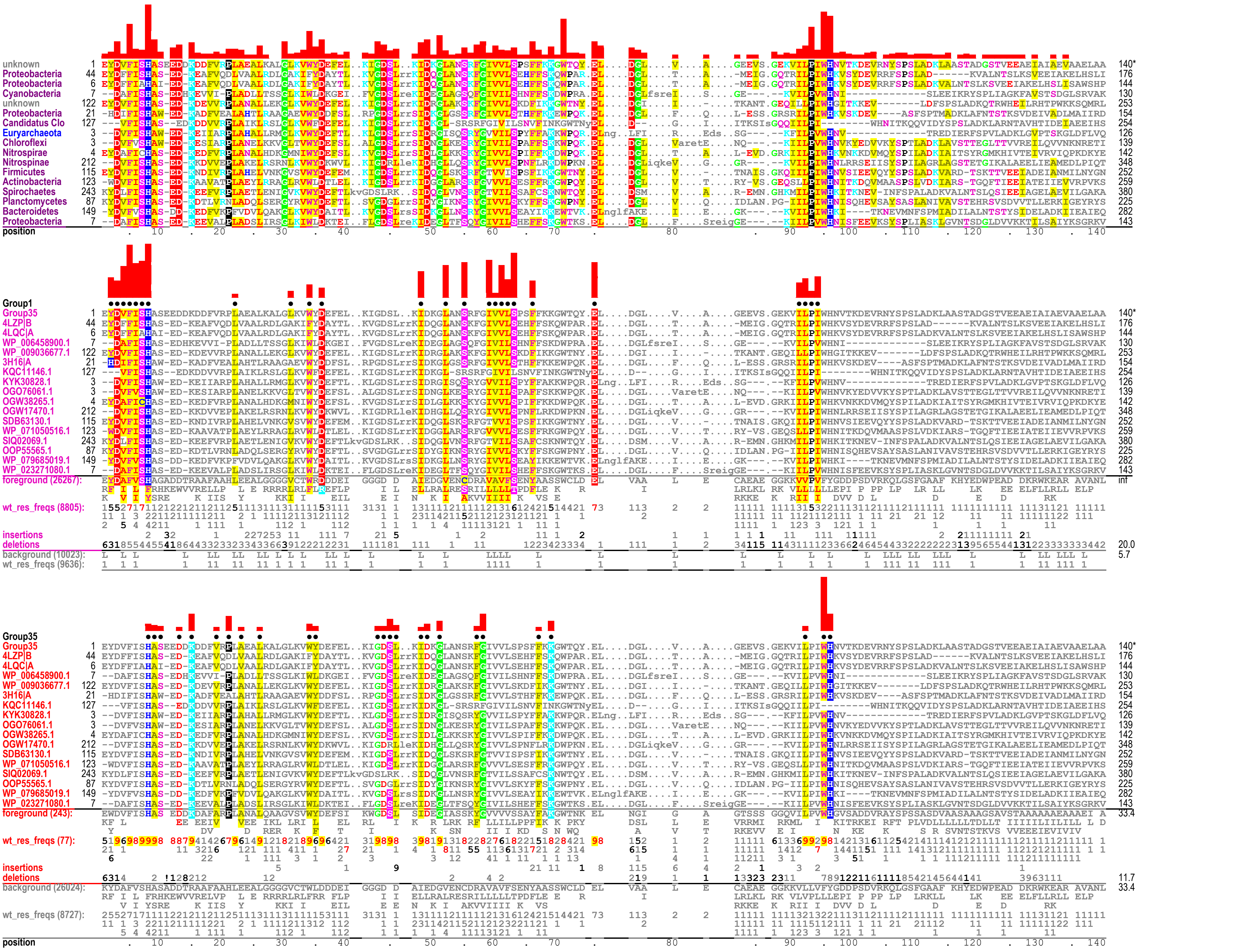

Group33 (bacterial TIRs)

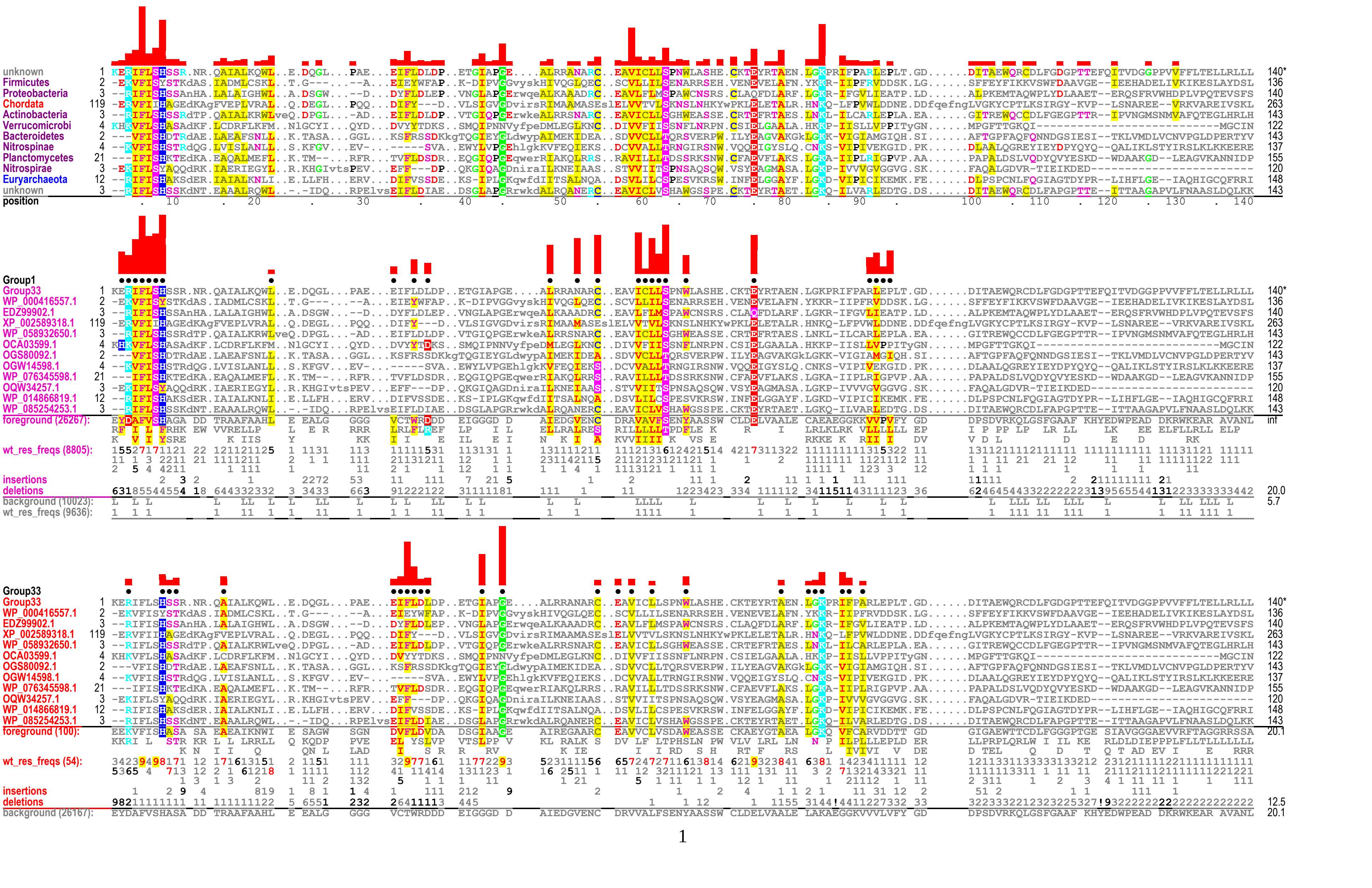

```
                     RF I L FSHK EW VVRELLP    L  E R   RRR   LRLFLREF  LP   I L    ELLRALRES  RILLLVLTPDFLE K    R    I    CRLKLRK KLLPLLL EP      I P PP LP  LRKLL    LK   EE ELFLLRLL ELP
                     K  V I YRRE      K IIS    Y          KK   I I    E  IL   E I    N  K I  A  KVAIIII K  VS E                  RKEE K  RII I   DV      V D L         D       E   D      RK
wt_res_freqs (8751): 15527171111 22 121121125  1 1131   113   11111531  113131 1    131111211  1112121612421514 4217311322 11111111311322 11      131121111211111 1111111111 11131121 11111
                     11 1 3 2211 21 1111211    2  1 1   111   21131211  12   1 1    231142115  2112133121121 1    1    2    1111111 1125112 11      1 1 11 21  21112      11    11 11111122 111
                     2  5 4 4211      1 111    1         11   2 1    1  12   1 1    1  1 4  1  1113121 1  11 1              1111 1  123 3   12      1 1 1         1       1    1      11
position                   .  10     .   20      .       30      .      40     .      50    .      60    .   70     .   80     .   90    .          100   .  110   .  120   .  130   .  140
```

Group32 (Arthropoda MyD88 TIRs)

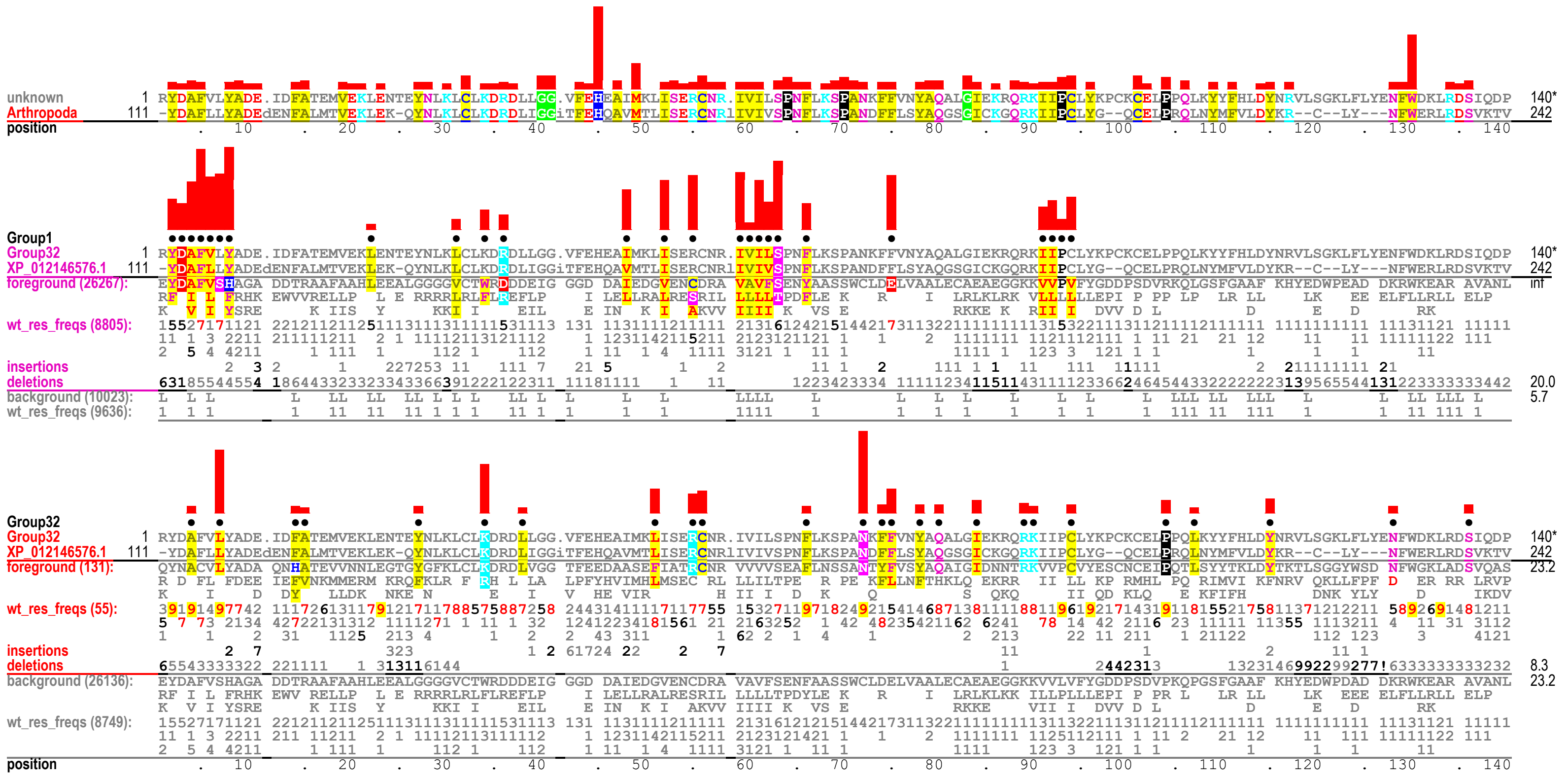

Group22 (bacterial TIRs)

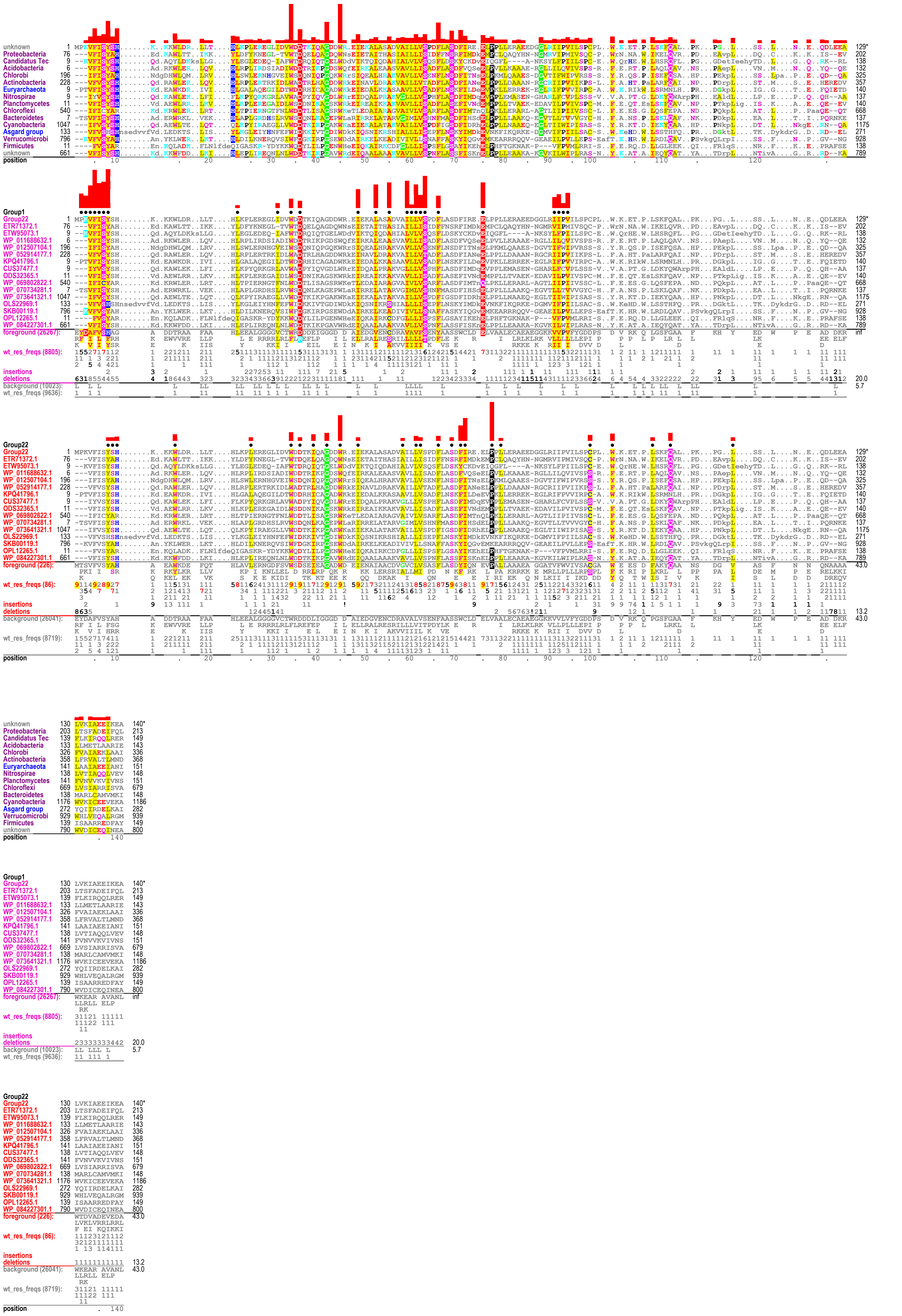

Group21 (plant TIRs)

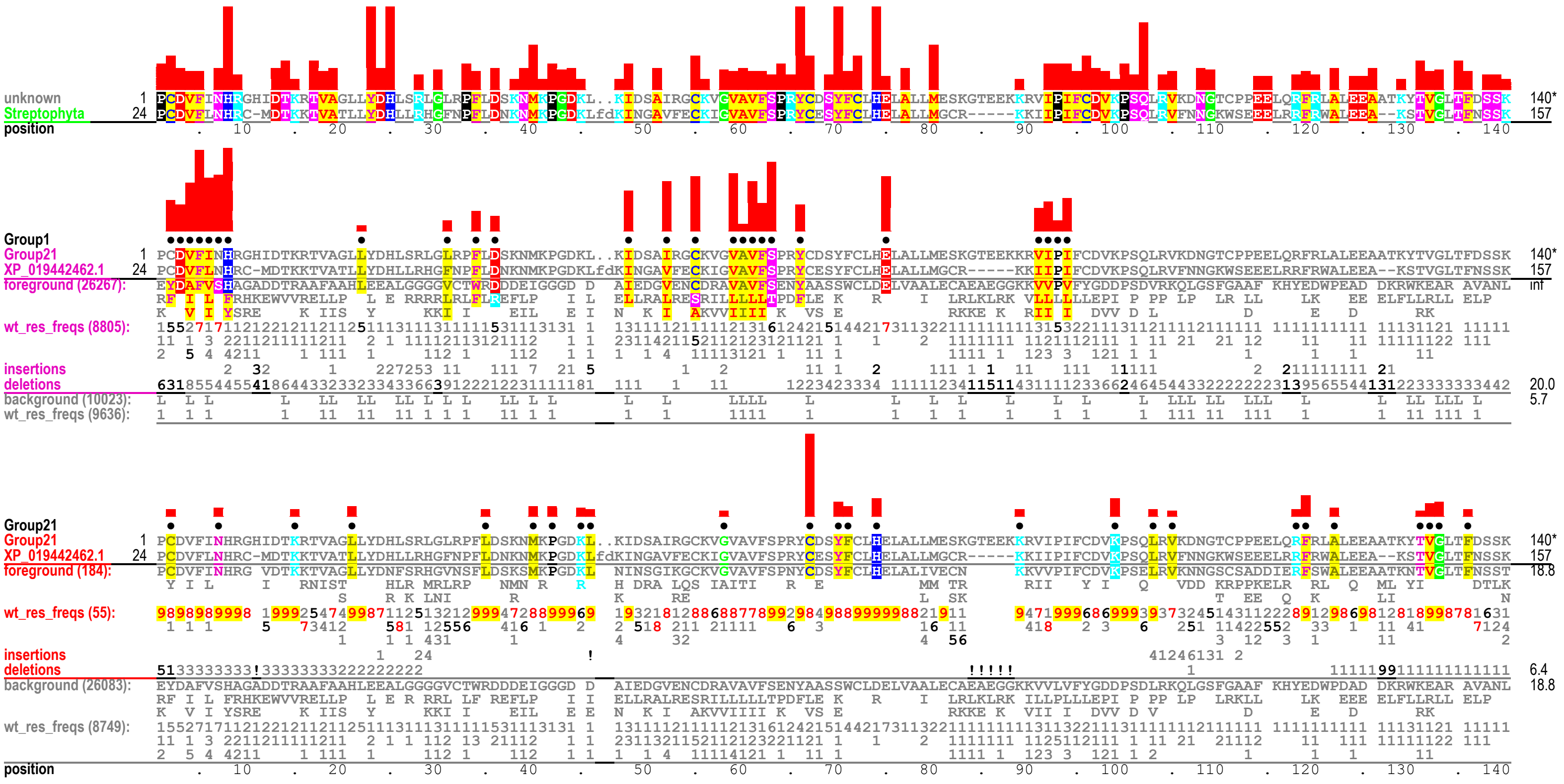

Group20 (bacterial TIRs)

```
unknown                 1 QKTVFLSHSHK......KDLAKGLADYLKNA.G..IDVYDVYYDD...LD.....M..PP...TTDTA.......RIKKRIRESDHFLFLATENSKKSRWVPW.EIGYADGKKGPK...IRI.ALLPLSDDTSPDYWGYLYLLYYIRDEV.D..VSLGKYGLALYI..TKGGG  134*
Fusobacteria           41 ---LFISHSFLd.....KKLILMLIDLFNNA.GysVYVDWINDKN...LD.....R..NN...ISPKTan.....VIKNRISNCKGLSYIATRNIVNSKWCPW.ELGLADGMLNG-...KSC.ILPVMEESSAFKGLEYLGLYPYIEYEK.I..--SGKSTYEFWV..IDQGD  173
Verrucomicrobi         86 -FDIFLSYS-SpdavavVGLYH--------W.L..VARRYRVYLD...RF.....D..RL...LNPAVvtratatIIRRRIVQSRSLFVATSPHALTSDWVPW.EMGFADGLMNKAavlFIA.PRPNVTFN---RQSYFELYPEVGPGPA.G..AAKGQDLILTDP..QAVPP  222
Proteobacteria         36 ----FISHSSKd.....EDLVAGAIEVIASHgG..VPYIDEVDPA...MP.....S..H-...--TSEetae...LLKKRIVECKRFVLLTSPNSKDSRWVPW.ELGVADGKKGI-...HKIaIFGASDSAGLDGWSSWEYMGLYPRIIW.G..--KLQGYSNELW..MVYSQ  166
Elusimicrobia           6 -KSIFLSHSHKd.....RDLAKGLINYLAPL.G..-LSLYVDWQD...ST.....MprIT...SRETAq......NIKRTISELDFFLILATPNALESRWV-PwEIGVADQAKSH-...AKM.AIMPVADSNG-NFQGNEYLLIYNRIEL.DslGGLLMRDSDNSW..TVGEV  141
Bacteroidetes          24 -KCVFLSHQKKd.....KEEAKKITDYLIKA.G..I---DVYFDE...YDkdlkiH..HQ...TNNAVgvtn...SIKMGINNSSHMIVLVSPNTLYSTWVPF.EIGYGY-DKTE-...VFT.LCLKGIT---KGTLPEYLKATKIIRDIyD..--FNIFCEN--ItgIKKEN  157
Actinobacteria         45 -FDVFLSHS-VrdarviLGV--------REW.L..QSKGLTVYVDwidDT.....Q..LDrekVSPATas.....QLRRQMDRSHTLIYATSQNAQASRWMPW.ELGYFDGRKGRErvsILP.IDNSSGGR---FTGDEYIGLYPRIEQQ.Q..ISPGNLGPIAIS..PSGTS  182
unknown                37 -FDVFLSHS-VrdarliLGLRNLLRKQGLSV.Y..VDWIEDPQLD...RT.....Q..V-...-SSATaa.....RLRDQMRVSRTMIYATSRAACESRWMPW.ELGYFDGLRNAErisILP.IESSVKYD---DFVGQEYLGLYKVIEK.V..EVLPYGTTEPYA..VLPSG  174
Firmicutes             41 ---IFLSHSYLd.....AKHIKLIRDDLISL.GysVYVDWIEDKS...LD.....R..TQ...V--TSetae...QLRIRMKQSKCLFYATTENYQKSNWMPW.ELGYFDALKDK-...VAI.LPVKSRPDSSEQYNGTEYLGLYSYITL.T..--EDTKQNECLW..VNNNK  173
position                      .   10          .   20    .      30    .             40       .          50    .   60    .   70     .   80    .       90    .  100    .  110    .     120    .    130

Group1                     ●●●●●●●                   ●           ●  ● ●                               ●   ●  ●   ●●●●●  ●         ●                   ●●●●
Group20                 1 QKTVFLSHSHK......KDLAKGLADYLKNA.G..IDVYDVYYDD...LD.....M..PP...TTDTA.......RIKKRIRESDHFLFLATENSKKSRWVPW.EIGYADGKKGPK...IRI.ALLPLSDDTSPDYWGYLYLLYYIRDEV.D..VSLGKYGLALYI..TKGGG  134*
WP_032836745.1         41 ---LFISHSFLd.....KKLILMLIDLFNNA.GysVYVDWINDKN...LD.....R..NN...ISPKTan.....VIKNRISNCKGLSYIATRNIVNSKWCPW.ELGLADGMLNG-...KSC.ILPVMEESSAFKGLEYLGLYPYIEYEK.I..--SGKSTYEFWV..IDQGD  173
WP_051945647.1         86 -FDIFLSYS-SpdavavVGLYH--------W.L..VARRYRVYLD...RF.....D..RL...LNPAVvtratatIIRRRIVQSRSLFVATSPHALTSDWVPW.EMGFADGLMNKAavlFIA.PRPNVTFN---RQSYFELYPEVGPGPA.G..AAKGQDLILTDP..QAVPP  222
WP_060839271.1         36 ----FISHSSKd.....EDLVAGAIEVIASHgG..VPYIDEVDPA...MP.....S..H-...--TSEetae...LLKKRIVECKRFVLLTSPNSKDSRWVPW.ELGVADGKKGI-...HKIaIFGASDSAGLDGWSSWEYMGLYPRIIW.G..--KLQGYSNELW..MVYSQ  166
OGR37722.1              6 -KSIFLSHSHKd.....RDLAKGLINYLAPL.G..-LSLYVDWQD...ST.....MprIT...SRETAq......NIKRTISELDFFLILATPNALESRWV-PwEIGVADQAKSH-...AKM.AIMPVADSNG-NFQGNEYLLIYNRIEL.DslGGLLMRDSDNSW..TVGEV  141
WP_076395552.1         24 -KCVFLSHQKKd.....KEEAKKITDYLIKA.G..I---DVYFDE...YDkdlkiH..HQ...TNNAVgvtn...SIKMGINNSSHMIVLVSPNTLYSTWVPF.EIGYGY-DKTE-...VFT.LCLKGIT---KGTLPEYLKATKIIRDIyD..--FNIFCEN--ItgIKKEN  157
WP_081821224.1         45 -FDVFLSHS-VrdarviLGV--------REW.L..QSKGLTVYVDwidDT.....Q..LDrekVSPATas.....QLRRQMDRSHTLIYATSQNAQASRWMPW.ELGYFDGRKGRErvsILP.IDNSSGGR---FTGDEYIGLYPRIEQQ.Q..ISPGNLGPIAIS..PSGTS  182
WP_084295204.1         37 -FDVFLSHS-VrdarliLGLRNLLRKQGLSV.Y..VDWIEDPQLD...RT.....Q..V-...-SSATaa.....RLRDQMRVSRTMIYATSRAACESRWMPW.ELGYFDGLRNAErisILP.IESSVKYD---DFVGQEYLGLYKVIEK.V..EVLPYGTTEPYA..VLPSG  174
WP_002597129.1         41 ---IFLSHSYLd.....AKHIKLIRDDLISL.GysVYVDWIEDKS...LD.....R..TQ...V--TSetae...QLRIRMKQSKCLFYATTENYQKSNWMPW.ELGYFDALKDK-...VAI.LPVKSRPDSSEQYNGTEYLGLYSYITL.T..--EDTKQNECLW..VNNNK  173
foreground (26267):       EYDAFVSHAGA      DDTRAAFAAHLEEA L  GGGGVCTWRD   DD     E  IG   GGD D       AIEDGVENCDRAVAVFSENYAASSWCLD ELVAALECAEAE   GGK KVVPVFYGDDPSDVRKQLGSFGAAF K H  YEDWPEAD DKR  WKEAR  inf
                          RF I L FRHK      EWVVRELLP  L E    RRRRLRLFLR   EF     L  P      I L       ELLRALRESRILLLLLTPDFLE K   R     I  LRLKL   RK  VLLLLLLEPI P PP LP  LR LL       LK   EE ELF  LLRLL
                          K  V I YSRE          K IIS  Y        KKI I       E     I  L      E I       N  K I  AKVVIIII K  VS  E            RKKE    K   RII I  DVV D L         D         E   D        RK
wt_res_freqs (8805):      15527171121      22121121125111 3  1113111115   31     1  13   131 1       1311112111112131612421514421 731132211111   111 1315322111311211112111111 1 1  11111111 111  31121
                          11 1 3 2211      211111211  2 1    1111211312   11     1  2      1 1       2311421152112123121121 1   1     2  11111   11  1121112111 1 11 21  21 12        11   11 111  11122
                          2  5 4 4211          1 111  1        112 1       1     1  2      1 1       1  1 4  11113121 1  11  1            1111    1   123 3  121 1 1         1         1   1        11
insertions                       2  3      2      1    22 7  253 11   1   11        7     21 5              1   2        11 1   2     111  1 1     1 1  111   11111          2   2  11111111 21
deletions                 63185544554      18644332332334 3  3663912221   22     3  11   11181       111   1   11      1223423334  11111234115   114 31111233662464544332222223  1  395655441312  23333  20.0
background (10023):       L  L L             L   LL  LL   L  L L L L      LL        L    L            L   L      LLLL    L         L   L  L       L     L  L     L  LLL LL  LLL      L       L    LL LL  5.7
wt_res_freqs (9636):      1  1 1             1   11  11   1  1 1 1 1      11        1    1            1   1      1111    1         1   1  1       1     1  1     1  111 11  111      1       1    11 11

Group20                       ●●●●               ●        ●  ●                                        ●●        ●   ●  ●●  ● ●●●● ●●●  ●
Group20                 1 QKTVFLSHSHK......KDLAKGLADYLKNA.G..IDVYDVYYDD...LD.....M..PP...TTDTA.......RIKKRIRESDHFLFLATENSKKSRWVPW.EIGYADGKKGPK...IRI.ALLPLSDDTSPDYWGYLYLLYYIRDEV.D..VSLGKYGLALYI..TKGGG  134*
WP_032836745.1         41 ---LFISHSFLd.....KKLILMLIDLFNNA.GysVYVDWINDKN...LD.....R..NN...ISPKTan.....VIKNRISNCKGLSYIATRNIVNSKWCPW.ELGLADGMLNG-...KSC.ILPVMEESSAFKGLEYLGLYPYIEYEK.I..--SGKSTYEFWV..IDQGD  173
WP_051945647.1         86 -FDIFLSYS-SpdavavVGLYH--------W.L..VARRYRVYLD...RF.....D..RL...LNPAVvtratatIIRRRIVQSRSLFVATSPHALTSDWVPW.EMGFADGLMNKAavlFIA.PRPNVTFN---RQSYFELYPEVGPGPA.G..AAKGQDLILTDP..QAVPP  222
WP_060839271.1         36 ----FISHSSKd.....EDLVAGAIEVIASHgG..VPYIDEVDPA...MP.....S..H-...--TSEetae...LLKKRIVECKRFVLLTSPNSKDSRWVPW.ELGVADGKKGI-...HKIaIFGASDSAGLDGWSSWEYMGLYPRIIW.G..--KLQGYSNELW..MVYSQ  166
OGR37722.1              6 -KSIFLSHSHKd.....RDLAKGLINYLAPL.G..-LSLYVDWQD...ST.....MprIT...SRETAq......NIKRTISELDFFLILATPNALESRWV-PwEIGVADQAKSH-...AKM.AIMPVADSNG-NFQGNEYLLIYNRIEL.DslGGLLMRDSDNSW..TVGEV  141
WP_076395552.1         24 -KCVFLSHQKKd.....KEEAKKITDYLIKA.G..I---DVYFDE...YDkdlkiH..HQ...TNNAVgvtn...SIKMGINNSSHMIVLVSPNTLYSTWVPF.EIGYGY-DKTE-...VFT.LCLKGIT---KGTLPEYLKATKIIRDIyD..--FNIFCEN--ItgIKKEN  157
WP_081821224.1         45 -FDVFLSHS-VrdarviLGV--------REW.L..QSKGLTVYVDwidDT.....Q..LDrekVSPATas.....QLRRQMDRSHTLIYATSQNAQASRWMPW.ELGYFDGRKGRErvsILP.IDNSSGGR---FTGDEYIGLYPRIEQQ.Q..ISPGNLGPIAIS..PSGTS  182
WP_084295204.1         37 -FDVFLSHS-VrdarliLGLRNLLRKQGLSV.Y..VDWIEDPQLD...RT.....Q..V-...-SSATaa.....RLRDQMRVSRTMIYATSRAACESRWMPW.ELGYFDGLRNAErisILP.IESSVKYD---DFVGQEYLGLYKVIEK.V..EVLPYGTTEPYA..VLPSG  174
WP_002597129.1         41 ---IFLSHSYLd.....AKHIKLIRDDLISL.GysVYVDWIEDKS...LD.....R..TQ...V--TSetae...QLRIRMKQSKCLFYATTENYQKSNWMPW.ELGYFDALKDK-...VAI.LPVKSRPDSSEQYNGTEYLGLYSYITL.T..--EDTKQNECLW..VNNNK  173
foreground (153):         QKCAFICHSSE      AGVAKGAADYGNSA G  VDGNDDDWDS   DT     D  AA   TGNGA       SIQNGINNCSRFVYVASDNAAASQWVPW EIGYGDGKDGKK   EAV AVIPASSSSSDGGGGSGGGEGKDKDEN D  GSGGKKGSAKED  TSGGG  50.2
                          RPKI LS KHL      KELILKLIELLKKL Y  IQPIIPPFLD   LP     M  PP   LPPDK       VLRKRMVRSKWMLLLLTPKTMK W M F  L IF ALKKPI   IVL LLL LEDKTYPDLLYYLLLLLLLRYII    VVLN L LKLPL  IMVLD
                          KFDV    QQK      SD V  IV VIEEV      VVYIVYQ    YE     E   Q   ITENE       H K KLRQADHLIVIV E SLD R C P    FA    SLV    RI II  I  D  K YWEEYYYYYVI RYV    R S  Y YEFYI  WK Y
wt_res_freqs (85):        33129319521      11132312121111 5  2411311221   11     1  11   11112       1711171121141124416111919495 959416421113   111 211511111111111111111111111 1  111211211111  11112
                          2112 68 131      21311141115111 1  4111111115   11     1  12   11111       1221311131113141511311 1 1 2  3 11 114111   211 132 11211111111211211111111    1111 1 11111  11111
                          2124    113      12 2  31 11111      1111131    11     1   1   11111       1 5 111112232211 2 311 3 3 1    16    111    13 12  1  1  1 11111211112 111    1 1  1 11111  11 1
insertions                          9                     1  15532  3 8    3     8  15   21118       4                          2    31 21   1        1   11                   1 2           1
deletions                 9541   1157       1111335919933 3  6133222564   22     4  18   91117       7                         1       452 117     1 211211111134533321442233232 4  553333333223  33444  13.7
background (26114):       EYDAFISHAGA      DDTRAAIAAHLEEA L  GGGGVCTWRD   DD     E  IG   GGD D       AIEDGVENCDRFVAVFSENYAASSWCLD ELVAALECAEAE   GGK KVVLVFYGDDPSDVRKQLGSFGAAF K H  YEDWPDAD DKR  WKEAR  50.2
                          RF I L FRHK      EWV RELLP  L E    RRRRLRLFLR   EF     L  P      I L       ELLRALRESRILLLLLTPDFLE E   R     I  LRLKL   RK  VLLPLLLEPI P PP LP  LRKLL       LK  EEE ELF  LLRLL
                          K  V V YSRE          K FIS  Y        KKI I       E     I  L      E I       N  K I  AKVVIIII K  VS              RKKE    K   RII I  DVV D L         D         E   D        RK
wt_res_freqs (8719):      15527471121      22121111125111 3  1113111115   31     1  13   131 1       1311112111112131612421514421 731132211111   111 1311322111311211112111111 1 1  11111111 111  31121
                          11 1 3 2211      211 11211  2 1    1111211312   11     1  2      1 1       2311421152112123121121 1   1     2  11111   11  1125112111 1 11 21  21112       11  111 111  11122
                          2  5 1 4211          1 211  1        112 1       1     1  2      1 1       1  1 4  11113121 1  11              1111    1   123 3  121 1 1         1         1   1        11
position                      .   10          .   20    .      30    .             40       .          50    .   60    .   70     .   80    .       90    .  100    .  110    .     120    .    130
```

```
unknown               135 KILNSL  140*
Fusobacteria          174 SSRYAS  179
Verrucomicrobi        223 WSATWD  228
Proteobacteria        167 KTDSAV  172
Elusimicrobia         142 KSWLAS  147
Bacteroidetes         158 LILESK  163
Actinobacteria        183 QRLSSF  188
unknown               175 LRGEPI  180
Firmicutes            174 DEYVKF  179
position                  .  140
```

```
Group1
Group20               135 KILNSL  140*
WP_032836745.1        174 SSRYAS  179
WP_051945647.1        223 WSATWD  228
WP_060839271.1        167 KTDSAV  172
OGR37722.1            142 KSWLAS  147
WP_076395552.1        158 LILESK  163
WP_081821224.1        183 QRLSSF  188
WP_084295204.1        175 LRGEPI  180
WP_002597129.1        174 DEYVKF  179
foreground (26267):        AVANL  inf
                           ELP
wt_res_freqs (8805):       11111
                           111
insertions
deletions                 333442  20.0
background (10023):       L L     5.7
wt_res_freqs (9636):      1 1
```

```
Group20
Group20               135 KILNSL  140*
WP_032836745.1        174 SSRYAS  179
WP_051945647.1        223 WSATWD  228
WP_060839271.1        167 KTDSAV  172
OGR37722.1            142 KSWLAS  147
WP_076395552.1        158 LILESK  163
WP_081821224.1        183 QRLSSF  188
WP_084295204.1        175 LRGEPI  180
WP_002597129.1        174 DEYVKF  179
foreground (153):         SSVNSA  50.2
                          YILLTL
                          K  Y
wt_res_freqs (85):        111111
                          111111
                          1  1
insertions
deletions                 411     13.7
background (26114):        AVANL  50.2
                           ELP
wt_res_freqs (8719):       11111
                           111
position                  .  140
```

Group31 (bacterial TIRs)

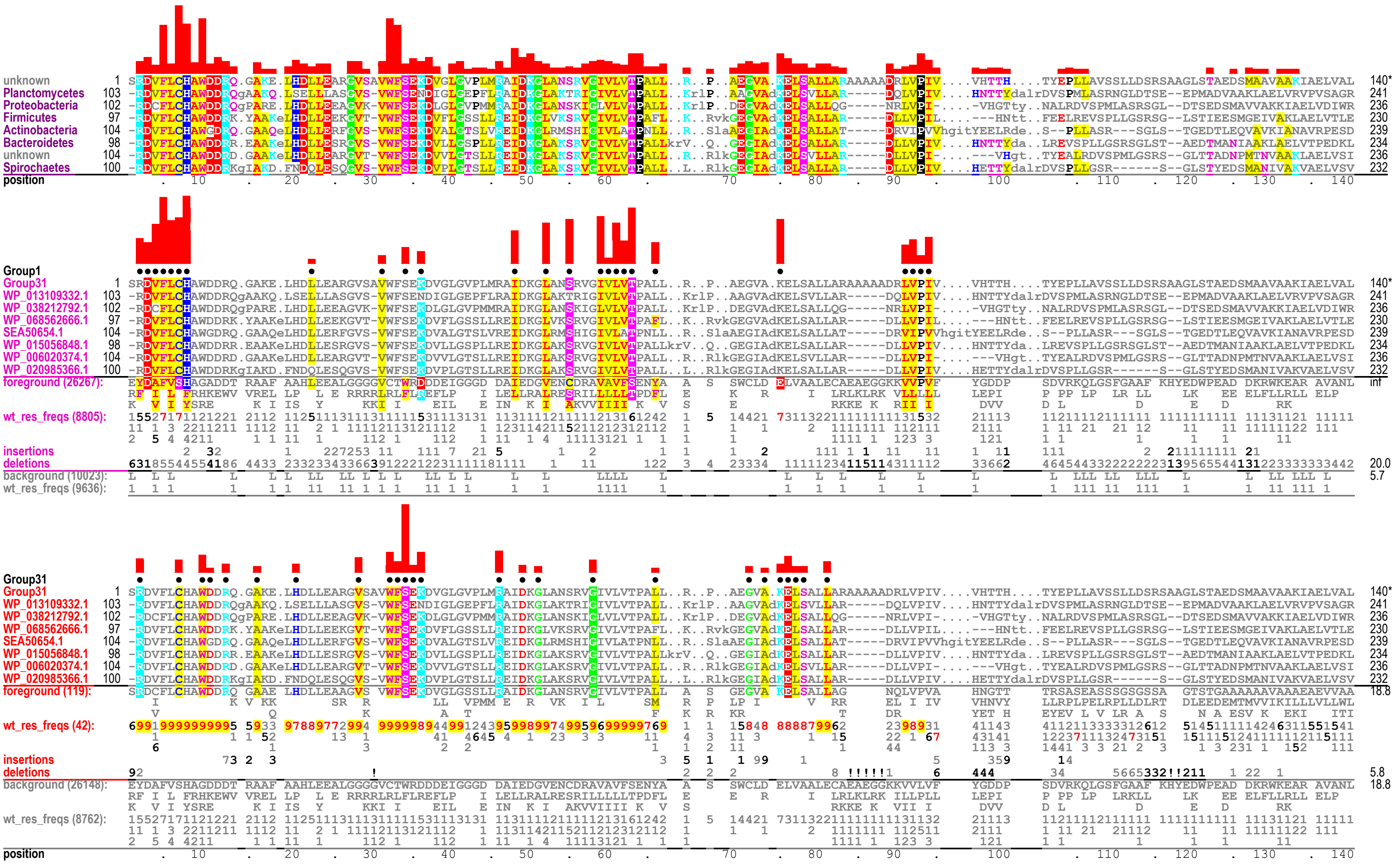

Group19 (bacterial TIRs)

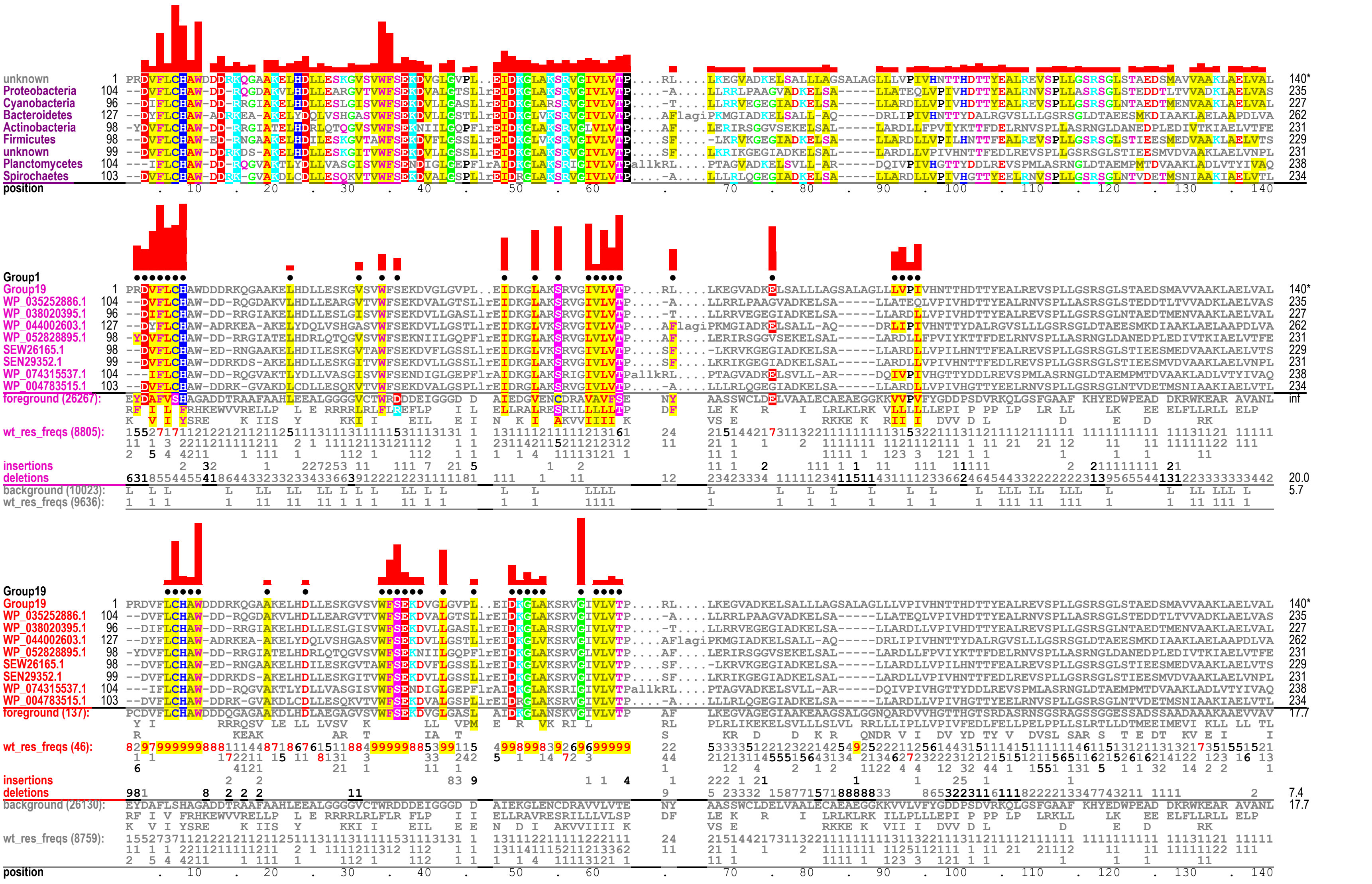

Group18 (TIRs of lower Metazoa)

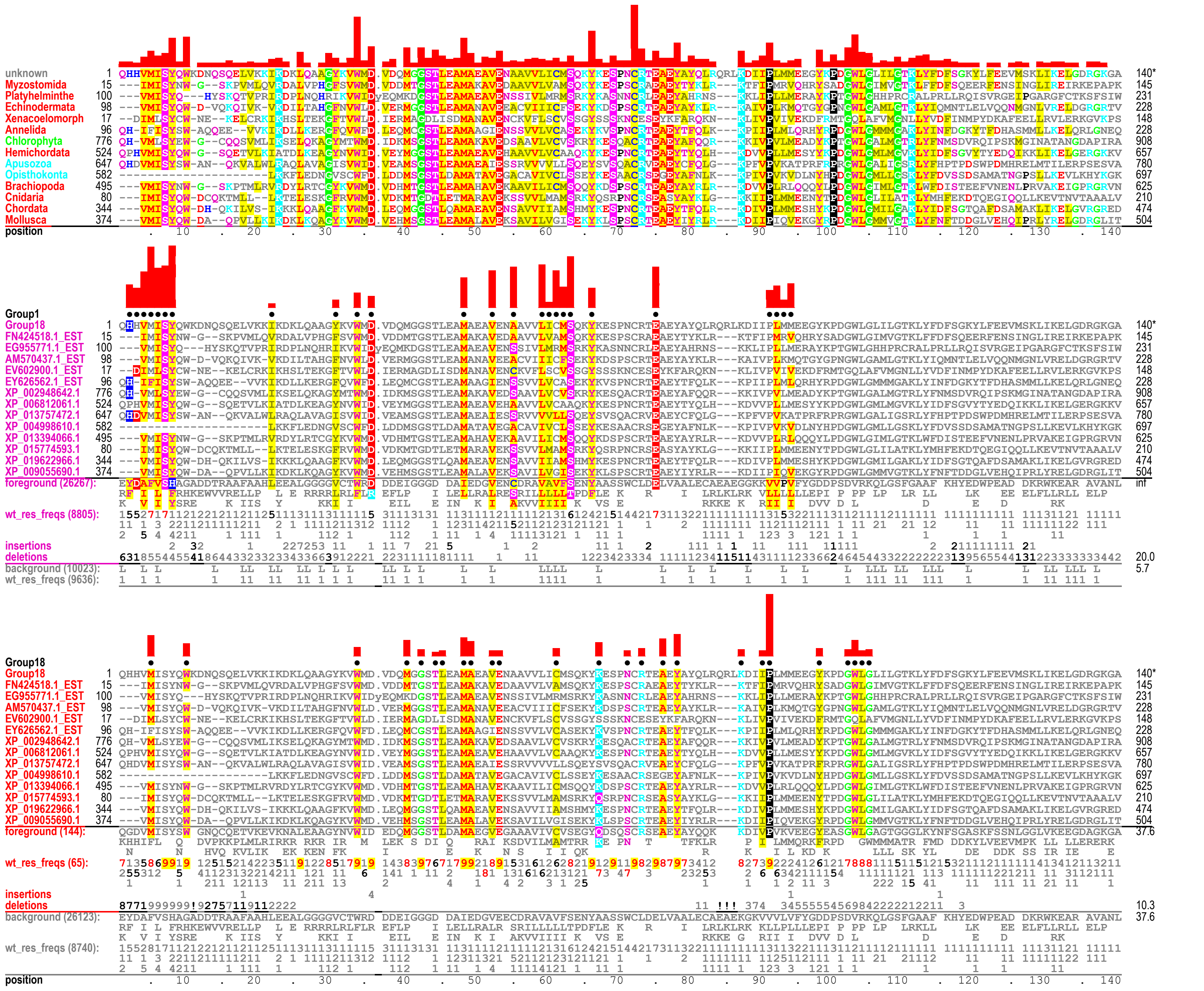

Group30 (bacterial TIRs)

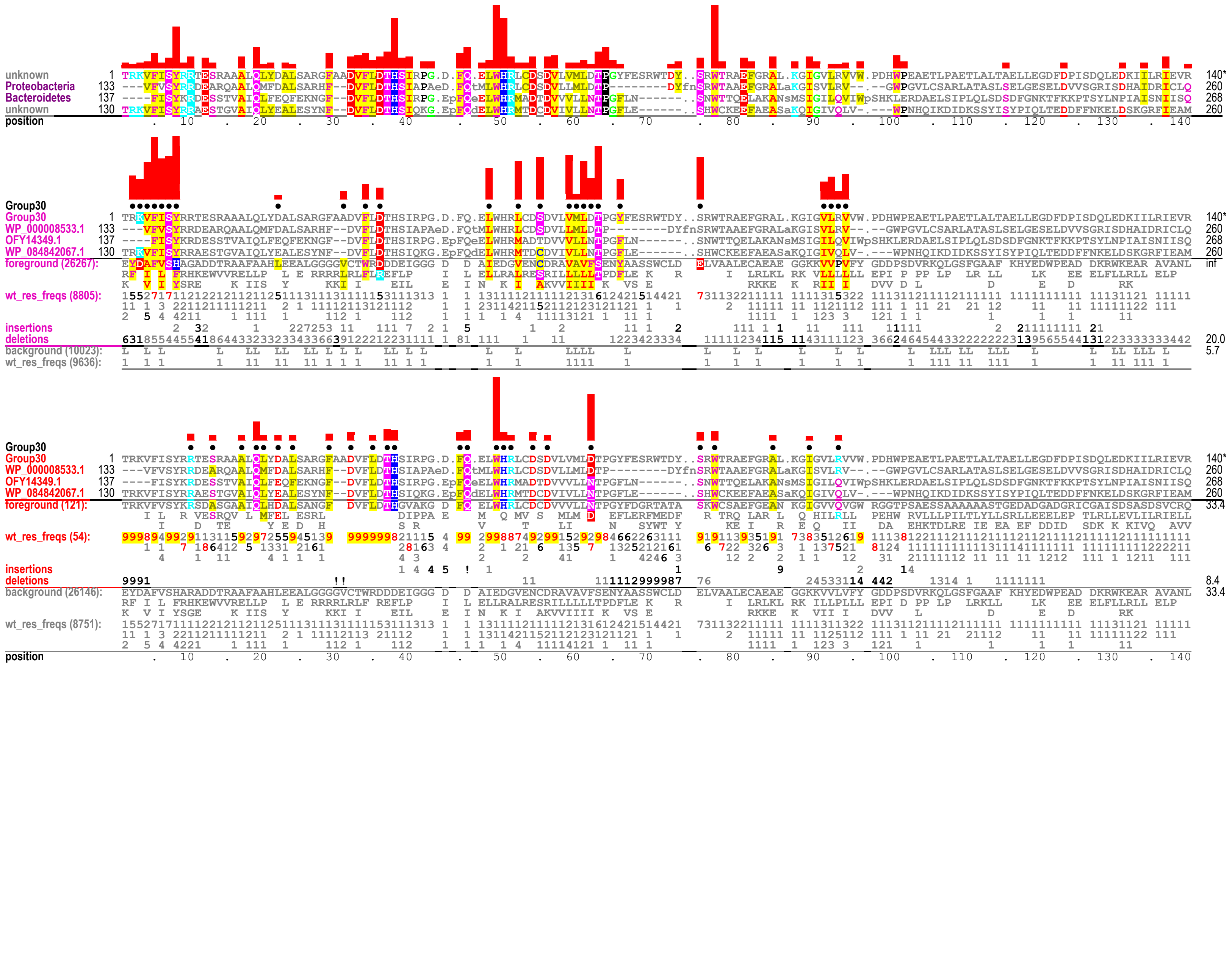

Group17 (bacterial TIR group with admixture of TIRs from Protozoa and lower Metazoa)

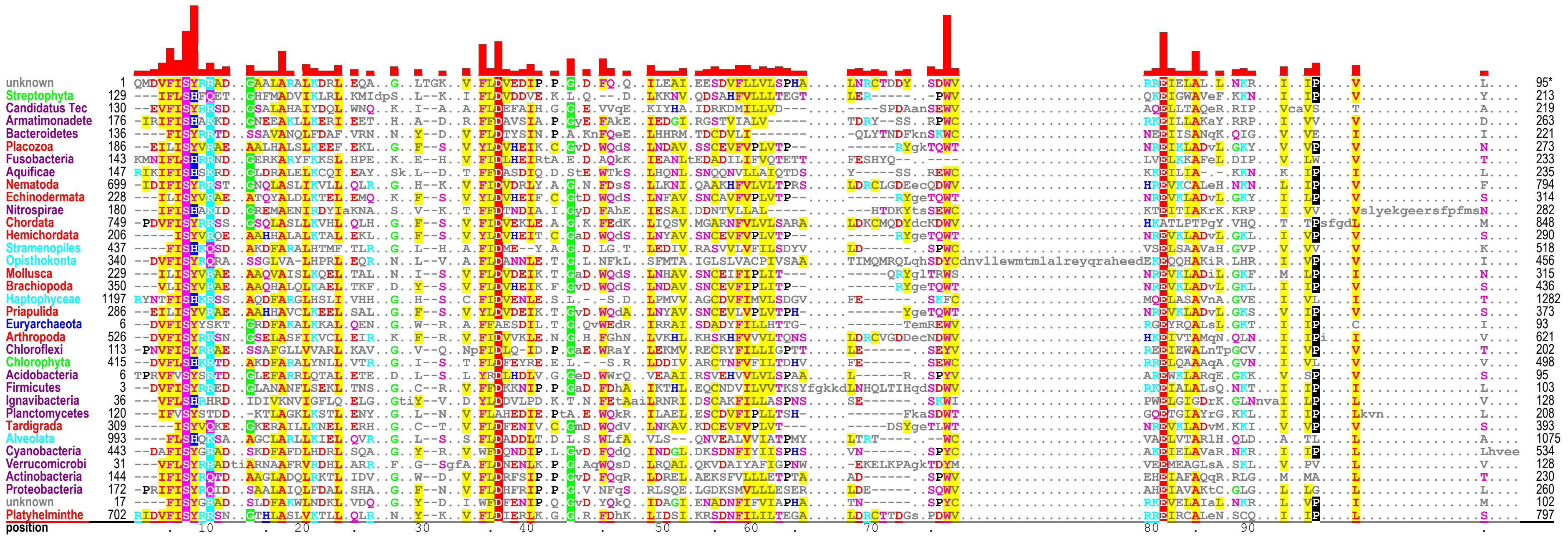

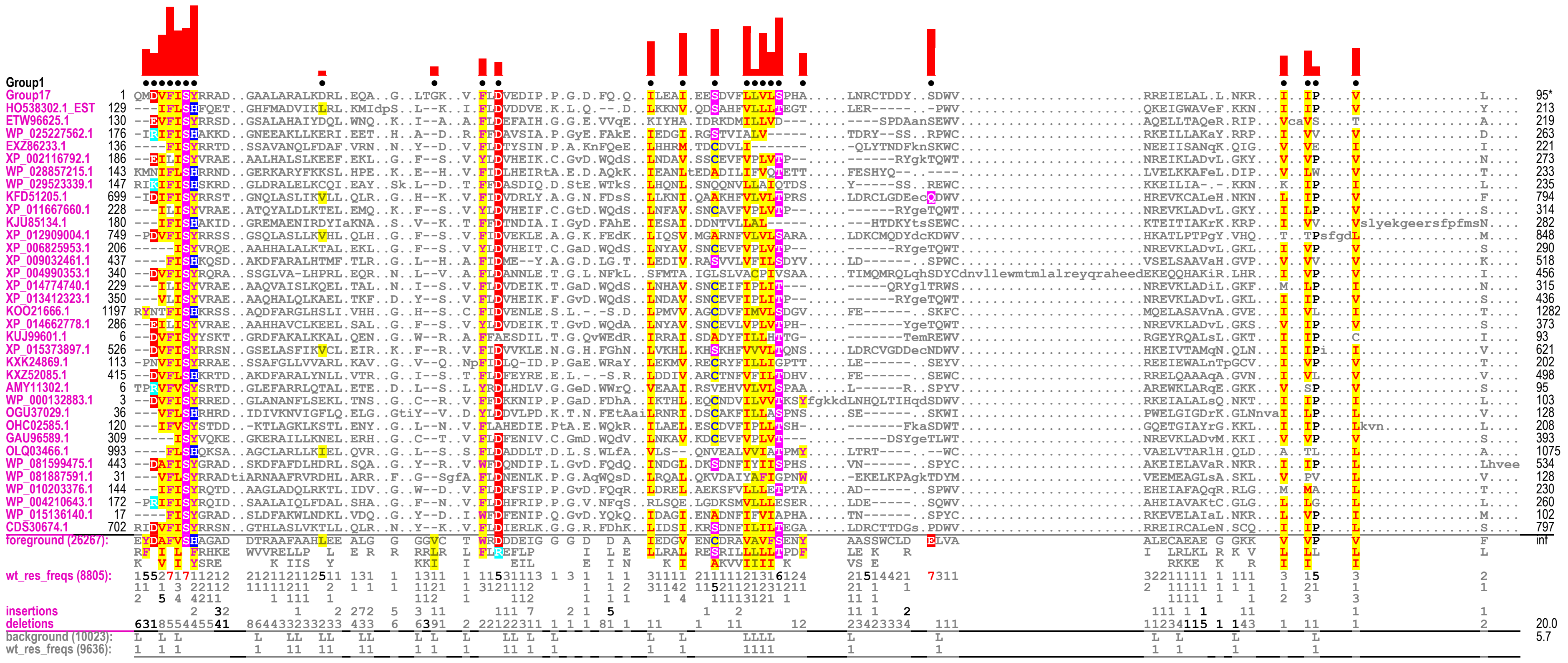

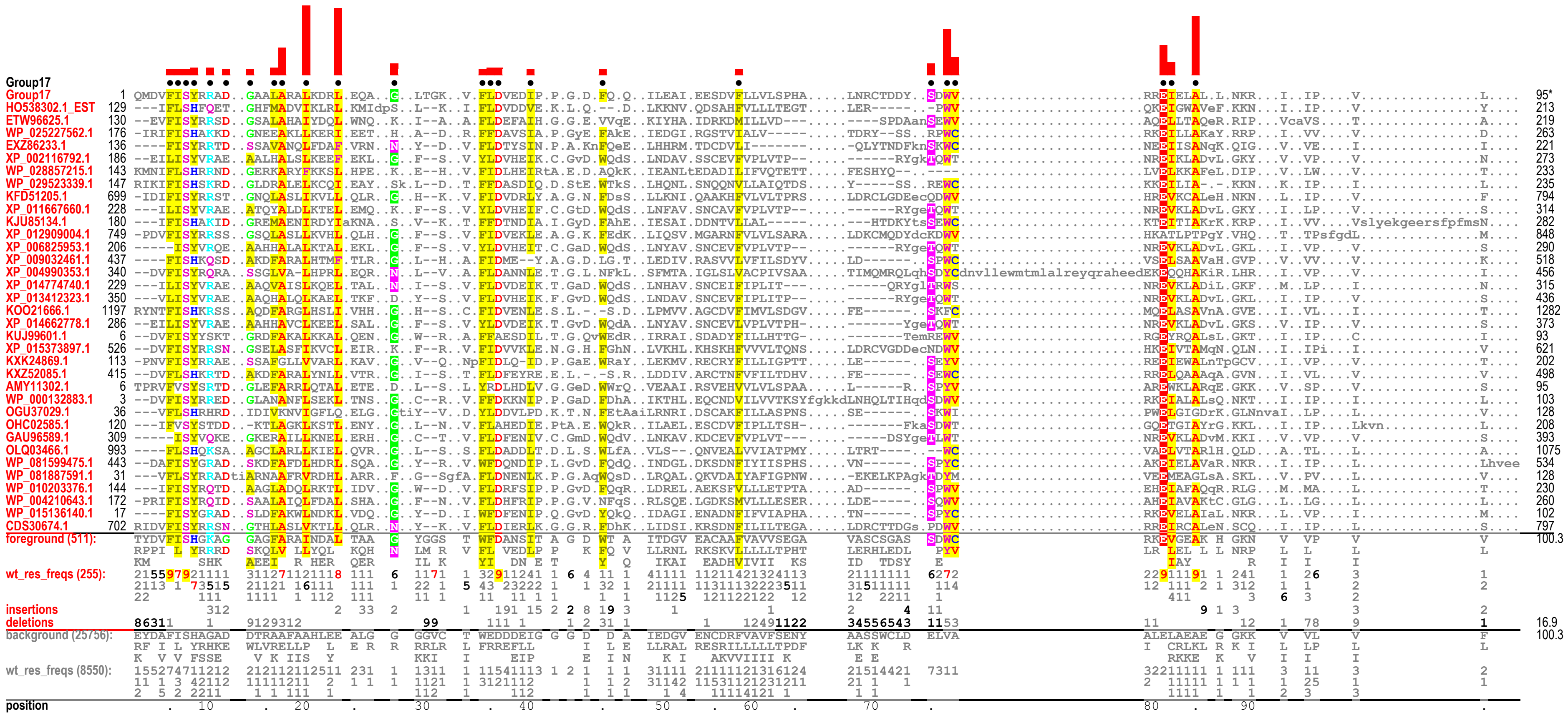

```
unknown            96 ...L.GGCW..PE..............P.................DD.............LP...EDLRKLVK....LNSVDWS.D.GYI.DA.......LRDELLELLKIN 140*
Streptophyta      214 ...F.K--QfpLV..............K.................DT.............YS...TEIKEALK....NRGIEYS.L.TYR.EQ.......AIDQILKRIPKN 258
Candidatus Tec    220 ...E.GVIH..PQ..............V.................RD.............VP...RLVWDDAK....QEQLLER.I.GLL.VS.......RCIASRDSLDLR 264
Armatimonadete    264 ...A.LEKE..EL..............R.................SF.............PY...LGNCRVLR....WTEDAYS.I.VLA.AL.......SETLRFEYQLRR 308
Bacteroidetes     222 ...W.PDCT..PD..............I.................HN.............LL...CEPIQLTQ....KMFMNED.F.HNG.SC.......CITEECIENIVN 266
Placozoa          274 ...F.LSDW..PP..............A.................CL.............AI...QFATTQFV....PWKTTEN.I.LKD.GN.......KDDISYWDKVYV 318
Fusobacteria      234 ...L.GLEK..NE..............Y.................RN.............LP...LHPVGAPH....FEIKKFT.A.DNV.NQ.......ITNYAFDMIRLR 278
Aquificae         236 ...I.LKAL..KE..............G.................ED.............RS...FPYMYNVP....TIKCKNE.R.IDI.DL.......LLMKILLEVLRF 280
Nematoda          795 ...D.PMFEw.PD..............E.................NQ.............IP...ADIRAITR....VNGVRWV.H.EYQ.EA.......CVDKLEKFINGE 840
Echinodermata     315 ...F.LETW..P-..............P.................RC.............LA...IQFATTQF....INWRKPK.P.GQP.AT.......PHSPSQELSMDS 358
Nitrospirae       283 ...V.KVFKyqSS..............K.................ND.............L-...EDIIGVAL....KETLKVK.Y.QEM.YI.......KYLLSINTMKGY 328
Chordata          849 ...E.GSLW..RH..............G.................EV.............I-...--------....-------.-.---.--.......-------GPWRL 864
Hemichordata      291 ...F.LSKW..PP..............P.................CL.............--...--AIQFAT....TQYVRWK.T.RDE.IR.......QAVERGDGDKAS 331
Stramenopiles     519 ...K.EGSRwrDP..............EapestarclnfpsyaelAK.............LP...PAARDVFQ....IKTVEHS.D.VYY.AS.......FIDKLFERLAEA 582
Opisthokonta      457 ...L.GSTW..HN..............A.................ND.............SG...MSGAEAGE....YDSRDNM.K.QLA.MT.......LPDAVSKQTAAA 501
Mollusca          316 ...F.LSEW..P-..............P.................SC.............LA...IQLATTQY....INGTRQS.A.CSA.LS.......HEAFPTIYPNQD 359
Brachiopoda       437 ...F.LDMW..PP..............K.................CL.............AI...QFASTQFI....SWKPKDI.I.EKE.LR.......SGELPENVYNIN 481
Haptophyceae     1283 ...K.EGSRwpNRngdmvevfpppeliE.................AL.............EP...PECRAAFA....RRAITHT.N.EYY.AAfshnlmqTVDKLIKEHRQQ 1350
Priapulida        374 ...F.VKKW..PP..............R.................CL.............AI...QFATTQFI....PWKTQEE.I.DLA.IK.......RGIEDAIDIKMW 418
Euryarchaeota      94 ...Q.ILGD..DT..............L.................GK.............IY...RDIETAFP....GISKIQA.I.TWK.FP.......SDLTSKVIWELK 138
Arthropoda        622 ...E.DFTW..P-..............-.................ES.............LP...EDIRKLQT....YDVIEWS.H.TNQ.DK.......FIDAIVTAMRGD 664
Chloroflexi       203 ...H.N-GF..QR..............D.................HS.............FP...EALAARQV....IEVRRES.A.EDY.EL.......AVIKLLNLLGYT 246
Chlorophyta       499 ...K.EGSRwrDA..............K.................GNkvcefppyellrtLP...ADAQKVFE....RKSVQHS.D.EYY.AF.......PDGQDVQQQQHP 558
Acidobacteria      96 ...G.PGAL..DF..............-.................SQ.............LP...RWMARAH-....------R.H.DIDiPE.......SLARLVQVLKGP 133
Firmicutes        104 ...FnGVPL..PD..............V.................SD.............IP...DDIRAVLK....RQYIKVSnD.DDW.DF.......LMNKIKNSLSQN 150
Ignavibacteria    129 ...E.NRDNwkER..............E.................YY.............QI...YGSIQISQ....QGNWCFF.T.PQK.SK.......GIKLTEWLTNSS 175
Planctomycetes    209 ...D.PFGF..IA..............I.................QA.............LNfstEDLESVRR....GILDIMA.K.KSD.KN.......NLFESLIKGFSQ 256
Tardigrada        394 ...L.IEHW..P-..............P.................SC.............LA...IQFATTQHvqwsRNSSPWK.D.DRR.SG.......SVFSPEEEKECV 441
Alveolata        1076 ...L.PHFFmpSD..............A.................FI.............DE...YESMAQSK....IDLASWG.F.GAT.DI.......TETIRWLRTLRS 1122
Cyanobacteria     535 ishD.TWQQ..RH..............P.................QG.............TP...EDWLAYTA....AGKHSVF.P.QMP.VS.......IGKVNWIYFRPG 582
Verrucomicrobi    129 ...E.RDRL..PP..............V.................SD.............TH...TIFASLRT....LQAHPLD.LgIYF.EP.......SVERLLSTTVLS 174
Actinobacteria    231 ...L.PDTR..P-..............A.................EL.............YP...--------....--AIEPS.S.RYQ.LD.......DTDVTGSAPEYV 264
Proteobacteria    261 ...L.PGGQ..PQ..............A.................SL.............EP...ENRRTVPL....ADFAGGQ.F.DKD.AT.......LLPAALERIVQE 305
unknown           103 ...H.VEEI..SR..............E.................TW.............QG...RNPNGTDE....QWEAYKT.E.GLH.SC.......FTNLHPELGKIN 147
Platyhelminthe    798 ...D.-RFDwsIT..............E.................-N.............IP...EDIRAITK....FNSVQWT.P.EFE.EG.......CIDRIIEFMRPK 842
position                    100                                     .                  110    .      120      .          130    .  140
```

Group1

```
Group17            96 ...L.GGCW..PE..............P.................DD.............LP...EDLRKLVK....LNSVDWS.D.GYI.DA.......LRDELLELLKIN 140*
HO538302.1_EST    214 ...F.K--QfpLV..............K.................DT.............YS...TEIKEALK....NRGIEYS.L.TYR.EQ.......AIDQILKRIPKN 258
ETW96625.1        220 ...E.GVIH..PQ..............V.................RD.............VP...RLVWDDAK....QEQLLER.I.GLL.VS.......RCIASRDSLDLR 264
WP_025227562.1    264 ...A.LEKE..EL..............R.................SF.............PY...LGNCRVLR....WTEDAYS.I.VLA.AL.......SETLRFEYQLRR 308
EXZ86233.1        222 ...W.PDCT..PD..............I.................HN.............LL...CEPIQLTQ....KMFMNED.F.HNG.SC.......CITEECIENIVN 266
XP_002116792.1    274 ...F.LSDW..PP..............A.................CL.............AI...QFATTQFV....PWKTTEN.I.LKD.GN.......KDDISYWDKVYV 318
WP_028857215.1    234 ...L.GLEK..NE..............Y.................RN.............LP...LHPVGAPH....FEIKKFT.A.DNV.NQ.......ITNYAFDMIRLR 278
WP_029523339.1    236 ...I.LKAL..KE..............G.................ED.............RS...FPYMYNVP....TIKCKNE.R.IDI.DL.......LLMKILLEVLRF 280
KFD51205.1        795 ...D.PMFEw.PD..............E.................NQ.............IP...ADIRAITR....VNGVRWV.H.EYQ.EA.......CVDKLEKFINGE 840
XP_011667660.1    315 ...F.LETW..P-..............P.................RC.............LA...IQFATTQF....INWRKPK.P.GQP.AT.......PHSPSQELSMDS 358
KJU85134.1        283 ...V.KVFKyqSS..............K.................ND.............L-...EDIIGVAL....KETLKVK.Y.QEM.YI.......KYLLSINTMKGY 328
XP_012909004.1    849 ...E.GSLW..RH..............G.................EV.............I-...--------....-------.-.---.--.......-------GPWRL 864
XP_006825953.1    291 ...F.LSKW..PP..............P.................CL.............--...--AIQFAT....TQYVRWK.T.RDE.IR.......QAVERGDGDKAS 331
XP_009032461.1    519 ...K.EGSRwrDP..............EapestarclnfpsyaelAK.............LP...PAARDVFQ....IKTVEHS.D.VYY.AS.......FIDKLFERLAEA 582
XP_004990353.1    457 ...L.GSTW..HN..............A.................ND.............SG...MSGAEAGE....YDSRDNM.K.QLA.MT.......LPDAVSKQTAAA 501
XP_014774740.1    316 ...F.LSEW..P-..............P.................SC.............LA...IQLATTQY....INGTRQS.A.CSA.LS.......HEAFPTIYPNQD 359
XP_013412323.1    437 ...F.LDMW..PP..............K.................CL.............AI...QFASTQFI....SWKPKDI.I.EKE.LR.......SGELPENVYNIN 481
KOO21666.1       1283 ...K.EGSRwpNRngdmvevfpppeliE.................AL.............EP...PECRAAFA....RRAITHT.N.EYY.AAfshnlmqTVDKLIKEHRQQ 1350
XP_014662778.1    374 ...F.VKKW..PP..............R.................CL.............AI...QFATTQFI....PWKTQEE.I.DLA.IK.......RGIEDAIDIKMW 418
KUJ99601.1         94 ...Q.ILGD..DT..............L.................GK.............IY...RDIETAFP....GISKIQA.I.TWK.FP.......SDLTSKVIWELK 138
XP_015373897.1    622 ...E.DFTW..P-..............-.................ES.............LP...EDIRKLQT....YDVIEWS.H.TNQ.DK.......FIDAIVTAMRGD 664
KXK24869.1        203 ...H.N-GF..QR..............D.................HS.............FP...EALAARQV....IEVRRES.A.EDY.EL.......AVIKLLNLLGYT 246
KXZ52085.1        499 ...K.EGSRwrDA..............K.................GNkvcefppyellrtLP...ADAQKVFE....RKSVQHS.D.EYY.AF.......PDGQDVQQQQHP 558
AMY11302.1         96 ...G.PGAL..DF..............-.................SQ.............LP...RWMARAH-....------R.H.DIDiPE.......SLARLVQVLKGP 133
WP_000132883.1    104 ...FnGVPL..PD..............V.................SD.............IP...DDIRAVLK....RQYIKVSnD.DDW.DF.......LMNKIKNSLSQN 150
OGU37029.1        129 ...E.NRDNwkER..............E.................YY.............QI...YGSIQISQ....QGNWCFF.T.PQK.SK.......GIKLTEWLTNSS 175
OHC02585.1        209 ...D.PFGF..IA..............I.................QA.............LNfstEDLESVRR....GILDIMA.K.KSD.KN.......NLFESLIKGFSQ 256
GAU96589.1        394 ...L.IEHW..P-..............P.................SC.............LA...IQFATTQHvqwsRNSSPWK.D.DRR.SG.......SVFSPEEEKECV 441
OLQ03466.1       1076 ...L.PHFFmpSD..............A.................FI.............DE...YESMAQSK....IDLASWG.F.GAT.DI.......TETIRWLRTLRS 1122
WP_081599475.1    535 ishD.TWQQ..RH..............P.................QG.............TP...EDWLAYTA....AGKHSVF.P.QMP.VS.......IGKVNWIYFRPG 582
WP_081887591.1    129 ...E.RDRL..PP..............V.................SD.............TH...TIFASLRT....LQAHPLD.LgIYF.EP.......SVERLLSTTVLS 174
WP_010203376.1    231 ...L.PDTR..P-..............A.................EL.............YP...--------....--AIEPS.S.RYQ.LD.......DTDVTGSAPEYV 264
WP_004210643.1    261 ...L.PGGQ..PQ..............A.................SL.............EP...ENRRTVPL....ADFAGGQ.F.DKD.AT.......LLPAALERIVQE 305
WP_015136140.1    103 ...H.VEEI..SR..............E.................TW.............QG...RNPNGTDE....QWEAYKT.E.GLH.SC.......FTNLHPELGKIN 147
CDS30674.1        798 ...D.-RFDwsIT..............E.................-N.............IP...EDIRAITK....FNSVQWT.P.EFE.EG.......CIDRIIEFMRPK 842
foreground (26267):      Y GDDP  SD              V                 RK             QL   GSFGAAF     KHYEDWP E AD  DK       RWKEAR AVANL inf
                         L EPI   P               P                 P              LP     LR LL        LK     EE  EL       FLLRLL ELP
                           DVV   D               L                                          D          E     D             RK
wt_res_freqs (8805):     2 1113  11              2                 11             11   2111111     1111111 1 11  11       131121 11111
                         2 111   1               1                 1              21     21 12        11     11  11       111122 111
                           121   1               1                                          1          1     1             11
insertions               1   11  11              1                                           2     211111  1 11  21
deletions                3 3662  46              4                 54             43   32222222    3139565 5 441 31       223333333442 20.0
background (10023):      L        L                                 L             LL    LL  LLL       L          L         LL LLL L    5.7
wt_res_freqs (9636):     1        1                                 1             11    11  111       1          1         11 111 1
```

Group17

```
Group17            96 ...L.GGCW..PE..............P.................DD.............LP...EDLRKLVK....LNSVDWS.D.GYI.DA.......LRDELLELLKIN 140*
HO538302.1_EST    214 ...F.K--QfpLV..............K.................DT.............YS...TEIKEALK....NRGIEYS.L.TYR.EQ.......AIDQILKRIPKN 258
ETW96625.1        220 ...E.GVIH..PQ..............V.................RD.............VP...RLVWDDAK....QEQLLER.I.GLL.VS.......RCIASRDSLDLR 264
WP_025227562.1    264 ...A.LEKE..EL..............R.................SF.............PY...LGNCRVLR....WTEDAYS.I.VLA.AL.......SETLRFEYQLRR 308
EXZ86233.1        222 ...W.PDCT..PD..............I.................HN.............LL...CEPIQLTQ....KMFMNED.F.HNG.SC.......CITEECIENIVN 266
XP_002116792.1    274 ...F.LSDW..PP..............A.................CL.............AI...QFATTQFV....PWKTTEN.I.LKD.GN.......KDDISYWDKVYV 318
WP_028857215.1    234 ...L.GLEK..NE..............Y.................RN.............LP...LHPVGAPH....FEIKKFT.A.DNV.NQ.......ITNYAFDMIRLR 278
WP_029523339.1    236 ...I.LKAL..KE..............G.................ED.............RS...FPYMYNVP....TIKCKNE.R.IDI.DL.......LLMKILLEVLRF 280
KFD51205.1        795 ...D.PMFEw.PD..............E.................NQ.............IP...ADIRAITR....VNGVRWV.H.EYQ.EA.......CVDKLEKFINGE 840
XP_011667660.1    315 ...F.LETW..P-..............P.................RC.............LA...IQFATTQF....INWRKPK.P.GQP.AT.......PHSPSQELSMDS 358
KJU85134.1        283 ...V.KVFKyqSS..............K.................ND.............L-...EDIIGVAL....KETLKVK.Y.QEM.YI.......KYLLSINTMKGY 328
XP_012909004.1    849 ...E.GSLW..RH..............G.................EV.............I-...--------....-------.-.---.--.......-------GPWRL 864
XP_006825953.1    291 ...F.LSKW..PP..............P.................CL.............--...--AIQFAT....TQYVRWK.T.RDE.IR.......QAVERGDGDKAS 331
XP_009032461.1    519 ...K.EGSRwrDP..............EapestarclnfpsyaelAK.............LP...PAARDVFQ....IKTVEHS.D.VYY.AS.......FIDKLFERLAEA 582
XP_004990353.1    457 ...L.GSTW..HN..............A.................ND.............SG...MSGAEAGE....YDSRDNM.K.QLA.MT.......LPDAVSKQTAAA 501
XP_014774740.1    316 ...F.LSEW..P-..............P.................SC.............LA...IQLATTQY....INGTRQS.A.CSA.LS.......HEAFPTIYPNQD 359
XP_013412323.1    437 ...F.LDMW..PP..............K.................CL.............AI...QFASTQFI....SWKPKDI.I.EKE.LR.......SGELPENVYNIN 481
KOO21666.1       1283 ...K.EGSRwpNRngdmvevfpppeliE.................AL.............EP...PECRAAFA....RRAITHT.N.EYY.AAfshnlmqTVDKLIKEHRQQ 1350
XP_014662778.1    374 ...F.VKKW..PP..............R.................CL.............AI...QFATTQFI....PWKTQEE.I.DLA.IK.......RGIEDAIDIKMW 418
KUJ99601.1         94 ...Q.ILGD..DT..............L.................GK.............IY...RDIETAFP....GISKIQA.I.TWK.FP.......SDLTSKVIWELK 138
XP_015373897.1    622 ...E.DFTW..P-..............-.................ES.............LP...EDIRKLQT....YDVIEWS.H.TNQ.DK.......FIDAIVTAMRGD 664
KXK24869.1        203 ...H.N-GF..QR..............D.................HS.............FP...EALAARQV....IEVRRES.A.EDY.EL.......AVIKLLNLLGYT 246
KXZ52085.1        499 ...K.EGSRwrDA..............K.................GNkvcefppyellrtLP...ADAQKVFE....RKSVQHS.D.EYY.AF.......PDGQDVQQQQHP 558
AMY11302.1         96 ...G.PGAL..DF..............-.................SQ.............LP...RWMARAH-....------R.H.DIDiPE.......SLARLVQVLKGP 133
WP_000132883.1    104 ...FnGVPL..PD..............V.................SD.............IP...DDIRAVLK....RQYIKVSnD.DDW.DF.......LMNKIKNSLSQN 150
OGU37029.1        129 ...E.NRDNwkER..............E.................YY.............QI...YGSIQISQ....QGNWCFF.T.PQK.SK.......GIKLTEWLTNSS 175
OHC02585.1        209 ...D.PFGF..IA..............I.................QA.............LNfstEDLESVRR....GILDIMA.K.KSD.KN.......NLFESLIKGFSQ 256
GAU96589.1        394 ...L.IEHW..P-..............P.................SC.............LA...IQFATTQHvqwsRNSSPWK.D.DRR.SG.......SVFSPEEEKECV 441
OLQ03466.1       1076 ...L.PHFFmpSD..............A.................FI.............DE...YESMAQSK....IDLASWG.F.GAT.DI.......TETIRWLRTLRS 1122
WP_081599475.1    535 ishD.TWQQ..RH..............P.................QG.............TP...EDWLAYTA....AGKHSVF.P.QMP.VS.......IGKVNWIYFRPG 582
WP_081887591.1    129 ...E.RDRL..PP..............V.................SD.............TH...TIFASLRT....LQAHPLD.LgIYF.EP.......SVERLLSTTVLS 174
WP_010203376.1    231 ...L.PDTR..P-..............A.................EL.............YP...--------....--AIEPS.S.RYQ.LD.......DTDVTGSAPEYV 264
WP_004210643.1    261 ...L.PGGQ..PQ..............A.................SL.............EP...ENRRTVPL....ADFAGGQ.F.DKD.AT.......LLPAALERIVQE 305
WP_015136140.1    103 ...H.VEEI..SR..............E.................TW.............QG...RNPNGTDE....QWEAYKT.E.GLH.SC.......FTNLHPELGKIN 147
CDS30674.1        798 ...D.-RFDwsIT..............E.................-N.............IP...EDIRAITK....FNSVQWT.P.EFE.EG.......CIDRIIEFMRPK 842
foreground (511):        D GGCW  TG              E                 DD             IP   EDIDAT E    LNGVDD  D GDI DA       LRNEVVEKRSIN 100.3
                         L VE I  PE              P                  E             L    L LRKL R     DPIKW    EL  EL        ILLLLLLLK
                         V DD     D                                                         I K      S       DY   S        DKII II
wt_res_freqs (255):      1 1111  11              1                 11             14   111111 1    111111  1 111 21       111111111111
                         1 11 1  21              2                  1             3    1 2111 1     11111    11  11        111111111
                         1 11     1                                                         1 1      1       11   1        1111 11
insertions               1    2  1               1                                 2                     1                 1
deletions                1 1198  57              5                 54             22   34445565    6464333 4 221 11       111          16.9
background (25756):      Y GDDP  SD              V                 RK             QL   GSFGAAF     KHYEDWP E AD  DQ       RWKEAR AVANL 100.3
                         L EPI   D               P                 P              L      LR LL     R  LK     EE  EL       FLLRLL ELP
                           DVV                   L                                          D          E     D    K        RK
wt_res_freqs (8550):     2 1113  11              2                 11             11   2111111     1111111 1 11  11       131121 11111
                         2 111   1               1                 1              2      21 12     1  11     11  11       111122 111
                           121                   1                                          1          1     1    1        11
position                       100                                     .                  110    .      120      .          130    .  140
```

Group16 (plant TIRs)

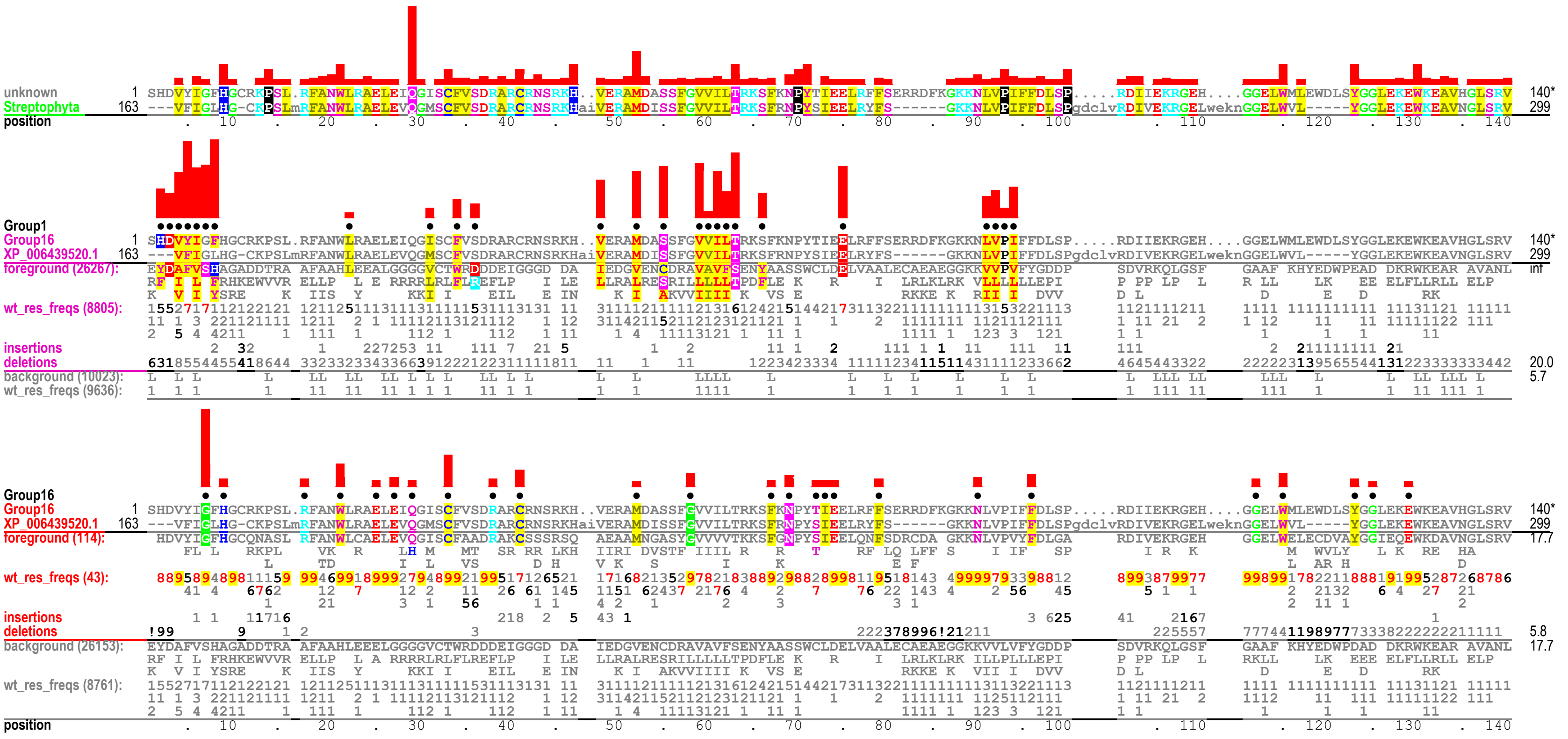

Group27 (bacterial TIRs)

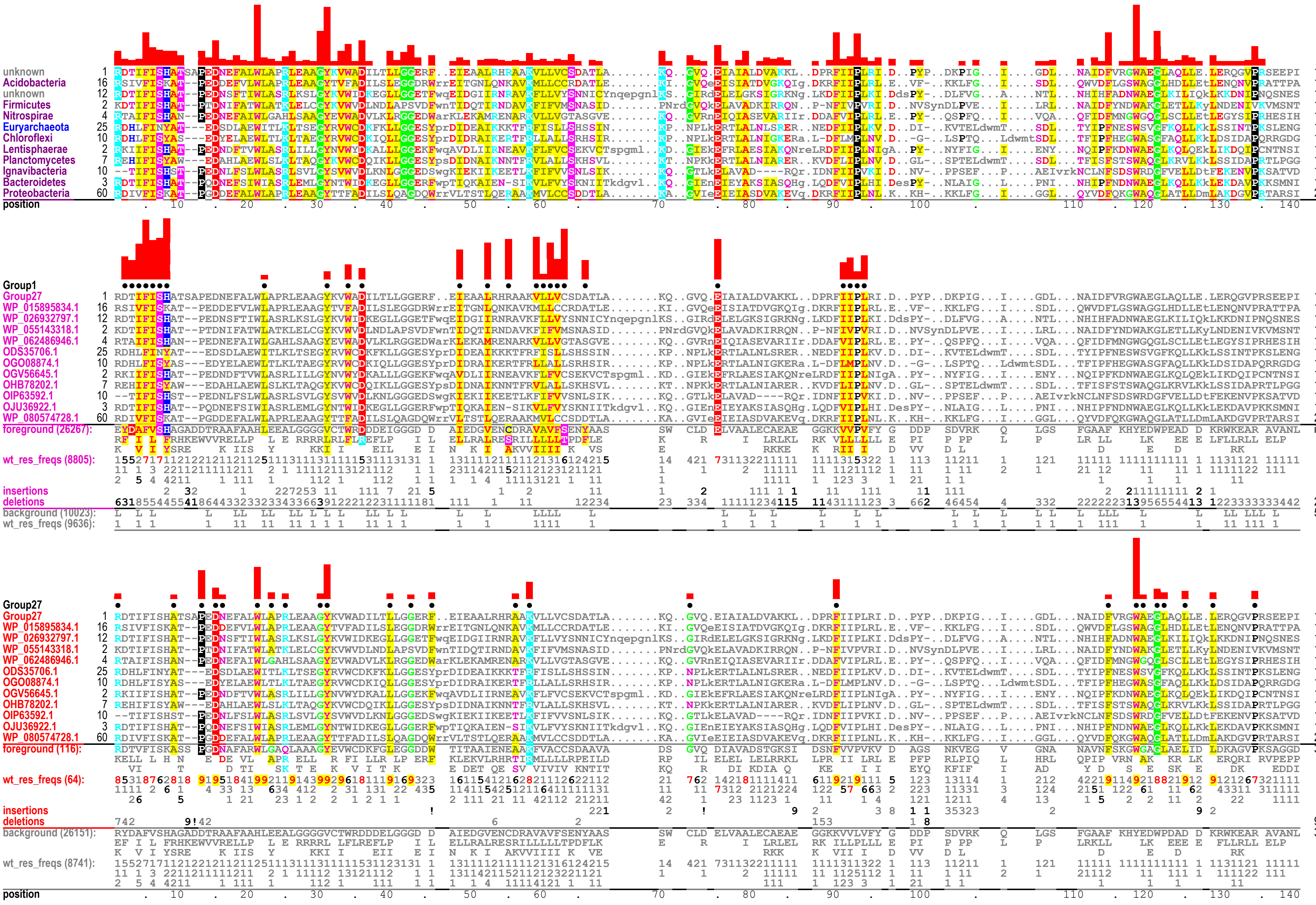

Group15 (bacterial TIRs)

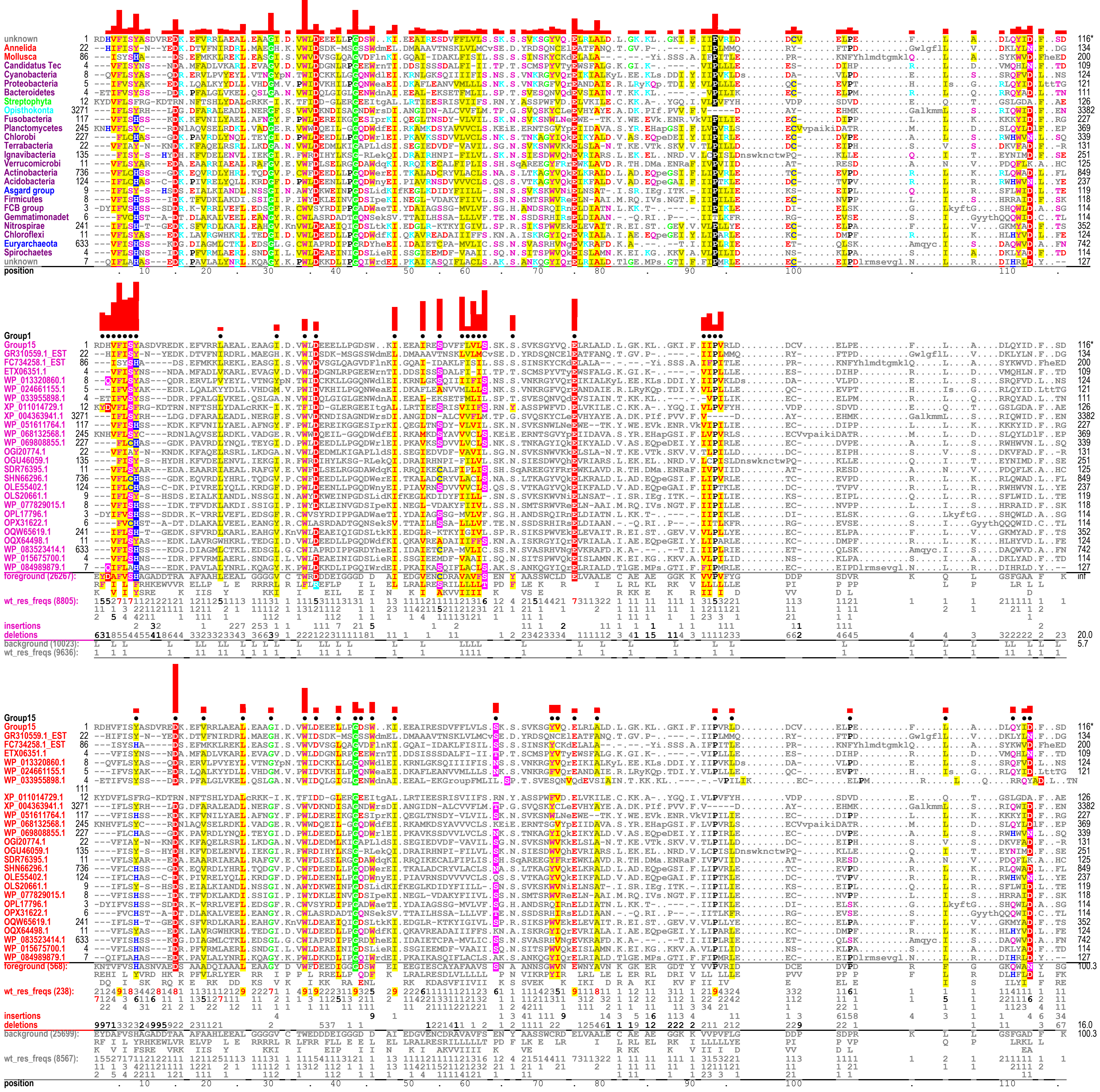

```
unknown                117 ..N....Y.EDGLARLLR..LKAQLEKLLVGPA 140*
Annelida               135 kfE....F.NEAFKMLLKe.MDQYIGQQGKKEY 161
Mollusca               201 ..K....I.EESFKELIDm.LHTELHQRQEEEE 225
Candidatus Tec         110 ..QrrgpW.GELIQTIND..TVFTLGQTDLILA 137
Cyanobacteria          125 ..S....-.-DSLLKIKQs.LDSQMENLGFEIS 147
Proteobacteria         122 ..A....W.IQVQASLQLa.AEQQSIEIKHGSN 146
Bacteroidetes          112 ..D....P.DKAIIDILT..FLGLKEKASTSLN 135
Streptophyta           127 ..L....-.ERNFKQKMDkvPRWRADLTSAANI 151
Opisthokonta          3383 i.P....Y.ETSLLNLIGq.VDRIRLEVGRNAV 3408
Fusobacteria           228 ..D....Y.NTALSFLYKs.L-----------I 241
Planctomycetes         370 ..G....-.--GYTNLLRs.LK-----ASTLRP 386
Chlorobi               340 ..Q....-.-NGFERLMRs.LCIRAESKQRLQP 362
Terrabacteria          132 ..N....D.SEFESEYSKi.LRTVGLPVSTNEA 156
Ignavibacteria         252 ..-....-.WRDKYIFRE..KMNSLIDGINLFY 273
Verrucomicrobi         126 ..T....R.LANGEPTPQ..FVEQVKRLLQAPR 149
Actinobacteria         850 ..P....G.A--YERLLKs.LER---------G 863
Acidobacteria          238 ..-....-.ANGYERLLAs.LLRRANDLGIGIT 260
Asgard group           120 ..N....Y.QEGIKKLINs.IHEIYDKPTIKNS 144
Firmicutes             119 ..N....Y.ETGLEEICEy.LKKDYGALSMFQN 143
FCB group              115 ..G....W.KEVRGRLLEs.LGNLPGIEPRTPP 139
Gemmatimonadet         115 ..N....Q.SNTPEIISA..LFSIAKTPVKLPQ 138
Nitrospirae            353 paK....Y.DESLGKLLRr.LK---------TL 370
Chloroflexi            125 ..-....-.KDGYEKLIRs.LQVRAKNVDTSVN 147
Euryarchaeota          743 ..G....I.DAQIAETIR..K-----AILSALN 761
Spirochaetes           115 ptK....F.DDSYQNLIRa.IKNHRLKNKPVEP 141
unknown                128 ..-....WeEDGFEQLERa.ITHQFKLEPEEPK 152
position                            120    .   130      .  140
```

```
Group1
Group15                117 ..N....Y.EDGLARLLR..LKAQLEKLLVGPA 140*
GR310559.1_EST         135 kfE....F.NEAFKMLLKe.MDQYIGQQGKKEY 161
FC734258.1_EST         201 ..K....I.EESFKELIDm.LHTELHQRQEEEE 225
ETX06351.1             110 ..QrrgpW.GELIQTIND..TVFTLGQTDLILA 137
WP_013320860.1         125 ..S....-.-DSLLKIKQs.LDSQMENLGFEIS 147
WP_024661155.1         122 ..A....W.IQVQASLQLa.AEQQSIEIKHGSN 146
WP_033955898.1         112 ..D....P.DKAIIDILT..FLGLKEKASTSLN 135
XP_011014729.1         127 ..L....-.ERNFKQKMDkvPRWRADLTSAANI 151
XP_004363941.1        3383 i.P....Y.ETSLLNLIGq.VDRIRLEVGRNAV 3408
WP_051611764.1         228 ..D....Y.NTALSFLYKs.L-----------I 241
WP_068132568.1         370 ..G....-.--GYTNLLRs.LK-----ASTLRP 386
WP_069808855.1         340 ..Q....-.-NGFERLMRs.LCIRAESKQRLQP 362
OGI20774.1             132 ..N....D.SEFESEYSKi.LRTVGLPVSTNEA 156
OGU46059.1             252 ..-....-.WRDKYIFRE..KMNSLIDGINLFY 273
SDR76395.1             126 ..T....R.LANGEPTPQ..FVEQVKRLLQAPR 149
SHN66296.1             850 ..P....G.A--YERLLKs.LER---------G 863
OLE55402.1             238 ..-....-.ANGYERLLAs.LLRRANDLGIGIT 260
OLS20661.1             120 ..N....Y.QEGIKKLINs.IHEIYDKPTIKNS 144
WP_077829015.1         119 ..N....Y.ETGLEEICEy.LKKDYGALSMFQN 143
OPL17796.1             115 ..G....W.KEVRGRLLEs.LGNLPGIEPRTPP 139
OPX31622.1             115 ..N....Q.SNTPEIISA..LFSIAKTPVKLPQ 138
OQW65619.1             353 paK....Y.DESLGKLLRr.LK---------TL 370
OQX64498.1             125 ..-....-.KDGYEKLIRs.LQVRAKNVDTSVN 147
WP_083523414.1         743 ..G....I.DAQIAETIR..K-----AILSALN 761
WP_015675700.1         115 ptK....F.DDSYQNLIRa.IKNHRLKNKPVEP 141
WP_084989879.1         128 ..-....WeEDGFEQLERa.ITHQFKLEPEEPK 152
foreground (26267):          H    Y EDWPEAD D  KRWKEAR AVANL inf
                                    LK   EE E  LFLLRLL ELP
                                     E   D        RK
wt_res_freqs (8805):         1    1 1111111 1  1131121 11111
                                    11   11 1  1111122 111
                                     1   1        11
insertions                   2    1 1111111 2  1
deletions                    1    3 956554413  1223333333442 20.0
background (10023):                 L       L    LL LLL L    5.7
wt_res_freqs (9636):                1       1    11 111 1
```

```
Group15                                    ●
Group15                117 ..N....Y.EDGLARLLR..LKAQLEKLLVGPA 140*
GR310559.1_EST         135 kfE....F.NEAFKMLLKe.MDQYIGQQGKKEY 161
FC734258.1_EST         201 ..K....I.EESFKELIDm.LHTELHQRQEEEE 225
ETX06351.1             110 ..QrrgpW.GELIQTIND..TVFTLGQTDLILA 137
WP_013320860.1         125 ..S....-.-DSLLKIKQs.LDSQMENLGFEIS 147
WP_024661155.1         122 ..A....W.IQVQASLQLa.AEQQSIEIKHGSN 146
WP_033955898.1         112 ..D....P.DKAIIDILT..FLGLKEKASTSLN 135
XP_011014729.1         127 ..L....-.ERNFKQKMDkvPRWRADLTSAANI 151
XP_004363941.1        3383 i.P....Y.ETSLLNLIGq.VDRIRLEVGRNAV 3408
WP_051611764.1         228 ..D....Y.NTALSFLYKs.L-----------I 241
WP_068132568.1         370 ..G....-.--GYTNLLRs.LK-----ASTLRP 386
WP_069808855.1         340 ..Q....-.-NGFERLMRs.LCIRAESKQRLQP 362
OGI20774.1             132 ..N....D.SEFESEYSKi.LRTVGLPVSTNEA 156
OGU46059.1             252 ..-....-.WRDKYIFRE..KMNSLIDGINLFY 273
SDR76395.1             126 ..T....R.LANGEPTPQ..FVEQVKRLLQAPR 149
SHN66296.1             850 ..P....G.A--YERLLKs.LER---------G 863
OLE55402.1             238 ..-....-.ANGYERLLAs.LLRRANDLGIGIT 260
OLS20661.1             120 ..N....Y.QEGIKKLINs.IHEIYDKPTIKNS 144
WP_077829015.1         119 ..N....Y.ETGLEEICEy.LKKDYGALSMFQN 143
OPL17796.1             115 ..G....W.KEVRGRLLEs.LGNLPGIEPRTPP 139
OPX31622.1             115 ..N....Q.SNTPEIISA..LFSIAKTPVKLPQ 138
OQW65619.1             353 paK....Y.DESLGKLLRr.LK---------TL 370
OQX64498.1             125 ..-....-.KDGYEKLIRs.LQVRAKNVDTSVN 147
WP_083523414.1         743 ..G....I.DAQIAETIR..K-----AILSALN 761
WP_015675700.1         115 ptK....F.DDSYQNLIRa.IKNHRLKNKPVEP 141
WP_084989879.1         128 ..-....WeEDGFEQLERa.ITHQFKLEPEEPK 152
foreground (568):            G    W AAGFAELVA  IKGALEKA   AA 100.3
                             D    Y EEALER LR  L RL   L   P
                             N      DD   K IK    AQ
wt_res_freqs (238):          1    1 113111511  11111111    11
                             1    2 111211 21  3 11   1   1
                             1      12   1 11    11
insertions                   3    4 111     5
deletions                    9    8 432211111  1689998866331 16.0
background (25699):          H    Y EDWPDAD D  QRWKEAR AVANL 100.3
                                    LR  EEE E  KFLLRLL ELP
                                     K   D        RK
wt_res_freqs (8567):         1    1 1111111 1  1131121 11111
                                    11  111 1  1111122 111
                                     1   1        11
position                            120    .   130      .  140
```

Group14 (bacterial TIRs)

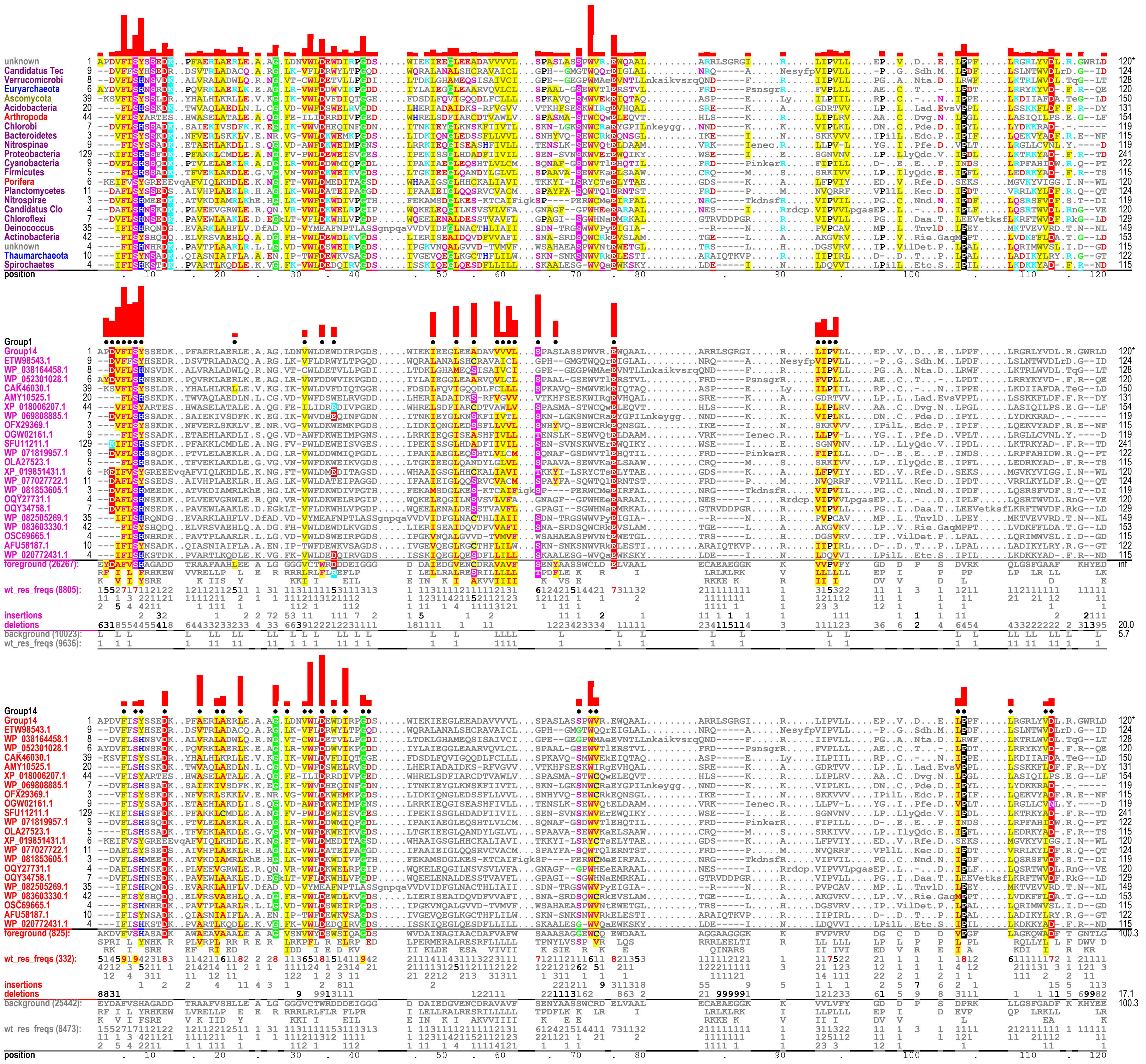

```
unknown               121 EDEA.LRLVRLLRAIRG......RARP  140*
Candidatus Tec        125 DVRQ.LD--ILINAIQH......QPPG  142
Verrucomicrobi        129 DNDAeLL--RLKAAILG......ERGT  147
Euryarchaeota         121 TPAA.FD--ELLAACRE......EAEA  138
Ascomycota            151 RDSV.RD--RLFRAVLG......DFDI  168
Acidobacteria         132 FGSL.AYVAGLIHDLPA......DAVE  151
Arthropoda            155 SVER.DLDRLVEVLKTD......RQWV  174
Chlorobi              120 ----.YSNKEEYMSLIK......YIKE  135
Bacteroidetes         116 DDGF.TELFRSLSIIAN......DYMG  135
Nitrospinae           120 YREG.LD--QLVDACYG......RSRK  137
Proteobacteria        242 FNQG.LH--DLINALRY......LSNK  259
Cyanobacteria         123 LEQY.NRILKACQPPAP......VDKS  142
Firmicutes            116 YDEG.FM--ALCAALGL......KHSE  133
Porifera              121 FFRP.NVDDYATSLLNL......IQGL  140
Planctomycetes        125 EEAF.EQIMADFTQPEA......PPQD  144
Nitrospirae           120 DSED.EMYRLVCGIQNE......PPCK  139
Candidatus Clo        121 DEEA.LL--KLLWGVTGenphkqGYRP  144
Chloroflexi           130 DADA.LR--CLIAGIKG......EEPG  147
Deinococcus           150 VLWT.KRLFTTRVAKSV......SPNS  169
Actinobacteria        154 AMGI.KE--RLARDMAA......HISP  171
unknown               116 VDRA.AR--ELVGVNSQ......ADLL  133
Thaumarchaeota        123 ERD-.RY--DLVESISG......KKPS  139
Spirochaetes          116 YQSG.LS--DLKEALIF......HNST  133
position                       .  130    .        140
```

Group1

```
Group14               121 EDEA.LRLVRLLRAIRG......RARP  140*
ETW98543.1            125 DVRQ.LD--ILINAIQH......QPPG  142
WP_038164458.1        129 DNDAeLL--RLKAAILG......ERGT  147
WP_052301028.1        121 TPAA.FD--ELLAACRE......EAEA  138
CAK46030.1            151 RDSV.RD--RLFRAVLG......DFDI  168
AMY10525.1            132 FGSL.AYVAGLIHDLPA......DAVE  151
XP_018006207.1        155 SVER.DLDRLVEVLKTD......RQWV  174
WP_069808885.1        120 ----.YSNKEEYMSLIK......YIKE  135
OFX29369.1            116 DDGF.TELFRSLSIIAN......DYMG  135
OGW02161.1            120 YREG.LD--QLVDACYG......RSRK  137
SFU11211.1            242 FNQG.LH--DLINALRY......LSNK  259
WP_071819957.1        123 LEQY.NRILKACQPPAP......VDKS  142
OLA27523.1            116 YDEG.FM--ALCAALGL......KHSE  133
XP_019851431.1        121 FFRP.NVDDYATSLLNL......IQGL  140
WP_077027722.1        125 EEAF.EQIMADFTQPEA......PPQD  144
WP_081853605.1        120 DSED.EMYRLVCGIQNE......PPCK  139
OQY27731.1            121 DEEA.LL--KLLWGVTGenphkqGYRP  144
OQY34758.1            130 DADA.LR--CLIAGIKG......EEPG  147
WP_082505269.1        150 VLWT.KRLFTTRVAKSV......SPNS  169
WP_083603330.1        154 AMGI.KE--RLARDMAA......HISP  171
OSC69665.1            116 VDRA.AR--ELVGVNSQ......ADLL  133
AFU58187.1            123 ERD-.RY--DLVESISG......KKPS  139
WP_020772431.1        116 YQSG.LS--DLKEALIF......HNST  133
foreground (26267):       WPEA D DKRWKEAR A       VANL  inf
                             E E ELFLLRLL E       LP
                             D       RK
wt_res_freqs (8805):      1111 1 11131121 1       1111
                             1 1 11111122 1       11
                             1       11
insertions                1111 1 21
deletions                 6554 413122333333       3442  20.0
background (10023):              L  LL LLL        L     5.7
wt_res_freqs (9636):             1  11 111        1
```

Group14

```
Group14               121 EDEA.LRLVRLLRAIRG......RARP  140*
ETW98543.1            125 DVRQ.LD--ILINAIQH......QPPG  142
WP_038164458.1        129 DNDAeLL--RLKAAILG......ERGT  147
WP_052301028.1        121 TPAA.FD--ELLAACRE......EAEA  138
CAK46030.1            151 RDSV.RD--RLFRAVLG......DFDI  168
AMY10525.1            132 FGSL.AYVAGLIHDLPA......DAVE  151
XP_018006207.1        155 SVER.DLDRLVEVLKTD......RQWV  174
WP_069808885.1        120 ----.YSNKEEYMSLIK......YIKE  135
OFX29369.1            116 DDGF.TELFRSLSIIAN......DYMG  135
OGW02161.1            120 YREG.LD--QLVDACYG......RSRK  137
SFU11211.1            242 FNQG.LH--DLINALRY......LSNK  259
WP_071819957.1        123 LEQY.NRILKACQPPAP......VDKS  142
OLA27523.1            116 YDEG.FM--ALCAALGL......KHSE  133
XP_019851431.1        121 FFRP.NVDDYATSLLNL......IQGL  140
WP_077027722.1        125 EEAF.EQIMADFTQPEA......PPQD  144
WP_081853605.1        120 DSED.EMYRLVCGIQNE......PPCK  139
OQY27731.1            121 DEEA.LL--KLLWGVTGenphkqGYRP  144
OQY34758.1            130 DADA.LR--CLIAGIKG......EEPG  147
WP_082505269.1        150 VLWT.KRLFTTRVAKSV......SPNS  169
WP_083603330.1        154 AMGI.KE--RLARDMAA......HISP  171
OSC69665.1            116 VDRA.AR--ELVGVNSQ......ADLL  133
AFU58187.1            123 ERD-.RY--DLVESISG......KKPS  139
WP_020772431.1        116 YQSG.LS--DLKEALIF......HNST  133
foreground (825):         DAAG LAEVAIVAGVRG       RARP  100.3
                          EEEA  RL LLLRAL A
                           D    E  R I  I
wt_res_freqs (332):       1111 111111111112       1111
                          1123  11 142121 1
                           2    1  1 1  2
insertions                2  1 1          1
deletions                 2221 1155  111134       5432  17.1
background (25442):       WPDA D DQRWKEAR A       VANL  100.3
                            EE E ELFLLRLL E       LP
                             D    K  RK
wt_res_freqs (8473):      1111 1 11131121 1       1111
                            11 1 11111122 1       11
                             1    1  11
position                       .  130    .        140
```

Group13 (bacterial TIRs)

```
unknown          1 AHADDDADDPNEGFVERLFRDLCDEVR.AL......PLGI.FMDRESIRWGEDW..RLSEALATCRVFVPLYSPRYFASE.ACGREWAAF.AQRAR....RL..RP.....EAIVPVLWVPVP........PS..P.DEV...LQFIHR.......RQYED.YALRGLYG.LS 127*
Armatimonadete  13 ADADADLLE---FR-SELCKKVYSNLS.MDrvpsadSLAY.F-DRD--RGSDNWkrEIGLALQRTRCFVCVLSGAYFAHErDCRKEFLTF.KARLEfakrADg.SP.....PVIIPVLWADPEfyre....EL..G.AGG...LLESVRgwdggfdSQYTKsGLLFLKGEaDK 159
Planctomycetes 159 -HKDNE--D---ASVTALVEHIRREHEqFVpne...PLKI.FFDLWGIESSDDWekRIYRGLIESKTMLAVLSPAYFGSP.WCRREYDTFvLQQKK....KLy.PG.....EPIHAIYIQEHAdfdr....DD..D.HPQrswFETLKR.......HQFLDaKPWWPDGQ.VA 295
Euryarchaeota   14 SHHDDNHDH---GYLTKLRDRLSGEVR.SHhak...EFPI.FQDRNSIEWGQNWieRITESLRGVTFLIPIITPNYFASE.ACLDELRQF.AEYGK....NVn.RN.....DLILPIYYIRCEqmet....NTtpD.DEL...VNLIKD.......HQYKDwRDLRGKSL.KL 150
Actinobacteria   9 ARGDEDAL------VQRLYEDLSAEVR.LIaglpr.DERVgFVDRT-ILLGERWpqRLVEALSTCRSFLALMTPRYFQSE.ACGQEWQIF.ADRTA....RFd.QQngvdsSLLKPLMWIPARpgr....IH..P.VAS...PLQYSShslgdl.YQRLGvRQLMRLQR.HS 152
Proteobacteria  12 -HADDEADG---QRIARLAWDVSAQFE.MLtge...SLEI.FLDKDAIRWGEEWrnKIDANLATIAFFIPVLTPRYFMSA.ECRRELQFF.AREAN....RLg.IR.....DLLLPVLYVDVSalhe....EN..PtDDL...ILLIRS.......FQWEDwRDLRFVDV.SA 146
unknown         17 --APDEYLY---RFYDDLCREIS--VR.SHfti...ESAG.FIDKNQPV-GGEWedSIGVALGTCDVFVPVYSPSLFGSS.NCGQEWHAF.GTRQS....RAgkLA.....PHIVPVWWVPTPgelptiadRI..Q.DTR...SQFGAE.......YQKYGlRYLMQLKE.NE 151
position              .    10   .    20   .         30   .    40   .     50   .    60   .    70   .    80        .         90   .  100                 .     110             .    120    .
```

```
Group1
                   ●●●●●●●                   ●             ●     ● ●                ●   ●  ●     ●●●●●   ●      ●                         ●●●●
Group13          1 AHADDDADDPNEGFVERLFRDLCDEVR.AL......PLGI.FMDRESIRWGEDW..RLSEALATCRVFVPLYSPRYFASE.ACGREWAAF.AQRAR....RL..RP.....EAIVPVLWVPVP........PS..P.DEV...LQFIHR.......RQYED.YALRGLYG.LS 127*
WP_025227563.1  13 ADADADLLE---FR-SELCKKVYSNLS.MDrvpsadSLAY.F-DRD--RGSDNWkrEIGLALQRTRCFVCVLSGAYFAHErDCRKEFLTF.KARLEfakrADg.SP.....PVIIPVLWADPEfyre....EL..G.AGG...LLESVRgwdggfdSQYTKsGLLFLKGEaDK 159
WP_011123202.1 159 -HKDNE--D---ASVTALVEHIRREHEqFVpne...PLKI.FFDLWGIESSDDWekRIYRGLIESKTMLAVLSPAYFGSP.WCRREYDTFvLQQKK....KLy.PG.....EPIHAIYIQEHAdfdr....DD..D.HPQrswFETLKR.......HQFLDaKPWWPDGQ.VA 295
KUK95354.1      14 SHHDDNHDH---GYLTKLRDRLSGEVR.SHhak...EFPI.FQDRNSIEWGQNWieRITESLRGVTFLIPIITPNYFASE.ACLDELRQF.AEYGK....NVn.RN.....DLILPIYYIRCEqmet....NTtpD.DEL...VNLIKD.......HQYKDwRDLRGKSL.KL 150
SCE68195.1       9 ARGDEDAL------VQRLYEDLSAEVR.LIaglpr.DERVgFVDRT-ILLGERWpqRLVEALSTCRSFLALMTPRYFQSE.ACGQEWQIF.ADRTA....RFd.QQngvdsSLLKPLMWIPARpgr....IH..P.VAS...PLQYSShslgdl.YQRLGvRQLMRLQR.HS 152
WP_074904325.1  12 -HADDEADG---QRIARLAWDVSAQFE.MLtge...SLEI.FLDKDAIRWGEEWrnKIDANLATIAFFIPVLTPRYFMSA.ECRRELQFF.AREAN....RLg.IR.....DLLLPVLYVDVSalhe....EN..PtDDL...ILLIRS.......FQWEDwRDLRFVDV.SA 146
WP_083795479.1  17 --APDEYLY---RFYDDLCREIS--VR.SHfti...ESAG.FIDKNQPV-GGEWedSIGVALGTCDVFVPVYSPSLFGSS.NCGQEWHAF.GTRQS....RAgkLA.....PHIVPVWWVPTPgelptiadRI..Q.DTR...SQFGAE.......YQKYGlRYLMQLKE.NE 151
foreground (26267): EYDAFVSHAGADDTRAAFAAHLEEALG GG      GVCT WRDDDEIGGGD D  AIEDGVENCDRAVAVFSENYAASS WCLDELVAA LECAE    AE   GG       KKVVPVFYGDDP       SD   V RKQ     LGSFGA      AF KH YEDWPEAD  D  inf
                   RF I L FRHKEWVVRELLP  L E R RR      RLRL FLREFLP    I L  ELLRALRESRILLLLTPDFLE K    R    I    LRL      KL   RK       VLLLLLLEPI        P    P P L    P  LR        LL       LK    EE  E
                   K  V I YSRE     K IIS   Y     K      KI I     EIL    E I  N  K I  AKVVIIII K  VS E           RKK      E    K        RII I  DVV        D    L                   D          E    D
wt_res_freqs (8805): 1552717112122212112112511131 11      3111 11531113131 1  13111121111121316124215 1 442173113 22111    11   11       113153221113       11   2 111     121111       11 11 11111111  1
                   11 1 3 2211211111211  2 1 1 11      1211 3121112    1 1  2311421152112123121121 1    1    2   111      11   11        1121112111       1    1 1 2   1   21         12       11    11  1
                   2  5 4 4211    1 111  1      1      12 1      112    1 1  1  1 4  11113121 1   11 1              111      1    1        123 3  121        1    1                   1        1    1
insertions                 2  32      1     2272 53       11     111 7  21 5        1    2        11 1      2         111 1      1              11    111  11       11   1                  2   2 11111111  2
deletions          631855445541864433233233433 66      3912 2212231111181  111   1   11        122342 3334 1111 12341      15   11       431111233662       46   4 544     332222       22231 39565544 13  20.0
background (10023): L  L L       L   LL  LL  LL  L       L L    LL L L       L   L     LLLL     L         L    L  L           L          L  L             L     LL   L LL       LLL    L        L  5.7
wt_res_freqs (9636): 1  1 1       1   11  11  11  1       1 1    11 1 1       1   1     1111     1         1    1  1           1          1  1             1     11   1 11       111    1        1
```

```
Group13
                     ●●         ●                  ●  ●   ●  ●   ●       ●      ●  ●    ●  ●    ●●● ●  ●   ●                ●● ●  ●                                        ●
Group13          1 AHADDDADDPNEGFVERLFRDLCDEVR.AL......PLGI.FMDRESIRWGEDW..RLSEALATCRVFVPLYSPRYFASE.ACGREWAAF.AQRAR....RL..RP.....EAIVPVLWVPVP........PS..P.DEV...LQFIHR.......RQYED.YALRGLYG.LS 127*
WP_025227563.1  13 ADADADLLE---FR-SELCKKVYSNLS.MDrvpsadSLAY.F-DRD--RGSDNWkrEIGLALQRTRCFVCVLSGAYFAHErDCRKEFLTF.KARLEfakrADg.SP.....PVIIPVLWADPEfyre....EL..G.AGG...LLESVRgwdggfdSQYTKsGLLFLKGEaDK 159
WP_011123202.1 159 -HKDNE--D---ASVTALVEHIRREHEqFVpne...PLKI.FFDLWGIESSDDWekRIYRGLIESKTMLAVLSPAYFGSP.WCRREYDTFvLQQKK....KLy.PG.....EPIHAIYIQEHAdfdr....DD..D.HPQrswFETLKR.......HQFLDaKPWWPDGQ.VA 295
KUK95354.1      14 SHHDDNHDH---GYLTKLRDRLSGEVR.SHhak...EFPI.FQDRNSIEWGQNWieRITESLRGVTFLIPIITPNYFASE.ACLDELRQF.AEYGK....NVn.RN.....DLILPIYYIRCEqmet....NTtpD.DEL...VNLIKD.......HQYKDwRDLRGKSL.KL 150
SCE68195.1       9 ARGDEDAL------VQRLYEDLSAEVR.LIaglpr.DERVgFVDRT-ILLGERWpqRLVEALSTCRSFLALMTPRYFQSE.ACGQEWQIF.ADRTA....RFd.QQngvdsSLLKPLMWIPARpgr....IH..P.VAS...PLQYSShslgdl.YQRLGvRQLMRLQR.HS 152
WP_074904325.1  12 -HADDEADG---QRIARLAWDVSAQFE.MLtge...SLEI.FLDKDAIRWGEEWrnKIDANLATIAFFIPVLTPRYFMSA.ECRRELQFF.AREAN....RLg.IR.....DLLLPVLYVDVSalhe....EN..PtDDL...ILLIRS.......FQWEDwRDLRFVDV.SA 146
WP_083795479.1  17 --APDEYLY---RFYDDLCREIS--VR.SHfti...ESAG.FIDKNQPV-GGEWedSIGVALGTCDVFVPVYSPSLFGSS.NCGQEWHAF.GTRQS....RAgkLA.....PHIVPVWWVPTPgelptiadRI..Q.DTR...SQFGAE.......YQKYGlRYLMQLKE.NE 151
foreground (325):  ARADNDADGRNEGRVTDFCDAVCGAYA AI      DAGG FRDRDGIGSGAGW  EIGDAVGGCTFFVAVYSPAYFASE ACGKEWAAF AERAK    AA   AA       GAIVPALWAGVD       DA   V AEE     VAFNAA      TQGEG RAAEGEGG GG  100.3
                   SHR DEHLRPR QFLRRLFRDLRQHLM LL      PLPL  M  ERMHL QL  RLDRELARTRVLLPLLT RFLRRP M RR FL    SR LR    RL   LP       ELLL I YIPMP       PS   P VPL     LRLLHR      LFYRR YELLLLYP LI
                       RVD  A IEQ YE IEDEIR R      RIEI  L   DLRW EE  K SE   TA    I II  S     Y  E LY   Q Q    EH   VT       DG I V  V  R                D V     IQIIRE      Y W D  DTRR DR  S
wt_res_freqs (168): 723824212266424113111111111 21      1121 81951141191 19  121141115226521367187283 195295149 11631    11   11       1453925611131       21   1 221     211111      15211 22112111 11
                   263 5111162 1311136224611121 12      2211  2   41211  11  461117411542175 13 511111 1 33 11   11 12    21   11       2333 2 11413       31   2 111     211121      11311 21312221 21
                         112    1 211 12 111412 1      1112  2   1223 31  1 22    21    2 21  1      1   1 11    2 1     11   11       11 1 3  3  1               3 1     121211      1 1 3  1121 21  2
insertions             2                11  7      1111              9                                6  1123    49   48       13        1  8       11   2    1     2 111        4 5     2 4
deletions          766544544999442221211222221 11      1113  11973                                                                     1      1211       23   3 546     86641          2        1 43  13.8
background (25942): EYDAFVSHAGADDTAAAFAAHLEEALG GG      GVCT FRDDDEIGGGD D  AIEDGVENCDRFVAVFSPNSAASS WCLDELVAA LERAE    AE   GG       KVIVPVFYGDDP       SD   V RKQ     LGSFGA      AF KH YEDWPDAD  D  100.3
                   RF I L FRHKEWVRRELLP  L E R RR      RLRL WLREFLL    I L  ELLRALRESRILLLLLTEDFLE K            I    LRL     KL   KK        ILLLLLLEPI        D    P P L    P  LRK       LL       LK   EEE  E
                   K  V I YSRE     K IIS   Y     K      KI I     EIP    E I  N  K I  AKVVIIII K YVS E                 CKK     E             KVI I  DVV             L                     D          E    D
wt_res_freqs (8637): 155281711212221111211251131 11      3111 31531113121 1  131111211111213162212151 442173113 22111    11   11       112153221113       11   2 111     121111      11 11 11111111  1
                   11 1 3 22112112112 11  2 1 1 11      1211 1121112    1 1  2311421152112123111121 1        2   111    11   11        1121112111       1    1 1 2   1   211       12       11   111  1
                   2  5 4 4211    1 111  1      1      12 1      112    1 1  1  1 4  11114121 1 411 1              111    1                133 3  121             1                     1        1    1
position              .    10   .    20   .         30   .    40   .     50   .    60   .    70   .    80        .         90   .  100                 .     110             .    120    .
```

```
unknown        128 KLYRRAVEYLAAR 140*
Armatimonadete 160 TSYRRVATAIASA 172
Planctomycetes 296 LQREVVTGRLQGL 308
Euryarchaeota  151 VKFNERLEELALD 163
Actinobacteria 153 DDYRTFLFELANQ 165
Proteobacteria 147 EAYRRGVAKLAVR 159
unknown        152 SLYRDFLVKFTMM 164
position           130    .  140
```

```
Group1
Group13        128 KLYRRAVEYLAAR 140*
WP_025227563.1 160 TSYRRVATAIASA 172
WP_011123202.1 296 LQREVVTGRLQGL 308
KUK95354.1     151 VKFNERLEELALD 163
SCE68195.1     153 DDYRTFLFELANQ 165
WP_074904325.1 147 EAYRRGVAKLAVR 159
WP_083795479.1 152 SLYRDFLVKFTMM 164
foreground (26267): KRWKEAR AVANL inf
                   LFLLRLL ELP
                     RK
wt_res_freqs (8805): 1131121 11111
                   1111122 111
                     11
insertions         1
deletions          1223333333442  20.0
background (10023):  LL LLL L     5.7
wt_res_freqs (9636): 11 111 1
```

```
Group13
Group13        128 KLYRRAVEYLAAR 140*
WP_025227563.1 160 TSYRRVATAIASA 172
WP_011123202.1 296 LQREVVTGRLQGL 308
KUK95354.1     151 VKFNERLEELALD 163
SCE68195.1     153 DDYRTFLFELANQ 165
WP_074904325.1 147 EAYRRGVAKLAVR 159
WP_083795479.1 152 SLYRDFLVKFTMM 164
foreground (325):  AAYRRAVAEEAAR  100.3
                   PL Y VLYYL KV
                   KE   R ERV E
wt_res_freqs (168): 1144233111523
                   12 1 11123 11
                   21   1 111 1
insertions         12
deletions          4              13.8
background (25942): QRWKEAR AVANL 100.3
                   LFLLRLL ELP
                   K  RK
wt_res_freqs (8637): 1131121 11111
                   1111122 111
                   1  11
position           130    .  140
```

Group12 (bacterial TIRs)

```
unknown          1 KKK..IFISHSSK.KELADALVD..LL.E.G.IGV.PSEDIFCTS..LEGY..GIPLGE........YLREE.INES.....ILVIFLLSDNYYESPACLNEMGAAWV..TQKDEHIPILLPPFDF.E.I....KGAIDPTKQGIKLND.....KDDRVLNELKDKLDIEEEF 133*
Synergistetes  457 KKK..ILISQTYPdKPFADIVYQ..ML.L.F.NNV.PADDILYTN..CDDEicRVPEGAsvyg....YLRDF.FVESysnqkIYVIFITSQNTKLSWGAITEVGASWI..TKID-HKIFNIPPFRP.E.-....-HPLDDESQWQSTNR.....EEK--TGKLWMTSLSSDIF 596
Actinobacteria   8 --K..LFLSHAYAdKAYATKFMDtiLI.Q.G.SRL.PSERILMTS..LRET..GIPTGKnlfe....VLRHT.AADS.....PLVVALISPTYLTRPTCLAELGAAWV..KEAL-FPIMIGGITRK.D.L....DGVL-QGIIIENFND.....EDT--LDELHGT--INREF 139
Firmicutes     120 KKKplIFISHSSKnRDQVAKIAD..LL.R.S.INLsPRRDIFCSS..LPGY..GIPNGAnifd....FLRERfLNYD.....LHIIFVHSPEYYESPVSLNEMGAAWV..LRAN-ATSLLLPGFDF.SgM....KGVIGSDCIAIKLDGdrsevKDR--LNQLRRE--LESEF 262
Euryarchaeota  167 QKK..VFISHSSKdKHFADELIE..IL.E.Y.IGV.PSENIFCTS..VEGY..GIPLGEnfle....TIKNE.LTPN.....TLVLFLISENFYGSPVSLCEMGATWI..QSNF-HIPILIPPMTF.EkI....KGVIPLTE-GLLINN.....KAK--INSLKMK--IEN-- 297
Chloroflexi      4 --K..LFVSHASAdKPVIDRLVT..LLdV.G.CGI.DHKSIFCTS..GDAT..GIPNGTpnyie...AIRSE.IDKT.....SLFLFVVSQNFLVSEFCLCELGAAWA..TQRS-WHLLVLPPLTP.D.K....VKNVLKVTQMGMIND.....AGD--LADLRDA--INQAF 136
Proteobacteria 118 --K..IFISHATKdKLIVEELIE..IL.E.S.MGI.DSTQIFCSS..FEGY..GIPLGAnfld....QIKQE.LSSD.....VLVLFVLTENFYDSKICLCEMGAAWA..LSKQ-HIPIVVPPLSYqD.I....QGVIPLT-QGLMIND.....ASK--LNSFKER--LETLF 248
Fibrobacteres    4 --K..IFISHSCSdKPVIEALQR..ML.QlA.CNL.KQQDFFCTS..ISGM..GCSGGDffne....TIRKS.LQEA.....SVVVAYLSKNYKQSEFCCAELGATWIsrESKL-FVPLLDSELDY.K.A....FGGVLNGTHLYKIND.....KAD--LLSTVQS--IIKTC 137
Verrucomicrobi   6 ---..LFLSHAVAnKDLADRLVV..LI.EtG.IGI.SDREIFCSS..LEGM..GIPAGAnfve....FIRSQ.IEEP.....KAVILLLTPDYYASEFCLCELGASWS..MAHR-AFPLLAPPLTFeE.I....KGVLTGTQI-LRLNV.....KED--LNQFYEE--LVEVM 136
Bacteroidetes    3 --K..IFISHSSKnSKFGQAIVD..LL.L.S.VGV.SSNQIIFTS..NDAF..GIPNGKnifn....WLKER.ITEK.....PHVIYLLSPEYYSSIACLNEMGAAWI..IENQ-HTIIFTPQFNS.S.SpefqNGALDPREMGFKINN.....VDR--ITEFVES--LKQYY 137
unknown          3 --K..LFISHASAdKKVCDAFVD..LL.E.E.LGV.PEEDILYSS..SSRH..GIPGGMdife....YLRNN.ISSG.....INVYYMLSDNYYKSAYCLNEMGAAWV..TQND-SSTFLLPEFTV.G.I....KGVIDSSKKAFTLSD.....PVE--LIQLKDK--ILAEY 133
Spirochaetes   455 KKK..LLISQTYRdKDLADIIYN..ML.V.F.NNV.PPEDIIYTNcdDEVS..RIPEGDvgksgiysYLRDF.FVDSystqkIYVIFVTSHNTKSSWGALMEVGAAWI..TQVE-HKIFNIYDFRP.E.H....PLDDEQQWHSSSRDD.....DDN--LYMSKLSVDIFAQK 600
Cyanobacteria    4 ---..IFISHSSQdAELARDLIS..LI.R.DaFNI.PPEDIRCTS..VDGY..RLPGGAdtde....QLRTE.VSEC.....EMFLGLLSNVSVRSTYVIFELGARWG..ASKK-LIPLLAPGFEP.S.S....LERPISNLNSLRCDS.....AAQ--LQQLLTE--LSIQL 134
position                   .  10    .   20       .   30    .   40      .           50    .         60    .   70    .   80     .   90    .          100    .  110          .  120    .  130
```

```
Group1
Group12                1 KKK..IFISHSSK.KELADALVD..LL.E.G.IGV.PSEDIFCTS..LEGY..GIPLGE........YLREE.INES.....ILVIFLLSDNYYESPACLNEMGAAWV..TQKDEHIPILLPPFDF.E.I....KGAIDPTKQGIKLND.....KDDRVLNELKDKLDIEEEF 133*
WP_005659100.1       457 KKK..ILISQTYPdKPFADIVYQ..ML.L.F.NNV.PADDILYTN..CDDEicRVPEGAsvyg....YLRDF.FVESysnqkIYVIFITSQNTKLSWGAITEVGASWI..TKID-HKIFNIPPFRP.E.-....-HPLDDESQWQSTNR.....EEK--TGKLWMTSLSSDIF 596
WP_037106651.1         8 --K..LFLSHAYAdKAYATKFMDtiLI.Q.G.SRL.PSERILMTS..LRET..GIPTGKnlfe....VLRHT.AADS.....PLVVALISPTYLTRPTCLAELGAAWV..KEAL-FPIMIGGITRK.D.L....DGVL-QGIIIENFND.....EDT--LDELHGT--INREF 139
CUQ61437.1           120 KKKplIFISHSSKnRDQVAKIAD..LL.R.S.INLsPRRDIFCSS..LPGY..GIPNGAnifd....FLRERfLNYD.....LHIIFVHSPEYYESPVSLNEMGAAWV..LRAN-ATSLLLPGFDF.SgM....KGVIGSDCIAIKLDGdrsevKDR--LNQLRRE--LESEF 262
WP_054834899.1       167 QKK..VFISHSSKdKHFADELIE..IL.E.Y.IGV.PSENIFCTS..VEGY..GIPLGEnfle....TIKNE.LTPN.....TLVLFLISENFYGSPVSLCEMGATWI..QSNF-HIPILIPPMTF.EkI....KGVIPLTE-GLLINN.....KAK--INSLKMK--IEN-- 297
KXK52492.1             4 --K..LFVSHASAdKPVIDRLVT..LLdV.G.CGI.DHKSIFCTS..GDAT..GIPNGTpnyie...AIRSE.IDKT.....SLFLFVVSQNFLVSEFCLCELGAAWA..TQRS-WHLLVLPPLTP.D.K....VKNVLKVTQMGMIND.....AGD--LADLRDA--INQAF 136
WP_071321075.1       118 --K..IFISHATKdKLIVEELIE..IL.E.S.MGI.DSTQIFCSS..FEGY..GIPLGAnfld....QIKQE.LSSD.....VLVLFVLTENFYDSKICLCEMGAAWA..LSKQ-HIPIVVPPLSYqD.I....QGVIPLT-QGLMIND.....ASK--LNSFKER--LETLF 248
WP_072801445.1         4 --K..IFISHSCSdKPVIEALQR..ML.QlA.CNL.KQQDFFCTS..ISGM..GCSGGDffne....TIRKS.LQEA.....SVVVAYLSKNYKQSEFCCAELGATWIsrESKL-FVPLLDSELDY.K.A....FGGVLNGTHLYKIND.....KAD--LLSTVQS--IIKTC 137
WP_075079977.1         6 ---..LFLSHAVAnKDLADRLVV..LI.EtG.IGI.SDREIFCSS..LEGM..GIPAGAnfve....FIRSQ.IEEP.....KAVILLLTPDYYASEFCLCELGASWS..MAHR-AFPLLAPPLTFeE.I....KGVLTGTQI-LRLNV.....KED--LNQFYEE--LVEVM 136
WP_076783456.1         3 --K..IFISHSSKnSKFGQAIVD..LL.L.S.VGV.SSNQIIFTS..NDAF..GIPNGKnifn....WLKER.ITEK.....PHVIYLLSPEYYSSIACLNEMGAAWI..IENQ-HTIIFTPQFNS.S.SpefqNGALDPREMGFKINN.....VDR--ITEFVES--LKQYY 137
WP_084669627.1         3 --K..LFISHASAdKKVCDAFVD..LL.E.E.LGV.PEEDILYSS..SSRH..GIPGGMdife....YLRNN.ISSG.....INVYYMLSDNYYKSAYCLNEMGAAWV..TQND-SSTFLLPEFTV.G.I....KGVIDSSKKAFTLSD.....PVE--LIQLKDK--ILAEY 133
WP_016524084.1       455 KKK..LLISQTYRdKDLADIIYN..ML.V.F.NNV.PPEDIIYTNcdDEVS..RIPEGDvgksgiysYLRDF.FVDSystqkIYVIFVTSHNTKSSWGALMEVGAAWI..TQVE-HKIFNIYDFRP.E.H....PLDDEQQWHSSSRDD.....DDN--LYMSKLSVDIFAQK 600
WP_023072283.1         4 ---..IFISHSSQdAELARDLIS..LI.R.DaFNI.PPEDIRCTS..VDGY..RLPGGAdtde....QLRTE.VSEC.....EMFLGLLSNVSVRSTYVIFELGARWG..ASKK-LIPLLAPGFEP.S.S....LERPISNLNSLRCDS.....AAQ--LQQLLTE--LSIQL 134
foreground (26267):      EYD  AFVSHAGA DDTRAAFAA  HL E E ALG GGGVCTWRD  DDEI  GGGD D        AIEDG VENC     DRAVAVFSENYAASSWCLDELVAALE  CAEAEGGKKVVPVFYG D D   PSDVRKQLGSFGAAF     KHYEDWPEAD DKRWKEA inf
                         RF   I L FRHK EWVVRELLP     L   E R RRRLRLFLR  EFLP     I L        ELLRA LRES     RILLLLLTPDFLE K    R     I    LRLKLRK VLLLLLLE P I    P PP LP   LR LL         LK    EE ELFLLRL
                         K    V I YSRE      K IIS     Y       KKI I      EIL      E I        N  K  I  A     KVVIIII K  VS E              RKKE K  RII I  D V V    D L         D           E    D      RK
wt_res_freqs (8805):     155  27171121 221211211  25 1 1 131 113111115  3111  3131 1        13111 1211     1112131612421514421731132 2  1111111111315322 1 1   311211112111111     1111111111 1113112
                         11   1 3 2211 211111211     2   1 1 111211312  1112     1 1        23114 2115     2112123121121 1  1    2    1111111 11211121 1 1    1 11 21   21 12        11    11 1111112
                         2    5 4 4211     1 111     1       112 1      112      1 1        1  1  4  1     1113121 1  11 1           1111 1  123 3  1 2 1    1 1         1           1    1      11
insertions                       2  3 2     1        2 272 53 11    1  11 7   21 5             1       2       11 1  2      11   1 1 1  11   111    1   1111           2     211111111 21
deletions                631  85544554 186443323  32 3 3 433 663912221  2231  111181        111    1     11       1223423334 111112  34115114311111233 6 6   246454433222222     23139565544131 22333  20.0
background (10023):      L    L L       L   LL     L L    LL  L L L     LL L  L             L    L         LLLL       L       L   L   L     L   L L        L  LLL LL  LL      L   L     L  LL L  5.7
wt_res_freqs (9636):     1    1 1       1   11     1 1    11  1 1 1     11 1  1             1    1         1111       1       1   1   1     1   1 1        1  111 11  11      1   1     1  11 1
```

```
Group12
Group12                1 KKK..IFISHSSK.KELADALVD..LL.E.G.IGV.PSEDIFCTS..LEGY..GIPLGE........YLREE.INES.....ILVIFLLSDNYYESPACLNEMGAAWV..TQKDEHIPILLPPFDF.E.I....KGAIDPTKQGIKLND.....KDDRVLNELKDKLDIEEEF 133*
WP_005659100.1       457 KKK..ILISQTYPdKPFADIVYQ..ML.L.F.NNV.PADDILYTN..CDDEicRVPEGAsvyg....YLRDF.FVESysnqkIYVIFITSQNTKLSWGAITEVGASWI..TKID-HKIFNIPPFRP.E.-....-HPLDDESQWQSTNR.....EEK--TGKLWMTSLSSDIF 596
WP_037106651.1         8 --K..LFLSHAYAdKAYATKFMDtiLI.Q.G.SRL.PSERILMTS..LRET..GIPTGKnlfe....VLRHT.AADS.....PLVVALISPTYLTRPTCLAELGAAWV..KEAL-FPIMIGGITRK.D.L....DGVL-QGIIIENFND.....EDT--LDELHGT--INREF 139
CUQ61437.1           120 KKKplIFISHSSKnRDQVAKIAD..LL.R.S.INLsPRRDIFCSS..LPGY..GIPNGAnifd....FLRERfLNYD.....LHIIFVHSPEYYESPVSLNEMGAAWV..LRAN-ATSLLLPGFDF.SgM....KGVIGSDCIAIKLDGdrsevKDR--LNQLRRE--LESEF 262
WP_054834899.1       167 QKK..VFISHSSKdKHFADELIE..IL.E.Y.IGV.PSENIFCTS..VEGY..GIPLGEnfle....TIKNE.LTPN.....TLVLFLISENFYGSPVSLCEMGATWI..QSNF-HIPILIPPMTF.EkI....KGVIPLTE-GLLINN.....KAK--INSLKMK--IEN-- 297
KXK52492.1             4 --K..LFVSHASAdKPVIDRLVT..LLdV.G.CGI.DHKSIFCTS..GDAT..GIPNGTpnyie...AIRSE.IDKT.....SLFLFVVSQNFLVSEFCLCELGAAWA..TQRS-WHLLVLPPLTP.D.K....VKNVLKVTQMGMIND.....AGD--LADLRDA--INQAF 136
WP_071321075.1       118 --K..IFISHATKdKLIVEELIE..IL.E.S.MGI.DSTQIFCSS..FEGY..GIPLGAnfld....QIKQE.LSSD.....VLVLFVLTENFYDSKICLCEMGAAWA..LSKQ-HIPIVVPPLSYqD.I....QGVIPLT-QGLMIND.....ASK--LNSFKER--LETLF 248
WP_072801445.1         4 --K..IFISHSCSdKPVIEALQR..ML.QlA.CNL.KQQDFFCTS..ISGM..GCSGGDffne....TIRKS.LQEA.....SVVVAYLSKNYKQSEFCCAELGATWIsrESKL-FVPLLDSELDY.K.A....FGGVLNGTHLYKIND.....KAD--LLSTVQS--IIKTC 137
WP_075079977.1         6 ---..LFLSHAVAnKDLADRLVV..LI.EtG.IGI.SDREIFCSS..LEGM..GIPAGAnfve....FIRSQ.IEEP.....KAVILLLTPDYYASEFCLCELGASWS..MAHR-AFPLLAPPLTFeE.I....KGVLTGTQI-LRLNV.....KED--LNQFYEE--LVEVM 136
WP_076783456.1         3 --K..IFISHSSKnSKFGQAIVD..LL.L.S.VGV.SSNQIIFTS..NDAF..GIPNGKnifn....WLKER.ITEK.....PHVIYLLSPEYYSSIACLNEMGAAWI..IENQ-HTIIFTPQFNS.S.SpefqNGALDPREMGFKINN.....VDR--ITEFVES--LKQYY 137
WP_084669627.1         3 --K..LFISHASAdKKVCDAFVD..LL.E.E.LGV.PEEDILYSS..SSRH..GIPGGMdife....YLRNN.ISSG.....INVYYMLSDNYYKSAYCLNEMGAAWV..TQND-SSTFLLPEFTV.G.I....KGVIDSSKKAFTLSD.....PVE--LIQLKDK--ILAEY 133
WP_016524084.1       455 KKK..LLISQTYRdKDLADIIYN..ML.V.F.NNV.PPEDIIYTNcdDEVS..RIPEGDvgksgiysYLRDF.FVDSystqkIYVIFVTSHNTKSSWGALMEVGAAWI..TQVE-HKIFNIYDFRP.E.H....PLDDEQQWHSSSRDD.....DDN--LYMSKLSVDIFAQK 600
WP_023072283.1         4 ---..IFISHSSQdAELARDLIS..LI.R.DaFNI.PPEDIRCTS..VDGY..RLPGGAdtde....QLRTE.VSEC.....EMFLGLLSNVSVRSTYVIFELGARWG..ASKK-LIPLLAPGFEP.S.S....LERPISNLNSLRCDS.....AAQ--LQQLLTE--LSIQL 134
foreground (266):        EKK  IFISHASA AAYGNAFYN  FI E G NGV KANDIFCSS  DDGS  GVPLGE        YIKDN FSSS     IYVFYVTSDNSKSSAGCICEVGAGWA  TSTDEHTIFNTPGFSY S I   KGAVNSGQQGFNITS     SSGGAINSFKDESAIEEEF 100.3
                         K    LLL  T K KKLVKILIE  ML L F LNL PKEQ LYTN  LPEY  RL  D         LRKF LVED     LL LLLLTPEFLK PVVMF M  T I  LKKKRVIPLLL PLDP E L   PLVPPPNWISLRLDE     PERIILDQLREKLLLIKLL
                         Q    V    S   EIAEEIVD  L  V S I I  EDE I     VE H  KI            EQ IQDN      F IFII K YYQ WFALN L  S V   QNE YF III N NF D     L  LLKTK AIK ND     KDKRV  E    D
wt_res_freqs (147):      178  78798351 112112311  11 2 2 173 111375548  1141  416272        33311 2112     217122172411181161199192  21124212211162411 1 2   242111113311311     1111111122211141213
                         6    111  1 3 512311311  17 1 1 212 2122 1351  1213  11  1          5411 2121     12 3134211121 21111 4  1 2  21213124132 3122 2 1   212111111111321     1111151141212121111
                         1    1    4   22311243   5  1 1 3 2  111 1     13 1  16              21 2111      1 4311 1 531 111163 2  1 3   211 11 412 1 13 2     1  22112 122 33     21133  2    2
insertions                 1        9        4    4 1 9 1 1 1 11    6   2 11  1     9            6347     2                      1  1 1        1  261 1 1     24  1  1    1 6     14  3 1
deletions                762  43222222 221111111  11 1 1     131      1  1  3  7 12                                            19     11111  1 2   323363272333111     111991111    88     13.6
background (26001):      EYD  IFVSHAGA DDTRAAFAA  HL E E ALG GGGVCTWRD  DDEI  GGGD D        AIEDG VENC     DRVVAVFSENYAASSWCLDELVAALE  CAEAEGGKVVVLVFYG D D   PSDVRKQLGSFGAAF     KHYEDWPDAD DKRWKEA 100.3
                         RF   A L FRHK EWV RELLP     L   E R RRRLRLFLR  EFLP     I L        ELLRA LRES     RILLLLLTPDFLE K    R     I    LRLELRK KLLPLLLE P I    P PP LP  LRKLL     R  LR   EEE ELFLLRL
                         K    V I YSRE      K IIS     Y       KKI I      EIL      E I        N  K  I  A     KVAIIII K  VS E              RKK  K  RII I  D V V    D L        ED         K    D      RK
wt_res_freqs (8658):     155  17171121 221211211  25 1 1 231 113111115  3111  3131 1        13111 1211     11121316124215144217311322  1111111113113221 1 1   311211112111111     1111111111 1113112
                         11   2 3 2211 211 11211     2   1 1 111211312  1112     1 1        23114 2115     2112123121121 1  1    2    1111111 11251121 1 1    1 11 21   21112     1  11   111 1111112
                         2    5 4 4211     1 111     1       112 1      112      1 1        1  1  4  1     1113121 1  11 1           111  1  123 3  1 2 1    1 1         11           1    1      11
position                   .  10    .   20       .   30    .   40      .           50    .         60    .   70    .   80     .   90    .          100    .  110          .  120    .  130
```

```
unknown          134 GLSIKDS 140*
Synergistetes    597 CQKIEHV 603
Actinobacteria   140 GIDTPPL 146
Firmicutes       263 DISDNED 269
Euryarchaeota    298 DLSIKNK 304
Chloroflexi      137 SLSPMPT 143
Proteobacteria   249 ELKNPLS 255
Fibrobacteres    138 RSRSDLP 144
Verrucomicrobi   137 GATRKAV 143
Bacteroidetes    138 SISTNPV 144
unknown          134 GGRISEK 140
Spirochaetes     601 IEYICDK 607
Cyanobacteria    135 ALTPNPP 141
position               .  140
```

```
Group1
Group12                134 GLSIKDS 140*
WP_005659100.1         597 CQKIEHV 603
WP_037106651.1         140 GIDTPPL 146
CUQ61437.1             263 DISDNED 269
WP_054834899.1         298 DLSIKNK 304
KXK52492.1             137 SLSPMPT 143
WP_071321075.1         249 ELKNPLS 255
WP_072801445.1         138 RSRSDLP 144
WP_075079977.1         137 GATRKAV 143
WP_076783456.1         138 SISTNPV 144
WP_084669627.1         134 GGRISEK 140
WP_016524084.1         601 IEYICDK 607
WP_023072283.1         135 ALTPNPP 141
foreground (26267):        R AVANL inf
                           L ELP
wt_res_freqs (8805):       1 11111
                           2 111
insertions
deletions                  3333442 20.0
background (10023):        LL L    5.7
wt_res_freqs (9636):       11 1
```

```
Group12
Group12                134 GLSIKDS 140*
WP_005659100.1         597 CQKIEHV 603
WP_037106651.1         140 GIDTPPL 146
CUQ61437.1             263 DISDNED 269
WP_054834899.1         298 DLSIKNK 304
KXK52492.1             137 SLSPMPT 143
WP_071321075.1         249 ELKNPLS 255
WP_072801445.1         138 RSRSDLP 144
WP_075079977.1         137 GATRKAV 143
WP_076783456.1         138 SISTNPV 144
WP_084669627.1         134 GGRISEK 140
WP_016524084.1         601 IEYICDK 607
WP_023072283.1         135 ALTPNPP 141
foreground (266):          GISIKSS 100.3
                           ELK  E
                           N E  D
wt_res_freqs (147):        1211111
                           131  1
                           1 1  1
insertions
deletions                          13.6
background (26001):        R AVANL 100.3
                           L ELP
wt_res_freqs (8658):       1 11111
                           2 111
position                     .  140
```

Group11 (bacterial TIRs)

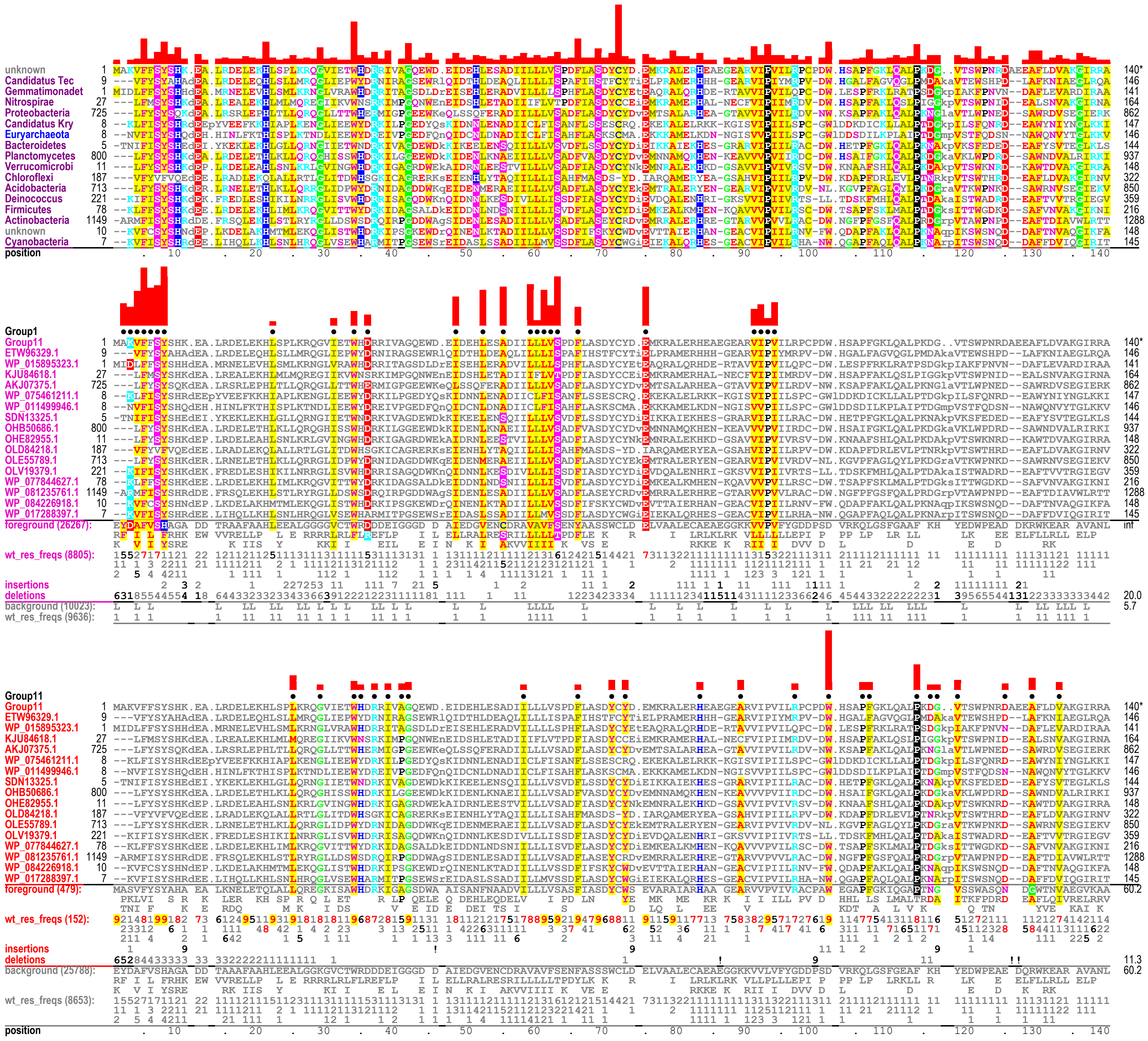

Group23 (MyD88 TIRs, except MyD88s of Arthropods)

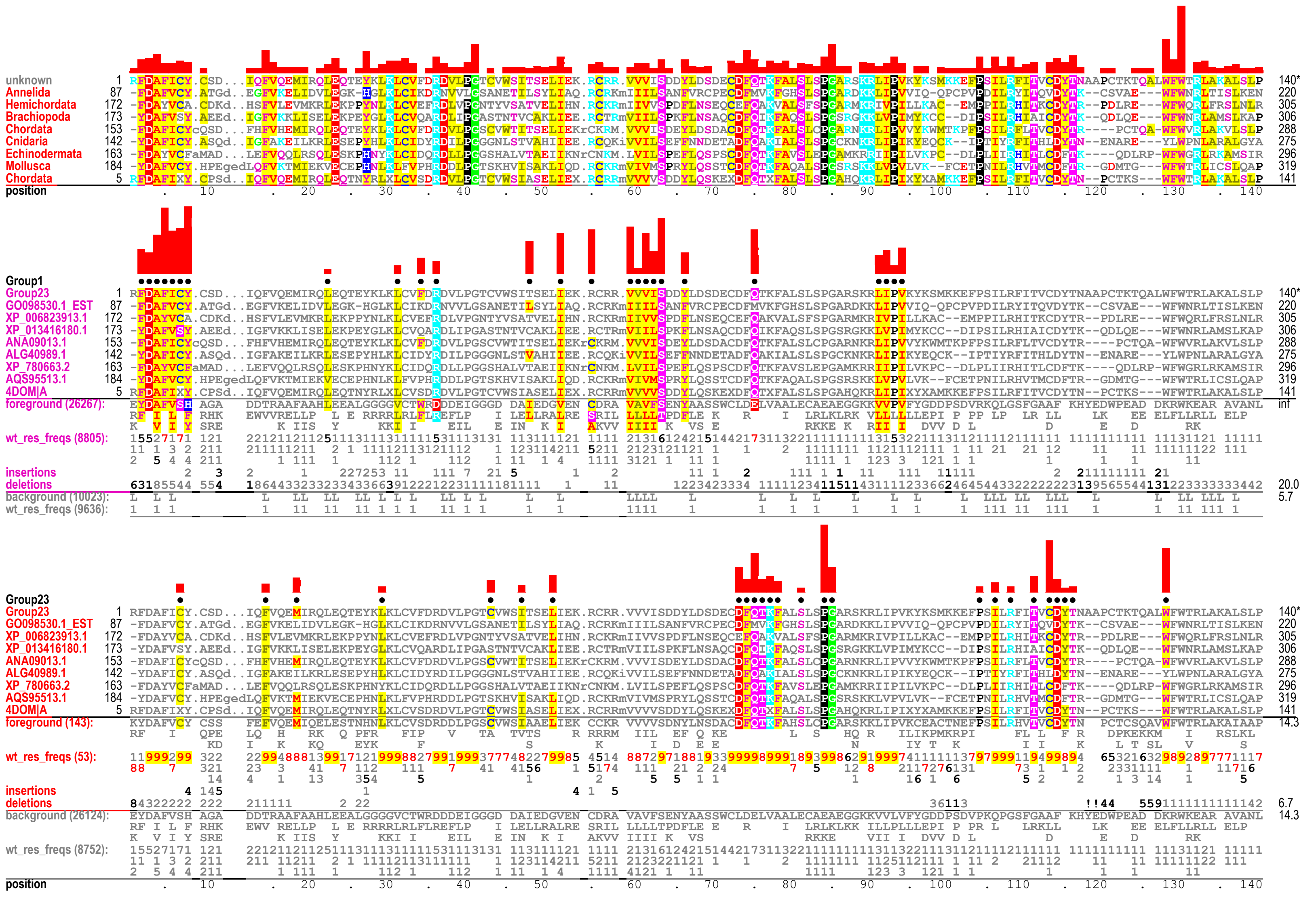

Group10 (TIRAP TIRs)

```
unknown          1  KYDVCVCH.EEDLEAAQELVSYLEGSPASLRCFLQLRDATPGGAI.VS.LCQALVSS.CRCWVLLITPGFLQDPWCKYQMHQALAEAPGANEGRTIPLLSGLDRAAYPPELRFMYYVDGRGNRQIPDGGFRQVKEAVVRYLQEL  140*
Chordata         9  -YDVCVXHsEEDLVAAQDLVSYLEGSTASLRCFLQLRDATPGGAI.VSeLCQALSSShCR--VLLITPGFLQDPWXKYQMLQALTEAPGA-EGCTIPLLSGLSRAAYPPELRFMYYVDGRG----PDGGFRQVKEAVMRYLQTL  143
Chordata        86  -YDVCICHsEGDLGLVEELVSYLEAQPESLRCFLQLRDAAPGSAVlTE.LCDAVRSShC--WVMLITPGFLRDPWCQYQMHQALAEAPMAN-GRTIPVLKDVDRKDYPKELRNIYYISMTE----NSFRQIRDTIVRYLQELCR  220
position                   .    10    .    20    .    30    .    40    .    50    .    60    .    70    .    80    .    90    .   100    .   110    .   120    .   130    .   140

Group1              ●●●●●●●               ●         ●  ●  ●              ●   ●   ●   ●●●●●  ●               ●                    ●●●●
Group10          1  KYDVCVCH.EEDLEAAQELVSYLEGSPASLRCFLQLRDATPGGAI.VS.LCQALVSS.CRCWVLLITPGFLQDPWCKYQMHQALAEAPGANEGRTIPLLSGLDRAAYPPELRFMYYVDGRGNRQIPDGGFRQVKEAVVRYLQEL  140*
4LQD|A           9  -YDVCVXHsEEDLVAAQDLVSYLEGSTASLRCFLQLRDATPGGAI.VSeLCQALSSShCR--VLLITPGFLQDPWXKYQMLQALTEAPGA-EGCTIPLLSGLSRAAYPPELRFMYYVDGRG----PDGGFRQVKEAVMRYLQTL  143
XP_009078222.1  86  -YDVCICHsEGDLGLVEELVSYLEAQPESLRCFLQLRDAAPGSAVlTE.LCDAVRSShC--WVMLITPGFLRDPWCQYQMHQALAEAPMAN-GRTIPVLKDVDRKDYPKELRNIYYISMTE----NSFRQIRDTIVRYLQELCR  220
foreground (26267): EYDAFVSH AGADDTRAAFAAHLEEALGGGGVCTWRDDDEIGGGD  D AIEDGVEN CDRAVAVFSENYAASSWCLDELVAALECAEAEGGKKVVPVFYGDDPSDVRKQLGSFGAAF KHYEDWPEAD DKRWKEAR AVANL  inf
                    RF I L F RHKEWVVRELLP  L E RRRRLRLFLREFLP   I  L ELLRALRE SRILLLLTPDFLE K   R    I  LRLKLRK VLLLLLLEPI P PP LP  LR LL    LK   EE ELFLLRLL ELP
                    K  V I Y SRE   K IIS  Y     KKI I    EIL   E  I N  K I  AKVVIIII K  VS E       RKKE K  RII I  DVV D L       D     E   D     RK
wt_res_freqs (8805): 15527171 121221211211251113111311111531113131  1 13111121 1111213161242151442173113221111111111315322111311211112111111 1111111111 11131121 11111
                    11 1 3 2 211211111211  2 1 11112113121112    1  1 23114211 52112123121121 1   1     2  1111111 1121112111 1 11 21  21 12    11    11 11111122 111
                    2  5 4 4 211    1 111  1    112 1    112    1  1 1  1 4  11113121 1  11 1              1111 1  123 3  121 1 1          1     1    1      11
insertions                 2  32    1     227253 11   111 7  21  5       1   2      11 1   2      111 1 1  11   111  11111          2  211111111 21
deletions           63185544 5541864433233234336639122212231111 1 81 111   1   11    1223423334 1111123411511143111123366246454433222222313956554413122333333442  20.0
background (10023): L  L L        L    LL   LL   LL L L L    LL L L       L    L       LLLL   L         L    L  L    L     L   L     L   LLL LL   LLL   L        L   LL LLL L  5.7
wt_res_freqs (9636): 1  1 1        1    11   11   11 1 1 1    11 1 1       1    1       1111   1         1    1  1    1     1   1     1   111 11   111   1        1   11 111 1

Group10                 ●  ●   ●  ●          ●         ●●●  ●          ●                             ●    ●●●  ●    ●  ●    ●  ●              ●●  ●●●    ●
Group10          1  KYDVCVCH.EEDLEAAQELVSYLEGSPASLRCFLQLRDATPGGAI.VS.LCQALVSS.CRCWVLLITPGFLQDPWCKYQMHQALAEAPGANEGRTIPLLSGLDRAAYPPELRFMYYVDGRGNRQIPDGGFRQVKEAVVRYLQEL  140*
4LQD|A           9  -YDVCVXHsEEDLVAAQDLVSYLEGSTASLRCFLQLRDATPGGAI.VSeLCQALSSShCR--VLLITPGFLQDPWXKYQMLQALTEAPGA-EGCTIPLLSGLSRAAYPPELRFMYYVDGRG----PDGGFRQVKEAVMRYLQTL  143
XP_009078222.1  86  -YDVCICHsEGDLGLVEELVSYLEAQPESLRCFLQLRDAAPGSAVlTE.LCDAVRSShC--WVMLITPGFLRDPWCQYQMHQALAEAPMAN-GRTIPVLKDVDRKDYPKELRNIYYISMTE----NSFRQIRDTIVRYLQELCR  220
foreground (203):   KYDVCVCH EGDLAAAEELVSYLEGSAAGFRCFLQLRDACPGGAV SS ECCAASSS CRCWVLLITPGFVQDDWCKYQMHQALAEGPGASSGCTIPVVSGVARAAYPAELRNYYYVDGAGNRQINDGGGRQVKNATVRCKQTL  37.6
                    R   FI    V  VLVQR    F   SPPRSL      V  S I VE LLQDLVQN SHR LM    N LR P   R M L   TQA MSME RI  LMKDLDHKQ   P    FM FIYMRLF   PEDSFF IVEKVMLYLLE
                              E  EE           AQTE        T     TT     V N    A      S            S    N       L N T SD   R    L     LS     K N  Y  RDT L  Y
wt_res_freqs (85):  59987699 71962362698879944131299997799718968 3 14 35353336 645676889959149199598959893819571191699313421733992998218656414777714251464313125511324
                    4   13    1  12351    1   115167           1  2 6 61 53415323 241 12    1 82 7  2 1 4   217 4218 71  51326 2121   2    64 1411322   42126 1 21315214523
                          4  41            1313            3     13    1 1   1     1   1        1           3     6      6 1 1 23   1     2     11      2 1  1  222 1  2
insertions                29                             1   19  4       6   2
deletions           82221111 111111111                       1 22 2111     153 224                      154491                        44429999 2211171             7.1
background (26064): EYDAFVSH AGDDDTRAAFAAHLEEALGGGRVCTWRDDDEIGGGD  D AIEDGVEN CDRAVAVFSENYAASSWCLDELVAALECAEAEGGKKVVLVFYGDDPSDVPKQPGSFGAAF KHYEDWPEAD DKRWKEAR AVANL  37.6
                    RF I L F RHKEWV RELLP  L E RRRGLRLF REFLP   I  L ELLRALRE SRILLLLLTPDFLE K   R    I  LRLKLRK ILLPLLLEPI P PR L   LRKLL     LK   EE ELFLLRLL ELP
                    K  V I Y SRE   K IIS  Y     KKI I    EIL   E  I N  K I  AKVVIIII K  VS E       RKKE K  VII I  DVV D L       D     E   D     RK
wt_res_freqs (8720): 15528171 12122121121125111311111111153111313 1  1 13111121 111121316124215144217311322111111111311322111311211112111111 1111111111 11131121 11111
                    11 1 3 2 211211 11211  2 1 11132113 11111    1  1 13114211 52112123121121 1   1     2  1111111 1125112111 1 11 2   21112     11    11 11111122 111
                    2  5 4 4 211    1 111  1    112 1    112    1  1 1  1 4  11114121 1  11 1             1111 1  123 3  121 1 1          1     1    1      11
position                   .    10    .    20    .    30    .    40    .    50    .    60    .    70    .    80    .    90    .   100    .   110    .   120    .   130    .   140
```

Group9 (bacterial TIRs)

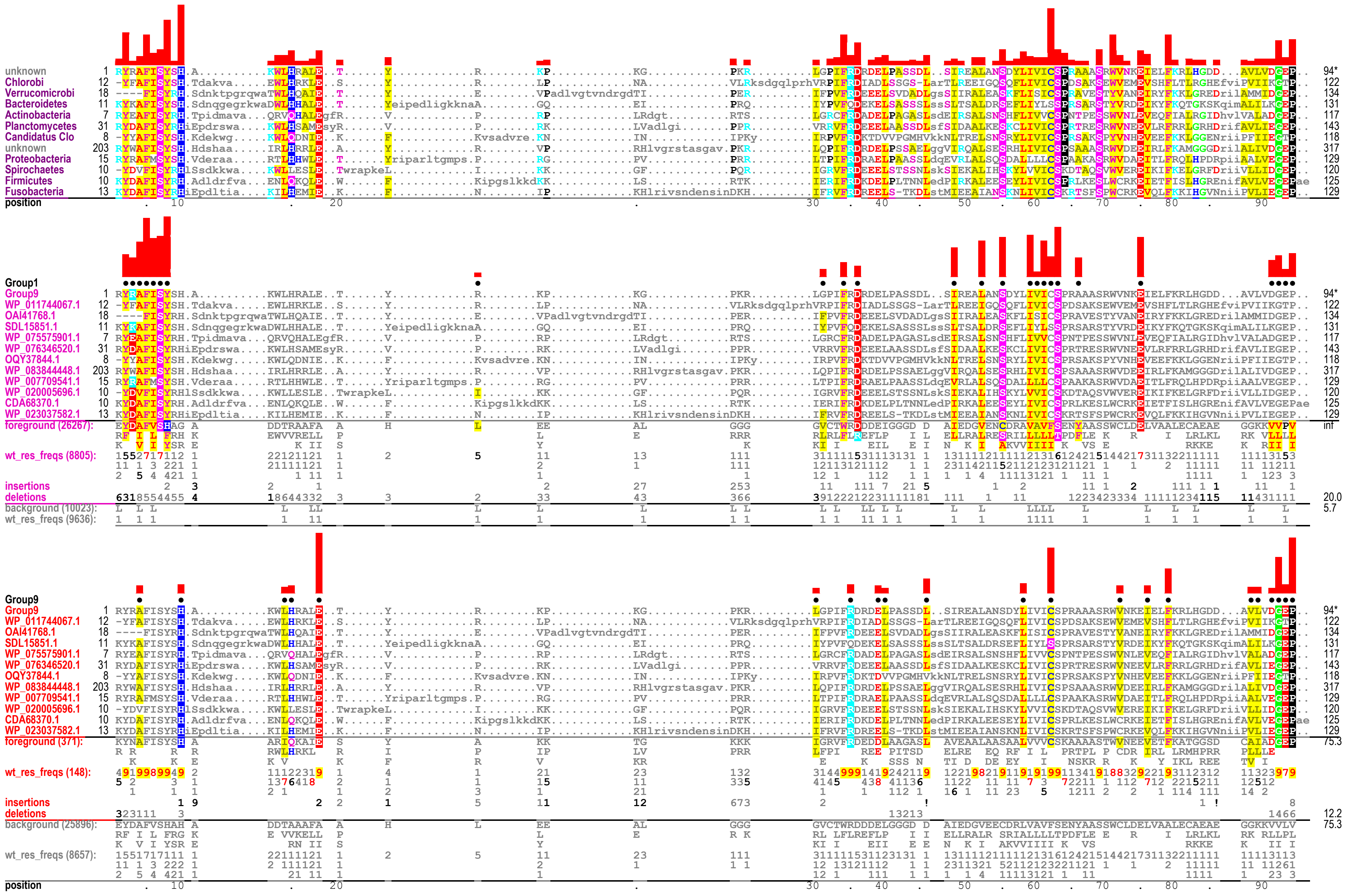

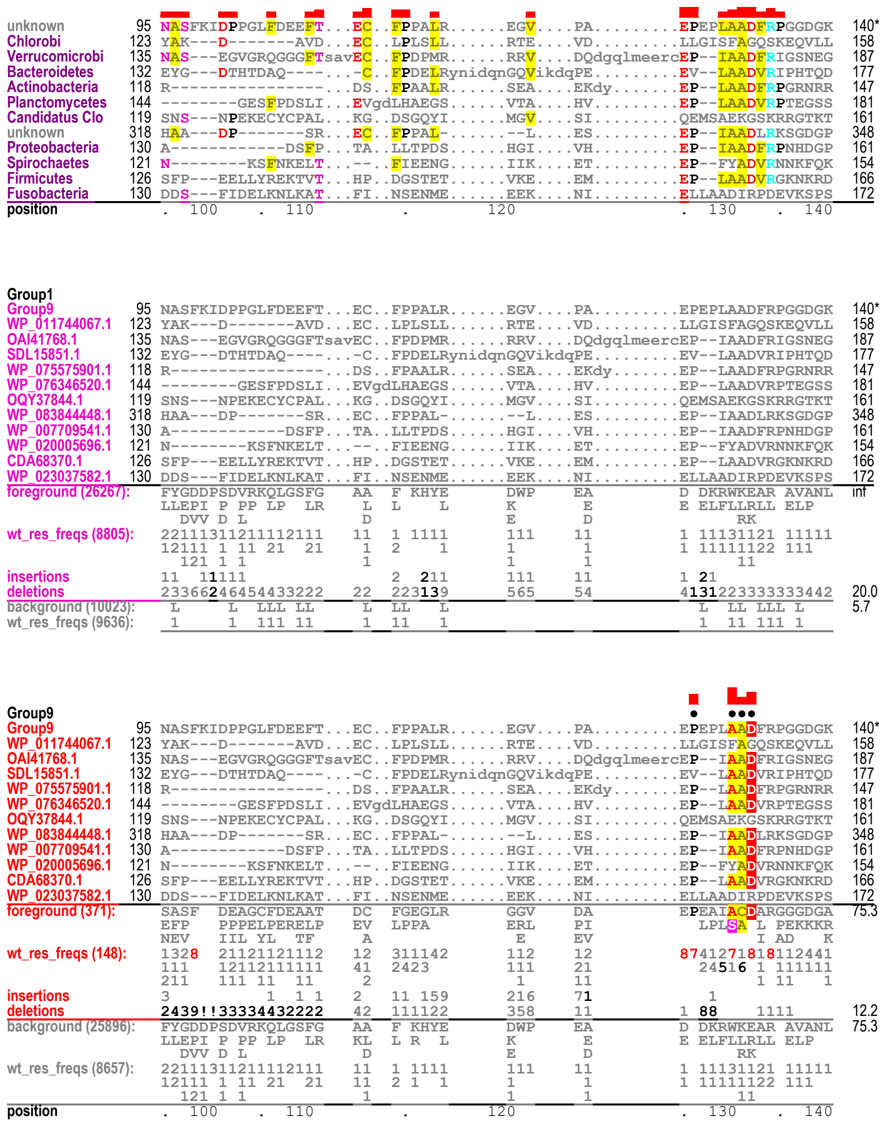

Group25 (bacterial TIRs)

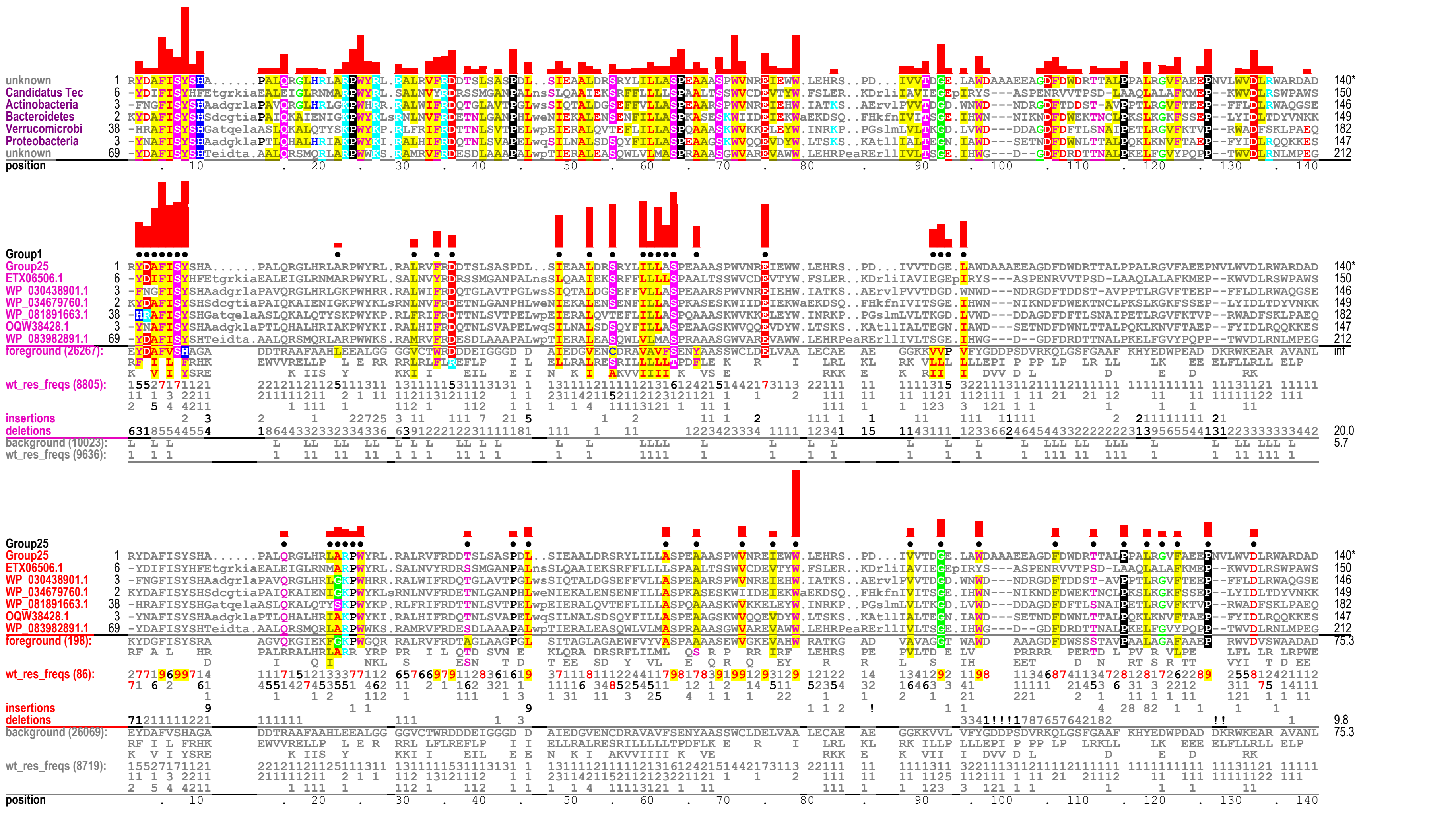

Group8 (bacterial TIRs)

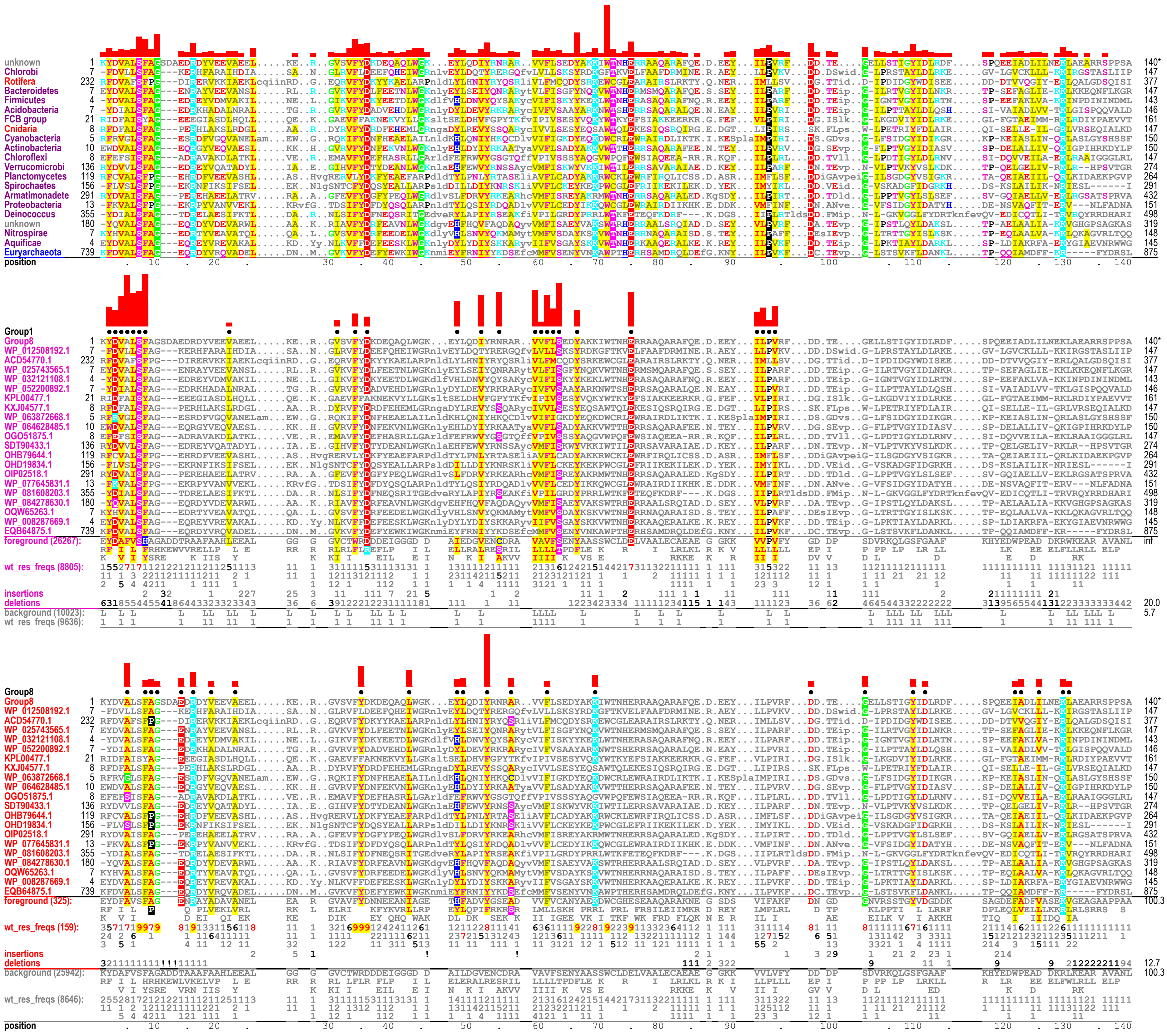

Group26 (actinobacterial TIRs)

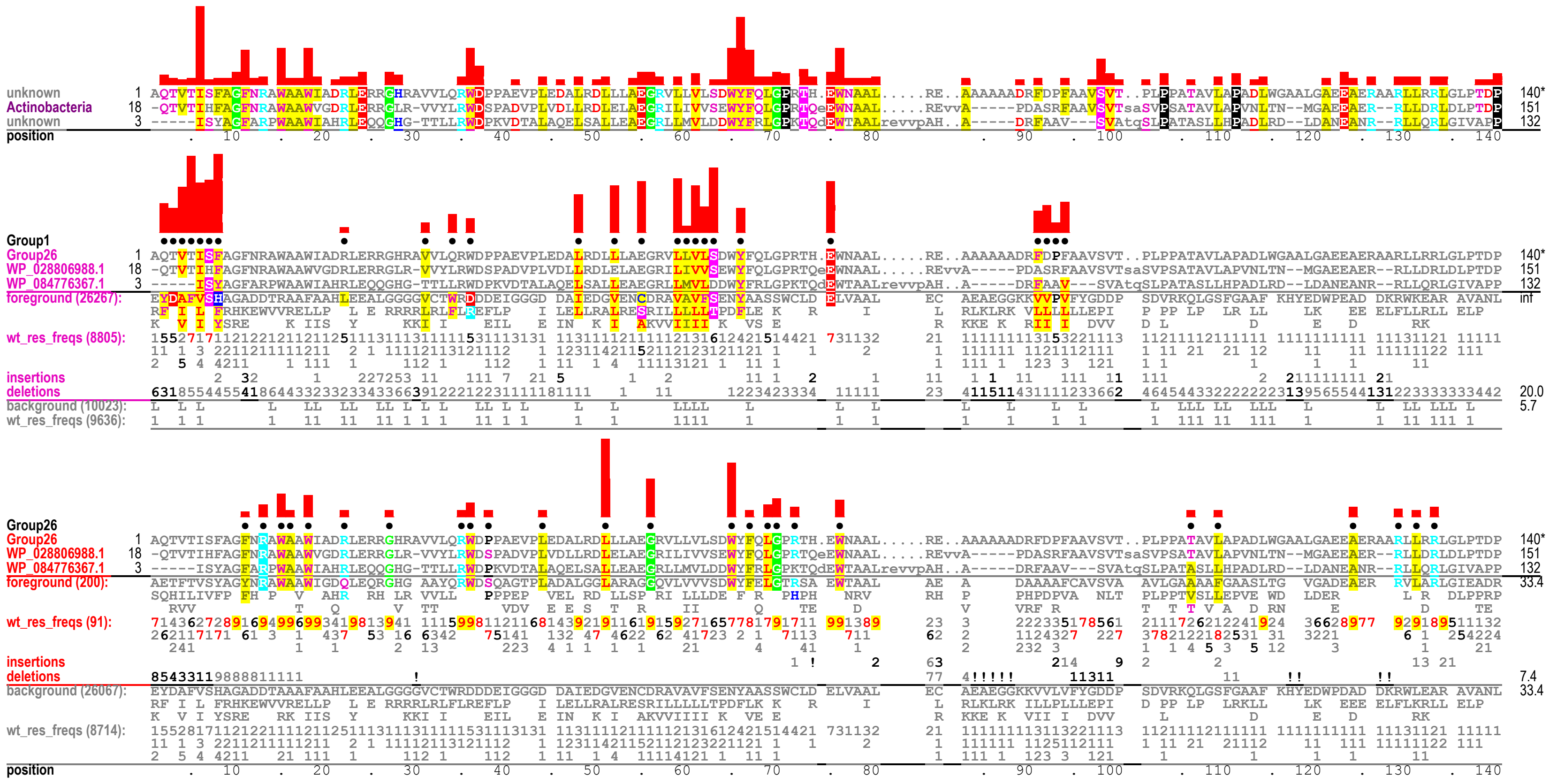

Group6 (bacterial TIRs)

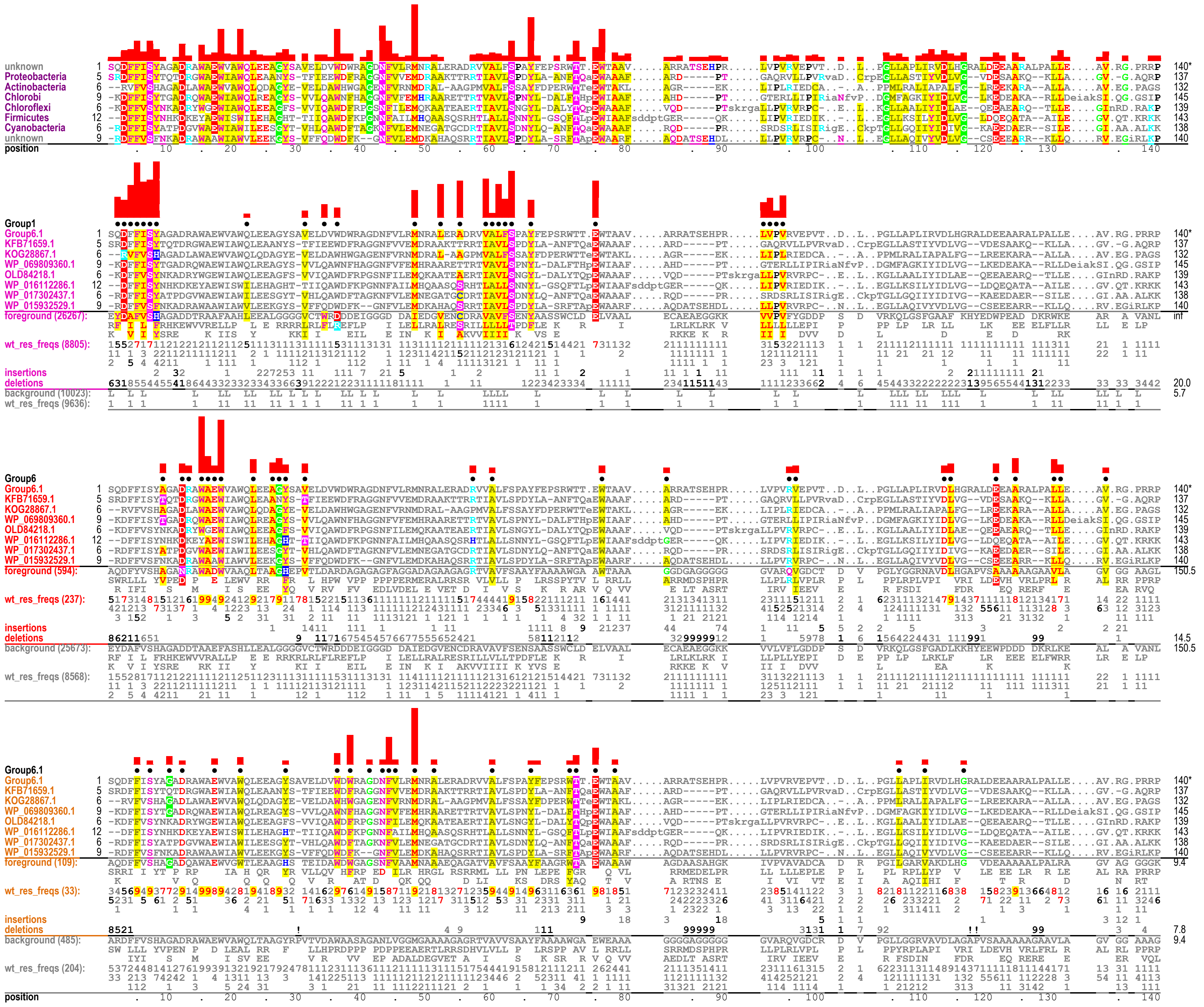

Group28 (bacterial TIRs)

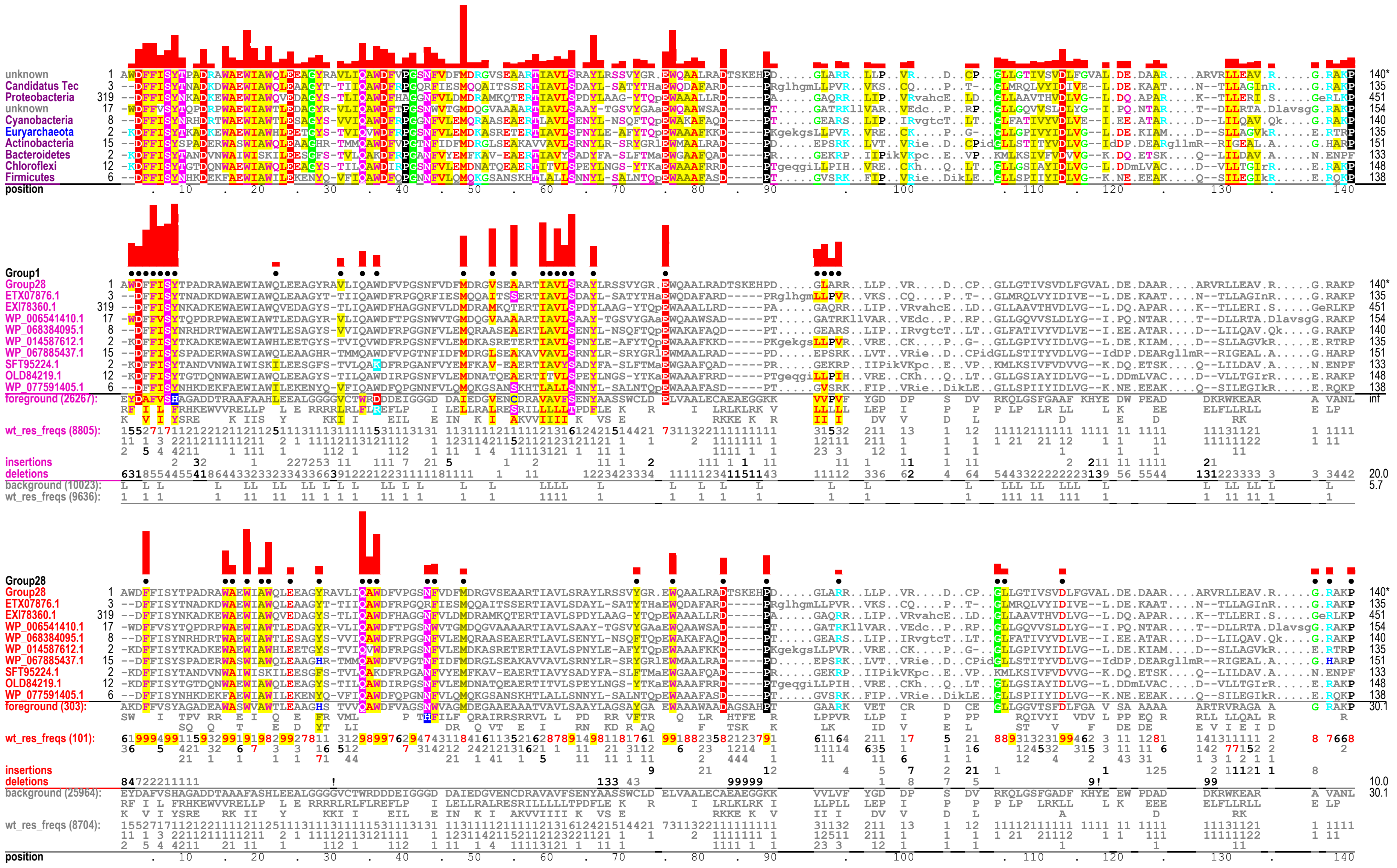

Group5 (bacterial TIRs)

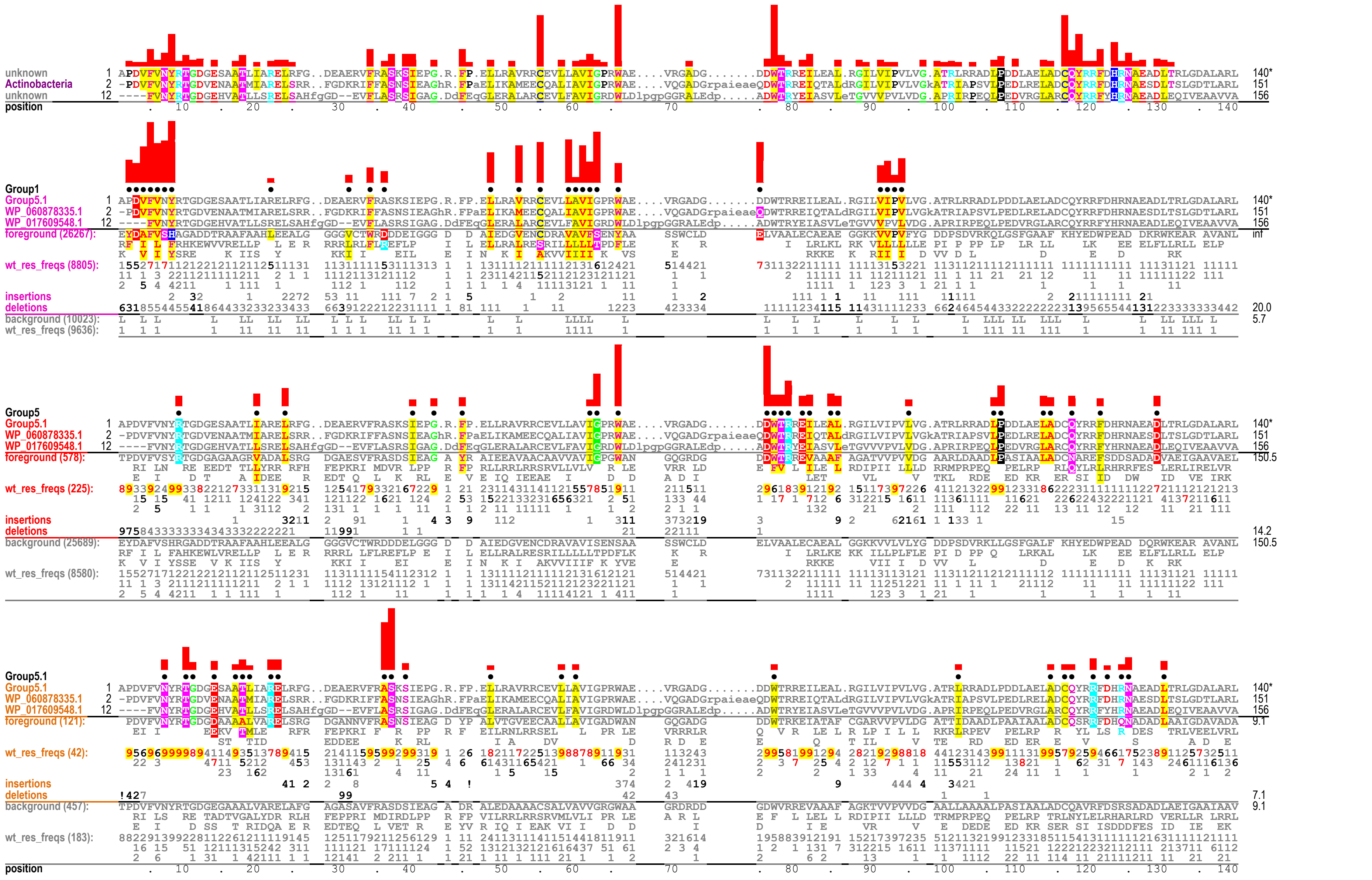

Group24 (SARM1 TIRs)

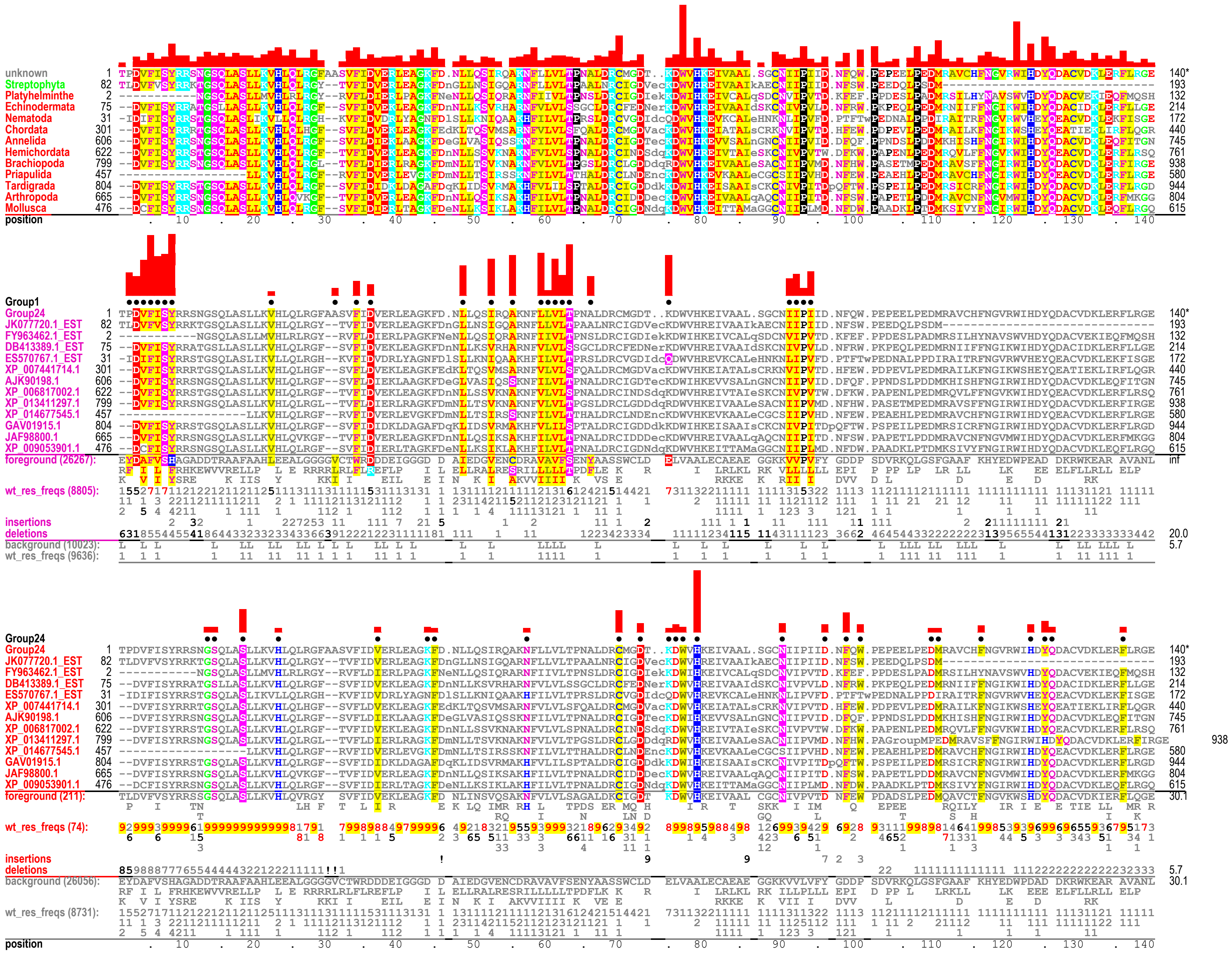

Group4.5 (IL-1R5 (IL-18R1) TIRs)

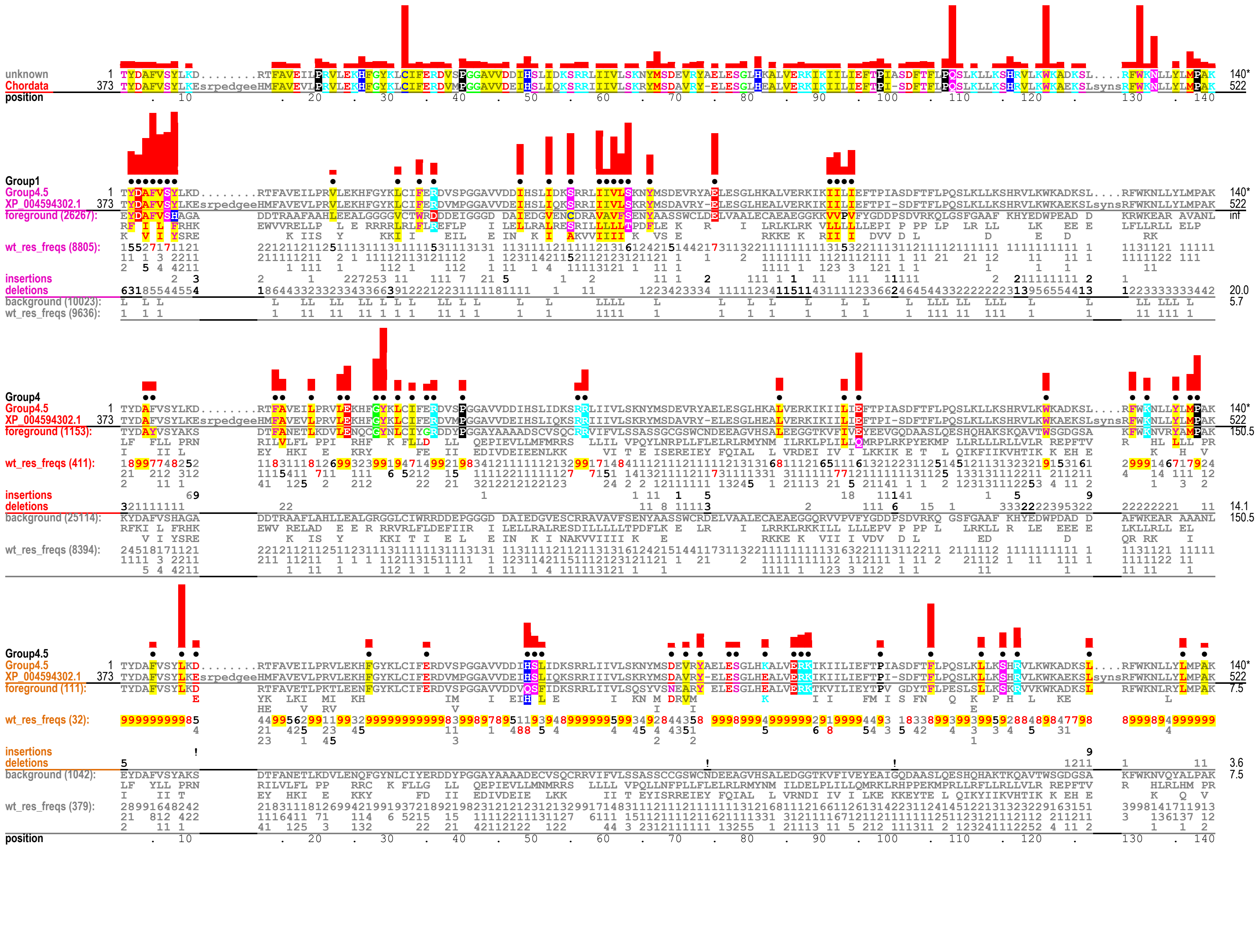

Group4.2 (IL-1R4 (aka ST2) TIRs)

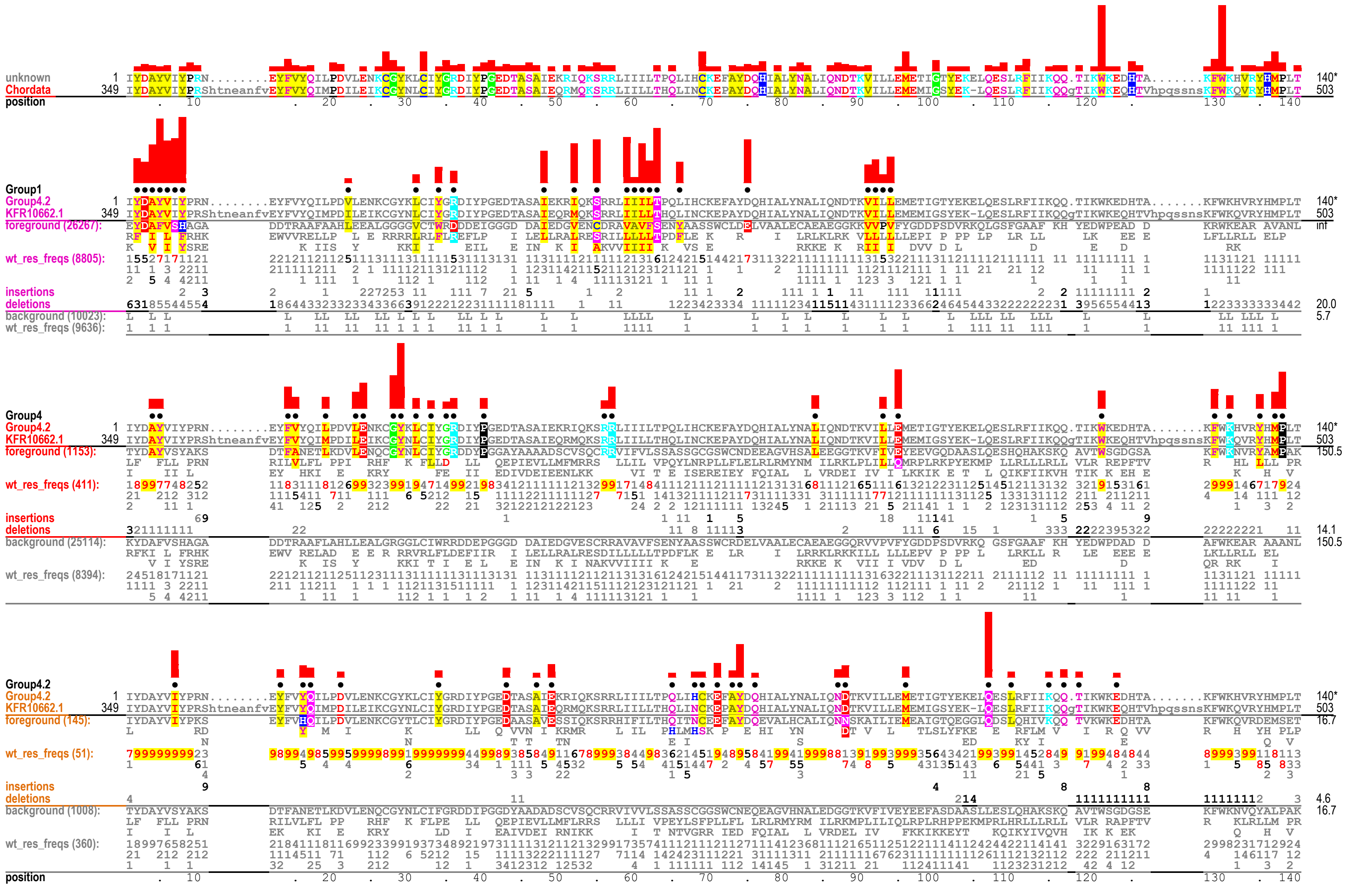

Group4.4 (IL-1R7 (IL-18RAP) TIRs)

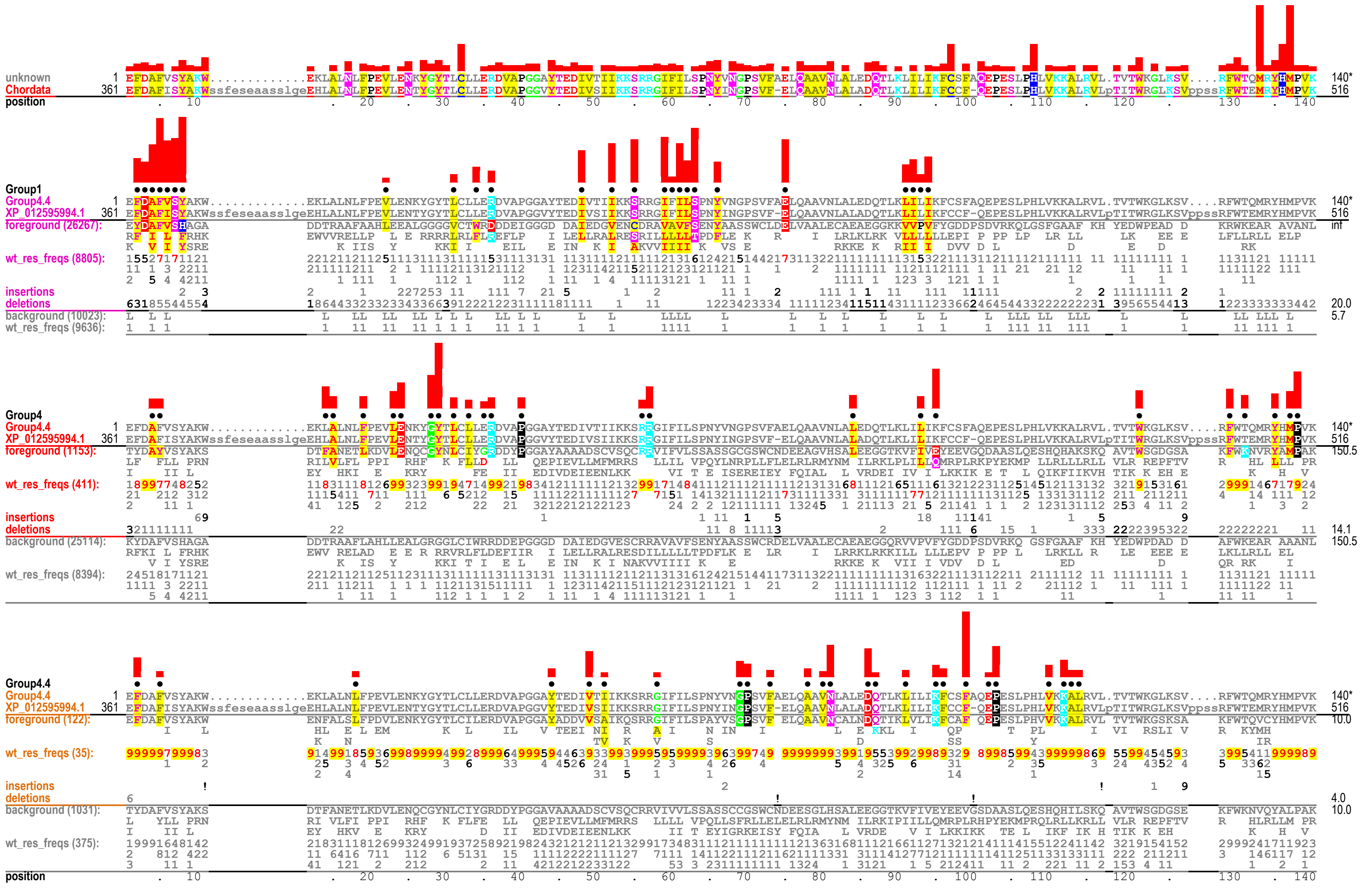

Group4.3 (IL-1R8 or SIGIRR TIRs)

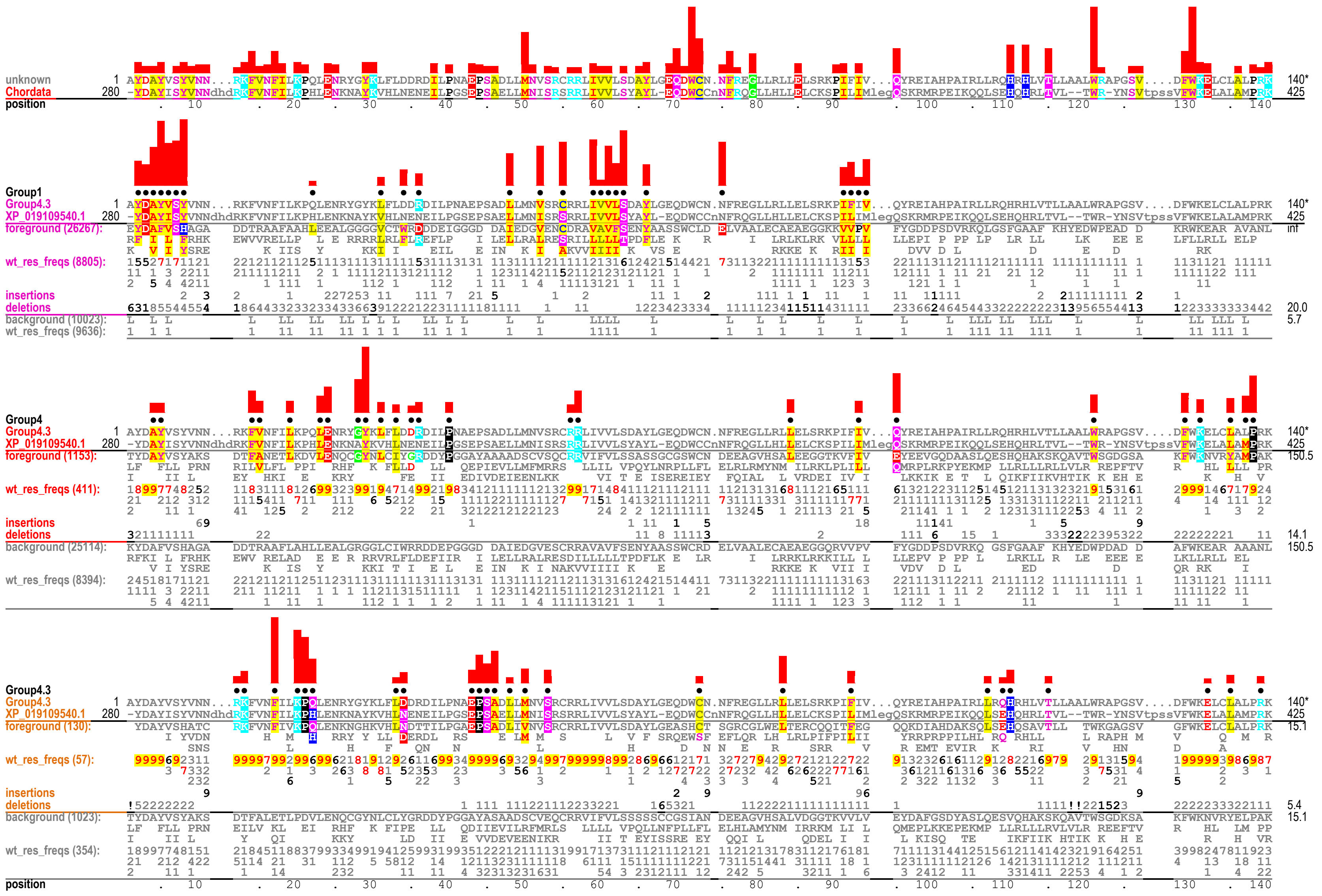

Group4.1 (TIRs of IL-1R1 and IL-1R6 (main chains of IL-1R and IL-36R))

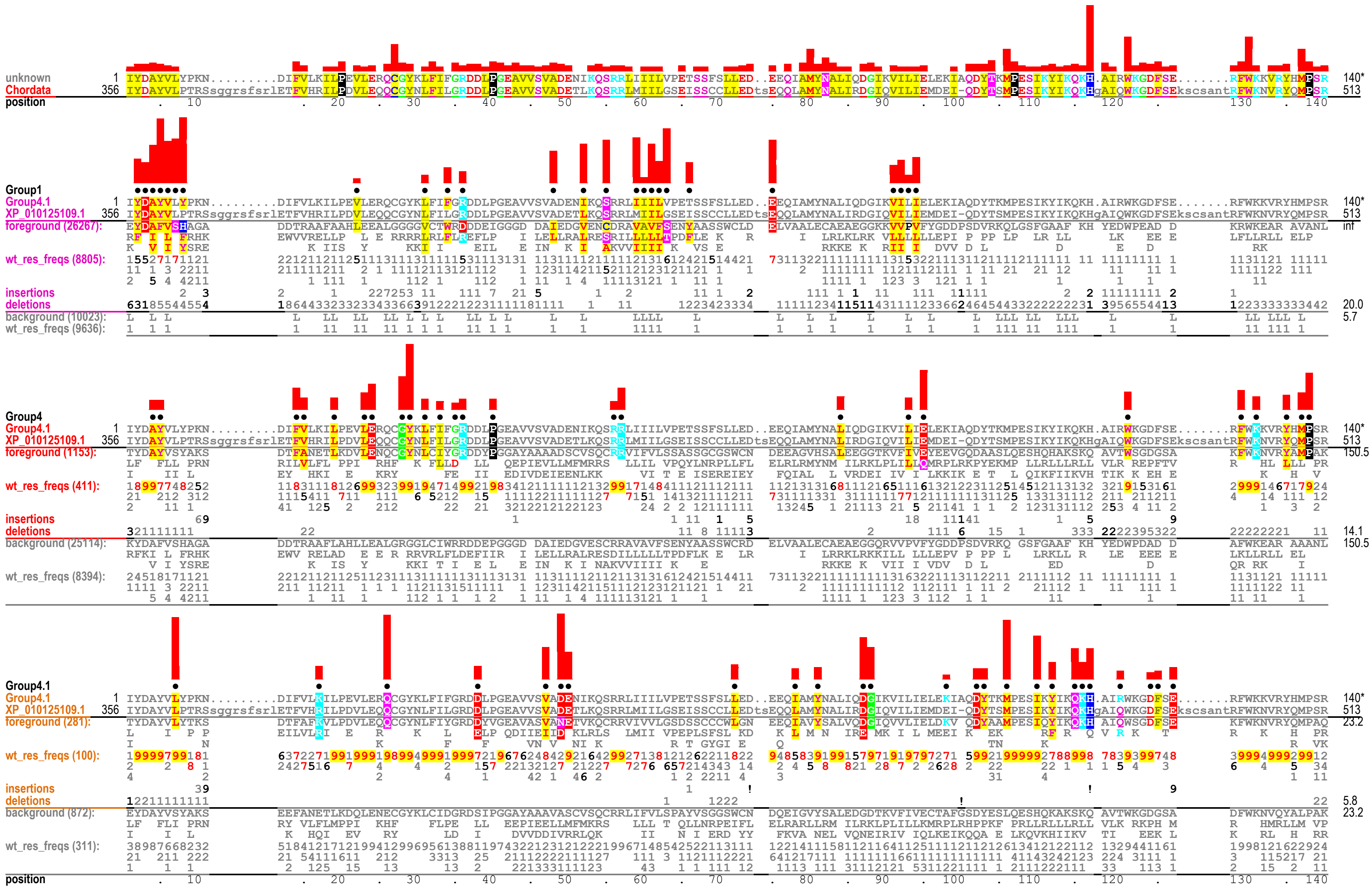

Group4.6 (IL-1R9 (IL-1RAPL) TIRs)

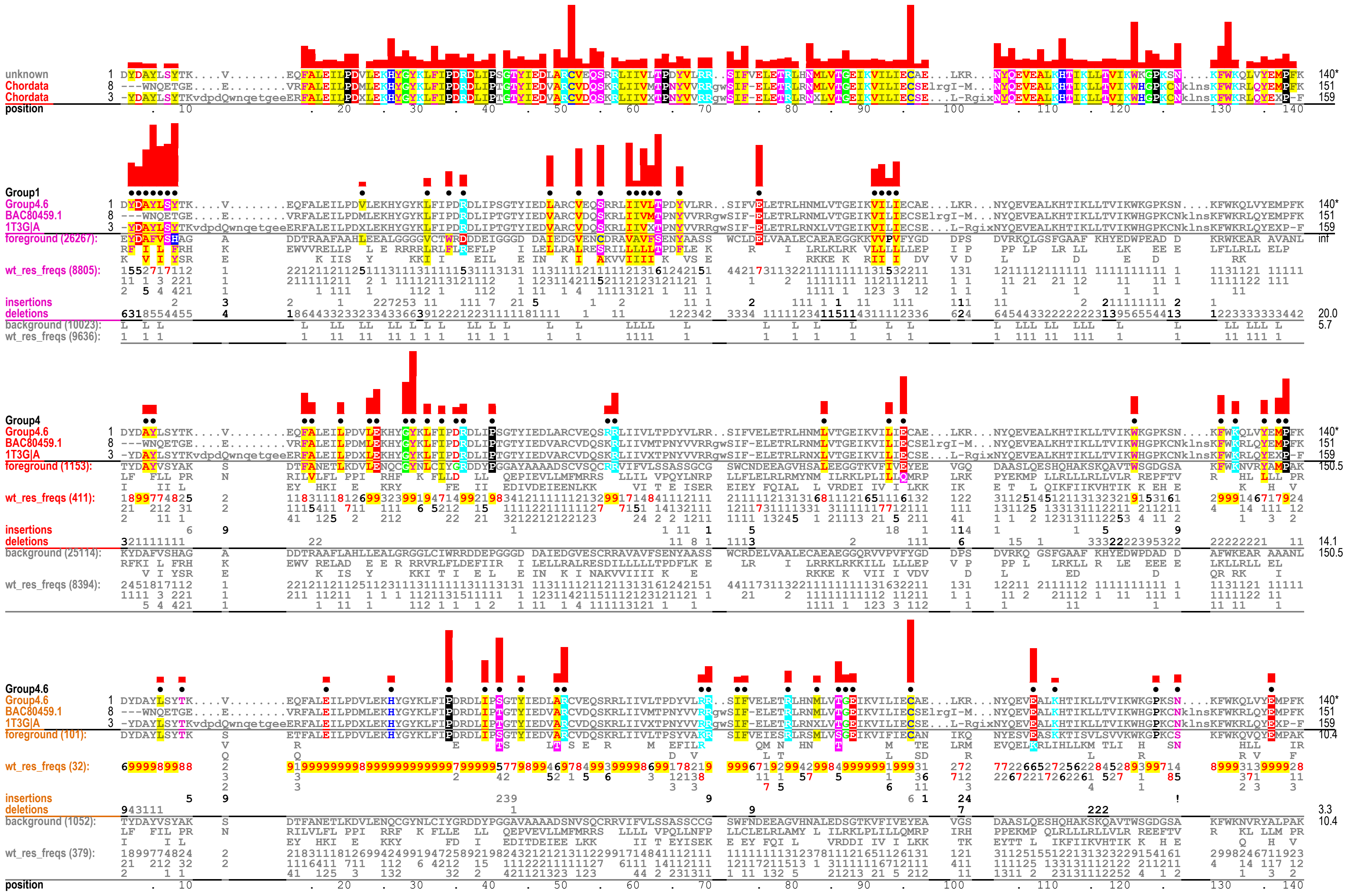

Group3.7 (TLR4 TIRs)

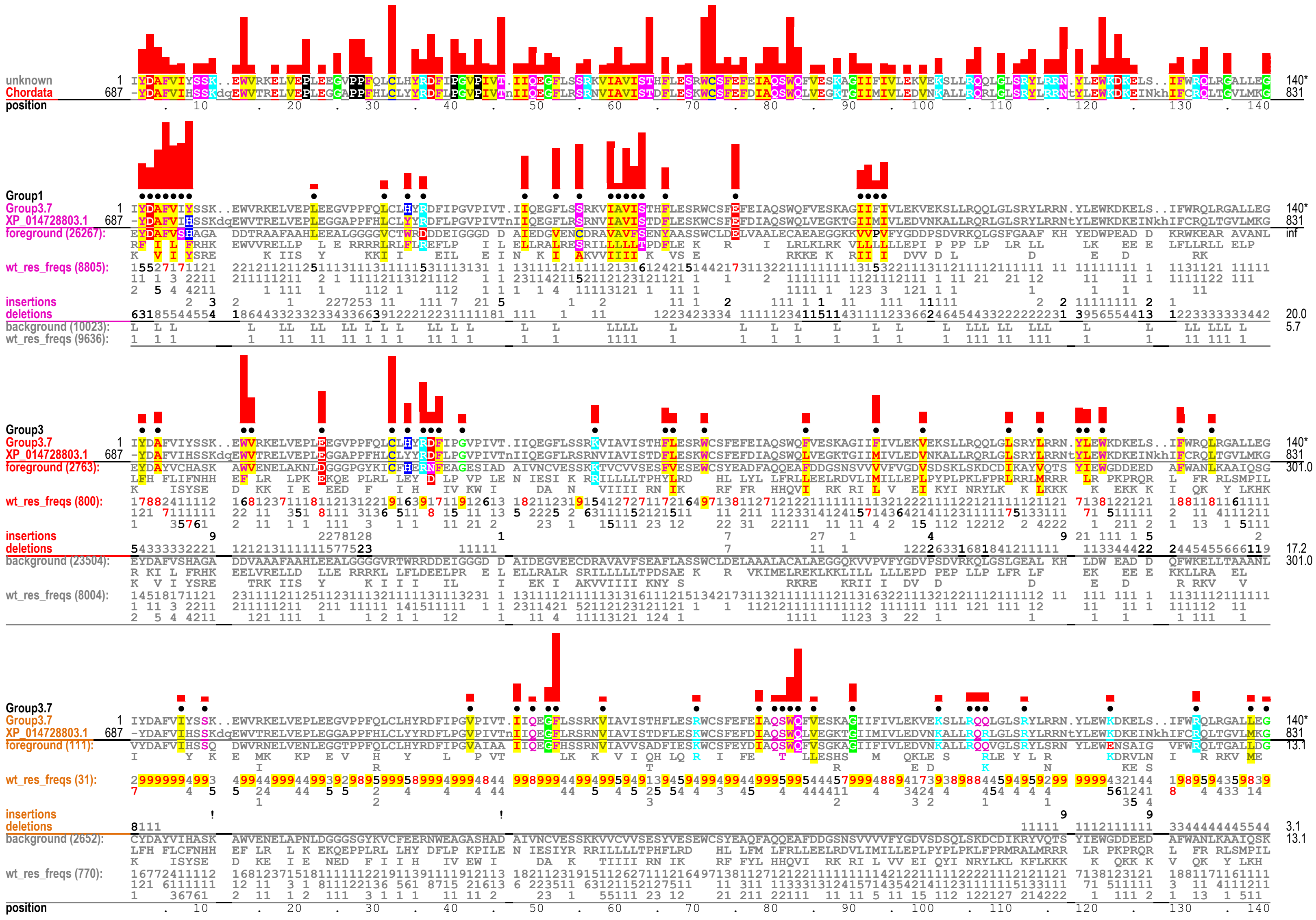

Group3.3 (TIRs from TLRs of vertabrates, except mammalan TLRs)

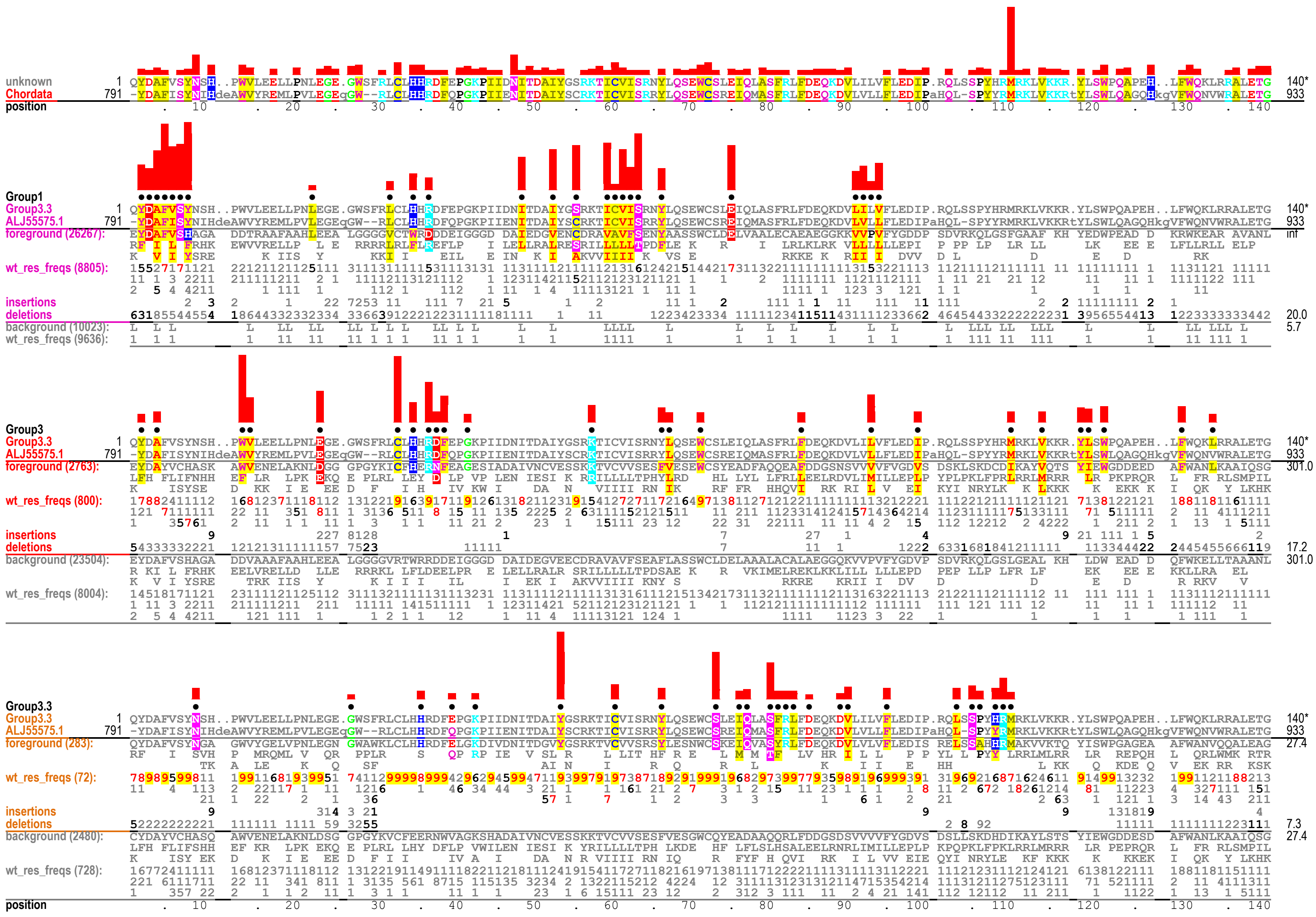

Group3.1 (Toll proteins)

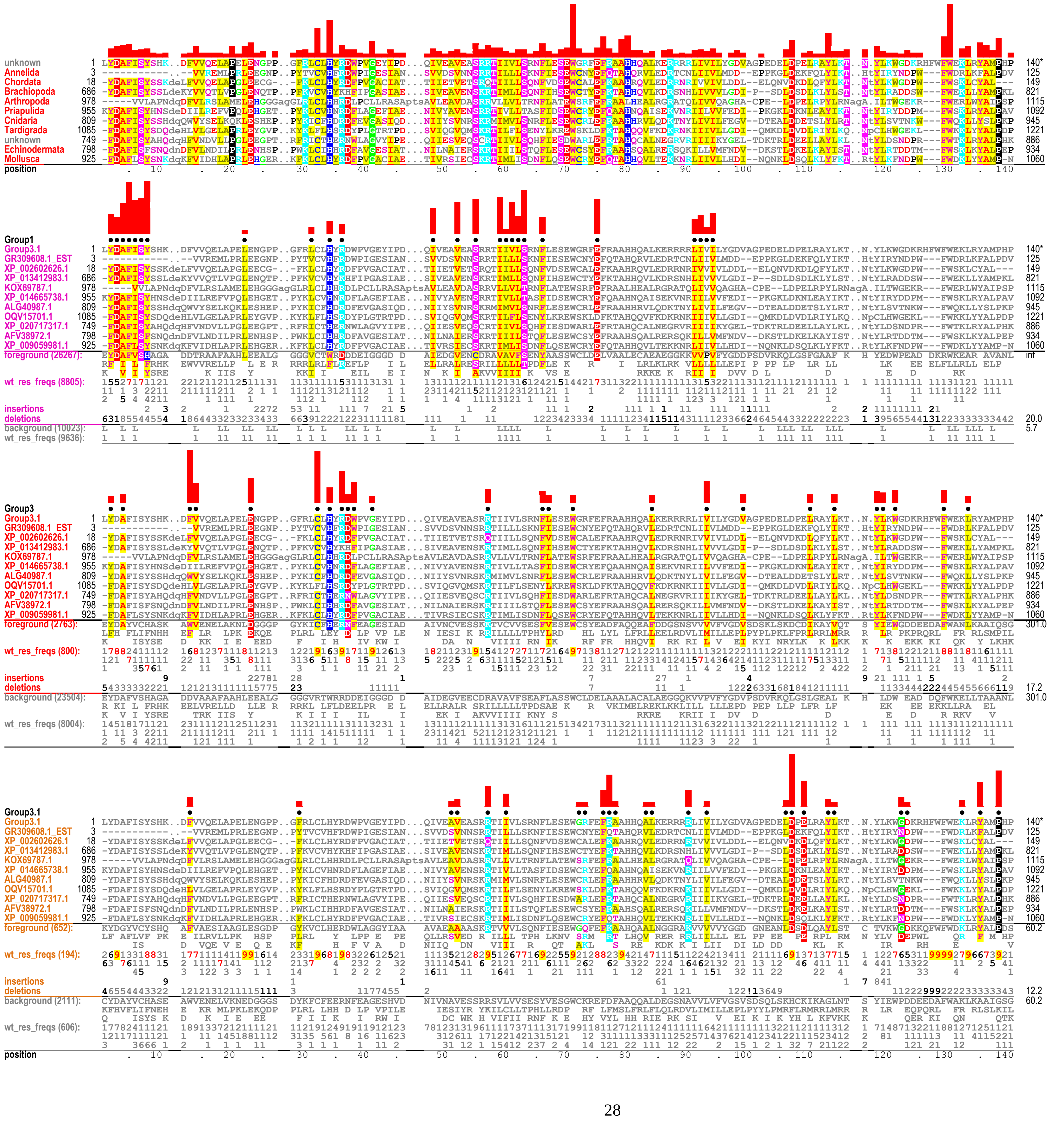

Group3.5 (TLR3 TIRs)

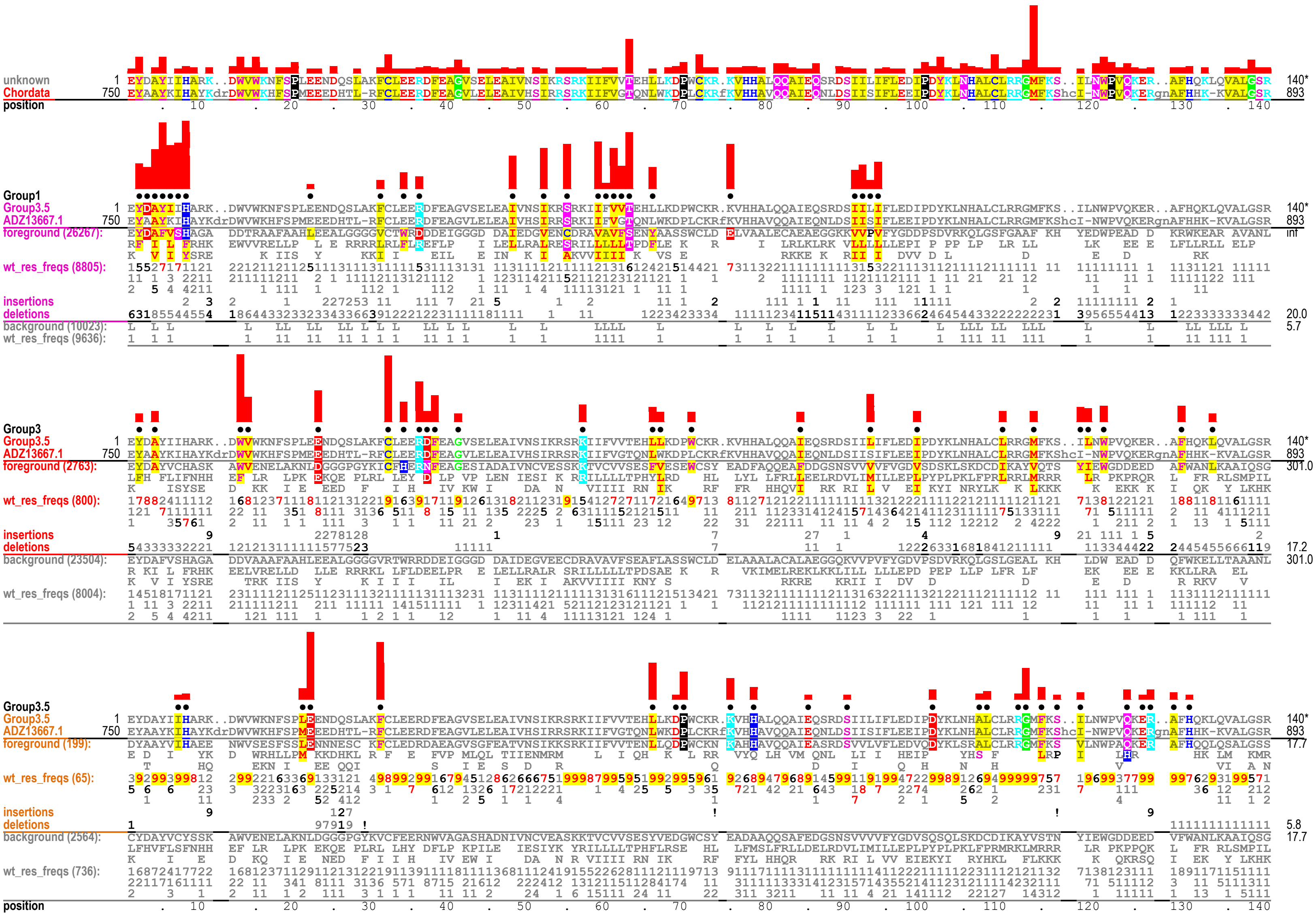

Group3.4 (TLR5 TIRs)

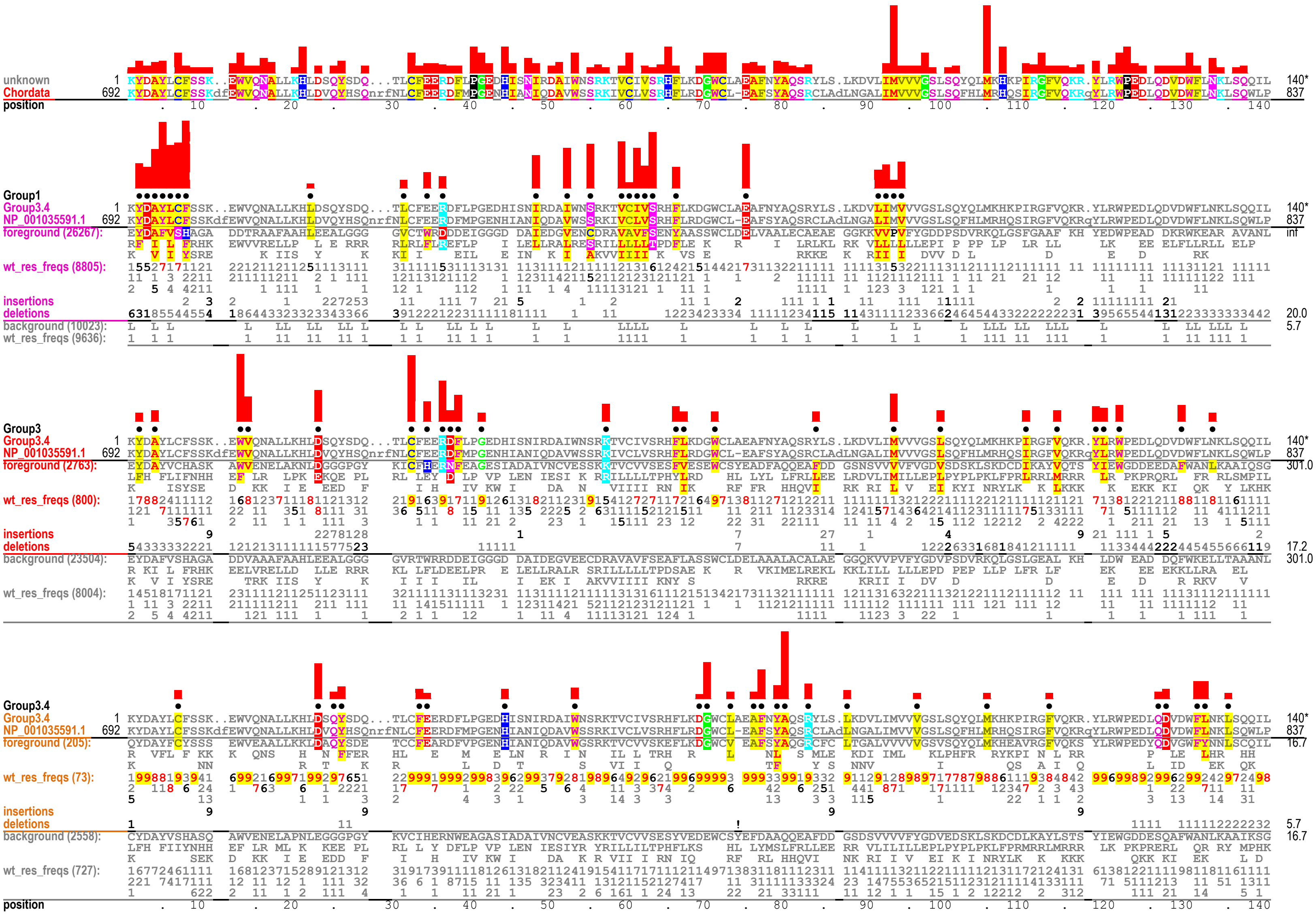

Group3.2 (TLR1, TLR2, TLR6, and TLR10 TIRs)

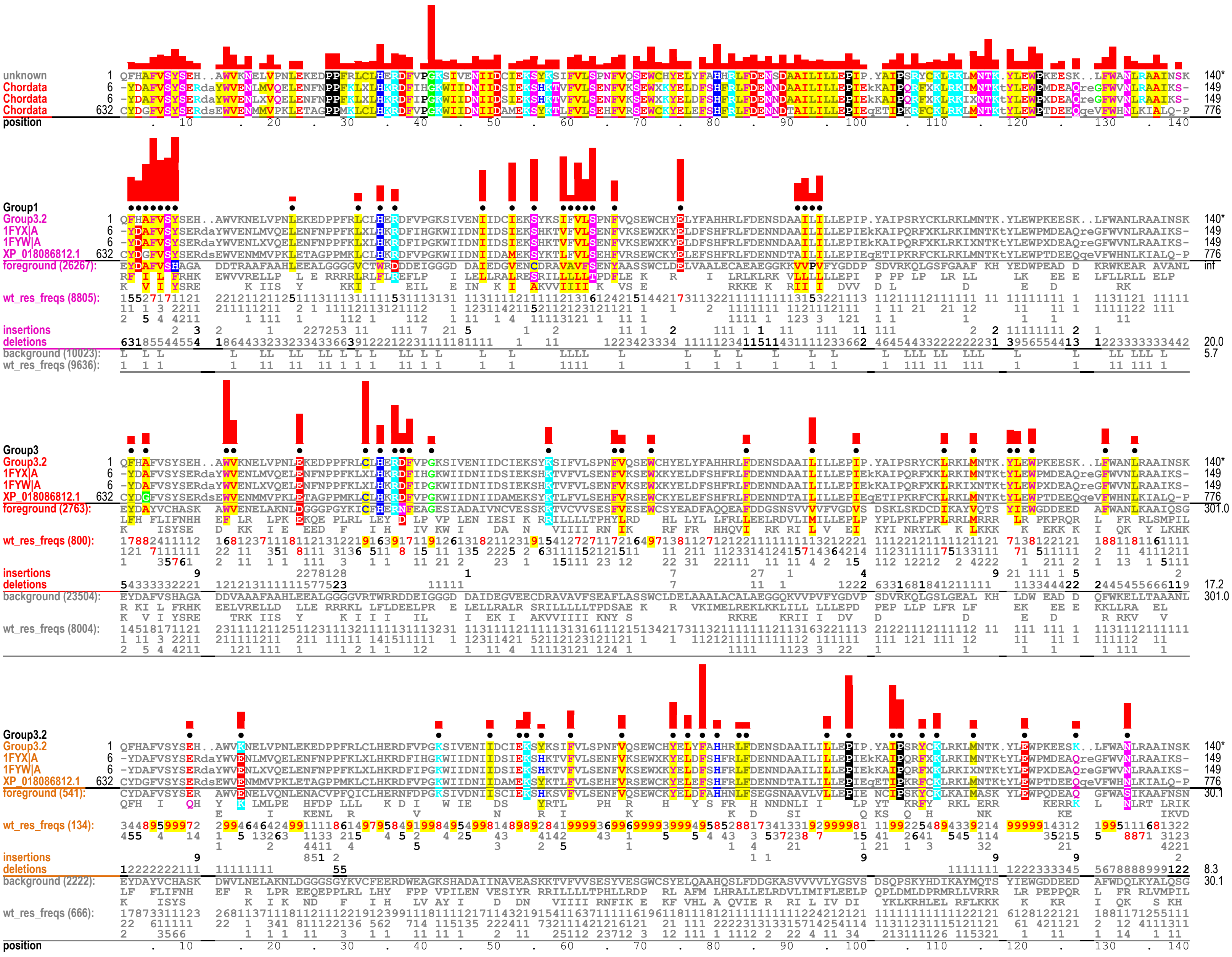

Group3.6 (TIRs from TLRs of cold-blooded vertabrates and birds)

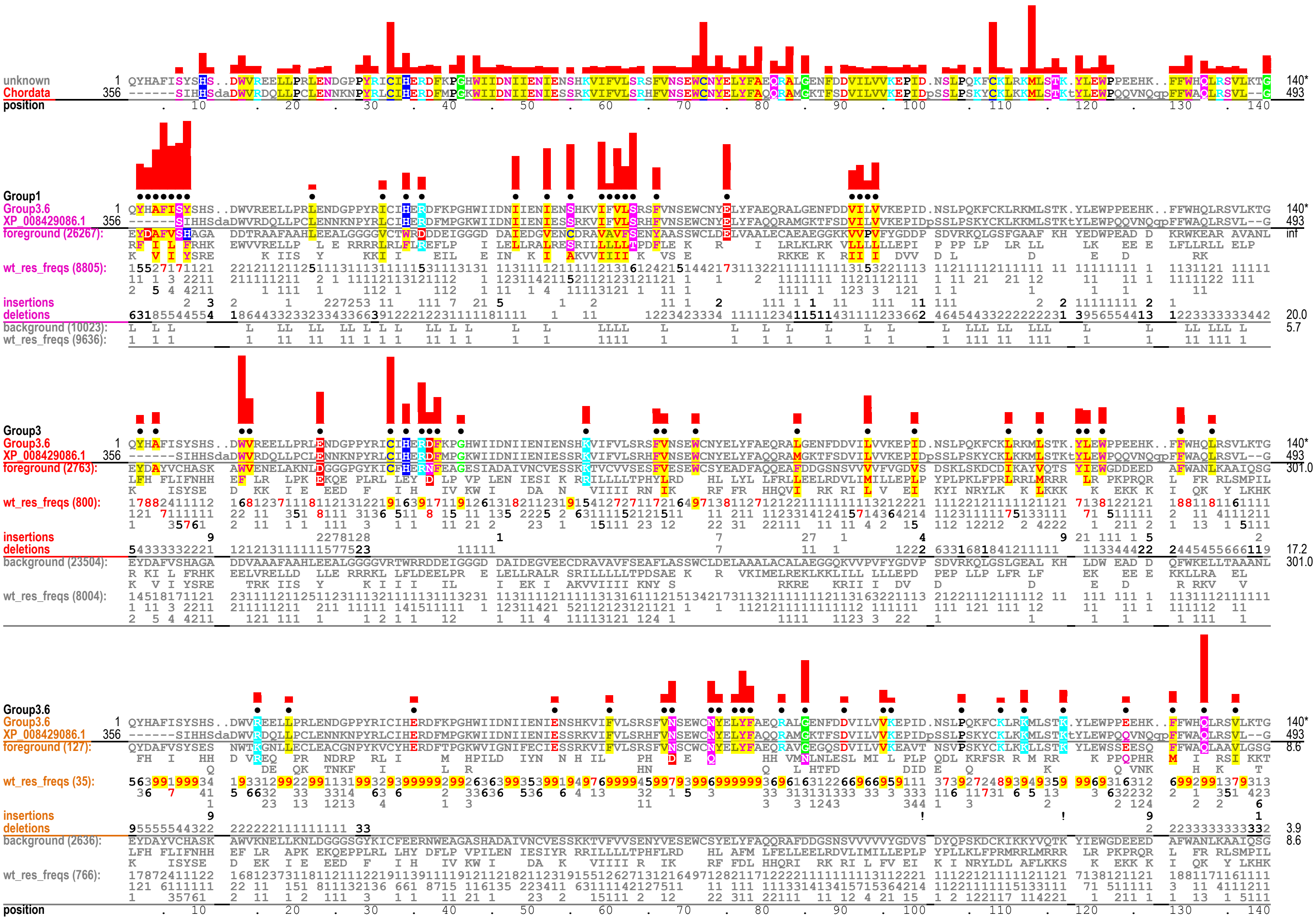

Group29 (TLR11-13 TIRs)

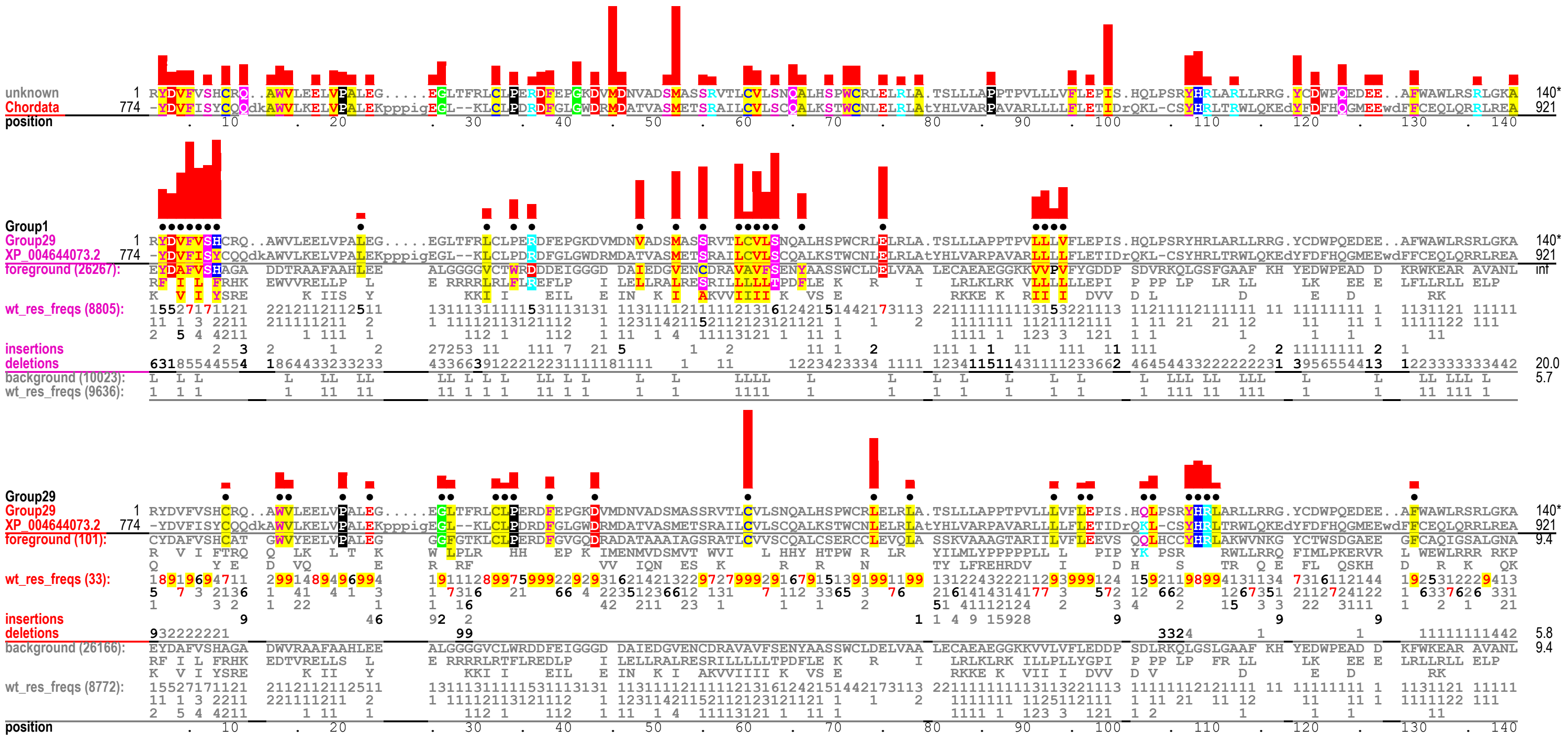

Group7 (TLR7-9 TIRs)

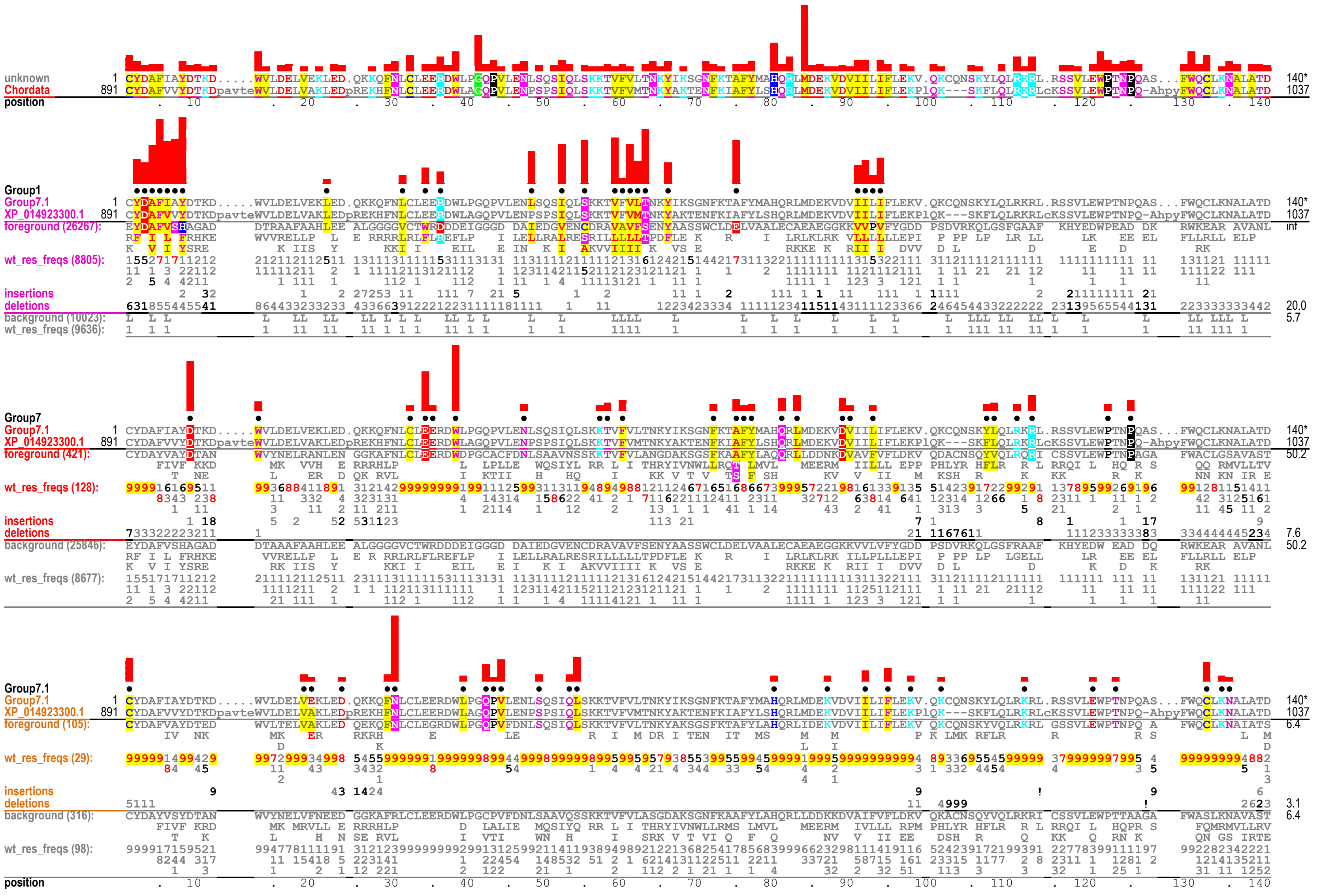

Group2.5 (NB-LRR TIRs)

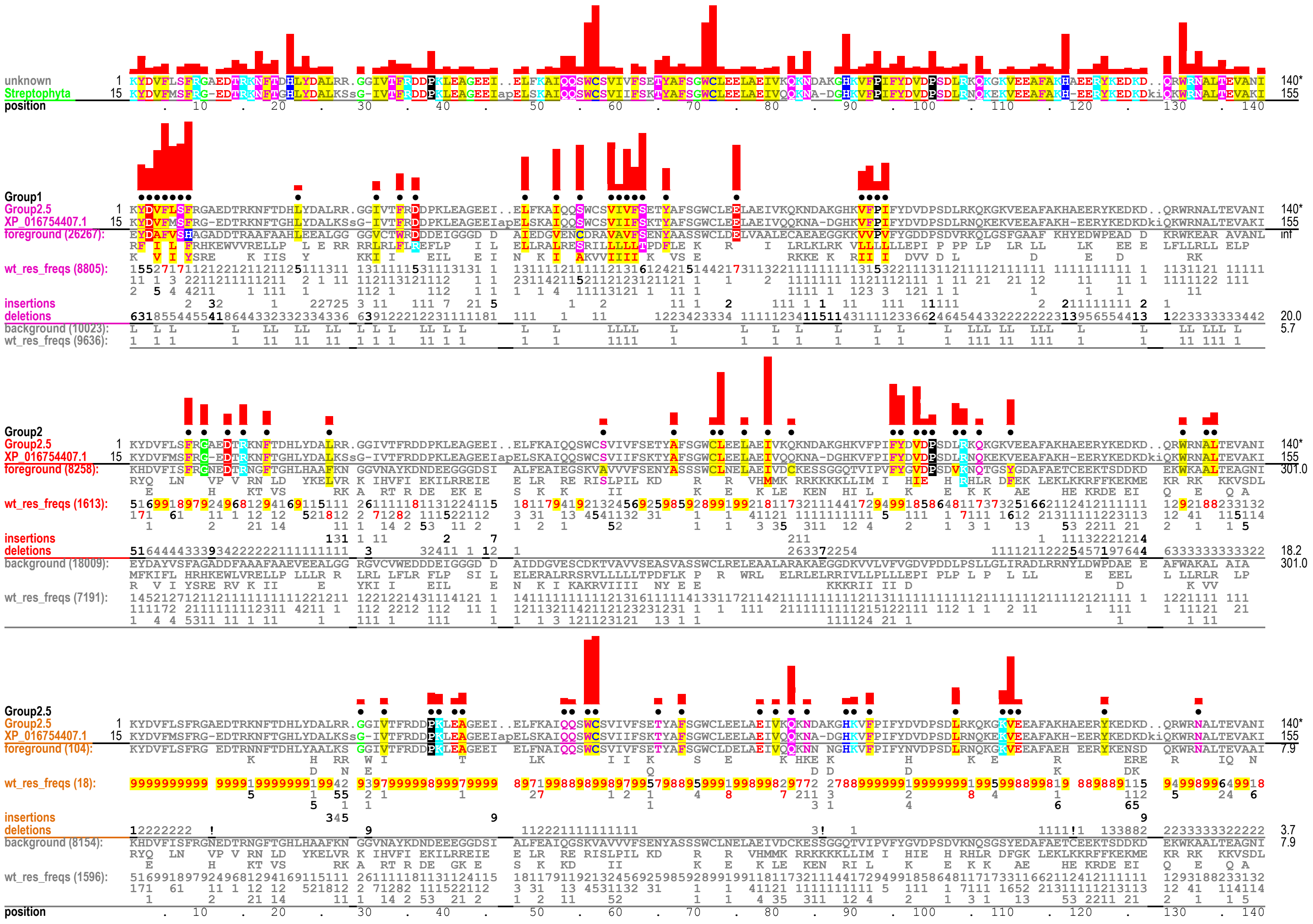

Group2.2 (plant NB-LRR TIRs)

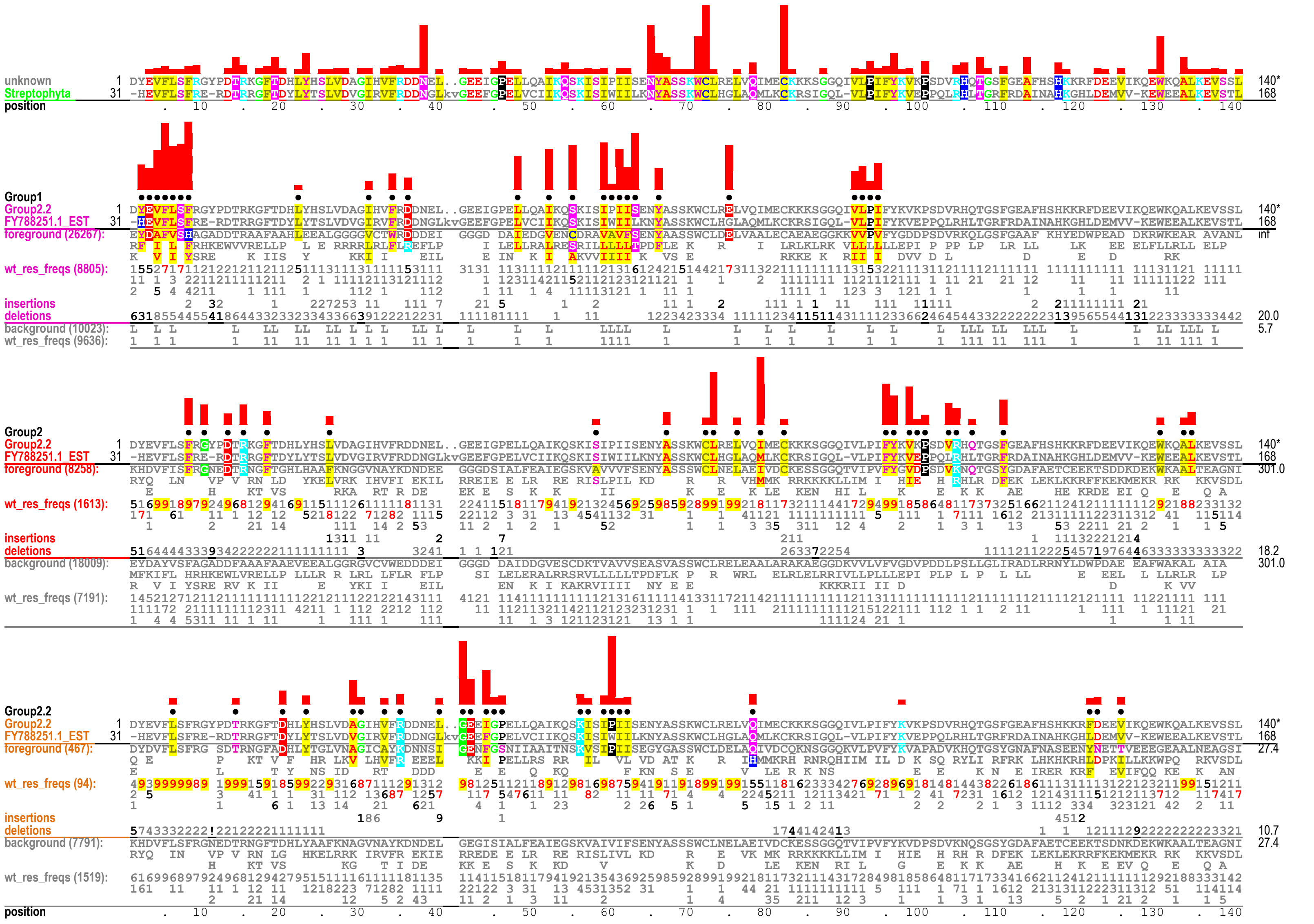

Group2.3 (plant NB-LRR TIRs)

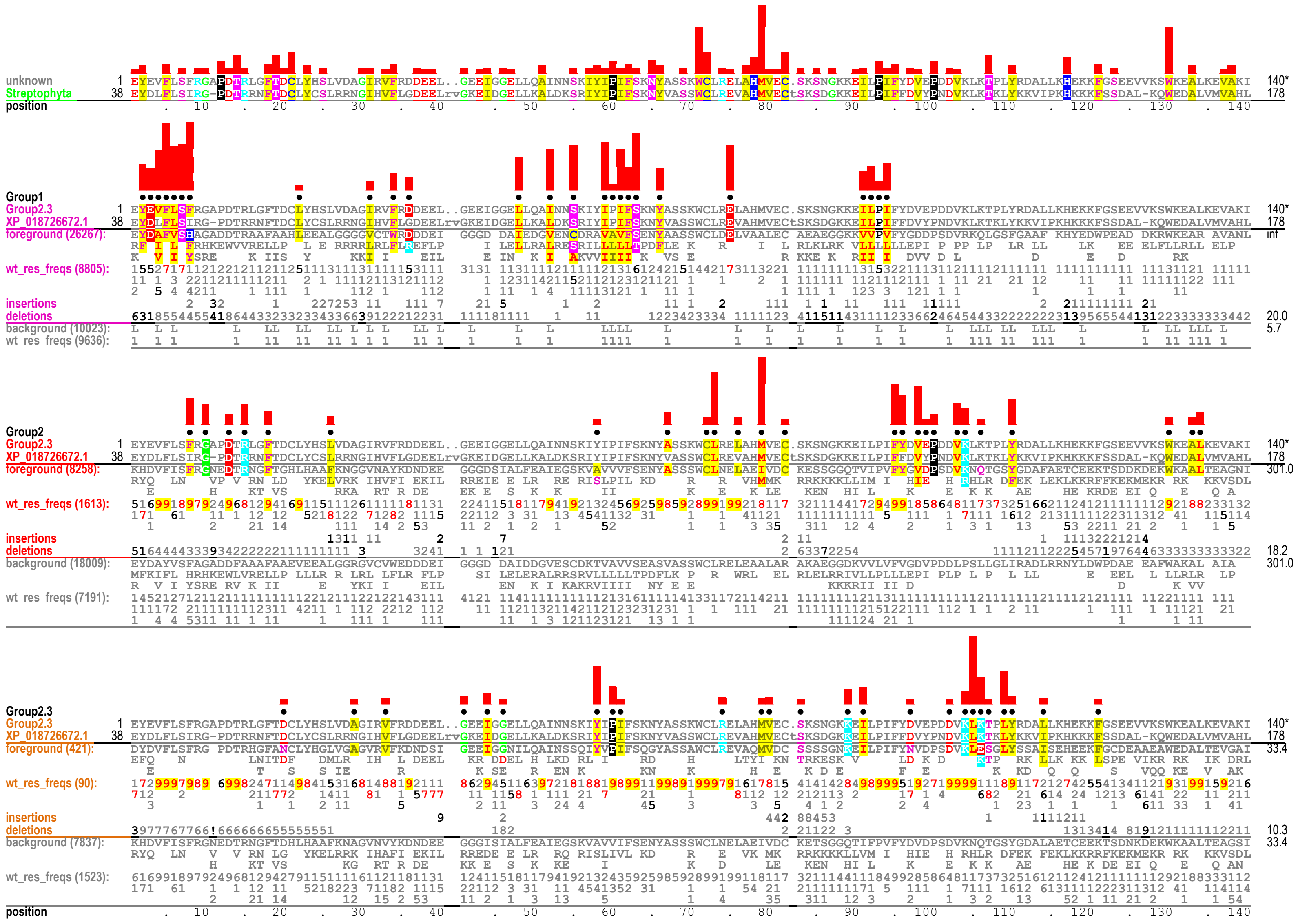

Group2.4 (plant NB-LRR TIRs)

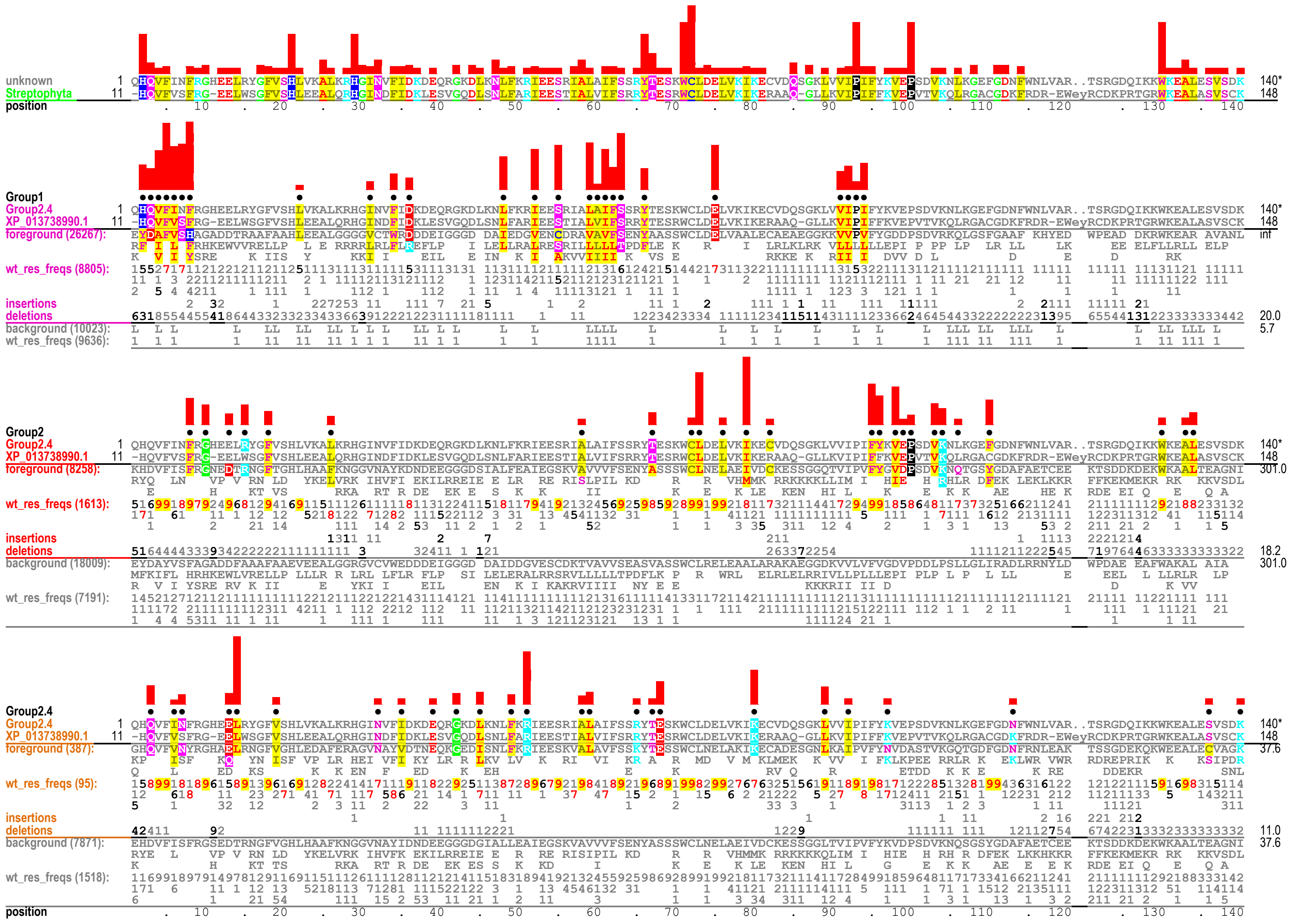

Group2.1 (plant NB-LRR TIRs)

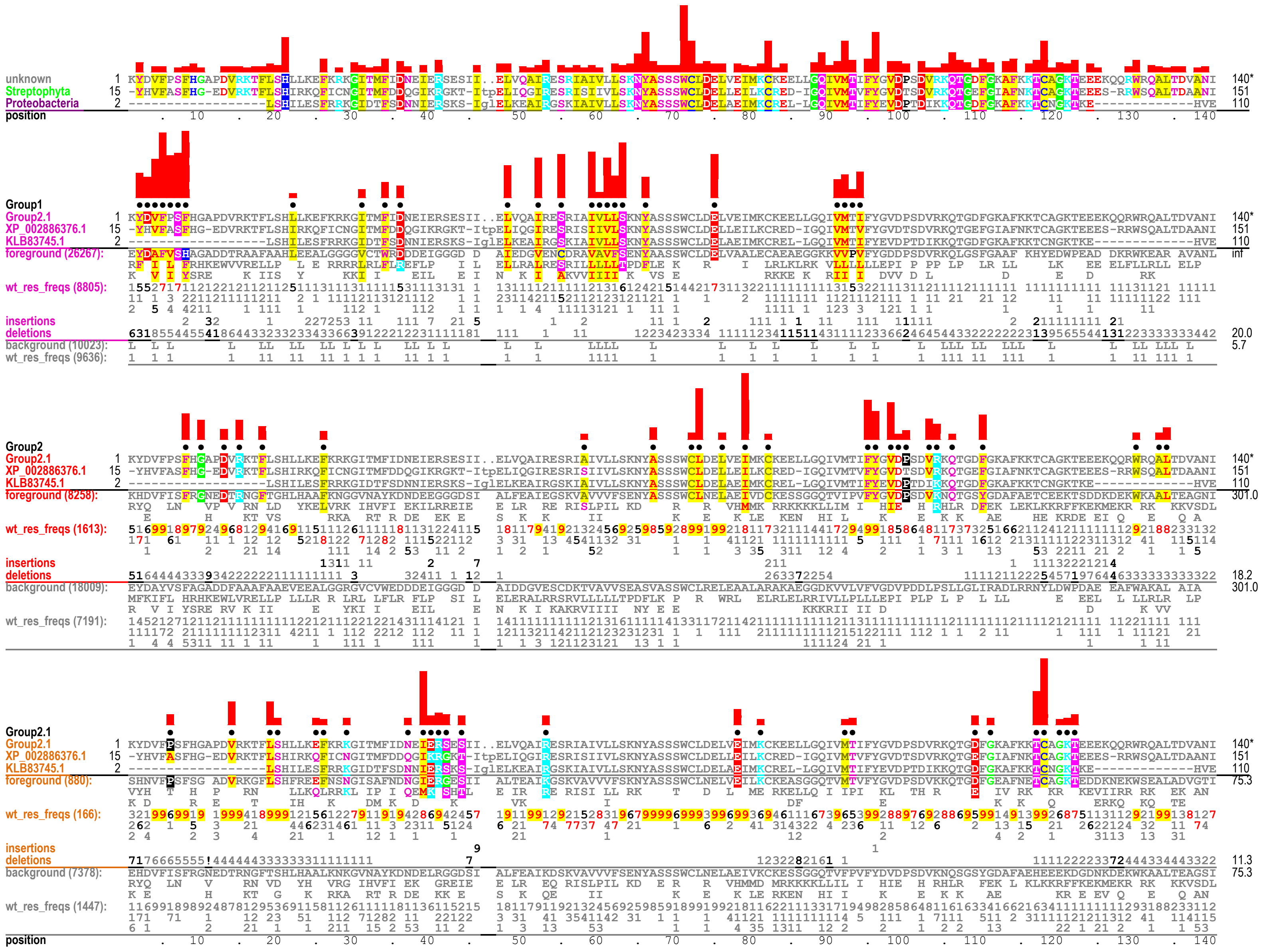

Group36 (bacterial TIRs)

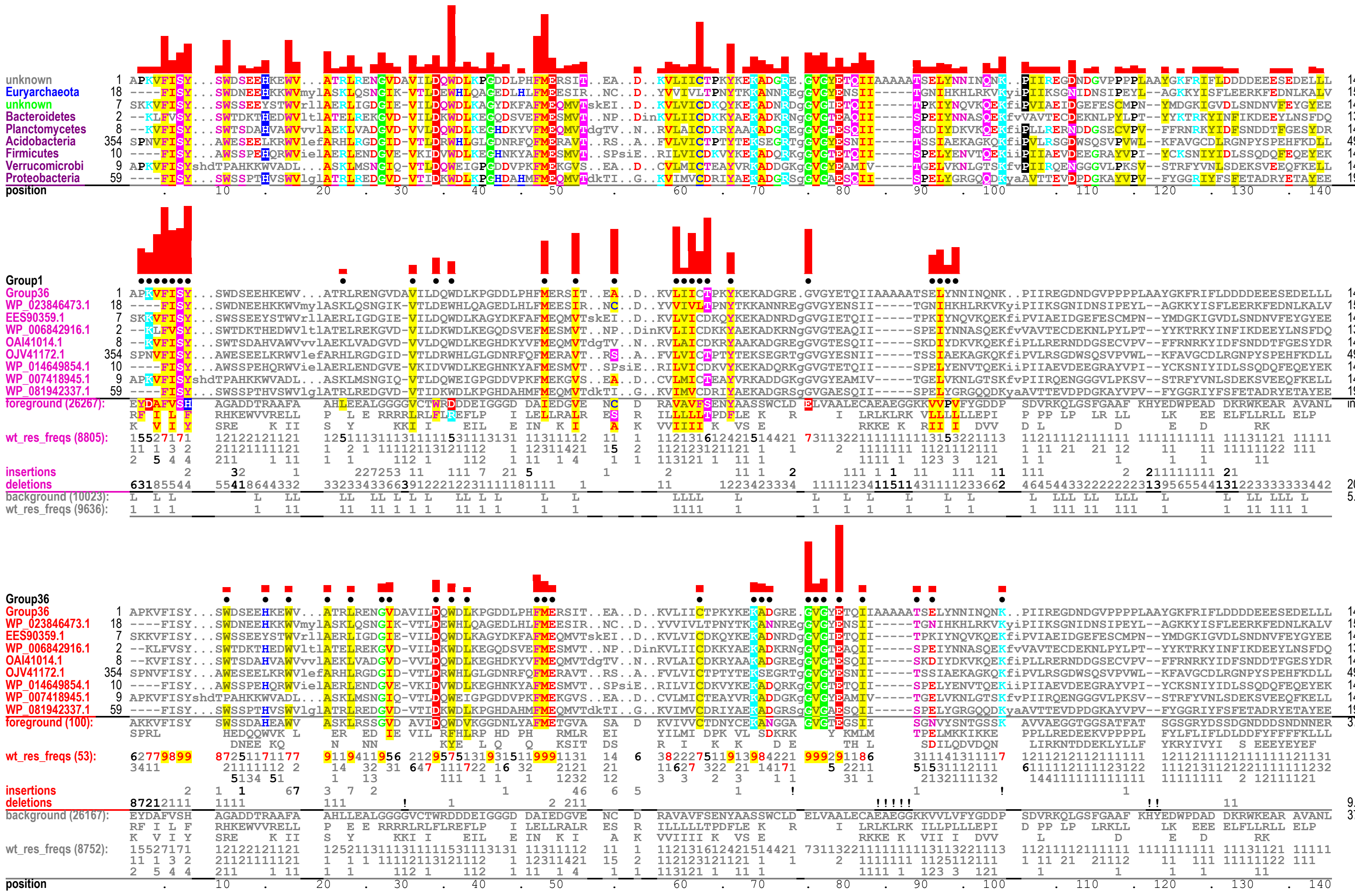

Group34 (bacterial TIRs)

```
unknown           1 APKVFISY........SHDSEEH..........KDWV...AT...RLRENGIDVVILDQW.DLKPG..QDWPAFMERQIT..EADFVLVVCTETY...........VR.......RAGGEG........E.........WEA.........ALA......R.........DL  84*
Acidobacteria     2 --RIFISY........SHDSEEH..........RRRVlalAD...RLRADGID-ATIDQY.LEPAGpaEGWPQWMEAQLR..TADFVLAVCSAKY...........RK.......RVEGDEapgaghgvA.........WEGrliyqhiynAAS......E.........N-  103
Chlorobi        330 APKAFIAY........SWEDDNH..........KKWV...ADlatRLRHSGVE-TILDQW.HAVPG..DQLPDFMEKEIR..NNDYVLIICTPKYrskcdkrdggagYE.......GDIMTAe.......V.........FTT.........K--.......N.........H-  425
Cyanobacteria   277 --KVFISY........SHDSEEH..........KDRVlslAD...RLRSDGID-CNIDQY.EESPL..EGWARWMLNQLE..WADFVLVVCTEAY...........NR.......RFRGLEe.......P.........KKG.........RGV......T.........W-  360
Bacteroidetes   246 APKVFISY........SWDSEAL..........KKWVldlAI...KLREKGID-AILDQW.ELELG..KPIPSFMGKSIE..ISDRVICIMTPSYktksn.........AE.......EPKGGVa.......Y.........ESS.........IMA......A.........ET  337
Planctomycetes   13 -RKVFVSY........AHTSEPH..........IEWIlafVA...DLQANGIE-VVVDQY.DLKEG..QDANTFMEKVVTdkTIDRVLIVSDENY...........AAkadg...RKGGVGt.......E.........SQI.........ITQklykdiE.........E-  109
Verrucomicrobi   13 ---VFVSY........THDSMEH..........DARVlalAK...ELRHRGFD-CDLDQ-.-FHAN..QSWPTWMENRIA..WAEKVIVVCTETY...........LR.......--RWQNn.......EkpgiglgaqWES.........LLT......RqwlyespevH-  109
Proteobacteria    5 --RVFISY........SHDSAIH..........KGWVldfAT...TLRNRGID-AVLDQW.DLTPG..DDLPHFMETELA..QCDYVIMVCSEIY...........VEkanagegGVGYEKm.......I.........ITS.........SLMkni...D.........G-  98
unknown           3 --RVFISY........SHDSREH..........ADCVldlSN...RLRADGVD-SHIDQY.EMSPP..EGWPRWMVNRIE..GADFVLVVCTETY...........ER.......RFRGRKg.......P.........GKG.........LGA......T.........W-  86
Chordata         26 -KKLFISYsmdpypvgTRSDRERrddrrtqrrrVRAL...AD...KLRQYGVD-AWIDQYqEYIPP..DSWTNWMEKQIT..KADYVLMICSPHY...........LE.......CVTGEKn.......E.........-VA.........VFG......H.........G-  125
Firmicutes        6 --KVFISY........AHKNQAY..........EDKVlelAN...RLRSEGID-AMIDQY.EEAPS..EGWPRWMEHQIM..ESEFVLVLCEETYnqkl.......YS.......EKKGRGv.......V.........WEAnivyqmlydSAA......E.........T-  102
Actinobacteria   18 APRVFVSY........AHESEEH..........KNQVlgfCR...LLRRHGVD-ARLDQY.DSGRR..WDWYAWMTDQIT..RADFVIVIASPQYrvagdgsp....GR.......MSKGVQt.......E.........--A.........ALL......R.........DL  110
position                 .         10   .              20         .   30    .   40         .   50       .   60   .                       70                .                80
```

```
Group1
Group34              1 APKVFISY........SHDSEEH..........KDWV...AT...RLRENGIDVVILDQW.DLKPG..QDWPAFMERQIT..EADFVLVVCTETY...........VR.......RAGGEG........E.........WEA.........ALA......R.........DL  84*
WP_011689320.1       2 --RIFISY........SHDSEEH..........RRRVlalAD...RLRADGID-ATIDQY.LEPAGpaEGWPQWMEAQLR..TADFVLAVCSAKY...........RK.......RVEGDEapgaghgvA.........WEGrliyqhiynAAS......E.........N-  103
WP_012509009.1     330 APKAFIAY........SWEDDNH..........KKWV...ADlatRLRHSGVE-TILDQW.HAVPG..DQLPDFMEKEIR..NNDYVLIICTPKYrskcdkrdggagYE.......GDIMTAe.......V.........FTT.........K--.......N.........H-  425
WP_013320651.1     277 --KVFISY........SHDSEEH..........KDRVlslAD...RLRSDGID-CNIDQY.EESPL..EGWARWMLNQLE..WADFVLVVCTEAY...........NR.......RFRGLEe.......P.........KKG.........RGV......T.........W-  360
WP_015782906.1     246 APKVFISY........SWDSEAL..........KKWVldlAI...KLREKGID-AILDQW.ELELG..KPIPSFMGKSIE..ISDRVICIMTPSYktksn.........AE.......EPKGGVa.......Y.........ESS.........IMA......A.........ET  337
WP_011119720.1      13 -RKVFVSY........AHTSEPH..........IEWIlafVA...DLQANGIE-VVVDQY.DLKEG..QDANTFMEKVVTdkTIDRVLIVSDENY...........AAkadg...RKGGVGt.......E.........SQI.........ITQklykdiE.........E-  109
WP_068768974.1      13 ---VFVSY........THDSMEH..........DARVlalAK...ELRHRGFD-CDLDQ-.-FHAN..QSWPTWMENRIA..WAEKVIVVCTETY...........LR.......--RWQNn.......EkpgiglgaqWES.........LLT......RqwlyespevH-  109
WP_068987332.1       5 --RVFISY........SHDSAIH..........KGWVldfAT...TLRNRGID-AVLDQW.DLTPG..DDLPHFMETELA..QCDYVIMVCSEIY...........VEkanagegGVGYEKm.......I.........ITS.........SLMkni...D.........G-  98
OGG46781.1           3 --RVFISY........SHDSREH..........ADCVldlSN...RLRADGVD-SHIDQY.EMSPP..EGWPRWMVNRIE..GADFVLVVCTETY...........ER.......RFRGRKg.......P.........GKG.........LGA......T.........W-  86
XP_019620788.1      26 -KKLFISYsmdpypvgTRSDRERrddrrtqrrrVRAL...AD...KLRQYGVD-AWIDQYqEYIPP..DSWTNWMEKQIT..KADYVLMICSPHY...........LE.......CVTGEKn.......E.........-VA.........VFG......H.........G-  125
WP_016296840.1       6 --KVFISY........AHKNQAY..........EDKVlelAN...RLRSEGID-AMIDQY.EEAPS..EGWPRWMEHQIM..ESEFVLVLCEETYnqkl.......YS.......EKKGRGv.......V.........WEAnivyqmlydSAA......E.........T-  102
WP_020667457.1      18 APRVFVSY........AHESEEH..........KNQVlgfCR...LLRRHGVD-ARLDQY.DSGRR..WDWYAWMTDQIT..RADFVIVIASPQYrvagdgsp....GR.......MSKGVQt.......E.........--A.........ALL......R.........DL  110
foreground (26267):    EYDAFVSH         AGADDTR          AAFA   AH     LEEALGGGGVCTWRD DDEIG   GGD DAIEDGVE   NCDRAVAVFSENY                AA         SSWCLD         E         LVA          ALE        C          AE   inf
                       RF I L F         RHKEWVV          RELL   P        L E RRRRLRLFLR EFLP      I LELLRALR   ESRILLLLLTPDF                LE         K   R                                I          L          RL
                       K  V I Y         SRE              K II   S        Y       KKI I    EIL      E IN   K I    AKVVIIII K                   VS         E                                           R          KK
wt_res_freqs (8805):   1552717 1        1212212          1121   12     51113111311111 5 31113   131 11311112   1111121316124                21         514421         7         311          322        1          11
                       11 1 3 2         2112111          1121   1        2 1 1111211312 1112      1 12311421   1521121231211                21         1   1                                2          1          11
                       2  5 4 4         211              1 11   1        1       112 1    112      1 11   1 4   11113121 1                   11         1                                           1          11
insertions                    2           32                1            227253 11    1 11 7    21 5          1   2                            11         1   2                            11         1          1
deletions              63185544         5541864          4332   33     2334336639122 21 22311   11181111    1     11          12              23         423334                    111          112        3          41   20.0
background (10023):    L  L L               L             LL           LL  LL L L L     LL L    L       L    L       LLLL                     L                                  L            L                     L    5.7
wt_res_freqs (9636):   1  1 1               1             11           11  11 1 1 1     11 1    1       1    1       1111                     1                                  1            1                     1
```

```
Group34
Group34              1 APKVFISY........SHDSEEH..........KDWV...AT...RLRENGIDVVILDQW.DLKPG..QDWPAFMERQIT..EADFVLVVCTETY...........VR.......RAGGEG........E.........WEA.........ALA......R.........DL  84*
WP_011689320.1       2 --RIFISY........SHDSEEH..........RRRVlalAD...RLRADGID-ATIDQY.LEPAGpaEGWPQWMEAQLR..TADFVLAVCSAKY...........RK.......RVEGDEapgaghgvA.........WEGrliyqhiynAAS......E.........N-  103
WP_012509009.1     330 APKAFIAY........SWEDDNH..........KKWV...ADlatRLRHSGVE-TILDQW.HAVPG..DQLPDFMEKEIR..NNDYVLIICTPKYrskcdkrdggagYE.......GDIMTAe.......V.........FTT.........K--.......N.........H-  425
WP_013320651.1     277 --KVFISY........SHDSEEH..........KDRVlslAD...RLRSDGID-CNIDQY.EESPL..EGWARWMLNQLE..WADFVLVVCTEAY...........NR.......RFRGLEe.......P.........KKG.........RGV......T.........W-  360
WP_015782906.1     246 APKVFISY........SWDSEAL..........KKWVldlAI...KLREKGID-AILDQW.ELELG..KPIPSFMGKSIE..ISDRVICIMTPSYktksn.........AE.......EPKGGVa.......Y.........ESS.........IMA......A.........ET  337
WP_011119720.1      13 -RKVFVSY........AHTSEPH..........IEWIlafVA...DLQANGIE-VVVDQY.DLKEG..QDANTFMEKVVTdkTIDRVLIVSDENY...........AAkadg...RKGGVGt.......E.........SQI.........ITQklykdiE.........E-  109
WP_068768974.1      13 ---VFVSY........THDSMEH..........DARVlalAK...ELRHRGFD-CDLDQ-.-FHAN..QSWPTWMENRIA..WAEKVIVVCTETY...........LR.......--RWQNn.......EkpgiglgaqWES.........LLT......RqwlyespevH-  109
WP_068987332.1       5 --RVFISY........SHDSAIH..........KGWVldfAT...TLRNRGID-AVLDQW.DLTPG..DDLPHFMETELA..QCDYVIMVCSEIY...........VEkanagegGVGYEKm.......I.........ITS.........SLMkni...D.........G-  98
OGG46781.1           3 --RVFISY........SHDSREH..........ADCVldlSN...RLRADGVD-SHIDQY.EMSPP..EGWPRWMVNRIE..GADFVLVVCTETY...........ER.......RFRGRKg.......P.........GKG.........LGA......T.........W-  86
XP_019620788.1      26 -KKLFISYsmdpypvgTRSDRERrddrrtqrrrVRAL...AD...KLRQYGVD-AWIDQYqEYIPP..DSWTNWMEKQIT..KADYVLMICSPHY...........LE.......CVTGEKn.......E.........-VA.........VFG......H.........G-  125
WP_016296840.1       6 --KVFISY........AHKNQAY..........EDKVlelAN...RLRSEGID-AMIDQY.EEAPS..EGWPRWMEHQIM..ESEFVLVLCEETYnqkl.......YS.......EKKGRGv.......V.........WEAnivyqmlydSAA......E.........T-  102
WP_020667457.1      18 APRVFVSY........AHESEEH..........KNQVlgfCR...LLRRHGVD-ARLDQY.DSGRR..WDWYAWMTDQIT..RADFVIVIASPQYrvagdgsp....GR.......MSKGVQt.......E.........--A.........ALL......R.........DL  110
foreground (226):      AKKVFVSY         AHDSEAH          AARV   AT     ELRASGVD AVIDQW DEGEG   DGWSAWMESGVS   DADKVVAVCSDAY                KA         GAGGGG         E         GTG          GGG        D          NA   60.2
                       SPRL I           SWENPE           VQWL   SE     R  ED LE VIL KY HLRPP   HDLPLFA RMLR   ES F LMIADPK                 LR         RVRYVK         I         WQS          LLL        R          EL
                                          SDK            KE     D        SN I      R E KLR    Q   H   KQIE     Y IL  TKE                  VE         KSE RE                   EA           AVA        E          DI
wt_res_freqs (104):    71579199         2555217          1117   72     19511946 312955 41115   124114851131   146181145 3118               11         122411         3         111          111        1          11
                       2631 7           631113           1141   11     3  11 11 436 12 14131   1523151 2121   22 3 6142131                 12         311111         1         111          121        1          12
                                          111            22     1        12 4     1 2 111      2   1   1231     1 21  411                  12         111 11                    23           211        1          11
insertions                    4         11 61             216   53     323 3       3            33      13 4       1                      71          33 29         4         154          567        3
deletions              76172                                    11                !    1 1  1       1             1                      21         234432         2         763          355        2          18   13.8
background (26041):    EYDAFVSY         AGDDDTA          AAFV   AH     LLEALGGGGVCIWRD DDEIG   GGD DAIEDGVE   NCDRVVAVFSENY                AA         SSWCLD         E         LVA          ALE        C          AE   60.2
                       RF I L F         SHEEWVV          RELL   P       E E RRRRLRLFLR EFLP      I LELLRALR   ESRILLLLLTPDF                LE         K   R                                I          L          RL
                       K  V I H         RRK   R          K II   S       Y      KKI T    EIL      E IN   K I    AKVAIIII K                   VS         E                                           R          KK
wt_res_freqs (8701):   15527174         1212211          1122   12     521131113111115 31113   131 11311112   1111121316124                21         514421         7         311          322        1          11
                       11 1 3 2         2112111          1121   1       1 1 1111211312 1112      1 12311421   1521121231211                21         1   1                                2          1          11
                       2  5 4 1         211   2          1 11   1       1       112 1    112      1 11   1 4   11114121 1                   11         1                                           1          11
position                 .         10   .             20         .   30    .   40         .   50       .   60   .                       70                .                80
```

```
unknown          85 LYRD.....EKFIPVVLP..GES..DLPTFLKGRIYIDL.......SDRSDDEYELLFERLLLRILDGPLPP  140*
Acidobacteria   104 ----.....RKFVPVLLG..EATreDIPVPLQGVSN---.......HR--ADEPEGYA--SLYGTLTGAPRV  150
Chlorobi        426 ----.....RKFIPVLAK..GSWdeSAPSWLKGKFYVDLs......TP--VEDERDYP--RLLAALLNSHPQ  476
Cyanobacteria   361 ----.....EGAVISQEL..YDAqvKSTKFIPVLFSSED.......ED--YIPIVIRGFSRYKLDSDEGYEM  412
Bacteroidetes   338 IKNIst...TKFIPLLRE..GNDnnAIPDFLIGRTYIDM.......RN--DNSFEDRLEELLRNIYNEPKDK  395
Planctomycetes  110 ----.....TRFIPILKErdDQGnaCLPVFLGGRIYIDF.......SN--PDAYATRLEELTRVVFDKPKRI  163
Verrucomicrobi  110 ----.....NKFVPMVFD..RSDlqYIPMPLRDVTRIVI.......TD---GYDGLVR--RLLDIPPAEAPP  158
Proteobacteria   99 ----.....NKIIPV-IR..QEAgpELPTFLKTKLYIDFsndsdveYS--LDE--LLR--TLLKAPLFEKPE  152
unknown          87 ----.....EGAIITQEI..YDAatNNVQFIPVLFSSQD.......SV--HIPIILRGATYYRLDTEEGYED  138
Chordata        126 ----.....VRFEGKVIY..GQIanGRNLNKFIPVFFDK.......IN--LNHVPQFLRGAHMYGPIEDPPE  177
Firmicutes      103 ----.....NKFIAAFFD..DDDqqYIPTPLKPYTYYNL.......SD--EKQYDRLYWRFRGVTNSKKPPL  154
Actinobacteria  111 LHSNratwlRRLLPVVLP..GRSvdDIPLFLQPSCADHY.......QL--SALNSASIAELLQVLTERPTHR  171
position             .         90    .   100       .  110             .  120    .  130    .  140
```

```
Group1
Group34              85 LYRD.....EKFIPVVLP..GES..DLPTFLKGRIYIDL.......SDRSDDEYELLFERLLLRILDGPLPP  140*
WP_011689320.1      104 ----.....RKFVPVLLG..EATreDIPVPLQGVSN---.......HR--ADEPEGYA--SLYGTLTGAPRV  150
WP_012509009.1      426 ----.....RKFIPVLAK..GSWdeSAPSWLKGKFYVDLs......TP--VEDERDYP--RLLAALLNSHPQ  476
WP_013320651.1      361 ----.....EGAVISQEL..YDAqvKSTKFIPVLFSSED.......ED--YIPIVIRGFSRYKLDSDEGYEM  412
WP_015782906.1      338 IKNIst...TKFIPLLRE..GNDnnAIPDFLIGRTYIDM.......RN--DNSFEDRLEELLRNIYNEPKDK  395
WP_011119720.1      110 ----.....TRFIPILKErdDQGnaCLPVFLGGRIYIDF.......SN--PDAYATRLEELTRVVFDKPKRI  163
WP_068768974.1      110 ----.....NKFVPMVFD..RSDlqYIPMPLRDVTRIVI.......TD---GYDGLVR--RLLDIPPAEAPP  158
WP_068987332.1       99 ----.....NKIIPV-IR..QEAgpELPTFLKTKLYIDFsndsdveYS--LDE--LLR--TLLKAPLFEKPE  152
OGG46781.1           87 ----.....EGAIITQEI..YDAatNNVQFIPVLFSSQD.......SV--HIPIILRGATYYRLDTEEGYED  138
XP_019620788.1      126 ----.....VRFEGKVIY..GQIanGRNLNKFIPVFFDK.......IN--LNHVPQFLRGAHMYGPIEDPPE  177
WP_016296840.1      103 ----.....NKFIAAFFD..DDDqqYIPTPLKPYTYYNL.......SD--EKQYDRLYWRFRGVTNSKKPPL  154
WP_020667457.1      111 LHSNratwlRRLLPVVLP..GRSvdDIPLFLQPSCADHY.......QL--SALNSASIAELLQVLTERPTHR  171
foreground (26267):     AEGG     KKVVPVFYG  DDP  SDVRKQLGSFGAAF        KHYEDWPEAD DKRWKEAR AVANL  inf
                        KLRK      VLLLLLE   PI   P PP LP  LR LL          LK   EE ELFLLRLL ELP
                        E K      RII I  D   VV   D L          D          E    D      RK
wt_res_freqs (8805):    1111     113153221  113  11211112111111        1111111111 11131121 11111
                        1111      11211121  11   1 11 21  21 12          11   11 11111122 111
                        1 1       123 3  1  21   1 1          1          1    1      11
insertions               1       11   111    11  111            2      211111111 21
deletions               1511     431111233  662  46454433222222       23139565544131223333333442  20.0
background (10023):       L        L  L           L   LLL LL  LL       L   L       L  LL LLL L    5.7
wt_res_freqs (9636):      1        1  1           1   111 11  11       1   1       1  11 111 1
```

```
Group34
Group34              85 LYRD.....EKFIPVVLP..GES..DLPTFLKGRIYIDL.......SDRSDDEYELLFERLLLRILDGPLPP  140*
WP_011689320.1      104 ----.....RKFVPVLLG..EATreDIPVPLQGVSN---.......HR--ADEPEGYA--SLYGTLTGAPRV  150
WP_012509009.1      426 ----.....RKFIPVLAK..GSWdeSAPSWLKGKFYVDLs......TP--VEDERDYP--RLLAALLNSHPQ  476
WP_013320651.1      361 ----.....EGAVISQEL..YDAqvKSTKFIPVLFSSED.......ED--YIPIVIRGFSRYKLDSDEGYEM  412
WP_015782906.1      338 IKNIst...TKFIPLLRE..GNDnnAIPDFLIGRTYIDM.......RN--DNSFEDRLEELLRNIYNEPKDK  395
WP_011119720.1      110 ----.....TRFIPILKErdDQGnaCLPVFLGGRIYIDF.......SN--PDAYATRLEELTRVVFDKPKRI  163
WP_068768974.1      110 ----.....NKFVPMVFD..RSDlqYIPMPLRDVTRIVI.......TD---GYDGLVR--RLLDIPPAEAPP  158
WP_068987332.1       99 ----.....NKIIPV-IR..QEAgpELPTFLKTKLYIDFsndsdveYS--LDE--LLR--TLLKAPLFEKPE  152
OGG46781.1           87 ----.....EGAIITQEI..YDAatNNVQFIPVLFSSQD.......SV--HIPIILRGATYYRLDTEEGYED  138
XP_019620788.1      126 ----.....VRFEGKVIY..GQIanGRNLNKFIPVFFDK.......IN--LNHVPQFLRGAHMYGPIEDPPE  177
WP_016296840.1      103 ----.....NKFIAAFFD..DDDqqYIPTPLKPYTYYNL.......SD--EKQYDRLYWRFRGVTNSKKPPL  154
WP_020667457.1      111 LHSNratwlRRLLPVVLP..GRSvdDIPLFLQPSCADHY.......QL--SALNSASIAELLQVLTERPTHR  171
foreground (226):       DSDT     NKFVPVVRE  GSS  DVPAYYGGKTYYDF       SNTSSDEEAGNYETDVRRATTGPKKV  60.2
                        LYRD     RRIL ILLP  PER  EL TFLRPRL I L       RDR P  YELLF RLLLWILDK PPP
                         HEN     E VI  IIR    E   I     KS I V          E    S   KR     I E LR
wt_res_freqs (104):     1211     133154211  211  11711112113141       21281211111211121111112111
                        3233     1212 2221  111  12 2451131 3 3       116 1  22111 135211111 111
                         112     1 14  111    1   2     12 1 1            1    1   11     1 1 21
insertions                 8     82  4 3     29  1     31 11   5      1   29  21      1   1
deletions               8888     1    381   11          1    3         3993 2212 1 331               13.8
background (26041):     AEGG     KKVVLVFYG  DDP  SDPRKQLGSFGAAF        KHYEDWPEAD DKRWKEAR AVANL  60.2
                        KLRK      ILLPLLLE  PI   P VP LP  LRKLL          LK   EE ELFLLRLL ELP
                        E K       VII I  D  VV   D L          D          E    D      RK
wt_res_freqs (8701):    1111     113113221  113  11111112111111        1111111111 11131121 11111
                        1111      11251121  11   1 21 21  21112          11   11 11111122 111
                        1 1       123 3  1  21   1 1          1          1    1      11
position                 .         90    .   100       .  110             .  120    .  130    .  140
```
