# Supplementary material for: A survey of TIR domain sequence and structure divergence
Source: Immunogenetics. 2020 Jan 30;72(3):181–203. doi: 10.1007/s00251-020-01157-7 (PMC7075850; doi:10.1007/s00251-020-01157-7)
Supplement: Supplementary file 3 — (PDF 424 kb) [file 251_2020_1157_MOESM3_ESM.pdf]

Supplemental Figure 3. Group 37 (TRIF-related TIR domains) contrast alignments **(a)** and the list of annotated sequences that form Group 37 **(b)**.

(a)

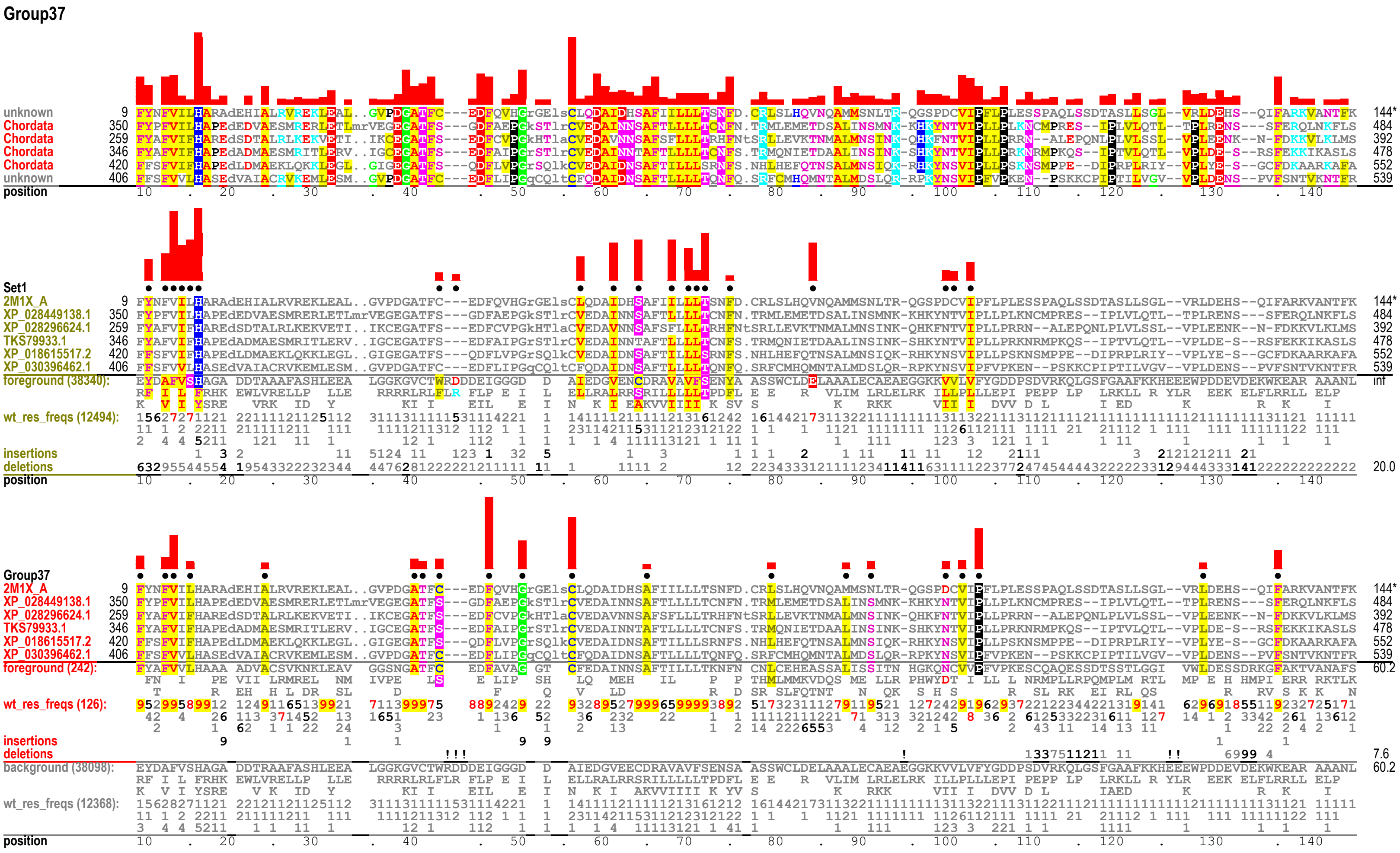

(b)

```
[0_(1)=1AD_fg(242){go=10000,gx=2000,pn=1000.0,lf=0,rf=0};BPPS=(R,6,4:0.8747):
(140)********************************************************************************
************************************************************

$1=154(158):
>2M1X_A Chain A, TICAM-1 TIR domain structure
{(MESSSEQK)FYNFVILHARAdEHIALRVREKLEALGVPDGATFC---EDFQVHGrGElsCLQDAIDHSAFIILLLTSNFDCRL
SLHQVNQAMMSNLTR-QGSPDCVIPFLPLESSPAQLSSDTASLLSGL--VRLDEHS--QIFARKVANTFK(PHRLQARKAM)}*

$2=293(297):
>XP_027015434.1  {|159(1234273)|<Chordata(M)>}TIR domain-containing adapter molecule
1-like [Tachysurus fulvidraco]
{(EHLAGLKVTPCLDTHGTSEIVTPKSPRIESSLEKTFLSSPSGDGKLGAQPVSTSQPHKASSTQPDSFSTEKRASQEKEDM)FY
AFVILHAQEdFEEADRLKTRLESLSSTTGATFA---EDFALPGfSNfrCVEHAIENSAYIMLLLTANFNTHLNETNTDSVLMNSI
EK-PHKYNTVIPLLPRTNCLTR--DQMRPILRTK--NPLDETKDRDIFEKMVRKVLD(SKNIERQKSMWRHAQLLKKQREHHQRL
LEERQYHMNIIRETTRVRELEEQVQRMKMQEQHLPPPNAHQSYSYQGFNG)}*

$3=296(300):
>XP_027403126.1  {|357(30522)|<Chordata(M)>}LOW QUALITY PROTEIN: TIR domain-containing
adapter molecule 1 [Bos indicus x Bos taurus]
{(FPSASTSPFPSPSTPPEAHSTPSKAHPTPSKAHPTPPKAHSTPPKAHPTSPKAHPTSPKAHPTIWNPEPPPPELESSEQK)FY
NFVVLHASAdEHIALRVRERLEALGVHDGATFC---EDFQVPGrGElhCLQDALDHSAFIILLLTSNFDCRLSQHQTNQSLMSSL
TR-HGWQDCVIPFLPLESSLAQLSPSTSSLLTGL--VLLDEHS--KIFARKVTNTFK(PQMLRARKAKWRKEQDARALREQSQQL
ESERQHAAAWGAAYSAYVHSYLAYQTQVEKLQVALANYMPFGTQLPFEGQGSL)}*

$4=296(300):
>XP_027442601.1  {|363(9704)|<Chordata(M)>}TIR domain-containing adapter molecule 1
[Zalophus californianus]
{(VLAAPKSLSLPSRNTCPDKDQPPLPLPGEDITSQVASPCPPAPSALRTSPPCPASSTPSSTGLASSSPCPPSPELESEQK)FY
NFVILHVAAdEHIALRVRERLEALGVPDGATFC---EDFQVPGrGElrCLQDAIEHSAFTILLLTPNFDCHLGQHQAGHSLMSSL
TR-PGWQDCVIPFLPLESSQAQLSSHTSSLLIGL--VWLVEHS--RIFARRVTNTFK(PQTLRARKAHWRKEQDVRALQEQRRHL
EGERQRVAALNAAYSADVQNCLSWQAQMETLRAAFGSHMPFGTQVPPGGPGPL)}*

$5=290(297):
>RVE61834.1  {|247(123683)|<Chordata(M)>}hypothetical protein OJAV_G00174280 [Oryzias
javanicus]
{(TKMAPGQPLLSLLQPTGSTLSDPERSHPTLPIGSLERHPDTPRSHTNPPSSTNICGSRCPIQKKIHDSKGSDEEEEEEET)FY
AFVILHAPEdEDVAESIKDKIEKVISSKGATFS---EEFAIPGkCPlrCVEDAINNSAFTFLLLTCNFNSNLLEMKANIALMNAI
KK-NHKFNTVIPLLPRENCMPEHRR--PMALSGL--VSLVENK--N-FEKKLQKSLS(QARIQSHKRKWKEEQALRLTERLSQLQ
LEEKQRLRTGLSLNQEESGEDGRTWQQKHPSIHIENANYIVIGNDSKMVL)}*

$6=296(300):
>XP_004441454.1  {|298(73337)|<Chordata(M)>}PREDICTED: TIR domain-containing adapter
molecule 1 [Ceratotherium simum simum]
{(ECTEVSAAPPSLPSPSVKDQTPPQLPAEDTPSPPAQPCPPTPSGPTTSPPGPAPCTPRLAHPAPVPLGATPPETESSEQK)FY
NFVVLHAGAdELIALRVRERLEALGVPDGATFC---EDFQVPGrGAlrCLQDAIDHSAFTILLLTANFDCRLSVHQVSQALMSSL
TR-HGWQDCVIPFLPLESSPAQLSPDTAGLLTGL--VWLDEHS--RIFPRKVASTFR(AQQLRARRASWRKEQGARALRAQSQHL
EGERRQGAAWSAAYSAYLQSYLAWQAQMGQLQAAFGSHLSLGTQGPYGGQVPL)}*

$7=293(299):
>EOA95601.1  {|333(8839)|<Chordata(M)>}TIR domain-containing adapter molecule 1, partial
[Anas platyrhynchos]
{(PTKDSSVPVSTSSSAPASTSACSFPPHTYSSSTPSPPLYATPYSLPHPSPFRSPPSPACPPPFQTAKAASAAEPDSGEGK)FF
TFVILHAGEdERVACRVKNLLEGMGVPNGATFC---EDFLVAGhGRltCFQDALENSAFIILLLTKNFLCHLCMFETNSALMESI
```

QR-PSKHNSVIPFVPKEN--PLERSEIPSILSVL--VPLDENS--PGFAKVVKNTFT(HRKINERKAMWCQIQQVREHKRRQQLF
KDHYQTLQNLGALNLGSFPQVPLSETPWQQLGQFVPHQLSQQSCPPAATYPP)}*

$8=296(300):
>XP_004464287.1 {|294(9361)|<Chordata(M)>}TIR domain-containing adapter molecule 1
[Dasypus novemcinctus]
{(PNSLPSPAPEPPENPRPANARTPLPLSVEDTTSQEAMLCPPTPSAPKTSPVSPPLPPPCSPAPFSSPCPPPPELEAWEQK)FY
NFVILHARVdEHVALRVRERLEALGVPDGATFC---EDFQVPGrGElrCLQDAIDHSAFTVLLLTSNFDCRLSLHQVSQALMGSL
TR-HGWHDSVIPFLPLETSRAQLGPDTAGLLAGL--VMLDEHS--PIFARRVDNTFR(ARTLRARRATWEREQEARAVREQSQRL
EGERQRAAALSSAYSAYCRSYLAWQGQMEALQAAFGAQMSFGTQVPSGVPGPG)}*

$9=295(299):
>XP_004595948.1 {|303(9978)|<Chordata(M)>}PREDICTED: TIR domain-containing adapter
molecule 1 [Ochotona princeps]
{(NPCCTPLRSLEPPQITGSPKEQEGPLQEPMGHFKSQDTEPCPPASCTPQTSSPLAPPPSSSSSSSPAPSPSPAPCKPEQK)FY
SFVVLHAKAdEDIAVRVRDKLEALGVPDGATFC---EHFQVPGyGElsCVQNAIDHSAFVIFLLTPNFDCQLGLHQVSHALMNSL
TR-RGWENCVIPFLPQGISQAQLAPHTVSILSSV--VWLREDS--KIFAKIVANTFS(TKTLQARKALWRIDQDSRALQEQNQQL
EAQQQRVAAMITSYATYMQNYMSFQAQMNKLPTAFASHVSLGAGAPADRYLG)}*

$10=296(300):
>XP_004619550.1 {|310(42254)|<Chordata(M)>}PREDICTED: TIR domain-containing adapter
molecule 1 [Sorex araneus]
{(APTGSENTSPNRKLRAGSVSPNPDRKSSQAPSADPPAPSAASSVPQSPSPWSSPESPSGAPLPSSAASPAPAPSEPQGQK)FY
NFVVLHTRAdEQVALRVRQRLEDLGVADGATFC---EDFQLPGrGElsCLQDALDHSAFTILLLTPNFNCRRSLYQLNQAVIGSL
TR-QGWRDSALPFLPLESSPDQLSPDTARLLAGL--VWLEERS--PVFAQRVSRTFR(SQLLRERREHWRQEQEMRALREQCQQL
QVEEQRARTWAAEYSAYIQRYMAFCQQMEQQQVELGKYTAWGARDPQTPPPAF)}*

$11=296(300):
>XP_004655027.1 {|313(51337)|<Chordata(M)>}PREDICTED: TIR domain-containing adapter
molecule 1 [Jaculus jaculus]
{(LSTLECTEKTSDASKQTPLQDSIREDTLQNSTLGAPAPPPPPQNFSSAPPSSFSSSCPVPPASSSHCPSPCHPDTSSEQK)FY
NFVILHARAdEHVALRVRDKLEALGVPDGATFC---EDFQLPGrGElrCLQDAIDHSGFTILLLTANYDCRLSLHQVNQALMNSF
TQ-SGRQDCAIPFLPLECSRAQLSRDTSGLLESL--VWLDEHS--PIFERKVANTFK(LQKLRIYRERWKKEQDARVLREHSQRL
DAERRQVAAMHAAHSAYAHSYVALQEQVQKLSAAFGDHLSLGTRMSYSGSQVP)}*

$12=296(300):
>XP_004689386.1 {|297(143302)|<Chordata(M)>}PREDICTED: TIR domain-containing adapter
molecule 1 [Condylura cristata]
{(CTEAPESLPSPSRSASSVADQTPLQGPVEDSTCRAAPPRPPAPQAQVPPPCSPSPAPSSAPPAPPEPCAPTSKVESQGQK)FY
NFVVLHARAdEHVALRVREKLEGLGVSDGATFC---EEFQVPGrGQlhCLQDALQHSAFTILLLTPNFDCHLSRYQVNQSVVSSL
LR-HDWHDSVIPFLPEESAQDQLSPKACGLLKGM--VWLDESS--PVFHRKVANTFK(PQQLQRRRELWKREQDAQALREQRQRL
EQERQHVGALSSAYAAYARSQQALQAQVEQLRAALANVLPPGAQGPAAPHVPP)}*

$13=296(300):
>XP_004771537.1 {|344(9669)|<Chordata(M)>}PREDICTED: TIR domain-containing adapter
molecule 1 [Mustela putorius furo]
{(PKSLSLASRNPCHDKDQTPLPLPVEDTASQVASPRPPAPPAPPTSSRGPPPSNLPPSTLPSPGLASSPCPPSPELESEQR)FY
NFVVLHVAAdEHIALRVRERLEALGVPDGATFC---EDFQVPGrGElhCLQDAIDHSAFTVLLLTPSFDCHLGRHQASQSLMSSF
TR-RGWQDCVIPFLPRESSRAQLSPHTSSLLTGL--VWLDEHS--QIFARKVANTFK(PQRLRARKAEWKKEQEVRALQEQRSHL
EGERQQVAKLNAAYSAYFQSCWSWQEQMEALRAAFGSHMPFGTQMPPGGLGPL)}*

$14=294(300):
>XP_012810436.1 {|242(8364)|<Chordata(M)>}PREDICTED: TIR domain-containing adapter
molecule 1-like isoform X1 [Xenopus tropicalis]
{(VYSVTGYSSISTNKQRKPEPVVFKTSIKSEIECSERQKPLYEAHPRNLNCDSNSVPSLKNNFFYPIPPQCCTYTVPDDSL)FF
NFVVLHVREdSEVARRVCDVLQSLGAGNGTTFC---EGFEIPGsNPltCIQEAVENSAYIILLMTEHFETRWAEFQSNAVLMNSI

ND-ENRTGCVIPFLPETNRLPIKK--MPLALKSL--IPLDELS--PVFRRAVCNTFK(QEKILSQKQHWERQQQKKKERQRMLEL
QRSEQANDSGQYLMHMCANEGNVNLSAQYMYQHPIPYAPVIHINNAENIQIGN)}*

$15=296(300):
>XP_004865772.1 {|306(10181)|<Chordata(M)>}TIR domain-containing adapter molecule 1
[Heterocephalus glaber]
{(SPDPEPTESLCPVKDQRAPQLSVEDTTSQDSKPSPPMSLAPETSLPLPPAASPSSGSEALPASSRPLCPSSFQSEASEQK)FY
NFVVLHALAdEHVALRVRERLEALGVPDGATFW---EDFQVPGrGElrCLQDAIDHSGFIILLMTPNFNSALSLHQVNQALTSSF
TH-HGRRDSVIPLLPLESAPGLQRSDTSRLLSGL--VWLDEHS--KIFARKVGNTFK(PQRLWEHRAHWQREQDARARLEARQRL
DSERQQAEALHAEHSAYLQSCLAWQAQMDKLKVDFGSHLSLGTQVPYPAQGLF)}*

$16=293(299):
>XP_027562012.1 {|348(114329)|<Chordata(M)>}TIR domain-containing adapter molecule 1
[Neopelma chrysocephalum]
{(STGESDIPAGIPCNSTSASISTCSLPPPPPTCSFSSTLPPPLQDPPSNFNPPPLHSSPSPAWPPPSLPAVDPESEPDGGK)FF
TFVVVHASEdELVAQRVKNRLESMGVSNGATLC---EDFSVAGrSRmgGFLEAMENSAFMILLLTKNFTCNLCLFQTDTALMQSI
QD-PSKHYSVIPFLPKEN--ALEHGQIRRMLSAL--VILDESS--PVFPRVVQNTFN(PKKISQKKAMWEQMQRRKLQLRWERQQ
AQQNLAALSLGRPSQVAPAATRPWPPEPSPQQWCPPNPTVPPFAGHPPPAQM)}*

$17=293(299):
>XP_027522403.1 {|349(415028)|<Chordata(M)>}TIR domain-containing adapter molecule 1
[Corapipo altera]
{(MGDSDIPAGIPCNSTSASISTCSLPPPPTCSFSSTFPPSLQESPSNLNPPPLRSSPSPAWPPPPLPAMDPESLSEPDGGK)FF
TFVVLHASEdEVVAQRVKNRLESMGVSNGATLC---EDFSVAGrSRmgGFLEAMENSAFMILLLTKNFICNLCLFQTDTALMQSI
QD-PAKHYSVIPFLPKENALEQ--GQIPRMLSAL--VILDESS--PLFSRVVSNTFN(PKKISQKKAMWEQMQRRKLQLCWEWQQ
AQQNLAALSLGRPSQVLPAATQPWPPEPSPQQWCPPNNPTLPPFAGHPPPAQ)}*

$18=293(299):
>XP_027580340.1 {|343(649802)|<Chordata(M)>}TIR domain-containing adapter molecule 1
[Pipra filicauda]
{(IGDSDIPAGIPCNSTSASVSTCSLPPPPACSFSSTLPPPLQEPPSNLNPPPLRSSPSPAWPPPSLPTVDPESLSEPDGGK)FF
TFVVLHASEdEIVAQRVKNRLESLGVSNGATLC---EDFSVAGrSRmgGFLEAMENSAFTILLLTKNFMCNLCLFQTDTALMQSI
QD-PSKHYSVIPFLPKENALEQ--GQIPRMLSAL--VTLDESS--PLFPRVVSNTFN(PKKISQKKAMWEQMQRRKLQLLWERQQ
AQQNLAALSLGRPSQAFPAATRPWPPEPSPQQWCPPNNPMVPPFAGHPPPAQ)}*

$19=296(300):
>XP_027625410.1 {|227(246437)|<Chordata(M)>}LOW QUALITY PROTEIN: TIR domain-containing
adapter molecule 1, partial [Tupaia chinensis]
{(ESPQNPCLVQDQTPLELSVDETTSQNTQPQPPASGSSSGPGSSASATEPSRASSTKSSACPAPSAPHPTSPAAPESSEPK)FY
SFVVLHARAdEHIALRVRERLEELGVPHGATFC---EDFQVPGrGElrCLQDAIDHSAFTVLLLTPNFDCRLSLHQINQAVMSSL
TR-HGWQDGVIPFLPLETSQAQLGPDAASLLGGL--VWLDQHS--PIVSRRVTNTFK(PQKLRARKAYWEKEQEARALRAQTQHL
EGARLQAAATHAAYAAYVQSYASWQAQMAQLQAAFGSHMALETQMPSGVQGPF)}*

$20=293(299):
>XP_027759422.1 {|336(164674)|<Chordata(M)>}TIR domain-containing adapter molecule 1
[Empidonax traillii]
{(AAGTDMPAGIPCNSTSASIPTRSFPPPPTQSLSSTVPPPLQEPPSNFTPPPLCSSPSPAWPPPPLPAVDPVSLSEPDGGK)FF
TFVVLHASEdEVVAQRVKKLLESMGVSNGATLC---EDFSVAGrSHmaGFLEAMENSAFMILLLTKNFTSNLCLFQTDTALMQSI
HD-PSKHYSVIPFLPKANALEQ--SQIPRMLSAL--VTLKESS--PLFPKVVHNTFN(PKKISQKKAMWEQMQRRKLQLRWEEQQ
AQKNLAALSLGLPSQVPPAVTQPWPPEPSQQWCPHTPTVPPSAGHHPQAQMG)}*

$21=293(299):
>XP_027706781.1 {|328(29139)|<Chordata(M)>}TIR domain-containing adapter molecule 1
[Vombatus ursinus]
{(PASVKPAETLVSKDSFRVLPELPPMSVPPAGEGKATPLPVQESDFPKKTTSPRPPISTQDPSPSPWSSLPSGPELDTGQR)FF
SFVILHAQEdEDIALRVRDTLESLGVPDGTTFC---EEFQVPGrFElrCLQDAIDNSAFTVLLLTKHFDCRMSLHQVHVALMNSL

TR-SEKENSVIPFFPRESTL--KSAKVSPLLKGL--VSLDESS--PVFSKKVSSTFN(PRRLQAQREVWKKQQEIRAIQEQTQQM
AEDRERVSQKESALSDLAYHYSLLQQQLQNLTLAFPNQASFAQGYRMPMPHP)}*

$22=296(300):
>XP_027808270.1 {|355(93162)|<Chordata(M)>}TIR domain-containing adapter molecule 1 isoform X1 [Marmota flaviventris]
{(AGPEPLPLPTVEATESPGLVKERTPPQLSGEETAPQKSKPSPPPASAPAPTASLPSPLPPTPASASPCPSSFESASSEQK)FY
NFVVLHARAdEQVALRVREKLEALGVPDGATFC---EDFQVPGrGElrCLQDAIDHSGFTILLLTSNFNCRLSLHQVSQALMSSF
TQ-HGREDCVVPFLPLESSLEQLGPDTASLLTGL--VWLDENS--KVFTRKVANTFK(PQKLRARKAHWRKEQDVRALREHSQRL
EGERRQAAAMGAAYWDSLQSYRAWQSQVDRLQAAFGSHLSFATRVPHPSQVPL)}*

$23=293(299):
>RXM93555.1 {|329(7906)|<Chordata(M)>}TIR domain-containing adapter molecule 1 [Acipenser ruthenus]
{(CTADPVSDQLVTSKTGLQEESHELSALPEKVSSEEEKTSPPSIPPKTSVEESHPETKPSEPPRNTTPAPTAEQEDETENK)FF
SFVILHAREdADNAIRVQETLKQLGAGEGATFS---VDFEIPGkSPltCIQDAVNNSAFTLILLTTNFNSHWAQYKTNSVLMDSI
ER-RHKYNTVIPFLPRRHCLPRES--IPFALKCL--IPMEENK--SFFPKKVKKTFN(QKMIENQRALWSQEQKIKQQKEKEEQL
KDQNKNYELLQRAIVNVQLQQYMALQYTQMLQGIIPPQQLPGDPNPQAQYPP)}*

$24=291(297):
>RXN20798.1 {|229(84645)|<Chordata(M)>}TIR domain-containing adapter molecule 1-like protein [Labeo rohita]
{(LNKSTNICTTKQLPTAEANDARNGSSQHMTSKNTNLDFSSTPNNGEKLKAQPLTANNHKPTTQPNSFRSPEPSESDVDET)FY
DFVILHEAEdADEAHRLRDKLERIIRGVGATFS---DDFAEAGrSTlrCLEDAIENSAFTLLLLTQNFNSNLSAASADSAIVNSL
EN-HHKMNSVIPLLPRENRLLRKS--FPLVLQTK--VPLDESN--RTFERNALKAIA(PEKVAAQKKIWMNKQRCKKLVEEQKRQ
REENAINAELRREAEKLARLRLESEMQQSNMFYPQTAPMHSPPLHGAMHP)}*

$25=290(297):
>QAU56515.1 {|230(75356)|<Chordata(M)>}TIR-domain-containing adapter-inducing interferon-beta [Mylopharyngodon piceus]
{(RRPTTNIHGNNQLQTAEVNHATNSKQCVDRRGSSQVVTSKSTNINFSSSPNDANAKVLKPPTQANSFCSPEPSQSDVEET)FY
AFVILHEAEdADEAQRLRERLESIISAPGATFS---DDFAQPGrSTlrCIEDAIENSAFTLLLLTQNFKSNLSETTADSAIVNSL
EN-HHKLNSVIPLLPRENRLSRES--FPLVLRTK--VFLDE-S--RMFERNVLKAIT(PDKVANQKKIWLMEQRKKKLHEEQKRL
REENVRNAQLQRETEKLARLKLESEMQQKKMFHTASAPPPFNGAMYTQPG)}*

$26=294(300):
>GCF56572.1 {|295(143630)|<Chordata(M)>}hypothetical protein parPi_0019126 [Paroedura picta]
{(TEPSAMVAAAVQDPQGGTPPNMRTACRLQAGLAAKGCVQTLPKDSPSPCPSPGPEHASSSSDLPPATAASSTDSPGNEDQ)FF
MFVVVHANEdERIACRVRDLLEKLGVRNGATFC---EDFLVPGhSQlaCFQDALDNSAFTLLLLTEHFKSHLCTFQTNMALMNSF
TH-FVKTNSVIPFIPKEC--PLKKEEMPMTLAGL--VPLVENS--RVFEKTVKNTFK(LSEFEKKKFTWGMKQQLRRQERLREES
QDHWQIQQQLLALGLQPNYPAQVPFPSSPNCFPGLCQVPPGPVPPPFLSPTSF)}*

$27=290(297):
>XP_028284193.1 {|236(210632)|<Chordata(M)>}TIR domain-containing adapter molecule 1-like [Parambassis ranga]
{(AVPFRPEESLLCEPQPKSTGSPLLEPMKDSHMTHNQTPSLTNPPSTKPKPSLPSTANISLPTKPVPKESHKSAEEEEEEI)FY
AFVILHAPEdEEVAEHIKEKLESATSSVGATFS---ENFAIPGkSTisCVEDAINNSAFTFLLLTRNFNTRMLEMKTNVALINSI
HK-THKFNTVIPLLPRENCMPKQS--IPIVLQTI--VPLHEN---KSFERKIQKSLS(PAKIEIQRKIWTEETERLKRLKLQQDR
ASLLMEEKLVLGSSAPPEQDGGDDRLRWQPPTNIHIEHAKYIMIGNDSQM)}*

$28=292(297):
>XP_028449138.1 {|269(8167)|<Chordata(M)>}TIR domain-containing adapter molecule 1-like [Perca flavescens]
{(SSEPPMFGAKKHSKMDGTLAAEGSKSDSLITRWETLNRTTKPTAEPNFALPTATTFLPPKMPVPNEMHESKDAEEEEKPT)FY
PFVILHAPEdEDVAESMRERLETLmrVEGEGATFS---GDFAEPGkSTlrCVEDAINNSAFTLLLLTCNFNTRMLEMETDSALIN

SMNK-KHKYNTVIPLLPLKNCMPRES--IPLVLQTL--TPLRENS---SFERQLNKFLS(PVKIKNQRKIWTAEQTVKMQLERQE
RLKNLNQYQKQLIKECSTAQLLEKENLSLLMAQKLIFNPIVPPEQDGGDSSV)}*

$29=290(296):
>XP_028296624.1 {|178(441366)|<Chordata(M)>}TIR domain-containing adapter molecule 1-like [Gouania willdenowi]
{(SGTLIVTRSGPCEAITSPPSYPSQLEISVPFTSSIKQEKPGGGGLPDMTTPLEPPGPTKENLLLPKETQEEEVEEEEEV)FY
AFVIFHAREdSDTALRLKEKVETIIKCEGATFS---EDFCVPGkHTlaCVEDAVNNSAFSFLLLTRHFNtSRLLEVKTNMALMNS
INK-QHKFNTVIPLLPRRN--ALEPQNLPLVLSSL--VPLEENK--N-FDKKVLKLMS(PATIRRQRITWTKERRSSTPHNILRL
SLEDTARGGARGGASQWPHQSNICIQNANYVMIGNDSQMTVRGAGGDGAE)}*

$30=296(300):
>TEA33334.1 {|307(103600)|<Chordata(M)>}hypothetical protein DBR06_SOUSAS8010206 [Sousa chinensis]
{(PAAPKSLPSPSRNACPVADQTPIQLSEEDTTYPAAQPRPPTPSAPKTSPPFSSPSTPPKAHPTVSKPEPPPPELESPEQK)FY
NFVVLHAGAdEHIALRVRERLEALGVSDGATFC---EDFQVPGrGElhCLQDALDHSAFTILLLTSNFDCRLSQHQANQSLMSSL
TR-HGWQDCVIPFLPLESSLTQLSPSTSSLLTAL--VWLDEHS--PIFAKKVANTFK(SQKLRARKAKWRKEQDVRALQEHSQHL
EGERQQAAAWSAAQSAYFHNYLSYQRQMEKLQVAFRSYMPFETLQPPVLQAPF)}*

$31=295(299):
>XP_028375832.1 {|304(89673)|<Chordata(M)>}TIR domain-containing adapter molecule 1 [Phyllostomus discolor]
{(VSPAPKSLPSPSRNTCPVKDQIPLQIPIEDTPSSTAQPRPPTPLGPKASPSCPSLSTPCSAHPASNPRPPSPESESSEQK)FY
NFVVLHAKAdEHIALRVRDKLEALGVPNGATLC---EDFQVPGrSKlnCLQDATEHSAFTILLLTASFDCSLSLHQVNHSLMNSL
TR-PGWQGCVVPFLPLESSQAQLSPDTSKLLTSV--EWLDEHS--EIFARKVANTFN(PRKLRARKANWKKEQDARALQEQCQHL
EGERQQAAALNAAYSTYLQTYLSWHSQMDQLRAAFENSMAFGTQLPSGAQGP)}*

$32=245(260):
>VFV19469.1 {|350(191816)|<Chordata(M)>}tir domain-containing adapter molecule [Lynx pardinus]
{(VLAAPASLPLPPKTTCPDKDQTPLSLPVEDTASRRHPPCPPTPSAPRTSAPCPSPSIPPSACSAAWNPCPPPPELEPEQK)FY
NFVILHAGAdEDIALRVRERLESLGVPDGATFC---EDF--------QDALDHSAFTLLLLTPNFDCHLGLHQAGQSLMNSLTR-
HECGDCVIPFLPLESSLEQLSPHTRSLLTSL--VWLDERS--QIFARKPPAPPQ(SPGLQPLIIHHAQMVQLGLNNHMWNQRGTQ
APEDKTQGAE)}*

$33=294(300):
>TFK00140.1 {|309(55544)|<Chordata(M)>}fibrinogen-like protein A [Platysternon megacephalum]
{(SAVHCPVECTDPPTMVTAELQELPSLCTPGPASGSIRVPVEDSCVLTEKADTASVSSSGGPPPPQAAATPTQELDCGEKR)FF
SFVVLHASEdVAIACRVKEMLESMGVPDGATFC---EEFLIPGqCQltCFQDAIDNSAFTLLLLTQNFQSRFCMHQMNTALMDSL
QR-RPKYNSVIPFVPKEN--PSKKCPIPTMLVGV--VPLDENS--PVFSKTVKNTFR(LRRINEQKTMWSQLQHIQEQHRKQQQY
QEHLLMLQQNLVGLNLGSQPGYPSQMPLPGLLPYPAGIQQLLQQLVSSLQLQT)}*

$34=296(300):
>AAC28630.1 {|17(9606)|<Chordata(M)>}unknown, partial [Homo sapiens]
{(LPLPILEPVKNPCSVKDQTPLQLSVEDTTSPNTKPCPPTPTTPETSPPPPPPPPSSTPCSAHLTPSSLFPSSLESSSEQK)FY
NFVILHARAdEHIALRVREKLEALGVPDGATFC---EDFQVPGrGElsCLQDAIDHSAFIILLLTSNFDCRLSLHQVNQAMMSNL
TR-QGSPDCVIPFLPLESSPAQLSSDTASLLSGL--VRLDEHS--QIFARKVANTFK(PHRLQARKAMWRKEQDTRALREQSQHL
DGERMQAAALNAAYSAYLQSYLSYQAQMEQLQVAFGSHMSFGTGAPYGARMPF)}*

$35=293(299):
>XP_015155511.1 {|358(9031)|<Chordata(M)>}TIR domain-containing adapter molecule 1 isoform X1 [Gallus gallus]
{(PIEDSYIPAGTSNSAPASTSVCSFPPQTYFSSAILRPLQSIPYNVPFPPPLHSSPSPTGPPPLKTVEASLAPEPNGEKKK)FF
TFVVLHAWEdEHIACRIKDLLENMGVPNGATFC---EDFLVAGhNQltCFQDAMENSAFLILLLTKNFLCHQCMFQTNSALMESI
QR-PSKHNSVIPFVPKEN--PLERSQIPSMLSVL--VALDENS--PVFARTVQNTFT(PEKINERKAMWCQIQQVQEQKRKLELY
QDHCQTLQNLGALTLGSLPQMSPSAMQLNQSSLEQLLEQLLPLQSSQQCHPP)}*

$36=293(299):
>XP_028569816.1 {|406(64176)|<Chordata(M)>}LOW QUALITY PROTEIN: TIR domain-containing adapter molecule 1 [Podarcis muralis]
{(EPRSLVASEPQGCAGCPLQNSQAPSTPLPGLEPSALFVPRTVEDSSSLKRSLETGSSSSSGSPPPHSSGPPSTDLPVEQP)FF
TFVVVHAPEdETVACRVRERLEALGVAGGATVS---EDFLVPGhCQlrCFQDALDNSAFTLLLLTENFKSRFCVFQANVALMDSF
QR-FCKTNSVVPFIAKES--PMKRREMPFLLAAI--VPLDETS--PVFARRVKKTFS(PAVIREKRALWNISRQIRNQERLQEQQ
SEYQQARQRLSALRVSLQAPMPLQRGFLGLQTPPDHPFFYSEMPQPQLGPDQ)}*

$37=293(299):
>XP_028672432.1 {|283(27687)|<Chordata(M)>}TIR domain-containing adapter molecule 1 [Erpetoichthys calabaricus]
{(NSPPVDSKYVIAKGNSQPWMKNGIPVHRPPVPTPSESDESLGALKNGKESATKSAPEAILTPGPPDLPSDAQGDEVLDAE)FF
SFVLLHAPEdAEQAEEVQETLERLGAGKGATFS---QDFEQPGkCRlsCLQDAINNSAFTVILLTRNFNSRWAAYKTNSVLTESI
EN-RHKYNTVIPFMPKKRCASR--DDIPYALRCI--NALDEKK--PGFEKKVKKTFD(KNTIEKQRSLWLQEQRISQKNQKEALM
KDSNRNLQSLCEADLRLAAETKRTHDLLNSWQQQMYLQFQRPPFPPSAHCPL)}*

$38=262(266):
>AAH33406.1 {|0(10090)|<Chordata(M)>}Ticam1 protein, partial [Mus musculus]
{(PTRPSLQASPKLPPSPLSSASSPSSYPAPPTSTSPVLDHSETSDQK)FYNFVVIHARAdEQVALRIREKLETLGVPDGATFC-
--EEFQVPGrGElhCLQDAIDHSGFTILLLTASFDCSLSLHQINHALMNSLTQ-SGRQDCVIPLLPLECSQAQLSPDTTRLLHSI
--VWLDEHS--PIFARKVANTFK(TQKLQAQRVRWKKAQEARTLKEQSIQLEAERQNVAAISAAYTAYVHSYRAWQAEMNKLGVA
FGKNLSLGTPTPSWPGCPQ)}*

$39=287(293):
>XP_028856297.1 {|247(299321)|<Chordata(M)>}TIR domain-containing adapter molecule 1-like [Denticeps clupeoides]
{(DKSTKMTSTPGLQPDRLPAKMFLPCPIRPASGELVTSKSRNIGPDIPPPGAKRETSHPPTPPLGPGAQEPALEESEEALQ)FY
SFVILHAQEdEDVAEEFKEKFEGMDISPGANFS---TDFAVAGkSPlaCLEDAIENSAFVILLLTRNYSSRMHELETNTALINAV
EN-PSRTNSVIPLLPRKNSLPREC--MPKVLRAI--QPLYEIR--KNFISSAKKAMD(SAMVAKRKRLWVQAQNKKKQAQCPNPW
LPYYPGPSNLPGCTNISINNAKCIMIGNNSTMTIGDPAVIEDEGGF)}*

$40=289(296):
>QCD16644.1 {|269(215358)|<Chordata(M)>}TIR domain-containing adaptor inducing IFN-Beta [Larimichthys crocea]
{(SEEPQLKSNEPKKLPRTDETFPAENIKLDSLTSPTKTQKPTTETNLVRPTATSIVLPMMPASNESPEEEDEEEEEKEEAQ)FY
AFVIFHAPEdADMAESMRITLESVIGCEGATFS---EDFAIPGrSTlrCVEDAINNTAFTLLLLTCNFSTRMQNIETDAALINSI
NK-THKYNTVIPLLPRKNRMPRQS--MPTVLQTL--VPLDE-S--RSFEKKIKASLS(PARIRKQKRIWSEEQTVKKQRERQERL
KQLCQHQKQLIKECKAARLLEKENFRLMKQQQQLLCPNVPPEQDGGDGR)}*

$41=293(299):
>XP_028906958.1 {|278(9258)|<Chordata(M)>}TIR domain-containing adapter molecule 1 [Ornithorhynchus anatinus]
{(PTPGTGAAGDLELSGEDLPRHQLASTPGPVGPSPLSPPASLPPSSPSAPDPSPLLPPSLSGPLPAVDPRPSSLPGDGERR)FF
SFVVLHAPGdEAIALRVQAKLERLGVPDGATFC---EDFLQPGkLElsCLQEAIDHSAFTVLLLTDNYNCRQSLFQVHTALHHSI
SR-RDKRDSVVPFLPRESQ--RRGSEVLALLSGL--VPLDERS--PVFQRKVTRTFT(AQRLREQRKIWSQEQAIRVLQERSLRL
EEERRREAQRREAEDGYLRQRSLRERVAGMSLEPQLNPSLAAPATPAAPPGH)}*

$42=289(297):
>XP_010882521.3 {|311(8010)|<Chordata(M)>}TIR domain-containing adapter molecule 1 [Esox lucius]
{(VSLSPGSSTPAHIKIGNPMSLNGHDKVCPTKNLVPPSNTSRPASHPTTLENSSTLGVPLTEQVSASAALIPPLLEEEEED)FF
SFVVLHAQEdVAMAEMLTEKLEATGVGKGATFQ---E-FAIPGkNLlmCVEDAINNSAFTILLLTSNFNSRLLEVEASSALMNAI
QN-RHKYNTVIPFLPRENCMP--NEKMPSVLRIY--IPLRE-D--KTFEKKAKRAMT(PAKIAMQRQLWLTEQAVRAEVKRRERL
QMEEKLRGDLIREQFVNQQVEMMRKHLSQQQVNELITPAGGHFAAQGLEN)}*

$43=196(200):

>ABH10662.1 {|0(66913)|<Chordata(M)>}TICAM, partial [Ictalurus furcatus]
{(KPPEASSIQEVGKHDSFSTEKRASQEEEDM)FYAFVILHAEEdSEDAVRLKSRLESISSTTGATFS---EDFAVPGqSTfrSV
EDAIENSAYVMLLLTPNFNTHLNETNADSALLNSIEK-PHKYNTVIPLLPRANGLTR--NQMPFILRTK--NPLDEmkDR--DIF
EKMAKKVLD(SRNIQRQKSVWRQAQLVKKQREKQQWLQEK)}*

$44=291(297):
>ABH10822.1 {|238(7955)|<Chordata(M)>}TRIF/TICAM1 [Danio rerio]
{(TNIFANSQAQTAVVNDTASSNRCRHDSRPSQFVTSKNTNFSSSPNDGAKPKAQPINANDRKPPTQDNSFYPTVSSDIDET)FY
AFVILHEAEdADEAQRLREKLEEIISANGATFS---EDFAQAGrSTlrCIEDAIDNSAFALLLLTQNFKSNQSMTTTDSAIVNSL
EH-HHKLNSVIPLLPRENRLSRK--NIPLVLQTK--VPLDESN--RTFERKALKALS(QDAVEKQRKFWMKKQTFKKLEEEYKRR
QEENFINANLRLEREKLARLKLESESPHNNMFHSSSAQPPSCGPMLQQHD)}*

$45=293(297):
>NP_001187154.1 {|165(7998)|<Chordata(M)>}TIR-containing adaptor molecule [Ictalurus
punctatus]
{(KFKFRQYPDKHDTSEIVTPKSPNIESGLEKTFLSIPSGGGKVGAQPVSTSKPPEASSTQEVGKHDSFSTEKQASQEEEDM)FY
AFVILHAEEdSEEAVRLKSRLESISSTIGATFS---EDFAVPGqSTfrSVEDAIENSAYVMLLLTPNFNTHLNETNADSALMNSI
EK-PHKHNTVIPLLPRANGLTR--NQMPFILRTK--NPLVEtrDR--DTFEKMAKKVLD(LRNIQRQKSMWTEAQLVKKQREKQQ
WLQEKKRYCKDFIQESPRVRELEEQIQQLKMQQQHLQPPYAQQTNSHQGFPG)}*

$46=293(299):
>XP_007488996.1 {|328(13616)|<Chordata(M)>}PREDICTED: TIR domain-containing adapter
molecule 1 [Monodelphis domestica]
{(ASGKPSEALISKESSGLLPGLPPMSVPPVGEGEPTQFSVQESNCSEEVSDLNPPLSTQDPSPSPLSSSLISGPELDTGQR)FF
SFVVLHAQEdEAIALRVRDTLESLGVPDGATFC---EEFQVPGhFElrCLQDAIDNSAFTLLLLTRHFNCAMSLHQVHMALMNSI
TR-KNKKNSVIPFRPRES--KLKSTEVSQLLSGL--VILDESS--PVFSRKVNNTFN(PRMLKAQREAWKKEQEIRAIHKQTQQI
VEDRENISKKHSALFDYAYNYNLLHQQLQTLNLAFPNQAPFVQGYNMPLMYP)}*

$47=294(298):
>TKS27112.1 {|307(9838)|<Chordata(M)>}hypothetical protein Cadr_00026562 [Camelus
dromedarius]
{(LEAQAPTSLPSPSRSDPITDQTPLQLPEDTTYPTAQPCPPTPPAPKASPPFPSSSTPPSAHETVSSPGPPPPELESLEQK)FY
NFVVLHAGAdEHIALRVRERLEALGVQDGATFC---EDFQVPGrGElrCLQDALDHSAFTILLLTPNFDCRLSQHQANQSLMSSL
TR-HGWQDCVIPFLPLESSLAQLSPSTSSLLSGL--VWLDERS--QIFSRKVANTFK(PQKLRARKAKWKKEQDVRALREQSQHL
EGERQQASVWSAAYSSYVHSYLSYQTQMEKLQGRALEHGVIYFKVTAGETF)}*

$48=289(296):
>TKS79933.1 {|265(240159)|<Chordata(M)>}TIR domain-containing adapter molecule 1
[Collichthys lucidus]
{(SEEPQLKSNEPKKLPRTDETFPAENIKLDSLTSPTKTPKPTTETNLARPTATSIALPTMPASNESPEEEDEEEEEKEEAQ)FY
AFVIFHAPEdADMAESMRITLERVIGCEGATFS---EDFAIPGrSTlrCVEDAINNTAFTLLLLTCNFSTRMQNIETDAALINSI
NK-SHKYNTVIPLLPRKNRMPKQS--IPTVLQTL--VPLDE-S--RSFEKKIKASLS(PARIRKQKKIWSDEQTVKKQRERQERL
KQLCQHQKQLIKECKAARLLEKENFRLMKQQQQLLCPNVPPEQDGGDGR)}*

$49=289(296):
>XP_028988652.1 {|254(158456)|<Chordata(M)>}TIR domain-containing adapter molecule
1-like [Betta splendens]
{(EPQSKSMEPAQSKAKKNPMPDAASGTVCVKLDSHTGPNQNPKPSIELKPPIPNQPNVLVPHEMHESPSADKEEEEEEEDV)FY
AFVILHAPEdVDLAESMRDRVEAAvgGGSRGAVFS---EHFAVPGrSAlrCVEDAINNSAFTFLLLTRSFNTRMLELKTNTALIN
AINN-EHKYNTVIPLLPRQNPMPSL----PMVLATI--VPLQEDN--RL-EGKVKIAMT(PAKLRRLKRIWTEEQRVKHQLERQD
ALKQLNQTQKQLIQECNRAKVLQMENMRLRGLLIGSEEPGDGPARPHAHSI)}*

$50=292(299):
>XP_018615517.2 {|339(113540)|<Chordata(M)>}TIR domain-containing adapter molecule
1-like [Scleropages formosus]
{(ISSSLAPKSNDSKSQLRMESSVKQINKTSSFAPIFSTENKNTETQTIENAFSDISKTVLSQNKTENEKDKEDEEEEDEPI)FF
SFVIFHAPEdLDMAESKLQKKLEGLGIGEGATFS---QDFLVPGrSQlkCVEDAIDNSAFTILLLSRNFSNHLHEFQTNSALMNSI

QK-RHKYNSVIPLLPSKNSMPPE--DIPRPLRIY--VPLYE-S--GCFDKAARKAFA(PEMIRKQKQLWSKDQLIRKQQEKQDRL
KEKCERDKILAYEANKTLYLEKMRLYWLQQLQIQCSAMSQFPIPGPPHTIGC)}*

$51=296(300):
>XP_024425012.1 {|305(9430)|<Chordata(M)>}TIR domain-containing adapter molecule 1
[Desmodus rotundus]
{(SPAPKSLPLPSRNTCPVKDQMPLQSPVEDTPSSIAQPCPPTPSAPKTSPPCPSLSTPCPAHPASLNLGPPPPESESPEQQ)FY
SFVVLHAKAdEHIALRVREKLEALGVPDGATFC---EDFQVPGrSKlsCLQDAIEHSAFTILLLTANFDCRLSLHQVNHSLMNSL
TR-QGWQDCVVPFLPLESSLAQLSPDTSRLLTSV--VWLDERS--EIFARKVANTFK(PHKLRARKANWRREQDARALREQRQHL
EGERQQAAALNAAYSTYLQTYLSWHSQMEQLQVAFGNSMTFGTQLPSGAQVPC)}*

$52=293(299):
>XP_029141819.1 {|155(103944)|<Chordata(M)>}TIR domain-containing adapter molecule 1
[Protobothrops mucrosquamatus]
{(LPFLSTVPRNNSGALASGRWELTPPRRSSPVSIDSSSPPDNPQPLRSTGSPISYISNLQINLASPSSEPSSVVPPDDERQ)FY
TFVVLHAAEdELVACRVKERLEALGVSNGATFS---EDFLVPGcGQlnCFQNALDNSAFTLVLLTKNFTGRLCDYQRDTALMDSL
TR-CCKRNSVIPLVPKEH--ALKSGVMPLLLASL--VPLVEKS--PVFDKRVKKTFT(SRVIQEKKATWDFMRQIQEREEERERE
RENLRLQQRLAVLNLQPSPALPSVVDFSRPPTSFMEPGPDQASFSQPFMFSV)}*

$53=290(297):
>QCV57275.1 {|254(173339)|<Chordata(M)>}TIR domain-containing adaptor inducing
interferon-Beta [Trachinotus ovatus]
{(SSEEPQPNEPAKLEVRKDSKMDAAVKLDHHIIQNESPNQTTKPSTEPKCVVPTATNTCVPKAPAPDEMHECEGAEAEEEE)FY
AFVILHAPEdANVAESMKEKLERVAGCTGATFS---DDFAIPGkSTlrCVEDAINNSAYTLLLFTRNFNTEWVEMKTDTALVNSI
NK-KYKHNTVIPLLPRENCMPREG--IPMILRPI--IPLDE-S--KNFEKKIKRFLS(PTNIETQKRIWTEGQKVKLQIERQEKL
KQLNQCKKQLIQECNKAELQHIENLNLSLRQNLHLGPGVPLQLDGGDGMA)}*

$54=290(297):
>XP_029308550.1 {|256(56716)|<Chordata(M)>}TIR domain-containing adapter molecule
1-like [Cottoperca gobio]
{(ECEAKDALGQSHKSQAQSKSSEPPMFGATKHSKIEETLATKPTTKPNFALPTATNVVLPKMPAPKEMHESIDTDVEEEEI)FY
AFVIFHAPEdADTAESMREKLEKVISSEGATFS---GDFTIPGrSTlmSVEDAINNSAYTVLLLTRNFDTRMQEMETDSALINSI
KK-KHKDGTVIPLLPRENPMPKE--MLPMVLQTI--NPLEEN---KSFERKLQRALS(PAKIKKQKEIWTAEQKVKLQKKRQETL
KNLKQHQKQLMRESARAKLLEEENLMLMAQRLKLVHIVPQAPDGGDDRVR)}*

$55=290(297):
>TNM97751.1 {|261(433685)|<Chordata(M)>}hypothetical protein fugu_013997 [Takifugu
bimaculatus]
{(EAEASVLLVSKVDVKNASEPCQTSEEPLKSSQFAADRLVSHSFPGSTPTTGQSVAAPSTCVPTNMSAAADLCCVEEEEAI)FY
AFVIMHAPEdADVADCVREKVEKVIGCKGATFS---DDFHTPGkSTlrCVEDAINNTAFTFLLLTRNFNTRMVELETNSALINSL
YK-KHKWNTVIPLLPMENCMPK--REIPLVLQTL--VPLEE-S--KSFERKLSKSLT(LAKIKRQEKIWMEEQRVKIQKAQLQHQ
KQLKHCEKVQWQETKKLGQTIFHNPNLLGEDSAALWQMPPNIHIENANYI)}*

$56=290(297):
>XP_029380951.1 {|245(173247)|<Chordata(M)>}TIR domain-containing adapter molecule
1-like [Echeneis naucrates]
{(SNEYAHIEAKEHSVIGAAFEGPCKNINNHIIQNKTQPQTTEPSTEPKYSMSTTTSISEPKAPASSETYKSKCTEADDEVT)FY
AFVILHAQEdADLAESLKERIEKVASCTGATFS---EDFAIPGkSTlrCVEDAINNSAFTLLLFTRNFNTTLLEMKTDTALVNSI
NK-EYKHNTVVPLLPCENRMPK--QDIPMVVQRL--NALDEN---RNFESKIRKFLS(AAQIEKQKRFWTAEQRVRREKERQGDL
KHLNHCQKRLNQELHNVHLGPQKDAADDRARWQNQGSIHINNANYVMIGN)}*

$57=292(300):
>XP_029470442.1 {|317(194408)|<Chordata(M)>}TIR domain-containing adapter molecule 1
[Rhinatrema bivittatum]
{(TDITRTSLLVMPKDELEKGKVQRTAKASVGVSSNTVTTQRCPYSTVSPVEEMTALQQDADSKPNSSATLPLTEIDSNERT)FF
SFVILHAKGdEAIASKVRDTLGGLGILDGTTYC---EEFEIPGhSPltCIEDAVDNCAFIILLLTNRFVSRWALLQTNTVLMNSI

NK-DHKFNSVIPFLPREDPL----QEIPMVLDSI--VPLNEKL--PSFSRTVQKTFK(PERIQKMKEIWKQEEKVRELKRRRKVT
AKQVKYNQKINDATLALWMQQQKLHQMMPYMHLNALPFHLLQPQQLFPPGTQP)}*

$58=290(294):
>EPQ03069.1 {|194(109478)|<Chordata(M)>}TIR domain-containing adapter molecule 1
[Myotis brandtii]
{(APTSLPSPCRNTGPAEDQVPLQIPVEDTPSPAAQPCPPTSSAPETSPPNPSLSAPCSAHPASSSSSPCPPSPEFEPSEQQ)FF
NFVVLHAKAdEHIALRVREKFEALGVPDGATFC---EDFQVPGrSSlrCLQDAIDHSAFTVLLLTANFDCCLSQHQLSQSLMSSL
TR-PGWQDCVVPFLPLESSLAQLSPDTASLLAGL--VWLDEHS--QIFARKTYAAWQ(AQMEQLQAAFASHMTFGTQLPSGAPGP
LGAQPPFPTWPSYQPPTLPPWLAGTPSPTFPQPPVFPQASPVSPQSP)}*

$59=296(300):
>XP_003788814.1 {|306(30611)|<Chordata(M)>}TIR domain-containing adapter molecule 1
[Otolemur garnettii]
{(SSPILEPVGHPRPDKYQAPHQLSKEDTTTPDTKSCPPTPSIPKMSPPKTSPPLPPSAPCSAHLDNSSPCPSSSEVTPEQK)FY
NFVILHAKAdEHIALRVREKLEALGVSDGATFC---EDFQVPGrGElhCLQDAIDHSAFVILLLTSSFDCRLSLHQVSHALMSSF
MR-LGWQDCVVPFLPLESSPTQLRSDAASLLTSL--VWLDEHS--PIFARKVANTFK(SQKLLARKANWRKEQDARALREQSQHL
EGERMQAAALNNAYTTYLQNYLAWQAQMEQLQAAFGSHMSFGTRMPFGGGPVP)}*

$60=292(299):
>XP_005060299.1 {|347(59894)|<Chordata(M)>}PREDICTED: TIR domain-containing adapter
molecule 1 [Ficedula albicollis]
{(DTAPAHRSIEDCYIPGIPCNSAPAYISTCSLPPPTYSFSSTLPPLQESHSNLSHLPPLCSSPSPGSPALQDPAVDPSEPE)FF
TFVVLHASEdELVAHRVKNLLESMGVSNGATLS---E-FLIPGrSQmtCFQEAMENSAFKILLLTKNFPCNLCLFQTGTALIQSI
QD-PSKQDSVIPFLPRSN--ALEHSQIPGILSVL--VKLSESS--PFFSRNVHRTFS(PERIREKRAQWEQMQRRRLQAQQRLAA
LSLGSRPWAPPAPWPAEPSAQPWCPPAPMDPPPAPQQLPPGQYSIPAGQGGI)}*

$61=293(299):
>XP_005155258.1 {|353(13146)|<Chordata(M)>}PREDICTED: LOW QUALITY PROTEIN: TIR
domain-containing adapter molecule 1 [Melopsittacus undulatus]
{(SMEEPHVSGGLPLNSASVSISTHSLPPPPTYSFASTLPPPAGPRSSSPYPLRSHSSPSLAWPPPLQTAEPAPPSELDGGK)FF
TFVVLHASEdELVAHRVKDLLENMGVPNGATLC---ENFAIAGrSHmtCFQDAMENSAFVILLLTKNFPCDLCMFQTNTVLMESI
MN-PSKQDSVIPFIPQEN--PLEWSQIPAVLTGL--TPLDENS--PGFSRTVQNTFT(TSRINKKKDSWDMLQRKKLQLQEERHR
MLQNVAALNLGSISSSATQPPLLEQPPREWWSPAGGGPRPARMGPVQPPSGH)}*

$62=293(300):
>XP_005281227.1 {|322(8478)|<Chordata(M)>}TIR domain-containing adapter molecule 1
[Chrysemys picta bellii]
{(TMGTAELQERVVQKPEQESLAELPSVCTPGPASGSIRMPVEDSCVLTEKAETASFSSSGGLPPPQAAAAPTPELDCGEKR)FF
SFVVLHASEdVAIACRVKEMLESMGVPDGATFC---EEFFIPGqCQltCFQDAIDNSAFTLLLLTQNFQSHFYMHQMNTALMDSL
QR-LPKYNSVIPFLPKENPS---KCPIPSVLAGL--VPLDENS--PVFSKTVKNTFR(LKRISEQKAMWSQLQHIQEQHRKQQQY
QEHLQMLQQNLVGLNLGSQPGYPSQMPLPGLLPYPAGIQQLLQQLVSSLQLQT)}*

$63=296(300):
>NP_001302667.1 {|309(9823)|<Chordata(M)>}TIR domain-containing adapter molecule 1 [Sus
scrofa]
{(APKSLPSPSRNASPDVTDQKPPLHLSEEDTTYPTAQPHPPTPSVPQTSPPFPSPSTLSSAPPTVSNPSPPAPELELSEQK)FY
NFVVLHAGAdEHIALRVREKLESLGVRDGATFC---EDFQVPGrGQlrCLQDALDHSAFTILLLTPNFDCRLSQHQANQSLMSSL
TR-PGWQDCVIPFLPLESSLAQLSPGTSSLLSSL--VWLDEHS--QIFARKVANTFK(PQKLRARKAKWRKEQDARALREQSQHL
EGERQQAAAWGAACSAYIHSYLSYQTQLEKLQMAFASYMPFGAQLPSAPPAPF)}*

$64=296(300):
>XP_005405837.1 {|307(34839)|<Chordata(M)>}PREDICTED: TIR domain-containing adapter
molecule 1 [Chinchilla lanigera]
{(LPHPEPTESLCPVKDQTTPPISVEDTTPQETNPGPPASLAPETSLPLPPAPSPSSSSSVLPAPSCPPCPPSLESEASEQK)FY
NFVVLHAMAdEDVALRVREKLEALGVPDGATFC---EDFQVPGrGElrCLQDAIDHSGFIILLMTPNFNSALGLHQVNQALTSSF

TH-HGRYDSVIPFLPLESTPGLRGSDTSRLLSGL--VWLDEHS--KIFARKAGNTFK(PQRLWERRAHWQREQEARARLRGSQRL
DSERRQAEALHAAHAAYLRSYLGWQAQVDKLQVDFGSRLSLGTQVPYPPQGPF)}*

$65=296(300):
>ERE72238.1 {|271(10029)|<Chordata(M)>}TIR domain-containing adapter molecule 1-like protein [Cricetulus griseus]
{(TTEGIGKQPSITDQMSPPGSVGDDSLQNTTSSPAAQLQSPQFSTTLPPPPLPSASFPSSCPVPPASMPPILDHLETSEQK)FY
NFVVIHARAdEHVALRIREKLETLGVPDGATFC---EEFQVPGrGElhCLQDAIDHSGYTILLLTTNFDCRLSLHQVNHALMNSL
TK-SGRQDCVIPLLPLECSQAQLSPNTTSLLHSL--VWLDEHS--PIFARKVANTFK(SQKLQAYRIRWKKEQETRALKEQSTQL
EAERQRVAAMSAAYSTYVHSYSAWQAQMDSLQMAFGKDLVQTLLILFIHQHFL)}*

$66=290(297):
>XP_029541726.1 {|322(8023)|<Chordata(M)>}TIR domain-containing adapter molecule 1-like [Oncorhynchus nerka]
{(EHRTGSEVRVSPLSPGNFTPVQTKFDNPESPNAHSIPCPKNNLVPPSNNNRPTSHPTTTEPVSASAPLKTPNQEEEEEEV)FF
SFVILHAAEdVDMAEKFKEELESIVGGEGATFS---QDFAIPGrNTlmCVEDAINNSAFTILMLTRNFNTRLLEVETSSVLMNAI
EN-LHKYNTVIPFLPQENRMPRE--NMPKVLRIF--IPLEE-G--NAFERKAKRAMA(PARIAKQRRLWLSEQAVRAQVRRQERL
RLEERLQMDLIRGQETERMQRYLLQQQLSNQPSTPAMPHFTTDPFLGSTP)}*

$67=296(300):
>XP_029773543.1 {|331(37032)|<Chordata(M)>}TIR domain-containing adapter molecule 1 [Suricata suricatta]
{(GQAAPPPLPAPSRNTCPDKDQTPLSRPVEDTYSQRASPCPPPPSAPRPAAPCPPPSIPPPASPASWSPCPPPPELESEQK)FY
NFVILHAGAdENIALRVRERLEALGVPDGATFC---EDFQVPGrGHlhCLQDAIDHSAFTLLLLTPNFDCQLGLHQAGQSLMNSL
TR-RECQDCVVPFLPLESSLEQLSARTRGLLTSL--VWLDERS--QIFARKVANTFR(SQRLRARRAHWRREQDVRALREQHQQL
EGERQRVSALNAAYSAYYQSHAAWQAQMESLRVAFGSHMPFGTPGPLGAPPPF)}*

$68=293(299):
>TRZ11119.1 {|333(364589)|<Chordata(M)>}hypothetical protein HGM15179_015978 [Zosterops borbonicus]
{(AVDTAPAHRPTEDSSIPQGISCNSVPASISTCSLPPPTYSFTSTLPPLQEPHSNLFYPPPLHSIPSPAWSPPLPEPDGAK)FF
SFVVLHAREdEIVAQELKTQLENMGVPNGATLS---EDFFIAGrSHltCFQDAMENSAFIILLLTKNFPCNLCLFQTDTALMQSI
ME-PSKQDSVIPFLPKAN--ALERSQIPKRLSAL--VVLDESS--AHFCRNVRNTFN(PRKIREKKALWEQMQSRKLQVCWEQQQ
AQQNLAGLRLGSPPCVPPAEPPAQPWCPPAPRGPPPGQAQLPPGHYNITPGP)}*

$69=290(297):
>XP_029931358.1 {|305(586833)|<Chordata(M)>}TIR domain-containing adapter molecule 1-like [Myripristis murdjan]
{(PILFKEGRESKTGSQGPAGLAAEYRRLDRHVAQNVQDQTAVKTTELKSAAPTAANTCLPKINDPKEMHEREGTEEEEEAT)FY
SFVILHAQEdAEMAERMREKLESIIGAEGATFS---GDFEIPGkRTlmCVEDAINNSAFTILLLTRNFHTRLLEVETDSALINSI
NK-KHKYNTVIPLLPQENCMSRHS--MPIILQTV--VPLVEN---KTFDKRIKKALS(PKAIESQRKVWAEEQKVKEQIRRQERL
KLQNKNDEQFIREMRKVKMLEHERQRLFLQQLNVRGSPNIPGFPYSCMGP)}*

$70=290(297):
>XP_029970829.1 {|247(181472)|<Chordata(M)>}LOW QUALITY PROTEIN: TIR domain-containing adapter molecule 1-like [Salarias fasciatus]
{(ERSAPCEPEPRSQESAQCSAKKDSNVDETSAAVSCSSNPAPQKTDTQPRRAQPAVPDAPPAKTFTTRDTQEAAEEEDDDT)FY
AFVILHAPEdVDVAESMKEKVEGVVGEDGATFS---GDFAIPGrSTlkCVEDAINNSAFTLLLLTRSFNTRMLDVKTNTALMNAI
NN-EHKYNTVIPLLPRENCMPRPS--IPLVLQTI--VPLEEN---RAFERKIQKFLS(PAKINVHRRKWSEEQRRKRQERLRVSQ
NTQTDPGRAAEPAPPVPERWPLPQSICIQNAKYIMIGNDSQMTVSLGDGA)}*

$71=293(297):
>TSK53730.1 {|161(175774)|<Chordata(M)>}TIR domain-containing adapter molecule 1 [Bagarius yarrelli]
{(EQVSELKLTQCQDTHGTSEVVTPKSPRVEPSLEKTFLSSPSGDGKVGAQPVSNIQPSKAPFTQQEGFKKENHASQETEDM)FY
AFVILHAQEdFNEAVRLKNRLESISSTIGATFA---EDFEMPGySTfkCIEDAIENSAYIMLLLTTNFNDHLNETNTDSALMNSI

```
DK-THKYNTVIPLLPRTNGLSRE--QMRPVLKTK--NPLDETKDREFFQRMVRKVLD(SKNIQRQKLMWKKEQLKKKQKKQERSL
LQERQYQRDLIQGAKRVQEIEKELQEMKMQQQHLPPPNVYPSYSYQGFNR)}*

$72=291(297):
>XP_030015455.1  {|265(375764)|<Chordata(M)>}TIR domain-containing adapter molecule
1-like [Sphaeramia orbicularis]
{(HCQSSEESQLKSNDPQNSKTVTGSAAGSSKSGGHSIQLQNPNQASVPATHPKCALPSASNVTVLEKKLESLCAEEEEEVK)FY
SFVILHAPEdADVADRIKERIESAISCEGATFS---EDFSIPGkSTlmSIEDAISNSAYTLLLLTQNFiTSGLLEMKANSALINS
IYN-KHKRDTVIPLLPRENSIPRDS--IPLVLRTL--VALEENN--R-FDTRIQKALS(RGRIETQREVWEKEQKVKRMREKQER
LKVLNRKQEQVNEESRRIQQLEQEHLELLRQQRSRFLRPTDVPPGHHREDG)}*

$73=295(299):
>XP_003938874.1  {|310(39432)|<Chordata(M)>}PREDICTED: TIR domain-containing adapter
molecule 1 [Saimiri boliviensis boliviensis]
{(PQSLPSPILESLKKPCPVKDQTAPQLSVEDTTSPNTKPSPSAPTTPKTSLPPPPPSAPCSAHLAPSSPFPSSLESPSEQK)FY
NFVILHARAdEHIALRVREKLEALGVPDGATFC---EDFQVPGrGElrCLQDAIDHSAFIILLLTSNFDCHLSLHQVNQALVSNL
MR-QGPSDCVIPFLPLESSQAQLSSDTASLLSGL--VRLDEHS--PIFNRKVANTFT(SHKLQARRAMWRKEQDTRALREQSQHL
DSQRVHVAAMNSAYSAYLRSYLSWQAQMEQLQAAFGSHMSFGTGAPFGAQMP)}*

$74=291(299):
>XP_030073778.1  {|328(1415580)|<Chordata(M)>}TIR domain-containing adapter molecule 1
[Microcaecilia unicolor]
{(TDPSSTSQHVMLTDEPENGSGQRTDVTSVGVSSSTEIAQSSPSSTVPPADNNSTLQHNIDSKPISSATPLLTETVDSEST)FF
SFVVLHAKGdEAIAIKVRDTLKNFGIPDGTTYC---EEFEMPGySPltCIEDAVDNSAFTILLLTSHFVSRWARLQTNTVLMNSI
NK-KHKFNSVIPFLPKEDPL----KEIPMVLNSI--VPLNEKL--PTFLRTARKTFN(PYRIQQMKENWKQEQAVRELKRRNKIT
EKQVIYNQQIKDATLALSSQHLKLLQTMYNTPTFHLPGPQQPFQPGMPQIPG)}*

$75=293(299):
>XP_009095163.2  {|339(9135)|<Chordata(M)>}TIR domain-containing adapter molecule 1
[Serinus canaria]
{(PALGSIQNPYIPAGIPCNTARASISTCSLPPPTYSFSSTLPPLQQSPSKLSNPPPLHSSPSPARPPAPPATDPSEPDGAK)FF
TFVVLHASEdEIVAHQVKDLLESMGVSNGATLS---EDFFIAGrSHmiCFQEAMENSAFVILLLTKNFPCNLCLYQTDTALMQSI
MD-PSKQDSVIPFLPKAN--ALESSQIPRMLSAL--VTLNESS--PLFSKNVQKTFN(PKKIMEKKAMWDQRRKLQARWEQHRAL
QNLAALSLDSPPWVPPAAPRPWPPGPAAQPWCPHAPMDPPPAQRGPPPSQAQ)}*

$76=293(299):
>XP_002194292.2  {|339(59729)|<Chordata(M)>}TIR domain-containing adapter molecule 1
[Taeniopygia guttata]
{(TAPALVSVQDSNNPAGIPSNSAPASTSTCSLPPPNSSSTLPPLQESPSRVSYPPPLHSSPSPARPPAPPAMDPSEPDGAK)FF
TFVVLHASEdEIVAHQVKNLLEGMGVSNGATLS---EDFFIAGrSHmiCFQEAMENSAFMILLLTKNFPCNLCLYQTDTALMQSI
VD-PSKKDSVIPFLPKAN--ALEDSQIPGMLRVL--ITLKESS--PLFSKNVHKTFN(PKKISEKKALWDQMQRRKLQERREQHQ
AQQNLAALSLGSPPRVPPAVPRPWPPGPPAQHWCPPAPMEPPPAQRGTPPPQ)}*

$77=296(300):
>XP_030148660.1  {|342(61383)|<Chordata(M)>}TIR domain-containing adapter molecule 1
[Lynx canadensis]
{(RYPVECSHVLAAPASLPLPPKTTCPDKDQTPLSLPVEDTASRRHPPCPPTPSPSPSIPPSACSAAWNPCPPPPELEPEQK)FY
NFVILHAGAdEDIALRVRERLESLGVPDGATFC---EDFQVPGrGHlrCLQDALDHSAFTLLLLTPNFDCHLGLHQAGQSLMNSL
TR-HECGDCVIPFLPLESSLEQLSPHTRSLLTSL--VWLDERS--QIFARKVANTFK(PQRLRARRAHWRKEQDVRALQEQRQQL
EGERQRVSALNAAYSAYFQSHSAWQAQMETLRVAFGSHMPFGTPGPLGAPPPF)}*

$78=293(297):
>XP_030220008.1  {|262(8049)|<Chordata(M)>}TIR domain-containing adapter molecule
1-like [Gadus morhua]
{(SEASSMLTSENSSNGSPCSIATTDVNSPQEDEKQTTTTPSIHSKSPPAAPKSPSPKFAVPKKTDTGKKTEEEEEEEEVDT)FY
AFVILHAAEdVNMAEAMRERVESLwgGTLVGATFS---EDFAVPGrSTmkCIDDAIENSAFTILLLTNHFvRNRRFQVEAEAALI
```

HSIEH-RLKNHTVIPLLPRDNGMAKAA--LPMVLKTL--ILLEENS---SFDKKIRRVLS(AERLRTQESVWSKAQEVKKELRRQ
ERMKEANKHRKLLCDMYAATAKEQLEGFRVLVEKGGASPFPLPVPGWQQPQNI)}*

$79=288(293):
>XP_030257680.1 {|244(8175)|<Chordata(M)>}TIR domain-containing adapter molecule
1-like [Sparus aurata]
{(MLPGNKNAAGPSKSSEGPQSNKPSEKLLDILAARNIKLDNLIPPTQPTKPTSEPTFAQPAADVSKAAEEEEEEEEEEEEET)FY
AFVILHAPEdEDIAESIKERLEGIIGSEfeGATFS---DHFAVPGkSTlkCVEDAVNNTAFTLLLLTPNYNTHMQEVQTNSALIN
SIEN-KHKYNTVIPLLPRENGMPRQS--IPLVLRTL--VTLQE-S--RNFERMTLKSMS(RVRIKTQWRKWTEEQRVKKQKNRQE
RLKELNQREKQMIKVSKEARELEEERIRLLMEHKLVLGAGGPPGQVGG)}*

$80=293(299):
>XP_030365526.1 {|350(0)|<unknown(X)>}TIR domain-containing adapter molecule 1
[Strigops habroptila]
{(VFTEESHVPGGIPSNSASASISTRSLPPPTYSFSSTLPPLQGPHSNLPYPPRSHSSPSLAWPPPLQTAEPGPASEPDGGK)FF
TFVVLHASEdEIVAHRVKDLLENMGVPNGATLC---ENFAIAGrSHmtCFQDAMENSAFIILLLTKNFPCDLCMFQTNTALMESI
MN-PAKRDSVIPFIPEEN-PLQWS-QIPAVLGGL--TPLDEKS--PGFSRTVRNTFT(TSRINEKKDSWDLLQRKKLQLREERYR
TLPNVAALNLGSIPPSATRPPLLEHPPHQWCPPAGGGHRPAHMGSPPGQPPS)}*

$81=294(300):
>XP_030396462.1 {|325(0)|<unknown(X)>}TIR domain-containing adapter molecule 1
[Gopherus evgoodei]
{(TAELQEPGEHVVQKPEQESPTKLPSLCTAGPAAGSIRVPVEDSYVLTEKADTASFSSSGGPPPSQAAAAPTPELDCGEKR)FF
SFVVLHASEdVAIACRVKEMLESMGVPDGATFC---EDFLIPGqCQltCFQDAIDNSAFTLLLLTQNFQSRFCMHQMNTALMDSL
QR-RPKYNSVIPFVPKEN--PSKKCPIPTILVGV--VPLDENS--PVFSNTVKNTFR(LRRINELKAMWSQLQHIREQHRKQLQY
QEHLQMLQQNLAGLNLGSQPGYLSQMPLPGLLPYPAGVQQLLQQLLQLQASSL)}*

$82=294(300):
>XP_005495270.1 {|342(44394)|<Chordata(M)>}TIR domain-containing adapter molecule 1
[Zonotrichia albicollis]
{(TAPALTSIQGSYIPAAIPCNSASISTHSLPPPTYSFSSTLPPLQESPSKLSYPLPLPSSPSPARPLAPPATDPAEPDGAK)FF
SFVVLHASEdELVAHEVKDLLESMGVPNGATLS---EDFFIAGrSHmsCFQDAMENSAFIILLLTKNFACKLCLYHTDTALMQSI
LD-PSKADSVIPFLPKANTL--EDNKIPKMLSAL--VILDESS--PLFSRQVQNTFK(PKRITEKKAMWEQLQREKKAMWEQLQR
EKLQARWEQHQAQQNLAALSLGSPPWVPPAAAGPPWPPGPSAQPWCPPAPLDP)}*

$83=291(297):
>XP_005476265.1 {|253(8128)|<Chordata(M)>}TIR domain-containing adapter molecule 1
[Oreochromis niloticus]
{(VTEPPQSSEAQPKTTGSPPFDPKQDSSKYGTPAAAASLVSNESDTKSDNQTKTPSPKLPKMSVPGEIHESKAVEEEEEEI)FY
AFVIFHAPDdSDMAESMKEKIEAAIGGEfeGATFS----DFSIPGkTTikSMEDAINNSAFTLLLLTRNFNTCMLDLKANAAFVN
SINK-AHKYNTVIPLLPRENCMPK--QDMPMVLQGV--NPLEERK--N-FEKKIQKSLL(PAKIEKQRRIWKQEKAQKQTMKHLN
KISNVTISVEPNLRPGPPTAAWHPQPNIHIENANYIMIGNDSRMNVDLVGNA)}*

$84=293(299):
>XP_005429516.1 {|341(48883)|<Chordata(M)>}PREDICTED: TIR domain-containing adapter
molecule 1 [Geospiza fortis]
{(AAPALVSIQDSYIPAGIPCNSASISTCSLPPPTYSFSSTLPPLQESPFKLSYPPPLHSSPSPARPPAPPAMDPAEPDGAK)FF
TFVVLHASEdELVAHQVKDLLESMGVSNGATLS---EDFFIAGrSHmtCFQEAMENSAFIILLLTKNFPCKLCLYQTDTALMQSI
LD-PSKQDSVIPFLPKAN--ALECSQIPRMLSVL--VTLNESS--PLFPKNVQKTFN(PKKIGEKKAMWELLQRRKLQARWEQHQ
AQQNLAALSLGSPPWVPPAAPQPWPAGPAAQPWCPPAPMDPPPAQRGPPPSQ)}*

$85=294(300):
>XP_005504021.1 {|255(8932)|<Chordata(M)>}TIR domain-containing adapter molecule 1
isoform X1 [Columba livia]
{(HTSMGDSYVPAGTFNSTSAAISTCSLPPPTYSSSSTLASPLRRAPSNFSCPAPLHPSPCPAWPPPQTVEPMPTSEPDSGE)FF
SFVVLHASEdELVARRVKNVLEEMGVPNGAMLC---EDFSIAGrSRitCFQDALENSAFTILLLTKNFQCSLCMFQTDTALMESI

LN-PSKRDSVIPFVPKEN--PLERSQIPSVLGTL--TPLDENS--PMFSRTVHNTFK(PSRIKQRKDMWDLMQRRKLQLYKEQTL
QKLAALNLGSLPQVPPSATQPGLLEQSPHQWGPPTGHPTPAPVGPSPFQPLQL)}*

$86=291(297):
>XP_005721268.1 {|251(303518)|<Chordata(M)>}PREDICTED: TIR domain-containing adapter molecule 1-like [Pundamilia nyererei]
{(AQNVTEPPQSSEAQPKTTGSPPFDPKQDSSKYGAAASLVSNESDTKSDNQTKTPSPKLPKTSVPGEIHESKAVEEEEEEM)FY
AFVILHAPEdSDMAESIKEKIEAVIGGEfeGATFS----DFSIAGkTTikSMEDAINNSAFTLLLLTRNFNTCMLDLKANAALVN
SINK-AHKYNTVIPLLPRENCMPK--QDMPMVLQGV--NPLEERK--N-FEKKIQKSLL(PAKIKNQRWIWKQEKAQKQTMKHLN
KISNLTISVEPNLRPGPPTAAWHPQPNIHIENANYIMIGNDSRMNVDLGGNA)}*

$87=292(299):
>XP_005812562.1 {|260(8083)|<Chordata(M)>}TIR domain-containing adapter molecule 1-like [Xiphophorus maculatus]
{(IHCTSPQHTSTEISQFGAKKSIKIEEASMRSNESAAQPLKPYTQTKSSLPAATHIILPSSTGLKSTHISKVDEEEDEEDI)FY
AFVILHAPEdSDVAESMKEKLEAVIGIEGATFS---GDFAVPGkSTlrCVEDAVNNSAFTFLLLTRNFNSQMVEMKTNIALINSI
NN-AHKFNTVIPLLPLENCMPKE--QMPITLQTL--MPLDERK--N-FERKLKKAFN(KAKIEKQRKIWVQEQRMKRMRLKEKER
QRLYMEQQLVLGAAVQREQDSGDPGPQRLAHSIHIENANYIVIGNDSTMTVG)}*

$88=296(300):
>XP_006206437.1 {|307(30538)|<Chordata(M)>}PREDICTED: TIR domain-containing adapter molecule 1 [Vicugna pacos]
{(LEAQAPTSLPSPSRSDPITDQTPLQLPEDTTYPTAQPRPPTPPAPKASPPFPSSSTPPSAHETFSSPGPPPPELESSEQK)FY
NFVVLHAGAdEHIALRVRERLEALGVQDGATFC---EDFQVPGrGElrCLQDALDHSAFTILLLTPNFDCRLSQHQANQSLMSSL
TR-HGWQDCVIPFLPLESSQAQLSPSTSSLLTGL--VWLDEHS--QIFSRKVANTFK(PQKLRARKAKWKKEQDIRALREQSQHL
EGERQQASVWSAAYSSYAHSYLSYQTQMEKLQVAFRNYMPFGTQLPSGTQVPF)}*

$89=296(300):
>XP_006982311.1 {|317(230844)|<Chordata(M)>}PREDICTED: TIR domain-containing adapter molecule 1 [Peromyscus maniculatus bairdii]
{(GTGKQLSITSQLSPPGSVGDDSLQNTTSNPPAQPPSSQVPSTLPPSSSSPPSSNSSSSGCSAPPASASPVLGHLETSEQK)FY
NFVVIHARAdEHVALRIREKLETLGVPDGATFC---EEFQVPGrGElhCLQDAIDHSGYTILLLTTNFDCRLSLHQVNHALMNSL
TQ-SGRQDSVIPLLPLECSQAQLSPSATSLLHSL--VWLDEHS--PIFARKVANTFK(PQKLQAHRIRWKKKQETRALQEQSIQL
EAERQRVAALSAAYSAYVQSCSAWQAQMDSLRGAFGKDLSVGTPTFPHWLGCP)}*

$90=296(300):
>XP_007169217.1 {|307(310752)|<Chordata(M)>}TIR domain-containing adapter molecule 1 [Balaenoptera acutorostrata scammoni]
{(PAAPKSLPSRSRNACPVADQSPIQLSEEDTTYPAAQPRPPTPSAPKTSSPFSSPSTPPKAHPTVSKPGPPPPELESPEQK)FY
NFVVLHAGAdEHIALRVRERLEALGVRDGATFC---EDFQVPGrGElhCLQDALDHSAFTILLLTSNFDCRLSQHQANQSLMSSL
TR-HGWQDCVIPFLPLESSLTQLSPSTTSLLNAL--VWLDEHS--PIFAKRVANTFK(SQKLRARKAKWRKEQDIRALQEQSKHL
EGEWQQAAAWSAAHSAYLHNYLSYQMQMEKLQVAFGNYMPFETQLPSVPQVPF)}*

$91=278(282):
>EFB21360.1 {|307(9646)|<Chordata(M)>}hypothetical protein PANDA_019591, partial [Ailuropoda melanoleuca]
{(ALAAPESLSLPSRNTWPDKGQSPFPLPEEDTASQVASASPPPPAPLKTSPPHPSPSTLPSTGLASSSLCPPSPELESEQK)FY
NFVILHVAAdEHIALRVRERLEALGVPDGATFC---EDFQVPGrGElhCLQDAIDHSAFTILLLTPNFDCRLGLHQASQSLMNSL
TR-PGRQDCVIPFLPLESSQAPLSPYTTSLLTGL--VWLDEHS--QIFARRVANTFK(AQRLRARKANWKKEQDVRALQEQRRHL
EGERQQVAALNAAYSAYFQSCLSWQAQMETLRMAF)}*

$92=296(300):
>XP_008149009.1 {|307(29078)|<Chordata(M)>}TIR domain-containing adapter molecule 1 [Eptesicus fuscus]
{(APKSLPSPPRNTCPVEDRVPLQIPVEGTTSPAAQPCPPTSSAPKTSPPNPSLPTPPSAHPASSSPCPPSPEFEFEPSEQK)FY
NFVVLHAKAdEHIALRVREKFEALGVPDGATFC---EDFQVPGrSSlcCLQDAIDHSAFTILLLTANFDCCLSQHQLSQSLMSSL

TR-PGWQDCVVPFLPLESSRSQLSPHAASLLAGL--VWLDEHS--PIFARRVANTFK(PHKLRARKANWRKEQDARALRERRQHL
EGERQRAAAVSAAYSTCVQAYASWQTQMEQLQAAFRNHMTFGTQLPSGGPGPL)}*

$93=258(265):
>XP_008280480.1 {|270(144197)|<Chordata(M)>}PREDICTED: TIR domain-containing adapter molecule 1-like isoform X2 [Stegastes partitus]
{(PRSKSSGSPLLGAKKDSRMSETLTDMSSKLDSQIVTPSQTNKPSSEGKVSLPSATNSCTTPKEILESKGAEEEEEEEEEV)FY
SFVILHAPEdADMAECMRDKLEKVTAREGATFS---EDFAIPGkSTlrCVEDAINNSAFTFLLLTRNFNTRMLEIKANSALINSI
NN-KHKYNTVIPLLPQKNCMPRQS--IPLVLNSI--VPLEEN---KSFERKVLKSLS(PAKIEKQRRIWTEEQRRKAQMERRQLQ
EEDALTLFKPEGGDGKVF)}*

$94=290(297):
>XP_008332072.1 {|259(244447)|<Chordata(M)>}TIR domain-containing adapter molecule 1-like [Cynoglossus semilaevis]
{(TTSSQLLDGSQVDSAEPKRDSKMAAAYRKLDNHMVQPETTKPSSAPKDVPSTPTNTFSTEVPDGDRMLESNDAEDEEEET)FY
AFVILHAQEdVDVAESMKIKMEMVTGLEGATFS---EDFAIPGrRTlqCVEDAISNSAFTFLLLTRNYNTRLLDMKTDTAIMHSI
NN-KYKHNTVIPLLPLENSMPETS--LPMVLNAL--VYLKEN---RNFEHKVKKVLT(PANFRKQLRIWREEQRVKREKKRQDEL
RRSNQCQRQFLKEHRMVKSLIRDNINLAMAQKCILNPSVPAGQGSSDGQA)}*

$95=290(297):
>XP_008431969.1 {|261(8081)|<Chordata(M)>}PREDICTED: TIR domain-containing adapter molecule 1-like [Poecilia reticulata]
{(LCASPQHTSTGTSRFGAEKDIKVEEASMRSNESATQPLKPYTQTKSSLPSATHIILPSSPGLKSTPISKVNEEEDEEEDI)FY
AFVILHAPEdSDVAESMKEKLEVVIGTXGATFS---EDFAIPGkSAlrCVEDAVNNSAFTFLLLTHNFNSQMVEMKTNIALINSI
NN-AHKFNTVIPLLPLENCMPKE--QMPITLKTL--VALDER---RNFERKLKKACN(RARIEKQRKIWVHEQKMRRXRLEEKEM
LRLCMEQQLVLGATVQXEQDGGDPRPQRLAPNIHIENANYIVIGNDSTMT)}*

$96=296(300):
>XP_008510112.1 {|75(9798)|<Chordata(M)>}PREDICTED: TIR domain-containing adapter molecule 1, partial [Equus przewalskii]
{(WAVARLYHLLSEEKLCPEPLRDVAYLAALRAFSSRDDHRLGELQDEARDRCGWDIVEDPVDFAPSNPGPASPEAQPSEQK)FY
NFVVLHAGAdEPIALRVRERLEALGVPDGATFC---EDFQVPGrGAlrCLQDAIDHSAFTILLLTANFDCRMSLHQVSQALMSSL
TR-HGWQDCVVPFLPLESSQAQLSPDTASLLAGL--VWLDEHS--RIFPRKVASTFK(LQQLRARRANWRREQDARALRAQSQQL
DGERLQLAALNAAYSTYFQSCLSWQAQMEKLQAAFGSHMSLGTQGPSGGQVPG)}*

$97=296(300):
>XP_008585959.1 {|312(482537)|<Chordata(M)>}PREDICTED: TIR domain-containing adapter molecule 1 [Galeopterus variegatus]
{(LPSPILQPIENHCSVKDPTPPPQLSVEDTTSQHTKPHPPMPSEGPRTSPLSPSPSSPYSSHPASSSPCLSSPDVTSSEQK)FY
NFVVLHARAdEHIALRVRERLEELGVPDGATFC---EDFQVPGrGQlqCLQDAIEHSAFTILLLTCNFDCRLSRHQTSHTLMSSL
TR-HGWHDCVIPFLPLESSLAQLGSDVASLLGGL--VWLDERS--PIFARKVANTFK(PQKLRARKATWRKEQDARTLREQSERL
HAEQQQAAAVSAAHSAYLQSYLSWQAQMESLQMAFGSHMSFGTQVPSGAQVPF)}*

$98=296(300):
>XP_008835192.1 {|311(1026970)|<Chordata(M)>}TIR domain-containing adapter molecule 1 [Nannospalax galili]
{(EDTGKLESVISQVSSQVSVGSDALQNTTSSPPAPPSPQQTSPHAPPPSSPSSASPSSSSHPPPMSSLSPPPGHSETSEQK)FY
NFVVIHARAdEHIALRVREKLERLGVPDGATFC---EDFQVPGrGElsCLQDAIDHSGFMVLLLTASFDCRLSLHQVNQTLMNSL
TQ-SGRQDCVIPLLPLECSRAHLNPDTVGLLTGL--VWLDEHS--SIFTRKVANTFK(PHKLQAHKARWKREQEAKILREHNQQL
DAERQRVASIHTAYSTYVQSCWAWQAQMDSLRMAFGKDLALGSQGPLGGPSPI)}*

$99=261(267):
>KFO63914.1 {|343(85066)|<Chordata(M)>}TIR domain-containing adapter molecule 1, partial [Corvus brachyrhynchos]
{(AHVSIEDSYIPAGIPCNSAPASISTCSLPPPTYSFSSTLPPLQESHSNVLYPPPLRSSPSPAWPPPALPPMDPSEPDGAK)FF
TFVVLHASEdEIEAHRVKNLLESLGVSNGATLS---EDFFIAGrSHmtCFQDAMENSAFMILLLTKNFPCNLCQFQTDTALMQSI

LD-PSKQHSVIPFLPKVN--ALECSRIPTMLSVL--VTLKESS--PLFSRNVHKTFN(PKKIREKKALWDQMQRRKLQACWERYQ
AQQNLAALSLGSPPWVPPAA)}*

$100=252(258):
>KFP06358.1 {|364(9244)|<Chordata(M)>}TIR domain-containing adapter molecule 1, partial [Calypte anna]
{(IPRKNSHVPVGISSNTVTASKSACFFPPPTYSFSSTLPCPPQGAPSNLSYPPPLYSFPSPVLPPLQTVEPMPTSEPGGGM)FF
SFVVLHAGEdEMVAHRVKNLLENMGIPNGATLC---EDFLIAGhSHmtCFQNAIENSAFIILLLTKNFPCDLCMFQTNTVLMESI
QN-PSKRYSVIPFLPKEN--PLEPSQIPRMLGGL--VHLDENS--LGFSRKVQKTFT(TSRIKERKARWDLMQREKLQQNLAALN
LGCQAQGPPAA)}*

$101=261(267):
>KFP14217.1 {|367(188379)|<Chordata(M)>}TIR domain-containing adapter molecule 1, partial [Egretta garzetta]
{(SMEDSYVPAGIPSNSASASISTGSIPPPAYSSFSALPHPLQGAPSNLSYPTPLHSSPSPAWPPPLQSVKPVPTSEPDDVK)FF
TFVVLHASEdEIVAHRVKDLLENMGVPNGATLC---EDFFIAGrSHltCFQDAMENSAFIILLLTKNFPCDLCMFQTNTALMESI
LK-PSKRDSVIPFVPKEN--PLERSQIPSALGGL--MPLDENS--PGFSRTVQNTFT(TSRINERKAMWDLMQRRKLQLYQEQYQ
TLQNLSALNLGSLPQVPLSA)}*

$102=256(262):
>KFQ17041.1 {|366(57421)|<Chordata(M)>}TIR domain-containing adapter molecule 1, partial [Merops nubicus]
{(SCVPAAIPSNAASASILNPSLPPPPPPYSFSSALPPPLRGAPSQTPYPPPFPSSPTPGCPPPLRTVEPVPTSQPDSAERK)FF
TFVVLHASKdEIVAHRVKNLLENLGVPNGATLC---EDFSIAGhSHltCFQDAMENSAFIILLLTKNFLCNLCMFQTDTALMESI
LN-PSKHHSVIPFVPKEN--PLERSQIPSTLGGL--IPLDENS--PLFPRTVQNTFS(ASRINAKKVLWDQAQRRKLQRHQEQNQ
TLEDLAALNLGSHPQ)}*

$103=261(267):
>KFQ89713.1 {|363(9218)|<Chordata(M)>}TIR domain-containing adapter molecule 1, partial [Phoenicopterus ruber ruber]
{(PAHMSRKDSYVPVGIPSNSASASISTCSPPPPTYSFSSTLPSPLQRAHPNLSYLPPLHSSPSPAWPPPVPTSDPNGGERK)FF
TFVVVHASEdESVAHRVKDLLENMGVPNGATLC---GDFFIAGrSHmtCFQDAMENSAFIILLLTKNFPCDLCTFQANTALMESI
LK-PAKRDSVIPFVPKEN--PLERSQIPSTLGGL--MPLDENS--LGFSRTVQNTFA(TSRINARRATWEQMQRKKLQLHQEQYQ
TLQNFAALNLGSFAQVPPSA)}*

$104=293(299):
>XP_009081957.1 {|335(57068)|<Chordata(M)>}PREDICTED: TIR domain-containing adapter molecule 1 [Acanthisitta chloris]
{(PRATVDTGPAHMSREDSYIPADIPSNSTSPSIPTHPLPPPRQEPPSNIHPPPLHSSPSPCWPPPLPAVDPAPTSEPDGAK)FF
TFVVLHASEdVTVAKRVKNLLENMGVANGATLC---EDFSIAGrSHmtCFQDAMENSAFVILLLTNNFTGNLCLFQTDTALMQSL
QD-PAKRYSVIPFLPKENALEQ--SQIPLVLRVL--VTLHESS--PLFHRTVHNTFN(SKNIRKKKAMWDEVQRRKHQLYWEQHQ
AQQNLAGLSLGSSPQVPPAATQSWPSQHPPPPGQMGPLPSQPPFPSAHNNIM)}*

$105=252(258):
>KFU84451.1 {|360(8897)|<Chordata(M)>}TIR domain-containing adapter molecule 1, partial [Chaetura pelagica]
{(EDSNVPIGISSNSAPASISACSLPPPTYSFSSTLPHPLNGASSNSSHPTPLHSSPSPVWPPPLQTIEPVPTSEPDGGEKK)FF
SFVVLHATEdEIVAHRVKNLLENMGVPNGATLC---EDFFIAGhSHmtCFQDAMENSAFIILLLTKNFPCDLCMFQTNTVLMDSI
QN-PSKRNSVIPFLPKEN--PLEPSQIPSMLGGL--VNLDENS--LVFHRKVQKTFS(TSRINERKAMWELRQRRKLPLNLAALN
LGSPAQEPPSA)}*

$106=296(300):
>XP_006904180.1 {|308(9402)|<Chordata(M)>}TIR domain-containing adapter molecule 1 [Pteropus alecto]
{(SPAPQSLPVPSTDACPVKDQKPLQLPTEDTTPPTAQPYVSAPSATKTSPPDPSASTPGPARLASPNLRPPPLELESSEKK)FY
SFVVLHAKTdEHIALRVRDKLEALGVPDGATFC---EDFEVPGrGElsCLQDAIDHSAFTILLLTANFDCRLSLHKVSHSLMNSL

MQ-HGRQDCVVPFLPLESSLAQLSPDTSSLLASL--VWLDEHS--QIFTRKVASTFK(PQKLRAYKATWKKEQDVRALREQRQHL
EVERQQAAALNAAYSAHLQAYLSWQAQMEKLQAAFGNHMTFGSQLPPGASVPH)}*

$107=293(299):
>XP_009282325.1 {|369(9233)|<Chordata(M)>}PREDICTED: TIR domain-containing adapter
molecule 1 [Aptenodytes forsteri]
{(EDSYVPAGISFNSAPASISGYSLPSPTYSFSSTLPPHLRGAPSNLSYPPPLHSSPSPAWPPPLQAVEAVPTSEPDGGERK)FF
TFVVLHASEdEIVAHRVKNLLENMGVRNGATLC---EDFLIAGrSHltCFQDAMENSAFIILLLTKNFPCDLCMFQTNTALMESI
LK-QPKRDSVIPFVPKEN--PLEWSQIPSTLGAL--MPLNENS--PGFSRMVHNTFT(TSRISKKKAMWDQMQREKLQRYQEQYQ
TLQNFGALNLGSFAQVPPSATRPQLLKQSPQQRCPPASTVPPATYPPPPAGH)}*

$108=293(299):
>XP_009316684.1 {|369(9238)|<Chordata(M)>}PREDICTED: TIR domain-containing adapter
molecule 1 [Pygoscelis adeliae]
{(EDSYVPAGVSSNPASASISACPLPSPTYSFSPTLPPHLRGAPSNLSYPPPLHSSPSPSWPPPLQAVEAVHTSEPDGGERK)FF
TFVVLHASEdEIVAHRVKDLLENMGVPNGATLC---EDFLIAGrSHltCFQDAMENSAFIILLLTKNFPCDLCMFQTNTALMESI
LK-QSKRDSVIPFVPKENPLEQ--SQIPSTLGAL--MPLNEKS--PGFSRMVQNTFT(TSRISKRKAMWDQMQRAKLQLYQEQYQ
TLQNLDALTLGSLAQVPPSATRPQPLKQSPQQRCPPASTVPPATYPPPPAGH)}*

$109=293(299):
>XP_009480176.1 {|355(36300)|<Chordata(M)>}PREDICTED: TIR domain-containing adapter
molecule 1 [Pelecanus crispus]
{(ISMRHSYVPAGLPSSSASASISTGSLPPPTYSSSSALPPPLQRVPSNLYAPSLHSSPSPVWPPPLQTVEPAPTSEPDSGK)FF
TFVVLHASEdEIVAHRVKDLLENMGVPNGATLC---GDFFIAGrSHltCFQDAMENSAFIILLLTKNFPCDLCMFQTNTALMESI
LK-PDKQDSVIPFVPKEN--PLERSQIPSTLGGL--MPLDENS--PGFSRTVQNTFT(TSRINKRKAMWDLTQRKKLQLYQERYQ
TLQNFAALNLGSLPQVPPSATGPQLLQRSPQQWCPPASTVPPATYPPPPVGH)}*

$110=293(299):
>XP_009470181.1 {|367(128390)|<Chordata(M)>}PREDICTED: TIR domain-containing adapter
molecule 1 [Nipponia nippon]
{(SMKDSEVPAGIPSNSASASISTSSLPPPPYSSSSTLPPPPQGVPSSFSYPPPLHGSPSPAWPPPLQTVESVPTSEPDSGK)FF
TFVVLHASEdEIIAHRVKDLLEKMGVPNGATLC---EDFSIAGrSHltCFQDAMENSAFIILLLTKNFLCNLCMFQTNTALMESI
LK-PSKRDSVIPFVPKEN--PLERSQLPSTLGGL--MPLDENS--PGFSRIVQNTFT(TSRINQRKAMWELMQRRKLQERYQTLQ
NFAALNLGSLPQMPPSATRPQLLEQPPRQWCPPASTVPPAAYPPPAADHPMP)}*

$111=293(299):
>XP_009511631.1 {|365(9209)|<Chordata(M)>}PREDICTED: TIR domain-containing adapter
molecule 1 [Phalacrocorax carbo]
{(HMPMEDSSVPAGIPSNSASASISTSSLPPPTYSSSSTLPPPLQGPHSNLYPPHLHSSPSPAWPPLQTVEPVPTSEPDGGK)FF
TFVVLHASEdEVVAHRVKNRLEEMGVPNGATLC---EDFFIAGrSHltCFQDAMENSAFIILLLTKNFPCDLCMFQTNAALMESI
LK-PSKHDSVIPFVPKEN--PLERSQIPSTLGGL--MPLDENS--PGFSRTVRNTFT(TSRINERKATWNRMQRRKLQLYQEQHQ
TLQNLTALNLGSLAQVPPSATWPQLLEQPQQWCPPALTVPPATYPPPPAGHP)}*

$112=293(299):
>XP_009561132.1 {|366(55661)|<Chordata(M)>}PREDICTED: TIR domain-containing adapter
molecule 1 [Cuculus canorus]
{(PREDSYVPAGIPSNSTSTSTCSLPPPPYSFSSTLPSPLPGAHSNLPYLPPLHSSPSSAWPPTLQTATPVPTLELHGGESK)FF
TFVVLHASKdEVVAHRVKDLLEKMGVPNGATLC---EDFFIAGrSHmtCFQDAMENSAFIILLLTKNFPCNLCMFQTNTALMQSI
FN-PSKHDSVIPFLPKEN--PLERSQIPSTLGGL--MPLDENS--LGFSKTVQNTFT(PRRINERKAMWDRAQQRKLQLNQEQHQ
TVHNLAGLNLGSPPQYSGQWCPPASTVPSATFPTPLAAHTVTAPVGPLPFQP)}*

$113=293(299):
>XP_009570504.1 {|366(30455)|<Chordata(M)>}PREDICTED: TIR domain-containing adapter
molecule 1 [Fulmarus glacialis]
{(MSMEDCYVPAGISSNSASASIPACSLPPPTYSFSSTLPPPLQEGPSNLSYPPPLHSSPSPAWPPPPPVAAEPISEPDGGK)FF
TFVVLHASEdEIVAQRVKNLLENMGVPNGATLC---EDFFIAGrSHltCFQDAMENSAFIILLLTKNFPCDLCMFQTNTALMESI

LT-PSKRDSVIPFVPKEN--PLERSQIPSMLGGL--TPLDENS--PGFSRTVQNTFT(TSRINERKAMWDHVMQRRKLQLYRERY
QTLQELAALNLGSFPQVPPSATHPQLLEQSPQQWCPPSSTVPPATYPPPPTG)}*

$114=293(299):
>XP_009676037.1 {|315(441894)|<Chordata(M)>}PREDICTED: TIR domain-containing adapter molecule 1 [Struthio camelus australis]
{(AVNQPVESSDVSSTVTAEPQAPKEGTAQKQEDLPTGLPDSRATAHTGPIHTSAEDIPAAYPPPVQNAEAASTSEPDEERK)FF
TFVVLHASEdVDIACRVKELLESMGVPNGATFC---EDFLTAGrGQlfCFQDAMENSAFTILLLTKNFLCQLCMFQTNSALMESI
LR-PSKHNSVIPFVPREN--PLERREIPALLSGL--VSLDENS--SVFSKTVKNTFT(PSKISELKAMWKQIQQIQEHKRKLQLY
QDHCQTLKNLADLNLGSVPQMPLSAPQPNLGSLQQLLEQLMPHQSPQQCHFP)}*

$115=293(299):
>XP_009808101.1 {|358(37040)|<Chordata(M)>}PREDICTED: TIR domain-containing adapter molecule 1 [Gavia stellata]
{(TVDTGPAHMSKEDSYVPAGISSDSASASISASVLPPPIYPFSSTLPPPSNLPYLPPLHSSPSPAWPPPLQVPPSEPDGGK)FF
TFVVLHASEdEIVAHRVKDLLENMGVPNGATLC---EDFFIAGrSHltCFQDAMENSAFIILLLTKNFPCDLCMFQTNTALMESI
LK-PSKRDSVIPFVPKEN--PLERSQIPTTLGGL--MPLDENS--PGFSKTVQNTFT(TSRIKERKAMWDLMQIRKLQAYQERNR
TLQNFAALNLGSLPQVPLSATQPPLLEQSPQQWCPPASAVPPATYPPPAAGH)}*

$116=293(299):
>XP_009707398.1 {|367(54380)|<Chordata(M)>}PREDICTED: TIR domain-containing adapter molecule 1 [Cariama cristata]
{(SMKDSCVPAGIPSNSAPPSISTCSLPPPTYSLSSTLPSPLQGSPSNLFYPPPLHSSLSPAWPPPLQTVEPVPTSELDGGK)FF
TFVVLHASEdEIVAHRVKDLLENMGVPNGATLC---EDFFVAGrSHmtCFQDAMENSAFIILLLTKNFPCDLCMFQTNTALMESI
LK-PSKRDSVIPFVPKEN--PLDRSQIPSMLGGL--MPLDENS--PGFSRTVQNTFT(TSRINERKAVWGLMQKRKLQLYQERYQ
TLQNLAALNLGSLPQMPPSATRPQLQEQSPRQWCPPTLTVPPATYPPPPAGQ)}*

$117=293(299):
>XP_009871442.1 {|373(57397)|<Chordata(M)>}PREDICTED: TIR domain-containing adapter molecule 1 [Apaloderma vittatum]
{(ENSYVPAGIPSNAAPASLITCSLPPPTYSFASTLPTPLWGAPANFSYPPPFPSSPSPAWSPPLRTAEPAPPSEIDDGERK)FF
TFVILHASKdEAVASRVKNLLEGMDVPNGATLC---EDFSIAGcSHltCFQEAMENSAFIILLLTKNFLCDLCKFQTNTALMESI
QN-PSKHDSVIPFIPKENRLDR--SQIPNTLGAL--IPLDENS--PGFSSTVKKTFA(SSRISQRKAMWDQMQRRKLQVYEEQHQ
PLPTFAALNLGSLPHVPPTHPQPPSPQQWCPPAATALPATYPPPLAGHPLPA)}*

$118=293(299):
>XP_009899013.1 {|361(118200)|<Chordata(M)>}PREDICTED: TIR domain-containing adapter molecule 1 [Picoides pubescens]
{(NSCVPAGIPSNSASASISTHPFPPPPAYSISSTLLPPPQGAPSNLSYPSPLPSSPSPAWLPPLKPADPVPTAQLDGGERK)FF
TFAILHASEdELVAHRVKTLLESMGVPNGATLC---EDFSVAGcSHmtCFQDAMENSAFIILLLTNNFLCNLCMFQTDTALMESI
QK-PSKRHSVIPFVPKEN--PLERSQIPSVLCGL--VPLDENS--PMFSRKVQNTFN(SRRIKERKAMWDQMQREKLQLQQEQYQ
TRQRLAALSLGLHPQEPLPAAQPQLLKPPPQQWCPPTSAVPPPTYPPPYDGH)}*

$119=293(299):
>XP_009893486.1 {|368(50402)|<Chordata(M)>}PREDICTED: TIR domain-containing adapter molecule 1 [Charadrius vociferus]
{(TEDSCVPEGTSNSASASISACPLPPPSCSFSSTLPPHFLGAASNLSYAPPLPSSPSPAWLPPLQSVEAVPTSELDGRELK)FF
TFVVLHASEdEIVAHRVKNLLENMGVPNGATLC---EDFFIAGrSHltCFQDAMENSAFIILLLTKNFPGNLCMFQTNTALMESI
LK-PSKRDSVIPFVPKEN--PLERSQIPSTLCGL--MPLDENS--PGFSRTVQNTFT(TSRINERKATWDMMQRKKLQQYQERYQ
TLHNLAALNLGSLPQAPPSAPRPQLLEQSPPQRCPPASAVPPAANPPPPAGH)}*

$120=293(299):
>XP_009934696.1 {|367(30419)|<Chordata(M)>}PREDICTED: TIR domain-containing adapter molecule 1 [Opisthocomus hoazin]
{(SMEDSCVPAGIPSNSASASISACSAPPPAYSFSSTFPSPPQGPPSNSSYPPPLHSSPSPAWPPPLQAVEPVPTSAPDGVK)FF
TFVVLHASEdEIVAQRVRDLLENMGVPNGATLC---EDFSIAGrSHmtCFQDAMENSAFIILLLTKNFPCNLCMFQTNTVLMESI

LK-PSKRDSVIPFVPKEN--PLERSQIPSTLGGL--TPLDENS--PGFSRTVRNTFT(PSRINERKAMWDLMQKKKLQLHQERYQ
TLHNLSVLNLGSPPQVPPSAAWPQQLDQSPQQWWPPTSTVPSAAHPPPPTSH)}*

$121=289(299):
>XP_010019457.1 {|360(176057)|<Chordata(M)>}PREDICTED: LOW QUALITY PROTEIN: TIR domain-containing adapter molecule 1 [Nestor notabilis]
{(VSTEEPHVPGGIPSNSASAAISTRSLPPPTYAFSSTLPPLHGPHSNLPYPPRSHSSPSLAWPPSLQTAEPGPTSEPDDEK)FF
TFVVLHASEdEIVAHRVKDLLENMGVPNGATLC---ENFAIAGrSHmtCFQDAMENSAFIILLLTKNFPCDLCMFQTNTALMESI
KN-PFKCDSVIPFIPKEN-PLQWSQ-----XGWL--TPLDEKS--PGFARTVQNTFT(TNRINKKKESWDLLQRKKLQLQEERYR
TLQNMATLNLGGSIPPSATRSPLLEQPPRQWCPPAGGGCRPAHMGSPPVQAP)}*

$122=293(299):
>XP_009979399.1 {|365(121530)|<Chordata(M)>}PREDICTED: TIR domain-containing adapter molecule 1-like [Tauraco erythrolophus]
{(STEDSYVPAGISSNSASASISACSIPPPTYSFSTTLPSPLQGAPSNSAYPPPLHSSPSPAWPPPLQGVEAVPTSEPDSGK)FF
TFVVMHASQdEIIAHRVKNLLESMGVPNGATLC---EDFFIAGrSHltCFQDAMENSAFIILLLTKNFPCDLCMFQTNTALMESI
LK-PSKRDSVIPFVPKEN--PLERSQIPLTLGGL--MPLDENS--PGFSRTVQNTFT(TRRITQRKAMWDLTQRKKLQFYQESYQ
TLQNFATLNLGSLSQVPPSATRPQLLERSPQQWCPRPSTVPPATYPPHPVAA)}*

$123=293(299):
>XP_009974479.1 {|372(56313)|<Chordata(M)>}PREDICTED: TIR domain-containing adapter molecule 1 [Tyto alba]
{(HVSMKDSYVPAGIPSNSASASNSTCSLPPPTYSGSTTLPPQVTASNLSYPLPLHSSPSSAWPPPLQTVEPAPTSEPDSGK)FF
TFVVLHASEdEAVAHRVKDLLENMGVPNGATLC---EDFFIAGrSHltCFQDAMENSAFIILLLTKNFPCDLCMFQTNTALMESI
LK-PSKRDSVIPFVPKEN--PLERSQIPSTLGGL--MPLDENS--PGFSRTVQNTFN(TSRINERKVMWDLMQRKKLQLYQERYQ
TLQNLAALNLGSLLQVPPSATQPQLLEQSPRQWCPPASTVPPATYPPPPAGH)}*

$124=293(299):
>XP_010137501.1 {|352(175836)|<Chordata(M)>}PREDICTED: TIR domain-containing adapter molecule 1 [Buceros rhinoceros silvestris]
{(EATVDTGHAHASMEDSSGPAGVPPKTASASILTSSLPPPLQRALSNSSYPPPPSSSCSPAWPPPLKTAEPVPTSEPEGGK)FF
TFVVLHASEdEIVAHRVKDLLENMGVPNGATLC---EDFFIAGrSHltCFQDAMENSAFIILLLTKNFPCDLCMFQTNTALIESI
MN-PSKRDSVIPFVPKEN--PLERSQIPSTLGGL--MPLDENS--PGFSKTVQNTFT(PSRIKERKAMWDQMQRRKLQLYRERYE
MPNLAALNLGSHPQMPPSATWPQFPKQSPRQWCPPASPVPPATYPPPLAAHP)}*

$125=293(299):
>XP_010159112.1 {|340(54383)|<Chordata(M)>}PREDICTED: LOW QUALITY PROTEIN: TIR domain-containing adapter molecule 1 [Eurypyga helias]
{(ADTGPAHVSMEDSCARTSNSASASIPTCSLPPPTYSFSSTLPTQGPPHNLSYPAPLHSTPCPAWTPPLQAVPTSEPDGGK)FF
TFVVLHASEdETVAHRVKDRLENMGVPNGATLC---EDFFIAGrSHltCFQDAMDNSAFIILLLTKNFPCDLCMFQTNTALMESI
LK-PSKRDSVIPFVPKEN--PLERSQIPSTLGGL--MPLDENS--PGFSRTVQNTFT(TSRINERKAMWDLTQRRKLQLHQERYQ
TLQSLASLNLAQASPSATRSQLLEQPPPLFFPPXPPAPPPPPAGLPSFQSIH)}*

$126=293(299):
>XP_010173154.1 {|357(279965)|<Chordata(M)>}TIR domain-containing adapter molecule 1 [Antrostomus carolinensis]
{(KDSHVPAGISSNSAPASMSARSLPPPTYSFSSTLPPAPHKPPSNLSYPPPIHSSPSPAWPPPLQTVEPVPTSEPDGEETK)FF
TFVVLHASEdEIVAHRVKDLLEKMGVPNGATLS---EDFFIAGrSHmtCFQDAMENSAFIILLLTKNFPCDLCMFQTNIALMESI
LK-PSKRNSVIPFVPKEN--PLERSQIPSMLGGL--MPLDENS--PGFSRTVQNTFN(STRINERKAMWDRMQKKKLQLYWDQYQ
TQQKLAALNLSSLPQVPPSTTRPQSLEQSPQQWCPPASTVPPATYPPPLTGH)}*

$127=293(299):
>XP_010072321.1 {|360(240206)|<Chordata(M)>}PREDICTED: TIR domain-containing adapter molecule 1 [Pterocles gutturalis]
{(GPAHTSEEDSYVPAGPSSNSASASMPACSLPPPTYSFSSTLPSLPGAPSNSSYPCALHSGPSPAWPPPLPTSEPDGEERK)FF
TFVILHANEdEIVAHRVKDLLENMGVPNGATLC---EDFFIAGrSHltCFQDAMENSAFIILLLTKNFPCDLCTFQANTALMESI

LN-PSKHDSVIPFVPKEN--PLERSQIPKTLGVL--MPLDENS--PVFSRTVQNTFT(TSRIKERKAAWDATQRRKLEERNQTLQ
NLAALNLGSLPPVPPRPRLPEQSPQQWWPGSVPQLIIQHARMVQIGNHNVMQ)}*

$128=293(299):
>XP_010177190.1 {|360(54374)|<Chordata(M)>}PREDICTED: TIR domain-containing adapter
molecule 1 [Mesitornis unicolor]
{(SKEEDSVPAGISSNSAPASSLASSLLPPPPPYSFSLALPPLQGPPSSLPHPTALHSSPSPAWPPLHAAAAVPTAEPDSGK)FF
TFVILHASEdEFVAHRVKDLLEKMGVPNGATLC---EDFFIAGrSHmtCFQDAMENSAFIILLLTKNFSCNLCLFQTNTVLMESI
LK-PGKRDSVIPFIPKEN--PLKRSEIPSTLGGL--MPLDENS--PMFSKTVQNTFT(SSRINERKATWDLVQERKLQLYQERYQ
TQQNLAALSLGSSPQVPPPPTQPQLPEPSPQQWWPPPSGVPPATYPLSHTAH)}*

$129=293(299):
>XP_010120978.1 {|367(187382)|<Chordata(M)>}PREDICTED: TIR domain-containing adapter
molecule 1 [Chlamydotis macqueenii]
{(CKADSGVPAGISSDSAPASISACSLPPPTHSFSSTLPPPHHGAPSNLSYPPPFHSSPSPAWPPPLQTVEPVPVSEPDGGK)FF
TFVVLHANEdELVAHRVKDLLENMGVPNGATLC---EDFFIAGrSHmtCFQDAMENSAFIILLLTKNFPCDLCMFQTNTALMESI
LK-PSKRDSVIPFVPKEN--PLERSQIPSTLGGL--MPLDETS--PGFSRTVQNTFT(TSRIKERKAMWDLMQSRKLQMHQERYQ
TLQNLAALNLGSLPQMPPSATRLQLLGQSPREWCPPVSTVPPATYAPPPGHP)}*

$130=293(299):
>XP_010202854.1 {|112(57412)|<Chordata(M)>}PREDICTED: TIR domain-containing adapter
molecule 1, partial [Colius striatus]
{(GPAHTPMDSSIPAGIAPNSAAAAVSTCSLTPTCSSSAFPPLHGAPSSLPHPPPFYSSPSPAWPPPLQRVPTAEPDAEEQR)FF
TFVVLHATEdEPVAHRVKQLLEAMGVPNGATLC---GDFSVAGrSHltCFEDAMENSAFIILLLAKHFLCNLCMFQTNTALMESI
LN-PSKQDSVIPFVPREN--PLQRREMPRTLSGL--IPLDENS--PGFSSTVRNTFT(SSKIERRKAKWEMMMKTRNLQLYQEQY
QASQNLAALNLGCSATGPLPAAWLEQPPQHWQPPTSTVPPATYPPPPAAQPR)}*

$131=292(297):
>AEX01719.1 {|250(94232)|<Chordata(M)>}TIR-domain containing adaptor inducing IFN
[Epinephelus coioides]
{(TSEPKSSQPPAFGANKHSKNESLTTESSLVARSETLNQITKPTTGPNFALPAAANIFLPMMPAMDEMHESRDAEEEEEAT)FY
AFVILHAPEdADMAESMREKLETILESDseGATFS---GDFALPGkSTlrCVEDAISNSAFTILLLTRNFNTRLLEMKTDSALIN
SINK-KYKHNTVIPLLTRENSMPRHS--LPMVLQTI--IPLVE-D--KSFTRKIQKVLS(PVKIRNQKKIWTAEQKVKMQIERQE
RLKHLNQHQRQLIRECTAAELLETEKLNLYMEQRLLLTQPQQDGGQWQQQQP)}*

$132=280(299):
>XP_010221010.1 {|94(94827)|<Chordata(M)>}PREDICTED: TIR domain-containing adapter
molecule 1 [Tinamus guttatus]
{(PELDAEAMVLLARIYSLLADENLCGREAVVRAYRRVIEACGASRDPQQEPLRSILAEAQEKCGAALGFVGSGSRFQPLRS)--
----------DLALRVKGLLEAMGVPDGATFS---GDFLTGGrGQlsCFQDAMENSAFTILLLTKNFLCQPCMFQTNSALMQSIE
T-PSKYNSVIPFVPREN--PLEDSEIPFFLRGL--VPLNENS--PMFSTRVRNTFT(RSKVSEQKALWKQRQQVQEQQRKLQLYQ
EHWRILRRLSELNLGSLPRAPWPSEPGDAQQVLEQLQALWSAPQCPFPTSA)}*

$133=293(299):
>XP_010288063.1 {|361(97097)|<Chordata(M)>}PREDICTED: TIR domain-containing adapter
molecule 1 [Phaethon lepturus]
{(IGPARMSREDSCVPVGISSNSASASISACSLPPTYSFSSTLPHNVSYLPPLHSSPSPAWPPPLQTVEPVLTSEPDGGETK)FF
TFVVLHASEdEIVAHRVKDLLENMGVPNGATLC---EDFFIAGrSHmtCFQDAMENSAFIILLLTKNFPCDLCMFQTNTALMESI
LK-PSKHDSVIPFVPKEN--PLERSQIPSTLGGL--MPLNENS--PGFSRTVQNTFT(SSRINERKAMWDLTRRRKLQLYRERYQ
TLQNLAALNLGSLAQVPPPATWPQLLEQPPRQWYPPASAVPPATYPPPTAGH)}*

$134=293(299):
>XP_010298283.1 {|365(100784)|<Chordata(M)>}PREDICTED: TIR domain-containing adapter
molecule 1 [Balearica regulorum gibbericeps]
{(HMSMKDSYVPAGISSNTASASMSTSLLPPTYPFSSTFPPPIQEPPSKLYPTPLHSSPSPAWPPPPQAVKAMPTSEPDGGR)FF
TFVVLHASEdETVAHRVKDLLENMGVPNGATFC---EDFFIAGrSRltCFQDAMENSAFIILLLTKNFPCNLFMFQTNTALMESI

LK-PSKRDSVIPFVPKEN--PLERSQIPSMLGGL--TPLDENS--PGFSRTVQNTFT(TSRINERKAMWDLVQRRKLQLYQDQYQ
PLQNLAALNLGSLPQGPPSATQGQQSPRLWCPPSLTVPPATYPPPTAGHPTP)}*

$135=291(299):
>XP_010774263.1 {|258(8208)|<Chordata(M)>}PREDICTED: TIR domain-containing adapter molecule 1-like [Notothenia coriiceps]
{(PESSEPPMFGAKKPLKMEGAAEGGELDSLITRKETFHQTSKPTTEPTFALPTATSDSLPKMPAPKEVHESEGAEVEEEPI)FY
AFVIFHAAEdADVAESMKERLEEVIHIEGATFS---GEFAVPGrSTlrSVEDAIDNSAYTVLLLTRNFD-RMQMMEADSALVNSI
YK-TNKDGTVIPLLPRENSLPKDS--FPMVLRTI--NTLDEN---RNFERKVRRVFS(PAKIKKQRDIWNTDQKMKMQMQRQETL
KNLNRSQKLFICERTKAELLQKDNLRFLLEQELHLGPRGPPEQPQIHINNAK)}*

$136=287(291):
>ELV09302.1 {|71(246437)|<Chordata(M)>}Dipeptidyl peptidase 9 [Tupaia chinensis]
{(ESPQNPCLVQDQTPLELSVDETTSQNTQPQPPASGSSSGPGSSASATEPSRASSTKSSACPAPSAPHPTSPAAPESSEPK)FY
SFVVLHARAdEHIALRVRERLEELGVPHGATFC---EDFQVPGrGElrCLQDAIDHSAFTVLLLTPNFDCRLSLHQINQAVMSSL
TR-HGWQDGVIPFLPLETSQAQLGPDAASLLGGL--VWLDQDP--PIFQVQKRSWAG(LRAIIHGSRKCSGLVANKAPHDFQFVQ
KTDEAGPHSHRLYYLGMPSGSRENSLLYSEIPRKVRKEALLLLS)}*

$137=296(300):
>XP_011371861.1 {|308(132908)|<Chordata(M)>}TIR domain-containing adapter molecule 1 [Pteropus vampyrus]
{(SPAPQSLPVPSTDACPVKDQKPLQLPTEDTTLPTAQPHVSAPSATKTSPPDPSASTPGPARLASPNLRPPPLELESSEKK)FY
SFVVLHAKAdEHIALRVRDKLQALGVPDGATFC---EDFEVPGrGElsCLQDAIDHSAFTILLLTANFDCRLSLHKVSHSLMNSL
MQ-HGRQDCVVPFLPLESSLAQLSPDTSSLLASL--VWLDEHS--RIFTRKVASTFK(PQKLRAYKATWKKEQDVRALREQRQHL
EVERQQAAALNAAYSAHLQAYLSWQAQMEKLQAAFGNHMTFGSQLPPGASVPY)}*

$138=290(297):
>XP_011485063.1 {|263(8090)|<Chordata(M)>}TIR domain-containing adapter molecule 1 [Oryzias latipes]
{(LLSVLQPTGCFLSEPERSHPALPAGTSQCHVMVQEETPDTLSIRSHINPPSSTNICGSRCPVQTKIHNSKGSDEEEEEET)FY
AFVILHAQEdEDVAERIKDKIEKIISNKGATFS---EDFAVPGkCPlrCVEDAINNSAFTFLLLTRNFKSNLVKMKTSMALINAI
NK-MHKYNTVIPLLPQENRMPK--DLIPMAVRSL--VPLEE-A--KNFEKKLQKLLS(RAKIQTQKRVWKKEQTLRVQEERLRQL
QLEEDKQRLLNERLCMGLNHQQEQSGEEGRTWQQRHPNIHIENANYIMIG)}*

$139=295(299):
>XP_012291832.1 {|309(37293)|<Chordata(M)>}TIR domain-containing adapter molecule 1 [Aotus nancymaae]
{(GPQSLPSPILETLKNPCPVKDQTPPQLSVEDTTSPNTKPSPSTPTTPKTSLPPPPSAPCSAYLAPSSLFPSSLESPSEQK)FY
NFVILHARAdEHIALRVREKLEALGVPDGATFC---EDFQVPGrGElrCLQDAIDHSAFIILLLTSNFDCHLSLHQVNQALVSNL
MR-QGPSDCVIPFLPLESSPAQLSSDTASLLSGL--VRLDEHS--RIFDKKVANTFT(SHKLQARKAMWRKEQDTRALREQSQHL
DSERVQVAAMNAAYSAYLQSYLSWQAQMEQLQAAFGSHMSFGTGAPFGARMP)}*

$140=296(300):
>XP_012370522.1 {|309(10160)|<Chordata(M)>}TIR domain-containing adapter molecule 1 [Octodon degus]
{(PNLEPTESFCPVKNQKAAPISAGEPTSQEGSSSPPVSLAPESSLPPPTPAPSPSSSPAAVLPASSCEPCPSSFSSDATEQ)FY
NFVVLHALAdEEVALRVRERLEALGVPDGATFC---EDFQVPGrGElhCLQDAIDHSGFVILLMTPNFDNALSLHQVNQALMSSF
TH-NGRYDSVIPFLPLESSLGVRHSDTSRLLSGL--VWLDEHS--KIFARKAGSTFK(PQRLWERRAHWQREQDARARLRGSQRL
DGERQQAEALHAAHTTYLRSYLAWQAQMHKLQVDFGSHLSLGTQVPCPPQGPF)}*

$141=296(300):
>XP_012416085.1 {|363(9708)|<Chordata(M)>}PREDICTED: TIR domain-containing adapter molecule 1 isoform X1 [Odobenus rosmarus divergens]
{(VLAAPKSLSLPSRNTCPDKDQPPLPLPVEDITSQVASPCPPAPSALRTSPPYPASSTPSSTGLASSSPCPPSPELESEQK)FY
NFVILHVAAdEHIALRVREKLEALGVPDGATFC---EDFQVPGrGElrCLQDAIEHSAFTILLLTPNFDCHLGQHQAGHSLMSSL
TR-PGWQDCVIPFLPLESSQAQLSPHTSSLLIGL--VWLDEHS--RIFDRRVVNTFK(PQTLRARKAHWRKEQDVRALQEQHRHL
EGERQQVAALNAAYSAYVQSCLSWQAQMETLRAAFASHMPFGTQGHPGGPGPL)}*

$142=296(300):
>XP_012501678.1 {|315(379532)|<Chordata(M)>}PREDICTED: TIR domain-containing adapter molecule 1 [Propithecus coquereli]
{(VGNPRPAKDQTPLQLSQEDTTCPDIKPCPAAPSIPETSCPQTPLPPPPPPSSCPAYLASSSHLASSSLWPSSPEVSSEQK)FY
NFVVLHAKAdEHVALRVREKLEALGVSDGATFC---EDFQVPGrGElrCLQDAIDHSAFVILLLTSSFDCRLSLYQVCQALTSSF
TR-HGWQDCVVPFLPLESSPAQLRSDTASLLAGL--VWLEERS--PVFARKVANTFK(PQKLRRRKDNWRKEQDARALRERSQHL
EGEREQAAALNAAHSAYLQSYLAWQAQMEQLQVAFGSHMSLGTGMPFWAPGPF)}*

$143=296(300):
>XP_020139539.1 {|356(30608)|<Chordata(M)>}LOW QUALITY PROTEIN: TIR domain-containing adapter molecule 1 [Microcebus murinus]
{(PQTPGPQTPPSAPPPPSPGSAYVASASPGSAYVASASPGSAYVASTSPGSAYVASASPGSAYVASASPWASSPELPLEQK)FY
NFVVLHAKAdERVALRVREKLEALGVSDGATFC---EDFQVPGrGElrCLQDAIDHSAFVVLLLTASFDCRLSLHQVCHALMSSF
TR-HGWQDCVVPFLPLESSPAQLRSDTASLLAGL--VWLEEHS--PVFARKVANTFK(PQKLRRRRDNWRREQDARALRARSQHL
EGERAQAAALGAAHSSYVQSCLAWQAQMEQLQGAFRSHMSLGTGMPFAAAGPF)}*

$144=291(297):
>XP_012680748.1 {|297(7950)|<Chordata(M)>}PREDICTED: TIR domain-containing adapter molecule 1-like [Clupea harengus]
{(RHSAAADKHRGVASAPVPAAPVEPGRPPVQCSGRDGTRQLVTSISTGMETLRLSEPVLPAFSRSVSAPETPAACEDERAV)FY
SFVILHSPEdAEEAEELRENLECVTSGTGATFS---QDFAIPGrPTlsCLDDAIDNSAFTILLLSRNFTSRMQEVLTDSALINSI
HN-THKYNSVIPLLPLNNPLPQDS--LPRVLRTI--VSLKEGS--RSFRQNAMKAIS(PQKVARQRVKWEREQEAKRLQERRQRL
LEENCWREQQMQQAELNQRLLRQAWDLHWRQQNMPSPTPAPPFHPPTPGT)}*

$145=292(299):
>XP_012714371.1 {|273(8078)|<Chordata(M)>}TIR domain-containing adapter molecule 1-like [Fundulus heteroclitus]
{(LRSAGASLFGAERDIKTDEASTKSGFQSNEGATKPNAPNAETKSPLPSATHIILPSIPGLKSPHESKGAEEEEEEEEEEM)FY
AFVILHAPEdSDVADIMREKLEAVIGTTGATFS---DDFAVPGkSTlkCVEDAVNNSAFTFLLLTRNFNSRMVEMKTNMALINSI
NN-EHKFNTVIPLLPLDNRMPTQS--IPVALQTL--VPLEER---RNFEKKLQKVFN(RAKIDKQRRIWTEEQSLRRLKRLRLRE
NERLKLHMEQHLVLGGSMPPGEDGGDPRSWWPAQSNIHIENANYVMIGNDST)}*

$146=294(300):
>XP_007072279.1 {|325(8469)|<Chordata(M)>}TIR domain-containing adapter molecule 1 [Chelonia mydas]
{(TAELQELGERMVQKPEQESPTELPRLCTPGPAAGSIRVPVEDSCVLTEKADTASFSSSEGCPPPQAAAAPTPELDCGEKR)FF
SFVVLHASEdVAIACRVKEMLESMGVPDGATFC---EEFLIPGqSQltCFQDAIDNSAFTLLLLTQNFQSRFCVHQMNTALMDSL
QR-RPKYNSVIPFVPKENSSKKC--PIPSMLVGV--VPLDESS--PVFSRTVKNTFR(LRRISEQKAMWSQLQHIQEHHRKQQQH
QEHLQMLQQSLAGLNWGSQPGYPPQMPLPGLLPYPAGIQQLLQLVASLQLQTP)}*

$147=294(300):
>XP_012863134.1 {|312(9371)|<Chordata(M)>}PREDICTED: TIR domain-containing adapter molecule 1 [Echinops telfairi]
{(QSLSPPTPEPPGDPSQDGTLLSGPGGDTASQKTRLKSPPPPAPQTPSPSPSSSLPSATPESTSSSLLPPGVASPEEEEEK)FY
GFVVLHARAdEAVALRVRQKLEALGVPDGATFC---EDFQVPGcSElsCLQDAIDRCAFTVLLLTQNFDCRLSLHQVTQALMSSF
GR-QGWQDSVVPFQPLET--PRLTSDVSRLLSGL--VPLDEHS--QLFDRKVKGTFK(KQRLQARRARWEKERQARAFRERRQHL
EAERQQAADVHAAYSALVQTQLAWQVQMEQLQAAFGGVSLHAQVPLGAQVPFG)}*

$148=295(299):
>XP_012878106.1 {|203(10020)|<Chordata(M)>}PREDICTED: TIR domain-containing adapter molecule 1, partial [Dipodomys ordii]
{(SSSASFPPTSSSANFPPASSSASFPPPSSSANFPPASSSSTTVPLASSSPASFPPASSSWASFPPVSSSSAFHADTTGQK)FY
NFVILHTRAdEHVALRVRERLEALGVPEGATFC---EDFQVPGrGElrCLQDAIDHSGFIILLLTAAFDSQLGLHQLNQALMSSL
TQ-LGRQDCVVPFLPIESSEAQLGPDTSSLLAGL--VRLDERS--AIFARKVANTFA(PRRLLARRAHWAQEQQARALRQQRPQL
QGHATYPTASLGAWGASQPQIHALQEALGSGLVLGTQTSQPLRVPLGGQVPP)}*

$149=293(299):
>XP_013050712.1 {|360(381198)|<Chordata(M)>}PREDICTED: TIR domain-containing adapter molecule 1 [Anser cygnoides domesticus]
{(VPTKDSYVPVSTSSPAPASTSSCFPPYTYSSSTLSPPLYATPYSLPHPSPFHSPPSPACPPPFQTVKAASASEPDSGERK)FF
TFVILHAGEdEHVACRVKNLLENMGVPNGATFC---EDFLVAGhGRltCFQDALENSAFIILLLTKNFLCQLCMFETNSALMESI
QR-PSKHNSVIPFVPKEN--PLERSEIPSILSVL--VALDENS--IGFAKVVKNTFT(ARKINERKAMWDQIQQVREQKRIQQLY
KDHYQTLQNLGARNLGSLPQVPLSGTPCLGQPLEQLMPHQSSQQCRPPWASY)}*

$150=294(300):
>XP_004378500.1 {|319(127582)|<Chordata(M)>}TIR domain-containing adapter molecule 1 [Trichechus manatus latirostris]
{(QSLPSRVLEPTDNATESGISLPLSVEDTAPQKSKLCPPPPSAPQMPSPFPPPSPSPAYAAPPASSSSAPPAPELESSEQK)FY
NFVVLHARAdEHVALRVRERLEQLGVPDGATFC---EDFQVPGcGElnCLQDAIDHSAFTILLLTPNFDCRLSLHQVTQALMSSF
VR-RGWQDSVIPFQPLETS--QLSSDVSRLLSSL--VCLDEHS--QLFERKVKNTFK(RQKLQERKANWKKEQEVRAVREHSQRL
DGERQQVAEMNVACSAYWQSYLAWQAQMEKLHATLRTQMSLGTQMPPGAQMPF)}*

$151=293(299):
>XP_013798144.1 {|362(202946)|<Chordata(M)>}PREDICTED: TIR domain-containing adapter molecule 1 isoform X1 [Apteryx australis mantelli]
{(IHTSVEDVPEGTSFHTTSPASACSLPPHSSSFSSAPPLQAAPPNLAYHPSLPSSSFPACPPPVQTAEAASTLEPDGEERK)FF
TFVVLHASEdVDIACRVKKLLESMGVPNGATFC---EDFLTGGrGQlsCFQDAMENSAFTILLLTKNFLCQPCMFQTNSALMESI
LR-PSKHNSVVPFVPREN--PLERRQIPTLLSGL--VSLDENS--PVFAKTVRNTFT(PSKISELKAMWEQIQHVQEQKRKLQLY
QDHCQTLKNLAELNLGSPPQVPLSAPQPNLGSLQQLLEQLMPHRSPQQCHFP)}*

$152=293(299):
>XP_013926540.1 {|295(35019)|<Chordata(M)>}PREDICTED: TIR domain-containing adapter molecule 1 [Thamnophis sirtalis]
{(QSPPTPNPASSGDLTRTFQSPSLQKPGSALSAGSGLPRSSAPAPITSFGPLLSSSSSTSDLASCSSEPSSSMVPPDDERQ)FY
TFVVLHAAEdELVACRVKERLEALGVSNGATFS---EDFLVPGrGQlnCFQNALDHSAFTLVLLTRNFTGRLCEYQRDTALMDSL
TR-CCKRNSVIPFVPKENSVNPV--QMPFLLASL--VPLCEKS--PVFEKRVKKTFT(PKVIREKKAAWDLLRQIQEREEARERE
RENLWLQQHLAVLNLQSSVQPPVVEMSRMQAQGGNDAQVFPPPATSFVKVGL)}*

$153=290(297):
>XP_013877135.1 {|256(52670)|<Chordata(M)>}PREDICTED: TIR domain-containing adapter molecule 1-like [Austrofundulus limnaeus]
{(KPPSPEPRSNESLFKSNKDSKPKETLHTVSSKLHDNIPQNGSXNQPITETFTLPSPQNLFLPKIPFLKTPHGTEAEEEET)FY
AFVILHAPEdEEQAERMKEKIETAIGREGATFS---EDFAIPGkSXlkCVEDAIDNSAFTFLLLTHNFNTKMLEVKTNMALINSI
NN-VHKFNTVLPLLPEDNCMPKQG--IPRVLQTL--VFLEE-K--RHFTKKLQKALS(PAKIEAQRKIWAKEQSVKRLHHLNHET
DRLSLYLQQQQFLRSSAMLEHEGANSMALWGPQPNIHIQNAKYIMIGNDS)}*

$154=290(297):
>XP_014025434.1 {|314(8030)|<Chordata(M)>}PREDICTED: TIR domain-containing adapter molecule 1-like [Salmo salar]
{(HRTGSEEVRVSPLSPVNFTPAHTKFNNPESPNAHSKPCPKNNLVPPSNTNRPTFHPTTTEPVSASFPLKTPNQKMEEEEV)FF
SFVILHAAEdVDMAEKLKEELESIVGGEGATFS---QNFAIPGrNTlmCVEDAINNSAFTILMLTRNFNTRLLEVETSSVLMNAI
EN-LHKYNTVIPFLPQENRMPRE--NMPKVLRIF--IPLEE-G--NAFEKKAKRAMA(PANIAKQRRLWLSEQAVRAQVRRQERL
RQEERFQMDLIRGQETERMQRYLLQQQRSNQPSTPAMPHFTAPFLGSTPI)}*

$155=293(299):
>XP_014133217.1 {|368(345164)|<Chordata(M)>}TIR domain-containing adapter molecule 1 [Falco cherrug]
{(MEDSSVPASIPSNSASASISASSLPPPPTYPFSSTLPYLLQGATSKSSYPPPLHSSHSPAWPPPLQSVEPAPTSEPDGGK)FF
TFVVLHASEdEIVAHRVKDRLENMGVPNGATLC---EDFFIAGrSHmtCFQDAMENSAFIILLLTKNFPCNLCMFQTNTALMESI
LK-PSKHDSVIPFVPKEN--PLERSQIPSILGGL--MPLDENS--PGFSRTVQNTFT(TSRINERKAMWDLMQRRKLQLYRQQYQ
TLQDLTALHLGSLSQLPPSATRPQPLEQLPQEWCPPASTVPPAAYPPPPAGH)}*

$156=293(299):

>KQK82830.1 {|353(12930)|<Chordata(M)>}toll-like receptor adaptor molecule 1 [Amazona aestiva]
{(SMEEPHMPGDIPPNSASASISARSFPPPPTYSFXSTLPPPQGPRSSLPYPLRSHSSPSLAWPPPLQTAXPAPTSEPESGK)FF
TFVVLHASEdEIVAHRVKDLLENMGVPNGATLC---EDFAIAGrSHmtCFQDAMENSAFIILLLTKNFPCDLCMFQTNTVLMESI
MN-PSKSDSVIPFIPQEN--PLEWSQIPAVLGGL--TPLDEKS--PGFSRTVQNTFT(TSRINEKKDSWDLLQRKKLQLREERYR
TLQNMAALNLGSIPPPAXQPXLLEQPPRPWWSLAGGGPRPARMGPPPVQPPS)}*

$157=296(300):
>XP_014389170.1 {|239(109478)|<Chordata(M)>}PREDICTED: TIR domain-containing adapter molecule 1 [Myotis brandtii]
{(APTSLPSPCRNTGPAEDQVPLQIPVEDTPSPAAQPCPPTSSAPETSPPNPSLSAPCSAHPASSSSSPCPPSPEFEPSEQQ)FF
NFVVLHAKAdEHIALRVREKFEALGVPDGATFC---EDFQVPGrSSlrCLQDAIDHSAFTVLLLTANFDCCLSQHQLSQSLMSSL
TR-PGWQDCVVPFLPLESSLAQLSPDTASLLAGL--VWLDEHS--QIFARKVANTFK(PHKLRARKANWRKEQDARATYVQTYAA
WQAQMEQLQAAFASHMTFGTQLPSGAPGPLGAQPPFPTWPSYQPPTLPPWLAG)}*

$158=293(299):
>XP_014460416.1 {|332(8496)|<Chordata(M)>}PREDICTED: TIR domain-containing adapter molecule 1 [Alligator mississippiensis]
{(LQRPQESVPKEPEKEPHVGLPGSSSSTIRLPVEDSYVPTGNRNMASSSSSICPPPSVQPPALAPAPVTAPSSEPDTGESK)FF
SFVVVHAMEdVDIACRVKELLESMGVPDGATFC---EDFLVPGhCQltCFQDAINNSAFTLLLLTENFQCRLCTYQMNSALMDSI
LR-LHKYNSVIPFVPKE--YPLKKGQIPGVLAGL--VPLDENS--RVFTKRVKNTFV(LSKIKEQRTKWSEIQQIQEAHRKQKQY
QEHLQAVQAALAALNIGSQPGFLPPMQLPDLTGLQQFLSSLSAQAALINQPY)}*

$159=293(299):
>XP_007899298.1 {|293(7868)|<Chordata(M)>}PREDICTED: TIR domain-containing adapter molecule 1 [Callorhinchus milii]
{(STYSPVKNSSAVGVKPIECTEETDSNQIVTSKTSTQDQDHLNSNLSDPHPTQNATLSEDPQSSRPRMNISEETEENVENK)FY
PFVILHADEdQEIAEKVRKSLEELGVMEGATFC---EDFSLPGkCPisCIQDAVDNSAFTILLLTNNFNTHWTELKTNIVLMNSI
QN-KHKYNTVIPMLTKENRLPKRK--IPFALQAI--NQLDETS--KHFERHVAKTFE(PAKLRTYKDKWLVEQKLQQIETQKAKV
AKDRKDLRDITRLTQQLGQNLQNYQKEGQHLASLRQYCNALYNFPNLPPIPS)}*

$160=293(299):
>XP_014742629.1 {|348(9172)|<Chordata(M)>}PREDICTED: TIR domain-containing adapter molecule 1 [Sturnus vulgaris]
{(DTAPARRSIEDSYIPGNPCNSAPASISNCSLPPPSYSFSSTLRPLQESHSNVLHPPPLRSSPSPASPAFQDPAVDPSEPE)FF
TFVVLHASEdELVAQRVKNLLESMGVPNGATMS---EEFFIPGcSHmtCFQEAMENSAFTILLLTKNFPCKLCLFQTDTALMQSI
LD-PSKKDSVIPFLPKEN--ALDCRQIPRMLSAL--VTLKESS--PLFSRNVDKTFS(PKRITEKKAQWMERRKFQTQQKLAALS
LGSHPWLPPGAPRPPEPSAQPWCPPAPTDPPGHYSITAGQGGIPPLIIQNAR)}*

$161=293(299):
>XP_014808293.1 {|357(198806)|<Chordata(M)>}PREDICTED: TIR domain-containing adapter molecule 1-like [Calidris pugnax]
{(LAHTTREDSYVPAGTSNSASSISASPPYSVFSTLPSPLQGAAPNLSYPSPLPSSTSPAWPPPLQTLEAGLASEPDAGDRK)FF
TFVVLHASEdESVAHRVKNLLESMGVPNGATLS---ENFFIAGrSHltCFQDAMENSAFIILLLTKNFLCNLCMFQTNTALMESI
MN-PSKCDSVIPFVPKES--PLERSQLPTTLNGL--MPLDENS--PVFSRTVQNTFT(SSRISKRKAMWDLTQRKKLEQYQTLQN
LAALNLGSHPQVPPSATQPQLLEPSPQQWCPPASAVPPATHPPHPMPGQMGP)}*

$162=292(299):
>XP_014840262.1 {|261(48701)|<Chordata(M)>}PREDICTED: TIR domain-containing adapter molecule 1-like [Poecilia mexicana]
{(HCASPQHTSTGTSRFGAEKDINIEEASMRSNESATQPLKPITQTKSSLPSATHILLPSSPALKSTHISKVDEEEDEEEDI)FY
AFVILHAPEdSDVAESMKEKLEAVIGTTGATFS---EDFAIPGkSAlrCVEDAVNNSAFTFLLLTRNFNSQMVEMKTNIALINSI
NN-AHKFNTVIPLLPRENCMPKE--QMPITLQTL--VALDER---RNFERHLKKAFN(KARIEKQRKIWDREQRMRRLRLEEKKM
LRLCMERQLVLGATIQREQDGGDPRPQRLAPNIHIENANYIVIGNDSTMTVG)}*

$163=292(299):

>XP_014898090.1 {|262(48699)|<Chordata(M)>}PREDICTED: TIR domain-containing adapter molecule 1-like [Poecilia latipinna]
{(CASPQHTSTGTSRFGAEKDINIEEASMRSNKSATQPLKPITQTKSSLPSATHILLPSSPGLKSTHISKVDEEEDEEEEDI)FY
AFVILHAPEdSDVAESMKEKLEAVIGTTGATFS---DDFAIPGkSAlrCVEDAVNNSAFTFLLLTRNFNSQMVEMKTNIALINSI
NN-VHKFNTVIPLLPRENCMPK--DHMPITLQTL--VALDER---RNFERHLKKAFN(KARIEKQRKIWDHEQRMRRLRLEEKKM
LRLCMERQLVLGATIQREQDGEDPRPQRLAPNIHIENANYIVIGNDSTMTVG)}*

$164=291(297):
>XP_018939138.1 {|240(7962)|<Chordata(M)>}PREDICTED: TIR domain-containing adapter molecule 1-like [Cyprinus carpio]
{(AKNQLPTAEVNSETKSKQCLDDNESSQLVTSKNTNLDFSSTPNDGEKLRAQPLSATDHKPTTQPDSFRSPEPSQSDVEET)FY
DFVILHEAEdADEAQRLRDKLESIIRGVGATFS---DEFAQPGkSTlrCMEDAIENSAFTLLLLTQNFNSNLGATSADSAIVNSL
EN-HHKMNSVIPLLPRKNCLSRKS--FPLVLKTK--VALDESS--RMFERNAQKAIA(PEKVAFQKTIWTNKQRCKKLVEEQKRQ
QEENAIKAELRQAEEKLARLRLASGMQQNNMFCAASAPMHPPPPHGPMHS)}*

$165=294(299):
>XP_015220763.1 {|329(7918)|<Chordata(M)>}PREDICTED: TIR domain-containing adapter molecule 1 [Lepisosteus oculatus]
{(SHARPLESTVSMGGHQTITSKEPSADFPVQPGKKLLEVKQTAAAESSDTDVQSETKDSKNNSPRSSPSILGFKEEDSGEK)FY
SFVILHAAEdSDIASELQLKLESLGAGEGATFS---EDFEVPGrHTltCIQDAIDNSAFTILLLTKNFNTRLLEHKTNSVLMNSI
EK-QHKYNTVIPFLPRENFLTRK--EMPLCLKIY--NHLDEgRT--RNFEKIARKTFA(PEKVERQRQLWKMEQSIKAAEERKEQ
LKGVNARHERLVKAASGTAELEQERLHLQQELLRQMYAGWMAGMPQPQFSNPN)}*

$166=292(299):
>XP_015242175.1 {|266(28743)|<Chordata(M)>}PREDICTED: TIR domain-containing adapter molecule 1-like [Cyprinodon variegatus]
{(QHTPAGRSQFGGENEIKTDEASMNTFQSTKSATYPYKANSGTKATQLASQSILPNISGLKSPSESKETKEEEEEEEEEEI)FY
AFVILHAPEdSDVADSMKEKLEAAIGTTGATFS---DEFAVPGkSTlrCVQDAVNNSAFTILLLTRNFNSQMLEMKTNIALINSI
HN-EHKFNTVIPLLPMDNCMPRRS--IPIALQTL--VPLEE-K--RNFEKKLQKVFN(RANIERQRKIWTEELKIRKLKQEKERL
RLHMEQRLVLGASVQREQDGGVWWPQHSNIHIENANYVMIGNDSTMTVGRGG)}*

$167=294(300):
>XP_015276078.1 {|309(146911)|<Chordata(M)>}PREDICTED: TIR domain-containing adapter molecule 1 [Gekko japonicus]
{(PNIMPAAALQDLKEDIPPNLRTACPLLADPATEGCIQTSVEYSCSPCQSTGPKDTSSSTDVPPVAASSSSSTDSTGDEDQ)FF
MFVVVHANEdESIACRVRERLENMGVSNGATFC---EDFLVPGhNQlaCFQDALDNSAFTLLLLTENFKSHLCTFQTNMALMNSF
TR-IVKANSVIPFIPKES--PLKKGEMPGVLAGL--VSLDENS--RVFERRAKNTFR(LSEFQTKKLTWSMNQQIRRQERLREQQ
QDYWQMQQRLLALSLQPGYPAQVPFPPTQMGLPGLPQVPPSHVPPPFFLPTSS)}*

$168=294(300):
>XP_007949252.1 {|313(1230840)|<Chordata(M)>}PREDICTED: TIR domain-containing adapter molecule 1 [Orycteropus afer afer]
{(PQSLPLPVLKATNNPTKDGTPLPLSVEDTASQKTKSCPPPPPAPQRSPPSPSPSPSPQCAAAPPSSSDPSPPELESSEQK)FY
NFVVLHARAdEHVALRVRERLEELGVPDGATFC---EDFQVPGrGEltCLQDAIDHSAFTILLLTPNFDCRLSMHQVSQALMNSF
GR-PGWLDSVIPFRPLETS--QLSSDTSRLLSSL--VYLDEHS--QLFNRKVKNTFK(KQTLQERKANWKREQETRAIREQSQRL
EGERQQLADMNAAYSAYMHSYVAWQAQMEQLHMAFGTQMSLGTHMPPRAHMPS)}*

$169=294(300):
>XP_014338163.1 {|313(72004)|<Chordata(M)>}PREDICTED: LOW QUALITY PROTEIN: TIR domain-containing adapter molecule 1 [Bos mutus]
{(LPSPSKNACLVTDQTPVQLSEEDTAYLSAQPRPPTPSMPQTSPSFPSASTSPFPSPSTPPEAHPTPSKPPPPELESSEQK)FY
NFVVLHASAdEHIALRVRERLEALGVHDGATFC---EDFQVPGrGElhCLQDALXK--FILLLLTSNFDCRLSQHQTNQSLMSSL
TR-HGWQDCVIPFLPLESSLAQLSPSTSSLLTGL--VLLDEHS--KIFARKVTNTFK(PQMLRARKAKWRKEQDARALREQSQQL
ESERQHAAAWGAAYSAYVHSYLAYQTQVEKLQVALANYMPFGTQLPFGGQGSV)}*

$170=271(282):

>XP_015399660.1 {|169(74533)|<Chordata(M)>}PREDICTED: TIR domain-containing adapter molecule 1 [Panthera tigris altaica]
{(VSPSGTRSLPKPIEGLSGWSRGCSLSSSSSPASLASNLEISQSPTMPLLSQHRGCRAPSKLCEEPRAGPGPEPPPTGCQE)--
-------PAdEDIALRVRERLESLGVPDGATFC---EDFQVPGrGHlrCLQDAIDHSAFTLLLLTPNFDCHLGLHQAGQSLMNSL
TR-HECRDCVIPFLPLESSREQLSPHPCSLLTSL--VCLVCASHMGLDPPAAARGLQ(AAVGRHLPLARNRSGCPAPWLRAANHL
PVLPPGPPAASSLTPSGHMGLPVNSFIRATNYRVS)}*

$171=294(300):
>XP_008120431.1 {|395(28377)|<Chordata(M)>}PREDICTED: TIR domain-containing adapter molecule 1 [Anolis carolinensis]
{(GSRCSSSDPSVSPERSVQCPEPVTGKHVSPRSLPHDPRISSADSTQYPRNSVADPYSQKPPSNSVSGPPPAVSSSSEPQE)FF
TFVVVHADEdEAIACRVKARLESMGIPDGATYS---EDFLVPGrCQlgCFENALEDSAFTLFLLTENFKSRLCAYQTNTALMDSL
TH-FCKANTVIPFLPKE--RPLKTREMPLLLKGL--VPLDENS--PVFAKRVKKTFR(EEDIRRKKADWSVRRQNRERERLQEQN
REYQQQLQRLSELSMNMTSLQGFPGMPQPPPGPDLTSLCPSFMFPQGFGGARP)}*

$172=293(299):
>XP_015741701.1 {|352(93934)|<Chordata(M)>}PREDICTED: TIR domain-containing adapter molecule 1 [Coturnix japonica]
{(PACIPIEDSYIPAGISNSVPASTSVFPFPPQTYFTSTITPAPPSVPYNIPFPSPPSSTGPHSLKTVEAALAPKPNGEGKK)FF
TFVILHALEdERIACRIKDLLENMGVPNGATFC---EDFLVAGhNQltCFQDAMENSAFLILLLTKNFLCNQCMFQTNSALMESI
QR-PSKHNSVIPFVPKEN--PLKRSQIPSMLSVL--VALDENS--PVFARTVQNTFT(PEKINERKAMWCQIQQVQEQKRKLELF
QDHYQTLQNLGALGSFPHTSPSAMQLSQLSLEQLLKQWQPLQSSQQCHPPVS)}*

$173=290(297):
>AMP81962.1 {|230(75372)|<Chordata(M)>}TIR domain-containing adapter molecule 1 [Squaliobarbus curriculus]
{(RRPTTNIRGNNQLETAEVSHSTNSKQCVDRKGSSQVVTSQSTNISFSSSPNDANAKDLKPPTQANSFCSPEPSQSNVEET)FY
AFVILHEAEdADEAQRLRERLESIISAPGATFS---DDFAQPGrSTlrCIEDAIENSAFTLLLLTQNFKSNLSETTADSAIVNSL
EN-YHKLNSVIPLLPRENRLSRDR--FPLVLRTK--VFLDE-S--RMFERNVLKAIT(PERVASQKKIWLMDQRKKKLHEEQKRL
REENIRNAELQRETEKLARLKLESEMQQKKMFHTASAPSPFNGAMYTQPG)}*

$174=290(297):
>XP_015812586.1 {|252(105023)|<Chordata(M)>}PREDICTED: TIR domain-containing adapter molecule 1-like [Nothobranchius furzeri]
{(NTPTERSLLGAESSSMTDETLKSMSSSSQSNVVQNERSVQPNESSTETKITLPCAKSTISPKISVPKSLYDTEEEEDEEI)FY
AFVILHAPAdAEQAEQIKEKLEKAISGEGATFS---EEFAVPGkSTlkCVEDAINNSAFTFLLLTRNFNTKMLEMKTNIALINSI
NK-THKFNTVIPLLPENNCVPR--PDIPIVLQTL--VPLEEKK--N-FEKKLQKALT(PARIKKQRSIWTEEQRVRRLNRLSLQE
VDRRCMEQQHLLSSREGGDGRALWPPQANIHIENANYVMIGNDSTMTVGG)}*

$175=295(299):
>XP_016000154.1 {|308(9407)|<Chordata(M)>}PREDICTED: TIR domain-containing adapter molecule 1 [Rousettus aegyptiacus]
{(SPAPQSLPAPSTDTCPVEDQVPLQLPTEDTTCLTAQPCTPAPSATKTSPPDPSTSTPGPARPASPNLRPPPPELESSEKK)FY
SFVVLHARAdEHIALRVRDRLEALGVPHGATFC---EDFQVPGrGElsCLQDAIDHSAFAILLLTANFDCRLSLHKVSHSLMSSL
TQ-HGRQDCVVPFLPLESSPAQLSPDTSGLLASL--VWLDEHS--KIFARKVASTFT(PQRLQACRAAWKRQQDARALREQRQHL
EGERQQAATLHAAYSAYLQSYLSWQAQMEKVQAAFGTHMTFGTQLPPGASVP)}*

$176=295(299):
>XP_016053353.1 {|305(291302)|<Chordata(M)>}PREDICTED: TIR domain-containing adapter molecule 1 [Miniopterus natalensis]
{(SPAPKSLSLPSRDTCPVKDQIPLQIPVEDTTSPTVQPCPPPPSAPKTSPPYSSQSTPCSAHPVPLAPCPPSSELELSEQK)FY
NFVVLHAKAdEHIALRVREKLEALGVPDGATFC---EDFQVPGrSKlhCLQDAIDHSAFTVLLLTANFDCRLSLHQVNQALMCSL
TR-QGWQDCVVPFLPEESSLSQLSPDTANLLTSL--VWLDEHS--SIFSKRVANTFT(PHKLRARKANWRKEQDARALREQRQHL
ENERQQAAALNATYAAYLQTYLSWQTQMEQLQLAFRNHMTIGAQLPSGAQVP)}*

$177=296(300):

>XP_007525002.2 {|254(9365)|<Chordata(M)>}PREDICTED: TIR domain-containing adapter molecule 1 [Erinaceus europaeus]
{(PAPTGHQEPGHPPPTKPAPRIPEPAPEAPENPGPAQAPTPSRPKPEDTPGASPPTLLPPRVPLSPSPWSWGATPSLEPPR)FY
NFVVLHAPEdEEAALRVRQRLEDLGVPDGATFC---EDFEVAGrGAlrCLQDALEHAAFTLPLLTPRFSCGLSLHQLNQALLCSL
TR-PGWGDSVVPFLPRDGCPSRLGPDAARLLAGT--VWLDERS--PVFERKVARTFR(PQRLRARQEQWERERGQRTRALNAGVS
GYLGSLQTWPGQLEQLQALWAAGLTLGTSPEPHLQPPASGIPLSPGQQPPGTP)}*

$178=291(297):
>XP_016085949.1 {|240(75366)|<Chordata(M)>}PREDICTED: TIR domain-containing adapter molecule 1-like [Sinocyclocheilus grahami]
{(AKSQLPTADINGATKSKQCLDHNGSSQLVTSKNANLDFSSTPNDGDKLQAQPLNATDHKPTTQPGSVSSPEPSQSDVEET)FY
DFVILHEAEdADEAQRLRDKLESIIRGVGATFS---DEFEQPGkSTlrCMEDAIENSAFTLLLLTQNFNSNLSATSADSAIVNSL
EN-HHKMNSVIPLLPRKNCLSRKS--FPLVLQTK--VPLDESS--RMFERNAQKAIA(REKVASQKTIWMTKQHRKKLVEEQKRL
QEENAIKADLRREQEKLARLRLESEIQQNNMFCGASAPMHPPPAHGPMHS)}*

$179=296(300):
>OBS82883.1 {|314(56216)|<Chordata(M)>}hypothetical protein A6R68_23101 [Neotoma lepida]
{(TTESIRKQPSITSQMSPPGSVGDDNLQNTSSSPPAQPPSSQASTTLPPSPLPSNSSFSSCPAPPASMSSILGHLETSEQK)FY
NFVVIHARAdEHVALRIREKLETLGVSDGATFC---EEFQVPGrGElhCLQDAIDHSGYTILLLTTNFDCRLSLHQVNHALMNSL
TQ-SGRQDCVIPLLPLECSQAQLSPNTTSLLHSL--VWLDEHS--PVFARKVANTFK(PQKLQAHRMRWKKEQEARALKEQSTQL
EAERQRVTAMSAAYSAYVQSCSAWQAQMDGLRVAFGKHLSLGTPTFFPAPQTP)}*

$180=291(298):
>XP_017277648.1 {|264(37003)|<Chordata(M)>}TIR domain-containing adapter molecule 1-like [Kryptolebias marmoratus]
{(SAEPEHKSNQSSLFGANKDSKTDETLESMSCKLHSRISQSESPTQPSTGTFTLPSPQHISLPKTPVLKNLHGTGEEDEEV)FY
AFVILHAPEdEELAETMKAKLETVIGSEGATFS---EEFAVPGkSAlkCVEDAIDNSAYTFLLLTRNFNTKMLEVKTNIALINSI
NK-VHKYNTVIPLLPLDNCMPR--QEVPRVLQTL--VPLDER---KSFMRKIQKTLS(PARIEAQRRIWTEEQRVKRLNRLKQEA
ERLSLYTEQHLRLGSGVPPLWAPQPNIHIENAKYIMIGNDSTMTVDLGRNA)}*

$181=291(297):
>XP_026119947.1 {|240(7957)|<Chordata(M)>}LOW QUALITY PROTEIN: TIR domain-containing adapter molecule 1-like [Carassius auratus]
{(TKDQLPTEEVNCTTKSKQSLDDKSSSQLVTSKNTNLDFSSTPNDGGKLRAQPLNATDHKPSSQLDSFRSLEPSQSDVEEM)FY
DFVILHEPEdADEAERLRNKLESIIRGVGATFS---DEFAQPGkSTlrCMEDAIENSAYTLLLLTQNFNSNLSATSADSAIVNSL
EN-HHKMNSVIPLLPRNNCLSRKS--FPLVLKTK--VPLDESS--RMFERNAQKAIA(REKVALQKKVWKSKQHCKKLVEEQKRQ
QEENAIKAELRKHEERLARMKLESKMQQNNMFYPTSAPMHSPPPHGTMHH)}*

$182=293(299):
>ANV78028.1 {|396(8839)|<Chordata(M)>}TIR-domain containing adaptor inducing interferon-beta [Anas platyrhynchos]
{(PTKDSSVPVSTSSSAPASTSACSFPPHTYSSSTPSPPLYATPYSLPHPSPFRSPPSPACPPPFQTAKAASAAEPDSGEGK)FF
TFVILHAGEdERVACRVKNLLEGMGVPNGATFC---EDFLVAGhSRltCFQDALENSAFIILLLTKNFLCRLCMFETNSALMESI
QR-PSKHNSVIPFVPKEN--PLERSEIPSILSVL--VPLDENS--LGFTRVVKNTFT(PSKINERKAMWCQIQQVREHKRRQQLF
KDYCQALQNLGALNLGSFPQVPLSETPWQQLGQFVPHQLPQQFCPPWATSCP)}*

$183=296(300):
>XP_017506473.1 {|315(9974)|<Chordata(M)>}PREDICTED: TIR domain-containing adapter molecule 1 [Manis javanica]
{(PAAAQALPLPSRNTCSDEDQTPLQPPTEDPASPMAQPCPATPAAQKACPPCPSPLTPSLGHPASSAPEPPSPQLESGEQR)FY
NFVILHARAdEHIALRVRERLEALGVPDGATFC---EDFQVPGrGElrCLQDAIDHSAFTILLLTASFDCRLSLHQVSQSLMSSL
TR-QGGQDCVVPFLPLESSLAQLSPDTSSLLTGL--VWLDEHS--KVFARKVANTFK(PQRLRALKAIWRREQGVRALQQQHRHL
EGERQQAVAWSAAYSAYVQSYQSYLALQTQMENLQLAFGSHVPFPAQVHYGGQ)}*

$184=297(300):

>XP_017576226.1 {|253(42514)|<Chordata(M)>}PREDICTED: TIR domain-containing adapter molecule 1-like [Pygocentrus nattereri]
{(AAKVTKCTDMLGTCQMVTSRSAVSGVEDAFSSITSGSGKLGAQPISKLASQPPKSASVQSTEQMDSFSSERKSAEDEEEM)FY
AFVILHAPEdVEEAARLKARLERTSSTTGATFA---EDFAEPGrSPfrCVEDAINNSAYVMLLLTPNFNTRLNEMNADSALINSI
EK-PHKYNTVIPLLPRDNSL--TSTELRMVLRTK--LPLKEND--RGFEimaRKVLDPDK(IRIQKKIWNQELLFRKEQEKKQQL
QDENRRHKDFIRECEKVVELEKQRMHLLMQQQRLLHPCASQQQWYPPFSGGAQPFG)}*

$185=294(300):
>XP_018109628.1 {|196(8355)|<Chordata(M)>}PREDICTED: TIR domain-containing adapter molecule 1-like [Xenopus laevis]
{(ADMVLTSSYDSTNNHTNMEPAVFEIRGDHSVKTSIQESEFSEFTERHKPNGNANTVPSSNNNSYRMPPQCSTYPPPDDSL)FF
NFVILHVREdTEVACRVCNDLQSLGAGNGTTYC---EGFEIPGsNPltCIQDAVENSAYIILLMTKHFETRWAEFQSNVVLMNSI
ND-ENRTASVIPFLPRSDRLPKK--NMPLALSTL--IPLDETS--QIFSRVVRNTFK(QDTILFRKKGWMQQQEIKIKRMVDAQR
SEQTRHYLHLNQMCGNVGSMSLGAPYMYPQPIPCQPQVIHINNAENVQIGNHN)}*

$186=294(299):
>XP_014341573.1 {|332(7897)|<Chordata(M)>}PREDICTED: TIR domain-containing adapter molecule 1 [Latimeria chalumnae]
{(TRRWCFAAQVEVSKPMDKKPIKEQGAQQILTPEYTTGVTNSTKVPEEDTYPKTEQSKSDHLSSQLPSELSETELLSSEKK)FY
TFVILHLPEdVTIASRVQDTLERHGIADGAT-S---SDFLIPGqSPlaCIQNIVDNSAFTILLLTSKFASKWAEYQTNMVLMNSI
HN-LHKYNSVIPFFPKEDSLSI--TVLPLALRCL--TGLKENieE--RVFQKILKTTFS(RKKIQQQKSLWEKEQRMKELQEKTL
QLNEEVHFHREYSEKVMLYNQQLCQLNFYQYLTAAHCLPLNHSQVPPGIPSEIP)}*

$187=290(297):
>XP_026170345.1 {|263(205130)|<Chordata(M)>}TIR domain-containing adapter molecule 1-like [Mastacembelus armatus]
{(QPKSNAPAVFEGKTDSKMDTAECRKLDSHVAQNKSSNQTNKPSTEHKFALPTATNIFLPKMPVPNEMHESKGAEEEEEAI)FY
AFVILHAPEdAEMADSMKEKLESVTGSKGATFS---DDFAIPGkTTlsCVEDAINNSAFTILLLTCNFKSRMLELETNSALINSI
NN-DHKFNTVIPLLPRENCIPRES--IPMVLQTI--VPLEEN---KSFEKKVQKALS(VAKIKKQRKIWAELQKMKLQIERQERL
KQLNHHQKQLILECKTAQVLVEENQNLLMAQKLLLCTSDPSKQDGGDGRA)}*

$188=289(296):
>XP_018532364.1 {|243(8187)|<Chordata(M)>}PREDICTED: TIR domain-containing adapter molecule 1-like [Lates calcarifer]
{(KSGQSQRSEEPQPKSNEPALPEAKKDSKTECRTLEETPNQTTKPFTEPKFALPTATMASKVSVPKEMHESKGAEEEEEEI)FY
AFVILHAPEdGDMAERIKERLESVTCCDGATFA---DDFAIPGkSTlkSVEDAINNSAFTLLLLTCNFNTRMEEVEADSALINSI
NK-KYKHNTVVPLLPRENCMPK--QNIPMILRTI--NPLEEN---KHFDKKAKQVLS(PANIKRQKRFWTEGQRVKRQIETQDEL
KQLNQNQKLLIQETKLVQSLVRETQSLLLAQKCLLGPSVPPGQDGGEGG)}*

$189=291(297):
>XP_018939140.1 {|227(7962)|<Chordata(M)>}PREDICTED: TIR domain-containing adapter molecule 1-like [Cyprinus carpio]
{(AKNQLPTAEVNEATKSKQCLDRNGSSQLVTSKNMNLNFSSSPNDGDKLHAQPLNANDHKPTTKPISFSSPELSQSDVEET)FY
DFVILHEAEdADEAQRLRDKLERIISGIGATFS---DDFAQPGrSTlrCLEDAIENSAFTLLLLTQNFSSNLSATSADSAIMNSL
EN-HHKMNSVIPLLPRKNCLLRKS--FPLVLQTK--VPLDESN--RMFERNAQKAIT(PEKVATQKKIWMIEQHRKKLVEEQKRL
QEENAINVELRREMEKLARLRQESEMKQNNMFYPPSVPMHASPSHEAMRQ)}*

$190=296(300):
>XP_019286450.1 {|348(9691)|<Chordata(M)>}PREDICTED: TIR domain-containing adapter molecule 1 [Panthera pardus]
{(ALAAPASLPLPPKTTCPDKDQTPLSLPVEDTASQRTPPCPPTPSAPRTSAPCPSPSIPPSACPAAWNPCPPPPELEPEQK)FY
NFVILHAGAdEDIALRVRERLESLGVPDGATFC---EDFQVPGrGHlrCLQDAIDHSAFTLLLLTPNFDCHLGLHQAGQSLMNSL
TR-HECRDCVIPFLPLESSREQLSPHTCSLLTSL--VWLDERS--HIFAKKVANTFK(PQRLRARRAHWRKEQDVRALQEQHQQL
EGERQRVSALNAAYSAYFQSHSAWQAQMEALRVAFGSHMPFGTPGPLGAPPPF)}*

$191=293(299):

>XP_019368973.1 {|328(94835)|<Chordata(M)>}PREDICTED: TIR domain-containing adapter molecule 1 [Gavialis gangeticus]
{(VPPKPQRPQESVPKEPKKEPHVGLPGSSSGSLRVPVEDSYVPTENRTMASSSSIFPPPLQAPAAAPVTAPSSEPDTGEGK)FY
SFVVVHAMEdVDIACRVKELLESMGVPDGATFC---EDFLIPGhCQltCFQDAINNSAFTLLLLTENFQCRLCTYQMNSALMDSI
LR-LHKYNSVIPFVPKE--YPLKKGQIPGVLAGL--VPLDENS--RVFARRVRNTFV(LSKIKEQRTKWSEIQQIQEAHRKQKQY
QEHLQMVRQTLATLTIGSQPGLLPPMQLPDLTGLQQFLSSLSAQTALINQPH)}*

$192=296(300):
>XP_019508889.1 {|305(186990)|<Chordata(M)>}PREDICTED: TIR domain-containing adapter molecule 1 [Hipposideros armiger]
{(VSPAPKSLPSPSRNPCPVKDQMPLQPPIEDTTSSTAQPSPPTPSAPKTSSPNPSPSTPLAHPASSSPCPPSPELESSEQK)FY
NFVVLHSREdEHIALRVREKLESLGVPNGATFC---EDFQVPGrGElcCLQDAIDHSAFTILLLTANFDRRLCLHQVNQSLMNSL
TQ-PGRQDCVIPFLPLESSQAQVSPYTSSLLRSL--VWLDEHS--LIFARKVANTFK(PQKLRACKTNWKKDQDTRALREQHQRL
EGERQQAAAWNAAYSAYLQSYLSWHEQMENLQAAFRSHTTLGTQVPYGGQGPL)}*

$193=293(299):
>XP_019406074.1 {|328(8502)|<Chordata(M)>}PREDICTED: TIR domain-containing adapter molecule 1 [Crocodylus porosus]
{(VPPKPQRPQESVPKEPEKEPHVGLPGSSSGSVRLPVEDSYVPTENRSMASSSFVCPPPFQAPAAAPATAPSSEPDTGEGK)FY
SFVVVHAMEdMDIACRVKELLESMGVPDGATFC---EDFLVPGhCQltCFQDAINNSAFTLLLLTENFQCRLCTYQMNSALMDSI
LR-LHKYNSVIPFVPKEC--PLRKGQIPGVLAGL--VPLDENS--RVFARRVKNTFV(PSKIKEQRTKWSEIQQIQEAHRKQKQY
QEHLQAVQRALAALNIGSQPGLLPPVQLPDLTGLHQLLSSLSAQTALINQPH)}*

$194=290(297):
>XP_026229899.1 {|266(64144)|<Chordata(M)>}LOW QUALITY PROTEIN: TIR domain-containing adapter molecule 1-like [Anabas testudineus]
{(SNEPTAFEGKKDLTIDAAFANKSEKLDSRTTQTHTPSQKTKPSTVQTFAVATATDDFPAKIPVSHEMHESQGAEAEEEEI)FY
AFVILHAPEdEDMADKIKERVEVTVNCKGAIFS---DDFAIPGkSHlsCVEDAINNSAFTFLLLTRNFNTRMLDMETNSALINSL
NK-EHKYNTVIPLLPRKNCLPK--QDMPIVLLTL--VPLEEN---RSFEKKIQKAMS(PAKIKEQKKIWSEERRVKIHNEKLERL
KQIKKQSTQDSKSVQLLLEENLRLQNLLLSRSEQHDGDGQARWPQHSNIH)}*

$195=290(297):
>XP_019712450.1 {|304(109280)|<Chordata(M)>}PREDICTED: TIR domain-containing adapter molecule 1-like isoform X1 [Hippocampus comes]
{(PTSSEAPGLEQSKTDAASGTERGKHQDRRFNMKQSCHQSITPTTGSKPSLPFSTNQVLPRRTVSVGAHLSQFGEEEDEVT)FY
SFVILHAPEdSEVAESMKEKLETIIGSKGAIFS---DDFAVPGkSTlrCIEDAINNSAFTILLLTQNFNTRMLEVKTNSALINSI
NK-QHKYNTVIPLLPKENRMPRHR--MPIVLQTI--VSLEEN---KSFETKIKKALS(PAKINRQRSIWLEEQRTHQRRLPQGCE
AAHMLENLNFLGSVDFSSAPSVLQQHPNIHIENASYVMIGNDSQMTVDYC)}*

$196=290(297):
>XP_019955201.1 {|255(8255)|<Chordata(M)>}PREDICTED: TIR domain-containing adapter molecule 1-like [Paralichthys olivaceus]
{(EEPKSNEPAPLETKIDSKRHTALGEHCRKLDIKILAQNQTTKPSSIPNFEPPTTANTLVPKVPVLDDMHKSEAADKEEAI)FY
AFVILHSPEdTDVAESMKEQMEMFSGCEGATFS---GDFAIPGkSTlrCVEDAINNSAFTLLLLTRNFNTRMLEMETETALINSI
NN-KHKHNTVIPLLPRENSMPRQS--MPMVLSTL--IPLEEN---RNFEKKVKRSLC(QVNVKKQKKIWEEGQRMKSEMERQEEL
KRINQYQKQLIQQCKEAQSLERDNLSLLMKQKLLLGQQDVGDGGDRWQQP)}*

$197=296(300):
>XP_020021442.1 {|308(51338)|<Chordata(M)>}LOW QUALITY PROTEIN: TIR domain-containing adapter molecule 1 [Castor canadensis]
{(LLSPIPDPVDTPCTSKXPTPPQPSIEDVTSQNSEPGPPAPSAIHTSPPARTSPPSPPNLVPPAPSSPXPFTFQTETPGQK)FY
NFVVLHARAdEHVALRVRDRLEALGVPDGATFC---EDFQVPGrGElsCLQDAIDHSGFTILLLTASFDCRLGLHQVNQALMSSV
TQ-HGRHDCVIPFLPLESSQGQLSPDTSSLLAGL--VWLDEHS--SIFARKVANTFK(PQKLQARQAQWRQEQEARALREQRQQL
EGQRQQVVAFHAAYSGYLQTWASLQAQMHRLQEAFRSGLSLGAQVPFGEQVPL)}*

$198=226(232):

>OPJ77699.1 {|0(372326)|<Chordata(M)>}TIR domain-containing adapter molecule 1
[Patagioenas fasciata monilis]
{(MPTSEPDSGELK)FYTFVVLHASEdELVAQRVKNLLEKMGVPNGAMLC---EDFSIAGrSRitCFQDALENSAFIILLLTKNF
LCSQCRFQTDTALMESILK-PSKRDSVIPFVPKEN--PLERSQIPSVLGTL--TPLDENS--PMFSRTVQNTFK(PSRIKERKDM
WALMQRRKLQLYEEQTRQQLAALNLGSLPQVPLSATRPLLLEQSSHQWGPPTGHPTPAPVGPSPVQQLQL)}*

$199=273(279):
>XP_020366239.1 {|0(259920)|<Chordata(M)>}TIR domain-containing adapter molecule
2-like [Rhincodon typus]
{(MSEDWGTGNEEHTNSISHDPSPEATKLLTSDQLNSSETMSRKFSLSAPPHLSDEEIFENK)FYSFVVLHAREdAEMAESVQLR
LESLIKMEGATFS---EEFSLPGqCPikCIEDAVNNSAFTILLLTHNFNSHWEEYETNSVLMHSIRN-EHKYNTVIPLLPKKNRM
CK--NNIPFALTAI--NSLDENS--RHFERHIRKTFT(WDVLERHKKSWLQEQRHKQIEEKTAQAKKDYQNAMKALSADRNYMQI
CSQLAQSYQHFQAMYPPHPGLAAVNGHAQPVL)}*

$200=290(297):
>XP_020471693.1 {|259(43700)|<Chordata(M)>}TIR domain-containing adapter molecule
1-like [Monopterus albus]
{(SFEEPQFKSNEPAVIETKRDSTMDEADCMKLVSHTTQPETSDQTTEPKSAPPNTLLPNMPVPNEMHESKGAEEEEEEEAI)FY
AFVILHAPEdADLAGIMRLKLEMVTGCNGVTFS---EEFAIPGrTAlsCVEDAVNNSAFIFLLLTHNFKTRMQQIEAESALMNSI
NK-EHKFNTVIPLLPRENGMPRHS--ISLVLQAI--VPL-EDD--GNFERKIRRVLS(PLKIQKQKEIWTREQRLKSLTKRQNSQ
KQMIQEHKACRERVNLRVCDGRAFCQPQPSIHIQNAKYIMIGDSSQMTVD)}*

$201=290(297):
>XP_020489648.1 {|260(56723)|<Chordata(M)>}TIR domain-containing adapter molecule
1-like [Labrus bergylta]
{(LKSIDHPVFDEKQNPKKVDKSAAESKKLDILIHPTTNKPTKPTTEPTSDLPVSTHINLPKMAAANAMQRSDSTEDEEEET)FY
AFVILHAPEdEDVAESMKEKLETVTGSDGAIFC---DDFDVAGkSTlrCVEDAINNSAFTLLLLTRNFNTPMKEVEADSALINSI
HK-KHKYNTVIPVLPRENFMSRE--DIPLVLRTK--TTLEENK--N-FEKRVKKAMT(PAVIKRQRKMWTEEQTLKMQKKKQERL
QQSNQHRKQLLEESKAVKLLEKEKLDLLLEQKLLLNPDLPPEQDDKARWH)}*

$202=293(299):
>XP_020649920.1 {|303(103695)|<Chordata(M)>}TIR domain-containing adapter molecule 1
[Pogona vitticeps]
{(NAVATGPQAPNKGLPENLGNPSVPQSGLPNPARWSPASTDGPSAPIPKKGEAEPQDYPGSAPSPHPPGSSLGAPAGDESQ)FF
SFVVLHAGEdEEVACRVKNELERLGVSDGATYS---EDFLVPGhCPlgCFQNALDNSAFTLLLLTENFQSRLCAYQTSVALMDSF
QR-LCKRNTVIPFVPKENPIPLK--EMPTLLAGL--VAMDENS--PVFRKRVQNTFT(ARAIQEKKAMWSTLRRAQEIRRQRERE
HEYQQMLRRLSELSPNERLRAPLGGFPGFQDPSGSPPPPAFAATPFPAPRMG)}*

$203=296(300):
>XP_020769932.1 {|352(9880)|<Chordata(M)>}TIR domain-containing adapter molecule 1
[Odocoileus virginianus texanus]
{(MPQTSPSFPSPSLSPFPSPSTSPKSHPTAPKAHSTPPKAHSTSPKAHPTSPKAHPTSPKAHPTVWNPEPSPAELESSEQK)FY
NFVVLHASAdEHIALRVRERLEALGVRDGATFC---EDFQVPGrGElhCLQDALDHSAFIILLLTSNFDCRLSQHQTNQSLMRSL
TR-QGWQDCVIPFLPLESSLAQLSPSTSSLLTGL--VWLDEHS--KIFARKVTNTFK(PQMLRARKAKWRKDQDARALREQSQQL
ESERQQAAAWGAEYSAYVHSYLAYQTQVEKLRVALANYMPFGTQLPFGGQGSL)}*

$204=288(296):
>XP_020778302.1 {|232(150288)|<Chordata(M)>}uncharacterized protein LOC110158200
[Boleophthalmus pectinirostris]
{(LASVPGKPMQPGQTESHIIVNPSCRVHGKNLNFTNPQPPADPTSRPIPKGSLPPSSVAAAAEEKEETEQEEEEEEEEEVR)FY
SFAILHAQQdAELAEALKEKLEGIIESEGATYT---EEFAVVGkAVlrCVEDAINNSAFTLLLLTTNFN-RFLELKTDSALVNSI
LN-PPKYNTVIPLLPQSNAMPRE--DIPMVLKTL--VSLDEKK--N-FERKVKQAMT(PARIKRQKVEWKKVQDMKSLKKKQEKL
KRSNQQQQKENEELRNMHVLEQERRMLLNERYHFVQEQTVGPDRIQLWQ)}*

$205=293(299):
>XP_020826901.1 {|328(38626)|<Chordata(M)>}TIR domain-containing adapter molecule 1
[Phascolarctos cinereus]

{(PVSVKPEETLVSKEGFRVVPELPPMSGPPAGEGKATPLPVQESDFPKKAINPRPSISTQDPSPSPWSSRPSGAELDTGQR)FF
SFVILHAQEdEAIALRVRDTLESLGVPDGTTFC---EEFQVPGrFElrCLQDAIDNSAFTVLLLTKNFDCRMSLHQVHVALMNSL
TR-SEKENSVIPFIPQENTL--KSAKVSPLLKGL--VSLDESS--PVFSKKVRSTFN(PRRLQAQREVWKKQQEIQAIQEQTQQM
AADRERVSQKESALSDLAYHYSLLQQQLQNLALAFPNQASFAQGYRMPMPHP)}*

$206=294(300):
>XP_003760653.1 {|317(9305)|<Chordata(M)>}TIR domain-containing adapter molecule 1
[Sarcophilus harrisii]
{(PPECVSTKPAEILESFGVLPELPPVSVLLAREEKATLLSVQEGDLPKKTSSPRSPVPPQDPSASLSSPLPSGPELDTGQR)FF
SFVILHAQEdEAIAFRVRDTLESLGVPDGATFC---EEFQLPGrFElrCLQDAIDNSAFTVLLLTKHFDCHMSLHQVHMALINSI
TR-KEKENSVIPFFPRESTL--KSTQVSPLLRGL--VSLDEKS--PVFSKKVKSTFK(SRKLQAQREVWKKQQEIQAIQEQTQQM
VEDRKRVSQKKNALSDYTHEYKRLQQQLQSINFVVQNQGPFAYGYSTPQLPPW)}*

$207=293(299):
>OWK51495.1 {|1223(299123)|<Chordata(M)>}Perilipin-3 [Lonchura striata domestica]
{(TAPALVSGQDSNIPAGIPSNSAPASTSTCSLPPPNSFSTLPPLQESPSRVSYPPPLHSSPSPARPPAPPAMDPSEPDGAK)FF
TFVVLHASEdEIVAHQVKNLLEGMGVSNGATLS---EDFFIAGrSHmiCFQEAMENSAFMILLLTKNFPCNLCLYQTDTALMQSI
LD-PSKQDSVIPFLPKAN--ALERSQIPRMLSVL--ITLDESS--PLFSNNVHKTFN(PKKIQKKKALWDQMQRRKSQERWEQHQ
AQQNLAALSLGCPPWVPPASPWPPGPPAQHWCPPAPMDAPPAQMGPPPSQAQ)}*

$208=296(300):
>XP_021488601.1 {|313(10047)|<Chordata(M)>}TIR domain-containing adapter molecule 1
[Meriones unguiculatus]
{(STTEGVRTQSISSQKTPQVSAGDDAPQNATASPPAQPPAPQASPMLSPSPPSSTSSSSSCPAPPASTAPVLGHSETSEQK)FY
NFVVIHSRDdEHVALRIREKLETLGVPDGATFC---EEFQVPGrGElhCLQDAIDHSGFMILLLTASFDCRLSLHQVNHTLMNSL
TQ-LGRQDCVIPLLPIECSRAQLSPNTTSLLHSL--VWLDEHS--PIFARKVANTFK(PQKLQAHWVRWKKEQEARALKEQRTQL
EAERRRVAATSAAYTAYFHSYKAWQEEMNRLGVTFGKDLSLGAPTPFPSWPGC)}*

$209=293(299):
>OXB70737.1 {|353(9014)|<Chordata(M)>}hypothetical protein H355_006521 [Colinus
virginianus]
{(GPACIPAGTSSSTPASTSVCSIPPQTHFSSTIPPPLQRVPYHVPPPAPLHSSSSPTFESSSAFKTVEPALAPEPSGGKKK)FF
TFVVLHAREdEHIACRIKDLLENMGVPNGATFC---ENFLIAGhNQltCFQDAMENSAFLILLLTKNFLCHQCMFQTNSALMESI
QH-PSKHNSVIPFVPKEN--PLERSQIPSMLSGL--VALDENS--PVFARTVENTFT(SGKINERKAMWCQIQQVQEQKRKLDLY
QDHCQTLQNLGALTLGSLPQMSPSAMQLNQSSLEQLLEKLLPLQSSQQCHPQ)}*

$210=290(297):
>XP_022057778.1 {|272(80966)|<Chordata(M)>}TIR domain-containing adapter molecule
1-like [Acanthochromis polyacanthus]
{(RSKSIKSLLGAKKDSRMSETLAAVSSKSDSQSATPSQTNKPSTEPKFALPSATNIFLPKTPFPKEIHESSKVPEEEEEEV)FY
AFVILHAPEdVEVAECMREKLEKVTSSEGATFS---EDFAIPGkSTlkCVEDAINNSAFTFLLLTRNFNTRMLEMKTNSALINSI
NK-KHKYNTVIPLLPRENCMPRQCIPLP--LQTI--VPLEEN---KSFERKIQKSLT(PAKIEKQRRIWTAEQRLKTQMERQEKD
NLTILMEQQLLLGPGGTTEQAGGDGKAWWLQQPSIHIENANYIMIGNDSQ)}*

$211=296(300):
>XP_022381312.1 {|347(391180)|<Chordata(M)>}TIR domain-containing adapter molecule 1
[Enhydra lutris kenyoni]
{(EELGGPKSSSLASRNPCPDKDQTPLPLPVEDKASQVASPRPPMPPAPPTSSPGPPPSTLPSPGLASSPCPPSPELESEQK)FY
NFVVLHVAAdEHIALRVRERLEALGVPNGATFC---EDFQVPGrGElhCLQDAIDHSAFTVLLLTPSFDCHLGRHQASQSLMSSL
MR-EEWQGCVIPFLPRESSRAQLSPHTSSLLTGL--VWLDEHS--QIFDKKVANTFK(QQKLRVRKAQWKKEQEVRALQEQCRHL
EGERQQVAKLNAAYSAYFQSCWSWQEQMEALRAAFGSHMPFGTQMSPGGPGPM)}*

$212=297(300):
>XP_007246244.2 {|271(7994)|<Chordata(M)>}TIR domain-containing adapter molecule
1-like [Astyanax mexicanus]
{(VSQCTDMPGNFQIVTSKNESTVEKAFSSITSSNGKTGAQPVLPSHPLTSRAGSAEYPMQNSEQLSSFSKEKANPEEEEDM)FY
AFVILHDQEdDEEAARLKARLEKISSTPGGTFA---EDFANPGrSTfsCVEDAINNSAFTMLLLTANFNTRLNEMNADSAIMNSI

VK-IHKYNTVIPLVPRDNSL--TNSEMPLVLRTK--VQMKERD--NAFElmaKRVLNPRK(IQKQKQIWKQEQQVRKQQEKQRLL
SEENRRHMDLIRETKRVQELERKLRELQLQQQHLSQPSAPQPQHFHPFSGQAQRFG)}*

$213=293(299):
>XP_026544024.1 {|293(8663)|<Chordata(M)>}TIR domain-containing adapter molecule 1
[Notechis scutatus]
{(GPTTPKTAPSGDLMCLFQAPPLQKPGLAAPSAGRAPPRSSAPAPSTSFGLLVSSLSSTSDLASGSSEPSSSMGPPDDEKQ)FY
TFVVLHAAEdELVAFRVKERLEALGVSNGATFS---EDFLTPGcGQlnCFQNSLDNSAFTLVLLTKNFTGRLCDYQRDTALMDSL
TR-CCKRHSVIPFVPREHSL--KSGEMPLLLASL--VPLCETS--PVFDKRVKKTFT(PKAIREKKATWDLLRQIQEREEERERE
RENLRLQQQLTLLNLQSSFRPPVVDMSRMQAQAGDETQGFPPPAASFMKLGL)}*

$214=290(297):
>XP_023124681.1 {|272(80972)|<Chordata(M)>}TIR domain-containing adapter molecule
1-like [Amphiprion ocellaris]
{(QSKSIESPLLGAKKDSRMSETLAAMSSKSDSQSLTTSQTNKPSTEPKFALPSATNIFLPKTPFPKAIHESSKVAEEEEEV)FY
AFVILHAPEdVDAAECMREKLEKVTSSEGATFS---EDFAIPGkSTlkCVEDAINNSAFTFLLLTCNFNTRMLEMKANSALINSI
NK-KHKYNTVIPLLPRENCMPRQCIPLP--LQTI--VPLEEN---KSFERKIQKSLS(PARIGKQRRIWTEEQRLKSQMERLKLQ
EKDNLTVLMERHLLLNPGGTTEQAGGDGKAWWPQQPSIHIENAKYIMIGN)}*

$215=293(299):
>PKU34580.1 {|410(1758121)|<Chordata(M)>}perilipin-3- hypothetical protein [Limosa
lapponica baueri]
{(SLDDLFEYVTHNIPLNWLVGPFRAIAKVSQDSRKQMKKDTPVPLLLLLIPSPQPFHLLFRELFPTYQAGLASEPDGGDSK)FF
TFVVLHASEdESVAHRVKNLLEKMGVPNGATLS---EDFFIAGrSHltCFQDAMENSAFIILLLTKNFPCNLCIFQTNTALMESI
LN-PSKRDSVIPFVPKEN--PLERSQLPSTLSGL--MPLDENS--PVFSRTVQNTFT(SSRIGNRKAMWDRMQRKKLQQYQEQYQ
PLQNLAALNLGSHPQVPPPATQPQLLEPSARQWCPPASAVPPATDPPHPMPG)}*

$216=293(299):
>XP_026576138.1 {|294(8673)|<Chordata(M)>}TIR domain-containing adapter molecule 1
[Pseudonaja textilis]
{(GPTTPKTAPPGDLMCPFQAPPLQKTGSAPPSAGSAPPRSSAPAPSTSFGLLVSSLSSTSDLASCSSEPSSSVGPPDDEKQ)FY
TFVVLHAAEdELVACRVKERLEALGVSNGATFS---EDFLTPGcGQlnCFQNSLDNSAFTLVLLTKNFTGRLCDYQRDTALMDSL
TR-CCKRHSVIPFVPREH--ALKPGGMPLLLASL--VPLCETS--PVFDKRVKKTFT(PKVIREKKATWDLLRQIQEREEERERE
RENLRLQQQLALLNFQSSVRPPVVDMSRMPAQAGDEAQGFPPPATSFMKLGL)}*

$217=290(297):
>XP_023261679.1 {|257(1841481)|<Chordata(M)>}TIR domain-containing adapter molecule
1-like [Seriola lalandi dorsalis]
{(EEPQPKSNELAQLEANKDSKMESRKCDHHIIQNMTPNTTTKPSTEPKFALPTATNTFVPKVPVPNEKHECKDTEVEEEVV)FY
AFVILHAPEdVDMAESIKDKIEAVAGCTGATFS---EDFAVPGkSTlrCVEDAINNSAFTLLLFTRNFNTQLVEMKTDTALVNSI
NK-KYKHNTVVPLLPRENCMPREG--IPLVVQRL--NPLDE-K--RNFEKKIKKFLI(PAHIERQKKIWTKGQEVNRQIERQDNL
KQLNECQRQLIQECKKAELEERENLNLSLQQNLLLGPEQGGGGGMARWQQ)}*

$218=290(296):
>XP_023699379.1 {|326(1676925)|<Chordata(M)>}TIR domain-containing adapter molecule
1-like isoform X1 [Paramormyrops kingsleyae]
{(ENNGTMTSRITNAKSKFNGGSPSKKPTEVLLSVTTALTENNKPGSATPGNVTNISRTTSPPLNSKDPEEQKEDEVEDEVQ)FF
SFVILHAPEdMETAERLQEKLKSLGIGEGSTFS---QDFLVPGkTKlrCIEDAIDNSAFSILLLSRNFNNQLQEIQTNAALMNSI
EN-VHKYNSVIPLFPSKNRLP--TEDMPKCLKIF--VALEESS--RSFDRMARMAMA(PERIKKQKELWRQDQYIRLQQKRKLRL
KEEIKKSKVLSNQEMEISQLEKEQRQLYMKMRQQNQTLQYYDTPGPISK)}*

$219=293(299):
>XP_023802262.1 {|336(156563)|<Chordata(M)>}TIR domain-containing adapter molecule 1
[Cyanistes caeruleus]
{(PSHESIQDSYIPAGIPCNSAPASTSTCSLPPPTYSISSTLPPLQESHSNLFHTPPLHSSLSPARPPALSAVDPSEPDGAK)FF
TFVVLHASEdEIVAHRVKNLLESMGVSNGATLS---EDFFIAGrSHmiCFQEAMENSAFMILLLTKNFPCNLCLFQTDTALMQSI

LD-PSKQDSVIPFLPKAN--ALECSRIPTMLSVL--VTLNESS--PLFSRNVHKTFN(PKKIREKKALWDQMQRRKLQARWERHQ
ALQNLAALSLGSPPWVPPAVPQPWPAEPAAQHWCPPAPMELPAQQGPPPSQA)}*

$220=293(300):
>XP_026510767.1 {|322(2587831)|<Chordata(M)>}TIR domain-containing adapter molecule 1
[Terrapene carolina triunguis]
{(TMVTAELQERVVQKPEEESPTELPSLCTPGPASGSIRMPVEDSCVLTEKAETASFSSSGGLPPPQAAAAPTPELDCGEKR)FF
SFVVLHASEdVAIACRVKEMLESMGVPDGATFC---EEFLIPGqCQltCFQDAIDNAAFTLLLLTQNFQSHFYMHQMNTALMDSL
QR-LPKYNSVIPFLPKENPS---KCPIPTVLAGL--VPLDESS--PVFSRTVKNTFR(LKRINEQKAMWSQLQHIQEQHRKQQQY
QEHLQMLQQNLVGLNLGSQPGYPSQMPLPGLLPYPAGIQQLLQQLVSSLQLQT)}*

$221=290(297):
>XP_024130230.1 {|248(30732)|<Chordata(M)>}TIR domain-containing adapter molecule
1-like [Oryzias melastigma]
{(KMSSGQPLLSLLQPTGSSLSDPERSHPTLPIGPLEHHPDTPRSHTNPPSSTNICGSRCPIQKKIHDSKGSDEEEEEEEET)FY
AFVILHAPEdEDVAERIKDKIEEVISSKGATFS---EEFAIPGkCPlrCVEDAINNSAFTFLLLTCNFKSNLLEMKANIALMNAI
HK-IHKFNTVIPLLPRENRMPRDC--IPMALKGL--VELDENK--N-FEKKLQKSLS(RAKIQTQKKVWKKEQTLRMTERQSQLQ
ETLRMGLNLNQEESGEDNRTWQQKHPSIHIENANYIMIGNDSKMVLGSGG)}*

$222=296(300):
>XP_026640203.1 {|314(79684)|<Chordata(M)>}TIR domain-containing adapter molecule 1
[Microtus ochrogaster]
{(PTEGVRRQLSVASHMSPLRSVGDDSRQNTTSSPPAQPPSPQTSSTLPPSPPPSTCSSSSCPAPPAPTCPVLGHSETSEQK)FY
NFVVIHARAdEHVALRIRDKLETLGVPDGATFC---EEFQVPGrGElhCLQDAIDHSGYTILLLTTNFDCRLSLHQVNHALMNSL
TQ-SGRQDCVIPLLPLECSQAQLSPNTASLLHSL--VWLDEHS--PIFARKVANTFK(PQKLQAHRMRWKKEQEARALKEQSTQL
EAERQRLAAMSAAYSAYVHSCSAWQAQMDSLRVVFSKDLSLGTPTFFPGWLGC)}*

$223=292(299):
>PWA25120.1 {|131(33528)|<Chordata(M)>}hypothetical protein CCH79_00005233 [Gambusia
affinis]
{(LSSLARHTSTGISQFGAEKGIKIEEASMRSNESATQTLKPYTQTKSSPPAATHIILPSSPGLKSTHISKADEEEDEEEDT)FY
AFVILHAPEdSDVAESMKEKLEAVIDTRGATFS---EDFAIPGkSTlrCVEDAVNNSAFTFLLLTRNFNSQMVEMKTNIALINSI
NK-AHKFNTVIPLLPLENCMPKE--QMPITLQTL--MPLDER---RNFERKLKKAFD(KAKIEKQRKIWDQERRMRMKKKERPRL
CMEQQLVLGAAVQREQDSGDPRPQRLVPNIHIENANYIMIGNDSTMTDCKDW)}*

$224=293(299):
>XP_025028501.1 {|308(176946)|<Chordata(M)>}TIR domain-containing adapter molecule 1
isoform X1 [Python bivittatus]
{(AAAAAAAAAAAGHLPPRFQAPPLQKPGLAAFAGASRPGANSPAPAQITRLGPLLSSFPSDLASSTSAGDSLVAPVAEERQ)FY
TFVVLHAAEdELVACRVKERLEALGVSNGATFG---EDFLASGcCQlnCFQNALDNSAFTLLLLTKNFKGRLCAYQRNTALMDSL
TR-FCKSNSVIPFVPEESSL--KPEEMPLLLASL--VPLNEKS--PIFAKRVKKTFT(PRVIREKQATWDIMRQIQEREQRQERE
REDLQMQQRLSALSLHSPAPTVDVSRLQAPPGRATQVFPTSTTPLMQPGPDQ)}*

$225=294(300):
>XP_006110580.2 {|303(13735)|<Chordata(M)>}LOW QUALITY PROTEIN: TIR domain-containing
adapter molecule 1 [Pelodiscus sinensis]
{(HCPVECTDPPTMGTAGLPESGQRLLQTPGQESPPESRVPIEDSYVLTEKEESESISSSRAPPPPQAAAAPAPELDCGEKR)FF
SFVVVHATEdVDIACRVKEMLESMGVPDGATFC---EEFLVPGqCQltCFQDAIDNSAFTLLLLTQNFASRLCMHQMNTALMDSL
QR-LPKYNSVIPFVPKE--YPLKKSQIPSILMGL--VPLDENS--PVFSRTVKNTFK(LKKINEQKMMWCQMQHVQEQRRKQQLY
REHFQQLQQNLAALTLGSQPGYPPQMLLPTLLPSPAGIQHLLQQLLQLQTPSL)}*

$226=289(296):
>AWP04170.1 {|247(52904)|<Chordata(M)>}putative TIR domain-containing adapter molecule
1-like [Scophthalmus maximus]
{(QSHSSEEPQTKPREPALLEAKTVSKMDAAFAAVCGKSAGPHVQIKTPNESTKLPAATKTFVPDSKEKHESKGAEEEEEAV)FY
AFVILHAPEdTDVAENLKERLETVSGWEGATFS---GDFAIPGrSTlkCVEDAINNSAFTILLLTRNFNTRMVETETDTALINSI

NK-KYKHNTVIPLLPRENPLPKQS--WPLVLHTI--VPLDEN---RNFERKVRSSLS(PAKLKNQKRIWEEGQRMKRQMERQDEL
KRLNEYEKQFIQECKLAQSQERENRRLLVTQRLLLGLSVEPEEDGGDGR)}*

$227=296(300):
>XP_025312834.1 {|366(286419)|<Chordata(M)>}TIR domain-containing adapter molecule 1
[Canis lupus dingo]
{(APSSLSLPSGNARPVKDQTPLPLPVEDTASQLPNPSPPPPSALRTSPPCPFPSTSPSTGPVPSHPCPPSPNSPELESEQK)FY
NFVILHAAAdEHIALRVRERLEALGVPDGATFC---EDFQVPGrGElrCLQDAINHSAFTILLLTPNLDCRLGLHQVSQSLMSSL
TR-HGWQDCVIPFLPLESSQAQLSRDTCSLLSSL--VWLDEHS--RVFARRVVNTFK(AQQLRARKAQWKKEQDIRALQQQRQHL
EGERQQVASLSAAYSAYLQSCSSWQAQMEALRAAFGSHMPFGAQGPYGGPGPL)}*

$228=296(300):
>XP_006754169.1 {|309(225400)|<Chordata(M)>}PREDICTED: TIR domain-containing adapter
molecule 1 [Myotis davidii]
{(TSLPSPCRNTCLAKDQVPLQVPVEDPTSPAAQLCPPTSSAPETSPPNPSPSAPRSAHPASSSSSSSPCPPSPEFEPSEQQ)FF
NFVVLHAKAdEHIALRVREKFEALGVPDGATFC---EDFQVPGrSSlrCLQDAIDHSAFTVLLLTANFDCCLSQHQLSQSLMSSL
TR-PGWQDCVVPFLPLESSRAQLSPDTASLLAGL--VWLEERS--QIFARKVANTFR(PHKLRARKANWRKEQDARALAAWQAQM
EQLQAAFASHMTFGTQLPSGAPGPLGAQPPFPTYWPSYQPPTLPPWLAGTPSP)}*

$229=294(300):
>XP_006896500.1 {|341(28737)|<Chordata(M)>}PREDICTED: TIR domain-containing adapter
molecule 1 [Elephantulus edwardii]
{(EDTASQKSKMCPPPPSIPQMSPPLGPQISPPSVPRSSSPSGLQTSPPSPPGAASPCQPPSTSSSSDLPCPELELESLEQK)FY
NFVVLHARAdEHVALRVRERLEALGVSDGATFC---EDFQVPGrGQlsCLQDAIDHSAFTILLLTPNFDCRLSLHQVSQALLHSF
RR-QAWQDSVIPFQPLETS--ELSSDTARLLTSL--VCLDEHS--QIFERKVRNTFK(KQKLQVRKAHWTREQEARAFRERSQRL
EGQRQQAAELHSAYSTYLQSYLAWQVQVQQLQVAFRTQMSLGMQMPPIGQVPL)}*

$230=294(300):
>XP_006868978.1 {|313(185453)|<Chordata(M)>}PREDICTED: TIR domain-containing adapter
molecule 1 [Chrysochloris asiatica]
{(SPSHPLPSPVLEPTENTSKDGGAPSLPVIKDTASQKTKLCPPPPSASQTSPPSPSSPCAAPSTTSSSDPSPPEVASSELK)FY
SFVVLHARAdEAVALRVRERLEALGVPDGATFC---EDFQVPGrGElsCLQDAIDHSAFTILLLTSNFNCQLSLHQVSQALMTSF
RR-HGWQDSVIPFWPLETS--QLSSDTSRLLCSL--TFLDEHS--PLFDKKVKNTFK(RQNLQARKAKWKTEQEARAFREQSRHL
ESERQQAVEMNAAYSAYLQSYLAWQAQMEKLQVAFGAQMSLGTQMPPGVQVPL)}*

$231=296(300):
>XP_025717171.1 {|363(34884)|<Chordata(M)>}TIR domain-containing adapter molecule 1
[Callorhinus ursinus]
{(VLAAPKSLSLPSRNTCPDKDQPPLPLPGEDITSQVASPCPPAPSALRTSPPCPASSTPSSTGLASSSPCPPSPELESEQK)FY
NFVILHVAAdEHIALRVREKLEALGVPDGATFC---EDFQVPGrGElrCLQDAIEHSAFTILLLTPNFDCHLGQHQAGHSLMSSL
TR-PGWQDCVIPFLPLESSQAQLSSHTSSLLIGL--VWLDEHS--RIFAKRVANTFK(PQTLRARKAHWRKEQDVRALQEQRRHL
EGERRRVAALNAAYSADVQNCLSWQAQMETLRAAFGSHMPFGTRVPPGGPGPL)}*

$232=296(300):
>XP_025875158.1 {|366(9627)|<Chordata(M)>}TIR domain-containing adapter molecule 1
[Vulpes vulpes]
{(APSSLSLPSGSARPVKDQTPLPLPVEDTSSQLPNPSPPPPSALRTSPPCPFPSTPPSTGPAPSHPCPPSPNSLELESEQK)FY
NFVILHAAAdEHIALRVRERLEALGVRDGATFC---EDFQVPGrGElrCLQDAINHSAFTILLLTPNLDCRLGLHQVGQSLMSSL
TR-HGWQDCVIPFLPLESSQAQLSPDTSSLLSSL--VWLDEHS--RVFARRVANTFK(AQQLRARKAQWKKEQDVRALQQQRQHL
EAERQQVASLSAAYSAYLQSCSAWQAQVETLRAAFGSHMPFGTQGPLGAPPPL)}*

$233=294(300):
>XP_003421709.1 {|316(9785)|<Chordata(M)>}TIR domain-containing adapter molecule 1
[Loxodonta africana]
{(PQSLPSPVLDPPDNPTKNGTSVPLSVEDTASQKSKQSPPPPSAPQTSSPSHSASASPACAAPPASSSGPAPPELESSEQK)FY
NFVVLHARAdEHVALRVRERLEQLGVPDGATFC---EDFRVPGrGElsCLQDAIDHSAFTILLLTPNFDCRLSLHQVTQALMSSF

VR-HGWQDSVIPFQPLETS--ELSSDATRLLSSL--VCLNEHS--QLFERKVKNTFK(RQKLQERKATWKKEQQVRAVREYSQHL
EGERQHAAEMSTAYSAYLQSYLAFQAQMERLQVALGTQMSLGPQMPPGVQMPF)}*

$234=293(299):
>RLV90980.1 {|325(44316)|<Chordata(M)>}hypothetical protein DV515_00014273 [Erythrura gouldiae]
{(AAPALLSVQDSNIPAGIPSNSAPASISTSSLPPPNSFSTLPPLQESPSKLSYPPPLHSSPSPARPPAPPAMDPSEPDGAK)FF
TFVVLHASEdEIVAHQVKNLLEGMGVSNGATLS---EDFFIAGrSHmiCFQEAMENSAFMILLLTKNFPCNLCLYQTDTALMQSI
LD-PSKQDSVIPFLPKAN--ALECSQIPRMLTAL--VTLNESS--PLFSKNVHNTFN(PKKIREKKAQWEQMQRRKLQARWEQHQ
AQQNLADLSLGSPPWVPPAAPRPWPPAQHWCPPAPMDPPPAQRGPPPFPAQL)}*

$235=299(300):
>XP_003460977.1 {|292(10141)|<Chordata(M)>}TIR domain-containing adapter molecule 1 [Cavia porcellus]
{(LPNLEPRESLHPVQDQTAPPASAEDTTSPESNPSPPASSAPETSLPRPPAPSRPSSTSAPPASSCPPCPVSFESEAPEHK)FY
NFVVLHAMAdEDVALRVQKKLEDLGVRDGATFC---GDFQVPGrGElhCLQDAIDHSGFIILLMTPNFSGALSLHQVNQALTSSF
TH-HGRQDSVIPFRPLESpsgCRCPEASDTSRLLSGL--VWLDEGS--PIFAKKVANTFK(VQRLSDRRAHWQREQDVRARLRAS
QRLDAETQQAEALRVAQAAYQHSLQAWWLQMERLRGDFGSHLSLGSQVPCPPPGPL)}*

$236=293(299):
>XP_025890944.1 {|243(30464)|<Chordata(M)>}TIR domain-containing adapter molecule 1 [Nothoprocta perdicaria]
{(EDPPASATGSRARPGPIHTSAEGAQASTSSHAXSPTPACSLPPHPPSFCPAAPDFACPPSLHAPCPAAASPSEPHAEQRK)FF
TFVVLHANSdADTALRVKELLEGMGVPDGATFC---EDFLAGGrGQlsCFQDAMENSAFTILLLTKNFLCQVCMFQTNSALMESI
LX-PSKHNSVIPFVPKENPLGE--SEIPIFLRGL--VPLNENS--AVFPSRVRNTFT(RSKISEQKVLWQQRQQAREQQRRLQLY
QEHWQTLRHLSALNLGSLPQAPWPPQPGDAQQVLERLLALWPAPGCPLPTAV)}*

$237=293(299):
>XP_025961666.1 {|358(8790)|<Chordata(M)>}TIR domain-containing adapter molecule 1 [Dromaius novaehollandiae]
{(THRSVEDVSAGKSFHTAPPVSACSLPPHTSSFSSAPPLHAAPPSXAYPPPLHSSSSPACPPPVQAAEAASTSEPDGEERK)FF
TFVVLHASEdVDIACRVKKLLESMGVPNGATFC---EDFLTGGrGQlsCFQDAMENSAFTILLLTKNFLCQLCMFQTNSALMESI
LR-PSKHNSVIPFVPREN--PLERRQIPTLLSGL--VSLDENS--PVFSSTVKNTFT(PSKISELKAMWKQIQQVQEQKRKLQLY
QDHFQTLKNLADLNLGSLPQVPLSAVPNLESLPLLLEQLMPRWSPQQCPFPM)}*

$238=293(299):
>XP_026720904.1 {|359(194338)|<Chordata(M)>}TIR domain-containing adapter molecule 1 [Athene cunicularia]
{(RMSREETYVPAVTPNSTSASISTCSLPPPTYPVSSTLPPHQVTPSSLSHPTPLHSSPSPAWPPPLQTIEPVPPSEPDSGK)FF
TFVVLHASEdEVVAHRVKDLLENMGVPNGATLC---ENFFIAGrSHltCFQDAMENSAFIILLLTKNFPCDLCMFQTNTALMESI
LK-PSKRDSVIPFVPKEN--PLERSQIPSTLGGL--MPLDENS--PGFSKTVQNTFT(TSRIHERKVMWDLMQRKKLQLYQERYQ
TLHSLAALNLGSLPQAPPSATQLQLLEQSPQQWCPPASTVPPTTYPPPPAGV)}*

$239=223(229):
>RMC01007.1 {|0(333673)|<Chordata(M)>}hypothetical protein DUI87_22694 [Hirundo rustica rustica]
{(MNSSEPYRAK)FFTFVVLHASEdAIVAQRVKTRLEDMGVPDGATLS---EDFFIAGrSHmtCFQEAMENSAFIILLLTKNFPC
NLCLFQTDTALMQSILH-PAKKDSVIPFLPETN--ALECSQIPMTLSML--VTLNESS--PLFSRNVSNTFN(PTRIMEKKALWY
QMQRRKLQACWERQQAQQNLAALNLGSAPWVPPAGPRPWSAEPSGQQRRPPAPRGPPPAQLPPDHYN)}*

$240=291(297):
>AYN78122.1 {|237(90988)|<Chordata(M)>}TIR domain-containing adapter molecule 1 [Pimephales promelas]
{(RQPTTNIHTNNQLQTAEANHATNSTQCVNQTGSSQLVTSQNPNIGFSSGLNAANANDCKAPIQANSFCSPEASQSDTEET)FY
AFVILHEAEdADEAQRLRDRLELITSASGATFS---DDFAQPGrSRlgCLEDAIENSAFTLLLLTQNFKSNFSATTADSAIMNSL
LK-HHKLNSVIPLLPRKNRLSRER--IPLVLKTK--FSLDESN--KMFERQASRAIT(PDNVEKQRKVWLIDQHKKKLQEEQKRL
IEENVRNAELQQAKLALLKLESEMRQNSTFYAPSAPTPLNGAMFQQHASW)}*

$241=293(297):
>XP_026783416.1 {|163(310915)|<Chordata(M)>}TIR domain-containing adapter molecule 1-like [Pangasianodon hypophthalmus]
{(ELKFIQCLDKHGTSEIVTPKCPSIDSGLERTFLSSPSGDGKVGAQPVSTSQPPKATSTQEVGKHESYGSEKRDSQEEEGM)FY
AFVILHAEEdSEEAMRLKSRLESISSTTGATFS---EDFAVPGqSTfrCVEDAIENSAFVMLLLTANFNTHLNETNADSALMNSI
EK-PHKYNTVIPLLPRANGLTR--DQMPYALRTK--NPLDEmrDR--DIFEKMAKKVLD(LKKIQIQKTMWRKAQLVKKEREKQQ
RLQEERQYCKDLIRESARVRELEEQVQQLQMQQQRLPPYAHQPNSYQGFPGR)}*

$242=298(300):
>XP_026888704.1 {|165(8005)|<Chordata(M)>}TIR domain-containing adapter molecule 1-like [Electrophorus electricus]
{(KRDQPTDSTSAHFNQCLDMFGNCQIVTSKSTSQEHSLEKSFSSFPRSTAKVGAHPVSPELNTELMDGFSKEKTLTGEEAL)FY
AFVILHAPEdTDEAVRIKARLEAISSTTGATFS---EEFAEPGrSVfrCVQDAIDNSAFSMLLLTQNFNTRLNETNVDSALMNSV
EK-VHKYNTVIPLLPQTN--ALTHDKLPLVLKTK--ISLDERkS--KNFEmmaKKVLNSKK(IQTQKEKWRQELHVKKQQEYQQR
LREENRHCRDLNREHRKVQELEQERMNLLRQKRCSPGPYYPQPFSDQGYFRGAQPHG)}*

_0].
