# Supplementary material for: A survey of TIR domain sequence and structure divergence
Source: Immunogenetics. 2020 Jan 30;72(3):181–203. doi: 10.1007/s00251-020-01157-7 (PMC7075850; doi:10.1007/s00251-020-01157-7)
Supplement: Supplementary file 4 — (PDF 87 kb) [file 251_2020_1157_MOESM4_ESM.pdf]

Supplemental Table 1. Frequency of occurrence of E75 in selected groups of TIR domains.

Group #	Frequency of E75	NADase activity
root	70-80%	
2 (TIR-NB-LRRs)	>90%	++
2.1	>90%	
2.2	>90%	
2.3	>90%	
2.4	>90%	
2.5	>80%	
16	>90%	
21	>90%	
24 (SARM)	>90%	+++
3 (Toll proteins and TLRs)	80-90%	Not tested in all subgroups
3.2 (TLR1, 2, 6, 10)	>90%	absent
3.4 (TLR3)	<10%	Not tested
3.6 (TLRs present in cold-blooded chordata and birds)	>90%	Not tested
7 (TLR7-9)	<10%	Not tested
4 (IL-1R family)	70-80%	Not tested
10 (TIRAP)	<10%	absent
23 (MyD88)	<10%	absent
32 (Arthropoda MyD88)	<10%	Not tested
8 (bacterial)	>90%	Not tested
9 (bacterial)	>90%	Not tested
11 (bacterial)	>90%	Not tested
12 (bacterial)	<10%	Not tested
13 (bacterial)	>90%	Not tested
14(bacterial)	80-90%	Not tested
35 (bacterial)	>90%	Not tested

E75 is critical for catalytic function of TIR domains of NB-LRR plant receptors and SARM1 (Horsefield et al. 2019; Wan et al. 2019). The frequencies of E75 in TIR groups, however, do not necessarily correlate with the enzymatic activity. Thus, E75 is present in more than 90% of TIRs in the groups with reported NADase activity, *i.e*. groups 2 and 24; whereas the E75 frequency varies widely in groups that do not catalyze degradation of NAD. Thus, E75 is frequent in group 3.2, but rare in groups 10 and 23.

Horsefield S, Burdett H, Zhang X, Manik MK, Shi Y, Chen J, Qi T, Gilley J, Lai JS, Rank MX, Casey LW, Gu W, Ericsson DJ, Foley G, Hughes RO, Bosanac T, von Itzstein M, Rathjen JP, Nanson JD, Boden M, Dry

IB, Williams SJ, Staskawicz BJ, Coleman MP, Ve T, Dodds PN, Kobe B (2019) NAD(+) cleavage activity by animal and plant TIR domains in cell death pathways. Science 365*:*793-799
Wan L, Essuman K, Anderson RG, Sasaki Y, Monteiro F, Chung EH, Osborne Nishimura E, DiAntonio A, Milbrandt J, Dangl JL, Nishimura MT (2019) TIR domains of plant immune receptors are NAD(+)-cleaving enzymes that promote cell death. Science 365*:*799-803
