# Supplementary material for: A survey of TIR domain sequence and structure divergence
Source: Immunogenetics. 2020 Jan 30;72(3):181–203. doi: 10.1007/s00251-020-01157-7 (PMC7075850; doi:10.1007/s00251-020-01157-7)
Supplement: Supplementary file 2 — (PDF 21511 kb) [file 251_2020_1157_MOESM2_ESM.pdf]

Supplemental Figure 2. A BPPS output showing annotated sequences of TIR domains that form individual Groups and Subgroups of the presented partitioning. Group 37 contents is shown in Supplemental Figure 3.

[0_(1)=Group1(5357){go=19,gx=2,pn=5.0,lf=0,rf=0};BPPS=(L,6,4:0.8694):(140
)**
****

$1=201(219):
>gi|138198410|gb|EBY35219.1| {|0(97)|}hypothetical protein GOS_6205733, partial [marine metagenome]
{(RLASTKSEPRLSLPPKCVSHS)----FLSHQwgtG-QDQSHTIVRQC--QLLVPGICFWLDVDNL---
DDLsKLEAAVR-LSATITVFlSKGYFASINCRRELYSALREE-----KTIIAV-------
HEADENKGGAPLAELKREAIEFCEEQTAVDMGGTELMQY(CVFDKEAPIVWVRVRDFQIQSLKAIVLRMLRVL
PETKGLRDELWIPGELGPLRLPSPV)}*

$2=189(220):
>gi|138441681|gb|EBZ69821.1| {|56(0)|}hypothetical protein GOS_4297142, partial [marine metagenome]
{(AFEPYRDIAIKNGVDGQTLLELDDEDLEDYIEKRPHRKQIRKKLDAIKASQPQVPKASSKDLTAAIFPSPT
DPSPTDGKV)--KLFLSYPRGDEttAFARKLKAGLEAKGFV-
VWMDEEDITGGVNFMNAIGSAIEASQGIVAVIDEK-FCGSTYCNnELAMAQA---------------------
-------TICSCFP--SSSVICALNLCPAGFDTCSPPX()}*

$3=155(156):
>gi|138621131|gb|ECA86447.1| hypothetical protein GOS_3176704 [marine metagenome]
{()MGEIFISYSASdTKMTEIIRKnHLK------
DFPTWSMNDANVGDDYIkVIDEKLKTCSSAILIITPYFFNSEFIRDvELPALLKRNQD-
DDFKLLPILLNKCEDQDLEKLGTINVFPSKNrpMSRFDSEEWNYYMKRFKSENLGE(LDFEPEIAKNVLWERS
)}*

$4=156(177):
>gi|139289121|gb|ECE46364.1| hypothetical protein GOS_6368438, partial [marine metagenome]
{(MISYCWSQQEEVKK)---------------------
IHGGLKRHGYEIWLDINHMavsGAGDGIleAMAAGVDSADVLVVCVSRQYSQSANCRLEAEYAHKRG-----
KKLIFVMLQKDFTNPSGWLGLLLGTKLWYNGFEGTEEQRLKTLVDAIQSRSS(THTAGGGRQKPAPLQRAKSV
GSI)}*

$5=209(220):
>gi|139266782|gb|ECE41066.1| {|34(0)|}hypothetical protein GOS_3079696, partial [marine metagenome]
{(AQLFGVDLSDASLVGVNCRTLVTFEGEGRFTILSRAIGLTQEQLDEMIGDRGVIIPAHLTYPEHWPEPPGE
AAVPVYTEG)-PFVFISYAHEnKAEVAKIVDYL----
RVQNINIWWDHDIVPGDSWRdQIRDKLAKCQAVLTL---------WTEQsVKSVNVIEEAE-
GGKRQGKLLHAKL-DSAPLPYGFGEVQYADLthWDRRSDTPESLRLLAALKQKLDX()}*

$6=217(224):

>gi|139290361|gb|ECE46770.1| {|20(0)|}hypothetical protein GOS_6353828, partial [marine metagenome]
{(VAAQAAAEAKAKAAAEAASAAEAAAAAEAAAAAEAAAVAAGLAAGLAEDEVRVEEEARAAPSLPSRVPRRS
SLEAERLPV)RYAAFLSHYKVeaateaRWMQQQLET-------TLSERVFLDSDDLNNLSRL--
QEFVRASRCLLLLQTRHVLTRPWCLLELLTAIDAGVPIlGVsIVSGQAPYD---
FAEASVLMTHLDRLLPDERAR-EVQA--LGVDLIDAAWKL(SCTL)}*

$7=127(156):
>gi|139618827|gb|ECG30391.1| hypothetical protein GOS_6316315, partial [marine metagenome]
{(VSYQWDVQATIIRVVE)-----------------------
ALKQRGFAVWFDLEAMSGSTLDAMAEAIEDACCALVCMTAAYKNSANCRLEGNYIHNI-----
KLPWVPLLLEEGYVATGCASVPPAWRLMPSHSIRGACMHTQKKVCLFASVSCW()}*

$8=250(240):
>gi|139720280|gb|ECH00896.1| {|62(0)|}hypothetical protein GOS_3487603, partial [marine metagenome]
{(FTNLNEYTCANINSNLVEMRRVSSVWWYDCNKPVLFSFLAVTITLFTVSSFIAYWKRDYLRYRCHRRRNIP
CAGLHHHHV)-FHAFVCYDRDdsDWIEYEMKKYASSESL--
KIAYVEDQVEIGQSYPVEIRKHLEKSRRVVLVLSPDFVHAnggDQFKYQAQMLEEKMMKTGRKSVLVVRLRPf
saVDfDQMPESRETLKSFLAHSqCLKWKENSHdAqmLFWTQLVGILKSP(LDELYSGSQEADDDPLLEXX)}*

$9=145(159):
>gi|139615964|gb|ECG29147.1| hypothetical protein GOS_6360537, partial [marine metagenome]
{(VCPPARRVPLATQ)-YHLFLSHHwgtG-QDQMRVVKQRLLEVLP--GARIFLDVDE--PGFEIgdL--
EGYVARSRAVLVFcSRGYFQSKSCMRELRAAVANG-----KPLIAL---LEPEAG----
HGGMAEEEVRRQLLHGHCHDESVRWPWVQHAQP(RSVPAS)}*

$10=163(164):
>gi|140250447|gb|ECK55311.1| {|0(91)|}hypothetical protein GOS_3412363, partial [marine metagenome]
{(NTMK)-KRLFVSYSRKdYELVNEFIENTKLFS----
FGVWIDkRDQEYGKKWRdSLTQAIRDSDGAILFVTINSLNSRAVKElEIPEFLSQKDL-RGEDFKLYIVILD-
YIPEKLLNEFHTADDEYifRERHIKNVSANRLDSEQKLPSEM(MSGARQKYWYKLCDEISEDI)}*

$11=164(175):
>gi|140633134|gb|ECM45602.1| {|0(78)|}hypothetical protein GOS_6353632, partial [marine metagenome]
{(F)----FLSHIW-K-TAQDQARVLQSRLQAVvpGLRVWLDVDDLSDLSALeqSINVA----
KAVIVLVTDGYFGSRNCMRELAQCVQTG-----
KPLIAVAEREAAHGGLTDEEAWQQCTAAQAKLAAWGFDATSPAAETMADALL(RRNLPIGFERSGVFQLAMLR
LIVQRLTPNREIYL)}*

$12=216(234):
>gi|140893047|gb|ECO21955.1| {|24(9)|}hypothetical protein GOS_6348591, partial [marine metagenome]
{(ADAKSMHVHKVNKAIEAIVAPASATRAKLAGAKAADEKLAEVAENAAVLGVKAKRRASVQTKPFGAFLSHH
KLACAMEAR)--------------------FLKQELERMlGVEVFLDSDDLH--
DLRQLGQHVKDADVLVLLQSSEVLLRPWCIFELVTAIENNVP-
IVTVAVASKNYDFAEAGKFMLHLDTMLDEANpgasKLLAEQGIDLKQAAWKISSTIPN(VISVSFNPSGSQNV
)}*

$13=162(162):

>gi|141062739|gb|ECP37615.1| hypothetical protein GOS_6348729, partial [marine metagenome]
{(EVGYASQQKIQAPIAPPAR)ATKLMISYCWAQQVPVRQIHSSLLKEGYQ-
IWIDIVHMSAaasGAGILEAMSAAIDAADFVLVCVSREYSRSANcRLEaEYAHTQGKQ-----
IRFLMLQPDfTKPtgWLGMLLGASLWYNFFdgqqG--VDEQVA----GLTAGLAPGAVA(GAV)}*

$14=183(181):
>gi|142343604|gb|ECX40556.1| hypothetical protein GOS_2544240, partial [marine metagenome]
{(MS)-KSIFISYSRKdALDVAEFRSIDAFRKFEILI---
DDEEIEFNKPWKqNIRNKINDSNGAILFLTKNALNPESpIRTlELPLIAKRYAN-KEdnFNFFPIFLEDI-
DEELLNNYSFTLLETKEevNFLEFFQLYGEQQKTTVRELTNR(KRKRFYKTANDHISHILEGGTLSPGETMLS
KLTRKQRIR)}*

$15=190(214):
>gi|142484117|gb|ECY41790.1| {|0(71)|}hypothetical protein GOS_2363893, partial [marine metagenome]
{(EQVFKEI)----------------------SRLHEAGYKIWYD-EGIEASTEWpeEIANAVLGCAA-
FLVFVTPRSTASVNCRnEINLALNENKP-----FLAVHLEDS-
TLPPGLrlrmGDLQAILKYKNTKDRYQRKLDDSLDLLLGKKVAK(GKALRSIPNDSSNALNSKTSKHKIPQKS
GWSKWVLPMVGLVFLGVWGAYEFIQSRGSEQAKVKQSEV)}*

$16=184(182):
>gi|142866708|gb|EDB13957.1| {|0(630)|}hypothetical protein GOS_1875508 [marine metagenome]
{(MFNMN)-KKVFISYSRKdTLDIAEFKTIKKF--RDFEIVI-
DDEEVPFNQLWKdNIRQKINDSDAAIFFISKNALNPESpIRSlEIPLISKRLRdpD-EDFNFFPIFLEDV-
EKELDSYTFTTLKDAKEvkFLDFFQIYDIEKQNTLENLSSRM(RKRFFRNINANISQVLKGGTLSPGQELITK
LSRIQKI)}*

$17=225(228):
>gi|143660086|gb|EDG12600.1| {|0(310)|}hypothetical protein GOS_836021 [marine metagenome]
{(MGMTITILNMPAIWNWPRISVPTLFTCSKEGAIPAKSPTKPRGNRMISSKDVPEPFESYSGDG)-
PYVFVSYAHAdKIMVYESIRYF----
RDAGVNLWYDEGIPPAGEWVeEIAHAIKRSVLFVVFVSPRSVDSRFVKSEVGYALSENKE-
ILTIFLEDTTLPA-
GLSLCLQQFQSILVSEKdwCERACSTMLAKQDEPSPKKEENA(VEEQDEIFDYGAQLWKCWERVIQSQ)}*

$18=174(178):
>gi|144078048|gb|EDI70986.1| {|0(81)|}hypothetical protein GOS_387171 [marine metagenome]
{(MENDVE)QKHIMFSYSW-EH--
KNDVNHIYSLVDNefKEIPKWIDTQSMSGNIIEAMSNAIENAFLVIIFLSKSYKNSKNCKIESELIIGKR---
KDYILVLNDNDFPYLENDEETNWVNDLYKDQFYIDLSDGMDETKILKLLDMINE(KIISYYDFDDKPTFTKKN
SNPKIPRMSLSPLK)}*

$19=191(220):
>gi|144016465|gb|EDI26580.1| {|22(0)|}hypothetical protein GOS_461491, partial [marine metagenome]
{(ELQDRMKFVEIDGYFESYGIPTDHQPSYYSKYVYVKEVLPKVKDEIVLEIAGELGIDHPAVSSIPILKEEE
ATFWKPSHF)--KLFISHLARfKKTIGILKKELEKYGIS------
SFVAHEDIEPTKewqdEIEKGLFSMDALCAVLMPGFKDSNWTDQEVGVAIGRNVL-IIPV-------------
----------RREL--DPYGFIGKFQGLQAVGKNVGQ()}*

```
$20=210(220):
>gi|144125878|gb|EDJ05145.1|  {|84(0)|}hypothetical protein GOS_1763310
[marine metagenome]
{(AIVGGRPVPLFQGALTEPQIRQYLDELLKLAAQAGVTGRIQPPPNLLPPIQPATPQFEVNRPDQSAPEAPS
TAPKTSCET)--RVFVSHASEDAGKAREIVEELSAQGID-
CWLATRDVQVGENYAAQIYSAILDSSHILVLLSQQSVE-SVHVQrEVNIALDQE-----KRILPILV--
TEnaDFMTTLPAEWRYWLGV--IQVAPYSNAHQAVSTVVQRINA()}*

$21=197(198):
>gi|144068207|gb|EDI63877.1|  {|0(145)|}hypothetical protein GOS_398725
[marine metagenome]
{(MYQEKKNM)--KVFISWSgERsKNMAGALKDWL-
PMALQYVVPWMSDKDIQPGERWQtSVGNELQSSDVGIVCITGENLNSDW-LNfE-AGALSKSLA-DSA-
VIPVLLDvEV-
RDVSGPFAQFQAKKMDIegILDITKAINARKGDQTLAESTLS(DAVKGLWTSLDEKLQAIPTNNKEQPQKRKS
DEILEDLVTNVRNLGQQMDH)}*

$22=235(236):
>gi|531009417|gb|EQD68600.1|  {|95(38)|}hypothetical protein B1A_07262,
partial [mine drainage metagenome]
{(GEYSTKLRELTEAAGHRFMPVQLTDSAWPIDKRLEDTNFLRAWAIDGVEERHKFIARRLVHLLIRRLRPRP
ENEDAPPVT)---IFLSHTK-MDLEHEpqVVKKLLAHLTATqPEKTWFDSGDISTGSKFahEIEKGIKDTAL-
LVIVTDSYSSRSWCRKEVLFAKHHQRP-
VVIVDAVQEREIRRFPYSGNTPVIRWKGQAQDAQGVVDLLLREVLRHAYSEESL(RQRKREGDLVLPAGPE)}
*

$23=240(230):
>gi|406533506|gb|EKC58428.1|  {|67(0)|}hypothetical protein LEA_13868,
partial [human gut metagenome]
{(SEAYWGIILCKYGIEYVEDPTTYKRVPTCHRTSYDAITADEDYKLAIQYADISQKIIYEAEAKAIDEIQKG
ILTISQNEK)PYDVFICYKE-
TDEsgKRTqdsvLANDIYHQLTQEGFKVFYAAITLedKLGQEYepYIFAALNSAKVMLVIGSkPEYFTAVWVK
NEwsryLKLMKADR-----
SKlLIPCYKDMDAYELPEEFAHLQAQDMGKIGFINDIVRGIKKVIQKRRAEVR(RKGNRLRQFR)}*

$24=163(166):
>gi|594814836|dbj|GAI85196.1| unnamed protein product [marine sediment
metagenome]
{(M)-EKVFISHSMKdSHLVRVLVKNLEIYSVEA----YIAERDY--
QPGKqlsqKIMQNIDTSNYFLVVYTINGEDSIYVQQEIG-
YWLGKKKFndFIPLVAKGINPRGFLHGLEFIEFDQQNPKLG--
IANVINYVKQQIEIKRKQLLI(DVGIGLGIIGLAALIVYLLSKLDED)}*

$25=206(214):
>gi|319910176|emb|CBI05199.1| hypothetical protein CARN5_1318 [mine
drainage metagenome]
{(MAVTRMYAFRGCPRSAESLPTIAGMRIYAETCFDEAKIAGLIEAAGTLGHELQFRDAAYANRRTVLEKPLA
)----FISHDSRDKEsIARPIALNLQRKICP-
VWYDEFSLQLGANLRESIEKGLKECKKCVLILSPNFLNNRGWGKkEFESIFTRE-----
ILEDSALVLPIWvgVTKEQVYQFSPSLLNV--KGIDWNALGEDEVCRQIYVAAT(SAV)}*

$26=210(221):
```

>gi|402684237|gb|EJX07214.1| {|0(892)|}Sel1 domain protein repeat-containing protein [gut metagenome]
{(MEQPY)---VFISYSSANKAIADATCHILEESGIS-
CWIAPRNIVPGNTWAGNIVQAIRNCSLMVLIYSQQSNNSSQ-VAnEVDKAFCRG-----
KVIIPFLVDDTPmnDDFEYYLSRKHWLVAY--PDYKEMLT--
PLVEAVAANLHF(SLDTLQQPKTKKEVTGSRLGAADGKATDVQGTQPPMYDKALENAQKALLTYDIDQAFAEL
IRPALDGCQEAQFTLM)}*

$27=189(187):
>gi|144208497|gb|EDJ65581.1| {|0(446)|}hypothetical protein GOS_1656784, partial [marine metagenome]
{(MEENNKLK)-KTVFISYSRKdIDLVQSFLD----
VGDNRFFKTKIDQaDKKVGADWRvTIRDMINSSDGAILFISKNALDPKSpINTeEIPLLIKRNND-
PEDKfeFFPVFLDYV-
DESTVDTYTFKEFGTDKeiRLFDKYDIWNIEANNPNDIGQEM(PSEMSENKLENFWSDLNLKLSDALKGKTVN
KLGEIKTWN)}*

$28=226(227):
>gi|594814172|dbj|GAI87083.1| {|0(216)|}unnamed protein product [marine sediment metagenome]
{(MNQKKKL)--
KLFIAYSHLdEEHINEFIKHITPFKNSGLIEGWYDRKIIPGQDFQdKIDNNLENADIVCLFISANFLSSNACK
KeE-KNALKLRRR-KGIVVISIILSPC-GWlDDKELSSLLALPTDGkpITDFTDSN--
KAWNNVYNGLKEA(IEKEIKIKQLKITEQFLFFLQDTELLAKAHSQKEKVSLDEIFIYAELGKYDDLREYEKK
ESSEKLIEDFCEYSKILIAGE)}*

$29=119(142):
>gi|604238738|dbj|GAH12922.1| unnamed protein product [marine sediment metagenome]
{(MA)----------------------
NALINEGLDVWYDEFTLRIGDSLrqKIDQGLANSRVGLVVLSSAFISKGWTNYELDGIV-
TRAVSGEQILLPIWHNITKQQV-VDFSPSLADKVARSTATHTVEEIAKEIAELLRSATQ()}*

$30=158(171):
>gi|604248995|dbj|GAG95454.1| unnamed protein product [marine sediment metagenome]
{(MEDKEIHN)---IFISHYHKDVEELSKL-KELLKGRGH--
EMRdssiddSDPNQAKNPDYIkSLIRpKIEWAGTIVVLIGPETHTRS------
WVNWEIEYANkLGKRIVGVYVRGAK--ESDVPKNLNMFGD-SLIGWDSD-------
KIIGAIEGK(HHDWLTPEGTPREGVWPVDRSSC)}*

$31=182(177):
>gi|594981935|dbj|GAH85935.1| {|0(55)|}unnamed protein product, partial [marine sediment metagenome]
{(MD)--KVFITHSRHdAPILTHITELVKTVGV--EPILY----
QYEYMHTAtawqEIRDDIRRSHAVFVVLSNNLNSSAHTQNwvgwEVGVAcafnkrvWVFEEL-
YRTVSFPIPYLTDYVAYELSNPQLRQLISAV--
ARGYNLEPQLMSTVGVGGLGA(LFFGWPGLVIGAIIGNIVGKPEQAPYIDLMCYHLD)}*

$32=236(241):
>gi|595032769|dbj|GAH51523.1| {|54(0)|}unnamed protein product, partial [marine sediment metagenome]
{(GIEFTGGKPLGPFYVDCDYRLAAAAEGVLPQGVFVTPVGTQGQYDFDVILHVKDDKEARIRELRKPGRSIK
IIAPIVPFA)--KAFLSHSNRnNEIVAELSKLLGNSGI------

LTYVAERVPKPGRkvqeEkIFKKLDRCDFVLALLTPSSLKSKWVKLELKYSKEKDYR-
IIPILRNGTIPKKKRIKDLYSAQLGEELAEY--
LDELDRVRLESSDISDIRKCA(QELISVLIKEKLQEYEDFAPR)}*

$33=208(220):
>gi|598764588|dbj|GAG06438.1| {|26(0)|}unnamed protein product, partial [marine sediment metagenome]
{(LQNTHRLYSELKQKVEISNIYIFANFQDKEQTAIEKEKIEEIFGEKTIALSAVKPESKEVLMNVIEQISNT
AISGKKFLA)----FVSYATAdSEFfeISKIAKKLKKHS-RIDDILYWEEA-----
LKDdiydYMNNNLESCDILLLFCSKNAKNSEPVQMEWKTALKIKKP-IIPIFqtedtIPPLLSTKLGV-----
QFKKYDIDAT--IEQIYKL--ILKKLDLT----()}*

$34=209(220):
>gi|604242096|dbj|GAG91803.1| {|57(0)|}unnamed protein product, partial [marine sediment metagenome]
{(KEKEVLKSVKRVNKEEKILISALNTIKKPSISSSTEGIVAPSCPIETSQSPRIGEIQQYTEEVSMFLEKDS
KIEKLTDKI)--RIFVSYATAdSDYfqVSNISNLLAenpDIG----KVFYWEED-----
LQDdiyeYMNKNLAKCDIFLIFCSANANQSEPVQMEWQAALKIKKK-IIPVFinesdIPPLLSTKLGV-----
QFIENNIKKT--TDQIYQL--VLKKLNL-----()}*

$35=209(220):
>gi|604243531|dbj|GAH00747.1| {|20(0)|}unnamed protein product, partial [marine sediment metagenome]
{(ILERLERVKKEEEIHISALNSIIKPAISSSTTGIGAPACPLETSKLPKMEDIQKLTQGMRREPREIPVKKK
VERKKKDYI)--KIFISYATTdSEYfqISRITNELTKYP-EIEKILYWEED-----
LKDdiynYMNTNVGACDVFILFCSPNALRSEAVQMEWQAALKIKKK-IIPIFieekdIPPLLSNKLGVVF---
------NEED--FNKTIEK--IHKL-ILKKLEV()}*

$36=158(175):
>gi|594862158|dbj|GAI53549.1| {|0(56)|}unnamed protein product, partial [marine sediment metagenome]
{()-----------DAEVALEIALGLEQAGYT-TWCYEVDSIPGPSYLIQTGQAVEQAKAVVVVISP-
HSVGSRQVTkEVVRAHESG-----KEFVPVLRGITHieFQNRQPEWREAMGAAA--
SIGIPLEGVAGILPRIVDGLKA(LGILPSLKAEAAHIARIRRALDELRGRGILEKGRE)}*

$37=220(230):
>gi|595056269|dbj|GAH40550.1| {|132(0)|}unnamed protein product, partial [marine sediment metagenome]
{(KITFEKISSKTGLDIEYLENLSENLIISKEIDAEINGDTLIFRKTIPESKIKEKEIASKKVEIEEDIKFKQ
LPEIKKNLI)---VFVSYATVDAEPfkVKEIAKNLATYKDI-
GDVLYWQEDMEDNIIKYMSDSVEKCDVMVLFCSPNALKSKP-IEkEWTTADIMN-----KPIIPIFVKP-
DHIPPLLKTRLGVEFDT--FDQQKTIDEVYNLILKKTEKRI(INIQDLKTDF)}*

$38=208(220):
>gi|595061946|dbj|GAH28162.1| {|1(0)|}unnamed protein product, partial [marine sediment metagenome]
{(EYFKNLPNLYDKISFDDLISRTSLSRTDLIKIIEKLVFDGEIEATISSNILEFKKEPASPSSYTSETSSVE
AREKREEML)---VFLSYATKD---AEMykipeLARMLEEYKDI-
GKVLYWQEDIHDSIIEYMNDNLGKCNGVLLFCSPNALESTP-VKkEWMAAEALK-----KPIIPIFFKP-
EHIPPLLSDRLGLEFDN--FD--FQKNVRGIHDIFLKKTRS()}*

$39=156(184):
>gi|598626917|dbj|GAG39828.1| {|0(92)|}unnamed protein product, partial [marine sediment metagenome]

{(DIALEITLG)----------------------LEEAGYT-
TWCYEVDSIPGPSYLIQTGQAIEQSKAIILVISPHSLGSRQITKeVVRASESNK------
EFIPVRRDITHveFQNRQPEWRQAVGAAA--
SIGIPRQGVASVLPRIIDGLKG(LGIHPRLKTDAGRIAQIRKAIGELQGRSMLEKAEE)}*

$40=147(149):
>gi|604255155|dbj|GAG97641.1| {|0(71)|}unnamed protein product, partial [marine sediment metagenome]
{(M)--KVFISYTHDgKEHAEKLEKYLNA----
QNIDIWIDkRCIRPGHIWLrEIDDALSQVDYVLGVITENYLNSVGGDEaYAIISKGLKEK-
RIKFISLFFVSPS-KVKSVIIPALQGFNFSKdyTQGFQSFVDFLKQEDVEKLTAEE(EIVTLNEA)}*

$41=169(171):
>gi|598798090|dbj|GAF86415.1| unnamed protein product [marine sediment metagenome]
{(MDQAQVPQPETISLQRGKQIMKKQSSTK)--QVFLSHAR---NDNDLANALAKELRSRGFKVW-
DDQSIGSGLDWsdQIKRALQDSDS-MIALLNQHSYSSSYVRnELEYALfeDEYKNRlLPVFVGSTSDTEF-
VRLPWVLTKLEYLKLSERGSLDAKAIKIADRFSALLRSKR(SDQ)}*

$42=165(165):
>gi|594927392|dbj|GAI16829.1| {|0(41)|}unnamed protein product, partial [marine sediment metagenome]
{(MQ)-YDIFVAYGDPdgvdfaEHIKSYLESLLSK-------
RVFVAKQEFAGGERVwpEIQQAIKESTDFILVLTPWALRSEWVKREVQYALRLRKYIipcKYASVTRENKA--
-KISSKLLALHDIDFSSKEELARKLYSSQSKATRNPENPVQ(TIQPYLLDGAILTFRRFVEMLKA)}*

$43=239(247):
>gi|816569274|gb|KKL74657.1| {|293(0)|}hypothetical protein LCGC14_2062690, partial [marine sediment metagenome]
{(TELNLSNNVIASLPESFVNLTSIRKLDLGYNQLSEIPTQLWALKELTKLHLSNNPLNDEDKNISQKVPDLI
REYLRKKAT)-IKVFISHAVIDFEPYriGELVDYLEKQNEI--SqVFFCEEDL-
AGNIDeWMLDAVQKCQLLLFIGTNKSVFNSVDCDN-----ELQLADkFSIPVIPLKGKDVDwpDLAE------
RNLSRELGLEYDTENFEDFCKNLYKYVENF(KREINLMEKSELRQGIIDIYERYRLML)}*

$44=264(271):
>gi|604370154|dbj|GAG65481.1| {|0(172)|}unnamed protein product, partial [marine sediment metagenome]
{(ANVSFSFSHSNLLFSGSNYQNLIYLQEAIEEVLKKIKVVDNITNNNQESNKIEENIK)EPKIFLSYSHEnK
ETADRVDKFFISKGIQltrdeRDAPAYSSLP----KFMDTIRDH----DYVILLISDAYLKSINCMYEV-
IQFIQEKK-YIEKTFPIIIDKQADIFIKEKHIKYISfWQDR--YKRFRNE--
INKLENTGTWPS(HVELDKIDKIQSNIGEFLNNIAELKCFPLDELENTNYKAILDKIGNKINIPQKEEIKSIK
DENIWDKEWIKKRH)}*

$45=183(173):
>gi|816460453|gb|KKK90556.1| hypothetical protein LCGC14_2721800, partial [marine sediment metagenome]
{(MTLI)---IFVSYAIKdSRIfkISKLAKLLLNYPEIEDVLYCEEYAR------
DdfikYMNEYLDRCDVLLLFCSPNSLNSEFVAMEWRAAISLKKS-
IIPVyldpkYIPPLLSPKLGVefdendpnltIDNIILTIKKNTSIDTSITHLEEK--
IPFRGKMVRYSD(YELLKELQKEINNLVEIFEVNKDSFLISL)}*

$46=264(272):
>gi|816538936|gb|KKL51844.1| {|80(0)|}hypothetical protein LCGC14_2291420, partial [marine sediment metagenome]

{(SLDLSNNKLISLPESFVNLTAIKNLNLSNNQLSEIPTQLWALKELTKLNLSNNPFNPEDATISQKVPDLIR
DHLRKKATI)--RVFISHAVIdFEpyRIKDLVDYLET-QKEISQVFFCEEDLAG-
NIDEWMLDAIQKCQLILFIGTQKSvFDSPDCANELQLA---DKF-SIPVI--
PIKGNEINWPDLAEInlSRELGLEFD--KENFDEF--
CSNLYEYILNFK(REIDLMDKEARQKGITDIYERFRLILEEKISGIKRKIDDIAERIAKLEEKAN)}*

$47=213(223):
>gi|816540015|gb|KKL52637.1| {|246(0)|}hypothetical protein
LCGC14_2283470 [marine sediment metagenome]
{(NLKILKQVTTDPDAYEILKILRTSIVTRNDLEKLGVYDIDDGLSKLWRNKILHVIEDIKGIEYYVLQSDFI
VKKSPVMFN)---IFISYSTKdTDYFNipKIAEKLEEYSEIENALYWTKERGM--
NNFEYMENVLNTSKVFVPFCSKNASKSKAVKNEWMAAFELKQL-DRLKIVPVYEKQKY--LPSLLRPF-
LNVAFD--SRNFEKF--IQNLYKEIMRDI(ESL)}*

$48=172(174):
>gi|816563937|gb|KKL70592.1| {|0(221)|}hypothetical protein
LCGC14_2103360 [marine sediment metagenome]
{(M)--KIFFSYASVdtAEYrIEDIVDFLEFQD-DIERVFYWDRDTKG---
GEsfddYMRNNIKNSDLIIVLFTSNTATSIPVFIEIGMSKAFQKQ-
VMPVFDQVDHIVADMQLSRGVQFNSNFRIFC--
EDLCFKITGKTAKFKESNPMV(DYRNELIESFKFNKSSYVEPEVRDVTDIYKEEE)}*

$49=129(145):
>gi|816583158|gb|KKL85382.1| hypothetical protein LCGC14_1955300 [marine
sediment metagenome]
{(MIKIQ)---VFLSYKWEdRSYADGMDGLLKNPNNRYRHL--
TDRERQDLRNkGEnawkkYLRDKIRDCDALICLIGKDTYNATGVIYELSVATSLGIK-
IIPVRIPNMHGNTPNIINKLKIlnwnAKEINDE---------------------LSKK()}*

$50=125(142):
>gi|816589419|gb|KKL90220.1| hypothetical protein LCGC14_1906880 [marine
sediment metagenome]
{(MA)-KKIFISFAIEdATY----RDLLKGQALNTDTPFtYTDLSA-
KEAWDEkwktNCRTRIKGCDGVIALISKN------TRNADGAKWEMQCaneE-
GIPMIGIHIHKDDKGQIPPQLAGKKVGNwtWDA--IKNFIDS--L-----------()}*

$51=164(167):
>gi|816611033|gb|KKM07521.1| hypothetical protein LCGC14_1733100 [marine
sediment metagenome]
{(MV)--KIFISHSTKdFRLVNVLGNYIRSFGID----VYIAERDY--
QPGKslsqKIFRNIDTSNYFLVVYTINGKDSHYVSEEIG-YWngkkGEGNN-
LIPFVEKGINPEASLCGVEYIEFDPLNPKLG--
MANVTNYVRQQIKLKKKEVIY(DVGITLGFAGIISLIFYGLYKWGEK)}*

$52=147(154):
>gi|816625728|gb|KKM20182.1| {|0(86)|}hypothetical protein
LCGC14_1648040 [marine sediment metagenome]
{(MVLIGQ)---IFISHSRKdKEIVNFFGTILGSTRVKgvFKEI-------
EGFKSEEefqEIQKDIQNSSAIFILLGENVQNLYHTRDWVVWESAIAST-
SGTEIWIFEPLELLGKINVIFPHLNHYLIYK--FDQDYQKYIRDIIESYDDADV(LPATVAGS)}*

$53=217(224):
>gi|816673284|gb|KKM64241.1| {|0(120)|}hypothetical protein
LCGC14_1503370 [marine sediment metagenome]

{(MGIL)---IFISYATKdAELfhIAEIAEKLTQYNEIDDALYW------
QEDVDGniitYMNKNLEKCDVILLFCSQNSLNSEPVQMEWTAAVAINKA-IMPIFI---
YAEYIPPILKPLLGIKFDPSNmhkN--IIELKKT--
IIKRIEKQKSEK(NRGKDLLQDYHGIPVLNNEYNVIMELEFLIGKTIPIKSVNGHVISLKLNNCKLKSIPESI
INLQSLSELDLSFNNLTSLP)}*

$54=264(269):
>gi|816674170|gb|KKM64988.1| {|272(0)|}hypothetical protein
LCGC14_1495820 [marine sediment metagenome]
{(TLDLSNNKLISLPESFVNLTAIKNLNLSNNQLTEIPTQLWALKELTKLNLSNNPFNPEDATISQKVPDLIR
KHLRKKATI)--RVFISHAVVdFEpyHIKDLVDYL----VAQKDIsqvFFCEEDLAG-
NIDEWMLDALQKCHLILFIGTQKSvFNSPDCANELQLADKFSIP-
VIPLKGQDINWPDLAEINLSRELGLEYDKEN--FNEFFEK--
LYKYILNFKQEI(DLMDKVSRQRGITDIYERFRLMLDETINDMKRKMDDLAKRIAKLEEKSN)}*

$55=162(170):
>gi|816679590|gb|KKM69572.1| hypothetical protein LCGC14_1449460 [marine
sediment metagenome]
{(MV)--KVFISHSTKdFKLVDVLGRYLEACAIK----VY--
IAERDIQPGKplsqKIEQNIEKSDYFLVVYTENGKSSEYVQQEIG-LW-KGRR-
HNINLVPFVEKGLDPGAFLCGLEYIEFDPFN--
LNLGFVKVKNFIEMKRKKERN(QLYLGVGVGIGFIGIISLALYGLSKLDD)}*

$56=213(221):
>gi|816466491|gb|KKK94756.1| {|0(41)|}hypothetical protein
LCGC14_2679680, partial [marine sediment metagenome]
{(FDRQYPNDHLDEVTTSPIAAGALPLGQRMAPSEIEVRRLWSDGRF)--
RLFLSHVSKHKVAVSSLKEALCLRGVD-AFVAHEDIEPSLDWQREIELGLRSMHALAAIITPD-
FRESRWTDqEVGWALGRG-----ILVVPVRLGADPhgLAGRFQAVSGSLQQPE--
ALAEDVVKALLANQQTHGEMQR(ALIDTFAKASSCDMAKVLCRLLESITDIADEERDVL)}*

$57=132(152):
>gi|604393924|dbj|GAG60629.1| unnamed protein product, partial [marine
sediment metagenome]
{(MPFKAYEGDKSF)---VFASYSHTdKL---QVYPIIDYLNKIGL-
NIWYDEGIPISENWKkSIVENLDKCSGFLVFITPHIIDSEYVRKEINYAMKMNKP-----VFTIYLK------
------ETKLPHELmfDMDEIQARQFIKEGKFEESLQLL()}*

$58=209(220):
>gi|816731197|gb|KKN14975.1| {|315(0)|}hypothetical protein
LCGC14_0990710 [marine sediment metagenome]
{(EIGTINRIKQTILTLGTKFTRLQLAEITERSGITDEILIESVISDMIRNKEIHGEYFTSTKFLALEAASPA
QIEERVGTH)--NIFMSYSTLdADYFEisKIVRRLELYPEINEVLFW---
EVDSKQNIVeFMEETLNKTDVFVLCCSENSIKSKAVKGEWQSAYQMVKK-GLMKIIPV-----------
YKDEDHIPKLLWqmLNVKYVKDDFEGFIQKLYEEILR()}*

$59=171(174):
>gi|816570356|gb|KKL75492.1| hypothetical protein LCGC14_2054340 [marine
sediment metagenome]
{(MIKNNNRILNKYLILLMSLPFKAYQGSE)-PYIFVSYAHKdKDV---IYNEIKRLNEEGC-
RIWYDEGILPANEWPdEIAVAINKCSTFLVFISPQSIESRNVRNEINYALSKNKR-FVAIFFKETALPP-
GLELQMGSIQAIMKFQMpdNHYYRKLISTFHPSIKRGYQPIP(EVGISE)}*

$60=173(176):

>gi|816589747|gb|KKL90476.1| {|0(71)|}hypothetical protein LCGC14_1904300 [marine sediment metagenome]
{(M)--KIFISYSHKdREFSRRLA---SLLQDKG-
IATWiDDRELNIGDSLIeKINEAIHQANVVLIVLSRNTSNSQS-QLsEIAMAIAARKK-STKQIVSIVLDKN-
IALPFFLKDRLYIDVSGkrGIEERVEQISQTLQSLPTASSSK(SEELSAIEKIKIEQEMLAFEKKAFEQKRLL
RTQTV)}*

$61=226(218):
>gi|816682423|gb|KKM71984.1| {|0(113)|}hypothetical protein LCGC14_1425050, partial [marine sediment metagenome]
{(MSLT)-
KNILAIYDSQdKDVLQHLLAHLRPLEKDFHIAILSDDAIHRGQTWKhQSISLLDHADVFLLLLSNTFMHSEFI
KQdEFKMVIDSYKE-GKATVIPILLDDC-PWDveftftdyNFNFKELLVFRQDEmqIGNWGPKD--
QVYTQVAYYVRGL(LTSSTKKRTLLEPVTTGVKAIPETSKEQQIAIDFLEEREANHKVEWERKDKKEAETKKR
VEEDNQLRDEAEAKE)}*

$62=150(175):
>gi|816693333|gb|KKM81378.1| hypothetical protein LCGC14_1330370 [marine sediment metagenome]
{(MTNCGMVRLAAGVLAGGVFLWQEPALVAREARPER)---IVLSYSHEdAEH-AAVIRH--RLIRAGC-
SVWWDGEIPAGSRyWGrAIERAIDGADVVLrLLGSP----CDYCRNE----TGRAGR-KGIRVVPTKLTSG-
ADTPIHLERLQRLP------------LNRLEEICTKRPPA()}*

$63=174(181):
>gi|816791219|gb|KKN67597.1| {|0(239)|}hypothetical protein LCGC14_0459900 [marine sediment metagenome]
{(MSTEI)----FIGYSLKdkEVLIEALIKKIQNSGFNVKSEL---
SFSIGDNIVEEVADNINTHNFGFIVISKNFLNMNWKKDELE--YLRSLEKTKGKLGLIVYNLE-
DDFINDFFMNISKIKILkIESNEEFENFQPNEILEYIIELY(KIYTYENEDNLRDTAEFEDVTKVILKQLVSK
INILI)}*

$64=186(185):
>gi|816706291|gb|KKM92669.1| {|0(157)|}hypothetical protein LCGC14_1216140 [marine sediment metagenome]
{(MPENGKDKPQVIEQKPVQSSSPL)---
IFISHDSRdADLAEAFSELLGSVSSGMLESFRSSDKkgTEGIEFGDdwykRVMLKLGDASDVVCLLTQRSLER
PWILYEAGVAKGASDT-PVFGIALGVPLRSVSTGPFYRFENCDDTEAD--
LMKLVRQLTSRVPKLKPHDNV(VKSQVETFKARADEILSGLAEP)}*

$65=129(145):
>gi|816720352|gb|KKN05201.1| hypothetical protein LCGC14_1089750 [marine sediment metagenome]
{(MIKIQ)---VFLSYKWEdKNYADGMDGLLRNPNNKYHHL--
TDREKQDLRNkGEnvwkkYLLGKIRDCEALICLIGHDTHSATGVIYELSVASSLGIK-
IIPVRIPNTHGGAPKIINKLKLinwdSREINDE---------------------LSRK()}*

$66=229(214):
>gi|816744907|gb|KKN27552.1| {|0(164)|}hypothetical protein LCGC14_0863490 [marine sediment metagenome]
{(M)--KIFFSYASIdQiDYkIEKIVDFLEFQD-DIERVFYWERDTKG---
GEsfddYMRNNIKASNLVIVLFTPNTKNSIPVLEEIGMAKAFQKP-
IMPIFdhtdkivagiqsirgvkyIPNFRmfcEDLYNKITGKK--AKFREADP--LFEFRNE--
IYETFKFNKTSY(VEPELRDVTEIYEDKDEIEKELPSISYRIFKFILFDPLNRGSLNVITAPSGSGKSILLNS
LKHDILHQEHLSS)}*

$67=172(174):
>gi|816750519|gb|KKN32763.1| {|0(221)|}hypothetical protein LCGC14_0810710 [marine sediment metagenome]
{(M)--KIFFSYASVdtAEYrIEDIVDFLEFQD-DIDQVFYWERDTKG---
GEsfddYMRNNIKKSELIIVLFTANTTTSIPVFIEIGMSKAFQKK-
IMPVFDQIDHIVADMQSSRGVQFNSNFRMFC--
EDLCFKITGKTAKFKESNPMV(DYRNELHESFKFNKSSYVEPEVRDVTVTYKEEE)}*

$68=172(174):
>gi|816754204|gb|KKN35833.1| {|0(221)|}hypothetical protein LCGC14_0779550 [marine sediment metagenome]
{(M)--KIFFSYASVdTtEYrIEDIVDFLEFQD-DIERVFYWERDTKG---
GEsfddYMRNNIKNSDLIIVLFTSNTATSIPVFIEIGMSKAFQKQ-
VMPVFDQVDHIVADMQSSRGVQFNPNFRMFC--
EDLCFKITGKTAKFKESNPMV(DYRNDLRETFEFKKSSYVEPEVRDVTDTYEKEE)}*

$69=195(195):
>gi|135236453|gb|EBG34486.1| {|0(68)|}hypothetical protein GOS_9448610, partial [marine metagenome]
{(MVDHGMIQNMETK)--DVFICHAS---ADKtDVVRPLARALQDEGISYWLD--EEEILWGEpivRKINDA-
MNKSRfvLVVISDAFVHRNWPQFELDAALNLEASGGGNRVLPLLVssDDGGidL-MANYPLLNGKKFIKW-
DGALDMVIKELNLLLSQDGDES(PVSIIDTIDRRKFYMPHIKKEFTQRDKDIFLKEAFGIVKSYF)}*

$70=183(181):
>gi|135288580|gb|EBG65154.1| hypothetical protein GOS_9397265, partial [marine metagenome]
{(FVAELYTRWMERFGNEISKASFPQKEQSSQFAPFED)-DAVFLSYAS---
EDRKAVLNLKTAMEDNGIPVWFDQRCLEAGDDYtaKILENIEKCSL-
FIPVISRHTNTDQkrcFFRlEWRKALEEAKFRpDSVpFILPVVIDDT-
PQDALYVLNEFKKKHWQWFEGGHPTPEFINIVKNRIRQLR(REKRG)}*

$71=152(152):
>gi|135818219|gb|EBK03673.1| hypothetical protein GOS_8798898 [marine metagenome]
{(MK)-PKIFISFSTKdMLFCKDFMQKLSSDHY----
DFWYQEEINVGEEYKkIIKQNIQSSVASILLLSNNFLNSEFITEqELPWIYDKDKT-SMIYeVFPVQIETC-
NWERIDhLKNKQVYPSRSssLDLNDNNQVKTISEQIYKYFKNA(GYIEKKGWFR)}*

$72=292(297):
>gi|135845425|gb|EBK20788.1| {|2(4)|}hypothetical protein GOS_8770510, partial [marine metagenome]
{(KIFYFPLFKDWLFYFSIVNLINFFSELLELPSNPLVTDYGVAITQILLRYIIFWGLPLFVRFKLKQRNLKK
EIINPGEKL)---IFVSFSTDdRLIVNKVITEL---
KEKTNINIWMQEHIHGGSDGPeVIKEKIRESFGALVFFSNNSFQSTFINEtEIPLIKQKQQE-
SEDyFVIPLRVSKT-DPEFLKqFSELQTIPSKSqtITRSNSIE----
FKEIINKIADS(IPDRAKFSEVKKERRDISKILTFFGWVLASLSLIATVLPQNQLNNLFNIFTSESNDDYVEY
IITSDPANDICTLWLQ)}*

$73=182(179):
>gi|136061375|gb|EBL64892.1| {|0(42)|}hypothetical protein GOS_8537137, partial [marine metagenome]
{()MKKVFISYSRKnDQDIFEFRSQEKN--REFEILI-
DDEDLKFDAPWKqSIIKKITEADAAILFISNDALKPESpIRTlELPLIAKriRDEN-DNFIFFPIFLEEV-

DRETLENYEFTIDGTNEkvNFLDFFQVLDITKQNKLKEARKR(ARNNFFRDINGNISSSLKGEGIYSNVLEIS
RIRQKQRAQ)}*

$74=264(255):
>gi|135991562|gb|EBL19280.1| {|155(103)|}hypothetical protein
GOS_8609108, partial [marine metagenome]
{(KADGKPYTDANVMQLVVLKGKRPDLPPAVARGGLLLKLMRKCWDGKPRNRPTFASIEAQLKPQLEQPVSAP
PPASASKPV)--VIFLSYRV--AADADLVERLHDKLAALGVKVWWDKKCLVSGQPWeeGFADGL-
ISAALFVPVLSKAALAPyanltpssrcDNVLlEHRMALEL-KAHdDllPRRIMPLLRGEM-
EVLGTLGECFGDFFAGGSMPASPDVVVDAVETKLAEHMKR(TGLGAPRTPAQSVKTVLGDLLAHQGVFLKGPR
ADA)}*

$75=170(168):
>gi|136858212|gb|EBQ87683.1| hypothetical protein GOS_7684339, partial
[marine metagenome]
{(METDDTRLRSLSMDSSIYNNE)QSHMMFSYSW-ENKDN--
VKHIYDLLDAEfpNISKWIDINEMEGNIIEAMSDAVENSFVVIIFLSRAYLNSKNCKIESELVIGKQ---
RKFLLVlleKGYPYIENNNENWLskmyKNQFYIDLSNG-MNQDSIDKLKQLINNEIIKTYG(EDSFQSS)}*

$76=213(202):
>gi|136905898|gb|EBR14844.1| {|0(74)|}hypothetical protein GOS_7645704,
partial [marine metagenome]
{(ASVMRAVEKQLELPRLRFKAKRNVAAPAPVPSDHWHV)----
FLSYTCTtsegytSTTEQDRMRIIKRLLleMFPTLSVFLDVDDLDTltgvsAKDLdeTIEENISRSRAVLVYC
SKHYFNSRGCMRQLILAVRMNKPIitlIEPDTSRGGLT---
CEEIHKELMNAEGRYDRWWLDDDTMWSIIHHGFDDDIPRA(ARLYDALCSADFIEWNQFGDFQRVT)}*

$77=195(193):
>gi|137607939|gb|EBV02375.1| {|0(32)|}hypothetical protein GOS_6968557,
partial [marine metagenome]
{(MMCFME)---VFISLKGTdRPFADSFIS--
DNLKTKYKFKYWYLFDLKkekTGDNTKeVILNKIDSSSGALLLVSKEFLKSEFINNyELPAIFRKKEE-
DSAfKVFPVFIENC-
DVSENKYLKNLEFYNSPgtNFQTLKNRSAKEYSEKVNEINKF(IDKNISRAGFLLSQLKRSRLIIASLLLTVL
FLLVNNTFYKNRDIDNS)}*

$78=219(234):
>gi|166104027|gb|FD534520.1_EST1 {<Streptophyta(V1)>}RR1F823TF RR1(CS)
Raphanus raphanistrum subsp. maritimus cDNA 5', mRNA sequence
{(GTLSDLRTKPMSSSSSSTCSLLAGK)KVDVFLSFCGGyrhEDLRDD--------
LLKKRIKTFISYRCfqTSVLP-InieTLKALEESKVAVVMASERKPCSVGFLEEIIAILEFQEK-
GSLTVLPFFLTA-----------FSFDLQEEICQEYPEKV-
PSWRDALIKLANI(TANYRFSGMSQVADLIKKIVKGINLPVLTFSSDEINNVLVAMDRHMKVVNDLLASEPNK
EVHTIGIWGR)}*

$79=229(246):
>gi|167498082|gb|FD980737.1_EST1 {<Streptophyta(V1)>}RR3GS24TF RR3(NY)
Raphanus raphanistrum subsp. raphanistrum cDNA 5', mRNA sequence
{(THRHKRIESNLLLSQEVNQTPMTSSYSFLLAGRDL)--DVFLSFSG-KKALDIDFGY---
DLSRNGIKSFKSECWKEKslKP-IdrHTLEALTESKVAVVMTSDEEASSIGFLEELLVILEFQEK-
RSLTVIPIFLTKHALDM------------EEVCRLFPERA-
RMWRTVIAKLENI(ATQYSLSRNLALIHGTHRIKQVADGTRLMFLSCASSDFKGLAGMDRHLKAFHELLALES
DKEVRTLGIWGC)}*

$80=132(159):
>gi|164391613|dbj|DB932480.1_EST1 {<Streptophyta(V1)>}DB932480 full-length enriched cassava cDNA library Manihot esculenta cDNA clone CAS01_035_F20 5', mRNA sequence
{(EAFFHHLSMEATSSTPPCK)-YDVFISFRG-KDIRGRFLSHLFDALQRKQINPFMDENLRKGEE-
IspALLETIQDSYVSIVVFSQNYADSPWCLDELVKILD--------------------------
AGNLGHAHPTIFPCRSNGCSRSHWEFRRGICGC()}*

$81=220(203):
>gi|170639961|gb|FE895658.1_EST1 {<Opisthokonta(E1)>}XYM9963.rev XYM Monosiga brevicollis rapidly growing cells Monosiga brevicollis cDNA clone XYM9963 3', mRNA sequence
{(PRVRPRVRRKEQTLNLDSCSLSRADVLDKMGSILPIQLQAEY)-YHVFISYRHEPDSTKGaatFANEL--
SLHTIGqtgdhISSFLDVVNLEHGKRFdiSFLKALAKSLVYVPLVSakalermegeVGLAQVDHFMLELMVAV
TLERARGFPRILPIFSGEIGEDDEGFPRMESFFSKYDFEHRLPDHINEHTVAAAREFLEQ(HMGIPLGKALTV
RGLLNEIKL)}*

$82=173(193):
>gi|167498979|gb|FD978379.1_EST1 {<Streptophyta(V1)>}RR3G025TF RR3(NY) Raphanus raphanistrum subsp. raphanistrum cDNA 5', mRNA sequence
{(AAKKL)------------------
ISLLVEALQRNEINFFTDKQEKKGEDLSNLFNRIEESKIALAVFSKRYTESRWCLDELVKIKERADL-
GKLKVLPIFYNVTTDNVKYLTEEFGSNLERH-
QSPHEQNKIGKWKEALACISCK(LGCPFIDNNGFSSGTEREFIDSIVKNVLEMLQDISSARTPNSSRSKPV)}
*

$83=182(215):
>gi|167440548|gb|FD945815.1_EST1 {|0(80)|<Streptophyta(V1)>}RS2G413TF RS2(RS) Raphanus sativus cDNA 5', mRNA sequence
{(F)---------------------------------
FIDSNEQVGRTLQHLLVRIEQSRLALAIFSKEYTTSAWCLDELVRIKERIDD-
GKLIGIPIFYKVDPSVVSNLEGDFGDKFRRLKRENRHELVKtHKWKEALTYMTTV(VGLHLREESGQTDNSFI
ESIVRKVQAVLADIPREVNADDSFMIYAKRIEIEHSEKPENWTWSSIHEAPNGKAID)}*

$84=195(219):
>gi|168496059|emb|CU683707.1_EST1 {|0(51)|<Mollusca(M1)>}CU683707 Crassostrea gigas mantle-edge subtracted library, sensitive line (oyge09) Crassostrea gigas cDNA clone oyge09b12m06 rprim, mRNA sequen
{()--HLFISCSTEdiTEV-NKICKELESR-
FFFKCMQHDRDFVIGKPLDDNIQSEMAKSVKVLILMSPAYITSRWCMSEADEAVRLSYSEREKlKIIPVLLRP
PDdG------LNLPPFLNRYrYID------------------
S(QKEKDIPAKIFEAYYHSDAVDYIKIQGEQELTEDEIKNQNGAKLLTKTFIQQRPYYEKGYVFETEQLTLNE
REYLKNLH)}*

$85=197(220):
>gi|164566761|emb|AM854726.1_EST1 {|56(0)|<Mollusca(M1)>}AM854726 cDN20 Crassostrea gigas cDNA clone cDN20P0001F21 5', mRNA sequence
{(SPDAWKGKPLLVFDRTKINCIWFNLYFVVGVSTASGILVLVVCVIIYKKRWWILYRCYQMQKLCMGSERSK
NGYQPINGY)VFDAYISNADEddKWVIEQLLPDIDSGEltevhGDFKLYFKARDAIPGKRDFYnIL-
ENIKVSKKVIIVLTEEYISSDLHQFEIDLAVSLKYKGEIADIIVINVGGVPYXRIPSSL--------------
----------------KS()}*

$86=204(219):

>gi|166897468|gb|FD731256.1_EST1 {<Streptophyta(V1)>}WS03736.C21.1_O13 SS-B-N-22 Picea sitchensis cDNA clone WS03736_O13 3', mRNA sequence
{(KEGQTKICSLCTFFICSSMADLEAQLVSISTPAPPHNTFASTSTSTATHLEDDTCG)-YDVFINHRG-PDVKKTFASHLYRRLLQRGLRVFLDEPELEAGLYLtwQIQRAIRTASVHVAIFSPRYAESKWCLDELVQMFESG-----APIIPVFYHVKPGQLRWTEGqGGVYSKDLQE----LEDK--K-----TYDSQT(HEEKPRHDPDTIQKWRGALSRLV)}*

$87=188(193):
>gi|164570707|emb|AM857789.1_EST1 {<Mollusca(M1)>}AM857789 cdn37 Crassostrea gigas cDNA clone cdn37P0002D10 5', mRNA sequence
{(KTGVTMGAYD)-YDAFVIYNPhgQdQEFVSLMTQVLTSPPYNLRLYVPWTDNNE-PFEAVATAENIEKRCKKvLVVISAVSLESDLFHFQLKVAHSMSPGARSRKIIPIRLDS--TEVPAVIRFTTSCDYYK--KELRV---FVWDRLNSAFCNC(DDNYKSRISKAVSLPEKRALPDLIPTSPLXRVQSWWRLKASVA)}*

$88=218(218):
>gi|164181876|gb|EB656641.1_EST1 {|0(25)|<Streptophyta(V1)>}3X6III_JF_D09 pAPO Cenchrus ciliaris cDNA 3', mRNA sequence
{()-------------RISDLRMRLEGEVRMQtgrPFPIFQDRNDIIWGEQWreRIKGAIDGISFLIPILTPSYFRSHMCREEFEAFQIRERTLgIRRLILPIYYVSADevddAAkyPNDMATILRERNWTDwGELRFPPLKSPIIREQIAQLAKT(IKRTMDDLRAEIDTATQIGLQTARPSPSMPSYTTEFATLNPRIVGNPLAIPVSRSEKYSTSALKRVQKQSYYIYTTKF)}*

$89=178(188):
>gi|167785377|emb|CU417245.1_EST1 {|0(119)|<Ctenophora(M1)>}CU417245 Pleurobrachia pileus library Pleurobrachia pileus cDNA clone SQ0AAA5YM21 5', mRNA sequence
{(MFSDESKI)--NIMISYSHQdSEIMQGIMDVLTA----HGFGVWVDTKLKGGANFFkDIGSAVIGCDIFLFLLSEHSISSKYCQDE----LSLARI-SNKRILP--LTYC-SFREITGVMEAGFRLILscVQWVCLDPKADEVDNNKRFLESL(NEVLQSELSEDARLANYTVVADENHEEEMYRSMGTLRRMN)}*

$90=136(140):
>gi|164582779|emb|AM866267.1_EST1 {<Mollusca(M1)>}AM866267 cdn37 Crassostrea gigas cDNA clone cdn37P0027D11 5', mRNA sequence
{()------------WVRGHLLSHIDERETEennvtvfgtYKIYCSDRDSHHGKSELENMWENIELSRVAVVVLSSSYTQKHLHTVELE----HLLYSKDisviQDIVIIMIEKLkFKQIPT---VLHHQIkHDKFLRWEADET-KekkFYGDLIRYLK--()}*

$91=137(157):
>gi|188292059|gb|EY732629.1_EST1 {<Streptophyta(V1)>}CS00-C3-704-064-G03-CT.F Sweet orange fruit, development stadium (5 of 6) Citrus sinensis cDNA, mRNA sequence
{(SIPMFLLIMASSYRNNK)KYDVFVSFRG-EDTRDNFTSHLYSALCRQNIPTFIDDQLNRGDQ-IseSLVYAIEASAISVIVFSEGYASSGWCLDELVKILECKKE-YAQIVIPV-------------------SIGLIIRLRTNWSLAFIRTEKVTNNDG()}*

$92=224(225):
>gi|188380810|gb|EY828232.1_EST1 {|0(66)|<Streptophyta(V1)>}PT11-C2-300-018-G11-CT.F Poncirus trifoliata bark, greenhouse plant Citrus trifoliata cDNA, mRNA sequence

{(LTSLFLFHLSISPVMVSSSSAPSSSSSASТ)-YNVFLVLLG-EDPHGGFTCHLYYNLneRTK-
IKTFIDDEGLGRGDEIsdALLNAIEGSKISVVIFSKDYASSKWCLNELVKILECQHT-
TGQLVIPVFYGGSALRCSRPTLKFRDTFYIS-
YEPPLDLPPDLTPEPRRSQATK(QPPPPRHSAPPQARTPPRNPRKDHPHTTPQTTRARARRPPPSPPHARERV
AQHPR)}*

$93=200(204):
>gi|190392771|dbj|BY892535.1_EST1 {<Streptophyta(V1)>}BY892535
Cryptomeria japonica male strobilus Cryptomeria japonica cDNA clone
CMFL041_C04 5', mRNA sequence
{(PFLSLFSNVLLVIISILELFAFSSQHRFSLMASSSTSGRKEQAHDPFLVLEPPSKKIKSSLSVK)QFDVFI
NCRG-
PDVQTTLAWQLYSSLKNAGISAYLDSQEIELGDYFpsATRNAIYSARVHIAILSPRYAESVWCLAELALMFQT
-----KARIIPLFYHVQPSDFRHIINGVADAFRKH-EKmgRYPIHDIQQWKECLQNV-TG()}*

$94=149(177):
>gi|192302656|gb|FG994894.1_EST1 {<Streptophyta(V1)>}GLLEG60TF JCVI-SOY1
Glycine max cDNA 5', mRNA sequence
{(HLSVSYLIWRPNKQPSTFILRFLIMASGSPSSSSSSN)-YDVFLSFRG-
EDTRLGFTGHLYNTLQSKGIHTFIDDEELPRGEEItpALMKAIQDSRVAITVLSEHYAS--------------
--------------VSISEATSMASGNKDVKQKKVQEVQIAKKPKRKVRRPAYLKDYA()}*

$95=178(202):
>gi|187062068|gb|FF487747.1_EST1 {<Hemichordata(M1)>}G708P5145RD1.T0
Acorn worm normalized gastrula pExpress1 library Saccoglossus kowalevskii
cDNA 5' end, mRNA sequence
{(FGRXPGIRYSSVMSASIPIEVEVVDDGMSHASAFHDGSVQHVITDTTRHDGLSTTAKEDATK)---
VFFSHSSKdsAWV-KETVERLEQNHSIECL-
FDERDFIAGRPIVDNIIRCIRNTDRTVLVLTPDFLDSPWCGYEAQMIFSEHLFREQKIMIPVLLSE-------
--CIVPDFMKHLtYL-----DVRdpQFW----------()}*

$96=199(203):
>gi|190131406|dbj|DC873722.1_EST1 {<Arthropoda(M1)>}DC873722 SMD06
(Embryos 96 hours after oviposition) Samia ricini cDNA clone
S06A01NCLL0006_G23 5', mRNA sequence
{(ARGEVQDYIPLLIATLGVFLLVLLITLMIFIYRQEMRVWCYSKFGVRLFYRASDVDREDSEKM)YDAFIC-
YSSKdeTWVTDELAPTLEHGDPSYKLCLYYRD--
VGGYVTEKIRQAVQTSRRTIMVLSENFLRSEWIRCELKSALHQVLTDRRKRLIVVLLRQV--
PQRDLDQDIRLYLKTNKCLYADDRLSWEKLQFALPDVR-()}*

$97=233(231):
>gi|194637950|gb|FK909216.1_EST1 {<Arthropoda(M1)>}EST_lsal_evj_955018
lsalevj mixed_tissue_mixed_stage Lepeophtheirus salmonis (Pacific) cDNA
Lepeophtheirus salmonis cDNA clone lsal_evj_522_298_fwd 5
{(GSSVVILSLIIIVIILAFIFRYEVRVWVYSKFGIRLFDKISQDQSIREKDM)LYDAFFAYSNNddVFVRQV
LAPELEHGSHSYKICLLYRDLPTQpSYIADTIVQASGESRRTIIILSDNFLKSEWSRYNYKSGLHQAFgtFKN
VYNLIVIILGNV--SLRDLDPDIRHYF-
SSSVIIRWGDKLFWDKLKYSLPDVE(NADESSVINLQDVRHSSTYSFNYDSCQQSNSSIRTMTIHI)}*

$98=197(195):
>gi|194637951|gb|FK909217.1_EST1 {<Arthropoda(M1)>}EST_lsal_evj_947338
lsalevj mixed_tissue_mixed_stage Lepeophtheirus salmonis (Pacific) cDNA
Lepeophtheirus salmonis cDNA clone lsal_evj_522_298_rev 5

{(FDKISQDQSIREKDM)LYDAFFAYSNNddVFVRQVLAPELEHGSHSYKICFFYRDLPTQpSYIADTIVQAS
GEFRRTIIILSDNFLKSEWSRYNYKFGLHQAFgtFKNVYNLIVIILGNV--FLRDLDPDIRHYF-
SSSVIIRWGDKLFWDKLKYFFPDVE(NADEFSVINLQDVRHFSTYFFNYDSCQQSNFSIRTMTIHI)}*

$99=186(205):
>gi|187082644|gb|FF438338.1_EST1 {<Hemichordata(M1)>}G142P60231RC4.T0
Acorn worm juvenile pCMVSport6 library Saccoglossus kowalevskii cDNA 5'
end, mRNA sequence
{(DNSLCIESVMSISMPIEVEVVGEGGSSNDSIYPTNRIHPRDEGYSADAGEQSQPASARAEEQVAK)---
VFFSYSSKdvKWV-EETVERL-
TESHGITCVYDERDFIAGKPILDNIIHCIRNTEITILVLSPDFINSHWCGYEAQMALIEHLGRGKKIVIPVLI
SEC---------IIPDFISHItYLDIRDPQ---FWEKFL-----E()}*

$100=199(203):
>gi|194633238|gb|FK903336.1_EST1 {<Arthropoda(M1)>}EST_lsal_evj_857557
lsalevj mixed_tissue_mixed_stage Lepeophtheirus salmonis (Pacific) cDNA
Lepeophtheirus salmonis cDNA clone lsal_evj_008_373_fwd 5
{(GESKNT)-
YDVFISYSHEdsDYVEGKLVDGLESPQDdpllKYKYLLAIRDFVPGEVISEQIVQAVDKSSRTIIVLSEAFLR
SDWAQYEFEIAH------SRRKVILLKIGDL-PSKEDIGPSIMNYISQNtYLDHDHPN---
FWRNIRYALPHK(GNSKSWRPTWLKPRKMVNQLHLMETPNSTPVPPFNDKIENTYRNTDNLKHNTSCLPV)}*

$101=163(188):
>gi|186771159|gb|FG057094.1_EST1
{|0(88)|<Stramenopiles(E1)>}CBOU6282.fwd CBOU Phytophthora capsici LT1534
Mycelium small Phytophthora capsici cDNA clone CBOU6282 5', mRNA sequence
{(THSHAEFAKKIRKA)------------------------
LEDANVTTWLDLMDpsgIGGGSVWreEIARGITNAAVVLCLLTEDYAQSEWCLKELALAKQVG-----
TPILAVSTEGVKIGEDLQVYLYTRQLIPFEPAITQTRRNSTNVRQIEYDYDE(AKFKSQFRLLLDGVRDEIEK
NRDAVQQKNIASSR)}*

$102=206(220):
>gi|187136163|gb|FF520753.1_EST1
{|17(0)|<Hemichordata(M1)>}G710P5238RF9.T0 Acorn worm normalized juvenile
pExpress1 library Saccoglossus kowalevskii cDNA 5' end, mRNA sequence
{(IKFCPEKEYRKGEKFWSDSTFAELLGQINYHAAPDPEKISDEYKNYVPQVDDTPIVKPKKKQQTETKEDSP
HNEENIEDP)--DVFISYQWGvqPQIKN-----
LYSKLTSLGYTCWLDIMQMGGGDALygKIDRGIRKAKVIVSSCTPKYALSTNCRREVSLADTLK-----
KPMIPILLE------SMEWPPEGPMSMPFAQLLYIDFTNNNSQKFDNDKFEE()}*

$103=215(223):
>gi|187204972|gb|FF524874.1_EST1
{|17(0)|<Hemichordata(M1)>}G710P5300RH10.T0 Acorn worm normalized
juvenile pExpress1 library Saccoglossus kowalevskii cDNA 5' end, mRNA
sequence
{(IKFCPEKEYRKGEKFWSDSTFAELLGQINYHAAPDPEKISDEYKNYVPQVDDTPIVKPKKKQQTETKEDSP
HNEENIEDP)--DVFISYQWGvqPQIKN-----
LYSKLTSLGYTCWLDIMQMGGGDALygKIDRGIRKAKVIVSSCTPKYALSTNCRREVSLADTLK-----
KPMIPILLESMEWPPEGPMSMPFAQLLYIDFTNNNSQKFDNDKFEELKHRLM(VME)}*

$104=201(198):
>gi|190367004|dbj|BY882771.1_EST1 {<Streptophyta(V1)>}BY882771
Cryptomeria japonica male strobilus Cryptomeria japonica cDNA clone
CMFL014_G18 5', mRNA sequence

{(GFSLPNFPSMASSSSPPEHYNFSTVFSSCSHKLGENPPAFSGIEPQIQRTKVSESTRL)-YDVFINHRG-PDVKETLALELYKSLEKLKIQAFLDSKEKVLGDSFpsTIQTAIHSAAVHIAIFSKGYAESAWCLAELVLMLRSR-----AKTIPVFYRVKPSGLRYIexgvygkafaeyENKERYMEKLEEWKQALQSI--SFIAGEEFN--R()}*

$105=181(171):
>gi|190373180|dbj|BY890919.1_EST1 {<Streptophyta(V1)>}BY890919 Cryptomeria japonica male strobilus Cryptomeria japonica cDNA clone CMFL036_M18 5', mRNA sequence
{(EFDLLMEEVFP)PYHVFINHRG-PDVKKTLASHIYYALETHGLRVFLDKQELQVGDFLtpAIQSAIRSASVQIAIFSETYAQSPWCLDELVWMLDSD-----PtpiKIIPIFYDIEPSELRHVEkeayatafekhrrKGRVTDERVESWIKALEKV--SAISGIPFRTEI(DDHGERLKEIVETVLVKVGR)}*

$106=148(173):
>gi|190375831|dbj|BY886927.1_EST1 {<Streptophyta(V1)>}BY886927 Cryptomeria japonica male strobilus Cryptomeria japonica cDNA clone CMFL025_M08 5', mRNA sequence
{(KNFLTLCSSLCYFSGLQYFVKVNDLLMEDVFPS)-YDVLINHRG-PDTKKTLASLIYRELSEHGLRVFLDERELEVGDPFppAIQSAIRGASVHIAIFSKTYAESVWCLDELLWMFDSH-----GNIIPIFYDIQPWELRHVGKGAY--------------------AGAFQDHRD()}*

$107=193(198):
>gi|190376063|dbj|BY879044.1_EST1 {<Streptophyta(V1)>}BY879044 Cryptomeria japonica male strobilus Cryptomeria japonica cDNA clone CMFL004_D19 5', mRNA sequence
{(GFSLPNFPSMASSSSPPEHYNFSTVFSSCSHKLGENPPAFSGIEPQIQRTKVSESTRL)-YDVFINHRG-PDVKETLALELYKSLEKLKIQAFLDSKEKVLGDSFpsTIQTAIHSAAVHIAIFSKGYAESAWCLAELVLMLRSR-----AKTIPVFYRVKPSGLRYIEXGVYgkafaEYENKERYMEKLEEW--K---QALQSISF()}*

$108=200(191):
>gi|190382567|dbj|BY887707.1_EST1 {<Streptophyta(V1)>}BY887707 Cryptomeria japonica male strobilus Cryptomeria japonica cDNA clone CMFL027_P06 5', mRNA sequence
{(EPTFKVLQPLLQMERGESSTSRANKEPGNPFHGITPQASASSSAVMKKG)PCDVFINHRG-CDTKHKLASTIYHTLDLIGLHAFLDEEELELGDSIpdEIQEAMRTAALHIAIFSERYAESPWCLAELSYMLKTG-----TPILPVFYHVKPVDLRWVGKGKGPYATafshhkrkrrylssiLEDWKAALERV--SNLSGHVLNSTT(GD)}*

$109=174(198):
>gi|190383099|dbj|BY895700.1_EST1 {<Streptophyta(V1)>}BY895700 Cryptomeria japonica male strobilus Cryptomeria japonica cDNA clone CMFL049_O11 5', mRNA sequence
{(GFSLPNFPSMASSSSPPEHYNFSTVFSSCSHKLGENPPAFSGIEPQIQRTKVSESTRL)-YDVFINHRG-PDVKETLALELYKSLEKLKIQAFLDSKEKVLGDSFpsTIQTAIHSAAVHIAIFSKGYAESAWCLAELVLMLRSR-----AKTIPVFYRVKPSGLRYIEKGVY-------------------GKAFAEYENK()}*

$110=182(164):
>gi|190383164|dbj|BY895737.1_EST1 {<Streptophyta(V1)>}BY895737 Cryptomeria japonica male strobilus Cryptomeria japonica cDNA clone CMFL050_A10 5', mRNA sequence
{(EPSKMASPSENQRTLCPSTK)PYDVFLNHRG-PDTKNTLASFIYHSLSESGVNVFFDKREFVPGQNLpgTIHSAIFSSSVQIAILSPRYAESNWCLDELSLMQKA

iqskR-----AELIPVFYDILPEDIDEPqnnegvyaeslgeleqkmsgNERRFKPTEIEEWKKALKEV--
KTTHWPLISYMC(NEDL)}*

$111=141(156):
>gi|190385163|dbj|BY887898.1_EST1 {<Streptophyta(V1)>}BY887898
Cryptomeria japonica male strobilus Cryptomeria japonica cDNA clone
CMFL028_H16 5', mRNA sequence
{(YASTSGGSMRPVKNLS)-CDVF--HRS-
PEVKqHTLVITLYKMFNAMGMDCFWDSGELKLVDHLpgKLEAA----
WLHIAIFPDQHAQPWLCLAdlstDLAFMFKTY-----VRIVSIIYHVKSEGVRYAKGVSADA-----
FHWSEKQS--RYT--PEKLQEW()}*

$112=209(198):
>gi|190388529|dbj|BY888260.1_EST1 {<Streptophyta(V1)>}BY888260
Cryptomeria japonica male strobilus Cryptomeria japonica cDNA clone
CMFL029_H09 5', mRNA sequence
{(GWNQDAPLTVFSLRSFEFDSIEMASSSTPWENQTQVENAFDSLAPDSAPKSRE)PWDIFINHYG-
LDVKYTLANTIYHKLHSMGLHVFLDSETLEAGDFFpaELQHVISSATLHIAIFSPNYAQSIWCLAELSFMLKT
G-----
AKIIPIFNHVDPSDLRWVNqgngkyayafsehekKGRYKSEKLDEWKRALHICSCLSGYLVSNNEDE(ATVLK
)}*

$113=203(220):
>gi|190388716|dbj|BY892042.1_EST1 {|14(0)|<Streptophyta(V1)>}BY892042
Cryptomeria japonica male strobilus Cryptomeria japonica cDNA clone
CMFL039_M22 5', mRNA sequence
{(GSGKQNLTLSNRHCLVKSNPIFFDWWPLLLHLPFFLHFFFIVSKKYYRSSYLWFREFHTSSTFMASSSTTQ
SRSIVSPKL)-YDVFISHRG-
PDVKDTLAKQLYDLLKERECKAFLDRKELQGGDSItsAIHNAICSSRVQIAIFSKGFAESSWCLDELVLMLQQ
T-----DAVfISVFYDVEPKELRSTERGSYAA-------------AFSDHPNKEKYRNN()}*

$114=196(186):
>gi|190389270|dbj|BY896450.1_EST1 {<Streptophyta(V1)>}BY896450
Cryptomeria japonica male strobilus Cryptomeria japonica cDNA clone
CMFL052_A09 5', mRNA sequence
{(GFRLVIQFNFMASTSISGLQLDTELSAAFEEIAPLSAPASGTSSML)PCDIFINHRG-
PDVKHTLANSLYDALHKTGLRVFLDVEEIELGDFLptTIEEAMRSASLHIAIFSKNYAQSPWCLAELSFMLnK
TD-----KQIIPIFYHVTPMDLRWVtkgkgifidafsdheQKGRYSQDKIDQWKLAL--
HTVSFYSGEIINNNE()}*

$115=174(173):
>gi|190389570|dbj|BY880980.1_EST1 {<Streptophyta(V1)>}BY880980
Cryptomeria japonica male strobilus Cryptomeria japonica cDNA clone
CMFL009_H20 5', mRNA sequence
{(FSFISLGMASSSTADQNHAFDQIVPYSAPKFTE)PYDIFINNRG-
CDAKHTLATTIYDKLRSMELKVFLDMEaTLEAGYGIppEAEHAIVSATLCIAIFSPNYAQSPRCLAELFFMLK
NG-----KRIIPIFYHVDPFDLRFirqgkyapafaehEKKGSYTPEKLDEWKSAL---------
HDISVLSG()}*

$116=140(146):
>gi|190392734|dbj|BY892526.1_EST1 {<Streptophyta(V1)>}BY892526
Cryptomeria japonica male strobilus Cryptomeria japonica cDNA clone
CMFL041_B19 5', mRNA sequence

{(MEEVFP)PYHVFINHRG-
PDVKKTLASLIYHRLEGHGLRVFLDKQELQVGDSLtpAIKSAICSASVQIAIFSKTYAQSAWCLNELLWIFDS
NS----SKIIPIFYDIQPSDLRHVDKGAY---ARAFQEHRGKGRVTIQQLDSWMSALN()}*

$117=203(218):
>gi|190396057|dbj|BY881308.1_EST1 {<Streptophyta(V1)>}BY881308
Cryptomeria japonica male strobilus Cryptomeria japonica cDNA clone
CMFL010_G05 5', mRNA sequence
{(RLTLVTCLLHFNDFLALPLPNIKPASIALLYNPLSLRPDSMASSSTTGSKGNAESIDAFHGIAPPAPTRAS
EPMKKPA)-YDVIINHRG-
PDVKSTVASVLYGILTGMQLSVFLDSKELVSGDFLprAIEAAMSSALLHIAIFSERYAESPWCLAELSFMLKS
G-----APIIPIFYYVNPSELRWLDQGN-EGKYAQSFKKHEEKG--RY-------LGR()}*

$118=205(207):
>gi|187048180|gb|FF436548.1_EST1 {<Hemichordata(M1)>}G142P60205RD10.T0
Acorn worm juvenile pCMVSport6 library Saccoglossus kowalevskii cDNA 5'
end, mRNA sequence
{(HADLRLLAESELSDVQLTARERAQLPKKGSY)-YDAILLSSTHdESELEELLSLFE--
GWQLKVCSFEGFKTGGKNVFENFKDAMYS-
CTYiVVVLTKNFLKDKWCEKHIHTSLITAIDDPEKHnfVVPIVYGLQDKEIPTELKSVACLKRSN--
RFYKEQLEKTITTLQREIKVE(EDEERYAEAEMKLKKQKLLKSLNKPRDELLLQQQYK)}*

$119=182(186):
>gi|189407868|gb|EW779313.1_EST1 {<Mollusca(M1)>}cg_GA0030.A06 Pacific
oyster hemocyte library Crassostrea gigas cDNA, mRNA sequence
{(GEITMDNQIN)QYDAYVIAGDteEdKLFLETMKSNLESSAFNLRLYIKSRDG-
PGDFDHQKESEIIMNSCEKiIVVLSRDFTQNEQCMYLLRVAISKSPGFHLRHIIPIHREECL--
APSCIRFLTANDYTK--EDIRT---
WFWPRVAACFKKN(VINIRTAKKNLPAVVCLPDIIVDSFSSEKQQEWRNN)}*

$120=232(235):
>gi|194609362|gb|FK876517.1_EST1
{|69(0)|<Arthropoda(M1)>}EST_crog_evp_905546 crog_evp Caligus
rogercresseyi cDNA clone crog_evp_511_362_fwd 5', mRNA sequence
{(KKESFTTRIVILWSRKPGTCVFSLFQALQCMDRPDVLQDYASSILEDCLSLQSNESPQGIDQALTLHDLEA
LSRNEPLTI)-YDAIVLFGDSnldVKFGMHVVERLESAGK--
KVFLYDRDLIIGAIEQEAAMRIVRARCRKvIAILTPTFHQNKKSLFLTSYALHLQnESEKMTRIIPVIYSSNY
LESEVMKNIAMYSKLKY--DKTNT---YFWKKLLAGLGLL(APHPELPAMSFPMPQ)}*

$121=187(219):
>gi|194609363|gb|FK876518.1_EST1
{|0(33)|<Arthropoda(M1)>}EST_crog_evp_900554 crog_evp Caligus
rogercresseyi cDNA clone crog_evp_511_362_rev 5', mRNA sequence
{()----------------------------
KVFLYDRDLIIGAIEQEAAMRIVRARCRKvIAILTPTFHQNKKSLFLTSYALHLQnESEKMTRIIPVIYSSNY
LESEVMKNIAMYSKLKY--DKTNT---
YFWKKLLAGLGLL(APHPELLQMSFPMPQDFDASNLEKTVASTSNPSISIEVPGIRASAVALDLPTIPSSPTD
DNSAESINSQTALILPNTPS)}*

$122=205(220):
>gi|194609426|gb|FK878979.1_EST1
{|69(0)|<Arthropoda(M1)>}EST_crog_evp_913388 crog_evp Caligus
rogercresseyi cDNA clone crog_evp_515_140_fwd 5', mRNA sequence

{(KKESFTTRVVILWSRKPGTCVFSLFQALQCMDRLDVLQDYASSILEDCLSLQSNESPQGIDQALTLHDLEA
LSRNEPLTI)-YDAIVLFGDSnldVKFGMHVVERLESAGK--
KVFLYDRDLIIGAIEQEAAMRIVRARCRKvIAILTPTFHQNKKSLFLTSYALHLQnESERMTRIIPVIYSSNY
LESEVMKNIAMYSKTQY--DKTNT---Y------------()}*

$123=203(219):
>gi|194609427|gb|FK878980.1_EST1
{|0(33)|<Arthropoda(M1)>}EST_crog_evp_916460 crog_evp Caligus
rogercresseyi cDNA clone crog_evp_515_140_rev 5', mRNA sequence
{()-----------VKFGMHVVERLESAGK--
KVFLYDRDLIIGAIEQEAAMRIVRARCRKvIAILTPTFHQNKKSLFLTSYALHLQnESERMTRIIPVIYSSNY
LESEVMKNIAMYSKLKY--DKTNT---
YFWKKLLAGLGLL(APHPELLQMSFPMPQDFDASNLEKTVASTSNPSISIEVPGIRASAVALDLPTIPPSPTD
DNSAESINSQTALILPNTPS)}*

$124=239(242):
>gi|194621526|gb|FK893299.1_EST1 {<Arthropoda(M1)>}EST_crog_evm_807717
crog_evm Caligus rogercresseyi cDNA clone crog_evm_504_261_fwd 5', mRNA
sequence
{(FTFFQVILWSRKPGTCVFSLFQALQCMDRLDVLQDYASSILEDCRSLQSNESPQGIDRALTLHDLEALSRN
EPLTI)-YDTIVLFGDSnldVKFGMHVVERLESAGK--
KVFLYDRDLIIGAIEQEAAMRIVRARCRKvIAILTPTFHQNKKSLFLTSYALHLQnESEEMTRTIPVIYSSNY
LESEVMKNIAMYSKLKY--DKTNT---YFWKKLLAGLGLL(APHPELLQMSFPMPQDFDASTWRKRL)}*

$125=206(221):
>gi|194621527|gb|FK893300.1_EST1
{|0(33)|<Arthropoda(M1)>}EST_crog_evm_810597 crog_evm Caligus
rogercresseyi cDNA clone crog_evm_504_261_rev 5', mRNA sequence
{(NL)----------DVKFGMHVVERLESAGK--
KVFLYDRDLIIGAIEQEAAMRIVRARCRKvIAILTPTFHQNKKSLFLTSYALHLQnESEEMTRTIPVIYSSNY
LESEVMKNIAMYSKLKY--DKTNT---
YFWKKLLAGLGLL(APHPELLQMSFPMPQDFDASNLEKTVASTSNPSISIEVPGIRASAVALDLPTIPSSPTD
DNSAESINSQTALILPNTPS)}*

$126=135(140):
>gi|194629012|gb|FK898650.1_EST2 {<Arthropoda(M1)>}EST_crog_evm_814622
crog_evm Caligus rogercresseyi cDNA clone crog_evm_512_062_rev 5', mRNA
sequence
{()--DAIVLFGDSnldVKFGMHVVERLESAGK--
KVFLYDRDLIIGAIEQEAAMRIVRARCRKvIAILTPTFHQNKKSLFLTSYALHLQnESEKMTRIIPVIYSSNY
LESEVMKNIAMYSN------SNTIRQTLTFGRNSLQGLDS()}*

$127=157(160):
>gi|194634656|gb|FK904512.1_EST2 {<Arthropoda(M1)>}EST_lsal_evj_936384
lsalevj mixed_tissue_mixed_stage Lepeophtheirus salmonis (Pacific) cDNA
Lepeophtheirus salmonis cDNA clone lsal_evj_516_096_fwd 5
{(GESLSRNEPLTT)-YDAIVLFGDSdLDlkFGMHVVERLEQSGK--
KVFLYDRDLIIGAIEHEAAMEIVRARCKKvIALLTPTFHKNKISLFLTSYALHLQNDpDKMTRIIPVIYSSHH
LESEVMKNIDMYSKLMY--DTSRP---YFWKGSFFHSVIL(MCIQTYYK)}*

$128=238(223):
>gi|193864679|emb|AM763998.1_EST1 {|0(37)|<Porifera(M1)>}AM763998
Oscarella lobularis whole organism adult Oscarella lobularis cDNA clone
IL0AEA6YF07RM1 5', mRNA sequence

{(SGSNVEILSSVVMTQGKGEMAPQLSVVEGPSSEKD)---VMLCYSRAdLSLLRKMKNALA----
ERGITAWYDTQdIDKGTYWTeSIAVAIKDCRAFVFLMSKNSLKSSYCMEELQMARAFGKpvfpvA-
TDSNLIPILptwaliiihiqwtfLSED-
AWNQSEFNKLCSAISRSigKELPTEMIEADVEDAEPSAQSEE(LTFEDFWSQSFALDTVFAVDLCKKLIVKCS
PQLSQHFGKDALHEAIDV)}*

$129=204(207):
>gi|187042051|gb|FF438570.1_EST1
{|0(5)|<Hemichordata(M1)>}G142P60235RA8.T0 Acorn worm juvenile pCMVSport6
library Saccoglossus kowalevskii cDNA 5' end, mRNA sequence
{(IHGNLINMNNPRVMFDSQEPIERSDDSGAALPSTTCTFPATEKTKEKRC)----FISHSSEDaeWIK-
EMITKLEK--
ENDIRCIYaERDFYPGKPILDNIVTSIRNSTIIIVALSPAFLDSRFCEYETQMALMEHLTTAPKQIVSIRLKE
CVIPdFISHLTYLDVEDsNFWEKFLLILKT-GTTGSVGSQFAST(LNKYNGTNVWCIPFSAYA)}*

$130=174(189):
>gi|187050950|gb|FF425620.1_EST1 {<Hemichordata(M1)>}G142P60042RG11.T0
Acorn worm juvenile pCMVSport6 library Saccoglossus kowalevskii cDNA 5'
end, mRNA sequence
{(IHGNLINMNNPRVMFDSQEPIERSDDSGAALPSTTCTFPATEKTKEKRC)----FISHSSEDaeWIK-
EMITKLEK--ENDIRCIYaERDFYPGKPILDNIVTSIRNSTIIIVALSPAFLDSRFCEYETQMA---------
---LMEHLTTAPKQiVSIRLKECVIPDfISHLTYLDVEDS-NFWEKFLLILKTG()}*

$131=187(190):
>gi|187054141|gb|FF437963.1_EST1
{|0(16)|<Hemichordata(M1)>}G142P60225RC2.T0 Acorn worm juvenile
pCMVSport6 library Saccoglossus kowalevskii cDNA 5' end, mRNA sequence
{(QEPIERSDDSGAALPSTTCTFPATEKTKEKRC)----FISHSSEDaeWIK-EMITKLEK--
ENDIRCIYaERDFYPGKPILDNIVTSIRNSTIIIVALSPAFLDSRFCEYETQMALMEHLTTAPKQIVSIRLKE
CVIPdFISHLTYLDVEDsNFWEKFLLILKT-AQQEGVGSQFAST(LNKYNGTNVWCIPFSAMP)}*

$132=195(200):
>gi|187100343|gb|FF425700.1_EST1 {<Hemichordata(M1)>}G142P60043RH9.T0
Acorn worm juvenile pCMVSport6 library Saccoglossus kowalevskii cDNA 5'
end, mRNA sequence
{(FSALKEKYETQQYLPKEVPVE)-YDVYLSHsESDSSSTDAIKQGLKKQKSNIKIF-
EERQDLNTDEVWQENIYQAMMkCAKIVTVLSPAYLSSTCCMEQYNIALCCNRKTKRDMLAPFYVESVPV-
LPTYMGLVQYVDCRP----
HAIQKIQVACVQLIDSLSK(LISLLPQNRSHVSQRLKFEYDVFLSYSHKNSNKASLLLN)}*

$133=210(220):
>gi|211386377|gb|FG541308.1_EST1
{|40(0)|<Arthropoda(M1)>}OtP007C08_012507d Onthophagus taurus pupal
library Onthophagus taurus cDNA clone OtP007C08 5', mRNA sequence
{(DVNAVATVMQKKILTNNILLGGNYIPLLAASLVTLIILFLIFSIIFAFRQDFKLWIHSKYGLRIFKKSSGE
VENFEENDR)LYDCYMVYSYKdeELANRIIGPDLE--AHGYTLCLHYRDL-
HLVGGASylvdAMVGAADASKRVIFVVSPSLFSNEWQRPEFRAALQTAITTRfRYKLICILYGE---
PLEPMDPELRALLRACSVtRWGERR---FWE---------()}*

$134=202(221):
>gi|208423310|gb|GE161086.1_EST2 {|0(77)|<Haptophyceae(E1)>}CAHP3937.fwd
CAHP Emiliania huxleyi CCMP1516 day 14 post innoculation L Emiliania
huxleyi cDNA clone CAHP3937 5', mRNA sequence

{(SFSQVGKTLYSMVRARLRLKRTGLAPRLGALPKKCHFHLYASHVWSS)---------G-
QDVTHTLVRQLQRLLP--GIRIWLDVDCLEDIDRLeeSVEDA----
AVFLLVLSQGYFQSRNCLREVYAAISAG-----
KPILTIKAAGDVTDMLAECRESSTHESVLEKYPDVGLEELMAKVFEREPMLW(ARIYAYQIESLKLVALRTLQ
HLPLYVKHPATLRG)}*

$135=213(220):
>gi|211701797|gb|GE554335.1_EST1 {<Streptophyta(V1)>}CCHT1280.b1_P07.ab1
CCHT Niger Seed Guizotia abyssinica cDNA clone CCHT1280, mRNA sequence
{(FSSSAIFPRSINHKHTFNSMASSSSYGHNKSY)QYDVFLSFSG-
EDTRKTFIDHLYVALDQQGICTLGGDERLakGEKINDELLQSIKDSRLYIIVFSKRYASSSWCLN-----
LNFCERLKRRLFIKQF---
ESLEVIYMKGLNTMELMDHIFTGHSNNSLLELVLTHYPGCIX(VPSSIGNLHKLVSLTIYNVNSLPGSICTLR
HLRTLDLTYSIIEELPED)}*

$136=226(228):
>gi|226745399|gb|GO502072.1_EST2 {<Streptophyta(V1)>}Mdrtc1032I02.g1
Apple_EST_Mdrtc Malus hybrid rootstock cDNA 5' similar to emb|CAD36200.1|
TIR-NBS disease resistance protein [Populus balsamifera s
{(NSVAVADIRESLTCQQMNTAGG)KYDVFLSFRG-E-
TRKGITDHLYHTLNDYKFNVFRDKDRLicGDVIKENLKQAIKESRLAAIMFSKGYAGSRWCLDELVEIMECSR
TQGQH-VFPIFFDVDTSDVRHQTGSFETAFKNHEQKFEEEMV-
RKWGNDFKATAEF(QRDEQEFKEKVRIWRDALHAAAGLSGKDFQVTDGHEGSFIREVVGQIATILKKPCLNVA
TNPVGID)}*

$137=196(195):
>gi|211765279|gb|GE603274.1_EST2
{<Streptophyta(V1)>}CCPX11423.b1_M24.ab1 CCP(UWX) Globe Artichoke Cynara
cardunculus var. scolymus cDNA clone CCPX11423, mRNA sequence
{(EVGDFWCVSAFVMMASSSSHSAHRWT)-YDVFVSFRG-
EDIRRNFVDHLFSDFRRKGIYAFRDDDrLKRGEE-
IssELYKAIEQSRFLIVIFSNDYASSTWCLRELVKILKCKetEK-
DKYEVRALYYDVTPPDWCYKKFKGIAICLVFTPRNPGRHKSSYGSIGFRFKNFD(GTPIGGEFPIPDSVFQYE
NIGIKSDQMLL)}*

$138=139(165):
>gi|223423482|gb|GO013203.1_EST1 {<Streptophyta(V1)>}LJGC723TF JCVI-LJ1
Lotus japonicus cDNA 5', mRNA sequence
{(G)--------------------------EG-
FKVFMNDEVSEEGDQIsePLITAIEKSRFSIIVFSENYAYSSLCLDVLAKILDWKKT-
KDQLVWPIFYKIEPSDLRHQRKSYDTAMTEH-
ENRFGKDSKkvKNWRSTLYNAANL(KGWYLKTGYEYEFIEKLVEMAIKI)}*

$139=175(166):
>gi|213599315|dbj|DB962324.1_EST1 {<Streptophyta(V1)>}DB962324 full-
length enriched soybean cDNA library, mixture of seedling, young plant,
adult plant Glycine max cDNA clone GMFL02-20-C23 5', mRNA se
{(FIMTNEPNN)KYDVFLCLVG-
PDTRYTFAGNLYNALRRNRINTFFTEDNhhdellLMNGDQIspFALRAIKESNLLIVVLSPNYASSPRNLDEF
VAIVRCIK-RKKQLLLPVFYKVERGEIMDAifSGPDQQALCVF-
EERFGdyKERVNEWKDALLEVYGW(TAMEYQNGSGYEYEFIR)}*

$140=206(218):

>gi|218453653|gb|GH288985.1_EST1
{|0(5)|<Streptophyta(V1)>}WS0472.C21_G07 SS-BD-32 Picea sitchensis cDNA clone WS0472_G07 5', mRNA sequence
{()---------G-
PDVKTSLASILYHSLQYMGLRVFLDSGEFELGDYFpdKIHHAICSASVHIAILSENYAHSPWCLAELALML--
---GTDAKIVPVFYHVPPSVPRHCTGSYAEAFRKH-
ElkGRYGKEQIDEWKAALHKASFY(SGCEFNEHNDDQGKLWKNIVDTVLTEVRRRSSLEVAEHPIGLDEAVDN
FNKRMAEFAQEREKNPNIVGIVGMGGSGKT)}*

$141=200(207):
>gi|219834208|dbj|DK954481.1_EST1 {<Streptophyta(V1)>}DK954481 TST39 Adiantum capillus-veneris cDNA clone TST39A01NGRL0020_I22 5', mRNA sequence
{(TREATGSSSEHPLSSLSGSDNVPAI)----FLSHSG-K--
QKPFVENLYNALRRVNHKAFFDQHSLPKGSTFpqRIKTAALGCELGVVVLSEDFLTSKWPMIELE--
ILVLQRGPKAKVLPLFYKLGPADLGSPANLtRWQQVWKE-
QFGASVDIIDNWSKALRSLKAF(HGEKFTQGTSETAYINTVVQSICKVVPSPVPEDLSQIQGLHR)}*

$142=230(244):
>gi|223752495|gb|GO126796.1_EST1 {|0(38)|<Annelida(M1)>}CAGA22532.fwd CAGA Alvinella pompejana Normalized library RN04 posterior end Alvinella pompejana cDNA clone CAGA22532 5', mRNA sequence
{(VAKMFPKISEDLNATPPNLDGTEKE)-YHVFIA-NSSdaEDISKELKDSLES--LKFSVYTDD-
DFEPGWLIIHSITEAVKKSQKIIFLVAKE--VSNWTVFNFASSKAKCIAENEDLLIPII-CDVDeSQIP----
---LFVKESnYL-----
KANdrDFWQHLIRKLTAK(IPLSESLPVGNIGHGIAWSYFYGFLNVVLPDMAKKMEDFEPESIPYKKCYKFII
IIPDSCKIPNDFNDKSIQKCGNLQY)}*

$143=160(157):
>gi|223780202|gb|GO157182.1_EST1 {<Annelida(M1)>}CAGA37557.fwd CAGA Alvinella pompejana Normalized library RN04 posterior end Alvinella pompejana cDNA clone CAGA37557 5', mRNA sequence
{(EMEREVQQCKQCS)-
YDAFVSCSDKglKFVEKQLIPKLEEPpegEDVFKLVFEQRDLPGGGDKETVIGEAINASRKTIVVIDNSYFQD
EWKLFEYGYAKSK-VARGENVLIIVWMEEVDfKDVP---
ASVVDFLGTRhSLRWTTEADGqeIFWRDNRDALR-R(PGII)}*

$144=114(140):
>gi|223854195|gb|GO233133.1_EST1 {<Annelida(M1)>}CAGB886.fwd CAGB Alvinella pompejana Normalized library RN05 posterior end Alvinella pompejana cDNA clone CAGB886 5', mRNA sequence
{()----------------
EFIPKLEEppeGEDVFKLVFEQRDFGIGGNKATDIEEWIEASRKTIVVIDNSYFQTEWKLLEYRYAKSKVARS
E-NVLIIVWMEEA--DFNDV---------PASVVDFFGSRWTRDLLAEISETL-()}*

$145=148(163):
>gi|222387924|gb|FG301412.1_EST2 {<Arthropoda(M1)>}MPOA052TF B. bovis infected B. microplus Ovary 4058 ESTs Rhipicephalus microplus cDNA, mRNA sequence
{()-------------
VSQVLAAELETCSGggkrrPLRLCLRHRDLAVPGCVAEAaVVEAVQCSHRTVVVISEQFLRSEWCRFELKAAQ
ASSTR----RLIAVLLDDA-GQ-DQWDVEARQCFRFAlILRWGERR---
FWQKLRYALPDP(QKGPPQRGGFPEPTARASAVKLV)}*

$146=139(148):
>gi|223773441|gb|GO149378.1_EST1 {<Annelida(M1)>}CAGA33864.fwd CAGA Alvinella pompejana Normalized library RN04 posterior end Alvinella pompejana cDNA clone CAGA33864 5', mRNA sequence
{()------------FVEKQLIPKLEEppeGEDVFKLVFEQRDF-
PGGGGKErVRREAINASRKTIVVIDNSYFQDEWKLFEYICAKSKVA-RGENVLIIVWMEEVDFKDVPDSV--
VDFLGTRhSLRWTTEADGqeIFWRDIRDALRRP(GIIQLTRT)}*

$147=203(207):
>gi|211597751|gb|GE477365.1_EST1
{|0(21)|<Streptophyta(V1)>}GQ0133.B3.r_M03 GQ013: Elongating ROOTS tips - saplings Picea glauca cDNA clone GQ0133_M03 5', mRNA sequence
{(RGRVSSSSPSASYA)PYDVFINHRG-
PDVKKTLAGLLYRDLKSKGLQVFLDQPELEPGHIIrsQIEAAIQGASVHIAIFSEKYAESNWCLDELVLMLDS
K-----
GTILPVFYKVKPSDLRWPDKGAYADALRGLEKNKRYDLQIIQGWKKALSSVA(DISGFELEACNGEKTGPKTP
VIRCSLGLSGPASISYSVDSTGSIAATASPGSH)}*

$148=188(188):
>gi|211599491|gb|GE476220.1_EST1 {<Streptophyta(V1)>}GQ0068.B3.r_O07 GQ006: Cambium and phloem from mature trees Picea glauca cDNA clone GQ0068_O07 5', mRNA sequence
{(PRATSSGASSSSTAADSNNSSDI)PYDVFINHRG--
EVKKTFASHLYRGLLSRGLRPFLDQQELQEGLPFpsQLQSAIRSASVHIAILSPNYAGSRWCLDELLLMQKSG

APIIPIFYHVTPDEVRWTDQNkegvyACSLNQLQMKKTYDAQMHQEKSRYDSSTIQN(WRDALSSVANISGFD
LVGAFNGDEK)}*

$149=218(223):
>gi|211602362|gb|GE480938.1_EST1
{|0(77)|<Streptophyta(V1)>}GQ0201.B3.r_N04 GQ020: Clean ROOTS systems - Diurnal cycle Picea glauca cDNA clone GQ0201_N04 5', mRNA sequence
{(ARIPNSAERIGSCFFIILYPMALTTSFASSSTSHSPGRV)-YDVFISHRG-
PDVKKGIASHIYHRLKDLGLAVFLDQQELQGGENLnlQLEGAIQTASVHIAIFSRGYANSRWCLDELSLMVDS
G-----
STIIPVFYDVQPSDLRWTRGGDRVYARALSIFLCILLSILLCIPTSFLLRFL(LCVPFSIMMCILFCTRGKNG
VYARALHELQNKTTVDSATKKKKP)}*

$150=203(203):
>gi|219831942|dbj|DK959781.1_EST1 {<Streptophyta(V1)>}DK959781 TST39 Adiantum capillus-veneris cDNA clone TST39A01NGRL0005_K01 5', mRNA sequence
{(VLSRMASRGTEYEF)--DVFWSHSG-E--
QKDFVIELDSRLCNLrgswkaGFNNFLDTKhnSLPAGQNFppNIDNAIEKSRIAVVIISKSFLRRWWPMQELS
AFQETR-----
AIILPVFYGVKPSELASHNSDTRVAEWRQLHPHLTDKTAIRWITALDDIRSK(SGYEIASSIGDVNSPKLQRR
LLSDIRLLLAAQVLKEERVDSENLGWACE)}*

$151=183(201):
>gi|222827317|gb|FE521728.1_EST1 {|0(76)|<Streptophyta(V1)>}li0678789 Radiata pine mature earlywood cDNA library Pinus radiata cDNA clone 48319 C3, mRNA sequence
{(I)----------------
TIASSLYWWLTSHGLSVFFDREEVEGGLVVgtQIKEAIASASVHVAVFSPRYAESASCLKELHLMTVSG----

-
KPIIPIFYNVKPADVRkARKHRGAYAADLDKHERKSFSTVLNWRNDLYNAARF(GGFELDRFNGDLGRLLFEVFTCVLKRLEKPRILLMPRPSRGIKEFNAGIEEFNGGIGFTD)}*

$152=177(186):
>gi|225245309|emb|FM920951.1_EST1 {|0(14)|<Rotifera(M1)>}FM920951 REH (sb103) Brachionus plicatilis cDNA clone sb103P0003P02 5', mRNA sequence
{(IFYYXMSKF)--DIFLSYQW-EIQDKV--KELCRQLTQSGLKVWMDIYQLAGG-NLhtEIVNGISNSKIFVCCITKKYSESKNCENELCFAYDSN-----QKIIAIMFEKVTLGDLMGVGPMLARLLRINAFDDERFPFNAESERFLELLTA(LTPDKPISTKSSSIKFLKNASISKQNSEQKKDDEXLN)}*

$153=260(267):
>gi|225913035|gb|GO365757.1_EST1 {<Streptophyta(V1)>}GQ03602.B7P1_K16 GQ036 - Shoot tip - Active growth Picea glauca cDNA clone GQ03602_K16 5', mRNA sequence
{(SETWTIRWAMNPEMYVGTHGGDLVKRKRSTKKKREKWVGALSDVTGINGFVLEHCNGDEGKLVGQVVERVLNMLKDHLD)-YDVFINHRG-PDVKKTLASHLYHGLVERGLRVFLDIEEMHEGDSLtsQIEGAIRTAFVHIAIFSDTYAQSNWCLNELVLMLESG-----RIIIPVFHNVKPSELTSKNGKY--DAQLRELEKKIDEEGKPRYDPATIGKWE(KALSDVDKDSFRFDLEQCDGDEGQLADEVIQRVLSEEVKVWKDGRHLR)}*

$154=169(189):
>gi|225913139|gb|GO365780.1_EST2 {<Streptophyta(V1)>}GQ03602.SP6.r_K16 GQ036 - Shoot tip - Active growth Picea glauca cDNA clone GQ03602_K16 3', mRNA sequence
{()-------------KKTLASHLYHGGVERGLRVFLDIVEMHEGDSLtsQIEGAIRTAFVHIAIFFDTYAQSNWCLKELVLMLESG-----GIIIPVFHNVKPSELPSKNGKY--DAQLRELEKKIvEEG--KPRYDPGTIGKW(EKALSEVDKDSFRFDQEQCDGDEGQLADEVIQRVLSEEVKVWKDGRHLR)}*

$155=212(222):
>gi|223766026|gb|GO145930.1_EST1 {|0(23)|<Annelida(M1)>}CAGA31893.fwd CAGA Alvinella pompejana Normalized library RN04 posterior end Alvinella pompejana cDNA clone CAGA31893 5', mRNA sequence
{(NEH)---IFISYAWSEQPVVKQINSRLKEAGYR-TWVD-FEQMSGSTL-EAMARGIEGASAVLICASDKY-KHSPNCRaEAEYTFQLK-----KDIVPLMMQRyyKPdgWLGMLIGAKFYINFDG--KyDFETAFQ--MLLEQLAGRGKI(KSSAVNSSSVIQPSSSTADTKTIPSVVNASYTRWTEKEVDEFFEKAGLTRIKSHFPDYNGKMLECLKKLKDEAPNFFYN)}*

$156=164(198):
>gi|212821540|gb|GE753883.1_EST2 {<Mollusca(M1)>}MUS16-O20.y1d-s SHGC-MUS Mytilus californianus cDNA 3', mRNA sequence
{()---------------------------IRVFFPERDDLAGDEKYVVTAEMFATkCRRVIVVLSRGFEDSEDCDFALKLAQSLSPGAKTKRIIPILLDNVK--IPLILNFVGTVNFTN----PMQRE-WVWPRVAATIKCP(LVPSIQDWQCTIDDLKNMKYDSKHVQHLLYGIGVACQEFPPEEEEDKNGKKGDKKKKT)}*

$157=190(192):
>gi|226262375|gb|GO412624.1_EST1 {<Arthropoda(M1)>}EST_lbra_evt_1076784 lbraevt mixed_tissue Lernaeocera branchialis cDNA Lernaeocera branchialis cDNA clone lbra_evt_002_336 5', mRNA sequence

{(DAFADCREAQNEGLGLSSEISNQALTIQDLEALAQGNPLP)QYDAIVLFgDSplDQSFGMHVVQRLESAGK
RIY--
IQDRDLIVGAIEHGASVDIIYQrCKKVIAIITPTFGQSQNNVFLTQIALDRQIrGKGLRLIIPVIYSAtnIND
ETIHSLNIYKKLKYSK------SLN-IFWTKLFEALGDL(TISEELKKMTFP)}*

$158=162(187):
>gi|254916595|gb|GR905421.1_EST1 {<Streptophyta(V1)>}312j_F12_012 ovule cDNA library of a seedless grape (Vitis vinifera var.Thomson seedless) Vitis vinifera cDNA, mRNA sequence
{(GKRNHASKSLLVVTQKEGKKTTMTSTNTQIISYSPSSSSSKSTHQYT)-YEVFLSFRG-
EDTRYGFTDHLYEALISSGIRTFRDDEELarGGVIASELLEAIEESKIFVIIFSENYAASRWCLDELVKISEC
GPTEGRL-IQQIFQYV------------------------GSVPWAGSKARSYEKGIF()}*

$159=152(168):
>gi|260524139|gb|GT144775.1_EST2 {<Streptophyta(V1)>}METCW11TR JCVI-MT3 Medicago truncatula cDNA 3', mRNA sequence
{(N)---------------------
LYKALTDKGIHTFIDDNDLqrGDEITPSLLKAIDESRIFIPVFSINYACSSFCLDELVHIIHRYKTKGRL-
VLPVFFGVEPTNVRHQKGSYGEALAEHEKRFQNdpknmERL-
QGWKEALSQAANL(SGYHDSPPGYLFFIFFLFNLNFTIISS)}*

$160=206(213):
>gi|295220943|emb|FN766068.1_EST1 {|0(3)|<Streptophyta(V1)>}FN766068 WZOAQPAA Quercus petraea cDNA clone WZ0AQPAH28YH09FM1, mRNA sequence
{(VFVFAIMTPSCAPSSSTSAL)RYDVFVSFCR-EDTHTSFTCHLFAALDRKKI------CACRAEF-
NrtELMKAIETSRMAVVVFSKSYATSDRCIDELAKIMGCNRV-
LNRRVLPIFYDVSPSEVRKEKGNFEEAANLAGFDLKANRPESEFIEEIVENILK(ILKQESSTSPSLRQNDAP
GQHSSATIPHNDASSGLDLENYFWTETLEEVTLKC)}*

$161=154(164):
>gi|260524937|gb|GT143006.1_EST2 {<Streptophyta(V1)>}METCA50TR JCVI-MT3 Medicago truncatula cDNA 3', mRNA sequence
{(FILFLSILMAMQSLSSSSSISSGF)KYHVFLSFRD-
IDTLYGFTGNLYKALIDKGIKTFIDDNDLERGDEStpSLVKAIEESGILIPIFSANYASS---------
SFAWTNL-STSFTATRQGVVRFCLFSMVRILLTYDIRLG-VMVSILLSMKISSKITRKTWRG()}*

$162=135(164):
>gi|262134614|gb|GT250676.1_EST1 {<Streptophyta(V1)>}PCO0117.CR_G07 Pco01-Bark-Wounded-Norm (PCO01) Pinus contorta cDNA clone PCO0117_G07 3', mRNA sequence
{()------------------------------
LQVFLDQQELKPGHIIrsQLKEAIQSASVQIAIFSKTYAESTWCLEELVDMLETTIS-
QRATILPVFYGVKPSELRRTdRGGYANAMRKH-
KKRNNLETIEKWKKALSDVAGI(SGFELEACNGFIKKRGLHLCKLWH)}*

$163=139(170):
>gi|238890938|gb|GR222351.1_EST1 {<Streptophyta(V1)>}GQ01303.T24.r_F14 GQ013 - Elongating ROOTS tips - sapplings Picea glauca cDNA clone GQ01303_F14 3', mRNA sequence
{(RGHRTPFLYLQDVIGIMEQDPEVQGRTPPA)-YHVFIRHRG-
PEKNKSPAILIYHQLSNCdRLPVFQNENDRRKGDTIsnAILTDSQSASLHIFIYTKRNAEARWCTEELCWILR
SYHKRNRNGI-PVFCDVEPADLRH--------------------------------TESG()}*

$164=220(244):

>gi|257173930|gb|GT033688.1_EST1
{|19(91)|<Streptophyta(V1)>}CLS_cLiFproots_13a2_1_g02cLibkit5LD_D01
CLS_cLiFproots_plant Festuca arundinacea cDNA clone 13g02 5', mRNA
sequence
{(ETIELTDPSDTQIDEIVFSSENTVNNIEPSSSLSETVDRYVENFGRVEQQVISTGNQIIATTSRHIMISYN
EFSFDICHK)---------------------
IYQGLRNAGYNVWMDEHNLHGRIFQGMADAIENASIILICYNENYAKSDYCYQEATYAFDNR---
IDFIPCRLQESYRPKGWLGNLIADRLYIDFSPPNTFESSFEKLLEEIKSIKTPS(QTYACSSPCSPTSIRNEN
MREQFD)}*

$165=189(199):
>gi|295247352|emb|FQ007481.1_EST1 {|0(74)|<Ctenophora(M1)>}FQ007481
Pleurobrachia pileus library Pleurobrachia pileus cDNA clone SQ0AAA78YH01
5', mRNA sequence
{(KLAQYPEAMDFGAVLEALIDQMGERH)---VMLSYQW-N--
SQELVKKVYTAIKEKGVNVWMDiEGGVSGNINEAMAEGVDGAAVVCPFMTQKYQESKNCSKELNYTN--D---
SSIVVVPCMAQTRTDNGNMYRARGWLGIITAGKLWIDFKGEDQMEYRVIQLLTE(CCNRLNKTEQPVGKMKIM
KEANAEKKKPEVKKK)}*

$166=194(183):
>gi|261431582|emb|FP219981.1_EST1 {<Haptophyceae(E1)>}FP219981 haploid
cell library (AAZ0AAA) Emiliania huxleyi cDNA clone AAZ0AAA35YI05
(AAZ0AAA35YI05FM1) 5', mRNA sequence
{(KGGGLQRAGRAYM)QYDCFLTHDW-
GEDEEGRSNHarvgkLCAALKAEGIRPWFDEECMRGDINDKMVDGIERSGAVVCFITDRYLEKAWgkganghn
dnCKFEFDYSLRRK---
GVEAMIPVVMEKRCRNPSDWAGTVGGKLGGKLYIDLSSDDSSAFDSGVKQLAAE(IRVVLESGRIRSGVACGN
KPGHPMAPSTAP)}*

$167=162(167):
>gi|260932851|emb|FP011576.1_EST1 {<Mollusca(M1)>}FP011576 Pascal Favrel
cDNA library hemocyte (wy0aba) Crassostrea gigas cDNA clone wy0aba4c11,
mRNA sequence
{(KFAFAFWYVAPRYVMAYDEGTDVDITH)--DVFLVHGNEpkedkVFALLKLKPLLERQNL--
KVLVDEDDFLGGETVFTNIARAVRRCRKSLIVLTEHSRESPWCSLELLLALEKSHRRNIMSVVVL-------
RKGNSVEQQLALDMLNdVPRIDIQNDDDIEGKLDDLVSKT()}*

$168=194(216):
>gi|262330591|gb|GT282780.1_EST1 {<Mollusca(M1)>}pmaximaP0021D16_652
Adult silver lipped oyster (Pinctada maxima) Pinctada maxima cDNA 5',
mRNA sequence
{(YISISNRSFEGIPNSLREMDADTEPLLNAYKTDTIDLRVLAEEENTNICDEYKNDTTSETECKRPIDAELP
NKK)-YHLYVIYSHEdlNEV-TELCEKLENI-
YQLKCMFAVRDFVPGVPIDENCKHKMEDSDKTLILTTPSTISSFWCDEEIGRAFKIVKSGKRDFVIPVLFKPL
SkED--KR--KLRPCLEFTtYID-------------------A(XR)}*

$169=224(224):
>gi|282175413|gb|GT986932.1_EST1 {|10(0)|<Arthropoda(M1)>}CCIN3182.b1
CCIN Tetranychus urticae London embryo (L) Tetranychus urticae cDNA clone
CCIN3182 5', mRNA sequence
{(EGSPSTFANEVSFFQNSLVQSYMPLTIGLIAGIVAIILIIVLITIYHKEIKVWIYSNYGFRFFQKSHYPPE
TER)LFDGFVSYCKKdeAFISQILAPELECGHPPYRLCLRYRDLPVTEYVAEAISEAIESSHRTIILLSDQYY
KSDSCHFELKVAHQECQVNLNHKIIVVVLDKN--
SLNHLDADSKLCIRSSPIIHWGDRRFWEKLRYAMPPGRG(LSKPLNCPDV)}*

$170=196(209):
>gi|260629050|emb|CU986476.1_EST1 {<Mollusca(M1)>}CU986476 Pascal Favrel cDNA library all stages (wy0aaa) Crassostrea gigas cDNA clone wy0aaa11i05, mRNA sequence
{(G)----------KddDYVTREFIPRLEN-
ELKYRLCVYYRDFPIGGTIAVTVASSINRSKRTILLVSKHFNDHEWRNTAFQHSFGGLFKQKDNHLIIVLLDD
A-KGM-KLDRQLKVLVKSHpVISYRDMC---
FWEKLQYMMGSS(KRSIVRNNTPDLILNHSYTQQQDADGYETPVSSSASNTETDKCRRSLDSINNIYEEIRSS
KLSDISLV)}*

$171=204(208):
>gi|260740072|emb|CU990465.1_EST2 {<Mollusca(M1)>}CU990465 Pascal Favrel cDNA library all stages (wy0aaa) Crassostrea gigas cDNA clone wy0aaa24f21, mRNA sequence
{(MLCSKKVPRRRGQS)-FDAFLAYSHKddDYVTREFIPRLEN-
ELKYRLCVYYRDFPIGGTIADTVASSINRSKRTILLVSKHFNDHEWRNSAFQHSFGGLFKQKDNPLIIVLLDD
A-KGM-KLDRQLKVLAKSHpVISYRDMC---
FLGEASLTNGPL(PAFPRFKKPPRFNTHPNFPHPQDAEGYKTPVISVPPPTRKQKMPRFPFKPLQNS)}*

$172=234(232):
>gi|268207929|gb|GT421884.1_EST1 {<Arthropoda(M1)>}DPO113.CR_J07 Dpo11-EAdult-MGFB-Fed-Norm (DPO11) Dendroctonus ponderosae cDNA clone DPO113_J07 3', mRNA sequence
{(VISKPGVSVQKRVSIRDAAVFYIDPNPFPWKQITIACLIVSTLIMSIIVLNRKYGPKVKALVKDNCYAIED
YDGKTN)--DVLIAYSAEdtELANGIMLPKLETRSY---
TCTSRKITCDITRCAAEISQYAESSRRIIVILSPAALNDIWTSAQLYQVLKHFQTINTCKIVYYVTVRSF-
PvsgaeVKNNSGESLRSLMSKMnIIQWNvskDAN---FWFRLCRELPPK(RSKYWVKSKSDGMLA)}*

$173=140(149):
>gi|254408949|gb|GR867683.1_EST1 {<Mollusca(M1)>}MYMAN01H11 Japanese scallop cDNA library from mantle Mizuhopecten yessoensis cDNA 5' similar to TIR protein [Salmonella enterica subsp. enterica serova
{(CLSTMSRES)-YDFYICNHEAdnDKAFQILETLEKKNG-L-
KGFHDERDAQPGQYVIRNIEKAIESSRFILLVLSEQSMEDGWFEMkLNTGLTHRLYSALRDSII---------
--PVYVAPSDGELTVPtaLNSIRGIDYSEDPRFFDKLSKIF()}*

$174=227(233):
>gi|241980956|gb|GO091840.1_EST1 {|0(4)|<Porifera(M1)>}DMPR7349931 Leucetta chagosensis DMP cDNA Library Leucetta chagosensis cDNA, mRNA sequence
{(AMLFYNANFVQKSWRNTAWPFPYLKASPHHSYSAASERRFQLRFSPSQRTKLMTNQNAK)--EIFLSYRH-
EEGVYEFASRLHKDLESEGFTVWFAPKGMSTGDKWrnKLPVAVDECKALIAVITPNYLESDNCTNELYRAFDK
K-----
KKIFPVVRRKTDWTSSKNATGVQFHIANIHRLNMREDIDDYDEALEKLINDL(RYDGLGQVNAHDSVGGKSPS
GSEPPSTCTPRAKR)}*

$175=189(217):
>gi|255780553|gb|GR954601.1_EST1
{|0(45)|<Streptophyta(V1)>}GQ03519.B7P3_E10 GQ035 - Needles - End of season Picea glauca cDNA clone GQ03519_E10 5', mRNA sequence
{()-----------------
FANQLYYRLRTYGLQVFYDQEELQKGNRInsVIENAIKVASVHIAIFSKGYAESEWCMDELALMVESE-----
SIIISVFHGVNPtkiWQNPVDGMNGVYAEALRK----LKEE--K-----

TFDPET(HQEKLRYNSNTLEIWEKALKEVTGRVGFELEKYDGDEGQLLDVIVREVKTKVKKDEAKLLTEPCQK
NNPSSSERERY)}*

$176=175(180):
>gi|257195335|gb|GT051888.1_EST1 {<Streptophyta(V1)>}GQ02803.B7P2_O14
GQ028 - Cambium / phloem scrappings (Normalized library) Picea glauca
cDNA clone GQ02803_O14 5', mRNA sequence
{(DQSDRGYNPNIWHTFHLYFFIVYPMANLLASSSNSSGNKA)PYKVFINHRG-
PDTKLSIAGHLYHRLHDLGLQVFLDQEEMEKGDFItsQIAAAIQAASVDIAIFSPGYADSEWCLNELLLMMES
KESGR-STIIPIFYGVEPTDLR-----WTLGKDGVYAKALTKLKEKKTADPQTQQRSR()}*

$177=198(205):
>gi|260925162|emb|FP007484.1_EST1 {<Mollusca(M1)>}FP007484 Pascal Favrel
cDNA library hemocyte (wy0aba) Crassostrea gigas cDNA clone wy0aba33f15,
mRNA sequence
{(HGFAVTELAKSHPPKSFSLPNFKQIVFNIYKFIVLKKDRMALQSEKVKGKLAMVTAGKVQ)-
YEVMISYDA-EDA--
AVASKLKDMIGKKGIHAWTADSEAIAGTDIfnAIGRIIVDCKIFVFIMSDSSVASKLCQDQLALAYVSK----
-KNIFPVQLNDREDLLAHMDNGMKLQLAGFEWFSLNNDLESESEEFVDRVWEE(VQSEI)}*

$178=207(220):
>gi|261232495|gb|GR308490.1_EST1 {|27(0)|<Annelida(M1)>}pl_xlvo_04e11
Pomatoceros lamarckii larval cDNA library Pomatoceros lamarckii cDNA,
mRNA sequence
{(ARPGETAGDERYWPTTKFQELLMQLRFTHAPEEGIVTDPYKDWWSPPEEIIVTKPKEINIANTTRSASKPK
NMEDEKKSP)--DVFISYQWDkqPQVKK-----
LFCTLSERGYHCWIDIMQMGGGDSLydMIDKGVRECKVVLSCVTSKYALSANCRREVSLADALK-----
KPIIPLLLEDITW-----PPTGPMNMPFTQLPYIEFCKPNVGIQIIGCSSMD()}*

$179=169(179):
>gi|261431288|emb|FP228583.1_EST1 {<Haptophyceae(E1)>}FP228583 haploid
cell library (AAZ0AAA) Emiliania huxleyi cDNA clone AAZ0AAA25YG11
(AAZ0AAA25YG11FM1) 5', mRNA sequence
{(RRRRRLRQVAPPGSAVSQRKGSA)-CDVFGSYKQ-NDGTDGLLFNLFYTLEMKGIDLWLDKK--
RGSERSeaGMRAGVRACKLFCAVISPKYFESAFCLLELRTALSHK-----KPIVVCF---
NSSKFTVQQAMSWIPEEFRGRLLSNEVVALHEDDEYMRVGVE(KVAAQLSLSSPEQSLS)}*

$180=195(204):
>gi|261451583|emb|FP200455.1_EST2 {|0(41)|<Haptophyceae(E1)>}FP200455
haploid cell library (AAZ0AAA) Emiliania huxleyi cDNA clone AAZ0AAA9YO07
(AAZ0AAA9YO07FM1) 5', mRNA sequence
{(NIATASASPTIRLVSSGREPALELSEGCEH)--DGFVSHVW-S-
TGQDQAATIVRRLQMLlpGICLWLDIENMQSVASL--ESAVGGSSVFIVFLSQGYFKSRNCRRELYAAHAAS-
----
KPVIAVRETEQAKGALSVQGLWTEGREGCAFEAGAEAPADAVPDGGAARGLS(QAGPLRHGRAAAAKLRLCRL
PRPIAVAIWPSSGG)}*

$181=145(158):
>gi|262150415|gb|GT265102.1_EST1 {<Streptophyta(V1)>}PCO025.CR_J11
Pco02-Xylem-wounded-Norm (PCO02) Pinus contorta cDNA clone PCO025_J11 3',
mRNA sequence
{()---------G-
PDTKATFASPLYRRLLSHGFRAFLDEQELEPGFDFpsQIVRAIRAASVQVAIFSPRYMESTWCLNELLLMLES
G-----

CPIIPVFYKVNPAELRYGKALHALEMKTTSDPESGMTRPRYDSATIQNWRNA(LSFVADISGFDLEGKFHG)}
*

$182=216(221):
>gi|262151471|gb|GT260812.1_EST1 {<Streptophyta(V1)>}PCO025.C21_J11
Pco02-Xylem-wounded-Norm (PCO02) Pinus contorta cDNA clone PCO025_J11 5',
mRNA sequence
{(QSVLPISNFFIYLYSFLKQLTMAYPMSASSFRQTNSEIEMEIHPATTAESSFASSSNATDPMV)-
YDVFINHRG-
PDTKATFASPLYRRLLSHGFRAFLDEQELEPGFDFpsQIVRAIRAASVQVAIFSPRYMESTWCLNELLLMLES
G-----
CPIIPVFYKVNPAELRYGKALHALEMKTTSDPESGMTRPRYDSATIQNWRNA(LSFVADISGFDLEGKFHG)}
*

$183=170(194):
>gi|262152580|gb|GT267455.1_EST1 {<Streptophyta(V1)>}PCO0213.CR.1_H16
Pco02-Xylem-wounded-Norm (PCO02) Pinus contorta cDNA clone PCO0213_H16
3', mRNA sequence
{()----------------------
YHRLNSFGSKVFLDREELQQGEYFnsQIEGAIRTASVHVAIFSARYAESTWCLNELLTMLESQ-----
GTIIPVFYHVKPSELRwTKGKDGVYARDLQKLEEKTTDDPQSSGKKPRHDSAT(IEQWRKALSTVSEISGFEL
ASFNRLEEMGLVKGWGDNMERFAWGSFALGDSPSP)}*

$184=198(201):
>gi|262157404|gb|GT266791.1_EST1 {<Streptophyta(V1)>}PCO0213.C21.1_H16
Pco02-Xylem-wounded-Norm (PCO02) Pinus contorta cDNA clone PCO0213_H16
5', mRNA sequence
{(DTPCSSASFQSQTEYYSSPSFTSLPFLCLFSKLEMAASSSTAASTATSVNPNSTGNS)PFDIFINHRG-
PDVKNTFASHLYHRLNSFGLKVFLDREELQQGEYFnsQIEGAIRTASVHVAIFSARYAESTWCLNELLTMLES
Q-----GTIIPVFYHVKPSELRwTKGKDGVYARDLQKLEEKTTDDPQSSGKKPRHDSAT(IETV)}*

$185=203(222):
>gi|270051621|gb|GT739361.1_EST1
{|0(45)|<Streptophyta(V1)>}GQ03519.B7P5_E10 GQ035 - Needles - End of
season Picea glauca cDNA clone GQ03519_E10 5', mRNA sequence
{(RCVPY)-------HRG-
KDVKKTFANQLYYRLRTYGLQVFYDQEELQKGNRInsVIENAIKVASVHIAIFSKGYAESEWCMDELALMVES
E-----SIIISVFHGVNPtkiWQNPVDGMNGVYAEALRK----LKEE--K-----
TFDPET(HQEKLRYNSNTLEIWEKALKEVTGRVGFELEKYDGDEGQLLDVIVREVKTKVKKDEAKLLTEPCQK
NNPSSSERERY)}*

$186=108(140):
>gi|238645225|gb|FL490891.1_EST1 {<Mollusca(M1)>}Mg_Nor01_62A16 Nor01
Mytilus galloprovincialis cDNA 3', mRNA sequence
{()-----------------
MARILEGPKYNLRLFLPWRDLqTTEDNETEVLAKIMVQRCRCaVVVISPNLLQSDVTSFLLSFVHSKYPSGRN
SKLIPILIENC--ELPTILQSVTVCDFTK--KDMRC---WVW----------()}*

$187=182(186):
>gi|260927085|emb|CU993827.1_EST1 {<Mollusca(M1)>}CU993827 Pascal Favrel
cDNA library all stages (wy0aaa) Crassostrea gigas cDNA clone
wy0aaa39a05, mRNA sequence
{(GEITMDNQIN)QYDAHVIAGDTeEVklFLETMKSNLESSAFNLRLYIKSRDG-
PGDFDHQKESEIIMNSCEKiIVVLSRDFTQNEQCMYLLRVAISKSPGFHLRHIIPIHREECL--

APSCIRFLTANDYTK--EDIRT---
WFWPRVAACFKKN(VINIRTAKKNLPAVVCLPDIIVDSFSSEKQQEWRNN)}*

$188=187(220):
>gi|282532291|gb|GW255061.1_EST1 {|46(0)|<Cnidaria(M1)>}CCHW5601.b1 CCHW Montastraea faveolata heat/dark/disease-stressed adult + 6 day old larvae Montastraea faveolata cDNA clone CCHW5601 5', mRNA se
{(LYFECRREPVLELISSYESKGKTISELLSLLEEMERFDVIEDLKEFVEATPTLEEKQREWERCQTEAAEDTKAEAISECH)--DVYIAYAKDdQVFVNEIVTTLERPPYCMKVCTDYRELS------
DTAAKAIETQCSKvLVVLSESFNRCPDADHMVNFALHLSRDLRKTILIPILYKSVK---------------------------YQESYNFYSSGLH()}*

$189=180(196):
>gi|261719121|dbj|FS085543.1_EST2 {<Streptophyta(V1)>}FS085543 library SmFL Solanum melongena cDNA clone SmFL28H04 3', mRNA sequence
{(KVEQENEFISKQSSSIRKRKQKIEKKEWHESKAIYQYVRGDFRFKLNSLISADKFN)---
IVLSFCKDdKQICDRILKYLQ------------
SETDFNILYGKifpePIAASVESSTIVILCLSSKYRKSYSCRLEAEYAKKRSKR-IIPVKVEN-
NYKVSGWLStvmaiDDEQILNIYEEDF--EKELMEK--IAQEFE-----A()}*

$190=226(226):
>gi|241974664|gb|GO085892.1_EST1 {|20(0)|<Porifera(M1)>}DMPR3134467 Heterochone reaggregate DMP cDNA Library Heterochone calyx cDNA, mRNA sequence
{(MPTLYKKVGRERSPVTQTPVLKEKKYQRTRDRDRRHSTRSDSSTGNLPRSDVIRAYAHPTRYHRDRDSHGSTLSRRKQYH)---VFVSHSTAdFQWaKVKVVMKLRNLKSHHVsvVASYHFR--
PDGDTYNqQaIRASMEEASIILLGLSQTYLNSDRCAVEWKLAAALADK-KQATVIMGAVKPC-S-
RPEPLKKLNLMYLDFvdPKKESVVWKVXVIHRHLKINSRV(VQELKF)}*

$191=165(193):
>gi|261629295|dbj|FS075200.1_EST1 {<Streptophyta(V1)>}FS075200 library SmFL Solanum melongena cDNA clone SmFL13G09 5', mRNA sequence
{(GLLSPNYFGQVRVCCDCLIPPFAMAEKKESGGFFNRNKGGDLLGETDKDLVGC)--
SVMISYSRKdKAFVKNIYDALAVDDRN----
IWVDwEDIPPSNDWLdEIHKGIEQSDCFIFVLSPDSIKSEVC-NwEVDHAV----K-NGKRMIPVVCRDV-
DY-------------------REVRRELASLNWIFFRAWG()}*

$192=142(149):
>gi|256984561|gb|GO001080.1_EST1 {<Cnidaria(M1)>}D015-G5 Acropora millepora Adult cDNA library Acropora millepora cDNA clone D015-G5, mRNA sequence
{(HNSKSSDSE)-EHIFISYQW--DYQKTMLL-VKTELESKGFQVWMDVNERT--
RNIpqRIARAIEKSSVVLIAMSRKYQSSKSR-SsEATYAYKLGKQ-----IIPLMMEEN-
YNPDGwlefiLATKPYMHFEKDPQEGIQQLLK--EVTNVTAADTC()}*

$193=191(218):
>gi|258674782|gb|GT086535.1_EST1 {|0(21)|<Mollusca(M1)>}dlsmcb0_004284 Japanese scallop adductor muscle normalized and full-length cDNA library Mizuhopecten yessoensis cDNA 5', mRNA sequence
{()-----------------------
ELSKTIKLFIPQDHLVGGAYEFEATAEVIEDrCDgRLVVLLSNHYTNSPACSFATQFAKTLDPDARRRRIIPVVTQEGVC-FPRILRGISTIKHYR----CASMT-
SFLTQVIASLSYK(PQPKSDTKMLNGPPRENKTISTSSSWSSVEYPRRSSGISTSSSVIALDCDRRSSLAVDKRNSNVSMASIISNESRIRN)}*

$194=200(199):
>gi|262329390|gb|GT277366.1_EST1 {<Mollusca(M1)>}hasininaP0045B16_602
Adult tropical abalone mantle (Haliotis asinina) Haliotis asinina cDNA
5', mRNA sequence
{(WVLTRTILRQVTNPLINMILGRRRINIQPIQGGGALAGLAPSDNTTV)KYDLYISYaDAQLSLVSEIAQAL
ALQGFRV--CFRDNDVLGNEIVTQAVSRLIVEsWKY-LVIVSQEYVASG---
FQNNFEIHLITQRHLDGFInnrsVALLELNPDLIPevfrsfPILNHYEKTDDTV----DDIMS-
WHGPSLFPNESCS(AVRIIERHSFGF)}*

$195=184(186):
>gi|294964365|gb|GW838168.1_EST1 {<Streptophyta(V1)>}Summer_Black_1565
Grape cv. Summer Black flower and fruit Vitis vinifera cDNA, mRNA
sequence
{(RGERVYVKVSGSSLTQSCACFLTMDSQISTTASSSSSISPTSISKD)EYDVFLSFSW-
EDIGTSLADRLYAALVRKGLRPFREAIGKpeSKEaIASESLMAIQKSKVFMVIFSENYARSRYNWDQLVKIVE
EYRrafltrpasfkedvratswssisfRWTQEVIVPVYYHVCQSDVREQKGSYGE------------------
----------()}*

$196=131(162):
>gi|308672082|dbj|DK510954.1_EST2 {<Streptophyta(V1)>}DK510954 full-
length kale cDNA library (capsules) Brassica oleracea var. acephala cDNA
clone KALE-144O10 5', mRNA sequence
{(KDFHRYSALMASSSSSSLRIWR)-YRVFTSFHG-PDVRKTFLSHLRKQFGCNGISMFDDQEIERSHT-
IapALEKRLRESRIFI-----------------------------
GGVNRKTMFLPSGVLKDFWEFSMSRKKWTERGASSREKPLILKKSRNLGGF()}*

$197=144(163):
>gi|308716806|dbj|DK483341.1_EST1 {<Streptophyta(V1)>}DK483341 full-
length kale cDNA library (flowers) Brassica oleracea var. acephala cDNA
clone KALE-072L02 5', mRNA sequence
{(WFSSQNLSSDSLPFMATSSSCNW)VFDVFPSFSG-
EDVRRTFLSHLLLSLDHKLITCFKDNEIERSQSIGlELVHAIRDSRIAIVILSKTYASSTWCLNELLEIVKCK
EEKGQM-VIPVFYGLEPSHVRNR------------------QASSERRFNDLQEQIR()}*

$198=204(215):
>gi|296561348|gb|GW943628.1_EST1 {<Streptophyta(V1)>}Dur25.seed.ADAA-
aaa55a04.b1 DUR25-seed Arachis duranensis cDNA 5', mRNA sequence
{(RWHAVARERLSYVLPSSQLVLPEFLFKFQLPNKSTDAMFKPGPEKPPNRYDIQNPNLV)-YDIFLS-SG-
SGTAESIIaytSCLHASLQKAGFSVFLDY----GHS-
IfsSPLQAMQRARVSVVALSTGYAGSPRDLELLELMLECRRN-
SGQFILPVFLGVTPRDMRKAFADFTKSVSE------KDKV-
ECWKVALERICQI(QSYQWVCLNNYKWYFYL)}*

$199=145(157):
>gi|296588874|gb|GW973268.1_EST1 {<Streptophyta(V1)>}Ipa.ADAB-
aad90c07.b1 IPA1-root Arachis ipaensis cDNA 5', mRNA sequence
{(RTKMAATSSSSSTNNGV)-HEVFLSFRG-EDTRKTFTSHLHAALKRFEIRTYIDYSLERGDE-
IsgTLLRAIEDAKLSVIVFSKNFASSRWCLEEVKKIIDCKKT-RGQMVLPIFYECGAFHLLGTK---------
-REALQLLSPDMRSGFEILIALTR()}*

$200=161(171):
>gi|296589701|gb|GW970415.1_EST1 {<Streptophyta(V1)>}Ipa.ADAB-
aad46f07.b1 IPA1-root Arachis ipaensis cDNA 5', mRNA sequence

{(PPSMSSTASSSSQKKEPGLMKFTTSSYRNWT)-YDVYVSF---
DRTTNKFVSDLYAKLSAARVRVFKDEMEPRS---
GdqILFNAIEDSRISIVVFSRSYGDSKWWLKELEKIMECRSS-KGQKVVPVFYGVDPSEA----
GAAFAYREPLREAACLPGFVTYISKERDEALNN()}*

$201=225(241):
>gi|308637913|dbj|DK482660.1_EST1 {|0(52)|<Streptophyta(V1)>}DK482660 full-length kale cDNA library (seedlings) Brassica oleracea var. acephala cDNA clone KALE-070O16 5', mRNA sequence
{(GQEDIVRTKPMYSSLLAGRDV)--DVFLSFCC-ETSdHRVLQDHLL----GSGIQTFESISWgeTRVRS-
VdqRTLKALEESK-AVVMLSKREPCSVGFLEELIVILEFQEK-GSLKVIPICLTPYPF----------
VDVEEENSQLYPENA-
PSWRAALTKLSNI(ATEYPFSRNIKSMFLPDWLREVARDICLQVLSSTSNDLNALVAMDRHMEAVYELLGVSS
DVNNNEVQTIGIWCSAGVGKT)}*

$202=189(220):
>gi|308699410|dbj|DK536374.1_EST1 {|0(73)|<Streptophyta(V1)>}DK536374 full-length kale cDNA library (flowers) Brassica oleracea var. acephala cDNA clone KALE-055I14 3', mRNA sequence
{()---------------------------RKRIKTYLFDELP-YEE-
RyeESLKAIEVSRVSVIVFSENFGDSKFCLDEVVAILKCKKR-
FGQIVIPVLYHVDHVDIENQTGSFGEAFAKRQDK--AEQI-
KEWKDGFTEAINL(PGWSTSHLRDEEMLVNEIALDIERKLLRASRTKVIIEWSLVITNLMLEIPSCVFDQISA
TQKPLYTLAAMSMSFLSCLLC)}*

$203=192(220):
>gi|308629273|dbj|DK497826.1_EST1 {|85(0)|<Streptophyta(V1)>}DK497826 full-length kale cDNA library (seedlings) Brassica oleracea var. acephala cDNA clone KALE-110I14 5', mRNA sequence
{(NYVKEKQLGVGDIVFLQRIFTDSSRLFIGFSRREAALDGTSSDKLKEVNSPLPLRSHVHSVTEFDPVLVLS
CAANQESGE)---------------RYFISYISTELCLRGFTPLIYDLTrGTLTG---
GLELPHRSRVGIIIFSNNYASSRQCQDNFVAIMDHSKA-
NSLVLLPVFFKVKVTDIRGQSGSFGRAFSQLENSIQASQV----------PTFT()}*

$204=130(159):
>gi|296601082|gb|GW987811.1_EST1 {<Streptophyta(V1)>}Ipa.ADAB-aae48e10.b1 IPA1-seed Arachis ipaensis cDNA 5', mRNA sequence
{(HIAAYLFVKNPKMAKSSEE)KHEVFICFRG-
EDTRNTFTSHLNAALRRLEIRTYIDHDLRRGEEIPErLLRAIKDAKLSVIVFSENFAASKWCLDEVVNILECK
KR-SNQIVVPVFYRVE----------------------------SGHGEEPDRKLWM()}*

$205=139(168):
>gi|285021097|dbj|DC601458.1_EST1 {<Streptophyta(V1)>}DC601458 Castanopsis sieboldii inner bark cDNA library Castanopsis sieboldii cDNA clone Cc_TUM05_06C13 5', mRNA sequence
{(GSDSKFWSLIQLLPFPSMATQTASSSTW)KYDVFLSFCG-
VDTRMNFTDHVYIGLKQKGIITFRDDGMLQHGAYIctELLKAIEESEYVISVLTRYYASSRWRLIELAKIVES
WKY-TGLNVLPIFYNVDPSHIRK-----------------------------PNWDFC()}*

$206=142(168):
>gi|293548133|dbj|FS349883.1_EST2 {<Streptophyta(V1)>}FS349883 LjFL3 Lotus japonicus cDNA clone LjFL3-016-AD11.r0 3', mRNA sequence
{(D)---------------------------KG-ILTFIDD-
GLPRPDDImsTVnSKAIESSRIAIVVISENYASSSFCLDELMYILKRREE-

KGLLIWPVFYQVEPFDVRYQKGGFGKAFASH-
EERFGSDSAkvRKWRNALKQVADL(KAFHFFKHGYHTSLINFTFDLFVVSCN)}*

$207=128(142):
>gi|296569720|gb|GW949847.1_EST1 {<Streptophyta(V1)>}Dur25.seed.ADAA-
aab28g07.b1 DUR25-seed Arachis duranensis cDNA 5', mRNA sequence
{(RA)-----------------FIDHLYKALDGiD-
IKTFKDDDKEEsttEEEETnpDLIEKIERSRISVVVLCKDFPFSTRCLDEVAKIMECYEK-
KTMQVVGLFYDgVTAADVRWQKEDraCGKAITQH-KERlgLGEESDkiKTWRSA-------()}*

$208=199(216):
>gi|301501975|gb|HO207515.1_EST1 {<Streptophyta(V1)>}xnzbca0_0016_F09
Phytophthora parastica infected Arabidopsis leaf Arabidopsis thaliana
cDNA clone xnzbca0_0016_F09, mRNA sequence
{(CQLCTKKL)-------------------
GFLYKELVGRDIRTFKDDKELENGQMIspELILAIEDSRFAVVVVSVNYAASSWCLDELVKIMDiQKNK-
GSITVMPIFYGVNPCHLRRQIGDVAEQFKKH-EAR-
EKDLEkvLKWRQALAALADI(SGDCSGEDDSKLVDVIADKISKELMIVTRISNGRNLVGIDKHMNELNRLMDL
NSNKGKRMVGIWARGG)}*

$209=120(150):
>gi|308649418|dbj|DK493836.1_EST2 {<Streptophyta(V1)>}DK493836 full-
length kale cDNA library (leaves) Brassica oleracea var. acephala cDNA
clone KALE-100B15 5', mRNA sequence
{(RPMASLSRVW)KYDVFLTFRG-
EDTRKNIVSHLHKELVNKGIVTFKADQRLEIGDSIseEIRRAIQDSRFAL-----------------------
-------GRTPFNHGSLLEEGDESSPHFLCLGVSL-WTSTTVQTRSQTGGKLSPRLPA()}*

$210=202(192):
>gi|292538316|gb|GW758392.1_EST2 {<Streptophyta(V1)>}PBA0212.C21_O04
Pba02-Xylem-wounded-Norm (PBA02) Pinus banksiana cDNA clone PBA0212_O04
5', mRNA sequence
{(SIMAIRQTSVSASTSNSTSASHACASTSSSTSITPLSSRNYS)-YDVFISLHR-
QDVKESFSKHLYSRLLSHGFRVFMGEEELDEGMELtpQIKEAIETASVHVPIFSPGYAGSKHRLDELVLMWKS
GGT-----ILPVFYDVYPDElecLRGEGGVYAQALHNL-
EKEstydsksqedkvlYEPSTIENWRVALSRVVEL(RGFELEACDG)}*

$211=151(178):
>gi|293522422|dbj|FS352217.1_EST1 {<Streptophyta(V1)>}FS352217 LjFL3
Lotus japonicus cDNA clone LjFL3-023-AF05.r0 3', mRNA sequence
{(E)----------------------------
GFVTFKDDESLEGGSPIeKLLDDIEESRFAIIILSKNYAESEWCLKELSKILECKdQEGKNQLVLPIFYEVTP
TEVRHVINRYADAMAKH-
EKNgIDSKTIQAWKKALFDVCTL(TGFEKPSDWTRDQFIGKIVNFACEHKHRLHIQSKYMN)}*

$212=140(164):
>gi|296601150|gb|GW987879.1_EST1 {<Streptophyta(V1)>}Ipa.ADAB-
aae49e08.b1 IPA1-seed Arachis ipaensis cDNA 5', mRNA sequence
{(NLPNTTVTMASSSSDQGPTPLSYF)KYDVFLNFRG-F-
TRFDFTDTLYHALIKARIETFRDSEGLRIGEELhgSLVEAIERSRMSVLVLCDMYPTSRWPLDELVKIMECSE
NGKTRPVLPIYFHVQRSDVQYEKNSYRTAM------------------------ID()}*

$213=149(162):

>gi|296604636|gb|GW983289.1_EST1 {<Streptophyta(V1)>}Ipa.ADAB-aab27d06.b1 IPA1-seed Arachis ipaensis cDNA 5', mRNA sequence
{(SLLFQLRLSLALSQITMAASYF)KYDVFLSFRG--
HTRREFTDALYHALVNKRIETFRDSENLRIGEELegALLEAIERSRMSILILCDEYPTSKWCLDELVKIMECS
GNGTKRPVLPVYFRVAKSDVQFQKNKYETAMAAH-QAK------------GRNNHQL()}*

$214=298(296):
>gi|313463365|dbj|FS994968.1_EST1 {|0(7)|<Streptophyta(V1)>}FS994968 Osmunda lancea leaf Osmunda lancea cDNA clone L1369 5', mRNA sequence
{(SSIFGGRVLLSLFAFSVCYSFGKNLLLAAMSKRKRDTDLPECSNADQEATPMERKRDTHLPECSNADQEAT
PMDTSIG)KHEVFISHRG-
LEMKRKFANHLFEALKQRGITAFLDLKELPEGNDVrdFMLEAARRATIQVPIFTKIYPESCWCLDELVEMIKS
TEKPSNAKVIPIFYDVKPEDLRLKDGPFAEAFEAH-
LEKrqYDEDRITQWKDALSEASWL(SGFRLETYNQREGELLKDVVYKIGHELTDRVPVLLDRVGIEKRVNDVV
GRFRLAEKTAVYFCLLWGMGGIGKTTLLEL)}*

$215=196(227):
>gi|308621004|dbj|DK528919.1_EST1 {<Streptophyta(V1)>}DK528919 full-length kale cDNA library (seedlings) Brassica oleracea var. acephala cDNA clone KALE-035P14 3', mRNA sequence
{(RLAQKWDQC)---------------------------------
FIDDKLEVGDNLTDLFEKIEESTVAVAIISSRYTESDWCLNELVKIKECVDR-
RTLRVIPVFYKLEISIVKKLKGSFGLQLWKL-
WRKenhcNRDDRILKWDAALQDVSGK(KAMRYLENSNEADFADKMAKHIQDYLNKDKPPREENPKPQGKREEN
VKPQGKGGDNPNPEEGSNMASSSIKPGKQCLK)}*

$216=225(235):
>gi|308715427|dbj|DK488220.1_EST1 {|0(25)|<Streptophyta(V1)>}DK488220 full-length kale cDNA library (flowers) Brassica oleracea var. acephala cDNA clone KALE-085H02 5', mRNA sequence
{(NATMGKPTKLKSPPPP)-HRVFISSHG-GDLllRGGFVHEIAKALRSIKVNAYAD--EPTGEEV--
VWTRIEKSRIAIVVFSSRYTESRWCFEELVKIKEGMEL-GELEVIPIFYKLDTS--AELEGGSGLN--PT-
MRDFDPQKVKIWNEALDSVSCM(MGYHLYKNSVESKFISRIVKDVKRILIQLPKEEQDDESHDSLDDSSKSET
LNGGSVLGEAESVEDDIFPNEKVDSVNDQ)}*

$217=138(152):
>gi|294401557|emb|FP944883.1_EST1 {<Cnidaria(M1)>}FP944883 Clytia hemisphaerica library Clytia hemisphaerica cDNA clone SA0AAB135YB20 5', mRNA sequence
{(KRIKEYKMPPKH)---IMISYNW-DI--
QDLVKKVVSGLKSRGIPVWMDLDAMSGDINSSMAEGVDGCKAIVCFMTQKYQDSKNCKRELTYADQQE---
KDIIP-----CMAQKDYKAQSWLGIVTAGLLWMNFGDDSQLETKIGSVAKEIIS()}*

$218=144(163):
>gi|313360140|gb|HS213843.1_EST1 {<Mollusca(M1)>}CCTS11825.b1 CCTS Crassostrea gigas mixed adult tissues library 4 normalized Crassostrea gigas cDNA clone CCTS11825 5', mRNA sequence
{(RIIIVRYKKSPNSCVMAEGKELK)KHDVMISYNW-EH--
QTTVLKIRDKLEENGISCWIDVENMSGSILEAMSAAVENCTIFLMCYSGKYSESKNCKKEAEYAENRD---K-
-IIIP-----------CKMEKGFDASGWLGILLGSKLYFEFSGKYPFDNKMK()}*

$219=156(164):

>gi|313360141|gb|HS213844.1_EST1 {<Mollusca(M1)>}CCTS11825.g1 CCTS
Crassostrea gigas mixed adult tissues library 4 normalized Crassostrea
gigas cDNA clone CCTS11825 3', mRNA sequence
{(RIIIVRYKKSPNSCVMAEGKELK)KHDVMISYNW-EH--
QTTVLKIRDKLEENGISCWIDVENMSGSILEAMSAAVENCTIFLMCYSGKYSESKNCKKEAEYAENRD---K-
-IIIPCKMEKGFDASGWLGILLGSKLYFEFSGKYPFDNKMKELLREIKIYLE(T)}*

$220=129(150):
>gi|313382324|gb|HS243202.1_EST1 {<Mollusca(M1)>}CCTS28504.g1 CCTS
Crassostrea gigas mixed adult tissues library 4 normalized Crassostrea
gigas cDNA clone CCTS28504 3', mRNA sequence
{(S)------------------
VLNIRDDLRKKGISCWMDIDNKDGSALMAMTTAVENCTVFLMCYSAKYSKSLNCRTEAEYAVRRQ---
KTIIPCRMQQDEFNPSSGGWLGSIIHDRPYYDFSGKYPFEQKIRELLSCIEAHL(HKQNQAETF)}*

$221=188(193):
>gi|308624574|dbj|DK469477.1_EST1 {|0(64)|<Streptophyta(V1)>}DK469477
full-length kale cDNA library (seedlings) Brassica oleracea var. acephala
cDNA clone KALE-036G24 5', mRNA sequence
{(RGQLK)-YSVFLCFRG-
EDVRVGFLSYLIGTLKRKSIKYYVDYEETRGEVMEIFLRRIAESRIVLVILSPKFMESDWCIDEVLKALETRE
A-G-TSIIPVFYKVSVADVKGWNIVKQGDRDKF-
INVTKRLGLRSKDYPTSEADFV(NKIVEELEKALTKLETEEEENKLKMKQAITTNTTMPFFGTNKRVKQLQ)}
*

$222=203(231):
>gi|283523261|emb|FN237832.1_EST1 {<Onychophora(M1)>}FN237832 dmp017cm
Peripatopsis sedgwicki cDNA clone dmp017cmP0054D03 5', mRNA sequence
{(RRLYWRMSSIFYVHGEIQNSC)--------------VDTILKSLRHQNHHCKQFM--
KDFPLGKNALDEIKEAIEISSHIIFILTDNFYNDYICWFPLSEALLQ-----
PKHIILVLTQSFEaEALFSNLEVLPKLCTYIsILDWRQQVHLedL-----------
S(TLLFEGSPIQVPYRCVQNCTRQLIHDELESASFTTFEFDYSQIPDVKVLNKLTLISDSSLGNKKGKKKGH)
}*

$223=141(147):
>gi|301540625|emb|FP541461.1_EST1 {<Annelida(M1)>}FP541461 Alvinella
pompejana pygidium library Alvinella pompejana cDNA clone MH0ADA92YA19
5', mRNA sequence
{(PE)---------
ENdeSWVEENLLPIIDsEYGQPWKVFYEKRDSMMNYCNIGEVVDAIYTSKRVILILTESYLKNSRRKYELETV
FTCLTEKEIDVSNFIIAVVVEpNTDVQVPRLLHPIFRNDqELNWTASEEGkeLFRRRLKD----
L(MPAHS)}*

$224=215(220):
>gi|301566922|emb|FP512038.1_EST1 {|42(0)|<Annelida(M1)>}FP512038
Alvinella pompejana gill library Alvinella pompejana cDNA clone
MH0ABA65YC12 5', mRNA sequence
{(HVVSKMNASQLVVTCRSMNLDMLPAHIFGERIVDLDLSGNHFKLPDWWRWTLRWYCYKVRYKRRLGKQNVL
PRMLQNDSV)-
YSSFVIYpQEGdeatRFVDEILLPEVEEELRKPRLYIKDRDKYVGATCLDEMVRAILSSQTAIWIVCPDFFRD
PYCEMAARFALHHLGPRKNQIVVLV--NQFEnYQEPQRFANLMNPTSRIrRVHFKEEGVArrVF---------
--()}*

$225=189(209):

>gi|301576203|emb|FP511480.1_EST1 {<Annelida(M1)>}FP511480 Alvinella pompejana gill library Alvinella pompejana cDNA clone MH0ABA67YL14 5', mRNA sequence
{(STTFCIA)--------NSSdaEDISKELKDSLES--LKFSVYTDD-DFEPGWLIIHSITEAVKKSQKIIFLVAKE--VSNWTVFNFASSKAKCIAENEDLLIPII-CDVDeSQMP-------LFVKESnYL-----KANdrDFWHHLIGKLTAK(IPLSESLPVGNIGHGIAWSYFYGFLNVVLPDMAKKMEDFEPDSIPDKKCYKFIIIIPDSCKI)}*

$226=191(220):
>gi|301588810|emb|FP491040.1_EST1 {|0(18)|<Annelida(M1)>}FP491040 Alvinella pompejana whole body library Alvinella pompejana cDNA clone MH0AAB59YM17 5', mRNA sequence
{(S)---------------KELKDSLES--LKFSVYTDD-DFEPGWLIIHSITEAVKKSQKIIFLVAKE--VSNWTVFNFASSKAKCIAENEDLLIPII-CDVDeSQMP-------LFVKESnYL-----KANdrDFWQHLIRKLTAK(IPLSESLPVGNIGHGIAWSYFYGFLNVVLPDMAKKMEDFEPESIPYKKCYKFIIIIPDSCKIPNDFNDKSIQKCGNLQY)}*

$227=128(146):
>gi|313305379|gb|HS161464.1_EST1 {<Mollusca(M1)>}CCBN21954.g1 CCBN Crassostrea gigas mixed adult tissues library 2 normalized Crassostrea gigas cDNA clone CCBN21954 3', mRNA sequence
{(RD)-------------------PRLEN-EAGFQLYVKDRDEPAGISCGEAIMEAIQKSKRVVCLVTKSFLKSTWQDYELNMSRMEAIEDREnlNFVHLILFPDVCnGSIS---IQTLDLVRQEcFSEYPEEECAfdDFWETLISHIK-E(LNRH)}*

$228=122(145):
>gi|313305889|gb|HS163534.1_EST1 {<Mollusca(M1)>}CCBN23026.g1 CCBN Crassostrea gigas mixed adult tissues library 2 normalized Crassostrea gigas cDNA clone CCBN23026 3', mRNA sequence
{(N)------------------------EAGFQLYVKDRDEPAGISCGEAIMEAIQKSKRVVCLVTKSFLKSTWQDYELNMSRMEAIEDREnlNFVHLILFPDVCnGSIS---IQTLDLVRQEcFSEYPEEECAfdDFWETLISHIK-E(LNRH)}*

$229=122(145):
>gi|313320417|gb|HS178286.1_EST1 {<Mollusca(M1)>}CCTP11021.g1 CCTP Crassostrea gigas mixed adult tissues library 3 normalized Crassostrea gigas cDNA clone CCTP11021 3', mRNA sequence
{(N)------------------------ELVFSCMFKDRDEPAGISCGEAIMEAIQKSKRVVCLVTKSFLKSTWQDYELNMSRMEAIEDREnlNFVHLILFPDVCnGSIS---IQTLDLVRQEcFSEYPEEECAfdDFWETLISHIK-E(LNRH)}*

$230=194(219):
>gi|313327188|gb|HS185835.1_EST1 {<Mollusca(M1)>}CCTP15099.b1 CCTP Crassostrea gigas mixed adult tissues library 3 normalized Crassostrea gigas cDNA clone CCTP15099 5', mRNA sequence
{(TAKMSADRLLRQSSLENLIESDSGGDQEMFNPVSVETSGDAEDLRHEIDEHVQRKKSLSLPPSKK)-YHLFISYSAEdrEDANKICEHMEKRFQ--MKCMNFERDFVPGKSIDENISYEMQRSVKVLLILSPNYTQSHWCVTEAREACQLSFKDLCDVsVIPLLLRPLEkQ--------LPPFLKSFvYID-------------------A(QKELDVPAKFMMLI)}*

$231=197(196):

>gi|313353733|gb|HS211686.1_EST1 {<Mollusca(M1)>}CCTS10664.g1 CCTS Crassostrea gigas mixed adult tissues library 4 normalized Crassostrea gigas cDNA clone CCTS10664 3', mRNA sequence
{(FHSSSFLSSIIIGITLVKNKWKIRYVVYKFKQRFRKHKGYFTNFQSGTPCYE)-
YDAMISYSPKelSFVLTDVIPRLEN-
EAGFKLYVKDRDEPVGTPFGETIMEAIQNSRRVVCLVTKSYLKSKLQDYELNMSRMEAIEDRGnlNFVHLILF
PDVCnVSIS---IQTLDLVRQEcFTEYPEEQCAldDFWERLRSII--S(KEFL)}*

$232=215(237):
>gi|313359632|gb|HS213335.1_EST1 {|24(0)|<Mollusca(M1)>}CCTS11551.b1 CCTS Crassostrea gigas mixed adult tissues library 4 normalized Crassostrea gigas cDNA clone CCTS11551 5', mRNA sequence
{(LFNVNGKSVSDIITSVVKEDAIKKWLIIVEEKETYFINQEETKLAEKKNNLCLKCRSPVIERQLSVFPDPR
EKPRSMKYP)-YDFYVIFSPAdrDWVCHILLETLE-
VSYGYKGAIAERDIRPGSTDIYERVKLIQNCSKILVILGQEFEGNLECEYELSHALKRKHEDNLENVLIPILR
NVD--------GLHQSLRTItFLDAVDD---------------C(DWTSLSSYGVNAMIYCR)}*

$233=115(141):
>gi|289140139|gb|GW564250.1_EST1 {<Arthropoda(M1)>}Ic600565643F1 Aphis gossypii cDNA Libraries Aphis gossypii cDNA, mRNA sequence
{()-----------------------------
QLCLHYRDLPITSMYHAgsspVVIEAAEASRRVIVVLSRNFIQTEWSRYEFRSALHEALKGKVYKLVLDRGEQ
H-RSRGGCRPRDQAVPEGGVQGALGRKTVLGTAPLPDAQQQP(P)}*

$234=291(292):
>gi|300755511|emb|FQ021028.1_EST1 {|2(21)|<Arthropoda(M1)>}FQ021028 Spodoptera littoralis cDNA library, male antenna Spodoptera littoralis cDNA, mRNA sequence
{(FVKKAGWYRSGIPGIFLPDNYFAVICGAIIIIVVILLIGAIFYAFRYEISDWLYTKYGVPLFKEQSCPNVD
HVLEGIPNH)MYDYYVICNTKdtSFVYHNIMSEIE-----YRKMSTKKFSPNEASLNI-
LTlesfsKSSKLSKRLIIVITTNFICNDLCDIHFKNLFFSYLKSLnrsdLNKVIFIKVLEN-
NQMSEELCFVLDKYKNVswN-----DPR--
FWDRFIALLNCT(DTIITVKGSEKSVFKKSLRQTPTLRYTTMPVSNDSCSKQNFTNTLQYCKRNEGESSQTES
SPSSEHNTYGEG)}*

$235=112(140):
>gi|312835703|gb|GW694263.1_EST1 {<Mollusca(M1)>}FPM_M_02631 Mantle of freshwater pearl mussel cDNA library Hyriopsis cumingii cDNA 5', mRNA sequence
{()----------EDYVVHDLVPVLEK-
NMNYRLCVHYRDFPVGAAIADNILSSVESSKRVIVVLSNNFVESEWCHYEFQIAHQRLLAEKKNRIIMILLGD
I--DKSKLVPELKDYIQTNTYVNQDDA---------------()}*

$236=191(202):
>gi|290518866|gb|GW643794.1_EST1 {<Arthropoda(M1)>}EST_lsaA_evu_1214658 lsaAevu mixed_tissue Lepeophtheirus salmonis (Atlantic Canada) cDNA Lepeophtheirus salmonis cDNA clone lsaA_evu_510_288 3', mRNA
{(RIYKL)--------
SHEdsDYVEGKLVDGLESPQDdpllKYKCLLAIRDFVPGEVISEQIVQAVDKSSRTIIVLSEAFLRSDWAQYE
FEIAH------SRRKVILLKIGDL-PSKEDIGPSIMNYISQNtYLDHDHPN---
FWRNIRYALPHK(GNSKSWRPAWLKPRKMVNQLHLMETPNSTPVPPFNDKIENTYRNTDNLKHNTSCLPV)}*

$237=179(197):

>gi|290526536|gb|GW656815.1_EST1 {<Arthropoda(M1)>}EST_lsaA_evv_1243686 lsaAevv mixed_tissue Lepeophtheirus salmonis (Atlantic Norway) cDNA Lepeophtheirus salmonis cDNA clone lsaA_evv_515_132 3', mRNA
{()-------------
VEGKLVDGLESPQDdpllKYKCLLAIRDFVPGEVISEQIVQAVDKSSRTIIVLSEAFLRSDWAQYEFEIAH--
----SRRKVILLKIGDL-PSKEDIGPSIMNYISQNtYLDHDHPN---
FWRNIRYALPHK(GNSKSWRPTWLKPRKMVNQLHLMETPNSTPVPPFNDKIENTYRNTDNLKHNTSCLPV)}*

$238=120(148):
>gi|313283112|gb|HS138663.1_EST2 {<Mollusca(M1)>}CCBN10252.g1 CCBN Crassostrea gigas mixed adult tissues library 2 normalized Crassostrea gigas cDNA clone CCBN10252 3', mRNA sequence
{(SAREEKPC)---------------------------
YKFFVPVRDLLVGDGKADSIIENMEKSKRVIIILSDKYDENEWCKFECQRA--EILELNNGRIIFIKYHPE-
ADEMIENEPWKSRVKGRkVFSPGEkkserRW---FWGKIKYELPVR()}*

$239=161(164):
>gi|312505039|emb|FP596995.1_EST1 {<Annelida(M1)>}FP596995 Hirudo medicinalis normalized library Hirudo medicinalis cDNA, mRNA sequence
{(VHPFDKDECKGENMI)-YDAFLSSSYAdRLFGIALVRLLES--
KGYRVCYHEKDFIGGQTIVENICQAVIHSKRVVCYITKTFLESNYCMYEFQKALHQNIMTKRRRLVALVDENl
V-INQSTLPDDIYQFLTTHtFIEIKANN---NWEKhLFYSLAVN(KLGNQLTNN)}*

$240=168(181):
>gi|312553866|emb|FP658314.1_EST1 {<Annelida(M1)>}FP658314 Hirudo medicinalis normalized library Hirudo medicinalis cDNA, mRNA sequence
{()----------DRLFAIDLVRTLESR--
GYCVCYHEKDFLIGPSIASNICRAVVYSKRVVCILSNDFLKSPYCMYEFQAALHRNIETKRKRLIALLCNSM-
TvNKEIMSDEMFAFLSSHtYVKITEGD---
KWlSQLCYSLAVN(KIRKADFDCSLLGGLETELIPQRNGSISPTDEPSLSSIYSI)}*

$241=136(149):
>gi|312565479|emb|FP641857.1_EST1 {<Annelida(M1)>}FP641857 Hirudo medicinalis normalized library Hirudo medicinalis cDNA, mRNA sequence
{()----------DRLFGIALVRLLESR--
GYRVCYHEKDFIGGQTIVENICQAVIHSKRVVCYITKTFLESNYCMYEFQKALHQNIMTKRRRLVALVDENlV
-INQSTLPDDIYQFLTTHtFIEIKANN---NWEKhLFYSLAVN(KLGNQLTNN)}*

$242=221(227):
>gi|292497641|gb|GW716760.1_EST1
{|0(55)|<Streptophyta(V1)>}WS02763.C21_D10 SS-IL-A-FL-14 Picea sitchensis cDNA clone WS02763_D10 5', mRNA sequence
{(INPPPPQ)------------
DLKNTFASHLYHRLLRDGLQPFLDREVLEKGELLapQIESAIRTASFHITIFSPTYADSRWCLDELVSILKSG
-----ATIIPVFYNVKPSELRwtgryaqslsiHEQKGRYDRQTLDSWRKALHDV--
SSIIGFELETYN(GDEGKLLDSVVQSVLKKRRQAALNVAKTTLNVARYPTGLQEKVEDFEKTVLLQKQISGEA
QVLGIVGLGGVGKTTLAKEL)}*

$243=208(216):
>gi|292503937|gb|GW721156.1_EST1 {<Streptophyta(V1)>}WS02821.C21_B08 SS-IB-A-FL-13 Picea sitchensis cDNA clone WS02821_B08 5', mRNA sequence
{(INPPPPRLAFSIAISPLSLISSLAIFAFIASTFLSILFLPISMFELAMAASSSIAAHYS)-YDVFINHRG-
PDVKKTFASHLYRDLSKYGLKVFLDDPEMQPGDSLirQIEGAVGTASVHVAVFSSRYAESRWCLVELLLMLKS

G-----KTIIPVFYDVKPADLRA---
GGKYAEELAKKGTCGGKRKRYDSDTIEEWRNA(LSHVAGISGFELEKCNG)}*

$244=174(193):
>gi|292512469|gb|GW730263.1_EST2 {<Streptophyta(V1)>}WS04812.CR_F04 WS-CRD-33 Picea glauca cDNA clone WS04812_F04 3', mRNA sequence
{(QLAMTYPTPASSIPQTHSEIEIEMENQRSPRATSNSASSSSTAADSNNSSSNI)PYDVFINHRG--
EVKKTFASHLYHRLFSRGLRPFLDQQELQEGLDFpsQLESAIRSASVHVAILSPKYAESRWCLDELLLMLESG
-----APIIPVFYHVQPAQLRWTD--------------PSKNGEYAVALRQLQKKKK()}*

$245=169(184):
>gi|292516409|gb|GW734753.1_EST2 {<Streptophyta(V1)>}WS0497.CR_M17 WS-CBM-34 Picea glauca cDNA clone WS0497_M17 3', mRNA sequence
{(GSLATSTSASTATSTATSTSASASTATSTSASASASAATGSYVF)--DIFISHRG-
PDTKKGFASHLYRALIRHSYRVFLDNEEMQRGENLmpQIEGAIQTASVYIAVLSPGYADSEWCLKELVLMLES
rsqrR-----STILPVFHGVRPSDLR-------------WAGGQKGIYGRALSELEQKKKKK()}*

$246=163(168):
>gi|292531183|gb|GW747305.1_EST1 {<Streptophyta(V1)>}PBA0114.C21_I11 Pba01-Bark-wounded-Norm (PBA01) Pinus banksiana cDNA clone PBA0114_I11 5', mRNA sequence
{(GDSNNNSC)-YDVFISHRG-
PDVKKTFASHLYRRLISYGLRVFLDYKELQEGEEFpcQIKDAIRTASVQIAIFSPTYADSQWCLDELVLMLET
R-----
AHIFPVFYHVNPTELRRTQGEGVYATSLRNLEKKRKYGSEPRYDSKTIENWR(NALSRVADISGFELDVGFNG
)}*

$247=229(234):
>gi|292548951|gb|GW768247.1_EST1
{|0(16)|<Streptophyta(V1)>}PBA0218.C21_J10 Pba02-Xylem-wounded-Norm (PBA02) Pinus banksiana cDNA clone PBA0218_J10 5', mRNA sequence
{(PFGIRIPNASKPTLKFKSNYMDASGASSSSAAAHPINNSGVDYR)-CGVFINHRG-
PDTKKTFASYLYRRLLIHGLRPFLDQEELEVGSNFptQIVDTIRSASVHVAIFSTSYADSPWCLRELVLMLES
E-----
APIIPIFYHVDPAALRWTDQPTKGVYAVALNQLQMKRTVDPQTHEKKPRYDS(ATLQKWRNALSTIADISGFD
LMGKFNGDEGLLLDEVVKRVLEIMSVNTQL)}*

$248=155(183):
>gi|300481023|gb|HO008422.1_EST1
{|0(43)|<Platyhelminthes(M1)>}PL08009B1G11 cDNA from juvenile hermaphodites Schmidtea mediterranea cDNA 3', mRNA sequence
{(EY)------------------------
LHRVGYTTWMDVGQMGGGDLLmhRIERAIRGCKVLIACISPRYIVSTICHREIVLADLLK-----
KPIIPVMVLPVPWPPPGGTGLMLAPYIYIDLFGVGGHGGTGLKSDLTSRFAE(IETQVSKYCSPICVNIESTA
MSCMNTISISPEFSSKPSTIS)}*

$249=214(206):
>gi|313257811|gb|HS117942.1_EST1 {|0(7)|<Mollusca(M1)>}CBSN3553.b1 CBSN Crassostrea gigas 2-12 hrs, 24 degrees Crassostrea gigas (L) Crassostrea gigas cDNA clone CBSN3553 5', mRNA sequence
{(PRVRKMCMIRTPERFATKINDLKDIVEDHIAGLEMMKSDHP)--DIFISYCW-
QNSQEAVnngtqARHgalgygdprkIKEFLEKKGLSCWLDIEQMgKYG--
LyqEIAIGLQNCKVIVACVSDEYVESDACMMELRFAVCNM-----

DLPLVVVVVGTGDKWKRSEVSLLMRRSDKGANKVYMQKENPEGFEALHKFVV(DVLPTYTDEILSEKKKKKKK
KGRPP)}*

$250=181(212):
>gi|313360674|gb|HS216041.1_EST1 {|0(39)|<Mollusca(M1)>}CCTS13021.g1
CCTS Crassostrea gigas mixed adult tissues library 4 normalized
Crassostrea gigas cDNA clone CCTS13021 3', mRNA sequence
{(LLCSSKTFWKENKRSRDDQLFSGTAPLLLRIKQAI)-------------------------
TEAGFSVWMDIEQMR--ENVydRMAEAIQDASVVLISMSYSYQRSEYCRREAAYASSLK-----
RARIPLLVDPGYTAEGWLALMTAGDYHYDFGREPFETKIVELIKELERRCLK(GQGEVTDGVVKQKVVSASKK
SPSLLELQEDLLRTEKR)}*

$251=184(190):
>gi|313386205|gb|HS237987.1_EST1 {<Mollusca(M1)>}CCTS24961.g1 CCTS
Crassostrea gigas mixed adult tissues library 4 normalized Crassostrea
gigas cDNA clone CCTS24961 3', mRNA sequence
{(IITSGNTSLSVDMLIAFLEKMNRDDVVEIIEKANDTNKD)PPQVFISYQW-DSQDEVKA--
LRDKMEKSGYSCWMDIGQMGGGDFLsaKIDQGIRNCKVVLACITPKYVVSHHCNRELSLADLLN-----
KTIIPIMFEEVPWPPPGGMSLIFSQLVYINMKGVGGSWRYWYPCRPEGQVQG(DCPETDELHCA)}*

$252=195(220):
>gi|300060093|emb|FP920692.1_EST1 {|95(0)|<Arthropoda(M1)>}FP920692
Acyrthosiphon pisum whole insect males Acyrthosiphon pisum cDNA clone
ACI0AAF58YM08 5', mRNA sequence
{(KIFKFCEAKTTIAHFTRCLERIDRYDVVEETEELFISDIKYFKEQCSLAKYPTPVVPNDFDINIITLDDIN
SSSNGLPLT)RYDAFLLYDKAdIQSAAAVVEKLE-
TDYKLKLCLKDRDLRPGVMFeYQSIIKLISDRCNWlIIIVSNDFSNRPWNRFIVIIYSRLALKNGYLKIIPVY
L--MNLDLPEKLKWLSSLKF-------------------------()}*

$253=185(192):
>gi|312528793|emb|FP652741.1_EST1 {<Annelida(M1)>}FP652741 Hirudo
medicinalis normalized library Hirudo medicinalis cDNA, mRNA sequence
{(ADSGAPEY)-FNAFVCYSPVdLTFVKNMMTELEDKQ-
QFKLCIPDRDFLPGPPKYEAITKLIQARCDKfIVVLSPDFLKSDECAFMLNYALALNPSARRKKLIPILYK--
RCEIPPILSFITLVDFTR--GDVIG---
WFWNRLSKSLITT(GCLNTISSSPSLPSLKDFQPTSQDTIVTLKTKPRPGEFLEVEKG)}*

$254=220(224):
>gi|313320736|gb|HS178605.1_EST1 {<Mollusca(M1)>}CCTP11190.b1 CCTP
Crassostrea gigas mixed adult tissues library 3 normalized Crassostrea
gigas cDNA clone CCTP11190 5', mRNA sequence
{(IARDIKNWLQSDAKEPNQPGADIPREQPPKLKIPPATPSNKQHQDYVSSPVSPTRSTGSDPTDHNDGALTL
DDLDGNQAS)-
YVACICCSDDdIEIAHTIIEAYREVTNTVQFFLPQEQLLGGKYEYESLAEVIEDRCDgRlLVIMSRTYVSSPA
CIFSTQFVKSLDPDAKRRKIIPI-IKDEDATYPRVLRGINAIKLQR--LKFSL---
PFWKLLSASLDIK(YIPQ)}*

$255=192(203):
>gi|294376969|emb|FP978085.1_EST1 {|0(61)|<Cnidaria(M1)>}FP978085 Clytia
hemisphaerica library Clytia hemisphaerica cDNA clone SA0AAB99YA24 5',
mRNA sequence
{(PNKMSAIM)-----ISYQWDSQPEAIRIKDALEADGYD-VWMDLDDMR--
GNIYQKMAEGVENAAVILVCMSTKY-ENSVNCNrELQFSQDKR-----
KQLIPIMIEaGYRpgGALGLiIAGLKYVDFSN--

PGDFDDKMVELRDEITHYCKPT(LQPTESAPKAASETATTGDYNMKLGVVEARAESWDGAANALVEKLRSRKL
KRGQV)}*

$256=196(209):
>gi|313336873|gb|HS195225.1_EST1 {|0(38)|<Mollusca(M1)>}CCTS2009.g1 CCTS Crassostrea gigas mixed adult tissues library 4 normalized Crassostrea gigas cDNA clone CCTS2009 3', mRNA sequence
{(PHLAFSRGLDVSPLFFVVQKLFGKKTKGHVM)-----ISYSQEQRPFLLRIKQAITEAGFS-
VWMDIEQMR--ENVYDRMAEAIQDASVVLISMSYSY-QRSEYCRrEAAYASSLK-----
RARIPLLVDPGYtaEGWLALMTAGDYHYDF--
GREPFETKIVELIKELERRCLK(GQGEVTDGVVKQKVVSASKKSPSLLELQEDLLRTEKRK)}*

$257=218(216):
>gi|301528359|emb|FP555227.1_EST1 {<Annelida(M1)>}FP555227 Alvinella pompejana pygidium library Alvinella pompejana cDNA clone MH0ADA34YA22 5', mRNA sequence
{(HESLDILEHHIKPLRTIPGVVLEQAYLNKMAEGLQPLAQLATSSSASELGRLHQNQDGQS)-
PRIFISYqwdmqSKVEDIRRQLEANsyvcssdispmmpLQQRGHSGINNRVHSLDAVP-
ESTQsKIQRNMRAASAVLCCITPKYLQSDSCIKDLLLADGLHKP-----ILPVMLRFC-PW-----------
PPEGapVQVRKILVKHPPVDLSNEKLFKQ(NIPQLLDKIRKMTCGR)}*

$258=221(220):
>gi|313350811|gb|HS206777.1_EST1 {|8(0)|<Mollusca(M1)>}CCTS7845.g1 CCTS Crassostrea gigas mixed adult tissues library 4 normalized Crassostrea gigas cDNA clone CCTS7845 3', mRNA sequence
{(AVTALLMNYSTNRQWRKFELDCQATEWLVFSIVLIVSTLITLIILAAIKKYHVHLEYVILRVKQRLMPVGD
HVCVEGDFQ)-YDVYISYNDDDtsWVANNLNPKLESLN--
IKAWFKVRDSIPGVSQSEEIIKCINESRKVMFIISESFLDIGWSSYAVQMAITHAFHNQRQrSIVVIIKDGLp
LERLPK---EIKHIWwCIEHLRWPEDET-NdemLLLNLSNVLVSE()}*

$259=191(220):
>gi|300467195|gb|GH566599.1_EST1
{|27(0)|<Porifera(M1)>}sd002_026d_c11.q1ca S. domuncula Library Suberites domuncula cDNA, mRNA sequence
{(EEIQRIRRVQVTAGIDPEQLAKGLAGISIAHSSPSRSPNSTNSTASPQTTPSGSFSSKQINHTSNKSKDLE
KCLESIQKQ)QFDVFLSFaEADERFAEEVRQRLIENA-
GVRVFVPSAGLMSDRAFHQEIADIIKQgCTRTVVILSPDYLSSSWCGYEANLAFTNSPDARRNTTIPILYRPC
--EVPAFLSHLHY----------------------------()}*

$260=189(217):
>gi|301589591|emb|FP504513.1_EST1 {|0(9)|<Annelida(M1)>}FP504513 Alvinella pompejana whole body library Alvinella pompejana cDNA clone MH0AAB22YM24 5', mRNA sequence
{()------------------LADNLED-
QNGFKLFIPNRNLTVGTAEYATISFIMENrCSSMLVVLSKDFRDCEKCNWMAKHWLSID---SFKITIPI-
ITSTEDDVPDYLAFIIPVRY-------CSDS-
WRWlERLKKSLLDC(STERMTRPNDDSTQRMTQPNDDSTQRVTQPNDDSTQRVTQPNDDSTQRMTQPNDDSTQ
RVTRPNDDSTPRMTQPNDD)}*

$261=209(220):
>gi|313321169|gb|HS179038.1_EST1 {<Mollusca(M1)>}CCTP11418.b1 CCTP Crassostrea gigas mixed adult tissues library 3 normalized Crassostrea gigas cDNA clone CCTP11418 5', mRNA sequence

{(IARDIKNWLQSDAKEPNQPGADIPREQPPKLKIPPATPSNKQHQDYVSSPVSPTRSTGSDPTDHNDGALTL
DDLDGNQAS)-
YVACICCsDDDIEIAHTIIEAYREVTNTVQFFLPQEQLLGGKYEYESLAEVIEDrCdGRLLVIMSRTYVSSPA
CIFSTQFVKSLDPDAKRRKIIPI-IKDEDATYPRVLRGINAIKLQR----LKFSL-PFWKLL-------()}*

$262=206(219):
>gi|313390121|gb|HS246169.1_EST1 {<Mollusca(M1)>}CCTS30169.g1 CCTS
Crassostrea gigas mixed adult tissues library 4 normalized Crassostrea
gigas cDNA clone CCTS30169 3', mRNA sequence
{(VLVKAIKEDLHVGSIKEGRIERPLDRLSSREPADFEH)--DAFLFYdDESCEIGNEIWKYLESKGTKLFI-
---EDQRAG-------
QRLISNldyvignCYWTIVILTRKALKDRTFTLQLISLLQSFLVDKKIRLIPVLLDTRYGDIPDAIRFVT---
YVG----VDENK-RYLERLRCTLKGK(EIPFDRDTLIPAGNVAYGLAWTYVLNYLAFVLQGLGDSIIRA)}*

$263=206(239):
>gi|324730282|gb|JG279681.1_EST1 {<Streptophyta(V1)>}LUSTE1NG-RP-
109_D05_06FEB2007_041 LUSTE1NG Linum usitatissimum cDNA, mRNA sequence
{(PCFSHSFFSTSFGDEIPNTQSSTMMTIQHLKLMPYPTPLIPLLCFPRWSM)--------------------
----
RDDDELrkGKEIGSNLLRAIDQSKIYVPIISRGYADSKWCLMELAEIVRRQDEDPRRTILPIFYMVDPSDVRH
QTGCYKKAFQKHANKFDEKTI-
QNWKNALKKVRNL(KGWHIGSNDEYVILILVFCYSVQLICFVLHLLTCLLTNFQIAYKHSSNI)}*

$264=119(147):
>gi|339687349|gb|JK184696.1_EST1 {<Streptophyta(V1)>}NRL_006587 Root
cDNA library of peanut (Arachis hypogaea) Arachis hypogaea cDNA clone
ocpnra0_0081_C09.ab1, mRNA sequence
{(NVAEMKY)KHDVFISFRG-TDVRRGLLSHLKKDLHREKIDFYVDDAKLkkGDKI-
LSLLTAIEESLILLVIFSKDYASSKWCLAELEKIIECAEKNEQIAVVPVFYKLDPSEVRHQRGDY--------
--------------------AA()}*

$265=195(205):
>gi|345063015|gb|HS063677.1_EST1 {<Streptophyta(V1)>}GR-TS-002-CN-006-
A08 Treated seedlings (EUGR-TS) Eucalyptus grandis cDNA clone GR-TS-002-
CN-006-A08 5', mRNA sequence
{(DNSLEMERKEPTPSEDPTHGASSSSTSPHRGDSGGGRQKPKGNDR)--DVFLSSVC-PMWSTLFDD-
LRHGLVIAGIDTFCSKDNADEWIPYNLPQ----CKISIPIFERGFVNDKSGLRELVHMWKCKRSSGQI-
VLPIFDEVGPLEVQLLEGESIEEFSRLEGRVDEMEL-
GEWRKALAEVGSL(RGWEAEKFAYGYIICHRNII)}*

$266=174(173):
>gi|345066664|gb|HS048666.1_EST1 {<Streptophyta(V1)>}GR-SE-001-GO-025-
G09 Seedlings (EUGR-SE) Eucalyptus grandis cDNA clone GR-SE-001-GO-025-
G09 5', mRNA sequence
{(PPTLSRKSMAGALASCVSRAGPLACGSTH)-YDVFLSFpGG-
SDTtPESFPGRLHACLAAAGVSICRPDESQaaGSLSSLLPDAAIWRSKICIPILSEDYVSSDRCLHELIQMME
-GKRSARQLVLPICNRVEPSHPRYLKGSFEKASHSDIEGVQRALVGVSMLHGWKITDGS(EDER)}*

$267=167(185):
>gi|345075843|gb|HS041347.1_EST1 {<Streptophyta(V1)>}GR-PU-000-MG-010-
D04.R Young leaves infected by Puccinia psidii (EUGR-PU) Eucalyptus
grandis cDNA clone GR-PU-000-MG-010-D04.R 5', mRNA sequence

{(FLIRATPQPPLFLFRGERYPEGISFLAMANSEAGTKSDVARELEV)EYQVFLSFRG-PDTRNEFTDFLYYGLEDVGVHVFRDEDELrvGEVIGKNILRAIDNSIIYIPIFSPTYASSKWCLRELVHIRQNlSQSEGQKCIFPIfFFHVEPDDIKLKTXRYIEDFSRHEK---------------------()}*

$268=212(211):
>gi|345077019|gb|HS069858.1_EST1 {<Streptophyta(V1)>}GR-XY-006-RS-006-H01 Xylem (EUGR-XY) Eucalyptus grandis cDNA clone GR-XY-006-RS-006-H01 5', mRNA sequence
{(VRCPRSRTDLVVGTDIPICIFVAFLPCSPTTERGRELLQLKFRMGISEAGTSSGSV)-YQVFLSFRG-PDTRHGFTDFLYHSLTDAGIYVFRDDEELrvGEKIDGALQRAIDDSRIYIPIFSRTYTSSHWCLRELRQIVDNtSKSNGNKEILHIFFDVEPLRWTNDGVRRSDTANGRRTNGGTPRLCLLPLSVSPSLSRF(LSLSHPFPDARVDPG)}*

$269=149(173):
>gi|345080434|gb|HS065390.1_EST1 {<Streptophyta(V1)>}GR-TS-002-CN-035-G05 Treated seedlings (EUGR-TS) Eucalyptus grandis cDNA clone GR-TS-002-CN-035-G05 5', mRNA sequence
{(VSEAQTIAAAKNY)--------------------FLYKRLLDSRVRVSRNDCALpvRKEKGAEqLVWAISRCKIAIPIISRGYASSTCCLEELAAIMDCRRRDDEL-VLPVFYKVDVVDLGRRCGNFDD-----EMKCSSEEV-LKWQEALTSVARI(RGWISQSTADGIWGKVGIQL)}*

$270=145(176):
>gi|332782242|dbj|FY440861.1_EST1 {<Streptophyta(V1)>}FY440861 Raphanus sativus GSK3-1 root Raphanus sativus cDNA clone RSCR04A21, mRNA sequence
{(G)------------------------------IDLFLDNDMERSKSIGpELVEAIRGSRIAIVLLSKNYASSTWCLNELAEIIKCRERFGQ-TVIPLFYELDPTDVKKQTGDFGKVFLKTCKGKAHEDIQ-RWKCALTEVAQI(TGYHSTNWLVVLFFPHMFLMFLTHVQHICTYFRSF)}*

$271=166(192):
>gi|342332245|gb|JG764085.1_EST1 {<Streptophyta(V1)>}VCDC11767 Daucus carota subsp. sativus B493_1-2kb Daucus carota subsp. sativus cDNA clone CAR02_027_E18 5', mRNA sequence
{(LGTCLFYAISVMFGSSAHPQKQLSVVKDKNEVTVEDTTTTQLAVVTSSPLW)--DVFLSFRG-QDTRGKFISHLYHALDQAGIVTFKDD----------------------LVISENYAASSWCLDELVEILERGLF-GIDLAVEEIYRELSMESNDVRALGICGMGGIGKTTIAKAFYHKYTHKFDISCFL(E)}*

$272=256(249):
>gi|326217590|gb|JG472271.1_EST1 {<Streptophyta(V1)>}EST11013984 CM-TEa library Cucumis melo cDNA clone TEa0002C01, mRNA sequence
{(SMASSAIMKRRDSITSLSSPPYSISLPLPPLR)RYDVFLSHRA-KDhranndTGRSFISDLHEALTsQG-IVVFIDKEDEEDGGKPltEKMKAVDESRSSIVVFSENYGS--WvCMKEIRKIRMCQKL-RDQLVLPIFYKVDPGDVRKQEGeSLVKFFNEH-EANPNISIEevKKWRKSMNKVGNL(SGWHLQDSHSEEGAIKEIVNHVFNKLRPDLFRYDDKLVGISQRLHQINMLLGIGLDDIRFVGIWGMGGIGKTTLARI)}*

$273=170(173):
>gi|339682409|gb|JK182079.1_EST1 {<Streptophyta(V1)>}NRL_003970 Root cDNA library of peanut (Arachis hypogaea) Arachis hypogaea cDNA clone ocpnra0_0049_B01.ab1, mRNA sequence

{(NLPNTTVSMASSSSDQCPTPLSYF)KYDVFLNFRG-F-
TMFDFTDVLYHALIKARIETFRDSEKLRICEKLegALLEAIERPMMSVLVLCDKYPTSKWCLDELVKINECWE
--NGRPVLPIYFHVQRSDVQYEKNSYRTAMIDQ-EKGRYKHRVEAWRSALSAVGSI(YGQNISRLT)}*

$274=195(219):
>gi|332780588|dbj|FY443171.1_EST1 {|0(10)|<Streptophyta(V1)>}FY443171
Raphanus sativus GSK3-1 root Raphanus sativus cDNA clone RSCR11E18, mRNA
sequence
{()-------------------------
LEDARVNVFIDTHELKGRSLENLFKRIDDSKIALVIFSDRFSESEWCLNEVARIDKRVKG-
GKLRVIPVFYRVNTTDVKSFTGKFGSCFEETVQRLSSEERNtaESWKSSVKSVSSN(TGFTSQRISIDSNLVD
AIVDDVKSQLPHMFTENKMSILEELYIAIVFAAFYKLVAPIFFSDTSFFQTRQWFVGVSCIVL)}*

$275=239(265):
>gi|318048853|emb|FQ669303.1_EST1 {|0(8)|<Mollusca(M1)>}FQ669303
Crassostrea gigas library (Genoscope - CEA) Crassostrea gigas cDNA clone
WY0AAA60YM16FM1, mRNA sequence
{(EWWCGKMDINPNHNQVTMESLERQRAMAMPRCDTPSDAPPLPVGKD)-YHAFFSFHGEnpdyNWV-
KVVTRELE--CRGFKCCVFPRDLDAGKSLSNNIKSVLTKCLRIVTVLSSAYIKNEWSQLEMEIISLGKLEEL-
-VFIPVLIEPCDI---PPFLKYYRCINATT---------------------
A(KETWWNAFHEQMLGNVPPLPPRKKHHVFFAHTSSDTKWVNDIVKRLEXPVNSIVCCFPARDFKPGASMQES
VDSAIKRS)}*

$276=147(162):
>gi|332318923|dbj|FY301428.1_EST1 {<Mollusca(M1)>}FY301428 full-length
enriched Mizuhopecten cDNA library Mizuhopecten yessoensis cDNA clone
Hotate0129-No15_D11_004, mRNA sequence
{(EDQIDWPPPDPEKYDVPGYHLE)-HDVFISYSSEdeEWVKTKLFKQLLNMG--
YNVNIDFKDFVPGMAIAENVMDSIYKSRKTIVLMSRNFLKSMWGQFELQQAHNKAIAQRKDVLILIKFDQ---
------CKVPAKLMGKtFLDWT-DKDIqpHFWQRL-------()}*

$277=161(195):
>gi|345207565|gb|JK359916.1_EST1 {|0(1)|<Arthropoda(M1)>}CTFHM53SA
Termite Coptotermes formosanus metatranscriptomic library Coptotermes
formosanus cDNA clone CTFH 5', mRNA sequence
{()--RPGG------------------------------
DFPVGAYIADTIVQAVESSRRTIMVLSENFIKSEWCRFEFKSAHHQVLRDRRRRLILVLLGEV--
PQKDLDPDIRLYLKTNTYLQWGDKLFWEKLRFALPDVPN(NQRARNATRNSNNTNNSNHHHVVHHHVHRHNNH
NRNHPNNTSGASNTTPRSVAIH)}*

$278=175(181):
>gi|325165763|gb|JG442667.1_EST1 {<Streptophyta(V1)>}04322 Klebsormidium
subtile cDNA library Klebsormidium subtile cDNA clone klebsor-C-a-
51c19.t7, mRNA sequence
{(QPCPSQAMARSQGFE)--DVFVSHRG-
LDTKRGFASWLRRDLERRLFTCFLDERSLRRGDDSwpTIMEALSSSKLVVIVLSKGFFESKWCLRELHLCRAL
G-----
KKVVPVFFDVSPDECGPADLAANIPPLPWAGGSGDWDGVGWEEDVQWIKGIT(GLRLEALDGFWDTCIDEIIQ
DEANAW)}*

$279=174(184):
>gi|318048277|emb|FQ663173.1_EST1 {<Mollusca(M1)>}FQ663173 Crassostrea
gigas library (Genoscope - CEA) Crassostrea gigas cDNA clone
WY0AAA50YG20FM1, mRNA sequence

{()------IYNPhgKdQEFVSLMTQVLTSPPYNLRLYVPWSDNNE-
PFEAVATAENIEKRCKKvLVVISAESMESDLFHFQLRIAHSMSPSAKTRKIIPIRLDG--
TEVPDMIRFSTPCDYYK--QDLRV---
FIWDRIFASFKDY(SRPKKSKVLKTVSFPPNRQVPAAVQTFSPLRRVKSWWRMRATVA)}*

$280=222(233):
>gi|315672489|gb|GW919379.1_EST1 {|95(0)|<Platyhelminthes(M1)>}NB482-F04 Neobenedenia melleni eggs, oncomiracidia, immature worms and adults cDNA library Neobenedenia melleni cDNA 5', mRNA sequence
{(IVRYSLELIWSLSFVNKNEELLRKFDEVRKWLDYFNRSSWPVTCRQNIRGILTNIEMNSSHLIQYMGRDRY
RKQKNGHVM)-----ISYNHKTQPVMLKVKEGLESFGFR-TWMDV-DHQKGS-
FVDAMAGSLEGASVVVLGLCQA-FKDSPHCRhELRYAYQLN-----
IPMIPLLLEDnfSPdgWLGFLLATIIYIPLVS--DDQCLRRFLRSRFVFRTFTTKQ(AMSTFPVLVTDDL)}*

$281=160(167):
>gi|317896087|gb|HS908258.1_EST1
{<Rhodophyta(E1)>}Porphyridium_cruentum_GCDJ7DB01EZFUA Porphyridium cruentum CCMP1328 cDNA library Porphyridium purpureum cDNA, mRNA sequence
{(FS)-------YaKEDKEAAHEMMNFLQSKNPAVRLYDAEpRDisVRQRGVVHAPVMEAI-
NqSRVFLSLPSDDYFHSVECTEAFSLAYCRDFDSQGNFIIPLFWRSCDLTPLARRLVATEGVDCR----
EASFESAKVLLEEILAALE(SPASRDGTGLDGDEEGRSLNSATRV)}*

$282=160(191):
>gi|355387771|dbj|FS942826.1_EST1 {<Streptophyta(V1)>}FS942826 Male strobilus SSH-cDNA Library (mature stage) Cryptomeria japonica cDNA clone Cj_ssh_male_m_068, mRNA sequence
{(HRIACXDFNFFIAVSVQSSLPFFSVTTLNMEDIKMEQYMTSATKIPFHSGK)EYHVFLSFRG-
KDVRNTLVRNLYEALTAAGLDVFLDSHKLkkGEIIEWSLKRAVESSAILIPIFSRGYASSAWCLREAADMLR-
----SRGLIIPLFYDVXPTHVRYPLNEY---------------------------SPY()}*

$283=118(148):
>gi|366034553|gb|JK649225.1_EST1 {<Streptophyta(V1)>}GL-XY-003_CN-006_F02 Xylem vascular tissue, differentiating (EUGL-XY) Eucalyptus globulus cDNA clone GL-XY-003_CN-006_F02 5', mRNA sequence
{(NNASSGNC)-YEVFSSFRG-
LDTRHGFTDHLYHGLLRAGIHTFKDDDELcqGEDIRPYLMAAITNSKILIPIFSLNYGTSSWCLDELVQIME-
HKNNNGQIVLPIFYKVKPAEVRYQTGRFG-----------------------------()}*

$284=129(156):
>gi|379470096|dbj|FY785164.1_EST1 {<Streptophyta(V1)>}FY785164 Eucalyptus camaldulensis leaf (dark treated) Eucalyptus camaldulensis cDNA clone EDL008H08, mRNA sequence
{(SRLLRAMAPVTNTSRM)KYEVFLSFRG-
PDTRDNFVSCLFCDMENMGIRVFKDDETLrvGEKIEGELLKGLDDSQIYIPIFSKGFATSSWCLCEIAYMRDC
TSKSDGKEIMPVFFDVTPNEVKCRTNMYRD----------------------------()}*

$285=279(277):
>gi|379472509|dbj|FY784674.1_EST1 {<Streptophyta(V1)>}FY784674 Eucalyptus camaldulensis leaf (dark treated) Eucalyptus camaldulensis cDNA clone EDL007E02, mRNA sequence
{(RERACTAMEDRKRKSDAEASSSTISKRQRSSDERESGSPLPDSPEKINEPSESEANSEPPASYADVIRNN)
-YHVFLSFRD-
PDTRKGFVDHLYHELIRVGLHlpnfVFRDDESLdfGEPIAENILSAIKYSKVSIPVISETYAASKWCLREIIR
IMECV-ESGEQKVYPVLYNVTLGDVRDMKGKFGEAFRNAENGFDEELK-

RKGLVALGKALAL(KVFESEKFAGYEGKLVKAVVEKIMREQQHDFPPSLPVDSVGIDDHVAKVMKLANTTSSE
TQIIVIYG)}*

$286=170(180):
>gi|379479410|dbj|FY811814.1_EST1 {<Streptophyta(V1)>}FY811814
Eucalyptus camaldulensis root Eucalyptus camaldulensis cDNA clone
Root_002_000058F09, mRNA sequence
{(RRRSSNSEAGKNQTETERTFARESPPKNMADTGGSSASAT)QYDVFLSFRG-
PDTRDTFTDCLYRTMVGQVIVAYRDNEELrpGEWIA-
DLLEAVSNSKICIPVLSRGYASSAWCLRELARVTELHESTGKPEILPIFFDVTPTDVKLRTEMYLKDLEKHEQ
RHGAEMR-QQWE---------()}*

$287=147(154):
>gi|379481399|dbj|FY786423.1_EST1 {<Streptophyta(V1)>}FY786423
Eucalyptus camaldulensis shoot (dark treated) Eucalyptus camaldulensis
cDNA clone EDS005B14, mRNA sequence
{(SEMATSSSSLRRLR)-YDVYLSFCG-
SDTQKSFTDHLYNrlvdarihtfrvneelhqtdhlynRLVDARIHTFRVNEELhqSEEINQANFAAITNSKIM
IPIISLNYGSNRRCLDELVQIIECKENNTEQIVLPIFYKVKTTHVRHQI------------------------
-------TK()}*

$288=145(160):
>gi|379527760|dbj|FY837189.1_EST1 {<Streptophyta(V1)>}FY837189
Eucalyptus camaldulensis stem Eucalyptus camaldulensis cDNA clone
Stem_005_000017H01, mRNA sequence
{(GPPWIERIPTLGKKSIKSYC)--QVF-PISS-
PATKKTFASHLPYYLERENISVFRGDDQYliGRDVDREIRNAMDHCKIFIPIISDDFASSPDCLDKLVQMFKC
VEENGQ-KILPIFYGVDLSQKKEFCNHIWTGMLQH--DVPQETR-QRW---------L()}*

$289=121(140):
>gi|354714649|gb|JK664564.1_EST1
{<Streptophyta(V1)>}VCOS42NG_T3_008_A11_09JUN2007_095 VCOS42NG Vaccinium
corymbosum cDNA, mRNA sequence
{()--------------RKTFIDHLYTALDHAGFLPFIDDDgIERGED-
IlsELEKAIRETRISIVVFSQNYASSTWCLEELVMILK-RRS-YGHVVLPVFYDVHPAD-----
EDWLSLVAFLSDSQLLGVPLRFGAGFRFVKRER()}*

$290=163(177):
>gi|379485334|dbj|FY816946.1_EST1 {<Streptophyta(V1)>}FY816946
Eucalyptus camaldulensis root Eucalyptus camaldulensis cDNA clone
Root_003_000044D05, mRNA sequence
{(KRMLAT)----------------TLVTHLYAALVHA-
GIYYFDVQRREKDEgNLmiTVPKMIEESDLAIIIFSEDNAYSRLCLEEVVKIMECKKQ-
RDLKVFPVFYRVELGRVSGLKGSYRTGIEYF-
QKKLGKDVRklMRWKDALQEAGSL(SGWYLDDEMSEAKIIEDIVKKISEVKMYDVF)}*

$291=157(184):
>gi|383097703|gb|JG719570.1_EST1
{<Streptophyta(V1)>}EDISC1NG_T3_012_G03_17JAN2007_019 EDISC1NG cell
culture cDNA library for Ephedra distachya Ephedra distachya cDNA, mRNA
sequence
{(SMTCSSVSGDLTHARVI)---------------------LYNGLQSrG-
IKAFKNDPDLEVGGAVspTLLKAIEESRFAIVVLSPDYASSTLCLEELAKICGCMEE-

DNNTILPLFYKVDPAE-----KSFGDAFTKH-ENSWRHRSEkvQQWRNA---
VANF(SGWDSNTAQCLFQFENLFVESALCWKE)}*

$292=161(167):
>gi|436407646|gb|JG847961.1_EST1
{<Streptophyta(V1)>}mhczaa0_015512.z1.scf cDNA Library of Shixiya I in the Whole-life Cycle Gossypium arboreum cDNA clone mhczaa0_015512.z1, mRNA sequence
{(TLHPHIFFFSYILMSLLLSTSSSISTK)KYDVFLSFRG-EDTRKSFTDHLYDALnRNG-
IVTFRDDPKLEAGEEIapELLKAIQQSWCSVIVFSETYALSGWCLEELAEIVKQKND-
KGHKVFPIFYHVDPSDLRKQKGKVEEAFAKH-EERYR-----KIKTRSKSGETL()}*

$293=220(225):
>gi|372729024|gb|HO399825.1_EST2
{|156(0)|<Streptophyta(V1)>}chaetospheridium_74953.1_c7722_c Chaetosphaeridium globosum EST library Chaetosphaeridium globosum cDNA 5', mRNA sequence
{(SLVPIQALLKRFDQRLDMTARAHVLVVTLEACSADGLVGAWQRRSQVPNAEMRANVAQQAQQLLGKSLFCP
KTVSGNEQV)--DVFISHAG-EDKLLGMA--LHAAVWQEhH-
SCFLDELSLQKAEDApaAMLRSIQTCRLAVFLLSAEFLKKEWPLKEVQQLAIVPGR-AMR-
ILPVFLGVTPDEVQGDVQWLTKIAGVRVKGEPMQSQAVVIEKVMAAIRGH(FARLL)}*

$294=179(181):
>gi|366020557|gb|JK680101.1_EST1 {<Streptophyta(V1)>}PE-XY-003_CN-017_E02 Xylem vascular tissue, differentiating (EUPE-XY) Eucalyptus pellita cDNA clone PE-XY-003_CN-017_E02 5', mRNA sequence
{(SGFPGRPT)--------RP-
RDVRKDFVNSLNEALVREGIKTFMDSENLMIGEEFpsKLKEAIDQSRMYIVVFSQNYAGSWWCLKELVQI--
LGEKNRTKKLLPIFYEVAPGAVRarnprkdgGKKQSYEEALNNH-
ESEFGKRKVKKWKEVLSKAANM(NGIELPDGNVPGSVPRIVKEIKEMMQRIGGRLG)}*

$295=202(210):
>gi|372714853|gb|HO376597.1_EST1
{<Streptophyta(V1)>}chaetospheridium_74953.1_c8775_c Chaetosphaeridium globosum EST library Chaetosphaeridium globosum cDNA 5', mRNA sequence
{(LSSLSLWADGHDLHGTEADSVSILDLLSDAECLAVWPKRLEQADAEASMGERRAR)---VFLSHRG-
PDTKRGFASDLRKDLHGAGMDAFLDKEDLRMGDHAdeRMMGELREADFVVVIISLHFFESPWCVDEL------
HEAVERGRAYPVFYDVEPSEIEDLAALGTGRLNSEQLKSVRQLGRVTGLCLSKYNAEL(ELRKAVVGALDEEV
G)}*

$296=192(198):
>gi|379526247|dbj|FY839733.1_EST1 {<Streptophyta(V1)>}FY839733 Eucalyptus camaldulensis stem Eucalyptus camaldulensis cDNA clone Stem_005_000042F07, mRNA sequence
{(GGWSSRKVAGDGRLVSLVVEKVYISSPPWIERIPTLGKKSIKSYC)--QVFpIS--S-
PATKKTFASHLPYYLERENISVFRGDDQYLIGRDVdrEIRNAMDHCKIFIPIISDDFASSPDCLDKLVQMFKC
VEENGQK-ILPIFYGVDLSQKKEFCNHIWTGMLQH---
DVPQETRQRWLESFDRVCSI(AGFVLENIGNGNG)}*

$297=181(197):
>gi|393846098|dbj|FY936593.1_EST1
{|0(123)|<Platyhelminthes(M1)>}FY936593 planarian head cDNA library Dugesia japonica cDNA clone Dj_aH_214_F10, mRNA sequence

{(TSEREPYFMLSYSHSN)---------------
KKIVMKIYEKLKNEGYKLWIDEYNINDHPnmMQAIARAIEKSKAFIMFLSKEYQNSKNCQSEAQYAYKLN---
KLQIPVLVEKQYKPSGWLGFCTDRSIYINLTKMTIEKAVTLIVKQISVIDVKVT(SFEEKSLQWPSDICPIDQ
VIKEKISSPIHTRSKDNTTNETH)}*

$298=190(220):
>gi|393832063|dbj|FY946308.1_EST1 {|17(0)|<Platyhelminthes(M1)>}FY946308
planarian head cDNA library Dugesia japonica cDNA clone Dj_aH_318_O22,
mRNA sequence
{(DDIEKRSIESSSTQIQMRNIPYKNPIKEFRISSEKESYIESDNERVNIKRGTNRLSARSMQDPGILMTLNQ
KSHKLNERY)KFHVFIIYHPAdrNLANG-
LVKRLESSPHEYKCCIDTRDFVNGQSQIQNIVCSLMLSERVIILFCKQFFMKLWPSIE---------------
-----------------ELLFNVTTPTfQMNSKLIGIYVEKVGVDYNLPNV()}*

$299=203(220):
>gi|393846098|dbj|FY936593.1_EST1
{|101(0)|<Platyhelminthes(M1)>}FY936593 planarian head cDNA library
Dugesia japonica cDNA clone Dj_aH_214_F10, mRNA sequence
{(GWLGFCTDRSIYINLTKMTIEKAVTLIVKQISVIDVKVTSFEEKSLQWPSDICPIDQVIKEKISSPIHTRS
KDNTTNETH)---AMISYCHEnkEFALKI---
YNELIAKGYKIWIDTHDWTPNQTVMEAMAQAVEQSLAVIVCISHCYKNSESCRAE---AHYAI--
DKKKPIIPIIIEE----NYRPDGWLGICIAGKqYL-----NFTGKYSFEESMSMLA()}*

$300=237(254):
>gi|372642145|gb|HO359215.1_EST1
{<Streptophyta(V1)>}chaetospheridium_74953.1_c8246_c Chaetosphaeridium
globosum EST library Chaetosphaeridium globosum cDNA 5', mRNA sequence
{(IPGMRSRLRVPLRYMVDLGDARSEPEFSETRDVVAQEDARQLT)---VFISHRG-
PDTKDSIAIPLKSEMGCAGMRALVDITGTTSGTNAqqVIFEXLEAAHVVVLVLSPHFWESEYCMAELEATRRK
G-----AnRALPVFFNLEPGDMVK-------QAESARGSWNVEEL--KNVVG--
FDCSD(DWQSRLLECVRYFTTIAGIRGSRFNLQDYHGRLDLRRQMVLYHNCWHSWLSFQFXXSTMDDWTCAVK
SWMM)}*

$301=127(155):
>gi|378875258|emb|HE618984.1_EST1 {<Streptophyta(V1)>}HE618984 Abies
alba, needles Abies alba cDNA clone WZ0AAACA1YN16FM1, mRNA sequence
{(PMADLLASSSGSNIS)PYDVFLNHRG-
PDTKSSIATHLYDCLARHRLRVFLDKPEMEKGDFFssQIAAAIRGASVHIAIFSPGYADSVWCLDELLLMVES
I-----K------------------------LRQSTVVLSQASHIDGFEYDGVEGELL()}*

$302=211(235):
>gi|393835802|dbj|FY958572.1_EST1
{|0(117)|<Platyhelminthes(M1)>}FY958572 planarian head cDNA library
Dugesia japonica cDNA clone Dj_aH_523_O20, mRNA sequence
{(XEATRSVLEQLIEINRTDAATIVENALIVAESMVSDDDPIEFVTPPLVFLSXXWGVQEEVKV)--------
----
LREHLQKAGYRCWMDIGQMGGGDKLyeAIDRGIRGAKVFISCVTKKYSESANCKRELNLAISLH-----
KAVIPLQMEKMEWPPIGTMGPLLTEYIFIRMFGRPGAEVVSGTFWPKDRFQE(LLMQVRYSCAPEYDTVDDMY
FNWWEPPETKTQY)}*

$303=171(199):
>gi|393865025|dbj|FY977190.1_EST1 {|0(36)|<Platyhelminthes(M1)>}FY977190
planarian head cDNA library Dugesia japonica cDNA clone
Dj_aH_523_O20.rev, mRNA sequence

{(QSRNIYFFQWGKQEEIKKLKEA)-------------------------
LTKLGYSCWMDIXQMGGGDSLydKIDKGLRNCLLMISCVTPKYSESPNCRREVSLCDNLK-----
KPLIPIMLERMTWPPEGPMSMIFSQLIYISFVDTYEEQQTWNGKATNELLGK(IRQIINPNTEQSTQLQNNDT
TSMKPEKSEKPKDNTVN)}*

$304=159(173):
>gi|407949219|gb|JZ079559.1_EST1
{<Streptophyta(V1)>}estPama_needle_BSC1-676-700_5 Pama-Needle-EST Picea
mariana cDNA 5', mRNA sequence
{(DYS)-YDVFINHRG-
PDVKKTFASHLYHGLSKHGLRVFLDEPELQPGDSLtsQIKGAVGTASVHVAVFSSHYAESRWCLDELRLMLES
G-----KTIIPVFYDVKPAELRSAKGN---------YAKALKElsT--
KETIGRYGRKRK(RYDSNTMEEWRYALSCVADISGFELEKCNG)}*

$305=219(224):
>gi|393867357|dbj|FY976560.1_EST1 {|44(0)|<Platyhelminthes(M1)>}FY976560
planarian head cDNA library Dugesia japonica cDNA clone
Dj_aH_521_M23.rev, mRNA sequence
{(DLIVYLKQLKRSDIIDNLKKELMEDAVAFFNKDDKKQMEFMSQEAFKEFVSTAEINEXKQYIEKVFTTDII
ETVNKKIDI)-YDVFISFeSESIDDIKVMwiiSSRLEEKGLTVakgsYSFVQ------
GICINEAFAKAIYNrSRKVILLISRAFINSESCLMQLNFARDADRACLKNKVIPVYIEDVKK--
PECIASINSFLYYN----EDIRK-FFFDSFIDSLKNS(LLVK)}*

$306=206(207):
>gi|695096732|dbj|HX952769.1_EST1 {<Streptophyta(V1)>}HX952769
normalized cDNA library Cryptomeria japonica cDNA clone Shoot-026-51,
mRNA sequence
{(DFNFYIAVSVQSCLPFFSVTTLYMEDFKMEQYMTSTTKIPFHSGK)EYHVFLSFRG-E-
LRETLVHLLYEALTAAGLDVFVDSHKLekGEIIESSLERALKGSAIRIPIFSQGYAQSAWCLREAATMV----
-NTKGLIIPLFYDVLPTHVRfplKESSPYKTAFEKHyaQSYHKGADI-
DGWKDALHQICSR(SGWSMDLSGGFLPEYHMVLELC)}*

$307=111(144):
>gi|627756276|gb|JZ481508.1_EST1 {<Streptophyta(V1)>}Mado-00-G2-K2-
002C_E03 G2-K2 Malus domestica cDNA similar to tir-nbs-lrr resistance
protein, mRNA sequence
{(ASLN)--------------------------REK-
FTIFKDDKGLEKGQPIspGIVKAIEESQIYLVTLSPDFASSTWCLDELVKMFECMET-SN------
SQRSLEDFTERKDASTVATGIKC-NTRDRKISKVRISVHYDGEWVS()}*

$308=165(165):
>gi|695097549|dbj|HX950903.1_EST1 {<Streptophyta(V1)>}HX950903 SSH12
(tester: shoots sampled at midday / driver: shoots sampled at midnight)
Cryptomeria japonica cDNA clone SSH12-8-93_010_E12, mRNA se
{(LEPPNKKIKSSLTAK)QYDVFINHRG-
PDTKTDLALPLYNSLQQAGIRTYVDAPETELGDYFpsAIKNAISSATLHIAILSPRYAESDWCLAELSLMFQT
-----RARIIPLFYGVQPSDFRYIKNEVADAFRKH-
EEKekegrYPKHDIQQWKECLQNVSGI(KGYEIKEQNE)}*

$309=222(221):
>gi|695097891|dbj|HX951479.1_EST1 {<Streptophyta(V1)>}HX951479 SSH24
(tester: shoots sampled at midnight / driver: shoots sampled at midday)
Cryptomeria japonica cDNA clone SSH24-8-59_006_C08, mRNA se

{(ILCFLSIVSKLFSFRSSSVMEQYNLMASTSTCNPQLEESGPSTSSTSGDSSVPDFPRYEPLTRKLAFHSNK)EYHVFLSFRG-PDVRKTLVDHLFEALSAAGLKVYKDDENLEKGDKIwpSLEGAIKSSAIRIPVFSRGYAESVWCLKEAAAMLSTTD-----LIIPLFYHVHPTHVRYplnELSPYKKSFQxhyEH-PDRHRRGEVDEWKYALFQICDR(SGWSTDSTQG)}*

$310=178(205):
>gi|695098521|dbj|HX951974.1_EST1 {<Streptophyta(V1)>}HX951974 normalized cDNA library Cryptomeria japonica cDNA clone Shoot-006-21_009_E03, mRNA sequence
{(CKERNASKTLPMDRTVTAQMKKLKSGEHSQAPRWTRETANFIHTHDSSQPNKPIRSEHGQKFLAK)--DVFLSHSS-K--QKYFVRQLYRDLTNQGVSCFFNEnrESLPVGED-SIVEAIKTCKAAVLLLSKDFLEEKWLMLQLSAFVEARdiiRTNPNLKILPFFFLISPDDLK--------------------------NIKPYTKKKK()}*

$311=154(160):
>gi|928554708|emb|FO986253.1_EST1 {<Streptophyta(V1)>}FO986253 Aeschynomene indica nodule Aeschynomene indica cDNA clone ADL0AAA13YE13, mRNA sequence
{(QTIIMADHGASSSSSSLSFI)-YDVFLSFGG--HTRHSFTDHLYNALVEKGINTFRDTESLRVGDEIgpTLLDAIQRSRMSIPVLCQNYASSSWCLDELVKIMECSGNGRNRPVLPVFYHVVPSDLAGKGG-----EDSAKLSILNHPPLELCIFLDEDAVGS()}*

$312=158(174):
>gi|673134162|gb|JZ593972.1_EST1 {<Nematoda(M1)>}H03_r_PDF_22_010 Panagrolaimus davidi 4 degree Panagrolaimus davidi cDNA, mRNA sequence
{(NS)-----------------NLFRPLENED--FTLCLLHQDGGAkrtytgnRHVIDDELIHQLDACQSLIMFVTKNFLENEWSSLQIKTXXXXXXX--SNKRIIGILDEH-VQANELDNELGQILRKNnCIRSNDAI---FWKLLFASMPQR(LHLQNSSGSTMLGDGSDIYSEAYGSIVPSEVV)}*

$313=202(212):
>gi|537575959|gb|JZ432368.1_EST1 {<Annelida(M1)>}KN-1127-B-217_B16_SP6 Platynereis dumerilii mixed stages library Platynereis dumerilii cDNA clone KN-1127-B-217_B16 5', mRNA sequence
{(ISFYTTSSIQEQKMGTGSSASRGPTPSPSSPSSPQKSPARHAVTTNASAGK)--DVMISYSH-AD--KEVMNKVRDGLESSGIRVWVDTVGLEPGVDFlsKIGEAIIDSKMFITLLSSGSVSSKYCQDELALAYVSN-----KPIFPV--GLEPEEDLFKIMDTGMKLQLVRYDWTMFTDQDHFEENLAHLIGK(METHKMTESQGNRRRTPSSKL)}*

$314=170(191):
>gi|537584942|gb|JZ423117.1_EST1 {<Annelida(M1)>}KN-1127-B-169_P03_SP6 Platynereis dumerilii mixed stages library Platynereis dumerilii cDNA clone KN-1127-B-169_P03 5', mRNA sequence
{(ISFYTTSSIQGKKMGTGSSASRGPTPSPSSPSSPQKSPARHAVTTNASAGK)--DVMISYSH-AD--KEVMNKVRDGLESSGIRVWVDTVGLEPGVDFlsKIGEAIIDSKMFITLLSSGSVSSKYCQDELALAYVSN-----KPIFPV--GLEPEEDLFKIMDTGMKLQLVRYDW-----------TMFTDQDR()}*

$315=213(225):
>gi|695095795|dbj|HX954068.1_EST1 {<Streptophyta(V1)>}HX954068 normalized cDNA library Cryptomeria japonica cDNA clone Shoot-058-19, mRNA sequence
{(LMLESMASSSSSQQQNQEFNAFSGIEPSGKRRKVSESSRLF)--DVFINHRG-PDVKETLATQLYNSLQQLGIRAFLDKEEKELGNSIpsTIETAIRSAKVHIAIFSKRYAESPWCLAELVLMLNS

Q-----AKIMPVFYEVKPSDLRHIEKGACADA----FKKYEEKG--
RYLDKHNQWKEA(LQSLSLIAREEFDRFSDCKNIIVAVQEEVKRKTCLHVAEYPVGL)}*

$316=147(144):
>gi|720250852|dbj|HX969228.1_EST1 {<Streptophyta(V1)>}HX969228 Pinus tabuliformis ovule Pinus tabuliformis cDNA clone F135, mRNA sequence
{(H)---VFINHKG-
DDVKETFARSLYRRLREKGLTAFFDEEEMEGGCEIypQLADAIRTASVHVAIFSPRYAESVWCLNELLLMLKS
D-----APIIPVFYRVSPANVRWMQglygealrthekKRRHDPSTIEEWRNALRQV--
ADNSGFELQKDN(DGE)}*

$317=135(157):
>gi|972907356|dbj|HX968752.1_EST1 {<Haptophyceae(E1)>}HX968752 Pleurochrysis haptonemofera EST library Pleurochrysis haptonemofera cDNA clone Phap2B03, mRNA sequence
{(TD)------------DKNDGIVRDLCHHLPPSGEH-WLD----
KYAEDVstKgMRMGIEEANAFLMVLSPKYFQSEYCCMEVHRALTLK-----KPIRPVYNN---
TAYTSRDALKWIPKELDRLKQINFLSVSEDFEHMKLGLKK(IQSEPMSYFDGQVPW)}*

$318=203(226):
>gi|972919093|dbj|HX962761.1_EST1 {<Haptophyceae(E1)>}HX962761 Pleurochrysis haptonemofera EST library Pleurochrysis haptonemofera cDNA clone P.hap15R_L24, mRNA sequence
{(GDAXTCSASPLSTQSLPRAASAARVSMSSCRGGSHMALRLCVLCCWVSTRSNGSHGVSASRPSTSSRTRRT
)-------------TRTTAVRDLCHHLPPSGEH-WLD----
KYAEDVstKgMRMGIEEANAFLMVLSPKYFQSEYCCMEVHRALTLK-----KPIRPVYNN---
TAYTSRDALKWIPKELDRLKQINFLSVSEDFEHMKLGLKX(IQSEPMSYFDGQAXW)}*

$319=129(158):
>gi|11958560|gb|AW688302.2_EST1 {<Streptophyta(V1)>}NF005G09ST1F1000 Developing stem Medicago truncatula cDNA clone NF005G09ST 5', mRNA sequence
{(SSFCCPAKKI)-----------------------------
IFSFRDDTKLkkGESIAPELLRAIEDSQIFVVVFSKNYASSVWCLRELECILQSFQLSGK-
RVLPVFYDVDPSEVRYQKGCYAEALAKHEERFQQNFENXAKMEGSTDTSGQ(SLWMGCTX)}*

$320=153(153):
>gi|1217318|gb|N65692.1_EST1 {<Streptophyta(V1)>}20732 Lambda-PRL2 Arabidopsis thaliana cDNA clone 229N15T7, mRNA sequence
{(KIKMSSHTAT)KYDVFLSFRG-HDTRHNFISFLYKELVRRSIRTFKDDkELENGQR-
FspELKSPIEVSRFAVVVVSENYAASSWCLDELVTIMDFEKK-
GSITVMPIFYGRGTESCEVADRSTRXTVLGNMRCRXGXXEVXNGGNIAQFXXLS(GXC)}*

$321=154(169):
>gi|6465701|gb|AW191200.1_EST1 {<Streptophyta(V1)>}T113367e KV2 Medicago truncatula cDNA clone pKV2-2A1, mRNA sequence
{(FGTVLILFLSILMAMQLPFYSSSFSYAFS)-YDVFLICKG-
TDTRYGFTGNLLKALIDKGIRTFHDDDDSDLQR-
RdkVTPIIIEESRILIPIFSANYASSSSCLDTLVHIIHCYKT-
KGCLVLPVFFGVEPTDVRHHTGRYGKALAEHENRFQNDTK-------------N()}*

$322=173(152):
>gi|7205687|gb|AW560261.1_EST1 {<Streptophyta(V1)>}EST315309 DSIR Medicago truncatula cDNA clone pDSIR-26H21, mRNA sequence

{()-----LSFCD-EDSH-SFATGIYTALTSNRIlnlnlhsdhrvYVFWDNNWLGSEyqtlE-
PsdSVLNAIEECEMAVIVYSKKYTESSWCLQELEKITECRRrtT-
DGLIVLPVFYdgvYSSYKRLWVRRDMYGEGFHnfmdrismeKKTSSEDEDKF-
MAWVAAISNEASI(HDGSDYLGDWHK)}*

$323=130(149):
>gi|13480075|gb|BG509418.1_EST1 {<Streptophyta(V1)>}sad13e09.y1 Gm-c1074
Glycine max cDNA clone GENOME SYSTEMS CLONE ID: Gm-c1074-210 5' similar
to TR:Q9ZS32 Q9ZS32 NL25. ;, mRNA sequence
{(FIMTNEPNN)KYDVFLCLVG-PDTRYTFAGNLYNALRRNRINTFftEDNHHDElllmnGDQ-
IspFALRAIKESNLLIVVLSPNYASSPRNLDEFVAIVRCIKR-KKQLLLPVFYKVERGEI-------------
------------MDAIFSGPDQQA()}*

$324=167(174):
>gi|5891600|gb|AW032844.1_EST1 {<Streptophyta(V1)>}EST276403 tomato
callus, TAMU Solanum lycopersicum cDNA clone cLEC11E10, mRNA sequence
{()-----------------FTDHLYTALINaG-
IRTFRDDDEIRRGENIesELQKGIRESKISLIVFSKDYASSRWCLDELVNILDRRKK-
EGHTVLPVFYTVSPEDVQNQSGSFAEAFVNH-
EKRGNAESgekrkewmEkmEKWRLALKEVAEL(EGMCLAKEVDGHEAKFIQKIIKEILKRLNRTVLS)}*

$325=221(217):
>gi|11909866|gb|BF644737.1_EST2 {<Streptophyta(V1)>}NF020A09EC1F1068
Elicited cell culture Medicago truncatula cDNA clone NF020A09EC 5', mRNA
sequence
{(SSSISYVF)KYQVFLSFRG-
SDTRYGFTGNLYKALTDKGIHTFIDDSELQRGDEItpSLDNAIEESRIFIPVFSANYASSSFCLDELVHIIHL
YKQNGR-LVLPVFFGVDPSHVRHHRGSYGEALAKH-
EERFQhntdhMERLQKWKIALTQAANL(SGDHRSPGYEYKLTGKIAFNQTPDLSSDCSQRYEYDFIGDIVKYI
SNKINRVPLHVANYPVGFKFRIQQ)}*

$326=139(150):
>gi|12715229|gb|BG227745.1_EST1 {<Nematoda(M1)>}kq12g02.y1 TBN95TM-SSR
Strongyloides stercoralis cDNA 5' similar to SW:TOLL_DROME P08953 TOLL
PROTEIN PRECURSOR. ;, mRNA sequence
{(I)------------MVCKTLCGPLDDDE--
YTMALLHRDGPRYNGnvhqISDELNRLLECSQTLLLVLTKNFLQHEWKMLQIKTSHQLFFTQslsKSKRVIAV
LEEDV-T-LNDLDDELGQILRKStYIRMRDPL---FWNLLRSALPLR(SGMGRMVGS)}*

$327=162(160):
>gi|9962659|dbj|AU084676.1_EST1 {<Streptophyta(V1)>}AU084676 Cryptomeria
japonica inner bark Cryptomeria japonica cDNA clone CC1684 5', mRNA
sequence
{(CRLQYFVKVNDLLMEDVFPS)-YDVLINHRG-
PDTKKTLASLIYRELSEHGLRVFLDERELEVGDPFppAIQSAIRGASVHIAIFSKTYAESVWCLDELLWMFDS
H-----GNIIPIFYDIQPWELRHVgkgayagafqdhrDKGRITIEQVDRWITALNKV--ADISGVVF----
()}*

$328=156(169):
>gi|14189188|gb|BG832546.1_EST1 {<Streptophyta(V1)>}NXPV_076_C09_F NXPV
(Nsf Xylem Planings wood Vertical) Pinus taeda cDNA clone NXPV_076_C09 5'
similar to Arabidopsis thaliana sequence At1g17610 dis
{(IQS)-YDVFLNHRG-
KDVKNTLASNLYYRLRHLGLRVFFDKEEMQKGSRIdfVIENVIKATPLHIAIFSAGYAESEWCMDELLLMIES

G-----SIIIAVYYNLNPAEMwsnPVDGTNGVYAEALR----LLEEE--K-----
AFDPQN(HQEXXRYKSSTIEKWKTALMXXTXXX)}*

$329=161(162):
>gi|14515713|gb|BI077056.1_EST1 {<Streptophyta(V1)>}NXPV_086_G09_F NXPV (Nsf Xylem Planings wood Vertical) Pinus taeda cDNA clone NXPV_086_G09 5' similar to Arabidopsis thaliana sequence At5g36930 dis
{(TTLLKAFSAMASSSSSSNKSHQ)---VFINHRG-
VDVKKTFVRSLYRRLREKGLTAFFNEEEMQGGCEIssQLADAIRTASVHVAIFSPRYVESEWCLNELLLMLKS
N-----GXIVPVFYRVKPQDVRWMLGlygealathemxGRHXXSTIEEWRNALNE------
VAXKXXXXL()}*

$330=152(162):
>gi|32163373|gb|CD668703.1_EST1 {<Streptophyta(V1)>}eec1c.pk005.l17 eec1c Eucalyptus tereticornis cDNA clone eec1c.pk005.l17 5' end, mRNA sequence
{(REETSNPGAAASGGDAPEPAGE)EYEVFLSFRG-
PDTRTGFTDCLYHDMLGAGIYVYRDDEELrvGREIGGELMQALDVSKIYVPIFSKDYASSSWCLCEVAHMVCC
-----------TLAGXSMXRCERNAPFRPECVYXGKXTNGGVTKGXXYKKXPRNKNRL()}*

$331=141(158):
>gi|22783173|gb|BM063055.1_EST1 {<Streptophyta(V1)>}KS01050F04 KS01 Capsicum annuum cDNA, mRNA sequence
{(TRRLGQNLYCPS)----------------------LKRLEDLGINAFRPDDVEsergEGEP-
IstKILEAIEESRIAITIFSKYYASSRRCLQELTKIMECVDN-KGMKFFSVFYQSTASQVL---
CNFDQVLLAQlgiygLNGSYMVEF-LRWKDILCKAFNI(AGLNCW)}*

$332=146(164):
>gi|24064209|gb|BU872685.1_EST1 {<Streptophyta(V1)>}Q045G02 Populus flower cDNA library Populus trichocarpa cDNA 5 prime, mRNA sequence
{(RQRHP)---------------
NNFTSHLYSNLKQRGIDVYMDDReLERGKTIEpALWKAIEESRFTVIIFSRDYASSPWCLDELVKIVQCMKE-
KGQTVLPVFYDVDPSEVAEQKGKY----
KKPSLSMKKNSRKIWTRSGAGKIVSL(RWPIYLGGTYGIDKSSIYL)}*

$333=138(155):
>gi|19820272|gb|BQ046286.1_EST1 {<Streptophyta(V1)>}EST595404 P. infestans-challenged potato leaf, incompatible reaction Solanum tuberosum cDNA clone BPLI14E4 5' end, mRNA sequence
{(SRSLVESAMEQAKFS)-YHVFLSFKA-HDTGKTFTDHLHTNLfRAG-
FHVFKceDDEEEEEDkiDELklKLKKGIEQSRMSVIVLSQNYASSERCLYELVVILEQRRN-
SGHIVLPVFFNVDPSDVRKQKGKF--------------------WERKSPDCQW()}*

$334=189(220):
>gi|18895834|gb|BM597731.1_EST1 {|47(0)|<Arthropoda(M1)>}17000687503235 A.Gam.ad.cDNA.blood1 Anopheles gambiae cDNA clone 19600449693148 5', mRNA sequence
{(GYSTLQCSDGQPINSATLNDLCAWPTTLKFYISAAVSLVLAACLLAIWLYMKYSLEIKVWLFKHNLLQWLV
TEEQIDMDK)RYDAFISYSHKdeEFVTDQLLPRLESEELNFKICWHVRDFMPGEMIATQITKAVEDSRRTIII
LSLNYLESVWGQMEFNTAYLQSLEDKRNRVIPIIYQDI-GDIDQLDP--------------------------
------()}*

$335=196(197):

>gi|30747109|gb|CB405382.1_EST1 {<Nematoda(M1)>}OSTR041A9_1 AD-wrmcDNA Caenorhabditis elegans cDNA, mRNA sequence
{(DAINQRRYKASSLNCSTSAGSSPLPVPLLS)-YHAFVSYSKKdeKMVIDQLCRPLEDED--
YQLCLLHRDGPTYCSnlhaISDELIAQMDSSQCLILVLTKHFLENEWKTLQIKTSHQLFAKNRAKRVIAVL-
GDG-VDANLLDDELGQILRKHtRIEMRSHL---
FWTLLHSSLPSR(LPLPSNSGDDSSQLYSDIYGIVPSXCC)}*

$336=150(156):
>gi|28343093|gb|BU775777.1_EST1 {<Platyhelminthes(M1)>}SJEBQA05 SJE Schistosoma japonicum cDNA, mRNA sequence
{(NCESKLKASIQ)PEHVMISYC---
HEQKEMASSLTTHLKKNGYKVWFDKSSMKDAEYTfsSLAEAIEKSYVVCILFSEDYKQSEHTEREAIYATNLR
-----KPIIWLRAQRDYKPDGWLGLITCRSNYIDISGKYPFETYFPDLCRRIDKYIS(QVRKV)}*

$337=161(178):
>gi|28516277|emb|BX252050.1_EST1 {<Streptophyta(V1)>}BX252050 Pinus pinaster differenciating xylem adult Pinus pinaster cDNA clone PP059A03, mRNA sequence
{(VRIPHLFCSVLHPMASSSASASASASTSTADGNTYYAF)--NVFLNHRG-SDGKQ-
FAIHLYDRLKSHRhLRVFLDRDEMEKGEELtsQIEGAIRASSVNIAIFSPQYCQSRWCLNELLLMLECKGTEE
KkgSTILPIFFHVEPSVLR------------------WTEGEEGVYAEALSSLQK()}*

$338=141(153):
>gi|31906940|gb|CD649668.1_EST1 {<Mollusca(M1)>}CvGil00373 Crassostrea virginica Gill Crassostrea virginica cDNA 5', mRNA sequence
{(RKTMAGASE)-CDIMLSYDP-EDRK--IVRKIYDELKNAGFNCWMDKHKPKGAVG---
TKAVDSCKVFLMCYSHEYFDNKDCKAEAEYANRNN-----
KTIIPCLLDRDYVPSGWLGAMKGSKQVFDFSGRYSFEEVMENLMEEIKTDEP(FLKL)}*

$339=261(265):
>gi|31904201|gb|CD648300.1_EST1 {|17(0)|<Mollusca(M1)>}AUF_102_G07_T7 Crassostrea virginica Gill Crassostrea virginica cDNA 5', mRNA sequence
{(EILRHITPKQRQNEVERFYYKLQEILNHQLNDRLNETVEVIMYDDEEKGERATASYQLLQQLTGAVTPDKP
QPPRKEDGK)RFDACLLYAKCdSKYALTVRVALENKQKKvLKVELPDQNM------HETEERLTSAA--
WvVMFMSSNamsMSKSECMPLWMANVLSSSLEDNELRVIPVLIDENPSAIPDFIRWVTYISIQE--
DGYQDRILDIINGKKISMKSL(SPVGNVAYGLAWAFFMNYLTHALQDLQNRCATKLIQIGAASTQCL)}*

$340=145(153):
>gi|27476824|gb|BU276054.1_EST1
{<Xenacoelomorpha(M1)>}Cr_Emb_04G07_TEXF1 Convoluta roscoffensis embryos from Eva Jiminez Symsagittifera roscoffensis cDNA clone Cr_Emb_04G07 5', mRNA sequence
{(GSVGAEGNGG)AKQIMLSYNWGTKQTVHKIKDRLESWGYK-TWID--
DNQMGDNVMLSMAEGVFNSDAVLICLTDNY-QNSFNCQrEASMTFDER-----
KPMIPLKLEGDVewKPWVKLLISGKRYIEM--ERSPSFDDFMQELKTYLERMEE(LKK)}*

$341=230(234):
>gi|42534951|emb|BX840868.1_EST1 {|0(56)|<Streptophyta(V1)>}BX840868 Arabidopsis thaliana Flowers and buds Col-0 Arabidopsis thaliana cDNA clone GSLTFB6ZE10 5PRIM, mRNA sequence
{(FKLALSMASASASHNW)VYDVFLSVRG-
EDVRVTVRSHCLKELERKRNTAFRDNESERSHALWpELEQAIKESRSAVVVFSKNYSSSSWCLNELLEIV---
-NGNDKRVIPVCYQVDPSQVRHQIGDCGKSFENTFKRQTdEEVKK-

QGKKALTLVAKM(PGFDSAKWNDEAKMIEEKAKEVLGKPLLTTPKDSEERVGIEKHIAEMSLPPQPESKEVRM
VCKTGSAGIGKTTISRAL)}*

$342=132(156):
>gi|32872031|dbj|BP129146.1_EST1 {<Streptophyta(V1)>}BP129146 MAT001
Nicotiana tabacum cDNA clone BY978, mRNA sequence
{(VFPAITYINMVAGAKP)KYDVFLSFRG-EDTRKDFVSHLYRALTKKGVHTFRDDRsVEKGKT-
IkdELIGAIEESRLAVIVFSANYASSRWCLEELVKIIECKIQ-KKQTVIPICYGIN-----------------
-------ESEKCSVLMVLLKLCPT()}*

$343=177(181):
>gi|45363338|gb|CK901807.1_EST1 {<Streptophyta(V1)>}EST0910 CM334 Root
cDNA Capsicum annuum cDNA clone RR45-76 similar to NL25 protein, mRNA
sequence
{(QNSKIPLMASQIQSVKKW)KYDVFLNFRQ-EDLDGYFVTYLYKHLEDIGINVFTPDF--EYER-
VttEISEAIEESRFGITIFSRDYASSRRCLEELSKIMECVDY-KGQEFISVFYRADTVQVSGIIDHV-
LDKHTVCLKASElEMT-QRWKNALCKAFEI(AGSEKDYVKMWSAAECIQKIVDK)}*

$344=169(180):
>gi|38589526|gb|CK105201.1_EST1 {<Streptophyta(V1)>}I076P87.5pR Populus
senescing leaves cDNA library Populus tremula cDNA clone I076P87 5', mRNA
sequence
{(VLEAK)-------------TPQEFTDHLYSAMsKAG-
IVTFRDDNSIQRGENIelEIEKAIQESQMSVVVLSKDYASSTWCLDELVMIMDRKRT-
AGHIVLPVFYDVDPSQVGEQTGNYAEAFAKH-
QDHFQDDMErvEKWKATLKEVAYL(GGMVLQDRHESQFIGDIVEEVGKRLDSTVLLAPYI)}*

$345=151(155):
>gi|45403199|dbj|AV779547.1_EST1 {<Streptophyta(V1)>}AV779547 Lotus
japonicus Pods (20-30 mm in length) Lotus japonicus cDNA clone
MPDL060d04_f 3', mRNA sequence
{(CV)-----VSFRG-EDTRNNFTDHLLGALYGK-
GFVtFKDDTMLRKGKNIstELIQAIEGSQILIVVFSKYYASSTWCLQELAKIADGIVG-
KRQTVLPVVCDVTPSEVRKQSGNYGEAFLKH-EERFKEDLEmvQRWRKALAEVADL(SGWDVTNKPEHEQ)}*

$346=199(220):
>gi|48485153|gb|CN993263.1_EST1 {|2(0)|<Streptophyta(V1)>}Mdfw2047m19.y1
Mdfw Malus x domestica cDNA clone Mdfw2047m19 5' similar to TR:Q9ZS32
Q9ZS32 NL25. ;, mRNA sequence
{(QTGSFAEAFARHEETYKDDPDKVRRWRAALTQVANLTGWVSRGRDESELIEEIVDVIRKLLHPTTKTSAPC
SSLFSSHKR)KYDVFMCFRG-KDTRLNFVGHTYASLnREK-
FTIFKDDKGLEKGQPIspGIVKAIEESQIYLVTLSPDFASSTWCLDELVKMFECMET-SN-------------
-------SQRSLEDFT-ERKDASTVATGIKCNTRDRKIS()}*

$347=229(230):
>gi|34493742|gb|CF476370.1_EST1
{|0(19)|<Streptophyta(V1)>}RTWW2_22_E06.g1_A021 Well-watered loblolly
pine roots WW2 Pinus taeda cDNA clone RTWW2_22_E06_A021 5', mRNA sequence
{(DPDTERVPSL)-YEVFISHRG-
ADTKNTLASLLYHRLKRDGLHVFLDKPEIAVGSRIssAIVSAIQSASVHIAIMSPGYADSVWCLDELSQMMES
CK-NHRSEIIAVFYDVEAADLRHiESKCYANAFKKH-
ERRHEIEEVNRWKKALKEAAEI(KGETYKGKESDQGKVSEVIDQGDVLEKIVEWVWKSVKFPPSEVASHQVGL
DQAADDFKNTIGYTSESKSTKIVTMAGNPG)}*

$348=148(174):
>gi|34624756|gb|CD073715.1_EST1 {<Platyhelminthes(M1)>}MA3-0001U-M274-G01-U.G MA3-0001 Schistosoma mansoni cDNA clone MA3-0001U-M274-G01.G, mRNA sequence
{(YSHNDYKIALK)---------------------
IKESLENEEIKFWIDKEhmVQHVKILDAMANAVENAAIVLILFSKSYQDSENTKAEAEYTRKLN---KPA----
IFFRVESKFVPSGWLGFIMGESRYIDFSGKYPFEEKFEELCTTIHNI(SRGSIIVKPKKPKKTQEYACVSM)}*

$349=187(181):
>gi|34360634|gb|CF402217.1_EST2 {<Streptophyta(V1)>}RTWW1_19_G05.b1_A015 Well-watered loblolly pine roots WW1 Pinus taeda cDNA clone RTWW1_19_G05_A015 3', mRNA sequence
{(DVILIMEIKRDLQ)-YHVFINHRG-
TDVKKGLASVVYNLFKIKdcRLQVFLDQKEIRCGVDVstAIEAAIKSSHVHIAIFSERYAESKWCLDELVSILGSK-----RKVIPVFYDVEPTALRYINSGcyqkafekhrrRVTVEKTEKWKKALLDA--
SLLKGELCNTKE(HDLWDFSQKIVDIVLKEFEHESLAVTKE)}*

$350=153(179):
>gi|37563989|gb|CF666634.1_EST1 {<Streptophyta(V1)>}RTCNT1_24_G08.g1_A029 Root control Pinus taeda cDNA clone RTCNT1_24_G08_A029 5', mRNA sequence
{(QRGLTIGHTHVLWDEGRLIHDIVKHVLSMKRVPKTPIQS)-YDVFLNHRG-
KDVKNTLASNLYYRLRHLGLRVFFDKEEMQKGSRIdfVIENVIKATPLHIAIFSAGYAESEWCMDELLLMIESG-----SIIIAVYYNLNPAEM---------------------WSNPVDGTNGVYAEAL()}*

$351=182(194):
>gi|48929790|gb|CO159249.1_EST1 {<Streptophyta(V1)>}FLD1_12_A05.g1_A029 Root flooded Pinus taeda cDNA clone FLD1_12_A05_A029 5', mRNA sequence
{(QRMLRVMKTFWLYLTGIWLYLTGKPHRDGQFARQIPSLSEMAESSSSAAANSDH)--DVFINHRG-
PDAKKNFGSHLHRSLRSHGLKVFLDQPELRRGDDFsvQIEHAIRSASVHVAIFSQGYADSSWCLDELLQMLDTK-----APIIPVFYHVKPDELRGTRG-----KKQRTYAEALDE-LARKTDYDGQTPRH()}*

$352=255(249):
>gi|48934315|gb|CO163774.1_EST2 {|0(9)|<Streptophyta(V1)>}FLD1_43_E05.g1_A029 Root flooded Pinus taeda cDNA clone FLD1_43_E05_A029 5', mRNA sequence
{(LEIIEGWRDALWDVSNISGFQLEDSNGGQEVLLGKVVDSVFKTCPL)PYHVFLSHRG-
TDTKEKFAQPLYDRLVSHGLRVFLDKPELVGAVKIetQIELPIQFASVNIVIFSPRFPESSSCMDELFLITKAT-----
GTILPVFYEVESSDLdgIYIETLYSHHREkqrqdtgtIEKWRDALANVPKSDEFMLAGEELK(EDLMDKIVERVRKYIPKPLEFMISSEEQRAWKSALKDVPKRSQAVPVKDTYVGLHEKLEDFEK)}*

$353=240(234):
>gi|48947982|gb|CO175110.1_EST1 {|0(25)|<Streptophyta(V1)>}NDL1_48_B06.g1_A029 Needles control Pinus taeda cDNA clone NDL1_48_B06_A029 5', mRNA sequence
{(HVFHFLKLGMMNQNSGLQ)-YDVFINHRG-
TDVKNSLASFIYHQLKTKdpNLRVFLDKNEIPYGVNFelAIETAIQSSSVHIAIFSERYAESKWCLHELDLIIKSG-----RKLIPVFCDIDPAALRriekgcyqkafEKHRGREPLEKTKRWEDALARA--
SYLQGLPFKMKE(SDHWEFVEKIVSIVLMNLKEGSSVDDKYPVAPEQAVEEFPASATELEDLGVCQEFMAEELAVAPEPDDDRPDLSDL)}*

$354=224(229):
>gi|49008207|gb|CO197032.1_EST1 {|0(5)|<Streptophyta(V1)>}GEO1_3_A04.g1_A029 Root gravitropism April 2003 test Pinus taeda cDNA clone GEO1_3_A04_A029 5', mRNA sequence
{(NSHDFFVAETERVHLKLLKSDEGRLVHDVVKHVLSMKHVHKTSSYS)-YDVFLNHRG-KDVKNTLASHLYYRLRHLGLRVFFDKEEMEKGNRInlVIENAIKGAPVHVAIFSAAYAESEWCMDELVLMVQSE-----SIIIPVFYHLKPTEIWWNSVDGRNGVYAEALHILKEEKLFDPESHQEKPRYE(SQTIEKWKEALIEVTGRDGFKLETYDGDEGELIDMIVREVVKY)}*

$355=212(217):
>gi|49008215|gb|CO197040.1_EST1 {<Streptophyta(V1)>}GEO1_3_B03.g1_A029 Root gravitropism April 2003 test Pinus taeda cDNA clone GEO1_3_B03_A029 5', mRNA sequence
{(TERVHLKLLKSDEGRLVHDVVKHVLSMKHVHKTSSYS)-YDVFLNHRG-KDVKNTLASHLYYRLRHLGLRVFFDNEEMEKGNRInlVIENAIKGAPVHVAIFSAAYAESKWCMDELVLMVQSE-----SIIIPVFYHLKPTEIWWNSVDGRNRVYAEALHILKEEKLFDPESHQEKPRYE(SQTIEKWKEALIEVTGRDGFKLETYDGDEGELIDMIVREV)}*

$356=138(162):
>gi|49023363|gb|CO210919.1_EST1 {<Streptophyta(V1)>}WS00919.B21_K12 IS-B-N-A-10 Picea engelmannii x Picea glauca cDNA clone WS00919_K12 3', mRNA sequence
{(TQIIMYTLWGIFVHKMIWLQFK)----FMNYF--ISQKKN------QCLSEHELRVFLEQEELQEGESIsaQMESVIRTVSVHIANFTPRYADSKWCLDELVLMMEleymlkSR-----STIILVFYRDKPSELR---------------WTRGKNGVYARSLCLFEEKNI()}*

$357=120(141):
>gi|49027129|gb|CO214683.1_EST1 {<Streptophyta(V1)>}WS00930.B21_J02 IS-B-N-A-10 Picea engelmannii x Picea glauca cDNA clone WS00930_J02 3', mRNA sequence
{(D)---------------------LCQCLSEHELRVFLEQEELQEGESIsaQIKSVIRTVSIHIANFTPRYADSQWCLDELVLMMEleymlkSR-----STIILVFYRDKPSKL---PWTRGKNGVYARSLCLFEEKKTFDGRPRYRSSTI()}*

$358=204(206):
>gi|49048764|gb|CO226449.1_EST1 {<Streptophyta(V1)>}WS01029.B21_L06 SS-R-N-A-11 Picea sitchensis cDNA clone WS01029_L06 3', mRNA sequence
{(SSRRKYSRSLQNIFDLAPMASSSTSPSHDDV)-YNVFINHRG-PDVKKGLASHIYRRLIGHGLKVFLDQPEMQEGEPItpQIKRAIRTASVHIAIFSKGYADSTWCLDELLDMldtVKSG-----SAILPVFYNVQPSDLRWTRGADRVNARALSIFLCILLSVLLCIPMSFLLRFL(LCVPFSIIMCILLCTRGENGVYARALHKLQKKKKK)}*

$359=163(169):
>gi|49056821|gb|CO234506.1_EST1 {<Streptophyta(V1)>}WS0262.B21_A02 WS-PP-N-A-12 Picea glauca cDNA clone WS0262_A02 3', mRNA sequence
{(VSMAARSTSTTASLPNIYT)--DVFINHCG-PDVTRTFASHLYRRRLSHRLRVSLEEDELQAGENIvsQRVDANRTASVHIAIFSETCAESSCCLDELRFMVESG-----STIIPVFYGVKPAGGKKDQCTLKPYTNLRRRRRRTLKLMKRSCDTVLTLLKL(REKLFLVLQR)}*

$360=235(240):

>gi|49057498|gb|CO235183.1_EST1
{|0(47)|<Streptophyta(V1)>}WS0263.B21_O09 WS-PP-N-A-12 Picea glauca cDNA clone WS0263_O09 3', mRNA sequence
{(QLAMTYPTPASSIPQTHSEIEIEMENQRSPRATSNSASSSSTAADSNNSSSNI)PYDVFINHRG--
EVKKTFASHLYHRLLSRSLRPFLDQQELQEGLDFpsQLESAIRSASVHVAILSPKYAESRWCLDELLLMLESG

APIIPVFYHVQPAQLRWTDPSKNGVYALALRQLQMKKTYDSQTHQEKSRYES(ATIQNWRDALSRVADISGFD
LDGTFNGDEGHMLEKIVERVVKLSKKG)}*

$361=200(200):
>gi|49061053|gb|CO238738.1_EST1 {<Streptophyta(V1)>}WS00722.B21_L03 WS-PS-N-A-8 Picea glauca cDNA clone WS00722_L03 3', mRNA sequence
{(GNRVRIPNILHPMGSTSNSTCASASASTSTPAGNNCY)PYDVFLTHRG-
PDVEETFAKALYDGLKAHGLEVFLDKKEMQTGEKLtdQIEGAIRTASVNIAIFSPDYCESRWCLDELVLMLES
mwekR-----
STILPVFFRVKASKLRWTEEGKGVYAQALLLLQNKRTSDSLPPRYNYNTIIE(WKNALSEVAEIIGFQLEESP
YNG)}*

$362=137(157):
>gi|56913361|gb|CB252736.1_EST1 {<Streptophyta(V1)>}19-E010698-019-002-F05-T7R MPIZ-ADIS-019 Arabidopsis thaliana cDNA clone MPIZp768F052Q 5-PRIME, mRNA sequence
{(SLMASSSSSSSSSHIRR)-HHVFSRFHG-PDVRKGFLSHLHSLFASKGITTFNDHNIERGQT-
IgpELIQGIKEARVSIVVLSKNYASSSWCLDELVEILKCKEA-
LGQIVMTVFFKLIRQMLRHRVECSGKLLRRLVKGK------------------M()}*

$363=158(190):
>gi|54621374|gb|CV628510.1_EST1 {<Streptophyta(V1)>}Mdlv3_4029m05.y1 Mdlv3 Malus x domestica cDNA clone Mdlv3_4029m05 5' similar to TR:Q9ZS31 Q9ZS31 NL27. ;, mRNA sequence
{()--------------------------------
TFIDTQLRRGENIQsELDQEIEGSRIAVIVFSKRYAESRWCLRELSKIMRCLKNQEGKIVCPIFYDVDPSQVR
KQKDSFGEAFQKHEKDEDPNVVK-
QWREDLKASAYL(AGWNLKTTADGREGVFIEKITGDIKGILKTIDLKAKHSVGISSSRARIQH)}*

$364=134(165):
>gi|56086916|dbj|BP562084.2_EST1 {<Streptophyta(V1)>}BP562084 RAFL9 Arabidopsis thaliana cDNA clone RAFL09-46-I17 5', mRNA sequence
{(SLXXSFLLLSLSLSMASSSSSRNWL)-YDVFLSFRG-
GDVRVTFRSHFLKEFDRKLITAFRDNEIERSHSLWpDLEQAIKDSRIAVVVFSKNYASSSWCLNELLEIVNCN
DK-----------------------------IYHTGFLRCGSFPSEASNRXLWKDL()}*

$365=137(164):
>gi|60707703|gb|DN497513.1_EST2 {<Streptophyta(V1)>}R007H07.5pR Populus root cDNA library Populus tremula x Populus tremuloides cDNA clone R007H07 5', mRNA sequence
{(KAYFYTYQAYTNMASSSSPTTPYL)KHEVFLSFRA-
TDTRNSFTSHLYDALQRNQIDAYIDNKLDGGEKIEpALLERIEESFISLVLFSENYADSTFCLRELSKILECM
ET-KQQMVLPVFYR-------------------------LIQARFKIDWELGDALC()}*

$366=171(194):
>gi|50320399|gb|CO515525.1_EST1
{<Streptophyta(V1)>}s13dSG52G0800068_418109 Glandular trichomes Medicago sativa cDNA, mRNA sequence

{(GLHD)---------------------------KG-
INTFIDDqEDVVKGNEIsqELSNLIQESRIAVILFSDRYVSSTYCLDELSCIVDNlyKNH-
AYRFILPIFNYIDPFSVREQSGRYAEAFAIH-
EEKFKDNKEkvLKWRNALSHVANL(SDKHFIHRDVDETELINQIVGVVSEKLDRVPLHIATWHKVSNWVSQTF
QN)}*

$367=222(221):
>gi|60697571|gb|DN487847.1_EST1 {<Streptophyta(V1)>}R008F07.3pR Populus root cDNA library Populus tremula x Populus tremuloides cDNA clone R008F07 3', mRNA sequence
{(NYPYIPSWESPTGHLIVASKETASSYIDSLANSSSYYQWM)-HDVFFSFRG-EHTSNNFT-HLYPALVQR-
GII-RGDTELEYLRVIesSLLRDIKESGLSIIIFARDYACSHWWFDELIKIVGfMKKM-
KPGTVFPistVSYNVEQSKVDARAESYTIVFEED-
EEDFGEDMEkvRRWMDILTEVAIS(SGSGSSRRVNGMDWPVGNRFRGTDVEHVQNFLKVLKERSEL)}*

$368=199(220):
>gi|68088520|gb|DR181834.1_EST2
{|0(81)|<Streptophyta(V1)>}RTMNUT1_41_G04.b1_A029 Roots minus micronutrients Pinus taeda cDNA clone RTMNUT1_41_G04_A029 3', mRNA sequence
{()----------------------------
YGLRVFLDQYVLCPGDKIenEIRRAISSSSVYIAIFSKNYADSRWCLKELCWMLTSSH---
EPWFIPIFCDVEPSELSFvEKGCYAKAFEKH-
FQRareeenttFKEVEVVEWKEALRKAAGI(SGKVFKGKEALGKAANNSDTVFEWKESDDYGKLVRNIQNTVM
EHVKRDPLELPPRPVGLDQAWKDLHDQIKKESLKKVVV)}*

$369=141(165):
>gi|56913852|gb|CB253227.1_EST1 {<Streptophyta(V1)>}06-E015331-019-005-K02-T7R MPIZ-ADIS-019 Arabidopsis thaliana cDNA clone MPIZp768K025Q 5-PRIME, mRNA sequence
{(KKTRNRRAIASSSPSSSSKESGIPT)GPQGFISFRG-
KKLPKGFISFLGPALKKKNITVFINDPEGRGKNLITLFRRIGESKIPWEIFSEGYPESKGGLDKLVQIKKCVD
Q-KKIIAIPIFYKLDPAGVKGLKGKFGDKFRDLI----------------------()}*

$370=148(146):
>gi|69358860|gb|DR466183.1_EST2 {<Streptophyta(V1)>}WS00937.B21_K05 IS-B-N-A-10 Picea engelmannii x Picea glauca cDNA clone WS00937_K05 3, mRNA sequence
{()-----------
RDVRRGFVDHLYNALTGAGFRVLIDTHQInRGEDISAsLQQAIEVSHIQIPIFSHNYAQSAWCLTEawfvALQ
VLLQGKE-ESSAIAVTLFDIlTPEQVRhpgtgtiwQQRGRYGSKEIGE-
WSDAVLQLCRVSGWSLQKDTNS(ACEARG)}*

$371=204(218):
>gi|69360068|gb|DR466562.1_EST1 {<Streptophyta(V1)>}WS00938.B21_L07 IS-B-N-A-10 Picea engelmannii x Picea glauca cDNA clone WS00938_L07 3, mRNA sequence
{(EASLLSFSAQAIMITFRSPHLRPTLPALPIFIVENVGRMPVRLPSRGVSNRLLEESAASIPIAIAMELGRS
RHRKFKI)-YDVFLSFKG-RDVRRGFVDHLYNALTRGGLHEFLDTHQInGGEDISAsLQNAVEVSRILI----
---AQSAWCLREASLIAQQVlfqgKE-ESTIAIPLFHSVTPELKKLVIGRIvssNIAVYLG-GHSKKTRI---
---------GG()}*

$372=165(175):

>gi|69365880|gb|DR468284.1_EST2 {<Streptophyta(V1)>}WS00944.B21_I08 IS-B-N-A-10 Picea engelmannii x Picea glauca cDNA clone WS00944_I08 3, mRNA sequence
{()---------G-RDVRRGFVDHLYNALTRGGLHEFLDTHQInGGEDISAsLQNAVEVSRILI-------
AQSAWCLREASLIAQQVlfqgKE-ESTIAIPLFHSVTPELKKLVIGRIvssNIAVYLG-
GHSKKTRIGGYSGSRRFPALVK(PAVVDVGKQMVHWPSVLCYCSPMAVGILNDESSSK)}*

$373=181(212):
>gi|55920767|gb|CO860960.1_EST1 {<Arthropoda(M1)>}LM_SM5_001893 Locusta migratoria solitarious phase's midgut cDNA library Locusta migratoria cDNA, mRNA sequence
{(STTPEEQITATWKIVVIVVSCVIVLMLGICYWKWRYIYQFGRAVRSVTMLRLLDDEKDKPNKAESNGQSNV
S)-YDVFISYSDPnrQWVLEELVPNFEnGSDL--
KVCLHERDFQVGLSILENIISCMDQSRILLLVISQAFLRSQWCQFELHMAQHRLLETRREDLILVLLEEIPtS
RRPK--------------------------------MLR()}*

$374=176(172):
>gi|57588546|gb|CX561517.1_EST1 {<Echinodermata(M1)>}ydb55e05.y2 Sea urchin EST Lib1 Strongylocentrotus purpuratus cDNA clone ydb55e05 5' similar to TR:Q9QX05 Q9QX05 TOLL PROTEIN. ;, mRNA sequence
{(LLKLAVLGYKEMQDARDHNDYE)-
FDVNIILYDEdeEWVREQLRPALEERLPQFQRNVFGDQDLVLGMHYLdSVDYVVTHSYKTIVVLSRAAVHDHW
FILKFRTAMDHVSDTLTEFVFVVFLEDIPDDEMPFLARLY-
LSDGRpYIHWTEDVRGqdYFWDELTKNLTIN(LRTNDLIPNE)}*

$375=231(225):
>gi|49448043|gb|CO366726.1_EST1 {<Streptophyta(V1)>}RTK1_29_E04.g1_A029 Roots minus potassium Pinus taeda cDNA clone RTK1_29_E04_A029 5', mRNA sequence
{(DVIVIMEIKRDLQ)-YHVFINHRG-
TDVKKGLASIIYNLFKIKdcRLQVFLDQKEICCGVDVstAIEAAIKSSHVHIAIFSERYAESKWCLDELVSIL
GSK-----RKVIPVFYDVEPTALRYINSGcyqkafekhrrRVTVEKTEKWKKALLDA--
SLLKGELCNTKE(HDLWDFSQKIVDIVLKEFEQESLAVTKELAAKDFPIGASELDDFKFIKEFITEEFALAHG
PDEDRLDLSVLL)}*

$376=204(217):
>gi|58028781|gb|CX715522.1_EST1
{|0(29)|<Streptophyta(V1)>}RTPQ1_34_E12.g1_A032 Roots treated with paraquat Pinus taeda cDNA clone RTPQ1_34_E12_A032 5', mRNA sequence
{(F)---------------------
IYHQLKTKdpNLRVFLDKNEIPYGVDFelAIETAIESSSVHIAIFSERYAESKWCLHELDLIIKSR-----
RKLIPVFCDIDPAALRriekgcyqeafKKHRGREPVEKTKRWEDALARA--
SYLQGLPFKMKE(SDHWEFVEKIVNIVLMNLKEVSSLDDKYPVTPEQAVEEFPTSATELEDLGVCQEFITEEL
AVAPEPDDDRPDLSDL)}*

$377=249(254):
>gi|67556773|gb|DR097418.1_EST1
{|0(18)|<Streptophyta(V1)>}STRR1_34_C02.g3_A033 Stem Response Resistant Pinus taeda cDNA clone STRR1_34_C02_A033 5', mRNA sequence
{(CAGLCIHNIRILVLLKSFCKLVADLLLLFQSVSIASTIQTLPNLSHFVSIFLLVMATSGNANNNYK)-
YNIFLNHRG-
SDTKDTIASDLYRWLTFFGLQVFLDLWEMKVGEKIttQIKEAIASASLHFAIFSPRYAESVWCLEELRLMFES
N-----

KPIIPIFYKVKPADLRLAQEHKGAYAAALKKHENEIAWDGQPLHNSTTVGKW(RNALSRAAGLSGLEVFNGNL
GELVFKAVICALKLVEKSRSLQISYEMR)}*

$378=239(243):
>gi|67679627|gb|DR110529.1_EST2
{|0(24)|<Streptophyta(V1)>}RTS1_11_H11.b1_A029 Roots minus sulfur Pinus
taeda cDNA clone RTS1_11_H11_A029 3', mRNA sequence
{(FEFYRSFASYYTVYQFIHFQISVSMANLTISAQGSAASSSASTSYT)PWDLFINHRG-PDGKMA-
ARYLYRRLTSNGLRrVSLDKEEMQKPQNIsrQIIEAIGGASLHIAIFSPRYAESWWCLDELVLMAESG-----
KAILPVFYKVDPFVVRHNDKAGAYADALRVLAKKTTPADPKTGEEKPRYSSD(TIQKWRIALSSVADLSGFEI
RDEDDELEELNNVVDRVLKMVPKPLLSVADHPTGLDL)}*

$379=134(161):
>gi|67958609|gb|DR162007.1_EST1 {<Streptophyta(V1)>}RTFE1_15_E06.b1_A029
Roots minus iron Pinus taeda cDNA clone RTFE1_15_E06_A029 3', mRNA
sequence
{(SMISLKHVLSMKRVPKTPIHY)-YDVFLNHRG-
KDVKNTLASNLYYRLRDQGLRVFFDKEEMQKGNRInfVIENAIKGTHVHIAIFSTGYAQSEWCMDELLLMIES
G-----SIIVPVFYDVKPTDM----------------------WSNGVYAEALRILKD()}*

$380=260(256):
>gi|68254862|gb|DR387264.1_EST1 {<Streptophyta(V1)>}RTHG1_20_C12.g1_A029
Roots plus added mercury Pinus taeda cDNA clone RTHG1_20_C12_A029 5',
mRNA sequence
{(SEIEIESPAPPPYHPQSANDFEASTSASTADDINVKNSKYYGR)--DVFINHRG-
PDVKKTFASHLYHRLLVHGVEAFLDIQGMQEGEDIsaQIKDAIATAAVHVAIFSPRYAESSWCLNELVDMLES
K-----APIIPVFYNVKPGDLRGKDGVYakaigelekkprHSSNTIENWRKALATV--
SKISGFELDACN(GDEGVLLEKLVELLLEKVKNKEKEVFVADHPTGLDEKLKHFEGFLCELPKNKPKVAGIVG
LGGIGKTTLAKEL)}*

$381=206(211):
>gi|68772889|gb|DR449732.1_EST2
{|0(57)|<Streptophyta(V1)>}WS00950.BR_O11 IS-B-N-A-10 Picea engelmannii x
Picea glauca cDNA clone WS00950_O11 5, mRNA sequence
{(PSSSTTPSQGNNNSYV)-YDVFINHRG-
PDVKKTLASHIYLRLKQQGLAVFLDQQELQEGENMtpQIEGAIRTASVHVAIFSPRYAESSWCLNELEQMLES
G-----
STIIPVFYKVEPSDLRWTRGGDRDYAQTLSILLCMLDLCIPLNIFVRILLCI(PLCIIPWTLGENGVYARDLR
RLQKNTTFDPQTNKTKPRYDSDTIEKWRKALYDVS)}*

$382=216(222):
>gi|69380881|gb|DR473078.1_EST1 {<Streptophyta(V1)>}WS00957.B21_P21 IS-
B-N-A-10 Picea engelmannii x Picea glauca cDNA clone WS00957_P21 3, mRNA
sequence
{(VGQEEDFHIDCIFFPICFSAASLTPLFFIFFVLCFCLHSNPMAHRSPSSTSTAASPSSNNSFR)---
VFINHRG-
EDVKKTFASHLYRRLCSaQRFRVFLDKPELRVGDDFpsQIKEAIRTAFIHVAIFSPQYAESEWCLNELLYMLE
SR-----
APIIPVFYHVLPANLRWTQSKHGVYSKALRKLEQKKTCDSESKEEKPRHDST(TIQKWREALSKWVRSPGDA)
}*

$383=219(199):
>gi|69415186|gb|DR483576.1_EST1 {<Streptophyta(V1)>}WS02811.C21.1_M01
SS-IB-A-FL-13 Picea sitchensis cDNA clone WS02811_M01 5, mRNA sequence

{(ETPTSSPPLQSRSESNSLPILHFSPISVSIFQLTMASSSSSTSANPNSTSNYS)-YDVFISHRG-
PDAKKTFVSYLYRRLVSCGLKVFLYEEGLLLGDYLitQIKGAIQTACVHVAIFSPRYAASRWCLDELLMMLES
K-----ATLIPVFYEVKPSDLRWtqgkdgvyaralrmleqkrsvfssptdKETPRHDPATIEKWRKALSDV--
SDISGFDLEACN(GDEDYL)}*

$384=207(222):
>gi|69436448|gb|DR489775.1_EST1 {|0(4)|<Streptophyta(V1)>}WS02912.CR_M13
SS-IB-A-FL-15 Picea sitchensis cDNA clone WS02912_M13 3, mRNA sequence
{(DTCG)-YDVFINHRG-
EDVKKTFASYLYRRLLRRGLRVFLDKPELEEGLSFalQIQRAIRTASVHVAIFSPRYAESKWCLDELLQMFES
E-----ASIIPVFYHVKPAQLRwTQDKGGVYSEDLRK----LEEK--K-----
TYDTLT(REEKPRHDPTTIQKWRGALSRVAEISGFELDANYTDEGELLDKVVERVVKQVKKRRLDEAAKDSEN
TALLEQHSGKPE)}*

$385=252(244):
>gi|69441307|gb|DR491334.1_EST2
{|0(36)|<Streptophyta(V1)>}WS02917.CR_D20 SS-IB-A-FL-15 Picea sitchensis
cDNA clone WS02917_D20 3, mRNA sequence
{(PARTIHASSASSSTAADPTTSGSCDYG)-YDVFINHRG-
PDTKKTFASHLYRRLLSHRFRPFFDQQELQEGLHFpsQLKSVIRTASLHVAIFSENYAKSQWCLEELLLMRES
G-----KPVIPVFYHVEPSSLRHgealndlegkrkrtdKTQPRYDPDTIQKWRNALSSF--
ADVSGYDLDGGK(FNGDEGEMLDKVVEHVVKILTKNTLFYVAKYPIGLNEKVKDFETVLRQEHTEKVQVVGIV
GLGGVGKTTLALEFFNR)}*

$386=224(204):
>gi|70222433|gb|DR496794.1_EST1 {<Streptophyta(V1)>}WS02910.B21_O17 SS-
IB-A-FL-15 Picea sitchensis cDNA clone WS02910_O17 5, mRNA sequence
{(DLPDEDDENFEVERVFPICTTYLQSYFFFIVYPMASSSTSTSHGNNDNYVF)--DVFINHRG-
PDVKQSLATDLYNSLCKHDLRVFLDQRELQEGEYItpQIDSAVRTASVHIAIFSPGYADSKWCLDELVLMVEt
mskSK-----STIIPVFYGVEPAELRWTQSKngvyaralcrlekkrtfdgqqRHDSSAIEKWRNALSQV--
ADIKGFdlkTYDLET(YNCVKTGVLKCLE)}*

$387=243(248):
>gi|70222901|gb|DR496951.1_EST1 {<Streptophyta(V1)>}WS02911.C21_G05 SS-
IB-A-FL-15 Picea sitchensis cDNA clone WS02911_G05 5, mRNA sequence
{(GFSPLDISLQISLGMFTCNLSRNLFALLKLLTKALIRDFIDLIDHPKPSHFSSIFHRAMAASTSSAVDSIN
NYC)-YDVFINHRG-
PDVKKTFASHLYRRLISYGLRVFLDYQELQEGEDLtcQIQGAIRSASVHVAIFSRTYAESNWCLDELLLMLES
R-----
APIIPVFYHVRPAEIRWTQGEGVYARSLHNLQKKRTYDAQPRYNSTTIENWR(NALSRVADVSGFDLDAFNGD
EGELLDKVVERVMK)}*

$388=208(198):
>gi|70229158|gb|DR499063.1_EST1 {<Streptophyta(V1)>}WS02917.C21_I04 SS-
IB-A-FL-15 Picea sitchensis cDNA clone WS02917_I04 5, mRNA sequence
{(QLAMAYLTPASSFPQTHSEIEMEIQRSSPARTIHASSASSSTAADPTNSGSCDCG)-YDVFINHRG-
PDTKKTFASHLYHRLFSQGFRPFLDQQELQEGLNFpsQIADVIRTAPVHVAIFSENYAKSQWCLEELLLMRES
G-----APVIPVFYHVEPSSLrhgealndlegkrkRKRTDDTQPrydPDTIQKWRNALSSF--
ADVSGYDLDGGK(FNG)}*

$389=181(178):
>gi|70254803|gb|DR507496.1_EST1 {<Streptophyta(V1)>}WS0272.BR_E15 SS-IL-
A-FL-14 Picea sitchensis cDNA clone WS0272_E15 3, mRNA sequence

{(LASSSNSSGNKA)PYEVFINHRG-
PDTKLSIAGHLYNRLHDLGVQVFLDQEEMEKGDFFtsQIAAAIQAASIHIAIFSPGYADSAWCLNELLLMMES
egskesdR-----
STIIPIFYGVEPTDLRWTLGKDGVYAKALNKLKEKKTTDPQTHQEKPRYNSD(IIEKWRNALFRVSEISGFDL
TGAYNE)}*

$390=183(198):
>gi|57583996|gb|CX556967.1_EST1 {|0(7)|<Echinodermata(M1)>}yda31d09.y2
Sea urchin EST Lib1 Strongylocentrotus purpuratus cDNA clone yda31d09 5',
mRNA sequence
{()---------KDQSSVDHIQTALRTIKPDVRIYLSNQKLD-
EDAIWQNDIYAVMTkSARVITVLSPNYLSSPDCLEQYNIALCCNRQSYRDILAPFYIA--
TVTLPTYMGLVQYSDCRS----
DPEVQISNTCKQLIKALPL(STQDDSPGNGMTTSTPVEEEDEDPFCYDVFVSYSHCDSEAATHIVGVLKELDP
SLRIF)}*

$391=196(220):
>gi|68761392|gb|DR443716.1_EST1 {|62(0)|<Arthropoda(M1)>}AR2019E09 A.
gomesiana hemocytes library Acanthoscurria gomesiana cDNA clone AR2019E09
5', mRNA sequence
{(LVEQMGFDYMDIKNFECESDSTAAVLDSWVLRRPKVTIQDLLDMLKNMDRYDVIEDVLQHFKNDANSYNEN
PIQAPAAVT)QYDAYLCYaEEDLHFVHKLAQYLENPGVNLKLCIKGRDILLGNIEFDSYVRLIENrCKALLLI
LTPQFVASKEFEFQMNYAAGLPVEQRHRKLILIVLKPC--EIPLILKFLTKLDFT------------------
------()}*

$392=191(222):
>gi|83668066|gb|DW018474.1_EST1 {|0(101)|<Streptophyta(V1)>}EST1227435
MTY Medicago truncatula cDNA clone MTYB705, mRNA sequence
{(RPG)--------------------------------
TFFDDGEIeiGTQITVRIHKGIEDSRIFIPILSENYASSSFCLDELVKILAEFQKGNGRWVFPVFYYVSISDV
KNQTGSYGQALTVLKNGVEHERF-
EKWISALTSIADF(RGCHMERAKGIYEFQYIYEIIQEVSKHVACSIGLDHRVEKVMRCLTSGSDDNRVYVVGI
CGVPGIGKTTLARGIYLFHG)}*

$393=211(227):
>gi|73884948|gb|DT487686.1_EST2
{|0(56)|<Streptophyta(V1)>}WS02534.B21_G04 PT-MB-N-A-15 Populus
trichocarpa cDNA clone WS02534_G04 3', mRNA sequence
{(RGRHPCC)-----------------FTSHLHAALERKNILTFIDNDLRRGEE-
IspSLVKAIEDSMLSVIIFSQNYASSKWCLDELLKILESRKV-
RGQIAIPVFYEVDPSDIRKQSGSFGDVFAQLVKRKAlkMEEE-
QCFRAALNEAANI(SGHDSRKIESESKFIEVIVEDILNKLCKIFPVHPTNLVGIDEHVRKIESLLDMETQDVR
IVGIWGMGGIGKTTIARAVYN)}*

$394=170(170):
>gi|83666351|gb|DW016759.1_EST1 {<Streptophyta(V1)>}EST1225720 MTY
Medicago truncatula cDNA clone MTYAL27, mRNA sequence
{(SPIFLFLKKSLSMSSSTSKTKWI)-YEVFINFRG-EDTRSNFVSHLHATLSNAGINTFLDDEnLEKGKE-
LgpELLRAIQGSQIIIVVFSKNYVQSSWCLDELEQIMECHKS-
TGQVVMPVFYGVTPSFIrEYASQTFGEAIVSKTNHFVLHNSDPRKNPRNKAQGDD(HYRYFFY)}*

$395=238(257):

>gi|83917564|gb|DW119644.1_EST1
{|0(43)|<Streptophyta(V1)>}CLRY8260.b2_G02.ab1 CLR(XYZ) lettuce serriola Lactuca serriola cDNA clone CLRY8260, mRNA sequence
{(NLSLFLDHFYLHFFKQLTTIAMANSSFDQSVANHKLS)-YDVFISFGD-DNIIQNFVNHLFSDFKRKGIHAFNeDNHLLKGED-RspQIYTAIEQSRFLSVIFSKSFTSSPSCLKELIKILECKNT-NpeKHQIRIIFHNFKLNGI-------------------EPEV-FEWKEALTMAAKL(PGWDLEDLVNGYESKFIEMISNHIFNELNNGPLHVGENLIGSHSRVDRMNLLEFTESNDVHMIGIYGSEGIGKTSIAKSI)}*

$396=140(147):
>gi|73931612|gb|DT514922.1_EST2 {<Streptophyta(V1)>}WS02426.B21_C11 PTxD-ICC-N-A-14 Populus trichocarpa x Populus deltoides cDNA clone WS02426_C11 3', mRNA sequence
{(FSIRQPP)-YDGFLSFRG-ADTPKKFSDPLFSAFQGGGIFSFGDWEEIKGGEHFgfKITKTIQGLKKSLVGFSRGFGFSKRGLEELLGFLKRREI-FGPLVFPVFFEVDPDDVSKPAGFFVGAFACP-EKNFMANRD-----MGERGGVF()}*

$397=169(184):
>gi|70353937|gb|DR543837.1_EST1 {<Streptophyta(V1)>}WS01042.B21_K04 SS-R-N-A-11 Picea sitchensis cDNA clone WS01042_K04 3, mRNA sequence
{(NPNLFPPLQIASIASTVTTILISVFRFAMAASSSTAAYSNNNQW)-YEVFLSHRG-VDTKETFARSLYLRLLEHGLKPFLDNPEMRPGYEIdsQIKNAIETASVHVAILSARYAESTWCLDELLWMLESHRT-KMVPLIPVFYGVTPAEVRWTLGKvgiYAQALREL-EKK----------------KKK()}*

$398=154(169):
>gi|70618291|gb|DR554084.1_EST2 {<Streptophyta(V1)>}WS03227.C21_O21 WS-MC-N-A-20 Picea glauca cDNA clone WS03227_O21 3, mRNA sequence
{(LQQYSHISIIIMSFTRYNPLERTVSSDTR)KYDVFLSFRG-E-VRKTFVDHLYTSL-ENaGLNAFLDSKKLEKGDEIspTIKNAIEKSAICIPIFSKNYAESHWCLEEVYYMCKSGG-----LIIPLFYDVSPTEVRYpDRGAFAEAFQKK-KGRYTKAKKKK----------K()}*

$399=264(263):
>gi|70632957|gb|DR568541.1_EST1
{|0(36)|<Streptophyta(V1)>}WS02622.BR_C01 WS-PP-N-A-12 Picea glauca cDNA clone WS02622_C01 5, mRNA sequence
{(ISLFGEALHSYSIQSLVIYLHLQEAIAMERVPDLQEAMTAMEGVPMPET)-FQVFINHRG-PDVKKTLASLIYHRLTSYGLSVFLDKDELRTGDYIslAISTAIHAASIHIVILSPGYAESTWCLEELYWILRSSHRRDTK-IIPVFFDVEPTDPRYiERGCYAKAFENYRsKGRVQIEVIQKWEIALNADSHI(SGLLFKTKESDYGECLEKIVKVVEEVFKSAPLEVAKYSVGLHQKAEDFETEIMKQRDSKSTLARFKILRHLDLS)}*

$400=193(191):
>gi|83402156|gb|DV996133.1_EST1 {<Streptophyta(V1)>}GQ02792.B3_C10 GQ099: Mixed spruce tissues Picea glauca cDNA clone GenomeQuebec_Id:GQ02792_C10 5', mRNA sequence
{(RRHRTPFLYLQDVIVIMEQEPELQGRTPAA)-YHVFINHRG-PDVKKSLASLIYNRLTNcHRLRVFLDENEIRTGDTIsnAILTAIQSASLHITIFSKRYAESWWCLEELCWILRSYHKRNRK-VIPVFCDVEPADLRHtDSGCYKDAFDKH-QSKgrVSMEVMEKWRKALSEASXX(SGIPXXXXXXLIHPLLISCXN)}*

$401=176(201):

>gi|84450134|gb|DW284730.1_EST1 {<Mollusca(M1)>}UI-S-GU1-aeb-a-01-0-UI.s1 UI-S-GU1 Euprymna scolopes cDNA clone UI-S-GU1-aeb-a-01-0-UI 3', mRNA sequence
{(QRSLQKQLTKATCRYGIFARRPKVFKQTFLNVFTCFYLFTMAARLIEKPEKDPKAGRKSKP)-YDGYAIFCDKdrAKIRNLLRV-----LKHLNLCIRCKDDaISGQGIFEaIVENGILASRCCLIFVSSAFKNDHLCVHFTKIAiLQYLRTDRKLRIIPICLDRTKITCLQifQEISLYK--------------------------SS()}*

$402=186(206):
>gi|70275848|gb|DR514555.1_EST1 {<Streptophyta(V1)>}WS02739.CR_D03 SS-IL-A-FL-14 Picea sitchensis cDNA clone WS02739_D03 3, mRNA sequence
{(SLANNLGVDTKIRAAKVLILCTRNLALLMLFVILYPMTNVNLTRDRDATSFASSTSPSLGNPNSHV)-YDVFLNHRG-PDVKNTLASHLYYSLKASGFRPFLDKQELQKGEKVkqQIEAAIQTASVHIAIFSPNYAESSWCLKELVLMLESR-----KTVIPVFYNVKPSDLR---------------WTWGGNGVYARSLEVLEKKKK()}*

$403=221(226):
>gi|70328483|gb|DR531934.1_EST1
{|0(5)|<Streptophyta(V1)>}WS02736.C21_O03 SS-IL-A-FL-14 Picea sitchensis cDNA clone WS02736_O03 5, mRNA sequence
{(NFEGKCKLPISRIHLILHPMASASISTPYGIGSHYFY)-CNVIMNHRG-PDVEKGFPRHLYRRLIVHELGVLLDAEELQRVDNLtsQIEGAIRTASVHIVVLSSGYAESSLCINELVFMSESG-----SAVLLLFYNVNPSELRWMEGGDGVYVRTLLILEKYSTFDFYPRYDSHAIEEW(RDFLSVVVEIRRFELNACNGGEGQLVCKFGQPELENVSKTPLNVAQYPT)}*

$404=231(226):
>gi|70334169|gb|DR533804.1_EST1 {<Streptophyta(V1)>}WS02741.C21_H04 SS-IL-A-FL-14 Picea sitchensis cDNA clone WS02741_H04 5, mRNA sequence
{(HRPLLHSFSVFHFAMAASTSTSAAANPNNKS)-YQVFINHRG-VDVKETFARSLYLRLLDKGLTSFLDKEEMRQGYQIssQIERAIRTASVHLAIFSPRYADSDWCLNELLLMLESN-----APIIPVFYRVKPSEVRWMlgfygealqtheRKGRYDPNCIEKWRNALHQV--ASNSGFELETCD(GEELVLELLDKVVKCLSEMMGKADLYVAEYPRGLDYKLTDFENTVPVAATAKRET)}*

$405=243(242):
>gi|70342527|gb|DR538086.1_EST1
{|0(30)|<Streptophyta(V1)>}WS02753.C21_P04 SS-IL-A-FL-14 Picea sitchensis cDNA clone WS02753_P04 5, mRNA sequence
{(QRLSGCSLDLIIHTKILRLSPHSRSLQNIFDPMADPSHPPRDATCFASSSTSPSHSAV)-YDVFISHRG-PDVKKGIASHIYHRLKDSGLAVFLDQQEMQEGEGItpQINGAIRTASVNIAIFSPGFADSVWCLNELDLMLESQRARPGaSTIIPIFYRVNPYELRWTRGQDGVYARALSILRCILLCTRDENEGYARALCM(LQKKTTIDSATNKKKPRYDSGTIEKWRKALSDVSVISGFELKKY)}*

$406=215(221):
>gi|70348136|gb|DR540911.1_EST2 {<Streptophyta(V1)>}WS01034.B21_I02 SS-R-N-A-11 Picea sitchensis cDNA clone WS01034_I02 3, mRNA sequence
{(DNISAASSSSGSASYAQYDVTAPRNIDDASAASNTASRNDGSDDASASSSTAPPTSYRHF)--DVFINHRG-PDVKTTFATSLYRRLISNGYQVFLDKPELEEGYTItsQLKDAIKVASVHIAIFSPTYAESKWCLDELVLMVESK-----KTVLPVFYNVKPSVVRWNVESGAYAEALRVHQRKTTYDSKTGKENPRYDSET(IEKWRSTLSYVSEIIGFELDG)}*

$407=227(240):

>gi|70613870|gb|DR549663.1_EST1 {<Streptophyta(V1)>}WS03216.C21_B20 WS-MC-N-A-20 Picea glauca cDNA clone WS03216_B20 3, mRNA sequence
{(NHLMSLGIFSSSANTSISVLTSDEGRLVHDIVKLSIKGVSKTPIH)-YDVFLNHRG-
KDIKKTLANQLYYRLRNYGLQVFFDQEELQKGNRInsEIENAIKVASVHIAIFSKGYAESEWCMDELALMVES
E-----SIIISAFLGVnrtKIWQNPVDGMNGVYAEALRK----LKEE--K-----
TFDPET(HQEKLRYNSNTLEIWEKALKEVTGRVGFELEKYDGDEGQLLDVIVREVKTKKKKK)}*

$408=116(140):
>gi|70623830|gb|DR559623.1_EST1 {<Streptophyta(V1)>}WS02617.B21_F11 WS-PP-N-A-12 Picea glauca cDNA clone WS02617_F11 3, mRNA sequence
{()---------------
RSLGTDLCQCLSEHELRVFLEQEELQEGESIsaQMKSVIRTVSVHIANFTPRYADSKWCLDELVLMMEleyml
kSR-----STIILVFY------------LLPRQAFRASLDAGQKWSLSSISLPLRREENI()}*

$409=170(186):
>gi|70773348|gb|DR684872.1_EST1 {|0(71)|<Streptophyta(V1)>}EST1074949 Normalized pine embryo library, Lib_D Pinus taeda cDNA clone PWAAX56 3' end, mRNA sequence
{(GE)-------------
VKKSFAGHLYRALLSGGFRPFLDQQDLQEGLPFpsQLEAVIRNASVHVAVFSPKYAESRWCLDELLLMRQSW-
----
APIIPVFYHIKPADLRRTAHNKTEGYGLSLHQLELKTTLDPRTQEEKLRYDP(ATIHKWRDALSSVADISGFD
LDDKYDGDEVHMIDEIVERVGKMI)}*

$410=232(226):
>gi|70776262|gb|DR687786.1_EST1 {|0(6)|<Streptophyta(V1)>}EST1077868 Normalized pine embryo library, Lib_D Pinus taeda cDNA clone PWABW53 3' end, mRNA sequence
{(NMMKQNSRLQ)-YNVFINHRG-
TDVKNSLASIIYNQLKTKDptLRVFLDKNEIPYGVDFelAIETAIQSSSVHIAIFSERYAESKWCLHELDLII
KSR-----RKLIPIFCDIDPAALRriekgcyqkafEKHRGREPLEKTKRWEDALARA--
SYLQGLSFKMKE(SGYWEFGEKIVGIVLMNLKEGSLVDAECPVALGQGVEDFPVSTSELNDLKACQDLIGDEF
VLSHGSDDDRPDLSEL)}*

$411=249(236):
>gi|70781534|gb|DR691182.1_EST1 {<Streptophyta(V1)>}EST1081268 Normalized pine embryo library, Lib_D Pinus taeda cDNA clone PWAD524 3' end, mRNA sequence
{(ETDSVASNILPITSISIFQSAMAQPSSSAAANSDH)--DVFINHRG-
PDAKKDFGSHLYWSLHSHGLRVFLDQPELRRGDDFsvQIEHAILSASVHVAMFSPGYADSPWCLDELVQMLDT
E-----APIITVFYHVKPDELwgegkkrtyaEALDNlasktdydgktPRHDPSKIQKWRDALRKV--
ANGSRFELEACN(GDEGELLEKVVKEVLERVKKPALPRDVPKYPTGLAEKAKDFENFCRGNSRGENIRSGGSW
G)}*

$412=173(186):
>gi|74162492|gb|DT632423.1_EST1 {|0(71)|<Streptophyta(V1)>}EST1147354 Normalized pine embryo library, Lib_D Pinus taeda cDNA clone PIMFJ83 3' end, mRNA sequence
{(RP)---------G-
GEVKKSFAGHLYRALLSGGFRPFLDQQDLQEGLPFpsQLEAVIRNASVHVAVFSPKYAESRWCLDELLLMRQS
W-----
APIIPVFYHIKPADLRRTAHNKTEGYGLSLHQLELKTTLDPRTQEEKLRYDP(ATIHKWRDALSSVADISGFD
LDDKYDGDEVHMIDEIVERVGKMI)}*

$413=179(190):
>gi|74163556|gb|DT633487.1_EST1 {<Streptophyta(V1)>}EST1148418 Normalized pine embryo library, Lib_D Pinus taeda cDNA clone PIMFV42 3' end, mRNA sequence
{(AGGIRPGTPPQLTKSPEQAKPDSQISVSIANLTISAQGSAASSSASTSYT)PWDLFINHRS-PDGKM-VARHLYRRLTSNGLRrVFFDKEEMLKAQNLsrQIIEAIGGASVHIAIFSPRYAESSWCLDELVLMAESG-----KAILPVFYKVDPSvvrhnvkagayadALRDLAKKTTPANDLKRY--------------------S()}*

$414=186(203):
>gi|76568509|gb|DV101176.1_EST1 {<Haptophyceae(E1)>}ppa01-19t3-e04 ppa01 Prymnesium parvum cDNA clone ppa01-19t3-e04 5', mRNA sequence
{(PYLVQFDDGSTYWYKKSEVLLTDSSVAASPPQEAGQEASMALNSPGKWKFFISYTQRNAEAEV)-----------------LASELYNGLKDLRDAVWLDVK-MQQCDSDAMMEGVNNSESVIAIFSggedigKRYLQREACVAELKQAIKAG-----KTIVPVVSVKDKEKVGEYINEGQAIGIDLSSCNFIEFIRSSPIFFQASMDSI()}*

$415=176(202):
>gi|77482139|gb|DV204197.1_EST1 {|0(23)|<Cnidaria(M1)>}HSAA-aaa03d12.g1 UCI-11 Hydra vulgaris cDNA 5', mRNA sequence
{(PTRPVLFYLDEYDLMISYSHKTKEVTWK)---------------------IHDWFEKRGFKIWIDKNYLDTE--LakEIRNGIDKSSVFIPCFSSEYEKSTWCKKELTYASEND-----KHIIPIIVENGYVLSNDIRFQITGLKYFTLEGDFDTAMNQVLKAVKKYTNKT(GTLSPERKELNCGSTQAVQAEQYVESPYSSKSKR)}*

$416=189(180):
>gi|83379677|gb|DV973655.1_EST1 {<Streptophyta(V1)>}GQ02774.B3_F05 GQ099: Mixed spruce tissues Picea glauca cDNA clone GenomeQuebec_Id:GQ02774_F05 5', mRNA sequence
{(RVSHPXXXXXXXXXHXXXRDXEELLKRVVESVFKVCPHPH)-YDVFISHRG-PDTKKTFAGPLYHRLISsarelcsRELRVFLDDPELVEGLPItsQIEVAIQFAFVHIAIFSPAFAQSRWCLDELFLMIKSR-----APILPVFYELKPPDLngdlngvydeaLVEKKRRYDSETIQRWRGAL----ADVPKSYDFVLDG()}*

$417=191(199):
>gi|84608325|gb|DN308359.1_EST1 {<Platyhelminthes(M1)>}PL05016A2H02 cDNA from sexually mature hermaphodites Schmidtea mediterranea cDNA 3', mRNA sequence
{(LTRDAMEFLKKTGKFLFEHVNQEAFKQITSPTELDEAKKTIGKLFTTDIIETANRQIDI)-YDVFISFeSESIDDIKIMwliSGRLESRGLTVakgsYSFIQ------GVCINEAFANAIYNrSKKVILLISKAFINSESCLMQLNFARDADRASRRNKIIPVYIEEVKK--PECIASINGFNYYN----NSIRE-MFFDSFINS---F()}*

$418=231(250):
>gi|90503848|gb|DY965706.1_EST1
{|0(59)|<Streptophyta(V1)>}CLSM16923.b1_E07.ab1 CLS(LMS) lettuce sativa Lactuca sativa cDNA clone CLSM16923, mRNA sequence
{(SLLSSFFQQLTTIAMANSSFDQSAANNKLS)-YDVFISFGD-DNIIKNFVNHLFSDFKRKGIHAFNeDNHLLKGED-RspQIYTAIEQSRFLLVIFSKSFTSSPSCLNELVKILECKNK-NpeKHKIRIISHNFKLNGI-------------------EPEV-FEWKEALTMAAKL(PGWDLEDLVNGYESKFIEMISNHIFNELDNGPLHVGENLIGLHSRVDRMNLLEFTESNDVHMIGIYGSEGIGKTSIAKSI)}*

$419=138(167):

>gi|91011331|gb|EB121749.1_EST1 {<Streptophyta(V1)>}010130AARA003845HT (AARA) Royal Gala partially senescing leaf Malus x domestica cDNA clone AARA003845, mRNA sequence
{(EGASSFASWSTSFTRSSSSSTSFTHPW)RYDVFLSFRG-
EDTRYNFVGHLYNNLSQMGIKTFMDEEaLKRGEE-
IslALLKAIEDSRISVVVFSENYASSRWCLDELVHIFHCKEQ-QQQMVFPVFFKGGSIGCEKPKREF------
-----------------------R()}*

$420=167(197):
>gi|89246424|gb|DY618211.1_EST1 {<Streptophyta(V1)>}AC4503 NOLLY Medicago truncatula cDNA 5', mRNA sequence
{(KRLD)------------------------------IRTYIDND-
LNSGDQIptTLIRAIEEPKLSIIVFSKNYAVSKWCLEELMKILEIKKM-
KGQIVVPIFYDVDPSDVRNQRGSYAEAFDKH-EKSF-
KKKIkvQEWRNGLMEAANY(AGWDCNVNSIISNINFHNISIHNKRTSSSMKEEENIVNEEQNWHWLRKLLWMY
)}*

$421=241(242):
>gi|90498971|gb|DY960829.1_EST1
{|0(42)|<Streptophyta(V1)>}CLSM11176.b2_O09.ab1 CLS(LMS) lettuce sativa Lactuca sativa cDNA clone CLSM11176, mRNA sequence
{(MVILNHTKQRSSSSSPIHGHKY)KYDVFLSFCS-RDTGRSFTDHLCNAIKHaN-
ITTFFDDDLIERGVYLkpGWESAIKASRASVVVLSKNYGHSQWCLNGLALILEQHS-----
IVIPIFYHVEPSFFEDAMAGYRLVMEamTN-
EDKRSRLAEkiDRWNKACTQVATL(KGMQLGFDRRETEFIQDFVKYIYHRLRISARTPLPLDRRFHREMKQPN
LFKIPFQAIKTCTQNFSERNFIAKGGYGRVYK)}*

$422=164(170):
>gi|93290316|dbj|BW992016.1_EST1 {<Streptophyta(V1)>}BW992016 Chamaecyparis obtusa cambium and surrounding tissues Chamaecyparis obtusa cDNA clone CO04789 5', mRNA sequence
{(ALESMASSSSSHKRSFSEYERETFTASKNL)-YDAFISHRG-
PEVKQTVAFALYDSLEEKGIRTFLDDQELQLGDSIepAIQNAIYSSAVQIVIFSPGYAKSPRCLNELLDMLKT
-----KALFLPVFCDVRPSDVRFPDxGVYAPAFAQHXkKGRFSNMTLQLWKEAL---HSS()}*

$423=154(170):
>gi|90671793|dbj|BW998485.1_EST1 {<Arthropoda(M1)>}BW998485 Bombyx mori embryo cDNA Library Bombyx mori cDNA clone ES4984, mRNA sequence
{(LFRDSLRVWLYTNCGIRVFSLPGTFEENQK)LYDAYVCYSPKdeESVVQSLVNELENGNPSYHLCLHYRDI
PHHGAQYMqcapPDVETAEASKRIIIVLTRNFMQTEWSRYEFRQGLHEALKGCIYKLVLIEECSV-
VADAMCDPDLRPYLR---------------------RDRG()}*

$424=213(210):
>gi|91199479|emb|AM228633.1_EST1 {|0(70)|<Echinodermata(M1)>}AM228633 RZPD MPMGp1174 Paracentrotus lividus EGG Paracentrotus lividus cDNA clone MPMGp1174M079Q 5', mRNA sequence
{(IEEACIQLSATLFISFHTELRVAESDPF)RYDIFVSYSHCdtEkanfFV--ELLRKL---
APDLKLFFDVQELKTGKSWQRALYQAIDGSRCMLALLSEPYLKSAVCQEEFSLAQAEHYAKGKQHlqLIQICL
EDL-
DSIQSEFTRIPMVKGTPdVFGDVVKTICPAVIQWLKGERMD(QIKSLGTLFDDQNIPTLSADVEMEEFRQTSF
HKEFGTKFGFP)}*

$425=220(221):

>gi|109395529|gb|EC431506.1_EST2 {|19(0)|<Echinodermata(M1)>}164036570 Sea Urchin Testis cDNA library Strongylocentrotus purpuratus cDNA clone TSP-5I17 3', mRNA sequence
{(QVHCKISGFLTKVNLTLSLATTEEHVVAVTDTTSVVVVVATLTILLSLVVLSWKRYGTDVKLWNHDHRATLETEEIDNKL)-YDAYIISCYEgqdKSFVDNILCPSLQN--QYKLFIRERDATFGSVESEEIVEALKKSRRCIVLLSADFYRHSSSSSsQSTSGRIETTAAQLTSSSSSSSSPSSPAFSXNXLSXNEXHXFX--LHHXXDTLQRNFIPLLYRDSG(P)}*

$426=229(235):
>gi|89466445|emb|AM200354.1_EST1 {|10(0)|<Echinodermata(M1)>}AM200354 RZPD MPMGp1172 Paracentrotus lividus MBSB Paracentrotus lividus cDNA clone MPMGp1172P2349Q 5', mRNA sequence
{(NSGSENADWLSLAYRLGYKKAKIQKLTEDEPNPALCILIDWFQTSGNTALSVEMLLSYLEQMKRDDVIEVIQKGQEVDKA)PPTVFISYNW-GIQEDVRA--LRDYLERTGFPCWMDIGQMGGGDQLynKIYEGIHNAKVFVCCMTPQYILSENCNRELSLADQLK-----KPIIPIIFKKVPWPPPGGMALIVSQLIYVTFVGVGGHGGSGVKADKEMKYQD(VIHPITRYTQPIKVK)}*

$427=192(185):
>gi|93285955|dbj|BW987656.1_EST1 {<Streptophyta(V1)>}BW987656 Chamaecyparis obtusa cambium and surrounding tissues Chamaecyparis obtusa cDNA clone CO01044 5', mRNA sequence
{(SSSLRIIFSIRLSLEYFMASSSSSNKRSFSEFERETLSVSEKI)-YDAFINHRG-PDVAGTVALALYDSLEENGYPTFLVDQELQLGDSIssAIRNAIYSSKVQIAIFSPRYAESSWCLDELLIMLETK-----ALVfIPVFCDVKPSDLRYPDKGIYAPafakheekgrfskERLQQWKEALYSS--SLISGYEFSTSS(DN)}*

$428=226(220):
>gi|93286225|dbj|BW987927.1_EST1 {<Streptophyta(V1)>}BW987927 Chamaecyparis obtusa cambium and surrounding tissues Chamaecyparis obtusa cDNA clone CO01265 3', mRNA sequence
{(PVNVFCFVFHRDGEGKNESLTM)--DVFIGHSG-K--QKILARELFKDLTNIGVSCFFDEDpqSLPIAEKFppRIFEAAEKCKIAVLVISKDFSRSKWPMLELSALLKAKISGKNpdMKILPLFFMVSPKSLseITEDNQTWKDlgitKETRtEWHRALREI--RSIKGLEFKEAE(DVVRFRDEVVKAIYSLLLSHSPNSIQGQVHQDQVTPLFNLPSEKSCFQVSARFFNFPL)}*

$429=255(261):
>gi|109401052|gb|EC437029.1_EST2 {|63(2)|<Echinodermata(M1)>}132603187 Sea Urchin Gut cDNA library Strongylocentrotus purpuratus cDNA clone GSP-1H8 5', mRNA sequence
{(NSGTENADWLSLAYRLGYKNAKIQKLTEDEPNPALCILLDWFQTSGNTALSVEMLLSYLEQMKRDDVIEVIEKGQDVNKA)PPTVFISYNW-GIQEDV--TNLRDYLERTGFPCWMDIGQMGGGDQLynKIYEGIHNAKVFVCCITPQYIMSENCNRELSLADQLK-----KPIIPIIFRKVPWPPPGGMALIVSQLIYVTFIGVGGHGGSGVKADKEMKNQD(VIHQITRYVKPIQVKPDVKIEDSAVEIITTNDSRGCSGRTR)}*

$430=221(228):
>gi|124677573|gb|EH772313.1_EST1 {|60(0)|<Streptophyta(V1)>}CNSM4849.b1_A14.ab1 CNS(LMS) yellow starthistle Centaurea solstitialis cDNA clone CNSM4849, mRNA sequence
{(GVQYQQESQLLYKLVKNHYENGNLEVIIDPNLQKQINSFSLSSFSTIAYKCLMKHREDRPTMSQVVEQLQKALIYQLAAS)--DVFVSFGE-PDAENTFVDHLYLALRQKGIHCYADDKTLdwDESaITRLHFKAIRESQIVLVVFSKKYASSSRCLNEIAYVRKKGKLC-----IPIYLDVLPMEVREQSGEFGEAFsTIHKSNI--NKV-ESWINALXXCTSV(PGFMTSAT)}*

$431=169(176):
>gi|124694085|gb|EH788821.1_EST1
{<Streptophyta(V1)>}CNSS28860.b1_G15.ab1 CNS(LMS) yellow starthistle
Centaurea solstitialis cDNA clone CNSS28860, mRNA sequence
{(AAS)--DVFVSFGE-
PDAENTFVDHLYLALRQKGIHCYADDKTLdwDESaITRLHFKAIRESQIVLVVFSKKYASSSRCLNEIAYVRK
KGKLC-----IPIYLDVLPMEVREQSGEFGEAFsTIHKSNI--NKV-
ESWINALKDATSV(PGFMTSATSRRIVIKNIVDTVSAKLIRSKSNLK)}*

$432=117(150):
>gi|124582980|gb|EH687353.1_EST1 {<Streptophyta(V1)>}CCIM1211.b1_E15.ab1
CCI(LMS) chicory Cichorium intybus cDNA clone CCIM1211, mRNA sequence
{(VLEALTLVIV)-----------------
FTNYLHKSFmEANNSSFFGNEEVETGKDLKpESESEIKASRTYVIVLSKSYVTLTWCLDELMLIFKKWMTSNH
IVVVPIFYHVEPILIMKQQSTFGD------------------KEKNANTRSQ()}*

$433=240(247):
>gi|124672432|gb|EH767172.1_EST1
{|19(0)|<Streptophyta(V1)>}CNSM13409.b1_B18.ab1 CNS(LMS) yellow
starthistle Centaurea solstitialis cDNA clone CNSM13409, mRNA sequence
{(KHAFDGTNEASFVEDIAYRVKYYLYDHTLGGFKLDSWPLQDQVKTTTSALEFPIEDTPLAAPSSPSSDFNT
PPSFQSSNH)--DIFVSFST-
EDTSEALVEHLCFALAQKRIYVYQDEGpIHSGLTIAgSRLKAIEESQIALVVFSKEYVNE-
WYLDELANIMKCKDERG-KVVIPIYHHVSPSEVRNQTGECGAALAKHEENKN---IE-
SWRKSLADISDL(AGWEIDGYEAQLVKEIVDTISCQFFPN)}*

$434=226(232):
>gi|124662349|gb|EH757091.1_EST1
{|0(40)|<Streptophyta(V1)>}CNSL1905.b1_B22.ab1 CNS(LMS) yellow
starthistle Centaurea solstitialis cDNA clone CNSL1905, mRNA sequence
{(GGGQWTRNQSIL)QYDVFFSCRR-KDTRNNIVEHLRSSLSDRGINVYEDNIAEFHHG-
KhtTILDAMERSRFVIVVISKNYLRSSRCMDELARIM--ATD-
TRRIVIPLLYNLNHMEVFSQIGPLGSTYNQMFHRYHWWK---
KWLLPLKEASRL(AGWKSNSYRQKSNEEIVDTILSGVSSSKANDGGNLIGVEPQMQHIVKDLLEIENRACRRS
SRRNKKTHHPPLPTVRSGGV)}*

$435=199(220):
>gi|124681250|gb|EH775990.1_EST1
{|57(0)|<Streptophyta(V1)>}CNSM8279.b1_M06.ab1 CNS(LMS) yellow
starthistle Centaurea solstitialis cDNA clone CNSM8279, mRNA sequence
{(YGEARPTMSQIVEQLEQALNDQLRASSVSGLQHGADDVVDNDLPAALHQGPSSSKDSRLMPSSSSSSSHST
LFSSSRLQN)-HDVFVSFRG-IDIDEKFVEHLRSTLLQKGIQTCMDDDHEtlhWGES-
IslSLCNKIKESKIVLIIFSKNYVNSWWCLNELAYVMENKDE-SGQIVVPIFYDVDPLDIQKHRG--------
--------------NLKLLKMSRT()}*

$436=158(178):
>gi|125420783|gb|EL432755.1_EST1 {<Streptophyta(V1)>}CHCS712.b1_P10.ab1
CHC(LMS) Texas blueweed Helianthus ciliaris cDNA clone CHCS712, mRNA
sequence
{(FLQKLPNLFVENSDNNRT)---------------------LYHALLEKGITTYKaDEKFEKEGR-
IdnQLIRSIQDSRFHIVVFSKNYASSSCCLDELVEIIKCRKT-
AKQTAYPVFFDVEPTEVGNQSGVVGEAFAKLVAKHEKGSGMFSFFKLFTRKRKR(TSEDVDLVTSLSLQLYLL
LM)}*

$437=221(220):
>gi|117711261|dbj|DB714058.1_EST1 {|0(20)|<Streptophyta(V1)>}DB714058
Solanum lycopersicum cv. Micro-Tom fruit Solanum lycopersicum cDNA clone
LEFL2015N21 5', mRNA sequence
{()--------RG-EDTRKNFTDHLYTALINaG-
IRTFRDDDEIRRGENIesELQKGIRESKISLIVFSKDYASSRWCLDELVNILDRRKK-
EGHTVLPVFYTVSPEDVQNQSGSFAEAFVNH-
EKRGNAESgekrkewmEkmEKWRLALKEVAEL(EGMCLAKEVDGHEAKFIQKIIKEILKRLNRTVLSVPSYTV
GLEARVKDINSWLQDESXEVGIGMICXLGGVGKTTXAKVX)}*

$438=132(162):
>gi|124652881|gb|EH747760.1_EST1 {<Streptophyta(V1)>}CNMS7144.b1_O10.ab1
CNM(LMS) spotted knapweed Centaurea maculosa cDNA clone CNMS7144, mRNA
sequence
{(FIMVVISEILEESSSSLSTLDH)RYDVFLSFRG-
FDTRSSFTDHLYNTLVKADITTFLDDEEIETGLPLkpELETAIKASRASIIVLSRNYASSTWCLDELVLILEQ
------------------------------QLRNL-VSCRAHRCQEATKQLRRSDGKA()}*

$439=227(238):
>gi|124654344|gb|EH749089.1_EST2 {<Streptophyta(V1)>}CNMS8435.b1_F22.ab1
CNM(LMS) spotted knapweed Centaurea maculosa cDNA clone CNMS8435, mRNA
sequence
{(TSIQFPHSLLFAVIFLHILSMDTETTFHERYSLRFSVSSWETPQR)KYDLYLNSHE-K----
TFTDLLYTALVGyG-THTFRADERP----SESSLKAIEESKISIVVFSKEYASCRSCLDKLVKIMDCKKG-
IGQSVLPIFHRVDPGDVRKQSGSFEADFVKH-
EECFDPPQNTPMAGVGTLGFSR(KTIQGHFGDKKNLDHFSICACHPCAGAMLIFSVSFQFYRMSPKGRLYLIC
SAI)}*

$440=192(220):
>gi|125385803|gb|EL399143.1_EST1
{|0(6)|<Streptophyta(V1)>}CFFS10465.b1_B01.ab1 CFF(LMS) safflower
Carthamus tinctorius cDNA clone CFFS10465, mRNA sequence
{()------------------------------IRTFRDTESIET--
DEDLKSQIERAKASIVVLSKNYANSTSCLDELMLIMERRKT-
FYQIVVPVFYHVDPSDVRKQQNSFAIKVntggegFQK-
SVKWTENKVERWKAALTEVANL(EGKVLSGPETKFIADIVNTIGDKLRLKLVCSPPHLTGMDTRAEHINFWVK
DKQDVEVLAICGMAGSGKTTLAKYIYDSNL)}*

$441=180(179):
>gi|121336994|gb|EB214072.1_EST1 {<Mollusca(M1)>}PEG002-C-012382-501
Normalized Pedal-Pleural Ganglia Aplysia californica cDNA clone PEG002-C-
012382 5', mRNA sequence
{(CQCVTWEFGGRQVSTDDRYFYYAAYILCKDREKTEEEAF)EFDVFISYASPdeQFVVRTVVPELTKREV--
KPHVHGLHFTAGNYIASNIVSAIKKSRKTWVILTEQMLDSYWCTYELQMANLESVYSGRRVLVFLLKDSIPrS
RLGMDflYH-----IQNKiYMAYPNTGSQrmnpevttAFWDK------MA()}*

$442=183(179):
>gi|121338450|gb|EB215528.1_EST1 {<Mollusca(M1)>}PEG002-C-012382-301
Normalized Pedal-Pleural Ganglia Aplysia californica cDNA clone PEG002-C-
012382 3', mRNA sequence
{(CQCVTWEFGDPQVSTDDRYFYYAAYILCKDREKTEEEAF)EFDVFISYASPdeQFVVRTVVPELTKREV--
KPHVHGLHFTAGNYIASNIVSAIKKSRKTLVILTEQMLDSYWCTYELQMAHLESVYSGRRVLVFLLKDSIPrS
RLGMDflYH-----IPNNtYMPYPNtgsqrmNPEVtpAFWDK---MARDL()}*

$443=184(179):
>gi|121362395|gb|EB239473.1_EST2 {<Mollusca(M1)>}PEG003-C-225451-501 Normalized Pedal-Pleural Ganglia Aplysia californica cDNA clone PEG003-C-225451 5', mRNA sequence
{(CQCVTWEFGGRQVSTDDRYFYYAAYILCKDREKTEEEAF)EFDVFISYASPdeQFVVRTVVPELTKREV--KPHVHGLHFTAGNYIASNIVSAIKKSRKTLVILTEQMLDSYWCTYELQMANLESVYSGRRVLVFLLKDSIPrSRLGMDflYH-----IQNNtYMPYPNTGSQrmnpevttAFWDK--MARDLF()}*

$444=171(183):
>gi|121472379|gb|EB349275.1_EST1 {<Mollusca(M1)>}CNSN01-F-105983-501 Normalized CNS library (juvenile 1) Aplysia californica cDNA clone CNSN01-F-105983 5', mRNA sequence
{(PTPEA)----------EKFVYSLLRPRLEDVG--FKLYLQARDQPAGEAISETILDAVTQSRRTILIINPDYVEREWSRFEFLIAQHETLKLKQRIIPIILKDLC-EEARKKDKTLKHIMESVkCLRYPHLKSEDSSTSPQSVSREQ(HSQVDVLKKEKNSKRKNVSSGRDWNLPCQKRESLLKKN)}*

$445=181(188):
>gi|110567894|gb|EC367041.1_EST1 {|0(55)|<Porifera(M1)>}G840P310RG19.T0 Oscarella carmela pSport1 Library I Oscarella carmela cDNA, mRNA sequence
{(RPMVDVLSRKV)---VFISYgRE-GEVTDFVLKLATD-LERGGFPVWLDQKDIPAGSDWheVIGNGLHNCQALIAVITRKYVDSRFCKSELYTADGDG-----KLIFPVFYEDVDLSVSERSRGIKYVISGINWTMFRPQKDVYDESLQKLFSGL(LNRGLGNSSTVTELFKQKEKEADLPSSGPLGDAPPPY)}*

$446=188(193):
>gi|211868816|gb|DV987547.2_EST1
{|0(105)|<Streptophyta(V1)>}GQ0205.B3_H24 GQ020: Clean ROOTS systems - Diurnal cycle Picea glauca cDNA clone GQ0205_H24 5', mRNA sequence
{(ASSSTPAHW)-YQIFINHRG-VDVKQTFARSLYRRLLQKGLKPFLDQDEMQPGYNFpsQIKHAITTASVHVAIFSPRYAEAYWCLHELQLMLESG-----SPIIPVFYRVTPAEVRWTQGKDGIYAQALKTLAEKRTDEGTLRYDSSTIGKW(RNALSQVADNFGFELKVAHNGEELELELLDKVVEYLSKMLKRPD)}*

$447=221(226):
>gi|211875267|gb|DV992541.2_EST1
{|45(0)|<Streptophyta(V1)>}GQ0256.B3_A13 GQ025: Annual flush SHOOTS diurnal cycle- trees Picea glauca cDNA clone GQ0256_A13 5', mRNA sequence
{(NSYFYDVNPNSYFYDVNPIPRIPLIPLNHRIPRIPLTSSASPSHGNPNSYPLHSLRILRTSSASSASSASPSHGNPNSYV)-YDVFLNHRG-PDVKTTLASHLYYRLEEHGLRAFFDKQELQKGEKMkpQIEGAIQTASVHIAIFSPNYAESSWCLNELVQMLESG-----KRILPVFYGVKPSDLRWTRGGHGVYARSLXVLEKKRTWDSQPLYDSNTIKKW(RDALSS)}*

$448=178(193):
>gi|211887142|gb|CO477523.2_EST2
{|0(21)|<Streptophyta(V1)>}GQ0133.B7.1_M03 GQ013: Elongating ROOTS tips - saplings Picea glauca cDNA clone GQ0133_M03 3', mRNA sequence
{()------------DVKKTLAGLLYRDLKSKGLQVFLDQPELEPGHIIrsQIEAAIQGASVHIAIFSEKYAESNWCLDELVLMLDSK-----GTILPVFYKVKPSDLRWPDKGAYADALRGLEKNKRYDLQIIQGWKKALSSVA(DISGFELEACNGEKTGPKTPVIRCSLGLSGPASISYSVDSTGSIAATASPGSH)}*

$449=214(209):
>gi|120462477|gb|DV962522.1_EST1 {|0(1)|<Rhodophyta(E1)>}GC00493
Gracilaria changii cDNA library Gracilaria changii cDNA clone 12-B09-T3,
mRNA sequence
{(ADILFQTP)PFQIYISHAD-VD-
KETIVRPLSQRLRAHGYTVFFDEEPYRPGDVCpeVMTNAMETAPVAVIILSPEFVAQQWPMHELIAFVrrydn
akernETP-----
PMLIPVFYRFSKHECTSPDIFSTYSSFFRRYRFFHRVWKGVLDLSDMMHYLG(TAASQWGIQNDRGITNSDTP
DMVRSREMYIEMLQTAIVGKLGSEGRESSDEFHPRIQNHPP)}*

$450=180(201):
>gi|120463195|gb|DV963952.1_EST1 {|0(1)|<Rhodophyta(E1)>}GC01935
Gracilaria changii cDNA library Gracilaria changii cDNA clone 155-G05-T3,
mRNA sequence
{()----------------------------
HGYTVFFDEEPYRPGDVCpeVMTNAMETAPVAVIILSPEFVAQQWPMHELIAFVrrydnakernETP-----
PMLIPVFYRFSKHECTSPDIFSTYSSFFRRYRFFHRVWKGVLDLSDMMHYLG(TAASQWGIQNDRGITNSDTP
DMVRSREMYIEMLQTAIVGKLGSEGRESSDEFHPRIQNHPP)}*

$451=133(146):
>gi|120464775|gb|DV967248.1_EST1 {<Rhodophyta(E1)>}GC05239 Gracilaria
changii cDNA library Gracilaria changii cDNA clone HCL_PLATE32_D09_07,
mRNA sequence
{(SSSQTP)PFQIYISHAD-VD-
KETIVRPLSQRLRAHGYTVFFDEEPYRPGDVCpeVMTNAMETAPVAVIILSPEFVAQQWPMHELIAFVrrydn
akernETP-----PMLIPVFYRFSKHECTSPDIFSTYSSFFRRYRF------------------F()}*

$452=190(211):
>gi|121454842|gb|EB331738.1_EST1 {<Mollusca(M1)>}CNSN01-F-137266-501
Normalized CNS library (juvenile 1) Aplysia californica cDNA clone
CNSN01-F-137266 5', mRNA sequence
{(HAPLDAMLRVMVSNALYWIRAGLPLKCIKLVLYTANPDRDRQFLYKSSIEQFGLIKKKWEFRNTQNEAKET
)-YDVCFCYaKSDEHWMTDVSRTLVSRDHSLKmykeLFIASTDD-----
SWQEVVFNILScSRRIIIILTPTFVSDPFCLEQFSLGLCCNRLKGDNVLIPFYIESV-TVFPS---
YMNLIQYTE----CGARY-------------D()}*

$453=163(186):
>gi|146271517|gb|ES444559.1_EST1 {<Streptophyta(V1)>}RWM_NL_0337_052
Arabidopsis thaliana Col-0 RACE (RWM_NL_2007) Arabidopsis thaliana cDNA
clone RWM_NL_0337 5', mRNA sequence
{(KPKDRSLLFQSCFLQYHSKNTFFTCKHLWSLMASSSSSSSSSHIRR)-HHVFSRFHG-
PDVRKGFLSHLHSLFASKGITTFNDQNIERGQT-
IgpELIQGIKEARVSIVVLSKNYASSSWCLDELVEILKCKEA-LGQIVMTVFFK-------------------
--LIRQMLRNRVECSGKLLRRXSR()}*

$454=127(145):
>gi|146271435|gb|ES444477.1_EST1 {<Streptophyta(V1)>}RWM_NL_0097_995
Arabidopsis thaliana Col-0 RACE (RWM_NL_2007) Arabidopsis thaliana cDNA
clone RWM_NL_0097 5', mRNA sequence
{(SSSQS)-YDVFPNFRG-
EDVRHSLVSHLRKELDRKFINTFNDNRIERSRKITpELLLAIENSRISLVVFSKNYASSTWCLDELVKIQECY
EKSDQM-VIPIFYKVDPSHVRXQTGEFGMVFGETLQGQNRE----------------()}*

$455=130(160):
>gi|151201434|gb|EV114480.1_EST1 {<Streptophyta(V1)>}0119792 Brassica napus Root library Brassica napus cDNA, mRNA sequence
{(SWYACRYRSRIPGSTHASGK)------------------------------
IIPFKDEEMKKGQSISpELIKAIEGSRISVVVLSHNFANSSWCLDELVEIMRCRVELG-
LIVIPIFYEVNPSHVRKQSGDFGEIFEETCKGRTEEDKLRSNHSRGGFQHLV()}*

$456=212(210):
>gi|156817996|gb|EX044250.1_EST1 {<Streptophyta(V1)>}BR028894 floral bud cDNA library KBFL Brassica rapa subsp. pekinensis cDNA clone KBFL-128H08 5', mRNA sequence
{(KVSVSSPATSNTTKLRSS)--DVFIGFHG-QNpnlVR--FCKWLKSELELQGIACFVADRAKYSDT-
QshEIADRVICSVTyGIVVVSCSSLLNYLSLEE-VRFFAQKKN-----
LIPIFYGTGPSEimgllncnaIDKECKEAIDGLIKSHEFKLEANE-
SNWRSCVGKTAMI(LRGKLGRKSVVDKEIVERIDELPFPRNRTFLGREKEIIEMEMAFFGSNGEFL)}*

$457=198(202):
>gi|156727372|gb|EV231483.1_EST1
{|0(2)|<Streptophyta(V1)>}VV_PEa21b11.g1 Vitis vinifera cv. perlette LibA Vitis vinifera cDNA, mRNA sequence
{(HIIQLWVIYILLSFVFHSWNFGNCINECGTLSFLQDNLDLRLETWSLYCSVMAAAAAFFFQR)RYDVFLSF
RG-EDPQNNFTAPLDKEFRtEG-IDCFIDEEKLERGKAIsfGLLAAIENS---
MFFYHCFVRKLCIFQVVLGEPGEDTGV-QTQFFTMWIPQMSEIIGGNSEKHWLNMKRIL-
RRIWGGCRFGGMLSLELPVYLV()}*

$458=199(220):
>gi|151293223|gb|EV199884.1_EST1 {|46(0)|<Streptophyta(V1)>}0101037 Brassica napus Cold acclimation - light Brassica napus cDNA, mRNA sequence
{(RMHCWSCFLSQRLLKDADKRFVRWRGALKSITDYPGLKYIKDSNQALFVNQIVETVKEMLGKVQSSDDVHD
RIRGLHIVR)QQKVFISFGGHdDDTRLGFISHLQAGLKRSGINFYINiENMTKGYDPEELIMNVRESRIALVI
FTESYLSSAWCLEELVEINKF--T-MSLVVIPIFYKGEPKYVRDGRLVEINNQLVL-----------------
---NCG()}*

$459=207(214):
>gi|154307479|gb|EV602461.1_EST1
{|0(42)|<Xenacoelomorpha(M1)>}COE00000278 Convoluta pulchra Library #1 Convoluta pulchra cDNA, mRNA sequence
{(PSDTHHSTDLFRAGRIFPQHKSETFSEETRTMSSSTSDER)-YDIMISYchRN-KDI----
VRKVKQRLLELGFKIWIDEEQIHGDVISNMREGVQRSKVVIGFVSKAYEESKACTKEYKAVDRYD---
KIFIPVIVEKGYKMQGDFEFLFGHLLHVDFEGGSKERFEPAMENLLRVLKQKSV(SPSKSGSEMPSTSTKSTT
NRHTPAAGSEEGERSL)}*

$460=197(204):
>gi|154307519|gb|EV602501.1_EST2
{|0(76)|<Xenacoelomorpha(M1)>}COE00000279 Convoluta pulchra Library #1 Convoluta pulchra cDNA, mRNA sequence
{(AWLLSRSPLQRFRRIQTETRTMSSSKSDEI)-YDIMISYchRN-KDI----
VYKVKRRLLELGFKIWIDEEQMHGDLISRMREGVQRSKIVIGFASKAYEESENCTLEYKAAKRYR---
KTFIPIIVEKGYKMQGDIEFLFGKDLYVDFEGGSEERFEPAMEKLLWILEQNSV(YPSKSGSGMPSTSTESTT
NRTTPAKDHLATQDWK)}*

$461=154(159):

>gi|151288116|gb|EV194777.1_EST2 {<Streptophyta(V1)>}0092263 Brassica napus Cold acclimation - dark Brassica napus cDNA, mRNA sequence
{(LWKRKRPPPSQTTRRLIMV)--EVYISFDRCeDKVRYSFISHLSAAFHRRGISSFVGGSDPGSNGSSKQC--LENSRACVVVFSEKYSSSKPCLEELVKVSErRGNE-GGLAVVPVFYRATKATGRKRSG--NRAILAHLYRSWKCVGRVFXPKSGPYLSXL()}*

$462=142(169):
>gi|125938940|gb|EL591134.1_EST1
{<Streptophyta(V1)>}65JKBNMO_UP_053_B06_17JUL2006_046 65JKBNM0 Brassica napus cDNA 5', mRNA sequence
{(LSIIGFNEFQSGPVSDASDTSATFPASKD)-YDVVIRHGG----NNDFISHLRAALSRRGISVHEDVEETTDA-------AVPECRVLIIFLTTTYVPS-----NLLNILQHQRQ-ESLMVYPIFYGISPSDL--------ITNSNY-YENFFPQDESERCQAALKEITQ()}*

$463=151(157):
>gi|146271534|gb|ES444576.1_EST1 {<Streptophyta(V1)>}RWM_NL_35_635 Arabidopsis thaliana Col-0 RACE (RWM_NL_2007) Arabidopsis thaliana cDNA clone RWM_NL_35 5', mRNA sequence
{(QSKHNFAMAASSEILPE)SWQVFINFRG-ADLRNGFISHLAGALTSAGITYYIDTEEVPSEDLTVLFKRIEESEIALSIFSSNYAESKWCLDELVKIMEQVKK-GKLRIMPVFFNVKPEEVREQNGEFGLKLYGE-GKSKRPNI-PNW--PXXXXXPX()}*

$464=153(182):
>gi|156906126|gb|EX107106.1_EST1 {<Streptophyta(V1)>}BR093396 whole plant cDNA library KFYP Brassica rapa subsp. pekinensis cDNA clone KFYP-010D12 5', mRNA sequence
{(TANKIRSSKKACISISSASKAYTRNKCERERDRQMERTNPHL)--QVFISFRG-KDVRGNILSSLKEKLKDEGVNVKTDEEMPRGRKIDeNLQKLIKDSKVAVVIFSENYPESPWCLDELVEIEKQIEE-TKLKPLPIFFKVRATHVALEDHnPFKDI---------------------------()}*

$465=209(195):
>gi|152789676|gb|EL566020.1_EST2 {<Amoebozoa(E1)>}Physarum01111 Physarum polycephalum starvation stress library Physarum polycephalum cDNA clone PpolyN1a15d12.t7, mRNA sequence
{(VLAKLPLICGTAGDSSRSCSLPQASQSHQHLHPVGEK)RVKIFISYawqpeKEDNTKLQAKLVKIKgdLGKAGI--EVMLDIHNLE-GEIGrYMVDGIQESDRVLLICTPRFRQRADEKSpkPNNLQKEFNAALeRQtqnpKFIIPIILEGDRstSIASALPDILSLDLSDPKXYCHNMISLNPKGLIPMILGLE(KIQLYPHLLDGYHAHLKS)}*

$466=232(228):
>gi|145888016|gb|ES393598.1_EST1 {|0(7)|<Mollusca(M1)>}MUS10-D02.x1d-t SHGC-MUS Mytilus californianus cDNA 5', mRNA sequence
{(KQSERKKKRTMKHCSYIENGGRRPFALPDGKT)-YHVFISYAtmEPdrQ-IAHTLFTKLSESNF--TCCLHEIEFQAGHLIEDNIKKCMKKSLKTIVLLSENFKNSYWCHIEMTEIVQLHMDNKGFPPIVLKLDscEVPaALKPYTYMLLQAGLDEKmPDIVDVINDMEITEFPGSMLTIS(FVPLPCINYNQDTPHSPCIWPHVCAKFILSDDGCKDMCTKHHVLNKESLEKLKKCG)}*

$467=196(221):
>gi|152814189|gb|EH645537.1_EST1 {<Mollusca(M1)>}AU_Cv_EST_008A_E04 Oyster mixed tissue normalized cDNA library Crassostrea virginica cDNA 5', mRNA sequence
{(NSKMSVEKLSLLSPAEDNNESDASLSHLSRDQDSDLEDRGNAGKSHSDVDVLPLPSAKR)-YHLFVSYSAEdkEGANKICGHMERRFK--

MKCMNFERDFVPGKSIDDNIADEMQKSVKVLLILSPNYIQSHWCVIEAREACQLSFKGLSDVNVIPLL-----
-LRSVEKELPPFLRSFvYID-------------------A(QKELDVPAKIYDAYLNPGSIDP)}*

$468=201(226):
>gi|152819121|gb|EH649201.1_EST1
{|11(0)|<Mollusca(M1)>}AU_Cv_EST_001B_C07 Oyster mixed tissue normalized cDNA library Crassostrea virginica cDNA 5', mRNA sequence
{(KYVLFSWPSTARPLFRNYRIIQEISMAEFVQTSTFGEEEIPLLLSENWTSDPDETDLKAMQESVEGQPLLL
TKPLPPGKE)-YHLFMSYSSEdrMDA-NEIRDQLEER-
FQLKCLYYERDFQIGKNIDENITEGMQKSMKALILLSPFYLQSHYCVTEAREACKMSFTDfENLNVIPVVLAP
LQkE--------MPAFLNSYvYID-------------------A(QKELDV)}*

$469=132(141):
>gi|145891905|gb|ES397487.1_EST1 {<Mollusca(M1)>}MUT05-L16.y1d-s SHGC-MUT Mytilus californianus cDNA 3', mRNA sequence
{(K)---------HThtAMVTRKLKKRXEEKKQE--
LFIPVINLQXGEEKADQIVKSMENSKRVIIILSDKYDENEWSVFECQQA--
EMLNPNDGRIIFIKYhpeaEDM-VQKEPW----KSRVKDRkVLAIGEkssehRW---FWDKLKYELPVK()}*

$470=211(221):
>gi|145891981|gb|ES397563.1_EST1 {<Mollusca(M1)>}MUT07-C20.x1d-t SHGC-MUT Mytilus californianus cDNA 5', mRNA sequence
{(RDKNPDREITNEKSATTLERSHTMSLFKDSYFNVNEAPSLPEDK)EYHVFFSYeyANSdrNWV-
KAVVRELEN--
KGFKCCVYEKDKNQSQSFSSNIQHFIDKSMRIVIVLSNTYINGDWANMEMEILSRGNVNDLV--
FIPVLIEPC-----NIPPFLXDCMFINA-TWQQVK---
WWESFLELIRGA(CPPLPPKEKYHAFFAHTPKNKSWVANVVKMLDRPKLE)}*

$471=143(141):
>gi|145896156|gb|ES401738.1_EST1 {<Mollusca(M1)>}MUT10-N04.y1d-s SHGC-MUT Mytilus californianus cDNA 3', mRNA sequence
{(S)-YDAYISFDDEniHirqWVTRKLKKRLEEKKQE--
LFIPVINLQIGEEKADQIVKSMENSKRVIIILSDKYDENEWSVFECQQA--
EMLNPNDGRIIFIKYhpeaEDI-VQKEPW----KSRVKDRkVLAIGEkssehRW---FWDKLKYELPVK()}*

$472=191(220):
>gi|156260760|gb|EW783937.1_EST1 {|73(0)|<Arthropoda(M1)>}G893P53RO13.T0 Ixodes scapularis Pooled Non-infected Standard Library Ixodes scapularis cDNA, mRNA sequence
{(PNQLVSLKRFMDLGQKDLCPSMMSKILSYGLPLLVLLIISLAASTAYLRHKRAIKVWLYARGVCSSLQCIK
EDDLDEDKI)-FDVFLSFSSKdsMWAYEQLIPGVE--
AHGFSVCTYDRNFKGGFLLQDIIHEAVSCSRRTLLLLTKNFVESEWCRWEFRVAHHQALEDR-----------
------------------SpSNPGPRDELRRAVTRLQLYMQHT()}*

$473=217(217):
>gi|145900886|gb|ES406468.1_EST2 {<Mollusca(M1)>}MUT11-L15.y1d-s SHGC-MUT Mytilus californianus cDNA 3', mRNA sequence
{(VCNNDGLLRSINEIEREDLGCSASVINVSLVNSSICTAFFAVITCAIIIYKLRFELFILTRTVITFFRKPL
SPTSVI)-HDIYIS--SNeddeliRKWLLSSLVPCLEKK-
GFDVFLFFRDSSIGEPREQETIDVISKSRNFIILLSDDTNGTQQWNKrEWKYAWNYYKWDLSRELIIINYDLL
TYDDV-VKHFFRGFFTLR--KVIDFSNFNNKIEEDIVALLK()}*

$474=166(180):

>gi|134275524|gb|ES227444.1_EST1
{<Streptophyta(V1)>}T01177_D21_A1A2_B09_047 AA - Norway Spruce Subtracted Library (Adaptability-cold) Picea abies cDNA 5', mRNA sequence
{(VVPKLVSSPWQFVSIATIIQSLPISVSIFLLAMAASISIS)-CDVFINHRG-
PDVKKNFASHLYYRLISHGLRAFLDQQELNKGENFesEIKGAIASASVHVAIFTRRYARSTWCLDELQLMLDS
G-----FPVIPVFYKVKPAVLRWTHeDKGVYARDLDNHEK----------KKAADGQPR()}*

$475=187(200):
>gi|138309791|gb|ES255048.1_EST1 {<Streptophyta(V1)>}WS03725.C21_A17 SS-B-N-22 Picea sitchensis cDNA clone WS03725_A17 3', mRNA sequence
{(NHLMSLGIFSSSASTSISVLISDEGRLVHDIVKLSIKGVSKTPIH)-YDVFLNHRG-
KDVKKTFANQLYYRLRTYGLQVFYDQEELQKGNRInsVIENAIKVASVHIAIFSKGYAESEWCMDELALMVES
E-----SIIISAFHGVNPtkiWQNPVDGMNGVYAEALRK----LKEE--K-----
TFDPET(HQEKQRYNSNTLEIW)}*

$476=188(194):
>gi|138325374|gb|ES260325.1_EST1
{|0(9)|<Streptophyta(V1)>}WS0383.C21_M16 SS-B-23 Picea sitchensis cDNA clone WS0383_M16 3', mRNA sequence
{(YGNISNNYVF)--DVFINHRG-
PDCKKTLAKYLYDRLIKEGLRVFLDVNELQDGETItpQIQSAIRTASVHIAIFSPGYAQSNWCLDELLLMFES
G-----
STILPIFYGVKPSDLRWGTQSETGVYARDLKTLERKRTFDSQPRYDSNTIQK(WRKALYDVAEIKGFDMETCN
GDEQQLLQQVVEGVLKKVKRPLHV)}*

$477=213(222):
>gi|139233505|emb|AM522153.1_EST1 {|0(14)|<Echinodermata(M1)>}AM522153 RZPD MPMGp1171 Paracentrotus lividus CLEB Paracentrotus lividus cDNA clone MPMGp1171N2322Q 5', mRNA sequence
{(YRNWIPQVEDTPVTTKMVSEVAKSSPKQQSQEESTKSPT)---VFISYQWDkqPEIKR-----
LFSRLTSLGYHCWLDINQMGGGDPLysKIDKGVRNAKVVVSCVTPKYALSANCRREVSLSDALR-----
KPIIPLLLEDMTWPLEGPMSMTFTQLVYIDFTKESTQESFNDQKFDELIEKI(KLHAQPTLEIVANPEENDVL
DDSDQGQSQTPTLQVEGRTESPR)}*

$478=253(259):
>gi|139239835|emb|AM535001.1_EST1 {|0(16)|<Echinodermata(M1)>}AM535001 RZPD MPMGp1171 Paracentrotus lividus CLEB Paracentrotus lividus cDNA clone MPMGp1171C23199Q 5', mRNA sequence
{(GTENADWLSLAYRLGYKKAKIQKLTEDEPNPALCILIDWFQTSGNTALSVEMLLSYLEQMKRDDVIEVIQK
GQEVDKA)PPTVFISYNWGihDDV-----
RALRDYLERTGFPCWMDIGQMGGGDQLynKIYEGIHSAKVFVCCMTPQYILSENCNRELSLADQLK-----
KPIIPITFKKVPWPPPGGMALIVSQLIYVTFVGVGGHGGSGVKADKEMKYQD(VIHQITRYTQPIKVKPAVKI
EDSAVEIIATNNSRSTDSTTP)}*

$479=190(198):
>gi|149970255|gb|ES870280.1_EST1 {<Streptophyta(V1)>}WS02831.CR_J01 SS-IB-A-FL-13 Picea sitchensis cDNA clone WS02831_J01 3', mRNA sequence
{(KTISTT)--NVFINHRG-
PDVKKTLASHLYHGLVERGLRVFLDIEEMHEGDSLtsQIEGAIRTAFVHIAIFSDTYATSKWCLDELVLMLES
G-----RIIFPVFHNVKPSELTSENGKY--
DAQLRELEKKIDEEGKPRYDPATIGKWK(KALFDVGKCSFRFDLAHCDGDEGQLADQVIQRVLLEEVKAWKDG
LHLGWSAN)}*

$480=236(242):

>gi|149979682|gb|ES879707.1_EST1
{|0(7)|<Streptophyta(V1)>}WS02941.CR_J12 SS-IB-A-FL-15 Picea sitchensis cDNA clone WS02941_J12 3', mRNA sequence
{(GEVRNPNLSHHFFIILHPMASSSNSTSTSTSTADGNNCYQYG)---VFINHRG-
PDVKKTFASHLYHRLSAHGLRIFLDQEVLQKGDNItpQIKDAIRTASVHIAIFSTNYCESRWCLDELVLMLES
G-----
SPVIPIFYNVKPSELRwTEGDNGVYTRVLRMFNRRSRTRGEIGVYARALLNLE(KKKTFDFETRRQMPRYNSG
TIRKWRKALSDVADIIGFELEAYNGRDEGQLLVDVVERVLE)}*

$481=180(188):
>gi|154307843|gb|EV602825.1_EST1
{|0(106)|<Xenacoelomorpha(M1)>}COE00000265 Convoluta pulchra Library #1 Convoluta pulchra cDNA, mRNA sequence
{(TRAIEPKPTMTDTD)PKDIMLSYCW---NEKELCRKIRDSLTEKGFTVWLDIERMAG--
NLnsDVANAVENCKVFLSCVSSGYITSKNCESEYKFARQKN-----
KLIVPVIVEKDFGMIGQIAFPIGNLLYVDFINMPYDIAFAELLRVLELKGVR(PSGEEQDCGGQTPAKPRQEF
AALEKLTSIADFKC)}*

$482=170(173):
>gi|145891895|gb|ES397477.1_EST2 {<Mollusca(M1)>}MUT09-D24.y1d-s SHGC-MUT Mytilus californianus cDNA 3', mRNA sequence
{(MGIMVES)-
YDAFVIHNEEekdLEFVQEMARILEGPKYNLRLFLPWRDLqTTEDNETEVLAKIMVQRCRCaVVVISPNLLQS
DVTSFLLSFVHSKYPSGRNSKLIPILIENC--ELPTILQSVTVCDFTK--KDMRC---
WVWPRIASSMRMT(QSRLTSGRRQFGPCSTRKSVLPDILH)}*

$483=215(228):
>gi|152799675|gb|EL575813.1_EST1 {|10(0)|<Amoebozoa(E1)>}Physarum14400 Physarum polycephalum starvation stress library Physarum polycephalum cDNA clone PpolyN1d80f03.t7, mRNA sequence
{(PTVSSHEPSPKGSTERKDVKAANTIETNETKVTKETSKPKEPKKDIPRETNEELTKRIEALASEKDEETLV
MLSVHCGTM)-----------LESANRLKQVLLKNGYDVFLC--DQMQSGDNYRYEIS--
LNAAQCHAfIPFMNAPWCKSKECEYEFNIALRMNfVKQGVPRIIPLILEdfPFYADYPTINGFLCSfngLRVT
P--AELND---ATWQKLLRALQKS(GTLFPPMP)}*

$484=209(218):
>gi|145884727|gb|ES390309.1_EST1 {|0(31)|<Mollusca(M1)>}MUS11-H15.x1d-t SHGC-MUS Mytilus californianus cDNA 5', mRNA sequence
{(IQLFEDLVCGQTKMEHKRMKAKLAMVTHLPAGNDVI)-----ISYDPEdRNYVIKIRDKLKEAGIAVW---
---VETYDLGGGAnvfsKVGQAVVDAKIFFYLISTDSVKSKLCQDQLALAYVSKKS-
ILPVAIDDEDDVSKIMDNGMRLQLAGFEWSF--
LDREIIEDGLPSLVARIKDML(ADYDSSNRDEETKKKTEEKKRYLRSAKSRSRIKASRIHLQDG)}*

$485=217(216):
>gi|145894983|gb|ES400565.1_EST1 {|0(53)|<Mollusca(M1)>}MUT02-O23.x1d-t SHGC-MUT Mytilus californianus cDNA 5', mRNA sequence
{(SRGKS)-FHIlFICQSQPSnvKIKENVKIQLQR--DGYTCG----
DCIPGLSTSENIVQTCKSAVTVIPLITDEFNKSTECKNKLEYAkqLHIDSGDKKQPFIIPLVDYLSTEkVPDT
IMTVECINmAEPVEIW-YPRL-
KkglKYALETYRNEWV(PIFDLVTDLPLEEREKLGAALINNRMRGLAPGKEKEIASDLMKDKTIDEKLSFFAN
LFETDIPQVKTLIRI)}*

$486=216(216):

>gi|145886797|gb|ES392379.1_EST1 {<Mollusca(M1)>}MUS01-L24.y1d-s SHGC-MUS Mytilus californianus cDNA 3', mRNA sequence
{(WQHASRVPFLLIVYSKRWEICVFLYQKFRNLIETKFKKLYT)-YDLYVSYETDSmyWIKTVLLPKVED-EWGLKVCLKDRDFLVGVSRFDIEAESIQQSRHVMFIITPSFKNSLDYLFEIERVKYEKM-MKNMANIIVIVKDIsISDIP---IELSYIWsNVSLVQWTENLE-QldiIWQRMKIWLSTD(CLGYFIQNTVNNDNTIPHARKMNAVTYGSISKQFR)}*

$487=165(171):
>gi|145900994|gb|ES406576.1_EST1 {<Mollusca(M1)>}MUT01-H10.y1d-s SHGC-MUT Mytilus californianus cDNA 3', mRNA sequence
{(KICLFFYGKFRKIVEKKYSRSFI)-YDVFVPYEGDSlpWIKSCLIPKLE-YEWNLKVCLKDRDFLPGDSYFDTEAECIENSRHVIFLLTPSFKSSRDYLFEVERVKHEKRIQNI-ENIIVLAKDItLKDIPS---ELAYIWnYVSFIQWPDDTD-DidiMWQQL------M(RWICQDFM)}*

$488=166(198):
>gi|145889511|gb|ES395093.1_EST2 {<Mollusca(M1)>}MUS07-L09.y1d-s SHGC-MUS Mytilus californianus cDNA 3', mRNA sequence
{()-------------------------HNIRLYVPGRDDLAGDEKYVVTAEMIANrCRRVIVVLSRGFEDSEDCDFALKLAQSLSPGAKTKRIIPILLDNVK--IPLILNFVGTVNFTN----PMQRE-WVWPRVAATIKCP(LVPSIQDWQCTIDDLKNMKYDSKHVQHLLYGIGVACQEFPPEEEEDKKGKKGDKKKKT)}*

$489=211(210):
>gi|145890803|gb|ES396385.1_EST1 {<Mollusca(M1)>}MUS08-I04.x1d-t SHGC-MUS Mytilus californianus cDNA 5', mRNA sequence
{(FLAKKISDEKIDINKDTMKSKWKNFLNLQDKE)-YHVFASYsEDDSKLVNGLLQPFE--NSNCLFYNPDRDEIPGQPVMRGQIEKGMEqSKITMLFITKNFLSDELCMFMANLAIWKYMKTKGiHRVFPIILQPCK--IPRYLKVLNCIhvwKYTH----TRSSH-MIYQtqaiaRLTKAMLEP(PPKFRRIRWNGIKTLTWTRNIVEKSKKSALKERHLISQ)}*

$490=215(220):
>gi|148315469|gb|ES575362.1_EST2 {|28(0)|<Mollusca(M1)>}FPS012.CR_P15 LSG10-01 Lymnaea stagnalis cDNA clone FPS012_P15 5', mRNA sequence
{(LVRGVCSGQMKSVFSISMGLRTPSNSGSRETLERSEEDEDDVALTMDEIKPVRINANIHRYNRNNDMLSIFDGPNNIRQY)-YSAFFCCaEEGMDsFGYSFHRMLENIGFKI--FLPPRDLVLTGQLYENMARALEErCNgKIIVILSKNYADSEECLLMTYFARVLDPDARCKNIIPVQIDKTIGQLPSVLQGMSIIRYNQ----AFRLG-WLKQKLIDAISA-()}*

$491=179(211):
>gi|161917127|dbj|DB887478.1_EST1 {<Streptophyta(V1)>}DB887478 full-length enriched poplar cDNA library, mixture of leaf, bud, stem, root Populus nigra cDNA clone PnFL2-071_K18 5', mRNA sequence
{(ATSVLSLQTCPATRVSLLFLRLFLHKTMAMFFLFLSIFLFVYIWRFIIRSRHISPSPSTPSTLTTAQPQVI)KYDVFLSFRG-EDTRFDFTSHLYAALKRKQILTFIDHQLVRGDE-IsaSLLRTIEEAKLSVIVFSENYASSKWCLEELAKIFERRXN-NGQIVIPVFYKVDP-------------------------------AISLPLSPH()}*

$492=180(204):
>gi|157717964|emb|AM742911.1_EST1 {<Streptophyta(V1)>}AM742911 Cucumis melo subsp. melo Piel de Sapo T-111 fruit 46 days after pollination Cucumis melo subsp. melo cDNA, mRNA sequence
{(NHLGKERKKKKKKEHFSIRIHFDSSQQSSHKPSMASSAIMKRRDSIKSLSSPPYSISLPLPPLR)RYDVFLSHRA-KDhranndTGRSFISDLHEALTsQG-IVVFIDKEDEEDGGKPltEKMKAVDESRSSIVVFSENYGS--

WvCMKEIRKIRMCQKL-RDQLVLPIFYKVDPGDVRKQEG-----------------------------
ESL()}*

$493=167(164):
>gi|158660684|gb|EX770575.1_EST1 {<Streptophyta(V1)>}RR4BT34TF RR4(PB) Raphanus raphanistrum subsp. landra cDNA 5', mRNA sequence
{(AHKMASSSSSSATPAP)KFDVFLSFRG-QDTRKNIVSHLYAALcRKG-
LYTYKDDEEMEKGDSIpdELIKAINTSRFFIVVISEKYDNSYWCLEELRAIMEvaavKRKE-
DISVLIPIFYRVKPGSINRE--NLTAAFSAM-KHPPMEETAmiDEWEKPLSQLANL(ASYLFSNL)}*

$494=199(218):
>gi|161929843|dbj|DB889181.1_EST1 {<Streptophyta(V1)>}DB889181 full-length enriched poplar cDNA library, mixture of leaf, bud, stem, root Populus nigra cDNA clone PnFL2-081_N14 5', mRNA sequence
{(ISSSFRRKTLRNVAASISSPKESREIFIHPTQKSLPLFIKPMAFNPVTLILFFLSLIPGAILTTRTCDPSF
RGPGLTT)-YDVFLSFRG-GDTRQHFTDHLYKALtRAG-
IPTFRDDNEIRIGENIelEIQKAIQESKSSIIVFSKNYSSSRWCLDELLMIMERRRT-
VGHLVFPVFYDVDPSEVGNKQGNLVK------------------NLLSLKYASR()}*

$495=260(260):
>gi|157636182|gb|EX437264.1_EST1
{|0(2)|<Streptophyta(V1)>}GQ04004.B7_M09 GQ040 - Shoot tip - Active growth (Normalized) Picea glauca cDNA clone GQ04004_M09 5', mRNA sequence
{(YSLPQNSKISETHLVAPKFLKLISSLPITQLAMAHTSDYG)-YEVFINHRG-
PDVKKGFAGHLYRALCSQQIRVFLDKPEMKKGDILnsQIVRAIETASVHVAIFSENYAESKWCLQELVLMLES
SK--SGATIIPVFHDVKPATVRwteDKDRGYGKSLHKL-
KVKTDEETIKQWKKTLSTVANI(SGFELEAYNGEEAELVDKIVEHVSKIVKKPRFSVPKYEIGLDAKVRDFED
KVLSRQHQSGKPQVVGIVGLGGVGKTTVAN)}*

$496=239(239):
>gi|158660273|gb|EX770164.1_EST1 {<Streptophyta(V1)>}RR4BO70TF RR4(PB) Raphanus raphanistrum subsp. landra cDNA 5', mRNA sequence
{(TSWSSAVPDFRYRTFLCSFILIFFVYFAVVCQQPS)RPQLFVSFRG-
EELRNGFVSHVVKALEDARVNVFIDTHELKGRSLENLFKRIDDSKIALVIFSDRFSESEWCLNEVARIDKRVK
G-
GKLRVIPVFYRVNTTDVKNFTGKFGSCFEETLQRLSSEERNtaESWKSSVKSVSSN(TGFTSQRISIDSNLVD
AIVDDVKSQLPHMFTEINVDSRELYIAIVSLLSIVVALYFLRHEFFRL)}*

$497=198(219):
>gi|158927307|gb|EX899339.1_EST1 {|0(10)|<Streptophyta(V1)>}RS2BR36JQ RS2(RS) Raphanus sativus cDNA 3', mRNA sequence
{()----------------------
VKALEDARVNVFIDTHELKGRSLENLFKRIDDSKIALVIFSDRFSESEWCLNEVARIDKRVKS-
GKLRVIPVFYRVNTTDVKSFTGKFGSCFEETVQRLSSEKRNtaESWKSSVKSVSSN(TGFTSQSISIDSNLVD
AIVDDVKSQLPHMFTENKMSILEELYIAIVFAAFYKLVAPIFFSDTSFFQTRQWFVGVSCIVL)}*

$498=207(218):
>gi|161295827|gb|EY625803.1_EST1 {|0(78)|<Annelida(M1)>}CAWW19243.fwd CAWW Capitella sp. I ECS-2004 Subtracted Late Library, Embryos >48hr after egg deposition Capitella teleta cDNA clone CAWW19243 5'
{(EFPGFRSSQREEIRRASRGCVYLVEGRLPPVRPLPLQKHSPDGA)---VVISFQW-S--
SRSIVGKIKERLRSAGYVVSTYTDSSGGPSIDSMLQHIESASIVLVAMSHKYKESPHCRTECEIAIRL-----
GLDLIPIRAQRRYSPEGWLSRLLSDKMCFDFTQDGNFDSMFTNLTREIANRGH(LNPAELETPGSRAVSTGSL
CQAQVQVHRALSGGP)}*

$499=179(185):
>gi|162048380|gb|FC240093.1_EST1 {|0(72)|<Cnidaria(M1)>}CAGH13138.fwd CAGH Nematostella vectensis Nemve Late embryo <24hrs Nematostella vectensis cDNA clone CAGH13138 5', mRNA sequence
{(YCSPLDVTMKH)---VMLSYQW-DI--
QDLVKKVYDGLRAYGIRVWMDiHGGIRGDINGSMAKGVDGAAILCCFMSTKYSESKNCKKELSYADGKT---
GceEVIIVPVMCEKGFKAKGWLGIITAGLLWIDFRDEANFENSVKSLAQEIMHAVG(DSSDITLAPDEKQEAA
SKSVVKPTAEVKKNPGRA)}*

$500=118(140):
>gi|157507223|gb|EX308454.1_EST1 {<Streptophyta(V1)>}GQ01308.T3_B15 GQ013 - Elongating ROOTS tips - sapplings Picea glauca cDNA clone GQ01308_B15 5', mRNA sequence
{()---------G-RDVRRGFVDHLYNALTMGGLHEFLDTHQInGGEDISAsLQNAVEVSRILI-------
AQSAWCLREDSLIAQQVllqgKE-VSAIAIPLFHNVTPELKKLVIGRIvssNIAVYLG-GHSKKTRI------
------GD()}*

$501=206(220):
>gi|157620768|gb|EX421850.1_EST1
{|5(0)|<Streptophyta(V1)>}GQ03719.B7_N22 GQ037 - Shoot tip - Dormant Picea glauca cDNA clone GQ03719_N22 5', mRNA sequence
{(QAQASLLSFSAQAIMITFRSPHLRPTLPALPIFIVEYVGRTPVRLPSRGVSNRLLEESAASIPIAIAMELG
RSRHRKFKI)-YDVSLSFKG-RDVRRGFADHLYNALTRGGLHEFLDTHQInGGEDISAsLQNAVEVSRILI--
-----AQSAWCLREAFLIAQQVllqgKE-ESTIAIPLFHSVTPELKKLVIGRIvssNIAVYLS-GHSKKTRI-
-----------GG()}*

$502=193(197):
>gi|161308964|gb|EY639862.1_EST1 {|0(61)|<Annelida(M1)>}CAWW4581.rev CAWW Capitella sp. I ECS-2004 Subtracted Late Library, Embryos >48hr after egg deposition Capitella teleta cDNA clone CAWW4581 3',
{(F)----FLSCGSSdqKRSI-EILEKLEKEGF--
KCCYPNRDFQPGMPVLKLIIEGIQNSQKVVLLVSKNFLKSSYMEFEVAEASRKMYSEKKVTLIPLLM-
DVDiVDVPTSIKAVNCIEMRKaGWFDRLIEGLKASSTVTDLITPG(DIGHGLAWSYYYGYLKLILPGLRQRVN
ESEFAKLGTPFLKKFIAILPETSICPSSF)}*

$503=164(161):
>gi|161310897|gb|EY642193.1_EST1 {|0(22)|<Annelida(M1)>}CAWW617.fwd CAWW Capitella sp. I ECS-2004 Subtracted Late Library, Embryos >48hr after egg deposition Capitella teleta cDNA clone CAWW617 5', mR
{(HALAPTLRGDH)QFHVFFVYHQNdrDWVLNVM-EKLEMPafGF--
KCCCHERDFDAQTSLKQTVHYGIKHSLKTVIVLSPDFVTETWCDSDSESQLLTDMDIALIQKDVITVMLQEcD
MPNSLMDVAWIDAAVEdWWGKLVSQLCASDDTPMTDVTNL(SGSSSLVGLN)}*

$504=246(269):
>gi|161273133|gb|EY608415.1_EST1 {|56(0)|<Annelida(M1)>}CAWU9478.fwd CAWU Capitella sp. I ECS-2004 Primary Early Library, Embryos <36 hr after egg deposition Capitella teleta cDNA clone CAWU9478 5', m
{(LNVKRKGYHEVRLKEDDSTQGEDTPVHAPKCTEDTHVVLDSRSVKDNTENKNEEFMLQHIINPKMKIFARD
LPPHLQFHV)---VFISHSNDlQWLSKVIQKA-QSPPYCFRCaCLHQDSFSKS-
SLLRSVHHSLKSASKTVLVLTRDFIRDVWLLSDMQLLSEMHVILTNPNLVLL---HTEsCDIPKALSEL----
CTiDtYnnLNW------------------
W(SELLTQLCAAADSPETEVMSMSSSFASSKPHIQSGPLLLSVGSTCGSCG)}*

$505=142(153):
>gi|161279764|gb|EY612526.1_EST1 {<Annelida(M1)>}CAWW11602.fwd CAWW Capitella sp. I ECS-2004 Subtracted Late Library, Embryos >48hr after egg deposition Capitella teleta cDNA clone CAWW11602 5', mRNA
{(PGS)------------
FVFQVLRPALEGEPaPTASLYLNGRNDFPGMAKSENVVDGMEKCGRVILLVTPEFSQDEMCEFALHMA----
LVKGINSVIILLKNWPDfASMSN---
TLRALLRPNsgvpCLEWPDDNSGqkLVLAELTEAIGAQ(RDGLQLNEIS)}*

$506=198(220):
>gi|160565005|gb|EY333845.1_EST1 {|33(0)|<Annelida(M1)>}CAWY5960.fwd CAWY Helobdella robusta Subtracted Early Library, Embryo st. 1-6 Helobdella robusta cDNA clone CAWY5960 5', mRNA sequence
{(DWLKSHVHVVRPEKMVCAYPEWHESKSLFEVDFCYSPPSNAAIISCVTVGIVVLIVAVTYVWYRLRFRSQK
IKDPPELTN)--DVFIFCSDEeqVALVKEIIRWLED-EHRFSTICGLRDF-
DSRPKADNIYEALTTSKRIIFIVSKDFLKDNWCILGTQTAFSSV--EDKRRFIVIFCDVD-
LQSANIPVELEIYIRTYtYL------------------SFT()}*

$507=222(225):
>gi|163315330|gb|FC565139.1_EST1 {<Mollusca(M1)>}CAWF5859.rev CAWF Lottia gigantea from mantle (H) Lottia gigantea cDNA clone CAWF5859 3', mRNA sequence
{(LSINVIDITEDNFKCNVQLKTYLYYLITILLLIIIFFVFLIIKCRYNAKVLLYTRLRIQYFAPPIVSEKLK
EN)--
DVFIVYDDEdiTWMKYVLLKRLEDIEKPgYKTVIKDRDFLPGLDENENVNDAISNSYCTLFIFSDNFLQNEWC
LYNFHCAYEHMLKEKDIK-IVIILKDK-IDVDKLNDDLKAYFKTHtYLEFNDTI---
FWDKLLYVLPKN(SHIIPHENYIIV)}*

$508=220(224):
>gi|160595889|gb|EY365781.1_EST1 {<Annelida(M1)>}CAXA10385.rev CAXA Helobdella robusta Subtracted Late Library, Embryo st. 7-11 Helobdella robusta cDNA clone CAXA10385 3', mRNA sequence
{(EEFCPKPSRLLISLLIPLLTGVLLLLALFVLIKKFKVELNTYLNIHLLDRDECIGENMI)-
YDAFVSCSYSdRRRGIELV-RLMEGKG-
YHVCYHEKDFIGGQSIAVNIVEAITFSKRVVCLLTSNFLKSTYCMFEFQTSLHRNIELKRKRLIVLLDESV-
EmDEDVLPKDVFNFLTTHtYIELSSNK---WTHQLFYSLPLN(PIQLKVVHQDTDYSVASDDASLITI)}*

$509=152(160):
>gi|160602622|gb|EY373723.1_EST1 {<Annelida(M1)>}CAXA14700.rev CAXA Helobdella robusta Subtracted Late Library, Embryo st. 7-11 Helobdella robusta cDNA clone CAXA14700 3', mRNA sequence
{(NIHPFDRDECTDEYME)-YDAFISCAFSdRGRAIELVSTLENR--GYKVCYPERDFIPNEP----
TTSFVSKSRRVIYLLTLDFVNTPRCLFEFQISLQRNLEVKHKRIIVLLDSSL-
KvDQKLLPNDMFNFLTTHhCIDLLKNN---WTNQLFYSLPIK(PLRN)}*

$510=219(195):
>gi|157510672|gb|EX311905.1_EST1 {<Streptophyta(V1)>}GQ02803.B7_O14 GQ028 - Cambium / phloem scrappings (Normalized library) Picea glauca cDNA clone GQ02803_O14 5', mRNA sequence
{(DQSDRGYNPNIWHTFHLYFFIVYPMANLLASSSNSSGNKA)PYKVFINHRG-
PDTKLSIAGHLYHRLHDLGLQVFLDQEEMEKGDFItsQIAAAIQAASVDIAIFSPGYADSEWCLNELLLMMES
KESGR-STIIPIFYGVEPTDLRwtlgkdgvyakaltkLKEKKTADPQThtekprydtdiIENWRKALSKV--
SYMNGFMYNGRE(LQLFVFLFFWVCFVW)}*

$511=246(248):
>gi|157514595|gb|EX315826.1_EST1
{|0(42)|<Streptophyta(V1)>}GQ02809.B7_L05 GQ028 - Cambium / phloem scrappings (Normalized library) Picea glauca cDNA clone GQ02809_L05 5', mRNA sequence
{(LQVGPNFLKLAPSLAIWQSLAIFLNSPHPLQFSETRPIPRRINHSKLSITHLAMASPTQNHSPA)-YDVFLNHRG-PDTKNGFAGHLYKSLCLHGIRVFLDKEEMEEGEEItqQIETAIKTASVHVAIFSKNYAESDWCLKELVLMLesrESG-----ATVIPIFYGVKPFSLRWTQGEDRQRLFLVSWMLTIPSFFFWLLSFFRQTEPT(YAEQLQKLKNKRVKNGERRFDGKTVAKWEKALKTAADLTGLELE)}*

$512=244(236):
>gi|157517103|gb|EX318334.1_EST1
{|0(54)|<Streptophyta(V1)>}GQ02813.B7_A17 GQ028 - Cambium / phloem scrappings (Normalized library) Picea glauca cDNA clone GQ02813_A17 5', mRNA sequence
{(LQEAMTAMERLSVPEIFQ)---VFISHRG-PDVKRTLASLIYHRLTSYGLSVFLDKDELRTGDTIslAISTAIQAASTHIVIFSPGYAESTWCLEELYWILRSGHKRD-TKIIPVFFNVEPTDPRYIKrgcyakafenyrsKGRVPIEVIQKWEIALNEA--SHISGLLFKTKE(SDYGECLEKIVKKVVEEVFKSAPLEVAKYSVGLHQAAEDFETEIMKQSDSIVVGIAGIVGVGKSTLARYLYELKRSGF)}*

$513=186(191):
>gi|157517672|gb|EX318903.1_EST1 {<Streptophyta(V1)>}GQ02813.SP6_L13 GQ028 - Cambium / phloem scrappings (Normalized library) Picea glauca cDNA clone GQ02813_L13 3', mRNA sequence
{(EGMVSNSD)-YHIFINHRG-PDVKRTFASHLYRRFQSLGYRVFLDKDELKVGENSdlQIKTVIKTVKVHIAIFSPKYAESDNCLDELNQMIESE-----APIISVFHKVKPAELRSTGGEEGWFYQVLRFLPWWTRGEDGVYAKALCNLKR(KRSYDAETHRKKPKYEPSTIEKWRNSLIHASNLTGLELAACNG)}*

$514=194(199):
>gi|157531201|gb|EX332432.1_EST1
{|0(10)|<Streptophyta(V1)>}GQ02904.B7_N19 GQ029 - Xylem scrapings - AT NITE Picea glauca cDNA clone GQ02904_N19 5', mRNA sequence
{(GGLASSQSGFHG)-YDVFINHRG-PDTKKTFSSYLYHRLLLHGFKPFLDKQELQEGLNFptQIVGAIRSASVHVAIFSPTYAKSQWCLEELLFMRESR-----APIIPVFYHVEPAELRWTDQNKNGVYARALHELQLKKTYDHQIHQEKSRYDP(ITIQNWRDALSSVADISGFDLDGAFNGDEGLMLEKVVESVLKVIKKP)}*

$515=177(187):
>gi|157531542|gb|EX332773.1_EST1
{|0(10)|<Streptophyta(V1)>}GQ02904.SP6_N19 GQ029 - Xylem scrapings - AT NITE Picea glauca cDNA clone GQ02904_N19 3', mRNA sequence
{()------NHRG-PDTKKTFSSYLYHRLLLHGFKPFLDKQELQEGLNFptQIVGAIRSASVHVAIFSPTYAKSQWCLEELLFMRESR-----APIIPVFYHVEPAELRWTDQNKNGVYARALHELQLKKTYDHQIHQEKSRYDP(ITIQNWRDALSSVADISGFDLDGAFNGDEGLMLEKVVESVLKVIKKP)}*

$516=160(154):

>gi|157537762|gb|EX338993.1_EST1 {<Streptophyta(V1)>}GQ03007.B7_I09 GQ030 - Xylem planings - daytime and seasonal Picea glauca cDNA clone GQ03007_I09 5', mRNA sequence
{(EVFSMEQMLEPGT)-YQVFINHRG-
TDVKGSLASLIYNRLAQHKLRVFLDQKEMPYGFDFatAIERAIGSASVDIVLFSERYAESTRCLHELCLILSS
shK-----RTIIPVFWDVEPTDLRYIERGCYAQafrkhetrqlkEKVKEWKQALRKA--
ADISGFAFKTRE(R)}*

$517=186(200):
>gi|157542546|gb|EX343777.1_EST2 {<Streptophyta(V1)>}GQ03101.SP6_P11 GQ031 - Xylem Scrapings (Normalized library) Picea glauca cDNA clone GQ03101_P11 3', mRNA sequence
{(SIAAHYS)-YDVFINHRG-
PDVKKTFASHLYHGLSKHGLRVFLDDPELQPGDSLtsQIKGAVRTASVHVAVFSSHYAESRWCLDELRLMVES
G-----KTIVPVFYDVKPAELRSAKGN---------YAKALKElsT--
KETIGRYGRKRK(RYDSNTMEEWRNALSCVADISGFELEKCNGDEGELVENVVRCVLKKWKRPRCM)}*

$518=193(198):
>gi|157546626|gb|EX347857.1_EST1 {<Streptophyta(V1)>}GQ03105.B7_H14 GQ031 - Xylem Scrapings (Normalized library) Picea glauca cDNA clone GQ03105_H14 5', mRNA sequence
{(NIDDASAASCSTPRNNDNAAAAAFSSTSPSTSYSR)-YDIFLNHRG-
PDVKATFATKLYRRLISSGYQVFLDKPELEEGHPItsQIEAAIQVAHVHVAIFSPTYAESNWCLDELVLMVES
K-----
AIIIPVFYNVKPDVLRWTAQKGAYAEALLKLQQKTTDDLETGEKKPRYDSQT(IKNWRKALSDVADISGFELD
ERV)}*

$519=231(211):
>gi|157576184|gb|EX377412.1_EST1 {<Streptophyta(V1)>}GQ03234.B7_J10 GQ032 - Shoot tip (Normalized library) Picea glauca cDNA clone GQ03234_J10 5', mRNA sequence
{(DLPDEDDENFEVERVFPICTTYLQSYFFFIVYPMASSSTSTSHGNNDNYVF)--DVFINHRG-
PDVKQSLATDLYNSLCKHDLRVFLDQRELQEGEYItpQIDSAVRTASVHIAIFSPGYAYSKWCLDELVLMVEt
mskSK-----STIIPVFYGVEPAELRWTQSKngvyaralcvlekkitfdgqqRHDSSAIEKWRNALSEV--
AEIKGFdlkTYDLET(YNGSTMDTPSCRRSQTRLGT)}*

$520=196(220):
>gi|157578993|gb|EX380221.1_EST1 {|95(0)|<Streptophyta(V1)>}GQ03301.SP6_E22 GQ033 - Terminal leader (Normalized library) Picea glauca cDNA clone GQ03301_E22 3', mRNA sequence
{(ITSRDKNVLARSGVQESSIYKLNGLNTEHSRELFCSYAFCQPHPLSGFEHMVDKFLRACHGLPLSLKVFGA
LLYGNNNYC)-YDVFINHRG-
PDVKKTFASHLYRRLISYGLRVFLDYQELQEGEDLtcQIQGAIRSASVHVAIFSRTYAESNWCLDELLLMLES
R-----APIIPVFYHVRPAEIRWTQGEGVYARSL-------------------HNLQK()}*

$521=148(158):
>gi|157584372|gb|EX385600.1_EST1 {<Streptophyta(V1)>}GQ03309.B7_B06 GQ033 - Terminal leader (Normalized library) Picea glauca cDNA clone GQ03309_B06 5', mRNA sequence
{(FYFWYQMASLDRDRAAPK)-YDIFINYlyED-VDVKRKFVSHIYHRLLPHGLRVFMEKEE-----
TTQIDEVIRTAPIHIAIFSPGYQNSPRCLNELLLMSEpST-----SSIIPVFYKVRPGQIR-
WAMNPEMYVGTHGGDLVKRKRSTKKKKGKMGGCSF()}*

$522=167(176):
>gi|157607323|gb|EX408547.1_EST1 {<Streptophyta(V1)>}GQ03602.B7_K16 GQ036 - Shoot tip - Active growth Picea glauca cDNA clone GQ03602_K16 5', mRNA sequence
{(FYFWYQMASLDRDRAAPK)-YDIFINYlyED-VDVKRKFVSHIYHRLLPHGLRVFMEKEE-----
TTQIDEVIRTAPIHIAIFSPGYQNSPRCLNELLLMSEpST-----
SSIIPVFYKVRPGQIRWAMNPEMYVGTHGGDLVKRKRSTKKKREKWVGALSD(VTGINGFVLEHCNGDEGK)}
*

$523=242(246):
>gi|157612694|gb|EX413918.1_EST1
{|0(35)|<Streptophyta(V1)>}GQ03617.B7_E14 GQ036 - Shoot tip - Active growth Picea glauca cDNA clone GQ03617_E14 5', mRNA sequence
{(RFSLQFFPEFLLFLASNILSIISISILQSAMAESFSTAASTSTAPNSD)-YDVFINHRG-
PDVKKTFASHLYRRLHSYGLRVFLDQPELQQGDYLtpQIEGAIESASVHVAIFSSGYAESTWCLKELVLMLES
N-----
SPIIPVFYHVKPAELRWAQGGYaRALRELEKKKASDPQSREEKLRYESSMIEN(WRDALFKVAGISGFELEAC
NGDEGELLDKVVEGVLKKVKKPALDVAKHPTGLDDKVND)}*

$524=218(223):
>gi|157616242|gb|EX417324.1_EST1
{|0(23)|<Streptophyta(V1)>}GQ03707.B7_I11 GQ037 - Shoot tip - Dormant Picea glauca cDNA clone GQ03707_I11 5', mRNA sequence
{(ARIPNSAERIGSCFFIILYPMALTTSFASSSTSHSPGHV)-YDVFISHRG-
PDVKKGIASHIYHRLKDLGLAVFLDQKELQGGENLnlQLKGAIQTASVHIAIFSPGYANSRWCLDELLLMVDS
R-----
STIIPVFYNVQPSDLRWTRGGDRVYARALSIFLCILLSILLCIPTSFLLRLL(LCVPFSIIMCILFCTRGKNG
VYARALHELQNKTTVDSATMKKKP)}*

$525=227(232):
>gi|157618190|gb|EX419272.1_EST1
{|0(49)|<Streptophyta(V1)>}GQ03712.B7_M03 GQ037 - Shoot tip - Dormant Picea glauca cDNA clone GQ03712_M03 5', mRNA sequence
{(KTSWIFCFFFLILYPMAHVNLTADRDAPSFSTTPSQGNTNSYV)-YDVFINHRG-
TDVNKTLASHIYLQLKQQGLAVFLDQQELQEGENMtsQIEGAIRTASVHVAIFSPGYAESSWCLKELVQMLAS
G-----
STIIPIFYKVEPSDLMWTHAGEDYARTLNILLCMLVLFIPLSIFVRILLFIP(LSIILWTLGENGVYARALRT
LQKKTTFDPQTNKKKTLYDSDTIEKWRKA)}*

$526=214(220):
>gi|157622547|gb|EX423629.1_EST1 {<Streptophyta(V1)>}GQ03805.B7_M15 GQ038 - Stem - Active growth Picea glauca cDNA clone GQ03805_M15 5', mRNA sequence
{(LFLAWAIGPTILHSVSISIFPSAMAASSSTAVNPDENYYS)--DVFINYRG-
PDVKKTFANYLYRRLLSHGLSVFLDQQEFQQGENLtcQIEGAIRSAFVHVTIFFAGNAHSKWCLNELLLMLDS
K-----
AYIIPVFYNVKPVDLRWTRGKDGVYAQALEKLEKKRTYALSLVAEEISGFEL(EACNGHERELLDKVVERVTK
KVKQPDLDVAKYPSMDEKIM)}*

$527=217(220):
>gi|158961766|gb|EX920251.1_EST1 {|26(0)|<Streptophyta(V1)>}lj40e09.g7 Cycas sporophyll (w/o ovule) (NYBG) Cycas rumphii cDNA 3', mRNA sequence
{(SSRDNDQNEKKIAPSSTTQLPSTGSATGGIGNFYTFLISSSVFWVFLRLGLPRLLNFFNSNSTHRSPSPPP
ASATADASP)PYDVCLSCHG-

SAVKKTLVSTIYYFIESKGLRVLFDKEEFEAADGLhpAIERAISGAYVQIPVFSRQYVESSDCLDELWLMLKS
G-----AKIIPVFYDIRPSDLRrigkwkgLVADAFAKHENSRRFSK------QRLHGWKAALRRV()}*

$528=255(264):
>gi|160599693|gb|EY368078.1_EST1 {|5(7)|<Annelida(M1)>}CAXA11604.fwd
CAXA Helobdella robusta Subtracted Late Library, Embryo st. 7-11
Helobdella robusta cDNA clone CAXA11604 5', mRNA sequence
{(SPWFSNDDVKKFATQSDPCMSLLDDWFACHTTREATHAVMRELKEMGRMDAYAVVERVVLNAEEVAGKDED
EEEASVQPV)---VFLSYQW-G--
KQDEVKLLKRHLEMSGYPCWMDVGQMGGGDKLfqKIDQGMRKVKVVVCCITQKYAESPNCNREVNLALNLN--
---
KTMIPLLMDKMEWPPRGGMGPIFGEFLFIRFYKRDKDEIMDGTYWPSSMFTQ(LLFQIRYQVAPLESLITEDS
PYKQWWVPKDQTMXNGGKKSDSNL)}*

$529=207(216):
>gi|160599878|gb|EY369134.1_EST1 {|0(61)|<Annelida(M1)>}CAXA12197.fwd
CAXA Helobdella robusta Subtracted Late Library, Embryo st. 7-11
Helobdella robusta cDNA clone CAXA12197 5', mRNA sequence
{(YFLIPFFENDHTSTEEVAGKDEDEEEASVQPV)---VFLSYQW-G--
KQDEVKLLKRHLEMSGYPCWMDVGQMGGGDKLfqKIDQGMRKVKVVVCCITQKYAESPNCNREVNLALNLN--
---
KTMIPLLMDKMEWPPRGGMGPIFGEFLFIRCLCLINFVIDLGISFCFLDCFF(HKFISVSIYIYVYVHRFYKR
DKDEIMDGTYWPSSMFTQLLFQIR)}*

$530=194(203):
>gi|161223211|gb|EY554614.1_EST2 {|0(9)|<Annelida(M1)>}CAWT2288.rev CAWT
Capitella sp. I ECS-2004 Primary Late Library, Embryos >48hr after egg
deposition Capitella teleta cDNA clone CAWT2288 3', mRNA
{(DTIQSANTSTNKASQPEASITAASPQ)---VFLSYQW-G--
RQSEVEVLYNKLTRLGYTCWMDIHQMGGGDSLydKIDRGIRGCCVTVSCVTPKYAMSANCRKEVTLAEALK--
---
KPIIPLMLDTIAWPPEGPMSMVLAPLAFIDCREEVNTWNGHGFQTLLKQISK(QMKPARTLGWPNLDQVNTKP
FDAKKQWRKVKNVGTVV)}*

$531=198(204):
>gi|161223212|gb|EY554615.1_EST1 {|0(97)|<Annelida(M1)>}CAWT2288.fwd
CAWT Capitella sp. I ECS-2004 Primary Late Library, Embryos >48hr after
egg deposition Capitella teleta cDNA clone CAWT2288 5', mRN
{(DVKKAAEEAENVVSDDTEMDVTN)PPPVFLSYQW-GHQEE--
AKMLKEHLEAAGFRCWMDLGQMGGGDKLfaKIDSGMRSAAVVICCVTHKYAKSDNCNREVNLAVTVG-----
KPIIPLLMEKMPWPPEGAMGPIFGEYLFIRFFARAKETVSPPEVFWPQAKLR(ELLYQLRLHIIPEEKLISKE
YEAWWLPPDQKPKPKIASIKP)}*

$532=196(205):
>gi|163052240|dbj|DC469633.1_EST3 {<Opisthokonta(E1)>}DC469633 Full
length cDNA Library, Monosiga ovata, MOC Monosiga ovata cDNA clone MOC-
028J12 3', mRNA sequence
{(LCHAADALTDAMAINAPITASESAAEQGSGPIGLSPSSGSAPDAIASAALHASKGDGASAHRA)---
VFISYCW-ANKSQVLQ--
LKQALEREGLACWMDTDVMRGGQNLfaEIDAGISSARVVVACLSSNYEMSTNCQREIQLAMDRQ-----
KLVVPVWVAELETWPPRGVMAPLLAGKLYINLSSKENFLTNLECLVTAVRDS(VS)}*

$533=203(192):

>gi|163081330|dbj|DC502671.1_EST2 {<Opisthokonta(E1)>}DC502671 Full length cDNA Library, Monosiga ovata, MOC Monosiga ovata cDNA clone MOC-076F04 5', mRNA sequence
{(PRTRWICIPRASSPCAAARWCASPTRKT)--DVFISYCW-
SNSytarqfnqvpalmgreqadPRVIANALQDGAK---
LKVWLDVKTMSQGGQLfeHIAAGLQNTRQAVVFISDEYAKSDNCRMELQFAAKSlK-----
KPIIPIICGTGDEWRQTVAGMLIQGLPTEPLDMRTAGTSADAVRFVLANLKD(MESQPPPYVAPKFSAPKKGD
HVLA)}*

$534=208(212):
>gi|161275252|gb|EY608920.1_EST1 {|0(101)|<Annelida(M1)>}CAWU9723.fwd CAWU Capitella sp. I ECS-2004 Primary Early Library, Embryos <36 hr after egg deposition Capitella teleta cDNA clone CAWU9723 5',
{(SGIENAPLVIPEPKVI)-YDVYFMKTTPdEPLFHLVLSVLSKLKSNLKFRTLA-
DVAVEDDTWKHDVHLVMQRCRRiVPLLTTEFLKDLSCVDQYNVALCCNRTTQRNILAPICFERM--
TLPTYMSMVQWINCRD--
RQPKQISSACEMLLEQTKKDK(SDERRRYDVFISYSHRNTDAAKIIHSSLLAIDKNLSIFFDNEELKSGDNWQ
KTLYE)}*

$535=229(229):
>gi|161275252|gb|EY608920.1_EST1 {|80(0)|<Annelida(M1)>}CAWU9723.fwd CAWU Capitella sp. I ECS-2004 Primary Early Library, Embryos <36 hr after egg deposition Capitella teleta cDNA clone CAWU9723 5', m
{(EFLKDLSCVDQYNVALCCNRTTQRNILAPICFERMTLPTYMSMVQWINCRDRQPKQISSACEMLLEQTKKD
KSDERRRYD)---VFISYSHRnTDAAKII--
HSSLLAIDKNLSIFFDNEeLKSGDNWQkTLYESIDSSRCMLALLSEDYFRSDVC-NeEFSIAMALHfaRE-
KNLELIVVLQDDA-ASVDSLTCLHPIRCSEKnfSDVAKANSAKICDWLXNGIATDI(LSPXESALC)}*

$536=154(157):
>gi|160911992|gb|EY442043.1_EST3 {<Annelida(M1)>}CBAY531.rev CBAY Tubifex tubifex Embryos and juveniles (L) Tubifex tubifex cDNA clone CBAY531 3', mRNA sequence
{(SYQWGIQEK)-------------------VLELEKRLANEGYSCWMDKHVMGGGDELyaEIDSGLRG-
AKVIICCLTDEYAASPACNsEVVEAATFRG-VDKKVIIPLMFAKS-
NWPpvgnmgPIMTKYVYIPFYketepdESWwqEDRFEELLVrLRKHKIPKTTPPG(PQGTCWQS)}*

$537=226(217):
>gi|163086170|dbj|DC509176.1_EST1 {|0(6)|<Opisthokonta(E1)>}DC509176 Full length cDNA Library, Monosiga ovata, MOC Monosiga ovata cDNA clone MOC-085L12 5', mRNA sequence
{(KKKKKKTPPKMAYKKNEDLVFMSDDRRAFTSFLEQTHLMLRARGL)RKKAFISYAWEsdpkanEVLQIWLK
RLQGDLQRAGIE-VFLDVADM--
TGDMaaRMRENIASSDVALVIGTPRflqraSDASTNVAFEVRNIIEGHAGGrlAVLPLVRVGTA---
ATSLVPELHRFPLRDLTTDDTSKYEKLLVEPGQQPQALLP(AILGLADTDKQWAALLAELEETEKMWRGELSE
)}*

$538=225(219):
>gi|163095509|dbj|DC514519.1_EST1 {|0(26)|<Opisthokonta(E1)>}DC514519 Full length cDNA Library, Monosiga ovata, MOC Monosiga ovata cDNA clone MOC-093G19 5', mRNA sequence
{(KKKKKTPPKMAYKKNEDLVFMSDDRRAFTSFLEQTHLMLRARGLKKK)---
AFISYAWEsdpkanEVLQIWLKRLQGDLQRAGIE-VFLDVADM--
TGDMaaRMRENIASSDVALVIGTPRflqraSDASTNVAFEVRNIIEGHAGGrlAVLPLVRVGTA---

ATSLVPELHRFPLRDLTTDDTSKYEKLLVEPGQQPQALLP(AILGLADTDKQWAALLAELEETERMWRGELSE
)}*

$539=207(228):
>gi|567194241|ref|XP_006405694.1|
{|0(351)|<Streptophyta(V)>}hypothetical protein EUTSA_v10027706mg
[Eutrema salsugineum] >gi|557106832|gb|ESQ47147.1| hypothetical protein
EUTSA_v10027706mg [Eutrema s
{(MASSRTWR)-YRVFPSFHG-PDIRKTFLTHLRKQFNSNGITMFDDQGIERSKF-
IasELIRAIIESRISIVILSKNYGSSSWCLNELVEILEC-----KNIVMPIFFQV--------------
VHPISCAGKTKEEE-
QRWSQALTDVANI(AGLHLLNWENEADMIEKIAKDISDKLNATPSRDFDGMVGLEVHIRELSSLLNLDCDQVM
MVGICGPAGIGKTTIARALER)}*

$540=206(225):
>gi|567167746|ref|XP_006398183.1|
{|0(394)|<Streptophyta(V)>}hypothetical protein EUTSA_v10001191mg,
partial [Eutrema salsugineum] >gi|557099272|gb|ESQ39636.1| hypothetical
protein EUTSA_v10001191mg [
{(MDKNEETE)-YPVHINYNE-NDSSVSpFISHLIPAIRRKVTSSF-----------YgfTLL---------
LVIFSRDYAYSVSCLEKLVKALESSRsdE-NSYVVVPVFYGVSRLAVKQQLATFSDAFTEHRRSYPADQV-
TKWRRALKEAAEF(LGHEYNDESSEESEFLEKIVEYVFEILYPTKEIGIYSRQMEIEDLLCKQAWGLRTLGIF
GEPHIGKTVLARAVFGRM)}*

$541=205(226):
>gi|567167818|ref|XP_006398201.1|
{|0(468)|<Streptophyta(V)>}hypothetical protein EUTSA_v10001152mg,
partial [Eutrema salsugineum] >gi|557099290|gb|ESQ39654.1| hypothetical
protein EUTSA_v10001152mg [
{(MAARNK)---VKISFdrSD-ETIRWSLVPHLSAAFGRKGISVLTDKHDE-----------
FSMSIASVLIFSKNYASSKESLDDFLKTIQRRHD-KGHVVTTVFCGVSRSNV---KANFAKALFEH---
WTSYQA-
SQWCNALEEIASL(PGHEDINFKSDCEFVEKIARDVYEKIFPKERIGVYSRMLPDIENLLCKQQWGVKTIGIW
GMPGIGKTTLANAVFDQMSGD)}*

$542=201(231):
>gi|567193711|ref|XP_006405542.1|
{|0(110)|<Streptophyta(V)>}hypothetical protein EUTSA_v10028295mg
[Eutrema salsugineum] >gi|557106680|gb|ESQ46995.1| hypothetical protein
EUTSA_v10028295mg [Eutrema s
{(MSSSSSLFAER)KVDVFLSFCN-TTT-----SH----------AIFK--EKTRVRP-
IdkQTLEALEESKVAVVMTSETKLCSVGFLEELIVILEFQER-GSLTVIPIFLTA-----------
FSFDVEEEIYQEYPENA-
PSWRIALTKLANI(AADSPSLSPSLAGMGQSDFLEQIAHIIDFLCLSSTSNDLNGLVAMDRHLKVVHDLLASQ
VNKEVSTIGIWGRTGVGKTTL)}*

$543=202(230):
>gi|567221320|ref|XP_006414289.1|
{|0(291)|<Streptophyta(V)>}hypothetical protein EUTSA_v10026940mg,
partial [Eutrema salsugineum] >gi|557115459|gb|ESQ55742.1| hypothetical
protein EUTSA_v10026940mg [
{(MASSSSRSWR)-YDVFPSFSG-EVVRKSFLSHLLKELDRKSINAFIDDGIERSRP-
IgpELLSAIRESRISIVVFSKSYASST-CVDP--------SE-----------------
VRKHTGEFGKVFKETCDGKTEDQK-

QRWMQALVDVANM(AGEDLRNWCNEASMIEKIADDVSNKLITTSDCYGDLVGIEAHLEAMSSILCLESEEARM
VGILGPSGIGKSTIGRALFKN)}*

$544=268(296):
>gi|567220684|ref|XP_006413971.1|
{|141(682)|<Streptophyta(V)>}hypothetical protein EUTSA_v10027276mg, partial [Eutrema salsugineum] >gi|557115141|gb|ESQ55424.1| hypothetical protein EUTSA_v10027276mg
{(QPPLNLSECKADDVIDFLRMQQASGDVEAIVDHLSTKCIKLKENPFRSLAVTRYVEGVTRESRCILVFFCN
DSHDVDDSH)-----------------FMKTILKELREREVTPLTYNISRRENL---
DTELLDRSRVGILVLSNNYACSSESLDHLVAIMEHWKA-N-----PVYFRVTLSNI-
ELEDPFEAVQLQCLNPIQADRV-
QNWKEAMAEPSPS(GFGRHHRPSMQLGDNIDGFPLIDDAAARPNKFHESLDDIESHSIGNLLPDEEDILTGMM
DDLVFTELPDADDYDLF)}*

$545=207(213):
>gi|567175108|ref|XP_006400333.1|
{|0(972)|<Streptophyta(V)>}hypothetical protein EUTSA_v10012485mg [Eutrema salsugineum] >gi|557101423|gb|ESQ41786.1| hypothetical protein EUTSA_v10012485mg [Eutrema s
{(MREMDYMKQAGARVTVTPPMMADYNHSVNIICE)-----------
ESVQYSIVSHLAAALRREGISVFVDScKLQETKSFSSV-
EQNEavtdgaNPRVSVVVISNMVELYDPWVVKFIKVIRRRRN-
SGHVVVPVFYGVDPLTRVYGWANRRLEAEKLTTHQSRISSNRILTDSELVEEIV(RDVYGKLNPAERVGIYSR
LLEIENLLYKQPWDIRRIGIWG)}*

$546=225(228):
>gi|567167722|ref|XP_006398177.1|
{|0(457)|<Streptophyta(V)>}hypothetical protein EUTSA_v10001238mg, partial [Eutrema salsugineum] >gi|557099266|gb|ESQ39630.1| hypothetical protein EUTSA_v10001238mg [
{(METLSPNQ)-HNVFIGFSM-KDTRYSFVSHLSAAFhRRN-ISTFLGEEDI-----
IssVTQSAIEGAKVFVVVFSENYAFSPLSLETLAKFLDLRRRENGPVVIPVFYgDVTPSIVEQQTEKFGKAFS
EH-
RSSFSGEDDkvERWRNGLVEAAKL(QGHDSNEQQNDSDLVEEIVSDVRERLYPTGKIGIYLRLLGIENLLCKQ
TPDIYRIGIWGMPGIGKTTIAQAAFNQMCQDF)}*

$547=175(187):
>gi|567878415|ref|XP_006431766.1|
{|0(463)|<Streptophyta(V)>}hypothetical protein CICLE_v10003578mg, partial [Citrus clementina] >gi|557533888|gb|ESR45006.1| hypothetical protein CICLE_v10003578mg [Ci
{(MKFSNQRVSSSVPSPSSYW)KYDVFLSFRG-KDTSNHFA-----------
FKIFEDHKELERGKAIspGLFKAIEESRFSVVVFSRNYAYSTWCLDELAKIVHSKNT-
SSAQMRHALTEVANPSGWHLKDRHEVEFIQE-
IVKEISRKKGPRTLGILDDLVE(MNSRLKKLRLLLDAESRDVRMIGICGMG)}*

$548=166(168):
>gi|567898374|ref|XP_006441675.1|
{|0(539)|<Streptophyta(V)>}hypothetical protein CICLE_v10018973mg [Citrus clementina] >gi|557543937|gb|ESR54915.1| hypothetical protein CICLE_v10018973mg [Citrus clem

{(MASSSSSRPSSSLSSMNNPRNN)KYDVFLSFRG-EETRDSFTSHLYSALcETN-IETFIDDQ-
LVRGNEIsqSLLDAIEASSISVIVFSEGCKVGGFVRSWFDNYLDWLEY-
SIVKDAAFCLYCYVFREENGSQGAGPCFTGE-GFRNWKKKEMLRQHVGGVNSAH(NIARRR)}*

$549=155(157):
>gi|567898854|ref|XP_006441915.1|
{|0(460)|<Streptophyta(V)>}hypothetical protein CICLE_v10023434mg,
partial [Citrus clementina] >gi|557544177|gb|ESR55155.1| hypothetical
protein CICLE_v10023434mg [Ci
{(MASSSRNNK)KYDVFVSFRR-EDTRDNFTSHLCSALcQQN-IQTFIDDQ-
LNRGDEIleSLANAIEVLAISVIVFSEGYDGLDDKEKNIFLDVACFFQ-
GEDVNLVMNFLNASGFYPEIGISVLVDKSLI-LISNNNKITMHDLKQEFGQEII(REESINPE)}*

$550=167(161):
>gi|567163727|ref|XP_006397133.1|
{|0(544)|<Streptophyta(V)>}hypothetical protein EUTSA_v10029401mg
[Eutrema salsugineum] >gi|557098150|gb|ESQ38586.1| hypothetical protein
EUTSA_v10029401mg [Eutrema s
{(MAESNRKIRERWS)-YDVFLSFRG-
SDVPDGFIAFLYVSLKLSGIYTFKDDQELRLGERgglgesIspELLKAIENSKFSLELMCFGSDDVQIIGIGG
MGGIGKMMLDKAAYNNYARQIEGTSFIENFGEYVKKLEWKVHIRQTLLSDILRRHKTVFNNTDH(AVKHRFRN
)}*

$551=136(159):
>gi|567862228|ref|XP_006423768.1| {<Streptophyta(V)>}hypothetical
protein CICLE_v10030396mg, partial [Citrus clementina]
>gi|557525702|gb|ESR37008.1| hypothetical protein CICLE_v10030396mg
[Citrus cle
{(MASTFTKVV)-HQVFLSFRG-EDTRNSFTNHLSQALVYTGIHTFKDDIELRRGEDI---------------
------YSERCFGEIVKTGNCKKHNNvRQTVLPVFFDISPSDVRNQNENFAEAFVKH-
EARFMLEKVKKWRAALTRAANL(SGWALHEAPK)}*

$552=234(232):
>gi|567167563|ref|XP_006398127.1| {|0(35)|<Streptophyta(V)>}hypothetical
protein EUTSA_v10001124mg [Eutrema salsugineum]
>gi|557099216|gb|ESQ39580.1| hypothetical protein EUTSA_v10001124mg
[Eutrema sa
{(MVDPTRARARARP)-PQVFINSGT-
TELPDDFMRHLVWGLSELGIlNIFIDRDEWWGRDLGHVFTCIEESTIALAIFSSCYPETEWCLDELVKMKERV
NE-
NRLFVIPVFYNVSKNDVRNLEGEFGDRFMEMRQKYveYKYDPFraQRWETSVTSISRM(EPSLTWEAHSSNIP
LASNIVKEVKRELDKALNPESSFPFELSNDSNSKMAIREVFVSAFLAAFLFSLFIARLVFTDVKF)}*

$553=174(190):
>gi|567167752|ref|XP_006398185.1|
{|0(881)|<Streptophyta(V)>}hypothetical protein EUTSA_v10001165mg,
partial [Eutrema salsugineum] >gi|557099274|gb|ESQ39638.1| hypothetical
protein EUTSA_v10001165mg [
{(MTDWKKEEQY)---VYISCV--DVARYSFVSHLSDALRREGISVFVDGDDVLSKELQE---
KVQGARVSVMVLPGNRT---VCLDKLVKVSEKA-----
QALVPVLYGVIPLNEEWLVKLDSRGLSPVHQSRKECSDSKLVEEIVRDVNER(LLHMGRIGIDSKVVDLENMI
NKQPLGIRCVGIWGMPGIGK)}*

$554=131(161):

>gi|567167574|ref|XP_006398130.1| {<Streptophyta(V)>}hypothetical protein EUTSA_v10001186mg [Eutrema salsugineum]
>gi|557099219|gb|ESQ39583.1| hypothetical protein EUTSA_v10001186mg [Eutrema salsugine
{(MSVSSSQAGDNR)-PQVFINYRK-EELRKTFIKSLLPELKRGRIKFFIDDNEEK---------------------------ESRWCLDELHKMKKLAEG-NKLVVIPVFVNVTTTDVKHFNGEFGKNFREM-CKKYVGQKVRKWREAVEYIADI(IGEVWDNLG)}*

$555=231(242):
>gi|567167589|ref|XP_006398135.1| {|0(37)|<Streptophyta(V)>}hypothetical protein EUTSA_v10000995mg [Eutrema salsugineum]
>gi|557099224|gb|ESQ39588.1| hypothetical protein EUTSA_v10000995mg [Eutrema sa
{(MEAGRLMSVLTSPPILFSSWGY)-------YRK-EELRDNFIPDLLRALKRDNIKFFIDDNLEKGRPIDDLLKIIENSKIVLALFSKRYPESKYCLDELHKMKELVVD-RKIFVIPVFVNVTTRQVKKFEGVFGKKFEEM--RAEHPHKINDWKEAVELISQR(SGVVWSDARLPSPGHPRYPQPCPKTDKYVVRETVKAIEEELIQSYGRAPKESNDGPFANFMASIIMAFGSGILLPRMLFT)}*

$556=202(220):
>gi|567187757|ref|XP_006403904.1| {|0(1021)|<Streptophyta(V)>}hypothetical protein EUTSA_v10010075mg [Eutrema salsugineum] >gi|557105023|gb|ESQ45357.1| hypothetical protein EUTSA_v10010075mg [Eutrema
{(MVESSD)---VYISFDRWeDTVRYSFISHLAAAFHRKGISSFVG-----GSDPEsNGFARvaMEKSRACVVVFSGKYSSSKPCLEELVNVSERRRN-NGLAVVPVFFPVTKLFVKKQ-------IWNL------GHVRSEWQSALLETAEL(PGHQLYDKQSDSEFVQEIVAHVREKLKITKNIGIYSKLLMIENLLCKQPWGGVRSIGLWGMPGIGKTTLAKAIF)}*

$557=232(249):
>gi|567162612|ref|XP_006396807.1| {|180(565)|<Streptophyta(V)>}hypothetical protein EUTSA_v10028393mg [Eutrema salsugineum] >gi|557097824|gb|ESQ38260.1| hypothetical protein EUTSA_v10028393mg [Eutrema
{(LSVHKDSHKIKRRHKVIHTTVSDFMNVVQRLTGDVRFPARPSMLMQEDELNIGFEESQSLYVDDGPFADASDVSAAIIKD)-YDVVIRYRR-GDISNeDFISHLRAAFCRRGISVNEEFHEV---------DAVPECRVLIIFLTSTYVPS-----NLLNILKHQRK-EKYLMSFHWSESELIDELVRDALKVLYSNDN-ELMIGMDTQVDEILSLLCIDSP(DVRSIGIWGTVGIGKTAIAEEIFRRISVQ)}*

$558=300(300):
>gi|567219836|ref|XP_006413547.1| {|262(38)|<Streptophyta(V)>}hypothetical protein EUTSA_v10027371mg, partial [Eutrema salsugineum] >gi|557114717|gb|ESQ55000.1| hypothetical protein EUTSA_v10027371mg
{(GSLKDYNKGFAFKPLLFPKKPQQFIGNKLIVSSQDIQDQRNSLQNLPDEDNNFDDVFTCFSLLCNFIKGFLMKISSPPSR)--RVSISFGE-KQLRENLVSFLKTELESNRISVCVEDEM---------KKRIKESKVAIIIFSAKYPESQHCLDELVEIKKLMDT-GEIDPFPIFYKLKAESVKVIKGWFRNRLLKIeekvRKNVNRGNDNsildteariWGWREALASLVSR(PGLSYQHSSDSLFVTDVVTKVKALFTFRERQKSSVSYMNTDLIVEKPLMHRHETEAATTAVKCLGDVLFHSLTSFLEALN)}*

$559=284(294):

>gi|674562939|emb|CDS22573.1|
{|193(17)|<Platyhelminthes(M)>}hypothetical protein EgrG_001173000
[Echinococcus granulosus]
{(RRGAIEDMVEDYKSFKSWILERGNLLPEVKAISPVDRRIKTALWMQSIFQRLQWEIHADDELHDRQRMGKV
EEKERAHVM)-----LSYSYVQKNIAHKIARAVSESGYR-
IWLDTEHMHTEPDIMDAIAIAVKRAFAVCILYSEAY-KRCPNTRmEAQYAQKLD-----
KPMIFCRVEQgyLPdgWLGVLTARRTCIDFSGK-FTFEDASS--
ELLHRLANIKMS(VRDNKAEIYDKSLVTYQTNEISLWDMEKVNEWFMNQGLPIVYRENISGKDLLFLKKMQSS
APESFFRFLSETWK)}*

$560=205(215):
>gi|557037748|gb|ESQ14735.1| {|0(56)|<Proteobacteria(B)>}hypothetical
protein N838_10910 [uncultured Thiohalocapsa sp. PB-PSB1]
{(MAH)---IFISHSSRDREFVvDQLKPALEAHDFT-
AWCSSTDLKSAANWEQQIRAALASSDWFIVVLSPDAQRSEWVQAeTHWALENLS-----
GRVIPVMVRNCRpiDLHLRLGTLQFVN-FQ--IDFQMAMR--
ELLALLQIHPDD(AIIRGGLLTAADGSEPTTLVLQSHQVSLLIRIVRPSAPEYQDHLQVHNSATIGRSPQAHL
QIDDRCVSRRHA)}*

$561=168(165):
>gi|557036403|gb|ESQ13661.1| {<Proteobacteria(B)>}hypothetical protein
N838_14430 [uncultured Thiohalocapsa sp. PB-PSB1]
{(MPRDLRY)----
FVSYAHQdKRQARRLLDLIEPrlaISRAFAFTGWIDDDIGLGSRWTdAIDDALADCDFGLLLLSPAFFASGFI
RRdELPTFIAhvRRPG-GGNRtrlrkpAIPVLLKPV-PLDgSADLAGLEQLQY-----FSDADG-
RAFNNTSGHISER(FADQLVAAMLRRLATCFP)}*

$562=191(220):
>gi|558847504|gb|AGJ74273.2| {|677(0)|<Chordata(M)>}Toll-like receptor 3
[Barilius bendelisis]
{(CNTPNAYFNRSVKDFDPLSCKDMTPFKVLYVFSSTAVLMLLFTAFLVHFQGWRIQFFWSIMVNRMLGSLKD
DSVTENRYL)-YDAYIVHSEEdkQWVERSLLPLE--DEKF-
NFFLEERDSTPGFSQLDTIVENMGQSRKIIFVITEMLLKDPWCRRfKAHHALHQVMEDNRDSLILIFLQDVTD
YSLNRSLHLRR----------------------------()}*

$563=196(191):
>gi|926405145|ref|WP_053732894.1| {|0(381)|<Actinobacteria(B)>}TIR
domain-containing protein [Nocardia sp. NRRL S-836]
>gi|557361911|gb|AGZ94470.1| hypothetical protein [Lechevalieria sp. NRRL
S-836]
{(MKGQQMTEPESHDG)-WDVFISYAREDyqkaRDLADGLHKCITSRGVP-
PRIfidvDREGFPPGVDWEEYLETALPRSRYFVALYSLRYFTKNVCQFeLQRAVdlnRAGR-----
IGFIPVLMEEAAkdKIWFTASKYNWVAVTH--
PNWFETLRTALGLRPARNRRQL(RFEGPPPSAVVNHTLPTVRVSVIGTDGTTVLESSEPI)}*

$564=231(232):
>gi|557869682|gb|ESS72700.1| {|233(0)|<Proteobacteria(B)>}hypothetical
protein MGMO_50c00160 [Methyloglobulus morosus KoM1]
{(LGRTMLRLVNDNRFYAKDTQEFLCPSVSDAGLSSFLTQYSPNTLGFEGQPEALILKLANRFNRAPLPQPQK
LQSNNQLAP)--TAFVSYAS--ENAEP-
ARQIADILLNLGFRdVWLDKKKLIGGDDWsdRIDEAIIKSDFFIPILSQQADSRREGVfWdEWKTALERARRI
kD-AYLLPVCIDKE-PPSKSRYRRISDGDTAVFFDKHLIHAPNGELRPEDRDALS(ERCRRFLEDSHG)}*

$565=213(230):

>gi|593263958|ref|XP_007134157.1|
{|0(439)|<Streptophyta(V)>}hypothetical protein PHAVU_010G024100g [Phaseolus vulgaris] >gi|561007202|gb|ESW06151.1| hypothetical protein PHAVU_010G024100g [Phaseolus
{(MEKKSKGNLC)-----------------LISILPSLLLASPLSFTTPNEDNPIPF---
QQPILNLCRVAIVVFTKFYSQSAWCLHQLQQIIKWHET-
YCRHVLPVYYEIQPSDVRLQKGDFGKAFKATAHQTFSGQelehgM-
SRWSHALIKAANL(FGWDESNYRSDAELVHKIVKTVLHLPVLSATQFPVGLESRVKDVIRIIKNRSTQVFTIA
ICGKGGSGKTTLAKAIYNQIQ)}*

$566=221(221):
>gi|551014647|ref|WP_022758995.1| {|31(0)|<Firmicutes(B)>}molecular chaperone Tir [Butyrivibrio fibrisolvens]
{(DSCIDPSEDWINDVEIFYDKYLKNHALGSRIETILFHRTSSAYTELVSCLRSISRDQDFINKMNGVSTVEV
PKYKAKTLP)EYDVFISHAN-KD-
KLSFVDELNESLKKLGVSIFYDKESLEWGDNWkeRILDGTKKAEFAIIVISENFFDREWTEKELSEFLNRQDR
NGQKLILPIVHNITNEDLRKKYPKVADIQAIDSKTYTCDQIALLFAKQLIKRLKT(M)}*

$567=129(160):
>gi|593264708|ref|XP_007134532.1| {<Streptophyta(V)>}hypothetical protein PHAVU_010G055300g [Phaseolus vulgaris]
>gi|561007577|gb|ESW06526.1| hypothetical protein PHAVU_010G055300g [Phaseolus vulgaris
{(MASNAIIQYTSSSSSLATHI)-YDLFVSFRG-
EDTRNTFTSFLFQALRTKGIIVFKDDEDLKKGESIapELLQAIQALHHFLSMVS-------------------
------------LCSLCFIYVLLVRSVKRLLLRARVCVVATATCVCATATCVVSFFRS()}*

$568=170(174):
>gi|593264064|ref|XP_007134210.1|
{|0(177)|<Streptophyta(V)>}hypothetical protein PHAVU_010G028600g [Phaseolus vulgaris] >gi|561007255|gb|ESW06204.1| hypothetical protein PHAVU_010G028600g [Phaseolus
{(MEFASSSSSSSSSFLRSEPQFT)-YDVFINSWG-
EEIGRKLVSHLHSFLLQAKVKTLIkEENLQEGMGLEEHMREIAASKIAIIVFSKSYTESYQNGSHVDVQENLL
---YNVLKSKLEVESVGMGRTMIENGLSRKKLMIVLDDVNGTEAIQGLSLKLHSTSR(DCFKAHAFREMK)}*

$569=246(247):
>gi|560121648|emb|CDJ93715.1| {|886(0)|<Nematoda(M)>}Leucine-rich repeat and Toll-Interleukin receptor domain containing protein [Haemonchus contortus]
{(NRTICVPVSSGFFGQEPQNSILTVIFLTSCVFLLCAMTFLGVSLVRKAHNDMSQRRYKASSSLNCSSTPGS
SPLPVPLLN)-FDAFVSYSKKdeKMVLEQLCRPLEDEE--
YALCLLHRDGPAYNSrlhaISDELISQMDAAQCLVIVLTKNFLESEWKTLQVKTSHQLFAKNRGKRVIAIL-
GEG-VDQNLLDEELGQILRKNtCIRQKDHL---
FWQLLHSALPAR(LASLPGSGDDGSQIYSDMYGIVPSAVI)}*

$570=208(209):
>gi|550968745|ref|WP_022717044.1| {<unknown(X)>}hypothetical protein [Rhizobium mongolense]
{(MTSEPQIGTCSTVSYQWDVFLSYPRKGHVPAWIE)---------------
RHFLPMFRGHLQGLlqeDPKIFVDSAQ-
PTGVQWpqNIRDALLRSRVMVAVWTPPYFKSDWCMAEWesmlqrEAFLKTQGKAhPNGLVYPVVFSDGRnfdp
RAQNTQSRDLSNMNY----
PWEAFRDSPKYldfHDEVKAIADE(VEERIAQAPQWDSLFPLADPSAVIVNNPQIGLPLL)}*

$571=217(220):
>gi|555261205|ref|WP_023243323.1|
{|86(0)|<Proteobacteria(B)>}hypothetical protein [Salmonella enterica]
>gi|677858641|gb|KFT73589.1| hypothetical protein SEEB0207_17235
[Salmonella enterica subsp. en
{(YFQASISYIHDYRWEWKEVDAKKINNIFIIYISDIDFPSQKLFYHNNKSLAEIDWAKLQAIFHQPYEIKND
VMLDNNDTH)-YDFFISHAN---EDKDtFVRPLVDELNRLGIIIWYDEQtlEVGDSLRRnidLGLR-----
KANygIVILSHDFLNKKWTQYELDSLINRAVYDDNKIILPIW--HNInaQ-EVSKYSHYLADKMAL-
QTSLYSVKEIARELAEIAYRRR()}*

$572=214(220):
>gi|550968749|ref|WP_022717048.1| {|69(0)|<Proteobacteria(B)>}molecular
chaperone Tir [Rhizobium mongolense]
{(AATTKRAAIAKDIANKNAELLKAQTALSKEEEKERVKVSKEIERKQAEQLRRQKDIDNRVVSAISSRRVAE
LQTDSSTES)-FDVFISHAS---EDKEeLVHELAEKSTAAGLSVFYDA--
KSIQWGEslrERIEHGLANSSFaVVVLSEAFFKKEWPKRELDGLFGLEM-DGRSRILPIwhKISKDEvlK-
NAPTLaGKLALTtaSLTV-DEIVEKLV-E--------IVKG()}*

$573=228(223):
>gi|550968750|ref|WP_022717049.1|
{|274(0)|<Proteobacteria(B)>}hypothetical protein [Rhizobium mongolense]
{(ALLRMKLHSARVHGAQNAAKLWLKTTDNPDHFWRHIEGWLAYISQVRGRDDPVTAKLCKMAVLSGLKGAEW
IEKYADMVR)EFDVFLSHAT---EDKPTVVKLKRKLESLGIKVFFDVDSIQ--WGDsivEKINHGLL-
KSSffVPFLSDTFSAKGWTNKELNSAIAMNINRKG-RILPICEANFSiaDNypLLNETLYQRWPEDPA-
LEaafvtsVADQIL--KIVQQEKSKPND(LSD)}*

$574=177(180):
>gi|545325196|ref|WP_021599471.1| {|0(124)|<Actinobacteria(B)>}TIR
domain-containing protein [Actinomadura madurae]
{(M)--KIFLSYAAYDQPVAHFLKESLSvEMGVS-FWMMSDDTPPGTVWSERIKMGIEKCDAIYSIVTPE-
SVGRPWITaEWASFWilgkPTT-----PLLIDVKVERLWepMRAFQSVILTDVSSVM--
RFLKALSGATGNSPVDGTRRLA(NEITQEIPLIRERQASGDIERAITRVGSQLRSGTPNIDP)}*

$575=290(298):
>gi|550968746|ref|WP_022717045.1|
{|243(96)|<Proteobacteria(B)>}hypothetical protein [Rhizobium mongolense]
{(DAPVEKLVQATTIPYVPFWSFGERISAVEDRSFDTSSINYSMETIAALLAHRLGQTRLLVESRDEYVGAAQ
RLGRPSENS)---VFISYSTENRDAARQITNSLMRRGVS-VLLE--
DLRqPGELEIDIAGRAMETATHFVLLFGRSdrdnkFMSDDARLFmRQAASDEVE-----
RQFIPFVLPNAVpqELPSFVRQYRYGILH----DPETAAD--
LIWERIRPPAAA(VPDNSQLSLRVTSDGDIPVVRAQVCALADNGTVIESTTDTDGNATLKLVTGTSYQILVAH
PEFSSKIIKDFTPGKQVN)}*

$576=191(182):
>gi|563456125|ref|WP_023681254.1|
{|0(25)|<Proteobacteria(B)>}hypothetical protein [Mesorhizobium sp.
LSJC277A00] >gi|562950142|gb|ESW73472.1| hypothetical protein X771_01545
[Mesorhizobium sp. LSJC27
{(MHVYQG)----
FFSYAHSdaaadpdlvEAFTRQLELRVSNKFTNAKFAIWRDTEnIRIGERWNaKIESAINSSDIMILLLSPKW
VESDYCRKeYLPfKAVELNKS-VGEYVAPLLVREI-
AGQKKHFTEEQNETVLEieQRQYVETLAASFVDLTKDQRTSL(VEQIADDIAGMVERMRDLPAGTRNDQGRSR
GRPALQ)}*

$577=179(183):
>gi|551333904|ref|WP_022953341.1|
{|0(1281)|<Proteobacteria(B)>}hypothetical protein [Leucothrix mucor]
{(MPTKPR)--HIFLSHAS--A-DDEFVALLRKKLEDHGLEVWVDSRNLRGGSKLspEIEEAIR-
TAQHFLVVISPKTINSDWVFdEIEIAEQVEKERpNYR-AIPLMLPGI-
TPKALKRYFSEEPAGEPIELEVGKLDEQLPHILAALGQRA(PDHDLATAQEVANKPIAELLLELTEPTLTCDE
SGKEI)}*

$578=207(220):
>gi|563522535|ref|WP_023728879.1| {|69(0)|<Proteobacteria(B)>}molecular chaperone Tir [Mesorhizobium sp. LNJC405B00] >gi|563076578|gb|ESY01335.1| molecular chaperone Tir [Mesorhizobium sp. LNJC405B00]
{(VPELFDWASSHSVSTNLHGLDEVASSEDLRFTGRVYLYSEVPVPESLKQQVRQEAASRGNFVEFRSVEYME
TRNRTEKPL)---AFISHDSRdkEEIAKKLALAL--SSRMCPVWYDE------
YSLRESIEKGLQECRKCIFILTPNFLANgGWAKTEYQSIFTRELVERQKVILPV-----
WAGVTSQDIYQYSPILADrVAAQWSEGLDTVAAKLVAAIGA-()}*

$579=189(196):
>gi|563916902|ref|WP_023808271.1|
{|0(669)|<Proteobacteria(B)>}hypothetical protein [Mesorhizobium sp. L2C085B000] >gi|563183192|gb|ESZ06343.1| hypothetical protein X735_31480 [Mesorhizobium sp. L2C08
{(MAAKV)----FLSLSHIdRDFVREVAKRLPRGLAYF----
YEESFDNGELLIAAMERAVADAAIFVLFASPATSKSPWVGFEIDQARLNDIQRANHRLLIFPTSLD-
ARIEELPSWLRQHWVAKaGWSPSDVARYIAITLLEPNTGVS(RGAVRIVGRGKSLDRFEQLIADHMQSRRKPP
NVYVIAGFRGIGRRTFAAYF)}*

$580=153(160):
>gi|565652285|ref|WP_023899509.1|
{|0(671)|<Proteobacteria(B)>}hypothetical protein [Cronobacter malonaticus] >gi|564119633|gb|AHB71954.1| hypothetical protein P262_05051 [Cronobacter malonaticus]
{(MI)--KAFLSHSSKdKEsYVRNVANWL-----GKESIVY---
DEFTFEEGEktldQIMDGLGESELFVLFISNSALESKWVKREITESKRLYDS-
GKVLKILPIIIDNSINHEDKRIPKWLREDYN--
LQPIMRARTAASRIKNHLYKI(SWQKHPKLEKINSLFVGR)}*

$581=192(200):
>gi|563315342|gb|ETA03629.1| {|0(803)|<Actinobacteria(B)>}WD-40 repeat-containing protein [Frankia sp. CcI6] >gi|631848803|gb|KDA43923.1| WD-40 repeat-containing protein [Frankia sp. BMG5.23] >gi|6678
{(MVA)--RVFVSHAGSDAELADRVHQTLTLLGHR-
VFLAqniRVGVQPGDMWKDRLHRELRAADAVVCVITNAY-NASPWCAyELGIAYEVG-----
SLLLPLRVEPTVtsP---LLEDRQHVHLHE--
NPGWADNLNIGLRRVEAGGGLG(WADDRSPFPGLRPFTRDMAKVFYGRGNELRRLTHRLRSLGEQRLLLVVGA
SGCGKSS)}*

$582=242(233):
>gi|564602211|ref|WP_023847114.1|
{|903(0)|<Bacteroidetes(B)>}hypothetical protein [Porphyromonas gingivalis] >gi|563395576|gb|ETA26681.1| hypothetical protein SJDPG2_06385 [Porphyromonas gingivalis S

{(RETNRKKRPIQDLYLSYAQADKDLTESHYIHLGTLDDESKTTAKIAAYPLKNGAIDKERVREVSTRPYKHL
SVNKNLSGV)-
KRVFISYSKHdREELEQCLLFFKPLVKSGHIELYYDELTKFETPIHsEIRRRITGADCIIALVSQNYLGTDYI
LDhELPVI----ED-YEKTLIPIQIKPC-TFyevetlsKHYFALKAKVVNLDSriftvpdgVTASALRE--
QNWCDVVREFKEK(ILGITKQEVNTDE)}*

$583=302(300):
>gi|565358519|ref|XP_006346074.1| {|84(583)|<Streptophyta(V)>}PREDICTED: uncharacterized protein LOC102592557 [Solanum tuberosum]
{(PPVSYCGSQSDDVPSTSYTPPPERYDFSDDPTGTKLKIVTCVPVSGPETDPRISFSFPVPRISFAKGSVSP
ASNAKLRSC)--DVYIGFHG-QNpnlVR--FCKWLKSELELQGIACFIADRAKYADN-
QshEIADRVICSVTFGVIVVTGCSFFNHL--SLEEIRFFAQK-KNLI--
PLFFNTDANEIaslSNCNGDTKKckealdVILKCHEFRLETDE-
SNWRSCVSKAAGI(LRAKLGRKSVVEKCTEGFEELPFPRNKSFVGREKEIIDIETTLFGCGDSFDQESSVPSV
KGGTPGQSEGLADDESEADVG)}*

$584=266(261):
>gi|565308917|gb|ETE62622.1| {|732(407)|<Chordata(M)>}Toll-like receptor 13 [Ophiophagus hannah]
{(LLDSYYCFGPGLTNRLFADEDLSFCFENWEMYLFIATTVTILLFLFPSLIYAKLGWTFRYAFYLLRGWGYN
QLHPKGRDY)QYDAYISCCSHdyKWVIDNILEKLEKQHdpcF--
RFCFGPRDFAPGQYYIDNVQSGISRSRKTLCLVSRNYLENEWCSLEIQLACSNIYYHGYDPLVVVFKEEIPnY
SPMPLDMDNGCQSNGHqRHGGAANSAGNGHRVGTLEEAKE(TILHLRETVVHQKEMILDQRETVRELTAKLSR
CETHSRRHS)}*

$585=248(257):
>gi|566009469|gb|ETI32380.1| {|839(20)|<Stramenopiles(E)>}hypothetical protein F443_20806 [Phytophthora parasitica P1569]
{(AIKSINDANKRKVMYEYDEERFAAQFRLLLDGVRDEIEKHREANIVGGSRRRLANGTSVAGFGENITDWDP
ATLDNIQFA)----FVSHG---DHHHSFVKRLHDRLQDNQVRCLLDGT--
QSYTDMteRIhaaKEAILKCEVFIVVLSRKTVNSELVKDQLAFAEDKG-----
KPILPVMLNDMEIPLDKHYTLSRLELLHFTPELGFNSSFMQLLGRVRDNMTS(ANASFNAPRASTRPTFRAVA
RMTAMGHARQNNSMAVN)}*

$586=276(283):
>gi|566009469|gb|ETI32380.1| {|645(186)|<Stramenopiles(E)>}hypothetical protein F443_20806 [Phytophthora parasitica P1569]
{(NVSVDLFECLVEHGADIDDGLSNGDTALHIAMKCEQKGTALALLDAGADVMETNRDGYRPVDCTTSTQLQF
EIKQAAGDR)--DVMISY---
THSHSEFALKIRDSLERANITSWLDLMDptgIGGGSVWreEIARGIKNAEVIICLYTEDYPGSEWCLKELALA
KQLG-----KPIIA----
VSPEGAPVTDEmqVYIYTRQMVPFESAIKSINDANKRKVMYEYDE(ERFAAQFRLLLDGVRDEIEKHREANIV
GGSRRRLANGTSVAGFGENITDWDPATLDNIQFAFV)}*

$587=236(252):
>gi|566017812|gb|ETI40723.1| {|766(195)|<Stramenopiles(E)>}hypothetical protein F443_13959 [Phytophthora parasitica P1569]
>gi|567955182|gb|ETK80815.1| hypothetical protein L915_13597 [Phytophthora pa
{(AGASLKTQVSGGNTALHIALQRENRQTAVSLIKCGSSMMITNDSGKRAVDCTTSTELQFLLKKEAGTRKIM
ISYTHTHAQ)-----------------
FASKVREHLeTQEQLTCWMDTMDpsgIGGGAVWreEIARGIHNCSLVLSIVCDGYTKSEWCLKELALAKLLR-
----

KPVLGLIVEEDHPASMAALESLIPYKNRIPFNDFIIARRQESPRTVIFDIDD(AAFARRWQVTRPLMISIMKD
GRQLDKEERENV)}*

$588=249(254):
>gi|566030786|gb|ETI53697.1| {|325(224)|<Stramenopiles(E)>}hypothetical protein F443_03408 [Phytophthora parasitica P1569]
{(ASRELVSELTTVGGDIGYQLPTTGDTALHVAVREENEASALAVLASGGDLMRMNNEGLRPLDCTASTRLLF
ELKRAAGQR)--DVMISY---
THSHLGFARKLRQSLEEASVTTWLDLMDpsgIGGGAVWreEIARGITNASVVLCILTEDYASSEWCLKELALA
KQVG-----
TPILAVSTEGAIVDANLQVYLYTRQIIPFEPAIVGLRHGEGRIEYDYDESRY(RSQFHLLLDGVRDEVEKRRY
FTKQKNVASSRNLD)}*

$589=249(254):
>gi|566030903|gb|ETI53814.1| {|663(214)|<Stramenopiles(E)>}hypothetical protein F443_03291 [Phytophthora parasitica P1569]
{(ASRELVSELTTAGGDIGYQLPTTGDTALHVAVREENETSALAVLASGGDLMRMNNEGLRPLDCTASTRLLF
ELKRAAGQR)--DVMISYTH-SHLE--
FARKLRQSLEEANVTTWLDLMDpsgIGGGAVWreEIARGITNASVVLCILTEDYASSEWCLKELALAKQVG--
---
TPILAVSTEGAIVDENLQVYLYTRQIVPFEPSIVGLRRDGGRIEYDYDESRY(RSQFHLLLDGVRDEVEKHRF
STRQNNVASSRMFT)}*

$590=249(254):
>gi|566030910|gb|ETI53821.1| {|665(224)|<Stramenopiles(E)>}hypothetical protein F443_03278 [Phytophthora parasitica P1569]
{(ASRELVSELTTAGGDIAYQLPTTGDTALHIAVREENHSSALAALSNGGDLMRMNNEGLRPLDCTASTRLLF
ELKRAAGQR)--DVMISYTH-SHLE--
FARKLRQSLEEANITTWLDLMDpsgIGGGAVWreEIARGITNASMVLCILTEDYASSEWCLKELALAKQVG--
---
TPILAVSTEGTIVDANLQVYLYTRQIIPFEPAIVGLRHDEGRIEYDYEESRY(RSQFHLLLDGVRDEVEKRRY
FTKQKNVASSRNLD)}*

$591=249(254):
>gi|566034528|gb|ETI57439.1| {|721(585)|<Stramenopiles(E)>}hypothetical protein F443_00259 [Phytophthora parasitica P1569]
{(TSVDMFKALVASGGDINHQLRLTGDTALHLAVKLDDQQTALALLSMGGDIMRTNHDGFRPIDCTTSTRLQF
EIKRAAGTR)--DVMISY---
THSHMEFALKLRKSLEDANITVWLDLMDpsgIGGGSVWreEIARGITNAAVVLCILTEDYAQSEWCLKELALA
KQVG-----
TPIMAISTEHAKISEELQVYLYTRQLVPFESAIVKVEQVNEKETRYKYDEDA(YKNQLRMLLDGMRDEIEKRK
EDNIKRNMRRAETN)}*

$592=227(230):
>gi|564595819|gb|ETC91992.1| {|0(88)|<Firmicutes(B)>}hypothetical protein T481_09175 [Enterococcus faecalis PF3]
{(MVIAERGVKMTVEKSVTIVYSWDSE)-------------EHCGWVKKLAEELERNSVQVTYDQNSLA--
LGDdinQ-FMEIAVSTASiiLAICTEQYKEKIdgregGSGYEGR-
MMAQLVKNRSASIIPITKDgykDSKiprafqG-LTSIDLELDIKK----
EKFARLLQAIHGFKIVKDVKKV(DPRKTIADALEVKLEEVKTDDELMLLNIGITNIISDKVTLPKMDGSQGSA
LYSIPFQLSESPTSR)}*

$593=214(220):

>gi|566569083|gb|ETJ46822.1| {|258(0)|<Proteobacteria(B)>}hypothetical protein X564_17555 [Pseudoalteromonas sp. NW 4327]
{(NLIEAEKRFIKVRYHAQETEIDMSKLDGSIYKRAVYGNLSFIRMVRGEDHSPYLTLCKKVLELDHNPPAFI
NKLKGIFEM)-YDVFICHAS---EDKESVAlPLYESLEKRNINAFIDCF--AINWGDslvAKINTALQ-
KSKyvIAIISEDSVKKSWPMKELNAVLSAEIAADKTRLLPLMVGDGEalvrqlpL-LADKlfVGYKD----
NI-EEITDMIE-SLLTE-------K()}*

$594=246(248):
>gi|566017813|gb|ETI40724.1| {|754(0)|<Stramenopiles(E)>}hypothetical protein, variant 1 [Phytophthora parasitica P1569]
>gi|567955183|gb|ETK80816.1| hypothetical protein, variant 1 [Phytophthora para
{(GVESSIFSNLVAAGASLKTQVSGGNTALHIALQRENRQTAVSLIKCGSSMMITNDSGKRAVDCTTSTELQF
LLKKEAGTR)--KIMISYTHThAQFASKVREHLET----QEQLTCwmdTMDPS-
GIGGGAvwreEIARGIHNCSLVLSIVCDGYTKSEWCLKELALAKLLRKP-
VLGLIVEEDHPASMAALEVGSIALLMIWLSA--
YTVTISECSHLSRTRTGSLST(TSSSHDVRRVPGRSFSISTMLRLPGDGK)}*

$595=250(258):
>gi|566034531|gb|ETI57442.1| {|896(485)|<Stramenopiles(E)>}hypothetical protein F443_00262 [Phytophthora parasitica P1569]
{(DPRIFQLLLDYGVDLHATRTSNGDSALHVALRRRNQTAAVLFVRAGLSLTAVNLLGQRAIDCARSPALRFA
VKKEAGQRD)---VMISYSHAhAPLARKIR---DALERQRV-
TTWIDTMgptgITGGSVWRqEIARGIQSSALVLAVLTKDYPQSQWCMKELAFA--------
KMQNIPVVAIQC-
EDMEISEELQVYLWTRQviDFRPAVKHNYTMETSEHAPYSLS(EDKGDGVKEARPMITSNSSHTIEDTDSRED
ESEFRIED)}*

$596=184(179):
>gi|564703928|gb|ETD04105.1| {|0(142)|<Firmicutes(B)>}hypothetical protein N568_0109805 [Lactococcus garvieae TRF1]
{(MATK)---VFISYAW--EEQKEdddkiklFTQWLAVYLKKWGFDVLLDLFENHPGSKLdkFMSEGI-
NSSKFVICICTETYIQKmynpkSGVYnEITL----LKEMsDSPFIIPIIEKGC-
FSDLPEFFEGKFVSELLFDTPYSQKNKDNIFELISTLRDE(SLSIKEVSSETRIDDYYKNVENFKFQAKTANL
MSF)}*

$597=256(257):
>gi|571029519|ref|WP_024124316.1|
{|261(343)|<Cyanobacteria(B)>}serine/threonine protein kinase [Thermosynechococcus sp. NK55a] >gi|564737507|gb|AHB87905.1| serine/threonine protein kinase [Thermosyne
{(ATAADALASLQQVEVPKSFWFRLGKFLRTPVQDLWRSPKAGEAPPRPLPPVSTLPPKATEPKPLSSPPAAT
AAFPNQGRR)---IFISHSS--
HGtDLQLASALQEALQKAGHQPFMASQSIRLGEAWaqRIDQELKQCDFFVLLL-
SQTAAQSEMVLeEVRTVKQLQAHRgDRRpFILPVRVNFP-
LDMPLNYELRGYLHRLQQRFWRSPADTDTLLQEILDLVNA(TTAPVATSSDAPQETIAEPDQKNTFVGDSAPV
PVAEP)}*

$598=220(230):
>gi|565306397|gb|ETE61120.1| {|114(0)|<Chordata(M)>}hypothetical protein L345_13129 [Ophiophagus hannah]
{(AGATLEQGILEMKQRALRSEQNSSLWGKLRGSFHRVSTHRDRLGQVVLCGRGGVGVGVTKNHHLPQGRLPH
NFYAMTSSF)QYDFSLWYcLEDDAIATAIHEMLVACGFH--GYVEHQDHVAGKPVLASVNEII-

QsSRLAIVLLTPHSVTDPWCQRILDWNLIHTVHQKGAKVIPVYVDITPEQMPPTLRHLTGLKY--------
RER-YFKKRLLDSLKKT(RATCCPSTTG)}*

$599=302(300):
>gi|568841619|ref|XP_006474755.1|
{|101(585)|<Streptophyta(V)>}PREDICTED: uncharacterized protein
LOC102621378 [Citrus sinensis]
{(SPPVSFRGSQSDDIPSSSYTPPSDQYEYSDDPTDSKLKFMTCVQVADPAPPRVSFSFPVPRISFAKGPVSP
VSNAKLRSC)--DVFIGFHG-QNpnlVR--FCKWLKSELELQGIACFVADRAKYSDS-
QshEIADRVICSVTYGVVVVTNSSFLNHL--SLEEIRFFAQK-KNLI--
PLFFDNGPNEILgllncnsidTECKEALDGLMKSHEFKLEANE-
CNWRSCVAKTAGI(LRAKLGRKSVAENDLEGFEELPFPRNRCLVGRDKEIMEIETAFFGSGDYLEQDYAMPIT
KGEASGQSEGLADEESDSVST)}*

$600=253(260):
>gi|675161839|ref|XP_008889804.1|
{|941(58)|<Stramenopiles(E)>}hypothetical protein PPTG_00186
[Phytophthora parasitica INRA-310] >gi|567972228|gb|ETK97216.1|
hypothetical protein L915_00199 [Phytopht
{(IEYDYDEAKFKAQFRLLLDGVRDEIEKNRDAVQQKNIASSRRNNGLKQTATGTILANTGGGFSRLFQQWDP
EASGAQKQF)---VFLSHG---DKHAGFVQQLYRELTDAGVVCYGDRN--
VEGQHFenRIhaaQEAILRCTCFIVILSKQTMNN-----
ELVRDQLAFAEDKGRPIFPItLNDLDPGLDKRYSLARNELFHFMGNGMSFKPSADRLIQGLRRYYIA(RDDNQ
GTMLDNSTRIASANTSFVSFTFSEPGTRDHDREAV)}*

$601=246(247):
>gi|915239376|ref|XP_013293219.1| {|949(0)|<Nematoda(M)>}leucine Rich
repeat-containing domain protein [Necator americanus]
>gi|568269666|gb|ETN70992.1| leucine Rich repeat-containing domain
protein [
{(NRTICVPASSGFFGQEPQNSILTVIFLTSCAFLLCAMTLLGVSLVRKAHNDMSQRRYKASSSLNCSSTPGS
SPLPVPLLN)-FDAFVSYSKKdeKMVLEQLCRPLEDEE--
YALCLLHRDGPAYHSrlhaISDELISQMEASQCLVIVLTKNFLESEWKTLQIKTSHQLFAKNRSKRVIAVL-
GEG-VDQNLLDEELGQILRKNtCIRQRDHL---
FWQLLRSALPTR(LASVPGSGDDASQIYSDMYGIVPSAVL)}*

$602=144(174):
>gi|568026170|gb|ETM00165.1| {|0(217)|<Stramenopiles(E)>}hypothetical
protein L917_03076 [Phytophthora parasitica]
{()------------------------------
VTTWLDLMDpsgIGGGAVWreEIARGITNASVVLCILTEDYASSEWCLKELALAKQVG-----
TPILAVSTEGAIVDENLQVYLYTRQIVPFEPSIVGLRRDEGTIEYDYDESRY(RSQFHLLLDGVRDEVEKHRY
VTKQNNLSRSRRLA)}*

$603=145(174):
>gi|568056644|gb|ETM53354.1| {|0(217)|<Stramenopiles(E)>}hypothetical
protein L914_03153 [Phytophthora parasitica]
{()-----------------------------
NVTTWLDLMDpsgIGGGAVWreEIARGITNASVVLCILTEDYASSEWCLKELALAKQVG-----
TPILAVSTEGAIVDENLQVYLYTRQIVPFEPSIVGLRRDGGRIEYDYDESRY(RSQFHLLLDGVRDEVEKHRY
VTKQNNLSRSRRLA)}*

$604=235(239):

>gi|567457888|gb|ETK11989.1| {|100(70)|<Bacteroidetes(B)>}hypothetical
protein T235_12455 [Tannerella sp. oral taxon BU063 isolate Cell 8/11]
{(TIQTLKHFSVGRKTVVHVLGKKDLSCIESEDLDIFDAALTYIDDTIVVISTRKNALILKDSPIVNKSLKKI
EDIFMTSKL)-KKVYISYKHDENCSKALEAI--KNGLKKNNIEY-SIDVQDIKYRDdiveY-
EKKIGTADRVIIFIIASYLKSIDCMFEMTEIFKKSDV-KERVFPIVDITPISRNREGLEEIKDYWKEQR--
VKAFAQMQTEAGISDYIINEL(YKINAIIKTLDEFWNYIVH)}*

$605=205(220):
>gi|567996851|gb|ETL46950.1| {|262(0)|<Stramenopiles(E)>}hypothetical
protein L916_03263 [Phytophthora parasitica]
{(SRELISELTTAGGDIAYQLPTTGDTALHIAVREENHSSALAALSNGGDLMRMNNEGLRPLDCTASTRLLFE
LKRAAGQRD)---VMISYTHShLEFARKLRQSLE----
EANITTWLDLMdpsgIGGGAVWReEIARGITNASMMLCILTEDYASSEWCLKELALA---------------
KQVGT-PILAVSTEGTIVDANLQvyLYTRQIIPFEPAIVGLRHDEGRI()}*

$606=261(263):
>gi|571783348|gb|ETR65959.1| {|53(72)|<Proteobacteria(B)>}hypothetical
protein OMM_13455 [Candidatus Magnetoglobus multicellularis str.
Araruama]
{(IVYADLSEMAPDFMDWHTFVDFSFSDEIPYLIKQEQHYRQLLTDPDLPLILRQDFDNQLRSVRQRLKQIRI
RKNASQYEY)--DVFIHYAVED-YSFASNLAYHLRLRAL--
SVYFDWDYPEDGVNPqQYDEAIENSKKMISVWSPSYFDQPSVLEiaVRFVntNHEIRTM--
NRPYIPILFRQCTPSPLFNQIPIIDFTNEDDFYIQLYNVIRALDIDTPFLDMD(TSFMEYQAEGIDLYATNHS
NQFINDIGKIFASLGFFVKRNVTF)}*

$607=148(173):
>gi|571579638|ref|XP_006572741.1| {<Arthropoda(M)>}PREDICTED: protein
toll-like [Apis mellifera]
{()-----M------------------
GNPSYKLCLHYRDFPVGSFIADTIVQAVESSRRTIMVLSENFIKSEWCRFDFKSAHHQVLRDRRRRLILVLVG
DV--
HQRDLDPDIRLYLKTNTYLQWGDKLFWEKLRFALPDVPN(NQRTTQRTRQPPPVRRQHNNRTSNGSRTVSVHI
)}*

$608=249(254):
>gi|570334432|gb|ETO82380.1| {|147(145)|<Stramenopiles(E)>}hypothetical
protein F444_03483 [Phytophthora parasitica P1976]
{(ASRELVSELTTVGGDIGYQLPTTGDTALHVAVREENEASALAVLASGGDLMRMNNEGLRPLDCTASTRLLF
ELKRAAGQR)--DVMISY---
THSHLGFARKLRQSLEEANVTTWLDLMDpsgIGGGAVWreEIARGITNASVVLCILTEDYASSEWCLKELALA
KQVG-----
TPILAVSTEGAIVDENLQVYLYTRQIVPFEPSIVGLRRDGGRVEYDYDESRY(WWSQFHLLLDGVRDEVEKRR
YFTEQKNVASSRNL)}*

$609=282(297):
>gi|570958520|gb|ETP23499.1| {|349(182)|<Stramenopiles(E)>}hypothetical
protein F441_03396 [Phytophthora parasitica CJ01A1]
{(ASRELVSELTTVGGDIGYQLPTTGDTALHVVVREENEASALAVLASGGDLMRMNNEGLRPLDCTASTRLLF
ELKRAAGQR)--DVMISY---
THSHLGFARKLRQSLEEASVTTWLDLMDpsgIGGGAVWreEIARGITNASVVLCILTEDYASSEWCLKELALA
KQVG-----TPILAV-----STEGAIVDEnlqVYLYTRQIIPFEPAI--------
VGLRHDEGR(IEYDYEESRYRSQFHLMLDGVRDEVEKRRYFTKQKNVASSRNLDLDGGGADRGAGSSSPGTIL
QTLGDARLSPVSSS)}*

$610=213(220):
>gi|657260836|ref|WP_029365257.1| {|321(0)|<Proteobacteria(B)>}DNA polymerase [Escherichia coli] >gi|571177320|emb|CDK82617.1| Retron-type RNA-directed DNA polymerase [Escherichia coli IS25] >gi|10015
{(WTDDKLKYALLFATEKGYQAKDNNHAIQIFRNHIYGRLSFIKMVRGKDYPGYLKLMSYMSHNDPLKTQEGL
RAMKETENF)--DVFICHAS-ED-
KKDIAIPIYDELTKLKISAFIDHVEIKWGDSLieKINAALVKSKYVIAILSANSVNKEWPQKELRAVLASE--
---ISSGDVKLLTLLKKEDEEVVNLSLPLLSDKYYMVYDNNPEVVANNIKSLLQR()}*

$611=200(188):
>gi|740708790|ref|WP_038494078.1| {|0(2)|<Proteobacteria(B)>}TIR domain-containing protein [Janthinobacterium agaricidamnosum]
>gi|571268846|emb|CDG84093.1| hypothetical protein GJA_3477 [Janthinobact
{(MDTVRYGCFFSY)AHGHYVFMN---
KFKNDLIEALQCYLEPHldnEHALFVDSEQLGGGDDLdqRIARALCESVCMILLYTPKYEAHPYTRREFAAMQ
lIELERRgwytlPSHLIIPVIMTRHPaglpRQITEPGMYLDFSGYTLASGDLKANPDYLPAIQKIVERIA(SH
YHLLKSSTPPGHDCSRFVMPDIPPEWRIVPPPHF)}*

$612=199(200):
>gi|570298189|gb|ETO53096.1| {<Firmicutes(B)>}molecular chaperone Tir [Staphylococcus aureus MUF256]
{(MKQKINLQKEQSRNQKKLSSMLDKNTKGNEEIMEKLLTNSDQINEISNQIKKAVNQKEII)EYDVFLSHSS
---LDKEdYVSKISEKLIEKGLKVFEDvKVFEI---
GKsqTETMNMGILNSRfvVVFLSPNFIESGWSRYEFLSFLNREINEEHVIILPIW-HKVSveD-
VRAYNPYlvdkyaLNTSDFSI-EEIVEKIY-QVIVNS-----KN()}*

$613=216(220):
>gi|571707757|gb|ETQ96784.1| {|51(0)|<Proteobacteria(B)>}TIR domain protein [Acinetobacter baumannii UH6507] >gi|587791287|gb|EXA84000.1| TIR protein [Acinetobacter baumannii 1267820]
{(ISDLQKKKTDIQNKLLKENQNLAKAEADERKKLDALTKKQQKEDIEHQRKLKREIESIKASTEYVNGTTTS
SYIDIAPEP)EYDLFISHAS---EDKEdFVRPLAETLQELGIKVWYDEFslKVGDSLRRkidSGL-----
KNSRygTVVLSEAFIKKEWTQYELDGLVAREMNGHKM-ILPIwhKISKTEvinyspN-LADKVA-
LNTSINTI-EEIahqlADVIL-EK-----------()}*

$614=197(206):
>gi|570550538|gb|ETO91794.1| {|0(39)|<Proteobacteria(B)>}hypothetical protein P857_971 [Candidatus Xenolissoclinum pacificiensis L6]
{(MKIN)---LFISHDSNdEKIACLIAKELKEYGIN------
CFVAHPDIHPMKdwknTIIEKLKSSDIVIALINDDYFNKVWTNQEIGFACKQETP-ILPIKLS--
DKNPEGMISNLQALRSDALKND--
EYNEYTQYNAKEDSEKIVLNL(PEIFPDININKLYILKFIHAKNYNDAIDYFKIMKKQDQKLDNDDVKLIIDG
FHNNNQLHPNY)}*

$615=177(175):
>gi|571789056|gb|ETR69856.1| {|0(32)|<Proteobacteria(B)>}hypothetical protein OMM_03650 [Candidatus Magnetoglobus multicellularis str. Araruama]
{(MGFKYSC)----FISYSSGqKELIQKFIYQIEEslnaeIELYMKDypVF---
LDRERLQIGDkfneSIAISLCQSICMVAVLTPAYYSNPYCLREYTAMENLEKE-
RFEKMQWSQSPEMGLIFPIVFRNSDRMPDII--
KESTQWVDFSKYSLSQPELLK(DTTFNETIQKIAQRIFDYFENFNELEIN)}*

$616=272(268):

>gi|570728751|gb|AHF00060.1| {|73(930)|<Proteobacteria(B)>}hypothetical protein THITH_08235 [Thioalkalivibrio paradoxus ARh 1]
{(AAEVGVNFSCKVSSERRGGAVNRRLWFEGTPRLAERAQRFSHAETGELNHLPATLRQRGRVLGPTIALDEY
RDGKPVARW)----
FLSYARDdNTMKERLLKLLQkrlACSRRFAFESWHDGDIVLGDDWHeSIEQVLDQCHFGLLLLSHAFLSSDFI
RDvELPRFVNTDGgiG-EEKRAVPVCLEKM-
NFGRVDLHGLGQKQIFRdqDDKSFKERKGHLQEAWVDQLVDR(IETMLERYGVAFPGGSEPIVQRVGRLSRSA
GLHRAIHRFPTADLMGLR)}*

$617=238(239):
>gi|570958652|gb|ETP23631.1| {|666(234)|<Stramenopiles(E)>}hypothetical protein F441_03263 [Phytophthora parasitica CJ01A1]
{(SRELFSELTTAGGDIAYQLPTTGDTALHIAVREENHSSALAALSNGGDLMRMNNEGLRPLDCTASTRLLFE
LKRAAGQRD)---VMISYTHShLEFARKLRQSLE----
EANITTWLDLMdpsgIGGGAVWReEIARGITNASMMLCILTEDYASSEWCLKELALAKQVGTP-
ILAVSTEGTIVDA-
NLQVYLYTRQIIPFEPAivGLRHDEGRIEYDYDESRYRSQFH(LLLDGVRDEVEKRRYFTKQ)}*

$618=213(220):
>gi|740887084|ref|WP_038672332.1| {|122(0)|<Firmicutes(B)>}hypothetical protein [Ruminococcus bicirculans] >gi|571259008|emb|CCO05393.1| hypothetical protein predicted by Glimmer/Critica [Ruminococcus
{(KQNEGQLIENIIKKYDLRKESSLGIHELFMEFCNRKFNMATIPPSFSKADITQMFNGAYFFCSKSDLTKKG
YNFNTHIRR)-KKVFISYCHAnKELVYEITE---ALEYSG-
IDLWIDKKdISVGSHLLeSILAGIEESDFAILFLSHATLSSNYG-QlELKTIM------SALTNKKKGWYIV-
KIDDVDVNNIMPSLGDYkyYDLSNGNKTELIKDIINAINAQN()}*

$619=127(143):
>gi|571781092|gb|ETR64874.1| {<Proteobacteria(B)>}hypothetical protein OMM_15210 [Candidatus Magnetoglobus multicellularis str. Araruama]
{()-----------------LLSHLTILKYDN-
IDIWFDDQIKTGEQWSpAILDAIQSAHMTVCLISKNYFNSDFIRErEIPAIMAKQKE-G-MYVFPVLMTDC-
LWNIVKWLKdMQIYPADGipIEDLPErtRE--KTLMKIAQAISEI(LIN)}*

$620=194(186):
>gi|571784400|gb|ETR66564.1| {|0(180)|<Proteobacteria(B)>}hypothetical protein OMM_12637 [Candidatus Magnetoglobus multicellularis str. Araruama]
{()MKQVVLTYQHSdEAIVDQLVFTLK----
HPDIELILNQSIAAGDDLYnNIGRKLSSADAMIVLLSRstgqcDWVSLDvarYLRDpEnliLPVLLKGYQE-
N--ILFSIIGDKY-
GLKFDDLGAKGIATLIHrvLGVATAENRFDLILPAKGPEADR(FDFEKVKRSTTVRDIAKSVIQGYYKEIRPS
KNFNVVDKISAEENER)}*

$621=164(167):
>gi|571785671|gb|ETR67355.1| {|0(238)|<Proteobacteria(B)>}hypothetical protein OMM_11694 [Candidatus Magnetoglobus multicellularis str. Araruama]
{(MTIKP)---LFISYSRKdKKEVELLVKTL----
HAAGIKTWQDVTSLDMEDTIpQIKKAINdECSGMVFYATRNSIRSDFIKAyELPPANDRSTK-
DNDFAGIYYVYNV-
DLKKAEQALRNISLPPLsnINGVMVEHAKGFKRLRENWLGNS(TKLLNAFKELRQKILARIFKSM)}*

$622=195(180):

>gi|571786223|gb|ETR67724.1| {|0(270)|<Proteobacteria(B)>}TIR protein [Candidatus Magnetoglobus multicellularis str. Araruama]
{(MTASKNA)---
IFISYAHVdDKgfgdndkgwvttLVDALQTKLAeYLGREEYFDLWQDKNKLHGNDlIQdTIMNTLDHTDILLI
ILSPAYINSEWCRLELNRFVEKMQS-HKeiTRVFVVEKNKT-
TPPDCLNGLLNYRFWEQkdAAKAPTVYGELKQHDKFYEMVND(IAYEMSQKLDLIKQMSPVQPQVLDNRPQIY
LAR)}*

$623=177(184):
>gi|571786228|gb|ETR67728.1| {|0(32)|<Proteobacteria(B)>}hypothetical protein OMM_04983 [Candidatus Magnetoglobus multicellularis str. Araruama]
{(MKTKEK)MNDIFISYARQdKTAARNICQALEKLGWS----
VWWDQNITTGATFReNIQNELNNSKCILVLWSEHAIKSDFVKDEADVGKEKK------ILIPVLIDNC-
EIPLGFRQVQTHRMDNAhsACKQSDLETLNSAISKILKQQVE(NRLPRFSIIPEQMSPMTAKFLIFFSVVLMS
FFTAVILS)}*

$624=204(204):
>gi|571786577|gb|ETR67973.1| {|0(75)|<Proteobacteria(B)>}hypothetical protein OMM_04845 [Candidatus Magnetoglobus multicellularis str. Araruama]
{(MLDYQSFNETKKTSHNWFSTTIRNEK)MAKIFISYSHQdKDFVQSIMDQINTTHHEIVIDN-QDFEI--
GSPINhTILKSIAEAEFILVFLSSNSVNSDWVLKeIYSSLYCELKD-KQTKLIPCIIDDC-
ALPKNLFKFKQYERINLdfKTNFEDSIQTLKKRLESTNPPIF(ENENYFVLKIPVSNLEIYMTGELYDWQKNS
QLKYVEMI)}*

$625=208(209):
>gi|571787342|gb|ETR68524.1| {<Proteobacteria(B)>}hypothetical protein OMM_04514 [Candidatus Magnetoglobus multicellularis str. Araruama]
{(MVFSQKHTENDVLYEEDIIDEKKIYTFVIRTSFVIPSRVDANQYNITPLKPAKKIIYTPDDFNTMH)-
KTIYISYSVEdREWLNRLLYHLMPLKHEN-IDFWFDHQ--
PEKNNFcATENSINRSHLIICLITQNFLNKESIRTkEIP-LIKIRQI-ENIPVVPILLEKC-
LWTiNTWLNSMTLYPTNKkpVAEFelDEQD--NLLMDIVSELFLR(LKN)}*

$626=152(175):
>gi|571787948|gb|ETR68980.1| {|0(314)|<Proteobacteria(B)>}hypothetical protein OMM_09993 [Candidatus Magnetoglobus multicellularis str. Araruama]
{(TDARA)--------------------------
KSQKIKIWRDNKLDGNQVFDqTISEKIKNSAVFLALMSNGYLASDYCRKELNqfYAYFKNHA-
SGIHVGDRSRIFS-
LLLTNISHENFPEGIAGtsSFAFHDALSDDEMGMGDPIDPCE(KLFRNELKIVANAIFRCLKQMQAPNETPIE
)}*

$627=183(194):
>gi|571789609|gb|ETR70312.1| {|0(75)|<Proteobacteria(B)>}hypothetical protein OMM_03335 [Candidatus Magnetoglobus multicellularis str. Araruama]
{(MVYD)---VFISYSKKdVNTARKLARILE----
KKELKVWWDKRLTPGIDYSkEIEHQLNTVNSVIVLWSENSIESRWVKDEALVAINRQ------VIIPVEL---
-
DGAQSPigFRQLQAAKLFTknTNSETYKNQIIAILKALKVRLNQ(KKLNNSVVDVKKESSHKTEPSHNTEPSH
KTEPSHKIEPSHKTEPPHTFGQ)}*

$628=256(257):
>gi|571790261|gb|ETR70874.1| {|225(11)|<Proteobacteria(B)>}hypothetical protein OMM_02917 [Candidatus Magnetoglobus multicellularis str. Araruama]
{(NENLVMKDLNTFRDAVWNLLLFSGYLTITEKRPDPENSNNNICCLKIPNTEIKNYFISEMSDLKAAQNIET
LIASENQSI)-KKVFISYNHNdLAFVKRLKNDLEQAGIQLIIDI---
ERMKFGDDIKeFIEFSVQTSDITLSVISKKSLASPWVMLeTLETFQQEDYM-KTMRYIPVIIDHN-
FQSPDFALQLIANIEKNidLIFDEISRLSKKYLATDSLDLKK(KRLVSLRSNIDQVLSRLNERLIADFSTQEK
YEMNFLR)}*

$629=202(203):
>gi|571791375|gb|ETR71847.1| {<Proteobacteria(B)>}hypothetical protein OMM_07856 [Candidatus Magnetoglobus multicellularis str. Araruama]
{(MQPIQTTESTDQTAKDI)---IYISYSPKdRSYLEIFIDYLMILKIKH-
IDYRFDHSNRTIDDWSdDVQQMISSAQMTICLLTNQYLSSALIQEkEFPAIQVRQKQ-G-MVLFPVLIKDC-
LWHIIPwFDNTQIYPKSGvpLQEMDDlsRE--
KTISQIMQDIPVW(IKTNNHVQMNPSVESSIYKTNGINYANGIYYKEKIGNPDLFSGRKK)}*

$630=119(143):
>gi|571792953|gb|ETR73283.1| {<Proteobacteria(B)>}hypothetical protein OMM_07034 [Candidatus Magnetoglobus multicellularis str. Araruama]
{(M)-----------------------PLKHEN-IDFWFDHQ--
PEKNNFrAIENSINRSHLIICLITQNFLNKESIRTkEIPMIKFRQTE-N-IPVVPILLEKC-
LWTiNSWLNSMTLYPTNKkpVAEYelDEQD--NLLMDIVGGIVPK(IL)}*

$631=252(247):
>gi|698820425|ref|XP_009843241.1|
{|0(532)|<Stramenopiles(E)>}hypothetical protein H257_16496 [Aphanomyces astaci] >gi|574101692|gb|ETV67253.1| hypothetical protein H257_16496 [Aphanomyces astaci]
{(MGAGASVDVVTKEQCQSLYGDLFNDAEWAKQAVDGVVTRDKLTTAFSALT)--
DAFLTHDWGtDGTTHKNVSVINTLLKARGITTWFDEEKMEGNVKKQMIHGIDNARAIVVFVTQRYIDkvggnn
aEDNCQLEFNYAARRK---TASKMIPVLIDPSPTlknTANW-
TGEVGFVLGGHLYLDLSSAFGNDALLASRIDELV(TKIVSVAGTPLSQRFQSAHHDAVPPPTGGPSTPRTASA
TISTVPLASLTQHQVGTLL)}*

$632=250(257):
>gi|698779595|ref|XP_009822841.1|
{|0(552)|<Stramenopiles(E)>}hypothetical protein H257_01374 [Aphanomyces astaci] >gi|574122502|gb|ETV87978.1| hypothetical protein H257_01374 [Aphanomyces astaci]
{(MGAAASVVTKGQCQALCGDLFDESEWDKQAVDGVVSRDKLTTVLSTLTDAFLTHDWGSDG)---------
G-THTKVSVVNHL---
LQARGITTWFDQEKMEGNVKKQMIHGIDNARVIVVFVTQRYiakvggpNAEDNCQLEFNYAARRK---
TASRMIPVLIDPTPALKNPASwnGEVGFVLGGHLYLDLSGTFDDDHLLASKVDELV(AKIVSIGGTPLARRFP
PESPSDAKEQAKYVPSPPVVATLQAAPVFSAPTVSLASLTK)}*

$633=266(263):
>gi|673053013|ref|XP_008880467.1|
{|23(389)|<Stramenopiles(E)>}hypothetical protein H310_14390 [Aphanomyces invadans] >gi|574463845|gb|ETV90902.1| hypothetical protein H310_14390 [Aphanomyces invadans
{(AYAAYAPSIVSNAVGGDVLSSLDIEQLPMLFATLGVTNTIHQAKLRVMLQQNRPSLLQRTLSQARAMDMAL
NQSGVAKTF)--

DCFLSHDWGvDGATHAKVGHVNAFLKARGIRTWFDQDQMTGNILQAMATGIERSILVLVFVTRRYmakvngsN
PNDNCQLEFGMAKCTQ---
TASHMVPVILDPTMKSVATWAGQLKMVLGNLLYVDLSADTADGFEQGCASLLQR(VQTLLGRTHVTSSKAGDT
YPAQLATEQLADVDEVAPTPVPTDR)}*

$634=252(247):
>gi|673030964|ref|XP_008869443.1|
{|0(536)|<Stramenopiles(E)>}hypothetical protein H310_06235 [Aphanomyces invadans] >gi|574474594|gb|ETW01595.1| hypothetical protein H310_06235 [Aphanomyces invadans]
{(MGAGASVDVVTKEQCQSLYGDLFDEGEWNKAAVDGVVTRDKLHAVFLELT)--
DVFLTHDWGmDGTTHKNVSAVNTLLKARGITTWFDEEKMEGNVKKQMIHGIDHARAIVVFVTQRYIDkvggnn
aEDNCQLEFNYAARRK---
TASKMIPVLIDPSPAlkNTGNWTGEVGFVLGGHLYLDLSSAFGNDQLLASKVDELV(AKIVSVAGAPLAERIQ
SVAQHDVMAAPPLGVATTGAGDPSPRASTISTVALATLTHH)}*

$635=140(153):
>gi|644972001|ref|WP_025392755.1| {<Proteobacteria(B)>}hypothetical protein [Desulfurella acetivorans] >gi|573479138|gb|AHF97550.1| molecular chaperone Tir [Desulfurella acetivorans A63]
{(MYYDYLRKKEKKR)---VFLSFASEdlDHVRGLRLLKdnpnfdLEFYDESIKEPIDSRN---
ADYIKRVIGEQISRASVTVCLISETTHQSKWVDWELEKS-----DEEGKTIIAMAIKGVErAILP-------
KLIKEKnLIFWSWDPE----HLAKLISEA-()}*

$636=269(257):
>gi|572311635|ref|XP_006621545.1| {|245(0)|<Arthropoda(M)>}PREDICTED: single Ig IL-1-related receptor-like [Apis dorsata]
{(CEVSNGVDEEITLPAHVYRQEPQFALGLQNGSWRRSFLVGVLVLVLLISAGAFYARCWLPLVLLCRDKFAP
LEENDGKES)--DALVCYHEKdsNLAIGIVIPTLESR-HRYK-
CTSLELSQLNQNWSLEIGAHVSSSRRIIVILSPACLGNVWNEASVGPILNQ-LSSLATRTIAIVTKDL-
PSATTIakrsnrgcNNSTELSLDRLkILHWDEAWNSSgsgdhnkFWYKLRLAMPPV(RPISTEGGCQSVAMIV
QANGKCGQQKARSRESLEVLV)}*

$637=274(280):
>gi|698814952|ref|XP_009840505.1|
{|723(183)|<Stramenopiles(E)>}hypothetical protein H257_14403 [Aphanomyces astaci] >gi|574104531|gb|ETV70062.1| hypothetical protein H257_14403 [Aphanomyces astaci]
{(GVGADVVSALVEAGADLAYTDMDGNTALHIALINENRTTAVSLLRNGADITATNRDGRRAVDCTTSTELQF
AVKKEAGAR)--DVMISY---
THAHAPVAKGVRDFLVEHaRMTCWMDTMDpsgIGGGAVWreEIARGIFHAKVVVAVVCDGYSRSEWCLKELAF
ARLAR-----TP--
AVVLVVDPHGMSSDVERYVASEYILPFESFDPTNPVLPDHVLAVIRQA(IQTKHIPPPPLLSSSLPPLSPPPN
STTTSTRSFPNNQQQQHRILSSPDASNTPNDDIPMV)}*

$638=238(246):
>gi|698789514|ref|XP_009827789.1|
{|797(0)|<Stramenopiles(E)>}hypothetical protein H257_04844 [Aphanomyces astaci] >gi|574117636|gb|ETV83118.1| hypothetical protein H257_04844 [Aphanomyces astaci]
{(HIDNTNPRNITYEYNEAKYNVQFRLLLDGIRDEIEKKRKKTAHLRSQLTMLSPTGSNTSSFFASPRASPSP
AQGLLDRKF)---VFLAHG---
DCHRSFSRRLYIELTKHKqLECYLDLKTSQEMSVRvhAAKDAILRCSAVIVILSAKSAKSELLNDQLAFAEDK
G-----

KPIFPVILHSDFTLPPSQMYSLSRTTLHHFAVDMGFAPSFEYLWTSLKSRVG(LGAIEEADEELNLLSSSSRV
VNGGMV)}*

$639=249(254):
>gi|698789514|ref|XP_009827789.1|
{|600(186)|<Stramenopiles(E)>}hypothetical protein H257_04844
[Aphanomyces astaci] >gi|574117636|gb|ETV83118.1| hypothetical protein
H257_04844 [Aphanomyces astaci]
{(VHPDLLRALVEFGGDINFQLKSSGDTALHIAIKHEKQDTATALVGCGASIMATNHAGFRAIDIATSTALQF
AIKSAAGHR)--DVMISYTH-SHAQ--
VALKLRQSLEAVNITAWLDTMDpsgIGGGSVWreEIAKGISNATLLVAIITEDYTRSEWCLKELATAKQVG--
---
TPVLPISTENVRLNEELQVYLYTRQIVPFEPAITHIDNTNPRNITYEYNEAK(YNVQFRLLLDGIRDEIEKKR
KKTAHLRSQLTMLS)}*

$640=274(280):
>gi|673046203|ref|XP_008877062.1|
{|727(181)|<Stramenopiles(E)>}hypothetical protein H310_11953
[Aphanomyces invadans] >gi|574467275|gb|ETV94299.1| hypothetical protein
H310_11953 [Aphanomyces invadan
{(GVSAKVVSALVEAGANLAYTDAQGNTPLHIALINENRTTAVSLLRNGADITATNMEGRRAVDCTTSTELQF
AVKKEAGAR)--DVMISY---
THAHSSLAKGVRDYLSEHaRMTCWMDTMDpsgIGGGAVWreEIARGIFHAKVVVAVVCDGYSRSEWCLKELAF
ARLAR-----TP--
AVVLVVDRHGMSSDVERYVPPEHILPFESFDPKNPVIPDHVLTVIRQA(MHTKQSNAVVALPSLQSSPPSPQM
QPLAPPPSSTTTTCSNSVHHASPHIRTGNATTDNPI)}*

$641=231(221):
>gi|738793592|ref|WP_036684781.1| {|0(48)|<Firmicutes(B)>}MULTISPECIES:
hypothetical protein [Paenibacillus] >gi|573555155|gb|ETT53911.1|
restriction endonuclease [Paenibacillus sp. FSL H8-237] >gi|11
{(ME)---IFLSYSYKdIEVAQYVSAQLKKNNIG------
TVIDYEFYEKKNnvldNLRYNIRKSDYVIFFLSKNFLSSNYSMLELSESFnelrIRKIN-
IIPILIErctipsdllefgiiDFSKSFEKGIEKLLAKITINKRID--IENLSPK--
IyEKLVEIFLKEY(GFQILQSNNNYDIGVDYICEYYAKDPFGNKIKERWMVEVKFYKNERFSINEIHKLYNYM
LDSLSKGSKLLMITNSILNS)}*

$642=259(263):
>gi|698778527|ref|XP_009822307.1|
{|654(222)|<Stramenopiles(E)>}hypothetical protein H257_01017
[Aphanomyces astaci] >gi|574121968|gb|ETV87444.1| hypothetical protein
H257_01017 [Aphanomyces astaci]
{(RPREAVLRLLRAYGGNIHARHDITGESALHVALKRGHVEMAKLLVQMGLDVTAVDAKGVRAIDCATSSEMQ
YAVKKAAGQ)-RDVMISYCHSHRSFALKVRQSLEDHHIT-
TWIDQLDPTGiggGSEWREEIARGIQSASVVLAVLSEDYPQ-SQWCMkELAFAKLHN-----
VPVVGITCGNVVvgDELEVYLWTRQAVDFR--
PSILSTAKAGNHVTFEFDNNQY(HVHIRQLLDGLRDEIEERRVVSAALKHQALELHVQHSHDGSRR)}*

$643=215(220):
>gi|698778531|ref|XP_009822309.1|
{|654(0)|<Stramenopiles(E)>}hypothetical protein, variant 2 [Aphanomyces
astaci] >gi|574121970|gb|ETV87446.1| hypothetical protein, variant 2
[Aphanomyces astaci]

{(RPREAVLRLLRAYGGNIHARHDITGESALHVALKRGHVEMAKLLVQMGLDVTAVDAKGVRAIDCATSSEMQYAVKKAAGQ)-RDVMISYCHSHRSFALKVRQSLEDHHIT-TWIDQLDPTGiggGSEWREEIARGIQSASVVLAVLSEDYPQ-SQWCMkELAFAKLHN-----VPVVGITCGNVVvgD-ELEVYLWTRQAVDF--RPSILSTAKAGNHVTFEFDNNQ()}*

$644=260(263):
>gi|698780121|ref|XP_009823104.1| {|617(188)|<Stramenopiles(E)>}hypothetical protein H257_01546 [Aphanomyces astaci] >gi|574122765|gb|ETV88241.1| hypothetical protein H257_01546 [Aphanomyces astaci]
{(APDLRPELLQALVLHGANINATVATGDTALHVAMKQGNRAAALALLGVGADINAVNGDGSRAIDCTTSTELQFAVKRAAG)SRDVMISYTHSHSEFAKKLRASLEQQHIT-TWLDLMDPSGiggGAVWREEIARGILNASCVVCVLTEDYPR-SEWCLkELAYAKQVG-----TPVLAISTEHVTitEELQVYLYTRQIVPFE--SAISAVDAANPRNITYEYNQDQ(YLTQFRSLLDGVRDEIEKKRCVARPTLDMPTTAMATWGPTLLQ)}*

$645=176(183):
>gi|571052605|gb|AHF26255.1| {|0(12)|<unknown(B)>}TIR domain-containing protein [uncultured bacterium Contig1777_n_1791_cl]
{(MAEQEN)--SVFISYSHKdREICDKID---SIIERQPGFRVWYDKGLLPGEVYRkRIAEAIRESSYFIILLSKTSVASDWVLDEVEYAKKLCK-----RILPIWLEDV-EIPDDLDMILQRFHSLFwhLRSSDEQFEESLMSMFGVTEKKP(VGXGAGGFRQRVFRKGQRPDEGTARTGKRKXASPRSI)}*

$646=254(259):
>gi|698824671|ref|XP_009845364.1| {|543(156)|<Stramenopiles(E)>}hypothetical protein H257_18052 [Aphanomyces astaci] >gi|574099551|gb|ETV65158.1| hypothetical protein H257_18052 [Aphanomyces astaci]
{(IDAGIFRLLRKHGLPEFGALENGDTPLHIAIKLCHQDTALALVRSGFSTTSTNAAGVRAVDCSQSTELQFIIKKEAGQRD)---VMISYSHAfSEFALQLRQSLE----DNFLTTWMDLMdptgIGGGAVWRdEIASGIKHAAVVLCVLSETYPVSQWCMKE----LAFAKA-HNVPVVGVLGETT-DMTDELQVVYLWSRQLVdfRQAITSTEPALVVNHDVYNTQLT(ALLDGLRNEVEKRRLVDRRDVLLSPLTVSVRQTEVEFAY)}*

$647=221(218):
>gi|738341677|ref|WP_036294295.1| {|0(1041)|<Actinobacteria(B)>}hypothetical protein [Microbacterium sp. C448] >gi|572850863|emb|CDK01174.1| hypothetical protein MIC448_50008 [Microbacterium sp. C448]
{(MVLDS)-YDAFISHR-StskaqaRRVKAALERFAQPKKGDEPFRVYLDESSLRVG-DLttEIETGLQRSRALIVLIARDTVGSRWVELEVRYWLSHGGsPDrlfMVrVDDIDLSWN---ET--SADWAHPDLVPSALHGLFSREQ--KFIDLASAAKGA(ERTALAPVFAAVRERPIESLLLEEADRARRRQRTIMVVAATMSVFFLLALAAGVWALISRNQAEAATREALGQ)}*

$648=222(221):
>gi|574460716|gb|AHG29002.1| {|0(826)|<Streptophyta(V)>}NBS-LRR protein [Cicer arietinum]
{(MASFSSSCRNKDSESIFNNDSNSNLI)-YDVFLSFCD-NDTDKSFASYLYTALTVAGVVVFRDDNNKlRNHD-QiitpSVLHAIEGSRMSIIIFSRNYADSARCLHELEKIMESYRT-

IGQMVLPVFYDVDPSDACHYEGMIGQAFKDFTQKIWKQDESSEGSNISGFVVDS(RNKSKDIKNLVEHAAHLL
DKTYLFVADHPVGVESRVQEVIQLLNNKPSDHTLLVG)}*

$649=242(247):
>gi|575412727|gb|ETW98420.1| {|99(0)|<Candidatus
Tectomicrobia(B)>}hypothetical protein ETSY1_18840 [Candidatus
Entotheonella sp. TSY1]
{(YLRDTQGLEDCIHFGPSTLDRRTLAHSGLLPLEFLRGYGLSDWEIEATKFYQQDLTQDDITTIGYNVINLK
TADPIQYQS)---VFLSHST-KD--ETFCHKLHDALQ-
KsGVRCWFSPEHIRWGDKLrpQLDEAIQQQDRLLLVLTKTSLQSPWVEYEIRRAIEREHQ-
EQRRILFPLKLVKFDVLRDWKLVDDDTGQDLAREVREYFIPDDFTSWQDDAAFE(AAFERRLANLRQEQRPGQ
PSGQRPGGA)}*

$650=205(220):
>gi|738040385|ref|WP_035999281.1|
{|66(0)|<Proteobacteria(B)>}hypothetical protein [Paraburkholderia
caribensis] >gi|944370332|gb|ALL67944.1| TIR domain [Paraburkholderia
caribensis MBA4]
{(TSTSEWVNQELRAFMRGPGRIIPLIIDDDGPAAIPEPLQQFQWADFRSEFDDAFYSLLTALHEVQGAESLA
KARFISRGY)---VFISYAYQDASFVEKL-KLHLKKRGY--
AYwdFRESERNYQVDYTlELEGVIQNAEATLSVISPNW-------------
KKSPTSMqEMHFSKEVGTPVFLLRLSDPGPTLSLAGLTCIEFTGSHANGFEKLDSKMKSFGL()}*

$651=206(204):
>gi|575421111|gb|ETX05290.1| {|0(245)|<Candidatus
Tectomicrobia(B)>}hypothetical protein ETSY2_23845 [Candidatus
Entotheonella sp. TSY2]
{(MIMSVLSLFKAIHSSHFEKITMREPLQK)QYDIFVSYSRLdrGWVQAELIPRLEKRGF--
QVCIDYRDFEIGIPTLHNIEYAVDNCRHTILVLTPSWLESEWTDFESLLVGTSDPAARRRKLIPLLLEPCAlP
PRITMLTHADFTRHQDhDTQFSRLLNQLQMTSINVNSPIS(QSKLSPFVAGPPIMYPRQFFGRERELRRLFNL
WRRH)}*

$652=186(186):
>gi|575410974|gb|ETW96999.1| {|0(244)|<Candidatus
Tectomicrobia(B)>}hypothetical protein ETSY1_24435 [Candidatus
Entotheonella sp. TSY1]
{(MHELSQK)QYDIFISYSRLdrEWVQAELMPRLER--
HGFQVCIDYRDFEIGTPILHNIEYAVDNCRHTILVLTPSWLESEWTDFESLLVGTSDPTARRRKLIPLLLKPC
-
ALPSRITILTYADFTQHqDHDTQFSRLLNQLQITSIDVNSP(ISQAELSPFVAGPPIMYPRQFFGRERELRRL
FNLWKRHP)}*

$653=249(254):
>gi|673016012|ref|XP_008861967.1|
{|604(181)|<Stramenopiles(E)>}hypothetical protein H310_00823
[Aphanomyces invadans] >gi|574483565|gb|ETW10556.1| hypothetical protein
H310_00823 [Aphanomyces invadan
{(VHSDLLRVLVEYGGDINFRLKSSGDTALHIAIKHEKQDTATALVGCGANIMATNHGGLRAIDIATSTALQF
AIKSAAGHR)--DVMISYTH-SHAE--
FAQKLRRSLEAVNITAWLDTMDpsgIGGGSVWreEIAKGISNATLLVAIITEDYTRSEWCLKELATAKQVG--
---
TPVLPISTEHVRLNEELQVYLYTRQIVPFEPAITHIDNANPRNITYTYNDTK(YHVQFRLLLDGIRDEIEKKR
KRASMMRSQMTLGS)}*

$654=235(243):
>gi|673016012|ref|XP_008861967.1|
{|799(0)|<Stramenopiles(E)>}hypothetical protein H310_00823 [Aphanomyces invadans] >gi|574483565|gb|ETW10556.1| hypothetical protein H310_00823 [Aphanomyces invadans]
{(ITHIDNANPRNITYTYNDTKYHVQFRLLLDGIRDEIEKKRKRASMMRSQMTLGSPTGSNNSSFFLSPHASP
NHGMLDRKF)---VFLAHG---
DCHRSFARRLYNQLTKHKqLECYMDLKTNQEISMRvhAAKDAILRCSAVIVILSAKSVKSELLNDQLAFAEDK
G-----
KPIFPVILHSDFALPPNQMYSLSRSTMHHFAADLGFESSCDHLLHTLKSRVG(LNAIEEADEDLGASSPLLVG
GVV)}*

$655=241(234):
>gi|575417649|gb|ETX02499.1| {|274(0)|<Candidatus Tectomicrobia(B)>}hypothetical protein ETSY2_35455 [Candidatus Entotheonella sp. TSY2]
{(ATELSSIAYVMERLSDLLASDFRDLEGALGSIARRPEGWQEVKFRVLSEGAKRLEQSSPSQWHYDIYVSYS
YQSSALTRW)--------------
IDVFIDSLSAYLALElpaPPRIFLDRERLDPNSVFseALSETMSRSKLLLAFLTPSYFASEWCHREWDTFERR
EQKMaPklrrssfvhssenHSLIKPVLLRGQKSRPE---
KLLRHMPWDAsQHVNLSDPEAairhPEMARLIQELATQ(IARTLEAIPDSDSM)}*

$656=206(209):
>gi|575417650|gb|ETX02500.1| {<Candidatus Tectomicrobia(B)>}hypothetical protein ETSY2_35460 [Candidatus Entotheonella sp. TSY2]
{(MSYTYDIFISYKRHRLAKKWIEE)----------------QFKELLEFYVDHAlgrTPAIFVD-
DQIDVGADWpsVLANNIQCSRVLVPLWSRNYFTSEWCCRELARMMARERGErVpgkdQRLVAPVIVNNYEqpa
kELPEPVRAIQAEPIHDYFNPYMQKEGKLAEGLAQVIAQRL(APGVTKSIEAAPDWKETWELRDEEVLFKALY
NGDAPVQVHVPGWSS)}*

$657=206(230):
>gi|575421999|gb|ETX05997.1| {<Candidatus Tectomicrobia(B)>}hypothetical protein ETSY2_19735 [Candidatus Entotheonella sp. TSY2]
{(MGAASNHVDAIEGYKWDVFISYRRREPVRSWVHEHFYKRLYEWLPEKMV)---------------------
-------REPTIFID-
EQIETGAPWrdELRNALQTSRFLVAVWSPSYFRSAWCTAEWHTMLAREKQLaLgahttpKGLIYPIV----
FDSKELFPATAATIQYRDFSPWNINFSGFEATEHYGHFIRE(IQQVAMELGALILQAPSWDPAWPVEMPSPDS
IQSVSLPRLR)}*

$658=149(149):
>gi|575419791|gb|ETX04245.1| {<Candidatus Tectomicrobia(B)>}hypothetical protein ETSY2_29910 [Candidatus Entotheonella sp. TSY2]
{(MSIQQDV)KYDVFVSYTA---SEIAFVKTLVLKLADRGLSVWFD---QGQLrLGDsvlRSLED-
GLEHSNyfLLIISPSFFQGPWTQFEAGVALSRV---
GKDHILPVYrgIDPDAikSHapLLADRVAISADQHSI-DEIADMVV-EIIQDERESKAEL(NP)}*

$659=239(219):
>gi|575408306|gb|ETW94876.1| {|0(48)|<Candidatus Tectomicrobia(B)>}hypothetical protein ETSY2_48990 [Candidatus Entotheonella sp. TSY2]
{(MQRLNS)---
VFISHSHPdSGLAAVFKEILEEVFAPSCTVLFSSDktPGGGIEGGTnwltWIHQRVTSSDETLLLLTPHSIQK
TWPMWEAGAVSGVAlareqspsssespqdqavtslpRS-VIPLTFQLSPKNMQGPFQTLQM-FNGNNEED--
LKKLLGD--

LLKRYHQSGDNS(VVVNALLEKYVPQLNSKLSEALRDTPHIVTEDVIQEWLRRLDDLRDQGRASEVRYYHRWI
SLMFDGHLVRNNL)}*

$660=259(263):
>gi|673014846|ref|XP_008861384.1|
{|654(223)|<Stramenopiles(E)>}hypothetical protein H310_00389
[Aphanomyces invadans] >gi|574482982|gb|ETW09973.1| hypothetical protein
H310_00389 [Aphanomyces invadan
{(HPRDNVLKLLRAFGGNLHARHELTGETALHVALKKGHVEMAKLLVQMGLDVTTVDSKGIRAIDCATSSELQ
YAIKKAAGQ)-RDVMISYCHSHRSFALKVRQSLEDHHVT-
TWIDQLDPTGiggGSEWREEIARGIQSASVVLAVLSEDYPR-SQWCMkELAFAKLHN-----
VPVVGITCGNVVvgDELEVYLWTRQAVDFR--
PSILSTCKLDNHVSFQFNEAQY(QVHVRQLLDGLRDEIEERRVTSAALKRQAIALHVQQSHDGSRC)}*

$661=207(224):
>gi|575418245|gb|ETX02997.1| {<Candidatus Tectomicrobia(B)>}hypothetical
protein ETSY2_34370 [Candidatus Entotheonella sp. TSY2]
{(MSRLRHAW)-----ITHAAKDYYVAAQLAEVLKRQRLR-
IGVDGTGRHAATLLSRERRAAISQSRVLVLLWSAAA-SQSRTVNvAWLAAFYED-----RFIVP-
CVLDVTPLPPCLQTTVYLDLRR--SSPHH-WE--
RLISAVRGAPNA(ANPIVHVVPRPPAALLRPLKPMMRIQQDICACWYRRDRAGMATLQRQLNGLITKHRLLSA
PDPGLASFQGYHLMHT)}*

$662=194(201):
>gi|738030820|ref|WP_035989732.1|
{|0(239)|<Proteobacteria(B)>}hypothetical protein [Paraburkholderia
caribensis] >gi|944368870|gb|ALL66482.1| TIR domain [Paraburkholderia
caribensis MBA4]
{(MLDDFIKMAQALQKRPN)--DLFISYGHPDKPIVDTIvRWLQHSAGLK-
IWHDDIGGDASSRTTALLERGIGSSRGSLFILSTN-WKASTWCEdEYNFALAER-----
RCNDAFLVVAVRidDVDIPPWFKLSNVLDF--
RQFDARSAAHLLRSLAPNPPVR(LDNDQDVYLAGPWKNTSTVAKAAMDAMHKMGWRIIGDSPDHPHF)}*

$663=172(181):
>gi|738461980|ref|WP_036412821.1|
{|0(327)|<Actinobacteria(B)>}hypothetical protein [Mycobacterium gastri]
>gi|574761181|gb|ETW24460.1| serine/threonine protein kinase
[Mycobacterium gastri 'Wayne'] >
{(MA)---IFISYSSQdRSSLEALTTALRRAQQQ----
VWFDQELGGGDAWWnKILEQIRASDVFIVALSNHWLQSK-PSQaELRYAQALQRP-----ILPVQVGPV-
DSVRVNPVSTLQIINYQnpTVDAGIQLVTAIHALRDKVPPLP(SPLPEEPPVPFGYLMRLGDALAEKELSPQQ
QLQMLVELR)}*

$664=189(184):
>gi|575404517|gb|ETW92260.1| {|0(187)|<Candidatus
Tectomicrobia(B)>}hypothetical protein ETSY1_44425 [Candidatus
Entotheonella sp. TSY1]
{()MPhNVFFCYSQKdEAFLQAFRVHLQPWEKADKLEIWRDKdKVKPGQRWDdEIKQALTEIDAAVLFLSPDF
FATDYIWDvELPALVKAREE-GRIQLSWLHLRHC-
NVDHADRAIEIALDGEAakSIKLTVYQGLHDKNIVIADPSTQ(RDAVYAKAAANLMALLSEPAPERSAWLTAG
RQSSRPKPARAPGN)}*

$665=158(159):

>gi|575415327|gb|ETX00559.1| {<Candidatus Tectomicrobia(B)>}hypothetical protein ETSY2_38840 [Candidatus Entotheonella sp. TSY2]
{(MKTQIQF)----FVSYAHAdQHSADQflrcLQEQLAPSKR-
YAYVLWRDTALLAGERWDdVIQQALADCQFGLLLISPMFLSRDYILTqELPRFI----N-
GDAKpFIPVMLKSV-NLQRHDLHGLDAYQMFRldHHLSFDDCGTARKQREFAEALFD(QIEHRLDRLVHM)}*

$666=185(184):
>gi|575415913|gb|ETX01042.1| {|0(223)|<Candidatus Tectomicrobia(B)>}hypothetical protein ETSY1_08955 [Candidatus Entotheonella sp. TSY1]
{(MRVSRYHF)----
FIAYASPdWRQARKLYFHLQEELRDEPDKVFFDRhQIQPGDTWPvRLSEALASSRAVVVLISSHTRAA-
FYEQeEIARAIQRARQQpGRHTIIPVLLETL-
PEGLPYGMSSLQVLDATrpSGLERVAVELVDWLAQQDAPEEK(LSHHSLSRANYQALGAASRLDRYPHWSRVL
EENQRP)}*

$667=138(145):
>gi|575419268|gb|ETX03834.1| {<Candidatus Tectomicrobia(B)>}hypothetical protein ETSY2_32325 [Candidatus Entotheonella sp. TSY2]
{(M)--
KVYLSYSQSdKEWADRLRTHLDTLEYSGEIDLWDARNVNIEAFTRdEITQALNSAQIVILLLSPDFLSSDLI-
NtELSEILQKLRS-KEIKLIPIVINYV-T------PKLLGEIENYkpInINYPLMT--
LSFEEAEKKLEEA(TTNS)}*

$668=149(150):
>gi|575420443|gb|ETX04763.1| {|0(162)|<Candidatus Tectomicrobia(B)>}hypothetical protein ETSY2_26965 [Candidatus Entotheonella sp. TSY2]
{()MSDIFLSYAREE--LERVRPLIQALEAQGW-
EVWRDEKIPTGRRYDqVIATALGEARCVLVIWSQASVNSDWVPDeAARGRTRGVLI-PAQIDDIELPLGF-
GMYQTAKLMGWNGDTADpeFRKITEEVTRLIGAPAQMEQTVP(PVEEPHIEEQ)}*

$669=117(143):
>gi|575423381|gb|ETX07102.1| {<Candidatus Tectomicrobia(B)>}hypothetical protein ETSY2_13180 [Candidatus Entotheonella sp. TSY2]
{(MPA)MPvKVIVTYSHHdVAYLgdDSLLGYLKELEQENV-
TFWTDRNVGVRESWDeVIKANIQDAHIALILVSQSFF-------------------------------R-
LLLLHECGNQLLSCSESpsYPYHSISLQLATLWVAAQSPDVA()}*

$670=206(189):
>gi|575424985|gb|ETX08406.1| {|0(298)|<Candidatus Tectomicrobia(B)>}hypothetical protein ETSY2_05595 [Candidatus Entotheonella sp. TSY2]
{(MSFTKH)---
LFISYAHIdNQpltpeqqgWITRFHATLEALLsmRLGsKAEIWRDEKLQGNDIFAdEITAQFPHTALLVSVLT
PRYLNSEWC-TrEVHEFCQSAEqnggvvvE-NKARVFKVIKTPV-
DTEASLHPVMQESLGYEffTFEDDTPLELDPAYGEKFAQDYN(RKVGKLAWDVAQLLKRLETYAPESRDTDGG
EQTATKSTVYLAE)}*

$671=190(197):
>gi|575425270|gb|ETX08630.1| {|0(853)|<Candidatus Tectomicrobia(B)>}hypothetical protein ETSY2_04250 [Candidatus Entotheonella sp. TSY2]

{(MEKYD)---VFISYRHQepdRSLAHTIAEAL----
RQAGYQVFTDTDIRWGMNWVkAIKEALEKSDYFFVLLSPEFAASEmAAVEiEIARELAYQQG-GHPVILPV--
RVC-----
YPLTELLPYPISAylSSIQQACWDSSDDTARLVQNLQN(TLANRVGWLADAPKPSPSKRVKHIGPPFHLDPRD
FIIPIPGGMVKRDSFAYI)}*

$672=183(169):
>gi|760088282|ref|WP_043770746.1| {|0(311)|<Proteobacteria(B)>}TIR
domain-containing protein [Roseivivax isoporae]
>gi|575506410|gb|ETX28843.1| hypothetical protein RISW2_04680 [Roseivivax
isoporae LM
{(MTAGAARRL)--
SLFVSYSHRdtapiraldtsrlAELMEKLAYKLGADNDYGRFRFLLDKQgmLRVGDVIDdRIGEHLREADIGL
IFLSRAYCESESC-EkELRELLALGKR-LCIVELDPFWDRE-
KTSRFEDLRERVKKILTcrFWAEDSGTVRLFGTPVPSAAKGD(SKADFTDALERLAAQIIDAA)}*

$673=205(206):
>gi|575417690|gb|ETX02531.1| {<Candidatus Tectomicrobia(B)>}hypothetical
protein ETSY1_03245 [Candidatus Entotheonella sp. TSY1]
{(M)-EPIILCHAF---
EDKKVVGRVYDALQEAGFEVWLDLESASDEQVWepDVADCLRQAACMLVFLSKNSVRKIGSTHhEFGQLIDTW
KDMqeGTVHTIPVRINDC-EIPEVLSRLDHIDLFE--
DEGLEHVIRcLRESETKQVLAPG(NDVPTSDQAVQGEALDDFQVAVDGRGEVVDAPTPLSDQPMASNRMSDSA
TFPVDPAPGADDAPNR)}*

$674=208(215):
>gi|644695641|ref|WP_025360962.1| {|0(415)|<Actinobacteria(B)>}TIR
domain-containing protein [Kutzneria albida] >gi|578012880|gb|AHI01139.1|
hypothetical protein KALB_7781 [Kutzneria albida DSM 43870]
{(M)-WDVFLSYSRSDAVPAQALAAELRRFGLR-
VFQDDADVHRFDSVSRTIMTELGRSRLLLAYYSAAYPTRRACQQeLTRAFLAGQ-----
HEGDPlRRVLVVNpaQGTGHIEPIELRDARY--WSPASALC--
HLLDALTRKLAE(VPTAMDEVALSPAPVWLPAPSRRPPPRFLGRTTQLWQLHSALHPQTAALTEGGGEPVAVV
HGFGGIGTTALVCE)}*

$675=178(182):
>gi|736410991|ref|WP_034433577.1|
{|0(1240)|<Proteobacteria(B)>}hypothetical protein [Candidatus
Contendobacter odensis] >gi|577002072|emb|CDH45646.1| hypothetical
protein BN874_2720008 [Candidatus Co
{(MAAPS)---IVVCHAP--P-DTAFARDLALALETCRLRVWRDIHELRGGDRIvpEVRWAI-
EKARQVIVVIGLNTGDPAGLRrEIELAQEVERGRmDAYRVIPLLLPGT-
DHSVLSRWFSPPPRTTPIQLTADGLGAALPTLLAILGEPL(PSDAATDRSPSPLTELTLTFSPADSPNAGAWQ
LVACL)}*

$676=192(203):
>gi|643541661|ref|WP_025225892.1|
{|0(290)|<Armatimonadetes(B)>}hypothetical protein [Fimbriimonas
ginsengisoli] >gi|663073387|gb|AIE85542.1| hypothetical protein
OP10G_2174 [Fimbriimonas ginsengisoli
{(MVVLLYKRN)--------AQ--PDDHLLAL-LEQSLRDAGHPVFVDRH-
LKIGVEWakAIEQTIRTADALVVLL-SESAWKSEMLEyEIETATDQFRKTgKPK-ILPILIGAD-
TKLEGAVAAQVGEFQFAVWKGPEDDEPIVAEVLAALEDSA(PIPKKVVPIEPAGGAVSASSPFYTWRETDAEF
DAALRNNESIVLVKGPRQIGKT)}*

$677=187(188):
>gi|502593087|ref|WP_012830664.1|
{|0(244)|<Proteobacteria(B)>}hypothetical protein [Haliangium ochraceum]
>gi|262082103|gb|ACY18072.1| hypothetical protein Hoch_5590 [Haliangium ochraceum DSM 14365]
{(MHFAR)---IFLSHSS-SD---
KELVHLVAAELAQrGVLSWLDIQELHAGATMsqVLARAIESQSLLALFLSPKAMSSAWVDEELATALRVEDE-
ARaaghaRPVLPIFLG-
DPHHLVAKHPRLRARWLHADGDRVDRLGIVPDAALPRSQQAA(HIAAKIADRVYDFLKLARADELGVILDQRG
KGQHRAEPPPLPE)}*

$678=243(253):
>gi|255083078|ref|XP_002504525.1| {|397(85)|<Chlorophyta(V)>}sterile alpha motif domain-containing protein [Micromonas commoda]
>gi|226519793|gb|ACO65783.1| sterile alpha motif domain-containing prote
{(HSSAAIWNLAFDESIREEIKACPGALDGLEEANKVGSEMTKAKARGALWMINSDAEEAVDHKISEDKVKLL
TSGGSTAAT)--QVMLSYEW-H--
SQAQAKALRDELVEEGFTVWMDVDRMMGSTLEAMAAAIESSDAIIMCVTNRYRESQACRTEAEYAY--T---
RKKLMIPAMLQKNYKPDGWLGILMGSKLYYNMHNQEDIRNAMPAIKAEVAKSMG(EKKAIGGEIDGANGAAQA
TPNLSSQAASKEIKV)}*

$679=222(230):
>gi|506282140|ref|WP_015801915.1|
{|0(96)|<Actinobacteria(B)>}hypothetical protein [Actinosynnema mirum]
>gi|255921515|gb|ACU37026.1| hypothetical protein Amir_3112 [Actinosynnema mirum DSM 43827]
{(MAGPPGLPGLLPLLDGYRFDVFLSYRRSGPGNAGAWVRDH)-----------------
FHEMLKDCLADElgsS-EVFLDLEA-
ETGVRWpsLLRDVLARSKVLVAVWSPPYFQSPWCLAEWRTMEARERALagRTRLVYPLRYSGGDnfseDARER
PSALVHAPREHQRPVAVVASGVGVRGVVALQANVG(GDHGVRAGTAGEISVQQGAHGAVGAVGGGQVAAADGG
AVGEGGGDRLLVL)}*

$680=179(179):
>gi|489552909|ref|WP_003457512.1| {|0(420)|<Firmicutes(B)>}hypothetical protein [Clostridium perfringens] >gi|170711706|gb|EDT23888.1| hypothetical protein AC1_0162 [Clostridium perfringens B str. ATC
{()MLNVFISHNYKdKPLARKISNELI----
KYGIKPWIDEsEIKLGDSLIeKIRAGLDNMDYLVVLISKNSVQSEWV-KkELDIAMNSEIE-
GKKVItIPILAGKC-
DLPGFLKGKLYADMSTNrkYNENIPILLRRFNVNNITDDINE(FTEYKLTTVQVIEKIEKAENENELIEFLES
FGYEEGRLF)}*

$681=240(243):
>gi|502006608|ref|WP_012690626.1| {|13(94)|<Actinobacteria(B)>}LacI family transcription regulator [Rhodococcus opacus]
>gi|226241330|dbj|BAH51678.1| putative LacI family transcriptional regulator [Rh
{(GVSRSTASLVLNDSPSVAPETTRKVREAMKALGYVYNRHAAMLRNQRSMTLGLVVTEVQNPYFAELAMTLE
DVADRSNYA)---VFIGYSRDdPARQRRLLDRFVERSIDGLI------
LLPAGDTQAaEIDELLASSGIPLVLLARHFhLSHDYVgaDNvEAGRLLGRHLR-AMGVRSVAMVGGP-
QHTSARREREDGFLEAVagSDVIMSPTEGLYGEATPDGGADA(TRRLLDRGEIPDAIVAYSDIVAV)}*

$682=168(178):

>gi|502643002|ref|WP_012879409.1| {|0(186)|<Actinobacteria(B)>}TIR protein [Xylanimonas cellulosilytica] >gi|269306117|gb|ACZ31667.1| TIR protein [Xylanimonas cellulosilytica DSM 15894]
{(MQG)-PRVFISYSSFdRPSVETMAKSL----
RNVDIDTWIDQELHGGGTWWsQILDQIEQSDVLVYALSQHSRRSKACRaErVYARALG----------
IPVLIVRL-
DDVELTRTPEALEQIFDydPQSVDSFITLLNEVKRLSARPRT(PVEPRPERPPVPGAYIHDFVVQLEADELSWTQQTR)}*

$683=246(255):
>gi|502711842|ref|WP_012946956.1| {|143(209)|<Actinobacteria(B)>}TIR protein [Geodermatophilus obscurus] >gi|284062577|gb|ADB73515.1| TIR protein [Geodermatophilus obscurus DSM 43160]
{(FEYLLRLGTAIRAEQLTPDQQGDFIRQLREVLETEDDEGVRDDARELLRALRRRQDITHRNAQAVDRLLADSTARETPVR)---LFVSYSRQdEVAVRDLVVYLDRAS----
LSVWADRDLQGGDRWWqEVLQQIRECDVFLFVLSDHSLASKPCVAELSYA----RA-LGLPILPVLIGDI-
KDLHLTPVADVRIADYRhrTLAEGMALLDAIRDAAQDRRPLP(DPLPKPPTVPFAYLLRLGSAIGAAHLTLDQQAEFI)}*

$684=205(221):
>gi|502711842|ref|WP_012946956.1| {|0(393)|<Actinobacteria(B)>}TIR protein [Geodermatophilus obscurus] >gi|284062577|gb|ADB73515.1| TIR protein [Geodermatophilus obscurus DSM 43160]
{(M)--WLFVSYSRKdETAVQELV---SDLERAHL-
SVWHDQELRGGDPWWqDILRRIRQCDVFLFLLSHRSLASKVCLAELSYA----RA-LGLPIVPV--------
QVGPVGNLRVTPVADmkVVDYQERT--
LAHaVELLNAIQQA(ASERGRLPEPLPEPPSVPFEYLLRLGTAIRAEQLTPDQQGDFIRQLREVLETEDDEGVRDDARELLRALRRRQDITHRNA)}*

$685=233(216):
>gi|489548087|ref|WP_003452728.1| {|0(245)|<Firmicutes(B)>}sefir domain protein [Clostridium perfringens] >gi|169297482|gb|EDS79589.1| putative sefir domain protein [Clostridium perfringens C str. JGS
{(MENSEVKK)---IFISYSwtT--
PKHEKWVLDLAERLVSDGVDVVLDKWNLKPGEDKysFMERMVTDEDInKVLIICDSKYKEKadgrnGGVGtETQIITPELYTKvNQRKYIPIIAEYG-
ESFEsyipayaksliGIDLASASDFEKNYEELLRDIYDKPRYVKPKLGKMP(SFLLDDENKTLKAITFKSREIKNHIINGRKDLVLSSLEDLNKLFFEQLDQYKLNSNDLKDPSYSIPMQ)}*

$686=268(276):
>gi|74852300|sp|Q54HT1.1|TIRA_DICDI {|741(327)|<Amoebozoa(e)>}RecName: Full=Protein tirA; AltName: Full=TIR domain
{(RFGNYEIATILLVRGCNRNLKDVQGQTPLHISVSQTNHQLSSLLCKHGAQSIPDKTMKNPLQYCSADIKSNLQNILFTNE)---VFISYAHKdSEFVNNLRSNFGLFS----
LRCWLDEyRLQAGCNWRaEITRGVVNSDVVVFVISETSINSLWCRKE----LKMSKK-LGKVIIPIFLQSV-
QIDPILYGLFTFIPKTTkkTFKEMSEKEVISNVSSISNLIHS(ILNGTQDSKLLTIQQQQQNFEKSVQSLSRKEFFDKCYLNDKNSGCYLLFNNADRI)}*

$687=179(182):
>gi|880905071|ref|WP_048671760.1|
{|0(1237)|<Proteobacteria(B)>}hypothetical protein [Candidatus Competibacter denitrificans] >gi|590104663|emb|CDI02052.1| hypothetical protein BN873_230067 [Candidatu

{(MMAAP)--QIFICHAQ--P-DAAFARDLGLALETFRLPVWNDSRNLRGGNRLapEVRWAI-
EQARQVIVVLGLNTGDPTWLRrEIEIAQEVERRRaDRYRVIPLLLPGV-
DEAMLPQWFTPVPRTAPLQLPSDGLGPILPTLLTVLGETS(PAGSATDRHAQPPTELALSFSQSTSSTASWQW
VARLN)}*

$688=196(227):
>gi|489614763|ref|WP_003519204.1| {|0(649)|<Firmicutes(B)>}peptidase S1
[Ruminiclostridium thermocellum] >gi|125714184|gb|ABN52676.1| TIR protein
[Ruminiclostridium thermocellum ATCC 27405] >gi|255990
{(MIYFCHAPND)---------------
RAIAEKIYFSLKQTGLTCWIPSQDIMAGQYYveAIANAIEKSDIVVFIFSSHSNTSIQVIDELQKASSLN---
--KTIIP-FC----------VDRAMPSEPIEHYLSSPYKV--
DATIGLPDDNIA(KLQNIIKQIYNQSSVHNANDINTVKDTFAPSNPLENNTNIIKDASDNNSQESIQFNGVIN
SANSQPPQNPNLPPQHQ)}*

$689=267(269):
>gi|119354459|gb|ABL65330.1| {|376(154)|<Chlorobi(B)>}Ankyrin
[Chlorobium phaeobacteroides DSM 266]
{(NNYRHCELFINFLISEINRDRVLLLVQPESIKHISSPPASKLNFHFLDADNPDKTWQKILQFVNIDYTDNT
LHELSIKEK)KYDIFLSYVS---QDAFLVRFIAEQLMAKGLSVWFAEYSV-----
EsfeLNFQEIINQgiaQSKkiICFSNKSYNQSEYCQIEAYISRNIISPENILEIELVEKDSSVA-
IDSIIGSKHKRFSTS-
SSDVRYILGMIADFIEEIRHEL(IPFYEVKMSTFIFTYKDLKFVLKIPNNWIVKENNIYKKTDIGIDNTVCD)
}*

$690=144(151):
>gi|499983502|ref|WP_011664220.1| {<Proteobacteria(B)>}TIR domain-
containing protein [Rhodopseudomonas palustris]
>gi|115518759|gb|ABJ06743.1| conserved hypothetical protein
[Rhodopseudomonas palustri
{(MPDSGE)--RVFISFSNADRAFARQLGALIQARGVS-
VTTNEATAARDRLIDDTLRHAIERATALVLLIPSQHVSDRNITWfEAGAAKALG-----
KPVLAVLppLRDAGstELPTDLAGLLVLDADR--RSLDSIAD--TLVQAMPEQDVH(VFAAQ)}*

$691=143(150):
>gi|499792369|ref|WP_011473103.1| {<Proteobacteria(B)>}TIR domain-
containing protein [Rhodopseudomonas palustris]
>gi|90106170|gb|ABD88207.1| hypothetical protein RPC_2657
[Rhodopseudomonas palustris
{(MSEGD)-KRVFISCAQEDRDFAHQLGDLLKQRGVS-VAGD-LDTAFGGDWADGLRRRIEDAAALVLLIPS-
--QNAPNRNtIWFEAGAAK-----ALGKPVLAVlpPSRdagatELPTDLAGLLLLDVDR--RSLESVAD--
TLVQAVPAEKLQ(LFAEH)}*

$692=245(242):
>gi|500065513|ref|WP_011744719.1| {|366(1257)|<Chlorobi(B)>}ribosome
assembly protein 4 (RSA4) [Chlorobium phaeobacteroides]
>gi|119354020|gb|ABL64891.1| ribosome assembly protein 4 (RSA4)
[Chlorobium
{(DPIFALWINSKNSDTLSSGAITFQGVGNRSIGQTRKEENVGNALKRGYSEITGYRSEEAVEIKAELPEPVG
ADDNKPRVD)---IFFSYAHGdsaikESFFDKIRARLKTSK-
SFRFSFSSDRDILIGEKWHdEIQEMIKRCDFGLLLLSTSFLGSDYIEEyELPKLLDKCLP-
VALDILDLERQNL-
KGLNEKQIFFFNGSKSFteVRENNRTRFINELAVQIENRVKK(QRCTEKTAEESELICCDPLGSF)}*

$693=192(205):
>gi|501452380|ref|WP_012475829.1| {|0(206)|<Chlorobi(B)>}hypothetical protein [Chlorobium phaeobacteroides] >gi|189496825|gb|ACE05373.1| protein of unknown function DUF323 [Chlorobium phaeobacteroides
{()MSDIFISYAREdERRVKPIVQHLQQMGW----
RVFWDRNIPPGQSWDeYIEQHLEASCCVVVVWSKHSVGSRWV-KaEAEEAKNRN------ILVPLLLDKV-K-
LSLGFRYIQAA---
DLTSWNHDDITHPQFRACIDAIAC(MVPQSGPLEPASPDVLVTPVEPPASRPQLPENFVLIRGGQFSMGSPED
EYGHQDNESLHEVKVSD)}*

$694=192(205):
>gi|501498514|ref|WP_012506765.1| {|0(206)|<Chlorobi(B)>}hypothetical protein [Prosthecochloris aestuarii] >gi|194312840|gb|ACF47235.1| protein of unknown function DUF323 [Prosthecochloris aestuarii D
{()MSDIFISYAREdERRVKPIVQHLQQLGW----
RVFWDRNIPPGQSWDeYIEQHLEASRCVVVVWSKHSVGSRWV-KaEAEEAKNRN------ILVPLLLDKV-K-
LSLGFRYIQAA---
DLTSWNHDDRTHPQYRACIDAIAR(MIPQSGPLEPASPDVFVTPVEPPASRPQLPENFVLIKGGQFSMGSPED
EHGHESDETLHEVKVSD)}*

$695=233(256):
>gi|501499747|ref|WP_012507986.1| {|98(0)|<Chlorobi(B)>}pentapeptide repeat-containing protein [Pelodictyon phaeoclathratiforme]
>gi|194308794|gb|ACF43494.1| pentapeptide repeat protein [Pelodictyon p
{(RGANLSGVYIAFTVFANIDLSSVIGLDTVQHRGPSTIGIDTLYKSKGNIPEIFLKGCGLPDTFIEYAHSFT
SKAIEFYSC)----FISHSTAdKAFADRLY---ADLQAKGV-
RCWYApHDMKGGKKIHvQIGEAIRQHEKLLLILSESSINSEWV-KqEITKAKKREdRE-GKQVLFPISL----
----IEFGKIQ--------EWEFPD--
SKGRDLAEEIRLY(YIPSFVGWEKDNEAYTREFGKLLNSFQAERDIEGKA)}*

$696=218(215):
>gi|501499772|ref|WP_012508011.1| {|0(742)|<Chlorobi(B)>}hypothetical protein [Pelodictyon phaeoclathratiforme] >gi|194308820|gb|ACF43520.1| WD-40 repeat protein [Pelodictyon phaeoclathratiforme BU-1]
{(MFDLMRNLLPKIAMGVGGSTWDSIASRLFG)-YDIFVSFALGrpprgtRNYASDLTRRLK----
DHDFRVFFSEEEAAPGEALdaALQKALFRSRALVVVANKETlNDPRWVQVEVEKFRERHPKRpviTINVEGAI
H-D---
PSIKDRTNKWLGHLGKIWLDETEGAVREGKASDDLVERLA(ITPRHARVNTLWKTLVFGVVFLLLTLTILSFK
SANRARSELKKEV)}*

$697=214(220):
>gi|521297494|ref|WP_020461762.1| {|0(744)|<unknown(X)>}WD-40 repeat-containing protein [Frankia sp. EAN1pec] >gi|158111433|gb|ABW13630.1| WD-40 repeat protein [Frankia sp. EAN1pec]
{(MSVSSNWGRAGSAHH)-
YDAFISYTEAdvaiaRELERAIASHVSPDPRLKSPRVFRDRTKLTTAPRLaaAIEQALDRSEYFVLVASPDVV
HSRWVRLEIEAWqRRDYRFErllVVLAG----------------
EDLRTSLPGPLRAFYFPERVDSTGDARHTPLEL(SPNYLSVKPVIRSLHKKHGRRPSRPGRLSQNATEEEIGE
FESQRQKCEEWDQNASSLITSHDEFA)}*

$698=226(223):
>gi|501498578|ref|WP_012506829.1| {|0(916)|<Chlorobi(B)>}hypothetical protein [Prosthecochloris aestuarii] >gi|194312905|gb|ACF47300.1| WD-40 repeat protein [Prosthecochloris aestuarii DSM 271]

{(MDSIPPNSETAKKDSLGSTGKNNRHDKQQKSFAVRLVG)-YDMFLSFSLGppprgtrnyaSDLARRLQD--
------
RNFTVFFSEEEAVPGDQLdsTLQQALFRSKCLVVIVNRGTlKDPRWVRKEVEEYRARHPGRpviIINIGGALQ
-D---
PELKDEAQQWLVYEDKIWLDESVSAVEQGRVSERLLDRLS(ITPKHIKRNLWWRGIKAGVFSLLTVFIVGMLF
FAWRYGNENQRAK)}*

$699=229(220):
>gi|497532902|ref|WP_009847100.1|
{|140(0)|<Proteobacteria(B)>}pentapeptide repeat family protein [Vibrio sp. MED222] >gi|85834151|gb|EAQ52308.1| pentapeptide repeat family protein [Vibrio sp. MED222]
{(AKLINVDLNNATLGSTDFADTDLTTAINLESMNHMSTSIFSIKTISDFGDNMPIKFLTKAGVSESLIERFY
GLEMTKEDY)-YSCFISYATAdeDKV-KSIYDAL--
WCAGVSCWFAPENLRAGKKVHEQLFEGIEGHEKLVIVLSESSMKSEWVKTELRKAFKLEQKTGERKLFPISLV
SFDtLRDWECFDSDFGKDLAVeLREYFIPSFHDVKSNADFSKVIerlvldlkkI()}*

$700=167(194):
>gi|154162926|gb|ABS70141.1| {<Proteobacteria(B)>}conserved hypothetical protein [Xanthobacter autotrophicus Py2]
{(MRLEARIGPN)--------------------------------
SYFYENNYVSQLARPsldTLLQNIYRDCLKliVVFIGVDYQRKDWCGIEFRAIREIIMERDHLRIMFFRTDEG
AveG-VFKTDGYIDARRFSP-AQIADFVG-
QRLKLLGQPCLPS(TSIRRRPMPVLPRFGGHPCQLLRASASKGDWCNTTAVATAWPRA)}*

$701=211(220):
>gi|497520978|ref|WP_009835176.1|
{|60(0)|<Proteobacteria(B)>}hypothetical protein [Marinomonas sp. MED121] >gi|86164831|gb|EAQ66100.1| hypothetical protein MED121_02775 [Marinomonas sp. MED121]
{(EVFEEWLNINYPDKHRIYNLFSHDLMKAIGNRSCNGMGLLDSEMLEYQMPSGTNEIFLHPDRPTYDATFPP
VLKEMTNYS)---VFLSHSSVdKELVDTFFNELQKSEVE------
SWFDRYEISPGDsitdRINDGLDKSKVGLLFLSKSFLsgKSGWTMNE----ANYFFQ-
KRMRQNEKNFIVVNIDLEHDEMPPLLQDYLY--LNYDDPECIPKIVNELRKINL()}*

$702=282(286):
>gi|85690536|gb|EAQ30539.1| {|115(189)|<Proteobacteria(B)>}hypothetical protein NAP1_07165 [Erythrobacter sp. NAP1]
{(RWLSRSEHGFEIWREWYYGRLEGLSHAFAEFDDEADQTFFRWIVEQDNEWWSREPAEVNADIKAFVDSLRK
RKSDDKPRV)--DFFISYASPDETDAREVAAILDQLGKS-YIVQYRDF-
PQANFVNAMNDAMDRADRLIPLYSRSYVA-SDHCNaEWNYYYNLD-----
PSSVERRIVGfkLDggDLKPLMQTVNYRDLTrysG--
FDREEAIREWIEWEPPAPTRES(VSDSVERLLSPQIAANAEGKLDTLPNPLTDVPVRESALETAVRELLLVLD
VIFASKHNLPSNMDRA)}*

$703=177(163):
>gi|502528798|ref|WP_012813825.1| {|0(337)|<Bacteroidetes(B)>}TIR domain-containing protein [Robiginitalea biformata] >gi|88784961|gb|EAR16130.1| hypothetical protein RB2501_04510 [Robiginitalea bifor
{(MAYVPGYEYD)---
IFISYAHVdnicfpdqeDGWIKQFYQNLNLMLarqfgRMDSVKIWWDTKKLDGSVlFDdSIADGIRKSAIIIC
LDSPGYNASDYCKQeLGTFRDSASAD-KLGLKVGDQSRIL-
HVLLNNIPHEKWLEPLNgtTGFPFHDAETADDFGETVETTSP(KFKAQMRHVKNAV)}*

$704=221(222):
>gi|589601100|gb|EXI73027.1| {|224(0)|<Proteobacteria(B)>}TIR domain protein [Candidatus Accumulibacter sp. SK-11]
{(LTSGPTPPQTSEGRAPRQLAERLVAACEQDLRGELLRAASEDEKKLLQRLFGRIDRRRPAAPERSDDVATT
SVEDDTGRH)-YDFFISYAHRqeREV-GEFVRALQESAPAARV-
FYDRESIPVGGQWLrIISDAVNRTRHFIAVLSPDYTASPVCWDEFQCAKLKEYNTRQSVI--
RTLRLYSESDLPPIIGIYSYIDCAeGDLDKLRQSATRIGSLPPGDDDA(SP)}*

$705=174(184):
>gi|589395114|gb|EXG79871.1| {|0(216)|<Actinobacteria(B)>}hypothetical protein CryarDRAFT_0921 [Cryptosporangium arvum DSM 44712]
{()-----------------
MVSRLYDSLRASALQVYLDAHEQasQSSEDFpdRVDRELRDCSGAILVVSPDVVGSAWVKHETSVLANRR---
--
KgmvtgqlPLIVLYVDgAKPDDLRGEHWNITRIPWLGSCTELSEAHDFAMTRLQPLMSRA(SRDLRKESIYLT
LDSTLRSLQLEELTAALAVLGLKAHVWSPTDA)}*

$706=205(220):
>gi|589608725|gb|EXI80265.1| {|143(0)|<Proteobacteria(B)>}TIR domain protein [Candidatus Accumulibacter sp. BA-92]
{(RWSALLMMEALVSAAIQWMRRGLPVSQLMIFERDPGRAPALLTLMKTLAENGEGSRTRGASLSAPPPVAPH
YDVFLSFSS)------HNA---DAADAFKRELAAISQSTT--
VFDFRLSIQKGKSYqdDIDRAIESCRKVVSILSPAYFASPECQEELNIARLRNKRAgF-GLLIPLCWKSV---
---SPALPLWLQTLNQSDCLEESLDKLTTAAKDVHATL()}*

$707=270(263):
>gi|589394742|gb|EXG79499.1| {|209(7)|<Actinobacteria(B)>}hypothetical protein CryarDRAFT_0540 [Cryptosporangium arvum DSM 44712]
{(AFSSLMRTNHGLYFDIDPALSVARYWQELVERISDSHGSDGLRGLAAATHEFAPDDPATMSIEAALRQLAD
EAARTPPGQ)SYRFFLSYSRRDvdqhrvlefwRALKQQTQTHARRAPkeYD-GYIDQ-
NMQLGSHWPSKLYHAATTSRVLIALCSRDYFERS-WCGlEWAVFneRARR-----
AGEDTVGILPLRwgDMGKADWPDGVRDVMH--
TTEALDSVYGGLSLADFGGDHS(YRDFVRILSQIIHSLPNSPDLPKLTVEEVNGLTPTFGNDPRPK)}*

$708=175(179):
>gi|668673094|gb|KFB68427.1| {|0(286)|<Proteobacteria(B)>}hypothetical protein CAPSK01_002281 [Candidatus Accumulibacter sp. SK-01]
{(MVS)---VYVSYAWKDEEqhrLVDRLGTACTARGID-FVRDTSHVGYGNSIREFMDR-
LAVAGHVVLVLSDTYFR-SEYCMyELRGIYEHR-----NfrkRVHPIVLSGTRlhKPKDRITWIAHWIKEK--
KELEEALEALEDPKHTLELRKS(LEDYADFHRLMDQLTSLLADMNTLTEDVHRDTDFAA)}*

$709=175(179):
>gi|668681928|gb|KFB74679.1| {|0(286)|<Proteobacteria(B)>}hypothetical protein AW06_004383 [Candidatus Accumulibacter sp. SK-02]
{(MVS)---VYVSYAWKDEEqhrLVDRLGTACQARGID-FVRDTSHVGYGGSIREFMDR-
LAAAGHVVLVLSDTYFR-SEYCMyELRGIYEHqnfR-----KRVHPIVLSGTRlhKPKDRITWIAHWIKEK--
KELEEELEALEDPKHTLELRKS(LEDYADFHRLMDQLTSLLADMNTLTEDVHRDTDFAA)}*

$710=194(178):
>gi|589610031|gb|EXI81518.1| {|0(242)|<Proteobacteria(B)>}hypothetical protein AW10_01055 [Candidatus Accumulibacter sp. BA-92]
{(MA)ALKLFISHssrlrpASADDAEAQG-NWQLlqdtckglkTEYGdkIE-
ILVDYEGLHPGDDWERRLNEWLAECHAAIILFSRRAIEESNWVRkEaailsWRAELEPE-----

FRLFPVLLDQQAtpEDLEKDFLGTLRITRT--
QCVAQVSTAAQILGGIRLKMGH(HPELLRGTVATPFERLLEAVESVIVEQVKLSSLERL)}*

$711=245(222):
>gi|668678045|gb|KFB72205.1| {|0(198)|<Proteobacteria(B)>}hypothetical protein AW09_002623 [Candidatus Accumulibacter sp. BA-91]
{(MP)ALRIFVSHssrlrddeTSAAQAQANWrllqeTCCALRDAypdDME-
VLVDYDGLQPGDDWEKSLDGWLYDCHAGVLLLSKCAVQ-SQWVRkEasilcWRAANEPD-----
FRLYPVLLDGQTpeDIDRdPFFRTLRLSRFG--VVQDVAcsksilaalRL--
RLDERVSLRRAP(RTPFEKIVAAVESIFAAEIDERTLEEHCRRLGEQSPPRCHLDRRRALARSLACALLRDGK
SAQGRFRQLIADIFPRPPRE)}*

$712=173(180):
>gi|1181405056|ref|WP_084700611.1| {|0(95)|<unknown(X)>}hypothetical protein [Cryptosporangium arvum] >gi|589397556|gb|EXG82313.1| TIR domain-containing protein [Cryptosporangium arvum DSM 44712]
{(MA)-ASVFLSYSRSdRGYAEQLVPFL----
RAADLTVWWDFELYAGEVWTrEIAQRINMCDVFLVILTPEAVKSPWVLRELHYA----SE-
SGKRIVPLLLDAC-
ELPLLLAGVHREDVRGGdlPGTQFLVDLGGVASPRRKRFSRR(ERSTNSALPQFAAQQALLVEEVRWLMAHDR
KSSVSITV)}*

$713=172(166):
>gi|668673755|gb|KFB69053.1| {|0(615)|<Proteobacteria(B)>}TIR domain protein [Candidatus Accumulibacter sp. SK-01]
{(MGSQ)---IFISYSRAdKDsydgqdFIAELLQALRTVpgIRD---
RLWIDEQgIEVGDRFDdVIQQAMVDSAIAILLVSNHFLNSDYVTRrELPFLLRRTEC-QGLKLGILYLGAV-
PDAALSSDRVDHDGKPYrlELLRTHSFNHPTKPLSAMKSRSD(RDLLYRKVVEWAGRQLPPVSLP)}*

$714=256(232):
>gi|589607528|gb|EXI79137.1| {|0(215)|<Proteobacteria(B)>}hypothetical protein AW10_02533 [Candidatus Accumulibacter sp. BA-92]
{(MAKANIKQIAGRR)---
IFVSYCHAdYSAACRLNEVLeAMCRRLGDSAVFldckGDNRLMAGDEWKaKIMAAVNSANVFVILMSTSFHAS
PFCRDvELKRMLERRRlE-PHVKVIGIALHKV-
NLKDFSvevdgqavaLEELQCLPQeltttaagqrpGLkpINRWRFAS--
DAWVTVGEQIEET(LLADNHPVFSARETMQLIEPALSAPKPIAIQASHLPYLCDRGEQHDALIATLLAWRKTN
FVRPLVLVTEGRTDDCLGKW)}*

$715=178(185):
>gi|589608213|gb|EXI79779.1| {|0(144)|<Proteobacteria(B)>}hypothetical protein AW10_02264 [Candidatus Accumulibacter sp. BA-92]
{()MKPIFISYSSQhRDLTRALV---------
GVIEaqygagsVWWDHELESRASYAeQIRAALEEARVVVVLWTAGAMISDYVY---AEAVGAQAQ-GKL----
VNVRPA-
DMRFSDIPEPFNIHHIDdaEDHARILATIAKVMTGEPIPTRV(PLHELYFRQHGHRLIDAKQRQLARDPREIA
PTELGLHEEALAASQ)}*

$716=155(162):
>gi|668683841|gb|KFB76261.1| {<Proteobacteria(B)>}hypothetical protein AW06_002683 [Candidatus Accumulibacter sp. SK-02]
{()MSDIFICYSHSdKTIATRLRDRLQ---REGW-
SVWMDLHIDAGHRWAdEIQAQLAAARAVVTLWSVRSVTSRFVMNEAHEAADRE------IIFPVRLENL-
RIPYGFRQFQTPDLIGWdgGEDCDGARQLVSSLHKHLTGATP(RRRRQSSLPSPRHTPAREHRLR)}*

$717=178(185):
>gi|589619388|gb|EXI90554.1| {|0(894)|<Proteobacteria(B)>}putative O-linked N-acetylglucosamine transferase, SPINDLY family [Candidatus Accumulibacter sp. BA-93]
{()MKPIFISYSSKhRDLTRELAGVIE--
EQYGAGSVWWDHELESRTSYSkQIKAALEEARVVVVVWTAGAMISDYAY---AEAVGAQAQ-GKL----
VNVRPA-
DMRFSDIPEPFNIYHIDeaEDHARVLATIAKVMTGVPIPTRV(PLHEIYFRQHGHRLIDAKQRRLARDPREIA
PTELLQAKFEVVGYL)}*

$718=290(300):
>gi|668679623|gb|KFB73246.1| {|104(308)|<Proteobacteria(B)>}hypothetical protein AW09_001506 [Candidatus Accumulibacter sp. BA-91]
{(APLDSSPHHVLPVALDQEGFGLGDAIHILPATLATGVSASDAKDRRLAEVSFHIAARSIQLLEHGKVPAIA
PDRMQAPVR)---
IFLSHAKAdldKQHHDDPVRQVRNLLNELPVEQWFDaQQIATGQDFAdAISAGIRDSSIMLAFQTDHYSSRPW
CRReVLE-----AKR-FGAHVLMV--------EALQSGEPRSFPYAGnvpTVRWQFRK--
DARVDAQRVIDRA(VLEALRFKHNRAVLDSTAAPDEWVLPAAPEALTLAIACADLDHDKTFLYPDPPLGREEL
QVLQQLRPKARFLTPLSKVAR)}*

$719=177(177):
>gi|668679628|gb|KFB73251.1| {|0(602)|<Proteobacteria(B)>}hypothetical protein AW09_001511 [Candidatus Accumulibacter sp. BA-91]
{(MDS)--KIFISYSSQdKPYCDELLPALKAVtgIRE---
RVWRDREeIDVGDPFHpTIQQALADSRLAILLVSANVLTSDYVIRhELPFLL-RQYE-GGALKLGIIYVSS-
VAKAALSVAVDGSTHPVdlTAIHNFNSPQQPLDRISERGERN(LLYARVADWAARHMTPLSPPARQPSAARHD
LAIF)}*

$720=212(220):
>gi|589607194|gb|EXI78820.1| {|485(0)|<Proteobacteria(B)>}E3 ubiquitin-protein ligase ipaH3 [Candidatus Accumulibacter sp. BA-92]
{(GLSIAEPDAVLTWRGRQSVAQFEVKVPGEVAGEKVIGALRIAVAGMPKSTLRFQVALLAAGASRPDADACE
IDARSYRRA)----FVSYSS---QDRGEVLRRVQAFRIAGMSVFQDILDLEPGERWekELYREIDNCDV-
FLLFWSQAAADSEWVGkEIDYAL--ARKHgDDD-RPPAIQPVP-
IEGPPIPLPPKKLSGMHFNDALLAHIAVAPGAARGSGSQP()}*

$721=159(158):
>gi|668674327|gb|KFB69584.1| {<Proteobacteria(B)>}hypothetical protein CAPSK01_000643 [Candidatus Accumulibacter sp. SK-01]
{(MAKNDSASEASTLPPTY)----FVSHS-SgddaivRQLRIALAELETPLTI--------
DSRAFHGGDPLesTIRDAITRSSGVLVLISPRAHQSTWVGKELKHALavqrQRGGVEhfpvvPLLLDGTPLGA
---FEVLFDEAPIHVALASAALDAAPHHILXXXXWKAGNMRMG(C)}*

$722=181(170):
>gi|589610317|gb|EXI81795.1| {|0(340)|<Proteobacteria(B)>}TIR domain protein [Candidatus Accumulibacter sp. BA-92]
{(MDPSET)-YDIFVSYAHAdnglalgastsvgwvtalaANLNEGPSALPK--------NIFIDHQ-
LRPGDEFndELLDKVRRSRLLVLLLSQNYVDSQWCGKELEHFVRSHGGSaekPSDVLVVEIWP---
FEDLTGVPPIIQALRKGTIHAKFWYKPVNAASPVLAGYPS(PKDCDEEGTRYYWRILNELRVPID)}*

$723=239(240):
>gi|668683846|gb|KFB76266.1| {|0(139)|<Proteobacteria(B)>}TIR domain protein [Candidatus Accumulibacter sp. SK-02]

{(MPGDDPKPLAASTAGGDDTV)--
DLFVSYAHAddevpagarkgwvTTLVGELRRVLRRKLGGSGAQVWMDHA-
LAANQPLtaTLRDRVSRSRTMLIVLSPGYVRSTWCQRELCDFvtaaQARGEAEnifVV------ELE---
PIDRD---SQPSALPAPIRELVPIA--------
FWERDGD(DEPRLAGYPTPNADEYSPYWKLVNRLAGQLARRLERALPAAPAAGAVAAPLSQQTAAIFAGPDSI
TTEPAGIPQAHGLSL)}*

$724=232(230):
>gi|594045719|ref|XP_006047327.1| {<Chordata(M)>}PREDICTED: TIR domain-containing adapter molecule 2 [Bubalus bubalis]
{(MGIGKSKMDPCHLSVPWGKSQSVDTSQSHHTSDSKQSEEISLHGDAVCSSTAEKPAGEQEGVEERPEEDTE
E)--
EVFLKFVilHAeEDTGEALrVQSLLENDFGIKPGIIFAEMPCGRQHLQNLDDAVNGSAWTILLLTENFLRDTW
CKFQFYSSLMNSVNRQHKYNSVIPMRPL-
NNPLPRERTPFALRTINaLEEESRGFPTQVERIFQDSVYRI(QQAIWKETRNMVQRQSVA)}*

$725=177(181):
>gi|695446946|ref|WP_032564561.1| {<Bacteroidetes(B)>}TIR domain-containing protein [Bacteroides fragilis] >gi|596042063|gb|EXZ17112.1| TIR domain protein [Bacteroides fragilis str. J-143-4] >gi|59604
{(MGRFF)-----LSHSS---KQKGYVEIIARSLGKQN--VVYDAwtFEEGN----
KTLDEIYKgiDSTGlfVFFISRDSLKSDWVKKEILKAEEYIKKGKIKKFLPVIIDkeikhsDPSipN-
WMKdeyNLRYVS----KP-TKVVDLIK-
QKQRLISWDLFPN(KKELDQLFIGRTEQLKQFQTRIYDIDQASPNCIIIS)}*

$726=172(178):
>gi|753189049|ref|WP_041496832.1|
{|0(159)|<Bacteroidetes(B)>}hypothetical protein [Nonlabens marinus] >gi|594836133|dbj|BAO56347.1| serine/threonine protein kinase [Nonlabens marinus S1-08]
{()MKRLFISYSRAdKEY----
VRHLVDVLRKQDFEIWFDEQIPVGQGWDdVLEAQIKKADAMILVLSCTSVASENVKD--EIAFAKHSN---
IPVVPIKIEEC-
NVPLRMVRTQFIDFTLGfdVGAQRLVDDLRVQFEKEAAKSGE(PGAAAIPTRKKKIKRVYTRKKPWKAYIIGA
AAAIILPF)}*

$727=192(191):
>gi|685236778|ref|WP_031605021.1|
{|0(129)|<Proteobacteria(B)>}hypothetical protein [Salmonella enterica] >gi|595748953|gb|EXX83916.1| hypothetical protein M568_19390 [Salmonella enterica subsp. enter
{()MTNVFISYAREdVLIARKLTEELS---
RYGYNIFLDQQSIRLGSHWQeDMLKNLKNADVFIALVTEHSDKS-YYFNiEVAGAISYvtNSN-
RSKLVLPVVLDKE-
ALNNSDLGVFQALFARSndMTNLVNKIVNAISRFEGARVAQE(ETKEIRAKVLKDESADYISETIEDLKAREK
DGRQLSSRWHLAGYGALVASV)}*

$728=210(220):
>gi|695522684|ref|WP_032591873.1|
{|0(564)|<Bacteroidetes(B)>}hypothetical protein [Bacteroides fragilis] >gi|596199696|gb|EYA47229.1| hypothetical protein M115_2933 [Bacteroides fragilis str. 3719 T6
{()MAKAFLSHSSSdKSIVRRIATQLG-----
NKNCTLDEISFDPGRKTLeEIFKELDSTDVFVLFISHEALDSSWV-KkEIIRAKENLSK-

EYMdRILPIIIdENI-KYSDVRIPKWIAKPYNLkyISNE---------
VIILKKIKQA(LREVSFKKNRFNQELEKVFVGRNSEMQKFETDINNLDNWTPTYIIAYNYFEGIGRRTFLRNA
LRKATLIDYLDSPTIITI)}*

$729=246(247):
>gi|597861248|gb|EYC10661.1| {|21(0)|<Nematoda(M)>}hypothetical protein
Y032_0054g2483 [Ancylostoma ceylanicum]
{(NRTICVPAASGFFGQEPQNSILTVIFLTSCAFLLCAMTLLGVSLVRKAHNDMSQRRYKASSSLNCSSTPGS
SPLPVPLLN)-FDAFVSYSKKdeKMVLEQLCRPLEDED--
YALCLLHRDGPAYHSrlhaISDELISQMEASQCLVIVLTKNFLESEWKTLQVKTSHQLFAKNRGKRVIAVL-
GEG-VDQNLLDEELGQILRKNtCIRQRDHL---
FWQLLRSALPTR(LASLPGSGDDASQIYSDMYGIVPSAVI)}*

$730=215(220):
>gi|738144532|ref|WP_036102025.1| {|39(0)|<Proteobacteria(B)>}molecular
chaperone Tir [Lysobacter capsici]
{(KAGRRYTYHGGHAKRSPVHLTVPRSGHWHLIVDLGGHRGTVKHSVRILPGPLKTLSDAPLSTVPSLIQGGA
EADASDDVR)EFDVFISHAS---EDKDkVVRPLAMALQGAGLKVWYDEFElkIGDSLRRkidKGLA-----
KSRfgVVVLSKAFLSKGWTNYELDGIITRAI-SGEQVVLPIWHEITKqeV-IDYSPSLADklarsTANYTV-
DEIAAEIV-EVIR--------T()}*

$731=224(215):
>gi|598999553|dbj|GAJ27552.1| {|0(148)|<Firmicutes(B)>}hypothetical
protein JCM15457_2555 [Lactobacillus sucicola DSM 21376 = JCM 15457]
{(MVESP)--KVFISYSHQsSEYNKKVLDFSNNFRLKGID---
TNIDQYVEAPSEgwpqWMEKQIYDSDYVLVCCSKSYIEKFRENTEgLGISWETSMI-
YQFIydnatknskIIPVFFDEKDKSFvprpLKKYTFYNLSKEAD--YVRLYNR--
LIGKTTNNKAPL(GQLKPLDEKKPKTLFVSTPIDIEKWNQARWRGIMYICIAGDVPVLGLVFKNYQRGLEIFD
QWRRLSTDRY)}*

$732=298(300):
>gi|918245464|ref|WP_052376465.1|
{|134(323)|<Proteobacteria(B)>}hypothetical protein [Chondromyces
apiculatus] >gi|599563810|gb|EYF02036.1| Hypothetical protein CAP_7515
[Chondromyces apiculatus DSM
{(EVAPGWFALEFVGFQVVRGPAAPSAHLARIDVTLPVLNTRDCCKAREEYVRNAWDLLPPEQLSAQAPCTHH
LDEMSTPLR)---
VVISYAEQdEAFLRELEKHLGPLKRQGVIDTWTKLRLLAGEELEkETRSRLEQAQVVILLVSVDLLASDFCYD
aELGRALERRQA-GHAAVIPILVRNC-SIEDTPLHDLLVLPRGGgtVARASSSD--
DAWTEVVKEIKEA(IRSFTAQRTEHQESPGAPFVPEHNLPSERACFGRLPILADLSRSLLTPSPARILLLGSG
GIGKTTLSLAALHHPDVAARY)}*

$733=231(234):
>gi|599564090|gb|EYF02299.1| {|315(0)|<Proteobacteria(B)>}Hypothetical
protein CAP_7228 [Chondromyces apiculatus DSM 436]
{(AVPAVPAVPAAPAVTPANATPTASTSPVIPDLAAPLPAVSTSTSPTAPSAPSASPAAPATPAATASAASPA
PGAAGPIDT)----
FICYAEGdADLFKRLQVHMAPMvKREKLIAPWHHGDILGGEEVEpQIQAHLDSAQLILVLISAELLASEEL-
DlQIARALERQTQ-GTARVVPILLRPC-TWEESRFGKLVVLPRSKipVTRSADRE--
EAFNEIAQELRTL(VKGLQSKGLSGLRR)}*

$734=228(230):

>gi|763389457|ref|WP_044246577.1| {|0(721)|<Proteobacteria(B)>}ATP/GTP-
binding protein [Chondromyces apiculatus] >gi|599565029|gb|EYF03182.1|
Hypothetical protein CAP_6158 [Chondromyces apiculatus DSM
{(MLAPARNPID)---
VFFTYASGdEDLRPKLEVHLDALKRQGFLRSYGGLPLGGGKEQReQAAARVREAKIILLLVSSDFLHSEPWQA
pELREAMGRDSL-GECHVIPVILRPC-PWDRTPLGRLSPLPRQAraVTSYPNQS--
EAFAEIAKGIRSA(VRDLSPVQQGSSPDLGDAAPEVKKLPPRNLSFTGRDQLIRELRTALTSGSPAALTQAIS
GLGGVGKTQLAAEYAHRYASQ)}*

$735=210(171):
>gi|1154069806|ref|WP_078452010.1|
{|0(657)|<Proteobacteria(B)>}hypothetical protein [Pseudomonas
aeruginosa] >gi|611852272|gb|EZO28636.1| hypothetical protein AJ60_05699
[Pseudomonas aeruginosa 3573]
{(MESLPQSKSARKVIFSSLYQHRDDVLTTSIR)---AFLSHSSKD---
KNLVGPIFNSLTAAHAHYDEATFEKGETSASEIYKAINNTEVFVLFLSRNSVRSPWVQTELVLAQQKIYSGKI
KKILVFLLDSFDhSDIPEWLLEYVYRKTSSpGIITTAIRSALFDVALAENANLsvfmgrdkeisvlkeslssl
sadspqviflagndgigrrtlakR()}*

$736=195(149):
>gi|612087829|gb|EZP46441.1| {|0(662)|<Proteobacteria(B)>}orc1/cdc6
family replication initiation protein [Stenotrophomonas sp. RIT309]
{(MEASLQGGL)VSKAFLSHSSMdkAFVSKV-ADSLGSA-----LC-
HYdeRTFEPAGESADEIIQALQNSDLFVLFLSENSLKSKWVQTEIRYARHLYFSGEIKSVAIFMMGDS--
LRERLDVWLRPFVVqtlmvpklvgfrirsllstadashvsrfigrddevarikdslrdlsasrpsSVLVSGPA
GIGRKRLLTRVYQDLYP()}*

$737=264(229):
>gi|617426084|ref|XP_007560189.1| {|275(0)|<Chordata(M)>}PREDICTED:
interleukin-18 receptor accessory protein-like [Poecilia formosa]
{(VTPQHLKHAYTCLAGNTVGNNNVTIKLKEKEQGKWRSLVGYPFGCFLLVVSLGIILHVKWVELQLIFRSRF
QFGKDDEEK)EFDVFVSYV-Wtgpsaeavkclaispkpysdagemqsre EPIEMRLT-
RVLEDQWGYRVCLLERDILPGGAYTNDVVLTINRSRMLICVLSAKYFaSSNAVF-
VLESGVKALLQNSALKMLLIWTrgtsaSFI-
QPEPPLPKLVQRALKVLpSLQWSTDAPSttarSFWISLQKAMPAD(GARPASRVQ)}*

$738=288(291):
>gi|894216136|ref|NP_001297720.1| {|360(86)|<Chordata(M)>}TIR domain-
containing adapter molecule 1 [Anas platyrhynchos]
>gi|614458436|gb|AHX37587.1| TIR-domain containing adaptor inducing
interferon-b
{(VPTKDSSVPVSTSSSAPASTSACSFPPHTYSSSTPSPPLYATPYSLPHPSPFHSPPSPACPPPFQTAKAAS
AAEPDSGEG)KFFTFVILHAGeDERVACRVKNLLEGMGVPNGATFCEDFLVaGHSRLTCFQDALENSAFIILL
LTKNFL-CRLCMFETNSALMESIQRPSKHNSVIPFVPK-ENPLE-RSEIPSILSVLvPLDENSLG---
FTRVVKNTFTPS(KINERKAMWCQIQQVREHKRRQQLYKDHCQTLQNLGALNLGSFPQVPLSETPWQQLGQFV
PHQLSQQCCPP)}*

$739=210(198):
>gi|612080455|gb|EZP39245.1| {|0(2)|<Proteobacteria(B)>}TIR domain
containing protein [Janthinobacterium lividum]
>gi|1104126172|emb|SFX09948.1| TIR domain-containing protein
[Janthinobacterium lividu
{(MAGPHGEQGDAMEIRYGCFLSY)AHGQYAFMN---
KFKNDLIEALACYLEPHldrEEVLFIDSEQLGGGDDIdlRVARAMCQSVCMIVLYTPKYEAHGYTRREFAAMQ

LIEQERrawyelPSHLIIPIIMTRHPdglpPQITESGLYVDFSGYTLASGDLKSNPQYLPDIERIVQRIA(TH
YHLLKRSTPPGHDCSRFVLPDIPPEWRAIPPPHF)}*

$740=219(220):
>gi|686254256|ref|WP_031838355.1| {|61(0)|<Firmicutes(B)>}MULTISPECIES: hypothetical protein [Staphylococcus] >gi|612612395|gb|EZS29343.1| hypothetical protein W603_02571 [Staphylococcus aureus VET035
{(GKQTELSKLYEEFSKIERKINETRAKLDKEHTKQQDKLEKTINNSRNDNHNIMTQLLSNSEQINALSEQVK
QSAKQKELI)EYDVFLSHSN---LDKEeYVSELSDKLTDKGVKVFEDEkvFEIGQS--
QTQMMNMGILNSRfvVVFLSRNFIESGWSKYEFISFLNREINEDNIIILPIW-HEISieE-
VKEYNPYlvdkhaLSTSKYTI-DEMVDKIN-SVVIKS-----KQ()}*

$741=219(220):
>gi|739736465|ref|WP_037589667.1| {|61(0)|<Firmicutes(B)>}hypothetical protein [Staphylococcus aureus] >gi|613112237|gb|EZX20459.1| hypothetical protein V070_01914 [Staphylococcus aureus C0673]
{(EKQKELSKLYKDYSKSETIIIETRTKIDKEQTKKLNQLEKTISNSKKDNQNLMTHLLSNSDQLNTLSEQVK
QSSNKKELI)EYDVFLSHSN---LDKEeYVSELSDKLTNKGLKVFEDEkvFEIGQS--
QTQMMNMGILNSRfvVVFLSRNFIESGWSKYEFISFLNREINEKNIIILPIW-HDISieE-
VREYNPYlvdkhaLSTSKYTI-DEMVEKIN-SVVIKS-----KQ()}*

$742=204(197):
>gi|612847386|gb|EZU60119.1| {|0(102)|<Firmicutes(B)>}hypothetical protein U985_02491 [Staphylococcus aureus 1110803248] >gi|930174113|gb|ALF44642.1| hypothetical protein [Staphylococcus aureus]
{(M)-KNIFVSYAW-DKENDEKViklAEMLEEYkELN-
VKFDKWDINKGQEIPLFMEKGIQQSDFVLVICSKRYKEntDKRIGGsGYEARLManeilkntDK-----
DRFLPVLLNENDkdHIPNFLTGKLWTSLYY--
QEGSEEYNIEINDLLTTIVGRS(KRNIKKSKSIYDQFENVSTKSENISEIKILGINQEEVTVPKMDGTRGSGL
YSIPFL)}*

$743=146(150):
>gi|738523133|ref|WP_036471610.1|
{|0(357)|<Actinobacteria(B)>}hypothetical protein [Mycobacterium triplex] >gi|620042074|emb|CDO90913.1| serine/threonine-protein kinase [Mycobacterium triplex] >gi|11
{(MA)---LFISYSSQdRSTVDALTTAL----
RRGQNQVWFDQELGGGDSWWaKILEQIRDCEVFIVALSSNWLQSKPSQAeLRYAQALGRPI-
LPVRIGEVDSMRV-NPLAALQIIDYREPTVDagIQLVTAVHALRSKPVPLPDPLPE(EPPVPFGY)}*

$744=195(196):
>gi|499779918|ref|WP_011460652.1| {|0(655)|<Firmicutes(B)>}hypothetical protein [Desulfitobacterium hafniense] >gi|89335048|dbj|BAE84643.1| hypothetical protein DSY2854 [Desulfitobacterium hafniense Y
{(MQDNNGPCRNFYQLVFEEGKF)MPRAFLSHSSAdKKSYVEMVA-----
KKLGYNTCVYDEfSFEEGMKSAeEIMKGLSRSDLFVLFLSDSALESNWVKNELNTAQELLNT-
GDMkRILPIIIDKN-
ITYKDNRIPQWLRDEYNlkYVSRPTVAARRITQKLREISWEQ(HPRLKEKDQIFVGRNNLTKTFEERIDAFDL
QTPTC)}*

$745=157(162):
>gi|1172283220|ref|WP_080666500.1|
{|0(685)|<Proteobacteria(B)>}hypothetical protein [Bordetella hinzii]

>gi|625310369|gb|KCB25847.1| TIR domain protein [Bordetella hinzii L60]
>gi|890876753|gb|AKQ539
{(MTKFP)--RAFLSHGHVdkhivREVARNLGRAA----------
VIYDEFEFSVGDEFrdAIIKGIDRSDVFILFASRASLQRDWVKLEIELASEALIKNalsKVTTYIVDQEL---
THDALPEWMRATLIRKEASPGLIATDIRRIISQRLGERIP(RYFVGRRAEIEQSLETI)}*

$746=173(185):
>gi|489298964|ref|WP_003206447.1| {<Firmicutes(B)>}hypothetical protein
[Bacillus mycoides] >gi|228763103|gb|EEM12011.1| hypothetical protein
bmyco0003_10580 [Bacillus mycoides Rock3-17]
{(MCTLLDSNDLDMHITPHQNHLFLFPDKETMEWIWKGGLKSYINRQ)--
KVFISHSSKdKHLIDKIFNEFQKNEIP------TFYDKHEIFPADnirdIINLNLQESKIGVVCVSSNF----
--NMELSEWLKHEVQ-YFKDKGILIIPLNINLEKNEVKRIVGDIRYI--
NLDKQGLAELVTITKKSLNKG()}*

$747=195(202):
>gi|302595926|sp|Q9FKN7.2|DAR4_ARATH
{|0(1418)|<Streptophyta(v)>}RecName: Full=Protein DA1-related 4; AltName:
Full=Protein CHILLING SENSITIVE 3 >gi|33200
{(MEPPAARVTPSIKADCSHSVNII)--------CE-ETVLHSLVSHLSAALRREGISVFVDAcGLQETKF-
FsiKQNQPLtDGARVLVVVISDEVEFYDPWFPKFLKVIQGWQN-
NGHVVVPVFYGVDSLTRVYGWANSWLEAEKLTSHQSKILSNNVLTDSELVEEIV(RDVYGKLYPAERVGIYAR
LLEIEKLLYKQHRDIRSIGIW)}*

$748=217(234):
>gi|15220796|ref|NP_173204.1| {|0(203)|<Streptophyta(V)>}Disease
resistance protein (TIR-NBS class) [Arabidopsis thaliana]
>gi|1063684329|ref|NP_001319029.1| Disease resistance protein (TIR-NBS
class)
{(MSTSYSFLLAGREL)--DVFLSFSG-KIALDVDFGY---DLSRNGIKAFKSESWKESSF-
KpiDLrtLEALTESKVAVVMTSDEEVSSVGFLEELIVIIEFQEK-RSLTVIPVFLTKHPLDVEKVSQIF----
--------PERA-
KIWRTAIAKLDNI(AAQYSFSRNLAVMHGTHRIKQIADDIRLMFLSSASSDFKGLAGMDRHMKALYALLALES
DEKVRTIGIWGSSGVGKTTLA)}*

$749=226(231):
>gi|15218619|ref|NP_177430.1| {|0(286)|<Streptophyta(V)>}Disease
resistance protein (TIR-NBS class) [Arabidopsis thaliana]
>gi|5903076|gb|AAD55634.1|AC008017_7 Similar to disease resistance
protein NL
{(MASSSSSSEHK)---VFLSFRR-EDTGRTFVSHLYRSLDQKEIRTykFENQQAGDGKR-
IssEVKQAINESRIAVVVISENYVSSVLCLDVLAKIIE--RL-
PLIKIETVFYEVDPGDLTRPTGKFADDFRRHEARENRRTV-
NRWRDALDQLVSI(TNSNFCSRNWEDDSKMIDDLIIRLINIYFSKYYTPDVDIIAETTVGLDASVLGLMTDID
MKYKLNSLSGSVESGFGLERY)}*

$750=238(230):
>gi|196016867|ref|XP_002118283.1| {|49(0)|<Placozoa(M)>}predicted
protein [Trichoplax adhaerens] >gi|190579114|gb|EDV19217.1| predicted
protein [Trichoplax adhaerens]
{(NKKNQEILKLVTRSQSPETYNIFITALREIPTRNNILADMVEKCNKKSVSYNSSSNTASANINADQTDSVG
VDKSSACGE)KWDVFLSYswNN-KDEVKKIKSYL-----
EQqKWTVWIDDDRVAGGQKLdsVLSNGIEACKVFVPCLSRSYANSDNCYSELSHAKD-----

LKKFIIPIVLgDVDPKNggLKGKNGDlnFKitgltyikiKDFEDKATTNKKLNDLNKAVHRRLIGYDE(EASS
VNTPYK)}*

$751=183(191):
>gi|196013599|ref|XP_002116660.1| {<Placozoa(M)>}hypothetical protein
TRIADDRAFT_60667 [Trichoplax adhaerens] >gi|190580638|gb|EDV20719.1|
hypothetical protein TRIADDRAFT_60667 [Trichoplax adhaerens]
{(MDTTDSQKKNQR)---VFISYSW-SDKAD--
VRYVKEFLESKNISCWIDEEKIYVGDELhpVIAKAIMSSEMFMPFLSKNYANSYNCYLE-----
FNYAHLHQKTIKPvIIKDIDPKKDLISSDGKRDFYFLISTKVYTKILRQKQTDDEFKKNLE(KLYESIVKPKP
ETKSSQQQLSPAKDEVDKMDTETAPGKL)}*

$752=213(221):
>gi|30694677|ref|NP_199318.2| {|0(1131)|<Streptophyta(V)>}Disease
resistance protein (TIR-NBS-LRR class) [Arabidopsis thaliana]
>gi|332007812|gb|AED95195.1| Disease resistance protein (TIR-NBS-LRR cla
{(MTESEQI)---VYISCI--EEVRYSFVSHLSKALQRKGVNdVFIDSDDSLSNESQSM---
VERARVSVMILPGNRTVS---LDKLVKVLDCQKN-
KDQVVVPVLYGVRSSETEWLSALDSKGFSSVHHsRKECSDSQlvKETVRDVYEKLFY(MERIGIYSKLLEIEK
MINKQPLDIRCVGIWGMPGIGKTTLAKAVFDQMSGEFDAHCFIEDYTKAIQEKGVYCLL)}*

$753=227(232):
>gi|196011585|ref|XP_002115656.1| {|47(0)|<Placozoa(M)>}hypothetical
protein TRIADDRAFT_59582 [Trichoplax adhaerens]
>gi|190581944|gb|EDV22019.1| hypothetical protein TRIADDRAFT_59582
[Trichoplax adha
{(LEKRGDRDFLKFCEALKNHDTDNAQELGNLLENQAKNAISGVQPSPVVTTPVTSVAPISHEAGKDTTDQAS
AVNDQTKYS)-YDVFLSHSW-DN--
KEDVIHVKKYLESKGITCWIDnEQLSGGMKLlPEISKAILASRVFVPFMSKKYIESKNCYSEISYASDKK---
KDMVAVIIEDIRPQGELVSSTGTDFYIYITGDLYIKILVNKQATAELEKKLNEL(YESIRKAKNSRK)}*

$754=301(300):
>gi|30686220|ref|NP_194082.2| {|262(72)|<Streptophyta(V)>}Disease
resistance protein (TIR-NBS-LRR class) family [Arabidopsis thaliana]
>gi|332659369|gb|AEE84769.1| Disease resistance protein (TIR-NBS-
{(QAGRNLLVLLGSLEYYNKGFTFKRIFLPKKSQQLPGYTVVAESNDNLRNHEVSYSDTVLTCFSFLCNILKR
SEMKIDPLS)-QRVFIGLGE-KHLGKFLVSSLKEELESNqILVYVEDETKSR----------
IKESGVAVVFFSKKYPNSEKCLDELVEIKKLMDA-
GKIDPLPVFYSLKDEPVKNLKGYFLNRLLKIenevRKNIKTRDDKsildteakiWGWRDALSSIASR(PGLSY
ELSTDDVFVSDIVTKVNELFASRERKKTEAATTVVKTLDADDLFYSRTSFLQAIDRDITDFESFTDIPNGLVL
LR)}*

$755=300(300):
>gi|1019104|gb|AAA79208.1| {|963(126)|<Arthropoda(M)>}wheeler
[Drosophila melanogaster]
{(GNLANGPDCSDLLDASASNISSSQDLAGAIGCPCWPAVLVLIFLVVVLIIVFVFRESVRMWLFAHYGVRVC
EPRFEDAGK)LYDAIILHSEKdyEFVCRNIAAELEHGRPPFRLCIQQRDLPPQASHLQ-
LVEGARASRKIILVLTRNLLATEWNRIEFRNAFHESLRGLAQKLVIIEETSV-
SAEAEDVAELSPYLKSVpsNRLLTCDRY--
FWEKLRYAIPIE(LSPRGNNYTLDHHERFKQPVSPGMIFRQAPPPPAYYCTEEMEANYSSATTATPSPRPTRP
GGAARIVDSMPMPMRPPSEH)}*

$756=166(200):

>gi|196010327|ref|XP_002115028.1| {|0(237)|<Placozoa(M)>}hypothetical protein TRIADDRAFT_59119 [Trichoplax adhaerens]
>gi|190582411|gb|EDV22484.1| hypothetical protein TRIADDRAFT_59119 [Trichoplax adh
{(MASNVPEHKHL)--DVMISY---SRVNQNVMRKLKDFLIENGLKAFL-----------------------
IILSKNSATSKYCRDELALAYISN-----
KPIIPVALEKKSEILSVLEFGMKLTLASMEWISLADDMQFDQQLSKIIAAVN(TFICDTDVSEHNNNSVKSQD
RLVNRQISKHKKLFHDHEEKDFIRAGEKF)}*

$757=241(249):
>gi|196010653|ref|XP_002115191.1| {|0(258)|<Placozoa(M)>}hypothetical protein TRIADDRAFT_59118 [Trichoplax adhaerens]
>gi|190582574|gb|EDV22647.1| hypothetical protein TRIADDRAFT_59118 [Trichoplax adh
{(MGSIASCGSSRKNVNNGISPVIDTEFPEVKNQAITSKDPREEDGDNQLQATDINTSITLDSFEDTSTAYKQ
SNEVD)-YDIYISYSP-DDTE--IVTPITAYLRNHGLKFW----
HLRYGsEKLkdNIDKgkAIVNAKVIIVVLSISYIKNKAKVDEVSLAYISN-----
KPIVPLSLRYFREINQEMGFALKLTLSKLNWVFFCKKERYDKEYATVLEGVK(REIRKISSHDEILENRRASF
IEDDQFNFQEETA)}*

$758=257(249):
>gi|196017583|ref|XP_002118576.1| {|35(0)|<Placozoa(M)>}hypothetical protein TRIADDRAFT_62605 [Trichoplax adhaerens]
>gi|190578705|gb|EDV18938.1| hypothetical protein TRIADDRAFT_62605 [Trichoplax adha
{(LLEARILTSYHQETIESESTSSKKVWTLLNILPERGDRAFDEFCNALTYCEITVENFNSGRKPKNSEDIKK
DTGMDTTDS)QKKnqrVFISYSWSdKADVRYVKEFLE-----SKNISCW-
IDEEKIYVGDelhpVIAKAIMSSEMFMPFLSKDYANSYNCYLEFNYAHLHQKT-
IKPVIIKDIDPkkdliSSDGkrdfyFLISTKVYTKIlRQKQT--DDEFKKK--
LEKLYESIVKPK(PETKSSQQQLSPAKDEVDKMDTGTTPGKL)}*

$759=176(182):
>gi|499421110|ref|WP_011108574.1|
{|0(583)|<Bacteroidetes(B)>}MULTISPECIES: hypothetical protein [Bacteroides] >gi|29348412|ref|NP_811915.1| hypothetical protein BT_3003 [Bacteroides thetaiotaomicron
{(MQSTVA)----FISHrsTHKD----FVRKIVDTVKRDNCIVDEFDFY--
PATKTVnEIVRNLNFAPIFVLLISKDALESDWI-KlEMSKARQLYDT-GVIReFMPFIIEEG-
VKLEELPSWMTRDWSLNikTITSPKIIGQFIIETQRKIRCVG(NNAYGNWINTFIGRSNELDEFQRAMYDSVY
MPHRSL)}*

$760=244(233):
>gi|499258888|ref|WP_010956428.1|
{|1022(0)|<Bacteroidetes(B)>}hypothetical protein [Porphyromonas gingivalis] >gi|34397792|gb|AAQ66853.1| leucine-rich protein [Porphyromonas gingivalis W83]
{(RETNRKKRPIQDLYLSCAQADKDLTESHYIHLGTLDDESKTTARIAAYPLKNGVIDKERVREVSTRPYKHL
SVNKNLATA)-
KQIFISYSKEdQTELETCLQFFKPLEKNGQIEIYYDKLTKFETPIHpEIRKRIVEADCIIALISQRYLATDYI
LDhELPVFREYNK-----TIVPILIKPC-TFEDDEFlrekyfaqkAQIINLGKEGktIKAYDSitasahRD--
ENWVAVVREFKEK(ILRITKQEVNTDE)}*

$761=202(228):

>gi|196018241|ref|XP_002118775.1| {|0(77)|<Placozoa(M)>}predicted protein [Trichoplax adhaerens] >gi|190578246|gb|EDV18739.1| predicted protein [Trichoplax adhaerens]
{(HRQPWTLNTKFEQPAIETETNELPVEKQNEQNRMQTNSPVYINDL)-----------------------
LVKEG-ISSWkIDEQFSERTEVLpAVAENIQLSQVFLPFISEKYIKSDYCLKEISWAI----K-
KEKWIIPVIIENI-
NgWKDSLIALGQDILKKMkeYHNIKFLGQGASTAKLKEELSEL(LKAIRFCIDIRKLPEKEYLQAYIKALMKGKCKNDFIRIMLIGP)}*

$762=264(274):
>gi|195998806|ref|XP_002109271.1| {|276(124)|<Placozoa(M)>}hypothetical protein TRIADDRAFT_53133 [Trichoplax adhaerens]
>gi|190587395|gb|EDV27437.1| hypothetical protein TRIADDRAFT_53133 [Trichoplax a
{(LWNYLFHDSVKLQVQRTTVINILQQVEEKFADYVVSGCLEGALWLLGAPVDIEPYIPLTILAKNLESGASDGLISHSSQF)--QIWISYHQ-NDLAIAI--
KIRSYLKYAGFRAFIDIDRntnvKNEFNLKSIAKNIDESNIVVVCLSHKYKQSPYCHSQ----AHFAVQ-
KKKKVIPILLQANY----
NLHGWLGDLFGSIPILDFSDGNDIDNNMKSLKQEVI(RSSGDRDQLYKNYAVSNSSSLNRWDSDSSLPREYSESVITNDVSERSTFMLTPS)}*

$763=160(169):
>gi|501493132|ref|WP_012501553.1| {<unknown(X)>}hypothetical protein [Chlorobaculum parvum] >gi|193085444|gb|ACF10720.1| Domain of unknown function DUF1863 [Chlorobaculum parvum NCIB 8327]
{(MDKSHN)---IFISHYGKDDKHV-QSLKARLKGQGY--
NIRnfsVDSTKHKDGRTPsKpvierlLRMRINWSSTFICLVGPKTHTRA------
WVDYEIQKAVeKGKRIIGVYTHGSK-ETALLPKGLEKVAS-NLIGWNS------
IDKIGEIISGK(NYPIENPDGKVITDAFDRPIIRC)}*

$764=243(238):
>gi|196008213|ref|XP_002113972.1| {|2208(363)|<Placozoa(M)>}hypothetical protein TRIADDRAFT_57945 [Trichoplax adhaerens]
>gi|190582991|gb|EDV23062.1| hypothetical protein TRIADDRAFT_57945 [Trichoplax
{(LVPCPHCLQNGSRDPSMFSSKRIRRAREVKAIFLQCDANFHTVSISELERILPPTSSDEFDVHLQNAMQGLDALSDSIMT)--DVYVSYCEDdlpaeensnetLHVKR-LMKSLEGEGY--KC--DARDYRENYSL-
ENIAVGMKQSRAIIIMLSDNYERNEQCRSEFSHAYELLKKPIILAVIGKTNNWTKtNLGFLVSSMLYIDFSNMqLYNEKLDEIIDQVEKKTHKKVQH(LQQTDCFISYCWKNSANA)}*

$765=300(300):
>gi|195124071|ref|XP_002006517.1|
{|968(149)|<Arthropoda(M)>}uncharacterized protein Dmoj_GI21098 [Drosophila mojavensis] >gi|193911585|gb|EDW10452.1| uncharacterized protein Dmoj_GI21098 [Drosophila
{(EKLLPSADCSDLLDASASNISSAQDLAHGYRLPLLAAVLVLIFLVVVLIIVFVFRESVRMWLFAHYGVRVCEPRFEDAGK)LYDAIILHSEKdyEFVCRNIAAELEHGRPPFRLCIQQRDLPPQASHLQ-
LVEGARASRKIILVLTRHLLATEWQRVEFRNAFHEALRGLAQKLVIIEETSV-
SSEAEDVAELAPYLKSVpsNRLLTCDRY--
FWEKLRYAIPIE(LSPRGNNYTLDHHERFKQPVSPGLIFRQAPPPPAYYCTEDMEANYSSATTATPSPRPMRPVVESLAMRPPSEHIYHSIES)}*

$766=220(245):

>gi|196008213|ref|XP_002113972.1| {|2390(204)|<Placozoa(M)>}hypothetical protein TRIADDRAFT_57945 [Trichoplax adhaerens]
>gi|190582991|gb|EDV23062.1| hypothetical protein TRIADDRAFT_57945 [Trichoplax
{(KTNLGFLVSSMLYIDFSNMQLYNEKLDEIIDQVEKKTHKKVQHLQQTDCFISYCWKNSANACELGIQQVDGAIGKVDPRD)---------------------LKDQLKAKGITCWMDIEQLgKAG--LfeEISIGLRKSKLFVACVSDEYVKSNNCIMEFRFAVTTL-----RLPTVLAIIGTGIKWQYSEVGMLAANLPMFNLQGNDSEMSSEKLLSFIVEKI(STLIKPEDNLLTTAPNIGKERKLQD)}*

$767=256(236):
>gi|196008339|ref|XP_002114035.1| {|548(0)|<Placozoa(M)>}hypothetical protein TRIADDRAFT_58085 [Trichoplax adhaerens]
>gi|190583054|gb|EDV23125.1| hypothetical protein TRIADDRAFT_58085 [Trichoplax adh
{(CYNNMKLSLRSILNDRSAVDFQHTPYNDAMDVLFFKLHSGSHLHGVYLQQDYIERVNDNMKTLQSIGSPEGQSFTDKDPV)---VFISYQW-DSQSKVV--LLKEQLEANGIPCWIDRPNItinknqmssHTGhnrtsgmasslsnnmtnivtDTIqsEIQRVMKNCKVVLCCMTPKYTRSDNFYKDLALADSLS-----KPILPVMLHFMPWPPEIAALHLRKNLASLEYIDISSEKLFRRNFATVINQIQ(RHQSQTMAVPKLPPLK)}*

$768=210(231):
>gi|196007256|ref|XP_002113494.1| {|536(269)|<Placozoa(M)>}hypothetical protein TRIADDRAFT_57729 [Trichoplax adhaerens]
>gi|190583898|gb|EDV23968.1| hypothetical protein TRIADDRAFT_57729 [Trichoplax a
{(IPTYITLVQYIDCRDNTEVCISKASEQFCEWHQRSSDELELIDNPYEKATIKYNYDLFISYCHENSNYAKLVLEKMKIFD)---------------------------EDANIFFDVQQLKTGTAWqkELYEAIDGSQSLVAIVTPAYVRSKVCYEEFSLALALDRERnTSEGKRFYFCPVILnsndDVPSTFRHANMMICKDGSFSNEFVSKVCQHLKIGQHLQDF(PMDKDEKSPKL)}*

$769=163(149):
>gi|501498312|ref|WP_012506567.1| {<Chlorobi(B)>}TIR protein [Prosthecochloris aestuarii] >gi|194312639|gb|ACF47034.1| TIR protein [Prosthecochloris aestuarii DSM 271]
{(MKMFWSY)ARLDDMEPK---RKVSKLRKAFENVLSQTqgtPCDVFFDRDSLHWGVAWreEIERSIRECDGIVAVVSPSYFNRRMCLYELQMAVEARKK-----IFPLYYRSCSelrsafkeDGDEAEINrglnsaslIITELQMMDfRELRNEKIGSKKVEDFLDRMAEV(VS)}*

$770=243(239):
>gi|502570176|ref|WP_012818500.1| {|275(0)|<Firmicutes(B)>}ATPase [Paenibacillus sp. Y412MC10] >gi|261280605|gb|ACX62576.1| ATPase [Paenibacillus sp. Y412MC10]
{(QAIRKYPEHRSSVNTVMEQGYLESFLNGEYAELFAECIHYEANTNVISVEDPKFVYYIRNIQWKKFVREVGYKTINFKSI)-YDFALSFAG---SDRDVAEGLFTRLCEEEVSVFYDKNEQHRILAEnveEYLGPIYRSEAQyvVVLLGSNYPRRIWTQFESQQFKDRF---GESSVIPIWFKDAPvgS-FDSTakIGGIEFDRdgdftLQI-NYITETLM-KKLADNRISSNVG(KITKSKNNKDDIGVQLTLF)}*

$771=263(268):
>gi|501500406|ref|WP_012508640.1| {|12(242)|<unknown(X)>}hypothetical protein [Pelodictyon phaeoclathratiforme] >gi|194309459|gb|ACF44159.1| protein of unknown function DUF323 [Pelodictyon phaeoclathr

{(PATIGEALIEAAKLPFRGEYKILFQQKRQKYIAGLSDYERPIWVGEMKNLLSLCESLFDGDPREVKRASTS DGVYRSWEF)KNDIFISYAQ---EDRQRVEPIVQELEKLGWCIFWDRNLRA---GHnwpIEISRA-LDESRcvLVfwsFASVDLNKHHWVREEAETGRKRGV------LVPLCIDDVEppL-GFRTIQAETLVNWKQ-NTSHPKFLQLIEAIASQVPLPG(DLQRRESAVRSPVPPPIQPKVFSDFVLIRGGIFPMGSPTGEVDRFDDE)}
*

$772=255(259):
>gi|501494260|ref|WP_012502637.1| {|0(1009)|<unknown(X)>}WD-40 repeat-containing protein [Chlorobaculum parvum] >gi|193086528|gb|ACF11804.1| WD-40 repeat protein [Chlorobaculum parvum NCIB 8327]
{(MVRSLSYILFTPFPEPFDHTIMPDSTPPNHPPASARLSQDASNVEQAMKPGYAEMAGYRSSEAVEIRATIK PRIE)---
LFLSYAHNdKSVKQRFTDELKArlaISRHFQFVLSSDNDLLCGDTWHdELQDRLSRCNYGILLLSNRFLASTY IQKhEIPLLQDK--------CFPVALKPI-
DIDNLETHGLDHRQIFFfeQTKPFQKVSGANLSQFINDFVEE(IEKSILKQREKHKEVACVETEEHEVEGIIR DEDGTQKFIKRNLE)}*

$773=275(279):
>gi|196007256|ref|XP_002113494.1| {|351(389)|<Placozoa(M)>}hypothetical protein TRIADDRAFT_57729 [Trichoplax adhaerens]
>gi|190583898|gb|EDV23968.1| hypothetical protein TRIADDRAFT_57729 [Trichoplax a
{(NNEPCSVAMPVLATGNQGYSQTRVFESLLNAAIRWIRAGLPLKSLLVCNFTKNPELDLLFTKIKATALLPQ PIAKVPEFD)-FDFYISYsPQDITLANRVKNELEnawaEEKKAIRLHTNFQEINPDISWQDEIYNAI--
CKsiRVIVILTPSYLQCEACKEQFNVALCVNQQSKWNLLAPLYV----IAIPDIPTYITLVQYID----
CRDNTEVCISKASEQFCEW(HQRSSDELELIDNPYEKATIKYNYDLFISYCHENSNYAKLVLEKMKIFDEDAN IFFDVQ)}*

$774=250(254):
>gi|493035746|ref|WP_006103609.1| {|827(13)|<Cyanobacteria(B)>}GTPase [Coleofasciculus chthonoplastes] >gi|196178152|gb|EDX73153.1| Miro-like protein [Coleofasciculus chthonoplastes PCC 7420]
{(EIECDNSYQMVDVRRLIDDVNLSPYPTDKLGIDSPFNKATDSRLQQELNQEREASFSHPHRQTSAKDSQMN DANAANFEK)--EIFISYawRG-ES--EEFVNRLDEVFQSQGITIVRDKRDLGYKG-
LikEFMERIGRGKCVIIVISDRYLKSPNCMYELVQIAKNGEF-YNR-
IFPIVLSDATIYDPVDRANYIIHWQEEINKLETKIKLIASAANLPSLQRS(INEYTEIRATIDGLTDTLQNMN TLTPDIHSESGF)}*

$775=284(282):
>gi|493030105|ref|WP_006099507.1|
{|124(315)|<Cyanobacteria(B)>}hypothetical protein [Coleofasciculus chthonoplastes] >gi|196182413|gb|EDX77399.1| hypothetical protein MC7420_536 [Coleofasciculus chth
{(GNLPMLRLRVMADAQRVQGGYSGSPVFDAETQAVIGLIAAFDHSQGALALPLTTISKKWSTLQEFLTPTPP HPIASPSPQ)TAKIFISYRsRDPDVSLAQQFYEALKAAGH--
EPFMAGASIRLGEnWAeRIDQELEHCDYFLLLLSSQSATSEMVTEevRR--
ARELRDRReEKKPIIlPIRVNFPMDSP--
LNYDLRgYLQRIQQREWHSPDDTPVIVQELLALISQ(EDGGEPWVVPLAETPEPAAPVVASPDFPPLPVAEPE LQREPGGAVSLKSGLYVERLPIETDC)}*

$776=221(222):
>gi|494629275|ref|WP_007387219.1|
{|95(276)|<Actinobacteria(B)>}hypothetical protein [Streptomyces sviceus]

>gi|197711682|gb|EDY55716.1| conserved hypothetical protein [Streptomyces sviceus ATCC 29083
{(APSGPSSTDVAAMRATAREHDGPVALLVYAPGGADPFSGLIIRRQRWGYVTSKAATVLPPPQRFEALGPLP
EGAVRRDGA)---VFISYRQSDGSPQADSIENLLRAAGL--
VVWRDRTDLRPGTTIdRLEQALtTGLSAAVLVVTPEIVYSDIVREreLPRLLQLDEDPAFSLCIANKIARAGS
DAQCDYNAPDRLLRLAPSRILADKKQSNMLQPSGESEIVR(DL)}*

$777=301(284):
>gi|198433982|ref|XP_002131336.1| {|360(0)|<Chordata(M)>}PREDICTED: uncharacterized protein LOC100175496 isoform X2 [Ciona intestinalis]
{(SDFGRYEVSLFQRTLNQTAAFEIQSNLQTFLLVRHATITCVAMALILLLLMFTAVLWWKRVYLFVYYKRKF
GTNEPDNKC)-YGAFLSYHfSTdmDKFAKQLtrdavsvvRDGLEG--
LGYRVYDEVKDGHSSGYQAPNIINTMRKCHKVIIIITPDYLKDHWCMFTLQKSFLEMI-
EARSKMIFILTPGS-SAFikqhrqDNESCRLvHRAIALNhALQWSDerrfdlKY---
FLLRLENVMPKL(VSQRFRHVSHTSSVSSQPCRYNSGYLRSVSNDTQTTELGSPRPESEKTFTELTVTMEKEN
ELRV)}*

$778=297(299):
>gi|198435396|ref|XP_002128758.1| {|32(330)|<Chordata(M)>}PREDICTED: uncharacterized protein LOC100182356 [Ciona intestinalis]
{(PSTNIHMLNTSSSFMNGRPNSEEKYKQQFTSKGFSHEDFYSDEKGNFPSYSYKQDKHDFGIPSSVSSYRSA
EPSSGDVGF)QHDVLLIYDPDtdYYQAEELRKAIE--
PQQLRIFDFAKDCPSGGAQSTFLESAFKTCKYTCIVVTRSFISNFWLKFKTDMAIQDMLQNQHKRYSVVVV--
V-MDLMLKSHDLPLDLQTLtPIFQSSRF---
FQNKISKAfqgMPFP(VPNSGNFSNSFKSVSMQDPTVHAVVETHKPQNFDSLTSWGTLNPQRKERLKSYSPIR
NTSSDPEMKRYLSHNDPRVNLC)}*

$779=276(260):
>gi|198419135|ref|XP_002122649.1| {<Chordata(M)>}PREDICTED: single Ig IL-1-related receptor-like [Ciona intestinalis]
{(MEKNVNNFPLVVGLSVGLSVLLIIVLAVILLACCFQVYTKVFWKKYFKAYTSETNDFG)---
AYLSYQYStemdifsqqqSRDALATTRDTLEK-
LGYRLFDENRDGQSNGYIAESSIQSMAKCHRVVIILTSEYIKDNwSVF-RLQQSFMKMIESGR-
KVIFILVPGI-
QEFVKQKANEgdacckmvcRALKLNHAIIWSDDKKFnlrKFKLQLDVAMPKI(NPHSTSLPTPMNENGNGKPN
GYHRGISTATDVTELNSPRYLSDGVDVYFPTVDENGTQDAQV)}*

$780=264(256):
>gi|198433980|ref|XP_002131320.1| {|388(0)|<Chordata(M)>}PREDICTED: uncharacterized protein LOC100177822 [Ciona intestinalis]
{(TYHVSVLYKGIPSDPATFTLVFDRTRMLVKMAAGLTVCLVVMVSLIVLWLVYRRYRFRIKLRWRRHFGKYQ
DDDKKNIVV)-----
LSYYYDcdladeTRKATETIITCIKQQLNsmqYKYYDSHEDLQAGGFSVSSLLQRLDDSHRMIIILTEEYIRD
RwSKF-EAEQGLINMENNR-TKLIFVRVKGvkkaL-
QGVEDFPSGLsDALKTARNIRWKpGMNSNLFKESVAYALPNL(KRSPTPTTNNPSVNYVKLASCDESLASPVN
GCASCV)}*

$781=256(256):
>gi|198423384|ref|XP_002129958.1| {|108(67)|<Chordata(M)>}PREDICTED: uncharacterized protein LOC100184671 [Ciona intestinalis]
{(YATEVTIYCMTGTLQNGDLCVQRFMYAEPTGLPIGIQGFLIFICVSVVAISSLFVAKKYKSQISLTCRDSC
SPTTDDDGK)EFDAYVSIAKTdsdKKFVQRNFLEALQKEYRYKFLVDEDNLLPRLEYSDLVQEKIHRCRRLMI
VFTAAYMTN-DWCMyCFSEGLRKLLEL-NIPLIFIFNDVTIVRELNSYQPLVGHVPKL--
LVSLVQTTTEQAATLRKIRLL(LPAARNGNHSITKGMRSPMLSDAEDMDHKSIRADNE)}*

$782=174(180):
>gi|494036319|ref|WP_006978445.1|
{|0(44)|<Verrucomicrobia(B)>}hypothetical protein [Chthoniobacter flavus]
>gi|196227369|gb|EDY21873.1| hypothetical protein CfE428DRAFT_1119
[Chthoniobacter flavus El
{(M)SREIFISYSTEDRAVADNLCEALEATGLT-CWIAPRNIEPGSSWTASIMNAISECRIMILIFS-
SKSNESDHVVrEILHAVEKR-----RQVFPIRIEDVIptGGLGYCLVGVQWFEAT--
AQPLSRHVGSLVTQIRQVLQNS(GETTAGPAIPASAPTSTPSPDIFFTCQCGQHLVVDASAA)}*

$783=240(226):
>gi|490052985|ref|WP_003955310.1|
{|159(0)|<Actinobacteria(B)>}hypothetical protein [Streptomyces
clavuligerus] >gi|197703992|gb|EDY49804.1| hypothetical protein
SSCG_02832 [Streptomyces clavuligerus
{(VPEGARSAWAVLAVPKGRMKQRVDFSVRYAVHSDKAMDYRPTQQPVSFTLTLSAPPQSRDEAELPTPNESA
ERPPASPPY)---VFISYAHDDarrKENALRFGELLRQCGID-
AHMDQWDCDERGDWSLWAIRHILAADFTIVLASPmckavgngnvdhrthRGLQSELGQLrELLHTDGETWV--
-PKILPVVLPGERveDLPLFLRPYTmdhyRVDEFA--CEEIEDLL--SVLTRRPRRTRP(PLGPGT)}*

$784=249(257):
>gi|493037599|ref|WP_006104765.1|
{|359(433)|<Cyanobacteria(B)>}histidine kinase [Coleofasciculus
chthonoplastes] >gi|196177222|gb|EDX72230.1| hypothetical protein
MC7420_8322 [Coleofasciculus chthono
{(LLKIIQQDAAYYNEHKILLTKALKWERQHRNPSILLRGYNLRHAEAWLKVAKQRTSYPPTRLQEEFISESL
RQPPASSLD)---VFISYSRAdSDFARQLNDALQM---QGK-
TTWFDQEsIASGTEFQlEIYRGIEVSDNFLFILSPRSVDSPYCADEVEYAAKLNK-----RVVTVLYRPI-
NSARLHPELAKVQRIDFnqNEHDFWANFNQLVRTLDTDRDHL(RVHTRLLTRAMEWDREGREPSFLLRGKDLE
ASEQWLK)}*

$785=251(256):
>gi|493035296|ref|WP_006103290.1| {|154(967)|<Cyanobacteria(B)>}type IV
secretion protein Rhs [Coleofasciculus chthonoplastes]
>gi|196178590|gb|EDX73589.1| hypothetical protein MC7420_3763 [Coleofasci
{(DLVKLLRSHHDYVEKHTRFLLKALEWERNKKQTNYLLTGEEKKQAEAWLKIRFKDEQPPCTPTNLHCEYIT
ESIKNANNL)MTQVFLSYADEdRTTMERIRNSL----
RQESITVWTNTsDIQTGEAFEeAIKRGIEQADNLVLLLSPESVNSTYC-QqELDLALSLHK-----
RIIPVLVREI-
EPNQVPTGLRDLQYIDLtdNVKEDDYLLDESQLLKTLHQDAA(YYNEHKILLTKSLKWQQQQKNPSILLRGYN
LHSAEA)}*

$786=265(271):
>gi|493031982|ref|WP_006101378.1| {|154(1015)|<Cyanobacteria(B)>}type IV
secretion protein Rhs [Coleofasciculus chthonoplastes]
>gi|196180111|gb|EDX75103.1| RHS Repeat family [Coleofasciculus chthonop
{(DLVKLLRSHTDYVQQHTDLLVKALEWERNQKQHSWLLIGEERQQAEAWLKIRFKDEQPPCLPTDLHGEYIT
ESIKNANNL)MTQVIIAYSDEdKATMEKIRRSL----
RRESITVWTNTtDIKTGEAFEeAIKRGIEQADNLVYLISPDAVNSTYC-QqELDLAVSLHK-----
RIIPVLVRET-SVEQIPLA-
LQGLQYIDltDNVQEDDYLLDESQLLRILHQDE(AYYREHKILLTKALKWKEQYGNPSILLRGYNLRSAEAWL
KVAQKRTHDLPI)}*

$787=261(266):

>gi|196180255|gb|EDX75247.1|
{|154(584)|<Cyanobacteria(B)>}tetratricopeptide repeat domain protein
[Coleofasciculus chthonoplastes PCC 7420]
{(DLINLLHSHQDYVDKHTRFLIQALEWERNQKQTKYLLIGEEKQQAEAWLSIRFKHEQSPCIPTDLHCEYIT
ESIKNANNL)MTQVFISYTDEdRTTMETIRNSL----
RRESLTVWTNTtDIQTGEVFEeAIKRGIEQADNLVYLLSPDSVNSDY-AQrELDYAVSLNK-----
RIIPLLVRET-
PPEQIPLALRDLQYIDLtdNVKEEDYYLDESQLLKIVQQDAA(YYNEHKILLTKALKWKQQQQNPSILLRGYN
LRSAETWLKVANKRTL)}*

$788=167(177):
>gi|493027485|ref|WP_006098139.1|
{|0(747)|<Cyanobacteria(B)>}hypothetical protein [Coleofasciculus
chthonoplastes] >gi|196183680|gb|EDX78663.1| hypothetical protein
MC7420_7316 [Coleofasciculus chtho
{()MADIFISYSSPdKDAIQRVYDALTQEKRN----IWLNWQESPLTQTEpEI----
EAAHTCIFFISPHAIQSERC-TqELEYAIQRCK-----RIIPIVLRDD-
QLKGVHPELARLNWVFFreTDDFNGSFESLLKTLDTDLDHIK(KHTRLQQLAKEWDKKQRNNHLLLRGSELEA
AEQWFTT)}*

$789=169(174):
>gi|493027603|ref|WP_006098194.1|
{|0(746)|<Cyanobacteria(B)>}hypothetical protein [Coleofasciculus
chthonoplastes] >gi|196183734|gb|EDX78717.1| hypothetical protein
MC7420_7370 [Coleofasciculus chtho
{()MKEAFISYSRRnKEFVLKLREAFKNANR----
EVWVDwEGIPLTADWRqEIYQGIEESNNIVFVISPDSVASKEC-GvEVDCSLKHHK-----RILPILYLDV-
DPDAIHPEMRELPQIDFreGTNFEQAFAELLQAIDNNKEDTQ(EHTRLLVQAMEWEKKGRNTSLLLSGSELTS
GESW)}*

$790=132(146):
>gi|503089785|ref|WP_013324628.1| {<Cyanobacteria(B)>}hypothetical
protein [Cyanothece sp. PCC 7822] >gi|306984706|gb|ADN16587.1|
hypothetical protein Cyan7822_4682 [Cyanothece sp. PCC 7822]
{(MSATSS)-
PQIFICYAHNdnesldpsKRWLDRLLEQLEPLQLNDQADIWSDQRIELGTDWHdEIQITLQKVSAAVLRV---
--------------------R-SKPVGHPVKRSPE-
NPWVKFLSAKYGCAHSYseIRALRSGTLRRQGVKSLSLRQSN()}*

$791=177(184):
>gi|506264376|ref|WP_015784151.1|
{|0(839)|<Cyanobacteria(B)>}hypothetical protein [Cyanothece sp. PCC
8802] >gi|256590412|gb|ACV01299.1| WD-40 repeat protein [Cyanothece sp.
PCC 8802]
{(MTNNSSKS)--SVFISYSRRdKTFVQKLHHALN----
EGKRDIWVDwEDIAPTEDWReAIAQGIQSADNFLFIISPDSVKSIEC-NkEIDHAI----K-
CNKRLVPILYREV-
EDNSVRPELAKHNYIFFqnEESFFENLEKLEQALDTDLAYIK(EHTYLLSRAILWQQKQRDPSYLLQGTALKE
AQEWIT)}*

$792=185(178):
>gi|490052101|ref|WP_003954434.1| {|0(230)|<Actinobacteria(B)>}TIR
domain-containing protein [Streptomyces clavuligerus]

>gi|197703142|gb|EDY48954.1| hypothetical protein SSCG_01982 [Streptomyces clav
{(MIR)-PRIFLSHSSSqcsrdgcgcREYLLALEDHL----
RAQGCEPVVDRHvLGGGDEWNpKLLTEIKKSHGLILLLSPHALDSFYVLQeAGAAAYEHAAN-
PRFLLLPVLLPGV-
RRRQLRGSRLEQLNLGRfdMVDWSAEAGPGEPPAKIADTLRP(LAECLGAEPHPEVTDFLAGRLREVSDTALE
RTAGI)}*

$793=171(173):
>gi|495378892|ref|WP_008103603.1| {|0(97)|<Verrucomicrobia(B)>}hypothetical protein [Verrucomicrobiae bacterium DG1235] >gi|198260134|gb|EDY84442.1| hypothetical protein VDG1235_4073 [Verrucomicrobiae
{(MGQSVL)---VFVSHSSEdKASL--IEPIIGDLESCY-
INVWYDkRKILPGDNLRkSIfRDGLDKADVALIFFTENSLKSSWV-DkEIKHVLREETK-
KGNNFDLSKIISI-
FDSKETYEEISDRYPELtdDLLHLMPEDYDKTQMGQLISAIW(SKYLSLQGGDIETQRQLLAKDKEIFKK)}*

$794=204(207):
>gi|493032658|ref|WP_006102053.1| {|0(41)|<Cyanobacteria(B)>}TIR domain-containing protein [Coleofasciculus chthonoplastes] >gi|196179727|gb|EDX74721.1| hypothetical protein MC7420_6199 [Coleofascicul
{(MNELNKQPQFD)---VFMAHNS---
KDKPQVLEIAEQLKQRGLKPWIDIEQIPPGESFqkVIQQAIpnvKSAAIFF-------
GLEGTGNWqEEEIPVILTQSRkANIPVIPVLLPGV-
NQLPDdllfLSERNWVAFASRLDDIKAMDLLEWGITGKKPGAEG(ENQTTSLSSSSTEQYSENELPEEPEDVA
LELLQRLRPPQFKRVLFYYKSVNEYDLP)}*

$795=179(183):
>gi|501737233|ref|WP_012630437.1| {|0(72)|<Cyanobacteria(B)>}TIR domain-containing protein [Cyanothece sp. PCC 7425] >gi|219867066|gb|ACL47405.1| hypothetical protein Cyan7425_5111 [Cyanothece sp. PCC
{(MSEVDSKK)---VFLSH-K--GVDKDKVREFHKILELLGYKPWLDEDAMPAGAALerAILKGMQ-
DSCGVVFFITPSFKDEGYLEsEVNYAIKEKRQKgDKFAIVTLQFPDD-
GRCGNIPDLLSQYVWKTPKTDLEALYEIIRALPVEPKLVD(WRENITGVVTSSILRSKTADLSDEAKLILREA
VSG)}*

$796=256(257):
>gi|501730360|ref|WP_012627646.1| {|269(352)|<Cyanobacteria(B)>}serine/threonine protein kinase [Cyanothece sp. PCC 7425] >gi|219864230|gb|ACL44569.1| serine/threonine protein kinase [Cyanothece sp. P
{(LHQPPSLLQRFGQFLRKPITELRGKPESDLPLDLPAEPYGNSPSGATPTIPPTHPQLPRSGSFPPSAGGSS
GQSPSQSAR)---IFISHHS--
QSPEiDLARQFFNALTAAGHQPFMSEQSMQLGEGWaqRIDAELKGCDYFLLLLSAQ-
SASSEMVLeEVRRIRQLQEERaDHRPIIlPIRVNFP-
LEAPLNYELRGYLHRLQQRFWQSPQDTPLLIQDVLDLLAS(TPLENTRQPTTGGTLPTVPPLLPDPNAPPLPV
AEPEL)}*

$797=248(252):
>gi|493501181|ref|WP_006455696.1| {|8(353)|<Cyanobacteria(B)>}hypothetical protein [Synechococcus sp. PCC

7335] >gi|196190978|gb|EDX85942.1| hypothetical protein S7335_3645 [Synechococcus sp. PCC 7335
{(RGFAEAMGVSVDTVRKFLKGKAISRSNFQDLCEGLELDWCEMAFADDSSVTTQASETAPLEGADRSKQSAD
SNQSANCRI)----FLSYNRNirpdelvaLDLHNAL---------RKQHNIFIDQ-
EMPIGTPWaeTIRKEIFQADALIVLLSEQSAQSEMVQQEIELARESAALRqglPLILPVRLRYT---
APFRYPLNEALDPLQWAFWNSEEDTPRLIAEIQNALVGKS(LPIETETAKRKLLSQSFTVRSQSDQPPMLPPD
)}*

$798=248(253):
>gi|494037053|ref|WP_006979179.1| {|973(67)|<Verrucomicrobia(B)>}TIR protein [Chthoniobacter flavus] >gi|196226150|gb|EDY20656.1| TIR protein [Chthoniobacter flavus Ellin428]
{(KAKFEAARRWCTAVTNWGKAGRWQFHVSKDPETLKLELKHLTAESEAFATRGGQKPEIQVIDEPTPSPMPS
TQRSSSPTV)----FLSHS-ShdkpfvRRLQHDFEKAE--------
IPVWLDEQEIGLGESIsrSIEVGLEKSDYLLLVHSTHSAGSEWVKREVEAAINMQVNHsgiAVLTAVMDDAP-
--
LPILLKDRIFADFRKSYEAGRDFLMRFFKREASSVVCNLS(SGDVPPGTVGMQLPGGGDDCPTRLSRLTHREL
R)}*

$799=214(220):
>gi|260793605|ref|XP_002591802.1| {|464(0)|<Chordata(M)>}hypothetical protein BRAFLDRAFT_83590 [Branchiostoma floridae]
>gi|229277012|gb|EEN47813.1| hypothetical protein BRAFLDRAFT_83590 [Branchiostom
{(AVLLPFSEDGYKKVPLKSRDGGTTWKGKARVPNGDDAERRYVFVVGTPNGTTEGQYLLEYDIELHDLRDSR
LKPLPDGFT)-YHIMISYSW-AQ--
QEVIIKVKEHLNKVGYRVWLDKDEMGGTMLESMARGVRSSAIVIIAFSEDYVISDNCKREANFASDQK---
REIVPLKMttYYPDN-WLGMLISGKYYVDFSSPGPLVDKLKELEFQIENRLKKLAQ()}*

$800=202(220):
>gi|260816487|ref|XP_002603002.1| {|87(643)|<Chordata(M)>}hypothetical protein BRAFLDRAFT_123976 [Branchiostoma floridae]
>gi|229288317|gb|EEN59014.1| hypothetical protein BRAFLDRAFT_123976 [Branchios
{(FKELTTDRKTCKDGFRMCADGIRVRDNALSEAAKVVVILLVALVFVTTGPWIKEKFLERDIRVTLENIQQM
ERSSEGSHH)-YDVFISYSSSdgSWVREDLLGNLESNGY--TVCLDTRDFQAGSH------------------
-
FGRIKSIDTQSPRLVVLGQSQDLRCHFEFNPLDIGLKLkFAWYKDSKRVSDTSDGRiTISENVVKVPSNISNS
CRVPKNT()}*

$801=252(256):
>gi|260801215|ref|XP_002595491.1| {|62(0)|<Chordata(M)>}hypothetical protein BRAFLDRAFT_69123 [Branchiostoma floridae]
>gi|229280738|gb|EEN51503.1| hypothetical protein BRAFLDRAFT_69123 [Branchiostoma
{(PVIVPDVSPIITFKQEQKVATDACPSMDGNSCAVNITSQIDSSFDAGTEFLGAVDGHVKELEEKIKATGNS
LEAPLKPRT)-YDCFIIYSRDneDVVYNKLIPFFKKNN--
IKYCEHQEHFDLGKNIFDNVNTCITCSRRVVAVLSRSFFDSGYCKMDLDAARGYAASSGyslQDFLISI---
KITdFVFPNKYSD----ISNStYADLSEDVNDerK-WLQIRKAL--
T(EFRLASVIMRIEMGSKYCTPKSKLAVPGANKIETVV)}*

$802=250(249):

>gi|241694652|ref|XP_002402229.1| {|976(0)|<Arthropoda(M)>}toll, putative [Ixodes scapularis] >gi|215504708|gb|EEC14202.1| toll, putative [Ixodes scapularis]
{(NATACTNFTAVTYFQAVFSDNYMPLLIVLPSVVVLLLFVLVLVLVYRKQMKVWVHSKYGVRLFRRSQYAPEVDR)LFDAFVSYCKKdeAFVAQILAPELECGShPPFRLCLRYRDLPMSGYVAEAITEAVECSHRTLVVLSEQFLKSEWCRFELKTAHHELRCNSRHRLVVVLLDDV--AVKEMDADARQCLRSAVLLRWGDKRFWEKLRYALPDAAR(AGRKQVVVNQASEVTVCPPHLMSSTRCPNNAIKLV)}*

$803=258(263):
>gi|260834789|ref|XP_002612392.1| {|4(816)|<Chordata(M)>}hypothetical protein BRAFLDRAFT_78250 [Branchiostoma floridae]
>gi|229297769|gb|EEN68401.1| hypothetical protein BRAFLDRAFT_78250 [Branchiostom
{(GAGHLLYTYLTWLYLTLLSQVCSHSLKGSRRISFRYWQRSYTVILIYFLPYFIPESRRRSSSRLSIFTGSSLSLTATFTR)--DVFVACADKdeAVVKSRVIQPLESREI--TCCYPQRDFIPGHSEYQSVVDAMETSRRTIVLLSKNFKEYKLFHAILGLSNQLIAQ---GRVIPVKIDED-TDLDGRFRDVVILDMMAiDETTFITRLEKAVNLEAKSPGIR(EDAAVSSEPYPADSTPASSETSSDISTANTDFQQVWDEHDFRH)}*

$804=231(235):
>gi|260809272|ref|XP_002599430.1| {|0(1195)|<Chordata(M)>}hypothetical protein BRAFLDRAFT_106573 [Branchiostoma floridae]
>gi|229284708|gb|EEN55442.1| hypothetical protein BRAFLDRAFT_106573 [Branchios
{(MELDINSKDTHEERADELIGLLQTRGRNAFSVFCEILQETYPDLADLLQEESETGPSL)----FIIHAG-ED-KKSFVRPLHKALLGQNLReeeIFFDEVSISAGENIseKIMStlASESLKLVTIVMSNHLLDKYWPRLEYELSLFHQ-----KKFYPIWLDQNTDNFEAFGKKVGNRFPMLKKTLAHRIQYDSIAEEITDVAVE(IGQLLLDQCHPDYVTEDMPGDVTNTDVLWVIPELKAR)}*

$805=255(257):
>gi|260797895|ref|XP_002593936.1| {|573(715)|<Chordata(M)>}hypothetical protein BRAFLDRAFT_98235 [Branchiostoma floridae]
>gi|229279168|gb|EEN49947.1| hypothetical protein BRAFLDRAFT_98235 [Branchiost
{(LFGMTEAASSNVDMGLMAINRDRYKEIQNMVLSILGDSSGLLFEMFAEELSKDGCKGEGKDLLSFICIAFLGRACGAEPR)---VFVIHAG-ED-KDSFVRPLVGNLQQQGLAekdIFFDDVSIKPGDVIrdRIMSTLSSESLEVAVIavsSSFLNKSYWPRLEYETCLKNN-----KHIFPIWVDANEDKFKAFSESVGRYSPTLKQMSARRVQRDDVTNELPNIAAE(IAQRLSTLSHGHGVARTAGPLCTVDDVINSLKDLYVT)}*

$806=220(226):
>gi|260802207|ref|XP_002595984.1| {|122(0)|<Chordata(M)>}hypothetical protein BRAFLDRAFT_96757 [Branchiostoma floridae]
>gi|229281237|gb|EEN51996.1| hypothetical protein BRAFLDRAFT_96757 [Branchiostom
{(LFVHEPMGFGTTTFHTEVKETETRDKKKEKEKDADSDGEEKKPGIKDQVKGSVSSLTSKSPTFEEVVTQMMKKTAVSDLG)PVEVFLSYQW-DHQKQVIQ--LHDMLEEMGHSCWMDIKEMGGGDQLyaAIDRGMRQAKVVISCVTPKYTESDNCRNEVALAHSLR-----KTIVPVLLENTAWPPEGPMAMPFAQLLYIDFSKESKQHPWQGPHFDELMASV(EQSLKN)}*

$807=253(261):
>gi|260826906|ref|XP_002608406.1| {|799(75)|<Chordata(M)>}hypothetical protein BRAFLDRAFT_127607 [Branchiostoma floridae]
>gi|229293757|gb|EEN64416.1| hypothetical protein BRAFLDRAFT_127607 [Branchios
{(GASSSSGKFWPDSSFYELLGQLAYHAAPDQSLITDEYRNWTPSVEKKEEKDSSTKSKSEKKEKTSSTPVED
KETEPVVNP)--EVFLSYQW-GHQPQVKA--
LYSQLTSRGFTCWMDIQQMGGGDALygKIDKGIRSAKVVVSCVTPKYTLSANCRREISLADALR-----
KPIVPLLLQDTSWPPEGPMSMAFTELLYIDCRDERTQNDWEGAKFEELLLQV(SRHATPTPKPARPSPQAKPQ
EKRGQTPTKAAAAKKEDGEAT)}*

$808=253(261):
>gi|260826908|ref|XP_002608407.1| {|723(70)|<Chordata(M)>}hypothetical protein BRAFLDRAFT_127608 [Branchiostoma floridae]
>gi|229293758|gb|EEN64417.1| hypothetical protein BRAFLDRAFT_127608 [Branchios
{(QMGGGDQLYAKIDKGMRGAKVVLAMVTPKYAESTNCNHEINLANLLNKPIIPLLVENTPWPPPGSMSMIFS
QVAELVLNP)--EVFLSYQW-GHQSQVKA--
LYSQLTSQGFTCWMDIQQMGGGDALygKIDKGIRSAKVVVSCVTPKYTLSANCRREVSLADALR-----
KPIVPLLLQDTSWPPEGPMSLAFTELLYIDCRDERTQDNWDGAKFKELVEQV(SIHATPNPKPERSSSQQPAT
NAPTVTQSVVAENNEKANEDA)}*

$809=255(261):
>gi|260826908|ref|XP_002608407.1| {|608(183)|<Chordata(M)>}hypothetical protein BRAFLDRAFT_127608 [Branchiostoma floridae]
>gi|229293758|gb|EEN64417.1| hypothetical protein BRAFLDRAFT_127608 [Branchio
{(FLAVRLGYGPEDIRAWATNADPTMAMLNEWYNTHRSSEATYAVLKSLQEMGRTDAAEIIETAMEEAGAAVP
KPAADEPTN)PPPIFISYQW--DIQDQV-
KLIKEHLQMAGYESWMDIGQMGGGDQLyaKIDKGMRGAKVVLAMVTPKYAESTNCNHEINLANLLN-----
KPIIPLLVENTPWPPPGSMSMIFSQVAELVLNPEVFLSYQWGHQSQVKALYS(QLTSQGFTCWMDIQQMGGGD
ALYGKIDKGIRSAKVVVSCVT)}*

$810=253(261):
>gi|260826910|ref|XP_002608408.1| {|830(18)|<Chordata(M)>}hypothetical protein BRAFLDRAFT_95419 [Branchiostoma floridae]
>gi|229293759|gb|EEN64418.1| hypothetical protein BRAFLDRAFT_95419 [Branchiosto
{(GASSSSGKFWPDSSFYELLGQLAYYAAPDQSLISDEYRNWTPSVEKKEEKDSSAKSKSEKKEKTQSMPVEE
KEAEPVVNP)--EVFLSYQW-GHQPQVKA--
LYSQLTSQGFTCWMDIQQMGGGDALygKIDKGIRSAKVVVSCITPKYTFSANCRREVSLADALR-----
KPIVPLLLQDTSWPPEGPMSMAFTELLYIDCRDERTQNEKADGDAEEPDGSL(DADGDNKTGNLSHPEPVVRN
VTNEKTAKTTQNVQPGSTHVE)}*

$811=255(261):
>gi|260826910|ref|XP_002608408.1| {|636(210)|<Chordata(M)>}hypothetical protein BRAFLDRAFT_95419 [Branchiostoma floridae]
>gi|229293759|gb|EEN64418.1| hypothetical protein BRAFLDRAFT_95419 [Branchiost
{(FLAVRLGYGPEDIRAWATNADPTMAMLNEWYNTHRSSEATYAVLKSLQEMGRTDAAEIIETAMEEAGAAVP
KPAADEPTN)PPPIFISYQW--DIQDQV-
KLIKEHLQMAGYESWMDIGQMGGGDQLyaKIDKGMRGAKVVLAMVTPKYAESANCNHEINLANLLN-----
KPIIPLLVENTPWPPPGSMSMIFSQLLYIMFHGASSSSGKFWPDSSFYELLG(QLAYYAAPDQSLISDEYRNW
TPSVEKKEEKDSSAKSKSEKK)}*

$812=238(261):
>gi|260826912|ref|XP_002608409.1| {|600(160)|<Chordata(M)>}hypothetical protein BRAFLDRAFT_127609 [Branchiostoma floridae]
>gi|229293760|gb|EEN64419.1| hypothetical protein BRAFLDRAFT_127609 [Branchio
{(WRFLAVRLGYGPEDIRAWATNADPTMAMLNEWYNTHRSSEATYAVLKSLQEMGRTDATEIIQTAMEEAGNYQWDIQGQVK)--------------------
LIKEHLQMAGYESWMDIGQMGGGDQLyaKIDKGMRGAKVVLAMVTPKYAESTNCNHEINLANLLN-----
KPIIPLLVENTPWPPPGSMSMIFSQEAEPVVNPEVFLSYQWGHQPQVKALYS(QLTSRGFTCWMDIQQMGGGDALYGKIDKGIRSAKVVVSCVT)}*

$813=253(261):
>gi|260826912|ref|XP_002608409.1| {|698(47)|<Chordata(M)>}hypothetical protein BRAFLDRAFT_127609 [Branchiostoma floridae]
>gi|229293760|gb|EEN64419.1| hypothetical protein BRAFLDRAFT_127609 [Branchios
{(QMGGGDQLYAKIDKGMRGAKVVLAMVTPKYAESTNCNHEINLANLLNKPIIPLLVENTPWPPPGSMSMIFSQEAEPVVNP)--EVFLSYQW-GHQPQVKA--
LYSQLTSRGFTCWMDIQQMGGGDALygKIDKGIRSAKVVVSCVTPKYTLSANCRREVSLADALR-----
KPIVPLLLQDTSWPPEGPMSMAFTELLYIDCRDERTQNDWDGTKFKELVQQV(SVHASPNPESGRSSSQGTKEADGAPNAGGDNKTENVPHPKP)}*

$814=255(257):
>gi|260831806|ref|XP_002610849.1| {|19(257)|<Chordata(M)>}hypothetical protein BRAFLDRAFT_94911 [Branchiostoma floridae]
>gi|229296218|gb|EEN66859.1| hypothetical protein BRAFLDRAFT_94911 [Branchiosto
{(LRFRDIRRRLLDSGTLNGENLDLIENQQTREDQAKALLDILPTRGPTAFAVFREALKHRYPHLARILKDEGQQGPPEAPR)---VFIIHAG-ED-
KESFVRPLVATLQQEGLAekdVFFDDVSIKPGEVIrdRIISTLSSQSlelVVVVVSTAFLNKPYWPRLEFETCLKNN-----
KRIFPIWVDANEDSFKAFSELVGKYSPTLKQMSARRVQRDDVTDELTNIAAE(VVQRLSTLRCSTPPPAAIQTLLTLTPFPSSDPSSSDD)}*

$815=202(226):
>gi|260793767|ref|XP_002591882.1| {|103(0)|<Chordata(M)>}hypothetical protein BRAFLDRAFT_89399 [Branchiostoma floridae]
>gi|229277094|gb|EEN47893.1| hypothetical protein BRAFLDRAFT_89399 [Branchiostom
{(ALITRNRVLLTKRLQVSSLFPHLIERGLLLIDEKEDINSKTTTQGKAEALLDLLSRQGKCTPDVFMEILKLGNHEHIVIQ)---------------------
LRDVLEKKGYSCWMDITKMGGGDKLhaEIDRGIRQAKVVISCVTPNYTKSDSCQNEVELAYRLK-----
KPLVPVLLEKTAWPPEGPMAMPFAQLLYIDFTKESKEHPWKGAKLNDLITSL(EQRLKQ)}*

$816=180(182):
>gi|260822747|ref|XP_002606763.1| {|0(114)|<Chordata(M)>}hypothetical protein BRAFLDRAFT_82405 [Branchiostoma floridae]
>gi|229292107|gb|EEN62773.1| hypothetical protein BRAFLDRAFT_82405 [Branchiostom
{(MGGPR)---VFIIHAG-ED-
KRPFVYPLFNTLRQQGLTnndIFLDQVSIYTGDVIrdRIISALSSESLELAVI----
VVSGWLLNkrywpklEYETCLKNN-----

KAIFPIWLDTNKDNFNTFGKHVGSYSPTLKQLSAWRVQRCDVSYQLPNIAAE(IIRRLSNVTNLQNKEVAFER
TLDCSPWIDHNDIAGVL)}*

$817=218(227):
>gi|260788662|ref|XP_002589368.1| {<Chordata(M)>}hypothetical protein
BRAFLDRAFT_77814 [Branchiostoma floridae] >gi|229274545|gb|EEN45379.1|
hypothetical protein BRAFLDRAFT_77814 [Branchiostoma florid
{(MVFLTQEAKKETEDVKSRAEHLKVAGRPQKQGDDVKKTQPEGAVAITKGDSPDPRTSALTARVEPMVRSEV
TGAQNA)---VFLSYQW-DHQDKVL--
LLHDRLQERGYSCWMDIKQMGGGDSLyeMMDKGIRGAKVVVSCVTPPYAESKNCRDEVALAHTLK-----
TPIIPVMLEKTTWPPPGPMSIPFAQLLFINMAKGHDEDPWKGALFEQLVRMV(DSFVKRPKAK)}*

$818=213(222):
>gi|260788664|ref|XP_002589369.1| {|656(0)|<Chordata(M)>}hypothetical
protein BRAFLDRAFT_77815 [Branchiostoma floridae]
>gi|229274546|gb|EEN45380.1| hypothetical protein BRAFLDRAFT_77815
[Branchiostom
{(VYQTLVQWREKEGEAATLSALEQHLRDLDFQQLADQLFRAPRPEGAVAVTKGDSSDSRTSALTARVQPMVR
SEVTRAHNA)---VFLSYQW-DHQDKVL--
LLHDRLQERGYSCWMDIKQMGGGDSLyeMMDKGIRGAKVVVSCVTPPYAESKNCRDEVALAHTLK-----
TPIIPVMLEKTTWPPPGPMSIPFAQLIYINMAKGQDEDPWKGVLFEQLKKKS(TT)}*

$819=249(230):
>gi|260788692|ref|XP_002589383.1| {|3(0)|<Chordata(M)>}hypothetical
protein BRAFLDRAFT_77825 [Branchiostoma floridae]
>gi|229274560|gb|EEN45394.1| hypothetical protein BRAFLDRAFT_77825
[Branchiostoma
{(AAETELERKFQERREKSARMKQQVSQQKDQTVERMDISKDEELCQAFNRLSLKVGQSQWRPIARQLGFSRQ
EIRDIEQNS)---VFLSYQW-DLQDKVL--
LLHDRLQERGYSCWMDIKQMGGGDSLyeMMDKGIRGAKeisvncllpvhlrtappsptlrfplenlVVVSCVT
PPYAESANCRNEVALAHTLK-----
TPIIPVMLEKTTWPPPGPMSIPFAQLLFINMAKGQDEDPWKGALFEELVRMV(DSFVKRPKAK)}*

$820=212(221):
>gi|260812916|ref|XP_002601166.1| {|490(0)|<Chordata(M)>}hypothetical
protein BRAFLDRAFT_75615 [Branchiostoma floridae]
>gi|229286457|gb|EEN57178.1| hypothetical protein BRAFLDRAFT_75615
[Branchiostom
{(SFKDVMARLMRELGDRGKGHTAAGAEVDTVGSKLEGLKLPRMRKEPEGAVVVKKGKSPRTAALTPRVEPMV
RSDETSAKA)---VFLSYQW-DHQEKVL--
LLHERLQERGYSCWIDINEMGGGDKLykMMDKGIRGAKVVVSCVTPPYTTSQNCQDEVALAHTLN-----
TPIIPVMLEKTTWPPPGPMSIPFVQLIYINMTKSQDDDPWKGALFEQLVKML(G)}*

$821=202(220):
>gi|260807523|ref|XP_002598558.1| {|409(0)|<Chordata(M)>}hypothetical
protein BRAFLDRAFT_66949 [Branchiostoma floridae]
>gi|229283831|gb|EEN54570.1| hypothetical protein BRAFLDRAFT_66949
[Branchiostom
{(QVEKLIPHFIERGLLDFEDKQVVMSKTTTPGKAEALLDLLADNGTCTPENFLEILNNGDHRHVAVQLKRIT
TRNENAGTQ)---VFICHAG-PD-
KARFVRPLVQKLQERGrlpmVEIFFEEISLSPGVDIaaEIKATLLSSSVKLVVFviSHHvLNDRYWPKLEFEL
TLRKN-----KKIFSIWLDQN------DDDFTAFGNRLRKYSPTLKE-----TVG------P()}*

$822=227(228):

>gi|260807741|ref|XP_002598667.1| {|20(0)|<Chordata(M)>}hypothetical protein BRAFLDRAFT_67068 [Branchiostoma floridae]
>gi|229283940|gb|EEN54679.1| hypothetical protein BRAFLDRAFT_67068 [Branchiostoma
{(LSKRLQVEKLMPQFIQKRLLDFPEKQVIMSKVTTPGKAEALLQLLSEKGTCTPEVFVEILKNGNHGHIVGQ
LTWPAKGPQ)---VFICHAG-PD-
KARFVRPLVQKLQEEGLpedKIFFDEISLSPGDDFaaEIKATLSSSsslKLVVFVIS-
HYVlnDRYWPKLELELTLQAN-----
KKIFPIWLDQNEDGFTNFGTRLRKYSPTLKEKVGRKVLVDDAGREMLSIAEE(IVRKLETS)}*

$823=255(257):
>gi|260833090|ref|XP_002611490.1| {|83(561)|<Chordata(M)>}hypothetical protein BRAFLDRAFT_63875 [Branchiostoma floridae]
>gi|229296861|gb|EEN67500.1| hypothetical protein BRAFLDRAFT_63875 [Branchiosto
{(NGGGVALSSREDQSVTTSYHIDYREESPCRSTTARADNVASTGDTSRTAAQHTGSTPAGGHSGQSTRLRGL
RQAPVQEPR)---VFVIHAG-ED-
KETFVRPLVGKLQEKGLDkndIFFDDLSIQPGDVIreRIMSTLSseSLKLAVVVVSASFlNKSYWPRLEFETC
LQNN-----
KHIFPIWVDANEDNFKVFTIQTRLDLVPSSYSSDSSDLDEERSIEEKVCGAE(SSNAQSVEQILKDEKEKQQD
LLLFGVTQLELLEKARG)}*

$824=252(261):
>gi|241833188|ref|XP_002414929.1| {|88(70)|<Arthropoda(M)>}conserved hypothetical protein [Ixodes scapularis] >gi|215509141|gb|EEC18594.1| conserved hypothetical protein [Ixodes scapularis]
{(RHFPYASFHGMRGYSYTDKELSRFVEEPNPGIALLRDWRESNGATRYCVDVLVSCLQQMGRHNVAKLIQDE
LEPESLVPP)---VFISYQW--DAQDVVL-
NIRQHLEFSGFPCWMDVGQVGGGDSLygKIYEGISRAKVVICCLTPRYAASASCAREVSLADVLR-----
KPIIPIMVEPTPWPPPGPLAIVMSSLVYVDLCGVGGHGGSGRRADWEMRFQE(IAGRVAQFLSAPGNVAVASG
SRPQSKAQKATLAPLRAGASG)}*

$825=277(299):
>gi|260821892|ref|XP_002606337.1| {|3673(107)|<Chordata(M)>}hypothetical protein BRAFLDRAFT_67580 [Branchiostoma floridae]
>gi|229291678|gb|EEN62347.1| hypothetical protein BRAFLDRAFT_67580 [Branchios
{(FLEHEDPQIQTIIALTLAYITDKENKQLVMSEETQRKVVSYILRRFREAVNVPNWRSDGFSALELASGIAQ
LATNDTEED)---------------ANKISKQLRSAGYR-VWIDETHMR--
GSILEAMGEAVSNAAVVLVCVSRQYF-LSKSCRaEGNACHVTN-----
TPVIPIKVEKtySPdgWLAVLVGAGLYFDFSDP-KGVKAAMK--
GLMKEIGSRGKA(DGHHDDDDDDEEGDEASEPDLRNWTKADVDKWMTEFDLEKDIRGVADITGAQLAFLRSLK
EQASGQCFGSADGLNTYNK)}*

$826=265(276):
>gi|260841544|ref|XP_002613972.1| {|316(207)|<Chordata(M)>}hypothetical protein BRAFLDRAFT_67462 [Branchiostoma floridae]
>gi|229299362|gb|EEN69981.1| hypothetical protein BRAFLDRAFT_67462 [Branchiost
{(SDEEEQLQAVGAIWQLSFHEDNRKKILEEPGLVEKLKNLKDSPYEEISDSAAGALFVLTGKGKDQPEQDIE
EDGDTRRRQ)-RHVMISYNWDHKKIAVKISKQLRSAGYR-VWIDETHMR--
GSILEAMGEAVSNAAVVLVCVSRQYF-LSKSCRaEGNACHVTN-----TPVIPIKVE--
RtcSPDGWLAVLVGAGLYF--

DFSDPKGVKTAMKGLVKEIGNR(NQTLGYISLGCWKDTRNRAIPTLEGTDPRLDGHVTSRNNAIEKCYQVALS
HGFPMF)}*

$827=287(294):
>gi|260822131|ref|XP_002606456.1| {|46(209)|<Chordata(M)>}hypothetical
protein BRAFLDRAFT_93247 [Branchiostoma floridae]
>gi|229291798|gb|EEN62466.1| hypothetical protein BRAFLDRAFT_93247
[Branchiosto
{(NNENEKLEGGSRRVPPRPRRQAVQNNENEKLVAKSQHVPPRPRRQAVQNNQNEKKEADTAKKGGQKALSRL
DSSSEFGQD)---VMISYSHQdMDFMRKMR---GSLEASG-
ISVWVDEQgLGAGVDFLnKIGHAIVEAKVFLSLLSPNSVTSKYCKDE----LALAYI-
SNKPIFPVAMatRDS-LRKNMDFGmQLTLAPMEWvdFSDRDSFD--
VSFAALVHKFIAA(TKAGDESKAPPSKAKPKLERPKTRQNLNKMTRLNSNPMDDESDFWERAFKDAKDVPWSK
FIEAFDSNYSSLLDG)}*

$828=265(270):
>gi|260816868|ref|XP_002603309.1| {|17(210)|<Chordata(M)>}hypothetical
protein BRAFLDRAFT_71397 [Branchiostoma floridae]
>gi|229288628|gb|EEN59320.1| hypothetical protein BRAFLDRAFT_71397
[Branchiosto
{(SDLRFKDIRRRLLDSGILNGENLDEIENQQSREDQAKALLDILPTRGPNAFGVFRDALKHRYPHLARILND
EGQQGPPEA)-PRVFIIHAGEdKESV--
VRPLVATLQQQGLAEkdiFFDDVSIRPGEVIRdRIISTLssQSLELAVVVVSTSFLNKPYWPKlEYETCL---
-K-NNKRIFPI-------
WVDANEDKFKAFSELVgkYSPTLKQMSARRVQRDDVTDELT(NIAAEVVQRLSTLRSGTPQPAAIQTLLYLTP
SPSSSDAGSSDDETSSKGQ)}*

$829=256(259):
>gi|260790887|ref|XP_002590472.1| {|621(444)|<Chordata(M)>}hypothetical
protein BRAFLDRAFT_86311 [Branchiostoma floridae]
>gi|229275666|gb|EEN46483.1| hypothetical protein BRAFLDRAFT_86311
[Branchiost
{(LRFKDIRRRLLDNGILTGENLGEIEKHQTREDQAKALLDILPTRGPSAFGVFQDALKYRYPHLARKLNDGG
QQEPSRDPR)---VFIIHAG--
EDKDSFVRPLVIKLQQLGLaekDIFFDEVSIEPGEVYrdKIISTLSSQSLELaVIVVSTAFLDKPGWPK----
LEYETCLkKNKRIFPIWVDAN-
EDNFREFRELVGKYSPTLKQMNARRVQRDDVTAQLTNIAA(EVVRRLSTLRSNMLQPTGMHAQALPSTSSDAL
SDTSIRY)}*

$830=184(187):
>gi|260811253|ref|XP_002600337.1| {|0(908)|<Chordata(M)>}hypothetical
protein BRAFLDRAFT_66570 [Branchiostoma floridae]
>gi|229285623|gb|EEN56349.1| hypothetical protein BRAFLDRAFT_66570
[Branchiostom
{(MAASHQCQ)--TVFICHAG---
ESKPFVRQLVDRLKTDGLeedQIFFDERSLDTGDELdSIVKVIESPSLKlFVFVVSKYCfdVDRTWIRtEWEA
ALENNKQ-----IFPIWLDDN-
NDDFKAFGNLVRKFSNKLRKMIAQRVNPQEISTTLSGLSE(KICSLVCGDQPSQQRPTRTADNAVVATRQNIM
PEVLPAK)}*

$831=224(220):
>gi|260786881|ref|XP_002588485.1| {|24(0)|<Chordata(M)>}hypothetical
protein BRAFLDRAFT_63433 [Branchiostoma floridae]

>gi|229273647|gb|EEN44496.1| hypothetical protein BRAFLDRAFT_63433 [Branchiostoma
{(LARKLGFKRGEIKAMADNNVQFPGPNEKCWEMLGKWINKPNRGEDPVRDLKQALMNIEERLTAEALEGIEGATATEAGAK)---VFISHAS--EDKEEFVTPLVQELLKPGrlqqSDVFYDKHSLRTGDHLwnEIDAALRDPALKlFVFVISRHVLsKKQWPQyEYQLAHGKGVRIfPIWLKRDGDGDFR-QKVSEYDTMREPDKLLAVPVHVNEVDEKLSGIADKIMEQL()}*

$832=220(220):
>gi|260821487|ref|XP_002606064.1| {|374(0)|<Chordata(M)>}hypothetical protein BRAFLDRAFT_92083 [Branchiostoma floridae]
>gi|229291402|gb|EEN62074.1| hypothetical protein BRAFLDRAFT_92083 [Branchiostom
{(RPSFDECEEQLRDVNSKSSKEDILDAITYVKMHTTTTKAKGAGSTFEGSEEFPSPTSMPTAFKAPQARSEPLLTAEASYA)-FDAYICYcTEDRDFVMQMEEKLENPPFGtgkkgLRLWYAERDALPAMPIYDTVREGIARSRHVVLVLTPEALISRKCTFEMEYAATLPPEARRQKVITVMYKP--TDLPSWLKSMTILDYAK----EErvRKD-WFWVKLAKAISNQ()}*

$833=239(244):
>gi|260795861|ref|XP_002592923.1| {|222(0)|<Chordata(M)>}hypothetical protein BRAFLDRAFT_65511 [Branchiostoma floridae]
>gi|229278147|gb|EEN48934.1| hypothetical protein BRAFLDRAFT_65511 [Branchiostom
{(GEKHSLQLQLTAKPPDGGFPELWSIVIAAGSFVFVFIVVSVPVTVFIYRRRRQWKHCPLNSESDMEEGDASESVDNLSKE)-YDFSALCcEKDKSWTKTdIVNKLEKTGR--KGYFNFRDEVGGGSVFQYLSVGIANSRNVIVVFSPSAVDDDWFHwfrkgYQTAVAEKLDRNF-TGQVIPVLLNGCKRsQLPVELKDTVALDAEE----PK-----FFERLQRSLSKD(SDYESARPSSASSEESIEDRISVV)}*

$834=294(298):
>gi|241613282|ref|XP_002407372.1| {|66(4)|<Arthropoda(M)>}myd88, putative [Ixodes scapularis] >gi|215502786|gb|EEC12280.1| myd88, putative [Ixodes scapularis]
{(QRGESPTEALIDAWVAQSRKERVTLHLLIQNLELLERFDVIDDVMPHLDHDVKLFRNKHSGDLPDSISDPLLKLPEGCPV)-YDAYVCYtAEDFPFVQTLVEKLERPELGIRLFLPERDLCAGVLKYNTFFQLMEKwCKKTIIIFSPEFLKSHECRMQQKFIESIEIEQAQQKLIPVVIKPCALDgMIRMISKINLCE-TA----SALSH-WSWNNLISSIKSS(GNGSFVQRAPSLCALRLAPSPPEVRAPSATRARTPVAAPKAVEVASHSHKGKAKWLSGIFSRKKNNSVSSASSGFQSM)}*

$835=250(254):
>gi|506437131|ref|WP_015956848.1| {|753(12)|<Cyanobacteria(B)>}GTPase [Cyanothece sp. PCC 7424] >gi|218174533|gb|ACK73266.1| Miro domain protein [Cyanothece sp. PCC 7424]
{(HRLKYKKLIPCNCKSCKDSQYPWFYDYEKLKKFIQDREPNIQCQESYQMVEVTGLIDDVINDFNPLIKTKEDIKLDSLEK)--EVFISYNW-GGESEEIVNQLDQAFQNKGITLKRDKRDLGYKG-LikEFMQRIGRGKCVVVVISDKYLTSHNCMFELIEVAK-HKN-FYKRIFPIVLDDAKIYKSVDRIKYIQYWKQEIEELDQAMKTVSSDNLQGFREDI(DLYTEIRKYIAQLVYDIKDMNTLNLSIHQKSGFE)}*

$836=276(279):
>gi|506437510|ref|WP_015957227.1| {|189(1191)|<unknown(X)>}hypothetical protein [Cyanothece sp. PCC 7424] >gi|218174918|gb|ACK73651.1| WD-40 repeat protein [Cyanothece sp. PCC 7424]

{(SLNQQIKDNKNYLRNHSYFLVKALEWEWYGKPINSLLIKDELAQAQEWLDQYEENLSLAKPTEIHREYIEE
SIKSSQAFF)--DGFISYGR-PDSL-
AFVNQVYQKLTKKGYRIWFDMQEIPYAVNYqkQIDNGIERAKNFIFFMSPHSVNSPYCGQEIDLAVKYK----
-
KRIIPVLHvpeiDEQTWKQRYPHKDWETAKQNREYTSDRNPNLHPALSYPNWIRYS(DHQDFEKLIEGLESLF
KTGSDYVTQHTYILDKALEWERHERRTEYLLVGEERQQAEKWL)}*

$837=242(237):
>gi|148907765|gb|ABR17008.1| {|0(411)|<Streptophyta(V)>}unknown [Picea
sitchensis]
{(MAASTSASAAANPNKKS)-YQVFINHRG-
VDVKETFARSLYLRLLEKGLMAFLDKEEMQQGYQFfsQIERAIRTASVHLAIFSPRYAESDWCLNELLLMLES
N-----APIIPVFYRVKPAEVRWMlglygeylqsheTKGRYDPNTIKKWRNALHQV--
ASSSGFELETCN(GEELVLELLDKVVKCLSEIMIKPDLYVAEYPTGLDCKLKDFEDTVLLQQQQSGEPQLLGI
VGLGGVGKTTMAKELFNRKK)}*

$838=210(215):
>gi|148910226|gb|ABR18194.1| {|0(369)|<Streptophyta(V)>}unknown [Picea
sitchensis]
{(MANVNLTPARDATSFASSSTSPSHANTNSYV)-YDVFINHRG-
PDVKKGLASHLYHRLKAYGLGVFLDQQELERGENMtpQIEGAIRTASIHIAIFSPTYAESSWCLKELVMMLES
G-----
STIIPVFYHVRPCELRWTRGGNGDGDGVYARALCTLEKKRTFDQEKPRHDSN(TIEKWRNALSNVAERSGFEL
TAYNGDEGQLVDDVVQQVLKKVRK)}*

$839=280(299):
>gi|506437510|ref|WP_015957227.1| {|416(960)|<unknown(X)>}hypothetical
protein [Cyanothece sp. PCC 7424] >gi|218174918|gb|ACK73651.1| WD-40
repeat protein [Cyanothece sp. PCC 7424]
{(GLESLFKTGSDYVTQHTYILDKALEWERHERRTEYLLVGEERQQAEKWLLTEFKNEQSPCEPSLLHCEYIA
QSKKNANNL)MTQVFICGGTKdEAIKEKI-TH--
SLMRKGFTVWTAKSDLRPGEKLEeGVNRGIDEADNFIYL-----LYDDNWAEaQLRT---RARS-
LNKRIFTLLV-----------GEVQELPSDIIIDFSNpqrYD--
ESFQSLIRGLNED(NSYYERHKLLLAKALKWKQQPDNVSLLLRGYNLREAENWLKEARLKPQQGATPDHEEFI
NASLRQPSGGSLDVFISYSR)}*

$840=221(218):
>gi|501632006|ref|WP_012606071.1|
{|0(498)|<Proteobacteria(B)>}hypothetical protein [Methylobacterium
extorquens] >gi|218525584|gb|ACK86168.1| protein of unknown function
DUF323 (plasmid) [Methylobact
{(MDFL)---AFISYKHSaglpfaARLASSLKSYARSPFRPPP-
RIFRDEDHLVPDNELpdTIKQALRRSKFLILVADPDSARSIWVKDELLFWCyELRRTNhliIVLAKGEIAFD-
--QETKEIQWENTDALPDYLKSGLRFIP--
FFIDLSHVDRET(QLDLRDPEFKKAINAISARLRNVSPEELMGEEIRLYKRTLWVRNIVLTTLAVLLVGVAFL
YQRSSSTLSEYERL)}*

$841=227(226):
>gi|223538080|gb|EEF39691.1| {|0(931)|<Streptophyta(V)>}leucine-rich
repeat containing protein, putative [Ricinus communis]
{(MASSFSASARIQNW)KYDVFLSFRG-EDTRNNFISHLHAALSRKSIRTFIDDELRRGDE-
ItrSLLKKIEESKIAVVIFSRNYASSTYCLDELEKIIEFHEC-
YGQTVIPIFFNVNPSDLLEPdTGIFAEALSRHEKDIMEklNKV-

QGWKKWWKDSVIK(AANFLMLHSQVIGSEQDQEKLDKVQRWKVALKKAGNLSGHDLQIIRRESELVDKIVSDV
WKRVKQVSPSISD)}*

$842=184(202):
>gi|223529897|gb|EEF31827.1| {|0(649)|<Streptophyta(V)>}conserved hypothetical protein [Ricinus communis]
{(MASSSSSSTTHQAKI)-YDVFISFRG-ADVRDGFLSHLHQSLDRNQVNAFVDEKLKRGKE-
ItsSLLEIIEKSYVSIVIFSKNYADSPWCLDELVKIFECYKK-----------------
MKQIVVRPDSRLIREIVSHVLEELDHLTPSDVCEDGLF(GIDSRSKDVRSLLCLESTDVQVIGIWGMGGIGKT
TIVYKLFSQIHKQ)}*

$843=194(210):
>gi|223535273|gb|EEF36950.1| {|0(448)|<Streptophyta(V)>}hypothetical protein RCOM_1122080 [Ricinus communis]
{(MASSSFSSSTCQWKK)-YDVFISFRG-KDIRDGFLSHLHEALRQSQINAFIDESIERGKE-
IssSLLKIIEESHVSVVIFTENYADSPWPDSKLIgeVVSQILEK-----------------
LNNVISSYNCADGLVGNDSSVKAVKSLLCLESTDDVRF(VGIRGMGEIEFLVGNRASYCSGSRVVITSRNKQL
LRNMDAKIYEVKKLNYLEALH)}*

$844=195(197):
>gi|224284598|gb|ACN40032.1| {|0(876)|<Streptophyta(V)>}unknown [Picea sitchensis]
{(MASSSTSPSHDDV)-YNVFINHRG-
PDVKNGLASHIYRRLIDHGLKVFLDKPEMQEGEPItpQIKRAIRTASVHIAIFSKGYADSTWCLDELLDMldt
VKSG-----
SAILPVFYNVQPSDLRWTRGGDTVYARVLSIFLCILLCTRGENGVYARALRK(LQKKTTLDSVTNKKKPRHDS
DTIEKWRKALSDVSLISGFELNAC)}*

$845=215(221):
>gi|491164545|ref|WP_005022917.1|
{|0(605)|<Proteobacteria(B)>}hypothetical protein [Helicobacter pullorum]
>gi|239524335|gb|EEQ64201.1| hypothetical protein HPMG_01658
[Helicobacter pullorum MIT 98-5
{(MI)-
KKIFYSYSHKdKAYLEKLQTHIKPIIKEYKIKEFVDEHIRGGEVLDeKIMTNIQESDLVISLISPDYLASEYC
-QkEMKIAIDAHKS------FPIIARTC-DWKNTSLRDIKVMPQDGkpIIDYDNDD--
KAYQYIAQELRKK(IQDSLQIIICNEGFSKYLEKIDFLHPNKAKITLADTFVYPNICSSKDEQITIKEILENE
KYKEILFFSPFYSGKTTLLK)}*

$846=184(183):
>gi|490324380|ref|WP_004213851.1|
{|0(222)|<Euryarchaeota(A)>}hypothetical protein [Natrialba magadii]
>gi|289529596|gb|ADD03947.1| uncharacterized protein Nmag_0357 [Natrialba magadii ATCC 43099] >gi
{(MTDPTG)--KMFVSYRRSqADIAEALITSL----
HEHGIRTWQDISDLPTEPTQpEIRNVLEveNPELAggIVLVSEDVTKSDIILElELPGLHNRWKN-
EEEFFVVVALCPG-
IDYDDANEILSESPTLLdfSDWNMIKLGSTADTPAAADDVAT(AVLTERVRLNHERLDEDHPFDISLDTYAPP
THASRPA)}*

$847=252(255):
>gi|506384084|ref|WP_015903803.1| {|66(74)|<Proteobacteria(B)>}TIR domain-containing protein [Desulfobacterium autotrophicum]

>gi|223691734|gb|ACN15017.1| hypothetical protein HRM2_19160 [Desulfobacte
{(IVNPELKKMSQELEAHSKGFLKMKLEDELPGKIVHPMEVDDDFDLLYFPCCNETIKASQHEHHFCIICGAE
IDMKENALK)--QVFLSH-K--GVDKDMVINFKKTLDLLGYSPWLDEDAMPAGTPLerGILQGMK-
SSCGAVFFITPSFKDEGYLEtEINYAIQEWREKkDKFSLITLQFIDV-
NGDAGVIPDLLKPYVWKTPKTELEALCEIIRALPVLPGIV(DWREKINGVVRLSTVKSTATELSIEAKTLLEA
AAA)}*

$848=172(176):
>gi|493912591|ref|WP_006857968.1| {|0(320)|<Firmicutes(B)>}TIR domain protein [Roseburia intestinalis] >gi|257201911|gb|EEV00196.1| hypothetical protein ROSINTL182_07906 [Roseburia intestinalis L1-82]
{(M)-FDAFISHSSKdkEKIVLELVQKLEKNNID--
VWLDANEILAGDTILDAVEKGVATALCTILIITPAFFESFWTPVEIGLAIGQ---NNNHKLIPVLCNVS-
VEDIAQRFPMLLSLKYIkLDPDNIDLCVDELCSNIIQIKKK(YEKDFPEISFHKAVKKFHSCDTPTANTISIL
LSEY)}*

$849=136(146):
>gi|493866469|ref|WP_006813113.1| {<Proteobacteria(B)>}hypothetical protein [Providencia rustigianii] >gi|282568227|gb|EFB73762.1| hypothetical protein PROVRUST_05034 [Providencia rustigianii DSM 4541
{(MNI)----FVSYTTRddN-
VNKKLLERVSEIVSLYGHCYIDLLHNTKKDKQQHVEFMLSQSNLLILIASSSVFTSKWVQWELDEA-------
KRCYIPIIMVEAK-SDMNNILKNLKSILATNaYLLSNHRSEKFKNSSENTMSLLG(GNV)}*

$850=217(236):
>gi|488237729|ref|WP_002308937.1| {|143(0)|<Firmicutes(B)>}hypothetical protein [Enterococcus faecium] >gi|257823169|gb|EEV50101.1| predicted protein [Enterococcus faecium 1,141,733] >gi|430564195|gb|
{(IHTSENYEEILKIAKKAKMSVVNDQGLEEKIEEMSYGYKTMFPIPLHSSEELILTLFGFGFSFPSLKEEQE
KGLIKNKKI)KKKVFISYCHKdKKKVHYIVDQLKSFGLNV------
WLDEQEIDFGDnileKVSMGLEECDLALVFLSNHTHKAKGARHELNALF---SK-II------
YQESLKSWILVRL------DEID--LNKIYFGLGNYKYFDYQLGAE(EMLYTIVDKKLKKVEA)}*

$851=221(221):
>gi|491794858|ref|WP_005603365.1| {|31(0)|<Firmicutes(B)>}molecular chaperone Tir [Butyrivibrio crossotus] >gi|292809170|gb|EFF68375.1| hypothetical protein BUTYVIB_01646 [Butyrivibrio crossotus DSM 2
{(RTLNENKKRHEVWMNDVDIFYNKYLKEHPLGQKIDTWLFHRKYDQLVAALESISEDKDFINKMNGISTVEV
PKYKAKMLP)EYDVFISHAN-AD-
KEAFIEEMYNSLNKLGVKIFYDKETLEWGDKFkdKILEGTKKSEFAIIVISTNFFGREWTERELSEFLNRQNQ
NGQKLILPILHNITIEQLKEKYPSIADIQAIDSSKYTCDQIALLFARQFIKRLKA(Y)}*

$852=253(260):
>gi|301112453|ref|XP_002997997.1| {|921(58)|<Stramenopiles(E)>}transient receptor potential Ca2 channel (TRP-CC) family protein [Phytophthora infestans T30-4] >gi|262112291|gb|EEY70343.1| transient re
{(IEYDYDEAKFKAQFRLLLDGVRDEIEKNRDAVQQKNIASNRRNNGLKQTATGTILATTTGGFSRLFQQWDP
EASGAQKQF)---VFLSHG---DKHAGFVQQLYRELTDAGVVCYGDRN--
VEGQDFenRIhatQEAILRCTCFIVILSKQTMNN-----
ELVRDQLAFAEDKGRPIFPItLNDLDPGLDKRYSLARNELFHFMGNGMSFKPSADRLIQGLRRHYTA(REDNQ
GTMLDNSTRIASANTSFVSFTFSEPGTKDLDREAT)}*

$853=225(220):

>gi|260763945|ref|NP_001159600.1| {|722(0)|<Chordata(M)>}toll-like receptor 2 [Ciona intestinalis] >gi|260162446|dbj|BAI43698.1| Toll-like receptor2 [Ciona intestinalis]
{(NLSESEYCPVVNLSLIGGLVGGFTALLIIIIIIVNIYLYKKRKKQERRDIVQGFKDLLEKEAMNEGDPETG
VGVAPVTYE)-YDAFVSYvsDSDdvEFVYKMLEEMEEKRER--
KMCIHERDFTPGRGIADNIVECISTSRRMVLVVSRKYASSAWCQYEVQIALTELHAKRRGRlLVPILLEDVTr
D---EQYAgSVTTILSAItAIQAPkaqDNDRTwaNFWNKLDKTL--T()}*

$854=282(291):
>gi|301090295|ref|XP_002895368.1|
{|646(176)|<Stramenopiles(E)>}conserved hypothetical protein [Phytophthora infestans T30-4] >gi|301111544|ref|XP_002904851.1| ankyrin-like protein [Phytophthora infes
{(NVSVDLFDCLVQHGADIDDRLPNGDTALHIAMKCEQKGTALALLDAGADVMETNRDGYRPVDCTTSTQLQF
EIKQAAGDR)--DVMISY---
THSHSQFALKIRDSLERANITSWLDLMDptgIGGGSVWreEIARGIKNAEVIICLYTEDYPVSEWCLKELALA
KQLG-----KPIIA----VSPEGAAVTEEmqVYIYTRQMVPFEPAIKSV--
NNTNKRKIMYEY(DEERFAAQFRLLLDGVRDEIEKQREANIVGGSRRRLANGTSVVGFGENITDWDPATLDNI
PFAFVSHGDVH)}*

$855=227(232):
>gi|301104228|ref|XP_002901199.1| {|0(177)|<Stramenopiles(E)>}conserved hypothetical protein [Phytophthora infestans T30-4] >gi|262101133|gb|EEY59185.1| conserved hypothetical protein [Phytophthora in
{(MCDTALHVAVREENEASALAVLASGGDLMRMNNEGLRSLDCTASTRLLFELKRAAGQR)--DVMISY---
THSHLAFARKLRQSLEEANVTTWLDLMDpsgIGGGAVWreEIARGITNASVVLCILTEDYASSEWCLKELAQA
KQVG-----
TPILAVSTEGAIVDENLQVYLYTRQIVPFEPAIVGLRRDGGRIEYDYDETRY(RSQFRLLLDCVRDEVEKHRF
VTKQNNLSRSRRLA)}*

$856=259(263):
>gi|301112453|ref|XP_002997997.1|
{|712(261)|<Stramenopiles(E)>}transient receptor potential Ca2 channel (TRP-CC) family protein [Phytophthora infestans T30-4] >gi|262112291|gb|EEY70343.1| transient r
{(DATREVFGVLTSAGADINYQLPTTGDTALHIAVREENEQSALAILANGGNLMKMNIEGLRPLDCTQSTRLL
FEIKRAAGQ)-RDVMISYTHSHAEFAKKIRKALEDANVT-
TWLDLMDPsgiGGGSVWREEIARGITNAAVVLCLLTEDY-AQSEWCLkELALAKQVG-----
TPILAVSTEGVGigEDLQVYLYTRQLIPFE--
PAITQTRRNSTNVRQIEYDYDE(AKFKAQFRLLLDGVRDEIEKNRDAVQQKNIASNRRNNGLKQTA)}*

$857=229(231):
>gi|260647869|emb|CBG70974.1| {<Actinobacteria(B)>}conserved hypothetical protein [Streptomyces scabiei 87.22]
{(MPFRRRHREAMPATSDPLCR)---VVITYARVeQHHRKTFRGHAADLERNGV-
EFFDDQDIPPGAPWEtTLFDRLDRADIIVLLLSSHYVASDYCMEvEFPRAMRRWQA-GECRLFPVNVAPF-
DlTQGSPLRQLQWIPS-GksVTEYgAAAA--
REWRKVTQELRRM(TEGLGGAPAAGTPAAPHRTGGGARTPGGGPATPGNSITGSVVHGPAVQIGEFTGNLSID
SAQQPPADGPRS)}*

$858=196(190):
>gi|262079346|gb|ACY15315.1| {|0(293)|<Proteobacteria(B)>}SEFIR domain protein [Haliangium ochraceum DSM 14365]

{(MADAIESDSEVPKL)----FISYSwtS--
EEHQNWVLQLANDLADNGVHVVIDKFDLRPGHDAfkFMEQMVTDPSIsKVLIVSDEAYATKsdermGGVGiEA
QIITpDVYKQAqQSKFVVLVRELKP-
DGQPWVPAYYNSKLYIDCSDLNEYEEKLEELIRWAFDKPL(HRRRPLGRPPAYLFETPSVDLGTRSAQQRCVK
AIRS)}*

$859=229(246):
>gi|227438285|gb|ACP30632.1| {|0(1009)|<Streptophyta(V)>}disease
resistance protein [Brassica rapa subsp. pekinensis]
{(MEPSLDSDEGNMDNCEPEQRLEAFISRFNSELRKQRQEHIITNEDLKASSQGSSSGGLPAVYI)-------
YCA-NTLQYSFASHLSMGFHRKGIYAS---------A-
NsnETLDVMEGASASVVVFSKNYLSSPSCLDKLVRVLQCRRK-
SGQLVVPVFYDVSPSNVEVQEQESVDRISALQELREFTGYQFREGCSECELVEE(IVKDVYEKLLPAEQIGIS
LRLLEIEHLLCKQPWGIRRLGIWGM)}*

$860=266(286):
>gi|227438197|gb|ACP30588.1| {|167(675)|<Streptophyta(V)>}disease
resistance protein [Brassica rapa subsp. pekinensis]
{(GVGDFVFFQSLYTDSSRLFIGFRRSQAAFGQYLDLTSLATSSKKLKEVETPPQLRSHRHRVTELDPVLVIS
CGENQDSEE)---------------RYFISYISKELCHRGFTPLRCDLTKS-TL---
TGGMLHRSRVGIIIFSKNYASSRQCQGEFVAIMDHSKA-
NSLVLLPVFFKVKVTDIRGQNGSFGRAFSRLEDSVQGSQVPTLTSINKYQYMKG(EEVILAKNIVSDVCLLLS
SESNMKLRGRLQMNSILSLLKFSQSSAPHIVGLWGMAGIGKTTISREI)}*

$861=267(286):
>gi|227438273|gb|ACP30626.1| {|84(925)|<Streptophyta(V)>}disease
resistance protein [Brassica rapa subsp. pekinensis]
{(GVGDFVFLQRLFTDSSRLFIGFRRREAVLGQCQDLTSLATASEKLKEVETPLQLRSPRHSVTGFDGILLVS
GGDNQDSEE)---------------RYFISYISKELCLRGFTPLIYDLTKSTLA---
GVEMLHRSRVGIIIFSNNYASSRQCLDKFVAILDYSKA-
NNFVLLPVFFKVKVSDIRGQSGSFRRAFSRLEHSVLSSQVPTLTAINKYQYMKG(EDVILAKSIVSDVCLLLN
SETNMKLRGRLQIQSILSLLNCSHFSAPHIVGLWGMAGIGKTAITREI)}*

$862=184(187):
>gi|227438183|gb|ACP30581.1| {|0(1048)|<Streptophyta(V)>}disease
resistance protein [Brassica rapa subsp. pekinensis]
{(MV)--EVYISFDRCeDKVRYSFISHLSAAFHRRGISSYIGGSDPKSDGLSK--
GDMEKSKACVVVFSEKYSSSKPCLEELVKVSErRGNE-
GGHAVVPVFYRATKSSVKKLIWKSSDLTSERRSALLEVVDLPGHESYVTQSESD(LVEEIVADVREKLNTTEN
IGVYPKLLRIENLLQPCGVCRIGLWGM)}*

$863=247(264):
>gi|227438247|gb|ACP30613.1| {|54(649)|<Streptophyta(V)>}disease
resistance protein [Brassica rapa subsp. pekinensis]
{(VPRLTGVDNSVLELEFISAFGPSASRGYQVVTIYRRGDISNDDFNKVVRRLTRNLNSVSDLSNSASEFSTP
AAASSALKE)-YDVVLRYRR-GCISDdNFITHLRAAFYRRGVSLREDIDEVD---------
AVPECRVLIIFLTSTYVPS-----NLLNIVEQQSK-KPRVVYPIFYGISPSDLISNRNYGRPFHQDE-
AKRLQAALEEITQMHGYILTDK(SESELIDEIVRDALNVLRSNEKKNMIGMDMQIKEILSLLCTESQ)}*

$864=187(193):
>gi|503814962|ref|WP_014048956.1|
{|0(656)|<Actinobacteria(B)>}MULTISPECIES: hypothetical protein
[Streptomyces] >gi|344318318|gb|AEN13006.1| TPR repeat-containing protein
[Streptomyces sp. SirexAA-E]

{(MIK)---AFFSYSTQdaAFVREVA-----
SEIGRAFAKIDYRAFSAGEHLLETMDETIRESAAFVLFLSKSALASSWVRHEIREARFQHAMGRIKNVLVVRM
DHT-
ISPEDLPDWLNRTKHITtISAKPAARKIRTLIDDLVRQGQS(PRFVGRAAEIAALQDVYSAPVPGAESQVACV
VGLSGIGRRATLTKMVRES)}*

$865=252(261):
>gi|269784895|ref|NP_001161599.1| {|927(48)|<Hemichordata(M)>}neuralized
PATS1 [Saccoglossus kowalevskii] >gi|268054195|gb|ACY92584.1| neuralized
PATS1 [Saccoglossus kowalevskii]
{(AGSEGSDWLALAEKLGYKKAKIRKFNDEVNPALAMLTDWIYSSGNTTLSIEMMLSYLEQMHREDVVDAIQQ
GQDSHTSAP)---IFISYNWGiqEEVKQ-----
LRDYLEKAGFPCWMDIGQMGGGDTLyeRIDDGMRNCKVVIACMTPKYIVSHNCNREISLADSIS-----
KPIIPIMYQRIPWPPPGRMSLIFSHLLYVQMKGIGGHGGSGINADTKAKYME(IVDQVQRHVTPLSSPKGILA
ISASKKTTTVSNPQQSEQTVT)}*

$866=209(199):
>gi|503054119|ref|WP_013289095.1|
{|0(187)|<Actinobacteria(B)>}MULTISPECIES: hypothetical protein
[Micromonospora] >gi|302573296|gb|ADL49498.1| hypothetical protein
Micau_5996 [Micromonospora aurantia
{(MTSERPALPGPPYFFLSYVPPPT)-HNDRTPGD---HWVRRFYHDLNLAIDGRpgdRLRRqgFADFM-
VRPPEDRteRKRTALAEAEVFVPLYSPDYLNRDDSRREREWFRQRLIRAgRpadGDNILPVLWIPSPaavhAP
DQARAVAMAADVDAYAANGMSALCRLQKFQHAYKEVLG(RLAQHIVDTAEQTPLQPAEPPTGPTDLPSSPTSE
VP)}*

$867=228(235):
>gi|502661766|ref|WP_012897794.1| {|0(83)|<Firmicutes(B)>}hypothetical
protein [Lactococcus lactis] >gi|281375416|gb|ADA64929.1| Hypothetical
protein LLKF_1235 [Lactococcus lactis subsp. lactis KF147]
{(MGNDKNVTILYSGDSE)-------------EHRQWVQSLANELESNQINVTYDQNSLE--FGDdvnQ-
FMEIAVDSAStiLAICSKEYKKKIdnrdgGSGYEGRMIADKI-KNKSARVIPVVLGGFEnvlpK-
MMVSLTGVDLgKGFED-KEFAKLLT---
LIHGYKINPSK(PVKNPRETIAKIMNISQENIEVDDEINFQNIFIEGIISEKVTSPRNDGTRGSALYNIPFQL
NYSPKHRWSEYFLHYWNN)}*

$868=149(161):
>gi|503189483|ref|WP_013424144.1| {<Actinobacteria(B)>}TIR domain-
containing protein [Frankia inefficax] >gi|311228171|gb|ADP81026.1|
hypothetical protein FraEuI1c_3001 [Frankia inefficax]
{(MGSGGESGGSPPARI)--DFFVSYTGADEAWATWVAAALEKAGRT-
VSLQVWDSPAGTNVVVWINEQMTAAKWTIAICSPAYF-DAHWSTyEWAGALAGM------KLIPLRV---
AdcPLPPILAAAKRRTIVR--MVTQVTWRYARAGALIFGGSRR(CSLLAR)}*

$869=222(230):
>gi|503188626|ref|WP_013423287.1|
{|0(420)|<Actinobacteria(B)>}hypothetical protein [Frankia inefficax]
>gi|311227313|gb|ADP80168.1| WD-40 repeat-containing protein [Frankia
inefficax]
{(MALDRDHDNGG)-RDFYISYVAADKEWASWVAWVILQEGNS-VEFGEW-
YPAGTQLPAKRSEGIERASWIIAIVSEEYqlddFELMVPRNvIQRDPRGHT-----TKLIPIRVEDCA-
LRRDLQSFVPIDLFG--LDKETAIL--
HLHDQIANARRG(TSQPVRSPPFPGPASTADDPAADLSPDLSTEPSFPGHASGLEHEKPLSASRPPPSETPLL
PPDQAPSPTPPPLSTPSPV)}*

$870=179(185):
>gi|282156859|dbj|BAI61947.1| {|0(161)|<Euryarchaeota(A)>}hypothetical protein MCP_1875 [Methanocella paludicola SANAE]
{(M)AHDVFISYSSRDKAIADAICNSLENRKIR-
CWIAPRDVLPGIPYPKAIIDAINSCKVLVLVYSASALESKD-IEsEVGRAYKKG-----
IPILPFKIEDVPlnDIMDYYIGNVHWLDAM--
TPPLEKHLQQLVDKIQVHISDP(NTIHEKITSEKIEPSVSALQNNVERTQHFVKYLKKTALISIFFG)}*

$871=217(225):
>gi|282157185|dbj|BAI62273.1| {|230(0)|<Euryarchaeota(A)>}hypothetical protein MCP_2201 [Methanocella paludicola SANAE]
{(TDQRYIIYLGFLLVVIVIISIVFGLNYYLKKRRALKVSKVESKPTQEAQAPAIKAQMNGAMAIPDAQKATS
EPGPRVVGH)--DVFISYSHDDKPTADAICAGLEAEGIK-
CWIAPRDVLPGAIFQEAIIDAIDSSSIMVVVYS-SKSNNSPHVVrELSRAVSKD-----
VIIIPFRIEDVPlsKTMEYLISVPHWLDAI--TPPVEMHISELVRVAKVNLDIK(RKKSN)}*

$872=235(239):
>gi|1160556245|ref|XP_020436019.1| {|724(231)|<Amoebozoa(E)>}Toll-Interleukin receptor domain-containing protein [Polysphondylium pallidum PN500] >gi|281209734|gb|EFA83902.1| Toll-Interleukin receptor
{(HTSSNESLLMLCEYGCDRNLKNAKGYTALHLATQDNKLAISMLLLKNGAQAITDRAMKTPLQYVNGKEKTL
LQNIMFTNE)---VFLSYAHKdIGFVRELRIAME----
QYSIRCWLDEyRLQAGCDWRaEITKGVANAQVIIYTISETSSISLWCRKELKMARRLGKT-
IIALYYHNVEIDP-
ILRGLFDYEFKTAAPLDpnEDIFITEMEKLAKITKNIIHGHK(LAKIYPTQKDLIEHFGNRS)}*

$873=210(211):
>gi|268324474|emb|CBH38062.1| {|0(61)|<unknown(A)>}hypothetical protein BSM_15390 [uncultured archaeon] >gi|268324859|emb|CBH38447.1| hypothetical protein BSM_19240 [uncultured archaeon]
{(MLIKYWDHRYSMGHMALFIYDNILIIGRYKLAQGVLGMQ)KNDFFISYSGPvdhsyaIDIQKRLNCLMLKS
VIS--------EDEYN-
NQDIteRIKKELESSEYQIIIISPDSLKTAWVHQELGYFLGLKDEQkqfLLIRGSKKHVP---
FNDPKKFGFLKDFHALRFVRKKRAKRSRRGIIDQSLEYCL(NTMVKSMIEKQIIKSKDRKIELCHDLPCHNDG
)}*

$874=191(178):
>gi|496491566|ref|WP_009200136.1| {|0(759)|<unknown(X)>}WD-repeat family protein [Actinomyces sp. oral taxon 848] >gi|269210623|gb|EEZ76963.1| hypothetical protein HMPREF0972_02551 [Actinomyces sp. or
{(M)KYDVFVSYRHvRpdadvaRAVARLVEGYVvagsvGGVVKRRGFRVFRDVEEL-
ASGELgvLIESALAESEFLVVVCSPRTAGSVWCMREVEFFSRVRGVDhvlAVVVEGDPGVV---
VGDVVGGDVLAVDVRAGAVRSEGFGGFEGCGSGVVDRLVR(QSVGVLRRDGIDRLMAGVVGVSLGDLRQRQRE
RRMRR)}*

$875=229(229):
>gi|493590380|ref|WP_006543281.1| {|50(0)|<Actinobacteria(B)>}TIR domain-containing protein [Frankia sp. EUN1f]
>gi|288347176|gb|EFC81475.1| hypothetical protein FrEUN1fDRAFT_5418 [Frankia sp. EUN1f]
{(MINDLLEKGILRDGRARLLAAAHDEWPANPAFEPRLFPDIGDTSAERVPPVDAVPLPSPAAPARTAGRPEP
RDATAEEYG)---
FFVSYVtrnEADRAWATWIAGELAQLGDASrvpptVCLPDADAPPVSGMVDRTKQDVPRSAWVVAVLSPAYLG

PDGDGAtEWGAVLnATR-----RQTDRVFTVIVAdcAPAGALAELPWVNLVG--LDEGKASR--
TLRGEVLRIVNV(ATNYPPACR)}*

$876=223(231):
>gi|502719841|ref|WP_012954825.1|
{|0(89)|<Euryarchaeota(A)>}hypothetical protein [Methanobrevibacter ruminantium] >gi|288541985|gb|ADC45869.1| hypothetical protein mru_0017 [Methanobrevibacter rumina
{(MSPYELIKDDGEVVNLGGSPDESQDFDVSLERLNDKSLADSDGDGKH)--
DVFISYSTKNSDIANEICYLLEKNGLE-CWIAPRNISSGKNYVDEIADGIKSTKIVVLVFSK-
YSQESKYVNnEVMMAFSYN-----KPIISFNIDQTEpnDIMGYYLKVAQWLPAY--
PNPKSQYETLVTDALKLCNERP(RTVITSLDGFIPEDISKQKKNWISLILLFTPIYWASFIYMGLVS)}*

$877=157(157):
>gi|493592925|ref|WP_006545745.1| {<Actinobacteria(B)>}TIR domain-containing protein [Frankia sp. EUN1f] >gi|288344510|gb|EFC78993.1| hypothetical protein FrEUN1fDRAFT_7887 [Frankia sp. EUN1f]
{(MDDPVA)-
ASLFVSWAHDdGAEKEALMARLAFGALSGVrLTWWEDSDLLLGADWNeEIQNRLAACDYGLLLVSPSFLASSY
IRNvELPSLLG---P-GRGRALPVMLKKV-
PLDGSFDLRGLATRQIFtgAGGRAFTQTARSGREQFALALTS(QIRSRVLRDAA)}*

$878=214(221):
>gi|502721159|ref|WP_012956143.1| {|254(0)|<Euryarchaeota(A)>}TIR domain-containing protein [Methanobrevibacter ruminantium] >gi|288543310|gb|ADC47194.1| hypothetical protein mru_1344 [Methanobrevibac
{(HKGEPVSDAIVSVGGIKGSTDENGECIFDDVEEGEISVKVNSNEYENQKYTITVSDNKTTFPINLGFTHLI
TPFPASDED)-PYIFASYSHDdAN-RVFLE-----
LKRFHDcgLNIWYDEGIESGLGWQgVVESKLKACTLFIAFISANAVES----INVRREIFLAIN-
KKIPVVPIYLEKT-ELQYGLDLQLSPVQAILkyAMTEEFYVERCRRAFVMYGLMDE(E)}*

$879=184(170):
>gi|260177231|gb|ACX33952.1| {|0(314)|<unknown(X)>}hypothetical protein [uncultured prokaryote EC6]
{()MSDVFVSYTHNdNLEFDAEKKgwvsrfHLVLLnyltsKLGRTAKVWRDAKLEGNDAFWdVIFKALAEAQT
LVAVVSPRYVQSKSCVKeVVEFNRHWQAA-GGVKVGDKPRLVR-
ILTEPVKLPELIDGKPVppELRELFKLSTGVEFYEVDPESGR(SRQLRSEFGAKYDAKFNLLVDDVALMLANL
)}*

$880=217(220):
>gi|493586397|ref|WP_006539367.1| {|290(0)|<Actinobacteria(B)>}TIR domain-containing protein [Frankia sp. EUN1f] >gi|288351171|gb|EFC85382.1| hypothetical protein FrEUN1fDRAFT_1529 [Frankia sp. EUN1f]
{(ELHTAWTNITTMSLDWLDRTYALGVPPGTMASAERFLTEIADLTETAAVFRLPHEAAGRRPLFDGPLRRRT
AAEGVGAPF)--TVVIAH-D--
ESDERAASLVEGHLTLAGLHPWRANVDLLGGADRrlEMRRTIRQSGFVLVCLSTRSVDQAGDLHrQLRLALDV
AEEMpEGRiFVVPVLLDeHC-ALPVRLDHLEPVRFYQ--PDGPERLLRaL--GVGARSARTS()}*

$881=223(225):
>gi|493586398|ref|WP_006539368.1| {|66(0)|<Actinobacteria(B)>}TIR domain-containing protein [Frankia sp. EUN1f]

>gi|288351172|gb|EFC85383.1| hypothetical protein FrEUN1fDRAFT_1530 [Frankia sp. EUN1f]
{(RLPSFHSTPEHAWFEVFQTLEAGVVAQPCRRLLAAVLVRYPANPVFLDLHDRHVAGRPGDATGSPIAPART
SSLLPAPPP)--TVFLSHDR--A-
DQGKVQALADLLTEEGLTPWVGSEQVTGGSRRdlAVRRAVLDCDFFVPCLSAAALARPSQLHrEIRIALDHAD
ELpDhEVFIVPARLDEC-DPPSALQEYQGVDLFR--PGGFRQLVRaLRAPTPSARNRRS(GPSGW)}*

$882=168(167):
>gi|503640530|ref|WP_013874606.1| {|0(176)|<unknown(X)>}AAA ATPase [Frankia symbiont of Datisca glomerata] >gi|334860243|gb|AEH10717.1| AAA ATPase [Frankia symbiont of Datisca glomerata]
{(MGHMGESLAAER)RYDAFLSYARAddpdfrQALVDGLDRAGR--------
RVWFDREAMPnCGTTFgqEIRQAIETSDRLILLAGPAAMSSGYVAQEWGYADDIGKPVvpvAHAVAFRDLPD-
--RLRHYHGVDARRRTVDAVVAELARLLAEPVRPLGPCFHLP(HRPPHARDRPELLEH)}*

$883=186(201):
>gi|502719842|ref|WP_012954826.1|
{|0(105)|<Euryarchaeota(A)>}hypothetical protein [Methanobrevibacter ruminantium] >gi|288541986|gb|ADC45870.1| hypothetical protein mru_0018 [Methanobrevibacter rumin
{(MSH)--DVFICYdEEDKDCAEAICRIFEE--NNIKTWIRSRDVSSKDAARNLTEAIRNSkC--
FVLVYSKNGKNTNYIINETDIAFS--------KEIPiILFKLDETSIPKDLEFILISKKKI----
VAYPHSKRQLKTLVKETSD(ILDRPTDDIKLDSNSVKTIERSNPKRKENNIKKAIGAAALIAAVLILIYLFVI
VPTGQ)}*

$884=182(187):
>gi|728048689|sp|C4B7M5.1|WR52W_ARATH
{|0(1191)|<Streptophyta(v)>}RecName: Full=Disease resistance protein RRS1; AltName: Full=Disease resistance protein RCH2; AltName: Full=Probable WRKY transcriptio
{(MTNCEKDEEF)---VCISCV--EEVRYSFVSHLSEALRRKGINnvvVGVDSDDLLFKESQA---
KIEKAGVSVMVLPGNCDPSDVWLDKFAKVLECQRN-
NKdQAVVPVLYGDSLLRDQWLSELDFKGLSRIHQSRKECSDSILVEEIVRDVYET(HFYVGRIGIYSKLLEIE
NMVNKQPIGIRCVGIWGMPG)}*

$885=171(199):
>gi|1084309429|ref|XP_018646029.1| {<Platyhelminthes(M)>}hypothetical protein Smp_014410.1 [Schistosoma mansoni] >gi|350645699|emb|CCD59674.1| hypothetical protein Smp_014410.2 [Schistosoma mansoni]
{(MKSPNDFTEGKNDQLKIISSRRKKKQMKQEIDSLNI)----------------------
DFLKELHENFWIDKEhmVQHVKILDAMANAVENAAIVLILFSKSYQDSKNTRDEAEYTRKLN---KPA----
IFLRVESKFVPSGWLGFIMGESRYIDFSGKYPFEEKFEELCTTIHNI(SRGSIIVKPKKPKKTQEYACVSM)}
*

$886=160(143):
>gi|495118267|ref|WP_007843082.1| {|0(604)|<Bacteroidetes(B)>}toll-Interleukin receptor [Bacteroides dorei] >gi|229436155|gb|EEO46232.1| tetratricopeptide repeat protein [Bacteroides dorei 5_1_36/D4]
{(MIK)---AFISHSSEqkQFVLD-LVEIL-GRDYCIVDCY---
NFNSAYQTINEIYDKIDKSTVFVLLLSKASLSSEWVKEEIRYVREKLEPGCLERFWPFIIDENLsIEECPEWM
KKDKCFNLKkFKSCKILAHDIEQKFRRIIWSKnpkrkqletlmvgrnkdidnfE()}*

$887=267(276):

>gi|502876294|ref|WP_013111270.1| {|35(21)|<Planctomycetes(B)>}TIR domain-containing protein [Planctopirus limnophila] >gi|296015600|gb|ADG68839.1| hypothetical protein Plim_3017 [Planctopirus limnoph
{(NTISGHSRLLRALNFGDDDYSGCAAEVLDRIFSANESAIDEVIDHFDIDLWYQQKSPEKYNRLFSAAASSS
ADFWKSGFL)--KLFVSHLSSnKTRMSALKAGLATWGISAFIAH---
EDIEASREWRdEVEAGLETMDVLAAVVEPGFKESDWCVQEVGYALGR-----KIDVIPLRagLDPF-GF----
FGKFQGIQIKGrlPEQVACDITQVLLKKPQYRERLI(QSISIAFSTLASEKKIENINTLDEWSILTDIQLKTV
VEQAAMSGAEKQAAKNLIAR)}*

$888=173(175):
>gi|283817256|gb|ADB39094.1| {|0(651)|<Bacteroidetes(B)>}TIR protein [Spirosoma linguale DSM 74]
{()MPKAFLSHSSVqKQFVTRIANELGS-----
STSIIDSRSFEEGMSNIeEIQNALDQTDIFVLFLSNEALNSKWVKDEILIAHENVKR-NIIkRIYPIIIDET-
ITHNDLRIPDWLKELNIktVLRPGKVTRLINRRLKEVSWQLH(PVLKEKELLFVGRTEQIKTLQERLYDFTKP
TPFCI)}*

$889=223(251):
>gi|490437512|ref|WP_004308543.1| {|0(366)|<Bacteroidetes(B)>}MULTISPECIES: hypothetical protein [Bacteroides] >gi|229449114|gb|EEO54905.1| hypothetical protein BSCG_01830 [Bacteroides sp. 2_2_4] >gi|
{(MKYKYDVFISYSRRDYVDENYNVIPGNAIAEIQNVFDEN)-----------------------------
GITYWFDKDGIYSGQEFieIITGAI-AESKMLIFISSKHSNESIWTAgEIFEALDGEKA-----
IIPVKIDNCQynKKFKlLIRPLDYIDYQENPQNALKDLLRaINKVKEDIAQKQR(EEEKLRQEKEAEAKKEKI
KEEISILAKDCQRLTLQQIDVVNQIFEKQTYIGNTTKICPICDKEVSISSNFCS)}*

$890=189(186):
>gi|502434045|ref|WP_012784477.1| {|0(776)|<Actinobacteria(B)>}TIR protein [Catenulispora acidiphila] >gi|256355685|gb|ACU69182.1| TIR protein [Catenulispora acidiphila DSM 44928]
{(MTAPVRDGGENEAV)-YDVFFSYSWKnlaEAL------
ILVAALEARRLQVFRDETGMRDFDDIgtEVFAALAGSRSFVALYTPQFPESAYCRLELAHamtaaYRLDGDVR
rilPVVRELPYEAV---
RPRALQDKRLPARSTGPDALAAVVAERLEEIDDRPFGIAS(EPGPPNWYPFPTVAEPAFHGRATELWDLHDAL
)}*

$891=221(221):
>gi|488942522|ref|WP_002853597.1| {|115(0)|<Firmicutes(B)>}molecular chaperone Tir [Ruminococcus albus] >gi|324106750|gb|EGC01042.1| hypothetical protein CUS_4504 [Ruminococcus albus 8]
{(ESIAEHNMPANVWMDDVRIFHAKYLSEHPLYSSMESLLFHRNFSRLVASLTSISKDRDFIDKMNGVEKVEV
PKYQAKTLT)EYDVFISHAN-KD-
KEELIEELYQSLQKLGISIFYDKESLEWGDNWkeRILNGTKKAEFAIIVISENFFDREWTERELSEFLNRQNR
NGQKLILPIVHNITMQQLQEKYPNVADIQAIDSSKYNCDQIALLFAKQLIKRLKA(N)}*

$892=206(210):
>gi|291528527|emb|CBK94113.1| {|0(54)|<Firmicutes(B)>}TIR domain [[Eubacterium] rectale M104/1]
{(MNNTKEN)-FDFFISH-S-
KETKQLIVIPLAQCLKSIGIDVWIDRKEILVGNHIysEIQDAITRSKFCIAVIDQSFLKRTWPMKELELF---
-NKKNNMRIIPIFVDLEKAIVYNEIPwLDGIAFEKmH-

TEPFDLNVHMSIFCRIISRLFK(DNNTNKNSIEKTFESITKYDFPCKETLSSLIYFSKYNSSNLRLATIDLCN
IGGIIYAISKTYS)}*

$893=220(254):
>gi|291235955|ref|XP_002737904.1|
{|253(1187)|<Hemichordata(M)>}PREDICTED: uncharacterized protein
LOC100371929 [Saccoglossus kowalevskii]
{(FKKIEHKKVVTGVEKFDETDAESKWQEMLKSKLLPQGTIKEMESKIKSSKKPAWSGKPLPSDEYEASIAAT
DEDMEYAEM)---------------------LAKILEKRGIKVTTDVD------------
KVHDSRVFVVIYSDSTARDENVVRHLTRAAERFNS-
GETIIVPVIRSGCTPRVLDKIPDNIAYSLSNTNFINMASFHDPLQKTLQTRVNE(LADVIRHHAREETDWNLT
GLWCCQAIVTDERSGQ)}*

$894=211(220):
>gi|291557627|emb|CBL34744.1| {|128(0)|<Firmicutes(B)>}TIR domain [
[[Eubacterium] siraeum V10Sc8a]
{(YQNYEKIKAAIEKYQLEIDMTQTLDSLYSQLYSRAYNTINFTSNCSKDQILLQMTAVYAYKDKDELARSGY
YVSEADKPN)---VFISYSHNdkIIVKDVKDRIVMCG---
LNVWFDENCIAVGKHIVNEFMDGITKSDYAIIFASKSTVSSVYSKAEIQNLFVKVLNNEIK--LFIVKLDD-
VDMNSIFPNLKNY---KyYDFYQEHNIDKLVMSIVDFVKKK()}*

$895=233(254):
>gi|291237078|ref|XP_002738472.1|
{|811(136)|<Hemichordata(M)>}PREDICTED: uncharacterized protein
LOC100375778 [Saccoglossus kowalevskii]
{(KGDQSLWRSKVFTELLGEVIFYVSPDFNEATKDYRDWMIALNDIPVRNLTDLSSSSSESMQSATDIPPVLI
SYQWSMQHE)------------------
AKQIYARLTKAGFSCWLDIFHLGGSECIynKIEKLISGFKVVVMCVSRNFLSSKACRQLGNLVKVLQ-----
VPILPIQLEDISWPEEDALATLIQMNRCIDFTEEFGSSTNINDDRFGELLTR(LREENFHKGDPSSPAVSRKA
KTLARVNKSFTYVQ)}*

$896=244(232):
>gi|291240346|ref|XP_002740090.1| {|444(0)|<Hemichordata(M)>}PREDICTED:
uncharacterized protein LOC100370582 [Saccoglossus kowalevskii]
{(LRPSVKAVLGECPAIDFETKMYAESMDVLEHQIKPLRKVPGVVLEQAYLNRMAEGLKPLQVLATSQAATLS
FGEELPEPK)---VFISYQW-DMQTKVLE--
IRRILESNGLPCWADiSPTMaQRGhssvssrssttsqmdgsvtETLqlNIQRNMKSSAVVLCCITPRYIQSDN
CIKDLSLAEYLN-----
KPIVPIMLRFVAWPPDTAQPGIKKMLARTVQIDLSNDKLFKQNFHLVLERIR(KHLAVNMKSSLM)}*

$897=249(257):
>gi|291242045|ref|XP_002740919.1|
{|1315(128)|<Hemichordata(M)>}PREDICTED: uncharacterized protein
LOC100370439 [Saccoglossus kowalevskii]
{(IKFCPEKEYRKGEKFWSDSTFAELLGQINYHAAPDPEKISDEYKNYVPQVDDTPIVKPKKKQQTETKEDSP
HNEENIEDP)--DVFISYQWGvqPQIKN-----
LYSKLTSLGYTCWLDIMQMGGGDALygKIDRGIRKAKVIVSSCTPKYALSTNCRREVSLADTLK-----
KPMIPILLESMEWPPEGPMSMPFAQLLYIDFTNNNSQKFDNDKFEELKQQID(GYGVEIHLEIIDNGEEDGEG
TEPISESQQSNTPTDDR)}*

$898=251(257):
>gi|291242045|ref|XP_002740919.1|
{|1125(316)|<Hemichordata(M)>}PREDICTED: uncharacterized protein
LOC100370439 [Saccoglossus kowalevskii]

{(RFLAIRLGYSSEDVRNWATAHDPTMSLLSEWYTMHKSSEATYAVLTSLKELGRNDCVTIIEDALKAADEVI
PEAPPDFTH)PPPVFISYQW-DHQSEV--
KTLKEHLEMAGYECWMDIGQMGGGDKLyeKIDEGLRGAKIVLSMCSEKYSQSKNCNHEINLASLLN-----
KPIIPLLLEEIDWPPPGPMSVLFSQLLYIKFCPEKEYRKGEKFWSDSTFAEL(LGQINYHAAPDPEKISDEYK
NYVPQVDDTPIVKPKKK)}*

$899=244(253):
>gi|291277999|gb|ADD91472.1| {|301(246)|<Rotifera(M)>}ankyrin repeat-containing protein [Adineta vaga]
{(RFGYSASELKHWATQFDPCMALLNEWYMTHKSDEATFGLLKILKEIGRQDAEVIVQNALRDAGETIPDNIL
DFDIQRLPP)---VFISYQW-NSQPMVIK--
LKEHLEEAGYSCWMDVGQMSGGQELqrRIDKGIRGAKVILCCINRDYATSDACIREVNLAISTG-----
KPLIPLQMEKQIWPPEGALGPLMSDYLFIRFFDRKATNDPNFWPDERFTELL(GQIRYYAAPNPDMIREPYTN
WFVPRAENLIFLK)}*

$900=236(260):
>gi|291277999|gb|ADD91472.1| {|535(20)|<Rotifera(M)>}ankyrin repeat-containing protein [Adineta vaga]
{(PRAENLIFLKDSDVNDKKMTKFEDSTSKFAYDFERFMIHYCLSIVMTVFLEPSLINTPLVVSHPQIMISYQ
WDRQTDIID)--------------------
LYKRLTELGYRVWLDIFQMGGGDSLfaKIDHGIRNAQCVLACITPKYTMSINCRREMSLADAIG-----
KPIVPLFMEQLPDSWPPTGPMSLVFADRHYIDFRSHQRNDMWSGKEYAQVLL(KLKELVPEIQIENPQRHLIE
MERPTSAVHLDKLVRVYPTR)}*

$901=215(220):
>gi|505370249|ref|WP_015557351.1| {|42(0)|<Firmicutes(B)>}DUF1883 domain-containing protein [Ruminococcus champanellensis]
>gi|291543335|emb|CBL16444.1| Domain of unknown function (DUF1883)./TIR domai
{(RRYHYTGGLAKQSPVRLAVPHSGHWHVAIDMNGLRGTVNASVRVLPTALPTIKQQPSLASMPSLVHNRGFG
IDVDIDNDP)EYDVFISHAS---EDKdEIVRPLANALVEKGVKVWYDEFEmkIGDSLRRkidKGLAN-----
SRfgIVVISKDFIKKGWTNYELDGIITKAV-SGEQIILPIW----HniT-KKEVLDFSPSLADKL-
ARNTAINTVDEIATEISELILQ()}*

$902=213(220):
>gi|291520739|emb|CBK79032.1| {|54(0)|<Firmicutes(B)>}TIR domain [Coprococcus catus GD/7]
{(YDVPQKNHVEKMIELFENENLILRNDLTTLLLTQELGNRRFNSCAFADAQEVDLRFESMFFFPEIEILEEK
GYCMRPFGK)EKQVFISHSSKdKKDVESIIPYLNGQELPVW------
FDKYSISVGDsiteSVQMGIEESDMVIFWVTDNFLVSNWCKVEMTA---YVKK-
LIEEKIRMIIVLDDGIGIKKLPLFLRDIKHI--RKENRTAIEVAEEIVKSIKRG()}*

$903=246(255):
>gi|291235955|ref|XP_002737904.1| {|1151(263)|<Hemichordata(M)>}PREDICTED: uncharacterized protein LOC100371929 [Saccoglossus kowalevskii]
{(AKDAEKMEGKLIPKSARLVGIREMSSGGQKIGQTRYEFDLDFERRKDYRFSFLIQDSDGLVAGLESLEEEA
VSQHFSEYD)---VMISYRSWdARWVDLLQEYL---EKHGVV-
VWRDRRMEVGVNWSeDITRAVRKSGCVISCISENYLASVLC-TkEVRMATEV-----GRSVIPLVLPET-
QTPKGQGQRMVSSHYKGkhLPHSVAHELSGITWIDFREVSKL(NEPDPTDLKSFEKKYGTHLQMLVDTVCEIN
GLHRV)}*

$904=162(165):

>gi|489892044|ref|WP_003795494.1| {|0(44)|<Actinobacteria(B)>}TIR domain-containing protein [Actinomyces odontolyticus]
>gi|292821131|gb|EFF80082.1| hypothetical protein HMPREF0970_00976 [Actinomyces
{(MQA)----FLSY-HEdEqgdAYASALQGELRIL---
GIDATMARHSIRPGADWAlTIRKHLEASSALLCIGTTGYTKSAWC-QqEIGWAIGRNLP-
VLWIRYDGEERSV-
GFLAARQDLEVLDTESSerTAQMVASWLLSETQTREETKTSL(LDALVHSSSFRNTRCIANVIAS)}*

$905=179(183):
>gi|258591494|emb|CBE67795.1| {|0(74)|<unknown(B)>}protein of unknown function [Candidatus Methylomirabilis oxyfera]
{(MGISDSKK)---VFLSH-K--GIDKDLVIDFKKSLKLVGYDPWLDEDAMPAGTSLerGLLQSMR-
DSCGVVFFVTPSFKDEGFLEtEVNYAIQEKRKKgDKFAIVTLQFCGA-
DGNVGEIPELLKTYVWKKPKTPLEAFGEIVRALPVVPGIV(DWREGITGVVTTPRTKSTTTQLCDEAKAILKA
AVA)}*

$906=262(253):
>gi|291521697|emb|CBK79990.1| {|280(912)|<Firmicutes(B)>}Protein kinase domain [Coprococcus catus GD/7]
{(RYSTRMLEDVRKSYDGHYLTRKNIQDMKCPPHAEEIIQHILEKALETDPKKRYHTAEEMLKDVSAALNVLR
PYHSQIAGM)KYDAYILYRSDnilhkhvaVTLQKLLEHFRTP-
GLKRIQRVFLDETELSADYQAdkQVMDALKASRYLIIIGQDSSGDDIWQSKEVKFFLKTHKPSevlTILAVQN
PKQS---
EISESSKAAEASNVSGTSEVAGIAAVSEYAVVKVGGRDRN(FPLAADIRGASEKEILKKLKKDGFLRIAAPIL
S)}*

$907=149(160):
>gi|629093179|gb|KCW59174.1| {<Streptophyta(V)>}hypothetical protein EUGRSUZ_H01813 [Eucalyptus grandis]
{(MASSSSKLRKS)-YHVFLNFRG-LDVRNYFLGHLYTALNQAGINTYKDDEELRkGEH-
IstELQKAIEESCITIVVFSKHYASSMWCLEEMSKIMECKEK-TDLMVYPMFYKSYFQDM---------
AKHENNVGKDLEKV-KRWKKALLDAGNL(SGWHFTDGY)}*

$908=277(279):
>gi|629093383|gb|KCW59378.1| {|0(324)|<Streptophyta(V)>}hypothetical protein EUGRSUZ_H02081 [Eucalyptus grandis]
{(MGPYSLSNWLPTTWLKKCLNCFEFVPFIARHVQSDIHMRDRAATRPDTEHSEEAHQTSSPLMAASSDLKGN
)-YDVFLSFRG-TDVRNNFLGHLYAALdQKG-
IYTYIDSEELRKGEQItpALIKAIEDSCIAIIIFSEDYASSPWCLQEVDKIMECKEQ-
RGLMVFPVFYKVEPKEVRTPRGSYREAMVKH-
ENKFGRDSEKEKLLSKILVGKE(LTVFSVDGGINLMRCRLCCKRVLLVLDDVDDVKQLNALAGGCEWFGKGSR
IIITTRDNHLLTVHGIDE)}*

$909=257(263):
>gi|492422836|ref|WP_005839431.1|
{|722(13)|<Bacteroidetes(B)>}hypothetical protein [Bacteroides vulgatus]
>gi|294450906|gb|EFG19383.1| conserved domain protein [Bacteroides vulgatus PC510] >gi|933248
{(IAIKSKDVLMEVTQYLRQLTQRGEMYKYLNRYIIGFEDKYYAAYSGISDINEAMRIADGGRLTKTEEEKAE
EIPWREDEG)---IYISYNW-
EGHSAHIVDYLGFVLENRGIPFKLDKKDcPYTVNIKEFMNAIRAGKTVIVVLSRPYLRSKNCMYELSGIMENV
NY--KDRMLPVVVDDTIRDDDFYVELVKHWKEKK-
DKQTEIVEKLRDLDPDMAEPEE(MKLKEIEQVYGLLKVIKEYIDWANADNLDALSSSRFKKIIDEI)}*

$910=164(167):
>gi|294462224|gb|ADE76663.1| {<Streptophyta(V)>}unknown [Picea sitchensis]
{(MAAASSSTTASLPNIYT)--DVFINHCG-
PDVTRTFASHLYRRRLSHRLRVSLEEDELQAGENIvsQRVDANRTASVHIAIFSETCAESSCCLYELRFMVES
G-----
STIIPVFYGVKTAELRWGQKDQCmlkPYTNLRRRLRRTLKLMKRGCDTAPSLLKV(GERFFLVLQR)}*

$911=177(184):
>gi|503422853|ref|WP_013657514.1| {|0(394)|<Firmicutes(B)>}TIR protein [Cellulosilyticum lentocellum] >gi|326542362|gb|ADZ84221.1| TIR protein [Clostridium lentocellum DSM 5427]
{(M)-QDVFISYSSKNKIEANKVCEQLEKRGIS-CWMAPRNIQPGKEWGEQIIRGIETSRVLVLIFSAA-
SNESPQVLrEVERAVSKR-----LKIINFRIENVSpsDSMEYFLSANHWLNAF--
DGNRETHIESLSITIKGLLEEQ(LLPQQAHPIVSKGLGKHTVYKKGLKFLAVGLIGIILIIIAYNL)}*

$912=219(223):
>gi|629109346|gb|KCW74492.1| {|0(470)|<Streptophyta(V)>}hypothetical protein EUGRSUZ_E03204 [Eucalyptus grandis]
{(MVR)-YQVFLSFRG-
PETRDNLVQYLYDQLKRNGVVVFRDSEEIedGQEISKTIEDAIESSDICVPVFSQDFASSAACLKEVAQMVKL
GKK-----IRPIFFFVEPCIVCHRLGTYGKSFAEHrrKNRYRESTI-
KEWEDALEKIGGI(LGPEFKQADLKAALRPALKAALRPALREIGHGLEFKKAALEVALESAVEAALQPALEAA
LQAAEQPALEAAQASLQEQTD)}*

$913=190(198):
>gi|629109631|gb|KCW74777.1| {<Streptophyta(V)>}hypothetical protein EUGRSUZ_E03511 [Eucalyptus grandis] >gi|629109632|gb|KCW74778.1| hypothetical protein EUGRSUZ_E03511 [Eucalyptus grandis]
{(MERKEPTPSEDPMHGASTSSTSPHGGDSGGGTQKPKGN)DHDVFLSSVC-PMWSTLFDD-
LRHGLVIAGIDTF----CSKDNADEWIPYVLPQCKISIPIFERGFVNDKSGLRELVHMWKCKRSSGQI-
VLPIFDEVGPLEVQLLEGESIEEFSRLEGRVDEMEL-
GEWRKALAEVGSL(RGWEAEKFAYGYIICHRNII)}*

$914=228(240):
>gi|702319232|ref|XP_010052395.1| {|0(929)|<Streptophyta(V)>}PREDICTED: TMV resistance protein N [Eucalyptus grandis]
>gi|629111400|gb|KCW76360.1| hypothetical protein EUGRSUZ_D00736 [Eucalyptus grand
{(MDAPYDSSSSSSASASSVQP)QHEVVLSFRG-
EDTLEHLADPLFTGLTAAGISVYTVERNLgpDRQIDPKLIEAIGQSKISIPIISSVYASSKSCLMVLAKMLD-
FMDTTNHAIIPIFYGLNPS------------FPDHKKRgSDGMLV-
HPWKSALHRIGQL(KGYRVDDASDRLIPEIVLYVTRQLKKAELVGASMPVGIDNQVCEIMTMLNIDYRNRQAV
DIRGNEVRMVVIHGIEGIGKT)}*

$915=248(283):
>gi|1091465733|ref|XP_018727692.1|
{|171(14)|<Streptophyta(V)>}PREDICTED: uncharacterized protein LOC104441500 isoform X2 [Eucalyptus grandis] >gi|629112148|gb|KCW77108.1| hypothetical protein EUGRSUZ
{(TCHEESAKWRKAMTFVAWVRGWTSQTIANGRDAELVKMVVERVSSELQTMWIERLPIFPMLMSNYYIRSAY
GCFSEKKRG)--EVFLAFHG-RDTRLGFAACLYSSLVGAGIRVLKDDHPYL---------------------
---------IDELAEMVDC-

KRQKGQKILPIFYKVKPSDVRNISGSFGEYIRQHKENIQPLDKIFGEYMRQHKENIQ(PLDEIGKDKDRKMSK
QALVEIGKYKDRKRWKQALVEIGKYKGLESEKIANGNEGELLKRVVEE)}*

$916=224(222):
>gi|629114177|gb|KCW78852.1| {|0(253)|<Streptophyta(V)>}hypothetical protein EUGRSUZ_C00292 [Eucalyptus grandis]
{(MGNNSGPSASYASVIGNN)-YFVFLSFRG-
LDTQKGFVDHLYHELVRVGLHlpnfLFRDDENLnpGEPIET-
LLNAIKCSKVSIPVISENYVTSKWCFREINQIMECV-
KSKKQKVYPVFYKVTTDNVKYMTGHFGDAFRRDEHKLDEEFSPVLLFDRHEGKLVK(KLVKKIMSEHQHEFLP
LPKDLVGIDDHVAKVMNLADSRTMKSANTAYSEAQIIVIYGIGGISKT)}*

$917=235(249):
>gi|629115894|gb|KCW80569.1| {|0(215)|<Streptophyta(V)>}hypothetical protein EUGRSUZ_C019281 [Eucalyptus grandis]
{(QIPEITMPQEEKSCQLNGIISSTSTSSGF)DYDVFLSFRG-
PDTRSGITDFLYTSLLAAGIHTYRDDDELriGDEIALSYSKQSTIQKFQFPYFPRA--------------
MLLCRKSRRQKIMPIFYDITPSEVRHQTGSYGDAFVSHEKRFDNKVI-
SKWKAALKEVGDL(KGWDINSMPTRREGELVKLIVQKVLAELKSSYLPVTNYLVGIEHHVKEINRMVCGDSKD
IQIVGIHETSQLKGIECLQNQ)}*

$918=199(212):
>gi|629116073|gb|KCW80748.1| {<Streptophyta(V)>}hypothetical protein EUGRSUZ_C02141 [Eucalyptus grandis]
{(MMNADSTSSGEGRSCRGSTEHECVM)----FLSFRG-
LDTRKGIADHLYIGLVDAaltvPISVFSEAEHLpmSSQFSSQPQVA--
GSEVSIPILSESYAASKRRLCELVQMTKN-RKSAGHVVIPFFHRVE----------
FDRAFCSHEMCFEQREV-
EAGQQGLRDLSIL(KGWESEKIANGVPKKVCAICFERDGTDEIVDQGCQKCKIDRKPWLKG)}*

$919=225(241):
>gi|629116582|gb|KCW81257.1| {|0(574)|<Streptophyta(V)>}hypothetical protein EUGRSUZ_C02627 [Eucalyptus grandis]
{(MAPEDVAAGRKRKCPELSSLV)------------------
TDHLHSRLTKAGIKVFRDDEELraGEEIHPKLKNAIKQSTISITVFSKDYASRRSCLMEAVQMWECRKSNGQ-
IIIPIFYNVRPHAVKNLEGDFATSFNKHDayKKVDSETI-
DKWKEVLREVVEL(SGFDRATINGGHVTDLLDKVMTRVWQELRKDDQDVTDKLVGIDLHVQEMMTKLGVVYSH
GKAMKVCGEDKRVVGICRMPG)}*

$920=194(220):
>gi|629116596|gb|KCW81271.1| {|0(1366)|<Streptophyta(V)>}hypothetical protein EUGRSUZ_C02641 [Eucalyptus grandis]
{()----------------------------------
MDDEELrvGEEIGGEILRAIDNSQFYIPIFSRNYATSKWCLRELARIVQNASKSEEKKILSIFYDVEPKDVDL
KTSLYTTAIKEHKEKHkefpnegESWEG-
KSWEEDLKTVDKI(KGWELPKCKNKAERMKLVVKEVLDRLKKKYKIVTEDLVGLDGPVQEVLKLFDVYCNDVR
LLGFHGMGGIGKSTLARVIYN)}*

$921=195(220):
>gi|629117895|gb|KCW82570.1| {|0(711)|<Streptophyta(V)>}hypothetical protein EUGRSUZ_C03970 [Eucalyptus grandis]
{()-------------------------
MIRVGICVFKDDEMLhvGEEIRGELLRAISNSKIYIPIFSKNYASSKCCLCELAYMVECRKRDKK-
VILPIFYGVDAFDLKLRTGSYGKALRKHERQSGEDTM-

KQWEEALREIAKI(KGYNLKDHGCCFLSDIRKSDILSLQKKLSRDILGIQHPKVIDVDEGRDVIKQKLGDKKA
IILAGKSSWFGSGSRIIITTR)}*

$922=137(152):
>gi|629123438|gb|KCW87863.1| {<Streptophyta(V)>}hypothetical protein
EUGRSUZ_A00258 [Eucalyptus grandis]
{(MAHSKARASEVR)-YDVFLSFCG-
VDTRYNFMGFLNeglrnakiRHLRNAKIRVFRDEDEIkvGDKISEKILRAIDNSTIYIPIISQTYPDRKWCLI
ELERMMNNvSRSKGRKKIFPIFFKVEPGDVKHRT------------------------PHPENPPSE()}*

$923=141(158):
>gi|629094780|gb|KCW60775.1| {<Streptophyta(V)>}hypothetical protein
EUGRSUZ_H03503 [Eucalyptus grandis]
{(MASPSQSKGP)-YDVFLSFRG-PDTRLGIVSHLYKDLDQRGIYTFKDDEkIKKGER-
IsdVIIKAIEESRIAVIIFSQRFASSSWCLDEVATIMDRKAA-KKIIVLPVFHNVDP---------------
RTKYGSGSEIV-KKWKENLEAAGCL(CGWEFNNW)}*

$924=143(156):
>gi|629097073|gb|KCW62838.1| {<Streptophyta(V)>}hypothetical protein
EUGRSUZ_G00432 [Eucalyptus grandis]
{(QRPPRNF)--DVIIIYKR-DDTRD-FVSHLKAALERRGIRTFVDYTLDEGQE-
IllAIEEVIKQSNIAIVVVSQNFHTSPWCLNELAKILRSQKK------------
RDAKELREQCSPFVENIGQGEEGFKQEKpclV-RRWKKALRALGMI(NGFTVSARY)}*

$925=154(182):
>gi|629097882|gb|KCW63647.1| {<Streptophyta(V)>}hypothetical protein
EUGRSUZ_G01286 [Eucalyptus grandis]
{()------------------------------MRTVVDYTLDRGEE-
IwpAIEEAIKQSNIAVVVVSQNYHCSPWCLDELEKILECRKK-
GGLIVLPIFWGIDARELREPSSSFVENIGQGEEGfkqKNPHLA-
QRWKKALQAVGRI(IGFTVNASLDRTEAEVIEALADEIAAELNRSASIFTSHPACG)}*

$926=193(221):
>gi|629102069|gb|KCW67538.1| {|0(222)|<Streptophyta(V)>}hypothetical
protein EUGRSUZ_F01287 [Eucalyptus grandis]
{(M)--------------------------------
AFQDDhhRDLQRGKCIkpEILRAIEQSRLVLVIFSENYSSSTWCLEEVAKAMECTNI-
NEGSVIPIFYKVDPSDVRKLRGNFGTGFAKT-
EETYSGDKGdiKRWKDALRKVADL(AGRELKDGHKTQFIRQIIGEIEDKLGPHPSYVVGNLVGMHSRVAKVVK
LLCLGESGARSIGIWGMAGSGKTTVARAVTDE)}*

$927=228(228):
>gi|629102100|gb|KCW67569.1| {|0(780)|<Streptophyta(V)>}hypothetical
protein EUGRSUZ_F01321 [Eucalyptus grandis]
{(MAGKEYEF)KYDVFLSFRG-EDTRKNFVDHLnYHLQRKG-
IVAFKDDHDRNllRGKHIkpEILGAIEKSKYVLLIFSENYPSSTWCLEEVAKAVECKDV-
NEGSVIPIFYKVNPSDIRKLRGNFGRGFAET-EEAYSSNKRdiKRWKDA---
VSDL(VGMYSRVANVVECLCLDESDVHSIGIWGMGGIGKTTVARVVHDKISGQFNDGCCFLANVREISKKNGL
VYLQNQFLGDIL)}*

$928=221(236):
>gi|629102099|gb|KCW67568.1| {|0(377)|<Streptophyta(V)>}hypothetical
protein EUGRSUZ_F01319 [Eucalyptus grandis]

{(MAPFPRLENKHSKYKY)KYDVFPSFRG-
EDKCKNFVDHLHDHLRRRGLVAFRDDHhqDLSRGKCIkpEILRAI---------------
EHVWKKLEVAKAVECKDINEG-LVIPIFYEVNPSDIRKLRGNFGTAFAKI-EEAYsdDKRDIKRWKDA---
VSDL(VRMHSHVANVVESLCLDGSDVRSIGIWGMGGIGKTTVAQAVYDKIHGQFDDGCCFLADVREISENGLV
YLQNQFLGDILQ)}*

$929=195(199):
>gi|629111645|gb|KCW76605.1| {<Streptophyta(V)>}hypothetical protein
EUGRSUZ_D00988 [Eucalyptus grandis]
{(MVVEKVSSILSPPWIEQIPMFRKKRKKS)KCEVFpLS--S-
PATKKTFASHLPYHLERENISVFRDDDQYLIGKDVdrEVRNAMDHCKIFIPIISEDFASSHDCLDKLVQMFKC
VEENGQK-ILPVFYEVDLSRKKELRDHFWTDMLRH---
DVPGETRQRWLESFDRVFSI(AGFVSENFGNGGELADVVRQHVIRLIADLKL)}*

$930=226(231):
>gi|759904371|ref|WP_043590698.1| {|234(0)|<Actinobacteria(B)>}TIR
domain-containing protein [Frankia sp. BMG5.23]
>gi|631848432|gb|KDA43569.1| family 3 adenylate cyclase [Frankia sp.
BMG5.23]
{(RQLLEQDRDAELALVLSSEFYHSTVAGRLRDLDPAQYRRVEVSVAGKYEGLAWITLPGRPFPVVRTPSNGS
DQGPVADGK)-WDFLISADPSDEAWGSWIARVLEGRGHS-
VHLAALDALPGEHEIGLLHEGISQSRRTLAVLSKSYLASEKMQAaWQADPNGVK-----RTLIPIRVETC-
DPAGLLRGISYIDLVDKKdgaAAELELVR--QIVRSLNGrGSRP(STPPPFPGQRS)}*

$931=225(226):
>gi|632869600|gb|KDB07236.1| {|47(0)|<Proteobacteria(B)>}TIR protein
[Burkholderia sp. lig30]
{(IAVTGATYVFKGLASVGLFVPDFLPEAKSMLRRVDSPAEIEFGPVLTVRTNAVHVSEHEIAQAFKVFPGRV
TIVSEDDLH)-QSVFISYGGLDEERAKEINEFLKAHNVK-
TWFFPDNSLPGQKLHRMMHDGVNTHDRVLLICSQSSLSRRGVLNelervfEREARQGGD-----
DILIPVTIDDYVfnEWTLARQDVVQQVRTR--VITTIPARELDPDGFLREMAKL(QKALKK)}*

$932=163(172):
>gi|736822626|ref|WP_034824251.1|
{|0(269)|<Proteobacteria(B)>}hypothetical protein [Hyphomonas sp. T16B2]
>gi|630770867|gb|KCZ52669.1| hypothetical protein HY2_07970 [Hyphomonas
sp. T16B2]
{()MSHVFISHSAKdFDALLELHEAL----RAESIPDWYVPNDGGSRDLA--
DQSIDDAFAMIILVSASSVRSKSVRKD----VERAKA-RGLKLIPYQIDKA-
RLNGFFKHEVQPHLRYSstMPDGMDKLVAETKAAYKRKCPVM(AVMNLKGGVGKTTVSSQVFGAWQGSLGGRI
LL)}*

$933=166(177):
>gi|737601943|ref|WP_035572608.1| {<Proteobacteria(B)>}TIR domain-
containing protein [Hyphomonas adhaerens] >gi|630937297|gb|KCZ83646.1|
hypothetical protein HAD_13629 [Hyphomonas adhaerens MHS-3]
{(MPIDLPPPIVDAHALVQQHEQRVFR)-TSAFLTYAHDdARYVDEVNSILKVL----
DVQTFIDKEILTVGRIDdQIIASLKQVEYQIIFCTEASNNSEYCRKEVDFSAGRRVK-Q----
IPIFVDPiekM-DWFHFHLSKQYAVI---VSGIEDRE--AATFAIAKQLARL(MQLDFGLYQPPK)}*

$934=208(219):
>gi|800957225|ref|WP_045984500.1|
{|0(80)|<Proteobacteria(B)>}MULTISPECIES: hypothetical protein

[Pseudoalteromonas] >gi|633471430|gb|KDC53056.1| hypothetical protein DC53_01840 [Pseudoalteromonas ful
{()MP-VFISYSHEnKEFVDQLAMQLV----
QQNVHIWLDRwELSLGDSIIdKVQDAADGASALLVILSNASVASEWC-KkELSAGLLRElEE-
KRVVVMPVLLEDC-KVPLFARGKLYA---DFR---SNFD--
DGLKTVIDGIAKV(TNPFMNRISAPEYHTDWSIDWGDVNGNFALIINFVEQAKDQPFTVLTNIELLASPAATLKYHQISQAENEEVARASIIT)}*

$935=171(188):
>gi|1054528854|ref|WP_066304125.1| {|0(41)|<Euryarchaeota(A)>}nucleoside 2-deoxyribosyltransferase [Haloterrigena mahii]
>gi|1032679846|gb|OAQ51137.1| TIR domain-containing protein [Haloterrigena mahi
{(MTGEQVYVSHAP)-----------DDLDLVQDLFSSVKNFPF--
GVHIALEEVESGRSRtRLEGRLANSDIVVAVLTEGAAD------
DEWINQEIGYAVaKGIPVVPLFDEATDRRGFVGDVEGVTIDRGDLSLTIFNLLCRLRSELAPLGALS(VPNWYIRFPCTIPDCGGPVTLELDYDQSKLWKLHAH)}*

$936=249(254):
>gi|635367116|emb|CCI44628.1| {|653(211)|<Stramenopiles(E)>}unnamed protein product [Albugo candida]
{(VGRELFTDLVNKGANINYALPTTGDTALHIAIRCENKNTALAILASGGMILTLNREGVRPIDCTTSTKFQYEVKQAAGHR)--DVMISY---
THTHIEFAKKLRKSLEDNHVTTWLDLMDpsgIGGGTIWraEIARGITNAALVVCILAEDYAESDWCLKELALAKQVG-----
TPILAVSTENVTIGEDLQVYLYTRQMIPFEPAIIATKRNILNSRLIEYEYDE(FQYAHQFRLLLDGVRDEIEKNRKNVLCRNMRLQQ)}*

$937=220(222):
>gi|635367359|emb|CCI44432.1| {|938(0)|<Stramenopiles(E)>}unnamed protein product [Albugo candida]
{(DEIEKQRKGQIQKQLSQNQTAMFETAVRGQTMFESLVGSHISFISNKEVTAGSGAEDPNSESTSKVSGEPHRSNLAERKY)---IFIAHG---
DCHRAFVRNLYERLSSCGYAVVVDGgnfNELPESSNMkeRIqagKNAILGCSACIVVVSERTAKNELVSDQLAFAEDKQ-----RPIFPILLnDVKLDTSVISLLSYRPLYHFTTNVGFTASLHQLMTRMREILPLE(GM)}*

$938=249(254):
>gi|635369136|emb|CCI42761.1| {|647(167)|<Stramenopiles(E)>}unnamed protein product [Albugo candida]
{(NTSKEGIKHLVNAGADINEQLPNGDTPLHIAVKKEHLETSLALLSCGADLMMTNHDGFRPLDCTTATNLQYEIKRAAGKR)--DVMISY---
THSHSEFAKRLRKDLEDASITTWLDLMDpsgIGGGAVWreEIARGITSAVLVVAIITEDYPKSEWCLKELAVAREVG-----
TPIVAVSTENAVIDSDLKAYIDSSAIVSFEPSIIDIDKSDKRNISYVYDEEK(YTAVIRLLLLRVQEHIAERTHERISISNHRRLKS)}*

$939=208(219):
>gi|738438186|ref|WP_036389435.1|
{|0(1212)|<Actinobacteria(B)>}hypothetical protein [Mycobacterium farcinogenes] >gi|633839469|emb|CDP85799.1| WD40 repeat-containing protein [Mycobacterium farcinogen
{(MS)--RIFLSHSSRDNRYAVALKHWLERvdpglAG-E-
IFLDldrASGIRTGTRWTDALWQANARCEAVICLVSESWVASKE-CHaEFRQAEGMR-----
KPIFCARLESVDgdD---VTRAWQSCDLFG--SGASTTI-------

AVDGGPEV(RLATDGLNRLLTGLRSVGIGADSFPWPPPGDPGRSPYRGWQPLESVDAAVYFGRDAQIVRALDE
LRGMREAGLERMF)}*

$940=194(155):
>gi|760105446|ref|WP_043787645.1| {<Actinobacteria(B)>}hypothetical
protein [Amycolatopsis rifamycinica] >gi|637494179|gb|KDN17385.1|
hypothetical protein DV20_35995 [Amycolatopsis rifamycinica]
{(MYE)-YDVFISYLR-TAgdisawIRHHFHPRLLEVLdnNRYeDVKIFFDDQ-
VRVGAAWpaELNSALQRSRVLVPVCSPKYFRDEWCLAEWASMAERetltaaAPG-EHRP--
PLIYPVifcdsrhfpdwaharRPRDLQQWNHPFEhfqtapayLDFNQQ-
IKQIAaelEELIERapeWRSDWPVRTPP(PEPPQPARMPRF)}*

$941=183(185):
>gi|636987402|ref|WP_024407195.1| {|0(54)|<Firmicutes(B)>}hypothetical
protein [Streptococcus suis]
{(MR)EFQYFISY-S-
RDIYYEFVNPLNNILNKIGLKTWIDKFEINIGDEIidNLYTVLDKSADYygiIFIFDETFFSKAWCLKEL---
--
NYALKKRISFFPVLYKIKKEDIPNEYALLKNYNLLTVSSLKEIDYIVDRFTLLHLNNII(CRNIPKFSIDSYV
GNFLLEQIRYSTVNSHDALFIIDNFSQYLR)}*

$942=224(222):
>gi|637499396|gb|KDN22192.1| {|454(0)|<Actinobacteria(B)>}hypothetical
protein DV20_09755 [Amycolatopsis rifamycinica]
{(SGSVETRDPVPVTLSGAQCPLLWVNLPDFFTRAATSYRRVAEPIMVLWLAAHGVDQLSLDQRNELLRALKG
VVGAKPPDF)EYDVYLCHAS-PDKPviREIKDRLWR---
DYGIRSWYDDVELLPGDTTrrTIEKGLMTCRFLLLCASSNLKKSEWANREIdsVLHLDVKRQGEPKILVLKLY
EQESNDEAVPLIVRGTKRHHFGRRGDFERLAEYIVDAREAGGRP(QT)}*

$943=161(165):
>gi|636809312|ref|WP_024335510.1|
{|0(345)|<Proteobacteria(B)>}hypothetical protein [Desulfotignum
balticum]
{(MSH)--KTFFSFSSQdS-----
FVpSLLSRIRAQALDVWVYADEGEEIPGGKridkYLHDRVRSSSLFIPVVSKNSMKSVYTRMEVACALACQAD
-TGLRIIPLVETSAVEIDVWPDPFSELRSIRY--
HEFNGKPGQHPQLENAVARIC(QDIAIPYVGIIADTPRLPLMQR)}*

$944=184(184):
>gi|639172304|ref|WP_024512036.1| {|0(208)|<Proteobacteria(B)>}TIR
domain-containing protein [Bradyrhizobium sp. ARR65]
{(MPGNGPL)---IFVSHADEdREVAILLKSLITRASGGRARVYVSSDISAK-
RFGEnwfrRIRENLSEAKAVIFLASPNSLKASWALLEIGGALFNTDrpK-
IIPICIAGLTIPELIDPLTGLDASEPSDVVR--
LQSVVRSVLERCRIPAKLRPT(LDWASEYIAFRDAQEEWVTIDHRIYSACSVNTLRHLE)}*

$945=118(140):
>gi|636820028|ref|WP_024346129.1| {<Firmicutes(B)>}hypothetical protein
[[Clostridium] methoxybenzovorans]
{()MINIFVSYCQKdKIFADNINLY---FKDEDICIHQDIRDI---
SQWKsirEYMQSIRDMDYAILIITDNYLKSFHCMYEVLEIM-KEKD-YQDRIFPAIVE---------------
---TKIYSAIGRIQYITYWEEKNTKHHS()}*

$946=197(181):

>gi|760105445|ref|WP_043787644.1|
{|0(126)|<Actinobacteria(B)>}hypothetical protein [Amycolatopsis rifamycinica] >gi|637494178|gb|KDN17384.1| hypothetical protein DV20_35990 [Amycolatopsis rifamycinic
{(MSEHDA)-PRVFFSYSHDsEEHKTQVRRFATFLhSRIGldvVLDQWHDNvRI----
DWSlWATRNLKAADFVIVVASPLYRAradgdapPDEGRGaQFETAIIRNkltkdiED-GTRRVLPVVLPGH-
SIDDIPEFLNPYSMTRYviSEFTDAEVADLLAAITGRGRHPK(PRRGVWRGGAGEPRRVLAGELPWTCTSPGV
RRGTA)}*

$947=213(220):
>gi|758539086|gb|KIU46716.1| {|4(0)|<Proteobacteria(B)>}hypothetical protein QU41_20065 [Bradyrhizobium elkanii] >gi|1052226640|gb|OCX29551.1| hypothetical protein QU42_17805 [Bradyrhizobium sp. UASWS
{(SQDFSIDASEQSVVWRGELCACPFSVTLSREPTGRSGQISANFFLDGVPIGILKFSVTVASPARDSRARVQ
LCGDSSRHF)EY-AFLSYSS-RD-RLEVLQCAQV-
LEGVGINFFLDAVSLrsGEIWQRRLFEEIDRSDMFMLFWSKNaeEAESKWIEREVTYALKRQVTSKDQ--
LPCF-----RSTFLGGDKPTRPAWLPGHIQFDNALRRHLLAARHDQDDQ()}*

$948=215(220):
>gi|639222357|ref|WP_024559727.1|
{|54(0)|<Proteobacteria(B)>}hypothetical protein [Franconibacter pulveris]
{(VNLQKDPVYFEEWLRNNYPSKIKRHNMFTKDIYTYNVTTLIDADMVDVLVTEEGNGFPFIYPEREYFEEKH
PQYRDIMKR)DSKVFISHSSKdKNsIVNPLNAYLQGNDIA------
TWLDSYEIDYGDniflKVNEGIQQADVGLFILTDNFFDS--QSGWPLAEFSTFFM-
SLMKSNKKVLMLDAGVSPDKMLEMMKAYKYI--SWNNGAGLPEVANAINRIITK()}*

$949=218(220):
>gi|639272693|ref|WP_024580505.1|
{|0(156)|<Proteobacteria(B)>}MULTISPECIES: hypothetical protein [Bradyrhizobium] >gi|758536960|gb|KIU44606.1| hypothetical protein QU41_27160 [Bradyrhizobium elkanii]
{(M)--RIFLSFPSELESQADSIAQSLRNCDHD-
VFFSHDDLPPGDSFDARVEKAIESSDFMVFLISPESVTRGRYTLsELAFARTkwpnPS-----
NRVLPVMMAPTPldQVPTYLKAVTILEPQG--SAAAetrAAVD--
RMLEAESAQSRG(RSIMPFLALALASAVACTLTVKYATGILQYSFVDTGPAGGVTVLPGLIFGLVVATCNFIF
GIRDRFQLALVVAITAAAW)}*

$950=165(164):
>gi|639382371|ref|WP_024607782.1|
{|0(139)|<Proteobacteria(B)>}hypothetical protein [Pseudoalteromonas sp. TAB23]
{(MKT)--DLFISYAWTSNEHKEWVhllASQLHLMGYT-VKIDeAVDY--GSSLSGFMQE-
VTDASHVLLVVDENYVEradnaptSGVGIEtEWISSVFES-----
KPNTWLSVIFVRnpNYRLPRWLKAKSPKGF--
DFNSNPDARRYPGSTQIDAVWR(WIEGLPADKQHAIPISVVHKR)}*

$951=212(220):
>gi|695371530|ref|WP_032552323.1|
{|59(0)|<Proteobacteria(B)>}hypothetical protein [Vibrio fortis] >gi|639231167|gb|KDN27531.1| hypothetical protein VFDL14_18740 [Vibrio fortis]
{(SEVFEEWINIHYPEKHRIYNLFSRDLMKAIGKRSCNGMGLLDSDMLEYQMPSSTNEIFLHPDRSIYDETFP
PVLKEMTNY)--SVFLSHSSVdKALVDTFFNELQ----

KSEVEAWFDRyEISPGDSITdKINDGLDKSKVGLLFLSKSFISGKSGWT-MNEAnyffqkRMRHSD-
KNFIVINIDLEH-----------DEMPPLLQdyLYLNYDDPECVTKIVNELRKLNT()}*

$952=178(165):
>gi|639278382|ref|WP_024582429.1| {|0(316)|<Proteobacteria(B)>}TIR domain-containing protein [Bradyrhizobium sp. OHSU_III]
>gi|1052225715|gb|OCX28628.1| hypothetical protein QU42_21440 [Bradyrhizobium
{(MRKL)--
KIFVSYSHAdTEPRpksdnSRLGLIMEDLKYElrcnsgrPRFEVLRDIDITrVSDNFRvEIDKAMVACDVAIL
FISEEFSRSEEC-EaEFARLIELGKP-LFLVEIEPVSLNE-
DEDRIRKYRNAVKDIIFvkFWGPDRNKRSVRYGYPLPNDTAT(RDEYGAALEQVVAGLKARALM)}*

$953=173(176):
>gi|639698123|ref|WP_024722024.1| {|0(159)|<Firmicutes(B)>}hypothetical protein [Clostridiales bacterium VE202-01]
{(M)--NIFISYSHIdKEFAFKLS---SALEADGY-
DVFIDNKIPIGNNIYkDIGKGIAKADAVIVVISKNSNGSHFVANeTISMLSFLGKG-RIPLVIPVVLGKD-
TLLPADLNRFNYIVIPYenKEDNSNDLSIEYGVHGPKVRLDK(PLEKDAIEKMRLILAAHDEKIKRGEEERRK
SEEKV)}*

$954=136(144):
>gi|640239012|ref|WP_024833497.1| {<Firmicutes(B)>}hypothetical protein [[Clostridium] josui]
{(MVKV)----FLSCSSHdQTYIRYIINALRTLPEKYKLFYFLPPYKKDIPI-
DDIIERLYEIDLFILFITNNSLNSQFVQRELRQAIYLNNLKRIKEICPVSLDES-INVNQ-
DSRIPEYIRNRiHLSESPSK---TVQIVRNFIMNY()}*

$955=240(239):
>gi|640331951|ref|WP_024866420.1| {|93(407)|<Firmicutes(B)>}TIR domain-containing protein [Butyrivibrio sp. FCS014]
{(DAPFKDVLKAAVDLGIRILPIAIEPGVSARFSEVYGHMQVLDYCGSEAKARLKHYIDGYVDLYTVIDDEGP
AIPELFSFH)---
IFMSYRKKnLVQLTELMKKIRRWPEHLDTSIWYDDALVEGENYDdQILEQLKGSDVILLVMTPDVLeEGNYVL
RcELPDARRFGKR-VITFVTEETDPEA-
LGKHSDFTGEVVYDLDAlrLILKEEHDKAAGAENNLTDEAAY(KLGLCYLAGNGTARDPMMG)}*

$956=223(221):
>gi|639277398|ref|WP_024582107.1|
{|69(0)|<Proteobacteria(B)>}MULTISPECIES: hypothetical protein [Bradyrhizobium] >gi|1052227126|gb|OCX30035.1| hypothetical protein QU42_16905 [Bradyrhizobium sp. UASW
{(ELIWKPESEVIVTAGLAGTAEKISNLDLRFSGRVLVYTPNITSSQQWSNLATQMKENGLHLLVRDGEYVAT
RATYEQPLA)----FISHDS--
RDKDPFVRELANKLASMLCPVWYDEFNLHPGDGLreNIERGLKTCPKCIIVLSKNFFGNPGWTKrEFD-
TVYTREILeGKRLMIPIWLdvdkqDVY-
DYSPILLDTLGIPAALGVEEVARKIVAaLNYVPPDDIRQPT(A)}*

$957=208(186):
>gi|636809315|ref|WP_024335513.1|
{|0(806)|<Proteobacteria(B)>}hypothetical protein [Desulfotignum balticum]
{(MKIRKHITY)RYFAFISYSRKdskaaKWLQKKLEwfRFPvklvdestSPGHPRYIRPVYRDKTSLEVDHAh
YwdNIKGAISQSRYLIVLCSPDSAASGPVDKEIRHFLSNTDRedalEsivPVILKGNVGSK---

DDKECLCDALLEQGSRITDRNLPSMVPDGDEAEKEGWENG(FTGVVSFLLRLKRETISDHCQKEERRRARRSR
ILSGL)}*

$958=201(196):
>gi|639258436|ref|WP_024578195.1| {<Proteobacteria(B)>}MULTISPECIES:
hypothetical protein [Afipia]
{(MTFLTEESMRQRAREWVQRAGQSADELLKRQSTQSLT)RYNVFLS-QTArdaeivLGVYSFLVE-------
-
KGLTVFCDwitarnETGAQVTPANaeTIRSAMSASDTLLFIDTEQAEQSRWMCWELGWFDGGKGLVgvlPVLP
EAKRSYR---GREFLGLYPYIELNEVGELMVVRPIVTNSRGITIIESPNS(TGFDHWRAGQSVSMRPRNF)}*

$959=278(270):
>gi|813219354|ref|XP_012211054.1|
{|28(138)|<Stramenopiles(E)>}hypothetical protein SPRG_16337, partial
[Saprolegnia parasitica CBS 223.65] >gi|641523354|gb|KDO18233.1|
hypothetical protein SPRG_16337
{(YAPYEAGILANGVDGDVVAHLQSEQLPMLFGTLGVTNTIHQTKLASLHSQHRKALLTRSLSMAGHMTAKLS
TSPRQPKAF)--
DVFLSHNWGhDELGrdnHARVANINAFLRQKYseIKTWFDQDKMHGNILKAMAHGIEESQIVLIFVTQLYQtk
vngdnANDNCQLEFGMAKTTQ---
TAQWMIPVIMDPCMKTPRHWCGQFKLVLANQLYVDLTSDEDDAFITGCNTLLQR(IDGLLLKLGRPSVLPPTH
RSSQETQAPSSPRTTDSASELVTLFATWQRAI)}*

$960=253(250):
>gi|813165446|ref|XP_012203743.1|
{|0(369)|<Stramenopiles(E)>}hypothetical protein SPRG_20633 [Saprolegnia
parasitica CBS 223.65] >gi|641531437|gb|KDO25509.1| hypothetical protein
SPRG_20633 [Saproleg
{(MGAAASVESGDSYSKEQCQAICGDLFDDAMWTMHASKESQTISGAKLHEVLSSIT)--
DVFLTHDWGvDGANHRHVSVINQLLIERGITTWFDEEKMEGNIKMKMITGIENSRCIVVFVTQRYmdkvggpN
GEDNCQLEFNYAARRK---
TNAKMISVVMDPKAKDTRAWVGPVGMTLGGDLYLDFSTVFDSHPELLPAKIDEL(MAKLMTIIGKPLKQCFAS
GGVAIPNATVPLESLSTDQVTTLLTALEYATYCDVFR)}*

$961=282(276):
>gi|813162719|ref|XP_012202659.1|
{|28(381)|<Stramenopiles(E)>}hypothetical protein SPRG_07917 [Saprolegnia
parasitica CBS 223.65] >gi|641532490|gb|KDO26515.1| hypothetical protein
SPRG_07917 [Saprole
{(YAPYEAGILANGVDGDVVAHLQSEQLPMLFGTLGVTNTIHQTKLASLHSQHRKALLTRSLSMANHMTAKLS
ASPRKPKTF)--
DVFLSHNWGhDELGrdnHARVANINAFLRQNEIKTWFDQDKMHGNILKAMAHGIEESQIVLVFVTQLYQtkvn
gdnANDNCQLEFGMAKTTQ---
TAQWMIPVIMDPCMKTPRHWCGQFKLVLGNQLYVDLTSDEDDAFIAGCTTLLQR(IDALLLKLGRPSVLPPTH
RSSQETQAPSSPRTTDSASELVTLFTTWQRGIEAHDVD)}*

$962=266(258):
>gi|813118116|ref|XP_012195854.1|
{|65(202)|<Stramenopiles(E)>}hypothetical protein SPRG_01898 [Saprolegnia
parasitica CBS 223.65] >gi|641539275|gb|KDO33084.1| hypothetical protein
SPRG_01898 [Saprole
{(RKILLLHLSKIKTVPTTSPSSLDTNDGRILVHRSTPSLAPAQVLSQLFAYQGIHLIDPDDLVSVVTKIVPL
VKPVSAHDE)-YDCFISYRVASEKEVAEKLYLHLKTKGY--
EPFLDRMSLKNAEpWKdGFLRGLGHSHLFLALVSEAGLEkaRDRTQNhhEDNLLLEYEVALeiataGRLNVLP

IYVAASNNGNFVKFQGFNPDLYAPTLDPEDATSRRTSVPVAALPGLS(DAIKRRQSEMPASRPLQPIPGEDAG
GVDASVNRKVSLS)}*

$963=230(238):
>gi|813227341|ref|XP_012212747.1|
{|387(0)|<Stramenopiles(E)>}hypothetical protein SPRG_17943 [Saprolegnia
parasitica CBS 223.65] >gi|641520303|gb|KDO16545.1| hypothetical protein
SPRG_17943 [Saproleg
{(NKKNPRSITYAYNDDAYNTQFRLLLDGIRDEVEKKRKQVALRPMETLSPTNGGAFENHSLAFGLSSPSGTS
PSTFQDTKF)---VFIAHG---
DCHRNFVKRLYHELTTtQGVPVYLDLKTKNDMAARvhAAKEAMLRCACVIVVLSSKSAKNEMLNDQLAFAEDK
G-----
KPIFPVVLHTEFTALPPNQMYSLSRAPMHHFALDLGFTTSFDQLLHDLRPHV(AAERPSLVNDDDDDYGIV)}
*

$964=249(254):
>gi|813227341|ref|XP_012212747.1|
{|188(180)|<Stramenopiles(E)>}hypothetical protein SPRG_17943
[Saprolegnia parasitica CBS 223.65] >gi|641520303|gb|KDO16545.1|
hypothetical protein SPRG_17943 [Saprol
{(VDPELLKLLVAKGANINYQMKSNGDSALHLAIKHEKQETAISLVSCGANIMCTNNASFRAIDIATSTALQF
AIKSAAGHR)--DVMISYTH-SHAE--
FALKLRKSLEASNITAWLDTMDpsgIGGGSVWreEIAKGIANATLLVAILTDDYTKSEWCLKELACAKQVG--
---
TPVLPISTEHVRLNEELQVYLYTRQIVPFEPAITAINKKNPRSITYAYNDDA(YNTQFRLLLDGIRDEVEKKR
KQVALRPMETLSPT)}*

$965=247(252):
>gi|813133452|ref|XP_012197825.1|
{|623(188)|<Stramenopiles(E)>}hypothetical protein SPRG_03838
[Saprolegnia parasitica CBS 223.65] >gi|641537367|gb|KDO31220.1|
hypothetical protein SPRG_03838 [Saprol
{(LSQELLSAIVQSGGNINDALADSGDAALHIAMKRGKKATALALLGAGADIMQVNGDGSRPLDCTTSTELQF
AVKRAAGSR)--DVMISY---
THSHMEFAKKLRTSLENNHITTWLDLMDpsgIGGGSVWreEIARGISNASCVVSILTEDYPKSEWCLKELALA
KQVG-----
TPVLALSTENVKIGEELQVYLYARQIVPFESAITAIEKSNPRNITYEYNEVA(FARQFRSLLDGVRDEIEKKR
QALAKTSGAHLG)}*

$966=273(279):
>gi|813126798|ref|XP_012197141.1|
{|687(139)|<Stramenopiles(E)>}hypothetical protein SPRG_03159
[Saprolegnia parasitica CBS 223.65] >gi|641538109|gb|KDO31943.1|
hypothetical protein SPRG_03159 [Saprol
{(GVGQDVIAALIAHGADLDFADDGGNTALHVALINENRVTAVSLLKCGANIMATNLDGKRAVDCTTSTELQF
AVKKEAGSR)--DVMISY---
THAHAPIAKRVRDHLVDHaRLTCWMDTMDpsgIGGGAVWreEIARGIFNAKVVVAVVCDGYARSEWCLKELSF
AKISK-----TP--
AVVLVVDPNGMSSEIERLVPKQCILPFPVSDAMLEDPTQWAAAFEAVL(GPVRDALRHAEPPPLLPLYNFDVH
APARRVFIYYTAQDPSFLRRIEVSVQAKGFVPLLA)}*

$967=223(229):
>gi|639171106|ref|WP_024510855.1| {|0(357)|<unknown(X)>}hypothetical
protein [Bradyrhizobium sp. ARR65]

{(MTTGYGRQYEY)--DFFLSRRGSVGRIAQEVADVLREKGYR-
VIVQDYDFQLSDSVIERMHEGVKTSRDLIILFTSDY-EKSAYTRkEFTSFEAQR-----
LRQPDERhtiILRCEdvSPDGLLADVIYQDLVG--IeDADERRR--
RIIAAAERQATA(TPPPPRPFIGVPPRIPSFTGREDQLSRLDAILIHNKPAVVTQAVQRVAVQGMGGVGKTSL
AVEYAYRYRSLYAGVCWC)}*

$968=182(186):
>gi|739912051|ref|WP_037762443.1| {|0(240)|<Actinobacteria(B)>}TIR
domain-containing protein [Streptomyces olindensis]
>gi|640934360|gb|KDN76873.1| hypothetical protein DF19_14140
[Streptomyces olinde
{(MG)--DVFISHSARGDALAMavlgKIEQGLRARTHT-
PLVDQSGIEPGDEWRPQLVDWLARCDAAVVLLNQAAL-DSTWVRrEVNILMWRR-----
ALGAPLLVVPVLldGLTTTAVKQAGLEELR--
PIQFARTAQGVEPDAESLADQV(LGRFARLPRATTGTDPMSVWLGRLALYLSEAQRRPDVLAEAARA)}*

$969=200(198):
>gi|640539674|ref|WP_024971921.1| {|0(78)|<Firmicutes(B)>}hypothetical
protein [Lactobacillus plantarum]
{(ME)---VFLSWSGDfsKALAEKFRDWL-
PLVLQDLDPFMSNKDLLLGSRWNeSINKHLDSSSVGLLLVTPENISSPW-LNyE-AGALTKTLK-
DEAKIIPIIFGDT-
NFDvllsSTPLKQFQSVTTPNkdGIFKLAENLNSNLDDSLSEDSLK(KTFEKWWPELDESLQEVSNNFKGQET
VGEGTSNTNNSQNSDNVSDELLMKVAQNVD)}*

$970=238(239):
>gi|813192196|ref|XP_012207980.1|
{|573(189)|<Stramenopiles(E)>}hypothetical protein SPRG_13216
[Saprolegnia parasitica CBS 223.65] >gi|641527005|gb|KDO21325.1|
hypothetical protein SPRG_13216 [Saprol
{(IDTAIFRLLRENGLGDFGTLENGDSPLHIALKLCHEKTAVALVRSGSSTTSVNDDGVRAIDCTSSTELQFI
IKKEAGQRD)---VMISYSHAfQPFALRLRQSLE----
DNFLTTWMDLMdptgISGGAVWRdEIANGIQNAAVVLCILSETYPASQWCMKELAFAKAHNVP-
VVAVMAQTTEMTD-
ELQVYLWSRQLVDFRPAivSASEGPLVVDNIIYSQQLASLLD(GLRNEVELRRMDRPAASVR)}*

$971=238(239):
>gi|813192204|ref|XP_012207984.1|
{|597(180)|<Stramenopiles(E)>}hypothetical protein SPRG_13220
[Saprolegnia parasitica CBS 223.65] >gi|641527009|gb|KDO21329.1|
hypothetical protein SPRG_13220 [Saprol
{(IDTRIFRLLRENGLGDFGTVANGDTPLHIAMKSGHEATAIALVRSGSSTTSVNHDGVRAIDCTSSTELQFI
IKKEAGQRD)---VMISYSHAfQPFALRLRQSLE----
DNFLTTWMDLMdptgISGGAVWRdEIANGIQNAAVVLCILSETYPASQWCMKELAFAKAHNVP-
VVAVMAQTTEMTD-
ELQVYLWSRQLVDFRPAivSASPTNGRSLIVDDVIYSQQLAS(LLDGLRNEVEKHRVDDKVV)}*

$972=116(149):
>gi|641820761|gb|KDO40623.1| {<Streptophyta(V)>}hypothetical protein
CISIN_1g045928mg [Citrus sinensis]
{(MASFSGNNK)KYDIFVSCSG-EDTCDSFTSHLYSALSRQNIQTFIDDQLNRGNE-IseSL--
AIKASAISVIIFSEDYASSRWCLDELVEILECKK------------------------------
CCARNAESSEPNNYRKINDTRITWF()}*

$973=199(200):
>gi|641820870|gb|KDO40705.1| {|0(281)|<Streptophyta(V)>}hypothetical protein CISIN_1g0060181mg [Citrus sinensis]
{(MASSSSSINMIPHP)KHDVFLSFRG-KDVRHNFVSHLNAALCREKIETFIDDKLNRGNE-
IspSLSSAIEGSKISIVIFSEGYASSRWCLNELVKILESKNK-
YGQIVVPVFYLVDPSDAGYCPSLGWMGIFDIPTSESVLIEGNVNDISKKLSDLF(PSDNKDQLVGVESIIKEI
ESQLLSGSTEFNTVGIWGIGGIGKTTIA)}*

$974=263(261):
>gi|641855169|gb|KDO73963.1| {|0(564)|<Streptophyta(V)>}hypothetical protein CISIN_1g003354mg [Citrus sinensis]
{(MTCVQVADPAPPRVSFSFPVPRISFAKGPVSPVSNAKLRSC)--DVFIGFHG-QNpnlVR--
FCKWLKSELELQGIACFVADRAKYSDS-QshEIADRVICSVTYGVVVVTNSSFLNHL--SLEEIRFFAQK-
KNLI--PLFFDNGPNEIlgllncNSIDKECKEAldgLMKSHEFKLEANE-
CNWRSCVAKTAGI(LRAKLGRKSVAENDLEGFEELPFPRNRCLVGRDKEIMEIETAFFGSGDYLEQDYAMPIT
KGEASGQSEGLADEESDSVST)}*

$975=222(234):
>gi|1173850604|ref|XP_012090050.2| {|0(1124)|<Streptophyta(V)>}TMV resistance protein N [Jatropha curcas] >gi|643705995|gb|KDP22127.1| hypothetical protein JCGZ_25958 [Jatropha curcas]
{(MKMASNVETFVPPSSNY)-YHVLLSYKL-EDTNKSLVSYLYKELERKGLLCFKANEkLPKEKS-
VpsEISNAIGLSRFAVVIISETYVSSNWCLDELEKIIQCKET-KGLSVLPIFYNVDPLHVKDRVDST----
LEL------EHV-
QRWREVLNKLALL(DGFNSRDWTDDRKLIEEVAKVILKEWTDKFSSEINGLIGIDSRIEQIQSLLTVESEGVV
FLGIWGMGGIGKTTIARA)}*

$976=299(297):
>gi|645215754|ref|XP_008246367.1|
{|108(592)|<Streptophyta(V)>}PREDICTED: uncharacterized protein LOC103344547 [Prunus mume]
{(PTTQPSPLVSLCRGSQSDDIPSSSYTPPSDQYEYSDDVSDPLKLKFDSAPPRISFSFPVPRISFAKGPVSP
ASNAKLRSC)--DVYIGFHG-QNpslVR--FCKWLKSELELQGIACFVADRAKYSDT-
QsqEIADRVICSVTygVVVVTSSSFI-NHLSMEE-VRFFAQKKN-----
LFPIFFDTGPAEILgllnyssidKECKEAIDGLMKTNEFKLEANE-
GNWRNIVSKAAGV(LRAKLGRQSVSQTDMEGVDELPFPRNKFFVGREKEIMEIETALFGSSGDYLEQECSMTI
IKGEASGHSEGIADDESE)}*

$977=178(196):
>gi|643731579|gb|KDP38823.1| {|0(657)|<Streptophyta(V)>}hypothetical protein JCGZ_04980 [Jatropha curcas]
{(MGSSFSSSASTSSQRTPSFIINTNAASSPSSTSSQQTHSELNN)-YEVFINFRG-
KDIRDGFLSFLFESLKEKGIDTFKDENLQKGTE-
ItpALKQTIQESYPTDVQELRGPFARALARLLKDHSDSSHNL-----------------
GGCCHALKTISNDLSAFVSNEIKNDQMLVQRIVNRVSE(MLSHMPSNASYHD)}*

$978=132(150):
>gi|641818142|gb|KDO39098.1| {<Streptophyta(V)>}hypothetical protein CISIN_1g0137244mg, partial [Citrus sinensis] >gi|641818143|gb|KDO39099.1| hypothetical protein CISIN_1g0137244mg [Citrus sinensis]
{(YEGVPTAIPS)-----------EDTRDNFTSHLYSALSQKSIETF-----
INRGDEIsqSLVDAIEASAISLIIFSEGYASSRWFFDKLVKILQCKRV-
YGQIVLPVFYGVDPAPVKWPTGSYGDSFLKL-EERFKENSEklQTWRNALKE----()}*

$979=125(151):
>gi|641821309|gb|KDO41022.1| {<Streptophyta(V)>}hypothetical protein CISIN_1g038213mg [Citrus sinensis]
{(LS)--------------------------RQN-IQTFVDDQ-
LSRGDEIseSLVNAIEASAISVILFSEGYASSRWCLDKLVKILEGKKE-
YAQIVIPVFYRVDPSDVRNQMGSFGILFSKL-EERLKVNTEklQSWRNALKEAASL(SSFHSLNMR)}*

$980=152(154):
>gi|643717437|gb|KDP28980.1| {|0(914)|<Streptophyta(V)>}hypothetical protein JCGZ_19531 [Jatropha curcas]
{(MSLILSSTVPPKTN)-YDVFLSFRG-
IDTRKNFTSHLYAALKQKHITTFMDDAGLERGEEIswSLLKAIEESKIACTDLSAIDSHINKLLSLLSIGSKD
VRF-IGIWGMGGIGKTTIAEVLLSRISDQFDNFCF-ESNVREKSEKDGLVQLRKCLFS()}*

$981=234(235):
>gi|645251987|ref|XP_008231917.1| {|0(922)|<Streptophyta(V)>}PREDICTED: TMV resistance protein N-like [Prunus mume]
{(MTTQPVSSSSSSSSS)-YDVFLSFRG--DTRFNFTDHLYKALRDKGIYTFIDGE-
LVRGEEIspALVKAIEESRISLIVFSENYASSRWCLDELVKILQCKQS-
KQQIVLPIFYKVDPSDVRKQTSKFGDAFEGL-
IKRkFKNDKEkvLIWREALTKVANL(SGHTFKDGEYETTFINNIVDGILSQVLSCTYWNVAKYPVGIQSRVQD
VERHLDVGGKDRRMVGIWGTSGIGKTTIAKAIW)}*

$982=138(160):
>gi|645299192|ref|XP_008245848.1| {<Streptophyta(V)>}PREDICTED: toll/interleukin-1 receptor-like protein [Prunus mume]
{(MALVKAADPQTSSSNASRYC)RYHVFLSFRG-QDTRKTFSDHLYTALVNaE-
FRTFRDDDEVERGEGIkpELQKAIKHSRTSVIVFSKDYASSTWCLDELVLI----------------------
REILLISEDFEEESTY-STILVGLGRATGSEAMKFTIFS()}*

$983=258(262):
>gi|641483972|gb|AIA66467.1| {|900(0)|<Mollusca(M)>}toll2 [Hyriopsis cumingii]
{(SNYGIIISHGITPKQIKDKTLLRYLKRGRFIDSRNRLFVEKVLYSMPIKNDNNHGWRRKPNAKTFIQREFS
IKEDHVEDN)RIHVFISYSDQdlEFVTKGLAPELERSG--
YRLCLPDRDFIPGASKEENILKAIDVSLRTVFVLSGPHIKDEWSLFTFRSACEKSLREKTNHLIVIVRAD--
ADIDTMAEEVKHYLKTYvSLHIEDRW---
FWSKLLNGLPPT(GQKRRSEFTSPLSMSQCAAPDAVTIEMKEVLQRSEKRVVTRF)}*

$984=215(240):
>gi|642063665|emb|CDQ90157.1| {|0(58)|<Chordata(M)>}unnamed protein product [Oncorhynchus mykiss]
{(MPVLRKSSLVCVFSCDLSLANE)--------------------------------
LYQDDTRLLRAYIEDVARCVDQSKRLIIVMTPNYVVRRgwSIF-ELETRLRNMLVTGEIKVILIECAEL-
RgimNYQEMEALKHTIKTLTVIKWRGPKSNklssKFWKQLQYEMPFC(RTEPMLSHEPAMDVSEQGPFGELQT
VSAISMAAATSTAMATAHPELRSSFHNSYHTTMRQKHYYRSYEYDLPPGGTLP)}*

$985=207(232):
>gi|642082739|emb|CDQ85328.1| {|0(56)|<Chordata(M)>}unnamed protein product [Oncorhynchus mykiss]
{(MLTCLCLGLANE)--------------------------------
LYQDDTRLLRAYIEDVARCVDQSKRLIIVMTPNYVVRRgwSIF-ELETRLRNMLVTGEIKVILIECAEL-
RgimNYQEVEALKHTIKTLTVIKWHGPKSNklssKFWKQLQYEMPFR(RTEPMLSHEPALDVSEQGPFGELQT
VSAISMAAATSTAMATAHPELRSSFHNSYHTTMRQKHYYRSYEYDLPPGGTLPTL)}*

$986=216(220):
>gi|794656979|gb|KJX74287.1| {|74(0)|<Proteobacteria(B)>}molecular chaperone Tir [Acinetobacter baumannii] >gi|942836908|gb|KQE07953.1| molecular chaperone Tir [Acinetobacter pittii] >gi|942897926|gb|
{(LSDLQKKKTDIQNKLLKENQNLAKAEALERKKLDALAKKQQKEEIEHQKKLKREIESIKASTQHITGATAS PYLDVTPEP)EYDLFISHAS---EDKDdFVRPLAETLQGLGLKVWYDEFsmRVGDSLRRkidSGLRN----- SRygTVVLSADFIKKEWTNYELDALVAREMNGHKM-ILPIwhKITKTDvmnyspN-LADKIA-LNTSFSTI-EEIAHQL--------ADVVLDK()}*

$987=212(220):
>gi|642956673|emb|CDR10393.1| {|2(0)|<Actinobacteria(B)>}predicted protein [Streptomyces iranensis]
{(QQLRSAQRKAEQDAKRAQRQFESQVEKAVTDYNRAAERHNRQQLAKLRRSSGSSVSFTVEEQHLVDRVQSA VAEQDEREY)--DVFLSYARVDGAdVAEQLRSELDSLGIT-VWFDEVAIRAGRSLSRQMDDGLTRARAGVTLLTKAYLGRRFWTErE-LGALLHK-----DTVIPVL-HGVSfgDVGKFSSFLGDQAGFS--TEYDSVEDIAQKIAEAVLLDEE()}*

$988=183(206):
>gi|643543994|ref|WP_025226629.1|
{|0(778)|<Armatimonadetes(B)>}hypothetical protein [Fimbriimonas ginsengisoli] >gi|663072602|gb|AIE84757.1| SEFIR domain protein [Fimbriimonas ginsengisoli Gsoil 348]
{(MLEAFRTF)------------------------
EANDLICLWHDNLTPVAINWDkHIIEQLVKAKIAIVLLTPEALRSDYIVKkELPMLKEMYDA-ERIQIIPLPCEPC-DLgTHSWLNGCQA-PNSLkpLDGDEEEKKRSLQELVVRVTQRL(TEIALEQGGISDRHTPLDPKWVSLDKLRLDGDRKRDVGRLIGR DQEIALLNLAFARSD)}*

$989=179(172):
>gi|647297653|ref|WP_025741734.1|
{|0(12)|<Bacteroidetes(B)>}hypothetical protein [Aquimarina pacifica]
{(MSY)KYDVFFSYK-----RNQLTETWHNKLVEqlefwlnmeiqdrN-VSIFYDKESIEIGDEWksKIGDAIRHSRCIVAILSPDYFKSTWCYAEILSFLKRQEI-IEDSTGLVLGARFHDGHSFPEKVKAIQQEDF-SDYASVSDSFWDTRKAAEFGDR(IKEFARVIAHQIKNAPEFQQDFPIVIPEE)}*

$990=248(254):
>gi|646874473|ref|WP_025605295.1|
{|1004(13)|<Proteobacteria(B)>}hypothetical protein [Vibrio parahaemolyticus]
{(IKAASPKLVEHLIDSISKTNHLDAKAIVEWFKGEPKEEKEKENRVEQEQKEPFSNIGKGALDPNRKPNVYF SYAWGKDEN)------------
DPKQKVCDEIFNKLKSDnSIQIFRDKDSMDSGDSIeAFEKQIGKADFVFIIISEKslYQSSH-CMNELRLVYERAQS-EKQEfvskVIPVIMDDAEIDSPIDRLKVVRNWTQKRDELDSIISEVGAEAAGAESTNQ(LQIMRSFINSTANA LTWIADLVINRTPELQAETA)}*

$991=249(254):
>gi|647233168|ref|WP_025685577.1| {|86(340)|<Firmicutes(B)>}hypothetical protein [Paenibacillus sp. 1-49]
{(INQLLAVNEKFPKKFMLYPIAIERNAFNLKNSLLKKNFIRSYEHDNDWIYIASMLTHEFCRLLYNVKRINE EDKNSRANG)PVKLFISHAK-ED-GVNFAKSLCSFISAETpLKTFFDANDIAFGYDFpqEIESHINK-SVFVAIHTDKYSSREWCRREVVLAKKHN-----

RPMVLINLFNEGESRSFPYMANVKTIRINQKGNKKNKNFKIILFTLAETLRF(KYHEMFMDYLVRKLPIPAKE
RNIFSYPPELLTLF)}*

$992=246(254):
>gi|647277272|ref|WP_025722975.1| {|86(339)|<Firmicutes(B)>}hypothetical protein [Paenibacillus polymyxa]
{(TEYLKKLIQLNGLTFIPIALDIFAFKHNIFNYQNFIRAYEFDQAYLNDLLFIAITHEIYRFSLNENFQRIA
NGKDTALKL)----FLSH-----
TKNGengiiLAKALKNFIDNSSMRNFFDATDIAPGYRFdeEIVGHIKESTI-
IAIHTDSYSSRYWCQREVISAKDND-----
RPIIAVDSLEEFEDRRFPFATNIPGIHINLDGGPSKRDLLRILSATLLETVR(YFYAKLLLKEYKTVGFIDDE
AEILSRPPEVSDID)}*

$993=178(182):
>gi|647129991|ref|WP_025645690.1| {|0(974)|<Firmicutes(B)>}hypothetical protein [Clostridiales bacterium VE202-26]
{(MMGYSGRQIAD)---VFLSYSHDdREIVEKIASNIQA---RG-
FECWKDKdRLRAMDPFNvLIEEAIDESQVFIAFLSKTYVSKDYCIHEFNRAIDKKKS-KIVVCIDDVNEYT-
NRKCSYMFSFSSGHNLLgfGSGFSTDEEFSAAVGDICDSVPM(VQLKRYQTSGEPSDLPPANTPDYIFSQLRL
Y)}*

$994=278(300):
>gi|240256009|ref|NP_193685.6| {|514(517)|<Streptophyta(V)>}nucleoside-triphosphatase/transmembrane receptor/nucleotide binding/ATP binding protein [Arabidopsis thaliana] >gi|332658790|gb|AEE84190.1|
{(KCSANDVIDFLRTRQVLGSTEALVDRLIFSSEAFGIKPEENPFRSQAVTSYLKAARDMTREKECILVFSCH
DNLDVDETS)-----------------FIEAISKELHKQGFIPLTYNLLGRENL---
DEEMLYGSRVGIMILSSSYVSSRQSLDHLVAVMEHWKT-
TDLVIIPIYFKVRLSDICGLKGRFEAAFLQLHMSLQEDRV-
QKWKAAMSEIVSI(GGHEWTKGSQFILAEEVVRNASLRLYLKSSKNLLGILALLNHSQSTDVEIMGIWGIAGI
GKTSIAREIFELHAPHYDFCY)}*

$995=272(263):
>gi|238872935|gb|ACR72645.1| {|76(339)|<Firmicutes(B)>}Hypothetical protein EUBELI_01654 [[[Eubacterium] eligens ATCC 27750]
{(AEGYWGLVLCKYGIEYADNASGKKVPVCHRISYDSVMDDEDFELVMENSDSESRAIFREEAKIIEENRKKY
IQIAESEQP)-YDIYISYRA-
KDDNgdktavSEIAGHLYNKLTSAGYRVFLSEAALkgKKRSECepYIYSALNSANVMLA-
LGTSYddYNDVWVKNEWNRYLEIAEKNKNKCLIPCYKDVDEYDIPKEFAGLKVCQLGNDDTFNNIMAEIANVV
KPESVNQP(APEPEKAEPAEEIELEEIEIIEPVNINKLLDEGFSAISDKNWK)}*

$996=171(177):
>gi|506338873|ref|WP_015858592.1| {|0(88)|<Euryarchaeota(A)>}TIR domain-containing protein [Thermococcus gammatolerans]
>gi|239910587|gb|ACS33478.1| Hypothetical protein TGAM_0976 [Thermococcus gammat
{(MEKDF)--EIFLSYYTRTGLDYAEELKKALRDNGW--
KAFLAHHNILPGEHWkDIIFNYVlpHIKYFILLYTPGA------
ETRKWTREEYKYAKrLGKTIIACVYMPVGESVGNILTRIEPAFPGISEKQVIEWRDKSELNSKVILTLE(RYQ
KRRLVIGHLSQTRDVIQPKGNSQNYHVDH)}*

$997=172(180):
>gi|648291321|ref|WP_026072470.1|
{|0(176)|<Cyanobacteria(B)>}hypothetical protein [Nodosilinea nodulosa]

{(MTEQQF)--DVFLAHSS-KD--KPLIRRIYRKLADLGLHPWLDEEEIVPGTKFqdEIQQAIGRIKTA-
AIFFGHGGLGRWQALELrSFISQCV-----
KRDIPIIPVLLPGVEEIPEELIFLQEFHAVSFQDSIEDEKALYFLEWGITQR(RPTRQYGTSQTSSDTSGQRP
AGSAPPISPPVALP)}*

$998=256(262):
>gi|1030081636|gb|ANF54270.1| {|68(33)|<Proteobacteria(B)>}hypothetical
protein DA69_05665 [Brevundimonas naejangsanensis]
{(QSTRAAMEARGNADVYIPFCDYSGSAIPASDSGRNTQYVYRKGWTGLNEIARDKRLLLHILVQIRRHLRKL
SADDRDEPL)----FVSHAKADGDTAAReIVDYVQSPGHD-VplhtFYDAMELNPGEDFKARFDAEISRGT-
LIAIVSDVY-DSRPWCVfELTTAKRHR-----RPIVLADIGGVRvsRTFPYGANLPRVRLDA--
GGPVGSWIDPLLVEAMSEGLRC(DVFLAQAAKVAAAASEVLVMPRPPELFDVIDAEVCPRVILYP)}*

$999=259(264):
>gi|815937173|gb|KKJ01712.1| {|160(653)|<Cyanobacteria(B)>}hypothetical
protein PROH_04335 [Prochlorothrix hollandica PCC 9006 = CALU 1027]
{(GLLALLDRDRDYVHDHTELLCRAMDWQRHQCQTRYLLTGDQLKTAESWLKTQFTDRQPPCLPTSLQCEFIC
ESLKNANNL)MTQVFIAYAETdRDLREKI--HCS-LMREG-
ITVWINRtDLAGGTEFQnQIDRGIEEADNIVVLISPQAVASPHCQQV----VAYGYS-LKKRIIPLLISST-
DLTYIPEDIRALQFIDFsqWENGTSYDIAINKLIKEVQNNAP(YHYQHKMLLTQALKWQRQQRNPSILLRGYG
LRQMESWYQIAKQN)}*

$1000=242(245):
>gi|815937173|gb|KKJ01712.1| {|364(466)|<Cyanobacteria(B)>}hypothetical
protein PROH_04335 [Prochlorothrix hollandica PCC 9006 = CALU 1027]
{(KLIKEVQNNAPYHYQHKMLLTQALKWQRQQRNPSILLRGYGLRQMESWYQIAKQNTVYSLVAAQAEFLDES
RKQPPHQTF)--NVFISYSRAdADFARKLNENLQI---
QGMFTWFDQENIDTGKDFQtEIYKGIENSENFLFIISPSSVSSPY-ASaEIEYASQLNKR-
FVAVLHREAPPSS-
LHPAIATVQWIDFRRRGsdFLSNFGELVRFLASDPLYIQNHT(RLLVKANEWEQSGRDDGFLLRGKDL)}*

$1001=225(212):
>gi|740851210|ref|WP_038636463.1| {|0(249)|<Proteobacteria(B)>}SEFIR
domain-containing protein [Pseudomonas chlororaphis]
>gi|647803483|gb|AIC18567.1| hypothetical protein EY04_06580 [Pseudomonas
chlo
{(MKDPK)---VFISYSwsS--
PSHERWVLDLAENLMASGIEVKLDKWDLKPGQDSvaFMESMVTDDSIdKVIIICDKTYSEKadgrrGGVGtET
QiISKKVYEQVnQERFIavIAEKDEEG-
KafTPTFYHSRLYIDLSepESYSDGFEALIRCIYGQPQYQRPQL(GKKPDFLTKEISISLGTTPLAKRAIAAI
KEGKTTATAAFEEYLSTYSENLERIRLKHQDAILFDEQV)}*

$1002=201(200):
>gi|648403839|ref|WP_026095590.1| {|0(303)|<Actinobacteria(B)>}TIR
domain-containing protein [Microbacterium sp. B19]
{(MVGAPGSTGYRFTMTTK)--HVFLSYIR---
EDSAQVDKLQRVLEAADFSVWRDTANLWPGDDWqqKIRQAIQGGTLVFLACFSSAlkARDTSYQFaELMLAAD
EYRLRplDTSWLMTIRFDEC-
EIPSVDLGGGRFLDRTIHRLDLFGEHESAQQARLITALHR(VMQTSPGSPSGNVLAAVAASKRADTSLDHLRD
LIRNPNLVMNY)}*

$1003=236(202):

>gi|649504665|gb|KDS12940.1| {|0(280)|<Bacteroidetes(B)>}formylglycine-generating sulfatase enzyme family protein [Bacteroides ovatus str. 3725 D1 iv] >gi|649508693|gb|KDS16860.1| formylglycine-genera
{(M)-------------------LQLMLEESGF--RkrVSFDKDNL-
NGRFDvELIRRIDECKDFIMFMVPETFTtiRPLNEEavEtgekatwdmeevafyermasltyeefeteikqis
htgeiDFVRIELGRALhrrsrnpKQINIIPIAPQESEsyDFATlQLPPDISGLKDFQAVFYSNSRVARFKDIK
GDLLKQM(LSKPSYVSAKWLVMTFIALSLIVAGSKTYTSIQRTAEQKLEFKDCRTYDDYSSFIKKHPDS)}*

$1004=187(193):
>gi|738757512|ref|WP_036651029.1|
{|0(575)|<Bacteroidetes(B)>}MULTISPECIES: hypothetical protein [Bacteroidales] >gi|649530244|gb|KDS37694.1| tyrosine phosphatase C-terminal region family protein [Par
{(MIK)---AFISHSSKqkDFALE-LVNRLGRDY----
CKIDCYNFESAYKSINEIYRAIEESTIFVLLISRDSLGSTWVEEEIRFAKEKLSKTEYERFWPFIIDDS-
MAIDDCPEWMKKDECFNlRRFKSPYVLARDIEQKFRKIIWS(ANPKIRLLDTTMVGRNEDIAKFEDKFQSGRG
MKLRALIISGRDGVGKETF)}*

$1005=234(240):
>gi|648486459|ref|WP_026178210.1|
{|1031(0)|<Actinobacteria(B)>}hypothetical protein [Streptomyces hokutonensis]
{(VRTQKGTARVHVPRLLIENTLTLAVGRVGQVRERAREIELPETASREPLFFLSYARPESDAGEPAGSPEVR
FFNELCAYV)-----------------------RELTRHrgeDVG-FIDRD-
ITMGEDWreQVNEAMVRAKVFVPLYSPQYFASAFAGSGWDIFWARRRSDdGPSsgmpAVVPVLWTPTSRDQRP
LSAMLAEFPYEElvsgyaeHGLRGLMESGPEdaYRRAVRRIAHQ(IVAATRDSPGGDRDPHGRAK)}*

$1006=199(187):
>gi|648662623|ref|WP_026354370.1| {|0(2)|<Proteobacteria(B)>}TIR domain-containing protein [Massilia niastensis]
{(MAIRYGCFFSY)AHGRHELMK---
RFKSALADALRCYLEPYfdnEDELFVDTEQLGGGDDLdrKIARAMCESVCMILIYTPKYEAHPYTRREYAAMR
QIEAERsRwyalpSHLIIPVIMTRHPdslpRQITESSLYVDFSHFTMATADLKSNPDFLPDIDKMVKRIA(TH
YEYQKKTTPPGHDCNQFVLPDVPPEWRELPELAF)}*

$1007=204(214):
>gi|648605076|ref|WP_026296827.1|
{|0(433)|<Proteobacteria(B)>}hypothetical protein [Hirschia maritima]
{(MAEDLKSEGSVAGSN)---VFVSYSRQDMDFANQIVSMLEDEGYD-
PKIDRDDIAATEQWEARLRELISSSDTVVFVLSDAYLASEN-CAwEIKEAVARK-----
KRLIPLVPRPITgDVPLELSRLNYIYFFN--
LKAGDGTGFYNGFKSLQRALRH(DLERLRLLRRFEERAKEWKTGDGELLSGEQLVQAASWKTETEKQDSVPKD
VLEYIAASK)}*

$1008=201(215):
>gi|651292219|ref|WP_026424083.1|
{|0(201)|<Actinobacteria(B)>}hypothetical protein [Actinokineospora inagensis]
{(M)--QVFVSHAPSDRDVAAALRADLAQLGRQ-VALDRGEP-TGAHWWDGVLHRVRRCDVFILVTSPAAVR-
SPGCLaTLRYARALR-----R---PVLLVSAAatGLPPEAETAPLFEPSG--LDISQRIP--
KLRGALLALPPA(PPLPDPLPLDPAVPYLDPIRQRLDEPQLDPTELRELVRQLRLRLRDEGERAAVWGLLVRM
RARTDLPPPLAAEV)}*

$1009=231(240):

>gi|651325538|ref|WP_026449845.1| {|52(31)|<Bacteroidetes(B)>}TIR domain-containing protein [Aequorivita capsosiphonis]
{(PILLLFLTKNKSFETNIRGLKILSALFVSAIVGVIILDALIGLEDIQVIKTSTYFLIPLEILLVYLIWYKK
GDLPPLPPC)---VFISYNHNDVDVALKIKEAIEAAKID-
VIIDTENMEAGTDIKTFIQTSLKVATVTVSLISNSSLE-SAWVGmETVDTLFLQ-----
AYLDHKKFIACYldNDFFQNDYTIKKVTKI--
EIKIKELELLILQSHQKDINTR(DLDSKLIRTRNLKNNMDGII)}*

$1010=167(167):
>gi|648571456|ref|WP_026263207.1| {<Proteobacteria(B)>}hypothetical protein [Chitiniphilus shinanonensis]
{(MAIPQSELLRAGQRRPLLKSQARASLQ)-KTAFLCHSHRdDKLAEGLQ---ALLAEQGVelyID-
WKDASMplQPNRETAsRIQHRIKTCDWFLFLATANSMASRWC-PwELGYA--DGQK-QPDRIAIVQTSDS-
TTTHGNEYMQLYRRVDMssAGELLWVPAGSNQGQFVRTVPFL()}*

$1011=253(256):
>gi|917036231|ref|WP_051642943.1|
{|102(105)|<Bacteroidetes(B)>}hypothetical protein [Bacteroides uniformis] >gi|649542618|gb|KDS49485.1| hypothetical protein M094_1913 [Bacteroides uniformis str. 397
{(FNIFKSKQDKYEPTFFAGFECNTFNEFPRKLKNAIDKSIETKDTKRLANKLSEVFLSKNIIPIDKVNNILK
ETISRKLPQ)-AKVFISHSSKdKPFVHKLKEELS---
KDNSLKFWIDEnEILAGDDIQkTISESLKEMDYLLLVISENSIKSPWV-NfEVSQFMGIAEN-K--
RIIPIILSKG-
QSFSEPIDNLVRRLNYLdfSDKKNWRKNITSIKSALLKGTGI(DCQSKEIKINFNNTVDNAVIGNNNKIVINQ
TRKKVV)}*

$1012=172(181):
>gi|651292218|ref|WP_026424082.1|
{|0(248)|<Actinobacteria(B)>}hypothetical protein [Actinokineospora inagensis]
{(MH)---VFISYARRdQASIASLR---ADFERFGN-
RVWYDREAHGGQDWWdDVLSHVRGCVVFTFALSPDSLRSKACLAELRYAAALRRP-----ILPVLVRPV-
DRGAIPDGLPAPVDYVNrtEENVIRLRKALSALPAARPLPAR(LPQEPEIPTSYLNAYLARIDSPELDDRAQR
ELLVQLRDR)}*

$1013=215(198):
>gi|651325675|ref|WP_026449982.1|
{|0(288)|<Bacteroidetes(B)>}hypothetical protein [Aequorivita capsosiphonis]
{(MALIPGFEYD)---
IFISYVHAdNEsetfeedgWIDQFYKYLdtKLNKHSRNIKIWWDSKnLDRSEVFDnSIAEAIDKSAIMICLYS
RLYPQSEYC-KkELEHFYEKVAK-EKIgmnvgnrsRIIPVFMSNI-P-
HTEWLEKLSGTSGFPfhDDNEYGDPLKNTSPQFGEQMIQL(RNALVRIFDDFSKELPEIQEPLIVEEEKEKGF
TIFMSEVNDSLYDRRD)}*

$1014=231(254):
>gi|648547933|ref|WP_026239684.1|
{|308(435)|<Actinobacteria(B)>}hypothetical protein [Frankia discariae]
{(SFEVIHVPGGGGDERCHGQVQISLGGLPVARIPLELAVSTAADPSTTPVTATGSAPMFREIYGSYAQADLD
IARRFREIY)---------------------------RALGITLFLDVLDVDGTREWkpELEAAI-
DRADLFALFWSPGSAAAGYVEkEWRRALEVaANRRpRGAFVFPFQWSRP-
APEPPLPLGDLPFREVDPWSAALNRFANPDRAGAGAGTGT(VPGPTPVPSGQTGTGRPGGPSGSSAPGGEGTA
RR)}*

$1015=223(225):
>gi|648619096|ref|WP_026310847.1| {|82(0)|<Actinobacteria(B)>}TIR domain-containing protein [Frankia elaeagni]
{(FRALEAGIVAQPCRRLLAAALARYPANGVFRDLHDRFLAGRPGDPAPSMTGAGRTSPPPPLPPPASPASPA
PTAPPPHAP)--TVFLAHDG--EEQSK-
VRALAELLTAAGFTPWVGSEMVTGGSRRdlVVRRAVLDCDFFVPCLSARVLGRPSQLHrEIRIALDRADELpD
HvVFIVPARLDEC-DLPADLQEYQWVDLFA--AGGIDQLLRaLRASTPSARNSRS(GPSGR)}*

$1016=183(192):
>gi|652322187|ref|WP_026720006.1|
{|0(144)|<Cyanobacteria(B)>}transcriptional regulator [Fischerella sp. PCC 9431]
{(MIFH)---VFLAHNS-QDKPK--IRIIYKNLQKLGLNPWLDEKQIRPGQLYqdVIQQAIQQIQC-
VAVFIGTTGLGRWQALELhgfiSQCVERD-----IPVIPVLL---
PGVKCIPDNLIFLRQFHQVCFECEIEEDFEAFKTLYWGITG(EELEIDNTIFSQPNKNTLDPLNSHPGIQPPT
QTDDLLSEKGIDYTRLR)}*

$1017=175(180):
>gi|651428036|ref|WP_026528891.1| {|0(537)|<Firmicutes(B)>}hypothetical protein [Butyrivibrio sp. VCD2006]
{(MSIYEGNESY)---IFISYSHKdNK---KVMPVIKRLQKDGY-
RVWYDDGIEVGSEWPeTVANHLLSCELFLVFVSNSYLDSFNC-KrEIDFSVSKRKS-TIAVILEETNMSP-
GTEMQLSSVQFLNKYLLkdEEFYSKLYQSELIKPCQIEAEEN(LPDEKTESRNTTDSTNRQKKRLNFSSKALG
)}*

$1018=213(226):
>gi|651428323|ref|WP_026529044.1| {|0(87)|<Firmicutes(B)>}hypothetical protein [Butyrivibrio sp. VCD2006]
{(MKPYSGAGNY)---IFISYSHKdT---DRVYPVIDHLINMGY-
HIWYDEGIDPGTEWDeNIAGHLNGCVGILAFLSENYLNSENCKDELNYA---RDL-GKDRVL-IYLESV-
ELpsgMAMRLNRIQALHK---YKYSDMG--
DFFTELTKAPLFG(KNDISSHENHEEVISGDNTDILPANQDNKNEVNPDEGIITKKDFLKKEEYRTAGYCIFG
SAISIYVFAIVLMFFGG)}*

$1019=262(258):
>gi|652295107|ref|WP_026707454.1|
{|1010(0)|<Bacteroidetes(B)>}hypothetical protein [Flavobacterium frigidarium]
{(INKLPDDLYISLDNEYFVKGTMLNSLIDGFRISSMSTIAEPVQKSPETGENYIIRTLSKVHSKELPIYKFQ
NFTNQNLKK)MKKIFISYSKDdFSLVKQFQDHLSSLKRDSIISTWYCTELIAGGEWDkDITKHFEEADIVCFM
ISSNLMRTDYIHKyEITKAFEKKKK-DDSfKIVPIILDFC-SWSTTnnDLSKFTALPYTAkpVCDFDNKN--
MAWYIIVECLRFM(IASDQQPTGDDWFKNDELPNNIYKIYKRIVEGLVDKNS)}*

$1020=274(279):
>gi|652320269|ref|WP_026719097.1|
{|141(309)|<Cyanobacteria(B)>}hypothetical protein [Fischerella sp. PCC 9431]
{(EFAVGFYDGLGADRSYEDAYEFGCSAIDLEGIPESATPVLKSRNATITHQNLQSDTPLDSETVRNTIRLKK
PEQIFTQTK)--HIFISYKrdT--QPDESVALQVFQALSKQ-HQVFIDQTMLV-
GTPWaeRIETEIRQADFLIIFLSA-HSINSEMVEaELSLAHDLLKKQgHPT-ILPVRL-AY-
REPFQYPLSVYLNHI-
NWAFWQGTEDTPHLIAELMQAVEG(GELSIGEQQDKADLIKVNEPSSFPRPFPSAQPARLEMPEGTMDCESAF
YVERPSDAIAL)}*

$1021=212(224):
>gi|652324150|ref|WP_026721468.1| {|0(79)|<Cyanobacteria(B)>}molecular chaperone Tir [Fischerella sp. PCC 9431]
{(MTVSV)----FLCHnKEDKPFVKQLARDLE--
NHGVKYWLDDAEINVGDSLIEKIRNGLDEVDYVAVILSPNSIASPWVQREMDVAMNQEIRGKRIKVLPIMYR-
-NCELPGFLLGKFYADFTE----ESRYE-
DAFAKLVRSIGVV(FNKKALESNLTGATLSQALDKAVILNLSLFSKPFHRPFQYIGMSIASASTAVGQEPNEV
GNIIIDNDDCHMLLEAEGNF)}*

$1022=227(221):
>gi|652487415|ref|WP_026881981.1| {|0(90)|<Firmicutes(B)>}hypothetical protein [Clostridium akagii]
{(M)--QIFLSYCW-KDEeRAN-----YIELKcseNY-IKLLRDKYDLEDFNSIkkYMEQ-
VKTSDKCILVISEDYMKSENCMYEILQVLESENT-KD-KVIPIIYYKEIYNTSeiakwiKYWDNKYHELKEN-
LEKLPENSqeslfEklQMYKTISLKIGKL(ISIIVDIYSPEEDTIEKMINNLKVGQKEDSNWNPDLYWFKLDA
LDADTIEISDYAENPEGEKTYKLISLMIDRDIHGSHC)}*

$1023=280(271):
>gi|652495928|ref|WP_026890390.1| {|76(191)|<Firmicutes(B)>}hypothetical protein [[Clostridium] aerotolerans]
{(AHWGLVLSKFGIEYVEDPKTHERVPTCHRVHSDSILTDADYLSAIENAEDLHTKDLYELEAKRIAEIQKGI
LVISAQEEP)-YDVFICYKetsegG-
TRTKDSaLAQDIYYELTEEGYKVFFARItlEDKLGQLYepYIFAALNSAKVMVVIGTKQeYFNAVWVKNEWSR
YLSLIKKDRRKLLIPCYSDIDVYDLPEELSMFQSQDMTKIGFIQDLIRGIKKVLSANEDKTD(SRLQSNSEIS
YLTPGVTQLLERTSLFLEDGNFQSAQEYCDRILDLEPRNAY)}*

$1024=265(255):
>gi|652441063|ref|WP_026836071.1| {|74(621)|<Firmicutes(B)>}hypothetical protein [Eubacterium xylanophilum]
{(AEAYWGLLLCKYGIEYVDDPVSGAKVPTCHRSSFDSIMEDSDFEMVMENADNISRNLYREQAKQIEEIRKG
IIEVSGKEE)PYDIFICYKE-
TDengdrtVDSVMAQNVYDMLTEKGYRVFFSRISLedKLGWEYepYIFAALTSAKI-
MLVFGTSYdnYNAVWVKNEwsryLKLMVKDKE----
KHLIPCFKGTDAYDIPNEFKHLQAQDMGKVGADQDLLRGIDKILKLNGAKSG(DINVEVSQALSANAGSNINS
LLERGFITLEDGEFD)}*

$1025=223(224):
>gi|652385278|ref|WP_026781206.1| {|131(0)|<Proteobacteria(B)>}TIR domain-containing protein [Polaromonas sp. EUR3 1.2.1]
{(REAGIDYVRDLNGFVDISDSEATNRSFAQLGFAPGFAAAGIFPAGSYEDDAKVAKATAGLEIAKLIQNWAE
KKRHNMENS)--RIFLSHKGANKPLIEKIDRALRLLNLK-
TWFDRDDLVAGDTLVRGVDNAFAGCAATVFFISGEYVDAGVIGNeIDRALHEAA-----
MRGGEFRIIPLvlaqhggSddRVPAPFKTLVWKTVDD--ADiVPTILK--ALPATVQGQVRY(AAPK)}*

$1026=229(228):
>gi|652398668|ref|WP_026794472.1|
{|26(301)|<Cyanobacteria(B)>}MULTISPECIES: hypothetical protein
[Planktothrix]
{(REVFIGSWEGKSYHEIYPLNPSYIEKYVGYKLWQKISQTFGEKVTKKTIKGAVERYQNQDNFVQLSQISGD
LLQLSPHCY)--RVFISHgnTELNINFAQQLEAILIQVGHS-
VFLAAWEVtrsqinQQETDFLAEIDTELKHCDYFILLLSPQSTLSEMVIEeLRQVRELQD-----
THSHRLPIIIPIwvNCPANLALNHDLRSYL--LGTVQRTWTGQQDTALIIQEIL(CLLQDSIP)}*

$1027=217(221):
>gi|652549797|ref|WP_026943447.1|
{|0(79)|<Proteobacteria(B)>}hypothetical protein [Helicobacter rodentium]
{(MI)-
KKIFYSYSHQdEKYLSELQKYLKPAIEDYQIEVFIDKDnIRCGEPLNeKIMQNLSNSDLIISLISANYLASEY
CLKEMSMALDNNKS------FPVILSHC-DWKNNELLKsIKVLPKDGkpIIDYTNSS--
EAYQFIASEFRQK(IQKDSNIIALKEDFKSKLEKINFLHPNKSKISLSDTFVAPNIKNSSDEKNKENIFKIEE
ILKNENYKEVLFFSPFYSGK)}*

$1028=162(171):
>gi|652743942|ref|WP_027067139.1| {<Bacteroidetes(B)>}hypothetical protein [Maribacter sp. Hel_I_7]
{(MGKEKGNN)---VFISHYGKDDDKV-QRLKERFAEKGY--
KIRnysVDSTKHKDGRIPqKkvleryLRRQISWSGSLICLIGPNTHKR------
DWVNYEIEEAHkQGKKIVGIYTHGNN-DSAELPDAFK---KYGGatIGWNS------
LDKLIDIINGD(VLPTETPSGSERKPLYKMATINC)}*

$1029=229(225):
>gi|652997159|ref|WP_027249626.1|
{|195(0)|<Cyanobacteria(B)>}hypothetical protein [Planktothrix agardhii]
{(STNSVFTKHLIAGLKGGITSNDGLIRIFDIFEYLQPKVTADQPNQHPIFKSDIEENFPLTLYLGGQKGVSP
ISPSVQEEF)RYDVYISYVDEdpdsTWVWDVLVPKLEAENL--
KVAVSGDVDLLGVARVINIERGVKFSKRTLVILSNLYLDNNMAEFENTLAQNLGIEEGKARIVCVEKEAVDaI
KLPERLKLGSMAKLNHpRRAEREFQKLIEQLKQPLPHRSA(SGLYE)}*

$1030=273(263):
>gi|652822721|ref|WP_027110902.1| {|74(511)|<Firmicutes(B)>}hypothetical protein [Lachnospiraceae bacterium NC2008]
{(AEAYWSLVLCRYGIEYVEDTLSNRRIPTVNRAQYTSIYADEDYKSAINYASDEQKIIYEQEAKAIDEIQKG
ILAISEKEE)PFDVFICYKE-
TDEngRRTqdsvLANDLYHQLTQEGFKVFFSRITLedKLGQEYepYIFAALNSAKVMVVLGTKQeYFNAVWVK
NEwsrfLALIRSgAQ-----
KTLVPAYKDMDPYDLPEDFSHLQAQDMSKLGFMQDLIRGIKKVIGSEKATEK(ETIVVQQGTTTGNVEALLKR
GYMALEDGAWDEADRFFEEALNY)}*

$1031=164(169):
>gi|652820547|ref|WP_027109462.1| {|0(220)|<Firmicutes(B)>}hypothetical protein [Lachnospiraceae bacterium NC2008]
{(MKPCDKNNNY)---IFVSYAHRdSEK----
VVHIINLLSNHGYNIWYDDGIDPGTEWDeNIARHIVDCSYFIAFVSKNYLESKNCKDELNYARDCEKK-
QLLVYLEEVELPI-
GMAMRMNRLQAIWWCKYedESAALDKLLGAKDIDIASIGTSS(YKRTGSDKESAFIANEAKS)}*

$1032=162(166):
>gi|652904365|ref|WP_027158598.1| {<Proteobacteria(B)>}TIR domain-containing protein [Methylobacter luteus]
{(MAKLIQNWAQARRRDVENSS)---IFLSH-K--
GINKPLVEKIDRALRLLNLKTWFDRDDLAASDTLvrGIDNAFAGCAAAVFFI-
SGEYVDAGVIRkEIDRAIHEAAMRsDSFRVIPLVLAQH-
GGSDAHVPAPLQTLVWKTIDDVEIVPTILRALPATGQGLI(QYAPPK)}*

$1033=193(199):

>gi|652402657|ref|WP_026798448.1|
{|0(672)|<Cyanobacteria(B)>}MULTISPECIES: hypothetical protein
[Planktothrix]
{(MPEPTT)-YDLFISYVEAdeAWVKGYLLDALTDAGV--K-
SLSEAAFRLGVPRIQEFQRAIEQSRRTLLVISPAYVADDFNQFVELLGQSYGQDTQTWPVIPLVLEPTP--
LP---
LRLKVLVGLDaTTPEDQDVAVARLCADLERSRAS(SSIPKPPCPYPGMMPFREADQERFFGREQEIDELVERL
RSHPFVTVIGPSGSG)}*

$1034=273(263):
>gi|653214721|ref|WP_027427360.1| {|73(437)|<Firmicutes(B)>}hypothetical
protein [Lachnospiraceae bacterium AD3010]
{(AEIYWDLVLCKYGVTYAKDPQTGRYIPTCNRTHTDSVLKDENYLRALELSVGDKAELYKRDAGIIENIQKG
ILEVSKREK)PFDVFISYKE-
TDangnrTKDSIeAQKLYEKLTELGYKVFFSRITLesKIGEEYepYIYAALSSSKVMITVCSSSENiQSAWVK
NEWSRYLTLRRKDTSKTLIPVYFDMDESGLPEEFALLSAQNMGAEDFEQELIRGIKKLIPTPIILKE(RRKKT
RKTLMIAGLVAIVVGIPLVLLITIPARKQAENEKKYTK)}*

$1035=273(263):
>gi|653214723|ref|WP_027427362.1| {|74(20)|<Firmicutes(B)>}hypothetical
protein [Lachnospiraceae bacterium AD3010]
{(AEVYWDLVLCRYGIEYVEDPRTKKRIPTVNRTQLQSVFDDHNYKLAIKYTSPEQCDVIESEARIIDNIQKN
ILDISNKEE)PYDIFICYKE-
TDDygKRTvdsvIAQEIYNELTREGYKVFFSRISLedKLGSEYepYIFAALNSAKIMIVVGTKvEYFNAAWVK
NEWSRyigLINSGEN---
KTLIPAYKDMDPYDLPDEFSYMQALDMGKLGSMQDLIRGIYKIINPVIQSSQ(YLRSNIDVAALKKRIEVFID
NGDFANAKSYCDKVLDLIPEDSN)}*

$1036=214(220):
>gi|646470884|ref|WP_025538704.1|
{|311(0)|<Proteobacteria(B)>}hypothetical protein [Vibrio
parahaemolyticus]
{(KDLIEAELTYLKNRYHAVDVNKSNLTGDIFKRAIYGRLSFIKMVKGQEHGPYLNLCRQALELDNNPPKSIA
KLKGVFEMF)--DVFICHAS-ED-
KEAIALPLYDELNTLGVKTFIDKVEIQWGDSIvgKINSALQKSKFVVAIISESSIGKAWPLKELhaVLGMEIG
-----S-GDTKLLPLIVGNEEELLMQLPLIKDKAYLPYTNNCSEIAKILKDKLDTSN()}*

$1037=174(181):
>gi|652325207|ref|WP_026722525.1| {|0(60)|<Cyanobacteria(B)>}TIR domain-
containing protein [Fischerella sp. PCC 9431]
{(MNEQFDF)--DVFLCHNS-KD--KPLIIKIADGLEQLELTPWIDRRNIDPGEEFqtAIEQAIPRTK-
SAAIFFGLHGVGQWQKEEINALYREKVN---
RKIILVPILLPEVNEIPSEFVFLQGHNYLKLETQEINEQFLNDLVKGVTRQN(IELPNTTTSEPNLNQAITND
QLFKLISTKMKLDE)}*

$1038=273(263):
>gi|653228152|ref|WP_027440651.1| {|74(150)|<Firmicutes(B)>}hypothetical
protein [Lachnospiraceae bacterium AC2031]
{(SEAYWGKCLCKYGIEYVDDTATGKKIPTCHRTIYKSIFDDPDYERALINADPVAEKQYHQEAKEIDNIQKR
ILEVASKED)PYDVFICYKE-
TDEKgdrtadSVYAQKIYDALTEKGLKVFFSRITLenKLGVDYepYIFSALQSSKVMLVVSTKyDYINSVWVK
NEwkrfIKLSEENKE----
KTLVPCYADMDPSDLPKEFKMLQGQDLTKIGAEQDVVRGVIKILEKTETTNV(PADSLKKYVDQSNKKLVKGM
IIGFIASLIVIAGSLFGIKKIRD)}*

$1039=225(227):
>gi|648535000|ref|WP_026226751.1|
{|251(0)|<Proteobacteria(B)>}hypothetical protein [Hoeflea sp. 108]
{(ARLRMKLHSIKRFGATEAAKVWLGPGGDKQKMWSHVAGNLAFIRQVRGMTDPVLAKLCKDAVIAGMTGPSWVLQMAEMVR)EFDVFLSHAS---EDKEKIRRLKDKLEECGVKVFFD--ETSIKWGDsivEKINNGLL-KSSyfIPFLSKTFSEKGWTNKELNSAISMNVSRKG-RILPIIDSNFSveE-NYPLLGETLYKKWPA-DESSEDAFIGEVADAILHLIEK(KVTSVAT)}*

$1040=297(288):
>gi|653177561|ref|WP_027418701.1|
{|104(0)|<Bacteroidetes(B)>}hypothetical protein [Crocinitomix catalasitica]
{(TKVYPVAISKNAYGFSKLLGQLNFLRADKIEEKREFLTHNENNLFRYIRLNITHELCRLMLNMKKAVDEQESFEHLPPPI)--KLFISHSkHDdSKYeANKFKDFI-----NSETQLKTFFDANDISFGSnfgdEIKRAIENSALVAFQ-SDSYAEREWCRIEVLTA--KKHG-CPVVIVNAIQNGEKRTFPymGNYPSIRLKDNYEDIicltleqvLYNkytrmLLDE--ITTLYGLRADSI(LSTTPELYSFLQLKDRKFEDNNDSAFKLIVYPDPPLGTEETELLLKMDNTYHFVTPTTIPSLILKKDD)}*

$1041=195(189):
>gi|652934595|ref|WP_027188235.1| {|0(212)|<Proteobacteria(B)>}TIR domain-containing protein [Desulfovibrio cuneatus]
{(M)---IFVSHARKdKPVTNPFVDKILRLGfeIRAEHIFYTSHPDTGISGGDyingSIFRKLLQSKLIVLFLSKNYFDSPYCLCEMGAARLLkeqgKAD-VFIFLFPGLSyKDMQGVFTENMVVGLDKSGTD--SFINMASSVCSAIISDRPAAY(TREADEFLKKLSSALLEIAPPKTVSIEEFSKLQTEQAALVAKLSQTGE)}*

$1042=218(220):
>gi|647556543|ref|WP_025837644.1|
{|153(0)|<Bacteroidetes(B)>}hypothetical protein [Porphyromonas cangingivalis] >gi|1152083862|emb|SJZ53096.1| TIR domain-containing protein [Porphyromonas cangingival
{(EYRKKEGVSTSRVVWYRELFSLLADEDIKIFISKLSEKVNASTNNKKRSDVCVDTTLFDNVEKISKPQEEKSELKVVPMI)---VFISYAWDENKEAvQRIAKHLKDANIE-VRIDE-DVPYGTDLVRFMNKEIRDCTKCLVFLTPKYKEK----AeLSTGGVAHE-----GRVisraiyqdqdttkfIPILLSGSFekCCPDFLIARKGFDFVN--HPFDEEIN--RLVRAL--TLQP()}*

$1043=159(164):
>gi|653204758|ref|WP_027424744.1| {|0(147)|<Firmicutes(B)>}hypothetical protein [Lachnospiraceae bacterium AC3007]
{(MDRDF)---IFISYSHKdS---DRILPLISRLENDG-IHVWYDEGIDPGTEWDeNIASHLKGCSGIIAFLSENYLASENCRDELNYARDLGRE-RLLVYLEDVELPA-GMAMRLNRLQAIHKYRYkaEDDFFAELIKAPMLQDFKAASAA(ALSAAAPTPAAPAPAAPAP)}*

$1044=175(183):
>gi|653223048|ref|WP_027435590.1| {|0(737)|<Firmicutes(B)>}hypothetical protein [Lachnospiraceae bacterium AB2028]
{(MAEINRKSKHD)---VFISYSSKnKNVADAIVADLES----HSIRCWYApRGIMPGQEWAtAISDAMAEASVFVLIYTEDSNKSKQVMNEVAMAFKL-----GLTIVPFKLTDD-LMNPELEYYLSRVHWLDalTPPLAESIESLRNHVAVILQKTD(EDALAEDTPENIREDISEIVRNAKTGDRATSA)}*

$1045=177(179):
>gi|643376740|ref|WP_025205136.1| {|0(121)|<Firmicutes(B)>}hypothetical protein [Dehalobacter restrictus]
{(M)--SVFISYSHKdSDFVDQLANAL--IERN--
IYFWMDRwELNVGDSLTdKIQTALTEASFLCVVLSKYSVDSDWC-KrEINAGLIRElEE-
KRVVILPILTEDC-
QIPLFLRDKLYADFRNDftTGLNALISALSKEINSNLGRVGT(PQYLTDWGLDWGLLTPDDSFVLYIDSISFA
KDQAWSIL)}*

$1046=169(165):
>gi|651291862|ref|WP_026423733.1|
{|0(31)|<Actinobacteria(B)>}hypothetical protein [Actinokineospora inagensis]
{()MPRVFLSYTHDsPEHRDEVRYFAERLVESGVdvcLDQWH---
LGGRQDWYvWALAEIPAADFVLVVGSPRYrIAGDGQAAaEDNRGLQAEAA-ILRDLLQHDRGVW-
TGKILPVLLPGHEPDEIplFLQPLGADHYRVGYDAEGMEDLL(RVLLGQPPYIRPELGRRPVLPARST)}*

$1047=205(218):
>gi|647484082|ref|WP_025804109.1| {|0(125)|<Proteobacteria(B)>}TIR domain-containing protein [Pseudomonas chlororaphis]
{(M)--KVFISWSGKrSKALAIALKDWLPLILQYA-
KPWvSDKDISGGDRWAqAVSSELETSNFGILCITPENLTSEWILFEAGAL--SKSM-LDAKVIPLLwgLELS-
DLSG-PLAQFQALKVD--------Q--
NGMLNVAKAINAV(AENKAADTTVEQLVPALWPQLQQKLDVIPDKEASDKHMRPQTEILEDLVSQVRGLGARM
RDFDPEFLDRDMRNYNMR)}*

$1048=205(201):
>gi|648272521|ref|WP_026053670.1|
{|0(527)|<Spirochaetes(B)>}hypothetical protein [Leptospira santarosai]
{(MHPSKI)-ASLFICYSSDqIDWARKIGNKVNTA-
YDGKISAWWDERKSTGDYWDsIIKQKLSEADSFLLILSPGFFKSEYIQNiELPIIIRRHES-
EKIRISPIIAEQI-
SFEqfnESWLNEIDVFPHRGfrTINQLLPHEINNWIKNLSIEPVW(RRIEKEFKQISIRTLGRPFQYDLRVPN
GSDYIIERNEIKEIEDWNLNANDQKIAF)}*

$1049=172(170):
>gi|653455948|ref|WP_027541754.1| {|0(340)|<Proteobacteria(B)>}TIR domain-containing protein [Bradyrhizobium sp. URHA0002]
{(MENLSYQKPFV)----FISYA-SedhaiaTVFEDALNAVSVELDL--
GLEVFRDVHSLERGLSLtdQIMAALERTDFLLIIYTEQLKKSHsFTGAEIGAFEMSIRRDvkeNKIRERRAIT
V---YLDEMPAVEGGVLGIKLDTSALSEEDLERQARALDPGQRL(AEFLRDVAYEALTRLFDLA)}*

$1050=219(220):
>gi|648525515|ref|WP_026217266.1| {|12(0)|<unknown(X)>}hypothetical protein [Streptococcus ovis]
{(KDIIDNEDTDNYWRNRFEKLNRKDDTILRGCFKELKEAGLISVTWADNMPYDLQVLKDGYSYERTKEMREV
NMSYKTTST)-YDVFLSHAN-KD-
KLDFVDELNRSLEKLGVNIFYDKNSLEWGDNWkqRILDGVKKATFAIIVISENFFDREWTEKELNEFLTRQTS
EGQKLILPIIHNISHDDLHDKYPEVGDIQVISSYSYSSDEIALLFAKQLIKRLKG()}*

$1051=166(175):
>gi|651612047|ref|WP_026606178.1| {<unknown(X)>}hypothetical protein [Methylocapsa acidiphila]

{(MGGSYGGGLGSLDIQSLSEKVKQKLTDAKSDASRH)---
VFISFNHEDLDEVnllrgqAKNDKVDLQFDDH--SVKEPFDSANADYIKrQIRDKIDRCSVTVVYLSEKT---
---ASSKWVNWEIEESIkRGKGVIGVYKGDK--APTNVPPAFQQ-NGCNSVKWEH---
AALMKAIEDASTKR()}*

$1052=224(222):
>gi|653554293|ref|WP_027582879.1|
{|0(90)|<Proteobacteria(B)>}hypothetical protein [Bradyrhizobium sp. Ai1a-2]
{(MIEEF)EYDVAFSFCA---IDEGVATQLNDRL-
ASRLKTFIYSERQREIAGTdgqESFSKVYGKTARlvVVLYRPEWGETPWTRIERDAIKNRSLDDGWDFTTFVP
TVDRPqmppW-LPKTRLYVGLERWGI-EAAAAVIE-
SRATERGSNPREE(SVAERAARLRRARELKDKQDQFQSSEHGVRSAIQAYEDFSRAIEQHAEQISGAGIKITA
QRSQEFRILTGLPPCNAI)}*

$1053=188(195):
>gi|654252930|ref|WP_027768395.1| {<Actinobacteria(B)>}TIR domain-containing protein [Streptomyces sp. CNQ865]
{(MGMAEFVLTAVGTLAGVVGTYFAHVSVRGRAARRRPPPAQVTAPEPERAAAGGAA)AYDVYVSHSPADADW
ARTFAGRLRAYGLR-VADDEVVRAPGGVRVHAVEQAIRDSAHGLLVFGAR-SADDGWALqEY-AALMER----
-SIESGRLFIPVLagDavLPEFARTRFSADFRD--VDDDAYTR--RLEQIVRALQHG()}*

$1054=249(256):
>gi|654237286|ref|WP_027752997.1|
{|81(153)|<Actinobacteria(B)>}peptidase [Streptomyces sp. CNH099]
{(STRRGLRLIPVLHSAVDIRELPFAATRLWVDFRDVTGAAFHDKVGELARVILGEEATGNGEAEALDLRDLP
ARSPDPPAE)-PSFVVCYARQeRDYARRL---LSTLSRAGL-
PVWSIGHLAWGDDVLrTIRTQIRHALGIIVLMSPEAESSEDVTReILEG--QRHSR-P---FFPILVRGD-
RHFLLGSTWHFDARTGSlpGHVELDLLRRLLDAGAPSAATDR(PAAGRTGVSAAVPPVPAAVRSAPPAPAAGL
PPGAPA)}*

$1055=182(197):
>gi|653575851|ref|WP_027589530.1| {|0(277)|<Proteobacteria(B)>}tRNA-ribosyltransferase [Pseudomonas sp. RL]
{(MAD)---VYIIYAK--ENRDTAVK--VHDLLSKSWSVWID-DRIVG--
DFatAIKENLKKAKCVVALYSEFAS--KPAVTdELRFATNYGKK-----IIPLQLDDS-
DPPYSFGNLSTVNFQHWSGEPDHEVFKLLQIKIQEVVPAM(RPAERLSSLESNTLPIPSIFMSVSSHETQFKP
AEALSVLKAHGTKSILVSAYDL)}*

$1056=150(148):
>gi|654626504|ref|WP_028090742.1| {<Cyanobacteria(B)>}hypothetical protein [Dolichospermum circinale]
{(MSKS)-YDAFMSHAS-ED-
KEQFVDPLFHALQTRRCRIWYDRFQIDYGDFFetRIYEGIHNSRFALFVITPNLirrPTDSWVWREFSRILEK
EEILRTNVLIPVFYDVTIQEFQQYIGNNPTLNTITERHSFKTLVDGGIYGVADKLQNL(FERR)}*

$1057=149(148):
>gi|654516874|ref|WP_027985620.1|
{|0(600)|<Proteobacteria(B)>}hypothetical protein [delta proteobacterium PSCGC 5451]
{(MSKP)TYDLFVSYNSNDVKP-AEALHKRLVTGGF--
SVWFDKARLNPGcKWHdEIEKGCEASRIVLPLLTPSWRKSEWTKFetYGAEAVIPCHYEGAWDEVATPPLMRY
QSMTFDCKQPEKADWPKFYKDIRKLLSRPPPKKAARITHL(RYRA)}*

$1058=200(188):
>gi|654643292|ref|WP_028104446.1| {|0(1)|<Proteobacteria(B)>}TIR domain-containing protein [Pseudoduganella violaceinigra]
{(MSIRYGCFFSY)AHGQHAYMS---
RFKDDLVDALKCYLEPHfdnETELFVDSEQLGGGDDIdrRIAMAMCQSVSMIMIYTPKYEAHPYTRREFAAMQ
MIEAERgawyelPSHLIIPVIMTRHPlclpRQISEPGMYVDFSRYTLATGDLKTNPDFLPDIRRIVDRIT(MH
YHCLKASIPPGHDCREFALPPVPPEWRPPAPPHFP)}*

$1059=182(184):
>gi|654647325|ref|WP_028108377.1| {|0(106)|<Proteobacteria(B)>}TIR domain-containing protein [Ferrimonas futtsuensis]
{(M)--RLFLSYSSRDFPLASAIVQGLGALGHS-
VWFAPEQIGASRCFATEISAAMKEAEVLLLLASrhsigDAAHAGSAEVKtEIKMAREKN-----
IPVVPLKVDASLdcGGAEGFDYLLKNFQWL--
NLEPMLAMEDNDAIAKLIDAAL(VSGVSADTQLFDQQLTEVEKLLRDKHWQKAKALLEALVMPADL)}*

$1060=126(146):
>gi|654580444|ref|WP_028047574.1| {<Actinobacteria(B)>}TIR domain-containing protein [Cellulomonas sp. URHE0023]
{(MIELVL)---VFISHAR---GDRAVVEALRDRVRAAPGAAWSPAEALKESPEWrgEFASLIRGCQA-
LLVVVSQHSRDSEHCRwELEQALE---------------AEV-
PIWAWFRQPVPWHPALSSASTLRVSPEVAPETWGRGALSR()}*

$1061=227(225):
>gi|654897548|ref|WP_028349091.1|
{|0(91)|<Proteobacteria(B)>}hypothetical protein [Bradyrhizobium elkanii]
{(MGTTMEEF)EYDVAFSFCA---IDEGVATQLNDLLSPR-
LRTFIYSERQREIAGTdgqESFSQVYGKTARlvVVLFRPEWGETPWTRVERDAIKNRSLDDGWDFTTFVPTVD
RPqmppW-LPKTRLYVGLQRWGI-EGAAAAIE-
SRATERGSNPHEE(TVAERADRLRRSRDLKEKQRLFQSTEVGVKSAIQAYEDFSRAIEQHSEAISNSGIGIAT
KRSQEFRILSGLSPCNAI)}*

$1062=214(220):
>gi|654925096|ref|WP_028375595.1|
{|0(109)|<Bacteroidetes(B)>}hypothetical protein [Leeuwenhoekiella sp. Hel_I_48]
{(MA)--RIYILHSREeDNFVRSLMKNLTDLG---HDLILSDFE-----
VGHdinlSLKNAMQSADIQVAIISDNAISSSYFMNEMmqlrnySVHTNLRKL-
FIPIFYPNVSFNELPSSIRNIQGIMLNGTTE--KDS--KT--
VAREMDIAINSF(YGKQIAIEEEEKEVKDKIESTAPEYIQQTLSDLKSKESNMKMYAVVWYTIGFIALLAGVA
ATIWLSDSGLDNFGEKEN)}*

$1063=230(220):
>gi|654799827|ref|WP_028253736.1| {|229(0)|<Proteobacteria(B)>}TIR domain-containing protein [Variovorax sp. URHB0020]
{(INKDRLSEKARREWLIEPVQAGDGLTSFVRIFSSGTEFHSGIPPAAFVAELHRRWLLERQADAAVPVQGET
WIPPTGSLF)----FISYSRLtDMAKAEAMV--
RSLMDLGVAEneIWFDRRaIEPGEEFGqEILEGIRNCRYFLPLLSEAAMAREeaFVFReWRAANYRRQSM-
QRRFVIPVIVDEdyepsryiassavV-DWNGLDFG-----FAPGgsPDEKMQSALRSLVRSARNPVGRP()}*

$1064=161(163):
>gi|654799893|ref|WP_028253802.1| {|0(335)|<Proteobacteria(B)>}TIR domain-containing protein [Variovorax sp. URHB0020]

{(MADGQPVDDKIR)---
VFISYASAdREIAQVLYDELTEIDRNRVECFFDVRTIESGAEWKqILKRELRQAEWLICIYTGE--
QSEFCGFeVGYFADANDSR-VAQDARVVCLHDV-
ADLPGIYSGYQSRRIISpsESPPGAPLPDESTFYKGAPLAKF(LNDFCKYRDLY)}*

$1065=211(193):
>gi|654844131|ref|WP_028296734.1| {|0(287)|<Bacteroidetes(B)>}TIR domain-containing protein [Olivibacter sitiensis]
{(MTFK)-TDLFISYAHIdDEslivnqkgWISEFHRALDIrlaqlLGRRPVI--
WRDPALQGNHLFDqQILDQFHEVAIMISILTPRYVKSEWCLREVDEFYEKCQqnigfniN-
NRARIFKIIKTPV-RIEQHP-
EKIQNVLGYEfyHVDPSTGRIKEYNQAFGQQSEMA(YWEKLDDLAHDICAFLESLSEPIAVSTSSQPNALKST
VSQKTKVFLAES)}*

$1066=227(226):
>gi|654915890|ref|WP_028366767.1| {|228(0)|<Proteobacteria(B)>}molecular chaperone Tir [Burkholderia sp. UYPR1.413]
{(AKNERLSARRETVEYLVDPQADRDPGLKAFLADFSPATRVVALDPTVFCAELRSRWEQRHPAGVASAASAP
AVQRAVDGA)---VFISYSS---
QDAAAVHGIAQALDAAGIDVWFDRSQLQGGDDWerQIRRGLEASSLFIPVISRatqDPARRRDFFWrEWMAAD
DRAAgMApDEPFLVPVVIDDT-PAYGGTVPDRFRRAQWMTLPGAQTTDAFISHVRGLYRQIS(SRSCRS)}*

$1067=222(221):
>gi|654893871|ref|WP_028345424.1|
{|0(309)|<Proteobacteria(B)>}hypothetical protein [Bradyrhizobium elkanii]
{(MSEFN)-
YDAFISYRRSdgiavaRWLRRTLVGFRLPAALRGAyakkLRVYLDTAYERGTSDFyqqSIKPALLSSRYLIVV
ATPDAMRRPggtedWIEREIRDFTAGPAAGnviVA------------RGAGDFDGP----
LPGGLKEAFPNIE--I-
VDLRGAGPLW(FLSFSRVVRLSAEKLKIVAPLLNIPPEEMPRLRQEEERRQQTQLGAVAGATLGTLVAVGSLS
VLALINQYKAIRAN)}*

$1068=232(233):
>gi|655077061|ref|WP_028525194.1|
{|96(59)|<Bacteroidetes(B)>}hypothetical protein [Runella limosa]
{(VYENKETDRKDMHRFIPVAISNAAYKISNSIRRENFINLYEYEKLEEQKDELIIRVTHELCRLIYSKPRIS
DIPTSYAGK)KLSLFLSYAR-FDGK-TITENIDNYFNQKtSLNTFFDKKDIPYGYRFdtVIDENLKESTL-
LVILTDAYASREWCRWEVLKAKKYSRP-
FVIVNAIKEKEDRSFPYLGNGPVCMYKQESIKIIFHTILFETLRFEYAKRLIQN(QIEAFDIEKKGLK)}*

$1069=202(205):
>gi|655058337|ref|WP_028506686.1| {|0(184)|<Firmicutes(B)>}hypothetical protein [Ruminococcaceae bacterium AB4001]
{()-MAVFISYRRGGSSeETAKTIYAELS-GEY--
EVFLDKESLGGGRFApEIERQIRECDDYLLIIEKTTFDRC---
NepGDWILHEAGIVReYEKNIIPVFVDGNSY-
KDKAPDELRFVYEYNGIKWTEEDPVVRLKRLLVSCRKY(DLYVDYDHHWFTLTEGSVEVLKEICRKYTEHYRV
KTPVNVHFNNIEGIVAGEMMTPDKAENKEQF)}*

$1070=203(192):
>gi|655059167|ref|WP_028507509.1| {|0(442)|<Firmicutes(B)>}hypothetical protein [Ruminococcaceae bacterium AB4001]

{(MEHNWR)---IFISYRGGTeGMAFAKRLYEYLREDPlyesvyG--
DVYFSEESAELGhDFIkDIPDIMKNVKYFVMPLTQGyfddFWDEANDKPdsYSVTYHEIVEALkVGCRFIRIV
FPGFKRDRDFLRKVFGNEFTAIDQSVIRDYTPESENDIMAQISNT(IIKKDHDVRGMSDIYSGITPNVFLSKK
RETEDTKMFPFYELLFDVH)}*

$1071=273(263):
>gi|655056551|ref|WP_028504931.1| {|73(268)|<Firmicutes(B)>}hypothetical
protein [Ruminococcus sp. FC2018]
{(AELYWSIVLCRYGIEYVDDPLTKKKIPTCHRTQYKSILEDADYLEALSHADSVQKEVYKQEAEYIDKVQKG
ILEISNKEK)PFDVFICYKE-
TDDmgRRTpdsvLAQDLYYGLVKEGFKVFFSRITLesKLGTEYepYIFAALNSAKVMVVVGTkPEYFNAVWVR
NEwsryLMLMQEDR-----
SRtLIPAYKDMDPYDLPDALSMFQAQDMSKLGFMQDLIRGIGKIANDQPVKSV(SAPAEASKPTNSNIENLLK
RGALALEDGDWSAATDFYEQVLNE)}*

$1072=213(213):
>gi|655267252|ref|WP_028677326.1|
{|0(168)|<Actinobacteria(B)>}hypothetical protein [Salinispora arenicola]
{(MSWTDPIGFGGRETYFFLSYAHSVPLSAAARPDTDYW)--------------
VTRFFQDLNAAVgRGAgnsDLDIgFFDGL-
VSPGADLrqVLTDALSLAHVFVPLYSPNYFSNAWAVGERAAFRSRLSRLqLGEAgrhVLPVLWIPFPpwdyRP
ELAEALDLLGGPVEYAENGLRALCKISAYRRQYDMVLS(ALAERIIAVAEGTPLGRSRAALVGSAAVDDPGDP
AL)}*

$1073=222(219):
>gi|655123840|ref|WP_028570955.1| {|0(69)|<Proteobacteria(B)>}TIR
domain-containing protein [Desulfonatronum lacustre]
{(MNHEIE)KPKLFISHATSdGEFANAVKAEIEKVFAKGVSVFCTSSPGS-
IAVGTdwltDIEQKLNKAQAVIAIVTPVSIERPWLWFELGATWSNgrsgDCH-
IYPLCVSEIDLNTLPPPLNRLQALSLGRAAD--LKMLFEA--
LIDQFGFGKISS(FRSSNITSRIPKYAKVEVKDVDLNERTLYSGKYSGYSNEELMEIIDTQLFHPDYDQERKY
GSIDTGREELVQQ)}*

$1074=206(218):
>gi|655242200|ref|WP_028653471.1|
{|0(162)|<Actinobacteria(B)>}hypothetical protein [Nocardioides
halotolerans]
{(MS)--RVFVSCSYRDRAVGAEVAGLVRALGHE-PA-
DDRDDAHGTAWWNEVVGRIEASDAFIAVASPAYADAHACRLaARHAAATGL-----P-
VVRVDLDERAvaDCHPVVASAVGVPFAP--EDPATAV---
RLGQALDGSPLE(DPPPSTGAARAPSAVDTAFTAVDTGVGAVTLLGAVGLVLVGLSILGHPEAATDQHHGTGA
RSEATRSADALLAEVR)}*

$1075=293(299):
>gi|655058810|ref|WP_028507155.1| {|97(378)|<Firmicutes(B)>}TIR domain-
containing protein [Ruminococcaceae bacterium AB4001]
{(TVMLPVALDSKVRILPVRTVGAGNIGSVFSKICGNLHLLDRNSPDYQSRLKEYYDEHVDPYYLWGISKEQN
ELREHLFRC)--
KAFISYRKKdIAQLRRLIDFIREIPELRDMAIFYDDSLIPGEFYDkRLSGEISEEDIVIFVVTPSLLeDGNYV
IReEYPQA----EK-EGKTMIPVLMADT-DIQEVK----
TIFPEFGeIYDFSDKDALrEKLIDVRKSIGEL(ASLTPEYKHFLALAYEKGEGTERNIRLSYQLLKEAFEAGN
ISSMAYMTIQHLEGRVADEYEGETEDMLTEAMCLFYSVI)}*

$1076=254(252):

>gi|655058869|ref|WP_028507214.1| {|90(834)|<Firmicutes(B)>}hypothetical protein [Ruminococcaceae bacterium AB4001]
{(MDHDYLFAREKHIPVLPILLDNGIREVYSRPDRFGEIQYLSPSNADKTKISYKEKLKKYLGSVLESNELRD
RIRKAFDAY)---
IFLSYRKTdRKLANDLMKMIHNNPECEDIAIWFDEFLTPGESFRdGIGRMLKDSKLFTLLVTPHLLDrqSDgt
pnYIMAqEYPEAVRL-----EKNIFPAEMMVT-
DYAQLIEAYPGLPDCTDtgDANTFNERLLDMLVGIAVSENNN(DPEHNYLIGLAYLEGIDVEIDIGRGVKLLT
KA)}*

$1077=178(186):
>gi|655228453|ref|WP_028641533.1| {|0(467)|<Proteobacteria(B)>}TIR domain-containing protein [Novosphingobium acidiphilum]
{(MEQTSPGPPDTTR)-PIVFLSYAHAD--RNRVTPLIAALTEAGQ-
QVWWDALIEGGDVFAdTIESALDLADAVVVAWSVHSVASDWVRDE-----ATHGR-DRGRLVPISIDGT-
LPPLGFRQYHFIDFARWdgSAQAPEFKALLSAVGSAGTVRAQ(ASRSAPMPRPARRVGRRAALIGMTTATLAA
GGG)}*

$1078=156(161):
>gi|655260201|ref|WP_028670691.1|
{|0(300)|<Proteobacteria(B)>}hypothetical protein [Saccharospirillum impatiens]
{()MSDVYMVYASEdRGIAEEVYGHL-----EKLWDVWWDDKIV--
GPFSrAIEDQIPKSKCMVLLLSESSRAKDTVLDELRLAKESELD-ILIARLDHCKAPY-
QYGGYSCCSMVDWDGDSdhAGFSQLMRKIATVVPPAMPPKRP(ISIADSELKLPRLFYSISSYN)}*

$1079=228(219):
>gi|655077063|ref|WP_028525196.1|
{|0(72)|<Bacteroidetes(B)>}hypothetical protein [Runella limosa]
{(MATKY)KYNAFLSYSWDdreiaEKLHDDIENSAKLLKIIPRLKIFKDQNDANNKMGLdEIYKGLDESEHLI
ILASPNSANSKNCGDEIKYWIeQKRGLKnikIILLKGKITIQEKIiEVEKQYILVSTSLHPVIQATLFKHRP-
-
IWTNLSTIENLA(KQDRNFGFYRANLLEIVAYILNTTSRNIEGKESRARWTVYFLAITFVIVVGFFGWFAYDN
ANEAERQKKKAEME)}*

$1080=186(185):
>gi|502434042|ref|WP_012784474.1|
{|0(159)|<Actinobacteria(B)>}hypothetical protein [Catenulispora acidiphila] >gi|256355682|gb|ACU69179.1| hypothetical protein Caci_0226 [Catenulispora acidiphila DSM
{(MSPQKTGVPPG)EFDIFLSLGG-PDRVE--
ANLLKEALVKEGLTVFIDEDDIRHFDGItrEIERCLRSSRALLAYYSREYPLRSACQFELtaALLAGRNEQDP
TRRIIVVNPEATEDHIQPAYISDARFAVRPGSDHERKALAALIKQKVDTLEGP(LSSIAFTRRPRWFAFRIAG
APGFVGRYRELWELD)}*

$1081=217(220):
>gi|302779565|gb|ADL70124.1| {|58(0)|<Firmicutes(B)>}hypothetical protein Tthe_2675 [Thermoanaerobacterium thermosaccharolyticum DSM 571]
{(LPNLREDIINQFKNLGCKLRTDLNLTDFFEAIGQRQFNFYSLIDSKNCDFIFDNYFYFSEKKYLKSKLYSN
QEMKPKNLI)----FISYSTAdeEEVDQL---
ALELNRRSISIWYAKNNIDYGESIVSKIEKAIDECVGVLFYITQNYLKSNWCELERETFYSRYASERNILMIT
IKDWNIDwNELPSFLRNLKYIDRKEfKSMQDVAVELVPTIKKHLNNVKR()}*

$1082=191(206):

>gi|494119687|ref|WP_007059466.1| {<Firmicutes(B)>}hypothetical protein [Clostridium carboxidivorans] >gi|255512669|gb|EET88942.1| conserved hypothetical protein [Clostridium carboxidivorans P7]
{(MAFFHLQGLKHFVVLRRQKYMRIPSKNKRRRKFLKEVYENQRFDIAAIPKELEYRIDNSKEQKIKE)-
YDFFISHSSAdfVEVQNMIKALNHDKKNVYCDWINDTDYlkrhLVGNSTKAVIEKRLEQSKNVLFVRSENSLK
SNWVRYELNYFYSlgkKIFEISKENVI-----------------------
KGrYGYKVLSDLWFYDENYNNMNLF-()}*

$1083=248(256):
>gi|502582364|ref|WP_012822467.1|
{|87(335)|<Proteobacteria(B)>}hypothetical protein [Pectobacterium parmentieri] >gi|261604047|gb|ACX86533.1| conserved hypothetical protein [Pectobacterium parmentier
{(SYLSKLQSKQNYYVIPIAINPHAYNITNNLNYVNFIRAYDFPIDFFNENLFISIAHEIYRITLNENFEEIK
KGTDTTIEF)----FLSHT--KDGKQgeNIAKQLKQFLDQSRLSCFFDATDIAHGYEFstEIENHIKNSSL-
IAIHSDPYSSRYWCQKELEYAKKHN-----
RPIIAVDCLEEYEDRRFPLFSNIPAIHVPHSQENKISDKDSYRIIITALLET(IRFFYSKVQFKKYKQRGWID
SDITGLYRPPELLDIL)}*

$1084=200(199):
>gi|502346436|ref|WP_012768216.1| {|0(171)|<Proteobacteria(B)>}TIR domain-containing protein [Dickeya chrysanthemi]
>gi|247536712|gb|ACT05333.1| hypothetical protein Dd1591_0446 [Dickeya chrysanthemi
{(MGLFRCLFFHFIIEGSSLDQQINSLNQETSHRRTNAL)---
VFISHDTRdAEIAEAFSRLLSSVSAGVLKSFRSSDKkgNQGIEYGVewypEIMKKLEASSDVVCLLTPNSINR
PWILFEAGVAKGKLDT-PVHGVALGIGLNIAATGPFAQFQNLDDDVDS--
LTRLVIQLVRRIPNSEPDHEV(VKTQVEAFKKKITAFLERKSEI)}*

$1085=213(220):
>gi|495979777|ref|WP_008704356.1| {|22(0)|<Firmicutes(B)>}MULTISPECIES: hypothetical protein [Clostridiales] >gi|251849664|gb|EES77623.1| hypothetical protein RSAG_00953 [Ruminococcus sp. 5_1_39BFAA]
{(YDIPKEEHVEKMIGLFENAGLVLRNDLTFFMFMNSMKDRRFNSCAFLGEQDIDLKFENMFDFVEIEDLKKK
GYYMQPFGK)EKQVFISHSSKdKKDVEMIIPYLNGQDLPVW------
FDKYSIPVGAsiteQVQRGIEESDMVIFWVTDNFLNSNWCQMEMKAyisrmiqeniriCIVMDDD-
IEIKKLPLFLRDIK---------------HI--RRDHRSVIEVAEEIAGIIKHM()}*

$1086=205(214):
>gi|506306633|ref|WP_015826408.1| {|0(419)|<Proteobacteria(B)>}TIR protein [Hirschia baltica] >gi|254041463|gb|ACT58258.1| TIR protein [Hirschia baltica ATCC 49814]
{(MAQDILSETSVAGSN)---VFVSYCRKDMDFANQIVSMLEDEGYD-
PKIDRDDIAATEQWEARLQELISSSDTVVFVLTDDYLASQNCGWEIQQAVARN-----
KRLIPLLPKPLTakDVPPELSRLNYIHFFS--
LNSGDGTGFYAGFKSLQRALRH(DLERLRLLRRYEERAKEWKSGGGVLLTGEQLVQAQNWQAQTEKIESVPQA
ITAYIDASQ)}*

$1087=172(181):
>gi|506306929|ref|WP_015826704.1| {|0(228)|<Proteobacteria(B)>}TIR protein [Hirschia baltica] >gi|254041759|gb|ACT58554.1| TIR protein [Hirschia baltica ATCC 49814]
{(MDYD)---IFLSYRRVdQELARALVT---ALEARGV-
GVWWDQKIEGGVDWRdAIVENLISSDMLVILFSEECNNS----KQLKKELALADD-MDKDVIPVLIEDT-

KPKGHFLYEMAARNWIQihPNPENKINELADRLTLLAEASPG(GLAGTKPEPIPEAAPVEAAAQPALSQQDAV
DTNQPDI)}*

$1088=119(140):
>gi|254265692|emb|CAX17041.1| {<Proteobacteria(B)>}hypothetical protein p1METDI0051 [Methylobacterium extorquens DM4]
{()MATVFFSYSHVdEGLRDRLEIALSALKRQGLIEAWHDRRLIAGDDFDrGVRSELERANVILLLVSPDFIA
SDYYYGvETTRALEFHER-DEARVIPVILR-----------------------
SAIGILCRSAMTLPPVLPSF()}*

$1089=176(179):
>gi|494124128|ref|WP_007063905.1| {|0(522)|<Firmicutes(B)>}hypothetical protein [Clostridium carboxidivorans] >gi|255508112|gb|EET84529.1| Tetratricopeptide TPR_2 repeat protein [Clostridium carboxidi
{(MN)MPKIFISYSWNdKVIADKIDKDFQML---
GIKLIRDERNLNFKDSIKQFMKSIRNADYVVMIISNSYLKSKNCMYeVLEFTKDENYK-G--RILPIILDDA-
EIYQISNRLKYMKYWEEqlNELKNEVSGLDVLNSIPIYEEIK(IIEQISRLIALFMDQLADMKAISLSKLIED
KYKQILD)}*

$1090=181(183):
>gi|503058226|ref|WP_013293202.1| {|0(650)|<Proteobacteria(B)>}hypothetical protein [Gallionella capsiferriformans] >gi|302581252|gb|ADL55263.1| Domain of unknown function DUF1863 [Gallionella capsife
{(MK)--RAFLSHSFLdkefvRAVALDLGRQFC---------LF-
DEQVFDTGESFkqSIEKHLDDSSIFVLFASTASLKSIWVDFEIEeaWYRRLEGRlSqtlVFITDTSISHD---
DLPPWLKRAKVSRVNVAKIVAREIRQHLEEMLRVEQHPYF(EGRTEDLGRLQKLMRPIAEPAPRVLAIYGLPS
IGRKTFIKK)}*

$1091=161(164):
>gi|503058488|ref|WP_013293464.1| {<Proteobacteria(B)>}TIR protein [Gallionella capsiferriformans] >gi|302581514|gb|ADL55525.1| TIR protein [Gallionella capsiferriformans ES-2]
{(MA)-FKVFVSYS-ShdlehvEQLQQQLSD--------
SPVEVFVAENSVAPSQELgsTISQAIEQCDLFILVWSKNAQESAWVSQEVG--
RAGALKKkilpLVLTEGMQLPG---
FIQNLKYLSVHKEPEKALAQARQIVVGAYEEKVKLESAHA(QAEKDKLALLAIGALLFWAFSK)}*

$1092=213(220):
>gi|655483674|ref|WP_028865373.1| {|166(0)|<Proteobacteria(B)>}hypothetical protein [Psychromonas aquimarina]
{(LKKFKTKETIIFFQKINDAERNTQIRNMAFEHLQSIGAYVQKRRGFKGKKKDYYHSTDKFIVTPADLADRL
KCGGIQSKK)EFDVFISHSYKdnKLVNEVMVELNKHGLSIYCDWTSDNDFlrrnLASEYTEIVLKARLSQSKY
VLFLQTDNSVDcggdflSKWVKMELDHA-------------------I-
KNGKDIFCINVSSQLPLfSEVTSSADKYKLTTDEANKLRIQ()}*

$1093=308(295):
>gi|655474381|ref|WP_028857219.1| {|64(226)|<Fusobacteria(B)>}hypothetical protein [Psychrilyobacter atlanticus]
{(IYELEKILKAAAEENIRMFPISTSTSNRKPLDDIDEYQSFDSITEKNIRGLDDNYIKIIGECFGREIIIHE
FSAIFNKKI)--KIFLSHRRKecEDVAKLFKDSLHD---KKENVF---
IDLHEVSTGEkaqeIIEENLENSDILIFIQTKTTFESEFQLKELKKAfelsipvlWItldlkenefKKMP-

LHPLGKPHFELDEITSIETNQIINYAFDMIK--LKKqrLLDK--
IIwKLKSLKENGI(DYKELCNRDSIYLIEQESLDPCFGTSIKNARLFKCLCREYQKEDLTNLKEHLLKIEKHN
QNCILSIKGEERELEN)}*

$1094=285(292):
>gi|655901079|ref|WP_028972867.1|
{|111(39)|<Spirochaetes(B)>}hypothetical protein [Spirochaeta
cellobiosiphila]
{(LDLSADRLNKLFDNRTCIPFLNEVQKFYDYGDYKGVSISKEILEKIEKLENRILAGKKLIGLKQIPKEPSI
TLTKNHSEL)--KVFISYSWSnENYADEL-----DILFNTKDIIL-QRDKRDIKYKDsikeFMGK-
IRIADYCLILISDYYLKSKNCMYEI-LEFIKDED-YINKILPIITNDAK-IFNAIDRAEYIrfwqDKYNE--
LNTSKEK--
ISALNQSDIIEE(MKFVENIQRNISEFLKIISDLQLIKLGNDVTLENFKTIYNIINPENKNIKLQNGYFLMNI
PRSINEHYFTWM)}*

$1095=240(244):
>gi|655495592|ref|WP_028876970.1|
{|0(71)|<Proteobacteria(B)>}hypothetical protein [Teredinibacter
turnerae]
{(MKVGNEQMPDEKTTKIYIDNFVGDDG)-----------
EEFAESLKERLYGLRLDapFKIFSFSDLDPSPDEEVDpDNElntvRAIENADIVIPVVTSSFIG--
YVTSdIESAYNEIINS-NDRYFFPVLLKAT-DWsSHEWLVRSQLTPRDAtpLYEiaRNEQE--
RAINDLVQTIKAS(VIKLSQKRHSDGAERIAQNVQRRDSVFISHDHDDADFAELLKLKLEKEGVESWLDTERL
KIGQNWREEIDQGIENSAA)}*

$1096=221(226):
>gi|655840527|ref|WP_028954775.1| {<Proteobacteria(B)>}hypothetical
protein [Sulfitobacter sp. 20_GPM-1509m]
{(MRRSEIQKILTMLIAVASAAAASFSLLAIENVSLSEFLFDDRQTLVMLLGVTSAIGAAFASALMLQVMRRN
RLRMRARRA)----FIIYAHEdAELAQTITSAL----
REGGIEPWLDiEQISAGEIWKdAISKGLEESAMAVVLLTPKTEKSDFAMSEIKAAIRNMEA-
ADKVTSPVIPVLF-EGASVPPQLAHIHYVDMtrDDAKHFLVKSVHRAMDRVVRETE(KVGERG)}*

$1097=209(220):
>gi|655993549|ref|WP_029035176.1|
{|224(0)|<Bacteroidetes(B)>}hypothetical protein [Salinimicrobium terrae]
{(NVLDRLSSIFNRTIDKHYILLPDHKFNFAEKKRLLLDKRLEVIEYKIDETHTQVDAFLSDLTEIFKEQQMP
EKENKKYDA)----FISYSHNnIKEIERLRSFLMDVR----
LSVWYDMELKPSQDIQnTLTEAINNSRFMIIVITKDYYDSEW-LKkELEIGLLKQVE-GKINVIPISIGPI-
DFQRLP-KELQNIHflqlnenfsiDDLkpLIKMMKRD--Q------------()}*

$1098=194(199):
>gi|655495572|ref|WP_028876953.1|
{|0(271)|<Proteobacteria(B)>}hypothetical protein [Teredinibacter
turnerae]
{(MR)---VFISHSS---
KDSLFARDLSKALQEAGIDVWIDEMSILPGDNIvkKIEEGLSTSNAIVVLMSKDSVSSKWAMQ--
ELSVFTARSIsEQSLrILPILLEEC-
DLPIYLRDKLYIDARSNKSEAINKAVNSILRFSEPSQQEE(VEFDRLEGKKSSFNYHRKKLASYLHNGELTLV
CGAGVSAASGVPTWSILLNELLSNL)}*

$1099=214(219):

>gi|656045383|ref|WP_029084041.1|
{|0(149)|<Proteobacteria(B)>}hypothetical protein [Bradyrhizobium sp. th.b2]
{(M)--RIFLSFPSELESQADSIAQSLRNCDHD-
VFFSHDDLPPGDSFDARVEKAIESSDFMVFLISPESVTKGRYTLsELAFARTkwpnPS-----
NRVLPVMMAPTPldQVPTYLKAVTILEPQG--SAAAETRA--
AVDRMLAAESAQ(SRGRASLPFVVLALASAVICSLIATYALNVLQYSFVVSDAGGITILPGVVFGMAVATCNVMFGARDRLQLALVVAITT)}*

$1100=172(174):
>gi|657015101|ref|WP_029259151.1|
{|0(260)|<Actinobacteria(B)>}hypothetical protein [Microbacterium sp. Cr-K29]
{(MSTSVDVLGP)---LFLSYRHNdGA---
EIVANLAWLLRAAGIPVWRDKdDLPPGDtaeRLAeAIGDGLSGA---
VFVITEDIANSAIVRGiEAPKLIDLHRS-DARFQLLIANDVA-
KESSASLDYSAPDRLLGrvKDDLKGVDQSGTSPEGLLSLVGK(AVAHRMAQHRPLVENNGRFDLSIQ)}*

$1101=232(228):
>gi|656045098|ref|WP_029083763.1|
{|0(758)|<Proteobacteria(B)>}hypothetical protein [Bradyrhizobium sp. th.b2]
{(MSQDALQIKTRKEGSGEAG)-DIVFLCHNS--A-
DKAFIRKLADALELEfGTKFFLDVFAIPTGEAFisWIEKALETCTACAIFLGGNG----
WGPThLWEAELALARYRrDPTLrIIPVALpglsqqDAA-KLGAGklFQEVNWADFTKGPND--
KDSLDkLEAALTGRKVLGY(RGPARLTPYQVRRDAERWERSGRKDRSILYGGRQLIDAARMLQDNPDAVVVADVASFLAASRERQSTFW)}*

$1102=219(219):
>gi|922340099|ref|XP_003624912.2| {|0(1865)|<Streptophyta(V)>}disease resistance protein (TIR-NBS-LRR class) [Medicago truncatula]
>gi|657379098|gb|AES81130.2| disease resistance protein (TIR-NBS-LRR
{(MASSGSSSISNNDINPSCI)-YDVFLSFCD-
KDTSESLASYLYTALTVAGIVVYKDEDKlLnhDQMITSSVLHAIAGSRLSIIVFSKLYAVSTCCRQELEKIMECRRTTCQ-
IVVPVFYDADPSGVFHQEDLLGEASKYLKQRILKKDKLIHEVCNISGFAVH(SRNESEDIMKIVDHVTNLLDRTDLFVADHPVGVKSRVQDIIQLLNSQESKSPLLLGVWGM)}*

$1103=258(255):
>gi|922350409|ref|XP_013451040.1| {|32(1223)|<Streptophyta(V)>}disease resistance protein (TIR-NBS-LRR class), putative [Medicago truncatula]
>gi|657381075|gb|KEH25080.1| disease resistance protein (T
{(KSIPGRSGKSCRLPWCNQLSPQVERGAFTPEEDEAIIRAHARFGNKWATIARLLPGQTDNAVKNHWNITLKHKCSSIIMD)--EVFLSFRG-
EDTRSSFTSHLHSSLQKAGIKVFMDDDSLqrGDHISTSLLQSIDRSQISLIVFSTNYAGSRWCLEELVQIMKCHRfdvpHSSSHELSSIFVYLPSLRSLWVECSSERQLSHDAAIILDALHTINRKELEPTATTS(QVSKHSLKSLLIQIGMNCQATNILKDIILQNIDVN)}*

$1104=223(235):
>gi|922349308|ref|XP_003619968.2| {|0(1055)|<Streptophyta(V)>}disease resistance protein (TIR-NBS-LRR class), putative [Medicago truncatula]
>gi|657382968|gb|AES76186.2| disease resistance protein (TI
{(MAMQSPSSSFSYGFT)-YQVFLSFRG-
SDTRYGFTGNLYKALTNKGIHTFIDDNHLprGSEITPSLIKAIEESRIFIPIFSTNYASSSFCLDELVHMSFT

ATRQRVASFC----------------SYGEALADHEKRFQNdkdnmERL-
QRWKMAMRQVANL(SGYHFSLGYEYEFIGKIVEDISDKINRVVLHVAKYPVGLQYRVQQLKLLLDKESNEGVH
MVGIYGTGGLGKSTLAKAIYN)}*

$1105=202(220):
>gi|922362274|ref|XP_003604856.2| {|0(1824)|<Streptophyta(V)>}disease resistance protein (TIR-NBS-LRR class) [Medicago truncatula]
>gi|657386748|gb|AES87053.2| disease resistance protein (TIR-NBS-LRR
{(MFSFTFSSKSDHKFEFSSTNPKRKR)-FNVYLSFCA-KDAG-
SFAMSIYKTLsIKAGFVVFWEEKRLgyGDRIVTPLE----------PV---------
RCLQELKKITECCRTTSGLTVLPLFHDhVYPSCGILKTCMFGDSFHNFVDRILMQETSHEGDKFISWVATI(S
KATTYSGPIDLVQIPPDRNKSEYIDNLVERVTRVISNKRGWLNCLNTMSINSRV)}*

$1106=134(165):
>gi|922324737|ref|XP_013441761.1| {<Streptophyta(V)>}disease resistance protein (TIR-NBS-LRR class) [Medicago truncatula]
>gi|657369255|gb|KEH15786.1| disease resistance protein (TIR-NBS-LRR class) [M
{(MRGF)--------------------------------MLSEMLRFNEEI-
IsqSLLKAIGWSRISIVVLSINYANSRWCMFELEKIMEIGRT-
GGMVVVPVFYEVDPSEVRHQEGQFGKALEDLMSTisIDESTK-
SNWKRELNIGGIA(GPFYLKVCSDTRSIFIVKVIP)}*

$1107=130(152):
>gi|922324741|ref|XP_013441763.1| {<Streptophyta(V)>}disease resistance protein (TIR-NBS-LRR class) [Medicago truncatula]
>gi|657369257|gb|KEH15788.1| disease resistance protein (TIR-NBS-LRR class) [M
{(MSSFTTDSKRKS)-YIVYFSFCD-KDA-ASFVSHLYTALRSEdGIVVFWVDYEMRGNG-
EipipiSMLKVIEHCQVAIIVFWVNSVFHPYIREEL-----------------------
NEEVMQRISRGGCPCSCSRVPNPIPANMLLYMTLGHFQGS()}*

$1108=229(229):
>gi|922328839|ref|XP_003626892.2| {|0(908)|<Streptophyta(V)>}disease resistance protein (TIR-NBS-LRR class) [Medicago truncatula]
>gi|657372197|gb|AET01368.2| disease resistance protein (TIR-NBS-LRR c
{(MDRRRYRPRRPIPSPPSTPSPPLHSSSSIPRNEWL)-YDVFINFRG-
KDTRNNFVSHLYAALTNVRINTFLDDEeLGKGNE-
LgpELLQAIQGSQMFIVVFSENYARSSWCLDELLQIMECRAN-
KGQVVMPVFYGISPSDIrQLALRRFGEAFNNNTDELDQLIYMALSDASYLAGWDM(SNYSNESNTVKQIVSQV
LTKLDKKYLPLPDFPVGLESRAEQSIRYLRHNSDGVC)}*

$1109=199(200):
>gi|922332186|ref|XP_013444084.1| {|0(685)|<Streptophyta(V)>}disease resistance protein (TIR-NBS-LRR class) [Medicago truncatula]
>gi|657372205|gb|KEH18111.1| disease resistance protein (TIR-NBS-LRR c
{(MSSSSSRSRPQWI)-HDVFINFRG-KDTRKTFVSHLYAALTDAGINTFLDDEnLKKGEE-
LgpELVRAIQGSQIAIVVFSKNYVNSSWCLNELEQIMKCKAD-
NGQVVMPVFNGITPSNIRQHSPVILVDELDQIIFGKKRALRDVSYLTGWDMSNY(SNQSKVVKEIVSQVLKNL
DKKYLPLPNFQVGLKPRAEKPIRFLRQNT)}*

$1110=149(173):

>gi|922330385|ref|XP_003630982.2| {|0(870)|<Streptophyta(V)>}disease resistance protein (TIR-NBS-LRR class) [Medicago truncatula]
>gi|657376258|gb|AET05458.2| disease resistance protein (TIR-NBS-LRR c
{(MLGSSSSAAAAISPPSQTQ)KYDVFLSYRG-EDTRDNFITHLYA----------------------
ELIHLYDESMIYVVVLSENYASSTWCLKFTSNGSWELGP-
NRRHVSFYRLKTNASFFFNYVTGQNTLIEDIVKDILIKLKLNCSFLNDYQGMIG(IDNHIEQIPLLHIE)}*

$1111=253(243):
>gi|922362270|ref|XP_003604853.2| {|0(267)|<Streptophyta(V)>}disease resistance protein (TIR-NBS-LRR class) [Medicago truncatula]
>gi|657386744|gb|AES87050.2| disease resistance protein (TIR-NBS-LRR c
{(MSDFTSSFYKIRKYKSISTTTTYQKKI)-YNVYLSFCN-KDA-
GSFALQLYTALSSeARVSVFWDNARLGSgdRQ-
IstSAVNIIGNCRVAVIIFSMKYFNSMWCLQEFEKITECCQrvT-
DGLTVLPVFFdGAYPCNGRLHKNMFGDPFdyyvdrilMVTETSKEQDIF-
MSWVAAVSDKAFK(YSASRYSLRKSVSGNEGEYIKDVVELVTYGGSTQEETPSVKSGAHDVIQLLKQSKSPLV
IGIWGMVGIGKSTIAKA)}*

$1112=244(243):
>gi|922365506|ref|XP_013454965.1| {|0(1142)|<Streptophyta(V)>}disease resistance protein (TIR-NBS-LRR class), putative [Medicago truncatula]
>gi|657386749|gb|KEH29013.1| disease resistance protein (TI
{(MSSFTSSSKICQEFESILTTDPKKR)RYNVFLSFCA-HD-KGYFLSSLEEALSlEAGINVFGD--
IKRFQH-VesVLNV-IQDCKVAVVLFSKNYTNSSSCIQELEKITQCCRT-
SDLVVLPVFYQgVGPFyhgdmFGGDTFHDFLDRISMEEISKEEDKL-
MTWVAAITKANKY(LGSRDLIPKPIYRYEHVSITDYIKDIVEHITCVINKNRDFCANSCTPSVKSGVQDVIQL
LKQSKSPLIIGIWGMTGIG)}*

$1113=259(247):
>gi|922362280|ref|XP_003604861.2| {|0(1572)|<Streptophyta(V)>}disease resistance protein (TIR-NBS-LRR class) [Medicago truncatula]
>gi|657386758|gb|AES87058.2| disease resistance protein (TIR-NBS-LRR
{(MFSFTSNSKRHLKLKSTPSIRTPTNPKRA)RYDVYLSFCD-
QDkDLHSFVFSIYNALSRKaGVDVFweneRDGYGDREKP-
TSVLNVIRDCKVFVIVFSRDYFNSRSCLHEFKKITECCRT-
KdDLMVLPVFYdGVDLSFGSWERGMFGGETLHDCVDkilmkktfKEEDKF-
MTWVASISKATIY(TGQSDLEDRNSSIYIDDVVECVTNVLRHWKDFSRAFCPVSIKSGVQDVIQLLKQSNSPL
LIGIWGMGGIGKSTIAQAI)}*

$1114=250(244):
>gi|922365547|ref|XP_013454984.1| {|0(1479)|<Streptophyta(V)>}disease resistance protein (TIR-NBS-LRR class) [Medicago truncatula]
>gi|657386775|gb|KEH29031.1| disease resistance protein (TIR-NBS-LRR
{(MSCFTSSSKIQQEIESRSTTGPNKM)RYNVFLSFCA-QDMGY-
FLSRLHTALSsQAGIVVFEDKRFQHGEQ-VesALNV-IQDCKIVIVVFSKNYANSSSCIQELEKITKCCRD-
SELIVLPVFYaAVNPTYGSLEGGMFgGDTFHDflnrismEEISKEEDKL-
MTWVAAITKANNY(LGSSDLIHKPIYRYEHINIKGYIKDIVEHVSCVINKNRVFSVTSCTPSVKSGVQDVIQL
LKRSKSPILLGIWGMTGIGK)}*

$1115=184(212):
>gi|922365115|ref|XP_013454803.1| {|0(891)|<Streptophyta(V)>}disease resistance protein (TIR-NBS-LRR class) [Medicago truncatula]
>gi|657386507|gb|KEH28834.1| disease resistance protein (TIR-NBS-LRR c

{(MKFLCWENGVLQ)----------------------------
KEIKAFVDDELKRGDEIPQSLVRgNEGSLISLIIFSQDYASSCWCLEEHVTIRQCREK-
YGQIVIPLDCKDSSRRRKTFRCWTARTFEKAEDILRAEGFKGCKLVELLGVAGS(KGKSSFCRIVTRWLGNMA
RLEVSVLRQSTGKIMAQWGRIVARWCVVTPVSMMILLVWIIA)}*

$1116=286(288):
>gi|922344514|ref|XP_013449176.1| {|0(856)|<Streptophyta(V)>}disease resistance protein (TIR-NBS-LRR class), putative [Medicago truncatula]
>gi|657378429|gb|KEH23203.1| disease resistance protein (TIR
{(MAAKFEIEKFNGRNFFLWKLKIREILREDNCLDAIDVRPADITDGKWKEMDDNVVANLHIDANDGLSIV)K
YDVFISFRG-PDTRNSFVDHLYDHLtRKG-
IFVFKDDKSLQKGESIslQLLQAIKDSRVSIIVFSKDYASSTWCLDEMAAIDESSRR-
LKQVVFPVFYDIDPSHVRKRSGAYEDAFVLH-TKQCS---
DkvDQWRTAMTYLAGS(AGWDVRNQPESVMIEEMVQAVIEKLGHTYSASIDDLFGIQPHIEALEGLLRLQSED
DGCRVVGIRGMDGIGKTALAKVL)}*

$1117=150(172):
>gi|922362184|ref|XP_003604567.2| {|0(667)|<Streptophyta(V)>}TIR-NBS-LRR class disease resistance protein [Medicago truncatula]
>gi|657386534|gb|AES86764.2| TIR-NBS-LRR class disease resistance protei
{(MSSATSSFSDEYIPDSGI)KHDVFLSFRG-EDTRTSFTS---------------------
QGDYIstSLLQGIYGSRVSVIVFSKNYAGPQWCQISGSSNSKHQLL-
LVFNKKRVAGSNGANIIISVIKMRILCWMCG-KTRQNMIITPIVEKIVETRLRW(FGNESEDIKDIVEK)}*

$1118=238(231):
>gi|922365526|ref|XP_013454974.1| {|0(1497)|<Streptophyta(V)>}disease resistance protein (TIR-NBS-LRR class) [Medicago truncatula]
>gi|657386761|gb|KEH29022.1| disease resistance protein (TIR-NBS-LRR
{(MSSFIPSSANSKSE)RYNVYLSFCH-QDAA-SFATGIYTALnrKSR-
FHVFWDDEKLGSGDRgIptSILNVIEDCKVAVIVFSRNYVNSRSCLQEFEKITECClTT-SGLIVLPVLY--
DGLNHYSSFGTVEETFHDF-
VDRIwikettSEEKDkfMSWVAAVTKATTY(SGVIDFADSYGREYVVDVVESVTRTVNKKRDLFGAFYTASVK
SGVQDVIHLLKQSRSPLLIGIWGMAGIGKSTIAEA)}*

$1119=128(150):
>gi|922375606|ref|XP_003601355.2| {<Streptophyta(V)>}disease resistance protein (TIR-NBS-LRR class) [Medicago truncatula]
>gi|657394500|gb|AES71606.2| disease resistance protein (TIR-NBS-LRR class) [M
{(MRRESASSNF)-FDVFINFHG-EDTRSKFTSHLNKALqKSG-
LWTFIDDSELEKGDEIssALIKAIEESDASLYI----HILKRLCFFKM-------KD-
HGQIVIPIFYEIDPSHVRNQIGSYKQAFAKH-KQNLKHNKDklQK-----------()}*

$1120=210(220):
>gi|657619973|ref|WP_029430966.1| {|99(0)|<Actinobacteria(B)>}TIR domain-containing protein [Blastococcus sp. URHD0036]
{(ESKISDLQAKIAREEDKLRKTEAQQEKRRDDVAHRRDRQLSQQLSKTGSAVDELSSRVANLESELLDRVRE
DVAADPVGR)EHDVFLSHAS-PDT--
DVAQELYDEMMARDLDVWFDGAELRLGEPLmrQLDRGIARSRCGVLLLTESFLSGRfWGEREMTALI-----
GSRRRIIPILFGIGFEDL-----ETYSPILSTFVGLDGDRHGMSEVAEQIANTLR()}*

$1121=274(236):
>gi|657574172|ref|XP_008290781.1| {|286(0)|<Chordata(M)>}PREDICTED: interleukin-18 receptor accessory protein isoform X3 [Stegastes partitus]

{(TPQHLNHTYTCSTSNAVGNSSITIKLKRRNKAKWPCLVGHAVVSFLVVAGLGGIVHLKRLELEIIYRSYFQ
YGKHDTEKK)QFDVFLSYA-
WsplladvtkswtlssrsgsfkneEddgeatQRPLEvLLPKVLEDQWGYRLCLLDRDMLPGGAYTNDVVLAIE
RSRMLICVLSADYLtNSNAVF-VLESGIQALLQKRALKLLLIWTsgasgSLM-
EADPPLPTLVRKALRVLpSLKWSSSKPArsksNFWGSLRKAMPNI(DSEAGFSHAAPMIKLC)}*

$1122=264(259):
>gi|657816543|ref|XP_008335367.1| {|623(49)|<Chordata(M)>}PREDICTED:
toll-like receptor 5 isoform X1 [Cynoglossus semilaevis]
{(LHDLPLLNYTTVVEPCEVDDEMSVQELKLALFVLSALLITAFTLGGIVYARLRGRIFVIYKKLVSRVVEGP
KVAPPLEEV)QYDAFLCFSNSdyMWVEAALLKKLDNNFSEEnifRCCFEARDFLPGEDHLSNIRNAVWGSRKT
LCVVSKEFLKDGWCV-
EAFTVAQSRMLEeLTDILILLVVGKVCMNYLTDQYLKPPTIPLVFSQGGSYCWRNISASSQSVSTEA(TGAAS
ICAGRLAHQKHGDESPHLHVFTKPKHTVKDHSSG)}*

$1123=210(207):
>gi|657726759|ref|WP_029523333.1| {|0(1186)|<Aquificae(B)>}hypothetical
protein [Persephonella sp. KM09-Lau-8]
{(MKNEIF)-----ISFDG---NKRDDVSLLAEKIKKiLNYDVFVDK-YKGDLLqkGNaksILRGKIFN--
ARkvILYLSKEYidnlPDKEYLQWELQAIRDRIKEEGRDFLVILKDDNkTKlndE-VIDDDVFELFDEFQK-
EEILFRYLRRSKLFNIEEIAKR(IRKDVESIGKRYLPFVKDKIENIEIKTLEKQISYLNKKDVDTIIKFLLNE
KFLNYLESYKK)}*

$1124=254(253):
>gi|657706421|ref|WP_029505083.1| {|44(22)|<Firmicutes(B)>}hypothetical
protein [Lachnoclostridium phytofermentans]
{(IIFGVQDNGKKLRVKGYKFNVDIDSIKEKLSDNVKLTFLDLPHKTEILKYISIEKSENVVFYNGAPYKIDD
NKNVTELYI)-KKVFLSYSHKdTCIADLVDERLNFIG--KGRLVITRDKRT-LEYKSdiekFM-
QTVSAHDYMISIISDSYLKSQGCMYEvselMRNRAFNDKL-LTIILSQSDESFYSTDKKPEQVEADVYSLNR-
-FKYIKYWEAEKEKVNKLNLEI(TDLALKQGLVDEIKQINVISMNVAELIEIFKKS)}*

$1125=194(184):
>gi|657844434|ref|WP_029559358.1|
{|0(7)|<Proteobacteria(B)>}hypothetical protein [Vibrio parahaemolyticus]
>gi|1014787216|gb|KYY61034.1| hypothetical protein AWQ14_13730 [Vibrio
parahaemolyticus] >gi
{(MARKT)----FISYKYSeskelrDKILDALGEDARYYKGETSESP--DRTD-----
QTtdTIKNALKNMIYqtsvLVVIASPDMNSSNWIEWEIKYALRsqkrgdtTSQPNgvvVVIKEenGSVEWF--
-
MNRVVGDDGCTFRRYNNHYLPEIIKANRFNRKIPEYSCEH(CATYSSTEGSYITIATQKEFLDDPDYYIENSY
NKSKNLD)}*

$1126=128(161):
>gi|658023355|ref|XP_008347087.1| {<Streptophyta(V)>}PREDICTED:
toll/interleukin-1 receptor-like protein [Malus domestica]
{(MDLVRTTQVTSSYSNSRRGYH)-YDVFLSFRG-
EDTRKTFTDHLYTALKSAGYRTFRDDDELetREGIKWGLKQAIQMSRTSVIVFSKDYASSRWCLNELVEIVDH
KRTSSDHVVIPV----------------------------------LLVFDFLIDSMEQ()}*

$1127=208(236):
>gi|657970492|ref|XP_008376996.1| {|0(282)|<Streptophyta(V)>}PREDICTED:
TMV resistance protein N-like [Malus domestica]
{(MAYPSSSSSGPARHWI)-YDVFLNFRG-EDTRKIFVSHLYKALVQNSIKTFIDAKKLRKGN-
DlsQLLSAINGSRLSIVVFIQNYASSTWCMKELVQILECMDT-QEQI-------------------------

EQDSNTGMEEL-
RSWKSALTRAANL(SGWDSRDYKDDAKLIEDIVQGIFKKLINRSSSEKNGLVGMDPHLQKMDLLLCPQVDGVR
LVGIWGMGGIGKTTIARAVYD)}*

$1128=207(225):
>gi|658021743|ref|XP_008346274.1| {<Streptophyta(V)>}PREDICTED: protein
PHLOEM PROTEIN 2-LIKE A8-like [Malus domestica]
{(MEKENKAENRRKRNRSGDDDDTDCEGSKARRKEVMDIASSSSSAADNPPRRKK)-YDVFISFRG-
EDTCLGITSHLHAALLQKKIETYIDYKLQSGEE-
IgpALLQAIEKSTISVIIFSQNYASSTWCLDDHSSAAPKIDS-----------------
HHSCGAAFPPPVGNGGYNAIFFFWEALLMVLGVWQLTK(LGISGSLSFGSGYFWVDWVFENLLSQTSELLR)}
*

$1129=189(207):
>gi|1039921466|ref|XP_017184121.1| {|0(508)|<Streptophyta(V)>}PREDICTED:
disease resistance protein RPP4-like [Malus domestica]
{(MASSSSSAAAISSXSRK)-YDVFLSFRG-EDTRNTFTSHLHAALLRKKLDTYIDYKLERGDE-
IgpALLEAIEKSKLSVIIFSKNYASSTWCLDELVHIKGCKEG-----------------
MDSLLYPFFTTSVHRMYENNRGVMQMHLPSLKNVSRTV(WTRCLSGGMLWRKLPTYLVMIIQTKPGIWGMGGI
GKTTLADVVYHRFSSK)}*

$1130=248(249):
>gi|657987708|ref|XP_008386017.1|
{|117(167)|<Streptophyta(V)>}PREDICTED: uncharacterized protein
LOC103448530 [Malus domestica]
{(QTGSFAEAFARHEETYKDDPDKVRRWRAALTQVANLTGWVSRGRDESELIEEIVDVIRKLLHPTTKTSAPC
SSLFSSHKR)KYDVFMCFRG-KDTRLNFVGHTYASLnREK-
FTIFKDDKGLEKGQPIspGIVKAIEESQIYLVTLSPDFASSTWCLDELVKMFECMET-
SNSQRSLEDFTERKDASTVATGIKCNTRDRK-
ISKVRISVHYDGEWVSTAYVGG(KAKEIVVSKDITCEELLRRVYRLVRIDQC)}*

$1131=228(229):
>gi|657900128|ref|WP_029603122.1|
{|95(266)|<Actinobacteria(B)>}hypothetical protein [Kocuria rhizophila]
{(VPSAPSSIDVAAIRAAAKVGDGPLALLVYTPGVAGGFHGLIAQRQRLGRTVTRTASVSVPASRFEPLGQLP
VGAVRGDGP)---VFISYRQWDGTPQAESLENLLRAAGL--
VVWRDRTDLRPGTTTdRLEQALTAGlSAGVLVVTPDIVDSVIVREreLPRLLQLDSDPAFSLCIANAVGRGNG
SKCDYDAPDRLLRLAPARTLADKKQANMLDPSGEIEIVRD(LLMHRIEQR)}*

$1132=194(195):
>gi|754792853|ref|WP_042156416.1|
{|0(1127)|<Cyanobacteria(B)>}hypothetical protein [Planktothrix agardhii]
>gi|658300488|gb|KEI68883.1| putative WD40 repeat-containing protein
[Planktothrix agardhii
{(MSK)QYSVFVSYADEddAWVEGYLLNALEEAGL--T-
CHSEAMFRLGVPRLIEFESAVKNSERTLLVISPAYLADNFVEFANLLFQFYGLDTNLWPVVGIILHPV-
QELPMRLKMLECLDATDgTKQQKAIQRICENFQKPVPTLVE(IPDCPYPGMVPFKPEDSDRFFGRQREVNDLI
QHLRLYPFVAVIGASGSGKSS)}*

$1133=195(225):
>gi|657930317|ref|WP_029631524.1|
{|0(1154)|<Cyanobacteria(B)>}hypothetical protein [[Scytonema hofmanni]
UTEX B 1581]

{(MAGTPQ)-YDLFISYAQAdyEWVEGYLQNALLQSGVHFH---
SERAFALGVPRIKEFENAIQRSHRTLLVISPAYLLDGFNLFVDLLSQSYGLESQTWPVIPLVYQSV--
QLPPRLNALVKL--------------------------
D(ATNPDQWESVIVRLCTDLQHPVPTVSTKPACPYPGMVPFSEADGDRFFGRDQEIEELLDKLRLQGFVTVIG
PSGSGKSS)}*

$1134=267(232):
>gi|658840589|ref|XP_008402693.1| {|275(0)|<Chordata(M)>}PREDICTED: interleukin-18 receptor accessory protein [Poecilia reticulata]
{(VTPQHLKHAYTCFASNAVGNNSVTIKLKQKKQGKWPSLVGYPFGCFLLVVSLGIILHVKWVELQLIFRSRF
QFGKDDEEK)EFDVFVSYV-Wtgpsaeavngltisskpysdagemqsre EPIEMRL-
PRVLEDQWGYRVCLLERDILPGGAYTNDVVLTINRSRMLICVLSAEYFaSSNAVF-
VLESGVKALLQNSALKMLLIWTrgtsASF-
TQPEpPLPKLVQRALKVLpSLQWSTDAPStaarSFWISLQKAMPAD(RARPASRVQCEA)}*

$1135=212(216):
>gi|657895843|ref|WP_029599086.1|
{|0(607)|<Fusobacteria(B)>}hypothetical protein [Fusobacterium nucleatum] >gi|929728678|gb|ALF21304.1| hypothetical protein RO08_03080 [Fusobacterium nucleatum subsp.
{(MGRA)----FLSHSSKdKILVEAVARKLGN--------
KYCVYDTFTFEEGQknldEIKKGLEDTDLFVIFLSNDSLKSEWVKEELEIAYNKlqkkeiSKI-
YPIIIDENVDYEDERIPEYLKIynLQKITKSER--IKKIILR--
LLKEIGWEKSDF(LKQKNTFFCGRNDLVEKLETDYHDFQKKINCFVASGFSLIGRKSLMKYTLEKLNLLSKAH
TPSIIKFRDNES)}*

$1136=212(217):
>gi|657866310|ref|WP_029571897.1|
{|0(223)|<Proteobacteria(B)>}hypothetical protein [Pseudomonas syringae] >gi|939980281|gb|KPW08220.1| Unknown protein sequence [Pseudomonas syringae pv. atrofaciens]
{(MSTAENWIKQVPAPRTLTGDDQ)-WSVFLSYRSADKAWVSNLYDVLRHYGHE-
VLLDQVALIEGDQFHERREGAIAASQAGVLVCctkdcnSKN--VVSLHQVmEATASLRTD-----
FKFVSVLVD-AGDLSGFISARTFLDFSS--
HPYGPNGGELLRLLHAVVGQPL(SDEAARIALDFDALSESAANQIGTAILDGSTERLNSLFAERGAPWRVAAV
LSCRT)}*

$1137=212(220):
>gi|746178941|ref|WP_039238564.1| {|59(0)|<Firmicutes(B)>}hypothetical protein [Clostridium novyi] >gi|658075942|gb|KEH89132.1| hypothetical protein Z965_03860 [Clostridium novyi A str. BKT29909]
{(AEKFEAWLIKNYRNKKRIYNFFIDDIVNSFFTRGYNVATFIDEQTVDSVITSEPNSTFLFPDKEGMKSLWK
SGIVEGSSM)---VFLSHSSKdKSKVDEIFNEVE----
KSDIRVWYDKyEIKPGDSITdKINDGLDSSDVGIICISNNFLNSSSGWTksELNYFIQRRMR-SGKK------
DFI-CLNFDVPHDELPPLVQDykYIDMEEKDAIDTLIGSLKNILNR()}*

$1138=190(190):
>gi|657881704|ref|WP_029586137.1|
{|0(296)|<Proteobacteria(B)>}hypothetical protein [Bradyrhizobium sp. URHD0069]
{(MVIPDAAPCL)----FLSHSG--A-
DTDGARELKRRLldspnaRAARLTIWLDKDDLGAGIGWqaQLEKAIIEKAtAFAVHIGAKGVV--
NWVDsEVRLALSRATGTaDYPF-IPILSREC-

AGAAALPPFAQQYQGVHDPLNDPEEFAKLLRAVLGRSPSE(KTVALENPFVGLKSMTEADADRFFGRNEEIAG
LVDKLKPH)}*

$1139=268(279):
>gi|736961623|ref|WP_034958108.1|
{|185(198)|<Proteobacteria(B)>}hypothetical protein [Erythrobacter
longus] >gi|660652051|gb|KEO91769.1| hypothetical protein EH31_03605
[Erythrobacter longus]
{(RELSASRQGFEIWREWYYGRIEGLPHAFADFDDAADEAFYRWIIEQDNDWWSREPAEVNADIKAKVDSLRT
PIERPEARA)--DFFISHAQGDTATLNDLITVLDLMGKT-YILPQRGGTKGQSY-----
KAIERADRVIALYSDGYVQSDE-CAdEWMhsyylDASTAE-----QRMLAFRLDR-
ADLKPLMNGLSFCDLTR--
LLPDRRKQAMAKWIEWKPRALT(RENVEQTLTTHLDPGVIASADGKLDTAPDAVLNAAELPSELASAMDALRM
MLDLARLNE)}*

$1140=231(218):
>gi|1172327440|ref|WP_080710719.1| {|0(248)|<Firmicutes(B)>}hypothetical
protein [Paenibacillus polymyxa] >gi|660636590|gb|KEO77622.1|
hypothetical protein EL23_16110 [Paenibacillus polymyxa]
{(MTIESKKPP)--TVFISYSwtT--
TDHEQWVYELATRLMSDGVMVKFDKWDLKDGNDTlaYMESMVTSAEIdKVLIICergyqAKADKRTGGVGtET
E--LITPKIYgDvgQEKFIPVvseRDEEG-
KpfLPTFCSSRMYVDLSNEYEKGYERLLRtLHQAPMHRRPALG(STPSFLFEQEVSHYESSNLIKQLNSAIEN
NPKRVRGITSNFTDTFLVELKQLVITSIDDPSHLDEKVKE)}*

$1141=211(220):
>gi|660647602|gb|KEO87400.1| {|31(0)|<Proteobacteria(B)>}hypothetical
protein EH30_07140 [Erythrobacter sp. JL475]
{(LTHGKHLENAGIVVQAKKDGETAYETISFFQHHKRTILSRRAVGAKAAPSDTIVKSSDSISRPKARRPKSK
PKQVEPNYD)---VFISHAS--EDKKPFVQGLADRLKSEGIKVWYDKFTLKWGDSLraQIDRGL-
ASSRFGIVVLSPAFFRKQWPQaELDalFALEMSNQTK---ILPI----W-
HNLSKEEVNKHSSLLAGRLALASDHPIEDLLMQLKDLLSE()}*

$1142=176(185):
>gi|655039493|ref|WP_028488244.1| {|0(1281)|<unknown(X)>}hypothetical
protein [Thiothrix lacustris]
{(MSSTTSGK)--HIFISHAT--A-DDAFVKELREKLELHHLNVWVDSRNLRGGDKLwpEVAEAIR-
TARQVLMVISTHTVNSSWVRkEVRLAEQAGVT-----VIPLLLPGI-
LPAALGMWFDEEPAGEKIELEVGKLQEAMPRILAALGERL(PDDLPPEQTIENKPLAELLLELTEPTLTRNAD
GGEQL)}*

$1143=180(179):
>gi|662338784|gb|KEQ37440.1| {<Firmicutes(B)>}hypothetical protein
SK667_1220 [Streptococcus mitis]
{(MSRKKKGKTNFIDNEIVTNRKGPEELKKRLYIDDLEKIK)KFDIFLSHNS-KDEEKIITFY--
KKLNKNGQVVYIDwvndkYDLKRewcnTSTVDIIKERIKQSDVFVLYFSSKTLNSQWCAWELGyadaigkKIC
iYFSEK-IDRTIIPKFY------------FKYPKLLFSEDFMVKNHGQSDIPFTKWKENKK()}*

$1144=199(188):
>gi|727219316|ref|WP_033680280.1| {|0(7)|<Firmicutes(B)>}molecular
chaperone Tir [Streptococcus mitis] >gi|662338781|gb|KEQ37437.1|
hypothetical protein SK667_1217 [Streptococcus mitis]
{(MVTK)---IFISFRFSDGKEikdeLVDLFDESTEVINR--
SEDVDRSQMSENIIQeYLYEKLKDTSVTIVLLTPEAVSYRKNwLGnyDDWLYDELRYSLeDRKNnrtngVIAV

YTDEAKykVLDDSTHYCQHCQQTKSCRSLKYFDNLARKNMLNIKSAYK(KNPCNDLYDDEHDSYISLVSLSDF
KENYSQYIQNAKDKRERLDE)}*

$1145=216(228):
>gi|662073768|ref|WP_030037749.1| {|110(0)|<Actinobacteria(B)>}TIR
domain-containing protein [Streptomyces resistomycificus]
>gi|918499009|gb|KOG34489.1| hypothetical protein ADK37_18320
[Streptomyces
{(GEVRYYALVEQLAKTIAESGGTELEPLGVTDARTLPPAFGSESAPPPGPLPEGEGGERWARRWLDARDPAA
RGHGPGSVA)-----ISYVGADQPWADWMMGVLEQRGHE-VEL--
WRWRAGrERLPDAVARARDSAESLLVIFSHNYFAAGDTAPtEWEEAFvPAS-----
DRPSPSVLVQIDaaPRPLLVRDAQVVVLAG--TDLAEAEQ--RIGEVLPRTRRQ(SQDRGGGR)}*

$1146=281(297):
>gi|662080555|ref|WP_030043163.1|
{|262(410)|<Actinobacteria(B)>}hypothetical protein [Streptomyces
resistomycificus] >gi|918498596|gb|KOG34097.1| hypothetical protein
ADK37_20440 [Streptomyces resist
{(LQVPYAPAYAFDEQVITDPGRAEERRSLANAYASLRGAITLLAPDDNPLASLRPASAPSRPGGGATEPVET
RYDPTSHFA)SADVFLSYRAADMETADQIRELLTGRGN----LQVAEPLTGQLW-----
DEIPRAKVNLVLVGPPG-ELGAYQRhELHTLSARE-----DaRLMPVLLPGVKRLPGALREFPYIDFRgG--
LDMDGLLQ--
TVQAGLRSPVPA(DQGPDTLPRSPYPGLAPFSEADAGLFFGREELVSAVLHSLGQDQMCTILGGPGSGKTSLV
NAGVLPALRAGLLPGSD)}*

$1147=199(193):
>gi|663143268|ref|WP_030183869.1|
{|0(510)|<Actinobacteria(B)>}hypothetical protein [Streptomyces
violaceorubidus]
{(MSTPDASAR)EWDVFISYSR-SDAGK--
VSALVSALQAHNLRVFVDDTAVDDFTSItaTITEALAHSRILLALYSADYPQRRACQWELTYAYLTGQREGdp
hrRTLVLnpePASDHVHPVELRDARHWPWPTTPEGLDRLAARVADHVDTVTTFMGDAA(DASIVPWLPAPART
GSFRFTGRLPEQWRIHTALHRHRAPLVDQA)}*

$1148=287(297):
>gi|684370363|ref|XP_009163436.1|
{|394(84)|<Platyhelminthes(M)>}hypothetical protein T265_12732, partial
[Opisthorchis viverrini] >gi|663056987|gb|KER32887.1| hypothetical
protein T265_12732 [Opistho
{(LWCLSFVPECVDHLVPGSELRRMIDQISEGKNWPADWRKAASGICWNLNRSRATPLVNPIQVDAMMATERL
QSRRGKHLM)-----ISYKHSTQQIMLKVRDELRCRGYI-VWMDVENMY--
GSFVDAMAKGIEQAAGVILGISQE-FKNSPHCRqEVKYAYSLG-----
IPLFPLTLEPdfFAdgWLGILLAALPRISLTDESmVPDAVAHL--
AKQLGVRGHMSE(CNSGSEAQFSRTLDERSAILSLLGRSTQSSSGHHNERINLPGSIDFKSDASRIALHHPTT
THHPGQECEKPVISSHS)}*

$1149=249(257):
>gi|663141029|ref|WP_030181685.1|
{|153(806)|<Actinobacteria(B)>}hypothetical protein [Streptomyces sp.
NRRL S-813]
{(EGDVTPGPRWRSVTWVLAWLDGQIGTSLFGPSFERSYWPLLPAGAGFVCGLVTMAARQAAERVGAARNPFA
DATGGAGGR)---IFLSYSRRdTDFARTLTAALQGRVR----
EVWVDwQAIKPSAKWRqSIADGIRASDALVVLLSRNALSSPYCWDECRQAIEQRK-----RILPIVIDPA-

LASGTGAALREAGWEELtaFQRLDMSRSDRSGPGVEQIAAFA(AQEHRWVAGHTRLGLQAHEWRESGRSDDFL
LREDELW)}*

$1150=194(186):
>gi|663361452|ref|WP_030359920.1|
{|0(433)|<Actinobacteria(B)>}MULTISPECIES: TIR domain-containing protein
[Streptomyces]
{(M)-WDIFLSYSR-RDADR--
VRPLHEALTAAGLKVFVDESAVAGFAGIsdSIRRALADSRVLLAYYSSGYPERPACQWELTAAFlaGLSEGDP
RRRVLVVnpepgtghIHPVELRDTRHGRAADLASDVIRHLRQVTGPMPLEAAPPRVHGTMP(PVMGEFTGRLP
ELWRLHSALHEQEAPLVTGRTMARPVQLRGMPGI)}*

$1151=176(183):
>gi|663695315|ref|WP_030472353.1|
{|0(755)|<Actinobacteria(B)>}hypothetical protein [Lechevalieria
aerocolonigenes]
{(M)----FLSFSG-DDRPR--
VMELYEALKVDGLRAFIDERGIRYGHGVsaEITTALRGSKTMLVYYSASFPQRSACQFELryAYLTALRAGQV
EQRILV----
VNPEDPATDHLLPLELSTNRYVRTWQSKRELAALVALVRQKAQ(ALDGPFPGMDFGAPPPVHHRKFEPTVSFV
GRYRDRWALHSAL)}*

$1152=241(232):
>gi|663666665|ref|WP_030450534.1| {|0(126)|<Actinobacteria(B)>}TIR
domain-containing protein [Herbidospora cretacea]
{(MNSTNAAGFWSYT)-HRDDLAEG---
GRLLRLAESVREEYALLtgnDLEIFVDREGIAWGEEWrqRIDQALQSTTFFIPIVTARYLQSVECRRELLRFA
GKAESLgVKELFLPIVYAPIRqldqpeDNDDEIVALVRRTHYQDwRELRLLDENSAAYRAAVHALAGR(LVKI
ADNLQENPPDLPTPEALAEDAWAAQDPGIVDIMVEAEKALPAWAKTIDDLLACMNQLVDVVAPMAARLQESDR
AG)}*

$1153=195(204):
>gi|663701110|ref|WP_030477976.1| {<Actinobacteria(B)>}hypothetical
protein [Lentzea albidocapillata] >gi|1175094405|emb|SMD23029.1| TIR
domain-containing protein [Lentzea albidocapillata]
{(MSYKYDFFISYCRYGSVQRWLLNH)-------------FLSKLKECLADQYA-PTPTVYVDRT-
MERGVHWpsSLQDALRHSKIMIQLLTPQYYGSRWCMAEQKSMRAREEMLgfaslqvPQGLIYPILYSDSEnfp
lEE--------
KERSWVDfKDVAHPDPVYQQSRKYLRFHTRV(NDLAADLVQLARQAPPWQPDWPDIDPPDPPLMPPPQIPRF)
}*

$1154=287(300):
>gi|663414139|ref|WP_030409546.1|
{|91(99)|<Actinobacteria(B)>}hypothetical protein [Streptomyces sp. NRRL
F-5917]
{(VLIGDAPLPPLAASRVWRDFRHVDGPGYEEKVDELVAAVRGETAPDGSRRSAAPGLQDNIAAAQPLPPRPL
TPPDQPAVV)-----LCYAAADAAYGQALAVQLRSAGLA-VWSVA-
DLKPGDSHVWSVQRQLDFATAVIVLMSPHSQESEDVTRMILEAMHHQ-----RPFFPILLEGQRN--
YLLAHTWYVDardgrLLR--EDEVGLLR--
DLDAASRNGASP(DAMSAVIPAPTLRPAVEAVRMPAAVSLGRLDSYLAERNFAYADLQTTAIVLEAAGRLDEG
WLSTRHVQRIPARVLARVDG)}*

$1155=232(230):

>gi|663690224|ref|WP_030467298.1| {|0(157)|<Actinobacteria(B)>}TIR domain-containing protein [Lechevalieria aerocolonigenes]
{(MSPVEIWRTCFPRARYLRKIGREPAMAASSDEAR)-TRVFLSYSRKdSDFVIRLAAELDA--RGYLVDFDQSSRdpnnvitgISAEDLWWiRLQEMIALADVVLFVVSPDSIRSAIC-DeEIAFAQNLKK-----RVIPVLCREI-DFAKAP-PRLSALNIKVkfAGGDAVVSADQLALLAAAVDQDV(VWLRGGTRLLAAAQRWDAGGRAEDALLRGVEHRDAEEWAVRRPGSAPELPELVLEF)}*

$1156=274(271):
>gi|664084607|ref|WP_030622787.1| {|91(688)|<Actinobacteria(B)>}TIR domain-containing protein [Streptomyces sclerotialus]
{(ARPESVRVVLEWLDGQAYGALFGPSYAQWYWPLLVAGAGLLAGGGSVALREWSARLAASRDPFDERLRISEVPEPVTRTE)--KVFLSYSRRdADLACRLVDRLQG--RVG--DVWVDwRAIKPSTQWReELSEAIRSSDALVALVSPDSLRSTYCWDECKQAIDL-----GKRILPVIVSPdfthgvtaalC-DAEWDELTAYQFLDMSTpqAFEAGVDAIARFVSREHDWVAFH(TRLGLQAHAWKTNGSSDGLLLRPHEVRLAESWRRNTPADPDFKAELTRLQE)}*

$1157=187(215):
>gi|663689310|ref|WP_030466388.1| {|0(256)|<Actinobacteria(B)>}TIR domain-containing protein [Lechevalieria aerocolonigenes]
{(MQALRED)-------------------------LERLHWPVWMDHE-IHGGEQWwrEIIETIQQTRV-FLFALSDASWRSRPCQeELDYA----KRLgIP--ILPVQVGPmL-HTRIPLMETHAIDYRERTPEAVLSLVAaLADLGRRQVRLPE(PLPEPPKVPFEYLYRMAERLGPKPIPADDQEMLIVQFRSKLKNEDDSVARADIVKLLKELRGRRELTV)}*

$1158=216(223):
>gi|663693640|ref|WP_030470694.1| {|0(600)|<Actinobacteria(B)>}hypothetical protein [Lechevalieria aerocolonigenes]
{(MYE)-FDVFVSYaSVDRAVVWPFVERLRADG--LRVWFDEHSMAGGLPAMQQLSEGIERsAH-LVACLTDAYLQRPWTGYELQVSRYLDPSGRAGRTIPVLFQRLTLPLPAEISFLAQCDLTC----TTTFE-AEYRKIVGALRRP(VPSAGADSLADACVAPFQHLDEPAVALFKTYQALRALLPMLEKLSSSGEVPAQLHASLATVQAFARERAEDVPVTGRTVA)}*

$1159=175(175):
>gi|664250543|ref|WP_030782050.1| {|0(442)|<Actinobacteria(B)>}TIR domain-containing protein [Streptomyces sp. NRRL S-920]
{(M)-WDVFLSYSR-KDAGH--VRRFHEELIAAGLKVFTDESAVAGFAGIseTIRRALANSRVLLAYYSLGYPERPACQWELTTAFlaGLGEGDPRRRVLVVNPEPDTAHIHPVELRDSRHGEPETLVPDILAHLRQVSGPMRPDVTP(PRVHGALPAAVGEFTGRLPELWRLHSALHVQEAP)}*

$1160=185(185):
>gi|664221717|ref|WP_030754590.1| {|0(233)|<Actinobacteria(B)>}TIR domain-containing protein [Streptomyces griseus]
{(MGE)---VFISHSARGDAYAldvlEKIEQGLEKRGHR-PLVDQAAIPPGEEWRTEVIKWVARCDAAVVLLNDKAL-TSHWVRrEvnilmWRRALGAP-----LLVIPVLLGDLTtsAVKAAGLEELRAVQFA--RNRPDAKAVADQVLERFAELPD(VLADSDPMREWISDLGGYLQEARANSQRLVKAARELGIEDAH)}*

$1161=187(186):
>gi|664409572|ref|WP_030935396.1| {|0(231)|<Actinobacteria(B)>}TIR domain-containing protein [Streptomyces sp. NRRL S-646]

{(MV)--KVFISHsTSSDEQTARLrdaIAKGLEERHHT-
VLLDVRLQEPGRPWQLKLAKFLGECDAALVLVNEAALGSS-WVRrEsnilhWRKTLHPG-----
MVLVTVLVGGVTagQLRASELRYLADDDVE--
RVRTWADEEVNRLVGQFPRIRR(LWDDAVSAWLDNIGIQLREIKDQSTLARIATELDIPAEEHEELI)}*

$1162=216(221):
>gi|664341804|ref|WP_030869947.1|
{|491(0)|<Actinobacteria(B)>}hypothetical protein [Streptomyces violaceoruber]
{(TANTVLHLAEARRKPIVPFEFLGGASRRAYQRRDWTAVYPALEHRRLMDRDAVPDAMKIADQLVTARLRTPHNYRWPPRR)---VFISRARPdAAFAQALETYLSGA---
GITALVGEKELRPDRMVNpTIEDAVLSADLFVVLWSRSYALSRFCYDEIELALRRHAV-GEIQL-WIINLDG-
SDIVPPEARALPQALARtpHALVALVRELLEHAEPARTVPGG(E)}*

$1163=163(161):
>gi|696506205|ref|WP_033043514.1| {<Proteobacteria(B)>}molecular chaperone Tir [Pseudomonas putida] >gi|665419543|gb|KEX92005.1| molecular chaperone Tir [Pseudomonas putida]
{(MP)---VFISYRH-MD--
RAYAININTRLIQANIKTYLDvlDAESQTTDDItgVITRNISECTHLLAVVSEKTALSWWVPFEIGEatitNRRICSFKTGPTELPLYLDKWPKLSSERDIEFFIDAYRNELTVKRSMSLESVTGSESVRSAN(RTNADRFHTDLKSRIIRGF)}*

$1164=225(226):
>gi|665505569|emb|CCH76157.1| {|90(270)|<Actinobacteria(B)>}conserved hypothetical protein [Tetrasphaera japonica T1-X7]
{(APSGPSPTDHAAMRAIAKVSHSPIALLVYARGKGDEFFGLIAGRGRLGRTVTRKATVSLPPPRFESLGPLPDGAVRGDGP)---VFISYRQSDGTPQAESLEDLLRAAGL--
VVWRDRTDLRPGTTTdRLEQALtTGLSAGVLVVTPDIADSDIVREreLPRLLQLDADPAFSLCIANKVARVGSESKCDYDAPDRLLRLAPARTLADKKQANMLEPSGEVEIAR(DLLMHR)}*

$1165=277(263):
>gi|665790896|ref|XP_008561155.1| {|242(0)|<Arthropoda(M)>}PREDICTED: single Ig IL-1-related receptor-like [Microplitis demolitor]
{(YECRISNGADEEMTLSAYIYRREPHFDFGLRSGSWRKALLFAVLGLVLFVSIGAFFIRCWLPMAVICHNKFQKSTENDGK)EFDAVVCYHEKdaALAVNTLIPTLKTK-YNYKcstleLTHLTRNW------
NLEIGSNASSSRRVIVILSPNALENEWTDTKVTQALKQ-LSLISNQVIVIILNEL-
SNVAAFAKSTGHCvdaacadgivLDKMtILRWkvdsekdtGGYK---
FWCRLRLALPPV(RSTPTQSLPDFQTRCQSVAMIVQNNNKSIPQKTQSRESLEVLV)}*

$1166=172(170):
>gi|664558082|ref|WP_031072887.1| {|0(284)|<Actinobacteria(B)>}TIR domain-containing protein [Streptomyces sp. NRRL S-118]
{(MGPYF)----FLSYAR-
VDDKDAYVRQFYDDLCDRIIELsdstaqvpvgFRDNSSIQLGEHWseQLERALGSCHSMVALYSPAYFRSEWCSREAGVMMRRA-----
GQYFARTNRGSSALIPVLWQPVEAPQEIAHIQYLTDGLGSWYADAGLQRLLQ(RDPTGRDYQNAVRLIAQRVLDVARA)}*

$1167=221(254):
>gi|664679262|gb|KES19356.1| {|114(339)|<Proteobacteria(B)>}hypothetical protein GASC598B02_014420 [Gilliamella apicola SCGC AB-598-B02]
{(TSEGRLKSLNCIRMFEWNEDFKKEYAIFYLSHELYRFGLKNINLLESGIDSSIRLFLSHAKKGDTGLKHAKAIKKFIDNS)-----------------------------NMQGFFDVNDISSGFKFdeEIEKHIKRSTL-

IAIESDAYSTRYWCQREILTAKREK-----
RPIILVNSLEEYEDRVFPAASNIPCVHITAEPLNDKDILRILIAALLETIRF(EHARKLLTYYRNQKWIDQNA
VIFARPPEIQQIVE)}*

$1168=189(190):
>gi|665611568|ref|WP_031174476.1| {|0(227)|<Actinobacteria(B)>}TIR
domain-containing protein [Streptomyces durhamensis]
{(MVMA)----FISHSSRGDANANAVRqavqAALEKNGWT-
VKVDMDALQPGDDWRSVLYHWVAECDAAVILLSSAAMK-STWVRrEanfllWRRALGSP-----
VTLIPVLLEDMApaDVRRSELSQLSALQLL--
VQRPGKRGGTPTRIASRVVGLL(PQIPDLVRDSAELSRMRNWTEDIICYLNGVPEDPLHAAARLLTAND)}*

$1169=163(163):
>gi|872693950|ref|WP_048545955.1| {<Actinobacteria(B)>}TIR domain-
containing protein [Tetrasphaera jenkinsii] >gi|665498219|emb|CCI53644.1|
conserved hypothetical protein [Tetrasphaera jenkinsii Ben 7
{(MGSHATAT)----FLSWAGKdRRLkaslLDRLEPHLKTL--
PGVkFTWWEMSHIQIGEAWRrDILARIDESDAILQLLSPNFFASDFIVQeEIPACIGPEAP-KRA--
LPVGLKPF-PLDPKRRLLQGVNEHQIfrLGDKFYSECSATEKDRFALELAN(AIQDSLTGKSPWRKL)}*

$1170=223(243):
>gi|664541483|ref|WP_031056943.1|
{|0(240)|<Actinobacteria(B)>}MULTISPECIES: hypothetical protein
[Streptomyces] >gi|973815176|gb|KUM86142.1| hypothetical protein
AQI94_24265 [Streptomyces pseudovenez
{(MSAPLLPADETDDKGHPSGSSFGERGLVFLSHSSRIRTERELGENDSLRDHQHRKNYLINLLTT)------
---------------LDTGLPRLGFQAWLDKKNLEEGEGFdtLIYDALARCRAAVILIDRDA-
LDSSYVLaEATI-LNFRRTVgDPIHILPVLLGDV-
TDNDVNASKLGRILRLDSLLPLRPTAGKTDDTSVEPTADE(IAQALDRSVTRCDTADSPTQRWIDNVSSYLAG
ASEDALL)}*

$1171=235(255):
>gi|297801410|ref|XP_002868589.1|
{|1083(138)|<Streptophyta(V)>}predicted protein [Arabidopsis lyrata
subsp. lyrata] >gi|297314425|gb|EFH44848.1| predicted protein
[Arabidopsis lyrata subsp. lyrata]
{(TISMEEAEDSSSSSKMDNFETESSSNRVDYYESGGNSNHYTDGDGNRDYKAEASKVSQVESTKTSKTYRML
ELVYEACQS)----------------------RESLICGTISAASLQEQSRQAK-
GsrRLMIgpAIRESSVSILVLPKKYASSRWCLDELVEIVKCKEG-
LGLIVMTVFQWISTAWCNCKELTLEKNVKSLLCRWLEFFWRPAGIGATTIFDYK(CLMGKLQGSYKIIVGVDV
YDSNFAVRGSLLKNHFC)}*

$1172=179(203):
>gi|297789741|ref|XP_002862805.1| {<Streptophyta(V)>}predicted protein
[Arabidopsis lyrata subsp. lyrata] >gi|297308538|gb|EFH39063.1| predicted
protein [Arabidopsis lyrata subsp. lyrata]
{(MLTGFITAKGRESEEDTSGFVFKNCEIKGSGKAFLGRVVFYETNMSSVIVHPGWDAWKYKGQD)-------
----
YLIAAFNQQGIIsAFVDGKSSHDEAVErEMgPEEFSKLRVVVVVFSKNYALHvSFLEKQILEY--
SYRNNNDFVVVPVFYGVSVSSVKQHMERFVEEFDAVQRS-----MI-KWRPGHEYDCKR()}*

$1173=289(299):

>gi|297794751|ref|XP_002865260.1| {|216(123)|<Streptophyta(V)>}predicted protein [Arabidopsis lyrata subsp. lyrata] >gi|297311095|gb|EFH41519.1| predicted protein [Arabidopsis lyrata subsp. lyrata]
{(VDLVAPLIANIDMDVSSGIKVLADRSLIRVSSNREIVMDSLPRRMGKEILHTESMLPGSSKDFGKIASMGLSSPHNQKCN)---VFLRYSR-QDIQINFLDYLIAALRKNVIPSSKRYGIKITQEVPESR-----STISIILFTINYASSSWLLNELVETAKSNEDVSHM-VIPIFHpDLDPLDVREQTGEFGRRFEETCKNKTEDEKQ-QWRRALTDISSI(GEYNITYCDSSDEEFIKSMVKEVKDLMARIRIKGKPSREVISQGVVFVVPARRLDITHSENPNLWTWGYVQSEADIEIA)}*

$1174=216(223):
>gi|297794767|ref|XP_002865268.1| {|0(374)|<Streptophyta(V)>}hypothetical protein ARALYDRAFT_916988 [Arabidopsis lyrata subsp. lyrata] >gi|297311103|gb|EFH41527.1| hypothetical protein ARALYDRAFT_9169
{(MDNNSNDEAKHT)---VYISFNK-NDSSVSsFISYLIAAFNRQGIIsAFVDGKSSHDEAVErEMgPEEFSKLRVVVVVVFSKNYALHvSFLEKQILEY--SYRNNNDFVVVPVFYGVSISSVNQHMERFGEEFDAIQRSR-----I-KWRPGHEYDCKR(RYSESEFLEEIANDVFEQLYPTEEIGIQSLLKEIVENYLCNQPWGIRTIGIFGEPGIGKTTLATAFFRRIS)}*

$1175=205(226):
>gi|297794773|ref|XP_002865271.1| {|0(507)|<Streptophyta(V)>}hypothetical protein ARALYDRAFT_917000 [Arabidopsis lyrata subsp. lyrata] >gi|297311106|gb|EFH41530.1| hypothetical protein ARALYDRAFT_9170
{(MAGQTM)---VRISFdsSE-ERLRWSFVPHLSAAFGRKGISVSTSMND--------------EFVASLLVFSEKYVSSKESLDEVVKTIQQRHD-KGHVVATVFYGVSRSDVQELKGNFGKVLLE---NGASDQV-TQWHNALAEIASL(PGYEASNTQSDYEFVEKITRDVYEKIFPKERIGIYSRMLPAIENLLCKQPWGVKSIGICGMPGIGKTALAKAVFDQMSGG)}*

$1176=209(234):
>gi|297794775|ref|XP_002865272.1| {|0(250)|<Streptophyta(V)>}predicted protein [Arabidopsis lyrata subsp. lyrata] >gi|297311107|gb|EFH41531.1| predicted protein [Arabidopsis lyrata subsp. lyrata]
{(MEARKMVKIINFDS)--------SE-ETLRCSFVPHLSAAFGRKGISVLTDKHDQ-----------SYKSIASVLIFSENYVSSKESLDEFIKTIQRRHE-KGHIVTAIFYGVSRSNVQELMGNFSKAFLEH---RDSDQV-NQWRNALAEITSL(PGYETSNNQSDYKSVEKIARDLYEKLFPNERIGIYSRMLPDIENLIQKQQWGVRSIGIWGMPGIGKTKLAKAVFDQMSCD)}*

$1177=224(248):
>gi|297809509|ref|XP_002872638.1| {|0(629)|<Streptophyta(V)>}predicted protein [Arabidopsis lyrata subsp. lyrata] >gi|297318475|gb|EFH48897.1| predicted protein [Arabidopsis lyrata subsp. lyrata]
{(MFVDRAFSDTSDVFGPLRTCARFSASED)-YDVVIRYRrG-DQINDDFISHLRAALCRRGISVFNE---------FDEVDAVPKCRVFIILLTSTYVPS-----NLLNILEHQQT-EYQAVYPIFYRLSPYDLISNSKNYERYFLQ-------NEP-ERWQAALKEISQM(PGYTLTDRSESELIDEIVRDALKVLCSGDKVNMIGMDIQVEEILSLLCIESLDVRSIGIWGTVGIGKTTIAEEIFRRISV)}*

$1178=278(300):

>gi|297804202|ref|XP_002869985.1| {|300(544)|<Streptophyta(V)>}predicted protein [Arabidopsis lyrata subsp. lyrata] >gi|297315821|gb|EFH46244.1| predicted protein [Arabidopsis lyrata subsp. lyrata]
{(CAKDVIDFLRMRQTSGMEAVEALAGYLKAMCEAHGIRPMDNPFRSLAVTSYLKSASEMTREKECILVFSCN
ENHDVDETS)------------------FIEAISKELHKREVTPLMYNLLSRENL---
DEKMLYRSSVGIMILSHSYACSRQALDHLVEIMEHGKA-
RNLVIIPIYFKATLSDICGLEGRFEPIYLQYMDSAQLSRV-
QKWKAAMAEIASI(DGHEWEKEKQVLLAEEVVRDACLNLYSKNSKNLISILAFLNHSQPSGVEIVGLWGMAGI
GKTSIAREIFGILAPKYDFCY)}*

$1179=181(203):
>gi|297807803|ref|XP_002871785.1|
{|0(893)|<Streptophyta(V)>}hypothetical protein ARALYDRAFT_909793 [Arabidopsis lyrata subsp. lyrata] >gi|297317622|gb|EFH48044.1| hypothetical protein ARALYDRAFT_9097
{(MVDFEKSSHSVNIICEETV)---------------RRSFVSHLSSALHREGISVcvFADTDFD---------
DQNQGARVTVVVFSENYAFPHPMLDNFAKILQLRSN-
SGHEVIPVFYGVDPSAVNPNHDWLPLHMEGHQSMNSSNVRSSDSQLVEDIVRDV(YGKICPTERIGIYTRLME
IENLLCEQSWDVRRLGLWGMPGIGKT)}*

$1180=236(228):
>gi|297794769|ref|XP_002865269.1| {|0(475)|<Streptophyta(V)>}predicted protein [Arabidopsis lyrata subsp. lyrata] >gi|297311104|gb|EFH41528.1| predicted protein [Arabidopsis lyrata subsp. lyrata]
{(METSRTVG)-YDVFIDYSS-KDTRHSFVSHLHAAFgRRG-
ISVFLaehctlSEATLKPGFELanEIQLAIERSKIYVVVFSKNYASSPLCLETLMTFMDLqRRK-
DGPVVIPVFYgDVTRSIVEQQTERFKEDFSKH-
RGFFSDEKDrvERWRKGLTEAAKL(HGHESIEQQNDSELVEDIVADVRERLCPTGMIGFYSRLLGIENLLFKQ
SHDIYRLGIWGMPGIGKTAISQESFNQMTKHF)}*

$1181=185(188):
>gi|506268793|ref|WP_015788568.1| {|0(80)|<Euryarchaeota(A)>}TIR protein [Halorhabdus utahensis] >gi|256690652|gb|ACV10989.1| TIR protein [Halorhabdus utahensis DSM 12940]
{(MTESKDDENR)--DLMISYSH-SDS-
SEIAEMLFEELTGYGLDVWYDEVDISIGDRIsnSIDRGLQTSNHAVVIVSPSYFEGMSDL-
ELGGLFKKQSRSDGKVILPVLYEISFEELEQQSWSLSDLHGVEISKDNVQEVASKVFQSIKDASEE(QEVNLE
DAPSNHFAEVQASFKDLADIEKGDKVRIDRWR)}*

$1182=162(179):
>gi|297791611|ref|XP_002863690.1| {|0(61)|<Streptophyta(V)>}hypothetical protein ARALYDRAFT_917378 [Arabidopsis lyrata subsp. lyrata] >gi|297309525|gb|EFH39949.1| hypothetical protein ARALYDRAFT_91737
{(MDEKEQPQP)QHQVFINFRG-KNLYQS--SH-----RYPG--------
EDEGQGINNLFKRIEDSRIALVAFSSRYTKSRWCLDELAKIKERQDQ-
GVLKSSFIWSIVSNVLKLLDKISSTEQTAKYQPNHLFEQGETSSALGYPKKSIL(TTPQENLKKTQPRKRIGF
NVGGSSIANPAN)}*

$1183=201(211):
>gi|297791263|ref|XP_002863516.1| {<Streptophyta(V)>}predicted protein [Arabidopsis lyrata subsp. lyrata] >gi|297309351|gb|EFH39775.1| predicted protein [Arabidopsis lyrata subsp. lyrata]
{(MLSLLVRRTFTKSRMHGEVKPPRNI)--QVFINFRG-
DQLHYNFVSYLVDALRRSEINVFIDNEEQRGEDLNTLFKRIEESGIAIVV------

QIIHLLTHITNFGNIVTL-YH-KVLPIFYKVTPTNVKRLKGEFGDHFRD-
KEYMYKSDEPmiKQWKEAIVSVSHK(FALALDEKSSLLEIDFVETIVKEVLKMLQAICKVESGQSSFCMEKA)
}*

$1184=180(187):
>gi|297791299|ref|XP_002863534.1| {|0(1180)|<Streptophyta(V)>}predicted protein [Arabidopsis lyrata subsp. lyrata] >gi|297309369|gb|EFH39793.1| predicted protein [Arabidopsis lyrata subsp. lyrata]
{(MADSEQL)---VCISCV--SEVRYSFVSHLSEALRRKGISSVIidvDSDDLLSKESQA---
KIEISRVSVMVLSRICEPTRVCQN-FVNVIECQRN-
KNQVVVPVLYGESPLLGEWLSVLDLRDLSPVHQSRKDCSDSQFVKEIVRDVYEK(LFYKGRIGIYSKLLEIEK
MVCKQPLGIRCVGIWGMPGIGK)}*

$1185=205(207):
>gi|297794745|ref|XP_002865257.1| {|0(1633)|<Streptophyta(V)>}predicted protein [Arabidopsis lyrata subsp. lyrata] >gi|297311092|gb|EFH41516.1| predicted protein [Arabidopsis lyrata subsp. lyrata]
{(MI)---VYISCV--DEVRYSFVSHLSESLCEKGINdVFVDSADNLSEEAQA---
KVERARVSVMVLPGNRklTTASACLGKLGKIIRCQRN-DDQVVVPVLYGVRKvnvewlSELKKITG--
LSHFHQSRKECSDSELVEEIARDVYEKLYH(IGRIGIYSKLLQIENMVNKQPLGIRCVGIWGMPGIGKTTLAK
AFFDQKSGKFDASCFIEDFDKVI)}*

$1186=215(223):
>gi|297794755|ref|XP_002865262.1|
{|0(761)|<Streptophyta(V)>}hypothetical protein ARALYDRAFT_916973 [Arabidopsis lyrata subsp. lyrata] >gi|297311097|gb|EFH41521.1| hypothetical protein ARALYDRAFT_9169
{(MNDCERAS)-PIVYISCVA--EVRYSFVSHLLDALSRKGINdVFVDSDDVLSEEAQG---
KVERSKICVIVLPGNRTVS---LEKLVKVLECQRN-NDQVVVPVLHGDGP-----
LQGEWLRELElrglSPVHRKECSDSKlvEEIVRDVNEKLFY(MGRIGIYSKVVEVENMINKQPLGIRCVGIWG
MPGIGKATLAKAVVDQMSGGFDAHCFIEDYDTSIHENRVYRILV)}*

$1187=180(187):
>gi|297794807|ref|XP_002865288.1| {|0(1157)|<Streptophyta(V)>}predicted protein [Arabidopsis lyrata subsp. lyrata] >gi|297311123|gb|EFH41547.1| predicted protein [Arabidopsis lyrata subsp. lyrata]
{(MADSEQL)---VCISCV--SEVRYSFVSHLSEALRRKGINSVIidvDSDDLLSKESQA---
KIEISSVSVMVLS-RICEPTRVCHNFVKVLECQRD-
KNHVVVPVLYGESPLLGEWLSVLDLRDLSPVHQSRKECSDSQLVKEIVRDVYEK(PFYKGRIGIYSKLLEIEK
MVCKQPLGIRCVGIWGMPGIGK)}*

$1188=180(191):
>gi|297794829|ref|XP_002865299.1| {|0(952)|<Streptophyta(V)>}predicted protein [Arabidopsis lyrata subsp. lyrata] >gi|297311134|gb|EFH41558.1| predicted protein [Arabidopsis lyrata subsp. lyrata]
{(MTESEQI)---VYISCVK--QVRYSFVSHLSEALRRKGIiDVFIDTDDFLSNESQS---
KVERARVSVVVLSGN---STVCLDKLVNVLGCQRN-
IDQVVVPVLYGEIPLQVEWDKALNSRGLSSVHQSRNKCTDSELVEEITRDVYEK(LFYMEGIGIYSKRLEIEN
IVCKQPFGVRCVGIWGMPGIGKTTLA)}*

$1189=223(223):
>gi|297800042|ref|XP_002867905.1| {|0(19)|<Streptophyta(V)>}Toll-Interleukin-Resistance domain-containing protein [Arabidopsis lyrata subsp. lyrata] >gi|297313741|gb|EFH44164.1| Toll-Interleukin-Resis

{(MAIQA)RPLVFINHGE-
NDLSDGFIRHLASALRDEGFNVFIDSDERRGRGMEHIFRAIDNSKVALVIFSDRYTASELCLHEAVRIYDRRR
E-
GKLVLIPVFYRVSTDDVNMFNGRFGESFLETLKIQGISDHPfaEHWMRNVNFICTD(TGYISEDYSSNDTSLV
VEIVRGIKRRLQGKKLPRRVQDENLPSIFKMFMPKQPNTQEGFGKELLYVAIPALLCYFILP)}*

$1190=226(225):
>gi|297800046|ref|XP_002867907.1| {|0(26)|<Streptophyta(V)>}hypothetical protein ARALYDRAFT_914667 [Arabidopsis lyrata subsp. lyrata] >gi|297313743|gb|EFH44166.1| hypothetical protein ARALYDRAFT_91466
{(MAMEA)RPHVFINHGG-
NDLSDYFVRELATALRDQGFNVFIGSDERGARrGIKYIFRTIESSDVALVIFSDMYAASEWCLHEAVRIYDRR
RE-
GKLVLVPIYYRVSKEDVNMFQGRFGECFVKTLKTRGINGHSfaDIWKANVNLICTE(PGFTSKDFCHIDVNFM
VAMVHWVKRRLRSRNFPSDIDMFIPKQLEPQEGFGKELLYVSIATVAALLCYFIISLVFTDLDV)}*

$1191=164(184):
>gi|297790138|ref|XP_002862976.1| {|0(120)|<Streptophyta(V)>}predicted protein [Arabidopsis lyrata subsp. lyrata] >gi|297308766|gb|EFH39235.1| predicted protein [Arabidopsis lyrata subsp. lyrata]
{(MSQE)-----------
EDVSKGLINFLEPILKNENINVFIDEETVRGKDLKNLFKRIQDTRISLAIFSESKCD----
FNELRKIKEPVDE-----
AIPIFYKVDAIGDLADLQNRCKKDLINSAVEEMSRLLANISVEVNREKEG(NHKCFMVPARKLQISHSEKLIN
WTWSSIYEAPNEAAIEIA)}*

$1192=210(232):
>gi|297790144|ref|XP_002862979.1|
{|0(163)|<Streptophyta(V)>}hypothetical protein ARALYDRAFT_920867 [Arabidopsis lyrata subsp. lyrata] >gi|297308769|gb|EFH39238.1| hypothetical protein ARALYDRAFT_9208
{(MASSSLAVKPTPT)GPQVFICFRG-
ADVRKHFISFLDPALRKANINVFIDEDEYLGSDLVNLLKRIEESEIALVIFSEEFTSSYWCLEELAKIKDCKD
Q----------------------
GKFGDHFRDQIRNLRHQPERtQKWEDALLSIPES(IGMPLAAQSDRTDKDFITSMVIKIQRLVEHMAVKGNQE
TEANLPGGSIVPAMKPKREANSQGGSMVLAMKPETEASHQG)}*

$1193=246(266):
>gi|297791261|ref|XP_002863515.1|
{|249(0)|<Streptophyta(V)>}hypothetical protein ARALYDRAFT_916997 [Arabidopsis lyrata subsp. lyrata] >gi|297309350|gb|EFH39774.1| hypothetical protein ARALYDRAFT_9169
{(TRLIGEGVAIDRIAVNLQKMPEYIPADGTENPRDTYTEIRTDKYEAKPERSSSAARLNADFLDRTFTNSRM
DGKFKPPNI)--QVFINFRG-
DQLRNNFVGYLRHALRISKINVFIDNEEQRGEDLNTLFKRIEESQIAIVVFSSRYTESKWCLDELVKIKERVD
Q-GL-----------------
LKGEFGDHFRDIEYMYKSDEPMiKQWKEAIVFVSHK(FALTLDEKSSLLEIDFVETIVKEVLKMLQAICKVES
GQSSFCVEKA)}*

$1194=163(179):
>gi|297794813|ref|XP_002865291.1| {<Streptophyta(V)>}hypothetical protein ARALYDRAFT_917039 [Arabidopsis lyrata subsp. lyrata] >gi|297311126|gb|EFH41550.1| hypothetical protein ARALYDRAFT_917039 [Arab

{(MAASSSVRQTPT)GPQVFINFRG-
KDVRNGFISFLEPAMREASINVFIDKDEVVGTDLVNLFVRIQESRVH---------------
HELAQVKDCIYQ-
GGLNVIPIFYKLAPSSVEELKGGFGDTFRVLKRKYKHDHERtQKWEEALQFIPKK(RGMTFSEQRLFSHLLLR
YSRLQIYIKL)}*

$1195=161(164):
>gi|297794865|ref|XP_002865317.1| {|0(585)|<Streptophyta(V)>}predicted protein [Arabidopsis lyrata subsp. lyrata] >gi|297311152|gb|EFH41576.1| predicted protein [Arabidopsis lyrata subsp. lyrata]
{(MAASSSSSSEILPK)SWQVFINFRG-
AELRDNFISHLEGALALAGIKYYIDTKEVPSEDLSVLFERIEQSEIALSIFSSKVTEYLNGISKASGAENVDT
F-SLGSYEFPYSSLEISVTDVDYGMTPLKRQAM-IDELCRVGGEHLNDISTLSSNG(NISSGHIEQS)}*

$1196=300(300):
>gi|297799638|ref|XP_002867703.1| {|496(83)|<Streptophyta(V)>}predicted protein [Arabidopsis lyrata subsp. lyrata] >gi|297313539|gb|EFH43962.1| predicted protein [Arabidopsis lyrata subsp. lyrata]
{(AGRILLVLLGSLEYYNNGFDFKRLFLPKKSQQLPGNTAVAESNDNLRNREVSYTDAVLTCFSFLCNILNRS
EMKIDPPAR)--RVFISLGE-KHLGKFLVNSLKEELESNqILVYAEDETKSR----------
IKESGVAVVVFSNKYPKSEKCLDELVEIKKLMDA-
GKIDPLPVFYSLKVEPVKKLKGCFLNRLLKIenevRKNIKTRDDKsildteakiWGWRDALSSIASR(PGLSY
ELSTDDVFVSDIVTKVNELFASRERKKTEAETTVVKSLDDDLFYSRTSFLQAIDRDNADFESFTDIPHGLVLL
RL)}*

$1197=256(259):
>gi|495930485|ref|WP_008655064.1|
{|81(318)|<Bacteroidetes(B)>}MULTISPECIES: hypothetical protein [Bacteroidales] >gi|256737582|gb|EEU50908.1| hypothetical protein HMPREF0619_02123 [Parabacteroides sp
{(CNTIWDEYIKELLVKQDNGALKIFAVKLSKYAFDINPLLQEEQFICLKNENIETDWHEFQIRLYDNILRYL
KSYKVGQKL)--KLFISHSKKdKDHlgestAISLRDYL-----
RSDTKLDSFFDVNDILDGHqfaqQIQSGIASSLLVI-IESDTYSEREWCRIE--AISGKKNN-
VPSILVNVLNGVSSRTFPYLGNMPKIRFNGK--
WDDVIILLLRTALDQYYEKEY(LEQLVMKCDLQNTSILPVPPELMNLINIEDNIKSILYPE)}*

$1198=199(198):
>gi|490759575|ref|WP_004621860.1| {<Firmicutes(B)>}protein of unknown function DUF1863 [[Clostridium] papyrosolvens]
>gi|325985325|gb|EGD46164.1| protein of unknown function DUF1863 [
[[Clostridium] p
{(MAKGINRAVNQSVIKSYSSIETASKTPC)---
IFLSHQSLdKELVKKIGDYITNAGIDIYLDIYDRDLQNANRTNNdlaiteCIEKGIKNSTHVMCIISRATKSS
WWVPYEIGYGKKNGNE-ISTLPLKELLASEHPSYLKITRQLTGIKSFN--
AYLVEILREKTGTSPTTFASY(QESAYKSFSNASILTESTLYHPLCTHLNQL)}*

$1199=154(163):
>gi|489709795|ref|WP_003613932.1|
{|0(342)|<Proteobacteria(B)>}MULTISPECIES: TIR protein [Methylosinus]
{(M)--KAFLSHSSKdKAVVRAVAD---RLGLAR-----
CEYDEYTFEFTLnseAIRNALKRSTLFVFFLSYNSIRSDFVAEELRIALESRSA-
GTLRRVLIFSLDVTSYKALPEWMREINVAFH--
IANAKSISRKIEASLIALEAE(TRSRLEVYIRRDDDERDLRRAL)}*

$1200=125(141):
>gi|489705989|ref|WP_003610126.1| {<Proteobacteria(B)>}MULTISPECIES: TIR domain-containing protein [Methylosinus] >gi|1040063710|gb|OBS53584.1| hypothetical protein A8B73_05135 [Methylosinus sp. 3S-1]
{(M)--KFFLSHGQDDRRYAASLAAEIERRGGT-VWRDA-
ALSPGSSWEDGLRKAIECSDAMILVLPQTGASGANFAVfEAGAAKAlgkkvfvvapnAD-----
GRDLPVNIADVAifdaankPLDEVVGALIR-------------------------YAS()}*

$1201=270(270):
>gi|257046427|gb|ACV35615.1| {|35(270)|<Proteobacteria(B)>}TIR protein [Candidatus Accumulibacter phosphatis clade IIA str. UW-1]
{(YDTIPQRGATADFVVVCVRRLLDRGCLGSRHALSILLDTVRSAAGNERKAEFQSLIDQLDQPCRQAAVTSS
GAVRPARDL)---VYISYRHRdREWHTQLRNVLDADSRLHD-
RVWDDTQIPSGRDFRkEIDDHVARARIMIMLASDDYFGPECgAAEcEIKPALEAQRK-GQLLILWLPVRAM-
AIATSPVAHLMAASGAGavPLAVLSPEARHATLVKVYHDVLR(HLGMAPLASLQLRPERTAPALSCPPEPTTT
PGKKRELPMLITRLPDSEAR)}*

$1202=171(164):
>gi|494177347|ref|WP_007115218.1| {<Proteobacteria(B)>}TIR [Enhydrobacter aerosaccus] >gi|257448891|gb|EEV23848.1| hypothetical protein ENHAE0001_1434 [Enhydrobacter aerosaccus SK60]
{(MTKSG)--
KIFISYTHVdKEYLQDLKKYIQPLLRfqHSEFEAWDDTTLITGQKLFdKINTQLDKFGLMICLISPDYLASTS
CLDELHIALERNKE-GnidQINIFPIIVREC-SWQFTPLKEFLLQPEDGtpIAtliqKGESKD--
KIYNEIVIELNKT(LNQIKQQAVTEEDSKKKAP)}*

$1203=228(227):
>gi|506247702|ref|WP_015767477.1| {|230(0)|<Proteobacteria(B)>}TIR domain-containing protein [Candidatus Accumulibacter phosphatis] >gi|257047112|gb|ACV36300.1| hypothetical protein CAP2UW1_3023 [Cand
{(QRRLSDTQRRREWLIEPLRPEGGLVRFLKTFSQETEILSDISPPEFVGQLHRRWLERHGGAEQSANPAAAT
APVSPSCMF)----FISYCR--
WTDLPAAEMFFESLLSAGVareEIWFDRTALEPGNDFrdRILEGIRTCRYFVPLISRPAdALEEKFFRrEWGE
AVDRSKGIqGRTFIVPLIVDRD-
YQPQAYERVPREWTDGLDFGHAPVGQPDERTGGLLKRLIR(AERQPRA)}*

$1204=187(173):
>gi|506246916|ref|WP_015766691.1| {|0(318)|<Proteobacteria(B)>}TIR domain-containing protein [Candidatus Accumulibacter phosphatis] >gi|257046312|gb|ACV35500.1| hypothetical protein CAP2UW1_2208 [Cand
{(MKFAD)--DVFISYAHLdnqavvEGQPGWISRFDRALKIRlgqllgREPRIWRDP-
KLQGNDFFadTLVERLPEVAVLVAVLSPRYANSDWCRREMSTFQaatEAHGGSrigNKSRLFKVIKT---
PLPLENHPPEVESLLGYEFFCVDPETGRAQELDQTFGQEM(QARYWQKLDDLAYDLASLLQVLDDQATP)}*

$1205=221(217):
>gi|506248220|ref|WP_015767995.1| {|0(279)|<Proteobacteria(B)>}TIR protein [Candidatus Accumulibacter phosphatis] >gi|257047638|gb|ACV36826.1| TIR protein [Candidatus Accumulibacter phosphatis clade I
{(MPADESL)--DVFVSYAHAdngvalgtsspvgwvtalaANLNEGPNVFRK--------RIFIDHA-
LKPGDEFdsDLLAKVERSRLLLVLLSQNYVDSSWCGREIEHFVRTHSGDpekpadvfVVEI------------
---FPFESLTAVPPiihALRKRLIHAK--
FWYQPATAASPV(LSGYPSPKDSGQEVAAHYWSVLNELRMAIDSRLRALRTTVLPASPAADTRRPDADGSARA
AVAPLGTVLL)}*

$1206=199(220):
>gi|667305007|ref|XP_008582525.1| {|717(0)|<Chordata(M)>}PREDICTED: LOW QUALITY PROTEIN: toll-like receptor 13 [Galeopterus variegatus]
{(PYLWSYHCQQPNTQSLLIDFNEAMCNFGLEKICFFCSFSLVLTTMIFSWFSAKMTLSLWYGLYIFRACCLD
EWYRTEKEF)IYGAFVSFIATdeQWVYEELVPALEEGsHXTFKFYLYHWDFEPGMDIFEnI-
QNAINTSQKTLCVVNNNYLHSXWCGLEVQLAIIKMFYEHEDIIILIFLEEIPNYKSP----------------
--------LLPTLQTVPMG()}*

$1207=215(220):
>gi|666610163|emb|CDH22046.1| {|335(0)|<Proteobacteria(B)>}RNA-directed DNA polymerase from retron ec67 [Xenorhabdus bovienii str. kraussei Quebec]
{(GKDRFQAAKKYHKIRYGKCDKSNQSAIISLRNHIYGRLSFIRMIRGAEWPLYLKLISRMANNDDSPTVEGK
RAMKIMEMF)--DVFLCHAS-ED-
KDDIVEPLYEELVNLRVNAFLDTKYIKWGDSLieKINGALAKSKFVIAVLSRNSVEKKWPLKELNSVLareit
gEDK-----KLLILV----KDGDEELIKDKFPLLVDKLYLVYKNNPEIIAGELLSLLAGKQ()}*

$1208=197(218):
>gi|667682888|gb|AIG77504.1| {<Actinobacteria(B)>}Hypothetical protein AJAP_23255 [Amycolatopsis japonica]
{(MALVYEYDVFISYRRGGGDAPDWVRNHFHPRLQRLLDDNVDY)----------------------------
-DVKIFLE-
DSVAVGGNWprEVREALQRARILIPVCSPKYFRDERCLAEWHTMAKREEivaRDgVTKagrLIYPVIFSDSDY
--
YPAWAHERRmRSFRDwNKPHPQYQKTPEYIEFEDELGRM(VKELVEIIEQAPPWSPEWPIETPPPEPPRPSKL
PRF)}*

$1209=143(149):
>gi|665970946|ref|WP_031336907.1| {<Proteobacteria(B)>}hypothetical protein [Rhodopseudomonas sp. B29]
{(MSDNG)-KRVFISYAHEDRAFARQLGALMEQRGVA-
VTSNEANATRGRRVDDVLREAIERATALVLLIPQQTVSDRNIT--WFEAGAAK-----
TLGKPVLAVlpPARtpgstELPTDLAGLLVLDADR--RSLDSIAD--MLVQAVPGEQMA(MATN)}*

$1210=238(224):
>gi|667682743|gb|AIG77359.1| {|151(0)|<Actinobacteria(B)>}Hypothetical protein AJAP_22520 [Amycolatopsis japonica]
{(RAGVRPGSYSAWIVLLTPAGRTEQKFRLAARFETDFADGEDFQPVPKSTEFGISLTGSSRSAAVEAEEHDE
PGVGLPRSS)-PRVFICYAHEgETHKKKVRDFADLLRENGL-----
DPRIDEGQegprtEWDhWALREIREADFVAVIASPTCRAvgdgtyrgnDRAGIRsELgviRNLLQKHPR-
WASHLLPVVLPGC-SVDDIPMFLHPVTMDHYivEALTETEIEYLLKTIRTTPPWPG(WDRS)}*

$1211=236(222):
>gi|665889706|ref|WP_031256912.1|
{|0(249)|<Proteobacteria(B)>}hypothetical protein [Curvibacter lanceolatus]
{(MTMSATTEHPK)---VFISYSwtS--
VEHEQFVIDLATSLRNHGVDAILDKWDLKPGQDKyvFMESmVVDTEVLKVLVLCdrmyqEKANNRAGGVGtES
QiISQELYGKVkQTKF-IPIVCEydddgqPC-LPVF-
MKGLIYIDVSSEerYGEGLDQLLRlIYEQPFHQKPRLG(GAPAFISNGGTSYVRELGAAVRAIQDGKPNRQGL
ETLFVKGVLTELNKLYVTPDGNDYDEGVYQAITGTAG)}*

$1212=210(195):

>gi|759695979|ref|WP_043412204.1|
{|0(1)|<Proteobacteria(B)>}hypothetical protein [Archangium violaceum]
>gi|667797540|gb|KFA87355.1| hypothetical protein Q664_47515 [Archangium violaceum Cb vi76]
{(MVGRLLEVRLPAMSGDYQ)-YDVFLSYRR-KWPALDWVqNHFYqrlegllgEALPHP-
PRIFMDTREIETGQEWplVLQEALKSSRCIVTVWSAEYFRSPWCVAELKSMLKREQL-
LGyrteshpdrRLVYPVLFSDGKLLPDYTQGILYRDLREW-
SIPDPVFAQSKKIIGFHKEMMK(LTEELSKLILEAPPWQDNWPIDIPEVSSATTIPVPRL)}*

$1213=199(187):
>gi|738257361|ref|WP_036212440.1| {|0(3)|<Proteobacteria(B)>}TIR domain-containing protein [Massilia sp. LC238] >gi|668949782|gb|KFC68528.1| TIR domain containing protein [Massilia sp. LC238]
{(MAIRYGCFFSY)AHGRHDLMK---
RFKTALADALRCYLEPHfdnEDELFIDTEQLGGGDDLdrKIACAMCESVCMILIYTPKYEAHAYTRREYAAMR
LLEHERsSwyelpSRLIIPVIMTRHPeslpPEITENTYYVDFSHFTMATSDLKSNPDFLPDIDKMVQRIS(TH
YHQQKKHTPPGHDCNQFVLPDVPPEWRAMPSLTF)}*

$1214=212(220):
>gi|759676198|ref|WP_043393472.1|
{|0(873)|<Proteobacteria(B)>}hypothetical protein [Archangium violaceum]
>gi|667803633|gb|KFA93056.1| hypothetical protein Q664_11580 [Archangium violaceum Cb vi76]
{(MA)--KVFISHVRSDRTPARRLAAALRQAGHE-PWLAEDALLVGESLSDALARGLKDTGTVVVLLSRNA-
QESGWTNfELSLIRSHgfaR-----ERLLPVRLDRI-SRPEGFADSPPIDLFpgE--VAWREGVE--
RLLRVLGGESPA(EARPPSSESSPDNLPPRTIPFVGREADLAWLRERLGPGRCALLWGRAGVGKTSLALEYAA
RHAADYPGGLWWVPGHEG)}*

$1215=131(159):
>gi|668921546|gb|KFC50965.1| {<Proteobacteria(B)>}hypothetical protein DK37_29100 [Halomonas sp. SUBG004]
{(MPELMGLKLPHSFEMS)----------------------LKVLGVA-
VWFDEVAIIPGRSQSLQMDAGLRKARSGITVLTPAYLTGRFWTErE-LGALLNK-----
STLIPVLHNVTFddVKQYSGILPDLAGFTT--QHDSVEDIAKKLLQQYLLKMMA(ELL)}*

$1216=156(165):
>gi|667798027|gb|KFA87795.1| {<Proteobacteria(B)>}hypothetical protein Q664_45430, partial [Archangium violaceum Cb vi76]
{()MAKIFISHSSKdKPFAIELKRRLESKGH----
DAWLDlSELLVGDEIRtKIDEGIERSDVVVFVLSPDAILSRW-FNyELRVAQTLLRS-
GKKRpLFPVLLRECTnlppDLEGVRFADFR----------RDHE--
QGWKELEQGLSQW(ERVQQPSSPHAPPSRSEPAQPPISG)}*

$1217=210(220):
>gi|667798350|gb|KFA88090.1| {|337(0)|<Proteobacteria(B)>}hypothetical protein Q664_43285 [Archangium violaceum Cb vi76]
{(GGATGGSSSLLIRLAEARGVPVIPFAFLGGSAGEAFLRHKDLYTRLGVAQELNRRNGVESLPKLIRSLARA
PIDPSARVG)--KVFVSRAIEdASIAKLFA---
SQLIERGLVPVFGDEPNEHGKDLQaVIHERLRHADVCAIFWSRHFAVSPYCYDELASALRRHRN-EGMRI---
----W-LFKLDETPVVHPDARRLplWSTLSEEALRDWCQDWLDTLRAP()}*

$1218=248(254):

>gi|670481351|ref|WP_031429699.1|
{|1000(13)|<Proteobacteria(B)>}hypothetical protein [Vibrio parahaemolyticus]
{(IKAASPKLVEHLIDSILKTNHLDAKATVEWLKGEPKEEKEKENRVEQEQKEPFSNIGKGTLDPYRKPNVYFSYAWGKGEN)------------
DPKQKVCDEIFNKLKSDnSIQIFRDKDSMDSGDSIeAFEKQIGKADFVFMIISEKslYQSSH-
CMNELRLVYERAQS-
EKQEfvskVIPVIMDDAKIDNPIDRLKVVRNWTQKRDELDRIISEVGAEAAGAESTNQ(LQIMRSFINSTANALTWIADLVIERKPELQAETA)}*

$1219=188(189):
>gi|671468342|ref|WP_031474885.1| {|0(335)|<Firmicutes(B)>}hypothetical protein [[Clostridium] aminophilum] >gi|1097215675|emb|SFR73606.1| TIR domain-containing protein [[Clostridium] aminophilum]
{(MEKKERNERTEFMKKNIHCEKVDYTKPY)---VFVSYAS---
ANwRTVFEKIVVKLHEKGLRVYCDNDFETnnDSWLNnvnKHLMD---
SKCHavLAFFSKEYILSYATMIELLISQDDNTVGYHWIDGERTPLEIIpiF-TENASSMGTYLEQAV-
DSFQEKCKIQKTEWDQIVKCIE(NGFTKSKIDNKELNIKDLQDL)}*

$1220=214(213):
>gi|671534001|ref|WP_031517760.1| {|0(247)|<Firmicutes(B)>}TIR domain-containing protein [Desulfotomaculum alkaliphilum]
{(MEQN)-KTIFLSYCWGnKKIADYLdskIQAFEGIEIKRDirEIYYS----------HniedFMKT-
IRKTDHVILLISKEYLMSVNCMFEVSQIvkdYNYVDK-
ITPIIIDrtsiyGPDNRLKYAQYWNKKYHSLNEEIR--KLNIEDN--
ISLIQELKKVKN(ICSAIDEILDILNKKKYHDLETHVENDFNDTFKAMNFKEIPNKKKIQLEEEVIEHFKYTLYKLEDVSTG)}*

$1221=174(173):
>gi|918368344|ref|WP_052420619.1|
{|0(343)|<Proteobacteria(B)>}hypothetical protein [Hyalangium minutum] >gi|670615350|gb|KFE61936.1| hypothetical protein DB31_4379 [Hyalangium minutum]
{()MARVFISYKRDvtpdQDLANALYKALKV-----
DHEVFIDTSMLVGEKWVeRIDAEIRQADFFIALLSPFSVNSEMARtEiELAHQLQ-VQR-
GRPVLLPIRVAYH-
ERLPPPLGGYLSPINWAlwEGPEDTPRLLEQLAKAMSGGTLP(GSASTPASPPRASAAPEPAPAAAATAFQAPLPS)}*

$1222=189(189):
>gi|736673206|ref|WP_034679764.1|
{|0(499)|<Bacteroidetes(B)>}hypothetical protein [Chryseobacterium formosense] >gi|671212147|gb|KFE97353.1| hypothetical protein IX39_20375 [Chryseobacterium formosen
{(MLKAIKKYIFG)-YDIFISYSRKdslgyaYSVAK--------
YFMKKGFNCYIDQlSNITPGTQLplNIKNAIERSTAFILIGSVGAQNSEAIEQEIKLFLTNSRNKpliPITIDGAINSN---
ARWYENIIGLALVNETLKNLQKSEPTQDVFDRIENALNFT(KKSKQLRIISLLIVVMIVLITSAATYYTFSAIQTAQIA)}*

$1223=271(262):
>gi|671567070|ref|WP_031547352.1| {|76(433)|<Firmicutes(B)>}hypothetical protein [Oribacterium sp. FC2011]

{(EAHWCAAISRFGIEYVEDPISTEYIPTCHRASFESFLDDIDYQAAVNNSEDITQKQYIREGNRIDEIQKAI
LATSSKESP)-FDVFICYKE-
SDkngqrTQESvLAQEIYYALTEKGYKVFFSRItlEDKVGTEFepYIFAALNSANVMVVVgTSKNNLDSIWVK
NEWSRFIALMKKDRKKVLLPCYKDMDPYDMPEALAVLQSYNMASIGFMQDLIRGIEKIINPTVTQRS(DSGFD
IDTLKKRICLFLENAEFNNAKEYCDKILDVRPEDTET)}*

$1224=211(202):
>gi|664379940|ref|WP_030906601.1| {|0(515)|<Actinobacteria(B)>}TIR
domain-containing protein [Streptomyces sp. NRRL F-5126]
{(MAPPAASSAVTAPVA)EWDVFLSYSR-TDADK--
ARALAHALRSQGLRVFLDDMAVDDFASItkAITEALARSKALLALYSAEYPRRRACQWELtyAYLAGQREGDP
RRRILVVnpetsaahVHPVELRDARHWTWPTAPNVLQCLTEQVGAHVRAIASPLGAAGHPM(DGREALSSPWL
PAPARTGSARFTDRLAEQWRIHSALVRHRAPLVAQP)}*

$1225=201(193):
>gi|663368538|ref|WP_030366585.1| {|0(517)|<Actinobacteria(B)>}TIR
domain-containing protein [Streptomyces roseoverticillatus]
{(MEGQGARS)-WDVFLSYSR-GDAHR--
IAPLRDRLLASGLEVFVDEVTVAGFAGIsgSIRAGLARSKALLACYSAAYPTRQACQWELtaAYLAGLAEGDP
RRRVMVVnpepgtghIHPVELRDAKHWALPAGTAGLDALVADVRAHVAALEGTIGATEAAP(ARWFPGPPPSP
APAFTGRLPRLWQVHSCLHQHDAPLTTGHSAPRP)}*

$1226=211(206):
>gi|664524063|ref|WP_031040597.1| {|0(515)|<Actinobacteria(B)>}TIR
domain-containing protein [Streptomyces olivaceus]
{(MSTPDASAPGAAEAPGAPGAPG)-WDVFVSYSR-SD--
AGRVGALVSALRARDLRVFVDDTAVDDFASItaTVTEALAHSRILLALYSADYPQRRACQWELtyAYLAGQRE
GDPRRRILvlnpePASDHVHPVELRDARHWPWPTAPELLDRLAARVAGHVDTVATFMGEAA(DASVVPWLPAP
ARTGSFRFTARLPEQWHIHTALHRHRAPLVDRT)}*

$1227=273(263):
>gi|671571968|ref|WP_031551719.1| {|78(535)|<Firmicutes(B)>}hypothetical
protein [Oribacterium sp. FC2011]
{(AEAYWGLVLCKYGIEYVDDPASGKKVPTCHRSSFDSVMDDSDFEQVMENADTVARAIYREQAKQIEELRKG
IIEVSGKEE)PYDIFICYKE-
TDehNDRTIDSVLaqdiYDALTSKEYRVFFSRISLedKLGVEYepYIFAALNSAKVMLAIgTSYDYYNAVWVK
NEwgryLKLMAADK-----
SKyLIPCYKDIDAYDIPKEFAKLQAQDLGKVGAIQDLLRGIEKLIPPEKTVEH(SQSSQTVVVQQSGGPNVES
LLKRGKQALEDEDWQKANEFFDRV)}*

$1228=273(263):
>gi|671585959|ref|WP_031562516.1| {|74(131)|<Firmicutes(B)>}TIR domain-
containing protein [Ruminococcus flavefaciens]
{(SESYWGLVLCKYGIEYVEDPATLKKIPTCHRTQLEPVQTDPDYLAALEHADFMQRAVYEQEAAEIDRIQKD
ILAIAQNEA)PFDVFICYKE-
TDAdgKRTqdsvIANDIYYQLTQEGFKVFYAAITLedKLGQAYepYIYAALHSAKVMLVLGTkPEYFDAVWVQ
NEwsryLKIVREDR-----
SKlIIPCYRDMDAYELPEEFAHLQAQDMSKIGFINDVIRGIKKVLTVDKSEKE(KNTSSFQGAIQGQSHLEVA
PLLKRVELFLEDGEFDRADDFCEQ)}*

$1229=273(263):
>gi|671585962|ref|WP_031562518.1| {|74(272)|<Firmicutes(B)>}hypothetical
protein [Ruminococcus flavefaciens]

{(AELYWSIVLCRYGIEYVDDPLTKKKIPTCHRTQFRSILDDPDYQTALEHADMLQRGVYESEAAYIDKVQKG
ILEISNKEQ)PFDVFICYKE-
TDEngRRTpdsvLAQELYYGLTQEGFKVFFSRITLesKLGTEYepYIFAALNSSKVMVVVGTkPEYFNAVWVR
NEwsryLMLMQEDR-----
SRtIIPAYKDMDPYDLPDALSMFQAQDMSKLGFMQDLIRGIRKLAAAEAPVKE(VQKVEVAVPANANVENLIK
RGKLALEDKEWEKADDFFEQVLNE)}*

$1230=236(226):
>gi|671571957|ref|WP_031551709.1| {|0(75)|<Firmicutes(B)>}TIR domain-containing protein [Oribacterium sp. FC2011]
{(MDDSDFEQVMENADTVARAVYREQAKQIEELRKGIIEVSGKEE)PYDIFICYKE-
TDenNERTIDSVLaqdiYDALTAKGYRVFFSRISLedKLGVEYepYIFAALNSAKVMLAIgTSYDYYNSVWVK
NEwgryLKLMAADK-----
SKyLIPCYKDIDAYDIPKEFVKLQAQDLGKVGAIQDLIRGIEKIVGHGDLSKK(DDYDSGDMAVSARNEIVAL
LKRVSIFLEDKDWQNADLYCEKIL)}*

$1231=273(263):
>gi|671571963|ref|WP_031551714.1| {|74(292)|<Firmicutes(B)>}hypothetical protein [Oribacterium sp. FC2011]
{(AEAYWGLVLCKYGIEYVDDPATGKKVPTCHRSSYDSVMEDSDFEQVMENADTVARAVYREQAKQIEELRKG
IIEVSGKEE)PYDIFICYKE-
TDENKertidsVLAQDIYDALTTKGYRVFFSRISLedKLGVEYepYIFAALNSAKVMLAIgTSYDYYNAVWVK
NEwsryLKLMAADK-----
SKyLIPCYKDIDAYDIPKEFTKLQAQDLGKVGAIQDLLRGIEKLIGPGNNAEQ(YKTSQTLLFQQNSGPNLEA
LIRRAYIFLEDKEWERADNYFEQA)}*

$1232=273(263):
>gi|671571966|ref|WP_031551717.1| {|74(593)|<Firmicutes(B)>}hypothetical protein [Oribacterium sp. FC2011]
{(AEAYWGLILCKYGIEYVEDPATNKRIPTCHRASYDAVTADEDYKNALKYSDTVQREIYEEQAREIDQLQRG
ILDIVHKEK)PYDVFICYKE-
TDEtgKRTpdsvIANDIYHQLTSEGFKVFYAAITLedKLGTEYepYIFSALNTAKVMLAIGTkPEYFNAVWVK
NEWSRFLKIM-----
KNdhtrlLIPCYRDMDPYELPEEFAHLQAQDMSKIGFINDIVRGIKKVIERNSDQPK(LQDNTVVQSVTSGTT
VNAQVKRGAMALEDHDWNKADEFFEEAL)}*

$1233=273(263):
>gi|671571985|ref|WP_031551733.1| {|74(246)|<Firmicutes(B)>}hypothetical protein [Oribacterium sp. FC2011]
{(AEAYWGLVLCKYGIEYVDDPASGKKVPTCHRSSFDSVMYDSDFEQVMENADTVARAVYREQAKQIEELRKG
IIEVSGKEE)PYDIFICYKE-
TDenNERTIDSVLaqdiYDALTSKGYRVFFSRISLedKLGVEYepFIFAALNSAKVMLAIgTSYDYYNAVWVK
NEwgryLKLMAADK-----
SKyLIPCYKDIDAYDIPKEFTKLQAQDLGKVGAIQDLIRGIDKLFGKGGTIIH(SQEIIQNKTNTTALLDRGK
MALEDGAYDNAKTFFDQVLNTDSK)}*

$1234=216(220):
>gi|671604353|ref|WP_031577228.1| {|41(0)|<Firmicutes(B)>}molecular chaperone Tir [Proteiniclasticum ruminis] >gi|1086286619|emb|SDJ15671.1| TIR domain-containing protein [Proteiniclasticum ruminis]
{(GRKYRYVGGLVTRSPIRLQTTHSGHWYVAIDLRGLKGIVRSSVRVLPTALPRVEQKPLSSMPSLLHEHGFG
VNNEDDFTP)QFDVFVSHAS---EDKDdVVRPLAFALKERNISVWYDEFElkIGDSLRRkidKGLAN-----
SRfgIVVISRDFIKKGWANYELDGIITKSI-SGEQILLPIWhnISKSEviN-YSPSLADKLARNtavNTI-
EEIADEIA-Q-------LVARV()}*

$1235=285(293):
>gi|739841532|ref|WP_037692448.1| {|274(778)|<Actinobacteria(B)>}ATP synthase [Streptomyces scabiei] >gi|672103307|gb|KFG00936.1| ATP synthase [Streptomyces scabiei]
{(RDPEEYWGRVEIPYRPIYSYEETLAPFREPSGNPTSLLAAYERLVEVVTEGQVSTMPRIDDNLRRETLDRFTRHRPPRIS)--DVYISFVPQDSSWANWASAVLESEGYK-VHLAQEGTIEGAPLREEIERGVEAASHTLIIYSEAYIRSARFRTtSEAVYAAGD-----LREVhqSVTMQVERqeGGQPFTDQLVDLTRCG--TGE-AARQ--ALLRGFGRTPES(LSGTNYAGDEPKLPRYPASEPPVWNLSLRTSSFTGRTELLEDIRERLWQEGNRALVLKGMGGVGKTLLAQEFA)}*

$1236=184(193):
>gi|1158441300|ref|WP_079021281.1|
{|0(265)|<Actinobacteria(B)>}hypothetical protein [Streptomyces lividans] >gi|672367170|gb|AIJ11052.1| transcriptional regulator [Streptomyces lividans TK24] >gi|672
{(MR)---IFLSHTT---RNKPLVREVRSGLPEH-INAWIDEKDLLAGDSIqnMIRNAIQTDTDFLVVFVDDSVPKSTWVAkELEWARAEENRLgRP-FIIPIIIEDA-E----LGDLDWLRERLFLRCHGYTEADTNRLANELSSALF(GWLSRDIERLRRPPEPDANDLALLGRADELLQATAQRVRAVVLPHRKSNPM)}*

$1237=302(300):
>gi|674234437|gb|KFK27202.1| {|81(583)|<Streptophyta(V)>}hypothetical protein AALP_AA8G348800 [Arabis alpina]
{(HNQPSPLLSYKSSEDVPSSSYTPPSDEFSDDPSDRKLKLSSACTPDPAPPRISFSFPVPRVSLAKVSVSSPATNTKLRSS)--DVYIGFHG-QNpnlVR--FCKWLKSELELQGIACFVADRAKYSDT-QshEIADRVICSVTyGIVVVSCSSLLNYLSLEE-VRFFAQKKN-----LIPIFYGTGPSEIMSLLNC--NAIDKECKEaidglikchefKLEANE-SNWRSCVGKTASI(LRAKLGRKSVVDKEIVDGIDELPFPRNRSFLGREKEIIEMEMALFGNGEYLESTAPSSKGEASGQSEGLADEESDVVSNR)}*

$1238=210(227):
>gi|674235836|gb|KFK28601.1| {|0(804)|<Streptophyta(V)>}hypothetical protein AALP_AA7G018700 [Arabis alpina]
{(MVDGNAL)----YMSFnRC-EDTiRYSFLSHLFADFRGRSISV--KNPASDHNR-VseELEAAITQSKVILVILSEQYVSSKWCLDELVKILG-----SNGPVVPVFYGLTKSALRKQCLKLKKTYPK-------DRV-ADWRRALLEIADL(PGHASSLDRSDSELVEEIVANVRQKLDRTSKIGVYSRLKKIENLLCKQPWGIRSLGIWGMGGIGKTTLANAAFEQLLSGT)}*

$1239=270(300):
>gi|674237481|gb|KFK30246.1| {|130(712)|<Streptophyta(V)>}hypothetical protein AALP_AA7G236700 [Arabis alpina]
{(GDFGNVFRETCKGKTKEETGRWSRALAQVATIAGYHSRNWDTEWHKMENIATEISNILKYSTPSRDLQRISNRLLHGVHI)-------YCA-DKLQSSFASHLSVDFSRKRIASFVN-------C-NRTLDVIEGAIASVVVISKDYLSSPSCLDKLVRVLQWRSQ-TGLQVVPVFYGISPLDVVV------------QENESAGRI-SVWSSALQELRDL(PGHQSREESSECEVVEEIVKDVYEKLFPTEQIGINSRLLEIEQLLCKQPWGIRRIGIWGMPGIGKTTLAKAVFDQVSGGY)}*

$1240=204(221):

>gi|674228163|gb|KFK21948.1| {|0(61)|<Streptophyta(V)>}hypothetical protein AALP_AAs41246U000100 [Arabis alpina]
{(MGIGATLKNFSDTLALRNLQRKELEITLIIYWNHGW)TYDVFPSFSG-
GDVRRSFLSHLLVKLHGKLINTFIDHGIERSRP-
IgpELLSAIRESTISIVIFSKKYASSTWCLNELVEIHKCYEE-----------------
LGQGKKNMKEMKFFTEYGDANRYQILQVIGKGSYGVVC(AAIDTHMEEKNMCDAGSNNDITSADYYFDSYSEM
LKDVVRTKSYR)}*

$1241=274(300):
>gi|674235845|gb|KFK28610.1| {|1233(504)|<Streptophyta(V)>}hypothetical protein AALP_AA7G020400 [Arabis alpina]
{(ELSRHWPGEIVSVAPSKKIKRKKESSEEQSDKQVEMSVKTQKIKDEESVLSSDSGSGSIIATNTGILVFSC
NDNDLDEAL)-----------------FIEAILSGLHEREVTPLKYDLSGRESL---
DREMLRRYSVGIMVLSNSFTRSSQSLDHLVAIMEHWKE-KNFVIIPVYINVTPSDIC----
RLEDWVEASVQQQYLNSF-
QQWTAATTELASI(DGHQWEEGTQFTLAEEVVRDAWLYLKNCKNVVRILAWLEHSQPSRAEIVGIWGMAGIGK
TSIAREIFGILAPQYDLCYFL)}*

$1242=208(225):
>gi|674233246|gb|KFK26011.1| {|0(884)|<Streptophyta(V)>}hypothetical protein AALP_AA8G191300 [Arabis alpina]
{(MVDSDETSRSVDIICE)-----------ESVRYSFISHLAAALRRQGISVsvFTDTDPD---------
DQNQGARVSVVVFSETYALSFPWLDKV--IERRRNN-
NGYVIVPVFYGVSPSDVNRFYDWLvfgDAGLRNSSNMIKSDSDLvEEIVRDVNRELSS(TERIGIYSRLLEIE
NLLCEQPWEVRTLGIWGMPGIGKTTLAKAVFDQMSNDYDASCFIENFDESLHKEG)}*

$1243=234(240):
>gi|672402570|dbj|GAK56719.1| {|437(62)|<unknown(B)>}hypothetical protein U27_03682 [Candidatus Vecturithrix granuli]
{(KTQSPNSPHRLFPVSMSLYAFNLYSRVPEVNFIRVHDLGKERQPAALVRSLTHELCRLLLAQERTVVPSGT
DVSFAPVKL)----FLSHAkrdG-LDIAEHFRDYLYR--KTA-LKSFFDANDIAPSYSFaeEIKAQIQDAAV-
LAIQTDAYASREWCRREILMAKQMERP-LVVVNALSNNEERSFPYLGNVATLRWKMDQI-
QENLELYDQAIESALDLLLYEV(LKDVYLRQYFEDLRQLFDLP)}*

$1244=169(160):
>gi|672403581|dbj|GAK56070.1| {|0(41)|<unknown(B)>}hypothetical protein Despr_1157 [Candidatus Vecturithrix granuli]
{(MPF)KYACFISYRrgA-EDMAKEMIADLHDALAGE-
IElafgtnaVFRDETRLHGGDFLndALARALCQSVCMILVFTRAYFDqnRTYCAREYKAMEKLEQE-
RLQLLPPEKRLHGLIIPIVFRGINYLPQELK-
QRLCLNFDRYLGSDGPMRRHPY(YASEIRKVVEYMENLYT)}*

$1245=192(221):
>gi|674251177|gb|KFK43942.1| {|0(155)|<Streptophyta(V)>}hypothetical protein AALP_AA1G194600 [Arabis alpina]
{(M)----------------------------K-
VRTFKDDIELKSGQRIssDLLVAIEDSKIAVVVVSKNYPASPWCLQELVKIMDVEKK-
GSLIVMPIFYNVKPAHVRRQIEKVAQQFTKH-EER--
ENHEtvVTWRQALTNLASI(SGHCSHDWEDDSKLLDEITKRITNILLFSATPTSDGINNQFGIDAHMKELYPL
LDLNSNEGLRVIGIWARGGNGRSALAR)}*

$1246=286(300):
>gi|674230275|gb|KFK24060.1| {|44(803)|<Streptophyta(V)>}hypothetical protein AALP_AAs46094U000200 [Arabis alpina]

{(GPSVIHSSPIDNVKSVLAAEALAMRSALVKARFLRIPKIIVKSDSQKLINAINIRSSIVEIHGIFEDCRIC SDFFAEIEF)---VFIP-RG-E----NFIAD------
GGGINVYVDQDDKlRGVDPNNsLVKKIEESKIALAIFSSKYTESSWCLDELVKIKERVEE-
GKLVVFPIFYKMNPSEVKGLEGAFGINLWNQ-
VKhTSEFDKLKKWKEALDFVSHK(AGLVWTEDSDENEFITSIEEEVKRMTCISEELELSKPDKDEFMDTRFSK SEELKRVDNETGVTLLDANPEEAERIQGHEA)}*

$1247=268(266):
>gi|674235863|gb|KFK28628.1| {|219(0)|<Streptophyta(V)>}hypothetical protein AALP_AA7G024300 [Arabis alpina]
{(EEVEKAFNHITKEEVDVADANRNSNDMVGSDDEHLKTKKVKEDSSKKRNVHTLVPPPPPPPPPLLRAPHPP PTPTPPLPS)KHEVFISFEA-
KDMGNGFLSSVKEAFINKHVNVYVDETELRSRDRQYLSKVIEESIVALVIFSHRYTGSEWYLNELVQINERMS E-VKLHVIPIFYKLDVENVKLLAGKFGFNLRET-
ARNCGGcggnwRKINKWEEALESIPQK(FGLSSFDYRCENDFINAIVEEVDKVLNHITKEEADAAEANRIVIS D)}*

$1248=183(192):
>gi|674238825|gb|KFK31590.1| {|0(770)|<Streptophyta(V)>}hypothetical protein AALP_AA6G132400 [Arabis alpina]
{(MTDSEKAEQI)---VYISCL--DEIRYSFVSHLSDALRRKGIVVFVDSDDLLSNEAQAMVKR---
ARVSVMVLTGNLEPTTACLIKLVNVIRCQRN-
HDQVVVPVCYGIGTHERGWLDSLLKFSGSSQGHQSRKECSDSKLVKEIVRDVYE(KLFYMERIGIYPKLLEIE NIIRKQPLGIRCLGIWGMPGIGKT)}*

$1249=244(249):
>gi|674238831|gb|KFK31596.1| {|142(0)|<Streptophyta(V)>}hypothetical protein AALP_AA6G133200 [Arabis alpina]
{(SIPQKVGLTLAGHRDESELVDSIVKEVKKVLIDISKKEIDNSKNPTNTEGQFTKYRTQNNNVSSFGPLMAE LDRQPPRPS)-PQVFLSFCK-DEVGDNFVSHLVWALRDSGLNVFTESYKLRE---
QEVFTSIELSNIALIIFSNKYSKSELCLNELVKIDKLANE-
GKLVVIPVFYNLKKNEVRRLEGEFGIHFADTKEKF-
AMEKLmiRSWEESLMFITIG(RIDLSLETHINEFALVAAIVRGVTRLQNR)}*

$1250=294(299):
>gi|674240285|gb|KFK33050.1| {|1632(44)|<Streptophyta(V)>}hypothetical protein AALP_AA6G323900 [Arabis alpina]
{(EVPLRYTPVQVTSPQPPITESPASLPEYGTGSNNLGGERHVPASDLDTQLSAEPQHLSTDLVKIPKVASSW QAGLKRSHS)--QVFVTFHG-AEMRRTFVRHLVSTMTDAGINVFTDNE--
NGIKLQHLYKRIEESKIALVIFSKRYIESIFHLNELVKMSELAKE-
GKLLVIPVFYNVRSNELKNLKGKFSKHFKEMRVR-
YKDEPQkvLKWETSVKSIAKL(IGIHSELHGVDAPLVEATVEAVQRALTKVSQGNPKSLGEGNIGIGHMTDFL LCLLMLIIYITYAKYFPTNWLVDFLVFV)}*

$1251=186(189):
>gi|674241635|gb|KFK34400.1| {|0(306)|<Streptophyta(V)>}hypothetical protein AALP_AA5G140200 [Arabis alpina]
{(MVD)---
VYISFDRWeDTVRYSFISHLSAAFHRKGISSYVGSDTETNGISKQSKSAIENSRASVVVFSEKYSSSKPCLED LVKVYERRRN-
DGLAVVPVFYPVTKSFVKKQILNLDQSESRSSLLATTDLPGHELYHKQSDSELV(EEIVADVREKLNITQHIG IYSKLLSIENLLSKQPWGVPRIGLWGMV)}*

$1252=208(227):

>gi|674238820|gb|KFK31585.1| {|0(107)|<Streptophyta(V)>}hypothetical protein AALP_AA6G131400 [Arabis alpina]
{(MAASTSSSSSVVGLISTR)-PLVFIDFKQ-
EDVSKSLISFLEPVLRSENINVLLDEEEVRDRDIDNLFHRIQDSRISLAIFS----
ESKCGLDELPKIREPVNK-E----IPIFYKVDAISFKKLK-------KDLVD--
LQNSERtKKDLINSVMEELS(RLLGNISVGVNKEREANSKCVMVPARKLQISHSEKLRNWTWSFIYDGSNESA
IEIAMLNEVYWLHISGN)}*

$1253=216(223):
>gi|672395965|dbj|GAK50503.1| {|3(0)|<unknown(B)>}hypothetical protein U14_01734 [Candidatus Moduliflexus flocculans]
{(QKAIPITKEQPLTLELPDATCRLTLRYEENSHSEKSISESKGNSIKPGGMQVRRYGGKYPEKEPAINFDKK
PTGSSPSTE)KLKIFVSYNTNDFPEVEQIIQKLKEHNQI--
KAWIDIEQIEPGDlIRkVLQQAILESICVLIFIG----------
TdiGRWQEIEIDLSIqqgveEQLHLIPVLLPGI----
DNIPQDLSVLKRWNYVSLKSMYDDNALKRLIRGIMKS(KSR)}*

$1254=215(224):
>gi|672394621|dbj|GAK52494.1| {|80(0)|<unknown(B)>}GUN4 domain protein [Candidatus Moduliflexus flocculans]
{(ALPRIVITGDPGGPIFSRFAPFGVSDVLIKSKFDRAHLAQAIENAITPRRATLLQEPKTTISPPASSAESG
NSGQTNAPY)--DVFFAYNAEERRQV-EIIADALKQRGI--
SVWLDTEQIAPGSWVkEVIQtAIPRAKSAAIFIGNAGLGRWEALE---LHTFISQCInARIPVIPVL----
LPGVKAIPEPLLFLKEFHAVSFSTPLDAEALNNLQWGATGK(RPAE)}*

$1255=241(223):
>gi|672403859|dbj|GAK56348.1| {|0(128)|<unknown(B)>}TIR protein [Candidatus Vecturithrix granuli]
{(MPCKQ)-PEIFISYsWNDEGRDVADDLERYLQEKGF--
TVMRDSRAIGYKGLIkEYMQRLGRGKCVILVLsdqyfkSPNCMYELLQIAqqSDF----------
RKRIFPvVLDNTLFYRPEDrlkylrywdeektrLQEEIRQmsnLANLHGITDDLDLYDdirRNIARLLDFLRN
L(NTYPLKGGNYEPLLEAIQANMPQAELDYSRLPIDFSSKIEFLTQYFVGRQDAMARIDQFIQTHRSGYLAIV
GEAGIGK)}*

$1256=222(223):
>gi|672400282|dbj|GAK59635.1| {|183(0)|<unknown(B)>}hypothetical protein U27_06620 [Candidatus Vecturithrix granuli]
{(QIQELWEKFCKIKESEKINYYGKPPIEYEFTKLKLCAIICKICQDHSWQNRPEWYENVEDAIQQTQKEREE
KQKKLQNEK)EFNVFLSYNSKdrDEVKD-IAKQLKDRGI--
KYWIDIKEIPPGRSIVDEIGNAMSTCATCAIFIGKNGLGEWQ-QTEIRNAIRRYSEE-
RNPVIPILLRGIErDQIPEGFDFLKNMNIVKcEDENMIDRLIWGITGKKPDNLES(SSF)}*

$1257=174(192):
>gi|672393258|dbj|GAK52876.1| {|0(10)|<unknown(B)>}hypothetical protein U14_04133 [Candidatus Moduliflexus flocculans]
{(MHEYFLPFLQGYLDNALNKAA)-------------------------------
QVFYDIDGISAGEDYpaRLKQALARSKCLIAIWSPLDFRSSWCQYECNVIRYREQQLqyrtlenPNGLIFPVV
VHDGEyfpdYAKRTQFFDCREYARVGSGFKKTKGYAKFQTEMIDWTTKV(ADGLHKAPEWIADMETDVWLNNP
LNQSLVID)}*

$1258=199(187):
>gi|738295702|ref|WP_036249286.1| {|0(3)|<Proteobacteria(B)>}TIR domain-containing protein [Massilia sp. BSC265] >gi|672863882|gb|KFI06750.1| TIR domain containing protein [Massilia sp. BSC265]

{(MAIRYGCFFSY)AHGRHDLMK---
RFKEALADALRCYLEPHfdnEDELFIDTEQLGGGDDLdrKIARAMCESVCMILIYTPKYEAHAYTRREYAAMR
LLERERagwyelPSRLIIPVIMTRHPeslpPEITENTFYVDFSHFTMATSDLKSNPDFLPDIGKMVQRIA(RH
YQEQKKHTPPGHDCNQFVLPDVPPEWRAMPSLTF)}*

$1259=202(207):
>gi|672401494|dbj|GAK58367.1| {|0(248)|<unknown(B)>}TIR protein
[Candidatus Vecturithrix granuli]
{(MDRYLQSMEIPE)---IFISYSWRDESLTitEALDAFLQAQGIT-VVRDNRDIGYKGLIKEYMQQ-
LGKGKYVVVVLSDAYFKSKscMFELlELSRQEGLY-----DRIFPVTVEGLSiyEAEDLLRYARYWDEKV--
EHLQQEIKSTPNIANLQGITDD(LNLYVEIRQNISRLIDFLRNINTHPLKGSNFEPLLQAIRTKMAQDRTIPV
SVEKE)}*

$1260=195(200):
>gi|672403993|dbj|GAK56482.1| {|0(585)|<unknown(B)>}TIR protein
[Candidatus Vecturithrix granuli]
{(MDTPE)---IFISYTwqGESLKITEALDTFLQARGIT-VVRDNRDIGYKGLIKEYMQR-
LGQGKYVVVVLSDQYFTSKDCMFeLLQLSRQEGFY---DRIFPITVEGISiyEAEDLLRYARYWDEKI--
AHLQQEIKNTANITNLQGITDD(LNLYVEIRQNIAKLIDFLRNINTHPLKGSNFDPLLKAIQSKIAQDQALSA
APEEV)}*

$1261=175(175):
>gi|672403636|dbj|GAK56125.1| {<unknown(B)>}hypothetical protein
U27_03087 [Candidatus Vecturithrix granuli]
{()MHSIFLSYSTKdVEFAQKLKETLK--EREVDVEI-
DSEKIESGDNVEtKIREAIEGMNVSITIVSQESLASTSVIWeFVETLKFQEVI-GREKFIPISLDKF-
GFEMENTGIVAVVGLIEefEQAGIHLHTILQSHQPAVFFNAA(IFADAQEDQAVNRALHRKIEFAPGHTRITQ
GNIPR)}*

$1262=159(159):
>gi|672996161|gb|KFI91296.1| {|0(11)|<Actinobacteria(B)>}calmodulin-like
3-like protein [Bifidobacterium stellenboschense]
{()MGYVFVSYSRRqSAAADVL---IDWMERNG-
IAVWKDTAsMPAGVAWRrQVTEAIRGADLVVMCVSRLWYGSRACRDeEDQAAHYHRPQ-VRVPVDEGRFDPA-
RAVAAIQAAWAAVTPEDrlAGELETAAGDWASRGGRSRDLAR(GRALKAYRRAFGGPARGAR)}*

$1263=225(223):
>gi|737767728|ref|WP_035735819.1|
{|0(619)|<Proteobacteria(B)>}hypothetical protein [Francisella
philomiragia] >gi|673477079|gb|KFJ43604.1| TIR domain protein
[Francisella philomiragia]
{(MNKDI)--
KIFLSYSHEnSNLLDNFEKHISLVKEKYCLDIYRDTKNLAGEEFWkNIITNLDNSDIVCLFISPDYLTSESCI
FEANKAIELQKE-KNIKIIPIILTPC-LWLDctviEGLPKKLAIPTDGkaVSSFSNEN--
DAWIDIIQNLKPH(FSKIQNLKNISLSGEISNFINSSEEFKKSHSKKEFLSLNDIFISPKLEIHNDNENVEEI
DFCEIDYNNHKKIIISGES)}*

$1264=180(208):
>gi|674897885|emb|CDY35031.1| {<Streptophyta(V)>}BnaC06g13550D [Brassica
napus]
{(MASSSHRIRK)-YHVFPSFHG-PDVRRKFLSHLHYHFASKGITVFKDQEIVRGQT-
IgpELKQAIRESRISMVVLSKNYASSSWCLDELVEILECKEA-CGQMVMTIFLRQ------------------
-------EVK-

QIWSKALTDVAEI(AGVHSLSWDDEAKMMQKIVADVSKNLNVTLSRDCDKVVGLGSHLRKVYSLLCLECDEV)
}*

$1265=119(152):
>gi|674907551|emb|CDY25539.1| {<Streptophyta(V)>}BnaC09g11640D [Brassica napus]
{(MASSSSFTSRNY)SYNVFPSFDG-PDVRKTLLSHMRDQFKRNGITMFDDQEIERCLT-
IspSLTEAIRESRISIVILSKKYASSIWCLDELVEILKCKET-IGQIVMTIF---------------------
-----------LLKPKEKPNLMSF()}*

$1266=205(233):
>gi|674907557|emb|CDY25545.1| {|0(476)|<Streptophyta(V)>}BnaC09g11700D [Brassica napus]
{(MASSSSSSPGTWR)-YRVFTSFHG-PDVRKTFLSHLRKQFNCYGITMFDDQAIERSQI-
IapALTQAIRESRISIVVLSKN-------------------------
AMPLLAVDPSDVQKQTGDFGKVFSKTCRRKREEES-
QRWSEALTDVGNI(AGEDFLNWFIALKSTVIENILSCGVLKFSGDFRVGGQLPQIKQNIYPNRYVINQSFTRS
PCLALVEIFFLPPPNLRSSLT)}*

$1267=265(272):
>gi|674924390|emb|CDY08921.1| {|997(363)|<Streptophyta(V)>}BnaC08g11510D [Brassica napus]
{(GHLYTFEVEVETGELTSNELTKVSSCAFHREGAAVGVVNGRHICSSQLSLYRKVSPVSSVASSSSLTLLAP
PSSLPHNWK)-HHVFPSFHG-ADVRTNFLSHIAKEFKSKGINLFIDNNIERSKS-
IgpELTEAIKGSRIVIVLLSKNYASSTWCLNELRK----NKE-GDTKMETCFDGSG-
SNCRKNESEMIEDIATDVSNKLNLSAPSSDFDGLVGMESQ(MTELEPLLQLDSDEVRKIGIWGPPGIGKTTIA
RYLFNRHSKDFQLNVFIDNI)}*

$1268=119(153):
>gi|674877803|emb|CDY54157.1| {<Streptophyta(V)>}BnaC02g47160D [Brassica napus]
{(MASSSSLSSRNWS)-YDVFKSFSG-
EDVRKNFHNHFLEDLERKWIIAFKDNEKERSELIAtELVQAIRDSRVVVVVLSNTFTSSSWCLNELLEIV---
------------------------------CVFHIPHVKGNCLSYKYRSNGEVFHMI()}*

$1269=248(268):
>gi|674877810|emb|CDY54164.1| {|35(134)|<Streptophyta(V)>}BnaC02g47230D [Brassica napus]
{(IAVIISTEINPKAKSYYLFDGYAHLSSGLACGLAGLSAGMAIGIVRDSGVRANAQQPKLFVGMILILIFAE
ATCNPLLQC)--------------------
HLFKEFDKKFIIAFKDNEIERSLSLDpELKQAIKDSRIAVVIFSQHYASSSWCLNELLEIVECRKECGQM-
VIPVLYRLDPSHVRKQTGGDIIFLIVRGNEAAMIELDAEEVRMVGLWGSS(GIGKTTIARVLFQRISQHFRGS
IFIDRAFVSQTMEIFNAANRDDYNMK)}*

$1270=234(253):
>gi|674958164|emb|CDX75556.1| {|0(1195)|<Streptophyta(V)>}BnaA01g01460D [Brassica napus]
{(MDSLLGSCAAAAIGFFTILVTLLLAFYRRFRSNRRNVTVASSSSTSTTLSSDFEAVPNGLRIRAVYIYCE)
-----------ETLQYTFASHLSVDFRRKRIAAFVNCD------LNpDVAE---
GASASVVVFSKSYSSSASCLDKLVTVLRCRRNTGQMVVVPVFYGISPSDVAWSNALRELRELPSHQCSEESDE
GQVVEEIVKDVYEK(LFPTEQVGINSRLVEIEQLLCKQPWGIRRIGIWGMPGIGKTTL)}*

$1271=149(152):

>gi|674859622|emb|CDY69718.1| {<Streptophyta(V)>}BnaCnng64950D [Brassica napus]
{(MASDTNSL)RYKVYFSFHkG-EDTiRYSFVSHLSASLRRKGLIPsaslFVDNCSNGTRT-EKH-----NFKAFVVVLSEKYVLSSECLDELAEIVDRQRD-NQNVVVPVFYSITQSEVMHKSRldILRDTFP--LESNSAERV-TRWIGALKKTVES(YKPR)}*

$1272=227(227):
>gi|923840238|ref|XP_013700683.1| {|0(215)|<Streptophyta(V)>}PREDICTED: disease resistance protein ADR2-like isoform X1 [Brassica napus]
>gi|923840240|ref|XP_013700684.1| PREDICTED: disease resistance
{(MSASFVP)QFDAFLSFSA-EEeATITFITDLYRLLSEKGVTTYYKDDKQEERI-PssSSDPMkyIRDSKLAVVVVSESYPTSVLCLNELQTILNLHDE-GQLSVLLIFYGVDTSNIRKQAAE-DEPFRNLGKEYSDEEV-QSWRMALIKLTSI(SGLDSRFWSKEAKMVDLIANEISHLISNKPNNPSKFKEDGPVAMDRHMQALYGLLDLNSNKEVRMVGICGPGGVGKTTLA)}*

$1273=202(232):
>gi|674897432|emb|CDY35457.1| {|0(733)|<Streptophyta(V)>}BnaC03g62030D [Brassica napus]
{(MSSNGLPHTVYI)-------YCA-DTLQYSFASHLSLAFHRKGIYASVN-------C-NETLDKIEEASGFVVVLSKNYLSSPSCLDKLVKALQCRRK-HVQLVVPVFYGLSPSDV------------AVQEQESDDRI-RDWISALQELRDL(PGILSREESSDCELVKEIVKDVYEKLFPTQQIGVNSRLLEIKQLLCKQPWGIRHIGIWGMPGIGKTTLAKAFFDQLSGRY)}*

$1274=267(297):
>gi|674897433|emb|CDY35458.1| {|466(259)|<Streptophyta(V)>}BnaC03g62020D [Brassica napus]
{(GSIIKFTYDALCVEDKERAHHYVSSLPDVELTERMETVLGKGLSEVRPKLTITNQDENVNTSERASRGMSSNGLVHTVYI)-------YCA-DKLQYSFANHLSLDFHRKGIYASVN-------C-NETLDKIEEASGFVVVLSKNYLSSPSCLDKLVKVLQCRRK-HVQLVVPVFYGISPSDV------------AVQEQESDDRI-RDWTSALQELREL(PGIHSRETEGKMIEDIVAHVSNKLYNSAPSSDFGCLVGIESHMTDMESLLQLDSDDVRRVGILGPPGIGKTTIARSL)}*

$1275=236(253):
>gi|674908259|emb|CDY24794.1| {|0(1565)|<Streptophyta(V)>}BnaA08g15160D [Brassica napus]
{(MDSSFFLLSTCAVIIGFFTIFVKVYKKFRSKPQIASSPSPNWKPQVSASSTPSNDFQGISNESQLHAVYI)-------FCA-DTLQYSFASHLSVDFRRKNFATFVN-------C-NRTLDVIEGASASVIVFSKDCLSSASYLDKLVTILQCQRK-NNMLVVPVFYGISPADVAVPEEHESADRISALKELRDLPVHQSREECNECQVVE(EIVSDVYEKLFSKEQIGIKSRLLEIEQYLCKQPKGNIRRIGIW)}*

$1276=137(147):
>gi|674910012|emb|CDY23151.1| {<Streptophyta(V)>}BnaC09g19050D [Brassica napus]
{(MEISSMN)QHDVFIDFSA-KDTRYSFVSHLSAAFRRRNILTFLGEDFSQAAT----QQAIEGAKVFVVIFSENYAFSPMSLEMLTKFLDlQRRE-NGPVVIPVFYgDVAPSVVEQQLERFGVAFQstEACSPKTTE-----WK---DGERSD()}*

$1277=130(154):
>gi|674911450|emb|CDY21739.1| {<Streptophyta(V)>}BnaA09g44900D [Brassica napus]

{(MSSSYSFLLAGREL)--DVFLSFSG-KKALDMDFGY---DLSRNGIKSFKSESWKEKsfKP-
IarQTLEALTESKVAVVMTSDEEASSVGFLEELLVILEFQEK-RSLTVIPIFLTKRPLDM-------------
-------EEEPCGDSRIAPDQAGG()}*

$1278=208(214):
>gi|674922155|emb|CDY11145.1| {|0(261)|<Streptophyta(V)>}BnaC06g23970D
[Brassica napus]
{(M)KNDVFLSFRG-EQTRKTFLSHLLSSLKKKDITT------STSSE-
ScpVSVQALQVSLVAIPIFSNDHASLDLWVDDLATIIDCDKK-
GTLTAVPVFFQLDPNDflkMLRTAGKDAQTMHEP-QRRSLEIV-
KKWLDAHIINKSG(FNSLDWYLDDSELVGKVTSFVSDILTSSTSRYQTSTGLSTYRSLKLLFKIKLSEEMHKP
LRRISVRDNTDIRR)}*

$1279=162(164):
>gi|674930653|emb|CDY02593.1| {|0(86)|<Streptophyta(V)>}BnaC02g08420D
[Brassica napus]
{(MASSSSLSRSWL)-YHAFLSFRG-EDVREGFLSHVLKELKSKGIIPFIDHEIKRGES-
IspMLLGAIRQSRVAIVLLSRNYASSSWSGYHSSNSGNEADL-
INKRSCQETFLWDTTEIADVLYENPGTGKVLGIFLDTSEGGEIQMSKSCFDGMN(NLQFLKISPDNL)}*

$1280=206(226):
>gi|674931871|emb|CDY01605.1| {|0(829)|<Streptophyta(V)>}BnaC07g35950D
[Brassica napus]
{(MVDGNA)---VCISFnRW-EDTiRYSFVSHLSAEFHRKSI----------SFS-
TcqNSDAAIAKAKVSVVILSEKSVSNYRFLNELVKVSACRSS-HGLLVLPVFYGLTKSVLRKYCLLLKKMY--
-----PEGPV-
AEWRNALLEIADL(RGYASSLERSDSELVEEIVADVRQKLDLEGATGVDSRLMKIQNLIHKQPLGIRSLGIWG
MGGIGKTTLAKAALKQYSGDF)}*

$1281=207(198):
>gi|674942119|emb|CDX91235.1| {|0(252)|<Streptophyta(V)>}BnaC02g26460D
[Brassica napus]
{(MDIHQLIYNIRREKSLINLPRRKAYNSSLSVSKRRKVGNRW)RYDVFLNFRG-
KELRSNFISHLYKSLQWKaalhdvagvmGFTMPKDDFIGSSES-EfvD-
KRNSQTTSYFILVFSNIWCwSRVHCINELMCFGLDDVF-
MIRICGMSGIGKTTLAKAAYNESAHLFEGTCFLENFGELLKKRRRDIVFNNTDD(PVKHRFQSKKVLVVVED)
}*

$1282=133(151):
>gi|674951342|emb|CDX82124.1| {|0(241)|<Streptophyta(V)>}BnaC02g21660D
[Brassica napus]
{(MDSSSSSSSK)KYDVFLSFRK-EDTGRTFVSHLYRSLHQKGIRTYRDQNQQTGE----------------
GRRMSDEEASLGYRGVSYGRRCDIGE-
LRLFRLELSRLSDLVPLCSLHCEDDSRMLDDLIIRIYSAMSQNITPSQMMIEAC()}*

$1283=131(154):
>gi|674951659|emb|CDX81841.1| {<Streptophyta(V)>}BnaC08g37600D [Brassica
napus]
{(MSSSYSFLLAGREL)--DVFLSFSG-KKALDMDFGY---DLSRNGIKSFKSESWKEKsfKP-
IdrQTLEALTESKVAVVMTSDEEASSVGFLEELLVILEFQEK-RSLTVIPIFLTKRPLDM-------------
------EEEPCGDSRNAPDQAGGG()}*

$1284=244(242):

>gi|674910223|emb|CDY22932.1| {|448(555)|<Streptophyta(V)>}BnaA01g18700D [Brassica napus]
{(MHNLLELLGLEISRKEYIDENRRRAREIGDVLADDTGYVSARGTDHVLTAIKDEISIDERANERTLDSLPA
LSPLPSNCT)-HHVFASFRG-VDVRKSFLRHMLMVLRNKGITLFTDIEIETGTS-
IvhELKEAIHRSRISIILISNKSVEdvekWKRALDEVSNIFRYVSR-
SWEMEEIIEKVANNLSDLLYEDVPSGDFDGLVGMENHIMQISSLLSLDSDDVRM(VGIWGPSGIGKTTTARAL
YRKL)}*

$1285=281(300):
>gi|674923541|emb|CDY09661.1| {|23(297)|<Streptophyta(V)>}BnaC07g34000D [Brassica napus]
{(KRSYSSRHCQKEAESMKASSVDIVEDTEDKLALSCNPSKQEEHAVAIFTTPVKELPLKPSATTADSKEIFV
TENSGSVWR)-YDVFPSFSG-EDVRKSLLSHLLKELHRKSINTFIDHGIERSRP-
IgpELLSAIRESRISIVVFSKNYASSSWCLNELVEIYKSFNE-----------------
LNQMTGEFGKIFEGTCNGKTEDQK-
QRWMQALAEVANM(AGEDSRNWSDESNMIERTANDVSNKLLIIPSNDFGDFVGIEAHLEAMNSVLCLESEDVR
MVGNVGPSGIGKSTIARALFS)}*

$1286=280(299):
>gi|674923983|emb|CDY09294.1| {|263(666)|<Streptophyta(V)>}BnaC03g63240D [Brassica napus]
{(DGGAALQLDPCQDPFPCHDLAPPTISFGDGGAAPRDCIYLLADSVTSSETLEQPRVSGYSITGVDPILVIS
CGYNQSSEE)---------------RYFISYIFNELRLRGFAPLGYDLSRNPvNG---
NQDLLDKSQLGIIIFSMNYVSSSECMDGFVALMDHSKA-
NELMLFPVFFEVEFCNVKDQAGSFEKAFAQFENSVQASQV-
LKWKAAVEELTST(DRYHQYKKGDEVMLAKNIVRDVQVNSKTGSNLTASLQLSHILSLLNSWQSSAPHIVGLW
GMAGIGKTTFSREIFRTQGE)}*

$1287=268(286):
>gi|674931866|emb|CDY01600.1| {|75(830)|<Streptophyta(V)>}BnaC07g35900D [Brassica napus]
{(NYVKEKQLGVGDIVFLQRIFTDSSRLFIGFSRREAALDGTSSDKLKEVNSPLPLRSHVHSVTEFDPVLVFS
CAANQESGE)---------------RYFISYISTELCLRGFTPLIYDLTrGTLTG---
CLELPHRSRVGIIIFSNNYASSRQCQDNFVAIMDHSKA-
NSLVLLPVFFKVKVTDIRGQSGSFGRAFSQLENSIQASQVPTFTFIDKHQYMKG(GEVILAKNIVSNVCLLLS
SENNMKLRGRLQMNSVLSLLYCSQSSAPHIVGLWGMAGIGKTTIAREI)}*

$1288=280(300):
>gi|674933915|emb|CDX99369.1|
{|259(1063)|<Streptophyta(V)>}BnaC01g11830D [Brassica napus]
{(FVSFQRDRKDSSKLFIGCRKSDESFPNPTSPGASIKTVEMRERLKGVETPFQPKLSEHSVTGSDPVLVIYC
GDNQVSEEI)----------------YLISYIFNELCRRGFSPLRYDmKKGTVTE---
NQKLLHISRVGIIIFSMNFAHFRECLDGFVAVMDHLKA-
NELVLVPVFFKVRVSDVRGQSGSFGRAFIRLRNSVKASEV-
LKWRAAMIELTSI(SGYAYKKGDEVILAKNIVRDVCLPLSSETNMKLRGTRLSLESILSLLNRSQPSSPQIVG
LWGMAGIGKTTITREFFRTQA)}*

$1289=280(300):
>gi|674934638|emb|CDX98624.1| {|77(653)|<Streptophyta(V)>}BnaA03g44090D [Brassica napus]
{(QTEEDSSLAAEGVKSGIASRDDAALNSTSADTYAKALEFRDKLKEVETPIQPKLSKHNSITGSDPVFVIYC
GDNQDSEEV)----------------YLISYIFNELRLRGFIPLRYDLtSGLVTR---
NPEMLYKSRVCVIIFSMNYAHSRECMDEFVENMRHLNK-
KNVVKIPVFFKVSFSEVRGQSGSFEEAFTRLGNLVEAAQV-

LKWREALLELPSI(NKYQYTKGDEVILAKKIVGDVCLMVKYEMDDKRRGTRSQLDSVMSLLKFSQSSAPRIVG
LWGMAGIGKTTITGDIVRTQA)}*

$1290=280(300):
>gi|674954464|emb|CDX78937.1|
{|124(1166)|<Streptophyta(V)>}BnaA01g10040D [Brassica napus]
{(QPQADVLSSGVDSLDRDDTGGVGRFFKEHASEKASEKEHGEMNLKEVETPFQPKLSEHCVTGSDPILVIYC
GDNQVSEEI)----------------YLISYIFNELCARGFAPLRYDMKKSTvTE---
NQKLLHISRVGIIIFSMNFAHSRECLDGFVAVMDHLKA-
NELVLIPVFFKVSVSDVRGQSGSFGKAFTRLGDSVMASQV-
LKWRAAMIKLASI(IGYAYKKGDEVILAKNIVRDVCLPLSSETNMKLRGTRLSLKSILSLLNRSQPSSSQIVG
LWGMAGIGKTTITRDFFRSQA)}*

$1291=219(229):
>gi|674938836|emb|CDX94478.1| {|0(985)|<Streptophyta(V)>}BnaC09g23580D
[Brassica napus]
{(MESLPSSSA)ELDVFLSFRG-
FDTRNNFTGHLQKALRLRGIDSFIDDKLRRGDDLTALFDRIEHSKIAIIVFSKNYSNSAWCLRELVKILECRD
R-NQQLVIPIFYKVDKSELKN----------
VPKKSFTEvKEEEtSTWEAALTTASNI(SGYVVNEFSTSEAKLVDEIAVDTFKKLNDLAPIGNEGLVGVDSRL
GTLEKLLCCDEVDSVHVIGIIGMGGIGKTTLADCL)}*

$1292=224(222):
>gi|674886148|emb|CDY46415.1| {|273(692)|<Streptophyta(V)>}BnaA08g20150D
[Brassica napus]
{(GHELMFILGQMNNKVILAAQGFRTQGPFEMDFEEHSKRFGEEEIQRWREAMKIVGNISGFLKMILQSEMET
DVMSKPHRL)KFDAFLSFRG-
EETRHNITKRIYDALNVKeKFRVFRDNDGLEgGGDETspNIVEAMKDSAASVVVSYALHVRNVSETPENSVDE
DMIEL-VVKSVLAQVSNTPEKVGEYTTTLAKAFYNKIIAEFEHRVFISNVRENSSDQDGL(VN)}*

$1293=156(157):
>gi|674961639|emb|CDX72045.1| {|0(770)|<Streptophyta(V)>}BnaC08g28290D
[Brassica napus]
{(MGEGRKRDRERWS)-HDVFINFRG--
ELRDNFVSHLEKAFKSNGIHIFKDDDALKAAEKIntELSKAIKTSKVHLVLLSKTEQVAEMEGSFNTSCKPVW
FCHGKLQVTSFFSPVCIYNFLIDDVTKEVGKMLSVSYLPLPTYAVGVRSRLHRMNEL(MCFG)}*

$1294=180(178):
>gi|918849998|ref|WP_052627645.1| {<Bacteroidetes(B)>}hypothetical
protein [Sphingobacterium sp. PM2-P1-29] >gi|672715831|emb|CDT01022.1|
putative toll-Interleukin receptor [Sphingobacterium sp. PM2-P
{(MKYFNKVELRAIPQMKIFSTSLKRVLNEAKLDE)QFDIFLSHSY-VDKDLIMA--
LYTDLTERGFRVYVDwliDPDLNrsvVTKDTaeVIRTRLNNSKSLVIAVSENSSINKWIPWELGYA---
DGHCGKCCILPMMEEQHSKKDFHGSEYLKLYPQIKKQISLGGGDTLYVEENQNKKTFR(IYLRE)}*

$1295=230(228):
>gi|922563523|ref|XP_013610046.1| {|0(36)|<Streptophyta(V)>}PREDICTED:
protein PHLOEM PROTEIN 2-LIKE A8-like [Brassica oleracea var. oleracea]
>gi|923656609|ref|XP_013646294.1| PREDICTED: protein PHLO
{(MVDPARPRP)-PQVFVNSGA-
TELPDDFIKHLVSGLTDLGIlNIFMDRDDWWGRDLDRLFTCIEESTIALVIFSPCYPETEWCLDELVKMKERV
KN-
NKLLVIPIFFNVSKNDVRNFEGEFGDRFMELRKRYtkYKYDPFrvQRWETSVMTISKL(EPSLAWETQSSSIS
LAMDIIKEVNKELDIASDAGFFLPLGFHKDSHSKLTNGEVFVSAFLVAFLFRLFIARLVFTDMTF)}*

$1296=129(150):
>gi|674906684|emb|CDY26387.1| {<Streptophyta(V)>}BnaA02g23830D [Brassica napus]
{(MTNTKRTEQP)---VYISCV--DEVRYSFVSHLSDALRREGISVFVDSDDLLSKEAQE---
KVEGARVCVMVLPGN---GRVCLEKLGKVLNSQRD-IDQVVVPVLYG---------
DSQWYDEWLDELHLRGFSAVYQSSFNFKFFLFNV()}*

$1297=230(228):
>gi|674920116|emb|CDY13080.1| {|0(34)|<Streptophyta(V)>}BnaA09g17370D [Brassica napus]
{(MVDPARPRP)-PQVFVNSGA-
TELPDDFIKHLVSGLTDLGIlNIFMDRDDWWGRDLDRIFTCIEESTIALVIFSPSYPETEWCLDELVKMKERA
NN-
NKLLVIPIFFNVSKNDVRNFEGEFGDRFMELRKRYtkYKYDPFrvQRWETSVMTISRM(EASLTWETQSSSIS
IAMDIIREVNKELNIASDAGFSLPLGFHKDMHSKLTNGEVFVSAFLVAFLFRLFIARLLFTDMTF)}*

$1298=202(215):
>gi|674960456|emb|CDX72956.1| {|0(256)|<Streptophyta(V)>}BnaC06g34040D [Brassica napus]
{(MT)-PQVFVSFRG-EDTRKTFVSHLLSSFKTSCITSFIASEPLEATDHEAMGK----
SLVSVPVITKSYSVSEWMVKDLSRMIECEKI-GTLRVVPIFFQISPLDIL-------SHVKKY-
AKVQGGnlEMVRKWLTAQVSRKPS(FHSSDWEDDSELVDKVTEFVSNILKSSGPSYYSTGLGPVRSLKLENEF
RLTPTTTCDIETNQRSFPLSFRFTP)}*

$1299=217(221):
>gi|674922427|emb|CDY10908.1| {|0(166)|<Streptophyta(V)>}BnaC01g15230D [Brassica napus]
{(MGKRKNR)-PQVFINFRG-KDVRKKLLPHLKHHLKDRNVNVFTDDD-
AFGEQVEKIFKYIRRSRTVIVIFSINYLESHWCLDELVEIQKCLEC-
KKVdFAIPIFYKVETSHVKKLSGKFGKKFVALQEKHR--
KSKvKRWKKAVRYVAKS(IGLTYEKRSSISELDFIKKIVERVDFTLTKISSKDKNSPETSKGEASSPTDVHHL
NEMIHTSRTLEALQLERSS)}*

$1300=201(204):
>gi|674882621|emb|CDY49780.1| {|0(1082)|<Streptophyta(V)>}BnaC02g47380D [Brassica napus]
{(MAKTGH)--QVFISFRG-
ADVRKNFLSFLTDGLKRACVNYYVDTKETKGEVLDILLQRIQESRLVLIILSENYMQSNWCIKELRTTTKDIT
E-SRRKVIPIFYNVQVADVkdKWKVGEQAEGVEGE-
ETVEEREKNVKEALMILTRHMG(MRSDEYGTDCEFIEHIVKEVKKVLTSIEQEEKVKASVNTTVRSEGEKQEV
SSLTGTNL)}*

$1301=245(264):
>gi|674922468|emb|CDY10734.1| {|110(795)|<Streptophyta(V)>}BnaA05g12020D [Brassica napus]
{(FSCIDKRGKSSHKQYSASKVSKLARGSVVDMQQDGLHNSVYNGVNLLQEDELSIIGFNEFQSGPVSDASDT
SATFPASKD)-YDVVIRHGG----NNDFISHLRAALSRRGISVHEDVEETTDA-------
AVPECRVLIIFLTTTYVPS-----NLLNILQHQRQ-ESLMVYPIFYGISPSDLITNSNYYENFFPQD-
ESERCQAALKEITQMPRYILID(KYESELIDEIVRDALKVIYSTEHEKMIGMDMQIKEIVSLLCIES)}*

$1302=275(299):
>gi|674942595|emb|CDX90813.1| {|25(504)|<Streptophyta(V)>}BnaA03g24930D [Brassica napus]

{(LEARFSGGNPSDQQDQRRLQMYVPRKKRPKYGKAQRKYIKTTASSFMNLVQQFTGDPSAVSESKSSNTGVV
AESSSYSTY)-YDVVIRYRR-GDIGNNdFISHLRAALCRRGLSVDEDFDEVDT---------
VPECRVLIIFLTSTYFSST-----LLNIIEDQRG-EYQAVYPIFYGLSPSDLISNSKVCDIF-FHQ-DEAKR-
-----
WQAAMKEITQM(PGYTLTDNSECDLIDEIVRDALAVVYSSYKVTMVGRDMQIKEILSLLCIESPDVRSIGIWG
TVGIGKTAIVEEIFRILS)}*

$1303=274(299):
>gi|674946560|emb|CDX86704.1| {|56(449)|<Streptophyta(V)>}BnaA09g20940D
[Brassica napus]
{(VPRLTGVDNSVLELEFISAFGPSASRGYQVVTIYRRGDISNDDFNKVVRRLTRNLNSVSDLSNSASEFSTP
ATASSALKE)-YDVVLRYRR-GCISNdNFITHLRAAFYRRGVSLREDIDEVD---------
AVPECRVLIIFLTSTYVPS-----NLLNIVEQQSK-KPRVVYSIFYGISPSDLISNSN------YVR-PSH-
QDEA-
KRWQAALDEITQM(HGYILTDKSESELIDEIVRDALEVLRSNYKKNMIGMDMQIKEILSLLCTESQDVRRIGI
WGAVGIGKTAIAEEIFHRIS)}*

$1304=274(299):
>gi|674946584|emb|CDX86728.1| {|229(536)|<Streptophyta(V)>}BnaA09g21180D
[Brassica napus]
{(GVNNSVSQLQFSASSASTDYEVIVIYRRGDITNDAFVKVVQRLTGIPNSVSVPLNSYVEIEKGSSKNLQDT
ELSTSAIRE)-YDVVIRYvRG-CISNGDFITHLRAALCRRGVSVREDIDEVDT---------
VPECRVLIIFLSSTYVPS-----NLVNIVEQQRK-KPRVVYPIFYGISPSDLISNSN------YGR-PSH-
QDEA-
KRWQAALEEITQM(HGYILTDKSESDFIDEIVNDALEVLRSNYKKNIIGMDTQIKEILSLLCIESQNVRRIGI
WGAVGIGKTAIAEEIFHRIS)}*

$1305=275(299):
>gi|674946586|emb|CDX86730.1| {|223(628)|<Streptophyta(V)>}BnaA09g21200D
[Brassica napus]
{(YEPPNTSQEKRIKKAVSLSNIVYPPRGISDIDLFSYKNLDPLSLTGFTSDYLDNLEFPDATRSRIASQPQL
LSGSSAPKD)-YDVVIRYRR-GDVNNeDFISHLLAALSRRRISVYGDFDEVDS---------
VQECKVLIILLTSTYVP-----PNLLSIVEQQRK-EPQVVYPIFYGISPSDL------ISNSKYYE-
GLLLQDEP-
ERCHAALREITQM(PGYILTDKSESELIAEIVRDALKVLCSYDDKKMIGMGTQIKDILSLLCIESPDVRSVGI
WGTVGIGKTAIAEEIFRRIS)}*

$1306=253(227):
>gi|674876714|emb|CDY55567.1| {|0(137)|<Streptophyta(V)>}BnaC09g41540D
[Brassica napus]
{(MERTNPHL)--QVFISFRG-
KDVRGNILSSLKEKLKDEGVNVKTDEEMPRGRKIDeNLLKLIRDSKVAVVIFSENYPESTWCLDELVEIKKQM
EL-
KKLAPLPIFFEVKASHVAlKEHNPFKDILLRLeddeRENARNGsrvgsgrmhcwscflSRRllkdadkrfVRW
REALKSITGY(SGLKYIKDSNQALFVNQIVETVKEMLDKVQSSDEVHGRIRGQHIVPQQKVFISFGGHGDDTR
LGFISHFQAGLKRSGIN)}*

$1307=306(300):
>gi|674922428|emb|CDY10909.1| {|257(116)|<Streptophyta(V)>}BnaC01g15240D
[Brassica napus]
{(LLSKKPQRFPGNKPVVSQEIQDQSYPSPVQVTNVLATVESNDNTRNRLGDDKNSDDDTLACFTFLCNIMKH
LSSLISPPP)-DRAFISFGD-KQLEKTLVSSLKIELEAKGISVYVEN---------
ETKERIKESKVAIVVFSAKYPESPQCLDELFEIKKLMDA-
REIDPFPIFYKMEnvsnlAQSVKLIQGWFLYRLLKIeqevRKDVNRKSVKsildteariWGWRQAIRSISSK(

PGFSNEYSSDPLFLTAVMTKVKEMFEFKDRPKKSSQMIEETPLMHQHDDLFYSLASLKRYPNLGLGMNHHSIS
RFEEPSW)}*

$1308=123(150):
>gi|674922430|emb|CDY10911.1| {<Streptophyta(V)>}BnaC01g15260D [Brassica napus]
{(MTNFSRSWKH)-DQVFISFRG-
KPLRKNFVSHLKEELKRSKINFFIDEDETRGRRIATLFETIRESGVALILLSNKYSDSEWCLDELVEIKKQME
T-GYLDAFPVFYEVKSASIKSQTGC------------------------LHSDQRH()}*

$1309=241(241):
>gi|675373566|gb|KFM66468.1| {|996(0)|<Arthropoda(M)>}Protein toll [Stegodyphus mimosarum]
{(SNSSCSNFSVTPFASAEFVEEYMPVMILVPMLLLLILGAVFFVCKYRDNMKMWIYNKYGIKLFEKNDYPAD
GEK)LFDVFVSYCKKdeAFVTQMVAPELECGSPPQRLCLRYRDLPVSRYIAETISEAVECSFCSIAVVSEQYL
KSEWCVFELKASHHESQCNRRHKVIIILLNKV--
DMKELDPDVRACFKSALIVHWGDRRFWEKLRYALPEGRN(HRHMNCSDMKLSTTLRRSSLSNSIKLV)}*

$1310=212(214):
>gi|675380009|gb|KFM72911.1| {|963(0)|<Arthropoda(M)>}Protein toll [Stegodyphus mimosarum]
{(NSSACSNHSALTYYKAEFASDYMPIMIIAPSIFILLIFSLVLFCVYNSEMKIWFYSRYGIRLFQRNNQAGE
SEK)LFDAFLSYCKKdeAFVSQMLAPELEDGTYPHRLCLRYRDLPMAEYVAEAISEAIECSRCTVAVISEQYL
KSEWCIYELKAAHHESQCNRRHRVVVVLLDKI--SFKELEPDVRICLRSASLVHWGDRRFWEKLRFAMPEP--
()}*

$1311=252(261):
>gi|675369844|gb|KFM62746.1| {|112(58)|<Arthropoda(M)>}hypothetical protein X975_26575 [Stegodyphus mimosarum]
{(ANTGQDWIALAKRLGYSEREIKHFEDANIPSVSLLNNWLDSNGRTRYCIDMLISCLEQMGRRDVADCIFLE
MGSDLPSPP)---VFISYQW-DSQESVL--
HLRRRIELAGYPCWMDVGHIFGGDKLygKIFEGINKAKVVLCCLTPRYVLSKLCTREMSLADLLR-----
KPIVPIMLEPTPWPPPGPLALILSSLVYINLCGIGGHGGSGRQADWESRFSD(IIERLSHFISPSFPSHQLAT
ISTERFMPPPAVTVIRNTEAY)}*

$1312=214(220):
>gi|675525687|gb|KFN20299.1| {|313(0)|<Proteobacteria(B)>}hypothetical protein JM66_05315 [Aeromonas salmonicida]
{(NLLEAEIRFIKARYNVSDSEMKNIKLDGSTYKRAVYGNLSFIKMIKGEEHSPYLTLCKQVLELDNNPPDFV
RKLKGVFDM)-YDIFICHAS-ED-
KDKVAIPLYTELKKRNINVFIDCFAINWGDSLvsKINTALKKSKFVIAVISDISVKKSWPMKELNAVLSSE--
---IGAEDTKLLPLMVGDGEALLKELPLLADKLFVAFDDNIEEIADKVEKLLIKI()}*

$1313=248(232):
>gi|676286004|gb|KFO37784.1| {|290(308)|<Chordata(M)>}Interleukin-1 receptor-like 1 [Fukomys damarensis]
{(DLSLKYDCLARNLHGVVRHTTRLIEKKPIGHQNVYYIAAGCSFLLMLINVMVIILKVFWIDLILLWRDLAR
PYKMRNDGK)LYDAYVIYPRDhtcgsegpnslEYFVHQILPDVLENKCGYNLCLYGRDLLPGEDVATAVEASI
QKSRRHLFILTPHVSHSKEFIYEQEDCEKISKSNLSYLKPILKKAEFKdqgyYSCVFSLLRNGKLYNITdTLN
ITILRERSDVIPVVLGPQLT(RIEVELGEDVEL)}*

$1314=262(261):
>gi|677369227|gb|KFP96458.1| {|366(0)|<Chordata(M)>}TIR domain-containing adapter molecule 1 [Leptosomus discolor]

{(MSMEDPYVLAGIPSNSAPASNSTCSFPPPTHSLSSTLPPPLRGVHPNLSYPPPLHSSLSPAWPPPLQTVEP
VSTSEPRDG)KFFTFVVLHASeDEIVAHRVKDLLENmgvPNGATLC--
EDFFIAGRSHLTCFQDAMENSAFIILLLTKNF-PCNLCMFQTNTALMESILKPSKRDSVIPFVPK-
ENPLERSQIPSTLGGLMpLDENSPGFSRTVQNTFTTSRINE(RKAMWDQMQRRKLQLCQERYQTLQNLAALNL
GSLPQVPPAA)}*

$1315=173(175):
>gi|754931967|ref|WP_042288783.1|
{|0(165)|<Bacteroidetes(B)>}hypothetical protein [Nonlabens ulvanivorans]
>gi|678229147|dbj|GAK94013.1| hypothetical protein JCM19298_488
[Nonlabens ulvanivorans]
{()MTKVFLSHSSAqKGFVREIAKKLS----KQDI-
VYDEYTFEEGEKTIsEIFIGIEQTCLFVLFISDESLNSSWVKSEIKEAKEKLDS-NSlLRIYPVIIDKN-
INYDDPRIPDWLKEEYNlkYVSKPTKISNRISSQLKMLNWNQ(YPKNQKRSNIFVGRTSQIPSYEERIFNFDK
PKPIV)}*

$1316=190(186):
>gi|740845627|ref|WP_038630880.1|
{|0(125)|<Proteobacteria(B)>}MULTISPECIES: hypothetical protein
[Yersinia] >gi|682105335|gb|AIN17211.1| TIR domain protein [Yersinia
kristensenii] >gi|755290399|gb|AJ
{(MDDKSNVA)---IFISYAW--GGSLEKKEWvrqqIVNSLKGK-
YSVFWDRDTIGFGESIdaCIHKALTHRPVKVFCICDADYLHSaktvgSGLHrELQILEKIAHE-
DQVKIIPLIFEYD-
CIRDLPAPLAGRAYLDLAELHKRNLFIGNEMYALAEGISQ(AEMSIWIKKKIASNDLVKLAKHHFQGLDIELY
GNGRTH)}*

$1317=156(159):
>gi|746261791|ref|WP_039309908.1| {|0(9)|<Proteobacteria(B)>}molecular
chaperone Tir [Pectobacterium atrosepticum] >gi|679634204|gb|KFX16439.1|
molecular chaperone Tir [Pectobacterium atrosepticum] >g
{(MSV)----FISYRHSdfalaISINKRLRDVNIETYLDKL------
DAESQTTDDItsVITRNIARSTHLIAVVSSQTAQSWWVPFEIGEATILGRRIssyQNGFLSLPEYL---
SKWPQMKSAEHLELFISAYQRDLFEHVTEGMEAYSTSAAG(RSLDSDRSAADEFHRN)}*

$1318=184(217):
>gi|685281513|ref|XP_009131664.1| {|0(119)|<Streptophyta(V)>}PREDICTED:
protein VARIATION IN COMPOUND TRIGGERED ROOT growth response-like
[Brassica rapa]
{()--------------------------------MFDDEGVERGHT-
IdpALTQAIRESTISIVVLTKNYASSSWCLDGLLEILKCRQA-
GKLIVMPVFYGVRPNDVQRQTGDFGKGFEKTCRGKTILDK-
GRWSQALNKVCSL(QGFMFSPRDDESELFEKIGREVTGILQRIEYLIAREDTLNPTTSTNKSRCSIDLVFLVV
IINLLLEITSAVADQLSS)}*

$1319=190(190):
>gi|739479129|ref|WP_037339009.1|
{|0(759)|<Actinobacteria(B)>}hypothetical protein [Amycolatopsis sp.
MJM2582] >gi|683981476|gb|KFZ81971.1| hypothetical protein ED92_17170
[Amycolatopsis sp. MJM2582]
{(MSSRTN)--DVFLCYSW-KNQASALA--
LHAALTASGLRVYLDQIGGEDWEPLgeSVLRELRRSRTLVALITPEFPLSPHCRDELHLALHasyyLDEG-
RTTRVLAVVQGVSPDHVRPRQLSSIRLARDGKQLDDVASSIARVVEQHAGTFGD(APEPATPRWFPEELAGDP
DFIGRHAELWELHEGLRSRAKTRDRG)}*

$1320=239(229):
>gi|685259768|ref|XP_009137076.1| {|0(449)|<Streptophyta(V)>}PREDICTED: LOW QUALITY PROTEIN: uncharacterized protein LOC103861120 [Brassica rapa]
{(MTNFSRSWKH)-DQVFISFRG-
KPLRKNFVSHLKEELKRSKINFFIDEDETRGRRIATLFETIRESGVALILLSNKYSDSEWCLDELVEIKKQME
T-
GYLDAFPVFYEVKSASIKSQTGCFGNTLLKTenkvRRKVDRGSYKsileteariWGWRQALVYIGGS(FGFSH
KKRRPDGPLIINIVVQLKKMLDEISYRRNYLFIIEKSLMHPQEALTSLLQALNIKKSDLEDLITRPFIQTRGT
D)}*

$1321=306(300):
>gi|685259768|ref|XP_009137076.1| {|287(95)|<Streptophyta(V)>}PREDICTED: LOW QUALITY PROTEIN: uncharacterized protein LOC103861120 [Brassica rapa]
{(LLSRKPRLFPGNKLIVSPQEIQEQRYFSPVQVSNVLAMAESNENTRNRLGVDKNSDCSLTCFSLLCNIMKR
FEMMINPPP)-PRVFISFGE-KQLKMNLVSSLKTEFESNEISVYIED---------
ETKERIKESKVAIVVFSDKYPESPQCLDELVEIKKLMDA-
GEITPFPIFYKLKdvprlAQSVKQLKGCFRNRLLKIeqevHKTVNRDNVNsildteariWDWRQAISSISSR(
PGLSNENSSDPVFFTDVVTKVKELFEFKAIPKKSSSYIITNQVIEEKPIMHRQKTAPAIPTVKSLDDDLFYSL
PSFLQAL)}*

$1322=194(215):
>gi|739484875|ref|WP_037344629.1| {<Actinobacteria(B)>}hypothetical protein [Amycolatopsis sp. MJM2582] >gi|683978385|gb|KFZ78976.1| hypothetical protein ED92_31575 [Amycolatopsis sp. MJM2582]
{(MYEYDVFISYRRGGGDAPDWVRNHFHPRLQRLLDDNVDY)-----------------------------
DVKIFLE-
DSVAVGGNWprEVREALQRARILIPVCSPKYFRDERCLAEWHTMAKREEivaregVAkAGRLIYPVIFSDSDY
--
YPAWAHERRmRSFRDwNKPHPQYQKTPEYIEFEDELGRM(VKELVEIIEQAPPWSPEWPIETPPPEPPRPSKL
PRF)}*

$1323=209(220):
>gi|739487678|ref|WP_037347429.1|
{|359(0)|<Actinobacteria(B)>}hypothetical protein [Amycolatopsis sp. MJM2582] >gi|683976341|gb|KFZ76990.1| hypothetical protein ED92_38415 [Amycolatopsis sp. MJM2582]
{(EDVATWTPDTYASRASGTGGESLIATRLLSEPELRQLLAGPQGQDWLTDHTLRRASVSFLITRQRLSRLDT
SEVTVRYDA)----FVSFAN---EDRPIVIDLVEALSHAGVRVFFD-GHIAPGERWqeVLDDRLGVADC-
LLYCVGETTPRATGQLrEYEM-----VSRrSEVTVIPILLpgaDAS-
LLPEFILARQWLDFRAGvSAEGIADLVStL-----RSKSRRA()}*

$1324=178(180):
>gi|297812009|ref|XP_002873888.1| {|0(169)|<Streptophyta(V)>}predicted protein [Arabidopsis lyrata subsp. lyrata] >gi|297319725|gb|EFH50147.1| predicted protein [Arabidopsis lyrata subsp. lyrata]
{(M)-YQVFPSFCG-GDVRRAFLSHILKELRSKGMTPFIDNEIKRGQL-
IgpELVLAIQESRIVIVLLSSNYASSSWCLDELVEIMKCKEK-
NQQTVMVVLDEADSLWQLNAMANQPGWLGRGSRIIITTEYRKLFKAHGINIQLE(MRLFKSSAYMLLTMESCA
RMHCLPQQMGREIVYKQSFDE)}*

$1325=228(230):

>gi|297846862|ref|XP_002891312.1| {|0(931)|<Streptophyta(V)>}predicted protein [Arabidopsis lyrata subsp. lyrata] >gi|297337154|gb|EFH67571.1| predicted protein [Arabidopsis lyrata subsp. lyrata]
{(MDSSFFVSSVVTAIVMLFGTIFPVVYRKFRSHQENNITVFSLPPSSSRSSPSRNWK)-HHVFPSFHG-ADVRKAFLSHILKELKSKGIDPFIDNDIERSKA-IspALIEAIRGSRITIVVLSRNYASSTWCLNELVDIMKCMDE-FGQIVMTISMKWIHQTDTEAVMVGKIATDISNKLNNSTPSRDFIGLVGMGAHME(KMKPLLCLESDEVRMIGIWGPSGIGRGLYKKEFL)}*

$1326=232(233):
>gi|297833100|ref|XP_002884432.1|
{|0(202)|<Streptophyta(V)>}hypothetical protein ARALYDRAFT_477682 [Arabidopsis lyrata subsp. lyrata] >gi|297330272|gb|EFH60691.1| hypothetical protein ARALYDRAFT_4776
{(MDSYFFLGTVVVVVALAIYTLLGMLSFMVYRKFRFHLDKKNIACFSFSSFTSFSSCSSLSSTSSPSR)KHQVFPSFHG-ADVRKSFLSHIMKEFKSKAIDIFIDKDIKRSKS-IgpELIEAIKGSRIAIVFLSRNYASSSCSLVGMEAHMKKLEL-MLYLDLNDVRMIGIWGPPGIGKTSIARVLFSKHSDSFDLSVFMENVKGRYTRPG(CSDEHSLKLHLHQQFLSQIFNQKDIEV)}*

$1327=220(241):
>gi|297840621|ref|XP_002888192.1|
{|0(540)|<Streptophyta(V)>}hypothetical protein ARALYDRAFT_893604 [Arabidopsis lyrata subsp. lyrata] >gi|297334033|gb|EFH64451.1| hypothetical protein ARALYDRAFT_8936
{(MAQPECVSVFSCNDSHDVDET)----------------QFMEAILKELHERGITPLTYNLSGRENL---NVEMLNRSSVGIMVFSNSYVCSKQSLDHLVAIMEHWKA-KDIVIIPIYFKVTLQHICGLKGMSEAAFLHLQSSVQEDRV-QKWKMALAEIESI(DGHEWTKGTEVMLAEEVVRNACLRLYSKNSKNLVRILALLNQSHPSDAEIVGIWGMAGIGKTSIAREIFGILAPQYDMCY)}*

$1328=220(222):
>gi|297850934|ref|XP_002893348.1| {|0(1268)|<Streptophyta(V)>}predicted protein [Arabidopsis lyrata subsp. lyrata] >gi|297339190|gb|EFH69607.1| predicted protein [Arabidopsis lyrata subsp. lyrata]
{(MMTTGASVSNPRSRV)KWDAFLSFQR--DTSHNFTDPLYEALVKKELRVWNDDLERGDNDELrpSLVEAIEDSVAFVVVLSPNYANSHLRLEELAKLCHLRSS-LELLVFPIFYEVQPWEVRTHNGPFEKDFEEH-SKRFGEEKMDESAASVIILSTN(YANSSWCLDELALLCDLRSSLKRPMIPIFYGVNPSDVRKQSGHFEEDFNDGEDTAMEESYEFSRKYP)}*

$1329=155(175):
>gi|297839821|ref|XP_002887792.1| {<Streptophyta(V)>}hypothetical protein ARALYDRAFT_895867 [Arabidopsis lyrata subsp. lyrata] >gi|297333633|gb|EFH64051.1| hypothetical protein ARALYDRAFT_895867 [Arab
{(MAISLLYLAIVVFVGLRRLLQFLRKSEDSPSRSLW)KYDVFLSFRG-TDVRKSFVSHLYEALNNGGVKTFHDDRELQKGTFIweGLEKEIDQSRFATVNFKK---------FEIEHPLKRCRVNAK---LCSFNHINLSVLKCKVG---------ENKIESSSTFVMMRSSERQNRRS()}*

$1330=160(168):
>gi|502991201|ref|WP_013226177.1| {|0(309)|<Actinobacteria(B)>}TIR domain-containing protein [Amycolatopsis mediterranei] >gi|300786216|ref|YP_003766507.1| hypothetical protein AMED_4332 [Amycolatopsi

{(M)------SFSG-EDRSVG--RRLEDNLRAFGVRVFFDEKiELFAG--
ItdSIREALSESKTLLAYYSADYADRAACQHELMLAFLAGEEEGDPCGRIMVLNPAPDTGHLRPVQLADAKFA
IRDDNDLVQAIAKRAAAVNTSIGS(VLPVSPPPWPAHRIGVVRRFLGRYREL)}*

$1331=214(215):
>gi|302817997|ref|XP_002990673.1| {<Streptophyta(V)>}hypothetical protein SELMODRAFT_131977, partial [Selaginella moellendorffii] >gi|300141595|gb|EFJ08305.1| hypothetical protein SELMODRAFT_131977 [S
{()-YEVFISHRG-
PDTKVSFVDSLNQELKRQQVHCFFDKEELQPGQGIpsQLLRAQRVANIYVIVFSPKFEESTWCNIEVLEAVKA
MEMQPDcKIIYPVFHGVNPGVVRH---
GYRDSFAALTaKGRATAEEVQEWIAAFTTVSMI(SGVDLNELNDDPSENGYVDSEGRVAGILSRFRKSGQMVT
SVGITGQGAIGKTRLAKEVFNRIQGNFEASCFVRVR)}*

$1332=199(212):
>gi|297839149|ref|XP_002887456.1| {|0(210)|<Streptophyta(V)>}hypothetical protein ARALYDRAFT_895140 [Arabidopsis lyrata subsp. lyrata] >gi|297333297|gb|EFH63715.1| hypothetical protein ARALYDRAFT_8951
{(MT)-HDVFLNFRG-KEMRKTFVSHLLSSFTTKNITSFI--VSDPFEDPD--
LKAMEQSLVAIPVISKNYVISNLWMDDLRKIIECEKI-GTLTAIPIFFQVSPLDILHATEKYADT------
QDETLEMVRKWLYAQVSRKPS(FHSNDWDDDSELVDKITSFVSDILESSTSSYQSTGLSPAPSLKLESAFRVS
KFMHWRSLSLPSSAFTPIS)}*

$1333=202(220):
>gi|297819854|ref|XP_002877810.1| {|0(1049)|<Streptophyta(V)>}hypothetical protein ARALYDRAFT_323710 [Arabidopsis lyrata subsp. lyrata] >gi|297323648|gb|EFH54069.1| hypothetical protein ARALYDRAFT_323
{(MVDCSD)---VYISFDR-REdtVRYSFVSHLSAAFHRRGVSSFTGEH---
GSDSEtNGFSKLEKSRASVVVFSEKYPSSKSCMEELLKVSEHRRK-NCLAVVPVFYPVTKSFVKKQ-------
ICNL-A-----
DVRSDWRTALLETVDL(PGHELYDTQSDSDFVVEIVADVREKLNMTDNIGIYSKLGKIETLIYKQPWGVRSIG
IWGMPGIGKTTLAKAAFD)}*

$1334=153(160):
>gi|502968997|ref|WP_013203973.1| {|0(671)|<Proteobacteria(B)>}hypothetical protein [Erwinia billingiae] >gi|299064370|emb|CAX61490.1| Putative TPR repeat-containing protein [Erwinia billingiae Eb661]
{(MI)--KAFLSHSSKdKEhYVRNVANWL-----GKENIIY---
DEFTFEEGEktldQIMEGLGESELFVLFISNSALESKWVRKEIIESKALYDA-
GVILKIFPIIIDSNINHEDKRIPAWLRKHYN--
LQPITRTKIAASRIKNHLYKI(SWQKHPKLEKINSLFVGR)}*

$1335=208(210):
>gi|495188065|ref|WP_007912855.1| {|0(95)|<Chloroflexi(B)>}hypothetical protein [Ktedonobacter racemifer] >gi|297553702|gb|EFH87567.1| hypothetical protein Krac_8903 [Ktedonobacter racemifer DSM 44963
{(MQMLTRSGKNYRLLC)------
CYAEEdRLIQARLETYLlDGLSREQVPATWQGLNLSPGRYEAvEAAVQITEADIILLLVSEHFKTLSLGNPrE
IQRILEREQR-GDVSVYPIVVRKT-
SWVTTSFGNLCPLPSQGgvLLRPESGKFDSAVLATILDELRA(VMTHPREGETDPEQPVPRFLFENELAQRAP
SGWATPTLTSEQLRTTDPSLPVVSL)}*

$1336=204(217):
>gi|503345653|ref|WP_013580314.1| {|0(103)|<Acidobacteria(B)>}TIR domain-containing protein [Granulicella tundricola]
>gi|321163290|gb|ADW68995.1| hypothetical protein AciX9_1949 [Granulicella tundric
{(MR)---VFLSWSgPKsRAVAELLKKYLPSL--
NNTVDPWlSSKEISTGARWSsEIAANLEAANIGVICVTAENQNEPWILFEAGAVSKLTTV-
GRAMVLRIGMKAT-DITG-PLSQFQSV-------GTERD--
DIWKLVVDINGAA(AGNAVPESTISLTFNAIWPTLQGELDQVTKQASADAPERSQDEIMQELLTLARRQEQSS
QLLLNQIVAVSERTIV)}*

$1337=197(202):
>gi|496147264|ref|WP_008871771.1| {|0(710)|<Proteobacteria(B)>}TPR repeat-containing protein [Desulfonatronospira thiodismutans]
>gi|298509173|gb|EFI33078.1| TPR repeat-containing protein [Desulfonatr
{(MK)---IFLSYGH--
DKNTPLVEKIKADLENRGHDVWIDTSEIKFGDDWrcSITDGILESDWMLSFLSQHSTRDPGVCLdELGIAL--
GSKGgIVKTVLVEKEKDV-KPPVSVSHVQWLD-
MSNWEEIKDRDPKDFEQWYKGKLDEI(VRVVESESNQRFAGEIEELQQKLKPVLSDARIWGLLKDGFVGREWL
FKMLEDWRINNQGQ)}*

$1338=120(150):
>gi|298571312|gb|ADI87656.1| {<Nitrospirae(B)>}tetratricopeptide domain protein [uncultured Nitrospirae bacterium MY2-1F]
>gi|298571413|gb|ADI87754.1| tetratricopeptide domain protein [uncultured Nitr
{(MTDKAAKPIR)---IFLSYGH--
DKNAELVKRIRADLEVRGHYVWIDESEIKSGDNWrrSITEGILSTDVVLAFLSKHSVRSPGVCLdEIAIA---
-------------------------IGEKHGNIKTILTESEKEVSPPLCRACVWRHLA()}*

$1339=126(152):
>gi|685327657|ref|XP_009102121.1| {<Streptophyta(V)>}PREDICTED: putative disease resistance protein At4g11170 [Brassica rapa]
{(MDVSSLASPQGG)-YDVFPSFRG-
LDVRQTFLSHLLKEFERKGINTFKDSQIKRGKYISpELKQAIRESRICLIILSKNYASSSWTLGELVEILESR
K-------------------------ASENRAGNLEWHLERLVRERQNIRSRDGSKL()}*

$1340=176(187):
>gi|685358402|ref|XP_009113874.1| {|0(2043)|<Streptophyta(V)>}PREDICTED: uncharacterized protein LOC103839134 [Brassica rapa]
{(MTDCEKAERF)---VYISCV--DEVRYSFVSHLSDALRRNVISsVFVDSGDLLSVEAQG---
KVERAKVSVVVLPANR---QVCLEKLEKVLNCQRN-
KEQVMIPVLYGDSKLHGEWLSAMNLRGLSPVFQSRNDCSDSKLVEKIVRNVNEK(LLYMGRIGMSWKLQEIEN
MVNKQPLGIRGVGIWGMPG)}*

$1341=286(286):
>gi|685358421|ref|XP_009113881.1| {|133(0)|<Streptophyta(V)>}PREDICTED: LOW QUALITY PROTEIN: disease resistance protein RLM3 [Brassica rapa]
{(ERVKKWKEALISIPQKVGLTLEGHRDESELVDSIVKEVKKVLIDVSNKKRGQSTNHRTQTDIISSFEPLVA
ELDRLPSRL)-PQVFVSFCK-
EELGDNFVRHLAWALRELGINVLMDSYNRRGKEDeqQQVFTNIEKSNIVLAIFSKRYSESDRCLNELVKMEEL
TKE-GKLVVIPVFYNVKTNEVRRLQGEFGIHFADSVKRFS-

MEPMmvQSWEEALNFIIKR(STGLSLERHRNEFALVAAIVKEVTRLLPSSXKNEKKKEIRDWRSFYQGFNSDF
SICSLHIPYTSYP)}*

$1342=275(299):
>gi|685360268|ref|XP_009114645.1| {|83(625)|<Streptophyta(V)>}PREDICTED:
probable WRKY transcription factor 19 isoform X2 [Brassica rapa]
{(VSQLQFSASSASTDYEVIVIYRRGDITNDAFVKVVQRLTGIPNSVSDLNNSVSVPLNSYVVKGSSKDLQDT
ELSTSAIRE)-YDAVIRYmRG-CISKGDFISHLHAALSRRGVSVREDIDEVDT---------
VPECRVLIIFLTSTYVPS-----NLLNIVEQQSK-KPRVVYPIFYGISPSDL------ITNSEYYE-
SFFLQDEP-
KRWQAALEKISQM(HGYILTDKSESDFIDEIVNDALEVLLSNYKKNIIGMDTQIKEILSLLCTESQDVRRIGI
WGAVGIGKTAIAEEIFHRIS)}*

$1343=253(227):
>gi|685377171|ref|XP_009121494.1| {|0(137)|<Streptophyta(V)>}PREDICTED:
uncharacterized protein LOC103846336 [Brassica rapa]
{(MERTNPHL)--QVFISFRG-
KDVRGNILSSLKEKLKDEGVNVKTDEEMPRGRKIDeNLQKLIKDSKVAVVIFSENYPESPWCLDELVEIEKQI
EE-
TKLKPLPIFFKVRATHVALEDHnPFKDILLRLedneRENARNGsrvgsgrmhcwscflSQRllkdadkrfVRW
RGALKSITGY(AGLKYIKDSNQALFVNQIVEAVKEMLGKVQSSDDVHDRIRGLHIVRQQKVFISFGGHDDYTR
LGFISHLQAGLKRSGIN)}*

$1344=263(261):
>gi|685833062|emb|CEF67983.1| {|1012(0)|<Nematoda(M)>}Toll/interleukin-1
receptor homology (TIR) domain and Leucine-rich repeat and Leucine-rich
repeat, typical subtype-containing protein [Strongyloid
{(MNILDSQLLLIILIIILFIGICLAIFCFCLSLLTRNRRCIDKKGYKKGTMSLNCSTTSPQSGCSPLPPSTLL
WSQGQSLLY)-FDLFVSYSKAdeIMVCKTLCGPLDDDE--
YTMALLHRDGPRYNGnvhqISDELNRLLECSQTLLLVLTKNFIQYEWNMLQIKTSHQLFFTQslsKSKRVIAV
LDEDV-T-LNDLDEELGQILRKStYIRMRDPL---
FWNLFRSALPSR(SGIGRMVGSPTPLSDCSSSQHYSDMYGTNTSTTGSVPSHLI)}*

$1345=214(201):
>gi|780098376|ref|WP_045471664.1|
{|0(212)|<Proteobacteria(B)>}hypothetical protein [Thioploca ingrica]
>gi|686203317|dbj|BAP54450.1| hypothetical protein THII_0153 [Thioploca
ingrica]
{(MPSYIPNY)KNDIFISYAHVdnEpfpgadagWVT-TLVSALKKSlaqklgrADGYTLWMDHQ-
LRGHQPVNSDIYAQLQESATLVFILSTGYLASKWCLLEFYTFLSQ-VNQDSGRLFMVEYEPV-RAEEQL-
PEVKKLLNYPfWQRDDNTGEPRTLGIPKPHPDRD(QGYYQQLNKLATELANKLKQLQAEAPPIEEVKDEFAEQ
LRLAEAKKSRFSDEK)}*

$1346=286(289):
>gi|686611256|ref|XP_009282325.1| {|368(74)|<Chordata(M)>}PREDICTED: TIR
domain-containing adapter molecule 1 [Aptenodytes forsteri]
{(MEDSYVPAGISFNSAPASISGYSLPSPTYSFSSTLPPHLRGAPSNLSYPPPLHSSPSPAWPPPLQAVEAVP
TSEPDGGER)KFFTFVVLHASeDEIVAHRVKNLLENmgvRNGATLC---
EDFLiAGRSHLTCFQDAMENSAFIILLLTKNF-PCDLCMFQTNTALMESILKQPKRDSVIPFVPK-
ENpleWSQIPSTLGALMPLN---ENSPG---
FSRMVHNTFTTS(RISKKKAMWDQMQREKLQRYQEQYQTLQNFGALNLGSFAQVPPSATRPQLLKQSPQQRCP
PASTVPPAT)}*

$1347=209(227):

>gi|844842893|ref|XP_012793687.1|
{|187(0)|<Platyhelminthes(M)>}hypothetical protein MS3_02105 [Schistosoma haematobium] >gi|685957859|gb|KGB33919.1| hypothetical protein MS3_02105 [Schistosoma haemat
{(LKLTLNSGTRDRIRRDFGVILKNLDISFIAQESETSNNIKKALNSILWQMSQDKNSLDYESNLNSSTQLEHLMISYSHKQ)--------------KET-AINLTKHLKEKGYKVWFDQDNLKYAANTfrSMAEAIEKSYAVCIIYSENYKNSEYAEMEATYAVCLK-----KPIICLRAQRDYKPKGWLGLITARENRIDISGKYPFDEYFSTLCRRINECIK(IKTDHCS)}*

$1348=262(235):
>gi|780101962|ref|WP_045473298.1| {|0(243)|<Proteobacteria(B)>}TIR domain-containing protein [Thioploca ingrica]
>gi|686204037|dbj|BAP55170.1| extracellular ligand-binding receptor [Thioploca ingrica]
{(MLIHAPKAYVPNCKHD)---IFVSYARVdDEpfagadkgWVTTLITGLKILlgKKLGspeLFSLWMDHELRGNTPTTpHICKQLETSALFVLILSPAYEVSQWC-QfELNTLLAIAGK-DSGRIFVVehdiveerspdlsdLLGYK-FWVRNEFGqpRILAIPKPNpeeFEYYQKLD--DLARQLVEKLKEL(KQEAQAKLATTPTLPSDYADLIAATTTLTTRLPADEADPTPTITSPPTTTVFLAKVSENLEKHRQEVKGYLEQQGVRIL)}*

$1349=251(259):
>gi|918440678|ref|WP_052491854.1|
{|210(1552)|<Proteobacteria(B)>}hypothetical protein [Thioploca ingrica]
>gi|686204738|dbj|BAP55871.1| TIR domain-containing protein [Thioploca ingrica]
{(VLDRHRSYVHQHTELLALALDWQQHQRATHYLIVGKERIAAEQWLLTHFQAGEQPPCQPNALQCEFICEARKNAENRLID)---VFVCYDSQdRAIRDPIVQALS----RYAITTWtHDQDIQKGTDFArAIEQGIENADNFLLLISPQSAVSEYV-QrELAHALKYHK-----RIIPLRIAPT-DPAHLHPTLRHLQSLTFqdGVIDELVTLLNQDKTYHEQHKIL(LARALKWLAEDKKPAFLLRGYNLENAKTWLRLNESRQLH)}*

$1350=226(222):
>gi|780105631|ref|WP_045474821.1|
{|0(744)|<Proteobacteria(B)>}hypothetical protein [Thioploca ingrica]
>gi|686204986|dbj|BAP56119.1| WD repeat-containing protein [Thioploca ingrica]
{(MTIKL)--KVFISYADPdQEFKQALLKQLAILQRQGAITTWDKSQLQAHEDEQqAIQTIIEKCQIALLLLSEDYLAADFTRSvVLPRLLARKIN-ENIRILPIIVRPC-EWESiAELKSLTMVPENCvpLNHFPqktgERA--EVWTQIAEQINHL(VQELQYTLHSHTRQKVDLQFLSPSATPFIGRIQQLTELDKAFNDKNINVIGIIGASGIGKSALVNEWLKRMEPSYNG)}*

$1351=285(287):
>gi|740682576|ref|WP_038467865.1|
{|91(279)|<Tenericutes(B)>}hypothetical protein [Candidatus Izimaplasma sp. HR1] >gi|685629583|gb|AIO18095.1| hypothetical protein KQ51_00192 [Candidatus Izimaplasma
{(FAYANYSFGSRRYLFDFYNHTVQIKSDAETIITHITEYSDVRDKSLVENFIARHNPEKIDAYRNKYTQKLIDEENYSSIP)RDV-FISFRS--TEIEIATEVLGV-LEKDGSACWISSRNLRPNDNenYwiNIEDAISKSKV-FLVISSHESMISRDVKrELDIANKLNKQR-----VEFKVDSS-RHTstfkYFFDGIKWIDATENKEDALIELKRRVYSMLTDTNANY(STSPDTKQNISDADSSDFIRKTNRAKIELIGSNYKDAANSIKDALGIDPESAEAWWLLFLSENNFQS)}*

$1352=280(276):
>gi|690429660|ref|XP_009316684.1| {|368(80)|<Chordata(M)>}PREDICTED: TIR domain-containing adapter molecule 1 [Pygoscelis adeliae]
{(MEDSYVPAGVSSNPASASISACPLPSPTYSFSPTLPPHLRGAPSNLSYPPPLHSSPSPSWPPPLQAVEAVHTSEPDGGER)KFFTFVVLHASeDEIVAHRVKDLLENmgvPNGATLC---EDFLiAGRSHLTCFQDAMENSAFIILLLTKNF-PCDLCMFQTNTALMESILKQSKRDSVIPFVPK-ENpleQSQIPSTLGALMPLNeKSPGFSRMVQNTFTTSRISKRKA(MWDQMQRAKLQLYQEQYQTLQNLDALTLGSLAQVPPSATRPQPLKQSPQQRCPPAS)}*

$1353=217(224):
>gi|738765330|ref|WP_036658598.1| {|62(0)|<Firmicutes(B)>}hypothetical protein [Paenibacillus wynnii] >gi|689314031|gb|KGE18424.1| hypothetical protein PWYN_28390 [Paenibacillus wynnii]
{(LEGHINTMKDLMLNAGLRYRPDITMDELFTEMGNRRFNSINLLDDIPIEDQINHLFIFPEKGELENNGYKFSAALGNQLP)---IFLSHASKdKSEIEDLIPYLNAAGLPvwFDKI--------NIDYGEsitkAIQRGIKKSAAVIFWITNDFLDSNWCDTELTNFLNRYSS-KKDVLIISVVSNDVSRDKLELFLTDLKYLVR--ENEAHLETVAKEIVPTLKRYI(DNIR)}*

$1354=222(225):
>gi|754821415|ref|WP_042183932.1| {|42(0)|<Firmicutes(B)>}MULTISPECIES: hypothetical protein [Paenibacillus] >gi|690620469|gb|AIQ21483.1| hypothetical protein H70737_00535 [Paenibacillus sp. FSL H7-07
{(REIKYVVNEIIKTKDWISNENDIYFIDLNKLGLAYPSIFNQCQWMNGLISVTPIYEDGLNDVRFFSLTISEDEIREKAPR)--KVFLSHKGAnKPLVRKYYQLLKELGFDP------WIDEEDMPAGAqlmrSIRQGFKQSCAVVFFITPEFKDEKYLEKEINYAineQLNRKQ-FTIITLQ--FEDEQG--NKGLIPEMLEDyvwktpRTE--LEAFKEI--IKSLPIKVGQTI(WKIER)}*

$1355=190(186):
>gi|727302838|ref|WP_033759703.1|
{|0(129)|<Proteobacteria(B)>}hypothetical protein [Pantoea agglomerans] >gi|687248644|gb|KGD71838.1| hypothetical protein ID10_18895 [Pantoea agglomerans]
{(MDDKSNVA)---IFISYAW--GGSLEKKEWvrqqIVNSLNGK-YSVFWDRDTIGFGESIdaCIHKALTHRPIKVFCICDADYLHSakkvgSGLHrELQILEKIAHE-DQVKIIPLIFESD-CIRDLPAPLAGRAYLDLAELHKRNLFIGNEMYALAEGISQ(AEMSILIKKKIASNDLVKLAKHHFQGLDIELYGNGRTH)}*

$1356=191(195):
>gi|757476740|ref|WP_042752052.1| {|0(122)|<Spirochaetes(B)>}molecular chaperone Tir [Leptospira interrogans] >gi|689454164|gb|KGE27659.1| molecular chaperone Tir [Leptospira interrogans serovar Lai]
{(MNPK)---VFISHAS--EDKDRFVINFSTKLRSKGIDAWLDKWEMRPGDSLidRIfEEGIKNADA-FIIVLSKNSVNKPWVReELNVA--YVKKInAQAKIIPVVIDEC-EVPECLKSTLWEKISRLDSYEENLNRIVQAIFGISEKPIL(GNPPKHIATAIDILPGLNKIDTILFNYSCEISLQKERKQINTSEVYGYLKE)}*

$1357=225(215):
>gi|696240837|ref|WP_032820385.1| {|0(238)|<Firmicutes(B)>}hypothetical protein [Oenococcus oeni] >gi|692691923|gb|KGH58614.1| hypothetical protein X288_06795 [Oenococcus oeni IOEB_9805] >gi|692709889
{(MADT)-KKVFISYSHTqQELATALAKDLQNNGIS------TIFDGWDLPAGYdldhFMEQNLKDSsiDYIIVLCDQDYKnkadkkdgsgvstESTIMTPEAYGS-ITSSR-

IIPLAI-DFNSENKAVVPtflATKKYINFNSKRRP-YNESLEE--
LERRIYGEPVLK(RPPLGLEPNFTAAEPQDDGIRKIQNISFQIENALMGNPLMAQDQFKTAYLPALQAFLTDW
DKQCAGKKTIA)}*

$1358=250(256):
>gi|759796467|ref|WP_043502796.1| {|173(819)|<Actinobacteria(B)>}TIR domain-containing protein [Streptomyces glaucescens]
>gi|692323429|gb|AIR99632.1| hypothetical protein SGLAU_18350 [Streptomyces gl
{(TGVLVVAYGVADPPQRHARLLDVLRWTDRQVQGTVFAPSVTQEYWPLLVAAAGLVAGGTAAVLRGAAPTRA
PAIGLHPRP)AGQVFLSYARADTATAERYVGALRAAGRT-
VWMDADGIRPSREWRTELSDAIRASDAVIVLLSRSSLR-SRYCWdECRQALEHG-----
KRILPVLIDADLaaGSVTGSLREADWDGIT--
RFQRLSMHRPEDFAPAVRETLA(FLAREHDWVALHTRLGEQAHAWQHHGHAPERLLRRH)}*

$1359=274(296):
>gi|738384448|ref|WP_036336499.1|
{|0(420)|<Actinobacteria(B)>}elongation factor G-like protein EF-G2 [Modestobacter caceresii] >gi|692530950|gb|KGH45937.1| elongation factor G [Modestobacter caceresi
{(MATHSGSSNRTGAGAPAPRPPEQVRNVALVGRAGAGKTTLAEALLAAAGALPRVGRVEEGTTCLDTEEVEV
RLQHSVSLA)-------------------AATVEHAGHR-VTLL--
DTPGAPDFVGELRAALRAADAALVVVS-AVAGVDAGTVqLWDecAAVALP-----
RAVVvtqvdrPRADVPAVvrSCRELLGAGVHP-LHV--LGDDGALT--
SLLEPLPGNPER(DRLRAELIEGVIAESEDETLMDRYLAGEPIGVADLVADLETAVARGDFHPVLCTAAPTGL
GVAELLDLLVSAFPSP)}*

$1360=197(191):
>gi|696225645|ref|WP_032806040.1| {|0(83)|<Firmicutes(B)>}hypothetical protein [Oenococcus oeni] >gi|692884670|gb|KGH87797.1| hypothetical protein X350_06820 [Oenococcus oeni S12]
{(M)--KIFLSWS------
GDLSKHIAecfheflPLVLQYTEPFMSSDDIARGDDWNnKINGELNNSRIGILFITPENINSNW-
LNfEAGALFswKNKDE-KQGTVMPVMINSL-
DitsvLSKSPLKQFQGTKSLSeeGVKSIFHSINLKADSDSLSEASF(QSTFDQWWPKINQEINQVLKRPSTKI
EPKKTDRKKDMWSAQDREVMVRTL)}*

$1361=168(158):
>gi|738285765|ref|WP_036239922.1| {|0(34)|<Proteobacteria(B)>}TIR domain-containing protein [Massilia sp. JS1662]
>gi|691633384|gb|KGF78639.1| TIR domain containing protein [Massilia sp. JS1662]
{(MAI)RYGCFFSYAHGrhelmqrfkATLADALRCYLEPYFDNEDE-
LFIDTEQLGGGDDLdrKIARALCESACMVLIYTPKYEAHPYTRREYAAMRLIEAERstwYTLPSQLIIPV---
IMTQHPVSLPPQISESTFYVDFSRFTMATADMKSNPDFLP(DIDKMVRRIVTHYQY)}*

$1362=261(280):
>gi|1079225530|ref|XP_018504677.1|
{|125(189)|<Streptophyta(V)>}PREDICTED: uncharacterized protein LOC103954249 [Pyrus x bretschneideri]
{(HTETYKDDPDKVKRWRAALTQVASLSGWDSRGRRESELIQDIVDDIRSKLHPKFPSTVPSSSILAETSKPY
CSHFSSQRR)KYDVFMSFRG-
RDTRLNIVDHIYHSLKQKGITTFRDDKGLDRGLSIssGLVQAIEESQISLVTLSPDFASSAWCLDELVKILEC
TET-SNLQ------------------RIHEAFTEH-

EEASSDMVGVKCNTRDRKISKI(RISVHYGGKWDNTAYTGGNATEIVVSKDITCEELLDRVYCIVGIDPDEYE
IIMKTTDESE)}*

$1363=240(240):
>gi|694451858|ref|XP_009351003.1| {|0(319)|<Streptophyta(V)>}PREDICTED: TMV resistance protein N-like [Pyrus x bretschneideri]
{(MALVRAAQGTSSDSNTHWGY)RYDVFLSFRG-EDTRRTFTDHLYTALNNA-
GFlTFRDDDELERGEDIkpGLQKAIRQSRASVVVFSKDYASSRWCLDELVMILERKRtT-
SDHVVLPVFYDVDPSHARKQTGSIGKAFARH-EK--
NQSPNkvRGWREALAELADL(AGMVLSNQADGYESKFITKIVKVIGDKLLRTPLAVESNLIGIQSRVKHINLW
LQDGSTDVGIVVVHGMSGIGKTTIAKYV)}*

$1364=253(260):
>gi|693582524|dbj|BAP69031.1| {|939(30)|<Stramenopiles(E)>}RxLR effector candidate protein [Hyaloperonospora arabidopsidis Emoy2]
{(EYDYDQAKFQSQFRLLLDGVRDEIEKNRDAVQRKNVASSRRNHGLKQTSMGTMIAASGPGGFSRLFQRWDP
DASFVQKQF)---VFLSHG---DKHTGFVQQLYSELQDAGVVCYGDHN--
VEGQDFedRIhatREAILHCTCFLVILSKQTMG-----
SELVRDQLAFAEDKGRPIFPIaLNDLDPGLDKRYSLARNELYHFMGNGMSFKHSADRLIESLRQQYTA(QDEN
VGWVVNNGTRIASANTSFVSFTVGESVSRDDNRSGD)}*

$1365=165(177):
>gi|694327209|ref|XP_009354479.1| {|0(105)|<Streptophyta(V)>}PREDICTED: TMV resistance protein N-like [Pyrus x bretschneideri]
{(MLHKNNWIPLYRNSKFNLEGCQEFVQKFKVQ)--------------
RKNLTDHLDCTLKTHGFKVFLDENELTSGEPItpELKQAIKDSRLAAIIFSRGYEGRFVQQIVEDIARKLKNT
CLAEPTYPVGIDSRVQEIGKYLDVGGSDKTTVANAIFNNYQDKFAGKSFLANVKR(NWLIRK)}*

$1366=215(224):
>gi|694335417|gb|KGJ88568.1| {|59(0)|<Proteobacteria(B)>}TIR protein [Colwellia psychrerythraea]
{(KDFEDVDRYLASKYPTTKRAYNRFVTDLMASMDNKSYNLISVSTPEDVDVYMTSKTNDYFTFSDRELFFEM
WGKPVTLDN)-FTVFLSHSSKdKEFVDEVFNELQKSE--
IKAWYDKEEIIAGESVTDNINNGLQKSNFGLIFLSKSFLNgqSGWTIAEANFFMQQRMRDASKKFVIV-----
--NIDLPHNEIPPLMQDYrYIDASDDG---FMVDIKKAINHH(QRRI)}*

$1367=286(289):
>gi|694857118|ref|XP_009470181.1| {|366(69)|<Chordata(M)>}PREDICTED: TIR domain-containing adapter molecule 1 [Nipponia nippon]
{(KSMKDSEVPAGIPSNSASASISTSSLPPPPYSSSSTLPPPPQGVPSSFSYPPPLHGSPSPAWPPPLQTVES
VPTSEPDSG)KFFTFVVLHASeDEIIAHRVKDLLEKmgvPNGATLC---
EDFsIAGRSHLTCFQDAMENSAFIILLLTKNFL-CNLCMFQTNTALMESILKPSKRDSVIPFVPK-
ENpleRSQLPSTLGGLMP---LDENSPG---
FSRIVQNTFTTS(RINQRKAMWELMQRRKLQERYQTLQNFAALNLGSLPQMPPSATRPQLLEQPPRQWCPPAS
TVPPAAYPP)}*

$1368=255(262):
>gi|694334771|gb|KGJ87957.1| {|65(130)|<Proteobacteria(B)>}TIR protein [Colwellia psychrerythraea]
{(LAEVIDDSIKRPDRTIYCFLSEDGGDTFSDNDCKILTTIGEQVAGNGGYNCRSLDGVVNLLNNDQRKKSFE
NSPVIDKHH)--DVYISYGR-NDSK-
TLAKHVSDSLTKKHYSVWYDNAAIPQEIAFlaQINQIIRQCHCFIFIFSAHAAHSEYCLRELEFARLLG----
-

KNIIFLFEHALPVEQQIEQATETSLFVDMNASFHIDLTKDANLNAVITYLST(SLEHLTPHTQWLNKTHHWQE
SQQDDDLLLYGRQRISACKWLS)}*

$1369=214(220):
>gi|694085670|ref|WP_032430481.1| {|78(0)|<Proteobacteria(B)>}molecular chaperone Tir [Klebsiella pneumoniae] >gi|960654491|gb|KSX46607.1| molecular chaperone Tir [Klebsiella pneumoniae]
{(SKKIADKTAELHRYETQLTKDEESERKKLEIAQKKREKEQQDLQKKIKEEMDSQIRTLHSRMLSSKTGIHD
AINDEDESP)QYDVFISHAS---EDKDlFVRPFAEYLKSQGVKVWYDEFSLA--WGDslrKKIDKGLAN-
SRfgIVVISKNFIKKQWTEYELNGLIASEI-EGTKRVLPIW---HEI-SKSEVIKFSPSLADK-
VAMNTAIQTYEEIADQLVTLLR()}*

$1370=259(263):
>gi|693582524|dbj|BAP69031.1| {|729(234)|<Stramenopiles(E)>}RxLR effector candidate protein [Hyaloperonospora arabidopsidis Emoy2]
{(DATRELFGILTSAGADINYQLPTTGDTALHVAVRKDNEQSALAILANGGNLMKMNIEGLRPLDCTTSTRLL
FEIKQAAGQ)-RDVMISYTHSHAEFAKKIRKALEDANVT-
TWLDLMDPsgiGGGSVWREEIARGITNAAVVLCLLTEDY-AKSEWCLkELALAKQVG-----
TPILAVSSEGVSigEDLQVYLYTRQIIPFE--
PAITQTRRNSTDTRQIEYDYDQ(AKFQSQFRLLLDGVRDEIEKNRDAVQRKNVASSRRNHGLKQTS)}*

$1371=185(194):
>gi|693460519|gb|KGI66953.1| {|0(331)|<Actinobacteria(B)>}hypothetical protein EU78_05235 [Mycobacterium rufum]
{(MAA)----FISYSSRdRTLVEGL---LKAMRRAHL-
PVWLDEELTGGESWWrEILTQIRDCEVFIVALSKNLLDSKAC-QaELRYAQAL-----GKPVLPVQVGPM-
DtLRINPLSAMQVIDYRTpdLESGIELISSVNALRAKTPPLPD(PLPEEPPIPFAYLMRLASTISDPTGLSAQ
QQTALVSELKAGLEEDGSDELA)}*

$1372=222(220):
>gi|696563545|ref|WP_033095382.1| {|0(68)|<Proteobacteria(B)>}TIR domain-containing protein [Colwellia psychrerythraea]
>gi|694333678|gb|KGJ86888.1| TIR protein [Colwellia psychrerythraea]
{()MSKVFISYSSSdRPAVTRLKEALEQLSY----
DVWFDQDaILPGGYYAkDIATALKDIDVFVLMTSANSIgdkANNIAGSsEVAKEVVLAQS-
SPAVIIPVKLDDC--WmettTHDEFTYLLAR-----TQWLDAgycnsS--
SDFMDVAQRIDSA(LVSGNFKFNHDTTLDEIDKLLKAGLFSQARSRMNANTFPDIASDRVVLINAILALSEQP
ISSLSKIKVDNLVRDLVQVRN)}*

$1373=171(173):
>gi|696563234|ref|WP_033095080.1|
{|0(96)|<Proteobacteria(B)>}hypothetical protein [Colwellia psychrerythraea] >gi|694335414|gb|KGJ88565.1| TIR protein [Colwellia psychrerythraea]
{(MGQSVL)---VFVSHSSDdKE---
GLIEPLVQDLEDCYINVWLDkRKILPGDNLRkSIfRDGLDKADVALIFFTEKSLKSSWV-
DrEIKHVLREESK-KGNDFDLNKIISI-
FDCEETYNQIAVRYPELtdDLLHLMPENYNRIQIGQLISAIW(SKYLSLQGGDVETQRQLLTKEKEIFQQ)}*

$1374=213(220):
>gi|725950570|ref|YP_009103093.1| {|67(0)|<dsDNA viruses(V)>}TIR protein [Enterococcus phage EFC-1] >gi|694818117|gb|AIS73994.1| TIR protein [Enterococcus phage EFC-1]

{(SLASKEKSATISSKLAKKRKELGDIQVKLSKQRTIENTQFQKNLKKTYDTQIMKIKETQTAALQNVQSELP
NDPSLVNKT)-YDLFISYAS-DDSEY--
VDKLTQAFTNEGFSIWRDKSDIAWGQSIrqSIDAGLSNSKFGLVVLSSKYIEKFWTNYELDGILNKESATGRQ
MILPLWHNITKDEIDKKSPSLSNRL-----ALDTRINSTNDIINAFKSLLE()}*

$1375=175(176):
>gi|918393389|ref|WP_052445664.1| {|0(123)|<Firmicutes(B)>}hypothetical protein [Bacillus sp. B-jedd] >gi|695916955|emb|CEG29450.1| TIR domain protein [Bacillus sp. B-jedd]
{(M)EYDLFISHAS---EDKEsFVRPLAQVLSKLKVNVWYDEFslRIGDSLSKsidIGLIN-----
SSfgVVVLSKAFMQKSWTDYELKSLLSKEIAYGKV-ILPIWHGISKddL-LNFSPFLADKYALNT-
NGMNENIIALELCKVVRPDILE(NITRQMIYESLYENKGEQLDIDINQIEDMFPLRHE)}*

$1376=217(224):
>gi|889537396|ref|WP_048827499.1| {|63(0)|<Firmicutes(B)>}hypothetical protein [Bacillus sp. B-jedd] >gi|695916959|emb|CEG29454.1| TIR domain protein [Bacillus sp. B-jedd]
{(GHVEVMKNLMRAEGLRYRPDITFNELSQEIGNRRFNSQTLVRESDVLEAMNNELLFPFVEKSKLEKMGYVI
DKPLGNQMP)---IFLSHSSKnKSEVEDLIPYLNGAGLPV------
WFDKVNIDYGEsivkAIQKGIKQSAGVIFWITNDFINSNWCDLELTKFSSRYAS-
KKDLLTIAVVHQDVDIDKIDFLFDDIKYLHR--KDENLETIAKKILPSLKRYKS(NNNT)}*

$1377=254(278):
>gi|748751414|ref|WP_040009529.1| {|27(27)|<Proteobacteria(B)>}TIR domain-containing protein [Francisella sp. FSC1006]
>gi|695168243|gb|AIT09566.1| hypothetical protein LO80_06040 [Francisella sp. FSC
{(AYFREHGVPTQDNYPHNSKKSYSKDMLAQVDEELLFKIAGELGLVDVPSGVEVVREVGFWKAGHFRLFLSH
LASFKVQTS)--------------------
HLQTALRKYAISSFVAHeDIEPTKEWQsEIEAGLKTMDALVAILMPGFQESKWC-DqEVGVAVGR-----
DILIIPIRkgLDPY-GF----
IGKYQGIQAQGktIGDVAEAIFETLIKSPKTRNKIL(ISLSGALSNATTSDEAIEKVSILKSLESVPSDILEN
LKQQVAENSHLIESKEFLDSLN)}*

$1378=232(240):
>gi|738963442|ref|WP_036847316.1|
{|805(0)|<Bacteroidetes(B)>}hypothetical protein [Porphyromonas cangingivalis] >gi|696209117|gb|KGL48220.1| hypothetical protein HQ34_06910 [Porphyromonas cangingival
{(ERRENPITDLYLSVYDPNLKVEDQLYVHLETLDDPEKTKSKIMGYPLTADGQIDKDRGQTLSTKRYKHLSV
NSELKSVRQ)---
IFISYSKAdREDLMELNKFLEDLKREEKIEVYYDETTTPGDMIHeVIRQKIIEADYVIALVSQDYISTEYIRD
vELPLIKEKNK-----CLIPIIIKPC-TWTMNEISNHYATLKGTEISFPDWN--
KDSHPGNYEACRA(QNWTRAVTEISQKLFNNKDN)}*

$1379=274(278):
>gi|695166275|ref|XP_009511631.1| {|370(79)|<Chordata(M)>}PREDICTED: TIR domain-containing adapter molecule 1 [Phalacrocorax carbo]
{(DSSVPAGIPSNSASASISTSSLPPPTYSSSSTLPPPLQGPHSNLYPPHLHSSPSPAWPPLQTVEPVPTSEP
DGGKFFTFV)-----VLHaSEDEVVAHRVKNRLEEmgVPNGATLC---
EDFFIAGRSHLTCFQDAMEnSAFIILLLTKNF-
PCDLCMFQTNAALMESILKPSKHdsVIPFVPKENPLErsqIPSTLGGLMPLDENS----
PGFSRTVRNTFTTSRINER(KATWNRMQRRKLQLYQEQHQTLQNLTALNLGSLAQVPPSATWPQLLEQPQQWC
PPALT)}*

$1380=221(222):
>gi|696575952|ref|WP_033107432.1|
{|94(281)|<Actinobacteria(B)>}hypothetical protein [Microbacterium profundi]
{(APSGPSPTDVAAMRAIAKSSDGPVALLVYAPGRTDPFIGLVAQRHRLGRITSTKADVSFPASRFAPLGPLP
DGAVRGDGP)---VFISYRQSDGTPHAESLEGLLRAAGL--
VVWRDNSDLRAGTTIdRLEQALTKGlSAGVLVVTPEIVYSEIVREreLPRLLELDNDSAFSLCIANAIPRVGN
GSKCDYDGPDRLLRLSPARTLADKKQSNMLAPTGEVEIVR(DL)}*

$1381=132(144):
>gi|697062825|ref|WP_033152426.1| {<Firmicutes(B)>}hypothetical protein [Streptococcus equinus]
{(MKNK)---VFVSYRADDeGSRYKNLLVAwsendrffpEISFHDT--
SVGTSINSVNATYIKsVIKDKIQKSDIVLCLVGRNTSD------
SDWVNWEIETAYqLNKKIVAVKIDRRYDTPRAL-------------YRKNVDWAFVFSKEAILRVLI()}*

$1382=286(289):
>gi|697018234|ref|XP_009570504.1| {|365(75)|<Chordata(M)>}PREDICTED: TIR domain-containing adapter molecule 1 [Fulmarus glacialis]
{(HMSMEDCYVPAGISSNSASASIPACSLPPPTYSFSSTLPPPLQEGPSNLSYPPPLHSSPSPAWPPPPPVAA
EPISEPDGG)KFFTFVVLHASeDEIVAQRVKNLLENmgvPNGATLC--
EDFFIAGRSHLTCFQDAMENSAFIILLLTKNF-PCDLCMFQTNTALMESILTPSKRDSVIPFVPK-ENPLE-
RSQIPSMLGGLtPLDENSPG---
FSRTVQNTFTTS(RINERKAMWDHVMQRRKLQLYRERYQTLQELAALNLGSFPQVPPSATHPQLLEQSPQQWC
PPSSTVPPA)}*

$1383=262(261):
>gi|697448309|gb|KGL88107.1| {|367(0)|<Chordata(M)>}TIR domain-containing adapter molecule 1 [Charadrius vociferus]
{(STEDSCVPEGTSNSASASISACPLPPPSCSFSSTLPPHFLGAASNLSYAPPLPSSPSPAWLPPLQSVEAVP
TSELDGREL)KFFTFVVLHASeDEIVAHRVKNLLENmgvPNGATLC--
EDFFIAGRSHLTCFQDAMENSAFIILLLTKNF-PGNLCMFQTNTALMESILKPSKRDSVIPFVPK-
ENPLERSQIPSTLCGLMpLDENSPGFSRTVQNTFTTSRINE(RKATWDMMQRKKLQQYQERYQTLHNLAALNL
GSLPQAPPSA)}*

$1384=215(215):
>gi|917502216|ref|WP_052108633.1|
{|0(71)|<Bacteroidetes(B)>}hypothetical protein [Polaribacter sp. Hel1_85] >gi|697005905|gb|KGL59091.1| TIR-like domain protein-containing protein [Polaribacter sp. H
{(M)KPKVFLSHSKKdKVIIEKIANDLRSCGI---DVWY---
DEWEIPPGEsirkKIfEDGIVSCDMFFVYLTKNSIPSYWVQKELDGALIHEIE-
TKNSFLVLFVDEDESrehlSLDLKALNIpKLNDDNY--LIPFGKL--
ISRTWNAHSKNQ(VRLKERDNKIQILELEKSNAELQKKILQIEKSSIIDVNGLKKALENIKYEFNGVETDLLK
FLIRLKNKFADGAN)}*

$1385=206(220):
>gi|696616860|ref|WP_033126788.1| {|199(0)|<Firmicutes(B)>}hypothetical protein [Eubacterium sp. ER2]
{(MYAISLEKSERPDYALTISNYQYYRLYISMCQQRSLFENKIRLLERKLDRLSLGETANEAACTKQSDVFPR
AYRGPEPFH)----FVSYAHLdKKTVYPLINRLQ----
ERGLHIWFDEGIEPGKEWPeEIALHIMQSRSVLVMLSGQSIHSPNVRRELSLALSENKK-----IIAVLLE--
---EVVLTAGMRLQLELQqmVMKYQYSNENFLEKLYTCINEEL()}*

$1386=214(221):
>gi|576249529|gb|AHH29253.1| {|401(0)|<Echinodermata(M)>}toll-like
receptor [Apostichopus japonicus]
{(SKQEEKAREVEIHLSRHANAIEEAFHGHGINCQGFECDLERFNYSGEERLGFVNILSEERHPLSPIGHPYM
SLPNHHSVY)KYDFFVSFSHIdhKW-TLFFFRKLER-
ELELKGCIRYRDYECGKAIAANISTSIRKSAKILVVISPDSLNDPWCKFEVQKSHAESLTRNHVSLIPIMLRM
C-KIPEG--------FELTtYIKCQDGQ---FsvddWHRLSKSLLKK(V)}*

$1387=246(230):
>gi|699250395|ref|XP_002126107.2| {|359(0)|<Chordata(M)>}PREDICTED:
uncharacterized protein LOC100180976 [Ciona intestinalis]
{(VKKTDLGVYELQVKTLDLQNNVSIALNFSQDKSSLVVGLAVSLGALFFLLLLVFILWWNRIYVSVIYKRKF
GPYTKDTKD)-FGAFLSYqftmdmdrfSQQqtRDVVSSTVTAL--NDIRFTVFDQHKD-
IGGEFMAEKAIQSMSECHRVIIILTSEYIKDNWSVFSLQQSFMKMIDSGRKVIFILVpgiqefTKQK-
GSENETCRMIDRAIKLNdSILWSDNK---HFNKnkfklmLEKAMPKV(RPNNRKGEKE)}*

$1388=232(252):
>gi|699656157|ref|XP_009897506.1| {<Chordata(M)>}PREDICTED: UPF0449
protein C19orf25 homolog [Picoides pubescens]
{(MSAKAKRVLSTRPEPPSVEQILADLQSTHPGDPIFILPTEPSQDHSPSSGEPLGFEERERLYQLSRSYVGM
NQQL)----------QdDAVASRISQRLRQEGF--RGYAEHQDQVAGTFLLLSTPQVLEAS-
RVaILLFSAKSLGDPWCQRVCEWSLCHAVRRGGTRMIPVCLGVEEAEVPAFLQHLTKLHY------HTD---
FFYDRLLASLRRA(CLGGSGGPLVGGEQVAVAAVLSFPSRDFPNPSKPPQL)}*

$1389=274(278):
>gi|699605013|ref|XP_009871442.1| {|378(76)|<Chordata(M)>}PREDICTED: TIR
domain-containing adapter molecule 1 [Apaloderma vittatum]
{(PAGIPSNAAPASLITCSLPPPTYSFASTLPTPLWGAPANFSYPPPFPSSPSPAWSPPLRTAEPAPPSEIDD
GERKFFTFV)-----ILHaSKDEAVASRVKNLLEGmdVPNGATLC---
EDFSIAGCSHLTCFQEAMEnSAFIILLLTKNFL-
CDLCKFQTNTALmeSIQNPSKHDSVIPFIPKENRLDrsqIPNTLGALIPLDENS----
PGFSSTVKKTFASSRISQR(KAMWDQMQRRKLQVYEEQHQPLPTFAALNLGSLPHVPPTHPQPPSPQQWCPPA
ATALP)}*

$1390=274(278):
>gi|699627684|ref|XP_009899013.1| {|366(77)|<Chordata(M)>}PREDICTED: TIR
domain-containing adapter molecule 1 [Picoides pubescens]
{(AGIPSNSASASISTHPFPPPPAYSISSTLLPPPQGAPSNLSYPSPLPSSPSPAWLPPLKPADPVPTAQLDG
GERKFFTFA)-----ILHaSEDELVAHRVKTLLESMGvpNGATLC---
EDFSVAGCSHMTCFQDAMEnSAFIILLLTNNFL-
CNLCMFQTDTALMESIQKPSKRhsVIPFVPKENPLErsqIPSVLCGLVPLDENS----
PMFSRKVQNTFNSRRIKER(KAMWDQMQREKLQLQQEQYQTRQRLAALSLGLHPQEPLPAAQPQLLKPPPQQW
CPPTS)}*

$1391=292(298):
>gi|1041926732|ref|NP_001316117.1| {|89(42)|<Arthropoda(M)>}myeloid
differentiation primary response protein MyD88 [Diaphorina citri]
>gi|698975880|gb|AIU34716.1| myeloid differentiation primary respo
{(KSSTLDRSVFNIATFLHFLECIDRFDVYDDCIGFIEEDCLYYENTRLGNSISKNGACMDDPSVITIEDVKS
LNLGCGLAH)-YDALLLYDEAdFDLAKQFNTQLNKMGFT--VCDKDNSFLTGQFEHSAFIQVM-
DRCTCvIVLMSTHFMNNSLLHSLTQYAEAKHIDHTiHSKIIPILCSDDYFSV-GFLQYCVKLKLNhY--
ASNME---

QFWDRLYYSLKGV(GKGVPEWDFASNMEQFWDRLYYSLKGVGKGSQKQAIQFQTTPTSMPVAPPDYTLSQQAV
APPDYTLPQQATNSQSITN)}*

$1392=163(168):
>gi|746383557|ref|WP_039426883.1|
{|0(64)|<Bacteroidetes(B)>}hypothetical protein [Porphyromonas gulae]
>gi|700234185|gb|KGN83678.1| hypothetical protein HR15_11750
[Porphyromonas gulae]
{(MKYMNNRLS)--KVYISykHDSKyNDVIDNIKNGLRKNGINY------SIDQESLQYKDnieaY-
EKEIGQADRVIMIMTPGYFKSQACMFEMTQIFKCGRV-EERVFPIVDMGDIPRNGDGLSEIKVYWQQEK--
EKKAFEITKEPGHSDFRISEI(TKIDDIIRLLDDLWKFIVH)}*

$1393=175(176):
>gi|700236816|gb|KGN86175.1| {|0(74)|<Bacteroidetes(B)>}hypothetical
protein HR08_04540 [Porphyromonas gulae]
{(M)--KIYISGCKEdREYIEKISQALKN-EAHEVFDLFSSISIF-
GKSIHkQVLDAIKESDVFIFLISKNSICTEYTSKeWFLILLGNTSN-RKSNIIPVLIDQA-
KIPKYLYGYNCIYLHKSleRGIRKILSFLSNYSHIQEAEKIK(VTREDNSKFISELSQALKNGKLTLVSGAGT
SIAAG)}*

$1394=234(240):
>gi|738904291|ref|WP_036791227.1|
{|789(0)|<Bacteroidetes(B)>}hypothetical protein [Porphyromonas canoris]
>gi|700242602|gb|KGN91769.1| hypothetical protein HQ43_06660
[Porphyromonas canoris]
{(KLERQKNPIPDLYISCYDPKLKPEGQLYVKLQKLDDPEQTKSTITAYPLTQEGEINREKELRVSTKRYKHF
SINPALKSV)-
KRIFISYSKSdIDDLNELNKFLTDMIREEKIEVYYDKTTTPGDLIHeVIKEKIIEADYVIALVSQNYISTDYI
RDvELP-LIKEHNK-P---LIPIIIKPC-TWTMNDISKHYAVLKGAEISFPDWN--
KGSNPDNYNACRS(ENWRKAVEELNEKIFTNKYN)}*

$1395=233(232):
>gi|702237570|ref|XP_010058955.1| {|0(236)|<Streptophyta(V)>}PREDICTED:
stress response protein nst1 isoform X1 [Eucalyptus grandis]
>gi|702237575|ref|XP_010059011.1| PREDICTED: stress response protei
{(MAHSKARASEVR)-YDVFLSFCG-
VDTRYNFMGFLNeglrnakiRHLRNAKIRVFRDEDEIkvGDKISEKILRAIDNSTIYIPIISQTYPDRKWCLI
ELERMMNNvSRSKGRKKIFPIFFKVEPGDVKHRTP--------HPENPPSEvEL-
RAFKEALAKVGRI(KGWKVEERQSQEKIVKLVVQKVLEELGRKDGSQTKCSIELDDLRQSLEGSLYRAKREEQ
GMRLGACNQIQEPDRTTRKTS)}*

$1396=193(223):
>gi|702283869|ref|XP_010046046.1| {|0(135)|<Streptophyta(V)>}PREDICTED:
TMV resistance protein N-like [Eucalyptus grandis]
{(MVP)------------------------------
ICVFKDDKSIpiGEEFGSQILDAITWSKISIPIISENYASSKWCLRELTHIMDC-
KKRTSHMVSPIFYKVEPSDVRYLKGSFGEAFHSHKNRFDKEEI-
QEVERALREVSGL(HGWESEKFANGHEGELVEKVVENILSKLRQDFQLDVTKYLVGIGDHVNKIRKWIDSPTS
VARMIGIYGMGRIGKTTLAKA)}*

$1397=213(208):
>gi|702285203|ref|XP_010046326.1| {|0(150)|<Streptophyta(V)>}PREDICTED:
TMV resistance protein N-like [Eucalyptus grandis]

{(MDRGQTQGKRKRAEEESTEGAATSSFIPPEATCVGSG)QYDVFLSFRG-
SDTRITFTDHLYYRLIKAGtvpFCVFRDENNIqiGEEFGSKILNAIAQSKISIPIISENYASSKWHEGKLiEH
VVETILSKLREDFQLDVPEQLVGLDHRMKEIMNWIDNSSISARMIGIYGMGGIGKTTLAKCI(YNQLSNKFSH
VSFLPDVRETSRCNGIMYLQS)}*

$1398=185(208):
>gi|702286331|ref|XP_010046571.1| {|0(287)|<Streptophyta(V)>}PREDICTED:
TMV resistance protein N-like [Eucalyptus grandis]
{()-------------------------
MKGAGIRVFRDDDELqvGQRIKEELPRAIQSSRIYMPILSRNYASSKWCLIKLTMMVTTaSKSDSTKEILPIY
LHVNSDDVKLKTPLYHDAFVQHERNFGADQV-
KVGRYGKLIKEIV(QEVLIKLNARYKNVTEHLVKDHVQIDAIMKLLDVDYSGVHFVGVHGIGGIGKTTLAKVI
YNKLYSRFD)}*

$1399=238(238):
>gi|1091460186|ref|XP_018726436.1| {|0(696)|<Streptophyta(V)>}PREDICTED:
TMV resistance protein N-like isoform X2 [Eucalyptus grandis]
{(MAKEGPNGGSSSASSASVTGNN)-FDVFLSFRG-
SDTRHGFTDVLYRSLVDHGVRTFLDEEELsdGEYLKKQLPESIKLSKIGIPILSENYASSKWCLRELAQMVE-
WRNEGRMIIMPIYFLITAWDVKMKdgKGPYANKIAAHQEKVNPDTI-
QKWVYALQEMTSV(KGRQLTEYTGQGEFANLMVSRVLKKLGKPKLNLSDKLVGIEDHLPELRRMLDTDSSDVR
MIVISGIPGVGKTTLAK)}*

$1400=243(242):
>gi|702298144|ref|XP_010048442.1| {|0(256)|<Streptophyta(V)>}PREDICTED:
TMV resistance protein N-like [Eucalyptus grandis]
{(MSQEGPHGVSGSASSAPVTGNN)-FDVFLSFRG-
SDTRKGITDVIYEGLVQAGVRTFRDEEEIsdGESISKELHGAIKWSKIGIPILSRDYASSKWCLRELAHMVE-
-QLNDGMIIMPVFFEIPVDHVKMKdgEGPYAAKIEAYKKDptVKPHMI-
QEWVDALKKVTSL(KGRQVDCGQGEFAKIMVSKVLKKLRKTKLNLSDKLVGIEEHLTKLRRMSDTDSSDVRMI
VISGIPGIGKTTLAKSVYNDI)}*

$1401=192(220):
>gi|702306388|ref|XP_010049923.1| {|0(685)|<Streptophyta(V)>}PREDICTED:
TMV resistance protein N-like [Eucalyptus grandis]
{()---------------------------------
MDDEEIpqGKEIRGEILQAIEDSKIYIPIFSTGYASSRWCLRELAHMVACAFPDSsggqtGHEILPIFYDVEP
SDLKLETKLYGDALRRHEEEQGRHVV-
NPWKEALRKVAQI(KGRKVKGQRHIVVIGDIKQEISRGMATRERNVPDDLVGITDRVEYIKKLLDPACDDIRV
VVIHGMGGIGKTTLANLVYNE)}*

$1402=240(239):
>gi|702311173|ref|XP_010050874.1| {|0(685)|<Streptophyta(V)>}PREDICTED:
TMV resistance protein N [Eucalyptus grandis]
{(MAKEGPYSGSSSASSASVTGNN)-FDVFLSFTG-
SDTRHGFTNVLYRSLVDHGVRTFLDEEELsdGEYIKKQLPESIKLSKIGIPILSEDYASSKPCLHELAQMVE-
WRKEGRMIIMPVYFLITAWDVemKDGRGPYADKITAHKEKgVDLATI-
QKWVKALEEVTSV(KGRQLTKDTRQGEFAIMMVSKVLKRLRKAKLNLSDKLVGIEDRLPELRRMLDTDSSDVL
MIVISGIPGIGKTTLAKS)}*

$1403=240(239):
>gi|702311185|ref|XP_010050876.1| {|0(99)|<Streptophyta(V)>}PREDICTED:
TMV resistance protein N-like [Eucalyptus grandis]

{(MAKEGPHGGSSSAPSASVTGNN)-FDVFLSFGG-
SDTRHGFTDVLYRSLVDHGVRTFLDEEELsdGEYIKKQLPESIKLSKIGIPILSEDYASSKPCLRELAQMVE-
WRKEGRMIIMPVYFLITAWDVemKDGKGPYADKIAAHQEKgVDPDTI-
QKWVDALKEVTSV(KGRQLTEYTRQGKFADMMVLKVLNKLRKAELNLSDKLVGIEDHLTELRRMLDTNSSDVF
LGNIGETTAEKDLVKLQN)}*

$1404=298(297):
>gi|702311204|ref|XP_010050880.1| {|98(299)|<Streptophyta(V)>}PREDICTED: putative disease resistance protein At4g11170 [Eucalyptus grandis]
{(IECERLEREVKMEKYLLASDKDKVRLIKKQCEEKSKSVADNERVITQLQAEIEARKQTVENAKSLRQFELS
SSASVTGNN)-FEVFLSFRG-
SDTRHGFTDVLYRSLVDHGVRTFLDEEELsdGEYIKKQLPESIKLSKIGIPILSEDYASSKPCRRELAQMVE-
WRKEGRMIIMPVYFLITAWDVemKDGRGPYADKIAAHQEKgVDPDTV-
QKWVDALKEVTSV(KGRQLTECTRQGEFAIMMVSKVLKRLRKAKLNLSDKLVGIEDRLPELRRMLDTDSSDVR
MIVISGIPGIGKTTLAKS)}*

$1405=203(198):
>gi|702312184|ref|XP_010051102.1| {<Streptophyta(V)>}PREDICTED: toll/interleukin-1 receptor-like protein [Eucalyptus grandis]
{(MANSDAGTSSGNAGGS)KYQVFLNFCG-
GDTRVGFTSFLYEGLRKNGIHAFMDDEEIpvGKKIGDEILQAIDDSQIYVPIFSEHYASRKWCLLELIRMVDN
ISKSKEKQIRPIFFYVEPNH--LKTESYKKAIgdlkEEHEKKQREKspyEL-
ESWEEAPTKVVES(KGSKLQKGESQAKFVDKIVEELLQELKIKYKSVTKDLVGLDH)}*

$1406=224(247):
>gi|1091468369|ref|XP_018728213.1| {|0(616)|<Streptophyta(V)>}PREDICTED: TMV resistance protein N-like [Eucalyptus grandis]
{(MKRKREGEGAALTASASSSCDDGSRKV)-YEVFLNFRG-
PDTRLTIVDCLYNDLICAGIRAFKDDEELrfGKKIEGGLLQAINDSRIYIPIFSKDYASSKWCLRELALIVEL
SRDNDE----------------------KDASQKHKNDSGEDLT-
KQWEEALRDVTRI(SGKKLKDYGLSELTKLMVQEVSNLLWTRRTDMSNHLVGIKDRMDHIMKLLDVGAFDVRF
IVIHGMGGIGKTTLAEAVFRQ)}*

$1407=242(235):
>gi|702415039|ref|XP_010066366.1| {|0(574)|<Streptophyta(V)>}PREDICTED: TMV resistance protein N-like [Eucalyptus grandis]
>gi|1091491209|ref|XP_018715289.1| PREDICTED: TMV resistance protein N-like [
{(MKNSVVGMSSGYEGGG)EYQVFLSFHH-
RDTHSGFTCILHRDLVNKGVRSFIDDEELrvGEEISGALLQAIDDSQIYIPIFSKNYTSSKWCLRELKRMVDN
KSNSAEKKILPIFYDVKPDDLVLENSLCTMAMEKHKKERSEEqkekflhEI-
ESWEKALEKVDKT(KGWELSKFETLAEFLTTVVGVVLHEWQKSVTKDLVGLDDRVAEVLEKLDVSCSNEVRLL
GIYGMGGSGKTTLARVVYNE)}*

$1408=220(223):
>gi|702427487|ref|XP_010068054.1| {<Streptophyta(V)>}PREDICTED: TMV resistance protein N-like [Eucalyptus grandis]
{(MIESEAEST)-YDVFLSFNS-GDSSFGFANNLYDRLKGDGIRVFKDEKVLR--
TSKELPLSIYSSKIYIPIFSVGYARSGQCLNALALMVEhTSRSGGKKEILPVFYQVQRSDVQLeKNSLYRDAL
NMHKQISGDEEV-
KQWKKALVEAGKV(KGWELSSYKSEKELIISIVGEISLKLMIKHNPVPGDHFSATWRVIHWLSNRTSVYFFPP
ILLSSREFSSKDSAG)}*

$1409=161(171):

>gi|702508704|ref|XP_010040268.1| {|0(217)|<Streptophyta(V)>}PREDICTED: TMV resistance protein N-like [Eucalyptus grandis]
{(M)------------
DTRKGFTDHLYNGLVDAGIHTFRDNNEIheGKKIGPNLLTAIKNSKILIPNLSVNYGSSNWCLNELVQIMECK
KSDREKWKQALVEVSSLKGWDANDGYEGELVKSVVRKVLRELKKKFELDIPKNLVRI(DSNVEKIMEFVDKSP
CDTLFIGIHGIGGIG)}*

$1410=281(281):
>gi|1091521741|ref|XP_018722327.1| {|0(200)|<Streptophyta(V)>}PREDICTED: TMV resistance protein N-like isoform X1 [Eucalyptus grandis]
{(MAFGAKAWTATILALVILSLTLAPSWSMAFLILFGMIGLAVEAAKERRNPESSSTSSSSSRF)KYEVFLSS
RG-
PNIRNRFADYLYVRLVDAGITVFGDHEDLhaEEEIKPELKEAIKQSRILIAILSKDYASSKSCLYKMVQMWEC
RKSNGQ-TIIPIFYDIIPSDVKGQAGDFERYFNQHEiDGVDSNTI-
ETWKEVLRQVGES(KGFHLKSINERHEHLLIHEVAARVLQELKKDDQIVTEALVGIDHHVQKVMRKLGVVYSD
GQAVKVPDKDVRVVGICGML)}*

$1411=222(222):
>gi|702388613|ref|XP_010065285.1| {|0(550)|<Streptophyta(V)>}PREDICTED: jacalin-related lectin 34-like isoform X2 [Eucalyptus grandis]
{(MSHPPW)KQDVFVSFVE-DDPGGTFISNLFKRLHQVGIGYFGDDKRESARE-
EnkAkLLRAICHPTIAfaLVVFSKKYVYSSPCLNELVEILYRPED-SKLVVIPIFYK-
DRAGVSNlvSNDKFKEAL--RSKNKTETDA-
QEKCSALEKGYVL(KAAKGDESKLIDVIVERLVNEIHSRYSGKDFTFGPFGGEGQNNWETVDIERAYRKGIHV
EYGNFIESVTFEVHEEI)}*

$1412=236(238):
>gi|702438006|ref|XP_010070416.1| {|0(121)|<Streptophyta(V)>}PREDICTED: TMV resistance protein N [Eucalyptus grandis]
{(MFLILMASSSSNSRASFK)---VFVSFKE-ENVGNNFVTHLYAALVHAGIYYF---DVQRREkdegN-
LmiTVPKMIEESDLAIIIFSEDNAYSRLCLEEVVKIMECKKQ-
RDLKVFLVFYRVELGRVSGLKGSYrsGIEYFQKKLDKDVRKL-
MRWKDALQEAGSL(SGWYLDDEMSEAKIIEDIVKKISELKMYDVFLSFRGKDVREYFVKGLNEALVQKGIKTF
MDSENLLMGQDFPSKLKEAIE)}*

$1413=150(169):
>gi|702454548|ref|XP_010026505.1| {<Streptophyta(V)>}PREDICTED: toll/interleukin-1 receptor-like protein [Eucalyptus grandis]
{(MSSSSSDAKSP)-FDVFLSFRG-EDIRRGSLDDLHRALLHSGINAYIDsENLRPGRE-
IplALTKAIEESRIVVLVFSEDYASSWWCLDELVKEVMEWKR-----------------LKGTPG-
VARVLQSGAEGDSRRER-ERVVARHWPGTWK(NWERRILRRGEEREESPD)}*

$1414=173(203):
>gi|702385232|ref|XP_010064473.1| {|0(28)|<Streptophyta(V)>}PREDICTED: disease resistance protein RLM3-like [Eucalyptus grandis]
{(M)--------------------------------
AFRDDhhPDLQRGKCIkpEILRAIQQSRLVLVIYYKNYASSTWCLEEVAKVVECKDI-
NEGSMIPIFYEVDPSDIRKLRANFGIGFAKI-
EEAYLCYKRDIKKYETEFIQQI(IGEVENKLGPRLFYVVGGLVGMHSHVANVVECLCLDESDVRSIGMWDIGI
KVLVDKFLIQIV)}*

$1415=206(220):
>gi|701780018|gb|KGQ13369.1| {|485(0)|<Ascomycota(F)>}hypothetical protein BBAD15_g905 [Beauveria bassiana D1-5]

{(QTASTLLHLAEAKGLPIIPFAFLGGVARRSYERRDWSRLNPGFDPSVLCDVDGIEHTIEIANRLILDRMQP
SSCPERPKV)---VFISFAH-QDS--VIANELRAAL-
VKeGIEVLVGDDEIRSDQMItaTIEQAVLRSDVCAVLWSRQYAQSPWCYDEMSL----------
AINQQEYGKIKVWLFNLDDSPIVPSQARKLPAISVRSGNAMHQCVHELLGKS()}*

$1416=146(158):
>gi|702312865|ref|XP_010051256.1| {|0(703)|<Streptophyta(V)>}PREDICTED:
TMV resistance protein N-like [Eucalyptus grandis]
{(MANLEAGLSSDAARTLGG)EYQVFLNFRG-PDTRYGFTDFLYHDLVDAGVRVFRDEDELRVGEVIepE---
----
DVKLKTPQYNDVLLEHENKFPDEVKAWRKALADVDEIKGWNVRKDQSQATIIKLVVKKVLEKLELKQKSVTEH
LVGLDDR()}*

$1417=241(244):
>gi|702315193|ref|XP_010051724.1| {|0(897)|<Streptophyta(V)>}PREDICTED:
TMV resistance protein N [Eucalyptus grandis]
{(MKKGKESADAAAMGGDVAWGSSGS)-YDVFLSW-G-
PDPYCfEFVDGVYRGLVEAGIRTFLEDKSksVQSGDSIseECLRMVDETKIYIIFLNAYYTS--
WCLPELVAIVESkWKSGGGKTILPVFFQIEPHDFELESSLYSVRLL----
ERIGPEKLKLWADALKEVVSL(HGWKVGSYSFDQVTGEIVEKVILELKEKGIDKQVEDMMDLLDLEVSDVRIV
GIWGKDGVGKTTLANVIYEKISSQFDSCS)}*

$1418=181(181):
>gi|702506872|ref|XP_010039851.1| {|0(270)|<Streptophyta(V)>}PREDICTED:
TMV resistance protein N-like [Eucalyptus grandis]
{(MASSNAGTSFGR)KYQVFLSFRG-
PDTRIGFTNVLYHCLTNAGIYVFQDDEELRVGEEIagSLAEAINDSIIYIPIFSQTYASSR-
CQGELIDLVVEEVMKKLKKKHRSVEGQVVGIDHQVEAVSKLLDINSGGVRFIKIYGMSGIGKSTLAKVV(FNR
VHTHFGKHCCFLENVREKSLRSDGLV)}*

$1419=190(191):
>gi|703217454|ref|WP_033383228.1| {<Actinobacteria(B)>}hypothetical
protein [Kibdelosporangium aridum]
{(MY)EYDVFISYHR-EGTmvpawvRNHFyprLSALLNEQLDYEAKVFFDGN-
VQTGGKWpvMLRDALRRARVLVPVCSPKYFMNEWCLAEwhsMARREEVARVGPQGLIYPVIFN----------
--DSKSFPQF-
AHDRRMHSLKEWNSPYPQFQDS(LQYLDFHREVERIAEELVDLVGQAPQWRPDWPLDMPEPGPPGPSHLPRL)
}*

$1420=217(219):
>gi|702427350|ref|XP_010068024.1| {|0(559)|<Streptophyta(V)>}PREDICTED:
jacalin-related lectin 11 [Eucalyptus grandis]
{(MSHPPWKQD)---VFVSFVK-DEPGKTFISNLFERLHQVGIGHFVDDKRE-
GATKNNEAERLHAIRrltfaFALVVFSKKYVDSSPCLNELVEILYCQKD-PKLVVIPIFY-
IDRAGVSNLasNNKFKEALRSR-RKTETDAQ-
KRWSALAKAGYEL(KAAKGDESKLIDIIVERLVNKIHSQYSGNDFTFGPFGGEGQNNWKTVDIEKIDEKGIYR
KGIHVEYGDFI)}*

$1421=191(225):
>gi|703478850|ref|WP_033431648.1|
{|75(0)|<Actinobacteria(B)>}hypothetical protein [Saccharothrix syringae]
{(HLPFTPGAKRALAAAVREAFALGHRRVDTEHLLLGLLAEDDGVAPDVLARSGVPTADVRDRAFAVLAGIEL
PPAERPARL)----FLSHRPQD-LHLAGRIADRLDDIGR--SVVGDV---------------

AQCDVLLAVIGPGWA------PTEAVLGHVEAARtRSVPVIPVLVDGAEPPADPLPGT-------
GCVTVRHETFREDVARLADAVRCA(RPGLG)}*

$1422=212(220):
>gi|703481775|ref|WP_033434547.1|
{|449(0)|<Actinobacteria(B)>}hypothetical protein [Saccharothrix syringae]
{(AGTLRTETNHELDGREFPIIWIDLHDLQVPPRTYTNLASPLASPKILRIDPKDVDELTGRQKDALFKVLSRVGRRSEDDY)RYDVFLCHSSEdKPVVLEVLKRLEEAR--
LECWYDDKDMWPGDTTLSTIEYGVKHSRRILIFASRSYSKSAWTALETAIFTHLQVKNPVDRAIQILVIDQ-
DfDVDDLPGSIR-------IKQRVEYLGPASIDRLIEVLRS()}*

$1423=262(261):
>gi|704236573|ref|XP_010159112.1| {|339(86)|<Chordata(M)>}PREDICTED: LOW QUALITY PROTEIN: TIR domain-containing adapter molecule 1 [Eurypyga helias]
{(TADTGPAHVSMEDSCARTSNSASASIPTCSLPPPTYSFSSTLPTQGPPHNLSYPAPLHSTPCPAWTPPLQAVPTSEPDGG)KFFTFVVlHASEdETVAHRVKDRLENMgvPNGATLC--
EDFFIAGRSHLTCFQDAMDNSAFIILLLTKNF-PCDLCMFQTNTALMESILKPSKRDSVIPFVPK-
ENPLERSQIPSTLGGLMpLDENSPGFSRTVQNTFTTSRINE(RKAMWDLTQRRKLQLHQERYQTLQSLASLNLAQASPSATRS)}*

$1424=214(239):
>gi|702777607|ref|WP_033261888.1|
{|0(137)|<Actinobacteria(B)>}hypothetical protein [Amycolatopsis vancoresmycina]
{(MTDFMSDQPAVQCFLSFTH)---EDDSFL---NFVTPLKNSLEKFCASAhgrKVEVFVDY-----------
----
FYESGACREELTAFYAKANELgLTQLILPLIVLGHElitgQSDDANIRIIEKLQYVDiKEAVLAGAGTAEWRRTIDVVADK(LIRAIRSAEAALEKLKKCDRLLLAGVAPERNGPGLQEYKENLTKIGELMERQFDAISHSLDELAEEMRQLTGDVAGLRPE)}*

$1425=265(284):
>gi|703141609|ref|WP_033357418.1|
{|0(140)|<Actinobacteria(B)>}hypothetical protein [Dactylosporangium aurantiacum]
{(MHPAYQAGATGQPTGQTYFFLSYAHLSPLQGLAPDPDLEVQQFFGDLSAQLRPLVGAAPEHRVG)------
----------------------------FLDTE-
LPPGTDWneARTLALSAAHVFVPLYSATYFKNAWTQSELESFRARLKATpvagSHRHIQPVLWDPLPpweqrpevAAALAVAPEIEAYQKDGlRALSRFSMYRDEYRNVLARVAAE(IADVATHHPLPPSPAPPPTPEPIPRDQAQFVVVVAAPVRAEVSSGADPEDYTDDATQWRPFHDADALPAAEYVASSAERL)}*

$1426=207(215):
>gi|703477225|ref|WP_033430033.1|
{|0(91)|<Actinobacteria(B)>}hypothetical protein [Saccharothrix syringae]
{(MS)EYDVAVSFSE---EQRSAVGKVVEAFEQRGLTVLHGPELTHEWWshkeGGdlpD-------
ARVRffVPFVSA--------VDDFTTAALRAVKAGDEHVLPVLLGDVSvppdL-LHPHVAYVRGTADRA-
DRLTEALA-
ARVEAAEAVGQAF(TPVTEVVGGVKGLASAEEAEPVVPATFSRYAEQDATLRYLGEQFAAALPRLERRGFVGTAHVGETRIAVRVER)}*

$1427=195(199):

>gi|703488014|ref|WP_033440624.1|
{|0(416)|<Actinobacteria(B)>}hypothetical protein [Saccharothrix sp. NRRL B-16314]
{(MA)--RVFLSYATEDGDTASKLAGLLRERDQD-
VFLFQNPATRGDRFVPLIAERIRDAGVVIVLMSPHYLA-SAWCRrESELAiqrQAAD-----
PGLVirVLQVAPTRheD-SGFLGAYDWIEFHP--
PYCHELPVPVLTALGSAEEQAP(GLRPVGFRNRDEEVAKVRHALELTTGQDFWVVVAPPRLGKTWFLRHLHDH
LAAASDG)}*

$1428=213(213):
>gi|703141613|ref|WP_033357419.1| {|0(5)|<Actinobacteria(B)>}TIR domain-containing protein [Dactylosporangium aurantiacum]
{(MPEVEPDLDATAPLFFLSYARMTRLRRGPIAQPDPNLRV)-----------
KQLFTDLSSHLNNLvyRETGADPGFMDLAMTGGEEWRpELLHAVGTCQVFVALLSPAYMKSEYCALEWNAFTE
RpiHRR-PGApghhetSVIPVVWLPS-
QSGTVPAAINKIQRFTPqrLPDENLAQQYADEGLYGLLTLGA(EGTDGIILRLAQQIRDLCWSHQVERRVLPD
TKNI)}*

$1429=172(179):
>gi|702925888|ref|WP_033313404.1|
{|0(120)|<Actinobacteria(B)>}hypothetical protein [Streptomyces iakyrus]
{(MAAADYT)-FDVFIA-GEPadsgteRAVRRRLHELGVRTEVA-------DRE-----TSLafHIEQ-
MDRCRNFLLLATPDSARSHPVREQVDYWRDHRGMRksfHILDVSGNTLR---
QETPGHPGGWHHLLDFPPFGPGDKKPAGIWLPAALDDPQH(AKKLAATLTAERAPVAPTDSPATATMRARSLL
)}*

$1430=184(180):
>gi|703477314|ref|WP_033430122.1|
{|0(12)|<Actinobacteria(B)>}hypothetical protein [Saccharothrix syringae]
{(MSGYQ)-FDLFISYARRgsvqKWLlnhfhQKLLECLADQLAPTP--KVYVDRT-
MPRAVHWpsNLRDALRHSKIMIQLLTPHYFQSQWCRAE---WYSMLERErmlGLASSGSSQGL---
VYPILYSDSDNFPPEGKLRSWLDFKEFAHPDPPYQQTSHF(VLFHREVNKVAADLVQLIQQAPPWQPDWPEVD
PPD)}*

$1431=257(261):
>gi|703556882|gb|AIW39919.1| {|172(0)|<Chordata(M)>}TIRD [Branchiostoma belcheri tsingtauense]
{(SSPLIAPDVSSRVKQEEIVSEDACSNRVGHSCVVNIISQVDSSFDAGPEFIGTVDGHVKELEEKIKASSYS
SEAPLKPRT)-YDCFIIYSREdeDIVYNKLIPFF--
ISNNIKYCEHQEHFELGNDIFDNANTCIAQSRKVVAVLSKSFFASGYCMMDLDAARGYAASNGysvQDFLISI
---KVTdFVFPTKYSD----ISNStYADLSEDVTDkrK-WLQIRKAL--
T(EFRLASVTMRIEMGAKYSAPKSRLTVQDLSVLNFCTTDSVV)}*

$1432=210(214):
>gi|703556878|gb|AIW39917.1| {|0(932)|<Chordata(M)>}TIRA [Branchiostoma belcheri tsingtauense]
{(MAEGRGSRNRSSSRLSILTGSSLSLNVPFTR)--DVFVACADKdqEVVKSRVIQPLESREI--
TCCYAQRDFIPGHSESQSAVKFMKTSRKTIVVLSKNFKGYRLSHTILGLTISNQLPQGS--VIPVKIDDD-
TDMDGRFRDVVTLDMTAvDETTFITKLEKAVVFEERSSGSF(VDVAAPPELYTPESTPSSEISTPSSETSSGS
STGNTDLHQELD)}*

$1433=220(226):
>gi|703556880|gb|AIW39918.1| {|81(0)|<Chordata(M)>}TIRC [Branchiostoma belcheri tsingtauense]

{(EPMAMEFHTTAFHTEVKDTGKEKTKKKEKDADYVVKEKKPGIKDKMKGFVSSSVSKLTSKPSPEEELTQQL
EKATVSDLG)PVEVFLSYQW-DHQKQVIQ--
LHDMLEEKGHSCWMDIKEMGGGDQLyaAIDKGMRQAKAVISCVTPKYTESANCRNEVALAHSLK-----
KPIVPVLLEKTAWPPEGPMATPFAQLLYIDFTKESKKNPWQGAHLDELMTSV(GQAMKQ)}*

$1434=278(278):
>gi|738120692|ref|WP_036078736.1| {|46(0)|<Firmicutes(B)>}signal transduction protein [Lysinibacillus boronitolerans]
>gi|704827321|gb|KGR83647.1| signal transduction protein [Lysinibacillus boronitol
{(IILGIKDDGMTLSIKKFAFKLNIGNILDLLDGAVKIKYNHFTFKGSNLFYISIDKSDELVKVNNIPYKINK
DGDLEEMAI)-KKVFISYAHKdSDLVNILEEEL---
KKHKNIKITRDINVTAYRDSLdEFMKTIKEHDFVISVVSSAYIKSLNCMYEVMHLMQDKDY-LEKLFF-
IIVNRY-DVEyyaeknryDGFEAKIYDVIDRLkyITHWRDKK-----
VELEQSINEA(GLSPELMVNLAVDMRKLNSVIPSMDDFIKLLSDKVGRSFKEMYEDEFKEIIDTINRSH)}*

$1435=276(276):
>gi|835189618|ref|WP_047386884.1| {|46(0)|<Firmicutes(B)>}signal transduction protein [Bacillus cereus] >gi|706245014|gb|KGT41976.1| signal transduction protein [Bacillus cereus] >gi|1003921063|gb|KXZ
{(IILGIKDDGITLSIKKFAFKLNIGDILDLLDGSVKIECSRFTFRGNNLFHISIDKSDELVKVNNIPYKINE
DGDVEEMTI)-KKVFISYTHKdSDLVDILEEELKQYEN---
IKITRDIKVTTYRDSLdEFMKTIREHDFVISVVSSAYIKSLNCMYEVMNLMKDKDY-QEKLFF-IIVSKD-
DVNyyneknryDGFEAKIYDVMDRLkyITHWRDKK-----
AELEQSINEA(ALSPDLMANLAVDMRKLNSVIPSMDDFIMLLSDKVGRSFKDMYEDDFKEIVDTIKG)}*

$1436=274(278):
>gi|704334326|ref|XP_010173154.1| {|362(80)|<Chordata(M)>}PREDICTED: TIR domain-containing adapter molecule 1 [Antrostomus carolinensis]
{(PAGISSNSAPASMSARSLPPPTYSFSSTLPPAPHKPPSNLSYPPPIHSSPSPAWPPPLQTVEPVPTSEPDG
EETKFFTFV)-----VLHaSEDEIVAHRVKDLLEKMGVPNGATLSEDFFIAGRSHMTCFQDAMENsA-
FIILLLTKNF-PCDLCMFQTNIALMESILKPSKRnsVIPFVPKENPLErsqIPSMLGGLMPLDENS----
PGFSRTVQNTFNSTRINER(KAMWDRMQKKKLQLYWDQYQTQQKLAALNLSSLPQVPPSTTRPQSLEQSPQQW
CPPAS)}*

$1437=234(239):
>gi|302840126|ref|XP_002951619.1| {|1276(209)|<Chlorophyta(V)>}TIR-containing protein [Volvox carteri f. nagariensis]
>gi|300263228|gb|EFJ47430.1| TIR-containing protein [Volvox carteri f. nagariensis
{(VQEVLKACLKREDLRVVDAARGTLLQLGELTDTAATRALKVSCADVAPPAAAGAAAGKGGVNGNDGAGSAG
GGGWTGPPL)-YDVFLSHKR-TDARD-FARALWNLLVSNGYTVFLDFEFKQE--
LGSLEEMVGHCTHFLFIMTDNVFKSEWCIKELVQQLAAAAAAAAAAGGGGAADPQQLQQQQSGGPHGHQHQHQ
QQQQGLTMPPSSGSMDAAQATAA(AAMLLPRPQLLFHQALSDP)}*

$1438=267(267):
>gi|302848474|ref|XP_002955769.1| {|130(0)|<Chlorophyta(V)>}hypothetical protein VOLCADRAFT_96659 [Volvox carteri f. nagariensis]
>gi|300258962|gb|EFJ43194.1| hypothetical protein VOLCADRAFT_96659 [Vo
{(QGAARANCDATSFGYSHHTHALQTAAMVEEEEDIPVEEQWSLIVWLKPGERGTTVDLRRFTAVVDKMANPA
FIKSADGSW)--DIFLSHFQGNAGNTVMSLKALLEsaSPGL--RCWLDKDEdaTEAG----
MRRGVAGSKYYLVFLTKGVFTR------
PFVQLEAREALaLGKPVILVHETDQRfgAAPgfndyvAEAPPDLKQIFSlANSIPWYREEEFRAGDTSEASAF
LS(TTSMARPFPASRVSMARHFPASRVSMARHFPASRVSMARHFPASPRY)}*

$1439=208(187):
>gi|302836445|ref|XP_002949783.1| {|0(271)|<Chlorophyta(V)>}hypothetical protein VOLCADRAFT_90161 [Volvox carteri f. nagariensis]
>gi|300265142|gb|EFJ49335.1| hypothetical protein VOLCADRAFT_90161 [Vo
{(MLKW)--DVMLSYRTADtgakefgGDGTVLQIKTYLEEHGY--
TVYLNENVLEGGHDWsKEIqQAVMNCMVFVMLCSQGQslrlampvhscleANAVLGRYgeNKWTFGEFQFAEs
ERKHIIPLWHSGTYPPPPLKVMLGNYTRVPKGGKPLVQCDFDTCMKQLESRLGQ(ALAKTAQEAAAARDATKP
STTPTSCIRKLIGHEDEVLVLCWSS)}*

$1440=209(214):
>gi|496367779|ref|WP_009076769.1| {<Actinobacteria(B)>}hypothetical protein [Streptomyces sp. AA4] >gi|302435302|gb|EFL07118.1| predicted protein [Streptomyces sp. AA4]
{(MLRGRIARTRKSTGSAAQVYHYDVFISYLRTADPIARWVRNH)-----------------
FHPRLREMLDGNldrEVRVFFDG-
SVRVGGKWpdELRAALQRTRILVPVCSPKYFYDEWCRAEWASMARREELAgGDRpatLIYPVIYCDSKnfppF
AHERRMQDLTRWNHPYEQFEVSTRYLGFHDEMNRIAAEI(EELLSAAPAWRPDWPVLTPLPETPPAASFPRL)
}*

$1441=222(220):
>gi|446288252|ref|WP_000366107.1|
{|72(0)|<Proteobacteria(B)>}hypothetical protein [Escherichia coli]
>gi|300454293|gb|EFK17786.1| hypothetical protein HMPREF9530_05663
[Escherichia coli MS 21-1] >gi|
{(YFQESISYIHNYEWKWKELDGKKIENTFYIYISNIEFPVHKIYYQRKEDNAEIEWAKLQAVFHQSYELQST
KMKENPEIY)-FDFFISHAS---EDKDkFVRPLVNELSRLGINVWFDEQtlEIGDSLRRnidTGL-----
KKASygIVVLSHAFFSKKWTQYELDSLINRAVNDENKVLLPIWhnIDATEvaE-YSHYLAdklALQTTIHSI-
DEIAKEL--SKIAYKRRSIATS()}*

$1442=212(209):
>gi|492842909|ref|WP_005996863.1| {|0(310)|<Proteobacteria(B)>}protein of unknown function DUF323 [Desulfovibrio fructosivorans]
>gi|302489350|gb|EFL49303.1| protein of unknown function DUF323 [Desulf
{(MADDKTTF)--DVFISFKSEDEKYAYKIFEFLQQHGFN-
PFFStiSVSFVGQSQYLKSIAKAISQCRHMVVVASKPEYIISGWVEyEWSIYIARRNDKKTeGNLLTLLTGDM
TvsDLPQELVPFQSIPLtrDG--
LEEMLQFLDTVPGPPTSVALRH(KGLSLAKPLAAMALVGLCVAAGFYAYEKTVTPRPTMVSHRQSQPSLPQTT
APAPSQKKTVP)}*

$1443=189(192):
>gi|494600207|ref|WP_007358460.1|
{|0(775)|<Cyanobacteria(B)>}MULTISPECIES: hypothetical protein
[Kamptonema] >gi|300332482|emb|CBN59289.1| conserved hypothetical protein
[[Oscillatoria] sp. PCC 6506]
{()MKEAFISYSRKdLEFVQKLFDSFCQEQR----
EVWFDQKnIPLTADWRkEMYIGIEESQNFVFIISPDSVTSKPCLEE----IEHAVL-HNKRLLPIMYRktPS-
STVHPAIRSLNFIPFPEdcDFDAHFKTLIAAIDTDLTHVKAH(TRLQLRAIEWDKKNRDVSFLLRGSDLREAE
SWIEESTNKEPKPTPLHTQYLI)}*

$1444=177(178):
>gi|493464689|ref|WP_006419879.1|
{|0(71)|<Proteobacteria(B)>}hypothetical protein [delta proteobacterium

NaphS2] >gi|300447912|gb|EFK11621.1| conserved hypothetical protein [delta proteobacterium Nap
{(MKY)--NAFISYSGVdVEIVENLRDHIN---
RYGVTAWVYSlDRTLAVDAWEEIQARIEESDLVIFVVSDNTPNAE-GQQkELKLALEKVEplA-
GTEKILPIVITGK-
NFSALPDELRYKNGLYFdaYKVKSVAWKIATRAFPSLLKAET(ENPWKFPIPGEWLEVSNLDEMIEQHFDIGDKLYFR)}*

$1445=214(219):
>gi|492836246|ref|WP_005990200.1| {|0(614)|<Proteobacteria(B)>}hypothetical protein [Desulfovibrio fructosivorans] >gi|302493262|gb|EFL53124.1| conserved hypothetical protein [Desulfovibrio fructosivo
{()MIKCFLSHSSSdK--IRYVKIVADNLGHDYCV--
YDEYTFEEGMKSLdEIISTLESSQIFVIFLSNSALNSNWV-KtELQLAKDNLSK-GTLRkIFPIIIdKDI-
DYTDARIPNWMRAEYNIkpILR---PK--
LAARRIEQKIREL(LWEDNPMQKDREQFFVGRNESIEEFEQRIYDYDRTKPVCIIASGWKEIGRSSFLKHCIRKLDLIKHSYLPPLIDMGELD)}*

$1446=177(177):
>gi|497762565|ref|WP_010076749.1| {|0(669)|<Firmicutes(B)>}molecular chaperone Tir [Clostridium cellulovorans] >gi|302578004|gb|ADL52016.1| TIR protein [Clostridium cellulovorans 743B]
{()MSKVFLSHSSCdKEFVRPIA------ELFGK-----
DRCIfdevtfEGGMKNIdEIFRNMDNTDIFVYFISDYSLNSDWV-KiELNNAQEKlysssHRL-KQ--
IFPIIIDPK-
IMYSDKRIADFIKAGFNgyNLRHINHYKLAYEKIISQMINLQ(MSIDSKFAEKKNLFYGRDAEIKKFKERYDDSSRNPMK)}*

$1447=259(257):
>gi|494600007|ref|WP_007358260.1| {|110(330)|<Cyanobacteria(B)>}MULTISPECIES: molecular chaperone Tir [Kamptonema] >gi|300332677|emb|CBN59089.1| TIR protein (modular protein) [[Oscillatoria] sp. PCC 6
{(IHLGDVIFSGNDVMGNGVNIAARLQTIAAPGGICLSQTVYDVVKNHISLSASYGGLRKLKGIDEPVSVYQITGFRNSLEN)RHKVFISYrAQ--
EPDKSLALEFYEALIKAGHAAFMAGESIRLGENWptRIEAELKQSDYLLLLLSEQSAI-
SEMVTeEVRRAKELRDSRpDrKPMILPIRINLP-
VTSPVNYNLYGYLDQIQQREWHSDNDTPNLIAEVLDLVKE(GRDLKEAPKNAPTILAAWENLDSPPLPVAGPELPEGQ)}*

$1448=266(264):
>gi|719999414|ref|XP_010255710.1| {|0(1297)|<Streptophyta(V)>}PREDICTED: disease resistance protein TAO1-like isoform X1 [Nelumbo nucifera]
{(MLSRIKNWVLVRSDSNVLESVGSSSRSGLPNELLLPEEIRGRVVL)-YDVFISHRG-SDGKI-
YARKIHESLKRLGVKSFLDTEGIlkGENLSAVIVEAINSCTVFLVILSKGFAESAWCLEELRLMLNKVKEEGNsAVLLPVFYGVEPDDLRDIRhGPFSKAFQMHKkgKEFSSELI-
RKWEEALKEVANV(LGPRLSDFEGKEKLLKSVVKDIIRKVKNVDLETPKFPVVVDERVHELDILVKNAKSLNATPSIVGIIGMTGIGKTTVAK)}*

$1449=214(220):
>gi|835234272|ref|WP_047410395.1| {|79(0)|<Proteobacteria(B)>}molecular chaperone Tir [Citrobacter freundii] >gi|721588335|gb|KGZ31609.1| molecular chaperone Tir [Citrobacter freundii]

{(KKIATKTGDLHRYQVQLLKEQDNEQKKRIAAQKKIEKEQLDHQKKITRELESQKKAISSSHNFNRQIINKP
DETEDIIES)-YDVFISHAT---EDKDsFVRPLAELLKAKGINVWYDEFSLG--WGKslrKTID--
YGlANSRfgVVVLSKSFIKKDWTEYELNGLTAREMSGEDQVILPIWheVSKSDilK-
FSPTLVdkmALNTSINTI-DEIAEQL----------ESLLK()}*

$1450=242(253):
>gi|723281691|gb|KHD03705.1| {|95(308)|<Proteobacteria(B)>}hypothetical
protein OT06_58835 [Candidatus Thiomargarita nelsonii]
>gi|1026317850|gb|OAD21044.1| Toll-Interleukin receptor domain protein [C
{(YVTQILLYQRYCLLDGLEKIPPTFNIFAAHWLRQFRDSRNVPQPPVEQPPVTTKSEDTEKKTKPSKGLRVV
LIYKRRAQP)----------DKQLLNLLEEHLSKAGYSVFI----
DRHLRIGDDWErQISAEIKQADAVVILLSSTSVYSEmLAYEvNVAYQTAQENT-GKPRLLPIRINFE-
EPLPQDLSNLLGNLQYAlwRSPDDDVPLVNELLEKLISPLPP(KKYELEMVGGAIPLDSKFYVKRQVDGDFQT
AIE)}*

$1451=213(220):
>gi|1180731377|ref|WP_084213759.1| {|289(0)|<unknown(X)>}hypothetical
protein [Pseudomonas sp. SHC52] >gi|727045993|emb|CDF94816.1| Retron-type
RNA-directed DNA polymerase [Pseudomonas sp. SHC52]
{(QDPKEAERKCFIDILKLNPDEVELTEDGRKLKHNIYGRLSFIAMVKGKDDPTYVKLILKMAEHDKTPPKFL
KSIKEEYKM)-YDVFLCHAS-ED-
KAAIVEPLYEALTKIGVHAFYDSKEIAWGDSLieTINKALIKSQYVIAVMTDNSVVKAWPQKEINAVLNGDIS
GGTKRLLPLIH------GNKEKILNNNFLMSDKLYVVWQDNPDEIAQKIKALLAS()}*

$1452=202(220):
>gi|703086932|ref|WP_033345740.1| {|779(0)|<unknown(X)>}hypothetical
protein [Catenuloplanes japonicus]
{(PRPECGYQLDEDLARRRLARGDAGMECPACSEAQISLRDRDRPVPAVAEEVSAMNASADLRRRRETATATV
RGKRAVGDF)--DVFLAYRTADRVTAERLRQQLLDEGLL-PWMHDEEPEADP-------
AELARIDVVVVVVGEN---GGPWDDeLQHAmindFITRR-----VRFVPVVLPVKEepELPGFLRDVPVVD-
FR--VTEPDPVR--RLIRRI--TRAH()}*

$1453=179(179):
>gi|726045554|gb|KHE92443.1| {|0(577)|<Planctomycetes(B)>}hypothetical
protein SCABRO_01795 [Candidatus Scalindua brodae]
{(MKNN)--SIFISHGMKyEGLVVTLRE---
AIENQGLNTRVVPESVAPGDELEaNIKEEIEQAGAFIAVIGPDTINSSQVVKEIKYALevnEKEAD-
DDYKVIPLLLAGV-
KPAEFNTHFANKSVETPikIGPGGINATTSQIVAVLNKSLSD(KRQSALFAGIENSLRRLSPGIREKIKPLGV
FQGGG)}*

$1454=205(207):
>gi|727485941|ref|XP_010418922.1| {|0(709)|<Streptophyta(V)>}PREDICTED:
probable disease resistance protein RPP1 [Camelina sativa]
{(MASSLSPSSGSPSVSRTLT)-HQVFLSFHG-ADVRNNLLTHILREFQRKGITPFIDNEIERGVS-
IgiELLRAIKHARITVVTFSRNYASSSWCLNELVEVMKYKDE-
LNQEVMIIFHQVKPADVRTSEANMVATIAIDVLRKLQLTQPRDFDDYIGLQVHV(TSIYSLLNLQTDEVRMIG
IWGPSGIGKSTISRVLLNELSPQFQLSARL)}*

$1455=214(223):
>gi|727416308|ref|XP_010514449.1| {|0(103)|<Streptophyta(V)>}PREDICTED:
uncharacterized protein LOC104790394 [Camelina sativa]
{(MVF)RDDVYI-FCR-DSSGTRFSSLLLAALLSRQIKVFED--ISNSPR-
QseHVDVqAIEESKVLVIVFSATLIKRRryWNLKKLETIIKGERT-AGQVIIPVFLDTEPCHVVA-----

DEVFARFKIGKEEAV---
RWVDAVTKIAAL(PGRMYARDESVFVGKIFLKVMKIVKPNKPIVKNRSRCSIAMTFLVIINLLVEIASVVADQ
CSSVGKPYFARLSLAMAIVS)}*

$1456=217(234):
>gi|727433827|ref|XP_010498131.1| {|0(201)|<Streptophyta(V)>}PREDICTED:
putative disease resistance protein At4g11170 [Camelina sativa]
{(MSSSYSFLLAGREL)--DVFLSFSG-KVALDLDLGY---DLSRNGIKSFKSESWKENtfKP-
IdqRTLEALTESKVAVVMTSDEEASSVGFLEELMVIVESHEK-RSLTVIPIFLTKHPLDVGQVFQLF------
------PDKA-
QIWKTAIAKLESI(AAQYSFSRNLPVIHGTHRIKQTADDIRLMFLSSASSDFKGLAGMDRHMKAFHALLALES
DKEVRTIGIWGGAGVGKTTLA)}*

$1457=204(213):
>gi|727488954|ref|XP_010420518.1| {|0(1421)|<Streptophyta(V)>}PREDICTED:
protein DA1-related 4 [Camelina sativa]
{(MEPPAARVATPMMADCSHIVNII)--------CE-
ETVLQSFVSHLSAALRREGISVFVDAcrlRESRSFS-VeqNQAALTDGARVFVVVISDE--------
RELndpkfSEVIKGWSI-
NSHVVVPVLYGLDPLIQVDGWANFWLEVENLTSHHSRISSNKILTDSELVEEIV(RDVYGKLYPTERVGIYKR
LLEIEELLYKQHSRDVRSIGIWGMPGIGKTTL)}*

$1458=276(300):
>gi|1109153897|ref|XP_019084085.1|
{|432(991)|<Streptophyta(V)>}PREDICTED: LOW QUALITY PROTEIN: probable
WRKY transcription factor 19 [Camelina sativa]
{(EGKHNHDLPAAKSRSHGSVIAENSANFVSFPPTSSKNLQDADLSVGFPRSQSLFVDEALTDTSDFFGPPST
SVTFSASKH)-YDVVIRYgRG-DISNDDFISHLRASLCRRGISVY--EELNEGD-------
AIPQCWVLIILLTSTYVPS-----NLLHILERQHT-DNLMIYPIFYRLSPNDFVCNSKNYDIFFFQ-------
DEP-
KRWQAALKEITQM(PGYTLTDKSESNLIDEIVRDTLKVLCYGDKVNMVGRDMQVEEILSLMCIESLDVRSIGI
WGAVGIGKTTIAEEIFRRISV)}*

$1459=220(222):
>gi|727492245|ref|XP_010422165.1| {|111(60)|<Streptophyta(V)>}PREDICTED:
probable WRKY transcription factor 19 isoform X4 [Camelina sativa]
>gi|727492247|ref|XP_010422166.1| PREDICTED: probable WRKY t
{(NDFYELEEVSFDGVTTLNSNELTFKQKELIDKLKLDAYENVTRFTEVDKQIFEHNPRDSVSESSDKSNVLP
PDTSRESEC)--DVFISYNE-KDVSSSFISHLHASLCQKEISVFdKFDVVPEYRV-
MieFIRSGDVSSKYSIFLNRKERGVSQMWYRLSQSSIGNKTN-
LERYSLQNKRMWSICDVLKEAPDYILTKESESELIGEIVRDALKELCSSDKDKM(IG)}*

$1460=195(200):
>gi|727504227|ref|XP_010428184.1| {|0(439)|<Streptophyta(V)>}PREDICTED:
putative disease resistance protein At4g11170 [Camelina sativa]
{(MASSLLSSSIQQ)KCDVFVSFKK-SDTGKCFVSHLYRSLDRKGITTFKEEL----KD-
QpfQLLQDIGYPKIALVVVSENYISSFWCMSQLTELLKFMKE-
GSLSVIPIFYEIDPSGLHSITGTTRLTWSRVLTLLASLPGEHTDNWDDDAGLID(KVTDDVCKKLTTSTTPSD
YSDLFGMDCHMKALYEMLDIGFHSQDVEKM)}*

$1461=177(184):
>gi|727510458|ref|XP_010431500.1| {|0(314)|<Streptophyta(V)>}PREDICTED:
disease resistance protein RML1A-like [Camelina sativa]

{(M)RYDVFLSFSG-EYIRKTFLSHLLRSLNRKGIRA------ATSAE-
SypMYNQAIEQCLVAISLISENYTSLDLWVDELAKIIKCAED-
RTLTAVPVFLQVHPSDVLRVVSLAEEFADRNGPRIIIILETVKHWLCDHLIRNSG(FDSRHWEDDSKLVDKITS
FVADTLMSSKISSPSPSLKWGTPKL)}*

$1462=214(232):
>gi|727519106|ref|XP_010435944.1| {|0(260)|<Streptophyta(V)>}PREDICTED: putative disease resistance protein At4g11170 [Camelina sativa]
{(MSSSSLLSGREV)--DVFLSFCC-ETSHEYFQEILF----QYGIKTFQSNRswETRYRP-
IdqQTLKALEESKVAVVMISETKPCFFGFLEELIVILEFHEK-GLLKVIPIFLNAHSFSA------------
EEICQHYPDKA-
PSWRTALAKFTNI(VAEYPFSQNLAGIGQSDWLKQIAHDIFLLLLGFTSNNLNDLVAMDRHMKVVCGLLASED
NKEVRTIGIWGSAGAGKTTLA)}*

$1463=219(237):
>gi|727519108|ref|XP_010435945.1| {|0(190)|<Streptophyta(V)>}PREDICTED: putative disease resistance protein At4g11170 isoform X2 [Camelina sativa]
{(MSSSSSSTSFLLLGREV)--DVFLSFCC-QTRHGYFQNILITY----GIKTFLSYRCLENRfgP-
IsqRTVKALEEARVAVVMTSGTKPCSIGFLEELIVILEFQEK-GSLMVIPIF----------VTDNYFNA--
EEICQQYPEKA-
PSWRTALTELTNI(AAEYQFSQNPAGMGLLDWFKKIAHDISLVSLYSTSNGLDGLVAMDRHMKVAYDLLDLEV
DKEVRTIGIWGSGGVGKTTLA)}*

$1464=202(237):
>gi|727522808|ref|XP_010437375.1| {|0(1353)|<Streptophyta(V)>}PREDICTED: disease resistance protein RRS1-like [Camelina sativa]
{(MMDDVNKATHVVYINCAYTL)--------------HYSFASHLSMEFHRKGINSFVD-------C-
NGVLDVIEGASSSVVVFSKNCLSSTSCLDKLVRVHQCWRK-NGQLVVPVFYDVSPSDVVVPP-----------
QHKSADRR-
MEWSSALRELREL(PGHQSREECTECELVEEIVKDVYEKLYPTESVGIDAKLLEIELLLRKQTWGIRSIGIWG
MPGIGKTTLAKALFDQIS)}*

$1465=214(232):
>gi|727533196|ref|XP_010441151.1| {|0(222)|<Streptophyta(V)>}PREDICTED: putative disease resistance protein At4g11170 isoform X2 [Camelina sativa]
{(MSSSSLLSGREV)--DVFLSFCC-ETSHEYFQEILF----QYGIKTFQSNRswETRYRP-
IdqRTLKALEESKVAVVMMSETKPCFVGFLEELIVILEFHEK-GLLKVIPIFLNAHSFSA------------
EEICQHYPDKA-
PSWRTALAKFTNI(VAEYPFSQNLAVVCGLLASEDDKEVRTIGIWGSAGAGKTTLARYIYAEISVNFQTHVFL
ENVENMKDKILKFEGEEYATV)}*

$1466=219(237):
>gi|727557118|ref|XP_010450687.1| {|0(218)|<Streptophyta(V)>}PREDICTED: putative disease resistance protein At4g11170 isoform X1 [Camelina sativa]
{(MSSSSSSTSFLLLGREV)--DVFLSFCC-QTRHGYFQNILITY----GIKTFLSYRCLETRfgP-
IsqRTVKALEEARVAVVMTSGTKPCTIGFLEELIVILEFQEK-GSLIVIPIFFTDNSCNA------------
EEICQQYPEKA-
PSWRRALTKLTNI(AAEYQFSQNPAGMGLLDWFKKIAHDISLVSLYSTSNGLDGLVAMDRHMKVVYDLLDLEV
DKVVRTIGIWGSGGVGKTTLA)}*

$1467=217(234):

>gi|727573537|ref|XP_010459355.1| {|0(200)|<Streptophyta(V)>}PREDICTED: putative disease resistance protein At4g11170 [Camelina sativa]
{(MSSSYSFLLAGREL)--DVFLSFSG-KVALDLDLGY---DLSRNGIKSFKSESWKENtfKP-IdqRTLEALTESKVAVVMTSDEEASSVGFLEELMVIVESHEK-RSLTVIPIFLTKHPLDVGQVFQSF------------PERA-QIWKTAIAKLDSI(AAQYSFSRNLPVIHGTHRIKQIADDIRLMFLSSASSDFKGLAGMDRHMKAFHDLLALESDKEVRTIGIWGGAGVGKTTLA)}*

$1468=216(231):
>gi|727580900|ref|XP_010463277.1| {|0(470)|<Streptophyta(V)>}PREDICTED: putative disease resistance protein At4g11170 [Camelina sativa]
{(MALSPTPSTGS)--DVFVSFRG-EEIRRVFVSHIYRLLEQKGIRTFKGETEQNLEP-SeIHLAAIRASKVAIVVVSENYVFSPSVLRELEEIL------NYLTISPVYYGVDDWDVNKRQA------EEVCTDQVElSAA-TAWRNALMELACI(PGEHSNNWRCLDREDDAELILKITNDIWDKLSASRFFGLVGMDLRMRRVYELLSLDAKYDEVREIGIWGRASLGKRTLAK)}*

$1469=207(216):
>gi|727587969|ref|XP_010466992.1| {<Streptophyta(V)>}PREDICTED: TMV resistance protein N-like [Camelina sativa]
{(MVF)RDDVYI-FCR-DSSGSRFSSLLLAALLSRQIKVFED--ISNSPR-QseHVDVlAIDESKVLVIVFSATLIQRRryWNLKKLETIIKGERT-AGQVIIPVFLDTEPCHVVA-----DEVFARFKIGKEEAV---RWVDAVTKIAAL(PGRMYARDESVFVEKIFLKVMKIVKPNKPIVKNRSRCSIAMTFLVIINLLVRLLQWLRTGVLSLASLTSQDSL)}*

$1470=216(231):
>gi|727596688|ref|XP_010471327.1| {|0(217)|<Streptophyta(V)>}PREDICTED: probable disease resistance protein RPP1 [Camelina sativa]
{(MSSSSSSSKTY)---VFFSFCS-EeDPSQTFVSDLGHLLEQKDITTNCNDDA----------FLAEESKLVVVVVSQGYPVSVLCLNQLEKILKPHNE-GRLSILPIFYGVDPSNVRKLTGELEEPFRKLGEEYPYDII-ETWRHFLIKLTTI(PALDSRYWRNEADMVELIADEIVSIVSDKKPVAANSVGLVGLDRQMKALDKLLDFKATEEVRLIGIWGPGGIGKTTLARY)}*

$1471=225(232):
>gi|727607617|ref|XP_010476917.1| {|0(204)|<Streptophyta(V)>}PREDICTED: putative disease resistance protein At4g11170 [Camelina sativa]
{(MSSSSTSLLVER)KVDVYLSFSG-EiSLAIDFNYHL----SQHGIKTFISDSWKekRCRV-LdqRILEALAESKVAVVMMSEKQASSVGFLEELVAILQVQEE-SSLIVIPIFITNNHLDVEEISQHY----LGPHEEMYPWKA-PLWRKALTKLKNI(AAQCSLSTDHSGVGGLNRIKNIADDIWLMFLSSSLSNFKGLVGIDRQMKEFHELLALESIKDVRIVGIWGKAGVGKTTLA)}*

$1472=243(261):
>gi|727621563|ref|XP_010481629.1| {|0(542)|<Streptophyta(V)>}PREDICTED: probable WRKY transcription factor 16 [Camelina sativa]
{(MLNSMLKSVSFPPSFVPTNKALISLKLPNLRFFQVMEARNM)---VKVNFdsSD-ETLRCSFVPHLSAAFGRKGLSVLTDNQDQ-----------SYMSIASVLIFSENYVSSKESLDEFVKTLQRRRE-EGHIVTAIFYGVSRSNVQERVGNVSKAFLEH---GTSDQV-SQWRNALAEITSL(PGHKTSHRQSDYESIENVAKDLYEKIFPNERIGIYSRMLPDVENLIYKQQSGVRSIGIWGMPGIGKTKLAKAVFDQMSGD)}*

$1473=204(226):
>gi|727621565|ref|XP_010481630.1| {|0(514)|<Streptophyta(V)>}PREDICTED: probable WRKY transcription factor 16 [Camelina sativa]
{(MAGQTM)---VRISFdrSE-ETLRWSFVPHLSAALGRKGIFVSTNMHD--------------ESVASVLVFSKNYLSSKELLDEVFKTNQRRHD-KGHVVTTVFYGVSRSNVQELKG-FGKTLFEH---GASDQV-TQWRNALTEISNL(LGYEASNCQSEYEVVEKIARDVYEKIFPKQRIGIYSRMLAEVENFLCKQPWGVKRIGICGMPGVGKTVLAEAIFDQMSGD)}*

$1474=231(227):
>gi|727648139|ref|XP_010494626.1| {|0(482)|<Streptophyta(V)>}PREDICTED: disease resistance protein RRS1-like [Camelina sativa]
{(MATSSAA)RCDVFIDFST-KDTRNSFVSHLSAAFRRRKISVFIGKDCTRTLE-PnfRLpeviQSAIDGSRVSVVVLSENYAFSPLCLETLVKFLERqRRK-DDLVVIPVLYgQVTRMMVEQQTEKFGEAISDHGKLYSHDRV-AKWRDSLIETASL(PGHKSNDQLNDSELVERIVADVREKLDPAGTVGIYSRLLDIENLLCKQSHAIHRLGIWGMPGIGKTAIALAAYHQMSHEF)}*

$1475=260(261):
>gi|727553214|ref|XP_010448962.1| {|0(1268)|<Streptophyta(V)>}PREDICTED: disease resistance protein TAO1-like isoform X1 [Camelina sativa]
{(MDSSPFIIGAAAAKDKEIDSSSSSSLSPSSVPPSSSSQKWI)-HDVFLSFCG-EDVRREFVSHILKEFQRQEITPpFIGSESKGGVF-NghELIiRAIRGSKIMIIVVSKDYASSKWHLDEMVEIMKCREE-SDQTVMVIFYKVDPSDLNKLTGDFGEVFLKTCVGESKEDT-EKWRKALAKVAKI(AGYCSRNCDNEAAMIEKFATNVKKELINYAPSSDFDVLETTKPFLDLESDDVNMIGIWGPSGIGKITKPCNDITEQQLHK)}*

$1476=278(300):
>gi|1109148028|ref|XP_019083130.1| {|73(6)|<Streptophyta(V)>}PREDICTED: protein SUPPRESSOR OF npr1-1, CONSTITUTIVE 1-like [Camelina sativa]
{(KGGEDYYKFQHIPFVKFSGVCTMETLSSSLGDHMASTSEVLPIRSHDVDESDSTKEMMTQESECILVFSCNDNHDVEESH)-----------------YMEAILKELQERGVTPLTCNLSGRESL---NTNMLERCSVGIMVLSTSYVCSTQSMDHLVAIMEHWKE-KNLVIIPIYFKVTRQDIFGLKGKSEAAFLHLQSSVQEDRV-QKWKMALAEIASI(DGHEWTKGTQVVLAEEVVRNACLRLYLKNSKNLVRILALLNQSQPSDADIVGIWGMAGIGKTSIAREIFGTLAPKYDLCY)}*

$1477=284(299):
>gi|727516074|ref|XP_010434401.1|
{|246(536)|<Streptophyta(V)>}PREDICTED: probable WRKY transcription factor 19 isoform X1 [Camelina sativa]
{(LSNRLPPLAISGCSAKDVIDFLRMRQTSGVEAAAGDLVGYLSATWETHGIRSIDNPFRSLDVTTYLRSASEMTGEKECVL)---VFSCNDN-NDIdEASFIEAISKELQKREVTPLMYNLLGKEKP---DEKMLYRSSVGIMILSNS---CRQSLDHLVEIMEHGKA-ANFFIIPIYFKATLSDICG----FEQSYLQYLDFAQLGSV-QKWKAAMAETASN(GHEWKKKKQVLLTEEVVRDVCLRLFLKNSKNLSRILQLLNHSQHSEADIVGLWGMPGIGKTSIAREIFGILAPQYDMCY)}*

$1478=281(300):
>gi|727516088|ref|XP_010434408.1| {|61(738)|<Streptophyta(V)>}PREDICTED: probable WRKY transcription factor 19 [Camelina sativa]
{(QNPFGSPVVSNFLKEHDEYGGKKKKKKKKKSVEADLGNSVTPSEEVETPLRLKLPGHSITGFESVLLVTCGGNDQESEE)----------------RYFISYIFNEFCLRGFAPLRYD--ITRSI---

VNgcpEMLYRSRVAVIIFSMNYARSTESLDGFVATMDYLKA-
NNLLLIPVFFKVSLEDVSGQSCYFEKAFSRLENSVQASQV-
LKWRAAMIKLTSI(NRYYYMKGHQVIMLAKDIFKDVCFLLSSETNGIKHPFEVILSLVNSSQSSSPHIVGLWG
MAGIGKTIISRDIFRTQAGKY)}*

$1479=276(295):
>gi|727529082|ref|XP_010439702.1| {|0(736)|<Streptophyta(V)>}PREDICTED:
probable WRKY transcription factor 19 [Camelina sativa]
{(MEEDWEAFSEFLSSDSGGKKKKKKKSVEASLGNSVTPSEEVETPLQLKFPGHSITGFEPVLLISCGGDNDQ
DSEE)---------------RYFISYIFNELCLRGFAPLRYD--ITRSI---
VNgcpEMLYRSRVAVIIFSMNYARSTECLDGFVATMDYLKA-
NNLLLIPVFFKVSLEDVSGQSCYFEKPFSRLENSVQASQV-
LKWRAAMIKLTSI(NRCHYMKRHQVIMLAKDIFKDVCFLLSSETNGIKHPFEVILSLVHCSQSLAPHIVGLWG
MAGIGKTIISRDIFRTQAGKY)}*

$1480=263(297):
>gi|727554196|ref|XP_010449344.1|
{|355(977)|<Streptophyta(V)>}PREDICTED: disease resistance protein RRS1-
like isoform X1 [Camelina sativa] >gi|727554199|ref|XP_010449345.1|
PREDICTED: disease resista
{(SSKFFIGCRRHNAGAAPASSSQSAALNPTSPATSKMKLELREKLKEVEIPFQQGLPEHSITGSDPILVISC
GKNQDSEET)----------------YFISCIYNELCRRGFTTCSYDLDrSTMTG---
DPDTLYRSRVCIMIITMNYARSRECLAKFTVIMGHLEA-INLALLPVFFNVIP-------GSFE-------
NSVHAFEV-
HKWRKAMNKLFVY(NGKQYIKGDECMLAKIIVRDVSLLIGFEAGRNLIGIKLLLGSILPLIHCHQPSAPQIVG
LWGMAGIGKTAITREIYR)}*

$1481=228(230):
>gi|727480411|ref|XP_010416054.1| {|0(203)|<Streptophyta(V)>}PREDICTED:
disease resistance protein RPP4-like isoform X1 [Camelina sativa]
{(MTSSSSSSTY)-FDIFLS-SA-
EDTGKGLASHLYRSLYLRGIPTYLrDAKLEERPSSSLVYDArkyIQGSKFAVVVVSQSYPTSVWCLEELQILL
NFHDQ-GRLYVLPIFYEVSFSDIRSQTGAVAEPFRKLGETYP---
DKvQAWRIALIKLTDI(PGLDLHHLSEVDVVLNRVLLYKSEAIYPRETKGVGLLGLDRHMKALYQLLDLKNST
EEVRLIGIWGPGGVGKTTLARFAY)}*

$1482=149(169):
>gi|727649338|ref|XP_010495260.1| {<Streptophyta(V)>}PREDICTED: protein
DA1-related 4-like [Camelina sativa]
{(MGDNETASSRSVNIICEETV)--------------RYSFVSHLSAALRREGISisVFADTDLDY-------
--RNQGATtLSVVVFSETNAFSLTWVDNFAKVLElRRNN-
NGHKVTPVFYGIDPSVIDQEWMLMLLETGYSAGNQSSDAQLVEDIVRDIYGKLC(PTXKSPPSL)}*

$1483=227(233):
>gi|727519104|ref|XP_010435942.1| {|0(340)|<Streptophyta(V)>}PREDICTED:
TMV resistance protein N-like [Camelina sativa]
{(MASQAASRVTNFM)-YDVFLSFRG-EDTRYSIVSHLYAALVSrG-
IITFKDDKRLEIGDHIsdELYKAIEGSDSDVVVLSGNYATSRWCLMELQLIMELQMK-
GRLGVFPVFNGVEPS--RHQLGSF--DLKRY-QRP--
EDADkvPKWREALKLIADL(SGVESGLCIDEATMVGKIVDDISRRKAMMQKIDFRNIVGVDTHLQDLKSLLDM
DSNNDEVRMIGIWGMGGIGKTAIAKCL)}*

$1484=270(299):

>gi|727534893|ref|XP_010441762.1| {|69(896)|<Streptophyta(V)>}PREDICTED: disease resistance-like protein CSA1 [Camelina sativa]
{(SVNKYEPPKIDYKEPDEKAYTYFLKSYMKNLEVERDGILTDNFQAEAKSRRDDDLPGEANPDATFIISLENKRAVEEKKL)------------DLSHGFVGHLVRALEKYGVNV----------------GGMEESRMALVIFSSKYAESRWCLSELLKIKALMEE-RKLLlVIPIFYKVEQSEVTQLKGDFGVRFWNS-WRRNRDLRIIEWKEALKSVASL(KGIYLNEEHCSESEFITLTVKEVLEKLILQKGEIPSLFLSTCEGTRPDLPTGRGEKHYLNKCQLFGMEQRMEQFERKLD)}*

$1485=156(161):
>gi|727621660|ref|XP_010481674.1| {<Streptophyta(V)>}PREDICTED: protein PHLOEM PROTEIN 2-LIKE A8-like isoform X2 [Camelina sativa]
{(MGAAFSQAGDRRP)--QVFLNYRK-EELLHTFVKRLLTELQRNNINYFIDDKVQKGKPITDLFEKIEESAIALAIFSKRYSESKWCLDELVRMKECVEA-GELVVIPIFVNVTPSEVKKFNGVFGVTFQRL-RNKYGKVQIQKWKEAVEYIAGI(SGMVWNDR)}*

$1486=236(237):
>gi|727528917|ref|XP_010439643.1| {|0(14)|<Streptophyta(V)>}PREDICTED: vesicle-associated protein 1-4 [Camelina sativa]
{(MERQAGPLTFLNQFRRKPL)-PLLFISFRG-EELRNSFVSHVVRALKEAGFNVFIDSHEVRGRDIRFLLRRIENSKVALVIFSERFAESEWCLREVTKIAEQVRA-GCLVAIPVFYRVSTNDVKMLQGKFGDCFVETLEKRVGVDHPlgPRWMNSVMFIAGM(IGFTSEVHSIDDDLVEEIVKAIKRQLPYQSPASKALEREFTRELLSALIVSCICYYLIPSLFTDLNFFASPKWFLLVL)}*

$1487=242(242):
>gi|727535035|ref|XP_010441808.1| {|0(97)|<Streptophyta(V)>}PREDICTED: uncharacterized protein LOC104724939 [Camelina sativa]
>gi|727535037|ref|XP_010441809.1| PREDICTED: uncharacterized protein LOC10
{(MADASPQGGSNPQPRPRCRLIP)RPQVFISFRG-EDVRNCFLRIVVMILGSRKINWFTDTRLPVGENLEELFTEIKKSKIAIVIFSKRYSDSKWCMEELSIIEELARD-GKLKVVPVNYKVSHSDVKHFQGEFGESFERLKDKYELKEPEniLKWKASVESIAKR(KGLLSYIRGKVPAPLHSQIVNAVIAYLNKMPGGKVKPLENKLTMIDVFILAFVAAFLCRYIISPLFFNNADFFNNKKWLF)}*

$1488=235(236):
>gi|727535051|ref|XP_010441814.1| {|0(7)|<Streptophyta(V)>}PREDICTED: protein PHLOEM PROTEIN 2-LIKE A8-like isoform X2 [Camelina sativa]
{(MAALTNRAQPNAQIRRG)-PQVFISFRG-DDVRNDLVKDLVNNLKSKRINVFIDTFLPGGRGLQELFSKIDKSKIAIVILSKKYPDSYWCMEELVKIDKRMKE-GKLIVIPVFYKVTTSDVKKFKGDFGKSFDKLKKCHAMGEPRkvKQWEASVKSIANI(KGLPGTVPPLVGQIVHAVEVCLNEMPAKDVKAVFTAIWLGVYLVVSFLSFLSYMIFNDLKPFKSGKWMLGFPTMAAILL)}*

$1489=214(226):
>gi|727558337|ref|XP_010451351.1| {|0(16)|<Streptophyta(V)>}PREDICTED: LOW QUALITY PROTEIN: protein PHLOEM PROTEIN 2-LIKE A5 [Camelina sativa]
{(MAMQARPL)-------------VSDGFIRNVARALRDEGFNVFIDSDERRGRGMEHIFRAIDNSKVALVIFSGRYAASELCLHEAVRIYDRRRE-GKLVLIPVFYRVSTDDVNLFNGRFGESFLETLKIQGIRDHPfaEHWMRNVNFICTD(TGFTSEDYSNDTSLVAEIVRTIKRRLQGKNISRRPQGEVLWSNFDMSXFMPKQPEAQEGLGKELLYVSIASLICYFMI)}*

$1490=298(299):

>gi|1109090373|ref|XP_019095941.1|
{|2729(33)|<Streptophyta(V)>}PREDICTED: uncharacterized protein LOC104760394 isoform X3 [Camelina sativa]
{(TEAAERSSDGGDKASSSEPPIAKSRFSWTKYRTVSNNSERERHIRALEVDAQLSEEPQHHIDDIIPMVASSSEAGIRQSH)-PQVFVSYHG-
AELHRTFVRHLVSTLTDARVSVFTDNDKGNGtKQLIQLYQRIEESKIALAIFSKRYVESNICLNELVMMDELAKE-
GKLLVIPIFYNVRSSDVRSLKGEFGQHFKEMRERYKDELEKvRKWEASVKSIAKT(IGIHSEVHGEDASLVEATVEAVQRALTKISEGSLKYLGSVNTPTDHVFALFVTFLSCLIMSPLLFIEAYSISSVMWLVN)}*

$1491=242(242):
>gi|727621651|ref|XP_010481670.1| {|0(106)|<Streptophyta(V)>}PREDICTED: uncharacterized protein LOC104760442 [Camelina sativa]
{(MADASPQGGSNPKPRPRRRLIP)RPQVFISFRG-
EDARNFFLRMLVMILGSRKINWFTDTRLPVGENLEELFTEIKKSKIAIVIFSKRYSDSKWCMEELSIIDELARD-
GKLKVVPVYYKVSHSDVKHFQGEFGKSFERLKDKYEINEPEniLKWKASVESIAKR(KGLLSYIGGKVPAPLHSQIVNAVIAYLNKMPGGKVKPLENKLTMTDVFILAFVVAFLCRYIISPLFFDNADFFNNKKWLF)}*

$1492=170(170):
>gi|727627988|ref|XP_010484830.1| {<Streptophyta(V)>}PREDICTED: protein PHLOEM PROTEIN 2-LIKE A8-like [Camelina sativa]
{(MAALTNRAQPNAQIRR)RPQVFISFRG-
DDVRRDLVKDLVNNLKFKRINAFIDTFLQGGRGLQELFSEIDKSKIAVVIFSKKYPDSYWCMEEVVNIDKRMKE-
GKLIVIPVFYKVTTSDVKNLKGDFGKSFHKLKKCHAMREPRkvKQWEASVKSIANI(KGLPQLTYIYILRF)}*

$1493=215(221):
>gi|727648022|ref|XP_010494567.1| {|0(1384)|<Streptophyta(V)>}PREDICTED: probable WRKY transcription factor 16 isoform X1 [Camelina sativa]
{(MADSEQL)---VYISC-A-EEIRYSFVSHLSEALRRKGIHdVFVDSyDDLLSDESQS---
KVERARVSVMVLPGDR--STACLDKLVKVLECERT-
NDQVVVPVLYGVRTLETDWHIALDSKGFSSIHQsRKGCSDSQlvKDIVRDVYEKLFY(TGRIGIYSKLLEIETMVCKQSLDIRRVGIWGMPGIGKTTLAKAVFDQMSSAFDASCFIEDYTKAIQEKGLYRLL)}*

$1494=200(212):
>gi|727504239|ref|XP_010428190.1| {|0(270)|<Streptophyta(V)>}PREDICTED: probable disease resistance protein RPP1 [Camelina sativa]
{(MT)-HDVFLNFRG-DEIRKTFVSHLVSSLKTKYISTFI--ASEPFEDLDH--
EAMEQSLVAIPVISKHYVISDFWMDDLRKLIECEKI-GTLTTIPIFFQVNPLDIlSHAVGKYADT------
QSETLEMVQKWLYAQVSRKPS(FNSNDWEDDSELVDNITSFASNILTSSTSSYHSTGLSPARSLKLESAFSPLNYRSPHKFKFRTWHCSLTL)}*

$1495=188(203):
>gi|727621545|ref|XP_010481619.1| {|0(1124)|<Streptophyta(V)>}PREDICTED: disease resistance protein RRS1-like [Camelina sativa]
{(MTNCENAEGFVCISCV)-----------DEVRLSFASHLSEALRRKGINnVVID--
VDSDDLCKESQEKVEKSRVSVMVLPGNCDYSEVWLDKFAKVLRKN---
KEQVVVPVLYGDNQLRGEWLSALDLRGVSPIHKSRKECSDSTLVEEIVRDVYEK(LFSMERIGIYPKLLEIEKMVNKQPFGIRCVGIWGMPGIGKTTLAKAV)}*

$1496=190(202):

>gi|727648108|ref|XP_010494612.1| {|0(1252)|<Streptophyta(V)>}PREDICTED: LOW QUALITY PROTEIN: disease resistance protein RRS1-like [Camelina sativa]
{(MTNCEKAEGFVCISCL)-----------DEVRLSFASHLSEALRRKGInNVVID--
VDSDDLCKESQEKVEKARVSVMVLSGNCDSFEVWLDKFAKVLEChqrN---
KDQVVVPVLYGDSQLRLKWLLALDLRGVSPIHKSRKECSDSTLVEEIVRDVYEK(LFSMERIGIYPKLLEIEK
MVNKQPFGIRCVGIWGMPGIGKTTLAKA)}*

$1497=148(157):
>gi|727421114|ref|XP_010435016.1| {<Streptophyta(V)>}PREDICTED: protein PHLOEM PROTEIN 2-LIKE A5-like [Camelina sativa]
{(MNQDKALAVKGTAPPQH)--QVFVSFRG-
SDVHHNFFSFLKDALIKNGINVVTDEDAPRGKPIDeNLLKLIRNSRIAVVIFSENYPEWTWCLDELVEIEKQM
DL-KMLDCCPIFFEVETSHVKPQAQRsiFDYNLLRLehkeRKKARQISKKA------------()}*

$1498=232(222):
>gi|727421236|ref|XP_010435629.1| {|0(198)|<Streptophyta(V)>}PREDICTED: uncharacterized protein LOC104719401 [Camelina sativa]
{(MAAE)KQVAFLSLYG-EDVPDSVPLYLKSGLERSGVSVLRRDNKV-
SEDLRTVLNKIRESSFVLVIFSVGFAKSRECLDELVLVKKRMDI-
GKLRTIPIYYKVEPEDVIRVQGLFGKNLLRCeaeeHKTAMRNNMEallrsgvrlINWREALKSVTTT(RDFMY
REEDDFIEKLTKQVNNAYAKDDPATMNQLSQITVKKRKEDLVSLMRSMGLGFEGNFSWETYPIDKLSKQCEEQ
)}*

$1499=302(300):
>gi|727515118|ref|XP_010433896.1| {|266(69)|<Streptophyta(V)>}PREDICTED: uncharacterized protein LOC104717956 [Camelina sativa]
{(VLLGSLEYYNDLIRIIALQGFTFDRLFLPKKPQVSYFDVVESNDNLRNPQVSYSDAALTCLSFLCNILKRS
EMKTDDHPS)-QRVFIGLGE-KHLGKFVVTSLKRELETNQILVYYLEDETRSR--------
IQESGVAVVFFSNKYPKSEKCLNELVEIKKLMDA-
GQIDAFPVFYGLKDEPVRKLKGCFHNRLLKIenevRNNIRTRDDKsvieteakiWGWREAISSIASR(PGLSY
ELSTDDVFVSDIVTKVKELFASREKQKTKAATTLVKSPDADLFYSKNSFLQALNRDITDFEGFTDIPNGLVLL
RL)}*

$1500=302(300):
>gi|727527636|ref|XP_010439164.1| {|257(69)|<Streptophyta(V)>}PREDICTED: uncharacterized protein LOC104722663 [Camelina sativa]
{(LVQARKILLVLLGSLEYYNNGFTFDRLFLPKKPQVSYSDVVESNDNLRNPQVSYSDAVLTCLSFLCNILKR
SEMKIEPPS)-PRVFIGLGE-KHLGKFLVTSLKRELETNQILVYYLEDETRSR--------
LQESGVAVVFFSNKYPKSEKCLDELVEIKKLMDA-
GKIDAFPVFYGLKDEPVRKLKGCFHNRLLKIenevRNNIKTRDDKsvieteakiWGWREAISSIASR(PGLSY
ELSTDDVFVSDIVTKVKELFASREKQKTKAATTLVKSPDADLFYSKNSFLQALNRDITDFEGFTDIPNGLVLL
RL)}*

$1501=302(300):
>gi|727552534|ref|XP_010448697.1| {|262(61)|<Streptophyta(V)>}PREDICTED: uncharacterized protein LOC104731123 isoform X1 [Camelina sativa]
{(ARKILLVLLGSLEYYNNGFTFDRLFLPKKPQVSYSDMVESNDNLRNNPQVSYSDAALTCCLSFLCNILKRT
EMKIEDPPS)-PRVFIGLGE-KHLGKFLVTSLKRELETNQIIVYYLEDETRSR--------
IQESGVAVVFFSNKYPKSEKCLDELVEIKKLMDA-
GQIDAFPVFYGLKHEPVRKLNGCFLNRLLKIenevRNNIKTRDDKsvietekkiWGWREAISSIASR(PGLSY
ELSTDDVFVSDIVTKVKELFASREKQKTKAATTLVKSPDADLFYSKNSFLQALNRDITDFEGFTDIPNDLVLL
RL)}*

$1502=232(222):
>gi|727566261|ref|XP_010455499.1| {|0(194)|<Streptophyta(V)>}PREDICTED: uncharacterized protein LOC104737097 [Camelina sativa]
{(MAAE)KQVVFLSLYG-EDVPDGVPSYLKSGLERSGVSVLRRDNKV-SEDLRTVLNKIRESSFVLVIFSVGFAKSRECLDELVLVKKRMDI-GKLRTIPIYYKLEPEDVIRVQGLFGKNLLRReeeeHKTAMRNNMEallrsevriKNWREALKSVTTT(RDFMYREEDDFIEKLTKQVNKAFTKDDPATMNQLSQITIKKRKEDLLSLMRSMGLGFEGNFSWETYPIDKLSKQCEEQ)}*

$1503=302(300):
>gi|729338907|ref|XP_010539360.1| {|98(579)|<Streptophyta(V)>}PREDICTED: LOW QUALITY PROTEIN: uncharacterized protein LOC104813467 [Tarenaya hassleriana]
{(PSPPVSYKSLEYEDIPSSSYTPPSDQYELSDDPVDRKLKFSACASDPAPPRISFSFPVPRVSFAKVSVSSPATNAKLRSC)--DVFIGFHG-QNpnlVR--FCKWLKSELELQGIACFVADRAKYSDT-QshEIADRVICSvTFGIVVVSCSSLLNYLSLEE-VRFFAQKKN-----LIPIFHGTRPEEimgllncnsIDKECKEAIDGLLKCHEFKLEANE-SNWRGCVAKTTAI(LQAKLGXKSVAEKQAVEGFDGLPFPRNRCFLGREKEIMEIEMAFFGNGEYLDSTTTSNKGETSGQSEGLEDEESDDVSTR)}*

$1504=302(300):
>gi|729356672|ref|XP_010545302.1| {|78(590)|<Streptophyta(V)>}PREDICTED: uncharacterized protein LOC104817726 isoform X1 [Tarenaya hassleriana] >gi|729356675|ref|XP_010545303.1| PREDICTED: uncharacter
{(TTTTVGYKGSECEDIPSSSYTPLSDQYEFSDDPVDRKIKFSACASDPVPPRISFSFPVPRVSFAKVSVSSPATNAKLRSC)--DVFIGFHG-QNpnlVR--FCKWVKSELELQGIACFVADRAKYSDT-QshEIADRVICSVTyGIVVVSCSSLLNYLSLEE-VRFFAQKKN-----LIPIFYGTGPAEImaLLNCNSIDKECKEAIDGlikchefKLEANE-SNWRSCVTKTTAI(LQAKLRRKSVADKEAVEGFEELPFPRNRSFLGREKEITEIEMAFFGNGEYLESTTPSIRGEASGQSEGFADEESDVVSIS)}*

$1505=302(300):
>gi|731433548|ref|XP_010644688.1|
{|106(588)|<Streptophyta(V)>}PREDICTED: uncharacterized protein LOC100246258 [Vitis vinifera]
{(SPPISYCGSQSDDIPSCSYTPPPERNDFSDDPTDPKLKFVTCVPVPDPAPPRISFSFPVPRISFAKGSVSSASNAKLRSC)--DVYIGFHG-QNpnlVR--ICKWLKSELELQGIACFIADRAKYSDN-QshEIADRVICSVTHgiVVVTSSTFLNH----HSLEEIRFFAQK-KNLI--PFFFGTDPAEimsllnhnsIDKECKEAIERLMKSHEFKLEASE-GNWRSCVSKAAGI(LRAKLGRRSVAEKEVEGFEELPFPRNRFFVGREKEMMEMETAFFESGDCLEQDGSVPIVKGGATGQCDGFADEESDAGTT)}*

$1506=202(206):
>gi|731438403|ref|XP_010646987.1| {|0(210)|<Streptophyta(V)>}PREDICTED: TMV resistance protein N-like [Vitis vinifera]
{(MDRRRASSTCTSTGSWD)-YEVFLSFKG-EDTRYNFTDHLYAALyQKG-IRTFRLDEI--RGEEVasALFKAIEKSRCILVVLSKYFAHSGWFLDELVKIMECRNQ-NGKVILPVFYHVDPFDVRKEEGWPEADYIED-ITRVILMRFSHKLLHVDKNLIA(MDYHLEEMEEIFPWMMDSISNDVRMVGIYGLGGIDSDTEEKKESLLTKR)}*

$1507=272(277):
>gi|731330452|ref|XP_010676140.1|
{|103(632)|<Streptophyta(V)>}PREDICTED: uncharacterized protein

LOC104892024 [Beta vulgaris subsp. vulgaris] >gi|870861377|gb|KMT12667.1| hypothetical protein BVRB_4g
{(QLPSPPVSYCGSLSEDIPSSSYTPAAAPDRSDFSDDENLTFVAENTSGAAPRISFSFPLPRLSFAKASISPPVLNSKLRS)-CDVFIGYHG-QNT--NLVRYckwLKSELELQGIACFL-ADRAKYADTQshEIaDRVICSVSFGIVVISSASLYNHFSTEEVRFFTQ-K-----KNLIPMFFDTEPTEIASLLNCTSDNKQCREAIEGLTRHHEFKLEAKDGDWRS(CISKAVGILCSKLGRQSTTAKDLEREAIELPFPRNKNFVGRLKEMMEMEVAFFGNGE)}*

$1508=206(219):
>gi|746238963|ref|WP_039287463.1| {|0(131)|<Proteobacteria(B)>}TIR domain-containing protein [Novosphingobium malaysiense]
>gi|733400608|gb|KHK89928.1| hypothetical protein LK12_18685 [Novosphingobium
{()MADIFISYSRSdRDRCLAIRKALEDLK----VSVWSDSGIGAGSSFDrEIEREIEASRALLVLWSGQSVDSDWVRNEARTGKER------SGLIAVQLEPC-Q-LPLEFRSVQAevLPEGAE---GTAN--STWLGILSRIGEL(VGRPGLADYARICSEGSLDDWKRWLAKHPEDPLAPDAIDGIAERAMPGMRQELASERTKRSALEAELAEHVEASKARST)}*

$1509=187(211):
>gi|734387500|gb|KHN25263.1| {<Streptophyta(V)>}TMV resistance protein N [Glycine soja]
{(MNGLAMFSFVFGVRIHRPVFQEIFII)------------------------LCEKGINAFMDDQGVrkEEEITPTLMKAIQESKIAIVIFSENYASSTFCLQELVKIMECFKHNGKL-VWPVFYKVDQVDVDHQKGSYVEALVKHETRISEKDKLKKMEVSFARSFKS(IWLAFQQRSFLVRVELEVVICFFELIEGFVYGFESIVECGCLAFR)}*

$1510=167(167):
>gi|734422387|gb|KHN41590.1| {|0(137)|<Streptophyta(V)>}TMV resistance protein N [Glycine soja]
{(MAVPSSSSSFT)-YDVFLSFRG-EDTRYGFTGNLYKVLHDSGIHTFIDDEELqkGDEITTALEKAIEESIIFIVVLSQNYASSSFCLNELTASSSAVWTRVKLLLDVGSRDGVHMVGIHGLGGIDYCQSLREIRGILPNIEHFSARNCKSLTSS(CRSSLLNQKLHEAGNT)}*

$1511=197(198):
>gi|734411172|gb|KHN35784.1| {|0(428)|<Streptophyta(V)>}Protein SUPPRESSOR OF npr1-1, CONSTITUTIVE 1 [Glycine soja]
{(MSKNNASQT)KYDVFVSFRG-VDIRRGFLSHLIGTFKSKQINAFVDDKLERGEE-IwpSLIEAIQGSSISLIIFSPDYASSRWCLEELVTILECKEK-YGQIVIPIFYHIEPTEGNEAIRSILLHLPTTKKENLSPRLFAKMNRLRFLEVSV(EDNYDCLDQLHILGTNLCWPKQQKTRIVDILAKGLKFLATELRFLSWKS)}*

$1512=130(155):
>gi|734353693|gb|KHN13475.1| {<Streptophyta(V)>}Protein SUPPRESSOR OF npr1-1, CONSTITUTIVE 1 [Glycine soja]
{(MASSSSFSFIPQETD)--DVFISFRG-EDTRHSFTAHLLAALGRLKIRTYVDYKLGRGDE-IspTLLRPIVESKVSVIVLSRNY------ICHFEVVLGRARE-----------------NYGMQKNKGSNRDTNFLSCRSVRCAEPDRKLCRCFCET()}*

$1513=141(171):
>gi|734337252|gb|KHN08379.1| {<Streptophyta(V)>}TMV resistance protein N [Glycine soja]

{(MSSFPRYDFDLHDDEMEMDFLRERYQEDNRN)-YDVFLSFRG-EDTRASFTSHLYTALHnEG-
IFVFKDDETLPRGNKIspSLR--IRDQ------ICAMYSLSR--------------------
LAEPNQYLSVVHKLCSQHPVSLSIPH-LNMRVIHPITSGHACDHDKLEL()}*

$1514=187(194):
>gi|734372750|gb|KHN19934.1| {|0(22)|<Streptophyta(V)>}TMV resistance protein N [Glycine soja]
{(MASKTTIHCC)-------FRG-
EDTRNSFTGFLFQALSRKgTIDAFKDGKDLKKGESIapELIQAIQGSRLfFIVVFSNNYAFSTIRKKGKSTL-
-FLSS-
LYAYIYPVQYLMKVIDFRGFHPKYGLQVLIDRSLITIKYELIHMRDLLRDLGRY(IVREKSPKKPRKWSRLWD
FKKISTKAADYLEAMVIKNKAPLQST)}*

$1515=190(217):
>gi|734335922|gb|KHN08040.1| {|0(35)|<Streptophyta(V)>}TMV resistance protein N [Glycine soja]
{(MALRSSSDAFTN)--DVFLSFRG-SDTRLGFFGNLYKALSDRGIHTFIDHEKLKRGEEItpTLMN------
-------------------LQPILDSVNGKKGLLVLPGFHNMDPSDVRRWKGSYGEALAKH-
KERAGYEYEFIGKIVERVSSKI(NHATLYVADHPVGLESQVLEVRKLLDDRSDDGVQMIGIHRMGGVEMECLR
EMLKVAVHAWCPEIR)}*

$1516=173(173):
>gi|734695776|ref|WP_034090799.1| {|0(546)|<Actinobacteria(B)>}TIR domain-containing protein [Streptacidiphilus albus]
{(M)-WDVFLSYSR-AD--
LGRVEPLVAALEAAGLRVFVDHAAIPGFGPIssTIRREMARSKVLLAYYSAGYPHRPACQWELttAYLAGWHE
GDPRRRVLIVNPERGSGHIHPIELRDSRHWPLPSSPAQFRAFAAEVEAYVAALSDS(LPLLSASGATTWSPGP
RPLPAASFTGRTAELW)}*

$1517=257(267):
>gi|831757577|ref|XP_012749073.1| {|565(216)|<Amoebozoa(E)>}hypothetical protein SAMD00019534_112900 [Acytostelium subglobosum LB1]
>gi|735849902|dbj|GAM28114.1| hypothetical protein SAMD00019534_1129
{(QSNSTESMRLLLEKGCDRNVKNTDGQTSLHLAVKSNNISLPMLLLRHGAQSMPDRFMKTPLQYATAQWKPA
LQKIMFDNP)---VFLSYAHKdIKFVRELRVGLETYS----
IRCWLDEyRLQAGCDWReEITKGVENAQVIVYTVSETSSISLWCRKELKMARKL-----GKTIIAIYLH---
DVEVDPILKGLFDFELKphQSPMDFGSIPSEQWLEHFERLSK(VIKSVLGGSKLQRKRLAQMEINSNYPHGTI
YFSFSNSDLKLFNLIRE)}*

$1518=287(294):
>gi|736425572|ref|WP_034447732.1| {|94(388)|<Firmicutes(B)>}hypothetical protein [Butyrivibrio sp. AE2032]
{(ARCLMLPEAMKKRVRILPVKSADSGRLGEEFSRICGKIHLLDRGTQTFKDDLKTYFDSVLEPYRMIDLSEE
QKRIRADLF)RCKAFISYRKKDIDQLLRLLDFIREIPEL--
RdmaVFFDSALVPGEDFNqRLKNEISGSDIIIFVVTPHLME----
DGNYVIREEYPQAVaEGKILIPVFMDDTDA------
SSVRSVFSAFEEVLDFDDKDALVEKLISVRSMF(GDIPSMTPEYKHLLAIAYETGDGTERNYILSNLLLREAA
SKGFPASVARLTIQHLEGLVNDEYEGETEDLLLSA)}*

$1519=294(299):
>gi|736428072|ref|WP_034450220.1| {|89(444)|<Firmicutes(B)>}hypothetical protein [Butyrivibrio sp. AE2032]
{(KDFDLNFAKERHIPVLPLLMESGIYEFYKKPENFGDMQYLKPYGDDATKIDYKDKLRNYLESVLISDELIQ
RIRKAFDAY)---IFLSYRKTDriyANELMKLIHRNPEFEDI--

AIWFDEFLTPGESFReNIEKILSDSKLFTLLVTPRLLERQ-EDGspNFIMREEYPAAKqLGKPILPAEME--
KTDEEALKRDYEGIPACS----
DTSDEEAFKKRLIDTLMGI(AVSGNNNDPEHNYLIGLAYLKGIDVEVDTDRGVALITRAAESGLPEAVEKLYL
MYKDGDHIKSDGERSLYWANRLYEIN)}*

$1520=213(209):
>gi|736306332|ref|XP_010794731.1| {<Chordata(M)>}PREDICTED: interleukin-18 receptor accessory protein-like [Notothenia coriiceps]
{(MILVFFMSVHHKSDEKEFDVFRRLYGAHQRFRGVGPFPAQKGPHWAACLSSWTFC)------
NTLKEGKSTLEvLLPRLLEDPVGVFACAAGEGVLSWRAYTNDVVLAIRRSQMLICVLSTDYLsNSNALF-
VLESGVQALLQNTSLKLLLIWTTrdsaaLI-
QPDPPLPALVKRALKVLpSLDWSSGKTArttsSFWRSLRKAMPSQ(RVNMVSLMQGQLTD)}*

$1521=293(300):
>gi|736210116|ref|XP_010776953.1| {|10(55)|<Chordata(M)>}PREDICTED: interleukin-1 receptor accessory protein-like 1 [Notothenia coriiceps]
{(GNALNLTCRAFFGYSGDVSTLIYWMKGEKFIEDLDEERIQESDSKVVKEHLGEQEVSISLTIDSLEEEDLG
NYSCFVENG)----------NgrrQATIQLFRRGLTHEHYQDETRLF-------
GAYIEDVARCVDQSKRLIIVMTPNYVVRRGWSIfELETRLRNMLATGEIKVILIECAELRgimnYQEVEALKH
TIKTLTVI--
KWRGPKSNklnsKFWKQLQYEMPFR(RTEPMLSHEPALDVSEQGPFGELQTVSAISMAAATSTAMATAHPELR
STFHNTYHTTMRQKHYYRSYEYDLPPGGTLPPL)}*

$1522=170(182):
>gi|736300890|ref|XP_010793061.1| {|0(126)|<Chordata(M)>}PREDICTED: uncharacterized protein LOC104965748 [Notothenia coriiceps]
{(MPSHIM)-----LSYQWDDQALVKKIYDRLKDDGLP-VWLDIEGGVTG-
NINDSMAAGVEEAVVICPFMSPAY-QASRSCKkELNYADSRE-----
VSIVPVMLTNNWeaSEWLGLITAGLLWVDF--
RNAEKGEEHFEMCLSSLEEEIM(FNVGHLLAVEEPQGEVVDQPEPSKPRLKKKPGRGFR)}*

$1523=179(184):
>gi|736099867|ref|WP_034232548.1| {|0(499)|<Firmicutes(B)>}hypothetical protein [Lachnospiraceae bacterium AC2029]
{(MERKIMTYEGNA)-PYIFVSYAHKdS---DRVMPILGRLAEDGY-
RIWYDDGIEPGSQWSeNIAEHLSRAEVVLAFGSKNYVDSKNCRQEIEFSIS--EE-KNLLVIWLEKTEL-
TAGLKMRLSTIQGINLYhmDEDSFFRKLDMTKNILLCRERTE(SAETEEPGGSISFTEEKPVGGSISFSEMPP
ET)}*

$1524=284(290):
>gi|736425670|ref|WP_034447830.1| {|91(626)|<Firmicutes(B)>}hypothetical protein [Butyrivibrio sp. AE2032]
{(MDFDYRFAREKHIPVLPLMMESGIHEIYSRPENFGEMQYLRPYNTDSTKIGYKDKLKNYLESVLISNELTE
RIRKAFDAY)---IFLSYRKTdRKNANELMK---
LIHRNpecEDIAIWFDEFLTPGESFReNIGKILGDSKLFTLLVTPRLLereadgNPNYVMKeEYPAAVKLGK-
-------PILPAEM-EKTDEEALKREF---EGipVCSDTSDE--
EAFNKRLLDALMG(IAVSENNNDPEHNYLIGLAYLKGIDVEIDIDRGVRLITKAAEADLPEAMRMLYDMYHDG
IGVELDYKRAV)}*

$1525=185(193):
>gi|736600580|ref|WP_034609345.1|
{|0(228)|<Actinobacteria(B)>}hypothetical protein [Cellulomonas sp. URHD0024]

{(M)---LFASYSSKdLAAVKRLVDDLGPTR----
FDVWFDQELRGGDDWWnEILGQIATCEVFIFALSDNSLRSKPC-GvELAYAQAL-----GAPVLPVQVGSV-
ANpRLNPFANLHIIPYVDraANAGIQLISALEQKSARPWTPTD(PPPARPPSPYAIFRDLSAQVAAPSLTYAE
QVDLFRQLRDGLDSERDESAGEE)}*

$1526=193(201):
>gi|736927230|ref|WP_034924616.1| {|0(318)|<Firmicutes(B)>}TIR domain-containing protein [Erysipelotrichaceae bacterium NK3D112]
{(MANYLGFEPTKRNNDYY)----FVSYSSAdAGYVGAVCGQLN--KR--
KIPLWYDYGIQYGNEWEvSITKRIAQCTAVILFFSPRILENKNSY--VLKELEIAKD-YEKPVYTIHLKAV-
NNKTVPAETHHYWVDIRryQGIEAFKLDRQTVLQELERALQN(TPVNLEADSDSTVKNTDSVKDKLALQKQAD
EEFERVLRQMEDDA)}*

$1527=270(255):
>gi|736098488|ref|WP_034231177.1| {|0(649)|<Firmicutes(B)>}hypothetical protein [Lachnospiraceae bacterium AC2029]
{(MLCGKCNSKEVEIFRDGIHGFCHRCAQMVPLVPGQEEEKQEM)--QIFFSYGH--
DNHAEFVVQLSQRIEQITdgrIRIWIDKTKIDEWTNWrqKITEGICGSYAVMAFLSKYSTRERGPCLdELAIA
--IASKHG--MIRSVLLEPegrI-
DAPAQTREYQWADMSdyriyldrggDEWDKYLeekaLQIIRmLDSDEVRQYNSEL(QTLREKLRLPEINLELT
KLDILAGKKLIGREWLRKRIDTWLDDPEGKRILLLYGKPGAGKSAFSAHLQHYNPR)}*

$1528=229(217):
>gi|736927469|ref|WP_034924855.1|
{|0(255)|<Bacteroidetes(B)>}hypothetical protein [Gillisia sp. CAL575]
{(MQSKEEKTIK)---IFISYSwK--
PVSNkDKVLTLAERLTNDGIHVILDDWDLKEGQDKyhFMEQMVNDPEVqKVLLICNKEYAKKanqkiGGVGiE
SLIVSDDIYSEaDQTKFIPVVMEYE-
NEKPCIPTfvksRIYIDLSNEdhYEENYERLIRNIYNKPISKRPPI(GKMPVHLEDDDPIFLPTAHKVAAIKN
SLINQKENSTLLIKDYLNTFVESLSLFEINFENINEENFIN)}*

$1529=263(275):
>gi|737134002|ref|WP_035121352.1| {|852(0)|<Cyanobacteria(B)>}GTPase [Fischerella sp. PCC 9431]
{(QNLHSYPANVLHRFLDARQYQIQCQKSFQMVDVRRLIDDVNLQSFDVKYERDLQVTSLQRELEQSRDKSLM
HQPQNNEQK)--EVFISYTW-RDQESKpLVEKIEQEFKTKGINIIRDTNAIGYKQ-
KfqEFMQRLSCGKCVILIISDGYLKSENCMYELVEIAKHGEF-YDR-IFPIILADAQIDKSVPRLKYIK----
----YWEDKI-
KELDEAMKEVSGA(NLQGFREELDLYTEIRNRIAELTNLLKDMNALTSDIHIQSNFETLLKAIEERLNK)}*

$1530=116(143):
>gi|737836940|ref|WP_035804239.1| {<Bacteroidetes(B)>}molecular chaperone Tir, partial [Lunatimonas lonarensis]
{(MS)---VFISYSTKdsEFVTRLSAELVKNR---
IQVWLDKWEMQPGDSLIDKIQSGLSDSSYLLVVLSNNSVDSEWCKKELNSGLMRELNEKKIVVIPILLDN---
------CKVPLFLQEKvYADFRTD---------------F(D)}*

$1531=182(182):
>gi|737133482|ref|WP_035120835.1|
{|0(168)|<Cyanobacteria(B)>}transcriptional regulator [Fischerella sp. PCC 9431]
{(MQEFEFD)---VFLAHNSKdKPQVRAIAEQLT----
RYNLKPWFDEeQIFPGDNIQqVVFQGISQSKVGAFFISQNGLGT-FQENlELgaiiQFFLKKHKN-
QGFRVIPILLGIN-

DIPDSLFYLSQWAWIQFtsFNDQAALKSLIRGIKGRGADVQA(PPVIQIPDIPLIIEPKQPQTDDLASEKGID
YTRLR)}*

$1532=148(151):
>gi|737636033|ref|WP_035606069.1| {|0(93)|<Verrucomicrobia(B)>}TIR
domain-containing protein [Haloferula sp. BvORR071]
{(MFAGRKL)---IFVSYSRKdRRVVNKLQKVLgigSAKPRQGLNSS------
MPGNDWGnEITPYLGEADVVLLCWSRHAAGDDSVRKdYLGAIGMGKQV-VPLCLDDLALPEE-
LAGFESISRETSPYEELvrVSRGAFSPEKLLRAVCGYITVAL(GAVL)}*

$1533=192(188):
>gi|737882146|ref|WP_035848654.1| {|0(216)|<Actinobacteria(B)>}TIR
domain-containing protein [Cryptosporangium arvum]
{(MTLS)---
IFISHGSLSDEAKLMVSRLYDSLRASALQVYLDAHEQasQSSEDFpdRVDRELRDCSGAILVVSPDVVGSAWV
KHETSVLANRR-----
KgmvtgqlPLIVLYVDgAKPDDLRGEHWNITRIPWLGSCTELSEAHDFAMTRLQPLMSRA(SRDLRKESIYLT
LDSTLRSLQLEELTAALAVLGLKAHVWSPTDA)}*

$1534=168(176):
>gi|737884494|ref|WP_035851002.1|
{|0(830)|<Actinobacteria(B)>}hypothetical protein [Kitasatospora azatica]
{(MVVVRPDGH)--DVFLCYAW-EDKEQ--
ADALRTELTALGLEVFQDHDGMRDYDCIpdRIDAALRGSHVFLALYSPAFLASEYCRQELHFALLSS-----
YHLHRSRARVLVLTQHLGIEQVRPERLKHWRLPAPNQSLDRLAASVAAQVRR(LGEQDRRRLGDAPNPPAPRW
YSEPMPD)}*

$1535=278(294):
>gi|738069945|ref|WP_036028830.1| {|127(296)|<Cyanobacteria(B)>}ATPase
TIR domain-containing protein [Leptolyngbya sp. PCC 6406]
{(MNQAIGDRAAIEFATSFYDALGAGETLEFAYRLGCNAMALAGVGGEQIPVLIQRSETAAPATPPGRVVIGY
KRGIEPDEP)---------------------LALALRAAlaqTHQVFIDQD-
MAIGTPWaqRIEAEIRQADFFIPLLSPQAVQSEMVVGEVETAHQHYQTQgHPQ-
ILPVRVANDIPFAYPLNAYLNGINWATwHSEADTEPLIAALQQAIAGTPLP(TPVTAVQNPPTPQYPNTPLPH
PSTPPLPHPAAQPHFLEMPEGTIDGDSQFYVQRQEDALALDTIQRQGVTITIK)}*

$1536=202(196):
>gi|738213180|ref|WP_036168843.1| {<Proteobacteria(B)>}TIR domain-
containing protein [Massilia sp. 9096]
{(MAIRYGCFFSY)AHGRHELMQ---
RFKATLADALRCYLEPYfddENELFVDTDQLGGGDDLdrKIARAMCESVCMILIYTPKYEAHAYTRREYAAMR
AIEAERskwyalPSHLIIPVIMTHHPdrlpP------
QIIESTFYVDFSRFTMATADLKSNPDFLPDIDR(MVRRIVTHYQLQKKTMPPDHDCNQFILPDVPPAWRELPN
PIFPKK)}*

$1537=209(222):
>gi|738226638|ref|WP_036182097.1|
{|0(699)|<Proteobacteria(B)>}hypothetical protein [Maribius sp. MOLA 401]
{(MNGTQVMPIT)---VFLSYARKDAAVMETLRDTLVAEGFD-
VLTDTKDILPGENWKARIGRMIRAADAVVFVMSDASLGSEICDWeVRVSAEYGK------
RILPVVIEDSIgaRVPQRLQELNY--LFM--RSEMERRE--
NLRRLTESLLLN(HRWIRYQTSLFERAMDWAERGRREDELLVGAPLEEAQGHVLDLPVDAPRPPDLLGEFVRT
SMRAETRRREEA)}*

$1538=222(220):
>gi|738200274|ref|WP_036156201.1|
{|0(76)|<Bacteroidetes(B)>}hypothetical protein [Maribacter forsetii]
{(MR)---VFISHSSKdKKFVRLLKDSL----
LENSIETWFDEDqLDLGDKLVnKLENALDDSSHLVIILSPSSINSDWV-NfELKRAIKNNRT-
GlNSKIIPIKYKECNvpdeisDLLHADLTDEVVLPDNDrvKFISSGFD--
SFFLKLVRTIRNS(AKTITKAEKEEILKSIKASKQAVDKHQKSIHRGNYKLIGYTTLESRKKFQNKLKGRVDK
VESIEDIKPLLLPYSLKTV)}*

$1539=221(216):
>gi|737391199|ref|WP_035372665.1|
{|0(234)|<Cyanobacteria(B)>}hypothetical protein [Dolichospermum circinale]
{(MSDNRLQFD)---VFLAHNS---
KDKPEVIKIANKLRERGLNPWLDKEQIFAGDNIqeVVFQGISRSKVGAFFISQNRL---
GAFQrNLELGAIIQLFLdKKETgfrVIPILLPDI-
SDIPDelfhLKQWAWIRFTNSNveqdEEALQDLIRGIRGRNFQYPSLN(NTEPKPEKFLDIPVTSFKFETAKL
ILKSGVFGLGKSSEIQRITKNANYFGEDLGNGVILEMVAIPGG)}*

$1540=181(182):
>gi|739395867|ref|WP_037256435.1|
{|0(712)|<Actinobacteria(B)>}hypothetical protein [Rhodococcus rhodnii]
{(MADGRGEL)-YNVFISYSG-DDIHQ--
IEKIKKRLDGLGISAFVDRTENRPSDSItaSIEHALRNSAMCLVYYSTRFAERHACQFELAQaFVASAKEGGP
SRLLVINPGPGRGHIAPVELQDRVFVEDDKSEAVLNQIIDGIQHMLATLTGT(FQDIDFQALPKSYFGSSGYE
PRVRRYSAIWALHS)}*

$1541=135(147):
>gi|738627208|ref|WP_036536763.1| {<Proteobacteria(B)>}hypothetical protein [Oceanobacter kriegii]
{(MIFGWKK)--DIFISYAYKTDKRYKNLLVawdkndlFDFEFYDG--
SVTVPVNSTQAGPIRrVISQKISQATHLLCIVGE--------EThkSAWVEWEINKAVeTGKKIIAVKTN---
-RSNTTPDALYGV----GATWAMSFTFAAIKK---AVDES()}*

$1542=202(206):
>gi|739409549|ref|WP_037269943.1| {|0(78)|<Actinobacteria(B)>}TIR domain-containing protein [Rhodococcus rhodnii]
{(MLHRIKAARPR)---VFLSFAG---NNRDQARLLAKEFKTRGFDAFVDDDSIP--LGRdvvIAINNALS-
QYDyfVLLWSRHAVDRRWVELEWAAALAREISERRSFVFVVLLDDTDppL-VLSPRRYLTARDGWA-
KVAKELVGAWQRDQRLDMPVLP(APYPQASEGLPMIVIHVRNRALKVAHVLPVLAGTTGAELLGMVRSALALP
QEEKA)}*

$1543=202(202):
>gi|738551607|ref|WP_036499792.1|
{|0(886)|<Actinobacteria(B)>}hypothetical protein [Nocardia aobensis]
{(MVDTVT)APQVFLSYAGSERSRAMAVRAELASRGID-VIMDD-
EFEPGRSVMVNIGRALHRATVVLALVSSQYL-DRHFTEiEVSTTLANR-----
DsNLIPIVIgadpepETEAgkDLLRVIKGRTYFRLEM--
TPNSFDKLVAAIRENYRHPARK(QAQVEVESISSADFTVVYDWEDSYLVDTVHERLVKLGVDDVKYYSAGVAT
SAGGED)}*

$1544=213(220):

>gi|739084257|ref|WP_036955394.1|
{|58(0)|<Proteobacteria(B)>}hypothetical protein [Pseudoalteromonas sp. A2]
{(DDSDFDHWLSIHHPNKSRTFNVLMDELTEAAFSRKGYSITTFCDSEMVEIVFSSEANELFLFPDRKQMYEIWGAPIMQKQ)--KIFLSHSSRdKDIVD---KYFSELQKSQ-IKAWYDKEeIVPGDSITsRINEGLNKCDLGVLFMSNNFLSKHSGWtEaESNFFISNRMK-KNKSLIVVNL-------GVKHEDIPPLLQDYlyIDGNKGSAINELIVATKKQLDRM()}*

$1545=252(262):
>gi|739181124|ref|WP_037044932.1|
{|407(352)|<Actinobacteria(B)>}hypothetical protein [Pseudonocardia autotrophica]
{(WLREGEVNPPSAAFRWEEAVHKVEFRIRASERAVGRSLSGGVRIFLGVVLIGEVTFRIAVTRGRQAATPPRERDVAVRYR)--KIFASYSHRdADIVRAVSSAVSVIGDRYIVDS---AALRAGERWEpRLRELIEEADVFQLFWSHNAMRSEHVRDEWEYALRLGRE-G-------FVRPV-FWEDPRPQDVAAGLPPEslDRLHWSWLHPPVPTAARRPAGPP(PRPALPPPEAAVTVPHSPPPPSAPAGAPLPPPTVRIGTPDLP)}*

$1546=183(193):
>gi|503086541|ref|WP_013321399.1| {|0(92)|<Cyanobacteria(B)>}molecular chaperone Tir [Cyanothece sp. PCC 7822] >gi|306981411|gb|ADN13292.1| TIR protein [Cyanothece sp. PCC 7822]
{(METSNS)-PQVFISYAW-QGDSEEFANQLEEAFQKRQIKIIRDKTDLGYRGLIKEFMeRIGQGKCILVVISDKYLKSENCMFELVEIAKHGD----------FYdRIFPIVLKDAKIYKDEDRIDYLHYWDQKIKNLEDKLKSGSILDV(TSIYKTLQQYADIKRNIDQLTDILKNINALTPELHRQSDFEQIIQAV)}*

$1547=246(247):
>gi|312082401|ref|XP_003143429.1| {|489(0)|<Nematoda(M)>}hypothetical protein LOAG_07848 [Loa loa] >gi|307761406|gb|EFO20640.1| hypothetical protein LOAG_07848 [Loa loa]
{(NTTICAPTASGVFGTEGTTNSFLIITALLAVALITIGLICLAVLFLRKTKSVIVQRRYKVPPSFTGTHTTPGSSPLPLIH)-FDAFISYSKKdeKLIIDTLYRQLESEE--YILCLLHRDSPNYSSrvhtISDELINQMECAQSLILVLTQHFLNNEWKTLQIKTSHQIFAKNRHKKLIALL-GDG-IEPNQLDAELGQILRKNtCIRMNDPL---FWNLLHSALPVR(IAPSSCSGGSSQIYSDCYGSIVPSDIV)}*

$1548=232(229):
>gi|490116792|ref|WP_004017374.1|
{|389(0)|<Actinobacteria(B)>}hypothetical protein [Mobiluncus mulieris] >gi|307615092|gb|EFN94305.1| hypothetical protein HMPREF9278_1141 [Mobiluncus mulieris FB024-1
{(SCVKQGWVFHRGFVVCTTAHSRPYGDKLWAIPYQALGEYPIARRGGSFDGKDGRESAPVASVSSPVGSVDVVVFMSCLPE)---DDAYLW---GAITALGLEVARAYRFQtgeHLEVLTDRDGTSWKENWnqSVRRAAGAQAFLIAAVTPGYLKSEVCRSELTAFASRLETAgY-QGILPLII--QPlgdsVPPSPARELVERYRFRDvpvADGWFS-PQSAEFRRLAADIAQH(IRAEITKPD)}*

$1549=222(217):
>gi|739444819|ref|WP_037304802.1|
{|0(321)|<Actinobacteria(B)>}hypothetical protein [Amycolatopsis orientalis]

{(MR)---VFISYAHDTeGRKVAQALAEALRKRGV--DVLWDRD-LPEGNpvsLPeWIVTSVT-
AYPVLCVLSPdyvlrfgagdGTAARKGVLFesRVLQRRIYDHTEsGGCPVIPVADLG-------
FSADLAPVVLKNLVISRFDaDTEDGLEQIVRRLLAL(GPVPPVTRPPVPPPEVGEELALVQDLEAAAPSSAAG
PALVHDWLGVVADLSRTDLVRALPAVERIVKAAGDAEAM)}*

$1550=165(166):
>gi|739771844|ref|WP_037624112.1| {|0(279)|<Actinobacteria(B)>}TIR domain-containing protein [Streptomyces aureus]
{(MALR)---VFISHGSGKDQGkdqcvedAVSLLVPELKRRGY--
EVFFDVESLRVGDvWAeELYMELFACDAAVVLLGPKTLEAP-----
EWVRRETEVLVSRHVMKDLPHVLPGLMGGLDPKQARKVGFGPLFRLQACIQDRVAPYNPPPSLDC(SVTVFVD
WMLAEFANFGEAARD)}*

$1551=273(263):
>gi|739484006|ref|WP_037343760.1| {|74(634)|<Firmicutes(B)>}hypothetical protein [Ruminococcus sp. FC2018]
{(AEAYWGIVLCKYGIEYVEDPTTLKKIPTCHRTQYESVVSDVDYLSAIENADAGQKELFEKEAAEIDKLQKN
ILEIVHKEK)PFDVFICYKE-
TDEngKRTsdsvIANDIYYQLTQEGFKVFYAAITLedKLGQEYepYIFAALNSAKVMLVIGSdPEYFNAVWVK
NEwsryLKLMKNDR-----
SKlLIPCYKDMDAYDLPEEFAHLQAQDMSKIGFINDVIRGIRKVLSKEKTSES(KAETKTVIKSSSGNVENLL
KRGNIALEDGKWNDAKGYFDQVLN)}*

$1552=194(215):
>gi|739446191|ref|WP_037306174.1| {<Actinobacteria(B)>}hypothetical protein [Amycolatopsis orientalis]
{(MYEYHLFISYRRGGGDAPDWVRNHFHPRLQRLLDDNVDY)----------------------------
DVRIFLD-
DSVPIGGKWpqEVRDALQRTRILIPVCSPKYFRDEWCLAEWHTMAKREELVgRDgsvkagRLIYPVIFSDSEn
FPNYAHERRMRS--
FRSwNQPHPQYQNTDEYLDFNREMERM(VQELVEIIDQAPRWRPDWPVETPPPDQPKPPKLPRF)}*

$1553=220(220):
>gi|739540500|ref|WP_037398858.1| {|41(0)|<Actinobacteria(B)>}molecular chaperone Tir [Solirubrobacterales bacterium URHD0059]
{(KYKRGGQYRFYGGLARKSPSHVGVPSSGRWYLVVDMQGLRGSTNASVRVLPRAAPPLRDINTDLSDIAQQL
AELPADERD)-FDVFVSHAA---EDKDFVAPLVAALAELEVNVWYDGFEIQ--WGDslrRKIDR-
GVSRSRfgVVVLSPAFFAKNWTQYELDGLVAREMHGGNNIILPLWhnVSKDEviS-
YSPSLadkAALRTSDSTI-TEIAEQVA-E--LVASPEDYRP()}*

$1554=283(296):
>gi|739796536|ref|WP_037648572.1|
{|259(515)|<Actinobacteria(B)>}hypothetical protein [Streptomyces avermitilis]
{(VPYKPFYAYEEILATVGDRPRQQDTVLAATERIVAHLTDHEITRLVTDLDEPGRRALLERFQRVGPLASDR
LTPDRRRQP)SSRVFISYSYDSIDHFEAVRelwFLLRGHGIN-ALLDR---
APGqrqPDWRPWQREQLLAADTVVVVPSPAYRRQPTDDVkDLREAYRTR-----
PRRFVRVVLPgGTheDLPDFLR------------GEASARS--
AEVRALTAEGVE(PLAELILRQVSAVRLSSSNLVTSRPTRNVRNTDPELVDAADALSYALIEEQHMEVRHRRL
TDPYPLPLRWSLNPFL)}*

$1555=297(289):
>gi|739903035|ref|WP_037753493.1|
{|81(107)|<Actinobacteria(B)>}peptidase [Streptomyces sp. CNQ-525]

{(STERGLRLIPVLHSDVDIGALPFAATRLWVDFRDAAGQAFHDKVGELARVILGEGARGDGESEAMALRDRA
ARSVDVPAE)-PSFVVCYARQeSDYARRL---LPALSRAGL-
PVWSISHLTWGDDVLsTIRAQIRHALGIVVLMSPEAENSEDITReILEG--QRHSR-P---
FFPILVRgdrhfllAS-TWHfdartgslpgHVELDLLRRLRDSGraVAPAPDRG--
AAAARPAAPPVAV(PDSPSPAPAPAPAPAASLLPDAPVRAQLGALLAEHEWEHADVLTTALILRAVDRLESGW
LRRGDGRRLP)}*

$1556=223(203):
>gi|739871433|ref|WP_037722151.1|
{|0(240)|<Actinobacteria(B)>}hypothetical protein [Streptomyces
mirabilis]
{(MSAPSLPAEETGDKGHPSGRSFGE)RGLVFLSHSS--
RIKTERELgendslrdhqtrkhYLSNLLTALdtslppiGFQAWLDKKNLEEGESFdtLIFDALARCRAAVILI
DRDA-LDSSYVLaEATI-LTFRRTVgDPIHILPVLLGDV-
TDNDVNASKLGRILRLDSLLPLRPTTGTTDDATVQPTADE(ISESLDRNVATCDKADSPTQRWIDHVSSYLAG
ASEDALL)}*

$1557=264(264):
>gi|740327603|ref|WP_038164470.1|
{|133(418)|<Verrucomicrobia(B)>}hypothetical protein [Verrucomicrobium
sp. BvORR106]
{(PATFRLLPVAVEPVAYNMPESMRKLNFLRHITPAGMVPEDAELIARITEVLCRDMRAWCHPSDKSLEKPAN
KRKAAAVPE)KIKIFLSHaKA-DDTEEAITLKQYIQRETQ-
CEAFFDETDIASGYDYttILDTAVtRESAGLIVIQGDNYADRPWCRKEIRDFLRpvedsCGAG-KGRQ-----
YFVAPAVVVQTMKGKQIARTIP-
ELGYSPCVRWQDAQSARFVITT(LLREILFGLFYRRLAAKYAALQPGSRKVFLNRSPDPVMIARIMA)}*

$1558=174(166):
>gi|740327647|ref|WP_038164513.1| {|0(309)|<Verrucomicrobia(B)>}TIR
domain-containing protein [Verrucomicrobium sp. BvORR106]
{(MAYTPGFV)-HDVFISYAT-ADDRATdglinyidgkLRIKLEARLSKR-
CKVYLDsNEGIAAGAPIqdQIIAHARRSAIFVIVYSEAYQRSDYCKRELHEYSSCDGN-
AICVLHLASTSNSQQDHSPLINSRRAHELFE-
TTHGGNEQRNFRHDAKPQIQRN(RDNRTLDEALDSLANEMA)}*

$1559=244(245):
>gi|740327584|ref|WP_038164452.1|
{|99(342)|<Verrucomicrobia(B)>}hypothetical protein [Verrucomicrobium sp.
BvORR106]
{(FVPVQLSGGAWPLDDRLRKVNFLRAFLSDADLELDEATRKLKELKVTAAVVRRVVVELCRFLAALTQGAGN
HSSKAPVRL)----FLSHTK-
LDMEKepKVTKQFIACLTEEqPIEAWVDSGNIDTGSEFakEIAEGVAESSGLLAILTDLYATREWCREEVLLA
KENHRP-
VVVVDALSGCEVRSFPYLGNVPRIRWDGDPQKGIDLLLKESLRHLHTGEVLKRA(SLATDTVFCRAPELATMV
GLPNGAS)}*

$1560=214(219):
>gi|740461490|ref|WP_038292918.1| {|0(161)|<Firmicutes(B)>}TIR domain-
containing protein [[Clostridium] clostridioforme]
{(MTKPT)---IFFSHSSKdKDVILSIKNKLDKSTGGVMDIFMSS-
DGQSIPFGTnwihKIEDGLQAAKIMFVFVTENSISSGWIYFEAGYAYSKGIQ-VIPVGI-
GVDIGALKAPLNLLQGFNILSEDS--LNNFITI--
INRTFDYRFESA(FEHGDYLEVISNIFSESFSTIKFEEVVGKAECEIYGEQTVNGESVKYDIEKLFNDIVSYL
ETNNIPFSRNDKYY)}*

$1561=235(240):
>gi|740492649|ref|WP_038323558.1| {|56(41)|<unknown(B)>}hypothetical protein [bacterium MS4]
{(TIHVKGFLKEFNMGEIMENVKHILSENPLEAFDNVQVDSKNIFVIKVKKAKTKICFEGKYYTYVDNRIEVIEDSKGYKFP)--KLFISYKECdAPIADILERAIKDKMDSQIEI----SRYTQINYKEsfklFMNS-IQDNDFVLSVVSDSYLRSQACMYEVGEIIKDHHY-QEKLLFVVLGENERKYYGKDSPEKISANIYD--GALGRLEYVNYWKRQYESLEK(MIKEINDYEATSNATHELKE)}*

$1562=262(264):
>gi|740327605|ref|WP_038164472.1|
{|300(82)|<Verrucomicrobia(B)>}hypothetical protein [Verrucomicrobium sp. BvORR106]
{(ESYWAQVEIPYVPFYAYEEILATVADRPELRSSLLAAMLNLTQAITEGQIKKLPSLSSREQHNLRARYATFSENRTVTRP)-PKFYLSYCQRdveEKTMRKIAGFLEMALGEESI-VW-DGKIPLAERWDlWLDRELEAATCVLFFVGPAWLESAN-TArELEIA---RSA-GKALV-PVIMHDtT-EWSDLPKPLREVRGRTIsqKTFKSDLEGLVQGLQPFTRSARQ(LKLASQPIDPDDPQRGRWGGRAMANGRRLTATVSAISKYWFEIK)}*

$1563=179(160):
>gi|740327648|ref|WP_038164514.1|
{|0(295)|<Verrucomicrobia(B)>}hypothetical protein [Verrucomicrobium sp. BvORR106]
{()MLNdVFISYSHKdnlplavrqrpedpEKWVDwfhlRLQTYLAQLLQRDV-KVWRDRRLRNEGELDdEIRNAVASSKVLVPLVSPCFAGSGWCSDEFQIFLDHLCD-IGEEKRAASLIHP-AVKLPTPEYELPSLLTNklGVPFFHKDEVSERVQQWDPTNPA(HAPLFAEALYGLCEDIRGAL)}*

$1564=226(218):
>gi|740399852|ref|WP_038233515.1|
{|0(544)|<Bacteroidetes(B)>}hypothetical protein [Zobellia uliginosa]
{(MNLT)-KNIFAICTGQdKGELQNLLRHLQSIEKDYETAIWSNDAIDDKQQWEhTDVERLNNTDVFLLLLSNTFMNSEFIENdEFETIVDRYKK-GEATLIPILLDDC-SWKneftfdhyNFNFGELQVFRKDAisIGEWNPTD--KVFTQVVYYLMGL(LSASTKKSTLDDTVNTVAQQTQNVGREGQMAIDFFEETVVDNKAEIQEEYKQEVEARESIEEYNKLDEENEVKE)}*

$1565=177(164):
>gi|740597706|ref|WP_038383250.1| {|0(316)|<Proteobacteria(B)>}TIR domain-containing protein [Bradyrhizobium elkanii]
{(MRKL)--KIFVSYSHAdARARpgfnvsrlgrimEELKYDLGCHSDRSRFEVLRDVEMThVSDNFRvEIDRAITACDVAILFISEEFSRSEEC-EaEFARLIDLGKP-LFLVEIEPILLSD-EEDRIRNHRKAVKDTIFvrFWGPDNNQKSVKYGYPFPDNIAT(RDKYDEALGQVVAGLKARVL)}*

$1566=253(238):
>gi|740327591|ref|WP_038164459.1|
{|0(546)|<Verrucomicrobia(B)>}hypothetical protein [Verrucomicrobium sp. BvORR106]
{(MNQNNPPQGTPASEAPPAPPA)-FNAFISYRRKdggraaRWLRSWLTQFRVPKAVKakmpghkfvEPLSIFIDEPFSRGVPDyYddNILPALQSSEFLVVIASPLSLvpaadgNANWVEREIGDFLQTPNQHhliVAATEEFAT-----------DPASVTRLPGGLLQLYPRLQ--ILClgSLSWLRAFR(HSWRWQPRDAALACAASLHHIPADLLPLLRREDQRRRTRTLVAFCTSAALIASALGWTTYQASQAEKREIETAITYS)}*

$1567=273(276):
>gi|740327613|ref|WP_038164480.1|
{|116(31)|<Verrucomicrobia(B)>}hypothetical protein [Verrucomicrobium sp. BvORR106]
{(SQQQEITDMSVLGISVSSAQAPQIRQNPMPSPVMLLGLDDLGERDLRQDMLCLYALDQAVWLLNRRQLAKS
PGSGGQPRI)----FFSHAKRdgvplaYSLRGWMNGLLKR------
FRYFYDTDHLDLKGDLsaQLQDAVAGSGSIIVVLRTEAFDSRyWCQKEV-YWAEHHGVPvlTVDARWNIRHS-
--
PAVIGFDGTPSVRVPDGSLTRILLAAYREALRVYLLRERV(RETALASQDSASRRTVVSFDDCSVLSRYPTLI
SLEAATKPNPAPCYLVYPNPTMPD)}*

$1568=214(210):
>gi|740327632|ref|WP_038164499.1|
{|0(383)|<Verrucomicrobia(B)>}hypothetical protein [Verrucomicrobium sp. BvORR106]
{(MTKPVPAPAAIAARNPFGHLIERLYG)-YDVFISYTRMddeqsaYplALQATLTQGSEKERRPPM-
RCFLDLRDLPHDDELrrAIEAKVRASRHVVFIAGPSAADHPWMCFEAEMARKHRRPMIVLDRG-IDWV---
NSKGKLRELVDNVLAIPSAPGQAQPGTEVEKLSSTIGSFR(VSKLRRRIWTGVVIALCAFLAAAVNWALVASR
ARDAAEKSRIAA)}*

$1569=224(198):
>gi|740327634|ref|WP_038164501.1|
{|0(505)|<Verrucomicrobia(B)>}hypothetical protein [Verrucomicrobium sp. BvORR106]
{(MSKDSNPVGGVRQPDGTAAGE)-
FSAFLSYCTHpdyklaKATKRFLETFHRwPPVRRqfakqewRPMKICTDDGSFRRSRAgteteeiIkaNIREM
LKRSAALILLTPGDPTAMKWQDDELAEFQHQNReagwARpvfVVVTQGDPLSE---
SKPFWTPLQIKEGLHEQIYFDFREWRPKEAARQGKVRDME(SERLALAAEVLKPEAKLANGGTRMLTADELVP
QWWAA)}*

$1570=223(215):
>gi|740327674|ref|WP_038164540.1|
{|0(766)|<Verrucomicrobia(B)>}hypothetical protein [Verrucomicrobium sp. BvORR106]
{(MRSEDPGAAASH)KYWAFISHS-
SkdrefaRWLASALGTFSIPsKYRKEyklpgcLSPVYLDVHEAPGSGDLgkPLRDALTASKALIVLCSPDSAK
SKWVNTEIEHYLSSSPNPivcWVVVGG--QFS---DGRGTAEY----
CMPPASESYFKYNEDGSIAEGSSALCDL(VRGDPEEKRGLVKRLIAAIGEGARGDLAREVSARMRRRVFMGGA
AAALAAMGGGAAYLQGREK)}*

$1571=222(221):
>gi|740599684|ref|WP_038385228.1|
{|0(309)|<Proteobacteria(B)>}hypothetical protein [Bradyrhizobium elkanii]
{(MSEFN)-
YDAFISYRRSdgiavaRWLRRTLVGFRLPAVLRGAyakkLRVYLDTAYERGTSDFyqqSIKPALLSSRYLIVV
ATPDAMRRPggaedWIEREIRDFTASPGAGnviVA------------RGAGDFDGP----
LPGGLKETFPNIE--I-
VDLRGAGPLW(FLSFSRVVRLSAEKLKIVAPLLNIPPEEMPRLRQEEERRQQTQLGAVAGATLGTLVAVGGLS
VLTLINQYKAIRAN)}*

$1572=220(224):

>gi|742483870|ref|WP_038938923.1| {|64(0)|<Proteobacteria(B)>}TIR domain-containing protein [Vibrio vulnificus]
{(VSSLAQNVTPIFDSISGVQIAIGNSITPTGSTVDSITMPFSGKIRLYTDGLKVPIQLVIAEFESFGHQIEVVDESNMYKS)---IFISYGG--P-DEDSVSMLNKKLKTVGVTTWFFPNDSIPGQKLhrMMHEGVNKHD-KVLLVCSKCSLTRAGVLnEIERVLEREAKEgGSDILIPVTLDQY-VFDGWKPDRTDIADQLKSRVIINVDPTDDNYDLSIEKLVK(ALKS)}*

$1573=176(179):
>gi|742413708|ref|WP_038892826.1| {|0(259)|<Proteobacteria(B)>}hypothetical protein [Vibrio campbellii]
{(MNK)--NIFLSYSWSdmkiaDQIEEDLSRLQ--------LNLRRDVRDLAYKNSIsdFMES-IRESDFAILLISDSYLRSKNCMKEVLHLLKDRNyEDkilPVIVKGTGIYS---TAGRLAYTRHWLEEQENLKEVINTLPATSVVSEISELKVV(ESITSQINDFLTYISDKKNLTFEELKAESYSSILDC)}*

$1574=206(220):
>gi|746224793|ref|WP_039273839.1| {|453(0)|<Proteobacteria(B)>}hypothetical protein [Enterobacter hormaechei] >gi|742986475|gb|AJB71109.1| hypothetical protein LI64_11370 [Enterobacter hormaechei subs
{(QTASTLLHLAEAKGLPIIPFTFLGGVARRSYERRDWSRLNPGFDHSVLCDVDGVEHTIEIANRLILDRMQPSFCPERPKV)---VFISFAH-QD--SVIANELRAALVKEGIEVLVgDDEIRSDQM-VtaTIEQAVLRSDVCAVLWSRQYAQSPWCYDEMSLAINQQEY-GKIKVW---------LFNLDDSPIVPSQARKLPAISVRSGNAMHQCVHELLGKS()}*

$1575=221(225):
>gi|743821506|ref|XP_011021441.1| {|799(0)|<Streptophyta(V)>}PREDICTED: TMV resistance protein N-like [Populus euphratica]
{(SFYYLKELKEWKHASFSNIELSFHSYEPGVKVKNCGVCLLTSVYITSQPSVHFIVTSKKAASLYKASLASSSSYHQWKAN)---VFPGIRV-SDTGDAFT-YLKSDLAQRFIIPF-EMEPEKVMAIrSRLFEAIEESELSIIIFARDCAYLPWCFEELVRIVGFVDE-MRSDtVFPVSYDVKQSMIDDQTESYTIVFDKNVENFRENEEKvPRWMNILREVEIL(TGSRR)}*

$1576=206(220):
>gi|743402893|emb|CDH98273.1| {|30(0)|<Echinodermata(M)>}Toll-like receptor [Paracentrotus lividus]
{(SSASLKPLRDKHLLEFNTNEFCSVNITLICLVSLVVVCLILVTALGYHNRWQLRYRLFLVRLAAIGYKEMREAREHNDYE)-FDLNIIFYDEdeEWIRDHLRPAMDERLPQFQRNVFGDEDLVLRMHYLdSVDYVVSHSYKTVVVLSRASVSDRWFMLKFRTAMDHVSDTGTEFVVVVFLEDIPDEEIPFLARLY-LSDGRpYVYW---------------TGR()}*

$1577=216(220):
>gi|746191850|ref|WP_039250003.1| {|73(0)|<Proteobacteria(B)>}molecular chaperone Tir [Acinetobacter baumannii] >gi|734951066|gb|KHO17358.1| molecular chaperone Tir [Acinetobacter baumannii]
{(KISELQKKKTDIKNKLIKENQNLAKAEAVERKKIDALVKKQQKEEIEHQKKLKREIESIKASTQLTGISTSPYIDMTPEP)EYDLFISHAS---EDKEdFVRPLAETLQELGIKVWYDEFslKVGDSLRRkidSGLRN-----SKygTVVLSADFMKKEWTNYELDALVAREMNGHKM-ILPIwhKISKTDvinyspN-LADKVALntsISTIQEIA-EQLADVIL-EK-----------()}*

$1578=226(218):
>gi|746331613|ref|WP_039377246.1| {|0(1118)|<Actinobacteria(B)>}hypothetical protein [Mycobacterium

setense] >gi|734974378|gb|KHO24852.1| hypothetical protein QQ25_03700 [Mycobacterium setense]
{(MS)--RLFLSHSSLDLRQAmalkQWLVNQEPALANE-IFVDV-DPevglQPGQRWKDALQRATARCEAVICLLSAN-WEASHECRvEYRVAENLN-----KQILCARLEPSAGD-HLTAEWQRCDLFGGPttkihVDdgppvefATAGLY--RLRDAIRGAGIS(AMSFVWPPPGDPERAPYRGWEPFEQVDAAVYFGRDAAIVRALDELRGMRLTGVRSLFVVLGPSGAGKSSFMRAGLL)}*

$1579=207(220):
>gi|746068110|ref|WP_039136038.1| {|141(0)|<Bacteroidetes(B)>}hypothetical protein [Flavihumibacter solisilvae] >gi|743029827|gb|KIC96259.1| hypothetical protein OI18_00365 [Flavihumibacter solisilvae
{(TGNQRGEAGNVFAALLDTAIFWLENDLPLNVIRFCVIDGDDADTAKNIFREKRNAYQKKLADTRDLAPAAAAPPPPGYDY)----FISYAHVhTSEVEEFVKAMKE-KNQGL-RIFYDRSsIPAGGQWIrLISDAIQQAESVIYVLSPQYSKSDVCWDEFQCAKLKEYR-LRKPVI-----------KTVYLYSDKELPPImgIYSYIDCTEGDLQKFRSAAQQIS()}*

$1580=213(214):
>gi|746214538|ref|WP_039266539.1| {|0(42)|<Proteobacteria(B)>}MULTISPECIES: hypothetical protein [Enterobacteriaceae] >gi|734997798|gb|KHO35312.1| hypothetical protein PI91_15160 [Enterobacter sp. FB]
{(MK)---VFFSY-S--QNDKEVVNRVKERLYKNGFNVFDQNNDVSTGSNLlsAINSAINDSDA-FLFFISKNTEKSHLMQqEISLAVS-NKLNgKEIKLIPIKLDEtV-EIPFFLKDYFYLDASgpSGFEVAMEQLITdLRKENKSSIKNDQ(AIKFEKLKIERELFLIESIQRDEYEKYKTRQISFITAMVVMVSLLIASLGVFGYFININNYYFELLVAFCIG)}*

$1581=284(295):
>gi|743935216|ref|XP_011011969.1| {|1363(90)|<Streptophyta(V)>}PREDICTED: TMV resistance protein N-like isoform X3 [Populus euphratica]
{(FYLSFDYLKELKEWKHESFSNIELSFHSYQPGVKVKNCGVCLLYSPSSQSSAHFIVASKEASSSYKASLSVSPSYRQWMQ)--DVFLSFRG-ADPTHDFT-HLNTALALRVI-VPDDEELEKVMAIrSRLFQAIEESGLSIIIFARDCASLPWCFDELVKIVEFMDE-MRSEtVFPVSY--------DRTESYTIVFDKDGEDFRENEEKvQRWTNILTEVAIS(SGSIRLHLTDAELVLYLLKRKICENSFKFDTIPYVDVYKWDPEELPELSPLEGRQWYFFGPRYRRYPRTGARLNR)}*

$1582=273(271):
>gi|743921575|ref|XP_011004852.1| {|1064(0)|<Streptophyta(V)>}PREDICTED: TMV resistance protein N-like isoform X1 [Populus euphratica]
{(LSFDYLKELKERENEACSELELSFRSYDRGVKVENCGVRLVNSPYTPSWQSPTGHLIVASKEAASSYMDSLANSSSYCQW)KHDVFYSFRG-EHTSYNFT-HLYTALAQR-GII-RGDTELEYLRVIesSLLRYIKESGLSIIIFARDYACSDWWLDELVKIVGfMEKM-KSDTVFPvstVSYNREQSLVDERTESYTIVFDKD-EEGFSEDKEkvQRWMDILTEVAVS(SGSESSKRVNDMDWPVGNRFHGTDMAFVQIFRKKVLEERSDLDFEYDVTLK)}*

$1583=138(143):
>gi|748150280|ref|WP_039724620.1| {<Cyanobacteria(B)>}hypothetical protein [Lyngbya confervoides] >gi|746962993|gb|KIF20510.1| hypothetical protein QP59_19410 [Aphanocapsa montana BDHKU210001] >gi|746

{(MQ)PYRVFFSH-G-
SDDKYVVANLLKPKVEATGAKVFLDAGEIEYGDDFrrKILDELAICDELLVLFTRSSLRRPWVLAEVGAILVR
N-----KRIVAIRYGPSESELQELGILSLLGTNNLLNLTDEDVDKYLSQLSDRVENHN(A)}*

$1584=267(275):
>gi|746957737|gb|KIF15276.1| {|365(748)|<Cyanobacteria(B)>}hypothetical protein QP59_33035 [Aphanocapsa montana BDHKU210001]
{(QLLNILQTDAGYYQRHKVALTQALKWDRQSRPPGLLLRGNTLRQFEGWLAVAQERSQSPALSLQTEFIEAS
RNQPEDLTL)--DVFLAYADEDVEFAAQLNAALQNQGKS-
TWFDEESIDAAADYQAELNEGIEQADNIVFIVSPAAIALTS-CLkSLSYAASLD-----
KRIIPVQLQPVEvtSLPPAIQALKAIDFQT--
YRSDFYEKYSELVRTLDRDRDH(IRGHTKWLNRSLEWEAEGRPNDMLLRGNELAVAEGWLQQAIALHKLPVPS
ALQKT)}*

$1585=287(288):
>gi|746957737|gb|KIF15276.1| {|154(939)|<Cyanobacteria(B)>}hypothetical protein QP59_33035 [Aphanocapsa montana BDHKU210001]
{(GLLAIFARDRDYVHHHTMLLQAALTWEAHQRQTPFLLTSEQRSQAESWLHQRFSDRQPPCEPTVLHCEYIT
ASIKQAQGG)MTAAFLSYADEdKAVTEQVRQ---LLMREGL-
TVWTNyTDLTTGEDFQsAIDRGIEATDTLVLVVSPAAIASAYCYHEVQYALSLHK-----RIVPLLYQPV-
AGEplVEDWAAIQSLQYIDVTHWsaatpvPQRD--
EPAVSQLLNILQT(DAGYYQRHKVALTQALKWDRQSRPPGLLLRGNTLRQFEGWLAVAQERSQSPALSLQTEF
IEASRNQPE)}*

$1586=167(173):
>gi|748153135|ref|WP_039727462.1|
{|0(60)|<Cyanobacteria(B)>}hypothetical protein [Lyngbya confervoides]
>gi|746957883|gb|KIF15422.1| hypothetical protein QP59_34005 [Aphanocapsa montana BDHKU210001]
{()MNFAFISYSREdEAYVNKLTKAL----
REHGIEFWIDLKASYGGSWSpEIQQQIEDCSVFILVMSPSSRQADWV-KaELIWARQRKK-----
TIFPISLQGE-
QWFEVAYVSAHMVAEDElpPLRFFKEIKREISKQSGASTKRK(NVKSRKKIYFYVGLATLIILFTGDLWMPAT
WKK)}*

$1587=248(254):
>gi|748150307|ref|WP_039724647.1|
{|35(772)|<Cyanobacteria(B)>}hypothetical protein [Lyngbya confervoides]
>gi|746962968|gb|KIF20485.1| hypothetical protein QP59_19250 [Aphanocapsa montana BDHKU210001
{(QQSLVNWLLRHYEAQAETIAQNINQSQQQVQASLDTLTQQGLLKTIDRQGTTFYRVKLAPKSGRQMPTDVW
QVLDRDTKQ)-ANVFISYSRRnKEFVQELHSALEATKR----
EVWVDwENIPVAVDWWqEIQLGIELADTFVFVLSPDSVASKVC-GqEIEEALKHNK-----RLVPVVCQDV-
QPDQVHPELARLNWIFLrtQDDFQQGFKNLLEALDQDLDYVR(THTRILVRALEWERNGLDTSYLLRGADLDR
ANHY)}*

$1588=246(232):
>gi|746968330|gb|KIF25679.1| {|0(68)|<Cyanobacteria(B)>}hypothetical protein QP59_12365 [Aphanocapsa montana BDHKU210001]
{(MPIQPISETQDRS)-
TNIHIAvlYAKAdQEAWEELEKQFTSINaRYPNVHIWTATDIDIGGSVErQLKDELKRADITLLLFSPEFVND
D-IVDeEVRDLLttyarfsdsDAYRK-EERFIMPILLNHlY-GWddiydKHFDIENLRVF--DKlpEAN-
EDRG--

KVYESIAQSLEEY(IARINAKSVQIALPTWIGFIPSIMYNGGFARNEDTPLFQKYRREVHFELNDHLDQLCED
FKAGQIDMMWCTIDRLPYVA)}*

$1589=277(279):
>gi|910241161|ref|WP_050046152.1| {|131(306)|<Cyanobacteria(B)>}ATPase
[Tolypothrix bouteillei] >gi|744447392|gb|KIE08230.1| ATPase,TIR domain-
containing protein [Tolypothrix bouteillei VB521301]
{(VSYTIGTNQTIGDNAAIAFSRAFYDALAAGKTIEYAFELGSACLVGLSEHQTPVLKKKSQDELSSLKKEIE
KLPDTKPTE)RKHIFISYKrnA--EPDEAVALELYHSLSQH-HEVFIDQRMVV-
GTRWaeCIEKQLRKSDFLIVFLSS-HSVHSEMVGlEIQKAHELAQLQgGRPAILPVRL-AY-
REQFQYPLSAYLD-
SINWAFWQDDSDTPRLIAELTQVISG(GELSIGEAQKPSLLRVSEASPFPRPFPSAQLEMPEGTMDSESQFYV
ERSCDAIALRTIE)}*

$1590=141(144):
>gi|746600302|ref|WP_039626495.1| {<Proteobacteria(B)>}hypothetical
protein [Vibrio sinaloensis] >gi|745638554|gb|KIE20081.1| hypothetical
protein SE23_14195 [Vibrio sinaloensis]
{(MKRI)----FISHSHKdsayiaDikgvrlndnhslSFHDR--SLADPVY-
NAFGHINRRSPEDPASKPVrdEILALLRQSDKLIVLVGNDTHSSLWVQWEIEQFSRIHGERaimLMRT-----
--------------PDNHRGGAPAIVRHLSVEGWDLQYLSRWARR()}*

$1591=177(166):
>gi|748152702|ref|WP_039727029.1|
{|0(320)|<Cyanobacteria(B)>}hypothetical protein [Lyngbya confervoides]
>gi|746957581|gb|KIF15120.1| hypothetical protein QP59_31970 [Aphanocapsa
montana BDHKU210001]
{(MTFAQ)--DVFISFTHidnqslnpDedgwiSSFHKALE-IRLSQLRGKESRIWRDRR-
LWGNEDLdeAIFDALTQSALLVSVLSPRYLQSEWCMRELQRFCQAADQGgglRVGEAKARLFK---
VIKTYLPIEQHPAVVANFLGYEFYEIDDAGRPQEFDRVYG(PEQERKFYAKLNDLAYDIHQT)}*

$1592=302(300):
>gi|747099276|ref|XP_011097686.1|
{|114(582)|<Streptophyta(V)>}uncharacterized protein LOC105176546
[Sesamum indicum]
{(PPVSYCGSQSDDIPSTSYTPPPERHDFSDDPANTKLKIVTCVPVSGPDTAPRISFSFPIPRVSFAKGSVSP
ASNAKLRSC)--DVYIGFHG-QNpnlIR--FCKWLKSELEVQGIACFVADRAKYADN-
QshEIADRVICSVTFGVVVVTNYS----LLNHlsLEEIRFFAQK-KNLI--
PLFFDTDaneiaslfnPHADNKECKEALDGLMRCHEFKLEANE-
GNWRSCISKASGI(LRGKLGRKSVAEKEVDMYEELPFPRNKYFVGREKEIMDIETAFFGCGDYLEQECGMPAT
KGGTAGQSDGLADGESEMDRI)}*

$1593=195(191):
>gi|754610457|ref|WP_042000071.1|
{|0(620)|<Actinobacteria(B)>}hypothetical protein [Streptomyces sp. AcH
505] >gi|747159731|gb|KIF67136.1| hypothetical protein HY68_36115
[Streptomyces sp. AcH 505]
{(MTPHTAH)-HDVFLCYTW-PD-
GKEWADAMYDALTERGLDVFQDDLSVPEwdEEEMlpTVRAALLGSRVMVPLLTPRFDESDHCWSELHLALSCA
KALGGTamcRVMPLTWNVRPKNVRPKALKRRKLTREGRSVEELAKHVAATVYAVLKADDR(RFGDAMGGGTSW
VTDALPGEFGFVGRRKELWALHDAFQELDGTL)}*

$1594=199(220):

>gi|748310699|gb|KIH42972.1| {<Nematoda(M)>}hypothetical protein ANCDUO_27036 [Ancylostoma duodenale]
{()-------------------------LKGY--
KVFIDVDKLYAGKFDsSLLKNIQAAKHFILVLTPNSLDRLLNDHngDDWIHKELKCAFeYQKNIIPIFDQAFE
fpQNEEAIPQDIRMITKYNGVKWVHDYQDACMGKVVRFIEGE(LNRTPSLPASSNSAPHSRRAVGGRFAASQS
SIRQTSTTSHRAIGRGGEHPPSTPTFTPASSTDKNRRKHYTSVTTVYDRN)}*

$1595=204(208):
>gi|748211897|ref|WP_039782585.1|
{|0(891)|<Actinobacteria(B)>}hypothetical protein [Nocardia cerradoensis]
{(MG)--RVFISHAGAETAQAlaikTWlIAQRPELEG-E-
IFLDRErmgGIPVGTRWKDALARANARCEAVICVVSQRWL-SLRECVvEYRTAETLG-----
KRILPVRLEPIPhgE--DITREWQACNLFG---
DPATAITVEGFAEPVLLNTDG(LHRLLIGLREASIGAETFAWPPDNDPGRSPYRGWNPFDDVDAAVYFGRDTQ
ILQGLNRLRGLRAAG)}*

$1596=249(257):
>gi|748281287|ref|WP_039822945.1|
{|180(774)|<Actinobacteria(B)>}hypothetical protein [Streptomyces xinghaiensis]
{(PLTDRIGDIERWTDAQIASSVFGPTFDRQWYWALLVGAGGLLAAGCCLLLRNAHLRATAGTDPFAEGSGGF
GAPAESTSR)---VFLSYSRKdSDYARRLCARLEG--RLGEI--
WVDwQAIHPSEEWReSIAEAIRTSDAVVVLLSRDALASPYCWDECRQAIKQRK-----RILPVVIDPE-
LERGSTSRLLRERGWGEltAYQNLSLADPDEDELAQGVQDII(AFVHQHHRWVAFQVRLGVLAHRWWESGCSD
GLLLRAD)}*

$1597=293(299):
>gi|696122434|gb|AIT40188.1| {|74(5)|<Arthropoda(M)>}Myeloid differentiation factor 88 [Rhipicephalus microplus]
{(ELLKERGKSPTASLLDEWDARPVATVSLLVQHLMCLERYDVLDDIEAELRQDIEFYRRKQSGGISDVIRPP
TVRSIRTPI)-YDAFVCYtSQDWPFVEVLIEKLESLG--
LRLFLPNRDLKAGVLQYSTFYELMEKwCRKTIIVFSPEFLESQECRVQQTFIESINNEQAQQKLIPVIVKQCv
LKGTIRMISKINLCDNT-----SKQAE-
WNWIKLIESVRCD(GSSLFVRTSATTTPLIPPSLVMSTAPPLGTKPARAVPMKTPVKDENESTSSTKKWWKIF
APRKKTSSMSSDSSGFQSMG)}*

$1598=182(187):
>gi|742510004|ref|WP_038964932.1| {<unknown(X)>}hypothetical protein [Vibrio vulnificus]
{(MAK)---VYLSYSG---SDRAAAITLSESLKDNGHEIIMDVD--SLLPGQdissTLFQNL--
RKSDfmIVLISDNSNNSKWVMSEIGAALSYSSERPQFSLIPVVLGEPEipD-TIKHKLYLQSISGNI-
EEVIPKIL-ESMSLLLGRREAR(KEKSDNIKRSTDDYIKSIISDLEIKTKKLKANLLPLERHWFFCS)}*

$1599=120(140):
>gi|749916457|ref|WP_040311810.1| {<Bacteroidetes(B)>}hypothetical protein [Bacteroides coprocola]
{()MKTIFIAFAIEdERMRDFLKGQ--
SLLTNSPFEYVDMSVKQAYDtDWKnKVRTRIRRSDGVIILVSKNSLTSSgqkweiQCAKeEGKKILGiwAYSD
-DRTNIVGVLTKTW-TWDN----------------------------IKNFINSL()}*

$1600=254(278):
>gi|749530641|ref|WP_040167126.1| {|29(27)|<Proteobacteria(B)>}TIR domain-containing protein [Klebsiella pneumoniae]

>gi|1030443206|emb|SBI26255.1| Uncharacterised protein [Klebsiella pneumoniae] >gi|
{(FRGHGVPTQDSYPHNSKRSYAKDMLAMVDENKIFTIAGELGLVDASNLTDALQQVDVGFWKPGHFRLFLSH
LASFKIQTS)--------------------
HLQTALRKFAISSFVAHeDIEPTKEWQnEIEAGLKTMDALAAILMPGFQESKWC-DqEIGVAIGR-----
DVLIIPIRkgLDPY-GF----
IGKYQGIQANGktIGEVAEAIFGTLVKSPKTKNKIL(LSLAGAVSNANSVEEAIEKISIFKTLEGIPISILDN
LKQQVMENQHLIESKEFLRDFN)}*

$1601=254(275):
>gi|750137698|ref|WP_040441776.1| {|12(0)|<Chloroflexi(B)>}hypothetical protein [Ktedonobacter racemifer]
{(NLLDSSNKAIHSLFLRDCCMERRSLARRCSLCLLESEVKKMLQVCCCYASEDTVSFSEVKGLFGRSEDAVS
FSEVKGLFG)-------------------------
RFEQLVSWHYREISPGTERYgEEATLLRKADMIFLLISPGFL-SAISESgLLDVVLERQRT-
QAARIVALILHPS-NWQESWLSQLPILpawPESGhpLTREHpryDRS--
LIQANVAIGIYKV(IRNLIAEKADRAWIPFPEFCPEDGGSQMIMLTTQQGEQIFGCDGCFQLWRGNPWM)}*

$1602=155(144):
>gi|749987449|ref|WP_040337140.1| {<Actinobacteria(B)>}hypothetical protein [Candidatus Blastococcus massiliensis]
{(MGNQ)-HNAFVSHRHEddalvgR-LKDLLKRHSCDV---------
RDSSVTSATPNQakSedyIKSILaDRIRWagkIVVIVSPQTKDHEWVDWEIEYTQKF-
PDKriiGVWapdadrcdlpealerhADAVVEWD---ADA--------
VVAALDGEDNWHGPDGAPVPPRAVNRIGC()}*

$1603=224(223):
>gi|751564589|ref|WP_041033491.1|
{|0(209)|<Cyanobacteria(B)>}hypothetical protein [Tolypothrix campylonemoides] >gi|750919329|gb|KIJ79202.1| hypothetical protein SD81_08350 [Tolypothrix campylonemoid
{(MIARI)--
KVLISHYHEeRALADAWKELLKSTSLGAIEAWYSSdsHAAGGMTIGIewrdQLHEKLAESNFILAIQTPTSAG
RPWVMWECGVASGIDKE-RgIIAIIYSMKPGDLANPISSYQLYQGEDENQ--VRQVCER--
LTVAADLHPEPH(VYTEPLKRYFNAIKLYCPRKAIHPEQIEIWRDRFEKLIQSGRVNEIFAMRQQMYASLGKP
FNPVDPSVHELLSKILLD)}*

$1604=188(197):
>gi|750505409|ref|WP_040787279.1|
{|0(234)|<Actinobacteria(B)>}hypothetical protein [Nocardia pneumoniae]
{(MA)---VFISYHSSKRPVVEHIATYFARHDIS-TWWAPRDVPPGSDWDEAISRAIKNSKALVLLFCASA-
DSSVHVKrEIHLADKAK-----IPIYWVRLEQVQpeRLGYYLSPRQWVDWLD--
RRDSTLDSLVKDLRRPGLSGRV(LDADATPRPAEPVPVSGWPAGVAVFENEREAAEAAAHAYFRIARDASDRS
VVLPT)}*

$1605=213(232):
>gi|750511647|ref|WP_040793517.1|
{|0(806)|<Actinobacteria(B)>}tetratricopeptide repeat protein [Nocardia paucivorans]
{(MGVANVVPMSAQPAPPGS)---IVVAYTSADTVWALWLQQLGTESGLE-FRLHR--
IEAGAGLPTRPEFGT-----LILVISDDFVRDIDFSPdDWAGYAADP-----RGLLAVIV---
AsaEVPPATHAIPAADLRL--LpDEKQARA--
RIRRALGRTDPT(VPRATLGQAGHMRVRYPNQQMFVDYRSTSVPQHQPNWFYGRERELEALRDRLDISGAVVL
TGEPGCGKTSIAAA)}*

$1606=188(197):
>gi|750559606|ref|WP_040841475.1| {|0(233)|<Actinobacteria(B)>}TIR domain-containing protein [Nocardia brevicatena]
{(MA)---VFISYHSSKRPVVEHIARYFQRRGIS-TWWAPRDIPPGSKWDEAISAAIKQASALVLLFCASA-DASVHVKrEVALADRAR-----IPIYWVRLEQVEpeGLGYYLSPTQWVDWLD--QRDSTLDNLVNDLKNPAVDGQG(LGPAQGPPVARQVVSGWPAGVVAFESEEMAAEAAAHTYFRSAQAAPHRSVVLPTG)}*

$1607=172(175):
>gi|750636469|ref|WP_040918334.1| {|0(159)|<Spirochaetes(B)>}hypothetical protein [Leptonema illini]
{()MGKIFLSHSSKdKDRIGQIANALGP-----DVVIYDELSFDDGQAIVdEIENGLDNSDMFVLFISDASLNSTWV-NrELAAAQTRFRD-NVLRFLPIIIDQK-VNYTDPRIPDWVRETYIlrYNGSLKKIIRLIKQKRIERSWQL(FPHLRQKDRLFVGRNAIIDNIESKLYHVTDKRPNT)}*

$1608=207(190):
>gi|751565624|ref|WP_041034523.1| {|0(278)|<Cyanobacteria(B)>}TIR domain-containing protein [Tolypothrix campylonemoides]
>gi|750917252|gb|KIJ77140.1| hypothetical protein SD81_10245 [Tolypothrix camp
{(MGFEDD)---IFISYAHIdNHPLTEgqegwisdfhraLQIRLAQL-RGETPKIWRDLKLQGNDYFGdTIVERFPKVALLVSVLSPRYVKSDWCVRELREFYKAAAttggvrvA-DKSRIFKVIKTPV-ERKQHPQEVQELLGYEFyqFDQSGRPIEFSKIFGAEAERNYW(TKVNDLAYDIKQMLDNFSSHGNGTGEQTPIAPTGTTIYLAETTS)}*

$1609=236(238):
>gi|750365168|ref|WP_040647084.1| {|67(307)|<Firmicutes(B)>}hypothetical protein [Ruminococcus callidus]
{(NLKKLLYLAKNKKATIYPIAMDKETRKPLEIIADKQSYDVWEQLRCRDLSDDYLPLAARCFARKVIANIMPTMYSESGLI)----FISHRRLdgeeitAQLCDTLS------VQFKACETFRDVTSVKVGEEAqsEIDKAMSESDAFIFIHTPESADSKWIQKELRYAILRNIPVlwvQIENADIHKLK---FVPSEKPHLSYSLDEFKDIKRLTEITDEIMERTFDLIMTK(SNVVFDCHNALEEMFEDK)}*

$1610=189(185):
>gi|750470224|ref|WP_040752097.1| {|0(607)|<Actinobacteria(B)>}hypothetical protein [Nocardia transvalensis]
{(MINIEGEDAAEKL)-FDVFLCYK-SedlavaLALHQAM--------KSRGLEVFLDKIEGEIWAPLtrSIEQALARSRTLVALITENFPISPHCREELhvalsaAYYLDAGETSrvmAVVQGISPDHV---RPAQLTMYRLPYGGAPPAELVETIVRNVQAHDGFIGDAPT(VTVRNWYPQELPGNRYFRGRFDELWDIHSGMR)}*

$1611=177(181):
>gi|751430475|gb|KIM06853.1| {|0(83)|<Proteobacteria(B)>}hypothetical protein KU38_11700 [Sulfurovum sp. FS08-3]
{(MTL)----FISHST-QD-KQTIALPLYQELLALNLDPWIDQEGIEPSDSLfqKINQAIAKSHYGVAIVSPAFIASEWTNRELQVLDNLRLEKRIKRLFLIYHEITREKVLCYYPLLSDDLALNSSEPIATLAQKIAEVIESKSKLE(RFVELKRENRAIDTQEFIEKLPDIQGAFYLLLNLQVDS)}*

$1612=191(184):
>gi|751605657|ref|WP_041073781.1|
{|0(134)|<Proteobacteria(B)>}hypothetical protein [Pseudomonas sp. W15Feb9B] >gi|751048339|gb|KIK83740.1| hypothetical protein OC71_23120 [Pseudomonas sp. W15Feb9B]
{(MTESDNRG)--SIFISYawgeSFEnKEWVRR---HIvSYLEWNHDV-
FWDRDNIDYGELPDvVISKQLAKRPLtVLCLCDQDYLQSakkeGSGLSrELQMLAEIIGQ-
PGVRVIPLILESG-
CTGHLPDPLINRIYLNLqpLIQRKIDIGMTLADLAEGCSQAV(VQSGINKTLAAFKLRERANTYPRKTPITLW
GNPQNH)}*

$1613=180(183):
>gi|751613453|ref|WP_041081385.1| {|0(169)|<Firmicutes(B)>}hypothetical protein [Clostridium botulinum] >gi|751087431|gb|KIL07591.1| hypothetical protein SR42_00655 [Clostridium botulinum]
{()MVDMFVSYCQRdSVYADNIDLY---FKNKGIVIHRDIRDI---
SSWKsirEYMGTIRDMDYAVLIISDNYLKSFNCMYeVLEVMKEKGYV-NK--IFPVVVETS-
IYSSTGRIPYIRYWQDKykEVQEQISQIDVVNAGNIIDDLKK(TQNIVLSMDEFLATVADMNNPNVSHVNKAI
EDKLQQNGLLNNN)}*

$1614=175(177):
>gi|751430477|gb|KIM06855.1| {|0(54)|<Proteobacteria(B)>}hypothetical protein KU38_11710 [Sulfurovum sp. FS08-3]
{(M)--KIFISYSTQdEKIVRGLEKYIS----
KEMIETWIDHKaIGGGSDLTkAIQKGIQRADIYFVFISLNSLASNWV-DkEIKWAMQREEK-
LKyEFVVPVVLEIE-
ALKRWDNKKLKDRKYITynGDFHAMAHEIKNTIVNKTIEKFE(YQCLLKKDILSNIGNVILTVFLAISFFTTP
SQKEHI)}*

$1615=218(220):
>gi|308490845|ref|XP_003107614.1| {|29(0)|<Nematoda(M)>}hypothetical protein CRE_13282 [Caenorhabditis remanei] >gi|308250483|gb|EFO94435.1| hypothetical protein CRE_13282 [Caenorhabditis remanei]
{(DYSSLDPSHVHVFLKSIDPQLCLYTYSMLKNGITLDILTASPNADDLMDTCGIKSKVHRLQLTTTLKKLWP
EPHKVIPKI)RLDFFISYRQDTGAELASLIKMHLELRGY--
RVRMDIDKNGEQDISeSLLRDVQAARNFLLVISPD--
NPYSKDIpcHTQLHKELRTAIqNEKNIIAVYHKDYdEtrDTICLLPYD----
KQYLRVNWVHDYQDACVQRIVKYM-YK()}*

$1616=234(230):
>gi|310706344|gb|ADP08783.1| {|699(0)|<Echinodermata(M)>}toll-like receptor Tlr1.2 [Strongylocentrotus intermedius]
{(SLASLEPLREKPLLDFDPNKLCGLNIILVCLTPLAGIFLVVIVAVLYHNRWLLRYKLFLLKLMVIGYKEMR
DARDHNDYE)-
FDLNIIFYDDdeEWVRQQLQPALEERLPQFQRNVFGDKDLILGMHYLdAVDYVVSHSYKTLILLSRAAVHDRW
FMLKLRTAMDRVSDTQTEFVVVVFLEDFPDEDIPFLARLY-
LSDGRpYQHWTEDVEGhvFFWNALVKNLTIN(LRTNDFIPNE)}*

$1617=182(203):
>gi|308274646|emb|CBX31245.1| {|0(30)|<Proteobacteria(B)>}hypothetical protein N47_E47570 [uncultured Desulfobacterium sp.]
{(MGYEYDVFISYRSTYKKWVKEVFLPIFEESLNEEVAIFQ)----------------------------
EKVTIFLDKDGITGGDNWnnRILNALSKSKCLIPIVSYQYLNSYNCRKEFVIMHHRQIQLgLQTINNPKGIII

PFaihdGEHFKKIITDIEFYDCTDYRIPECGIEAFLKSNAFLELGR(KMNNWAKAVAKSILYVNNNNPWNP)}
*

$1618=219(225):
>gi|489810063|ref|WP_003713919.1| {|72(0)|<Proteobacteria(B)>}TIR domain-containing protein [Neisseria lactamica]
>gi|309378744|emb|CBX22694.1| unnamed protein product [Neisseria lactamica Y92-1009] >
{(PTSFIQTLGIGNIKLNLNEIDRTNPNMIRKELFSLLNLKKRDAIFLFDNTNNYSNWNADLERDLKENFLQH
KMVMNMEPM)--KIFLSHKSInKDKVRNFKTILELLGFSP------
WLDEEAMSAGAnlerSLHSGMVDSCAAIFFITPDYKDEKYLATEIDYAISRKRQ-
DEKFSIITLVFSDENGEKGVVPNMLQPYVWK--EPANDLLALAEILKALPLEVG(DIRFK)}*

$1619=156(158):
>gi|308272306|emb|CBX28912.1| {<Proteobacteria(B)>}hypothetical protein N47_B20580 [uncultured Desulfobacterium sp.]
{(MIISQTADDSYWSC)----FISYSSKdAEFVKYLKERL----
RGELISVWLDfDQIVHGGQLTeQIKSAISKHDKLLIVLSKNSLESDWV-EkEIKEAL---KK-
DTKVLFPIRITSV-DeirkhdfiwdtFKDVPILDFEFDPEHEavNASFED-----
SFWKLVKSLKKA(KGSS)}*

$1620=174(173):
>gi|308274655|emb|CBX31254.1| {|0(216)|<Proteobacteria(B)>}hypothetical protein N47_E47660 [uncultured Desulfobacterium sp.]
{()MKPVFISYSREdLHEVKEIVSILQVAG----
IPVWQDINSLDVGLTEdQIRTAIrEDCAALVFYATSNSIVSDFIKDiELKEAYQKfKSD-
NKFSIVPIFNESI-
DKVNAALKGVIDGNISRfnGIVIEPGKPLMEEISQKLRRNLL(SIVLGQSQEKRVRIAVMTYSPTPDDAKPML
NLD)}*

$1621=191(223):
>gi|2853079|emb|CAA16929.1| {|0(1524)|<Streptophyta(V)>}resistance protein RPP5-like [Arabidopsis thaliana] >gi|7268748|emb|CAB78954.1| resistance protein RPP5-like [Arabidopsis thaliana]
{(MVD)------------------------EFQRKGVSVFASEDSASDDR-
FaeESDAAIAKARVSVVIFSENFASSKGCLNEFLKVSKCRRS-KGLVVVPVFYGLTNSIVKKHC-------
LELKKMYPDDKV-
DEWRNALWDIADL(RGGHVSSHKRSDSELVEKIVADVRQKLDRRGRIGVYSRLTKIEYLLCKQPGCIIRSLGI
WGMAGIGKTTLARAAYDQLSR)}*

$1622=202(220):
>gi|15230522|ref|NP_190724.1| {|0(1051)|<Streptophyta(V)>}Disease resistance protein (TIR-NBS-LRR class) family [Arabidopsis thaliana]
>gi|6572077|emb|CAB63020.1| disease resistance-like protein [Arab
{(MVDCSD)---VYISFDR-SEdtVRYSFVSHLCAAFRRRGISSFIREN---
GSDSEsNGFSKLETSRASVVVFSEKYSSSKSCMEELVKVSERRRK-NCLAVVPVFYPVTKSFMKKQ-------
IWNL------
GDVRSDWPSALLETVDL(PGHELYDTQSDSDFVEEIVADVREKLNMSDNIGIYSKLGKIETLIYKQPWGVRSI
GIWGMPGIGKTTLAKAAFD)}*

$1623=229(226):
>gi|21655199|gb|AAM28914.1| {|0(119)|<Streptophyta(V)>}TIR/P-loop/LRR [Pinus taeda]

{(MASSSSSSNKSHQ)---VFINHRG-
VDVKKTFVRSLYRRLREKGLTAFFNEEEMQGGCEIssQLADAIRTASVHVAIFSPRYVESEWCLNELLLMLKS
N-----GPIVPVFYRVKPQDVRWMlglygealatheMKGRHDPSTIEEWRNALNEV--
ANKSGFELQKDN(SLLWMELLDKVVKRLLEMVQQQDRKPQILGIGGLGGVGKTTLLKAFLYRLLLYVAYTSIS
CLLPEELPSLETL)}*

$1624=258(244):
>gi|21655205|gb|AAM28917.1| {|0(729)|<Streptophyta(V)>}putative
TIR/NBS/LRR disease resistance protein [Pinus taeda]
{(HEATLSSMASTSYGSSNTNNSFVF)--DIFLNHRG-
PDSKKTLAKHLYDGLSKHGLRVFLDEDELQAGEYIkpQIESAIRTAWVHIAIFSKGYADSPWCLNELLLMFQS
G-----STIIPVFYDVDPADLRwvthsekgvyarslqtlekkrkADSTPRYDPDTIQKWRKALTDA--
AEIKGFDKKTYN(GSSLEQVVKGVLEKVKRPLNVSTYPTGLDEKVAEFERAMLLNQQSSRETRVIGIVGLGGA
GKTTLAKEIFNRRRSNYKQI)}*

$1625=216(220):
>gi|21655261|gb|AAM28907.1| {|0(75)|<Streptophyta(V)>}putative
TIR/NBS/LRR disease resistance protein [Pinus taeda]
{(LPSIFIILFHRCIQSLMASSSASTSTSRRYA)-YDVFINCSS-
SDDSSKFAAHLCRRLISYGLRAFLDNREMFDSDGLpklQTEIAIRTASVCVPIFSPRYAESIRCLNELLLMLE
SG-----
ATIIPVFHHMTPAELRWTRNGDGVFAQAPCNLEEEKVYDPQTHDMRQRYESN(TIEHWRKSLSHVSSLSGFEL
DAFNGDEGELLDKLVNYVLRKGKRLLNVA)}*

$1626=229(210):
>gi|754287224|ref|WP_041846909.1| {|0(95)|<Firmicutes(B)>}hypothetical
protein [Bacillus thermoamylovorans] >gi|752646512|gb|KIO60327.1|
hypothetical protein B4064_3666 [Bacillus thermoamylovorans]
{(MRKEY)-PTVFISYSW-
DsEEHENWVVFLAAKLRENGVDATIDKFEMQSKTVnlnRMMIEKIKNSDKILLILTENYAkkadafqggvgyE
TNLLIryikdnpDKIILIMRHKGN-YK-EAIPFyldgFYFIDFSD----
DGQFDIKFNELLHRIFQVDIIDVPKVGAKPKLEP(KRINNDRITNETSIIIPNFKEITDLDKNKYMKRSYSEI
LSHLTDFAEQTRQKNSNFDYDKDIIHN)}*

$1627=275(273):
>gi|752611252|ref|WP_041280635.1| {|64(16)|<Proteobacteria(B)>}TIR
domain-containing protein [Desulfococcus oleovorans]
{(AIPESIYLKIVNGKADLQSEIREGLNKIHDIQNEFIEEVFFEMEVPEDHDWRKDSGLLITHNRIIPDPVAS
RIWDKNCFR)---VFLSHKSEVKKETAGL-KDKLKLYGI--
SCFVAHEDIHPTtEWQnEIENALHTMDSFVALLTDGFHDSL------
WTDQEVGFAFgRGIPIISVklgkdpygFIGKFQalTSTwDNAPFEITKILIKKEPMLNAYINAIQECPNYDSS
NKL(SELLPFIERLTESQVEKLIKAFNENGQVHDSYGFNGKNPKYYGDGLPYYLKRT)}*

$1628=173(175):
>gi|752587932|ref|WP_041257320.1|
{|0(63)|<Bacteroidetes(B)>}hypothetical protein [Fibrella aestuarina]
{(M)--KIWITYSYEdKEFVDKIKYFLN----
QSDLEILDiENEILPGDNIVvAIAGAIEKADLILIIILSKNS-GERQWFStEVGLIIsEIRRD-
NHKKIIPIIKDKG-
SVIPPFINQYQFLDLSDekDIDIRLEKLLKTLQIRKISEISD(RKLYLESEIVLSRDELLEYEKYEYEKQRNH
KQGL)}*

$1629=220(224):

>gi|753203691|ref|WP_041510037.1| {|64(0)|<Proteobacteria(B)>}TIR domain-containing protein [Shewanella sp. cp20]
>gi|752458201|gb|KIO36759.1| hypothetical protein DB48_09635 [Shewanella sp. cp20]
{(VASVAQNLKAIFDVFAGLYIGMGNNLNPTGSSVDSVTMPYAPKILLYTNGLKVPIQSLISEFQNMGHQIDV
VDESSMYQS)---IFISYGG--P-DEEAAGTLNKALKSVGVSTWFFPDDSIPGQKLhrMMHDGVNKHD-
KVLLVCSKDSLTRNGVLnEIERVLEREAKEgGSDILIPVTLDDY-
IFTEWNPERTDIADQIKARVIINVDPESENYKQDIDKLVK(SLKS)}*

$1630=248(245):
>gi|754122800|ref|WP_041756049.1| {|277(382)|<Proteobacteria(B)>}TIR domain-containing protein [Bradyrhizobium sp. ORS 278]
{(HDPSAISLPRFLDGIKLDAKMQPVVFWTTPAEAAHQIFDTTGRILSFLNSLTGNGAETIGTGPSAQRYFEQ
YWKRAERGT)---VFISFAG-SGGAAKLADRLARIFRfQN-
LRCFqyRNEDstsggRLESGEDIerGLRKRIDDADIVVYLIEENFAASAFCMEELAQGTKLRDQ-
GLIELRAYSLDGLKARLPALAGTSIHNFRRP-
DWLQREVEEKIVSDVESSADSI(GWALRHEQREQLEEWLKQDGRDNVE)}*

$1631=179(183):
>gi|753954081|ref|WP_041659903.1| {<Cyanobacteria(B)>}TIR domain-containing protein [Acaryochloris marina]
{(MGMEGFY)----FISYSKVDSTDLARKLANDLQriPPSI--
PVWLEERECQGGiDWDeQLVEALRTCSGVLFLMT---ADSVHPRSecKrEWTR-----
ALrYKKPIIPLLvePNVEMPLDSILIFRATMTRHWQHCVAIYNGACRLKGYYLVFKNVS(TMLNGTCPGHQMK
RNRPEFKMRWTRLGKRLLRSRRR)}*

$1632=229(232):
>gi|754225720|ref|WP_041832992.1|
{|436(0)|<Actinobacteria(B)>}hypothetical protein [Actinoplanes sp. N902-109]
{(TEGPGGAAEVAGRRLTGTEFPLLWVQVPDVAVLPGRSFRIDRRPVKILHIDAQGFFRLEPEVCGALHAAVR
LAARDCFEY)--DAFISYNSGDVE-VARRWAEELEADGL--RVFRDEPR-
RGAEFPrRLWLAIRQSRALVPLISP----
RVILRDqrDNWVRREIDAHKhYfgeESRIYPVILAGATPEQIADGFHPIEIGGDEPASIRELAAELKKLRDGS
ELPPV(AMSVRPESPPRV)}*

$1633=121(142):
>gi|754049744|ref|WP_041696675.1| {<Actinobacteria(B)>}hypothetical protein [Pseudopropionibacterium propionicum]
{(ME)----------------
QLVEALRREARQVlddGFEVFFDRHDLRTREEWrtQLAWAVREAEVLMACVSKPYFESDFCLWEFQEYEVKPT
GPdTGEGLVPVLLEDtseQDqpdqEHRDWH-ARVTEMQGQDlR------------------CVHP()}*

$1634=320(300):
>gi|753951921|ref|WP_041658876.1| {|139(262)|<Cyanobacteria(B)>}ATPase TIR domain-containing protein [Acaryochloris marina]
{(AIEFASGFYDGLGANRTYEDAFEFGLSAIDLEGIPETSIPQLKKPDSTDSAPKTPSIQPEPSPSAPDQPGN
SNLSPIENA)--RIFISYkrdATPDEQLALEICQHLNQR-HQ-VFIDQS-
MPIGTYWAERIEEELRQSDFLITLLSEQSVGSemvqGEIETaHRLSKEFKK-----
PTILPIRVkyrEPFAyplsayldpinwafweqeeDTPGLLAeleqAIAGQDLSI--NSDTAKRS--
LLLPAVGAAAIP(QPFPSAQFLELPEGTMELESKFYIERQLDRIALETIERQGVTVTIKGPRQMGKSSLLNRT
MQAARKAGKQVVFLDFQLFE)}*

$1635=200(204):

>gi|754095317|ref|WP_041734609.1|
{|0(1246)|<Proteobacteria(B)>}hypothetical protein [Burkholderia sp. CCGE1002]
{(MVSFPSAVNCEPAVRGAMP)--RIFISHASANNALALAIGHWLADNG--------
WDdhfldlepvkgFAPGERWQQALKAAADRCEAVLFLISPAWC-DSRSCLaQLSLARFLG-----
KTIFGVLVEPTPpdALPKEMTAQWQLCDLA--
TGTQRRSFRVWHDPLVPQTEVS(FAKAGLAKLELGLRRAGLAASSFSWPPANDVERPPYPGLRALEAE)}*

$1636=224(224):
>gi|753216630|ref|WP_041521915.1|
{|0(627)|<Proteobacteria(B)>}hypothetical protein [Gilvimarinus agarilyticus]
{(MDTKPL)--
KLFYSYSHEdDELRRGLEKHLTTLVRNDVIEQWFDGEITAGSDWKsEIVSNLEKSDIFIFLITANWLNSDACt
EEwELAKKISIKDT-GK-RLIPIIGSRC-PWTDFDnMSKYLALPYDGkpVADWTSTD--
SAWLNIYEGIKNV(AMDLKKNFQVRPEFLSDLTSIEFCSKNTTKITLKDIFVFPNLATYDESERIEENLIETI
DQLMKYDYQIIHGEDQCGK)}*

$1637=276(276):
>gi|755165728|ref|WP_042514425.1| {|46(0)|<Firmicutes(B)>}signal transduction protein [Bacillus cereus] >gi|753356550|gb|AJG91367.1| divergent AAA domain protein [Bacillus cereus]
{(IILGIKDDGITLNIKKFAFKLNIDNILDVLDGTVKIECNCFTFEGNNLMHISIDKSDELVKVNNIPYKINE
DGEVEEMAI)-KKVFISYAHKdSDLVNILEEEL---
KQHENIKITRDINVTAYRDSLdEFMKTIRDHDFVISVVSSTYIKSLNCMYEIMHLMQDKDY-
QEKLFFIIVSRdDV-DYynEKNRYKDFEAKIYDvmdrLkyITHWRNKK-----
VELERSINEA(KLSPELMVNLAVDMRKLHSVITPMDDFIKLLSDKVGRSFKEMYEDDFKEILDTINR)}*

$1638=262(286):
>gi|754558746|ref|WP_041952399.1|
{|696(0)|<Bacteroidetes(B)>}hypothetical protein [Spirosoma spitsbergense]
{(GELADWLNDNEDARIKSKQGNKVPVKGFAYLIGKEPIKEEIPIIDPVEPPAIIPTMSTIPDINTPEIFFSY
AWGDEHETG)------------ESREKIVNDLYESLKKDGYRVVQDKKNLGYRKSIsdFMKR-
IGKGNLLIVAISDKYLKSPYCMFEMYKAYRDCKFERDILIEKIFpIRVESLNLAL-TGPKRTEYRQH------
------WREA-
KKAADE(YMGEFLHETTAKEKVEHERLTSTYSKIGDLFEILHDLNADTKDLLSADNFAKIKEAIKERIDSLKS
)}*

$1639=166(175):
>gi|754503332|ref|WP_041913996.1| {<Proteobacteria(B)>}hypothetical protein [Geobacter sulfurreducens]
{(MGGGGGGSISSSDMRTLEDKAKEKLSQAAADTSRH)---
VFISFAYEDVDEVnllrgqAKNDKTDLQFDDH--SVKEAFNSNNADYIKrQIREKIDRCSVTVVYLSGNS---
---SKSDWVNWEIEESFkRGKGVIGVYKGD--SPPKSLPSAFSE-NKCKAVKWSHE---
ELTKAIEAASKKR()}*

$1640=233(238):
>gi|754894525|ref|WP_042253135.1| {|46(49)|<Firmicutes(B)>}hypothetical protein [Leuconostoc mesenteroides]
{(IKDTGTYLEYKGYSFRVPDQTKIQQYLGGFKEFKIDKQLLENKCLIEIIVDSASQSILFKGKLFKFMSDYH
NDTKQLEAL)--KIFISYNHKvSDFADILENQLNTMFGP--QILIS--RDTSLKYKDnidlFMES-
IKTHDFIISLISDSYLKSEACMYEITELMRDEHY-SDKLAFITFDEEDNKLVNTSVEIEDLVPIIH--
GELRFDYQTFWNKRINIYQKK(LDELGSNRPETHLEILKT)}*

$1641=263(269):
>gi|755000182|ref|WP_042353594.1| {|45(13)|<Firmicutes(B)>}signal transduction protein [Bacillus rubiinfantis]
{(IILGIHDDGRTLRVKNYSVPFQVENARKLLNVNVNCIFEPFQFEGKKLSFISIDKSIDLVKVNDIPYIINT
DGDLLEMKT)-KTVFLSYCHAdADLANIVEK---
SLNKHVDISVSRDINITQYRDDLdEFMKTIRNHDFVVSIVSRKYLESLNCMYEITELMKDPNF-NEKLLF-
IIVNK----
DDAKFYKEQNIYDNFeaGIYDFNKRLSYITYWNNKGKEME(RSLKSADLPSEMIAEFALEKRKLVSIIPSTSS
FMSLLQNKIGRAFKDIK)}*

$1642=274(279):
>gi|754986618|ref|WP_042341884.1| {|121(308)|<Cyanobacteria(B)>}ATPase TIR domain-containing protein [Calothrix sp. PCC 7507]
{(HQHINHVVGMNQAVGDRAAIEFAVGFYDALGAGRTYDDAYEFGRIAIDLENIPEYATPVLKSRKPPVAQPE
IFPPSPCKR)---IFISykrHVT--P-DEPIALQVQQALAQH-HTVFIDQIITV-
GTRWaeRIEAELCQADFFIVFL-SEHSIHSEMVEaEIATASRLAKEQnGKPMILPVRL-AY-
REPFQYPLSAYLDAI-
NWAFWRDEEDTPRLIAELMQAISG(GELAIAQANQADLLQPREASTLPQPFPSAQPISLEMPEGTMDGESQFY
VERDRDVLALN)}*

$1643=161(154):
>gi|756159272|ref|WP_042607791.1| {<Proteobacteria(B)>}MULTISPECIES: molecular chaperone Tir [Pseudomonas] >gi|755303826|gb|KIP95098.1| molecular chaperone Tir [Pseudomonas fluorescens] >gi|929886600|
{(MP)---VFISYRH-MDRAHAIA--
INTRLTQAKIRTYLDvlDPESQTTDDItgVITRNITECTHLLAVVSERTALSWWVPFEIGEatisNRRICTFK
TGPAELPIYLDKWPklstaSDLDFFIEAYRDEVSNQRSMTLDSISESVKGAYKRDAEMF(HEQLKNRIRRGF)
}*

$1644=220(228):
>gi|754522458|dbj|GAM96840.1| {|771(0)|<Proteobacteria(B)>}high-affnity carbon uptake protein Hat/HatR [alpha proteobacterium U9-1i]
{(SCGESVDNPRAINARRKRGATQIPCAFCDAAIDLVDVLHEHFAEPASIAAARNIESNIERARSLSISNTIR
EAKEAIGEH)--DVFLAYNSSDRQEVAA-IAAQLRARGL--
NPWFDRERIAPGRaFQtALEEAIRLSKAAAIFIGAKGPAKWQQME---IRAFVSQRVeRDLPVIPVL----
LPGVAEPPQELLFLREFTCVKFADGVEDEsVIDELEWGITGQ(RPQRGHLS)}*

$1645=141(147):
>gi|749954128|ref|WP_040333354.1| {<Nitrospirae(B)>}hypothetical protein [Candidatus Magnetobacterium casensis]
{(MKE)--QIFISSAMQDSEWVSLFFDELRQTGVT-
VWFFTKDknIVQGSSIVDLMEKALRESNVMVVIISKNSI-TSQWVFfEIGVAIADE-----
KRIIPVVIDDVPyeSFPVRLKAAACFHEKS--PSKAAIKVASFLKSESKTGHHK(QLIH)}*

$1646=207(220):
>gi|754522186|dbj|GAN00112.1| {|170(0)|<Proteobacteria(B)>}hypothetical protein U91I_03777 [alpha proteobacterium U9-1i]
{(RIGPTPGTFADLLAGIDAFGVRYGLPGGVTMWLFLAAILVMFGAIAASVASMFTRPRAGSGAAASDAEPEE
APAPPQDDA)---VFVSYARANSDKVMPVIEAAKAEGRK-
FWVDQQGIAAGDGWAGEIVRAIRSAKGVLVMCSKAAFESDHVKReIYLADRYHK------
KLVPVFVEDATppeDFEYFFAGVQFLKLFE--TPEAerpAAVL--RLLEA------A()}*

$1647=198(206):

>gi|754523734|dbj|GAM98116.1| {|0(82)|<Proteobacteria(B)>}hypothetical protein U91I_01747 [alpha proteobacterium U9-1i]
{(MA)--DVFISYPQRERDLMLPLKEKLQRLDLD-LFVDidgRLDSSP--
NFPDALDAGVRDAKAVLACWSPWALTRE-WVKnEC-AIARDL-----
NKLVAVELAPLSvsDVPAEFYRVERKSLVG--
FDGDDAHPGWGAVLSALAIKFR(IWASMHGADPDVERVLEKAARLDRMALAARPSPPNGAGEVRMGPVRERGR
TSRPASIRWTITSS)}*

$1648=169(170):
>gi|749954815|ref|WP_040333829.1| {|0(826)|<Nitrospirae(B)>}hypothetical protein [Candidatus Magnetobacterium casensis]
{()MAKIFISHSSTdkEEIATPLFNHLKTNH-----
TVWYDSeRIAPGDSIAsKIAEGMDQSDCFVLLISDDYNQRAFC-QrEQDAIIYRHIN-KRKRLLIVKINNA-
RISSIIEDLSCIEYTSDsiNIQSICDAIDRSCKTTVTRVDEP(DFYIDKLVSDILQFRSGCMELETYTYITSG
)}*

$1649=165(171):
>gi|754520586|dbj|GAN00561.1| {|0(82)|<Proteobacteria(B)>}hypothetical protein U91I_04227 [alpha proteobacterium U9-1i]
{()MADVFISYKKSdADRVRPLVEHL----RATGLDVWWDEGIQPSTSWRgEIAKQLAAAKCVVAV-----
WTTDSIDPeQGAWVMEEATH-GQARKILAPVRLD-
LVSPPLGFGETQYADLAawSGGADDPRLQHFLGVVQAIVRSE(PIPLAPRPTHFEAPGIKLQGVTMENVNIHV
G)}*

$1650=172(177):
>gi|754521217|dbj|GAM99142.1| {|0(276)|<Proteobacteria(B)>}chromosome (plasmid) partitioning protein ParA [alpha proteobacterium U9-1i]
{()MTNAaFISCSGEdLEQAKVLEN---LLDHAGL-PAWVYTKPLTGSQFPtEITEAITTSSCVIVLVSPSS-
ASSAWVGrELHFAQE-----NGIPIVPVLVKPT-
PTNSALHLRIAGMSRIDatKGFAGEAFDELCRAVKAHYRRLN(PIVAVMNMKGGVGKTTLSANIFGGLFKWRH
KNVLLVD)}*

$1651=160(172):
>gi|754522186|dbj|GAN00112.1| {|0(217)|<Proteobacteria(B)>}hypothetical protein U91I_03777 [alpha proteobacterium U9-1i]
{(M)--TVFIAHAPKnQDAAEGLEKFL---ERRGHFSELDDGQTAL--------
RPLQSSDVFILLLSQDFVFSPHRLRlE-QRALDAWTE-NRLILVKLDHAFA-
PVGLRDLPAVDASFEAQreFKWNELANQIQTLLRAPRPQGAP(PASPPQPTARRSGMGGALLTIAMILPGLLA
L)}*

$1652=189(199):
>gi|754522604|dbj|GAM96986.1| {|0(641)|<Proteobacteria(B)>}protein of unknown function DUF323 [alpha proteobacterium U9-1i]
{(MNGRADLSE)---IFLSYARED--RDRVAPIVEALKAEGLPVYW-
DRQLEYGGNWLqEINAHLDAAPAIVVIWSQYSIASPFV-QaEAMKGFDRQ------VLVPISIEAN-
LRPPAPFGMVQGADFAAgvDRNDGEWRILVDRLKSLVRKAAA(DGVSASPHALSDTVRAARTAHQEKIAGIAG
PLRGIAAGSDVAQPEIARSI)}*

$1653=216(219):
>gi|754523663|dbj|GAM98045.1| {|0(358)|<Proteobacteria(B)>}high-affnity carbon uptake protein Hat/HatR [alpha proteobacterium U9-1i]
{()MADIFVSYKKDdKA---RVEPLVRALESQG-
FTVWWDYEIETGGDWLqQILKEIDVARAVIGVWSQQSVTADGCFAvSRGSTIsyvdREHAR-AQTKLAPVLL-
---DRVRTPFeyEKIQAA---DLTDWgGDAN--

HpGFRQVVARIEQL(ALPLWVERKLLAANAAADTERRRREAAEANAAARESEIERLTRGQVDLSIAQARVRAL
EEQLAGAGMQPKGDRVIARDG)}*

$1654=171(178):
>gi|754524523|dbj|GAM96539.1| {|0(97)|<Proteobacteria(B)>}protein of unknown function DUF323 [alpha proteobacterium U9-1i]
{()MADVFISYKREsLEQVERLSGVLRDLN----
LSVWFDASIHLGEAWAqRILHELDQASAIVVCWTPDALLSDWVLREAQAGIDRE------VLAQVKLEPC-
TPPAPFNAQQIGDLIDWegGDLTHPALKALLARIEKLTGVSN(LVRNAHLRAGGQHDELVAMLRALLVDRARA
GAIPMTYT)}*

$1655=210(202):
>gi|754520778|dbj|GAN00401.1| {|0(97)|<Proteobacteria(B)>}hypothetical protein U91I_04067 [alpha proteobacterium U9-1i]
{(MA)--DVFISYAREdrtraDQIARGLQAMG--------LECFWD-
TEIPPGQTWadYIEGKLAACKAVIVLWSAHSTGSQWVREEA---
RMGRDKGkliPALLDASPapfgfgevqaanlaNWN---
GQTNHPDWvRFSNAVDHAVRGPNAAPRTAPPPQPSFTAAMG(AMTASASPAEENTPIGYIKKCLRLYVNGKGR
ARRSEYWWWTLFVVGVSVVATIIDIAVSG)}*

$1656=177(207):
>gi|749955486|ref|WP_040334288.1| {<Nitrospirae(B)>}TIR domain-containing protein [Candidatus Magnetobacterium casensis]
{(M)-YDVYLSYMAEDkaIVGE--IARLLKDEYHLEVWLDKWNIVPGDSIHGAILKGLKEA-
KTCAIFTGKSTPGGWFKEEISVALSRLAEDTSFRVIPVLLPGGkNDNIPD-FLQSR-----------------
----
S(FVDFSKGIYSEYEMYRLVCAIKGIQPVRGSVHDKFTGLKEFKTLGIITEEIYLETQRRIIDKYIKH)}*

$1657=207(208):
>gi|756977895|ref|WP_042666002.1|
{|0(67)|<Euryarchaeota(A)>}hypothetical protein [Haloferax sp. ATB1]
{(MTEEPATGESAEEREE)EYDAMISYSH-EDS-
KPVANGLYDELTAYGLDVWYDGVELGIGDNIrsSIDRALTESEHAVILISPSYFQG-
MSEWELDGLVQKHKKTDENVILPLLHGMEFEELQEKSPSLANIIGDEVTEDNIDEITAKLYSAIESPDET(PK
LDDVEGVDRVELNVTMEDLVDISKGSKVRVEEWRTSGTPHTSGVTAVKLT)}*

$1658=193(199):
>gi|759016311|ref|WP_043075269.1| {<Proteobacteria(B)>}molecular chaperone Tir [Methylobacterium sp. C1] >gi|758502164|gb|KIU27110.1| molecular chaperone Tir [Methylobacterium radiotolerans] >gi|76716
{(M)------SYSHMdADARRKLETHLAPLKGDNV-
STWFDGDMNAGDKLDtAIARALRNAHVFVGLLSPDYLASHYCWKiEYRRAMNRRAR-GTMRVVAVVVRPC-
DWKSTPAAGFKLLPEDGreVTRWRSAD--
AAYLNVAQGIRRV(VAAVRREIAAASQPAKPKRAAAKATAKAAAKGSAPPKAKKPRARANVARKPSKAGTKT)
}*

$1659=146(150):
>gi|759734824|ref|WP_043445551.1| {<Proteobacteria(B)>}hypothetical protein [Halotalea alkalilenta]
{(MGLVPMK)---IFLSHKG--A-DKPLVREFKQTLALLGFDPWLDEDAMQAGAELerSLLKGF-
NDSCAAVFFVTPNYKDENYLAsEVDYALQEKRKKgDKFAIITLVLDKN-
GSEAVVPQLLHRYVWKEPSNHLQALREVIKALPVQTGSVY(WKQ)}*

$1660=190(185):

>gi|759991413|ref|WP_043675067.1| {|0(443)|<Actinobacteria(B)>}TIR protein [Streptomyces xylophagus]
{(M)-WDVFLSYSR-DDVER--
VRPLAQMLRDGGLDVFTDETGVASFAGIsdTIRRELARSKALLAYYSAGYPEREACQWELttAYLAGLGEGDP
RGRVMvvnpePTSDHIHPVELRDARHAPADDPATLVADVREHVARLDGPMAIQERAVR(RWLSGSPEPRLDFL
GRLSELWQVHSALHAHSAHLVTGSAAPRAV)}*

$1661=219(220):
>gi|764495342|ref|WP_044392675.1| {|61(0)|<Firmicutes(B)>}hypothetical protein [Bacillus subterraneus] >gi|761634947|gb|KIY22540.1| hypothetical protein UB32_07920 [Bacillus subterraneus]
{(KYEKEMTQLMEQVRKNKDDINRYKERVDKEQKREYDKMLKSIQSQTSMNENYMNSVSEVSEKINQLAVDVK
HTAAEKEVI)EFDVFLSHSS-LD-
KEIFVTELSEKLSEKGLNVFEDVKVFKIGQSQtdMMNMGILNSRFVVAFLSPNFIKSGWSDYEFKSFLNREIN
EKRIIILPVWHDVSVEDVRQYNPYLVDKFALS-TNKFTIDDIVEHIFDVVSMSKE()}*

$1662=248(238):
>gi|762154736|ref|XP_011415447.1| {|379(0)|<Mollusca(M)>}PREDICTED: uncharacterized protein LOC105319554 [Crassostrea gigas]
{(KHHIKSKHPVIYIFKRHSTLHGSNFNISLFCDALSLALIVAIPLLGVSIAIVVCINCVYKEDIYLLSRRLK
FPRIDKNGY)EYDAYVCLCESnigivTWSLQTLLPYLESYGY--
SIFFPPRDEAFGGFREEEIIMKLKKCRNYIFLMTEDFEIEGdsenalWCGLMWRNA-
WKNFRERSNDLNIIIVNCDYlRWKNSKNKVLKAYLRQQlAVEFSNKKHEVLDAIRKRMGVPK(ALASVFSATA
SKYNNCNN)}*

$1663=248(273):
>gi|762078880|ref|XP_011456278.1| {|0(220)|<Mollusca(M)>}PREDICTED: uncharacterized protein LOC105348508 [Crassostrea gigas]
{(MARICQNDKIEEIQLIPSSLNLDSDETEVKTVLNSENKGELSTIVTKPLPPGKE)-
YHLFMSYSSEdsDDA-NTLRQHLEER-
FHLKCLYYERDFQIGKNIDESITEGLRKSVKTLLLLSPFYIQSHWCVTEAREACKLSFADmENHNVIPVLLRQ
LHkE--------LPPFLNSYvYID-------------------
A(QREMDVAGKINKAFNQPGYLDPLQMDKSALSCNGTLLIRKRAIKADFYNHGLAYRFLPLEAFEEKKICQNG
VDLVGCKK)}*

$1664=274(299):
>gi|762078887|ref|XP_011456314.1| {|0(82)|<Mollusca(M)>}PREDICTED: uncharacterized protein LOC105348522 isoform X3 [Crassostrea gigas]
{(MAEECSPLLSTSLEADAVEEIHLMSVSPNSEPNETEVKTIVVNNFEERISNETEVKTVAVYEDRDEVVTSD
TTPLPPGKE)-YHLFMSYSSEdrDDA-NTIRQHLEERFY-
LKCLYYERDFQIGKNIDENITDGLQKSVKALILLSPFYIQSHYCVTEAREACKLSFTDmENHNVIPVLLRPLP
kD--------LPPFLNSYvYMD------------------
A(QREMDVAGKIYEAFNQPGFLDPLQMSKSGLQCNGTVLFRKVGSRAKYLDHGLVYRFSPLEPYEEEKISQNG
MDQDADFL)}*

$1665=222(249):
>gi|762096593|ref|XP_011430837.1| {|0(193)|<Mollusca(M)>}PREDICTED: uncharacterized protein LOC105330678 isoform X1 [Crassostrea gigas]
{(MEKNGAHSFLSDTPVTSLTMSVPVLRHPHV)-YHAFISYC-AdaD--
TSHARTILDSVEsRGFTCCFAERDFLPGECTSDVVVDAIHCSKNVILVISPASLQSEWSKFEMLMAVDDSHQR
NNVCLVPVLLGGVKvDDLPPP---LRPLTC------------------------
I(ELMDDFRNTDDIIQAISKPEDTWESLLPVGNLAHGFAWGYYYGYLKIILPDLDKTVRQWRRVNNAEGRMSE
KLFLFFPQ)}*

$1666=249(278):
>gi|762111346|ref|XP_011438385.1| {|0(229)|<Mollusca(M)>}PREDICTED: uncharacterized protein LOC105335941 isoform X2 [Crassostrea gigas]
{(MAETEEIPMSEIQRQSSIRESTSGIDNEEKCDFLTDIPEDTCDGEHKSPILYELPPGKK)-
YHLFIAHSAIdgQQALKIC-KELESR-
FLLKCMFFDRDFMPAKRIDENIQQEMEKSVKVLLLLSPDFLNSHWCDMEARLAVQMSFDRSSDlRIIPVLLRD
LDpENDLPPFLK-----------------------------
P(YVCIDAQKEYDCTAKIHEAFYYTGVVDAVHQAELESKFAEDNRSQNGVRLLEMEGKLEKSACFNASHYVFL
DLSDQDRA)}*

$1667=226(224):
>gi|762111726|ref|XP_011438584.1| {|525(0)|<Mollusca(M)>}PREDICTED: toll-like receptor 7 [Crassostrea gigas]
{(KFNFSDADNSLKLLKKNCKSYLVIYIVTSVAVAIMFSIIFGIFLLKNKWKIRYVIYRFKQRFKTYTGHSII
PQADSPSYE)-YDIMISYSAKelSFVLTDVIPRLEN-
EAGFQLYVKDRDEPAGISCGEAIMEAIQKSKRVVCLVTKSFLKSTWQDYELNMSRMEAIEDREnlNFVHLILF
PDVCnGSIS---IQTLDLVRQEcFSEYPEEECAfdDFWETLISHIK-E(LNRH)}*

$1668=226(224):
>gi|762111728|ref|XP_011438585.1| {|688(0)|<Mollusca(M)>}PREDICTED: toll-like receptor 4 [Crassostrea gigas]
{(KFNFSDAKNSLVLLKKNCESYLVTYIVSSVAVAFILSIIIGIVLVKNKWKIRYVVYKFKQRFKKYACHSTI
PQAESPSYE)-YDIMISYSPKelSFVLTDVIPRLEN-
EARFQLYVKDRDEPLGIPLGGAIMEAIQKSKRVVCLVTRSYLKSTWQDYELNMSRMEAIEDREnlNFVHLILF
PDVYnGSIS---IQTLDLVRQEcFSEYPEEECAfdDFWETLIAHIK-E(LDRC)}*

$1669=240(228):
>gi|762132629|ref|XP_011449505.1| {|0(267)|<Mollusca(M)>}PREDICTED: putative GPI-anchored protein pfl2 isoform X1 [Crassostrea gigas]
{(MSGRVNNKE)-
YDCLMIYNHArsRLSRvddNELsLRIVERADQQLTAWgyrnnhYYDRDSIPGRNVFTELFRVVDSSQFVLLIL
TRGFLNNCWIRYCQMAAFKSLIDESTRPecvashrLIPILI-NISeNEIPQELGQLQHIcfMN----
DWDTNERE--WRKLKNAL--
D(GHPMQESTQRSIGADIPPVTRQHFSQGISESPNTPAVPSDTGASPISNAPSSRSARTAIVTPNSSGNYNAQ
SDQGRLSS)}*

$1670=211(221):
>gi|762153138|ref|XP_011414595.1| {|542(0)|<Mollusca(M)>}PREDICTED: uncharacterized protein LOC105318958 [Crassostrea gigas]
{(LYSNGKTMSDIITSVVKEDDIEKWRVTVEEKEKYFRSQEETKLAKKKKEVLCSRCKGPVIERQLSVFPDPL
EKPRSLKYP)-YDFYVIFSPTdrDWVCHILLETLE-
VSYGYKGAIAERDIRPGSTDIYQRVNLIQNCSKILVILSQKFEGNPECDYELSHALKRKHEENLENVLIPILR
NVD--------GLHQSLRTItFL-----DAVddCDWNKLINALESK(P)}*

$1671=270(299):
>gi|762157843|ref|XP_011417085.1| {|87(243)|<Mollusca(M)>}PREDICTED: uncharacterized protein LOC105320730 [Crassostrea gigas]
{(QKESDCPAKINEAFYQIDYERTCARNKTPSMNQIRHSARKSTSKSKIDEITNLLESDTQNNTCDEEQYHPI
FYELPPGKK)-YHLYISHSDKdeTQALKIGRDL--
ESRFFFRCMISGRDFLPGTWICKNIHHEMRKSVNVLLILTPSFLKSLWCDVEARLAVQMSDDPNFNlQIIPLK
LRDLGaDSDLPPFLK-----------------------------
P(FEYIDAQRESDIPARINKALYHIGSELTSSKNSHSMLNDLVHWSRMRKRRKNCLFMEPDAQEEVCDKNRKN
ELLYKLPP)}*

$1672=254(280):
>gi|762162442|ref|XP_011419484.1| {|0(223)|<Mollusca(M)>}PREDICTED: uncharacterized protein LOC105322450 isoform X1 [Crassostrea gigas]
{(MDEVYRPLLSTSYQDDDIDLEEIQPMPSSPNLNPDETEVKIIVSSDELSAGDTKPLPPGKE)-
YHLFMSYSSEdrDDA-NTIRQHLEER-
FHLKCLYYERDFQIGKNIDDNITDGLQKSVKALILLSPFYIQSHYCVTEAREACKLSFTDmENHNVIPVLLRP
LPkD--------LPPFLNSYvYI--------------------
Y(AQREMDVAGKIYEAFNQPGFLDPLQMNKSGLPCNGTLLFRKIGTRAKYLDHGLVYRFSPLEPHEEEKISQN
GMDKLACR)}*

$1673=198(224):
>gi|1139855520|ref|XP_019921450.1| {|0(121)|<Mollusca(M)>}PREDICTED: uncharacterized protein LOC105325212 [Crassostrea gigas]
{(MSNQE)-FDLFIVYSSEntQAAKTIMKDLEKRFK--
IKCCFADRDFLPGTEIAVNISFAIEKSDKVLMLISPDFIKSGWCWYEQQEAFRKCTQDKRHCIIPVMLETVDE
--------MPKLLRNLtYVDF------------------
L(KEADPVERIYKAFIHHIDQEEACQDKKIKRQKGDLGFLSQRWPVSRHFNDNGDFVKAVSRHFYDCDAFVED
KERIEFLK)}*

$1674=276(299):
>gi|762170428|ref|XP_011423642.1| {|1(222)|<Mollusca(M)>}PREDICTED: uncharacterized protein LOC105325678 [Crassostrea gigas]
{(LSVQEDTLTERDVLLTVEESQDTNCDINNLSKSYSGSETCLKTPIGQDHYEANVERGHDTLTPENTLRQRR
VAKLPPGKK)-YHLFISCSTEdiTEV-NKICKELESR-
FFFKCMQHDRDFVIGKPLDDNIQSEMAKSVKVLILMSPAYITSRWCMSEADEAVRLSYSEREKlKIIPVLLRP
-----PDEGLKLPPFLNRYrYID-------------------
S(QKEKDITAKIFEAYYHSDAVDYIKIQGEQELTEDEIKNQNGARLLTKTFIQQRPYFEKGYVFETEQLTLNE
REYLKNLH)}*

$1675=291(298):
>gi|762077307|ref|XP_011450129.1| {|925(15)|<Mollusca(M)>}PREDICTED: protein toll [Crassostrea gigas]
{(AIVVSYDVNLKEIPDKDLQQYIKRGRYIDANQSFFQEKLLYCMPQLTGDDMRVKSLPDLKYLIQNMYGNDV
TDAEMYERH)---AFLSYCESdmGYVMNELRPIIEDNG--
FTLCLPDRDFIPGAPKEENVLKAIDNCLHTIFLLSGEQLDNEWSVFTFRSASEKSMREKCNHLIVVL-GDD-
VNVETMGEEVRAYVKTHvSLKVSEKG---
FIRRLLNALPDI(ENHELYIKHDDNNFVDFENLHKTKMEDGLSRIVADMIENEEKKNGIQIQNGKMNGHVQLT
DIHLNGTQHYQNRAFQKD)}*

$1676=294(297):
>gi|762077310|ref|XP_011450148.1| {|929(161)|<Mollusca(M)>}PREDICTED: toll-like receptor 6 [Crassostrea gigas]
{(YVVTVLYNVRFRKTTNKEMARFAKNSNIIKWNDRLFGQKLLYCMPVHKGFSDIDVKDKKLPNIQNCVMNMY
GDITLRQEV)-YDrhVFISYSETeiNCMLNDIKPVLD--
EIGYTLWLPDRDFIPGASKEENILKAIDSCLHTLFFVSENHLLDEWSVFTFRTAFEKSLRSKSNHLIVILSEN
V--DIDELDEEIKNLVSTHvILQIGEEW---
FVQKLLNSLSDA(KPCVRCVEDEAQIVEIFNFENDIQSVTDSMLFESSEDSDTDVDNDDDVIENNIDEINEDE
ELKEEADNAQINIVVEN)}*

$1677=225(220):
>gi|762106534|ref|XP_011435861.1| {|460(0)|<Mollusca(M)>}PREDICTED: protein toll [Crassostrea gigas]
{(MQCTFENEDVYKIREVSESLLNCTYDVAIGLTIGFSILLALVIVAVIWAKKCPYETKVILYRIFRYHPWDK
YRVDNELLA)EHDAYVSFDDSniHirqWVLRKFAKRLEEEKPCYKFFVPVRDLLGGDSKADSIIENMEKSKRV

IIILSDKYDENEWCKFECQRA--EILELNNGRIIFIKYHPE-
ADEMIENEPWKSRVKGRkVFSPGEkkserRW---FWGKIKYELPVR()}*

$1678=255(254):
>gi|762111591|ref|XP_011438513.1| {|0(277)|<Mollusca(M)>}PREDICTED:
uncharacterized protein LOC105336039 [Crassostrea gigas]
{(MEEDHESLVKSDFTGVCEENSCISHYDSDDASFVDCIHFDPPEDSLTPTESVEVDIFYTAPPLKDLY)RYH
IFFSHSLEdsKWISG-LVHRLESEPYSYKC--
HYNDSVTQtkQNVHQTLMSAVILSERVVIVLSPSYVKSNWN--EFQELLQNltTCSLYQQRMLVVLLKDC-
NIPDHLQPLGFLDARECdFFDCLVRHLRSERLPRSSESFSS(EMSSLLYRPASVGNGQVLSTAHISFCGAWNA
HLRYTEDDDVPTSLCC)}*

$1679=282(274):
>gi|762115355|ref|XP_011440485.1| {|495(17)|<Mollusca(M)>}PREDICTED:
protein toll-like [Crassostrea gigas]
{(NASKTTTIYCITSSGIVPISKFNFKDLNCKSGFDYKSLAIGIAVLLLITSGILVTAHYFRFEIFLLTKRLH
VARKHIAQY)EYDVYISVRENhglliSWITLTLHPFL--
VAKHYNVYFHSINGDVGVSKEKEIVSNISKSRNFLIILSSDYTKededSIWTNIEWKHAWNLFKEDSRVRNII
IVnYDQL-
QTRVFPVGPLKAYLRLGlDIDFANRKHSLLREVRDRLGLPN(HEHRFPKLGPAKPIFLPKHNPFETGFHSEFG
FHESQTGLDEIDTIELGPGPQTG)}*

$1680=228(223):
>gi|762132925|ref|XP_011449657.1| {|421(0)|<Mollusca(M)>}PREDICTED:
protein slit-like [Crassostrea gigas]
{(CEYDDGSERYFIRDITDSLLKCSFENLIIIISVVVGILIAIIIGAVVTARRCPYETKVLMFRLFGVHPADK
YRVDLEEPH)QYDIYVSHYEHdlqarQWVKSKFLRKLEENQKrKFKVFYFERDLNAGTDMYDELVTHMKQCRR
IVFILTNDFFNDEKNIFEADQAEieHRASDNLHGRVIYLLWNES-VREKIkLDP-WKSRMEGKrVLCPDDKF-
--FWSKMRYELPIK(GVP)}*

$1681=223(221):
>gi|762153188|ref|XP_011414620.1| {|305(0)|<Mollusca(M)>}PREDICTED:
uncharacterized protein LOC105318979 [Crassostrea gigas]
{(KCLTKNNTEPARTYLQNIDCNVHTSDSIYLTLTSMCLALLVLLLFLTAAWKRKCEIRLLIRETKLGKLLRS
RVTLDQDRV)---VFISFdgsNHCihSFIFQKLEPFLVTNG--
FHVFIPSRDLAVGSVRSEEAAWQISVSRYYITFLSLSYLDEDVFetRSEWRYIWNGYLSDNRKELLVLNYDLL
-KPSDVPCSKMRAVLRAGnVVDFDAGENTIFSKIVKLFHTSS(F)}*

$1682=203(210):
>gi|762102286|ref|XP_011433635.1| {|0(231)|<Mollusca(M)>}PREDICTED:
uncharacterized protein LOC105332663 isoform X2 [Crassostrea gigas]
{(MALQSEKLKGKLAMVTAGKVQ)-YDVMISYDA-EDA--
AVAIKLKDMIGKKGIHAWTADSEATAGTDIfnAIGRIIVDCKIFVFIMSDSSVASKLCQDQLALAYVSK----
-
KNIFPVQLNEREDLLAHMDNGMKLQLAGFEWFSLNNDLESESQEFVDRVWEE(VQSEIEQEKADGSKKAARKT
FKRQKTLTNLNKSLSKTKMSRLHLEDAST)}*

$1683=245(253):
>gi|762114104|ref|XP_011439822.1| {|1151(214)|<Mollusca(M)>}PREDICTED:
uncharacterized protein LOC105336991 isoform X1 [Crassostrea gigas]
>gi|762114106|ref|XP_011439823.1| PREDICTED: uncharacterized
{(RPGEETNDDRIWPAAKFTELLMQMNCLKIMPDEGNIAPRYKNWWMPVVEEIVIPKRNEKKGENKAASSGQQ
NTESQVVSP)--DVFISYQW-GKQKEIIA--
LFQRLTSLGFTCWLDIRQMGGGDSLydKIDRGVRGCKVMLSSVTTKYALSANCRREVSLADALK-----

KPIIPMLMEKIDWPPTGPMSMVLTQLLYVNFSNDESVQLSWDGKYFDELLSK(IRQQIPTDESNDYVNVESES
GVQSLKNSKYEKS)}*

$1684=241(248):
>gi|762080744|ref|XP_011418724.1| {|289(0)|<Mollusca(M)>}PREDICTED:
uncharacterized protein LOC105321929 [Crassostrea gigas]
{(EKLREIDIQLHIKRFYYTLGGLLDDRSFTRTYDRARVITYDEQQSGFVLADVVLNEVKKDLCSPNEKKIPP
QHFQHAEYE)-FDAFVFIHEGdDNTSDEITRFLEGKGVKLM-----RNDRIGQSILNISD--
VIKRCRWiIPILSKTSLKDPVFAHCCLMLVGDILETKELRMIPVLTgGAAYEDVPEVIRMVTLTVADN--
PEYLNRLHQTIKGNTIPFETE(SLLPAGDVVYVLAWNYAVNYLEKVMPGP)}*

$1685=191(210):
>gi|760152301|ref|WP_043833410.1| {<Actinobacteria(B)>}hypothetical
protein [Amycolatopsis orientalis]
{(MYHYDVFISYERRAEAISKWVRNHFHPRLCELLDGNLD)----------------------------
RDVQVFFDG-
SVRVGGKWpdELRSALRHTRILVPVCSPKYFYNEWCLAEWASMERREELAgRDRpatLIYPVIYCDSKnfppY
AHQRRMKNLTEWNHPYEQFQVSAKYIGFHDQMNRIATEI(EELLSAAPAWRPDWPVLTPRPDVPPASRFPRL)
}*

$1686=245(258):
>gi|762101113|ref|XP_011433026.1| {|310(108)|<Mollusca(M)>}PREDICTED:
uncharacterized protein LOC105332211 isoform X1 [Crassostrea gigas]
{(ATKVLAHLAFDDDNKKTFVSDTQTDVVTTLFDLARSSRYPDVRESAKGVLWTLSEQLHSSTKYADLVQELF
GKKTKGHVM)-----ISYSREQRPLLLRIKQAITEAGFS-VWMDIEQMR--
ENVYDRMAEAIQEASVVLISMSYSY-QRSEYCRrEAAYASSLK-----
RARIPLLVDPGYtaEGWLALMTAGDYHYDF--
VRKPFETKIVELIKELERRCVK(SQGDTTDGIIKQEVSPQQSSQAVSSHGVPSYNVPSAHG)}*

$1687=245(258):
>gi|762110998|ref|XP_011438204.1| {|128(110)|<Mollusca(M)>}PREDICTED:
uncharacterized protein LOC105335805 [Crassostrea gigas]
{(ATKALAHLAFDDDNKKTFVSDTQTDVVTTLFDLARSSRYPDVRESAKGVLWTLSEQLHSSTKHADLVQELF
GKKTKGHVM)-----ISYSREQRPLMLRIKQAITEAGFS-VWMDIEQMR--
ENVYDRMAEAIQDASVVLISMSYSY-QRSEYCRrEAAYASSLK-----
RARIPLLIDPGYtaEGWLALMTAGDYHYDF--
VRKPFETKIVELIKELERRCLK(GQGEVTDGVVKQKVVSQQSSQAVSSQGYSSAPCAHGDY)}*

$1688=256(257):
>gi|759987997|ref|WP_043671751.1|
{|228(258)|<Actinobacteria(B)>}hypothetical protein [Streptomyces
xylophagus]
{(LKLIHDPPDGRYVPQPSKLVSGAALRAAEPADRITVRWSHPEELAGRVQDLVARFYQPRLEFETYEEGVGY
FRSLGRAQG)--SIFLSNARGdDALAQRIGRSLE-
LQNLSYFHYLRRNTIELGADWRsQLYANVAACRMFLPLISRHYWESEYCREEYDIAERLRAD-
GRLVILPYFLGPG-
VARKVSFQGRAVGHLSQdeQVAVISRDVDNEFVERRRLEDRG(VTAGERGAAARTSTPGRRCDIALVTLLPEA
HDSLRRH)}*

$1689=145(149):
>gi|760090473|ref|WP_043772927.1| {<Proteobacteria(B)>}hypothetical
protein [Roseivivax isoporae]

{(MTAG)-ARVFLSFSSKdAAAADRIVAAFAagaPEVRVFDFR----
RDIEIGVAYQrEVDAALKRSDRALCLVSPDYMASGECLEELMVARLRNKR-SGFGFLW----PV-
YWRNVPDGLEEWIQILNyaDCRECDSVRLETAVRALAAQLAS(RQGVR)}*

$1690=228(238):
>gi|762111222|ref|XP_011438320.1| {|0(243)|<Mollusca(M)>}PREDICTED:
uncharacterized protein LOC105335884 [Crassostrea gigas]
{(MGSKPSTGRHESKPEHEKPSVQPQSKATGTVGSGDTFSPHEVPEMSDKYDIKRQESVSNRDLM)-----
ISYSHVdKEMMAKLRE---SLEKNG-
ISVWVDVvGLGAGVDFLsKIGQAILEAKLFISLLSTSTVKSKYCQDEVALAYISQKA-----IFPVAIQSQ-
EEIYAIMDTGMKLQLASleWTFIDPENFDEGFKELLPKLKAE(LEDQEKEVNQGHQVKERLKERQKKFKREVS
LHLDP)}*

$1691=292(281):
>gi|762116499|ref|XP_011441087.1| {|310(280)|<Mollusca(M)>}PREDICTED:
uncharacterized protein LOC105337863 [Crassostrea gigas]
{(NMAICCTRRKGKDFLVPLYFETIQIIPVSISLVQFLDCRVRKEGDTPALKMESVCTRLMLTETEGGESNVS
QADDNVNHD)---VFISYAHKdPKQAQLL---
LDEFEKQYpDMEVFFDRQdLKVGGMWQkGLYYSLDTVKCVVALVSPSYLSSVVCQEEFNIAIYRsqNKA-
DNLLLIPVCIEdipsvPC-
LYGQHRLVDFRGnqyselIPSLVkdVASWVKANKRPNYCSNTASKVAL(FDFKRMSEIRKKEVYDSYNIKTNS
VERKSSFSVPPLDQSENHVVVSVSQNDLSLATSLLYQ)}*

$1692=235(225):
>gi|762111730|ref|XP_011438586.1| {|635(0)|<Mollusca(M)>}PREDICTED:
toll-like receptor 4 [Crassostrea gigas]
{(SQKFNFSDVEASSVLLKENCKSNLIKYICSAASVVLFFSVVIYILVKKKWELKDVFFKILNIFKDVFCYED
NQSEDESFH)---AMISYAEKDlpIVLKYVVPNLEAQH-
NFMLFVRHRDDPHAGMTINAIRNSIKNSGKAVCLVTKNYLKSKRQCFELAMIRKHMESHIDQEEVLINNQEnh
dnnqenflcriirFpNVSIPDEISNL----DNNIIECPVEDG-NndhFWRELRDAL--G(GNINL)}*

$1693=219(232):
>gi|762167670|ref|XP_011422173.1| {|607(0)|<Mollusca(M)>}PREDICTED:
toll-like receptor 3 [Crassostrea gigas]
{(IESKNPIVELFEDQTWRKFELDCQSKEWLTFSAVLLVLTMLTFAITVAIKKYRVHLEYVILRLKNKWKGIP
LRKPDNNFL)-YDVYVSYSDLDylWVTKTLYPILESLN--VKPCLDE-
DIYGGRTILEGIVNRINECRKVLFVVSESFLDIGQSSIEVRTAITHAVHNQFQGYIVVLIkDGFpLERLPD--
-ELKNIWwCIEYFGWSEEEQ-NE----------V(ILKKLTDIFKPE)}*

$1694=221(220):
>gi|762167688|ref|XP_011422182.1| {|616(0)|<Mollusca(M)>}PREDICTED:
toll-like receptor 4 [Crassostrea gigas]
>gi|762167690|ref|XP_011422183.1| PREDICTED: toll-like receptor 4
[Crassostrea gigas]
{(IESKTAIVELFNENMWRKFELDCQTTEWLIFSVGLLVLTLLTLIIIAVINRYRVHLEYVVLRIKQRWKGVY
LPTQGDMFL)-HDVYVSYCDEDlsWITDSLYPRLQILN--
LKAWFKEKDSIPGGWVSEEIVNCINDSRKVIFIVSESFLDKGWQSYAVQMAITHAFRNQRQkSIVVIIKDGLp
LERLPKEF---KHIWwCIEHLRWPEDDT-NddmILSELSNVLKTE()}*

$1695=221(220):
>gi|762167692|ref|XP_011422184.1| {|612(0)|<Mollusca(M)>}PREDICTED:
toll-like receptor 4 isoform X1 [Crassostrea gigas]
>gi|762167694|ref|XP_011422186.1| PREDICTED: toll-like receptor 4 isoform
X1 [Cr

{(GSKNATDELFNESNWRKFELDCQATEWLIFSIVLIVSTLITLIIIAAIKKYHVHLEYVILRLKQRLIPVGD
HVCEEGDFQ)-HDVYISYNDDDtsWVANNLNPKLESLN--
IKAWFKVRDSIPGVSQSEEIIKCINESRKVMFIISESFLDIGWSSYAVQMAITHAFHNQRQrSIVVIIKDGLp
LERLPKEF---KHIWwCIEHLRWPEDET-NdetLLLNLSNVLVSE()}*

$1696=223(222):
>gi|762101967|ref|XP_011433473.1| {|66(0)|<Mollusca(M)>}PREDICTED:
uncharacterized protein LOC105332541 [Crassostrea gigas]
{(NPDSACGNTRSPNSKQPNATRTRTSHRQTAYPEGNTTVPDERNDNEDIEVDSGPITSNLSLVEDAVPATPIA
CIPPHIETH)--DAILLYaEEDRPLVQQIQQQLVSNlgtdidDLNIVLY---
EEFAPDVQSQFKTMEQLFCrCRFLFVVVTSNFRKDSLTRYQHEIALTDSIrnPSRNERVIPLWAEAGAQNFLH
ELSVLKGIDYSR----GEVDFRMFRNVFQHGRRTI(NL)}*

$1697=273(278):
>gi|762116499|ref|XP_011441087.1| {|154(455)|<Mollusca(M)>}PREDICTED:
uncharacterized protein LOC105337863 [Crassostrea gigas]
{(KNQSTTIITPLLSTGNQHSDEITVLQSIVTGAISWIQAGLPLKKLKIVLYEDNNKELNSKFAKLKQTHLKS
QRKRQAVKE)-YDIFLMHqPTDATIVSSLKQNLLAKNDKLSIYYdPSKFDQKGV--
WQQEIFDLMMgSKRIVPVLTPNFVKSDECLEMFNMAICCTRRKGKDFLVP-
LYFETIQIIPVSISLVQFLDCRV----
RKEGDTPALKMESVCTRLM(LTETEGGESNVSQADDNVNHDVFISYAHKDPKQAQLLLDEFEKQYPDMEVFFD
RQDLK)}*

$1698=238(223):
>gi|762132633|ref|XP_011449507.1| {|0(99)|<Mollusca(M)>}PREDICTED:
uncharacterized protein LOC105343734 [Crassostrea gigas]
{(MARRDL)-YDCLLIYnHEDSSFSdggtredsSLLFSYVDFVDRKLNLCKYrihhlDRDSIPGRSVF-
YELERVIEsSQVILLILDKGFLKNSWKCFSQEAAIKNLIDTSTdpkcttsNRLIPVLIHLEKNEIPSALKTLT
PIEIME----ESDID-
KKWKNLKKAIDSH(FEQERIPRNIVAKNPQLTPEPQYAPAVSKEPRVNPKSNGPSCRTLRLQSTVETDAPQSM
TSTTAATVQQSVASPQNH)}*

$1699=179(181):
>gi|763125562|ref|WP_044005466.1| {|0(69)|<Firmicutes(B)>}hypothetical
protein [Lactobacillus salivarius]
{(MEDT)---VFFCFSG-QD-RLKYAQSLNFHLKNFGVKVWYDYEQLflGD-
IGDtvNIEEGLHNSRIFILFFSKRLFQSTGATLEIDEIKKKIDNNQNLVIIPVFCDNSIPQIPPKYSWLLKYI
FGELTESTGTYDLAIDVLKRLLHEKL(KPVEYKNLLELSNLNLGDFINNLIRIYMSIDKNNIHV)}*

$1700=222(225):
>gi|763431393|gb|AJQ21543.1| {|0(500)|<Mollusca(M)>}stimulator of
interferon protein 2 [Mytilus galloprovincialis]
{(MATGRDPNGKDDEDPWLKAVENA)--DVFVMYNNDdeYEISKYIFTIFQNNKVTY--
VSPEEHIMPGFRTYQAIDQAISASKKMLLILSKESIENFSFSLETLLTLEKSLQTNKLRLIILLKDGL-GE-
ENIPKLPMLQLASHvILERNLHEKCIMNVIEMIRADVT(IDSVLPAGNLAAGMAWSHFTGYLELVLPELAQGI
KKSDWFNDNPGHMPLCLFMLLPHSGRST)}*

$1701=257(260):
>gi|764522889|ref|XP_011457652.1| {|2(289)|<Streptophyta(V)>}PREDICTED:
L-type lectin-domain containing receptor kinase IX.1-like [Fragaria vesca
subsp. vesca]
{(EIMSLSYITDLTNILPEWISVGFSASTSQYGERHKILSWVFDSSLDGVLPPNKKFTEVLRILAGSRLFGTT
FKCNKTQRW)KYDVFLSFTG-E-TRKGFTHFLYKALQRRGLKVFRDQELGRGTN-
IssNLLAAIEESRFAIVL-SPKYASSTWCLEELTKIMDCMEV-

KGTTILPIFYKKKPEKEEEAPDSVLAVTGLTATGNLAPIIEEISEGEAAPGRFT(CQNIATATNNYSNDNKLG
EGASGTVYKGDFTDINITVAVK)}*

$1702=142(150):
>gi|764548175|ref|XP_011459771.1| {|0(209)|<Streptophyta(V)>}PREDICTED:
uncharacterized protein LOC101301450 [Fragaria vesca subsp. vesca]
{(MASLIASAPW)KYDVFLSSSS-QDA-GNFSYLLHKALKDEGILTLRDSQEVEGAE-StaSLEVGIR-----
-LEGRPSYYLAGLPPLGSWNNLCRFLK-
QTGIQVVSSSQAFTLSTPQMSENKKELLEQASPSMNKNKTRTRLNGSNSAYNDI()}*

$1703=226(245):
>gi|764539627|ref|XP_011459010.1| {|0(129)|<Streptophyta(V)>}PREDICTED:
uncharacterized protein LOC101311535 [Fragaria vesca subsp. vesca]
{(MVLKKRRVSIPFPLRISIPSNSIWAV)--DVFLSCDH-ED---IFCSHLYLKLQSrG-ICTF---------
---QFFDAIEESRFAIVVLSQNYASSPRRLNELSKILERMKD-RNR-
ILPIFHDVNLFHVQKQKGTFEKAFAKH-
EEMFQDDLAkvEAWRDALTKVCNF(AGWTSNDRNEVQVLEEITEAVWNKIHPRPRPTERVHPTSTHNLVAHTS
IKVNEGNRNHPMHTTDLVDTSAEWNFLIANL)}*

$1704=228(229):
>gi|764567130|ref|XP_011462117.1| {|166(0)|<Streptophyta(V)>}PREDICTED:
uncharacterized protein LOC105350848 [Fragaria vesca subsp. vesca]
{(AFANYEKKYKDEPQKVERWRAALTKLGNLAGWDSKHYWPEAKLIREVVDEIHRKLYSSFTKPAIKNLPSTP
SWSSCCQRN)KHDVFLSFRG-KDTRLNFTNRLYDALIQcR-
ISTFRDDKDLPKGMSIrkQLDKSIEESRVWVVIVSPNYASSKWCLDELLKILQCRKA-
SKVAKKNGTLMKASGNDEDQQAHTSVDGDKT-HKKKASLRNKENISIHDNRYVR(YSHLQFHAC)}*

$1705=135(156):
>gi|764642047|ref|XP_011470936.1| {<Streptophyta(V)>}PREDICTED: TMV
resistance protein N-like [Fragaria vesca subsp. vesca]
{(MASISPKTDASKARRW)KYHMYLSFRG-K---GKFASRLYDALQDERIKTFMDNRDTS-
GNASypTLKATMEDSWFAIVVLSGKYADPTRSLDELAMICECMNT-GQDRIFPVFDQVEP-------------
---A-GYHASTMFVNSVSFIYWLSVCL()}*

$1706=148(159):
>gi|764567745|ref|XP_011462250.1| {<Streptophyta(V)>}PREDICTED:
toll/interleukin-1 receptor-like protein [Fragaria vesca subsp. vesca]
{(MASSSQIAFASPSSSDPCW)KYDVFLSFRG-
PDTRKGITVDIYDGLNRRGIKTFMDDPDLKVGDVIspTLLEAIKKS------------
WTHKELVKVIVEFVCTKVVPDAIESTGDFEVFEVTRRAMYEVMKAFTDDEVTAIGVYSMGGVGKTTLCRIPKP
()}*

$1707=202(198):
>gi|764639827|ref|XP_011470666.1| {|0(845)|<Streptophyta(V)>}PREDICTED:
TMV resistance protein N-like [Fragaria vesca subsp. vesca]
{(MAMSIQVRASTGSALPW)KYDVFLSFRG-
ADTRKGFTDYLYKELQRQGIMTFRDDPQLERGTVIspELLTAIEQSRFAVVVLSPKYATSTWCLLELSKILDY
CRYETeliRDIVHVLWSRVHPSLTVFGSSEKLVGMDTKLEEIDVLLDKEANDVRFIGIWGM(GGLGKTTLARQ
VYEGVSHLFEVCIFLANVREVSATQGLVHL)}*

$1708=148(154):
>gi|764642050|ref|XP_011470938.1| {<Streptophyta(V)>}PREDICTED: TMV
resistance protein N-like [Fragaria vesca subsp. vesca]

{(MCLIL)-----LSFRG-
PDTCKGIVVDIDERLKRSGIKTYMDEEGLQVGDVItpALLVAIKESRFASFVSLTKYASSTWCLEELRQICLS
MENN---RILPLFYQVDPTVFDISRGVSKKAFSKH-EtyERHESEKVNQWRDKEWPISLG(GTQMVITYM)}*

$1709=167(168):
>gi|765290911|ref|WP_044618623.1|
{|0(239)|<Proteobacteria(B)>}hypothetical protein [Gynuella sunshinyii]
>gi|764038433|gb|AJQ96667.1| hypothetical Protein YC6258_04635 [Gynuella sunshinyii YC6258]
{(MTDTSGIC)----FLSYKRECSDQ-AALIVDALRDHGV--
AVWQDVSDLPAGITEsEIRTRLNDqetACAVILVTPEVRNSPMIRDVevEGIFRRVSQQDGFFAQLVLADGVV
DYKDADEILGTRTSGILPSSKNCLKFNGKVDADIARKVAEVV(LKNRLIKLNETSQDAGPIRI)}*

$1710=261(255):
>gi|765349851|gb|KJE20634.1| {|412(0)|<Actinobacteria(B)>}TIR domain [Frankia sp. CpI1-S]
{(VQSLELVAPRDDRAVSVVRRLVAELAARRAGGPEPSADPGVRAAGPQFVSWPLGDGVAPSAPPPPRGPAGA
AAYYFFTSH)AHR---HDR---
DRVVAFHRELEAELRRKvrrRVEPagFLDAERMGGGEHWptALRDAARTAPVLVALWCDDYFESDWCGREFGV
FQERIRRAtRPGGKPPAGIIPVPwlvrEADVPEAARELHIARIDLGRKYDNLPVLDLMRHPAAFAEY(VSLLA
YRVMDVARDELPPLAADATELVRSPFHPES)}*

$1711=254(253):
>gi|764110719|gb|KJD39620.1| {|44(22)|<Firmicutes(B)>}hypothetical protein QD46_13445 [Paenibacillus polymyxa]
{(IVFGVQDDGRKLRVKGYKFNVDIDSIKAKLSVNVNLTLFDLPHETAALKCINVEKCENVVVFSDTPYTLDH
NRNVAELNI)-KKVFLSYSHNdTCIADLVEERLNFFGKGRLAI--TRDKRT-LEYKSdiqkFM-
QTVSSHDFMVSIISDSYLKSQGCMYEvselMRNRAFNDKL-LTIILSETDENFYPKDKKPKQVKADVYSLNR-
-FEYLKYWETEKGKVNKLNSEI(SDLALKQGLVDEIKQINTISTNISELIEIFKKS)}*

$1712=163(158):
>gi|764431563|ref|WP_044362045.1| {<Proteobacteria(B)>}hypothetical protein [Vibrio fluvialis]
{(MEFEN)---IFISYSWDNaehKQWSKYLADRLEEISeLT-
VSFDQYDLDSLSDKNHFMEKSVFETDLILAVITDNYktkandrvggvgIETSMVTSrHWEESLSlGK-----
SNIIPILRE-GDSVPNYLKNKFYIDFRN--NDEfDKSFE--RLLEHIKGTSKS(SRPQKKYSIKKSL)}*

$1713=304(297):
>gi|313225394|emb|CBY06868.1| {|437(407)|<Chordata(M)>}unnamed protein product [Oikopleura dioica]
{(YSSEDASGIQKWLSSIGREFSQYTFHLIQAGVDRNFLPHLTDEHLKVDCQITNGVHRTKILEAVRQNEAEH
DKDGGLEAK)--DVFVCAWRE-GQLLSESLCAYLRLRRF--
SVATNWGLIPKSEEVtlRHETNIAHAGHFLLVLTPGCLENLIQNacedenTlaSDPFYRWIVTAFaKGCNIIP
mVAPGWKWPLPDSVPEAIRDLCFMNGVRWRWGYQGEIINRLQRFMTDH(GSLQSLTSVNRAQSKISIATTNHG
EYILPTPAENLSRQASQRSITSVTSRPSLRSYGGSPQADTGSQQSARSRHSSR)}*

$1714=214(224):
>gi|525314207|ref|NP_001266226.1| {|289(0)|<Porifera(M)>}IgTIR1 precursor [Amphimedon queenslandica] >gi|313757061|gb|ADR78338.1| IgTIR1 [Amphimedon queenslandica]
{(RTIELMVHENASSSPTSSALERKRQNQLIIAATVPVILVAIAVALVLIVSMSVVLWRRSRRSQNSAAELEK
KFGDYKEAT)-YDVFISFSEEdrETAEKDIKSTLESNG--
YQVAWHHEVFIPGCSILENMETFIFQSRITLVLLSGHFLKSEFCNKELYIALRKEKQADRRSIIPILLSNS-
KD------DVPDMLRGRtYLSLKDNN---FSKKLLRTLGRP(LSSC)}*

$1715=223(225):
>gi|525314197|ref|NP_001266224.1| {|138(0)|<Porifera(M)>}myeloid differentiation primary response gene 88 [Amphimedon queenslandica] >gi|313757059|gb|ADR78337.1| MyD88 [Amphimedon queenslandica]
{(PSLKPYVDPVNLQHRNGSASTNSSDCEHVQKQLSSLSLDHQNGDYNKKEGIGDDGGGIEVMIRDRSLSDLDRSLLIDGSD)-FDIFLSFaPADAEFADEMRLRLINRA-GISVYIASEGLMPGQSFIDEVADKIKRgCRKTIIILSPDYNQCSWCNYEARLAHHKNPDPKRHTLIPIVYR--KCEVPDFMSHLFYLDFsrYK----DDHRqceK-YFWDRLYKSVRHQ(QPGHT)}*

$1716=202(197):
>gi|766767592|ref|WP_044841689.1| {|0(387)|<Proteobacteria(B)>}TIR domain-containing protein [Thalassomonas viridans]
>gi|765433529|gb|KJE42919.1| hypothetical protein SG34_26925 [Thalassomonas virida
{(MSG)--EIFVSYRwGEQSKAAAWQLYSALQqkLGKD--RIVIDVRSEFTGENiRtKILQDLKAAKILLVVIHPDWIEGIPQLHheDNYIKLEIEAAIeLGLRIIPVlLEGAEMPGKEVLPDSLFTVADTGAFAITASEAYDQAVNRLALTLKA(HLPGKTVALLEWFADKNSAGNLVFGLMIAAAFLLFISRMLDIHMLYFTTSVRTD)}*

$1717=194(215):
>gi|766792856|ref|WP_044850676.1| {<Actinobacteria(B)>}hypothetical protein [Amycolatopsis orientalis] >gi|1039682971|gb|ANN17071.1| hypothetical protein SD37_16405 [Amycolatopsis orientalis]
{(MYEYHLFISYRRGGGDAPDWVRNHFHPRLQRLLDDNVDY)-----------------------------DVKIFLD-DSVPIGGKWpqEVRDALQRTRILIPVCSPKYFRDEWCLAEWHTMAKREElvvrdgSVkAGRLIYPVIFSDSEnFPNYAHERRMRS--FRSwNQPHPQYQNTDEYLDFNREMERM(VQELVEIIDQAPKWRPDWPVETPPPDQPKPPKLPRF)}*

$1718=214(219):
>gi|765408479|ref|WP_044687399.1| {|0(197)|<Firmicutes(B)>}TIR domain-containing protein [Streptococcus suis] >gi|995223377|emb|CYV02485.1| Uncharacterised protein [Streptococcus suis]
{(MNKPT)---VFFSHSSLdKEIAKYLKDKLIEYTGGTVRIFVSS-DGESIPFGKnwlsTIEHGLEESRIMFLLVTPNSINNLWLSFEAGFGYSKNIR-VIPLCFKVSVSDLKYPLGML-QGYDLQSADS--FNNIISI--LNSEFDYSHKLS(FTESDFSEISSIGITDDVNQEFAKIVKEVTWYKDFQPNIIESLEGEELTLYNKICSGLLYPTEEFLQENQIKYS)}*

$1719=213(214):
>gi|766747742|ref|WP_044832734.1| {|0(86)|<Proteobacteria(B)>}TIR domain-containing protein [Thalassomonas actiniarum]
>gi|765431676|gb|KJE41141.1| hypothetical protein SG35_12320 [Thalassomonas actin
{(M)--KIFISHASSeQILADAWKHLLEDLGANIEA-WYSDdpDPDGGIGPGKwreKIEKELQKADLIFALFTPEsknrpwiYFESAYVLG-MG----KNQT-IIPIVYYMVKDELLSPMQDLSI-FCGDDEAS--LTELYHR--LVKKYKGTPVNP(KLLKLAFEDYLTAVDRYNQSRLAQSLFHGHFHNQHTAAFLQGNWYSKWTQIAEDGSQTPFEVHPVKIWTTPER)}*

$1720=162(164):
>gi|766746281|ref|WP_044832048.1|
{|0(283)|<Proteobacteria(B)>}hypothetical protein [Thalassomonas

actiniarum] >gi|765432443|gb|KJE41881.1| hypothetical protein SG35_08790 [Thalassomonas actiniarum]
{(MSE)---IFISHSHAdHRLAERLHNFLTDISDNKFEVHRSSDIS-AIPTGTpwkkWIDDNIVNCDVAIVLLTESSLRGRWVLWEAGAVAGVQYY-RDKAKAPKQEAGEQQAVEGRVRVLNFGISSS--DMGPFAGDQAQDGRNPESVSQ(FAFELLETFQQKLGKTYAKSL)}*

$1721=171(178):
>gi|767058853|gb|KJF81575.1| {<Proteobacteria(B)>}hypothetical protein UB36_11055 [Photobacterium damselae subsp. damselae]
>gi|767121719|gb|KJG40827.1| hypothetical protein UA35_11705 [Photobacterium
{(MVDMIITPVHMDKVFHFPERELFETGNPQYKAMFKNAS)--RVFISHSSKdKEqIVNPLNSYLQANDIG------TWLDSYEIDYGDniylKVNEGIENADLGLFILTDSFFDSAsgWPLTEFSAFFmemMKNNK-KVLLVNAGVDESKLHALMKVYKYIMWDNGNG--LPEIANA--INRRLN-----A()}*

$1722=285(293):
>gi|765549141|gb|KJE89762.1| {|1131(130)|<Opisthokonta(E)>}hypothetical protein, variant [Capsaspora owczarzaki ATCC 30864]
{(AGEKQMAEYLESTGQLRTSATGSTTATTATTATTHRPLHSLLSAIRSRTRGAGHSHGHSIAAGIMAGGNLGNSLAAKLKP)-KEVFVSWAHAdAEFVRE---RLWPDLEARQLDCWIDFlQLEAGEFWRkAIGAAIKHCRVCLFVLTPTSVRSQYCQEELLYA----SK-CGKRIVVVMVKPC-KKELADTLDVKAV-IDAsaIVDFSDSD--LYHINVNMLSSLI(SAQLNLSQAAQAKREAGTAPGTAPVRDKRASSVAVTTMRALTARMSRGVSLQRSTDLSSDSSSAEALSRPPSL)}*

$1723=244(254):
>gi|765549141|gb|KJE89762.1| {|868(434)|<Opisthokonta(E)>}hypothetical protein, variant [Capsaspora owczarzaki ATCC 30864]
{(LYDDGRYLLAALRSTTTIAAAGWIVSVIFGDQLSVSTVRAIESVCFLAAGALLVGYILGPKMHSIHARSWKTSGAGREAV)-----ICYTFEdRAFMKKLGAGLA----HYGIDAWFDEmRLEASAFWRkEVAQTIQACRVFIFIVSPHSVTSNYCLEE----IQFAYD-HGKVIFPVIKDRN-FDDEIDPGLDMLLKRFQwiFFDGVEQSAKVGGDDELAPTPAA(VESAGDRTLATPTFDEQAARLAKAIVKQLQESGS)}*

$1724=250(256):
>gi|765551381|gb|KJE92002.1| {|1262(568)|<Opisthokonta(E)>}hypothetical protein CAOG_008660 [Capsaspora owczarzaki ATCC 30864]
{(RATTLFNEAKLLSWVVWVVTVFGVGGAVLVLALSSAVSATGLQVFINISFFVSGCALTFIIFLPKIYSIISGSWKAGGLG)-KEVVLCYTIEdKSFMKRIEAMLT----EAGIEVWFDElRQHADKTRRrEVAQTVMGCQSLIFIVSEHSVKSPYC-QtEVQFAF----D-SGVRVFPVIISNK-FMEVIDPGLEMLLKRFQwvIFDEPNKPESALDASSDDLSAAS(HKQKKNLRAERMLQLKNAVSNHLKMKSDQRSKPASH)}*

$1725=250(259):
>gi|765553236|gb|KJE93857.1| {|232(700)|<Opisthokonta(E)>}hypothetical protein CAOG_004582 [Capsaspora owczarzaki ATCC 30864]
{(HSIQPGFPASSGFEPNVLGGSIASGISRSETRQFTPPPIVAADSDGLEEDTSISFWTTLRSMMPTMLTSGELPQVSDKEF)----FLSYSRKdKATAARLVDMFT----EANVDVWVDwTSIPPSADWMsEICKGIENSSAFLFLLSPNSVSSLVC-NqELDYAIQ-----CGKRVIPIVIEDI-

DASIVVRTEISSRNYIFmrQTDNGAESFKTIIHALSQNLDYV(HEHTRILKLAHTWNDNHRKPDFLVFGHVAN
AAQQWLTDS)}*

$1726=273(275):
>gi|765551381|gb|KJE92002.1| {|1473(334)|<Opisthokonta(E)>}hypothetical protein CAOG_008660 [Capsaspora owczarzaki ATCC 30864]
{(AASHKQKKNLRAERMLQLKNAVSNHLKMKSDQRSKPASHVAEAGNANQEGYAGAPSKTQTHAQAPNTPNEL
AAELKPKDL)----FISWAH---IDHALVRErLWPDLEARHIDCWIDVSALVAGDFWrrAIGQAIKHCKV-
FLFVMTPASIKSQYCQeELLYAASCKKLI---YVIHTKsckreLDEF-
PDIRAvIADAVKTDFSDTVLYLSHVDSLAEQIAKEFATIRE(QEQAVALKKQQDREQQQLRARSRLPNGSSSV
RSASNTKWKPLAIISSALSRTRMH)}*

$1727=194(191):
>gi|766752714|ref|WP_044835061.1|
{|0(283)|<Proteobacteria(B)>}hypothetical protein [Thalassomonas actiniarum] >gi|765429088|gb|KJE38758.1| hypothetical protein SG35_24130 [Thalassomonas actiniarum]
{(MNTPK)KVRVFLSY-LSednhvaEVIKKELVGYDERI------
EVFKADDSLRAGVNYktQLRDSLNDSDWLLLIYTNQGKDWQWPIFEGSYFLAANSagnTDsrlCCLYDSD-
EWP---
KPFSDYQSYKAEVYKPQIGDDERTNKYASDQFYHNSALFN(FLVNFLSYPDDNPIRSNPLSSHENLIASASRI
AEAFLENRSNTVEK)}*

$1728=173(172):
>gi|766758010|ref|WP_044837248.1|
{|0(304)|<Proteobacteria(B)>}hypothetical protein [Thalassomonas viridans] >gi|765438888|gb|KJE47749.1| hypothetical protein SG34_04070 [Thalassomonas viridans]
{(MNTPR)KVRVFLSY-LTednhvaEVIKKEL------
IGYDERIEIFKADDSLRAGVNYktQLRDSLNDSDWLLLIYTNQNKDWQWPIFEGSYFLAANSTEnedSRLCCL
YDSDK---
RPKPFSDYQSYKAEVYKPKLDDDEQINKYASKEFYHNSAL(FKFLVKFLSYPDEHPIRSNPLKCQDNL)}*

$1729=291(292):
>gi|768432417|ref|XP_011557532.1| {|992(58)|<Arthropoda(M)>}PREDICTED: slit homolog 3 protein-like [Plutella xylostella]
{(GDPMVSRNEIHSNSIFLPDNYFAIICGIIIIVVVICLIGAIFYAFRYEVSDWLYTKYGVPLFKEQSCPNVD
QVLEGNLNH)MYDYYVICNTKdtNFVNHNIMSELE-----FRKMASKKQIVSESSLNI-
LTldsfsKSSRLSKRLVIVITTNFLCNDLCEIHFKNIFYSYLKSLnrsdLNKVIFVKLVDN-
NQINDDLGFLLDKFKNIswD-----DPR--
FWDKFVALLNST(DTIITLKSTEKSVFKKSLRQTPTLRYTTMPVTGDSCSKQNFTNSLQYCKGRDEDDSPCES
SPSSDNNTYGDG)}*

$1730=246(247):
>gi|768188761|gb|KJH45275.1| {|747(0)|<Nematoda(M)>}leucine Rich repeat-containing domain protein [Dictyocaulus viviparus]
{(NLSICVPLSSGFFGQEPQNSILTIVFLASCLFLLCAMVFLGISVVRKAQNDMSQRRYKASSSLNCSSTPRS
SPLPVPLLN)-FDAFVSYSKKdeKMVLEQLCRPLEDEE--
YTLCLLHRDGPTYHSrlhaISDELISQMEASQCLVIILTKNFLESEWKTLQIKTSHQLFAKNRSKRIIAIL-
GDG-VDQNLLDEELGQILRKNtCIRQRDHL---
FWQLLHNAMPTQ(LSSIPSSGDTNSQIYSDMYGIVPSAVI)}*

$1731=253(262):

>gi|768441150|ref|XP_011562284.1| {|550(0)|<Arthropoda(M)>}PREDICTED: protein toll-like [Plutella xylostella]
{(CRASIFLNELREMDTKGADSNQLNCKFMQTFYCDGHELTYFLVLPMLIALTLLGIIYLELKERWQAKLDFSGKYRQIELT)-----VAYSEKdeAFVLKELLPGLR---ERINVNCNLRPLKPtrrNKTIVEHIKNYIKKETVTVVVFSPNYLTECYCDIEIRK-IRRIMRKQRNNIDVFL--DV-IDDNSLYEYLKSVANPMsSAFWSDSN---FWDHLLPLIEEK(QSLMKYAGVFPPRKTQKSKSKRSRIMKYTSRPAPVAVTHSQV)}*

$1732=247(246):
>gi|768936000|ref|XP_011608354.1| {|246(0)|<Chordata(M)>}PREDICTED: interleukin-1 receptor type 2-like isoform X2 [Takifugu rubripes]
{(SEFEEVFWLSGDSFVDTNESLPVYYNYTSDSTGTEIKMTASLVFREVSAEDLSKRYTCKLESESVPSTFVTVTLEQKGGK)SYDALLVSYKNeedtgmmeLDR--KWMKSILEERFGYA--VYDCGIMRGKG--DALSERIQQSRTVILVPTSSDGCLESG---LLSHNHIALMKQSSRVVLIR-SES-SASPRSEELQHLAKDGHCVTWKGTSSRlsssSFWKELQCYLPVP(QQTKRRPLLSTFTKHDNCETMDTHYC)}*

$1733=263(225):
>gi|768936011|ref|XP_011608359.1| {|300(0)|<Chordata(M)>}PREDICTED: interleukin-18 receptor accessory protein isoform X1 [Takifugu rubripes]
{(TPQHLHNTYTCTATNINGSSTVTIRLKQRHKVKWPSLVQYPIGPLLLVAGLGIILHMKWLELQLIFRSYFQFGNHSRAEK)EFDVFLSFVwprksSPsppgthdeagctnSNLLNWekggaterpleaLLPQVLE-EWGHRLCLLERDVLPGGVYANDVVLSIQRSRMLICVLSADYLsNTDAVF-ILESGIQALLLKSTPKLLLIWTnkasaDKI-KPDPPLPLLVQKALKVLpSLDWTSGNSErrtgDFWRSLKKLLPSD(NMSGY)}*

$1734=262(225):
>gi|768936013|ref|XP_011608360.1| {|300(0)|<Chordata(M)>}PREDICTED: interleukin-18 receptor accessory protein isoform X2 [Takifugu rubripes]
{(TPQHLHNTYTCTATNINGSSTVTIRLKQRHKVKWPSLVQYPIGPLLLVAGLGIILHMKWLELQLIFRSYFQFGNHSRAEK)EFDVFLSFVwprksSPsppgthdegctnsNLLNWEkggaterpleaLLPQVLE-EWGHRLCLLERDVLPGGVYANDVVLSIQRSRMLICVLSADYLsNTDAVF-ILESGIQALLLKSTPKLLLIWTnkasaDKI-KPDPPLPLLVQKALKVLpSLDWTSGNSErrtgDFWRSLKKLLPSD(NMSGY)}*

$1735=186(192):
>gi|951064793|ref|WP_057569386.1| {|0(269)|<Firmicutes(B)>}hypothetical protein [Paeniclostridium sordellii] >gi|770675677|emb|CEP48201.1| Uncharacterised protein [[Clostridium] sordellii]
{(MDS)--NIFISYCW-ND--NETVNEIDNYFKSKKIIFQRDKRDIGSwESIKDFMNRIRKSSYAILVISDTYLKSLNCMYEVLEVMKDEGY-RSRILTVVLDNAKIYDALGKAKYISYWNQKY-KELSEEISKIDDCESTIILSEE(LRKINEIKNNVGEFMKIVSDMNNPNVDNIKEEIRKKLCDKGLYTTLNLI)}*

$1736=156(157):
>gi|772636377|ref|WP_045274410.1|
{|0(277)|<Actinobacteria(B)>}hypothetical protein [Microbacterium ketosireducens] >gi|770659794|gb|KJL44934.1| hypothetical protein RS81_00402 [Microbacterium ketosir
{(MPDTAINPRDP)---VFVSYRHSDGIALAAELTWLLRAAGI--PVWRDVDDLPPGDTDaRLQQAIDEGISGaVIIITPQIADSRVVREveAPRLLRLHRSSPQFALGIVNAIQTSTGVVDYDAPDRVLGMERPELRSVDQKSASRLGLVTMARQML(WHRIAA)}*

$1737=218(220):
>gi|769220765|ref|WP_044980468.1| {|168(0)|<Firmicutes(B)>}molecular chaperone Tir [Streptococcus suis]
{(SKYDSKEVRKFFYAINGHIRNQSLKIEVQLYFQSLSLPFRLSRKKKGKKNFIDNEIIKNKKGPDELKKRLY
INDLEKIKK)-FDVFLSHNSRdeEKIIEFYKKFNKKGQVVYIDWINDkydlrREWC-
NASTVDIIKERIKQSDVFVLYFSGETLNSQWCTWELGYA-----
DALGKKICVYFSEEINrKGIPKFYVAYPELqMSKNiYVKIKGNEDIllEEWKKL-------()}*

$1738=175(180):
>gi|769124636|ref|WP_044906684.1| {|0(94)|<Firmicutes(B)>}hypothetical protein [Lachnospiraceae bacterium MC2017]
{(MSFP)-KYVFISYSSKdIVKVNKIVELLESMNVE-----YWKAPE-
KIPAGSsyanEIVNAIENCSLFLVILSENAQSSIWVEKEIDCALSSAKE-
VIPYNIDNCVLVNAFKFYLNNVQMIHDTPGS--
PEGIDKLKGFLRPLQPAESKS(SGTNVNIPMAPEQKIPAPKAPDPKAAAKKSNLKTAT)}*

$1739=223(226):
>gi|769142545|ref|WP_044919620.1| {|0(195)|<Firmicutes(B)>}hypothetical protein [Lachnospiraceae bacterium MA2020]
{(MFSPNY)---VFLSYSSKdQEFVKKIVELIESMGIE-----YWKAPE-
KIPAGSsyakEIVNAIDNCSLFLIILSENSQSSIWVEKEVDCALSSSKK-
VIPYNIDGssLNKAFKFYLNNVQMIIHTPNSkksiDE--LIGFLRP--
LQPEFKKMNVKK(IVSTKMKLQNYVYSLNSGTNKLPNDNTNPISTPTSAANTSLNTKTERKQSDSEKIEKNIP
IEHRVPIMSRVPIIPKVTIV)}*

$1740=277(290):
>gi|772751383|ref|WP_045314170.1|
{|695(72)|<Actinobacteria(B)>}hypothetical protein [Lechevalieria aerocolonigenes] >gi|770208201|gb|KJK45591.1| hypothetical protein UK23_25525 [Lechevalieria aerocol
{(ARWKAEDQAAQQVLDAFDRNLAKLPRDRALQHALREVADRHPADWACWSLYGDAGVQTKAGPLRRRLRKNG
EPVPLETRP)--KVFLSFAGKDRPHAEQLRAELEARNVN-
AYLDEHEIAPGANVVASLSDALATSDYYVLLWSAN-TPSREWVTeEWTAAftLQLT-----
RKRAFLFIVRLDeePLPPLLAPRKHIDLV-------EAAD--
RLVSTWRADRKS(ELPVFPQPVPPQPGGPTVAISVRSHDLGVTHVVMTPLHLTGAELYRAVFKAMQLPTEQAT
FNGTIGMRFS)}*

$1741=228(230):
>gi|769241998|ref|WP_044986083.1| {|0(728)|<Proteobacteria(B)>}ATP/GTP-binding protein [Sorangium cellulosum]
{(MVALATSPAD)---
LFFSYSPRdEDLRPKLETHLDALKRQGVIRTYQGFALGGGRERReAFAARLREARVVLLLVSADFLNTDAWLS
pELREAIARHEA-GECRVIPVILRPC-AWEKTALGKLQPLPNRArpVTSYPNQS--
EAFTEIAKGIRSA(VRETEPPTQRPAPRRQSSSPDAEPHIKKLPPRNPSFTGRDRLLNELRSALTTGGPAALT
QAISGLGGIGKTQLAAEYAHR)}*

$1742=234(230):
>gi|780045836|ref|XP_011668830.1| {|215(0)|<Echinodermata(M)>}PREDICTED: toll-like receptor 13 [Strongylocentrotus purpuratus]
{(SSDSLEPFRDEYLIDFDPKKFCSINIALVCLPTLAIICLIFIVALVYHYRWPLRYRLFLVKLAVLGYKEMR
DARDHNDYE)-
FDLNVIFYDDdeEWIREHLRPALAERLPHFQRNVFGDADLVLGMHYLeSVDYVVSHSYKTIIVLSSAAVLDRW
FILKFRTAMDHVSDTLTEFVVVVFLEDIPDDEMPFLARLY-
LSDGRpYIHWTEGLRGqeYFFDKLTTNLTIN(LRTNDRIPNE)}*

$1743=214(218):
>gi|889717077|ref|WP_048836610.1|
{|0(48)|<Proteobacteria(B)>}hypothetical protein [Acetobacter orleanensis] >gi|775236022|dbj|GAN69491.1| hypothetical protein Abol_037_008 [Acetobacter orleanensis JC
{(MTT)---VFISHITReNKIASSLKSEIDDSLLGGVQVFQSSDPNS-
IGMGDnwlkVIDQNLNTCDALILLVSPESVDRPWINFEFGAAWARRIS-
VIPIVHSGMTLGKLPIPMSLQQGFELTNTQG--MQGLIGK--
LAGLANLRAPVR(NYEKFCSDVKRLEDISYKYREALIEIKSIDEEVYKFLCELKVEAGQFFPRQYPERIFEKM
RESFDALKSGGFIEY)}*

$1744=175(178):
>gi|778708803|ref|XP_011656285.1| {|0(872)|<Streptophyta(V)>}PREDICTED: TMV resistance protein N-like [Cucumis sativus]
{(MA)-FDAFLSFNR-DeedDGYRRFIKDLYETLSEWGIKMFMDEEV----N-
LsdNIVKAIEGSITSMVVLSEGYASSKWCLRELVKIIDQKKK-
TKHQVLPLFYGGRVAPPPPPRPLFKQWEGRSLRSLSRNRETDAYSEVGSMTSRQ(VKFRYQHAFEKFINSEAK
KDYLKEVDKWWLSVLEVS)}*

$1745=257(290):
>gi|780030598|ref|XP_011666611.1|
{|303(70)|<Echinodermata(M)>}PREDICTED: uncharacterized protein LOC105439381 [Strongylocentrotus purpuratus]
{(LKKIRETTENSDVKDACSGALFVINDVIDIYDAFKGHTPTRGDPGHSSAKSEVSSGHVEGHIMISYQHASQ
ERMLRVKSY)-------------------------
LEELGYNVWMDVDKMKGDILDSMAKAVQRASVVLVCMSRKFNESYHCRTEATYAYTLK---
KDIIPLKLQNQFYPED--WLGALMG--MKKY-
YSMFSDDLMKQYLPNLVRELGD(RGKREKLDLVVPLLDQVTGASNPTAHAQRQGDGTAEDDVDCSGGIKHDKP
TGNEDEGNSRSQTAVDLVKW)}*

$1746=256(289):
>gi|780030608|ref|XP_011666614.1|
{|302(69)|<Echinodermata(M)>}PREDICTED: uncharacterized protein LOC105439383 [Strongylocentrotus purpuratus]
{(LEKIKETTEDYDIKDACSGALFVINDVLDIDDEFKGHIPTRGDGGHSSVKNEVSSGHVEGHIMISYQHASQ
ERMLQVKCH)-------------------------
LEESGYNVWMDVDKMKGDILDSMAKAVQRASVVLVCMSHKFKESQHCRTEATYAYTLK---KD--
IIPLMLQDQFTPEDWLGALMG--MKKY-
YPMFSDDLMRQCLPDLVSELSD(RGKREKLDLVLPLDDEVTGASNPTSHAQRQRDGTAEDAVDCSGGNKDDKP
TGNGDEGNSRSQTTVDLGK)}*

$1747=240(273):
>gi|780030628|ref|XP_011666616.1| {<Echinodermata(M)>}PREDICTED: uncharacterized protein LOC105439384 [Strongylocentrotus purpuratus]
{(MDSSCSELLDAINEMFNLDSSDDSDDSDESEGHTPTRGDPGHCSVKSEVSSGHDKGHIMISYQHASQQRML
RVRNY)-------------------------
LVERGYNVWMDVDNMEGDILESMAKGVERASVILVCMSHKFKESKNCKAEAGYAHALG---KH--
IVPLMLEDKFVPTAWLGPLVG--MKKY-
FPMFSDDLMKQQLPKLLKELGD(KGKHGGKGASNPTAHDQIKWKGPAKGKEIDWLQGLDSWDEKEALGWLIKN
GVDTSMW)}*

$1748=256(289):

>gi|780098271|ref|XP_011674898.1|
{|303(71)|<Echinodermata(M)>}PREDICTED: uncharacterized protein LOC105443448 [Strongylocentrotus purpuratus]
{(LKKIRETTENCDVKDACSGALFVINDVFDIDEEFKGHTPTRGDRGPSSVKREDSPGHVEGHIMISYQHASQ
ERMLQVKRH)------------------------
LEESEYNVWMDVDKMKGDILDSMAKAVQGASVVLVCMSHKFKESQNCRTEATYAYTLK---KD--
IIPLMLQDQFTPEDWLGALMG--MKKY-
YPMFSDDRMKQCLPDLVSELSD(RGKREKLDLVVPLLDQVTGASNPTAHAQRQGGGTAEDDVDCSGGIKDDKP
TGNEDGGNSRPQKAVDLVT)}*

$1749=252(285):
>gi|780098334|ref|XP_011674906.1| {|0(64)|<Echinodermata(M)>}PREDICTED: uncharacterized protein LOC105443453 [Strongylocentrotus purpuratus]
{(MVFGMALKKIKETTKDSDVKEACSGALFVINEVFDIDDEFKGHTVKSEISSGHVQGHIMISYQHASQEQML
RVKSY)------------------------
LEESGYNVWMDVDKMKGDILDSMAKAVQRASVVLVCMSRKFKESQHCRTEATYAYTLK---KD--
IIPLMLQDQFTPEDWLGALMG--MKKY-
YPMFSDDRMKQCLPDLVSELSD(RGKREKVDLALPLLDKVTGASNLTTHGPPENDVDCSGGIKHDKPTANEDE
GNSHSQKVVDLVPGDEDVG)}*

$1750=233(236):
>gi|780005012|ref|XP_011661326.1| {|688(0)|<Echinodermata(M)>}PREDICTED: toll-like receptor 3 [Strongylocentrotus purpuratus]
{(SLTSLPSLAGQPLLNFKPRYSCAPKIALYIGISILCVSFLLGMATAYQKRWWISYKCFHIKLWILGYEEFQ
DGREHVDFK)-
YDVNIVFDEDdeEWVQDTLRPGIQEHLPHLnRVVFGDKDLPLDMFYMDAVIYVMENSFKTVLVVSKSAINDHN
FITKLRQAVDLMNEEEIERALLVFKDDISqGRMP---YLVRLLLSqNKpYLLWNKERYAkrLFWEK-------
F(TKNMLVNKIMNDLLPV)}*

$1751=234(236):
>gi|780158611|ref|XP_011682738.1| {|362(0)|<Echinodermata(M)>}PREDICTED: toll-like receptor 3 [Strongylocentrotus purpuratus]
{(SESSLDPLRAKPLLQFDPDELCKRSIVLFYSLPLATICLIVIIVLVHHYRWKVKYKIFLLKLAALGYNELR
DARDHSDFE)-
FDLNIIFYDDdeEWIREHLRPALKAQLPQFqRNVFGDEDLVVGMHYLDSVDYVVSHSYKTIILLSRAAVHDRW
FMLKLRTAMDHVSDTQTEFVVVVFLEDIPdEEIP--FLARLYLSDGRpYLYWTDDVRGhgYFWHGL------
T(KYLTINLRTNDWIPNE)}*

$1752=246(243):
>gi|780020158|ref|XP_011665006.1| {|318(0)|<Echinodermata(M)>}PREDICTED: LOW QUALITY PROTEIN: toll-like receptor 7 [Strongylocentrotus purpuratus]
{(SPASLKPLQNKPLLDFNPSELCSLSFPLVCLLPLAVISLVVIVVLVYHYRWQLRHRLFLLRLAAIGYKEIQ
DAREHDDYE)-FDLNIIFYDDdeEWVREHLrlairVPLXQYQRKRHKV---
MRSSYLGMHYLESVDYAMRGSYKTVVMLSRPAVGDRWIMLKFRTAMDQVSDTQTEFVVVIILEDIPdEELP--
FLARLYLSDGRpYLHRPEDVRGqeYFLDELTKNL--T(INLKTNDLIPNALYPFPRCYTTE)}*

$1753=285(299):
>gi|780058132|ref|XP_011670043.1|
{|24(130)|<Echinodermata(M)>}PREDICTED: uncharacterized protein LOC100890087 [Strongylocentrotus purpuratus]
{(KVATAEKEKLLEGEDTHMSSFLTDWDTEADQGGGRMVTADSGDDHSVDSLPMSPDEANSNQNADMLVRGMA
PVLVGEETS)--HAFFCFAWEddEWAINVIRK-
LESSAYGFSCDNSNHKWDPGVAKMDKIINSISTAKKLVLVVTPDFMRSPWCMYENLRAL-
RGHYTNTAKVIVVVLGEIE---------LPDFLEGI----PTINAMsrNFWQRFVAALR-

L(GSSLDAHDASLNNNVPALYNSAHLATIQSSTDCCCKARFDTIYCPDELARKGVQIPQQDYTAAISSILDAPKMRWYTLW)}*

$1754=201(229):
>gi|780102704|ref|XP_011675464.1| {|655(0)|<Echinodermata(M)>}PREDICTED: slit homolog 3 protein-like [Strongylocentrotus purpuratus]
{(GTVLPGNTSLRLDISNNPFSCTCSLAWFRQWLDSADIDVKHADQTLCSGTSLKGLSKQPILSFHPEDHCGVNIFLIAGIN)-----------------------------
IIYGDRDLHLGMFYINAINDALDNSFKTVLLISNQSISDAWCMTKLRMALEHLNDTGLDKIILIFLEDIEdENLP---YLVRLFMSRnKpYMLWTDDEDGqeLFWAQFERSMRAN(KAINNAIPL)}*

$1755=215(229):
>gi|780102708|ref|XP_011675465.1| {|391(0)|<Echinodermata(M)>}PREDICTED: toll-like receptor 4 [Strongylocentrotus purpuratus]
{(SADIDFKHADQTLCSGTSLKGLSKQPILSFHPEDHCGVKYLLIATVVXYEEMAEDFDADNYEFHLNFMFLKRKKNGLIXF)-----------------
MKPALEERFPHLQnIIYGDKDLHLGMFYINAINDALDNSFKTVLLISNQSIRDAWCMTKLRMALEHLNDTGLDKIILIFLEDIEdENLP---YLVRLFMSRnKpYMLWTDDEDGqeLFWAQFERSMRAN(KAINNAIPL)}*

$1756=233(229):
>gi|780106994|ref|XP_011675979.1| {|193(0)|<Echinodermata(M)>}PREDICTED: toll-like receptor 4 [Strongylocentrotus purpuratus]
{(SGTSLKEFVNKPILSFHPEDHCGVNVVLIVVLSFLGVLVGVMAMLVYNKRWWLNHKIFLLKLAIVGYIEMEEDFNAGNYR)-
HHLNLMFHETeeEWVNQVMKPALEERLPHLQnIIYGDEDLHLGMYYINALYDAIDNSFKTVLLLSNQSVNNAWTMTKLRMALEHINDTGLDKVILIFVEDIEdDNLP---
YLVRLFLSRnRpYMLWTEDEDRqeLFWAQFEKSTRAN(RAINNAIPL)}*

$1757=234(232):
>gi|780142240|ref|XP_011680821.1| {|774(0)|<Echinodermata(M)>}PREDICTED: toll-like receptor 4 [Strongylocentrotus purpuratus]
{(SLASLEPLRGKPLLNFDTSKLCGLNIALVCLTPLAGIIVVVIVVVLYHNRLQLKYKLFLLKLMVIGYKEMRDARDHNDYE)-
FDLNIIFYDDdeEWARQQLQPALEARLPQFqRNVFGDQDLIPGMHYLDAVDYVVSHSYKTLILLSRAAVHDRWFMLKLRTAMDHVSDTETEFVVVVFLEDIPdEDIP--FLARLYLSDGRpYLHWTEDVRGhdFFWNKLVKSL--
T(INLRTNDLIPNE)}*

$1758=233(231):
>gi|780159497|ref|XP_011682851.1| {|689(0)|<Echinodermata(M)>}PREDICTED: leucine-rich repeats and immunoglobulin-like domains protein 1 [Strongylocentrotus purpuratus]
{(SQASLSKFKNQPILNIDPRGVCGPRIIVYVISTFVIVTCIVVIIVAYKRRWLINYKCFHFKLLFVGQHGDQDGRERLDYE)-
YDINLAFDEDdeQWVRGILKPGLEERLPDFdRIVCGDDDLPLGMYYIDAISEVVEQSYKSILIVSNRAVDNHEFISKLRLAVDQMNEVELEKVILIFKEDIPdGRLP--YLVRLFLSKNKpYFRWSEDEYGqkLMWEKLVKEL--
A(WNKKMNDVLPI)}*

$1759=209(214):
>gi|780100827|ref|XP_011675231.1| {|458(0)|<Echinodermata(M)>}PREDICTED: toll-like receptor 3 [Strongylocentrotus purpuratus]
{(PLRWKPLHDFDPCKLCGCNFVLVGLTPLAGIFLAVIVAVIYHNRWSMKYKLFLLKLMVIGYKEMRDAREHNDYE)FDLNIISYDNDeEWVRQLLQPALEERLPQFQRNVFGDqDLIIGMHYLDAVDYVVSHSYKTIILLSRAAVNDRWFMHKFRTAMDHVSDTQTEFVVVVFLEDI--PDEEIPFLARLYL-SDGRPYQHWTEDVKEDTIFLE----
()}*

$1760=261(258):
>gi|780033455|ref|XP_011667032.1|
{|149(236)|<Echinodermata(M)>}PREDICTED: uncharacterized protein LOC105439572 [Strongylocentrotus purpuratus]
{(KACLEQYNIALSCNRRALRDMLVPIYVDSVEMMPTYMGLFQYVDCRPHDHLKIGEACSQLAASLTDTFHTE
LRVAEFDPL)RYDVFLSYSHCdaEKANR-
LVGLLQKLAPDLKLFFDLKELKAGKSWQRTLYHSIDGSRCMLALISQPYLKSAVCQEEFALAQAKHCAKGKQH
lqLIPICLEDL-
NTIQPEFTHIPMVKGTPdVFDDMVKTVCPAVIQWLKGERVD(QTETIKTLFDDQNITTLSADADMEKFRQCHF
QKEFGTQ)}*

$1761=252(251):
>gi|780024433|ref|XP_011665744.1|
{|457(235)|<Echinodermata(M)>}PREDICTED: uncharacterized protein LOC100890762 [Strongylocentrotus purpuratus]
{(NACIEQYNIALCCNRRALRDMLAPFYVDSVEMMPTYMGLVQYVDCRPHDPSKIQDACSSLSVSLSITLHPE
LTVAEFDPL)RYDVFLSYSHRdtEKAN-
CFVDMLQKLAPNLKIFFDVQELKAGKSWQRTLYHSIDGSRCMLALISEPYLKSAVCQEEFALAQAKHFQKGKQ
HLQLVPISLE-
DLDTIQPEFTHIPMVKGtPDVFEDTVNTICPAVVHWLTGGK(VDTSEALTKMFDEDNIPCASPNDEMASFHHA
)}*

$1762=299(289):
>gi|779988119|ref|XP_011665199.1|
{|211(13)|<Echinodermata(M)>}PREDICTED: X-linked interleukin-1 receptor accessory protein-like 2 isoform X2 [Strongylocentrotus purpuratus]
{(TSEGNYTVVCSTPLGNINNTLKLKVQPRWSLPKWEILVSVMMAFIVMTVLMSALIYRTKRTEIQLLYKDQF
GKQEQDDGK)DYDAYISYywsdgesSHNpDRLYALDLKQRL-
TKLGYTVCLAETDFLPGNETSEEIVQSLTRSRRYIILLSPQFLTYRYMSLETEQCLD-
IIKQNKKEIIPIFIREISPDVIKAHVALDHIVSThprL--
DWqifKDSRSQEPLRTLSMENGNH(IEMEARESIQVAPKLKRAETRVWSTLRLQMPPVPRQRSSSSSTTEQPL
SSVEADSLIENAVLQEDSMPG)}*

$1763=234(230):
>gi|779998168|ref|XP_011677123.1| {|91(0)|<Echinodermata(M)>}PREDICTED: toll-like receptor 13 [Strongylocentrotus purpuratus]
{(SSASLEPLREKLLLDFDPNTLCGLNIALVCLTPLAGIFLVVIVVVLYRKRWQLKYKLFLMKLMVIGYKEMR
DARDLNDYE)-YDLNIIFYDDdeEWACQQLQPALEERLPQFQRNVFgDQDLIPG-
MHYLdAVDYVVSHSYKTLILLSRTAVHDRWFMLKFRTAMDHVSDTETEFVVVVFLEDIPDEEIPFLARLY-
LSDGRpYLHWTEDVRGhdFFWNKLVKSLTIN(LKTNDLIPNE)}*

$1764=234(230):
>gi|780011842|ref|XP_011663867.1| {|258(0)|<Echinodermata(M)>}PREDICTED: toll-like receptor 3 [Strongylocentrotus purpuratus]
{(SSASLEPLREKPLLDFEPNELCIFNNGLLFLIPLASISLIIIIVLLYHYRWQLRYKLFLLKLAVVGYKEMR
DARDHNDYE)-
FDVNIIFYDDdeGWIREQLQPALEERLPQFQRNVFGDEDLVLGMHYLdSVDYVVSHSYKTIIVLSKAAVHDHW
FILKLRTAMDHVSDTLTEFVLVVFLEDIPDDEMPFLVRLY-
LNDGRpYIHWTENVRGqdYFWDELTKNLTIN(LKTNDLIPNE)}*

$1765=234(230):
>gi|780018721|ref|XP_011664815.1| {|30(0)|<Echinodermata(M)>}PREDICTED: toll-like receptor 4 [Strongylocentrotus purpuratus]

{(CSSASLEPLREKPLLDVDPNQLCIINGLLFLIPLASIGLVVISVLLYHYRWQLRYKLFLLKLAAVGYNEMR
DARDHNDYE)-
FDVNIILYDDddVWIREQLRPALEERLPQFQRNVFGDEDLVLGMHYLdSVDYVVSHSYKTIVVLSRAAVQDRW
FILKFRTAMDHVSDTLTEFVVVVFLEDIPDDEMPFLARLY-
LNDGRpYIHWTEDARGqeCFWDELTKNLTIN(LRTNDLIPNE)}*

$1766=234(230):
>gi|780019712|ref|XP_011664946.1| {|648(0)|<Echinodermata(M)>}PREDICTED:
toll-like receptor 3 [Strongylocentrotus purpuratus]
{(SPASLEPLRGKPFLDFDPNKLCGLNIVLVCLTPLAGIFLVVIVAVLYHNRWQLKYKFFLLKLMVIGYREMR
DARDHNDYE)-
FDLNIIFYDDdeEWVRQQLQPALEERLPQFQRNVFGDQDLVIGMHYLdAVDYVVSHSYKTIILLSRAAVHDRW
FMLKFRIAMDHVSDTQTEFVVVVFLEDIPDEEIPFLARLY-
LSDGRpYQHWTEDVRDhdFFWNALVKNLTIN(LRTNDLIPNE)}*

$1767=234(230):
>gi|780030817|ref|XP_011666641.1| {|490(0)|<Echinodermata(M)>}PREDICTED:
slit homolog 3 protein-like [Strongylocentrotus purpuratus]
{(ELCIINGQLFLIPLASIGLVVISVLLYHYRCRCRCPCQCPCRCRCRCRCQWQLRYKLFLLKLAILGFKEMQ
DARDHNDYE)-
FDVNIIFYDDdeEWIREQLRPALEERLPQFQRNVFGDEDLVLGMHYLeSVDYVVSHSYKTIVVLSRAAVHDHW
FILKFRTAMDHVSDTLTEFVVVVFLEDIPDDEMPFLARLY-
LNDGRpYIHWTEDMRGqeYFWDKLAKNLTIN(LRTNDLIPNE)}*

$1768=234(230):
>gi|780032046|ref|XP_011666822.1| {|384(0)|<Echinodermata(M)>}PREDICTED:
toll-like receptor 3 [Strongylocentrotus purpuratus]
{(SSASLEPLREKPLLDFYPNELCMLNNSLFSLIPLISISLVVISVLLYHYRWQLRYKLFLLKLAVVGYKEMR
DARDNNDYE)-
FDVNIIFYDDdeEWIREHLRPALEERLPQFQRNVFGDEDLVLGMHYLdSVDYVVSHSYKTIIVLSRAAVHDHW
FILKFRTAMDHVSDTLTEFVVVVFLEDIPDDEMPFLARLY-
LSDGRpYIHWTENVRGqeYFWDELTKNLTIN(LRTNDLIPNE)}*

$1769=234(230):
>gi|780079041|ref|XP_011672549.1| {|435(0)|<Echinodermata(M)>}PREDICTED:
toll-like receptor 3 [Strongylocentrotus purpuratus]
{(SSASLEPLRGKHLIDFDPREFCSINIALVCLPTLAIICLIFTVALVYHNRWQLRYRLFLFKLAAVGYEEMR
DARDHNDYE)-YDLNVIFYDDdeDWIREHLRPALTEQLPQFQRSVFgDADLIPG-
MHYLdAVDYVVSHSYKTIVVLSRAAVRDRWFILKFRTAMDHVSDTLTEFVVVVFLEDIPDDEMPFLARLY-
LSDGRpYTHWTEDVRGheYFFDKLTKNLTIN(LRTNDRIPNE)}*

$1770=234(230):
>gi|780094232|ref|XP_011674438.1| {|99(0)|<Echinodermata(M)>}PREDICTED:
toll-like receptor 4 [Strongylocentrotus purpuratus]
{(SSASLEPLREKPLLDFDPNELCIMNYGLFSLIPLASISLIVISVLVYHYRWQLRYKLFLLKLAAVGYKEMQ
DARDNIDYD)-
FDLTIIFYDDdeEWIREQLRPALEERLPQFQRNVFGDEDLVLGMHYLdSVDYVVSHSYKTIIVLSRAAVHDHW
FILKFRTAMDHVSDTLTEFVVVVFLEDIPDDEMPFLARLY-
LSDGRpYIHWTENVRGqeYFWDELTKNLTIN(LRTNDLIPNE)}*

$1771=234(230):
>gi|780125049|ref|XP_011678465.1| {|285(0)|<Echinodermata(M)>}PREDICTED:
insulin-like growth factor-binding protein complex acid labile subunit
[Strongylocentrotus purpuratus]

{(TGTSLKPLREKPLLEFDPNELCIINNGIFSLIPLVSISLVAISVLLYHYRWQLRYKLFLLKLAILGYKEMR
DVRDHNDYE)-
FDVNIIFYDDdeEWIREQLRPALKERLPQFQRNVFGDQDLVLGMHYLeSVDYVVSHSYKTIIVISRAAVHDHW
FILKLRTAMDHVSDTLTEFVVVVFLEDIPDDEMPFLARLY-
LNDGRpYIHWTEEVRGqdCFWDKLTKHLSIN(LRTNDFIPNE)}*

$1772=269(264):
>gi|780160023|ref|XP_011682927.1|
{|66(741)|<Echinodermata(M)>}PREDICTED: toll-like receptor 4
[Strongylocentrotus purpuratus]
{(SFLSMKPFGGEALESIQPNDLCTANFPVYFSAVFFAVIFVIFIIFVYHFRWQLRYKHFLLRLAILGYREIL
DKNDREDYD)-
FDVYVISTDDdeNWIHDQLKPSFQRFLPYYKRSrnVFTEDDLPLGMHRTeAVDHVLTRSFKILVLVNKAACAD
DWFLTCFRMAMDQVADTQTENIIVVFLENIEEDEMPLNEIRPHNEHGIrTIAGHFSGIRKSFACNVDIPEKM(
ADCSDKQLKDVPSNLINDLRSLDLSSNIIRSLWNTSFLRYSLLE)}*

$1773=234(230):
>gi|780167008|ref|XP_011683911.1| {|653(0)|<Echinodermata(M)>}PREDICTED:
toll-like receptor 3 [Strongylocentrotus purpuratus]
>gi|780167013|ref|XP_011683912.1| PREDICTED: toll-like receptor 3
[Strongy
{(SSVSLKPLRGEHLIDFDPREFCSINIALVCLPIIATICLIFIVALVYHNRWQLRYRLFLLKLAALGYEEMR
DARDHNDYE)-
FDLNVIFYDDdeNWIREHLRPALAERLPQFQRNVFGDEELVLGMHYLeSVDYVVSHSYKTIVVLSRAAVHDHW
FILKFRTAMDHVSDTLTEFVVVVFLEDIPDDEMPFLARLY-
LSDGRpYIHWTEDVRGheYFFDKLTKNLTIN(LRTNDWIPNE)}*

$1774=234(230):
>gi|779992947|ref|XP_011671170.1| {|320(0)|<Echinodermata(M)>}PREDICTED:
toll-like receptor 13 [Strongylocentrotus purpuratus]
{(NNSSLQPLREKPLLDFDPNELCIMNYGIFSLIPLASISLIVISVLLYHYRWQLRYKRFLLKLAVLGFTEMR
DARDHNDYE)-
FDVNIIFYDDdeEWIRRQLRPAFEERLPQFQRNVFGDEDLVLGMHYLnSVDYVVSHSYKTIVVLSRAAVHDHW
FILKFRTAMDHVSDTLTEFVVVVFLEDIPDDEMPFLARLY-
LSDGRpYIYWTENLRGqeYFWAKFTKNLTIN(LRTNDLLPNE)}*

$1775=267(264):
>gi|780022556|ref|XP_011665334.1|
{|135(252)|<Echinodermata(M)>}PREDICTED: uncharacterized protein
LOC752660 [Strongylocentrotus purpuratus]
{(QTEFVVVVFLEEIPDDEIPFLARLNIVLYCVLSLAAICFVVAVGFVFHNRWQIRFKLYLLKLAVLGYNELR
DARDHNDYE)-
FDMNIIFYDDdeHWIREQLQPAFGERLPQFERNVFGDDDLVLGMHYLdSVDYVVSRSYKTVIVLSRAAVRDRW
FMLKFRIAMDHVSDTQTEFVVVVFVEEIPDDEIPFLARKLSALQELsIEFCDISNLHHLTFVGLISLQKL(SF
LSLANNKFTSFNQSTFQPIKPSLSSIDLSNNPMNCNCDLKWL)}*

$1776=237(230):
>gi|780023546|ref|XP_011665570.1| {|31(0)|<Echinodermata(M)>}PREDICTED:
toll-like receptor 4 [Strongylocentrotus purpuratus]
{(SLESLEPLREKPLLDFNPNELCIKNYGLFSLIPLVSISLVVIIVLLYHYRWQLRYKRFLLKLAVLGYKEMR
DARDHNDYE)-
FDVNIIFYDDdeDddeWIREQLRPALEERLPQFQRNVFGDQDLVLGMHYLdSVDYVVSHSYKTIIVLSRAAVH
DHWFILKFRTAMDHVSDTLTEFVVVIFLEDIPDDEMPFLSRLY-
LSDGRpYIHWTENVRGqqYFWDELTKNLTIN(RRTNDLIPNE)}*

$1777=234(230):
>gi|780030637|ref|XP_011666618.1| {|612(0)|<Echinodermata(M)>}PREDICTED: uncharacterized protein LOC105439386 [Strongylocentrotus purpuratus]
{(LHLKVYLLNHERYKLRSDVEALYAKPDMNRKTMKSISSLVVISVLLYRYRWQLRYKVFLFKLAVLGYKEMRDARDHNDYE)-
FDVNIIFYDDdeEWIREQLRPALEERLPQFHRNVFGDEDLVLGMHYLdSVDYVVSHSHKTIIVLSRAAVHDHWFILKFRTAMDHVSDTLTEFVVVLFLEDIPDDEMPFLARLY-
LSDGRpYIHWTENVRGqqYFWDELIKNFTIN(LRTNDLIPNE)}*

$1778=234(230):
>gi|780030642|ref|XP_011666619.1| {|102(0)|<Echinodermata(M)>}PREDICTED: toll-like receptor 4 [Strongylocentrotus purpuratus]
{(IPTGIFQQLSVLQELNLDYCDITNLHPLVFSGLESLRNLVVISVLLYRYRWQLRYKVNLLKLAVLGYKEMRDARDHNDYE)-
FDVNIIFYDDdeEWIREQLRPALEERLPQFQRNVFGDEDLVLGMHYLdSVDYVVSHSYKTIIVLSRAAVHDHWFILKFRTAMDHVSDTLTEIVVVVFLEDIPDDEMPFLARLY-
LSDGRpYIHWNENVRGqeYFWDKLTNNLTIN(LRTNDLIPNE)}*

$1779=236(230):
>gi|780052757|ref|XP_011669383.1| {|77(0)|<Echinodermata(M)>}PREDICTED: uncharacterized protein LOC105440655 [Strongylocentrotus purpuratus]
{(QINPSGSSWKIDLRELDLSENNLQMLSPFAFKGFSVVIVVIVIVIVYHYRWQLRYKLFLLRLSILGYREILDEYDREDYD)-FDIYVISTEDdeNWIQDKLKPYFQ-
GLPYYKRSrnVFTEDDLPLGRHRMeTIDRVLQKSFKILVLLSETACADGWFLDCFRMAMDEVADTQTEKIVVVFLQNIEEDVMPFYVRWY-MGGQGpYVEWvEDDDEGqkYFWKRLEKCLSVN(RKRNHLIPAE)}*

$1780=234(230):
>gi|780079956|ref|XP_011672655.1| {|208(0)|<Echinodermata(M)>}PREDICTED: toll-like receptor 4 [Strongylocentrotus purpuratus]
{(DFTFVCHTDTLDLRLNPIQSVDHDVIASLHVSSLMLCGYRLSYEVLANIILRISKSDIEWLTIQEGYKEMRDARDHNDYE)-
FDVNIIFYDDdeEWIRDQLRPALEERLPQFQRNVYGDQDLVLGMHYLdSVDYVVSHSYKTIIVLSRAAVHDHWFILKFRTAMDHVSDTLTEFVVVVFLEDIPDDEMPFLARLY-
LSDGRpYIHWTDNVNGqdYIWDKLTKNLTIN(LRVNDLIPNE)}*

$1781=237(230):
>gi|780133686|ref|XP_011679662.1| {|74(0)|<Echinodermata(M)>}PREDICTED: toll-like receptor 4 [Strongylocentrotus purpuratus]
{(SSASLEPLREKPLLKFDPNELCLMNNCLFVLIPLASIILVVISVLLYHYRWQLRYKLFLFKLAAVGYYEMRDARDHNDYE)-
FDVNIIFyddDEDdeEWIREQLRPTLEERLPQFQRNVFGDDDLVLGMHYLdSVDYVVSHSFKTIIVLSRAAVQDRWFILKFRTAMDHVSDTLTEFVVVVFLEDIPDGEMPFLVRLY-
LSDGRpYIHWTEDVRGqeYFWDKLTTTLTIN(LRTNDLIPNE)}*

$1782=234(230):
>gi|780174769|ref|XP_011661189.1| {|404(0)|<Echinodermata(M)>}PREDICTED: toll-like receptor 3 [Strongylocentrotus purpuratus]
{(SSASLEPLRGKHLIDFDPREFCSINITLVCLPTLAIICLIFIVALVYHYRWPLRYRLFLVKLAAVGYEEMRDARDHNDYQ)-
YDLNVIFYDEdeEWIREHLRPALAERLPQFQRNVFGDEDLVLGMHYLeSVDYVVSHSYKTVVVLSSAAVLDRWFILKFRTAMDHVSDTLTEFVVVVFLEDIPDDEMPFLARLY-
LSDGRpYIHWTEDVRGheYFFDKLTKSLTIN(LRTNDRIPNV)}*

$1783=234(243):
>gi|779984918|ref|XP_011665045.1| {|620(0)|<Echinodermata(M)>}PREDICTED: uncharacterized protein LOC105438652 [Strongylocentrotus purpuratus]
{(FYNDKEYVRGEKFWEDAKFSELLGQISYHANPDESMISDEYRDWIPQVEDKPVTVKKVSEEAKSSPAQQNR
TMESTEAPS)---IFISYQWDkqPEIKR-----
LFSRLTGLGYHCWLDINQMGGGDPLysKIDKGVRNAKVVVSCITPKYSLSANCRREVSLSDALR-----
KPIIPLLLEEMTWPPEGPMSMTFTQLLYIEFTKETTQASFDDEKFEELIEKI(KEHAEPTLQLVPASEQDEMK
VTS)}*

$1784=251(257):
>gi|779984918|ref|XP_011665045.1|
{|428(175)|<Echinodermata(M)>}PREDICTED: uncharacterized protein
LOC105438652 [Strongylocentrotus purpuratus]
{(FLAVRLGYSGEDIRNWALSPDPTMAILAEWYTTHKSSDATYAILSALEDMGRTDAAEIITQSLEEAELLVP
QAPPDESEK)PPTVFLSYQW-DHQSEVKA--
IKKHLEMAGFPCWMDIGQMGGGDQLfaKISQGMRSSKVVLCMVTEKYSNSENCNKEVNLANLLN-----
KPIIPILIDHTQWPPQGAMSMLFAQLLYIQFYNDKEYVRGEKFWEDAKFSEL(LGQISYHANPDESMISDEYR
DWIPQVEDKPVTVKKVS)}*

$1785=251(257):
>gi|779990206|ref|XP_011667905.1|
{|95(278)|<Echinodermata(M)>}PREDICTED: uncharacterized protein
LOC100888223 [Strongylocentrotus purpuratus]
{(FLAVRLGYSGEDIRNWALSPDPTMAILAEWYTTHKSSDATYAILSALEDMGRTDAAEIITQSLEEAELLVP
QAPPDESEK)PPTVFLSYQW-DHQPEVKA--
IKKHLEMAGFPCWMDIGQMGGGDQLfaKICQGMRSSKLVLCMVTEKYSNSENCNKEVNLANLLN-----
KPIIPILIDHTQWPPQGAMSMLFAQLLYIQFYNDKEYVRGEKFWEDAKFSEL(LGQISYHANPDESMISDEYR
DWIPQVEDKPVTVKKVS)}*

$1786=252(261):
>gi|779990206|ref|XP_011667905.1|
{|287(85)|<Echinodermata(M)>}PREDICTED: uncharacterized protein
LOC100888223 [Strongylocentrotus purpuratus]
{(FYNDKEYVRGEKFWEDAKFSELLGQISYHANPDESMISDEYRDWIPQVEDKPVTVKKVSEEAKSSPAQQNQ
TMESTEAPS)---IFISYQW-D--
KQTEIKRLFSRLTGLGYHCWLDINQMGGGDPLysKIDKGVRNAKVVVSCVTPKYSLSANCRREVSLSDALR--
---
KPIIPLLLEEMTWPPEGPMSMTFTQLLYIDFTKETTQASFEDEKFEELIEKI(KEHAEPTLQVVPAGEQDQTK
VTSQEQSPTPTPKDEGTAETS)}*

$1787=192(201):
>gi|780086544|ref|XP_011673485.1| {<Echinodermata(M)>}PREDICTED: uncharacterized protein LOC105442751 [Strongylocentrotus purpuratus]
{(MQQALKYTPGTYFFYPRYRGTIGMGRGSSHGEEGGGKGLPIATLESPS)---IFISYQW--DKKPEIKR-
LYSRLTDLGYHCWLDFKQMGGGDVLfsEIAKGVRDAKVVISCVTSNYALSPNCQKEVSLSHDLG-----
KPIIPLLLEKIWPLEGPMGMIFTGVLYIDCSKESTQARFEDEKFDELIEKIK(QHVQRNVDSVGSD)}*

$1788=235(251):
>gi|780101986|ref|XP_003731320.2| {|33(0)|<Echinodermata(M)>}PREDICTED: uncharacterized protein LOC100890789 isoform X2 [Strongylocentrotus purpuratus]
{(GFEDADIGRANDENTRQGGNYMGTYGLLKKWRRRQTSLTEKAALRSALVHAGFHDLASKYLSTPITEPIVP
QTPSDESGK)PPTVFLSYQW-DHQPEVKA--
IKKHLEMAGFPCWMDIGKMGGGDKLfaKIDDGIRSSKVVLCMVTEKYSKSENCNKEVNLANLLK-----

KPIIPV----------
LIDNTQWPPEGAMSVLFAELLYIRFFNKKEYVRGEK(FWEDAKFSELVDQISCHATPDEVSDGQAREK)}*

$1789=255(261):
>gi|780101991|ref|XP_783754.3| {|114(230)|<Echinodermata(M)>}PREDICTED: uncharacterized protein LOC578497 [Strongylocentrotus purpuratus]
{(FLAVRLGYSGEDIRNWALSPDPTMAILAEWYTTHKSSDATYAILSALEDMGRTDAAEIITQSLEEAELLVP
KAPSDESGK)PPTVFLSYQW-DHQPEVKA--
IKKHLEMAGFPCWMDIGQMGGGDQLfaKISQGMRSSKMVLCMVTEKYSNSENCNKEVNLANLLN-----
KPIIPVLIDQTKWPPEGAMSMLFAQLLYIQFFNEKEYVRGEKFWEDAKFSEL(LGQISYHATPDESMVSDEYR
DWIPQVEDDPVTVKKVSGDAE)}*

$1790=255(261):
>gi|780101991|ref|XP_783754.3| {|304(40)|<Echinodermata(M)>}PREDICTED: uncharacterized protein LOC578497 [Strongylocentrotus purpuratus]
{(IQFFNEKEYVRGEKFWEDAKFSELLGQISYHATPDESMVSDEYRDWIPQVEDDPVTVKKVSGDAENSPVQQ
QSHVTETSE)PPSVFISYQW-D--
KQTEIKRLFSRLTGLGYHCWLDINQMGGGDPLysKIDKGVRNAKVVISCVTPKYALSANCRREVSLSDALR--
---
KPIIPLLLEEMTWPPEGPMSMTFTQLLYIDFTKETTQAGFEDEKFDELIEKM(NELAQPMIEVMPANEERKVD
ENLQTTTPKAEGKETTLTGND)}*

$1791=274(274):
>gi|780011203|ref|XP_011663792.1| {|4(76)|<Echinodermata(M)>}PREDICTED: uncharacterized protein LOC100892679 [Strongylocentrotus purpuratus]
{(YVITGNYTLNGAYKGDSIKIIVSQAPDTMIEITVGVVVSLTVIFLVVTVVTVSCHKYHLTLQLYWKNRFGK
IEEHDGMEH)--DAFISFSdsPDDGRFAIAVAQRLQQQGYD-
VWMREMHAIPGEALIAEEIVSMKRSRRCILIISPDYSTSSHSEVlTDLAAdqtLRKR-----
SRIIPILYRDVTrehfKRHRRLAEIVRVCPCI--
TWQEKEDAGSSENQLSNNISGS(GPEEVHGNGSSGSGLGEMPNNDVSGSGSEELPSNVSGCGTGEMPIDSNNS
INST)}*

$1792=182(182):
>gi|780794462|gb|KJS00872.1| {|0(91)|<Proteobacteria(B)>}hypothetical protein VR65_10705 [Desulfobulbaceae bacterium BRH_c16a]
{()MIKCFLSHSSEdKGLYVEIVA--SKLEKESII--
YDTKSFEEGMPPLdEILRGLNQTGIFVLFLSDSSLNKEWV-KrEIIEAKKLHEN-GKIKqIYPIIIdKSI-
DYKDPRIPDWLRDSYNLkqILRPTVAARRIKQRMRELSWDFH(PRLKEREDIFVGRNELISSFELRIDDFAKK
KPNCIIASGIEG)}*

$1793=198(187):
>gi|780831072|gb|KJS33792.1| {|0(286)|<Proteobacteria(B)>}hypothetical protein VR64_00180 [Desulfatitalea sp. BRH_c12]
{(MGYVPNYKND)---
IFVSYAHVdDVpvpfvqtgWVTTLIGCIKTrlaqrLGRSDAYTLWMDYELAKHVKLTpQIMDALQQTAIMIVV
LSPGYLASDWC-QrEKATFMQLIKA-HDSRVF-IIERDM-
IEAEDCPAEFQDLIPFRfwVREREGKPPRILGDPIPDKEDRA(YYSGVDEITYELANALRALKKKAHTGTDDE
GHQREHL)}*

$1794=173(176):
>gi|786014643|ref|WP_045725210.1| {|0(176)|<Firmicutes(B)>}hypothetical protein [Clostridium baratii] >gi|782618631|gb|KJU71828.1| hypothetical protein UC77_07610 [Clostridium baratii]

{()MVNMFVSYCQKdSVYADNIDLYFKG--KNVVIH----
RDIRDISNWKsirEYMHTIRDMDYAILVITDNYLKSFNCMYeVLEVIKEKNY---ESRIFPVVVETS-
IYSSEGKIKYVKHWEEKfyELKNQISQIDIVNAGNLIDDLKR(TQNICSSIGEFLSKVSDMNNPNVQDINMAI
ENKLNE)}*

$1795=193(197):
>gi|780796427|gb|KJS02694.1| {|0(120)|<Proteobacteria(B)>}hypothetical protein VR65_04455 [Desulfobulbaceae bacterium BRH_c16a]
{(MDSPK)---VFISHSW--DDKARFVLDFGKKLRARGIDAWIDEWEMMPGDSLvkKVFcEGIKNANA-
FLIVLSQNSVQKKWVNeELDLA--VVKKLeEGTRIIPIIIDDC-
KIPEPLKSTVWVKINDLQNYHNELEKIVMAIFGQSDKPPL(GSSPNYLTNPTGKISGLNEIDSITFNAIFEEA
LKQNKQYQIPLDQVFRNIKE)}*

$1796=291(293):
>gi|980983012|ref|WP_059401471.1|
{|81(238)|<Proteobacteria(B)>}hypothetical protein [Alicycliphilus sp. B1] >gi|781561994|dbj|GAO25281.1| hypothetical protein ALISP_5101 [Alicycliphilus sp. B1]
{(ELGHNPSIFVFVCTEALNAADKDRAHELIAKKYGWILDLYDLERLRVQLVSPQRHLLAQHPSIFAPPFFPQ
SGGQSLAES)RDTILIDHV---
DANHALATWLSRRLTLAGYRTWCRGTAPPAGEDPdaSVRKLIDVRGCQYLPVVSEESLADGLFLE-
RCALAAAKE---SFVLPcscISGSDP-
RLPSRLRSVAAANFADSWSNGLAQALFrLTTLGIRPDLDGD(IGKQIALRDYLPSRVTVAKPEPVYANMFALH
LPSAMLITELTRSLNVAETNELRSRWAFVELSPGRLISFSPH)}*

$1797=273(277):
>gi|780033455|ref|XP_011667032.1| {|0(373)|<Echinodermata(M)>}PREDICTED: uncharacterized protein LOC105439572 [Strongylocentrotus purpuratus]
{(MGLGCGEISMMKGIVEAAMKWMKRGLPPRILKLVPYAKVIDGDVQSHSLRRFSEVLKKFTGLKKRYEMQLL
LPKAVPLE)-
FDVYLSFsEEDEKVAKVIREKLTEAKDGIRIYDSSHQGINKDTVFQKDVYSIMMkSARVVAVLSPNYLNNKAC
LEQYNIALSCNRRALRDMLVPIYVDSVEM-MPTYMGLFQYVDCRP----
HDHLKIGEACSQLAASLTD(TFHTELRVAEFDPLRYDVFLSYSHCDAEKANRLVGLLQKLAPDLKLFFDLKEL
KAGKS)}*

$1798=260(266):
>gi|780011262|ref|XP_003724802.2|
{|160(90)|<Echinodermata(M)>}PREDICTED: uncharacterized protein LOC100892172 [Strongylocentrotus purpuratus]
{(YVITANYTLSGAYKGASIQITVSQAPDTMTEITVGVVVSLAVIFLVVTVVTVTCHKYHLTLQLYWKNRFGK
IEEHDGMEH)--DAFISFsdsPDDGRFAIAVAQLLLQRGYNV--WMRDMHSTPGEAL--
IAEEIVsMKrSRRCVLIISPEYSTSSHSEVLTDLA-
ADQTLRKRSRIIPILYRDVTREHFRRHQRLAEIVRVC----
PCITWQEKEEAGSLEDQLS(NGINGSGLGEIQSNDVSGSGLEEMRSNNVSGGGTEELPSNVSGCGT)}*

$1799=233(236):
>gi|780024433|ref|XP_011665744.1|
{|105(606)|<Echinodermata(M)>}PREDICTED: uncharacterized protein LOC100890762 [Strongylocentrotus purpuratus]
{(ETPQGADEVAMLKGMVEAAVNWMKAGLPLRRMKLVLYVQLKDGEVQGHSLKRFEDVTKTFDDLKERYEMQL
LLPKAVPLE)-
FDVYLSFsKEDEEVAKVIKEKLSSAKEGVVIYDSSQQAMNTDNVFQQDMYSVMMkSARIVTVLSPNYLKTMAC
IEQAVPLEFDVYLSFSKEDEEVAKVIREKLSSAKEGVVIYDSSQQ----
AMNTDSVFQQDMYSVMMKS(ARIVTVLSPNYLKNNA)}*

$1800=274(278):
>gi|780024433|ref|XP_011665744.1|
{|186(484)|<Echinodermata(M)>}PREDICTED: uncharacterized protein LOC100890762 [Strongylocentrotus purpuratus]
{(DVYLSFSKEDEEVAKVIKEKLSSAKEGVVIYDSSQQAMNTDNVFQQDMYSVMMKSARIVTVLSPNYLKTMACIEQAVPLE)-
FDVYLSFsKEDEEVAKVIREKLSSAKEGVVIYDSSQQAMNTDSVFQQDMYSVMMkSARIVTVLSPNYLKNNACIEQYNIALCCNRRALRDMLAPFYVDSVEL-MPTYMGLVQYVDCRT----
LVRHCLSPSRSPSTPSSPS(PSLIPFAMMSFSHSHRDTEKANCFVDMLQKLAPNLKIFFDVQELKAVLSPNYLKNNAC)}*

$1801=232(239):
>gi|786062986|ref|WP_045742925.1|
{|0(143)|<Actinobacteria(B)>}hypothetical protein [Actinoplanes rectilineatus]
{(MTRAVSFFLSYAHSVPLSDRRGTDDTDIW)--------------
VRRCFDDLKREVTALtgaPSadTGFVDYQA-
GPGADWkaMLDEQLGRADVFVPLYSPGYFNKSWPQQERAAFLDRVERAfpgeperGQEHIVPVLWIPLLgpgATPVP----------
RDlARAVSLGTAIPEYAENGLRALLM(LTSYATQYHRILVRLARRIVEVAEASVLPPGHSVSIGDIPRLEPAPGTTPFVVGVLGPAGPWKPFGKALD)}*

$1802=226(220):
>gi|786067005|ref|WP_045745395.1|
{|0(162)|<Actinobacteria(B)>}hypothetical protein [Actinoplanes rectilineatus]
{(M)----------------
RFFKDLSAEVRVLagaDPHEevgFLDAHSIELGELWrsRLTTALSESRCFVALCTPRYVMSPMCTAEWAAFEDKMHRYqQSTGVLPgalmplswCALEDIPditeRYQRTHQILGPEYSAEGvRQLVRLSINRDQYRRMVSALAGR(IVRLARSHDLPPLPADRTLESYSPPGTTTTAGTKNSWSAPDTAEGSVGLVHFVVAAAARTDMLGQRESLEYYGESALDW)}*

$1803=144(146):
>gi|783325848|gb|KJU84244.1| {<Nitrospirae(B)>}TIR domain-containing protein [Candidatus Magnetobacterium bavaricum]
{(M)KDQIFVSSAIQdSEWVSLFFDKLTQIGVNVW--FFTKDKN--
IVQGSsiveLMEKALRESNIMVVVISKNSITSQWVFFEIGVAIADEKR-
IIPVIIDDVPYESFPVRLKSTACFHEKSPDE--AAIKVASFLQSESKTGHLQAI(SSLSG)}*

$1804=224(224):
>gi|785927158|gb|KJV06884.1| {|34(0)|<Proteobacteria(B)>}hypothetical protein VZ94_08415 [Methylococcaceae bacterium Sn10-6]
{(KEILEKNKVNWIGENKGIWWINIEKLYEFIPNLHRIDWLHGVVNSTVEIEYETRKVFDKEREETLIQLIVTKEEMERSLP)-KKIFLSHKGVdKPLVRKYSQLLSNLGFEP------
WLDEDAMVAGVplerAILQGMKNSCAAIFFVTSNYEDENFLASEVNYAISEKREKkerfsiITLVMRNKTDSTKVTVPDLLKQYVWKEPEDD--IMAINEI--IRALPIRLINIE(WKEI)}*

$1805=142(154):
>gi|783326797|gb|KJU84603.1| {<Nitrospirae(B)>}TiR protein [Candidatus Magnetobacterium bavaricum]
{(MAKYKNI)---VFISHSGADTWVARQIADKVTSCGAQ-
PFLDEADVDVGTEFEEEIRNMLGKASEFLVLFTPWAFE-RPYIWvEIGVSWY--------
RRIPIVVLLYGltKEEFLSNPKTPSFLKR--RDMISINNIDKYLDQLQQRISN(VKRKDKK)}*

$1806=174(176):
>gi|783327223|gb|KJU84778.1| {|0(815)|<Nitrospirae(B)>}Toll-Interleukin receptor domain protein [Candidatus Magnetobacterium bavaricum]
{()MAKIFISHSSIdKSEIAIpLFNHLK-----
KDHTVWYDSDqIRISDNIPtKIAEGLDNSDYFVLLISEDYNRSGYCRME-QNAIFHQYA-
GNTEKRPLIIRIN-
NANIDIMLESFRRIDYYsgRTNMQEIYDTLDNALKTPIAHVN(QADSDMDNLIEDILKFNQGLIRLKPSLSSS
DTIRDK)}*

$1807=150(180):
>gi|783329064|gb|KJU85566.1| {|0(825)|<Nitrospirae(B)>}hypothetical protein MBAV_002242 [Candidatus Magnetobacterium bavaricum]
{(MFNHLKKDHT)--------------------------------
VWYDTeRIWLGDSIVsQISEGIDQSDCFVLLISEDYNQSAFC-QrEQNVIIYRHIN-RRKRLLIVKINNA-
KISNIIEDLRYIEYSSNsrNMQPIYDEINRVCEATVTRIDEP(DFDRDKLIDDILQFKSGCMELETYTYSTSG
)}*

$1808=285(297):
>gi|786064104|ref|WP_045743624.1|
{|56(77)|<Actinobacteria(B)>}hypothetical protein [Actinoplanes rectilineatus]
{(GQTEAAAQLLDEARQRRESAARFDSDVREGTIATTVIALVTALLAVLGARPVLRHRRDSLYRDVVTGPPPV
TTAAAAGPS)--TVFLSYSRKdAATVRPLHDALVARGR----
EMWVDwTGIAPTADWMqEIVRAIERADAVLFVVSPDSLDSRTCREELAHAV----A-AGKRIIPVLVRDV-
DGAGVPehLGRLHWVTcRAGepL------D--
TAVDDIVEAIDLD(FSWVQLHTWLYVRAREWDANGRDPGSLLGGADLNRARSAVLVANGRHPALTELHQEYVA
AAEQNAVARTRKERGGAY)}*

$1809=178(186):
>gi|783323397|gb|KJU83195.1| {|0(104)|<Nitrospirae(B)>}protein containing DUF1566 [Candidatus Magnetobacterium bavaricum]
{(MGYD)---VFISYDS---
RDLTFADKLVSRLESRGYKCWIDRGEIVAGKEFdeEIFTAItENPGLLLVVLLSEKTLQSKYVKdEIRLA---
---YdNNLHIIPVFIEDI-
KLTGAYALRLNGRHRIDAFNLDIDTVADKICVAVKQQKPD(LPTPTPIPTPIPSERFVDNDDQTITDNSTILM
WTKDANLAGR)}*

$1810=152(165):
>gi|797195327|ref|WP_045856325.1| {<Proteobacteria(B)>}hypothetical protein [Alteromonadaceae bacterium Bs12]
{(M)-PDVFISYSVKD-EKLAKFVREHLLQKEL--
DVFLASISLNPGEHWtPQIFDALKSSDWVFLLASKNAMESPN-----VQQELGAALsHGKKVVPIMWD---
LQPSDLP---RWVADFQGLILSGATMENINLQMSQLAERV(KSNKVKGQLVAGAVFAGLLYFLAN)}*

$1811=286(298):
>gi|797150185|gb|KJY54284.1| {|8(40)|<Actinobacteria(B)>}uncharacterized protein JF68_00380 [Bifidobacterium coryneforme]
{(SECNLVDVNLLLHEMHLDEVVENEWECGVFERSYEERFGVVLSKIRNLPRSDVEEMVPAVNELFEVTVQTD
PQQSARPLY)---LFASHLNSqKALVGQVSDYLKGWGIK---LF---
VAHEAIEPDRewhnEIEKALRDCDAGVVFLSQGFNESKWCDQEVG--WLLGRE-
IPCYPLKFQDEDPYGPFGK-KQAFPVRADMT--AGKIGDE--
IIRWLSTKAELE(KGFYASMVEALKRSRSFHRTDLVWRRLQTATGLDSDEVASILAAIRDNDQVYNAHNGIGE
ETGEYKELAFKLALEQPG)}*

$1812=210(184):
>gi|797194732|ref|WP_045855774.1| {|0(301)|<Proteobacteria(B)>}TIR domain-containing protein [Alteromonadaceae bacterium Bs12]
{()MPannEIFISYAHTdNEnpigagWVEQFHKTLKLRIKQilgarqpdDMPDIWRDKELQGNDELSdILVDKINHVDLLVSILSPSYINSDSCLKELNTfsAIAAQNG-GltvdnKARIFKVIKTPV-ELEQQPAPVRGQLGYPFfyFDEDEEITREYTLMPGDKHTAQA(LQEINDLAHNIIKTLRTWREHKPGAAVGRNVNNEPSPLKPAANE)}*

$1813=140(144):
>gi|800993147|ref|WP_046019265.1| {<Proteobacteria(B)>}hypothetical protein [Marinomonas sp. S3726] >gi|798761698|gb|KJZ09578.1| hypothetical protein TW85_21795 [Marinomonas sp. S3726]
{(MRTV)----FISYDHDdQNAKRDMESIKANTNNSvsfhdnsldePVRNVYGDVNrrmpEDPASKEVrdAIERKLKESSKLLVLIGRDTHSSKWVKWEVETFKSLKRSPdilFMRVKGD--------------------YGSGLPSFANSHEIKNWSMSELISWLK()}*

$1814=175(181):
>gi|802219574|ref|WP_046054946.1|
{|0(365)|<Proteobacteria(B)>}hypothetical protein [Pseudomonas fluorescens] >gi|799220041|gb|KJZ64364.1| hypothetical protein VD17_17940 [Pseudomonas fluorescens]
{(MKQFP)-YDIFLSHRI-GDSAHELRD----QLVSCGCSVWFDENRPlvDRQLFASIRKAHAESRVTIAYLSPSHSPSPWTLLEMLTALRREQQFAIRCLF--FYGSAEAFECSHAWSLPDELSSAFADLDRRLETQVTLFASRMSRLN(REMMVHGSRTPLSRARCIARLRRSIDGYRCSANGFA)}*

$1815=214(219):
>gi|910779487|ref|WP_050085149.1| {|0(197)|<Firmicutes(B)>}TIR domain-containing protein [Streptococcus pneumoniae]
>gi|799961345|emb|CEX86201.1| Uncharacterised protein [Streptococcus pneumoniae] >gi
{(MNKPT)---VFFSHSSLdKEIAKSLKDKLIEYTGGTVQIFVSS-DGESIPFGKnwlsTIERGLEESRIMFLLVTPNSINNLWLSFEAGFGYSKNIR-VIPLCFKVSVSDLKYPLGML-QGYDLQSADS--FNNIISI--LNSEFDYSHKLA(FTESDFSEISSIGITDDVNQEFAKIVKEVTWYKNFQPSIIESLKGEELTLYNKICSGLLYPTEEFLQENQVKYS)}*

$1816=214(220):
>gi|803576788|ref|WP_046082745.1| {|321(0)|<Proteobacteria(B)>}DNA polymerase [Escherichia fergusonii] >gi|984378861|gb|KWV98482.1| DNA polymerase [Escherichia fergusonii]
{(KWREDKLTSALLFVTKKGFKATNNEHAISIFRNHIYGRLSFIKMIRGEDFPLYLKLMAEMSHHDPLKTKEGLRAMKETET)-YDVFICHAS-ED-KTSIAIPIYEELIKLNISTFIDHVEINWGDSLiqKINSALVKSKYVIAILSANSVDKHWPKKELHSVLARE-----ITEGEVKLLTLVKEADEAIVAESLPLLSDKLYMTYKDNPAEVADKVRALLNK()}*

$1817=211(220):
>gi|805396887|gb|AKB47719.1| {|379(0)|<Euryarchaeota(A)>}hypothetical protein MSKOL_1942 [Methanosarcina sp. Kolksee]
{(LDQINASIKKINVSKQIPCNCSKNCPERYSYDKLLKAEKNNIEIIQCHESYESISISSLLDGYKKREERLRNLGETDNLF)EYDVFICHSS---KDKPIVDSLIKDLKKKSVTYWVDK-EQINF-GDpvtQKIEEGLKN-

SKyvVPCLSKNFTISGWTRAEYGSILNREFSSKSKRiIIPLKLDNCEddD-IPN---------LLY-
DKKRVTYSNKTEFNEFIEFLKN()}*

$1818=194(196):
>gi|851271805|ref|WP_048142333.1| {|0(235)|<Euryarchaeota(A)>}TIR domain-containing protein [Methanosarcina horonobensis]
>gi|805384361|gb|AKB80238.1| hypothetical protein MSHOH_3755 [Methanosarcina h
{(MIE)EKQIFLSYSHKnMDLANEIDNTLRIRGIRvTRDIR-------GVKCSQgfkeFMKS-
IGSHDLVLMLISDQYLKSQACMFEV-IEFMKESN-YkqrvILIVNDDVNFDLKGILYLKYWEEKGKLIEE--
TIKKHHREKVKPLEEESDEIK(LIENNIIEFIATIRDLKYIAFKELNKSYKELYERIGLKIDKSEANYDTITE
ED)}*

$1819=292(298):
>gi|851232027|ref|WP_048120546.1|
{|294(81)|<Euryarchaeota(A)>}hypothetical protein [Methanosarcina vacuolata] >gi|805392987|gb|AKB44085.1| hypothetical protein MSVAZ_1816 [Methanosarcina vacuolata Z-
{(SHVLQIIGESNIKINYLTFANFIDIHDKKYVIETKNPKGKLIRIPSNRDIGESIKYVCHVKAYDDRLTISN
SLKSSKNID)---IFISHAHFdQELVNNINKWLLNIWPSLK--IYCSRPEDRIPLFQfvyeASSK----
SKCVLYIATPVSIQRPMVGIELSG----NKS-IITVLAQTSYNDIKHIRDKDlyckidmEKIVDIYDKNG--
WETLAKM--
IAKVLGLNLPNN(IPKGPWINNATMPSHNIKKLNEDFTKLYVEFVTNFGMGKKQTRVEAEQLLYLVEEELIKL
GEKDGTVISRRMLPQLNT)}*

$1820=248(237):
>gi|919157218|ref|WP_052712850.1|
{|0(211)|<Euryarchaeota(A)>}MULTISPECIES: hypothetical protein [Methanosarcina] >gi|805405294|gb|AKB55100.1| serine/threonine kinase [Methanosarcina barkeri MS] >gi|8
{(MSTLKEQEFPITDPSLNEIHYNNVPKSS)--RVFISYSHdsnKhKKSV-----LLLSNRLRREGIDCN-
IDQYETSPPEgwfcWMSNQIEEADFVLIVCTENYEKRFKGKDEvgkgMGAKWegaiitqelyysGANNK-
CIPIVFSPQDSEYIPSILKSATYYVLNMEEI--NDEIYDE--
LYARLTYQKLVI(KPDIGKLRPITSKYCSASVSTQKKKGGIVESHQKPMIGELRKITTEPQSSKIHETFTSPS
TGMEFVLIP)}*

$1821=183(189):
>gi|806483607|gb|KKC02761.1| {|0(67)|<Actinobacteria(B)>}hypothetical protein WU83_22480 [Mycobacterium nebraskense]
{()MP-LFISYSSQdRATVDALTTAL----
RRGQQQVWFDQELGGGESWWnKILEQIRSCDVFIVALSNNWLQSKPSQSELRYARALNRP-----
ILPVRIGDI-
DsMRVNPLAALQIIDYREptVDAGIQLVTAVHALQSKPVPLPD(PLPEEPPVPFGYITRLGDTLAEKELSPQQ
QTQLLIELRQGLDEDGDDPS)}*

$1822=208(220):
>gi|919172317|ref|WP_052727948.1|
{|669(0)|<Euryarchaeota(A)>}hypothetical protein [Methanosarcina vacuolata] >gi|805393132|gb|AKB44230.1| hypothetical protein MSVAZ_1961 [Methanosarcina vacuolata Z-7
{(INASIKKINVSKQIPCNCSNNCPERYSYDKLLKAEKNNVEIIQCHESYESISISSLLDGYKRREERFRGLG
KTDNLFEYD)---VFICHSS---
KDKPIIESLVKDLKMKNVTYWVDKEQINFGDPVtqKIEEGLKNSKYVVPCLSKNLTIL-

GWTRaEYGSILNREFSGnSKRIVIPLKLDNC-EDA-DIPDLLY-------
DKKRVTYSNKTEFNEFIEFLKN()}*

$1823=155(152):
>gi|806833960|ref|WP_046182404.1| {<Actinobacteria(B)>}hypothetical protein [Mycobacterium nebraskense] >gi|806487345|gb|KKC06262.1| hypothetical protein WU83_03910 [Mycobacterium nebraskense]
{(MADQ)-HNAFISHRHEddslvgQLI----DLLAKNGANVRDSSI---
TSDIPNNANAesYIKQILAEriqwAGKIIVIISPETKNHEWVSWEIEY-ANRFPDKriiGVWAPGAVGAD---
MPPELDEYADAVVGWNSEAIIEALNGKDNWQGPDGTPVPP(RSIKRLAC)}*

$1824=184(189):
>gi|806777968|gb|AKC01903.1| {<Mollusca(M)>}truncated myeloid differentiation primary response protein 88-2 [Crassostrea gigas]
{(MDNFE)-YDAFVIYNPhgQdQEFVSLMTQVLTSPPYNLRLYVPWSDNNE-
PFEAVATAENIEKRCKKvLVVISAESMESDLFHFQLRIAHSMSPSAKTRKIIPIRLDG--
TEVPDMIRFSTPCDYYK--QDLRV---
FIWDRIFASFKDY(SRPKKSKVLKTVSFPPNRQVPAAVQTFSPLRRVKSWWRMRATVA)}*

$1825=216(222):
>gi|806823845|emb|CQD20979.1| {|0(720)|<Actinobacteria(B)>}WD40 repeat-containing protein [Mycobacterium lentiflavum]
{(MPRSAKT)--KVFISHSREDLDFVGPLETQLRDAGYS-
VFIDQNDIAGGEPWRSRLDAIIVNCDAVVFVLSPHSVKSKA-CAdEIERTIQLG-----
KRLVPLILEPVPkeSVPAALASPNWMTMKkgfagG--LD-ASSLA--
RLRYAIDTDIQW(VREHSRLTELAEGWRVSDSADRLLRGEEIAAALNWLDRTPAHDLTITGLVQSFIDASVER
DRAEELAEKREAFRR)}*

$1826=195(187):
>gi|808104871|ref|WP_046262249.1| {<Actinobacteria(B)>}hypothetical protein [Streptomyces sp. WM6386] >gi|807122256|gb|KKD04009.1| hypothetical protein TN53_32025 [Streptomyces sp. WM6386]
{(MDYE)-WDVFISYSHRGHV-KDWVKNHFQEELTK-SL---
WDVLPddprifvdyeipnGTHWPEKLEDALLKSRCLVAIWSPPYFR-SEWCMaEWvsmrmrQAALQEA-----
NGQAPTLIYPIRfaDGDHFHPDAKATQQCR--
DLSKYGHDREQFRDTPDYMDFQ(KEMRTVAEEIAELLACTPEWQAGWPVDRPDAFTKVPQSRLPRL)}*

$1827=292(296):
>gi|808103689|ref|WP_046261088.1| {|124(87)|<Actinobacteria(B)>}TIR domain-containing protein [Streptomyces sp. WM6386] >gi|807123447|gb|KKD05179.1| hypothetical protein TN53_26060 [Streptomyces sp. W
{(DGLDDQILTGHFGPDYAESGLFHLVPEENEPKSKEYKKIVLAVAKRLATVLYNGADLPHLEVTEARALTPL
FGPERLRPV)--DVVIVYTDldDDSEWGLWAIDELGKQGYD-VDAEVV-
SAADRSTVERLRRALHRADRVLVLVSGSFFSHGDMSAaALDLALSEG-----
SsdwlRMIPVFLDPPEprAMPVSFRQFPGLALRD--LDEGSARD--
LLVQSAEALAQT(SGRPTGMGSRFPGGRTPGTGYPGGITAGTPTTPAQLHIPLVNALLKADSISDGIIRPIWV
RQTGIDVGSLDVGGLP)}*

$1828=146(150):
>gi|806823884|emb|CQD21097.1|
{|0(354)|<Actinobacteria(B)>}serine/threonine-protein kinase [Mycobacterium lentiflavum]

{(MA)---LFISYSSQdRSTVDSLTTAL----
RRGQNQVWFDQELGGGDSWWaKILEQIRECEVFIVALSSNWLQSKPSQAeLRYAQALGRPI-
LPVRIGEVDSMRV-NPLAALQIIDYREPTVDagIQLVTAVHALRSKPVPLPDPLPP(EPPVPFGY)}*

$1829=256(257):
>gi|808104630|ref|WP_046262013.1|
{|228(253)|<Actinobacteria(B)>}hypothetical protein [Streptomyces sp. WM6386] >gi|807122477|gb|KKD04225.1| hypothetical protein TN53_30830 [Streptomyces sp. WM6386]
{(LKLIHEPPDGRYRPQPSKLLSGEALRAAEPADRIAVHWSDPEELADRLRDLVARFYQPRLEFETYDEGVGY
FHSLGRAQG)--SVFLSNARGdDSLAQRVGRSLE-
LHNLSYFHYLRRNTIELGADWRsQLYANVAACRLFLPLISQHYWASEYCREEYNIAERLRAD-
GRLVILPYFLGPG-
VARQVTFQGRAIGHLSQdeQVAVISDDVDNEFVERRRLEERG(VTPGESAAAPGRRCDIAIVTLLPEAHDALR
RHLETSG)}*

$1830=238(221):
>gi|1177666306|ref|WP_082755965.1|
{|0(1008)|<Proteobacteria(B)>}hypothetical protein [Bradyrhizobium diazoefficiens] >gi|806927798|dbj|BAR58800.1| hypothetical protein NK6_5642 [Bradyrhizobium diazoe
{(MRMSSYD)-
YDAFLSYRRRdatrlaQWIRRRLQRFRippeilrelsseRRELHERTPRIWLDTSyEKSSDDFLvkKIFPALD
NSARLIVILTPAAleqvygregeAHENWLVREVDHFL-----------GSAAAD---
QASRPIDVIFgPGATEGKYPGRLSEKP--
RwdWIDFRSFSSWR(LRTFTGELDDGLIKLVAALYEVPDRFLPILRREERRERNRRTVVAAFGALTIFALITA
LAIWGLIQQENAAGAL)}*

$1831=161(170):
>gi|496504512|ref|WP_009212795.1| {<Actinobacteria(B)>}hypothetical protein [Actinomyces sp. oral taxon 180] >gi|315312828|gb|EFU60907.1| hypothetical protein HMPREF9006_1340 [Actinomyces sp. oral tax
{(MSTYRLYILKALED)---------HKdADSFLTLTKQrLKTIK-
SHKFAWWALSLLLPGDDREaEINKHLSEASYVIPLLSPSLLAA---MKdEKDREVPNIAK-
IQDKLLPVMLVDV-
LLDGTVECFGIDARKIYrgDQPEPRSYNSLDSVYQRNCFVDG(FVSQMIRRIEGGGGWR)}*

$1832=161(158):
>gi|496504513|ref|WP_009212796.1| {<Actinobacteria(B)>}TIR domain-containing protein [Actinomyces sp. oral taxon 180] >gi|315312829|gb|EFU60908.1| conserved hypothetical protein [Actinomyces sp. oral
{(MSDIR)---
LFLSWAHNdAEAKDSFLKLLKPrlgIAQNHVFTWWVDSFILPGEEWKeEIRAQLEKADYIVQLISPSFLASDF
IRDyEIPGVGEAPLK-KTLPVMLVDVPLD-
GTVEFHQIDRRQIYCGNsiEPHSYVSRETDYQRNQFVDGFFS(RIIDRVEGKGGYR)}*

$1833=252(231):
>gi|808878496|gb|KKF26737.1| {|273(0)|<Chordata(M)>}Interleukin-18 receptor accessory protein [Larimichthys crocea]
{(TLQHLNNTYTCMASNTVGNSSVTIKLKKKIKANWPSLVRYPIVSLLVVSGLGIVLHVKWLELQLIYRSHFR
HGQLDTEKK)EFDVFLSYV-Ctseelegvskeac ETTPKPLevlLRQVLEDQWGYRLCLMERDVIPG-
VYTSDVVLAIQRSRMLICLLSAEYLsNNNAIF-VLESGVQALLQKSALKLLLIWTNkapAA-
LIHPDLPTQVQRALKVLpRMYWTSGQPVratsNFWKSLRKAMPDH(RMKLVSLIKPQ)}*

$1834=223(226):
>gi|810858942|ref|WP_046349839.1| {|66(0)|<Proteobacteria(B)>}TIR domain-containing protein [Janthinobacterium sp. B9-8]
>gi|984916743|gb|AMC33881.1| hypothetical protein VN23_04330 [Janthinobacterium
{(IYAVAQSMDKIFLELNSSVYVKIRNSLKPTGHEIDTNQMVFAPRVILYTNKLCIPSERVIQAFNSVNALID
IVDENEMYK)--TLFVSYGG--P-DESTVAEINKRIKSKGIKTWFFPDDANPGDKLhrMMHEGVNNHD-
HVLLVCSKVSLTRHGVQnEIERVLEREAKEgGSEILIPVTLDDY-
VYGDWAPKRPDIADQIRSRVIIKLEIDSDQFDSSIDKLAR(VLKSNG)}*

$1835=183(205):
>gi|815811432|ref|XP_012226637.1| {<Arthropoda(M)>}PREDICTED: protein toll-like [Linepithema humile]
{(MSFYDFCPLDTTAIIIISVLIILTGFIICILGLYYKYQEHIKIWLFAHEWCSRFVTEKQLDEEKL)-
YDAFVSYSYNdhSFVINELVPKLENDPTPYKLYLHYRDCSIDKCISDIIARFVQNSRRTIVILSPNFLGSVWG
KMEFRVAHCQALSEGRAKVILIFYDDI-ELINDLDAELKAIIS----------------------IKN()}*

$1836=177(182):
>gi|851256179|ref|WP_048133839.1|
{|0(182)|<Euryarchaeota(A)>}hypothetical protein [Methanosarcina sp. 1.H.T.1A.1] >gi|814923505|gb|KKH95924.1| hypothetical protein EO95_11540 [Methanosarcina sp. 1.H.
{(MDNIREEY)QYRIFISYSHKdKDRVEKLAKILTENGLNP-------
VYDKHLEGGFgftdQIKKFIAHAHVFVPLISKSSSQRGWVHQEIGYAAALNIP-
IMPVMIDKVPSAMLQQVQALHWDDNEQNLKK--
IMSFERFRNLIEDAQKSMSQK(DISPLFECGLLREDRTKLMVRYASDISKLGFYGH)}*

$1837=176(177):
>gi|817677513|ref|WP_046642627.1|
{|0(137)|<Proteobacteria(B)>}hypothetical protein [Neorhizobium galegae]
>gi|816159505|emb|CDZ31069.1| Hypothetical protein NGAL_HAMBI490_59420 [Neorhizobium galegae
{()MAKVFISYSNRnREYADEFA--------
QGLIEyghqpLYDGTLLSPGAELYkVLDQALDEADAVVFLVSRESLESAWVLAEIGRAKALASG-
GGKMLLPVIIDDV-
HAPEILRQWLVVMGRGRpaKEVAAEIATSISNFEIKRRHQEI(RLREVEHDLSRFVDEAIEAQKTERANNKRA
AYACYVG)}*

$1838=204(209):
>gi|919183667|ref|WP_052739265.1| {|0(89)|<Euryarchaeota(A)>}TIR domain-containing protein [Methanosarcina mazei] >gi|814790111|gb|KKG69845.1| hypothetical protein DU43_18400 [Methanosarcina mazei]
{(MD)---IFISWSG--DISKIVAEALKDFLETvlQATKPWVSSIDLASGDRWneEISKKLSDSKYGIICL-
TKYNINAPWILfE---AGALAKSVdKKTYVCPYLVD-F-
DSSELKPPLSLFHARKADMEGTLQLIKDINFILEKNYHDS(HLKDKTLEKLFKNMWPAFESTINNLPNIESEE
VPKRDLEDMVKEILEITRSIDSPETKGVISLAKKV)}*

$1839=198(194):
>gi|816981361|gb|KKO20972.1| {|0(50)|<Planctomycetes(B)>}hypothetical protein BROFUL_00307 [Candidatus Brocadia fulgida]
{(M)EYDIFFSYPH-KDAKE--
VQHILQALQAEGLNVWIDKSEIRDYESItrSIVGGLAHAKVLLAYYSLNYWRSRACQWELTAgfLAAQREGDA
RRRILVInpeekAGHIHP--

VELRDELFQKAPADAEALRKLVQSVKAHVSGIKNTIGA(IYALTQPRWYGRKGAGSNRFVGRLPDMWRIHSAL
HSSGVPVITGAVASAVVQV)}*

$1840=211(202):
>gi|818266165|gb|KKP52428.1| {|0(154)|<unknown(B)>}SEFIR domain-containing protein [candidate division TM6 bacterium GW2011_GWF2_33_332]
>gi|1082543796|gb|OFY78389.1| hypothetical protein A2281_11885
{(MEKEN)-PKVFISYSHDSSEQqdKVLQLSNKLRSEGI--
DCSLDqYEDSPPEGWPkWMDRQVKNSNFVLVVCTETYYNRAMGTDekGMGIKWESSLiyqhlynAGaNNTKFI
PVIFNDG--
NFKFIPEPLQAATFYNVDNSNEYEKLYWRLRGVKTAKSE(LGKLRELPIKERKTFFISRLIDQEKWDKAKWEN
GVSYLYSSNGDLPPIFTFLFEKLE)}*

$1841=284(299):
>gi|817130476|ref|WP_046502995.1|
{|84(108)|<Actinobacteria(B)>}peptidase [Streptomyces sp. NRRL B-24891]
{(RGLRLIPVLCGDADVPGFAATRVWVDFRKLSGPAFDAKAAELAGFLRGEEQVRKAHTDSELLDLARPRTQP
PPTESAQPA)---FVVCYARADAGYGERLVDQLSGSGLP-VW-
SLGHLKWGDNYISTIRERLRHALAVVVLMSPEA-EESEDVTrEILEGQRHS-----REFFPVLLR--
GERNYLLASSWYLDARngD--LPGPDAL---
RLLRRVHDSHRS(HGSRASHRQPPAPRTLPVLSLPAGRPSVPAVRSPARRSLLTLEALLAEHETEHADLLTTT
LLLEAVDRLETGWLRRSDG)}*

$1842=224(202):
>gi|816977478|gb|KKO17952.1| {|0(278)|<Planctomycetes(B)>}hypothetical protein BROFUL_03360 [Candidatus Brocadia fulgida]
{(MAYIPGFEHD)---
IFISYAHVdNcaiqgkscwvnQFHEELEIALAKCIgRMGLVKIWRDMRLEGNQLLDnTIQSAIDKSALFLALT
SHGYFHPDsYC-PkELQLFHTKAQA-EKyglqmgdrSRIYNVLLSniPY-
DKWPKEFSGMSGYPFHDaeKTDAVGMPTDPADKRFHDQLRKL(VDSLHTMLQAFKTKAEGTATRKPGKETVSL
KDIPCVFVSDVSDSLRSVRSRV)}*

$1843=216(220):
>gi|818545171|gb|KKR96941.1| {|177(0)|<unknown(B)>}hypothetical protein UU48_C0021G0006 [Candidatus Uhrbacteria bacterium GW2011_GWF2_41_16]
{(AKYKADKTVKFLQKVNAKETNYSLKQEAFKSLQQLNEKVILRRKKMGKKKESVEAIYNTTDSPDNLIEKLN
DNTLEKIKS)-FDVFLSHSS-SDRKK--
VILLYKNLNSKKLHVYIdwvnDSDALkrnLSNRNTanVIIERLKKSKMLIYCHTEASMKSQWASWEIGYFHGI
GRqifvFNPDNFELPDFLKVYPKLIEKDSEYYI-------ENKSEKITLNNW------IFNK()}*

$1844=208(223):
>gi|818351650|gb|KKQ33289.1| {<unknown(B)>}hypothetical protein US45_C0012G0003 [Candidatus Nomurabacteria bacterium GW2011_GWA1_37_20]
>gi|818357008|gb|KKQ38128.1| hypothetical protein US55_C0014G000
{(MYRISA)----FISYSSKeKVIGGKFKSCLENFCgyeafIAHDDILGSTI------
WEDEIIKSIKNADFFMPLISKEFKESPFTDQETGIAVCLKKK-IIPIKLSEINP--YGFI---
EKYQALQYKND--VNNLALT--IAQI-
GLIYEPK(SSYHQKALNSIVYAFCESMSFEVANATIQILCKCNDLSPNQLTQIVKAIKTNSQIENAYGLNALK
ECLRKNYKISID)}*

$1845=199(209):
>gi|818388455|gb|KKQ67849.1| {|0(26)|<unknown(B)>}hypothetical protein US89_C0013G0019 [Candidatus Peregrinibacteria bacterium GW2011_GWF2_38_29]

{(MLNEIGIGIGIKSFR)---IFFSYSTVdKLIVGEIKKHLELLGFEVFLAH---
EDIEPCIDWQeEIKKNLLRCDVFIPLYSKNFRDSKW-TDqETGIAI----A-SEKFIIPLHmdLAPY-GF---
-
MGKTQGLKIDEklLALTANEIVKIIKSKSKFKDDMK(NFTINALARSSSFDEAKFRARQLSGFDTFSEEEVLR
VYSAVLSTNQISGSFGARSV)}*

$1846=171(186):
>gi|818644976|gb|KKS91834.1| {<unknown(B)>}TIR protein [Candidatus Collierbacteria bacterium GW2011_GWC2_43_12] >gi|818728164|gb|KKT71636.1| TIR protein [Candidatus Collierbacteria bacterium GW2011_GW
{(MTNSGLILRNATSALHGKKVVNLRVAFVVAEFDRDVSD)--------------------
QLIWLFRENGIKCWTERDILPGSNHFgAIRRAYKEADFILVFLSKASVARSGEYQkYINLAIDASDempE-
GGIKLIPICLEQC-EVPFSLQKFYSVAATQPdaLLRLVLAWAKEWQRRHGADDWPD(VVYQSVWE)}*

$1847=197(196):
>gi|1175579326|ref|WP_082120414.1|
{|0(602)|<Proteobacteria(B)>}hypothetical protein [Pseudomonas sp. CCOS 191] >gi|820684580|emb|CRI57077.1| hypothetical protein CCOS191_2541 [Pseudomonas sp. CCOS 19
{(MDATVELAKGRRFSARLFG)-YDVFVSFAlG-
EPPRGNFsyASDLARRLQDAGLVVFFCEEELGYGDKLtpELERALAKSKILVVVInSEVLKEPRWIKTEVQQF

RKYHPDRPVIPVSLDGCLPTIVGQNAATEDWHGFDAQKYIVEPWEEGVPNSASALTVE(SIVISVGRLRANRK
WRALVALIMLLLITLTFVSWRMK)}*

$1848=163(173):
>gi|821171256|ref|WP_046819849.1| {<Proteobacteria(B)>}molecular chaperone Tir [Pseudomonas putida] >gi|820907029|gb|KKX58242.1| molecular chaperone Tir [Pseudomonas putida] >gi|1137703934|gb|OMQ33723
{()-MPVFISYRHMDRA-YAININTRLMQANI--
KTYLDVldaESQTTDDITgVITRNISECTHLLAVVSEKT------ALSWWVPFEIGEATiTNRRI-------
CSfkTGPTALPLYLDKWPKLTSERDIEFFIDAYRNESTLKRSMS(LESVNGNESARSVNRSNADRFHTDLKSR
IIRGF)}*

$1849=197(206):
>gi|818844698|gb|KKU82470.1| {|0(16)|<unknown(B)>}hypothetical protein UY10_C0032G0006 [Microgenomates group bacterium GW2011_GWA2_47_8]
{(MDRIAA)----FISYSSEeKEIGGKLKQlVIRYCGYDAFIA--HD-
DIPGSEVWEkEILRAIRNADFFIPLVSEHFKNSSY-TDqETGCAVCLNKK-----IIPIklgLINPY-GF---
-
INKYQALQYRKdpVDNLFELAITIAHIGLHYQPKTI(HYKKSLQSLVYALCNSSSFEATNAIIRILTKCNHQF
SEDQVAQIVNAIKTNLQITGAFGL)}*

$1850=174(175):
>gi|820799677|ref|WP_046760993.1|
{|0(647)|<Proteobacteria(B)>}hypothetical protein [Acinetobacter sp. Ag2] >gi|819910589|gb|KKW76757.1| hypothetical protein AAV97_15325 [Acinetobacter sp. Ag2]
{()MIKCFLSHSSSdKSsYVRHIAEHL---KRDSIV--
YDEMTFEPGMDPAeEILTNLEKSSLFVIFLSNESLNSNWVVKEIEIAKKLEEL-GSlKRIFPIIIDKN-
ISYTDPRIPQWMKDTKNiqPILSHKVAIRRIKERIRELSKSI(HPNLKDKDEIFVGRNDNEKQVEIRLDDFYK
DPVIC)}*

$1851=177(178):

>gi|818881409|gb|KKW17062.1| {|0(238)|<unknown(B)>}hypothetical protein UY58_C0012G0002 [Candidatus Magasanikbacteria bacterium GW2011_GWA2_50_22]
{(MRNLIRQLQTQVFG)-YDFFISYRHNsaggyaANLAQSLEE--------
RGYAVFIDRRETPAGSDLplAIRWALWRAKILIPLLSHDAPNSEWMRNEISQFLPSGRTVlpiDFASLLSSEL
I---LPDQFLALKKRHYIVDTTEHLPDGKPTTRVLDAIVEGLNH(IRVRTAARWALAAAATLLVIAALI)}*

$1852=189(168):
>gi|821533651|ref|WP_046855532.1| {|0(178)|<Proteobacteria(B)>}molecular chaperone Tir [Pseudomonas sp. CCOS 191] >gi|820684576|emb|CRI57073.1| molecular chaperone Tir [Pseudomonas sp. CCOS 191]
{(MAYVGTPFA)-
HDLFISYSHGydeksgigflqawsTAFTTELERELRADRRFRHdLAIFLDGDHRPGQgidpmSPLteQLRQEI
GNSALLVILMSPDYLASTWCADEREWWSTQQASFglpCDERIAVVRIW---
PTDTAWPKLLTDSQDQPLVGFRFHAPLSGKEYPLGWVDMP(GPFQRDFRKALLDIVTRVY)}*

$1853=217(220):
>gi|821476374|ref|XP_012401634.1| {|333(0)|<Chordata(M)>}PREDICTED: interleukin-18 receptor accessory protein isoform X2 [Sarcophilus harrisii]
{(TKKDLLRNFICFAQNSVGNSTRTIKLKQKRGVFFIYIIFGTTITLVTILMTSAFIYRYWVEIVLLYRTYKA
KDETLGDRK)EFDAFVSYAKQdsfdnldpsflneGSFALDLLPKVLEKKYGYTLCLFERDVTPGGAYTEDIVK
IIKQSRRVICVLSPRYrdrfFTSPS--------------------EASPCCTP-
HYHLEKFKINTSKFPILeLRPLPYACEKPWGFRRKRVQDFS()}*

$1854=234(225):
>gi|822068493|gb|KLD40728.1| {|0(105)|<Firmicutes(B)>}hypothetical protein WU67_13745 [Lactobacillus plantarum] >gi|822087713|gb|KLD58638.1| hypothetical protein WP50_24715 [Lactobacillus plantarum] >
{(MVSKSL)---VFVSYAKEdAEFALRIKQcLLSTASLTKYDVFC-
SEDGTSLTAGVdyvtEIKRAINSAYCHLLVLSPAFMNSPFCLAELGAMWGQPAN-
HpyeiFALLTPGTRDDIlNGTPLEHIQTMKIENAvnwcNR--IFHFLEK--
NipgIHDFDLVGKDA(NKLFEEKQDLTKNIQMNNRLQAHIDHGSDKMTAEAAKQNLEQLLISKGSTEISVAQF
GISHAGKLFMETKYSSNYRKAI)}*

$1855=268(278):
>gi|822611924|ref|WP_046994519.1| {|13(29)|<Proteobacteria(B)>}TIR domain-containing protein [Arcobacter butzleri]
>gi|822318044|gb|KLE06027.1| hypothetical protein AF77_03020 [Arcobacter butzleri L35
{(ALKLQEEMKYSEIDMYLPSFGINCKDYQPSTNSKRVYVQELLANESNETILNIAYDLELTTITQNKDKENL
ATFWEANYF)--KIFISHLAKhKKQATNLRSVLKSYGISG------
FVAHVDIEPSKewqsEIEIALHTMDAIVVILMEGFKESNWCDQEVGFAVGKDTL-IIPIKK---
DLDPYGFIAK-YQAINGNNKNV--
GEVAQEVFTAIIKHSKSKNKM(LSILTNLVGKSTNVSDAIKRINILINISNIPEELLVQMLEQIYNNSILKNS
NIFISNL)}*

$1856=193(198):
>gi|920110098|ref|WP_052949884.1|
{|0(34)|<Proteobacteria(B)>}hypothetical protein [Dyella japonica]
>gi|822106705|gb|KLD63765.1| hypothetical protein Y882_10255 [Dyella japonica DSM 16301]
{(M)AHDLFVSYSQPDRQAAFALVQRLEARGIS-VWIAPRDVSPAADWAAEIIDAITSARLMLLIFSAS-
SNDSGQVRrEVERAVHKQ-----LPILPFRLENVLpskSLEYFLSTQHWMDAFP--

PPLEPHIDRLCAYLESALGKVA(VHERPVELHASQHPAPPAITHPTATLDAAHLRQLEVELARHIGPVAAHLV
RRATQGG)}*

$1857=199(204):
>gi|822676616|ref|WP_047018635.1| {<Actinobacteria(B)>}TIR domain-containing protein [Streptomyces sp. CNQ-509]
>gi|822596506|gb|AKH85401.1| hypothetical protein AA958_27760
[Streptomyces sp. CNQ-509]
{(MNVAGLVLTIVGTLATLAGTYVAVREYLRRRPRITAPSPEPAPAPAPAPAAAADPAADPPY)--
DVFIAYADTDAPWVRDFAAFLTARGVR-VAYDELLAQPGGVRVHTLERAIRDAAHGLLVFGP-
GMRDDGWVRrEYAALMRrslenEA-----QRFVPVLIgDPGEslDLPEFAEVLYPADFRG--ADE-AAYD--
RLCEQISRAVLT(NRA)}*

$1858=217(222):
>gi|822676617|ref|WP_047018636.1| {|0(282)|<Actinobacteria(B)>}TIR domain-containing protein [Streptomyces sp. CNQ-509]
>gi|822596507|gb|AKH85402.1| hypothetical protein AA958_27770
[Streptomyces sp.
{(MA)--DAYVAYAADDAEWVGVLTKRLRAHGVS-
ALGDRHHLLPGSVVVHEVEDAIRSASTGLVVFSPAAMAQ-AWLRqEYAAlfqASADR-----
DlRLVPVLLGN-AGPPPFAATRLPMDFRQ--LPAEGAafedRV--
ALLARIIRGEQP(PDELLRKVEDVQDDLSVVRSGPVRPLTGPAEPSVVICHAHADLEYVRGLGRRLADAGLPV
WTAGRLTWGDDWVWAIRRQL)}*

$1859=197(185):
>gi|821528845|ref|WP_046850767.1|
{|0(284)|<Proteobacteria(B)>}hypothetical protein [Nitrosomonas communis]
>gi|821348500|gb|AKH38731.1| hypothetical protein AAW31_14425
[Nitrosomonas communis]
{(MRGATRPTPENA)---
VFISYARAdneqppdkkaEGWVSFFWNQLRFeLTDRGAAEAklWLDRyEIEPHEAFTpKIEQALKQAKLLLPI
LSRNWIQREWC-QrELELFVEQHAD-DDDCYIPVFKHRL-
ERKHLPSLMQNSNARTGyqFFEIDPTGKTREFYWRGLKDETA(YYDLVRTIAEVISDKLGIVRPVTMASSVTP
ASQ)}*

$1860=227(229):
>gi|822064833|ref|WP_046917994.1|
{|0(5)|<Actinobacteria(B)>}hypothetical protein [Streptomyces stelliscabiei] >gi|908300702|gb|KND46113.1| hypothetical protein IQ64_02955 [Streptomyces stelliscabiei]
{(MSVTPDPLCR)---VVITYSRAeQHHRKTFRRHVADLERSGV-
EFFDDQDIPPGAPWEtTLFGKLESADIIVLLLSSNYVSSDFCMEqEVPRALRRWKA-GECQVFPVNVAPF-
DlTERSPLRQLQWIPS-GksVTEqGAAAA--
REWRRVAQELRKL(VEGLGGATGGAASAANPAVAPASAANPASYAPGAGGKAPGHRPPTVTNSVEGSIVSGTV
VQAGEIAGNVIIGSPRHPPA)}*

$1861=156(159):
>gi|822015796|ref|WP_046877333.1| {|0(10)|<Proteobacteria(B)>}molecular chaperone Tir [Klebsiella michiganensis]
{(MSV)----FISYRHSdfalaISINKRLRLRDANIETYLDKL------
DAESQTTDDItsVITRNIARSTHLIAVVSSQTAQSWWVPFEIGEATILGRRIssyQNGFLSLPEYL---
SKWPQMKSAEHLELFISAYQRDLFENVTAGMEAYSTSGAA(GRSLESDRSAADEFHR)}*

$1862=281(282):

>gi|825996485|ref|WP_047156808.1| {|37(309)|<Cyanobacteria(B)>}molecular chaperone Tir [Trichodesmium erythraeum]
{(LAEIEAESGVGSDTISKIRQGRVGADLSKIQQLFAAFDLTLDETDYRSGKQSSSEMDSEPLSQLLPLLNDEQIVTKNSFQ)---IFISYNRQDpDQELARQFETALAAAGH--QVFMAGDHIRLGEnWFrRINIELKRCDYLLLFLPPQSAQSELLTYqvQ--LARELQFSRpERKPVIlPIRVGFSLTQP--LNHDLRGYLYRiQQREWRSPEDTIIILKEVLTLLAA(PPGNIALEIEEKKEISLSEEISSIASANFPPLPSAEPEIPGGQIDVASNFYIERPPIEERCY)}*

$1863=233(227):
>gi|823411102|ref|XP_012418625.1| {<Chordata(M)>}PREDICTED: TIR domain-containing adapter molecule 2 [Odobenus rosmarus divergens]
{(MMGIQKSKIDPCPLSLSWGKSPRVEMSQRHQESASKTPDDLPLRDLAEPGPMAPASAGEHEGAEDREDT)--------SEEdeVFLKFVILHAEDDTDEALRVqNVLENDFgikpgiifaemPCGRQHLQNLDDAVNGSAWTILLLTENFLRDTWCKFQFYTSLMNSVNRQHKYNSVIPLRPL-HNPLPRDRTPFALRTINaLEEESRGFPTQVERIFQESVYQI(QQALWKESRSSGRQQALS)}*

$1864=286(257):
>gi|826459184|ref|XP_012533855.1| {|24(0)|<Arthropoda(M)>}PREDICTED: interleukin-1 receptor accessory protein-like 1-B [Monomorium pharaonis]
{(CEVSNGVDEEITLAAHVYRQEPQFVLSLPNGSWRKSLLLAVLVLVLLLSAGAFYARCWLPLALFCRDKFGRLEESDGKEC)--DALVCYHEKdsSLVIGIVIPTLESR-HRYKCAALELSQLSHNW-SLEISPHVNTTRRVIIVLSPASLDNIWTDASVGTVLKQ-LSSLPSRTIVVTLKRL-PSTtatiakrsrwDRRDVDCTTSFDRLtILSWQDggggggggdggsssngtttygsggRK---FWYKLRLAMPAI(RPVASETGCQSVAMIVQANGKCGQQKARSRESLEVLV)}*

$1865=247(257):
>gi|825325611|ref|WP_047139822.1|
{|88(143)|<Actinobacteria(B)>}hypothetical protein [Streptomyces sp. KE1]
>gi|824325379|gb|KLJ03681.1| hypothetical protein WQ59_07720 [Streptomyces sp. KE1]
{(FIPVLIGDAALPPFAANRVWRDFTNVDGWEYEAKVEELSTVIRGEPVPALPGGSGTPPEENLAAALPSPPRPLTAPDRPA)---FVLCYAPADAAYGAALAEQLRTAGLT-VW-SAADLRPGDSQFWTIRRELSFAAAVVVLMSPHSQDSDDITSmILEGIL-HQ-----RPFFPVLLEGRRnyHLAHTWYVDARDGRLL--RPAELDLLRELDAARDPGLTGT(PSPAFPSPSAHPTVGAVRVPPASSLERLDAYLGAGEL)}*

$1866=233(244):
>gi|823473435|ref|XP_012426725.1| {|0(108)|<Chordata(M)>}PREDICTED: uncharacterized protein LOC105758947 [Taeniopygia guttata]
{(MKACTPPSAPEAAATSPVSGTSSAP)-YDFSLWYvPEDDHVASRIAERLREEGFR--GFTEHQDQAAGTSAILTAMEAIEASRVAILLLSARSVGDRWCQRVSQWNLFRSIQQRGPRVLPVCVGLSRAQLPPFLQHLVPLEYQN--------E-FFFQRLLASLRAR(PRGGHRAERWGVWVFPTLTGHITVFQNISNLRHVFYKEMSKLGDPQGRNPSPTLQEVEVTVMDAQKCINSRFWHGEIGP)}*

$1867=220(224):
>gi|823779196|gb|AKI82220.1| {|213(0)|<Proteobacteria(B)>}putative caspase-1 [Sorangium cellulosum So0157-2]
{(LRHDANSLFTKHLLAGLRGAAASKDGFVRIFELFEYVARAVRDAGGRQRPVFKCQTEDNFAVALGPGAPAAREKGEELDD)--DVYVSFaPEDLRFVYEtLIPYLERAGLRVVTTDSGTLDLGGYPVANAARGI--ErSKRTLLVVSEAYLREHYAALETTLAIQLGIDQGKYRLIPLtLRRPRDLEMPLLLRNLQAADYAH----PYTgGE-KALERVVQAIKLP(VRSM)}*

$1868=263(250):
>gi|828322102|ref|XP_012572957.1| {|0(1347)|<Streptophyta(V)>}PREDICTED: LOW QUALITY PROTEIN: uncharacterized protein LOC105851158 [Cicer arietinum]
{(MSSFTSSSKSHRVFEFESTTSSNNTNPKKKKN)-YNVYLSFCN-EDA-NSFVSNLYTALISEpRIVVFCDDNNKKFEN-GdrEIVPtsslnAIENCEVVVIVFSKNYVNSRLCLQELEKITECCRN-SDLIALPVFYnnGVYPCHGRLQRGMFGEGFHEfvdkisldmEESSLEEDKF-MTWVAEITKATAY(SKSRNLVPNYRKESKHIKNVVERVTHVINNKKRVSFGAFYTESVKCGAQEVIQILKQSKCPLLLGIWGMVGIGKSTIA)}*

$1869=205(221):
>gi|828195166|ref|XP_012566869.1| {|294(0)|<Cnidaria(M)>}PREDICTED: uncharacterized protein LOC100209303 isoform X1 [Hydra vulgaris]
{(SDGLWVVLIGFLLLLFFFLKIKSKWCKSLKKSGKIQEDLTVVVNDHRNENELHFMSSITNVEQSMTCFKQNITNPDQRTN)--DVFISYSSQnrEWVNNTLLFELEKRN--ISVCIDYRDFVLGEYISKNILDGMHNSRKTIVVISSDYLKSNYCKEELQTASQ------GHSVIFIM---IEkCKLP-RY--LKQQLSKNqVFEWEDNER--FWKNLVLAI--H(S)}*

$1870=291(292):
>gi|827536589|ref|XP_012543930.1| {|1017(57)|<Arthropoda(M)>}PREDICTED: LOW QUALITY PROTEIN: slit homolog 1 protein-like [Bombyx mori]
{(SDPTMSRDEVISKSVFLPDNYFAVICGIIIIVVVICLIFAIFYAFRYEVSDWVYTKYGVPLFKEQSCPNVDHVMEGNPNH)MYDYYVICNTKdtGFIYHNIMSEIE-----FRKMCSKSFSLNEATLNI-LTldsfsKSPKLSKRLLIVLTNNFICNDLSDIQFKNIFFGYLKSLnrseSNKVIFINIVDN-IQLSDELCFALDKFKNIswN-----DPR--FWDRFMVLLNST(DTIITVKGSEASVFKKSLRNTPSLRYTTMPVPNDSCSKQNFTNSLQFCKRYDGETSLNESSPSSDNNTYGEG)}*

$1871=168(194):
>gi|828195858|ref|XP_002159036.2| {|0(321)|<Cnidaria(M)>}PREDICTED: uncharacterized protein LOC100211260 [Hydra vulgaris]
>gi|828195860|ref|XP_012567101.1| PREDICTED: uncharacterized protein LOC100211
{(MDGYDLMISYSHKTREVTWK)---------------------IYDWFEKRGFKIWIDKNyldtEMAKEIRL----GIDKSSVFIPCFSSDYEKSPWCNKELTYASVNN-----KRIVPIIVENGYELSSDIKFKITGLKYFTLEEDFDTAMNEVLKAVKKYTNKT(DTQLPERKESTSASTQCVQAEQYVENPYAKNRES)}*

$1872=167(176):
>gi|828230847|ref|XP_012564995.1| {|0(78)|<Cnidaria(M)>}PREDICTED: uncharacterized protein LOC105849207 [Hydra vulgaris]
{(ME)-YDLMISY---SHTTREVTMRIYDWFEEKGFKIWIDKNHIDT--DMarEIRMGIDNSLVFISCLSSAYEQSTYCKSELTYAYESK-----KNIVPIIVENGYQFSGDIRFKITGIKYFTLEEDFDIAMNNVLKAVQKHIKKK(EISIINEITDPTPPIQTVNYVFEVNGWFRLVSAV)}*

$1873=213(220):
>gi|828216972|ref|XP_012560505.1| {|47(0)|<Cnidaria(M)>}PREDICTED: myeloid differentiation primary response protein MyD88-A-like [Hydra vulgaris]
{(EVNGLSIACDPTMELFKYYEVKYDKKVTLCYVIESLQYIGRKDVVDDIFEYINNNNFIENDNQGLQYPITHVGNFTLNDN)-YDVFISYAKDdISFAKLIKNRLEKEN-

GFKVCIDYCDFLPGYNPFEQVCSVIIEKCKKvIVILSPNFNKSSYCQTHAKIASSSLLEGNKHKVIPILYE--KCEIPLVLKYFSRLDCTD--PHTSS---YFWPCLIKAINVL()}*

$1874=173(179):
>gi|829459751|ref|WP_047329819.1|
{|0(206)|<Actinobacteria(B)>}hypothetical protein [Mycobacterium sp. EPa45] >gi|828181754|gb|AKK26989.1| hypothetical protein AB431_10215 [Mycobacterium sp. EPa45]
{(M)AHDVFISHSSVDKPAADAVCAALEAKGVR-CWIAPRDIKPSESWAAAIVAAIRGAQIILLVFSQNA-HDSEQVRrEVELAASAA-----KQILVLCVEDSIshRALTVSTPHWLDAATP--PFEAHLEKLVVQCRRLLGRPDR(GPAWRDERRQVKIASIAVVIVILGVGVGVAEGFWRSSP)}*

$1875=220(224):
>gi|1129016983|ref|WP_075335886.1|
{|0(1098)|<Proteobacteria(B)>}hypothetical protein [Archangium gephyra] >gi|827420482|gb|AKJ00378.1| Hypothetical protein AA314_02004 [Archangium gephyra]
{(MGLVG)----FCSYARKdRKHLLQLERHLSVLERLGLLYLWFDEKVQVGTARDsEIERQLNEADVILLLISSDFLHSEVCQNQLARALERHEA-GEALAIPILVRPV-DISGTSLDALQVLPKGRraVTQWKPPD--AAWQLVAVEIRQL(VETILRRNETRTASDSASALDVVPLRALLKGIRELPNDIANRVENFLRSYVGPGTAPFGGRTDDLRALDQWLDEPQQPP)}*

$1876=174(173):
>gi|836726669|ref|WP_047858732.1|
{|0(355)|<Proteobacteria(B)>}hypothetical protein [Archangium gephyra] >gi|827425215|gb|AKJ05111.1| WD40 repeat protein [Archangium gephyra]
{()MARVFISYKRDvtpdQDLAQALYKALKATH-----EVFIDTSMLVGEKWVeRIDAEIRQADFFIALLSPFSVNSE-MAQtEIELAYRLHtQK-SQPVLLPIRVAYH-ERLPPPLSGYLSAINWAlwEGPEDTPRLLEQLGKAMSGGLQR(GTAPAASPTGTAFQEHPPELLEQLARAMSGGSP)}*

$1877=230(229):
>gi|827039368|gb|AKJ63927.1|
{|217(0)|<Kiritimatiellaeota(B)>}hypothetical protein L21SP4_00658 [Kiritimatiella glycovorans]
{(RLIKNAPINSHETQGLFLGGREAHDTSFCHFVTRYCADAGLYEGSAGEFVRELRDRWRETSRKGAAGPGGPRRAPMTEGA)---IFLSYAR---EDLEAARAMRDALDEWGLEVWLDEAKLAPGCAWvpEIVRNIHKAAAFVPLISANteAASQDRFFYrEWKEARDFVERNaErrPGFIFPAVIDDT-EPYEAKNLPLAFRSLQMTSCPAGLPAQALVRELHKADRNK(QKELMGVPA)}*

$1878=252(255):
>gi|827435020|gb|AKJ10148.1| {|287(514)|<Actinobacteria(B)>}hypothetical protein ABB07_08965 [Streptomyces incarnatus]
{(VLDQFRCVPDRQDVIWLEQLEALRFDLTYVGSSADATCRGRIEVNINGLPVAEVPLVIQVTVPAARRELRQLSSAPMLRR)---VFASYAH---EDEQFVRRCKAAYRALGINLFVDREDLESGQPWrsVLREMIAQHDLFQLY-WSQSAAASTHVAeEWDFALKVATGRaaGQNFIRPLYWTRP-MAAAPAALKDLHFRYVDPQSLGLTEVAQTTCEAVSTPGLP(PHFDVVFPVLACTPDPSGSTLEAIRHALPSVVPFI)}*

$1879=205(220):

>gi|828195156|ref|XP_012566864.1| {|308(0)|<Cnidaria(M)>}PREDICTED: toll-receptor-related 1 isoform X1 [Hydra vulgaris]
{(FLLLLLLFLKIKSKWYDGMCKGSLKKVQGKIPENSIAIVNDKNYEKEMYLQKKCISSITNVEQNMTCFEQNITNPEQKNN)--DVFISYSSQNreWVKNTLLFELEKRN--ISVCIDFRDFVVGEYISKNILDGMYNSRKTIVVISSDYLKSNYCKEELQAASQGH------SVIFIM---IeKCKLPRYF---KQQLsKNQVFEWEDNER--FWKNLILAIRS-()}*

$1880=194(220):
>gi|831281684|ref|XP_012671936.1| {|770(0)|<Chordata(M)>}PREDICTED: uncharacterized protein LOC105890458 [Clupea harengus]
{(QPKFLEVPFICVAQNSMGLTRSQILLTQANHRSFYWLVVVVSLCGLVMLGVLLYRFFKLDLVLFYRSHCPRLHQRSDGKQ)-YDAYVCYPCEgqgismaMTFALRILPEVLENKHGYKLFIRGRDDSLGEEVFDVIEDALKKSR---------------------------------SYKRSGGKNCA-LAAGDSGAGLTHLAHIWpGPGPDQSQTRARVACRVCLAGGC()}*

$1881=192(220):
>gi|831325514|ref|XP_012695834.1| {|235(0)|<Chordata(M)>}PREDICTED: toll-like receptor 4 [Clupea harengus]
{(IGELFHFQNLKEIIVVEGIMATIPGLNLSHLPNLKKMVFKMAFDAVMLLLFLVYKFQFYLRYCCILLRGYRTSRQDECSH)--DAFVIYSSKdeAWVRDELVERLEKGIPPIQLCLHERDFEAGKSITSnIVDEGLIGSRNVIVVVTQHFIDSSWCRFEFELAQSWLVLGRNTSIIIIILEDVEDKKIKRVFR-----------------------------TS()}*

$1882=278(273):
>gi|831297813|ref|XP_012680748.1| {|297(85)|<Chordata(M)>}PREDICTED: TIR domain-containing adapter molecule 1-like [Clupea harengus]
{(RHSAAADKHRGVASAPVPAAPVEPGRPPVQCSGRDGTRQLVTSISTGMETLRLSEPVLPAFSRSVSAPETPAACEDERAV)-FYSFvILHSPEDaeEAE-ELRENLEC--VTSGTGAtFSQDFaIPGRPTLSCLDDAIDNSAFTILLLSRNF-TSRMQEVLTDSAlinsIHNTHKYNSVIPLLPLNNPLPQDsLPRVLRTIVSLKeGSRSFRQNAMKA-ISPQKVARQRVKW(EREQEAKRLQERRQRLLEENCWREQQMQQAELNQRLLRQAWDLHWRQQNMPSP)}*

$1883=272(231):
>gi|831555873|ref|XP_012729865.1| {|287(0)|<Chordata(M)>}PREDICTED: interleukin-18 receptor accessory protein-like [Fundulus heteroclitus]
{(TPQHLKHTYTCFAGNVIGNNSVTIKLQNKIRARWTSLIGYPVACFVLVAGLGIILHIKWLELQLIYRSHFQFGKHKEGEK)EFDVFVSYV-WtvpsaeapnglarfskpysdaegclstvkmqsseEPIEM-LLPQVLEDRWGYRVCLLERDILPGGAYTNDVVLTINRSRMLICVLSAEYFASdNAVF-VLESGVKALLQTSALKMLLVWTGgtspSF-IQPEsPLPKLVQKALKVLpSLQWSTDVSStavgSFWISLRKAMPVQ(ASRLASHLERS)}*

$1884=199(187):
>gi|836676139|ref|WP_047824053.1| {|0(3)|<Proteobacteria(B)>}MULTISPECIES: TIR domain-containing protein [Massilia] >gi|833720573|gb|KLU37207.1| TIR domain containing protein [Massilia sp. WF1] >gi|94
{(MAIRYGCFFSY)AHGRHELMQ---RFKATLADALRCYLEPYfdnEDELFVDVEQLGGGDDLdrKIARAMCESVCMILIYTPKYEAHAYTRREYAAMRQLEIERsRwyalpSHLIIPVIMTRHPeqlpPQIAESSFYVDFSRFTMATGDLKSNPDFLPDIDKMVRRIV(AHYQCLKKYMPPGHDCNQFVLPDVPPPWREITDTTF)}*

$1885=222(224):

>gi|835310777|ref|WP_047447819.1| {|0(112)|<Bacteroidetes(B)>}TIR domain-containing protein [Alistipes sp. ZOR0009]
{(MSQNF)----
FISHYSKdKHIAELFSTALSRITLEQISPWFSSDNASnsGLKPGDiwfnQILSIITQSKAVVALLTPNSINRP
WIYFECGIGQALEKcE-VIPVCI-GINRDTIIPPLGLYQCYQLNDYRS--VVDFFSK--
LLTLFNIRFDEE(MARVVIEKLVSEISKVKFEEQDKKTDKSLMNIELVLENFKNHVDKRFIEVLEKQNYHINN
STIINGNNFNKKNNNTNTE)}*

$1886=240(241):
>gi|835402048|ref|WP_047494007.1| {|0(108)|<Bacteroidetes(B)>}TIR domain-containing protein [Chryseobacterium sp. OV705]
{(MEEQKQEKQIETIKHSKSESPF)---
VFISHDTRdAEIAEAFSKLLKSVSAGMLKSFRSSDNkgTQGIEYGIewypKIIDSLQDSSDVVCLLTELSINR
PWILFEAGMA--KGKL-NTPILGIAVGVSLSNVsTGPFAQFQNCDDNIvS--LTKLVKQ--
LVRKLPNSEPDE(DVIKQQVEIFKAKSQEIIQSRLEKSQIAEVEKNVDNNESAKLFEEVKLMFKELPSRIENV
SAKKLNRPNRNLHPGMIDE)}*

$1887=284(279):
>gi|831511846|ref|XP_012714371.1| {|273(23)|<Chordata(M)>}PREDICTED: TIR domain-containing adapter molecule 1-like [Fundulus heteroclitus]
{(LRSAGASLFGAERDIKTDEASTKSGFQSNEGATKPNAPNAETKSPLPSATHIILPSIPGLKSPHESKGAEE
EEEEEEEM)-FYAFvILHAPEDsDVADIMREKLEAV-
IGTTGATFSDDFaVPGKSTLKCVEDAVNNSAFTFLLLTRNF-
NSRMVEMKTNMAlinSINNEHKFNTVIpLLPLDNRMPTQsIPVALQTLVPLEeRRNFEKKLQKVF-
NRAKIDKQRRIWT(EEQSLRRLKRLRLRENERLKLHMEQHLVLGGSMPPGEDGGDPRSWWPAQSNIHIENANY
)}*

$1888=217(195):
>gi|505937537|ref|WP_015724203.1| {|0(273)|<Proteobacteria(B)>}TIR protein [Desulfobulbus propionicus] >gi|320122116|gb|ADW17662.1| TIR protein [Desulfobulbus propionicus DSM 2032]
{(MYVPHC)KYDIFISYAHVddlpfpgssagharGWVTTFvgclktrLAQKL-GRSDAYSLWMDHEmeSWP--
-YISQQLLSNIRDSASLVVVLSPGYVASPWCQRE-RNAFLALLEEHVARPVFLVererVEDE-
FPAELMDRKIFRFWVQErEGKAPRILGVPCPDPADHEYYNQ(IDDLCQDIATNLKRLRGAGGAVTALAASSPS
SLAPAMAPRGRVYLAQVT)}*

$1889=195(200):
>gi|321530320|gb|ADW94527.1| {|0(1295)|<Streptophyta(V)>}putative TIR-NBS-LRR protein [Pinus monticola]
{(MAASTSNAAHSNNNSC)-YDVFISHRG-
PDVKKTFASHLYRRLFSFGLRVFLDYQELQKGEDFncEIKGAIETASVHIAIFSPTYADSPWCLDELVFMLET
T-----
APIIPVFHKVKPTDLRRSQGEGVYATSLLNLEKKRKRDSDQPRYDSTTIENW(RDALSRVADISGFDPDVGFN
GDEGELLNQLVEVVLKKGKKKLNV)}*

$1890=227(231):
>gi|321530322|gb|ADW94528.1| {|0(588)|<Streptophyta(V)>}putative truncated TIR-NBS-LRR protein [Pinus monticola]
{(MTMAASTSNAAHSNNNSC)-YDVFISHSG-
KDVKKTFASHLYYRLVSFGLRVFLDYQELRKGEDFpcEIKGAIKNASVHIAVFSPTYAASQWCLDELVLMLET
R-----APIIPVFYHVEPTELRRSHGDgvyARSLSNLEKKRKHDNEP--
RYDSGTIENWRN(ALSQVADKSGFVLDLDLGFGGDEGKLLDQLVEVVLKKGKKKLDVARYPTGLGDLVKDFET
KLSLQQHGERVQF)}*

$1891=158(188):
>gi|497429877|ref|WP_009744075.1| {<Actinobacteria(B)>}MULTISPECIES: hypothetical protein [Actinomyces] >gi|319977918|gb|EFW09553.1| hypothetical protein HMPREF9005_1456 [Actinomyces sp. oral taxon 17
{(MSDSKLYILNAREDQELAGSFLALAEPRLKSV)-------------------------
RDHAFAWWSPSSLLPGEERRtQIDDRLAESDYVILLLSPDLLV--YLRDeDAPKISDSY-----
AKLLPVMLVDV-
PLDRSMELFGIDDRQIYcgDTVEPHSYVSLDSAYQRNRFVDG(FITRMIRRINGKGGYQ)}*

$1892=162(159):
>gi|497429879|ref|WP_009744077.1| {<Actinobacteria(B)>}MULTISPECIES: TIR domain-containing protein [Actinomyces] >gi|319977920|gb|EFW09555.1| hypothetical protein HMPREF9005_1458 [Actinomyces sp. oral
{(MSGIQ)---
LFLSWAHDdAEAKDSFLKVLRPrLKsaRGHTFTWWKDSFILPGEEWKrEILTQLAKADYIVQLISPSFLASKF
IRDyEIPGVGEAPLK-KTLPVMLVDVPLD-
GSMEFHQIDRRQIYCIQqgRSRYSYISRETDYQRNQFVDGFV(ERVLARVEGKTGYR)}*

$1893=273(228):
>gi|835880846|ref|XP_004543461.2| {|279(0)|<Chordata(M)>}PREDICTED: interleukin-18 receptor accessory protein isoform X1 [Maylandia zebra]
{(TPHHLTYTYTCIASNTFGNISATIKLKRKIKVQWPSLVGYLVTAFLPVGGLIIIVRVKWLEIQLIYRCYFK
NGKHNGDEK)EFDVFLSYV-
WspaseevlggeiissrsplndeegdlcitdllneEVKATKkplevLLPQVLEDQWGYRLCLLERDVIPGGAY
TNDVVLALKKSQMLICVLSDDYFtNSNAVF-ELESGVQALLQNSVLKLLLLWTtkaspSLI-
QPDPPLPTLVQRALGVLpSLDWRSGEPShsNFWRSLRKTMPKN(KVKLVSVR)}*

$1894=249(228):
>gi|835880851|ref|XP_012772515.1| {|279(0)|<Chordata(M)>}PREDICTED: interleukin-18 receptor accessory protein isoform X2 [Maylandia zebra]
{(TPHHLTYTYTCIASNTFGNISATIKLKRKIKVQWPSLVGYLVTAFLPVGGLIIIVRVKWLEIQLIYRCYFK
NGKHNGDEK)EFDVFLSYV-
WspaseevlggeiissrsplndeegdlcitdllneEVKATKkplevLLPQVLEDQWGYRLCLLERDVIPGGAY
TNDVVLALKKSQMLICVLSDDYFtNSNAVF-ELESGV--------------------
QPDPPLPTLVQRALGVLpSLDWRSGEPShsNFWRSLRKTMPKN(KVKLVSVR)}*

$1895=158(163):
>gi|821526535|ref|WP_046848462.1|
{|0(2)|<Proteobacteria(B)>}MULTISPECIES: hypothetical protein [Rhizobiales] >gi|816371127|emb|CEJ88028.1| conserved hypothetical protein [Hyphomicrobium sp. GJ21]
{(MPI)--KVFISHKKEDTEQAGAIAAHLTANGLF-VYLDVIDAQlgkSGPDLADYIRAQMEKCTQLLAVIS-
AQTQASWWVPwEIGVATEKE-----RFLASFVSGNATvpEFLQKWPYLRTRADLD--
AYVRESKRAELLVENRVQKGDR(LTARAQGFRSFHTSLKASLG)}*

$1896=226(210):
>gi|920978098|ref|WP_053065571.1|
{|0(253)|<Proteobacteria(B)>}hypothetical protein [Escherichia coli] >gi|838820949|gb|KLY03121.1| hypothetical protein SK85_00865 [Escherichia coli]
{(MTPK)---VFISYSwsS--
PAHKERVKAIADRLLSDGVDVVIDIYDLKEGNDKnaFMEKMIVDKSITNVLVmcdsvyAKKADNKQSGVGtES
QiISQQVYTKVeQSKF-IPIvcefdeNSEPC-LPVF-MSSLIWIDLStpekenQNW-

ERLVRLLNGKPADIKPKLGKR(PSYLDSGQYDPVSEPHAKFNSLKQAVLQNRAGVSYYRRDFVDSVVKYLDGM
RIRARPEQNDLQSMG)}*

$1897=221(222):
>gi|849042999|ref|WP_047980181.1| {|61(0)|<Firmicutes(B)>}hypothetical protein [Ornithinibacillus contaminans]
{(KYEKEMTQLMERVRKNNDDINRYKERVDKEQKREYEKMMKAINSQTNTNENYMNRLSEVSERLNELAIDVK
HTAHEKEII)EFDVFLSHSS-LD-
KEVFVTELSEKLSEKGLKVFEDVKVFKIGQSQtdMMNMGILNSRFVVVFLSSNFIQSGWSEYEFKSFLNREIN
EKKIIILPIWHDVSAEQVRQYNPYLVDKFALS-TGKFTVDEMVEHISQVIFQSKE(AN)}*

$1898=198(205):
>gi|849054416|ref|WP_047984592.1| {|0(80)|<Firmicutes(B)>}hypothetical protein [Ornithinibacillus californiensis]
{(MKGS)--RVFISYNNQdYSKASIIKERLESVGLEV------SIEE--
IETSNdiinDINYKISSSDYFIILLSEHYVESKWTEFEMNKIRSDFYY-
RNIVIIPVLISKVKKPYsLRNFKSFKLYEDFD--
NNLEYLSSYLKNIKYLDFDLI(SEYEFMNLVSELLVKLKFKIDNKEIRQLDYGVDIIASSINRNQLVDSFTTN
WLIDCRFYKS)}*

$1899=241(242):
>gi|851346826|ref|WP_048190682.1|
{|9(57)|<Euryarchaeota(A)>}hypothetical protein [Methanobacterium sp. SMA-27]
{(ILELLFKLRLDNKMDLALSNFIIEKLSLTLTAYEYNIDYLIEKEFIEPKMYTDGFMTHTYYIISVKGIDFI
DNNIKLNKK)MLKVFISY-------NNVEKHIGAIIKEMLesfgIECFmaHD-
DIGVSEEWKqRIFKELNDADIFIPILSDNFRGSEWC-SqEAGIACFRNIL-FIPLSLDKKNRIP-
YGFMSHRQGKLISKYNIplEYLVNPIVDNFPKINILANLIDE(LFMVRGFREAERLMSNLEPYFN)}*

$1900=241(239):
>gi|852748327|ref|XP_012871538.1| {<Chordata(M)>}PREDICTED: TIR domain-containing adapter molecule 2-like [Dipodomys ordii]
>gi|852748330|ref|XP_012871539.1| PREDICTED: TIR domain-containing adapter m
{(MGTGKSKIDACPLSLSWHKRRSVETAAGRRESAAERAGEAASCDTAGPGGGGTEAGEPEEARAQEKVPEDE
AGQ)--DVFLKFvilhAEDdTDEALRVLERLQN-
DFGIRPGLIFAEMPCGRQPLQNLDDAVNGSAWTILLLTENFLRDTWCNFQFYTSLMNSVNRQHKYNSVIPMRP
L-NKALPRARTPLALQTINaLEEDSSGFSTQVERIFKDSVYKT(QQTIWRETRHAGRRQFTGAGKVAQW)}*

$1901=174(177):
>gi|857774677|emb|CRL66237.1| {<Proteobacteria(B)>}TIR domain protein [Proteus vulgaris]
{(MSFKKKLTEELKAQKRAIENTPQIIQKQDKNYPLDIP)EHDVFISHAS---
EDKEnFVKPLADLLSKQGINVWYDEF--SLTWGSslsRSIDKGLAN-
SRygIVVLSKAFlSEKPWTDYELQGLISKEMSGDTPVILPIWheVSKSDviK-YSPTLAdklALNSSTHTI-
EQIAEQLA-RLL--------SK()}*

$1902=151(166):
>gi|857973002|emb|CEP00789.1| {<Rhizaria(E)>}hypothetical protein PBRA_008101 [Plasmodiophora brassicae]
{(MPDDGLSM)----FLSFSHDDLDLAQGLHRALQAGRGQ-VWAD-
WETVAATAaWTDEIHRAVARADLFVALISPEYLLNAS-CQeEIALAAHHR-----KRVLAFCTKSLPrdCLP-
--AGIADADIAV--LKPPRADA--DLHDAAPLLLPF(LRRHRRAPATTLTYATPT)}*

$1903=205(211):
>gi|857971213|emb|CEP02444.1| {|0(63)|<Rhizaria(E)>}hypothetical protein PBRA_009028 [Plasmodiophora brassicae]
{(MAEKNVVRNFFLNKSTNLADAVRDGQN)-ASVKVAFARQdKSFVQMLVSTLKAQSRN----VWVDwEDVPPSTDWLeEINRAIERSDVFLLVLSPDSIRTEVI-GwEIEHAV----K-NGKRIVPIVCRDV-EYSEVPPEVAKLEWIIFqeGPENVEAAMKLLQKALDAELHHL(KYHTVILQRALVWSNRDFDKFSLLVGDDLERAKTWQAAAALGKD)}*

$1904=281(284):
>gi|844287310|gb|AKN04685.1| {|83(0)|<Mollusca(M)>}MyD88-1 [Mizuhopecten yessoensis]
{(NDPEPTVGNLISFLFQLERFDVISECRQMIERDVKKWKQNQQSLKKIYDDPTFRDPSSPSSPPPDKIETVSDAGSQHEIY)-YDCFVIYnPDasdQLEFVKEMSRILEGPEYNFRLFIPWRDDLVGTAMHSVSAAIIEKkCRRCIVIVSKSFNNSPAADFQLKFAHALSPGARQKRVIPIVIE--NVEVPSILNFVAPAPFYN----VGIRH-WQWPRVAATIKSE(LRPDPETWMPTVDAWNIALDTSDLTKRELWGITVATQVYPEEKPVDIPDVDAQRKKHKSKKSGK)}*

$1905=291(298):
>gi|844287318|gb|AKN04689.1| {|85(214)|<Mollusca(M)>}MyD88-4 [Mizuhopecten yessoensis]
{(LGNLVGYLLQIERFDCLTEIWQKLLTDANKWAKRSRTRESSCTAVSFNGNVPSTESSSDDVTSSTDFLTIDDVNAGEIQM)-YSACVCCaDDDLGLARDIVRELSKT---IKLFIPQDHLVGGAYEFEATAEVIEDrCDgRLVVLLSNHYTNSPACSFATQFAKTLDPDARRRRIIPVVTQE-GVSFPRILRGISTIKHYR----CASMT-SFLTQVIASLSYK(PQPKSDTKMLNDPPRENKTISTSSSWSSVEYPRRSSGISTSSSVIALDCDRRSSFAVDKRNSNVSMASIISNESRISN)}*

$1906=220(220):
>gi|871212789|ref|XP_012946829.1| {|691(0)|<Mollusca(M)>}PREDICTED: uncharacterized protein LOC101858387 [Aplysia californica]
{(NYTCVTERGHLSSISAFRDIHGMWRWCYSGYFYWISLLLVAGMLIVFLMAALVLRHGNYFRAAILRLLDNKFKMKSRADY)QICVFIGYAEKdyRFPCLRLLPYLESLGM--TVYVRDRDSLPCQDVPSSIMDAMQSSWRIVLVVSKAFLtDDQWSEFTMRSAVYCQSPSNPARVVLLV-EERNrNRLP--LLLLGAVADDNiVCLGRLLMCYELRQRLQVLLKHN()}*

$1907=227(220):
>gi|871227828|ref|XP_005096872.2| {|875(0)|<Mollusca(M)>}PREDICTED: toll-like receptor 4 [Aplysia californica]
{(IYKDGSQKNIFDGYQGIFMLLERECISNFPLFLWVLALTILLMCVIIGVLVYRFRWKIRYFYYAAYILCKDREKTEEEAF)EFDVFISYASPdeQFVVRTVVPELTKREV--KPHVHGLHFTAGNYIASNIVSAIKKSRKTLVILTEQMLDSYWCTYELQMANLESVYSGRRVLVFLLKDSIPrSRLGMDllYH-----IQNNtYMPYPNTGSQrmnpevttAFWDKMaRDLLN-S()}*

$1908=221(220):
>gi|871253759|ref|XP_012941792.1| {|1078(0)|<Mollusca(M)>}PREDICTED: toll-like receptor 8 [Aplysia californica]
>gi|871253762|ref|XP_012941794.1| PREDICTED: toll-like receptor 8 [Aplysia californica]
{(LNCSMPRSWHRHRSKQFPHFCADLPLIQLGCRSQDVFLAMVVLTVVFASTLILLAAAHHYRWRLQYLFYVGQPRNRLPSV)--DVYLTYDEEdirirTLVLAQILPRLEREGL--RVTVGERDFSGGDEARE-ITRAVVDSKKVMVLLSEDIFDQSLREFEVNMAILYEMKNRCRLLLPVFIEQLednfPrNS-PEIHSFLDSQWERCfYL------ADteTFWEGLLTAIRQQ()}*

$1909=220(220):
>gi|871263045|ref|XP_012943559.1| {|917(0)|<Mollusca(M)>}PREDICTED: uncharacterized protein LOC101850511 [Aplysia californica]
{(WHRHKLKQFPDFCADQALIRQGCHSQDTFVATIVLFAVLTSALVLTGVAQLYGWRMRYLYYMGLHGSRFLELPYATDPRI)--DLFLTFDGDdtrirCLVLSYILPRLEREGL--GVLVGERDFEAGHELT-LICRVLVDSRKVLVLMSRDLFQDSLREFEVNMAIIHEVKNRRRFLVPVFvdrVEEIIqNC-PREIH--------AFLDSQHERcllySEnqRFWERL-LDDVRH()}*

$1910=222(222):
>gi|871273104|ref|XP_012945486.1| {|696(0)|<Mollusca(M)>}PREDICTED: uncharacterized protein LOC101855917 [Aplysia californica]
{(CLTENGTLTTTADFSDVRALWRKCQGQTALLISVILLCLMSLGFVVTILCWKMKTRLKSFIYRVFLQGFVLHGPRDYRLG)---VFIGYADAdtRLACFTLRDFIQSRlGL--SVYVRDINLLPASDMAESIVDAVNSSWRIVLLVTSDYLDRElWSYFTMKTAAY-TVSPSNPGLIVVLLEETVrHRIPACLLRALEEDCILyIPPSRHLSRDIEDRLARLLLPSH(SY)}*

$1911=185(185):
>gi|871260157|ref|XP_012943033.1| {<Mollusca(M)>}PREDICTED: toll-like receptor 2 [Aplysia californica]
{(MFLLTSVMVGFLFVALAMRYSNFYKAFLFNVIKGQFRFKRRQDYQ)YGVYLG-YAANdhWFPCHIVRAYLEESLGD-TVCLADRDFPVNQDMATSIMEGIHRSWRTVLVITEDFLrRDQWSEFTMKYSVYAQGPENPSRIVVIvedrlrhliptqLLGAV--SDENIvsVPELRMC--YELGQRLKFLVSSN-----------()}*

$1912=332(300):
>gi|871236273|ref|XP_012938631.1| {|378(30)|<Mollusca(M)>}PREDICTED: uncharacterized protein LOC101856027 [Aplysia californica]
{(YTVEDLYCNCEHDWVGGRCEMTKPDPEREAKILAGGLTAALVIVVAIAVALPVLLWRLRVVFIMKVVNIFKDYEDDDGKV)-YDAYVSMSPtpEaeKFVYSLLRPRLEDVG--FKLYLQARDQPAGEAISETILDAVTQSRRTILIINPDYVEREWSRFEFLIAQHETLKLKQRIIPIILKDLC-EEARKKDKTLKHIMESVkCLQYphlksensstspqsvsreqhsqvdvlkkekkfkkKECK---FWERLELTMPKK(RKSSEKKLSFLEKLKMCKKSRDKEQSASEHSTKSSKAKVLENKKKADELEAEFTNPNILGLAYKNILNAHVVDMVDLKDP)}*

$1913=218(222):
>gi|871230232|ref|XP_012937530.1| {|6(0)|<Mollusca(M)>}PREDICTED: uncharacterized protein LOC106011663 [Aplysia californica]
{(CIDDRGQDRWTVDLVTTKALWRRCVGKTALLVSLSLALSLIVGFLAVYVWRRFRTAVWAFFLNILAPGFRKTKMEDYGIH)---VFVGYADDdFRFVRFVLRRYLEEDLNLTTYIHQRD-LGAGYLDQQLLDAIQNSWRLVLVLSTNFLRRYEKANlVMKMCTSAVTPVNPDRMLVLVEQGQGRHVPDYVLAVLDEEKLV--RLHSLNEELEYEQKQRIKHLI(LS)}*

$1914=246(254):
>gi|871212845|ref|XP_012946843.1| {|935(45)|<Mollusca(M)>}PREDICTED: uncharacterized protein LOC101858605 [Aplysia californica]
{(ELLMQLLCLGVEPQTERVRTKYQRWWEQTVVGEPKTTGLTVGPSRPRAISTKTTGSSVETSRPKVVSRKIAEQAAVAKTP)--DVFISYQW-SSRKHAL--HLTDILTSRGLTCWTDVQQMGAGDSLfeKAETGLRTCQLVVACVSQKYAVATNCRREISLARTLE-----KRIIPVAVEEITWPLEGPLRDIMSKLSCFELWKDNLGQEQWNGPHLQKILTE(IEKYTRRSDRGSKSLGSGTRDKETSKEGTAALSP)}*

$1915=251(257):
>gi|871212845|ref|XP_012946843.1| {|723(252)|<Mollusca(M)>}PREDICTED: uncharacterized protein LOC101858605 [Aplysia californica]

{(RLLSSRLGYSKQDVKSWAQLADPSTAMLNKWYSDHKTSEATTALLAQLQEMAREDAAVIVENAMKNAEAVV
ESEDFEYTS)PPHIFVSYQW-AHQAEV--
KQIVQHLEMAGHTCWLDIGQMGGGDKLfeKIDSGIRRARVVLSCVTDKYAKSPNCNREVNFAASIN-----
KPTIPLLLESCEWPPKGSMGPLFSQLLYIRFFKRAEERGERTLWPPEKFYEL(LMQLLCLGVEPQTERVRTKY
QRWWEQTVVGEPKTTGL)}*

$1916=215(243):
>gi|871223978|ref|XP_012936376.1| {|1089(426)|<Mollusca(M)>}PREDICTED:
uncharacterized protein LOC106011403 [Aplysia californica]
{(RIGKQESMVMIKNKSRYSSRVEEMVTGVKAKLGSIHSQVQQHPSVFISYCWTNSQHAKSRGTNCPPEALGW
GDPREIKEK)-------------------------
LEERGLTCWLDFDQPAAGKGLfkNITEGIRNSKVMVACVSDEYVKSDNCMMELRFGVLNM-----
GLPTVICVVGTGKEWEYSEVGILMHRAKAAKVFFQEENPEAMDVLEKFIRER(LPENSDQLIQKQTAEIAMRT
LQE)}*

$1917=253(261):
>gi|871231866|ref|XP_005098116.2| {|1005(164)|<Mollusca(M)>}PREDICTED:
uncharacterized protein LOC101850562 [Aplysia californica]
{(QPGEELDDQRYWPGDKFQELLMQLSTFGLTPDEGKIQPVYRNWWMPKVEEIKIDKSKTRDGGNGSQKQQEE
FDKQAAETP)--DVFISYQW-GKQPNIIK--
LYERLTSLGFTCWLDIKQMGGGDSLydKIDRGLRGSRVVLSCVTQKYSLSANCRREVSLADALK-----
KPLVPLLLEKMTWPPAGPMSMVMTQLLYIDFSQDDAEESTWTGPKFRELEAK(IREHVKGAPSDFSGGSSSSD
QVNDQVAAIVTTHPKGSRTST)}*

$1918=247(253):
>gi|871231866|ref|XP_005098116.2| {|810(365)|<Mollusca(M)>}PREDICTED:
uncharacterized protein LOC101850562 [Aplysia californica]
{(RLLGSRLGYSNDDIRSWAQQADPCMAMFNEWYATRKTSEATFAILTQLQEMDRMDAAIVVENAMKNAEAVV
EDEDFEYAS)PPPIFISYQW-GHQEEV--
KLLKQHLEMAGYECWLDIGQMGGGDKLfeKIDNGIRAAKVVISCVTEKYAKSPNCNREVNLSVGLN-----
KSLIPLLMEKCVWPPPGSMGPIFSEFLYIRFYQQPGEELDDQRYWPGDKFQE(LLMQLSTFGLTPDEGKIQPV
YRNWWMPKVEEIK)}*

$1919=253(261):
>gi|871231870|ref|XP_012937829.1| {|1157(304)|<Mollusca(M)>}PREDICTED:
uncharacterized protein LOC101855561 [Aplysia californica]
{(RAGEELDDQRYWPGDKFQELLMQLSMLGLPPDEGKIQPVYRNWWMPKVEEIKIDKSKTRGGGKGSQKQQEE
LDKQAAESP)--DVFISYQW-GKQPNIIK--
LYERLTSLGFTCWLDIKQMGGGDSLydKIDRGVRGSRVVLSCVTQKYSLSANCRREVSLADALK-----
KPLVPLLLEKMTWPPAGPMSMVMTQLLYIDFAQEKAQTEWSGPKFQELLDKI(NEHAPELAAKTEEKKETTSE
SDKKPGPTSSDTGKSVQFKSG)}*

$1920=247(253):
>gi|871231870|ref|XP_012937829.1| {|962(505)|<Mollusca(M)>}PREDICTED:
uncharacterized protein LOC101855561 [Aplysia californica]
{(RLLSSRLGYSNDDIRSWAQQADPCMAMLNEWYATRKTSEASFAILTQLQEMNRMDAAIIVENAMKNAEAVV
EDEDFEYAS)PPPIFISYQW-GHQEEV--
KLLKQHLEMAGYECWLDIGQMGGGDKLfeKIDNGIRAAKVVISCVTEKYAKSPNCNREVNLSVGLN-----
KSLIPLLMEKCVWPPPGSMGPIFSEFLFIRFYQRAGEELDDQRYWPGDKFQE(LLMQLSMLGLPPDEGKIQPV
YRNWWMPKVEEIK)}*

$1921=244(254):
>gi|871243312|ref|XP_012939950.1| {|634(80)|<Mollusca(M)>}PREDICTED:
uncharacterized protein LOC101861518 [Aplysia californica]

{(PENQGWIALAQHMGYKQGRIDLLNEDLNPSLALLTDWIVSSGNTAMSVDVLLHHLQQLPAPDIVELITRAK
ETELERPQL)----FLSYQW--DVQDEV-
SALRDHLEHAGFSCWMDIGQMGGGDQLsaKIDQGIRNCKVVVACVTPKYTASHLCNRELWLADLLH-----
KPIIPVIMETVSWPPPGGMAVILSQLVYINMKGVGGHGGTGIHADLMDKYTE(VVQRVSLHAEPDLTPCLDTT
VVVPVEAPTHMSDH)}*

$1922=223(254):
>gi|871245205|ref|XP_012940310.1| {|2190(321)|<Mollusca(M)>}PREDICTED:
uncharacterized protein LOC101848126 [Aplysia californica]
{(DINLILADEVFVKLTQPERYPSKFKELLEHIQFRCSKKKIEYPECFISYAWANSKTAVELGSATVEGALGW
GDPRKIKEF)------------------------LQSHGVNCWLDVEQMGQ-
EGFfeDLADGLRKAKVMVAFVSDEYAKSKNCRMEFRFAVSTL-----R--
IPIVLAVVGTGYEWERSEIGLLTVGHSQSCPKVNFQYENEAGLMDILKE(VQRFLPKSVASSDEPSGGTIDNA
QAVAFQEVLEL)}*

$1923=248(240):
>gi|871265966|ref|XP_005109462.2| {|1920(363)|<Mollusca(M)>}PREDICTED:
uncharacterized protein LOC101845043 [Aplysia californica]
{(EACCNLFKYARITLKKPVLLVAIGDNLDWKKSNMGILLADEVFINMMKASRYPHKFEELEIMLKEKHALKT
DKRKQKVFP)PC--FISYCW-QNSAHAVARgtrasdgavgfgdprEIKDFLQDNGIKCWIDVE--
RVGVHgLfeDIGEGLLNCKVVVICMSDQYAQSSNCIIEFGYAAKTL-----
NLPIVLAIVGSGNKWRALEVGIMSMDYPLVSFQEGSKNAMNKLLDMVKVHIP(NDAELVRRQEEEERKEKEAR
)}*

$1924=286(291):
>gi|871239361|ref|XP_012939183.1| {|1262(0)|<Mollusca(M)>}PREDICTED:
uncharacterized protein LOC101858280 [Aplysia californica]
{(NQYGFKDGSSPIMFHAEPGSSAAYHNSLMMNQDHPDFDIAMVNGTIPMETSQLLANHHSDSQPLLPHQDTV
SAPPIQDLY)RYHVFFSHCPEdREWVEHMVAHLEAPPFN-YTCayasLQDEADPGTLQQRILCAAMLSER--
VVLVLSSKYVQDTWFSFEKTLKQLTQMSLHNQRIMGVLLEDC--EIPESLGELYFLDSSD--PDFFH---
VFTKRLKTSRIPR(SSASVSSDLGQNAVAPASPDEVNGPPVGRGYINRPTTPSVGNDEVSHTDLNPLGSVELL
RQLYLIIRGTDA)}*

$1925=224(248):
>gi|871257510|ref|XP_012942474.1| {|323(417)|<Mollusca(M)>}PREDICTED:
uncharacterized protein LOC101852264 [Aplysia californica]
{(QYTECRARYDGETVEGKITSACAELVTTLHTDTVTSDPADDQGDVSVVKESPIYDVFVSYSHRNPEMARAL
VELMGRKYP)-----------------------------
ELYVFFDRSELKSGNVWqqTLYEAVDRTRCFVAMLTDSYLKSSVCQEEFNIAL--
ARYTaRDQLVFTSVCLPELsslpASHRRGHHVTVSDSGQLEVLCDKMAAAAVKAQQEEKEHLQ(IGKIKDTLS
RHRMAEFNEKYFFDKGKVV)}*

$1926=216(223):
>gi|872716253|ref|WP_048566961.1| {|63(0)|<Firmicutes(B)>}hypothetical
protein [Bacillus cereus] >gi|859501794|gb|KMP26054.1| hypothetical
protein TU50_21670 [Bacillus cereus]
{(DHIDVMKRLMLGEGLRYRPDITFHELQQEIGNRRFNSQTLVRDCDVERAMKSGFLFQFSEKSEIEKLGYII
DKPLGNQMP)---IFLSHSSKnKSEVEDLIPYINGAGLPV------
WFDKVNIDYGEsivkAIQKGIKQSAGVIFWITNDFINSNWCDLELTKFANRYAS-
KRDILTIAVVHQDVDIEEIDFLFDDIKYLKR--TNEDLEIIAKEIIPTLKRYMD(SIK)}*

$1927=221(221):

>gi|921064986|ref|WP_053078814.1| {|42(0)|<Firmicutes(B)>}hypothetical protein [Bacillus cereus] >gi|859501797|gb|KMP26057.1| hypothetical protein TU50_21690 [Bacillus cereus]
{(KELLPIVRTFTSDIHWIKWNAESLFLDLSKMKEAFQDLFYNCDWMKGVVKVDPQFDVELRTEVPIVTYQMTKEQIELTFP)-KKIFLSHKGSnKEFVKKFYEVLKELGFDP------
WLDDEDMPAGMdpnrGILKGLQESCACIFFITPEFKDERYLTFEIDCAQkqkMNKGE-
KFSIITLQFTNEegKKGVIPDILQISLWKLPKN--DMEALKYILRGLPIKVGTVDW(K)}*

$1928=193(215):
>gi|859296498|gb|KMO98801.1| {|0(776)|<Actinobacteria(B)>}ATP-binding protein [Streptomyces roseus]
{(M)-----------------WIAHRLEEHGHR-
VALARWDPQSAPGLAPALDDLARSGSRVLLILSERYFSAAAHTDeEWNTALrtvtdAHP-----DRFAAVCL-
--TdaPLPSAVAVLEKTDLWG--LDAYEAEY--
RVLRRLELPTDR(VGAETGRRGPRFPNDPPEIWGRVPRRNPRFTGRNDVIGRLRTALCDAPPGASTVTLLGLSGVGKTQVATEYAYR)}*

$1929=223(222):
>gi|861604741|gb|KMQ84259.1| {|0(112)|<Arthropoda(M)>}molecular chaperone tir [Lasius niger]
{(MTVQA)----
FLSYAIKdKEGVLALLKHCAPFLKEKQLNIVQDANDVRDVALTsENKRKIEQSDIFIALFTQDYVYNGACRDELYIALQLHLA-GKLEILPIAWKEC-HWTSIS-GSIQrlVLPKDKriVTypeGWkHNPT--
EPWDQILNRLSLY(IQKAECLAQNPERKNAPSPLIQTSQEILQRKPAPRPANEAAENPEFSEKLEMVSQNLSTATITAQKNLIAQASLTIA)}*

$1930=216(220):
>gi|871225524|ref|XP_012936652.1| {|705(0)|<Mollusca(M)>}PREDICTED: uncharacterized protein LOC101847182 [Aplysia californica]
{(NLDPSTSPGEKRNLDPVTSPVRVAPPGGADASASGLDEEEGREEQARRPLGPEAGQRQMAPPAGRQPYLAIHDQEYGRQC)-
YSAYLCVaPGDLTDFGIPFHRLLEHTLGFRVFLPERDFLAGGFAYEELSRVVEErCRgKIIVVLSKNYHTSGECRFLTYFARNLDPDARKRSIVPVKIDKNMEPIPNVLRGITIVHYNH----AFRCG-WLNEKLRDAIRA-()}*

$1931=217(225):
>gi|871230302|ref|XP_012937543.1| {|689(0)|<Mollusca(M)>}PREDICTED: uncharacterized protein LOC101860409 isoform X2 [Aplysia californica] >gi|871230305|ref|XP_012937544.1| PREDICTED: uncharacterized p
{(IDDRGQDRWTADLVTTKALWRRCVGKTALLVSLSLALSLIVGFLVVYMWRRFRTAVRAFFLNILAPGFRKTKMEDYGIHV)----FVGYaDDDFRFVRFVLRRYLEEDLNLTTYIHQRDLGAGYLDQQLLDAIQDSW-
RLVLVLSANFLRRYEKAHLVMKMCTSAVTPMNPDRVLVLVEQGQGRHVPDYVLAVLDEEKL----
VRLHSLNEELGYEQKQRIK(HLILS)}*

$1932=272(280):
>gi|871257510|ref|XP_012942474.1| {|134(558)|<Mollusca(M)>}PREDICTED: uncharacterized protein LOC101852264 [Aplysia californica]
{(TPILATGCQHAPLDAMLRVMVSNALYWIRAGLPLKCIKLVLYTANPDRDRQFLYKSSIEQFGLIKKKWEFRNTQNEAKET)-YDVCFCYaKSDEHWMTDVSRTLVSRDHSLKmykeLFIASTDD-----
SWQEVVFNILScSRRIIIILTPTFVSDPFCLEQFSLGLCCNRLKGDNVLIPFYIESV-TVFPS---
YMNLIQYTE----
CRARYDGETVEGKITSACA(ELVTTLHTDTVTSDPADDQGDVSVVKESPIYDVFVSYSHRNPEMARALVELMGRKYPELY)}*

$1933=216(221):

>gi|871271800|ref|XP_012945226.1| {|711(0)|<Mollusca(M)>}PREDICTED: toll-like receptor 4 [Aplysia californica]
{(LEERQFLSPVPSSQDIRALWRRCVGKTSLLLSLMTATLLFTGFLLVVLFSRHKRYIKAFAMRIFLHDYHLKTRADYNIGL)----FIGYaEEDYQFPCLVLRAFIEDELGLTCYVRDRDQIPNQPMANSIAEAI-NsCWKTLLVVTPRFLTSDEWGLFTAkmsvYAQ--GPENPHRLLILVDHMTVDRLPTDLLCAVSEDNIIC----LPRLRMCYELRQKLRTRLL(N)}*

$1934=221(225):
>gi|871275908|ref|XP_005112701.2| {|4(0)|<Mollusca(M)>}PREDICTED: uncharacterized protein LOC101847551 [Aplysia californica]
{(GNYTCTDYRGYSINISDSAAVKAIWRRCVGRAALLISICFALAMAFGFLALYKWRHFKTALRKVFLNILHSVRMKTMREY)RYQVFIGYaDDDFRFIRHILRRYLEDDLNLSTYIHQRDLGPGYLDQQLLDAIQDSW-RLVLVLSVNFLRSYDKAHLVMKMCTSALTPLNPDRILVLVQKDQGRYIPDYILAAVDEGQL----ITVTNLQEQLNYEQKQRIK(HLILS)}*

$1935=220(238):
>gi|75180565|sp|Q9LUJ8.1|CHL1_ARATH {|0(239)|<Streptophyta(v)>}RecName: Full=Disease resistance protein CHL1; AltName: Full=Protein
{(MSSASSSSAASLLLGREV)--DVFLSFCC-QTSHGYFQNILI----RNGIRTFLSYRCleKRFGP-IgqRTLKALEESRVAVVMTSTTKPCSVGFLEELLVILEFQEK-GSLMVIPIFLTDLSFNV-----------EEICRQHPEKA-
PSWRTALTKLTNL(AAEYPLSQNLAGMDQSDLLNQIARDISLVVFYSGSNDSNALVAMDRHMKVVYDLLALEVNKEVRTIGIWGSAGVGKTTLA)}*

$1936=274(282):
>gi|499304969|ref|WP_010995744.1|
{|156(617)|<Cyanobacteria(B)>}histidine kinase [Nostoc sp. PCC 7120]
>gi|17135395|dbj|BAB77941.1| alr1575 [Nostoc sp. PCC 7120]
{(LELFLRHQDYVHQHTYFLAKALEWEQKQQQSQYLLIGEEREQAENWLKLRFRDEQPPCEPTDLHCAFICQSIKNANNLMT)--QVFLSYSENERLFMEKIAKILMRESFT-VWTNKTDIQSGADFTESINRGIEEADNVVLLLSTASLQ-SEYCQmELSYAFSLK-----KRIIPLLIEPVDieQIPVELSILQFIDFTD--
QEEQGKESADTDRLINVLRRDE(AYYEQHKILLAKALKWERQKRNPSILLRHHNLRHAEDWLKIAQQNTQHPPIPLQAEFIAESL)}*

$1937=248(256):
>gi|499304969|ref|WP_010995744.1|
{|359(440)|<Cyanobacteria(B)>}histidine kinase [Nostoc sp. PCC 7120]
>gi|17135395|dbj|BAB77941.1| alr1575 [Nostoc sp. PCC 7120]
{(LINVLRRDEAYYEQHKILLAKALKWERQKRNPSILLRHHNLRHAEDWLKIAQQNTQHPPIPLQAEFIAESLKQPPQAALD)---VFISYSRAdSDFARKLN---DTLQIQNK-TTWFDQEsIASGTNFEqEIYRGIESANNFLFIISPKSLSSPYC-TtEVEYAMNLNK-----RIVTVLYREI-KGATLHPGLAKVQWIDFsqHNTDFLTKFGELTRTLDSDPEYV(RSHTRIFLKAKDWEEHNRDDSFLLRGKDLLASEEWL)}*

$1938=301(296):
>gi|499369722|ref|WP_011057300.1|
{|260(299)|<Cyanobacteria(B)>}serine/threonine protein kinase [Thermosynechococcus elongatus] >gi|22299003|ref|NP_682250.1| serine/threonine protein kinase [Thermosyn
{(FATAADALAGLQQVEVPKSFWFRLGKFLRTPLQDLWRSPKTGEVLPPPLPPVSTLSPQASEPKPVSSPPAATTALPNQGR)--RIFISHSShgTdLQLASALQEALQKAG---HQPFMASQS---IRLGEawaqRIDQELKQCDFFVLLLSQTAAQSEMVLEEVRTVkqlQAQRGD-RRPLILPvrvnfpldmPLNYELRGYLHRLQQRF-WRSPAD--TDTLLQE--

ILDLVNATTAPV(ATRSDAPQETIAEPDQRNTFVADGPPVPVAEPELPEGQVEVASAFYIERPPIEQRCRETL
LQPGSLIRIKAPRQMG)}*

$1939=248(230):
>gi|1169637369|ref|WP_080511473.1|
{|0(65)|<Proteobacteria(B)>}hypothetical protein [Burkholderia thailandensis] >gi|584100541|gb|AHI72177.1| TIR domain protein [Burkholderia thailandensis 2002721723]
{(MTNEQEKIPA)----FISYAwggpYDkKDWLRS--
DIITNLEGAGFSVFWDRDSIPIGHSIFqEIENTLNKAPLnVICICDSDYIASankeNSGLHvELEMLQHRNRK
-SGVKIIPVIIEES-ISHLLPqplagrlYVDLISLHKEGitlgptVTSAvlgASQS--
QIRVEIAARLREA(AIWEKARLYFQHRPYEIHGEASSHRVFCSPGELLLPPRWMYKDPRWSYRVLDCQDKYHP
AHGIWHWDWWTPGASMCTLGA)}*

$1940=196(195):
>gi|1181273366|ref|WP_084579375.1| {|0(719)|<unknown(X)>}hypothetical protein [Kutzneria sp. 744] >gi|585090958|gb|EWM12899.1| LigA protein [Kutzneria sp. 744]
{(MTSICPFSAVAVGDSVLVTQS)-HDVFLSFTW-SDIVE--
VEKLDKAMRAAGLRVFRDSLGIKSFDGItaELRTALAESKVLLAYYSRRFPTRYACQWELTSAFLAAQSrgiD
PRRRVLVINPEQNGDHIGPVELQDAAYVQRPRKDAEMAEIVARVAEVVHATASP(LGTAHSAAPMLPRLLHPR
RFVGRYRELWQIHSAL)}*

$1941=243(252):
>gi|585646078|ref|XP_006811668.1|
{|351(224)|<Hemichordata(M)>}PREDICTED: uncharacterized protein LOC102806257 [Saccoglossus kowalevskii]
{(VDIDQEIDSEDDDDVIIRPGSAKRTGTTEVDLQLKSVAPVPPPPPPPPAAAPPPPPPAAPAVFSHGPLPPP
PPTQRKEGH)---IMLSYNW-DN--
QKLVLKINEKLKEHGYKTWIDVEHMSDSILDAMAHAVQDADIVLLCFCQSYQNSTNCRTEGEYVFSRK---
KPLIPLRMKSYSPQNWLGALIGNQLYVDFSDGEVVFNDKMKDLLEQIKRRQSKL(ADQTVNYGYHAVYMLKYI
SGMSYSEILQPSAT)}*

$1942=215(220):
>gi|585723538|ref|XP_006813661.1| {|655(0)|<Hemichordata(M)>}PREDICTED: uncharacterized protein LOC102804436 [Saccoglossus kowalevskii]
{(DSVSISDLSVATGNKKVLLSVTEDVDSNNFIYKAPDGTEHIKTNICPDTISQILSQIEKEYPEKINIPPAP
KDAKNKHGK)KYDFFAVFSEgDdaDWVWYTLLPKLEKElEL--
KGCITQRDCTPGKNKLENIIDAIENSVFVIFILTPAFF--NCLQHCVC-ALKQTLATEEYQIIPI-----
RKQNYDTSMKTAKLLKAIeHVEFCKHYDWQQIEQALREGLEN()}*

$1943=253(269):
>gi|585644595|ref|XP_006813898.1| {|0(214)|<Hemichordata(M)>}PREDICTED: uncharacterized protein LOC100366292 [Saccoglossus kowalevskii]
{(MSASIPIEVEVVDDGMSHASAFHDGSVQHVITDTTRHDGLSTTAKEDATK)---VFFSHSSKdsAWV-
KETVERLEQNHSIECL-
FDERDFIAGRPIVDNIIRCIRNTDRTVLVLTPDFLDSPWCGYEAQMIFSEHLFREQKIMIPVLLSE-------
--CIVPDFMKHLtYL-----DVRdpQFWDKFLEIL--
L(LDDTNVEIHDSMLEQFSSGSETVKFNGKSILPVNMNLTLDTTRIYESLASKGIRVPQDNIHAAVEILTRSS
PVRFSCCY)}*

$1944=225(230):
>gi|585688239|ref|XP_006820644.1| {|0(22)|<Hemichordata(M)>}PREDICTED: uncharacterized protein LOC102809950 [Saccoglossus kowalevskii]

{(MSSRMSSGSFE)-YDVNLIYHEThlDYALTYFMPVLEQR-
WRLRVCAKERDHLPGLPIANNIIQSAEESRVSVILFSKQFIeDGGWSTYESLVALQKMVtgsGRHRHAVIPVF
VSSNVkDSNFRPFVHLNI----------TDSMErqYFWKRMRDSL--
N(ICDRDVDLITVPPLVEDYYAAPGGPSTHGLHHHIVSHHRPSSLPLQESGEQNQEISSSTGAVQPNHQESLV
DDIITWVT)}*

$1945=254(231):
>gi|585675351|ref|XP_006818761.1| {|0(26)|<Hemichordata(M)>}PREDICTED:
X-linked interleukin-1 receptor accessory protein-like 2-like
[Saccoglossus kowalevskii]
{(YTTFSLCFFLGKT)-YDVFISHigSEKtvQF-CKQLLEYLENKW-
NFKTCLVERDLLAGAATCDNIVESVKDSRCFLLVLTPDYIqvpsdcdqivsespeyvkETHWRPYEFQIALEE
SLK-LQTRIVIIKLEKL-PANINMDhmKTLKHVIGVTrCLKWepnvrrKQDK---
FWKNLLYQLPTS(SKWRPTFGQLVQPGTRLSQHKSADDEKPLNRQFSDESGYGEGSPTEETSAPFYSISNELG
NSREGHSYSSSHGDIQVS)}*

$1946=224(238):
>gi|585719586|ref|XP_006826089.1| {|10(0)|<Hemichordata(M)>}PREDICTED:
toll-like receptor 2 type-2-like [Saccoglossus kowalevskii]
{(QVESTDLPSEPLEDYDAESPVLDWELQDTHTEHFFVQTEEEHHVESDSTEGGIEISPVVSTTPDSICSMAP
PLVGKETSH)---VFFCYSPSdnEWVANV-
VRKLESPAYGFKCYNHELVCEPEKSKADLILSCTCCCKKVVVVLSSAFLESAWCQLDNAYVIQQLIKD-
TNKIIVVALNDC-NAP--------HYLQHFvPVNAMSKS---
FWPRFIASLKQE(IFVDVVNSFSNEHAEQKS)}*

$1947=282(259):
>gi|585721090|ref|XP_006813208.1| {|179(0)|<Hemichordata(M)>}PREDICTED:
interleukin-1 receptor-like 1-like [Saccoglossus kowalevskii]
{(YGKYIAKAANGHGDVYVPLTLKYGDPNAEPQSPYLYILIIIGVIIIFILPLGLLIRYKFGLDIQLLWKYKI
RKVDTDGKT)-YDVFISHigSEKtvKF-CKKLLEYLENKWG-
FKTCLVERDLLAGVATCDNIVESVKDSSCFLLVLTPDYIqvppdndqivlespeyvkETHWRPYEFQIALEES
LK-LQTRIVIIKLEKL-PANINMDhmKTLKHVIGVTrCLKWepnagrKQDK---
FWKNLLFQLPTS(SKWRPTLGQLVCAGLSVLQHKSADDEKPLNRQLSDERSR)}*

$1948=223(239):
>gi|585711587|ref|XP_006824766.1| {|0(8)|<Hemichordata(M)>}PREDICTED:
uncharacterized protein LOC100368451 [Saccoglossus kowalevskii]
{(MASHQVLHEGDDAPDFFVTGDPA)-----------
EHISKWIDEITTHMENNFRVvgAFGDRDFIGGPPILVQITEFIDTCKRTIVVFDG---
RSRWANYAAQMVIHRmfqeNAEKCNERLIIVLRNK------EAAKQVPTFLRNNhTLNADAKD---
FYNKLFYSVKGY(SSKERYNLNKQNEILPKQETENPGACLLELLPPQDELYLPPEPNSDKPGKRGKFYHDLDF
CPCTGDKRSKLSVISL)}*

$1949=173(186):
>gi|585656394|ref|XP_006815659.1| {|0(62)|<Hemichordata(M)>}PREDICTED:
uncharacterized protein LOC100368881 [Saccoglossus kowalevskii]
{(MAEPGESRHSAM)-----ISYSW-ND--KTKVDKIANEMTANDIRVWRDVNDM--
GNEIysSMGGGVVNSLVFIACISDGYQSSINCKQELRLAADKK-----
KCIIPIKIQDVKFDNEVALICAGKLYIDFSDDSKFEEKIKNLINRVKTHMDQ(PDSAEPINNVAVSTEVSTDN
IEFVVNEITKEWKP)}*

$1950=244(253):

>gi|585666745|ref|XP_006817171.1|
{|540(158)|<Hemichordata(M)>}PREDICTED: uncharacterized protein LOC102803728 [Saccoglossus kowalevskii]
{(PPPGPMSAIYCQFPCVQLYEFSANNQSGRKYWPHSNFVDLLAKICYHTSPNVDKITKEEYRNWIHNIESFPENSEKTSNA)--DVFISYEW-SQIEE--AMVLYSVLIKFGFTCWLDVLNL-GAKPIinKVDRVIRVAKVMICLLTPQYAQSTNCCREVTLATVLK-----LPIIPLVMEKMFWPPRGPASTTFTQLPYIDFTLEESELFYRGKKLQELYNHL(KEIVPSKVVERCHEFLFTSETEFCDFSSGEVLD)}*

$1951=214(220):
>gi|916876826|ref|WP_051483882.1| {|0(89)|<Firmicutes(B)>}hypothetical protein [Clostridium bornimense] >gi|584457812|emb|CDM70069.1| Hypothetical protein CM240_2952 [Clostridium bornimense]
{(M)--KIFISWSGEfsQKVASALKEWL-PCVIQSIEVFFSTED---IEKGEkwdsKISGELSESEFGIVCLTSQNVSAPWIHFEAGAlSKALDDK-VSALMLNVNPSDIKGPL-SRFQATKFT-KED--FYKLLET--INNNNDTKLEES(ILKRTFEVMWEKINVTINNLIKEYDSESIKNPKKTTEINENREAIEEILQILRNQNNILSNPEKLLPMEYFEYLMRNSN)}*

$1952=178(177):
>gi|760046937|ref|WP_043729913.1|
{|0(19)|<Actinobacteria(B)>}hypothetical protein [Kutzneria sp. 744]
>gi|585091574|gb|EWM13515.1| hypothetical protein KUTG_03819 [Kutzneria sp. 744]
{(MSGYEY)--DIFISYAreGSAQKWllnhflsklKECLADEIAPKP-R-VYVDR-EMERGVHWPSSLQRALLHSKIMLQVLAPPYFE-SDWCRaEWESMRARQ-----ERLGLASVDTPQglIYSVLYGDSDSFPDAA--REVAWVDFKEFANPDMAFQKTK(KFGAFHWKVRDLARDLVVLLPQVPEWQPDWP)}*

$1953=209(215):
>gi|918330843|ref|WP_052395648.1| {|0(338)|<Actinobacteria(B)>}TIR domain-containing protein [Kutzneria sp. 744]
>gi|585094029|gb|EWM15970.1| LigA protein [Kutzneria sp. 744]
{(M)AHDVFLSYCRSDTTPAEALAAELRARGLR-VFQDEVGVHRFDSVSRTILAELGRSRLLLAYYSQTYPTRRACQQeLITAFLAGQ-----REGDPLR--RVLvvNPERGTGHIEPIELRD--ArfwSPSAELR--HLTEAVTRKVDE(LPTTIGAVDHGRVPLSLPAPGHRPPPKFVGRTAELWRLHSALHPQSATLTEGGGEPVAVVHGVGGIGTSSLVAE)}*

$1954=262(271):
>gi|585666745|ref|XP_006817171.1|
{|367(313)|<Hemichordata(M)>}PREDICTED: uncharacterized protein LOC102803728 [Saccoglossus kowalevskii]
{(DWRFLAVYLGYASDNIKLLSEMDNPSLCLFNDWYTSHKGREATYAVYKALQVIGRNDCMQTIDSSIPPKDPLIPENEKLI)--DICISYHTDDQVDANVLKFHLELAGFQ-CWMFTGTTLHLDTLYHVVDEAMRSADVVLCMCSRVYVNSRTCCKqA-NLAQQLN-----KPIIPVIIGPLSwPPPGPMSAIYCQFPCV--QLYEFSANNQSGRKYWPHSNFV(DLLAKICYHTSPNVDKITKEEYRNWIHNIESFPENSEKTSNADVFISYEWS)}*

$1955=191(195):
>gi|585646269|ref|XP_006811698.1| {|0(267)|<Hemichordata(M)>}PREDICTED: uncharacterized protein LOC102803167 [Saccoglossus kowalevskii]

{(MGVTTSSPATKAGGGRSKPKNDTSRRPSFAVGQGKD)---IMISYSHTdVEWMRKIKERLESAG----ISVWVDEAyLGAGVEFLsKIGQAIVDAKLFLSLLSTTSVKSKYCKDELALAYVSNKL-IFPCALQTREELS-NDMDFGMKLTLAPMKWIyfSSDRPIKDSCDELIIEIKLKLKE(LKEEEEEVDSQENSTLSPH)}*

$1956=277(281):
>gi|585664867|ref|XP_006812094.1|
{|525(267)|<Hemichordata(M)>}PREDICTED: uncharacterized protein LOC102802839 [Saccoglossus kowalevskii]
{(SVGARVIDTTNPVTDQKKDHESACFDDESLSRPTEEVAANNRKSMSSGQHADLRLLAESELSDVQLTARERAQLPKKGSY)-YDAILLSsTHDESELEELLSLFE--GWQLKVCSFEGFKTGGKNVFENFKDAMYSCTYIVVVLTKNFLKDKWCekHIHTSLITAIDDPEKHNFVVPIVYGLQDKEIPTELKSVACLKRSN----RFYKEQLEKTITTLQREIK(VEEDEERYAEAEMKLKKQKLLKSLNKPRDELLLQQQYKQEKKKKQSQYQKDSSQLRKHEQL)}*

$1957=275(283):
>gi|585688407|ref|XP_006820675.1|
{|144(403)|<Hemichordata(M)>}PREDICTED: uncharacterized protein LOC100375438 [Saccoglossus kowalevskii]
{(GDQGYSETVMLKGMIDAAVNWMKAGLPLRLLKIVVFTRSVKEGKMDSANKKHFKGILSLFTALKEQYESKEIEEKNDTIE)-FDVYISYsEKDLGVVDTIQGLLMKEKKDIKIFSEQQDLDT-TCVWQENMYKVMVkCAKIITVLSPAYLTSATCTEQYNIALCVNRKTNRDRLAP-FYVEQIELLPS---YMGLVQYVN----CSPHDKLKLATACHQVIVA(LTLEVTMVTQKGQSIKKRSDLNYDVFISYSHKNSQQATAVLNKLTNLNPDLKIFFDKEELKTE)}*

$1958=252(267):
>gi|703153355|ref|XP_010110659.1| {|1373(536)|<Streptophyta(V)>}TMV resistance protein N [Morus notabilis] >gi|587940914|gb|EXC27504.1| TMV resistance protein N [Morus notabilis]
{(LETVPRFYDFFGTEVPLHDKRSSTSPSRDSSTTHEEAVCLNFDTEDSCYERINCFDFCCWSNFFSKILASSSSFVPTSYK)-YDVFISFRG-EETRMNFTSHLYAALRRNAIETYIDDKsLKRGDE-IspSLLKAIEESQLLLVVFSKDYASSIWSESEFIEkiVKDILEK-----------------LENTTPSTAADHFQGQLVGISKRLKKLESLLCVDSPTV(RIIGIWGMGGIGKTTLAEALYKKFSSKFDSCILINVKEDVNKHGLCS)}*

$1959=139(151):
>gi|703150887|ref|XP_010109975.1| {|0(1274)|<Streptophyta(V)>}TMV resistance protein N [Morus notabilis] >gi|587938214|gb|EXC24971.1| TMV resistance protein N [Morus notabilis]
{(MAASSSAPKQT)-YDVFISFRG-EDTRHNFTGHFYAALrRKK-VRAYIDEDRLEKGEEIslSL----------SPYQKRLFLGTVPHKGFRSRLSTAGMV-ELNNMIELRLDRGMDIRNVKPLCVTNELIPQ-SLVMAASSSAPKQTYDVFISFR()}*

$1960=163(165):
>gi|739599316|ref|WP_037456832.1|
{|0(479)|<Proteobacteria(B)>}hypothetical protein [Skermanella stibiiresistens] >gi|587639644|gb|EWY38668.1| hypothetical protein N825_12715 [Skermanella stibiiresist
{(MRKTTMGES)-YDIFFSLAH-AD-RAT-AAPVVEALRDRGLKVFHYETEIADARSIteRVVTGLGHSRILVAWYSKTYPTRRACQWELTSAIIAAQRDVPDGRAVERRVVVLNPETGVGHLQPAEIIGNKFIDASAGRAVELAHRVAAGLDG(LVGEFGEIQVLGGPLW)}*

$1961=162(164):

>gi|739593726|ref|WP_037451310.1|
{|0(816)|<Proteobacteria(B)>}hypothetical protein [Skermanella stibiiresistens] >gi|587641644|gb|EWY40658.1| hypothetical protein N825_34965 [Skermanella stibiiresist
{(MGES)-YDIFFSLAH-AD--
REAAAPVVKALRDRGLKVFHHETEIAGARSIteRIVNGLGHSRMLVAWYSKTYPTQRACQWELTRAIIAAQRE
VPDGGAVERRVVVLNPETGVGHLQPADVLASKFIDVSAGRADDLARRIAAGLDE(LTGEFGEIQDLRGPSWHG
GN)}*

$1962=211(220):
>gi|691025394|ref|WP_031983652.1|
{|64(0)|<Proteobacteria(B)>}MULTISPECIES: hypothetical protein [Acinetobacter calcoaceticus/baumannii complex]
>gi|588201919|gb|EXE74979.1| TIR domain protein [Acinet
{(FKFWIKERYSSKANIMQLKYCFSDYFFHIQNKGYNAFSLTDAKSVDVFFTDIGNDMFLFHSKQVMNKILPP
IDDYLRNAT)---IFLSHSSLdKDKVDSVYNYL-----NSKDIR-
AWYDKYEIDFGEsitdKINKGLDNSNLGLIFLSKNFLkaESGWTLNEM----NFFFS-
RRMRDTQKKFIVINLDLKQSELPPLLQDYKY--MDFNSISFYQDLYNTLKRLDF()}*

$1963=178(184):
>gi|1180883506|ref|WP_084328078.1| {|0(211)|<unknown(X)>}hypothetical protein [Intrasporangium oryzae] >gi|586966045|gb|EWT01980.1| hypothetical protein N865_20480 [Intrasporangium oryzae NRRL B-24470
{(M)SHDVFICHSSLDRAVANAVCATLEAKRIR-CWIAPRDVVPGREWAASIIEAIHGSKLVILVFS-
AHSNESQHVHkEIERAVNAG-----LPVLPFRMEDVTpsPALEYFISDAHWLDAL--
TPPLEKHLDYLAETVQLLLDRQ(APLPDAEKPAPPPPPPPGRRRTALWAGLAALAVVLAVVVGVFV)}*

$1964=164(161):
>gi|880969652|ref|WP_048697958.1| {|0(2)|<Actinobacteria(B)>}TIR domain-containing protein [Tetrasphaera australiensis]
>gi|586945201|emb|CCH72541.1| conserved hypothetical protein [Tetrasphaera austr
{(MKRE)---IFLSWAHAdqpavERLMTKLRPELASLKDMTV-
TVWADSEVHIGDEWRrEILGRLEVCDYALALVSPQYLTRRFIVHeEIPLIVGSGAV-TSVLPVGLKPVAL-
DGSREWHGLDVRQMHLRrqPSGADRFFNQLRGSAAQDEFAAT(LATRIRQRVLRDVAAGA)}*

$1965=185(187):
>gi|882568148|emb|CJD59502.1| {|0(63)|<Firmicutes(B)>}TIR domain [Streptococcus pneumoniae]
{(MEDR)---VFFCFSG-KD-
RLVYAQSLNFHLKNLGIEVWYDYEKLylGDDGDYvNIEEGVEQSKIFLLFISTQLFQSTGAMLELDAIKTKID
KYQEIVIIPILCEITSSQIPKKYNWISKYIYGEMKLDISSGTYDLSIDILKRLLYE(QLKSVEIKNLLTLENI
TTDKFINAIVDKYNYIDKNCFSIRITM)}*

$1966=219(220):
>gi|880996239|ref|WP_048718340.1| {|61(0)|<Firmicutes(B)>}hypothetical protein [Bacillus sp. 522_BSPC]
{(KYEKEMTQIMEQKRKNNDDINRYKERVDKEQKREYNKMLKSIQSQTSMNEDYMNSVSEISEKINQLAEDVK
HTAAEKEII)EFDVFLSHSS---LDKEdYVTELSEKLSEKGLKVFEDvkvfKIGQSQTDMMnmgIL-------
NSRfvVVFLSANFIKSGWSDYEFKSFLNREINEKRIIILPIW-HNISveD-IRQYNPYlvdkfaLSTNKFTI-
DEIVEHI------FGVVNMSKE()}*

$1967=197(207):

>gi|929022462|ref|WP_054046225.1|
{|0(193)|<Actinobacteria(B)>}hypothetical protein [Alloactinosynnema sp. L-07] >gi|886828559|emb|CRK56231.1| hypothetical protein [Alloactinosynnema sp. L-07]
{(M)--QVFVSHAEPERDLAAKLRADLAGMGNQ-VAFDR-GVSTGPTWWDGVLRRIQRCDAVIFVLSPDSLR-SPGCMaSVRYARQLR-----RPVLTVRAHPMAV-PAELVDTEVFDpvVAG--ADRARALRMALLSLPPAPHLPD(PLPRPPLVPYLDAYFELLDQPSIERAEQHAMLAELRQRMRDQSEQAAVWSLLAQLRGRSDVAPGVA)}*

$1968=179(188):
>gi|929022464|ref|WP_054046227.1|
{|0(235)|<Actinobacteria(B)>}hypothetical protein [Alloactinosynnema sp. L-07] >gi|886828560|emb|CRK56232.1| hypothetical protein [Alloactinosynnema sp. L-07]
{(MH)---VFISYARRdQASISQLR---ADFERLGHY-VWFDRESHGGQDWWdDVLAHVRGCVVFVFAISPDSVRSKACLAELRYA----EA-LKRPVLPVVVRRV-PKSEIPDGLTAGLVDYSdrSAETAIALLKAFNALPMAKPLPQ(RMPAEPEIPTSYLNAYLDRIDADEIADADQVELLAALKVRLGDEQE)}*

$1969=182(178):
>gi|929024723|ref|WP_054048486.1|
{|0(771)|<Actinobacteria(B)>}hypothetical protein [Alloactinosynnema sp. L-07] >gi|886830010|emb|CRK57682.1| putative ATP/GTP binding protein [Alloactinosynnema sp. L
{(MVEGSLG)-YDVFLCYK-SedavaaEELRLAL--------VAKGLTVFRDEISGGDWAPLgaSIEQALLRSRTLVALITPHFPISPHCREELhmaltaAYHLDAGDTSrvmAVAQGVSPDDI---RPRQLTRFRLPRSGLPTAELVASIAAGVHRHDRRTFGDAP(RPREPKWYPRELPGDSYFRGRYAEMWELHEG)}*

$1970=292(299):
>gi|503373380|ref|WP_013608041.1| {|65(3)|<Spirochaetes(B)>}TIR domain-containing protein [Sphaerochaeta globosa] >gi|324027434|gb|ADY14193.1| hypothetical protein SpiBuddy_2378 [Sphaerochaeta globosa
{(VPEIIYPNNADDKTELQDQITKAINKVHDIQNELIAEVFIETQEGSEVIDWRKESGLLLPNKRVISAPVTERIWGDKGFR)---VFLSHKTEVKKETAQL-KAKLKLFGI--SCFVAHEDIDPtQEWQtEIENALNTMDSFIALLT-GSFH----DSN-WTDQEVGFAFgRGVPIISVRLERdpygfigkFQalSSTWiGAPDKIVQILIKN---------DAMVNAYIEAIKDC(TNYDESNKLSELLPYIEKLSQNQIDNIIDAFNSNSQINESYGFNGRKPTKYGEGLVYHLNRVSKIDFKLTQWNQIERLN)}*

$1971=155(149):
>gi|503366593|ref|WP_013601254.1|
{|0(279)|<Actinobacteria(B)>}hypothetical protein [Pseudarthrobacter phenanthrenivorans] >gi|323469643|gb|ADX73328.1| hypothetical protein Asphe3_21760 [Pseudarthroba
{(MTSGSA)TPDpvdpVFISYRQNDGTDVAAELAWLFRAAGI--PVWRDRDDLPPGDTEaRLKQAIAvGISGGVLVTTPDVVNSRVVKTleAPQLLDLHADHEVFALGITNSVKTEDGGTDYNAPDRLLELRPGTLSGVDQQPAERNGLLALIRGFV(WHR)}*

$1972=249(254):
>gi|325180151|emb|CCA14553.1| {|654(167)|<Stramenopiles(E)>}conserved hypothetical protein [Albugo laibachii Nc14]

{(NTSKEGIKHLVNAGADINEQLPNGDTPLHIAVKKEHLETSLALLSCGADLMMTNHDGFRPLDCTTATNLQY
EIKRAAGKR)--DVMISY---
THSHSEFAKRLRKNLEDASITTWLDLMDpsgIGGGAVWreEIARGITNAVLVVAIITEDYPKSEWCLKELAVA
REVG-----
TPIIAVSTENTAVDNDLKNYINSSAIVSFEPSIINIDMSDKRNISYTYDEGK(YTAVVRNLLVRVQEHIAERT
HERVSLSNRRRLKS)}*

$1973=249(254):
>gi|325184228|emb|CCA18688.1| {|652(203)|<Stramenopiles(E)>}conserved
hypothetical protein [Albugo laibachii Nc14]
{(VGRELFTYLVNKGANINYALPTTGDTALHIAIRCENKNTALAILASGGMILTMNREGVRPIDCTTSTKFQY
EIKQAAGHR)--DVMISY---
THTHIEFATKLRKSLEDNHVTTWLDLMDpsgIGGGTVWraEITRGITNAVLVICILTEDYADSDWCLKELALA
KQVG-----
TPILAVSTENASIGEDLQVYLYTRQIIPFEPAILANKRNAQNSRLVEYEYDE(FQYANQFRLLLDGVRDEIET
NRKNVLWRNMRLQQ)}*

$1974=220(222):
>gi|325185057|emb|CCA19549.1| {|938(0)|<Stramenopiles(E)>}conserved
hypothetical protein [Albugo laibachii Nc14]
{(DEIEKQRKGQIQKQLSHNQTAMFESTLRGHTMFDSLVGSHTSFISNREVIVGSELDYPNSESTSQISGDSY
RSDPAEKKY)---IFIAHG---
DCHRAFVRNLHERLSSCGYAVIVDggtLKDLPEASNMkeRIqvsKNAILGCATCIVIVSERTAKNELVSDQLA
FAEDKQ-----RPIFPVLLnDVKLNSSVLSLLSYRPLYHFTTNVGFTASLHQLMTRMREILPQE(GM)}*

$1975=311(300):
>gi|676385937|ref|XP_009036247.1|
{|1028(265)|<Stramenopiles(E)>}hypothetical protein AURANDRAFT_63685
[Aureococcus anophagefferens] >gi|323453255|gb|EGB09127.1| hypothetical
protein AURANDRAFT_63685
{(ELLGAWKRRGVPLVARRESAGRGALADGDRAGDAATPPRETPRVATPPGAADSPTEIKLAHGPTLHVTTPV
RALERTKRR)-KRVFISFRvSEALEEAKALAAHLETLGFA-
PFVCEADIMNAEDWVSVITEALRTCCVFVALASDTYGAPGgdiLGTFhEFACALkRNA-----
TRGQPgILIVRLSrqWPPEtelelLLGTRnftpweAGVDTLGTMVEEMAAAQ--
AFAGGVASRAPP(LSSEQEHLHARVFSRFGLQRPQTRALLLAATRRIEHRGAELTTQGTTVTSLGLLVEGAAS
VHVDGARVATINPFKFVGEV)}*

$1976=249(257):
>gi|330844922|ref|XP_003294357.1| {|690(370)|<Amoebozoa(E)>}hypothetical
protein DICPUDRAFT_159346 [Dictyostelium purpureum]
>gi|325075197|gb|EGC29117.1| hypothetical protein DICPUDRAFT_159346
[Dictyo
{(VNFLVTEFLLLQGCNRNPKNKVGETPLHVSVKTGTNISMSSLLCRNGAQSIPDRVMKTPLQYCTNMEEKQN
LQNIIFTNQ)---VFISYAHKdSVFVNNLRSIfgLSSLR------
CWLDEyRLQAGCNWRaEITRGVENSQVIVFVVSETSVQSLWCRKE----LKMSKS-LGKTIIPLFLQPN-
VYIDPSLYGLFTIIPKQtkESYQDMKEEEVLSNVKTLSRLIN(SLLKNGLGGRLNESSSKSLVEKYNIEKTIY
FTFNQAD)}*

$1977=175(178):
>gi|503410045|ref|WP_013644706.1| {|0(145)|<Euryarchaeota(A)>}TiR
protein [Methanobacterium lacus] >gi|325330293|gb|ADZ09355.1| TIR protein
[Methanobacterium lacus]
{(MNKH)---VFISYSHEdKYFANRIA---EDLMKNG-
IEVWFDKwEIQVGDSLIeKIfSEGLSNSDYFLILLSKDSINSKWVKEELNNAIIRRME-GVTKLIPLLTESI-

EIPQPLKALKWLDMYSNydKGLRSLINTIYNISEKPPVGKIP(EHIVSLRNSVGGLSRQASTIGSLLIFKQEE
EPGF)}*

$1978=207(220):
>gi|268564087|ref|XP_002647086.1| {|179(0)|<Nematoda(M)>}Hypothetical protein CBG03616 [Caenorhabditis briggsae]
{(DYSKIDVTNLEKRLEAVSPALTVYTYQMLSNGIDSKVLPSLTYEIMTKCGIVNPVHQLKLAKFYGIKEREA
MKTPSIEDI)---VYISYRNSPGAELASLIKLYLEKYGY--
RVRMDIDNTEANDLEeSLLRDVSSCKHLVLILTTNSLE------
PDQMPKELDFAFqFDKNIITVNHKDFEqTAEWQ-----ETLRTAFSVNWVHDYQEACIQKIINCFQEN()}*

$1979=246(249):
>gi|499432251|ref|WP_011119715.1|
{|0(487)|<Planctomycetes(B)>}hypothetical protein [Rhodopirellula baltica] >gi|32472902|ref|NP_865896.1| hypothetical protein RB4097 [Rhodopirellula baltica SH 1] >gi
{(MPVDISKPYGISAIRLKDLASNPRISNRICLVTKGDIVKGEFD)---VFLCHNS--A-
DKQEVRKVATELAANGLTYWLDEEQLRPGLPWqrTLERAivdIRAAAVFI----GGSGL---
GPWqEMEQEALLRRLVkDDCPVIPVILPSC-
QSTPDLPvfleGMNWVDFRSTTADPLRRLIWGITGRAPGETHSD(FPTGPRIKELRLREKLTQAQLADLIAVS
PATVSKWETGKQIAPQSQARKICAVLEKEQVQETVCDV)}*

$1980=140(144):
>gi|499539938|ref|WP_011220721.1| {<Proteobacteria(B)>}hypothetical protein [Photobacterium profundum] >gi|46915734|emb|CAG22505.1| hypothetical protein PBPRB0632 [Photobacterium profundum SS9]
{(MKRI)----FISHNHRDSKTI---
NLIKSIYcnpnhnlnfanKSLAAPIFNDHQHInrrpptdSASKPVkqEISRLLNESDKLLVLIGPDTHSSLWV
QWEINAFTSKHSSSnilMMRI--------------------
PDNQTAGAPANARHLSIHNWDLKLLTQWVK()}*

$1981=202(206):
>gi|901814990|gb|KMZ69347.1| {|0(610)|<Streptophyta(V)>}hypothetical protein ZOSMA_216G00170 [Zostera marina]
{(MSKEPCFGYRYDLNGGSLSSSPTES)-YDVYIGFlgRR-
KHLLRRFVKWLQAELQIQGITSFISYTNRRSNE-EdrNVSMAAMISAYVILIIIITEDTFSDPCwIDELGGFV-
-----
NERNFLPIFFNFGKFPDLVEYIKEAKEGKKVWNGLGGFHSENWQSVADWIQMLK(LKLEAYAGNWRDCISEAV
IFAGKKLGRKKVVKKVKQWKKWS)}*

$1982=155(149):
>gi|918699453|ref|WP_052568749.1| {<Actinobacteria(B)>}hypothetical protein [Mycobacterium abscessus] >gi|899017740|emb|CPV35659.1| MTH538 TIR-like domain (DUF1863) [Mycobacterium abscessus] >gi|89902
{(MAD)QHNVFISHRHEDDALVGQ-LKTLLSKNGA--
DVRdSSVTSNTPNDANsEsyikqiLADRIKWAGKIIVIISP--------
DTknHEWVSWEIEYANrYpDKRIIGVWAPgaaGADmpPELDEYADAVVGWNSEAIIDALNGKDNWQGPDGATV
PPRS(IKRLAC)}*

$1983=218(220):
>gi|900989636|gb|KMZ20523.1| {|72(0)|<Proteobacteria(B)>}hypothetical protein ABN54_08420 [Haemophilus influenzae]
{(FDDKYSLHNIFLGGVSEGGNIATFINEDTLIFFDRLFIFSESESELFTRNKLLPTLRFGERSVFETKYPQY
KNAKYQIPA)---IFISHSS-LD-

KDKIALPISDYLCAKEIPIWLDRNEMTLDDNLseklihkKIYDGISICKTGIFIVTSNFIEkSKWTKVELSIC
KELD-----KNILLLIFDENEERVEKLKKDYSLNDSSIVLMNGIEGNYETIRKKLSENNFI()}*

$1984=194(220):
>gi|902937317|ref|XP_013056672.1| {|217(0)|<Chordata(M)>}PREDICTED:
toll-like receptor 4 [Anser cygnoides domesticus]
{(FARSPQWNSSGVGAIAPRLDRLLLQDISNPDVLRFDWTFTWLSGVAALSIINVNFNYVPCDAWAEMRSVEF
LNISRNRLE)--DAYI-YNQRCH-
YHGVIRRLESAEPPYRLCIHERDFMPGRWIIDNIVENIENSAKVIFVLSRSFIDSEWCNYELYFAHQRAVGLG
YQDVILVVKEPIDaRGLPRRFARLRKMLGSK----------------------()}*

$1985=216(221):
>gi|902773231|ref|WP_049644328.1|
{|0(621)|<Proteobacteria(B)>}hypothetical protein [Candidatus Rhodobacter
lobularis] >gi|893673369|gb|KMW58755.1| kinesin light chain-like protein
[Candidatus Rhodoba
{(MPDQAQ)-RDFFISFTIDDLEVATVLNDALNAAGFS-
TYFHPKDIPSGGHIAYWMETGLDLARQLIAVCSTKYFAEEKvYSHaErlakfWQDPLNAN-----
PLIVPVQIEDCT-FPSLMGPLKRIDLRG--KQAPEMGA--
ALVAALQEDGAK(QARLQAGGLTLDKRRPEFWNVEGQPNPLFTGRDTEMADLHERIRAGGATAITAVAGMGGV
GKTTLAREYALRHGN)}*

$1986=209(218):
>gi|902962233|gb|KNB52500.1| {|0(497)|<Actinobacteria(B)>}hypothetical
protein AC230_11100 [Streptomyces caatingaensis]
{(M)------SYSRTDADKARVLARILREQGLR-
VFLDDMAVDDFASITASISEALARSKTLLALYSAEYPRRRACQWeLTYAYLAGQ-----REGDPRR--
RVLviNPERAAHHVHPVELRD--AkhwiwpAVPDALQ--
RLAAQVAAHVAA(IDSPLGAAGQLTGTQEPRPIPWLPAPARTGSARFTNRLAEQWRIHTALVRHRAPLVAEPG
SGSGRTAQLRGMPGIGK)}*

$1987=261(260):
>gi|1160913334|ref|WP_079754850.1|
{|275(8)|<Proteobacteria(B)>}hypothetical protein [Acinetobacter
baumannii] >gi|902757213|emb|CRX63689.1| hypothetical protein
ABR2090_0756 [Acinetobacter baumannii]
{(IWNPWHTTNKNKENNPFIPNDLYISLNEEHYIKYINLIKQDKSQTQIEAFYFNNNLVPINSDYPIPIQPFS
IFSDKEFIS)MKKVFISYSRMdVEYKNELVTFLKPLTSSGH-EVWDCGELEVGR-
WDeQIQEKLEQADLVICMLSINFFNSNYILEkELIPVLQAAEE-DKKQIMCIVVKHF-
PWASFHHLGNKAKLSTQnlNLIENTLQSSDIVSARATITNYQ(FLPYKFHDASSINNENIQRITPLSNMSPAE
RDDVYVDIFE)}*

$1988=223(226):
>gi|902715403|ref|WP_049630832.1| {|66(0)|<Proteobacteria(B)>}TIR
domain-containing protein [Cellvibrio sp. pealriver]
{(IYDIALHMDKLFSDLDSSVYVKMGNSLKPAGNEIDTNQMIFSPRVILYTNKIITSMENVFQTFNSVNTLID
LIDESKIYK)--TLFVSYGG---TDEISAIKINKKIKAKGIKTWFFPDDANPGDKLhrMMHDGVNNHD-
RVLLICSRESLNRPGVLnEIERILEREAREgGSEILIPVTLDDY-
VYTEWAPNRSDIANQIRSRVIIKLETESDQFDNSIEKLVK(ILTQNG)}*

$1989=265(265):
>gi|923134075|ref|XP_013760503.1| {|853(14)|<Apusozoa(E)>}TKL protein
kinase, variant [Thecamonas trahens ATCC 50062]

>gi|906552963|gb|KNC46206.1| TKL protein kinase, variant [Thecamonas trahens ATCC
{(LAAGSYPYKGTGLDERGIRQAVLQDEIRPEFEPSMGASEVYVALARKCWSASPAQRPGMTAVVDKLEIIEA EEQKHSLKR)-WDVFICHGG-GD--KGAALDLYDDLTARGLCVFLDSKSL-DGErcEYpdDLADHVRASRVVIVLVSKHMMVTHWAIAE-AKAALAEADAGRCKLLPVFicSDISPNTLRHAADVDTADPQHLVLRIMDASFLSPDHAEQYGLWRH(PELTPA RKADHVATVYALENYGAETHNWHTTSPSRALADVANRAA)}*

$1990=241(247):
>gi|923128986|ref|XP_013757959.1| {|0(972)|<Apusozoa(E)>}hypothetical protein AMSG_05240 [Thecamonas trahens ATCC 50062]
>gi|906556002|gb|KNC49245.1| hypothetical protein AMSG_05240 [Thecamonas trahen
{(MAKRGKSASKGKETSLNHEPDVGPSNNTGDNRSDSLPPL)-YDVFLCH-------GSFTKAwclaLYDMLSERNIKTFVDNVSLIGDCVEgpkDLVAKVLRSRVVVVIVSKYLFVSNWAIAEAEAVPR EK---IDAKLVPVF-GLDPVMTYDQLGKWNRAHVAN-DPIQSHPLIKYMSDHAFPDAVD(NAVYKKAREIWTDVLKSEEKLQQRVAVARRLKSRGLFLCELNKTSVQEIL KKLRDAVVMAVEKSRAES)}*

$1991=276(287):
>gi|923138867|ref|XP_013762898.1| {|912(0)|<Apusozoa(E)>}TKL protein kinase [Thecamonas trahens ATCC 50062] >gi|906552667|gb|KNC45910.1| TKL protein kinase [Thecamonas trahens ATCC 50062]
{(EILARQLPYPETPVGANELAVAVAAATQGKRPSLDAIDAPLAIVDLVTRCWDVDPAARPEFSDILTVLAVT LDALPPIKR)-WDVFICHGG-GD--KGAARELYDNLIARELRVFLDVHSLDGERCECpddLAAQVRASRVVVVLVSKFMLVSRWAVAEARAALEAADS -GHVVIFPAFIG---QDVTLET----LAHYGE-VKTDDPHH-PLLSLMDTFFHAP(HQQTLHDTWRHSDLDAEKKAHHVAVAYDLKRYGLATYDYQTISPTRALVNIADRVFNLA DSPQASSS)}*

$1992=221(217):
>gi|923129094|ref|XP_013758013.1| {|0(640)|<Apusozoa(E)>}hypothetical protein AMSG_05301 [Thecamonas trahens ATCC 50062]
>gi|906556060|gb|KNC49303.1| hypothetical protein AMSG_05301 [Thecamonas trahen
{(MSTDTSIAGEATEAKKLFH)---VFISYRVATEAGLAESLCDKLQqavigkdKVRV--RCFLDRNNLVEGEDWeRgFLNGLHGSMLFIPLISAKAiANIASLGPddKDNVLLEYEKALalrdaGRMEILPL LVGHV--------DDARAYHRFSAWSPFPDIVSLTTRQAASDAGGE(LKTVAQTMAELFSIQGVFVDPRSLKDKIPTIVKRLDSYV WPRYRAMWHARDELGPEET)}*

$1993=233(240):
>gi|923122238|ref|XP_013754589.1| {|43(37)|<Apusozoa(E)>}hypothetical protein AMSG_08833 [Thecamonas trahens ATCC 50062]
>gi|906560091|gb|KNC53334.1| hypothetical protein AMSG_08833 [Thecamonas trahen
{(LEVLVLNNSSAITDDGLLAVVRNCRHLRTVLIKYQSAGSSPTSSPPPTSSQTNEESASGGYMASPSFIQPP ATNISGRKW)--DVFICHGG-GD--KGIARDLYNNLTGRDLRVFLDIKSL-DGERCatPRDMVahVKASSVVLVLVSKFLIVSRWAMAEAKAAVDAA-----DANRGKVFSAIIGSDITSTTLRNMKEHDVNSIDHPLIHAMDNFFQTDAHHDQ(WSNWRHEKFTPAHKTDHVAT)}*

$1994=225(230):

>gi|923136312|ref|XP_013761621.1| {|0(675)|<Apusozoa(E)>}hypothetical protein AMSG_01571 [Thecamonas trahens ATCC 50062]
>gi|906561478|gb|KNC54721.1| hypothetical protein AMSG_01571 [Thecamonas trahen
{(MAASSTIAAVVAAAATAAAAAVATTQTLTAAHVPTRRPGTSESGDSW)--DIFICHGS-CD--
KRMARSLYHRLTAMGLRVFLDVYSLDGRYCGneEALARMARCStVVIVLVSKYMLVTTWAVaeAEAALDPASQ

CVVLPVLADAEMSHIALRRWMGTPAPAGEPHFLIQYMDSAFSALTENGSENM(DKWAKADKDARVDLVRRLRQ
IGLAAYNTQDYVLDDILDDIAER)}*

$1995=243(229):
>gi|913097552|ref|WP_050375384.1| {|0(75)|<Actinobacteria(B)>}TIR domain-containing protein [Streptomyces acidiscabies]
>gi|908277571|gb|KND24334.1| hypothetical protein IQ63_42840 [Streptomyces acidi
{(MTGRPPFFFT)SYAERRADS---
DLVEQFHARLQREVEIKrgrSApnEGFLNTGAVRLGQGWrrKIGTALGRTRFLVVLLTDDYLASPWCAREWAV
MKERVRLAePAEpvALLPLFWTGVsRelpaEVADLQLRmATLGAAYTDrCLVDLMREDPRGYEKFVISLTDH(
MVKESSTELPEMDGELAASYPPAFGLAAPEGEEEAAQIMRVAPELKTAQATPVETFRKVDLIDAFVRSPISGP
RETYDL)}*

$1996=192(191):
>gi|920287944|ref|WP_052976183.1|
{|0(129)|<Proteobacteria(B)>}hypothetical protein [Shigella sonnei]
>gi|905785317|emb|CSZ12191.1| TIR domain [Shigella sonnei]
>gi|934392253|emb|CTY61630.1| TIR domai
{()MTSIFISYAREdfliaKKLTEELNRH--------
EYKVFLDQQsIQLGAHWQeDILKHLKNADVFIALVTEHSDKS-YYFNaEVTGAISyvAHSN-
RDKLILPVVLDKE-
VLNDSDLGAFQALFARSddMTYLVHEIVNAISRFEGARLAQE(EIKEIRAQVLKDESADYISETIEDLKAREK
AGRQLSSRWHFAGYAALILSV)}*

$1997=162(164):
>gi|906299670|emb|CRZ16781.1| {|0(539)|<Actinobacteria(B)>}lipoprotein LpqN [Mycobacterium neworleansense]
{()MRQLFISYAREnRADVEALLRDLHALGY----
ETWVDSSLRGGQSWWhEILERIADSDVFVAIVSGHTLNSVAC-KrELAWALALNKP-VLPLAVEQLPDAL-
PRTLSMRQIIDYSQSGReaAFALAGALGTLPPAPPVPDQLPE(PPGAPLSYLSDLFEQVGQAEPLTQ)}*

$1998=247(255):
>gi|923127903|ref|XP_013757418.1| {|1046(135)|<Apusozoa(E)>}hypothetical protein AMSG_06249 [Thecamonas trahens ATCC 50062]
>gi|906556698|gb|KNC49941.1| hypothetical protein AMSG_06249 [Thecamonas tra
{(AALAASVATAEPPFGEYVDAQYVGRIPIENDSLEAWRYEFECQFTARSLRYCFFLTDGGKIVRAMAQADRT
AHTRASQHD)---VMISYRSTdAGFVDVLQAYL---EDHGV-
SVWRDRRMEAGTDWSsTIAAASRGAsSILISALSRNYLASSLC-TkEVRLAAEL-----GKPILPIILPAT-
AEPGEGGRRMVAAPYEAsgPPATIARALANVTMVDLRGAYSG(PDPEDGRALRRAYPTQMSALLRQIRHSLAS
GITWD)}*

$1999=232(238):
>gi|908422379|ref|XP_013072562.1| {|311(102)|<Mollusca(M)>}PREDICTED: uncharacterized protein LOC106059484 [Biomphalaria glabrata]

{(LRHTDPREQSSAAECLWTLAFDKTVRQTIVESPDLVPALEELKNSENSLVKNNVQGALWMIKGENDPAASV
SRPNSGNAI)KNHIFISYSW-AE--
KEMVKKIHNSLLSEGYKIWVDYEQMGGSTLQAMADAVENAAVVLICMSEKYKQSPNCRTENSKFVFKP---
IQKNGKKKYRPDGWLGAILGAKLFFDFTGKYPYEKPLQGLLKELRGRGKTAQIQ(GNKNPSVDEIDSNIEANH
)}*

$2000=188(192):
>gi|908638873|ref|WP_049782894.1|
{|0(281)|<Bacteroidetes(B)>}hypothetical protein [Odoribacter
splanchnicus] >gi|1124972380|gb|OKZ42254.1| hypothetical protein
BHV82_03450 [Odoribacter sp. 43_10]
{(MKNQTDM)EHDVFISYSR-KD--
EELVKKIRAALTQANISYWIDkEEIKRGTSYAKAIsQAVYNSKILLFIWSEHSNNSENTANEISLALEFE---
KNIVPFKISKKLGPSDMTYHLLKLDRIDACPFESHHIQELVASIAQCLGRGLTP(SSTTSGTSGSTPISNTFT
SSSTASNTSKMETHKFDNGSYTGEMAD)}*

$2001=291(292):
>gi|909565928|ref|XP_013138305.1| {|994(59)|<Arthropoda(M)>}PREDICTED:
slit homolog 2 protein-like [Papilio polytes]
{(MNDPDVSKNEIISTSLFLPDNYFAIICGVIIIVVLVCLIGAIFYAFRYDVSDWLYTKYGVPLFKEQSCANV
DMLEGNPNH)MYDYYVICNTKdtQFIYHNIMSEIE-----FRKMTAKKLSLNESTLNI-
LTldsfsKSPKLTKRLLIVLTTNFICNDLCDIHFKNMFYSYLKSLnrsdLNKVIFIKIVDN-
NQINDELGFLLDNFKNLswN-----DPR--
FWDRFVALLNST(DTIITVKSSEKSVFKKSLRHTPTLRYTTMPVSNDTCSKTNFTNSLQYCKRNDGESSHNES
SPSSDNNTYGEG)}*

$2002=216(220):
>gi|908411610|ref|XP_013066915.1| {|719(0)|<Mollusca(M)>}PREDICTED:
toll-like receptor 4 [Biomphalaria glabrata]
{(TCMNTDGERKYTLDYSNLDSLWRECWGSFFLYFALIMLCLYCIGVFAVFITMRNKNFIVSFFLQLFGGFKL
HSRRDYPVG)---VYIGYSDKdyQFPCKELRSFIESS-
LKLKTFLIDRDLIASVDKASGIIEALNASWRILLVCSKSFLkEDDWSMFTMRSAIYTQTPANPARVVVLVHKD
C-LP---LLPPALLSSVNDeNICAVSEWAMNYEMMQMLTTRLH()}*

$2003=220(220):
>gi|908418738|ref|XP_013070687.1| {|342(0)|<Mollusca(M)>}PREDICTED:
toll-like receptor 8 [Biomphalaria glabrata]
{(RNYTCMNKDGERTNTLAFSDLESLWRQCSGELFFYIALITLCIYVIGVVILFLVLKNKNFLVSYFLQLLGN
FKLHIRSDY)KTDVYIGYSDEdyRFPCIELREHLEH-
VLKLSTFIIDRDLLASIDKASGIVNAMNSCWRILLVCSERFLKSEdWSMFIMRTAVLSQSPANPTRIVIIVHN
SC---LSLIPTELLSAVNDEnIIVVSEWKINYILSEKLRTRLFD()}*

$2004=219(222):
>gi|908432185|ref|XP_013076246.1| {|59(0)|<Mollusca(M)>}PREDICTED:
uncharacterized protein LOC106062513 [Biomphalaria glabrata]
{(RNFTCINSEGVFTYTLVYLQLEDLWRQCWGRWFLNFSFILLFVFVIGHLLVFLWVKNKTVIISNVFQFFTN
LRLKKVSDY)RYGVFIGYCNFdyQFACVTLREYIEEK-
LKLTTFLQDRDLLPSSAVSEGIMEAMTSSYRILLVVNESFVkQNDWFLFTVRSAVYSVTPNNPFRVVIIVEE-
--AQVKQLPTELFNCIPEDnVIRINEWL---LDYKLKESLRTR(LQ)}*

$2005=217(226):
>gi|908435781|ref|XP_013077467.1| {|765(0)|<Mollusca(M)>}PREDICTED:
toll-like receptor 5 isoform X1 [Biomphalaria glabrata]

{(FTCMDRNGERSYTMRYSHVDTLWRECWGQYFLYLAIIILCFYVTGVFLLVLVQRNKTFLVSFFLQLLGNFK
LLKRGDYPI)--DVFVGYSDEdyHFPCRDLRLYLED-
VIKLKTFLNDRDLLASLSKASGIVDAINSSYRILLVCSESFLkDDDWSLFTMRAAMYAQSPANPSRVVVVVHE
SC-LH--LLPTELLSVVNEEnILVVSG------W-KLNYEITEM(LRTRLQ)}*

$2006=218(220):
>gi|908461373|ref|XP_013085514.1| {|201(0)|<Mollusca(M)>}PREDICTED:
toll-like receptor 4 [Biomphalaria glabrata]
{(FTCTNKDGKTTYTLAYSDIEYLWRECTGFIYFYIVLIVFCLYLIGCSIVFIMMKNKHFITVYILKRIFGIE
THTRRDYPI)--DVYIAYSDTdyQFPCNELRQFIEQ-
SLGMTTFLIDRDLNASFDLALGIVNAINKSWRVLLVCSESFLrEGGWSMFTFSSAIYAQSPANPARIVALVHR
DC-LPLLPM--ELFGCINEDnILYVSEWAMTYEMNQMLTTRLNG()}*

$2007=223(221):
>gi|908466823|ref|XP_013087194.1| {|140(0)|<Mollusca(M)>}PREDICTED:
toll-like receptor 4 [Biomphalaria glabrata]
{(GNFTCINEKGERTYILFHSDLESLWRECNGILFLYLSVIIMCLYFIGLCIVFIIYRNKQFLISYLLQTFVG
FKISTRKDY)KIDVYIGYSDRdyKFPCKDLREFFENS-
LGYKTFLIDRDLIASVDKASGIVDALNDSWRILLVCSESFLkEDDWSMFTMRSAIYIQSPANPARVVVLVHKD
C-LHLLPTTLIGSVNEEKIiVVSEWKINYEMKQKLTTHLSGDK(I)}*

$2008=216(220):
>gi|908466827|ref|XP_013087196.1| {|202(0)|<Mollusca(M)>}PREDICTED:
toll-like receptor 5 [Biomphalaria glabrata]
{(TCINNKGDRTYTLIYSELDALWRDCWGSFFLYLALIILCLYMIGISLAFIMIKNKKFIISYFSQILGLFRV
HPKEDYPIG)---VYIGYSDTdyLFPCTDLRKFIESS-
IKLKTFLIDRDLIASVDKASGIVDALNASWRILLVCSESFLkEDDWSMFTMRSAIYTQSSANPARVVVLVHKD
C-LP---LLPTAVVSSVNDeNICVVSEWAMNYEMMQMLTTRLT()}*

$2009=216(220):
>gi|908466829|ref|XP_013087197.1| {|708(0)|<Mollusca(M)>}PREDICTED:
toll-like receptor 7 [Biomphalaria glabrata]
{(TCINHKGERTYTLIYSELDALWRDCWGSFFLYLALIILCLYMIGISLAFIMIKNKKFIISYFSQILGLFRV
HTKQDYPIG)---VYIGYSDRdyLFPCTDLRKFIESS-
IKLKTFLIDRDLIASVDKASGIVDALNASWRILLVCSESFLkEDDWSMFTMRSAIYIQSPANPARVVVLVHKD
C-LH---LLLTALLSSVNDeNICVVSEWAMNYEMMQMLTTRLT()}*

$2010=217(228):
>gi|908491854|ref|XP_013095644.1| {|463(0)|<Mollusca(M)>}PREDICTED:
toll-like receptor 3 [Biomphalaria glabrata]
{(TCLNKDGEKAYTLFYSDLDSLWRECWGQYFLYVAVIIVCLYVIGFFVILLLLLRNKHFLVSYFLKILGNIKL
LKRTDYPIH)---VYIAYSDIeyKFSCSDLREYIE-
GTLKLNTFLNDRDLISSLNSAADIVKAMNSSWKILLVCSASFLNGDWAMLTLRSAIYAQSPTNPARIMVLVHQ
---NDLLLLPHDLLSVVDDEnMLIISE------W-KVDYVMREW(LRTRLTDK)}*

$2011=216(220):
>gi|908508337|ref|XP_013064747.1| {|702(0)|<Mollusca(M)>}PREDICTED:
CD180 antigen-like [Biomphalaria glabrata]
{(TCLNNKGERTHTLLYPELGALWRECWGIFFLNMAVILMCFYLIGSFVTFILIKNEKFIASYFLQLIGNFKQ
HTKEDYPIG)---VYIGYSDTdyLFPCTDLREFIENS-
LRLKTFLIDRDLIASVDKASGIVDALNASWRILLVCSESFLkEDDWSMFTMRSAIYTQSPANPARVIILVHKH
C-LP---LLPPALLSSVNDeNICVVSEWAMNYEMMQMLTTRLT()}*

$2012=219(222):

>gi|908434526|ref|XP_013077006.1| {|657(0)|<Mollusca(M)>}PREDICTED: toll-like receptor 5 [Biomphalaria glabrata]
{(TCMNIKGILSSTLAYSNLDGLWRECWGQFFFNLSMALLCFTLLAYILFFTWIKNKTVILSSILQIFTDFKLKKPSDYQSG)---
VYLGYAESeYKFPCSELRQYIEDELCLNTFIRDRDLLPSLDIAQGVMDAINSSWRILLVINERFLHQDDWFLfTIRAAIYSISPANPSRVVVLVEKNKVHSVPTELLSSVPNENII--VVSQMQLTYKLKQALKTRLLS(LK)}*

$2013=265(254):
>gi|908410061|ref|XP_013066085.1| {|573(368)|<Mollusca(M)>}PREDICTED: uncharacterized protein LOC106054655 [Biomphalaria glabrata]
{(KSNAHLLYTMDTLQKDCTIVVIGESLDWQMTDLGMRIGKQEQMIMVRNKSRYDASRVEQMVSVVKGKLMQNNYQIQKHPS)---VFISYSW-
SNSKHaqergTVcppgaigwgdPRQVKQELEKRNISCWLDCDQPAAGKGLfkNITEGIRNSKVLVAFVSDEYAKSDNCMMELrfgVLTLELP-----
TIIVVVGTGREWKESEVGLLMQRSKAAKIYMQRENTEAINVLEQYILERLPK(HADQLIARDAVLQDIKALKQQEKSKQEKKMPESV)}*

$2014=255(261):
>gi|908426207|ref|XP_013074148.1| {|964(359)|<Mollusca(M)>}PREDICTED: uncharacterized protein LOC106060709 [Biomphalaria glabrata]
>gi|908426209|ref|XP_013074149.1| PREDICTED: uncharacterized protein
{(RLLSSRLGYNNEDIRGWAQQADPCMALLNEWYTTHKTSEATLAVLTQLQEMDRLDAAAIVENAMKNAEAVVEDEEFVYPS)PPPIFISYQW-SHQAEV--
KLLKQHLEMAGYECWLDIGQMGGGDKLfkKIDTGIRAAKVVISCVTSNYAKSPNCNREVNLSVSLN-----
KPIIPLLLEQCPWPPPGSMGPIFSEYLFIRFFQRTGEEMPDQRYWAKDKFQE(LLMQLNVVGVPPDETKVQPEYKKWWIPVVQEITIDKSKTKN)}*

$2015=253(261):
>gi|908426283|ref|XP_013074178.1| {|1162(174)|<Mollusca(M)>}PREDICTED: uncharacterized protein LOC106060740 isoform X1 [Biomphalaria glabrata]
>gi|908426286|ref|XP_013074179.1| PREDICTED: uncharacteri
{(RTGEEMSDQRYWAKDKFQELLMQLNVVGVSPDETKVQPEYRNWWIPVVQEITIDKSKTKNGGTVAEVVQSDLDKIASESP)--DIFISYQW-GKQKNIIK--
LYERLTSLGFSCWLDIKQMGGGDSLydKIDRGIRGCKVVLSCVTQKYSLSANCRREVSLTDSLK-----
KPMIPLLLEKMTWPPSGPMSMVMTQLVYINFSKDESIQMTWDGSEFNDLLNQ(IKQHIPNKMTTMTTSSNNSTEVAKSTTIIVSPSTKTTQNTL)}*

$2016=164(176):
>gi|908449813|ref|XP_013081907.1| {<Mollusca(M)>}PREDICTED: uncharacterized protein LOC106067287 [Biomphalaria glabrata]
{(MQEQVKSTLELHWPTASNKFFFYSLKLFSSIQKHPS)---VFISYSW-
SNSKHaqergTVcppgaigwgdPRQVKQELEKRNISCWLDCDQPAAGKGLfkNITEGIRNSKVLVAFVSDEYAKSDNCMMELrfgVLTLELP-----TIIVVV----------------------
GTGREWKESEVNIIHRNVHYIYT()}*

$2017=253(261):
>gi|908458467|ref|XP_013084591.1| {|293(138)|<Mollusca(M)>}PREDICTED: uncharacterized protein LOC106069471 [Biomphalaria glabrata]
{(RTGEEMPDQRYWAKDKFQELLMQLNVVGVPPDETKVQPEYRNWWIPVVQEITIDKSKTKNGGTISELMQSDLDMIASDSP)--DIFISYQW-GKQKNIIK--
LYKRLTSLGFSCWLDIKQMGGGDSLydKIDRGIRGCKVVLSCVTQKYSLSANCRREVSLTDALK-----
KPMIPLLLEKMTWPPTGPMSMVMTQLVYINFSKDETIQMTWDGPEFNDILSQ(IHHHIPNKVTTKTSSKPSTNNIFQSNKTKQNTLKPASPLQK)}*

$2018=252(261):
>gi|908483686|ref|XP_013092857.1| {|741(80)|<Mollusca(M)>}PREDICTED: uncharacterized protein LOC106076577 [Biomphalaria glabrata]
{(AADNQGWFALARLMGYKQNRIDLFNEDLNPSLALLTDWIVTSGNTTMSVDVLLHYLQQLGCVEVLDVISRAKEFEFDRPQ)---VFLSYQW--DMQDEV-SLLRDYLERCGYSCWMDIGQMGAGDQLsnKIDQGLRGCKVVIACITPKYIASHLCNRELWLADMLH-----KPIIPVVLEMVSWPPPGAMAVILSQLVYINMKGVGGHGGTGIHADLMDKYSE(LRQRISLYAKPDLTPCVDSTFWPPQQKPVSDVTSERISGYR)}*

$2019=253(261):
>gi|908485473|ref|XP_013093445.1| {|219(171)|<Mollusca(M)>}PREDICTED: LOW QUALITY PROTEIN: uncharacterized protein LOC106077222 [Biomphalaria glabrata]
{(RTGEEMSDQRYWAKDKFQELLMQLNVVGVPPDETKVQPEYRKWWVPVVLEITIDKSKTKNGGTVAEVVQSNLDKIASDSP)--DIFISYQW-GKQKNIIK--LYELLTSIGFSCWLDIKQMGGGDSLydKIDRGIRGCKVVLSCVTQKYSLSANCRREVSLSDALK-----KPMIPLLLEKMTWPPSGPMSMVMTQLVYINFSKDESIQITWDCPEFNDLLNQ(IKQHIPNKFTTITTSSNNSTEVAAKSTTIIVSPSSKTTKNT)}*

$2020=195(201):
>gi|758852426|gb|AJP06331.1| {|0(306)|<Streptophyta(V)>}NB-ARC [Pinus tabuliformis]
{(MAASSSTAAQSNNHQWF)--DVFLNHRG-PDVKETFARPLYLRLLSNGLRAFLDKDEMQPGYNFpcQIEHAIKTASVHVAIFSPRYAESSWCLNELLLMLESK-----APIIPVFYRVTPAEVRWTQGKDGNYAQALQQLAEKKTKDPRTHKMKRRYDSK(TIENWRNALSRVADNSGFELEVRNGEELELELLDKLVECLSKMV)}*

$2021=291(296):
>gi|908403907|ref|XP_013081141.1| {|560(48)|<Mollusca(M)>}PREDICTED: uncharacterized protein LOC106066613 [Biomphalaria glabrata]
{(NNNKINHFIKDGVPVSYDGMSPVEYAFSNQHYHHSDCCGELSNGGVPLEELRLLSHQDGHASALLQHQDTVSAPPIQDLY)RYHVFFSHCPEdREWVEHMVAHLEAPPFN-YTCayasLQDEADPGTLQQRILCAAMLSER--VVLVLSKRYVEETWFSFEKTLKQLTQMSLHNQRIMGVLLEDC--DIPESLGELYFLDSSD--PDFFL---VFTKRLKTSRIPR(SSASVSSDLGQTALTPANVTNGKILTFCRLQVKVGWDSYLTPADGPDLDIPPSLSAHGIGMEKTEFGEIVTKVSGI)}*

$2022=228(230):
>gi|913452561|ref|WP_050431970.1| {|0(724)|<Proteobacteria(B)>}ATP/GTP-binding protein [Chondromyces crocatus] >gi|908357400|gb|AKT39975.1| ATP/GTP-binding protein [Chondromyces crocatus]
{(MLAPAKNPID)---VFFTYAPRdEDLQPKLEIHLDALKRQGFIRSYKGWPLDALKERQeQATQSLHEARIVLLLVSSDFLHDGSWRLpELREALQRDGK-GECRVIPVILRPC-SWEKTSLGRLSALPARAraVTSYPNQS--EAFAEIAKGIRIA(VRDLSPAQKRPPGSSPDLGEAAPEIKKLPPRNPSFTGRDRLLSDLRAALTSGGPAALTQAISGLGGVGKTQLAAEYAHRY)}*

$2023=183(176):
>gi|908420144|ref|XP_013071410.1| {|0(308)|<Mollusca(M)>}PREDICTED: uncharacterized protein LOC106058510 [Biomphalaria glabrata]
{(MK)-KTIFISYSPDTGFLERkfIVETVKQLKENDLgDDIWFDRDEKNSSTpcWFsTRIEAVEKCHAAVLFISNCYLTNSLSLTEMKILLDRHRNIlNSLKLFPILFDKL-

NVSLIDKQKELLDQLTMsvDLTGTHNCSKSVAEKVSIVVGSL(MDDLEKVALVLSKTKTVTPLSSEFNDEFRK
KIIF)}*

$2024=213(209):
>gi|908580928|ref|WP_049756613.1| {|0(521)|<Chlorobi(B)>}hypothetical
protein [Chloroherpeton thalassium]
{(MRNESENKRASPMNLTVSAHDTVHDKKPKQSFASRVFG)-YDIFLSFAlgpppRGtqsytaNLAHRLQER-
----
NFSVFFSEKEAPPGEHLdsTLQKALLRSKTLVVIANRGTLEApRWVRKEVEEFRKNHPKRpiiIINIGNALQQ
L---
SDIAAPWLQYKDKIWLDETEQAAIEGIASDEVVERLATAP(TRVKSNVRWRWLMGGIIAILVALGISLGLSA)
}*

$2025=226(223):
>gi|908457519|ref|XP_013084288.1| {|806(0)|<Mollusca(M)>}PREDICTED:
toll-like receptor 3 [Biomphalaria glabrata]
{(STGETVSLGDLMERAQQVQRACASKAIILVISISSTVVVTLMVSLATLYRFRWKLRYLRNIALTKYAGFRP
KKGTGKKFQ)-HDAYILYEDQTikFVFVRDFIQELE-
VKRGHRLLLVDRDIMPGTIMTTAILSAVQNSYKTIPVVTPYFFDVWYSEYAVQMAIMEEHYEPRQILHLCLYQ
ATdPKDMPKDLLSVMKR--NRYTEFPpetdLNEE-MvkqFWDQLSSTI--Q(QRE)}*

$2026=226(223):
>gi|908501020|ref|XP_013062262.1| {|637(0)|<Mollusca(M)>}PREDICTED:
toll-like receptor 3 [Biomphalaria glabrata]
{(NTGEIVSFGDLQERAEQVQRACASKAIVLVISISSAVVVTLMVTLALVYRFRWKLRYLRNIALAKYAGFKP
KKVTGKKFQ)-HDAYILYEDQTinFVFVNDFIQELE-
VKRGHRLLLVDRDIMPGTYMTTAILSAVQNSYKTIPVVSPYFFDGLYSEYAVKMAVMEEIYEPRPVLHLCLYQ
PTdHEGMSK---DLLSIMqRNHYTEFPPDPD-MneelvkqFWDQLSNVI--Q(QRD)}*

$2027=187(192):
>gi|908410699|ref|XP_013066429.1| {<Mollusca(M)>}PREDICTED: myeloid
differentiation primary response protein MyD88-like isoform X2
[Biomphalaria glabrata]
{(MDDVEVSQLTSGTTSSRNTSPQNHEVETLSDPFLHTNDMLSIFDGPGKFRMY)-
YSAYLCCaQKDLSsFGLPFHAMLEKIGFKI--
FLPPRDLVITGPMYENMARALEErCNgKIIIVLSCNYLDSEECLFLTYFARVLDPDARYRNLIPVQIDKEIGD
VPSVLKGMSIIRYNQ----AFRLG-WLKQKLIDAIAA-()}*

$2028=202(220):
>gi|908429121|ref|XP_013075226.1| {|632(0)|<Mollusca(M)>}PREDICTED:
leucine-rich repeat-containing protein let-4-like [Biomphalaria glabrata]
{(PRISHLILSKNRFFSIPITMSQVPNLRFLDLCGNSLIYLDNKEMSSVSQHRDAVPDFQLALAENSLTYLTL
IRDTNYTFD)-------------------SYLEE-DLKVSTFVHHRDLGPGYTDQQMFEAMRESW-
RILLIITKHFLNNyDLSDIIMKFASHSVTLANQGRVVALIQQSQLHNIPGYLydvledsRIIVLSDLT-----
SYLDYVQRQAMKECLRDIQ()}*

$2029=214(220):
>gi|908455994|ref|XP_013083821.1| {|666(0)|<Mollusca(M)>}PREDICTED:
uncharacterized protein LOC106068863 [Biomphalaria glabrata]
>gi|908455997|ref|XP_013083822.1| PREDICTED: uncharacterized protein LO
{(QSSSGRATSPATRQKSPQSRSESPASRPSSPGKKHTDLEDTNPTIQDVQVKRNEPADINFAEPANDALSIF
DGPSGYRQY)-YSAFLCCaPEDLQhFGLSFHSMLEE--
WGFKIFLPPRDLVLTGCNFDNMSRALEErCNgKIIVILSVNYEASEECSFLTSFARVLDPDARKRNIIPVQID
K-NVEIKNVLKGLSIIRYNH----DYKCG-WLKQKLVDAIAA-()}*

$2030=273(278):
>gi|908464191|ref|XP_013086380.1| {|137(78)|<Mollusca(M)>}PREDICTED: uncharacterized protein LOC106070936 [Biomphalaria glabrata]
{(LATGGQGHSSLKVLSGMFHAACEWIRAGLPLKCLKLVVYSANPSKPTSIVLECMKLFTKLKSKWQELENMD SIAELEGGP)-YDVCLSWaPENRDWVGKVCHQLLQENSKLKIY-
SDEFIFNHEDVWQKHIFHIMKnSKRIIVLLTPSYITNSECLEQFSIALCCNKLKKEETLVP-
FYLETIDSIPSYMILVQYIDCRV----
RKADQTQDLLLQQASSKVL(KPSENSVSESLLFGSWTQDKDNDVVDGNRNITYDIFVSYSHSHWQQPKELVEI LQRNH)}*

$2031=218(222):
>gi|908500163|ref|XP_013061949.1| {|417(0)|<Mollusca(M)>}PREDICTED: toll-like receptor 4 [Biomphalaria glabrata]
{(YTCMNKDGERTNTLTFSDLESLWRQCWGECFFYVAMITLCLYVTGAVLIFLMLKNKNFLVSYFLQIFGNFK LHTRSDYKT)--DVYIGYsDEDYRFPCIELREHLERNLKLSTFIIDRDLLASLDKASGIVDAI-
NsCWRVLLVCSKSFLKDeDWSIFTMRSAMYAQSPANPAKIVLMVHTSCLSLLPADLLSVVNDENIL----
VVSEWKINYILSEKLRTRL(MA)}*

$2032=207(225):
>gi|910073332|ref|WP_049990430.1| {|0(9)|<Euryarchaeota(A)>}nucleoside 2-deoxyribosyltransferase [Halopiger salifodinae]
>gi|1094582984|emb|SEV88916.1| TIR domain-containing protein [Halopiger salifod
{(MTGEQ)---VYVSHAPAD-LELVQDLFSTVKNFPF--
GVHIALEEIESGRSRkRLEGRLANSDVVVAVLTE--------DGagNTWIDQEIGYAVaKGIPVLPLRDEG--
VSRGGYVGDVE------
GVTIDRRDLSRTIFDLLSRLRGE(LAPLGALSVPNWYIRFPCTIPDCGHPVTLELDHGQPKLWKLHAHGKHLT TSCERCESTYYFNPATIGFRGRADGNENGTD)}*

$2033=172(182):
>gi|909677636|ref|WP_049920990.1| {|0(31)|<Euryarchaeota(A)>}nucleoside 2-deoxyribosyltransferase [Halopiger djelfimassiliensis]
{(MTGEQ)---VFVSHAPGD-LELVQELFSTVKNFPF--GVHIALEEVESGRSRkRLEGRLANSDIVVAVLT-
---DRASN--
SQWVNQETGYALaKGIPVLPLYDDEHHRGGFISDVEGVKIDRENPSVTIFNLLCRLRSELAPLGALS(VPNWY IRFPCTIPNCDHPVTLAIERDQTKLWKLHRHG)}*

$2034=273(263):
>gi|910007505|ref|WP_049962373.1| {|74(464)|<Firmicutes(B)>}hypothetical protein [Oribacterium sp. FC2011]
{(AEAYWGLVLCKYGIEYVDDPATGKKVPTCHRSSFDSVMDDSDFEQVMENADTVARAVYREQAKQIEELRKG IIEVSGKEE)PYDIFICYKE-
TDenNERTIDSVLaqdiYDALTSKGYRVFFSRISLedKLGVEYepYIFAALNSAKVMLAVgTSYDYYNAVWVK NEwsryLKLIAADK-----
SKyLIPCYKDIDAYDIPKEFAKLQAQDLGKVGAIQDLIRGIDKLFGKDNKPEN(PLHSQIVQQVVTGGSNVAA LLKRGYDSIEDGEFEEANKFFNDA)}*

$2035=273(263):
>gi|910007971|ref|WP_049962839.1| {|74(322)|<Firmicutes(B)>}hypothetical protein [Ruminococcus sp. HUN007]
{(AELFWSIVLCRYGIEYVDDPVTKNKIPTCHRTQYKSILEDPDYLTALSCADTMQRDIYEREAKVINDIQKK ILNISYREE)PFDVFICYKE-
TDangrrtVDSVLAQDLYYGLTQEGFKVFFSRITLesKLGTEYepYIFAALNSAKVMVVVGTkPEYFNAVWVR

NEwsryLQIMQKDK-----
NRIlIPAYRDMDPYDLPNELSLFQSQDMSKIGFLQDLIRGIKKITGQDNAAPA(IQEKQVIREEVSDQDESSS
EKDESEKKDSSVLALIIGSICIIG)}*

$2036=200(198):
>gi|913430227|ref|WP_050412215.1| {|0(1)|<Proteobacteria(B)>}TIR domain-containing protein [Massilia sp. NR 4-1] >gi|909845404|gb|AKU24519.1| TIR domain containing protein [Massilia sp. NR 4-1]
{(MTIRYGCFFSYAHGQHAYMSK)-----------------
FKNDLADALRCYlepHLdtekELFVDSEQLGGGDDLdrKIASALCESVCMIVIYTPKYEAHPYTRREFAAMQL
IEDERrhwyplPSHLIIPVIMTRHPlslpPQISEPGMYVDFSRYTLATSDLKTNPDYLPDIERIVQRVV(AHY
HYLKYCIPPEHDCSRFVLPAMPPEWRAIPPPHFP)}*

$2037=212(216):
>gi|909693074|ref|WP_049936322.1|
{|0(58)|<Euryarchaeota(A)>}hypothetical protein [Haloplanus natans]
{(MSTG)EYDVFISHAN---EDKDaVVDALAQQLEALGVDVWYDDDQIqiGDSLMDsldEGL-----
SGSQygVIVLSENFIDKNWPESELKALMQR-FQQDDVGIMPLRY---EipH-QAIVDSYPLLSDLVS-
RPIApDMIQ-
DRVQEIYNVVKQE(PEQLSGDWAGGANSEEDSRTEFESITVTIQDRPQIDIGNKFTVKKWKTSDRPPISNISA
TELYDPERDLTYS)}*

$2038=214(220):
>gi|919993823|ref|WP_052929801.1| {|321(0)|<Proteobacteria(B)>}DNA polymerase [Salmonella enterica] >gi|911328089|gb|KNL84063.1| DNA polymerase [Salmonella enterica subsp. enterica serovar Litchfield]
{(KWREDKLTSALLFVNKKGFKAENNEHAISIFRNHIYGRLSFIKMIRGDDFPLYLKLMAEMSHHDPLKTKEG
LRAMKETET)-YDVFICHAS---EDKtSIAIPIYEELTKLKISTFIDHVEIN--WGDsliQKINSALV-
KSKyvIAILSANSVDKHWPKKELHSVLAREIAEGEVKLLtLVKEADEAivAAslpLLSDKLYITYKD-NP-
AEIADKVR-ALL--------NK()}*

$2039=176(177):
>gi|910017790|ref|WP_049972497.1|
{|0(253)|<Euryarchaeota(A)>}hypothetical protein [Haladaptatus cibarius]
{(MTNPQG)--KMFISYKHSqQPLAEGLEMAL----REHGVPVWRDvHEIGP-
DPLEqQIREDLADPNLAggIAVVSEDVSESSIILEvELPALRERCDD-EDFFVVVALCPGL-
GHTEAKTILAQADSPRDfsHWFMESIEVESNGEEPTSILDHV(VDAVTSVVPGTQRTNNQSPAFDQVVTAVLE
R)}*

$2040=192(204):
>gi|910083677|ref|WP_049998784.1|
{|0(220)|<Euryarchaeota(A)>}hypothetical protein [Halococcus sediminicola]
{(MTEITG)--NVFLSYKHEqKDVATVLQ---ESLEDHG-IPVWRDiFDLRP-
EPLRdEIRDKLTSPDIAcgVALISEGVAGSEVILEdELPGLRRRWDA-GDGFFI-VLVPHP-DLSAT------
----
DAkpILEKASGPINLKSWKMVPLEAAT(SEAARSISKTVLQERLRQADEELSSDEPIECSLDTRDSPAYSIDP
LVVIDWEKHFDVG)}*

$2041=229(225):
>gi|913632429|gb|KNZ31194.1| {|0(309)|<Proteobacteria(B)>}hypothetical protein AD742_18490 [Methylibium sp. NZG]
{(MATGSL)----FVSYRSGDLHQGVAKMHSTLSaaFGEP--
AVVRDQDaFVPGADWLpQLRASVAASDLVVSAIGPAWIASFSvaKGNagVDFVREELLIAReLGKPVLPVRL-

GVKpeLVRSVLPQELRWLDGLQQVAWRgPSDTPALVRGI-
RSLSGL(VPRFQPDGPTASFVRSYAISVLSLAWRRLNATLWRPTGAAAASLRPGSGNLAVALAEACLALGLVI
LYVLIDRGDLGLY)}*

$2042=292(293):
>gi|913298140|ref|XP_013191197.1| {|994(58)|<Arthropoda(M)>}PREDICTED: slit homolog 1 protein-like [Amyelois transitella]
{(GDPTISKDEIVSKSIFLPDNYFAIICGVIIIIVFICSIGAIFYAFRYEVSDWLYTKYGVPLFKEQSCPNVD
HVLEGNSNH)MYDYYVICNTKdtNFVYHNIMSEIE-----YRKMASKKYSPNEPSLNI-
LTldsfsKSSKLSKRLLIVLTANFISNDLCDIHFQNIFYSYLKSLnrsdINKVIFIKIVDN-
NQISDELGILLDKFKNIswN-----DVR--
FWERLVSLLNST(DTIITVKSSEKSVFKKSLRQTPTLRYTTMPVSNDSCSKQNFTNSLQYFRRSDGESSQNES
SPSSDNNTYGEGD)}*

$2043=272(273):
>gi|913322286|ref|XP_013192348.1| {|533(0)|<Arthropoda(M)>}PREDICTED: protein toll-like [Amyelois transitella]
{(LSTKIPDLSTFQCSIWTKVSCYTRYTIFIIIIIILVSLYLVMIVFFIFYRKNIKLAIKRKLWKSKNKNSKP
ARKVKTGIM)-----VNFCENdeEFILKDFVPQLKS--QNFNLFIH--------
PVQNEIPIYVRKSGRrvkdictrkddgalkydtTLVVFSPNYLMTAYKNVDIKKIHGEMLKAKDT---
IYIFVDI-GPENSIYAFLKDQRDINtSVLWSDVD---
FWKKIIDMLSVD(HDLGVVSAAEKDDIKTPVKLKISKAKCSRNISFSKLPDWSTQYNVNPIAHSHV)}*

$2044=249(250):
>gi|913327126|ref|XP_013195005.1| {|229(0)|<Arthropoda(M)>}PREDICTED: uncharacterized protein LOC106138412 [Amyelois transitella]
{(TTSNVTVHDRFARLIDGGGPWRGAAPVSWRAVAGALSGAALLALSLAALRRRCCPRLQLALRQARARAATA
AHHARVLEK)EFDVLVCYTSVdGELVRGALLPTLSLKYKYRL----
NSLLLNTGPDNwynEIMGEMSHCRSVVVVVSPAqYSAQQLLtALrQLRALPLPpvLVLLQDLPKLKREAKESG
ETLVELVRRSR----LV--
PWRHVHERAFWTQLRLELPLP(PPVALTQVKTSASETIEDKSQRTSSLTALV)}*

$2045=191(185):
>gi|920706524|ref|WP_053052659.1| {<Proteobacteria(B)>}hypothetical protein [Vibrio harveyi] >gi|913481819|gb|KNY44646.1| hypothetical protein AKG93_07135 [Vibrio harveyi]
{(MAGINRALPQPIVEKYAFSKTAPSTGENKPC)---
IFLSHISIdKKAVQDIADYIMEkadidvyLDIHDEEL----
QEAVGKGNAEqitaLIERGIDKSTHTMCLISQKTKSSWWVPYELGYAKRSGKK-
ISSLKLKGDvelpDFLKVGEIIYGTKSLNDYIKNE--
IRDFKSLKSHASINESLESHT(DANHPLDGILDWRN)}*

$2046=236(240):
>gi|913632233|gb|KNZ31012.1| {|236(0)|<Proteobacteria(B)>}hypothetical protein AD742_19725 [Methylibium sp. NZG]
{(KLGDSEPRQLLVERFATEDPFVGFLRDYSPYTEVLSELDPVQFVAELHRRWVEQAPANAATTAAGAAPAAA
PTGQPQSAL)---FFISYSRGDLASAQKLHQVLLDLGvrhHE-
IWFDQEELKPGDEYKQRIFDGIANCKHFLPLVSRSTTeRDEGFVFsEWEAATNRL-----
PKLNRPPEQPFLvpVMLDAENKPHLLGRPS--
SLVAWKERNINFGHAPAGEPDE(TMRSFLRGLVRQIRLSGAGS)}*

$2047=251(256):
>gi|914397765|gb|AKV65434.1| {|1022(15)|<Cyanobacteria(B)>}hypothetical protein VL20_199 [Microcystis panniformis FACHB-1757]

{(IPGLLKALEDSEGYVRRYAAEALGNIGTETAMTELIKCLKNPDFVTLNNGDTLSQARKALDTIQNKLKYYH
PLSQPMNQQ)---VYISYNW-QEDSNEMANQLVQAFAAKGIEIIRDKTHTTYKD-
SikNFMQQIGQGKCVVAVISDRYLKSENCMFELVEIARNGDF-Y-
QRIFPIILPDAIIHKDFERIDYLKYWEDEKAKLQAKYKQIDLAKTNSILETV(NLYDEIRGNIDNLTNILKDM
NTLNIDLHRQSEFAAM)}*

$2048=291(292):
>gi|914568939|gb|KOB72728.1| {|992(59)|<Arthropoda(M)>}putative toll
[Operophtera brumata]
{(NDPTISRDEIVSKSIFLPDNYFAVICGIIIIVVVICLIGAIFYAFRYEVSDWFYTKYGVPLFKEQVCPNVD
HVLEGNPNH)MYDYYVICNTKdtNFIYHNIMSEIE-----FRKMTSKKFSISESTLNI-
LTldsfsKSSKHSKRLLIVLTTNFICNDLYDIHFKNIFYSYLKSLnrsdLNKVIFIKVVDN-
EQINDELCFALDKCKNISW---SDPR--
FWDKFVALLNST(DTIITLKGSEKSVFKKSLRQNPTLRYTTMPVTNDGCSKQNFANSLQYCKRSDGESSHNES
SPSSDNNTYGEG)}*

$2049=210(220):
>gi|914631935|ref|WP_050623610.1| {|57(0)|<Firmicutes(B)>}TIR domain
protein [Clostridium phoceensis]
{(PKEEHLDRMFRLLEDAGLRQRKDLTARNIMRAMADRRFNSASLLDEDSVPTIMEHLFFFPEIRVLEEKGYR
MTPFGREQM)---VFLSHSSLdKPEIEEIIPLLNGMDLPVW------
FDKYSIALGDsitdKVQEGIQQSSIVLFWITEHFLDSRWCKFEMRA---FIKK-
LIEEDVRVISVLDRGIQISRLPLFLQDIKCL--MREERTIYQVAQQIASALRPN()}*

$2050=234(239):
>gi|914861232|ref|WP_050739617.1| {|72(49)|<Firmicutes(B)>}hypothetical
protein [Acetobacterium bakii]
{(DLPVEKDKEIAFLYQLLKYGQEAFGNYTAFTHGYGSGNKFQDHMDAFNNQVVKHLVDYIREYLEDLIIDSD
DQLLNNDSQ)EKRIFISYSWAnKDIADIIDEDFLKLGYPLTR------DERDIKYKDsikeFM-
QQIGKHDFVIMLISNSYLKSENCMYEVMEVIRDREY-KKKILMIVLEDEDKKFYKNYEKLMAEEPVFN--
ALKVGAEIYSSSGRIGYTEYW(ETCEDEMIEQIARIQNDIN)}*

$2051=217(227):
>gi|914284572|ref|WP_050565111.1| {|48(0)|<Actinobacteria(B)>}TIR
domain-containing protein [Salinispora arenicola]
{(EIEADNRRIEAHNRKVINDYNQAADQHNRRADAHNKKVIDEINRRLAVASRPQVQVRYTPAEQELVDRVQA
ALPIDDREH)--DLFCSYARIDGALVtDELYAALTALGLD-
VWRDKAEMKPGRSMARQLDNGLRLARAGVVVLTPAYLAGRFWTErE-LGALLHK-----DTVIPV-
LHNVTfaDVKEYSSFLG--DLVG--FDTAEDSIAEIAAKIASALIGP(TVIDPTA)}*

$2052=124(147):
>gi|914603313|ref|WP_050597740.1| {<Proteobacteria(B)>}hypothetical
protein [Enterobacter asburiae]
{(MNKPNGA)--KIFISHSSLDKPFAKMLCMDLEENGYA-
PWLDEWDIKVGGSIPERISDGLEEADFVIVILSENTVSSkwveREWQTkYWNEIQHNC-----
IHVLPLLLKDCKipEL----------------------LKTKNTQILEMILTVD()}*

$2053=176(177):
>gi|914410231|ref|WP_050587805.1|
{|0(645)|<Proteobacteria(B)>}hypothetical protein [Pseudomonas sp.
LAIL14HWK12:I12]
{()MMRAFLSHSSKdKAHY--VERVADWLGKDNIHyDAWTFEE---
GERPLdEILRGLNRSEIFVLFLSSNALSSDWVIREIVEAEVRLEA-SLIeKVFPIIIEDG-

LTYEDKRIPDWLRDNYNvkPIKRPYVAARRIHNKLRELSWSR(HPELKKRQEIFVGRNDKQEEFEIRIHDFDK
IKPTVIV)}*

$2054=179(179):
>gi|916526295|ref|WP_051137495.1|
{|0(384)|<Actinobacteria(B)>}hypothetical protein [Amycolatopsis alba]
{(MSDTI)-YDVFLSFSG-DDRK--
FGRDLAEQLRSEGMDVFLDEDGITLTDSLteRIGLAVQGSKTLVAYYSEGYASRHACQHELMTAFLAAQREGt
vRQRILVINPEPGTDHIHPIELADAKFAHPQAGVTDLARWIAGRARELNGPFGR(PPSFEERLWPVWLARHVR
GFVGRHRELWTLHTEL)}*

$2055=215(205):
>gi|916566757|ref|WP_051173848.1|
{|0(723)|<Actinobacteria(B)>}hypothetical protein [Amycolatopsis
orientalis]
{(MTDPDPGLSDARSSDR)-FDVFLCFTR-AD-PDGAARVVEIeaALRDAGLRVFRDTVIDEFA-
PItrELADAIAGSRVLLVYYSRVLPTRYACQWELTAafVAARRHGAPADRVLAINPDpdsahlapVELADAKF
FTGPVTRETLPALVERVVRKVAEAGHPLGARNRVP(GRRPPARTVGRYRELWAVHSGLHAAEYPGMTPYSRPV
VMVRGLPGVGKT)}*

$2056=213(220):
>gi|922045847|ref|WP_053321247.1|
{|58(0)|<Proteobacteria(B)>}hypothetical protein [Vibrio
parahaemolyticus] >gi|916473685|gb|KOE14595.1| hypothetical protein
ACS85_06110 [Vibrio parahaemolyticus]
{(DDCDFDHWLSVHYPNKSRTFNVLMDELTEAAFSRRGYSITTFCDSEEVDLIFSSEANELFLFPDRGLMYEV
WGAPIMQKQ)--KIFLSHSSRdKHVVDD---YFSELQKSQ-
IKAWYDKEeIIPGDSITsRINEGLNECDLGVLFMSNNFLSKHSGWtEaESNFFISNRMK-KNKSLIVVNL---
----GVEHEDIPPLLQDYlyIDGRKDTAIDELIFAAKKQLERI()}*

$2057=225(225):
>gi|514685473|ref|XP_004990315.1|
{|1006(189)|<Opisthokonta(E)>}hypothetical protein PTSG_08524
[Salpingoeca rosetta] >gi|326431857|gb|EGD77427.1| hypothetical protein
PTSG_08524 [Salpingoeca rosetta]
{(PRTLLHMLYNTITTAVTQRYPELKATLLLPFNDTRLLFFEDVHTHHTNKKALRVGRSQLLSPGDLVAKYGP
LLPVMGQQD)KYDVFISYRQ-RGNR-
GLVMTLYPKLDQHGLVTFVDANNLetGVNFKHACMTALHNSTVACPVVSVGAIHQMRSLSHTDTCDNVLLEWM
AMLELQQLARQHPDNIRLRRVVPLFVGSGWHDSSHHVMSAAEVSEYDSFDRLK(RLATK)}*

$2058=177(177):
>gi|514697051|ref|XP_004996095.1|
{|0(551)|<Opisthokonta(E)>}hypothetical protein PTSG_02597 [Salpingoeca
rosetta] >gi|326436342|gb|EGD81912.1| hypothetical protein PTSG_02597
[Salpingoeca rosetta]
{(MAGVVSTDGV)-FDVFISLRF-SEARNAGRA-LERALEERGIKTFL-
CDVAPGDDIaSAVIRALHGCRMAVILGTRTYgkrtATKFSTFEELRYIVNRSKP-
FFLVKMCDDFAEPEAQFRLSEDISYFPWTPDEGAPHVEVPAALVDEIVAKLKTI(PETAAPADAAPFERGRHG
YGALPPPNV)}*

$2059=188(188):
>gi|514697619|ref|XP_004996379.1|
{|0(424)|<Opisthokonta(E)>}hypothetical protein PTSG_02868 [Salpingoeca

rosetta] >gi|326436626|gb|EGD82196.1| hypothetical protein PTSG_02868 [Salpingoeca rosetta]
{(MFRCCHKPTATVPVAAHDAAA)-FDIFISLRF-SEARNAGKA-
LQRALEARGLKVFLCEVPPGSNIAKVVIRALHGCRMAVILGTRTYGketdSSFSTFDELQYIVDRRKP-
FFLVKMCDDFEEPEAQFHLRSNVAYFAWTPKNEDTNISVPPPLVDDIVGKLKEQ(TPNTLTNASGQQLAATTS
FDSPLSVSE)}*

$2060=226(247):
>gi|514681184|ref|XP_004988178.1|
{|1424(229)|<Opisthokonta(E)>}hypothetical protein PTSG_10643 [Salpingoeca rosetta] >gi|326434818|gb|EGD80388.1| hypothetical protein PTSG_10643 [Salpingoeca rosetta]
{(DELQAGELLSCDGVPLRKDETYVYQLATLANLCDMGEEHETKDGNDKKEEKGENGEKEGKGKAMCFLSYSW
SHKRRFQKL)-----------------------
DTYLENtGLKVWRDENEIFKKASMleSMAGGIHGSACVIIDYSPEYAKSHNCRAELHYANKLRKP-
LFFVKTTRKNPPPASALDFIQAGHLYVDYSKVSSTKSVKNLNKQLRKQIGARLK(LPPFKDDAADADEDEEEE
KDALNTFLY)}*

$2061=223(223):
>gi|514693530|ref|XP_004994337.1|
{|165(215)|<Opisthokonta(E)>}hypothetical protein PTSG_05022 [Salpingoeca rosetta] >gi|326427736|gb|EGD73306.1| hypothetical protein PTSG_05022 [Salpingoeca rosetta]
{(QPRAMLHMLHTMISEAARERYPELKSSLLLPYDDKTLLFFEDVLHHHKSKQDMWVDEQLLPPDDLVAKYGR
LLPVLGLQD)KYDVFISYRQ-RAS-
SGLVMALHPRLEQRNLVAFLDANNLETGLNFklSFMTAIGLSLVACPIVSAATIMQMRQLQHSDYCDNVLLEW
MTMLALHEHQRKHGGRGEGEATDLIRLRRVVPVIVGSAWHNVNDSSMSRAEMSE(YDS)}*

$2062=223(223):
>gi|514683933|ref|XP_004989547.1|
{|1033(188)|<Opisthokonta(E)>}hypothetical protein PTSG_12865 [Salpingoeca rosetta] >gi|326432654|gb|EGD78224.1| hypothetical protein PTSG_12865 [Salpingoeca rosetta]
{(QPRAMLHMLHTMISEAARERYPELKSSLLLPYDDKTLLFFEDVLHHHKRKQDMWVDEQLLSPDDLIAKYGP
LLPVLGLQD)KYDVFISYRQ-R-
ANSGLVMALHPRLEQRNLVAFLDANNLETGLNFklSFMTAIGLSLVACPIVSAATIMQMRQLQHSDYCDNVLL
EWMTMLALHEFQRAHEKDDEKEQHAKIRLRRVVPVFVSSAWHNANDSSSMNKGGAS(QYD)}*

$2063=223(223):
>gi|514682472|ref|XP_004988819.1|
{|906(216)|<Opisthokonta(E)>}hypothetical protein PTSG_10437 [Salpingoeca rosetta] >gi|326434021|gb|EGD79591.1| hypothetical protein PTSG_10437 [Salpingoeca rosetta]
{(QPRAMLHMLHTMISEAIRERYPELKSSLLLPFDDKTLLFFEDVLHHHKRKQDMWVDEQLLSPDDLIAKYGP
LLPVLGLQD)KYDVFISYRQ-RAS-
SGLVMALHPRLEQRNLVAFLDANNLETGLNFklSFMTAIGLSLVACPIVSAATIMQMRQLQHSDYCDNVLLEW
MTMLALHEHQRKHGGGGEGEAADLIRLRRVVPVVVGSVWHNTSDSGSTSRAEMG(EYD)}*

$2064=235(235):
>gi|514677868|ref|XP_004987724.1|
{|436(94)|<Opisthokonta(E)>}hypothetical protein PTSG_11226 [Salpingoeca rosetta] >gi|326435619|gb|EGD81189.1| hypothetical protein PTSG_11226 [Salpingoeca rosetta]

{(TGLIIILLNTVIVGGCFSMVFSPQPSILFEDKPQRNVTPLLAGPLTYMFYPLWAVTFLLTFGSAILAALVPGFNKDGEGFA)---VFISYRhG-AD--DNLAGVLYTGItfqsreylsETQSLRVFLDVAKLKGGEDWmeGFLQALTTSKVYVPIFS------WWRLSESMAAEGEAAPPECVGGSLGTLGGTGRDQDNFLLELIVAYELLEQGHLAAVVPILSGGISGTDSYY(CDMLGSTGPKPGGSA)}*

$2065=243(252):
>gi|514689695|ref|XP_004992422.1| {|796(89)|<Opisthokonta(E)>}hypothetical protein PTSG_07010 [Salpingoeca rosetta] >gi|326429207|gb|EGD74777.1| hypothetical protein PTSG_07010 [Salpingoeca rosetta]
{(GHADDVSDNSDDDDDDNSEDDEMTNAAVKETDVGGVITSTDMSSLHKPAPVVREVSHTQVAEAAPEAGELQQEQQLRPGH)---IMLSYPW-KH--QSFMLRVRNALESHGYKVWMDVDHVAGSILESMSDGVEKADVIVMCLCEAYKTSTPCRTEGEYAYKLG---KPIVPIRPEVYVPDGWLGALCANLLYVDFTTDDAMFWAAQQHMLLQQVGRYTQP(HTPASSGVHPGEGSLTTTTTTTTSADGSGGKN)}*

$2066=206(199):
>gi|514700748|ref|XP_004997942.1| {|0(131)|<Opisthokonta(E)>}hypothetical protein PTSG_11812 [Salpingoeca rosetta] >gi|326433416|gb|EGD78986.1| hypothetical protein PTSG_11812 [Salpingoeca rosetta]
{(MS)EFDVFLTHDW-GVDGEGRNNHDRVAmvntmLKAYGFNTWFDEERMSGHIVQQMCKGIDGSQVVLVFVTKRYiskvnaEHDDNCKKEFNYAVMRR---TTTRMIPIVMEDAALDTRKWEGPLGMELGSHLYVRMTSDRHMRDNLEELVEKLD(KLGVKPKTGSAPTPAQHEAAKVVPRETSFVDHDGDAIAFQVTPSGLSKFVNKQFRKA)}*

$2067=197(199):
>gi|514692715|ref|XP_004993930.1| {|0(400)|<Opisthokonta(E)>}hypothetical protein PTSG_05359 [Salpingoeca rosetta] >gi|326428079|gb|EGD73649.1| hypothetical protein PTSG_05359 [Salpingoeca rosetta]
{(MPPTTTHNF)--DVFISLRFGEARHAARGLEKALQAHGL--RVFL-CDIAPGGDIArAVIRSLHGCKMAVLL----GTRTYGQQTasKFSTYEELRYIIdQEKPFFLVkMCDDFVepETKFHLPSNVAYFPWLPEGGDVPDMDVPVELVDAIIAKLK(TLPDAPKAPAAPLQRHQHGYGALPPPNIPLMGREEAEVLLLDRDDCAFGD)}*

$2068=275(260):
>gi|514655654|ref|XP_004999115.1| {|1546(158)|<Opisthokonta(E)>}hypothetical protein PTSG_11612 [Salpingoeca rosetta] >gi|326426976|gb|EGD72546.1| hypothetical protein PTSG_11612 [Salpingoeca rosetta]
{(TLLHMLYDTITAAAKQRYPDLKAKLLVPYDESTLLFFEDVLLHHTNKRVLRVGRSDLLPPDDLVAKYGPLLPILGLQDKY)--DVFISYRQRGNRGLALALHPKLEHHGL--VAFMDANNLETGlNFKhACIQAIQHSVVASPVVSVAAIhqMRSLDYDdaCDNVLLEWMAMLeLQqlvhqqsdkirlRRIVPLFLGSGWHDSSSHAMSAAEVSEYDSFDRMKRLATKLPDVVSEKTAAA(LDEYFTHVLHMPPPRQHKSVREVVLSLFDVDGVVSFQSEH)}*

$2069=275(260):
>gi|514693715|ref|XP_004994429.1| {|1161(121)|<Opisthokonta(E)>}GTPase [Salpingoeca rosetta] >gi|326427036|gb|EGD72606.1| GTPase [Salpingoeca rosetta]

{(ALLHMLCDIIVEAIRQRYHDLKPYVLLPHDDKTLLFFEDVRRHHNNKRALRVGRSQLLSPDDLIAKYGPLL
PVLGLQENY)--DVFISYRQRGNRGLVLTLHPKLEHHGL--
VAFVDVNNLETGvNFKhACLTALQHSVVACPVLSVAAIhqMRSLGDTdtCDNVLLEWMAMLeLQqlahkhpdk
irlRRIVPLFLGSGWHDSNHTMSVASASEYDSPEYMKRLAESLPDVVSEKTAASL(DQFFTQVLHLPPPQQHK
TVRETVLSLFDIDGVMSFQRRVD)}*

$2070=275(260):
>gi|514692889|ref|XP_004994017.1|
{|1011(139)|<Opisthokonta(E)>}hypothetical protein PTSG_12317
[Salpingoeca rosetta] >gi|326428166|gb|EGD73736.1| hypothetical protein
PTSG_12317 [Salpingoeca rosetta]
{(TLLHMLHDTITTAVTQRYPELKATLLLPLNDTRLLFFEDVHTHHMNKKALRVGRSHLLSPGDLVAKYGPLL
PVMGQQDKY)--DAFISYRQRGNRGLVMTLYPKLDQHGL--
VTFVDANNLETGvNFKhACMTALHNSTVACPVVSVGAIhqMRSLGHTdtCDNVLLEWMAMLeLQqlarqhpdn
irlRRIVPLFVGSGWHDSNHHVMSAAEVSEYDSFDRLKRLATKLPDVVSKNTAAE(LDQFLSQVLHLPPPQQH
KSVREVVLSLFDIDAVVAMDATV)}*

$2071=232(233):
>gi|514688392|ref|XP_004991772.1| {|1213(251)|<Opisthokonta(E)>}TKL
protein kinase [Salpingoeca rosetta] >gi|326430281|gb|EGD75851.1| TKL
protein kinase [Salpingoeca rosetta]
{(MLRLVEGAVHRVLAQHYRQLRSHTLVPLRDCSAFLRLDEVTTHYRERRAMWAEQVRLEIADLERHFARWLP
SLGLQTQYH)---VFLSYRQSANSAFVMNIRARLEERGL--
STFLDANNLEIGvNFKmAFMEAISRSVVACPIVSAAALERMRRLGeqDACDNVLLEWMTMISLIRHAHHARST
DSQARGHRTSSSVGRTMSRGEQMTESTTCTGSHSGEGETK(HVLLCRVAPLLLG)}*

$2072=266(255):
>gi|514687485|ref|XP_004991319.1|
{|1097(371)|<Opisthokonta(E)>}hypothetical protein PTSG_07523
[Salpingoeca rosetta] >gi|326430834|gb|EGD76404.1| hypothetical protein
PTSG_07523 [Salpingoeca rosetta]
{(NATDCLSCDPVSPYPVLHRLSTGRLACTTRSSADSASRTLTPGQTAAVLVVLLVLLLVGALAWLYHVYRKR
YAKRKTFHF)----FISYRRDVHDELAQELCSRLQRlflttgHRV--
VCFLDRQDIREGdDWRqRFRKGLMRSCAYLPLLSHESLAEVREGTfkhtPDNFLTEMELALqQQrsrrIAVLP
LLVSSGGGDVPCDDTGTGIMETKTLEGGESDVRVLGKPVLGRTPTTA(TTTTIVSDDSEDENSQSSDDSDDSA
DDAQQQQQQQ)}*

$2073=275(260):
>gi|514684300|ref|XP_004989730.1|
{|1008(137)|<Opisthokonta(E)>}hypothetical protein PTSG_08933
[Salpingoeca rosetta] >gi|326432484|gb|EGD78054.1| hypothetical protein
PTSG_08933 [Salpingoeca rosetta]
{(TLLHMLYDTITTAVTQRYPELKATLLLPFSDTRLLFFEDVHTHHMNKKALRVGRSQLLSPGDLVAKYGPLL
PVMGQQDKY)--DAFISYRQRGNRGLVMTLYPKLEQHGL--
VTFVDANNLETGvNFKhACMTALHNSTVACPVVSVGAIhqMRSLGHTdtCDNVLLEWMAMLeLQqiarqhpdk
ihlRRIVPLFIGSGWHDSSHHVMSRAEVSEYDSFDRLKRLATKLPDVVSEEMAAE(LDQFFSQVLHLPPPQQH
KSVRKVVLSLFDIDAVIAMDAGV)}*

$2074=278(260):
>gi|514700330|ref|XP_004997733.1| {|869(190)|<Opisthokonta(E)>}GTP-
binding protein [Salpingoeca rosetta] >gi|326433207|gb|EGD78777.1| GTP-
binding protein [Salpingoeca rosetta]
{(RAMLHTLYTTICQAVARRYADLQSRLLLPHDDATLLYYDDVLQHHKNKKDVWVDEDLVSAKMLATRFGPLL
PMLGLQDKY)--DVFISYRQQANSGFVMSLHPMLEQHGL--

VVFLDANNLETGlNFKlSFMTAIGLSLVACPVVSAGALARmrtlatedycdnvlleWMTMLalRDHVHAHGdD
PAQvRLRRIVPLFLGSAWLNSNSTSASSDSGSRLAGEGSTGDGGGDSFGAIRDVAMTL(PDVVSKETAASLDQ
YFAQVLHAPPPSKHRSVRETVLALFD)}*

$2075=274(260):
>gi|514694998|ref|XP_004995070.1|
{|944(142)|<Opisthokonta(E)>}hypothetical protein, variant [Salpingoeca rosetta] >gi|326437996|gb|EGD83566.1| hypothetical protein, variant [Salpingoeca rosetta]
{(RAMLHMLHTMISEAIRERYPELKSSLLLPFDDKTLLFFEDVLHHHKSKQDMWVDEQLLSPDDLVTKYGSLS
PVLGLQDKY)--DVFISYRQRANSGLVIALHPRLEQHNL--
VAFLDANNLETGLNFkLSFMTAIGLSLVACPIVSAATIMQMTQLqeADYcdnVLLEWMTMLaLGevelnheli
rlRRVVPVMVGSAWHDMKISSNMSRVEASKYDSLEPMKRLAESLPDVVSKETAA(ALDQYFSHVLHLPPPQQH
TTVRQVVASLFDIDAVVALDAT)}*

$2076=241(251):
>gi|514693679|ref|XP_004994411.1|
{|1150(167)|<Opisthokonta(E)>}hypothetical protein PTSG_04323 [Salpingoeca rosetta] >gi|326427018|gb|EGD72588.1| hypothetical protein PTSG_04323 [Salpingoeca rosetta]
{(QRRADSARDEALDMNEDDPDSQPEHESDRDSLQAGELSSRDGVPLSKFETYVYQLAELAKLCAEDEADTAK
AVAAGTRIC)----FLSYSW-KH--
KTRFKQLEHALKQAGFTVWRDENEMGKKDSIleSMGDGIHGSACVIIDYSPEYAESHNCRSELHYAHKKR---
--
KPLFFVKTTRANPPDGSSLDFAHGSHLYVDYSKVNSGTDYRRLNQQLCNQIE(ARLKLPPSEITDAGWGFEET
LENFLSYAGVT)}*

$2077=222(231):
>gi|514692523|ref|XP_004993834.1|
{|359(0)|<Opisthokonta(E)>}hypothetical protein PTSG_05259 [Salpingoeca rosetta] >gi|326427982|gb|EGD73552.1| hypothetical protein PTSG_05259 [Salpingoeca rosetta]
{(NAGTGPSAVYTATVRTTTPAAHPPSTKAAAPAPPQPASSDDNNDETSAGIGSHASRVAASQAARSDGDSGR
DGATKKEGT)---VFLSYTW-KYQERVRA--
VRDKLADEGYATWMDIQQLSGGDNLlaQIDHGIRTSDVVVSFVSSEYSKSDYCRKEVMLAENLQ-----
KPILCVLVEPAPRIPWPPTGPMGPVFAGALYIEYFNEETIAAVVRGVTDRIK(QARALKDAAKD)}*

$2078=244(254):
>gi|514682813|ref|XP_004988989.1|
{|244(307)|<Opisthokonta(E)>}hypothetical protein PTSG_10079 [Salpingoeca rosetta] >gi|326433938|gb|EGD79508.1| hypothetical protein PTSG_10079 [Salpingoeca rosetta]
{(GGVHWHLGRITRVRSENGERVYDGCHLKGADDGKWITFKGYAPTFEGLRLKDMRVSPNVLNMLTSGMSSAA
QQAAGAVDK)-CDVFISYCR-ANCLEDITA---RDLEARGFTCWM---
NMDHPNASqeQVALAIKHAKLVVACVSNEYADDSDCRSQFQFAKKlLR-----
KTTVPVVVGRGSWEWQQTVVGLLIAGDLYIDFQNMSNYSAKFEELVGSIKTV(LNTQTSGNQSATTQAAAISD
DVAKRVFISYCWTN)}*

$2079=294(291):
>gi|503426714|ref|WP_013661375.1| {|96(291)|<unknown(X)>}hypothetical protein [Marinomonas mediterranea] >gi|326546250|gb|ADZ91470.1| hypothetical protein Marme_2229 [Marinomonas mediterranea MMB-1]
{(YIEQLPLSSAFPAIPVAINREGLGHGSDGCLKNLNFLRAYQWQGETRIQQALLCMAHEIYRHGLVEIKKED
HGKDSSIKL)----FLSHAK-AgDTGRLHAEAIYRYIDNTNMSRFFDATEIAPGFEFdkEIINHIKDSTL-

IAIGSDAYSSRYWCQREILCAKHDQ-----
RPILAVDCleefedRVFPAGsnvpcVHVSADANLSERDILRILIAtlLETI--
RHTHAQKMLAYY(QSQEWIPSDCAITSRPPELRQVIDITKNGQTSICYPEPSVYMEESDWLCHFGVKAFTPLW
NKKEDKVLENK)}*

$2080=259(254):
>gi|514688849|ref|XP_004992000.1|
{|810(130)|<Opisthokonta(E)>}hypothetical protein PTSG_06613 [Salpingoeca rosetta] >gi|326429973|gb|EGD75543.1| hypothetical protein PTSG_06613 [Salpingoeca rosetta]
{(PDTPDDNAFHCQCRSAFAGRFCEKDRAVVSRGVDPARLRDGLIGAAFAAAAVVLLAWWIQRERLKKSYHVF
VSYRQATDA)----------------
PLAERVVRALQACtvdgqPLVVFWDRLSIEVGDNWqlKFLSGVHRSCMFVPIVSNagvaniervSVFDSKP--
DNVLLEYETALSLhRKRRigIFPLFVQGGGggggGGMSSDVGAGMSSDVGSHQVGGGGGGDGGGAMKRGCGAG
G(GELFNFDYDRFPNGPSKTNKNVPVCETMKQLFAI)}*

$2081=240(242):
>gi|514696888|ref|XP_004996014.1|
{|654(145)|<Opisthokonta(E)>}hypothetical protein PTSG_03428 [Salpingoeca rosetta] >gi|326437208|gb|EGD82778.1| hypothetical protein PTSG_03428 [Salpingoeca rosetta]
{(AQDNIFTETADKSTFLHFAARSPMAQAIENVLRLNRISRCFSGHQLAEQFARRDANRKTVLDVATNARSVK
SALRQHAGV)-KQVFLSYKHR--VCDEFADKLcRDLQAKG-
ISVWWDRDIPSGEEWErAVDWQARYCHAVIFLFSKPYLQSPFCMHELEVNQNRLGI-HPPISPEQRGEPD-
QLPDYVSSRQLFNFENAatYEQDLEDLVQRIHDMMECDPDTI(VPAEPALVERDDHVLIINPMGG)}*

$2082=225(226):
>gi|514685735|ref|XP_004990446.1| {|650(41)|<Opisthokonta(E)>}TKL/DICTY4 protein kinase [Salpingoeca rosetta] >gi|326431988|gb|EGD77558.1| TKL/DICTY4 protein kinase [Salpingoeca rosetta]
{(WLPKPRGSSEEGEEEGDKSGNSDHLADGGSDGNARTQAAHEEAQAEGSSSGGSSGEEGKQDKQEKEKEKDE
NGEEWPEYE)-YDVFISYRKEcdrvaaEGVADQLQHAQ--------
LRVFLDADSILPSHDWqqTFQDALRRSRLFLPLMSHRSLCETGPCNGLHNVTPTSPVDnvlLEYEMAVMLAS-
--KGRMSIFPVGLAEGVDVPGRGRAILPFGDFDFPSRCPETG(TPTAPD)}*

$2083=180(182):
>gi|916614914|ref|WP_051222005.1|
{|0(161)|<Proteobacteria(B)>}hypothetical protein [Neptunomonas japonica]
{(MSCK)--DVFICHAS-ED-
KDSYIKPLTERLKDNDISFWIDSEDLRVGDHLdqSIIQALNSARFVLVIFSKDFLSKEWTNRELQLIRDREET
ENEIILLPILEDVSQELILTKYSFLRGRKFLKWTELDKLIHDLQHYLQGTAFPNN(NLLFSDFLSHMSRFNAV
MHWTARPVPQKETEQFFSALQ)}*

$2084=222(220):
>gi|916896085|ref|WP_051502798.1|
{|129(0)|<Cyanobacteria(B)>}hypothetical protein [[Scytonema hofmanni] UTEX B 1581]
{(GMNQAIGDRAAVKFAKGFYQAIGRSKSYADAFEFGCNAIDLQGIPESSIPVLKYKTEELAPRFDEVNHRQN
VPDQNQKNF)QYDIYISYVDQkpdtTWVWDVLLPRLEQANL--
RIAVSGDIESPGVARVVNIEQGIRQAKRTIIVLSPAYLANYEATFENILGQTIGIQEGTYRLLPVKALPFDsS
QLPTRLSMLTTL--DLaHPRRAEREFERLIHALGESLPQM()}*

$2085=219(220):

>gi|916712708|ref|WP_051319799.1|
{|206(0)|<Cyanobacteria(B)>}MULTISPECIES: hypothetical protein
[Planktothrix]
{(KNSLFTQYLLEGLQGGVIAPGGVIRILDLFSYLQPKVTQNFPNQHPILKAEIEENFPIALYLGGKAATPLP
TTLPNDDYA)-YDVFISYLAKdaDktWVRKTLLPALES--QGLRVAIDSR-
FPLGVPLITSVEKAIQQSRYTVVVLSPHYLESSYGEFENLIAQHLGLEESKYRLIPVMREEC-TPRLGL-
RILFLLDMADeDEFDTNIERLVYQLKQLSGGRQG()}*

$2086=185(192):
>gi|916708537|ref|WP_051315628.1|
{|0(191)|<Bacteroidetes(B)>}hypothetical protein [Arenibacter certesii]
{(MQKSSYVPSIY)-----ISYAwgS---ESEAIAEAVEKEFQKRGLRIIRDKNdlgYKGRI--KdfmEQIG-
----RGKyvILIISNKYLRSENCMFELLQIFE--NRDFYERIFPVVLDEVKiaK-AVDRLDLLKYWENET-
ENLNNKIKELKELSNIQGVTDD(LNLYTAIRGNIARLTHILKDINTLNTDRHIKSDFKQLYELV)}*

$2087=188(189):
>gi|916784561|ref|WP_051391617.1| {|0(146)|<Proteobacteria(B)>}TIR
domain-containing protein [Rhodoferax saidenbachensis]
>gi|1133322821|gb|APW44217.1| TIR domain-containing protein [Rhodoferax
saiden
{(MADEKLEQIAKSANPL)---
VFISHDTRdAELAEAFSNLLKSVSAGVLKSFRTSDRRgnQGIEYGVewypEIIKNIQGASDVVCLLTKRSVDR
PWILFEAGMA--KGKL-DTPILGVALGIELKDASSGPFAQFQNCGGDE--
DSLTKLVFQLVNRIPNSEPDI(DTIKFQVGKFRQAVAEILKRLDGTSTDGAAKSS)}*

$2088=231(238):
>gi|916798075|ref|WP_051405131.1| {|47(46)|<Firmicutes(B)>}hypothetical
protein [Bacillus cihuensis]
{(VFGIRDDGKRLEIKNFPFRFDLERAQNLIEGNIDIKSEYFTLKGIKLYYISINKAEELVKVNKTIYKIGDS
GVAEQMKNQ)--KIFISYCWNdI----AFADLIDN---DFKNIGYTLTRDVRnIEFKDsikdFMKT-
IGKHDFVISIISDQYLKSVNCMYEISEVMRSHEY-KEKMLLVILKDSDKEFLPSLSEIPSTIAANI--
YSFEQRIGYISYWEEKEKAYS(EMIRKITQDTNKIEPLQE)}*

$2089=243(244):
>gi|916920732|ref|WP_051527444.1| {|0(101)|<Proteobacteria(B)>}TIR
domain-containing protein [Balneatrix alpica]
{(MTSENNSKAESKEKAELPKAINPL)---
VFISHDTRdAEIAEAFSKLIGSVSAGVLKSFRSSDRKgvQGIEYGVewypTIMGQLDNASDVVCLLTPMSLER
PWILYEAGVA--RGKL-NTPVhgvaIGVPLSKAATGPFAQFQNC--GDDEES--LTSLVMQ--
LVDRIPNSEPDR(EVVKFQVAAFKAKIETILSARKESSQVSSPDEVEDNNVVKMFEEVKVMFQDLPRHIERRL
DPEVREEEFHRRSKRFRLHP)}*

$2090=212(220):
>gi|916986079|ref|WP_051592791.1| {|130(0)|<Firmicutes(B)>}hypothetical
protein [[Clostridium] saccharogumia]
{(NNDELTKVLEIAKKYNLIFKTDKGIKDFFADMGNRMWNSVDFHCNESKEIFDLIFNSMFYYKTTSQLQKEG
YNFSSESVR)-KKVFISYSWNdDVFVENFVNQLKYSGISVW------
IDKQSIDFGDnileSCLSGLNECDAALIFISKNYSTSAMAKQELITFWSkaikLKKK-MYVIKIDDVNPD---
----EIFFGLSDLKYF--VPGKDSIELLAESLKRNLNNN()}*

$2091=216(222):
>gi|916584449|ref|WP_051191540.1| {|35(0)|<Actinobacteria(B)>}TIR
domain-containing protein [Microbacterium luticocti]

{(VEAYNREVNRVNQHNARVNAEIRQANAYNQQVAAQAGRAVDEINRKLRTSASTSRVVYTPAEQALTERVQH
ELVGHDDRE)-WDVFLSYARIDGAkVATELKDELEDLRVR-
VWFDEVAIIPGQSQALQMDKGLRKARSGVVLVTPAYLTGRFWTErELGVLLG-K-----
TTLIPVLNGVTFadVAEYSGFLPDLAGFET--ARDSVPVIAEKIAGAVLSPGDA(GD)}*

$2092=217(222):
>gi|916761034|ref|WP_051368090.1| {|0(282)|<Actinobacteria(B)>}TIR
domain-containing protein [Streptomyces sp. CNQ329]
{(MA)--DAYVAYAADDAEWVGVLTERLRAHGVS-
ALGDRHHLLPGSVVVHEVEDAIRSASTGLVVFSPAAMAQ-AWLRqEyaalFQASAGRD-----
LRLVPVLLGN-AGLPPFAATRLPMDFRR--LPAEGAafedRV--
ALLARIIRGEQP(PDELLRKVEDVHDDLGVVRSGPERPLTGPAEPSVVICHAHADLEYVRGLGRRLADAGLPV
WTAGRLTWGDDWVWAIRRQL)}*

$2093=213(221):
>gi|916860550|ref|WP_051467606.1| {|137(0)|<Actinobacteria(B)>}TIR
domain-containing protein [Actinomadura oligospora]
{(DLAVAYSERIFEDAIRQGHTRLPAEELREVRVVNKEFDQPAWVWVPGHDAHALSLPDTVRPAEETAAPFPD
LATAPEGRV)--DAVICFAEQDVTLAERLALRLRDRGLA-VYIDPW-
AEIGSVILHERERAIAAAPNGVLLFSRAALANAK-VMdDYAALLSRvhapgG-----GRFVPVLVEDV-
SLPELAAIRQPLDLTGA-HAEDESRI--DLLARALRAEPS(V)}*

$2094=264(276):
>gi|916959408|ref|WP_051566120.1|
{|326(104)|<Proteobacteria(B)>}hypothetical protein [Variovorax sp.
URHB0020]
{(FVDRLLAELPPWMPQDSPVTMVMPRAPSSESASSGPGPLPAEPSTPPAAPIGVPQPGTPAPQDPFAGVQPS
SKAPGPAKG)-KRVFISYAPSDIAVAQKADLMLREHGYE-VFLDR-
DLKSGVNFQGAIADALDASDVVIVFWSKASIASDFVKLeASEG--GRR-----GVLVPVLL--
DGSYPPLeYRGYQAIDLSR--
DSRRDWDALLSQVESVAGRRFV(SQPGGAVLVSAPPSRSLWWLLAAIGMSVAILGAWAAFQSARTGPRGEASY
PSTAPN)}*

$2095=254(278):
>gi|916612803|ref|WP_051219894.1| {|7(27)|<Proteobacteria(B)>}TIR
domain-containing protein [Rheinheimera baltica]
{(AYFRGHGVPTQDNYPHNSKRSYSKDMLAKVDEELLFKIAGELGLVDIPNGTEVVREVGFWKAGHFRLFLSH
LASFKVQTS)--------------------
HLQTALRKYAISSFVAHeDIEPTKEWQsEIEAGLKTMDALAAILMPGFQESKWC-DqEVGVAVGR-----
DVLIIPIRkgLDPY-GF----
IGKYQGIQAQGktIGDVAEAIFETLVKSPKTRNKIL(ISLSGALSNATTSEEAIEKVAIFKSLEGVPSDILEN
LKQQVSDNSRLVESKEFLDSLN)}*

$2096=174(178):
>gi|916653921|ref|WP_051261012.1|
{|0(1199)|<Proteobacteria(B)>}hypothetical protein [Desulfovibrio
inopinatus]
{(MRA)----FISYNSIdQKVADKLNKAL-
MKVLPGFASYFAPRSNIGGAYWLpQLGEEIERADIVLLLLGSHV---GTWQElEYYEALRLNRT-
NEKpHIIPILLGEE-
SPGLQFLHQLHRLRFDDnhFDRVIDQISAVVQNADVGAARQP(LWREINPYCGLQAMGSADAAHFFGREDLTD
QIIAQ)}*

$2097=230(223):

>gi|916679882|ref|WP_051286973.1|
{|0(328)|<Bacteroidetes(B)>}hypothetical protein [Algoriphagus mannitolivorans]
{(MSS)-KQVFISYAWG-
GLSEEVAKELESLLTQHKIDLTRDKTDLGFKGLIrEFMQQIGKGDVVILIISDKYLKSKNC---
MFELLEAEKK-GEfyKRIFPIVLPDA-DIYDSlgmvnylkyWDQKIKELNQSVkeLENLADTR--
KIQEDInlYTDIRGA(FDDLASRLSNMNTLTLELMRSKEYQPLLSALKEGINPPPAPTPSKKEGKVLYHIPGM
MQVDTWTRCTVRLAWDEILLQEN)}*

$2098=200(186):
>gi|916755648|ref|WP_051362704.1|
{|0(194)|<Actinobacteria(B)>}hypothetical protein [Amycolatopsis thermoflava]
{(MLYRFPVVSTV)--TVFISYAHDdEPHRAAVREFAASLRAQGIdadIDGWADTDRRDWQAWAtE---
HILSADFVLVVASKvyremgDGLGpADRHRGvQAEAALLRdllsADR-GKwtKKILPVILPGH-
RIDEIPLCLQPHNVDRYdvSSPAGMEYLLRTLTRQPEHVRPP(LGPPVVLPPRSGPGAVPVGRSPQWRRLARP
ATVLW)}*

$2099=245(256):
>gi|916761034|ref|WP_051368090.1| {|92(162)|<Actinobacteria(B)>}TIR domain-containing protein [Streptomyces sp. CNQ329]
{(LLGNAGLPPFAATRLPMDFRRLPAEGAAFEDRVALLARIIRGEQPPDELLRKVEDVHDDLGVVRSGPERPL
TGPAEPSVV)-----ICHAH--A-DLEYVRGLGRRLADAGLPVWT-AGRLTWGDDWvwAIRRQL-
ASAVAVVVVMSPEAQAAQDVSrDIL----EGQRLgRPFFPVLLRGERH-
YLLASSWYFDARGGALPGPAELRQLQRLLKRSGGGLRSEH(PVFTPVPRPRYRAAARPPVDASLATLHALLAA
GEVE)}*

$2100=186(180):
>gi|916959406|ref|WP_051566118.1|
{|0(28)|<Proteobacteria(B)>}hypothetical protein [Variovorax sp. URHB0020]
{(MSSGF)KYACFISYSHGeqalvkSFIdqfKEALKAYLEPYME---
EEVYVDSDRLKASYMYneALAQAICQSVCMVVIYSPVYERRPYCGREFEAMVRLEARRlSLLAKGGEPRG---
LIIPVVLRGFKDLPARIQQGRQAIDFSHFTLATQEMSRNP(DFVARIQEIAQQIYEHFQAFSTAGDDPCKECM
AFS)}*

$2101=216(205):
>gi|917067392|ref|WP_051674104.1| {<Actinobacteria(B)>}TIR domain-containing protein [Streptomyces sp. URHA0041]
{(MPEGLLPRRSFQNPYGFLSYGRTPQVKD)SHRPPDTGL---IRLhDRLSEDVMALTDLQmgrT-
PVYLDRR-
TRLGAGWkdELKRHLADCQVLIPVLSPRLFTSTWCTVewkcfELRQQLQRDKGTfTRNAIVPVLWTPLRhrdi
PSPYADLQYTHEGMGHDYAAGGLFSLLSNGRHTTFNRVVY(QLAMTVVDVAVSARLEPCDPALFDDIFDSLDS
SSGQE)}*

$2102=211(221):
>gi|918010431|ref|WP_052303017.1| {|0(258)|<Firmicutes(B)>}hypothetical protein [Clostridium botulinum]
{(MS)KKIIFLSYCWAnMDSANEVDEILQSYeGIKIKR------DRRELNYGQniqeFMNT-
VRQSEHVILLVSEKYLCSVNCMYEVSQIikdYNYSDK-ITPIILENV--SIYGSENRLKYAKYWNDK----
YCNLNEE--IRK---
LAIEDN(LSLLNELKKIKSICSTIDEILGLLNKNQYYTLEQHKQNNFNDIFKALNMDKVPNKVLSIDKIGNKH
EGSDEVKEIEYNY)}*

$2103=252(233):
>gi|917130598|ref|WP_051737310.1|
{|0(179)|<Actinobacteria(B)>}hypothetical protein [Pseudonocardia autotrophica]
{(MRRCSSTSGGPSAWEAAGGFAAALIGRGEVGSSVVDDRGDR)SVRVFISYAHgvpeheADvrRLW-----ELLRHNGVD-AVFDRVADEEPQDWSTWTREQVRTADHVLLIASPAYrrrFESDVGEEdEagpglgvqWEARFirselyrdqaGAR-----RKFLTVLLPGTStaDVPYTLQPGVVTEYRV--ESFTVEGVTRLLRFLTGQPAIV(SPELRPVPRLAPDDPTPRIVLHITVSGGSPWTRRRAAEVLTTKAPADAEVVT)}*

$2104=219(227):
>gi|917136660|ref|WP_051743372.1|
{|89(0)|<Proteobacteria(B)>}hypothetical protein [Burkholderia sp. K24]
{(RVENKPNAIFDVGATLLTSPREFLSSELHYTSRLFLYCESRLDESTIGQLMDEAKSINVNLQIRTPDYEKTRSNMESPLA)----FISHDSRDKSdVARPIATTLQRMMCP-VWYDEFSMSVGDSLRESIEKGLRECKKCVLVLSKNFLANTGWGKkEFDSIFTRE-----LIFEQRLVLPVWygVSKQDVYNYSPSLVDT--LGLDFNALGIDEVCRRLYNKIT(AEDKPSV)}*

$2105=211(220):
>gi|917138813|ref|WP_051745525.1|
{|0(822)|<Actinobacteria(B)>}hypothetical protein [Streptomyces yerevanensis]
{(MVRE)--KIFISYQRDSAAVAQRIHDALEAAGFD-AWVDTERIRHTDRWATSIDQALRAADRLVVLLTAKA-AESHEVFnEWFFFYDHR-----KPIHVVRIDGCElhYQLVPFQRLEWTTEAD--EDEWAARmS--ALVTALHGSFSW(PDGASPDDAVVTTPFAPARTLPGAFAALEEAIRSPDRSVALTDHQLHDIRDHPSRSVREYLLSCYARWCSPRYQLD)}*

$2106=298(275):
>gi|917154790|ref|WP_051761502.1|
{|462(179)|<Actinobacteria(B)>}hypothetical protein [Microbispora rosea]
{(DGAIPRPSGRRPFALLSVEALRTLGDLDIPVRGTVRRPRPAAVRQASPQTPAAAREPAAGVQDWSAKGATSRRPRPPDDR)APYFFLSYAPippydatsknDPNRWVVKLFGDLcthimqmtdAPAGRA-GFMDL-EPRAGDHWPDRLAEALATCRVFVPLYSPRYFESEQCGREWAAFQRRvdfsegpdgwmP-----PAIVPALWVPVRndDLPGVARSIQFMDDAM--GTRYQDQGFYGLSKISSMRRHY(QQATYALARRIIEVAMSTHVSVLDRPPRFDELRSAFHDSPSRRPLRVTVVAPDLD)}*

$2107=192(217):
>gi|917054450|ref|WP_051661162.1|
{|0(369)|<Proteobacteria(B)>}hypothetical protein [Bosea sp. 117]
{(MAICSAI)--------------------------EKRGVGCWISSLDVGPGENFqdSIYRAI-GQTRVMVLVFSQNANNSDEIKkEIALAG-----QHKLVVIPLRIEDV-LPSsafaYELATRQWINLFEDWNASIERVIEqIDKVLAVEPPTRG(GGDGGGQESPSPRPPIKRDEERSVAKYAIVAMMLVALAVIGGGAYAWHRSTEDARLAAEEKARQERLAAD)}*

$2108=225(230):
>gi|917263493|ref|WP_051870205.1|
{|134(0)|<Actinobacteria(B)>}hypothetical protein [Streptomyces resistomycificus] >gi|973923880|gb|KUN92146.1| hypothetical protein AQJ84_34575 [Streptomyces resistom
{(VHLGGTGINSLDAKPFCEAKGIRHESPSPVDSRTVVATYPHPQLQRFMTDCGVPDIFATYMIVCAHALNEADLRSLMQST)----FISYGG--P-DEPFARKLYDSLRSHGVATFFFPENATLGERIdtEVYNNLQKHD-

RIVLICSAASLNRPGVLhEIWETLGREARDgGATYLLPITLDEY-
VFTDWKKKEPVLAERVGRRIVGDFRGTINDSAVFDTAVKR(LIDALMKKRP)}*

$2109=213(222):
>gi|917774364|ref|WP_052288305.1|
{|635(0)|<Cyanobacteria(B)>}hypothetical protein [Lyngbya confervoides]
{(QTLDRFLAKGRTDVTCEKSALDVPIRSLTAVIPTGELFADLRDRYEQAELLPGLEKQATQDKFDRLKQQHT
EYYPANQFY)--DVFLAHNSQDKP-FVRQVYQQLKKTGL--
QPWLDEEDIAPGtLFQdEIQKIIQQTKTAAIFIGPTGLGPWQNVEQ---RAFISQCVsREIRVIPVL----
LPGVHAIPEELIFLQEFGFVSFETNDDETAIDRLRWGITRE(KS)}*

$2110=144(153):
>gi|917774455|ref|WP_052288396.1| {|0(173)|<Cyanobacteria(B)>}TIR
domain-containing protein [Lyngbya confervoides]
{(MTEQQF)--DVFLAHSS-KD--KPLIRQIYQTLKGRGMRPWLDEEEIAPGQHFqqEIQRAIGTVKAA-
AIFIGEDLGKWQELELGALIQRCV-----
SKSVRIIPVLLPGIESIPEDEYFLSQYQFVKFGETLQNQKAFDLLEWGITDV(KPQNLDS)}*

$2111=216(220):
>gi|917622899|ref|WP_052191965.1|
{|227(0)|<Proteobacteria(B)>}hypothetical protein [Pseudomonas parafulva]
{(DNPVEAERKYYIDILGLKVEDAGLTGDGSKLKRNIYGRLSFLAMVKGSDDPTYVKLILKMAKHDPSPPKFI
KPLKEQYQM)-YDVFLCHAS---EDKaEIVEPLYNELNKLNIHAFYDAKEIG--WGDsliGTINKALL-
KSRyvIAVMTDKSVGKAWPQQEINSVLNGDISGGTKRLLPlIHGDKETilGNnfLMNDKLYVEWNN-NP-
AEIAQKVQ-ALLA-----QKAE()}*

$2112=178(171):
>gi|917479452|ref|WP_052085869.1| {<Firmicutes(B)>}hypothetical protein
[Clostridium sp. HMP27]
{(MN)-KNIFLSYCWKdEEVVNEIDNYFKSRQVIFQR------DKRDIESWEsikvFMKR-
IRKSSYAILVISDNYLKSVNCMYEVLEVMKDENY-
RERILTVVLNDALIYDALSKAKYIKywkekyselydtikeIDDNQS--TIKLLEE--
LKKTKEIMNNIG(EFMAIVSDMNNPSINNVCEEIGIKLKNNS)}*

$2113=215(220):
>gi|917471712|ref|WP_052078129.1|
{|152(0)|<Bacteroidetes(B)>}hypothetical protein [Porphyromonas gulae]
{(IEYRKKEGVSTSRVVWYRELFSLLSDEDVKIFISKLSEKINISINNKRSDVCVETTLFDNVEKISKPQEEK
GELRVIPMI)---VFISYAWdENKEEVQRIAKHLKNANIE-VRIDE-
DVPYGADLVRFMNKEIRDCTKCLVFLTPKYKEK----AeLSTGGVAHE-----
GRVisraiyqnqdttkfIPILLSGTFeeCCPDFLIARKGFDFVN--HPFDEEIN--RLIRAL-----I()}*

$2114=212(220):
>gi|917543842|ref|WP_052150259.1| {|37(0)|<Proteobacteria(B)>}TIR
domain-containing protein [Pseudomonas aeruginosa]
>gi|956799801|gb|KSM47577.1| TIR domain-containing protein [Pseudomonas
aeruginosa]
{(VEAHNRQIAAHNRKVQAQINTHNHKVEVHNRRAAQHNAKLIADLNRSLRSSTGSLQYTDSERRLADRVHQA
VALADPREY)--DSFLSYARIDGAqVAGELRAYLEELGVA-
VWFDEVAIVPGRSQSLQMDAGLRKARTGIALLTPAYLTGRFWTErE-LGALLNK-----STLIPVL-
HGVTfeDVKQYSGILPDLAGFT--TAHDSVADIAEKIAAAVLPIEE()}*

$2115=202(210):

>gi|917581145|ref|WP_052172665.1| {|0(252)|<Actinobacteria(B)>}TIR domain-containing protein [Nocardia sp. BMG111209]
{(M)--RIFISHAGTDVAAALALKQWLvrqrpELAGGI-
VLdTEVAGIRAGKHWRDAMRAAVTDADAVICLISPRWLRSAE-CRaEFRTAEVLG-----
KRILCAGIEPAEDD--TMREWQRFELFG--PGPSVAIA--
ARPYPDPVRFTE(SGLRLLISTVEAPPAPPAAEHDAAIPGQQAIVSGEENITFQSNRDAEVIINHAPSNAAVRSGALIIGGT)}*

$2116=232(236):
>gi|917495342|ref|WP_052101759.1|
{|1010(0)|<Bacteroidetes(B)>}hypothetical protein [Porphyromonas cangingivalis]
{(ERRENPITGLYVSVYDPNLKAEDQLYVHLETLDDPEKTKRKIIGYPLDKDKQINKDSGQTISTKRYKHLSVNSELKSVRQ)---
IFISYSKAdREDLMELNKFLEDLKREEKIEVYYDETTTPGDMIHeVIRQKIIEADYVIALVSQDYISTGYIRDvELPLIKEKNK-----RLIPIIIKPC-
TWTMNEISNHYATLKGTeiNFPDWNKSSHPGNYEACRAQNWT(RAVTEISQKLFNNKDN)}*

$2117=178(175):
>gi|917538455|ref|WP_052144872.1| {|0(656)|<Firmicutes(B)>}TIR domain-containing protein [Bacillus okhensis]
{()MKRIFLSHSSKdKIfYIENIVKNLTKLIGKDKF-
VYDAITFEEGQETSqEISKWLKNTEIFVLFISETALESPWV-KkEILEAKDLFDR-NDLRkIYPIIIDEQ-
ITHEDKRIPNWMRETYNirIISRPAVAARKIHNRYIELVWEK(NPKIKEKNTFFVGRNSLIDNIEERIDTYDKPVPTS)}*

$2118=207(217):
>gi|917631835|ref|WP_052200894.1|
{|0(763)|<Acidobacteria(B)>}hypothetical protein [Terriglobus sp. TAA 43]
{()MAEVFISYSRRdRGFAHKL---LAAFQVEGR-
EVWLDEKeIEVTSDWLnEIFSNIESADNFLFIISPDSIAS-AIAQqEIDHA----AA-NNKRIVPVYYRPV-
PDADIP----QAVARfHRidFTSAADFD--
SSFATLAKALDTD(LDWKRTHTRLLTRAKEWERREKDHSFLLRGKDLKEAEQWVVSSTARDPRPTSLHSQYVLAGRQATANTLRMVIAAIA)}*

$2119=174(180):
>gi|917774456|ref|WP_052288397.1|
{|0(157)|<Cyanobacteria(B)>}hypothetical protein [Lyngbya confervoides]
{()MSFIFISYSRQdQAYVSTLVQALQS----
HRLPIWIDDRIDYGTDWPdVIEEHLEQSAVFLLVMSPRSKRSHWV-NcELTRAIELDKP-----
IFPLLLEGR-
QWLQVATKQSVDVIGGKlpEARFFDALRKCLPLPIETADALS(VQEAANNKSSKFAGALLASHENLLTTNSTNFEDDLSSEKW)}*

$2120=230(216):
>gi|917538221|ref|WP_052144638.1| {|0(243)|<Firmicutes(B)>}hypothetical protein [Bacillus okhensis]
{(MSERTLINP)--TIFISYSwtT--
PDHEEWVVDLATRLMEVGIKVKLDKWDLKEGQDIfaFMESMVNSDEIdKVLIICdegykNRADGRQGGVGtETQiISPEVYKDVkQEKF-IPVvaeRDESG-
NDFIPsyIASRLYIDLSSmdSYEENLERLIRNIYKVPLHQKPKL(GKPPEYLFKSEIDHSGLRSIIRKMRVALEKQPSRLKYIQREFNDLFEETLNEYEIEKFTNIHEGDQQ)}*

$2121=155(150):

>gi|917622879|ref|WP_052191945.1| {<Proteobacteria(B)>}hypothetical protein [Sphingomonas sp. Ant H11]
{(MAQSGS)-
YIAFISYSHSdrywaEWLHRTLERYRPPRHIgadlkkpTRLRPIFRDQAELSAGPDLgdSIRSALDRSSHLIL
LCSPSALASRWVNAELAHFLTTHSVDrvlCVLVG-----------
DSQPEKSLLECLPQDCARLCLSDKSLWPPICVLAGTGD(GWPG)}*

$2122=221(228):
>gi|919011232|ref|XP_013390051.1| {|1210(0)|<Brachiopoda(M)>}PREDICTED: uncharacterized protein LOC106158546 [Lingula anatina]
{(EKLLSSKLTWLLAFQRDNKQVMRDDENLVAMLEQVEHDTCKELRHYAQGASFELHKTQEKINKVLEEHFRP
RGSPRPSGW)RYDVMLSYHP-ESRP--VTKLVCEALHQEGLRVWMAEDEMSFG---
AATEAIEQSTMVLMAMDHRYHTSARCRSEAEYAFDLQKS-
VLPMKMKRSFKPCGWMGRFVAGKMYVDFSKCQEDAGFSDLVKELIEEIRMVLRY(ANEKPVFL)}*

$2123=195(204):
>gi|918572281|ref|WP_052517652.1|
{|0(333)|<Proteobacteria(B)>}hypothetical protein [Archangium violaceum]
{(MAR)---VFISYKRDvtPDQDLAQALYKALKASH---
EVFIDTSMLVGEKWVeRIDAEIRQADFFIALLSPFSVNSEMAQTEI----ELAYRLqTQKR-
QPVLLPIRVAYHERLPPPLSS--
YLSAINWALWEGPEDTPRLLEQLGKV(MSGGLQRDSAPPAPAPTSFQEHPPELLEQLARAMSGGSPPGSVVLS
QPQPSASLELDETLE)}*

$2124=196(188):
>gi|919000392|ref|XP_013404162.1| {|0(508)|<Brachiopoda(M)>}PREDICTED: uncharacterized protein LOC106169291 [Lingula anatina]
{(MAEETADY)EYDVFISYSFKdsREV-EQLSEDLKEEGV--
KCVLAERDFDPGKPVLESIINCFEHSRRSLLYITTHSIKSNWCRFEKSLSLAHAVKSEKQQVIPVYanitdee
LEEVPgHLRALDPIDSRKERFFDtLLSRIKAPLVGLKKALPTGNVAH(GLVWGYFYGYLNLILPGLKQRFQEW
EDFDPHSRKGKYIPK)}*

$2125=196(188):
>gi|919036603|ref|XP_013401739.1| {|0(508)|<Brachiopoda(M)>}PREDICTED: uncharacterized protein LOC106167497 [Lingula anatina]
{(MTEKAGDY)EYDVFISYSFEdrREV-EQLSEDLNKEGV--
KSVLAERDFEPGKPVLESIINYFEHSRRSLLYITTHSIKSNWCRFETSLSLAHAKKSEKQQVIPVYanitdee
LEEVPgHLRALDPIDSRKEGFFDtLLSRIKAPLVGLKKALPTGNVAH(GLVWGYFYGYLNLILPGLKQRFQEW
EDLDPHSRKGKYIPK)}*

$2126=251(249):
>gi|919055086|ref|XP_013409484.1| {|137(0)|<Brachiopoda(M)>}PREDICTED: uncharacterized protein LOC106173051 [Lingula anatina]
{(PLPTAIPSVRPALNIIIPCASVGIVIIVSAPIVFIWALRKGIILRVKLTARTRNFEGDNDLKEGDAEDEWR
ETGEIKGFY)-YHAFISYNEyakEdkAFVKGRLIPELERRGF--RIYKPDERVGDIARQKEDI--
GIEASSRFIMIVSPRYFEMEGVNDQCGRA-
RKKLNDDKIRIIPIILEDFDrKlKQNKQYASVRELMKQPrFEKPNEDAMDKFFNDIVDFMPML(ENQAERADR
DGLHQEQIELDVEINGNGGE)}*

$2127=208(222):
>gi|918278356|gb|KOF62973.1| {|220(0)|<Mollusca(M)>}hypothetical protein OCBIM_22021016mg [Octopus bimaculoides]
{(PDWQDKQLLDFTRSKIDCTDYTLYYILGGVGGGFLLTVVIVLFAYTKRWYIRFKIFKLYQYVQKAGNKKEY
EAIPGDDMY)-

FDGYISYSDKdaDWVEKYLMHTFDNgengedkNNGNFKLCFRNRDFAYGKYIIGMIESSLAVSKKMIMVLTPY
YKKDKRCEFELQLGIMKL---NIKNVMPIVLKNLQpNQIPN---SLKEIFETNkFIEW---------------
---D(NN)}*

$2128=225(249):
>gi|919004400|ref|XP_013379346.1| {|89(0)|<Brachiopoda(M)>}PREDICTED: uncharacterized protein LOC106150873 [Lingula anatina]
{(KFKTIQGIKAGLTAQGREKLCVIAAERYANVAKVITDMSWKRYESLREDLSTAQFMFQELPIPALEAQSQE
ISMEHESFQ)-YDAFLIHSGEdkPKVEEVL-ELLESRD--
IRCCYAPRDFLPGRSILSCIEDAVELSRCTVVMVSENFMKSTWCQHERDVAEVKAKDTEGYSVIPVLL-
GFKpSDLPSAY-KVKKFILADS---------------------
Q(DFIPTLVKAIEGIFLSRSLYLMGFISLHY)}*

$2129=226(252):
>gi|919008387|ref|XP_013388956.1| {|192(0)|<Brachiopoda(M)>}PREDICTED: uncharacterized protein LOC106157757 [Lingula anatina]
{(AIKNNCTNIRDLKVGLQAANFPRLANAAGDCYAGVVTEILNMTPDEFEERCRTLVTARHMHTSRSTTTNQQ
DVRLPLDQY)CYDAFIAYSGEdeETIEPVLHQLKE---
LGVKLWYAKEDLIPGTQVITSISEAIIASKHTVAFLSKSFVQSRWCQQELQYAMQEALNAKRDVLIPVVM-
NISpEEIPAEIKGLKFIL-------------------------
F(DDEELIPKLSQAIKGPQDTPTYGELRDEVEVL)}*

$2130=212(233):
>gi|919019524|ref|XP_013393805.1| {<Brachiopoda(M)>}PREDICTED: toll-like receptor 1 [Lingula anatina]
{(MASDEFEALCRTLVTAKHMYISGATAADQQDTSSSLEPIR)-YDAFIAHSSEdkA-VVEPVIEQLEEQG--
VQCCYGIRDFHPGKSLVTSMGEAVYESKRTVVFLSKSFMESPFCAKEREFAIHKASESGEDVVIPVLM-
DLLpQQIPVEFKPLKYILVSDrEL--------------------
I(PKLVQIIKGPTESPTYGNLRNALRDAHEQIAFLRDENRELQAKIRQNLQQHVI)}*

$2131=257(284):
>gi|919023034|ref|XP_013395417.1| {|468(0)|<Brachiopoda(M)>}PREDICTED: ankyrin-3-like [Lingula anatina]
{(NFKTIQEIKAGLLAQGREELCELAAEQYATVVMKIINMSTEHYQGFCEGLSTAQFMLQERQPAALETQSQE
IGMELESYK)-YDAFLIHSGEdnSKVEEVLEQ-LESRG--
IRCCYAPRDFQLGRSIFSCIDDAVESSRCTVVMVSENFMKSTWCKHERDVAEVKARDTEGYLMIPVLL-
DFEpSDLPRTY-KARKFVR------------------------
A(DCQDFIPKLAKAIEGPTAAPCYGQLRRENEALKQEMEGMSQQLEDSNSTIRQLRANLESFTLYY)}*

$2132=233(228):
>gi|919024457|ref|XP_013396099.1| {|619(0)|<Brachiopoda(M)>}PREDICTED: chondroadherin-like protein [Lingula anatina]
>gi|919024460|ref|XP_013396100.1| PREDICTED: chondroadherin-like protein [Lingula a
{(ERRGMPLLQFDPDAIDCVNRWPIYLASAVGGTLALLVVVFISLYRWRWFLKLRYYRFKRLALKRDVPHGYE
RVEGDDIVS)--DAFVSFCvDAdrEWVAVELLARMDSRPPNagrFNL-
VCDLNFLPDKSELESVVEAIECTRKAIVVLSDAYIGDPRCvQFELEQIVYESSVERPQRyeMILVLKGLNPnG
KIPKV---LRQRLERGeFLEWTEDANGqqLFWDQLGEKL--E(QRPHNINV)}*

$2133=173(182):
>gi|919030835|ref|XP_013399071.1| {<Brachiopoda(M)>}PREDICTED: toll-like receptor 2 [Lingula anatina]

{(MTQKDLILDNKKFECCDSNSESIPPPPCNAKNKKGKL)-YDFYVIYSEEdsDWVSKHLLTTLEGTKG-
FKGCVKERDFIPGTTITDSILESIKNSLKVLVIISGREPKDEWYDFEVRHAiHHRHDSGQNSFVIPICRENAN
kSDVP---LTLRDV--NPcYMVSAHD-----WEKLERAL--D(DDNSQ)}*

$2134=208(236):
>gi|919034798|ref|XP_013400955.1| {<Brachiopoda(M)>}PREDICTED: toll-like receptor 2 type-2 [Lingula anatina]
{(MSPGDFEELCRTLITARHMCITCSATTNQQEINSSTEPLR)-YDAFIAHSSEdkA-VVEPVIEQLEELG--
VQCCYGVRDFHPGRPVITSMGEAVYGSKRTVVFLSKSFMESPWCAKERELAMHKAVESREDVVIPVLM-
DIPpEQIPDEFKPLKYI-------------------------
P(FEDKELIPKLVQTIKGPSESPTNGELRIALQNAQEENAFLRDQIRELTTKLRPKCQ)}*

$2135=242(268):
>gi|919037978|ref|XP_013402450.1| {|574(0)|<Brachiopoda(M)>}PREDICTED: ankyrin-1-like [Lingula anatina]
{(KNNYRLTNIRDLRAGLEAANLPRLSNAAGDYYAGVVTEISDMTPDEFEALCKTLVTARHMHTSRSTTTNQQ
DASVSLDQY)CHDLFIAYSGKdeATIEPLLRKLKEM---
GFKLWYAKEDLILGTLVIPSICKAVSASKHILAFLSKNFFQSGWCQQELQYAMQKAIDSKRDVLIPVVM-
NISpEEIPTEIKVLKYIL------------------------
F(DDEELIPKLLQAIKGAQNTPTYGELRDENEVLRDEVRLLTSERRLQSQ)}*

$2136=225(251):
>gi|919038223|ref|XP_013402582.1| {|178(0)|<Brachiopoda(M)>}PREDICTED: ankyrin repeat, SAM and basic leucine zipper domain-containing protein 1-like [Lingula anatina]
{(VNKNNYKNIRDLRVGLQSANLPRLSDAAGDCYAGVVTEILNMTPDEFEERCRTLVTARHMHTSHSATTDQQ
DASVSLDQY)CYDAFIAYSGEdeATIEPVLHQLKE---
LRVKLWYAKEDLIPGTQVISSISEAIIASKHTVAFLSKSFVQSRWCQQELQYAMQEALYAKRDVLIPVVM-
NISpEEIPAEIKGLKFIL------------------------
F(GRRPRCITDIANKVELKSSSVSGKPPGESVL)}*

$2137=200(226):
>gi|919042882|ref|XP_013404710.1| {|0(331)|<Brachiopoda(M)>}PREDICTED: uncharacterized protein LOC106169678 [Lingula anatina]
>gi|919042884|ref|XP_013404711.1| PREDICTED: uncharacterized protein LOC10
{(MIAQPHW)--DVYVASSEEddHLVEPLVNEIQSHSYVCFRA---
HKDIIPGQNEIQARDVAMKEARKVLLYLTNNFLKDGWCEMERGVAMIKAYDKmenpSNDIVLIPVIKDLNpHE
IPETLKALR---------------------------
C(IDVDKDDRYMDRIIAALQDPQLCWAENFTECPNCSRACTKSGSALDLHIWFNCQETQVCCPTGHCPTVLRR
SQMKIHVQ)}*

$2138=243(274):
>gi|919046730|ref|XP_013406542.1| {|0(258)|<Brachiopoda(M)>}PREDICTED: uncharacterized protein LOC106170997 [Lingula anatina]
{(MEVSDLSTGTDNLSLSEMDISLSDDNTTPKVDNDTNGSLEDGNATEPMCVLLREK)-
YHAFLAHSNTdaKFVESIM-DTLEKRG--
ISCCYSKRDFRLGTSTVSCINEALDRSKFVICFLSKDFLTSPWCKHERELALHKAAETSEDIVVPILM-
DASvEDLPLNIRRL-----------------------------
T(HIRREDIDFEKLFSSLGFPEGNSRPTYIDLETANAELKTKLEDAQIKLKDAQIELEDAQKLIKIKDDALIQ
QQMHIKQC)}*

$2139=262(278):

>gi|919047355|ref|XP_013406871.1| {|129(0)|<Brachiopoda(M)>}PREDICTED:
uncharacterized protein LOC106171199 [Lingula anatina]
{(LADVIKGPADAPLYGQLRNENENMKREIAALRQQLEDSTALNRRLQAEVMTADAPRQFVCEPNKLYRLAHS
SMETDSYQC)--DVYLAHDSDdeE-VVDEVRGKLE-----
FSGVRCSEPALAGTSIIKSDNDPVKTAKRTVPFFSKNSLESKLFMMTCDVALIEANERKEDVMIPVTI-
DVDpKSLPPPYGTLNCI-----------PYKsqDFIQKLIDAIK-
G(PTDPPFYGQLRDENENLKRELAALRQQLEDSTSLNRRVQAELDAAAALMQFVCESEGT)}*

$2140=257(285):
>gi|919047417|ref|XP_013406902.1| {|44(0)|<Brachiopoda(M)>}PREDICTED:
uncharacterized protein LOC106171216 [Lingula anatina]
{(AYGIKSLQELKRRLAEAGCLNLAAAAAKHYAEIVKEIMEMPDECYKAFCQDPMSQRHMISSAASPTEQRCE
ISMETESYQ)-YDAFLVHSSDdgKTVGGVCEQ-LEARG--
VRCCYAPRDFTYGKPIPASTEDAFEKSKFILVVFSENFMKSSWCQYEMHMAVQKTVDSRQDVVIPILL-
DFPeENLPLGFKTMKYI-------------------------
S(VRQHDFLPKLVQTIEGSGTEPLYGQLLAENQKLKAELEDTRQRLADSTTLVKRLQAAMPATGVPS)}*

$2141=246(261):
>gi|919047419|ref|XP_013406903.1| {|35(0)|<Brachiopoda(M)>}PREDICTED:
uncharacterized protein LOC106171217 [Lingula anatina]
{(TNGVKTIQELKVRLSNVGRHNLAVAAANHYAKIVQAIMDMSAEYYETFCQDPMSNRHMITSTVCPTEQRCE
ISMETDLYQ)-YDAYLAHHSDdeE-VVDKVRGELKSSGVR---CSETA--
LAGTSKVNSDNDPVKTAKRTVPFFSKNSLESKLFKMTCDVALIEADERKEDVMIPVTI-
DVDpKSLPPPYGTLNCI-----------PYQsqDFIPKLVNAIK-
G(PTDAPLYGELRDENENLKREIAALRQQLEDCTALIRRLQAE)}*

$2142=243(240):
>gi|919058757|ref|XP_013411236.1| {|669(0)|<Brachiopoda(M)>}PREDICTED:
toll-like receptor 3 [Lingula anatina]
{(ELCFGPEWAQKTPIKEFRPSWFACDDHSVYLAAISGGCVFFVIFLSAVLYSFRWDILYIYAIYRASGKKSK
LGRREPHKT)-
YDAIAMYSPTsvTWIKKHLIPNLERGEDIrFKLCINDRDYIVGDPLVDNVETNMEKSRRILFLLTREYFESQL
HETEINLAQVKLFDGFVDKIIFVFLEEVPkTTFKEP---LKTMMRHGnCLHWPRKKRVrertIFWKRLKLAL-
-L(EARYPVRPGEKTPLLVNENN)}*

$2143=231(235):
>gi|961115991|ref|XP_014780765.1| {|908(0)|<Mollusca(M)>}PREDICTED:
protein toll-like [Octopus bimaculoides] >gi|918303988|gb|KOF76688.1|
hypothetical protein OCBIM_22033064mg [Octopus bimaculoides]
{(IILILHNIKRNVVKNKNLKKYLYSGRFIKSTDHMMEKKLLYMLTGRNLYNNHDIPLRRRKSYKEFFPEVLG
SANTLNEQK)KYDIFISYPDQtyQFATGPLLSTLQSRG--
YSICLPDRDFVVGSAKEENILRAIKSSVRTLIVITKSHVEDEWQLFTLRTAVQCSLKKPFNYLLCIL-DGI-
VDKSKLDLETQAYVTSHvVIDKDDPL---LWKKLFRSIPPA(RTIENSTSAFDSRKS)}*

$2144=286(288):
>gi|961082283|ref|XP_014768952.1| {|1147(0)|<Mollusca(M)>}PREDICTED:
protein toll-like [Octopus bimaculoides] >gi|918328025|gb|KOF93357.1|
hypothetical protein OCBIM_22004649mg [Octopus bimaculoides]
{(FDVQLHCLNITPVTNLSSHVNTQIDSKVICALAIFSAVFLTLIIAVISIACYREELKVWLFTQYGWRIGDP
WAKLDDSNR)RYDVFVAYTSKnaMFVEHELTPRLERREPPYQVCLTYRDYDVDISYAQNTINCIQNSKRTIML
VSNDFFQTEWFRYDFQINNHDILKTLSERLIVILMEKV--DRKKLECDLMFYAKTKkFLKYQDTH---
FWDKLYYMLPKV(RGLPLLPQTPESAVSSGELRDQCCNNIAFSKTSLESGYEEINFVRKNVFPRSDHQTVYLA
PVCQRNCK)}*

$2145=212(220):
>gi|919038243|ref|XP_013402593.1| {|239(0)|<Brachiopoda(M)>}PREDICTED: uncharacterized protein LOC106168180 [Lingula anatina]
>gi|919038245|ref|XP_013402594.1| PREDICTED: uncharacterized protein LOC10
{(NCVKGNIQNSHQYDPVSWSSSGNYQNGHTSGLEREMSLIENGQNGHVNGLPRREQLQNGALEREGAQMQGD DNPAPPVRY)KYDLFLVHSSEdeEEVLE-LAERLEE-
EHGIKCCYADRDFQLGKRVMNNVEDLFKECRHIALCLTRNYLASSWCQHEQSMALGKATENGGQGMIPLLFSG F-PESD-VPPTLKAY------SYFKLTSDPQSLTQLARHIKG()}*

$2146=158(159):
>gi|919056846|ref|XP_013410238.1| {<Brachiopoda(M)>}PREDICTED: toll-like receptor 5 [Lingula anatina]
{(MQGDDNPVPPVRY)KYDLFLVHSSEdeEEVLE-LAERLEE-
EHGIKCCYADRDFQLGKRVMNNVEDLFKECRHIALCLTRNYLASSWCQHEQSMALGKATENGGQGMIPLLFSG F-PESD-VPPTLRAYSYFKlTSDPQSLAQLARHIKGNNKEKSV(CFGGNL)}*

$2147=188(218):
>gi|919004253|ref|XP_013422169.1| {|0(296)|<Brachiopoda(M)>}PREDICTED: uncharacterized protein LOC106182077 [Lingula anatina]
{(M)-------------------DTLE--
KRGVSCCYSKKDFRAGTTTVTCIDEALEKCTYVICFLSKDFLTSPWCKHERDVALHKAAETSEDIIVPIL--- ----MDASLKDLPRNMRRLtHIRYNEFD---
FEKKLFLSLGVP(EGNSRPTYSDLETANAELKTKLEDAQEVIRMKDEALVQQEMHIKQCEAKLNQYEAVPEGN SRPTYSDLENANAELKT)}*

$2148=206(216):
>gi|919025296|ref|XP_013396456.1| {<Brachiopoda(M)>}PREDICTED: toll-like receptor 1 [Lingula anatina]
{(MEVSDLSAGTDNLSLSEMKISLSDDIATPKVTNDTEKSLEECDATEPIGVLYRD)KYRAFLTHSDIdAEFV ESVMDTLE--
KRGISCCYSKKDFRPGTSTVSCINEAMDRSKFVICFLSKDFLTSPWCKHEREMALHKAAETSEDIIVPIL--- ----MDASLEDLPMNMRRLtHIRYEDID---FEKKLFFSLGFQ(EGNSRPTYDDLEAANAKLNTEL)}*

$2149=252(257):
>gi|918223978|ref|WP_052357110.1|
{|115(12)|<Bacteroidetes(B)>}hypothetical protein [Prevotella brevis]
{(ESKILLVLSNFYEHFANISQADVLRLHSFLSKCKQCRLWVCGDGEWYKGKHSVYLQLYRKLDPMYLPYYEA IKKGEKQPT)---VYISYKW-KGESMDAADNLCDLFKRKQIYYRRDKEDCRYGDSItaFMDE-
IREGAFVVVMFSEAYLKSFNCVYELAGVLnhpDYM-----
KRVFPIVIDETVREENKYKELANYWEKKKNDTTFIIKQIISEDKTVILPLEK(KIRLIEEYIHQLQKLTEVTD DINSYSFESLKERSYEP)}*

$2150=248(257):
>gi|918305945|gb|KOF78130.1| {|518(61)|<Mollusca(M)>}hypothetical protein OCBIM_22031247mg [Octopus bimaculoides]
{(NGVENLDWVCLANYLGYKKPKIDKFNNDLNPSLALLTDWILGSGNTQVSVDVLHTFLEKMSRYDIIDLLKQ SREQSHCPQ)---VFISYQW--DSQDAV-
KALWDRLERAGISCWMDIGQMGGGDQLynKIEQGIRNCKIILACVTPKYVISHLCNRELSLADGLR-----
KPILPVMFESIVWPPPGGMALIFSNLVYIDMNGIGGHGSSGMTADLADKYKE(IIHRISHYFINDENSKSTTE SKSFVPSSVKSNQTSNE)}*

$2151=212(220):

>gi|918391731|ref|WP_052444006.1|
{|52(0)|<Proteobacteria(B)>}hypothetical protein [Aeromonas dhakensis]
{(QGDPIKFDAWLKDKYPEKYRRHNIFLSDIYKYNVMTFIDEFMVDLVLIPEGQGVLFHWPEQELLETMNPQYKCLKKNESR)---VFLSHSS-KD-
KEKIVNPIYAYLQSEGIGTWLDSAEIDYGDNIyiKVNEGIQASDLGVFVLTDNFFDarSGWPVTEfstfFMSMMESK-----KNIILINAGVSPSNMHALMKPYRYLE------WSGLDSLPEIGNTIQRKLNS()}*

$2152=231(239):
>gi|919022552|ref|XP_013395161.1| {|0(242)|<Brachiopoda(M)>}PREDICTED: uncharacterized protein LOC106162415 [Lingula anatina]
{(MGSGSSIQTGKNSSASSPSKTSKTGQKETKSDYIVE)PKDVMISYSH-AD--
KDMMNKLKDALEKHGISVWVDTIGLKAGVDFlsKIGQAIIDAKLFISLLSEKSIASKYCQDELALAYISN-----KPIFP--
CGLKTPEELFPLMDTGMKLQLARFEWNLFTEEDAFESNFDKLANK(MDAEISKMNIESHVPPADEASSSKQAEPSEKGDGNPESRQILKRQDSVRMNQRFDKNMMGRLD)}*

$2153=228(254):
>gi|919027806|ref|XP_013397728.1| {|1137(40)|<Brachiopoda(M)>}PREDICTED: probable serine/threonine-protein kinase pats1 [Lingula anatina]
{(IGLLVAGEVYVNMQQKDKYTSKLEELAKKLDMNTLLKKDEAIDYPPVFISYAWANSARAVELGTPEKEGAAGFGDPRDYK)-----------------------DFLEKNGIKCWLDV-
EQVGANGLfeDIAVGLQNAKLVVACISDQYAESTNCNMEFRFAkTALN-----
LPIIVVTVGRGQMWRKSEVGMLSINYPVVSHDDTDQLLKLVKENLPSEVQQP(VSNETKEKKKEKEQANQQQKNTSFQELYELAQRK)}*

$2154=135(145):
>gi|918316692|gb|KOF85958.1| {<Mollusca(M)>}hypothetical protein OCBIM_22019451mg [Octopus bimaculoides]
{(MTSNE)-YDFFLHFSKEdYETAEEVLHILEQeyPHRKLKgyICDRDFDCIGSKFELDEVIKDI----
DIiFVIFSQNSERDKEFMYMNSLIMKIHLENeETKKTIPI----------YIDEDVVDKCIFK--
TLQGINYNSKNFLSKMKTIFR()}*

$2155=296(300):
>gi|919036603|ref|XP_013401739.1| {|287(121)|<Brachiopoda(M)>}PREDICTED: uncharacterized protein LOC106167497 [Lingula anatina]
{(VLYCQVLKQALDSALPEGAKDTYLLLKYKDEIADGSKKYPIADLIVKAIRDGLNTEASGNTTQQEHGLPTEFEKVKVGEE)-YDAFVWHSDDdGEEVDRLVEKLESYGDR-
QFRIIHRGKFEVDKMETKSIEEALKNSRNtILYVTKRFLESTpyLNTLLMHERLADDIQQGRLTLVPVLVDVEPNELPSVLTnviYIEKCKMTE--EE-------
CFGRLVKALEAP(ILKLKSAQPAGDIATGMAWSYYYGYLKLILPGLKQRIEASSYWDQGGSNTVVIPKLVLLLPQTCYVWDKLQDSDQKIQYV)}*

$2156=263(266):
>gi|919044293|ref|XP_013405319.1| {|273(0)|<Brachiopoda(M)>}PREDICTED: uncharacterized protein LOC106170122 [Lingula anatina]
{(KNNRTTIKDIRDGLLAANLPRLSSAVAQYYAEMVTNISNMSPDEFESLCRTLTTAKSMCQNTPSSSTRGLDDTSLPLDPY)RHDVFIAYSG---EDEATIEPVLHQLKECGIECFYAKEDLA--LGTyviTSIIEA-
VNSSRhtIVFLSKSFIQSDWCQLELQYIIQNARKSKGHVVIPVVMNMApeEipG-ELSIWKYI---LFDD-
EELIPMFL-EAIKGPRDTPTYG(QLRHENEVLRDENADLREKNEILRDENEVLRDEVRHLTTKLRPQIR)}*

$2157=243(217):
>gi|918386125|ref|WP_052438400.1| {|0(214)|<Actinobacteria(B)>}TIR domain-containing protein [Streptacidiphilus jiangxiensis]

{(MASESWAEGASRPY)---FFLSYAHTPRPRAErhprdqdiWVRRLFDDLSET-
VMdltgVDSREAagfmdqglVLGEGWSDRLSDALATCRVFVPLYSPRYFR-
SQWCGkEWAAfasrstfslrgtdgAETAS-----RAIVPIQWVPMSpgSLPRTAAELQYLhDLFG--
PDYVTEGLYALSKLNYFRNAYE(FAVHRIAQRVVELAETTVTPVGPRRGSESFPDAFESARSPRRRLRIAVVA
PTVAALPADRSSD)}*

$2158=152(156):
>gi|918409801|ref|WP_052461067.1|
{|0(190)|<Bacteroidetes(B)>}hypothetical protein [Psychroserpens
mesophilus]
{(MKERLT)---VFISYSWDSDEHKEWVlklaNYLIEKAGCN-
VILDQFDLAAGKELTHFMENGLEKADKVIIILTKDYKKRAENRQgGTGFEYSLI-----
SQGLYDLQSKNDkfIPILRQGTKSTSAPAY--LGTKIYHSMKDDTKFENDAFNL(SRLVYEKPEI)}*

$2159=210(217):
>gi|918704120|ref|WP_052573153.1|
{|0(261)|<Verrucomicrobia(B)>}hypothetical protein [Haloferula sp.
BvORR071]
{(M)AHDVFISYSSKNKQVADAICSALEQRKMR-CWIAPRDVPPGGNYGEAIVHAIAGCQVVVLVYSAA-
VNLSAAVMrEAERAMHHA-----KPIIPFRLEDVPmqpGLEFFLASCHWLDAIH--PPMEEHIQ--
RLGDAVESLMGR(EVANPVPFPAAAAHIPVRKARVAWLPILLGGTLVAGGAALVAMKPWQGAPPAAAPPAANT
SPTEAAKSPSLAIALA)}*

$2160=270(270):
>gi|918729645|ref|WP_052588471.1|
{|112(146)|<Cyanobacteria(B)>}hypothetical protein [Leptolyngbya sp. PCC
7375]
{(ACYSDTQATAIYQHIDYVVGMGQAIGDTAAVQFAAGFYRALGAGRSYEDAYGFGCNAIALENIPEAQTPVL
RKKPAAVEP)-QQIFISYRSAepDLSLAGELHQALTAAGHS-
VFMAGESINWGENWVERIDQELKRSDYFLLLLSEQSVTSDMVAGeVQTAKRlqnkLGK-----
PIILPIRLNLPLddPLNPELRSGLQTIQQK--
FWQTPGDTPNLVDDILALIASG(QMPAAAAENSQQVVKPSTRKHPPLPVADLELPGGTMPLESAFYIRREPWD
)}*

$2161=238(239):
>gi|919027806|ref|XP_013397728.1| {|952(215)|<Brachiopoda(M)>}PREDICTED:
probable serine/threonine-protein kinase pats1 [Lingula anatina]
{(DYLMPCPDCSKAHARDPHMFSASSIRRATQLKAPFLQCTKYFHVIPLTDLHSMMPPESSTDYDLQLVQTVR
GLTDLRKDM)SADIFISYCAKDVPKKSEkrvapvdVCRDLEEAGYR-CWFQE---
DPSSQSLEVTAIALRDARVFLACISDNYIEDQDCCAmFKYACTTLR-----
KSIVLVVLGDSMnwQTSQIGLLVAGEVYVN--
MQQKDKYTSKLEELAKKLDMNT(LLKKDEAIDYPPVFISYAW)}*

$2162=211(222):
>gi|918244913|ref|WP_052375915.1|
{|0(95)|<Euryarchaeota(A)>}MULTISPECIES: hypothetical protein
[Methanobacterium]
{(MNY)-PKVFMSYSYLdKEFATKIANDLKKAE----
IDIWNDKwEIQEGDSlIYkIISEGIANSDFFLILLSNASVNSKWVKEELDAAIIQNIE-SITHIIPILIEPI-
D---------IPLPlRNLiwIEMYSNYD--
NAIKSLIKIIHED(YENYSLNIISKYFISSIGGLSKNASSVGSILLLRPDDKTGFEKSFSAKELQSLVPLTSQ
ELNDAINELEEYGLVKTLNY)}*

$2163=270(268):

>gi|918329650|gb|KOF94076.1| {|291(465)|<Mollusca(M)>}hypothetical protein OCBIM_22002797mg [Octopus bimaculoides]
>gi|918329651|gb|KOF94077.1| hypothetical protein OCBIM_22002797mg [Octopus bimaculoi
{(ALCYRRQAEDVILVPLYTETMENLPPVISNIQYMDCRLNENENIKEKFKSVSVDINKKLLPPLLSPTTAQAQTSKKTTYD)---IFVSYPTKnTEEMKIVYEILT--
EHNSDLKIFYDKmELETGSDWQqSIYKALECSKCCVILIAPAYFESLMCQEEFSIAIALsiseEST-
NPMVLVPVCIADV-
PSTPQWFGPLHMIDARGdcLVPVLSCLSPLLNKCYKNQTLQH(EFSWMMSKSKLNNENISLLDQLSLLRQIQLEDNYELKGKKITAKVNYD)}*

$2164=226(229):
>gi|918570515|ref|WP_052515886.1|
{|39(0)|<Bacteroidetes(B)>}hypothetical protein [Phaeodactylibacter xiamenensis]
{(HFPDAAETQIAILLTGEMHELRHREMKGVISNADRSLARNQWVDKFLNLLGDAKNMVANNQSGKQSDAATTPESTPAPLR)---
LFLSYAEAdQDLKNRLDNHLTALKRGNLIDLWSDEQVLAGHDWGaETKRNIQDADIVLLLVSASFIASEHIWKsEVEKALARRRE-G-VIVVPVALKPV-DWEGLPFAGLQGLPRSGkpVTTYNDLD--
LGLLEVAKGVREV(VEYVHRQKA)}*

$2165=218(221):
>gi|919032457|ref|XP_013399883.1| {|384(0)|<Brachiopoda(M)>}PREDICTED: uncharacterized protein LOC106166029 isoform X1 [Lingula anatina]
{(ALVDIPTNALHRLINIKSLCLNSNRLPCEVVKGIQVIQESSPFVKVSLRENCKYFIEIRDAFRAKLHQYFEKDGKIDFES)--DVYVMHADhaEDyaLVDQEIVPHLEERN--
LKVTVNIQALRPGLPVSDQLVHFIESSRKILVVFTKNDVFEHTCKetlTKVKAALEKRKKEVSETSPIVLVEWCpKSKVPHEFKDLYVIHrR--------TPT-HekhFWPNIINAI--T(Q)}*

$2166=271(278):
>gi|918329650|gb|KOF94076.1| {|135(620)|<Mollusca(M)>}hypothetical protein OCBIM_22002797mg [Octopus bimaculoides]
>gi|918329651|gb|KOF94077.1| hypothetical protein OCBIM_22002797mg [Octopus bimaculoi
{(LLGTGNQGFGYIPVLNTMVESACNWMKIGLPLKCLKIVLHTSDINEISNSNKKCLERFKVLKKTWEKSTLSKISKKNHGN)--DVYISYcNTDSKKANLLKASLEKNNPNISIFNKrikvDQDVWPEK-VYSCMFG----
CKCIVIILTTSYLSSKFCQDEFNMALCYRRQAEDVILVP-LYTETMENLPPVISNIQYMDCRL----
NENENIKEKFKSVSVDINK(KLLPPLLSPTTAQAQTSKKTTYDIFVSYPTKNTEEMKIVYEILTEHNSDLKIFYDKME)}*

$2167=296(300):
>gi|919000392|ref|XP_013404162.1| {|287(121)|<Brachiopoda(M)>}PREDICTED: uncharacterized protein LOC106169291 [Lingula anatina]
{(ELYCQVLKQALDSALPEGAKDTYLLLKYQDEMEGGSKKYPIADLIVKAIRDGLNTEASGNTTQQEHGLPTEFEKVKVGEE)-YDAFVWHsDDDGEEVDRLVEKLESYGDR-QFRIIHR----
GKFEVDKMeTKSIEEalknSRNTILYVTKRFLEStpYLNTLLMHERLTDDIQQGRLTLVPVLVDVEPNELPSVLTNVIYIEKSK----MTEDE-CF-
GRLVKALEAP(ILKLKSAQPAGDIATGMAWSYYYGYLKLILPGLKQRIEASNYWTQGGSNTVVIPKLVLLLPQSCYVWDKLQDSDQKIQYV)}*

$2168=262(275):
>gi|919024826|ref|XP_013396261.1| {|102(0)|<Brachiopoda(M)>}PREDICTED: uncharacterized protein LOC106163266 [Lingula anatina]

{(ISLGIRTIREFKARLAGAGHQNLTVAAANHYAEIAKKITDMSADCYGTFCQAPLAHRHMISTAGCPTEQRC
EMSMETDSY)QYDAYLAHaSDDEEAVDEVRGQLESNG--VRCCEPP---
LPGSIKIKSMCESVEMAKRTVPFFSKTSLSD---
NVFLNICDVAEFEAKKRKedvVIPVTMDVGPESLPPVVRVRNCINFQS----Q-----
NFIPSLVDAIKGP(ACAPLYGQLRDENKNMKQEIAAKDDMIRELRQQLEESRALNRRLQAELEVTAPPK)}*

$2169=215(205):
>gi|1039682967|gb|ANN17067.1| {|0(723)|<Actinobacteria(B)>}hypothetical protein SD37_16385 [Amycolatopsis orientalis]
{(MTDPDPGLSDARSSDR)-FDVFLCFTR-AD-PDGAARVveLEAALRDAGLRVFRDTVIDEFA-
PItrELADAIAGSRVLLVYYSRILPTRYACQWELTAafVAARRHGAPADRVLAINPDpdsahlapVELADAKF
FTGPVTRETLPALVERVVRKVAEAGHPLGARNRAP(GRRPPARTVGRYRELWAVHSGLHAAEYPGMTPYSRPV
VMVRGLPGVGKT)}*

$2170=175(177):
>gi|919146726|ref|WP_052702358.1|
{|0(83)|<Proteobacteria(B)>}hypothetical protein [Marinomonas sp. S3726]
{(MRK)--DIFICHAC-ED-
KSSIIRPLVHSLSERGISCWVDEAEIFAGESIseKINEGLRESDFVVVIISRSFMAKNWPRKELSAVLNLEAK
SGRAIIIPILVGTQNEIDSVVEEFPLLNDKLFLLWNGDANRVANTVQMTVLKSKG(QLIEPQIKMHTCGHCST
PFQHGVHVCLGCHGTVI)}*

$2171=220(225):
>gi|919191879|ref|WP_052747380.1| {|53(0)|<Firmicutes(B)>}hypothetical protein [Salinicoccus sediminis]
{(LNSAIRDFDNANRKLNRSQGEKAELSKRLAKKERTLNDKRDRLMTLHYKHMREDIDEIESFYTNKEAEIQR
KIDYSKQKP)-FDVFISH-A-
TDDKDDFVTQLTDGLKDEGIRLWYDSDEIGWGQSIrqRIDRGLTNSRFFIVVLSTSYINSHWTNYELDGILQR
VAGSEENILLPIWHNISKEEI--------DAFsltlsDKF-ALNTGQHDVEEMIDALVSLVKH(NSVNV)}*

$2172=243(240):
>gi|919075229|ref|XP_013418738.1| {|163(0)|<Brachiopoda(M)>}PREDICTED: uncharacterized protein LOC106179580 [Lingula anatina]
{(PLPTAIPRERPPWNIIISCVSVGIVIIVSAAIVFIWALRKGKILRANLDARSRHFEGDNVLKEGDPKDEWK
ETAEIKGFY)-FHAFISYNEsvpEdrAFVKGRLIPELERRGF--RIYKPDERVGEIARQKEDI--
GIATSSRFIMIVSPEYFD-MDGVNDLCGRVRKKLNDNKIRIIPIILKNFDrNlKLNKDFASVRKLM-
KQpCFEKPNEDAMdkFFNDIADFMPKLE(NQAERAGGDGLHQEQIELDV)}*

$2173=255(248):
>gi|919075267|ref|XP_013418756.1| {|137(0)|<Brachiopoda(M)>}PREDICTED: uncharacterized protein LOC106179590 [Lingula anatina]
{(PLPTAIPRERPPWNIIIPCVIVGIVIIVSAAIVFFWALRKGKILRADLNAWSRHIDGDNVLNEGDPEDELG
ATLEIKEFY)-YHAFISYNEsakEdkAFVKGHLIPELERRGY--KIYKPDERTGKVVKRPENI--
GINASSRFIMIVSSQYLD-
MDGVNDLCGRVRNRLKDNKLRIIPIIMEnfDFDhYLLqrndGTDYTSVRKLLEQPrLKKPNEDAMDKFFDDIV
DYMPKL(KNKADGEGLDQERIELDVENDGNVHKRY)}*

$2174=211(220):
>gi|919083201|ref|XP_013378957.1| {|202(0)|<Brachiopoda(M)>}PREDICTED: cortactin-binding protein 2-like [Lingula anatina]
{(NVQKLKKWAISRNCTTIRCLQAKLRTANLPRLSSAALDYSAMVVMQISDMTSNEFEALCRTLTTASGLSDT
SSSLDHRRC)--DALIVYSGNdkD-VVEPVVQQLEEQGV--
RCCYSGHDFRLGNTGKESFEEALCESKCTIVFLSKSSWNNQFCKKKWIMALSRAIHKREYAVIPVIF-
DLPrHQIPS-------YLKYIkPISAESKNLIPKLVEAVKDKISN()}*

$2175=205(232):
>gi|919061975|ref|XP_013412637.1| {|0(273)|<Brachiopoda(M)>}PREDICTED:
uncharacterized protein LOC106175270 [Lingula anatina]
{(MENRKRPSLTADH)-YDVFLAHSFKdqYAVEQTL-QMLEERG--
LNCCYSRRDFLPGYSTIACIEDAVLAARFTLVFVSREFLTSPWCQRERQIA-
EEKAAETSDVVVIPILVDVHkKELPISFKAL------TYVE-------------------
M(ADPGAFIKLCLRLGVPDLPLRPTYKQLEDQITELKTEVRYLSDNAQHHQRLYDTLQCRLQDSNMMVKRLQR
ELHNLRKV)}*

$2176=217(225):
>gi|919062297|ref|XP_013412800.1| {|763(0)|<Brachiopoda(M)>}PREDICTED:
uncharacterized protein LOC106175383 [Lingula anatina]
{(NQDSPPTPPASTSSCEESSSAPAPSTEDKKTKDPGKVTDFEENVIQKDLNLENKKVERCDSNSESIPPPPC
KAKNKKGKL)-YDFYVIYSEEedsDWVSYHLLTTLEGTKG-
FKGCVRERDFIPGTDYVDSIVESIKNSFKVLVIISGKKTNDKWYNYEVDQAIHQKLDSELDSfVIPICREDAN
kSDVP---------LKLKtVIACSMVSAHD-WEKLERAL--D(DDNSQ)}*

$2177=241(268):
>gi|919066846|ref|XP_013414964.1| {|72(0)|<Brachiopoda(M)>}PREDICTED:
uncharacterized protein LOC106176922 [Lingula anatina]
{(TKNNCTNIRDLKVGLQTANIPKLSRAAGDYYARVVTEILNMTLNEFEALCRTLVTARHMHTSRSTITNQQG
ASLSLAQCL)-YDLFIAYSGEdeATIEQVLHQLKE---
LGVKVWYAKEDLVPGTQVITSISKAVIASKHTLAFLSKSFVQSQWCQLELQYAMQEAVKSKRDVLIPVVM-
NMSpEEIPTEIGGLKYIL------------------------
F(DDEELIPKVLQVIKGPQDTPTFGQLRGENELLRDEVRLLTSKLRLQSQ)}*

$2178=190(220):
>gi|919071902|ref|XP_013417256.1| {|737(0)|<Brachiopoda(M)>}PREDICTED:
protein toll-like isoform X3 [Lingula anatina]
>gi|919071905|ref|XP_013417257.1| PREDICTED: protein toll-like isoform X4
[Lingula
{(RNLTFIATCNDTTLTNFDFTEQCDKTKFILNVVALAVVLVVLAVFIALVAITFFYRTEVEVLIYNRYKQFS
RDDSDTDKD)-YDIFISYSNDdsVFVRNVIIQKMET-
EWGYKLCIHERDFLPGEYIADNIANAVEKSRRTLTLLSDSYLHSEWCVFEFAMAHQQSLKDRCRRLVVVKLSD
------------------------------LDSNLLAKENMG()}*

$2179=287(282):
>gi|919076315|ref|XP_013419237.1| {|550(0)|<Brachiopoda(M)>}PREDICTED:
uncharacterized protein LOC106179956 [Lingula anatina]
{(QTASSNVSVEIRELLVSEPLGTNEPFHIDNRAAIATITGLALLFVVTVVVLPWVVYQKRFIIKLRAKRYFG
KYEDTDDKL)-YDVFVSYCpdSVdeIFVVRTLVPKLEQT-
YNMRVCVDYKEFLPGEVLSVSVIEAVESSRRTILVLSPRFIENGWCRFHFLSAQLEMLQSKHS-
IIPLIYEDI-SKISSTDKDLRNILKIAkPVRWpserrprTENN---
FWDHLHLLLPKK(RKPASGFRVNFSRWVFASNKVDYVSTDLSYDNSRPKSRSERSNGVISPADFTDLKLHIED
IV)}*

$2180=256(259):
>gi|919083192|ref|XP_013378953.1| {|73(0)|<Brachiopoda(M)>}PREDICTED:
uncharacterized protein LOC106150601 [Lingula anatina]
{(KNNRTTIKDIRDGLLAANLPRLCSAVAQYYAEMVTNISNMSPDEFESLCRTLTTSKSMCQNTPSSSTRGLD
DTSLPLDPY)RHDAFIAYSGEdeAIIEPVLHQLKECGIE----
CFYAKeDLALGTFVITSIIEAVNSSRHTIVFLSKSFIQSDWCQFELQYIMQNARKSKGHVVIPVVMN---

MAPEEIPEELSIWKYILfDDEGLIPMCLEAIKGPRSTPTYG(QLRHENNVLRDENKVLRDENEVLRDEVRHLT
AKLRPQSR)}*

$2181=231(237):
>gi|919083194|ref|XP_013378954.1| {<Brachiopoda(M)>}PREDICTED:
uncharacterized protein LOC106150602 [Lingula anatina]
{(MVTNISDMSPNEFESLCRTLTTAKSMCQNTPSSSICALDDTSLPLDQY)RHDAFIAYSGEdeATIEPVLHQ
LKECGIQ----
CFYAKeDLALGTYVITSIIEAVSSSRHTIVFLSKSFIQSDWCQLELQYIIQNARKSKGHVVIPVV-------
MNMAPEEIPGELSIWkYILFDDeeliPM---
FLEAIKGPRDTP(TYGQLRHENEVLRDENADLREKNEILRDENEVLRDEVRHLTTKLRPQNR)}*

$2182=249(259):
>gi|919083196|ref|XP_013378955.1| {|188(0)|<Brachiopoda(M)>}PREDICTED:
BCL-6 corepressor-like [Lingula anatina]
{(KNNRTTIKDIRDGLLAANLPRLSSAVAQYYAEMVTNISNMSPDEFELLCRTLTTAKSMCQHTPSSSARGLD
GTSLPLDPY)RHDAFIAYSGEdeATIEPVLHQLKECGIQ----
CFYAKeDLELGTYVITSIIEAVSSSRHTIVFLSKSFIQSDWCQLELQYIIQNARKSKGHVVIPVV-------
MNMAPEEIPGELSIWkYILFDDEE---
LIPKLLEAIEGP(RDTPTYGQLRHENEVLRDENADLREKXLGYDYLQFKEIK)}*

$2183=213(220):
>gi|919090461|ref|XP_013382283.1| {|711(0)|<Brachiopoda(M)>}PREDICTED:
uncharacterized protein LOC106153060 [Lingula anatina]
>gi|919090463|ref|XP_013382284.1| PREDICTED: uncharacterized protein
LOC10
{(RAYKMQTLQSVAMEAKITPSQYIGVNQETERAVTRRNDPQQSTALLTNELSVCDSITVPAEISYSIPDQPA
QARNAELEN)--DFYVVYADGdrDWVCYHLLTTLEVS-
LKFRGCLRDRDFLPGSKVLENITAGMANSMKTIVVISHEAVgDSNW-
QYELHQAVYEGLTRKASYVIPIVRETK-GQGEpSIPKELQVYVPSRrPTKDHD------WHKIECSL-
LE()}*

$2184=210(220):
>gi|919096060|ref|XP_013384746.1| {|279(0)|<Brachiopoda(M)>}PREDICTED:
protein LAP2-like [Lingula anatina]
{(ICVSENCLQDIPAEKLAKLPKLRLCCLHSNRLGQAVFQTLKKAKFHVRFGDNRLVDPKIAKDKIEAGCHMT
DVPSGKMPI)-YDVFILYSGgsEEdeKLINDVFLPGLEE-
ENELKVCVAFRDYIPGQYVSEEAISNMKKSRKIIALLTEHFDEQA-
KAVEINQAVGADQGRQSCSVIPVVSGNP-DYI-----KIPAQFEKIvPLRA--PV---DWDKLLTA-
IKA()}*

$2185=141(151):
>gi|919096074|ref|XP_013384751.1| {<Brachiopoda(M)>}PREDICTED:
uncharacterized protein LOC106154804 [Lingula anatina]
{(MTDVLSRETPV)-FDVFILYSGgsEEdeKLITDKFLPQLEDKA-
ELKVCFASRDYIPGHFELKEALNNMKKSRKIIALLTEHFDEQV-KAVEINQAVDADLARQSCSVIPVVWGNP-
DHV-KMPAQFKKIVPLRrPVDWD----------RLLTAIKE()}*

$2186=247(249):
>gi|919100085|ref|XP_013386468.1| {|0(698)|<Brachiopoda(M)>}PREDICTED:
uncharacterized protein LOC106155965 [Lingula anatina]
{(MDNFFLKGTKKRSTNGGGCHTPEVGNRSGSMRGFQVLSQNAYAGF)KYDLFICYTEEddDMVDHKLISFLd
qERSGGGQNTCTYEKkNFLPGRHNVENVCQAMKESRKTLLVLTSNFVKTEWCKY---
VAGYRFEEEFKNRILIVQLEEC-----

NISKRLRQYIHGVeILKYGEPPDDFYRTLQFFIDHDI(DTSTSSSFGRHLHMEGKKYDTGTEPNNDYYGEQFK
AALSDANYCSQNNTDLVDKAVAEGSLCSF)}*

$2187=224(252):
>gi|919066799|ref|XP_013414940.1|
{|2455(365)|<Brachiopoda(M)>}PREDICTED: uncharacterized protein
LOC106176900 [Lingula anatina] >gi|919066801|ref|XP_013414941.1|
PREDICTED: uncharacterized protein LO
{(QVAEKVYLNFSRPSLFEESMKELVSKLRLKTKKKKGVSVASDCFISYCWTNSASALKMGQVFRPKEGAIGN
GDPREIKEF)-------------------------LEKNGINCWLDVE--
RVGENgLfeDIAEGLKNTKVVVACVSDEYALSANCQMEFRFAhVALR-----
KPIILAVVGTGFQWAATEVGMLSLGYHKFNFQAPSETSHDQLLSAVREQIQA(TKAEEAVSHVPTSSKTSANP
SSAFQELYELAQ)}*

$2188=210(237):
>gi|919068623|ref|XP_013415784.1|
{|2552(335)|<Brachiopoda(M)>}PREDICTED: uncharacterized protein
LOC106177527 isoform X1 [Lingula anatina]
{(IGLLVAGELYVNMQQKDRYNSKLEELARKLDSNTLLKKDESVDYPPVFISYSWANSARAVQLGTRQVEGAT
GYGDPREYK)-----------------------DFLESHGIKCWLDI-
EQTGANGLfeDIAVGLQNAKLVVACISDQYALSANCNMEFRFAKTAL-----
NLPIVIVAVGSGLNWRQSEVGMLSINYPVIAHDQMEKLLALVKENLPKDDDQ(KTLIKKDGNEKKKHKEQ)}*

$2189=255(261):
>gi|919079936|ref|XP_013421118.1| {|1720(56)|<Brachiopoda(M)>}PREDICTED:
uncharacterized protein LOC106181321 [Lingula anatina]
{(LNGGSEGRDWTALAKRLGYKKNKIEMFNNDLNPALSLLTDWIISSGNTALSVDMLVTYLEQMHCDDVVDII
LKGQEADVD)PPQIFISYQW--DIQDEV-
KLMRDRLEKSGYHCWMDIGQMGGGDQLnaKIDEGIRSAKVIIACVTPKYIVSHLCNRELSLGDVLR-----
KPIIPVMFDKVPWPPPGGMALMFSQLVYINMKEVGGHGGSGVHADLEDRYSE(IMQQVSRFSTPVLPKVRASS
VKTNSDKSHNSQSTVGSESHF)}*

$2190=214(220):
>gi|919147016|ref|WP_052702648.1|
{|276(0)|<Proteobacteria(B)>}hypothetical protein [Pseudoalteromonas
piscicida]
{(QHKSQRQEVTGLVVNDGVNVNRKFIRKTRAMINEWRKDILAAEIRFIKERYHVDEAEIDVLKLDGNPPDFI
KKLKGIFNM)-YDVFICHAS-ED-
KEAVATPLYESLTKLGINTFIDSEAIKWGDSLvsKINTALKKSKYVVAVISEESINKSWPMKEINAVLSLE--
---IGEENTKLLPLMVGDGDALLKQLPLLGDKLFESFNNNAEEIALKLAERLQKN()}*

$2191=214(220):
>gi|919141535|ref|WP_052697167.1|
{|317(0)|<Proteobacteria(B)>}hypothetical protein [Vibrio vulnificus]
>gi|948523235|gb|ALM70781.1| Retron-type RNA-directed DNA polymerase
[Vibrio vulnificus] >gi|103
{(GLAQAEIRFLKQRYHLTDSEIKTDGLDGSIFKRAVYGHLSFIRMVKGEEHSPYLKLCEQVLALDSKPPEFI
RKLKGVFDM)-YDVFICHAS---EDKAEVAlPLYDALAKREIKAFIDCFAID--WGDslvSKI-
NTALKKSKyvIAVVSDESVKKSWPMKELNTVLAAEISSEETKLLPLMVGDGeellkEIPLLGDKLFVNFND-
NI-DEIADKVE-KLLQRS-------()}*

$2192=168(175):

>gi|919129030|ref|WP_052684662.1|
{|0(146)|<Actinobacteria(B)>}hypothetical protein [Lechevalieria aerocolonigenes]
{(MT)---VFISHSFDnQPEFENVTDKLDQLAVPYW------
HPSEIKTGSAslsdQLRAAVQQCSVCIFVATRASVKSSWCGAELGAFWGAGVP-
IIVYLAEASLPEQELPPILQGGVWERKLSVV--
ASRAAELRSHGRNISPRPSTL(VSTMNAQQLEKVVTGAVSLVLAQQAKEPAQFDV)}*

$2193=202(220):
>gi|919196777|ref|WP_052752278.1|
{|58(0)|<Proteobacteria(B)>}hypothetical protein [Nitrosomonas communis]
{(RLESNGWGEFTIYIDIAYKDGKHQQLSHDLRLTSEIDSEDNTEQEGKELISDIGKVMPVLKNTAQAFSRKAIDVTVKHLP)QKMIFISGGAAdAEVLGHLRESLKKLNL-------
CIKDATDVPAGIplqtYIDSLIAKADVVVFVISGR--PSLWLCQEIESAKHSNKP-LIPILV-
GQDSHLPASLSK-YQYFRIDNLNN--ITDLAHK--LLKY-------L()}*

$2194=213(217):
>gi|919937893|ref|WP_052914348.1|
{|0(67)|<Actinobacteria(B)>}MULTISPECIES: TIR domain-containing protein [Frankia] >gi|1139649149|gb|ONH37477.1| hypothetical protein BL254_03315 [Frankia sp. BMG5.30]
{(MCV)--KIFISWSGdTsKQIAEIFRDWL-PILIHEIDVFVSSQD---
IGGGTraldVLADELDATSYGLIVTTKATQSAPWINFESGALakHVAKSR-VVPILIDLGVSDLKNPL-
AQFQAIDSRQSDA--MVKLVRE--
INAEASPRPVVD(EILRKRFEAFWPELDEKLATVRSTLGKTSGEPAITEKEMMAEIRAEVRRQSHQIFEISARISDALPKGERQIIF)}*

$2195=213(195):
>gi|919165467|ref|WP_052721098.1|
{|0(65)|<Actinobacteria(B)>}hypothetical protein [Actinoplanes rectilineatus]
{(MPRDEGP)ASRAFISYAHDSGAHRDRVRafWHFLRTVCN-V--
DAWcDFSAEasrTEWTRTMESAMDAAEFILVVASPQYrlragpdlpptLESGRGVAyEasllrdrsYRDPVGAL-----RQILPVVLPGCSadDIPGFLLRHSTTHYPV--
SSFTVAGAEQLVRVLTHQPAEI(VPDLGGRTPELSARPVPDGERDRLVAALRTLPVMSSPEAREYFATMVA)}*

$2196=164(172):
>gi|919473029|ref|WP_052832920.1| {|0(46)|<Proteobacteria(B)>}TIR domain-containing protein [Bradyrhizobium sp. LTSP885]
{(MSDAMVWKANRL)--RVFISHLSSKQAEAASLKVALAEYGID-
AFVAHADITPTSRWENTIEDALRTCHSLVALLHP-GFHKSNWTDqEVGFVMGRN-----
MPAFAVHLGEAPygFLAKFQAFPLKGGDLS--
KLAHTLFKAYCRHEQTAGRLAT(YTALRFADSSTFAAAKSRFS)}*

$2197=180(186):
>gi|920158799|ref|WP_052960249.1|
{|0(237)|<Actinobacteria(B)>}hypothetical protein [Mycobacterium sp. EPa45]
{(MRAL)AHDVFISHSSVDKPAADAVCAALEAKGIR-
CWIAPRDVKPSQSWAAAIVAAIREARIFLLVFSRNANESDQ-VQrEVERAANSG-----
KQLLTLRIEDVApeAALEYYLNTPHWLDAI--
TPPFEAHLEKLADACVSLLAWL(DSADDASAAGGGQVGGAGSRRQSKFTADRPPVRWWHRQRRSV)}*

$2198=285(297):
>gi|919066022|ref|XP_013414543.1| {|307(191)|<Brachiopoda(M)>}PREDICTED: uncharacterized protein LOC106176622 [Lingula anatina]
{(KTARHLLAPFYIESIPTFPTYMCLVQYMDFSLVQYMDFRTIDTKPQSVVQGCKTFVSLLSAKDITGQQNHQ
QAHTNYDYD)---VFISYSHKnTDKANFFLNTLTRL--
NPHLRIFYDRnELKAGTSWQqMLYHSVDGSKCVIALFSPTYLSSAVCTEEYNLALLKHMG-
ETFdgQLIPVLIEPV-G----------EVPKTHssVKAIDAMG--
DAFQSVAVAICKS(VVSWLKTEEMNDLVFNKVPVSLNVSKILETRRLHYFKERLELFNNQTKAKYNEKSEQPQ
SDSKENVPPKAEKTSPED)}*

$2199=246(249):
>gi|919077524|ref|XP_013419885.1| {|299(284)|<Brachiopoda(M)>}PREDICTED: uncharacterized protein LOC106180448 [Lingula anatina]
{(NLAVCCNRKTARHLLAPFYIESIPTFPTYMCLVQYMDFRTIDTKPQSVVQGCKTFVSLLSAKDITGQQNHQ
QAHTNYDYD)---VFISYSHKnTDKANFFLNTLTRL--
NPHLRIFYDRnELKAGTSWQqMLYHSVDGSKCVIALFSPTYLTSAVCTEEYNLALLKH-M-
GEAIDAMGDAFQS-
VAIAICKSVVSWLKTEEmnDLVFNKVPVPLNVSKILETRRLH(YFKERLELYNNQTKAKYNEKSEQPQSDSK)
}*

$2200=244(219):
>gi|919121471|ref|WP_052677109.1| {|0(56)|<Actinobacteria(B)>}TIR domain-containing protein [Terrabacter sp. 28]
{(M)--KVFISWSGQrSNKTARLLyDWLPQLINE--
IEPWmSDEDIKTGGDWSdAIKVALNEAAFGIICVTPG-NRSREWLLfEAGALSRQVSD-STAHVAPFLIGFE-
DKSELryPLARFQAtmptksdmrkllmsintlaqMPRTDkqIDNaldvfWPRFE--
EEFTVIRDSVRTQ(PSHRRSDKDLLQEILILVRGLDKRVGESPEDRFNPYQAALRDRLRTRTVTPAYTEAVSL
SDFFSSSNAIRVKLDEMAH)}*

$2201=230(229):
>gi|919589807|ref|WP_052887560.1| {|156(0)|<Chloroflexi(B)>}hypothetical protein [Thermogemmatispora carboxidivorans]
{(ESGCVCGMIQYSLGLNSERGGQGLQARVILAQLPDLVNHQLVAHRQNRRWLELLSVEQRQRLGQCCPQYQP
LLQQNSKAL)--KIFLSCSsdRRdRQLRKELEQQLSIFKNEHLIESFHSEQLGPGQERIkS-
QRQLEEADIILLLISPAYVASEQCYNkEMQQAMRRHEA-GTARIIPIILRPT-
AgLASTPFGKLQALPRYSpsITESSNRD--ATLAEIADELLQI(IQELKSKQT)}*

$2202=233(231):
>gi|919589886|ref|WP_052887639.1|
{|0(1293)|<Chloroflexi(B)>}hypothetical protein [Thermogemmatispora carboxidivorans]
{(MQSGAAASRSLH)---
VYLSYAGAdQSWGERLERRVRLAAREAGLEAprlSGPRQILAGSDYQqARQAALKSADLILLLISPDFLDSSD
CLDEMEAALQRRRA-EGVVVVPIIVRPV-AWEHTALGELEALPpgQPPkaLSLWRARD--
EGLHRVEEGLRRL(FSERLRFGRQASGARQFSPEQEQALHALLIDHRPFIRSRLEGFVGRSAELVAIRRQIAG
LQPYGGYLVISGQAGQGKSS)}*

$2203=221(233):
>gi|919590149|ref|WP_052887902.1| {|0(373)|<Chloroflexi(B)>}hypothetical protein [Thermogemmatispora carboxidivorans]
{(MEASQSTAREILCL)-------YAIEdKRGFEELSKHLYNL--NGIRDI---
QNTPPGKPPKqEQSKLINQAKLIIMLISPDLLSS---LReEASTALKQAKE-GNAELIPILYKTV-
DLGGTDLAELQLLPRNGeaLAqrEGDNRN--

KAWKEIAQEIRKI(WEALDAGSGTEEKEKTTVNAGQSDAHRSQQQEQDPTRPATVFISSAGDAGKNREQLELM
LRLRGLKLWHRDSEDLPFLG)}*

$2204=225(229):
>gi|919592668|ref|WP_052890421.1| {|0(218)|<Chloroflexi(B)>}hypothetical protein [Thermogemmatispora carboxidivorans]
{(MTVNLFYLCAR)--------
SDDdERLLDQLKTFLRPLRQAGLINDWGPRQLRAGQLIReELRRRLNEAQIILILLSPDLLvDSLYPELrDLA
QARYLQEP-ERVRLFPVKLREC-PYELEPFADLVIFPSNGraLHALPPaqRE--
RELKNLALQIRAL(AEELATGEGGQERPGPAAASAATAFGPASHAGGSQTAESRRSGSTLSAQRCDVLLVTAT
DVEVRAVREVAVNEWGARF)}*

$2205=221(238):
>gi|919592691|ref|WP_052890444.1| {|0(336)|<Chloroflexi(B)>}hypothetical protein [Thermogemmatispora carboxidivorans]
{(MERPCAIAVLVSWSGTVT)----------DGTWHERLLRLLRPLAAQG----W----
IALGDDGPpSIDSAggPSGAALFLVLLSPDYLAAPE-RVaELEQVQQRHAS-GELVLIPVVLRAC-
AWRESAVGDLTPLPADGqaLTSRDDPE--
EGLRQVERGLRCA(LGSLLFARARQHDASAAELLRFCSCLAAAPVPESLLTEGLARLPHPLATTAADPSALTA
ALAALEADGLLTRDPQSGNLQ)}*

$2206=225(227):
>gi|919593360|ref|WP_052891113.1|
{|0(1004)|<Chloroflexi(B)>}hypothetical protein [Thermogemmatispora carboxidivorans]
{(MTGAETKTAL)---
VFLCAAARdRRFVEELRNHLQAPALRSRLHCFASLDVPAGRVPSqEVEQVARQAQVAVVLVSAELLSGRKEEQ
kVLNVLWRRGQV-GGLLLIPVRARAC-SLEGTPLEKLKFMNEKGcaELRRAERD--
QLWVRVAKAICAM(LEADEEGQAEAAREMGKEGLEALCSRVEERYRRALLGDRSLTLLQVLGMRTAFPIERIY
IRLRLHEKPEIRYLGPEE)}*

$2207=183(191):
>gi|919942886|ref|WP_052915573.1|
{|0(320)|<Actinobacteria(B)>}hypothetical protein [Mycobacterium haemophilum]
{(MA)---LFISYSSQdRLTVDALTTAL----
RRGQQQVWFDQELGGGDAWWsKILEQIRSCDVFIVALSSNWLQSK-PSQaELRYAQALKRP-----
ILPVRIGAV-
DsMRVNPLATLQIIDYTNptVDAGIQLVTAVHALRATPVPLPD(PLPDEPPVPFGYITRLGNTLAEKELSPQQ
QIQLLVELKAGLAEDGDDPS)}*

$2208=152(153):
>gi|920137676|ref|WP_052954931.1| {|0(132)|<Proteobacteria(B)>}TIR domain-containing protein [Microvirga vignae]
{()MADIFISYARED--RDRIIPFVRLLEAQGW-
TVWWDRKIPVGKTFEqVIKEQLATARCVIVVWSTEALGSGWVIDeAGEGRKRDILL-PVLFDRVEPPFGF-
RQLQSANLSDWHGQADHpeCQQLIADISGLLGGGSKSSYPLP(EPRAEDYYTSMDD)}*

$2209=263(221):
>gi|920566099|ref|WP_053002631.1|
{|746(0)|<Bacteroidetes(B)>}hypothetical protein [Kordia jejudonensis]
{(KSVGITKEENIVIGKELFTRNAFPKNIKVSLDGDYYISISKLEVCIDSNEYHIQLANTKKRTKLFPLNQFQ
LFTEKTIKK)MKKIFISYSNAdIYYKKELEKFLKPFQKFQLAKSWSCEEITPG-
LWDdQIQEELESSDIVIFMMSMNFASSDYILKdEVyNTFQQMAEK-PEKKIVCVLVKQF-

PWNFFSSSSLKnifnikdgdisdadkagfalaNLPTNqfipfytdtssenksdhkryLkpINQWKFEE--
EAYTQIIEILGKI(I)}*

$2210=141(140):
>gi|920673302|ref|WP_053046437.1|
{|0(435)|<Proteobacteria(B)>}hypothetical protein [Thalassomonas
viridans]
{()MKKVFISYSHEsDKHKAWVAK-LAAKLRQQGIDAMIDRYAIPEEPWPqWMQNQILNSDITLL-
VCTKEYRLRFEGKsQAKGVTWEGLI-ANQILYDNRCKNK-
KFIAVLPSSEDIEFIPIpyKGYPYFVLYEQFKSLLRYLDGKA()}*

$2211=275(279):
>gi|919066022|ref|XP_013414543.1| {|142(366)|<Brachiopoda(M)>}PREDICTED:
uncharacterized protein LOC106176622 [Lingula anatina]
{(IAPLLASGVQSFDPKMMLKAMLDAAVNWIKAGLPMSCLKLVVFAHRPDEINLEKHELWNVFESMKKKYETV
TMMPEEPQI)RYDIYLSYsIEDEDKVKQIEGVLKEEKKDIRIF-
SQVQALREDQVWQQEIYEVMItCVRVVTFLSPAFLASPECLEKYNLAVCCNRKTARHLLAPFYIESIPT-
FPTYMCLVQYMDFSL----
VQYMDFRTIDTKPQSVVQG(CKTFVSLLSAKDITGQQNHQQAHTNYDYDVFISYSHKNTDKANFFLNTLTRLN
PHLRIF)}*

$2212=325(293):
>gi|919073813|ref|XP_013418114.1| {|242(55)|<Brachiopoda(M)>}PREDICTED:
uncharacterized protein LOC106179130 [Lingula anatina]
{(RMSNGIGRGTEVKARLEYGEENPQMPFPIWICIVPATTLVLLLIIVSLIWVKYNLEFRLCYRSIKIRVLKE
KDADADRHD)--DIYICTcPDstnERQFAETILkEKLEKEGYSVRL---
DQDVLGFQSENREFTKAFDECRRYVILISPELLNpvPDYFDFFIDNAL-----
NQHTRIIFVIfgeiniddwnrfaetqIKNCKNPqlLPKVLKmpFLNCLQYYP----
DAEREErpksrWryickkskrqknerFWKKLQLCLPPP(SEKKDPSGHDVVDGRRKDLSSESTERPLLEEDRP
DPVPVDVGQTRIEVILDDEAEDDVFNEASDQNAVASNTG)}*

$2213=275(279):
>gi|919077524|ref|XP_013419885.1| {|142(412)|<Brachiopoda(M)>}PREDICTED:
uncharacterized protein LOC106180448 [Lingula anatina]
{(IAPLLASGVQSFDPKMMLKAMLDAAVNWIKAGLPMSCLKLVVFAHRPDEINLEKHELWNVFESLKKKYETV
TMMPEEPQI)RYDIYLSYsIEDEDKVKQIEGVLKEEKKDIRIF-
SQVQALREDQVWQQEIYEVMItCVRVVTFLSPAFLASPECLEKYNLAVCCNRKTARHLLAPFYIESIPT-
FPTYMCLVQYMDFRT----
IDTKPQSVVQGCKTFVSLL(SAKDITGQQNHQQAHTNYDYDVFISYSHKNTDKANFFLNTLTRLNPHLRIFYD
RNELKA)}*

$2214=256(264):
>gi|919083222|ref|XP_013378964.1| {|278(0)|<Brachiopoda(M)>}PREDICTED:
serine/threonine-protein phosphatase 6 regulatory ankyrin repeat subunit
B-like [Lingula anatina] >gi|919083224|ref|XP_013378965.
{(KNNYTTIKDISDGLLAANLLQLSRAAVEYYAEVVKKISNMSPDEFEALCRTLTIAKYMYTPSSTTCGLKST
SVPLDQCHQ)--DAFIAFaEEDEPTIEPIVRQL--KKLDIQCWYSEEDLIPGRYEYKET--
VMAIrSSRIsILFLSKSFIQNLGCNYVMENII-RNATEKKHGVIPVVMNITPEEIPDGLRSWKHFFFDD----
QELVSNILAAIKGPENTPT(YGQIRHENKVLRDQNEVLRDDNEVLRHKNKALLEENEVLRDAVR)}*

$2215=241(247):
>gi|919083251|ref|XP_013378977.1| {|213(0)|<Brachiopoda(M)>}PREDICTED:
E3 ubiquitin-protein ligase HACE1-like [Lingula anatina]

{(KNNYTTIKDIREGLLDANLPRLSRAAVQYYAEVVENISNKSPDGFEALCRTLTTAKYMYMCTPSSTTRGLKGKSVPLDQY)RQDTFIAYaEEDEPTIGAVVREL--KKLEIQCWFAEEDLIPGKYVNDETVKAISTSRFSILFLSKSFIQSRGCKYVMENI----IENAKEKehgVIPVVMNISPEEFPEGLRNWEPLFFDD----EELIPKIVATIKGSRDIPT(YGQLRDENEALRDEVRRLTAKLCLQSQ)}*

$2216=207(222):
>gi|1044539366|ref|XP_017435463.1| {|0(1132)|<Streptophyta(V)>}PREDICTED: disease resistance protein At4g27190-like [Vigna angularis] >gi|920686826|gb|KOM30809.1| hypothetical protein LR48_Vigan01g036
{(MASS)---VFVSFND-EETRD-LTAFVLDPLRSRGIQVFV----KGESTTFDLFQVIERSRLFIVVLSKNYASSICCLRELVAIVNAVESSPR-FLLPIFYGF------HQ---YGQAISKHEERFREhkermEEV-QRWREALTRVAGF(PGLLMENAKGFGGFDFVQYAVDILSREFSTPQNETIFNRYGNFEDMVQYKDGDEVSYKELRGLIRLRDFISLVLTYPG)}*

$2217=274(300):
>gi|922547690|ref|XP_013601762.1| {|737(123)|<Streptophyta(V)>}PREDICTED: disease resistance protein RPS6-like [Brassica oleracea var. oleracea]
{(LESFPHISRNISNLVLDETKIEEVPERIEDISGLNYILMTGFSNLKHFSSNISKLKHLEMMLFAVATTTDNSGIVHNRAN)--DVFLSFHR-EDVHKAFVSYLQKEFM-------------RDQD-LstNVVNEIRSSRIAIVILTDNYMGSKRCLDELVEIIKCSEE-IGQKVIPVYYGVDTVILSKQIQDI---------VKTESYP-KKLRQAFTAVTQL(QGYNFSTWDPENAVSQMLADDISFAPKELSDVEALKLQFNGLMLMEKLLFCHIACFFNNETYENVTQLLKDSLFDVGGSL)}*

$2218=133(164):
>gi|920683776|gb|KOM30433.1| {<Streptophyta(V)>}hypothetical protein LR48_Vigan1693s000100 [Vigna angularis]
{(MGKHGEEEEYF)RYDVFISFRG-EDIRHTVLRQLREGLGRRGILSFHDDRDMRiGTG-LslALREAIAESNVFIVVFSENYASSTWCLDELDKIIQRTHM-N------------------------------KFGKESQRV-QAWRSALSEAANL(PGMHITPGDSNQS)}*

$2219=226(227):
>gi|922527916|ref|XP_013596731.1| {|0(481)|<Streptophyta(V)>}PREDICTED: probable WRKY transcription factor 16 isoform X1 [Brassica oleracea var. oleracea]
{(METSSVN)QHDVFIHFSL-EEFRYSFASHLSAAFSRSSISVFVAEDTSGAAV-TsntdfqlpdATQSAIEGTKFIVVVFSKKDAFSPLFLETLVKFLERRKD-GLVVVIPVFYgDVTLSMIEQKMESFGEAFSNHLQG--------RWRNGLIEAANL(RGHKSSDQRNDSELVDEIVADVREKLYPTGTIGFYSRFLGIENLLRKQSHDIYRLGVWGTPGVGKTTIAQTAFNLMSQDF)}*

$2220=217(234):
>gi|922544487|ref|XP_013600025.1| {|0(203)|<Streptophyta(V)>}PREDICTED: putative disease resistance protein At4g11170 [Brassica oleracea var. oleracea]
{(MSSSYSFLLAGREL)--DVFLSFSG-KKALDMDFGY---DLSRNGIKSFKSESWKEKsfKP-IdrQTLEALTESKVAVVMTSDEEASSVGFLEELLVILEFQEK-RSLTVIPIFLTKRPLDMEEVSHLF------------PERD-RMWRTVIAKLENI(AAQYSLSRNLAVIHGTHRLKQVADDVRLMFLSCASSDFKGLAGMDRHLKAFHELLALDSDKEVRTVGIRGCAGVGKTTLA)}*

$2221=199(206):
>gi|922560612|ref|XP_013608586.1| {|0(1087)|<Streptophyta(V)>}PREDICTED: protein DA1-related 4-like isoform X1 [Brassica oleracea var. oleracea]
{(MEFMKPKAARVMPPMMSEYRHSVNII)--------CE-
ESVQYHIVSHLCAALRREGISVFVDSCgSQETKP-FsvEQNQAVtDGARVSVVVIS-NKAESygPWVV-
KFLKAIKRRRN-
SDHAVVPVFYGVDPLTQVYGWSHGRLEAEKLTSHQSRISSNRILTDSELVEEVV(RDVYEKLNIAERVGIYSR
LLEIENMLYEQPMGIQSIGIWG)}*

$2222=186(203):
>gi|922565344|ref|XP_013610932.1| {|0(543)|<Streptophyta(V)>}PREDICTED: probable WRKY transcription factor 16 [Brassica oleracea var. oleracea]
>gi|922565346|ref|XP_013610933.1| PREDICTED: probable WR
{(MTACNK)---VKISFdrSD-ETIRCSLVPHLSAAFGRKGISVLTDKHDE-----------
FCKCIASVFIFSKNYVSIKESLDDFFKTSQRRHD-KVHVVATVFYGVNRSELE---
GNFAKALFEHPTSYQASQLRSAEEIITLPGHEK(INKQSDCEFVEKIATDVYEKIFPKERIGIYSRMLQDISN
LLCSQQWGVKSIGFWGMP)}*

$2223=190(195):
>gi|920681523|gb|KOM28317.1| {|0(506)|<Streptophyta(V)>}hypothetical protein LR48_Vigan521s000500 [Vigna angularis]
{(MASLFSLSFFLKLV)SGEVFIHCLE-DDLHENFVSHLMSGLLQAGVEPSLVAVGVLSEK---
FMPSITRFQIGIVVFTKAYIESSRCVEDLLRIIECHET-
YGLMIIPVFYDTDQSFLVRVSEENFRNARRAQRRAEAQAKIRITADSGEIYDKR(MRPKAKTMQNFSLSRALN
RVRNLPTWDESKHRDDAELVEEI)}*

$2224=208(211):
>gi|920719229|gb|KOM58426.1| {|0(142)|<Streptophyta(V)>}hypothetical protein LR48_Vigan11g146000 [Vigna angularis]
{(MEFASFSSSSSSSSFLKSEPQFIY)--DVFINFGG-EDIGRKFVSHLHSALLLAQVKTFISKENLQEGI--
-KleehMRAIASSKIAIIVFSKKYTESTCSRLELEKIIECRQT-
FGQIVLPVIYDIARLDEHLQKVVRVLEKAAPGSYSGEQLDHAMSRHDAEFVKEI(VRRIQTLLDYTDMSITQF
SVGLESHVEKGTKAIQGLSLKLHSTGRDY)}*

$2225=280(300):
>gi|922415867|ref|XP_013597384.1|
{|263(1031)|<Streptophyta(V)>}PREDICTED: disease resistance protein RRS1-like isoform X1 [Brassica oleracea var. oleracea]
>gi|922415869|ref|XP_013597387.1| PREDICTED
{(FVSFQRDRKDSSKLFIGCRKSDESFPNPTSPGASIKTVEMREKLKGVETPFQPKLSEHSVTGSDPVLVIYC
GDKQVSEEI)----------------YLISYIFNELCRCGFSPLRYDmKKGTVTE---
NQKLLHISRVGIIIFSMNFAHSRECLDGFVAVMDHLKA-
NDLVLIPVFFKVSVSAVRGQLGSFGRAFIRLRNSVKASEV-
LKWRAAMIELTSI(SGYAYKKGDEVILAKNIVRAVCLPLSSETNMKLRGTRLSLESILSLLNRSQPSSPQIVG
LWGMAGIGKTTITREFFRTQA)}*

$2226=280(299):
>gi|922571643|ref|XP_013614149.1|
{|286(669)|<Streptophyta(V)>}PREDICTED: probable WRKY transcription factor 19 isoform X1 [Brassica oleracea var. oleracea]
{(PAPAALQLDPCQDTFPCHDLAPPTISFGDGGAAPRDCIYLLPDSVTSSETLEQPRVSGYSITGVDHILVIS
CGYNQSSEE)---------------RYFISYIFNELRLRGFAPLGYDLSRSPvNG---
NQDLLDKSQLGIIIFSMNYVSSRECMDGFVALMDHSKA-

NELMLFPVFFEVEFCNVKDQAGSFEKAFAQFENSVQASQV-
LKWKAAVKELTST(DRYHQYKKGDEVMLAKNIVRDVQVNSKTGSNLTESLQLSYILSLLNSSQSSAPHIVGLW
GMAGIGKTTFSREIFRTQGE)}*

$2227=216(222):
>gi|920686825|gb|KOM30808.1| {|0(929)|<Streptophyta(V)>}hypothetical
protein LR48_Vigan01g036300 [Vigna angularis]
{(MAFA)---VFVSFKD-EDIRDITAFVLDPLRSRG-IQVF------
VRGESTtfDLFQAIQRSRLLIVVISKNYASSIRCLRELVAIINGVES-
SPRIVLPIFNGVHQSNVLSQNGCYGQAFSKY-
EERFREHKqrmEevQRWREALTRVACF(PGWRMEKMKGVGGRGLINYAVDILNREFSTPQNETIFNRYGKFED
IVQYKDGDEVSYKELRGLIRLRDFISLVLTYPV)}*

$2228=188(220):
>gi|920703297|gb|KOM46522.1| {|118(0)|<Streptophyta(V)>}hypothetical
protein LR48_Vigan07g022600 [Vigna angularis]
{(QLVWPIFYGVEPTDVRFQRGRYEQAINRFEERYYSPERMNKWRAALAEVSNLSGWFYQKGHKYEYKFIRKI
VEAAVQCLS)RYDVFLSFCG-EDTRYTLTGFLYNALRrEG-
FKIFMDDEELEGGNQIsqKLMGAIESSRVSIVVFSENYGYSTW------------------------------
--FHHESIERIVERLI-NIEDEEAYNNSFPCSNDSYGEQ()}*

$2229=198(192):
>gi|921219383|ref|WP_053164553.1|
{|0(506)|<Actinobacteria(B)>}hypothetical protein [Streptomyces
ahygroscopicus]
{(MDRHTGEE)DWDVFVSYSR-SD--
AELLELLAPALRHRGLRVFVDDTAVEDFAGItsTIARALARSKVLLALYSQQYPRRRACQWELTYAFLAgqRE
GDPRRRILVVnpeqsADHVQPLELRDARHWPGIRDESAAARLADRIASYVGGLAGPLGEAP(GPAHPRWLPAP
IPSGSRHFTGRLAEQWHLHTALHRHRAPLVSQT)}*

$2230=209(220):
>gi|922428117|ref|XP_013620083.1| {|101(0)|<Streptophyta(V)>}PREDICTED:
vesicle-associated protein 1-4-like isoform X2 [Brassica oleracea var.
oleracea]
{(ISYSENVTISNVRPESGTEERSPAQRTATSYQPPTTESRVSPHVYGTSDMDPLLSGEPQQYRADLQTLIHS
QAGLGRSHS)--QIFVSFHG-
GDLRRTFVRHLVSTLTDAGISVFTDNDKGNGIKLQNFYKRIEESKIAVAIFSERYIESHFCLDELVAMDKLVR
E-NKLLVIPVFYNVKPSDVKHLKGEFGRRFKEMRVR-YKDEAEkvLKWET--------()}*

$2231=175(194):
>gi|922525314|ref|XP_013596127.1| {|0(341)|<Streptophyta(V)>}PREDICTED:
protein DA1-related 4-like [Brassica oleracea var. oleracea]
{(MDTDVNNNNDKANHC)---VYISFDN-NDQSISsFINYLKATFNHHGIYQLDDQSDRLAD-------RI--
-KILVVVFSKEdtFSAPENLVNQLPK--------
KDLVVVPVLYKVTILSARQNAMLDQVLLDSSAVLPGHIYNEERSEYEFTKEIAR(DVCEKLYPTAEIGIHRRQ
KEIENLLCKQPWGVRTLGIFG)}*

$2232=295(296):
>gi|922531299|ref|XP_013597564.1| {|1611(0)|<Streptophyta(V)>}PREDICTED:
disease resistance-like protein CSA1 isoform X2 [Brassica oleracea var.
oleracea] >gi|923609423|ref|XP_013743749.1| PREDICTED:
{(HEANHYGAYSKNAISNVRRETETMDMRLDGGHDIVELPNEMIAHGTANTSQRPNNKSPVTTLKQTRRETQV
RALSVRLTG)KPRVYISCHG-
GDLCITLVKHLVNNLTNAGVNVFIDNDERMWIKMHQLYNRIEDSRIAVVIFSKRYLASRLCLNELAKMDELAK

E-GRLLVISVFYQVISSDMKNLKGECGRCFREMRKRHED-
EPEnvQKWETSLMSMAET(TGVHSEIHGIDAALVKATVKAVHRELTKIAGGKFKSLGRGVMLSARVFIFALFA
ALLFSLFVSPLLFADAAFAAFW)}*

$2233=176(187):
>gi|922565338|ref|XP_013610929.1| {|0(2032)|<Streptophyta(V)>}PREDICTED: disease resistance protein RRS1 [Brassica oleracea var. oleracea]
{(MTDCEKAERF)---VYISCV--DEVRYSFVSHLSDALRRNGISsVFVDSGDLLSDEAQE---
KVERARVSVVVLPANR---QVCLEKLEKVLNCQRN-
KEQVVIPVLYGDSILHGEWLSAMNLRGLSPVFQSRNDCSDSKLVEKIVRNVNEK(LLYMGRIGMSWKLREIEN
MVNKQPLGIRGVGIWGMPG)}*

$2234=200(218):
>gi|922554071|ref|XP_013605067.1| {|0(1031)|<Streptophyta(V)>}PREDICTED: disease resistance protein RRS1 [Brassica oleracea var. oleracea]
{(MVEF)----YISFDRCeDKVRYSFISHLSAAFHRRGISSFVGGSDPEADGSSKPC--
LEKSKACVVVFSEQYSSSKPCLEELVKVTErRRND-GGLAVVPVFYRATKSSVKKL-------IWK------
SNDLTNEWRSALLQVVDL(PGHESHATQSDSDLVEEIVADVREKLNATENIGMHSKLVKIENLLRKKPCGVRR
IGLWGMAGIGKTTLAKAIFD)}*

$2235=275(299):
>gi|922454181|ref|XP_013629539.1| {|24(617)|<Streptophyta(V)>}PREDICTED: LOW QUALITY PROTEIN: probable WRKY transcription factor 19 [Brassica oleracea var. oleracea]
{(LEAHFSGGNPSDQQEEKRLKICVPRKKRRKYDKGQQKVIQTTASSFMNLVQQFTGDASSVSESKSSNTGVV
AASSSSSTY)-YDVVIRYRR-GDIGNNdFISHLRAALCRRRISVDEDFDEVDT---------
VPECRVLIIFLTSTYFSST-----LLNILEHQRR-EYQVVYPIFYGLSPSDLISNSKVYDIF-FHQ-DEAKR-
-----
WQAALKEITQM(PGYTLTDKSECDLIDEIVRDALSVVYSSYKVTMVGRDMQIKEILSLLCVESPEVRSIGIWG
TVGIGKTAIVEEIFRIIS)}*

$2236=275(299):
>gi|922569301|ref|XP_013612964.1|
{|231(626)|<Streptophyta(V)>}PREDICTED: probable WRKY transcription factor 19 isoform X1 [Brassica oleracea var. oleracea]
{(GANNSVSELQFSASSPSTDYEVIVIYRRGDITNDAFVKVVQRLTGIPNSASLPLNSYVEIEKGSSKDLQDT
ELSTSAIGE)-YDVVIRYmRG-CISKGDFTSHLRAALCRRGVSVRQDIDEVD---------
AVPECRVLIIFLTSTYVPS-----NLVNIVEQQSK-KPRVVYPIFYGISPSDL------ITNSEYYE-
SFSLQDEP-
KRWQAALEKITQM(HGYILTTDKSESDFIDEIVNDALEVLRSNYKKNIIGMDTQIKEILSLLCIESQDVRRIG
IWGAVGIGKTAVSEEIFHRI)}*

$2237=252(246):
>gi|922418530|ref|XP_013609179.1| {|288(0)|<Streptophyta(V)>}PREDICTED: LOW QUALITY PROTEIN: uncharacterized protein LOC106315887 [Brassica oleracea var. oleracea]
{(LLSRKPRLFPGNKLIVSPQEIEEQRYFSPVQVSNVLAMAESNENTRNRLGVDKNSDCSLTCFSLLCNIMKR
FEMMINPPP)-PRVFISFGE-KQLEMNLVSSLKTEFESNEISVYIED---------
ETKERIKESKVAIVVFSDKYPESPECLDELVEIKKLMDA-
GEITPFPIFYKLKdvprlAQSVKQLKGCFRNRLLKIeqevRKTVNRDNVNsildteariWEWRRAISSISSR(
PGLSNENSSDPVFFTDVVTKVKKLFV)}*

$2238=150(147):

>gi|922528349|ref|XP_013596835.1| {|0(219)|<Streptophyta(V)>}PREDICTED: uncharacterized protein LOC106305001 [Brassica oleracea var. oleracea]
{(MVFQMK)KPQVFISFRG-KDERKKLLPHLKHHLEaSPsNVRVFTDDDNI-
AQRLDRLYNHIRNSWLVIVIFSKGECGAKFRTLEKSCPDKVKKW-
KKALGFVANYSGLTYQEQSSVSELDFIKTVVkkvdQIFAKSPPNEIFDKSQLHLSKTL(E)}*

$2239=253(227):
>gi|922565751|ref|XP_013611148.1| {|0(137)|<Streptophyta(V)>}PREDICTED: uncharacterized protein LOC106317888 [Brassica oleracea var. oleracea]
{(MERTNPHL)--QVFISFRG-
KDVRGNILSSLKEKLKDEGVNVKTDEEMPRGRKIDeNLLKLIRDSKVAVVIFSENYPESTWCLDELVEIKKQM
EL-
KKLAPLPIFFEVKASHVALEEHnPFKDILLRLeddeREIARNGsrvgsgrmhcwscflSRRllkdadkrfVRW
REALKSITGY(SGLKYIKDSNQALFVNHIVETVKEMLGKVQSSDDVHDRIRGQHIVPQQKVFISFGGHGDDTR
LGFISHFQAGLKRSGIN)}*

$2240=299(300):
>gi|922571713|ref|XP_013614187.1|
{|287(113)|<Streptophyta(V)>}PREDICTED: uncharacterized protein
LOC106320376 [Brassica oleracea var. oleracea]
{(FAPLLFRKKPQKFTGNKPLFSSQEIQDRSHSLPVEVTKSNDNPQNLPGQDTNSDDTFNCFSLLCNIMKNLA
MIIIPPYRG)---VSISFGE-QQLEHNLVNLLRTEL-TSnRFSVPEEGEM---------
KERIKESKVAIVVFSSKYPESQQCLDELVEIKRLMDA-
GEIDPLPIFYQLKDKSVRELKGWFLHRLLKIedqvRKKVDRGSEKsildaeakiWGWRQALLSISSR(RGLSS
QHSNIAFVRDIVTKVTKMFAYRERKPSPNSSYVTTDLLVVEETPMHRQEMAAITTVQCHDNDLFYSPNSFLQA
LN)}*

$2241=219(223):
>gi|921140533|ref|WP_053090739.1| {|72(0)|<Proteobacteria(B)>}TIR domain-containing protein [Neisseria lactamica]
{(PTSFIQTLGIGNIKLNLNEIDRTNPNMIRKELFSLLNLKKRDAIFLFDNTNNYSNWNADLERDLKENFLQH
KMVMNMEPM)--KIFLSHKSInKDKVRNFKTILELLGFSP------
WLDEEAMSAGAnlerSLHSGMVDSCAAIFFITPDYKDEKYLATEIDYAISRKRQDekfsIITLVFSDENGEKG
VVPNLLQPYVWKEPAND--LLAL-AE--ILKALPLEVGDI(RFK)}*

$2242=175(177):
>gi|921969862|ref|WP_053263412.1| {|0(647)|<Proteobacteria(B)>}TIR domain-containing protein [Escherichia coli]
{()MIKCFLSHSSNdKDY---VRVVASGLRKETLI--
FDEQTFEKGMSPSeEILNGLDDTSLFVLFLSDFALESDWVKEEVRLAKKKVED-GHLhRIYPIIIDDK-
ITFSDPRIPSWMKDGFNiqPIRKPNVAVRKINARLREIAFKT(HPALKERQKIFVGRNEKTNNVEQRFDDFDK
SPPIVFI)}*

$2243=217(221):
>gi|921064984|ref|WP_053078812.1| {|50(0)|<Firmicutes(B)>}TIR domain-containing protein [Bacillus cereus]
{(DNGTKGLVSSSVVDFTPITLGVEELNYENIEKVREKIRNDFQLVPRDSVVILKMNGENLSSELENLLKRHE
IVMGVVGMK)---IFLSH-K--GIDKEIVRNYKNTLELLGFDPWLDEDAMHAGVELerGIKQGF-
NESCAAIFFVTPNFVDEDYLGtEVNYAIAEKRKKkGQFSIITLVFEAN-
GRKGNVPELLESYVWKEPKNDLEALREIIKALPLKLGKPL(L)}*

$2244=179(181):

>gi|896161041|ref|WP_049176328.1| {|0(67)|<Firmicutes(B)>}hypothetical protein [Lactobacillus salivarius] >gi|1173435304|gb|OQQ82951.1| hypothetical protein B6U58_07750 [Lactobacillus salivarius] >gi|
{(MENT)---VFFCFSG-QD-RLKYAQSLNFHLKNFGVDVWYDYEQIflGD-
IGDtvNIEDGLHNSRIFILFFSKRLFQSTGATLEIDEIKKKIDNNQNLVIIPVFCDNSIPQIPPKYSWLLKYI
YGELTESTGTYDLAIDVLKRLLHEKL(KPVEYKNLLELSNLNLGDFINNLIRIYMSIDKNNIHV)}*

$2245=205(220):
>gi|895916565|ref|WP_049014334.1|
{|453(0)|<Proteobacteria(B)>}hypothetical protein [Klebsiella quasipneumoniae]
{(QTASTLLHLAEARGLPIIPFAFLGGVAKRSYERRDWSRLNPGFDPSVLCDVDGVEHTIEIANRLMLDRMQP
SRLLERPKM)---VFVSFAH-QDAE---
MANALRAVLVKeGIEVLVGDDEIRSDQMItaTIEQAVLRSDVCAVLWSRQYAQSPWCYDEMSL----------
-AINQQEYGKIKVWLFNLDDSPIVPAQARKLPTISVRNVQAMHNCVHELLGR()}*

$2246=216(220):
>gi|896155787|ref|WP_049172248.1| {|66(0)|<Firmicutes(B)>}hypothetical protein [Lactobacillus casei]
{(SAQAKEAKNQASISKELQRKNEKLGVLQTQLSKEMQAEQNKSAAKIQRDYQNQLNQLRNQQISILNEQLSS
ANDAEQVQR)KYDVFISYAH---SDSGYVTRLREDLEEADIKVWIDSDEIV--WGSslvESINKGFQN-
SRftILVLSKDYFTRFWTQQELRAAFVKESKRKDETILPIYL---NitA-DEVSDYNFSLGDIMA-
MNAAIVPNKDIVSAIKRALKKI()}*

$2247=170(169):
>gi|896087829|ref|WP_049126103.1| {<Proteobacteria(B)>}hypothetical protein [Enterobacter cloacae]
{()MAKIFISYAREdSPIAQRLAGEL--LKNNHDV-
FLDQQSIRLGSHWQeDILHHLKDAGVFLALVTKNSDKS-YYFNaEVTSAISyvAHSN-RDKLILPVVLDQM-
SLDDSDLGSFQALFGRAddISYLADEIEDAISRFEGYRLAQE(EIKALRGQTLKDESEDYISETIEDLKSRE)
}*

$2248=156(159):
>gi|895911202|ref|WP_049010435.1|
{|0(7)|<Proteobacteria(B)>}MULTISPECIES: molecular chaperone Tir [Klebsiella] >gi|949924994|gb|KRR39938.1| molecular chaperone Tir [Klebsiella pneumoniae] >gi|1030763
{(MTV)----FISYRHTdraqaMAINKRLTEEGISTYLDVL------
DVESQSTDDItsVITRRISESTHLIAVVSSQTAQSWWVPFEIGEATITYRRIcsyRTTVSELPEYL---
SKWPQMTKPSEIELFIQSYKNEISGKRQITLESFNGSEAM(RRVDKVNADTFHADLK)}*

$2249=230(213):
>gi|896055027|ref|WP_049104460.1|
{|0(593)|<Proteobacteria(B)>}hypothetical protein [Klebsiella michiganensis]
{(MIKC)----FLSHSSSDkeSYVRIVAEKIRK--ENRI--
IDEEAFEEGMVTAEEIIKYLDKSSLFVFFISNKSLDSEWVKLELDRARELLDENTIKRVYPIIIDPKIEYkdk
ripewmrdrlniqlISSPTHAARKINaRLIELTWadhPRLKE-
RrdiFVGRNDQIKLIE(ERLDDLQRQMPTVLIASGLPSIGRRSLMTHSLRKANLVTDSYDFSVVSLTSMDSIE
DFIIKCCDLGVVE)}*

$2250=183(185):

>gi|896633605|ref|WP_049514396.1| {|0(54)|<Firmicutes(B)>}MULTISPECIES: hypothetical protein [Streptococcus] >gi|1080211780|gb|OFK07388.1| hypothetical protein HMPREF2832_02285 [Streptococcus sp. HMSC
{(MR)EFQYFISY-S-
RDIYYEFVNHLNNMLNEIGLKTWIDKFEINIGDEIidNLYTVLDKSADYygiIFIFDETFFNKAWCIKELDYA
MK-----
KRIPFFPVLYKIKKEDIPSEYTFLKNYNLLTVSSLKEIDYIVDRFILLHLNNIV(CRDIPKFSISSYVGNFLL
EQIRCCTVNNIDAIFIIDNFSQYLR)}*

$2251=155(166):
>gi|896485767|ref|WP_049397231.1| {<Proteobacteria(B)>}hypothetical protein [Stenotrophomonas maltophilia]
{(MKSR)--NVFVSHIQEDEAHIQK-LYDLLSRSGF--
QCkdsSITSQTPNNAQSQdYIKYQVISpqiewAGVVVVLITP--------
DTrtSDWVNWEVERANaLGKRIVGIWVHGE--GGCDLPESL--------
VKYADSVVGWNSERIISAIEGK(DSWECSDGSEMGDREIKRIKCQ)}*

$2252=218(220):
>gi|896605150|ref|WP_049488533.1| {|168(0)|<Firmicutes(B)>}molecular chaperone Tir [Streptococcus parasanguinis]
{(SKYDSKEVRKFFYAINGHIRNQSLKIEVQSYFQSLSLPFRLSRKKKGKKNFIDNEIVKNKKGPDELKKRLY
INDLEKIKR)-FDIFVSHNSRdeEKIIEFYKKFNKKGQVVYIDWINDkydlrREWC-
NASTVDIIKERIKQSDVFVLYFSGETLNSQWCAWELGYA-----
DALGKKICTYFSEDINrKGIPKFYVAYPELqMSKNlYVKIKGNEDIllEEWKKL-------()}*

$2253=213(220):
>gi|922842366|ref|WP_053388461.1|
{|275(0)|<Proteobacteria(B)>}hypothetical protein [Pseudomonas sp. 10-1B]
{(KDPKEAEKHYYIDVLGLKPEDISLTDDGSRLKHNLYGRLSFIAMVKGKDDPTYVKLILKMAEHDKSPPKFL
NAIKEEYEM)-YDVFLCHAS---EDKEtIVEPLYNELKKLGVHAFYDTKVIA--WGDsliETINKglI---
KSKyvVAIMTENSVIKAWPQKEINAVLNGDISGGTKRLLPlIHGDKEKilSSnyLMSDKLYVEWKE-NP-
EEIAKKIK-DLLA--------S()}*

$2254=194(195):
>gi|896488890|ref|WP_049399055.1|
{|0(145)|<Proteobacteria(B)>}hypothetical protein [Stenotrophomonas maltophilia]
{(MAEDVQVGDSGDSSSMRTNNPF)---
VFISHDTRdAELAEAFSNLLKSVSAGVLKSFRTSDRRgnQGIEYGVewypEIIKSIQAASDVVCLLTERSVDR
PWILFEAGMA--KGKL-DTPILGVALGIELKAASSGPFAQFQNCADDE--
ESLTKLVFQLVNRIPGSEPDR(DTIRFQVGKFKAAVDEIIGKLALPASQEKKKAA)}*

$2255=201(202):
>gi|896657380|ref|WP_049535792.1|
{|0(620)|<Proteobacteria(B)>}hypothetical protein [Vibrio harveyi]
{()MIKAFLSHSSKdKA--SYVRNVANWLDKEQIV--
YDEYTFEEGERSLdEILLGLDKAELFVLFISEHSLNSEWV-KtEISEAKQRLDT-
QALfKVYPIIIDSSIkfddpripDWLRT---------
YNMkpITRAPVAARRIHTKLREICWKKH(PKFKLRDNFFAGRNTETDHFEGRLLDFFKDKPLAIFCSGFPGVG
RRTLLHYVLKKTDVTQDP)}*

$2256=201(197):
>gi|896609667|ref|WP_049492500.1| {|0(649)|<Firmicutes(B)>}toll-Interleukin receptor [Streptococcus constellatus]

{(MSK)---IFIAHAW--SDDREyndkqlsFIRLLQLELEHKGLEVVFDDSTINKDSLNrfMIENV-KNSDI-IIAICDEYYLKKSedlgsGVSfEL-------KEIrDHNFldkVIPLKISEC-DLPYDFGTMQYISFKNEFENQLTDFETSASLRELFVRIAD(FLSKEELYPKDPIKPEILSEINSLGLLSNVLNNSDLTLSDIYTYPEFSIESEKE)}*

$2257=257(289):
>gi|923504471|ref|XP_013644649.1| {|0(1184)|<Streptophyta(V)>}PREDICTED: disease resistance protein RRS1-like [Brassica napus]
{(MDSLLGSCAAAAIGFFTILVTLLLAFYRRFRSNRRNVTVASSSSTSTTLSSDFEAVPNGLRIRAVYIYCE)-----------ETLQYTFASHLSVDFRRKRIAAFVNCD------LNpDVAE---GASASVVVFSKSYSSSASCLDKLVTVLRCRRNTGQMVVVPVFYGISPSDVAVRV------------HGSADRIR-EWSNALRELREL(PSHQCSEESDEGQVVEEIVKDVYEKLFPTEQVGINSRLVEIEQLLCKQPWGIRRIGIWGMPGIGKTTLAKAVFDDISGG)}*

$2258=253(256):
>gi|922987278|gb|KOP23490.1| {|142(10)|<Cyanobacteria(B)>}GTPase [Hapalosiphon sp. MRB220]
{(QEPYSYSLDNLYRRFNAGRYQIECDKSYEMVDVRRLIDDVNLQSFDITQGRELPVNPLQRELEESRDKSLTHQPQNNEQK)--EVFISYTW-RDQESKpLVEKIEQEFKTKGINIIRDTNAIGYKQ-KfqEFMQRLSHGKCVILIISDGYLKSENCMYELVEIAKHGEF-YDR-IFPIILLDAKIYKFTERMEYIRYWQNEITQLREAIKGVDITISEKSLQTL(KLYEEIKDKFDELANLLNDINTLNPNIHIQSNFETL)}*

$2259=254(266):
>gi|923073959|ref|WP_053457373.1| {<Cyanobacteria(B)>}hypothetical protein [Hapalosiphon sp. MRB220] >gi|922989091|gb|KOP25238.1| hypothetical protein AMR41_17705 [Hapalosiphon sp. MRB220]
{(MDDQEALQKTLNRARRVLQILEEQKASYGIRVPVDLQIELEEKQKEVASLEARLNHSQGVSQATDNMTTKQK)--EVFISYTW-RDNHSEpFVQQLEKAFESKGITIIRDKNAVGYKQ-RfqEFMQRLSRGKCVIVVISHEYLKSENCMYELVEIAKNGNL-YERIFPIVLADAQIYDPLKRIEY---------VEYWEEKI-QKLDDAMKKVSGA(NLQGFREDIDLYTEIRKMVANLTNLLRDMNAMTPDIHIKSEFEALFKAIAKSLE)}*

$2260=219(226):
>gi|923837215|ref|XP_013699552.1| {|0(480)|<Streptophyta(V)>}PREDICTED: probable WRKY transcription factor 16 [Brassica napus]
{(MAACNK)---VKISFdwSD-ETIRCSLVPHLSAAFGRKGISVLTDKHDE-----------FCKCIASVFIFSKNYVSIKESLDDFFKTSQRRHD-KGHVVATVFYGVNRSDL---KRNFAKALFEHITSYqiatdvyekifPKERI-GIYSRMLQDISNL(LCSQQWGVKSIGFWGMPGIGKTELAKAVFDQMASDYEVTCFLQNFHETFETKGLYSLLQEHFKNLTNQNIPKRVLLVLDD)}*

$2261=153(154):
>gi|923884114|ref|XP_013713728.1| {|0(153)|<Streptophyta(V)>}PREDICTED: uncharacterized protein LOC106417475 [Brassica napus]
{(MASSSLTW)KHDIFPSFCG-GDVRKKFLSNLLEKLRSNGISPFIDNDIQRGQS-IgpELKQAIRESRVAVVLLSRNQLALLGKPLDLEDQLEFIVE-GLPDEYKQVVDQIQGRDVPPSITEVHEKLLNQEVRLQDITPNSVLPISANVAQS(RPPSNN)}*

$2262=268(286):
>gi|923608952|ref|XP_013743593.1| {|156(820)|<Streptophyta(V)>}PREDICTED: probable WRKY transcription factor 19 isoform X1 [Brassica napus]

{(NYVKEKQLGVGDIVFLQRIFTDSSRLFIGFSRREAALDGTSSDKLKEVNSPLPLRSHVHSVTEFDPVLVLS
CAANQESGE)---------------RYFISYISTELCLHGFTPLIYDLTrGTLPG---
GLELPHRSRVGIIIFSNNYASSRQCQDNFVAIMDHSKA-
NSLVLLPVFFKVKVTDIRGQSGSFGRAFSQLENSIQASQVPTFTFIDKHQYMKG(GEVILAKNIVSNVCLMLS
SENNMKLRGRLQMNSILSLLYCSQSSAPHIVGLWGMAGIGKTTIAREI)}*

$2263=188(206):
>gi|923780422|ref|XP_013681815.1| {|0(1163)|<Streptophyta(V)>}PREDICTED:
protein DA1-related 4-like isoform X1 [Brassica napus]
>gi|923780430|ref|XP_013681816.1| PREDICTED: protein DA1-related 4-like
{(MSCEKNSHSVNIICSETV)--------------
RYSFVSHLSGALRRQGISVFVFAGTDGLESYSFPDDQNQEARVSVVVFSETYASSGRWF---AKQLELHKK-
NGYVIVPVFYGVDPSVVNRDLEFLGTMKRDGNLTRHPSSDSELVEETVRNVFAK(LSPTERIGIYSRLLEIEN
LLCEQPWDIRSLGIWGMPGIGKTTLAKALF)}*

$2264=168(172):
>gi|923615975|ref|XP_013745861.1| {<Streptophyta(V)>}PREDICTED: disease
resistance protein LAZ5-like [Brassica napus]
{(MEVHSHHLGPPL)QHQVFVNFRG-LDLRFGFL--
LVEALQRHGINVFIDSLERKGEDLINLFARIEESTIALVIFSKRYTESIWCLDELLKIKRRGKE-
GDEINNILRRSPRSEEAVVWRWQKKLPGVVEVEPSKKKKIGGDIKLAVVVEEKE(RRPRRESLVDTAMEEDGD
DE)}*

$2265=175(187):
>gi|923543350|ref|XP_013734065.1| {|0(1985)|<Streptophyta(V)>}PREDICTED:
disease resistance protein RRS1-like isoform X1 [Brassica napus]
{(MTNTKRTEQP)---VYISCV--DEVRYSFVSHLSDALRREGISVFVDSDDLLSKEAQE---
KVEGARVCVMVLPGN---GRVCLEKLGKVLNSQRD-
IDQVVVPVLYGDSQWYDEWLDELHLRGFSAVYQSRKECNDSELVEEIVRDVNEK(LSHMGRIGIYSKLLEIEN
MVNKQPLGIRCLGIWGMPG)}*

$2266=176(185):
>gi|923567855|ref|XP_013738985.1| {|0(507)|<Streptophyta(V)>}PREDICTED:
disease resistance protein LAZ5-like [Brassica napus]
{(MNNNDEIKQT)---VYISFNI-NDSDVSsFIRYLVAALHREGIDVAS---EKSGHDLnKGWFSRI---
KLFVVVFSKPCTYSVACLEKLVKLLEFLRE-
EDNVVVPVFNDAMAKQMDRRNFLERLRHWESLILQISEAAELIEEIAKNVFQKL(NPTENIGIHTQLRVQENL
LCKQPWGFRSIGILGKP)}*

$2267=200(216):
>gi|923712681|ref|XP_013662912.1| {|0(1034)|<Streptophyta(V)>}PREDICTED:
disease resistance protein RRS1 [Brassica napus]
{(MV)--EVYISFDRCeDKVRYSFISHLSAAFHRRGISSFVGGSDPGSNGSSKQC--
LENSRACVVVFSEKYSSSKPCLEELVKVSErRGNE-GGLAVVPVFYRATKSSVKKL-------IWK------
SSDLTSEWRSALLQVVDL(PGHQSYVTQSDSDLVEEIVADVREKLNATENIGMYSKLLRIDNLLRKKPCGVRR
IGLWGMAGIGKTTLAKAIFD)}*

$2268=187(205):
>gi|923756964|ref|XP_013676053.1| {|0(898)|<Streptophyta(V)>}PREDICTED:
LOW QUALITY PROTEIN: protein DA1-related 4-like [Brassica napus]
{(MDPMNPPPPQNNSHSVRIFCEGTGQ)---------------YSFVSHLSTALHREGISFSVDACE------
---FPEINSFsdqnhSISVVVTSEVVKVNdPWVIIKFVEVIERpRDK-
GMQVMVPVFYDVDPLTRVYGWANRWLEAGYITMHQSRISSSKILTDSELVEDVI(KDVYEKLHRTERVGIYSK
VLEIENLLYKQPWDVRSIGIWG)}*

$2269=275(299):
>gi|923710011|ref|XP_013662164.1|
{|251(625)|<Streptophyta(V)>}PREDICTED: probable WRKY transcription factor 19 isoform X1 [Brassica napus]
{(GVNNSVSQLQFSASSASTDYEVIVIYRRGDITNDAFVKVVQRLTGIPNSLSVPLNSYVEIEKGSSKDLQDTELSTSAIRE)-YDVVIRYmRG-CISNDDFISHLRAALCRRGVSVREDIDEVDT---------VPECMVLIIFLTSTYVPS-----NLLNIVEQQSK-KPRVVYPIFYGISPSDL------ITNSEYYE-SFFLQDEP-KRWQAALEKITQM(HGYILTDKSESDFIDEIVNDALEVLRSNYKKNIIGMDTQIKEILSLLCIESQDVRRIGIWGAVGIGKTAIAEEIFHRIS)}*

$2270=274(299):
>gi|923836169|ref|XP_013699220.1| {|54(625)|<Streptophyta(V)>}PREDICTED: probable WRKY transcription factor 19 [Brassica napus]
{(VPRLTGVDNSVLELEFISAFGPSASRGYQVVTIYRRGDISNDDFNKVVRRLTRNLNSVSDLSNSASEFSTPAAASSALKE)-YDVVLRYRR-GCISNdNFITHLHAAFYRRGVSLREDIDEVD---------AVPECRVLIIFLTSTYVPS-----NLLNIVEQQSK-NPRVVYPIFYGISPSDL--ISNSNYVRPFHQ-DEAKR------WQAALEEITQM(HGYILTDKSESDFIDEIVNDALEVLRSNYKKNIIGMDMQIKEILSLLCTESQDVRRIGIWGAVGIGKTAIAEEIFHRIS)}*

$2271=275(299):
>gi|923887081|ref|XP_013714873.1|
{|231(626)|<Streptophyta(V)>}PREDICTED: probable WRKY transcription factor 19 isoform X1 [Brassica napus]
{(GANNSVSELQFSASSPSTDYEVIVIYRRGDITNDAFVKVVQRLTGIPNSASLPLNSYVEIEKGSSKYLQDTELSTSAIGE)-YDVVIRYmRG-CISKGDFISHLRAALCRRGVSVRQDIDEVYT---------VPECRVLIIFLTSTYVPS-----NLVNIVEQQSK-KPRVVYPIFYGISPSDL------ITNSEYYE-SFSLQDEP-KRWQAALEKITQM(HGYILTTDKSESDFIDEIVNDALEVLRSNYKKNIIGMDTQIKEILSLLCIESQDVRRIGIWGAVGIGKTAIAEEIFHRI)}*

$2272=143(148):
>gi|923722495|ref|XP_013665453.1| {<Streptophyta(V)>}PREDICTED: protein PHLOEM PROTEIN 2-LIKE A5-like [Brassica napus]
{(MERTNPHL)--QVFISFRG-KDVRGNILSSLKEKLKDEGVNVKMDEDMPRGRKIDeNLLNLIRDSKEAVVIFSENYPESTWCLDELVEIKKQMEL-KKLAPLPIFFDVKASHVAlKEHNPFKDILLRLeddeRENARNG-KKALE------AGPA()}*

$2273=253(227):
>gi|923729516|ref|XP_013667593.1| {|0(137)|<Streptophyta(V)>}PREDICTED: uncharacterized protein LOC106372012 [Brassica napus]
{(MERTNPHL)--QVFISFRG-KDVRDNILSSLKKKLKDEGVNVKTDDDMPKGTKIvENLLKLIRDSKVAVVIFSENYPESPWCLDELVEIEKQIEE-TKLRPLPIFFKVRATHVALEDHnPFKDILLRLedneRENARNGfrvgsgrmhcwscflSQRllkdadkrfVRWRGALKSITGY(AGLKYIKDSNQALFVNQIVEAVKEMLGKVQSSDDVHDRIRGLHIVRQQKVFISFGGHDDDTRLGFISHLQAGLKRNGIN)}*

$2274=214(220):
>gi|922868099|gb|KOO34573.1|
{|679(0)|<Haptophyceae(E)>}dimethyladenosine transferase (rRNA methylation) [Chrysochromulina sp. CCMP291]

{(HIRELCVLQEDDFFPRQPFLSKLILVTARDGGRTKIAVEQARPNAQISFALPNALIVGAAAEPSVPLRTQP
GLRNEGAPW)--DFFMSHKQNESGRDAALITRDLEQAGK--KVWLDVNM--
KDFSTdAMMEGVEHSKTFVLVLSHGYFDskFCVM--------
ELSRAVdLRKNIVLCHKEGVNvgAILQKMPPEFRTISDhasLQLVTSDATYREVAVKRLI-SLSST()}*

$2275=212(226):
>gi|922868300|gb|KOO34724.1| {|0(914)|<Haptophyceae(E)>}apoptosis
inducing factor [Chrysochromulina sp. CCMP291]
{(MGCGSSSPPGAKYAESPPIKGDDLIKSYTERIRESDLRKAQ)-
WDTFVSAAPAETALAKRVRDHLRAAGYK--------IPNPRKEPDAQKQGIRDSRRVVSFISQTYF-
DSPACCaEFCEAVING-----VEVLPVSVEGSSwrGMPFPALTDVPETAQT--
DEGRLVKPRDAAAAVFGHTIAL(DHMDAYLDAFLENLGKRLGGPGEASSAGRRPRETSTDFLLGERAI)}*

$2276=113(140):
>gi|923073831|ref|WP_053457245.1| {<Cyanobacteria(B)>}hypothetical
protein [Hapalosiphon sp. MRB220] >gi|922988928|gb|KOP25075.1|
hypothetical protein AMR41_16785 [Hapalosiphon sp. MRB220]
{()MKYLFVSYASQdSLKAKELVSNLKEVQVSG----
WLDQaDIATDEEISsVLRDSIRKGNAVLVLISPASVNNRWV-EfEVSAGQAL-----GKTIIPVIIS------
---------------GENVEEKLPSSMPDIMYIDARK()}*

$2277=218(229):
>gi|924369197|ref|WP_053492234.1| {|60(0)|<Firmicutes(B)>}hypothetical
protein [Bacillus sp. FJAT-18019] >gi|923174311|gb|KOP67250.1|
hypothetical protein AMS62_19850 [Bacillus sp. FJAT-18019]
{(QEGHINNMIDKLSGLGLRYRPDITYDELLSELGNRRFDLVTISNGDEVEFKFSSGFFVFAEKEYIQSQGYV
IKPLGNKVP)---VFLSHSSRdKPDIEDLIPYLNGAG----
LPVWFDKiSIDYGESITeAIERGIDKSGAVIFWITKDFLNSNWC-KtERRNFLSRHSgD-
DDVLIISIISDDI-DI-RTEIPRFL---RDLkgLVRNKNQD---IYL-IAKEIIPV(LKKYLEGIS)}*

$2278=260(265):
>gi|935599684|ref|WP_054465572.1|
{|154(922)|<Cyanobacteria(B)>}hypothetical protein [Planktothricoides sp.
SR001] >gi|923471551|gb|KOR37311.1| hypothetical protein AM228_07815
[Planktothricoides sp.
{(GLIQVFQHHADYVSQHTELLVKALEWVRGKKQSSYLLIGEAKQGAERWLQVRFKHEQPPCLPTDLHCEYIC
ASIKNSNNL)MTDVFLCYAEAnREMMEQITRTL----
RREKLTVWTKiTDMETGINVQeQIHRGIAAADNFIYLISPESLQSKYC-QaEIKLALAYHK-----
RIICVIVELT-
DQQMIPLELINLPFLNLtpREKKENYGLVMAQLIKQLNTDKN(YYQQHKILLVKALKWQEQKYNQSILLRGYN
LQHFQAWLKVAKTRQ)}*

$2279=249(257):
>gi|935599684|ref|WP_054465572.1|
{|359(728)|<Cyanobacteria(B)>}hypothetical protein [Planktothricoides sp.
SR001] >gi|923471551|gb|KOR37311.1| hypothetical protein AM228_07815
[Planktothricoides sp.
{(LIKQLNTDKNYYQQHKILLVKALKWQEQKYNQSILLRGYNLQHFQAWLKVAKTRQQHPPIPLQVKFIEKSL
SQSTIFSLE)---VFISYSRVdSDFARKIN---DALQLQGK-
TTWFDQEsIAAGSDFQqEIYRGIEQCDNFLFIISPSSVNSPYC-GdEVEYAKKLNK-----RLVTILYRPV-
AAGDLHPILAQVQWINFsqHGGDFYANFSELIRTLDTDREYV(HSHNQLLQRAIAWYQKKQNPDLLLRGSELA
IAETWLK)}*

$2280=291(290):

>gi|923075257|ref|WP_053458671.1| {|118(298)|<Cyanobacteria(B)>}ATPase [Hapalosiphon sp. MRB220] >gi|922987323|gb|KOP23535.1| ATPase [Hapalosiphon sp. MRB220]
{(EAICQHIDYVIGMNQAIGDRAAIKFAVGFYDGLGAGRSLEDAFESGCIAIDLESIPESATPILKKRSSSAE
TVSLTPQRR)---IFISYKrnT--ETDEKVAMQVFQALRQE-HEVFIDREMLV-
GTPWaeRIEAELRQADFLIVFLSERA-VNSEMVEaEIATAYHLGKEQgGNPTILPVRLD-Y-
REPFQYPLSVYLNGI-
NWAlwqtdEDTPSLIEeLKRAIAGDTLSIN(TEKSKADLLQISPQPSLPRPSPSAQPIPLEIPGETMDSESRF
YIERPQDAIAIATIQRQGVTLTIKGPRQ)}*

$2281=216(214):
>gi|1028092391|ref|WP_063849613.1|
{|0(111)|<Cyanobacteria(B)>}transcriptional regulator [Hapalosiphon sp. MRB220] >gi|922990009|gb|KOP26130.1| transcriptional regulator [Hapalosiphon sp. MRB220]
{(MEQFEFD)---VFLAHNS---
KDKPQVRAIAQELTRYNLKPWFDEEQIFPGDNIqqVVFQGISQSKVGAFFISQNGL---
GTFQeNLELGAIIhffLKKHkNQGFrVIPILLginDIP-
DSLFYLSQWAWIQFtSSNDEAALKSLIRGIRGRGAEVQAPP(VIQIPDTPLIIEPKPPQTDDLASEKGIDYTR
LRDLLKAGKWKEADKETYQVMIQVVGKKDGDWFTSD)}*

$2282=216(214):
>gi|922990371|gb|KOP26456.1| {|0(126)|<Cyanobacteria(B)>}transcriptional regulator [Hapalosiphon sp. MRB220]
{(MEQFEFD)---VFLAHNS---
KDKPQVRAIAEQLTRYNLKPWFDEEEIIPGDNIqqVVFQGISQSKVGAFFISQNGL---
GTFQeNLELGAIIqffLKKHkNQGFrVIPILLginDIP-
DSLFYLSQWAWIQFtSSNDEAALKSLIRGIRGRGAEVQAPP(VIQIPDTPLIIEPKPPQTDDLASEKGIDYTR
LRDLLKAGKWKEADKETYQVMIQVVGKKDGDWFTSD)}*

$2283=202(205):
>gi|923072338|ref|WP_053455752.1|
{|0(126)|<Cyanobacteria(B)>}hypothetical protein [Hapalosiphon sp. MRB220]
{(MSHQAEFD)---VFLAHNS---QDKPEVREIANQLKRLGLNVWIDEEQIPPGKPFqdIIQQAIPN--
VKAAAIF----LGSGGLGkwqTMELRTLTSQCVsANIPVIPVLLPGV-
DKIPDdllfLKELNWVNFNEGVNDSEALYKLQWGITQQKPQISQ(NEEIISASEEDVNYYTQLETCLSQGKWQ
EADQETKKILLFSSGKKSDEKLGVEEIRN)}*

$2284=287(291):
>gi|922864605|gb|KOO31829.1| {|193(3)|<Haptophyceae(E)>}hypothetical protein Ctob_010733 [Chrysochromulina sp. CCMP291]
{(SDTAENEAAALAALVNNLSAAEAFKRGKELFRKLPFSRLTPEDLPGTSKVLGGSSNGMSAAAQPTKELSEK
TEKAILHEV)--DAFISHSWSddgdaKYAQM------
KEWAAGRDVSIWLDKACLDqrdITTSLaGLPVFLAGCKQLLVLAGPTYASRLWCVMEIFVFVRMGGntKQdmv
VRLLSGATDLA---AALKKFDAGKAETYDPN-----DRER--
LWAVIEASFGTF(DPFNKVVRDMFDKKLQRGQSSHFKLPQLKNRISSSSLNPRPSADVLPACLITVKPDDDAA
TTPSHSGPAPS)}*

$2285=247(252):
>gi|922866205|gb|KOO33107.1| {|458(0)|<Haptophyceae(E)>}hypothetical protein Ctob_009523 [Chrysochromulina sp. CCMP291]
{(MSAAAQPAKEFSKKTEKAILHEVFKRGKELFRKLPFSRLTAEDLPGTSKVLGGSSNGMSAAAQPTKELSDK
TEKAILHEV)--DAFISHSWSDDGDA---

KYARmtEWAAGRDVSIWLDKACLDqrdIKTSLaGLPVFLAGCKQLLVLAGPTYASRLWCVMEIFVFVRMGGnR
EdmvVRLLSGATDLA---PALQKFDAGKAKTFDPT-----DREM--
LWAVIEAAFGTF(DPFNKIVRAMLTDKIQRGRSAHLASLQLPVSV)}*

$2286=259(266):
>gi|922868300|gb|KOO34724.1| {|132(735)|<Haptophyceae(E)>}apoptosis inducing factor [Chrysochromulina sp. CCMP291]
{(FPALTDVPETAQTDEGRLVKPRDAAAAVFGHTIALDHMDAYLDAFLENLGKRLGGPGEASSAGRRPRETST
DFLLGERAI)RYDAFLSHKRSeaqdivARVHDRLSDFG--------YRAFIDRNDLIELPSL--
KLAVRDTQTLVVFLTPAYFASAWCCLE--FVEAVRHGVsilFIHVDGS-TWG---
ASGAQFPSLSDVPVTIKVEDVTIHVREAAAKVFPPAQLVE(HTRSYFNPFVDALCKKLGPPPAISSIEGETRG
VWSAAGGGATVSWA)}*

$2287=197(209):
>gi|918438876|ref|WP_052490052.1|
{|0(183)|<Cyanobacteria(B)>}transcriptional regulator [Tolypothrix campylonemoides]
{(MTYAQPQF)--DVFLAHNS-QD--KPLVRAIANQLKQRGLNPWLDEEQIPPGRAFqdEIQQAIPLSK-
SAAIFLGLQGLGSWQSWELkALISQCV-----KKNIPVI----
PVLLPDVRNLPEHLLFLEQFRWVSFSDRIDDENALYLLEWG(ITGQKPEPTVKVIPQSQSDDISYEPNKEVPI
TQERRKLEVIETQNTQQNQRDDSGDDLSTE)}*

$2288=160(162):
>gi|917130602|ref|WP_051737314.1|
{|0(324)|<Actinobacteria(B)>}hypothetical protein [Pseudonocardia autotrophica]
{()MADIFFSYKRED--RGRVEPIVRLLEREGL-
KVWWDPDLAAGERFDeVIAEEIDAALCVMVAWSVHSVRAPWVRDEASRGMRRGVL-VPLSLDGTEPPLG-
FGVIHTPDLQGWQGDEAdpRIRHLVTDVVGRVTGTVPEPGPD(LVPEPPRVSRRTLLQVGVGTGA)}*

$2289=221(216):
>gi|1174066383|ref|WP_081424266.1|
{|0(199)|<Cyanobacteria(B)>}hypothetical protein [Anabaena sp. WA102]
>gi|924290368|gb|ALB42132.1| hypothetical protein AA650_18225 [Anabaena sp. wa102]
{(MSDNRLQFD)---VFLAHNS---
KDKPEVIKIANKLRQGGLNPWLDKEQIFAGDNIqeVVFQGISQSKVGAFFISQNRL---
GAFQrNLELGAIIQffLEKkEKGFrVIPILLPDI-
SDIPDelyhLKQWVWIRFTNSNveedEEALQDLIRGIRGRKEVILTPQ(PDPIINPEIKPIPKTAEYFAQDLG
NGVILEMVAIPGGTFIMGSPENEEGRTSDESPQHQVTVPPFFM)}*

$2290=138(148):
>gi|918434907|ref|WP_052486083.1|
{|0(335)|<Cyanobacteria(B)>}hypothetical protein [Scytonema tolypothrichoides]
{(MVNQPEFD)---VFLAHNS---QDKPQVKAIASELKRRGLKVWIDEEQILPGRPFqdVIQQAISN------
-VKSAAIFIGSQGLgKWQVMELRILISqCVDADIPVIPVLL-
PGVEGIPADLLFLHQFNCLSFASGIDDVGVLDKLVSGITQ()}*

$2291=194(196):
>gi|503476079|ref|WP_013710740.1| {<Firmicutes(B)>}hypothetical protein [Carnobacterium sp. 17-4] >gi|328673752|gb|AEB29798.1| hypothetical protein CAR_c11060 [Carnobacterium sp. 17-4]

{(MKKIFEQGEFKNYSQHNLNEARASIKKSYQFSETKTT)---IFISHKHDELENLKDILGFLEKNFDV--KVYIDSKdpslpTITSGETAsRIKKRINDCDKFILLATNGAIE------SKWCNWELGFgdAKkFEKNIALFPMKPAETSDWSYKGSEYMSIYPYITFFDGSETDIDGNFVPKGYYIF(TDKKDGTYITSFKEWLLEK)}*

$2292=195(200):
>gi|496510309|ref|WP_009218592.1| {|0(262)|<Firmicutes(B)>}hypothetical protein [Lachnospiraceae bacterium oral taxon 500]
>gi|330411724|gb|EGG91129.1| hypothetical protein HMPREF0491_00310 [Lachnospi
{(MNYDEYIERMKDNKYKELVVTKSGEDKPY)---VFISYRS-DSWKKVLTEIVYKLQKEYGLRIYFDKDFASETNTWveQFQKNMNSpfCKAFICFFDEGYVTSYATLIELMHAMNPK-----SKLKDSIFSINFNIDWKKLDDNGNNTGLGVKQDDNPGWEKEKEAFEDEFELI(KDDNEYKAVKAYYKRNDELRVCDCKDIMNII)}*

$2293=212(213):
>gi|493107781|ref|WP_006140010.1| {|0(177)|<Actinobacteria(B)>}hypothetical protein [Streptomyces griseoaurantiacus] >gi|329303519|gb|EGG47405.1| hypothetical protein SGM_2329 [Streptomyces griseoaura
{(MSHFYLSHVRRVDEEW)--------------VAAFFRDLSAAVAALtgrPAETVGTRGD---PQSLevPLER-LSTSTVLVPLLSPRYFSQSYCVAEWNYFQQRLdvhwSRTgRRaDAVVPVVWEPDPdhvlpaDQPYTPLKVPSQAYQRDGVLHLLRLRTRYQEDYTSLLTAL(AGRIVAHAERLPEVHGFRTAPAGVGAASTTRPGRRTVDFVMASMPVGELPAERRAHH)}*

$2294=234(239):
>gi|495779566|ref|WP_008504145.1| {|192(6)|<Bacteroidetes(B)>}molecular chaperone Tir [Mucilaginibacter paludis] >gi|373888703|gb|EHQ24600.1| hypothetical protein Mucpa_0406 [Mucilaginibacter paludis
{(FRNSLFSDTTLYLCAFIDCVLANKTIIAQKTYLHPNYASLDFQTILKSDLLDPIILKNYFNIQAPDVKEKVKDISSEIHF)-KKIFISYSFKdKAFATILNGVLNKNGVK------TFLWENDAPGGQyledIMRKNVHEHETILFIASESSLRSKACQFELSEGRRKQEE-TWSNVFFPIHIDHYLFEVLENQIRPIDKAKE--YWENILELKRTNSKDFSAYAN(VDATNMEKFERAVQTDILQ)}*

$2295=215(220):
>gi|497356983|ref|WP_009671196.1| {|58(0)|<Proteobacteria(B)>}hypothetical protein [gamma proteobacterium IMCC1989] >gi|330418234|gb|EGG92842.1| hypothetical protein IMCC1989_2444 [gamma proteobacteri
{(TPKDSKIQVMKEKMLSVGARFRVDIIFDEMTKLSTKKRFNSFVVSTSDQIGFAVENTFQFADRAYVSSKVPMGPLGAELP)---IFLSHSTIdKPFVEELIPYLSRHGL---AVWY---DKLNISYGEsitsAIMNGIDDSGAVIFFITESFLSSNWCKEEMesflsRAAEGDKIL-PISVVFPNIEHSQLPRFIKNKKYLKLNN-----GTAPSIVANELVPTFKDFFSL()}*

$2296=235(239):
>gi|470242492|ref|XP_004354895.1| {|636(262)|<Amoebozoa(E)>}Toll-Interleukin receptor domain-containing protein [Dictyostelium fasciculatum] >gi|328868115|gb|EGG16495.1| Toll-Interleukin receptor doma
{(ACEKNYFDSALLLLEFGCDRNVKNTKGSTPFHFAVKNYPVGVLMAQKGAQNIPDRSMKTPLQYAVGEEKAFLQSLLFTNE)---IFLSYAHKdSVFVKDLRLSLE----ESSLRCWLDEyRLQAGCNWRsEITKGVQGSNVVLFTISPTSINSLWCRKELKMAKRLCKP-

VVALYYFNLDIDP-
VLHGLFDYEIRFADNKAwaDLDAPQRRVELERVGKIVKSIDN(RTPCNAKNQFDKVDTYNKF)}*

$2297=205(193):
>gi|504850206|ref|WP_015037308.1|
{|0(217)|<Actinobacteria(B)>}hypothetical protein [Streptomyces venezuelae] >gi|328886174|emb|CCA59413.1| hypothetical protein SVEN_6127 [Streptomyces venezuelae ATCC
{(MVRR)-PRIFLSHSSSkctvesgcqcRRYLESLESHL---KDSG-
FDPVVDRAfLNGGDEWHqEVLKEIMTSHGMVILISPHALVSHYVLEEAIVATARKVA-
SEdaFLILPVLLpgvRRS-
HLADSGLKKLNLGRFDMvdWSAKAGPAKPPPKIAASLLPLAE(RLGAVPYPEVTAFVAGRMSDVPSVVLEEAATVLGVPTLAHATDHSRHRV)}*

$2298=250(257):
>gi|504850212|ref|WP_015037314.1|
{|198(795)|<Actinobacteria(B)>}hypothetical protein [Streptomyces venezuelae] >gi|328886180|emb|CCA59419.1| hypothetical protein SVEN_6133 [Streptomyces venezuelae AT
{(PELRTLEDFYRSLGGRIADTPFGVAYDRPYWPVLAAGAGLIAGGAAAAARETVARTALAGRVFEELSEAQGQGPPRETTG)--KVFVSYSRRdAAFARRLHEELAASLR----
EVWVDwLDIEPSAKWReSIDEAIRGSDALIVLLSRDSLRSKYCWLECERALAL-----GKRVLPVVIDPS-
LEQGAGAALRENGWEPLadYQFLRMTRPEQFTPGVGRIHSFV(KSRHRWTAFHTRIGLRAYEWHSGGRSGALLLRGHELA)}*

$2299=214(238):
>gi|493107778|ref|WP_006140008.1| {|0(192)|<Actinobacteria(B)>}TIR domain-containing protein [Streptomyces griseoaurantiacus] >gi|329303517|gb|EGG47403.1| hypothetical protein SGM_2327 [Streptomyces g
{(MPGPHVFLSRSEPLDCPCNCWPARRAMEEL)------------------------
LRAEGCAPVVVDREsLVPGQEWMeAISDGMGSAHGMLLIVSVHALRSEHVQNELAMAELRNRT-
DGFPVILLMLPEV-DLEALERSGLNSLNPTRrqTVEWPERG--
DLEAVRRDIAPQL(ELMRAGLNDSRVHHQVVRHLREVPDRSLDRASATLGVPLAGLSPLKQHRLASGLLAERPSEQEADADP)}*

$2300=180(185):
>gi|926394600|ref|WP_053722992.1|
{|0(302)|<Actinobacteria(B)>}hypothetical protein [Saccharothrix sp. NRRL B-16348] >gi|925588650|gb|KOX13005.1| hypothetical protein ADK67_45660 [Saccharothrix sp. NR
{(M)------SFSG-ADRESGRA--
LAVELRALGSRVFLDEDSIECFSGItdSILNGLRASKMLVAYYSADYAVRAACQHELMLAFLAGQRDGDacSRIMVVNPEADTDHVRPIELADAKFVLPSTDVHTLAQQLAKHAAAVDRSIGE(VRPVERPRWSVRRSGHVRKFVGRYRELWDLHSALHAIDFPLVNE)}*

$2301=194(186):
>gi|926319237|ref|WP_053653587.1| {|0(458)|<Actinobacteria(B)>}TIR protein [Streptomyces sp. MMG1121]
{(M)-WDVFLSYSR-GDVER--
VRPLVRALRDGGLTVFTDETGVASFAGIsdTIRRELGRSKALLAYYSAAYPEREACQWELttAYLAGLGEGDPRLRVMVVnpepatghIHPVELRDARHASADDPAALVADVREHVARLTGPMTIREHGGQGRH(RAPEPHPEFVGRLSELWRVHSALHAHAAPLTVGRAATRTVQIRGM)}*

$2302=194(186):
>gi|926417365|ref|WP_053744925.1| {|0(445)|<Actinobacteria(B)>}TIR protein [Streptomyces sp. NRRL WC-3618]
{(M)-WDVFLSYSR-SD--
ADLVRPLVRALSDGGLSVFTDETGVASFAGIsdTIRRELSRSKALLAYYSAGYPEREACQWELttAYLAGLGE
GDPRRRVMVVnperttdhIHPVELRDARHASFDDPAALVSDVRAHLARIDEPMTITEPGVRRWL(LGTPEPRP
DFLGRLSELWQVHSALHAHAAPLVTGRAAALAAQIRG)}*

$2303=323(257):
>gi|925682696|gb|KOX79342.1| {|396(0)|<Arthropoda(M)>}hypothetical protein WN51_09144 [Melipona quadrifasciata]
{(RILEEIVPGGRVGAASADLGRGVLRALLATPGAALPRQIRPPRGECEPQCIRARNDWALMHWAGWTAIDAV
WLTHDGKEC)--
DALVCYHEKdsSLVIGIIIPTLESRHRYKCTALelsqlnqnsHTEPFLSPAFsiteviietqqsrvarvivan
vqigkIKKNIIASFVRvlcdfegrgaeigshavsSRRVIMILSPASLGNVWNETNVGTVLNQ-
LSSLTVPIIVIATKDL-
SNTSTIakrsnrgcNDSSEFSLDRLkILHWNENHGTSgfgsqkFWYKLRLAMPPV(RPISSESGCQSVAMIVQ
ANGKCGQQKARSRESLEVLV)}*

$2304=196(205):
>gi|926406586|ref|WP_053734335.1| {<Actinobacteria(B)>}hypothetical protein [Nocardia sp. NRRL S-836]
{(MSGYKYDFFISYCRYGSVQRWLLNH)-------------FLDKLRECLADQYA-PTPTVYVDR-
SMERGVHWpsNLQKALRHSKIMIQLLTPQYYQSRWCMAEQRSMRAREEMLglasldiSQGLIYPILYADSEnf
ppEE--------
RERSWVNfKDVAHPDPVYQQSKKYLRFHTRV(NNLAADLVKLAKVAPPWQENWPDIDPPNPPLLDPPQIPRF)
}*

$2305=205(215):
>gi|926393704|ref|WP_053722096.1|
{|0(92)|<Actinobacteria(B)>}hypothetical protein [Saccharothrix sp. NRRL B-16348]
{(MS)EYDVAVSFAE---EQRSAVEEVVEAFKQRGLTVLHGPEQNHEWWahkeGGdlpD-------
ARVRffVPFVSE--------VDDFTTAALRAVKAGDEHVLPVLLGDVAvppdL-LHPHVAYV--RGTAP-
DQLTEALV-
ARIEAAEAVGRER(APVTEVVTRVKAVVPVAEEAPAEPVVPVTFSRYTEQDATLRYLGAQFAAALPSLERRGF
VGTAHVGETRIAVR)}*

$2306=280(300):
>gi|925431272|gb|KOU64069.1| {|263(416)|<Actinobacteria(B)>}hypothetical protein ADK57_20185 [Streptomyces sp. MMG1533]
{(LQIPYEPAYAFDEQVITDPGRAEERRGLANAYASLRNAIALLAPDGNPLAALRPALTDDDPLGGGPGPVET
RYDPTTRFA)SPDVLLSYRHTDRETADEIRELLTGRG---------
NLLVAEALSESPPEEARRAKVSLVLVSPPG-DLSMYQRyELAALAARE-----
DAsLLPVLLPGVSSPPEELRGYAFLDFsMG--VDAE------
RLLETVQAGLRP(PVSSRPGSGRTERSPYLGLMPFSEADADVFFGRDELVSRVLMSLDQDRMCTIIGGPGSGK
TSLVQAGVLPALRRGALPGS)}*

$2307=186(185):
>gi|926413482|ref|WP_053741042.1|
{|0(252)|<Actinobacteria(B)>}hypothetical protein [Streptomyces sp. NRRL WC-3618] >gi|925560404|gb|KOV86250.1| hypothetical protein ADL01_07135 [Streptomyces sp. NRRL

{(MN)--KVFISHSTRGDAYATAVRSQvvhgLRERGYD-
VLVDADRLLPGEKWHPRLYEWLLECRAAVVLLNRAALASG-WVHrEvdilmWRHHFNRS-----
FFVLPAPVGDVTpeDMTAAGFEDLMSLQLV--
QRTGAGDAGTEPSDPAEAAAVI(VRAFPPLPALPDDDDPVRRWAEDITAQLSQVRTMSRLARMGRA)}*

$2308=182(181):
>gi|926301126|ref|WP_053636820.1| {|0(245)|<Actinobacteria(B)>}TIR domain-containing protein [Streptomyces sp. XY152]
{(MT)--QVFISHSSRADSLArqvrERIAAGLETKHYE-
VKVDTDALRPGEDWCPTLYQWLAECDAAVILFNQEAL-DSFWVRrEvnilmWRRALNPG-----
LLVVPVLIGDLKtaVLKENGFSDVLPVEFA--
RTQAAVQDESAIEDLVKQVLQE(FAELPDLSTEPDSMRKWIGLVADYLERTRKKKALTEAVR)}*

$2309=245(238):
>gi|926399886|ref|WP_053727998.1| {|0(348)|<Actinobacteria(B)>}TIR domain-containing protein [Streptomyces sp. WM6378]
{(MTPAGQDAAPAAGGVGAAPPSAAGQ)-WDVFISYAREDYIQAKDLHDALSACvtAHggapR-
IYLDVSrtsGTPLGADWQSFLEEALPRSRHVVALYSQTYF-DKPVCQwELHEAYklnpPEG-----
GRLVPLLIDPGAaaKVPYIVNRINWIptsRPHW--IDEVRGAV--
GLLPAGTRTTLR(FDTSVTEAVVGHTLAPVCVTGSVPEGTPPWPVDGTVTVSAEPAGAELTGTLTAPVVRNAA
VFTDLAFRTAAAE)}*

$2310=177(175):
>gi|926404478|ref|WP_053732227.1|
{|0(20)|<Actinobacteria(B)>}hypothetical protein [Nocardia sp. NRRL S-836]
{(MGYE)-WDVFLSYTrsgGGDvwvrehfyRALREWLGNEMADEP-R-IFVD-
WNQDGGTSWPESLERALLRSKVMVAVLSPPYFR-SAWCLaEWESMWARH-----
RVLGYGTADHPRhlVIGVRFADGQHFPPEA--
QATLLRDFTPWNKPLAYEVYSR(SAQYEHFYTAVQKLAEEVAKRIEEVPPWNPE)}*

$2311=222(231):
>gi|926432372|ref|WP_053759136.1|
{|227(0)|<Actinobacteria(B)>}hypothetical protein [Streptomyces sp. AS58] >gi|925539648|gb|KOV66629.1| hypothetical protein ADL00_17725 [Streptomyces sp. AS58]
{(LNLIHEAAGRGTGRSTPDLVVGESLRDATDSRQPVLWWTDPAKLAVAVERQLERFDLPRQQFRSLEEGIGY
IRSTGREEG)--SIFLSTTGAdNELSREVGRALK-
LQNFTFFHYVYNNTFPKGSKWQdRLEEQLAATQVFVPLLSESYWRSDWCRLELETARRLSDE-
GQLTIIPYFLDGS-SGDS--------VPEQGtdITDLPQHDRVARIVGDMDRHLAR(RPAADDSGVRP)}*

$2312=163(157):
>gi|926410186|ref|WP_053737935.1| {<Actinobacteria(B)>}hypothetical protein [Nocardia sp. NRRL S-836] >gi|925553793|gb|KOV79860.1| hypothetical protein ADL03_35375 [Nocardia sp. NRRL S-836]
{(MTIQTVR)---
IFLSWCRRdKVHKEALLHDLRPALRLFtdlRVEWWGDDRLMVGEEFTpAIVDRIDECDFGLLLTSTHYLVSPY
IRKhELPRFAGPAAD-KGALpvmlgVLPPFGSEI-DFGG--LAELNMFSREGksysATTGPRRA--
EFAIDLAAEIRRR(ALGQNGYTAA)}*

$2313=178(173):
>gi|926393708|ref|WP_053722100.1|
{|0(18)|<Actinobacteria(B)>}hypothetical protein [Saccharothrix sp. NRRL B-16348]

{(MSGY)KFDFFISYARRgsvqKWLlnhfhQKLLECLADQLAPTP--KVYVDR-
EMARAVHWpsSLQHALRHSKIMIQLLTPHYFESRWCMAE---WYSMLEREkvlGMANPGQPMGL---
VYPILYSDSDNFPPEGKERSWQNFKEFANPDPPYQETREF(VYFHREVNRVAADLVDLLKQVPPWRPDWP)}*

$2314=214(220):
>gi|926405027|ref|WP_053732776.1|
{|0(612)|<Actinobacteria(B)>}hypothetical protein [Nocardia sp. NRRL S-836] >gi|925560912|gb|KOV86714.1| hypothetical protein ADL03_08380 [Nocardia sp. NRRL S-836]
{(ME)QFDVFVSYaTVDRALVRPFVERLRADGFRI--WFDEQRMAGGRPAMQQLAEGIENsAH-
LVACLTDAYLQRPWTDFELQVSRYLDPSGRLGRTIPVLFDRLTVPLPAEIRFLATCDLTA----VATYD-
AEYRKIAEALRRQ(VPATGGESLQDACVAPFQHLDEPGVALFKTYQAVSALRRHQGTVGGPTLDQLADSGEVP
PHLHAPLATVQAYARSHED)}*

$2315=251(257):
>gi|927673620|gb|KPA10420.1| {|277(7)|<Proteobacteria(B)>}Toll-Interleukin receptor domain protein [Candidatus Magnetomorum sp. HK-1]
{(MAVYQSLSISTDGRLSFEDLQEKNLHIDDIDNSLNVLSFHGIINDDDPDEPEIAGTMFKEWFLRNYLKKSK
HRHSEKQGI)----FISYAW-EAHSEKIVDELESMFKEKGLTIIRDKSELSYKD-
NikTFMETLGKGMSIIIVISDKYLRSHNCMRELVEILKNGNF-YDR-
IFPIILKDADIYEPIKRIAYVQFWDKKKQELDEQIGCVPSSSSKGFRDEI(DLYKEISDSISELTEILKNMNT
LTPDMHQRSGFNQLL)}*

$2316=240(226):
>gi|927685441|gb|KPA17229.1| {|0(135)|<Proteobacteria(B)>}hypothetical protein MHK_002549 [Candidatus Magnetomorum sp. HK-1]
{(MSLSSY)KYDIFLSYAN-KDmIKVDFVT---EELKSNKLSVFEAHntlQKKIGHS-
FieSLQEAIINSKDFVLVWTKNAVSSDWVKKEYETfINKCNIS-
DRENRRIIIYVTKEEDIKLLLPPFLADYqsckslkdLVYCLTYTEdikshfEKK-
EREKQKLIEKVNI(KNKEIDKFQVTLSSLKEEQTRFERSIAKRDEIISIQRSKINEFINKIEIKKETILQLND
KIDTERNECISLNRLLQKKEN)}*

$2317=227(238):
>gi|927678604|gb|KPA13704.1| {|0(197)|<Proteobacteria(B)>}Toll-Interleukin receptor domain protein [Candidatus Magnetomorum sp. HK-1]
{(MLDYKIPSFFVSYCHRNNERKPGQTAGWVTN)----FVNTLK-IDLGEKFAKH-------
ENYHLFIDENEIKGNNQidDKIFNELKNTDLLIIILTNAFLESQYCQEELnffVKNMKTQDF-
ERIFVVEkeiIKENKKPNSIKNLKG-----FRFI-
NAENNKDRLLGGNDNNFEQGWY(DQILTIAETMKNKILQIHPPNNNKGIESSKNKVYLAQVTEDLHEKYRDLK
NFLEDNGYQVVPESPIF)}*

$2318=239(239):
>gi|926641619|ref|XP_013786951.1| {|1033(0)|<Arthropoda(M)>}PREDICTED: protein toll-like [Limulus polyphemus]
{(VSACGNLTTITIFQAAFTVHDYMPFIIVGCIVFLFLVIIMVLLCIYRQEIKIWFYTKYGIRLFHKNHYTPE
AEK)LFDAFVSYCKKdeAFVAQILAPELECASPQQRLCLRYRDLPMVGYVAEAITEAIECSHRTVVVLSEQFL
KNEWCRFELKSAQHEARCNRHHRLVVVLLDNT--
SFKHMDADTRSCLRSAVVVRWGEKRFWEKLRYSLPDGRG(SSKHITCPETQLEERGLPGNSVKLV)}*

$2319=256(261):
>gi|926618539|ref|XP_013774478.1| {|864(90)|<Arthropoda(M)>}PREDICTED: LOW QUALITY PROTEIN: uncharacterized protein LOC106459406 [Limulus polyphemus]

{(IRKLFPFSSLNGAIPGGVRVTENELXRFQDEANPSQAMIEDWLKINGRTRYCVDLLVSCLSQIGREDISEK
ILANLEPEN)PCpSVFISYQW-DAQELVIE--
LRRRLELAGYPCWMDVGQLGGGDSLygKIYEGISGAKVVLCCLTPRYVTSLTCSREISLADVLR-----
KPVIPVMVEPTPWPPPGPLAIVLSSLVYIDMCGVGGHGGSGKQADWEQRFNE(IVDKVFPYLASEMTQRAPHT
QVKPYTPSRAILISWDEYVNE)}*

$2320=208(205):
>gi|927685930|gb|KPA17546.1| {|0(520)|<Proteobacteria(B)>}1-alkyl-2-acetylglycerophosphocholine esterase [Candidatus Magnetomorum sp. HK-1]
{(MKEIFFNYARSDNKINSEITGEP)--------D---
QIISRFKSLFEEIYRRGsgikDLSnlIFMDVNDIEDGQIVknVLDHEIKHCSVMFSFYSPTYFESEFCYDEWR
LFNNyKENDKkKRKLLIAIEVDPVSkineDAFQEGKNWFDEITKNIHRNISSNDLRDNSNQNLTEKIKN(LVE
NVKKHISKNKGIIENRELSSNILIIDKKIKDSIKMPEIE)}*

$2321=221(221):
>gi|928969890|ref|WP_053994327.1| {|44(0)|<Firmicutes(B)>}hypothetical protein [Lysinibacillus macroides] >gi|927331324|gb|KOY83079.1| hypothetical protein ADM90_07210 [Lysinibacillus macroides]
{(KELLPIIKKYSGDLNWIKFHGNKSVVNLNHLKNSFSDLFKNCEWMNGEYEVHPEFDQEIGSKVPIVSFSLS
KDVIELNSP)-KKIFLSHRGSnKEFVRRFYDILKTMGFEP------
WLDDEDMPAGSnldrSILKGLKDSCACVFFITPEFTDEQYLADEVDYAHIqkrkKQEK-
FSIITLQFENEDgVKGTIPELLERFVWKSPKN--ELEALNQILKALPIKANVIDW(I)}*

$2322=165(164):
>gi|927301317|ref|WP_053805200.1|
{|0(139)|<Proteobacteria(B)>}hypothetical protein [Vibrio parahaemolyticus] >gi|926596511|gb|KOY38515.1| hypothetical protein ACX08_03985 [Vibrio parahaemolyticus]
{(MKT)--DLFISYAWTSNEHKEWVhllASQLHLMGYT-VKIDeAVDY--GSSLSGFM-
REVTDASHVLLVVDENYVEradnaptSGVGIEtEWISSVFES-----
KPNTWLSVIFVRnpDYRLPRWLKAKSPKGF--
DFNSKPDARRYPGSTQIDAVWR(WIEGLPADKQHAIPISVVHKR)}*

$2323=199(207):
>gi|928986816|ref|WP_054011048.1| {|0(113)|<Actinobacteria(B)>}TIR domain-containing protein [Arthrobacter sp. ERGS1:01]
>gi|927294424|gb|ALE05875.1| hypothetical protein AL755_11020 [Arthrobacter sp.
{(MVNGQQPEPDRP)-RRFFVSYARADRENLAGLLFGLETMHHQ-VWIDS-
KLDGGQAWWDEILASIRACDAMVVAVSNS-LINSEAARlERAYAQRLG-----
KPLVPVVVAPVNydFLPPEIAAVQLIDHTH--
PDSASSFRVASALNALPLAPPL(PNPLPAPPPVPVSYMSGIAQKLRKPVLSEDEQRVIVGQLRDRLARPRDPA
DRAGA)}*

$2324=222(233):
>gi|927671741|gb|KPA09236.1| {|450(0)|<Proteobacteria(B)>}ATPase AAA [Candidatus Magnetomorum sp. HK-1]
{(IDRQAEKLEGDIFLIPVQLEKCDLPHALEKYQAVDLDGFEGFERIISTIKYQFKKTLNAHYEDNIKSQIVN
VSLSIPDLK)-KKIFISYYHEdQQWLDKFLNYLSPLEYEGI------------
EYLDiEIQNSIADSQIFVCFISNNYLGAKFV-KkELPEILNKNKE--EILIMPILVAKC-
AWKvVAWLKKPQLFPIDSipLVKknEEEQE--EILIDIVNEIGGV(TIHSSEVIADKNQ)}*

$2325=184(177):

>gi|927672139|gb|KPA09463.1| {|0(323)|<Proteobacteria(B)>}hypothetical protein MHK_010295 [Candidatus Magnetomorum sp. HK-1]
{(MAYVSSYEYD)---
IFISYAHDdNAsgWVDSFYEYLKktlitVFGKKQPINIWIDSSLDGNQMFNeAICNTLEKSALFISLMSKNYL
ESDYC-KkELDWFCEHvHKK-GENLCHGQSHHRI-
IHGLLVNIPPDDWPDALkgKAGFCFYDENFCESSGLSLDLED(VRFRNVFRKLIDGIYKTLTAMKKNENN)}*

$2326=250(251):
>gi|927674046|gb|KPA10686.1| {|0(1)|<Proteobacteria(B)>}Toll-Interleukin receptor domain protein [Candidatus Magnetomorum sp. HK-1]
{(MKNIHLNGSGSIACGKNSIAVGERGVAAKGNMYGNVIITGDQNYIDNGSQDTVTSKDPHTFIENHS)-
KSVFISYSHEdSEFALKLKHHLELVGIECLMDV---
NSLSVGSDISvFIENAVYKTDYTIAIISKNSLRSAWVIKeYLETMMHEHVH-RSKKFIPIYIDKS-
LFDDAFFIETLKSIQKEmdKLKHFMNDDIVKDVKIPNLITKY(DRLMTLKQGLGEFFQQLNNTLVADFATDVL
FNENVDQLVRLLKGN)}*

$2327=205(205):
>gi|927674112|gb|KPA10728.1|
{|0(110)|<Proteobacteria(B)>}serine/threonine protein kinase [Candidatus Magnetomorum sp. HK-1]
{(MSNT)-AKIFISYSRKdLHSVDELADDLAFLGHS----
VWFDQNLQGGQSWWdNILAQIVDSDFFVCILSQWTVQSTAC-KkELQYA----KD-LGIAIIPILISEK-
NLSMNllppELSKIQYLdyrNDDRktILKFAKTFCEKVPAKKVHEKLPP(PPSIPISYLGSIAEKLSSSITLS
YEVQSSLLIDIKKGFNEPTTEKDAFQLLKNLRKRRDLF)}*

$2328=193(197):
>gi|927675029|gb|KPA11320.1| {|0(204)|<Proteobacteria(B)>}hypothetical protein MHK_008470 [Candidatus Magnetomorum sp. HK-1]
{(MQTNPKETFSPGP)---VFISYSRKD--LEEVKQIVKALKTAG-
ISIWQDIESMDVGMTEsQIRKAiLEECSGLLFYVTENSMDSIFIKEiELCEAYKKFRE-DETFsIIP--
FSDI-
PWDKADRDFNGVFMGKIsrFNGVNTLQYKSITDALNTLRKRI(LSSIVNRINESCIKISVMTHITTPKNFRSI
LDLDWSYFSKKDII)}*

$2329=168(175):
>gi|927678044|gb|KPA13337.1| {|0(642)|<Proteobacteria(B)>}radical SAM protein [Candidatus Magnetomorum sp. HK-1]
{()MIDIFISYSRFdSEKMNQLATVL----
RDRRLNVWSDQKIHGGAKFDdEIEKQLNLSKCVIVLWSENSISSDFVISEASRA-----R-
KQNKLIPILLDQV-
SIPIEFERLHYINLVDWciDASNPEFSKLIDSIDIYLADGQL(TNTQTLNINNSDIKINNAKKLSINNKNLSL
YKYSS)}*

$2330=253(255):
>gi|927678725|gb|KPA13770.1| {|349(10)|<Proteobacteria(B)>}ATPase AAA [Candidatus Magnetomorum sp. HK-1]
{(ENLVMKELKTFRDAVWNLLLFSGYLTITEKKPAPDHSDSHICCLRIPNTEIKNYFINEMMILKSTQEIDTL
MASENHVIQ)--KVFISYNHKdLAFVERLKNDLENAGIQLTIDI---
ERMKFGDDIQeFIEHSVEFSDITLSVISENSLASPWVMLeTLETFQQEDYI-KAMRYIPVMIDQH-
FQSPDFASTLIAHIEKNidLIFEEIARLSKKYVSTQSLNVKK(ERLINLRSNIDHVLLKLDERLIADFSTPKK
YEINI)}*

$2331=209(221):

>gi|927678834|gb|KPA13835.1| {|131(0)|<Proteobacteria(B)>}Toll-Interleukin receptor domain protein [Candidatus Magnetomorum sp. HK-1]
{(SALEHAKNITGSYKTIEADISLHGIMTQKDYNKLIHIKIKRGDSCNPTAIGPLGPIWSDNVPSWWDRAGLDKFDELATVL)-PHVFISYSHEdQVFVNELSDLLAL---SGVTCFKAERDIEPTSDWDeAIWNAIRGCSVFVSVLTPSFLKSRW-LDlEVGAA----KA-TNKIILP-ILRGV-TFDDlpHPFDRIQSV---AVETQPHMD--ELLKKLKSFTVDV(I)}*

$2332=219(217):
>gi|927681218|gb|KPA14923.1| {<Proteobacteria(B)>}Toll-Interleukin receptor domain protein [Candidatus Magnetomorum sp. HK-1]
{(MSKYTKD)---IFISYSRQdNK-KNIISIFLESLwdsmqHHVGRVPPFflDTADINTGDEWEsKLLQTVDTCRLLIAFLSPSYFRSSYCIKeW----KRMHKR-NYTAIFPVTLIPY-QPADTPLKLGQELNAEQtkVFNQANdfQN--LNWFNVQDNKQEE(ELKKFSFLIHNKLIQETDLKDQLPRDNHHIIDESIEENLNDSKKGKITKTILEVNQKRKFNHEPVCVIYT)}*

$2333=211(219):
>gi|927681345|gb|KPA14975.1| {|0(908)|<Proteobacteria(B)>}TIR protein [Candidatus Magnetomorum sp. HK-1]
{()MTKIFISYSREdEKFAQKLFNNLK----QDKMDPWlDDQKIITGDKWEeKIDKEINTTHCFLPILSKASINNNRYFQtEVDIAIQvSESR-DNNFIMPVRNEEC-DPGHTKLKDYNIL-----DLFPSFE--HQYKKLLKHIKEH(NRKFDFKKITDISLKTVDFLSKFATISYVASQFMSDDVQPPKYNMDFASYQFPKEGGKVQLLGPDVADQLRTIRLYARE)}*

$2334=175(163):
>gi|927681540|gb|KPA15069.1| {|0(169)|<Proteobacteria(B)>}hypothetical protein MHK_004723 [Candidatus Magnetomorum sp. HK-1]
{(MAYTTGYEYD)---IFISYSHVdnetpdksEGWIDQFHKKLNVqlsqfVGKANVVKIWRDIELKGNEEFDsVISHRIKTSALFITILSTGYFKSKYCQDeLQCFHDFAQSS-SLGLSVDKMRRIV-NVKIADIDYNTFSKNLGrtSCFKFHDEKGRPMRTKLDEFDDQ(IWELAKNIHSTME)}*

$2335=195(183):
>gi|927685114|gb|KPA17007.1| {|0(320)|<Proteobacteria(B)>}Toll-Interleukin receptor domain protein [Candidatus Magnetomorum sp. HK-1]
{(MAYVSSYDFD)---IFISYAHVdNIsssdkesgWINRFHKELqtKLDKRIGrpkIIKIWRDNKLDGSQVFNqTIVEKIKKSAIFLAFMSNGYIESEYC-QkELNQFHTHANN-KDPGLIVVDRSRI-FNLLLTNIPYESFPDEItgTSGFVFHDADSDDDIGVGEPTDP(DEKIFRKQLKPLTNAILSCLKKMNVQNQGEKQD)}*

$2336=120(140):
>gi|927685890|gb|KPA17523.1| {<Proteobacteria(B)>}molecular chaperone Tir [Candidatus Magnetomorum sp. HK-1]
{()--KVFISYSHNdKNFVLKFAKHL----RKKQISVWLDERIRSGNSWKkEISDTIKFTDYLIWIASPDSIKSK-MSQwETNLA----QI-EKKVILPII-------------YKQGKLPSWvnNIQWINYDNDFEKLVNKVSERIK()}*

$2337=185(185):
>gi|927686969|gb|KPA18252.1| {|0(351)|<Proteobacteria(B)>}protein containing Toll-Interleukin receptor [Candidatus Magnetomorum sp. HK-1]
{()MSKIFLSYSRKnEAFVEKLYKRLS---RDG-IDCFFDKEsIAWGANWYlELEKGIDEADCIVLILSPDFCKSEW-ASiEQSSARLKDPS-

GvKRRIRPLLLEPC-
GNHIPPFLQTIQQIDVSsnDLFENNYPKICKDLGGTLTKPDE(ISAPTNHLNSNAPEKSPCSIVTKSHEIPKQ
KPQNKSQSNHINPYD)}*

$2338=201(220):
>gi|927688418|gb|KPA19204.1| {|104(0)|<Proteobacteria(B)>}adenylate
cyclase [Candidatus Magnetomorum sp. HK-1]
{(LWTKRISLQEDISLPSSLIKVCSAIYAPIMFKSKKIGIIHLDSISNKNSFSNTDLQLITMIANTLSPALMI
RNHDNHEAY)-PKVFISYSHKdKNFVFKFAKHLR--KRQ--
IRVWLDERIEAGNSWKkEISDAIKSIDYLIWIASPDSIKS-----EMsQWETILAQI-
EKKVILPIIYKKCKlpSWAD----DIQWINYD---------NDLEKLVHKVSERVK()}*

$2339=200(220):
>gi|927689062|gb|KPA19605.1| {|104(0)|<Proteobacteria(B)>}Toll-
Interleukin receptor domain protein [Candidatus Magnetomorum sp. HK-1]
{(IWSKKVSLNQDLSLPSSLIKVCSAIYAPVIIRNQKIGMIHIDSISDNNAFSHKDLQLLTMIANTLAPSLVV
RNSCDHDMY)-PKVFISYSHKdKDFVLKIARHL----
RKKQIRVWFDERIEVGNIWKqAISDAIKYADCLIWVASPDSIKSEvTLWEvNLARI-------
EEKIIIPIKYKNC--------------KLPTwaLDIQWINYDQNFESLINIISARV()}*

$2340=225(215):
>gi|927672287|gb|KPA09562.1| {|0(260)|<Proteobacteria(B)>}Toll-
Interleukin receptor domain protein [Candidatus Magnetomorum sp. HK-1]
{(MPCYTIKHE)-HDIFVSYAWVddkpmfdqqkgwvSTLVDALQtKLAQELGRKDSFDLWKDER-
LSLNDKItpEIEKEVTNSAVLLVILSPGYMKSEWCRIERDHFLRANPNHkifIV------------
ERNEVLDNERPEALKDIIGYKFwhrekKSEPVIILGDPKPDVNDK(EYYNKINRLVYDLVVEMKRLQTINQKP
DDSSIHAISTISDDSPAVFMAEPTDDVHEIWEDLQDKLK)}*

$2341=290(298):
>gi|926621896|ref|XP_013776308.1| {|88(42)|<Arthropoda(M)>}PREDICTED:
myeloid differentiation primary response protein MyD88-like isoform X1
[Limulus polyphemus]
{(LFDLLEEIERTDVIDDVKQYAEIDAHNYLERKQKWCDKESPLQDPNVSSCTRSAFPADHPNHLTVDDVTNS
EYKGQEVSL)-YDAYVCYtDEDIECVHILSRQLESEG--
IRLFIRDRDLLLGQMEYEAFARLIDErCNRVLIVLSPEFLKSVECEFQTRYATSLAVEQQQRKLIPIIYRSC-
-DVPHLLRYLSKIDFTK----LHIHD-
WVWHRLIHSIKGE(NGREFTSSVSEQSSISCSIPPTYVTSVPFSAKNNPPYVQSQNDRESCCDDKEEVSPVPG
TSSVSSLHTSGKWAFHTGL)}*

$2342=135(150):
>gi|927262067|pdb|4W8G|A {<unknown(x)>}Chain A, Crystal Structure Of The
Tir Domain Of The Toll-related Receptor Trr-2 From The Lower Metazoan
Hydra Magnipapillata (crystal Form I)
{(MGKT)-YDVNICYaPEDREWViNTLVFKLERAG--
IKTFVNIRDDTPGNFFAENIMDAIENSNRTIVVMSPDFFKNNICD------KTLQIGLSHQ-IIPILYRPC--
EVPYFLNHMTYLDWCD----KDVRP-VFWRNLFRDIRNN(HHHHHH)}*

$2343=220(225):
>gi|927687165|gb|KPA18376.1| {|0(1305)|<Proteobacteria(B)>}repeat-
containing protein [Candidatus Magnetomorum sp. HK-1]
{(MISQY)KYDVFIAYaEADMKHASKLSERLGNAGVKV-FFDQWEVESSGD--
RSKMIKAGLKfSKKLVMIWSPDFFEDSAClSLLTSFIKDQNstLIQKDRLLIPALFKE--
TIIPDKYENLIIVDFRE----KADFE-

LKIRQLIEAMDLP(DLMYHVTRSSESTKTKTDDTLVFDTIGSRLKGFITEISDVYSLLNFEVHANERIEGLPN
SFTIKREVSGIGGFTISAVVV)}*

$2344=281(240):
>gi|928020727|ref|XP_013858449.1| {|244(0)|<Chordata(M)>}PREDICTED: interleukin-18 receptor accessory protein-like [Austrofundulus limnaeus]
{(TRHLNHTYTCVARNAVGNRSVTIVLKLRKQVRWPSLVGYPFACFLLVAGLGLVLHLNRLEIQIIIRSRFQT
EKDTAEETT)-FDVFLSHV-
WscqsnqaegsnsvsrsglhnneEEEMLNstaqtsqspvevLLTQVLEDQWGYRVCLLERDILPGGAYTSDVV
LALKRSRMLVCVLSAEYLSDrNAVF-VLESGVKALLQSSALKLLLIRTxgpsaSLV-
QPDPQLPELVQKALKVLpSLDWSPDPSSrtacGFWTSLRRAMPDR(RAAASHVKRGNIFPSRRVKK)}*

$2345=165(172):
>gi|1011880746|ref|WP_062697560.1|
{|0(657)|<Bacteroidetes(B)>}hypothetical protein [Chryseobacterium indologenes] >gi|929461565|gb|KPE51901.1| hypothetical protein AOB46_06655 [Chryseobacterium indol
{(MK)---VFLSHSS-KQ--
KNFVEIIANKLGKNKIIYDKYSFEAGSITLDEIYKGIELSGIFVFFISKESLNSEWVQKEILRAEEYIKN-
GKLKKFLPIIIDSEITHDNQQIPDWIKDSYNLKYFSKPTKVYDLIRQTLVLVNW(ELYPNKKELNQLFIGRTL
QIKDFEQRIYDF)}*

$2346=334(227):
>gi|929263195|ref|XP_014008084.1| {|329(0)|<Chordata(M)>}PREDICTED: interleukin-18 receptor accessory protein-like isoform X1 [Salmo salar]
{(TQLHLDSTFTCLAQNSVGNSSVTIRLIRKSAVSRVLVIVCPVVSLLFVAGLGITVHVYWLELSILYRIYLP
YKQTTTDDK)EYDAFVSYVPSssseevggrgrgvreEPLEV-
LLPRVLEGQWGYRLFLLERDLLPGGAYAEDVVCAIRSSRRLVCVLSPDYLASTCLF-
ELETGIRALQQDPHLRLILIWTSPN-
Psspsttspsttspkpsipsttspnpsnpspsttspnpspsttspnpnpspritspnpspspsttsptssrpl
slplshtlglvaPASPLPPLVRRAIRVLpALHWtpqdqgRMITSSnsgsRFWRSLHKAMPAQ(SGTGTDT)}*

$2347=262(261):
>gi|929431119|ref|XP_014133217.1| {|367(97)|<Chordata(M)>}PREDICTED: TIR domain-containing adapter molecule 1 [Falco cherrug]
{(SMEDSSVPASIPSNSASASISASSLPPPPTYPFSSTLPYLLQGATSKSSYPPPLHSSHSPAWPPPLQSVEP
APTSEPDGG)KFFTFVVLHASeDEIVAHRVkDRLENMgvPNGATLC--
EDFFIAGRSHMTCFQDAMENSAFIILLLTKNF-PCNLCMFQTNTALMESILKPSKHDSVIPFVPK-
ENPLERSQIPSILGGLMpLDENSPGFSRTVQNTFTTSRINE(RKAMWDLMQRRKLQLYRQQYQTLQDLTALHL
GSLSQLPPSA)}*

$2348=212(220):
>gi|1033081258|ref|WP_064391224.1|
{|56(0)|<Proteobacteria(B)>}hypothetical protein [Pseudomonas sp. RIT-PI-r] >gi|929898337|gb|KPG94471.1| hypothetical protein AK821_19875 [Pseudomonas sp. RIT-PI-r]
{(IQNDPVGFESWLRAKYPEKLQRHNVFLNDVIFYNVITFLEDGMIDLVFTSEPNSMFIWPERELLEMMNPQY
KKMNRNESK)---VFISHSS-KD-
KELIVGPLNAYLQAEDVATWLDSYEIDYGDNIylKVNEGIENAKVGVFILTNNFFDSAsgWPLAEfstffMDL
MRANK-----KVL-------MVNAGVDQDKIHAMMKAYKYLNWDDGKGLPEIANAIKRKLMS()}*

$2349=194(180):
>gi|930049850|ref|WP_054153120.1| {|0(337)|<Proteobacteria(B)>}TIR domain-containing protein [beta proteobacterium AAP51]

>gi|929675303|gb|KPF58146.1| hypothetical protein D621_07075 [beta proteobacte
{(MPLTKSFERD)---IFISYCHAdnedpmgdgwvELFHKNLRVRLTQLlgaraaHEQPVI--
WRDISLQGNDEFAgVLAEELQKVALVISVLSPSYVRSEWCRReIDTFSAAARAR-GGMVVGANKGRIL-
KVLKDEVPQDEHPEVLRgqIGFPFYVKDPDRQRPVPFTLTKG(DDTIALAKKVIDDLARSIMDTVKALNELQP
)}*

$2350=190(191):
>gi|931406861|gb|KPJ94720.1| {|0(18)|<Proteobacteria(B)>}hypothetical protein AMJ53_04710 [Gammaproteobacteria bacterium SG8_11]
{(MHYH)---VFLSYNSHDQSE-ASLIFQYLKDSNY--
KTFFEKDALKPGDiWQkKIEDALLSSDVIIILLGSQGVGR-------
WQEIESRIyqiistqCIeEKKPIIPVYLPRVNeaSDREKFPIFLKQFHEFRFKESIDNEIINLLTHLTCDTSS
L(NNYERPILSYQQDILLKNTTRCYNKNAEKYFETWKDIISPSLYAFAD)}*

$2351=291(292):
>gi|943952978|ref|XP_014359365.1| {|957(59)|<Arthropoda(M)>}PREDICTED: slit homolog 2 protein-like [Papilio machaon]
>gi|930674012|gb|KPJ14418.1| Protein toll [Papilio machaon]
{(MNDPDVSKNEIISKSLFLPDNYFAIICGVIIIVVLVCLIGAIFYAFRYDVSDWLYTKYGVPLFKEQSCANV
DMLEGNPNH)MYDYYVICNTKdtQFTYHNIMSEIE-----FRKMTTKKLSLNESSLNI-
LTldsfsKSPKLTKRLLVILTTNFICNDLCDIHFKNMFYSYLKSLnrsdLNKVIFIKVVDN-
NQINDELGFLLDNFKNLswN-----DPR--
FWDRFIALLNST(DTIITVKSSEKSVFKKSLRATPTLRYTTMPVSNETCSKPNFTNSLQYCKRNDGEPSHNDS
SPSSDNNTYGEG)}*

$2352=198(201):
>gi|931592575|ref|WP_054288167.1| {<Actinobacteria(B)>}hypothetical protein [Kibdelosporangium phytohabitans] >gi|930828988|gb|ALG06191.1| hypothetical protein AOZ06_03960 [Kibdelosporangium phytohabi
{(MSGYKFDLFISYSRYGSVQKWLLNH)-----------------
FHRKLSECLADQfapPLKVYMDRTmprAVHWPSN--
LRTALRHSKIMIQVLTPHYFTSRWCMAELRSMQAREEMLglggaeiSQGLIYPILYSDSEnfplEE-------
-
RQRSWVDlKEFAHPDPvyqQTPDYilfHRKVNELASD(LVQLVRQVPDWQPDWPIVEKPEPVLIPPPPLPRFD
P)}*

$2353=210(219):
>gi|931592586|ref|WP_054288171.1|
{|0(88)|<Actinobacteria(B)>}hypothetical protein [Kibdelosporangium phytohabitans] >gi|930828992|gb|ALG06195.1| hypothetical protein AOZ06_03980 [Kibdelosporangium ph
{(MGEVAT)-YDVAVSFAE---EQRSAAEEAVNVWRQRGLTVLHGPDQTHEWWahkeGGdlpD-------
ARVRffLPFVS--------TLDDFTAAMLHAVKTGNEHVLPVLMGDVAvpADlVHPHVTYLRATEQWP-
DQITEALI-
ARVEEAESAGWNR(APVAEVVTGARDLTPVATVPVTFSRYAEQDATIRYMGEQFAAALPGLDQRRFVGTVHLG
DSKISVRIEHRGDI)}*

$2354=218(225):
>gi|931357437|gb|KPJ49785.1| {|0(116)|<Chloroflexi(B)>}hypothetical protein AMJ38_02780 [Dehalococcoidia bacterium DG_22]
{(MSQQKPV)---VFISHAEFeADLIRKLADYLQEA-LPDVFFFVSSSDS--
LEPGAiwtqEIRRALQDARIVLACISRQSVDRPWVLFETGFGAGRGNR-
VIPLILDDLPFSALTPPLSMFQAVR-PDQEQ--FVKLVES--

IAKETNTQPDTA(GLDRAAASRLVEALEPGGSSPGVYLGDAWNNIRIGWSRYDGDPRTFEAHKGYVSIGRSFTDGFRYPPRDTLMGPWHYF)}*

$2355=147(149):
>gi|931383817|gb|KPJ73915.1| {|0(762)|<unknown(B)>}hypothetical protein AMJ52_02610 [candidate division TA06 bacterium DG_78]
{(M)--KVFISYTHDgKEYADTLERDL----RAQDIDIWIDkRCIRPGHIWLrEIDDALSQVDYVLGVLTENYLTSVGGDEaYAIISKGLKEK-KVRFISLFFVPPH-EVKSVIIPALQGFNFSKdyTQGFSSFLDFLKQEGGKKLVIEK(EIILPKET)}*

$2356=169(176):
>gi|931442982|gb|KPK27527.1| {|0(334)|<Nitrospirae(B)>}hypothetical protein AMK69_10430 [Nitrospira bacterium SG8_3]
{()MKDIFISYAAEdRAIAQRLA---AGLEGSGL-SVWWDRQIQVGSDWDkTIEDALDGAKCAVVLWTGHAKSSRWV-----RAEARQAL-KEEKVVPVMLEAN-AIPLAFTGIQALRFLGWggAAGSQEFEILLGVIRAKIEGKSI(ELPEASSTKASVVGKLVALLGIKAMVGSVLALLLLA)}*

$2357=208(220):
>gi|931400465|gb|KPJ89121.1| {|59(0)|<Spirochaetes(B)>}hypothetical protein AMS17_03205 [Spirochaetes bacterium DG_61]
{(TKTAARPSTKKSTGKSTGTRKTAIKKGKTTQKRKTGTKITPARQKRKLQAAARKSVKVSQTHKPPVKSPFPAYKGGNPFM)----FTSYAH---EDMKTVFRVIKKLNTSRYRIWYD-EGIEPGNEWpeVVGKAIIKCSQFLVFMSSYAALSR-NVRnEINLAFNENKEI-----LVVFLDET-QLTEGMKLQIGTVQFINKYEFTEKEFFNKLKKVINSRIRS()}*

$2358=170(173):
>gi|936565656|gb|KPM51731.1| {<Actinobacteria(B)>}hypothetical protein ACG83_33475 [Frankia sp. R43]
{(MTRPTSEGPEGLEGTGSAGSVQGAAGAPDLP)-YDVFVSYRHRepAlsWVRHRLVPGLRQRGL--RVCLDVDSFRLGAPLVLEMERAVVESRCTLAVLTPAYLDGTFTELENVLAEHLGLETAQRRLIMALREPCEpK------LRLRARMWLDlTADDELEAGCDRLAGEIRSLQPP(PR)}*

$2359=225(225):
>gi|936565657|gb|KPM51732.1| {|0(1140)|<Actinobacteria(B)>}hypothetical protein ACG83_33480 [Frankia sp. R43]
{(MADGD)--RIFLSYAEADR---EWVAGYLTPAlGLR-AdqVLTADQFPPGEPVVTAFEQAVLGSRATVVVLTPAYLTDRWALLaELLASYqdiavdgtgavdGAA-----ARVVPVLREPC-DIPLRVGFRVRLDFTD--RSRWEAQA--ARLRAFLAQPEP(PVAEEIRCPYPGLAPFGRTHTRFFHGRDREISDLRRRVRSQPFTLVVGPSGSGKSSLVLAGLVPRLGADPDRWLVRTLRP)}*

$2360=170(175):
>gi|931511574|gb|KPK89715.1| {<Chloroflexi(B)>}hypothetical protein AMJ88_17185 [Anaerolineae bacterium SM23_ 63]
{()MTDVFISYSRKdIAFARILHDSLV----ENRLETWIDwQDIPPSVDWLaEVYEAIEKSETFVFIISETSVTSEIC-KlEIEHAVKHNK-----RVIPIVINDI-DPGVVPPALAALNWIFFrdDDPFKRAVNDLIEAIHTDYEWVQ(EHTRILNRALDWERGGGEAGALLRGRDLQEAELWL)}*

$2361=182(186):
>gi|931515087|gb|KPK92981.1| {|0(891)|<Chloroflexi(B)>}hypothetical protein AMJ88_09410 [Anaerolineae bacterium SM23_ 63]

{(MSNTEVTTTDA)----FISYSRSnLDFVEQLASELAA---SGK-
EPWFDksdvplRGIPAGSEWWhEIEQGIEAADNFVFIISPQSVVSPYC-NaEIAHAVEHSKR-K----
IPIMLYGE-
DEASALGEITQAINAIPdeELIPEAIFVPERDLKKLVHRNWQ(RISAVQYIPFKSHDDWMKPTQELISALEQD
IERVR)}*

$2362=223(224):
>gi|1011229687|ref|WP_062148835.1|
{|0(754)|<Proteobacteria(B)>}hypothetical protein [Beggiatoa
leptomitiformis] >gi|933513640|gb|ALG66876.1| hypothetical protein
AL038_03035 [Beggiatoa leptomitiformi
{(MATDL)--
KLLICYAHQdATFKDTLLAHLQDLRQQKAVTVWEDTALHEGQLRIsVLEAELQQAGVILLLVSPDFMQSRFVA
MpALLRLFRQLSL-AGKRVVPLILSAT-NWGVLDFCKTQALPKGDipLERVLYPA--
EEWQAIVKRISRW(CAEQLSAPNEVSIAVDLQHLPATNTPLVGRRQASKDLTNAFDNKNTFIISITAPAGVGK
SALVNAWLEKIAPHYLGASH)}*

$2363=241(245):
>gi|935598342|ref|WP_054464996.1|
{|341(1054)|<Cyanobacteria(B)>}hypothetical protein [Planktothricoides
sp. SR001]
{(NELIKQIKDDEAYYHQHKILLVQALKWQEQNHNPSILFRGYNLQHFQAWLKIAETHDHPPTDLQKEFLTAS
SQHQAKSLE)---VFISYSRTdSDLARKINDALQF---QGK-
TTWFDQEsIAAGTDFQqEIYRGIENSDNFLFIISPHSVSSAYCSDEVEYAEKLNKR-FVTILYRQVEPKT-
LHPALAKVQWIDFTQHEgdFYANFSELVRTLDTDRDHVQSHT(LWSQRALEWEEKSQTKDLLLRGSEL)}*

$2364=250(252):
>gi|935598342|ref|WP_054464996.1|
{|155(1231)|<Cyanobacteria(B)>}hypothetical protein [Planktothricoides
sp. SR001]
{(LIEVFHSHEDYVRQHTEILAKAREWEQNKKPSRYLLIGQERQAAEAWLDIRFKEEQAPCQPSDLHCEYICE
SIKNANNLL)-TQVFIRYAEPdREVMEKLTR---TLQRE-
KITIWTERsDIKKRMSFQeKLHRGIAIADNFVYLVSPESLTAHYC-QtEIDLALSYHKR-
IICFIVPNKDTET-
LPEFSYEYQANELIKQIkdDEAYYHQHKILLVQALKWQEQNH(NPSILFRGYNLQHFQAWLKIAETHDHPPTD
LQ)}*

$2365=251(259):
>gi|935599679|ref|WP_054465569.1|
{|358(993)|<Cyanobacteria(B)>}hypothetical protein [Planktothricoides sp.
SR001]
{(ELIKQLREESAYYQDHKLLLAKALKWQEQNRNPSILLRGYNLQHFQAWLKLGKTRDRYQPIPLQGEFIEVS
SQYQFSYLE)---VFISYSRTdSDLARKIN---
DALQSQGKFTWFDQESIAAGTDFQqEIYRGIENSDNLLFIISPSSVNSPYCAAEVEYAQKLNK-----
RFVTILYRPV-
DPETLHPVLAKVQWIDFnqHGGDFYANFSELVRTLDTDRDHL(RSHTKWSQRALEWQEKGKSADLLLRGSELS
VAEDWLQES)}*

$2366=292(299):
>gi|935599679|ref|WP_054465569.1|
{|154(1156)|<Cyanobacteria(B)>}hypothetical protein [Planktothricoides
sp. SR001]
{(GLIQVFDTHINYVRQHTKFLADALNWQENHQQTRYLLIGEQRQAAEQWLDRKFVDEPAPCQPTDLQCEYIC
ESIKNANNL)MTQVFIGYADSdRDIME----

HLTRTLRREKITVWQNtRDIKTGADFQeEIKRGIVGADNLVYLISPDSLASQYFHSEIELALSYQK-----
RIICLMVQPI-EVKTIPetLRSLQFI---ELTDWQNY---
EIYQkqsnELIKQLREE(SAYYQDHKLLLAKALKWQEQNRNPSILLRGYNLQHFQAWLKLGKTRDRYQPIPLQ
GEFIEVSSQYQFSYLEVFISYSRT)}*

$2367=250(252):
>gi|935599682|ref|WP_054465571.1|
{|155(935)|<Cyanobacteria(B)>}hypothetical protein [Planktothricoides sp. SR001]
{(LIDLFYKHQDYVRQHTELLAKALEWAENKKPNRYLLVAEERQKAEDWLEIRFKGEQAPCQPTDLHCEFICE
SIKDANNFL)-TQVFIRYAEEdRGTMEKLTR---TLQRE-
KITIWTERsDIRKRMSFQeKLNRGILVSDNFVYLVSADSLQSDYC-QkEINLALSYHKR-
VICFMVPSPEMEA-
LPEFSYEHQANELIKQIrdDDKYYEYHKILLVQSLKWQEQNR(HPSILFRGYKLQNFQAWLKVAETRLDHPPT
AL)}*

$2368=250(252):
>gi|935607869|ref|WP_054469341.1|
{|155(815)|<Cyanobacteria(B)>}hypothetical protein [Planktothricoides sp. SR001]
{(LIEVFHSYEDYVRQHTEILAKAREWEQNKKPSRYLLIGQQLQAAEAWLDIRFKEEQAPCQPSDLHCEYICE
SIKNANNLL)-TQVFIRYAEGdRAVMKKLTR---TLQRE-
KITIWTERsDIKKRMSFQeKLHRGIAIADNFVYLVSLESLTADYC-QtEIDLALSYHKR-
IICFIVPSPEIEA-
LPEFSYESQTNELIKQIkdDELYYHQHKILLVQALKWQDQNH(NPSILFRGYNLQYFQAWLKVAETRQDHLPT
DL)}*

$2369=249(257):
>gi|935607869|ref|WP_054469341.1|
{|342(629)|<Cyanobacteria(B)>}hypothetical protein [Planktothricoides sp. SR001]
{(ELIKQIKDDELYYHQHKILLVQALKWQDQNHNPSILFRGYNLQYFQAWLKVAETRQDHLPTDLQKEFLKAS
SQHQAKSLE)---VFIAYSRRdSDLARKIN---DALQIQGK-
TTWFDQEsIAAGTDFQhEIYRGIKNSDNFLFIISPDSVFSPYCSDEVEYAEKLNK-----RFVTILYRPV-
KSKTLHPALAKVQWIDFtkHQGDFYANFSELVRILDTDRDHV(QSHTKWSQRALEWEEKSQSKDLLLRGRELT
GAESWLR)}*

$2370=223(224):
>gi|931517513|gb|KPK95339.1| {|0(363)|<Proteobacteria(B)>}hypothetical protein AMJ94_00130 [Deltaproteobacteria bacterium SM23_61]
{(MSDEH)-
YDIFISYaRDDSTWVREqLYRPLLEYTLadgklpRIFLDISELGIIQGENYLIALGRAIQKSQKVILVYSKTY
FHKEMTSWELTKALQLDPSGKKLKVNPILINPEaESEIPFEINHIQYLDTRN----P---D-WF-
SRLVKNMGLV(RIKESEPPSLSFLTHPEDIAVHNTLPRMEVALSNRGEKNFREEAVSLSAPGAELQGTLTVKT
SGGVAFFSDLSFKSKAA)}*

$2371=217(236):
>gi|931538564|gb|KPL14411.1| {|0(221)|<Bacteroidetes(B)>}hypothetical protein AMS26_10720 [Bacteroides sp. SM23_62]
{(MEVMMTNYKELSSKIHVFLSHSWGERRKQH)------------EFAKKICEILKQQD-
DLKPILDDEYFTPGTS-WKNIYENIVECGKAVVIFmfSKEFLLSPNCQFELNFARN----
AFLTKGVPLIaVKIENVNIPEMMKDDIFVDMTR----
CYELFIENVVDIESEIKSK(VDSLVIGIRDRIKEYENKLHITRVIHPDDRLFLESRELFTLYPRDYRDDFESV
QQWIAECSDKLLE)}*

$2372=225(229):
>gi|934161629|gb|KPM09999.1| {|55(0)|<Arthropoda(M)>}myeloid differentiation primary response protein MyD88-like protein [Sarcoptes scabiei]
{(YAEIAWLEKQIDPFGSLLGLEKTCKLSVYNLFEFLVTIGRFDVLDDMIPLILEDLNTLEIRDKQNFSLRSAPIENETTIW)-
YDAYVCYaDQDLDFVQQLVNYLESPTIGFRLFIRDRDLLVGNWIYETFARLIEKqCRRMIIILSPDFLQSPECKFQSMFAAGLAIEKCSRILVPIIYKPC-TQLPSMLRLMSKIDMSG----PKSLpE-
WSLKRLIIPIGIK(LFYEYPLQQ)}*

$2373=231(222):
>gi|939177603|ref|YP_009173511.1| {|0(21)|<dsDNA viruses(V)>}Toll-like receptor [Chrysochromulina ericina virus] >gi|936628159|gb|ALH23162.1| Toll-like receptor [Chrysochromulina ericina virus]
{(MTHK)--DIFLSHawgvddnNRNNHTRVKNLSKL-LQEKGY--NIWLDENDL-
LGNIDnSIMKGISNSMVVLVCLTEKYCNKinnSVYDNspNDNCYKEWNFCLfKQKKIIPIIMEKKMEEVFLNTDGVIQMYLNNVMFINyYDDENNDFDILCKTLRLY(NVYTHDEKIFMNIKPNSSFDKLKDLLQLSPKRLKTPSPLTITIPERKNNNLLRSILHRLLIKINISKQSKPLLFRKQK)}*

$2374=215(220):
>gi|983591535|ref|WP_060741281.1|
{|48(0)|<Proteobacteria(B)>}hypothetical protein [Pseudomonas fluorescens] >gi|937318233|gb|ALI09095.1| hypothetical protein AO356_20480 [Pseudomonas fluorescens]
{(VVNLQGDPVRFEEWLRNKYPEKLKRHNVFLNDVLFFNVITFIEETMVDMALVSEPSFVFLWPERELFETLNPQYKQMKRN)DSKVFISHSSKdKElIVGPLNAYLQAEGIG------
TWLDSYEIDYGDniylKVNEGIENSKVGIFILTNNFFDSAsgwpiteFSTFFMDL--MKNNK-
KVLMVNAGVDQDKIHAMMKAYKYLNWDDGEG--LPELANA--IRRK-----LKS()}*

$2375=187(200):
>gi|938950335|ref|WP_054729798.1| {|0(184)|<Proteobacteria(B)>}TIR domain-containing protein [Sphingopyxis macrogoltabida]
>gi|938157999|gb|ALJ14360.1| hypothetical protein LH19_15930 [Sphingopyxis ma
{(MT)--DVFISYKREERGRCVAIYNALVDLKLS-VWFDA-HIEPGTDFDREIEREVRSAKAVLVLWS-
ALAADSDWIRaE-ARTGRQN-----ERLVAARLDDC--LPPLeFASVQAVDLFD--
RRDFQTGEGWRQVVGRIGRLVG(RPGLGDYVRCEQAADAGLWRGWIAANPDDPLVETARQRAAALAHRAPPDPGRAAPADP)}*

$2376=192(191):
>gi|937079472|ref|WP_054544775.1|
{|0(129)|<Proteobacteria(B)>}hypothetical protein [Aeromonas hydrophila]
{()MAKIFISYAREdSQIAQRLTKELHQRKHDVFLD---
QQSIRIGAHWQeDIKRHLKDADVFLALVTNHSDKS-YYFNvEVTSAISyvAHSN-RDKLILPVVLDQA-
SLNDSDLGAFQALFGREedFDFLIAEIENAISRFEGFRIAQE(EIKDLHGRILEENSADYIKETIEDLTAREKRGRKLASRWYLAGYATLVASV)}*

$2377=204(217):
>gi|937369206|ref|WP_054587341.1| {|0(167)|<Proteobacteria(B)>}TIR domain-containing protein [Sphingopyxis macrogoltabida]
>gi|937289404|gb|ALH79950.1| hypothetical protein AN936_06090 [Sphingopyxis m

{()MTDVFISYKREeRGRCVAIYNALVDLK----
LAVWFDAHIEPGTDFDrEIEREVRSAKAVLVLWSALAADSDWI-----RAEARTGR-QNERLVAARLDDC-L-
PPLEFASVQA-----
VDLFDRRDFHtgEGWRQVVGRIGRL(VGRPGLGDYVRCEQAADAGLWRGWIAANPGDPLVETAQQRVAALAHR
ALPGPARAAPANPVAAEAARGGPARYWPLF)}*

$2378=217(220):
>gi|941082384|ref|WP_055061019.1| {|306(0)|<Firmicutes(B)>}hypothetical
protein [[Eubacterium] rectale] >gi|937566625|emb|CRL33184.1|
hypothetical protein T1815_05241 [[Eubacterium] rectale]
{(NDIYDNDTEYLISAPTRLSDEDNIREFYDKCIYENCWKLKEEISDVRTKIQIIGLDSIRQFVKRWEGAPKE
NSNIYFGKS)-PFLFISYAHTdKDI---VYPIIERLQKKGV-
RIWYDTRLRPGDEWPeEIGWNIIDCALFIVMLSDAAVLSIHVREELNMANSRHKN-LFGVKVEPLKDIK-
LSPGMELQLSISQMIDDalSEDQMVDSIFSIITTKYPELIEK()}*

$2379=192(198):
>gi|938901167|ref|WP_054685098.1| {|0(44)|<Firmicutes(B)>}hypothetical
protein [Ruminococcus champanellensis]
{(MRDVLVSAINNGYYEYESKIKQIIEELKLEKAVKPT)---VFISYQW-SKTSSEIADRIQNELKDI-
AAVRRDTTNLVTGDSLsEFMKTIRSQDFAILIITDEYLTRRNCMYEVAQLLRDYDN-
HETKFWERVLLFVTAKIYAAEEKAAIIKFWD-
DKVNELDAKIKDLHGETTEKLA(KELKECRYISMEIGKLLEYASD)}*

$2380=222(221):
>gi|938974435|ref|WP_054752563.1| {|31(0)|<Firmicutes(B)>}molecular
chaperone Tir [Ruminococcus faecis]
{(NEYNRPSEVWINDVEIFYNNYLKEHPLGDRMKTILFHRNLDAYKNLVGCLESISKDQEFINKMNGISMVSV
PKYQAKTLP)EFDVFISHAN-KD-
KEDLIEELYQSLNKLGIKIFYDKESLEWGGDNwkDrILNGTKKAEFAIIVISENFFDREWTERELNEFLNRQN
RNGQKLILPILHNITVSQLQEMYPSIADIQAIDSSKYSCDQIALLFARQLIKRLKS(E)}*

$2381=204(222):
>gi|938302879|gb|KPQ36388.1| {|0(3)|<Cyanobacteria(B)>}TIR domain
[Phormidesmis priestleyi Ana]
{(MNQQPASNDYQYDVFLSYSREPIIYEWVHEHFLDLFRFHLQGGLGYSP)----------------------
----
TIFVDTLSIYDGDAWplRLKMALAYSKCLVVLSSPSYFESEWCMKECHVMLHREKEAgfgiegnGRGLVLPVI
ISDGEsrpqYMKNIQSTYFDQFVKRGSGFTKTELYIDFQVKMSEWTKEV(ARAIKNAPDWNSDWINERIINIP
KLEIPDFDTPP)}*

$2382=175(179):
>gi|932922585|emb|CUQ77187.1| {|0(432)|<Firmicutes(B)>}Uncharacterised
protein [Lachnospira pectinoschiza]
{(MADIDELKKKLEDGWIKGEPY)---VFVSYASKdKEKVYEFVYELRKRGIN---------
VYIDIELQEniskNWLQNIKErlydvdCVAMISFLSLNYFRSYACLIEQLVTRSEDLK-
MEKGKYLENFYIALDDNMSTIQKMEDAVYNN--
TVAKESQSMAIKMVPEEVNVL(KDVMEDNIAVKSKLKKDV)}*

$2383=217(225):
>gi|938510798|gb|KPQ43778.1| {|0(68)|<Euryarchaeota(A)>}TIR domain
protein [Candidatus Methanoperedens sp. BLZ1]
{(MTENKKRKP)--
RIFLIYSFADRFVAQTIESRLHAGGVDFIRAQYEDVAAGDSIAVAVRQAIATSDYILVLLSPSSVE-
SKWVQyELSAAYAleLE-----SRGIGILPVIIEdcQIPSLLAERRWIDL-S--SDFDANLD--

RLVQQIQAISDL(DFSRLDGHLFTKLVADLLIELGFKIVQEADVSGGRIDITAEIERTDPFGNTRTEIWLVETKFYRKERADLSALHQL)}*

$2384=200(195):
>gi|938908146|ref|WP_054691037.1| {|0(57)|<Proteobacteria(B)>}TIR domain-containing protein [Desulfosarcina cetonica]
{(MTEPKP)-PKIFISHSAKsKAHESRLMQFAEQLSNSGL-DPWVDRqRIGVGDAWNpAITKALQDCHGAVVLFSQEALQSDFV-KyEVSClAYRKRTQ-SGFALFPLILDET-PIQtilDGFFGAIQFGDYQIgtIDEKEQEVIGKLRALDLKSCRPV(SAIEGALLEIFRRMAPEAIREAAVNLGWATWPHVTLADAESLHFVRRLL)}*

$2385=172(177):
>gi|938951455|ref|WP_054730918.1|
{|0(141)|<Proteobacteria(B)>}MULTISPECIES: TIR domain-containing protein [Vibrio] >gi|1065522953|gb|ODZ54440.1| hypothetical protein BBM41_01640 [Vibrio parahaemolyti
{()MTKICLSYAQDvKDEVQEVKELLLDLEYE-VIDLVED--ILPGDDISdAIEAFMKASDVIVHFVSQASVSSKW----FSRELSLSEA-LSKRVIPVICHPN-IPIPPQLESRLALHLDAteLDQVALGIDRASTKYRVKDSLDT(EIKIETSQRVEKNISEYISVSIKDLNRKEKSFKYQAY)}*

$2386=193(220):
>gi|941045712|ref|WP_055052870.1| {|140(0)|<Firmicutes(B)>}hypothetical protein [Blautia obeum] >gi|933098954|emb|CUN67354.1| TIR domain [Blautia obeum]
{(AEGKRKMIVPLVWAGGAAQSSYERRIYQLTDIFGDDLYLLNSRTHYNKIIEKIGIEKQGIPKSIKERKIFISYARKRPYE)------------------ADYIETLLRRRGISVFRDESEFGAGKEIpiQIEENIHKANTFIAVYCAEYACSPWCYDELELALDLGE-----------KKQLDIWLICVDDTRIVSKRARNLVFYQVHNREDIEGRILELIMK()}*

$2387=179(181):
>gi|929027474|ref|WP_054051237.1|
{|0(693)|<Actinobacteria(B)>}hypothetical protein [Alloactinosynnema sp. L-07]
{(MTIMNRPGDQEGDRSVSNDVDDGVEL)-FDVFVSYAG---ADRDVVAPFVELLKKGGWKVwFYDA---HMSAGPalkPQIASAIR-RSAymIVCLTDSYLASDWTEFELLNGTHADPAGKSGTTIVVKFKPMTgkV-PAHAAHLFVYDFSDQ-RDYDRHFARITNLIKRRKANPV(AETYDAPLSAEPVEA)}*

$2388=275(280):
>gi|941910527|ref|WP_055238764.1| {|470(0)|<Firmicutes(B)>}hypothetical protein [[Eubacterium] rectale] >gi|933089367|emb|CUN29096.1| Uncharacterised protein [[Eubacterium] rectale]
{(NSDEIMAINKLIASCFDAILRWNDITMANEVCEVLYLLQKNEMLDKYYIPEISNNHSMYKYLSKCMENKKLIIGEIVMGR)-KTIFISYTDAdEKVVDVIDSYLSSCGYDvKRDIR-------NIGDYEsideFM-NEIRNQDFVVPVVSDTYLRRNNCMYEITQLlkddnFTKRTL-PVIISLPKTKDRTYSFFDPRYRIEIISfWENE--AKTLKEA--IEK---LSLENK(VELSMEYRRINNYSQDVSKFMNWFKSKLVGVIPSDTSASKRKQVALEIADKIDNIISGKQ)}*

$2389=175(177):
>gi|939326653|ref|WP_054873386.1| {|0(96)|<Firmicutes(B)>}TIR domain-containing protein [Oxobacter pfennigii] >gi|939119878|gb|KPU46123.1| hypothetical protein OXPF_02330 [Oxobacter pfennigii]
{(MDKPT)---IFISHIAEeKALAGSLRKIVISSFRDMVNVFVSSDPEC-IPAGQdwykKVMKNLKNCKMMIILCSTKSIKRSWINFEAGAGCVRGIP-

VIPLCHSGMKPSQLPTPLSSLQALEASEPLI--
KERIFQVVAGAVGSSIPEGAD(FTGFIEELKKFELGNGISSSVIYINYEGLDIY)}*

$2390=172(174):
>gi|933167176|emb|CUQ12528.1| {|0(28)|<Firmicutes(B)>}TIR domain [Blautia obeum]
{(M)---LFFCFSSKdRHsIVESILFHLT----
NYGLPVWYDRhKMLLGDERDnKnFDEGVKACNYSIIILSANTIASE-CANeEIDLIYQRYKQ-
HKMYVFPIFFNIK-
TSQLPEKYCWMKRLVYKelTVANDSRSACNHIICKVTLDELQ(KYKIKTINEYLKLYKNNKAFSYLTELIDSYCKI)}*

$2391=215(222):
>gi|933309595|emb|CUQ05393.1| {|16(0)|<Firmicutes(B)>}Putative beta-lactamase hcpC precursor [Roseburia hominis]
{(DYRIAFSWFKKAADKNHAGACNWMGDCYENGYGVNKNLDFAIKWYKKAKQNGYDAKKCDKKINEIIKKKNNFLEPYEGHD)-PYIFISYCHKnQDMVMDI---LNNLSRLGY-
RFWYDKGINVGSSWNdNIASHIDNASHFIFFLSNDFIQSKYCLDE----LEYAKS-EDKQIIPVCIEET-
KISGGLKLSINRLQVFNkyQFSESYFYDQIAQIQNIHKCNKN(TE)}*

$2392=256(259):
>gi|942080933|ref|WP_055290005.1| {|81(120)|<Firmicutes(B)>}hypothetical protein [Catenibacterium mitsuokai] >gi|933362078|emb|CUP39905.1| Uncharacterised protein [Catenibacterium mitsuokai]
{(YARDFELKYALENNIPVLPILIEEIEYQLYIQFFMNAQYIVRESLDTTERLYEHKLKEKIIEYIGSASLKEKIKHECTSQ)---
IFISYRKKdRQYANKLMENIHSIPELRDTIFWYDEYIITGKDFEkEIQNELNEADFMILLVTQNILeNKNYILEtEYPYA----KN-KNKKIIPIEMKST-
DHELVHKHYPKLDHIIDgnNLQYLKDVLLPVQKKKNRNNPSH(LYLMGLANYYGIYMLRNQKKGIELLEQSAQKNNYKACVK)}*

$2393=192(220):
>gi|933362081|emb|CUP39999.1| {|484(0)|<Firmicutes(B)>}Putative beta-lactamase hcpC precursor [Catenibacterium mitsuokai]
{(DYRIAFSWFKKAADKNHAGACNWMGDCYENGYGVNKNLDFAIKWYKKAKQNGYDAKKCDKKINEIIKKKNNFLEPYEGHD)-PYIFISYCHKnQDMVMDI---LNNLSRLGY-
RFWYDKGINVGSSWNdNIASHIDNASHFIFFLSNDFIQSKYCLDE----LEYAKS-EDKQIIPVCIE--------------------ETKISGGLKLSINLNTHNLQVTI()}*

$2394=186(183):
>gi|933404726|emb|CUQ17238.1| {|0(296)|<Firmicutes(B)>}SEFIR domain [[Ruminococcus] torques] >gi|1052966505|emb|SCH21428.1| SEFIR domain [uncultured Blautia sp.] >gi|1052978429|emb|SCI35545.1| SEFIR d
{(MQIDKIPK)---IFISYSW--SSD-
ALVLELANRLVFHGVDVVLDKWDLKEGNDKyeFMERCVNDSSItKVLIICDKAYAQKandrtGGVGdETVIISSEVYGNaRQEKFIPIIAERD-
EEGKEYVPTYIKTRIYIDLSNPEKYEEEYEKLLRNIYEKP(QFVKPPLGKKPEWLDEEKTNFFPVKDLIRQIRGGN)}*

$2395=180(187):
>gi|495464861|ref|WP_008189552.1| {|0(1201)|<Cyanobacteria(B)>}type IV secretion protein Rhs [Moorea producens] >gi|332349907|gb|EGJ29515.1| WD-40 repeat-containing protein [Moorea producens 3L] >gi|1

{(MTTFF)--DAFISYGR-ADSK-AFATKLEGRLTQLGLTVWFDQKDIPPAVDWqhQIDNGIERTHNFIFIISPHSVNSKYCLKEIELAIKYN-----KRIIPLVHVKSPREKIPTIIRKLNWIYFQDKINDFEASFKQLMNSIRKHADY(VEQHTRFLVKALEWDRNQQQTNHLLIQSERIQAESWLKVRFQ)}*

$2396=225(228):
>gi|503533203|ref|WP_013767279.1| {|512(0)|<Bacteroidetes(B)>}hypothetical protein [Haliscomenobacter hydrossis] >gi|332335641|gb|AEE52742.1| hypothetical protein Halhy_4913 [Haliscomenobacter hydross
{(DQLRRKKGSEFCTSFVAFLDAQNDILHLEREVIGFLELLSGWGSNETIKKGIVSFRTQQPKISEAFPPLEDMLLTREKKF)--KIYIIYSSAasdNEYLRLLISHIDSSLRPYEFEILYAEILEAGLETIkAIEENISEANLFLVLNSSHLLALED-KNnELNLVI----A-TGKKMIPINCRPC-DI-DQRLAIFEAIPSKTtfLSQLskPRRE--SAMVKLVHTIKDQ(IKRQSTVH)}*

$2397=227(227):
>gi|503533536|ref|WP_013767612.1| {|360(0)|<Bacteroidetes(B)>}Fis family transcriptional regulator [Haliscomenobacter hydrossis] >gi|332335976|gb|AEE53077.1| putative two component, sigma54 specific,
{(KPIEVEIRLEEPDHRLKEAHEFLHKINIAMNQNENNFESAAEALNKDVSIIKKQLKHYFDLVPSAFDKDRYASIFGFLEF)--NIFFCYAPEsEDIYNELKKHLSSLSKHPLCKVTHNQKPFLGSNLVnQLKTQLSQSKIICFIICKDLFSSESSKCrVPLEYIPRKKE-NQD-VVPIIGKSY-AWEELPeFKDLEALPRNGqpIMHNNSfSD--EILTQVAKEIKAL(FNERLFR)}*

$2398=220(219):
>gi|503534797|ref|WP_013768873.1| {|0(224)|<Bacteroidetes(B)>}hypothetical protein [Haliscomenobacter hydrossis] >gi|332337255|gb|AEE54356.1| hypothetical protein Halhy_6540 [Haliscomenobacter hydross
{()MKKLFIVYAKEdLSYLNAAKTNLALLERQGLIRIWDETKLMPGEVRKtIIEQELQSADIVLLLFSSSLIADDFIWDvPMQQVLEKAKK-GIVQLIPTIIRPC-SFSDTPFGEYETVPSQEkpISNWSNAD--DAWKLVVDKIKKT(VQPVQAQPTPLPSDSPALKHLKDSVNQFTHQAKTREALTAIKTYAHQNNLEKLKRDTLDVEESWLGLEQEKTLGQVSEE)}*

$2399=268(271):
>gi|495464861|ref|WP_008189552.1| {|126(987)|<Cyanobacteria(B)>}type IV secretion protein Rhs [Moorea producens] >gi|332349907|gb|EGJ29515.1| WD-40 repeat-containing protein [Moorea producens 3L] >gi|
{(QLMNSIRKHADYVEQHTRFLVKALEWDRNQQQTNHLLIQSERIQAESWLKVRFQKDQAPCVPTDLHCEFICESIKNANNL)MTQVFISYSDKdQDIKDKLGK---TLMREG-ITIWKpETDIKTGIDFQaAINQGIEEADNVIYLISPNSIQSKYC-QqELEHAFAHNK-----RIISLLIEDT-EIDQIPpkIRSLQFLDFTKsnHEANYRFMAAQLLKELNQDALYY(EEHKLLLVKALKWLIQNRNSSILLRGHNLNYYENWLNVNRQRLEHPPLGLH)}*

$2400=281(294):
>gi|493898233|ref|WP_006844073.1| {|15(16)|<Bacteroidetes(B)>}TIR domain-containing protein [Dysgonomonas mossii] >gi|332885256|gb|EGK05507.1| hypothetical protein HMPREF9456_02708 [Dysgonomonas mossi
{(YSIAVELQSRMTYDEINVYLNEYGIKKPSDGYGSKRVYVQKALESVPSSKIVKIAEELGLYKSDDLTLTEEPQCWKLGFF)--KMFISHLTAnKSSATNLKICLEEYAISGFVAH---

EDIEPSKEWMtEIEKALFTMNGLCAIVTPDFIKSAWC-DqEVGVAIGRR----------VLVAPI-RKGADPyglFGKYQGIQSKNKDSHKIAE--
EIFKIVTTNEKSK(SIYAEMIRNLVLNSKNTEEGLKWITLLERIPNIEVAYISELQSKFYSNDNLDNESVFEIANRIFKKYNLALVDH)}*

$2401=239(239):
>gi|503534747|ref|WP_013768823.1|
{|31(416)|<Bacteroidetes(B)>}sulfatase-modifying factor protein [Haliscomenobacter hydrossis] >gi|332337205|gb|AEE54306.1| Sulphatase-modifying factor protein [Halisc
{(VHPKDQTASKIQHVLLVNAGKFRGLNQERVLGILKREDEQTTTAQVQAAMLYVIEELPEKVWQTKTESDKTSVPHSEDFD)-FDIFLSFS-SkdresaRLI----------
WEMLRGLgfRIFLSDEALKAHAGMsfldRIDWALRRSQHFLLLCTPTAVQSQWVKLECETFFTRFHLAkpsQRRFFIFERGG---
KKWLGDVYQNLQTLDEIQTLVEILRAQLGQAGSVIPMAIG(HAFDAQNSNLQQTKPSIEQ)}*

$2402=167(166):
>gi|493854223|ref|WP_006801119.1|
{|0(36)|<Bacteroidetes(B)>}hypothetical protein [Dysgonomonas gadei] >gi|332827297|gb|EGK00062.1| hypothetical protein HMPREF9455_03586 [Dysgonomonas gadei ATCC BAA-2
{(MARKT)----FISYKYSearglrDTIIKRLGEDAKYY-----------
QGETSASPDLtdAsterIKEHLKDMIYdtsvTIVVISPNLKESDWIDWEIEYSLKEVKRGdrcSKSNGVVGVIM---KYNGGYDWLVSQNRNNDGCVSRQIDSSKLYGIINNNRYNL(NTDNKYACSTCKTFNQLDGSY)}*

$2403=167(167):
>gi|940415752|ref|WP_055005061.1| {<Proteobacteria(B)>}TIR domain-containing protein [Pseudomonas syringae group genomosp. 7] >gi|940282513|gb|KPY89133.1| hypothetical protein ALO44_00515 [Pseudomonas
{(MPIRQSDLYTAGYSRYLQGQFSKRSGQ)-PTAFLSHSHTdAVLALGLQELLKKAGWDVYID-WQDRTMPetPDAETAfNIKVAIVQADWFLLLATQHSTASRWC-PwEIGFADGRKAH-DRIAIVP--TQDN-QGNFYGNEYLNLYNKIDvpRITGTPGLALFDTRGNGTWVRGL()}*

$2404=213(220):
>gi|940687037|ref|WP_055020688.1|
{|315(0)|<Proteobacteria(B)>}hypothetical protein [Pseudoalteromonas sp. P1-26] >gi|940466497|gb|KPZ74681.1| Reverse transcriptase (RNA-dependent DNA polymerase) [Pse
{(NDLISAEARFIMQRYNVSDSEVGNDRDGSIFKRAVYGNLSFIKMVKGEEHSPYLNLCRKVLELDNEPPSFISKLKDVLDM)-YDVFICHAR--
ENKNEVAIPLYEALKSKGINTFIDSSAINWGDSLvsKINTALQKAKYVIAIISDDSLNKSWPMHEL-RSVLST-----EIRSGEIKLLPLMVGDADALISQIPLLADKRFIHFEDNIDDVVEEIQKLLKD()}*

$2405=274(283):
>gi|940687038|ref|WP_055020689.1| {|12(10)|<Proteobacteria(B)>}TIR domain-containing protein [Pseudoalteromonas sp. P1-26] >gi|940466498|gb|KPZ74682.1| hypothetical protein AN394_00399 [Pseudoalteromo
{(ISSELQAQMTFSDIENYFLIHGVPLEDTSSYSSKRVYSKKMLGQVSEELLFKIANEIGLKNLPRNIVNSSEVYIWEGGYF)--KLFLSHLSSfKNQTSNLQLALKKYAISGFVAH---
KNIEPTKEWLnEIEGALRNMDALVAILTPGFEESRWC-DqEVGVAIGR-----DVLVIPIKkgLDPY-GF----

IGKFQGIQAENktVAQVANAIFEVLVKSPKTKYKIL(ISLSEALSKAQSVEEVREKLSILNSLEGIPTDILEK
LKLQILDNSWLMSSWEQIESINLMLSK)}*

$2406=147(148):
>gi|943648270|ref|WP_055476240.1|
{|0(569)|<Actinobacteria(B)>}hypothetical protein [Gordonia sp. HS-NH1]
{(MSGH)---VFISYSRSDGSGPATALADRLRAQGH--
KVWIDQRSIPVTvPWMaEIKSGIWGAAVVVVVDTPGWRSSENCQIeaRFAQDISAPQIMVSPTAHPDHSAAEV
TGVVRQLPHEERTRSELFERADHWNNHGRPPRSLASGRSL(TKYR)}*

$2407=261(253):
>gi|943930797|ref|WP_055564272.1|
{|286(4)|<Actinobacteria(B)>}hypothetical protein [Streptomyces
atriruber]
{(ERVDLLLDTVFEQEDPEFVLALADTVAAETDAAGTGRQMADSVRAIAEELAARWQGSRQASPAEPAEVPSC
FFFTSWHNR)-----DVDF---DEALKFHNLLEKRVTAKlggRVTRsgFFDKMMQH-
GATWepKLIEAIRTTRLMVPLITEGYFQREWCRREWAVMVQRMANLnADPgqepiAILPVFWVRPLdgwdMPA
DFGPYQHSVHSGGRPEYDGDVYDLVADDKEELNNYVS(TLAQSMVALSRTRLPHLDRDLVKKLPLAFRDVN)}
*

$2408=287(300):
>gi|943670412|ref|WP_055497238.1|
{|91(99)|<Actinobacteria(B)>}hypothetical protein [Streptomyces
flocculus]
{(VLIGDAPLPPLAASRVWRDFRYVDGPGYEEKVDELVAAVRGETARDGSRGSATPGLQDNIAAAQPLPLRPL
TPPDQPAVV)-----LCYAAADAAYGQALVAQLRSAGLA-VWSVA-
DLKPGDSHVWSVQRQLDFATAVIVLMSPHSQESEDVTRMILEAMHHQ-----RPFFPILLEGQRN--
YLLAHTWYVDardgrLLR--EEEVGLLR--
DLDAASRSGASP(DAMPAVIPAPALRPAVEAVRVPAAVSLGRLDSYLSERNFAYADLQTTAIVLEAAGRLDEG
WLSTRHVERIPARVLARVDG)}*

$2409=299(296):
>gi|944008662|ref|WP_055600329.1|
{|213(1110)|<Actinobacteria(B)>}hypothetical protein [Streptomyces
aureus]
{(PAVINDEGTRIRYRNVNTTPGSSGGPVVDLRGRLVGIHHYAAGALNQGVPASAIARSVASGAHARILDPRP
PEPSRPSGA)APAVMLSWVPQDRPWADLVHDVLAASGHQ-VLMGPSADRPG---
HRSLREHVDGGGKVLALVSPAYLAD-PGMTdERDHIVhdfdhaSAP-----DRLVPVLVE--
GEASGSLALLSPVDLrempvapgpgqHG--RDRmREASA--
RLSSALDASPAE(RAGPPTRASRESSLGFRPGDPLPRLRLLSDRARHSAGTVSGTVFADGQESQFAAGLYVTR
DLEEELLERLETDALT)}*

$2410=172(181):
>gi|943986850|ref|WP_055581483.1|
{|0(334)|<Actinobacteria(B)>}hypothetical protein [Mycobacterium
gordonae] >gi|943375529|gb|KQH75628.1| hypothetical protein AO501_25485
[Mycobacterium gordonae]
{(MA)---IFISYSSQdRTSLDALTT---ALKRAQQ-
KVWFDQELGGGDAWWnAILEQIRSCDVFIVALSNNWLQSkPSLAElRYAQALNR-------PILPVKVGPV-
DSVRVNPVSTLQIIDYQnpTVDAGIQLVTAIHAMRDKAAPLP(SPLPEEPPVPFGYIMRLGNDLSEKELTPAQ
QLQLLLELR)}*

$2411=124(141):

>gi|941350328|gb|ALK44209.1| {<Proteobacteria(B)>}Toll-Interleukin receptor [Colwellia sp. C1]
{(M)--SIFLSYAYRdNDVIQKVRNHLV----
HNQIDVFDDRnDIAAGASLIrSINDAIDHSDTLLFFISGNSEKS-
IWMSqEISLAINKKMNGGEATLIPILLE----------------
KNAKtpFFLKDYLYLDLTDESNFDAVMDK()}*

$2412=236(251):
>gi|943612533|ref|WP_055442426.1|
{|186(0)|<Bacteroidetes(B)>}hypothetical protein [Lacinutrix himadriensis]
{(SDSIIENCTLSLNQFSACTFGLNTFKNQNSSPGSEFNMIDIRSILNSPPINDDNLKTLFGIHNSEIKEYLF
ELTSEIKFQ)--SIFISYSFEdHEFAKAINEFL---
LRKGVFTFLWENNSTAGELLNhIMESNINEKDRILFIASENSIKSKAC-QfELTQGQKKQEiT-
WEKILFPIHIDDF-LFKVT---KEQIRPS------EKQN--
EYWSNISELKKHN(SLDFTEYINKENRKSQEFEKLMFRLLKGLQK)}*

$2413=167(161):
>gi|943996067|ref|WP_055589367.1|
{|0(240)|<Actinobacteria(B)>}hypothetical protein [Streptomyces griseoplanus]
{(MS)RPRIFLSRSE--PEACDCSCWPARRamvrlLEAEGCEpVVVDKQTLVAGEEWmqAITDGM-
ASAHGLLLLILSVHALASPHVQnELAMAELRHRSGdFPIVIVTLPEVSR-
DELENGRLGSLSPTRRQLVAWSTGTEEEEVRRELGPQLEL(LRAGMNGARVQQYMAHRLE)}*

$2414=168(179):
>gi|944216583|gb|KQK82487.1| {<Chordata(M)>}hypothetical protein AAES_70358 [Amazona aestiva]
{(MGSVPWSPEGGMSSAPSCCGFT)-YDFSLWYCPEdDHIASRISERLRKEGFQG--
YAEHQDQVAGTTLILTASKVIEAS-
RVaILLLSAKSLGDPWCQRVSQWNLCHIIHCHGTKVIPVCVGVKKAEVPPFLQHLTELEYQN------E---
FFFDRLVESLRTS(RLAARRAQQALSSKEKR)}*

$2415=234(239):
>gi|948024870|ref|WP_056684132.1| {|46(42)|<Firmicutes(B)>}signal transduction protein [Bacillus solani] >gi|944254829|gb|KQL19089.1| signal transduction protein [Bacillus solani]
{(IILGIKDNSITLSIKKFPFILNIENILELLDGGVKIEYNFFTFEGNNLFYISIDKSDELVKVNNIPYKINN
DGAVEEMAI)-KKVFISYAHKeSDLVNILEEELN----KYENI-EISRDIKAIEYRDslddFMKT-
IRDHDFVISVVSSAYIKSLNCMYEVMHLMQDKDY-QEKLFFIIVSRDDVDYYNEKNRYDGFEAKIY--
DVMDRLKYVTHWRDKKAELER(SISEAALSPELMVNLAIDM)}*

$2416=221(230):
>gi|944538085|ref|WP_055749575.1|
{|0(847)|<Actinobacteria(B)>}hypothetical protein [Frankia sp. AvcI1]
{(MTVGGEGDTL)--DCFVSFAEDGRAWAEWIAGALEGAGLX-
VLVESWDRVPGSNWTALLDRATRQARYTIAVVSDGY-VNSEYAVaEWGAAWSPRVA---
DGDRRLLVARVTdrPVPGLLGQIAPINLFD--RGVLAGET--ALLAAVR-
PDRP(DRADHGDHGEGGTPGGRGDGAGTVGFARRPHPAIHPGELPAVWNVPTPPAAFIGRHEALNRLDAALKR
SPLVTVTGLAGI)}*

$2417=156(157):

>gi|946948799|ref|WP_055870635.1| {<Actinobacteria(B)>}hypothetical protein [Frankia sp. CpI1-P] >gi|944472877|gb|KQM02791.1| TIR domain [Frankia sp. CpI1-P]
{(MGERVQV)--
SMFLSWCHSdADAKQALLGQLHLDGLAGVrAEWWEDSHLQIGEDWRrXTLDQLAKCDYGLLLLSPGFFASRFI
LDvELPRLLG---P-DVDKVLPVMLKRV-
PIDGSWDLHGVDTRHIFtsKGRCFTETRGPSRERFALDLATE(IRRRVLSDAA)}*

$2418=184(206):
>gi|944565012|gb|KQM17240.1| {<Proteobacteria(B)>}hypothetical protein ASE50_07725 [Sphingomonas sp. Leaf5] >gi|944636449|gb|KQM88132.1| hypothetical protein ASE73_09675 [Sphingomonas sp. Leaf24]
{()----------------------MAQLHREGV-
RTWFDGDITPGAELDpNIRRALKQADIFVALASPNYLASRYCFEtEYGFALRKAAR-KKLFVVVVLLRQC-
QWRHTRMARYKLLPRDAkpIDQWARRG--
DAWENVVEGIRAV(VKQFRLVPQAPDAPKKRKAPAPTRTTKTKPTSVRPSGKAAPAAKPKPKRSVKPTGKRTR
PFKPRGS)}*

$2419=214(220):
>gi|1011552489|ref|WP_062445116.1|
{|94(263)|<Proteobacteria(B)>}MULTISPECIES: hypothetical protein [Rhizobium] >gi|944578542|gb|KQM30669.1| hypothetical protein ASE62_17985 [Rhizobium sp. Leaf202] >g
{(GARLRDANIKSTDHGYPAQVDFSSGKGLTQQQISSAAGDTGVILPVGLVHPEEWAHWEKVAASNEDDAAVA
TKPTVSSKF)---VFLSYSNKdSG----LATHVvAALKLSG-ISVWWDKEIPSGAIWRkTIDEKLEEADVVL-
-TIWTENSVKSLAVvEEASRAQRDGN-LLHIKTDSSALPY-
GFSEIQYTNLRGWNGDAndPRIRVLVQSILDRFSPLSQEAKE()}*

$2420=189(220):
>gi|947603767|ref|WP_056266995.1|
{|468(0)|<Proteobacteria(B)>}hypothetical protein [Methylobacterium sp. Leaf87] >gi|944808957|gb|KQO59600.1| hypothetical protein ASF22_08150 [Methylobacterium sp. Le
{(IVPFAFLGGAARRSYERRDWARLNPGFDPSILHKDEGIDQAVSIANQLLVDRVKRSASGGARPKTIFISVA
NQDAAMGEA)----------------------LSRALHTQGLeAVFGDNLLEPGQM-
VpaAIEQGIRRSEVTAVMWSRAYAQSAWCYDEL------------
SLALSLEALGMMKIWLFNLDDSPIVHAGARKLAAMSIRSPESLKNAVTELLR()}*

$2421=221(250):
>gi|947752825|ref|WP_056414804.1|
{|0(662)|<Proteobacteria(B)>}MULTISPECIES: hypothetical protein [Acidovorax] >gi|944772244|gb|KQO23170.1| hypothetical protein ASF11_23885 [Acidovorax sp. Leaf76] >gi
{(MCPDIATIFEWGVQHKAQISNIGGVMANFAK)---AFVSHSSIdkPLVE-QVVKAVSAARWE----
IDSHTFEEGNYSAGEITAALGRSDLFVLIASAHSMSSEWVKSELEIAQHLLYAGKLGGVVAFIVDGTQaEALP
E-WLRMRVFVRT------------------------
S(NPVRITNLIRSKLVELDTKRGVKPKPYVQRSKLQGEMERRIADLRLPIQAIYLSGVDGIGKRTVGTRTLEP
LFPGRDVS)}*

$2422=214(220):
>gi|947640264|ref|WP_056303185.1| {|72(0)|<Proteobacteria(B)>}molecular chaperone Tir [Methylobacterium sp. Leaf100] >gi|944885927|gb|KQP35951.1| molecular chaperone Tir [Methylobacterium sp. Leaf100]

{(RADLSSQMASKSKDLRSYLEKQAKEDQSALKKAADTQKKMADEQKRMIREREDHDRRVRSDLHRRAISFPT
SATDPNTEG)-FDFFISHAS---EDKEsFVRSLAQELTTLGAKIFYDEatLRVGDSLRRkidDGLA-----
RSRfgVVVLSEHFFRKEWPQRELDGLVAREIQ-GVSRILPIwhKVSKDEvaA-NSPTLadkVALNSSTKSV-
SEIAKELY-DML--------GT()}*

$2423=201(213):
>gi|947178212|ref|WP_055949220.1|
{|0(508)|<Actinobacteria(B)>}hypothetical protein [Agreia sp. Leaf244]
>gi|944755511|gb|KQO06512.1| hypothetical protein ASF06_15765 [Agreia sp.
Leaf244]
{(MGVDIVM)--DFFISYAREDAIFARALVGRLKEAGWD-
VWIDQEDIPPSVPWMTEIQHAIDESMMMIAIASPDWLASAACLIE--VDLAEK-----
GRVPVVRVTPDSDRLDDIVGRVVAAIRA--
LPEQRAVALQANSAAAIWDSAG(RTKPLLVRGQPLRLMRATLTNHPVDFSDTARVFIATSRQASIRRWILGLA
LGFIAPVLGLGIWVSL)}*

$2424=213(220):
>gi|947914714|ref|WP_056574918.1|
{|26(0)|<Actinobacteria(B)>}hypothetical protein [Aeromicrobium sp.
Leaf272] >gi|944876657|gb|KQP26776.1| hypothetical protein ASF38_07105
[Aeromicrobium sp. Leaf272]
{(EKASRTFEREVNREIDKANRSNKRIVDAHNRKADAHNRKVIADLNKQLRASSPSVTYRPAEEALVERVHGA
INFDDGVEY)--DVFISYARIDgETTATALADALRERGVT-
PWLDQVAIEPGKSQSLQMDAGLRRAAAGIAVLTPAYLTGRFWTQrE-LGALLHK-----
DVLIPVLDGVTFadVAEYSGILPDLAGFTT--AEDDIETIADKIAPAVLPAAAS()}*

$2425=192(199):
>gi|947771272|ref|WP_056433150.1| {<Proteobacteria(B)>}hypothetical
protein [Methylobacterium sp. Leaf91] >gi|944835568|gb|KQO85989.1|
hypothetical protein ASF32_09380 [Methylobacterium sp. Leaf91]
{(M)------SYSHAnMAQQAKLQVHLAQLKRDEV-
ETWFDGDMEAGDALNtEISRKLRKAHAFVALMSPEYIHSHWC-QlEYRRAMGRRAR-GAMRVVVVVLRHC-
DWKDTGASALKVFSRDGrvVSNWRTAD--
EAFHDVTQGIRGA(VRAVRASLAEDAKARPARAARSKASIGNAKTAGARRMKGATGPKRTKDGPPTRSRVSG)
}*

$2426=266(276):
>gi|946727853|ref|WP_055753718.1|
{|15(11)|<Proteobacteria(B)>}hypothetical protein [Brevundimonas sp.
Leaf280] >gi|944896730|gb|KQP46674.1| hypothetical protein ASF31_05560
[Brevundimonas sp. Leaf280
{(ADHMESDDWTMIDLVLKQFGLRTSDQWSNDRKSYVIEMVSEASDDDLSELAEHFGMEVKAKDADPALPPNL
PYWADGQVR)---VFISHLTDeRQQAADVQ---
AALSRFGMSSFVAHNDIHPTVEWQiEIESALATCDLLVALIHPKFVESRWC-DqEVGYALGRG--------
IPVFTVRC-
GADPHGFvSRFQAFNGNGktAFQIATNIFEAAIAHKKLQAKMA(DVVISLFENSGSFASAKSRIGFVEQLKVW
DKTYSSRLVKAVEKNDQVSGSWGVPEQ)}*

$2427=225(212):
>gi|948147093|ref|WP_056805391.1|
{|0(246)|<Proteobacteria(B)>}hypothetical protein [Pseudomonas sp.
Leaf58] >gi|944708523|gb|KQN59780.1| hypothetical protein ASF02_21720
[Pseudomonas sp. Leaf58]

{(MKTPK)---IFISYSwsS--
PTHERWVLDLGESLMESGIEVILDKWDLRPGQDAvaFMESMVTDSTIdKVLLICDKVYSEKadgrqGGVGtET
QiISKKVYEQVnQERFIavIAEKDDEG-
RayTPTFYHSRLYIDLSdpEAYSNGFDELIRCIYNQPQHTKPKL(GKKPEFLTNEVIISLGTSSLAKRATTSI
REGKGNATATFNEYLSLYSENLERIRIERKPDIPFDEQV)}*

$2428=200(188):
>gi|947496363|ref|WP_056160526.1| {|0(1)|<Proteobacteria(B)>}TIR domain-containing protein [Duganella sp. Leaf126] >gi|944975754|gb|KQQ31943.1| TIR domain containing protein [Duganella sp. Leaf126]
{(MTIRHGCFFSY)AHGKHAHMK---
KFKDDLVEALKCYLEPHfdnEEELFVDSEQLGGGDDLdgRIARALCESVCMIVIYSPKYEAHPYTRREFAAMQ
lIEAERRqWYDlpshLIIPVIMTRHPlnlpPQIGGPGMYVDFSSYTLASGDLKNNPDYLPHIARIVDRVA(AH
YHKVKHSIPPGHDCGRFVMPDIPSEWRAIPPPHFP)}*

$2429=170(160):
>gi|947670595|ref|WP_056333200.1| {|0(32)|<Proteobacteria(B)>}TIR domain-containing protein [Massilia sp. Leaf139] >gi|945040836|gb|KQQ96645.1| TIR domain containing protein [Massilia sp. Leaf139]
{(MAI)RYGCFFSYahgKHAlmqrfkTALADALHCYLEPYFDTE-
EELFVDTEQLGGGDDLdaKIARAMCESVCMVLIYTPKYEAHAYTRREYEAMRLIEAERskwYTLPSHLIIPV-
--IMTQHPDRLPPQISNSTFYVDFSHFTMATADLKSNPDFLP(DIGKMVKRIATHYQYQK)}*

$2430=190(210):
>gi|947412796|ref|WP_056078672.1| {|0(207)|<Bacteroidetes(B)>}hypothetical protein [Chryseobacterium sp. Leaf394] >gi|945233901|gb|KQS92092.1| hypothetical protein ASG21_06460 [Chryseobacterium sp. Le
{(MSLITRLQRFFTG)-YDFFISYSRSDCSEYASNLANKLIEEKY--
SCYLDQwGSLPGKELPlSLKRKIKNSSVLILLGSDASL--------------ASIAIeEEINIFT------
PTKRIIIPVELCDISKANWYKSIEGIALSNEKLNFKNNQIS(TNLLRRLKNSLNYTRQSVRIRNSIFSFLSLI
LISIIALIILFLERKKSQSLNHALND)}*

$2431=200(188):
>gi|947158483|ref|WP_055931556.1| {|0(1)|<Proteobacteria(B)>}MULTISPECIES: TIR domain-containing protein [Duganella] >gi|945499075|gb|KQV54525.1| TIR domain containing protein [Duganella sp. Root336D2
{(MSIRYGCFFSY)AHGQHAYMS---
HFKDDLVDALKCYLEPHfdnETELFVDSEQLGGGDDIerRLALAMCQSATMIVIYTPKYEAHPYTRREFAAMQ
mIEAERSgWYElpshLIIPVIMTRHPlnlpRQISEPGMYVDFSRYTLATGDLKTNPDFLPDIRRIVDRIT(MH
YHCVKASIPPGHDCRQFVLPPVPPEWRPPAPPHFP)}*

$2432=195(208):
>gi|947412779|ref|WP_056078655.1| {|0(313)|<Bacteroidetes(B)>}hypothetical protein [Chryseobacterium sp. Leaf394] >gi|945233895|gb|KQS92086.1| hypothetical protein ASG21_06425 [Chryseobacterium sp. Le
{(MKSSSY)---VFISFKT--QE-RELALILKEGLNSSGYKVWW-QEEIQCGQEWhgEIDKAI-
EQAGAIVVLWSTRSMKSPWVRhEASQAT------IRDVYTPVRIEIM-EVDSPYNRIQATDII-
NWDGNLTHPGFQNLLVRLNELMPP(PVPFWRQTKRFLWKQRAVLVISVIAFYALFLLLRQSTVLKQQIKKQDE
TFKNVQQQRQVLQS)}*

$2433=168(158):
>gi|947779976|ref|WP_056441720.1| {|0(34)|<Proteobacteria(B)>}TIR domain-containing protein [Massilia sp. Root335]
>gi|945489513|gb|KQV45032.1| TIR domain containing protein [Massilia sp. Root335]
{(MAI)RYGCFFSYAHGrhelmqrfkATLADALRCYLEPYFDNEDE-
LFVDTEQLGGGDDLdrKIARALCESACMVLIYTPKYEAHAYTRREYAAMRLIEAERstwYTLPSQLIIPV---
IMTQHPDRLPPQISESTFYVDFSRFTMATADLKSNPDFLP(DIDKMVRRIVTHYQY)}*

$2434=236(234):
>gi|950165835|ref|WP_057215384.1|
{|99(61)|<Proteobacteria(B)>}hypothetical protein [Ensifer sp. Root127]
>gi|945617563|gb|KQW72349.1| hypothetical protein ASD03_31790 [Ensifer sp. Root127]
{(TGSTGLSTRLFPVAMAPGIVAALKVDEQILRWADWKGSIDDLTATLIGHLTFQFCRMLRHYLEHLRRPNETTEALLGYLK)KVDIFLSHSK-NDT-NDAGVHVAMAIRKK-
LTqsdgmgSFFDVYDIPPGLKFqdVLLTQISVSAV-IAIHTDSFSSREWCRLEIIEAKRHNRP-
LVIANCISDKDERGFPYLGNVPIIRLDQKKT-KRIDVVIRALLDEILRNFLWQC(RVELMRPKATSGVT)}*

$2435=200(188):
>gi|947732928|ref|WP_056395068.1| {|0(2)|<Proteobacteria(B)>}TIR domain-containing protein [Massilia sp. Root418] >gi|945642634|gb|KQW97320.1| TIR domain containing protein [Massilia sp. Root418]
{(MSTIRYGCFFSY)AHGRHPHMI---
RFKNDLAEALKCYLEPHfdnESELFIDTEQLGGGDDLnrRIATALCESVCMIVIYTPKYEAHGYTRREFAAMQQIENERrLwyplpSHLIIPVIMTRHPfslpPQITDPGMYVDFSRYTLASGDLKTNPDFLPDIEKIVQRIA(AHYHYLKSSIPPEHDCSRFVLPPIPEEWRATPPPHF)}*

$2436=232(238):
>gi|948163152|ref|WP_056821283.1| {|0(1191)|<unknown(X)>}hypothetical protein [Nocardia sp. Root136] >gi|945777361|gb|KQY31404.1| hypothetical protein ASD42_22495 [Nocardia sp. Root136]
{(MNADRSAEREGDPRSARAHSGKIA)--RLFLSHSSRDSREAVALrAWLIEAEpGLA-
GEIfldldQDTGIPAGVRWKDALRRAGDRCEAVICLLSDN-WDSSHECKvEYRHAESMN-----
KPIFTVRLEPLTgrD---MTSEWQRCDLFG--EGPKTAIS--
VDGETVEFLTEG(LQRLRYGLRAAGIGADTFAWPPPGDPGRAPYRGWEPLESVDAAVYFGRDAQIGRAMDAVRGLRSLGGGHLFVVL)}*

$2437=215(220):
>gi|945744545|gb|KQX98736.1| {|97(260)|<Proteobacteria(B)>}hypothetical protein ASD36_21760 [Rhizobium sp. Root1334] >gi|946158680|gb|KRC10644.1| hypothetical protein ASE23_24140 [Rhizobium sp. Root73
{(RTNLTEAFLLDADIRSGPSPTDVTRLNHTTGLTQAQVEQMVGDTGVALPDSMAFPPHWPLWEGLPIDDQEGLRADFSTFA)----FISYAHGdRDIANLIRSQFEVSR----
IPTWWDQELLAGESWReTIGERLNSAAAIVTLWTEKSSTSKAVVEeASRAQKDAKLV-HVRLDSAEIPYGF-
SETQYADLRKWDEELDHpeMQKLIQAVRDKLFPPSREDIAAR()}*

$2438=228(220):
>gi|947613754|ref|WP_056276901.1|
{|0(122)|<Proteobacteria(B)>}hypothetical protein [Hydrogenophaga sp. Root209] >gi|946144804|gb|KRB96841.1| hypothetical protein ASE11_15670 [Hydrogenophaga sp. Root2
{(MNR)---IFISYRSSDGKKDADRLCADLSRlyGED--
QVFFDKQDLRGGHSWrSAIMAALGTQPVVLLLvTPDLLgmahpEGGRRIDrdDDPIRGEVLTAQsHGATIIPL

LtEGVLMPATGDIPAPLRFLKESHALKLRTEDWAGDLNKIVTDLASH(GISPLVPRSEPDVQAKRGWMQRMQR
GFTWIGGIFVGLVVIGLLLPEDEDAVPAPAPLVSPTPAPVPGQNTRDVSGMW)}*

$2439=204(217):
>gi|947683833|ref|WP_056346356.1|
{|0(193)|<Proteobacteria(B)>}hypothetical protein [Sphingopyxis sp.
Root1497] >gi|945911425|gb|KQZ64713.1| hypothetical protein ASD67_09705
[Sphingopyxis sp. Root1497
{()MTDVFISYKREeRARCVAIYNALVDLK----
LSVWFDAHIEPGTDFDrEIEREVRGASAVLVLWSERAADSDWI-----RAEARSGR-QTNRLVAIRLDDC-L-
PPLEFASVQA-----
IDLFNHRDFQasEGWRQIVSRIGRL(VGRLGLGDYVGCEQETDADRWRAWIADNPGDPLVDKARQRLALLIRP
EPALSLAQATAPAADIAPPATSIPTPAPAE)}*

$2440=160(162):
>gi|947607018|ref|WP_056270184.1|
{|0(157)|<Proteobacteria(B)>}hypothetical protein [Hydrogenophaga sp.
Root209] >gi|946152311|gb|KRC04297.1| hypothetical protein ASE11_04380
[Hydrogenophaga sp. Root2
{()MSDVFLSYASEdRERAGRLAAALGHMGWS----
VWWDRKIIAGQAFDqAIEHELETAGCVVVLWSVNSIASEWVKNEAAAASERDVL-VPALIDNVKLPLE-
FRRKQAADLLGWQGNELhpGFQALCQGVAHAMRGAAPSPRPR(PRPRRAAQPAPSRPRWVVPALT)}*

$2441=222(223):
>gi|947680902|ref|WP_056343454.1|
{|95(280)|<Actinobacteria(B)>}hypothetical protein [Arthrobacter sp.
Soil762] >gi|946425633|gb|KRE76013.1| hypothetical protein ASG77_20545
[Arthrobacter sp. Soil762]
{(APAGPSHIDVSALRAIAKTSDGPIALVVRSTSHSTPFHGVTAQRQRFGRVTSEKVQVISPEPKFQPLGPLP
PTAVRGDGP)---VFISYRQTDGTDHAARLEHLLRATGL--
VVWRDYRDLRAGTTTdRLEQALTQGlSAAVLVVTPDIVYSEIVREreLPRLLQLDEDPDFSLSIANTVPRRGS
SSKCDYEAPDRFLRLSPKRTLADKKQSNMLDSMGDLDIAR(DLL)}*

$2442=215(220):
>gi|946388194|gb|KRE38866.1| {|74(0)|<Actinobacteria(B)>}hypothetical
protein ASG73_00380 [Janibacter sp. Soil728]
{(AIANLQKKIDKEGDAAQGRRDRTDARRERDRARRDQVVDRDLDLLVQTTSALDARLTEVEASSLNTLALAV
ASDLVDRQY)--DVFLSFATPDETAASNLRDELQARGLD-
VWMVDAQVGLGQSLVTGIDNGIGSSRCGITFVTPAYVDPSRfWPRqELAALIMGR-----KRVIPV-
ISGLEfdDVEkfsSLLGDRkgLSTDIHG--LDE---IADLIVHSLSDETPRQ()}*

$2443=185(194):
>gi|950025622|ref|WP_057151189.1|
{|0(338)|<Actinobacteria(B)>}hypothetical protein [Mycobacterium sp.
Soil538] >gi|946374706|gb|KRE25489.1| hypothetical protein ASG82_18180
[Mycobacterium sp. Soil538
{(MAA)----FISYSSRdRALVEGL---LSALRRAHL-
PVWLDEELGGGESWWrEILAQIRGCEVFIVALSKNLLDSKAC-QaELRYAQSL-----GKPVLPVQVGPM-
DtLRINPLSAMQVIDYRNpgLESGIELISSVSALRAKNSPLPD(PLPEEPPIPFAYLMRLASTISDPTGLSAQ
QQTALVAELKAGLDEDGSDEMA)}*

$2444=194(184):
>gi|947939287|ref|WP_056599356.1|
{|0(1140)|<Actinobacteria(B)>}hypothetical protein [Nocardioides sp.

Soil774] >gi|946447308|gb|KRE97552.1| hypothetical protein ASG76_02260 [Nocardioides sp. Soil774]
{(MAAIDPV)--
TIFVSYKKDdEvgdgrgrEFVKALREAVEDSARtQGELipEVWFDDDIESGAEWEeEIERRLRSAEVTVLVYT
PQFFAPDgYISKnELPIVLKR--L-KDTQVVQIVRRPV-
APGTSFFSAEAVPKALRklDPEHDGQPFDVLNAGEFATRLGM(TADNIVAAAKRAYALRQTTHVRPVLEEYIA
KVNKRFA)}*

$2445=204(216):
>gi|503592166|ref|WP_013826242.1|
{|0(374)|<Euryarchaeota(A)>}hypothetical protein [Methanobacterium paludis] >gi|333826081|gb|AEG18743.1| Tetratricopeptide TPR_1 repeat-containing protein [Methanobac
{(MNNVESP)-YEIFIAYETTTGRSTAQNLYKAFKKTNY--
KSFYDREILLSSDNWrDIIDsAIEEAQFFVVIFTSGTFN------SDEVKRECNKALkLNKSIITC-------
RSSDIPiSDTQELSRFQRITFEDKYDLADKLVVNIRKLKE(RHSKIRAEEDFEEICKRGDLFYELNQFEKALE
SYDKVSELKPEMEVVWNNKGVLLDKLKRYEEAIKAYD)}*

$2446=167(176):
>gi|503627708|ref|WP_013861784.1| {|0(152)|<unknown(X)>}hypothetical protein [Microlunatus phosphovorus] >gi|334684314|dbj|BAK33899.1| hypothetical protein MLP_08850 [Microlunatus phosphovorus NM-1]
{(MTA)----FISHSFSnKAEFRNVADFLTQVNVPY-------
WEPTSIRSGEglrnQLRSTIRECSVCVFIATHDSVNSAWCGAELGAFWGAGIK-
IVTYVADSSLQPDDLPPVVQGDVWERELRRV--
AETARRIHDSARPRAGTDPAP(QTPLTNLTAMELTSLIASAVSLSRAQEQSPAGT)}*

$2447=275(274):
>gi|493683525|ref|WP_006633668.1| {|32(315)|<Cyanobacteria(B)>}molecular chaperone Tir [Microcoleus vaginatus] >gi|333458057|gb|EGK86676.1| toll-interleukin receptor [Microcoleus vaginatus FGP-2]
{(IRGTLTEIEAESGVGADTVSKIKQGREGADLSKIRQLFAAFEMTLAASDHRSAKLEEPENLDELDNLPPAE
DTATSSRIV)--KIFVSYRSADpdRELGKELETALSAIGHT-
VFTAENNIRLGDNWFDRINLFLQECDYLLILLPPTGNQSELLTYqVQQAKELQD-----
SRpdrkpiILPIRVGFPLnaPLNHDLRGYLYRIKQR--
EWKTTADTPIIIEEVVNLLAEK(PAPVTEQTEDLTQKQLLEISSDEIPLPAAEPELPGGQVDVASQFYVERPP
IEEH)}*

$2448=248(258):
>gi|334858160|gb|AEH08634.1| {|94(44)|<Actinobacteria(B)>}hypothetical protein FsymDg_1135 [Frankia symbiont of Datisca glomerata]
{(NALVGAGWNDYLAELLRAVGAGPGNWLLPFALTSNFARVPVAVDVDVVDLHTLPAHFREQVLLNHVIHRVC
RQVGASDPV)--RVFISYASADGgAIAREVARHLRfETDVD-DFLDAHHISGGDPFADVLDAAATSS-
ALLAVNSDSY-GSRPWCQrEF---LGAK-----RASMPVVVLDALtdRSVRAFPYLGNVPTVR--
WHRDSRRVLDHLVGVLLTETLR(HRYFPLRAAEMCGRHGVALPSFLLARPPELLTLLDLPR)}*

$2449=265(268):
>gi|505827702|ref|WP_015713070.1| {|83(1282)|<Spirochaetes(B)>}cell surface protein [Treponema azotonutricium] >gi|333735302|gb|AEF81251.1| cell surface protein [Treponema azotonutricium ZAS-9]
{(RKPFIPVWLDSLADIQQDYQNRECDPQLLSALEMLTARYSCTSIGKLIAELERFTPDDTPYTPSTPQICDK
PCEAYEGDE)-PYIFISYAHDdAI---QVYPKIKELYESGW-
DLWYDEGIKVTERYLpVIADHVRHCSVFVLMLTNRCLDRPFVMNyELEYARQR-----
GIPIVPVLLEeltPQ-

PWSMENAAQLVKTAIAPdaLQMSISSVDLKNCGKRTAVPPAI(KQNVVYDVNLPLDMPGFNILVQGDKITITR
YVGNDRDVIIPSTATSAE)}*

$2450=266(269):
>gi|505825123|ref|WP_015710500.1|
{|83(89)|<Spirochaetes(B)>}hypothetical protein [Treponema
azotonutricium] >gi|333735861|gb|AEF81810.1| hypothetical protein
TREAZ_1515 [Treponema azotonutricium ZAS-
{(RKPFIPVWLDSLVDIQQDCQNIDGDRHLLSALEMLTAKYSCTAIDELIAELERFTPDNTPYTPSTPQICDK
PCEAYEGDE)-PYIFISYAHDdAI---QVYPEIKELYESGW-
DLWYDEGIKVTERYLpVIADNIRHCSVFVLMLTNRCLVRPFVMNyELEYARQR-----
GVPIIPVLLEeltPQ-
PWSEENAAKLLKAAIAPdvLIEHISRASLTNRGTRIAVPPAI(KQNVVYDVNLPLEMPGFKILVQGDEITIVR
YVGDDRDVVIPSTVTSVDG)}*

$2451=169(176):
>gi|503582989|ref|WP_013817065.1|
{|0(144)|<Proteobacteria(B)>}hypothetical protein [Methylomonas
methanica] >gi|333806123|gb|AEF98792.1| Domain of unknown function
DUF1863 [Methylomonas methanica MC0
{()MSDIFISYASAdKVQASLLAEKLS---EQGWL-
VWWDRQIQAGQSFDeVIEEALNSAKCIIVLWSQNSTTSRWV-KtEAAEGVAR------GILVPVLIEKV-
NIPLEFKRIEAADLSDWrgKSHHIEFNQLLMTIEKLLNGTVS(ISVPPKPLSTTQKFKKWWKRILIGAAVAIG
LASAVF)}*

$2452=173(179):
>gi|496100840|ref|WP_008825347.1| {|0(608)|<unknown(B)>}hypothetical
protein [Haloplasma contractile] >gi|543105211|gb|ERJ11785.1| Toll-
Interleukin receptor protein [Haloplasma contractile SSD-17B]
{(MQ)-KQVFISYAYEdRLIADSIVIDLE----
KNDIECWIApRDIEAGMDWVeAISRAIMEVKIFILIVTEKSNVSEFV-KkELHSAVEYNK-----
LIIPFFVEDV-
KLRYSLNFHLQGIQHLKdlDTKMSVRLDNLVNYVSRRLENNT(VNVRKFSNQTGSFTKQSRINRFSKKELTKF
LENKGIS)}*

$2453=231(232):
>gi|503584176|ref|WP_013818252.1| {|250(0)|<Proteobacteria(B)>}TIR
domain-containing protein [Methylomonas methanica]
>gi|333807329|gb|AEF99998.1| hypothetical protein Metme_1579
[Methylomonas methani
{(RAMLRLFNDNRFYSQEKFTSEFLLPSADDSGLKAFLSQYSPNTLGFDDPTEAFIEKLALRFPSATPTTQPK
PNARASGGP)--TVFVSYAS---
ENAEAARRIADALVNLGFsDVWLDKKKLIGGDDWadRIEDAVETCDFFMPLLSAEANSRREGVfWeEWNMAVR
RARRIqD-AFLLPVGIDNE-
AASKTRYARIDDRETRVFFDKHLFHAPGGFFDTDQQDALQ(ERCRRFREAAND)}*

$2454=225(224):
>gi|503585160|ref|WP_013819236.1| {|254(0)|<Proteobacteria(B)>}TIR
protein [Methylomonas methanica] >gi|333808330|gb|AEG01000.1| TIR protein
[Methylomonas methanica MC09]
{(EINLAEFLLHFSTRTKIFPLQAVEFVDELYRRWTAKHPGDANCPEPAQSVVEAIPSSDNHTKPDQDAVDDG
GSERMRSGA)---VFLSYAH---
EDESAVSSIKNAFEKAGIEVWFDKDsgALRVGDKFksRIKTNIEQCSLFVPIISRNTlTQERRFFRfEWKYAE
ECADFYpDSrRFIIPLVIDDT-PPDDPNLDEYIRELHWERLQNGQCSAAFLTEIKHLQRAYR(RSME)}*

$2455=231(214):
>gi|503618263|ref|WP_013852339.1| {|0(93)|<Firmicutes(B)>}hypothetical protein [Streptococcus pasteurianus] >gi|334283390|dbj|BAK30963.1| SEFIR domain-containing protein [Streptococcus pasteurianus AT
{(M)KTKVFISYAW--EEDNEndrkvksFTQWLAVYLKKWKFDVLLDVYENHPGTRLdkFMSEGI-NSSHFVLCICTKTYVEKmqntnTGVYnEISL----LKEMaDSPFIIPViekgmfaNLPSF-FEGKFVSELNFeTPDSQDNKDGIVQLLCtLRDETVSAKDVKS(ETSIDRYFNSVEKMKFQTTVSNLMNFQCQSEGKITFQYLLNGGDFKLGIPPMEFLTHWSTCGSDSIYSCKKVQ)}*

$2456=223(235):
>gi|947058998|gb|KRH08404.1| {|0(486)|<Streptophyta(V)>}hypothetical protein GLYMA_16G147000 [Glycine max]
{(MAMRPSSSSFSYRFT)-YDMFLSFRG-EDTRCGFSGNLYNALQDRGIHTFVDYKELqrGHEITSALEKGIEVSRIFIIVLSQNHASSSFCLNKLAYILNF---------------------IKGKEVVFGEALANHEKKFNAnkmgfkhnmEKT-EAWKMALHQVANL(SGYHFNGGGYKYEFIKRIVDLISSKINHAHLHVADYPVRLESQVLNVMSLLDVGSDDVAHMVGIHGLGGVGKTTLALQHL)}*

$2457=167(167):
>gi|947089740|gb|KRH38405.1| {|0(274)|<Streptophyta(V)>}hypothetical protein GLYMA_09G134400 [Glycine max]
{(MAVPSSSSSFT)-YDVFLSFRG-EDTRYGFTGNLYKVLHDSGIHTFIDDEELqkGDEITTALEKAIEESIIFIVVLSQNYASSSFCLNELTASSSANLVTIHESIGFLGKLKFLDAVGCSKLRCHSLESFPEILGKMEIITELVLEASAIKELP(FSFQNLIRLQILQLRC)}*

$2458=250(254):
>gi|947062995|gb|KRH12256.1| {|0(679)|<Streptophyta(V)>}hypothetical protein GLYMA_15G162700 [Glycine max]
{(MDTRLRVIDDPMNEVQWQNGAEEKSMSINTAPQI)KFDVFVSLRG-KDIRDGFLSHLTEAFKRNQVHAFVDDKLERGEE-IwpSLVTAIERSFILLIIFSQDYASSRWCLEVLVTILECRDK-YERIVIPVFYKMEPTNVQHQLGSYTK--MHLLSMKEDTKA-RNDAEVVKEIVNL(VLKRLAKPKIKSKGLHLTIKESPLRKEKNEGNFLPCLLTPPPQTKPFKIIELVLTALILTYTISLNDAAVVKEIVNLVLK)}*

$2459=233(244):
>gi|947090852|gb|KRH39517.1| {|0(775)|<Streptophyta(V)>}hypothetical protein GLYMA_09G202800 [Glycine max]
{(MAFIYAAAVVGCASSSSSPAVSEN)-HDVFISFRG-EDTRGDFTSHLHAALCRNGIQTYIDYRIQKGYE-VwpQLVKAIRESTLLLVIFSENYSSSSWCLNELVELMECKKQ-GEEDV----------HVRKQSGSYhtALAKHKKDWKVSKEKM-QKWKDALFEAANL(SGFHSQTYRTEPDLIEDIIIDVLQKLNHRYTNDFRGLFISDENYTSIESLLKTDSGEVRVIGIWGMGGIGKTTLTAAIFH)}*

$2460=128(154):
>gi|947097247|gb|KRH45832.1| {<Streptophyta(V)>}hypothetical protein GLYMA_08G295400 [Glycine max]
{(MASPSTKSNDSQKE)-HEVFLSFRG-EDTRNTFTGHLNAALKRYAIRTYIDHNLKRGDE-IshTLLNAIEKANLSVIVFSKKtFATSKWCLDEVVKILECKEK------------------KGANCGAY---------FLSCRSIYATKSDRKLRRSIC()}*

$2461=211(237):

>gi|947055482|gb|KRH04935.1| {<Streptophyta(V)>}hypothetical protein GLYMA_17G197100 [Glycine max]
{(MTSAVASSSNSFSMLSPKIMMF)----FLSFRG-EDTRRNsTTSHLYEALlHKK-IESYIDYQ-LEKGDEITLIQAIKDSAY---------------LFSFSQRTMLPQS--GQIVIPVFYNIYPSHVRKKTGSYDQAFAKH-V-----GEPrcNKWKAALTEAANL(AGWDSQTFWTDSELLKDIIGDVLRKLPTRYPNQVKGLVGIEENYKQIGSFVGIFNKPYKIPNEHQNTLRQIIKRF)}*

$2462=282(279):
>gi|947077073|gb|KRH25913.1| {|0(403)|<Streptophyta(V)>}hypothetical protein GLYMA_12G138400 [Glycine max]
{(MIGPNLGPWAQKPTLRFIRTLGPSLAALAQSSWSLLLMALVTDPFLGRIASIAMASNTIIQCSPSTHTKR)KYDVFVSFRG-EDSHNNSTGFLFEALqKKG-IDAFRDDAGLNKGESIapKLLQAIEGSRLFVVVFSKNYASSTWCLRELAHICNCIEI-SPRHVLPIFYDVGPSKflVMRFSKINLLSLFKL-KVRVVEEATtiCREIEEIVQNILK(ILGPKFSSLPNDHLVGMESCVEELVKLLELEISGMCGIGNTTLDRALYERISHHYDFCCFIDDVRKIYQ)}*

$2463=201(232):
>gi|947097376|gb|KRH45961.1| {|0(161)|<Streptophyta(V)>}hypothetical protein GLYMA_08G303600 [Glycine max]
{(MTLSLIILDSKD)--------------------------KEK-VQTYIDDQ-LEKGDEIspALTKAIEDSHVYILIFSENDASSKWCLGELIKIMECKNE-KGQMVIPVFYNVDSSHVRKQTGSCEQAFEKH-E-----GEPrcKKWKAVLTEAFNL(AGWNSRTYRTDPELLKDIVGDVFRKLPPRYQNQRKGLVGIEEHCKKIEYLLKIGQSEVKTLGIWGMDGIGKTTRATTLYH)}*

$2464=186(186):
>gi|947076996|gb|KRH25836.1| {|0(434)|<Streptophyta(V)>}hypothetical protein GLYMA_12G133000 [Glycine max]
{(MACNSIQSSSSLCTRN)-FDVFLSFRG-TDTHNGFIDHLFASLPRKGVAAFRDDQTIKKGNSWslGILQAIEGLRVYIVVFSKDYALSTWCMKELAKIVDWVEETGRKIEKIVEEVINILSHNQILSLGDDLVDMLSCVKQMEEFLDLDANKDIRVVGI(CEMAIKVLGSFLFDRDVSEWRSALTRLKEN)}*

$2465=156(160):
>gi|947077405|gb|KRH26245.1| {|0(366)|<Streptophyta(V)>}hypothetical protein GLYMA_12G163000 [Glycine max]
{(MASKTTIHCC)-------FRG-EDTRNSFTGFLFQALSRKGtIDAFKDGKDLKKGESIapELIQAIQGSRlFFIVVFSNNYAFSTIRKKLQYAEIEDLEKITNILGHKFSSLPNDDLVGMESCVKELAKLLRLGSVNDIQVVGMSGIGGIGKTTL(GHGFYAMLAR)}*

$2466=216(220):
>gi|951143542|ref|WP_057624472.1|
{|70(0)|<Proteobacteria(B)>}hypothetical protein [Candidatus Berkiella cookevillensis] >gi|946811813|gb|KRG18482.1| TIR domain protein [Candidatus Berkiella cookevill
{(FLARSNTIKVTSGFISDIHLGNVGSDFAAFSNKVYFYLDNNLNPTDLVHLDGIAKSKNLAITIRDNTYLTKKSEVEKPLA)----FISHDS-RD-KKQVALPIAIGLQRLLCPVWYDEFSLQVGDNLreSIEKGIKETKKCVLIISKNFiSNSGWTKTEFDSvftkeIIEKS-----NVILPVWVDVSPAE-VYDYSPSLANKMALNWSQGEEKVINELYRAIAFDKAQ()}*

$2467=195(220):

>gi|946942178|ref|WP_055864075.1|
{|0(470)|<Actinobacteria(B)>}hypothetical protein [Frankia sp. CpI1-P]
{(M)-------------------AWTLEAAGHE-ALIQAWDFGPGSHFVGEMQRALSDDRRTVAVLSAAYLE-SAFATeEWQAAWAAD-----PvgteqRLLVVRAEDC-DRPGLLRQVVSVDLFG--VSREEARI--RLLEMVRLGRRK(PTVEPDFPAGLPAQGSPAGPARSAAPARSDGPARSAGSAGGSAEAAPTFPAELPEVWNVPPRLGRFVGREQQLAAVAKE)}*

$2468=227(240):
>gi|947713783|ref|WP_056376127.1|
{|0(117)|<Proteobacteria(B)>}MULTISPECIES: hypothetical protein [Sphingopyxis]
{(MMAAMTDAGPPPIKPKRLSA)----FISHHSSQVETARRLKRLLAVRGID-GWLAPDDIAAGTAFDQSIVDQIDRSDAIVLLFCPQ-SDQSRHVKrELMLGEDSN-----KIILPVRLEDVRaqGLAYWLKDYQWIDWFA---GEEQAVE--RIVAAMSPGTAP(AVSPGTTARQMSAPLAPRRKRLWMVIAAGVALVGLIGGGIWWQSRTPTAGDPGAAVADNPIEAGIWTTKREVVEILFPEM)}*

$2469=178(182):
>gi|949009938|gb|KRO20519.1| {|0(75)|<Firmicutes(B)>}hypothetical protein IV88_GL001580 [Pediococcus argentinicus]
{(MSNES)---VFLCFSS---KDRDpYIHAVAYHLKSFGLSIWYDYDNLHlgnDRNKKnmiEPF-----KKSNysVIFISNNLFNSKCAVEELNKIMS--LKDKGMYIIPVFLDYLPqtL-NPNLSWIVNLIYQEA-SKTDDAMNLTLKIVDAVLQNEL(GKLTDIDTSFNALIADISNSADWKLKSIATLLSDYQN)}*

$2470=218(223):
>gi|950999967|ref|XP_014506669.1| {|0(751)|<Streptophyta(V)>}PREDICTED: uncharacterized protein LOC106766455 [Vigna radiata var. radiata]
{(MASSA)----FVSFHD-EDIHF-ITDFVLDPLRSRGFHVFV----KGGSTTFDLFQAIERSRFHIVVFSKNYPSSICCLRELMAIINAVESSPWsTNFLPIYHGVQQSEVLFQTGCYGQVFSKYEERFREhkqrmEEM-QRWREALTRVAGF(PGLNMENVTRHGGFDFVQYAARILGREFLTSQNETISSRYGKFDDIVQYKDGDEVSYKELRGLIRLRDFISLVLTYPV)}*

$2471=197(204):
>gi|951032832|ref|XP_014515507.1| {|0(844)|<Streptophyta(V)>}PREDICTED: TMV resistance protein N-like [Vigna radiata var. radiata]
{(MDVSSSLSIFSKFIVAG)--EVFIHFLG-DDIHRNFVSHLSSSLLQAGVKPsLIAVEMLSEKF----MPSITRFQIGIVVFTKAYIESSRCVEDLLRIIECHEN-HGLIVMPVFYDIDPSDLLDLKMDSSSYVLYPMKGELKKNSSPEQRSAKRKVKTT(VKWTQMKGERLNYPIILYTSAGSRALNRVANLPTWDESKHRNDAELV)}*

$2472=190(196):
>gi|951032839|ref|XP_014515509.1| {|0(960)|<Streptophyta(V)>}PREDICTED: TMV resistance protein N-like [Vigna radiata var. radiata]
{(MASVSSSLSFFSKLV)-SEVFIHCLE-DDIHENFVSHLRSALLQAGVEPSLVAVGKLSKK---FMPSVTRFQIGIIVFTKAYIQSCRCVQDLARIIECHET-HGLMVIPVFYDIDPSYDQKADVDPSHVPDQKRDIDPSHVDDQKRDVDPFHISSV(STNPFRELEEAETKISDKVNIEKIYNKTMQNFSLSRALNRV)}*

$2473=243(245):
>gi|951032865|ref|XP_014515518.1| {|0(733)|<Streptophyta(V)>}PREDICTED: disease resistance protein TAO1-like [Vigna radiata var. radiata]
{(MEFASFSPSSSSSSSFLKSEPQFIY)--DVFINFGG-EDIGRKFVSHLHSALLLAQVKTFISKENLEgikLEA---

YRRAIASSKIAIIVFSKTYTESTYWRLELEKIIECRQT-
FGQIVLPLIYDIVRLDERHQMvvRVLEKATPGSYSGEQLERpM-
SRWRRALTKAAGI(TGWDFRDFRHDAELVEEIVRHIQTLLDYTDMSITQFPVGLESHVEKLIGMIEEQSTEVC
MIGIWGMGGSGKTTIAKAIYN)}*

$2474=234(237):
>gi|951033974|ref|XP_014515875.1| {|0(791)|<Streptophyta(V)>}PREDICTED: TMV resistance protein N-like [Vigna radiata var. radiata]
{(MASASSSSSFSKFRIEG)--DVFIHCLG-YDIRRNFVSHLSSALLQAGVKPCLLAVEMQQEQ---
FVASIEGFQIGIVVLNKSYFESFQCVDELVRIIECHET-
HGLTVMPVFYEMDRSDFGNMLKATAREVMKVEHIIWSGEyqktwF-
QRWNVALTKAGTL(PTWEESEHRSDAELVEKIVKSVLAKLDCPLAITKFPIGLEPHVKNVIGFFENQPTKVCM
IGIWGMGGSGKTTVAKAIYNQ)}*

$2475=261(256):
>gi|952921455|ref|WP_057935115.1|
{|1057(0)|<Bacteroidetes(B)>}hypothetical protein [Pedobacter ginsenosidimutans] >gi|951541376|gb|KRT13215.1| hypothetical protein ASU31_25785 [Pedobacter ginsenosidi
{(LYTKIYNADRLYKVEECRPDDMLISVDGRKFASYKEICSDNAGTMIKVCLADEDGELTGETMIVPIYPFQP
FTPNDLKRK)-
KKVVVSYSKYdYALVNRFLNHITPLVDDEIIEkPWLCAELIAGSEWNeEITRRFAQADIIFFMISDHLMQVDY
VKEfEIRNAIDRYNK-DGLpKIIPIILSPY-LWQrkgDYDLGRFSALPFKAvaVTSFPDQN--
EAWKFITQAVKLM(IMENAQPTEEGMRMNDEMRKICERLMDNNTIYYPKN)}*

$2476=249(254):
>gi|953489636|emb|CEG47002.1| {|709(584)|<Stramenopiles(E)>}transient receptor potential ca2 channel (trp-cc) family protein [Plasmopara halstedii]
{(TSVDMFKVLVASGGDINHQLRVSGDTALHLAVKLDDQQTALALLSLGGDIMRTNHDGFRPIDCTTSTRLQF
EIKRAAGTR)--DVMISY---
THSHMEFALKLRKSLEDANITVWLDLMDpsgIGGGSVWreEIARGITNAAVVLCILTEDYTQSEWCLKELALA
KQVG-----
TPILAISTEHAKISEELQVYLYTRQLVPFESAIVNVEHVNGKEVRYEYDEFA(YKRQLRMLLDGMRDEIEKRK
EDNIKRNMRRAEIN)}*

$2477=248(257):
>gi|953500249|emb|CEG38890.1| {|816(20)|<Stramenopiles(E)>}ankyrin-like protein [Plasmopara halstedii]
{(AITKIDNSNKRKIVYDYDETLFAGQFRLLLDGVRDEIEKQREAKFHGENRRHLANGTSVTGFADNISDWNA
ATLDNIQFA)----FVSHG---DHHHSFVKRLHDRLNDNQVRCLLDGTQTYA--
DMteRIhaaKEAILKCDVFIVILSRKTVNSELVKDQLAFAEDKG-----
KPILPVMLNDMEIPLDKHYTLSRLELLHFTPELGFNSSFMQLFNRVRDSMTP(SNASFIKPRTSARTNFRAVA
RMTAMGHGRQNTNLAVT)}*

$2478=249(254):
>gi|953500249|emb|CEG38890.1| {|622(213)|<Stramenopiles(E)>}ankyrin-like protein [Plasmopara halstedii]
{(NVTAGLFDCLVEHGADIDDRLPNGDTALHIAMKCEQKGTALALLDSGADVMETNRDGYRPVDCTTSTQLQF
EIKQAAGDR)--DVMISY---
THSHSMFALKIRDHLERANITSWLDLMDptgIGGGSVWreEIARGIKNAEVVVCLYTEDYPVSEWCLKELALA
KQLG-----
KPIIAVSTENAKVTDEMQVYIYTRQIIPFESAITKIDNSNKRKIVYDYDETL(FAGQFRLLLDGVRDEIEKQR
EAKFHGENRRHLAN)}*

$2479=222(224):
>gi|953252423|emb|CUS31302.1| {|232(0)|<Nitrospirae(B)>}hypothetical protein COMA1_10043 [Candidatus Nitrospira nitrosa]
{(GAAHGLGLPTIRMVSAASGPIELPWILKGGPGGFENDIVMWSKPEDVLALVDPRIAAMDRISEALSDGDGF
DYLQSKRYA)KFFVFLShtLKG---ADRALVEQIYRLLKERYVIPF--
EYHQANTAGMdwrAALDESLKKTTHfVTLLDPMYEQSETCEYELREILKR---RNEVAILPFMINGRDrpN-
PDLKNMHNELLSNPN-PSANAKSVVQQVMQELDRALSG(SSQI)}*

$2480=259(263):
>gi|953489639|emb|CEG47005.1| {|723(250)|<Stramenopiles(E)>}transient receptor potential ca2 channel (trp-cc) family protein [Plasmopara halstedii]
{(DATREVFSILTSAGADINAQLPTSGDTALHIAVREENEQSALAILANGGNLMKMNIEGLRPLDCTTSTRLL
FEVKRAAGQ)-RDVMISYTHSHAEFAKKIRKALEEANVT-
TWLDLMDPsgiGGGSVWREEIARGITNAAVVLCLLTEDY-AQSEWCLkELALAKQVG-----
TPILAVSTEGVRitETLQVYLYTRQLIPFE--
PAILQTKRNSVNARQIEYDYDE(IKFQAQFRLLLDGVRDEIEKNRDAIVQKNIASSLRVNRLQQTT)}*

$2481=209(205):
>gi|952988862|gb|ALO25075.1| {|0(1307)|<Spirochaetes(B)>}TIR domain protein [Leptospira borgpetersenii serovar Ballum]
>gi|1137166990|gb|ANH00069.2| TIR domain protein [Leptospira borgpetersenii str.
{(MESFVHPSKI)-ASLFICYSFDqIDWTREIGSKINTI-
YDGKINAWWDERKSTGDYWDnIINQKLSEADVFLLILSPGFFKSDYIQNtELPIIIRRHES-
EKIRVIPIIASKI-
NfeqFKESWLNEIDVFPHRGlqTINQLQPDEIDNWIRNLSIEPVW(RRMEMEFRRISIRTLSRPFQYDLQLPD
GSGYIIERNEIEELENWNVRENDQKIAF)}*

$2482=178(186):
>gi|953253496|emb|CUS33372.1| {|0(134)|<Nitrospirae(B)>}conserved hypothetical protein [Candidatus Nitrospira nitrosa]
{()MPSVFLSYSREdLPLIEQLAARLRASSPE--VSLWRDQeKIYGGQKWPkVLGEAIADQDVFLLAWSKH-
AASSHFVElEWNTAVALKK-----TIIP-----C-
LLDETPLAPSLRAFHGYplQDVAGLTTSLAAALPADQSRRTP(VLAKLDDITSRNETVVLAQAKAVFAQQQWT
VQGNVYQAGGDIHIHN)}*

$2483=172(176):
>gi|953253740|emb|CUS33832.1| {|0(291)|<Nitrospirae(B)>}hypothetical protein COMA1_11364 [Candidatus Nitrospira nitrosa]
{()MHEVFVSYSSVdE---NAVRKRLQLLKSRG-
IPYWFapDAEIPGGEDFAsFIETAISNSKVFLLMASESALGSE----EVKKELALADN-EKVPIIPLFLETE-
LSFPPGFRYRLLSRQYIsaTEEENEEWVYRLLKTLRYHGVAI(SNDEVQTSAAGSREKPKISSGLMPYLADRD
QQEALI)}*

$2484=250(258):
>gi|953489633|emb|CEG46999.1| {|893(435)|<Stramenopiles(E)>}Ankyrin [Plasmopara halstedii]
{(DPRIFQLLLDHGVRLHDSRTVNGDSALHVALRRQNRTAAVLFVRAGLSLTAMNFLGQRAIDCAQSPDLRFA
VKKEAGHRD)---VMISYSHAhASLacqiRDALECH--------
RVTTWIDTMgptgITGGAVWRqEIARGIQSSALVLAVLTKDYPQSQWCMKELAFA--------
KMQNIPVVAIQC-

EDMEISEELQVYLWTRQviDFRPAVKQLYAIKYNENQSYPPS(DEQIGESAKKKECVTMRNSSHTVEDSESHG
VYSDEINS)}*

$2485=251(251):
>gi|954203609|gb|KRX13459.1| {|1160(0)|<Nematoda(M)>}Slit -like protein
3 protein [Trichinella nelsoni]
{(GNNNNNNNDNSRLHTALIAVLVLCTVVAGALLTLMLRRMYCSRKPTAAGRRSYCRYVHDSTPAKEADDGCS
VPPSTQQLL)QYDLLVCNSAAdqPFVEEALLGRLEEEPPYYRVVSLHRDLttPTSGTSGEQLLKAFDRSQRVL
LLLSDGFVEADWQQPDLRAAYQLLLRDKHRKLVLVVRDRA-VPVET-DPVLGHYVRTNrCLFWTDPM---
FWDKLHDAVPSP(LPFNVCQSNASQPNNEDYSDMYGTIVPSHFV)}*

$2486=200(219):
>gi|930611664|ref|WP_054263783.1| {|0(3)|<Proteobacteria(B)>}TIR domain-containing protein [Janthinobacterium sp. CG23_2]
>gi|955160519|emb|CUI04571.1| hypothetical protein BN2497_3919
[Janthinobacter
{(MAIRYGCFFSYAHGRRDGLMNTFKIALSDAIRCYLEPYFDNED)---------------------------
----
ELFIDTEQLGGGDDIdrRIARALCESVTMILIYTPKYEAHGYTRREFAAMQAIEAERsRwytmpSHLIIPVVM
TLHParlpPQIAASSFYVDFSHFTMATGDLKSHPEFLPDIKKIVSRIV(TQYEYQKKYTPVGHDCNQFVLPDV
PPVWRDLPDLTF)}*

$2487=280(281):
>gi|654351605|ref|WP_027844770.1|
{|125(316)|<Cyanobacteria(B)>}molecular chaperone Tir [Mastigocoleus
testarum] >gi|959134837|gb|KST69847.1| molecular chaperone Tir
[Mastigocoleus testarum BC008]
{(LPMLRLRIKANSQNVQRGYSGSPVFDAENQRVIGIIAAYDNTEGALAVPLSTVQDKWVNLEKFMNLQPFVP
PIEANKSAR)---VFISYRSQDpDLSLAQYFYESLKADGH--
DPFMAGESIQLGeKWTqRIDQELEQCDYFLLLLSPKSATSEMVIEE--VRRAKELAdlrPeKKPVILPI--
RVQFPMNSPLNYDLRGYLRqIQQREWKSSADTAKILEEVLNLLAR(EDSNNYSETDKTEDLSVIPTLQPSNIT
STECFALPLPVAEPEIPGGKVNLASKFYVERSPI)}*

$2488=292(291):
>gi|957818139|ref|XP_014668414.1| {|686(40)|<Priapulida(M)>}PREDICTED:
uncharacterized protein LOC106809736 [Priapulus caudatus]
{(LDDPDALSCTFSDQSIALRKVHIQEDDCGLPVINIFAIVMASLILLAIVVVTYRRRRYIAYYFHVAAVRLK
RYEPAVGEY)EYDAFVGYSTStgfelNWIINFLLPKMENDEVNPyRLFLEERDMPAYGMQVSNIVAFMDKSHT
VILVITQTFLTDVYCNFMLKTAAMR------NNVIIIFLETIAtEEFPA---
ELRVLQLHStCLHWSENRNSqeRFWKAIEYAMPLP(QRDHYAPILASSNYSAVQAIWTVDRAAALPSCMPCVT
TQTETDNRSNTTGNAICDNECYTYNDQWPINDDL)}*

$2489=248(251):
>gi|954595041|gb|KRZ72243.1| {|1053(0)|<Nematoda(M)>}Slit -like protein
2 protein [Trichinella papuae]
{(SSNDNDNNNDNNSRLHSALIAVLVLCTVVAGALLTLMLRRMYCTRKPNSGRRSYCRYVHESTPTKEASSVC
DPAPSTQLL)QYDLLLCNSVAdqPFVEEALLGRLEEEPPYYRVCSLHRDLST-
FSTGEQLLKAFDRSQRVLLLLSDGFVETDWQQPELRAAYQLLLRDKHRKLVLVVRDRA-VPVET-
DPVLGHYVRTNrCLFWTDPM---FWDKLHDTVPSP(LTFNICQSNVSPNNNEDYSDMYGTIVPSHFV)}*

$2490=255(261):
>gi|957818494|ref|XP_014670082.1| {|944(47)|<Priapulida(M)>}PREDICTED:
uncharacterized protein LOC106811067 [Priapulus caudatus]

{(IAQQARNTESVVTDLPHWVRGAAKMLNAATPGADWSALAKKLGYRKEKVRDFEDDINPGLALIVDWVHRGG NNVACADVQ)PAQVFLSYSW--DVQDQV-KVLRTRLEQAGFTCWMDLGQMGGGDYLysKIELAIRDAKMVVCCITPKYVSSYMCIKEANLADVLR-----KPTIPVMFKKTQWPPPNGLALVFPHLLYIDLFGLGGHGGSGIHADFEARMAE(VIYQVAQYSQPALPRKHLAG YKPVHFELQEKMNVFNVEAAT)}*

$2491=270(269):
>gi|654353731|ref|WP_027846549.1| {|119(317)|<Cyanobacteria(B)>}hypothetical protein [Mastigocoleus testarum] >gi|959132352|gb|KST67515.1| ATPase [Mastigocoleus testarum BC008]
{(EAIHQHIDYVIGMNQAIGDKAAIKFAKGFYDALGAGRTIEDGFQLGCTLIGLEGIPESYTPVLKSKSSQNL ASTSNTKDN)-KRIFISYKRDvcpdEQVASEIVQAMSPHH-----EVFIDKKILVGTCWAeLIETEIRQADYLIVLLSSRSVCSE-MVEtEIRMAHEFAqvQG-GNPQILPVRLNYS-DPFQYPLGAYLNKINWAfwQDASDTPRLIEELKQAVAGNQLT(IDKLPRKAELSQESESEPSSVPRPFPSAQP LPLEMPSGTMDTSSAFYVK)}*

$2492=274(279):
>gi|654353794|ref|WP_027846597.1| {|120(308)|<Cyanobacteria(B)>}hypothetical protein [Mastigocoleus testarum] >gi|959134084|gb|KST69116.1| ATPase [Mastigocoleus testarum BC008]
{(IYQHINYVIGMNTAIGDNAAIKFAVGFYDALGAGRSIEDGFEFGCASLDLEKIPESSTPVLKSKNIKNKNV TFKESTGKR)---IFISYKR--DVnpDEPVALQILQALSSQ-HQVFIDQNISV-GTPWaeMIEAEIRQADFLIVLL-SEHSVHSEMVEtEIRMAQQFAQEQsGKPAILPVRL-AY-RQPFQYPLSAYLNRI-NWAYWNDEQDTADLIAQLKQAIYS(NNLSLPRKNQVDLLTLDKSSDLPRPFPSAQPGALEIPEGTMKLKSAFY IERRADNIALS)}*

$2493=173(179):
>gi|960876932|ref|WP_058349054.1| {|0(322)|<Proteobacteria(B)>}hypothetical protein [Caulobacter vibrioides] >gi|955711478|gb|KSB88943.1| hypothetical protein AS593_21410 [Caulobacter vibrioides]
{(MA)--DIFISYRMDdstvlaRELARLLRKVGwTVWFAP----------ELAIGDELeeKIDPELAAARCVIGLWSRTAAASPHVEAEFVYAKQHRKLLATITDATPIPE---HFKTAIIGNLSKFSMMTYGPDLPQLLRGVAGMIGLPAGFE(DVETLIASAVDEEFGFYRLGNITVPAKYLFGS PREPF)}*

$2494=213(222):
>gi|959924525|ref|WP_058183658.1| {|782(0)|<Cyanobacteria(B)>}hypothetical protein [Mastigocoleus testarum] >gi|959132328|gb|KST67491.1| hypothetical protein BC008_30315 [Mastigocoleus testarum BC008]
{(DKCNTPVSELLVRRRRERMYQWITCGVCDSRVWLFDREERLKAIKLESSFIPEMDKAADTKRNEERATTII QGKIETQDF)--DVFLAHNNRDKSQI-EIIGNFLKQRGL--NPWLDTEQIPPGRWFqDVIQqaipHVKSAAIF---ISSNGLGKW-EEVelRAFTRR----CIeAKIPLIPVL----LPGVKGIPENLHFLKQLNAVYFKKIDDPQALNDLQWGITGK(IP)}*

$2495=214(220):
>gi|1011933643|ref|WP_062742354.1| {|321(0)|<Proteobacteria(B)>}DNA polymerase [Enterobacter lignolyticus] >gi|959990941|gb|ALR78680.1| DNA polymerase [Enterobacter lignolyticus]

{(KWREDKLTSALLFVTKKGFKATNNEHAISIFRNHIYGRLSFIKMIRGDDFPLYLKLMAEMSHHDPLKTKEG
LRAMRETET)-YDVFICHAS-ED-
KISIAIPIYEELTELNISTFIDHVEINWGDSLiqKINSALVKSKYVIAILSANSVDKHWPKKELHSVLARE--
---IAEGEVKLLTLVKEADEAIVAASLPLLSDKLYITYKDNPAEVADKVRALLNK()}*

$2496=187(180):
>gi|1173476867|ref|WP_081262107.1|
{|0(131)|<Proteobacteria(B)>}hypothetical protein [Ralstonia
solanacearum] >gi|959205079|emb|CUV11833.1| conserved protein of unknown
function [Ralstonia solanacearu
{(MSEKE)--KVFISYAwggalERkEWLRDDVVLHLSS----
QFSVFWDRDSISFGKDCDeVISEVLAQRPITVLclcdneyIVSASRVGSGLHR-ELTMLAEIANS-
DGVRVIPIILDNE-
CRSNLPRPIAGRTYLDLsaVHAKGLSLGWAVLAVASGATQAQ(VAGLLSDQLRTAEVRSKAIEYFSNSPMALH
GSART)}*

$2497=178(175):
>gi|960275620|ref|WP_058204626.1| {|0(657)|<Firmicutes(B)>}hypothetical
protein [Lactococcus lactis] >gi|959249019|gb|KST82945.1| hypothetical
protein LK337_1801 [Lactococcus lactis subsp. lactis]
{()MRRVFLSHSSKdkEKYLRPLLEKLS--
KDAGKEKFVYDEvTFEVGLENEeLITDWLSKTDLFVLFLSDESLNSPWV-KkEILEASDLQNL-
GQIkKIYPIIIDSS-
ITHEDYRIPDWMRDTYNlrFVSKPAVAARKIISMMREISWKQ(TPKLKEKQQFFVGRNDLINTLESRLDNYDV
TSPNT)}*

$2498=152(155):
>gi|960882599|ref|WP_058354089.1| {|0(216)|<Firmicutes(B)>}hypothetical
protein [Acetivibrio ethanolgignens] >gi|959611912|gb|KSV57681.1|
hypothetical protein ASU35_15705 [Acetivibrio ethanolgignens]
{(MER)-PTVFISYKRDkSDVVNELEKQIngrAYIKR-------
DTREIGAWESITEFMNSIRKQDFAILVISENYLKSVACMYeVLQLLKDDNWL-EKTMIMLADDINI-
FGTKEQITYIKYWNDEYtnITELIKTLPPETVSSQAEDLKKI(SRIKDEIGSFLH)}*

$2499=248(257):
>gi|959925049|ref|WP_058184115.1|
{|42(141)|<Cyanobacteria(B)>}hypothetical protein [Mastigocoleus
testarum] >gi|959129662|gb|KST64921.1| hypothetical protein BC008_19090
[Mastigocoleus testarum BC008
{(EIVTGQSHAQVIFKLIQWAEANGKVEELLIAACEPEVGRPNNLKLKPFCQRLNQTQLVVEQPPIRKEPLEQ
TINTSQQFD)---IFLCHNS---QDKDEIRKVREALQQQGFQCFLDEY-
LSGGELWrqSIARAIRNSRYVVIFV-GGYGLG-QYQRkEIEYCDGI---FfDGIKVIPVLLSTA-
TKESIQTFHSQDVNWIWRNQRVDFRSPRDSDPIGKLIQAI(SGKNSSNQPPVNTPPEDDLSSEKGVDYTRLRD
YLKAQ)}*

$2500=234(222):
>gi|960293904|ref|WP_058213862.1| {|0(234)|<Firmicutes(B)>}MULTISPECIES:
hypothetical protein [Bacillus] >gi|959343087|gb|KSU73477.1| hypothetical
protein AS035_07630 [Bacillus cellulasensis] >gi|1049
{(MDMTNPK)---IFISYSW--
TTDDheDWVLDLAKRLLEDGVDVTLDKWDLKEGHDKyaFMEQMVSSDEIdRVLIICDsgykiKADNRSGGVGd
ETQIITpELYTDIkQGKF-IPIvseRDSSG-NDFVPayIKNRIYIDLSNidTFEDNYEQLLRtLHK--
VPKHKKPA(LGKVPSYLSEQEINHYKTQNVLKQMRSALDKNPNRLRVMSIKFQEAFIEELASLHFETVKPHEL
DDKIVDSIEKS)}*

$2501=233(219):
>gi|960302076|ref|WP_058218169.1| {|0(97)|<Firmicutes(B)>}hypothetical protein [Lactococcus lactis]
{(MEDSVGTK)---VFISYAW--EEQKEddekiksFTQWLAVYLKKWGFNVLLDVFENHPGSKLdkFMSEGI-NSSKFVVCICTKTYIQKmdnpkSGVYnEITL----LKEMsDSPFIIPIIekgcltdLPEF-FKGKFVSELLFdTPYSQKNKDNVFELIStLRDESLSIKEVNS(ETRIEDYYKNVEIFKFQAKTANLMSFECQTEQTVSFQYLLNGGDFVIGIPPMNFTTHWSTAGASSIHSYNK)}*

$2502=225(223):
>gi|961138788|ref|XP_014788673.1| {|597(220)|<Mollusca(M)>}PREDICTED: leucine-rich repeat-containing G-protein coupled receptor 5-like [Octopus bimaculoides]
{(HPNGTVWKLKGNQLVRIVSFLNHTCSALIGIIIAISFGVVILIIFLVASLIYHFRWKLRYLYYMIREKYSYKQLTPDEYI)-YDAFVIYADEnrDFALNYLIPELEgKESL--RLNIHHRDFPVGKLITENILSAIQSRRKCIILLRRHEDIYMAMKGIFILAFAYFMKTFIKVTANCRTDCPDvCNCTEIIFNRCTVICTDqNLTQIPDTFPKNATSIDLSRNRI(KSI)}*

$2503=228(224):
>gi|961142268|ref|XP_014789884.1| {|296(0)|<Mollusca(M)>}PREDICTED: toll-like receptor 13 [Octopus bimaculoides]
{(PDWQDKQLLDFTRSKIDCTDYTLYYILGGVGGGFLLTVVIVLFAYTKRWYIRFKIFKLYQYVQKAGNKKEYEAIPGDDMY)-FDGYISYSDKdaDWVEKYLMHTFDNgengedkNNGNFKLCFRNRDFAYGKYIIGMIESSLAVSKKMIMVLTPYYKKDKRCEFELQLGIMKL---NIKNVMPIVLKNLQpNQIPN---SLKEIFETNkFIEWDNNEYAtnFIKRKLENFL--N(ERNF)}*

$2504=181(177):
>gi|1107016533|ref|WP_071879379.1| {|0(649)|<Firmicutes(B)>}hypothetical protein [Enterococcus silesiacus] >gi|961352303|gb|ALS02057.1| hypothetical protein ATZ33_11875 [Enterococcus silesiacus] >gi|1
{()MKRIFISHSSSdKAFYAEhLVKALNNSLGKDKI-VFDAHTFEEGAKINlEISEWIEKTDLFVLLLSNKALESEWVQKeIMQsEFLELSNP-DEERIFPIIIDPN-INHEDNRIPEWLKQYNIrmITSPKKAARQIISRAVEISWMIN(PRIKEKNNLYVGRNDKMEEFEDRMDNFNSARPNILIA)}*

$2505=219(240):
>gi|965616434|dbj|BAU01584.1| {|0(843)|<Streptophyta(V)>}hypothetical protein VIGAN_11084900 [Vigna angularis var. angularis]
{(MMCSSVFEGKIPAKVSQEIS)----------------------TNLCGRGIHTFIDDHHLprGDQISSELENAIQDSRIFIIVLSQNYASSSFCLNELHYILRFIKHKGRL-VFPVFYGVDPSHIRHHTGTFREALAHHQNNsyYNWEKF-ETWKMALHQVANL(SGYHFKHGEGYEYEFIERIVELVCSKINRAALHVADYPVGLESQVLEVKLLLDVGCDDVVHMVGFHGLGGVGKTTLAAAV)}*

$2506=197(202):
>gi|1044551651|ref|XP_017442189.1| {|0(836)|<Streptophyta(V)>}PREDICTED: TMV resistance protein N-like [Vigna angularis]
>gi|965610885|dbj|BAT97024.1| hypothetical protein VIGAN_09036500 [Vigna angula
{(MDVASSLSIFSNFRV)SGEVFIHCLG-DDIHRNFVSHLSSSLLQAGVKhSLVSAEMLSEKF----MPSITRFQIGIVVFTKAYIESYRCVEDLLRIIECHEN-HGLIVMPVFYDIDPSDLPDLKMESSYVRDPITGGFKKNPKVWSVKTTVKVTQME(TERFNYPFILYTSAGSHAVNRVANLPTWDESKHRDDAELVEEIVKSV)}*

$2507=221(222):
>gi|965665224|dbj|BAT80712.1| {|0(110)|<Streptophyta(V)>}hypothetical protein VIGAN_03031000 [Vigna angularis var. angularis]
{(MA)RYDVFLCFRG-KDTRHTLTGNLYAALQQaR-FRTFMDDDELKGADQIayTIV--
LEASRISIVVLSEHFAFSSWCLDELANILDCMNT-KNQAVFPIFYEVDPFYVRHLKGSFGEAMVAH-
EARFGKDSEkvEKWRSALAQVANL(SGWCFGRGRCQYEYEYEFIERIVQHVTKLLPRYSIFLSFSGKDTRSFT
GFLYNALSRSGYHTFISDGEHSSQSIVGVIEK)}*

$2508=206(220):
>gi|1044585295|ref|XP_017429756.1| {|118(0)|<Streptophyta(V)>}PREDICTED: TMV resistance protein N-like [Vigna angularis]
>gi|965665225|dbj|BAT80713.1| hypothetical protein VIGAN_03031100 [Vigna angula
{(QLVWPIFYGVEPTDVRFQRGRYEQAINRFEERYYSPERMNKWRAALAEVSNLSGWFYQKGHKYEYKFIRKI
VEAAVQCLS)RYDVFLSFCG-EDTRYTLTGFLYNALRrEG-
FKIFMDDEELEGGNQIsqKLMGAIESSRVSIVVFSENYGYSTWC--------------
ALSQITNLEGEHLSENEFHHESIERIVERLI-NIEDEEAYNNSFPCSNDSYGEQ()}*

$2509=223(233):
>gi|965665508|dbj|BAT80996.1| {|0(3)|<Streptophyta(V)>}hypothetical protein VIGAN_03063200 [Vigna angularis var. angularis]
{(MLSGESSSNSSRW)-------------TYHVFTDHLYVALvRKG-
MITFRDDKNLEKGDKIdkELFKAIKESLGAIVILSENYASSSWCLDELNKILESNRA-
LGRKVFPVFCGVSPSEVQHQtTKSFEEAFQKH-
ERRFEKDTEklQQWRDSLKEVSQI(AGWESKNYQHQTELIENIVESVWTKLRPEMPSFNDGLVGINSRVKKMD
SLLRIESKDDARFIGIWGMGGIGKTTLARVVF)}*

$2510=179(185):
>gi|970536265|ref|WP_058805135.1|
{|0(148)|<Proteobacteria(B)>}hypothetical protein [Sphingopyxis sp. H115]
>gi|962319054|gb|KTE02328.1| hypothetical protein ATE71_20205 [Sphingopyxis sp. H115]
{(MA)ATQVFISYSSKDGAVANMVCALLEERGHR-CWIAPRDIVPGKEWGESIIGGIKGATLFVLIFSRHA-
NTSPQILrEVERAVSHG-----LPIIPFRIEDVLpaGSMEYFMSVPHWFDAL--
SPPVEDHVVRLAAVIEQLTSGD(ADARAYRPAVPRGAGRIDWNNGLVRALAGGTGLALLLLAGWLG)}*

$2511=217(225):
>gi|962337485|gb|KTE20562.1| {|25(0)|<Proteobacteria(B)>}hypothetical protein ATE67_09955 [Sphingopyxis sp. H050]
{(ELQSRYTYADIDAFFAALDISTINVDSTNTNSKRVYAKAVLSCVTEAVLLQVASELDIIPHTPIVAPIGLP
KNWEGTKDF)--RLFISHISKHKARATKLKQALARYGIS-
GFVAHEDIQPTLSWQDEIERALQTMDALVAVRTP-GFSASIWTQqEIGYALGRG-----
AMVISFKMGEDPtgFIGKHQALPHRDRSAE--EIAEEIDKLLAADPRTEARLLE(AKASA)}*

$2512=219(232):
>gi|970542795|ref|WP_058809416.1|
{|0(121)|<Proteobacteria(B)>}MULTISPECIES: hypothetical protein [Sphingopyxis] >gi|962355365|gb|KTE38231.1| hypothetical protein ATE62_11630 [Sphingopyxis sp. HIX] >g
{(MSDAQTTPDPARRLSA)----FISHHSSQVAVARRLKTLLKERGID-
GWLAPDDIAAGTAFDQAILDQIARSDA-IVLLFCAQSDQSRHVKrELMLGEDGS-----
KQILPVRLEDVPaqGLAYWLKDYQWIDWFA---GEEQAVE--

RIVAAMTGAVAS(APAAPPSRPPVAVKPWLRRPLTLGVGGLALAGLFAGGLWWTGGTSGGGGDPSAAVADNPI
EPGIWTSKREVVKILF)}*

$2513=177(183):
>gi|962357205|gb|KTE40047.1| {|0(149)|<Proteobacteria(B)>}hypothetical protein ATE62_08055 [Sphingopyxis sp. HIX]
{(M)AAEVFISYSSKDRTVANMVCALLEERGHR-CWIAPRDILPGTEWGEAIITGLKGAQVFVLVFSQHA-
NTSPQILrEVERAVHLG-----LPIIPFRIEDVVpeRSLEYFMSVPHWLDAL--
SPPVEDHIARLGDVVSQLVNGV(TDAREYRSAKPQGAAKLSWNNALVRAGTGGAVLALLLLAAWL)}*

$2514=177(185):
>gi|962374479|gb|KTE57184.1| {|0(147)|<Proteobacteria(B)>}hypothetical protein ATE66_18425 [Sphingopyxis sp. H107] >gi|962398786|gb|KTE81115.1| hypothetical protein ATE63_07550 [Sphingopyxis sp. H067]
{(MPA)--QVFISYSSKDRTIANMLCALLEERGHR-CWIAPRDIVPGTSWGEAIIDALKSSTLFVLVFSRHA-
NESPQILrEVERAVHLG-----LPILPFRIEDVMpeRSLEYFMSVPHWFDAL--
SPPVEDHVDKLADVVAMLIAGP(KTGYVPGKPRGAKRLSWNNGLVRAVAGGAALAVLLLAAWLGG)}*

$2515=291(285):
>gi|966503637|ref|WP_058520296.1|
{|84(6)|<Proteobacteria(B)>}hypothetical protein [Legionella tucsonensis] >gi|962289531|gb|KTD73239.1| hypothetical protein Ltuc_1086 [Legionella tucsonensis]
{(QHFFSNMRSRDQAITWLTALEKKYNKIFQVLKVDKPNSLQPQKSTAWVTQGALSDEDLYPLFQQTLYNVRK
ESLKAECLP)-PKVFISYAwplpeHTnEYWVKEYIIQLaKDLKSAGVIVYLDEMDSRYGYSLQdHI-
NHLKESD-AVLLIGTPSLKTKYetpanlhMINdEFTEII--HQS-KRLKIIPLLLE-----
GRYELSFPRAITRNRsIEDWTNGDYINHFMALLQHLTGL(PNHLYEPLWRSVLKAYIRNMEGCLALQTQPTVT
DDSSTSKQSDNLNRWSLFNIFASTSITEDQAP)}*

$2516=152(153):
>gi|970543257|ref|WP_058809726.1|
{|0(280)|<Proteobacteria(B)>}MULTISPECIES: hypothetical protein [Sphingopyxis] >gi|962351411|gb|KTE34319.1| hypothetical protein ATE62_16155 [Sphingopyxis sp. HIX] >g
{()MTDVFISYKREeRPRCVAIYNALVDLK----
LSVWFDGRIEPGTSFDrEIEREVEGAKAILVLWSELARNSDWIRNeARTGKQGERLV-AARLDDCLPPLEF-
RSVQTVDLFDAGNFQSGegWCQIVSRIGRLAGRPGLGDYVRC(EQAAEPGLWHDWL)}*

$2517=108(140):
>gi|1056966892|ref|WP_068379873.1| {<Cyanobacteria(B)>}hypothetical protein [Leptolyngbya sp. NIES-3755] >gi|965630414|dbj|BAU09986.1| RHS Repeat family protein [Leptolyngbya sp. NIES-3755]
{()MADIFISYSRKdKLFVQTLHAALGQAKY----
DVWVDwEDIPFTSDWWeEIQRGIEGVHSFIFVISPDSIASKVCRREIDHAVQLNK-----RLIPLVLR-----
---------------------EGFAMEQVHGTLAGSAI()}*

$2518=186(194):
>gi|1056971567|ref|WP_068383831.1|
{|0(255)|<Cyanobacteria(B)>}hypothetical protein [Leptolyngbya sp. NIES-3755] >gi|965632547|dbj|BAU12119.1| RHS Repeat family protein [Leptolyngbya sp. NIES-3755]
{(MTQTMTHLSSEPISQSHD)---VFISYSRKdQAFVRSLDAEFRKLDR----
VPWVDwDSIRKGEEWWaAIQHGIEGANTFVFVISPDSIDSPICQDEIKYA----AL-NNKRFLPIVYREG-

FDPSKVHPEISSHNWLFfrDTDDFQSTFQELLKAIDTDLEYV(RMHTRLLVRAIEWQGNSKDSSYLLRGSDLE
QAEQWA)}*

$2519=185(191):
>gi|1056971726|ref|WP_068383990.1| {|0(753)|<unknown(X)>}hypothetical protein [Leptolyngbya sp. NIES-3755] >gi|965632636|dbj|BAU12208.1| RHS Repeat family protein [Leptolyngbya sp. NIES-3755]
{(MQAGSLNLQGKA)MFEAFVSYSRKdKFFVQRLHAALTQQDRKVWVD-WQDIPLTAG--
WRqEIYNGIDASNNFIFILSPDSIGSTVCQEELERAI------DSKRIVPIVWRDV-
DPSKVHPALAKLNWIFFreTDNFDQALKSLTTALDTDLDHVR(AHTRFLTRALEWDQKRQEKSLLLSGRDLRN
AEDWLVHAA)}*

$2520=135(140):
>gi|1056972461|ref|WP_068384725.1| {<Cyanobacteria(B)>}hypothetical protein [Leptolyngbya sp. NIES-3755] >gi|965633052|dbj|BAU12624.1| RHS Repeat family protein [Leptolyngbya sp. NIES-3755]
{()MSDVFISYSRKdKAFVERLHQALKQQNR----
ETWVDwEDIPLSADWWqEIERGIEGTNTFVFVISPDSVASEVC-DrEINHAVKHNK-----RLVPIVYREG-
FKQTHPALAKHNWIFLRetDEFDRGFANLPRQLATARLSMAR()}*

$2521=180(185):
>gi|1056973830|ref|WP_068386078.1|
{|0(740)|<Cyanobacteria(B)>}hypothetical protein [Leptolyngbya sp. NIES-3755] >gi|965633636|dbj|BAU13208.1| RHS Repeat family protein [Leptolyngbya sp. NIES-3755]
{(MQGSTAFTN)MSDVFISYSRKdQDAVQKLHYMIEAKGR----
EAWVDwQDILPTAKWWqEIQRGIEGADTFVFVLSPDSIASKVC-EqEIDHAVLHHK-----RIVPIVYREG-
FEFKLTSNAHQVLKEHNwlYFTDPSKVEQAFQTLLEIMDTDL(EEVRLHTRLIVRSVEWENNARNDSFLLRGD
DLNLAE)}*

$2522=174(179):
>gi|1056974989|ref|WP_068387237.1|
{|0(428)|<Cyanobacteria(B)>}hypothetical protein [Leptolyngbya sp. NIES-3755] >gi|965634110|dbj|BAU13682.1| RHS Repeat family protein [Leptolyngbya sp. NIES-3755]
{()MRDVFISYSRKdKPFVERLHQALEAQNR----
DTWVDwDDIPLTAEWWqEIQRGIEAANTFVFVISPDSLASQVC-GrEIDHATQHNK-----RLIPIVYRAI-
DGLAVHPALQPMQWIFFqdPERFESQLQRLLTEMDRDLDYVR(SHTRYQLRAIEWQNQKRNESFGLKGDELRS
AEAWLSQGE)}*

$2523=172(178):
>gi|504279326|ref|WP_014466428.1| {|0(667)|<Proteobacteria(B)>}TIR domain-containing protein [Zymomonas mobilis]
>gi|335933083|gb|AEH63620.1| hypothetical protein Zmob_1828 (plasmid) [Zymomonas mobili
{(MRA)----FLSHSSKdKGYVQAVKDNLRPGNYEFDSQTFDEGGLNS----
DEILKALNRCDLFCLFLSKDALNSGYV-NfEIAFSRELIAS-GKIsRVLTICLDED-
VFNSAKDFIKHYNMVRRssSPESTARLIEGKLISSNHSKRIK(SHPFVGRETELKILEKQANNLDKPRIKALY
VSGNS)}*

$2524=187(192):
>gi|503711693|ref|WP_013945769.1|
{|0(75)|<Proteobacteria(B)>}hypothetical protein [Hyphomicrobium sp. MC1]

>gi|337757536|emb|CCB63356.1| protein of unknown function [Hyphomicrobium sp. MC1]
{()MADVFISYSKAeRKLTEDLAKILTGAG----
LTVWWDTELLPIQRFRtEIDAQLDDCSAAVIIWSATSANSDWVRSEADHAMRQGKL-FNAHHFDILPEHL-P---
KPFGQIHAVPLTDadRILRAIKLITAGKPPRATAVSSV(ETSAIEFITDAIQDALRYALQLRDLADREDWEDGEAYPKELLKLAEKVLTPL)}*

$2525=214(220):
>gi|338804382|gb|AEJ00624.1| {|0(740)|<Proteobacteria(B)>}Sulphatase-modifying factor protein [Nitrosomonas sp. Is79A3]
{(MTATTNQLAKLR)---IFISYAR---GGNAHT-
WserIQSHLEQQGAEVWRDERSIEEGnDNWyrSIQRGIEKSDV-VVCIVGKDTDQCGWQErEMLYSDKLKKQ-----VVALRIDNV-
SLPFYIQEKQPIEAHSDPQDTLDRLTRkLAEILPALQNRTP(NIAPLSENNYTQTSASPQRRDELTYLNDLINKDLSDLESRYVALEACERRSLSPERVMKSLRVDTDAI)}*

$2526=229(215):
>gi|339279082|emb|CCC20830.1| {|0(99)|<Firmicutes(B)>}hypothetical protein STH8232_2193 [Streptococcus thermophilus JIM 8232]
{(MNKS)---IFISYAW--EQDEPldkkvknFSQWLAIYLQSWGFRVHLDVLENHPGTKLdeFMKNGI-
DSSHFVICICTQTYLKKTndsatGVYnEIELI----KKQaNSPFIIPIIdIESF-
TDLPNFFKGKYIsqlkfdtPFSQENKKGLFELIStIRDELFLNIKVET(KPDAQDRIERYYNSVEKIKFLNDSANLMNFKPQSEGQISFQYLLNGGDFKLGIAPMDFITNWSVAGTDTIH)}*

$2527=157(159):
>gi|969922560|ref|WP_058631463.1|
{|0(288)|<Actinobacteria(B)>}hypothetical protein [Microbacterium oxydans] >gi|968269536|gb|KTR77404.1| hypothetical protein NS234_07275 [Microbacterium oxydans]
{(MNIPVNDDDIDVRGPL)----FLSYRQSDGTKTVSSLGWLLRAAGI--
PVWRDVDDLPPGDTAdRLEQALAdRLSGGVVVITPDIANSPVVKSieAPALIRLNRGDNRFQLLVANAIERSPGKVDYSAPDRLLERVRRELEGINQNPVTDDGLFEIVRAAV(AHR)}*

$2528=220(220):
>gi|966704365|gb|KTG36606.1| {|261(0)|<Chordata(M)>}hypothetical protein cypCar_00040580 [Cyprinus carpio]
{(YLPHLYEEKQNIWWTVDNKTVEQLADPRFRSPEATLFSYDYGVTLALMLMMFVIYHVFWLELLLLYRSWFGSDERYTDDK)QYDVYISYARNseeEEFVLSTLRRVLEAEFGYSVCIFDRDSLPGGTITEHTLRFVGQSAAGGGCESVQ-RCPGTQALlELQAGLTSMLHGGSLKVVLIQFKTgRRKSWVKELRQARLALTLI--CWKGEN--
RPLCPHASGNSCS()}*

$2529=266(262):
>gi|966701118|gb|KTG33359.1| {|232(807)|<Chordata(M)>}hypothetical protein cypCar_00035415 [Cyprinus carpio]
{(DQYMVPVVRSSIQCEDEEDETSVEKLRLVLFIFCSTLIIIFTASAIIYVRRRGYIFKLYKKLITKLVDGKQQELDLDRFL)-
YDVYLCFSSSdiKWVERALLKRLDSQFSEQntlHCCFEERDFIPGEDHLTNMRNAIQNSQKTLCVVSEHFLKDGWCL-
ETFSLAQKRMQVdLEDILVVLVVGNITQYSDLRMATRHTLSLILLGLYISSHIVKCTSVCSVDGSVV(LCTDRGLQEVPELPTHVTYVDLSNNSINDLHETSFSHVEGLR)}*

$2530=211(219):

>gi|966787060|ref|WP_058554036.1|
{|0(52)|<Proteobacteria(B)>}phosphopeptide-binding protein [Thiohalocapsa sp. ML1]
{(M)-KNVFISHSSKDLDFVfANVKGVFDELGYC-
AWCSATSVRLASDWEQQIRRALLQADWFVVVLSPDAVKSEWVQAeTHWAFENKR-----
GRVLPIMARSCDpaDAHLRLGTLQFLDFRD---DPEAGRR--
QLCDLLRGHAPR(LTTALGRHATATAPVQEQTTLISKRREARLRLRIERADGTASEHSMQICNWAVIGRATDA
DLPLADDCVSRKHARIAV)}*

$2531=191(183):
>gi|1179979042|ref|WP_083501396.1|
{|0(124)|<Proteobacteria(B)>}hypothetical protein [Ralstonia sp. NT80]
>gi|966733705|dbj|GAQ26660.1| hypothetical protein SAMD00023378_0343 [Ralstonia sp. NT80]
{(MDEKG)--TAFVSYAwggelERkEWLRDDIVQVLSY----
DFSVFWDRDSIPFGETPDeAISSNLAKRPvVVLCMCDQAYIASaskaDSGLRsELAMLAQIAGS-
KDVRIIPIILdREC-
IPNLPTPLAGRTYLDLSalYERKLQLGDAVLAVAAGGTQAQV(AALLSLRLRQADMRDRARAYFHDSPKEFLG
SGRTHVVR)}*

$2532=206(220):
>gi|966787438|ref|WP_058554414.1|
{|36(0)|<Proteobacteria(B)>}hypothetical protein [Thiohalocapsa sp. ML1]
{(ELDFPGERFAPGGTPLGDWLATVKAANAGMSSTAGFRADAVARVVERVADRLPGNPELSRLFFDLGSAGAQ
AAPAAGERG)---IFLSYASAnRHQVDALYDALAAAAP-
GLALFLDHRSIGPGQRWLdVIRDAAGSASVLACWVTPDFLQRTFCAYEIGVADSR-----
GAAIVPIWVdgldpnSAA-SYLSSRQGRMSAAPHDFadLAAW--------------LVAGV()}*

$2533=265(271):
>gi|966028675|gb|ALT22131.1| {|358(425)|<Cyanobacteria(B)>}transmembrane sensor-like protein [Anabaena sp. XSPORK2A]
{(LINVLTQDAAYYEQHKILLIKALKWERQKRNSSILLRGYNLRQAEAWLKIAQQKTQHQPIPLQVEFITESL
NQPPLVALD)---VFISYSRAdSDFARKLN---DTLQIQNK-
TTWFDQEsIASGTDFEqEIYKGIESTNNFLFIISPDSISSPDC-QvEVEYAAKLNK-----RIVTVLYREV-
NFGDLspELAKVKWIDFCQhgGDFLTKFGELTRTLDADPDYVRS(HTRLFLKAKEWDDTKHDDSFLLRGKDLV
ASEEWLKQSDNKEPVPTNLQLEY)}*

$2534=188(181):
>gi|966791059|ref|WP_058558035.1|
{|0(1283)|<Proteobacteria(B)>}hypothetical protein [Thiohalocapsa sp. ML1]
{(MLRL)----FLSHAHEnkalaANLQRVLEQQSSAEHPVH---
VWLDKRELRAGDGLtpEIERAITAADALLVLVTPAALQSVWVGKEVAMARRVkaereqaGGAYriiPIRVDGA
PLGL---
LETLFGDDKPLEVRLHSGANGIFAALPALRAALGLEAPSG(TEPGPRADARPMDELVLELTDLSIHVADNNVR
RPRAR)}*

$2535=302(300):
>gi|970038407|ref|XP_015080544.1| {|81(583)|<Streptophyta(V)>}PREDICTED: uncharacterized protein LOC107024147 [Solanum pennellii]
{(PPVSYCGSQSDDVPSTSYTPPPERYDFSDDPTDTKLKIVTCVPVSGPETDPRISFSFPVPRISFAKGSVSP
ASNAKLRSC)--DVYIGFHG-QNpnlVR--FCKWLKSELELQGIACFIADRAKYADN-
QshEIADRVICSVTFGVIVVTGCSFFNH--HSLEEIRFFAQK-KNLI--
PLFFNTDANEIaslfnhNGDTKKCKEALdviLKCHEFRLETDE-

SNWRSCVSKAAGI(LRAKLGRKSVVEKCTEGFEELPFPRNKSFVGREKEIIDIETTLFGCGDSFEQESVVPSAKGGTPGQSEGLADDESEADVG)}*

$2536=194(186):
>gi|972460326|ref|WP_059047494.1| {|0(433)|<Actinobacteria(B)>}TIR protein [Streptomyces acidiscabies] >gi|970389803|dbj|GAQ56504.1| hypothetical protein a10_06360 [Streptomyces acidiscabies]
{(M)-WDVFLSYSR-ADVES--
VRPLARALRGEGLRVFTDETGVAPFEGIseTIRRELARSTALLAYYSAGYPEREACQWELtcAYLAGLAEGDPRRRVMVVnpeptaahIHPVELRDARHADPGDPAGLVADVVARLARLDGPMPLPRTAVRGPH(AEFLGRLPELWRIHSALHAHAAPLTAGRVPARTVQIRGMAGIGKT)}*

$2537=223(223):
>gi|42572961|ref|NP_974577.1| {|0(18)|<Streptophyta(V)>}Toll-Interleukin-Resistance (TIR) domain family protein [Arabidopsis thaliana] >gi|332658845|gb|AEE84245.1| Toll-Interleukin-Resistance (TIR) do
{(MAIQA)RPLVFINHGE-
SDQSDGFIRHVERALNDEGFNVFIDSDERRGRGMEHIFRAIDNSNVALVIFSDRYTASELCLHEAVRIYDRRRE-
GKLVLIPVFYRVSEDDVNMFNGRFGESFLETLTIQGFRDHPfaEHWMRNVNFICTD(TGFTSADYSSNDTSLVEEIVRAIKRRLQGKKLPRRVQDEKLPSPLDMFMPKQPNAQEGFGKELLYVAIAALLCYFIIP)}*

$2538=271(261):
>gi|970888396|ref|XP_015109355.1| {|251(0)|<Arthropoda(M)>}PREDICTED: single Ig IL-1-related receptor-like isoform X1 [Diachasma alloeum]
{(CIVSNGADDEMTLSAHIYLHDERYVLGLRNGSWRKPLMLGVVFFVLVASMIASYIRCWLPLAVLWRNRFSRLEENDGKDC)--DALVCYHEKdaVVGLGVLVATLES---KYHLkCTTLELSHLNRNWSMeispH----
AASARRVIILLSPNSIQQPWTEGHVGLVLKQI--SQLTTKSIVVSLTD-
LPSITsfaktgscVDPasDGIVLDKMTILRWDEKerenssdyK---
FWCRLRLLLPVV(RPSSKNSDDLRCQSVAMIIQSNGTNSLQMKARSHGNFEVLV)}*

$2539=299(300):
>gi|42569093|ref|NP_179297.2| {|1043(13)|<Streptophyta(V)>}disease resistance protein (TIR-NBS-LRR class) [Arabidopsis thaliana] >gi|330251486|gb|AEC06580.1| disease resistance protein (TIR-NBS-LRR cl
{(ECGVYAITENVDQTNLDFRGPSFALLPPYKKRKRSFSGSEDIEMENQRLNISKTKQGDVPEKISQADTAYLTSPSLLQRR)SHQVFLSFSE--
DVPRYFVSYLIKKLKWIGITVVYSGFMGGKSMSRpEVTQAIEESSISVVILSKDYVSSSKCLDELVEIIRWREENLGNRVMPIYYEMGTSDVMKQAKTIGNRLVETyLGKVVEKPE-
LRWMRALAYIVNI(VGESSQYWVDKAKMIEKTVVDVSNQMNILESNEAGLLFIYQEEENMENFKRNVYDEMNGVRIIPIWHVYAFLLIYLFLLI)}*

$2540=197(202):
>gi|970635443|gb|KUF80331.1| {|0(222)|<Stramenopiles(E)>}tirA protein [Phytophthora nicotianae]
{(MNNEGLRPLDCTASTRLLFELKRAAGQR)--DVMISYTH-SHLE--
FARKLRQSLEEANVTTWLDLMDpsgIGGGAVWreEIARGITNASMVLCILTEDYASSEWCLKELALAKQVG-----
TPILAVSTEGXIVDXXLXVYLYTRQIIPFEPAIVGLRHDEGRIEYDYDESRY(RSQFHLLLDGVRDEVEKRRYFTKQKNVASSRNLD)}*

$2541=250(258):
>gi|970655129|gb|KUF93337.1| {|257(456)|<Stramenopiles(E)>}hypothetical protein AM588_10004085 [Phytophthora nicotianae]

{(DPRIFQLLLDYGVDLHATRTSNGDSALHVALRRRNQTAAVLFVRAGLSLTAVNLLGQRAIDCARSPALRFA
VKKEAGQRD)---VMISYSHAhAPLARKIR---DALERQRV-
TTWIDTMgptgITGGSVWRqEIARGIQSSALVLAVLTKDYPQSQWCMKELAFA--------
KMQNIPVVAIQC-
EDMEISEELQVYLWTRQviDFRPAVKHNYTMETNKGDGVKEA(RPMITSNSSHTIEDTDSREDESEFRIEDDN
EVTNMVGK)}*

$2542=253(250):
>gi|971075425|ref|WP_058885543.1|
{|922(0)|<Bacteroidetes(B)>}hypothetical protein [Tenacibaculum dicentrarchi] >gi|970869494|gb|ALU75402.1| hypothetical protein AUW17_09040 [Tenacibaculum dicentrarch
{(NSFENYKRSFEKLKKPNDLYISVDNKYFVHYNTLNDEEKTHENIVAYELPNQKNDLDKTNTRTQRSYGYRN
FTSNKNIKK)MKKIFISYSREdVEYKDELRKHLNMLKLFDVADNWSCEDITIG-
KWHnQIQQELEESDLVIFMLSINFFNSKYILEqEVQKTMdEIANG-SDKNVYSIIVSDF-
PGLELFNKEDMNDKQKSvlELGSYQYGMYTKTENRITGNKKE(EIISLKEASNRGIIDTQLTKIVTKIMKDIS
)}*

$2543=259(264):
>gi|75704107|gb|ABA23783.1| {|154(635)|<Cyanobacteria(B)>}possible Sensor with Chase2 domain [Anabaena variabilis ATCC 29413]
{(GLLELFRRHQDYVHQHTYFLAKALEWEQKQQQSQYLLIGEEREQAENWLKIRFKDEQAPCEPTDLHCAFIC
QSIKNANNL)MTQVFLSYSEKeRMFMEKIAK---
ILMRESFTVWTNKTDIQSGADFTeSINRGIEEADNVVFLLSPASLQSEYC-QlELTYAFSLKK-----
RIIPLLLEPV-
DLEQIPVELRILQFIDFtdQEEEGKESADTDRLINVLRRDAA(YYEQHKILLAKALKWERQKRNPSILLRNHN
LRNAEAWLKIAQQN)}*

$2544=249(257):
>gi|75704107|gb|ABA23783.1| {|359(440)|<Cyanobacteria(B)>}possible Sensor with Chase2 domain [Anabaena variabilis ATCC 29413]
{(LINVLRRDAAYYEQHKILLAKALKWERQKRNPSILLRNHNLRNAEAWLKIAQQNTQHPPIPLQTEFITESS
KQPPEAALD)---VFISYSRAdSDFARKLN---DTLQIQNK-
TTWFDQEsIASGTNFEqEIYRGIESANNFLFIISPKSISSPYCVTEVEYAMNLNK-----RIVTVLYRDI-
GEATLHPGLAKVQWIDFspRDTDFLTKFGELTRTLDSDPEYV(RSHTRIFLRAKEWEDHNRDNSFLLRGKDLL
ASEEWLK)}*

$2545=229(230):
>gi|499608591|ref|WP_011289325.1|
{|233(0)|<Proteobacteria(B)>}hypothetical protein [Dechloromonas aromatica] >gi|71848833|gb|AAZ48329.1| TIR protein [Dechloromonas aromatica RCB]
{(LVNEDPLFSKAKQEFMTDFEGDTSLSTFVSRFSPNSLVFPGTPKEFVRELAERWQKWRLTHPQQDKKAPPI
AAVKAADSP)--QVFISYAS---
ENADAARAIADRLLQLGAgDVWLDKKKLRGGEDWssKLEQAIASCDYFLPLLSSEADQRREGVfWdEWGSALE
RARKVaDT-FILPTLIDAS-
PDSRNNYQRIGKQLGTERFWTLQLLNAPSGTFDAKSESDL(VDCFANFRGH)}*

$2546=210(199):
>gi|499259522|ref|WP_010957062.1|
{|0(188)|<Spirochaetes(B)>}hypothetical protein [Treponema denticola] >gi|42527322|ref|NP_972420.1| hypothetical protein TDE1816 [Treponema denticola ATCC 35405] >gi|

{(MK)---VFISYAW---
TNNEYTskiLLLAERLMSDGVETIIDKWDLSAGQDKyaFMEQMIQDSSIDHVLIMlnreykQKSDERKGGVGt
EAQiISSDLYNKVdQTKF-IPIVIEKD-
DsgrpyLPIYLNNRIYIDLSENENCEYRYEELLRLIYKRPQFQKP(KLGTAPLWIFEDKENISNINIKERELI
TSLEKNHKSRIIKVRDYIEIIQSELINAEI)}*

$2547=168(159):
>gi|490649321|ref|WP_004514316.1|
{|0(68)|<Proteobacteria(B)>}hypothetical protein [Geobacter
metallireducens] >gi|78195452|gb|ABB33219.1| hypothetical protein
Gmet_3004 [Geobacter metallireducens GS-
{(MAHS)-YQVFISFKN-
SDeqglpTRDSqLADLLYREFTSRGLDVFYSNLSLprlGEsEYKKCIDDALDSSTVLIAVgTSRENLESKWVR
YEWDSFYNDVLSGVKPQGRLFVYYEKMSTKELPRTLRQNQAFPHGLDHLNELSEYIMRALPASPVPK(FQDLA
ICTNCGHQFD)}*

$2548=193(195):
>gi|499625041|ref|WP_011305775.1| {|0(235)|<Euryarchaeota(A)>}TIR
domain-containing protein [Methanosarcina barkeri]
>gi|72395452|gb|AAZ69725.1| hypothetical protein Mbar_A0747
[Methanosarcina barkeri
{(MK)EKRIFLSYSQKnMDLANEIDNALhiKGIGVTRDikNVKCA----------QsfkeFMKS-
IRDHDFALMLISDYYLKSQACMFEV-IEFMKKPN-YkqriIAVVNDDINFDLKGITYLKYWEEKGKLIEG--
AIKEHHQEKIKPLKEERDEIK(LIEDNIMEFISTIRDLKYIPFKELYKSYLDLYDTVGLKINKSEEIYDIVTE
ED)}*

$2549=201(188):
>gi|490647058|ref|WP_004512053.1|
{|0(948)|<Proteobacteria(B)>}hypothetical protein [Geobacter
metallireducens] >gi|78194257|gb|ABB32024.1| SEFIR domain protein
[Geobacter metallireducens GS-15] >gi|3
{(MNG)-KRVFISYSHDsAEHRDKVLGLGACLLRDGCdcrIDLYKDTD----
EDWPlWMTRQLTEADFIVCVVTETY--ARRFNDkELPdrglgvgwesglirRLLYQKKL-
HNDRIFPVFFDKS-
DDHHIPLELVGYDYFILdnHTGYETLLRKVLNRPLHLKPSIG(SEPELRTSTATPLFARPSEEPQSDRIDAPA
VESPLESKYHGKQRR)}*

$2550=222(220):
>gi|499352664|ref|WP_011041615.1| {|0(68)|<Proteobacteria(B)>}TIR
domain-containing protein [Colwellia psychrerythraea]
>gi|71143795|gb|AAZ24268.1| hypothetical protein CPS_0766 [Colwellia
psychreryth
{()MSKVFISYSSSdRPAVKLLKEALEQLSY----
DVWFDQDaILPGGYYAkDIATALKDIDVFVLMTSANSIGDKVNNiagSsEVAKEVVLAQS-
SSAVIIPVKLDDC--WmettTHDEFTYLLAR-----
TQWLDAGYCdspTDFMDVAKRIDSA(LVSGNFKFNHDTTLDEIDKLLKQGFFGQATSRMNANAFPDIASDRVV
LINSILALSARPISSLSKITVDNLVRDLVQIRN)}*

$2551=201(198):
>gi|499353734|ref|WP_011042621.1|
{|0(745)|<Proteobacteria(B)>}hypothetical protein [Colwellia
psychrerythraea] >gi|71144452|gb|AAZ24925.1| hypothetical protein
CPS_1796 [Colwellia psychrerythraea 34H

{(MLEC)----FISYKV--
KADDNVALQLSRFLQDNDFKVFVDKKSLLPGQNWvdELQHSVTQSDYVFAIftqdyiarVVEGNCNGDNFIIe
ELNWALKDNKLIpIG---IGVTVEEI-
KTCFDLvPSLKPIQFQMFPTDLSEQCFHIILNKLYKRIYSK(DSKAKSILPVSHSVNVSGTALSSEDAYLKLD
MPWNDSDAIEYKNTLKRKVLKSA)}*

$2552=233(229):
>gi|13384079|gb|AAK21261.1| {|509(0)|<Echinodermata(M)>}toll-like
receptor Tlr2.1 [Strongylocentrotus purpuratus]
{(SGTSLKGLSKQPILSFHPEDHCGVNIFLIAGISFTCIFLFLITLLAYNRRWWLNHKFFLLKLAVVGYEEMA
EDFDADNYE)-
FHLNLMFLEEeeEWVDRVIKPALEERFPHLQnIIYGDKDLHLGMFYINAINDALDNSFKTVLLISNQSIRDAW
CMTKLRMALEHLNETGLDKIILIFLEDIEdENLP---
YLVRLFMSRnKpYMLWTDDEDGqeLFWAQFEKSMRAN(KAINNAIPL)}*

$2553=234(230):
>gi|47550995|ref|NP_999671.1| {|699(0)|<Echinodermata(M)>}toll-like
receptor Tlr1.2 precursor [Strongylocentrotus purpuratus]
>gi|13430191|gb|AAK25762.1|AF335483_1 toll-like receptor Tlr1.2
[Strongylo
{(SSASLQPLREKPLLDFDPNKLCGLNIGLVVMTPLAGIFLGVIVAIFYHNRLQLKYKLFLLKLMVIGYKEMR
DARDHNDYE)-
FDLNIIFYEDdeEWVRQQLQPALEERLPQFQRNVFGDQDLILGMHYLdAVDYVVSHSYKTLILLSRAAVHDRW
FMLKLRTAMDHVSDTQTEFVVVVFLEDFPDEDIPFLARLY-
LSDGRpYQHWTEDVGGhvFFWNALVKNLTIN(LRTNDFIPNE)}*

$2554=149(151):
>gi|971566747|ref|XP_015168401.1| {<Streptophyta(V)>}PREDICTED: TMV
resistance protein N-like [Solanum tuberosum]
{(MASQFRSVNKW)KYDVFLNFLR-
EDFNENFVTDLYERLEDIGIKTFKHDDRSarGVPISTEISGAIEESRIAITIFSGKYALSRRCLEESTKIMEC
VDSHGQEFFPD--YRTEPSVVSLEfdYGNYDDALSK---ASDIAGFNKYYYYYGFEPICW()}*

$2555=149(154):
>gi|971566751|ref|XP_015168403.1| {<Streptophyta(V)>}PREDICTED: TMV
resistance protein N-like [Solanum tuberosum]
{(MAFQIESAQKW)KFDVFFNFRL-QDLDKSFVADLYQRLKDIGTNAFKPDvNSEWGEQ-
IstEILEAIEESRIAITIFSKDYTSSPRCLEELTKIMGCVDN-KGQEFFSVYYKCEPLDCLRSIVNFGAD---
-LKGSDLRKV-QRWKDALRKADDM(EGS)}*

$2556=237(203):
>gi|971566743|ref|XP_006356543.2| {|0(893)|<Streptophyta(V)>}PREDICTED:
uncharacterized protein LOC102582774 [Solanum tuberosum]
{(MNIFEAQTQIDCFKYTYAEKETKLCWDIRGLPRN)------SLRQ-
QDLDKSFVADLHKRLKDIGinvFKP--DVNSEWGEQ-
IstEILEAIEESRIAVTIFSKDYTSSPRCLEELTKIMECVDN-
KGQEFFFVFYKVKPSDGLSPLETFEADIKGFdlekvqrwkdalriaddmagsrdyfagaqfshwedgkciaEI
TNKICQVPRIAKYPVGIKSRV(EEVKSLLKVEFGGVSFIGIWGRGGVGKTT)}*

$2557=179(184):
>gi|971511663|dbj|GAQ86531.1| {|0(982)|<Streptophyta(V)>}disease
resistance protein (TIR-NBS-LRR class) family [Klebsormidium flaccidum]
{(MTAPHRVG)KYDVFISHCG-
KDCKKGFADLLRDDLERAGVLCFFDEHSLEVGDAApaEMLKAMEEATYGVVILSPGFWEREWCMKELETFVR-

-----
RGRFVPVFYEGFQAVSAAAEAAMAKRVWRGFQQSKWDEEAYRRLAQESASFTG(IRLEEESWWLSCIRRVRYE
VLRLLGKVGGGIQISED)}*

$2558=187(187):
>gi|971513003|dbj|GAQ85215.1| {|0(267)|<Streptophyta(V)>}putative TIR-
NBS type disease resistance protein [Klebsormidium flaccidum]
{(MDIHEERAESSGAGPSHRAAGVA)-YDVFISHCG-
ADCKRDFAVQLKNELERAGLCCFLDDRDLRLEDDAakTICAAMETTKFGVVLVTEGFFKREWRIKELETFLRR
GNCTPVFLERSMERQGEIVEECRSSRVWEGFGRFLWSEEEYLGLVKGVFGFVGLRLDA(SDGFWDTCFAKLKQ
ELLLQLGRIE)}*

$2559=229(231):
>gi|971460613|emb|CUV66812.1| {|410(0)|<Mollusca(M)>}toll like receptor-
1 [Mimachlamys nobilis]
{(YDEETQYTIIDVTSTLLDCSYDLLIGLAVGFGVLLVVVLIAMVWAKRCPYETKVLLYRFFGFHPRDRYIVD
REFGRESNI)----YVSFDDQniHirqWVSKKLVAKLE---
PTYKLFIPERDLPIGNDRSEETITHLENSKRIIIVLSDGYDENQWCAFECQRAEILDFNEG--
RIIYIKYHDS-VSELLREEPWKTRVHNRkMFspggtKSERRW---FWDKLRYELPIE(TKKDRKYCCAV)}*

$2560=286(289):
>gi|971436375|ref|XP_015155511.1| {|357(92)|<Chordata(M)>}PREDICTED: TIR
domain-containing adapter molecule 1 isoform X1 [Gallus gallus]
{(IPIEDSYIPAGTSNSAPASTSVCSFPPQTYFSSAILRPLQSIPYNVPFPPPLHSSPSPTGPPPLKTVEASL
APEPNGEKK)KFFTFVVlHAWEdEHIACRIKDLLENMgvPNGATFC---
EDFLVaGHNQLTCFQDAMENSAFLILLLTKNFL-CHQCMFQTNSALMESIQRPSKHNSVIPFVPK-ENPLE-
RSQIPSMLSVLvALDENSPV---
FARTVQNTFTPE(KINERKAMWCQIQQVQEQKRKLELYQDHCQTLQNLGALTLGSLPQMSPSAMQLNQSSLEQ
LLEQLLPLQ)}*

$2561=176(185):
>gi|971514235|dbj|GAQ83946.1| {|0(1895)|<Streptophyta(V)>}disease
resistance protein (TIR-NBS-LRR class) family [Klebsormidium flaccidum]
{(MADLAAAYG)---VFISYCG--
DVRRDFAVMLRAELERVGVHCFLDVSSLMPGDNVdeRLLFAMQTASIGIVILSPRFFEGEWCMMELHTFMTRG
-----NAV-
PVFFASLEDLKEARNKAVADRIWERFGRRFGEITEEQYLGAVDGVLRT(TGLRLEALDGWWDECVEKIVPVVL
RKLDSSARLSNH)}*

$2562=163(168):
>gi|499817892|ref|WP_011498626.1| {<Euryarchaeota(A)>}hypothetical
protein [Methanococcoides burtonii] >gi|91711537|gb|ABE51464.1| Protein
with Toll-Interleukin receptor domain [Methanococcoides burto
{(MSF)--KIFVSYSTKdVEEIKPILQQIQTI--KDTEFFFADETLN---
PGDhisqRIINYITNCDVFIFFYSKFAYDSTYVQHEIGVAKANDKL-
IIPILLDETKPDGMLQGINYLNFYNQNKKQQ--
EFVRLYNYINQNVQRKNRNSA(LSTLALLGLEYFLLKSDDEQEEDVF)}*

$2563=207(217):
>gi|499813610|ref|WP_011494344.1|
{|0(224)|<Proteobacteria(B)>}hypothetical protein [Paraburkholderia
xenovorans] >gi|91693921|gb|ABE37118.1| Conserved hypothetical protein
[Paraburkholderia xenovoran

{(MGY)--DAFISFSSKDCIAADKLRHGLELRGLS-CWISSRDVAPGADFADSIVKALETSSVMVLVFSANA-NNSDEVKkELVLAGEYK-----MPVMPVRIENVLpsGAFRyQLTIRQYLDLFE---DWDTNLA--KLAEQVKRMVES(RMAFVGDASADTKPATAGAADTREQPAARPAARASAPARTGAPARTAPSASTPGAQPQTERGRRPSRVVPVVAG)}*

$2564=213(213):
>gi|499829837|ref|WP_011510571.1|
{|0(165)|<Proteobacteria(B)>}hypothetical protein [Nitrobacter hamburgensis] >gi|91800517|gb|ABE62892.1| hypothetical protein Nham_2095 [Nitrobacter hamburgensis X14]
{(MK)---IFLGYPS--ERlSDARAIW--DFLTNLGLTVWFDKEHVLVGEDWraAREQAQDEADLVIHLISPEMQSRAGEVQrEIKRSLDKAKDIppGNLFLIPILVGGA-AVPREIASYQYVEYKKGdWPYRVAKSIArKHEQLCSCGPEEL(TNYIKAQATAGGRIDLKIHDVDSVRDLQADYFKYRFSNRFFDFINAEISAHVLAAYYDARRREAEMPESPE)}*

$2565=225(248):
>gi|972463605|ref|WP_059050355.1| {|35(58)|<Firmicutes(B)>}fructokinase [Paenibacillus sp. SIT18]
{(LENGTYHRYFGGSPANIAMNTKKLGIRPLVASAVGEDGLGQFLVEHLNRAEMDTSCVQRVNESTSLVVVNKSQSTPIPIF)-------YRG-ADYRLGFTEELRAALHRTKIVHFSCWPLSR-QP-VrsAVEQVIQEARQNgtLVCFDPNYHPALWEEGEDGVQYLKTIL-----------------PLVDIAKPSEDDAERLFGKDTPENQLQKFLDLGVKLVI(LTLGKDGALVSNGQETLQFDTLATEVTD)}*

$2566=181(186):
>gi|973765230|ref|WP_059160792.1|
{|0(127)|<Actinobacteria(B)>}hypothetical protein [Mycobacterium sp. IS-1496] >gi|972084026|gb|KUI26050.1| hypothetical protein AU195_13780 [Mycobacterium sp. IS-1496
{(MR)---VFLSWSG--DLSHSIAELMHEWLPSV-IQTvdpFLSSSSIEKGSRWgaQLTAGLTGSGFGLVVLTRENLSSPWMTFEAGALSEG--L-SNGRVCPVLVDVEPGDVAGPLAEFQGCRITK-PDMFKLIGSMNRASGEGSIEPN(RLARLFEAMWPDLETSVNNILMTHADQTGSGVKEAPSSRTFKLL)}*

$2567=209(209):
>gi|973233891|ref|WP_059095101.1|
{|0(1060)|<Actinobacteria(B)>}hypothetical protein [Mycobacterium sp. IS-1742] >gi|972086971|gb|KUI28923.1| hypothetical protein AU196_14555 [Mycobacterium sp. IS-174
{(MIRKGRQMSTSLSSHTAELPYY)----FVCYSREqRGFAEKIERKLQPRRARGELEVWRDvRNLDMWEEFTpKILEALRNAAGAIVLVSDSWYASDYIQTyEWPTVKACRER-DPSFQIFLLAYNA-LDDDDPLRKRNFVNDIQreLLVKSRPAMRDTILTRLSNLVGE(HARMRCPRVTPPVTAPTERTVSGQPSTGEGIAEPDIASDTDGALNDV)}*

$2568=185(193):
>gi|973226538|ref|WP_059087865.1|
{|0(321)|<Actinobacteria(B)>}hypothetical protein [Mycobacterium sp. GA-2829] >gi|972097371|gb|KUI39174.1| hypothetical protein AU194_14145 [Mycobacterium sp. GA-2829
{(MA)---VFMSYSSRdRAL---VENVLKALRRAHE-QIWLDEELGGGEAWWrEILEQIRGCEVFLVALSKNMLESKACAAELRYAQQL-----GKPILPVQVGDL-DnMRINPLAAMQVIDFRRpsIDSGIELVSSVHALRARAHPLPD(PLPEEPQIPFAYLMRLATTISDPSPLSAQQQMTLVAELKAGLDEDGDDVSA)}*

$2569=111(140):
>gi|972371445|ref|WP_059025566.1| {<Bacteroidetes(B)>}hypothetical protein [Gabonibacter massiliensis]
{()MGRIFLSHSSKqKGYVEVVARNLG--KQKVVYDAWTFEE---GNKTLdEIYKGIDVTGLFVFFISQESLNSDWV-KkEILKAEEYIKN-GKIKqFLPVII--------------------------DENIFFFGNKCIIRYPLA()}*

$2570=264(260):
>gi|973393843|ref|WP_059136441.1| {|909(0)|<Bacteroidetes(B)>}hypothetical protein [Chryseobacterium greenlandense] >gi|972590616|gb|KUJ56479.1| hypothetical protein AR686_07920 [Chryseobacterium gree
{(HIDLYNFSSNEDFIEKIKEKIIKINKEKKYNVEEYVSKDGDNFIPLKLINDNEGINSTIFYKGIYYKLYDFKKYFKNKNI)MKKIFISYSKDdLTLVNEFIDHLSALKRDGLVGSWYCTELLAGGYWDkDIQKHFDESDIICIMISPNLMKTDYIHKyEITKAFEKLKE-NEnFKIVPIILNYC-RWttKNNNLSQFTALPYTAkpVNDFKDRN--MAWFIIIECMRIM(IEKQVQPKGEDWYSNNDLLPKDIRKIYERIVNGLIDNNSI)}*

$2571=175(175):
>gi|1011899083|ref|WP_062715761.1| {|0(478)|<Actinobacteria(B)>}TIR protein [Streptomyces regalis] >gi|973253391|gb|KUL20495.1| TIR protein [Streptomyces regalis]
{(M)-WDVFLSYSR-GDVER--ARPLARALRDGGLEVFTDETGVESFAGIsaTIRRELGRSKALLAFYSAGYPEREACQWELttAYLAGLGEGDPRRRVMVVNPERAADHIQPVELCDARHAAPDDLPALVADVRAHVARLDGPMTIA(EPGVRHWLLGTPQPRPDFLGRLSELWQVHSALHA)}*

$2572=185(184):
>gi|1011893241|ref|WP_062709946.1| {|0(520)|<Actinobacteria(B)>}hypothetical protein [Streptomyces regalis] >gi|973258549|gb|KUL25480.1| hypothetical protein ADL12_35025 [Streptomyces regalis]
{(MSASSERPAS)EWDVFVSYSR-SATGH--AETVVAALRSQGLRVFVDDTAVEDFTSIttTITRALAHAKVLLALYSSDYPQRRACQWELtyAYLAGQREGDPRRRILVVNPEPSADHIHPVELRDARYWPWRAGADEANRLATRVLAHVATLSEP(MGSHTASPVVPWLPAPARTGSARFTGRLPEQWHI)}*

$2573=178(179):
>gi|1056203145|ref|WP_067690505.1| {|0(737)|<Actinobacteria(B)>}hypothetical protein [Actinoplanes awajinensis] >gi|973269480|gb|KUL34494.1| hypothetical protein ADL15_15540 [Actinoplanes awajinensis
{(MSQKV)-YDVFMSLAG-PDrTR---VENLVRAMEKAGLSVFYDQ-RLPVYRGItaEIESALRNSTTLLAYYSRDYTNRAACQYELTTAFlaGLREGDPIRRIMVINPHRETDHLAPAVLADEKFDRLPFPGDDAETSRVVREIKAKVRTLT(GRIGDHPVSGEQTWHARRQPGTPDFTGRYHQIWE)}*

$2574=203(206):
>gi|973143299|gb|KUK86470.1| {|0(108)|<unknown(B)>}GUN4-like family protein [candidate division TA06 bacterium 34_109]
{(MVKLK)--DIFICHASEdkESVVKPLLETLDKESISY--WYDEAEIKWGESIPNKINEGLRISRYVIVVLSNVFLSKNWPQKELTSALNIESSAGEVKVLPLI---V-GNKEVRDKILLQYpiLNDKrYIIWEKDVKKIVEELKRCLGRSN(KKIEHKNLDNSESNLDIPIPKIPKEFSQLDKDRFIKNTFSIVKQYFRNALNKLKIRYQEIE)}*

$2575=256(242):
>gi|973215945|ref|XP_006642979.2| {|359(0)|<Chordata(M)>}PREDICTED: interleukin-18 receptor accessory protein isoform X1 [Lepisosteus oculatus]
{(TEQHLEATFICFAQNSKGNETSILKLKKKPEVPGLLYIIIGPIVTVVTISVFSVVVYYYWIEILLIYRNYF
TFNEARGGK)EYDAFVSFASNssseeaigvteETFALHHLPEVLEQQCGYRLCLLERDVLPGGAYTEDIVSCI
QRSRRAICILTPFYFLSPCLF-ELESALKTLLKDNQFKVIFIKFKSL-
HEIETLPPLVRKALKVFpALEWNPVESFhsdsKFWRDLKSAMSVK(KISFSHKGVQLHDASRTVGKLY)}*

$2576=204(220):
>gi|973396770|ref|WP_059139346.1| {|356(0)|<Chlorobi(B)>}hypothetical protein [Chlorobium limicola] >gi|973254829|gb|KUL21865.1| hypothetical protein ASB62_07815 [Chlorobium limicola]
{(AYAQITLVVISIVVSTGVSWFALRRGLEKKLIAPPEEQSASGGKEMTEYDVFISYARTPENSAWVKAQVYE
RLLRLRKAD)--------------------------
GSPLRVFFDQRNIEPGEDWygKLALSIQGSRFFLPVYTADYFSRKFCEFEMLRAAPRHVELgdffiaiaRDDV
TVPTQYNHIQyldvRTDADFMDRIAER---IRKRDSGSGNGQENNQNSQTKGTE()}*

$2577=242(239):
>gi|1056220278|ref|WP_067703689.1|
{|0(96)|<Actinobacteria(B)>}hypothetical protein [Actinoplanes awajinensis] >gi|973258488|gb|KUL25421.1| hypothetical protein ADL15_40885 [Actinoplanes awajinensis s
{(MIFFLSHANTARGSGTLDPW)--------------VKRFFDDLSDGVRARtgaDETGFYDGL-
LEPGADRraRLTDEIGRARVFVPLYSPQYFGNSWAAAELDSFRSRLRGLgRAEAarhVVPVVWTPLPpwenrr
efDEAFAVVGKSVEYTENGlRALCMLSVYRDAYRDLVAAFTDR(IAGVARDSVLEPSPPAPLVPRAAGGGEPA
LIVSALGAGLDARRDQVIEAAERLGVPAQPLPADQLRRQAARRPGVLLID)}*

$2578=231(228):
>gi|973093975|gb|KUK53508.1| {|69(0)|<Firmicutes(B)>}hypothetical protein XD78_1134 [Desulfotomaculum sp. 46_296]
{(RIIDDKAGINKKIAEKRKKRADTAVKLQREEAAETQKANKAQQAIQNGYERRIADLSAQIRKQLSNTKPPR
HMYEESGHE)EYDVFISHAS---EDKaEFADALCEALENSGVKVWYDALSIG--WGEslrAKIDD-
GLKRSRfgIVILSKDYIRKGWTQYELDGLLQIEMT-NGKTILPIWhnITKDEvqA-
FSPTLAGrkaLDTAMFST-REIADELV-KVLPGTGNTEEAN(EMEDIANG)}*

$2579=225(222):
>gi|973109696|gb|KUK66637.1| {|0(85)|<unknown(B)>}GUN4-like family protein [Parcubacteria bacterium 34_609] >gi|973158395|gb|KUK99333.1| GUN4-like family protein [Parcubacteria bacterium 32_520]
{(MVELK)--DIFICHAS---EDKEIiVKPLLVALDKENISYWYDEAEIK--WGDsipNKINEGLRI-
SRyvIVILSNNFLSKNWPQRELNSALNIESSTGEVKILPIIVGNDEvkAKilhqypILNDKRYIIWKD-DV-
NDVVEELK-
RCLQRSSKKIKHK(NSVTKKTNLDIPIPKIPKEFSQLEKDRFIKNTFSIIRQYFRNALNKLKNQYPEIEFDID
DIDNFKFICAIYKNGKLL)}*

$2580=186(197):
>gi|973083537|gb|KUK44979.1| {<Euryarchaeota(A)>}Uncharacterized protein XD72_0685 [Methanosaeta harundinacea] >gi|973153578|gb|KUK95169.1| Uncharacterized protein XE07_1907 [Methanosaeta harundinacea
{(MIT)--KVYITHSEEdEPLARELADALWRVGLESFVAIY--
RRARGISPGERVRFAIRHSDCVIALLTLDGVVSPRVNQEIGFASGIDQL-VIPLLETGV--EMPALIRHLKP-
ITFSRETY--SDALGEV--
ILTIRDLTSLEW(LKIKCPLCGEEMTQYLPPLEVVERAVLAETGLETICSYCETPLTLDPRTFKPVP)}*

$2581=175(184):
>gi|973085926|gb|KUK46688.1| {|0(174)|<Chloroflexi(B)>}TIR protein [Anaerolinea thermophila]
{(MEIGSYPY)--RIFIAYSHEDRNLLENAVRVLEENGYH-PLCDK-
NIMVGKPFTDEIKRMIAHAHIFLPLLTEN-SSLRPWVHqETGYALAMN-----
IPVLPVAVGTAPgeMTAQLHAVAVCDDFSD--
FPQKVSLQDIENLVMHNPSEEI(TNLWVTAWAEERTKLLGEYAFWVSNLHYYGRVRQRA)}*

$2582=144(166):
>gi|1056191921|ref|WP_067683635.1| {<Actinobacteria(B)>}hypothetical protein [Actinoplanes awajinensis] >gi|973279761|gb|KUL42345.1| hypothetical protein ADL15_00175 [Actinoplanes awajinensis subsp. m
{(MMTIDARWSMWRVFRHAGDVP)---VLLSYATADALWAQWINRSLQAAGHA-VQM----
LPAGVDFVQRIADALSGSDQVIILVSAEHRAS-------TSDWSEV-----
PLVPGLVVIRLDaaELPAPLRAANCKSVHE--LDEEEALE--VLMVAVGGPQNP(FSRTA)}*

$2583=210(197):
>gi|1011849111|ref|WP_062667799.1|
{|0(841)|<Actinobacteria(B)>}MULTISPECIES: hypothetical protein [Streptomyces] >gi|973316169|gb|KUL76170.1| hypothetical protein ADL34_13100 [Streptomyces sp. NRRL W
{(MVSDAGMRP)--QVFVSYAWEDEKHKSAVlllARLLLNAGAD-
VWLDAMAVERGPEWAAETAREIADADFVLVIASPAYKRRMEGAEePgqgrgvrWEArhivefAFTHEQDWT-
DRVLNVVLPGGSveDIPTILLPNSRHRYEV--
PALTPQGVSELTRRLRTGLHPT(EAGVAPRETGRPRLRIGELPPTAAWFQEREVMAALRETVQENGTTVVC)}
*

$2584=217(223):
>gi|973591629|gb|KUM35245.1| {|37(0)|<Actinobacteria(B)>}hypothetical protein AR689_14425 [Arthrobacter sp. EpRS71]
{(AVDDYNRKVRAHNQQVVNQFNRRVDAHNQDVRRANDRNSRVIADLQRQLRSGGSGPRYTPAEQALADRVQH
SLVPRDERE)-WDVFLSYARIDGAqAAAALRDALEAFKVR-
VWFDEIAIVPGRSQALQMDQGLRNARAGLVLLTPAYLVGRFWTErE-LGALLHK-----
NTLIPVLHGTTFkeVAEYSGILPDLAGFET--ARDDIETIAEKIAAAVLPADSE(IED)}*

$2585=229(240):
>gi|973264710|gb|KUL30797.1| {|148(0)|<Chlorobi(B)>}hypothetical protein ASB62_03505 [Chlorobium limicola]
{(RGANFKRASLGYTIFGAVDLSNVIGLDTIKHRGPSTIGIDTIYASKGNIPEIFLRGCGVPDNFIEYAHSLT
SKAFDYYSC)----FISHST--A-DKDFADRLHADFQSKGVRCWYAPEDMKAGEHVdtQIDHGIKSH-
EKLLLILTESSINSNWVKrEITKAV-KREEAeGKRVLFPVSLVDY-------
STLEEWEFVDSKGRDLAEMIRkFNIPSFKGWEHDN(SIYTTEFPKLLKAFQDNSKL)}*

$2586=174(177):
>gi|1011885938|ref|WP_062702752.1|
{|0(670)|<Actinobacteria(B)>}hypothetical protein [Streptomyces regalis] >gi|973274857|gb|KUL39170.1| hypothetical protein ADL12_15565 [Streptomyces regalis]
{(MIK)---AFFSYSTkDadfvrevaSKVGRAFARI-------------
DYRSFAAGGDIlgSIDDALKESAAFVLFLSKAALESPWVAHEIREARfqtAMGRIKntlVVRLDQRISPE---
DLPDWLNRSKHITATAARPAAREIGGLIDDLVRQGQSPRF(VGRATEIAALQDAYSAPGPGAEAQIACVTGLA
GI)}*

$2587=194(186):
>gi|1011223463|ref|WP_062142789.1| {|0(449)|<Actinobacteria(B)>}TIR protein [Streptomyces curacoi] >gi|973810753|gb|KUM81968.1| TIR protein [Streptomyces curacoi]
{(M)-WDVFLSYSR-GDVER--
VRPLARALRDGGLEVFTDETGVASFAGIsdTIRRELARSKALLAFYSTGYPERAACQWELttAHLAGLGEGDP
RRRVMVVnpepatdhIHPVELRDARHASADDLAALVADVRAHLARLDGPMPIAEPGVRRWL(LGTPQPRPDFL
GRLSELWQVHSALHAHAAPLVTGRAAAHAVQIRG)}*

$2588=228(211):
>gi|1055327401|ref|WP_066992364.1| {|0(430)|<Actinobacteria(B)>}TIR protein [Streptomyces cellostaticus] >gi|973827727|gb|KUM98295.1| TIR protein [Streptomyces cellostaticus]
{(M)-WDVFLSYSR-GDVER--
VRPLVRALRDGGLTVFTDETGVASFAGIsdTIRRELGRSKALLAYYSAAYPEREACQWELttAYLAGLGEGDP
RRRVMVVnpeaatghIHPVELRDARHASADDPTALvadvREH-
VARLDgpmtlpEPAVRRWLPGGPEPHPE(FLGRLPELWLVHSALHAHTAPLTSGRAAPRAAQLRGMAGIGKTL
LAQEYAARFEAAYPGGVFRLNGQAYD)}*

$2589=194(186):
>gi|1055465407|ref|WP_067125618.1| {|0(456)|<Actinobacteria(B)>}TIR protein [Streptomyces yokosukanensis] >gi|973833507|gb|KUN03913.1| TIR protein [Streptomyces yokosukanensis]
{(M)-WDVFLSYSR-GDVER--
VRPLVRALRDGGLTVFTDETGVASFAGIsdTIRRELGRSKALLAYYSAAYPEREACQWELttAYLAGLGEGDP
RRRVMVVnpeaftghIHPVELRDARHASADDPAALVAVVREHVARLAGPMTLPERGGGRGR(YRAPEPHPEFL
GRLSELWRVHSALHAHAAPLTVGRSAARAVQIRG)}*

$2590=194(186):
>gi|1011903575|ref|WP_062719128.1| {|0(449)|<Actinobacteria(B)>}TIR protein [Streptomyces caeruleatus] >gi|973935894|gb|KUO03973.1| TIR protein [Streptomyces caeruleatus]
{(M)-WDVFLSYSR-GDVER--
VRPLARALRDGGLEVFTDETGVESFAGIsaTIRRELGRSKALLAFYSAGYPEREACQWELttAYLAGLGDGDP
RRRVMVVnpepdadhIHPVELRDARHASAQDIPALVVDVRAHVSRLDGPMTIAESGVRRWL(LGRPQPRPDFL
GRLSELWQVHSALHAHTAPLVTGRAAAHAVQIRG)}*

$2591=194(186):
>gi|1055350816|ref|WP_067015252.1| {|0(435)|<Actinobacteria(B)>}TIR protein [Streptomyces sp. RV15] >gi|973955435|gb|KUO22800.1| TIR protein [Streptomyces sp. RV15]
{(M)-WDVFLSYSR-GDVER--
VRPLARALRDGGLEVFTDETGVASFAGIsdTIRRELARSKALLAFYSTGYPEREACQWELttAYLAGLGEGDP
RRRVMVVnpepgtghIHPVELRDARHASADDLAALVADVHAHVARLTGPVTVQERDVRRRP(PGAPRPRPDFL
GRLSELWQVHSALHAHAAPLVTGRAAAHAVQIRG)}*

$2592=224(226):
>gi|1011121310|ref|WP_062043171.1|
{|0(347)|<Actinobacteria(B)>}hypothetical protein [Streptomyces ciscaucasicus] >gi|973842101|gb|KUN12334.1| hypothetical protein AQI96_15940 [Streptomyces ciscaucasi
{(MTTTDRATD)QWDVFISYAREDYIQAKDLHDALLECvtadgRAP--
RIYLDVSRTGtpvGADWQnFLEDALENTRHVVALYSPTYFDK----
DvcQWELHEAYKLRLtRNIGLVPLLMD--PNAAKKVPYGL------

NRINWIPTSRPRWIDEVREALGLS(TAVARPDLRFDTPAGDVVAGHTLPPLTVTAAPAGAAAGPAQGTVTLAA
QPSGTELTGTLTVPLTDGAAVFGDLAFPA)}*

$2593=206(220):
>gi|974064967|ref|XP_015229843.1| {|697(403)|<Chordata(M)>}PREDICTED:
toll-like receptor 13 [Cyprinodon variegatus]
{(HQYICSFPVNEQGHFLLDFDINYCLNNGSFFYFVSSTFLVVLTLLISFIHHFLKSHLVYAFYLFKAYLYDS
RKRMKEGPH)KFDAFISYNAHdeDWVYSKMVPVLEGEqGW--RLCLHHRDFQPGKLML---------------
--
YLFYKVKHEIKLSDPTAFKQRPRPIHPQDVDAVRYYRRfVEGFSSVVKPLNELTAGyPPCEIADKQRTDSVIQ
HIIAQLE()}*

$2594=271(234):
>gi|974059266|ref|XP_015226725.1| {|286(0)|<Chordata(M)>}PREDICTED:
interleukin-18 receptor accessory protein-like isoform X1 [Cyprinodon
variegatus]
{(EVMPQHLKHTYTCFASNIIGNKSVTIKFKPKHQEPFLVWIPFACFVLFAGAVVILHVKWLELQLIYRSHFQ
LGGSDGDEK)DFDVFVSYV-WtapstevangltlsskpysdakgcvsnirqiseAPIEM-
LLPHVLEHQWGYRVCLLERDILPGGAYTNDVVLTIKRSRMLICVLSAEYFaSSDAVF-
VLESGVKALLQTSAHKMLLIWTSGtsasfI-
QPDLTVPKLVQRAVKVLpSLQWDAGASYsAFWIPLQKALPAQ(EAWQISHMQRPQLD)}*

$2595=167(154):
>gi|974232771|gb|KUO72870.1| {|0(60)|<Firmicutes(B)>}hypothetical
protein APF81_26710 [Desulfosporosinus sp. BRH_c37]
{(MGR)--
KIFISYKYRdNSvqnlvgkfgnaIVRDYVNILEDLFSNSDHIFKAEHDDEDLSNlsDEtiweSLKDRIYDSSL
TIVLISPNMKEKGSSENQQWIAQEVSYS-LKAISRTNKSSKYITSRENAIIAVVLPDKMG--
SYSYFINAQSCCSNGCTKYHS(DVTFEIIAKNI)}*

$2596=218(226):
>gi|1011236017|ref|WP_062155163.1| {|0(703)|<Actinobacteria(B)>}ATP-
binding protein [Streptomyces curacoi] >gi|973799450|gb|KUM70755.1| ATP-
binding protein [Streptomyces curacoi]
{(MTTGTG)-QRFFISYAGADRLWAEWVGWHLERDGHL-
VELDVWDWRTGDDFVRRMDEGLEKADAVVALFSKSYFEDGRWTQeERSAVVARR-----
DRIIPLAVEPLSnhDVDPMLASKVRKKLHG--LDETAAIA--
ALRDAVGGGGRP(TAAPAFPGAGVAVEAVVPEGPQPRLPGTTDRPDVWNVRRGNRDFSGREAEIVQLRDGLLS
GRHSAVYALHGMGGIGKTQL)}*

$2597=260(275):
>gi|1011090514|ref|WP_062025546.1|
{|262(809)|<Actinobacteria(B)>}ATP/GTP-binding protein [Streptomyces
phaeopurpureus] >gi|973804233|gb|KUM75488.1| ATP/GTP-binding protein
[Streptomyces phaeopurpureu
{(WGAVEIPYKPFFAYEEVLATVADTPRQPASLLGAYERLTSVITDGRVTALRPVEEPVRRQLLSRFQRVPVP
GGRLVPRVR)-----IDYAKRDGAWADWIAAQLQAAG---VTVTQRGSSPSEPLPGGAEEG---
ADVVVALLSPDFATTPVADEvRALVAASEG-----PRIIGLRTREHQpePALRGLPGFTLVDASA--
EQVLGKVFAMLEIDASATAVAT(GGTGFPGRVPGIFNVPRRNNSFTGRDLQLERLRTKLSAESLRVDTPVSRA
FLYGL)}*

$2598=268(276):
>gi|973855130|gb|KUN25114.1| {|143(792)|<Actinobacteria(B)>}hypothetical
protein AQJ23_17260 [Streptomyces antibioticus]

{(RGVLPVAYGSEDLPAHYERFLRMLREADRQLQNTVFGPSVVQWYWPLLVAGAGLLAGGVAAALRGAPTDRT
PSEAVGTRP)AARVFLSYARADGAIARPYVEALKGAGQD-
VWVDWEGIKPSREWREELARAIRSSDALVVLISPASLA-SPHCWdECRQALEQG-----KRVLPVVID--
AtiDAPAAMASADWRELTE--
FQLLSMRGPEEFETAVRETVAF(VGDEYAWVAQHTRLGEQAHAWQQHQHAPGWLLRRHELTSAEAWLAYRPAA
TDFHAE)}*

$2599=260(275):
>gi|1011328993|ref|WP_062243237.1|
{|262(810)|<Actinobacteria(B)>}ATP/GTP-binding protein [Streptomyces
griseorubiginosus] >gi|973892751|gb|KUN61818.1| ATP/GTP-binding protein
[Streptomyces griseorubi
{(WGSVEIPYKPFYAYEEVLATVADTPRQPASLLGAYERLTSVITDGQVTALRPVDEPVRRQLLARFQRAPVP
GGRLLPRVR)-----IDYAKRDGAWAEWIEAQLRSAGIT-VGLR------
GSSSSEPLPSGSESgADVVVALLSPDFATTPVADEvRALVAAPEG-----
PRIIGLRTREHQpePTLRGLPGFTLVDAPA--
EQVLGKVFAMLEIDAPTSAAVT(GSTAFPGRVPGVFNVPRRNNSFTGRDVQLERLRSKLSAESLRVDTPVSRA
FLYGL)}*

$2600=286(300):
>gi|973934525|gb|KUO02649.1| {|77(1129)|<Actinobacteria(B)>}hypothetical
protein AQJ67_19490 [Streptomyces caeruleatus]
{(SDSGGAHIHYRNVRTARGSGGAPLVDVQGRLVGIHDDMAGAVGRGLTAAEIASAVKNGPHPWIADLAAVVP
GPTHGPAIM)-----LSWVPEDRSWARRVHAILAEAGHR-VFL---
EPDSGTPGHRTVREHVEGGGRVFAVVSPAYLADSAKTAerdfvvhDLDHATA-R-----SRLVPLLVE--
GEATGSVALLSPVDLRG--TPDPDLSA--
RVTSAAPPARAP(LPAPTSPATAETARDAVLTFRPDDPLPRLQRLSARARRAAGTVSGTIFADGEHSQFEAGL
YVTRDLEEELLELLDADRDT)}*

$2601=234(240):
>gi|1055608374|ref|WP_067249974.1|
{|157(0)|<Actinobacteria(B)>}hypothetical protein [Streptomyces sp. DSM
15324] >gi|973943450|gb|KUO11283.1| hypothetical protein AQJ58_14710
[Streptomyces sp. DSM 15
{(HSGKVLAGSHSGCFALVLPAECEEVQVTADGRYDIELDPDLRLRPSSRDDAFRVRLPPASTGRTATAAPTG
TRSRPRGSG)-RRVFLSYAHESEAHkaaVRQLYELLVANGLD-
VDFDQERLHRRRNWETWVNRRILRADFVLVIASPSYRRAGDGTLpE-GTRLGLR-----SEY--
RRLVDLQhaDPDRWMEKILPVVLPG--RSPEEIPLSFTPWTADHYVVDS(ITQDGAADLLEVLLHEDTAS)}*

$2602=237(221):
>gi|973951247|gb|KUO18757.1| {|0(196)|<Actinobacteria(B)>}hypothetical
protein AQJ91_23045 [Streptomyces sp. RV15]
{(M)----FISYSHdsEDHRKAVlRLAHRLQKAGLD-
VGFDRFvEHEPPASWPSWMRQELNRADFVLLVVTEVYRKrflgesvvdrgfGVRWEgtliAsELYYTTSEK--
---ARFLPVVL---RaeDRPLIPSPLDQTSWFV--IGESAdadiaellrvLRR--
EPLAGVHRGADP(VGWFDGITPALEIGETIGDGDLAAIEDALPHLRGPQWARAVHFAGVLHRQRGELMQAITC
FTRVRETTLHAHLHQAATDQ)}*

$2603=168(166):
>gi|1011236339|ref|WP_062155485.1|
{|0(897)|<Actinobacteria(B)>}hypothetical protein [Streptomyces curacoi]
>gi|973799630|gb|KUM70935.1| hypothetical protein AQI70_29855
[Streptomyces curacoi]

{(MSQGAIGEPDAADARDGDAGR)-PHVFISYAWEnEKHKSSVGE-LARLLRNGGADVWLD-DIEAerGPQWArETADEIETADFVLVIASPAYKRRAAGEEePGTGRGVVWES-RLLMEFAFTHEQD-WPDRVLNVVLPGGTAEDipFFLLPNSRHRYELRALTHQGVRP(LLNRL)}*

$2604=250(257):
>gi|1011228023|ref|WP_062147171.1| {|199(798)|<Actinobacteria(B)>}hypothetical protein [Streptomyces curacoi] >gi|973806878|gb|KUM78119.1| hypothetical protein AQI70_11535 [Streptomyces curacoi]
{(AEGIADVLRRLDAHFSGSAFGPTYERHQYWAALVAGAALLAALVSRLLRDLASRTGPVEDPFSGSGSGQRSDAPLAQPTG)--KVFLSYSRRdTELARRITRSL-----EGRLqDIWVDwEAIEPSEQWRrAIREGIRRSDALIVLLSENSLRSAYCRDECEYAIEL-----GKRVLPVLADPS-LDGRTTAVMREHDWDRLlaYQRLPMIEEGDEAFERGIEGIVS(FVTQEYRWVAFHTRLGNLAHEWWDSGCKRGLLLRAEE)}*

$2605=250(257):
>gi|973806883|gb|KUM78124.1| {|135(801)|<Actinobacteria(B)>}hypothetical protein AQI70_11560 [Streptomyces curacoi]
{(TYGAVTPGPPWRSATWVYAWLDEQIGGSLFGPSFEQPYWPLLPAGAGLVSGLLAAATRQAADRAGGARNPFAQAEHGIGG)--KVFLSYSRSdTEFARALTESLRGRVR----EVWVDwQAIKPSARWRkSISDGIRESDALIVLISRKSLASVYCWDECLQAI----A-GGKRILPVVIDPE-LSSGAGAALREAGWEELtaFQRLDMSDPDRFEEDTRHIVAFT(AQEHRWVSGHTRLGLQAHEWRESGRSDGFLLREDELR)}*

$2606=160(161):
>gi|974600508|ref|WP_059190338.1| {|0(116)|<Actinobacteria(B)>}hypothetical protein [Streptomyces antibioticus] >gi|973859483|gb|KUN29435.1| hypothetical protein AQJ23_01260 [Streptomyces antibioticus
{()MPGhVFISYSRRdRAYVAELKARL----RQEGIPFWVDDELDYGDRWAsTVQRQIDQAIVLLVVVTPAALDSEWV-DrELSYAQELGLP-VLPLLRRKVNSLR-LVNLQFEDVTDGRMPSPrfFERVHALVTSTSADATTRRAPYT(ASHPPRQRGTRRFVGPINRTP)}*

$2607=183(176):
>gi|1011909543|ref|WP_062724228.1| {|0(227)|<Actinobacteria(B)>}hypothetical protein [Streptomyces caeruleatus] >gi|973924932|gb|KUN93180.1| hypothetical protein AQJ67_38965 [Streptomyces caeruleatus]
{()MAITFISHSsaetEEdQANVDSVRGELR--KRLPDFgwEVRVDDQLPPGVDWRwVLHQWLEECDAAVVLVSRDWPKSPW-FKrEVEILLHRRAA-GaGLTVVPVVLRGY-TTGELRRSEISRLADPQciVQGDMAGEAVAARVAERLGELGA(CASTAAMRSMTVKIEHCLGEVNDRFPLRNMARALGS)}*

$2608=178(175):
>gi|974004429|dbj|BAU29587.1| {|0(166)|<Firmicutes(B)>}TIR domain protein [Aneurinibacillus soli]
{()MANIYISYSQKdNKVVDIMINRLKELGHTILTANYHlKLEIsLPRSIVYeDIRELVHKSEVAIVVFTENYIDSD--HRfESNTIISYAKN-ENKLIIPIVIGDV-LPPNSYFEINWARISSNriDELHECIKKIEHVISVFESKITE(IKEQEETQKEEKQKVVKRVIESQVLKDVLFGLRMR)}*

$2609=266(276):
>gi|974210966|gb|KUO52701.1| {|14(9)|<Proteobacteria(B)>}hypothetical protein APF82_02015 [Sphingomonadales bacterium BRH_c42]
{(IAEHLEGEEWTSLDLTLKQFGFPVSDQWSGDKNSYVIHMIEDGNDDELVELAQHYGINQAGADVASPEPETPYWADGHLR)---VFISHLTGeRELAAHIQTSLS---
RYGMSSFVAHNDIHPTLEWQtEIETALATCDLLVALIHPDFGASKWC-DqEVGYALGKG--------
IPVFAVRC-
GADPHGFvSRFQAFNGNGksAPQIAKELFEAAIDHKKLQQKMA(DIVIDLFVNSGSFATAKERMTYVERLKSWDESYSPRLKKAVKTNSQVSGSWGVPEQ)}*

$2610=136(140):
>gi|974237474|gb|KUO77093.1| {<Firmicutes(B)>}hypothetical protein APF81_09905 [Desulfosporosinus sp. BRH_c37]
{()MITIFLSHSSKdIKDFETILNNLKPLNDYCHIRCYrsaIEKNITM----
EKIIKSLPEVDLFVVFISDNSLSSANV-QkELSEAIRLSKL-GNLReICPIII------
DITANSDSRIPEYIKknIYYAASPIRAAQIIEENSRKLMC()}*

$2611=190(182):
>gi|1055608356|ref|WP_067249956.1|
{|0(788)|<Actinobacteria(B)>}hypothetical protein [Streptomyces sp. DSM 15324] >gi|973943444|gb|KUO11277.1| hypothetical protein AQJ58_14680 [Streptomyces sp. DSM 15
{(MRSDARHVED)-FDVFFCYSWKdksraDALVRLL---
TEQSLDGRPLRVFQDHRALEDYDTIseSVQEGLRRSRCLVVLYSEKTLKSYYCRGELRYALSaaqhlDGSTErvmPILHHTRYEDV---
RPQALASGRLPDTRTSSDLDLVGSVVARVRQTDARRFGDA(PAPREPDWYPSPLVGNRAFRGRDVELFEVHDA)}*

$2612=284(279):
>gi|974088108|ref|XP_015242175.1| {|266(13)|<Chordata(M)>}PREDICTED: TIR domain-containing adapter molecule 1-like [Cyprinodon variegatus]
{(QHTPAGRSQFGGENEIKTDEASMNTFQSTKSATYPYKANSGTKATQLASQSILPNISGLKSPSESKETKEEEEEEEEEI)-FYAFvILHAPEDsDVADSMKEKLEAA-
IGTTGATFSDEFaVPGKSTLRCVQDAVNNSAFTILLLTRNF-
NSQMLEMKTNIALINSIHnEHKFNTVIPLLpmdNCMPRRsIPIALQTLVPLEeKRNFEKKLQKVF-
NRANIERQRKIWT(EELKIRKLKQEKERLRLHMEQRLVLGASVQREQDGGVWWPQHSNIHIENANYVMIGNDS)}*

$2613=214(220):
>gi|979844339|ref|WP_059353805.1| {|321(0)|<Proteobacteria(B)>}DNA polymerase [Enterobacter cloacae complex sp. GN03842]
{(KWREDKLTSALLFVNKKGFKAANNEHAISIFRNHIYGRLSFIKMIRGDDFPLYLKLMAEMSHHDPLKTKEGLRAMKETET)-YDVFICHAS-ED-
KTSIAIPIYEELTKLNISTFIDHVEINWGDSLiqKINSALVKSKYVIAILSSNSVDKHWPKKELHSVLARE-----IAEGEVKLLTLVKEADEAIVAASLPLLSDKLYITYKDNPAEVADKVRALLNK()}*

$2614=200(201):
>gi|1056280320|ref|WP_067747853.1|
{|0(131)|<Proteobacteria(B)>}hypothetical protein [Novosphingobium sp. Fuku2-ISO-50] >gi|975155519|gb|KUR75192.1| hypothetical protein AQZ50_16360 [Novosphingobium s
{(M)--KVFISWSGErSKALAVALKEWIPLILQYA-
KPWvSEKDISAGERWAqAISGELESSNFGILCITPENISSEWILFEAGAL--SKSM-LDAKVVPLLfgLELS-DLSG-

PLSQFQALKVDQqgIMDVIKAINAVSEAKASEATINQ(LVPALWSQLQQKIDDIPGKAPAGKHMRPQGEILEE
LVSQVRGLGSRMRDLDPDFVDRDLK)}*

$2615=174(175):
>gi|979435299|ref|WP_059327187.1|
{|0(650)|<Proteobacteria(B)>}hypothetical protein [Escherichia coli]
>gi|975303700|gb|KUT18692.1| hypothetical protein AWE58_21540
[Escherichia coli] >gi|975644104|gb
{()MIKAFLSHSSKdKEHY--VRNVANWLGKENII--
YDEFTFEEGERTLdQIMEGLGESELFVLFISNSALESEWV-KkEITESKKLLDE-GKIlKIFPIIIDNS-
INHEDQRIPDWLRKDYNlqPITRARTAASRIKNHLYKISWQK(HPKLAKISSLFVGRNDKLEEFEERINDYTK
KKPTV)}*

$2616=212(220):
>gi|446852145|ref|WP_000929401.1|
{|175(0)|<Proteobacteria(B)>}hypothetical protein [Vibrio cholerae]
>gi|340045196|gb|EGR06143.1| hypothetical protein VCHE48_3972 [Vibrio
cholerae HE48]
{(KKYNTTKVIEFFQKLNDSERNNQVRRMAFEHLQKLGASVRLRKNFKGKVKSYNIETDRFIVSPEDLYQRLE
SNTIQNKKK)-FHVFISHSYKdaD-
LLRQVKSCLNNHDlSVYCDWTSDNDFLKRNLVSDytKMVlkKRIEQSRCVVFLQTKNSVntdnsfTSPWVSLE
IEHA--------------------LsIGKPIYSFNFTNDSDIFkQVKYFINNGIEIDDAEANKIRT-()}*

$2617=285(220):
>gi|340372497|ref|XP_003384780.1| {|44(429)|<Porifera(M)>}PREDICTED:
mRNA cap guanine-N7 methyltransferase-like [Amphimedon queenslandica]
{(KVQPFNLDSIDPSELSLPPLPPPPPPSLPPLKHTPEEGYTDKHSTSSSSSPPDDSLTIHNHLPSPAPTLDH
TYLPRHKSI)--HLFISHSTSdiQIVKESLIVVLRDSGRSVFACYH---
FMPDSTRynDHEIKDSMRSSCVVAIGLSKAYLHSDRCKREYDYALQ--LYNNKDiTNILVLAFGTCsR-----
-----PPVLKRFlYLDF-
MDATKrsSVWSKMEENIDKavavaneqyprterpkerespksggrspaasegsspeeimeyqgafedalvymk
ekgevtsddedkyghykmkD()}*

$2618=195(220):
>gi|492916790|ref|WP_006044937.1|
{|97(0)|<Bacteroidetes(B)>}hypothetical protein [Prevotella pallens]
>gi|339613409|gb|EGQ18164.1| hypothetical protein HMPREF9144_1247
[Prevotella pallens ATCC 700821
{(TILLDIMRNHIERRTILHLEGEKDLSSFIYEDLCKLKTSLDYSSHPIIIINKPKHAIGIKKDPNINIITFQ
ENNFMEKRT)-EKVHISYKHDtAYKADILA---IKTGLQENNIPY-SIDEYDILYRDniddY-
EKEIGSSDIVIMFVIPNYLTSLECMFEM----------------------TQIFKNGNIKTRLFPVVDM--
GEIKRNGDGLIKIKDYWSKEK()}*

$2619=225(230):
>gi|976514793|gb|KVE15016.1| {|6(0)|<Proteobacteria(B)>}hypothetical
protein WS66_10955 [Burkholderia sp. LA-2-3-30-S1-D2]
>gi|1061575053|gb|AOI98149.1| hypothetical protein WS66_21090
[Burkholderia s
{(GELTDDVVVDIPTLVDLNDDRWQLRQVLHTGNFVDPVAILLPPIQESELAHIPADVTFETVQILLNKEQEV
HDQVQEQAR)PKKIFLSHKS-ID--
KAIVRDVCLTLKEIGFDPWLDEDRMTAGANLerEIKHGMVESCAAVFFITPSFSDKDWIASEVdHAMSEKR--
---KKNSRFAIVPLLLTSPDGKRGNVLEIFEQYLHKDVEYHQIVREILRAIPIRM(TQSVEWKDGV)}*

$2620=210(201):

>gi|976141379|ref|WP_059300965.1| {<Actinobacteria(B)>}hypothetical protein [Streptomyces canus]
{(MKIISYEWSMGKRERERRRQREQRGPL)---
IFISHATAdsdKPLVEALCTFMKGAGLGSGDYFCSSQTPYWFDPGKndasQIYGLLDRAVVFLQLTTKVFLER
PNCLLEVGYAISRRRrdqadrrahrlQ-MLPVVVPPVDFE---PINMMKLGTAISLDDD--
TKNVKLFAAQLRKLLEQSGQP(IHDDSWTAAAGQFVSNDWPKALSDYRRQSDDKVA)}*

$2621=215(220):
>gi|977899068|emb|CUZ33987.1| {|335(0)|<Proteobacteria(B)>}Retron-type reverse transcriptase [Serratia marcescens]
{(GKDKVTAAKKYHKIRYGNDVQDVNYLVAHFRNHIYGRLSFIRMIRGSEWPLYLKLSAWMASNDDNPTSEGK
RAMRAMEMF)--DVFLCHAS-ED-
KDDIVMPIYDELCKLDVHAFVDTKYIRWGDSLteKINGALAKSKYVVAVLSSNSVEKEWPLKELHSvlareIA
EGD-----KKLLP---LVKEGDEELIKAKLPLLADKLYSVYKGNPIKIAQDLQVLLSDGE()}*

$2622=193(197):
>gi|1119291855|ref|WP_072270020.1|
{|0(630)|<Proteobacteria(B)>}hypothetical protein [Serratia marcescens]
>gi|977079587|emb|CUY40260.1| Uncharacterised protein [Serratia marcescens] >gi|977256321|emb
{(MK)---AFLSHNFSdknfvKTIYDNL---------TATHAVF-
DEKTFPSNSHLvdEIRSSMLDCDVFVLFLSKSALASDWVNGEINLAKELafvKSIKkimVFLLD-DTKWS---
ELPATFQQYRVESVPNPIQISIIIKNELYTLFNNKMDECY(GREDDVKRITSDLVTMDDKPKFIFLSGPDGIG
RKTLGREIYRKLYRFVSGVIELY)}*

$2623=174(177):
>gi|981539722|ref|WP_059743752.1|
{|0(81)|<Proteobacteria(B)>}hypothetical protein [Shewanella frigidimarina] >gi|979439876|gb|KVX03221.1| hypothetical protein AWJ07_01215 [Shewanella frigidimarina]
{(MR)---VFLSHSYEdNDIASKVRNYLA----
HHDLTVFDDKaDIATGASLNaSISQAISDSDVVLFFVSKNSEKSQWI-QqEMSLAVsNRLKG-
KDVKLIPIVLDRN-
AEIPFFLKDYLYLDMSKskDFDISMKKLVDGIKSNSKTSVKQ(DLEATVANIEIEKELLKIKSLEHEEFKKFK
TRQMF)}*

$2624=220(226):
>gi|980312339|gb|KWF01345.1| {|31(0)|<Proteobacteria(B)>}hypothetical protein WT55_30285 [Burkholderia pseudomultivorans]
{(ALAQNSVEWLRLDRGAWLFRSDKFKDFFPGYEAGSWLSGDFELEMDHDIRRVPVFDREEDRTYVKFVITPE
IVREKMERK)---IFLSHKG-VDK--PVIRRFKAALeVVG-
FEPWLDEEAMVAGAELerALLAGFKSSCAAVFFVTPNYADSGYLASEVNYAIQEKRR-
KGDRFSIVTLVLADSEGKVGEVPELLKSYVW-KSPATELEAFIEIVKALPLQLG(PTVWRK)}*

$2625=226(234):
>gi|980317943|emb|CUU60127.1| {|0(831)|<Actinobacteria(B)>}WD domain-containing protein, G-beta repeat-containing protein [Frankia sp. G2]
{(MTGVIGGQNGGTGTPATR)-RAVFISYAAADEPWALWVAWQLQQAGYQ-
PRLQAWHAVPGRNLVEWTVQELTAASCVVVILSPAYLR-SEWPRaELNSALADsvsgR-----
RLLVPVRVVPCES-PPLIREIARISLVD--RGEDDAHR--
VLIEGMRAAFGG(QATTPKISPAFPGDSGEVRGQPAPPFPGPDRDALTAQLLELAEEACRLRHPEATIRRGRA
RGLSGYLDVAGERDGE)}*

$2626=168(180):

>gi|980319858|emb|CUU59482.1| {<Actinobacteria(B)>}TIR domain-containing protein [Frankia sp. G2]
{(MTCSGVQPSTLRAWTLAPLSDSDCNTAGITQLGTVERAGA)SPDFFISYTGADEDWAKWVAWcwrTRTDANYS-VVIQKWDFQPGNNFIQKMDEGLQQAARTVVILSARYMKSSFATAeMRTAwqrdPLGRR-----RGLLVFRVEDCRrpGLPG----EVPLDEFG------------KPGNPGAACWPL()}*

$2627=222(225):
>gi|980329097|emb|CUU56273.1| {|0(1146)|<Actinobacteria(B)>}WD40 repeat [Frankia sp. G2]
{(MADGN)--RIFLSYAEADR---EWVAGYLTPAlGLR-AdqVLTADQFPPGEPVVTAFEQAVLSSRATVVVLTPAYLTDRWALLaEllasYQDIAAAD-----SAGPSARVVPVlrEpcDIPLRVGFRVRLDFTD--RGRWEAQA--ARLRAFLAQPEP(PAAEEIRCPYPGLAPFGRTHTRFFHGRDREISDLRRRVRSQPFTLVVGPSGSGKSSLVLAGLVPRLGADPDRWLVRTLRP)}*

$2628=223(225):
>gi|980324290|emb|CUU58166.1| {|69(0)|<Actinobacteria(B)>}TIR domain-containing protein [Frankia sp. G2]
{(SFHSTPEHAWFEVFQTLEAGVVAQPCRRLLAAVLVRYPANRVFLDLHDRHVAGRRADATGSRPVPAGASTPLPAPTPSPP)--TVFLSHDR--A-DQGKVQALANLLTDAGLTPWLGSEQVTGGSRRdlAVRRAVLDCDFFVPCLSATALARPSQLHrEIRIALDHADELpDhEVFIVPARLDEC-DPPSALQEYRWVDLFT--ADGFRQLTRaLRAPTPSARNRRS(GPSGR)}*

$2629=217(220):
>gi|980324291|emb|CUU58167.1| {|290(0)|<Actinobacteria(B)>}TIR domain-containing protein [Frankia sp. G2]
{(ELHTAWTNITTMSLDWLNRTYDLGVPPGTMASGERFLTEIADLTETAAVFRLPHEAAGRRPLFDGPLRRRAAAEGVGAPF)--TVVIAH-D--ESDERAASLVEGHLTLAGLHPWRANVDLLAGADRrlEMRRTIRQSGFVLVCLSTRSVDRAGDLHrQLRLALDVAEEMpEGRiFVIPVLLDeHC-ALPVRLDHLEPVRFYR--PDGPERLLRaL--GVGARSARVS()}*

$2630=267(258):
>gi|1055677832|ref|WP_067308623.1| {|0(120)|<Actinobacteria(B)>}hypothetical protein [Micromonospora rifamycinica] >gi|983631834|gb|KWV31983.1| hypothetical protein AWV63_14695 [Micromonospora rifamyc
{(MSWPDVVPAGVAGQETYFFLSYAHSVPLSDAARPDTDYW)--------------VGQFFHDLVEAVRGHprrAAEIeagFFDGQ-VSPGVDLrrMLTDALSLAHVFVPLYSPNYFRNAWALGERESFRSRLVRLsAARAqrhVLPVLWMPLPswedRPETVHALELVgdpddRADYADnglRALCKLSAYRAQYRSLLGALADR(IVTVAEHEPLAPSRAAALTSGPVPDGPGTALVVTTLAADRARWRPYGDQHQLAVADYVAATAERLGLPTRVVELAEARD)}*

$2631=180(177):
>gi|982922824|ref|WP_060398127.1| {|0(652)|<Firmicutes(B)>}hypothetical protein [Bacillus subtilis] >gi|982206191|gb|AMA51428.1| hypothetical protein AN935_03805 [Bacillus subtilis subsp. inaquosorum]
{()MKRVFLSHSSAdKKsYIEIVVEQLK--KELGIQRIVYDNLTfEEGMGNLeEIEKGLDKSDLFVIFLSNSSLESEWV-KkELDSAFDLYQS-GKlKRIYPIIIDHR-ITHLDSRIPSWLRKEYNlkPVNRPTVATRRILQRLREISWMD(NPRNKEKEKLFVGRNELIAQFEERLDSFEKQSPICVI)}*

$2632=255(257):

>gi|983633677|gb|KWV33773.1| {|229(224)|<Actinobacteria(B)>}hypothetical protein AWV63_05140 [Micromonospora rifamycinica]
>gi|1052677499|emb|SCG64877.1| TIR domain-containing protein [Micromonospora
{(VRLVCHRPDDAVRQLPRLAVSHALQASSAVNRHAIWWSEMDVLLPELGREIGRLQMPRLPFVGLPEARRYVWSLGRDQGP)---VFLSSAQGdNDFAQLIAQELD-LRNVEHFHYLFNSDLPRGKAWEhRLLAKVGECRIFVPLVSRHYLDSAWCRKEMEIALELSGR-GRMRILPYFLDHH-AGASVPVQGASVVHLDAaaRVRRVVDDVDRALTDLTDRRHRP(RIGITVAGGRTALDVDVVVLATGPTEYAAAARTLRHP)}*

$2633=256(260):
>gi|1019395992|emb|SAL78528.1| {|907(1)|<Proteobacteria(B)>}Archaeal ATPase [Caballeronia telluris]
{(CAQNVSALGERALALAGLAVCGDKAKMPLAVQAYKVAFERASSSCMRKRLIRRFNALVKADAGEIGLDLMDMIFKQESQK)---VFISYPG-EDAGNALVDKIEQDMP-LELHLIRDRNRMrvGDSIDRF-MQQIGKARLTIVVISDAYLRSEYCMTELLYMWEGSKRDCAGFMNRMVLLVVPPLEISSDENRRVYSQIWSDKHKDALTHARDNEDADVVATQ(KKLKDLAENTVSMLKCIANTLMADSTKTTIEAVISRLLDS)}*

$2634=271(276):
>gi|1019487491|emb|SAL04297.1| {|789(0)|<Proteobacteria(B)>}Archaeal ATPase [Burkholderia arationis]
{(DALAGKALALAGLAVSMPDYRMEVARRAYIAIADPEFGAGTLARCGRRLHLLGKFDSSNGIRRLLAELGDRFAPLPDSGK)--EVYVSYRH-EPASTALVDEIELRMPSQFIF-FRDSNELLFGDSIdaYMMR-IGRAEHIIVVLSLSYPRRITCMTELRYVWERRDKAMEKRIVPVVTDSLRLDKRKTRDEYG-AFWTS-EYENLKASIPEQRLKRGTMEST(ELENIKFIRDHITEMLLWLSDILAPAKRRHGEKTVDHVISMLKHRSRKAGRHERRK)}*

$2635=183(185):
>gi|1019488246|emb|SAL00737.1| {|0(1100)|<Proteobacteria(B)>}TPR domain-containing protein [Burkholderia arationis]
{(MEYA)RQSIFLSYRCGNRPW-AVAIFQHLERRGH--DVVFDFLDVGDGEFEgVALAHIKACTHFVVLLAPSALVQCRK-PDDRLRREIEAALdSGCHVVPLIAGGFEFASPAIGAQLTGKLALLADHDPLIVQADRFDEAMAHLNER(CLHFPPRDAPVVEANGFAHEAAMNEQAAARSAPAVVEDELA)}*

$2636=260(260):
>gi|984746394|gb|KWW24258.1| {|126(0)|<unknown(B)>}hypothetical protein F082_1877 [bacterium F082]
{(IFRFLWYPLGKNAEGKGKGFLLGNKHEYDNSLEEEILFKKNMSFMEFLERINYEKSEQMYLLLDAVLKDVKSQYSGKYSQ)THDFFISYASED-RELVCKIKDFLANMGF--DVWLDKERPKdlDGKYWtGLEQAIANSRYIIPIITKNYIQRFFG-KKRVVQNIISALEeEARRFIETLDRKYADEDKMLSRIIPIIRYGDTAKVWLRNKEVDKKVTIDNINNW(SEMGNPCFWIFNDINFKIYSETAPEFDTFHTFDWTIYKKK)}*

$2637=222(217):
>gi|1019487492|emb|SAL04302.1| {|0(270)|<Proteobacteria(B)>}TIR domain protein [Burkholderia arationis]
{(MPSVPG)-YDIFISYAHAddEvpqgagtpsgWVSS-LAYNLNVGPNvrKKKIYIDHR-LKPGDNFSAQLIDKVCACKLLIIILLSQNYIDSEWCGKElerFVASHTND-PEKPTDVFVVELAPY-EELVKVPDNIAQLRKRNI----HAK---FWDHPINAKSPI(LAGYPTPQGCSREIEQLYWSMLGELQRAVDERLREMGDGREESSRQPLVFPGTDAKPLSMAKPNLGTILLA)}*

$2638=215(223):
>gi|1019401269|emb|SAL19058.1| {|88(0)|<Proteobacteria(B)>}TIR domain protein [Caballeronia telluris]
{(QLQVKNVDPFSIHYRLFGDPSWRDLFSIHTNKRVFLYSEANLSQSNIEKLQAESNTIAHDLQFRSSEYASGRSQFEKPHA)----FISHDSRDKEsIARPIALNLQRKLCP-VWYDEFSLKLGENLRQSIEKGLKECKKCILILSPQFFANSGWGKkEFESIFTRE-----ILEETQLVLPIWcgVTKHQVYEYSPSLLNV--KGVDWNSLGEDEVCRQIYLAVT(SSP)}*

$2639=229(233):
>gi|1019512681|emb|SAL88751.1| {<Proteobacteria(B)>}translation initiation factor IF-2 [Caballeronia choica]
{(MASSNVAEFAAELQMPTNLLLEILQEAGVQKAGLADVVSETDKARLLHHLRRKHGSNDEGKRTTRYSTL)----FISYGGCDELIARQINESLKSRGIK-TWFFPDDSVPGEKLHRVMHVGVNSYDRVLLVCSRTSLGRSGVLNelervlEREAREGGG-----DILLPITIDDFVfsDWKPQNEDVATQVRSR--VICRIPDPGTDSIIFEKTMDRL(EAMSLYTTSLSRHDSEKARLLSMT)}*

$2640=242(245):
>gi|984668207|ref|WP_060771651.1| {|0(4)|<Proteobacteria(B)>}hypothetical protein [Methylobacterium sp. AMS5] >gi|984354649|gb|AMB47689.1| molecular chaperone Tir [Methylobacterium sp. AMS5]
{(MAARRMGGMSAKAPRTGATPEPPAPVR)---VFISYSHRdEAARGRLDVHMAQLKREGV-SVFFDGDMIAGTELDpRIRRELRLAGIFVALASPAYLHSRYCFEiEYGYALRKARR-GKVHLVVALVEEC-QWRHTRMARYKLLPKDGkpVSKWRPYG--EAFEDIVNGLRGV(VKAVRAEQLPPPAKPERKPKAAPAPAPNRAAAASPKAKRVPSSSAKGTAKGAAAATRSAKPKAPARKPAARTRRGRPV)}*

$2641=267(271):
>gi|984536341|dbj|BAU41321.1| {|10(733)|<Cyanobacteria(B)>}translocation protein TolB [Leptolyngbya sp. O-77]
{(QQSVVRWLLRHNVVSLSETAAHFQQTEAEMQAFLLPLLEKRIVRQLEQDGAIAYEVKLAHKSNRRMPKDIWKILDVSSET)-ANVFVSYSRRnKPFVQTLVQALQKRGR----EVWVDwDSIPSGADWWeEIKLGIEVADTMIFVLSPESAASEVC-GrELEHALQHQK-----RLLPVVCKDV-DpaLVHPELGKINWIFLRDtdNFEVGFRSLVAALDADLSYVRTH(TRLLVRATEWNARGKDRSLLLRGSEIQEARRWLAESDRKSPQALQVQKEYI)}*

$2642=234(232):
>gi|1019399954|emb|SAL40866.1| {|231(0)|<Proteobacteria(B)>}hypothetical protein AWB66_02102 [Caballeronia telluris]
{(ATRDRLRVAQRAYFSAGEATPSESTLPEFLRRFGRETEINLYAGTAAAFVAELHERWLERNPETSRVEASRPDADSRGAS)---IFISYGR--ENLNE-VERLHACISALGGDVWFDKVELEPGDYWerKILPQIQRDVRLFVPVISERTAQRlrtseGYVFqEWSEAFKRKRRMpGRKFIMPVVIDAD-YTGDLKRYQALYDVFPDLQEVQPGHAPGGKPDAALETSLV(EEIRAMRREDAR)}*

$2643=234(232):
>gi|1019493861|emb|SAK45332.1| {|231(0)|<Proteobacteria(B)>}hypothetical protein AWB81_00370 [Burkholderia arationis]
{(ATRDRLRVAQRAYFSAGEAAPSDSALPEFLRRFGRETEINIYAGTAAAFVAELLERWLERNPETSRTETSRPDADSRGGS)---IFISYGR--ENLNE-VERLHACISALGGDVWFDKVELEPGDYWerKILPQIQRDVRLFLPVISERTTNRvrtseGYVFqEWSEAFKRKRRMpGRKFIMPVVIDAD-YTGDLKRYQALYDVFPDLQEVQPGHAPGGKPDAALEMSLV(EEIRAMRREDAR)}*

$2644=225(224):
>gi|985431185|ref|XP_015387869.1| {|0(420)|<Streptophyta(V)>}PREDICTED: putative disease resistance protein At4g11170 [Citrus sinensis]
{(MASC)KYDVFLSFRG-EDTRDNFLSHLVVALQEKKIKTFIDEELTRGDQ-IspALVKAIEESMISVIIFSKNYASSKWCLDELVKILKCHKK-NIQLVIPIFYNVDPSVVRNQTGSFKDAFVKHDKqfnKKMPEKV-QKWRAVLTEASNL(SGWDSNTTRPDAKLVKEITADILEKLEKLSAAISDSDGLIGLESRVEQVKSLLAIGLPDVRLVGIWGMGGIGKTTIAGVV)}*

$2645=158(151):
>gi|985416869|ref|XP_015373891.1| {<Arthropoda(M)>}PREDICTED: sterile alpha and TIR motif-containing protein 1-like [Diuraphis noxia]
{(MSLYKENTDKP)-YDAFISYRQSTDSMLASLIKVYLDIEGH--NVFMVVKSSNDVKXRhNVLQTIKQTKNFIIVLTPGSLDKHTMnssasDIdaSGLIAEEVKTAKqSQCNIIPVLSSNFTwPPTALLPDDMKNLCVYNSIRWVHEYQEACVKKLMTFMNQL()}*

$2646=221(222):
>gi|985856286|ref|WP_060904950.1|
{|95(276)|<Actinobacteria(B)>}hypothetical protein [Streptomyces scabiei]
{(APSGPSSIDVAAMQATAREHDGPIALLVYAPGGADPFSGLIARRQRWGRVTSKTATVLPPPQRFEALGPLPEDAVRRDGA)---VFISYRHSDGSPQADSIENLLRAAGL--VVWRDRTDLRPGTTTdRLEQALtTGLSAAVPVVTPEIVHSDIVREreLPRLLQLDEDPAFSLCIANKIARAGSNTQCDYEAPDRLLRLAPSRTLADKKQSNMLQPSGESEIVR(DL)}*

$2647=268(269):
>gi|985411950|ref|XP_015371458.1| {|994(0)|<Arthropoda(M)>}PREDICTED: slit homolog 1 protein [Diuraphis noxia]
{(QNATECSSFFGSIVENRIVRDVLPTALTAVCIILALVLILYLVVVYREECRAWVYYKCGFRICHKTVPFEDDRM)FDAYVT-YSLKddGFVAQMLAPGLEQGNPRYRVGLHYRDFNVSSFVADTIVEAIESSKRTILVVSKNFVESEWCRFEFKSALHEGLKDKKGRLIVVALGEI--QPKDVDPELRVYMKNSIQVNWGDRMFWEKLKFAMPDVRK(CQSLMSRSRNGVNIYATPLRTSYSFGSQQPRTASVQSSKYYLSPAYVDSSQHLWA)}*

$2648=200(205):
>gi|985610095|ref|WP_060849590.1| {<Proteobacteria(B)>}hypothetical protein [Methylobacterium aquaticum]
{(M)----FVSYCHAnAVQQAKLQIHLAQLKRDEV-ETWFDGEMEAGDKLNtEISRKLRAADIFVALMSPEYIASRWC-QmEYKRAMGRRAR-GSMRGVVVVVRPC-AWKDTGASDLKVLPHDGrtVSDWRSMD--HAFANVAEGIKGA(VKAVRSTLPEAVPARLAKPARSAAAPGPKKRPAPVKGGRKASPKATPGARTTKGKRPARSRAAG)}*

$2649=221(221):
>gi|992355847|ref|WP_061058793.1|
{|0(626)|<Proteobacteria(B)>}hypothetical protein [Vibrio vulnificus]
>gi|991844438|gb|AMG12715.1| hypothetical protein AL549_15515 [Vibrio vulnificus]
{(MKL)--KLFYSYCQKnDTERAELEKHLNTLVSNNSIDEWYDGKIVAGENWKaQIDANLKEANIVVFLITAEWLASDAC-KqEWEDAKKWANC-QSVKVlIPVIARPC-SWmDYSDMSDYQALPNSGkaVLLWEHQE--DAWMDVYRGIKNV(ITALKKKSGLRPDFESELREIEFCSKGDGKIDIEDVFIFPNLKSYTNNAEKISLESDIKTLSTLLKHSHQFIIGDTQA)}*

$2650=238(220):
>gi|992223742|ref|XP_015463803.1| {|281(0)|<Chordata(M)>}PREDICTED: interleukin-1 receptor type 1-like isoform X1 [Astyanax mexicanus]
{(VLPEFFEVPFECTILSPRGMDMGVVWLKHEKPYTIQATLTILLALIAVVVVNIVLFVFFRTDIILCYRQLC
GKSRTGQTK)VYNAYVC--SChgntpgstaEDLALRILPDVLEQKHDLKLFIHGRDDAENVAdMAGVLSQS--
---KAVLLILPGSRS----ANqqEEESAiplnpgqetldvshcseLYSVIAQSSVPVIVVEDGEN-
ADYSLLPESIQSIIQRDrVLRWSPAVQPngRFWKHLRYH--IT()}*

$2651=147(148):
>gi|992010689|ref|WP_060994171.1|
{|0(175)|<Proteobacteria(B)>}hypothetical protein [Aliivibrio wodanis]
{()MSKIFISYSQQdREIREKLSDALLAEGHEVLgIET-----
LTVGSDLNkEISKLLYEADAVIALLTENSISSKNVLNeITISQAQVSAM-NNKLFLPLVIGSI-
EIPNFLRDTLLEMVPDLstEYLQKVILNINRAVAHNKAVKVE(RQRVKELK)}*

$2652=177(178):
>gi|992035828|ref|WP_061013522.1| {<Proteobacteria(B)>}hypothetical protein [Photobacterium leiognathi]
{(MRKY)--
KLFISYSSEdKETKENIIQYLKPLVKSNKIEIWHDGELRAGDLFDnVIKNKLLESDIVVFLLSQSFLNSDYCQ
NvEAELTFNKFDD-NDddaLRIIPIVVKNC-HWKLSEFQRFNM-PFKAnpISEMD--D--
NVYFKISEEIMSV(IDLLDSSRSYKLVKFEEKKTPFDDLTPDYKKYII)}*

$2653=171(175):
>gi|147771828|emb|CAN62508.1| {|0(201)|<Streptophyta(V)>}hypothetical protein VITISV_028499 [Vitis vinifera]
{(MGYRVRKLSTSYLGLP)-----LGFKG-
EDTRNSFTAHLYKQLCQKGINVFIDDKKLkvGQAITPALVTAIENSTASVGLLSEIYASSSWCLGELVKMSAG
EQRDRGFFQFSIRSIHQMWGIKGANLEKPWLNMKRIWRIGRWCGFGGMLLDKLPIYLW(LGFKELGPVVRFFK
KMSEF)}*

$2654=260(263):
>gi|147845221|emb|CAN81612.1| {|107(534)|<Streptophyta(V)>}hypothetical protein VITISV_003348 [Vitis vinifera]
{(LHVRGQIQSFGEALADHERNVGHEEGRRKIEKWRAALTMVAGITGWWLKNRLNQELLHVEKNLVWMDRQRV
TSSSIGPSE)-YEVFLSFWD-EDARHNFIDHLYVALHRKGIRTFRIDDL-RGGD-
IalGLLHAIEKSRLVLVILSDNYVXXSXCLDELVKIMKCRKE-
MGKIVFPVFYHVDPSHMINPLSNDVCMVGIYGFGGIGKTTIAKVLYNGIAAQFM(ITSFLANVREDFKSQGLL
PLQKKLLNDILPRRKNFISNVDEGI)}*

$2655=164(166):
>gi|147780471|emb|CAN66815.1| {|0(34)|<Streptophyta(V)>}hypothetical protein VITISV_034308 [Vitis vinifera]
{(MAAAAASFSQR)RYDVFLSFRG-EDTRNNFTAHLDKELRaQG-
IDSFIIEEKLERGKAIssTLVAAIENSKL-IMQLCFKYGKNESLLIQEIVKDILNKS-
LSTSIDDTKNPVGIGARIQEMEMQLCLESND-VQMVEIWGVGRTGKTTITKAIY(RKISSQFEACSFFEK)}*

$2656=199(203):
>gi|147838520|emb|CAN69856.1| {|0(336)|<Streptophyta(V)>}hypothetical protein VITISV_041231 [Vitis vinifera]
{(MDRRRASSTCTSTGSWD)-YEVFLSFKG-EDTRYNFTDHLYAALyQKG-IRTFRLDEI--
RGEEVasALFKAIEKSRCILVVLSKYFAHSGWFLDELVKIMEXRNQ-

NGKVILPVFYHVDPFDVRKGGRVLHIKGLKT-
QSHADETQDRRPKFLDETLKPE(ADYIEDITRVILMRFSHKLLHVDKNLIAMDYHLEEMEEIFPWMMDS)}*

$2657=241(248):
>gi|500342659|ref|WP_011928006.1| {|0(5)|<Actinobacteria(B)>}TIR domain-containing protein [Arthrobacter sp. Rue61a] >gi|403311694|gb|AFR34536.1| putative TIR domain protein [Arthrobacter sp. Rue61a]
{(MAIDRSYLRPRTRHPAKNNGHFANGTRLAVSGWSRIQRDRVSRHKVLLMTSLF)-----LSHSS---
DDKQLVRQVMSELTLMGAAPFFDEVGVAN--GAsipGWIESALAVTDKfvI-
FWSSNSAESDWVKRELEAAQW-AFKSEPERLLIVILDDTPrpP-MLAPKKYIDGREGVD-
AVAQELLDLVRPAKMLMAIQQT(LETWGIETTLIPGYGPIVGCPFCGADLRHIKASSEYIFDDEYVTVYCTNS
PCTWS)}*

$2658=167(172):
>gi|500432539|ref|WP_011930912.1|
{|0(2)|<Proteobacteria(B)>}hypothetical protein [Bradyrhizobium sp. BTAi1] >gi|146411270|gb|ABQ39723.1| hypothetical protein BBta_p0026 [Bradyrhizobium sp. BTAi1]
{(MFFQWEGGTMPV)--KVFISHKKEDTEQAAAIAAYLKSNGLF-
VYLDVIDSQlakTGPDLADYIRGQLDQCTQLLAVISAR-TQASWWVPwEIGVATEKE-----
RFLASFVSGGATvpEFLVKWPYLRTQADLD--
KYIAESRRAELLVESRLQKGQG(ATARAQGFRSFHTSLKASLG)}*

$2659=160(162):
>gi|493887062|ref|WP_006833179.1| {|0(382)|<Proteobacteria(B)>}adenylate cyclase [Erythrobacter sp. SD-21] >gi|148830822|gb|EDL49257.1| adenylate cyclase cya3 (Sinorhizobium meliloti) [Erythrobacter s
{()MADIFLSYSSKdKARLAPLVDELRARDRS----
VWWDRELVAGPAFAdRIAEELSKAECVLVAWSEHSVASHWCRDEAAVGLERNVL-VPFRIDNVLPPLG-
FRSSHTPDLAGWPNREGeiGQLLAGVAECLKVAIPVPHRAAK(TGATEKTLLILPFRQPAGEATD)}*

$2660=166(175):
>gi|344172880|emb|CCA85543.1| {<Proteobacteria(B)>}conserved hypothetical protein [Ralstonia syzygii R24]
{(MGGSSGGGLLGSDIRNLEDKVKQRLAEAKADTSRH)---
IFISFDHEDLTDVnllrgqAKSDKVDLQFDDY--SVKEPYDSTNADYIKrQIREKIDRCSVTVVYLT------
--DKSasSKWVNWEIEESIkCGKGVIGVYKGDT--PPAKLPSVFQQ-HGCKAVKWEH---
VGMMKAIEDASTTR()}*

$2661=169(177):
>gi|343392766|gb|EGV05328.1| {<Proteobacteria(B)>}TIR domain protein [Haemophilus pittmaniae HK 85]
{(MKAF)-----LSHSS---KQKTYVEQVAKILGKDNIA--YDAwtFEEG---GKtldE----
IYKgiEKSGifVFFISIDALNSDWVKRELLIAKKFVNEHESVYFKPIIIDDNIdyK-HEKIPNWMRTYNIKY-
IINPGRSAFLIKNELFSLSLEK(KPRLKKEEQLFIGRNEEKNVLKSFMEILNRALL)}*

$2662=272(258):
>gi|494361738|ref|WP_007195323.1|
{|0(625)|<Proteobacteria(B)>}Sulphatase-modifying factor protein [Thiocapsa marina] >gi|343798265|gb|EGV16224.1| Sulphatase-modifying factor protein [Thiocapsa marina
{(MPETDPITSTESAEDRTSDPAPTSETERPSDRASPRRSK)-
PLVVVSYAREdENLKTQIDLYLQSEVNMGRFDYWSDRSIDAGRNYQhEIERHLREADIAILLVSPYFFASSFV
TRhELPMLCRRAED-GSCVLIPVQARPM-DRESTgclYDGRIQYFSPNGviqwgarpTWAYADLN--

EqqrpyFLKDLVKTVRET(LERRRIADRHISSDDERRQEHSNSPERPSREGAGRSEPSGDDGRPGPSGMPPGG
SGPDGVLSAPSPQSNTRPRHRPSPY)}*

$2663=207(216):
>gi|494098437|ref|WP_007039235.1| {<Proteobacteria(B)>}TIR protein
[Thiorhodococcus drewsii] >gi|343922613|gb|EGV33312.1| TIR protein
[Thiorhodococcus drewsii AZ1]
{(MKKSKPLKAIDSIITLMILMAFFYAALVFLSGTNYADLIQGVIGILAALIGLIILSAFSDTRRRKKPRR)-
--VFILY-S--EEDLDFVSKLYKALKVTPYRILWDRKEIKVGDNVieTLEQ-MQNESDDFIFVISKHSVDS--
-EETKMSLETARNQnKR--ILPVLIDDS-
ELPEELQEIMFADFRESFDDGYFSLRDaLKTTNQQNKPVAP(PRRKAAS)}*

$2664=276(267):
>gi|152074159|gb|EDN71044.1|
{|276(249)|<Proteobacteria(B)>}Extracellular ligand-binding receptor
[Beggiatoa sp. PS]
{(SRSLFGLEYFFEKDGTKIERSHGYTVGKIELKRETGTLTFLPRRETRLQGGEKDVVPEYSIKLTNEHTIPK
EFQLLKPYH)--DIFVSYASAdnQalsgtekgWVN-TLITGLKNklgtklGRDNYSLWIDDES-
RGNGTVPLNIMTQLENSAILVVILSHHYLASSWCQEELST---FLAKNGRDRVFIVERNSV-
PRPKELNELVSYPFGTTdELDYQKLGDLARDLSQKVTELTQ(PAQIEKVVPASATISSQTTPKELQTTLFLAK
VSDDLEEQRDEVKNFL)}*

$2665=218(223):
>gi|501031802|ref|WP_012083879.1|
{|554(0)|<Proteobacteria(B)>}hypothetical protein [Sulfurovum sp. NBC37-
1] >gi|151425548|dbj|BAF73051.1| hypothetical protein SUN_2111
[Sulfurovum sp. NBC37-1]
{(LKEKTININALTLKYSKGTMDFCKYMIINENIQDVIDDLYINLNFDINKDALLYRHASINAHQRVSEKDKL
NIKTDNSEF)--DFFISHSS-VD-
KFPFVDEFVSELKDLGFNVFYDRMNIKEAENIvqEINEGFIRLKYGViAIISPNFIEGSWCNEELAigysLKI
EKE-----KILIPILLNISLKE---VKDTYPILRNIKMFDNYVSSKSIAIEITRYKHEIS(NNV)}*

$2666=190(191):
>gi|501093958|ref|WP_012144082.1| {|0(154)|<Proteobacteria(B)>}TIR
domain-containing protein [Shewanella sediminis]
>gi|157319254|gb|ABV38352.1| hypothetical protein Ssed_3748 [Shewanella
sediminis HA
{(MADDMGKKKQIDNSNASL)---
VFISHDARdAKLAEAFSGLLKSVSAGVLKSFRTSDRkgNQGIEYGVewypEIIKNIQNASDVVCLLTERSVDR
PWILFEAGMA--KGKL-ETPILGVALGISLTDISSGPFAQFQNCGDDE--
ESLTKLVFQLVDRIPNSEPDE(DTIKFQVGKFKEAIAKILKEDNNKLLDLPKVVD)}*

$2667=254(262):
>gi|501031804|ref|WP_012083881.1|
{|61(309)|<Proteobacteria(B)>}hypothetical protein [Sulfurovum sp. NBC37-
1] >gi|151425549|dbj|BAF73052.1| conserved hypothetical protein
[Sulfurovum sp. NBC37-1]
{(DLLIAKTYANSDIQLKLLHKWQENKRVVFVALTENYGQMKLWGDEINFIRAFQKEDLLNFLYTEITHDILR
FILRKDKLQ)---IFISHAKKdGRNIAKLIKTFIDNDIKLDNFF----DETDIQNSSswkkVLEKNVNDS-
LFLFVYSDNYGHTVWTQQEL--IWAKQKQ-IPIVGIDTLSKEDKRMFPYLGNVKMVKLLHS--
VTNIEHLCNETFSVKSNHNLR(LIINALLKEALEHYLFIDMYQNRQNCKNYKILSRPPELLDVC)}*

$2668=210(222):

>gi|501047042|ref|WP_012098814.1| {|0(268)|<unknown(X)>}hypothetical protein [Anaeromyxobacter sp. Fw109-5] >gi|152030408|gb|ABS28176.1| hypothetical protein Anae109_3998 [Anaeromyxobacter sp. Fw109-5
{(MV)--DVAILYSREDRDTVELLDRALSARGWS-VW---WDDRIDqGDFPAEIERTLSSAKAVVPVWSK-HSRDKVWVLdEARYASEAN-----VPLFPVRVDGCT-LPIGFGRLHTQDLrgwHG--EDGHIGLR--RLVRALEAKVRP(QNKPHPAEVTQLTVGKKLIRLPCFLRSVSSFETLLRPDASLKALALLRTPSVLISAYDLAHDRTASAMVRETRNLLRGGS)}*

$2669=171(179):
>gi|495528562|ref|WP_008253203.1| {|0(796)|<Proteobacteria(B)>}hypothetical protein [Limnobacter sp. MED105] >gi|149822781|gb|EDM82035.1| hypothetical protein LMED105_11730 [Limnobacter sp. MED105]
{(MNSTP)---IFISYAREdSEWVQAISESLA----DKGIQAWVDTKaLDYASLWQeEIEDAILRAQLIVVLVTPDFLDSLACRFEVERAERLRK-----RLLPVIDSRV-EEHRIPAALSAYQWFRFdrDTDHANTVSAWIERSIHLDPDWL(KRHTDLTSRAQQWVNNHKDPSLLLRGKDLASAQA)}*

$2670=226(218):
>gi|501031800|ref|WP_012083877.1| {|0(1826)|<Proteobacteria(B)>}hypothetical protein [Sulfurovum sp. NBC37-1] >gi|151425546|dbj|BAF73049.1| conserved hypothetical protein [Sulfurovum sp. NBC37-1]
{(M)--KIFLSYSSKdHVYKDEIKITLSVLKRENKIHLWvDDEKLKVGDIFDsIIKKEIIDSDIFLLLLSRNFWASDYIQEqELPLILKQSAE-KNAIIIPIILKDTGdLLNHDEVKNINAIPRDEkddkrllpIDTFESQE--KAYNSILSSIQNL(ITTSKNYIPYLELSRYSLKTLSPYNDLEKIELREIVHKNETTFEIEFIEHYESQKKVDKILIFSYSKKEKEFLIAKF)}*

$2671=224(227):
>gi|488720650|ref|WP_002644373.1| {|104(0)|<Planctomycetes(B)>}pentapeptide repeat family protein [Gimesia maris] >gi|148847439|gb|EDL61773.1| pentapeptide repeat family protein [Gimesia maris DSM 879
{(EVDFNGARSQNTLWVNVDLSRAVNLEMVIHSGPSTLGMDTLMSSKGHIPAEFLQGCGLSNELIEYIAGHFEQTALDFFSC)----FICY-S--PEDAAFAERLYSALQARGIRCWLDEKQTLHATGTSIQRGMKILD-KVLLCTSRYSLQSDWVNlEIERAFDnEDREFkRDTNVIhsllPLNLDQ--HLFEGWEHQQKETLCNRVAGDFQGNDSDPAVFDREVERLV(SVLQSNP)}*

$2672=189(186):
>gi|493472418|ref|WP_006427459.1| {|0(296)|<Firmicutes(B)>}hypothetical protein [Dorea longicatena] >gi|149751795|gb|EDM61726.1| SEFIR domain protein [Dorea longicatena DSM 13814]
{(MLIMEDDKTPK)---VFISYSW--SSD-ELVLDLAKRLVAHGVDVVLDKWDLKEGNDKyeFMERCVNDSEItKVLIICDKIYAQKandrtGGVGdETVIISSEIYGNaRQEKFIPIIAERD-EEGKEYVPIYIKTRIYIDLSDPAKYEEEYEKLLRNIYEKP(QFIKPKLGKRPEWLDEEKSDFFPIKDLIRQIRGSN)}*

$2673=257(242):
>gi|152071689|gb|EDN69089.1| {|209(99)|<Proteobacteria(B)>}NB-ARC domain [Beggiatoa sp. PS]

{(IEFARHYQDQNSDLKIIPIFLLQAKIDELVPVLLELKPLKFQTEFDEEALWQLERIIKGLPTDQCEIEELE
ISYADDKET)KHDIFVSYARvdneplpGadkgwvTTLINGLKNLlGQKLGRRDAYSLWMDYESRGNDPATpeI
INQ-LENSIILLVILSPAYLESAWCRLELNTFLANICEEsecLFIIERSEVEH---
PKPINHLMGYKFWIKNERGQSRTLAIPKPTSEEIEYYQKL(DDLACDLSDNIKTLRKKASENK)}*

$2674=202(220):
>gi|501031807|ref|WP_012083884.1|
{|66(0)|<Proteobacteria(B)>}hypothetical protein [Sulfurovum sp. NBC37-1]
>gi|151425552|dbj|BAF73055.1| hypothetical protein SUN_2115 [Sulfurovum sp. NBC37-1]
{(FKEIKHSLNEYAIKELTFNTTRSLMNQMKNRRFNILTTPCTKEGIEMAFHSEKIFLIDERTKLKDRGFEFF
NTNLYTDTV)----FFSYSNKDliDVFDKTINMLNA--
DAYPVFLDRKSLYVGNQLNSSLDGAIKDAKSIIFFIDKEFKTSEYCLKELELAKKY---------------
NKNMLmIVNNDTEIDSEYlFKKLDFNTLTSD-QLYKIIREYLIGK()}*

$2675=221(220):
>gi|1006218839|ref|WP_061745197.1| {|167(0)|<Firmicutes(B)>}molecular chaperone Tir [Streptococcus pneumoniae] >gi|995206663|emb|CYO51297.1| Uncharacterised protein [Streptococcus pneumoniae]
{(LSKYDSKEVHNFFYAINGHVRNQSLKKEVQSYFQSLSLPFRLSRKKKGKTNFIDNEIVTNRKGPEELKKRL
YIDDLEKIK)KFDIFLSHNS-QDEEKIITFY--
KNLNKNGQVVYIDwvndkYDLKRewcnTSTVDIIKERIKQSDVFVLYFSSKTLNSQWCAWELGyadgigkKIC
iYFSEK-IDRTIIPKFYSI------------YPELHLSENITIEVDKQSSIIFNDWKNRSS()}*

$2676=248(253):
>gi|156360785|ref|XP_001625205.1| {|329(87)|<Cnidaria(M)>}predicted protein [Nematostella vectensis] >gi|156212026|gb|EDO33105.1| predicted protein [Nematostella vectensis]
{(KAEIQVLTKELEQYKSKLHSFPPPPRDKSKEVWVPLPSPEQLRTNDTENLRFMQINYTITRMTFDPPKPLF
DKYGLPIEQ)KFDIMLSYnWG-IQ---
SFVREVYMELSQQTFQTWMDIwGGMQGNINEAMATAVENSTMLVAFLTEKYQQSVNCNLELKYATMKE---
KPIIFIKAEKDLVLKDWIQQLVDKSVVFEMSSSGDFKVEDKGVPKAKRLGEAAR(NIVQWAAKQPKLIRKDVS
EEVYKLQDMLEDARC)}*

$2677=153(168):
>gi|357499843|ref|XP_003620210.1| {<Streptophyta(V)>}disease resistance protein (TIR-NBS-LRR class) [Medicago truncatula]
>gi|355495225|gb|AES76428.1| disease resistance protein (TIR-NBS-LRR class) [M
{(MGNLLSWFPSSFT)-YDVCLVFCG-DDLHDSFPRNLLKALNERGIRTFIDKNQES------
TLKAIEESRTVIIIFAEDICGLPL---DFYSIDRYG---EHAIITPVLINTNPSQVAEAAYGFYDPYNPE-
EHPSPYDEYFEVDCYHRCCYTA(EKPGWYFKDGYLPDH)}*

$2678=181(179):
>gi|490429579|ref|WP_004301715.1|
{|0(258)|<Bacteroidetes(B)>}hypothetical protein [Bacteroides ovatus]
>gi|156107457|gb|EDO09202.1| hypothetical protein BACOVA_05060 [Bacteroides ovatus ATCC 8483] >g
{(MKYK)-YDVFISYSR-
RDyvdesynvIPGNAIAEIQNVFDENGITYWFDKDGIYSgqEFIEIITGAIAESKILIFISSKHSNESMWTAG
EIFEALD-----GEKAIIPVKIDNSQYNKKFKLLIRPLDYIDY-
LENPKNALKDLLRAINKVKEDI(AQKQREEEKLREEREAEAKKEKIKKEISVLAKDCQ)}*

$2679=229(232):

>gi|156390934|ref|XP_001635524.1| {|270(0)|<Cnidaria(M)>}predicted protein [Nematostella vectensis] >gi|156222619|gb|EDO43461.1| predicted protein [Nematostella vectensis]
{(EDNTMAGRMGSTSQTRSDKSLVIGLSLGVLLFIVLLVLSALVLRRRQQHHGVDFKTLLFADPKRFIDQTEL
LAPEETHHE)-FDVFISYSSQdrDLVKNVFYPEI---
TRTHKVCIDFKDFEAGLYITDNIANAIFKSRKTLLMITRNFLRRegGWTYFEMQIAQGRL-
QHGHDVLVPVMLENIPlNELPSTLQHYR---SRRtWLEW-FNEDVrpHFWERLRVAL--
E(PSLFEHQGEGAV)}*

$2680=215(224):
>gi|156400969|ref|XP_001639064.1| {|1288(0)|<Cnidaria(M)>}predicted protein [Nematostella vectensis] >gi|156226190|gb|EDO47001.1| predicted protein [Nematostella vectensis]
{(EADEKQLYEQSFLVLRRWHEINASAATLDNLGRALVKVGRGDLGRKHCGDALQGAEACAQDSTDSAKAPSP
VASPSSSPP)---VFLSYQW--DMQET-
ALELGRQLREAGIECWMDVGQMGGGDSLyaSIDAGVRGAKVMLCCVTSRYCASENCQCEATLGGLLK-----
KPIIPLLFEEIPWPPAGQLGPVFAKLLYIDMTQCSENIPSQKFQELVQRIEK(YCSK)}*

$2681=220(226):
>gi|156405972|ref|XP_001641005.1| {|25(0)|<Cnidaria(M)>}predicted protein [Nematostella vectensis] >gi|156228142|gb|EDO48942.1| predicted protein [Nematostella vectensis]
{(VEALIRLWEFRDNDATIKRLKSCLKSMEREDILQDLKPFEASHCLSIHQSQTPSTGGWDLKFLRSKLFFLT
TSDLPDDEM)-
YDAFVCYAPEdVEFVKQLLQKMEGPPYNLRLCVDYRDILPGASELHGIAHIIEERCRKiIVILSPNFNRSAMA
DFQAQIGLSLSPDCKQRRLIPVKIEHC--TIPIIYRFITHVDMTR--TEVVE---
FIWGNLARSLGER(ADIVAL)}*

$2682=247(250):
>gi|501631997|ref|WP_012606067.1| {|0(2)|<Proteobacteria(B)>}TIR protein [Methylobacterium extorquens] >gi|218525580|gb|ACK86164.1| TIR protein (plasmid) [Methylobacterium extorquens CM4]
{(MAVRRATSDVLARGSRKGARSDEAPAVAAPIR)---VFISYSHMdDAARRRLDIHMAQLKREGV-
SVFFDGDMIAGAELDpRIRRELRLADIFVALASPAYLHSRYCFEiEYGYALRKAKR-GKVHLVVALIEEC-
QWRHTRMARYKLLPKDGkpVSKWRPHG--
EAFEDIVNGLRGV(VKAVRADRATSSLDKRVRTARAAQPAKRPSAPEPPSRKRKTVPVGPSKAAASPSRPAKS
RTPARKPAGRPRQGGPGTR)}*

$2683=259(259):
>gi|156372884|ref|XP_001629265.1| {|293(315)|<Cnidaria(M)>}predicted protein [Nematostella vectensis] >gi|156216261|gb|EDO37202.1| predicted protein [Nematostella vectensis]
{(NMAMCISRKKHKEVLAPLYITTIPTLPTYMGLIQYIDCRPRDTEKLEKAMYAIKEWLSSNKVKQEVSPLEL
SQLSGDKHD)---VFISYSHCnDA---
EAMKVLQLLKEHNPsINVFIDTSgLNTGASWQqALYHALDNSRSVVALLSPDYVKSKVCKEEYSLALALHMdp
Q-RDFRLVSIVIEPV-
DQLPVWCCKPLAVDCSAedADGTSHLSVVVRSLSAPEEEIAD(LQYRLSLLKSSDVTIDAITTKYRQIQFVRR
QKLLPEPVP)}*

$2684=189(197):
>gi|992053035|gb|AMH40424.1| {|0(1222)|<Cyanobacteria(B)>}WD40 repeat-containing protein [Leptolyngbya sp. ISBN3-Nov-94-8]
{(MKRYSLNTSNTNTSCIMTHYQDA)----FISYGRAdsKAFATKLNDQL---VDQGL-
DVWFDfEDIPLGVDYQkQIDTGIDRADNFLFIISPHSVNSSYC-SlEIERAISR-----GKRIIPILHVEE-

ISREIWQQRNPQGADADweGYKAAGKHSSFLNMHAAIAKINW(IYYREGIDDLDQSFQGLLAILQKQKKYVHR
HTIL)}*

$2685=284(297):
>gi|156372884|ref|XP_001629265.1| {|139(444)|<Cnidaria(M)>}predicted protein [Nematostella vectensis] >gi|156216261|gb|EDO37202.1| predicted protein [Nematostella vectensis]
{(LTSGDQGYSEQLVLRQVMNAAIHWMKAGLPLRTLKVVIFARNPEKEDKSHKPLFELFKEYKDKYEDKNIDE
EIQDPADSK)--DIYLSHaSEDHNTAVEVRKYLESKG-DISIFDQVQELNANES-
WQDEIYTAMArCARVVTLLSPEYLSSVSCIEQYNMAMCISRKKHKEVLAPLYITTIPT-
LPTYMGLIQYIDCRP----RDT------
EKLEKAMYAI(KEWLSSNKVKQEVSPLELSQLSGDKHDVFISYSHCNDAEAMKVLQLLKEHNPSINVFIDTSG
LNTGASWQQALYHAL)}*

$2686=117(140):
>gi|156373773|ref|XP_001629485.1| {<Cnidaria(M)>}predicted protein [Nematostella vectensis] >gi|156216486|gb|EDO37422.1| predicted protein [Nematostella vectensis]
{()----FICYsSKDIKFAKELQERLENAG--
FKCCIDLRDFMPGEAIVVNIANAIYHSRKTLAILSPDFIESEYCKHELHQALNRSIRAHQ--VVAILYRECN-
-VPLELRHKTYLDYLN----CYVKP-HFWE---------()}*

$2687=257(262):
>gi|999973807|gb|KXJ12981.1| {|346(75)|<Cnidaria(M)>}hypothetical protein AC249_AIPGENE22024 [Exaiptasia pallida]
{(DKADIAALTKQIDSVKPKLDEWPPXQRQETKERSLPPHPDTLITQDKENLRFNYIKYEVTRMTFKPPKPLF
DDHGRPIEQ)KFDIMLSYQW-DI--
QNFVRDMYMELDMKTFKTWMDVwGGMQGNINEAMATAVENSTLMISFLTEKYQKSVNCNLELQYAKQQQ---
MPIIFIKVEXELELKDWIQDLAEKSLVYNLRSTSDFGVMDNGVPKINRIAEAAR(NLIKWSAXQPKILRNDVS
EEVYRLQNLLEDALCAQAEKENRS)}*

$2688=216(231):
>gi|999970546|gb|KXJ09720.1| {|403(0)|<Cnidaria(M)>}Hemicentin-1 [Exaiptasia pallida]
{(WNSSFVCLHLSGSEDLISKLHQSYSPPSKPVSTVWYTTLPIFLVCTVVCMTGFIIGLLVRRKKAKNSDYWV
SEDNXEGYQ)-YDVFITFSSKdfHWVKSELLPLIEKHNLNY--
CIHHRDFEPGKTIVENMADSVYKSRHILAVLSKNYMSSTFCRGELDIALGRNTQMKDSSLIAIRVDEIEkKCL
PKA-------LRSQmFLDFYNHEEKeaL-----------E(TQLTKELVKNI)}*

$2689=219(250):
>gi|999971401|gb|KXJ10575.1| {|247(0)|<Cnidaria(M)>}Toll-like receptor 6 [Exaiptasia pallida]
{(KQLQQGLKYEISXTTDIQYDKAEFQLXVKNLSSSDSGIYKLAVFTNIPPPAEAWIKLEVRQPDKKDVATNK
AGLKKEIPK)-FDVFISYSTSdfDWVRQQLIPLL--
AKHHIRYSIHSKDFEIGKTIVDNMADSVYMCKHIIAVLSDRYLESAFCHTELDMAICRSCIDKKPALTIINL-
--NCDLPKKYSKLSK---------------------------
Q(NVHFQSVQXGDDRWKEELMHYVKSCITEKV)}*

$2690=189(221):
>gi|999971417|gb|KXJ10591.1| {|322(0)|<Cnidaria(M)>}Toll-like receptor 2 [Exaiptasia pallida]
{(SNELITGNAQQVESRLTIKKLVAXDADSPRQVKDHFSSLEVSMLVVAICLLAGLATGVLLRYMKRKKSLRR
HSKGYGNYL)-YETFLTYSTKdyDWIKSKLIPLLEK--

YLVKYCIPHRDFEPGKALIENITDSIYQSRTVIAVISKNYMSSKKCQDELDVALYRVAERNENSLIVIRVDSI
E-SMSCRCHK------------------------------A(N)}*

$2691=256(260):
>gi|999973795|gb|KXJ12969.1| {|311(0)|<Cnidaria(M)>}Titin [Exaiptasia pallida]
{(DNGNYTCKVYVNDETVSDTILLTVAKKEPNKPDLPDDSKMLFKVVIPVAVSVFILVVVILVFYRCRSKADP
PKIPKGPFK)-YGAFITYSSQdyPWVKANLLTFL--
NENGIXTCIHYRNFEAGVLLEQNIVNCIYXSRVVIAVISKNYMKSKYCRSELDYALHRSIEEpGFNPLIALKI
DESVkR------KKLPLGIRRKtFLDATSDVEKDTWkQRLLMHLK-
G(KEDDQNQNVLNGGINHENNPGDDVHVPLMDPEQXRENCCV)}*

$2692=268(255):
>gi|998509265|ref|XP_015515051.1| {|235(0)|<Arthropoda(M)>}PREDICTED: single Ig IL-1-related receptor-like [Neodiprion lecontei]
{(KCEVSNSIDQLIVITASVLPEEVPIRGMQVGSWRKVVLLVALVMICTLSLGAFYIRCWLPVTLFFRDRFSP
LEENDGKDC)--DALVCYHEMdaSLAIGVIIPTLES-----RYC-----
YKCASLELSQITRNWsleigpksKTARRVIVIVSPAAVQgSTWNDATLRTVFCQlaSLHLPLNRVIVVALKDL
--PISLTAPKLAS--SRHvYeegagigrvktLRWCDKdettgfvK---
FWCRLRLCLPPI(RPGARKTQQSVALIVQMKSGAQTTTRSRESLEVLV)}*

$2693=276(280):
>gi|999969398|gb|KXJ08572.1| {|199(3)|<Cnidaria(M)>}Toll-like receptor 13 [Exaiptasia pallida]
{(QKRAQTPRPSSMYWLFQYGSNSTIFSSSTDPXNTKYDTSIIFSSKDEKFIVKKLLPFLEGEKHFKCCVHFR
DWEPDPMNT)KYDTSIIFSSKdeKFIVKKLLPFLE-
GEKHFKCCVHFRDWEPGEVVPTSMAKMVYNCKKVIAVISKHFLASNFCQEELNMALGRLAHQGDSSLIVINL-
DG-VAVEELPPAL----
QTRtFLDLTSSTEKKTWRTRLERQISA(ENSRIVYSDIFEDDNKRNSERLLEREETSSENTVGRLYISGGYSH
XNDCSTSSNSESSIX)}*

$2694=181(185):
>gi|999970220|gb|KXJ09394.1| {|0(145)|<Cnidaria(M)>}Toll-like receptor 2 type-1 [Exaiptasia pallida]
{(MEII)-YDVFLSFSNNdhEF-GQTLQSTLE--
EQGYKCCFHYRDFEPGQWIVQNITDAIYKSKVTLVVLSPSYLASRFCKQELRQALTAVAEQ--
RRVLPVIYKXY-
LPNVTSKQLNRDVVEGDnHVEIPCKIESNPPACVTWLFNGK(SIRKLIKSNFYVQNPIPHIDYSPXYSTLKLY
SIKPDLHRGQ)}*

$2695=214(220):
>gi|999966624|gb|KXJ05798.1| {|337(0)|<Cnidaria(M)>}hypothetical protein AC249_AIPGENE15127 [Exaiptasia pallida]
{(VGRALGLNEADLDEIEIDEDQLYERCYAVLRRWTESHGXSANYEALSSALTHPAVEMSDLAWKYCGSVEID
APGISSDDT)PPEVFISYQW--DIQSTVielASLL----
KENGFNCWIDIGQMGGGDSLyaELDKGIRSAKVVLSCVTDRYCKSENCQREATLADSIK-----
KPIIPLLLEPIPWPPAGQLGLIFAKLLYIDMTQGYQAKMDELVRKIKVHTSK()}*

$2696=212(220):
>gi|999972724|gb|KXJ11898.1| {|1333(0)|<Cnidaria(M)>}Tyrosine-protein kinase PR2 [Exaiptasia pallida]
{(VGRALGLHEADLDEIEIDQDQLYERCYAVLRRWTEIHGSSANYEALSSALTHPAVEMSDLAWKYCGSVGMD
NPEIISKEP)--EVFISYQW--DIQST-

ALELSSLLKENGFSCWVDIGQMGGGDSLyaELDKGIRSAKVVLSCVTDRYCKSENCQREATLADSIK-----
KPIIPLLLEPIPWPPAGQLGLIFAKLLYIDMTQGYQAKMDELVRKIKVHTSX()}*

$2697=217(220):
>gi|1011833859|ref|WP_062652788.1|
{|96(0)|<Bacteroidetes(B)>}hypothetical protein [Chryseobacterium
kwangjuense] >gi|998182341|gb|KXH81613.1| hypothetical protein
AU378_18150 [Chryseobacterium kwangj
{(GRRKEYEDLKSNALEAQEKTQHFNCVVQMVISLDEQLKILDSVKILLAILKTKKLYLLEESIINNTFVKKE
IILQPSILE)EYDVFISHAS---EDKNgFVKDFCNELKLENIKFWYDEYEIG--
WGESVLRKINQglEKSKfaIVVLSKSFINKKWTNAELEAVLNIETNTGDVRVLPLMLGDSnDikE-
ILSHYPLLSTKRY-----LKVSDGNDLIIQNLKKVLSK()}*

$2698=177(181):
>gi|998616174|dbj|BAU52107.1| {|0(353)|<Bacteroidetes(B)>}lipoprotein
NlpI [Mucilaginibacter gotjawali]
{(MADNLN)-PNIFLSYCWAnVSIADEIDN---DFKRIG-
IQFKRDVRDLTYRNSIkEFMQQVGKSDFVLMIISDEYLRSENCMYEVMELLNTHEF--EKRILPIIIDNA-
TRIFKPINQATYYEYWTdKKDAEKFNRKHGNEVSIEYFKKC(RNIEDNLPKFFQIITDFNASSFKRLKDGNY
RDLLN)}*

$2699=219(221):
>gi|998619489|dbj|BAU55422.1| {|0(102)|<Bacteroidetes(B)>}TIR domain
protein [Mucilaginibacter gotjawali]
{(MNNN)---IFISHAS--EDKDEVARPLAALLQADGFNVWYDEFQLKAGDSLiqSIDKGLNTCDFAITIL-
SQSFFSKKWTQrEL-VGLATRELYnDSKIILPIwHNLTL-
KDVLSYSAPLADKFALNSSIGLEALAKaIKSAIAPKNISVA(SSDLDKPIIYINGHNRYTIKDSIGHLATAKK
TNWFYTTQADTRVLRDGGMSGTGKIHNVRSNLGRVEYVKEGGTNAA)}*

$2700=236(241):
>gi|999981185|gb|KXJ20359.1| {|290(0)|<Cnidaria(M)>}Toll-like receptor 2
type-2 [Exaiptasia pallida]
{(TKTTTDPAGPLTVVVTATIVTLXXAVXIIVAILLYRKKHSRTPGIIQDGNHPMDPSADRLINVDVGIPLVG
NFDCNDKVV)-FDVFISYSSQdrDFVKNILFDEL---
STTHSVCIDFKDFEAGLYIGDNIWNSIFRSKKTLLVVSRNFLSGVWTHFEMQIAQGRL-
AQGQDVLIPVIIENIAfDELPKP---LQYYRQTKtYLEY-FNEDVrpHFWNRLRSTL--
G(PSLREYQDNSQQAEVDASNNV)}*

$2701=272(254):
>gi|999987004|gb|KXJ26129.1| {|371(142)|<Cnidaria(M)>}hypothetical
protein AC249_AIPGENE15838 [Exaiptasia pallida]
{(FQYAKKTARKPVIPVVVGSSFEWMMSVVGLLIAGELYIHFKDQNVLQSKMNELQKAIRKNIPHENNEIDGS
VPNVPKGKA)--DVFISYCW-
SNSNKALqanqvtklignpfadARXIKEDLIARNkdLAIWLDIERLETTNqsdetmSMfqQIATGINQAKVVI
MFVSEEYAKSDNCRAEFQFALTSlK-----
KPVIPVVVGSGDNWRSTVIGLLLSSQANVEILDMQQITTEQEYQNSLLKIEK(SMSRHLSHLTTKGQHVFSAP
MLGDHVVCHHQRWA)}*

$2702=169(177):
>gi|999988712|gb|KXJ27782.1| {|0(130)|<Cnidaria(M)>}hypothetical protein
AC249_AIPGENE9065 [Exaiptasia pallida]
{(MSE)-CHVMLSYQW-DS--QDLVDKVYHALKAQGLRAWMDVHGGVSG-
NIndSMAEGVDNAGVVCCFMTQKYSDSKNCKKELNYADAQD-----

VPIVPVMVENGFKAKGWLGVVTAGLLWINFRDPSNFSSSVESLAKEIMHAAE(NDITLSADETKEAEKQAPSE
CPTKVVEKKPGRAF)}*

$2703=176(181):
>gi|1000275021|gb|KXK37201.1| {|0(317)|<Bacteroidetes(B)>}TIR domain protein [Bacteroidetes bacterium OLB9]
{(MRHS)---IYISHAW-GGESDQIVSAIDKRFEKEGIEVILDKKNLGYRESItsFMQNLGR-
AEAIILVISNKYLHSEYCMFELLQIYENKDML--
DRIFPIVLDEVEISKSTDRIQLVKYWEEQVNTLENQIRQLSSINNIEGITDD(LNLYHRVRNEIAKLTTILSD
LNTLNVNRHRETDFSEL)}*

$2704=251(255):
>gi|999981668|gb|KXJ20842.1| {|53(61)|<Cnidaria(M)>}S-phase kinase-associated protein 2 [Exaiptasia pallida]
{(TNFKYSSSSNIWTLEVTKCNYLGISSKEEMASFDMTDELVNEQELRRLNEAVTREKLRDYLEDNILPIVIR
KIKYTRFEP)QFRFFLSHKSKdKPLMRTFQNGLKFLGYK------
TWLDEXNLPIMTdlkgGLKVSIDKCDCLIAWLNXEYFESDYCKAELLYARSPNHK-
QKKIILPFGKYSEIKDYLTGDLEFLKDRLIF--
DPSKASFFEILRRIDEALFND(GVQITAESLAAITQKLLLQHLSISRCYNIQPQAFR)}*

$2705=224(230):
>gi|1000287946|gb|KXK49422.1| {|48(0)|<Chloroflexi(B)>}hypothetical protein UZ13_03066 [Chloroflexi bacterium OLB13]
{(EIETFLEAEVKMHTWLLLIETENANNSRWVRFEIERAKHYGKALYAIEAQRFTEDTDKTRQEIETDEKFRS
CIHKLSKGF)--RIFLSYTRVDTPFASRLRDELRVGGFE-V-
YNPESMSLGTNWIQALEDAIREVTDTgifVTLISKTSLRNDFW-LkELTFAQENH-----
GLVLPVIIEPIDktAIPSHLQRIQWLDVSN--EDNPYKMIEQILSAITRIRHED(FVRTCGRIIT)}*

$2706=250(253):
>gi|999989354|gb|KXJ28392.1| {|92(51)|<Cnidaria(M)>}hypothetical protein AC249_AIPGENE23243 [Exaiptasia pallida]
{(PDHLSYRRVLCFEGGFPATSPPRVIGLPLRTLKIVAFSTYPDKENHAFQPLFDLFENYCVVEKEPEQESFK
EEDAGNSYN)--NIFISHCHTdNSTAIKLKDYLeeKGLRVSNNIQEVNNK-----
ESWQdGIYKTLXKCSWIVLLLSPDYLASESCREQYNMALLISSR-MXTKVIAIGEQSA-
NLPTYVGQIHFQFGDFRpnDETKLKETADTIYRQLLITHKTM(HRNDVQAAIEESQPPTTAFKNLALSNKVDI
PYS)}*

$2707=223(230):
>gi|999989430|gb|KXJ28468.1| {|266(0)|<Cnidaria(M)>}hypothetical protein AC249_AIPGENE23311 [Exaiptasia pallida]
{(CISRRKHKEVLAPFYVQSVEYLPTYMGLMQYVDCRPNDLEKLKGASDSVQLWLKTNNVKSVHQEQXAAVEK
PLTSSQEYE)---VFISYSHCnDDKARQLYKALKTFAPNLNVYIYTAG-
LKTGTSWQqSLFHALDDSKVVIALLSEDYFKSKVCKEEYSLAIXLHldSE-RDFKLVTVLIE---
DVEEVPVWCSKPHPVDC-TSLDQAE--HVISTVAKGIVNS(LKGEKSKKYL)}*

$2708=131(147):
>gi|1000248626|gb|KXK11786.1| {<Chloroflexi(B)>}TIR domain protein [Chloroflexi bacterium OLB14]
{(MTESKKS)--TVFISYSRKnKAFVRKLD---AALEEQG-
INAWVDwEGIPLSTDWMvEIAKGIVAADAFLFVISPDALDSEYCLKELELAISENKK-----IIPIV------
----YSEPEKRKKIHPrlASANWVFLRPKKRKIYRRYSQNY()}*

$2709=168(179):

>gi|1000250750|gb|KXK13840.1| {|0(362)|<Chloroflexi(B)>}serine/threonine protein kinase [Chloroflexi bacterium OLB15]
{(MR)---LFLSYAREeKTFCVQIAEALRV---HGV---WYDSRLVAGSDWWqEICRRLEWCEGFVYLLSPRSTASEYCLREMQMALDM-----GKMIFPAVIAPG-TIAPEEIRHIHHADLSNgmNISGVSDLLNAIYEAELEMLRRK(NAQQQQPPHARRNSGAYTNNHRDEPPPAAQPAPEPEE)}*

$2710=196(220):
>gi|1000255995|gb|KXK18876.1| {|278(0)|<Chloroflexi(B)>}hypothetical protein UZ15_CFX003002129 [Chloroflexi bacterium OLB15]
{(QFNPPSASIMLLEDWHRFDFKLRAATAPVNQAANGKMTFSVEGVIVADIPLSVFVSPQTQRAGSAASAGQLSAGVSPYRA)---VFCSYSHKdTAIVERVERAIKLLGYTYLRDA---TTLRSGEDWNaALMKMIDQADIFQLFWSSNSSQSKYVRQEWEYAL-KNKG-SKANFI----RPV-YWE-------------EPMPNVPDELGMLHFEFEPTLDDF()}*

$2711=249(257):
>gi|1000289265|gb|KXK50651.1| {|39(375)|<Chloroflexi(B)>}NACHT domain protein [Chloroflexi bacterium OLB13]
{(MTKVKWDMKGAVAFSAHFVDELVLFDDVVSVCAVLRHIATLVGEPTVTRIADLIREIEGGTPRPKPESPARPEKPTTTDS)-PHAFISYSRKdTAFVERLE---AALNKRGV-ATWRDaTSIPGGDDWYqSISEGLKRAYAVVCIVTVNADESKWVRReQL-------RA-DEDGIPPIAVHPT-KYRNPLHLQEVQPILMDedHFDAGLDAVHDALERCKREARPA(PRTDDAVQEFLRWVLTEAHADLRDSLYVDLAATPERT)}*

$2712=272(278):
>gi|999989430|gb|KXJ28468.1| {|107(110)|<Cnidaria(M)>}hypothetical protein AC249_AIPGENE23311 [Exaiptasia pallida]
{(TGDQGYPKKLVLRQILKAAVNWIKTGLPLRTLKIVLFSRNPQKEDKSFVPLFQLFEEYKAEVENKDLVEESEKEEGAIHL)-YDIYISHcDKDHNTALQVKEYLQEKD-ELKVFDEIQNLDADKS-WQDDIYDIMVkCTRVVTLLSPNYLSSESCIEQYNMALCISRRKHKEVLAPFYVQSVE-YLPTYMGLMQYVDCRP----NDLEKLKGASDSVQLWLKT(NNVKSVHQEQXAAVEKPLTSSQEYEVFISYSHCNDDKARQLYKALKTFAPNLNVYIYT)}*

$2713=202(220):
>gi|999989444|gb|KXJ28482.1| {|583(0)|<Cnidaria(M)>}LINE-1 retrotransposable element ORF2 protein [Exaiptasia pallida]
{(TGDQGYPKKLVLRQILKAAVNWIKTGLPLRTLKIVLFSXNPQKEDKSFVPLFQLFEEYKAEVENKDLVEESEKEEGAIHL)-YDIYISHcDKDHNTALQVKEYLQEKD-ELKVFDEIQNLDADKS-WQDDIYDIMVkCTRVVTLLSPNYLSSESCIEQYNMALCISRRKHKEVLAP-------------FYVQSVEYLPT----YMGFGIKKSSKFILIELYI()}*

$2714=225(226):
>gi|1124567931|ref|WP_074850683.1| {|95(286)|<Actinobacteria(B)>}hypothetical protein [Tsukamurella tyrosinosolvens] >gi|1001212666|gb|KXO99708.1| hypothetical protein AXK58_00325 [Tsukamurella tyrosi
{(IPSAPSPIDIAAIRAAAKGSDGPLAMLVYSPGATGEFRGLIARRTRFGRTATRTASVSVPPSRFKPLGPLPSGAVRGDGP)---VFISYRQSDGTPQAESLENLLRAAGL--VVWRDRIDLRPGTTTdRLEQALTAGlSAAVLLVTPDIVDSIIVREreLPRLLQLDRDPAFGLCIANAVARGNNSKCDYDAPDRLLRLAPARTLADKKQANMLEQAGQIEIVRD(LLMHRI)}*

$2715=198(198):

>gi|1001637157|ref|WP_061215017.1| {|0(600)|<Firmicutes(B)>}hypothetical protein [Syntrophomonas wolfei]
{(MEY)--QVFISYSRYNRAVADKIIEELESAGIR-CWMAPRDIAPSRQYAEEIVKALEECTVFVLVLSPR-SDSSWHVQrELDRAIGEK-----LVIIPFLIEDFTfskamnyyvcTCQYLYATIPPLDPHI--LKLRNLVCSILNEGTQMQVAVD(ASYLVAADIASPRELINHEIQSLLYDYFDRPNWEEQLRSSIKQYRKVFIYGAAGV)}*

$2716=205(201):
>gi|1001626337|ref|WP_061206052.1| {|0(1303)|<Spirochaetes(B)>}TIR domain protein [Leptospira santarosai]
{(MHPSKI)-ASLFICYSSDqIDWARKIGNKVNTA-YDGKISAWWDERKSTGDYWDsIIKQKLSEADSFLLILSPGFFKSEYIQNiELPIIIRRHES-EKIRIIPIIsaeINFE-QFNESWLNEIDVFPHRGlrTINQLLTHEIDNWIKDLSIEPVW(RRMEKEFKQISVRTLGRPFQYDLRLLNGSGYTIERNEIKEIEDWKINANDQKIAF)}*

$2717=265(277):
>gi|1002993193|ref|WP_061437680.1| {|1275(0)|<Bacteroidetes(B)>}hypothetical protein [Bacteroides intestinalis] >gi|1002419318|gb|KXT43667.1| hypothetical protein HMPREF2531_04231 [Bacteroides intesti
{(IAIKSKDALMEVTQYLRQLTQRGEMYKYLNRYIIGFEDKYYAAYSGISDINEAMRIADGGRLTKTEEEKAEEIPWREDEG)---IYISYNW-EDNSAHIVDYLGFVLENREIPFKLDKKNcPYTVNIKEFMKAIRAGKTVIVVLSRPYLKSKNCMYELSGIMENADY--KDRMLPVVVDDTIRD-----DDFYVELVGH-WKEKKDKQ-TEMVEKLRDIDPD(MAEPAEIKMKEIEQVYGLLKVIKEYIDWANADNLDALSSSQFKKIIDEIYKRKGNRA)}*

$2718=211(220):
>gi|1002730419|gb|AMN34795.1| {|132(0)|<Firmicutes(B)>}hypothetical protein JFP838_03185 [Clostridium perfringens]
{(CVDNEEDKVKKLLEEFNLTSRVDRGLEEVIRELKYKMWNIKGLPPYGKDELREAYRSMFFFSSNEELEKKGYKLEVELKR)-KQVFISYSHKdKNIVRTFADELRDTGINA------FIDARSIDYGEnilkSIMEGMEQSDLNIIFISNNYKESNYAKTELYNAFngiiQRNSN-WIIVKLDDVNPDDVIFGLGGYKYFEwENNSNE--LINvIKNK--LRK--------I()}*

$2719=237(238):
>gi|1004135123|gb|KXZ43192.1| {|0(552)|<Chlorophyta(V)>}hypothetical protein GPECTOR_98g776 [Gonium pectorale]
{(MGNAASKSFLCSNDRSADAAAPQPQATASQSSAIQARSLAPSQEAEEELDDVESQ)---IMLSYRIADtgakelgGDGTVMAMKAFLQSQGY--TVFVGESSLQGGqKWSqRIQDAVVNCKAFIALCSPT--------YgaTEWTFREFQLADnEKKPILPVWHSGPYPPPRLKIFLSGMQRVPQGNKPLVESDFGDSMQQDGWTPLH(IATSNGHMKVVRALLQAGANKEAIEEDGWTPLYIAAMNGHMEV)}*

$2720=212(220):
>gi|1004042816|ref|WP_061498043.1| {|57(0)|<Proteobacteria(B)>}hypothetical protein [Enterobacter sp. FY-07] >gi|1003371961|gb|AMO50751.1| Hypothetical protein AKI40_4376 [Enterobacter sp. FY-07]
{(QKDPVYFEQWLRDNYPEKIKRHNMFVKDILPYNVVTLLNAEMVDLFITANGGGHPFLWSERDVLEKEYPQYIDFKKREAK)---VFLSHSS-LD-KNNIVFPLHSYLQANDVATWLDTYEIDYGDNIvlKVNEGIDKADIGLFILTDNFFakRSGWPLAEftttfFMSLMNSD-----KKILMIDAGVSPENM------LPMMKSYRYLRWDNGSGLPEIANAVKRIMAK()}*

$2721=216(221):
>gi|1005564639|ref|WP_061660157.1| {|63(0)|<Firmicutes(B)>}hypothetical protein [Bacillus cereus] >gi|1003794671|gb|KXX93945.1| hypothetical protein AT266_07665 [Bacillus cereus]
{(DHIEVMKNLMLGEGLRYRPDITFHELQQEIGNRRFNSQTLVGECDVEKAMKSEFLFPFAEKSEIEKLGYII
DKPLGNQMP)---IFLSHSSKnKSEVEDLIPYINGAGLPV------
WFDKVNIDYGEsivkAIQKGIKQSTGVIFWITNDFINSNWCdleLNKFANRYASKRD-
VLTIAVVHQDVDIEKidFLFDDIKYLKRTNE-D--LETIAKE--IIPTLKRYMESI(K)}*

$2722=205(218):
>gi|1003224634|gb|KXV78999.1| {<Proteobacteria(B)>}hypothetical protein AD954_00765 [Acetobacter cerevisiae]
{(M)--KIFISWSgPRsKALAVALKEWL-PLILQYA-
KPWvSDKDITAGERWAqSIAGELEAANFGIICVTPENINSEWILFEAGAL--SKSM-
LDAKVIPLLfgldLRD----LSGPLSQFQALKVD--------Q--
DGMLNVIKAINNA(AEHKAAEATIEQLIPALWSQFQQKIDAIPLPSKSGNKVRPQAEVLEELVTQVRGLGARM
REVDTEIIERDIRRGFLT)}*

$2723=183(191):
>gi|1004718933|ref|WP_061557199.1|
{|0(317)|<Actinobacteria(B)>}hypothetical protein [Mycobacterium simiae]
>gi|1004315897|gb|AMP23221.1| hypothetical protein VC42_11325
[Mycobacterium simiae]
{(MA)---LFISYSSQdRAKVDALTNAL----
RRGQNQVWFDQELGGGDSWWaKILEQIRECEVFIVALSSNWLQSK-PSQaELRYAQAL-----
GRPILPVRVGDV-
DsMRVNPLAALQIIDYREptVDAGIQLVTAVHGLRSKPVPLPD(PLPEEPPVPFGYITRLGNTLAEKELSPQQ
QLALLVELRSGLDEDGDDPS)}*

$2724=217(218):
>gi|1177725156|ref|WP_082814666.1|
{|0(183)|<Proteobacteria(B)>}hypothetical protein [Collimonas fungivorans] >gi|1004065512|gb|AMO94538.1| hypothetical protein CFter6_1841 [Collimonas fungivorans]
{(MHDQDPP)--QVFLSYAK--PDQHRVTPFFES-
LEKSGFNVWYDCKRLKPGQNWdfEIKRALDKASIVIAFLSENSINRRGYAQrEIKIALDkLSEKLiDDIYLIP
VMLDDG-ILIPD--
QLQGIQCISaNASNCMIGVVEaLNLQLNKLGVETQ(KLQVKEELSWSFQTKKEICDGLPGYETELQFIRLQST
KYPALYEAGEYIQGRLLESLFSSRSSMLDQAPSQ)}*

$2725=220(224):
>gi|1003585358|emb|CUR52643.1| {|0(241)|<Thaumarchaeota(A)>}protein of unknown function [Candidatus Nitrosotalea devanaterra]
{(MSSDFE)-YDIFLCHnSLDKPWVQKLAESIENEDYNnrkLKVFYDDWDIIPGENNVLK-
IEYAMKkSRFVGVTLSENSINAEWPNMEWTMAVYNDPSGRRGFVIPIWLG--NCEIPSSLKIRTVLYCRN---
-NIEYN-
KSYTKLLALLRNQ(KLPRGQMRPSTLNSLPTEMLFPIEYADEVEEQLASNLLPVLKIPRHIWSGPVASLTYRE
VFDHLNRKVKGAHPTFMIK)}*

$2726=242(247):
>gi|1005451088|ref|XP_015762744.1| {|641(0)|<Cnidaria(M)>}PREDICTED: hemicentin-1-like isoform X2 [Acropora digitifera]

{(GRSVIHLFQNLTLMKQITDKKDWLGTNENSPDTLSLIAAGSAAVGFAMGVIFVAYKMCQSRGYTWKSFFDV
PVDSLSCQF)QYDVFITFSSQdsNWVKKELIPLVEKHNL--
NYCIYDRDFEVGKPVVDNAAHSVYTSRKVLAVMSHNYMSSEFCRGELEMALYRCAKMGDPCVIVIRIDSIDlR
KMPKA-------LRNRtFLDYYDLIERKTWKERLIKYLKP(PPFVETVISSQKIHGFTENNFQNYASV)}*

$2727=209(227):
>gi|1005433436|ref|XP_015754266.1| {|598(0)|<Cnidaria(M)>}PREDICTED:
uncharacterized protein LOC107333916 [Acropora digitifera]
{(GDNTTSEAAEATTVSTTDALDVLQSVQDKPTCSLMIALIICAVCGGVFALVLRVAWHVKKKRMLKNIKSYL
EEGRQKENL)----FISYSSKdfSWVFENLISPLKKHSIPFSI-----------
HSIENTSDIVHNSRQVLIVLSNNYLASNFCREELPMALRRRADAGDSSLIVVTIYRLTeKQLPCA-------
LSEKnLLDFEKHQKKQDWeEKLIKALQ-V(SEETANV)}*

$2728=218(228):
>gi|1005433458|ref|XP_015754278.1| {|379(0)|<Cnidaria(M)>}PREDICTED:
uncharacterized protein LOC107333928 [Acropora digitifera]
{(TEATTVSTTEATTVSTTDAPDVLQSVQGKPTRSLMIALIICAVCGGVFVLVLRVAWHVKKKRMLKNIKSYL
EDGMQNEKL)----FISYSSKdfGWVTDNLISPLEKHAIPYS--
IHSRDFELGRPIVQNMADSVYNSRQVLIVLSNNYLASNFCREELHMALQRKADTGDSSLILVTIDKLKkKQLP
-------RALTEKnLLDFEKHQKKQDWEkKLVNAL--H(VTENTTYV)}*

$2729=210(226):
>gi|1005447261|ref|XP_015760917.1| {|227(0)|<Cnidaria(M)>}PREDICTED:
toll-like receptor 6 [Acropora digitifera]
{(SYFEPGLGKEKKEITATTVQQQVTSPSGSQVERRQWLVPAVCAAVLFVILAVVFALKKRIFLRKRPFQGKS
REDVLNNTL)----FISYSSKdfSWVTENLISPLEKHSIPYS--
IHNRDFELGRPIVQNMADSVYNSRQVLIVLSNNYLASNFCREELQMALQRGVDTGYSAVTLVTIEKLKkSQLP
SA-------LRDKnILDFEKHQKKQDWEK-------T(LLGVVL)}*

$2730=261(284):
>gi|1005449784|ref|XP_015762099.1| {|324(0)|<Cnidaria(M)>}PREDICTED:
hemicentin-2-like [Acropora digitifera]
{(VYICGVDSTLGQANDKITLTVKDKERNGIWIILCVSVAIFVVVLIVALYCYLISKHRRRQRKMAMVAEKYT
RDGSGEHFH)-HDVFVSYSSKdyDWIQENLQPVFDQNNINY--
IIHSRDFIPGKAFFDNMADSVYSSRKVILVMSKNYLASGFCKDEMHMALVRaARNDEAHSLIVVKIDDVKpQN
IPKS-------LRHKtFIDY------------------
A(SREESTTWKTRIVEFVLSGNRSLSVSAVAGEGETSDTISLFLSKFRSKRKKKEDVWRELPYQSC)}*

$2731=226(240):
>gi|1005465293|ref|XP_015769649.1| {<Cnidaria(M)>}PREDICTED: toll-like
receptor 2 [Acropora digitifera]
{(MYPFGPTEVAPASHRSIQGLHHYNVAFVPISIGIVFGVILVGCLMWYFSHRKKEYPPLPYDFR)-
YHAFIIYSHQdsRWMRKQLLPLLEEKH-
HLTCCIHYKDFIPGKPIRDNMTDSVYTSYKVIALFSQNFINSSCCMYELDVAVNRLVQKRDNSLAIIRIDGAD
wKRLPAE-------LRGRcFIDY-HYPQErPFW---------
G(KRLLKFLDLPTEIRRQSTSEEHITDNNNDESNCNDEK)}*

$2732=257(271):
>gi|1005477610|ref|XP_015775571.1| {|531(0)|<Cnidaria(M)>}PREDICTED:
uncharacterized protein LOC107353721 isoform X1 [Acropora digitifera]
{(SNGWEYPDELSLNVTVFPSRNTVKNMTNFYPPGSSLSDKTTKFVTIVLPIVAGVVAASVLLFLYRRWRQRE
HKNVLKTFK)-YHAFLIHNTSdtEDVKTIL-SVLES--
YGLKCCIHWRDFIPGKPYVENIVDSVQNSFKVIAILSRSSFSSKCLSFEIQQTLKRLVDNGDDCLILIALDRD

VvSNLPQA-------IRDRsYIDFT--ERAyrSNWEK-------
R(VVEILSKGVVGEDDSLSDTASSSESHYNGDSQRPISEGSSTGSEGSALLYL)}*

$2733=147(162):
>gi|1005490284|ref|XP_015747855.1| {<Cnidaria(M)>}PREDICTED: toll-like receptor 2 [Acropora digitifera]
{(MGDVVENEK)---VFISYSSKdfSWVTENLISPLERHSIPYS--
IHNRDFELGRPIVQNMADSVFNSRQVLIVLSNNYLASNFCREELKMALQRGVDKRDSSVTLVTIDKLKkTHLP
SE-------LRKKnLLDF--EKHQrrQDWEK-------K(LIKTVNWSELVSA)}*

$2734=212(223):
>gi|1005438518|ref|XP_015756660.1| {|57(0)|<Cnidaria(M)>}PREDICTED: uncharacterized protein LOC107336116 [Acropora digitifera]
{(HSASDAWTLEVVKCNYLDISGKEKMDSFDMEDKLVNKQELYQLNECQGSQEELRKYLERNILPLVMKKIKY
TKFLPQFRF)----FLSHKS-KD--KPLMRTFRNGLKFLGYQTWLDEEDMPLG-
RLdgALKVAVEKCDCFVAWLNQEYFESDYCKAELLHAQRNE-----
KIILPFGVHHEIREHLTGDFEFLVHLNIYDTTTSSFFEVLRRIDDSLFNFES(LTK)}*

$2735=164(172):
>gi|1005473585|ref|XP_015773614.1| {<Cnidaria(M)>}PREDICTED: protein tirA-like [Acropora digitifera]
{(MKGEEVKKKGIEDGKMNIHVEPVVRA)--DVFISYQW--DHQDTV-
KLLKSKLEEAGFHCWMDISRMGGGDALytEIDAGIRAAKVIISCVTQRFCQSEMCQRESTLADSLR-----
KPIIPVLLELVKWPPPGQLALIFAKLLYIDMSLNPGTFPAEKLQELFQKVRG(HVESGH)}*

$2736=149(157):
>gi|1005473587|ref|XP_015773615.1| {<Cnidaria(M)>}PREDICTED: protein tirA-like [Acropora digitifera]
{(MNIHVEPVVRA)--DVFISYQW--DHQDTV-
KLLKSKLEEAGFHCWMDISRVGGGDALytEIDAGIRAAKVIICCVTQRFCQSEPCQRESTLADSLK-----
KPIIPVLLELVKWPPPGQLALIFAKLLYIDMSLNPGTFPAAKLQELFQKVRG(HVESGH)}*

$2737=220(225):
>gi|1005494614|ref|XP_015749936.1| {|157(0)|<Cnidaria(M)>}PREDICTED: uncharacterized protein LOC107329778 [Acropora digitifera]
{(CCRSILLQWIKRTGTTQAVYSQLAQALLHQALKKRKIVQKYCVMHQEEQSASSVEQVPLPGENPESILMDT
DGGIGVVPA)---VFISYnwgKG-KENQKTV-
KHLCSKLTEAGFHCWLDIEQMGGGDNWnaKINEGIRAAKVIICCLTEEYCKSDSCLREATLADQLK-----
KPIIPVLFEPIEWPPQGQLSVILAEKLYIKMLHRDNTFPEEKLQELFKQVTS(KLRLE)}*

$2738=220(224):
>gi|1005437055|ref|XP_015755967.1| {|159(0)|<Cnidaria(M)>}PREDICTED: myeloid differentiation primary response protein MyD88-like [Acropora digitifera]
{(LPGSGCSQLELSAKAIEQKCRKVLVIMSENLTNYDVTDFQAKVAFSLSCRGHLPACPKLLTFLDFVLADKQ
GPPETLCEE)-
YDVFICYAPPdKPFADEVLRKLEQWPYRFKVCIDYRDFLPGSgcSQLELSAKAIEQKCRKvLVIMSENLTNYD
VTDFQAKVAFSLSPAAKTRILIPIKYKPC--TVPPWLRHLFYLDYTT--EEARL---
HFWKKLLISLGYE(DSSS)}*

$2739=263(277):
>gi|1005437055|ref|XP_015755967.1| {|41(75)|<Cnidaria(M)>}PREDICTED: myeloid differentiation primary response protein MyD88-like [Acropora digitifera]

{(QKIVYLESFTSTDRGPVFHLIKDYESNEKTVSELISLLEEMERRDLIEDLQGPIEESKKRKARQSSAADKQGPPETLCEE)-
YDVFICYAPPdKPFADEVLRKLEQWPYRFKVCIDYRDFLPgsGCSQLELSAKAIEQKCRKvLVIMSENLTNYDVTDFQAKVAFSLS---------------CRGHLPACPKLLTFLDFVL--
ADKQGPPETLCEEYDVFICYA(PPDKPFADEVLRKLEQWPYRFKVCIDYRDFLPGSGCSQLELSAKAIEQKCRKVLVIM)}*

$2740=229(230):
>gi|1005437141|ref|XP_015756012.1| {|109(0)|<Cnidaria(M)>}PREDICTED: myeloid differentiation primary response protein MyD88-like isoform X3 [Acropora digitifera]
{(LGFNRQFIDWLGSTNEPVVRLLTHIESIKITEVISHLEKMGRQDAAEDLHQFEELTHSPGELEEHRRRIGDTEDRPTTHK)RYDAFVCFAAQdKQFLKKLVTTMEAQPYNLRLCYTERDFLGGGCYLENAALVIEQMCKKfVVLLSKNYDGSDGAVYESQIAITLEPGAQQQRIIPILLEDEyRKTIPKPMAHLTYLDYPRW-QENIP---
KFWEKLAVSLGWN(QSQTSCRTGE)}*

$2741=246(236):
>gi|1020049448|ref|WP_063197968.1|
{|0(145)|<Proteobacteria(B)>}hypothetical protein [Bradyrhizobium sp. AT1] >gi|1005523607|gb|KYG22373.1| hypothetical protein SE92_20900 [Bradyrhizobium sp. AT1]
{(MDENKSFNGFLSYCRH)---DAETDP---
GVVEAFATDLERRINAKllntRFDIWRDINNIRVGEKWnqQIESALSCADVFIVLVSPRWIESTFCRREYQFFEERVEsKIeVGEYVAPILirsIERQKdfltAEQLEVYDGLFERQFFKaDAVAFMQLSPAERAAKIDLLADD(IVGMLQRLRDPSVTAGHSATSARRKRRSEFEGRSESYAEVDFLRSYEVLIDPARGDAARGVYAQVDFVERLYVKGRKALI)}*

$2742=148(167):
>gi|1005520803|gb|KYG19569.1| {<Proteobacteria(B)>}hypothetical protein SE92_04255 [Bradyrhizobium sp. AT1]
{(M)--------------WADWIGWTLLQERHD-
VFVHEWEIGAGENIADWMSRRLKDTEKVLGLFSPAYIEAiyskSEYLAaYWADPVGRD-----
GFLVPIVVEPCADLPKLVQPLRRLHLYG--LPEHMAAA--
ELVKFLTPPTAP(IIKPPFPDASTSTGASSPGEGEQYNG)}*

$2743=209(220):
>gi|1005525317|gb|KYG24083.1| {|42(0)|<Proteobacteria(B)>}hypothetical protein SE92_30685 [Bradyrhizobium sp. AT1]
{(PTFSPPEVRVTDRANNFRLESSGWGEFRIQADVRMTNGQNLQLSHWLRLGRSVPSKEEMAAAGTEERDAPMAIEPGERQP)--KVFLSYTGSDARLAGAVAKELKRHHVD-VAIDV-
DIPSGVNLRDWVSERIAQSDATVFLLPNAasgALSNSSIGYELQLAQKSD-----RPIIPIL---
RSgaDVPDQLKSFKFITVEG----GPSAEIDVQAIARSISNVVR()}*

$2744=166(189):
>gi|1011950005|ref|WP_062757720.1|
{|0(239)|<Actinobacteria(B)>}hypothetical protein [Streptomyces sp. WAC04657] >gi|1005796106|gb|KYG52317.1| hypothetical protein AWI43_23985 [Streptomyces sp. WAC046
{(MNVLRKVVEE)----------------------LEAKGHR-
PLVDQSDIPPGEEWRAEVVDWLARCDAAVVLLNEKALK-STWVRrEvnilmWRRALGAP-----
LLVIPVLLEGLGtrAVKDAGLEELGPVQFA--
RTPAGAEHDALEVAGKVLRRFS(ELPSAAVGDDPMRGWLARIGLYLKEARTDPRLLAEAAKA)}*

$2745=189(196):

>gi|1004826775|gb|KYC51865.1| {|0(301)|<Euryarchaeota(A)>}lipoprotein NlpI [Arc I group archaeon U1lsi0528_Bin055]
{(MYLCFYKGEYVVNNSPSTT)----FLSYSHKnRDIAEEIDNFcLSI----
GKILTKDDRDIRYKDSISgYMKK-IRQSDFAILLISDDFLKSINCMYEVYELI-KEED-YKDKILPILIEPI-
NIFTYEGILNYINYWDEkeKECRNKLNEKDPLKTIKLREDHK(IIDNIGHEIGDFLEILKGIKCKTYEEIKKS
GYIDIIN)}*

$2746=228(230):
>gi|1005181517|gb|KYF59210.1| {|0(696)|<Proteobacteria(B)>}ATP/GTP-binding protein [Sorangium cellulosum]
{(MVALATSPAD)---
VFFSYSPRdEDLRPKLETHLDALKRQGVIRTYQGFALGGGRERReAIAARLREARVVLLLVSADFLSADAWLT
pEIREAIARHDA-GECRVIPVILRPC-AWEKTLLGRLQPLPNRArpVTSYPNQS--
EAFTEIAKGIRSA(VRETEPPTQRPAARRPSSSPDAEPHIKKLPPRNPSFTGRDRLLDELRSALTTGGPAALT
QAISGLGGIGKTQLAAEYAHR)}*

$2747=226(223):
>gi|1005336257|ref|WP_061620025.1|
{|0(227)|<Proteobacteria(B)>}hypothetical protein [Sorangium cellulosum]
>gi|1005188270|gb|KYF63643.1| hypothetical protein BE11_35445 [Sorangium cellulosum]
{(MNER)-
PVVFIASSEKdACWRERLISQLQIHVHQGTLEIWDERRVMPGDELRqRIREAMDAASVLVLLITADFLCSWLF
QEeILVRLAERRHG-EPPLVFPLLVRPC-DWTSIrALAGLACYPSDNrfISSGtpPQID--
ADLARVASSVRER(VAPAARGALGGRPPLQGARPLEIPKEHTSTRTDDPWRPRHTSELEDGSACVAIVSETER
GSEHDPLPSCYLLQQTASIG)}*

$2748=224(220):
>gi|1005203283|gb|KYF75481.1| {|729(0)|<Proteobacteria(B)>}hypothetical protein BE17_33675 [Sorangium cellulosum]
{(LLMLKDTAPALATPLRRFEEEQWMQTLQASYPELEMFLFYDPSMHRITIDDPQLAFYLRSVHLEEIAREAG
VLKQQGGSR)---
IFISYVLSdLRWLERLQVHLGPLTKRGlVLDIWEESRLTRHAGAYsELRQVIEAAAMSIVLVSADYLVSEFSG
NpDrggIDVLLGVAAE-NGASVRPVVLKPC-
LLTGSGLDRFPVLNPDAalSEMGESAVESTLVRLAEEIARLL()}*

$2749=228(230):
>gi|1005333161|ref|WP_061616929.1| {|0(728)|<Proteobacteria(B)>}ATP/GTP-binding protein [Sorangium cellulosum] >gi|1005203467|gb|KYF75626.1| ATP/GTP-binding protein [Sorangium cellulosum]
{(MVALATSPAD)---
VFFSYSPRdEDLRPKLETHLDALKRQGVIRTYQGFALGGGKERReVIAARLREARVVLLLVSADFLSADGWLT
pEIRDALARHDA-GECRVIPVILRPC-AWEKTLLGRLQPLPNRArpVTSYPNQS--
EAFTEIAKGIRSA(VRETEPPTLRPVTRRPSSSPDAEPHIKKLPPRNPGFTGRDRLLDELRSALTTGGPAALT
QAISGLGGIGKTQLAAEYAHR)}*

$2750=216(218):
>gi|1005330954|ref|WP_061614722.1|
{|0(73)|<Proteobacteria(B)>}hypothetical protein [Sorangium cellulosum]
>gi|1005211188|gb|KYF81284.1| hypothetical protein BE11_22200 [Sorangium cellulosum]
{()MYRIGILYAPAdEPYVLQLHKHLTPLMRDGV-
DVWHAGQIAAGSDRSaELRAAIHRADILLVLVSADYISTNECYSnEIYGALASH-----TKILVAVVRSC-
DWRHAPFGHLpHALHKEsDtpIAAWINPD--

DAWARLAAIIRST(LEEDTTDLTPTFIDKYDNRSERCRPHERAALEIANHHRPRTIQLREFNTIGRSSRSNIT
LIHPSVSRLHCIIFLHEGG)}*

$2751=216(226):
>gi|1005223917|gb|KYF90182.1| {|0(716)|<Proteobacteria(B)>}hypothetical protein BE17_35255 [Sorangium cellulosum]
{(MKLLLL)-------YCQEdADGAMALRPQLDQVCRRESVELL-
DTCTPAGEYVErALDERLRAATHVVILVTARLLAWSAWTRvVHPALTSRKPA-EVSLVRW---
RACVDDQDEVLRRFGPIP-DPvpVATAADPD--
TAAVAVARRVGEA(LRAARLGPRCVALGRLPSPERGIVGREQEKAELNGALVNPAVHVVVVIGEGGCGKTQLV
RTWLDGLQPTYGEVDAVISCS)}*

$2752=228(230):
>gi|1005226849|gb|KYF92439.1| {|0(709)|<Proteobacteria(B)>}ATP/GTP-binding protein [Sorangium cellulosum]
{(MVALATSPAD)---
VFFSYSPRdEDLRPKLETHLDALKRQGVIRTYQGFALGGAKERReVIAARLREARVVLLLVSADFLNTDAWLS
pEIREALARHDA-GECRVIPVILRPC-AWEKTPLGKLEPLPNRArpVTSYPNQS--
EAFTEIAKGIRSA(VRETEPPTQRPVVRRPSSSPDAEPHIKRLPPRNPSFTGRDQLLNELRSALTTGGPAALT
QAISGLGGIGKTQVAAEYAHR)}*

$2753=214(222):
>gi|1005239345|gb|KYG01657.1| {|0(676)|<Proteobacteria(B)>}hypothetical protein BE21_56335 [Sorangium cellulosum]
{(ML)-------
HSSRdDALAQDIERLFAVLERQGMVETWSPRRIPAGQEVMsATQQQIQRADILLLLISSDFLAAED-
TEaWIQLAQQRRRL-EGTRVIPVLLRP--AVLPDELERLAPLPRNRraISTWADRD--
EALLEVVDGVRTA(LAYRPALAPAVQPAPAAPSAPPDNNAAEAPQGSAPGTPPTPIQSIFRLNGPPDITFVEP
SQFPALQVELDTLGSGLIVEG)}*

$2754=221(223):
>gi|1005244773|gb|KYG04540.1| {|0(496)|<Proteobacteria(B)>}hypothetical protein BE21_46265 [Sorangium cellulosum]
{(MSDPVR)---
LFISAVPKdAALRDELDVCLKPHERGGRIDVWHEGRVGAGEASAaEARARLEAADIVVLLVSPDFLASDRHHH
eEAARALARSAA-GEVVIVPVPVRHC-DWSWTGFGRLKPLPAGGrpVNAWPDRD--
EPWNEVTRGLLEV(ADQVARRRAAGGRPRAGAAGLPDVGRCIGRDAEVEALAAAVSRDLPGRVLLLGAAGIGK
STVSRAVLHRPEVVARFG)}*

$2755=215(230):
>gi|1005245281|gb|KYG04927.1| {|0(715)|<Proteobacteria(B)>}hypothetical protein BE21_43925 [Sorangium cellulosum]
{(MQLLLLHGPD)----------DDDSAKALLHQLEPVCRKEGARVMD--
AIAAGEHVErGLAECLHAAAHVVVLVSAQLLAWPAWIRVVHPALARKAR-GDVSL--
VRWRACIDDGDDVLRRFGPIP-DRepVATAADRD--
AALVTVACRIGEA(LRAARLGPRWVALGSLPSPEQGIVGREPQKRLLDEALASHAIHAAVVVGEGGAGKTQLV
RTWLDDQQPTYAGVDAVISCS)}*

$2756=316(300):
>gi|1005459261|ref|XP_015766740.1| {|282(90)|<Cnidaria(M)>}PREDICTED: uncharacterized protein LOC107345532 [Acropora digitifera]
{(PIVVGSGSFEWMMTVVGLLIAGETYIHFHSKKVQHAKMAELLRAIKKSVPELSVPDEMENIQDLQRSLEVT
DNRPVGPAD)---
VFVSYcwSNSeKAFKSKQAKQLvgsafadprkvhSILAQSDKFSLWIDTDQLKTTNQNdEslgmfaqIADGLG

KAKVVLAFVSKQYANSENCRMEFQFAIKSLKK-P---VVPVIVgEGN-EWESTVVG-
LLVAGQENqpInIQDVNTE--
EELQLKALEIQKA(ILPLLGLEERTRSYRAPVVGDHVISHHNQWAFFGATIASFNHKTMTYTVNWDDGDPTDR
VQSYRDLGLDEIPTEDQVGLD)}*

$2757=212(217):
>gi|1020046244|ref|WP_063194925.1|
{|0(872)|<Proteobacteria(B)>}hypothetical protein [Bradyrhizobium sp.
AT1] >gi|1005521131|gb|KYG19897.1| hypothetical protein SE92_06140
[Bradyrhizobium sp. AT1]
{(MGNGMAERTPGKPEHRAASENDACRQ)-TRVFISYSRKdLTFAETLVTSLVAL---
GFEAYLDKKDILPGELWReRLTALIARADAVVFLISPDSVLPTSVCDwELNET----ER-
MGKRLLPVVMRRT-
ENAPSRLSRLNWIFMDAgeVLEPGLNTLASALMRDIAWIREH(TRIGELAFHWANARNKDEHALLRGKDIDDA
ELWISSQPSLSPETPEVTADQ)}*

$2758=221(225):
>gi|1055561762|ref|WP_067206053.1| {|42(0)|<Firmicutes(B)>}hypothetical
protein [Sporosarcina psychrophila] >gi|1005379448|gb|AMQ05222.1|
hypothetical protein AZE41_04310 [Sporosarcina psychrophila]
{(FLLLIKEKTEHIDWISFKVSGVFINLDILKKNFVNLFADCDWMTGDLLVLPQYDNAMHTNIPVVSFEMSKE
EIMMKSPKK)---IFLSH-K--
GSNKVFVKRYYDVLKTMGYEPWLDDEDMPAGSNLdrSIFKGFKDSCACIFFI-
TPEFRDEDYLAdEIDYAHSENRKKkEKFSIITLQFENE-
KGETGVIPELLERFVWKNPKHELEALSQILKALPIEANLI(DWKKE)}*

$2759=208(215):
>gi|1004327646|gb|AMP34890.1| {|0(102)|<Proteobacteria(B)>}hypothetical
protein pSinB_023 [Sinorhizobium sp. M14]
{(MTK)---IFISH-T--GDQGLLVKKLVNELRRAGQHVFVD-
LDMLPDDDVtqQITDGLEAAEVFVLVISGEI---PEWTAnEIEVARVYGRSRR-MLIIPVLIGDG-
RVPPQLKYSKAVKAgEREPSAYIADILAiIEGDKKRSEIFAA(RKVEVEKDLAVFVNEAVVRQQEHEAKNRRA
AQLWHGAGIVFLLLVLGFAGASSYTALTMPSNTTTYTGVLII)}*

$2760=295(300):
>gi|1005426517|ref|XP_015751075.1| {|138(38)|<Cnidaria(M)>}PREDICTED:
uncharacterized protein LOC107331059 [Acropora digitifera]
{(IMPLLASGDQGYSRSLMLSLILDSAIHWIKAGLLLKNLKIVVYSRSPQNYDKSFEELFKCFQEFKCNYDDK
ELVFETEII)-YDVYMSHsTKDRDIALSVVKFL-QSQRNINVFHEFQELN-
ENQSWQEEIFNIMGkCSRVVALLSPTYLETETCIEQYNMALCINRRSKGTFLAP-FYISSVDNMPT---
YMSLIQY-E----DCRPM-
DEWkmrkasDKVAESLLKP(QKNEVSESEVIQTNKGYDVFISYCHQNKAHADKLLTTLRSVDSDLNIFIDSAG
LNTGTSWQQLLYSALGMLDKNRSQESP)}*

$2761=278(284):
>gi|1005426519|ref|XP_015751076.1| {|138(749)|<Cnidaria(M)>}PREDICTED:
uncharacterized protein LOC107331060 [Acropora digitifera]
{(IMPLLASGDQGYSRSLMLSLILDSAIHWIKAGLLLKNLKIVVYSRNPQNYDKSFEELFKCFQEFKCNYDDK
KLVFETEII)-YDVYMSHsTKDRDIALSVVKFL-QSQRNINVFHEFQELN-
ENQSWQEEIFNIMGkCSRVVALLSPTYLETETCIEQYNMALCINRRSEGTVLAPFYISSM-
DNMPTYMSLIQYEDCSS----
PDSHYFALNSHASCLGELM(SHTCSNHKFACYALHHIFLFSFSDSSKSSIALLSPDYIKSKVCYEEFCLSYAL
FADKDRSLDLV)}*

$2762=219(220):
>gi|1006542180|ref|WP_061793167.1| {|61(0)|<Firmicutes(B)>}hypothetical protein [Bacillus firmus]
{(KQDKDITSLVEQIRKNTEGMNRYKGRLAKEQEKQFKTMLKSMESQSSTNLEYLNNYSEMSEKLNELSLDIKDSVKDKEII)EFDVFLSHSS-LD-KDIFVSELSEKLSNKGLKVFEDVKVFkiGQSQTDMMNMGILNSRFVIIFLSQNFIESGWSQYEFKSFLNREINEKRVIILPIWHEVSVDEVR-QYNPYivdKYAFNTSKQTLDEMV---NQIHQVVLDSLN()}*

$2763=269(220):
>gi|1007736798|ref|XP_015808949.1| {|288(0)|<Chordata(M)>}PREDICTED: interleukin-18 receptor accessory protein-like isoform X2 [Nothobranchius furzeri]
{(TTHHLNHMYTCVASNTVGNSSVTTRLKQKKQVKWPSLLGYPIACFLLVAGLGITLRVKWLEVQLICRSHFLFGTYRKEDT)DFDVFLSHVwKPssseverdlffstisgpyhdaeECLSsvemlgaaeanatqrpiEVMLTEVIEDQWRYRLCLLERDLLPGGAYTDDVVMMIKRSRTLICVLSADYLtDSNAVF-VLDAGIKALLQDSALKLLLVWTngasaSHI-HPDLPLPKLVQRALKVLpSLDWCSDTSSadtsRFWTSLRRILPKD()}*

$2764=252(261):
>gi|1005949882|ref|XP_015783257.1| {|696(70)|<Arthropoda(M)>}PREDICTED: uncharacterized protein LOC107361056 [Tetranychus urticae]
{(SSPGRDWLALARRLGYTDKDVARLVDDVSPSLALIHDWFESNGRTRYCIDVLMSCLQMISREDVRQIIEVDLQPIGSAPP)---VFLSYQW-DSQKLVL--DLRRRLEMAGFPCWMDIGLMGGGDSLygKIYEGISKARVIIACLTPRYASSRICAREVTLADVLR-----KPILPVMLEPTPWPPPGPMAVVMSSLVFVDLCGVGGHGGIGKSGDSETRFRD(ILNTISRCIAGNPDAAIPSRNQTLQHLFYPRSGDPRLSLTT)}*

$2765=140(159):
>gi|1007667094|gb|KYH45386.1| {<Actinobacteria(B)>}hypothetical protein AZH51_01410 [Branchiibius sp. NY16-3462-2]
{(MD)---------------ERLLPQLSIIG-SYSIEWWEFSLLTAGDTVDpTIQRRVSEADLGLLLLSPGYFSSSYIMTkELPQLIERN------LFVPVALRPF-PHLDGGRTLGGLEKAWViyGPNQRCYNELSGQAAKDRFALTV(SNEVLRRLNGDGGWRSL)}*

$2766=293(298):
>gi|1005956508|ref|XP_015786667.1| {|81(75)|<Arthropoda(M)>}PREDICTED: myeloid differentiation primary response protein MyD88-like [Tetranychus urticae] >gi|1005956510|ref|XP_015786668.1| PREDICTED: m
{(NVEKELKICESGSKNPYSINDLIGFLESIERFDVIDDCMELFYQDALFHSNKMKKLSIKGRKHDLLTINDSLGDNDEDIH)-YDAYVCYaDEDIDFVTFLSDYLESPQIGLRLFIRDRDLLLGTMKYDAYTKLIEQrCNYVIIILTPDFVESEECKYQTLFATDLAIQDRERKLIPIILKSCN--IPPFVRLMSKIDFSR----RDSKWEWVWRKLIFSISSK(PMILTSPPSYPCITYSSVPQEKHSTNENSQLHDQLYLHNHSSRHLNHQSPLTAPYKPDSPINETSISTKTLSSNISIK)}*

$2767=172(174):
>gi|1009167800|ref|XP_015902312.1| {<Streptophyta(V)>}PREDICTED: TMV resistance protein N-like [Ziziphus jujuba]
{(MAASSSSFQE)KYEVFLSFRG-EVTRYGFTGHLYNALRQKHINTYKDDENLqsGHKIS-EIIDAINESKICIIVFSKDFASSTWCLDEVIRILE--SKRDGNDIIPIFYAWKDALNKVPAIKGHVSKNKGPEYKFIDEIVEDILLKLSRYPST(CDHSKQGSACDHSKQGLIGIENRI)}*

$2768=136(156):
>gi|1009172836|ref|XP_015867491.1| {|0(140)|<Streptophyta(V)>}PREDICTED: disease resistance protein TAO1-like [Ziziphus jujuba]
{(MTSETCSNSSFSPSRG)-YDVFLSFKE-EDTGKSFADHLYNALIQKGIFAFRDDDQKLV------------------
ESRKNGTDHLQKRLYKESFKIQGYVRNDGPGKNLLRRRLHSKRVLIVLDDVDSSKQIQDLVGNWKVENWLGRGSKVIVTS()}*

$2769=276(300):
>gi|1009133136|ref|XP_015883744.1|
{|332(316)|<Streptophyta(V)>}PREDICTED: TMV resistance protein N-like [Ziziphus jujuba]
{(NKHILTSHGANEIYEVQRLDYHKAIELLSWNAFKRKLPVEDYLLLSNCVVDFAGGIPLDIEILEEKIAAKISLAIFAKLC)--------------------------SKREFRLFIDDkELRRGEE-
VspSLIKAIRGSKISIIIFSKNYASSSWCLDELVEILHCRGS-
LGQLVWHVFFDVDPSDVRNHTGSFGEALAqyEESSNKKKEKL-
PNWKLALNKASNL(SGWHLADGDQSQLIQKIVEASLSKLNRTPLQVAKCPVGIEERLQYLKTLINVSKEHVHFIGLYGMGGIGKTTIARAIYNV)}*

$2770=231(235):
>gi|1009172834|ref|XP_015867490.1| {|0(178)|<Streptophyta(V)>}PREDICTED: TMV resistance protein N-like [Ziziphus jujuba]
{(MTSETCSNSSFSSPW)KYHVFLSFKE-EDTGKSFTDHLYNALNKKGIITFRDDDQKLGSL-VP-
SKAIEESRFTIVVFSENYASSTRCLDQLVKIAECKKD-
LRLEaIYPVLYDVVPTQLIVECGKACFAERQQVFKDIVEKV-
KKWEGALTEVINL(SRSRWDLKDRHETDFIKEIVTDIWEKLNQTCPRGDNELLVGMGSRMEKMDSLLDIESDDVRTVFERVKHKFDATAFIANV)}*

$2771=145(158):
>gi|1009159261|ref|XP_015897716.1| {<Streptophyta(V)>}PREDICTED: disease resistance protein RPP4-like [Ziziphus jujuba]
{(MAVSSSSSFPNAISPQEK)-HDVFLSFRG-
LDTRDQFTSYLYEALCRKQIYVEGKPVEVCFKRSVpsLWLVS------------
KDFRPEYKLVQRVVEDISWKLH-
KNLSSNDSSKGCLIGIEKRIKEIESLLCIGSTDVCIVVGIWGMAGIGKTTLAKG()}*

$2772=174(175):
>gi|1009155955|ref|XP_015895987.1| {|0(662)|<Streptophyta(V)>}PREDICTED: disease resistance protein LAZ5-like [Ziziphus jujuba]
{(MASSSSSSSQFSSISSEKKHEIQE)KYDVFLSFRG-EDTRNTFASYLYAALsANQ-
ISTFMDDHELERGDEIspTLRKAIEESNIWVIILSENYASILIHKSLIESSDSPLDN-
ELRMHDLLRQMGQAIVRNEHKEPGNCSRLWD-AKDICQILERSTGTAAVEGISL(NLSQINRDVK)}*

$2773=192(220):
>gi|1009173779|ref|XP_015867994.1|
{|0(1138)|<Streptophyta(V)>}PREDICTED: TMV resistance protein N-like [Ziziphus jujuba]
{(Q)---------------------------KA-
IDTFRDDEGLVRGEAIspGLLKGIEESRFAIIVLSEKYASSSWCLDEVVEIVKCHRV-
RGQTIIPVFYHVEPNHVRNQTGTFGEPFTHK-HK--
DQDVNrvNRWREALSLVANV(HPCWELKADGWHDSVVIKEIVNEIFKELYQIASKDFVGMDSRIKKIHKLLDKCPRDDVCTIGIWGIAGIGKTTLARAFY)}*

$2774=178(177):

>gi|1009162670|ref|XP_015899558.1| {|0(91)|<Streptophyta(V)>}PREDICTED: TMV resistance protein N-like [Ziziphus jujuba]
{(MNRSWSLISTSSSSSSSNPQT)KYDVVLSFRG-KDTPNTFTSLLYAALSQKGISTFLEDNKLEKGKSIslELLKDIEDSSCSIIAPSKNYASFAWCLDELRDEAKFIQVFIREVSSKLDVALFNILEELVGTDSRLEKLNLRVSTSSLDHVRFIGICGMAGIGK(ITLAKYYYQWMSSQFK)}*

$2775=224(229):
>gi|1009167049|ref|XP_015901914.1| {|0(608)|<Streptophyta(V)>}PREDICTED: TMV resistance protein N-like [Ziziphus jujuba]
{(MKRSYSLISATSSSKPRT)QYDVFLSFRG-EDTRYGFTGHLYEALCQKGIYTSLDDDKLERGKSIspELLKAIETQA-LPSSFSQKtmlLPLGAW--MNLSRFFNAGRPTNR-----LLCPFSITWIHLMVESRLEVLEKH-SRDMNKNLVTTWKMNEAKFIKD(LIVEVTSKLVVARLNILEDLFGMESRLEKLNFCVCNSSLDDAFAVRFIGICGMGGIGKTTLGRAYYEWMSS)}*

$2776=209(221):
>gi|1009170876|ref|XP_015866434.1| {|0(840)|<Streptophyta(V)>}PREDICTED: TMV resistance protein N-like [Ziziphus jujuba]
{(M)----------------NFTNHLYKALRRKDILTSMENEDQCdKGKDIppKLLQAMKHSRLFIIILSTNYVASNWCLDQLVHILECGGTSVTRKVFPIFYNVDSSMFQHQTNSYENAFAAH-EENYEhnPEKVERWRSALAEVGNL(PVSWDLTNGSEKEFITDIVGKITCELNKSPTTPDPKPVGIDSRVQELLSILDTSVMDVRIIGICGMRGTGKTTVARFIYE)}*

$2777=242(242):
>gi|1009555659|ref|XP_015913090.1| {|994(0)|<Arthropoda(M)>}PREDICTED: slit homolog 2 protein-like [Parasteatoda tepidariorum]
>gi|1031608185|gb|ANG08901.1| toll family protein LongTollB2 [Parasteatod
{(SNSSCSNFSVIPFTSAEFVEEYMSIVILVPMLIVLMLTAIFFAWKYRNNMKMWIYNKYGVKLFEKGDYPAENEK)LFDAFVSYCKKdeAFVTQMVAPELECGVPSQRLCLRYRDLPASRYMAETISEAVECSSCSIAVISEQYLKSEWCLFELKACHHESQCNRRHKIIIILLNKV--DFKELDGDVRACFKAAVIIHWGDRRFWEKLRFSLPEGRS(HKHLSDYSDNKLSTTLRRSSMSNSVKLV)}*

$2778=219(220):
>gi|1009571258|ref|XP_015917704.1| {|947(0)|<Arthropoda(M)>}PREDICTED: slit homolog 2 protein-like [Parasteatoda tepidariorum]
>gi|1009571260|ref|XP_015917705.1| PREDICTED: slit homolog 2 protein-like
{(YLTAFNMSSCSQPNYRNYFELTFHLYYIAIPIISLLLIIMILICLCRHNIKVWLYTKYGIRLFKRNTYTAESEK)LFDAFVSYCNKdeTFVLQFLTPELECGEPSYRLCLRYRDLPLSEYVAEAVTEAIECSQRTIIVLSEHFLRNERCRFELKTAYRESQCNHKHKLLIVVVGKI--SFKSLDSDAKQCL-SNAHIIHWGDKRFWQKVKFIMPNVN(PRTVLR)}*

$2779=241(241):
>gi|1009595997|ref|XP_015926689.1| {|961(0)|<Arthropoda(M)>}PREDICTED: toll-like receptor 13 [Parasteatoda tepidariorum]
>gi|1031608178|gb|ANG08900.1| toll family protein LongTollB [Parasteatoda tepid
{(NSTACSNHSALTYYKAEFSGDYIPVMILAPSLFILLVFCLILFFIYNSEMKIWFYSRYGIRLFQRNNYPAENEK)LFDAFLSYCKKdeAFVSQMLAPELEDGTYPHRLCLRYRDLPMAEYVAEAISEAIECSRCTVAVISEQYLKSEWCIYELKAAHHETQINRRHRVIVVLLDKI--SFKQLEPDVRICLRSATLVHWGDRRFWEKLRFAMPEARL(HKDSVSCKDFKTSSLRRSSITNSVKLV)}*

$2780=258(254):

>gi|1008904165|emb|CZQ50134.1| {|788(0)|<Annelida(M)>}membrane pattern recognition receptor TLR [Eisenia andrei]
{(VRCADGRYLWRLNLTLDCSLGLPTLSGNGLMSPVHGVLLAVCVLAVASAVAVLILRRVRASASRRSRGTWRKIENQDELI)KQDVFISYSDSdeAWVTGTLLEIIWNESSQYSVCLQMQqhqleDYGSSLHLeSDTIRRHIDNSKVTIVVLSRPFLKNVWAVDSYRSTVLEKLESQRHQFIFVYLEQV--DVEEQDRDLHGFLPEDkTLKVADPL---FAEKLIYHLPPP(LKISRNGINTSDPSRGLLVELTDEISPEYHSQKY)}*

$2781=246(247):
>gi|1009342499|emb|CRZ23727.1| {|566(0)|<Nematoda(M)>}BMA-TOL-1 [Brugia malayi]
{(NTTICAPTASGVFGNEGTTNSFLIITVLFAVALITTGLVLLVVLFLRKTKSAIVQRRYKVPPSFTGTHTTPGSSPLPLIH)-FDAFISYSKKdeKLIIDTLYRQLESEE--
YILCLLHRDGPNYSSrvhtISDELINQMECAQSLILVLTQHFLDNEWKTLQIKTSHQIFAKNRHKKLIALL-GDG-IEANQLDAELGQILRKNtCIRMNDPL---
FWNLLHSALPIR(IAPSSCSGGSSQIYSDCYGSIVPSDIV)}*

$2782=241(243):
>gi|1009531170|ref|XP_015930917.1| {|114(0)|<Arthropoda(M)>}PREDICTED: protein toll-like [Parasteatoda tepidariorum]
{(FEEDRENMRGRVIITLSEIELCPKLIRNYILMGVGGAVILMMTVASILLYFRYRYHLKVWLYSRGWRCFKKRDKRDDGKL)-
FDAYISFTDEdaDSVRKHFLPELEEKHPFYKLFVPQRDIKEDNFEINYLMERVVDSKRIIILLTKHYLLNEFCMEMLRMAFANSLEEKLHQVILVKIGPL-PPIKEMDQSLKIVMESSrCLKFGTRL---
FWEMLRYEMPEK(SPNIDDYELLDDQPDDVPLIQEL)}*

$2783=244(244):
>gi|1009531181|ref|XP_015930921.1| {|723(0)|<Arthropoda(M)>}PREDICTED: protein toll-like [Parasteatoda tepidariorum]
{(YRDGKESLRGRLIMNLSEIEFCPELLRNYILMGAGSLMTLMMTVTSFVVYFRYRYHLRVWLYSRGFRCLKNRDKRDTGKL)-
YDAYISFSDEdaEMVRHHFLPELEEKHPFYKLFVPQRDLKAGNFEINNLMEQVLDSKRAIILLSRHYLKNEFCMEIFRVAFANSLEEKLHRVILVKFGPL-PPMnhEPSTHEPSTMMESSrSLKFGTRL---
FWDMLQYEMPEK(RPRSTNNYELLDDKSGDVPLIEEF)}*

$2784=242(244):
>gi|1009535639|ref|XP_015904642.1| {|510(0)|<Arthropoda(M)>}PREDICTED: protein toll-like [Parasteatoda tepidariorum]
{(SSEEGRKSLRGRVIITLSEIELCSKSQNYILMGAVGSVILIMTVIFILLCFRYRYHIRVWLYSRGLRCLKKRGERDVGKL)-
FDAYISFSDVdaSDVRKHFLPVLELKHPFYKLFVPQRDIKTGNFEINNLMRRMVDCKRTVVLLTKNYLENEYCMGIFRMAFANSLEEKLHRIIPVKFGPL-PPLKEMDQSLKTVMESTiWLEFGDKL---
FWDMLRYEMSEK(SPDTIIDYEMQEDSSDDVLLIQEF)}*

$2785=242(244):
>gi|1009563790|ref|XP_015914794.1| {|571(0)|<Arthropoda(M)>}PREDICTED: protein toll-like [Parasteatoda tepidariorum]
{(FEEDRESLRGKVIITLSEIDLCPKLIRNYNLMVAEVSVILIMTVAFILLCFRYRYHIRVWLYSRGLRCLKKRSERHVGKL)-
FDAYISLSDEdaSEARKHFLPVLELKHPFYKLFVPQRDIKTGNFEIDNLMRRMLDCKRTVFLLTKNYLENEFCMEIFRVAFENSLEEKWHRIILVKYGPL-PPLKEMDQSLKTVMVSSrCLKFGDKL---
FWDMLRYEMSEK(RPDTSIDYELQDDSSDDVPLVQEF)}*

$2786=234(236):

>gi|1009563797|ref|XP_015914798.1| {<Arthropoda(M)>}PREDICTED: protein toll-like [Parasteatoda tepidariorum]
{(MRGRVIITLSEIELCPKLIRNYILMGVGGAVILMMTVASILLYFRYRYHLKVWLYSREWRCFKKRDKRDDGKL)-
FDAYISFTDEdaARVRKHFLPELEEKHPFYKLFVPQRDLKAGNFEINNLMERVVDSKRIVILLTKRYLRNEFCMEILRVAFANSLEEKSHRIILVKFGPF-PPMKEMDQSLKIVMESSrCLKFREKL---
FWEMLRYEMPEK(RPNIDDYELLDDQPNDVPLIQEF)}*

$2787=232(235):
>gi|1009572668|ref|XP_015918338.1| {|717(0)|<Arthropoda(M)>}PREDICTED: protein slit-like [Parasteatoda tepidariorum]
{(QDIKCGDNGSLKNQVIINLSYGNFCPEKSEKDTYIVIIAIFSVFLLMTCFILYLYRKLLVAIFFECGCQCLKTEEDYDYS)-FDAFLLFADEddEFALGVIEEGLETDSRKYRLFVPSRQVLLSSS-
PLSINSSVADCCKVIVILSSNFLRNSQCMQLMKTAMACSIEKTAQRFIFIITDSI-
PPELKLDAVLNCLVKRStLIKWGECL---FWTKLKFSLPKK(SKTVTSNEEESERLI)}*

$2788=252(261):
>gi|1009582665|ref|XP_015922543.1| {|940(43)|<Arthropoda(M)>}PREDICTED: uncharacterized protein LOC107451071 [Parasteatoda tepidariorum]
{(NTGHDWIALARRLSYNEREIKRFGEEAIPPVSLLEHWINSNGRTRYCIDLLVSCLEQIDRIDVANYITLELGQQETPSPP)---VFISYHW-DSQEAVLQ--
LRHRIELAGYTCWMDVGHLMAGDALygKIYEGINKSKVILCCLTPRYILSPLCTREMSLADVLR-----
KPIVPIILEPTPWPPPGALALILSSLVYVDLCGIGGHGGSGRQADWESRFSS(IIERLTHFITPSFVSPPLSNMPSERHVPSPILIRSPEEFLD)}*

$2789=210(215):
>gi|1055538975|ref|WP_067191967.1|
{|0(199)|<Bacteroidetes(B)>}hypothetical protein [Mucilaginibacter sp. PAMC 26640] >gi|1008900852|gb|AMR32517.1| hypothetical protein A0256_14330 [Mucilaginibacter s
{(MSY)--KIFISFTEEdKALAILLDKFLNN---
QYGGKVHTSFSKLDVKFGEdwkkWIRTQATECDAIVVLLTKNSLKKPWIYIEWAAFWYNDRE---
SLVLKTDEVGINEMINPmlSKQFGDITDEEM--FNSFISR--
IQELCGADRIPY(RAVPQFLTDARAAIEEQKVSGLNKLAAEIEKSLSAIPADDAEKKKIAESLLKGGKKGAALQRIITSIGSPGV)}*

$2790=167(183):
>gi|1055538204|ref|WP_067191530.1| {<Bacteroidetes(B)>}hypothetical protein [Mucilaginibacter sp. PAMC 26640] >gi|1008900647|gb|AMR32312.1| hypothetical protein A0256_13230 [Mucilaginibacter sp. PAMC
{(MTTKTEYQ)--KVFISFSFKdKRFAKRLDDEFT---
KRGIRTFFWVKDAPPGETLKdIMRSGVRANDRIIFIASANSIRSEAC-QfELSEGRRKQAE-
LwENIIFPVHIDDY-LFT---VQKDQIRPVD------KME--EYWLNI-
EEIRRI(NSADFTNFNKDRVNKVAFADAVDKIVAQLKNINND)}*

$2791=212(219):
>gi|1009556149|ref|XP_015913302.1| {<Arthropoda(M)>}PREDICTED: myeloid differentiation primary response protein MyD88-like [Parasteatoda tepidariorum]
{(MTDVRLNDFEILTKDDSKTGICTL)-
YDAFVCYsDEDIDFVLLLAKYLETDEVGFKLYFRHRDELAGHSEHATNLKIMEErCKRVIVVVSPSFLKCPADEAQAGYAAVTGLNERYRKLIPILYKPC--EMPKMFRFFTKIDFTN----GIRKE-EV-
DKLVASLRDG(HGTSSFTSPEDPPLHNPLPQFGPRNNFLPPTRISGNLFLADLRMVDYQTPESYKC)}*

$2792=291(298):
>gi|1009556157|ref|XP_015913306.1| {|80(61)|<Arthropoda(M)>}PREDICTED: myeloid differentiation primary response protein MyD88-like [Parasteatoda tepidariorum]
{(MGQLIQYLENMGRHDILQDLQPKLELDAKSYIQRYIQAQSQESPLQVDEISSCRSVSSDVRLDDSGILTREDAYTGEVTV)-YDAFVCYaDEDIEFVRELARFLEAVGF--
KLYIRERDMLAGQSEYETSLQLIHErCKRVLIILSPDFLRCPAYEAQASFAAGLGIDEKCRKLIPIVHKPC--
DIPMMLKFISKIDFTK----PgCVVE-
WIWDKVISSLRDG(HGPSSSFVSTERRYPAVLPQSFQQIQPGSPDPAYPSITYPPSTSSIINTSCSISPPQMIFQEESLSDSAVNLNRVASK)}*

$2793=140(170):
>gi|313769426|gb|ADR81932.1| {<Chordata(M)>}Toll-like receptor 20 [Heteropneustes fossilis]
{(LVGDYLLAFFHIARTWVEEAMRANRKGRYD)-
FDVFVSYSGKdeHWVTDELLPNLGKRGPPFlRLCLHSRDFELGKDIVENITDSLYRSRHTLCLVSRNYLRSNWCSLEMRLATYRLLAEHRDVLVLVFLENVPhQLLNVHH----------------------------------
()}*

$2794=136(166):
>gi|313769428|gb|ADR81933.1| {<Chordata(M)>}Toll-like receptor 20 [Clarias batrachus]
{(YLLAFFHIARAWVEEAMRANRKGHYH)-
FDVFVSYSGKdeRWVMDELLPNLGKRGPPsLKLCLHSRDFELGKDIVENITDSLYRSRHTLCLVSRNYLRSTWCSLEMRLATYRLLAEHRDVLVLVFLEKVPhQLLNVHH----------------------------------
()}*

$2795=176(177):
>gi|1011460687|ref|WP_062357423.1|
{|0(146)|<Proteobacteria(B)>}hypothetical protein [Pseudoxanthomonas mexicana]
{(MNPL)---
VFISHDTRdAELAEAFSNLLKSVSAGVLKSFRTSDKRgnQGIEYGIewypEIIKNIQSSSDVVCLLTERSVDRPWILFEAGMA--KGKL-NTPILGVALGIELKNASTGPFAQFQNCADDE--
ESLTKLVFQLVDRIPGSEPDR(DTIRFQVGKFKTNVEEILKKLAASGMAAQPKRQ)}*

$2796=207(218):
>gi|1011656253|ref|WP_062505559.1|
{|0(1158)|<Actinobacteria(B)>}hypothetical protein [Nocardia pseudobrasiliensis]
{(MLA)--RVFISHASADREWATEIRGWLIADGHE-
VFLDlhpVDGLIVGEEWERELYRRLRWADAVVCVITATYLR-SVWCAaEVGIAKANG-----ARLLPVRV---
AadITHPLLKSLQQSN-AA--ADPAVARE--
KLAVALRRLDMG(GGVGWSDDLPPYPGLEAFGTDRRRVFFGRTREAAEVAELLRKPATRTEPAIQLVIGPSGCGKSSLVRAGVIPLIA)}*

$2797=267(276):
>gi|1011266307|ref|WP_062185257.1|
{|47(29)|<Bacteroidetes(B)>}hypothetical protein [Dysgonomonas macrotermitis] >gi|1110217983|emb|SHG42516.1| TIR domain-containing protein [Dysgonomonas macrotermiti
{(GIDYYRVEIFISPAKYAELEQDKKIEKIESAISSAFDNATKGDESVVFNNIIIVPSATIEDNLSNADTLVDFSFWNFGYY)--NIFISHLTKdKISASNLKTALFDYGISCFVAH---
EDIEPTKLWAnEIENALKTMHCLCAIIAPEFINSRWC-DqEVGYALGR-----GVLVIPI-------

RKDSDpyglLGKIQGIQSKGksANKLAKEIFQILCSNSLSKKLYI(KNLAGLFLNSKSNIEADRWIELLNSIG
TLDYEVVEFIYSNYLTNENLKDDTILNKA)}*

$2798=258(252):
>gi|1011345534|ref|WP_062259409.1|
{|0(1201)|<Proteobacteria(B)>}hypothetical protein [Caballeronia
megalochromosomata]
{(MEEDRYLATSPGDGDREIPLYARLGLQVGAAEWLHVALEQDWVQGSS)-
PTVFISYCTKdESWRFQLERFLGPElatlscpSTLEPYRPWSfaslERGTATGTQFPvELASQLWRCRAAIVL
LSQDYLNSAICAAtELPFLAWRHVA-QGM---PLYVLQV-GVVSAAAAEI-PLPQvDG--
QWPDFDLKKQTSDRFPGLDHR(EQKAVRQPNIFELRKRTERDRRLAAFSELVRRNLAEQHKRTVVSTSPSARE
LPNRAESVDPPLFR)}*

$2799=176(176):
>gi|1012023344|ref|XP_015950394.1| {|0(844)|<Streptophyta(V)>}PREDICTED:
LOW QUALITY PROTEIN: TMV resistance protein N-like [Arachis duranensis]
{(MQQRP)-YDVFLSFQG-
KETRSKLISHLYASLQNAGIYVFKDENGLarGEKLSISLLKAIGECKTSIIILSPNYAFSRWCLQELEDIMIC
CKNKTQKCLRCRNESEDIKNIVEQVTHMLEMKELFIANHPVGIESRVEEVIQLLKDQQ(QENPLLLGIWGMGG
SGKTTIAKEVYNKIFRE)}*

$2800=198(203):
>gi|1012079243|ref|XP_015954105.1| {|0(224)|<Streptophyta(V)>}PREDICTED:
TMV resistance protein N-like [Arachis duranensis]
{(MADHDAESCSSYFI)-YDVFLSFRG-F-TRYGFTDRLYHALCERGITTFRDDENLrvGDT----
LLEAIERSKISIAVLCKDYASSTWCLDELVQIMKCWNNGKNQPVLPIFYQVEPSDLLHKISSIYSNFTSFFDG
VSEKLPPEPLYIKHPIGFDS(HFEVVESLWNIESDNTICMLVIYGDGNKTTFVGELFNKFRHQFEAASFL)}*

$2801=242(264):
>gi|1012348186|gb|KYP59377.1| {|973(311)|<Streptophyta(V)>}TMV
resistance protein N [Cajanus cajan]
{(LMIRRLLGSQPSESHVSQRENIFHTRCNILDKACSLIVDSGLCCNCCSTRLVEKLNLTSIPHPKPYQLHWL
NEDRDIIVD)-----------------------KQVKVKGIHTFiDDEELQSGEE-
ItpSLMKAIQESRIAIPVFSKNYACSPFCLDELVHILACAKE-
KGQLVLPVFYDVEPSHVRDSAETLELLSWSAFGIDKVDPCFMTILNRVVTYAAG(LPLALEVIGSNLFRRSIE
ECESDLDQYERIPHKKIQDILKVSFD)}*

$2802=169(169):
>gi|1012322177|gb|KYP34530.1| {|0(287)|<Streptophyta(V)>}Putative
disease resistance protein At4g11170 family [Cajanus cajan]
{(MACITNNSGSSSRTMN)-FDVFVSFRG-
EDTRNNFTDHLFAALRRKGVVAFRDNQnINKGQLLEpELMQAIKRSRLFIVVFSKNYASSSWDLILEGCINIT
RIHPSIGILRELFYLNLTNCKNLFHNLNMFFGLKSLKMLILSGCSKLLNNRLLKKPRE(VEHLENVDKNTSV)
}*

$2803=205(211):
>gi|1012023007|ref|XP_015950312.1|
{|0(1630)|<Streptophyta(V)>}PREDICTED: uncharacterized protein
LOC107475200 isoform X1 [Arachis duranensis]
{(MSSTASSSSQKKEPGLMKFTTSYYRNWT)-YDVYVCF---
DRTTNKFVSDLYARLSAAGVRVFKDEMEPRS---
GdqILFKAIEDSRISIVVFSRSYGDSTWWLKELEKIMECRSS-
KGQKVVPVFYGVDPSEAGAAFAYREPLREAACLPGFVTYISRERDEALDNIVEH(ITAFLGGKDHWFTLPNVE
KLQPRVQDLIQVQLLNYDKVLILGI)}*

$2804=223(227):
>gi|1012023055|ref|XP_015950321.1| {|0(280)|<Streptophyta(V)>}PREDICTED: putative disease resistance protein At4g11170 isoform X2 [Arachis duranensis]
{(MSSQNQE)-YDAYIS-SG-LQFLHPFISGLYDALKSVGLHVLADRTEPKTNK-ILLSGAIERCRVSIIVFTTAYAESTLYLQELVKIMECHRR-KDQKVVPVFYCLDPSQVCNLSGYFGEILSRTLQGilRDENRM-LSYETALRQAASI(LPRKNRNVESHIVGRVTSLIDSTKLFIAKHPVGVDSRVKDLIQLLNNQKADGVPIIAIWGMAGIGKTTIAKALYNQISHN)}*

$2805=219(221):
>gi|1012023340|ref|XP_015950393.1|
{|0(1296)|<Streptophyta(V)>}PREDICTED: LOW QUALITY PROTEIN: putative disease resistance protein At4g11170 [Arachis duranensis]
{(M)-YDVYISFEP-HYPSHPFISLLSGALKRAGVHLFLDNYMKP-KS-KdlILSSVIKGSRVSIVVFTTDFACTTWSLKELEKIMEYRSS-RGQQVVPVFYEVDPKEVFNQSGHFGEALLATlaRTSTNQAKV-VSYMTALKEAAAI(SPRFLTNFRDRSKTIDSIVGHVTSLLDSTALFVAEHPVGVNSHVQDLIQLLDNKKSDSVFIMAIWGMGGIGKTTVAKVLY)}*

$2806=155(173):
>gi|1012236665|ref|XP_015939841.1| {|0(9)|<Streptophyta(V)>}PREDICTED: uncharacterized protein LOC107465377 [Arachis duranensis]
{(MSSSFCTTK)-YDVFISFRR-EDTRKTFTVYLFNLLANKTIRAYMDCLLRRGDE-VwpALEKAIESSLISIVVFSENYALGRARQDSSMEGISRRFS-----------------LAVSVTGFACGIGSGLCSVVLHVVAVLYAVYPQRCISS(MRRQQGMPLDERYVSYLQMAGLHY)}*

$2807=138(157):
>gi|1012350318|gb|KYP61507.1| {<Streptophyta(V)>}Putative disease resistance protein At4g11170 family [Cajanus cajan]
{(MSGSSSSTIVSPPQIRR)--DVFLSFRE-EDTHDSFISHLYAELQRKKIDTYIDYKLGRGEE-IspALHKAIEDSMIYVLVFSQDYASPTWCLDELINDKTIVLI-----------------LWMLHGLRSLTMHIGLCSFTFQGDQFLINSLVMARDIQ()}*

$2808=249(253):
>gi|1012365080|gb|KYP76262.1| {|134(0)|<Streptophyta(V)>}TMV resistance protein N [Cajanus cajan]
{(AGVFNFFLGSLMLAVMKWSLKFALAAAVFCYVLINIRIHSDSDSDGGDMEETDVALIENGGRRRNASEVQVPPELRPPVM)-YDVFISFRD-KDT-SDIPEYLACALLKKSLRVYAHPELTGDHVPQTVIEEIGKAQVSVVVFSKSYLETNWCLHEFHFIMACRAR-YARTVIPVFYNVHRTAIQDYSSNYVSIKKLCDDNGFSQIYAEDLSSDLAEAASL(QGWEMLRASRPYELLESVSDHVLSHCKLNLDHS)}*

$2809=198(200):
>gi|1012120365|ref|XP_015962215.1| {|0(147)|<Streptophyta(V)>}PREDICTED: TMV resistance protein N-like [Arachis duranensis]
{(MADEGAAGSSSSSTPWRWASSSLSEEGMC)--DVFLNFRG-EDTRFAFTDYLYYALSEigK-LKVFRDDPGLELGDEIksTLMEAIKRSRIHIVVMSEKYVCSSWCLLELEQMLKYSDD-GNKRYETQVIGEIAEQVFAKHRKIKQLLDKF-DSEFEAVEPLLNLESCDTVRMV(GIYEDPKIGKSYITTFAFELYYKIKYKFRAA)}*

$2810=301(300):

>gi|1012223701|ref|XP_015936439.1|
{|125(1130)|<Streptophyta(V)>}PREDICTED: uncharacterized protein
LOC107462388 [Arachis duranensis]
{(FDLHREKFKEEPDKIYKWKSAMTYLANLSGFALRDPAEAEGIERIVEAVRDTLVYKFQSSRSTHKNPESTE
RRPSLQEFN)KYDVFLSLRR-THTPYTFIDYLYHYLtKKG-
FSTFTSEEETERGDSIpsQDLQVIKDSRILIVVFTRDYADSTCSLEEMATIVDCHRE-
LNQTVIPVFCGVDRRDVQRQTGPYEKAFVSY-
TKEFKQDPLkvQKWREALKYMTNL(QDISLKDWSEIEAIGNIIQIVERLCPKFSRLSSHSPVGIQSSLAMLEK
LLKLEDDCVLVLGVWGMGGSGKTTHALALYDR)}*

$2811=220(220):
>gi|1012236635|ref|XP_015939832.1| {|0(185)|<Streptophyta(V)>}PREDICTED:
TMV resistance protein N-like [Arachis duranensis]
{(MSSSSYTT)KYDVFISFRG-EDTRETFTVHLFNALANKTIRAYMDC-
LLQRGDEVwpALEKAIESSLISIVVFSEKYATLKWCLEELVKILQWREY-
HGQVVISVFYRTDPSDIRNQSGSFEKAFAKY-
ERDFAESLVgiEEIRKIVKVNMKQ(HRVIGIWGMRGIGKTTIAKMLFAKSFPHYDHVCYAENAKEYTPQRLLS
ELLRERITTDATGFVNSMSSLSNK)}*

$2812=206(220):
>gi|1012248900|ref|XP_015976270.1| {|291(0)|<Chordata(M)>}PREDICTED:
interleukin-18 receptor 1 isoform X2 [Rousettus aegyptiacus]
{(FSYNCTVAGKEGIDTRSFILLKKEDPVDIPGHIFTRGMIIAVLISVVVVCLVIVGIIYRVDLALFYRHFMG
RDETLTDGK)TYDAFVSYLKEcqpengeeRTFAVETLPRVLEKHFGYKLCIFERDIMPGGAVVDEIHSLIKKS
RRLIIVLSKSYMSNEVSS-GL--------------------KQK-
PESRKGQIWKGKQTDLRTFVYREKHISKAKDISSWDMYNF()}*

$2813=205(220):
>gi|1012248902|ref|XP_015976271.1| {|291(0)|<Chordata(M)>}PREDICTED:
interleukin-18 receptor 1 isoform X3 [Rousettus aegyptiacus]
{(FSYNCTVAGKEGIDTRSFILLKKEDPVDIPGHIFTRGMIIAVLISVVVVCLVIVGIIYRVDLALFYRHFMG
RDETLTDGK)TYDAFVSYLKEcqpengeeRTFAVETLPRVLEKHFGYKLCIFERDIMPGGAVVDEIHSLIKKS
RRLIIVLSKSYMSNEV------SGLKQKPESRKGQIWKGKQTDL-R--TFVYREKHISKAKDISSW-------
-------DMYNF()}*

$2814=250(261):
>gi|1016955008|ref|WP_063155316.1| {|15(44)|<Proteobacteria(B)>}TIR
domain-containing protein [Enterobacter cloacae]
>gi|1014932855|emb|CZX82043.1| Uncharacterised protein [Enterobacter
cloacae]
{(KLQSRMTFSDIDIYFRGHGVPTQDNYPNNSKRSYAKDMLANANENLLLVIASELGLLEQVNDHETKDVEVS
FWQPGYFRL)----FLSHLA------SFkvqTSHLQSALRKfA-
ISSFVAHEDIEPTKEWqnEIEAGLKTMDALVAILMPGFPESKWCDQEVGVAVGR-----
DILIIPVRRGLDPYGFIGKYQGIQAQGKKT-
GEVAEAIFNTLVKSPKTRNKIL(LALSGSLSNAITVKDAIEKIEIFNSLTLVPPDILESLKQQI)}*

$2815=189(191):
>gi|1012572146|ref|WP_062810857.1|
{|0(69)|<Proteobacteria(B)>}hypothetical protein [Alcanivorax sp. NBRC
102028]
{(MKK)--DIFICHAS-ED-
KVSVARPLSSCLAQRGLTYWIDEAEIAPGESIteRVNEGLKVSEFVVVILSRAFMAKHWPKRELwaVLNLEAK

SgkvIIIPILVGTQTEVNRIIDEFPLLNDKLFLLWNGDANRVANAIQTTVLKSQG(VSQEPQFEMHTCGHCAT
PFQHGVHVCLGCQGTIKYGLTAQERLRIRQI)}*

$2816=292(299):
>gi|1013934629|gb|KYQ88341.1| {|653(265)|<Amoebozoa(E)>}Toll-Interleukin (TIR) receptor domain-containing protein [Dictyostelium lacteum]
{(QNNIIVAKVLLKYHCNRNIEDAKGLTAMHIAVINKYFNFSILLAKNGAQAIPDKNMKTPVQYLHDADSKSK
LQSAIFTNE)---IFLSYAHKdSAFVSQLRLQLESMS----
LRCWLDEyRLEAGCDWRsEIIKGLENCQFVFFVISEVSVNSLWCRKE----LKISKK-LGKVVIPILYQQV-
PIDPVLLGLFDIPPvayHDGqtLIPFCDTE--
ENAYLVHKEMKKI(SKLIKTGWFTERKEFPQNSKLIEKYTRSKMVYLSFSNIDRALNDLIKESLISRGVPTIN
RDDIHGRPSAKDDEFSKQNI)}*

$2817=290(298):
>gi|1015450360|gb|AMV32143.1| {|92(227)|<Planctomycetes(B)>}hypothetical protein VN12_08470 [Pirellula sp. SH-Sr6A]
{(LSTAVFTPSERDEAVQFIQDTYGWPLELYGLERLSSMLRSSHRELVSQHPQIFTPLFFPFAGGLSLSPSPD
HLVVDHVEA)--------------
DAGLAHWLSRRLTLAGFHVWCRGLAPMAGSSVndAVRGLLTQRAFRYIPILSPEALSDPEFTaRRNMALAVGG
SRGSQFLIPAIAKPF-
DSArldQETRSITAASFHVSWALGLKSIEEaLSSTACPRKSEGA(RDLAIRSYFPGEIIINEPEDLASNLFKV
THVPEVIRRFHSSTPITDEDGPLAGQWSFRKVSPTHFLSFHYPPAELTVD)}*

$2818=240(238):
>gi|1016640075|ref|XP_016043099.1| {|206(0)|<Chordata(M)>}PREDICTED: uncharacterized protein LOC103112541 [Erinaceus europaeus]
{(VNKAHPFPTWMLRMGVGKSRIHPCPFPCFWGKSPRADRNQTHLESGGQRPGDISLCNATVTAEDGQEGPEE
MPREEEDED)--
DVFLKFVilHSeDDTQEALrVQHLLQSRFGIQPGIIFAEMPGGRQHLQNLDHAVNGSAWTILLLTENFLRDTW
CHFQFHTSLMNSVNRQHKYNSVIPMRPL-
NRPLPRERTPFPLRTINaLEEQSQGFATQVQRIFRDSVYQT(QQNIWRETRGAPSGPPAV)}*

$2819=170(175):
>gi|1016282950|ref|WP_062967308.1|
{|0(131)|<Actinobacteria(B)>}hypothetical protein [Nocardia gamkensis]
{(M)--RVFLSHTSYdAGVVRYLRSLLEhDLGV---EFFLLPDDA---
PHGLpwieQIKLGIENSDELFTVITPESVHRPWVSAEWACFWMQGKP-
TTPLLLEVGVDQLWEPMRAFQTVDLLDVSSL--
TRFLRTIADRTGCEPTSGVTP(VAHQMVKEIPRIRERQAVADLERTLERLKRTVTA)}*

$2820=188(197):
>gi|1016384510|ref|WP_063052695.1|
{|0(234)|<Actinobacteria(B)>}hypothetical protein [Nocardia arthritidis]
{(MA)---VFISYHSSKRPVVEHIATYFARHDIS-TWWAPRDVPPGSDWDEAISHAIRQSTALVLLFCASA-
DSSVHVKrEVHLADRAK-----IPIYWVRLEQIQpeRLGYYLSPTQWVDWLD--
QRDSTLDSLVRDLKRPGSSGRA(PDNAPVPRSTEPVPVTDWPAGVAVFENDRVAAEAAAHTYFRIARDASDRS
IVLPT)}*

$2821=262(270):
>gi|1057121962|ref|WP_068517196.1|
{|79(28)|<Proteobacteria(B)>}hypothetical protein [Thalassospira sp. MCCC 1A02491] >gi|1016020393|gb|KZB69316.1| hypothetical protein AUQ42_10750 [Thalassospira sp.

{(YLENSHNVQNTERSVEVLLESVIPRRLSIPLDISLFPKSFLNANDSHQWRSNPQYHFREALQTVSPEMVEN
IWGTASSLH)---VFIS--
HKADKKKRAHKLKEGLSRYGISSFVAHDDIKPAEEWQvAIEKALFSSDALIALICDEFSASDW-
TDqEVGIAIGR-----QIPIFPVKLSDT-
ANPYGFIGKIQAFKVTPdtLEDFISRLLSDQRTSTTAINSLL(EALKSTEEKSIQNNLLKQLVKSPKVSWSTK
AITELTKIGKSKKLTKSSKE)}*

$2822=175(177):
>gi|1016549270|ref|WP_063117870.1|
{|0(647)|<Proteobacteria(B)>}hypothetical protein [Escherichia coli]
{()MIKCFLSHSSKdKDY---VRVVASGLRKETRI--
FDEQTFEKGMSPSeEILKGLDDTSLFVLFLSDSALESDWVKEEMRLAKKKVED-GHLqRIYPIIIDDK-
ITFTDPRVPSWMKEGFNiqPIRKPNVAIRKINARLREIAFKT(HPALKERQKIFVGRNEKTTNVEQRFDDFDK
SPPVVFI)}*

$2823=200(192):
>gi|1016807613|emb|CVI70770.1| {|0(203)|<Firmicutes(B)>}hypothetical
protein BN3660_02014 [Eubacteriaceae bacterium CHKCI004]
{(M)-YDFFISFKC-TDSGEltedrTIAEELYIRLQSAGYQVFFSEQILEK-
EGItkyisEINQALNDARIFILVCSKKeYILNGWVAQEWSSFLNLMMTNENKKFFSYLIEMKEFELPALLGPF
QSFQHRNGMDKAIKYFIAANPISQTRGKIE(SLQTVQEILDGEFGIFGHLKILNKSSVEYFSDKYPEMMAFVQ
AHYFFRQGE)}*

$2824=273(261):
>gi|1016779089|emb|CZT57473.1| {|74(365)|<Firmicutes(B)>}hypothetical
protein BN3661_02004 [Eubacteriaceae bacterium CHKCI005]
{(DAYWGGLLCKYGIEYVLDQQSGQRIPTCHRVMYTSILDHPYYKKAIELAKDGKEILIQDANEIARVQKDIL
SVSQKENPF)--DVFICYKesidgtneRTQDSVLAQDIYKELTRDGY--
NVFFARKTLAEmlGSEYeAKIFAALNSAPVMLVIGTQ-
KEYVTAPWvkNEWMRYLEQIQQgKKKTIIPLyqeMSPYEFPSELSSFQALDMSKIGAMQELTDSIMRILGKKD
STNAQSL(VDNLVLRGEQELSHSNWETAHEYFRKAVDESATCAAGWWGL)}*

$2825=273(263):
>gi|1016799163|emb|CVH78999.1| {|74(191)|<Firmicutes(B)>}hypothetical
protein BN3662_03158 [Clostridiales bacterium CHKCI006]
{(AEAYWGLVLCKYGIEYVDDPATGKKIPTCHRTSFDNVMDDGNFEIVIKKADILARQVYLDEGQKIENLRKD
ILTVSSKEK)PYDIFISYKE-
KDDngERTIDSVLaqdiYDQLTAKGYRVFFSRISLedKLGQEYepYIFAALYSAKLMLVIgtdyehFNAVWVK
NEWKRYLQLMTRDHT-----
KVLIPCYKDIDAYDLPKEFQKLQAQDLGKVGATQDLLRGIDKICGRATEQIK(QDGTNNYQPIIQEGMLYEIR
RHFNGPDLIEKVLSYGTKDPHEL)}*

$2826=180(178):
>gi|1016914521|gb|AMY06953.1| {|0(33)|<Acidobacteria(B)>}TIR domain
protein [Acidobacteria bacterium DSM 100886]
{(MTDTLHAC)----FISYRH-
PATagsreEKLIAhvfKAISDHVEmyTHTHQVYFDQKRLVPGYQYdeKLAEAICRSACMVIVYWPAYLESDYC
LQEIEAMLEIE-----
KRRRKKLGSELHGCRLIIPFIVRGRFEDLPNAVRDGCQYLDYSRQATNPHFN(IGEDEETSAKLFEIANYIKS
LCDKLQRVGA)}*

$2827=245(235):

>gi|1020137755|ref|WP_063261065.1| {<Firmicutes(B)>}N-acetylmuramoyl-L-alanine amidase [Bacillus cereus] >gi|1017076379|gb|KZD66192.1| Phage lysin N-acetylmuramoyl-L-alanine amidase family 3 [Bacillus
{(MARYSLHGGHNSIVQGANYGNRKEHIM)--------------
DRQVKDAVVAKLRALGHTVYDDTDEVGTTQAQnlnNIVSKTNSHDVDlvVSFHLNSYDTKAngvevlyydqqa
lsakiaaqlskdigWSN---RGAKER----KDLYVL----SNTKapA-ILIELGFIdneaDMAKWDP-
DKIANSIV-
YALTGqSGGTTPPA(KQNIIQSGAFSPYETPDVMGALTSLKMTANFILQSDGLTYFISEPTSDAQLKGMTDYL
DRKGWWYEVK)}*

$2828=202(220):
>gi|1018291207|gb|KZF36902.1| {|75(0)|<Proteobacteria(B)>}molecular chaperone Tir [Escherichia coli APEC O2]
{(SDINKNIAKKNNELNRLQTQLQKEQENELKKRNVAQKKLEKEQLEYQNKISRGLDIQKNIISNTSHPIPIN
NSTNSAMIE)KYDVFISHAT---EDKEaFVRPLAELLKNRGIKVWYDEFSLG--WGSslrKTID--
YGlVNSRfgVVILSRSFIKKQWTEYELNGLTAREMNGENKVILPIW-----------------HEVSK-
SDIIQFSPSLADKVALNSSMYT()}*

$2829=185(171):
>gi|1016916874|gb|AMY09306.1| {|0(302)|<Acidobacteria(B)>}TIR domain protein [Acidobacteria bacterium DSM 100886]
{()MSEVFISYAHAdDlpfaedtpgwvtALADRLEKSL-
MMRRGGsRVAVWMDHRLEPEKQVDgALRDRVRRAECFVAVLSPRYLESPWCRKELDAFIAHAGE-
QGERVFLLEMSPT-
SREEWPRGIRNLSARKFwaQGFDDPTAMPLGWPVADPAQDRP(YWRALNELSHFVSARLQAAGAVTRDDDARC
V)}*

$2830=233(242):
>gi|1016917036|gb|AMY09468.1| {|169(0)|<Acidobacteria(B)>}Bacterial DNA-binding protein [Acidobacteria bacterium DSM 100886]
{(GCRFNPPRATFLWLKDWHRVEFDVQADPELPRKNGAVNGRVAFYVGPVLVGEMEIQAHLVPVSGDAATAAA
TSVTATPYR)--KIFVSYSHDdAQIVEALE---
AAAEALGDVYLRDVRTLRAGEPWGpALLELIERADVFQLCWSHAAKRSRYVRHELTHALAQRRP-KP------
FIRPT-
YWERPMPAPPRSLKGIQfsYLAITHAGRHAADLSASRPGYPP(AAESRLGGPAAAGRSRTSGDAG)}*

$2831=185(185):
>gi|1028199958|ref|WP_063943878.1|
{|0(639)|<Proteobacteria(B)>}hypothetical protein [Enterobacter cloacae]
>gi|1017289106|emb|SAF52731.1| Uncharacterised protein [Enterobacter cloacae]
{()MIKCFLSHSSSdKERYVRIVA--EKIRRENRII--
DEETFEEGMVTAeEIIKYLDTSSLFVFFISNNSLNSEWV-QlELDRARELLDE-NTIkRVYPIIIDPkI-
DFKDTRIPEWMRNQLNIqlISNPTLAARKIHARLIELTWSDH(PRLKERRDIFVGRNDQIKLIEERLDDLQRQ
MPSVLIASGLPSIGR)}*

$2832=229(229):
>gi|1016918402|gb|AMY10834.1| {|245(0)|<Acidobacteria(B)>}hypothetical protein LuPra_04077 [Acidobacteria bacterium DSM 100886]
{(RLFSDQRTKKEFFAGDRGMADVNFTVFLERFSQDSRSYDMDARAFVAELHRRWVMRNPDEAAPAPAAAQPG
ESAATSSSG)--TIFVSYSS---
EDLGAAKQLVAELEEIGGDVaWFDKSALKPGDRWdeRLKSAVQRCSLFLPLLSgNTERRTEGYFRlEWSEAAD
RARKIqGRKFIFPTVIDPD-YAGAMQAYGLVPEAFKAFQFAHAPAGRLSDALKSELRDQL(RSLRRGKAQ)}*

$2833=198(212):
>gi|1028103108|ref|WP_063858745.1| {|0(263)|<Proteobacteria(B)>}tRNA-ribosyltransferase [Enterobacter cloacae] >gi|1017917838|emb|SAG37779.1| 7-cyano-7-deazaguanine tRNA-ribosyltransferase [Enterobact
{(MAD)---VYIIYAN---ENRDTALKVRDILSSS-WSVWLD--DLIVGD-FnsVIKENIQSTKCVIALYSSFAS--KPAVTgELSLANKYDKK-----VIPLILDDS-DPPYPYGHFSSIDFRAWQGDVDAEAVKmLQVKIGEVVKPQC(PPSRLEVVNNGLLPIPNIFMSVSSHETQFTPAAALSVLKAHNTSSILVSAYDLVKSKERKKMVKEIQAY)}*

$2834=215(222):
>gi|1020050625|ref|WP_063199145.1| {|329(0)|<Proteobacteria(B)>}RNA-dependent DNA polymerase [Serratia plymuthica]
{(GKDKVQAANKYHKIRYGKCDKNDEFAVESFRNHIYGRLSFIRMIRGAEWPLYLKLSAWMSSNDDNPTIEGRRAMKAMEMF)--DVFLCHAS-ED-KESIVMPIYDELCKLHIHAFVDTKYIEWGDSLteKINGALARSKFVVAVLSSNSVEKRWPLKELHSVLARE-----IAEGDKKLLTLVKDGDEELIKAKLPLLADKLYAVYKGDPIEIAHGLSSLLTG(KK)}*

$2835=209(219):
>gi|1020049811|ref|WP_063198331.1|
{|0(112)|<Proteobacteria(B)>}hypothetical protein [Bradyrhizobium sp. AT1]
{(MYH)---VFISYAQKnGEIAASLASQLDDLGLR---VF---LAERNLNAGEnwepTIRSALKNSKLVLCLITPESKTSGWVHAEAGAAWVLEKP-LLPVFRHVDPRELID-ILRMPLGRRIETSAQ--IGILLDD--ICKMFSVSRCER(SNGSQQSEEFTSPMGWQNLLKIGRWYRRDEDEALIGGGMHNYVLSHGHYSRGISIDAVLRFMDLAPVNRLDAVNAG)}*

$2836=238(243):
>gi|1020045582|ref|WP_063194263.1|
{|138(0)|<Proteobacteria(B)>}hypothetical protein [Bradyrhizobium sp. AT1]
{(EFRGVNLSKSILDGTLFADVNLSSAIGLATCVHRGPSIIDHITLQVSGELPKQFLTGIGLQEALILALPQIRTSLEHYSC)----FISYSSKDEKFASQIHKDLTRARIK-CWFAPHDMPIGGKVLDEIDGAISKRDKVLLILSEASLA-SEWVEdEVTKAFAEE-----RRrntkiLFPIRLDDAVelSQEPWAAKLRVRHIGD--FRRWTVRKWYKEGFQRVLRDLM(RQPEQDATSAPALKTETDEGASS)}*

$2837=212(221):
>gi|1020049125|ref|WP_063197645.1|
{|0(173)|<Proteobacteria(B)>}hypothetical protein [Bradyrhizobium sp. AT1]
{(MA)--DIFLSYTHVDRPRARPIVDLLEEQGWT-VW---WDrgIEPGMPWRPELDIELANCRAVVVLWSQTSTASEWVCHE-ARAGLKK-----GALVPILLD-DDEFPTEFAAVQATDLsrwngeTD--RTEVEALL--RRLARLVPPSRI(DTVRPGFDPDFLDETRPISLPGVNGAVAVLRYLHFTVVMNPARRLAHYVAYNCDGSQFVDEPRGADKWSPDPLLPPSLQ)}*

$2838=238(243):
>gi|1020516781|ref|XP_016097698.1| {|0(105)|<Chordata(M)>}PREDICTED: toll/interleukin-1 receptor domain-containing adapter protein-like [Sinocyclocheilus grahami]
{(MEENHMSSIMGWFRQRFGKQRNDLDQVQPGKCSQTCSSGHSSISAKPSASDQPLPSVLSSPYRSSL)RYDVFVCHnDEDSDLAQSLASFLEAPSSGLRCYLHERDCPAGGAVsTELLQAVQDShC--

WLLLVTPNFVKDDWCLYQMHQVLSEGP--MSQRIIPAVVNISSSSSSISAKPSASDQPLP----SVLSSPYRSSLRYDVFVCH(NDEDSDLAQSLASFLEAPSSGLRCYLHERDCPAGGAV)}*

$2839=220(227):
>gi|1021481482|ref|XP_016183884.1| {|0(1615)|<Streptophyta(V)>}PREDICTED: uncharacterized protein LOC107625704 isoform X1 [Arachis ipaensis]
{(MSSTASSSSQKKEPGLMKFTTSSYRNWT)-YDVYVSF---DRTTNKFVSDLYARLSAAGVRVFKDEMEPRS---GdqILFNAIEDSRISIVVFSRSYGDSKWWLKELEKIMECRSS-KGQKVVPVFYGVDPSEAGAAFA-YGEPLREAACLPGFVTYISRERDEALDNIVE(HITAFLDGKDHWFTLPNVEKLQPRVQDLIQVQLLNYDKVLILGIWGMAGIGKTTIAMAL)}*

$2840=230(241):
>gi|1021481491|ref|XP_016183888.1| {|0(1614)|<Streptophyta(V)>}PREDICTED: LOW QUALITY PROTEIN: uncharacterized protein LOC107625707 [Arachis ipaensis]
{(MFKPGPEKPPNRYDVQNPNLV)-YDIFLS-SG-NGTAESIIaytSCLYASLQRAGFSVFLDY----GHS-IfsSPLQAMQRARVSVVALSTGYAGSPRNLELLELMLECRRN-SGQFILPVFLGVTPRDMRKAFADFTKSVSE------KDKV-ECWKVALERICQI(QSYQWVYLNNYKWSDNDMHIKNVVGYVGGLLGTRKLFIAEYEVEVGYRIQDATKLLVHSKSKEVLLLGIWGMVGIGKTAI)}*

$2841=219(223):
>gi|1021485634|ref|XP_016185920.1| {|0(205)|<Streptophyta(V)>}PREDICTED: TMV resistance protein N-like [Arachis ipaensis]
{(MSSQNQE)-YDAYIS-SG-LQFLHPFISGLYDALKSVGLHVLADGTEPKTNK-LLLSGAIERCRVSIIVFTIAYAESTLYLQELVKIMECHRR-KDQKVVPVFYCLDPSQVCNLSGYFGEILSRTLQGisRDEHRM-LSYEAALLQQAAS(ILPRFLPDIWNNRKVMSDIVGHVTSLIDSAKLFIAKHPVGVDSRVQDLIQLLNNQKADGVLIIAIWGMAGIGKTTI)}*

$2842=149(156):
>gi|1021485650|ref|XP_016185929.1| {<Streptophyta(V)>}PREDICTED: disease resistance protein RLM3-like [Arachis ipaensis]
{(MSSQNQE)-YDAYIS-SG-LQFLHPFISGLYDALKSVGLHVLAD----RTEP-KtNISDAIERCRVSIIVFTIAYAESTLYLQELVKIMECHRR-KVQKVVPVFYCLDPSEVCNLSGHFGEILSRTLQGisRDENRM-LSYGTALRQAASI(LPRFLPDIW)}*

$2843=290(291):
>gi|1021561219|ref|XP_016171850.1| {|94(683)|<Streptophyta(V)>}PREDICTED: disease resistance protein RPS6-like isoform X1 [Arachis ipaensis]
{(SNIREGFGLIGDEDSSSYHSADSNMSQVSALSDDEDSSSYHSVDSNISEVSSLCEEDIAPVQFQDSNNSEECVDSHKQVY)RYDVFLSFRG-PDVRNNFVDHLFERLTKKGIFTFKDNvRLKRGKR-VkpELMDAIRDSRLAIVVFSSTYPTSTWCLDEMSAIADLHRQ-EKQIVVPVFYGITSSDVGREGGPYDVHFNSEQYSKIPNRV-DRWKFDAKYLAKA(YGFPINPERPETKQLEEIIAFVTKELNHKFTIDISDLIGIQPRVADLERRLKLTSEDVPFQILEIVGMSGI)}*

$2844=145(165):
>gi|1021569823|ref|XP_016176109.1| {<Streptophyta(V)>}PREDICTED: TMV resistance protein N-like [Arachis ipaensis]

{(M)-------------------SHLQQALNKQQIKVLIDHKLPKGHN-
IwsSLLNAIERTHVSVVVFSQNYASSKWCLDELVKILDTMKQ-
QGQVVLPVFYGIDPTHVRNQLGSFKQVFETHVQKFDPIVV-
QDWKIAITEAANL(ISWDSKSPYIRDDDDLIQQLVKMF)}*

$2845=125(154):
>gi|1021573032|ref|XP_016177601.1| {<Streptophyta(V)>}PREDICTED:
toll/interleukin-1 receptor-like protein [Arachis ipaensis]
{(MNY)KHDVFISFRG-TDVRRGLLSHLKKYLQHKKIDFYVDD--------PKLNKGDKISSI----------
---------LTIIECAEK-NEQIaVVPVFYKLVPADVRHERGDYAAAFVKHEAREDKDKV-
QKWRSALAKAAEI(SGFTYPLDSEL)}*

$2846=221(220):
>gi|1021481474|ref|XP_016183880.1|
{|0(1367)|<Streptophyta(V)>}PREDICTED: disease resistance protein TAO1-
like [Arachis ipaensis]
{(MTSSTTRNQYRVNPYRTRM)-YDVFISVsRQ-HPHRQHFASRLCGALIDA-
GVHVCDEILLRTGDFVfsqsvGVPAGIEGSRVSVIIFTKEYLDSAWCFQELLKIMECRSS-
KAQVVVPVFCGVDPSEVRDYSGHFGRVMTSL-
DGKSTPRPCHVTTLQEAADISP(SFVTVSGKEMESIKSIVEHVAFLIDSRDLFVAQHPVGIKSRVQDLIQILN
IHEKPNDVLVL)}*

$2847=173(203):
>gi|1021554510|ref|XP_016168641.1| {<Streptophyta(V)>}PREDICTED: disease
resistance protein RPS6-like [Arachis ipaensis]
{(MTHATTSQAIFVLLLTKARY)-------------------------------IQVFIDNR-
ISKGDENssSVFKAIRDSNVSVVVLSKDYASSTWCMHELNKILKQKRhG-
KGHIMVPVFYKIDPSHIRKQIGTYKKAFEKH-
ERDVKHNILLKWKAVLTEVCNM(ESKHRVKESKHLVEMAKSITTRTVGVISKIDQAAAEPKALAAV)}*

$2848=239(232):
>gi|1021555972|ref|XP_016169384.1| {|0(192)|<Streptophyta(V)>}PREDICTED:
TMV resistance protein N-like [Arachis ipaensis]
{(MANHGVASFDYT)-YDVFLSFR--RGVHRAFIDHLYKALDCiD-
IKTFKDDDKEEsttEEEETnpDLIEKIERSRISVIVLCKDFPFSTRCLDEVAKIMECYDK-
KTMQVVGLFYDgVIAANVRWQKEDtaYGKAITQH-
KErlRLGEESDkiKTWRSALSRVCDL(IALNCDKKYETELISMIVKGVSARLPPLRLQMKDVVGLDSRFEEVK
SHLDIGNNDAVQMVAICGPPGIGKTTFAAYIYNN)}*

$2849=301(300):
>gi|1021558566|ref|XP_016170584.1|
{|237(549)|<Streptophyta(V)>}PREDICTED: TMV resistance protein N-like
[Arachis ipaensis] >gi|1021558568|ref|XP_016170585.1| PREDICTED: TMV
resistance protein N-like
{(FDSHRERFKEEPDKIYKWKSAMTYLANLSGFALRDPAEAEGIERIVEAVLDTLVYKFQSSRFTHKNPESTE
RRPSLQEFN)KYDVFLSLRR-THTPYIFIDYLYHYLtKKG-
FSTFKSEEETERGDSIpsQDLQVIKDSRILIVVFTRDYADSTCSLEEVATIVDCHRE-
LNQTVIPVFCDVHPRDVRRQTGPYERAFVSY-
TKEFKQDPLkvQKWREALNYLTNL(QGVSLKDGSEIEAIGNIIQIVERLCPKFSRLSSHGPVGIQSSLAMLEK
LLKLEDDCVLVLGVWGMGGSGKTTHALALYDR)}*

$2850=226(226):
>gi|1021502829|ref|XP_016194625.1| {|0(808)|<Streptophyta(V)>}PREDICTED:
disease resistance protein RPP13-like [Arachis ipaensis]

{(MAASYF)KYDVFLSFRG--
HTRREFTDALYHALVNKRIETFRDSENLRIGEELegALLEAIERSRMSILILCDEYPTSKWCLDELVKIMECS
GNGTKRPVLPVYFRVAKSDVQFQKNKYETAMAAH-
QAKgRNNHKLEAWKSALSEVGKI(YGQLCDQKTAWGEAIDNIVEEVTTRLPPPPLYIDRPLGCDSELEEAKSI
LEIGSHATCFMLGIHGDGDEISKFVAELYNK)}*

$2851=158(158):
>gi|1021505853|ref|XP_016196337.1| {<Streptophyta(V)>}PREDICTED: TMV resistance protein N-like [Arachis ipaensis]
{(MADSSDHDSTPLSYF)KYDVFLSFRG-Y-
TRFGFTDTLYHALINNRIDTFRDSEELRIGEELegALVEAIERSRMSILILCDEYPTSKWCLDELVKIMECSG
NGTKRPVLPVYFRVAKSDVQFQKNKYETAMVAH-QEKgRNNHKLEAWKSALSQVGKI(YGQ)}*

$2852=136(167):
>gi|1021505857|ref|XP_016196339.1| {<Streptophyta(V)>}PREDICTED: TMV resistance protein N-like [Arachis ipaensis]
{(MKGRGQIVTTGGSSSSDKGAALSSHFR)-YDVFLSFRG-Y-TRLKFTDTLYHALINKRIDTF---------
-
EGSLLEAIERSMMSILILCHKYPTSQWCLDELIKIMECSENGRKPPVLPVYFNVAKSDVQFQLNEYGKAMAAH
-EEK-----------------GR()}*

$2853=187(186):
>gi|1021513817|ref|XP_016200399.1| {|0(448)|<Streptophyta(V)>}PREDICTED: TMV resistance protein N-like [Arachis ipaensis]
{(MATQQASNSCSFPSLREW)EFDVFINFRG-
PETRYGFTGYLHKAFCEKGIRTFMDAEDLISGNKIleTFDRAIETSRIGIIVFSAHYADTDFLLRELVKLLEC
SRRKGQDEFEYEFIERTSKQVLAFLKEDTLPVVADYPVRAESQVKSSSSFSNPRQYQH(HVFLNFRGCDTRYR
FTGSLYKALQDNKI)}*

$2854=171(177):
>gi|1021777509|ref|WP_063380863.1|
{|0(653)|<Proteobacteria(B)>}hypothetical protein [Pseudoalteromonas luteoviolacea] >gi|1021382144|gb|KZN67519.1| hypothetical protein N478_01850 [Pseudoalteromonas
{(MI)--KAFLSHSSKdKDhYVRNVANWL-----GKDDIIY---
DEYTFEEGEkpldEIIEGLDRTEIFVLFLSENALKSEWVIREISEAKIrLDSN-
QISKIFPIIIDEKVQYTDDRIPDWLRDNYNL--
KPIKRACISARRIHNKLREIS(WKKHPELKIRESYFVGRLRELDQFEERIHDFAKEK)}*

$2855=269(290):
>gi|1021006547|gb|KZM73892.1|
{|429(1018)|<Actinobacteria(B)>}hypothetical protein AWN90_35780 [Nocardia terpenica]
{(AVTVQMLAERFVGIVQAVVADVSVRLSALPVVAGAEWTDVVVERNRTAVGSPETGTVVSLLEGWADRAPDA
VAVVCGQRR)-----LSYRELD-AWANGVAWRLRDAG----------
VGPETVVGVCVERGVAAIVAIVAVLKAggAYLPLDPaYPTdRLAGILA-------
DVAVPVVVTDSStsSVVPVAAGVGLVDVGE--FEHASDRG--
RLAPTAGPENMA(YVIYTSGSTGRPKGVVVEHHSLSNLGSFLASIYPDAGVSRSTVGLFASLVFDSSVKQLLP
MMCGHVLHVI)}*

$2856=270(270):
>gi|1021007757|gb|KZM75099.1|
{|25(1261)|<Actinobacteria(B)>}hypothetical protein AWN90_24300 [Nocardia terpenica]

{(GTRAAKTASLNRVVAAVRACSADVELDTTGARHPEGRTIGFVAERATESEPRLAELGTVGTRFVDGQTATVSRDGKPLVR)---VFLSYSAEdERRAGELWKRLSefaAIDNRFQFEFWDfkSESLLPGDDWHrKVLDAVDRGDLGIFAVSRHFLASEYIHRhELPSFVERNAA------VPVLLEKM-DLDKVDLKGLEGRQIFRpaGRAFDQLRGNAAKNTWVQALRDA(VHNVLDCRSVAARPTDGTRDHRSDRKRSARARLTDLDSVPFLTRVLGRPG)}*

$2857=200(200):
>gi|1021719640|ref|WP_063364102.1|{|0(624)|<Proteobacteria(B)>}hypothetical protein [Pseudoalteromonas luteoviolacea] >gi|1021359302|gb|KZN45397.1| hypothetical protein N476_05105 [Pseudoalteromonas
{()MIKAFLSHSSQdKEHY--VRNVANWLDKDEII--YDEYTFEEGERPLdQIIDGLDKSEIFVLFLSDNALNSEWVIKEITEAKIRLDS-NQInKIFPIIIDDkI-QYTDSRIPEWLRDNYNLkpIKRANVAARRIHNKLREVSWTKH(PELKIRENYFVGRLNELDSFEERIHDFSKEKPTVLICSGFNGVGRRSLIHKGCLKTNISE)}*

$2858=176(180):
>gi|1021776591|ref|WP_063380144.1|{|0(234)|<Proteobacteria(B)>}hypothetical protein [Pseudoalteromonas luteoviolacea] >gi|1021384105|gb|KZN69456.1| hypothetical protein N478_12550 [Pseudoalteromonas
{(MHVN)-KQVFISYSWLdMKIADQIEQDLSMFQLTLVRDV---RNL----EYKqSISsfmERIRDADFAILLISDSYLRSKNCMKeVLHLLEERNYE-EK--VLPIMVKGT-QIYTTQERLKYTTHWVEerENLSALINSMPVTAIVNEIAELK(TIETIASKTNEFLAYISDKKNLTFDELKDEGYVSIL)}*

$2859=200(180):
>gi|1021715468|ref|WP_063360741.1|{|0(343)|<Proteobacteria(B)>}hypothetical protein [Pseudoalteromonas luteoviolacea] >gi|1021366708|gb|KZN52569.1| hypothetical protein N476_10930 [Pseudoalteromonas
{(MNNYIR)--YIWVSYAGSQkdWVYEltkifdqspdtniKLLTYLRE--EDSEGyskpCSQEEDtaptyipFLKPTQRIEDLIQAIGASFRKIIVLSPDYLDSPYCMQEL--AC-ILIHRHSNAPFIILSGFTDLLhAFEAREYKFLENnSYTLGQALAHIY-LERQSADVSKDCV(DFKLIDCPDPRDDKELALTFAINFFEKKLVELNQ)}*

$2860=208(215):
>gi|1054545884|ref|WP_066315340.1|{|0(131)|<Bacteroidetes(B)>}hypothetical protein [Aquimarina sp. RZW4-3-2] >gi|1023039388|gb|KZS39660.1| hypothetical protein AWE51_08390 [Aquimarina sp. RZW4-3-2]
{(M)--KIFISWSGKlsNEFSTILGDWLNCI-LPNIPTFISSSS---LSPGDrwsdKLAKELEQCNAGIICLTNENLMSPWINFEAGALskHINNSM-VIPLLINIKDSEISKSPLSQFQTLTIDKS------GLKKL--LIKLFDSLDLPN(NKIDIIFDKWWDDLEVKLNSLNFPDKRKSDNEKYENLLLNYNDVNEGLKIRVNQIEKILSNLTEQNNESIDSNL)}*

$2861=206(220):
>gi|1023039248|gb|KZS39521.1| {|167(0)|<Bacteroidetes(B)>}hypothetical protein AWE51_25660 [Aquimarina sp. RZW4-3-2]
{(SIAMPILATGDAMFPIQKMLSVILESSINWLKLGLDLDTIKIVAYNEQQAKTAKLIFNDYKEKEQLKHTHKIKRDFKYDY)----FISYSHKnSDEAKILLDSL--

LKKSSSTKVFFDKNsLKAGHAWQqELFEALDDCKQIFTLLSPEYLDSKVCKEEYHIAYYRQRE-
SDLNImLPVYL-------------YSANLPTYmkMIQYHDCREGATDKLFDLVNNHI()}*

$2862=224(215):
>gi|1027836546|ref|WP_063733912.1| {|0(148)|<Firmicutes(B)>}hypothetical protein [Lactobacillus plantarum] >gi|1023773876|gb|KZT93046.1| hypothetical protein Nizo2259_3192 [Lactobacillus plantarum]
{(MVENP)--KVFISYSHQnSDYNKKVLDFSNNLRLKGID---
TNIDQYVEAPSEgwpqWMEKQIYDSDYVLVCCSKSYIEKFRGNTEgLGISWETSMI-
YQFIydnattnskIIPVFFDEKDKSFvprpLKKYTFYNLSKEAD--YIRLYNR--
LIGKTTNNKAPL(GQLKTLDEKKPKTLFVSTPIDIEKWNQAKWRGIMYICIAGDVPVLGLVFKNYQRGLEIFD
QWRNLSTDRY)}*

$2863=242(238):
>gi|1025148140|ref|XP_016467568.1|
{|0(1164)|<Streptophyta(V)>}PREDICTED: TMV resistance protein N-like isoform X1 [Nicotiana tabacum]
{(MASSSSSFASNSQYCPRW)KYDVFLSFRG-EETRKTFTGHLYEGLRNrG-
IFTFQDDKRLEHGASIseELCKAIEESQIVVIIFSKNYATSRWCLDELVKIMECKTQ-
FGQTVIPVFYDVDPSHVRNQRESFAEAFSKH-
ESKFKDDVEgmqkvQRWRSALTEAANL(KGCDIRNRIESDCVQQIVDQISKLCKFSLSYLQDIVGINPHLEEV
KSLLQIEINDVRIVGIWGMGGVGKTTIARAIFDTL)}*

$2864=156(159):
>gi|349858612|gb|AEQ20328.1| {|0(760)|<unknown(B)>}putative toll-interleukin 1-resistance protein [uncultured bacterium EC5]
{(MVK)--DVFISYRH---
TRKAEVIAFCEQLEAAGITVFRDEERVNEDSLIqrGIEHGLGNCKLLLAWFDPGYLDSRACTWELGRALIAGR
RLGDTFEHALQRIVCIDPTGSFSHIPEEIWNRRVLDSKDPGACVAHVREQLAGITG(TFGRVPADFTGNWYER
)}*

$2865=233(230):
>gi|695368935|ref|XP_009513925.1|
{|0(851)|<Stramenopiles(E)>}hypothetical protein PHYSODRAFT_470533 [Phytophthora sojae] >gi|348686836|gb|EGZ26650.1| hypothetical protein PHYSODRAFT_470533 [Phytophth
{(MHGGGSVTRLEGDAGEPGSQPAATACVHTLQYTPSARLK)---IFLSYGHDRFQALAFHLKAQLQRRGH--
VVWLDIEKLSAGiDWEeGIARGLSwvrdagQDGRVLLVMTPHALRR--PDGycLNEVAR--
AASLrLNIFLVMVAESAPPPSIAMLPfFDMRDCVPGEAEQVHAKDSPEWRDLMKACLETP(NFLEKAERLYTM
LELVRGSSCRLLTLTLGSFTLIICFDSMTSYGVPAVAGL)}*

$2866=171(188):
>gi|493182769|ref|WP_006180498.1| {|0(41)|<Euryarchaeota(A)>}TIR domain-containing protein [Natrinema pellirubrum] >gi|433306235|gb|AGB32047.1| hypothetical protein Natpe_2222 [Natrinema pellirubrum D
{(MTGEQVYVSHAP)-----------DDLDLVQDLFSTVKNFPF--
GVHIAHEEVESGRSRtRLEGRLANSDLVVAVLTEGAAD------
DEWINQEIGYAVaKGIPVLPLFDEGIDRRGFVSDVDGVTIDRGDLSATIFNLLCRLRSELAPLGALS(VPNWY
IRFPCTIPDCRGPVTLELEHDQSKLWKLHAH)}*

$2867=249(254):
>gi|695391778|ref|XP_009520132.1|
{|325(134)|<Stramenopiles(E)>}hypothetical protein PHYSODRAFT_481651

[Phytophthora sojae] >gi|348685029|gb|EGZ24844.1| hypothetical protein PHYSODRAFT_481651 [Phytoph
{(ASPELFSELTAAGGDIRYQLPTTGDTALHLAVRQENEASALAALASGGDLMRMNHEGLRPLDCTASTRLLFELKRAAGQR)--DVMISYTH-SHLE--
FARKLRQTLEEANVTTWLDLMDpsgIGGGAVWreEIARGITNASVVLCILTEDYASSDWCLKELALAKQVG-----
TPILAVSTEGTIVDENLQVYLYTRQIIPFEPAIVGLRRDEGRIEYEYDESRY(RSQFHLLLDGVRDEVEKHRYFTRQKNVASSLKPG)}*

$2868=281(290):
>gi|695394378|ref|XP_009520869.1|
{|621(180)|<Stramenopiles(E)>}hypothetical protein PHYSODRAFT_257795 [Phytophthora sojae] >gi|348685766|gb|EGZ25581.1| hypothetical protein PHYSODRAFT_257795 [Phytoph
{(NVSVDLFECLVQHGADIDDRLPNGDTALHIAMKCEQKGTALALLDAGADVMETNRDGYRPVDCTTSTQLQFEIKQAAGDR)--DVMISY---
THSHSEFALKIRDSLERANITSWLDLMDptgIGGGSVWreEIARGIKNAEVIICLYTEDYPVSEWCLKELALAKQLG-----KPIIA----VSPEGAAVTDEmqVYIYTRQMVPFEQAIKSV--
NNANKRKITYEY(DEERFAAQFRLLLDGVRDEIEKQREANVTGSRRRLANGTSVVGFGENITDWDPATLDNLSFAFVSHGDHH)}*

$2869=248(257):
>gi|695394378|ref|XP_009520869.1|
{|814(20)|<Stramenopiles(E)>}hypothetical protein PHYSODRAFT_257795 [Phytophthora sojae] >gi|348685766|gb|EGZ25581.1| hypothetical protein PHYSODRAFT_257795 [Phytopht
{(QAIKSVNNANKRKITYEYDEERFAAQFRLLLDGVRDEIEKQREANVTGSRRRLANGTSVVGFGENITDWDPATLDNLSFA)----FVSHG---DHHHTFVKRLHDRLQDNSVRCLLDGTQTYA--
DMteRIhaaKEAILKCDVFIVVLSRKTVNSELVKDQLAFAEDKG-----
KPILPIMLNDMEIPLDKHYTLSRVELLHFTPELGFNSSFMQLLGRVRDNMTT(ANASFNAPRASARPTFRAVARITAMSHARQNNSMAVN)}*

$2870=221(220):
>gi|351592811|gb|AEQ51148.1| {|0(92)|<Proteobacteria(B)>}TIR protein [Pelagibacterium halotolerans B2]
{(MKNEFD)-YDVAFSFTA---QDEALATQLND-
LVSDRLKTFIYSEQQKALAGTdgqTAFSDVYGKMARivVILYRKEWGQTRWTGVEMNAIRTRAFEHGFDFTVFIPVEPSPstpAWLPPTRLYVGLERWGV-EGAAAVIE-
QRVIDAGGVSQPE(SVAERAARLKRATDLKNAQHQFERSQEGTALANGAYEEFSDKLSAGTKTVQASGVKLEYKTSQHFRIVTCYPVN)}*

$2871=195(201):
>gi|349858617|gb|AEQ20333.1| {|0(732)|<unknown(B)>}formylglycine-generating sulfatase-like enzyme [uncultured bacterium EC5]
{(MTKNIAAVPAIP)----FISYSNTDWDAAVELRERLRGAN-----
IECTELRLPGRWMTGLQQVLTECSAFIALIGRDGVQ--RYVGaECEVALsrhlslrGDG-----
PRLPmhPVLLPSVTpeDLPPFLALFQAERWAP--
GTDLPPGLCKALHDGVKRLQQA(VRFEGCPFRGLGAFRRKHAALFFGRQRETLEALAGLGDQRPAGTLQSLA)}*

$2872=231(227):
>gi|504590641|ref|WP_014777743.1| {|432(0)|<unknown(X)>}TIR domain-containing protein [Thiocystis violascens] >gi|390426194|gb|AFL73259.1| hypothetical protein Thivi_1236 [Thiocystis violascens DSM 19

{(NIGLLGRVQQNAASGGEFQYFRKPFEFDWNQKEILVEKAIYLLHPGLTSSICRERGMNLNRVNIIGIDRKW
LSKGEHNGV)-
PKIFLSHSSIdKLFLEKILPLLEHrLNLKFPSDFWLDKwKIRPGDDIHqKVELGVAGSDIVILFASANSLASG
WVEKeWRTKHLEEISN-GEIRVIVAIIDGT-
SPKKLPEFLKSKLAIVLseTDYEYSVGDLAESIGYHASTHLA(RLFPSGC)}*

$2873=188(189):
>gi|504592600|ref|WP_014779702.1|
{|0(76)|<Proteobacteria(B)>}phosphopeptide-binding protein [Thiocystis
violascens] >gi|390428233|gb|AFL75298.1| FHA domain-containing protein
[Thiocystis violascens D
{()MPRVFISYSSKD--LDFIRTQLQPILDELGMVAWcsaTDMRIAA--
DWErQIRVALAQADWFVVVMSPDAQTSDWV-QaETHWALE-HKR-G--RVIPLMVRSC-
TPEeiHLKLGKLQYIDYRHdpAEAAARLRALIGEPASNAITRMT(APIPMHDACDATVIRERLAAEVLLFVEP
DSQPGYEQRLRIQSSAMIGRM)}*

$2874=243(218):
>gi|493799656|ref|WP_006747598.1| {|0(125)|<Proteobacteria(B)>}molecular
chaperone Tir [Thioalkalivibrio paradoxus] >gi|570726950|gb|AHE98258.1|
molecular chaperone Tir [Thioalkalivibrio paradoxus ARh
{(MSYVGPPFS)-
HDIFVSYSHGddgngqSYLQSWSVAFAAELGRelkvdrryREPLRIFLDQEHRPGHGvdplaPLtdQLRREIE
ASALLLVLMSPDYLASPWCEDEREWWCKRQTatglpiEGrvvIVqILPTEEVWP---
DAFSDQRGHRLKGFRFHTSGKIPARP--
LGWSVLPGPRPF(GQDFDEALLDIVGRLHLKLDDMQRVLEEQLRARAGAERLAHTDGQVLYLHGRVDQARRWE
DTAIALTQE)}*

$2875=198(223):
>gi|1025371766|ref|XP_016514882.1| {|0(195)|<Streptophyta(V)>}PREDICTED:
TMV resistance protein N-like isoform X2 [Nicotiana tabacum]
{(MVK)--------------------------KRG-
IHAFKDDDRLETGKSIsyELLKAIEESRFAVVIFSKSYASSKWCSEELAHIIKCQSE-
LEQTVIPIFYDVSPSDVRHQNPPFAESFSQH-
EEKYKDDMEkvQRWRDAFAEAGKL(SGHDLKNYKDEVDCINKVVDYILPKSLQAIPTSSGSLVGVEPQIEKII
SLWDMESNDVRSIGIWGMSGIGKTEIVSIIYE)}*

$2876=257(267):
>gi|1025641724|gb|OAA29507.1| {|92(46)|<Actinobacteria(B)>}TIR domain-
containing protein [Frankia sp. EI5c]
{(ALVAADWGSYLDSLKSRIFADPGSWIIPVATTRNHLNLRLIADLSVLELQGADLEFWDQVLVNRVMHRAYR
LLLDSAPPL)--QVFVSHAVRD---GLEISHRFcEYLNRR-TdltyFISQRDIASGDQFAEVIEKAVGEC-
ALLAVLTDAY-SARPWCQrE---VLAAK-----DNMLPVVVLDALsdGTARLFPYLGNAPVVR--
WREDSPETMEKIVGKLLDTALR(RTYFPIRAAQVCGAHRLPVPDGLVWQPPELATALEHSRRVTTRDDTA)}*

$2877=188(191):
>gi|1057443012|ref|WP_068788738.1|
{|0(782)|<Cyanobacteria(B)>}hypothetical protein [Phormidium willei]
>gi|1025909835|gb|OAB58561.1| hypothetical protein AY600_10545
[Phormidium willei BDU 130791]
{()MTDAFISYCRRdKEFVQKLYQAFCDAKR----
EVWVDwENIPLNSDWRdEIYRGIEAGDNFVFVLSPDSVKSQVCREEVDHAV----A-NNKRLVPIVHKEV-
SYDevHPELAKLNWIFFYDdpETFDTAFPKLLSTLDTDLDHVKA(HTRLQKRALEWDSKNRNPSYALRGDDLK
QAEQWLGIAEGKDPKPTPLQTQY)}*

$2878=257(264):
>gi|1180479882|ref|WP_083986249.1| {|0(742)|<unknown(X)>}hypothetical protein [Frankia sp. EI5c] >gi|1025641848|gb|OAA29631.1| WD40 repeat-containing protein [Frankia sp. EI5c]
{(MVPDTGADMPTTGNEASLRAGHTSARVAANGSTPSTGETAGIPAKEQRIF)RYDAFISYSHRqkelaMELR
RALHRLGRRWYERPRLNVFRDTDNLPAVGPLppALDRAVRESRYLVVLASEEAARSRWVELECQTWLAAGGDl
DrlvIVLANGSP----------------PTSLPPCLRNLIDEDW--
LLTDVRAPTPGP(KRGQIFDAAAAVSARIQGIDKTDLLSEDHRNRRRAVGLFATLAALALVFAVAAGVSASYY
AVERADAVRQRNAA)}*

$2879=232(231):
>gi|1026009098|ref|XP_016556283.1| {|0(12)|<Streptophyta(V)>}PREDICTED: TMV resistance protein N-like [Capsicum annuum]
{(MDSQIPTVKKW)KYDVFLNFRR-QDLEGNFVTQLYKRLEDMGINVFKPEfEYERKEE-
PiktEISEAIEESMIAITIFSEYYASSRQRLEELSKIMEWVDN-
QGQEFYSVFYCVTASQVSGIIDQTLAQYiaDDIIFSYTVE-L-
QRWTDAFSKAFNI(AGFKRDSFPLELIRWNAEECIQEIVDKISLVTSIAKYHVGIKSRIEKVKSLLNVESGGV
YFVGLWGLGGNFLVGTPLNKE)}*

$2880=235(231):
>gi|1026009162|ref|XP_016556315.1|
{|0(1476)|<Streptophyta(V)>}PREDICTED: TMV resistance protein N-like isoform X3 [Capsicum annuum]
{(MASQIQSVRNW)KYDVFLNFRP-EDLGRIFIAHLYKRLEELRINAFRPDDVEsergEGEP-
IstKILEAIEESRIAITIFSKYYASSRRCLQELTKIMECVDN-KGMKFFSVFYQSTASQVL---
CNFDQVLLAQlgiygLNGSYMVEF-
LRWKDILCKALNI(AGLNCWNVEECIQQIVDKISPFPRIAKYHVGLKSRIEEVKSLLNVESGGVYFVGLWGLG
GVGKTTIARGIFDEISCQFEG)}*

$2881=228(225):
>gi|1026079158|ref|XP_016538801.1| {|0(536)|<Streptophyta(V)>}PREDICTED: TMV resistance protein N-like isoform X1 [Capsicum annuum]
{(MATEF)KSQVFLSYYG-----NTFADHLYEALVGA-
GFVTlRGGDGNEGGEQIkfELQKGVEESGVSVIIFSNDYVFSSLCLDELVMVLDWKRI-
SRGAVLPIFYHVDPSDVRKQKGRIGEAFDRH-
EEELGRNqsgnewKAkvRKWKEALKQVADL(GGMVLQNQADGHESKFIQKILKVVENKLSRPVLYICPHLIGI
ERRVEKINSWLEDGSTDVDTLVICGIGGIGKTTMAKYV)}*

$2882=209(204):
>gi|1057215916|ref|WP_068589075.1|
{|0(199)|<Bacteroidetes(B)>}hypothetical protein [Ulvibacter sp. LPB0005] >gi|1025932918|gb|OAB80846.1| hypothetical protein ULVI_01920 [Ulvibacter sp. LPB0005]
{(MTSENKT)-PTIFLSYSW-SN--
KKEATLLTKDFDEIGIPLIKDTITLKYKDsLTDYMQSIRNTDFAIILLSDEYLKSQNCMFEAIEIlKEQNHK-
EKILPIlinnPIIFK-
AQDRIKYIKYWRNKRdLLKAeleeLDVTSAIDSYNDLKIIEIIYSSI(DSFLKTIGDLKTSTLEELKEENYKS
IIEYLGFEDISFVLDLLLIMRIENLVIKEYAL)}*

$2883=227(226):
>gi|1026781548|gb|OAE39996.1| {|271(0)|<Proteobacteria(B)>}hypothetical protein A7J42_14580 [Ochrobactrum intermedium]
{(LKRTIRDRPDHRGSVGQVVSKGYLETLMRDKADILSEFFHYQPENSVFTVEDPKLIFYLRSINWLNFVKEV
GFSTAEFRG)KYDIALSFAG---

RNRDLAELLFEKLTQREVSVFYDLDHQHRIIAQqveDYLAPIYSSEAAyvVPLLSVEYPTRIWTKFESDHFKD
RF---GKNAVIPIRYHDAPdgY-FSSSYGFGSLSfdpNKPI-EPQAELIV-
ETICKRLEEDRIA(SGNQPL)}*

$2884=185(172):
>gi|1027760826|ref|WP_063692917.1|
{|0(321)|<Proteobacteria(B)>}hypothetical protein [Bradyrhizobium stylosanthis]
{(MTDNRVQF)----
FMSYAwndnrlppgeHPdKqpfvsALARQIEWHFNYAKPNSPLLWWDRDNIDDGTQFRpAIRAAIEASSFLVI
VLSEHWLASEYC-QlELKMFRERWQH-ESDFDFKHRIILA-
HKSHVPKERCPELLPEQrgFQFFSKAGPHYTLWRPSEEFYSV(ANDLAQILIKRAKFEYPPPPPPPP)}*

$2885=229(226):
>gi|1028065043|ref|WP_063833169.1|
{|0(280)|<Bacteroidetes(B)>}hypothetical protein [Aureispira sp. CCB-QB1]
{(MHLKATYTL)--NIFIAYHPKdQVQFEELLHNLEGLDKKNagyvVNKVWYDGT-
DLSESGRaSIRELTEAADLVLLLMSDNSILSPFFISqELRETLQQHDN-EQSIVVPIILNTC-
WWEDTVFRNLDVLPRAGlpIYDSSNiKN--
ELFDQVVQELDRK(LEKVRQRKEELEETFKSKITEAEAIFNNWQKRPGRLRAALPLYKEAVKLWREGFAPDIK
IIEAHVDICHREIDFRHY)}*

$2886=118(141):
>gi|1028946866|ref|XP_016716734.1| {<Streptophyta(V)>}PREDICTED: toll/interleukin-1 receptor-like protein [Gossypium hirsutum]
{(M)-YDVFLSFRG-EDTRDSFVSHLYNDLdRKN-
IHIFKDNEKVGRGDEIsgALLTAIEGVRVSVIVFSKHYASSRWCLDQLVKIMDCKKL-NGHnFVFPI------
----------------Q-RDSNMLPSTSSSSSSAAANMIM()}*

$2887=196(222):
>gi|1028973567|ref|XP_016730247.1| {|0(291)|<Streptophyta(V)>}PREDICTED: TMV resistance protein N-like [Gossypium hirsutum]
{(MG)---------------------------KG-
VHTFRDDPKLEAGEEIspELFKVIQQSRGSIIIFSETYAFSSWCLEELAEIVKHKNE-
RRQEVFPIFYNVDPSDLRKQKGKVEEAFVEH-
EERYKEDKNkvQKWRNALTQVANI(KGWHLNNRHEAEFIGDIVKKLSVKLCETYPIAATDELIGISSRLKELY
SKMEIGEDDVRVIVICEMGGIGKSTLARVMYD)}*

$2888=197(221):
>gi|1028960181|ref|XP_016723536.1| {|0(127)|<Streptophyta(V)>}PREDICTED: TMV resistance protein N-like [Gossypium hirsutum]
{(M)KHQVFLSL-G-EDTRLNFTNHLVNAL-
EKvGINVFPDNETLKKGEKLppTYSRAISASNLSILVLSADYASSKSCLVELSDI-----------MI-----
-------RNGGHFKTSFEEH-
ESEQPADRVEQWKTAFEEVGKL(KGWHIEGGKFDRPETAYIKDVEYVIKKLTNIGFESASEELVGIEYQKNAI
LKLIKQKDCRVIGLWGMGGQGKTTLAEAVY)}*

$2889=172(181):
>gi|1028862983|ref|WP_064031576.1|
{|0(54)|<Proteobacteria(B)>}hypothetical protein [Methylomonas koyamae]
{(MDCYT)----FISYQTADKQIAGRIKELLESIGIA-
SFLAHEDIDVSEEWRLKILEEIRKASVFICILSKSYFESSWCVQeSGIAAFRAD-----
MTIIPLSLDGSIpqGFFSHIQSTKISLENL--
ALPVLFPALIQHDCTGTINEII(RIIGRSKSFRGAEANFKMILPYLDDLTPEQNKDLLE)}*

$2890=184(188):
>gi|1057235375|ref|WP_068606006.1| {|0(563)|<Firmicutes(B)>}hypothetical protein [Paenibacillus swuensis] >gi|1028463531|gb|ANE46454.1| hypothetical protein SY83_09400 [Paenibacillus swuensis]
{(MDFSKVMDMGTGVP)---IFISYASLdVDPVKELV---ANIEERGY-SCWYaNRDIRSSAQYNtVINEQINNCEIMIVMVTPNVEHSEFV-PkEISLAYEKKNE-KKPTVLPILWNVD-SYPSNVSLFLTNNQHLNrkAFEDLNSFYNVLAEDIEEIYAKR(EDRVSDERIDRKPARINNYFIKNLNTISTDWLNQ)}*

$2891=169(170):
>gi|1028415353|gb|OAI45624.1| {<Planctomycetes(B)>}hypothetical protein AYO44_12605 [Planctomycetaceae bacterium SCGC AG-212-F19]
{(MALGHNDVVEMIRARYNVLV)RYSCFISYGG--P-DKDFARMLYESLTKQDVQAFFFPTHAVPGKRLhkVMRKGVNQYD-RIILICSKASLDRPGVLnELTETLQREAREgGKDYLIPITLDNY-VFKDWAPNEPQLAQAVRDRVVADFTGTIDDKVKFKKAFKK(LMSALDKPLT)}*

$2892=200(201):
>gi|1029083745|ref|XP_016702041.1| {|0(365)|<Streptophyta(V)>}PREDICTED: TMV resistance protein N-like [Gossypium hirsutum]
{(MVRFDNETSSSSSSSSSSLESSSSVSRG)KYDVFLSFRG-EDTRRNFTDHLYDAFKRrG-ITTFRDDEKLETGEPIapELFKVIHESWCSVIVLPEGYCFSNWCLQELSEIIQQKND-KGYPVYCKARVGALDSLGCCMLTMASTGVNS-YTYLKQSTWTLNADAFLTMNRL(RLLRFFNVPNSRDFKYLSNELRLLEWHGYPFKS)}*

$2893=196(223):
>gi|1029083865|ref|XP_016702107.1| {|0(73)|<Streptophyta(V)>}PREDICTED: TMV resistance protein N-like [Gossypium hirsutum]
{(MMR)----------------------------G-IITFRDDEKLETGEPIapELFKAIHESWCSVIVLSEGYCFSSWCLEELSEIIQQKND-KGHNVFPIFYYVDPSDLRKQTGKVQEAFLKH-ENNFQDNKErtQRWRSALTQVANI(KGWHLSNRHESELIGEIIKKVSTKLCQTFASAPTDMIGLESRLEELNCKIGIGEEDDSVQIIGICGMGGLGKTTLARFVY)}*

$2894=214(227):
>gi|1031860543|ref|WP_064178606.1| {|321(0)|<Proteobacteria(B)>}RNA-dependent DNA polymerase [Klebsiella pneumoniae]
>gi|1030343056|emb|SBH94031.1| Retron-type reverse transcriptase [Klebsiella pneumo
{(KWREDKLKASLLFASKKGIKTNSNEHAISVFRNHIYGRLSFIRMIRGGDFPLYLKLMAEMSHNDPVKTKEGLRTMKVTET)-YDVFICHAS-ED-KATIAIPIYDELNKLNISAFIDHVEIKWGDSLieKINSALVKSKYVIAILSANSVDKEWPKKELnaVLAREIA-----EG------DVKLLTLVKEEDEKIVAESLPLLRDKLYMA---YTDNASEVAAR(VHALLDR)}*

$2895=214(220):
>gi|1030340658|emb|SBG96834.1| {|329(0)|<Proteobacteria(B)>}Retron-type reverse transcriptase [Klebsiella pneumoniae]
>gi|1170234784|emb|SLW21882.1| Retron-type reverse transcriptase [Klebsiella pneum
{(RWKEDKVKYALLFTTEKGYQTENNTQAIEIFRNHIYGRLSFIKMVRGEDYPGYLKLMSYMSYNDPSKTKEGVRAMKETEN)-FDVFICHAS---EDKkDIAMPIYEELSKMKISAFIDHVEIH--WGDsliEKINSALV-KSKyvIAILSADSVNKEWPQKELRAVLASEISEGKTKLLtLVKKNDEEivtkalpL-LLDKL-YM-VYDNNP-DMVADNIK-KRLQ--------S()}*

$2896=153(160):
>gi|1031851023|ref|WP_064173416.1|
{|0(671)|<Proteobacteria(B)>}hypothetical protein [Klebsiella pneumoniae]
>gi|1030591266|emb|SBJ72162.1| MTH538 TIR-like domain (DUF1863)
[Klebsiella pneumoniae]
{(MI)--KAFLSHSSKdKEsYVRNVANWL-----GKENIVY---
DEFTFEEGEktldQIMDGLGESELFVLFISDNALKSKWVKKEITESKRLHDS-
GKILKILPIIIDNSINHEDKRIPKWLRDDYN--
LQPIMRAKTAVSRIKNHLYKI(SWQKHPKLEKINSLFVGR)}*

$2897=192(191):
>gi|1031844851|ref|WP_064168336.1|
{|0(129)|<Proteobacteria(B)>}hypothetical protein [Klebsiella pneumoniae]
>gi|1030401799|emb|SAU68196.1| Uncharacterised protein [Klebsiella
pneumoniae]
{()MINVFISYAREdVLIAKKLTEELSRYDYNVFLD---
QQSIRLGSHWQeDILKHLKNADVFIALVTEHSDKS-YYFNiEVTGAISyvANSN-RDKLVLPVVLDKE-
ALNDSDLGAFQALFARSddMANLVNEIVNAISRFEGARVAQE(ETKEIRAKVLKDESADYISETIEDLKAREK
DGRQLSSRWHFAGYGALVASV)}*

$2898=185(185):
>gi|1032702348|ref|WP_064279005.1|
{|0(639)|<Proteobacteria(B)>}hypothetical protein [Klebsiella oxytoca]
>gi|1030551287|emb|SBM35798.1| Uncharacterised protein [Klebsiella
oxytoca]
{()MIKCFLSHSSSdKERYVRIVA--EKIRRENRII--
DEETFEEGMVTAeEIIKYLDTSSLFVFFISNSSLDSEWV-QlELDRARELLDE-NTIkRVYPIIIDPkI-
DFKDTRIPEWMRSQLNIqlISSPTLAARKIHARLIELTWSDH(PRLKERRDIFVGRNDQIKLIEERLDDLQRQ
MPSVLIASGLPSIGR)}*

$2899=204(220):
>gi|1055112899|ref|WP_066861072.1|
{|454(0)|<Proteobacteria(B)>}hypothetical protein [Sphingobium sp. TCM1]
>gi|1032082075|gb|OAN54973.1| hypothetical protein A7Q26_22765
[Sphingobium sp. TCM1]
{(TANTLLHLAEAKGLPIVPFAFLGGAALRTYQRRDWKRLHPGFDASILANDEGIEQAVAIANRLLLDRVKRS
ISGGDRPKA)---VFVSVAS-SDAAMaSALGNELKSLGVEVIL--
GDNEIEAGQMVPaSIDQAIRRSDIVAVLWSRSYAQSAWCFDEL------------
SIALNQEALGAMKVWLFNLDDSAIVPAQARKLPVISVRSPEGLRSAVMELLA()}*

$2900=207(204):
>gi|501380412|ref|WP_012411978.1|
{|0(218)|<Cyanobacteria(B)>}hypothetical protein [Nostoc punctiforme]
>gi|186468234|gb|ACC84035.1| hypothetical protein Npun_F5735 [Nostoc
punctiforme PCC 73102]
{(MDDSLCSNEFFSPIGSKFLYRILENLKKMN)--QIYISYAW-
KDnkselgsQREELVDKICTALISERYNLIRDrNYLTLGKSIHDFMQEIGRGNYVIVVVSDKYLRSEYCMFEA
IEIMKR-KD-
YEQKVFPVVLQDANVYTKEGKTEYIKYWKQQSEKLKQLMGSELSAHQDSAMYNV(AEKIMEISQKIDEFMFFI
SDKLSIDPSQNFNGFI)}*

$2901=210(223):

>gi|1055648383|ref|WP_067287998.1|
{|0(625)|<Proteobacteria(B)>}hypothetical protein [Sulfitobacter sp. EhC04] >gi|1032105978|gb|OAN78420.1| hypothetical protein A8B82_08520 [Sulfitobacter sp. EhC04]
{(MSEETR)---IFISYSPKDQEKVATLCRSLADEPGL--
TVFLDTeDDLPKQESRpRLEKLIRESDMILFALSP----RSAESQdcA----WQLGLADgLNKRIIPVVVDH-
--VDGKLPPALAGLNHIYAT--
DHDDLTTAIDSIRSAIGSD(IDWIREHTRLAALAHSWSAAARPRAQALRGAELQAAQNWLAGQPKDTPQPTAE
QRRFILDSQRAAARRQRSTASGAV)}*

$2902=193(205):
>gi|501377021|ref|WP_012408587.1| {|0(126)|<Cyanobacteria(B)>}GUN4 domain-containing protein [Nostoc punctiforme]
>gi|186464771|gb|ACC80572.1| GUN4 domain protein [Nostoc punctiforme PCC 73102]
{(MEQF)--DVFLAYNSLDKPEVQD-IAAALRRRNL--
EPWIDDEQIRPGRpFQdEIQQAIplvKSAAIFFGVLGLGRW-------QSWeLRSLINQCVnRGIPVIPVL--
--LPGVNQLPDN---
LIFLNEFRWVCFSQSIDDGRALSLLEWG(ITGIKPESQPIIHPPVFERPSKPPKTIPEAPTQTDDLSSEKGIH
YTRLRDLLVAKNWKEAD)}*

$2903=212(214):
>gi|501376032|ref|WP_012407598.1| {|0(311)|<Cyanobacteria(B)>}molecular chaperone Tir [Nostoc punctiforme] >gi|186463775|gb|ACC79576.1| TIR protein [Nostoc punctiforme PCC 73102]
{(MPTLPAASKSARF)----FISYRSQDpDLSLAKDFYEAIKTAGH--
EAFMASESIRLGeKWPqRIDEELKQCDYFLLLLSERSATSEMVTEevRQ--
AKELRDGSdEHKPVIlPIRVNLTWSTP--
LNYDLRgYLQQIQQREWKSATDTAKILQEIFSLLAN(GDVERLADGEMSSDFFPSSPPHPLTLSPPLPVAEPE
LPEGQVDLASAFYIERLPIESRCYE)}*

$2904=174(187):
>gi|501377820|ref|WP_012409386.1| {|0(197)|<Cyanobacteria(B)>}TIR domain-containing protein [Nostoc punctiforme]
>gi|186465594|gb|ACC81395.1| conserved hypothetical protein [Nostoc punctiforme PCC 731
{(MNARQNEVCLL)-------YSESdKAIASLLEDALNYYS----
IDVWQAQNISIGSKIInQTTEMLDKAKFVIVLWSNNSVKSALLKSL---ALEAKKN-NK-ILIPVLIENI-
KVPGEFLDIQPAYLLNWdgDHENEQIVKLWRLIQDKVLKYRS(KKVGFNQFIAIFGLVLTGLSVIIAIATPEV
RCFLKL)}*

$2905=239(241):
>gi|499999101|ref|WP_011679819.1| {|49(39)|<Firmicutes(B)>}signal transduction protein [Leuconostoc mesenteroides]
>gi|116097023|gb|ABJ62174.1| Predicted signal transduction protein containing TIR dom
{(QDRGNIIECKGFSFPLPKREEIVDFLDGFAGFEIFDASYYKQRIAVINVPPSFEKIAFSKNKFYKFDSNYT
NELSEKKPV)--KLFISYNHEvSEIADFIETKMKQLFHYDLIIT-RDTSLVYKDDIDkfmlSIKKH----
DIVLSLISNSYLESEACMYEISELMKDSEY-SKRLAFIVLTEKD-
NELLEKPISIEKLVPSIysDNRFKYVTFWNDKIDYYQSVLKD(TKHHPETSLEIIDTLRRITNI)}*

$2906=225(222):

>gi|1031614493|ref|WP_064119060.1|
{|0(102)|<Proteobacteria(B)>}hypothetical protein [Pseudomonas fluorescens]
{(MTDEEKRR)--TIFISYAWGdgfenkEWVRQHIVSHLD---WNYDV-
FWDRDNIGYGELPDaAISKQLAKRPLtVLCLCDQDYLRSakkeNSGLSrELQMLAHIVDQ-
PGVRVIPLILEaDC-AKELpAPLTKRIYL--DLrpLLQ-RNID---
IWVTVACLAEGR(SQAAVQSGINEQLAGFELRERAENYLGQFPLMIWGNGRNHEVTVYSDEAAPYLLKPPQWM
WESDRWNYMLNDDG)}*

$2907=264(259):
>gi|1134139726|ref|WP_076382638.1|
{|1085(0)|<Bacteroidetes(B)>}hypothetical protein [Filimonas lacunae]
>gi|1031979684|dbj|BAV05085.1| internalin [Filimonas lacunae]
>gi|1132074917|emb|SIT34247.1| C-
{(IYKQLNNCELLFEREACRPLDLYITLNNKNFIKYQDLCNEDNVLLINSWELTNERTFTENSKFTSAFPFQP
FTNKELKRR)-
KKVVISYSKKdLKLINKFKDYLIPLYDDELIDTpWYCSELMPGSDWDeEIGKRFSEADIIFFMVSENLMSTKY
VKQkEIKYAIDRWEK-DQsVKIIPILLVPY-HFQRNgkyNLSRFTTLPYTLkpVVDFPNQL--
TAWHLISHIVRKV(IESDLNPENDHEIVTSEIRMYFERIASDDQSMRFQNTGF)}*

$2908=202(207):
>gi|501381238|ref|WP_012412804.1| {|0(309)|<unknown(X)>}toll-interleukin receptor [Nostoc punctiforme] >gi|186469070|gb|ACC84871.1| toll-interleukin receptor [Nostoc punctiforme PCC 73102]
{(MLNSQAKR)---IFISYkrnAS--P-DEPVALQVFQALSQQ-HKVFIDQTMSV-
GTRWaeCIEAEIRQADFLITFLSALST-SSEMVVaEIETAHHLAKSQsGRPIILPVRL-AY-
QEPFLYPLSAYLNGI-
NWAFWKDAEDTPRLIAELLQAVYG(GALAISEEKSKADLIQKSQPSLLPRPFPSAQPVSLEMPEGTMESQSAF
YVERSSDALTL)}*

$2909=207(233):
>gi|1032280716|gb|OAO95044.1| {|0(546)|<Streptophyta(V)>}hypothetical protein AXX17_AT5G43450 [Arabidopsis thaliana]
{(MEARNMVKIHFDS)--------SE-EELLYSFVPHLSAAFGRKGISMFTDKHDQ-----------
SYKSIASVLIFSENYVSSEESLDEFIKTIRRHEE--GHIVTAIFYGVSRSNVQELMGSFSKEFLEHCA---
SGHV-
TQWRNALSEITSL(PGHEINNNQSDYESLEKIARDFYEEIFPNERIGIYSRMLPDIENLIYKQQWGVRSIGIW
GMPGIGKTKLAKAVFDQLSGD)}*

$2910=225(236):
>gi|1032284330|gb|OAO98657.1| {|0(103)|<Streptophyta(V)>}hypothetical protein AXX17_AT4G04840 [Arabidopsis thaliana]
{(MTKHFEMILTELVMVF)RFDVYIFCRG-S-TSTRFASSLLAALLSRNIEVFEDIDYVR--Q-
FehRDILAIEESKVLVVVLSANELRRRWTLSKLEKII-NAER-TEQVIIPVFLDTEPYDV---
ISDNVLAILTTLEDKKE--A-
NRWISAVTKIAAL(PGRTFARDENVFVGMTFLKIMKILNSSKPIVKRKSRCSIAMTFLVIISLLVEIVSVVAD
QFSSVRKPYFARISLVMSIIS)}*

$2911=278(300):
>gi|1032282495|gb|OAO96822.1| {|111(468)|<Streptophyta(V)>}hypothetical protein AXX17_AT4G22930 [Arabidopsis thaliana]
{(REKSKKGKEKFVLDDEFLPREDEFLPKEDTYSEMLSPTSNWEPSEDDVDSGDEVAREMAQERECILVFSCY
DNLDVDETS)-----------------FIEAISKELHKREVTPLTYNLLGREKL---
DKEMLYRSRVGIMILSNCYVSSRQSLDHLVAVMEHWKK-

TDLVIIPIYFKVTLSDICGLKGRFEAAFLQLQRSLQEDRV-
QKWKAAVTEIVSI(GGLEWTKGSQFMLAEEVVRNASLRLSLKNSTNVAEILALLNHSQSLDAEIVGLWGMAGI
GKTSIAREIFEILAPHYDLCY)}*

$2912=211(214):
>gi|1032279433|gb|OAO93761.1| {|0(349)|<Streptophyta(V)>}hypothetical protein AXX17_AT5G43480 [Arabidopsis thaliana]
{(MAAFSSSSSVEELPP)QHQVFISFRG-
TDLREGFVIPLVKALLSNGIKVFIDTDASVCPSGEILSKRIEGSRIALVIFTDDYTESKWFLRVLAKIKGYVD
E-GKLVAIPIFYKLDPSIVRGMRLRFGDVFKDV-
RSISYRVGINVKKFSPSDNVIE(NVVEEVKKELNTMPSQRSKDVEEVKKESNTIPSEGSHKIKNVVEEVKKES
NTIPSDGSH)}*

$2913=296(296):
>gi|1032277601|gb|OAO91929.1| {|960(16)|<Streptophyta(V)>}hypothetical protein AXX17_AT5G43200 [Arabidopsis thaliana]
{(SEPERIKKWKEALISIPQKIGLTSAGHRDESELVDSIVKEVKKVLIDVSRKDRNNSQNSTNIKGQSFEPLM
AELDRLPTR)RPQVFVNFCN-
DELGDNFIKHLVWALRDSGINVFKDSFKLIGSgQKQEVFMSIENSNIALAIFSKRYSESYRCLNELVKMEELA
KE-GKLVVIPVFYSVKTNEVRRLEGEFGIHFRNTKERF-
AMEPMmvESWEKSLKSSTVT(GRIGLSLEAHMNEFALVGAIVKEVTRLLPNSPRRRRRRKLGIGEVFLRALTA
TCIFAVFIYPMPMPDVNFLKIGKL)}*

$2914=275(299):
>gi|1032283710|gb|OAO98037.1| {|216(928)|<Streptophyta(V)>}WRKY19 [Arabidopsis thaliana]
{(SNGETSEGEKDEDEPEPKRRITEVQVSELADASDRTVREPRVIFQTTSEVDNLDDGYRWRKYGQKVVKGNP
YPRFSSSKD)-YDVVIRYGR-ADISNeDFISHLRASLCRRGISVYEEFNEV---------
DALPKCRVLIIVLTSTYVPS-----NLLNILEHQHT-EDRVVYPIFYRLSPYDFVCNSKNY-ERFYLQ-
DEPKK------
WQAALKEITQM(PGYTLTDKSESELIDEIVRDALKVLCSADKVNMIGMDMQVEEILSLLCIESLDVRSIGIWG
TVGIGKTTIAEEIFRKIS)}*

$2915=297(298):
>gi|1031608107|gb|ANG08895.1| {|999(214)|<Arthropoda(M)>}toll family protein LongTollC [Parhyale hawaiensis]
{(ILSNTPGHCDHFVTTAHMNNLMVNDYIMFSSISVGIILVIIVFVFIGVMYRRRIKLWASNQYSKTLFEKST
SYVEESEKL)-FDVFISHSNKdsNWVYSVLAPELE--
LRGYRTCAVYRNCSSPTapFVAQAMSEGIACSKRLLLVLSRGLVDSEWCKYDFKSTHVDAIRKLSKKSIILVN
LDD-IPRGELDSEMICVIKKAsFsFKPRDPR---
FWERLRRALPSL(RHRRRYDDGLSKSVSSPLVVADVPPSEVSKFTCHPNKNLTLNPYWETAITSNVPAWNMKK
PDLGAPPWVQVPSAKNNQ)}*

$2916=218(219):
>gi|1056066184|ref|WP_067591847.1|
{|0(77)|<Actinobacteria(B)>}hypothetical protein [Amycolatopsis sp. M39]
>gi|1032308069|gb|OAP21307.1| hypothetical protein A4R44_07825 [Amycolatopsis sp. M39]
{(M)------SFAG---
AQRALARQFARECESLGLVVFFDENVTEEMWGRdfiTEFRRVYGGKlARyvVPFLSVEYFDSGYPMDEFYAAI
AYAIKRgDDPYVLPITVGDVEipEElLRSSIGYLRSENYSV-EQLAQIAA-
KRVSGSGDAMPTP(APTPIAAVQLPKVAPTDFSQFGTLESGLATLSEHFRRAESSLAPFGYSCHVSTGGSSLE
VRVESHGRQVYGLKVQLQD)}*

$2917=146(141):
>gi|1032816077|ref|WP_064322632.1| {<Proteobacteria(B)>}TIR domain-containing protein [Ochrobactrum pseudogrignonense]
>gi|1032547094|gb|ANG98671.1| TIR domain-containing protein [Ochrobactrum pseudog
{(M)---IFISYAK---
EDAAYAHKVFLALHAQELSPWMDKppsphtkEGLQVGQKWevVLQEKLKIADYIILILSPRSVAKRGYVQvEF
RTALDRMNYLpDDDvLVLPILIEPC-DVPSLSVGNINLRDLHWDIIPHEHIDEFAIGLAERIKEPV()}*

$2918=208(204):
>gi|1057527622|ref|WP_068823633.1|
{|0(40)|<Bacteroidetes(B)>}hypothetical protein [Pedobacter sp. CCM 8644]
>gi|1032666539|gb|OAQ38240.1| hypothetical protein A5893_15705
[Pedobacter sp. CCM 8644]
{(MNIIPKAF)---ISYSHDT--QEHKKWALQLATRLRNNGIDAILDQFELQPGADLphFMETHL-
ASANKILMICTdkyvaKANKGQGGVGyEKMIITSSLMKNiDANKIIPIikQSGTL-
EVPTFLRSKLYINFSKEDDFEFSYDELVRSIHNSPLFEKP(AIGNNPFKPIVQEKLEDDSKLLDSILSVIIEK
QGIELYVLSKFIINELKISPMLFK)}*

$2919=186(178):
>gi|1055508257|ref|WP_067167006.1|
{|0(235)|<Actinobacteria(B)>}hypothetical protein [Streptomyces sp. ERV7]
>gi|1033213108|gb|OAR23277.1| hypothetical protein A8W25_27685
[Streptomyces sp. ERV7]
{(MAR)-PRIFLSHSSQctpesgcdcRAYLQALHAHLEDLGCQPVVDS---
GILNGGDPWYgKVLTELKNCHGMIILLSPHALDSYYVMVeAIVADAERAAT-DDAFVVlPVTLPGV-
RRRQLPDSGLGKLNLGRldMVDWRKQYGPGRPPAKIARTLRP(LIERQGSLPYPEVTDFLVGRISDLSDSALE
DTAEI)}*

$2920=189(183):
>gi|1035778196|ref|WP_064587890.1|
{|0(131)|<Proteobacteria(B)>}hypothetical protein [Pseudomonas koreensis]
>gi|1033857736|gb|ANH98584.1| hypothetical protein A8L59_14565
[Pseudomonas koreensis]
{(MLKEEEKDA)---IFISYAwggslHQkEWLRQRIIEPLDWKYR----
VFWDRDSIGFGESIDkVIARALEQRPINVFCICdSDYLVAaqrvNSGLHrELQMLTQIADH-
VGVRIIPLIFDAD-
SSADLPEPLAGRAYLNVhpLHERGLDLSSLLLGVADGATQAQ(VNTHIAERLKADDLRQLAKAYFRRQNMELW
GSAR)}*

$2921=230(227):
>gi|1035836579|ref|WP_064628484.1|
{|140(0)|<Actinobacteria(B)>}hypothetical protein [Mycobacterium immunogenum] >gi|1034216988|gb|OAT69689.1| hypothetical protein AWB85_20075 [Mycobacterium immunogen
{(FGSGSFIATDLSSAIDLITALHRGPSAIDTSSLQLTCSGLEGQPEYVVDDMFKFLSATGLHDDLSAVVRTW
VGQPIEYYS)---VFISHSS---LDKTFARKLYRDLQGLGVKCWMDEKNILPGDSIldSIDQGIKVHD-
RVILVCSQNSLGRKTgwwVDeEIERALAKERELrraGEKFgvLVPVTIDNY-
VFDGWNSGFQATVLQKNVGDFKNHQDVQSYAETLDKLAQA(LNRRRDI)}*

$2922=261(238):
>gi|1035425095|ref|XP_008465659.2| {|0(559)|<Streptophyta(V)>}PREDICTED: TMV resistance protein N-like [Cucumis melo]

{(MNPPSTSPTSSSPPNTCQ)---VFISFAT-AD--SKFVSELAKEFRNIRLRTF----MSSGDD-
GeiIHEDAMKKSRHFVVVLTKDYVNSIKCLRELSRIMEYKDE-GYEEVIPVFYQ-
DPSCLVKELQDFGKRledyeeiikeegliefthcwFLKTDLAKrlnksFSEKeenksnllmeV-
PRWKFALERINNL(PKSIFVGEESLESDVIKEVTEIIYKVKASPKLVGISPPLHQMDNLLNLGSNDIRFIGIV
GLGGIGKTTIAKVLYEKLEHK)}*

$2923=206(225):
>gi|1035912574|gb|OAY42168.1| {|0(938)|<Streptophyta(V)>}hypothetical protein MANES_09G158500 [Manihot esculenta] >gi|1035912577|gb|OAY42171.1| hypothetical protein MANES_09G158500 [Manihot esculenta]
{(MADAG)---------------------LSQSLERDGFHTFRDDDgIVRGAN-
FvaEVAKVMQHSKICIVVFSKNYASSIWCLDELVKIMELRKT-
VGLVVFPVFYDADPNQVWEQSGSYAEAFarHEELFKGQMDKV-
QGWRAVLREVTDL(SGMDIQERHEADFIQDIVKKVGKRLDSSVLLHVPSYLVGIESRLKDINSWLQDVSNDPA
IAIISGIGGIGKTVIAKKVYN)}*

$2924=234(234):
>gi|1035397522|ref|XP_016899476.1| {|0(721)|<Streptophyta(V)>}PREDICTED: TMV resistance protein N [Cucumis melo]
{(MVDVPSSSTTRRWM)-YDVFLSFRG-EDTRQNFTKHLYDALdTAG-
VNTFRDDVELRQGDAVgsELVVAIKKSRIAVVVFSDGYADSQWCLGEIAEIMDCRTV-
EGQLVLPIFYEVDPSDVRKQKGRFAAAFEKH-
EKRFGVDSVevLRWRAALREAASL(SGWDLRQLADGHEGKFITKIVERVQSELRVTYLEVAIYPVGIDVRLKH
LISLMAISTNHSTLVLGIYGMSGIGKTTLSKA)}*

$2925=264(225):
>gi|1035270729|ref|XP_016888205.1| {|292(0)|<Chordata(M)>}PREDICTED: interleukin-18 receptor accessory protein-like [Cynoglossus semilaevis]
{(VTPQHLNHTYTCFANNTAGNVSVTVGLEEKVKWPSLVVYPVSSVLLVVVVGVIVYLMRLELQIIYRSHFHC
GRHDEDEKM)-FDVFLSYV-WspekeaanfyinteeeeeeeenteeeeeedevsqRSLSL-
LLHQVLEGEWGYRLCLPERDIIPGGAYTKDIVLALQKSQMLICLLSAHYFcSSNAVF-
VLESGIQALLKNSGLKLLLIRTrrdsaSMF-QEAQ-
LPSLVQRSLKVLpSLMWTGDQPDrvscNFWRSLRKNMPKY(QVKMK)}*

$2926=161(163):
>gi|1036453121|ref|WP_064691317.1| {<Proteobacteria(B)>}hypothetical protein [Pantoea agglomerans]
{(MPILQSDLFNASQRGQFAKRFGQ)-PTAFLSHSHKdAQLALGLQ---
ALLNDQGwdVYIDWQDQTMPdkPDTETAlNIKSAIVRADWFLFLATQHSMTSRWC-PwEIGFADGKKVY-
DRIAIIP----TQ-DYLGNFYGNEYLNLYNKidVSSGHTGLAFFDTRGNGKWVRNL()}*

$2927=212(229):
>gi|357469487|ref|XP_003605028.1| {|0(1128)|<Streptophyta(V)>}disease resistance protein (TIR-NBS-LRR class), putative [Medicago truncatula] >gi|355506083|gb|AES87225.1| disease resistance protein (TI
{(MANHYEDFT)-HDVFLSFRG--
GTRYSFTDHLYRSLLRQGINVFRDDQNLkiGHEIGPSLLQAIEASRISIVVLCKEYASSTWCLDELVKIVDCY
ENNGKSKN-----------------SYEDAIRKHEKRFGreSEKV-
KAWKLALNRVCAL(SGLHCKDDVYESEFIEKIVRDISTKLPTVPLQIKHLVGLNTRFKQVKSIIDINSSERIC
MLGIYGAGGIGKTQFALHIYN)}*

$2928=153(155):
>gi|922324745|ref|XP_013441765.1| {<Streptophyta(V)>}disease resistance protein (TIR-NBS-LRR class) [Medicago truncatula]

>gi|657369259|gb|KEH15790.1| disease resistance protein (TIR-NBS-LRR class) [M
{(MSYN)-YKVFLSICA-QD-RDHFIWHLNTVLRSKaGTALF---GVEERLQ-YesELDAIVGYCKLAIVVFSSNYNKSISCVQELEKITECCRT-SDLVVFPVFFdGVYPLHERSEGGMFdGNAFHDfiDRIGKDKDKF-LSWVVGVTKATEY(FGPSDLIYRYF)}*

$2929=188(184):
>gi|357499417|ref|XP_003619997.1| {<Streptophyta(V)>}disease resistance protein (TIR-NBS-LRR class), putative [Medicago truncatula]
>gi|355495012|gb|AES76215.1| disease resistance protein (TIR-NBS-LRR
{(MAMQPPSSTSSSGLSNNFS)-YDVFISFRG-TDTRHGFTGNLYKALCDKGIRTFIDDKELQRGDEItsSLHKKIEDSRISVIVFSENYATSSFCLDELVHITHCSKE-KGRLVLPVFYGVEPSHLRHLnNNSYGEALAKH-EERFqnnKENMErlQKWKIALNQAANL(SGYHYSPSEYPQLTSIFFPLYISFL)}*

$2930=144(152):
>gi|357499857|ref|XP_003620217.1| {<Streptophyta(V)>}disease resistance protein (TIR-NBS-LRR class) [Medicago truncatula]
>gi|355495232|gb|AES76435.1| disease resistance protein (TIR-NBS-LRR class) [M
{(MANLQSSSFSTT)-YDVFLSFQG-HDTCKGFAGDLCKVLsKGR-ISTFMCDEELFKGDEIppVLYKVIHESRIAIIVFTANYASSSFWLEVLGHIVENKSY-G--LILPVFYDVDPFHVLNQSG----TSTQL-YPLLLNLIRLQQFNPRNLAPKL()}*

$2931=240(235):
>gi|357493433|ref|XP_003617005.1| {|0(1251)|<Streptophyta(V)>}disease resistance protein (TIR-NBS-LRR class), putative [Medicago truncatula]
>gi|355518340|gb|AES99963.1| disease resistance protein (TI
{(MSCWPELNSNGTSHR)KYDVFISFRG-EDTRFGITDHLYDALiHKS-IKTYIDYQ-LNRGEDVwpALSKAIEDSYISIIVFSENFATSKWCLEELVKVLECRKD-HGQIVIPVFYKADPSHIRNQKASYETAFAKH-ERELGtkdsiSNKSkvLKWKAALTEAANI(SGWDSHTYEKESILILKIVNDVLEKLQLRYPNELEGVVRNEKNSECVESLLKKFRILGIWSMGGMGKTTIAKVFFAKHFA)}*

$2932=231(249):
>gi|357494463|ref|XP_003617520.1|
{|70(0)|<Streptophyta(V)>}toll/interleukin-like receptor-protein [Medicago truncatula] >gi|355518855|gb|AET00479.1| toll/interleukin-like receptor-protein [Medicago t
{(NQLVWPIFFKVEPWEVRNQKGRFGEAMLKLEKWLQNYPYRILQWKKALHQVSLIAGWNYGAEFEYELFKKIVESTVQSLP)RYDVFLSFCG-EDTRYSFTGFLYHDLRhEG-FKIFMDDEGLKGGNQIsqTLLKAIEKSRLSIVVLSENFGYSTWCLDELVKIME--------------------YVSNQTKSYGKAMAAH-EDRFGKECEnvQKWKSALSEVALL(KADHIKENEYEYEFIKKIVERAIEAENHM)}*

$2933=266(273):
>gi|1084293347|ref|XP_018649774.1|
{|0(17)|<Platyhelminthes(M)>}hypothetical protein Smp_194540 [Schistosoma mansoni] >gi|353230733|emb|CCD77150.1| hypothetical protein Smp_194540 [Schistosoma mansoni
{(MLTYLLCLYFIDTMKLIRKYITSSSRKVIFPNRTINDNNNTTNIVETSLNEQVDNKTEWLMNTESNIGLVNTIPH)---ILVSYSHQEKDMAKYLVEQLQNKGYK-VWIDYKDMMYETDVMEGMAKAVENSFVVCILYSKSY-KESLHTKcEAQYAYHEK-----KPILFLRAQRgyVPdgWLGFMMGTRLYIDISG--

KYPFEDKFTELCKRIDVYKNQS(IDQLNNHCQNKSTEVLNLSNCMLLMDRNQQHPLKSSYSQNILNLFLLKQM
KMFELSTK)}*

$2934=185(189):
>gi|493769526|ref|WP_006718070.1| {|0(263)|<Firmicutes(B)>}TIR domain-containing protein [Desulfitobacterium metallireducens]
>gi|570737646|gb|AHF08677.1| hypothetical protein DESME_14190 [Desulfitoba
{(MGYAEHNENKCNMNK)-PYLFISYSSKdKDI---VLKDVEQLRELGV-
NIWVDTQMRTGTNWQkEAFSAINEVDckVIVLYVSEYSLASDNVLKE----LEYSRS-EEIRVThPIIDIQI-
LPIQLIKFGEENNINNWynTYILQTYKGEKEKLNTIYKIKKD(FLHNGDIIMDNGVDEHALKYYEGLLRRLQD
FGVV)}*

$2935=201(218):
>gi|353671245|gb|EHD12952.1| {|0(163)|<Proteobacteria(B)>}hypothetical protein CIN_21180 [Commensalibacter intestini A911]
{(MLPFYDY)------------------------
LEHHGINVWMDCKRLKGGQQWdfEINKAFRQAALVIIFLSKNFIEKRGYNQrEVKMSLkNLEEKLdDDIYIIP
VLLDDIEqEQIERPVALQHIQMINGYlSDGKEQILDsIKTQFEKMKIELK(HVQEETNVFWTVKPIEKSWDGF
PGYKVNFDFIEFQSNHYKDIDYIADIINGKLRERALETMSYRFEQDINY)}*

$2936=214(220):
>gi|1037406455|gb|ANM29143.1| {|121(0)|<Acidobacteria(B)>}hypothetical protein ABI59_05350 [Acidobacteria bacterium Mor1]
{(ISNIPTRTVAMPLIGAGDQGYGAGRMMAAVLRAAVGWFRRGMPLDVLKIVAYSEADARLAKDRFLEIQRED
AEPREREAN)-WDVFLSYSS-KDQSA--
AKLIGDLLTSSaeRLRVFRDKPSLQKGQSWimQIADAIDTSRCIVSLYSPSYWDSVVCRDELsAAYVRQLKA-
EKRLLYPIFY------RDTQIPSFFEAMHYDDCREADHERLAEICQDIAGSVRS()}*

$2937=138(142):
>gi|1037407467|gb|ANM30155.1| {<Acidobacteria(B)>}hypothetical protein ABI59_12095 [Acidobacteria bacterium Mor1]
{(MD)PYRVFLTHGG-ADT--
WLAReTLKPRLEESGAEVFLDNVNLEYGDDFreIILSELRRCDELLALLTPSSIRRPWVYAEIGAMVvrEQG-
----DRVIAVQYGPEEQEL-QELGVLSLLGTRTLLPLDDFDTYLAQLKGRVESKHA()}*

$2938=222(220):
>gi|1037381794|gb|ANM05252.1| {|0(152)|<Proteobacteria(B)>}hypothetical protein AMC78_CH03183 [Rhizobium phaseoli]
{(MTNRDF)EYEVAFSFAA---QDEAIATQLND-
LISDRLQTFIYSERQKEIAGRdgqEAFSEVYAVKARlvVVLYRATWGQTPWTRVEMDAIKNRSLNDGWDFTVF
I---PTEakpsvppW-LPKTRLWVGLERWGA-EGAAAVIE-
ARATECGADIREE(TIKERAARHERKEALRQKQRQFVNSDVGVNAARGAYASVKAANPARTMSVRPRWHNAKT
ARRHRHAQPLHSLTF)}*

$2939=173(167):
>gi|1037407488|gb|ANM30176.1| {|0(309)|<Acidobacteria(B)>}hypothetical protein ABI59_12230 [Acidobacteria bacterium Mor1]
{(MSKPL)---
VFISHKHSdTDIGGAIARFVRNISGGNVDVHLSSDPNYeGPRVGKkindELRTALSRTGLVILIYTSridqng
EYKDWDYCMWECGLAEEPNSA-RTEVLVLQCLPDKPIVFQGKLQVLAWERDSV--
ISFARRFREPDFYPAYEVMDE(KTGESKTIAPGPLTGLSEREID)}*

$2940=204(215):

>gi|1037115783|emb|SBT10760.1| {|0(53)|<Proteobacteria(B)>}hypothetical protein PROAA_640016 [Candidatus Propionivibrio aalborgensis]
{()MADVFISYKRSeRKRVERI----
AELLRDYKMTVWFDAQLEAgrGEGFDaEIEREVTSAHCVVVCWTKDALKSMYV-KaEAKKGLEREA------
LVPVFLENC-Q---------LPVPFNGvdtidLTDWNgDED--
EpVWKRVVEIVQQR(VESSRADESRRRVHSLAAYERVSERLYPGILALLVPRITAIGDWDTRDYGEDIDAILS
WIGAIAEKERAYHAYGY)}*

$2941=206(219):
>gi|1037116535|emb|SBT09702.1| {|0(92)|<Proteobacteria(B)>}hypothetical protein PROAA_340025 [Candidatus Propionivibrio aalborgensis]
{()MADIFLSYSREdEARIKSLVTEF---EAQGWTVFW-
DRRIPAGETWRsYIGTALQDARCIVVAWSQSSVESQWVA-------EEADE-GKARkiLVPVLLDQV-P---
APrgFREIQAA---DISGWQPgqAS--
ERFTELIGDLRRI(LGKPSASAREMPPAGQPFTPERKAEGQAGGRSMHGRRTVLVALLAVFAIGIAGYFVVES
LQTGKQHVPSPSIPMQVARD)}*

$2942=183(191):
>gi|1055063878|ref|WP_066813559.1|
{|0(323)|<Actinobacteria(B)>}hypothetical protein [Mycobacterium sp. 1100029.7] >gi|1037466993|gb|OBA64778.1| hypothetical protein A5647_25260 [Mycobacterium sp. 110
{(MA)---LFISYSSQdRSKVDALTNAL----
RRGQNQVWFDQELGGGDSWWaKILEQIRDCEVFIVALSSNWLQSK-PSQaELRYAQAL-----
GRPILPVRIGDV-
DsMRVNPLAALQIIDYREptVDAGIQLVTAVHGLRSKPVPLPD(PLPEEPPVPFGYITRLGNTLAEKELSPQQ
QLQLLVELRSGFDEDGEDPS)}*

$2943=228(234):
>gi|1037406541|gb|ANM29229.1| {|157(0)|<Acidobacteria(B)>}hypothetical protein ABI59_05940 [Acidobacteria bacterium Mor1]
{(FGGADFSEANLNNNRFINCEMGQAKHLHTVRSYGHSAVDHRTLERSPNLPKAFLRLVGLPDAFIDNIPAIF
GSPFEFYSA)----FISYAL--P-DEEFARRLSADLRDNGVRCWFAPEDLKAGDRImnTINDAIRVQD-
KLIVILSKHSIERDWVEhEVKQALKAEVERGRDLLVPIRLDNA-
VFECGFGWAKEIRAAHRPSGRHIADFTDWKNHETYGSAFE(KLLRDLEHRESGDS)}*

$2944=221(217):
>gi|1037126581|emb|SBT05820.1| {|0(264)|<Proteobacteria(B)>}TIR protein [Candidatus Accumulibacter aalborgensis]
{(MDSAEPD)--DVFISYAHAdngialgtsspmgwvtalaANLNEGPNVFKKC--------IFIDHQ-
LKPGDQFsdDLLVRVGQSRLLVLLLSQNYVDSQWCGKELEHFVRTHAD-----DPEKPAD---
VYVVEiFPFERLTSVPPiiqALRKRLIHAK--
FWYQPANAASPV(LSGYPSPKDSGPEGAAYYWSVLNELRVAIDSRLRALRAAGVEARAAVARLGTVLLADTTE
DLERQRMTVK)}*

$2945=260(246):
>gi|1037408307|gb|ANM30995.1| {|0(677)|<Acidobacteria(B)>}hypothetical protein ABI59_17570 [Acidobacteria bacterium Mor1]
{(MWTMLWRTFRSGDAILSPEISGGNPIVENRQGK)RYRGFISYSQEdislaKRLHLALSfwrapkgvKLAGL
APDRGLGSFFRDEEEMGAADSLrdSLKRALDNSDNLIVLATPRSAQSDWVGMEIRHFRE-
RGHGrvfAVVARGPL----------GFRVSRSACFHPALLERVeldrspagePEEP--
LAPDLGREGLRK(VATRLAAGLLDVSFDTLWKHEQRRRTKRLLVSSASLFVGAALAIASGIYLVGTLTERVAD
ERATRFAVAAERL)}*

$2946=147(145):
>gi|1039191584|gb|OBQ33653.1| {<Cyanobacteria(B)>}hypothetical protein AN487_19905 [Anabaena sp. CRKS33]
{(M)-YDAFLSHAS-ED-
KEKFVKPLFNALEDRGCCIWYDQFQIDYGDFFetRIYEGIHNSRFALFVITPNLiqrPTNSWVWREFSRILEK
EEILRTNVLIPVFYDVTIQEFQQYIGNNPTLNTITERHSFKTLVDGGIYGVAKKLSKL(FGRR)}*

$2947=189(200):
>gi|1063736199|ref|NP_001330203.1| {|0(121)|<Streptophyta(V)>}phloem protein 2-A7 [Arabidopsis thaliana] >gi|1039019283|gb|ANM68446.1| phloem protein 2-A7 [Arabidopsis thaliana]
{(MAASTFSSSSSFVGLIPSR)-PLVFIKSQE-
EDVSKGLINFLEPVLQNKNINVFIDEEEVRGKGLKNLFKRIQDSKISLAIFSESKCD----
FNDLLKNNESADE-----
AIPIFYKVDATGDLADLQNSVKCKKDLINSAVEEMSKLLANISVGVYREK(EANSKCFMVPARKLQMSHSEKL
INWTWSSIYETPNDAAIEV)}*

$2948=185(186):
>gi|1056610967|ref|WP_068041845.1|
{|0(1223)|<Actinobacteria(B)>}hypothetical protein [Mycobacterium sp. E2733] >gi|1038227813|gb|OBH95466.1| hypothetical protein A5678_02780 [Mycobacterium sp. E2733]
{(MS)--RIFLSHSSKDNRQAvalkQWLVDQRPELANE-
IFLDIdagSGLRVGARWKGQLFESNSRCEAVLCLVSAA-WDGSHECRtEYRTAEGLG-----
KQILVVRLEDTGdgDITSEWQRCDLFAEGA--
LTEIPVPGGGPVRFNTAALYAL(RKAIEGAGVGPQTFVWPPKEDPHRAPYRGWEPFEDIDAGVFFGR)}*

$2949=183(191):
>gi|1056592338|ref|WP_068023461.1|
{|0(329)|<Actinobacteria(B)>}hypothetical protein [Mycobacterium szulgai] >gi|1037941979|gb|OBF23821.1| hypothetical protein A5725_00260 [Mycobacterium szulgai]
{(MA)---LFISYSSQdRSSLDALN---AALKRAQQ-
HVWFDQELGGGDSWWnAILEQIRACEVFIVALSNNWLQSK-PSQaELRYAQALNRP-----ILPVKVGPV-
DsVRVNPVSDLQIIDYQNptVDAGIALVTAIHSLRAKAAPLPS(PLPPDPPVPFGYIMRLGNTLAEKELSPQQ
QLQLLVELRSGLDEDGDDPS)}*

$2950=146(148):
>gi|1055629467|ref|WP_067269494.1|
{|0(363)|<Actinobacteria(B)>}hypothetical protein [Mycobacterium scrofulaceum] >gi|1038218249|gb|OBH86356.1| hypothetical protein A5681_15985 [Mycobacterium scrofula
{()MP-LFISYSSQdRATVDALTTAL----
RRGQQQVWFDQELGGGDSWWnKILEQIRSCDVFIVALSNNWLQSKPSQAeLRYARALNRPI-
LPVRIGDIDSMRV-NPLAALQIIDYREPTVDagIQLVTAVHALQSKPVPLPDPLPE(EPPVPFGY)}*

$2951=206(220):
>gi|1056631832|ref|WP_068061689.1|
{|116(0)|<Proteobacteria(B)>}hypothetical protein [Rheinheimera sp. SA_1] >gi|1039000761|gb|OBP16510.1| hypothetical protein A5320_03650 [Rheinheimera sp. SA_1]
{(WRGGVPGPAMPIHNAFSLFKILTFVGEKTKIDPNCAGCRASKVPQQGPYGHLMLHFQAIRQLSVYGANQLF
MLQDKMVQK)-KTVFFSHCHRsAAPLVEAVANL--LEPEALC--

WWDTKVMPQNRWYnAnllldmLSDGIRQARIFIAFVTPDYFDSQW------------------
ARVEWDKAQQ-TPGLLVLAVMLGGTTPEsvKRVIQAQLGTDAPKISDFIRQYF()}*

$2952=265(271):
>gi|1039157740|gb|OBQ03204.1| {|359(426)|<Cyanobacteria(B)>}histidine kinase [Anabaena sp. AL09]
{(LINILHQDAAYYQQHKLILTKALKWERQKRNPSILVRNNDLRNAEAWLKVAKPKTQQPSLPIQEEFIAESL
KQPPNAVLD)---VFVSYSNAdADFARKLN---DTLQIQNK-
TTWFDQEsIISGTDFEeEMYKGIELANNFLFIISPNSISSNYCATEVEYAIKLNK-----RIVTVLYLPV-
DIDslHPSLAKVQWIDFRKhgGDFLTNFGELTRTLDADPDYAKN(HTRLLLKSKEWEQQKRDDSFLVRGKDLA
ASEEWLKESENKQPSPTNLQLEY)}*

$2953=173(181):
>gi|1039157740|gb|OBQ03204.1| {|0(877)|<Cyanobacteria(B)>}histidine kinase [Anabaena sp. AL09]
{(MNDFQGA)----FISYGRAdsKAFATKLHKRL----
REEKLEIWFDQnDIPLGVDFQkQIDDGIETSDNFLFIIAPHSINSPYC-GkEIDLALKRNK-----
RIIPLLHVEQ-
ITEEIWQQRNPGKDPSEweIYKSKGLHSSFPNMHPAIGKINW(VYFREGIDDFEQSFTGLLELLTRHQEYVHQ
HTYF)}*

$2954=259(264):
>gi|1039160597|gb|OBQ05999.1| {|154(637)|<Cyanobacteria(B)>}histidine kinase [Anabaena sp. LE011-02]
{(GLLELLTRHQEYVRQHTYFLAKALEWEREQQQSSCLLIGDERVEAEKWLKIRFKEEQPPCEPTDLHCAFIC
QSVKNANNL)MTQVFLSYSDKeREFMEKIAK---
TLMRESFTVWTNKTDIQTGTDFQeAVNRGIEEADNLVFLMAPSSLASEYC-QqELTYALSLKK-----
RIIPLLIQPL-
DLDQIPQELRNIQFIDFtdKEEEGKESADTDNLINILHQDAA(YYQQHKLILTKALKWERQKRNPSILVRNND
LRNAEAWLKVAKPK)}*

$2955=249(257):
>gi|1039175694|gb|OBQ20215.1| {|358(764)|<Cyanobacteria(B)>}type IV secretion protein Rhs [Anabaena sp. WA113]
{(LLQELKKEPFYYEKHKILLVKAQKWKRQNENPSLLLHGYNLQHFEAWLKVAKQREDYPSLPLQEEFIIASL
NQSEASSLE)---VFISYSRTdSDLARKINDALQELGK----
TTWFDQEsIATGTDFQqEINRGIASSDNFLFIISPKSVNSPYCASE----VEYAKN-LGKRFITVLHRPL-
SDDDKQKLPAALASVQWldFNHHGGEFSANFNELVRTLDTDR(DHVRSHTKWSQRALEWDEKGKNADLLLRGS
EFAIAEE)}*

$2956=263(269):
>gi|1039175694|gb|OBQ20215.1| {|156(952)|<Cyanobacteria(B)>}type IV secretion protein Rhs [Anabaena sp. WA113]
{(NAINKQQDYVEQHTNFLVQALEWSRNQQQTNYFLVGEERGKAEQWLKQRFIEEQPPCIPTDLHCEFICESI
KNSHNLLSQ)---VFLAAADKdEVIKEKIRK---
TLMRESVTVWTNKTDIKTGTAFPeAITQGIEGADNFIYLISLDSLKSEYC-QqELNQAL----A-
NNKRIIPLLITET-
DFKLIPleLKKLQFIDFTQseSETEYRFSADKLLQELKKEPFYY(EKHKILLVKAQKWKRQNENPSLLLHGYN
LQHFEAWLKVAKQREDYPSLP)}*

$2957=211(217):
>gi|1056259545|ref|WP_067735236.1|
{|0(297)|<Actinobacteria(B)>}hypothetical protein [Mycobacterium sp.

852014-50255_SCH5639931] >gi|1037577237|gb|OBB69794.1| hypothetical protein A5758_03045 [Mycobac
{(MPL)----FISYSS---QDRAAVDALTAALRRAQQQVWFDQE-LGGGESWwnKILEQVRSCDVFIVALSNNWLQSKPS-QaELRYA------RaLNRPILPVRIGDV-DsmRVNPLAALQIIDYREptvDAGIQLVTAIHaLQSKPVPLPDPLP(EEPPVPFGYITRLGDTLAEKELSPQAQTQLLIELRQGLDEDGDDPSARGDIGQLLRMLRLRHDVTYRTRTEIDN)}*

$2958=155(152):
>gi|1039516733|ref|WP_064849431.1| {<Actinobacteria(B)>}hypothetical protein [Mycobacterium fortuitum] >gi|1037537211|gb|OBB31268.1| hypothetical protein A5763_12655 [Mycobacterium fortuitum]
{(MADQ)-HNVFISHRHEdDALVGDLKNLLSKNGSDV-----RDSSVTSATPNNanSesyIKQILaDRIRWagkIIVIISPDTKNHEWVSWEIEYANRY-PDKriiGVWAPGAAGAD---MPPELDEYADAVVGWNSEAIIDALNGKDNWQGPDGATVPP(RSIKRLAC)}*

$2959=199(226):
>gi|1039890174|ref|XP_008377871.2| {|0(390)|<Streptophyta(V)>}PREDICTED: putative disease resistance protein At4g11170 [Malus domestica]
{(MPLCAG)---------------------------KK-IETYIDYQ-LERGEKIapALVEAIEKSKLSVIIFSKNYASSTWRLDELAHILRCKES-KGQVVIPIFYDIDPSDVRKQQGTYADAFAQL-EQRFKDSLDkvHKWRDALREAAKI(SGFDNSNKAGQESDLVQKVVKDILTKLNREASSDLKGLVGIESRIEEIESLLCIDSLDVCSVGIWGMGGIGKTALADAIF)}*

$2960=166(168):
>gi|1039916265|ref|XP_017183022.1| {|0(486)|<Streptophyta(V)>}PREDICTED: TMV resistance protein N-like [Malus domestica]
{(MDTAMTACEAASSSSSMSKLWN)-HDVFLSFRG-EDTRSGFTGHLHAALTDR-GyQAYIDEEDLKRGEEIkeELFWAIEESKISIIVFSKRREAKVIREIVDKIIKEWLPN-XXKLHVAKHQVGIXXRIQNIITHLSSGGLND-VVMIGIWGMGGLGKTTVAKAIY(NQIXSK)}*

$2961=244(245):
>gi|1039893309|ref|XP_017189733.1| {|0(227)|<Streptophyta(V)>}PREDICTED: TMV resistance protein N-like [Malus domestica]
{(MDTSSSARGAGSAASSSSSHSSNRW)KYEVFLSFRG-EDTRNTFTDHLNIAFRKAGINTFIDYQ-LRRGEDVqrELDREIEGSKIAVVVFSKRYAESRWCLKELSKIMRFREEQEGKKVYPIFYDVDPSQVRKQSGSFGEAFPKH-ERDEDPNEVKQWRKDLKASADL(GGRGSXKPPRTEKIVEDIRGLLKTTDLKQPKCLVGMDFRMKEFNTEYFDVGGSPNVQIIGIWGMGGTDIKVSSVAEGTKE)}*

$2962=183(165):
>gi|1039798154|ref|WP_064967020.1| {<Bacteroidetes(B)>}hypothetical protein [Tenacibaculum ovolyticum]
{(MAVNRKYINEHYNTRNDFLRQRIKC)---VFISYQNNDKSHAKKVADYLLK-AGI--NVYFDeydsdlklsNQSNNPGQVTnALTKGINNSSHMLVIVSPTTM------KSNWVPFEIGYGYdKTdlsvltlkgipKNAIPDYIKTANiiRDIWDLNtliSNLRGIDKevlinknsiENSSNIYHplnSYMDKCI---------T()}*

$2963=172(181):
>gi|1039980123|ref|WP_065045317.1|
{|0(346)|<Actinobacteria(B)>}hypothetical protein [Mycobacterium gordonae] >gi|1038430172|gb|OBJ81962.1| hypothetical protein A9W97_25190 [Mycobacterium gordonae]

{(MA)---IFISYSSQdRTSLDALTT---ALKRAQQ-KVWFDQELGGGDAWWnAILEQIRSCDVFIVALSNNWLQSkPSLAElRYAQALNR-------PILPVKVGPV-DSVRVNPVSTLQIIDYQnpTVDAGIQLVTAIHAMSQKATPLP(SPLPDEPPVPFGYIMRLGNDLSEKELTPAQQLQLLIELR)}*

$2964=180(189):
>gi|1039970738|ref|WP_065036672.1|{|0(339)|<Actinobacteria(B)>}hypothetical protein [Mycobacterium asiaticum] >gi|1038469082|gb|OBK19055.1| hypothetical protein A5635_27210 [Mycobacterium asiaticum]
{(MPSIEETAVA)---IFISYSSQdRTALEALTT---ALKRAQQ-KVWFDQELGGGDAWWnAILEQIRSSEVFIVALSNNWLQSK-PSQaELRYAQALNRP-----ILPVKVGPV-DSVRVNPVSTLQIIDYQnpTVDAGIQLVTAIHALREKAPPLP(SPLPEEPPLPFGYIMRLGNTLSEKELTPQQQLQLLVELR)}*

$2965=172(181):
>gi|1040836110|ref|WP_065137699.1|{|0(329)|<Actinobacteria(B)>}hypothetical protein [Mycobacterium gordonae] >gi|1039781625|gb|OBR97802.1| hypothetical protein A9W98_05595 [Mycobacterium gordonae] >g
{(MA)---IFISYSSQdRTSLDALTT---ALKRAQQ-KVWFDQELGGGDAWWnAILEQIRSCDVFIVALSNNWLQSK-PSQaELRYAQALNRP-----ILPVQVGPV-DSVRVNPVSTLQIIDYQnpTVDAGIALVTAIHALVAKAPPLP(SPLPDEPPVPFGYIMRLGNTLSEKELTPQQQLQLLVELR)}*

$2966=220(247):
>gi|1040835887|ref|XP_017231629.1|{|0(1546)|<Streptophyta(V)>}PREDICTED: TMV resistance protein N-like isoform X1 [Daucus carota subsp. sativus]
>gi|1040835890|ref|XP_017231630.1| PREDICTED: TMV resi
{(MTTHVKNGSGLQGRPRGIAYNKEICSG)---------------------------RYGIRTFKDDRELctGDVISDSLLKAIQGSKTYVVVFSENYASSTWCLDELVEIMGCYKSMGR-SIFPVYYNIEPSAVRHQTGSFDEAFREHGTQFHMDRL-SKWRITLTSMAGL(TGYNVHENMSQTDIINKIVDRILLEINPLTLDVAKYPVGLDPRVKALTTLLSSGTEGVIRIGIHGMGGVGKTTLAKAVYN)}*

$2967=249(247):
>gi|1040896889|ref|XP_017256202.1| {|83(0)|<Streptophyta(V)>}PREDICTED: vesicle-associated protein 1-4-like [Daucus carota subsp. sativus]
{(KSYISPPLSPASSQRLSPNPHFYSPLSPPLSPASSQRFSPNRHFSPNISPTLSRASSQRLSPPHFNSRLSPPLSPPSPLV)-YDLFLSFRF-KDTGNNFTSHLYKALSEARIRTAGsPRVPLssTENISSEFHHAIRASEMYIIIFSENYAHSIWLLQELRHIIEHSELTGR-PVIPLFYNVEPHYLQLEAERLGDAILESYEYSDNSTthfM-EKCRVALSTAAEL(PGVVIDDTTSEARIVEDVVRAVLQHIA)}*

$2968=199(231):
>gi|1040908981|ref|XP_017217223.1| {|0(398)|<Streptophyta(V)>}PREDICTED: TMV resistance protein N-like [Daucus carota subsp. sativus]
{(MASYPLTSTPT)QWDVFLSFRG-IDTRHTFTDHLYTALHRTGIRTFRDNPELcsGEVISDTLLQVIQESKTYIVVFSKNYASSPCC---------------------------------GTSRSFEEAFKKHHTRFSTDTL-DKWRNALTMVGKL(SGKVCMNMSEADIVNEIVDDILLKINPNILDVAKYPVGLDSRVKVITSMLSSGTQGVTKIGIYGMGGVGKTTLAKALYNQ)}*

$2969=198(202):
>gi|1040852927|ref|WP_065139617.1| {<Actinobacteria(B)>}hypothetical protein [Mycobacterium asiaticum] >gi|1038435016|gb|OBJ86613.1| hypothetical protein A5640_09590 [Mycobacterium asiaticum]
{(MTDGYDH)--DIFLSFPRAGTAF-DWVCNHfhpiLEktltdELGTE-PEIYVYTEQeTGVAWPENLAASLRRSRYLVAVWAPTYFR-SPWCLaEWES-MRQR-----ERQLGLR----TeeQPRGLVYPVVYRDGVS--FPEQAKQIFTGKGDLSRWAYPS(PIFEKTAEYVKFYKAMRVIAEELAGWLTVAPEWQPDWPVVRPDPGPAPPLPQPRL)}*

$2970=218(224):
>gi|1040806222|ref|WP_065127971.1| {|67(0)|<Actinobacteria(B)>}hypothetical protein [Mycobacterium colombiense] >gi|1038509463|gb|OBK57044.1| hypothetical protein A5653_10855 [Mycobacterium colombiens
{(RHNEEVQRQIEKANRENQRQLDAYNRHVTSVNQHNRQVDASNKAAIADLTRRLNATRYSPQEEQLAERIAGVVAAHDPRQ)-WDVFLSYARIDgSEIGRELRSQLDALGVR-VWFDEVAIQPGKSQARQMDQGLAKAHCGIALLTPAYIAGRFWTErE-LGALLSK-----NTLIPVLHGVTFdeVKEYSGILPDLAGFET--ARHSVAEIADKIADAVLRQGSL(PGGS)}*

$2971=155(155):
>gi|1040953162|ref|WP_065170059.1| {<Proteobacteria(B)>}hypothetical protein [Photobacterium damselae] >gi|1040612725|gb|OBU46062.1| hypothetical protein AYY27_00240 [Photobacterium damselae]
{(MSF)--KLFYSYCQKnDKERTELAKNLDLFVENKLLDEWYDGQLIPGENWKaKIDAKLKDANIVVFLITTEWLASSACKEewELAKKWAKSQT-NK-VLIPIIACPC-AWTDySDMKQYQALPKAGkaVLLWEHRE--EAWLDVYQGVKAV(IDDLKKKVGIES)}*

$2972=201(199):
>gi|1040853813|ref|WP_065139827.1| {|0(95)|<Actinobacteria(B)>}hypothetical protein [Mycobacterium asiaticum] >gi|1038434907|gb|OBJ86508.1| hypothetical protein A5640_11120 [Mycobacterium asiaticum]
{(MADHEDFQ)---VFISWSgPLaKSIASALDNWLPTMFDH--IDVWFsDTDIPAGTAWFgDIQQRLNSSDYGIVVVTTENLNRPW-MNfESGALSKKLKQ-DVARVTPVLVNFT-DitqLTGHPLQQYNAVLLDEdgIRKLCDSIARSAGRNTASVMLRF(RNMWPDLEAEIDKAKKSAGTQPEPPDVSEPEVLESLSASVRSLESTVARLV)}*

$2973=194(203):
>gi|1040888799|ref|WP_065145135.1| {|0(324)|<Actinobacteria(B)>}hypothetical protein [Mycobacterium asiaticum] >gi|1038474762|gb|OBK24365.1| hypothetical protein A5634_03250 [Mycobacterium asiaticum]
{(MRYRRIADLPYQLPMPSIEEIALA)---IFISYSSQdRTSLEALTT---ALKRAQQ-KVWFDQELGGGDAWWnAILEQIRSSDVFIVALSNNWLQSK-PSQaELRYAQALNRP-----ILPVQVGPV-DSVRVNPVSTLQIIDYQnpTVDAGIQLVTAIHALATKDAPLP(SPLPEEPPLPFGYIMRLGNTLSEKELTPAQQLQLLVELR)}*

$2974=165(197):
>gi|1040926648|ref|XP_017225294.1| {<Streptophyta(V)>}PREDICTED: TMV resistance protein N-like [Daucus carota subsp. sativus]
{(MTLVTFAALGLALSYAISVMFKPSADPQQQCLVVKDKNEVPVDTTTSQLAVNSSPLW)--DVFLSFYG-KDTRRNFISHLYYALDQAGIVTFRDDpALEKGQD-

IpsALRHAIKHSKMFVVVISENYARSSWCLDELVEILSCKRT-KTQVV-PVFYYVDPSDYDTTRG-----------------------------VLV()}*

$2975=272(237):
>gi|1041138843|ref|XP_017294202.1| {|288(0)|<Chordata(M)>}PREDICTED: interleukin-18 receptor accessory protein-like [Kryptolebias marmoratus]
{(TARHLNQAFTCVASNAVGTSSVTMRLRARRPVRWRSLIGCPLACFLLAAGLGILLRAKWLEIRLICRSRFQRGNDTGEEK)EFDVFLSHV-
WspgsptwaggsdfisteaeEKLNAQrpgevLLSQVLEDRWGYRVCLLERDILPGGAFTNDVVLALKRSRMLLCVLSADYLaDANAVF-VLETGVKAFLQNAALRLLLVRTrgtggtspSLP-
RTDPPLSKLLQRSLKVLpGLDWSSDPSSstadRFWTSLRKALPDR(RAAAVSSETSFPQRRFK)}*

$2976=228(235):
>gi|1055015074|ref|WP_066766730.1|
{|0(191)|<Proteobacteria(B)>}hypothetical protein [Caulobacteraceae bacterium OTSz_A_272] >gi|1042022627|gb|ANP44523.1| hypothetical protein ATE48_00585 [Caulobacter
{(MSVMASTRGFTADNKV)--RVFVAAARADASFADQIGAFLHNAGFQ-PLLDRYESG-
GHGWQDRLAPLIDDADALVLVVSEA-SASSDQCVwEVEEAQRLG-----
KRVVLVLPAPLRGVARELAGLNTIyfysdpNIPN--SGFYDGQR--
RLEAALRGAERV(RNGVGPSRPSAQDQQRDARRAEAHARKEAHRLGKAEAKELKREIRRATGRRFPMLRVAFLTVVAAVVIGVVMKPELLTQ)}*

$2977=208(224):
>gi|1055023071|ref|WP_066774384.1|
{|0(215)|<Proteobacteria(B)>}hypothetical protein [Caulobacteraceae bacterium OTSz_A_272] >gi|1042025958|gb|ANP47854.1| hypothetical protein ATE48_19100 [Caulobacter
{(MAIAA)----YLAFEPEDANAAHAVSAELEQHGWD-VLRSGAEYRVQDKL-
GALVEAVGKAAIVVVLASPAA-QESAWIRrEVAAALNNG-----
RSIIVVKTNDLAaeSwINQTLDPSSFIDL------RQGAQS--
EVLAQIVDRARS(ANGGGRVIAMLNIKGGVGKTVLAANLFAAAHLADKRAISFIDLDPQHNLTQYFLSPGERHRIRDRNHTLYGILNARGEH)}*

$2978=174(177):
>gi|1057571746|ref|WP_068863892.1|
{|0(491)|<Proteobacteria(B)>}hypothetical protein [Porphyrobacter dokdonensis] >gi|1041149940|gb|OBV11241.1| putative adenylate cyclase protein [Porphyrobacter dokdo
{(MVTRNDAEEMPDGPR)-PTVFLSYSRAD--LDRSRPVIGLLEDHG-
FDVWWDGRLEGGENYLaTTEGALEAADCVVVLWSKTSVASHWVRDEAQRGRERGCL-VPLSLDGTMAPLG-
FRQFQLLDIAGWSGDPAasQAQKILAAVTRQAGEAPIAGGNA(SAGAALAPSPSGTVQAAKGITL)}*

$2979=211(219):
>gi|1041848390|ref|WP_065220250.1|
{|0(559)|<Bacteroidetes(B)>}hypothetical protein [Butyricimonas sp. Marseille-P2440]
{()MSKAFLSHSSKdKELVRRIATELG------------
DQCIldeisfEPGRKTLdEIFRTMAESDIFVLFISGNSLDSPWV-KqEITRSEKLSSE-
KLTHVLPLVIDPTISHEDKRIPDWLAKPYNLryIS---NVK--
IILQKIENALRKI(NVLKFPRNQELMNLFVGRNDEIARFEFDINNLDNWMPTYVVAYNYFAGIGRRTFLMNALKKVNLLDVLHRPTFISIDAK)}*

$2980=211(220):

>gi|1041881796|gb|OBX25080.1| {|318(0)|<Bacteroidetes(B)>}hypothetical protein A9996_11825 [Gelidibacter algens]
{(FSLLFKYITPLKTKPKTLAVLAFVISLIFFVLYGMLIYTFDRIIPIGLTLLGMLIAVLLTWRFAYKFLVTG VAEGAEKYD)---
VFISYSHGnSAWvKQNVYEPLKTFKKENgeSLDIFFDEKsIGVGEAFTsKYMWGIVDSKVFIPVISEEYYGKN HCKNEMDLAYKRSVE-KLLKILPIAFSyncvPQ-IYTHINFLDITVNPK--------------
FMEAIKESLLK()}*

$2981=181(189):
>gi|1055017475|ref|WP_066769090.1|
{|0(84)|<Proteobacteria(B)>}hypothetical protein [Caulobacteraceae bacterium OTSz_A_272] >gi|1042023673|gb|ANP45569.1| hypothetical protein ATE48_06385 [Caulobactera
{()MSDIFISYKREdKARVEALYTLLLDL----DAPVWFDAGIEVAMEWEaRILEQIEKARAVI--
VCWSFAAvSSHWVIrEATIGLER------GILVPVTIHPC-A-
LIPPFDALQSADLTQwdGSPDHPEVQKVLLRLGLLIGKKN(LARNGRLRAGGQKEAMVDLIRALLVERALSGG DPFTYKEAEEALRAAAA)}*

$2982=208(218):
>gi|1055022486|ref|WP_066773815.1|
{|0(126)|<Proteobacteria(B)>}hypothetical protein [Caulobacteraceae bacterium OTSz_A_272] >gi|1042025665|gb|ANP47561.1| hypothetical protein ATE48_17445 [Caulobacter
{()MSDVFISYKREdEPRVGRLVQ---ALEKAGL-
KLWWDRGLPGGESWRaNIQGSLDAAKCVVVAwthqsTSP---AGDFVRDEAGQAKAR------
GILVPVLLERG-VRPPLGFGEVQAI---DLSHWRgsQSD--
PFFQDAVAAIRAK(VEGRAVPPARGPMRRLLRRLTIGSVASAGMAGLVGFGMNLLQVQDQVCTIDVGQPYLSD VCGAVNLGNRPTQAERVAF)}*

$2983=234(220):
>gi|1054439015|ref|WP_066256876.1|
{|0(246)|<Bacteroidetes(B)>}hypothetical protein [Bizionia sp. APA-3] >gi|1041874635|gb|OBX18059.1| hypothetical protein BAA08_15605 [Bizionia sp. APA-3]
{(MNETNPK)---IFISYSwsS--
PNHEDWVINLAERLVSDGIEVKIDKWDLKEGHDMfdFMESMVNSSDIdKVLIIldekySSKANSRKGGVGtET QiISPEIYKNVsQEKFVpIVRQRDDS-
NEAFiptFLKSRIYIDFSEDehFEKSYESLLRnLYKRPVYNKPKLG(KAPSFLFEETPMNHKTSILLRSFDKK LAQSPNSLNSMARDFFDKFYDNLKDYSITFSSRDYKIIGKEIIDNLN)}*

$2984=210(220):
>gi|1055022217|ref|WP_066773602.1|
{|186(0)|<Proteobacteria(B)>}hypothetical protein [Caulobacteraceae bacterium OTSz_A_272] >gi|1042025558|gb|ANP47454.1| hypothetical protein ATE48_16835 [Caulobacter
{(PTPGGWPELLAGIDGFGVRYGLPSGVTLWLFVVSIGLFFAVLGLIVRGFFVRKPSTDAAPRQPRNPHKPRA RPRGATPSA)-DAVFVSYAR--ANDM-MVLPLVEGAKRAGRKFWLDQQGISAGDGWagEIVRAIK-
AAGGVVVMCSQAAFESDHVKrEIYLADRYKKRL-----VPVFIEHA-TPPED--
FEYFFAGVQSLNLYDTPEEERSEALVRILGAVA()}*

$2985=176(177):
>gi|1042877777|emb|SBU96122.1| {|0(552)|<Actinobacteria(B)>}TIR domain-containing protein [Streptomyces sp. Ncost-T6T-1]

{(MTRFPSSPGVRDSSTGRPPQT)EWDVFLSYSR-SDAEK--
TGVLVSALRDRGLRVFVDDTAVDDFASItaTIIRALAHSKVLVALYSADYPRRRACQWELTYAYLSGQREGDP
RRRTLIVNPEHGTDHVHPVELRDARHWPWPTVPEEADRLADRVASHTAALA(DPMGHAPTGPTVPWLP)}*

$2986=205(238):
>gi|1043411941|gb|OCA24574.1| {|401(0)|<Chordata(M)>}hypothetical
protein XENTR_v90020057mg [Xenopus tropicalis]
{(WVQQQKDKLLKQSYQMFCKTPNTLNGLPLTDFTPNCSMTALISETVLGILCPSLIAILLASLSVISAKEIM
IITMYMMHL)---------------QQLVPKLENENG-FIICLHYRDFVPGVPIVTnI-SNSILNSQKT----
---FLESKWCNFEFEMAKAWQSLEGSFQII---------TQLKKALGLHKYLAKTtYLELNKDLF--
FWLRLQNSLKNG(QCRTNQHNYHKGMPENVL)}*

$2987=216(222):
>gi|1042711766|ref|WP_065286077.1|
{|68(0)|<Proteobacteria(B)>}hypothetical protein [Haemophilus
parainfluenzae] >gi|1042700476|gb|OBY50647.1| hypothetical protein
BBB48_07970 [Haemophilus parainfluen
{(KNSPFSNVFSFSYDQLSPNDIGFNEITSNNIEQVRKYFYSNLNLSLDDGIFILCRDLSNLSTEIKIICETH
KQYMGLLPM)--KIFLSHKSKdKSNVREFKNILQKLGFSP------
WLDEEQLKAGDklnrSLLKGMQESCAAIFFITDNYEDEQYLSDEIDYAKNRAME-
DPNFKIITLVFNNASHNIPQLLRHYVWKNVN--SDLSALGHIIDALPLQVGEIR(YK)}*

$2988=285(298):
>gi|1043351540|ref|WP_065367952.1|
{|97(20)|<Bacteroidetes(B)>}hypothetical protein [Prevotella scopos]
>gi|1043184048|gb|ANR73780.1| hypothetical protein AXF22_09950
[Prevotella scopos JCM 17725] >gi
{(TILLDIMRNHIERRTILHLEGEKDLSSFIHEDLCKLKTSLDYSSHPIIIINKPKHAIGIKKDPNINIITFQ
ENNFMEKRT)-EKVHISYKHDtAYEADILA---IKTGLQENNIPY-SIDEYDILYRDniddY-
EKEIGSSDIVIMFVIPNYLTSLECMFEMTQI-FKNGN-IKTRVFP--------VVDMGKIKRNGDGLIK--
IKDywSKEK--
IKKSEQIKTEPG(GSSFLIKEIQKIDDILKTMDDLWTFLCRENSGSYHKLIDNNAKQLMEEIKKYLCLKLAPD
TENFTPTTATKPTGTRVI)}*

$2989=230(223):
>gi|1056322053|ref|WP_067773934.1|
{|0(120)|<Verrucomicrobia(B)>}hypothetical protein [Akkermansia
glycaniphila] >gi|1043326102|gb|OCA03548.1| hypothetical protein
AC781_04145 [Akkermansia glycaniphil
{(MCK)KHKVFLSHASAdKFLCDRFLKFMNlGCYIQYDDVYYTDKSSMQIPNNVyfpeDMLEGLKNCDIVVFI
ISSNFLNRPNCSIELGAALAHKKP-IISLLVPPITyGNMPGFTTGKQIMGCINEEEA--LLNFAEE--
VMQKlygevKNLSNTNK(EVKSFLEDCKSIIEDLKSDGLITKDEYESAKKSAARYKQISNEREIDLEKLREQY
EKVKNAKDVDEVREIEWESMDESKK)}*

$2990=220(224):
>gi|1056327747|ref|WP_067777569.1|
{|0(6)|<Verrucomicrobia(B)>}hypothetical protein [Akkermansia
glycaniphila] >gi|1043324799|gb|OCA02269.1| hypothetical protein
AC781_11325 [Akkermansia glycaniphila]
{(MADNIRNNLQNVSIGGNFVGGNQTVHHHHHRETASTAAEKNAAATPKE)-
ATVFLSYCRAnSDEADSIERRLS---
DAGLIVKRDIRDLPPWQSILAFMQSIRDQDVAVLLISDAYLKSHACMYEVMEIM-KEKD-
YHDRILPVVIERG-

IYNRAKQIEYIAHWQHEaeQYKTAMQTIESENQNDMPKALKQ(MKQIAASMEEFLKAVASKNNPDISDAGDAI
IAHLKH)}*

$2991=234(235):
>gi|1044528428|ref|XP_017408760.1| {|0(792)|<Streptophyta(V)>}PREDICTED:
TMV resistance protein N-like [Vigna angularis]
{(MASASSSSSFSKFRI)DGDVFIHCLG-YDIRRNFVSHLSSALLQAGVKPFLLAVEMQQEQ---
FVASIEGFQIGIVVLTKTYFESFRCVDELVRIIECHET-
HGLMVMPVFYEMDRSDFENTLKATAREVMKVEHIIWSGEyrktwF-
QRWNVALTKAGTL(PTWEESQHRSDAELVEEIVKSVLAKLDCPLGITKFPVGLEPHVKNVMGLFENQPTKVCM
IGIWGMGGSGKTTVAKAIYNQ)}*

$2992=243(244):
>gi|1044549979|ref|XP_017441288.1| {|0(630)|<Streptophyta(V)>}PREDICTED:
TMV resistance protein N-like [Vigna angularis]
{(MEFASFSSSSSSSSFLKSEPQFIY)--DVFINFGG-EDIGRKFVSHLHSALLLAQVKTFISKENLQEGI--
-KleehMRAIASSKIAIIVFSKKYTESTCSRLELEKIIECRQT-
FGQIVLPVIYDIARLDEHLQKvvRVLEKAAPGSYSGEQLDHaM-
SRWSRALTKAAGI(TGWDFRDFRHDAEFVKEIVRRIQTLLDYTDMSITQFSVGLESHVEKVIALVEEQSTEVC
MIGIWGMGGSGKTTIAKAIYN)}*

$2993=196(194):
>gi|1043621089|emb|SBW21443.1| {<Actinobacteria(B)>}hypothetical protein
FDG2_2009 [Frankia sp. Dg2]
{(MSGYQ)-FDVFLSYRR-AGTvlpwvhnhfHRMLVKCLTDELERRpKI--
FIDVDGVEVGDHWpdTLAQAHSRSRLLVAVWTPSYFGSHWCVAEWRSMQHREAM-FDlstapdacRLVYPI--
-------RLKDG---TRFPSD-
AKKTQQQDMKEWNVSSPAFEQT(PAYAGFEKQVGTVAEELAAKLELVPDWRPDFPVLRPDPEPLHRADVPRL)
}*

$2994=246(254):
>gi|1043621411|emb|SBW20496.1| {|74(48)|<Actinobacteria(B)>}hypothetical
protein FDG2_1711 [Frankia sp. Dg2]
{(DAWLMSGGWGDYVSDLVGMVSTSSRHWLLPVLLTRKVDRTSSISDMNCLRLYETDLELRDQVFVNKILHRL
FRHVTDDDS)PLTVLLSHAS-TD-GRGVAlavrKYLHDY-TDIG--DFLDVHSIAAGERLveAIDRAI-
SASTFLAIVTDSYSSRGWCRHEV---VKAK-----
RELGPIVLLDAVEDGDERRSPYLGNIPTLRWKPDSSLVMERLLGLLLAESFR(HRYFPRQVAWLSRLQRVDLP
YYCLSRPPEPLTLL)}*

$2995=206(200):
>gi|1148481395|ref|WP_077268868.1|
{|0(36)|<Proteobacteria(B)>}hypothetical protein [Klebsiella pneumoniae]
>gi|1044261585|emb|SCA38361.1| Uncharacterised protein [Klebsiella
pneumoniae] >gi|104427874
{(MSKHAM)---LFISHINEeKEIALQFQELVDNAFLGLIDTFVSSSPD-
AISMGSkwltKVSDALNNCDIAIVICSPQSVKRPWVNFEAGATWIRNID-
VIPLchsgmtpaTLPIPLSELQGAIATDPSSIRLVLSAL--
SSKLGSKQSSVDVTPFTNFMV(EYEKEYLFKKNLMCAINTISDVAPQLKIAFKNKRPIEIIVPTDRQGEIYKH
FDF)}*

$2996=225(224):
>gi|1043621088|emb|SBW21440.1| {|249(0)|<Actinobacteria(B)>}putative
ATP/GTP-binding protein [Frankia sp. Dg2]

{(RLLGFLTIPYVSYWSFGEDLPALEEEDPTPEKIGYSLETLAATIAHRLDRSELLVESRDSFVDAATRLGLR
SRNDDGYEI)--DVFISSSRKDDRLATSLEDLLARRSLR-
VAgpsrLRQLGVGISTDWQREMDTLLARSRHLVALFDGPYLDRAQSYEiDRFIRQTLD-----
DRsnrmVLPVLTSDAEpdHLPSLLASRQYLRITG--EEDLSDVADGIVDTITRGARGT(SGRG)}*

$2997=312(299):
>gi|1158995234|ref|WP_079165048.1|
{|874(378)|<Actinobacteria(B)>}hypothetical protein [Streptomyces lincolnensis] >gi|1043641797|gb|ANS70744.1| hypothetical protein SLINC_8520 [Streptomyces lincolnen
{(AMAGAPVFSEGFLLGFVIGRSAGSRLAVAGISTLVNEGDFTEFCRPYMPGIPPLQRLPATPTSPAEGDRTN
DTSARHPPP)--RVFISYTHEDDngAHAEQIHnlvEVLSAAGIN-
VRLNAGELEGSRDWAARMRHEMEAADVILAIASPEYkrhadTAQSDTASqVRVASEARllrnelahgpenlS-
----RRILPVLLPGSTteDLPAFLRPLRPLAVDT--VT-HAGIA--
QLLDRIPGYARE(DSAPAREVDPARVHALIVGVERYDAGTPWDLPGPARDAVRFHRLLREAGVPETQLRLHLA
PLPPYVPDVRYEPADQATL)}*

$2998=212(220):
>gi|1043694134|gb|ANS32650.1| {|46(0)|<Actinobacteria(B)>}hypothetical protein R1CP_40350 [Rhodococcus opacus]
{(VDNYNREVARRNQKVVDKYNRQVDAVNAANRKADSHNRTVIASLNSQLRSASQGPRYTPAEQELADRVHEA
LIVQDEREY)--DAFLSYARIDGGDAgNLLREHLDELGVN-
VWFDEIAIIPGKSQALQMDKGLRKARCGIALLTPAYLTGRFWTErE-LGALLHK-----STLIPVL-
HNVTfeQVAEYSGILPDLAGFE--TSRDPIPVIAQKIAAAILASDE()}*

$2999=220(224):
>gi|1053926140|ref|WP_066104409.1| {|43(0)|<Firmicutes(B)>}hypothetical protein [Bacillus sp. FJAT-27986] >gi|1043532063|gb|OCA86160.1| hypothetical protein A8L44_07020 [Bacillus sp. FJAT-27986]
{(EYLLKVSGLSKEFEWINVEDDAFIFDLEKIKRNFSVFSECKWMKSYITLKPNYEPALDNSKFIVSFSATKK
EIEVNAPKK)---IFLSHKG--A-
DKPLVREYFQLLKELGFDPWLDEDAMAAGTKLnrGIYQGFKESCAAIFFV-
TPSFKDEDFLEdEIDYAIDEQKEKgDKFAIITLALKDS-
NHKTGEIPGLLRKYVYKEPKSQLEAFREIIRALPLETGKK(VWKN)}*

$3000=228(229):
>gi|1043783044|gb|OCB14885.1| {|229(0)|<Actinobacteria(B)>}hypothetical protein A5717_09030 [Mycobacterium vulneris]
>gi|1043836285|gb|OCB65655.1| hypothetical protein A5729_15530 [Mycobacterium vulne
{(SNTRLSLERNQFFFAGCTSSRESSLASFFATYCRKTLVQQLEMEPTEFVAELRARWAEHAVPRPQPSAYSC
GAPTTEAPS)---IFISYMR---
EDADAARRLCNAISTLGGDVWLDERRLLPGDDWerEVLTGIRKTVRLFVPIISANTerEGEGYAFrEWREAAE
RSRSIlGRRFIVPVIVDND-YAGNPSQYRRMPDSFRDVHFGHAPAGDPDAGLQLMLTEEI(RAMRRTDAA)}*

$3001=137(166):
>gi|1045369720|dbj|GAU43039.1| {|0(1475)|<Streptophyta(V)>}hypothetical protein TSUD_329370 [Trifolium subterraneum]
{(MASSGSSSTNQDSEPIFSHDMNRSSI)-YDVFLSFYD-VDTGDSFPSYLYTALREAAIYV-----------
----
KLIEVFRVGFSSVSSSSAEQSFSFTLLIFMASSSSSSTNQDSESIFNNDMNQSWTYDVFLSFCDKDTADSFAS
YLYTALT()}*

$3002=156(156):

>gi|1045365674|dbj|GAU46096.1| {<Streptophyta(V)>}hypothetical protein TSUD_95680 [Trifolium subterraneum]
{(MSSSILSNTVADPTLK)RYDVYLSFCE-EDSL-SFVLGIYTALtSQHGPVVFLEtqrfgsDDDQTLLR-
PseSTLNVIGECKIAILVLSKNYTNSRWCLDELEKITECCRTSDGLVVVPVSY------
DGVYSPNRRLQGVCMERLFLIFWIKSQWRKRPIWKISL()}*

$3003=154(159):
>gi|1045375692|dbj|GAU38595.1| {<Streptophyta(V)>}hypothetical protein TSUD_266340 [Trifolium subterraneum]
{(MSYGH)-YDVYLTYNG-KHACKSFALDLDSALRQAGLNVYlnHDNLMARGDyT-
VtdTNFSEIEGSRTCIIIFSINFDASTGFLQELEKILERRKT-RGLTVVPVFYDVDRSELRHQEGVF-----
ARKSWFSEDKA-MRYRAALIEVANI(MPGFRVWDSMWDFR)}*

$3004=166(162):
>gi|1045382710|dbj|GAU33019.1| {<Streptophyta(V)>}hypothetical protein TSUD_358860 [Trifolium subterraneum]
{(MSSFTTNPKTES)-YDVYLSFCD-EDA-GSFAMDIYKALSSEdGIVVFCDDE--RLGN-
GdrEILTSslnvIGECKIAIIIFSRKYVNSIYCLQELEKITECCQT-TaGLIVLPVLYdGLHPSSGS----
IFGEAFHDfldrismVETSKVEDKF-MTWVAAISNKAFQ(YSGSSDLVHR)}*

$3005=215(233):
>gi|1045385524|dbj|GAU30758.1| {|0(528)|<Streptophyta(V)>}hypothetical protein TSUD_354830 [Trifolium subterraneum]
{(MASSSSSSSNSRWI)-HDVFINFRG-QDTRKNIVSHLYAALSNAGINTFLDNEkLRKGRE-
LgpQLLRAVEVSQIAIVVFSKSYTESSWCL---------------------------
VLMNYQKGAFGRSLKSTAKRRisgLPEFK-
LRkdymdsgWRNALTVAANF(SGWDVNNCRTEGELVKKIVGGVFTKLEGSFLSIAEFSVGLEPRMLKVVKFIE
NQSSNVCMIGIWGMGGSGKTTAAKAIY)}*

$3006=205(196):
>gi|1045386899|dbj|GAU29719.1| {<Streptophyta(V)>}hypothetical protein TSUD_264550 [Trifolium subterraneum]
{(MSSFTSSSKRRHKLKSTSTSSTTTSTNPKRK)RYNVYLSFCD-EDA-
TSFATGIYTALNRKaRFDVFWDDKKLGSgdQE-
IptSVLNVIGNCKVAVIVFSRNYVNSRSCIQEFEKITECClTT-GGLIVLPVLY--
DGLNHYSAIGTSGEAFHdfvdriwikeKETSSEEEDKF-
MTWVAAITRATTY(SGLIDFADSYGREYIEDVVETVTRA)}*

$3007=135(148):
>gi|1045381583|dbj|GAU33918.1| {|0(62)|<Streptophyta(V)>}hypothetical protein TSUD_357170 [Trifolium subterraneum]
{(MSTSNKK)-YDAFISFRG-EDTRDIFAIPLHDALENQNIDTYIDislkeVKIERNRK-
VfiVLDNVDNIAQLKSLCGALNGLGANTNSRLVITTRDKRTL-----------------
KGNVDEIHEVTKWSFQDSLKFFIGKAFEQGNPQQGYDS(V)}*

$3008=273(300):
>gi|1045392539|dbj|GAU25082.1| {|94(127)|<Streptophyta(V)>}hypothetical protein TSUD_257760 [Trifolium subterraneum]
{(FKVIAGTSDKPMIVVNYKNEEKHFTVEEISSMVLIKMRQIAEAYLGSAVKDVVVTVPAYFNDSQRQATKDA
GVIAGLNVM)---------------------------QG-
FKTFIDDEALEGGSPIdKLLEAIEESRFAIVVLSENFADSKWCLKELVKILDCRKR-
KKQLVLPIFYEVEPTDIRHLKGRYGEAMAEH-
EKKLAKDSKtvLEWTLALSDISQL(KGEHFRISNFKGEHNERRTSQHFQLQTRALRKALEAIEKLQKTVNTLI
VIPNDHLLDIADEQMPLHDAFRLADDVLRQGV)}*

$3009=271(300):
>gi|1045407264|dbj|GAU12315.1| {|8(877)|<Streptophyta(V)>}hypothetical protein TSUD_252660 [Trifolium subterraneum]
{(QRRVTTLMELVMTRKRDGVFKHMPLDLLLLGLEVDKTRDTPYSYGCFWTLLYTCFSLSQVWFCLLEMSMAK YKEQSHRFT)-YDVFLSFRG-EDTRHSFIGYLRDALRQrG-
INAFFDDKNLSIGEDIspALLKAIEESKISVIVFSENYASSRWCLGELMYDIKERAT-E-----------
KPWLLIKDSG-----------------
NIKAWTAALSEVADL(KGHHIHTGFEIDHIKEIVDKVHAKVTPKPLLSGDDPVGLDHHTENVKSLVDDTDHSV CMLGIYGLGGIGKTELAKALYNK)}*

$3010=213(212):
>gi|1158966064|ref|WP_079137784.1|
{|0(527)|<Actinobacteria(B)>}hypothetical protein [Streptomyces sp. PTY087I2] >gi|1044666436|gb|OCC11573.1| hypothetical protein A3Q37_02770 [Streptomyces sp. PTY087
{(MSARRREPLREGLIVVPPAASDGAPALVA)EWDVFLSYSR-TDADK--
ARSLAHMLRDRGLRVFLDDMAVDDFASItaSISEALARTKVLLALYSAEYPRRRACQWELtyAYLAGQRDGDP RSRVLVINPELSTDHVHPVELRDAKHWIWPSVPDALLRLAGQVAAHVAGIDSP(LGAAGHAAGGREAPSVPWL PAPVRTGSARFTDRLTEQWRIHTA)}*

$3011=199(193):
>gi|1045374377|ref|WP_065476577.1|
{|0(514)|<Actinobacteria(B)>}hypothetical protein [Streptomyces sp. PTY087I2] >gi|1044668938|gb|OCC14066.1| hypothetical protein A3Q37_00339 [Streptomyces sp. PTY087
{(MVDHAGADA)EWDVFVSYSR-SDAQKIAA--
LVPALRTAGLRVFVDDTAVDDFASItaTITEALSRSKVLLAVYSTEYPQRRACQWELtyAYLSGQHEGDPRRR ALVInpeqsADHVHPVELRDARHWPWPVTQAAIARLAERVAAHTEVITAPMGNLA(HAPFVPWLPAPARTGSV AFTGRLAEQWLIHTALHRHRTPLVAQA)}*

$3012=174(174):
>gi|1045385526|dbj|GAU30760.1| {|0(299)|<Streptophyta(V)>}hypothetical protein TSUD_354850 [Trifolium subterraneum]
{(MSFSSSSSDHPWI)-HDVFINFRG-
EDTRSTFVSHLYAALSNAGIYTFLDDEELKKGENLgpALKRAIEGSHIAIVVFSVNYAESTWCLNELLSSWRK ALTQVAHISGWDANNFRTEGELIQEIVADILEKLDISLLSITEFPVGLESHVQQFTKF(IHDQSRKVCMIGIW GMGGSGK)}*

$3013=218(221):
>gi|1054406387|ref|WP_066246792.1|
{|0(84)|<Proteobacteria(B)>}hypothetical protein [Arcobacter porcinus] >gi|1047018495|gb|OCL86696.1| hypothetical protein AAX30_01421 [Arcobacter porcinus]
{(MNE)--EIFISYTTRDKLLVDKTLEVLNQNKIS--
CWFAPKDIAPGEKHTdVIIPAIKNAKLFIVFLSKYTRPYGENNKdvSDWVKDEISVARkFGKYIIPIkLDSTV DlpT------
DDLLFERLGNYIDFNSNSFELASKQLVDFVKYL(LEDNNYLKKENPYLEIIKEQEFEFIKKIANLIKSGSINL AEEDLKNNIGLKDKYKEQIKLYETIILMSKKPLKDLNSY)}*

$3014=218(221):
>gi|1054618610|ref|WP_066386376.1|
{|0(84)|<Proteobacteria(B)>}hypothetical protein [Arcobacter thereius]

>gi|1047028592|gb|OCL96722.1| hypothetical protein AAX27_00354 [Arcobacter thereius]
{(MSK)--DIFISYTTRDKLIVDKTLEVLNQNKIS--
CWFAPKDIAPGEKHTdVIIPAIKNLKLFIVFLSKYTRPYGENNKdvSDWVKDEISVARkFGKYIIPIkLDSTV
DlpT------
DDLLFERLGNYIDFNSNSFELASKQLVDFVKYL(LEDNNYLKKENPYLEIIKEQEFEFIKKIANLIKSGSINL
AEEDLKNNIGLKDKYKEQIKLYETIILMSKKPLKDLNSH)}*

$3015=219(228):
>gi|1046642413|ref|WP_065707071.1|
{|0(640)|<Proteobacteria(B)>}hypothetical protein [Agrobacterium sp. 13-626] >gi|1046081922|gb|OCI98479.1| hypothetical protein A6U85_32255 [Agrobacterium sp. 13-626
{(MHTTAIKGT)---IFLSHSNKdKTLVEQIV-----AGLDPVNTFY---
DIRSMDAGQptldAMKHGINATAVFVLFHSPASQSSWVDL-EKALAEVQSivNP-
ATKILVCPINGADHHTLPSwMSKYFTTTSSFK--PNDIIRT--
IRHLYQKYIDDA(YPETVRNHPGREVLQRQISLELMRSSATTGKVVNAVVLTGVQGMGRGTLAADLVRESYRN
MRQAGPVFEIPPGGDGTDW)}*

$3016=229(219):
>gi|1054162112|ref|WP_066173958.1|
{|0(586)|<Proteobacteria(B)>}hypothetical protein [Arcobacter porcinus] >gi|1047013585|gb|OCL81891.1| TIR domain protein [Arcobacter porcinus]
{(MI)--KAFLSHSSAdKErFVRIVADKLR----NKMNIHY---
DEYTFEAGNktidEIFLGLEQTDLFVFFISDNSLKSEWVKKELLIAnnnRTKTKQ-
FYPIIidknikynderIPNWikEYNLKYVAKPTIVARRIEQKLR--EISLskHPK--
LMKKNNLCIGRN(KILEEFEQRLDDFNKDKPKCIITTGLESIGRRTFLNFALQKTDITDQNYAINDIYLNTDD
SIEDFIFKINDLGFLDI)}*

$3017=174(178):
>gi|1054177110|ref|WP_066178558.1|
{|0(1693)|<Proteobacteria(B)>}hypothetical protein [Arcobacter porcinus] >gi|1047024792|gb|OCL92941.1| NACHT domain protein [Arcobacter porcinus]
{(M)--KVFISYSSTdRKIVREIYQLLK-----
DEFEVWIDEEsLLAGEEFErEIPLELSNSDVYILMMSKKFWESDFIYNnELPKIFQQAKL-GK-
FIVPFVLDDV-
FCLEDPNGTYLLHKSVNhlNSLPRCKNGHIKSYTELLKTETK(EHLLKTLKDEIKKLPFSYSYFRFSLDILKL
LHYNCKV)}*

$3018=221(227):
>gi|1046230246|ref|WP_065620753.1|
{|147(0)|<Proteobacteria(B)>}hypothetical protein [Legionella jamestowniensis] >gi|1045921128|gb|OCH98060.1| hypothetical protein A8135_12925 [Legionella jamestownie
{(IYIGWIKQQVEHINIQINHHNLQLKSISQQLHRKAQQRKEKISNLLSDLGIPVHKNEKEVLSVNVPKKISN
QSNQYYSTA)-----ISYGG--P-DKPIATMLNEFLRNKGFDTWFFPVHAKPGEKLhrEMYS-
MANDYERVLLICSKNSLTRAGVLnEIERILEREADEgGSNILIPISIDDY-
IYTWESPKRDIANQIRSRVIISLDPCDFSSNKNQIEMSKI(LNALTKS)}*

$3019=204(220):
>gi|1051748526|ref|WP_065947587.1|
{|453(0)|<Proteobacteria(B)>}hypothetical protein [Pseudomonas sp. 1 R 17] >gi|1048646075|emb|SAM32948.1| 5'-methylthioadenosine/S-adenosylhomocysteine nucleosidase

{(TASTLLHLAEAKGLPIIPFAFLGGVADRIYRRRNWARLNPGFDASILESEGSIEKTVSISNHLVRDRAKRS
IAPDTQPKT)---IFISVAR-QDAAMGMA--LDHVLKSYGIePVLGDREIVSDQL-
IppTLEQAIRRSDIVAVLWSRFYAQSPWCYDELA-LALSMEA-LGHMKVWLFNLDDSSIVPTQARKLPS----
------FAVRSEEGLRRALRDLLS()}*

$3020=277(300):
>gi|1048491581|ref|XP_017523574.1| {|84(20)|<Chordata(M)>}PREDICTED:
single Ig IL-1-related receptor [Manis javanica]
{(VNLTSAENFGAFTCSVQNVSSFSFTLWRAGPAGHVAAVLASLLVLLALLLAALLYVKCRLNVLLWYQDVYG
DVEMNDGKL)-YDAFVSYSDSpedRKFVNFIL----------------------------
LVNLSRCRRLIVVLSDAFLGRAwcSHSFrEGLCRLLELTRRPIFITFEGQRRDSVHPALRLLRQHRHLVTLL-
-LWR-
PGSVtpssDFWKELQLALPRK(MQYRAMEGDPQTRLQDDKDPMLIVRGHAPEGRALDPELDPDPEGDLGVRGP
IFGEPSAPPHASGVSLGGGRGSEVDISDL)}*

$3021=198(211):
>gi|356546339|ref|XP_003541584.1| {|0(237)|<Streptophyta(V)>}PREDICTED:
disease resistance protein ADR2-like [Glycine max]
>gi|947071799|gb|KRH20690.1| hypothetical protein GLYMA_13G194600
[Glycine ma
{(MVTFNLSVEPKPFTN)--DVVI-ISD-KDTRWGFGGTLLKAFQLCGFRAVL-------VG-
AgnELgRKEIEESMVVIPVFSMDLVSSPDHLEELATVVD-EKR-MCQMFLPFLYKLELKDVRYLMG--
GKLFEKFYEVLTKVTDLTGFRFGDGVTYEY(QCVEKIVQVSAKHAASTIGVIPRVTEAMLLLSPESDNGVNVV
GVVGPGKETITRKV)}*

$3022=230(230):
>gi|504090732|ref|WP_014324726.1| {|25(0)|<Firmicutes(B)>}hypothetical
protein [Leuconostoc mesenteroides] >gi|356644557|gb|AET30400.1|
hypothetical protein MI1_04750 [Leuconostoc mesenteroides subsp.
{(RLAKYKQKLAPTNYLKAQKDMAKKSSDLGATQQKLTKERKRENDKFQKDQDKQLKQMKAAQKEARRWVTSG
ITLLENKNK)KYDVFISYVQ-SHS-
LEYVDKLEIALRNKDVSVSRDKTDMIIGQSMtkAIENGLAKSKLAIVVLSPNYLHKYWTNFELNGLFSKQGIT
GHQMILPIWKNVSAEEIAVKRPSLANLLAWNVANDTTEEIANTINQLLDKPVIE(GSDENGTTIN)}*

$3023=184(187):
>gi|493831348|ref|WP_006778637.1| {|0(55)|<Firmicutes(B)>}hypothetical
protein [Hungatella hathewayi] >gi|356699780|gb|EHI61290.1| hypothetical
protein HMPREF9473_00652 [[Clostridium] hathewayi WAL-18
{(MNL)----FLSFSG--
EARETFAVKFLNFFNNYGIHCWYDQHELFLGDNLknSIIKEgVETANYCIIIINKSFLSRPWPCEEAKRLYES
LESRKHNAIFPILLDIEKEEVRNSELDFLLNIKYQFLHTGESIDKIGLQILNRIFHDI(AMQTKFATLNDALN
YFKRLTLTNSIDIYNALNVVNNFDHTNYKE)}*

$3024=291(292):
>gi|357604744|gb|EHJ64305.1| {|958(57)|<Arthropoda(M)>}putative toll
[Danaus plexippus]
{(NDPTVSKNEIVSKSLFLPDNYFAIVCGSIIIIVVICLIGAVFYAFRFEVSDWLYTNFGIPLFKEQSCPNVE
HVIDGNPNH)MHDYYVICNTKdtQFIYNNILSEIE-----FRKMSAKKLTINESTLNI-
LTldsfsKSSKLSKRLLIVLTTNFICNDLCDFHFKNMFYSFLKSLnrsdLNKVLFIKIVDN-
NQINDELCFVLDKFKNLswN-----DPR--
FWDRFVTLLNST(DTIITVKSSGRSVFKKSLRQTPTLRYTTMPVTNDSCTLQNFSNSLQYCKRNDGATSQNES
SPSSDNTYGEGN)}*

$3025=240(243):

>gi|491383357|ref|WP_005241238.1| {|13(95)|<Actinobacteria(B)>}LacI family transcriptional regulator [Rhodococcus opacus]
>gi|356667082|gb|EHI47153.1| transcriptional repressor purR [Rhodococcus opacu
{(GVSRSTASLVLNDSPSVAPETTRRVREAMKALGYVYNRHAAMLRNPRSMTLGLVVTEVQNPYFAELAMTLEDVADRANYA)---VFIGYSRDdPARQRRLLDRLVERSIDGLI------
LLPAGDTQAsEIDDLLASSGIPLVLLARHFdLSHDYVgaDNvEAGRLLGQHLR-SMGARTVAMVGGP-
QHTSARREREQGFLAAIdgSDMTMYSTDGLFGEATPDGGADA(TRRLLDRGEIPDAIVAYSDIVAV)}*

$3026=143(140):
>gi|503953628|ref|WP_014187622.1| {<Firmicutes(B)>}hypothetical protein [Desulfosporosinus orientis] >gi|357216202|gb|AET70820.1| hypothetical protein Desor_5445 [Desulfosporosinus orientis DSM 765]
{()MLGIFFAYSHKdKEKLYNLIENLNSLK-
NYCELIYYQKTLPEKNKKEdFFLKLLSEIDFFVVLLSDSSLESKYV-QrEISEAIRLCQC-
KPgniKKIFPLIIDD--TIDSSDLRIHEYIKQQNvfRPTSMKSAAKIIEEEVIHELSNM()}*

$3027=293(299):
>gi|357622568|gb|EHJ73995.1| {|70(26)|<Arthropoda(M)>}putative Myeloid differentiation primary response protein MyD88 [Danaus plexippus]
{(TDKVLNIWISRNDGTATLGRLMDFLQILDRFDVSDDILELARDNKLIGNHNQIACNPAIVLDGADDDFITNDDRLYGVPQ)RYHAYVLFaREDRDFVDELLRRMRQAGF--
KLCTEDDLIAGHATPFEPVSRLIAErCRNIVLVYSPDFLQSPAIAFYMNLAQADAISKKQLKIVPVMYREC--
SLPRHLAYYTNLRYAP----HSRVpY-
DFWERLSQRLRIF(DGPRITMPNSTSNTDIRITETSHSLSNGHQVNGAIKTLALPELPKETSSLTNLHRLSDETPTSYETKSLNGSRDSDDGK)}*

$3028=276(284):
>gi|1049318739|gb|OCQ96938.1| {|156(614)|<Cyanobacteria(B)>}histidine kinase [Nostoc sp. MBR 210]
{(LELFSRHQDYVHQHTYFLAKSLEWERQQKNSQYLLIGDERLQAENWLKIRFKDEQAPCEPTDLHCAFICQSTKNANNLMT)--QVFLSYSEKERDLMEKIAKILMRESFT-
IWINKTDIQSGTDFVESIYRGIEEADNLVFLMSPASLQ-SEYCQmELSHAFSLK-----
KRIIPLLIEQVDieQIPVELRTLQFIDLTE--
PQPQQANIDKLINVLTQDVTYY(EQHKILLVKALKWERQKRNSSILLRGYNLRESEAWLKIAQQKTQHLPIPLQVEFITDSLKQPPL)}*

$3029=218(221):
>gi|1051610909|ref|WP_065916839.1|
{|1512(0)|<Actinobacteria(B)>}hypothetical protein [Lentzea guizhouensis]
>gi|1050302133|gb|ANZ38492.1| hypothetical protein BBK82_22940 [Lentzea guizhouensis]
{(RRVRRCKPCDYVVDEVIVRRKAHLGHTAMKCPICETTVISLVDHETADVRPVVVEMNTNADAQRDQDVAAVKLKGKRDEG)AYDVFLCHNVKDKQQVVEIARRLEARGIL-
PWLDVDAIQPGTRWQQEMAKGIEKSQTAAVLLGPS--GHGPWHQaEmelvndWSVRTGK-------
KVIPVILTGTNgdLEVLQGFTKVWSTVDM--RMPDPDPFEQLVWGITGEHPRR(A)}*

$3030=173(178):
>gi|1049318739|gb|OCQ96938.1| {|0(873)|<Cyanobacteria(B)>}histidine kinase [Nostoc sp. MBR 210]
{(MNSF)-KNAFISYGRAdsKAFATKLYQQLT----
EAGMEIWFDQnDIPLGVDFQnQIDDGIEKADNFLFIIAPHSINSPYC-GkEIELALRRHK-----
RIIPLLHVEQ-

ITQETWQERNPQREASEweQYRVKGLHSSFPNMHPAIGKINW(VYFREGIDDFEKSFAGLLELFSRHQDYVHQ
HTYF)}*

$3031=248(256):
>gi|1049318739|gb|OCQ96938.1| {|356(442)|<Cyanobacteria(B)>}histidine
kinase [Nostoc sp. MBR 210]
{(LINVLTQDVTYYEQHKILLVKALKWERQKRNSSILLRGYNLRESEAWLKIAQQKTQHLPIPLQVEFITDSL
KQPPLVALD)---LFISYSLAdSDFARKLNNTLQI---QNK-
TTWFDQEsIASGTDFEqEIYKGIESANNFLFIISPDSINSSDCQNQLEYAVSLHK-----RIVTVLYREI-
NTADLHPGLAKVQWIDFcqHGVDFLTKFGELTRTLDADPDYV(RSHTRLFLKAKEWEDAKHDDSFLLRGKDLV
ASEEWL)}*

$3032=259(257):
>gi|1049321092|gb|OCQ99278.1| {|110(330)|<Cyanobacteria(B)>}molecular
chaperone Tir [Oscillatoriales cyanobacterium USR001]
{(IHLGDVIFSGNDVMGNGVNIAARLQSIAAPGGICLSQTVYDVVKNQISLSASYGGQRKLKGITEPVSIYQI
SGFRNLVEN)RHKIFISYrAQ--EPDKSLALQFYEALTKAGNAAFMAGESIRLGENWpsRIEAELKQSDY-
LLLLLSEQSATSEMVTeEVRRAKELRDSRpDRKPIIlPIRVNLP-
LASPVNYNLYGYLDRIQQREWHSEADTPNLIQEVITLVKE(NRALENNPKNTPIITPGWENFDTPPLPVAGPE
LPEGQ)}*

$3033=198(199):
>gi|1050612289|ref|XP_017628843.1| {|0(368)|<Streptophyta(V)>}PREDICTED:
TMV resistance protein N-like [Gossypium arboreum]
{(MASSSSSSSSSTLM)KYHIFLSFRG-EDTRLNFTTHLLQALKDeG-
LDVFFDEEKLERGEQLsqALSRAIAVSNISIIVLSEDYASSKSCLAELSDIMDRKHA-
QGHIVLPIFYHVDPSDLTETQYIEDIVVYVM-
QKLKHRVFLSLGEDTRLNFSNH(LVKGLEKVGINVFPDNETLGKGEKLLPTNVRAISASNLSILVLS)}*

$3034=177(175):
>gi|1051611941|ref|WP_065917871.1|
{|0(20)|<Actinobacteria(B)>}hypothetical protein [Lentzea guizhouensis]
>gi|1050303171|gb|ANZ39530.1| hypothetical protein BBK82_29265 [Lentzea
guizhouensis]
{(MGYE)-WDVFLSYTrngGGDvwvrehfyRALREWLGNEMADEP-R-IFVD-
WDQDGGTSWPDSLERALLRSKVMVAVLSPPYFR-SAWCLaEWESMWERH-----
RALGYGTTDHPRhlILAVRYADGQHFPAEA--
QSTLLRDFTPWNKPLAYEVYSR(SAQYEHFYTAVQQFAEEVAKRIEEVPPWNPE)}*

$3035=194(217):
>gi|1051614182|ref|WP_065920111.1|
{|0(1105)|<Actinobacteria(B)>}hypothetical protein [Lentzea guizhouensis]
>gi|1050305417|gb|ANZ41776.1| hypothetical protein BBK82_43410 [Lentzea
guizhouensis]
{(M)-------------AFAVEVSEWLRADGHE-
NFLDRApvdGVQVGEDWRERLYGELRQVDAIVCLVSRGFLT-SFWCSaEVGIADALG-----
CRILQLRLDDV--VHPLLHRLQYAD-FA--ADPHRARE--
QLLRAVRGLDLG(REKWCEGQNPFPGLKAFTAELSGLFFGRADETRELSSRLRAATTSRLLAVVGPSGCGKSS
LVRAGLLPVLAQESDW)}*

$3036=208(206):
>gi|1056432683|ref|WP_067874523.1|
{|0(1203)|<Actinobacteria(B)>}hypothetical protein [Mycobacterium sp.

E2699] >gi|1038134215|gb|OBH07228.1| hypothetical protein A5696_02355 [Mycobacterium sp. E2699]
{(ML)--RIFLSHSSLDSRQAvalkQWLSLRRPQLANE-
IFLDidtRTGLQLGDRWKGQLFASNARCELVICLLSRN-WEASHECKaEYRIAEGLG-----
KRILCVRLEDVAdtDISSEWR---RFDLFadgphtE--
LDVAAAPELARLGDVGEPVRFN(TTVLEEVRRAVEGSGVGPENFVWPPDEDRTRAPYRGWEPFESIDAAVFFG
RDAAIVRGVDELRA)}*

$3037=308(299):
>gi|942419177|gb|JAN91518.1| {|1022(90)|<Arthropoda(M)>}Toll receptor 13 [Daphnia magna]
{(NGNGRLSPSHGYIPLFAAVLVTIITSALLIALVCIFRQDVKLWAFSKYGLRFGGDGTSKNSNEKINRGVVA
GAGDDNEKI)-YDAYFVYSINdtDLLTQILLPELQNLGYNISMYNHHnkptmlRDNYQ---
ITNylidTLRNGTDLSHKMVIVLSFNFLQTEWCDANFRCALQalidSLINQRRNKNLILILTVP-
IQIIQMDPVLQLLMRTCTvICWGEKR---
FWTKLRYALPDV(THDKKFIQGPDNNMTHRYTAAPTSTLQRGPMMMAMERGGGGSNNNDWYNLSPVVCSGYSM
PINQYQQQQQNHQPQQYY)}*

$3038=302(300):
>gi|942419175|gb|JAN91517.1| {|698(33)|<Arthropoda(M)>}putative Toll receptor 13 [Daphnia magna]
{(KRELDFNSSSACNDFYAEGSSGLPNLISNGYIPLMITLLATICLLVLFAILFVFREPLRLWLFAHYGVRVF
GPPCEDNDK)LYDAVFLHSAKdtEYIIKHIANELEsgNGRPAFRLCLQHRDLAEDASYIQ-
LLETICASRKIVILLTRNFLQTEWSRYDVKRAIHESLRGRPQKLVIVEDPDV-
LLDAESDIELLPYMKTSavKRIRRNDRQ--
FWDKLRYALPVE(VVYRGNNYTLDHQRPHYQSHTMPHIAMHHERFKHANGNGTINGGMIYHHHHXXXXXXXXX
XXXXIIIIIDKRHRPLITSL)}*

$3039=299(298):
>gi|942383683|gb|JAN73771.1| {|1032(97)|<Arthropoda(M)>}Toll receptor 13 [Daphnia magna]
{(QILIANESCPTSDGSSVLLSPYQPADWWLLMAATFSLALVILSAGICLCCCRRQLRLLLFSQCGVRMCYNH
DEESADSKP)-FDALVIYSAKddVLLKDTLA-RLER--KTYQLCLQHR---IADH-
DQDLNAAALASRRLLILLTKSFVETEWGKANVRALLRSTWQpaaaavgvRSRRDRVIILLPEP-
QIVREIDADLDLHLllKHSqVVEWNARF---
CWSRLLLALPPP(RGVLAPADCHQPAISHPQQSTMPSAYSGIMPSAGNANGAGLGCTTASRRLAMRTVLVLAA
RRPPATTPRVRLLLPGTI)}*

$3040=209(199):
>gi|1038981866|emb|SBT63699.1| {|0(187)|<Actinobacteria(B)>}hypothetical protein GA0070622_0657 [Micromonospora sediminicola]
{(MSPERPVLAGARYFFLSYVPPPT)-HSDRTLGD---HWVRRFYHDLNLAIDGRpgaRLRRrgFADFT-
VRLSEDRaeQKRVALAEAEVFVPLYSPDYLNRDDSRREREWFRQRLLRAgRpadGDNILPVLWTPSPaavhAT
DQARALAMAADVEAYAANGMSALCRLQIFQRDYQEVLG(RLAQHIVDTAEQTPLQPAEPPTGPTDVPSSPTSE
VP)}*

$3041=209(223):
>gi|1056513412|ref|WP_067948632.1|
{|0(293)|<Actinobacteria(B)>}hypothetical protein [Mycobacterium sp. E2238] >gi|1038262857|gb|OBI28051.1| hypothetical protein A5711_02560 [Mycobacterium sp. E2238]
{(MPL)----FISYSSQDRAAVDALTAALRRAQQQ-VWFDQ-
ELGGGESWWNKILEQVRSCDVFIVALSNNWLQSKP-SQaELRYARALN-----
RPILPVRIGDVDsMRVNPLAALQIIDYRD--PTVDAGI---

QLVTAVHAlQSKP(VPLPDPLPEEPPVPFGYITRLGDTLAEKELSPQAQTQLLIELRQGLDEDGDDPSARGDI
GQLLRMLRLRHDVTYRTRTEI)}*

$3042=227(218):
>gi|1056862480|ref|WP_068279928.1|
{|0(1174)|<Actinobacteria(B)>}hypothetical protein [Mycobacterium sp.
E787] >gi|1038288400|gb|OBI49302.1| hypothetical protein A5705_13615
[Mycobacterium sp. E787]
{(MS)--RVFVSHSAEDRRAAialrNWLCEHRPQLVNE-
IFLDLGpDSVTGSaaRWKRELFKNDSRCEAVICLVSAA-WQASAQCRaEYRTSEGLG-----
KQILVARLEDTGpsDI---TSEWQRCDLFA--VGPqtpievpgapplrfnSAALD--
QLRRAIAGTGVG(PENFVWPPAEDPLRAPYRGWEPFETLDAGVFFGRDAAIVRGLDEMRAMRLAGAKPLFVVL
GPSGSGKSSFLRAGLI)}*

$3043=183(189):
>gi|1056624958|ref|WP_068055412.1|
{|0(326)|<Actinobacteria(B)>}hypothetical protein [Mycobacterium sp.
E342] >gi|1038160173|gb|OBH31041.1| hypothetical protein A5692_17760
[Mycobacterium sp. E342]
{()MP-LFISYSSQdRATVDALTTAL----
RRGQQQVWFDQELGGGESWWhKILEQIRSCDVFIVALSNNWLQSkPSQAElRYARALNR-------
PILPVRIGDI-
DsMRVNPLASLQIIDYREptVDAGIQLVTAVHALQSKPVPLPD(PLPDEPPVPFGYITRLGNTLAEKELSPQQ
QTQLLIELRQGLDEDGDDPS)}*

$3044=183(191):
>gi|1055132861|ref|WP_066880641.1|
{|0(328)|<Actinobacteria(B)>}hypothetical protein [Mycobacterium sp.
1245801.1] >gi|1038360198|gb|OBJ16975.1| hypothetical protein A5622_24295
[Mycobacterium sp. 124
{(MA)---LFISYSSQdRSTVDALTTAL----
RRGQQQVWFDQELGGGDSWWnKILEQIRSCDVFIVALSNNWLQSkPSQAElRYARALNR-------
PILPVRIGDV-
DsMRVNPLAALQIIDYREptVDAGIQLVTAVHALQSKPVPLPD(PLPEEPPVPFGYITRLGNILAEKELSPQQ
QTQLLIELRSGLDEDGDDPS)}*

$3045=226(223):
>gi|1052340209|emb|SCF74959.1| {|91(275)|<Actinobacteria(B)>}TIR domain-
containing protein [Streptomyces sp. Ncost-T6T-2b]
{(HSHPAPSGPSPTDLAAMRAMAREYDGPIALLVYAPGGTDPFSGLITRRQRWGRVTGKAATILPPPQRFKEL
GPLPKGAVR)-PDgaVFISYRQSDGSPQAESIENLLRAAGL--
VVWRDHTDLRAGTTTdRLEQALTQGlSAAVLVVTPDIVHSDIVREreLPRLLQLDEDPAFSLCIANKTARAGS
DTRCDYGAPDRLLRLAPSRPLADKKQSNMLQASGESEIVR(DLL)}*

$3046=226(246):
>gi|1052263503|emb|SCF18279.1| {|0(9)|<Actinobacteria(B)>}TIR domain-
containing protein [Micromonospora purpureochromogenes]
{(MSHVGRERQVTEEEFRVDPGAPVFFLSYARTKNRVAAPPRDTNSKVFQLFVDLSDHVVELLGLPPGRPAG)
----------------------------------FMDRM-
IDGGQLWteDLAFAAGHSQVFIPLISPQYLNSAWCAREWDAFARRrftlrpgAAATaGETPIIPVNWSVVErq
qvPPVVSQRQMFTPTRLPSDIAPQYHDEGIYGLLSLGKNGKD(AYDAVVWRLAQRVAKAYHTHWVQAQVPADM
QQLRAT)}*

$3047=217(216):

>gi|1052291012|emb|SCG19526.1| {|0(186)|<Actinobacteria(B)>}hypothetical protein GA0070610_5902 [Micromonospora echinofusca]
{(MTWSDPAPPRGAPRRETYFFLSYAHSVPLSAGVRPDTDYW)--------------
VNRFFNDLATAVRhaATrggDLDAgFYDGQ-
VSPGADLrqTLTDALSLAHVFVPLHSPNYFRNAWALGERESFRSRLTRLaPAQAeqhLVPVLWLPLPswgdRP
ETTRALDLVPDAGDRADYAENGLRALCKLSAYHDQYLR(VLGALAKRIVTVAERQPLARSRAAMLSSHPVPDS
VG)}*

$3048=210(211):
>gi|1052304369|emb|SCF39773.1| {|0(209)|<Actinobacteria(B)>}FxsC C-terminal domain-containing protein [Micromonospora mirobrigensis]
{(MSPYGADPGTYFFLSYAHSAPPQGARTDTDVWVRR)-------------
FFTDLSAAVAERARPTpgmRIGFFD--
QHIPVGADWkaALAEAISGAQVFVPLYSPGYFRRPWALGEQESFRARLAAAgaagdPAGHVAPVLWIPFPyge
sGPELAAAALERGRRIPGYAENGVRALCMLSAYADPYQDLLE(GLARHIVDVAERHPLAPSRAPGLDEVARPVP
GDPDL)}*

$3049=229(229):
>gi|1052355919|emb|SCF48593.1| {|0(159)|<Actinobacteria(B)>}hypothetical protein GA0070216_12759 [Micromonospora matsumotoense]
{(MTWSDTPSPAGVVGQETYFFLSYAHSVPLSDAARPDTDYW)--------------
VGQFFHDLARAVRDHprrTAEIevgFFDGQ-
VNLGADLrrTLTDALSLAHVFVPLYSPNYFRNAWALGERESFRSRLARLaVGRAerhVLPVLWMPLPsweDRP
ETVQALELVRDPDDRADYADNGLRALCKLSAYRTQYL(SLLAALADRIVTVTETEPLARSRAAALTSRPIPDG
PGTALVVTTLTAER)}*

$3050=230(225):
>gi|1052249287|gb|OCX45680.1| {|274(0)|<Proteobacteria(B)>}ATPase [Acidiferrobacter thiooxydans]
{(NVHREADRHPQQRSSVSQVASKGFLQDLVVASEDIQRVIYYDAANATLVVQDPQFVFYIRNLSWSRFAQDI
GYISIEFPS)QYDFALSFAG---
SDRDIAEAIFNELSERELEVFYDKNEQHRILAEdveEYLGPIYSSDAAfvICILGPDYPKRVWTKFESDQFKK
RL---KSGEVISIVLSTAPlgM-FDDaaKVGHYAWDRdgnfgDQL-QEISELLV-
RKCREVRSRHADD(AKQHA)}*

$3051=219(197):
>gi|1052260071|emb|SCF29240.1| {<Actinobacteria(B)>}TIR domain-containing protein [Micromonospora chokoriensis]
{(MSTVDAPSGSRAPLF)----
FISYSRApigratgkpaeyvSRFFEDVSVHVSELVGpvTGVDAGFLDTSIAGGERWSpELLQAAGTCQVFVPL
VSSALLGSEWC-GrEwdafsRRRIVPRHSSAspHETAIVPVTWSPT-
NGTALPrvLRDIQRFTPTAmpDPDIAVHYQRDGVYGLLTMQWEN(AYRAVVWRLAQRIVAIHRSYLVEPWVPA
SVDELCNRFAEEPG)}*

$3052=195(179):
>gi|1052287435|emb|SCG15949.1| {|0(215)|<Actinobacteria(B)>}FxsC C-terminal domain-containing protein [Micromonospora echinofusca]
{()MPLFFLSYAHEdnrngqvQEFFGDLSEAVARLAGVG----
WDqagfvDGQMPLGATWSqELADALATSSCLVALTSPRYVVSDYC-GrEFRVFADRVDA-
HEREfrrrppaVLPIRWIPC-
DRVPGALTGFQHKNFPAgaVYAQDGLLTLKQRRRHHDEYQDF(VDALAKHIVQTDRDHRLLRPARRPVLAEVT
SAFETTRRR)}*

$3053=210(188):

>gi|1052291013|emb|SCG19527.1| {|0(15)|<Actinobacteria(B)>}TIR domain-containing protein [Micromonospora echinofusca]
{(MAEETLWEDESAPVF)----
FLSYAHKkNQvaaprdtnqkvfqLFVDLSDHVVELLGLGPGRTagFMDRMLDGGQVWTdDLAFAAGHCQVFIP
LISTHYLNSDWCAReW--DAFTRRpiltrpgadpSA-GETPVIPVNWSLV-
DRRRLPAAVSRRQMFTPtrLPPEIAPQYHEEGIYGLLSLGDT(GKAAYDAVVWRLAQRIARAYQTHWVQAQVP
ADV)}*

$3054=219(220):
>gi|1052774377|emb|SCH10434.1| {|62(0)|<Firmicutes(B)>}Uncharacterized protein containing a TIR (Toll-Interleukin 1-resistance) domain [uncultured Faecalibacterium sp.]
{(GNENRYIQGDNSVFPAPYRSSIPSEFEKLRMYGMISSMTFWLGGWEATLTPQGLSYFDDKEKAVAASTSAE
AIPTKPTRK)EFDVFISHAN-AD-
KSDYVDELYLTLRKLGINIFYDSEVLSWGDNWkqVILNGTEKSEFAIIVISENFFGREWTERELDEFLKRQNV
NGQKIVLPLLYNITFEQLKDKYPTLGE-IQAIRTEGRSKEDIAILFAKELIKRYK()}*

$3055=221(221):
>gi|1052879522|emb|SCI11970.1| {|31(0)|<Firmicutes(B)>}Uncharacterized protein containing a TIR (Toll-Interleukin 1-resistance) domain [uncultured Clostridium sp.]
{(NEYNRPSEVWVNDVEIFYNNYLKEHPLGDRMKTLLFHRNLDAYKNLVGCLESISKDQDFINKMNGISMVSV
PKYQAKTLP)EFDVFISHAN-KD-
KEDLIEELYQSLNKLGIKIFYDKESLEWGDNWkdRILNGTKKAEFAIIVISENFFDREWTERELNEFLNRQNR
NGQKLILPILHNITVLQLQEKYPSIADIQAIDSSKYSCDQIALLFARQLIKRLQS(E)}*

$3056=187(189):
>gi|1052945086|emb|SCH81413.1| {|0(68)|<Firmicutes(B)>}TIR domain [uncultured Blautia sp.]
{(MKKFSD)--KLFICFSA-KD-RYVIAQPIVYHLKNYGINVWYDRYEMIMGD-
DRFKKNIEEgagkSKYAIIILSHNTITSFCAREEIQTLKLRYFK-
GEVTVFPVLYELKPSDIPPVFAWIKKLIFKEADRSTGTLEICNHIVCKITCDML(NNCKYKNINEIIQSKLPL
PRSIRHLFENYIRVDNSNLNSKITL)}*

$3057=190(219):
>gi|1052856254|emb|SCJ93258.1| {|0(153)|<Firmicutes(B)>}Uncharacterised protein [uncultured Ruminococcus sp.]
{(M)---------------------------GY--
KVFYDIESLSSGKFNaKLLEVIDECNDVLVILPPNSLERCVNED-DWLRLEVAYAIrQGKNIIPIMMKGFEW-
GKNIPEDIKELKNYNGVIVTFDFFEGVLARIIKYLTNS(SKEKMITEKKSHILFWADFDDAIIEKIVNKLKLD
DYFIEIMSEPVEILSKNLQEIYAIILIVTDCTKFSSNIYAAKRI)}*

$3058=210(211):
>gi|1052671048|emb|SCG48639.1| {|0(209)|<Actinobacteria(B)>}FxsC C-terminal domain-containing protein [Micromonospora siamensis]
{(MSPYGAAPGTYFFLSYAHSAPPQGARTDTDVWVRR)-------------
FFTDLSAAVAERARPTpgmRIGFFD--
QHIPVGADWkaALAEAISGAQVFVPLYSPGYFRRPWALGEQESFRARLAAAgatgdRDGHVAPVLWIPFPyge
tGPELAAALERGREIPGYAENGVRALCMLSAYADPYRDLLA(GLARHIVDVAERHPLAPSRAPGLDEVARPVP
GDPDL)}*

$3059=222(249):
>gi|1052681104|emb|SCG40729.1| {|37(23)|<Actinobacteria(B)>}TIR domain-containing protein [Micromonospora coxensis]

{(VRLSILWAVEDVHTGRERRVTEEDFRVDPGAPVFFLSYARTRNRVAAPPRDTNQKVFQLFVDLSDHVVELL
GLPAGRPAG)---------------------------------FMDRM-
LDGGQVWtdDLAFAAGHCQVFIPLISPQYLNSAWCAREWDAFARRRVTLrPGAVTTPGETPIIPvnwsVVERR
SMPPVVSQRQMFSPTRLPSDIAPQYQEEGIYGLLS(LGKNGKDAYDAVVWRLAQRVARAYHTHWV)}*

$3060=167(175):
>gi|1052726822|emb|SCH72787.1| {|0(331)|<Firmicutes(B)>}MTH538 TIR-like domain (DUF1863) [uncultured Eubacterium sp.]
{(MGYA)----FISYSSKdQEKANAIRSIFL-----NNDISCWMAP-
YDIPAGTkyayVINDAIKDAACLVLLFTNASQESIFVEKEVERAVANGKP-
IITMELERVVLNSGYQFYLGNQQIIATPKIN--
EDDENFQRALQGIAAFTGRTD(TLKKKTQLSAANETATVQNAGGNKKTETVSR)}*

$3061=224(227):
>gi|1052764898|emb|SCJ67484.1| {|0(101)|<Firmicutes(B)>}flagellar operon protein [uncultured Eubacterium sp.]
{(MVTNMKEL)-KSVFISYSSKdTEIVNEVVNMLETSGIS-----YWKAPEM-
IPAGSnyakEIPRVICECNIFLLIISEDSQRSIWVEKEIDFAINNRKV-
IVPLkLTEGKLTDMFSFYLNNIQMVNYNGNLKKTLRVLRTR--
IETLLSKeSLKDN(TRNIADGREIANSNNRELDDNNETTSNRNIENTGNGENSDKDIEGAENREEHPEMEKTD
RENIRRSKIKAFSYNPQPEF)}*

$3062=171(176):
>gi|1052894444|emb|SCG98079.1| {|0(88)|<Firmicutes(B)>}Uncharacterized protein containing a TIR (Toll-Interleukin 1-resistance) domain [uncultured Ruminococcus sp.]
{(M)---IFLSHNSKdKRIVEPFANKLAEV-FDRENVFYDS---
WSIQPGDgiidKMESGLTDAKYFFFFVSENSLKSNMVKLEWQNAIIKATN-
GKIKFIPVRLDKSRMPTLLTQTLYLDVYQNG--
FDVVLRQMIDVVNGVNTYRGT(AQNYENVKADVKISEKEAEILFYAETYMEPISRYG)}*

$3063=213(220):
>gi|1052899552|emb|SCH89256.1| {|54(0)|<Firmicutes(B)>}TIR domain [uncultured Blautia sp.]
{(YDIPRENHVEKMMELFENAGLISRNDLTFSMLMKSMGDRRFNSCTFLSEKEVDLQFESMFIFSEIEVLEEC
GYYMRPFGK)EKQIFISHSSMdKKDVEMIIPYLNGQDLPVW------
FDKYSIPVGTsiteQVQKGIEESDMVIFWVTDNFLKSNWCKMEMKA---YIGR-
MIEENIRMFIVMDDEIEIKTHPLFLRDIKHL--RREHRSVIEIAEEIAGIIKQM()}*

$3064=187(196):
>gi|1052726825|emb|SCH72904.1| {|0(184)|<Firmicutes(B)>}Uncharacterised protein [uncultured Eubacterium sp.]
{(MNKKTSIDSVDVNKGNY)---VFISYSSVNQQMADSVRLLLKENNIS-
CWMAPYDIPAGSRYAYVINDALENCSCVLLLLTNA-SQLSQFVErEIERAITYK-----
KPILPMQLEDLKlnSGFKFYIGSSQIVAVP--
EIRADVPEFIRILAGIKELLGC(SDCGYAGTVCSDVRQNEDVIMARENQERQALDKTDDIRS)}*

$3065=219(197):
>gi|1052693119|emb|SCG39158.1| {<Actinobacteria(B)>}TIR domain-containing protein [Micromonospora zamorensis]
{(MSTVDAPSRLRAPLF)----
FISYSRApvgratgkpaeyvSRFFEDLSVHVSELVGpvTGVDAGFLDSSIAGGERWSpELLQAAGTCQVFVPL
VSSALLGSDWC-GrEwdafsRRTIVPRHSSAspHETAIVPVTWSPT-

NGTALPrvLRDIQRFTPTAvpDPDIAVHYQRDGVYGLLTMQWEN(AYRAVVWRLAQRIVAIHRSYVVQPWVPA
SVDELCNRFNEEPG)}*

$3066=182(191):
>gi|1052855185|emb|SCI18714.1| {|0(474)|<Firmicutes(B)>}Internalin-A precursor [uncultured Roseburia sp.]
{(MRDEFNLYEGRENY)---IFVSYSHKdK---DRVFPVLKKMIESGY-
RIWFDKGIHPGSQWPeIVAGHLARCTVFMVFISDHYMQSQNCIRELHFAVARNKS-----LISIMLQPV-
LLTPGVEMQLCVSQAVRhyEFPTEEQFYKVLNTSAVLEQCQR(REELFTAEELPVTKEKEGQPEQIVPQKMKA
GDASGEV)}*

$3067=187(184):
>gi|1052722253|emb|SCH40940.1| {|0(296)|<Firmicutes(B)>}SEFIR domain [uncultured Clostridium sp.]
{(MLQGDEIPK)---IFISYAW---
ESSDLVLELAQRLVSHGVDVVLDKWDLKEGQDKyaFMERCVNDSEItKVLIICDKAYAQKanertGGVGdEtV
IISSEIYGNTkQEKF-IPIIAERD-
EEGQPYVPTYIKTRIYIDLSNQETYEVEYEKLLRNIYEKL(QFVKPKLGKRPEWIDDAKTDFFPIKDLIRQIR
GSN)}*

$3068=273(263):
>gi|1053018625|emb|SCJ67582.1| {|74(354)|<Firmicutes(B)>}Uncharacterised protein [uncultured Clostridium sp.]
{(AEAYWSLVLCKYGIEYVEDPDTHKRIPTCNRTRLESVFADEDYKAAIKNADVNARVVYEAEAEAIDGIQKE
IIRISSQEE)PYDVFICYKE-
TDSsgKRTqdsvLAQELYFELEKMGYKVFFSRITLedKLGTSYepYIFAALNSAKVMVVIGTKaEYVNSPWVK
NEwsryLTLIKQgDR-----
KILIPAYRDMDPYDLPEEFSHLQAQDMSRLGFMQDLIRGIQKMLEEPAAERV(KTEPAAGDLVNIESLLDRAF
ICLEDLDWNKADEVLERVLDQDP)}*

$3069=273(263):
>gi|1053018626|emb|SCJ67603.1| {|74(347)|<Firmicutes(B)>}Uncharacterised protein [uncultured Clostridium sp.]
{(AESYWSLVLCKYGIEYVEDPKSHKRIPTCNRTHFESIFADENYKAALQYADESAKSIYEAEAKVIDEIQKE
ILKISSQEA)PYDVFICYKE-
KDAsgKRTqdsvLAQELYYELTEAGYKVFFSRITLegKLGTAYepYIFAALNSAKVMVVIATRvEYVNSPWVK
NEwsryLALINQgSK-----
KTLIPAYRDMDPYDLPEEFSYLQAQDMEKLGFMQDLIHGIEKMLVTPSAGKE(EEEQVNNGNINTNITALLDR
VFICLEDADWNKADELLEMVLNQ)}*

$3070=222(249):
>gi|1053000356|emb|SCG68914.1| {|0(23)|<Actinobacteria(B)>}TIR domain-containing protein [Micromonospora halophytica]
{(MRLSILWMAEDVHNGREWRVAEEDFRVDPGAPVFFLSYARTRNRVAAPPRDTNQKVFQLFVDLSDHVVELL
GLPAGRPAG)---------------------------------FMDRM-
LDGGQLWtdDLAFAAGHCQVFIPLISPQYLNSAWCAREWDAFARRRVSLrPGAATTPGETPIIPvnwsVVERR
SMPPVVSQRQMFSPTRLPSEIAPQYQEEGIYGLLS(LGKNGKDAYDAVVWRLAQRVARAYHTHWV)}*

$3071=282(299):
>gi|1054593311|ref|WP_066361714.1|
{|252(764)|<Actinobacteria(B)>}ATP/GTP-binding protein [Herbidospora mongoliensis]
{(EERTMYWGAVEIPYKRFYAFEETLATFGDTPGSPTSLLAAYERLTGAITNGEVISLPPMDEALRARWLDAF
TRRQPADQA)--DIMLSYVPEDRMWADWIGSVLERTGCR-VM-------

PKSLAQASLESAPDPHTQVMIVLSPAYLR--AWNGeDSGPRLSPDLLTM-RQIIPLRVAEVR-
MTSAFGDQPPVDLIR--LSPEQATE--
AVIRAAGRDPRD(YPADQFQLGIRFPGSVPPIWNVGPRNNTFTGRSNALETLRDQLVGIGQGVVLPQQATALH
GLGGVGKTQVALEYAHRFR)}*

$3072=237(234):
>gi|1055840356|ref|WP_067427056.1|
{|0(343)|<Actinobacteria(B)>}hypothetical protein [Streptomyces
lincolnensis]
{(MNPHGQAAPPADHQ)-WDVFISYAREDYIQAKDLQDALlecvTAAGTtpR-
VYLDVSrtGTPVGADWQIYLEEALRGSRHFVALYSKRYFEKDVCQWeLHEAlklPLTES-----
GGFIPLLMEPAAaeKVPYVANRINWIPMTRPRwIDEVRGAL--RLR---
TSAARP(VLRFETPPADAVVGHTLPPLRVTGSAPQGTPVGSAGGTVTLSAQPPDAGLTGTLTVPFTGGTAVFE
DLAFASAASEVRLD)}*

$3073=251(257):
>gi|1055271735|ref|WP_066939405.1|
{|98(812)|<Actinobacteria(B)>}hypothetical protein [Streptomyces sp. NBRC
110611]
{(PEGRAAVVGELLGWFDRQVSAGLFGPSFAQWYWPLLVSVVALVIGGGCAALREWPARRARLRDPFAGTPEG
EGLPQLRNT)-ARVFLSYARTdEEFATRYVRALEQHVR-GVWVDWQG--
IRASEKWRwKIFDALRASDAVIVLVSSNSLRSEWClREcEEAIAL-------GKRILPVLISPE-
PAGSTTAALRAAGWEPLaeYQRLTMSGAADFDRGVRETLAFV(AQEHVWVDLHTRFGEQAHAWRASGFSEGLL
LRPHEVR)}*

$3074=184(170):
>gi|1055029858|ref|WP_066781097.1|
{|0(146)|<Proteobacteria(B)>}hypothetical protein [Sphingomonas sp. CCH5-
D11]
{(MSALGDTYD)-YDVFFSYAWAgdigdqhmrewsrkiaDTIKALLsQRFNRGDVR---
LNAFLDRDVLRGGQDLdrELKSAVQRSGILVAMVSDHYA-SDYCQKELNWFCEGLTEAnfaDHVCILRMQEV-
--SDGLWPAALQGTDAKPRLFIDFCDERGQPINMAGFILSGS(LADLVTPINRVVVEIADKITA)}*

$3075=180(179):
>gi|1056116761|ref|WP_067637327.1|
{|0(700)|<Actinobacteria(B)>}hypothetical protein [Actinomadura latina]
{(MAESG)DFDVFMSLGG-CDrpavTR------
LVRAMRERGLRVFLDTDRIAHFEGVtaTIESALNSSKALVAYYSAGYPSRPACQAELTAAFLAgqREGDPGRR
IIVVNPEPDTGHIMPVELSDARFAVAPSGTRNTATLADLILDRVRGIDGR(IGAVHFADRPRWYPGRVPGASR
FVGRYRELWDLH)}*

$3076=229(254):
>gi|1055922221|ref|WP_067465381.1|
{|0(590)|<Actinobacteria(B)>}hypothetical protein [Nocardia amamiensis]
{(MPRSPKNEPAHTVSRDTAPHEYRMTNPPPKTAGDF)-YHVFLCYKSEdlA-
IAHALYSELVDRGmRVFLDVITGEDWAP---
LSASISDALAHSRTLVALMTENFPISPHCREELHVALlaSHYLDDGDTSRVMAIARGISaDEVRPKQLTVHRL

P(FGGLPPGELAGTIIANVDAHYGVFGDAPTGMPQPDWYPRERPTERFFRGRYAELWDIHSGLRARQRTRDRG
LPVVSVTG)}*

$3077=203(222):

>gi|1056061646|ref|WP_067587344.1|
{|0(85)|<Proteobacteria(B)>}hypothetical protein [Endozoicomonas ascidiicola]
{(MPI)----FISYSHEnkEFVDKLATQLV---
NRNVNIWLDRWELSLGDSIIDKVQDAVEGASALLIIMSKASIASEWCKKELTSGFLRELEEKRVVVMPVLLED
---------CNLPLFARGKlYADFRGD----FDDGLRTVL--
E(GIAKVTNPKLARSKEPEHHIDWSLDWGDINDHFSIILNFVEQSKDQPYSCLTNIQIVSSPEATKKYQKMCS
NFSQDHAQ)}*

$3078=171(192):
>gi|1055917092|ref|WP_067460467.1|
{|0(290)|<Actinobacteria(B)>}hypothetical protein [Actinomadura macra]
{(MAEVYLAYGRADAAWVRK)---------------------LWRHLDRLGIDVFFDELVL-
PGDGVvhTLEQAICDTTSGIAVFGPGLASDRWALTEYAALLRSctekG-----
LRFVPVTFGGAPIPPFARDWGWIDFADGGDFDTRADEVARALRDEAPADGMP(EERVLAEPPRRLTEPRRRSV
VVCYAPADAEYGRR)}*

$3079=275(265):
>gi|1056011642|ref|WP_067542448.1|
{|67(156)|<Firmicutes(B)>}hypothetical protein [Emergencia timonensis]
{(AESYWSLVLCKYGIEYVEDPSSHKRVPTINRTQYTSIFLDSDYASALKYADKSQRAIYEFEAKQIDEIQNN
IIMISKNEE)PFDIFICYKdlS-
ADGRRSedsvIAAELYHELTEAGYKVFFSRITLddKMGIEYepYIFAALNSAKIMI-
VLSTRaeYFNSPWVKNEwsryLALIRQgAD-----
KMLIPVYRDMNPYDLPVEFSHLQAMDMSKLGFMQDLVYGIQKILQRNSSNEM(KIKSVIHSQPTAESLIKRAF
LFLEDEEWERADIYCEKVLDMDPEC)}*

$3080=174(190):
>gi|1056351498|ref|WP_067795246.1|
{|0(291)|<Actinobacteria(B)>}hypothetical protein [Actinomadura formosensis]
{(MAEAYLAYGRRDAEWT)-------------------RMLWQQLTRHGLRVFFD-
ELMLPGDGVvhTLEQAIRDTTAGIAVFGPGLAGNgndGWALTEYSALLQScaerG-----
LPFVPVTFGGAPIPPFARDWVWVDFGKATGQAFDDKAADLARTLLGEQTAGV(PEETVLAAPPRPLTEPEQRS
VVVCYAPADAEYGR)}*

$3081=144(148):
>gi|1056051028|ref|WP_067577879.1| {<Actinobacteria(B)>}hypothetical protein [Amycolatopsis sp. M39]
{(M)------SFAG---
EDREFVEAVVREVAEAGYQVFSDQDEQVALWGEdltEYLPKIYEERARyaVMFVSRHYAAKPWTRFERAGAGD
RSAGTVFASGALGRDPARRgpG-QHQLPGRKPRRDERR-RERDLHETGSAAAAGSPATFRA(TSTKPRC)}*

$3082=122(141):
>gi|1055867754|gb|ODA34862.1| {<Planctomycetes(B)>}hypothetical protein A6X21_04210 [Planctopirus sp. JC280]
{(M)------SHATAdKWIATTICEKIDAIGA-------
TSFrDDRDINGGDsipqSIRTEIQISRELVVLLTPDSIERPWVLLEVGAAWGRRKD-
YRIVpVLCHVTFDAIPDIIEGKKAFHINDFDKY-LAELKRR--VKK---------()}*

$3083=206(220):
>gi|1057552519|ref|WP_068846355.1| {<Planctomycetes(B)>}hypothetical protein [Planctopirus sp. JC280] >gi|1055867755|gb|ODA34863.1| hypothetical protein A6X21_04215 [Planctopirus sp. JC280]

{(MTATVTPTFD)---VFVCHSSHdREFATDVADRLKAEGLQP-------
FHDASVPIEQeissAIWDAIAECHAFIVIVSPDSAPDAMGMIELGAATAWNKP-IFILLNGPASTRVPEALG-
---GYQVYPRNR--IDEVLSQ--
IRRNLEPITDEE(RQALLDTYRDLSIPADRLSQSPRELQKLVDNFSQKTHKQISGTRLLSELLRMRKQSKLPR
VSSIRRKTNG)}*

$3084=188(186):
>gi|1056460034|ref|WP_067901303.1|
{|0(411)|<Actinobacteria(B)>}hypothetical protein [Actinomadura
chibensis]
{(MTEPETHDGWD)---VFISYAREdYRKARDLADDLQKCvtakGVPPR-
IFLDVDRDGGTPVGVdwvhYLEKALRGSRYIVALYTPRYFSKGICLSELHEAVRLTDA-
KKAQLIPVLMDEAAQDSIAFTANRINWLPVT--
HPKWFDHLRDALDLRPAASGR(RLRFETAVPDAVVNRTLPTVRVTVVGADGTPVPES)}*

$3085=292(300):
>gi|1055917092|ref|WP_067460467.1|
{|74(95)|<Actinobacteria(B)>}hypothetical protein [Actinomadura macra]
{(AALLRSCTEKGLRFVPVTFGGAPIPPFARDWGWIDFADGGDFDTRADEVARALRDEAPADGMPEERVLAEP
PRRLTEPRR)-RSVVVCYAPADAEYGRRLVERVHEAGLP-VW-
SVRSLRPGDRLVWTTRQQLRYATVVVVVMSPAAQDSEDVTRmILEGQWHER-----P-FFPVLL--
AGNRHYLLANLWWADARtgG--PLDDGALA--
QLValheAALAGRPLP(QPEVVTEPSRAAPSVHVPTSVSLDRLRAALAEHETEHADLLTTSLLLEAADKLDFG
VLGRRDAGKLSADLLAGVDALWAA)}*

$3086=204(208):
>gi|1055946123|ref|WP_067488511.1|
{|0(1177)|<Actinobacteria(B)>}hypothetical protein [Actinomadura hibisca]
{(MN)SCSVFISYAPTDAAWVRALTPPLLEAGVA-IAYDALVLGPGAVAVHRLEDAIRDSAAGLLITSRAAL-
SEAWVReEYAALMRRsiegG-----RRFVPVLIEDV-ELPEFAANRYCCDLRN--
ADgEEFARRVSELARVLRGDGPE(LPQPGPPVRPGTGVRPEGPQRFVLRVSEAAVELRREDTGALLAEHVPAG
FTHRLDDLRRHLENARG)}*

$3087=263(275):
>gi|1056351498|ref|WP_067795246.1|
{|78(124)|<Actinobacteria(B)>}hypothetical protein [Actinomadura
formosensis]
{(ALLQSCAERGLPFVPVTFGGAPIPPFARDWVWVDFGKATGQAFDDKAADLARTLLGEQTAGVPEETVLAAP
PRPLTEPEQ)-RSVVVCYAPADAEYGRRLVERIGRAGLP-VW-
SVRSLRPGDRHVWAIRRQLRYATVIVVVMSSAAQDSDDITRmILEGQW-HG-----RLFLPVLLE--
GDRHYLLADLWWADART--
GGPLDDGALAQLGRLHEAALSG(RPVAQADVLPEPSTVVPSVHVPAAVSLERLRAALAEREIEHADLLTTSLL
LEAAD)}*

$3088=162(166):
>gi|1056072580|ref|WP_067598126.1|
{|0(470)|<Proteobacteria(B)>}hypothetical protein [Altererythrobacter
ishigakiensis]
{()MPQIFISYARSaEA---EVRTVVGWLEGAGY-
DVWWDRHLLANRSFSdAIEEQLDASDMVLVLWSKDAAASEWVRAEADFA--RHNK-KLVQASIDGTLPP-
LPFNQFHCEQIRISRNGdnSEGWKKLLDAFAHRGAEAPYQSR(SIPTQRISRRSALAMGGVGSLVIAAG)}*

$3089=175(185):

>gi|1056090947|ref|WP_067615125.1|
{|0(469)|<Proteobacteria(B)>}hypothetical protein [Novosphingobium sp. FSW06-99]
{(MTMVPSGQRGRI)---LFISYARAdRERIDPLTRVLTEAGHQ----
VWWDALIEGGEAFAkSIESALDLADVVIVAWSVHSVASDWVRDE-----ASHGR-DRGRLVPISLDGT-
LPPLGFRQYHFIDFSKWrgSAQAPEIAALVSAIAAGGTARAT(PSPDARARPSALRATLGRRALLFGTGSAVA
ALG)}*

$3090=226(207):
>gi|1056361812|ref|WP_067805560.1|
{|0(243)|<Actinobacteria(B)>}hypothetical protein [Nocardia beijingensis]
{(MLGERLRSDSATPSTSLAPVGASALAPAPSNR)--
KLFISHSSRirdadElrpgdsinrhVHRKRFLAHLlgdlkARLTDLGY-
DVWVDRDeIQPGMEFEpMTHLALQNCGTAIVLIDLDSLDSSYLRKEATILMWRRWT-CGINVVPVLLGSV-
SERDLAANPLGNPIGLStlSVLRSPTRKTNKTAAQTVSEYVT(EHLLAAAPTYERSSPLERWVEDFSDLISAV
NDERL)}*

$3091=309(299):
>gi|1056749435|ref|WP_068172770.1|
{|460(122)|<Actinobacteria(B)>}hypothetical protein [Rothia sp. ND6WE1A]
{(KELYPDDFHRGFLVSHNPEVMRVSEDIWAIPIEYLGSRDSWEGDFALQIPPQIADGEEAQDEIEMLGEDMS
DTQIFMSYT)-HQDaDSVVG---
GDMRQFGRDIAEALESLfgrSVDLFLDTDKARWGEHLwnRLDAEIQRSTFFMPFITPRYLNSKACVDEFQKFL
DAAERAgAeKQLMLPLVWITPPafkrnTGNSLIVERVKQTLYQDvSEIRTLDRSASGYRQKVEELAGY(INEM
IEEREQQGIEISESTSTEEEEVAKGAFDYLAEIEETYPQVESSLESFMSAFTQLGLGFQESGGKLNEQALSNP
FQ)}*

$3092=202(217):
>gi|1056582173|ref|WP_068013575.1|
{|0(807)|<Actinobacteria(B)>}hypothetical protein [Nocardia mexicana]
{(M)AEPIDVVHAPEDTVWALWLQQIGTEAGFdiR-TRL------VGAEWEL---
TGAAGAGSAILVISGSFVHAAALAPeDWRKAETDP-----AGLLAVIVG--TaaEVPSEAGRIAAVDLRL--
LpDEAQARS--
KVLQALGADPAA(AGERLRQAGRTPVRYPRQEMFVDFQSKSLPRYQPAWFYGRDRELDRIRSQLDTAGAAVLT
GVAGSGKTWLAAAYVH)}*

$3093=268(290):
>gi|1056585193|ref|WP_068016594.1|
{|255(10)|<Actinobacteria(B)>}transcriptional regulator [Nocardia mexicana]
{(TLVSGRSVVLSCTPIDGVDGSLVDIVSDDAFRSGTGAGVTARWPLLVIGEIGSGRSTEAYRAAGADAIVLD
ATEVVRQGE)------------RAWATAVAMLLEQDGPA-VVVENIQL-
LSERLSTLLAQCLREARRHIVLTST--PDHSEGVHaPLAAMCNAR-----RTLLPLR---RRrhEIP-
YLAQRMLADLDG--TDRlrftADALR--
VLAAQPWPGNLA(ELHRVVRSVAEVRSAGDIIPSDLPASYRGGPAPGSPLREAEREVIVAAIEAAGGNKLRAA
RALGVSRSTL)}*

$3094=213(220):
>gi|1056904102|ref|WP_068318489.1|
{|37(0)|<Actinobacteria(B)>}hypothetical protein [Janibacter anophelis]
{(DAANRKIAAQNKAEVDRVNKANQRAVDAHNRKADAHNRKVIAGLSRRLTGPTRVVSYRPAEQDLVERVHNA
ISFDDGREY)--DVFISYARIDgEPTATALAAALTDRGVS-
PWLDQVAIEPGKSQSLQMDAGLRKAAAGVAVLTPAYLTGRFWTQrE-LGALLHK-----
DVLIPVLDGVTFadVAEYSGILPDLAGFTT--AEDNLETIADKIAPAVLPAAQQ()}*

$3095=202(202):
>gi|1056655591|ref|WP_068083719.1|
{|0(139)|<Proteobacteria(B)>}hypothetical protein [Novosphingobium rosa]
{(MGCEHRSTFGIDKPTRPIVCAQKGRKQ)MAKIYVSYSRKdGDFMRALVNRL---
KQEGHEITVDDDVLTAGRDWRsTLDEGLKSADVFLTLLSHNTPTSAYTMMELGAARAFAgAT-
GTPLVIPIAIDQV-
EVPLVLQDIQILFASDRdvDAIVAEISKSISSQHGLSLAREV(KAEVAATRIKQNAPQYIDEAIKAQDAIKRR
NACAA)}*

$3096=192(179):
>gi|1057329116|ref|WP_068689861.1|
{|0(1000)|<Bacteroidetes(B)>}hypothetical protein [Culturomica
massiliensis]
{(MNETPH)-YYAFISYSHEdknlvAAFHDRLEnyeipfyfRKKRKKIPATLTPIFRDETDLNIGI-
MteEIGKALKNSDSLIIFCSKASQHSSWVEKEIELFKKFHHKGeccIVVIVVSENGE---
KFSGTEYYPSNLSKDIYYVDSCCIDQEWERIICKLVGVDY(TKISRRKRKDKHLKNNWIVFILCMFGVLGGGG
Y)}*

$3097=261(258):
>gi|1058042589|gb|JAS24956.1| {|225(0)|<Arthropoda(M)>}hypothetical
protein g.15899 [Clastoptera arizonana]
{(VCRISNAGDQIIRLSTVLREEEWVFTKPDNSVPWSSLILVTCACILSSLTVLMIYKRFALSVVLFLKQNLC
KTDDSDNKE)-YDVMVCYSEKdaAFVLGVLKPALQHkydySTIGFQI------------
SAEsvtpELKTAASLSRRLLVVLSPSLVDNQWTPATLYQTLNKLSNLHTNIICLFI--KP-
LPQRELyktddGIKLKTLLqKTKVIQWENstplerRR---
FWTSLRLYLPIP(TKKPVMETNVLTAINTSATEETIHTPFSKSNESLEILV)}*

$3098=208(219):
>gi|1057510733|ref|WP_068809228.1|
{|0(1027)|<Proteobacteria(B)>}hypothetical protein [Thauera sp. ZV-1-C]
{(MSG)---IFISHSSRDRQAAEEMAARLRAHGYQ-
SLFLDFDPadgiPAGRDWEREIYAGLRSCSGVVVLCSAHSMA-SDWCFaEITHARALG-----
KRLFPVLIGECTlrAL--LLDTQV-VDLRA---DPDDGYA--
RLWRGMRGAGLD(PADSFAWESGRAPYPGLAPFQAADAAVYFGREEEQRRCLDALAQMRRYGGERLLVVVGAS
GCGKSSLVRAGVLPRV)}*

$3099=171(176):
>gi|1057521516|ref|WP_068818473.1|
{|0(598)|<Cyanobacteria(B)>}hypothetical protein [Phormidesmis
priestleyi]
{()MTDVFLSYSRKdKAFVQSLH---DALHREQR-
EIWVDwQAIPLTADWWqEIERGIEGTNTFVFVISPDSVASDICRQEIDHAVKHNK-----RLVPIVHREG-
FEAAKVHPMLSKHNWLFfrQTDDFAHGFEQLTTAIDTDLDQV(RTHTRILEKAIEWKKEGRNPSFVLRGEDLV
RSQAWL)}*

$3100=187(193):
>gi|1057522811|ref|WP_068819683.1|
{|0(31)|<Cyanobacteria(B)>}hypothetical protein [Phormidesmis priestleyi]
{(MAALNPSTTDLNPNNLN)-PEIFVSYCRRdKELVRSLCDALQHHQRS----
PWVDwQDIPPSADWRrEIRTGILTAHTFLFVISPDSIVSKECLVELDYAIQHHK-----RLLPVVCREV-
KAEQVHPALASLNWIFFreKDDFDRSLTTLLQALDTDLEYVQ(AHTRLLLRAADWENNGRDQSLLLHGRSLEA
VEQWLV)}*

$3101=171(176):
>gi|1057523442|ref|WP_068820314.1|
{|0(387)|<Cyanobacteria(B)>}hypothetical protein [Phormidesmis
priestleyi]
{()MTDVFLSYSRKdKPFVQSLY---DALQREQR-
ETWVDwDAIEYTEDWWqAIERGIEGTSTFVFMISPDSVASDVCRREIDHAVKHHK-----RLVPIVHREG-
FEISKVHPMLSKYNWLFfrQTDDFAHGFGQLMTAIDTDLEQV(RTHTRILEKAIEWDKEGRNPSFVLRGEDLV
RSQAWL)}*

$3102=261(257):
>gi|1057603217|ref|WP_068887322.1|
{|907(0)|<Bacteroidetes(B)>}hypothetical protein [Pedobacter
panaciterrae]
{(IEIYKMKNSLNDEFLDKIDQQLLQLTKGMNVHKAVTVNGENFVPIYILEEMAKKELWVFEYEDNTYRLSNF
KKHLNLQKK)MKNIFISYSKDdLVLVDTFIRHLAALRQDGKVANWFCTELTAGKEWEpEIKEHFDQSDIVCFM
VSPNFMKTKYILDhEVKWAFEKKAK-NPVfRIVPIILDFC-RWTtvENNLGDFTALPYTAkpVVDFKNQN--
MAWYIIIELIRIM(IEKDLDPKEDDFYAGDNVLPKDVMAIYKRIVTGSVDA)}*

$3103=225(234):
>gi|380877377|gb|EIC19470.1| {<Proteobacteria(B)>}TIR domain-containing
protein [Thiorhodovibrio sp. 970]
{(MSWVWRPLIAMKKKEQPIVTHFVAVILGLMVVFYLALVFLGGVDYAAGLELVLYVLTGLAAVMIFGLSLAT
LRKNDIRK)---IFILYDVQDARFVAKL-YEALKIAPF--
RVLWDKKEVQVGDNVeDKLKELfDSSSDIIFVISHQSIA------
ANWANPAIAKAVqKNKRIFPVLLDDAEPPEALQGIKAADFRLSFDDGYFSLREALQTRREEAPPPSA(SANVA
PNAPPSAGTD)}*

$3104=163(165):
>gi|490174283|ref|WP_004072918.1| {|0(321)|<Proteobacteria(B)>}TIR
domain-containing protein [Desulfobacter postgatei]
>gi|389401457|gb|EIM63679.1| TIR domain-containing protein [Desulfobacter
postgat
{(MSGNET)KRKIYVSYCRKdghaKRLIDALIKTAPGTGCS----IC--YDETSLQLGEsitkFMAK-
VSEADRIVFLLSQNYFQSRSCMNELLLAYEKQAS-ELQPAVMMLDNFLPDVEQEQSSQNNREKKDA--
VDLTTCCENPGVVPVILAWLF(GKYKPEFKYRDRLVLVQDD)}*

$3105=213(218):
>gi|358245884|dbj|GAB08293.1| {|0(246)|<Actinobacteria(B)>}putative
transcriptional regulator [Gordonia araii NBRC 100433]
{(MLMVG)--RIFISYSTRDRDIADLVCSYLESEGKT-VWIAPRDVPPGLSYPEAIIGALRDSEVGLLVLS-
SHSNLSPHVLrEVERISADG-----KRLYVLRTEDVKlsDGLSYFASMVqwieaPRDRLL--RDPASVLR--
PIIAAPTVSTEP(ATVARAAAVPTQSSEVSFSVLAEQQRRMLTTFGVAVLDYICQQPNPAKPIPQRELLSRLL
EVSPGVFDGLTQR)}*

$3106=158(165):
>gi|498362012|ref|WP_010676168.1| {<Proteobacteria(B)>}MULTISPECIES:
molecular chaperone Tir [Aeromonas]
{(MP)---VFISYRHKDRDAALGVAAVMHQYGIK-TYLDVLDPqsQTTDQITDVITTRMKDCSHLIAVVSEN-
TEGSWWVPfEIGEATIID-----NRIATFQVGYVTlpEYLKKWPKMTTMEHLK--
LFAIAYKADYPLAFNRKLGGES(VSTSSRSSEQFHYNLKRNLGQIY)}*

$3107=196(200):
>gi|489898954|ref|WP_003802394.1|
{|0(1234)|<Actinobacteria(B)>}hypothetical protein [Arthrobacter

globiformis] >gi|359305589|dbj|GAB14230.1| hypothetical protein ARGLB_059_00060 [Arthrobacter globifo
{(MS)--RIFLSHSSRDTRQAialkRWLAEQDPALAQE-IFLDldpVTGIAPGERWKEALLRANERCEAVICLLSAA-WEASRECLaEYRTAENLG-----KLILCARLEALQdtEITGEW---QRCDLFG--PGATTEVTVNDGGSPVVFLTEG(LQRLRLGLLRAGIGAEFFPWPPPQDQHRAPYRGWEPLEEIDAAVFFGRDGQIVRGLDT)}*

$3108=193(202):
>gi|494531192|ref|WP_007320643.1|
{|0(335)|<Actinobacteria(B)>}hypothetical protein [Gordonia araii] >gi|358245480|dbj|GAB08566.1| hypothetical protein GOARA_013_00100 [Gordonia araii NBRC 100433]
{(MTDSTTIPVRAVTDLLEAHEGTEEY)---LFISYSHVnTDEVYGILHQLQDLK----FRFWYDDTMETGEDFRrELHRKIDDCTAVVLFMSKASMNSKYC---GMEIITAHQL-GK-RIYPILLEPD-VEVPAPLKLILDTLQHVkaHTGEQRYVDRLIKSLPPETMKRL(EIEDGVVLSCEDNGADITVPDGVHSIADDAFRECLQL)}*

$3109=290(290):
>gi|498158141|ref|WP_010472297.1|
{|142(297)|<Cyanobacteria(B)>}hypothetical protein [Acaryochloris sp. CCMEE 5410]
{(FAVGFYDGLGADRSYEDAFEFGLSAIALEGIDETTTPKLKKPGSGLSSDTAQQSIPSSTKKPNLQGKTAHTSLSQGKKAR)---IFISYKrnA--EPDDQLALSIYHHLSQS-HEVFIDQSMAV-GTKWaeKIEQELRQSDFFITLLSAQ-SVGSEMVQgEVETASRLASEFgQPA-ILPVRV-NY-RELFTYPLSAYLNPI-NWAfweqqEDTPQLLAeLERAIAGESLSID(SDRAKQDLLQLPTADKSEIPQPLPSAQPPTLEMPEGSMDPESQFYVERQGDEIALGAIQRRGVTVTIKGP)}*

$3110=199(194):
>gi|1059277257|emb|SCK39827.1| {|0(508)|<Actinobacteria(B)>}TIR domain-containing protein [Streptomyces sp. WMMB 714]
{(MTARDHSEAT)-WDIFVSYSS-SDART--IGVLVPALQAAGLRVFVDNTAVDDFTSItaTITEALARCKVLLALYSADYPQRRACQWELTYAYLTgqHEGDPRRRILVInpepsADHVHPVELRDARYWPWPTGQENVDRLAERAAAHIASITTPMGDRA(HAPIVPWLPAPARTGSVHFTGRLSEQWRIHTALHRHRAPLVAQT)}*

$3111=179(176):
>gi|1060042843|ref|WP_069070925.1| {|0(213)|<Cyanobacteria(B)>}DNA transporter [Nostoc sp. KVJ20] >gi|1059439343|gb|ODG96884.1| DNA transporter [Nostoc sp. KVJ20]
{(MN)--QIYISYAW-KDnqselgrQREELVDKICAALVSERYNLIRDrNYLTLGKSIQQFMQEIGRGNYVILVVSEKYLRSEYCMFEAVEIMKH-KD-YEQKVFPVVLQDADIYTKEGKIEYITYWKQQREKIEKIINSELLSHEYSAMHNI(AAKIIEISEKIDEFMFFISDKLSINPTNNFNEFI)}*

$3112=213(215):
>gi|1060043498|ref|WP_069071434.1| {|0(314)|<Cyanobacteria(B)>}molecular chaperone Tir [Nostoc sp. KVJ20] >gi|1059438710|gb|ODG96274.1| molecular chaperone Tir [Nostoc sp. KVJ20]
{(MPTLPAASKNARF)----FISYRSQDpDLSLAHNFYEALKAAGH--EAFMAGESIRLGeKWPqRIDEELKLCDYFLLLLSERSATSEMVTEevRQ--AKELRDGSnHYKPVIlPIRVNFPWSKP--

LNYDLRgYLQQVQQREWNSPADTPRILQEIFSLLTD(GDTKRLANGEMGREESSYSFPSSPFHPLTLSPPVPV
AEPELPEGQVDLASAFYIDRPPIESR)}*

$3113=228(220):
>gi|1059966064|ref|WP_069046769.1|
{|0(121)|<Proteobacteria(B)>}hypothetical protein [Hydrogenophaga sp. RAC07] >gi|1059493689|gb|AOF86805.1| TIR domain protein [Hydrogenophaga sp. RAC07]
{(MNR)---IFISYRSSDGKKDADRLCADLSRlyGAD--
QVFFDKQDLKGGHSWrSAITGTLGTRPVVLLLvTPELLgmehpEGGRRIDrdDDPIRGEVLTAQaHGVTIVPL
LtEGMLMPATADIPAPLRFLKESHALKLRTEDWADDLDKIVADLASH(GIHPLKQRGNEPTPRSWMQRVQRGF
MWIGGIFVGLIVIGLLVPDDEEAMPPSAPPYAAATPPPAPAQGTRDVSGLWW)}*

$3114=268(259):
>gi|1059570292|emb|SCL59759.1| {|0(135)|<Actinobacteria(B)>}hypothetical protein GA0070608_2198 [Micromonospora peucetia]
{(MTWSDPAPARGAPRRETYFFLSYAHSVPLSAGVRPDTDYW)--------------
VNRFFNDLAAAVRHAatrSGDLdagFFDGQ-
VSPGADLrqTLTDALSLAHVFVPLHSPNYLRNAWALGERESFRSRLTRLaPTQAerhLVPVLWLPLPsrgdrp
ettRALDLVRDADDRADYADnglRALCKLSAYHRQYRRILAALAER(IVTVTETQPLTRSRAAMLSSHPVTDG
GGPALVITALTDHVGWRPYAGQHELAVADYVAATAERLGLPTRVVDLAVARAL)}*

$3115=235(237):
>gi|1060045467|ref|WP_069072833.1|
{|0(414)|<Cyanobacteria(B)>}hypothetical protein [Nostoc sp. KVJ20] >gi|1059444684|gb|ODH01878.1| hypothetical protein A4S05_26825 [Nostoc sp. KVJ20]
{(MSDSVHASLESSVVQISQSSNSSSKTAKVSSEFE)---
VFISYRtSPdQGLAVALKELIEGAIEPQPHVFVAGAGGLR-
PSNIgykpQIQAAAQSSRAFIAIITQQSKEREWLFFEAGAAWGRNQL-YAPLLV-
GTSPSELASTMAEYQTVRASDREE--
VKHLLIAVAQAVGGNIKTRFN(QRYQAFSKQIEAYQKIEIIQENIEQSSSLLEIVEENLEQSSSLLEIVEENL
EQSSSLSQAIKL)}*

$3116=190(193):
>gi|1059956990|ref|WP_069038752.1|
{|0(683)|<Proteobacteria(B)>}hypothetical protein [Methyloversatilis sp. RAC08] >gi|1059487252|gb|AOF80369.1| TIR domain protein [Methyloversatilis sp. RAC08]
{(M)--KIFLSYASEIRARAEGVNAALQAAGHD-
VFFDREDLPAGLNYDDRIRSAIESSDLFVFLLSPAAVAEGHYTRtELKIARRRwpdpA-----
GHVLPVMVEATPfsALPPYLGSVTVLVPEG--
NLAAEVVLAVADIERLPSHAAT(EPDAVTPPPDSPDATPTYASLKVRFSYAEAGRYTVDINSPQAVDGFSGDC
AI)}*

$3117=172(177):
>gi|1059966946|ref|WP_069047651.1|
{|0(778)|<Proteobacteria(B)>}hypothetical protein [Hydrogenophaga sp. RAC07] >gi|1059493715|gb|AOF86831.1| TIR domain protein [Hydrogenophaga sp. RAC07]
{()MSEVFISYSRKdIGPVRRMHAALAARERQ----
TWVDwEGIPPTAEWMqEIRGAIDAAQAFAFVLSPDSLASRVCREE----LEHAVA-QGKRLIPVVCRDV-
VASEVPEALARLNWVYCreQDDFEAAMSLLVQAVDTDLAHVQ(AHTQLLLRAVDWQSRGEDTSLTLRGAALAA
AERWLAE)}*

$3118=195(179):
>gi|1059572579|emb|SCL71216.1| {|0(215)|<Actinobacteria(B)>}FxsC C-terminal domain-containing protein [Micromonospora peucetia]
{()MPLFFLSYAHEdnrngqvQEFFGDLSEAVARLAGVG----
WDeagfvDGQMPLGATWShELADALATSSCLVALTSPRYVVSDYC-GrEFRVFADRVDA-
HEREfrrrppaVLPIRWIPC-
DRMPEVLTGFQHKNFPAgaVYAQDGLLTLKQRRRHHDDYQDF(VDALAKHIVQTDRDQRLARPARRPVLAEIT
SAFEAPRRR)}*

$3119=151(163):
>gi|1054947937|ref|WP_066702721.1| {<Proteobacteria(B)>}molecular chaperone Tir [Curvibacter delicatus]
{(MA)--DVFISHATSELGFAQFLKRHLETEGHS-
VYVAALDMKPGEKWMPAIMENLKSSQWVLCLASRSACA-SPWVMqEMGAAIGSN-----KKLVPI-
VWDQRpsELPAWMRQYQAVEL-G--HDEATAKA--AIARIAEQIKAD(KQRGLLILALLAAGLLIFGGK)}*

$3120=212(220):
>gi|1060727313|ref|WP_069123264.1|
{|323(0)|<Proteobacteria(B)>}hypothetical protein [Candidatus Thiodiazotropha endolucinida] >gi|1060271774|gb|ODJ88141.1| adenylate cyclase 1 [Candidatus Thiodiazotr
{(SGDMRDYTAIGTAVNLSAAFESNARNGKRILCDQVTYRNAKDIIVAADDAVDFLLQKPGQDVGVKYKCYNI
RELDSDKSV)--SVFLSHSHEdSHFIEK--KLLEPLKKRG-
IRTWYSTaDIPKGALWTaEIRKAISESNWMAVIVSRNSAQSKWVRReIDLGVASGHLD-N--RIIPIIIDD--
----TSPGDVNDYLAAMqaIDLPSSPDAEKILADRFSKLDPT()}*

$3121=307(298):
>gi|1061486349|gb|ODN03450.1| {|1177(177)|<Arthropoda(M)>}Leucine-rich repeat-containing G-protein coupled receptor 4 [Orchesella cincta]
{(ARNDSDAGGSGNSSFYIFLLTCIIAIVLVAVVALLIFKRGVFSKLGFGGGISAKCLSAENEAGGVDGNADY
YYSDSENAY)-YDVYFISSQKdsEIVEKIV-----QNSANLRFCLHYRDL-
CLSPANPwnceAMMTACNASKRVVIVLSNNLLNCEWSNPNFRTSVLSAISHQKLIVITLppvndqgvysaIEA
TM-QQQRQRVPDVTNKLSVPSITkleWGEKD---
FWPKFRQLLPIP(QPSATGNNNSNFATISSNATLPYATNLNSNGMNFINVNNAAMLPYQMLAGHPHYMSQQQA
PLITMNWQQGISPLPMSH)}*

$3122=265(257):
>gi|1061124408|ref|XP_017887473.1| {|230(0)|<Arthropoda(M)>}PREDICTED: single Ig IL-1-related receptor-like [Ceratina calcarata]
{(CEVSNGVDEEITLPAHIYRQEPQFALGLQNGSWRKSFLVGILVLLLLISAAAFYARCWLPLVLLCRDKFAP
LEENDGKEC)--DALVCYHEKdsSLVIGIVIPTLES---------RHR-
YKCTGLELSQLNQNWgleisphaATSRRVIVILSPSALGNVWNETSV-AAVLKQLSTLATRTIVIATKDL-
PDATTIakrssrDDTGELALDRLkILHWqetgkptaGSQK---
FWYMLRLAMPPV(RPVSADNSCQSVAMIVQANGKCGQQKARSRESLEVLV)}*

$3123=167(189):
>gi|1061023893|gb|AOH41075.1| {|0(42)|<Bacteroidetes(B)>}hypothetical protein BCB71_08065 [Tannerella sp. oral taxon HOT-286]
{(MEAIKN)-------------------------GLKKNNIEY-SIDLQDIKYRDdiveY-
EKEIGTAVRVIMFITASYLKSIDCMFEMTEIFkksdVMERV-
FPIVDITPISRNRKGLEEIKDYWQKQKVEAS--
AQMQTEVGSSDYIIDELRKIN(AIIKALDEFWYYIVHINTGVFKTLLENDAALLMEEIQKTTPYR)}*

$3124=238(239):
>gi|1062965798|ref|WP_069349754.1|
{|443(28)|<Cyanobacteria(B)>}hypothetical protein [Scytonema millei]
>gi|1061902146|gb|ODN57958.1| hypothetical protein QH73_028640 [Scytonema millei VB511283]
{(GLVAQEKVPIPGHSNIVVDYQNLLIYEQKGLPIIPEGLTETFDARQLLNGIEPEEQRRERQREDKFMGASKSTRSSKPEF)EKEIFISYTWRdehsKPYVEQLEQTFQAKGIKI--IRDTNAVG---DYKArfqeFM-QRIGRGKWIIAVISDQYLKSENCMFELVQIAKNGEF-YKRLFPIILSDANIYDSIERIKYVEYWEDKK--NKLDEAMKRVSGAKLQGFRED(IDLYTDIRNTIAELTNLLR)}*

$3125=219(221):
>gi|1061374573|ref|WP_069178431.1|
{|0(572)|<Chloroflexi(B)>}hypothetical protein [Anaerolineaceae bacterium oral taxon 439] >gi|1061026038|gb|AOH43219.1| hypothetical protein BEQ56_06865 [Anaerolinea
{(MAFFDRFKRQNKTNSKTDINIVPQAEESPVHLQETMIDQAPAQPRKNLTGVLNTQGADSSLVPLVGRIPFPAYRGNE)-PYIFISYAHAdA---DTVFPIIKQFYDQGY-HIWYDEGIAPGNEWTnEIADALEGAAVFLVFLTPDSEKSINVRDeINFAINDNKPF-LGIHLKETDLTGG-LKLRVGTKQAILKYNMSeeEFLYKYTFAFEHLGLPVPDAIKA(YKER)}*

$3126=213(220):
>gi|1062965999|ref|WP_069349826.1|
{|174(0)|<Cyanobacteria(B)>}hypothetical protein [Scytonema millei]
>gi|1061902064|gb|ODN57884.1| hypothetical protein QH73_028900 [Scytonema millei VB511283]
{(LVEIDEEKAKELNNVFEKFKQEQLWTVDTGPIDSIDSLVEEPEIVREPPRAIETMAPPRVEKISQPGPAIAPPIANDQFD)---IFISYAHKnKEHVQYFIESFKT--KHPSVRLFYDTEtIPKGGQWLkEIARCIDRSNKVLVFLSREFDESPACWDEFQCAKVRQNR-TGKNVI-----MN-IYLHHHPELPTIFEIFNwiDCREGDRLKLEKAIDEVIGSLDK()}*

$3127=286(289):
>gi|1061684632|ref|WP_069207725.1|
{|22(105)|<Proteobacteria(B)>}molecular chaperone Tir [Sphingomonas panacis] >gi|1061229515|gb|AOH87155.1| molecular chaperone Tir (plasmid) [Sphingomonas panacis]
{(KAIALGMQAIEEKARGAAASIVDPETGHHADVFVDRLPGNKVAIRTTGSPAFARLIEKRLGVDPGKVQVMNAADGAAHPK)---VYLAHAS---EDKARVRPVAEYLMANGIEVWFDEWEIEPGDSLrqKMEEGL-GAMTHFVVVLTETSIAKPWVAkEIDV--GMVQQVgGKSRFVPLvvDLDPA-KLSPFMQAMLFLKIDPASEVDLKGLVDrLHGVSRKPALGEA(PRYVQNAPKGLEGWSAAAVAIGRHIVETSGNAMLVDPAVTLEDLQGSLDLASDDLRIALLDLKDGGYLR)}*

$3128=277(279):
>gi|1062958703|ref|WP_069347358.1| {|120(310)|<Cyanobacteria(B)>}ATPase [Scytonema millei] >gi|1061904699|gb|ODN60362.1| ATPase [Scytonema millei VB511283]
{(SKVQAEAISQHIEYVIGMNRQIGDKAAINFAVAFYDALAAGEEVEFAYELGCSQLFGLKEHETPVFKRKSLNNPIPQKTT)RQRIFISYKRDVEPDEPVALEVFQALSQQ-HEVFIDQRMLV-GTRWaeRIEGEIRQADFLIVFLSAR-SVYSEMVEqEIKMAHELAQVQrGRPAILPVRLG-Y-REPFQYPLSAYLN-DINWAFWQSERDTPRLIAELTQAISG(GELPFDAAQAKAALLETSEPSPLPRPFPSAQPVALEMPEGTMDTESAFYVERPSDAIAI)}*

$3129=222(221):

>gi|1061974198|ref|WP_069280161.1|
{|0(309)|<Proteobacteria(B)>}hypothetical protein [Bradyrhizobium elkanii] >gi|1061444552|gb|ODM70543.1| hypothetical protein A6X20_08030 [Bradyrhizobium elkanii] >g
{(MSEFN)-
YDAFISYRRSdgiavaRWLRRTLVGFRLPAALRgvygKKLRVYLDTAYERGTSDFyqqSIKPALLSSRYLIVVATPDAMRRPggaedWIEREIRDFAAGPGAGnviVA------------RGAGDFDGP----
LPGGLKETFPNIE--I-
VDLRGAGPLW(FLSFSRVVRLSAEKLKIVAPLLNIPPEEMPRLRQEEEKRQQTQLGAVAGATLGTLVAVGGLSVLALINQYKAIRAN)}*

$3130=234(242):
>gi|1061903208|gb|ODN58941.1| {|83(0)|<Cyanobacteria(B)>}hypothetical protein QH73_006360 [Scytonema millei VB511283]
{(SSSSDSSAPDSPVQPSKLEPTASQSNPIDLPDINLAAQSSTSDTTTSAGNQINSANQNLAVQQSTQSSKRKSKRDSEKVR)--DVFLCYnSKDYDLVKSICFDLE-NKWNVKCWIDKRDVGGG--
IWDEILNKQAKtVKSVAIFLSKNGIgprQIDEIHVFREQAR-----
IRSLVLIPVLFpGFDKSQIPPNLSIYNYVDFHR----
KDYDPVEELVRVFAKVSPR(KIAKLIPDKATLGSRQETYPNY)}*

$3131=181(184):
>gi|1062675344|ref|WP_069329912.1| {|0(68)|<Firmicutes(B)>}hypothetical protein [Paenibacillus sp. TI45-13ar] >gi|1062493984|gb|ODP26052.1| hypothetical protein PTI45_04605 [Paenibacillus sp. TI45-13a
{(M)---IFFCFSS-KD-RHNIVEAI---
LYHIrNYRipVWYDRDQMLLGDERnykNFVEGVEASSYGIIILSPNSIQSYCANEEIDLMYEQYLK-
GNMHVFPLFFNLSASEIPPKYDWLKNLVYKEITPSIDVRSTCNHIICKFLSDDL(SKHESRNFHALEKRMKKRECDPYILILLRSYLELDEKNFNSRI)}*

$3132=219(220):
>gi|1062536182|ref|WP_069297349.1| {|61(0)|<Firmicutes(B)>}hypothetical protein [Bacillus cereus]
{(KQDKDITSLVEQIRKNTESMNRYKGRLAKEQEKQFKTMLKSMESQASTNLEYLNSYSEMSEKLNELSLDIKHSVKDKEII)EFDVFLSHSS-LD-
KDIFVSELSGKLSNKGLKVFEDVKVFKIGQSQtdMMNMGILNSRFVVIFLSKNFIDSGWSQYEFKSFLNREINEKRVIILPIWHEVSVDDVR-QYNPYivdKYAFNTS-KQTLDE--MVNQIHQVVLDSLD()}*

$3133=252(255):
>gi|1062662236|ref|WP_069325784.1| {|14(0)|<Firmicutes(B)>}signal transduction protein [Paenibacillus sp. TI45-13ar]
{(IILGIEDDGETLTIKNHLNSFNLDKALALLNIKVEIRSESFNYDGHNLYFISIDKSTELVESEGIPYIINLNGELTEMRR)-KTVFLSYSHKdADLADIIEDRLEEYE---
DISISRDIKITQYRDNLpQFMRTIRNHDFVISIVSSEYLHSINCMYEITELM-KDDN-FKDRLLFIIVSHE-
DAKYYSDNNKYNKFEAGlyNVTNRLNYIKHLNEVNTDIEQQI(IDAALPTEMLVEFVREKRKVASIIQPASIFMDELK)}*

$3134=199(222):
>gi|1063501045|ref|XP_017976186.1| {|0(41)|<Streptophyta(V)>}PREDICTED: TMV resistance protein N [Theobroma cacao]
{(MT)-------------------------
LVHVGIHTFRDDDEIerGEKRRDEIERAIYESKISIIIFLKNYTSSTWSLNELVKIME-
HRKFSKHIVLPIFYDVNPSQVKEQTGSFAEAFARHEESFksDMDTV-
QRWRAALREVADL(GGMLLEDRDMRRNSTRQESPDLAKRSRLWQKDAFDALREKIGTKIIKCLTIDLQRLLKEKYGKTIANQKNPLLMSNEVDI)}*

$3135=302(300):
>gi|1063517373|ref|XP_017980722.1|
{|107(588)|<Streptophyta(V)>}PREDICTED: uncharacterized protein LOC18593165 [Theobroma cacao]
{(SPPVSFRGGSQSDDTPSSSYTPPSDQYEYSDDPADPKLKFVTCVPVPDPAPRISFSFPVPRISFAKAPVSPASNAKLRSC)--DVFIGFHG-QNPNLaRFCKWLKSELELQGIACFVADRVKYSDS-QshEIADRVICSVTYGVVVVTNSSFLNHL--SLEEIRFFAQK-KNLI--PLFFDTGSAEImgllncNSINKECKEALDgliKSHEFKLEASE-GNWRSCVAKAAGI(LRAKLGRKSVVETDFVGEGFEELPFPRNRFFVGREKEIMEIETALFGHADSLEQDCCSRPIIKGEASGQSEGLADEESDH)}*

$3136=108(141):
>gi|1063964496|gb|ODS76516.1| {<Proteobacteria(B)>}hypothetical protein ABS43_01395 [Bordetella sp. SCN 67-23] >gi|1113424583|gb|OJW86745.1| hypothetical protein BGO71_25705 [Burkholderiales bacterium
{(M)---------------------------------
FRDESDFGAGHSIptLIREAIFEADVFVVLWSSAYACSPWCFDEMELALDRH-E-EGKLKLWILRVDDTRIVPKRARDLLFYDSSSRAEVEGRMLQLLAQYVPGGGLLK()}*

$3137=202(216):
>gi|1063906825|gb|ODS34682.1| {|0(748)|<Planctomycetes(B)>}TIR domain protein [Candidatus Scalindua rubra]
{(MTNPS)---VFISYSHKdeKWVKDYLLTNLEKNGIP--CHIDYRDFEIGTASLINMEQAVEQCTKTVLVFTPDWVNSEFANFEGLMLQTDDPAGLKKRIFPIMLHKC-TPPKRLG--------IFtYADFTDKA---EWDTQLERVIKQ(IKKDYEQTEPEKKTYPPLEDHYIDIKRLPQTGFELFGRQKELTLLNEAWESGATNIVSFVAYGGVGKSTLV)}*

$3138=208(212):
>gi|1063913236|gb|ODS36219.1| {|0(210)|<Euryarchaeota(A)>}hypothetical protein BEH94_05180 [Candidatus Altiarchaeales archaeon WOR_SM1_SCG]
{(MKRIKSF)-----LSYSS---ADSVLADKIFRWLQNQAISVWFDKVKL-----RsgdSLLQKIATgiSDSDflIVLITENSVNSHWVMYELETALSREMSGKGPKVIPLRVRNTEipT-IIANKIYITIDENlsGI-DEIIPAIFRESFILDISLSSND(LNLMVNDLKEKLHEYYRSDYKDLKVRICSHGFNEKITKIAESMIEEVIQLGKNDPGGYPPQVATQ)}*

$3139=163(172):
>gi|1063913422|gb|ODS36390.1| {<Euryarchaeota(A)>}hypothetical protein BEH94_12080 [Candidatus Altiarchaeales archaeon WOR_SM1_SCG]
{(MI)--KVFISASIQdKKIIDLIQNTLQNIGIE------TISPLYFPAATQpieeEIKNAIQTSDIIFLILTPYTLspDSVGNINyEIGFAQSINKP-VYVFVERGTNIDVN---MKATAYFTF-DRIN--MVELKER--IKVVTEQADMIK(MEEEKKQALIAAGIMVGVPLFIWLLKNMKK)}*

$3140=153(162):
>gi|1063916047|gb|ODS38749.1| {<Euryarchaeota(A)>}hypothetical protein A7316_07280 [Candidatus Altiarchaeales archaeon WOR_SM1_86-2]
{(MM)--KVFVSHSVKdRNIVSTLKSNLDLAGIQT----YIADEDYQ--PGTdiweKVTRNIDDSDIVIAILTRDGDRSSYVNQEMGYASKANIQ-IIPIVEDNVKPV--GAL-EGMEYIRFNRLSP--LDAIDLTTQYLERHKTKSNDI(VSLVITIIFFAFILRILSKK)}*

$3141=246(254):

>gi|1063969641|gb|ODS81307.1| {|101(47)|<Bacteroidetes(B)>}hypothetical protein ABS44_19460 [Chryseobacterium sp. SCN 40-13]
>gi|1113302826|gb|OJV67288.1| hypothetical protein BGO42_11570 [Flavobacter
{(RIFPVSLCNKAYSIGCGLSTLQFTDGTKFFKKTLDLQNEKDLEKSIKKIKTELLHDCSRLILNFHPKQKDK
EHHRIGSPV)--KLFLSHAKKdgENTTIKFKQFIEN-NLKLDVFF----
DTVDIANGYdfaeQFEQEIKHSALVVF-HTDEYSNREWCRRE---ILIAKKH-
KSPIVVVHNILNGEKRAFPYLGNMPTTVISK--
KGIVGFYKIVNLTLYQVLNNL(YQNRLLESYSTIKGDNNSIIKVITSPPELFNFLD)}*

$3142=180(188):
>gi|1063903806|gb|ODS31757.1| {<Planctomycetes(B)>}TIR domain protein [Candidatus Scalindua rubra]
{(MPTKP)--KVFISYSTTDRSIAEALYKDLLDAGAE-
VFQFERSATPGTSAWDEVLGWIESSDVFIVLVSKSALKSRPVNAeVVHAHYQYL-----
NSNQPFKLIPIVleEefrPHRVIRRFAQLPLD----DYATGLQ--
RLIAELGLERFS(KSANELRDNRDLLVSNFAEGVFRSNRAKAHKDSHLFERFASSP)}*

$3143=224(229):
>gi|1063918699|gb|ODS41067.1| {|285(0)|<Euryarchaeota(A)>}hypothetical protein A7315_07045 [Candidatus Altiarchaeales archaeon WOR_SM1_79]
{(FCAFFGELFSAFSRVEAVIVYALIYAIPLSILNFISLLITRNHGRELTIKGDIAHKKELIVPEIEGETTGL
EEKIDLHPP)ARDVFISHVEEDYDVASQIANGLEAAGYT-
TWYYERDSFGGPSYLLQTKKAIESSQAFLVVISPHALSSTQMTKeVIRAHESGK-----
HPIPILYGVTDVeyKNRQPEWQEAMGAATS--LYIPKEGVSAIIHRIVGGLKEL(GIQPGGKVR)}*

$3144=156(162):
>gi|1063918700|gb|ODS41068.1| {<Euryarchaeota(A)>}hypothetical protein A7315_07050 [Candidatus Altiarchaeales archaeon WOR_SM1_79]
{(MTDRKKKLDTPPP)SRDVFISHVEEDYDVASQIANGLEAAGYT-
TWYYERDSLPGPSYLLQTGQAIDESKSFVLIISPQSLGSQQVTGeVVQAHESGK-----P-
FIPVLSNITHveFQRRQPEWRRAMGAST--SINIPKEGVSAIIHRIVGGLKE(LGIQPGGKA)}*

$3145=215(220):
>gi|1063918702|gb|ODS41070.1| {|816(0)|<Euryarchaeota(A)>}hypothetical protein A7315_07060 [Candidatus Altiarchaeales archaeon WOR_SM1_79]
{(YASVVTNVSNAWWIEKKIRLSAELEEVVTPPSTQAASSPGNNPNMTSTVIGIVIIFLLIVFISIYFTRRKG
KEEEPESLL)-RDVFISAASADYDYARQVYDFLTENGVS-
TFYSEESIPNSNdiNFRMEITKALGEAQHMIVVVSSVEYAKSFWVEtEWGFFIKEK-----
RsgrktgNVITLTAGSIQpsDLPPGLR-------GY--EVMPFGEESFEKLLSYVERKVD()}*

$3146=215(220):
>gi|1063918704|gb|ODS41072.1| {|136(0)|<Euryarchaeota(A)>}hypothetical protein A7315_07070 [Candidatus Altiarchaeales archaeon WOR_SM1_79]
{(WALNDKKKIIYLACAGAIAGASAVWGITEGSVEDAEVLIAPLIPIPIALIMLGIAWILTPKYTKKPEKDII
YEKESGSTQ)-RDVFISAKSADYKYARQLYDFLTSHGIS-
TFFSEESLPelADADFRREIDRALDEVQYMIVVVSSAEHARSPWVEaEWGFFVNEK-----
RsgrkkgDLITLTVGSIQpeNIPPGLRS-------C--EVMPFGDESFEKILSYVKRKVD()}*

$3147=215(225):
>gi|1063546485|ref|WP_069407639.1|
{|0(265)|<Actinobacteria(B)>}hypothetical protein [Mycobacterium holsaticum] >gi|1063309037|gb|ODQ85141.1| hypothetical protein BHQ17_24405 [Mycobacterium holsaticum

{(MNCAVTTRPERSSIVA)---VFISYSSRD---
TETVGHLAAALRRGHHDLWMDDELSGGDAWWrAILGKIRSCDVFLAALSQNMLQSKAC-QaEMRYALAL----
-GKPILPVLVGPV-DsMRTNPLAGMQVIDFQQrsIEAWMELD--
EAVRAKQASAPAL(PDPLPAEPPMPFGYLMRLGGQISGSQLTPQQQTVIVAELRAALDDDGDDAGARRDICAL
LGQLRDRQDV)}*

$3148=172(181):
>gi|1063562290|ref|WP_069422026.1|
{|0(342)|<Actinobacteria(B)>}hypothetical protein [Mycobacterium
intermedium] >gi|1063322260|gb|ODQ97656.1| hypothetical protein
BHQ20_25920 [Mycobacterium intermedi
{(MA)---VFISYSSQdRAALEALHTALKRAQQQ----
VWFDQELGGGDAWWsAILEQIRNCEVFIVALSNNWLQSK-PSQaELRYAQALRRP-----ILPVRVGPV-
DSVRVNPVATLQIIDYQnpTVDAGIQLVTAIHALRAKAEPLP(SPLPEEPPVPFGYITRLGNDLAEKELSPQQ
QIQLLVELR)}*

$3149=176(186):
>gi|1063903850|gb|ODS31799.1| {|0(142)|<Planctomycetes(B)>}hypothetical
protein SCARUB_03085 [Candidatus Scalindua rubra]
{()MSDIFISY--DSFDLERTKILAEVLQQQGW-
SVWWDRKIPPGKSFDeVITKALNVAKCVVVLWSRHSVKSDWVKEEASEGAQRK------ILVPALI----
DYVSIPLGFRRIHVAQLvdGKGQMPHPEVNELIGAITTILGH(PRVEKKKPITTEKTDVELLEQDERPEIPKR
KLRSQPIEDLTEDEVK)}*

$3150=238(239):
>gi|1063827423|ref|WP_069437973.1|
{|0(166)|<Proteobacteria(B)>}hypothetical protein [Methyloceanibacter
methanicus] >gi|1063650356|gb|ODR98521.1| hypothetical protein
AUC68_08850 [Methyloceanibacter
{(MRGAGRVVTDMTLKPPGTIEWERGAGMK)---IFLGYPA--EHEN-
IAREVFGLLTAKGDDVWFDKERLVPGMDWdaERDKGQREADL-
AIHLCSDAIWERRGvVNrEIRQSLRLAEDQpfGARYIIPIRIGSF-
NLPVELTRFQYFDFGDGWEEQLSSGVEkRREQISVGAPERS(RQAVTAGRSHEGLRKVEFENITEDHDCRGEY
LNYENEGLYWTYLNGAIAALVLEGYFSALSDFKRLYPDNM)}*

$3151=179(180):
>gi|1063903241|gb|ODS31229.1| {|0(568)|<Planctomycetes(B)>}hypothetical
protein SCARUB_03650 [Candidatus Scalindua rubra]
{(MK)KDYIFISHCT---KDAEFAKTLRKGLEARGLYTRVDSRVSALGEELapEIKQAI-
EQACLFVVIIGANTKNSIHVLkETKYALEVRKRGgDDYKVIPIILEGV-
EPAALSVCFGKEPVRTKIQIGSGGISEAIPQILAAMGERL(SDKQKRSLFADIELSLRRLSPGIREKIKSLGV
FQGGGS)}*

$3152=217(219):
>gi|1064960541|ref|WP_069471538.1|
{|0(249)|<Proteobacteria(B)>}hypothetical protein [Candidatus Marithrix
sp. Canyon 246]
{(MSVYVPQY)QYDIFVSYAHVdnEifagvnkgWVT-TLVDTLKiflaqkiGSANAYSLWLDQ--
ILPGHLAPtYNSIQYLENSAVFLLVLSPAYLKSEQCRLELN-AFLAQVGVDSGRVFIIEYQAA-ERIESL-
SDLRGY-----------T---
FWEADPINARPR(SLSFDSKQYHWKTEDLAIDIVEKLDVLKTDIVPVSSEYTIFLAEVSDDLFFQRDELKRNL
ELQGLRVLPNK)}*

$3153=209(215):

>gi|1064089748|gb|ODT91332.1| {|0(154)|<Bacteroidetes(B)>}hypothetical protein ABS85_12865 [Sphingobacteriales bacterium SCN 48-20]
>gi|1113378396|gb|OJW41660.1| hypothetical protein BGO56_17535 [Sphi
{(MLRK)RFDVALSLAA---ADLEIGEAIKAELAKRRISCYLYTDHD---
NSGEHLMKLtlkYYLRAKliLLIRSENSTGAYWSDIEDKIAKSKR-PSWGSPVLFLRVGNVEvqQ-ADR--
VYLVWSN-NA-EEIADKIR-
SRLWIRQRWLIKM(WMLLLLLISASVGIGFIVKEHITTLPKGVYVPAQTLQRRFWCGGNPVPVPAFRISNTEI
TVRQYRNFCDST)}*

$3154=205(214):
>gi|1064132946|gb|ODU32150.1| {|0(766)|<Proteobacteria(B)>}hypothetical protein ABS97_18140 [Xanthomonadaceae bacterium SCN 69-320]
>gi|1064223726|gb|ODV19010.1| hypothetical protein ABT27_12285 [Xant
{(MSERADSEVAETGDATAEPS)---VFISYSRKDGDRMQALAEGLRTAGFR-
VLLDTTGIRPAEIWRERLAQLIRQCDSVIFLISPDSVRSSVC--EWELDEAER-----
RakRLFPVRIRETAdaDIPGRLLQLNHIVACQ--
DELLQPALADLVESLRTDISWL(REHTVVEEMAQRWRDSDESPARLLRGADIDAVERWRDRRPRTAPNLTEIQ
KRFI)}*

$3155=173(181):
>gi|1065017450|ref|WP_069474527.1|
{|0(127)|<Cyanobacteria(B)>}hypothetical protein [Microcystis aeruginosa]
>gi|1064241403|gb|ODV38443.1| TPR repeat [Microcystis aeruginosa NIES-98]
{(M)--SIFISYSRQdQAYVNKLLEALT----
KHELPWWLDNKIDYGDKWPrVIEENLRKSQVFMLVMSPRSKNSHWV-NcELALALELKKP-----
IFPLLLEGS-
RWLEVGIIQTVDIQGGKlpPASFFDRVRSELNNKVRSKLNHK(TVQLTAEEWFNSAYNKGESGDYQGAIADYN
QAIKIKPDYA)}*

$3156=222(223):
>gi|1064135578|gb|ODU34738.1| {|240(0)|<Proteobacteria(B)>}hypothetical protein ABS97_06045 [Xanthomonadaceae bacterium SCN 69-320]
>gi|1064220552|gb|ODV15872.1| hypothetical protein ABT27_21545 [Xant
{(VPDRRVTYLADCADKLGAPMVFFFDKLLRSTRVIDGSPIEFAVELEARWRSQYGEPDPATVQSEIDAFIDN
LPDEAPPSC)---VFISYSRDD--IARLLPYLQAL-
RSVHVPIWFDKKgLHAGDTYSqEIERAIkERSALFCAFVSRATEAdgTRYVHRERAWAAERHVS-
GRHYYLPFIVDLP-LSEMPRREPDRVATVHYdrLSLMDPAHFAEHVRLRLADKRSQ(AHG)}*

$3157=278(297):
>gi|1064149805|gb|ODU48485.1| {|42(8)|<Proteobacteria(B)>}hypothetical protein ABS92_08400 [Thiobacillus sp. SCN 63-374]
{(ITDHPRLLRSLGFNDDDYPYCVAEVISAIFESDNSLIDEVVDHFDIDLWYQQKEPEKYQRVFSPATLRSAD
FWKDGYLRA)----FVSHLSSnRERMSALKSSLAHWGISAFIAH---
EDIKASREWRdEVEAGLETMDVLVAVVEPGFKESDWCAQEVGYALGR-----KVDIVPLRagLDPF-GF----
FGKYQGIQIKGK----LPD--
QAANEIAQALLNK(PHHRMRLLRCIAKAFASLPSKRKIELINALDSWSTATGEQLKELLEQAALSEFERKSLE
GLIARVGAFTQPEPATQI)}*

$3158=196(184):
>gi|1064963266|ref|WP_069472651.1|
{|0(271)|<Proteobacteria(B)>}hypothetical protein [Candidatus Marithrix sp. Canyon 246]
{(MPVYVPNCKHD)---
IFVSYAHVdNEplidgdhgWVTTLINSLKVLlgKKLGSADAfsfWMDSKLQGNSPATaDIVKQLEGAATLLLI

LSPAYIQSQKCMLELNIFLAQIKE-VNNRVFVVEHDVV-
EEKPAGLDDLLGYKFWLldDYGNPRILALPKPNPEEYQYYQR(LDDLSRQLANQLKILKQEYGNKPVSTSEKT
IFL)}*

$3159=191(179):
>gi|1064963273|ref|WP_069472654.1|
{|0(74)|<Proteobacteria(B)>}hypothetical protein [Candidatus Marithrix sp. Canyon 246]
{(MTYKND)---
IFISYAHvdnepfpgAdKGWVTTFVNGLKnelgrQLGRSDAYKLWMDYDLRGNQPVTaDIDNQLSSCATIVLI
LSRGYLASSWCVLELNTFLAQVGN-GSGRIFMVEHDFV-
PFEERPPEFNDLLGYPFwiKNPDSGRIRTLGIPKPNPDREPE(YYQTLNELARDITTTLNKLKSETDKEPETG
GLA)}*

$3160=183(174):
>gi|1064971692|ref|WP_069472898.1|
{|0(271)|<Proteobacteria(B)>}hypothetical protein [Candidatus Marithrix sp. Canyon 246]
{(M)------
SYAHVdNEplidgdhgWVTTLINSLKVLlgKKLGSADAfsfWMDSKLQGNSPATaDIVKQLEGAATLLLILSP
AYIQSQKCMLELNIFLAQIKE-VNNRVFVVEHDVV-
EEKPAGLDDLLGYKFWLldDYGNPRILALPKPNPEEYQYYQR(LDDLSRQLANQLKILKQEYGNKPVSTSEKT
IFL)}*

$3161=304(299):
>gi|1067073821|ref|XP_018020409.1| {|783(79)|<Arthropoda(M)>}PREDICTED: toll-like receptor Tollo [Hyalella azteca]
{(YFDLSHISAIVKENCIEELENITWLTPSALGVIIACCCGTAVVLFTLMLVFVYRLRYSTSKVNQFKSLPYS
TKNSGDSEY)-FDLYVCHSSEdlPFVRDVLVARfkevspltkifLSSNPESF--------
CSRGSLNIFLTKKNISNSCRSMIVVTKNLLEHEWKSKNFAESHIDGFRALNNSIISLYLDDI-PD-
SLLKEDIRLIISESiKIYWGSDK---
CWNELLelivsYRLPFS(RTSSISPSNDRRSNSSARFYSLVSHIPPSPDKVFRKDYSSKVRHNSGAFDFNDSE
NDYSARPLARRSVVVISDDRKFST)}*

$3162=297(298):
>gi|1067080840|ref|XP_018024173.1|
{|1005(218)|<Arthropoda(M)>}PREDICTED: toll-like receptor Tollo [Hyalella azteca]
{(VLSNSPGHCDHFVATAHMNNLMVNDYIMFSSISVGIIIVILVLVFIGVVYRRRIKLWASNQYSKNLFEKSS
SFVEEREKL)-FDVFISHSNKdsTWVYSVLAPELE--
VRGYRTCAVYRNCssPTASFVAQAMSEGIACSKRLMLVVSRGLVDSEWCKYDFKSTHVDAIKKLSNKNIILVN
LDD-IPKNELDSEMINIIRKAsFsFKPRDPR---
FWERLRRALPSL(RHRRRYEDSTGIGKSVSSPLVVADVPTSDLAKYNCHQNKNLTLNPYWETAITSNVPEWNM
KKPDLGAPPWYQVTSVKC)}*

$3163=189(218):
>gi|1066949192|gb|AOP46147.1| {|0(86)|<Actinobacteria(B)>}hypothetical protein SL103_07755 [Streptomyces lydicus]
{(ME)---------------------VLRALHHV-
PWVDRElESEGGKPWWDRILAEIEACDGIVVVLSPQLFDSVPAERerEYADALGKP-----P--
LPVMVEAVTpdILPSELSSRQFVD-FT--RGGPLAGA--
MLASAVAGLPPA(PPLPDPMPKRPDVPSSYLGDLRKRVRAPALSLEEQIHLITELRLRLDLERERQAVVTLLR
ELQQRRDLCHATWQLL)}*

$3164=199(200):
>gi|1067128540|ref|WP_069488468.1|
{|0(623)|<Proteobacteria(B)>}hypothetical protein [Vibrio parahaemolyticus] >gi|1065343985|gb|ODX82954.1| hypothetical protein BBM12_03155 [Vibrio parahaemolyticus]
{()MIKAFLSHSSAdKDGYVR-----
KVAEWLGKDNIHYDEwTFEEGEKPLdEILSGLGRTDLFVLFLSQNALKSEWVQReILEAKALLSED-
KISKIFPIIIDDmT-TYEDEKIPEWL-
RNYNLkpIKRAVVASKKIQNRLRELSWSKH(PELKKRHNMFVGRNDKQDEFEERIHDFCLDKPTAIFATGLSG
VGRRTFLHRALVKTNVSE)}*

$3165=233(225):
>gi|1067160215|ref|WP_069501204.1|
{|172(0)|<Proteobacteria(B)>}hypothetical protein [Vibrio parahaemolyticus] >gi|1065620255|gb|OEA48001.1| hypothetical protein BBM62_05085 [Vibrio parahaemolyticus]
{(SDCSVYDTKLLDIDLEPFCSNNQNEVSSSYVDWKSVARSLSQPQLSEFLISTGMPELVSLYLIDSVKTLDP
EMLFKLMQK)---VFISYGG--P-DADFARMLKSDLNRNGVQTWFFEDDAEFGQKLhqMMRQNIGKYD-
RVILICSEQSLSRNGVLnEIEQALAKEAREgGSSRILPLATDSF-
VFSEQfkstevgqeLNDRVIGDFSnpENYERNLVRLLStLRIKKPDSIGVYE(NIVPN)}*

$3166=156(159):
>gi|1067137236|ref|WP_069490138.1| {|0(2)|<Proteobacteria(B)>}molecular chaperone Tir [Vibrio parahaemolyticus] >gi|1065200539|gb|ODW44751.1| molecular chaperone Tir [Vibrio parahaemolyticus] >gi|1065
{(MTV)----FISYRHSdrnvaFGINEYLQRSGIPTYMDVLDP------
ESQSTENIteVITKNIAKSTHLIAVISQETAQSWWVPFEIGEATISATRIasfRTGYSQLPEYL---
EMWPQMSQQSHLELFVKEYRGEKSLAVNNRGVFDSARTSK(SYAADFHRNLKNRISR)}*

$3167=223(229):
>gi|1070291040|ref|WP_069713010.1|
{|37(0)|<Actinobacteria(B)>}hypothetical protein [Curtobacterium sp. ER1/6]
{(AVDDHNRKVRAHNQKVVDQFNRQVNSHNQEVRRVNVHNARVVGDLQKQLRSTGSGPRYTPSEQALADRVQE
SLVSRDERE)-WDVFLSYARIDGAdAAEALRSSLEVLGVR-
VWFDEVAIVPGKSQSLQMDQGLRKARAGIVLLTPAYLVGRFWTErE-LGALLRK-----
STLIPVLHGVDFkaVEEYSGILPDLAGFET--ARDDISSIAEKIAAAILPADAT(IEAGSGSDE)}*

$3168=182(187):
>gi|1069981015|gb|OEH91345.1| {|0(593)|<Firmicutes(B)>}hypothetical protein BFG57_05625 [Bacillus solimangrovi]
{()MGNIFISYSSAdTKQMLELVNRLESYE----
LKCWYaKRNIGPGENYAvEIIEQIASCKVFIILISRKAFESRHVATEISLAYRR-----DMLIIPLLIEDL-
AHDNSEWPKDFRYFLETaqMFNGVHLTEEDWQQIVDKICEQL(ELEDNVTITSSKIPEFKLESHKVNGADFLF
SPMEKTKLDFIESVYEP)}*

$3169=225(228):
>gi|1070090465|ref|WP_069705134.1|
{|61(0)|<Proteobacteria(B)>}hypothetical protein [Burkholderia seminalis]
{(SNELEWLMTIHPKIIHMARNEILVEALPAKGQWRKTKDFVVAPVLTLYTDRLAISLEEVVRKFDEVGMSVD
ILSESEQHQ)--TVFISYGG--P-DQPVVDRINEFLVSRGVKTWYFPINKLPGQKLhrMMSEGVKTHD-
RVLVVCSKNSLSRNGVLnEIERVLEREAREgGSEILIPLAIDDH-
MFSDEFRPARLDVDQLKSRVAAIVPDPYTDAAGFEREMSL(LLTALRRG)}*

$3170=172(179):
>gi|1071376734|ref|WP_069808967.1| {|0(196)|<Chlorobi(B)>}hypothetical protein [Chlorobaculum limnaeum] >gi|1070918831|gb|AOS83243.1| hypothetical protein BIU88_03240 [Chlorobaculum limnaeum]
{()MSDIFISYAREdRPKAQQLAE---VLERQGW-
SVWWDIAIPAGQKFGdVISEKLSQADVIVVLWSAISVKRDWVLDEAQIGKKRN------ILIPVFIESV-
DPPLGYGQIHAANLVGWngSPSAPEFIRLVRDIERIAGSPKG(SPASTNVPGDGSAKRGGASVDPIEIKIPQR
MVNLPVLSS)}*

$3171=204(220):
>gi|1071377017|ref|WP_069809250.1| {|0(56)|<Chlorobi(B)>}hypothetical protein [Chlorobaculum limnaeum] >gi|1070919121|gb|AOS83533.1| hypothetical protein BIU88_04870 [Chlorobaculum limnaeum]
{(MSC)--KVFVSYSWSnSAERRALQAEIGELE--
GISVLVDKNYIKPGDQIHaRVAQMIDAADYVVVLLTTEGLSSP----EVRDELSRCHD-RGKRIIPVAM----
--EGTPLEDLPWFLRDVrVIKYNHRN----
FDAVVEELLAL(LHSLVDPKEQIDLTRLPPAIKCKLEKGCRLIVVGNESRESGLEGLLLFELHMRHSDAIFVI
ASDPKFSVELVAEYLA)}*

$3172=219(221):
>gi|1071377697|ref|WP_069809930.1| {|101(0)|<Chlorobi(B)>}hypothetical protein [Chlorobaculum limnaeum] >gi|1070919507|gb|AOS83919.1| hypothetical protein BIU88_07000 [Chlorobaculum limnaeum]
{(SEAILSYATASLTVFGNVDLSTVKGLETISHSYPSTIGIDTLYRSKGNIPESFLRGCGVPDQMIEYARSLT
ATPIQYYSC)----FISYSHKdEELAQRIHNDLQA---SGV-RCWfapHDLKI--
GDKIRpVIDESIRVHDKLLLILSEHSVQSDWV-EhEVEHALDRERiE-KKNILFPvrldesVIDNTA-
GWAGNV--RRQRLIGD-FSRWKDHDSYmRAFARLLRDLKAE(K)}*

$3173=233(234):
>gi|1070919589|gb|AOS84001.1| {|364(0)|<Chlorobi(B)>}hypothetical protein BIU88_07525 [Chlorobaculum limnaeum]
{(LHTEMRMQPVEELELTGEGEQWISVRSLREVEQPENPTQKLPVQPDGTAEVNVPQELDKLVPAEARAIDRD
PATAPIPVR)---
LFVSYAHDdERQLKRLDLMLDVLEQQHHLTSWRDTRLIAGDEWDeEIRNRLEAMDIFLFIASQTSLVRPYIKD
pELKRAKERREA-GEIEIVSVKLEPC-ACdDDAYLGKLQRLAPTFksVAQAPLKS--
TAWEQVRKDLLPV(IQRVRARKNAAMNR)}*

$3174=178(178):
>gi|1071378178|ref|WP_069810394.1| {|0(143)|<Chlorobi(B)>}hypothetical protein [Chlorobaculum limnaeum] >gi|1070919787|gb|AOS84199.1| hypothetical protein BIU88_08680 [Chlorobaculum limnaeum]
{()MSQIFISYSHKdREFVKRLTNDLNV--
NLPDAQVLYDMLIQPGTSWAeTLASQIEQADIVLAVLSPDYLAS-AWAEqELNVALERQLK-
NESFLLPLLIRPC-
NPTGFLSQLTWVDFTEDygAGLANLIWGITGKRPKAAKGEVP(GAPIKAVDPIEVESLRNQLQEAVELFKSRA
AEPLPHQQ)}*

$3175=221(238):
>gi|1071369616|ref|WP_069802243.1|
{|0(338)|<Chloroflexi(B)>}hypothetical protein [Thermogemmatispora onikobensis]
{(MERPCAIAVLVSWSGTVT)----------DGTWHERLLRLLRPLAAQG----W----
IALGDDGPpSIDCAggSSGAALFLALLSPDYLVSPACLAELQQVRQRHDA-GERLLLPIMLRAC-
AWRESVVGDLTPLPADGlaLVSRDDPE--

EGLRQVERGLRCA(LGRLLFERIRQRDAVAAELLLFCSCLAPAPVPESLLTEGLARVSHPLAAMAADRSALMA
ALAALEADGLLARAPQSGNLQ)}*

$3176=225(229):
>gi|1071369663|ref|WP_069802290.1|
{|0(218)|<Chloroflexi(B)>}hypothetical protein [Thermogemmatispora
onikobensis]
{(MAVNLFYLCAR)--------
SDDdERLLDQLKTFLRPLRTAGLIDDWEPRQLRAGQLVReELRRRLSAADIILVLLSPDLLNDPF-
YPeLRQLAWERHQREpGRVRLFPVKLREC-PYELEPFADLVIFPPNGraLHALPPaqRE--
KELKNLALQIRML(AEELAAGEGGQERPGPAAAPVATSFGPVSQAGGSQTAESRRSGSTLSAQRCDVLLVTAT
DVEVRAVREIALREWGARF)}*

$3177=228(231):
>gi|1071370195|ref|WP_069802822.1|
{|0(456)|<Chloroflexi(B)>}hypothetical protein [Thermogemmatispora
onikobensis]
{(MDRTEQPDREANII)---
VFIICAREdYRLLYSLLPLMSYLEVLAGIVLRTSTDLRAGAHIAaELRRHIESADVLLLLISPDLLASP-
TYErAISLGLERAQR-GELQLIPILARPI-PLEGTPLASLQLLPRNGraLSEEIARE--
RALQEIVHEVLLI(CRELRNRRRISPSSPAASSESRTRGLTVPGLPLTSYRLDGVFKRTGLPTVTFVPPSIFE
PLKLALVEPGRGVVIEGP)}*

$3178=221(233):
>gi|1071370311|ref|WP_069802929.1|
{|0(368)|<Chloroflexi(B)>}hypothetical protein [Thermogemmatispora
onikobensis]
{(MGASQSIAGEILCL)-------YASEdKRGFEELSKHLYNLNRIRDI-----
QDTLPGKPPKqEQSKLINQAKLIIMLISPDFLFS---MGeVASMALKRATS-GEAELIPILYKTV-
DLGGTDLAELQLLPRNGeaLAqrEGDNRN--
KAWKEIAEEIRKI(WEALDAGSGTEEKERTTMDAGQSEAHRSQQEQDPTRPATVFISSAGDAGKNREQLELML
RLRGLKLWHRDSEDLPFLGL)}*

$3179=225(227):
>gi|1071373604|ref|WP_069806018.1|
{|0(492)|<Chloroflexi(B)>}hypothetical protein [Thermogemmatispora
onikobensis]
{(MTAAETKTAL)---
VFLCAAARdRRFVEELRNHLQTPALRSRLRCFASLDVPAGRVPSqEVGQAARQAQVAVVLVSAELLSGRNEEH
kELNVLWRRGQL-GGLLLIPVRARAC-PLEGTPLEKLKFMNEKScaELRRAERD--
QLWVRVVKAICAV(LEADEEGQAEAAREMGKEGLEALCARVEERYRRALLGDRSLTLLQVLGMRTAFPIERIY
IRLRLHEKPEIRYLGPEE)}*

$3180=233(231):
>gi|1071373639|ref|WP_069806053.1|
{|0(1293)|<Chloroflexi(B)>}hypothetical protein [Thermogemmatispora
onikobensis]
{(MQSGAGASRSLH)---
VYLSYAGAdQSWGERLARRVRLAAREAGLEAprlSGPGQILAGSDYQqARQAALKSADLILLLISPDFLDSPD
CLAEMEAALRRRRA-EGVMVVPIIVRPV-AWEHTALGELEALPpgQPPkaLSLWRARD--
EGLHRVEEGLRRL(FSERLRFGGPASGAGRLLPEQEQALRAMLIDHRPFINSRLEGFVGRSAELAAIRQQIAG
LQPYGGYLVISGQAGQGKSS)}*

$3181=184(185):

>gi|1072771122|ref|WP_069960991.1|
{|0(918)|<Verrucomicrobia(B)>}hypothetical protein [Lacunisphaera limnophila] >gi|1070532368|gb|AOS43662.1| invasion protein regulator [Lacunisphaera limnophila]
{(MSDPGKA)---VFLSYAS---
QDAEAAKRICDALRAAGVEVWFDQNELVGGDQWdgKIRGQISSCALFVPLISANTqARLEGYFRlEWKLAAQR
THTMaDEmTFLLPVLIDGT-
RDAEARVPAEFRAVQWTKLPAGEATPAFCVRVRKLLDGTA(AGVVDPGLPGSTSPATSRPVPPRRGASKWWWV
LPIFGV)}*

$3182=228(215):
>gi|1079811443|ref|WP_070214200.1| {|0(253)|<Firmicutes(B)>}hypothetical protein [Listeria monocytogenes] >gi|1071570815|gb|OEP32286.1| hypothetical protein AJM39_09480 [Listeria monocytogenes] >gi|10
{(MTQIKNP)--HVFISYAQ--
EEDTKInVEHFATKLMSDGVNVTIDFWDLKEGQNMyrFMEKCVSDPSIdFVLLLCTPLYTKKandnaGGVGtE
STIIMPEIYNNaEQTKFLPIIFsrdesNNF-
QKPYFLNSLIHFDLtNNNYEKEYQKIIRtLFGKPEQQKPEIG(NPPTYLNTQKKQLKTDKVRLSYSNINYSNI
ENRLQFFYDLKDDLDIFDIEYDKIKEQDLYTLLNQKYI)}*

$3183=208(203):
>gi|1071378623|ref|WP_069810829.1| {|0(375)|<Chlorobi(B)>}hypothetical protein [Chlorobaculum limnaeum] >gi|1070920225|gb|AOS84637.1| hypothetical protein BIU88_11130 [Chlorobaculum limnaeum]
{(MNQKT)-YRAFISYSHAdekqaSWLQKKLESYTIPAQIRKAYgeiipkhlrPVFRDKTDLRPG-
NLgkLISSELDDSGYLIVVCSEASKQSTWVNREIEHFRQAGNADriiPVVTG------------
CKQQRDITDCYPAALDKSNQLGISLHTLGKTRTLLSI(IATILDIRFDDLYKRHQKAQKKKLVLLVLLSLLIL
LIASAPAREIFRLWQQQQALTSA)}*

$3184=209(207):
>gi|1071969265|ref|WP_069911875.1|
{|0(531)|<Proteobacteria(B)>}hypothetical protein [Devosia insulae] >gi|1071465544|gb|OEO28841.1| hypothetical protein VW23_002835 [Devosia insulae DS-56]
{(MAETVTETTGGGAARNGWRAIARALSRAVRTVRQIPRAIADLIFG)-
YDFFISYAHAdgheypRLLRERLEDLPAR------
YRVFYDKTGFVAGGSLtfETRRRIRMSTHLILLARPAAlNTSHWVGKEVELFSKLDRNMftvEFGDLISGLSP
---SRPIAAFLQDPITIRETPGANSPSESVIADLNRSFEGVRQ(ETRRVRFLALAALFFLLLASFA)}*

$3185=274(300):
>gi|1072967959|ref|XP_018434070.1|
{|743(123)|<Streptophyta(V)>}PREDICTED: disease resistance protein RPS6-like [Raphanus sativus]
{(LESFPHISRNISILVLDETKIEQVPGRIEDISGLNYLLMTGFSNLKYISSNISKLKHFEVMLFTVDTRTDD
SGIVDNRKN)--DVFLSFHR-EDVRKAFVSYLHKEFM-------------RDQN-
LatNVVNEIRSSRIAIVILADNYLSSERCLDELVEIIECSEE-TGQKVIPVYYDVDTVNITRQIQDI------
---VKTESYP-
QKLRQALTTAAEL(QGYKFSTWDPETAVRQIRADDISFAPKELFDMETLKEQFNGLEWKEKVLFCHIACFFNN
ETYENVTQLLKDCGSDVGGSL)}*

$3186=261(294):
>gi|1072921432|ref|XP_018471327.1|
{|0(1196)|<Streptophyta(V)>}PREDICTED: disease resistance protein RRS1 [Raphanus sativus]

{(MDSSFSLLGICAATIGFVTILVKLLRPVYRRFRSNRRNETVASSPQSSPNFSTPSSDFEAVPNRLLLRAVY
IYCE)-----------ETLQYTFASHLSVDFRRKRIAAFVNCDR------NpD---
ATEGASASVVVFSESYLSSTSCLDKLVTVLQCRRKTGQ-VVVPVFYGVSPSDVAMP------------
EHGSADRIR-
EWSSALKELRDL(PSHQCSEESNECQVVEEIVKDVYEKLFPTEQVGINSRLLEIEQLLCKQPWGIRRIGIWGM
PGIGKTTLAKAVFDDISGG)}*

$3187=206(226):
>gi|1072940067|ref|XP_018480581.1|
{|0(1241)|<Streptophyta(V)>}PREDICTED: disease resistance protein RRS1
[Raphanus sativus]
{(MVDGNA)---VCISFnRW-EDTiRHSFISHLSAEFRRKSIYV----------S-
TcqNSDEAIAEAKVSVVILSEKSVSNYRFLNELVKVSACRSS-HGLLVVPVFYGLTKSVLRKYCLLLKKMY--
-----PEDPV-
ADWRNALLEIADL(RGYASSLERSDSELVEEIVADVRQKLDLEGTIGVYSRLTKIQNLIHKQPLGIRSLGLWG
MGGIGKTTLAKAALKQYSGDF)}*

$3188=217(234):
>gi|1072947127|ref|XP_018484189.1| {|0(199)|<Streptophyta(V)>}PREDICTED:
putative disease resistance protein At4g11170 [Raphanus sativus]
{(MTSSYSFLLAGRDL)--DVFLSFSG-KKALDIDFGY---DLSRNGIKSFKSESWKEKslKP-
IdrHTLEALTESKVAVVMTSDEEASSVGFLEELLVILEFQEK-RSLTVIPIFLTKHALDMEEVCQVF------
------PERA-
RMWRTVIAKLENI(ATQYSVSRNLALIHGTHRIKQVADDIRLMFLSCASSDFKGLAGMDRHLKAFHELLALES
DKQVRTLGIWGCAGVGKTTLA)}*

$3189=222(235):
>gi|1072991244|ref|XP_018446358.1|
{|0(1000)|<Streptophyta(V)>}PREDICTED: protein DA1-related 4 [Raphanus
sativus]
{(MEFMKPKAARVLPPMMSNNSHSVNII)--------CE-
ESVQYSLVSHLSAALRREGISVFVDScGFQETKP-FsvEQNQAVtDGARVSVVVISnMVELFDPWVL-
KFLKVIKRRRN-SGHMVVPVFYGVDP-----LNRVYGWANRRLEAEKLTSHQ-
SRISSSRILTDSE(LVEEVVRDVYGKLNIAERVGIYSRLLEIENMLYKQQRGVQSIGIWGMPGIGKTTLAKAV
CDHMSTDYDA)}*

$3190=205(226):
>gi|1072999950|ref|XP_018450892.1| {|0(842)|<Streptophyta(V)>}PREDICTED:
disease resistance protein RRS1-like [Raphanus sativus]
{(MGHANA)---VYISFNlC-EDTiRHSFLSHLAAGFHRRGISFNEPDRVPEVND-A----
AIVKSKVSLVILSKKYASSKGCLAELVKIFKCQES-NGLVVVPVFYGLTKSGIKKECLKLKKMCRVA------
----
RQALLELGDL(PGHASSPDISDSELVEDIVADVRQTLDLTGKVGVYSRLTKIESLLRKQPWGVRSLGIWGMGG
IGKTTLAEAAFEQQLPSG)}*

$3191=199(219):
>gi|1073009441|ref|XP_018455893.1| {|0(532)|<Streptophyta(V)>}PREDICTED:
disease resistance protein RRS1-like [Raphanus sativus]
{(MAACDK)---VKISFdrSD-ETIRCSLVPHLSAAFCRKRISVLTDNHDE-----------
FCECIASVMIFSKNYATSKECLEDFLKTSQRRHD-KGHVVATVFYGVSSR--SDLKRNFAKALFEHC---
TSYQT-
SQWLSAEEIITLP(GHEKISNQSDCEFVEKIATDVYEKIFPKERIGIYSRVLQDIGNLLCNQQWGVKSIGFWG
MPGIGKTELAKAVF)}*

$3192=293(291):
>gi|1073010275|ref|XP_018456318.1|
{|84(476)|<Streptophyta(V)>}PREDICTED: uncharacterized protein LOC108827425 [Raphanus sativus]
{(TTHKPSSPPPEDVPSSSYTPPSDHYEFSDDPSDRKLRLSACTPDPALPRISFSFPVPRVSLAKVSVSSPAT
TNTTKLRSS)--DVFIGFHG-QNpnlVR--FCKWLKSELELQGIACFVADRAKYSDT-
QshEIADRVICSVTyGIVVVSCSSLLNYLSLEE-VRFFAQKKN-----LIPIFYGTGPSEIMGLLNC--
NAIDKECKEaidglikghefKLEANE-
SNWRSCVGKTAMI(LRGKLGRKSVVDKEMVERVDELPFPRNRTFLGREKEMMEMEMAFFGSSGEYLESTTPPS
SKGEASGQSEGL)}*

$3193=246(263):
>gi|1073017644|ref|XP_018460124.1|
{|429(864)|<Streptophyta(V)>}PREDICTED: disease resistance protein RRS1-like [Raphanus sativus]
{(IIKFTYDALSGEDKKLAHHYISSFRDVELTQRMEEFLRKSLSEVRPKLTITNQDEKGNVNTSERPSKGTSS
NGLLHTVYI)-------YCA-DTLQYSFASHLSVDFHRKGI---------EASV-
NcnETLDVIEGASAFVVVLSKNYLSSPSFLDKLVRVLHYRRK-
NEKLVIPVFYRISPSDVAAALQGTIALQELRDLPGIHSREESIDCELVEEIVKH(VYEKIFPTQQIGINSRLL
EIKHLLCKQPWGIRRIGIWGMPGIG)}*

$3194=267(285):
>gi|1072921693|ref|XP_018471465.1|
{|176(103)|<Streptophyta(V)>}PREDICTED: probable WRKY transcription factor 19 [Raphanus sativus]
{(AGDFVFLQRLYTDSSRLFIGFRRRQASALGQCQDLTSLATSSEQLKEVETPPQLRSHGRHVTELDPVLVIS
CGENQDSEE)---------------RYFISYISKELCLRGFTPLIYDLTrSTLTA---
GLEMLHSSRVGVIIFSKNYVCSRQCQDEFVAIMDHSKA-
NNLVLLPVFFKVEVTDIRGQSGSFGRAFSRLEYSVLASQAPYLTSINKYQYKKG(DEVLLAKNIVRDVCFLLN
PDTNMNLRGRLQMKSILSLLNFSQFSAPHIVGLWGMAGIGKTTIARE)}*

$3195=283(285):
>gi|1072940069|ref|XP_018480582.1|
{|70(911)|<Streptophyta(V)>}PREDICTED: probable WRKY transcription factor 19 [Raphanus sativus]
{(VLTKGWSNYVKEKQLGVGDLVFFQSFYTDSSRLLLIGFRRSQAALGQCLDLTSLATSSEQLKEVEILPQLR
SHGHRVTEL)-NPVLVISCG-ENQdseERYFISYISKELCLRGFTPLIYDLTrSTLTA---
GLEMLHSSRVGVIIFSKNYVCSRQCQDEFVAIMDHPKA-
NNLVLLPVFFKVEVTDIRGQRGSFGRAFSRLEYSVLASQAPYLTSINKYQYKKG(DEVLLAKNIVRDVCFLLN
PDTNMNLRGRLQMKTPHIVGLWGMAGIGKTTIAREIFGTQAERYEVC)}*

$3196=206(209):
>gi|1073027134|ref|XP_018464664.1| {|0(11)|<Streptophyta(V)>}PREDICTED: disease resistance protein RLM3-like [Raphanus sativus]
{(MLSKVLRPSQTPYSSLTDQTSAVSDPCWRFE)-WDIFLSFQR--DRSHSFTNRLYVALI-
KaQVQVWKGDVE-RKNQELgpSLVEAMEDSVSFVAVLSPGYDKSYWCLEELAKLCDLRSS-
LARPLLPIFYEVLSKVLRPPQTPYSSLTDQFDRSRECLTMNEQTKAVTEAFVRL(HKEGLIYEEIGLFIVAVN
FSETALSDRKVDHIDIKRRT)}*

$3197=203(187):
>gi|1072829347|gb|OEU43702.1| {|0(9)|<Euryarchaeota(A)>}hypothetical protein BGV40_02710 [Methanosarcina sp. Ant1]
{(MMPF)KYSCFISYCH-GqyDLVNAFIEQIKEALQ-
CSIESYSDqevyiDERLGPGYHYneELAQAICQSTCMIVIFTPRYKSHSYCLREYIAMerlekkrleLLADKS

NNMGMIIPIIFRGDESDIPPRIRDCIHYYDFRDFALSTLEIKRNPKYEPEIEKIA(KIIHRFSKLFKEQNINP
CECDSFKLPSEREIESESWGEKSSNS)}*

$3198=207(189):
>gi|1072856451|gb|OEU58226.1| {|0(5)|<Proteobacteria(B)>}hypothetical protein BA862_08560 [Desulfobulbaceae bacterium S3730MH12]
{(MSL)KYSCFISYCH-
GqgNLMKSFIDQLKRALEDYlepwlDEKVFIDEDRLQPGYHYndVFANAICQSICMIVVYSPKYERHSYCLRE
YQAMesieknrlelLGDKSFPGQGMIIPIIFRGEKEDIPLKFENCIHYSDFSKFTLASIDINTNPEFIIQIEK
IA(KYIYKCYKNLNEQTDICSECNSFKLPTDEEIQPWREQPKKQMVFPG)}*

$3199=156(161):
>gi|1072937211|ref|XP_018479161.1| {<Streptophyta(V)>}PREDICTED: protein PHLOEM PROTEIN 2-LIKE A8-like [Raphanus sativus]
{(MENRP)--QVFISYRG-KDLRLTLVPHIKHHLKDSNVNVFTDSNA-
AGEQLKELFNHIKNSRIVIVIFSISYLESRWCLDELAEIRNCLLR-
KQLdFVLPIFYKVKTSHVEKQSGDFGKPFPSL-
QKKHPRPGADPHRHWVWHVPHT(NSIYCFMKVYLTIRIV)}*

$3200=294(295):
>gi|1072944923|ref|XP_018483051.1|
{|2369(0)|<Streptophyta(V)>}PREDICTED: uncharacterized protein LOC108854054 [Raphanus sativus]
{(GANHYGAYSKNVTISNVRREKETMNRRLDGGHDIVELPNKMISHGIASTPKRPNNKSPVTILKQIGRETQV
RALSVRFTG)KPRVYISCHG-
GDLCITFVKHLVNNLTNAGVKVFIDNDERMWIKMNQLYNRIEDSRIAVVIFSKRYLASGLCLNELAKMDELAK
E-GKLLVIPVFYQVISSDMKNLKGECGRCFREMRKRHED-
EPNnvRKWETSLMSMAET(IGVHSEIHGIDAALVKATVKAVHREITKIAGGKFKSLGRGFMLRARVFIFALFA
ALLFSLFVSPLIFTDATFAAF)}*

$3201=175(187):
>gi|1072971109|ref|XP_018435762.1|
{|0(2022)|<Streptophyta(V)>}PREDICTED: uncharacterized protein LOC108808059 [Raphanus sativus]
{(MTNWKETEQT)---VYISC-T-DEVRYSFVSHLSDALRREGISVFVDSDDLFSEEAQE---
KVERARVSVIVLPGNR---NVCLEKLTKVLSCQRV-
NDQVVVTVLYGDSKWHHEWLNALDLRGLSPVYHQSRKECNDSELVEEIARDVSE(KLSHMGRIGIHSKLLEIE
NMVNKQPLGIRCVGIWGMP)}*

$3202=230(228):
>gi|1073008026|ref|XP_018455182.1| {|0(34)|<Streptophyta(V)>}PREDICTED: protein PHLOEM PROTEIN 2-LIKE A8-like [Raphanus sativus]
{(MVDPARPRP)-PQVFVNSGA-
AELPDDFIKHLVSGLADLGIlNVFMDRDEWWGRDLDRIFTCIEESTIALVIFSPCYPEAEWCLDELVKMKERA
NN-
NKLLVIPIFFNVSKNDVRNFEGEFGDRFVELRKRYtkYKYDPFrvQRWETSVMTISRM(EPSLTWETQSSSIP
LAMDIIREVKKELDIASDPGFSLPLEFHNDSHSKLTNGEVFVSAFLVAFLFRLFIASLVFTDMTI)}*

$3203=187(199):
>gi|1073009432|ref|XP_018455889.1|
{|0(1990)|<Streptophyta(V)>}PREDICTED: disease resistance protein RRS1-like [Raphanus sativus] >gi|1073009435|ref|XP_018455890.1| PREDICTED: disease resistance prote

{(MTDCEKGEQF)---VCISCV--EEVRYSFVSHLSEALRRKGIDDVIevdDSDDLFSQESQA---
KVEKAKASLLVLPGN---STICRDKFVKVLEcCRRN-SDQVVVPVLYGDSP-----
LQGEWLTALGLRGLSPLHQSREECSDSVLVEEIVR(DVYEKLFHMGRIGIYSKLLEIETMVCKQPLGTRLVGV
WGMPGIGKTTLA)}*

$3204=206(205):
>gi|1073004917|ref|XP_018453491.1| {|0(262)|<Streptophyta(V)>}PREDICTED: probable disease resistance protein RPP1 [Raphanus sativus]
{(MT)-HEVFVNFRG-NDMRKTFVSHLLVSFKTSCITSFIASEPLEDTDHE----
AMENSLVAVPVISKSYSVSEWMVKDLSRMIEYEKI-
GTLRLVPIFFQISPLDILKQIKKYvevqGGNFemvrrWMY-
AQVSRTSSFHSSDWEDDSELVD(KVTEFVSNILISSGRSRSYYSTGLSPVRSLKLENEFRSTPTTTCHIDTNQ
RSFPIGFRFMPDD)}*

$3205=276(299):
>gi|1072915255|ref|XP_018468151.1|
{|572(625)|<Streptophyta(V)>}PREDICTED: probable WRKY transcription factor 19 isoform X1 [Raphanus sativus]
{(VNRVFPRVKINTSLSDFKNLVHQLTGLHSSVSTISNRASLQEDRLNIDLNKSPYINVNDAPFSDVFAPIST
CLTYSASKD)-YDVVIRCGR-AVIRNNdFTSHLRAALSRRGISVHEDFDEVD--------
DAVPRCRILIIFLTSTYVPS-----SLLNILQHQCN-EPRVVYPFFYGISPSDL-----ITNCKNY-E-
RFFLQYQP-
KEWQAALKQIAQM(PGYILTAKPETELIDEMVRDALKVLCSNDKEKMIGMDKQVDEIVSLLCIESPDVRSIGI
WGTVGVGKTAIAEEIFRIIS)}*

$3206=272(297):
>gi|1072918838|ref|XP_018469962.1|
{|35(614)|<Streptophyta(V)>}PREDICTED: probable WRKY transcription factor 19 [Raphanus sativus]
{(SRPRNRRPPVFIEANASDFRDVVHQLTGSHISVNLQERELGIIGFNESQSINVNDDLFAPPPPITTSATAT
ATFPASSKD)-YDVVIRHGR-N--NYDFITHLRAALSLRGISVHEDFEEET--------
DAVPGCRVLIIFFTPTYLPS-----NLLNILQHQRK-ESLVVYPVFYGISPSDL-----ITNSKYYES-
FVPQEDEP-
ERWQAAVRQIAHT(PAGYILIDKYESELIDEIVKDALKVILSTDNKKMIGMDMKIKEILSLLCIESPDVRSVG
IWGAVGIGKTALAEKIFR)}*

$3207=280(299):
>gi|1073002768|ref|XP_018452361.1|
{|69(625)|<Streptophyta(V)>}PREDICTED: probable WRKY transcription factor 19 [Raphanus sativus]
{(RLTGVDNSVLELEFISAAFGPASRDYQVVTIYRKGDISNVDFNKVVRRLTRNHNNSASWPLNNSCKYLQEA
VLSTPVASE)-YDVVLRYRR-GGCisNDNFVAHLRAALYRRGVSV---REDMDDE----
VNAAVPECKVLIIFLTSTYLPS-----NLLNIVEQQGK-EPPRvvVYPIFYGLSPSDVISNSNNY-ERLFHQ-
DEAKR------
WQAALEEITQM(HGYILTDKSESELIDEIVRDALKVLRYNNKKNTIGMDMQIKEILSLLRIESPDVRIVGIWG
TVGIGKTAIAEEIFHRIS)}*

$3208=266(256):
>gi|1072987432|ref|XP_018444379.1| {|0(149)|<Streptophyta(V)>}PREDICTED: uncharacterized protein LOC108816297 [Raphanus sativus]
{(MNYFKNFKGWRMFHPACCLRRTGPGDRQMERTNQDLK)---VFISFRG-
EDVRRTILSLLKNKLKDEGVNVMTDEDMDRGKKIDeNLLKLIRDSKVAMVIFSENYSQSHWCLDELVEIEKQM
EL-
KNLDPLPIFFEVKASHVALERHnPFKDILLRLeddeRETARTVGRKalkdadkrfVRWRRALKSITGI(SGLK

YIKDSNEALLVKDIVKTVKKMLGEVESTDDVHDQIRAQHIVPQQKVFISFGGHDDDTRPGFISDLQAGLERSG
IN)}*

$3209=266(256):
>gi|1072990358|ref|XP_018445913.1| {|0(138)|<Streptophyta(V)>}PREDICTED: uncharacterized protein LOC108817662 [Raphanus sativus]
{(MNYFKNFKGWRMFHPACCLRRTGPGDRQMERTNQDLK)---VFISFRG-
EDVRSYIFSFLKEKLKDEDVNVMTDEDMDRGKKIDeNLLKLIRDSKVAVVIFSENYAQSPWCLDELVEIEKQM
EL-
KNLDPLPIFFEVRASHVTLEEHnPFKDILLRLeddeREKVRKDGKKaledadkrfVRWRGALKSIAGH(TALT
YKKDSNQALFVNDIVKAVKKMLGEVRSSDDVHDRIWAQHIVPQQKVFISFGGHDDDTHPCFISYLQAGLERSG
IN)}*

$3210=200(220):
>gi|1072990360|ref|XP_018445914.1| {|0(149)|<Streptophyta(V)>}PREDICTED: protein PHLOEM PROTEIN 2-LIKE A8-like [Raphanus sativus]
{(M)----------------------------------
TDEDMDRGKKIDeNLLKLIRDSKVAMVIFSENYSQSHWCLDELVEIEKQMEL-
KNLDPLPIFFEVKASHVALERHnPFKDILLRLeddeRETARTVGRKalkdadkrfVRWRRALKSITGI(SGLK
YIKDSNEALLVKDIVKTVKKMLGEVESTDDVHDQIRAQHIVPQQKVFISFGGHDDDTRPGFISDLQAGLERSG
IN)}*

$3211=178(177):
>gi|1072862861|gb|OEU63400.1| {|0(212)|<Proteobacteria(B)>}hypothetical protein BA867_13150 [Desulfobacterales bacterium S5133MH16]
{()MKPVFLSYSRKnVKDVQEIADTLCA----
GGIRIWQDIESLTAGNTDeQIRKAIqEECAVFFLYVTDESVKSEFIREvEIKEALNKSKK-
DETfHIVPLIDMPL-
EKANQKLQGSMLGDISRynGVIIHNGETLFEASSKIRKILLK(AIIKRPADQDFTISLMTYSRTPGNIQPQLD
LDWSRLN)}*

$3212=186(178):
>gi|1073592749|ref|WP_070017317.1|
{|0(227)|<Actinobacteria(B)>}hypothetical protein [Streptomyces nanshensis] >gi|1073062348|gb|OEV11102.1| hypothetical protein AN218_14570 [Streptomyces nanshensis]
{(MAR)-PKIFISHSSGkcgdEgcecaAYRDALAAHLEELG----
CDTVVDRDvLRGGDEWHrKLMVELLRSQGAVVLLSRHALDS-YHVMeEalLANALREANQ-
QRFLLLPVVLPGV-
RRADLQGSRLDGIGLGRldMVDWSPAAGPAVPPPEVAGIVRP(LVERHGALPHPRVTEYVASRIEGLSDHALR
QVAQE)}*

$3213=250(257):
>gi|1073591621|ref|WP_070016250.1|
{|190(789)|<Actinobacteria(B)>}hypothetical protein [Streptomyces nanshensis] >gi|1073063865|gb|OEV12299.1| hypothetical protein AN218_09070 [Streptomyces nanshensis
{(RQPAPRVGSLVRWIDAQIASSVFGPTYDRQWYWAFLVGAAALLAGGCSLLLRDALLRAGTAADPFGAASGP
YEAPSEPTS)--KVFLSYSRKdTASARRLSAGLE--ERLG--
ELWVDwQAINPSEEWReSIARAVRGADAFVVLLSRDALTSTHCWDECRQAIELRK-----RILPVVIDPE-
LERGSTSALMRERGWGEltAFQKLSLAEPDEEQLAQGIEDIL(AFVHQHHRWVAFHSRLGVLAHQWWESGRVD
GLLLRAD)}*

$3214=175(182):

>gi|1072886546|gb|OEU83319.1| {|0(61)|<Proteobacteria(B)>}hypothetical protein BA865_04350 [Desulfobacterales bacterium S5133MH4]
{(MSLV)----FISYSHAdsdlaDSICEVLSDCGVEH--------
FRDVKDIPWGGSIpsEVRDALERSIAVLVILSEHSLDSHWVPYEIGFAAALR--KrllPFVSESSLRLP---
LYISDLSHVTDLAQVREFFGEGGLQDAAIATSPGVPPTLE(AVFSKLLALMPALFAEMKADLNQEPLVREFVI
SPNKNV)}*

$3215=187(195):
>gi|503985601|ref|WP_014219595.1|
{|0(47)|<Bacteroidetes(B)>}hypothetical protein [Niastella koreensis]
>gi|361060690|gb|AEV99681.1| hypothetical protein Niako_3372 [Niastella koreensis GR20-10] >gi|1
{(MSSF)----FISYSRKdLAFAETLRKHIQLMD-SGHDVF---
LDLYKIKTGVrwkqYLLSSVRKNDFFILILSNNSAISTFVQQEV--KWVQQDE-
LKTGVRKLFIIRIDDVPIPTYMSTFQVLNAT--
GNFVIDFYKLMEGINGKASYF(KIMHEKESDSPTGYWIKLYVDAPLPFLQKIEKVEYRFDYEFQCSKFVDAVE
)}*

$3216=158(162):
>gi|497222183|ref|WP_009536445.1| {|0(661)|<Firmicutes(B)>}hypothetical protein [Oribacterium asaccharolyticum] >gi|361958951|gb|EHL12248.1| hypothetical protein HMPREF9624_00555 [Oribacterium asaccha
{(MAN)---IFLSHSSKdKErYVRIVAEKLEK-EFDEHSIHY---
DEHTSEAGIktleEIKKSLNKTDLFVIFLSRASLNSEWVQKELFLARESHII-
KRIYPIVIDVNLDCKDEEIPDWLKEYNLRYI--
SKPSKAVQLIRKRLIEITWEQ(TPEMVYRDEVFVGRNTQIQ)}*

$3217=154(158):
>gi|503983358|ref|WP_014217352.1| {|0(123)|<Bacteroidetes(B)>}TIR domain-containing protein [Niastella koreensis]
>gi|361058447|gb|AEV97438.1| hypothetical protein Niako_1059 [Niastella koreensis GR20
{(MGIQSGIQTDIKNE)---IFISYSRRdMDAANRINTVLT----
EHKIAVWYDQLISPGVNWReAIVSHLSQAKVMLILLSENAQKSNELKReLALASSLSVPL-
LGVRLTNVSLTGA-FAYELTGLNWFDVFEDEekGYRQLAIFLEKLVNDTSTVRSDS(EMLI)}*

$3218=177(184):
>gi|503986017|ref|WP_014220011.1| {|0(77)|<Bacteroidetes(B)>}TIR domain-containing protein [Niastella koreensis] >gi|361061106|gb|AEW00098.1| hypothetical protein Niako_3804 [Niastella koreensis GR20-
{(MLNKY)----FISHSTAdNRFIEE---
EIIPLLRAMNIETWYSKnDIRSATEWErSIKYGLEHSDGVILIMSHNSSKSDYVKDEVSWAIDKR----
PGKLLPVMISDC-
DPIDFHIRLPRIQYINYkeDERTARINLIGLLKSLENAQKRL(FTIKGKWTGHAFQIFGNEALESPLTLEVWQ
IDNNSISGN)}*

$3219=155(161):
>gi|492832124|ref|WP_005986078.1|
{|0(25)|<Actinobacteria(B)>}hypothetical protein [Actinomyces graevenitzii] >gi|365258742|gb|EHM88748.1| hypothetical protein HMPREF0045_00961 [Actinomyces graevenitz
{(MTPIQLVTLKACED)---------HKdAQSFLALaETRLKTIKNRD-
FAWWDRTLILPGEDREsETAKHLSEAHYVILLVSPNLLAAMKDRNvPRIGEIEHKLL-PVMLVDVPLDGSR-
EFHQIDRRQIYRGLPGEacSYDCLENDPQRNRFVNGFVSSMI(RRIDKEH)}*

$3220=162(159):
>gi|492832126|ref|WP_005986080.1| {<Actinobacteria(B)>}TIR domain-containing protein [Actinomyces graevenitzii]
>gi|365258743|gb|EHM88749.1| hypothetical protein HMPREF0045_00962 [Actinomyces graeveni
{(MSDNIQ)---
LFLSWAHDdAKLKESFLKLLKPrLRnaRNHVFTWWEDSFILPGEEWKdEILTQLAEADYIVQLISPSFLASDF
IRDyEIPGVGEAPLK-KTLPVMLVDVPLD-
GSREFHQIDRRQIYRVLpgEARSYDYLENDPQRNRFVNGFVD(AIVARVEGKGGYR)}*

$3221=208(205):
>gi|496523771|ref|WP_009232054.1| {|0(13)|<unknown(X)>}hypothetical protein [Actinomyces sp. oral taxon 849] >gi|365266223|gb|EHM95935.1| hypothetical protein HMPREF0975_00034 [Actinomyces sp. oral ta
{(MTPV)--NVFLSY-NEdKVGRQvatELKEALSTVHINAVMAR---
HNIAPGAEWEpTIRHYLEESSALLCVATSGFSTRPWC-QqEIGWALARH-----APILWIQYEakevPC-GF-
---
LASQQALipdePKDqsqvAlsVMEWLADKSNTRDDLADTLLTAL(EKAEHYDDARSAAELLAILKSLSAEEWC
RIERAANNNRQVGNAYVWTGNYFVPMEQQPRVL)}*

$3222=212(220):
>gi|504098191|ref|WP_014332185.1|
{|72(0)|<Proteobacteria(B)>}hypothetical protein [Sinorhizobium fredii]
>gi|365183685|emb|CCF00534.1| conserved hypothetical protein (plasmid) [Sinorhizobium fredii H
{(SKRAGYAKDIAQKNTELLKARAALSKEEGKQRSKVAKDIERQQASQLRRQKDLEDRVVNGMVAKMVPGGRT
NETPLEQYD)---VFISHAW--
EDKDDFVRELAAKAIAADLKVFYDEKTLKWGDTLrrRIEAGLANSRFAVVVLSA-
AFFKKEWPQrELDGLFNL--EIeGKSLILPI---WH-
KISKDEVIKNAPSLSGKLALNTASLSVDEIVERLVEIVRN()}*

$3223=250(246):
>gi|496523774|ref|WP_009232057.1| {|0(690)|<Actinobacteria(B)>}molecular chaperone Tir [Actinomyces sp. oral taxon 849]
>gi|365266163|gb|EHM95876.1| hypothetical protein HMPREF0975_00037 [Actinomyces
{(MKHDLAKNMTSMFKAQWMRWKHRRVGN)-
FDVFISYSHDpsktlaFNLHDSLEKISRNWYSFRALNVFLDRSSLGPTMKLekELESIVKRSEWLVFICSENA
AESAPVIKELNWYIEHNGVEkliLIVDDGAIRWD---RNTSSFSKT-TTNIPGDILEKIKDEP--
LWIDLRWRNQAQ(ESFENHPDYLDAVLSISSRVQNTSKKSLLDRENKRQNRLVRFRRVSFSILTILLTISVAV
SWYAVYARNEALSNLNVAT)}*

$3224=241(238):
>gi|496321107|ref|WP_009030285.1| {|0(539)|<unknown(X)>}TIR domain-containing protein [Bradyrhizobium sp. ORS 375]
>gi|365287824|emb|CCD95369.1| hypothetical protein BRAO375_4180024 [Bradyrhizobium sp
{(MRFALTHSRSTAMSWPVGTKQVSASPISRQLGLFFVKIGLTWIAAQRWIDAWWG)-
YDVFIAHRRAdaagyaSALHSQLE--AEKISS------
FVDRVVYGPGDSLlvATERHVAKSAILLLVGSPELLKPRqpvdWVEKEIETFLRTHRADpkvIIVDFGNAIAD

ALVGRCTTVMPDHPILKQVASFLRVSEPLSGLQLSPSGEV(FAAIRQKLDGRRRDRTRLWFFEAVSGALALLL
VLAIVLGVVAEF)}*

$3225=291(292):
>gi|1073547783|gb|JAT91003.1| {|16(21)|<Arthropoda(M)>}hypothetical protein g.4324 [Pectinophora gossypiella]
{(NDPTVSKDEIVSKSIFLPDNYFAVICGVIIIIVVICLIGAIFYAFRYEVSDWLYTNYGVPLFKEQSCPNVD
HVLEGNPNH)MYDYYVICNTKdtNFVYHNIMSELE-----FRKMSSKKFSPNEPTLNI-
LTldsfsKSSKLSKRLIIILTTHFICNDLCDIHFKNIFYSYLKSLnrsdMNKVIFIKLVDN-
NQINDELCFVLDKFKNIswN-----DPR--
FWERFISLLNST(DTIITVKGSENSVFKKSLRQTPTLRYTTMPVSNDSCSKQNFTNSLQYCKRTDGESSQSES
SPSSDNNTYGDG)}*

$3226=236(239):
>gi|1073708485|gb|JAT97299.1| {|941(0)|<Arthropoda(M)>}hypothetical protein [Amblyomma aureolatum]
{(FDASSCGANGTGWAEAPASPSPPHPLPGLVPLLIAAPSALVLLLLGAVLAACYRRQVKLWLHSRCGLRLFD
RASEAPERL)-
FDAFVAYCPKdePFVAQVLAAELEGCGGkkrPLRLCLRHRDLAVPGCVAEAaVVEAVQCSHRTVVVISEQFLR
SEWCRFELKAAQASSAR----RLIAVLLDDG-GQ-DQWDLEARQCLRAAlILRWGERR---
FWQKFRYALPDP(QRPQRGSEPAARANAVKLV)}*

$3227=273(263):
>gi|1073552015|ref|WP_069986747.1|
{|74(288)|<Firmicutes(B)>}hypothetical protein [Massilioclostridium coli]
{(AEAHWCLALSRYGIEYVEDPTSHERIPTFHRMSYDLIMNDADYQAALQYSEGITRSLYEKEGNRIAEIQKG
MLAISQQED)PYDVFICYKE-
TDEKgertqdSVIAQDIYYQLVNEGYKVFFSRITLegKLGQKYepYIFAALNSAKTMLVVGTkPEYYNAVWVK
NEwgryLQLMKKDK-----
NRlIIPCYRDMDPYDLPEELSYFQSQNMGKIGFIQDLIHGIKKILSDKQEPVQ(ATVQQAVSVQNTVNDQLRN
IVALGFMDLEQGKNESAEQTFRDA)}*

$3228=273(263):
>gi|1073555763|ref|WP_069989307.1|
{|74(293)|<Firmicutes(B)>}hypothetical protein [Massilioclostridium coli]
{(AEAYWSLILCKYGIEYVEDPSSHKRVPTCNRTQFTSIFDDVDYQSALKYADPNQKRIYETEAKAIDEIQKE
ILEISQKEE)PFDIFIcyketdNHgrRT-PDS--
VLATELYHELTEIGFKVFFSRITLedKLGSAYepYIFAALNSSKVMVVLGTrAEYFNAVWVKNEwnryLALIR
QgAK-----
KTLIPAYKDMDPYDLPEEFSHLQALDMSKLGFMQDLTHGIQKLVEPEKEQED(DTATNGVTVNSLLKRAHIFL
EDEDWASADEYCEKVLDMDPEFS)}*

$3229=199(187):
>gi|1079850086|ref|WP_070250336.1| {|0(2)|<Proteobacteria(B)>}TIR domain containing protein [Duganella phyllosphaerae]
>gi|1078247107|gb|OEZ96527.1| hypothetical protein DUPY_39350 [Duganella phyllosp
{(MTIRHGCFFSY)AHGKHAHMK---
KFKDDLVEAMKCYLEPHfdnEEELFVDSEQLGGGDDLdgRIARALCESVCMILIYTPKYEAHAYTRREFAAMQ
lIEAERRqWYDlpshLIIPVIMTRHPfnlpPQISGPGMYVDFSTYTLASGDLKNNPDYLPEIARIVDRIA(AH
YHHLKRCTPPGHDCGRFVMPDIPPEWRAIPPPHF)}*

$3230=199(187):
>gi|1079869991|ref|WP_070268607.1| {|0(2)|<Proteobacteria(B)>}TIR domain containing protein [Duganella sp. HH101] >gi|1078255607|gb|OFA04902.1| hypothetical protein DUGA2_16450 [Duganella sp. HH101]

{(MTIRHGCFFSY)AHGQHAYMS---
KFKNDLVDALKCYLEPHfdnENELFVDSEQLGGGDDIdqKLARAMCESACMIVIYTPKYEAHAYTRREFAAMQ
LIENERkhwyplPSHLIIPIIMTPHPdglpPQISGPGMYVDFSRYTLASADLKTNPEFIPDIKKVVARIA(EH
YHYLKRCTPPEHDCGRFVMPDIPAEWRAIPPPHF)}*

$3231=219(220):
>gi|1079300466|ref|WP_070111249.1| {|61(0)|<Firmicutes(B)>}hypothetical protein [Staphylococcus haemolyticus] >gi|1078419697|gb|OFB46132.1| hypothetical protein BBG12_05505 [Staphylococcus haemolyticu
{(EKQTELSKLYEDYSKSERKINETRAKLDKERTKQQEKLEKTINNSKNDNHNIMTQLLSNSEQINALSEQVK
QTANQKELI)EYDVFLSHSN---LDKEkYVSELSDKLTDKGLKVFEDEkvFEIGQS--
QTQMMNMGILNSRfvVVFLSRNFIESGWSKYEFISFLNREINEDNIIILPIW-HEIGveE-
VKEYNPYLVDKHALN-TSKFSIDEMVEKISAVVEKSKQ()}*

$3232=215(220):
>gi|1074968207|ref|WP_070079572.1|
{|54(0)|<Proteobacteria(B)>}hypothetical protein [Acidihalobacter prosperus] >gi|1073959501|gb|AOU99221.1| hypothetical protein BI364_15905 [Acidihalobacter prosperu
{(VNLQGDPVKFESWLREKYPDKVKRHNIFLKDTFNYNVQTFIDDFMVDLVLTEEGGNRLFIFPEKNAFEKAN
PQYECMKRN)ESKVFISHSSKdKElIVNPLNAYLQANDIA------
TWLDSYEIDYGDniylKVNEGIENSDVGLFILTDHFFDSA--SGWPMTEFSTFFM-
ELMKNNKKVLMLNAGVSPENMHSMMKAYKYI--SWKNGAGLPEVANAIKRVISA()}*

$3233=174(182):
>gi|1079873365|ref|WP_070271866.1|
{|0(42)|<Proteobacteria(B)>}hypothetical protein [Duganella sp. HH101] >gi|1078251232|gb|OFA00570.1| TIR domain protein [Duganella sp. HH101]
{(MGV)--DVFLSHSHVDKVFADAICHRLEAADIR-CWVAPRDIRPGDDWAESIIDAMDQAKMLVLIFSASA-
NNSPQVRrEVERAVNKG-----LMVLPFRIENVPlsKSLEYFISTQHWLDAI--
TGDLEQHLDELCHCVAMLLERL(PQVAAASVPASAPAPAAAIAPDALAAIERALAQELGPIA)}*

$3234=190(191):
>gi|1074964519|ref|WP_070078268.1|
{|0(134)|<Proteobacteria(B)>}hypothetical protein [Acidihalobacter prosperus] >gi|1073958172|gb|AOU97892.1| hypothetical protein BI364_07870 [Acidihalobacter prosper
{(M)--KVFISWSgKRsKALANALREWL-PMVLQYV-
EPWvSDKDISAGDRWAqAIAGELEASNFGIICITPENLHSEWILFE-SGALSK-SM-LDAKVIPLLfgLELS-
DLSG-
PLSQFQAQKMEQsgVMEIVKAINTLSETKTSEQIANQ(LVPALWSRLQEAVENIPDSEPSEKHMRPHHEILEE
LVTGVRGINTRMREL)}*

$3235=291(299):
>gi|1073708217|gb|JAT97165.1| {|82(14)|<Arthropoda(M)>}putative tir domain protein [Amblyomma aureolatum]
{(EQGKSPTASLLDLWEVRSSQHSATVGWLVQHLICLERYDVLDDTRAELRQDIEFYRKKQSGEAADIIQETM
SRSSMRKPI)-YDAFVCYtSEDWPTVEVLVDRLESLGFRL--
FLPKRDLKAGVLQYSTLYELMEKwCRKTIIVFSPEFLQSEECRVQQRFIESINIEQRQQKLIPVIIKPCELDg
TTRMFSKISLCD-------DRVAE-
WGWNKLIESIRCR(SDSLFVSAPHSCILPATPSSPLVVPPSLVRSAVMPRKPGAMQARTRSDGQAAVTTTAVN
KSGVFRRLGNFFGRKKANSV)}*

$3236=284(292):

>gi|1073712808|gb|JAT99331.1| {|57(0)|<Arthropoda(M)>}putative tir domain protein [Amblyomma sculptum]
{(EQGKSPTAALLDQWEMRPSQHTATVGRLVEHLMCLERYDVLDDTSAELRQDIEFYRKKQSGERSGVIQMSMSRSSVRIPI)-YDAFVCYtSEDWPAVEELVYRLESEGFRL--
FLPMRDLKAGVLQYSTFYELMKNwCRKTIIVFSPEFLQSEECRVQKRLIESINLEKMQQKLIPVIIKPCKLDgTTCMISKISLCD-------DTVAE-
WGWNKLIASVRCS(DSVFVPSPRPKPEKKPRPVEPMTPVEDSRAVVTKTAGNKNRVLDVLRPLFSRKKGSSVSSASSGFQSMAQLQ)}*

$3237=174(190):
>gi|1079237567|ref|XP_009374571.2| {|0(807)|<Streptophyta(V)>}PREDICTED: TMV resistance protein N-like [Pyrus x bretschneideri]
{(MTSREPSSSKLW)SYDVFLSFRG-EDTRNGFTNHLHAALQNWGFDAFIDEDdLKRGGE-
IkpELLRAIEESRISVIVFSKSYAESTWCLDELVKIMEFFRP-----------------
EANVIKTIVEENIVELLPRTDELQVAKYPVGIDSRVQP(IISYLFSGGLNDVKMVGIWGMGGLGKTTAANAIYDKIH)}*

$3238=274(298):
>gi|1079439548|ref|XP_018510167.1| {|3(570)|<Streptophyta(V)>}PREDICTED: probable WRKY transcription factor 19 [Brassica rapa]
{(PNLTIFHVKSHLQKYRMVRYKYLMDKRNLQEEEVLPKSNTLNDIQRWLQSLVLPSCDNPVQPCVCVISCGGHHVDAGEVL)------------------FIDEIIIELQKRAITSLRYDLVTDTGT---
RMPGLHtLYGVFLLFISKNYT-SGESLDKLVTLTEYQKA-NGLLLIPIFYKVTPSEFPKFFT--
EERLLQLDDSVQIRRV-
QKWKEAMNELALS(DDCKWIFGNDSILPEEIVRYARLRLLCISSQNVTRMYSSLNSWQPTDMQMIGVWGMPGIGKTTVAGEVYRRLAPGYDS)}*

$3239=180(190):
>gi|1079777493|ref|XP_018558737.1| {|0(172)|<Chordata(M)>}PREDICTED: uncharacterized protein LOC108901666 [Lates calcarifer]
{(MPSH)---IMLSYQW-DD--
QALVKKIYNRLKDDGLPVWMDiEGGVTGNINDAMAAGVEEAVVICPFMTMAYQASRSCKKELNYADSRE----
-
VIIVPVMVEKNWDASEWLGLITAGLLWVDFRNAENDEEHFEMCLRSLEEEIM(FNAGHLLAVEEPPREEVDEPEPSKRCPKRKPGRGFRHAFSDLYIHD)}*

$3240=286(237):
>gi|1079739782|ref|XP_018539223.1| {|296(0)|<Chordata(M)>}PREDICTED: interleukin-18 receptor accessory protein-like isoform X2 [Lates calcarifer]
{(TQQHLKHTYTCIASNTVRNNSVTIKLKKKTTVKLPCLVGYPLVSFLLVSGLGIILHVKWMELQLIYRSHFQHGKHDEDNK)EFDVFLSYV-
WsppsaegergwtfssgsgpntdeegcppsmdplnseEGKATQkplqvLLPQVLEEQWGYRLCLLERDVLPGGAYTNDVVLTIQRSKMLICLLSADYLcNSDAVF-VLESGVQALLQNSAIKLLLIWTsrdsaSLI-
QQDPPLPTLVQRALKVLpSLNWTSGKPAratcNFWRSLRKAMPHH(RVKLVSDMQDQSLNGYI)}*

$3241=254(252):
>gi|1080063879|ref|XP_018574042.1| {|228(0)|<Arthropoda(M)>}PREDICTED: X-linked interleukin-1 receptor accessory protein-like 2 [Anoplophora glabripennis]
{(VISSPGNPPIERRVSVQETVNKEVYINPNPVPVGRMILIMSAILFAGLALIIILYLRYGLKAQVHIKDYFTPVEENDGKSN)--DVLIVYSPKdSDIALGVLMETLEKRYNYKCT--
SRELPPNSNMwYSDLREEIQKCRRIIVVLSPA-ALNGNWDTnNVQQALKQLQSL-

DAKLVCVSLKtlpknqnEIKDSQGETLASLTHSIGVI--MWE----
RkqddKFWYSLRLRLPAK(RRTECEETKKLTQGENNLRLNSDSQESLDNLV)}*

$3242=252(261):
>gi|1078807964|ref|XP_018496002.1| {|880(86)|<Arthropoda(M)>}PREDICTED: uncharacterized protein LOC100902364 [Galendromus occidentalis]
{(EAARLVGSDWVALAKRLGYTDRELSRFSEEPAAGLALLRDWRESNGATMYSTDVLVSCLQQMERDDIAKLI
QSDEAPQPI)---VFISYQW-HSQEAVLS--
IRQRLEAAGLPCWMDVGQLGGGDSLygKIYEGISRSKVVLCCLSARYAASASCARELSLADVLR-----
KPIVPVMVEPTPWPPPGPMAVILSSLVYVDLCGVGGHGGYGRDADWNSRCQD(IIERANSLIHSPPLAPGPHL
PGAPHRKLPQGMSPPKGRECS)}*

$3243=273(263):
>gi|1079631621|emb|SCW43565.1| {|74(485)|<Firmicutes(B)>}TIR domain-containing protein [Ruminococcaceae bacterium YRB3002]
{(AESYWSIVLCKYGIEYVEDPATHKRVPTVNRAQFTSVIADEDYKKALEYADIEQKIIYEAEAKAIDEIQKG
ILEISQKEE)PFDVFICYKE-
TDSngRRTqdsvLANDLYHQLTQEGFKVFFSRITLedKLGTAYepYIFAALNSAKVMVVLgtkaefFNAVWVK
NEWSRY-LALIRKGE-----
KKmLIPAYRDMDPYDLPEEFSHLQAQDMSKLGFMQDLIRGIKKIIGDSQPKAA(AQTIVNNNYSSNVTALLKR
GQMQLEDGEWEKADEFYEEVLNQD)}*

$3244=221(219):
>gi|1094001771|ref|WP_070999084.1|
{|0(93)|<Proteobacteria(B)>}hypothetical protein [Brucella sp. 141012304]
>gi|1079704789|emb|SCD25480.1| hypothetical protein BR141012304_21021 [Brucella sp. 1410123
{(MENDF)EYDVAFSFNQ---QDEALALKLNDLLSDR-
MKTFIYTERQKEIAGRdgvQAFSDVYGKKARivVVFYRKEWGETKWTTVEMNSIRTRAFDIGYDFTLFIPTED
EPvmptW-VPKTRLYFGLKRFGL-EGAAGAIE-
QLVTEAGGRPHEE(SLEEYAARASRASKFKQDQKRFQNSDYGVKAATEAYAAFGTALSQKALAIAPHGLNITV
RQNQTFWISSLYPIH)}*

$3245=214(220):
>gi|1096235621|ref|WP_071180251.1|
{|317(0)|<Proteobacteria(B)>}hypothetical protein [Vibrio cholerae]
>gi|1079954141|gb|OFJ29157.1| hypothetical protein BFX32_13220 [Vibrio cholerae]
{(GLAQAEVRFLKQRYHLADSDIKTDGLDGSIFKRAVYGHLSFIRMVKGEEHSPYLKLCEQVLALDSKPPEFI
RKLKGVFDM)-YDVFICHAS---EDKEVVAtPLFEALVKREIKVFIDNDAID--WGDslvSKI-
NTALKKSKyvIAIVSDESVKKSWPMKELNTVLAAEISSKETKLLPLMVGDGeellkEIPLLGDKLFVNFNN-
NI-EEIADKVE-KLLQRS-------()}*

$3246=295(286):
>gi|1079336676|ref|WP_070138133.1|
{|104(0)|<Bacteroidetes(B)>}hypothetical protein [Crocinitomix sp. 0182]
{(TKVYPVAISKNAYGFSKNLGQINFLRADKITGEKECQTQDENSVFRYLRLNITHELCRLMLNMKKAVDEQE
SFEHLPPPI)--KLFISHSkHDdSKYeAMKFKDFI-----
NSETQLKTFFDANDISFGSnfgdEIKRAVENSALVAFQ-SDSYAEREWCRIEVLTA--KKHG-
CPVVIVNAIKNGEKRTFPymGNYPSIRLKDNFEDIicltleqvLYNkytgmLLDE--
ITTLYGLKADSI(LSTTPELYSFLQLKDRKLDVSNNSEYKLIVYPDPPLGTEETELLMKMDESYHFVTPTTIP
SLILKQ)}*

$3247=156(165):

>gi|1080126817|ref|WP_070325475.1|
{|0(585)|<Proteobacteria(B)>}hypothetical protein [Acetobacter pasteurianus] >gi|1079991300|gb|AOW50687.1| hypothetical protein A4R89_14485 (plasmid) [Acetobacter pa
{(MEIMEKIIRGN)---IFLSHSSSdKEYVEKVYKLLDPT-ITFYDIR-------
TIQPGQetieAMESGIKQASVFVLFHSNESRKIWVDFEKSLARLHVINN-
TSVQILVCPINGSNHHSLPPWMTNYMTTSEL--FLPNDIARSIKYLYEKSLNKA(YPETIRTFPGREVL)}*

$3248=174(174):
>gi|1080114851|dbj|BAV79651.1| {|0(66)|<Firmicutes(B)>}TIR domain protein [Streptococcus sp. NPS 308]
{(M)---IFLCFSIKdrETIAEKILNHLLSFG----
FEVWYDRKdIFLGDNrFDvNLKNGAGGSNIkyAVIIISESFQKGDYC-NrELNILWQRKLK-
NEVSIFPIFYNMP-
INQIPKNFQFLLEFVCKfiSNSDESIFTAYHIVSKITDDLLT(KTYDKTFQYYANNCQDNYITEIIILYLSMD
RSN)}*

$3249=219(223):
>gi|1092701975|ref|WP_070633338.1|
{|72(0)|<Proteobacteria(B)>}hypothetical protein [Neisseria sp. HMSC069H12] >gi|1081010520|gb|OFR66722.1| hypothetical protein HMPREF2872_10035 [Neisseria sp. HMSC06
{(PTSFIQTLGIGNIKLNLNEMDRTNPHMIRKELFRLLNLKKRDAIFLFDETNNFSNWRTDLERDLKENFLQH
KMVMNMEPM)--KIFLSHKSInKDKVRSFKTILELLGFSP------
WLDEEAMSAGAnlerSLQSGMTDSCAAIFFITPDYKDEKYLASEIDYAISRKRQDekfsIITLVFSDENGEKG
VVPNLLQPYVWKEPAND--LLAL-AE--ILKALPLEVGDI(RFK)}*

$3250=260(272):
>gi|1080993855|gb|OFR50867.1| {|12(34)|<Actinobacteria(B)>}hypothetical protein HMPREF2883_07955 [Actinomyces sp. HMSC075C01]
>gi|1128422918|gb|OLO55249.1| hypothetical protein BKH27_02060 [Actinomyce
{(AAILECACRLEELDGTVVQTVLAEYGLDTVTPTVDYIPHERLIPVLRAADTRTLLSLANFLRRPRTLLLDE
ELPFRGQGL)--RLFFSHLSRSHEFVESVSAVLEDLSIE-VLIAHQDIRPSRDWLAFLANGLDQCDGLVA-
FVETGFRESEWCDqEVGWALGRH-----RPILSL----FReqTVPYGLAGRVQGLAFE--
NDAPSTVAEKIISWAGTEELLR(HRLLESLVQTLENSPNFDTTREVVKMLREVPATALSTDLADRLSTAATSN
PQ)}*

$3251=250(251):
>gi|1092524083|ref|WP_070508378.1| {|46(26)|<Firmicutes(B)>}signal transduction protein [Staphylococcus sp. HMSC072H03]
>gi|1080544261|gb|OFN24314.1| signal transduction protein [Staphylococcus sp. HM
{(IIFGIKDDGVKLTVKKFAFNLDLKHIQNLIEGSIKIKIKYNNLLFNGNNLFYISIDKSDDLVKVNNIPYKISK
YGELEEMMI)-KKVFISYSHKdSDLVDILE---
SELDRYNNIEITRDINITKYRDSLeEFMKTIREHDFTIAVVSSAYIMSLNCMYeVMQLMQDKNYQ-
EKLFFIIVNSNDE-
IYYNEKNRYNGFEANIYdfENRIKYLAHWENKRKKLNQSIEC(ANLPVESMSNLSLDVRKLNSIISTMDDFIN
T)}*

$3252=181(177):
>gi|1127346162|ref|WP_075226996.1| {|0(647)|<Firmicutes(B)>}hypothetical protein [Streptococcus sp. HMSC061D10] >gi|1080606945|gb|OFN83290.1| hypothetical protein HMPREF2728_03005 [Streptococcus sp. H

{()MRKVFISHSSRdKDMFVRpLVEKLK--
KNLGEDRLVYDELTfEAGAKIIeEIRRSIEKTDLFVLLLSKNSLSSEWV-
KqEIIWAQGLDSDFvDEQRIMPVIIDDV-
TKYNHEEIPDWLRNYNIkkIKSPSKLSRIIFSRVTEISWENS(DFLNKKTNLFVGRNSEMESFESRINDFTID
KTNFIIT)}*

$3253=221(220):
>gi|1080768318|gb|OFP36444.1| {|144(0)|<Bacteroidetes(B)>}hypothetical protein HMPREF2992_08575 [Prevotella sp. HMSC069G02]
{(DFIEHLKNTNESTSRIKWYRELFKKIKDEDFGPFLDQLSDAINQKTDSESNDASSFLNNTLQQTSKGSLNS
SIIKDSPNK)RVTVFISYAW--
DKHKETVLQIATRLQEAGIRILIDQKVPYGADIVnFMNKEIKNCDKCLVFLTpnykAKAELTKGGVAhEGRiI
SKEIYKDQdTTKFIPVLLVDSF-ESccPDFLFARKGFDFVNN------SFDEEINRMIEELKTLK()}*

$3254=244(254):
>gi|1081467782|emb|SCX50290.1| {|67(50)|<Proteobacteria(B)>}TIR domain-containing protein [Nitrosospira sp. Nsp1]
{(IPEQLFLSYVEKKSDIQDQITADLNKLHNVHSEFIARAFLEMEDAANQEWRNESGLLIDGKRQAPPDATKR
IWGDASFRL)----FLSHKTEVKKETTT-VKDGLRLFGI--
SCFVAHNDIHPTKaWQeEIENALASMDGFVALMTEGFHDSV------
WTDQEVGYAVaRGVPIIPVRLGKDPYGFIGKFQALSSTWPAVVVDLMKILIKNGQALNAYINALH(NCPSWNA
GNVLAEILPSIEKLSSSQIDALIATYN)}*

$3255=218(220):
>gi|1092504815|ref|WP_070491043.1|
{|72(0)|<Proteobacteria(B)>}hypothetical protein [Neisseria sp. HMSC15C08] >gi|1081409189|gb|OFV45083.1| hypothetical protein HMPREF3136_00925 [Neisseria sp. HMSC15C
{(FGDKYSLHNIFIGDVSKGGNIATFKDAYTLSSFDKLFIFSESESESFIRNKILPTLRFGLRSSFEAKNPQY
KSAKYQIPA)---IFISHSS-LD-
KDKIALPISDYLCAKEIPIWLDRNEMTLENNsseevIyeKIRHGISICKTGVFIVTPNFIeESKWTKVELSIC
KELD-----KNILLLIFDKNEERVEKLKKDFSLDDSPIVLMNSIEVDYETVRKKLLKNNFI()}*

$3256=246(254):
>gi|1092504820|ref|WP_070491047.1|
{|91(347)|<Proteobacteria(B)>}hypothetical protein [Neisseria sp. HMSC15C08] >gi|1081409191|gb|OFV45085.1| hypothetical protein HMPREF3136_00935 [Neisseria sp. HMSC1
{(YINSLPEQDNYHIVPVAIEREGLNQTSSGRLKNLNCIRAFDWPTALKKEYAILFLSHELYRFGLKDTTENN
LGIDSSIRL)----FLSHAK-KgDTGLKHAEAIKNFLDNSNMQRFFDANEISPGFKFdqEIENHIKNSTI-
IAIESDAYSSRYWCQREILSAKRER-----
RPIILVNSLEEYEDRVFPAASNIPCVHITAEPLEEKDILRILIAALLETIRF(EHAHKLLRYYQEQGWIDKSA
EIFARPPEIQQIVD)}*

$3257=225(219):
>gi|1092457079|ref|WP_070447128.1| {|0(89)|<Firmicutes(B)>}hypothetical protein [Lactobacillus sp. HMSC24D01] >gi|1081161473|gb|OFT08260.1| hypothetical protein HMPREF3094_03815 [Lactobacillus sp. HMS
{(MKT)---VFISYfrSEVNKEKLDQLIELLEESDIR-
VIIDERNLEIGGDLPLFMEKNLVESDFIIVILTSGYKKRADdreggvgYEAaIMAASLSKR-----
EtikKFLPVTFETYRdeIVPVFLASKLASDLsDN--LDSITfkrAVT--
KLKRTILGISMT(SQPTPKAGPSKVVRVADEADTTLISDNDNKNTQIMLISIDSDEVTQPRNDGTPGSALYEV
PFRLNHDPNRTWQQLF)}*

$3258=242(241):
>gi|1081454695|emb|SCX31656.1| {|17(40)|<Firmicutes(B)>}TIR domain-containing protein [Ruminococcaceae bacterium P7]
{(RTVRIDVPAADRYHIPILPIIVEQIPDELFERVFGKRHYLDSTGIDNTQISFEKKLTDYLNRIASADTWSQQIRDAFDAY)---IFLSYRKKdRKAAQRLMNliHKTPFCRD--IAIWYDEYLVPGESFTdAIKDAMHRSSLFALAVTPNLLeQGNYVMMhEYPEALKNNMN-ILAAELEKTDREQ-LIQHYNGLPPTVDLDDIviLGQALKNTHAKKVNNEPRHLFFI(GLAYLYGIDVEVNRERALNLI)}*

$3259=181(184):
>gi|1081455611|emb|SCX24041.1| {<Firmicutes(B)>}TIR domain-containing protein [Ruminococcaceae bacterium P7]
{(MQYLDKNNSDPTAIPYEEKLTKYLDAIIVGDKLAKKVRAAFDAY)---IFLSYRKKdRRYAQELMR----LIHQNDfcrdIAIWYDEFLTPGEDFNdAIKAALEKSGLFALAVTPNLINEiNYILNiEYPMAQEM-----GKNILPAEMEPT-DREKLREMYPDIPDVVVrsDKQTLCDRLMTSLKGTRNRRKRQ()}*

$3260=234(221):
>gi|1081484762|emb|SCX80943.1| {|0(987)|<Proteobacteria(B)>}KAP family P-loop domain-containing protein [Nitrosospira sp. Nsp13]
{(MIMAPG)-KRIILVYASRdEDWRHRLEDRLvvRTNDRERFpaglsVEAWSDEYLAESARAPdTLRDEVENVAIMLFLVSDDLLGSPCVRDeSIASVLQsilLEEQ-SESHVFRVLVR-----LPTLFLGWLALPpkiamTGDkaLADFPPslSD--AELDQVADTVATL(AGTPAKVETAPNGNLNSSNIDGRNEAAATDPATGPATGQASAQTPEQSNRPLTKGASRKSRQQPESPVPPDTGTV)}*

$3261=227(227):
>gi|1081458913|emb|SCY11941.1| {|0(262)|<Firmicutes(B)>}TPR repeat [Lachnospiraceae bacterium XPB1003]
{(MQVNLRDNDPD)---IFISYSS--ANKN-IADNIVSYLEKQGHRCWYAPRDILPGTRWvsAINTAI-NSSRIFLLIFTDKSNDSRQVAnEVALAFNAGKIIvpFKCNEIPMNpeLEYY-LTRVHWFDATRYPYREDILDDLGTYIDkLMKDGDPSSHTVS(RVHKGSYTEKSFWSRPTALIAAITVAVLIILPGTLYAGTLGKKHSAQQLSKAVSLYYGEADTKKDHAAAFEIFEKL)}*

$3262=216(191):
>gi|1082385594|gb|OFX41874.1| {|0(467)|<Bacteroidetes(B)>}hypothetical protein A2X03_09990 [Bacteroidetes bacterium GWA2_40_15]
>gi|1082441288|gb|OFX87067.1| hypothetical protein A2X06_03335 [Bacteroi
{(MSSILPGFE)-YDIFISYRH-KDNKYdgwvtEFVDNLKRELEAtfKeEVSVYFDinpHDGLLETHDVdaSLKEKIK-CLIFIPVISQTYCDSksfawqheFCAFNKLSKENHFGRD-IKLTsgniasrILPVkIHDLDPEDKMLLENELGGVLRSIEFIYKSAGVNRPLRANEDHPQDN(LNKTYYRDQVNKIANVIKEIIGCLKNPSSSIGISTKKDSSNI)}*

$3263=315(298):
>gi|1084277912|ref|WP_070366724.1| {|97(197)|<Firmicutes(B)>}site-specific DNA-methyltransferase [Pediococcus acidilactici]
>gi|1082254593|gb|AOW75251.1| DNA methylase N-4 [Pediococcus acidilactici]
{(SDKVKLIYVDPPYNTGKDFVYKDNFEDSLDNYLEQTGQVDSEGNNFSVNPETNGRYHTDWLNMMYPRLKLARNLLTEDGV)---IFIS---VDDAEQANLKKIADEIFGE--KNFLAQVIWERAYSPvNLKKNFSESHDYILVYGKDttkivsnglkrsqeSNDRYSNADndsrGPWTSGDLSVGPaIEKNIYPIItpggrevlpPSGYSwrLSESTLQEYIED----NRIWFGEDGNNT--PRIKRFLSEV(KQTIVPMTIWKYEDVGHSQSASQYLKRLFDDKAYFSYPKPVPLIQRIVELYSNPDSIVLDFFSGSATTAEAVMRMNVE)}*

$3264=210(218):
>gi|1084331930|ref|WP_070395989.1|
{|0(1229)|<Cyanobacteria(B)>}hypothetical protein [Moorea producens]
>gi|1082414013|gb|AOX03618.1| hypothetical protein BJP34_33055 [Moorea producens PAL-8-15-08-1]
{(MLGNCITISSETCEPKFLSNSYIESLLHYPTSMKTFF)--DAFISYGR-ADSK-
AFATKLHQRLTELGFRIWFDQQDIPLGVDFqnQIDDGIEKSHNFLFIIAPHSVKSAYCLKEIQLAVQLN----
-KRIIPLLH-
VEPKNCWDKMHPIIQKINWVYFRDNTEDFETSLAGLLQVIHHQ(SDYVRQHTEILVKALEWSRHQKQTNYLLI
GEERKKAQSWLK)}*

$3265=179(187):
>gi|1084331933|ref|WP_070395992.1|
{|0(1243)|<Cyanobacteria(B)>}hypothetical protein [Moorea producens]
>gi|1082414016|gb|AOX03621.1| hypothetical protein BJP34_33070 [Moorea producens PAL-8-15-08-1]
{(MNTFF)--DAFISYGR-ADSK-
AFATKLHQRLTELGFSIWFDQQDIPLGVDFqnQIDYGIEKSHNFLFIIAPHSVKSAYCLKEIQLAVQLN----
-KRIIPLLH-
VEPTDCWDKMHPIIRQINWVYFRDNTEDFETSLAGLLKVIHHQ(SDYVRQHTEILVKALEWSRHHKQTNYLLI
GEEQKQAQSWLKV)}*

$3266=224(223):
>gi|1082371034|gb|OFX29504.1| {|0(115)|<Bacteroidetes(B)>}hypothetical protein A2X08_10620 [Bacteroidetes bacterium GWA2_32_17]
{(MNSSDK)KYDIAISLCK---QDVDFAKKLVSQLNPS-
LSVFFYENNQEELINKmgpEVFARTFKEQSRivVILSRNEWSKTVYTEIEANAIIEKFSKDGYGFLFVIPMEP
NQipA-WYPSIrIYADPRRFTI-EDLAKFIE-
FKVSEEGGNIKQL(TVEDRYQNLLDKIDAKKYVIELQQNEEAIAQANNEIKILKDCFNQKIKFLQQSIFDGIS
MFLFSEHVSTAHFGIGHY)}*

$3267=177(187):
>gi|1082169568|gb|OFW13813.1| {|0(583)|<Acidobacteria(B)>}hypothetical protein A3H29_12820 [Acidobacteria bacterium RIFCSPLOWO2_02_FULL_67_21]
{(MNTL)----FISYASDDQAVALEVCAQLEAQGVR-CWIAPRDVAPGAKWDEALLQAIDAAGAFLLLLSEA-
SNESPFVQnEVNRAFGKK-----KPIFTFRIEDIPpsGSLEFYLARHHWTDGF--
PPPLEPKVAQLAVAVSGLLGVS(DLSGTTGTAVATGAAAAPPRAKKRAGAAPAWRRTALIGALCAL)}*

$3268=209(220):
>gi|1082211704|gb|OFW38760.1| {|0(547)|<Acidobacteria(B)>}hypothetical protein A3F70_00560 [Acidobacteria bacterium RIFCSPLOWO2_12_FULL_67_14]
{(MSTL)----FISYASDYQAIALEVCAQLEAQGVG-
CWIAPRDVAPGAKWDEAIVDAIHSAGAFLLLLTEAA-NESPYVGnEVNHAFAAK-----
KAIFTFRVEDVQpnKSLGFyLARHHWTDGFP--PPIETKVA--
QLAAGVSGLIGV(SALAGVAGTTTAAAAAQPKARKKAGAQPAWRRTALVGAAGALAGSLLVGAAWYATHSAAL
TGAGALPVNRVTIPTA)}*

$3269=174(182):
>gi|1082529087|gb|OFY65638.1| {|0(482)|<Bacteroidetes(B)>}hypothetical protein A2V64_12175 [Bacteroidetes bacterium RBG_13_43_22]
{(MPH)--KVFISYSSIDKNSADIVCSVLEQNGIP-
CWIAPRDITPGVSFAEAIIDAIKESRVFILIYSQNSNHSSQ-VIkEVDRAVHHG-----

LAVITLRMEDVPmsKQLEYYISDVHWLDAM--
APPLEEHINRLSRVVKMLMSKD(EGKDDDIEKAIRKGTLRLGRTGKPGSGTGRYRSPWKNAV)}*

$3270=268(271):
>gi|1084331203|ref|WP_070395262.1| {|126(987)|<Cyanobacteria(B)>}type IV secretion protein Rhs [Moorea producens] >gi|1082413260|gb|AOX02865.1| type IV secretion protein Rhs [Moorea producens PAL-8-15
{(QLMNSIRKHADYVEQHTLFLVKALEWDRNQQQTNHLLIQSERIQAESWLKVRFKKDQAPCLPTDLQCDFIG
ESTKNANNL)MTQVFISYADKdKYIKDKIGK---TLMRKG-
ITIWKpETDIKTGIDFQaAINQGIEEADNLIYLISPNSSQSKYC-QqELEHAFAHNK-----RIISLLIEDT-
DIDQIPpkIRSLQFLDFTKytNEKNYRFMAAQLLKELNQDALYY(EEHKLLLVKALKWLRQNRNSSILLRGHN
LNYYENWLKVNRQRLEHPPLGLH)}*

$3271=241(245):
>gi|1084331203|ref|WP_070395262.1| {|331(809)|<Cyanobacteria(B)>}type IV secretion protein Rhs [Moorea producens] >gi|1082413260|gb|AOX02865.1| type IV secretion protein Rhs [Moorea producens PAL-8-15
{(LLKELNQDALYYEEHKLLLVKALKWLRQNRNSSILLRGHNLNYYENWLKVNRQRLEHPPLGLHEEFLAASR
NQQHESYLD)---VFISYSRTdSDLARKLNEALQL---QGK-
TTWFDQEsIPPGSDFQqEIYRGIEQSDNFLFIISPASVNSPYCAGEVDYAQTLNKR-FVTVLSIQVPAKM-
LHPGLASIQWIDFNRHRgdFYPNFSELVRTLDTDREHVRSHT(KWLQRAREWQQSKQDTDLLLRGSEF)}*

$3272=258(264):
>gi|1084331930|ref|WP_070395989.1|
{|161(1020)|<Cyanobacteria(B)>}hypothetical protein [Moorea producens] >gi|1082414013|gb|AOX03618.1| hypothetical protein BJP34_33055 [Moorea producens PAL-8-15-08-1
{(LLQVIHHQSDYVRQHTEILVKALEWSRHQKQTNYLLIGEERKKAQSWLKVRFDDQQPPCVPTDLQCELICE
STKNSNNLL)-TQVLICYADQdKSVMQKISK---
TLMRKSFTVWTNKTDIETGTPFQdVINQGIEGADNIVYLISPASIKSDYC-QqEITHAL----D-
NNKRIIPLLIEST-
ELEGMPPQIRGLQFLDFttYKDEDKYRIEADKLIKVLHQDEP(YYSDHKTLLVKAIKWKKQNRNPSILLRGYN
LRYYETWLTINQQR)}*

$3273=250(256):
>gi|1084331933|ref|WP_070395992.1|
{|129(1043)|<Cyanobacteria(B)>}hypothetical protein [Moorea producens] >gi|1082414016|gb|AOX03621.1| hypothetical protein BJP34_33070 [Moorea producens PAL-8-15-08-1
{(LLKVIHHQSDYVRQHTEILVKALEWSRHHKQTNYLLIGEEQKQAQSWLKVKFTDQQPPCIPTDLQCELICE
STKNANNLL)-TQVFICYADQdKPVMRKIAK---
TLMRKSFTVWTNKTDIQTGTRFQdVVYQGIEGADNIVYLISPASLKSDYC-QqEITHALV-----
NNKRIIPLLIEST-
ALECIPPHIRALQFIDFttYNDEAKYRIDADKVIKVLHQDQP(YYEDHKRLLVKAIKWKNQNRNPSILLRGYN
LRYYQN)}*

$3274=230(242):
>gi|1082212137|gb|OFW39167.1| {|167(0)|<Acidobacteria(B)>}hypothetical protein A3J28_10920 [Acidobacteria bacterium RIFCSPLOWO2_12_FULL_60_22]
{(QEAKLMGCLFGYTILADCDLSGAIGLETAHDAYPCTIGIDTILKSQGKIPEAFLRGVGVPEQFIQYALDLR
NNPIKYYRC)----FISHA---
DEDKEFTEHLHSSLRERGVQCWYYPKSARAGRGVqeQVGRAIGYDYDKVIVICSQNSLNSSGVIdEIERAIQR
EDAEkRRDILVSVRIDDY---VFGKWDKQ------
NWEIAYKARLKSKHIEDFTGWQDP(NKYCSALDRLLLELNRQQANES)}*

$3275=214(220):
>gi|1082478873|gb|OFY23783.1| {|72(0)|<Bacteroidetes(B)>}hypothetical protein A2W98_02655 [Bacteroidetes bacterium GWF2_33_38]
>gi|1082554964|gb|OFY88910.1| hypothetical protein A2236_02300 [Bacteroid
{(ILEDISKLGSYTIESNIPNERVCISNDLIFCNCIYLYFENILTTPEKADLYKILSKNKIIGELRDEAWMLE
VIKCKKPLA)----FISHDY--
NDKEKIARPFAQKLAHNSYSaIWYDEFSLTPGDKMrkEIEKGIKESQYCILLLSKTYLNNESW-
GKIEFESIFTKELieKKDCIIPIWVDItkeelY-EFSPSLVNILGINWNIGVDKVVSEILRrFAK--------
-K()}*

$3276=217(210):
>gi|1082135446|gb|OFV91559.1| {|0(275)|<Acidobacteria(B)>}hypothetical protein A3H95_01010 [Acidobacteria bacterium RIFCSPLOWO2_02_FULL_64_15]
>gi|1082198786|gb|OFW31700.1| hypothetical protein A3G76_
{(MSFDT)--DAFISYAHLdnvelieghkgwvANLHRALEvRVGQLLGKKP--HIWRDP-
KLTGNDVIaeTLIDRLQRVAALVSVVSPRYVRSEWARRELSEFSK-----AAEQQGGVSWH---
KKARIFkvmkTPVPPEMHPPELQALLGYEFFTVHPETGRIRELD(EVFGPDAQRDFWIKLDDLAHDICCLLEM
VEGSPSPDAERATAVVFLAETTSDLREQREVIKRDME)}*

$3277=175(164):
>gi|1082179141|gb|OFW17972.1| {|0(329)|<Acidobacteria(B)>}hypothetical protein A3F69_04185 [Acidobacteria bacterium RIFCSPLOWO2_12_FULL_66_10]
{(MQFDG)--DAFISYAHLdnvelvegrkgwvANLHRALEvRVGQLLGKTP--HIWRDP-
KLSGNDLFadTLIDRLKHVAVLVTVISPRYLKSEWTIRELNEFWHAAEDQggvHVSNKARVFKV---
LKTPVPLDKTPPELRGILGYEFYNVDPETGKVRELDEIFG(PEAHRDFWIKLDDLAHDVV)}*

$3278=175(164):
>gi|1082179947|gb|OFW18720.1| {|0(368)|<Acidobacteria(B)>}hypothetical protein A3H97_17820 [Acidobacteria bacterium RIFCSPLOWO2_02_FULL_65_29]
{(MQFEG)--DAFISYAHLdnvelvegrkgwvANLHRALEvRVGQLLGKSP--HIWRDP-
KLSGNDLFadTLLEKLKQVALLVTVVSPRYLKSEWTIRELNEFWHAAEDQggiQVNNKARVFKV---
LKTPVPLDRTPPELRALIGYEFFKVDPETGKVRELDEVFG(HDAHRDFWLKLDDLAHDIV)}*

$3279=165(154):
>gi|1082532407|gb|OFY68468.1| {|0(575)|<Bacteroidetes(B)>}hypothetical protein A2V64_00125 [Bacteroidetes bacterium RBG_13_43_22]
{(MASLIEGY)EYDVFISYRQ-
SDNKydgwvKTFVNNLTIELeatIKDKISVYFDidpRDGLLETHSVdrSLEDKL-
KCLIFIPILSKTYCDKNAFAWQNEFCAFNRLAKEDRFGRDIRLLKGNFTSRILPVRIHELDHEDSMLIENELG
GSLRSIDFVYKSPG(VNRPLR)}*

$3280=167(165):
>gi|1082560489|gb|OFY93702.1| {|0(730)|<Bacteroidetes(B)>}hypothetical protein A3K10_17170 [Bacteroidetes bacterium RIFCSPLOWO2_12_FULL_31_6]
{(MKE)--EVFISYAwikattG-AKTQDGIVSDIEKAL-
NKNFNVVIDKKYIKYKDNIKEFEERlgKGDKIVLVI-SDNFLKSKHCMYEVLKIQEKGNV-
YKRIFPIVLSDAKIYDSLAAADYIEHWENEAEKLNKKLKSLKTQANTTAIRIDL(DNFTKFRAMFDDFRGLLC
NMNT)}*

$3281=248(258):
>gi|1082700934|gb|OGA22891.1| {|204(29)|<Proteobacteria(B)>}hypothetical protein A3I02_05765 [Betaproteobacteria bacterium RIFCSPLOWO2_02_FULL_67_26]

{(LSLIVPVDLFARLGDQVRPIEEQIGKRITGVLRGPDRHHVSAVVIQPSLLTRTSAELADVVVARSERPIPQ
FWAPGQFRL)----FISHVTSF-KQRATALRHELSRYHI--
TGFVAHETIDPGELWqREIEAALRSMHaMAALITP--------
DFhvSNWTDQEIGWALgSGVYVLPVQRGADPYGFLGEVQGIQGMGKKVPEVADEIFMTLLRLPATSDALLE(A
LVVGFERSGSYREARENIALLERARSIPESLLRRIEV)}*

$3282=199(210):
>gi|1082592961|gb|OFZ23559.1| {|0(224)|<Proteobacteria(B)>}hypothetical protein A2202_04315 [Bdellovibrionales bacterium RIFOXYA1_FULL_36_14]
{(MN)-YDIFISYSSQdlSLSMD-ICKKLEETGL--
TCWMAPRNIDTGISFAKAIIEGIKSSQNILLIYTAHSNISEQVLREVDRAVH--FEKK----
LLVFKLDDQrYSESLEYY-----
LCKAdSIDATKGKYQQYLKQIENKVRLL(TKAKPKPSTLIELKPKLTNIQTKKRMPSFFIIFIIFLLLIFSGV
YFQKLQHHQPSIEKRKNTFAPLPK)}*

$3283=226(230):
>gi|1082765165|gb|OGA83268.1| {|0(989)|<Proteobacteria(B)>}hypothetical protein A2711_14525 [Burkholderiales bacterium RIFCSPHIGHO2_01_FULL_63_240]
{(MQAKEDNTPT)--DVFLSYReG---ETR--VPEINAWLKQRGVRTHY--FHDTVGLGEpidE--ARALR-
ESRcmVVFLG----DRGWGNTQRSQVAE--FQASGRRVLPVLIGNPseteklDfdG-
LFSQRLYLDMRahdDRKL-EELLHAIR-
SAVQNTGAEPERS(ESDRMSAQASSDANMDHPTSVRLAQLLHILKDGSDRERLDALRQINGLHTNDRGALAHI
LRRHLRGDFAVGTEDNFAAAV)}*

$3284=143(144):
>gi|1082552429|gb|OFY86605.1| {<Bacteroidetes(B)>}hypothetical protein A3F72_09025 [Bacteroidetes bacterium RIFCSPLOWO2_12_FULL_35_15]
{(M)--KYFISHESYsIKDAVKLKELLTEIN-NEHSIFLTS-DWESLPSGNvwmgDIFSALQDCDELIVLIT-
--RKEAFCnlwiNFEVGSAIGRKKK-PKILIWGGIDLAEMKYPIKGIHCIGTGDTNR--
WEKELGSLGYKFENKKPFAEL(FKQ)}*

$3285=174(180):
>gi|1082531941|gb|OFY68076.1| {|0(683)|<Bacteroidetes(B)>}hypothetical protein A2Y71_06885 [Bacteroidetes bacterium RBG_13_42_15]
{(M)AHDVFISYSHIDKSVADAVCSIMEKNGIR-CWMAPRDITPGAPFAEAIIDGIKGSKVFVLVYSSN-
SNHSNQVIkEVDRAVHHG-----LAIIPLRLEDVPmsKQLEYYVSDVHWLDAL--
TPPLEKHIDKLCKVIQMLLTID(EVDSDDIKEAFRKETIKQVEPGKPGRRFKLRNLLIPAVV)}*

$3286=212(219):
>gi|1082582081|gb|OFZ13134.1| {|0(258)|<Proteobacteria(B)>}hypothetical protein A2X86_09955 [Bdellovibrionales bacterium GWA2_49_15]
{(M)SEKFFISHSSQDAKWANQLVEILEGKGKT-CWVAPRNIPPTQNYAQCILDALEGSTALILLYTKSA-
ENSVQVQrEVDRAINAK-----LTIAVLMLENITptGPMEyYLCNTQWISAYG--NNWDSGVA--
NLLQALDGKASL(PTERPKRKLKFVSLMLVSFVALALGAWYHIHSGQKNQTLHIEGAVQTDFIRAETLDDKDP
LDKLSTGLTKMLEKDDGP)}*

$3287=178(184):
>gi|1082606776|gb|OFZ36873.1| {|0(397)|<Proteobacteria(B)>}hypothetical protein A2385_04510 [Bdellovibrionales bacterium RIFOXYB1_FULL_39_21]
>gi|1082613703|gb|OFZ43410.1| hypothetical protein A2485_1
{(M)AHQIFLSYSSFDRKIVDLVCQRLEGAGHK-CWMAPRDIVPGEIYAKSLLAAINSAELFVLIFS-
GHSNESEQVQkEIDRAVFAK-----LPILPFKIENVKpsEAMEYYLCNTQWMDAY--
DGGCEKYIPKLVETCRAKLTNT(QREQVREEKVVTPQEEFKHESSIAIEIRPLPSGERKLRVIETAS)}*

$3288=184(190):
>gi|1082979505|gb|OGC84065.1| {|0(57)|<FCB group(B)>}hypothetical protein A2W07_02975 [candidate division Zixibacteria bacterium RBG_16_43_9]
{(MAKENKETRVFRGK)SLRIFVSYSSEDKQLAGAIKEGFEHYGMD-VFLAHEDITPSAEWQETILKRLKQTDVFIPLLTNN-FKESNWTDqETGIAVANR-----KLIVPLRVDINPygFIAKVQAFKFKYDTSR--GSNGSKTYSCWDSCLEITKLIL(NKKKLRENLKDCLIRSFAKSNSFEDANKKTEMLENY)}*

$3289=209(223):
>gi|1082582238|gb|OFZ13288.1| {|0(279)|<Proteobacteria(B)>}hypothetical protein A2X86_16190 [Bdellovibrionales bacterium GWA2_49_15]
{(MGHQ)---IFISYSSKdiketELIRDELES-------QGLPCWMAPRDIRPGEIYAsAIIDAIESAKIFVLVLTEHSNISPQV-QkEVDRAVNA-----KLKILALILRKV-ELSKA----LNYYLCDShwIDCHDGRE--VAVAAVVQACRDQ(PAPVKVSRKKSKWWLMGLFLPLLLFLFAVLWHFLMRPVEKFSYPVRPLPQDSLGTWSERIGGRYQSYHAPQTMRSAPGI)}*

$3290=180(170):
>gi|1082789788|gb|OGB06578.1| {|0(316)|<Proteobacteria(B)>}hypothetical protein A3E25_20365 [Burkholderiales bacterium RIFCSPHIGHO2_12_FULL_69_20]
{(MRFKRH)---LFISYTHKdnkpllndaEGWVSRFHKTLGVVLEQRLgrmPEIWRDERLQGNDVFTpEIMARLPESAVLLAVVSEGYVASKWCRDEAQAFCEAAEQ-TGGLTRDNKSRVF-KVFKAPPATLDPLPAVMreMTGYNFFERNADNAPKELDPDFG(KESRAKYIDQIMTLAFHLTSLIND)}*

$3291=247(256):
>gi|1082917562|gb|OGC25406.1| {|50(0)|<unknown(B)>}hypothetical protein A2247_07915 [candidate division WOR-1 bacterium RIFOXYA2_FULL_41_14]
>gi|1082917909|gb|OGC25743.1| hypothetical protein A2291_00
{(YFYTLYLEVPVELFASIESNLTKHEKAIESKLQLALRENLNHILNDVVIQPILAEISSLNKPISSSNKDLERIWGKNQIL)--KLFISHQHLdKIFATQLK---SLLAKYGIAAFVAHQDIEPTFEWQkEIKRALNTMDVFVALLSPNFRTSNW-TDqEIGYAVAR-------NVLMIHVRDG-QDPYGFINETQSLSMDIsnVDELAEKIVDIICKNPYFENKMK(KKLLDVLKNPYGYRIVCSILRITSVPSLKGSKRCNA)}*

$3292=233(233):
>gi|1082531301|gb|OFY67530.1| {|0(1219)|<Bacteroidetes(B)>}hypothetical protein A2Y71_14390 [Bacteroidetes bacterium RBG_13_42_15]
{(MYSKKRYLYICEDCNNEFKDEKTTPSHLR)---IFLSYSH--DQNEELVLIIKSDLEKRGHDVWFDKSGmkehgIKPGEDWrrSITDGIVSSNRVVTFLSKHSTRDPGVCLdEISIAIGVK--GGNIQTILVESEkEV-QAPPSISHIQWLD-MHDWKEKMAEGAEIWGKWYNKKFIEI(LEVIESDESRRFAGEIENLAGYLKPISSDSRISQLLRKGFVGRKWVLDSIEQWRTRSDRSSRLF)}*

$3293=209(220):
>gi|1083460054|gb|OGF63817.1| {|63(0)|<unknown(B)>}hypothetical protein A2Y62_03475 [Candidatus Fischerbacteria bacterium RBG_13_37_8]
{(PFSNKSGHILQEVKWAIQAKKTIIILRLEDIALSKPLEMLIKNSLSLKGSLPLTQEILESLTKMLLKLLFNKDNVDKLGK)DFDVFICFRS-ND--FSYAKQLFDFLRAKGLRAFFSSETLrsPEFI-

EEISRAIEHSRnMAIVTSSANNVNSKWVKHEWNCFLNEILSGRKEGIL----------
LTVATEEVNINELPINLRSMEVIRFNPEGFNKMLKFIK()}*

$3294=227(236):
>gi|1083336700|ref|XP_018619020.1| {|3(0)|<Chordata(M)>}PREDICTED: uncharacterized protein LOC108941064 isoform X2 [Scleropages formosus]
{(SEDYRIRLFATKLIWQLAFNCTIREELESNSTLMELLDRLSKDTNKEVCNSTHGALWVIQKQSKEKARPPS
AAVTGPPGH)---IMISYQW-N--
SQGTMLKVNRALQDAGIKVWMDVEQMAGSTLQAMAEAVEKASVVLIGVSAKYKDSPNCRTEPALVTWTK---
ADVSAWLKDNALHFCLNSFAEYDGSLIYQLQKLRKEAPEFFYSSLRKDLAFTSL(HEILQFLKALEKINSL)}
*

$3295=173(180):
>gi|1083460054|gb|OGF63817.1| {|0(99)|<unknown(B)>}hypothetical protein A2Y62_03475 [Candidatus Fischerbacteria bacterium RBG_13_37_8]
{(MA)-YDIYISYATPDNN-IAHSIYEYLERETI--KCYIAHQDLAHGKNiSeELFKAIQTSRAFLLLLTP--
------
FSnkSGHILQEVKWAIqAKKTIIILRLEDIALSKPLEMLIKNSLSLKGSLPLTQEILESLTKMLLKLLFNK(D
NVDKLGKDFDVFICFRSNDFSYAKQLFDFLRAKGLRA)}*

$3296=194(194):
>gi|1083126842|gb|OGE22842.1| {|0(766)|<unknown(B)>}hypothetical protein A3J42_07600 [Candidatus Dadabacteria bacterium RIFCSPHIGHO2_12_FULL_53_21]
{(MTKE)EYDVFFSYVHKdkDSAIR-IVEAL--
RASGIKVWFDENEIKDYDGIIRHLTKGLSHSKALVAYYSAKYPKSRFCQWEFTSAFLASQREGDpTKRIMVLN
PEK-
GHEHIMPSEFQGALFKRvPENVDDAALNVIVESIKERVSRI(ANPIGHISTFDKPTWYGRNGIFYPNFVGRLS
DMWNIHTKLNSYETILITS)}*

$3297=251(233):
>gi|1083419227|ref|XP_018583942.1| {|311(0)|<Chordata(M)>}PREDICTED: interleukin-18 receptor accessory protein-like [Scleropages formosus]
{(IRHLAASFTCFAQNSRGNVTSTLRLTRRPEALQRLEVIIAPVVVLILVTGASVVVQLYRMEIFLLCRAYLP
FQVSTAAGK)EFDAFVSYTMSssleeevgdvtgEALGLCYLPRVLEQQWEYKLCLLERDLDPGGSYTEEVVRS
IQRSQRVICLLNASYLMSPCLF-ELETALKTLQADVHLKVILVWIStppAH-
LRSLPLPRLVRAALRVLpTLHWSPADSTcsetRFWKVLRVAMPKT(KVLHCTSSTSGHS)}*

$3298=156(163):
>gi|1083438783|gb|OGF45507.1| {<unknown(B)>}hypothetical protein A2533_01260 [Candidatus Falkowbacteria bacterium RIFOXYD2_FULL_35_9]
{(MVFG)---IFISYSTYdSDTAKRIENYLRQI--TNTEIY---
LYETQVKTGQiseAIIQKIRQCDLFIVLYSKNSIQSTYVQQEIGVAKGNHKI-
IIPILLDSDAKPDALLTGINYLSIYDEKKRN--
EQLPKLYQYVANESQKKASAQ(ALLVIGGLATLAYLASKNK)}*

$3299=174(180):
>gi|1083128103|gb|OGE23969.1| {|0(283)|<unknown(B)>}hypothetical protein A3J42_05120 [Candidatus Dadabacteria bacterium RIFCSPHIGHO2_12_FULL_53_21]
{(M)AHDVFISHSGKDKPIADAVCATLEIGGVR-
CWIAPRDIVPGTQWGEAIIDAISSSRVMVLVFSSNANESQQIIREVERAVSKG-----
IPVIPFRVEDVDpnKSLEYFISAPHWLDAL--
TPPLEKHLEQLLSTIKLLLSRQ(GARTEPPSDGDTARASGATPSGPERAGRESDTRIETIRP)}*

$3300=206(219):
>gi|1083252108|emb|SCZ86812.1|
{|0(1880)|<Proteobacteria(B)>}hypothetical protein NSMM_800006
[Nitrosomonas mobilis]
{()MRDIFVCYSRKdLAIADRLIQHL----
HEAGWTVFIDRQIHVGHRWHhDIEKELHAAKAVVVLWSVSSRESDFV-------LEEAEY-
GKRnnLLFPIYIE-----QVEPpygFSRIQTA---DLTGWRNgtDN--
SGLTKLIEALRQH(LNKTASPLPIGENPYKGLAVFKEEDAERFFGRDEEIEKFLRQFLSLTTPLNDQTPVRIL
PVLGPSGSGKSSLVQAGLLP)}*

$3301=131(144):
>gi|1083462604|gb|OGF66222.1| {<unknown(B)>}hypothetical protein
A2Y62_02695 [Candidatus Fischerbacteria bacterium RBG_13_37_8]
{(MIRC)----FISHTwR--
EGEHHFAMRLASALKKRQIKVWIDEEKISPGSFImaAIQRAIKHEVDIFIFIMSPASLRSRWCRiELKLALS-
QEEYcIP--IIPVLIKPS-AIPRSLKNIKYADFTK--------PSCFDSAMDYLIAEVH()}*

$3302=188(178):
>gi|1083512294|gb|OGG12895.1| {<unknown(B)>}hypothetical protein
A2773_01650 [Candidatus Gottesmanbacteria bacterium
RIFCSPHIGHO2_01_FULL_39_10]
{(MNDEPIEEITSQEINYEEVVVGTIPDPKIE)-YELFLS--HVtdkntpQ--
QKLITEICRQLEEIHGLPTFLDKNKITSNPVNIINKAMNRSRIMIPICTPEYInlfnneSTSWPSRELGAFIi
yeRQQRLDkiiPVLLDITRDKF---
QEPEGGIPTILTKRNVVIPSNYTEDIEIVKLKIEEIVKIY(KDYLKQLN)}*

$3303=275(277):
>gi|1083273051|ref|XP_018615517.1| {|342(85)|<Chordata(M)>}PREDICTED:
TIR domain-containing adapter molecule 1-like [Scleropages formosus]
>gi|1083273053|ref|XP_018615526.1| PREDICTED: TIR domain-cont
{(SLAPKSNDSKSQLRMESSVKQINKTSSFAPIFSTENKNTETQTIENAFSDISKTVLSQNKTENEKDKEDEK
EEDEPIFFS)----FVIFhaPEDLDMAEKLQKKLEGLGIGEGATFSQDFLVPGRSQLKCVEDAIDNsA-
FTILLLSRNFSN-HLHEFQTNSALMNSIQKRHKynSVIPLLPSKNSMppeDIPRPLRIYVPLYESG----
CFDKAARKAFAPEMIRKQK(QLWSKDQLIRKQQEKQDRLKEKCERDKILAYEANKTLYLEKMRLYWLQQLQIQ
CSAM)}*

$3304=218(221):
>gi|1083974892|gb|OGJ90245.1| {|52(0)|<unknown(B)>}hypothetical protein
A2350_07010 [Candidatus Raymondbacteria bacterium RifOxyB12_full_50_8]
>gi|1083977932|gb|OGJ93178.1| hypothetical protein A2248_
{(HTGTRAFQKSDGLGELSSTTLDVSDITIDNCDQVRSKLENFLSLKQQDGIIFIKQDIAPLAEFQKLLERHE
LAMGGGAMK)---IFLSHKG-ID--
KPMVRDFSDTLLLLGFQPWLDEDAMTAGVELerGILQGFKESCAAVFFITPDF-
KDESCLaseinYAIAQKREKG-----
NRfsIVTIVFSVNDKKGSVPDLLKQYVWKEPSSHLAALQEIIKALPIKIGNPKW(K)}*

$3305=208(214):
>gi|1083611746|gb|OGH04189.1| {|0(14)|<unknown(B)>}hypothetical protein
A2W22_02970 [Candidatus Levybacteria bacterium RBG_16_35_11]
{(M)----FFSYSIDdKKIVGMLKEQLEYMG------
FETFLAHEDLEPCVewqeEIKKNLNMCDIFIPLLTESFKKSDWTDQEIGLAIAADKF-
IIPLQVDfppyGFIGKIQGLKIDTYYLRNEDVQTV--RNHLKDI--

AIKIFQTLRTKS(KFKDDMKDFILYKFINSASYNEGIARAKLLEDFSNVTQVELSQILKATKSNNQIHEPSTARKALRKFFEIHKG)}*

$3306=292(294):
>gi|1083974013|gb|OGJ89393.1| {|107(79)|<unknown(B)>}hypothetical protein A2248_22460 [Candidatus Raymondbacteria bacterium RIFOXYA2_FULL_49_16] >gi|1083978768|gb|OGJ93984.1| hypothetical protein A235
{(IANKLNRDNLYSTVTIEEIEFEDKSDKERFSPHWTFMRGAGLLNNESTGGILEATYKFWEYYDDMTNTSKNDIKSPYRYD)---IFIVHADEdKDFTEAFGKQIQKIfNIPSDRIF-TASDSKSIPLGEqwysYIVDAHKFSKIGIVLLTPNSVSRPWVNFEVG-GFVLRNT-SSPISICCCKEtvDKIEFPISGLQYRKLYDKSD--REAIIEK--LAKLLECPKPTY(NDSEFEQIISNIRLLPPKINHEVLVRAWIGDRPTHVSPSFNKNDYYWMEITNKSTTPLHIESAGIIDCNGYILF)}*

$3307=158(164):
>gi|1084152580|gb|OGL48150.1| {<unknown(B)>}hypothetical protein A2161_12835 [Candidatus Schekmanbacteria bacterium RBG_13_48_7]
{(MSY)--KVFLSHSSHdQGLVRALAHLLTQYGI----IVF--VADWYLEPGAsidkKVFKHIDDSDCVVVFLTHNGIRSHWVNQETGYAFGAKKL-IIPLVEKGTQKNELAALNSKDYIEYDPYFPQ--DALYKTSTFIKSLKLKKEEQN(NTLLVIGSILSFLLLLSGADR)}*

$3308=190(190):
>gi|1083738204|gb|OGI20773.1| {|0(93)|<Terrabacteria group(B)>}hypothetical protein A3B68_09565 [Candidatus Melainabacteria bacterium RIFCSPHIGHO2_02_FULL_34_12]
{()MPKVFISYSSKdK----EFVEHLTNKLKKDNIDYWlDEKDLNIGDVLIeEISAGIMKSEYVAIIISENSNKSNWV-KkELSIAINREvNE-GKKVVLPIIIHDC-PFPDSVADKLAANFKDKskYENEYKRLLKAMNIVANTKELSY(DSKNEPAQVPGEYVNLADTFEDIKIIGIDKSKISKNDDGTFNIYFKLSDE)}*

$3309=189(196):
>gi|1084021615|gb|OGK28477.1| {|0(22)|<unknown(B)>}hypothetical protein A3C31_02775 [Candidatus Roizmanbacteria bacterium RIFCSPHIGHO2_02_FULL_40_53] >gi|1084022568|gb|OGK29368.1| hypothetical protein
{(MRRI)--KAFIAYADEdSDVGENIKKCLERCY--GIIAFFSPEDTWTSEDFEeKIYEELPTSDLFIPLISTHFHKSIY-ANqEVGMALAYKRS-----IFPVSL----DGKKPEgfISKKQAYPLFKknYDPPFQAATAIYLALLNSKKYRE(LRAFTKNSLVHALCTSNDFYDSIKIISMMIRIKDFNRTQLTKIVHSIKNERC)}*

$3310=223(226):
>gi|1084150617|gb|OGL46415.1| {|0(637)|<unknown(B)>}toll-Interleukin receptor [Candidatus Schekmanbacteria bacterium RBG_16_38_10]
{(MNKNEKL)--KLVISYSHPdKDHIEKFTKHITPLSD--LIDIWYDGKIIPGQDFQdNIDKNFENADIVCLFISADFLSSNSCLDERKSVIELKKK-KGIAVIPVILSRC-GWlDDKEISTLKALPTDGkpISDFTDSN--EAWNTVYHGVKEV(IEKEIKIKQLKITEQFSSFLQNIDLLTKAHSQKEEVLLNDIFIYPELEQYNDLREYDKKNSSKKLIEDICDYPKILIAG)}*

$3311=181(186):

>gi|1084672330|gb|OGP20053.1| {|0(90)|<Proteobacteria(B)>}hypothetical protein A2054_01250 [Deltaproteobacteria bacterium GWA2_55_10]
{(MK)---VFISWSG-EK-----SKHLGEAFREwlpsviqA-
VKPYFSPDDISKGSRWntEISKELEDSKIGLIMLTPDNLEAPWVMFECGAL--SKNI-
DKSKVCPILFEVKPVDIQGPLVQFQASLFTK-
QDIRKIVKMINDILGEHSLASH(VFDSVFEMWWPKLNEKVESILKRDPAVEKELRTEKDLLIEILNI)}*

$3312=178(186):
>gi|1084510014|gb|OGN72211.1| {|0(233)|<Chloroflexi(B)>}hypothetical protein A2X25_00685 [Chloroflexi bacterium GWB2_49_20]
>gi|1084518169|gb|OGN80192.1| hypothetical protein A2X26_09540 [Chloroflexi
{(MTH)--DVFISYSSKDKPIADGICANLEASGIR-
CWVAPRDIGPGEEWPRAITNAITKSQVMVLVFSSQSNSSSDVGReIVLAA-NHK-----
LIIIPFKIENIEpePGKQYYLAQTHWLEAM--
NPPTAEQIQKLVERVRAIVPPL(DSNANVQPVLVPAPIIEQPPTPSPVVKKNWFHPEYLWIGIALL)}*

$3313=178(184):
>gi|1084555278|gb|OGO13165.1| {|0(371)|<Chloroflexi(B)>}hypothetical protein A2Y53_08175 [Chloroflexi bacterium RBG_16_47_49]
>gi|1084607043|gb|OGO60548.1| hypothetical protein A2032_01830 [Chloroflex
{(M)AHDVFVSYSNKDKPVADAVVAGLENKGIR-CWVAPRDITPGSSWGDAIISAIEGSRFMVIILSGN-
SNRSKQVVrEVERAVANN-----VIIIPFRIENIDptGAMAYFLSTEHWLDAI--
TPPLERHIEKLQTTLQLFMSGG(DEAALRGNLSPPIAQPTAPVLRWRIKTILPVLFALVILAVVAV)}*

$3314=163(172):
>gi|1084563475|gb|OGO20588.1| {<Chloroflexi(B)>}hypothetical protein A2144_02775 [Chloroflexi bacterium RBG_16_50_9]
{(M)AYNIFLSHSVVDQRWVQWIADNAHGIDIN-VYMYEHDIQPGTSIAVKVQSAIENCQALVVLLTPN-
SEYSPYVQqEIGFAQAKG-----KPIIP-LVQPGVspRCMAMLDGVEYISFNP--ENPEAALS--
HLLTYLGKLKNA(RDTDQAILMGLGTLFILALLGKRDQGKRFLS)}*

$3315=177(183):
>gi|1084576144|gb|OGO32365.1| {|0(502)|<Chloroflexi(B)>}hypothetical protein A2Z29_02080 [Chloroflexi bacterium RBG_16_56_11]
{(M)AHDVFISYSSEDKAVADAVCATLEGRKIR-CWIAPRDVLPGIAYAEALIDGITRSRLIVLVFSRN-
SNKSQQVMrEVERAVNKG-----IPIIPFRIEDVTpsKAMEYFLSAPHWLDAL--
SPPIEKHLQKLADTVEVLLGTE(TPLKVETEARPKIKESPAQKRNAKPVYIVAGIVVLALVAFGV)}*

$3316=223(224):
>gi|1084579257|gb|OGO35295.1| {|0(200)|<Chloroflexi(B)>}hypothetical protein A2W35_01460 [Chloroflexi bacterium RBG_16_57_11]
{(MTETDMAN)---VFLSYSPETEDFVEGIARKLfGDARLT-FWFRPWHSQPGKPVQEQMEDALYNAQA-
IAVFVGGAEGVEGWQNeQMRVAIQTR-----VeddpayRVIPVLLPGAhkpArrGLPALLRRYEMVEFRE--
MDDPQAFQ--
RLLAGVLGLPPI(DVEGYVQERKQQQKQQRPPEASFVQGHALLVGIANYLSVRSLPESVLNDARATGTLLADP
AACGYPAGQVQILLDE)}*

$3317=158(164):
>gi|1084612046|gb|OGO65050.1| {|0(343)|<Chloroflexi(B)>}hypothetical protein A2Z45_05190 [Chloroflexi bacterium RBG_19FT_COMBO_55_16]
{(M)-RDVFISHVEEDETIAMQIAQGLEVAGYT-
TWYYERDSIPGVSYLLHTRQAIEQSQAIILIISPQSLGSQQVTReVVRAHESSK-----

YFIPVLRNVSHLefQKRQPEWEEAIGAATS--
IRLPVEGATAILPRIIAGLKAL(GIDPSGNASLSGQEEILKTSPYD)}*

$3318=172(177):
>gi|1084510052|gb|OGN72249.1| {|0(1064)|<Chloroflexi(B)>}hypothetical protein A2X25_00880 [Chloroflexi bacterium GWB2_49_20]
>gi|1084515444|gb|OGN77534.1| hypothetical protein A2X26_02220 [Chloroflexi
{()MSDVFISYSRKdIAFARLLQESLQ----
HNQIDIWIDwERIPVGERWWdEICQAIENANAFMFIISKNSMGSPVCRDEINHAL----K-
NNKRIIPIIVDDL-
KPEAIKDFAPDLPQFNWiiFERNQLFRIEENPEVRSDKPEDS(QVALPKLPQYEEALEKLSKAIHTDWDWVKYHTRLQVD)}*

$3319=171(179):
>gi|1084530758|gb|OGN91847.1| {|0(220)|<Chloroflexi(B)>}hypothetical protein A2Z70_01175 [Chloroflexi bacterium RBG_13_48_17]
{(MAHE)---VFICYANQdRKIADEVYAAL----
RSNGITCWmAPQDIMAGEDWDkAIMDAIPVCRIVVMIFSASSNDSPYCISEIRTAFELKKE-----
ILPILIDST-
LPHGRIALYLGSKQWLYanTPPLQQHLKKLVDEVKRHLSQIK(TREEASAREREKAKEVEPEVVAEPEVKKPPVKEKP)}*

$3320=149(148):
>gi|1084526585|gb|OGN88209.1| {<Chloroflexi(B)>}hypothetical protein A2Z74_02680 [Chloroflexi bacterium RBG_13_46_9]
{(MPSPQLR)---VFISYPT--
SDGLDKARQAANLLKQVGLQVWIWHHSKTAGALTwrEISNCIVNESDLVLYICTASSCQSWGQGqEAGYALNHRRKvivVaIDAAMVPLELTA--RCYERVPSAQFVEKLRGIAINLPEITNrIQKLDDSVAATDL(R)}*

$3321=204(221):
>gi|1084725918|gb|OGP69976.1| {|0(16)|<Proteobacteria(B)>}hypothetical protein A2169_05070 [Deltaproteobacteria bacterium RBG_13_47_9]
{(M)--DTFISYA-SDIRRTAGRIKEYLDQFEF--
NCFLAHEDIQPQtVWPaEILRALKRCDLFLPLLTSGFIESF------FCQQETGFAYcRGVEILPVMISR---
------
EPMGMIANMQAVRFNGNEFDSSCWKIVKHVAKR(GSLSEPVLDALVSWFGESHSYDIAAERAKKILNEFDFTPRQVKNIRRRIRRNSQIHETKEARDSIFRFIDKYGDYFDKGF)}*

$3322=224(222):
>gi|1084720917|gb|OGP65432.1| {|0(91)|<Proteobacteria(B)>}hypothetical protein A2170_02190 [Deltaproteobacteria bacterium RBG_13_53_10]
{(MTYRAF)KFDVAFSFLK---GDEDLVSRIDTLLHDR-
FKTFVPSRREEFIAHTdfeRTVHRVFGREARivTVLYRGSWGRTGCTLLEETTLRNRAHEEGYDFILLIPVDipPSLppW-IPKKQTWLGLDRWGA-EGMAAVID-
ARVQQAGGTVHAE(TPLEHAHRIEHDMAFEEARRRFVYSQEGVRSAQSELAKLFDEIERISKDIAQTTQKILLRLDRDEKHLVLSTRGIS)}*

$3323=170(178):
>gi|1084706548|gb|OGP52270.1| {|0(1050)|<Proteobacteria(B)>}hypothetical protein A2Y79_13375 [Deltaproteobacteria bacterium RBG_13_43_22]
{(MS)--RIFLSHVERDLPIMLQIAQGLEAAGYS-
TWYFERDVLVGTSYLIQITQAIEDCEGLVLIASPNAFSSDQVTKeVVGGFERGK-----P-

FFPVLINITPpeLKERQPEWRHALGGTA--
MITVGPEGIPAAVTRIIEGLKA(KGIMPGEAQSSPGSSVLSTSMASAPKHVVERVLAVR)}*

$3324=174(180):
>gi|1084881808|gb|OGR09007.1| {|0(270)|<Proteobacteria(B)>}hypothetical protein A3K23_03430 [Desulfobacca sp. RBG_16_58_9]
{(M)AHDIFVSYAHQDRTVANAVLATLEAHGIR-CWIAPRDILPGMDWGEAIIDALQEAKVMVLVFS-
SGSNDSEQIKrEVERAVHQG-----IAVVPFRIEDVLpnKTLEYFISTQHWLDAL--
TPPLEDHLNHLAETMTVLLAKK(GGKERPPHLGGEEPAPPQPQSRPEVAAPEPPLPPIMARG)}*

$3325=177(183):
>gi|1084910093|gb|OGR30565.1| {|0(350)|<Proteobacteria(B)>}hypothetical protein A2139_00785 [Desulfobacca sp. RBG_16_60_12]
{(M)AHDVFVSYSVKDKTTADAICASLEASGVR-VWIAPRDVMPGSDWGESIIDAIHESRVMILVFSAN-
SNASPQIKrEIERAVNKG-----VTVVPFRIDDVLpsKTLEYFISTQHWLDAF--
TPPLEKHLDNLVSILRSILTKK(GEEVAEPRLKPHPPEEDEAPERPAPQAAPPEADATITLKVSK)}*

$3326=172(180):
>gi|1084731967|gb|OGP75429.1| {|0(339)|<Proteobacteria(B)>}hypothetical protein A2Y80_01065 [Deltaproteobacteria bacterium RBG_13_58_19]
{(MEYE)---VFIAYAHEdIKAAQSVVAYLAAAG----
LHCWWDDRLVPGTpEWEaEIERAIRHTGVFIALISPHSVMSRHV-KtEMTLA---GNA-GKA-IIPVFLSEH-
VDLPNGWGYRLALHQHLyaLPSLEAVMPKLAAAVDQVLDSHR(HAAAYRDEKEVRQRANFSWQTRFAEDTAGL
YVGESE)}*

$3327=246(253):
>gi|1084923674|gb|OGR41947.1| {|54(33)|<Elusimicrobia(B)>}hypothetical protein A2X35_11610 [Elusimicrobia bacterium GWA2_61_42]
{(TYYYTLSLEIPVDIFARIESALSKHEGEIGAKLQLVVRETGNDILNRVIIRPQMVESVSELAVEHSGNERV
QAIWKNKPV)--KIFISHQNSdKVAAAKLSAFLSKFNMAAFVAH---
EAIEPTIPWQsEIKIALNTMDVFLALLTSNFRNSKW-TDqEVGFAV----A-KKAVIIHVRDGQD-
PYGFIGETQSLSMPIEEneNLGMEIISILLKNPVLKSRVRET(LFVTLENIGNFTYACKTMKLLQKAGAFSEQ
EIQ)}*

$3328=172(170):
>gi|1085014894|gb|OGS23007.1| {<Elusimicrobia(B)>}hypothetical protein A2252_06325 [Elusimicrobia bacterium RIFOXYA2_FULL_39_19]
{(MAISQSLLINKSASFQEPLHGSMQKSASKG)MKSAFLCHSHKdEA----
IVKGLIVLFQEAEIDIyidWKDHTMpdVPNVETAkKIQQKIKTCDFFFFLASLNSKVSRWC-
PWEIGYADSNKR-NIYIISTSDGRDT-YGNEYLQLYPRIDQGLIetREVLAIFEAGAKKGQLLSVDALS()}*

$3329=182(174):
>gi|1085061857|gb|OGS61954.1| {|0(62)|<Euryarchaeota(A)>}hypothetical protein A3K59_00220 [Euryarchaeota archaeon RBG_19FT_COMBO_69_17]
{(M)----
FISHSKRdAELTSNISIALSNIGAEAVAMEYVAEKPEGGQEWERIRKEVSLADFVMLFktdnaISTDYTKSWI
IFEvGLAAAMSKRllvfeRR-GSPITFPIPXXXD-
CMLFDPAKVPDVLQVQRvaKVLQDELASRGKQQPKEKSDEGW(FWLALFMPEVFASLLVLGFVAATAAGIAAV
LTG)}*

$3330=218(220):
>gi|1084866497|gb|OGQ95658.1| {|195(0)|<Proteobacteria(B)>}hypothetical protein A2521_17340 [Deltaproteobacteria bacterium RIFOXYD12_FULL_57_12]

{(PYEAECILHLYALCLSYEKAYIDRSGSTDPGYWASRGVMIGETSAQDVIARLVPAQGNEYSFLDGIFPSRKLIDISREKQ)-YDAFISY-FSgdrdfvAQLERDLA--------
LREFHIWLDEREIEVGDSMtdKIQSGLSNSYTFIIVLSPEALNRPWVKEELraAYAQRLGGNFkilPVL--
HRECDI---PPFLADYKYADFRETRRYQEQLELLERAIRNAVRDVRGKR()}*

$3331=220(221):
>gi|1085291437|gb|OGU68858.1| {|87(0)|<Ignavibacteriae(B)>}hypothetical protein A2X62_05475 [Ignavibacteria bacterium GWC2_38_9]
>gi|1085306952|gb|OGU82751.1| hypothetical protein A2279_05485 [Ignavib
{(YLSEDSVNPFDINNIQYSYEFSHYAKEDGELLLSMDKIPKHIQQIIVDIHKYVNEEMYKLFYNSFSFEDSQIKINTKKKK)--ELFISYR-SNNYESAISLFHLLGDYNNK-
NIFtprMDKIDLSSGSWIqQLIDLIIKCQVFIPILSKDYLNGPISKPelEQALRKNFTD---
NMKIIPVLIEGDFIDYENHFIGNYQILDARNGLDNELIDTIYHMVFALSRNPY(E)}*

$3332=261(269):
>gi|1085369025|gb|OGV40215.1| {|320(0)|<Lentisphaerae(B)>}hypothetical protein A2X48_20675 [Lentisphaerae bacterium GWF2_49_21]
{(FGINIKYNPKPHIKECMIEKAINTIGLSHLWVLSRGNETLKLSNNTTVIPIKKFLSGQMKPGSSTAASNKLPKLSLKAKQ)---VFISYCHRddIFVNK-LVPILESS--
KINVIIDHKTLRLGDKIEEFIKKAVRNTEWTILIVSENSIKSPWVMAEFlETVFFEKFT-NQSRLIPI----C-
LDKSIFNPDLPIEIDKElEIKIAEVDDRIRTALDRKMGLDP(FVNVRDRLRILQFNVGKAVERLSSVLMGDFSDPKLFNHEIKKILNALQK)}*

$3333=160(167):
>gi|1085217202|gb|OGU01184.1| {|0(716)|<Proteobacteria(B)>}hypothetical protein A2X82_09160 [Geobacteraceae bacterium GWC2_55_20]
>gi|1085244521|gb|OGU26645.1| hypothetical protein A2X85_17125 [Geobac
{(MAT)---LFISYSRKnKEQVYPFAEALTKAGV---DIW---
IDREEIDPLNdfpaRIRDGLARSHALLAWYSPEYAQSSYCQKELIAAWICTQR-
LTRDVLSRILILNPLENVAHIALGDVERQNY--
LSAPKDETTQNTCIRLLGERL(ADLADDFTALRQFKLPIWRPSAQQ)}*

$3334=179(187):
>gi|1085252130|gb|OGU33602.1| {|0(126)|<Ignavibacteriae(B)>}hypothetical protein A2057_04550 [Ignavibacteria bacterium GWA2_35_9]
>gi|1085269230|gb|OGU48857.1| hypothetical protein A2080_15635 [Ignavi
{(MRKNI)--KIFISYSWKdKKIVNKVDDYFKSLAI---TII---RDERTVKYKEsikeYMK-
RIRTTDYVLMIISDSFLKSSNCMYEV-IEFIKDVN-YNDRILPILLDDTKIYDPIESIEYSKYWDRE--
YDRFYGKIKGLPVTSVESYII(ELKHLQNIRDSIGPFIHSIKDMKIVLYNEVKKTGYREVLNHI)}*

$3335=133(147):
>gi|1085465960|gb|OGW30109.1| {<Nitrospirae(B)>}hypothetical protein A2X56_00345 [Nitrospirae bacterium GWC2_57_13]
{(MTQQKIT)---IFISHAKDdETLAILLKDFLENIFLNAN--FFVS--
GSDLGGGEvwieELRDKLESATAIIALITPYAVENKWVYFEAGAGFA-QRR-
SIPLIADGMTFKTLPIPLSLLQGRTF-DISG--LKALTSD--I-----AILMVS()}*

$3336=212(224):
>gi|1085218293|gb|OGU02177.1| {|0(154)|<Proteobacteria(B)>}hypothetical protein A2X82_01445 [Geobacteraceae bacterium GWC2_55_20]
>gi|1085243628|gb|OGU25854.1| hypothetical protein A2X85_16595 [Geobac
{(MPPRNDSV)---FFVSYAGEDLKWAQWIVWELQNAKprYR-CIAQFKDFTPGMSFIQKMREAAE-
SDCTIAVFSPHYF-SSRYCQqELDASLTGDV----TRLLPVRVEPC-DPGQFLQNRIYIDLVN--

KSIDDARN--
SLLSGVEAYLTS(TRKLSDKPAFRQRPVFPGPMQDETSHNKPVIPTVAEGPLKVLFLAPQVGGLSPSSQLQKM
KRCLEQARFPKSIIFK)}*

$3337=247(240):
>gi|1085205717|gb|OGT91256.1|
{|61(1009)|<Proteobacteria(B)>}hypothetical protein A2514_10820
[Gammaproteobacteria bacterium RIFOXYD12_FULL_61_37]
{(NSFRSFRNRLNAAAKEAGIALQLEADNQKRSAPEARECWFVGEDTKVADIERYSAEEASDSDSANLVPARG
RPVDGKRSI)--
KLFVSYARKdNEkgdvdrFLEKFEEYARVYAGDCRFQIWADWHIAIGQDWNrEILDALKKCDFGLLFVSVSFL
DRQYIREhELPQLLATERA-LPLGFRKLDIHKA-
DLLGVKERQIFRLKDKQkfYSECRGEDKRGEFFSELIAQIKT(RIEAIPTEPPRNPPHPPFCE)}*

$3338=209(220):
>gi|1085218622|gb|OGU02478.1| {|676(0)|<Proteobacteria(B)>}hypothetical
protein A2X82_01805 [Geobacteraceae bacterium GWC2_55_20]
{(RLSGRGLTIDPPEQELIWQGGRSLLDFDVKVADDAGEGTIAVKFDAAIEGIIVARLRLELEITAKPKKRGQ
ATASGEPAR)--TAFASYSSKD--
RLRVLDRVDAIKISAGIDVFQDcLDLNPGEEWKsRLDNEIRQRDLFLLFWSKD--ASESkWVGwELETAL--
KEK-GE-------QALQ-LHPLDPGVKPPPGLEKLniGSNAMWVRKGYEALLAERKAAAP()}*

$3339=281(294):
>gi|1085385048|gb|OGV55529.1| {|51(0)|<Lentisphaerae(B)>}hypothetical
protein A2X49_14285 [Lentisphaerae bacterium GWF2_52_8]
{(FYTLTIEVPISVYAELDETRTSIEKSIHRRVSELTRTEAGVSITEVVISPELADDVSSNDETSGEQAEAPP
LFWAPGHFR)---LFITHVSAmKNAAHSVKVALAPYQIAGFVA--HD-
DIEPTKEWQaEIESALRTMDTLIAIITPEFMESKWC-NqEVGIAIGR-----GKLVVPIRVGAD-P--
HGFLGKYQALTIEKsATVNDIAR--
EIFDIVVKHHQSY(ARMADALVERLAQSSSFDMAKRTMTLIEQVPNMNHDQVARLVRTIDENLQVEKAFGVPE
RIKSIIGRLGQPDAS)}*

$3340=152(153):
>gi|1085489379|gb|OGW51407.1| {|0(6)|<Nitrospirae(B)>}hypothetical
protein A2Z50_07035 [Nitrospirae bacterium RBG_19FT_COMBO_42_15]
{(ML)---IFLSWSgHKsKAVAEALKTWLTQVIQA--
VEPWISSDIDKGSRWNqEVSAKLEESKFGIICLTRNNLDSKWILFeAGALSKTKNTK-VCTLLLDITPSEV-
EQPLSEFQHTTIGKKDMlkLMHTINKSIISAGKRGLPDKVLD(STFETFWPPAS)}*

$3341=231(217):
>gi|1085129526|gb|OGT22754.1| {|0(251)|<Proteobacteria(B)>}hypothetical
protein A3C55_06080 [Gammaproteobacteria bacterium
RIFCSPHIGHO2_02_FULL_42_13] >gi|1085180339|gb|OGT68741.1| hypothetical
protei
{(MKGSTAQPK)---VFISYSwtS--
PQHEQWVLDLAERLSGDGIVVVLDKWDLKEGQDKhvFMEQMVNDESIKKVLVVcdrvyqTKADVRKGGVGtET
QLISKEVYENtVQEKFIPI-VREY-
DDtgkpciPHYMASRIYIDLSSDekFEENYQKLIRnLYDKPLIKRPPLG(TVPAYIVEEEQITLKTSHKVVEI
KNALLNDRRSANGLIADYLDTVISSLEDFRLSGGSVTDFDDRVVE)}*

$3342=225(226):
>gi|1085650047|emb|SDH22935.1| {|94(270)|<Actinobacteria(B)>}integrative
and conjugative element protein, VC0181 family [Microbacterium pygmaeum]

{(PAPSGPSTIDRNAMRAAAKAADGPVALLILTPGDTAAYFGLIARQRLGRVVTRDAHVTIPPPRFGQLDPLPDGAVRGDGP)---VFISYRQSDGTQQAGSLENLLRAAGL--VVWRDRVDLRPGTTTdRLEQALTEGlSAGVLVVTPDIIDSDIVREreLPRLLQLDEDPAFSLCIANRIARPGSESKCDYDAPDRLLRLSPARTLADKKQANVLDRGGELEIVR(DLLMHR)}*

$3343=223(220):
>gi|1085515024|gb|OGW74069.1| {|0(58)|<Nitrospirae(B)>}hypothetical protein A3J72_03435 [Nitrospirae bacterium RIFCSPHIGHO2_02_FULL_40_19]
{(MSEHQIK)KPKLFISHASSdGEFAQVLQTEIGKVFADGIEVFCTSSPG-AIKASKdwlaSIEEKLDNAQAVIVIVTPVSIERPWLWFEVGAFWLRARKDqtaIYPLCAPEIDLGNLPSPLDRLQALSMGKSQD--LKLLFEG--LIKQFGFGKISS(FRAANVTKRIPKYKNVKLSEIDIQERVLYSGEYTGYSDEELKEIIFSEYLSNAFDEIKDDPSLYEERESSIYS)}*

$3344=267(253):
>gi|1085867226|emb|SDT15793.1| {|136(0)|<Actinobacteria(B)>}Predicted nucleotide-binding protein containing TIR-like domain-containing protein [Actinoplanes derwentensis]
{(RMRWRHTATTAAGVPVGSHTGWLVVETPPDVREMAVRFSADYRLEPKDTMGLRAGSRPVDRTVALPAPAPGPRAEPDADR)SRRVFLIH-GRDDVFAGRMRDLLSNLGLR-VL--EWDPlvrtagrGASPHVLEVIHHGLNSAQAVVALLTPDdvvSLHPDLWAPrEdahetvpmmqprpnvlmelGAALLAFR-----ERTVMVKVGEIRP-LGDIAGVNYIDFHE--TD--AARG--KLVSRLKAAGCT(VDDTGTEWRRPARFEGLAVFDRRPPVTGPAGTP)}*

$3345=213(197):
>gi|1085620094|emb|SDB38088.1| {|0(291)|<Bacteroidetes(B)>}TIR domain-containing protein [Flavobacteriaceae bacterium MAR_2010_188]
{(MTGVTGFKYD)---IFISYAHVdNEmafnqkdpYVEQFYKNLQLILtkrfgRPNMVTTWIDHKKLVGnTVFDkSIEDNLKNSAIMICLDSKPYQSSDYC-QkELKMFYDKANQ-EeqgliigyESRIVHVLL------NNIPFAKWSKEFRGTsgFPFHDSTDDQNLGDPFETTTEEY(KKSMLKLRDALIPLLESFQINPTSTEVFKKLDEKEKDEKFCIYLAEV)}*

$3346=163(161):
>gi|1086088434|emb|SDV14837.1| {<Proteobacteria(B)>}TIR domain-containing protein [Pseudomonas vancouverensis]
{(MP)---VFISYRH-MDRAH--AIHINTRLMQANIRTYLDvlDPESQTTEDItgVITRNISECTHLLAVVSERTAQSWWVPFEIGEatitHRRICSFKTGPTELPLYLDKWPKLSSDRDIEFFIDAYRNEVTHKRSMSLESIHGSESARSYN(RSNADHFHADLKARVLRGF)}*

$3347=161(171):
>gi|1179585862|ref|WP_083354071.1| {<Proteobacteria(B)>}molecular chaperone Tir [Pseudomonas moraviensis] >gi|1086009422|emb|SDU73730.1| TIR domain-containing protein [Pseudomonas moraviensis]
{()-MPVFISYRHMDRAH-AIAINTRLTQAKI--RTYLDVldpESQTTDDITeVITRNITECTHLLAVVSERT------ALSWWVPFEIGEATiSNRRI-------CTfkTGPAELPIYLDKWPKLSTASDLDFFIEAYRDEVSNQRSMT(LDSISESVKGAYKRDAEMFHEQLKNRIRRGF)}*

$3348=279(278):
>gi|1086176003|emb|SDJ07433.1| {|75(254)|<Firmicutes(B)>}TIR domain-containing protein [Lachnospiraceae bacterium G41]

{(ILWYRTLCEYGIEYVPDPISDKYFPTLHRINDESILDCSFFKNCIELCEGEQKETLLKEAKYIDEVQTKYL
NIAANEDPY)--DVFICYKETDldtgeeteDVHLCEELYKKLKMMGY--
KVFFARETLEqklSIEYEpYIFAALKSAKVMV-VLGTKA-EYFSSVWvkNEWGRFLKLMAKnPEKQIF-----
FACDDPEELP---
RAFAYKQAQILGEAGAINNLAGNIDEYLKG(SPQKTKVITNQEKFDQAISAKSLEYADRLDKTQFGDREKHLR
QDIWQEYGKAELTNKI)}*

$3349=254(250):
>gi|1086174488|emb|SDJ71400.1| {|73(46)|<Firmicutes(B)>}TIR domain-containing protein [Lachnospiraceae bacterium G41]
{(ADILWSETLCDYGIEYVEDPVSGKYLPTLHRIKDESILASPYYLEALDLCDDEQKEKITAAAEQIYKIQEE
YLNIASNEK)PYDIFICYKE-
TDNgKQTedseVGMKLYDRLNAMGYKVFFSRVTLkdKLGIEFepYIFAALKSAPVMVVIGTkPEYFEATWVKN
EWSRFLRLR-----
EKDPNKQIFFACDDIEDLPKAFARRQAQILKSDESIVNLANNIFDYLKPNGG(AQQVNAKCPFCNKLQKINPV
NETAICIHCN)}*

$3350=218(221):
>gi|1086194040|emb|SDL06077.1| {|0(201)|<Firmicutes(B)>}TIR domain-containing protein [Halanaerobium congolense]
>gi|1086199485|emb|SDN16510.1| TIR domain-containing protein [Halanaerobium congolense]
{(MSAKKF)--KIFLSHIHEeREIAIFLKQKIEEEFLRAVEVFVSSDDSC-
IGGGDlwlsRISEELEECNLVLILASPKALKRPWINFEAGAGWFMNKK-
VIPVCIKGVTPLTLEQPLKSLQVRNIEIKDD--LRGLLFD--
IANNSNLDEPKS(NINELFSKVKEKINSIDTIKSVEIDSNENENKIFEKKERKNDELPSLKQKSTVFFSNRIE
SAFPGVRGTQWFTKS)}*

$3351=174(182):
>gi|1086176430|emb|SDI94771.1| {|0(749)|<Firmicutes(B)>}TPR repeat [Lachnospiraceae bacterium G41]
{(MANDNF)--DVFISYSSKNKNVADAIVAEFESSGIK-
CWYAPRDIMPGEEWVTAITKALEDCKLQVLIYTED-SNTSRQVMnEVAMAFNAG-----
KTIVPFRLSEEKmsSEFEYYLTRVHWLDAI--
TPPLQEKIEDLREYVDIILSGV(DTSNLSRNAVKKQKSEKIEPKKKSKKKVILPVAIIG)}*

$3352=254(254):
>gi|1086236401|emb|SDB16471.1| {|81(24)|<Firmicutes(B)>}TIR domain-containing protein [Eubacterium oxidoreducens]
{(LPADEKELVSYGYQLLEYILDGPKELFGLCMGYTDSRKFSDNIEAFIRKSIEPFVVALRTYLEIEFIEADN
DKPSTDKEI)-KNIFLSYCQKdTDIANYLEEKLQPVI-
EGKATISRDiRDVEYHESFKRFMNSIEKHDYVITLISDRYLKSRNCMYEMLEVVKDSNF-
SKRLVFVVISEKD-
RSLYKAEQDLLIEAGVYtaSGQAEYSKYWTAEKHKLEREIEE(IGNPVYAISQIKEVKVLEKILLDLSDFLEF
IRDN)}*

$3353=213(224):
>gi|750288594|ref|WP_040570580.1|
{|0(219)|<Actinobacteria(B)>}hypothetical protein [Microbacterium paraoxydans] >gi|1086342694|emb|SDG59401.1| TIR domain-containing protein [Microbacterium sp. 77mfts
{(MPDPVINPHDPV)----FVSYRHSdgivlaAELTWLL--------
RAAGIPVWRDVDDLPPGdtdARLqqAIDEGISGA---IIIITPQIADSR-
VVREIEAprLLNLHRAApafALGIVNAVHAD---SGAVDYDA------PDRVLGMEPPEL--

RSVDQKSATRDG(LVAMARQMLWHRIAAIRPLVSASGGELRMSLQTRNTPQVYDRTDADLDIRIRPSTHEKLP
SAAGLQDLAQTA)}*

$3354=188(190):
>gi|1086249705|emb|SDG82223.1| {|0(585)|<Bacteroidetes(B)>}TIR domain-containing protein [Psychroflexus sediminis]
{(MN)--RAFLSHSSQ---QKELiKKIASNLGKSNC--VF-
DEYEFESGMPLfeEILASLEKTELFVLFISDDSLNSKWVQKEITLARRNLEIDenkrifPILIDKSIDVV---
QDSRIPDWLKDYLMKPYQDHFIITKKIRQRLREISFDQNP(LFKAKENLFVGRNSLFEDFEAKIFSLNDVKPN
SIIVSGLEGIGRRTFL)}*

$3355=223(221):
>gi|1086152351|emb|SDL35210.1| {|0(101)|<Firmicutes(B)>}SEFIR domain-containing protein [Clostridium cochlearium]
{(MEKKPS)---AFISYswdsEEHKEWVVGLVNLLRDNGVDASadFFEIQR---
GTTNLYSMMVENIRDKDYIIIVLTPEYAKKADSLqggvgFETSMLIPL-
IQENLQKIIPIIRCNENDSkaIPFYLKGVHYIDFSN----PYSfKE-
KFYELLHRIFKVD(LIEQVPLGKRPDLKPKKVARPDRVAIEGIDDLVPDFREITDADKNKFMKSSFQQIREGL
IQLLESTREKNPNFDF)}*

$3356=221(221):
>gi|1086373587|emb|SDI57180.1| {|29(0)|<Firmicutes(B)>}TIR domain-containing protein [Pseudobutyrivibrio sp. 49]
{(DEYNKPSQDWINDVEIFYNKYLKNHALGTRISTLLFHRNLSAFNDLRSCLDSINRDKEFVFKMNAGTTVEV
PMYKSASLP)EYDVFISHAN-KD-
KLQFVDELNDSLTKLGISIFYDKNSLEWGDKWkkKILEGTQKAEFAIIVISKNFFDREWTEKELIEFLNRQNK
NGQKIILPIVHGISNDDLRNMYPEVADIQTIDSKDYSCDEIALLFARQLIQRLKS(Y)}*

$3357=217(214):
>gi|1086415411|emb|SDN96831.1| {|0(1426)|<Bacteroidetes(B)>}TIR domain-containing protein [Prevotella sp. BP1-145]
{(MENY)--DIFISYKVDTNQTDAKKLYDVLENKGY--
SVFLDKEKLQGGKeWNpQLEYTLNTAKCCFVFTTPESLNAFQDGWdkED------
SDTYsYYCHEINLIvkrlnDNSCTKKPLCIPlftEPIETFCKGNEYLRNNPNAEQCILTLAQVQGYQ(YSKIQ
NNLTFAPEFIDKICKQLSNEGIYPFGKKGKHISAKEFFYDEGRATKAQGFREERQYYVERDIDNT)}*

$3358=199(202):
>gi|1086455882|emb|SDM43189.1| {|0(3)|<Euryarchaeota(A)>}TIR domain-containing protein [Haloarchaeobius iranensis]
{(MAEEQ)---VFVSHAPSD-LDLVQELFSTVQNLHL--
DIHIALEQIEPGRNRqDLEGRLTNSDLLVVVLTEA--------
SatNRWVNQEIGYAVaKGIPILPLsadgveLGGYLQRHEEvSLDPDEMEVTVFNLLARLRSELAPLGTLSTPN
WFVR(FPCNFENCGTTVTLDVEEIQKKLWQMFEHNQALVAGCEECDAQYFFNPATLGYVRRE)}*

$3359=188(199):
>gi|1086415413|emb|SDN96923.1| {|0(411)|<Bacteroidetes(B)>}TPR repeat [Prevotella sp. BP1-145]
{(MDT)KYDVFLSYSTFdKLIAEAVCGYLES--
HQVRCFVAYRDIPKGTQWPKIIPSAIKSSLLMVAIFSLEFNRSE------QTDNEISIADKRH--
IPILTFKI-ADEDF--
DGIKEYFLTKsNWIEAFPNPEKHFGQLLNSVRLL(LGIKCEDTRIYTHTIITPEQKIADGYLQKGLHLRNGID
GQCDFALSAYYLQKSAKL)}*

$3360=175(183):

>gi|1086384019|emb|SDG80526.1|
{|0(269)|<Bacteroidetes(B)>}Formylglycine-generating enzyme, required for sulfatase activity, contains SUMF1/FGE domain [Prevotella sp. BP1-148]
{(MNH)--DVFISY---
SSQNKEAAQAICHALEQNEIRCWMAPRDIPYGAQYgdVIDDAIKTCKVVVVLFSETAASSQWVNGELNVAFEE
Q-----
KTIIPFRLDQTPLKGQTRVMLNQRHWIDAYPDYKTKFNDLVIAVAKSIDKEV(ITDSPVPTSKFSTTTGKGNK
RKLGCHITIGFVSITIIAAF)}*

$3361=207(210):
>gi|1086466085|emb|SDB14051.1| {|0(166)|<Firmicutes(B)>}TIR domain-containing protein [Ruminococcaceae bacterium FB2012]
{(M)---VFISYKS--DPDYDIALKVSDLLKGEGVNVWMAPKSID--
DNFvyEVPHAINTCEIFVIIISKNTVHSEQVMNELLLAVKHN-----KKVIPLIFE-
KKSEFRMPDSyeyqlgqkqwRYFSLEDQNMVNWLVEQC--
KMGEPIIEMSVD(KFNDKKITLMKGDYQENMDYVINNYPEKLEHIAFAMGIDCSSSLKISSKKGILKWVCKYL
DEKYGIKID)}*

$3362=170(173):
>gi|1086383177|emb|SDH26649.1| {|0(188)|<Bacteroidetes(B)>}Sel1 repeat-containing protein [Prevotella sp. BP1-148]
{(M)KYKIFISYSRKdSGKVEAIKKEIeQATGVE------
CWMDMEGISYDSpdfvdVIVKAIDNARVFLFMLSEHSQKSRIARGEITLA-QKKNK-
HISIKNIDKCEMTDNFTVLYSQNNLCDYSEE--
NQKKKLFHEIRKWLGLEDFNS(GVFYQKGNITQDTFLEVAEQLYKRKAYAEAYN)}*

$3363=218(240):
>gi|1086464243|emb|SDB50838.1| {|0(543)|<Firmicutes(B)>}WD40 repeat [Ruminococcaceae bacterium FB2012]
{(MPQEPGCFLTAAERGGNKVDH)---IFISYSRKyQESVTALAEVLRERGLTVwQDI---
SGKGFGIPYSLkwfeVIKEAIFTASAAVIITSPEWYKSHVCKDE----W--------------
FLIEETGLFTTVIPAAEIQDNLG--RQKAADK--
IISDISKVGEDL(IQMHTWALSGAFQLLNKRSPIILLPNVADSLKTFGKLTVLKESREYFEDNKTAEFKPEIR
ENIFRYIGLVKRRVISDFV)}*

$3364=215(220):
>gi|1086542452|emb|SDO78449.1| {|61(0)|<Firmicutes(B)>}TIR domain-containing protein [Clostridium gasigenes]
{(PKDGDINFMKEVMTKAGAIYRPNITNINLISEFGNRRFNSININAANQVDGIFEQMFVYPEKGILQKKGYP
IRPMIGKAS)---VFLSHSSEnKDYIEELIPYLNASNLP---VWYS---
EFCIDYGDsivnEVQKGINSSATVVFLITKEFLESNWCKTEMetfltRLAYKDDIL-
IISLIDSNIDEKNIPIFISNKKYLRIKSPYD--YKSIANK--IM-PTIKNKYYN()}*

$3365=208(224):
>gi|1086565517|emb|SDQ06156.1|
{|0(334)|<Bacteroidetes(B)>}Tetratricopeptide repeat-containing protein [Chryseobacterium soldanellicola]
{(MNQSKV)EKTIFISYSWQdFEVADKIENDLNCSGIK-------IVRDVKLEYKDnifdFMK-
RIKNEDYALLLISNSYLNSVNCMREV--------L-EIITNQQEFSKKILPVILPNTKLFKAEDRID--
LIKNWEK--
RIEDLNIKLKSL(NNLSNTEEIIKTIGDYSKIRDSIDIFTQTITQLNCKSFQELDNANYQPLLEHIGILKDDL
INLIIRISQIKDLLEREI)}*

$3366=129(147):

>gi|1086334217|emb|SDK14616.1| {<Euryarchaeota(A)>}TIR domain-containing protein [Halovenus aranensis]
{(MTDPTGR)---VFLSYKHEqTDVANFLQTEL---ERHGV-PIWRDiFDLKP-EPLRdEIIDQLENPETAsgIALVSEGVADSDIILNdELPGFNSRWDR-DDEFFVVVV--PC---------------PDIsvGEAKSILNEAPILHDFSAWKMLP()}*

$3367=174(179):
>gi|1086464053|emb|SDB53440.1| {|0(577)|<Firmicutes(B)>}TIR domain-containing protein [Ruminococcaceae bacterium FB2012]
{(MRRCSVKPYEGNEGY)---VFISYSHGdK---DRVYPILERMAADGC-RLWYDEGITPGEEWPeAIGRHLADCSAFISFVSKRSADSDNCRREIIFAQRKHKP-FFSVILEEFELSP-GLEMQLSAYQSLYMYLLrdEDEFFEKFYKTDFADSFRTISAK(PEKAPAPETAAPERPAAPSEKAPN)}*

$3368=259(259):
>gi|1086465340|emb|SDB30161.1| {|79(201)|<Firmicutes(B)>}Sel1 repeat-containing protein [Ruminococcaceae bacterium FB2012]
{(TAHCPGREFDLVCAREKNIPILPILCEAGLETLFSRVCGHYHLLDARKEEFSYELSEYLTSRFDDYIRLAEEKDPVRYRA)----FLSYRKKD--RSEAVKLLGMIQSYPVCldtAVWFDDFLTKGENYDrNIDAALRGADFVLLAVTPSVLeasggAPNYVESiEYPHA----QA-YGKPIIAVEMQPV-DRREFARRFPKAVLVSPreLGNVLGQLYGRPCTAPPVIDAET(ACNLGKLYLKGERVYKNPGLGCRLLQEASRHGSWEADWR)}*

$3369=255(253):
>gi|1086465420|emb|SDB31491.1| {|88(477)|<Firmicutes(B)>}TIR domain-containing protein [Ruminococcaceae bacterium FB2012]
{(ADEDLGFAVSNGIRLLPICTEDISDCLNEYSRLFGTMQYLDRSVSDDTAVDYRKKLKDFLDSTLLDDKTAELIRSQFDAY)---IFLSYRKKdRRSANKLMRQIHRNRHFISLAVWYDEFLVPGEKFDeSISKAMDSSKLFMLLVTPSLLEKDdfgeknYIQRiEYPRAREL-----ELPVLPVELIKA-DRELLEKDFIGLPVIVSadDEEQLYCVLTESVSGCISDLSGC(AERLYYLGLAYLNGCDVEVDRDLGIELLTSAGE)}*

$3370=227(212):
>gi|1124276338|ref|WP_074573138.1| {|0(248)|<Firmicutes(B)>}hypothetical protein [Selenomonas ruminantium] >gi|1086360621|emb|SDP63366.1| SEFIR domain-containing protein [Selenomonas ruminantium]
{(MIDK)RPKIFISYAwgN--KEVKDRVKELAERLRHDGVDVVLDIWDLKEGQDKyaFMEQSVNDETIdRVLIICdkvykNKADNREGGVGdETVIISPEVYKHnQQSKYIPVIFErdengET-YIPTYISSRMYIEFSSGNEDDYEQLLRmLYEKPLNKKPPLG(EMPSWLLEDNSENIKMEGEVRKLKKANENGRGDFAKNNARKFTDIIINALNEQQNVETFKKADAIDKV)}*

$3371=224(248):
>gi|1086415414|emb|SDN96942.1| {|0(258)|<Bacteroidetes(B)>}Formylglycine-generating enzyme, required for sulfatase activity, contains SUMF1/FGE domain [Prevotella sp. BP1-145]
{(MEQADLKRHVFISYSRRDYVNERGEVKPGNSVSQIMDA)------------------------LEKAHISYWIDKEGIYHGDKFkgVLASAIEAADV-FIFVSSESSNKSEWTAkEIDVAVYEKK-----IIIPVRIDDS-KynnsVKIDLVGLDFINYQSNPALALEELVKsIKKHIKAPVVDVK(KIKDEIKTLEKEGCQLYSQQKRIYDEVANKKRQIGEGTMNCPVCGETNDISSLYCPRCGWAFVPFTSKKL)}*

$3372=176(177):

>gi|1124323565|ref|WP_074615851.1|
{|0(82)|<Actinobacteria(B)>}hypothetical protein [Microbacterium enclense] >gi|1086382085|emb|SDB95345.1| TIR domain-containing protein [Microbacterium enclense]
{(MKI)----FISWSGNasrlvaEALRTWL-----
PTVLAGRVECFVSSQDIRRGERGmeVIAGELQDRDYGIVVLTRDNLNSPWVNFEAGALGKSLGLGkvaPLLVDVSRADV---
EGPIAQFQSTLLTERDDMRQFVRDLALLVPGLPEATIDTM(FGAKWDELDSAVASAGVVAASPKTTRTAESMLEE)}*

$3373=254(260):
>gi|1124525577|ref|WP_074809626.1|
{|7(40)|<Actinobacteria(B)>}hypothetical protein [Cellulosimicrobium cellulans] >gi|1086620411|emb|SDF96915.1| TIR domain-containing protein [Cellulosimicrobium cell
{(RITMIREAATLLGMQEWGEIDLVLDQFGLPTTPMWEGTKDEYCRSFLQRASDDALDQVHAYLTTNGRPGPRPGGGPWSGE)KLRVFFSHLA-E---
HKRVVGEVARVLENyGVEPFVAHDSIDPSKEWqlVIEGALSDCDAMVVFLHPGFRESNWCDQEVGWAL----G-
RQRPILALNYGVHPHGFMSKFQDQPCSTAHVGQVAAYIMDWLIKTPSLHGRLTR(GLVDAFVESRGWDLTRRLVPYLDALSSVDDDDLTRMERAA)}*

$3374=238(240):
>gi|1086782845|emb|SDE59353.1| {|204(30)|<Bacteroidetes(B)>}SIR2-like domain-containing protein [Pricia antarctica]
{(WYFQLFFQLFLQIMDFDDDSDILNSNNLDPKKLNTKNFIMNEFSMDFFEDDASKAIKVLNEAVKSGKISWEKEDAFDGPP)-PKVFISYKW-GGESEKLANELKDTLTEKKIVVKIDKEELpyKGN-
IEEFEKHIGQADGILVVVSHAYLESEHCMYELSEIFNNGNF-
VERIFPLILSDAKTFKSTDLGIYKDFWNEKIKDIGKEIAKDPGKAGTFMDDINK(YNQILKDFDDITGVLKKSNL)}*

$3375=216(206):
>gi|1086827765|emb|SDB53387.1| {|0(300)|<Proteobacteria(B)>}TIR domain-containing protein [Bauldia litoralis]
{(MAFVPGYEN)--DVFISYAHADNEvdvfggawvdKLAKYLGIALKQRlgrgdEV--
RIFFDSKRLRANHHLdEILQNVRGSAVFLAIGSPSYINRQ------
WTRDELAAFVdCSKGDSRRLFAVERlpLDPgDRYPDPLNDYYRVNFWQRDPADDIPLTVTQQLNSDLY(YVLLQKLAEQIRSQLVAMRKQGDAPDLAPISIAAASRHQAENPVAEPAAVPRHPSVA)}*

$3376=172(193):
>gi|1086673428|emb|SDK69488.1| {|0(31)|<Euryarchaeota(A)>}TIR domain-containing protein [Natronorubrum texcoconense]
{(MTGEQIYVSHAPADLE)---------------LVQELFSTVKNFPF--
GVHIALEEVGSGRSRkRLEGRLANSDVVVAVLTGGSAD------
SRWINQEIGYALaKGIPVIPLSDDERRQGGFVGDVDGVTIDRRNPSYTIFNLLSRLRSELAPLGALS(VPNWYIRFPCTVPDCGHPVTLEIERDQTKLWKRYEHG)}*

$3377=209(221):
>gi|1086705714|emb|SDD47898.1| {|0(126)|<Actinobacteria(B)>}TIR domain-containing protein [Geodermatophilus telluris]
{(M)--RIVISYAREDRAVVEELVETLRLLGHD-PWTDA-GAHSGSRWWDEIVLRIQQCDLLLAVTSPASLV-
SRACTlERRYALELG-----RLLLPVQVAPVNmqGLPSEFAQIQFFDYRT--RD-SASTG--
RLYRALAQLPPP(RPLPRPLPPPPPPPLSYLNQISDLVSALVPDAYLQDRIVTDLTTGLRSADPEERTTAADLLRRFAEHPQSLQGPAERARQ)}*

$3378=212(221):
>gi|1086828102|emb|SDB48180.1| {|0(120)|<Proteobacteria(B)>}TIR domain-containing protein [Bauldia litoralis]
{(M)--KIFLSYASERRAAAEPIAFSLRDRGFE-
VFLDRDDLPPGRSFDEQIEMAVARADLFVFLISPEAVDPGRFTLtEVEFARRrwrkAD-----
GYVLPVMIAETQlaSIPSFLKSVNILQPAG------NAAA--
EVSSAVAAMARP(PPAARIVPLAIAMGVVTGLLTTFVPLPLDAIPGEPVSAGYVIHYPVAAPLGAPVFFGIGL
TGLVAYFERPSFARLLAILL)}*

$3379=153(152):
>gi|1086658462|emb|SDC93556.1| {|0(175)|<Proteobacteria(B)>}TIR domain-containing protein [Sphingomonas sp. YR710]
{(M)--KVFISWSgSRsKDLANALRQWL-PMVLQYV-
EPWvSDKDISAGERWAqAIAGELESANFGIICITPENINSEWILFeSGALSKSMLDG-KVIPLLFGLELSD-
LSGPLSQFQAQKVEQAGimEVVRAINKVAEKPASQQIVDQLV(PTLWPQLQETL)}*

$3380=172(174):
>gi|1086666436|emb|SDJ23226.1| {|0(116)|<Proteobacteria(B)>}TIR domain-containing protein [Ferrimonas sediminum]
{(MH)---LFLSYSSAdYDIAAPIQQALVQLGH----
TVWFApQRIGAGQCFAaEISDAMKSAEALLLLASEHSVgNATRPGSnEVKTEIKMARE-KGIAVIPLKVDAS-
LESGGATGFDYLLKNFQwvDIEAMLLSRRFDVIAQRIDGEIQ(GGLKPDFSGFSAQLEEVETLLKAKRCNDAR
QV)}*

$3381=226(218):
>gi|1086782795|emb|SDE80590.1| {|0(506)|<Bacteroidetes(B)>}TIR domain-containing protein [Pricia antarctica]
{(MNST)-
KNIFAIYANEdKDVLPSLLLHLKPLEKDFNIAIWSDDVINRGQPWTpRNVSRLNHADVFLFLLSNTFMYSEFI
KQdEFKMVVDRYKE-GKAIIIPLVLDDC-PWNveftfndyNFNFSELLVFHKDEkqIDDWNPTD--
KTFTQVAYYVRGL(LASSTKKRDLEESAKRGGKKILNTKKEEQIVIDFFEEAEVNNNEEKERKVKKETETKIR
VEENSTFSEEAEATE)}*

$3382=227(228):
>gi|1086814883|emb|SDM66277.1| {|0(74)|<Actinobacteria(B)>}TIR domain-containing protein [Geodermatophilus siccatus]
{(MAACERPPAGAVVVGRAGASPGLRFRARASGPREGPQMR)---VFISWSGDRS-
RRAAGKLYEWLKRvlPGQIDPWmSTEDLEPGGRWEeELDERLEETNAGICVLTPENLASTWLHYEAGAI--
GKAV-GHSRVMTVLigLSPA-DVPR-
PLGKFQHTMPTRdgVSRMVISLNKLMGEPIESSVVNY(LFEIQWPWLEDSLVEITESAPAAALPRRSEYEMLE
EILTLIRDLPRSPL)}*

$3383=187(195):
>gi|1086705705|emb|SDD47562.1| {|0(339)|<Actinobacteria(B)>}TIR domain-containing protein [Geodermatophilus telluris]
{(M)--TIFLSYAR---DDEDLVKTMARVFEAARREVWFD-HDLRGGDVWwpTILDHIRSCSVFVFAL-
SDRSLESRACLsELDYAE-----RLERPVLPVRVGPV-
VKQQGnpLAQRHFVPFRADDAVAAIEVLAAAESSAARCPPLP(EPLPPEPPIPGAYVRQISQQVESTELTPKA
QLDVIAELRRTIAEETDRAVRQEV)}*

$3384=192(207):
>gi|1087137581|emb|SDM20372.1| {|0(32)|<Bacteroidetes(B)>}TIR domain-containing protein [Kriegella aquimaris]

{(MES)---IFLSYHFDqNVESLAGSIKRLIHSHDL--EV-IDGHRLEGQQLTdAVAERIQGADAMVVLLSK-
-----RGEGktNDWVQHERTTAFtHKIPFIALIE-------EGLPNN-
GPFQTFEYIRYKPDDLVEALLKVSETIFKW(KLRLGERVEAYLEPEEIVALVRSNYNQKDIVKYRFFSSKTGW
STWKEALIKPQSGGLSLFLEGV)}*

$3385=199(187):
>gi|1087230092|emb|SDA42014.1| {|0(2)|<Proteobacteria(B)>}TIR domain-containing protein [Janthinobacterium sp. 551a]
>gi|1099645330|emb|SFA87659.1| TIR domain-containing protein [Janthinobacterium sp.
{(MDIRYGCFLSY)AHGQYAFMN---
KFKDDLIEALACYLEPHldrEEVLFIDAEQLGGGDDIdlRVARAMCQSVCMIVLYTPKYEAHGYTRREFAAMQ
liEQERRTwYDlpsHLIIPIIMTRHPdglpQQITESGLYVDFSGYTLASGDLKSNLQYLPDIERIVQRIA(KH
YQLLKRSTPPGHDCSRFVLPDIPPEWRAVPPPHF)}*

$3386=221(220):
>gi|1086948693|emb|SDL72908.1| {|267(0)|<Actinobacteria(B)>}TIR domain-containing protein [Glycomyces sambucus]
{(TRAWGGVQSGHNLQIADRLISSFDEKEGGWLGPSRSVGKASSWASSLGVLNTYLLARDLVAAKISAEEFLE
LTQSEHSRR)RFDIVISFGG---
PDRAVAQEVRDRLVTAGLRVFFDFDFRHNLLGEdlaVTLQDIYFRRSRyaVAILSRSFLKSKWAgNWEWRAVL
ARMNSQQQGYLLPYFLENVEvpG-LNPTIGFLSSDHVSP-REFAEIVV-QKVTGA-FDAQFR()}*

$3387=221(227):
>gi|1087119317|emb|SDB74441.1| {|56(0)|<Proteobacteria(B)>}TIR domain-containing protein [Belnapia rosea]
{(KHTGIKAVMNFAEIGELSDAVIGANTIDAASMEPIRKRLEAHFDLGPRDAVVFERNGGFTQHEIDYMLQKH
EEAMGLGSV)--RIFLSHKGAdKPLVRRFKETLSLLGFDP------
WIEDEDAMPAGTnlerGLLKGFEDSCAVVFFITPRFVDENYLATEVDYARQQLRG-
KGEKRFRIITLLFDGADEKHVPQLLRSYVFN--RSYDDLEALRHILKALPVELG(PVHWKQP)}*

$3388=292(290):
>gi|1087113978|emb|SDE17659.1| {|0(436)|<Proteobacteria(B)>}KAP family P-loop domain-containing protein [Belnapia rosea]
>gi|1087119510|emb|SDB73997.1| KAP family P-loop domain-containing protein [Bel
{(MTAMGGYDAALDDAADDLLERRHVARRLHRLLTGTPADWSVRVGLLGDWGEGKSTVAGWTKALVEGDGHLV
AWFAPTA)------SATAEDL-WAGLASAVLKAADARKiepqatTRLRA--
FVTGRDAARRLAEAAKASKATQAIQSAAtkFLEIGPGHLkEVRAALGPD-----RRVV-
VILDDLDraDprlLPGLLLALRDVlDLPGYSfllpFDEDVVAA--
SLQEYNKAWGDG(RRFLDKILDFRVRLPAIGLAERRRLFEAETARACPFVPHGAAAGLEEVLPPNPRQLKALA
RGLSLMEEEARR)}*

$3389=173(180):
>gi|1087135992|emb|SDM86413.1| {|0(326)|<Bacteroidetes(B)>}TIR domain-containing protein [Kriegella aquimaris]
{(MESNTL)--KIFISYSREdSLFINELTAGLEACGFSILID---
KEDIVFGEEWQkRLKNIIHSVDIVILIISSESVKSSHVIWELQEALKLNK-----RIIPIVFTEI-
KNIKIPTFISKLNYCFFtnKSFGEALKNLSITIKIDIEWVRE(HTRLNILAKNWLQKNKPDELLLRGQELENA
RIWR)}*

$3390=179(187):
>gi|1087136038|emb|SDM87732.1| {|0(163)|<Bacteroidetes(B)>}TIR domain-containing protein [Kriegella aquimaris]

{(MLELGHYLKDM)--KIFVTYSRStQREVASLVKDLEELGH----
EVWFDRDISGGQSWWnNILSQIRDCEIYVFAIAKDAIDSTACIRELKYAEALGKA-P----LPVLIKND-
VSLALAPRYISNVQYADytKSDQKNAIIDLIKSLKGITPTTS(LPDPLPEPPEIPISYLDTLKEKVDAPTMTR
NEQIGL)}*

$3391=231(224):
>gi|1087138415|emb|SDL83403.1| {|0(815)|<Bacteroidetes(B)>}Protein of unknown function [Kriegella aquimaris]
{(MRRKR)--
NIFAIYAGQnNEQLLYLLLYLKQLEDEFNVAIWHDDPIYPEQQWKpKNASRFNRADIFLLLVSEAFLHSEFIK
QdEFKLVINRYRR-GKAIVIPVLLDDC-KWdidfefddHNFNIKELKVLPNEGkpITDWNSAD--
QVFMQVVDHLKEV(MASFTENHEQMKSKDKIKKKMAVSNRKEQIEINFFEENKINAITEEDKRLKEEAEAKKK
LQKENKRMEEAKAIRIAQEE)}*

$3392=222(219):
>gi|1087140044|emb|SDL41530.1| {|0(249)|<Bacteroidetes(B)>}TIR domain-containing protein [Kriegella aquimaris]
{()MKEIkmFMAYASEdEEYKVQLSKHLSALKEEGYISEWTAGEILPGANVMsDLRKRMSRCDIIALLLSADF
VGSDDGIElE-EMAFALKAK-KGTRLVPIKIRAC-MLSDA-YAQFSMLPDQSkpVDDpaWGSRD--
NAYMNIAEGLKKL(ALSMRDEDARGVREKIKQTMQSTAAAPSRPVSSSGSSEKSSSNNKTLLISIGVLAALLI
GLAGFIFMNNDRLKDDETTI)}*

$3393=213(220):
>gi|1087137579|emb|SDM20322.1| {|309(0)|<Bacteroidetes(B)>}sensor domain CHASE2-containing protein [Kriegella aquimaris]
{(ALLFALFFKYFKRLQTKPLLIAFLSFFTSIALLLGVGAAILAMGQVIPMGLPLMAILLSAILSWRFAYKFL
VTGVAEGSQ)KYDVFISYSRGnsdwviKNIYEPLKAFRKPNGDK--
LNIFFDRDSIGIGEAFtaKYMWAIVDSRFFIPVFSEEYYGKNHCRNEMDLAYK-RSVEkliTVL---------
------PVAFSYDAVPPIYTHINFMDITVNLNFIKDIEKVFE()}*

$3394=187(177):
>gi|1087382095|emb|SDC44283.1| {|0(306)|<Proteobacteria(B)>}TIR domain-containing protein [Variovorax sp. CF079]
{(MPLALPADLDNRSEP)-FDVFVSYaRA-
DDTAldgkmgwvTRFVLNLDRQLTMQmgnGASIFMDHQLAAGVAVTPLLERTASSARTLLLFlSRSYQQSPWC
ATELITFLQKNAA-
AARPASVFIVEMFTTNRSTWHPQLQSLKTVQMWAKDPRATAPKLLGDPMPKDDE(SSPYWINVNELAFALMNR
LDGA)}*

$3395=234(238):
>gi|1087382099|emb|SDC44470.1| {|44(122)|<Proteobacteria(B)>}TIR domain-containing protein [Variovorax sp. CF079]
{(VVFAEHGVDWSSLDLDTFADLACRDRRVLPVIDVAANATRIPSALTKYNAFLTSPWRTGWPTGLVDEVLSH
AWLRRRERK)---VFVSYKR-TDSAA-LANRLYDELT-
RhGYVTFLDDVSIDKGTDFqsDLKWWLNDADLLLVLLSPNFTSSKWCLEEIAFARSRSIG-
LLAVEWPDSVYAQKMTRVFPGAAAGSLRPSVLQATDPDQRLQLREKDLEGLRDD(PLCDRPLSPIGMERLLTA
)}*

$3396=204(192):
>gi|1087260317|emb|SDF44286.1| {|0(2)|<Proteobacteria(B)>}TIR domain-containing protein [Duganella sp. OV458] >gi|1087277617|emb|SDI82447.1| TIR domain-containing protein [Duganella sp. OV510]
{(MKGDIMTIRHGCFFSY)AHGQHAYMS---
KFKNDLVEALKCYLEPHfdnENELFIDSEQLAGGDDLdsKIARALCESVCMIVIYTPKYEAHGYTRREFAAMQ

LIEEERkhwytlPSHLIIPVIMTRHPvslpPQITGPGMYVDFSRYTLASGDLKANPDFIPDIEKIVHRIA(EH
YHYLKQSTPPGHDCSRFVLPEIPPEWRAIPPPHF)}*

$3397=237(238):
>gi|1087549885|emb|SDN76032.1| {|0(159)|<Actinobacteria(B)>}FxsC C-terminal domain-containing protein [Streptomyces guanduensis]
{(MSRFYLSHIRGVDEEWTAA)-----------------
FVRDLSAAVAARtgeAADrIAVRADPHVPGAAVEQ---
MAAATTMVVLHAPGYFGQSYCAAEWTYFQQRAQvlwsRTgRTaDAIIPVVWepTGQPlpaaVRRQAPLAVDSA
AYARDgvlHLLRLKARYAEDYRRVVAALADR(VAATHGRPLPEVPGFDAGSSGSFSFPVLPAEPGEGQRAVRF
VMAAAAAADDLPAERTATEFYGRSPTDWTPYAPGQPGVL)}*

$3398=214(217):
>gi|1087590906|emb|SDH16969.1| {<Actinobacteria(B)>}TIR domain-containing protein [Lechevalieria fradiae]
{(MPTFLSFIGKWSHLLFLPVSGYKYDFFISYCRHGSVQRWLLNH)-------------
FLDKLRECLADQYA-PTPTVYVDR-
SMERGVHWpsNLQDALRHSKIMIQLLTPQYYESRWCMAEQRSMRAREEMLglasvevSQGLIYPILYSDSDnf
ppEE--------
KERSWVNfKDVAHPDPvyqQSKKYLRfhtRVNQLAED(LVRLAKQAPPWQPDWPDVDPPQPPLMPPPQIPRF)
}*

$3399=149(164):
>gi|1087590910|emb|SDH17060.1| {|0(100)|<Actinobacteria(B)>}hypothetical protein SAMN05216553_11741 [Lechevalieria fradiae]
{(MRAHPAAGREAVREVGVTESAT)-YDIAVSFAE---EQRAVADEVVEACRQRGLTV---------
LESNdlpE-------AEVRflVPFVTSADEVGTTDEHVLPVLTGEDDPSSPHLRANGHLVDTLvsR-
VEEAETAGLARVPVA-DLVAALKQPEPEAAAVQELETG(QE)}*

$3400=196(207):
>gi|1087478705|emb|SDF73729.1| {|0(250)|<Bacteroidetes(B)>}SEFIR domain-containing protein [Mucilaginibacter pineti]
{(MASEN)KTKVFISYSwdnEDhKKWVITLADLIDANG--GAAI----
VDRNNLPFGGhiksFMLSSIMNSDIVLMILTPNY-KRKADAYEGGAGY----E-YLII-----
NDDLFKIITKNEKYIPIIRQGD--
LHSSATNFLQGFNCLDLRDGK(DYEENLEKLIKQILKPATVPITNPTPKIQLMENKYKDVSLVSPEIINKSLE
YFKQLFITDDL)}*

$3401=201(216):
>gi|1087255070|emb|SDC17443.1| {|0(192)|<Actinobacteria(B)>}Protein of unknown function [Alloactinosynnema iranicum]
{(MH)---VFVSHSVPDRDAAARLRADLAQLGRQ-VVLDRGEPV-
GPRWWESVLQRLQRCAVFVLLVSPSSVR-SPGCLaQLRYARALR-----R---
PVLLVPVRpaPLPPDAEDAPVFDPAG--LAGPERLT--
KLRRALMSLPAA(PPLPDPLPPDPPVPYLDPLRERLADPGLDQTDLREIFDELRGRLRDEHERPAVWGLLVRL
RARPGLPPSLLAEV)}*

$3402=206(217):
>gi|1087350440|emb|SDC67039.1| {|0(742)|<Proteobacteria(B)>}WD40 repeat [Cupriavidus sp. YR651]
{(MS)---VFISYSRRNTDFVRRLHEGMVAHGRE-
TWVDWEGIPPTADWMNEIHAAIDAAEAVAFVLSPDSIK-SAVCLqELEHALAQN-----
KRLIPLVCQEVNpgETPPALARLNWVFFTG--DDFDKALQ--

TLLSAVDTDLDW(VKAHTRLLVRAAEWDRKAREPSLALRGADLKAAEQWLTEGPTKSPPPTELQTRYVIESRR
LAVRRRTFLLSGVGV)}*

$3403=209(221):
>gi|1087428053|emb|SDI74324.1| {|0(174)|<Actinobacteria(B)>}Protein of unknown function [Alloactinosynnema album] >gi|1087439736|emb|SDP88889.1| Protein of unknown function [Alloactinosynnema album]
{(M)--QVFISHAAPDRAVATRLRADLAAMGRQ-VTIDGGEP-NGPRWWDGVLARIERCEAFLFVLSPTSLR-SPGCMaELRYAKQLR-----RPILTVRTQSM-PVPADLAGTGLYDpaIAG--ADRVRLLR--AAMLTLPAAAPP(PLSLAPRPLIPYFDGYRAQLEQPKLDRTAQHEMLGELRRRLRDEVDRTAVWSLLVRLRAR
PDVAPGVAEELEKLLAPGWR)}*

$3404=255(262):
>gi|1087449979|emb|SDC81242.1|
{|0(921)|<Proteobacteria(B)>}Tetratricopeptide repeat-containing protein [Burkholderia sp. TNe-862]
{(MSGVNGTQGQNANSLSSACPYCGSADLVFKSKVALWECQQCEQRFPGSAGTPANRSDSQATRPQY)---IFFSYAHdENRELVEMFKADLEKRGHR-IWFDAKDIGTWDNWKGSITRGIDSSNMMVAFMSRHALRDPGVCRnEIALGLNRF-----GTVYPVVLEAALenDIPLTLRELQWPDLHE--WRDIRAGNVPGVEWNRWYESHL(IELIGKIEGEAGAYASEIEALRDVLRPTGFESRIAEHLEDFVGRQWVFDA
YQHWVDH)}*

$3405=195(197):
>gi|1087549891|emb|SDN76223.1| {|0(581)|<Actinobacteria(B)>}hypothetical protein SAMN05216259_105437 [Streptomyces guanduensis]
{(MKSTCTYA)-----VSYlHGRDEAW---VARFLDDLNTR-LA-----ALPGATFVRWTARtpgpaspGVATADLVLALCSPAYFTEGP-ADaDW-AVLAHR-----TSLErargrsPRIVLPLLwePmaqrLPDVVAEADGFTARQ--PPEYRSLGLALLTMQAARHRAV(LDEVLADLTRWFAESRATAPELPGGTDPAPSVGELPRALAADDARFTAL)
}*

$3406=165(190):
>gi|1087423084|emb|SDK96659.1| {<Bacteroidetes(B)>}TIR domain-containing protein [Catalinimonas alkaloidigena]
{(MFKKDFEANVGNLPFAD)------------------------------IQVYTDEQIPLGENWHeALQTEIHACDFAILLVSDQFMHSKYIKEeEVAKLFTRKEK-EGILVVPVYFYSCRfyDWKVLSKNQLFKPLGADygRADRDPKKRFCYADLVRLDSVNG(VRIPQPNPDRGNYM
MDFVEKLEPQLKALANSKK)}*

$3407=179(188):
>gi|1087428054|emb|SDI74339.1| {|0(235)|<Actinobacteria(B)>}Protein of unknown function [Alloactinosynnema album] >gi|1087439737|emb|SDP88894.1| Protein of unknown function [Alloactinosynnema album]
{(MH)---VFISYSRRdQASITQLR---ADFERLGH-QVWFDRESHGGQQWWdEVLHHVRSCVVFVFALSPDSIRSTACLAELRYAAALRRP-----ILPVIVSRV-PRSDIPEGLGAGAVDYTerSAETAISLLKAFNALPMAKPLSS(RLPLEPEIPATYLHAYLDRIDADELGEEDQ
VELLDQLRTRLTETDE)}*

$3408=185(201):
>gi|1180224260|ref|WP_083743100.1| {|0(36)|<unknown(X)>}hypothetical protein [Pseudomonas gessardii] >gi|1087294682|emb|SDR38577.1| TIR domain-containing protein [Pseudomonas gessardii]

{(MAD)---VYIIYAK--ENSKTALK--VRDLLSASWSVWLD--DLIVGD-
FsvAITENLKEAKCVVALYSSFA--HKHTITgELSLAEKYQKK-----VVPLILDDS-DPPYPYGHFSSVDF-
RSWQGEADHSCFQLLQKKIGLVVPP(QGKPVRLATIADGALALPNVFMSVSSHETQFDPVEALEALTVYPTRS
ILVSAYDLVNS)}*

$3409=225(212):
>gi|1087419682|emb|SDB02204.1| {|0(248)|<Proteobacteria(B)>}SEFIR
domain-containing protein [Pseudomonas sp. NFACC13-1]
{(MKEPK)---VFISYSwsS--
PSHERWVLDLAENLMASGVEVKLDKWDLRPGGDAvaFMESMVADKTIdKVLIISDKNYSEKaderrGGVGtET
QiISKKVYEQVdQERFIavIAEKDEEG-
KayTPIFYHSRLYIDLSspETYSEGFEELIRCIYGQPQYQRPKL(GKKPDFLNKEVAINLGTAPLARRAITLI
KEGKNIAGAALEEYLMVYSENLERIRIKLNSEVLFDDQV)}*

$3410=222(219):
>gi|1087351567|emb|SDB98162.1| {|0(1268)|<Proteobacteria(B)>}WD40 repeat
[Cupriavidus sp. YR651]
{(MATDTGGD)RYDAFISYKRTdiafaRLLEKALNAYAPPKalaVPQRRLRIFRDEGDLTGT-
EYykSIDGYLARADKLIVICSPAARQSTFVDDEIRRYIAHHGPEtliPILLAGVPNNE---ATPGQAAEQ---
AFPQALTEALEM-P--
LAVNYHSFDPAH(DKVDRGKFEGSWYTLLANLYGLPRDEIEQRDRVRRQRRRNLLIGVMGSVTLVVSALAVTA
WMQKLAADEQR)}*

$3411=221(218):
>gi|1087590676|emb|SDH33797.1| {|0(200)|<Actinobacteria(B)>}TIR domain-
containing protein [Lechevalieria fradiae]
{(M)RFWAFLSYSWSdrpkaVALHRRLEKFVIPRHarhaadpegpaGRRLRPIFRDDDEMAPSGPLaeRLRTA
IDEAPAMILLASPASARSHYVNLEVEHFLSGHDPKrlvIIVTG--------------
ADRPAKPELPPALDRV--EDP--
LWLDARGARKLT(RRNLVRVAAVVLDVGFEALWRRHVRYRRQLTALWCALALVVGTVVGMAIVRQLDAEKRSP
EQQRAQFVEWMTNNTNA)}*

$3412=228(254):
>gi|1087830032|ref|XP_018668765.1| {|438(145)|<Chordata(M)>}PREDICTED:
uncharacterized protein LOC100182474 [Ciona intestinalis]
{(KIPGIKIKDVSHPATGAGVMDLYSEQGVGVTGKPVISGTADIFFSYCWNNSHDAHQKKQVSSYQGGEWSDP
RLIVKKISE)---------------------------
ELGIASWIDVERLEsanEGMGMfdQIATALKECKVAVVCISTEYANSDNCRMEIQFAVKSlK-----
KPVVAVIVGTGDDWKEGVIGNIVDEGIPTVDLQSVTSAGELEVKILEISKAI(QDRISLSTNPSMTSQNQVLA
TKGSPERNRAPKCG)}*

$3413=220(236):
>gi|1087599302|emb|SDP90092.1| {<Actinobacteria(B)>}TIR domain-
containing protein [Lentzea jiangxiensis]
{(MPTFLSFIDRVPHLPLFLVSGYEFDFFISYSRQGNVQKWLLNHFYNK)-----------------
LKDCLADECA-PTPRIYMDR-
HMPKAVQWahNLQTSLRRSKIMIQVMTPHYFTSGWCMAELYSMRAREEMLgfatteiPQGLIYPILFSDSEnf
peEERM--------RSYHDfKEFAHPD---
PVYQQTVHYFQFH(QEVKRLARDLVELVNQVPDWQPDWPIVELPDPIVTPRPPLPRFKPCPEP)}*

$3414=202(188):
>gi|1087630051|emb|SDM56940.1| {<Actinobacteria(B)>}TIR domain-
containing protein [Lentzea violacea]

{(MSGYQFDFFISYSRQGSVQKWLLNHFYNK)-----------------LVECLADECA-PTPRVYMDRfmpKAVEWSHN--
LETSLRRSKIMIQVMTPHYFTSGWCMAELYSMRARENMLgfastqiPQGLIYPILYSDSEnfpmEermrkwhdfkeyaHPDPVYQQTVHYfQFhkevkQLaRDLVELADQVPDWRPDWPIVERP(DPIVTPRPPLPRFKPCPEP)}*

$3415=227(223):
>gi|1087608081|emb|SDG78075.1|
{|0(146)|<Bacteroidetes(B)>}Formylglycine-generating enzyme, required for sulfatase activity, contains SUMF1/FGE domain [Chitinophaga filiformis]
{(MRKPF)KYDVAISVAE---
EDKDVAKQIVAALKKKSIPYYYYEDDTAGNWGEyvmNLTKDAYGKHARyiLLITSQIYVEKYWSGVELQVAVS-SFVRGKPRVLQLRLDNTPvdS-ISKHQVYLEWNN-NP-EEIAGILE-
EKIkrqkrAALRRELPLF(ICIVLLSITAAVSYHISWPPGGRPLMTPPKEEKVLITGMPALTENGGNIQRDINSFYISNTEVTITQYRKFCESQQKE)}*

$3416=171(199):
>gi|1087630047|emb|SDM56772.1| {|0(28)|<Actinobacteria(B)>}hypothetical protein SAMN04488074_123103 [Lentzea violacea]
{(MTESAT)-YDIAVSFAE---EQRAVVGEVVEACRQRGLTVLEDN------DLPE-------
ARVRffVPFVSS---------------VDELAVNGDEHVLPVLTGDAPapSSfLRTDDGLVDALVSRV-
EAAETAGRERVSVAEMATTAET(EQPATTSPLRSLAEQFAAGAKRDLTGTLHLGDTTIAVRVERAGDTVYAVEVRD)}*

$3417=212(220):
>gi|1087609791|emb|SDG15723.1| {|62(0)|<Bacteroidetes(B)>}TIR domain-containing protein [Chitinophaga filiformis]
{(DMAFDNWLSIYYPNKKVSYLFNYSMDEIVANLLNKKQYTSYSFSFFEQVQKVLTEDSNEIFIFPGQKKVEEKWGKPEKIF)KYKVFLSYSSKdKEIAEAIFKELQKEEVK------
TWFDIIEVKYGDsildRLNEGLAQSDLGVLCFSKNFLAANWPKREMNyfLHERMSSN-
KINFLILNLDLDLSDFppLFRDYKHINLNQENW--LEELINR--IREL------SR()}*

$3418=200(217):
>gi|1087605654|emb|SDO10117.1| {|0(1001)|<Actinobacteria(B)>}WD40 repeat [Lentzea jiangxiensis]
{(MA)--RVFISHATQDVAIAAQIRDWL-DPDHD-
TFLEVHpddGIQVGEAWRERLHHELRRADAVVCLVSGASVVSS-WCTaEVAIADALG-----
CLLLPVQAEDV--IHPLIRHLNHI-------PHATARE--
HLEKALRQFDAG(GRKLAKDQEPFPGLDAFSTALSAVFFGRDQETRALAREVRRGPGLLAVVGPSGCGKSSLVKAGLLAQLADDPRWL)}*

$3419=278(292):
>gi|1087635286|emb|SDJ90300.1| {|667(70)|<Actinobacteria(B)>}CHAT domain-containing protein [Lentzea violacea]
{(ARWQAEETAARQVLDTFDRHLAKLPRDRALQHALVEVADRHPADWACWSLHGDAGFQTSAGPLRRRLRKNGDPVPLETRP)--KVFLSFAGKDRAHAEQLRAELESRNVN-
AYLAEDEIGPGDNAATAVDETLATSDYHVLLWSANTPRREP--AaEWTAAftLEMT-----
RRRAFLFIVRLDeePLPPLLAPRKHIDLVD-------AAD--
RLVATWRSDRKS(ELPVFPQPVPPKPDGPTVAISVRSHDLGTTHVVMIPLHLTGASLYRAVFDAMRLPTEQITFDGTIGMRFSYE)}*

$3420=187(186):
>gi|1087697392|emb|SDM35522.1| {|0(231)|<Actinobacteria(B)>}TIR domain-containing protein [Streptomyces sp. cf386]

{(MV)--KVFISHsTSSDKRTARLrdaIAQGLEERQYT-
VLLDVRLQQPGRPWQLKLARFLGECDAALVLVNEAALGSS-WVRrEtnilhWRKTLQPG-----
MVLVTVLVGGVSagQLRDSALRYLADDDVE--
RVRTCAEEEIAQLVRQFPRIRR(TRDDAVSAWLDNIGIQLAEIKADSTFARIARELDIPDEEHTELI)}*

$3421=200(196):
>gi|1087598538|emb|SDP95943.1| {|0(372)|<Actinobacteria(B)>}TIR domain-containing protein [Lentzea jiangxiensis]
{(MTEPESHDGWD)---
VFISYAREdyqkaRDLADGLHKCITSRGVPPRIFIDVDREgIPPGVDWEeYLETALPRSRYFVALYSLRYFTK
NVC-QfELQRAVDLNKA-GKIGFIPVLMEEA-
AKDKIWFTASKYNWVAVthPNWFETLRTALGLRPARSRRQLR(FEGPLPSAVVNHTLPTVRVSVIGADGTTVL
ESSEPITLTTVPPDS)}*

$3422=188(173):
>gi|1087670806|emb|SDR64120.1| {|0(331)|<Proteobacteria(B)>}TIR domain-containing protein [Rhizobiales bacterium GAS113]
{(MAV)--KIFISYARLdDEappglsqaeayvtfLRTQLQFQLCSLG-
EPCPYIWVDRReVEASDQFDdVISQAISESSILVVVLSRNWLGRPYCLKELDWFAQRWgKE-
GQNRVKKRIIVLN-
KNLVKSEQRPPLLQGQVghNFFAVQGKAELGREEEFFVLGRP(KDERYTKCIVELASLLWRRAQAPTADFESG
)}*

$3423=183(176):
>gi|1087691718|emb|SDO92929.1| {|0(227)|<Actinobacteria(B)>}TIR domain-containing protein [Streptomyces sp. cf386]
{()MAITFISHSsaktEEdQANADSVRTEL--
MKRLPDLgwEVRVDDTLTPGAAWRsDLYTWMADCDAAIVLVNTDGLTSTW-LKrEVNILLWRQSV-
GSRLVIvPVLLSGL-
TPRELRRSELSHLADLQciVQDDTGGEDVVSVIVRRLGRLGT(CADTSAMRSMTAKIEHCLDGVTDRFTLRAM
AGALGA)}*

$3424=218(224):
>gi|1087829764|ref|XP_002121702.4| {|218(0)|<Chordata(M)>}PREDICTED: myeloid differentiation primary response protein MyD88 [Ciona intestinalis]
{(QDVLTDMEPWFARDSNMAVERSHSSMSSNFDLSPSRLLSSPTSLPIQQNAVTSSWNFGSTFPAQEKPPIDQ
GTADIQNKS)-
YDAFLLYdAADSKFIREEFLPKVETDAKIKLFVPDRDLDAGVQAFQSQLEFMLRrCSKLVVILSDEFLKNPDS
QWSLNVGVSLDPGSKGKKIIPVIYKKITE--PNLLAGINPCNRTR----VLEDS-
WFWNSVIRSLGGV(QDNK)}*

$3425=212(220):
>gi|1088573453|gb|OHD97099.1| {|291(0)|<Proteobacteria(B)>}hypothetical protein A3E21_07205 [Sulfurimonas sp. RIFCSPHIGHO2_12_FULL_36_9]
{(GLEEAEKKHLETKKLKMDISSIKNDGKYLKNVVIGKLAYLKMIRGENDIVFIKLALKLASLSSDNLPKFIK
EIKLKSEEF)--DVFICHAS-ED-
KEKVARPLYRELTELGINAFLDEEYIEWGDSLtaKINHALGRSKYVIAILSNSSINKEWPLKEI-NSTLAR--
---EISGDKKLLSILVGDEQEIFKVLPLLADKLYKPWNNNAKTLAIEIKTMLIPK()}*

$3426=151(164):
>gi|1088312591|gb|OHB64813.1| {<Planctomycetes(B)>}hypothetical protein A2Y76_13340 [Planctomycetes bacterium RBG_13_60_9]

{(MATA)----FISYSSQdEAVAKQLHAALSHAGIPA------
FLAAISIEPGEkwteEIFGNLREATWVFFLASRHSCASPSVQQELGASLVQKKV-
IIPVLLDITAEELPGWVDRHQ---AIDPKKA--PEVIHET--
IDSIAEKIKVDK(FWAGVIIGAIVLWLVISPGE)}*

$3427=192(191):
>gi|1088609447|gb|OHE25360.1| {|0(177)|<Proteobacteria(B)>}hypothetical protein A3J94_00420 [Syntrophus sp. RIFOXYC2_FULL_54_9]
{(MAARKTPRIVTTPTDATVETPRVARANPT)---VFISHDSRdADLAETFGNLLTDVsgGILKP--
FCSSDRKGirGIEFGEewfkTIMSKLGETTDVVALLTQHSIDRPWILYETGVAKGKSGT-
PAFGVAIGVPLEKASTGPFAQFQNCAADDDS--
LTKLAIQLIRRNPDASPREEA(VRRHVAAFLESVTSLMKKRGAT)}*

$3428=178(187):
>gi|1088541856|gb|OHD70404.1| {<Spirochaetes(B)>}hypothetical protein A2177_15355 [Spirochaetes bacterium RBG_13_68_11]
{(MKKPAVRFPKAPTGTAKPAKRPSLLARLIAPRKPLVKPPFQAYRGTK)-
PYIFASYSHKnMREVFGIIRKIS----
DSRYRIWYDEGIEPGNEWPeEVGRALTACQLFLVLMSPEAIESRNVRNEINMATSENKA-----IMVVFLQP-
--TELSEGMKLQLGTVQFlnKHEMAEVEFNAKMEQVLDPGLRG()}*

$3429=211(220):
>gi|1088546853|gb|OHD74983.1| {|23(0)|<Spirochaetes(B)>}hypothetical protein A2V99_06145 [Spirochaetes bacterium RBG_16_67_19]
{(KSGASPRPKTRLAIRRKRAPARAADKAPRRVPRAPARAARVVGARRAAKKASVRKVARRKPAVPRGLQLKP
PFEAYRGTK)-PYIFASYSHRdMREVFAVIKRLA----
DGRYRIWYDEGVEPGNEWPeEVGRALLGSNLFVAFMSPSAIDSRNVRN------EINLA-
SSENKNILVVFLQ-LADLSEGMKLQIGTVQYmnKYEMTEKEFYEKLKKTLDPALRG()}*

$3430=216(221):
>gi|1088560764|gb|OHD86482.1| {|67(0)|<Proteobacteria(B)>}hypothetical protein A3I60_01365 [Sulfuricurvum sp. RIFCSPLOWO2_02_FULL_43_45]
{(LKEVINCHLEDEPSQTIGYVDSPEKYNLEDFQFTKRIYIYIDEVLTNQQIQDIENHGKYFGFVIILRDKNY
AIEKSELAK)-PLAFISHDSRdKDdLVKELVKEMYSL----
ACPVWYDEyTLKGGDNLReSIERGLKEAKKCIVILSPNFLANEKWAKaEFETIFIREiHK-QENIMIPI----
---WHHVSEEEIYEYSPQLlnKMGLSSAMGVKELAKKLVAIING(E)}*

$3431=139(144):
>gi|1088766850|emb|SDX99300.1| {<Firmicutes(B)>}TIR domain-containing protein [Ruminococcaceae bacterium YAD3003]
{(MISK)EYDVFVSHAS-KDKKK-
YVDGLVDALKKEGLSVFYDTECISWGEHSsaIIEEGLQNCKRAIVVISKNFFGRSWTEHELRTLLHRQNKEGK
KIILPILYNITKKEL-----IQHYPELSDIQYKHSKSCKCEVMARIIASELK()}*

$3432=201(183):
>gi|1088971473|emb|SDZ75620.1| {<Proteobacteria(B)>}TIR domain-containing protein [Thiothrix caldifontis]
{(MTY)EYDVFLSYR-----RYGmwprwvsgiFLEIFKHWLgeeLGRDVTIFFDQDM-
ETGTNWpsRLANAHAKSRVLVPLWSHQYFTSDWCLTELSLMRAREEqcgfssrENPKGLIIPaIIHDCEdlPS
DIKCIQSISLHGYTNPLITINSPTAEKLSDAISDRWAKD(IAHAINSAPEYDASWLNLSHEKFKATYSRNNAK
QTQVPRI)}*

$3433=247(252):

>gi|1088971694|emb|SDX96045.1| {|104(42)|<Proteobacteria(B)>}TIR domain-containing protein [Roseicitreum antarcticum]
{(RTREAIVARRNVDFIIPFGSPSGDPALPSDAAANVQYVYRKKWANEGMPVAARDSRLLLHTIYQIRRQLALLGGGDGQER)---IFVSHAK-ADgdaTASAVVAFVNDKTQDVPLHTFYDAKELNPGEDFshRFEEEI-GAGTLLAIVSDAYDSRPWCVFELTTAKRKR-----RPIVLADVGQRRTSRTYPYGANLPKVRISPTPGGTEWIEPLLVEVLSEGLRC(DMFERHAARVIDDPDKALMLPRPPELFDVVDA)}*

$3434=223(221):
>gi|1088978676|emb|SEB68506.1| {|246(0)|<Proteobacteria(B)>}TIR domain-containing protein [Nitratireductor sp. ES.061]
{(KRLRMKLYSAKKYGLDQAAELWIGPRAKPEAFWAHVEGWLGYIKQVRGEGDAVLSKLCKQAIEANPNSSDWIQEHAYMMH)EFDLFLSHAS---EDKPRVRKLYEKLVGLDVKVFFD--ESSITWGDsivDQI-NIGLMKSNffVPFLSSTFAAKGWTNKELNSAISININRKG-RILPICDHDFHvgQNypLLADILYESWPGPDA-DEEAKLneIA-DKLLAAAERAKHP(Q)}*

$3435=222(227):
>gi|1088867224|emb|SDY95688.1| {|0(350)|<Proteobacteria(B)>}TIR domain-containing protein [Nitrosomonas sp. Nm58]
{(MNKNTNSTE)---VFIVYQKHeEKLAENLSKLFELWGLNS---FYCrdSSKAHAHKPYRdYLAEELFRSVLVVLLVSREFEWSKYCQSEAGATITMGKP-RIIITIPPVVNDDVSKIAPILEGFNCFNPSP--FNAnlLRES--IKGTLKATLNTL(SFKKRNVSNQKEGIEAALNRVEDYKSGSEDERALERRIDQTIKDITDAYAIIPSRRYIVKTWPSIKEESCLQARSSIV)}*

$3436=203(207):
>gi|1088967820|emb|SEB14340.1| {|0(513)|<Proteobacteria(B)>}NB-ARC domain-containing protein [Thiothrix caldifontis]
{(MDNEFSNKMH)--DIFISRAGEDKEISINISHILNKAGYR-TYLQDEDFGH-TSFMARMEQGfqiIENGGRLIALLSSSY-QKKEHCMaEAHYPLIDD-----PqnkkqRLIVFRIDECAPS-GFLKPIPYIDLVP--ILHNVERLEKVVESALTDEKTN(QIDSAKFYRRSGNQIMHSNIHANKDFISREIEFEALNKLFSIENKENYNSETLSVAI)}*

$3437=234(233):
>gi|1124490894|ref|WP_074776090.1|
{|0(1005)|<Proteobacteria(B)>}hypothetical protein [Nitrosospira multiformis] >gi|1088819316|emb|SEA72697.1| KAP family P-loop domain-containing protein [Nitrosospir
{(MDVDISEAPQVPKRRL)-PLVFISFNIVdSTFRDKLSLLLTREPISSLISV-SDWPIENGKEPSpLFQAAVERADVAVLLISPEYLKSQA-SSaEGLEFVKQREH-RGLRLLPILLRPS-NWREVrWLSETQVFPLDGvpISAYvgEEQE--YLWAQAASHIMSV(ATNHLPLETGARASNEDVTEQATSETVSSASGDPIDPSSEGLAKYLARYQLSDGANSIVHRARVLATFKEDKAGCVT)}*

$3438=197(185):
>gi|1088867620|emb|SDY80764.1| {|0(284)|<Proteobacteria(B)>}TIR domain-containing protein [Nitrosomonas sp. Nm58]
{(MTEATRPTSENA)---VFISYARAdneqppdkkaEGWVSFFWNELRFeLTDRGAAEAklWLDRyEIEPHEAFTpKIEQALKQAKLLLPILSKNWIQRDWC-QrELQLFVELHAD-GSERYIPVFKHRL-ERKLLPPFMQGNNARTGyqFFEIDLTGKTREFYWRGLKDETA(YYDLVKTIAEAISDKLGIVRPAAMASSVTATGQ)}*

$3439=181(182):
>gi|1088869426|emb|SDY04404.1| {|0(1146)|<Proteobacteria(B)>}WD40 repeat [Nitrosomonas sp. Nm58]
{()MTAIFISHSSAdSAAAADMKR---
WLEAQGHTSLFLDFDpeagIKGGSDWEqTLYQKLRQCQAVIALLTPNWLTSKWCFAELVQARER-----
GKVIFPVKIQPC-
EVHDVFNDTQHIDFTVEpeDGYQRLRISLLERGLDPLDVFDW(DPSRPPYPGLLAFQEQDAAVYFSRNAAIQG
TMETLNRLQRLG)}*

$3440=209(197):
>gi|1124375558|ref|WP_074664989.1|
{|0(272)|<Proteobacteria(B)>}hypothetical protein [Nitrosomonas communis]
>gi|1088912768|emb|SDW11514.1| TIR domain-containing protein [Nitrosomonas communis]
{(MTETTQPTPENA)---
VFISYARAdneqppdkkaEGWVSFFWNQLRFeLTDRGAAEAklWLDRyEIEPHEAFTpKIEQALKQAKLLLPI
LSRNWIQREWC-QrELELFVQQHAD-DNDCYIPVFKHRL-
EREHLPLLMQNNNARTGyqFFEIESTGKTREFYWRGLKDETA(YYDLVRTIAEVISDKLGIVRPAAMASSMAT
TGQTVFIALAASDLR)}*

$3441=227(227):
>gi|1088968079|emb|SEB09865.1| {|1100(0)|<Proteobacteria(B)>}Leucine Rich repeat-containing protein [Thiothrix caldifontis]
{(EYANKQQSTQGRLHFQLRGCQPERLVYALRKLVSETSPHRRYQEYLSKVGAERTLLPEFKDEAMQHPSKLD
ASKPADKTI)--
KLFISYSHAdEAHKERLEKHLKAISRTLPLTAWSDRHLFAGEAVDeQIFQQLNTADIVLLLVSPDFINSDYCF
TkEMERALERYQD-EGNVVIPVIIRHTASWRNHKIGQIVALPKDGkpLAKWEDAD--
EFWGSVENGIHDQ(VKKRLQA)}*

$3442=229(227):
>gi|1088970058|emb|SEA38734.1| {|620(0)|<Proteobacteria(B)>}Leucine Rich repeat-containing protein [Thiothrix caldifontis]
{(CAYRDKKQSTQGQLSFHLRGNQLAPLVYALRKLVSEISPHHRYQEFLRKGSEERAQPPECEENPQMPTLLD
EIKPTSKTV)--
KLFISYAHEdEKpYREELDKRLKAIKRQYpMLEPWHDRNLLAGEQVHdKILQQLEVADIVVLLISPDFVDSDY
CFShEMEIALKQYEQ-NQNIVIPIIIRDTADWHTYQIGNHVALPTDGkpLCDWSSPD--
KFWADVQQGIRKQ(VENLLKT)}*

$3443=187(175):
>gi|1088971249|emb|SDZ86502.1| {|0(178)|<Proteobacteria(B)>}TIR domain-containing protein [Thiothrix caldifontis]
{(MAYTAGYDCD)---
IFISYSRDdnvvlpgetAGWVTQFRDYLEnwLVKRRGLkgLNIWFDDQgLRGSTAFDaEIQQAIEKTAIFLVL
HSHNYQGSAYCRKELGWFMAHNRR-FRNGIMVGNDSRL-
FNVLISNIHHQQWPEELagTVGFKLHDAPDKTTQFGYPLAMQ(AAAFDKTMRQLVEDATDLMDALGKQ)}*

$3444=195(197):
>gi|1088971439|emb|SDZ74769.1| {|0(770)|<Proteobacteria(B)>}SEFIR domain-containing protein [Thiothrix caldifontis]
{()MTRVFISYSHDsDAHRD-FVRGLSDRLRTDGLECLIDQYINgfPPEGWQcWMENQIEAADFVLLV-----
-----CTEtYLRRYRKQETE-GgrgvtfEGVVISQVLYDH-YYHNTKF--
IPVVPQQGkpEHVPIPLKQYSTYRLPEDYETLY(RLLTNQAQFLPTPLGEIRPPSLLSRWFGGKKPAPTSAAN
TAIAVYDQRFPVTDNNAM)}*

$3445=179(183):
>gi|1088971153|emb|SDZ83691.1|
{|0(1295)|<Proteobacteria(B)>}Tetratricopeptide repeat-containing protein [Thiothrix caldifontis]
{(MSGQHQ)--HIFISHAS--A-DDAFVKQLREKLELHHLNVWVDSRNLRGGDKLwpEVAEAIR-TARHVLVVISPQTINSDWVFdEIELAEQVEKEGaDYR-VIPLMLPDI-TPKALKRYFTEEPAGEKIELEVGKLQEAMPRILAALGERL(PDDLPTEQTIENKPLAELLLELTEPTLTRNADGSEQL)}*

$3446=189(174):
>gi|1088968481|emb|SEB02142.1| {|0(264)|<Proteobacteria(B)>}TIR domain-containing protein [Thiothrix caldifontis]
{(MPP)--DIFVSFAHVddqpvaNQQQGWITHFTndlrgelnRRMGRKDNYHFWKDFR-LQGNTAVtpEIEQQVRHARTLLILFSTGWLASPWCQKELELFLQSHPDvnGrifVVELERFQQDH---KPPIIQDLLNYPFWRVTDKDRVRQLGYPVPQATDSAYYER(LVDLSHDLANAIKLLQASTAEPVDAPKATVY)}*

$3447=163(160):
>gi|494888681|ref|WP_007614727.1| {<Proteobacteria(B)>}hypothetical protein [Bradyrhizobium sp. WSM471] >gi|374426240|gb|EHR05773.1| TIR-like domain-containing protein (DUF1863) [Bradyrhizobium sp. WS
{(MSK)RRNVFISHHHADDEHVGGLTK-LLDRQGF--QIRnssirakpsnqarLDKKQIPEGTLKrLLRMKMSWASTVIVLIGKDTHQR------PWVDWEIRKANeLGKRIIGVFTRG--GTEADIPPAFEEYGDAL-VNWNSDSVIQAIEGTNSPFETP(GSAAPRELVHASRTARC)}*

$3448=136(144):
>gi|504080312|ref|WP_014314306.1| {<Firmicutes(B)>}hypothetical protein [Clostridium sp. BNL1100] >gi|373946026|gb|AEY66947.1| hypothetical protein Clo1100_2789 [Clostridium sp. BNL1100]
{(MVKV)----FLSCSSHdQTYIKYIIDDLRTLPEKYKLFYFLPPYKKDIPI-DNIIERLYDIDLFILFITNNSLNSQFVQRELSQAIYLNNLNRIKEICPIsLDNSI-DVFK--DSRIPEYIKNRiYLSES---PLETVQIVRNFILNY()}*

$3449=249(254):
>gi|489081732|ref|WP_002991661.1|
{|100(45)|<Bacteroidetes(B)>}hypothetical protein [Myroides odoratus] >gi|373912088|gb|EHQ43937.1| hypothetical protein Myrod_3121 [Myroides odoratus DSM 2801] >gi|40
{(SNRIFPIKLFEHAYNINCGLNKLQFTDAIKYFGDPLNINKSADQEKCINKIKSELLHDLSRLIWNFQDKQEDKESQRIGS)PVKLFLSHAK-KDGEE--IALKFKRFIEHNLKldVFFDTVDIANGYEFdaQFEQEIT--KSALVVFqTDEYSDREWCRREVLLAKKSK-----SPIVVVNNIRKGERRAFPYLGNMPTTVLIDDEVVSFYRIVNLTLYQVLNNRY(QLLLLEQYSKLINTDQYSVLGISSPPELFNYVHI)}*

$3450=157(163):
>gi|504121056|ref|WP_014355042.1| {|0(334)|<Firmicutes(B)>}hypothetical protein [Acetobacterium woodii] >gi|375301305|gb|AFA47439.1| hypothetical protein Awo_c06430 [Acetobacterium woodii DSM 1030]
{(MSD)---IFISHSSKdKtNIVRELVAELRALRldvwVDEDNILC----------GDnildEIERGIKNAVCVALILTPAFFQSNWASLEIGLSRSGKEG-TLIIPVLAGITVEEVAKKYAFLIAQKYISLD--SSDMSVGARELAKAVEGQKNR(KRNDEPLDYQSAIRRLNNFD)}*

$3451=164(157):
>gi|487748693|ref|WP_001830713.1| {<Firmicutes(B)>}MULTISPECIES:
hypothetical protein [Staphylococcus] >gi|374825068|gb|EHR89017.1| SEFIR
domain protein [Staphylococcus epidermidis VCU125] >gi|3833584
{(M)-KNIFISYAW-DKENDEKViklAEMLEEYkELN-
VKFDKWDINKGQEIPLFMEKEIQQSDFVLVICSNRYKEntDKRIGGsGYEARLManeilkniDK-----
DRFLPVLLNENDkdHIPNFLTGKLWTSLYY--
QEGSEEYNIEINDLLTTIVGRS(KRNIKKAKVFMISLKM)}*

$3452=213(223):
>gi|488858524|ref|WP_002770834.1| {|0(35)|<Spirochaetes(B)>}TIR domain-
containing protein [Leptonema illini] >gi|373873844|gb|EHQ05838.1|
hypothetical protein Lepil_1143 [Leptonema illini DSM 21528]
{(MSKKNPDTTFTTEHQVPADAIQRIWGDSKFR)---IFITHLAKdKELAEKLKTDFKDFGVAGFVAH---
TSIEPALEWQsEIEFALSSMDALVALMTDGFHESRW-TDqEIGFALGRK--------VPIVsirlgTDPY-
GF----
IGKYQALPPSKsnSVDVLSALMKEQKMLNCFIQKME(NCRSFNAGNRLAQLLPALTTMPDWALRRLISAFNDN
PQIIDSFGFSGKKVRE)}*

$3453=227(225):
>gi|490178682|ref|WP_004077302.1| {|0(639)|<Euryarchaeota(A)>}TIR
protein [Methanoplanus limicola] >gi|373907443|gb|EHQ35547.1| TIR protein
[Methanoplanus limicola DSM 2279]
{(MQTSKL)--
KLFISYSHVdnlnEPYIDNFYNYISPFEKNGLIEIWYDKKISPGDDFQnIIDSNLETSDIICQFISKDFLISN
SCISEKNKAIELKNK-KNVKILPIILSTC-LWKeDNEISKLLALPTDGkpVTDFEDQT--
KAWDNVSTRLKEI(INIENYIRNVKVKDNFKDFLNNAEMLTKAHRNKEEVYLNDIYIHTELEEFDRLKDYCDT
INSKDILDVLLKEEKITISG)}*

$3454=254(236):
>gi|497239162|ref|WP_009553421.1| {|0(193)|<unknown(X)>}TIR domain-
containing protein [Burkholderiales bacterium JOSHI_001]
>gi|374668750|gb|EHR73535.1| hypothetical protein BurJ1DRAFT_4749
[Burkholde
{(MPMPTVVQTRPPGTVRH)---
VFISYSRDdVQPGGDLPGLFAQALRDlaskrpelGLADerIFFDRhRLMPGDVWGeAILQAVERSQLFVLLVS
ASSLKSTVCMQkELAVAAGR-----GIPIVPLLLEDC-
EWAGFavpgdargrKLGDFDAQPKNSqgrlqpVRTCADPA--
AVLAQTVAAIGER(LQRDALDLGPAPGTPPRPMVSPLLAYHCNEEKIERQFDRGLMRWDSQALLVLARGRFDD
NVPSFWDRLRLKNLSDIVDE)}*

$3455=213(220):
>gi|1089081823|emb|SDU66733.1| {|0(44)|<Proteobacteria(B)>}TIR domain-
containing protein [Desulfobacula phenolica]
{(MS)--DIFLSCSEKD-KKIAKIISDHLEKEGL--
TTWYYYrDFIPGVKNLqSTGKEIDTCKAVIVLVSESSLN------EDFVFPEILCAVeKNKNLLPVLI-
GISfdELKALKPKWINAFGYTVSIEWDSTNSFDILGKITHGLKKI(TILNNPLSERFQHSSNSLIDLNKAETL
PTFFNISCPVDLAVFVDGFCLAEIRHADNSGDALVDTTDYDIKISPTTPFT)}*

$3456=212(209):
>gi|1089148102|emb|SEB22191.1| {|0(136)|<Bacteroidetes(B)>}TIR domain-
containing protein [Pedobacter hartonius]
{(MSPRKKATNPITQSENETISSNINPL)---
VFISHDTRdAELAEEFSNLLKSASAGALKSFRSSDKkgTQGIEYGMewypAIMDKIDEASDVVCLLTQHSVDR

PWILYEAGVAKGkLDKK-VIGIALGiPFSSAITGPFALFQNNSGDSESIT--
KLVLELVMKVPGLDPDKSLVR(LLVDKFVEKVKEITEKKTEDGGEIEKAESVDENSVAKLFEEVK)}*

$3457=164(163):
>gi|1089167281|emb|SDZ19460.1|
{|0(285)|<Actinobacteria(B)>}Tetratricopeptide repeat-containing protein
[Geodermatophilus africanus]
{(MI)--RIFVSHAHAdAPLARALDALITTV-
FDEVEVDYSSdvSAGGGIEAGTdwqrWILDKLRSCDMAVVVLTPESLSRPWLMWESGAVNGIALA-
LEQQPQIVPLLFRVNREDVAGPLSHLQAELG--
ESEDGVRRVAQAVWRRRGQKP(AADKLEVRLTLALKTYLDGVR)}*

$3458=206(219):
>gi|1089167863|emb|SDZ00080.1| {|0(1130)|<Actinobacteria(B)>}WD40 repeat
[Geodermatophilus africanus]
{(MPSL)----FVSHCSADRAAAERMRDWLQREEFS-
ALFLDFDPalgiPAGRNWERELYARLRSADAVVFLASPASVASS-WCFaEVSLARSLG-----RPVFPVRVE-
AGVRMPLLEDVQWVDL-G--EGEP-ALA--
RLRGGLRRAGFH(AADAFDWDVTRPPYPGLEAFTAEDAAVFFGRDPEIERLMDLLQPTLQRGSGRFVGIIGPS
GSGKSSLLRAGLLPR)}*

$3459=189(201):
>gi|1089169790|emb|SDY14542.1| {<Actinobacteria(B)>}TIR domain-
containing protein [Geodermatophilus africanus]
{(MSPPRVRWWERPESPTA)----FISHASADATQAAEIVRVLEAARLR-
CWLAPRDIPVGSEWATAIVDGIRRSRLLVVVFSVRSCA-SPEVYrEVELASRER-----KPMLTVRID--
RtaELTGPLAFFLSSIQWF--
EATARPLRPQLGRLPSTVAGLL(DVPVTPPPPPTAAPVSGAQPEESIRSVSLDDLTRSGPIFRPWAH)}*

$3460=156(149):
>gi|1089333015|emb|SEB11734.1| {|0(116)|<Bacteroidetes(B)>}SEFIR domain-
containing protein [Chitinophaga terrae Kim and Jung 2007]
{(MNL)-
KQVFISYAHEnDAYRNEVREFVEWLRVEGkgvvkVVCDFDHEKKAPKQGWPvWMEDMIEESDIVLLLCTPLYL
KRFRKKEePGKGAGVTFEG-AIITQDLYSANMV-
NNKFVPIIPDTGHHNHIpkMLLAFNNGFKFPSNQDKILECIL(LDDSTV)}*

$3461=189(208):
>gi|1124560162|ref|WP_074843053.1|
{|0(80)|<Proteobacteria(B)>}MULTISPECIES: hypothetical protein
[Pseudomonas syringae group] >gi|1089273420|emb|SDY77269.1| TIR domain-
containing protein [Pseudomonas
{(MNDSKLWITYAW)--------AN--NQNGDF-
DYLAQELEAAGIKTHYDKVALTTGRRLwdQIAEKINSPETKAwAYLVTAESLASAPCReELAYAIDRA-----
-----LR-EKG-
DEFPLIGILHGVPFSEVPPVLKVRLCVNLESPDWKEEIRS(GLTGKPPERAKKEIPQYVWKMHISESPTQGIL
EIRPRFGSVSPWVIAIPKDGPQPL)}*

$3462=173(177):
>gi|1089417498|emb|SEB22623.1| {|0(377)|<Proteobacteria(B)>}hypothetical
protein SAMN05444680_116105 [Variovorax sp. YR216]
{(MKHFP)-YDVFLSHRI-GDNAHELRD----
QLVACGCTVWFDQNRPllDRQLFPLIQKAHAESRVTIAYISPGLAPSPWTVLEMLTALRSEQLFGIKCLFVYG

PSDALELLRTWSLPEELGSALADPERRLEAEVTLFASRVGGLNRE(LLPHSSVAPLSRARCIARLRRSIDGYR
RRPVE)}*

$3463=176(177):
>gi|1089636062|emb|SEF29528.1| {|0(285)|<Actinobacteria(B)>}NB-ARC
domain-containing protein [Amycolatopsis pretoriensis]
{(MRP)-HDVFLSFTW-SDTAE--VNALESALRAAGLRVFRDRGIDRF-
DGVteKLVSALAESKVLLAYYSRRFPTRYACQWELTSAFLAAQrfGDPRRRVLVVNPEPDGNHIAPVELADAA
YFGRPRTERDLTRIAERVAEMSREADTP(LGAVPSTAPLLPRLLRPRRFVGRYRELWAVHSAL)}*

$3464=194(186):
>gi|1089660447|emb|SED23533.1| {|0(439)|<Actinobacteria(B)>}TIR domain-
containing protein [Streptomyces sp. 3213]
{(M)-WDVFLSYSR-GDVER--
VRPLARVLRDGGLDVFTDETGVASFVGIsdTIRRELARSKALLAYYSAGYPEREACQWELttAYLAGLREGDP
RGRVMVVnpeattdhIHPVELRDARHAPAHDLTALVADVREHVARLDGPMAIQERAVRRWL(LGSPEPRPEFL
GRLSELWQVHSALHAHSAPLVTGRAAARAVQIRG)}*

$3465=251(260):
>gi|1080112963|gb|AOW69249.1| {|343(82)|<Cnidaria(M)>}hypothetical
protein [Actinia tenebrosa]
{(EKADIAALTKKIEEVKGKLDEWPPKPRNQEEQRSTPPPPEKLVTQDTENLPFNYIKYEVTRMSFNPPKPLF
DKHGIPIEQ)KFDIMLSYQW-DI--
QDFVRNVYMEMDMKTFKTWMDIwGGMQGNINEAMATAVENSTLMISFLTEKYQKSVNCNLELMYAKQKQ----
--MPI-
IFINVEPNLQLKDWIQELVDKTVVFNMSSISDFGEMDKGVPKINRLA(EAARNLMKWSAAQPKKIRTDVSEEV
YRLQNLLEDALIALA)}*

$3466=175(184):
>gi|1089417528|emb|SEB22708.1| {|0(441)|<Proteobacteria(B)>}TIR domain-
containing protein [Variovorax sp. YR216]
{(MGQLEEWLKLAPPPPPLESGQKWH)---VFLSYRS-TERQWVLA--
LYDVLTQLKYQVFMDQFVLVAGEGLasSLGENLNASQSGILVWSARSADSAWCKKEYNSFEGRH-----
ANGDFSFVAVRVQDVPMPPFVQGALWIDASDQRDGPRGTSLLRMLYGLQGKP(LPPEAVRLAASIDDTTRKDL
)}*

$3467=212(220):
>gi|1080112973|gb|AOW69254.1| {|1126(0)|<Cnidaria(M)>}hypothetical
protein [Calliactis polypus]
{(LGLSDADLDEIEVDEEQLYERCYAVLKRWTEMYGSSANYEVLSAALVHPAVQMGGLAWKYCGTACQETTDA
SQSGAQPLP)--EIFLSYQW--DIQSTaleLASLL----
KENGFTCWVDIGQMGGGDALyaQIDKGIRSSKVVISCVTNKYCKSENCQREATLADTLK-----
KPIIPLLLEDIPWPPEGQLGPIFAKLLYVDMIHGHQAKIEDLVKNIKPYLAK()}*

$3468=198(204):
>gi|1089535789|emb|SEC48984.1| {<Actinobacteria(B)>}TIR domain-
containing protein [Amycolatopsis lurida]
{(MALVYEYDVFISYRRGGGDAPDWVRNH)-----------------FHPRLQRILDDNvdyDVKIFLE-
DGVPVGGKWpqVVRDALQRARILIPVCSPKYFRDEWCLAEWHTMAKREEivagsgSAkPGRLIYPVIFSDSEn
FPGYAHERRM--
HSFRDwNKPHPQYQNTPEYLDFDRELALM(VEDLVEVIEQAPPWSPEWPVETPPPPGPPRPSKLPRF)}*

$3469=195(218):

>gi|1089574878|emb|SDW33844.1| {<Actinobacteria(B)>}TIR domain-containing protein [Amycolatopsis xylanica]
{(MTRVYEYDVFISYSRAGGGHAPAWVRTHFYPLLVDLLQDNVEHQAK)--------------------------------IFYD-
DNVGVGGKWprQVCDALQRTRVLVPVCSPKYFHDEWCLAEWHSMAEREKVSgIADherLIYPVIFSDSVnfpg
YARERRMRSLKRWNQPYPQYQQSLEYHDFHREVQNMVEEL(VEIIQRVPEWQPDWPVRTPMPEQPPGSKLPRF
)}*

$3470=221(229):
>gi|1089605749|emb|SEC88672.1| {<Actinobacteria(B)>}TIR domain-containing protein [Amycolatopsis tolypomycina]
{(MVLSSFPPFFVAQTRPRMGRCLAGSGEGAKARVYEYDVFISYQRAGRDISAWVKNH)-----------------FRPRLAEVLDNNfyrDVRIFCD-
DQVRTGASWagEVRTALLRTRVLVPVCSPKYFRDEWCLAEWHSMAEREALTaRAQpLIYPVIFCDSRtFPAWA
HERRLRDLQQ-
WNLPFEHFQTAPAYldfHQQIKQIAAE(LEELIERAPEWRADWPVRTPAPEPPKRAQKPRF)}*

$3471=189(209):
>gi|1089639180|emb|SEF21499.1| {|0(1)|<Actinobacteria(B)>}TIR domain-containing protein [Amycolatopsis pretoriensis]
{(MYEYDVYISYQRSGHDVPSWVRNHFHPRLTEVLDNNFY)----------------------------
RDVRVFLD-
DRIPTGSNWpaQLRSALQRTRVLVPVCSPKYFKDEWCLAEWHSMAKREELTgehPRQLIYPVIFCDSWnfpdY
ANQRRMRDLHKWSQPFEHFQTAPAYLDFNEQIGHIAKEL(EELIEGAPDWRADWPVLTPAPEPPKRARMPR)}
*

$3472=218(220):
>gi|1089509928|emb|SEE23584.1| {|0(152)|<Proteobacteria(B)>}TIR domain-containing protein [Bradyrhizobium erythrophlei]
{(M)--RIFLSFPSELESQADNIAQSLRNCDHD-
VFFSHDDLPPGDSFDARVEKAIESSDFMVFLISPESVTKGRYTLsELAFARARwpnpS-----
NRVLPVMMAPTPldQVPAYLKAVTILEPQG--SAAAetrAAVD--
RMLAAERAQSRG(RSSWPFVVLALFSIVGSFITVRYATGVLQYSFVDSTPTGGITILPGVIFGLVVATCNFIF
GVKDRFQLALVVAVTAAAW)}*

$3473=219(226):
>gi|1089511652|emb|SEF02547.1|
{|0(644)|<Proteobacteria(B)>}Tetratricopeptide (TPR) repeat [Rhizobiales bacterium GAS188] >gi|1089592485|emb|SEE71804.1| Tetratricopeptide (TPR) repeat [Rhizobiales bac
{(MSSPTEFLY)--DFFVSRRGSVGSAAAEVAAVLEREGYR-
VKVQDYDFGRGGDYVGDIHDALVAARHLFVLHTTDY-DENYWTRkEFTnflATLPES-----
QGMRRICILRCDesMPRGILANVVFGDLVG--VANAEERR--
KVILATAAGQPL(QARSEPRIFGGSMPGKNANFTGRGRVLAQVENLMRGEPGSTSLVVVAICGLAGTGKTSVA
RACVDALAPEHSGVWWV)}*

$3474=136(145):
>gi|1089538309|emb|SED40903.1| {<Actinobacteria(B)>}TIR domain-containing protein [Amycolatopsis lurida]
{(M)--RMFVSYSRADNSLERLVALRARLAGsAD-VYVDDLDWAAsGFDREDAVHRELVRASIFVAVVSVTY-
RKTPWTAwEFDVAL--------KRGIPklaylpdGRLVDERsdEWP-----FASADLTG--SVPSVPPR--
VLRGSTRGRHRI(SGSS)}*

$3475=222(221):

>gi|1089546484|emb|SDZ51763.1| {|0(205)|<Actinobacteria(B)>}TIR domain-containing protein [Asanoa ishikariensis]
{(M)--RFFISHsarTEQAKAVLRQLRVRLRAAGHT-VFVDA-DVLAGLQWRSVLYHELAICDAAVVLLDQTSL-SRRWVQrEadvllWRRAFDER-----LTIVPVLLDDTKidTVRGsgfgefTEAQFLLAD--G--LGPATVAE--RVADRFAASERP(DPPDLPGTRMRQWFDDIETVLTRIGHQNKLRLAALELGVSPQDADQVLLGVAGGYRFLAHQFLARSVDTAAVDAMSRILY)}*

$3476=260(275):
>gi|1089556028|emb|SEE92518.1|
{|262(810)|<Actinobacteria(B)>}Tetratricopeptide repeat-containing protein [Streptomyces sp. Ag109_O5-10]
{(WGAVEIPYKPFFAYEEVLATVADTPRQPASLLGAYERLTSVITDGQVTALRPVEEPVRRQLLARFQRVPVPGGRLVPRVR)-----VDYALRDGAWAEWIAAQLQAAGIS-VALR------GSSPSVPLSSApPEGADVVVALLSPDFATTPVADEvRVLAAAPEG-----PRIIGLRTREHTpePALRGLPGFTLVDQSA--TQVLSRFFGLLELETRIPAVSG(DLTYPGLAPRIFNVPRRNNAFTGRDVQLDRLRSELNAESLRPDTPVGRVFLYGLG)}*

$3477=217(208):
>gi|1089416905|emb|SEB24313.1| {|0(129)|<Proteobacteria(B)>}TIR domain-containing protein [Variovorax sp. YR216]
{(MEPSQRSKAFLPKFEHD)---IFLSYRHirKpDEWLEKLHECLRTelVARLNNVDIWRDRlDLRAGDEWReEIEKALSSAAIFLAVVTETYFDSPVCAHEFDLYVGRRMG-AAAgatrgVVIPVLRHRF-DVPPDDFEPFQAVNFCDpvTGREYHESLAEDPRGGFYAAVGE(LALILRERLRDLAGAISAQMPAIFLAETGFPEHERRQIVRRELLQRDDLVV)}*

$3478=249(240):
>gi|1089417525|emb|SEB22698.1| {|0(190)|<Proteobacteria(B)>}TIR domain-containing protein [Variovorax sp. YR216]
{(MLQVKLHTPTHSEATGQVRGV)---LFISYAHAdVQDVLPFIEQLQPLVERERLELFFDaRELKAGDVWNdKLLSALSRCELFVLMATPKCLTSRFCIErELLMALNRQRS-NQCRIVPVVLRDC-DWKGklLPdgsgrcLSEFQALPAGGaaVaaSAGAERD--RVWLAIVNALAEV(LADPATTAPPRRAQSAVPVLLPYLCDQVMPESGVRELLMRWRAQTGPLVVVLRAAATDCPDHFINRIDERHLRKLLPRL)}*

$3479=200(187):
>gi|1089503367|emb|SEB87881.1| {|0(330)|<Proteobacteria(B)>}TIR domain-containing protein [Bradyrhizobium erythrophlei]
{(MYEEQFHIPPGPDDPVQ)---IFVSYAHDdnsvppglepeKGFVAVLISHLEyILTRRGHPRPkiwWDQRRIDKADRFDnIIQEAIKRSSLLLVVMSTNWLSRPWCRRELELFAKRQDS-TEHMVLVRANHVE-PAEHPGELQNIEGYNFFslDDWREPGNERFFVDQGEICDKLF(TKHVSELGSVVWRRASNVRRKILPPQPPLA)}*

$3480=240(243):
>gi|1120069923|ref|WP_072942625.1| {|13(95)|<Actinobacteria(B)>}LacI family transcriptional regulator [Rhodococcus koreensis]
>gi|1089616626|emb|SEC82092.1| transcriptional regulator, LacI family [Rho
{(GVSRSTASLVLNDSPSVAPETTRKVREAMKALGYVYNRHAAMLRNQRSMTLGLVVTEVQNPYFAELAMTLEDVADRSNYA)---VFIGYSRDdPARQRRLLDRFVERSIDGLI------

LLPAGDTQAaEIDELLASSGIPLVLLARHFhLSHDYVgaDNvEAGRLLGRHLR-AMGVRTVAMVGGP-
QHTSARREREDGFLDAIagSDMTMSPTEGLFGEATPDGGADA(TRRLLDRGEIPDAIVAYSDIVAV)}*

$3481=197(181):
>gi|1089639179|emb|SEF21498.1| {|0(127)|<Actinobacteria(B)>}NPCBM/NEW2 domain-containing protein [Amycolatopsis pretoriensis]
{(MSDREV)-PRVFVTYSHDTPEHKELVGRFAAFLRATIgldvhLDAWYDN-IR--
RDWSaWAVEHLDRADFILVIASPEYKrrADGAAMPhEgrgaqFEAAIIRNnltrdlRR-ETERVLPVILPGR-
SVDEIPTFLNGHSTTRYhvEEFTEAGVADLLAAITGQGQYPM(PRRGVWRGGGARDREPVRAGELPWACASQG
VRRDA)}*

$3482=208(205):
>gi|1089417519|emb|SEB22684.1| {|0(557)|<Proteobacteria(B)>}TIR domain-containing protein [Variovorax sp. YR216]
{(MLVDDTNRPDRVAKADPSGIDGAAAAPQ)-FDAFVSYASAqcrqaRAIQRYLQR--
RVPGVVEPMTVYLDETDIRGG-
DLqiELGRALDSTRQLIVCCSPEAADSRWVSTEIARFLRHHPPNdivLAWLDGPLARA---
VPAELDPDALRVHDLRHWWFGRWPGPRTGLELARMLARLA(DVPMRQTVDWERRRMSARLLAGAVVTVVVGAG
SAAAL)}*

$3483=222(221):
>gi|1089508897|emb|SED92071.1| {|0(309)|<Proteobacteria(B)>}hypothetical protein SAMN05444164_6291 [Bradyrhizobium erythrophlei]
{(MPEFN)-
YDAFISYRRSdgiavaRWLRRALVGFRLPAALRdvygRKLRVYLDLAYERGSSDFyqqSIRPALLSSRYLIVV
ATPNAMRRPggvedWIEREIRDFATGPGAGnviVA------------RGAGDFDGP----
LPGGLKETFPNIE--I-
VDLRGAGRFW(FLSFSRVVRLSAEKLKIVAPLFNIPPDEMPRLRQEEEKRQQTQLGAVAGATLGTLLAVGSLS
VVALINQHKAIRAN)}*

$3484=253(223):
>gi|1089525952|emb|SED47035.1| {|0(116)|<Proteobacteria(B)>}TIR domain-containing protein [Burkholderia sp. WP9]
{(MAYLGEPFT)-
HDVFISYSHGsgnqdgrgpharwsEAFAKQLESEVRVHPSLRQYEHFLDVDyrpdrSVDPMVPLsdGLRSHIQ
KSAVLMVLMTPHYLESQWCKDERQWWCEHHEVpvsvdhDriaVVRVFPTdrtwpPELV---
DSRGEQLVGFPFHVPVEGRNLAAIRP--
FgWHDMRNPDGEF(SKALVHIAGHLSTKLFAIRERLEAEQRQQAETERLGKPDGLTIYLHGHAEEENAWQEAG
VHLGDSGFVVLPGSP)}*

$3485=155(161):
>gi|1091439172|ref|XP_018730908.1| {<Streptophyta(V)>}PREDICTED: toll/interleukin-1 receptor-like protein [Eucalyptus grandis]
{(MAHSKARASEV)QYDVFLSFSG-
ADTRFDFTGFLNRALNNAEIRVFMDKDAIkvGDKIGETILQAIDNSEIYIPIISQTYLDRKWCLIELERMMNN
vSQSEGRKKIFPIFYKVKLGDVKHRTPRL-------KNLSSEVEL-EAFKEALEKVGKI(KGWKVEKRQR)}*

$3486=155(183):
>gi|1091439517|ref|XP_018719542.1| {|0(358)|<Streptophyta(V)>}PREDICTED: TMV resistance protein N-like [Eucalyptus grandis]
{(M)-----------------------------
GIRVFRDNEELlvGEMIKPSLIEAIKQSEIAIPIISKNYASSKSCLMELQQMLAC-
RENNEQMIIPIFYDVATRDALLELRYAWGWQNYSCKDVYNRLYHLFDGCAYLENVRE(KYTQVEGLKTLRCNL
VSQLVKRESREFSTSDEAMQFIKHRFS)}*

$3487=275(275):
>gi|1091440071|ref|XP_018724713.1| {|52(0)|<Streptophyta(V)>}PREDICTED: TMV resistance protein N-like [Eucalyptus grandis]
>gi|1091440073|ref|XP_018724715.1| PREDICTED: TMV resistance protein N-like [
{(PGSTVPVIRRADSDEVHPVNLVYSRGSPSSSPCALPRKHSKNLSSLPGQATPHQVTNLFFSSGEGERGLED
MAPSKARAF)EFDVFLSFSG-
ADTRFDFTDFLSRDLNNAGIRVFRDEDAIevGDKIGQTILQAIDNTKIYIPIISQTYPDREWCLIELEHMMYN
vFRSNGKRRIFPIFYKVEPAHVKHRTPR-PKNLSSEEALAKIGQI-
KGWKVEEGQSQAK(IVDLVVRKVLEELGRKDESQTEHPIELDDLTQSLEDSYNHAVMRYAGRESLTEEV)}*

$3488=291(291):
>gi|1091440378|ref|XP_018725435.1|
{|52(126)|<Streptophyta(V)>}PREDICTED: uncharacterized protein LOC104446064 [Eucalyptus grandis]
{(PGSTVPGIRRADSDEVHPVNLVYFRGSPSSSPRALPRKHSKNLSSLPGQATPHQVTNLFFSSGEGERGLED
MAPSKARAF)EFDVFLSFSG-
ADTRFDFTDFLSRDLNNAGIRVFRDEDAIevGDKIGQTILQAIDNSKIYVPIISQTYPDSEWCLIELEHMMHN
VFTaNAKKKIFPIFYKVEPAHVKRRTPR-PKNLSSEEALAKVGQI-
KGWKVEEGQSQAK(IVDSVVRKVLEELGRKDESQTEHTIELDDLTQSLEDSYNHAVMRYAGRESLSRGSIGRG
TSLSSTSTERRW)}*

$3489=273(299):
>gi|1091445000|ref|XP_018718792.1| {|0(200)|<Streptophyta(V)>}PREDICTED: TMV resistance protein N-like [Eucalyptus grandis]
{(MVGDQDFHSISATDHADPDLETTLSQNVSSMSVDLHFLAKEDNSDVAAVQSNGEVNHDGWVAEDSENEDND
LYLSECSG)QYGVFLSFSS-
LDTRKTFTDHLYHSLLKAGtVPVFRDENSIsiGENFGSKILDAIAQSKISIPIISE--------------
IMLRANGASTSSF-------------HPEGNFKNAFELSRKHFNEKDI-
QEVQLALNEVGYL(NGWESEKFANGHEGKLIERVVKTVRSILWEDFLLDVPKQLVGLDGKTTLAKCIYNRLLN
KFGHVSFLPDVRETARRHNIT)}*

$3490=240(240):
>gi|1091457243|ref|XP_018725460.1| {|2(784)|<Streptophyta(V)>}PREDICTED: TMV resistance protein N-like [Eucalyptus grandis]
{(RLIFGYSFAELVAPILLLGLTLYFLYKKKASVRGNPDDADTGTYDSMIVPTDTNSVGSSSSPTETNNNESS
SLTVSTGNH)-YEAFLSFRG-
LDTRHGFADHLYHGLRRAGIHTFRDDDELqqGEGIRPELMAAITNSKVLIPIFSVNYGTSSWSEAELVKLVVR
EVLNELKKKFELIISENLVGIDSHVKNVMELMDDKSHALLFVGIHGMGGIGKTTLAKT(IYNKLSNQFEYRSF
IEDIRE)}*

$3491=189(194):
>gi|1091457245|ref|XP_010046835.2| {|0(522)|<Streptophyta(V)>}PREDICTED: protein SUPPRESSOR OF npr1-1, CONSTITUTIVE 1-like [Eucalyptus grandis]
{(MDRRLKKKKTNASANAEDVDTGASGSMAVLTKTNFVGSDSSPKENNNNASTGNC)-YEVFLSLRG-
LNTRHGFPDHLYPGLLRTGIHPFKDEDELcqDPN-GTDKIEAIDLSK----
GNLEGPGKIAERDGDNYTAKQFKKLTSLRFLFMKGAHLIGDFEDSMEELKWLQWQECPKSFEVNNFHMQELAV
LELKYSK()}*

$3492=263(258):
>gi|1091457387|ref|XP_018725522.1| {|0(854)|<Streptophyta(V)>}PREDICTED: TMV resistance protein N-like [Eucalyptus grandis]

{(MGNYLSKQRPKRPRDTEASSPTTPKRRRREKSVELQAGECESESPHLLPSTDSLEQIDVPSASGANSDPSA
SSTSVNGN)DYYVFLSFRG-
PDARNGFVDHLYHRLKDVGLpfhpnFVFRDDENLpfGQKIGENLISAIKHSKVSIPVISEKYAASEWCLRELI
QIMEC-
EERGEQKVLPVLYKVKQQGLVALRKALDLRVLESEKFANGHEAELIKELVRVIMDEQ(QHDFLPPLPGNLVGI
DHHVAEVMKLADTDPSEIRIIGIC)}*

$3493=178(196):
>gi|1091457678|ref|XP_018725656.1| {<Streptophyta(V)>}PREDICTED: TMV
resistance protein N-like [Eucalyptus grandis]
{(MIAYAFQVLYGASGVIVLVF)-------------------
DSLYDALLNKGIYVFLDRKGIdvGEEIGLKIFQATNDSRICISIFSRGYASGSLCLRELGHMMECRKIN-
ELDVIPIFYDVDPFDVKLESEVYRDALTLDEEKWGSISCSIVPRCLRKLLQSR(DGTPRTRDYNLKSTAFSVF
RTKNCDGISHGTLRSII)}*

$3494=264(254):
>gi|1091457893|ref|XP_018725746.1| {|0(429)|<Streptophyta(V)>}PREDICTED:
TMV resistance protein N-like [Eucalyptus grandis]
{(MLARNVQREIERDLNLQSKAAMTNSDAGTSSGSAGGG)KYQVFLSFCG-
QDTRDGFTSFLYEGLRKNGIHAFMDDEEIrvGKKIADEILQAIDGSEIYVPIFSENYASRKWCLLELVRMVDN
ISKSQEKKIRPIFFYVEPDD--LKTESYRKAIgdlkEKHEKKQQEKlpheleswKL-
ESWEEAPTKVVGR(KGSTLRKRESQAKFVDKIVEELLQELKIKYKSVSKDLVGLDHRVEEVLKLLDQVRLLGI
YGMGGIGKTTLAKVVYNK)}*

$3495=239(239):
>gi|1091460184|ref|XP_018726435.1| {|0(700)|<Streptophyta(V)>}PREDICTED:
TMV resistance protein N-like isoform X1 [Eucalyptus grandis]
{(MAKEGPYGGSSSASSASVTGNN)-FDVFLSFRG-
SDTRHGFTDVLYRSLVDHGVRTFLDEEELsdGEYIKKQLPESIKLSKIGIPILSEDYASSKPCLRELAQMVE-
WRKEGRMIIMPIYFLITAWDVemKDGRGPYADKIAAHKKKENPDTI-
QKWVDALEEVTSV(KGRQLTGYTGQGEFVKMMVSKVLKRLRKAKLNLSDKLVGIEDRLPELRRMLDTDSSDVR
MIVISGIPGIGKTTLAKS)}*

$3496=142(165):
>gi|1091461255|ref|XP_018726633.1| {<Streptophyta(V)>}PREDICTED:
toll/interleukin-1 receptor-like protein [Eucalyptus grandis]
{()-------------------------
MLGAGIRVYRDDEELrvGREIGGKLMRALDVSKIYVPIFSKDYASSSWCLREVAHMVDCVaRSKGNKEILPVF
FNASPADVKLRTGLYRNAMTKHRKKFGDEEV-
KRWEDALVEVAEL(KGWDLKTRGLHFSSQQPAFRTNEPG)}*

$3497=146(171):
>gi|1091463504|ref|XP_018727172.1| {<Streptophyta(V)>}PREDICTED: TMV
resistance protein N-like [Eucalyptus grandis]
{()-------------------------
MIRAGIRVFKDNEELhvGEEIGGELLRAISNSKIYIPIFSKNYASSKWCLRELAHMVE-
HRKRGEKVILPIFYEVDASDLKLRTGSHDKALENHEKQSGKIVA-
KQWEEALKEVAGI(KGLNLKDHRRDKLIALALEEVFSKLTPMQRN)}*

$3498=273(279):
>gi|1091464857|ref|XP_010052538.2| {|121(0)|<Streptophyta(V)>}PREDICTED:
protein PHLOEM PROTEIN 2-LIKE A5 [Eucalyptus grandis]
{(RGEFGKGFSKLAKHHSPEQVEKWKEALRSVTRIKGCSLREDADGRQGQLVEMVFKTVSSILRRPWIERIPM
FRKKRKKYI)-YEVF-PISS-

PTTTKIFTSHLARHLECKNISVFRDDDRYliGKDSDLEVRNAMDHCKIFIPIISEDFASSPDCLDKLAQMFKC
VEKKGQ-NILPIFYGVKPSQEKKLR-RFWMDMLRH--KVPQETC-
QRLLESVNWVWSI(GGFVSENIGNGDKGELVEVVFQQVIKLMRTRSCRSWSIPIDSETNPATAKVAKEPLIVF
)}*

$3499=247(250):
>gi|1091468307|ref|XP_010054648.2| {|0(154)|<Streptophyta(V)>}PREDICTED:
uncharacterized protein LOC104442975 [Eucalyptus grandis]
{(MYGLQIVLLLALFVCFYILLGQKKARDPQT)-YDVFLSFRG-
YDTRKSFTDHLYNGLKAAGFHVFRDNEGIraGEKIDIKLEQAIRSCRIAIPIISKNYVESKWCLRELTEIMNY
RKKH-NIRVFPVFYEVDVLDLCGPSGRFAVDLANHKAKYRKES--
QKWAKALTSVARI(RGWTSQTIADGHDGELVKMVVERVSSELQTMWIERLPIFPMLMSNYHIRSAYGYFSEKK
RGEVFLTFHPDTRPGFAAYLY)}*

$3500=199(199):
>gi|1091470931|ref|XP_018728798.1| {|0(836)|<Streptophyta(V)>}PREDICTED:
TMV resistance protein N [Eucalyptus grandis]
{(MLNASSNNQEPEKLSGY)DYEVFLSFRG-
PDTRNDITDYLHTSMIDAGLRAYIDDEELriGEEISGQLLQAIEQSKISIPIFFKGYADSTWCLRELVKMVE-
SKNTRRHKIMPIFYNVTPSEAALNEVGALKGWDFQSMPNRGKGEFVKEIVNNVLTELK(TAYLEVSDCLVEVD
SHVDEIMRMIRTHDHEIKIVGIHGIGGM)}*

$3501=181(181):
>gi|1091470933|ref|XP_018728799.1| {|0(56)|<Streptophyta(V)>}PREDICTED:
disease resistance protein LAZ5-like [Eucalyptus grandis]
{(MEKSLGY)DYEVFLSFRG-
PDTHLDIVDYLYVNMIDAGIRAYRDNEELciMEEISGQLLQAIQHSKILIPIFSKGYADSPWCLRELVKMVE-
SKNTGDKRSCPISILSRHPRGKGEFVKEVINDVLTELKTAYLEVSDCFVEVDNHVDEI(MRMIGAHSCETKIV
GIHGMGGNQLLSDILKKKWL)}*

$3502=197(220):
>gi|1091470950|ref|XP_018728805.1| {|0(188)|<Streptophyta(V)>}PREDICTED:
TMV resistance protein N-like [Eucalyptus grandis]
{()-------------------------
MIDAGIRAYIDDEELriGEEISGQLLQAIEQSKISIPIFSKGYADSTWCLRELVKMVE-
SKNTRRHKIMPIFYNVTPSEVKYQTDRYGNAIVSHanKKRFDDETI-
DIWKSALNEVGAL(KGWDFQSMPNRGKGEFVKEIVNNVLTELKTAYLEVSDCLVEVDNHVDEIMRMICTHNHE
TKIIGIHGIGDMGKTTLAQIV)}*

$3503=205(229):
>gi|1091471158|ref|XP_018728895.1| {|0(243)|<Streptophyta(V)>}PREDICTED:
TMV resistance protein N-like [Eucalyptus grandis]
{(MASSLKSER)KYHVFLSFRG-
EDVRNNFLGHLYKALDQKGIYIYVDNEELrkGEQIDLALMKAIEESQIAIIVFSKDYASSWWCLEE-------
-------------------ELRTPRGSYRDAMVKHESKYgkDSKEV-
KRWEKALSYASYL(SGWPLKDGDESVFIQRIVTEISTHLDRTLLHVAMHPVGIDSQVDKLKSMLNLESDDNVL
MVGLWGQGGIGKTTLAKAVYN)}*

$3504=197(220):
>gi|1091472253|ref|XP_018729309.1| {|0(96)|<Streptophyta(V)>}PREDICTED:
TMV resistance protein N-like isoform X1 [Eucalyptus grandis]
{()-------------------------
MIDAGIRAYRDDEELrtGEEIDGQLLQAIQHSKISIPIFSKGYADSPWCLRELVKMVE-
SKNTGGQKIMPIFYNVTPSEVKYQNEHYGNAIVSHlnKKRFDDETI-

NNWKAALKEVGEL(KRWDLHSMLNRGKGEFVKEVVNDVLTKLKTAYLEVSDCFVEVDNHVDEIMRMIDAHSCE
TKIVGIHGMGGVGKTTLAKIV)}*

$3505=177(177):
>gi|1091473321|ref|XP_018729581.1| {<Streptophyta(V)>}PREDICTED: TMV resistance protein N-like [Eucalyptus grandis]
{(MQPYKGIVYRDQINMLNASSNNQELEKLSGY)DYEVFLSFRG-
PDTRNDITDYLHTSMIDAGIQAYKDDEELhiGEEISGQLLQAIEQSKISIPIFSKGYADSTWCLRELVKMVE-
SKNTRSHKIMPIFYNVAPSEAALNEVGALKGWDFQSMPNRWEEFMELEVSKQYVSSLM(KYLNTP)}*

$3506=208(208):
>gi|1091476156|ref|XP_010057624.2| {|0(15)|<Streptophyta(V)>}PREDICTED: putative disease resistance protein At4g11170 [Eucalyptus grandis]
{(MIFYFLNPTEDAAPTPSRQKPERDRENFCERSPPKNMAAIVGSSASAT)QYDVFLSSRG-
PDTRDTFTDCLYRDMVGQGIVAYRDSEELhaGDRI-
DDLLRAVGLSKFCVPLFSSGNASSAWCLRELARIMGLDEARTREALEHDLKQVVLKLSGNNRPLGSKFLSWEV
GQTSGVDDKSVRRRITSWQKGE(MLGRGSFGDVYKGFSDDGFF)}*

$3507=290(294):
>gi|1091491693|ref|XP_018715393.1| {|0(618)|<Streptophyta(V)>}PREDICTED: disease resistance protein TAO1 isoform X3 [Eucalyptus grandis]
{(MEYAVYDWERGEDEPERLDQSTKSNSEDEPLKEEMIESLVGSSVAEYVRTERWKAKAEQARRNQDTIKSEA
AESN)-YDVFLSFSG-SENPFRFADSLYCRLKAGGCLVFRDDVPIrtPK----
ELPTWVHSSKVYIPIFTGTYGYSPWCLESLALMVERaSRSGGKEEVLPVFYNREPSDVKLETSLYKGTLDMLR
RTWSADRV-
ERWKKALVEAGNV(KGWELRSYKSEGELITTIVEEISPKLKIEHKYAPGDLSTGAEPLKTNVALQDRFTGAGS
LEAKGKTYEVHTSHSKMAHV)}*

$3508=286(300):
>gi|1091493946|ref|XP_018715980.1|
{|133(750)|<Streptophyta(V)>}PREDICTED: TMV resistance protein N [Eucalyptus grandis]
{(RRLSIYEWFALMVTWKRRRVQYFLDTKAFQTLFPDSHFDFLLDSDLKEKLRAKKDSFAAFVAPVVLSMIAY
EQLHRKKAC)ECEVFLSFSG-KGALKNFADSLYKGMVDAGIGVFRNDYWLprHEKIG---
LKAIKSSKISILILSRKYDSSRRCLDELVQVMKCTNDSSGHVVLPIFYRVKPADVRCEVDV-----------
CDPMVA-
EERKQVLKEISSL(KGWESDEIAAGDEGQLVESVVRKVLIELKKALEPVITENSVGIKSPLEKIMKFLDSCSS
TQYVGIHGEGGVGKTTLAKII)}*

$3509=212(218):
>gi|1091507629|ref|XP_018719104.1| {<Streptophyta(V)>}PREDICTED: toll/interleukin-1 receptor-like protein [Eucalyptus grandis]
{(MDNALALSFSHAGPVGSGSTR)-YDVFLSFpSG-SNAaRESFSGRLHASLTAAGVSVCGPDESQvaGS--
SSQLPDLAWRSKISMPILSEDYSSSERCLRGLVQMME-
GKKSGQQMVFPIFNRVKPSDVRYLRGSFGKSFHSRE----ADDI-
EGVRRALKEVSML(HGWKVTDGYQSHLHSNLVYFLSPMMFEKYYLVGENENGSISFLRSRTYILVIGSRDG)}
*

$3510=233(241):
>gi|1091511206|ref|XP_018719919.1| {|0(137)|<Streptophyta(V)>}PREDICTED: disease resistance protein RLM3 [Eucalyptus grandis]
{(MAGALALCVSRAGPVACGSTH)-YDVFLSFpGG-
SDTTLeSFPGRLHACLAAVGVSICRPDESQaaGSSSSLLPDAAIWRSKICIPILSEDYVCSDRCLHELVQVME
-GKRSARLMVLPIFNRVEPSHLRYPKGIFEKACHS-----DTEGV-QR---

ALFEVSML(HGWKITDGSEDELLKTVVQTVLRCLQPDIQNVSQSARYKVFLNFRRADTRHGIAYHLYQGLMNA
GVSVFKDDIDLPIGEE)}*

$3511=250(258):
>gi|1091520031|ref|XP_010039926.2| {|0(580)|<Streptophyta(V)>}PREDICTED:
TMV resistance protein N-like [Eucalyptus grandis]
{(MDRKEPTPSEDPIHGASTSSTSPHRGDSGGGTQKPKGN)DHDVFLSSVS-SMWSTLFAD-
LRHGLVIAGIDTF----CSRHFADERILYSLQQPKISIPIFGRGFVDDESGLRELVHMWKCKRSSGQI-
VLPIFDGVGSSEVQLVEGRSIEVFSRKEGRVDEMEL-
GEWRKALAEVGSL(RGWEAEKFAYGNKGALVELVVARVVSLLRTTFKFPTELVGIGDHVKSIMNSIDHEYNDK
RIIGIHGVHGSGKTTLAKVLW)}*

$3512=197(220):
>gi|1091520262|ref|XP_018721897.1|
{|0(1009)|<Streptophyta(V)>}PREDICTED: TMV resistance protein N
[Eucalyptus grandis]
{()-------------------------
MDGAGIHVFRDNEEIrkGEVIKGELERAIKNSTIFMPIFSKNYASSPWCLRELAFRLDCSRnRDDNAMILPIF
FDVDPDDVKLKTGLYHDAFQKHEQKFGSNLV-
QQWKEALMEVARI(KGWDLKDTGHGELIRSIIAEVLIKLNKREKILPDHLVRIQDRVEDVMHLLDEGSPDVRY
LVIHGMGGIGKTTLAKVVFNQ)}*

$3513=201(220):
>gi|1091486578|ref|XP_018732484.1| {|21(0)|<Streptophyta(V)>}PREDICTED:
protein SUPPRESSOR OF npr1-1, CONSTITUTIVE 1-like [Eucalyptus grandis]
>gi|1091486580|ref|XP_018732485.1| PREDICTED: protein SUP
{(LQSSQLPVVSSNRTTTMAAHGTESAIGISETFAKLKKMGKVAPQSMAILSSNNLILKFGVERFMSTMNDAG
VHGQRPPRV)-YDVFISYKQ-EDMLHT-MSHLKSTLERREMKTFVDFTLDGGVP-
FrpAINEAVEWSKSAVVVVSQNYHLSPWCLNELVKILECQKR-WGLVVLPIFWEMSARDLREQGGRF------
----------RGEYWPRRKGFQAG()}*

$3514=177(179):
>gi|1091502801|ref|XP_010029908.2| {|0(247)|<Streptophyta(V)>}PREDICTED:
PYK10-binding protein 1 [Eucalyptus grandis]
{(MREGVMPLAVVVD)--DVFVSFRG-GDIRKNFAAHLFHALKQAGIYYFADNEKEETGI-
LiegKLLDAIRHSRVSLVVFTANYADSRRCLNELVEILESQQE-
VQRSSRSCVVSQSHKRRTCLINFNWVHLDYPTEYLTSVSGYTREDSCHVINSLT(FHSNKRAHGPIGEEKGKF
FSFPQVNG)}*

$3515=259(255):
>gi|1091504367|ref|XP_010028041.2| {|0(461)|<Streptophyta(V)>}PREDICTED:
TMV resistance protein N-like isoform X1 [Eucalyptus grandis]
>gi|1091504369|ref|XP_018718440.1| PREDICTED: TMV resistance prot
{(MAQTLRNLLLVLLGRPPQPLPPPPPSPPPPPPPPSPQWE)-YDVFVSFRG-
EDLRKNFIAHLFYALSQASIRCFRDNaREDIGEQ-
IksKLFKAIGRARNALIVFSENYADSKWCMSEMVEILECGKrfrN-
HGHLVIPIFHKVKAADSSNLTsgSEFAQGFERLCGSQRDDgQI-
QKWRDALIEPKHL(TGFDLRNDAGGDEGRLVDIIVKHLVEKIHETWPLYFVKNAIGVEFLVDEVISLIRMGQN
KDVCVIGICGVEGIGKT)}*

$3516=233(255):
>gi|1091504371|ref|XP_018718441.1| {|0(461)|<Streptophyta(V)>}PREDICTED:
TMV resistance protein N-like isoform X2 [Eucalyptus grandis]

{(MAQTLRNLLLVLLGRPPQPLPPPPPSPPPPPPPPSPQWE)-YDVFVSFRG-
EDLRKNFIAHLFYALSQASIRCFRDNaREDIGEQ-
IksKLFKAIGRARNALIVFSENYADSKWCMSEMVEILECGKrfrN-HGHLVIPIFHK----------------
-------RDDGQI-
QKWRDALIEPKHL(TGFDLRNDAGGDEGRLVDIIVKHLVEKIHETWPLYFVKNAIGVEFLVDEVISLIRMGQN
KDVCVIGICGVEGIGKT)}*

$3517=188(189):
>gi|1091486148|ref|XP_018732405.1| {<Streptophyta(V)>}PREDICTED:
toll/interleukin-1 receptor-like protein [Eucalyptus grandis]
{(MSTINDAGVHGQRSPGV)-YGVFISYKQ-NDMLHT-VSHLKGALERRGIRTFVDFTLDGGVQ-
FqrEINDVIEQSEIAVVVASQSYHFSPWCLNELVKILECQKK-
GGLILLPIFWGINARELREQSILFMENIGQGEKGFKQDnphQI-
QRWRDALRALGMI(KGWSASASLHKTEAEVIEELADKISAKLDHLE)}*

$3518=138(154):
>gi|1091490680|ref|XP_018715197.1| {<Streptophyta(V)>}PREDICTED:
toll/interleukin-1 receptor-like protein [Eucalyptus grandis]
{(MLHP)------------------VSHLKGALERRGMKTVIDYILDGGKE-
IwpAIEEAIEQSNIAVVVVSQNYHFSPWCLNELVKILECRKK-
GGLILLPIFWGIEARELRKPSSPFVENIGQGEEGFKQEnlhQV-QRWRKALQAVGRI(IGFPVNPSRN)}*

$3519=145(168):
>gi|1091493598|ref|XP_018715835.1| {<Streptophyta(V)>}PREDICTED:
toll/interleukin-1 receptor-like protein [Eucalyptus grandis]
{(MSSSSSSSPSSSEAGWP)-FDIFLRFRG-EDARPSFLPSWRHrrLHRRRWPKA-------RGAR-
SrrALTKAVKVTRIAVVVFSEDHALSRWCLDELVKEGNGVEE-----------------
AQGIAGAPVLRGGAEGDLMAERWRGLWQGIGQSPVFPG(GIIMTMGNQCC)}*

$3520=155(181):
>gi|1091471072|ref|XP_018728856.1| {<Streptophyta(V)>}PREDICTED: TMV
resistance protein N-like [Eucalyptus grandis]
{(MARGTINFKAGLGFRAHKHLKTGLQVAVRPTSSSSILALDL)---------------------------
KG-IYTYIDNEELRKGDQIvsVLMKAIEESRIAVIVFSEDYASSSWCLEEVAKIMKCKEE-
RDLRVFLVISKVEPREVRTPRAQYREAMVKH-ESKFGENSKkvKRWKKLVQGSLSG()}*

$3521=245(245):
>gi|1091471405|ref|XP_018729006.1| {<Streptophyta(V)>}PREDICTED: TMV
resistance protein N-like [Eucalyptus grandis]
{(MGNPGSEAAEHLREQSPLCFCFKPLIFLARLFLSLCSEAWHSREAHVPSALLTVASSNMKRN)-
YDVFLSFRG-TDVRQGFLSHLYAALdQRG-
ICTFMDSEELRKGEEIslKLMRAIEESRIAIIIFSEDYASSPWCLEEAVKIMECRER-
MGLIVFPVFYKVEPREVRGGRESYRRAMAKH-
KSKFRKDSEkvKRWKKALSEAGSL(SGWVLNDRDEAKLIQCIVEELLIRLNRKLLHVAKYPVYGDLEA)}*

$3522=193(221):
>gi|1091479527|ref|XP_018731041.1| {|0(963)|<Streptophyta(V)>}PREDICTED:
TMV resistance protein N-like [Eucalyptus grandis]
{(M)--------------------------------
AFRDDhhQDLPRGKCIklEILRAIEQSRLVLVIFSNNYASSTWCLEEVAKAMECKNI-
HEGSVIPIFHRVDPSDIRKLRENFGIAFAKT-
EEAYLGDKRdiKKWKDAVSKVASL(AGIELKDGYETKFIQQIIGEVENKLGPRLSYVVGDLVGMHSRVANVVE
CLCLDESDVRSIGIWGMGGIGKTTVARAVYDK)}*

$3523=237(234):
>gi|1091479567|ref|XP_018731061.1| {|0(97)|<Streptophyta(V)>}PREDICTED: TMV resistance protein N-like [Eucalyptus grandis]
{(MTSFPRPENQQSQF)KYDVFLSFRG-EDTRKNFVDHLnYHLQRKG-IVAFKDDHDRNllRGKHIkpEILGAIEKSKYVLLIFSENYPSSTWCLEEVAKAVECKDV-NEGSVIPIFYKVNPSDIRKLRGNFGRGFAET-EEAYSSNKRdiKRWKDAVSKVADL(AGIEFKDGEISKKNGLVYLQNQFLGDILHDDNLRIRDDHRAFRSNHPPAEFDELVEQVIGYANGLPLALDVLGSFLACRS)}*

$3524=267(267):
>gi|1091494463|ref|XP_018716138.1| {|0(1195)|<Streptophyta(V)>}PREDICTED: uncharacterized protein LOC104457139 [Eucalyptus grandis]
{(MATESKPVQSDIHMRDGAATRPDTEHSEEAHQTSSPLMAASSDLTGN)-YDVFLSFRG-TDIRKSFLSHLYAALDQK-GFyTYIDSEELQKGDFIkpALMKAIEDSRIAIIIFSENYASSRWCLEEVAKIMECKKQ-RDLTVFPVFYKVDPREVRTPRESYEKAMVEH-ERKFGRDSDevKRWKNALSDAGSL(SGWDLKDKDEAEADLIKRIVQEISMQLDRTPLHVAKHPVGIPRQVVKLKSMLNLESRDDVLMIGLWGQGGIGKTTLAKAL)}*

$3525=232(252):
>gi|1091457566|ref|XP_018725605.1| {|0(7)|<Streptophyta(V)>}PREDICTED: disease resistance protein RML1B-like [Eucalyptus grandis]
{(MAIEFGILPNCERSVGVYSTTRGGDEVSAQTA)---------------------LHHfTLLFTGIVAYSDSEELHTGDWIdNLLRVVCDSKICILVLSRGYALSAWCLRKLTRVIELNESTGKPEILPIFFDVTPSNVKLKMKLYVEDLDKH-EQRPGGESRERWEAAVSKVGKI(KGWKVQGRGIVAYSDSEEIHTGDRIDNLLRVVCDCKICILVLSRGYALSAWCLRKLARVMELNESTGKPEILHIFFDVAP)}*

$3526=219(249):
>gi|1091457895|ref|XP_018725747.1| {|0(763)|<Streptophyta(V)>}PREDICTED: TMV resistance protein N-like [Eucalyptus grandis]
{(MTSVAANQEPPMPTSAAGTSSGGTALGAG)-FQAFLRFRG-PDTRRGFTDTLYHALVDAGVRVFRDDDELRVGETIggALLRIHDPSR----------------------------SRGNKKILPIFFDVEPDDVKLKTSLYSDALVEH-QKKF-PDEVNAWRESLVEVGKI(KGWSLKNYGGQGELMKLVVEEVLDELKTKHRYVTEHLIGMDDRVAAIKKSLDIHAGGVRLVGIHGMGGIGKTTLAKVIFN)}*

$3527=141(149):
>gi|1091493602|ref|XP_018715837.1| {<Streptophyta(V)>}PREDICTED: TMV resistance protein N-like [Eucalyptus grandis]
{(MASSSSNSF)--KVFVSFRE-ENVGN-FVTHLYAALVHAGIYYF-DVQERE-KDEGNLmitdLKMIEESDLAIIIFSEDNAYSRLCWEEVVKIMECKKQ-KDLKVFPVFYRVELGRVSGLKGSYrtGIEYFQK-KLDKDVRKLMKWKDTL------()}*

$3528=198(197):
>gi|1090783130|emb|SEG42418.1| {|0(165)|<Acidobacteria(B)>}TIR domain-containing protein [Bryocella elongata]
{(MRVRKQTQKPINGQTVRGEDLVAIAQRSPL)---VFVSHDSRdAALAEAFSKLLSGISAGMLKSFRSSDKkgTQGIEFGVewypEVMSRIDEATDVVCLLTHRSIERPWILYEAGVAKGKANT-PVYGVALGISLSKASTGPFAQFQNCGDDAES--LTKLVKQLLCRIPGAEPDTDT(ITMQVEAFRTKSAALAQEGQAQGPLTK)}*

$3529=199(212):

>gi|1093638953|ref|WP_070904573.1|
{|0(257)|<Bacteroidetes(B)>}hypothetical protein [Elizabethkingia meningoseptica] >gi|1091761256|gb|OHT31629.1| hypothetical protein BGC12_04850 [Elizabethkingia men
{(MEGKTNKGYLDKTVPENL)--KIFISYSwdtSEhQKWVLSLADKINTRG--GNAI----
VDRTHLKYGGhiktFMLKSILEADIVLIILTPNYKRKADGL-EGGAGY----E-YNII-----
NDELFKIIDSNEKFIPILREGT--
FEKSTTHFLQGFNCVDLRDGD(SHNRHLKELLDQLFNTNINQPLNKDIKIANMEEEYNDIPLIVKEVKERAFKYFQ)}*

$3530=196(198):
>gi|1090840506|emb|SEG40333.1| {|0(141)|<Actinobacteria(B)>}TIR domain-containing protein [Actinomadura echinospora]
{(MRGDVKTY)---FFTSYApGEDRPWVRRFHSDLERE-LR-
IRQGPYAGGAMAAPPADPVRVAATAPAMVALISDRYLRDLGCGRqW--AIFAER-----
SRpdagardhcLIPIRWRPLRgPLPDAVRPMPDFWASV--
TPEPYRTSGLYELMRTHQLRDE(GGYYGMVRRIAEQVFAAERVRLRQLDPAAAAAVRPAFGSQEPGDDRRAGA)}*

$3531=243(245):
>gi|1090826007|emb|SEG48235.1| {|65(534)|<Actinobacteria(B)>}TIR domain-containing protein [Saccharopolyspora hirsuta]
>gi|1099904034|emb|SFE57524.1| TIR domain-containing protein [Saccharopolyspora j
{(TVLVLSGHKRGLVVVVPAEALRSLIPLVTSADASISVLAVINLGLLGYVLLRSDELDDAVPAPRARGTGAAVGREDGSGK)-PDVFLSYSRRqFHFAESL-----
MLRLEGRaVSTWFDThRIRAGEDWReSIERGLSVCTSLLLVASREALASPEV-DyEWRTALERGKK-
LYVVLFEAVELPP-
ELRGEAVAIIDMRTRFEpkAETLAELLAQPRHHRDEIPRRKP(FGLPLPQPAGTSLIAAALLLILLVS)}*

$3532=209(213):
>gi|1090777041|emb|SEG87188.1| {|0(241)|<Proteobacteria(B)>}TIR domain-containing protein [Marinobacterium lutimaris]
{(MK)---IYLTY-S--DKDNDTASVISSALKKARHKVIEDPLSTTPGENVfkKLEESSSSSDAYVVIL-
STHSLASDALKhELS-ALALNELSkSTLRVIPLLIDKS-
QAPSYLIQDFIIDMSDNLDLGISKLLEiLQDEERFKDDRVS(AKPSITEKYANQIQSIAESLHAGQLTLFCGAGVTIGAGVPSWNKLLLTLLESMMRRVSNDHSISLEGSDAG)}*

$3533=179(187):
>gi|1094117730|ref|WP_071107229.1|
{|0(1243)|<Cyanobacteria(B)>}hypothetical protein [Moorea producens]
>gi|1092225738|gb|AOY84080.1| hypothetical protein BJP36_33295 [Moorea producens JHB]
{(MKTFF)--DAFISYGR-ADSK-
AFATKLHQGLTELGFSIWFDQQDIPLGVDFqnQIDDGIEKSHNFLFIIAPHSVKSPYCLKEIQLAVQLN-----KRIIPLLH-
VEPTDCWDKMHPMIRQINWVYFRDNTEDFETSLAGLLKVIHHQ(SDYVRQHTEILVKALEWSRHHKQTNYLLIGEDKKQAQSWLKV)}*

$3534=261(270):
>gi|1092264539|gb|AOY73623.1| {|5(23)|<Actinobacteria(B)>}hypothetical protein ARZXY2_4123 [Arthrobacter sp. ZXY-2]
{(LEGLGTLEWDPTRVNVLLREFELQPFTGQFDHPSLDEIIADVTDEKLTELYSVVMGVDIDAVVDVVESVAGDGVWKPDYA)--RVFLSHSAKhKKFVGEVADELALVGIHG----FVAHDTM--

AFSKpwqsQIEQSLRSMQAFVAIIHPEFNSSPWCQQEVG--WARGRR-V-
PFYAIRYGTDPAGFIGRDQWPSATNQTPA--
QVATIISSWVSALPELGAAVT(EGLFESLRTAPSFIDAGTAAKRIAALGSLTADQWKRLDATYWSNDQVYGG)
}*

$3535=301(292):
>gi|1094069730|ref|WP_071062262.1|
{|340(376)|<Actinobacteria(B)>}hypothetical protein [Frankia sp. NRRL B-16219] >gi|1092322831|gb|OHV34478.1| hypothetical protein BBK14_15270 [Frankia sp. NRRL B-162
{(ILGGTQKYWEQVALPHTPRYAFQEGPPHGIDRDRLQRACEFLAQNIGRDDTIRLNPPSRTAWAKMVAGFDR
PARKLQYDE)---FVISYAegGRDPLWAAWVYEQLCRISAR-VR-QHNASRDGTGF---
LDDRLRDAGgdpgtdidggklCVLILLSPRYVESD-FGRaTWRWAASRY-----
TRadpadrgssrwsLLPVLISSHApeTAP--FAEIQPVSLVK--LDEETART--
TLLAELADTWDE(RESAVLTPVEFPGSPEDLVRRYQERADELERANAVTAERVATLRQLAQEKLEHHGTTSTS
QAVQHLREAHRL)}*

$3536=302(297):
>gi|1094061295|ref|WP_071054645.1|
{|399(382)|<Actinobacteria(B)>}hypothetical protein [Frankia sp. BMG5.36] >gi|1092331343|gb|OHV42743.1| hypothetical protein BCD48_30355 [Frankia sp. BMG5.36]
{(VGKDDRDYWDRAIVPYSPRYAYTETPPREASSDYDEVLKAVEFLAENVTLSEPVKFTVPRDQWKTFTDSFE
RPPIDADYT)--RVVLSHSAEDEAVAVWVEWLLKEHGLS-VRRQR-AYHLDTRI---
LEQELRTAHgnptntrsggtlCVMPLLREDYisldLAKEAWEWaRPRVTKGGR-----
EVLLPVRTEQFARLPPFDRPVECVELasalaFG--RRE-EARA--
TLLRSIADRYDV(PEEQNEPEPEFPLPPVVLDDREAAERRTARRKATDGDSVTRFQYLLALANQQKEEPAAVG
QLKEAVEVARDLGDALK)}*

$3537=241(245):
>gi|1094116978|ref|WP_071106477.1| {|331(809)|<Cyanobacteria(B)>}type IV secretion protein Rhs [Moorea producens] >gi|1092224921|gb|AOY83263.1| type IV secretion protein Rhs [Moorea producens JHB]
{(LLKELNQDALYYEEHKLLLVKALKWLSQNRNSSILLRGHNLNYYENWLKVNRQRVEHPPLGLHQEFIAAST
NQPKESYLD)---VFISYSRTdSDLARKLNEALQL---QGK-
TTWFDQEsIPPGSDFQqEIYRGIEQSDNFLFIISPASVNSPYCAGEVDYAQTLNKR-FVTVLSTQVPATV-
LHPGLASIQWIDFNRHRgdFYPNFSELVRTLDTDREHVRSHT(KWLQRAREWQQSHQDTDLLLRGSEF)}*

$3538=268(271):
>gi|1094116978|ref|WP_071106477.1| {|126(987)|<Cyanobacteria(B)>}type IV secretion protein Rhs [Moorea producens] >gi|1092224921|gb|AOY83263.1| type IV secretion protein Rhs [Moorea producens JHB]
{(QLMNSIRKHADYVEQHTRFLVKALEWDRNQQQTNHLLIQSERIQAEFWLKVRFQKEQAPCVPTDLHCEFIC
ESIKNANNL)MTQVFISYSDKdKDIKDKICQNL---MREG-
ITIWkHETDIKTGIDFQaAINQGIEEADNLIYLISPNSIQSKYC-QqELEHAFAHNK-----RIISLLIEYT-
DIDQIPpkIRSLQFLDFTKynNEANYRFMAAQLLKELNQDALYY(EEHKLLLVKALKWLSQNRNSSILLRGHN
LNYYENWLKVNRQRVEHPPLGLH)}*

$3539=258(272):
>gi|1094117730|ref|WP_071107229.1|
{|129(1035)|<Cyanobacteria(B)>}hypothetical protein [Moorea producens] >gi|1092225738|gb|AOY84080.1| hypothetical protein BJP36_33295 [Moorea producens JHB]

{(LLKVIHHQSDYVRQHTEILVKALEWSRHHKQTNYLLIGEDKKQAQSWLKVRFHDQQPPCVPTDLQCEFICE
STKNANNLL)-TQVFICYADQdKPVMQKIAK---
TLMRKSFTVWTNKSDIQTGTRFQdVVYQGIDGADNIVYLISPASLKSDYC-QqEITHALV-----
NNKRIIPLLIEST---------
ELQCIPPHIrgLQFIDFTTYNDEAKYRIDADKII(KVLHQDQPYYEDHKTLLVKAIKWKNQNRNSSILLRGYN
LRYYQNWLTLNQQR)}*

$3540=157(165):
>gi|1093671855|ref|WP_070920201.1|
{|0(668)|<Actinobacteria(B)>}hypothetical protein [Mycobacterium
chelonae] >gi|1092037227|gb|OHT81080.1| hypothetical protein BKG69_03420
[Mycobacterium chelonae]
{(MSSKRF)----FISHAS--PE-KPFAIELKAALEG---DAWVDLHEIKSGDILlrEISDGI-
EAATDFVLLWSAHSAASNWVAfEINLAFIRYIEEqSIQIHVVCMDATA-
PVLTLRPFLQIRGSQTAQEVATKMLSPSVPAEPRRHFLNR(NLEVEKIESALYDPATAAL)}*

$3541=191(184):
>gi|1093956777|ref|WP_070956822.1| {|0(278)|<Firmicutes(B)>}hypothetical
protein [Clostridium perfringens] >gi|1092199822|gb|AOY53892.1|
hypothetical protein FORC25_1477 [Clostridium perfringens]
{(MENNQK)--TVFISYCwtT--
LEHEKWVIELAEKLLSVGVDVKLDKWDLVPGNDTyaFMEQMVTNPDIdKVLIICDKGYKTKadgreGGVGtET
KIITpEIYRSVnQEKF-IPIIVEKD-
EDDFMKYVPTYARNMLGINMTSEECFGESFEELLRCIYKK(PRYKKPALGNNMPSFLKDDEEIIMSVVLKLKN
EEIKKY)}*

$3542=231(254):
>gi|1094073406|ref|WP_071065764.1|
{|369(461)|<Actinobacteria(B)>}hypothetical protein [Frankia sp. NRRL B-
16219] >gi|1092311981|gb|OHV23897.1| hypothetical protein BBK14_23850
[Frankia sp. NRRL B-162
{(SFEVIHAPGDGGDERCRGHVQISLDGLPVARIPLDLAVSTAADPSTTPVTVTGSAPMFREIYGSYAWADID
IARRFREIY)---------------------------RALGITLFMDVLDVDGAREWkpELEVAI-
ERADLFALFWSPASAAADYVEkEWRRALEVaANRRpEGAFVFPFQWSRP-
VPEPPLPLVDLPFRQVDRWSTALDRLANLARAGAGAGPGA(AGARAGAAADAAPVPPGPTASGGPHGPNGSGG
PH)}*

$3543=223(225):
>gi|1092322383|gb|OHV34037.1| {|73(0)|<Actinobacteria(B)>}hypothetical
protein CC117_22295 [Frankia sp. Cc1.17]
{(TSEHAWFEVFRALEAGIVAQPCRRLLAAALARYPANGVFRDLHDRFLAGRPGDPALSMTGAGQTSPPPPPA
QPAPPPHAP)--TVFLAHDR---
EDQSKVRALAELLTTAGFTPWVGSEMVTGGSRRdlVVRRAVLDCDFFVPCLSARVLGRPSQLHrEIRIALDRA
DELpDHvVFIVPARLDEC-DLPSDLQEYQWVDLFA--AGGVDQLLRaLRASTPSARNSRS(GPSGR)}*

$3544=171(192):
>gi|1093236741|emb|SEH14238.1| {|0(32)|<Euryarchaeota(A)>}TIR domain-
containing protein [Natronorubrum sediminis]
{(MAGEQIYVSHAPADLE)---------------LVQELFSTVKNFPF--
GTHIALEEVESGRSRqRLEGRLANSDVVVAVLT--------
EESstSRWVNQEVGYALaKGVPVIPLADSERHLGGFLENADSVAITRENPSRTIYDLISRLRSELAPLGALS(
VPNWYIRFPCTIPDCGHPVTLEIEQDQSKLWKRYEH)}*

$3545=292(279):

>gi|1124432048|ref|WP_074719043.1|
{|76(356)|<Firmicutes(B)>}hypothetical protein [Ruminococcus
flavefaciens] >gi|1093239669|emb|SEH86800.1| Tetratricopeptide repeat-
containing protein [Ruminococcus f
{(SYWGLVLCKYGIEYVEDPATLKKIPTCHRTQLEPVQTDTDYLAAIEHADTLQRSVYEREAVEIDRLQKDIL
EIVHNEKPF)--DVFICYKETDADGkrtqdsvIANDIYYQLTQEGF--
KVFYAAITLEDkiGQAYePYIYAALHSANVMLVLGtkPEYFDAV------
WVRNEWSRYLkivrqdRSKLLIPCyrdMDAYELPEefAHLQAQDMSKIGFINDVIRGIKKVIAPENISTKIIG
IS(KPVNSEIEPLLQRIELFLEDGEFERADEFCEKVLNIEPTNAEAYIFKLLIEYKCRTKDQ)}*

$3546=273(263):
>gi|1124432040|ref|WP_074719035.1|
{|74(622)|<Firmicutes(B)>}hypothetical protein [Ruminococcus
flavefaciens] >gi|1093239665|emb|SEH86745.1| TIR domain-containing
protein [Ruminococcus flavefaciens]
{(AELYWSMVLCRYGIEYVDDPITKKKIPTCHRTQFRSILNDPDYQAALEYADVTQRSVYEEEATYIDNVQKS
ILEISNKEQ)PFDVFICYKE-
TDEngKRTqdsvLAQELYYGLTQEGFKVFFSRITLesKLGREYepYIFAALNSSKVMVVVGTkPEYFNAVWVH
NEwsryLMLMQNDKE----
RTLIPAYKDMNPYDLPDALSMFQAQDMSKLGFMQDLVRGIRKILNTDKDDIS(IKNRSHQQADIKGSRGINVE
NYIKRGYLCLEDEKWNDAVNFFE)}*

$3547=273(263):
>gi|1124432046|ref|WP_074719041.1|
{|74(203)|<Firmicutes(B)>}hypothetical protein [Ruminococcus
flavefaciens] >gi|1093239668|emb|SEH86786.1| TIR domain-containing
protein [Ruminococcus flavefaciens]
{(SESYWGLVLCKYGIEYVEDPVTLKKIPTCHRTQLEPVQTDTDYIAALEHSDASQRSVYEREAAEIDRLQKD
ILAIVHNEK)PFDVFICYKE-
TDAdgKRTpdsvIANDIYYQLTQEGFKVFYAAITLedKLGQAYepYIYAALHSAKVMLVIGTkPEYFDAVWVR
NEwsryLKIVREDR-----
SKlLIPCYRDMDAYDLPEEFSHLQAQDMSKIGFINDVIRGIKKVLNTEKNEPK(ASKGSVVHKTTSTNVENLL
KRGNLALEDEKWEEANNFFEQVLN)}*

$3548=247(255):
>gi|1093243050|emb|SEI17122.1| {|12(53)|<Actinobacteria(B)>}TIR domain-
containing protein [Leifsonia sp. CL154] >gi|1097495786|emb|SFM09264.1|
TIR domain-containing protein [Leifsonia sp. CL147]
{(AADALLAQFDDALLVLGQFGIDTEGWSWYDSDDSRRKFLLGTIQTATDTQLIDLHDFLIGSDQAPVSVSAD
DDPWTELPI)--RVFLSHKWEDAEWVTALRSNLAKYGIS-
AFVAHKDITPSKHWREVIKAGLRSCHMMVAVLHDD-FHTSQWCDqEVGWALGRG-----
IPIATVRRTLEFdrGEDGFLEEVQDIQLDP--
GKASGEMRAAQEIFRAAIRTVK(PPELVRRAIAEALVTSPSFENSRNLFPAIQRQDQW)}*

$3549=227(240):
>gi|1093263234|emb|SEH18589.1| {|0(117)|<Proteobacteria(B)>}Protein of
unknown function [Sphingopyxis sp. YR583]
{(MMAGMTDAGPTPITPKRLSA)----FISHHSSQVETARRLKRLLAARGID-
GWLAPDDIAAGTAFDQSIVDQIDRSDAIVLLFCPQ-SDQSRHVKrELMLGEDSN-----
KTILPVRLEDIRaqGLAYWLKDYQWIDWFA---GEEQAVD--
RIVAAMTSGAAP(SLPPGASARQISPAPAPRRKRLWMMVAAGVALVGLIGGGIWWQSRAPAAGDPGAAVADNP
IEPGIWTSKREVVEILFPEM)}*

$3550=202(209):

>gi|1093958929|ref|WP_070958578.1|
{|0(359)|<Proteobacteria(B)>}hypothetical protein [Hyphomonas sp. Mor2]
{(MGQLLGDRESKTEQL)--KVFISYSRAQVAFADELELALTDKGHE-
VLIDRHSIAKGEAFRERLAEMILACDSVVFILSDE-SAASEVCQwEVTEASRLL-----
KRVLVVSIAELSksiSAPEDLASIDWIHCWN--
NPKVPGSNQTKGIIELDRALRT(DLSWLRQRTRLHEQAVFWSSRSKKDNHSSSLLRGELLQEAMDWASKKPAA
ESLP)}*

$3551=212(220):
>gi|1093960020|ref|WP_070959669.1|
{|229(0)|<Proteobacteria(B)>}hypothetical protein [Hyphomonas sp. Mor2]
{(AVWDGRLSWSQAVLIGGGVLVAFMFIGLLVSLLSGDTPPASKRSSSPPRAPVARPAAPAPKLAERSLSTPI
PAIAASAAS)SHEVFVSYAHDNATTVLPIVTQVEKGGVS-
IWIDREEMRAGQNWAAQIVRAIKAANRFCIMCSEQAFASDHVRReVYLADKYGK-----D-
MVPIRLDGAEmpeDIEYFLIDRQWVDLTS--LEDDEQAS--AVREAL-KNEES()}*

$3552=202(207):
>gi|1093960804|ref|WP_070960453.1|
{|0(844)|<Proteobacteria(B)>}hypothetical protein [Hyphomonas sp. Mor2]
{(MDMTIIRDRNPDD)ALKVFISYSRAQVHFADELELALSRWGYT-
VLIDRHSIAKGEDFQDRLGQMILNCDTVVFVLSDD-SAQSDICAwEVEEARRLT-----
KRILVITIADLSdgvSCPPHLAGIDWIHAWR--
NPKIPGSSQTKGFIELDTALNT(DVDWLRDKTVLQEQAERWAQRAPDSTVDEFGDNASSLLLRGNVLQEAMAW
LQRK)}*

$3553=213(220):
>gi|1094158176|ref|WP_071146827.1|
{|92(0)|<Bacteroidetes(B)>}hypothetical protein [Bacteroides ihuae]
{(KQDLQKEEIKEKDKLKREQEIFRKQQEDFQRNQLRFQQEQERLQQKLQSDISSQKEILNVLVHQIHSTNAD
REVIEQKEY)--DFFISHATEDKEsFVRPLAELLTNNGYK-
IWYDEFQMKIGDSLRKKIDEGLKFSKYGIAVLSRDFFR-KNWTEyELNGLVARE-----
MNGIKVILPIWHnvTRDEVLSFSPTLADKV--ALNTAIYSLQEIANELGKLIEK()}*

$3554=192(191):
>gi|1093279172|emb|SEH97323.1| {|0(129)|<Proteobacteria(B)>}TIR domain-containing protein [Rheinheimera pacifica]
{()MAKIFISYAREdSQIAQRLTTELHQRKHDVFLD---
QQSIRIGAHWQeDIKRHLKDADVFLALVTNHSDKS-YYFNiEVTSAVSyvAHSN-RDKLILPVVLDQA-
SLNDSDLGAFQALFGRTedFDFLVGEIENAISRFEGFRIAQE(EIKDLQGKILEENSADYIKETIEDLTAREK
TGRKLASRWHLAGYATLVASV)}*

$3555=206(205):
>gi|1093958926|ref|WP_070958575.1|
{|0(807)|<Proteobacteria(B)>}hypothetical protein [Hyphomonas sp. Mor2]
{(MKIKKRIDEALSGLEGSDAPLR)---
VFISYSRRdAEFMRRLADALITVPFEVDFDQADhgdhnsDFGISAEDDWWnRLQEMISVADVMVFIVSPNSSK
SKVC-DeEIAYA----RA-LGKRIIPVLAEHL-
DFSTAAPRLAALNVEIDfsNETGDFKIAFDQLCVALNDNIHW(HRKGRRLSDRASEWDRLNQPGHLLLRKGAI
TDAEEWITERPQL)}*

$3556=172(179):
>gi|1093960150|ref|WP_070959799.1|
{|0(1565)|<Proteobacteria(B)>}hypothetical protein [Hyphomonas sp. Mor2]

{()MADVFVSYKREdKDKVARLVDLL----
RDSGLSTWWDHDIEAGAPWEtTIEEKLADASVCIVVWSSTSVSS-----ENVKAEARRAR-
TDGKLIQVFIEDC-
DPPIFFGERQGINLTEWrgETNAPGPQDVIDAALAIRSGRKP(KPGTGYVPKRNRFRWAFLLTPFVGFLGLPI
HLDLALVFY)}*

$3557=262(272):
>gi|1094308735|emb|SEW23501.1| {|42(32)|<Bacteroidetes(B)>}TIR domain-containing protein [Prevotella sp. khp7]
{(GGIDTYNIVLKIPTDLFKSLKRRNAIEQAEAELYKCYADAMRGEGESLQIASVIIRPTTADVTQLGNNYND
RMWKPGYFR)---LFISHLSEhKESASNLKECLQNFGIDGFVAH---
EDITPSKEWEqEIEAALFSLDALCAIVAPAFKNSNWC-DqEVGIALGRKK-----MVFPIKKgrNPY-GF---
-
FGKYQALPNQRnaNDMAQELTKAICVNEFTRDEYFT(KLVNLVLNASSETTALKFIQVLSEAVYMDKQYVSLL
HDNFASNLTIMKENVL)}*

$3558=200(210):
>gi|1094074469|ref|WP_071066826.1|
{|0(225)|<Actinobacteria(B)>}hypothetical protein [Frankia sp. NRRL B-16219]
{(MA)---VFISYHS--SK-
KAVVQHLAAYFARHTITTWYAPRDVPPGSDWdaEISRAIKNSTALVLLFCSSAD-SSMHVKrEILLAD----
KArIPIYWVRLeKVEPE-KLGYFLNATQWID----
WLDKRDTTLDtLVQDLKSPVSPGR(VLEPNEIDRTALPATEAPVEGWPAEFIAFENERVAADAAAHSYFRIAQ
ESPDRSLVLPTGRAATQLFR)}*

$3559=195(198):
>gi|1094380992|emb|SEQ78859.1| {|0(293)|<Proteobacteria(B)>}TIR domain-containing protein [Nitrosomonas sp. Nm51]
{(MPTIPEGKIIPKTGIKKTRMGHEIH)---IFLSYRQ-
KNPDDIKARDMLAALCTKhGFKLIYDENTLGEGENLtRFMNDLGSARCVLILLSPEYFKSAYTLYELIRIYEN
ADL-
DRRFIFPLRATADMSAYVKTGAKNDCWLKSEAVRNELCSRLKENDESAVWECVL(DAWDAIVGKNLDTVTPSL
EQNGPEEVLEAKLAT)}*

$3560=271(262):
>gi|1094499282|emb|SEP75778.1| {|75(296)|<Firmicutes(B)>}TIR domain-containing protein [Lachnospiraceae bacterium NE2001]
{(EAYWGLVLCKYGIEYVDDPKTGKKIPTCHRSSFDSVMDDGNFDLVMENSDTVARGIYREEAKQIEELRKGI
VEVSSKEEP)-YDIFICYKetaddG-
QRTIDSvIAQDVYQELTDKGYRVFFSRItlEDKLGQEYepYIFAALNSAKIMLAFgTCYDYYNAVWVKNEWSR
FLQLMEAGQKKTLIPCFKDIDAYDMPKEFARLQAQDMGKVGAMQDLIRGIDKIFGRDKNDVI(QDSSVDLHNF
QNSTDEKVSSVGVGGPNSPSIDSLIKRAQGFV)}*

$3561=156(165):
>gi|1124462444|ref|WP_074748298.1| {<Bacteroidetes(B)>}hypothetical protein [Myroides marinus] >gi|1094606590|emb|SEJ40221.1| MTH538 TIR-like domain [Myroides marinus]
{(MGKKHN)---IFISHYYKDDDKVQDLKRRLIDS-GY--
DVRnysVDSTKHKDGRTPsKavieryLKARIAASSTFVCVIGDKTHTR------
PWVDFEIKEAIkQGKKIIGIYNHGDK-DKVEIPEALKEIN-ANIIGWNS------
VDKIGDLIEES(NDNNPNSNSTNYAIQRVKC)}*

$3562=151(173):

>gi|1094343971|emb|SEP43869.1| {<Proteobacteria(B)>}TIR domain-containing protein [Nitrosovibrio sp. Nv6]
{(M)-PDVFISYSVKD-AALADLVHENLRTHDL--
DVFYAPISLTVGsQWApQILEALRNSEWFFLLASKNALDSAN------VQVEVGGAMsQKKKVVPIMWDI---
QPNELP------------
HWIADYQGLVLTNANMGDISQ(QVTKLAANIKASKEKAQLIVAAVFFGLLVFSK)}*

$3563=204(210):
>gi|1094485891|emb|SEN70245.1| {|0(42)|<Proteobacteria(B)>}TIR domain-containing protein [Sphingomonas sp. S6-262]
{(MAGRISIF)-----VSYAY---
DDKPLVERLVEALRARtGDAVDYVLDEQFLEAGEsisESVAtRIFNSQGVllVL---
PSNPQGLWLQAEMAFALAKAQKDPNFKLLPVVTGNGAvpQ-LISDRLYVDIR--TP-
EKFAAGVEALGRAVLLDRQSSP(DSVQQAAKEMTMRAQSQALQAAKLEWEATKSGNQKSIGFAFAGGLTAALI
SIVPLAAEIDHL)}*

$3564=215(224):
>gi|1094513505|emb|SEM20919.1| {|0(71)|<Proteobacteria(B)>}TIR domain-containing protein [Syntrophus gentianae]
{(MTSRFS)---VFISHVSEdEAVAVMLKNSIERVFLNS-DVFVS---
GRDLKGGEiwikEIREKLRQSSVIIALVTEFSRDSNWVLFEAGSGFI-ENK-
SIPLCVPPLLISNLTPPLSLLQARE-YNKEG--LKKILQD--
IANVTGLRIPQE(IPGISEEIAELKKYLSLRNKGTDEKRAVGPNNVPSNKRGKVCKEDADIKSKISEIERRLK
EFLISQLLTVDPTYEIPR)}*

$3565=210(217):
>gi|1094450770|emb|SET52731.1| {|0(1541)|<Proteobacteria(B)>}TIR domain-containing protein [Nitrosomonas marina]
{(MAT)---IFLSHSSIDKAAAQQLKAWLEAAerGHQ-
VFLDydiQTGIKAGADWERSLYEQLRLCRVFLPILTQNWL-DSKWCFaEMTHARAQG-----
KLILPVKLDTALdsSNLFADLQHTPIDLSG---DDQSGYQ--
RLQRALGEEFSW(NPERPPYPGLPAFQEEDAAIYYGRNPEIISVIELLEGLRRRGYSTPHFVLLLGASGSGKS
SLARAGIMPRLRLK)}*

$3566=219(222):
>gi|1094485890|emb|SEN70215.1| {|62(0)|<Proteobacteria(B)>}TIR domain-containing protein [Sphingomonas sp. S6-262]
{(SRAQEHVLKGEEDERKKTSDLYEKQRKADEAARKRLTDDNRALSKDLASLRAQLTAAISTQASSTQPFVVE
NAEGRETPY)--DFFISHAWKDKEdFVNGLVSAAKEAGLD-
VWDDQSAIVWGDSIRQKIDDGLRRSFFGVVVLSPNFFE-RPWTQyELdgivQRDLSGA-----
GRLLPIWHRLTQddVAAKAPSLAGRLALPT--STYSTAQIVDELLEMRDRFKAV(AR)}*

$3567=179(179):
>gi|1124552559|ref|WP_074835820.1| {|0(167)|<Firmicutes(B)>}hypothetical protein [Ruminococcus albus] >gi|1094507886|emb|SEL35701.1| TIR domain-containing protein [Ruminococcus albus]
{()MKKLFISYSHDdVAKVKRFALLLSL----
HGFDLWLDEKnISNGDYYTtKILNGIHESDAYLLFLSNSSIKSPWV-NaEIDFALrEKIER-
NRLTIIPVLLEDV-
DIPVPISNIDYLDARFSmqKAVSEFAEMFQKKEQQINDLTVS(SISFTISEDTSVEIGPFVESITSSDLKEDR
EHILQDLRK)}*

$3568=220(221):

>gi|1094620053|emb|SEQ87517.1| {<unknown(B)>}TIR domain-containing protein [bacterium A52C2]
{(MPETKNTV)--
NVFVSYSHKdEVHWDKLRTHLAPLKRQGLISLWWDHDILEGGELTpEIRKALRQSNVFVALVSPDYLASHYCF
DiEYQYALRRRQR-KTMHVVAAIIRPC-EWKHTEMVRYRSLPKDGksVTEWKNRD--
SAYEDIAAGLRRV(VAAVVAEQASKPPLSRETDKRRQRPKSSGLGKPAGQVGAKPTSPRKGIAGAPTKTASKR
SAATRTQRPNSKTA)}*

$3569=291(295):
>gi|1173666873|ref|WP_081349232.1|
{|92(495)|<Firmicutes(B)>}hypothetical protein [Ruminococcus flavefaciens] >gi|1094417370|emb|SEL53092.1| Regulator of chromosome condensation (RCC1) repeat-containi
{(DPVAGKRVPTVNRAQFTSIFDDDNYKSALKYADLDQRKLYEAEANAINEIQKGILAISQKEKPFDVFICYK
ETDSNGRRT)-------------
QDSVLANDLYHQLTQEGFKVFFARITLedKLGTAYepYIFAALNSAKVMVVLGTRPEYFNAVWVKnEWSRYLS
IVRQSnGKKILIPAyrDMDPY-
NLPEEFSHLQAQDMSKlGFMQDLIRGIRkIVKADEPKPTPRE(AISGASVNVSALIRRAFLFLEDGSWNEADT
YCEKVLDQDPENAYAYLGKMMVEMQIRKKTDLADCEKPFDDNNYY)}*

$3570=191(195):
>gi|1094471869|emb|SES25585.1| {|0(122)|<Proteobacteria(B)>}TIR domain-containing protein [Halomonas subterranea]
{(MAPK)---VFVSHAS--EDKERFVLGFAEKLRQNGVDAWLDKWEMLPGDSLveKIfEEGLKEAQA-
VIVVLSSYSISKPWVKeELN--TSIVNKIsNGTKIIPVVIDQC-
DIPESLKSTVWERVQDTNNYQDNLDRIIASVFGHSTKPSL(GSPPSYTQHALADIPGLTQIDNLVLKKSCDYD
LENNAHIINPGKLFIENDK)}*

$3571=239(221):
>gi|1124552556|ref|WP_074835817.1| {|0(573)|<Firmicutes(B)>}hypothetical protein [Ruminococcus albus] >gi|1094507885|emb|SEL35673.1| TIR domain-containing protein [Ruminococcus albus]
{(M)--QVFFSYGH--
DTHKDFVHRLAEEIESLSkgeIKVWIDTYGIKFGDHWrdKITEGILESQSVIAFLSKYSAREESVCRdELSIA
LVSKHGMirtilldavppKGDFIPSSRLSEY--
QWRDLSDYQSVQkqgeeaFSEYISTEAKQIIElLNSDEIKKYNDEI(KRLRKAYCLPEFDDWTKFDHLLKEEM
IGRNWLFDKIKKWEEDPKGSKVMMLYGQPGAGKSMFSAHLQMRDPAVVAAFPCD)}*

$3572=250(255):
>gi|1124459457|ref|WP_074745311.1|
{|267(1180)|<Proteobacteria(B)>}hypothetical protein [Nitrosospira multiformis] >gi|1094431637|emb|SEN38872.1| WD40 repeat [Nitrosospira multiformis]
{(LPYLPEFAVEEQLVGDDFVNADRTSAVSGAFERLADALVLLANAGDPLYRLRNEALARLRGQVSPEITLLH
ADVSRATSG)-FDFFIDYGQSdrdvaLFLSRELET--------RGMKVFID-
ASLNMGALWseEIERALDYSAH-VLVCFGHSSDSPW-RAKLLNFVRRSDKKqiiPIFLPGAQLSA---
LQSYGLDNYVGINFREEIHEDGLREIILLAKRTVAANKLG(NGMDINIQCPYPGEHAYLEDDTRFFYGRDEEI
GVL)}*

$3573=194(190):
>gi|1094567787|emb|SEO16438.1| {|0(589)|<Proteobacteria(B)>}TIR domain-containing protein [Acinetobacter sp. yr461]
{(MEKGKSKIDMLG)-YDFFISFTLGpypRGTQSYASDLARKL-
REKNFTVFYSEEEAPPGANLsnVLLKALRKSKVLLVIVNEGAlLQSTWVKKEVEYFHSQNPKRlivPINIGNT

LEKY---
GDVIEADNWLNYKNNIWIVEDELAVEIGIANKIVIDRIAV(SYHFLKKKNQLIIILTSFISILILLSVFLWINMREAKI)}*

$3574=173(183):
>gi|1094714604|emb|SEQ05521.1| {|0(39)|<Euryarchaeota(A)>}Nucleoside 2-deoxyribosyltransferase [Natrinema salaciae]
{(MTGEQ)---VYVSHAPGD-LELVQDLFSTVKNFPF--
GVHIALEEIESGRSRkRLEGRLANSDVVVAVLTEDAVENR------
WINQEMGYAVaKGIPVLPLYDDGIGRRGFVSDIEGVVIDRHNLSFTIFNLLCRLRSELAPLGALS(VPNWYIRFPCTVPDCGHPVTLELEQGQTKLWKLHSHGK)}*

$3575=206(219):
>gi|1094701292|emb|SET95117.1| {|0(1124)|<Actinobacteria(B)>}WD40 repeat [Geodermatophilus poikilotrophus]
{(MTSL)----FVSHSARDWAPTQQVREWLRSEGYA-
ALFVDFDPdsgiGAGQDWERELYAQLRRCDGVVFLASSS-STASRWCFaELSLARALG-----
KPVFPIRVEPATrmD---LLDDVQWVDLAE--GD--AALD--
RLRGGLQRAGLD(PADSFSWDPIRSPYPGLAHFEAADAAVFYGRGPETTRLLELLQPTILRGPGRFVGIVGPSGSGKSSLLRAGLLPR)}*

$3576=181(185):
>gi|1094766117|emb|SEO74728.1| {|0(234)|<Euryarchaeota(A)>}TIR domain-containing protein [Halorientalis persicus]
{(MTDPTGR)---VFLSYKHEqTDVANFLQTEL---ERHGV-PIWRDiFDLKP-
EPLRdEIIDQLENPETAsgIALVSEGVADSDIILNdELPGFNKRWDG-DDEFFVVVV--PC-
PDISVGEAKSILNEAPIlhGFSAWKMLPLEETTSDKATEIVQ(AVLSERIERINGYLPDGEPLECSLDTYESPAHDIDPAI)}*

$3577=195(190):
>gi|1124374633|ref|WP_074664272.1| {|0(206)|<Firmicutes(B)>}hypothetical protein [[Clostridium] clostridioforme] >gi|1094899879|emb|SEU15320.1| TIR domain-containing protein [[Clostridium] clostridiof
{(ME)---IFISWS-
KdKSRLLALETKKFILNTLGnKIEVFFSPDMYKGTSVDnEIHTNLLKSDkCIVCITSDNFKNPWLMYEaGVVYGAHFAEP-GGSIVIPILFEHipdwS-
SWIDKPLNRYVPIRLSGenNNSKKSREEFKCFLQELANDLNT(DIKNFSSYWNTYIKEVKKILQREQMIPDTCRDLVDQIMKDSYENFTIV)}*

$3578=171(174):
>gi|1094955018|emb|SEU10384.1| {|0(268)|<Firmicutes(B)>}Cellulose biosynthesis protein BcsQ [[Desulfotomaculum] guttoideum]
{()MISVFLSYCQKdSRYADEIDFHF----RNTNITI--
KRDIREISSWKsirEYMKTIRNMEYAILIIITENYLKSFNCMYEVMELM-KEKN-YQNRIFPVLYTID-
IYETTGKILYIKYWENEycSLKRQIRQLKMENAISVINKLKE(IQSIQSSIDEFLSLVTDMNNPDVKNVNKEIEKHL)}*

$3579=184(170):
>gi|1094651908|emb|SEJ88969.1| {|0(146)|<Proteobacteria(B)>}TIR domain-containing protein [Sphingomonas sp. OV641]
{(MSTLGDAYD)-YDVFFSYAWAgdtgdqhmrewsrkiaDTIRTLLsQRFNRGDVR---
LNAFLDRDVLRGGQDLdrELKSAVQRSGILVAMVSDHYA-SPYCQKELNWFCEGLTEAnfaDHVCILRMQEV---SEGKWPAALQGTDAKPRLFIDFCDERGQPINMAGFILSGS(LADLVTPINRVVVEIADKITA)}*

$3580=209(220):
>gi|1094999948|emb|SEM05948.1|
{|0(633)|<Proteobacteria(B)>}Tetratricopeptide (TPR) repeat [Bosea lupini]
{(MAKLP)--RAFLSHS-StdkgivGEVAKILGRAA----------
VIYDAFEFDVGDEFrdAILSGLANSDLFVLFASRHALQRDWVKFEIAAAQEavtRQALSrvvTFIIDPDLDLK
---DIP---EWMRSTLIVRQSHAGL------
IALDIRRLISQR(LKERLPSYFVGRRAEMDQALDLISSFTDPSHRPPLLIYGLKGIGRRSLVQAIARDNLSYATTIPLPLKAGDLLPE)}*

$3581=189(187):
>gi|1095235350|emb|SEP08819.1| {<Bacteroidetes(B)>}TIR domain-containing protein [Mucilaginibacter gossypiicola]
{(MSLLNKQDFKNRSSQKHGLNSRLKSIVNLNESSKSFSYLN)KYDIFLSHSF-LDAED--
VDTLRQDLQDAGFSVYVDwiDDNHLTRENVtsYtatvIRNRMKQCKSLIYAFSVNSVLSKWAQWELGYF---
DGIDGKVSIAPIFETSQSGEEYKGAEFLGLYPYVTKTLSSFYVHEASDIWVSYSGWLN(NSMPVKR)}*

$3582=184(201):
>gi|1095124004|emb|SEK28869.1| {|0(64)|<Firmicutes(B)>}TIR domain-containing protein [Paenibacillus sp. OK003]
{(MISKFDFFFSYSRSSYE)----------------
GFSKHLIKDLSTYGIKLWVDIHNVPFginvlEHLNEVLIETKESIGAILIISDSYFFKEWCQIEF----
QYFVN-NNIPVFPILFQMTKKDIPMEYSFIKSLDRVT-
IHSINDDSYEYAINKILTVYIE(KNKKKYNLTKIENTIFESLINSYIETQYKESNVISANNIGRYIN)}*

$3583=199(187):
>gi|1095024080|emb|SEL60789.1| {|0(2)|<Proteobacteria(B)>}TIR domain-containing protein [Duganella sp. CF402]
{(MTIRHGCFFSY)AHGQHAYMS---
KFKDDLVDALKCYLEPHfdnEDELFVDSEQLGGGDDLdeKIARALCESVCMIAIYTPKYEAHGYTRREFAAMQLIERERkqwytlPSHLIIPVIMTRHPdglpPQIAGPGMYVDFSRYTLASGDLKANPEFLPDIEKIVHRIA(EHYHCLKRSAPPGHDCSRFVMPDIPPEWRVIPPPHF)}*

$3584=200(188):
>gi|1095174478|emb|SEN29778.1| {|0(1)|<Proteobacteria(B)>}TIR domain-containing protein [Duganella sp. CF517]
{(MTIRHGCFFSY)AHGQHAYMS---
KFKNDLIDALKCYLEPHfdnENELFVDSEQLGGGDDLdeKIARAMCESVCMIVIYTPKYEAHAYTRREFAAMQLIEDERkkwytlPSHLIIPVIMTQHPlslpPQISGPGMYVDFSRYTLASGDLKTNPEFLPDIKKIVLRIA(QHYHYLKQCTPPGHDCGRFVMPDIPPEWRAVPPPHFP)}*

$3585=218(229):
>gi|1095205341|emb|SER39786.1| {<Actinobacteria(B)>}TIR domain-containing protein [Actinokineospora terrae]
{(MTGYKYDVFLSYSRRGTAQRWLLNH)-----------------
FYERLVDCLTDQlahTPEVYMDRMSIARGVHWpsDLQQALLHSKIIVPLFTPPYFRSPWCMAEFRSMQERQRKLgigrgqvPQGLIYPILFADSDnfpsEA--------REYSWWDfKDVSNPD---
PVYRMTRDYPRFH(ERVTKFAADLARLLEQVPQWEPDWPLLVTPDPVLMPPPLIPRFGDPAQRAEQQLLNDTQWLDEL)}*

$3586=196(217):
>gi|1095206713|emb|SER09165.1| {<Actinobacteria(B)>}TIR domain-containing protein [Actinokineospora terrae]

{(MYEYDVYISYDRESETVSPWVRHHFHPRLRELLDDNLV)----------------------------
DKVRVFFDQD-
VGGGTKFpeALYRALHRTKVLVAVCSPKYFYNEWCLAEWHSMAGREEHMgLHDtgLIFPVIYSDSYSFPDSVL
RR-
NPVDLRDwNEPRPHYQDSPAYfdfRKQVGRLAED(MVRVVLEPPPWQRDWPILRPVPGVLPTMRLPGRGAWPV
R)}*

$3587=232(242):
>gi|1095205345|emb|SER39860.1| {|0(87)|<Actinobacteria(B)>}hypothetical protein SAMN04487818_103154 [Actinokineospora terrae]
{(MTDPATHDDATHDAQRSGGPVGAITDQDH)--DIAISFAE---
DQRTTAEVLVRACEQRGLIVLHGPEQTHEWWdrkgGGDLP----GARVRyfVPLVSA--------
IDEFTEAALRAVKAGDEHVLPALVGDVSvpADlLHPHVTYPRGDADQP-DRFVDALA-
TRVEAAETAGTDR(ALVADVVGRVKARVEVAGTPVTFSRYAQQDAVLRYLGEQFAAAMPGLDRSRFTGTVHIG
DSRIAVRVEQAGDT)}*

$3588=185(193):
>gi|1095202204|emb|SES32554.1| {|0(495)|<Actinobacteria(B)>}TIR domain-containing protein [Actinokineospora terrae]
{(MTTA)--RVFLSHAPEDEPQAELVEIALRARGIP-VARGR-
DLTLFDGITDKLRQAVDEASVFVAVCSPHYLVRHACQWeFTRAVLAAQ-----
RSGDPAdRIVAVGcPDPTGLAEWARANEIA--
DVVAAKVDKAGRVPLCGTRDPQ(PERLARPRRFVGRYATLWELHRATGPVVLTGKAASGKTTLAEQYGALFR)
}*

$3589=174(186):
>gi|1095202940|emb|SES19054.1| {|0(226)|<Actinobacteria(B)>}Protein of unknown function [Actinokineospora terrae]
{(M)--QVFVSHAPSDRDVAAGLRADLAQLGRQ-VSLDRGEP-
TGARWWEGVLQRVQRCDVFVLVASPAAVRSPGCLAaLRYARALRR---------
PVLVVTAVatALPADAETAPVFAPAG--
MEISERVPKLRGVLLALPAAPP(LPDPLPADPAVPYLDPIRQRLDEPGLEPAELRELLRQLRLRLRDE)}*

$3590=169(179):
>gi|1095214279|emb|SEO17693.1| {|0(53)|<Proteobacteria(B)>}TIR domain-containing protein [Bradyrhizobium sp. OK095]
{(MLA)----FMSYQTADREIAAGVAQLLDTLGVQ-
SFMAHEHIEVSVEWRGEILRQLSLADLFVPILSQRYYA-SIWCKqESGIAAFRR-----
MTLIPLSIDGSIpeGFISHVQSSLLQGGQA--
TYHQLMRGVASANIGFAIDAGI(RLIAGARSFRQAEYHFEAVRSHLNGATDQQIIQLLT)}*

$3591=231(240):
>gi|1173347066|ref|WP_081203660.1|
{|11(32)|<Bacteroidetes(B)>}hypothetical protein [Niastella yeongjuensis] >gi|1095274162|emb|SEP04633.1| TIR domain-containing protein [Niastella yeongjuensis] >gi|1
{(LAGNKHDKVFSTLLEITRHDTELQNQLLVLMARYKEIKKLELIGAISSGDSFMHRANLNMSVLTLISLIEE
QYPNQETGG)---VFISYSRADAATANLIKKHLTANNID-
VVIDEDNLIGGRDIAAFIRESIRKTGVTLSVISKKSL-LSPWGAmETLTTFQQE-----
KITNSHRFIGCYveKDFFNHSFTDDAITTI--
NEELSQLSEYKRKRIGEGLSTR(DLDNEFIRWSELKQNIDEII)}*

$3592=196(188):

>gi|1095287864|emb|SER70706.1| {|0(2)|<Actinobacteria(B)>}TIR domain-containing protein [Lechevalieria xinjiangensis]
{(MGYEY)--DVFLSYTrrgGGDvwvkehfyRALKEWLGNEMDKDP-D-IFVD-WNQETGVAWPTNLERALLRSKVMVAVFSPPYFR-SPWCRaEWESMLHRH-----EKLgygsadHPRqLVLPVRyaDGRHYPPEAQATQQAD--FTAWNKPLAYEVYSRSASYEHF(LTAVQQLAKEVAERIEYAPPWDPQWRVVRPALDPASLPPGPLP)}*

$3593=290(300):
>gi|1095289104|emb|SER23643.1| {|679(55)|<Actinobacteria(B)>}CHAT domain-containing protein [Lechevalieria xinjiangensis]
{(ARWAADDDAAGKVLAAFDRHLAALPRDQALQRALGEVSDRHPADWACWSLHGDAGVQTAAGPLRRRLRKIGEPVPLETRP)--KVFLSFAGEDRAHAEKLRADLEARNVN-AYLDEDGTAPGDMAAATVAEALAPSDYHVLLWSAN-TAHRRFVTeEWTTAFAlelTR-----RRAFPF-IVRLDeePLPPLLVPRKHFDLF----DAAERLV--ATWRADRKSELP(VFPQPVPPAPHGPAVAISVRSHDLGVTHIVMTPLHVTGAELYRTVFDALRLPTEQATFDGTTGMRFSYELYRQNESIPAD)}*

$3594=173(175):
>gi|1095191226|emb|SEO91370.1| {|0(607)|<Bacteroidetes(B)>}TIR domain-containing protein [Mucilaginibacter sp. OK283]
{()MAKAFLSHSSSdKPLIEKIATQL------GKNNCHYDRfTFEIGKQTLeEIIRALDTTDIFVLFISEKALESKWVKDEISTAKKNIDK-GIIeRVYPIIIDKN-ITYQDLRIPKWIRKPYNlqKLDNEVLILRKIKQILREVEYKK(NSTIHELSDLFVGRYDLSAEFEKKIINIDNKKPTC)}*

$3595=179(188):
>gi|1095202941|emb|SES19070.1| {|0(239)|<Actinobacteria(B)>}Protein of unknown function [Actinokineospora terrae]
{(MH)---VFISYARRdQASITTLR---ADFERFGN-RVWYDRESHGGPDWWdDVLGHVRGCVVFAFALSPDSLRSKACLAELDYAKELRRP-----ILPVLVRPV-DRGDIPDGLQVPVDYVNrtEESVIRLRKALSALPAARALPAR(LPQEPEIPTSYLNAYLARIDAPKLDDREQIELLGQLRERMVLADER)}*

$3596=210(223):
>gi|1095258885|emb|SEM74556.1| {|0(358)|<Bacteroidetes(B)>}TIR domain-containing protein [Chitinophaga rupis]
{(MEKVG)---VFLSYSHAnKELRDEIINILSS---DDNIEIYYDEKnILIGYPIHiRISTMLNASHIVIAIATKEWLGSTETREEFTRAHERKKH---------LVCFA-D-ESTNLGALPQFIRDArqIRFSPNHkQ--QALQKLVPAIRGL(SLTKDFAKQIMYKRLREIEDEVEQRNPEFYRLNLANDHIEHVLEETKNILGDGPYSIDIGNENGYLLKAISIFESASN)}*

$3597=216(220):
>gi|1173671925|ref|WP_081354004.1| {|141(0)|<Firmicutes(B)>}hypothetical protein [Lactococcus garvieae] >gi|1097036477|emb|SFL08317.1| TIR domain-containing protein [Lactococcus garvieae]
{(KQNFDEILLLTKKYGYEIDYSFGFYELLKGASGIRYNFTDLGIEATLDSVIDAVERIVLFVSKDTLSNQGYIFNKPEKKK)---VFISYSHKnkDEVTH-IVNELQSSGL--QVWWDMQDIEFGDSIMENIQQGISESHLYLIFLSKNTMESMNAREELKTFYNKIIQSRdTMKKWFIVRLDT-VDPSELFLNLGDY-KYFdMPEHTSDELGKAISKKLKKIKPS()}*

$3598=202(218):

>gi|1095369220|emb|SER86630.1| {<Actinobacteria(B)>}TIR domain-containing protein [Lentzea flaviverrucosa]
{(MSGYQFDFFISYSRQGSVQKWLLNHFYNK)-----------------LVECLADECA-PTPKVYMDRfmpKAVEWSHN--
LETSLRRSKIMIQVMTPHYFTSGWCMTELYSMRARENMLgfasakiPQGLIYPILYSDSENFP-------
IEERMRSwHDFKEYAHPDPVYQQTVHYFQFH(KEVKQLARDLVELADQVPDWRPDWPIVERPDPIVTPRPPLPRFKPCPEP)}*

$3599=213(216):
>gi|1095380484|emb|SEQ52261.1| {<Actinobacteria(B)>}TIR domain-containing protein [Lentzea albida]
{(MPTFLSFIERVAHLPLCAVSYKYDFFISYCRYGSVQRWLLNH)-------------FLNKLKECLADQYA-PTPTVYVDRTmerAVHWPSD--
LQDALRRSKIMIQLLTPQYYESRWCMAEQKSMRAREEMLgVasldvsQGLIYPILYSDSEnfppEE--------
KERAWVDfKDVAHPDPvyqQSRKYLRfhtKVNDLAAD(LVRLAKSAPPWQPDWPDVDPPEPPLLPPPQMPRF)}*

$3600=218(220):
>gi|1095370767|emb|SER21811.1| {|269(0)|<Actinobacteria(B)>}TIR domain-containing protein [Lentzea flaviverrucosa]
{(TRHWGGVQSSHNFQISARLIESFDRTESGWKGPSHSPGRASSWASSLALLNVYLLARDIVTSGLDVTEYQNTMRSMSARK)RFDVVISFGG---
PDRAVAREIRDHLVAAGLRVFFDTDFRHDLLGEdlaILLQDIYFERSRfaIAILSRSFLKSDWAgNWEWKAVLARMNKQRQGYLLPYFLENVEvpG-LNPTIGFLSADKVSP-CEFADIVV-RKVQAGRR----P()}*

$3601=217(220):
>gi|1124514692|ref|WP_074798936.1|
{|124(0)|<Euryarchaeota(A)>}hypothetical protein [Methanobrevibacter olleyae] >gi|1097028399|emb|SFL81530.1| TIR domain-containing protein [Methanobrevibacter olleya
{(DGFEEYSLFRKKFLTCNYFKDMPIFLKRNPKHFRYYMQSQGGYKERRKIISEEFNKLFSIIEGSNFNSDSNNKNKSINKK)EYDIFVSHSS---EDKEdFVKEFVNLLKQKGLSVWYDDDIVK--IGHnlrKRISKgI--
KSSNyaVVIFSEDFFKSKWTNYEYDNIFLDFYDE--EKVLPILHDLTiedleKfdG-SIPLIRALSTKKFTV-EEIIHEIL-ERI-----NEEKS()}*

$3602=297(294):
>gi|1124675910|ref|WP_074954909.1|
{|181(761)|<Proteobacteria(B)>}hypothetical protein [Myxococcus fulvus] >gi|1095411399|emb|SEU13675.1| NB-ARC domain-containing protein [Myxococcus fulvus]
{(ARLPGSLRLPSPPSGSVPEWMPWVAPEGLERIRLGVKMSPLSLTLDPRNESHEDFIELPRTNPLVVEVAEADADDEFDEP)-RRIFISYGLAERgvqRWNHRIRDALRRTPHQ-
VFPEDEVPQPGTSWRETLEKELAGCDAAILLF----FAPTRYsgfQEeFLLERQRKD-----
PEGFTLIVVNIRpeNdselLPPVVLPGGFVPLFM--FEasagqEQQVIR--
RILEALKRSRGA(PRFGRRFDFSPPGTARIAHSGQALRLRTLVGDEYIIEPARLDPRHVPPPEPGPIAHPQAFKTLLEEFDRSPVIA)}*

$3603=251(259):
>gi|1096252349|ref|WP_071190345.1|
{|357(439)|<Cyanobacteria(B)>}histidine kinase [Trichormus sp. NMC-1]
{(LINVLHQDAAYYEQHKTLLAKALKWERQKRNPNVLLRGHNLRNAEAWLKVAKQKTQHPSLPIQEEYIAESLKLPPNAVLD)---VFISYSRAdSEFARKLN---DTLQIQNK-
TTWFDQEsIASGTNFEqEIYQGIESANNYLFIISPNSLNSSYCATEVEYAMNLNK-----RIVTVLHREV-

DSATLHPGLAKVQWIDFrkHGGDFLTNFGELTRTLDTDPDYV(RSHTRLLLRAKEWEQQKRDDSFLLRGKDLA
ASEAWLKQS)}*

$3604=173(181):
>gi|1096252349|ref|WP_071190345.1| {|0(874)|<Cyanobacteria(B)>}histidine kinase [Trichormus sp. NMC-1]
{(MKGFQDA)----FISYGRAdsKAFATKLHNRL----
REQKLEIWFDQnDIPLGVDFQkQIDDGIETSDNFLFIIAPHSINSPYC-GkEIDLAIKRNK-----
RIIPILHVEE-
ITEEIWQQRHPGKEASEweTYKAKGLHSSFPNMHPAIGKINW(VYFREGIDDFEQSFAGLVDLLARHQEYVRQ
HTYL)}*

$3605=219(222):
>gi|1098555318|ref|WP_071236590.1|
{|66(0)|<Proteobacteria(B)>}hypothetical protein [Vibrio rotiferianus]
>gi|1095536097|gb|OHY90373.1| hypothetical protein BI375_23205 [Vibrio rotiferianus]
{(LSLIAQDLDSIFSELDSSLYVKVSNDQKPTGHEIDTVQMTFAPRVLIYTSKLCIPVANVINIFNSVSTLVE
IVDESEMHK)--TLFISYGG--P-DEDCVIEINKKIKSKGVKTWFFPEDSLPGDKLhrMMHEGVNSHD-
RVLLVCSENSLTRPGVLnEIERVLEREAKEgGSEILIPITLDDY-
VYGEWAPQRSDIADQIRSRVITKFTSDNEDESIEKLVKVL(VN)}*

$3606=220(220):
>gi|1097390936|emb|SFO64300.1| {|218(0)|<Bacteroidetes(B)>}SIR2-like domain-containing protein [Prevotella sp. tf2-5]
{(SFSDWLSRFICFSLKGKIDNQPMGMVVDPIAEDSFLQFMKRIDAFTQRDAEEVVNKIESLIAEKEAEMQKT
RFNMPQQGT)--DVFISYSRADAEITAK-LYEAMSERGI--
NVWYDRNSISmGGNFMnEIIAGIKSTKLFVPIMTHNIQEQHNEYHpyRTEWKTAIDLASgYGRTfIMPISEKD
FDfyGS--NIPDALKACNAYLYDTDNPD-FEPFIDEIQKLLANI()}*

$3607=275(273):
>gi|1097409644|emb|SFU69185.1| {|64(16)|<Bacteroidetes(B)>}TIR domain-containing protein [Porphyromonadaceae bacterium KHP3R9]
{(AIPESIYLKTVNEKADLQSEIRDGLNKIHDIQNEFVQEVFFEMEVPEYHDWRKDSGLLITHNMVIPDPVAS
RIWDKNCFR)---VFLSHKSEVKKETAGL-KDKLKLYGI--
SCFVAHEDIHPTtEWQnEIENALHTMDSFVALLTDGFHDSL------
WTDQEVGFAFgRGIPIISVklgkdpygFIGKFQalTSTwDKAPFEITKILIKKEPMLNAYINAIQKCPSYDSS
NKL(SELLPFIERLTESQVEKLIKAFNENDQVHDSYGFNGKNPRNYGDGLPYHLKRI)}*

$3608=220(229):
>gi|1120176774|ref|WP_073045294.1|
{|0(324)|<Bacteroidetes(B)>}hypothetical protein [Prevotella ruminicola]
>gi|1097427121|emb|SFC45859.1| TPR repeat [Prevotella ruminicola]
>gi|1109947488|emb|SHM5583
{(MENGNITMRAN)--DIFISYSTKN-QDIALNVRRYLEAHGL--
TCWMAPESIPAGsNYTkEIPYGILNSKIAVLILSDAAI------
HSIWVNQEVTYLLnADHIVIPYLVASHKRNLLNIAPPFSTVIQRQ-
PSIEHTCDTNSLENLLKTVQHQ(LGRLTTFTLPDDSDDLLQLGIKEIGEKGGLLYDMGQAEYYLTKAADMGNA
VAMRHLARLLTDEKDYEKDARPWWEKAA)}*

$3609=204(210):
>gi|1097323613|emb|SFD86998.1| {|0(62)|<Proteobacteria(B)>}TIR domain-containing protein [Nitrosomonas sp. Nm166]

{(MSDTVLETEYSEGI)SSDVYISYARSDISFVRRLVNQLRDEGLS-
VWFNEESFLLGSNLTHDITKAISSARVVLVVLSER-SEDNEFINfESALALESK-----
KLVIPVISTKDSkiSLPYILRDIKKIDLSD--
DASFKQDVHFLAQSIKQHGSAL(RKVLPLGNEDLEAKKKRIDLESKILESEIEEYSHIQLSKNFRLILSTGIV
IIVFSM)}*

$3610=214(190):
>gi|1097248306|emb|SFR34163.1| {|0(315)|<Bacteroidetes(B)>}TIR domain-containing protein [Robiginitalea myxolifaciens]
{(MAFVPGYEYD)---
VFISYAHVdnicfpdqgDGWIKQFYQNLNLMlaKRSGRldsIKIWWDTKKLDGSVlFDdSIAAGIEKAAIMIC
LNSPGYKASKYCAQELD-LFHKKAA-GEpgglkagqqTRIFHVLLNnipHT-
DWPDELQGSTGFPFHDGedQHDFGDTVETTSAQFKAQMRHLK(DAVFRALETMQKEAGSASAPTKPKKLAGKK
PSTAKASAAE)}*

$3611=226(228):
>gi|1097292711|emb|SFM78163.1| {|312(35)|<Proteobacteria(B)>}TIR domain-containing protein [Nitrosomonas nitrosa]
{(HKKLLNLSKAHRNAQPQYKLVELAFKLTEACHGTESLIDQLQGVNEYLAWMEKTNVPEKQSIVDYLNRNKT
LIDEFIRGG)--TVFISYNHTdAPIVNDLAQRL---
KEAGITVILDSQAMLAGTEIKsFITKSILSSQAIISIVSASSLLSGWVGMeTINTLAYKEFF-
PNTKFIPCNIDNE-FFDDNFVANSVTKIQGRinVIDGIIAAQNKLGIDSLNFNEEK(TRLHYLRS)}*

$3612=214(220):
>gi|1120176801|ref|WP_073045321.1|
{|595(0)|<Bacteroidetes(B)>}hypothetical protein [Prevotella ruminicola]
>gi|1097427112|emb|SFC45587.1| Type I restriction-modification system, DNA methylase subunit
{(VAKVMSESLAELLSRTSNPYQIELFLLDRFGEKKEFDYLSVFEYFGVQEKLIQKNRNYLFDDESEVIKELE
IPNMVQDYP)---VFISFSDSdWDIVKKLI--LQVLNKEG-
IECFaYKKENYAGANYQdKIIHAINHAKVVIFACSNKSMASD----EVRNELFYSRS-
RKKLIIPVKLdsSST-DWYDYNFMQI-DYIDPKhmPNAIHKLIKRVKFEIKRNSSLQE()}*

$3613=186(185):
>gi|504797225|ref|WP_014984327.1| {|0(759)|<Actinobacteria(B)>}molecular chaperone Tir [Nocardia brasiliensis] >gi|407307571|gb|AFU01472.1| TIR protein [Nocardia brasiliensis ATCC 700358] >gi|10455148
{(M)-YDVFLCYN--WD-
YKSWADELHAALRARNVSVFQDDKDIRPNRSLapALEAALNGSRMLVPLIGPTFHQSPSCRVELHTALRAAyy
lEEGRTERVLPITWRVSPGELRPSQLKRARLTKRETLDTDAQADLIAERVTELARLDS(RRLGDAPAGRDPEW
LPLPLPGNKQFYGRELELWQIHDALEVKAK)}*

$3614=231(228):
>gi|505732123|ref|WP_015694093.1|
{|0(262)|<Bacteroidetes(B)>}hypothetical protein [Saprospira grandis]
>gi|378577506|gb|AFC26507.1| hypothetical protein SGRA_3791 [Saprospira grandis str. Lewin]
{(MYIKATYTL)--
NIFIAYHPDdRSKFEELYQHLKELDEQNetyqVNKIWYSKTKLESSDYEQLAEMMEAADLLLLLLSEASILSP
LFSGrNLKTALALHRK-EKTLVVPIILNTC-WWEDTAYRELAVLPRAGlpIYDSENvKN--
ELFDQLVQDLNQK(LLKVRQQKFDLEDTFKDLLQEADALFANWEQQPERLRSALPLYKDALEHWREGFVPHRE
LLEAKIDRCHREIDFRHYAR)}*

$3615=223(217):

>gi|505732140|ref|WP_015694110.1|
{|0(369)|<Bacteroidetes(B)>}hypothetical protein [Saprospira grandis]
>gi|378577524|gb|AFC26525.1| hypothetical protein SGRA_3809 [Saprospira grandis str. Lewin]
{()MVNVQILAVDRqREHQATLEQFLAPLKASGQIQIVGEQEENPFEAYEeK-DLAVN---ILILLLSPAYLEDAQLEAaPLAQLLARKAQ-KRREywVQPILLEAC-DYSHKPYADYPVFPmpngqKTAlpVLdpSWSSTA--EAWKRAVEGVELG(VTYIEQQRSNAPTAAGQQQKLLLLTANPKNTQALDLNIEINAIKNALRRSDYRDNFEVELSLDIQKKALLETLLQEK)}*

$3616=292(295):
>gi|105922810|gb|ABF81438.1| {|905(327)|<Streptophyta(V)>}TIR-NBS-LRR-TIR type disease resistance protein [Populus trichocarpa]
{(IPGWFNHRKLKEWQHGSFSNIELSFHSFQPGVKVKNCGVCLLYYSSSKSSARFIVASKEASSSSFTSSLSVSSSYRQWVQ)--DFFLSFRG-ADTSNDFI-HLNTALALRVI--IPDDKeLEKVMAIrSRLFEAIEESGLSIIIFARDCASLPWCFDELVKIVGFMDE-MRSDtVFPVSYDVKQSKIDDQTESYTIVFDKDEEDFRENEEKvQRWTNILTEVLFS(SGPRRLHLTDAELMLYLKRKICENSFKFDTIPDVDVYKWDPEELPELSPLENRQWYFFGPRGRAPRGERTDWEMQ)}*

$3617=291(295):
>gi|105922867|gb|ABF81441.1| {|1021(66)|<Streptophyta(V)>}TIR-NBS-LRR-TIR type disease resistance protein [Populus trichocarpa]
>gi|105922933|gb|ABF81445.1| TIR-NBS-LRR-TIR type disease resistance pro
{(GWFNHQKLKEWQHGSFSNIELSFHSYERGVKVKNCGVCLLSSVYITPQPSALFTVTSKEAASSYKTSLAFSSSYHQWTTY)---VFPGIRV-TDTSNAFT-YLKSDLALRFIMP-AEKEPEKVMAIrSRLFEAIEESGLSIIIFASDWASLPWCFGELVKIVGFMNE-MRLDtVFPVSYDVKQSKIDDQKESYTIVFDKIGKDVRENEEKvQRWMDILSEVEIS(SGSKRRSRKAITSCQPSLSTAQPPCHQSPSHLQKREHLSEQQLQLEQLQQRMQQLQQLQRRRRRQRPEKLQQRQE)}*

$3618=235(240):
>gi|105922831|gb|ABF81439.1| {|943(0)|<Streptophyta(V)>}TIR-NBS-LRR-TIR type disease resistance protein [Populus trichocarpa]
{(SFDYLKEVKEWKHGSFSNIELSFHSYKRRVKVKNCGVCLLSSIYITSQPSAHFIVTSKEAASSYKASLAFSSSYHQWMSN)---VFPGIRV-TDTSNAFT-YLKSDLALRFIMPAEKEQEKVMAIRSRLFEAIEESGLSVIIFSRDCASLPWCFDELVKIVGFMDE-MRSDTIfPVSYDVEQSKIDDQTESYTIVFDKNEENFRGNVEKKREQRRLLERLRL(EQRLLEQRLSEQLHEFWLRI)}*

$3619=274(283):
>gi|105922434|gb|ABF81416.1| {|651(0)|<Streptophyta(V)>}NBS-LRR-TIR type disease resistance protein [Populus trichocarpa]
{(WFNHRKLKEWQHGSFSNIELSFHSSQPRVKVKNCGVCLLSSLYITSQPSSAHFIVTSKETASSYKASLTLSSSYHHWMAS)---VFPDIRV-ADTSNAIT-YLKSDLARRVIISLNVKAIR-----SRLFKAIEESGLSIVIFSRDCASLPWCFDELVKIVGFMDE-MRSDtVFPVSYDVEQSKIDDKKESYTIVFDKIGKNLRENKEKvQRWMDILSEVEIS(SELSSSESSTYQPLQCKKKIRKKILGNLPVRVLDLKQPEKIRSHWNKKKRKGLSKLVTKIPEE)}*

$3620=291(295):
>gi|105922849|gb|ABF81440.1| {|925(12)|<Streptophyta(V)>}TIR-NBS-LRR-TIR type disease resistance protein [Populus trichocarpa]
{(FDYLKELKEWQHESFSNIELSFHSYERRVKVKNCGVCLLSSLYITPQPSSAHFIVTSKEAASSYKASLTFSSSYHQWKAN)---VFPGIRV-TDTSNG-VSYLKSDRSRRFI-IPVEKEPEKVMAIrSRLFEAIEESGLSIIIFSRDCASLPWCFGELVKIVGFMDE-

MRLDtVFPVSYDVEQSKIDDQTESYKIVFDKNEENFRENKEKvQRWMNILSEVEIS(SRSRSLKRNGKRNVWIQKSEHLSEQLLLFEQLVLEQLPLEQLSLEQLQLLEQQLSEQLSEQQLLEQQLAVQQLLL)}*

$3621=291(295):
>gi|105922631|gb|ABF81427.1| {|984(61)|<Streptophyta(V)>}TIR-NBS-LRR-TIR type disease resistance protein [Populus trichocarpa]
{(FDYLKELKEWQNESFSNIELSFHSYERRVKVKNCGVCLLSSVCITAQPSSAHFIVTSKEAASSYKASLAFSWSYHQWKAN)---VFPVIRV-ADTSNSF-SYLQSDLALRFIMS-
VEKEPEKIMAIrSRLFEAIEESGLSIIIFARDCVSLPWCFEELVKIVGFMDE-
MRSDtVFPVSYDVEQSKIDDQTESYTIVFDKNEENLRENEEKvQRWTNILSEVEIS(SGSKSGIGAPATQQQQRIQHLQLLKWRQGSAAAAPRAAAAAAPRAAAAPRAAAAPRAAARAAAEARAAARAAAAA)}*

$3622=257(270):
>gi|105922306|gb|ABF81409.1| {|1521(0)|<Streptophyta(V)>}TIR-NBS-NBS-LRR type disease resistance protein [Populus trichocarpa]
>gi|105922325|gb|ABF81410.1| TIR-NBS-NBS-LRR type disease resistance prot
{(NESSSELELSFHSYDQGVKVENCGVRMVNSGHLIVASKEAASSYTPSWQSPTGHLIIASKEAASSYIDSLANSSSYSQWM)-HDVFFSFRA-LFQRGII------RYKRQ-IKYLK---------
KIesSLVSDIKESGLSIIIFARDYV-STLGFGGFVKIDEfMKKM-
KSDTVFPVStvsYNVEQSRVDEQTESYTIVFDKD-
EEDFSEDKEkvQRWMDILTEVAIS(SGSESSKSKDKNKVQRLMGILTEAAISSGLESSGREGKEDRKLYSYEETK)}*

$3623=273(300):
>gi|105922722|gb|ABF81433.1| {|14(1159)|<Streptophyta(V)>}TIR-NBS-TIR type disease resistance protein [Populus trichocarpa]
{(WTRSASDIEIEAAALRIFIIGRIENLFYEFAFGVQAMVATLSKSVNLPVRVLDLKEPEKNSTSLGLARSCASLANTYHTR)----------------------------G-
IDVYMDDRELERGKAIepALWKAIEESRFSVIIFSRDYASSPWCLDELVKIVQCMKE-
MGQTVLPVFYDVDPSEVTERKRKYEEAFGEH-
EQNFKENLEkvRNWKDCLSTVANL(SGWDIRNRNESESIKIIAEYISYKLSVTMPTISKKLVGIDSRVEVLNGYIGEEGGKAIFIGICGMGGIGSCFLENVREDF)}*

$3624=281(300):
>gi|105922664|gb|ABF81429.1| {|14(812)|<Streptophyta(V)>}TIR-NBS-TIR type disease resistance protein [Populus trichocarpa]
{(DDRGASSLVKVSDKEGASAAQCAFDIPFISLFVFSPPKLHLLYSVNLPVRVLDLKEPEKNSTSLGLARSCASLANTYHTR)----------------------------G-
IDVYMDDRELERGKTIepALWKAIEESRFSVIIFSRDYASSPWCLDELVKIVQCMKE-
MGQTVLPVfydvdpseFYDVDPSEVIERKRKYEEAFVEH-
EQNFKENLEkvRNWKDCLSTVANL(SGWDVRNRNELESIKIIVEYISYKLSITLPTINKKLVAIDSRVEVLNGYIGEEVGKAIFIGICGMGGIGKTTVARVVYDR)}*

$3625=220(221):
>gi|67511366|emb|CAI68016.1| {|33(0)|<Porifera(M)>}Toll-like receptor adapter protein [Suberites domuncula]
{(EEIQRIRRVQVTAGIDPEQLAKGLAGISIAHSSPSRSPNSTNSTASPQTTPSGSFSSKQINHTSNKSKDLEKCLESIQKQ)QFDVFLSFaEADERFAEEVRQRLIENA-
GVRVFVPSAGLMSDRAFHQEIADIIKQgCTRTVVILSPDYLSSSWCGYEANLAFTNSPDARRNTTIPILYRPC--EVPAFLSHLHYLDYprfkdDR----KNCQD-YFWKRLYKSVTHH(I)}*

$3626=181(185):
>gi|1097638906|emb|SFL26870.1| {|0(149)|<Proteobacteria(B)>}TIR domain-containing protein [Shimia haliotis]

{(MK)---VFISWSG--
DRSKEMAKALRDWLpmALNYVEPFVSDKDIQAGDRWaqKIAGELESANFGIICITPENISSEWILFESGALSK
SmlD-----
GKVIPLLFDLELSDLTGPLSQFQAQKLEASGIMEVVKSINEIAEKKTADDII(ARSVPALWPQLETALTEIPS
IAPTAKHRRPTPEILEELVTDVR)}*

$3627=217(227):
>gi|1097712021|emb|SFL02959.1| {|135(0)|<Proteobacteria(B)>}Pentapeptide
repeat-containing protein [Nitrosomonas aestuarii]
{(NFTGCKFHGDSFDATDIKRGQIDSKLESYLAKKGVNFELVRQPYTDDWIDWENVQLPKDSREFGVIIYNYE
AYWITENRW)--DVFISYQT-A--
QKGFANNLADEIKMRGLKVWFDSATLVANDSLlqCINVGLNACPFGVVILSEEYFGRKWTEYELKKLL-SK--
---KMII-ILHDIQLDKAVSFYPGLDDKIMLTSELGAKSLSHMIAETIRRPPRNL(LSGVADQ)}*

$3628=230(256):
>gi|1097714136|emb|SFK26739.1| {|134(345)|<Proteobacteria(B)>}TIR
domain-containing protein [Nitrosomonas aestuarii]
{(AMLDTSIGNTQEATERRLAEVSFHIAARAIQLLTQSKVPADAPDKMKAPVTIFLSHAKADLDKQQREDPVR
QTRAVLNEL)----------------------------
PVEQWFDAQQIATSQDFadAIQAGIRGSTIMLAFQTDQYSSRPWCRREVL----
EAKRLgAQILIVDALESGEPrsfpYSGNVPTVRWRFGEPDVDARRVIDRAVLEALRLQHNRAVL(ETVAEPGE
TVLAIAPEAVTLAYAYQENESEKTFLYP)}*

$3629=223(225):
>gi|1097519496|emb|SFQ42399.1| {|282(0)|<Proteobacteria(B)>}Retron-type
reverse transcriptase [Roseivivax halotolerans]
{(DIQRLRMKIYSTRKYGQAEASLLWLGDSSKSISMHLVGWLAYIRQVRGQSDPVLAKLCQQVVAAGISDIEW
IVRLADMTK)EFDVFLSHAS---DDKSKVRPLYESLNSAGVKVFFD--ESSIKWGDsivERVNHGLL-
KSRffVPVLSSTFSRKGWTNKESNSAIAMNISRKG-RILPIKFSEFDldG-RYPTLNDTLYKTWPE-
DSGKEDDFIREVTDDLIALVEQ(DKQLG)}*

$3630=209(217):
>gi|1124643502|ref|WP_074924073.1|
{|0(29)|<Proteobacteria(B)>}hypothetical protein [Proteus mirabilis]
>gi|1097636589|emb|SFG96640.1| TIR domain-containing protein [Proteus
mirabilis]
{(MHDSF)-----ISYAE---EDNDFVSDVAFGLKRNGLSIWF-----
APMSLKvgeRLLDSIEKglNESRtgILVLSPAYLSKNWTSYEMD-ILIRQNIDGSKKILPIWFNVTKedI-
DNKHIGLSGIVGITD-VTDASKVV-
SRLVEVLSDGAPY(RGVIPSWENPEYRFLNGLGEVNLQTIDGKTATIFELLIYSKDDQFPFWLAGKSYSKREL
LMEVSRIIGADPT)}*

$3631=219(220):
>gi|1097713102|emb|SFK69604.1| {|290(0)|<Proteobacteria(B)>}TIR domain-
containing protein [Nitrosomonas aestuarii]
{(KFGLIEAEKTYISCYAKPTLDISSDNLNGAYYKSVVYGRLAFLKMVRDEEDEVYMKLCLKVAEIDSNAPRK
ILETKEMFN)KFDIFISHAS---EDKDDVAlPIYEACKKIGVNAFLD--VKYIRWGDsltEKINHALG-
QSKfvLAILSENSVGKTWPSKEINAALAREI-EGKQKILPLIVGSPDlsS-VSLLVDKLHFpWEKDA-
SKVAHRIK-ELTYEKYQISNCK()}*

$3632=221(213):
>gi|1097727054|emb|SFC94419.1| {|0(75)|<Bacteroidetes(B)>}TIR domain-
containing protein [Flexibacter flexilis DSM 6793]

{(M)--RIFISWSGDvsKQIGEAIKDWLPAVLQNVKPYF----
TPNDIEKGArwnaDISKELEHCKLGIFIYTKDNIDSQWMLFEAGAIskMLDNSK-
VCPILFGLDNADFKGPLtqfqtsqfqktDFRKLVYSINNSLS--EQKLEEK--
VLDEVFEMWWPK(LENKVNKLLDANKVENIAVRNDRELLEEILAISRITAKRTIETGKSNSKRRYNTEVYEKL
VSSFIFGTETIF)}*

$3633=213(220):
>gi|1097584437|emb|SFI37302.1| {|173(0)|<Proteobacteria(B)>}TIR domain-containing protein [Nitrosomonas sp. Nm34]
{(VTRSDTQVLDALQLFSEKKAEYLSQGNLIQSDKLNLHNKIIQGDKVIRSHDKLTLRNEIIQGGKLLTGDLI
EKALRDEYD)---VFISYSRNnANDSEAFEKALREL-RPG-
IRIFVDRKaLNIGSAWQpEIFETLDKCRKVVALLSPDYLSSKVCKEEFNIAwIRSRET-DENIIFPVYL----
---YTTQLPTYMKYRNYfdCREGDRSKIREASMELLNTLTGA()}*

$3634=229(230):
>gi|1097714104|emb|SFK35135.1| {|230(0)|<Proteobacteria(B)>}TIR domain-containing protein [Nitrosomonas aestuarii]
{(TKQGRLLDTQRKREWLIENNCLDKNLVYFLESYSTGTKLISDISPTAFISELHQRWLARNKAVEHASSSKT
GTVPRGALF)----FVSYCRGtDSLSAT--
TLMESLQSQGVssNEIWFDKNsIEPGNDLRnRILEGIQTCRYFIPLISknADSLDEKY-
FRrEWHEAIERSKAIqGRTFIFPIIVD-----
QDFKLETYRCIPHEFkqLLIGHAPVGQPDEQTNAALKKLV(RSERLTQNHA)}*

$3635=190(203):
>gi|1097714135|emb|SFK26717.1|
{|0(200)|<Proteobacteria(B)>}Formylglycine-generating enzyme, required for sulfatase activity, contains SUMF1/FGE domain [Nitrosomonas aestuarii]
{()MSDIFLCYSKKdAAIANRLVEQLQA-------
ESWSvfiDRNTHVGRRWHkEIERELHTARAVVVLWSAQSRDSDYVLEE-----AEYGK-RKDILFPALIESI-
ELTY-GFGRIQ----
TAkLTDWEDGADHVGLTDLLTPLRLH(LNGHAPEPSIIAVQPQQIVTSSLPEPGRIFRDKLKIGGEGPLMVGI
PAGRFLMGSPEDELDRY)}*

$3636=178(179):
>gi|1097714474|emb|SFK13064.1| {<Proteobacteria(B)>}TIR domain-containing protein [Nitrosomonas aestuarii]
{(MAQILEAVQPPPVSPTIVMSKPNGHGP)--TIFVSYAS---
ENADYVRMIADRLPGLGFSgVWLDQKKLIGGDDWsnRIDEAIEKCDFFMPVISREADAQREKVfWgEWQAAIN
RSKRIkD-VFLIPVGIDDD-
FPAKASYQRIADGETAVFFDKHLIHAPQGILSLKDCDNLI(ERCRRFQQALYD)}*

$3637=173(178):
>gi|1097750765|emb|SFS06187.1| {|0(657)|<Firmicutes(B)>}TIR domain-containing protein [[Clostridium] populeti]
{(MRV)----FLSHSSKDkeFYVSKVAKRI----
GRDRVVYDELTFEEGLKSIDEINRTLKASDLFVVFLSLNSIESDWVKYELFKAHDLLDEGGKIQQIYPIIIDN
KLKyNDPKIPDWLRDNnLQLVVSSNKASD-
LILQRLRELSYKQ(HPRLEEKNKIFVGRNDIINMFEMRINDFEKVVPFS)}*

$3638=180(182):
>gi|1097889277|emb|SFG66594.1| {|0(161)|<Proteobacteria(B)>}TIR domain-containing protein [Neptunomonas qingdaonensis]

{(MSHK)--DVFICHAS-ED-
KDSYIKPLTELLKDNGITFWIDSQDLrlGEHLDQSIPQALNNARFGLVIFSKAFLSKEWTNRELQLIRDKEEA
ENEIILLPILEDISQELILTKYGFLRDRKFLKWTELNKLIDDLKHYLQGTAVPNN(NLLFSDALSHMSRFNAV
MHWTARPVPQKETKQFFTALQ)}*

$3639=251(254):
>gi|1097891459|emb|SFG01605.1| {|545(16)|<Proteobacteria(B)>}internalin
A, partial [Neptunomonas qingdaonensis]
{(IDIKTAGRNAEKLICHLLDSIKGVQHLEQKSRIEWQKGQPDSSQKHKGLSPESELTPFGALEPASLPPSTR
SAVYFSYAW)---------G-
EDTdeRQQTCDQIYNKLSEEnELNIYRDKNATKPGDSIaAFERKIGQADFVLLIMSEKSLYSVHCMNELRLIY
ERSQQ-
QQSdfvgRVIPVVLSDAKISTRIDKFKVVEHWVSQGQELEDLINKVGLEAAGAESANE(RQMIRSISNCTMNA
LTWIGDLITERQPELQVDAT)}*

$3640=201(220):
>gi|1124624228|ref|WP_074905563.1|
{|43(0)|<Proteobacteria(B)>}hypothetical protein [Nitrosomonas communis]
>gi|1097911214|emb|SFM37977.1| TIR domain-containing protein
[Nitrosomonas communis]
{(FPNPVREIHTRKGGFRLESNGWGEFTIYIDIAYKDGKHQQLSHDLRLTSEIDSEDNTEQKGAELTSYMGKV
DVTVKHLPP)-KMIFISGGAAdAEVLGRLRESLKKLNL-------
CIKDATDVPAGIplqtYIDSLIAKADVVIFLISGR--PSLWLSQEIESAKHSNKP-LIPILV-
GQDSHLPASLN-EYQYFRIDNLNN--ISDLAHK--LLKY-------L()}*

$3641=233(238):
>gi|1097917033|emb|SFL61305.1| {|47(46)|<Firmicutes(B)>}TIR domain-
containing protein [Gracilibacillus orientalis]
{(IYDDGCKLHVKGNAFDIPKMQDLVKILNGFDRFNIMETKVKDKSILQIDVQQKVLGVKCHNILYTFYSEYH
NRMQEIKPV)--KIFISYNHLvSELADIVEENINKTYGPKVLI----SRDTQLQYRDnidkFM-
ETIKENDVIISLISDSYLKSEACMYEIIELMRDPEY-HQRLAFIISSECDLKLFHNQPARDNLVPKIY--
GAQRFDYIKYWTSKLEDYIER(LNELQAHYTSTLELNGAI)}*

$3642=230(234):
>gi|1098067287|emb|SFP93126.1| {|237(3)|<Bacteroidetes(B)>}SIR2-like
domain-containing protein [Parafilimonas terrae]
{(PDWLMRFLIRLIAAERYTQSNQKLISDAGTLSRIEFENFLQQYQGQVITQNEETFKSPGDFVQALHKAVQN
NPENIVQRY)KEKVFISFISEdKNIAQLLNNAFAEKGVP---VFF---
DGNEIFSGTnfnnVIKPEIQNCDFFLPVITARSVKDPIDVKRYVYKEWTLAG-
FRRMAGEDAAATTTFIKPYTIGTDAINMSVY--ETYFDGIGMEKIPDTSREALN(AFVESFITRNNLTP)}*

$3643=205(218):
>gi|1097820652|emb|SFT99052.1| {|0(878)|<Actinobacteria(B)>}WD40 repeat
[Arthrobacter sp. ov118]
{(MTSL)----FVSHSSRDRVPTEKVRDWLRAEGYV-
ALFVDFDPddgiRAGRDWERELYAQLRKCDGVVFLASAAS-TTSRWCFaEVSLARALG-----
KAVIPVRVEP-GARLDLLDDVQWIDLAD--GDP--ALS--
RLRRGLLAAGLD(PFESFAWDPNRAPYPGLRPFEAEDAAVFFGRGPEVGRLLELLQPTLQRGTGRFIGIVGPS
GSGKSSLMRAGLLP)}*

$3644=181(185):
>gi|1097851803|emb|SFL63737.1| {|0(234)|<Euryarchaeota(A)>}TIR domain-
containing protein [Halogranum rubrum]

{(MTDPTGR)---VFLSYKHEqTDVANFLQ---SELERHGV-PIWRDiFDLKP-
EPLRdEIIEQLENPRTAsgIALVSEEVSDSDIILNdELPGFKNRWDS-DDDFFVVVV--PC-
PDIKVSEAKSILNEAPIlhDFSAWKMVPLEATTSDEAADIVD(SVLSERIERIDGYLPEGEPIECSLDTYESP
AHDIDPAI)}*

$3645=219(227):
>gi|1097965123|emb|SFF17494.1| {|49(0)|<Firmicutes(B)>}TIR domain-containing protein [Bacillus sp. OV194]
{(KDTGSNAVMASSVREFNSGNLGVEEINNQNIETVRSKLTSDNRLSPRDSVVILKMNGENFESELNKLLQKH
EVVMGVVGM)--KIFLSHKSIdKEKVRSFKNTLELLG----
FDPWLDEDaMHAGVELErSIKQGFSDSCAAIFFVTPNFMDEDY-LGtEVNYAIseKRKKK-GLFSIITLVLE-
-------EDGKKGTVPELLepyVWKEPKTDL-EALREIVKAVPLK(LGQPFYK)}*

$3646=197(196):
>gi|1097985001|emb|SFN10343.1| {<Proteobacteria(B)>}TIR domain-containing protein [Dokdonella immobilis]
{()MSSVFLSYVHEnTHVVKELCESL----
RAHDIEVWIDRDnIAPGVRWKdAIREAIQRGSYFVACFSSEYGSkSKSYMNeELVLAvdeLRQHSA-
NKPWFIPVLLSEC-EVPALSIGGGRTL-LDFqwVSLWVDWD--
LGIKKILQVLKAG(QIQEIKELIDQLGYDYHKRIESRRDSETPRSFYVRKVHELRDVYGVRYDPLKLNFS)}*

$3647=170(169):
>gi|1098066541|emb|SFQ07821.1| {|0(61)|<Bacteroidetes(B)>}TIR domain-containing protein [Parafilimonas terrae]
{()MANFFISYSRKdAAIADNIRNHI------
YKLDAGHDvfldiKSIHVGANWKaELQRKIKACDFFILIHSNNSINSPYVKEELKWISESELK-
SGMRKLIVYRLNY-
AEIIPEIASYQILDSTDnfTIDFFRLMQGVLAENSFYNVEYE(IMLEDEYWYKGKIWIEAPGQFLEKIQMAE)
}*

$3648=218(214):
>gi|1124625943|ref|WP_074907229.1|
{|0(44)|<Proteobacteria(B)>}hypothetical protein [Nitrosomonas communis]
>gi|1097909325|emb|SFN13283.1| TIR domain-containing protein [Nitrosomonas communis]
{(MK)---VFFSHAS---EDKPLVEQVFLRVSNKYPKIqgWLDKYEILGGDQLidKVHAGI-
ESAEKFLIFLSPDSIDKPWVRtELRKALMDEIKGiKPEFIIPIRAGHI-
SQfPPFLEGRFYIDIVSktedEWLTDIYSAITrEKKQNQAPAENLQ(ISIALAHDDPNAAMVVFEPQFWANEI
GFKITTSEKIENSLWTYAGFKGMRQISITELKGDYEYGVKIDDHTI)}*

$3649=185(187):
>gi|1097946372|emb|SFB90285.1| {|0(209)|<Proteobacteria(B)>}Protein of unknown function [Polaromonas sp. OV174]
{(MTDHEQPL)---VFFSYAN---
KDQDRVKPFYEELDRQGFSVWMDCFRLKAGQNWdfEIKRAQEKSEFVVTFLSRNSVTRRGYVQrEMKEAVDKL
KeKLiDDIYLIPMLLDDD-
IDIPDDVKGIQCIRASSPNFMSELTDAVIFQIGKRGVKIS(KAQKDQDFSWSSSVRKEEWDGLPGYEVTLHLP
ELRSEKY)}*

$3650=302(300):
>gi|1098728548|ref|XP_018814110.1|
{|97(582)|<Streptophyta(V)>}PREDICTED: uncharacterized protein LOC108986050 [Juglans regia] >gi|1098728551|ref|XP_018814113.1| PREDICTED: uncharacterized protein LOC

{(VTLPSPPVSYRGSLSDDIPSSSYTPPSDQYEYSDDPADAKLKFVTCDPAPPRLSFSFPVPRISFAKGPVSPASSAKLRSC)--DVYIGFHG-QNpnlVR--FSKWLKSELELQGIACFVADRAKYSDS-QshEIADRVICSVTYGVVVATNSSFLNHL--SLEEIRFFAQK-KNLI--PLFFHTGPAQImgLLNCNSIDKECKEAIDGlikcnefKLEANE-GNWRSCVTKTAGI(LRTKLGRNSVAEKDGVVEGFEEIPFPRNRFFVGREKEISEVEILLFGCGDCQEQVCSAHAIKGEATGQSEGLADEESDVV)}*

$3651=167(186):
>gi|1098732814|ref|XP_018832642.1| {<Streptophyta(V)>}PREDICTED: E3 ubiquitin-protein ligase COP1-like [Juglans regia]
{(MATRNLQNKKADGKSQVTSHGLQRKDALSASESQHINQSGLSVA)--------------------------RKK-QVLAQDEDELPKGENIseKLINAIHGSRISIVDFhSKGYASSRWCLDELVEIMDGTKT-RGHNLIPTIYHVEPrmSENRKGLLPVHSLGVKN-TKDMRlsfKQMDrvKRWKIALCYSENC(TS)}*

$3652=256(256):
>gi|1098740622|ref|XP_018835825.1| {|0(373)|<Streptophyta(V)>}PREDICTED: TMV resistance protein N-like [Juglans regia]
{(MLKLHAVDQAIHTHVHRLISSMASSSFSSSKTSPWP)-YDVFLSFRG-EDTRDTFTAHLYHHLTQKGIITYLDEDGLRRGDEIspALLQAIEDSRISIIVVSKNYASSTWCLDELLQIFECKKS-KQQIVLPVFYHVDPSDIRHQRGSFGEALAKH-ADKLNVDHMklQLWEAALQKVSNL(SGYHLEKNGRNESKFIEKIVQDVSRMANNHLYLHVAEHPVGLEPHVEHVNLLLNVETKDIRMIGIFGVGGIGKTTLAKAI)}*

$3653=192(194):
>gi|1098802299|ref|XP_018806977.1| {|0(588)|<Streptophyta(V)>}PREDICTED: TMV resistance protein N-like [Juglans regia]
{(MAYSPTTQTSPSSSSSSPP)-WDVFLSFHG-EDTRKSFTAHLYTALKQRSLSTFRDDKELERGKYIsqELLNAIENSMYAVIVLSPNYAFSRWCLTELAKIMERMKK-TRDESMVIQEIVGKIHDGKLNSIVSAVSKKL-VGMGSRVEKMMNSYLNIGPDDV(RFVGILGMGGIGKTTLARAVYDRISSQFEARSFIA)}*

$3654=174(200):
>gi|1098194316|emb|SFB63531.1| {<Proteobacteria(B)>}TIR domain-containing protein [Azotobacter beijerinckii]
{(MGNKYIFISYKRADPTTMVAQQLFKRIVVNLKGHGFEDP)----------------------------------FFDRKSIDAGDSWepMIDQALDRTTHFIALLSDDYWLSEQCQRELVGAVTRWEQHgVPKLLFVLTERMDPsalvIERGQGGAAVQKKFPQVERLSQINFLGPYDAAGRLIRLNC(TDHNILGDQLFSLIQDIRKIT)}*

$3655=199(187):
>gi|1098358633|emb|SFC21330.1| {|0(3)|<Proteobacteria(B)>}TIR domain-containing protein [Massilia yuzhufengensis]
{(MSIRYGCFFSY)AHGRHELMK---RFKTALADALRCYLEPYfdnEDELFIDTEQLGGGDDLdrKIARAMCESVCMILIYTPKYEAHAYTRREYAAMRLLEAERaawyalPSRLIIPVIMTRHPdslpPQIAESSFYVDFSHFTMATADLKSNPDFLPDIDKMVKRIA(THYQYQIKHTPPGHDCNQFVLPDVPPEWRELPEPTF)}*

$3656=227(224):
>gi|1098209470|emb|SFI34552.1| {|0(91)|<Proteobacteria(B)>}hypothetical protein SAMN04515618_11867 [Collimonas sp. OK307]
{(MTIPKYD)-YDVAISFGY---KDEPFANKLHSLL-EGRLKLFLYSKEQEKLAGTdgeAKFNEVFGKTAKlvVILYREAWGHTPFTRFEETAIRNRAYSEGYDFAVFIPMDDSEkqkvpAWVPKNRLYIGLERWGI-EAACAVIE-

ARFSELGGEIHEE(SVAEMAVKVAEAIGYEERRQKHLNSHEGVQAQRLSFDEVREKLIEGVATIQKTLPAINL
HEKHIGESGGAIIFLSDP)}*

$3657=221(220):
>gi|1098374310|emb|SFE52485.1| {|0(158)|<Bacteroidetes(B)>}TIR domain-containing protein [Thermoflexibacter ruber]
{(MT)EYDIFISFAD---ENEAYAVKLCEALQKEGIKAWCSA-KNVGIAGNiyGEISHA-
LHHAPffLPLISKHYQRN-WHKIEFE---NGCYNKAPQYIIPVaygvtykYIENNPifT-
LIRNIRLVEANNISV-SKLAKLIA-
ARVRNPEAPPPPP(KKLLMSFIPLFLLESKFLLSLVAAVLALSLYKLLNVPSENHSQPALPPIKKDTLTIADS
FKEITKFLEVTIDFDNPNV)}*

$3658=211(216):
>gi|1098153947|emb|SFS20838.1| {|0(57)|<Acidobacteria(B)>}TIR domain-containing protein [Granulicella pectinivorans]
{(MDDKSCS)---IFISHAAVdEALAVSLKGSIEKA-LPGHKVFVSSDP-
TDLKLGDewipKILRSLETAQFVLVLATERGLSRKWVWFEAGRTWFTGVT-
MLPCCVGRLRKSQLPAPFSSRMGANIDDPAD--LKSVFES--
LRLHFGELAELP(DYEDLAKTMIRLDVRAEERNKILDDPFMVERLRDLNDTMSRLSPAERETIRQFVIHRELS
TAGVKMKVK)}*

$3659=252(254):
>gi|1098157053|emb|SFS15447.1| {|92(70)|<Acidobacteria(B)>}TIR domain-containing protein [Granulicella pectinivorans]
{(AWLREQGASVVDEQLKRVQGMVENLCSSFVGSSAQQHSKYLDAASGEAALMKARIESSITIHLLEQQQTVL
PKPSRSEEH)-WDVFICHASEDKPYVETLFQKLTEAGIR-
VWLDRTAIDWGNDLRTTIDHGLKNCIYGIVVFSKAFLAKKKWTEyELSSLFAleepGR-----
TRILPIWHGITEaeMREYSPGFSLRLAKGT--
TRDSYQGIVESLKQLLNKTSTE(GAEAPSATTQYPKREHIGPAAADAHYEMVGKAEP)}*

$3660=170(177):
>gi|1098373554|emb|SFE75529.1| {|0(200)|<Bacteroidetes(B)>}TIR domain-containing protein [Thermoflexibacter ruber]
{(MKN)--DIFISFADEDKSFALELCDALQAEGLK-VWCSAKDVPIGADIHAEVSKALPDCLYFLPIISPNY-
-GRFWHEkEFHSASHNR-----PNhlIIPVTYLINYdyIKNNAMFSLISSIRVV--
ATENKSPQAIANEIADRIRQKS(EPAFPEITFESQTLSINLPSIWLKRKTWIASSFF)}*

$3661=211(203):
>gi|1098385664|emb|SFP06336.1| {|0(416)|<Actinobacteria(B)>}TIR domain-containing protein [Yuhushiella deserti]
{(MQARAAR)-WDVFVSYSRADDAgPALAVARALERHGLR-
VFVDEHGVAPFQAISATILDALRAARVLLAYFSAAYPRRRACQHeLTTAFLAGQ-----
AEGRPSRrvlvinperdsdhIHPVQlrDAKHVPAPRSRRDLAT--
VADLVTAHLADIDTPIGTGEPG(APPRWAAGSAPARPARFVGRLAELWRLHSILTPEDAPLTAEPSAPVAVVH
GAPGIG)}*

$3662=174(178):
>gi|1179674839|ref|WP_083413310.1|
{|0(261)|<Proteobacteria(B)>}hypothetical protein [Pseudomonas composti]
>gi|1098289382|emb|SFP51131.1| Tetratricopeptide repeat-containing protein [Pseudomonas comp
{(MN)-KNIFLSYSWSdMEIADQIEKdlsHLQLNLRRDVRDLAYKCSIS------
SFMESIRESDFAILLISDSYLRSKNCMKeVLHLLKDR--N-HEDKILPIIVKGT-

GIYSTTDRLAYTKHWLQeqEKLKEIINSLPATSVINEISELK(TVEAITSSINNFLTYISDNKNLTFDELKAE
SYSSIL)}*

$3663=234(219):
>gi|1098179545|emb|SFN21268.1| {|0(245)|<Bacteroidetes(B)>}SEFIR domain-containing protein [Chryseobacterium oleae]
{(MQKK)---IFISYSwgT--
NEHQEWVLNLAKRLMNDGVEVALDKWDLQGGHDVfeFMESMVKSEDIsKVLIICDKNYqiksnNRDGGVGaET
QIITPEIFSNqKQEKFIPVvaeRDENG-
KviLPIYLASRKYFDLSKEeyFEDGYEELLRniLEAPALPKPKVGK(IPSYITESSSNNNQTNFILRRLENQL
TKNPEKINSSSKEFIEIFLDNLWNYEFKSTSRDINVFGVELVNNLHSYK)}*

$3664=126(150):
>gi|1098929222|gb|JAU13739.1| {<Streptophyta(V)>}Putative disease resistance protein [Noccaea caerulescens]
{(MASSSSHIKR)-YKVFTSFHG-PDVRRGLLSHLHNHFETKGITTFKDQEIEGGHS-IgpSI--------
ISIVLLSKKYASSSWCLDELVEILKCKED-QGQIVMTVF--------------
SRRNREEISDSLTRKPVKAKLRKTSRDGSKL()}*

$3665=119(147):
>gi|1099100357|gb|JAU52862.1| {<Streptophyta(V)>}Disease resistance protein RPS6 [Noccaea caerulescens]
{(MCSSSRL)-YDVFPSFSG-
EDIRKTFLSHLLKELDRKLISVFKDSEIQRSHSIApELVQAIRGSKIAVVVFSNKYASSSWCLKELLEIVKCK
EEFG---------------------------SSGDTHFLRLGSFSYQETNRRLWRSL()}*

$3666=130(156):
>gi|1099238143|gb|JAU80023.1| {<Streptophyta(V)>}Disease resistance protein RPS6 [Noccaea caerulescens]
{()-------------------------
LERKLINVFKDTEIERGQHIGpSILPAIRGSRISVVVLSKNYCSSTWFLNELVEIMSCRENMGQ-
RVIPIFYYVDPSDVRKQTGEFGLAFQKVCENKTEDVKQ-RYCRALYDISTI(SGYCSQTWDSEADMIA)}*

$3667=231(235):
>gi|1099246047|gb|JAU81947.1| {|0(326)|<Streptophyta(V)>}putative WRKY transcription factor 16 [Noccaea caerulescens]
{(QKTDKKHQLVISKPTRNKRFLSSFDAQDNCMDNNKND)--EVFLSFDH-
GDSSVSsFISYLIAAFNRQGISAFFDGKSSHVEAVErEEEERFSRLKVFVVVFSDNYVSHVPCLEKHL--
YSC-
RNNDDFVVVPVFYRVSTSSVKQHIENSGDVYEAVQISMPKLRRGHEYDGKKTECEFL(EEIARDVFEELYPTE
EIGIRSSVLMIESLLCKQPWGVRSIGIFGEAGIGKTTLARAVF)}*

$3668=141(147):
>gi|1098941481|gb|JAU17280.1| {<Streptophyta(V)>}TMV resistance protein N [Noccaea caerulescens]
{(IG)-------FSS-EVIRYSFASHLSAAFRRSSISVFVGEDTKRKSaavtskidfR-
LpdATQSAIEGTKFFVVVFSESYAFSPLFLETLVMFLERRKD-
GLFVVIPVFYgDVTPSIVEQQTENFGEAVSR-------DLL-ARWRDGLTEAANL(RGHNS)}*

$3669=208(226):
>gi|1098958170|gb|JAU23923.1| {|0(555)|<Streptophyta(V)>}putative WRKY transcription factor 16 [Noccaea caerulescens]
{(MEARNM)---VKISFdrSD-ETLRCSLVPHISAAFGRKGISVLNDKHDE-----------
FCESVASVLIFSKNYVSSKESLDDFLNTIRRRND-KGHLVTTVFYGVSISNLQELKGNFAKRFFEH---

RTSYQA-
SQWHNALAEIGNI(PGHEQSNNQSDCEFVEKIARDVYEKIFPKERIGVYSRMLPDIENLLCKQQWGVKRIGIW
GMPGIGKTTLAQAVFDQMSGD)}*

$3670=273(288):
>gi|1098960090|gb|JAU24615.1| {|175(647)|<Streptophyta(V)>}putative WRKY transcription factor 19 [Noccaea caerulescens]
{(NQKELTHKLKHEAYENVDRFVPVYRAGFVDQDPPLWESNYVEPDFSASGSECSSYTSGSESSQEDSSEIFP
PDPSRVSSD)KYDVIIRYNK-RDISNGFISHLLAALCQKGISA--------SGA-
SifKRPDGVPECRVVITFLTHRCVS-----YNLLEFCDRLPS-
KELGVSQIFYRVSPSNLISNTKKLERFLLQHQKSIWWNVL-
QQVAQMSDYILTD(KSDPELMHEIVKDVSKVLLSSDKEKMIGIDSQVEEILSLLGIESPDVRSIGLWGTAGIG
KTAIAEKIF)}*

$3671=273(288):
>gi|1099003222|gb|JAU35042.1| {|308(319)|<Streptophyta(V)>}putative WRKY transcription factor 19 [Noccaea caerulescens]
{(SLRSELLTSNQKELTDKLKHEAYENVDRFVPVYRAGFVDQDRLLWGSNYVEPDFSASGSESSQEDSSEIFP
PDPSRVSSD)KYDVIIRYNK-RDISNGFISHLLAALCQKGISA--------SGA-
SifKRPDGVPECRVVITFLTH-----RWVSYNLGEFCERLPS-
KELGVSQIFYRVSPSNLINNTKKLERFPLQHQKSMWWNVL-
QQGAQMSDYILTD(KSDPELMHEIVKDVSKVLLSSDKEKMIGIDSQVEEILSLLRIESPDVRSIGLWGTAGIG
KTAIAEKIF)}*

$3672=208(208):
>gi|1099026779|gb|JAU42705.1| {|0(1029)|<Streptophyta(V)>}Disease resistance protein RRS1 [Noccaea caerulescens]
{(MVESI)--DVYISFdRL-EDTvRYSFISHLSAAFHRRGLSSFFDgENGSDPET-
SgiSTLAAVEDSRACVVVFSEKYSSSSSCLEDLAIVSERRRN-
NGIPVVPVFYAVTKSFVTKQIWKLGDVRSELRTGLLETVDLPNHESSNNQSDSE(FVEEIVADVREKLDKTEK
IGIYSKLLRIENLICKQPWGVRSIGLWGMPGIGKTTLAEAFFDQM)}*

$3673=239(240):
>gi|1099033881|gb|JAU44530.1| {|0(628)|<Streptophyta(V)>}putative WRKY transcription factor 16 [Noccaea caerulescens]
{(MSSSPSSSSSSSKATRPTTN)--DVFIGSGR-
DNTRYAFVSHLSAAFRRKHISVIeeddKDDTLPseNSEY-
LskETQSAIKSSKVCVVVLSEEFSFFKSSLSTLVEvIYRQHDK-
NCVAVVPVFYRVSRSTVEQQSEKLDEAFL----SEPKEHV-
IKWRNALKEVSHL(EEALESKDEQSDLELVEKIVKYVNEKLFLPNKVGIKWRLLEVENLISKQPLGIRRVGIW
GMAGIGKTTVAEAVYSQMKGD)}*

$3674=200(232):
>gi|1099040967|gb|JAU45965.1| {|0(1135)|<Streptophyta(V)>}putative WRKY transcription factor 16 [Noccaea caerulescens]
{(MGDDNKATHTIYINC)-----------EDTlRYSLASHLSMAFRRKGLSAFVN-------S-
KGTQDVIEQGSTFVLLLSINFVSSALCLNKLVRVLEWRME-NGLLVVPVFYGVSPSDVVAP-----------
EHESADRR-
REWTNALQELIEL(PGYHSREEHSDCELVEEIVDDVYEKLFPTEQIGISSRLLEIELLLCKQPWGIRRLGIWG
MPGIGKTTLARAFFDQVS)}*

$3675=225(233):

>gi|1099122620|gb|JAU57298.1| {|0(296)|<Streptophyta(V)>}Protein VARIATION IN COMPOUND TRIGGERED ROOT growth response [Noccaea caerulescens]
{(KKKESIISSLSIIMDSPPS)---VFLSYRR-EDTGRTFVSHLYRLLDQKRIRTYKDENQQaIDGR-VspEVARAIRESNVAVVVISENYASSVRCLNVLAEIIQRSGS-----VKAVFYEVDPGDLTRSTGKFADDLRRHEESETPETV-TRWRNALEGLEST(DPKFCSRNWEDDSKMLDDLIISISSPGFPRFQLRGRRYRESDTYMRAGSSSSTDPRGSELMSDTYMKHIPKSLS)}*

$3676=183(206):
>gi|1099132641|gb|JAU59299.1| {<Streptophyta(V)>}Protein VARIATION IN COMPOUND TRIGGERED ROOT growth response [Noccaea caerulescens]
{()------------DIRRTFVSHLIHSLDRKGI---------RSVT-SaaSLseDQAVSQSLVAVSIISKTCTSLDLWVVQLARII----A-GQTKAVPVFLGVDPqNILRVLNLAEGSAGVNESWRNTLETV-KHWLDGHVTSNSG(FNSTDWEDDSQLVDQITRFISDTLTEDSISHRWCPRIDWRTSPWDDPSPVSRGWRMVQLNIKPGLG)}*

$3677=297(255):
>gi|1099025507|gb|JAU42339.1| {|2(497)|<Streptophyta(V)>}Putative disease resistance protein [Noccaea caerulescens]
{(FYMRTKLSQKRCDLYLLSIILMFSSFSFLRTCAAAIGFFTFLCTLLFLLYKKFRFNPQNETTASSSTPSSAPSSSSQRWN)-YHVFLSFSS-VDVPISFRRRISKELRRKGFDPFIDDEIERSVS-IgpELVKAIRGSRIVLVMLSRNYASSSWSLNELVEIMNCKEE-MGQRVVTFFYELDPVDVKAQTGDFGKNFNKTckgktkediekwrqaleevativdyhssdwdnetamiekiatdiSKKLNNTTRSSGFECLVGMEPHI(EKMEHLLCLDSDEVRMIGIWGPPGIGKTTIARVLF)}*

$3678=289(247):
>gi|1099279548|gb|JAU90061.1| {|0(159)|<Streptophyta(V)>}Putative disease resistance protein [Noccaea caerulescens]
{(RLNFYQVSIILMDSSFSFLRICAAVIGFFTFLCTLLFMVYYKKTRYNPQNETIASFSPPSSAPSSSSSQAWN)-HHVFSSFSS-VDVPISFRSRIFKELRRKGYDPFIDNEIERCVS-IgpELIKAIRGSRIVLVMLSRNYASSSWSLNELVEIMNCKEE-MGQRVVTFFYELDPVDVKAQTGYFGKIFNKTcegktkediekwrqaleevativdylssdwdnetamiekiatdiSNKLNNTTRSIGFECFVGMEAHI(EKMEHMLGLDSDEVRMIGIWGPPGIGKTTIARVLF)}*

$3679=281(300):
>gi|1098897725|gb|JAU07351.1| {|22(25)|<Streptophyta(V)>}putative WRKY transcription factor 19 [Noccaea caerulescens]
{(FIGCRRREGGKSSRNDALVSSRNAVLNPTSPATSAKTLELREKLKEVETPLQLELPEHSIPVVDDPVSVIYCGEYQDSEE)---------------RYFISYIFNDLCLHGFTPLRYDL--TRSI---VTgnpKMLYRSRVGIIIFSMNYARSRGCLDGFVAIMDHLKA-NNLVIIPVFFKVSLLDVRGQSGSYEKVFTRLKNSVQASQI-LKWRAAMLELCSI(NRYQYMKGDEVMLAKNIVKDVCLVLNSETHRNLQGMRLQLKSILSLLSCSQSSAPHIVGLWGMAGIGKTTISRDIFRRQA)}*

$3680=277(300):
>gi|1098950406|gb|JAU20900.1| {|185(1)|<Streptophyta(V)>}putative WRKY transcription factor 19 [Noccaea caerulescens]
{(CRAEDAIAFLRGRNTSGDSKALVSRRLSAACEVYGIKPKNNPFRSLAVITYLKMEREMTREAECVLVFSCHDKFDVDETN)-----------------FTEAILKELHERELTPLSYNLSGRENL---DVDMLYRSTVGIVVVSNCLAHSGQSLDHLVTVMEYWKA-KDLVIIPVYLKVTPSDI-VLRGLLEVGFPEFQSSIQADRV-

QKWKAALTETASI(DGYQWSKGTQFMLAKKVVRNICLRLQLKNSKNLIRILALLNHPQPSEAEIVGIWGMAGI
GKTSIATEVFEILAPQYDLCY)}*

$3681=274(280):
>gi|1098984889|gb|JAU28783.1| {|6(31)|<Streptophyta(V)>}putative WRKY transcription factor 19 [Noccaea caerulescens]
{(GDFVFFQRHLTDSSRLLVGFRRRGNGGAALGQRGYIRRQDSTSPTTSSKTLELTEKPKEVETPLQLRIPGH
CITGFYPVF)---VFYCGHK-QDSEEtYLISYISNELCLRGFTPMIFDLTRSTVT---
GSlEMLQRSRVGIIILSKNYAFSTQCLDEFVAIVDHSKA-
KNIVLLPVFFKLELSEIRGQSDSFGKAFSRLENSGQAFRVPDIFLYMKGNEVIL(AKNIVRDVCLLLNFETNR
KLKGRLQLNSILSLLNVSQLSAPRIVGLWGMAGIGKTAISRE)}*

$3682=279(300):
>gi|1099319289|gb|JAV01593.1| {|208(535)|<Streptophyta(V)>}putative WRKY transcription factor 19 [Noccaea caerulescens]
{(CRAKDAIDFLRLGMTTLGNSTALVGGLIAACEAYGIKPKDNPFRSLAVVSYLKETREMTRETECIFFFSCN
DNFDVEETS)-----------------FNKAILKELYEREVTPLTYNLTGREDL---
DSEMLYRASVGIMVVSNScFARGSQYLDNLLVIMEHWKA-
KNLVLVPVYFKVTLSDICGLEGHSEEVLLQYSSSVQADRI-
PKWKAALTEMASI(DGYQWSKGTQFMLAKKVVRNICLRLQLKNSKNLIRILALLNHPQPSDAEIEGIWGMAGI
GKTSIATEVFEILAPQYDLCY)}*

$3683=238(243):
>gi|1099127142|gb|JAU58185.1| {|0(318)|<Streptophyta(V)>}Protein DA1-related 4 [Noccaea caerulescens]
{(SSLTQKTDKKHQLVISKPTRNIRFLSSFDAQDNCMDNNKND)--EVFLSFdHG-
DSSVSSFISYLIAAFNRQGISAFFDGKSSHVEAVEREEERFSRLKVFVVVFSDNYVSHVPCLEK--
HLYSCRNN-
DDFVVVPVFYRVSTSSVKQHIENSGDVYEAVQISMPKLRRGHEYDGKKTECEFL(EEIARDVFEELYPTEEIG
IRSSVLMIESLLCKQPWGVRSIGIFGEAGIGKTTLARAVFCRMS)}*

$3684=138(155):
>gi|1098896270|gb|JAU06624.1| {<Streptophyta(V)>}Toll/interleukin-1 receptor-like protein [Noccaea caerulescens]
{()---------------NNITERLYEAL-VKeHVRVWNGDAE-
RENFELgpSLVEAMEDSVAFVVVLSPDYAKSHWCLEELAKLCDLKSS-LGRWMLPIFYGVDPWNFRRQS-
PFEKDFKEH-LKIFGEEEIQRWRSAMNLVGNI(TGLVHRDDDSVDEDE)}*

$3685=131(151):
>gi|1099168133|gb|JAU66612.1| {<Streptophyta(V)>}TMV resistance protein N [Noccaea caerulescens]
{()-------------------ERLYEALI-
KeHVVVWNDDVERGYDHELgpGLVEAMEDSVASVVVLSPDYAKSHLCLEELAKLCDLRSS-
MGRWVLPIFYGVDPWTFRKQT-PFEKDFEEH-SKRFGEEEIQRWRGAMNSIGNV(SGFVHSKDSVD)}*

$3686=187(190):
>gi|1098996407|gb|JAU32683.1| {|0(849)|<Streptophyta(V)>}Disease resistance protein RPS4 [Noccaea caerulescens]
{(MDPPT)-HQLFLCFRG-
KDVREGFLSFLVKRLKENKsLKYVVDYEEMRGEVVHNLLKRIEDSRIVLVIFSENFMESKWCTKEVLKAMETR
GP-GK-
TIIPVFYKVSVEQVKDNWSAVQEGQIDNSSGLRVTDYFGLKSSDYRANEAD(FVEKIATEVEITLNKLETEEK
KKRVSKGEAEKKQEITNTLPFFGI)}*

$3687=212(216):
>gi|1099011978|gb|JAU38292.1| {<Streptophyta(V)>}Vesicle-associated protein 1-4 [Noccaea caerulescens]
{(MADYDETRSQPP)--QVFLSYRG-
AELPQSFVAHLEVALRRSGVNVYVDNVALKGKGLALGFEGINESRIALVIFSKRYTESRWCLDELVKITKQVK
Y-GKLMAIPIYYDVKVSVVERLDGDFGSNLWNH-
AKgNSEFYKLKQWKKALDFISQT(VGLSFTEKSSESVFINDIIFHVHDLLRSDDGLTLRRDDGVNLRRDDDDD
DDDADADDDDDDDDD)}*

$3688=196(201):
>gi|1099111217|gb|JAU54943.1| {<Streptophyta(V)>}Protein PHLOEM PROTEIN 2-LIKE A8 [Noccaea caerulescens]
{(KRSKLLFEMEKRP)--QVFISYRG-KDLRLTLVPHLKHHLNDSNVNVFTDSNA-
AGERLKNLLNHIKNSRIVIVIFSISYLESRWCLDELAEVRNCLLR-
KKLdFLLPVFYKVRTYQVQKQTGDFGKPFRNL-
KRRHPHHRVIRWKKALRFAAKS(IGLAYRENSSISELDFIQNIVEVVNGLLGRIALKDSNNNNNTQEISTG)}
*

$3689=213(226):
>gi|1099290705|gb|JAU92902.1| {|0(495)|<Streptophyta(V)>}Protein DA1-related 4 [Noccaea caerulescens]
{(QMSNNHEIKQT)---VYISFND-DDPHvSSFISYLDAALYREGIDVVLDKS---GHELEK--
DRFSDIKLFLVVFSNLYAFSVACLENVVKLLEFLQQ-KDHVIVPVFYD---EKVKQPMEVFGDKFAVL-
EKSHSADQVTKWRRVLEETMKL(QGHEYSDELRDESKFIEDIAKDVFDKLHPTEHIGIQKPLTELENLQCKQA
WGFRSIGIVGKPGIGKTTLAKAAFR)}*

$3690=197(219):
>gi|1098958453|gb|JAU24022.1| {|0(34)|<Streptophyta(V)>}Vesicle-associated protein 1-4 [Noccaea caerulescens]
{()-------------------------
TDIGIfNVFIDRDEWWGRDLNHIFTCIEESTIALAIFSPGYPETEWCLDELVKMKERANE-
KKLLVIPIFLNVSKKDVRTFGGEFGERFMEMRKKRYtdYKYDPFriQRWETSVMTISRM(EPSLTWEAQSSNIH
LAEEIIRAVKRELDMASDPGFSFPSEFSNESHPKMTIGEVFVSAFLAAFLFSLFIAPKIFTDVKI)}*

$3691=283(296):
>gi|1099083067|gb|JAU52429.1| {|166(16)|<Streptophyta(V)>}Vesicle-associated protein 1-4 [Noccaea caerulescens]
{(EALISIPQKAGLTLAGHRDESELVDSIVKEVTKVLIDISSKESDDSNNSTNSQGQSTNQQTQNKSFEPLMA
ELDRLPPRL)-PQVFVSFCK-EELGDNFVKHLVWALRESGVNVFTESYKLRGREVEQD----
QETNIALAIFSKRYSESGLCLNELVKMGKLAKK-GKLVVIPVFYNVNKNEVRRL-------FIDTKMRF-
AMEPVlvQSWEESLTSLFAG(RISLSLEAQINEFALVGAIVREVTRLLPNSSRRRRRRRKLGIGQVFVRALLA
TFIFALFIFSMPPTDVNVLKVGNF)}*

$3692=283(296):
>gi|1099226359|gb|JAU77400.1| {|165(17)|<Streptophyta(V)>}Vesicle-associated protein 1-4 [Noccaea caerulescens]
{(EALISIPQKAGLTLAGHRDESELVDSIVKEVKKVLIDISSKESDDSNNSTNSQGQSTNQQTQNKSFEPLMA
ELDRLPPRL)-PQVFVSFCK-EELGDNFVKHLVWALRESGVNVFIESYKLRGREVEQD----
QETNIALAIFSKRYSDSGLCLNELVKMGKLAKK-GKLVVIPVFYNVNKNEVRRL-------FTDTKMRF-
AMEPVlvQSWEESLTSFFTG(RTSLSLEAHINEFALVGAIVREVTRLLPNSSRRRRRRRRKLGIGQVFVRALQ
ATFIFALFIFSMPPTDVNVLKVGN)}*

$3693=183(195):

>gi|1099234510|gb|JAU79142.1| {|0(633)|<Streptophyta(V)>}putative WRKY transcription factor 16 [Noccaea caerulescens]
{(MTDCKN)-PEQFVCiSCV-EEVRYSFVSHLSDALRRNGISVFIDTDDLLSKEAQE---
KVGRARVSVMVFPGNR---KVFLEKLVEVLKCQKD-DDQVVLPVLYGDSPS----
LRGEWLSALELSGLSPVYQSSKECSDSQLVDEIVR(NVNEKLLFVGRIGIYSKLLEIENMINKQPLGIRCVGI
WGMTGIGKTTLA)}*

$3694=230(228):
>gi|1099314825|gb|JAV00066.1| {|0(28)|<Streptophyta(V)>}Vesicle-associated protein 1-4 [Noccaea caerulescens]
{(MVDPTQQRE)-PQVFVNSGA-
TELPDDFIRHLVWGLTDIGIfNVFIDRDEWWGRDLNHIFTCIEESTIALAIFSPGYPETEWCLDELVKMKERA
NE-
KKLLVIPIFLNVSKKDVRTFGGEFGERFMEMRKRYtdYKYDPFriQRWETSVMTISRM(EPSLTWEAQSSNIH
LAEEIIRAVKRELDMASDPGFSFPSEFSNESHPKMTIGEVFVSAFLAAFLFSLFIAPKIFTTAKW)}*

$3695=225(239):
>gi|1099222071|gb|JAU76529.1| {|0(280)|<Streptophyta(V)>}Putative disease resistance protein [Noccaea caerulescens]
{(GGNPLGKFSLIIEGIETFLWLFLLIIIMT)-HEVFLSFRG-EAMRKTFVSHLLSSLKSHDIKIF--
TASEPFEDVD--LQAMKQSLVAITVVSQNCATSDRWVDDLARIIECEQK-GTLTAIPIFFQVHPLDmLRYVD-
-------KH-
GlQNESSETARNWLYSHAPRKSS(FHSSDWKDDSELVDKIISFVSSILVSSSASYQSDDSLIPKHSKLTTKLT
LAVHKPLKDTTKSTSTLLQGS)}*

$3696=155(182):
>gi|1099013067|gb|JAU38589.1| {<Streptophyta(V)>}Protein DA1-related 4 [Noccaea caerulescens]
{()-----------------FIGYLDAALYREGIDVVLDKS---GRELEK--
DRFSDIKLFLVVFSNLYAFSVACIENVVKLLEFLQQ-KDHVIVPVFYDEK---VKQPMEVFGDKFAVL-
EKSHSADQVTKWRRVLEETMKL(QGHEYSDELRDESKFIEDIAKDVFDKLHPTEHIGIQKPLTEL)}*

$3697=228(241):
>gi|1099182028|gb|JAU69169.1| {|0(495)|<Streptophyta(V)>}Protein DA1-related 4 [Noccaea caerulescens]
{(YRHFCLIFHNVPKSLQMSNNHEIKQT)---VYISFNN-DDPHvSSFISYLDAALYREGIDVVLDKS---
GHELEK--DRFSDIKLFLVVFSNLYAFSVACLENVVKLLEFLQQ-KDHVIVPVFYDEK---
VKQPMEVFGDKFAVL-
EKSHSADQVTKWRRVLEETMNL(QGHEYSDELRDESKFIEDIAKDVFDKLYPTEHIGIQKPLTELENLQCKQA
WGFRSIGIVGKPGIGKTTLAKAAFR)}*

$3698=199(210):
>gi|1098956716|gb|JAU23358.1| {|0(116)|<Streptophyta(V)>}Uncharacterized protein PHLOEM PROTEIN 2-LIKE A7 [Noccaea caerulescens]
{(MAASTSSSSSVEGLIPTR)-PLVFIDFKQ-
EDVGKSLISFLEPVIKKENVNVLIDEEEVRDGDLENLFQRIQDSRISLAIFS----
ESKCGFDEVPKIKEPVVE-G----
MPIFCKMDVISYNKVKGDMADLQNSEKSKEDHIKSAVEELSRLLVNISV(GVSVKREADSKCFMVPARKLEIS
HSEKLRNWIWSFIYEAPNEAAIEIAMLNE)}*

$3699=248(264):
>gi|1098954341|gb|JAU22275.1| {|372(387)|<Streptophyta(V)>}putative WRKY transcription factor 19 [Noccaea caerulescens]

{(ISPSEGSVTVNMFSSDLFSSDSRFLDTSVASRSSKNMQDTESSIGFPGSSVDERSQITNLSGCDSGLRSTRVGSSDSKDY)-YDVVIRYER-GDISNDdFTSHLRAALCRRGISVDQDFYDV--------LYKVPECRVLIIFLTSAHVPS-----SLLHILEHQGK-ERRVVYPIYYGISPSDLIGNHKNRKRWQAAW-KEITQTPGYVLTDNSESELIDE(IVIDALKVLRTNDNEKMIGMDMQVEEILSLLCIESPDVRSIGIW)}*

$3700=240(257):
>gi|1099015452|gb|JAU39260.1| {|147(683)|<Streptophyta(V)>}putative WRKY transcription factor 19 [Noccaea caerulescens]
{(SVTVSPGYDASPSDLCSSDARFLDTSVASRSSKNMQDTESSIGFPGSPVDERSPISNTNLSGCDSGLRSTRVGSSDSEDY)-YDVVIRYKR-GDISNDdFTSHLRAALCRRGISVDQDFDDVYT---------VPECRVLIIFLTSAHVPS-----SLLHILEHQGK-ERRVVYPIYYGNRKNYERFFLQDQRKRWQSA-WKEITQTPGYILTDNSSESELI(DEIVIDALKVLRTNDNEKMIGMDMQVEEILSLLCIES)}*

$3701=275(299):
>gi|1099104713|gb|JAU53685.1| {|314(633)|<Streptophyta(V)>}putative WRKY transcription factor 19 [Noccaea caerulescens]
{(ISPSEGSVTVNMFSSNLFSSYSRFLNTSVASRSSKNMQDTESSIGFPGSSVDERSQITNLSGCDSGLRSTRVGSSDSKDY)-YDVVIRYER-GDISNDdFTSHLRAALCRRGISVDQDFDDVYT---------VPECRVLIIFLTSAHVPS-----SLLHILEHQGK-ERRVVYPIYYGISPSDLIGNRKNY-ERFFLQ-DKRKR------WQAAWKEITQT(PGYVLTDNSESELIDEIVIDALKVLRTNDNEKMIGMDMQVEEILSLLCIESPDVRSIGIWGTVGVGKTAIAEEIFRNIS)}*

$3702=198(204):
>gi|1099150379|gb|JAU63694.1| {|0(362)|<Streptophyta(V)>}Disease resistance protein RPS4 [Noccaea caerulescens]
{(MAVTDH)--QVFISFRG-ADVRRSFLKFLEEGLKRGCVDYYVDTKEMKGEVLEILLQRIQESRVILIIILSQNYMESKWCIKELLEATKDMKG-SRRKVIPIFYNVRVEDVKD-NWKLGKQARGE-EGEEIGEEREKEVKEALRTLTR(HMGIRSAEYRTDYEFIEDIVKEVQKVLTSIKMEEKMKASGSTSDRAEGEKHDMSFMRS)}*

$3703=240(257):
>gi|1099301453|gb|JAU95973.1| {|347(672)|<Streptophyta(V)>}putative WRKY transcription factor 19 [Noccaea caerulescens]
{(SVTVSPGYDASPSDLFSSDSRFLDTSVASRSSKNMQDTESSIGFPGSPVDERSQISNTNLSGCDSGLRSTRVGSSDSKDY)-YDVVIRYKR-GDISNDdFTSHLRAALCRRGISVDQDFYDVYT---------VPECRVLIIFLTSAHVPS-----SLLHILEHPGK-ERPVVYPIYYGNRKNYERFFLQDQRKRWQAA-WKEITQTPGYILTDNSESELID(EIVIDALKVLRTNDNEKMIGMDMQVEEILSLLCIESP)}*

$3704=296(300):
>gi|1099288348|gb|JAU92256.1| {|290(76)|<Streptophyta(V)>}Vesicle-associated protein 1-4 [Noccaea caerulescens]
{(RLLPKIPQLFPASNLLVSFQESQYQSYSVPDCTGVVANRSQTAESTDNLRNFRGDDSLTCFSFLCNIMKRCSMMIRPPLH)--RVFISFGE-EKLDKTLVSSLKKEFISNRISVDVKDE-------------IKESKVAIVVFSIKYPESQQCLDELVQIKKLMDA-GEIDPFPIFYTLKAEPVKDLKGCFLNRLLKIedevRKKVNRENEKsildteariCGWREALLSIASR(PGLTYQHSSDPVFVTDVVTKVKELFAVRERPKDSISYISPDLVVENPPMHRQETEAAITRVESFDDDLFYSLTSFFQALN)}*

$3705=215(220):
>gi|1100218297|ref|WP_071359111.1|
{|55(0)|<Proteobacteria(B)>}hypothetical protein [Raoultella

ornithinolytica] >gi|1098931285|gb|APB04114.1| hypothetical protein BK817_03660 [Raoultella ornithinolyt
{(VNLQKDPIFFELWLKDNYPGKLKRYNIFTKDIPQYNVQTFINADMVDFAITPEGGGHLFLWPERELFEKQHPQYKNINEK)KSKVFISHSSKdKlNIVNPLNAYLQANDIA------
TWLDSYEIDYGDnifiKVNEGIEESAVGLFVLTDNFFNS--ESGWPLAEFSTFFT-
DLMKSNKKVLMLDAGVSPKNMLAMMKAYRYI--SWNNGASLPEIANAINKIIKQ()}*

$3706=194(192):
>gi|1099913005|emb|SFJ96095.1| {<Actinobacteria(B)>}TIR domain-containing protein [Amycolatopsis sacchari]
{(MYE)-YDVFLSYRR-KGgtvpawVRTHFhprLAELLDDYLDHEAKIFFDDQVPAGTA-
WplEVRNALQRAKVLVAVCSPKYFSSEWCLAE-WHSMARREE-LTGLATPerpqgLIYPVIFCD--------
SENFPKYAHERRMLDL-
KRWNQPYQQFQDS(PAYLDFHRNLQVVATELAGLIAHAPEWRPDWPVETPLPDPPPTSRLPRF)}*

$3707=187(187):
>gi|1099913093|emb|SFJ97882.1|
{|0(690)|<Actinobacteria(B)>}Tetratricopeptide repeat-containing protein [Amycolatopsis sacchari]
{(M)-HDVFLCYVW-EARP---
TAVLLQRALEErGLAVFRDETGMRDHDHIetRIDAALRASRTLVALTTPPFLTSEYCRQELHFALSRAHR-
LgpgRARVLSVLSEVPPADVRPQPLKAWRLPAPDQEIGTTADGIAAHVAALRAEDDR(LLGDAPDLPAPRWFPRPPPPGPQLCGRELELWDLHEALRAESSVVT)}*

$3708=175(176):
>gi|1099573271|emb|SFD27196.1|
{|0(733)|<Actinobacteria(B)>}Tetratricopeptide repeat-containing protein [Verrucosispora sediminis]
{(MT)DHDVFLSFAG-PDRRS--AQRLAEALGR-RVDVWLD-
EKLPVGRGItaEIEEHLNRSKIMVTLYSAAYPSRSACQFELTAAFlaGAREGDPLRRILVVNPEAYEHHLQPVQLADVKFARWPQPSNARDMAALVKAVAAKAASVE(GTFGDIGFTARPTWYPYGVAGVPSFVGRYREQWE)}*

$3709=181(187):
>gi|1099886971|emb|SFG24211.1| {|0(672)|<Proteobacteria(B)>}TIR domain-containing protein [Ensifer sp. OV372]
{(MKKV)----FLSLSFVdADFVRKVHARLPKGLAYF----
YERSFDNGEALLDAMGRTVPDSALFVFFASPSALASPWVGFELDAARTKSIVDSNHRVLVFpTDPNV-
SHSDLPVWMKDYWVPKAgMTVGDIARYITATLLELETGASG(SVGKVVGRGKTADRYEQLIADHLVRTHRTPNVYLLGGFRGIGR)}*

$3710=200(188):
>gi|1099770435|emb|SFM06813.1| {<Proteobacteria(B)>}TIR domain-containing protein [Rugamonas rubra]
{(MAIRYGCFFSY)AHGRHAFMS---
KFKNDLVDALKCYLEPHfdsERELFVDSEQLGGGDDLdsRIATALCESVCMIVIYTPKYEAHPYTRREFAAMQLIEDERkqwyalPSHLIIPVIMTRHPlslpPQITGGMYVDFSRYTLATGDLKTNPDYLPDIERIVQRIAA(HYHCLKRYMPPGHDCSRFVLPDIPPEWRAIPPPHFPR)}*

$3711=236(234):
>gi|1099573269|emb|SFD27151.1| {|36(0)|<Actinobacteria(B)>}hypothetical protein SAMN05216284_11417 [Verrucosispora sediminis]
{(VDPGRLRRFARRWLDAKTAELWERARATQTYRIWAETAGPEQVIEADDLATALLRKGVPGEIAAPAAVAMHRDEVARAPV)-YDVAFSCAE---
RQTEYVQLAVSAVRATGLCVFFEKEMTYEWWGKnfiVEGRRVYGRSARyfVPFFSEEYLSEPRPRDALEWAMS

AAVERGDDYILPVVVGDVSvpANlLGPNIGYLRADEHSP-EQLAAALR-
AKVAASRASDVEP(RDIGVIVRGVRGSQ)}*

$3712=240(244):
>gi|1099566132|emb|SFF20875.1| {<Proteobacteria(B)>}TIR domain-containing protein [Methylobacterium sp. 13MFTsu3.1M2]
{(MTVKSGTNKPKPPARRAAPGTRALVPAKATAPKPKKAKVR)---
IFVSYCHAnEIQQAKLQIHLAQLKRDEV-ETWFDGDMEAGDRLNtEISRKLREADIFVALMSPEYIASHWC-
QlEYRRAMGRRAR-GSMRIVVVVVRPC-AWKDTGASDLKVLPRDGrtVSDWRSMD--
HAFANVAEGIKGA(VKAVRVALPPAVPARPAKAMRSAAAPVAKKRPVSAKGGRSASTKVSPGAGAAKGKRPAR
SRATG)}*

$3713=247(250):
>gi|1175400872|ref|WP_081948660.1| {<Proteobacteria(B)>}molecular chaperone Tir [Methylobacterium sp. UNCCL110]
>gi|1099726214|emb|SFU94575.1| TIR domain-containing protein [Methylobacterium sp. UNCCL
{(MAARNPVRSGKPPPSRRTAAPRKAATRPSTVARTAAPGKPEVR)---
IFVSYSHVdADERARFETHLAPLRRDNV-
TTWFDGDLNAGDALDaNIARELKKAHVFVALLSPSYLNSHYCWQiEYKRATGRRRK-GTMRVVAAVDKPC-
DWKATDAAGFKLLPKDGkpVINWRSAD--
LAFLDIAQGIRGV(VKDVRKHMAVDPQPPSAAKAPAVAPRTKPPAKPKPGKVANARTRTGAATKRTAKPASSR
PKVTRSRP)}*

$3714=206(210):
>gi|1099812696|emb|SFM23134.1| {<Proteobacteria(B)>}TIR domain-containing protein [Methylobacterium pseudosasicola]
{(MKAKVR)---IFVSYCHAnAVHRAKLQVHLAQLERDEV-
ETWFDGRMEAGDKLStEISRKLRAADIFVALMSPEYIASHWC-QlEYKRAMGRRAK-GSMRVVVVVVRPC-
AWKDTGASELKVLPHDGrtVSDWRSMD--
HAFANVAEGIKGT(VKAVRASLAQASLVRPAKTGRSTVSPKVKKPPVAAGGIPKAAAKARSGVGSTKARQSAR
NRAAR)}*

$3715=225(229):
>gi|1099813111|emb|SFM08931.1| {<Proteobacteria(B)>}TIR domain-containing protein [Methylobacterium pseudosasicola]
{(MATRNRTRSAKTAVPGKPESAVTRAKATAKANVRTATSKPEIR)---
IFVSYSHVdAAARQKLETHLAPLLKDGV-KTWFDGRMDPGDALDaGISKALRQAHVMVALLSPEYIFSRYC-
QlEYDRAMARRAR-GTIRVVGVVVRPC-GWKETKAAGFKLLPEDGrpVQDWRSAD--
AAYLNVAQGIGKL(VKTLRAEMAGSPIKPSAHPPKAKPAVRMPSAALEAVMNLWRVERPF)}*

$3716=155(156):
>gi|1099736025|emb|SFH74742.1| {<Actinobacteria(B)>}TIR domain-containing protein [Actinopolymorpha cephalotaxi]
{(MTISNEAAGHA)----FISYVR---
EDVDRMQDIVSALKAADIPVWTDKMKLSPGEDWklTIRKAIQRNALAFLAVFSSSSqaRDQSYQHeELLLAVE
QFRQHppGRVWLIPVRLDDC-KLPDYDLGAGRTLDSLQRVDLFGEDAKDETIRLGRVLRIF(IAGAG)}*

$3717=141(160):
>gi|1100112721|emb|SFR05875.1| {|0(779)|<Actinobacteria(B)>}TIR domain-containing protein [Lentzea waywayandensis]
{(ME)---------------------
LYEALKDEGLRAFIDERGIryGHGVTAEITTALRESKTMLVYYSASFPQRSACEFELRYAYLTALRAGEVEQR
ILVVNPEDPNTGHLLPLELSTNRYVRTWQSKRDLATLVALVRQKAEAL(DGPFPGMDFGPPPLTHHR)}*

$3718=171(165):
>gi|1099972128|emb|SFH22491.1| {|0(31)|<Actinobacteria(B)>}TIR domain-containing protein [Amycolatopsis regifaucium]
{(MIERVSDYK)-YDVFISYsrHG-S-APKWLLNHFYPKLEEYLIdevypspKIFIDKGMSRGVA-WspELKSALRYSKIMIAVLSAPYFGSNWCMAEWGSMAAREKM-LGLASDDRPQGLIYPVRYSDSVHFENEGRLRSWWDFKGLDGADRAFHGSKDWHA(FQLKVRDCARDLAELL)}*

$3719=191(179):
>gi|1100068742|emb|SFQ75634.1| {<Actinobacteria(B)>}TIR domain-containing protein [Amycolatopsis rubida]
{(MYH)-YDVFISYlRT-ADPIARWVrNHFHPRLCelldgnlDREVRVFFDG-SVRVGDRWpdELRNALQRTRILVPVASGQYFYNEWCLAEWASMAHRENlvggARPATLIYPVIYADSKNFPPFAHQRRWQNLTDWNHPYEHFQISPKYIGFHDKMNEV(AQELEELISAAPPWQPGWPVLTPVPDDPPTSRVPKF)}*

$3720=177(177):
>gi|1100053575|emb|SFO75855.1| {|0(700)|<Bacteroidetes(B)>}TIR domain-containing protein [Chitinophaga sp. YR627]
{(MRRSFSLSKVLPFFFSD)--DIFISYSRKDGNAYTASLARQLTSRGY--KVFFDHYGTPPGReLPkEVINKALSARMLVFVASAEALQSVHVRSevELFVDKRHVRNKGIFQCINFGAFQQTKWVQEKNIDTISFISEDATFIREEKASPQVIDFISDSFLY(NKSNYQLRRAAGITGVILLT)}*

$3721=200(188):
>gi|1100015680|emb|SFV07007.1| {|0(1)|<Proteobacteria(B)>}TIR domain-containing protein [Massilia namucuonensis]
{(MGIRYGCFFSY)AHGQHAYMS---KFKNDLADALRCYLEPHfdnESELFVDTEQLGGGDDLdrRIALALCESVCMIMIYTPKYEAHAYTRREFAAMQLIEEERrawyplPSHLIIPVIMTRHPlclpPQITNPGMYVDFSRYTLATGDLKTNPDFLPDIEKIVQRIA(AHYHYLKHCIPPGHDCSRFVLPPIPEVWRAEPPPHFP)}*

$3722=195(204):
>gi|1100108105|emb|SFR24984.1| {<Actinobacteria(B)>}TIR domain-containing protein [Lentzea waywayandensis]
{(MSYKYDFFISYCRYGSVQRWLINH)-------------FLGKLKECLADQYA-PTPTVYVDRTmerAVHWPSN--LQDALRHSKIMIQLLTPQYYGSRWCMAEQKSMRAREEMLgVaslevsQGLIYPILYSDSEnfplEE--------KERSWVDfKDVAHPDPVYQQSRKYLRFHKRV(NDLAADLVRLSKQAPPWQPDWPDVDPPEPPLMPPPQIPRF)}*

$3723=255(265):
>gi|1099972132|emb|SFH22580.1| {|0(84)|<Actinobacteria(B)>}hypothetical protein SAMN04489731_10372 [Amycolatopsis regifaucium]
{(MTETGGQRDTGIHTLCTSGPHATSMLRRSDQGDRRQAGDEQKGAPVEEIPS)-YDLAISFA----EDRQSgTHELEDASRQRGLTVLHDPERTHEWWARksaGDLPDV---HVRffVPVISA--------LDDFTTAMLRAVATGDRHVLPVLADDVVvppeL-LHPHVSYLRDGDHLI-EELAGR-V-ERAEAAGWERGRL(ADVVTRAREIAPPAAVPVTFSRYVEQDAALRYLGEQFAAAIPRLRSRGFAGTTHHGDERIGLRIERAGDIVYAL)}*

$3724=134(149):
>gi|1100108109|emb|SFR24993.1| {|0(76)|<Actinobacteria(B)>}hypothetical protein SAMN04488564_108179 [Lentzea waywayandensis]

{(MTESAT)-YDIAVSFAE---EQRAVVDEVVEACRQRGLTV---------LESNDLP----QAEVRflVPFVSTADEIVNDHEHVLAVLTGEPPTSSDVTYLRADEHLVNtlV-ARIEAAETSGHDRVP-VADLVAALKQPETLAPAQSTAL(RTL)}*

$3725=189(188):
>gi|1100861768|ref|WP_071420192.1| {|0(91)|<Firmicutes(B)>}hypothetical protein [Oenococcus oeni] >gi|1100333707|gb|OIK85192.1| hypothetical protein ATW79_09680 [Oenococcus oeni] >gi|1100362194|gb|OIL
{(M)--KIFLSWSGDlsKHIAECFHEFLPLV-LQYTEPFMSSDD---IARGDdwnnKINGELNNSRIGILFITPENINSNWLNFEAGAlfSWKNKDE-QQGTVMPVMinSLDITSVLSKSPLKQFQGTKSL--SEEGVKSIFHSINLKADSDSL(SEASFQSTFDQWWPKINQEINQVLKRPSTKIEPKKTDRKKDMWSAQD)}*

$3726=200(217):
>gi|1100109627|emb|SFR20800.1| {|0(491)|<Actinobacteria(B)>}AAA ATPase domain-containing protein [Lentzea waywayandensis]
{(MA)--RVFISHATQNVAIATHIRNWLE-GDHE-IFLEVHpedGIQVGEAWRERLHHELRRADAVVCVISQASVTSS-WCNaEIAIADALG-----TLLLPVQLDGV--IHPLIGHLNHI-------PHTTARE--HLQKALRRFDTG(GRKLAKDQEPFPGLDAFSTALSAVFFGRDAETRALARKVRRGPGLLAVVGPSGCGKSSLVRAGLLAQLADDPRWL)}*

$3727=263(259):
>gi|1099961845|emb|SFE40088.1| {|885(0)|<Bacteroidetes(B)>}C-terminal of Roc, COR, domain [Chitinophaga sp. CF118]
{(PDFDDEGIDWSSDYFSVYISLDGVFYTSWSKLIENVDNDILQIKLSSVHRTSEIPENIILTKTTSVFDFNKFLPKKSKGQ)MKKIFISYSKEdLRMVNKFQEHLSALKLDGKVTTWYCTELSAGGEWNfEIQSHFDDADIVCFMVSPNFMKTKYIHEyEIKKAFKRKSE-NpDFMIVPIILDFC-RWttNHNNLGDYSALPYSAkpVADFSNQN--MAWYIVQECLRFI(IDNKDSNRGDALYNNPGLPKDVLQIFERIMEHKVDNNSN)}*

$3728=297(297):
>gi|1100135510|emb|SFF06121.1|
{|293(442)|<Proteobacteria(B)>}Serine/threonine protein kinase [Nannocystis exedens]
{(CLCKDREQRFASCAELRAALEPFEGIYAASSPDNSFPRTERSGLLTLGELVAQAPAEAETPAGRRADVSAPRPVAPPGDH)---VIIAHGRGdRRWYEHLREHLRTHRDTAQLEFWNAGDSSSRASRIdVLADAFARARAVILLISPRFVAEELAPGgELYSLVLMAQE-RGLPVMWLMVSET-PYEGTDLLDFRTLGDPSqpLDRLsvADAD--RVLEQICDQLVVS(LGLPQTAPPRGGTKIGLPVGDTRLDTIRDLEQRIEAARARKARLEALGEDTREIHEEIKMLRRQLRMGRPLHRGDVL)}*

$3729=212(220):
>gi|1100997071|ref|WP_071502763.1|
{|237(0)|<Bacteroidetes(B)>}hypothetical protein [Arsenicibacter rosenii] >gi|1100700749|gb|OIN59964.1| hypothetical protein BLX24_09005 [Arsenicibacter rosenii]
{(NDFHYLLVERGRLNEFEKKRLALDKRIFTIEYNNHDGTHSEVYDFLFHLNELTSLDGKYRREYESRFYTLPVSQTNVTRI)--SAFISYSGHdKEYAVNIAELLS----ENGIHVWmDDFNVKVGDTVVsTIESGIKESDYFIVLLSNNSINSDWVKQeIELAYITNHER-NKPKIIPIV-------FDSLIRNKIPKLLMDirWLVVNESNIFQKIQRLIEQLKAS()}*

$3730=183(191):
>gi|1101005637|ref|WP_071510629.1|
{|0(311)|<Actinobacteria(B)>}hypothetical protein [Mycobacterium

malmoense] >gi|1100804621|gb|OIN80825.1| hypothetical protein BMG05_10835 [Mycobacterium malmoense]
{(MA)---LFISYSSQdRSTVDALTAAL----
RRGQQQVWFDQELGGGDAWWnKILEQIRSCDVFIVALSNNWLQSKPSQSELRYAQALNRP-----
ILPVRIGDI-
DsMRVNPLAALQIIDYQNptVDAGIQLVTAVHSLQAKPQPVPF(GYITRLGNTLAEKELSPQQQTQLLIELRQGLDEDGEDPSARSDIAQLLR)}*

$3731=192(196):
>gi|1100020729|emb|SFF32128.1| {|0(69)|<Bacteroidetes(B)>}TIR domain-containing protein [Spirosoma endophyticum]
{(MR)---IFISYAL---
EDQEFVNQLAEKLRGVDFQPISTNLRYSLGEDVhsKIEEGTFFSKHVIIIFSKNYIQDKWLMNkELLACFVLEKSRKPGYIIPIQIDDC-
ELPNHLLSRPPIEFRKDFTNTVKEIQSAIRSRRQAFIVMK(LGDKTLDSAHEGVIIPTLDNFGFSAIRIDSFENSGKINNQILEEIDRSEIVLAD)}*

$3732=232(224):
>gi|1099948923|emb|SFN89789.1| {|0(795)|<Actinobacteria(B)>}TIR domain-containing protein [Saccharopolyspora antimicrobica]
{(MTGQ)RYHAFISY-HSparreaKRLKHVLHAIAKRHDAGADFAVYLDTSDLRPGS-
LggEIRSALAQSRCLIVLLDRTTVRSSWVAQEIELWLAGGGsPQrlfLVRLDPELNlaWD---ADRGTFADA--EALPAPLREVFPDEQ--
KWIDLTGpaqRRDHE(ALAGLCAALMDIDANEYLLEEARYQRGRRRTITAVAVVMVLLFAVAAVGAVAAVSGQRMAVRKAELAEAQADASEALLTA)}*

$3733=221(226):
>gi|1100034198|emb|SFO94197.1| {|141(0)|<Proteobacteria(B)>}TIR domain-containing protein [Variovorax sp. OK605]
{(NMLLLLAEGRRKPLLPFPFLGGAAQIAFERRRYELTDRLGSKNLLQLHHASCASSAMKYVDLLSNNSSLSAKPTLISPRI)----FISYSRErqaEADH------
VETLFRRRNMRVFRDETDFGAGHELptVIHEALFSSNIFVALWSAEYACSPWCFDEFEMALNRKAQGgleLWVLRIDETRV---VPVRARSIISYHTRTREELEGRIISLIEGIECADSIVKPL(PNSSNF)}*

$3734=191(199):
>gi|1101260208|gb|OIQ05130.1| {|0(102)|<Euryarchaeota(A)>}hypothetical protein AUK59_05035 [Candidatus Altiarchaeum sp. CG2_30_32_3053]
{(MNKVNK)---VFISYETKTGKSYAEHLKEALEEGNY--
SSFLADLSIDKGtKWKeEINLALNNCKYFVVVIT--SLTLRSKE----VIKEYEKAMgLKKRIIPCRYS--
QlpVTETGTMSELQQLEFSDKYELANKVIIELNKINKREEKQI(PIEKDANEFLNRGILFYNIFNFRKAIDEYEIALEIFKEIGDKAGESACYTNLG)}*

$3735=223(223):
>gi|1101203001|gb|OIP53328.1| {|228(0)|<Fibrobacteres(B)>}hypothetical protein AUK31_01655 [Fibrobacteres bacterium CG2_30_45_31]
{(GLRQGSLFEYIPPRELIADDHSVLDPDFLCFLSRCNADLYPNGDAVAFVRELGKRWREKHPQGFQTQSLSATKKDPEKKA)---VFLSYASED-KTIARQTRDALEAEGV--
DVWFDEKKLESGDQYeGVIaQNIQDASFFVPIVTPNTL------
IqqRRFFHFEWNKAIeEAKfrpvgipFILPLVSGDIDVDNMLIPEAFRKVHWSRFCSPDNLGFFVSECRQKVRNLNR(LSH)}*

$3736=230(226):
>gi|1101142496|gb|OIO98310.1| {|192(0)|<Chloroflexi(B)>}hypothetical protein AUK03_01465 [Anaerolineae bacterium CG2_30_64_16]

{(LPGDTNSLFTQHLLAGLRGGIASDDGLIRIFDLFEYLQPRVTGDQPNQHPVFKAELEENFPVALYLGGQKG
VVVKDEQGF)RYDAYISFvdREPdaTWVWDVLAPRLEEAGL--
RVAVSGDVEEPGVARVVNVERGIRQAKRTVIVLSDAYLADHMADFENVLAQTMGIQEGAYRLLPVQIAPLDeS
QLPVRLSMLTTLNLSAlLKAGLIHPRRAEREFARLVQALQ(GPLPRR)}*

$3737=312(299):
>gi|1101347297|ref|XP_018899477.1| {|1001(45)|<Arthropoda(M)>}PREDICTED:
toll-like receptor 6 [Bemisia tabaci]
{(KASAPASDPINTTGVNNTAFFATSILNVDYIPFISSSLVILIIIILISIIIVIFSKEMLLFLYVKYNVRLF
SPSCDASDK)LYDGYVIYSIKdeEFINEIIYKQLQNF--GFQLCLHHKDI--
NVRSNQylteSMIEITESSKRLILVLSLNLIQNELETFnHFHKALKANLDRiqnsqNKNKLIIIILLTS-
TNLINhntlVNIELQKLLynlknKIVVLKYNDKH---
FWNKLRYYMPDV(KFSSQTNNKVNKSINCDFKKIIVNSNGKITTALGNGARYTAAPMVMATEPWYKMSPQSSV
AVTQSTSVSEESSQEDDPT)}*

$3738=217(219):
>gi|1101141599|gb|OIO97486.1| {|0(65)|<Armatimonadetes(B)>}hypothetical
protein AUJ92_03715 [Armatimonadetes bacterium CG2_30_59_28]
{(MSEDL)QFDMALSYSD---SDAWLAKDLANLVLRYGYEV-
YCADLQPDQARGflrANLREIYRSACVnvVIW-SKDYAAKEkdsIVGTEMRLIINRHIEKDDARALFVL----
AidG-TPPQDDLQEVLFHKL-KDIGITGA-
EKCVLSRLKDLAT(FRTAGKVCHPTGTEHDRGQLHPCSFFIQEGFQKDCLGRWRRLADVEIRFPRPSPVNTPH
VYLIPSGKVMNLLSH)}*

$3739=198(204):
>gi|1101142495|gb|OIO98309.1| {|0(354)|<Chloroflexi(B)>}hypothetical
protein AUK03_01485 [Anaerolineae bacterium CG2_30_64_16]
{(MAQGEQRY)--DVFVSYNPADREWVdEWLLPRLRAAGLR-VITD-
FDFTAGAPRLAEVERAVDSSRRTLAVITPDWLAS-----EWNnfetllvqtlDPAVRR-----
RKLIPVLLKP-TDLPKLLDALVKVDLTA--
EKHWAGQLRRLVRDVEDVIPVA(LPWQAGQRMRDWTQWGRWVRRYRRELRRGVAALLALWVAAALLLAWWPFQ
RRDVWL)}*

$3740=174(190):
>gi|1101028201|gb|OIN92585.1| {<unknown(B)>}hypothetical protein
AUJ42_00295 [Candidatus Collierbacteria bacterium CG1_02_44_10]
{(MSEAKMDDPQAPMKMIQLAGKTAPNLRVAILAHEIDRTEARK)---------------------
LVQLFRKNKILCWTEDELLPGQDWRlETERAYREADFILVLISKKSVHLESVYQkHIKTALEAGSE-
KpesGIKTIPVRLDEC-NIPFVLREIYSVNWWEKnaSLRLILAWAKEWQRRYDANDWPK(IKYVVRWK)}*

$3741=173(177):
>gi|1101160358|gb|OIP14739.1| {<Proteobacteria(B)>}hypothetical protein
AUK53_04945 [Betaproteobacteria bacterium CG2_30_59_46]
{(M)--KVFISYSAKdTH---RVRIIADKLKKDSCAEVhwWEDSQ-
RPGKAAWgQIFQWIEDADLVVVVISGSVLRRALAVGnEIGCA----KA-KGKVIIPLVwESPS-
LWAKLLRNLGAKVGIGTddLGCIGDLVHIRINERNPEDGFQR(LNEELRLIAAQKDEETRKALLALAGIIALG
VLAAKG)}*

$3742=179(182):
>gi|1101177968|gb|OIP30489.1| {|0(227)|<unknown(B)>}hypothetical protein
AUK22_02070 [bacterium CG2_30_54_10]
{(MATFSTEGLPFEAYKGSD)-PYIFVSYSHRD--LDSVYAVIANLKNQGY-
RIWYDEGIDPGNEWPdEVAQALAKCGQFLVFISPNSVDSPNVRNEINFAINRAKP-FVAVHLVESVLPP-
GLELRMGDIQAIMKFRMddEDFFRKLQKSLPKTFMQGSISGK(PEVAGVLPRADVSVQQGRPTPKGA)}*

$3743=158(163):
>gi|1101187246|gb|OIP39007.1| {<unknown(B)>}hypothetical protein AUJ95_06095 [Candidatus Desantisbacteria bacterium CG2_30_40_21]
{()MYQVFISHSTQdRGLVIALANVLTKFEIKVLVAEWY---
LTPGEPVSkKVFDEIEKSDCIVALLTRDGMRSNWV-NqEIEYALNA-----GKPLIPIVEKGV-
NVNELVALQGKEYIEYDppQYQNALIKTASFVKSLKLNKQKQ(RKALLVAGGILAFFLLLSSRGEK)}*

$3744=163(160):
>gi|1101203006|gb|OIP53333.1| {|0(42)|<Fibrobacteres(B)>}hypothetical protein AUK31_01680 [Fibrobacteres bacterium CG2_30_45_31]
{(MNLYYNNNDKTPKHKT)----
FLSFHHKdEGYRDRFEQEYSEDRGDYVSRSVKDGDIDptwPTERIRqKIRDEfISDATVTLVLISPDTWKRKH
VDWeISSSIRDTKNN-PRNGLLGILLPNY-
QYTMDGCSYADIKRTESgrLYNPFTIPPRLFENVQCGYAKIY(SWPA)}*

$3745=172(177):
>gi|1101230309|gb|OIP78021.1| {|0(661)|<Cyanobacteria(B)>}hypothetical protein AUK48_02560 [Oscillatoriales cyanobacterium CG2_30_44_21]
{()MTDVFISYSRKdKEFVSTLY---AAFERSQR-
NIWVDwNNIPLTSDWWaEIEKGIEAADTFVFVISPDSIASPVC-TkEVDHAVLHNK-----RLFPIVRRDA-
NNFEVGNVAHDKIKQHNwlMFREQDDFEKSFETLTETISLDL(EHLHRHTRLLVRAIEWGNGRSDSLLLRGDD
LANAEQW)}*

$3746=205(220):
>gi|1101154882|gb|OIP09750.1| {|80(0)|<Armatimonadetes(B)>}hypothetical protein AUJ96_04775 [Armatimonadetes bacterium CG2_30_66_41]
{(DSLRASIEISKTDVAQRQAAIGRANKTLNQALQTYRRSLEGEVSRALFADVSGHAAEGNQDPAPGPDGPTP
AAEADAPFI)----FVSYARDerqrllPHV----ERMAQ-WG----CRVWYDR-
GIPGTAEWvaVIEDRIRRCQLFMVFLSQRAAESRWVRREVLY--ADKQKKpilTV-------KL---
GDVELGAGLDLVLSPYQMISEEAHEFSEELKRAIEYVRLL()}*

$3747=222(204):
>gi|1101203003|gb|OIP53330.1| {|0(1309)|<Fibrobacteres(B)>}hypothetical protein AUK31_01665 [Fibrobacteres bacterium CG2_30_45_31]
{(MSEQNQ)-
YDAFISYSRKdievaAWFQKEMEKLRVPKAlpypagmepLPRKLRLFRDQTDLGVrSNSFfeGLSANLAKSRD
LILLCSPHVLQNnseglNYIDWEIREFIRIHGVEyaknhilPVIIYGKIQSG---D-------
SREACIPTALNDLGVDFRQRNLPVLGSPEISR(VSKNDKEHVLLQCLSYLARVETNIVDNRFQKERRRTMQRW
LLLAGAVILALSGLAIWA)}*

$3748=280(300):
>gi|1101634635|ref|XP_018980893.1| {|124(58)|<Chordata(M)>}PREDICTED: interleukin-1 receptor accessory protein-like 1-B [Cyprinus carpio]
{(DAVEESDLGNYSCYVENGHGRRQANIQLSQRAELMYTVELAGGLGAILLMLIFLVSLYKCYRIELMLFYRN
HFGSEDVDG)--------------------------
GFSSDHFQDDTRLLRAYIEDVARCVDQSKRLIIVMTPNYVVRRgwSIF-
ELETRLRNMLVTGEIKVILIECAklrGI-
MNYQEVEALKHTIKLLTVIKWPGPKSSklnsKFWKQLQYEMPFK(RPEPKLSHEQVLDVSEQGPFGELQAVSA
ISMAAATSTAMATTHPELRSTFHNTYNTQVRQKHYYRSYEYDIPSSGTLPPL)}*

$3749=302(300):
>gi|1111036309|ref|XP_019225008.1|
{|119(587)|<Streptophyta(V)>}PREDICTED: uncharacterized protein

LOC109206624 [Nicotiana attenuata] >gi|1102286095|gb|OIT32954.1| hypothetical protein A4A49_28525 [Ni
{(PPVSYCGSQSDDVPSTSYTPPPERYDFSDDPTDTKLKIVTCVPVSGPETDPRISFSFPVPRISFAKGSISP
ASNAKLRSC)--DVYIGFHG-QNLNLaRFCKWLKSELELQGIACFIADRAKYADN-
QshEIADRVICSVTFGVVVVTNCSFFNHL--SLEEIRFFGQK-KNLI--
PLFFNTDAneiaslfnrnADAKKCKEVL-DAILKCHEFKLETNE-
GNWRSCVSKAAGI(LRAKLGRKSVAEKTAEGFEEFPFPRNKSFVGREREIMEIETTLFGCGDSFEQESVVPSV
KGGTPGQSEGLADDESEADAS)}*

$3750=179(187):
>gi|1102724261|ref|WP_071592889.1|
{|0(136)|<Cyanobacteria(B)>}hypothetical protein [Pleurocapsa sp. PCC
7319]
{(MSSFF)--DAFISYGH-TESQK-
FVTKLYERLTEVGLKVWIDQRDIDDSVKWqkEIDRGIETAHNLIFIVTPHAVKSPYCLIELELAVKYN-----
KRLIPLLH-
VEPSDCWDRVHPAIAEIQFIFFQDGIDDFEDSFERLVSSIKHQ(ADYIEQHTRYLVKALEWERHQKQTNYLLI
GEDRNKAESWLRV)}*

$3751=208(219):
>gi|1102824061|ref|WP_071605292.1|
{|0(81)|<Proteobacteria(B)>}MULTISPECIES: hypothetical protein [Serratia]
{()MP-IFISYSHEnKDFVDKLAIQL--VHRN--
VNVWLDRwELSIGDSIIdKVQDAVDGASALLVILSKASIASAWCKReLSSGVLRELEE-KRVVVMPVLLEDC-
DIPLFARGKLYA---DFR---TNFD--
DGLRTVVEGIAKV(TSPFLARAKEPEYHVDWSIDWGDVNGLFAIILNFVEQAQNQPYTCLTNIEVLCSPVATE
KYEARKESIGEEHARAYIVR)}*

$3752=246(242):
>gi|1103290379|gb|OJA03477.1| {|60(92)|<Proteobacteria(B)>}hypothetical protein BGC33_04140 [Bathymodiolus thermophilus thioautotrophic gill symbiont]
{(FVKRVRDAIDNAQAEDGDLTGRKILESIEVNIVKASKTQPTRLQLTTRYVAPIVVQPEANRQYDESNFQEN
TVKDIGNDI)--
KIFISYSKEdEALVNnfkELFTSQAPYINERRVEIWTMQELTFGNTFDwQIQQNLHSADFGLAALSQNFLESE
YIKKeEASYLLDRNCL-LLFGLDAKIDGKG-
VNVGEFFTKMKKTLPTKdiHQQTLQQQVCYLDDRVGNFFGDC(KDKDSKIKFVDKVINDLKKVIE)}*

$3753=243(240):
>gi|1103290380|gb|OJA03478.1| {|66(90)|<Proteobacteria(B)>}hypothetical protein BGC33_04145 [Bathymodiolus thermophilus thioautotrophic gill symbiont]
{(DAIDNAQAEDGDLTGRKILESIEVNIVKASKTQPTRLQLTTRYVAPIVVQPEANRQYDESIFQKNIAKDAG
KKIDANDII)---
IFLSHSKEdERLvhdFQELFESRSPCINERSVKIWTMQELTFGGSFDqQIQQNLRSADFGLAALSQNFLKSEY
IKEkEVPYLLDNNRL-LLFGLDAKIDGKD-
ISADDFFTQMKEALPEGdlHQQTLQQHVCYLNDRKGNFFGDC(IKEESKIKFVDKVISDLTKV)}*

$3754=206(226):
>gi|1103823303|ref|XP_019057126.1| {|0(839)|<Streptophyta(V)>}PREDICTED: probable WRKY transcription factor 16 [Tarenaya hassleriana]
{(MATKES)---VFISCGG-EDTRYSFVSHLCMAFCRRNISINLSE--------------
IEEANASVVVFSHDYAFSEPSLDDLVKVWRLRLE-
NGHLVVPVLYGVAQSDVEQLNGSFGEAFSEHEGSDSAHRV-

PEWRKAVVEIARL(PGHECNAQISESKFVEDIVTDVHEKLFPTDKLGIQSILLEIEDLICQQPWAIRCVGIWG
MGGIGKTTVAQAAFARMSEHY)}*

$3755=278(300):
>gi|1103823476|ref|XP_019057328.1|
{|323(1159)|<Streptophyta(V)>}PREDICTED: protein SUPPRESSOR OF npr1-1, CONSTITUTIVE 1 [Tarenaya hassleriana]
{(VGLVDDIYELCGSNFTNDDQSYHVCAPKGHMTKPSMGGKGETHQERKQAIPEEVNELHMRKTDHISVFFCS
DKQEADEAS)-----------------FMEDILRELRKHSITPLTYSLTGRKIL---
NLGMQRRFGVGIIIFSNNYAFSRECLDQLVTIMEHRKA-
NDLLIIPIYFKVTPSDIRECQGSFKEAFLQLANSYQAEQV-
HKWRTAIIETASV(DGYEWTKGNHFTLVKKIVRNACLRLYSRTSENLTNILSLLEHSQTSDMHTVGIWGMAGI
GKTTIATEIFEILAPRYDSCY)}*

$3756=222(228):
>gi|1103823476|ref|XP_019057328.1|
{|0(1538)|<Streptophyta(V)>}PREDICTED: protein SUPPRESSOR OF npr1-1, CONSTITUTIVE 1 [Tarenaya hassleriana]
{(MDVKELVT)QHQVYISFRG-
SESRHGFVSHLVRNMKRAGINVFIDSDLQRGEDLENLWKRIEESRVALVILSSGYLDSNWCLDELVEIMKLVK
E-GKLVVIPIFFKV---DVRRLKGDLGNKFRNL-
ERASPEDRITKWKDALEYVVRK(MGLTLEPQRSEADFIDTIVEAVKRTLIGVSSEDGRVDISRGKGENTEMEQ
WLKQLEEKLEFQQDTTRLVGVVGGHGRIQL)}*

$3757=273(275):
>gi|1103823338|ref|XP_019057147.1| {|156(0)|<Streptophyta(V)>}PREDICTED: uncharacterized protein LOC104825027 [Tarenaya hassleriana]
{(LESVSDIMGLTCAKDRYCPFTCLYMEGDAAGRERHQDSIADLRKPTALRSSRQTTQSRYQAPFVDEVVERV
KEMLANIPS)EHQVYINFRG-
GDVRKSFLSCLVDALRRNGIGVFIDVEMLiKGEDPRDTFNRsIREARVALVIFSTKYAQSRLSLDELVEIKNL
MAT-KKLQVIPIFYNVKSSDVRRRMRELSDKL-RY-
RTSRNEDRIMSWREALECVMTT(TGFTYGAYGSKEADLVDIIVEKVKKVLARIPPRETENPPACDDSPNGGST
MGEQT)}*

$3758=210(223):
>gi|1103732866|ref|WP_071690214.1|
{|0(561)|<Proteobacteria(B)>}hypothetical protein [Nioella sediminis]
{(MTQRSR)---IFISYSHKDRDR-ADFLWEELRSRGH--
EVFIDREGIlETELITaRIRDMMQGSDVVVLVLGPNW----ITSPAC--
RMEMQFALeLNKRMVPAAFEDVGP---DLPPEIREI---
NYVRFYGEDgeWSQAIERLDSALDRD(IEWIRDHTRFGEQAARFLAGVGAPPRGAELRAMRRWIERNPRNAPG
PTRDHWRYFKAGMSRRKWFQIGSAVATTVAI)}*

$3759=211(220):
>gi|1103712975|ref|WP_071675593.1|
{|0(560)|<Proteobacteria(B)>}hypothetical protein [Nioella nitratireducens]
{(MANRS)--RIFISYSHRDRDRADFLWESLLARGYD-
VSIDREGILETELITTRIRDMIHEADVIVLVLGPNWL-TSPACRqEMQVSLEMN-----
KRVVPAAFEDVGpDLPDEIRDINYVRFYGRNRDWDQALD--
RLEASLDRDIAW(IRDHTRFGEQAGRYLSGIGAPPRGRELRAIRRWIERHPRGAPDPTTDHWRYFKAGMRRRK
WQQITGSILATVAVC)}*

$3760=276(276):

>gi|1119391652|ref|WP_072367585.1| {|46(0)|<Firmicutes(B)>}signal transduction protein [Bacillus sp. ru9509.4]
>gi|1104029072|emb|SFX36840.1| Putative DNA-binding domain-containing protein [Bacillus s
{(IILGIKDDGITLRIKKFAFNLNIENILDLLDGTVKIEYNSFTFEDYNLFYISIDKSDELVKVNNIPFKINKDGDLEEMKI)-KKVFISYTHKdSDLVNILEEEL---
KKYEDIKITRDINVTKYRDSLdEFMKTIRDHDFVISVVSSAYIMSLNCMYeVMHLMQDKDYQ-
EKLFFIIVSEEDI-DYYDEKNGydgfeaKIYDVMNRLkyVTHWRDKK-----
AELERSINEA(SLSPELMVNLAVDMRKINSVIPPMDDFINMLSDKVGRSFKEMYEDDFKEILDKINR)}*

$3761=175(174):
>gi|1119429672|ref|WP_072404904.1|
{|0(145)|<Proteobacteria(B)>}hypothetical protein [Pantoea ananatis]
>gi|1104041576|emb|SFY16392.1| TIR domain-containing protein [Pantoea ananatis]
{()MSNIFISFASEdRDLAQQIAQHLMQYDHRIYLDVSDE---
LAQKGIKnALTRQIKNSDVFLSLVTKNSIKSEF-FKiEFDIAssLRNLEN-KNSLIVSVIFDRD-
IFHNMGIRAGLALYADEndLNKTVEDIIAEIEVYYDRQSAES(EKLELREKVLKEESSDYINETVDDLKARETRGRI)}*

$3762=221(220):
>gi|446443303|ref|WP_000521158.1| {|167(0)|<Firmicutes(B)>}molecular chaperone Tir [Streptococcus mitis] >gi|383340966|gb|EID19242.1| TIR-like domain protein [Streptococcus mitis SK616]
{(LSKYDSKKVHNFFYAINGHVRNQSLKKEVQSYFQSLSLPFRLSRKKKGKTNFIDNEIVTNRKGPEELKKRLYINDLEKVK)KFDIFLSHNS-QDEDKIIT--
FYKKLNKDGQVVYIDwvnDKYDLKREWCntSTVDIIKErikqSDVFVLYFSSKTLNSQWCAWELGYADAIGKkiciyfsEKINKSIIPKFY------FKYPKLIFSEDFMVK-NHGQSDIQFTKWKE-----NKK()}*

$3763=171(192):
>gi|494237227|ref|WP_007141756.1|
{|0(35)|<Euryarchaeota(A)>}hypothetical protein [Halobiforma lacisalsi]
>gi|445781627|gb|EMA32479.1| hypothetical protein C445_10197 [Halobiforma lacisalsi AJ5] >gi|1
{(MPGEQLYVSHAPGDVE)---------------LVQELFATVKNFPI--
GVHVALEEVESGRSRkRLEGRIANSDVVVAVLT--------
ERSehNQWIHQEIGYARaKGIPIVPMYDSEKRRAGFVDDAEGVAIDRENPSVTVFNLLSRLRSELAPLGALS(VPNWYVQFPCTVPNCEQPVVLEIDRDQTKLWKLHRN)}*

$3764=176(186):
>gi|496043096|ref|WP_008767603.1|
{|0(237)|<Bacteroidetes(B)>}MULTISPECIES: hypothetical protein [Bacteroides] >gi|382983500|gb|EES68945.2| hypothetical protein BSIG_1270 [Bacteroides sp. 1_1_6] >gi|5
{(MDH)--EVFISYSS-ANIQT--
AQAICHALESNRIKCWMAPRDIRPGAEYgdIIEEAIVTCKVFLIVFSETSQISRWVRSELNIGFSSN-----
KPIIP--
FRIDPTDLKGSMKLMLNDKHWLNAYPNPEEKFSELAAVILDLLQH(PAVDTISPPGFQPASNVASADGSNAGAQGLFGKLLNQLFVKRR)}*

$3765=195(200):
>gi|496030361|ref|WP_008754868.1| {|0(262)|<Firmicutes(B)>}hypothetical protein [Lachnoanaerobaculum saburreum] >gi|383303305|gb|EIC94763.1| hypothetical protein HMPREF9970_1875 [Lachnoanaerobaculum s

{(MNYDEYIERMKDNKYKELVVTKSGEDKPY)---VFISYRS-DSWKKVLTEIVYKLQKEYGLRIYFDKDFASETNTWveQFQKNMNSpfCKAFICFFDEGYVTSYATLIELMHAMNPK-----SKLKDSIFSINFNIDWKKLDDNGNNTGLGVEQDDNPGWEKEKEAFEYEFKLI(KDNDEYKAVEAYYKRNDELRVCDCKDIMNII)}*

$3766=251(256):
>gi|1107046132|ref|WP_071879726.1| {|863(15)|<Cyanobacteria(B)>}hypothetical protein [Microcystis aeruginosa]
{(IPGLLKALKDSNKYVRSQAAEALGNIGSEAAMTELIKCLKNPDFVTLNNGDTLSPVREALDTIQNKLKYYHPISQPMNQQ)---VYISYNW-QEDSNEMANQLVQAFAAKGIEIIRDKTHTSYKD-SikNFMQQIGQGKCVVVVISDRYLKSENCMFELVEIARNGDF-Y-QRIFPIILPDARIYNDFERIDYLKYWEDEKAKLQAKYKQIDLAKTNSIMATL(NLYNEIRSNIDNLTNILKDMNNLDINLHGQSEFAAM)}*

$3767=243(245):
>gi|1180097185|ref|WP_083616913.1| {|0(310)|<Cyanobacteria(B)>}hypothetical protein [Planktothrix serta] >gi|1108765824|emb|CUR15672.1| TIR protein [Planktothrix serta PCC 8927]
{(MSFIASTIANVVSTIINSWVFKQGHTDESKRLIGRKKEIPNAR)---VFISDRsQEPDLSLAEKLYESLTKAKY--QVFMAHKSLKVGdKWPqRLDEEINACDYFLVIFSPQSANSEMIMEevRR--AKELRDGRkNRKPVIlPILVNCEM---NSLNHDLRGYLYgIQYLKWRSPDDTSPILQQILEKLLE(QGDTTSLEATVEALPLIEPIQINPDTRPSPAAEPELPPGKPTDIYIERPPIEQQCYDAILNP)}*

$3768=229(225):
>gi|1108370434|emb|CUM59808.1| {|195(0)|<Cyanobacteria(B)>}WD-40 repeat protein [Planktothrix agardhii]
{(STNSVFTKHLIAGLKGGITSNDGLIRIFDIFEYLQPKVTADQPNQHPIFKSDIEENFPLTLYLGGQKGVSPISPSVQEEF)RYDVYISYVDEdpdsTWVWDVLVPKLEAENL--KVAVSGDVDLLGVARVINIERGVKFSKRTLVILSNLYLDNSMAEFENVLVQNLGIREWKARLVCVRKEDVDpSKLPERLQLEKPRKLDDsRRGERDFKHLIDELKKPIPGHTA(SGLYE)}*

$3769=194(195):
>gi|1108370435|emb|CUM59809.1| {|0(1122)|<Cyanobacteria(B)>}putative WD-40 repeat protein [Planktothrix agardhii]
{(MSK)KYSVFVSYADEddAWVEGYLLNALEEAGL--T-CHSEAMFRLGVPRLIEFESAVKNSERTLLVISPAYLADNFVEFANLLFQSYGVDATLWPVVPIILNPV-QELPMRLEMLGCLDATDaTKQQEVIQRICEDFKKPVPTLTT(IPDCPYPGMVPFKQEDSDRFFGRQREVNDLIQHLRLYPFLAVIGASGSGKSS)}*

$3770=229(225):
>gi|1160835180|ref|WP_079680959.1| {|195(0)|<Cyanobacteria(B)>}hypothetical protein [Planktothrix sp. PCC 11201] >gi|1152215638|emb|SKB14892.1| WD-40 repeat protein [Planktothrix sp. PCC 11201]
{(STNSVFTKHLIAGLKGGITSNDGLIRIFDIFEYLQPKVTGDQPNQHPIFKSDIEENFPLTLYLGGQKGVSPVSPSVQEEF)RYDVYISYvdEEPdsTWVWDVLVPKLEAENL--KVAVSGDVDLLGVARVINIERGVKFSKRTLVILSNLYLDNSMAEFENVLVQNLGIREWKARLVCVRKEDVDpSKLPERLQLEKPRKLDDsRRGERDFKHLIDELKKPLPGHTA(SGLYE)}*

$3771=194(195):

>gi|1160835181|ref|WP_079680960.1|
{|0(1113)|<Cyanobacteria(B)>}hypothetical protein [Planktothrix sp. PCC 11201] >gi|1152215639|emb|SKB14893.1| putative WD-repeat protein [Planktothrix sp. PCC 11201]
{(MSK)KYSVFVSYADEddAWVEGYLLNALEEAGL--T-
CHSEAMFRLGVPRLIEFESAVKNSERTLLVISPAYLADNFVEFANLLFQSYGLDTNLWPVVGIILHPV-
QELPMRLKMLECLDATDgKKQQKAIQRICENFQKSVPTLVE(IPDCPYPGMVPFKQEDSDRFFGRQREVNDLI
QHLRLYPFLAVIGASGSGKSS)}*

$3772=225(221):
>gi|1180106976|ref|WP_083626519.1|
{|195(0)|<Cyanobacteria(B)>}hypothetical protein [Planktothrix serta] >gi|1108762960|emb|CUR24229.1| WD-40 repeat protein [Planktothrix serta PCC 8927]
{(SNNSVFTQHLIAGLKGGITSNDGLIRIFDIFEYLQPKVTADQPNQHPIFKSDIEENFPLTLYLGGQKGVSP
VSPQAQEEF)RYDVYISYvdEEPdsTWVWDFLVPKLEAENL--
KVAVSGDVDLLGVARVINIERGVKFSKRTLVILSNLYLDNNMAEFENVLVQNLGIKEGKARLVCSQKEDLDlS
KLPERLQLEKPIDLNHpRLFKKLVKQLKDPLPGRTASGLY(E)}*

$3773=194(195):
>gi|1119824708|ref|WP_072717248.1|
{|0(1134)|<Cyanobacteria(B)>}hypothetical protein [Planktothrix tepida] >gi|1108768419|emb|CUR36136.1| putative WD-40 repeat protein [Planktothrix tepida PCC 9214]
{(MSK)QYSVFVSYADEdgAWVEGYLLNALEEAGL--T-
CHSEAMFRLGVPRLIEFESAVKNSERTLLVISPAYLNDNFAEFVNLLSQIYGRHTDIWPTVVIILHPV-
QELPMRLEMLGYLDATDaTKQQEVIQRICEDFKKPVPTLAT(IPDCPYPGMVPFKPEDSDRFFGRQQEVNDII
QQLRLFPFLAVIGASGSGKSS)}*

$3774=229(225):
>gi|1119824709|ref|WP_072717249.1|
{|195(0)|<Cyanobacteria(B)>}hypothetical protein [Planktothrix tepida] >gi|1108768420|emb|CUR36137.1| WD-40 repeat protein [Planktothrix tepida PCC 9214]
{(ANNSVFTQHLIAGLKGGITSNDGLIRIFDIFEYLQPKVTAEQPNQHPIFKSDIEENFPLTLYLGGQKGVSP
VSPQPQEEF)RYDVYISYvdEEPdsTWVWDFLVPKLEAENL--
KVAVSGDVDLLGVARVINIERGVKFSKRTLVILSNLYLDNSMAEFENVLVQNLGIKEKKARLIYTQKEDVDlS
KLPERLQLEKPRKLDDsRRGERNLQNLIEQLKQPLYHRTE(SGLYE)}*

$3775=191(223):
>gi|1180106973|ref|WP_083626516.1|
{|0(1124)|<Cyanobacteria(B)>}hypothetical protein [Planktothrix serta] >gi|1108762959|emb|CUR24228.1| putative Histone acetyltransferase [Planktothrix serta PCC 8927
{(MSKK)-YSVFVSYADEdgAWVEGYLLNALEEAGLT---
CHSEAMFRLGVPRLIEFESAVKNSERTLLVISPAYLADNFAEFANLLSQYYGFDTHLWPVVGIILHPV-
QELPLRLKIL----------------------------
E(CLDATDATKQQKVIQRICEAFQKPAPSSVEIPDCPYPGMVPFKPEDSDRFFGRQREVNDLIQHLRLYPFLA
VIGASGSG)}*

$3776=303(300):
>gi|1108479788|emb|CRK92032.1|
{|959(107)|<Arthropoda(M)>}CLUMA_CG005612, isoform A [Clunio marinus]

{(REIDFNSSSACSDYYADSSSGLPNLILTNGYIPLMITLLATICLLVLFAILFVFREPLRLWLFAHYGVRVFGPPCEDSDK)LYDAVFLHSAKdtEYIIKHIATELENGngRPPmFRLCLQHRDLAEDASYIQ-LLETICASRKIVILLTRNFLQTEWSRYDVKRAIHESLRGRPQKLVIIEDPDV-LLDAESDIELLPYMKTSavKRIRRTDRQ--FWGKLRDALPVE(PLFRGNNYTLDHQRALPHYQSHTMPHHMVMHHERFKHANGNGTMNGGMMFHHHRQAPPPAYISSAPEMDDTNYSSATTAT)}*

$3777=222(240):
>gi|1108472977|emb|CRK98857.1| {|647(0)|<Arthropoda(M)>}CLUMA_CG012345, isoform A [Clunio marinus]
{(REIVKDFNIIRCSNSKYFLEVEVDSSCAPSVLIAIFATLGLVSVAISIYGIRKNAEVISEWTFRNDKLHLIERMNERMKF)-FDAIIIATEYdkIF-GKYVTAKLMNKPNQFKIGMVIKDWAENEEIPDHVLKTVRSSRRVIVILSEYFEENNWKRWNF--------YRTKSRIIFVLKGKS-NVSNI-------EISNKiSVNFSDPW---FWDKLKHAMNNC(KELEIDNQQNSVELQSLVSV)}*

$3778=260(264):
>gi|1108484963|emb|CRK86691.1| {|1031(0)|<Arthropoda(M)>}CLUMA_CG000524, isoform A [Clunio marinus]
{(PNQTRGEYFNIMNETNFCSEKLALSNMFFFEKNLPIFLIVFLSCLFFLIIIFVFHQRIMFCCYKKFNLRFCKKRNDKNKF)-FDGFLIFSKKdeNFLIDDFLKHFETTEGNfYKFFLFFHDFSI---FNEQILKNCENSQKIVLFLSENFLKNEWRKIEFKKIFYEILKTKKRKKLIIVFLDN-FDNKNFDPELKLILKTNfILRWSEKL---FWEKFKFFLPSL(SRNAQLQHQPQAAPRDPENFCQNFHLNNLHNNQNQQRNLTSIHI)}*

$3779=208(221):
>gi|1110744628|ref|WP_071969786.1|
{|0(621)|<Proteobacteria(B)>}hypothetical protein [Sulfitobacter sp. AM1-D1] >gi|1108550672|gb|APE42294.1| hypothetical protein BOO69_01845 [Sulfitobacter sp. AM1-D1
{(MADRI)--RIFIAHSPEDRHGTDRLrGWLATAAEMD-VTGETSDAGPDGD----ADRLIHACDTVVVAMTPRSTMSDHCARdLALAATLNK------RIVTVMLEHHGgRLPGPIATAVPIPATP--HhDPEQART--ALLAAIRADQGW(VTTHRALATRAEDWEKASLARASLLRGRALREAETWLADQPDRAPPPTALHRRFILASREAAGRRQRRITGALIAA)}*

$3780=229(228):
>gi|1180102518|ref|WP_083622246.1|
{|26(396)|<Cyanobacteria(B)>}molecular chaperone Tir [Planktothrix serta] >gi|1108765266|emb|CUR17420.1| conserved hypothetical protein [Planktothrix serta PCC 8927]
{(REVFIGSWEGKSYPEIYPLNPSYIEKYVGYKLWQKISQTFGEKVTKKTIRGAVERYQNQDNFVQLSEIPRELLQLSPHCY)--RVFISHgnTELNIHFAQQLEAVLIQVGHS-VFLAAWEVtrsqlnQQETDFLAEIDTELKHCDYFILLLSPQSTLSEMVIEeLRQIRELQD-----THPHRLPIIIPIwvNCPANLALNHDLRSYL--LGTVQRTWTGQQDTTLIIQEIL(VFLQESIP)}*

$3781=261(254):
>gi|1119827411|ref|WP_072719788.1|
{|26(364)|<Cyanobacteria(B)>}molecular chaperone Tir [Planktothrix tepida] >gi|1108771459|emb|CUR33145.1| conserved hypothetical protein [Planktothrix tepida PCC 921
{(REVFLGSWDGKSYLEIYPLNPTYIEKYVGYKLWQKISQALGDKVTKKTIRGAVERYQQKENIIQSSQISGELLQLSTPVY)--RVFVShgYAELNLRLAQQLEAVLTQVGNS-VFLAAWEVirsplnQQDSDFLAQIDKELKQCDAFILLLSPQSSHSEMvieelRQVrELQDTHPHRL----

PKLIPIWVNCPAnlPLNHDLRSYLWGTMQY--
TWSGQQDTVLLLQKILSVFQEL(IHESPPQYSPLDWDKSELENQPNLVIQSHKSPSI)}*

$3782=176(176):
>gi|1109051193|ref|XP_019090082.1| {|0(211)|<Streptophyta(V)>}PREDICTED: disease resistance protein RPS6-like [Camelina sativa]
{(MDLSSRNW)VYDVFPSFSG-
EDVRVTFLSHFLKELDRKLISVFTDGEIKRSQSLDpELTQAIRDSRIAVVVFSKNYASSSWCLNELSEIVKCR
EEFGQNSDSCFQRFGSFSLVLDALASRTPWFGCGSRIIVVTQDKQFLRAHGIDLIYQ(VGLPSDKLALEMLCR
SAFKQKTPPDGFT)}*

$3783=219(237):
>gi|1109033485|ref|XP_019087025.1| {|0(239)|<Streptophyta(V)>}PREDICTED: putative disease resistance protein At4g11170 [Camelina sativa]
{(MSSSSSSTSFLLLGREV)--DVFLSFCC-QTRHGYFQNILITY----GIKTFLSYRCLETRfgP-
IsqRTVKALEEARVAVVMTSGTKPCSIGFLEELLVILEFQEK-GSLIVIPIFLTDSSFNA------------
EEICQQYPEKA-
PSWRTALTKLTNI(AAEYQFSQNPTGMGLLEWFKKIAHDISLVSLYSTSNGLDGLVAMDRHMKVAYDLLDLEV
DKEVRTIGIWGSGGVGKTKLA)}*

$3784=283(290):
>gi|1109079418|ref|XP_010471325.2|
{|39(147)|<Streptophyta(V)>}PREDICTED: disease resistance-like protein CSA1 [Camelina sativa]
{(HPKERIFGKSLLVKTEYQRKVDLTSLAVIVFVHGRSADHVIYLIERAYTPKNKSETWSATNFDKIVIHPLC
EKGKSFVIM)RYDVFLSFSG-EYIRKTFLSHLLCSLDRKGIRA------ATSAE-
SypMYNQAIEQCLVAISLISENYTSLDLWVDELAKIIKCAED-RNLTAFPVFLQVHPSDVLRVV-
SLAEDFADRNGPRiiILETV-
KNWLCDHLIRNSG(FDSRHWEDDSKLVDKITNFVADTLMSSNISSPSPSLKWATPKLPSSSGRLKDIGTKKKG
FVHHSKLSGAT)}*

$3785=191(213):
>gi|1109051110|ref|XP_019090058.1| {|0(247)|<Streptophyta(V)>}PREDICTED: probable WRKY transcription factor 16 [Camelina sativa]
{(MSRSSFLVDLYGAESKNPILNSSVNIICEETA)--------------
RYSFVSHLSAALRREGISisVFADTDLDY---------
RNQGATLSVVVFSESNAFSLTWVDNFAKVLELRRN-
NGHKVTPVFYGVDPSGTNQGCWRHRRDISLDTSSLIFDVNPDAFKKMVSLRFLK(VYNSYSENAPRLNFPKGL
NSLPCELRLLHWENYPFEYLPQG)}*

$3786=228(231):
>gi|1109226247|ref|XP_019187760.1| {|0(991)|<Streptophyta(V)>}PREDICTED: disease resistance protein RML1A-like [Ipomoea nil]
{(MDGEQLQSPPSS)-YHVFLSFTSQEESVNTFTDHLYTALEQaG-FSCFRQEDGGTDG-----
QKAIQESKVYIVVLSENYARSRSCLDELAIILERKRG-FGHycAVLPVFYRVDPSDFRKLRGKIGEALNWI-
EENGGRQwKEnvKKWKQALMEVADV(GGMILHNQANGHESKFIQKILRVVENKLSRPALSTSNKLIGIHRRTE
NINSWLQDRSTDVDILVICGMGGIGKTTVAKY)}*

$3787=194(222):
>gi|1109256031|ref|XP_019151936.1| {|0(825)|<Streptophyta(V)>}PREDICTED: TMV resistance protein N-like [Ipomoea nil]
{(MQ)--------------------------HRG-
IYTFKDDEKLERGKSIspALEKAIEESSMAVVVFSEHYADSTWCLEELVKIMEVK----
GQMVVPIFYGVDPSTVRKQKEKYGEAFEGH-

EKRFEKEGEkvKKWRKVLDDVSNL(SGWDQNNTENGHEAKFIQKIVEDIVTKLGSMRSIDDPKLVRMEPRIQK
LYPLIGMGSSDVRFVGIHGMSGIGKTTIARAM)}*

$3788=225(226):
>gi|1109154804|ref|XP_019084318.1| {|125(0)|<Streptophyta(V)>}PREDICTED: vesicle-associated protein 1-4-like [Camelina sativa]
{(FDYNLLRLEHRERKKARQINKKAWEDAEERFDGWRRALVSVASRLGLTYKKNSNQATFVNEIVDKVKAMLD
NVSSSDIVP)QHQVYICYRG-
QEMRSKFSSFLRAALQKSGINVLADDEEDITRiesrdELDNFFVKIQESRIVLMIITKNHTSSAWFLEEMLEM
NKRAEM-KMLRVIPIFYEMNPNDMiphDKLMELFGST-------
EDGIKGYEEYKDALDALTSF(ISTQHK)}*

$3789=178(189):
>gi|1109038884|ref|XP_019088163.1| {|0(988)|<Streptophyta(V)>}PREDICTED: LOW QUALITY PROTEIN: probable WRKY transcription factor 16 [Camelina sativa]
{(MAESEQI)---VYISCV--EEVRYSFVSHLSAALRRKGIhDLFIDSDDLLSDESK---
SKIERAKVSVMILPSNRT---VCLDKFVKVLECRTN-
KDQAVVPVLYGVRSIENLWVSALDSKGFSLVHQTRKECSDSQLVKEIVRDVYEK(LFSMERIGIYPKLLEIEN
MVSKQPFGIRCVGIWGMPGIGKTT)}*

$3790=203(202):
>gi|1109090255|ref|XP_019095914.1| {<Streptophyta(V)>}PREDICTED: vesicle-associated protein 1-4-like [Camelina sativa]
{(MSALFPPLLYFRFTQLLYICCISGQSKDHQTQNNIISSFEPLMSELDRLLG)RSQVFVSFCK-
DELGDNFVMHLVWALRDSGINVFTDTYNPREKQQQQqvLTSSIEKSNIALAIFSKRYSESNWCLNELAKMNKL
AKE-GKLVVIHVFYNVKPNEVRKLQGEFGIHFRDTKERF-
AMEPMmvQSWEESLKSPIMT(GRIDMSLEAHM)}*

$3791=235(236):
>gi|1109103251|ref|XP_010495687.2| {|0(8)|<Streptophyta(V)>}PREDICTED: protein PHLOEM PROTEIN 2-LIKE A8-like [Camelina sativa]
{(MVALTNHAQPNAQIRCG)-PQVFISFRG-
DDVRNDLVKDLVNNLKSKRINVFIDTFLPGGRGLQELFSKIDMSKIAIVILSKKYPDSCWCMEELVKIDKRMK
E-
GKLIVIPVFYKVTTSDVKNLRGDFGKSFDKLKKCHAMGEPRkvKQWESSVKSIANI(KGLPGRTVPPLVGQIV
HAVEVCLNEMPAKDVKAVFTVIWLGVYLVVSFLSFLSYMIFNDLKPFKSGKWMLGYPTMAAIL)}*

$3792=233(233):
>gi|1109103369|ref|XP_019097270.1| {|218(0)|<Streptophyta(V)>}PREDICTED: uncharacterized protein LOC104771740 isoform X2 [Camelina sativa]
{(HKIVELPNKIIAPRIPSSPQPPITTPLFPLKYSTGSKNLEGERHVQTSDMDTRLSEKPQQPYPSRISKVAS
SSQAVLQRS)RSYIYVSFHE-
GELRKSFVNLLLRTLRERGFIVLTNNYKGNGtRQLEQLYKRIGESKIALAIFSKRYVESNTCLNELVMMDKLA
KE-GKLLVIPVFFDVRSSDVKNLEGEFGRHFKKMSET-
YKDEPEkvQKWKVSVTSITKT(IDKHSELYGFQRR)}*

$3793=277(271):
>gi|1109105048|ref|XP_019097648.1| {|107(0)|<Streptophyta(V)>}PREDICTED: disease resistance-like protein CSA1 [Camelina sativa]
{(ALISIPKKVGLTLAGHRDESELVESIVKEVKKVLIEMSSKERDYFPSSSNRKGQSKDHQTQNNIISSFEPL
MSELDRLPG)RSQVFVSFCK-
DELGDNFVMHLVWALRDSdsGINVFTDTCNPREKQQQQqqqevLTSSIEKSNIALAIFSKRYPGSNGCLNELV
KMNKLAKE-GKLVVIPVFYNVKTNEVRKLEGEFGIHFRDTKERFS-

MEPMmvQSWEESLKSPIMT(GRIDLTLEAHINEFALVGAIVREVSRLLPNSPRRRRRRKLGIREVLYSSGL)}
*

$3794=193(218):
>gi|1109105048|ref|XP_019097648.1| {|0(191)|<Streptophyta(V)>}PREDICTED: disease resistance-like protein CSA1 [Camelina sativa]
{()------------------------
MRDAKINVFTDEIEVKGRDLHNLFRRIEESRVAVAILSERYTESSWCLDELVKIKERMDQ-
ENLVVIPIFYRLDATNCKRLLGAFGDNFRNLEREYRSEPERiKKWKEALISIPKK(VGLTLAGHRDESELVES
IVKEVKKVLIEMSSKERDYFPSSSNRKGQSKDHQTQNNIISSFEPLMSELDRLPGRSQVFV)}*

$3795=211(220):
>gi|1108889245|ref|XP_019138598.1| {|303(0)|<Chordata(M)>}interleukin-18 receptor 1 isoform X2 [Corvus cornix cornix]
{(QNFTCMLQADESTQIKIVKLKKGKTQDLPVHIFTTGMVLALLFPFVVVAVVFVFVMFRVDFVLFYRDTCRR
DDTAGDGKE)-
YDAFVSYLKDcaspieeeREFALKILPMILEENFGYKLCIFERDVFPGGAFVDDINSFIDKSRRLIIILSQNY
VSDRAIY-ELESGLHKALVERKTKIILIEYMPI---------------
VMNAGDLQKGWRMEGTLRLLVMLQSN()}*

$3796=229(210):
>gi|1120156848|ref|WP_073026820.1| {|0(95)|<Firmicutes(B)>}hypothetical protein [Lutispora thermophila] >gi|1109923474|emb|SHJ22568.1| SEFIR domain-containing protein [Lutispora thermophila DSM 19022]
{(MSKVH)-PTVFISYSW-
DsEEHENWVAFLAAKLRENGVDATIDKFEAQSKTVnlnRMMIEKIKDSDNIILILTENYAkkadtfqggvgyE
TNLLIRYINdnpdKIILIMrhKG-NYRKAIPFylegFYFIDFSD----
DGQFDIKFNELLHRIFQVDIIDVPKVGVKPKLEP(KRITYDRITNETSNIIPNFKEITDLDKNKFMKRSYSEI
LSHLSDFAEQTRQKNSNFDYEKDIIHS)}*

$3797=223(228):
>gi|1120504671|ref|WP_073365130.1|
{|76(0)|<Bacteroidetes(B)>}hypothetical protein [Flavobacterium fontis]
>gi|1109816026|emb|SHF76694.1| TIR domain-containing protein [Flavobacterium fontis]
{(EGEKESLRIVDIPPLGGKDFCVQIKLPEDIERQTLDMRIELWSPARRTWSRSSIDTIALFYRSNWGNGIDI
IKKEDLIAT)---
VFISYSWFSDEHVKWVSKLAQELARRQIKATLDQkDLELGQPITKFMEKGIKSSICICVCSEAYTKKANDESS
YGGVEYEI---
SILSNQVLKGRLRSTIIPIIKDNPEKLLPDFLGSAKYINMDTEQWDGIPLTELA(RTIIKLSS)}*

$3798=234(239):
>gi|1120261173|ref|WP_073127574.1| {|72(49)|<Firmicutes(B)>}hypothetical protein [Asaccharospora irregularis] >gi|1109744346|emb|SHH42935.1| TIR domain-containing protein [Asaccharospora irregularis D
{(DLPVEKDKEIAFIYQLLKYGQEVFGDYTAFSNGYGSGTKFQDRMDAFNNQVVKHLVDYIREYLEDLIIDSD
DHLLNYESQ)EKRIFISYSWAnKDIADLIDEDFLKLG-------YTlTRDERDLKYKDsikeFM-
QQVGKHDFVIMIISDSYLKSENCMYEVMEVTRDREY-KKKILMIVLRDEDKKFYKDYEKLMVESEEFR--
NLKVGAEIYSISGRISYTEYW(EKREIETTEEISRIQTDIN)}*

$3799=218(222):
>gi|1120189746|ref|WP_073057968.1|
{|0(316)|<Fibrobacteres(B)>}hypothetical protein [Fibrobacter sp. UWB8]

>gi|1109901126|emb|SHG56767.1| TIR domain-containing protein [Fibrobacter sp. UWB8]
{(MD)EKKVFISYSRDdKEIVFPLVERLKK---DVGDVFWIDLD--
GVECGAqyekIIINAIDKAEIVLLMYSDNIINSKWVEKEATYSEDSEKK-LVPVIING--
DKMKGWPkfrYASIDSINIKDSNQ--YKKLVRD--
LIKWLGLNKEQK(NGSISTSTGTSHSIARNQNDKYIYITKKPHSQCNRSDSISYSTSYGFNNPQKSTSKTIYTTSNNENKQSNQFNSSSDSIK)}*

$3800=264(269):
>gi|1120248535|ref|WP_073115345.1|
{|49(32)|<Fibrobacteres(B)>}hypothetical protein [Fibrobacter sp. UWCM]
>gi|1110018567|emb|SHH62282.1| TIR domain-containing protein [Fibrobacter sp. UWCM]
{(DYDDYDGGIYYHKLILTVPFETYKLIGISSKTIENTIQQDINDIGKISGEEICCVSILVNEASFPNEKKNVDESMWEEGF)--RVFISHKVEdKSKATELKKSLAKYGITA------
FVAHEDIDTNKewlaTIENALLSMDAFVALITKGYNEKFWTQQEIGFAYCMNKlK-
GTPFVSIRMGDDPKGFFGHVQAYSPHTDSYA--
DSLCEQWINHPRMIDSLIVAL(KKSECFSDSARYFELLKRTTNITELQIKNLIAVYNKNESVYRCFKMNGQ)}*

$3801=208(220):
>gi|1119938671|ref|WP_072820411.1|
{|0(1227)|<Proteobacteria(B)>}hypothetical protein [Halomonas ilicicola]
>gi|1110037725|emb|SHE74737.1| WD40 repeat [Halomonas ilicicola DSM 19980]
{(MSTL)----FISHSSADAQAAAELTAWLERQGHH-
SLFLDFDvehgIPAGHDWERELYRRLRTCQAMIVLCSPAAMA-SCWCLaELSHARALG-----
KRILPVRIAPCT-LRPQLAELQVIDLV---KEPETGYT--
RLHRALASVFAW(DSRRPPYPGLMAFDEQDAAIFFGRDDEIQQALDRLHQLRRFGGPRLLMLLGASGSGKSSLVRAGLLPRLRAAPESW)}*

$3802=172(188):
>gi|1120252188|ref|WP_073118627.1|
{|0(412)|<Bacteroidetes(B)>}hypothetical protein [Reichenbachiella agariperforans] >gi|1110299415|emb|SHJ46653.1| WD domain-containing protein, G-beta repeat-contain
{(MS)--DIFISYSRED-KLLARRLGQAIRSVGW--SVWY--EQVMDGESRdEA-RKYAKAQIVI------
WSEASVSD--DVVHREVQEAYdR-KQLLAVAFDATTP-
PDSLITRHLFHLGDWTDDNDKAFQILVRELEELAGPPR(KTIRWKSYLIAAASIIGLALLITGVVLVKRMNDENMADHLAWQQAY)}*

$3803=199(187):
>gi|1120348726|ref|WP_073212912.1| {|0(3)|<Proteobacteria(B)>}TIR domain containing protein [Massilia sp. CF038] >gi|1110413298|emb|SHG53275.1| TIR domain-containing protein [Massilia sp. CF038]
{(MAIRYGCFFSY)AHGRYELMN---
KFKAALSDAIRCYLEPYfdnEDELFIDTEQLGGGDDIdrRIARALCESVSMILIYTPKYEAHGYTRREYAAMQMLEQERgqwypmPSHLIIPVIMTMHPgrlpPQITNSSLYVDFSHFTMATSDLKSNPDFLPDIKKIVERIV(IHYQYQKRYTPVGHDCNQFVLPDVPPEWRDLPDLTF)}*

$3804=217(220):
>gi|1120429514|ref|WP_073292201.1|
{|96(0)|<Bacteroidetes(B)>}hypothetical protein [Chryseobacterium

polytrichastri] >gi|1110363508|emb|SHK84408.1| TIR domain-containing protein [Chryseobacterium poly
{(DQVQSYEKIISNALDAQSKTRRFNCVIQMVQTLQDQIKILESVLILIAILKTKDLYLLEERVINKTMSAVSKNSNTIESD)EYDVFISHAS---EDKGtFVETFCEHLSAENIKFWYDSNEIG--WGEslvRKINQ-GLSKSKysIIVLSKSFVDKKWTNKELEAVLNIETNTGDVRVLPLMLGNSdEikS-ILDQYPLLSTKRYlKV-SDGISAIV-ENLKK----VLSK()}*

$3805=258(272):
>gi|1120383744|ref|WP_073247480.1|
{|102(35)|<Bacteroidetes(B)>}hypothetical protein [Flavobacterium flevense] >gi|1110107921|emb|SHM21020.1| TIR domain-containing protein [Flavobacterium flevense]
{(RIFPISLCNKAYSIGCGLSTLQFTDGTKFFEKDLNLQNKKDLEKSIKKIKTELLHDCSRLILNFHPKQKDKEQHRIGSPV)--KLFLSHAKKdgENTTIKFKQFIEK-NLKLDVFF----DTVDIANGYdfaeQFEQEIKHSALVVF-HTDEYSNREWCRRE---ILIAKKH-KSPIVV------VHNIIDGEKRAFPYLGNMP--TTVIGKKEIIGFYKIVNLTLY(QVLNNLYQNRLLEWYSNMGTNDNNEIKVITSPPELFNFLDIKKYKENLGSKK)}*

$3806=169(171):
>gi|1120325708|ref|WP_073191067.1|
{|0(295)|<Actinobacteria(B)>}hypothetical protein [Tessaracoccus bendigoensis] >gi|1110155081|emb|SHJ89838.1| TIR domain-containing protein [Tessaracoccus bendigoens
{(MDSSA)---VFVSYRHGmEG--DRLVSRVAALLRCSGFRPWIDNRDVGAGYIEqRITEALDNCEAGVLVVTDDLVNSDYVKLkELPHMIRQVAK-KRLPMMVVNTYRD-PETGRVDIRKPDEIIDSvtSIPLIDITQADVQSGQGQEEFVY(GLLRRRGERWVKSHTTHLTLFIQTDP)}*

$3807=150(155):
>gi|1120324414|ref|WP_073189773.1|
{|0(883)|<Actinobacteria(B)>}hypothetical protein [Tessaracoccus bendigoensis] >gi|1110155612|emb|SHJ67239.1| TIR domain-containing protein [Tessaracoccus bendigoens
{(MRPFDPGREGRFA)----FISHSHAdRGTGFEF---LTPLAREG-FQLWYDEGLTPTSDWErELRTTIAGSALFILLVSGVSVHRDEVVKeTLIAREAGVPI-LALHLGATPLPAD-IGFLGAVQGIEVVGRTYddVLGKVRDQLEAHQVTKLTSETPS(DE)}*

$3808=233(223):
>gi|1120101860|ref|WP_072972667.1|
{|1011(0)|<Bacteroidetes(B)>}hypothetical protein [Flavobacterium saccharophilum] >gi|1110315318|emb|SHM11471.1| internalin A [Flavobacterium saccharophilum]
{(YPLVSPDDLYISTDNKFFVKHKDLQNEDKTFSKIISFGLSIKKEVQNDKEIEIRRLNKNLVVEKSTGLFKNFTSNKNTEK)MKKIFISYSKFdDDYKREFVKHLITLKDENLIDPFNCDEIDLGDDSHeVIQRKLAECDYMVALVSVDLLNTKYIRDfEVDKAEEL-----GKKIIPIIIKPC-DWETSKLGKHHAslrgtnisldkklyLDGDIkeVSKI-ERA--AFWTAIIKEFREK(LFK)}*

$3809=194(195):
>gi|1120046959|ref|WP_072920895.1|
{|0(300)|<Actinobacteria(B)>}hypothetical protein [Geodermatophilus obscurus] >gi|1110350805|emb|SHN88467.1| TIR domain-containing protein [Geodermatophilus obscurus

{(MSFPTSSTPGHA)----FISYVR---
EDAEHVDRLQELLEAAGVAVWRDTENLWPGQDWriEIRRAITTGSLAFIACFSENSqaREQSYQNeELLLAVE
qLRRRApGKQWLFPVRFAEC-
ELPAFDLGAGRTLDDLQRSDLFGDRRDVTTTRLITAVVRL(LDSSPSTGNSQKAYADDPAGFMKTTLLDPKKR
IQLDDFVTTKV)}*

$3810=257(256):
>gi|1111059575|ref|XP_019236475.1| {|0(220)|<Streptophyta(V)>}PREDICTED:
TMV resistance protein N-like [Nicotiana attenuata]
{(MASSFASTSTSQFPHEVFLSSSSSTSTSTSQFPRWN)-YDVFLSFRG-
EDTRRTFTGHLFKCLENSGIFTFQDDKrLEHGSS-
IsdELLKAIEQSQVAVVVFSKNYATSRWCLDELVKIMECKDQ-CGQTCSSMMWN---
SHMFETRGRaLAKPLTNTETRYKDDdegmqKV-
QRWRNALTAAANL(KGYDIVDQISKLCNRATLSSLRDIVGTDTHLEKLKSLLKVGINNVRIILGIWGMGGVGK
TTIARAIFDNLSHQFEASCFL)}*

$3811=199(187):
>gi|1119895703|ref|WP_072780839.1| {|0(2)|<Proteobacteria(B)>}TIR domain
containing protein [Duganella sacchari] >gi|1110500157|emb|SHM34140.1|
TIR domain-containing protein [Duganella sacchari]
{(MTIRHGCFFSY)AHGQHAYMS---
KFKDDLVDALKCYLEPHfdnEDELFVDSEQLGGGDDLdeKIARALCESVCMIAIYTPKYEAHGYTRREFAAMQ
LIEYERkqwytlPSHLIIPVIMTRHPvglpPQIAGPGMYVDFSRYTLASGDLKTNPDFIPDIEKIVHRIA(EH
YHCLKRSTPPGHDCSRFVLPEIPPEWRVIPPPHF)}*

$3812=210(219):
>gi|1120977270|ref|WP_073484184.1|
{|0(88)|<Actinobacteria(B)>}hypothetical protein [Streptoalloteichus
hindustanus] >gi|1110515861|emb|SHF83125.1| hypothetical protein
SAMN05444320_105152 [Streptoall
{(MSEVAT)-YDVAVSFAE---EQRSTVAEVVEACRQRGLRVLHGPEQTHEWWahkgGGdlpE-------
ARVRffLPFVSA--------VDDFTADMLRAAKAGDEHVLPVLVDDVAvpTAlLHPHVAYLRVSGRRT-
DQLIETLI-
VRIEAAEAAGWER(APVTEVVTRAKGLAPVAVMPSTFSRYLEQDAVLRYLGEQFVAAVPGLSQRGLVGTVHIG
DSRITVRVERAGDT)}*

$3813=191(200):
>gi|1120401840|ref|WP_073265276.1|
{|0(611)|<Actinobacteria(B)>}hypothetical protein [Cryptosporangium
aurantiacum] >gi|1110574495|emb|SHN47430.1| TIR domain-containing protein
[Cryptosporangium auran
{(MPLT)---FLLSVAAADAVWASRITDVLDQLGYS------WRHVSGRN-
ELALNPSLDAVRAdapaiLIMALSPTYFTDSQtndlWPVaE-AMAGRGR-----LRLLPVKI---
GwtSLPPPLRDYPYLDLSL--
IRSPLTAVEQLRTELRRLGEAQ(PAPVADRRTGPLRVAVAGAGGELTDRVESAVRGLRAELVDRGTQNPPPAE
ADVVLL)}*

$3814=194(220):
>gi|1181446717|ref|WP_084742229.1| {|0(103)|<unknown(X)>}hypothetical
protein [Cryptosporangium aurantiacum] >gi|1110574793|emb|SHN47123.1|
GUN4-like [Cryptosporangium aurantiacum]
{(MR)------------------LAHHLRVHDLS-VWIDA-DIRWGTSFTRAINSHIRQALAILVVMSPEA-
EKSEWVErEILEGQRSD-----RQFLPILLR--GDRFFLLAATHYFDargdrLPG--RAEVEQLR--

ELSEKALTGAND(APTIVLPQAPADDLVRTPASGAALRRIEAHLAGGETAHADILTTALLLDSVGRLSHGWLR
QDDGDALPRALLDEVDAL)}*

$3815=175(175):
>gi|1120415342|ref|WP_073278448.1| {|0(671)|<Firmicutes(B)>}hypothetical protein [Anaerocolumna jejuensis] >gi|1110490887|emb|SHK99195.1| TIR domain-containing protein [Anaerocolumna jejuensis DSM 159
{()MARVFLSHSSTdKTY---VRKVAELLGYDNCICDEYEFEI--
GTKTInEVFKSLDKTDIFVYFISNDSLNSEWV-KiELNNAIEKlgiYSN-RIFQIYPLIIDTN-
IRYDDERIAYFLRKGFLsyNLRHIDNPKIAFRKIKSRLINIL(MESDTKYENKYNFFYGRDKDKLAFKSRIDD
LLEGP)}*

$3816=173(176):
>gi|1120409378|ref|WP_073272527.1| {|0(174)|<Firmicutes(B)>}hypothetical protein [Anaerocolumna jejuensis] >gi|1110494471|emb|SHJ58935.1| TIR domain-containing protein [Anaerocolumna jejuensis DSM 159
{()MTEIFISYCQKdSSYIDLMDSYFNakdiVLRRDiREISPW--KSI------
KEYMQTIRNTDYAVLLISDKYLKSFNCMYeVLEVMKEQNYR-N--RIFPAVLETG-
IYDTSCRVEYVKFWQDKykELGNQMSQIDMVNASGLIDDLKR(TQNIAASMSEFLALVADMNNPSISDINTAI
ENKLNE)}*

$3817=196(193):
>gi|1120976153|ref|WP_073483244.1|
{|0(169)|<Actinobacteria(B)>}hypothetical protein [Streptoalloteichus hindustanus] >gi|1110516569|emb|SHF61766.1| TIR domain-containing protein [Streptoalloteichus h
{(MDTATPDGR)-TRVFLSYSRKdSDFVHWLAAELTG---
RGCVADFDqstadpsnvDTGISAEDAWWaRLEEMITLADVVVFVVSPDSARSPIC-DeEIAFAQNL-----
GKRIIPVLCRPI-
DFATAPPRLSALNIKISfvDSDNTADHLARLHSAIQLDVTWL(RTGARLLSAARRWDASGRLDDGLPRGSEQS
DAEDWAARRPTSAP)}*

$3818=244(244):
>gi|1120548979|ref|WP_073406961.1|
{|866(0)|<Bacteroidetes(B)>}hypothetical protein [Mucilaginibacter sp. OK098] >gi|1110526584|emb|SHN31664.1| Ran GTPase-activating protein (RanGAP) involved in mRNA
{(ESKRETQRSTDKNSQELKERKFDDKRLGGDLELSIDGQNYADIEEINNAIDQSALKVRDTTGGYMSIDSMF
YVLLNKTTV)MPKkIFFSYSRYdKGYREELDVHFATLKRQGLIATWYDGEIEAGMDANaKIKTELENADIVLF
LLSPHFIANDYIVNiEMKIAKEQHK-----TVVPITIRAC-
DYKKFFAEYFEEDKQGFltEKQFYKGQGNCETRERDIIWIAS(EQHAAKRDEYYNFIVEGVKRIIER)}*

$3819=223(224):
>gi|1110561887|emb|SHH28766.1| {|196(0)|<Bacteroidetes(B)>}TIR domain-containing protein [Chryseolinea serpens]
{(TTAILYLDKEGELVSVSNPITIRTTTEPSEIELDGYDYYIVLTGPSAGKKLYSHTGGLTTPVYKPKPVAPA
PKPKSSAGI)--
KIFISYARKdEKFKDALAEHLSGLRNNKIIEDWTDKELLAGDVWNdAICQVLEESKIILFLVSSTFMNSNYIN
EvEIKRALERNKE-GDAIIIPIIIRPC-DFKSLPLQKFAALPKNLkpISDWTSRD--
KAWLDVVDGLKKV(IATL)}*

$3820=168(177):

>gi|1181446138|ref|WP_084741652.1| {|0(123)|<unknown(X)>}hypothetical protein [Cryptosporangium aurantiacum] >gi|1110577783|emb|SHN75698.1| TIR domain-containing protein [Cryptosporangium aurantiacum]
{(MSRR)---VFISYAREdRSGVDEMASDL----
RHAKMDVWYDARLAGGDPWWtEILRNIRECDTFVFALSEWSMASEACRREYGYARDLRR-----
RVLPVRMGEL-
PVVGPELDDLSGLHQTPyrTGDKTATFDLLAALEALPPAGPL(PSPLPSPPPRPRPEDSGRVSAVRTETTAVP
YST)}*

$3821=268(274):
>gi|1120388836|ref|WP_073252401.1|
{|284(374)|<Actinobacteria(B)>}hypothetical protein [Cryptosporangium aurantiacum] >gi|1110582370|emb|SHM71659.1| von Willebrand factor type A domain-containing prot
{(VIDGIQCYPSTPQLVVWENALEHREFTLLADSTAGVREGTVEVSVEQDGMRLVAARIPVSIQVGESQAAWE
TRTVRMVSK)---VFASYAR---EDNRIVERFWAAYRALGIELFVD-
LDMPPGVVWepFLREQIDDSDAFQLF-WSDESAGSPNVAaEWQHALATASAKrGASFIKPVfWADTC-PAVP-
-
PELNHLHFRKFDPADLGLLPDGESRTHAEKPSPS(RLQLRVPVLSFVTDTAAAITASRAESALRGIVPFLESL
TELRYYPPVTCLVDAA)}*

$3822=178(173):
>gi|1120977276|ref|WP_073484190.1|
{|0(19)|<Actinobacteria(B)>}hypothetical protein [Streptoalloteichus hindustanus] >gi|1110515865|emb|SHF83215.1| TIR domain-containing protein [Streptoalloteichus hi
{(MSDY)KYDLFISYSRHGSVQRWLLNHFFKKLREcladqiaPAPRVYVDQT-
MPRAVHWpsNLQYALRHSKIMVQLLTPQYFVSPWCMAE---WRSMQAREralGLAGLKIPQGL---
IYPILYSDSDNFPMEARERSWWDFKEFSQPDPVYQDTRDY(VKFHRKVTELACDLVQLLQQVPEWRPDWP)}*

$3823=219(214):
>gi|1119634508|ref|WP_072555533.1|
{|0(885)|<Chloroflexi(B)>}hypothetical protein [Dehalococcoides mccartyi] >gi|1111251717|gb|APH11914.1| hypothetical protein ASJ33_01470 [Dehalococcoides mccartyi]
{(MIKTM)-----ISHA---EEDDAFVDYLYEKLERAklGLDIFVDHKRNKTGDKAqnMIDEA--
KRSVVFIPIFSKQWQGSEFLRnELAAAQG---SLnTNIF--PIKLDlnfsdlsagikiEF-
KTPDKVDGLICTDFSDkhRWELGTQDLLKaIQDKLIQLNIFQK(DEFFYHDVEHIDKIIARENPSPSEIKIMI
DVFLLKDGYQHYFFSRLKQLNWLAFLKLYGFFDRNPWPVE)}*

$3824=168(169):
>gi|1113298443|gb|OJV62992.1| {<Firmicutes(B)>}hypothetical protein BGO41_09820 [Clostridiales bacterium 38-18]
{(MS)-YDVALSFSM---
DEVDLVKDVYFYLKAEGYTVFFAPECQEALSGKnqrEVFYNIFGLSSNyvVLFVSESYLRKKVTMEEAHIAFS
KLGK--TGRVLPIYTDHAElpI-ELLNPSEFNYFKESR-
AAFIASHISGKIALNKHKKGSP(SVEQKEGSMNNINNQATNQVFINKINI)}*

$3825=283(293):
>gi|1113191455|gb|OJU61780.1| {|47(11)|<Armatimonadetes(B)>}hypothetical protein BGO01_12745 [Armatimonadetes bacterium 55-13]
{(YTLSLEVPFELFNEIEGEVENLQAFLTPKAEKFTKDCSDGRITNVSISLDLEAPLDWRSSANPKAVSASDA
ERIWKQGYF)--RLFISHESVhREKISTLKTSL------
AKRFVDGFVAHRDIQPSLewqkEISLALASCHAIVAIHTDNFCNSVWCMQEVGWGLGRGVL-VQPIKV---

DIDPKGFLASTQALIgSLDSDAS--IRELRDH--
LISTFFRNHQTR(RQMLEPLILSFEHTIHEQAFKEVVELLEKAPDLPGEVLRRLQASSKKKESFPKELELRIN
ALLARHNVSLSQS)}*

$3826=196(197):
>gi|1113206802|gb|OJU75839.1| {|0(145)|<Bacteroidetes(B)>}hypothetical
protein BGO09_13490 [Bacteroidetes bacterium 47-18]
{(MIDIKNIQQEIKEEQIPSRGVKPL)---
VFISHDTRdAELAEAFSKLLSSVSAGVLKSFRSSDKRgnQGIAYGVewypEIMKKLGDASDVVALLTQNSVDR
PWILYEAGVA--KGKL-NTPLLGIAIGIPLNKANNGPFAQFQNSGDDI--
DSLTKLVVQLVSRIPNSEPDT(EVIKMQVEAFKKQAEVILKKQPEVELKDRELME)}*

$3827=166(173):
>gi|1113186631|gb|OJU57380.1| {<Actinobacteria(B)>}hypothetical protein
BGO04_15010 [Microbacterium sp. 70-38]
{(MRSAASGPRYTPAEQDLADRVQQRAAQHPERDH)--DAFLSYARIDgSEVGVALRAELELLGAS-
VWFDEVAIIPGKSQALQMDKGLRSARCGIALLTPAYLTGRFWTErE-LGALLHK-----
ETLIPVLHNVTFeqVAEYSGILPDLAGFET--ARDSVQDIAAKIAAAILPTSTT()}*

$3828=181(188):
>gi|1113139008|gb|OJU13801.1| {|0(454)|<Firmicutes(B)>}hypothetical
protein BGN88_01690 [Clostridiales bacterium 43-6]
{(MKLPAAYKGSE)-PYIFVSYSHVD--REEVLKDIIWMVGLGY-
RVWYDEGIEFGQDFPtELAIAIKNCSQFIIYISPNSVISKYV-NrEFDYANFL-----GFKVFPVYLEHT-
ELPERLEFLMSTTHRLYrfRYPWQDFCEYYEKGIYEQCRYIP(DATKEKPKSSESGKPLKSAKTLDSSLPEST
EQACKTE)}*

$3829=256(257):
>gi|1113152386|gb|OJU26357.1| {|9(6)|<Bacteroidetes(B)>}hypothetical
protein BGN92_02325 [Sphingobacteriales bacterium 41-5]
{(TMIGAGNSNAAITILLKAVDSASDFCSQITMQAGKLKEITKKETLGIISFDDAMMARNQINYALLEIIKEL
EPVLAKTNQ)-PKVFISYNHAdAFTANKIKDRL---
EQEGIAVTIDSEAMMPGENIQaFIEKCVRESNIVLSLVSKKSLLSAWVAMeSINTFFQEKSA-
DPKQFIPCYIEPD-
FFDQKFTDDAINEIDQRlnALNTIMEERIKQNLGIDDIDDER(SRLLDLKHYIDKIVGRLRNNLCIDVRDENF
ETGIKKI)}*

$3830=237(253):
>gi|1113290585|gb|OJV55330.1| {|196(0)|<Bacteroidetes(B)>}hypothetical
protein BGO31_20580 [Bacteroidetes bacterium 43-16]
{(NSRLVNSVLFKSAFIGCVLDNECFMMDKELQYDSFASIDFQTILKSGELSEGILSTYFKINSKTNIKKTVS
RMTNQVKFS)--TVFISYSFKdSLIAERINT---
SLNREGIRTFLWKKDAPGGKPLEeIMSAGISSHDKLLFIASENSIKSKAC-QfELTAARKKQEQ-
SwQNVFFPIMIDDF-IFK---VKKSQIRPIEY------AD--DYWKNI-
EEIRNV(NMLDFTEFTTDVYDEDMLGISLRKIVEGLSLDL)}*

$3831=196(198):
>gi|1113288323|gb|OJV53118.1| {|0(577)|<Bacteroidetes(B)>}hypothetical
protein BGO31_05025 [Bacteroidetes bacterium 43-16]
{(MN)--RAFLSHSSK---QKQLvQQIANNLGK--ANCVF-
DEFEFESGMPLfdEIKKSIGQSDVFVLFLSDDALNSEWVKLeitEIKNLIDNGLDKqffPILIDKSLDVS---
NDERIPNWIKKFLLKPISEHFLITKKIRQRLLELRLDTNP(IFKAKASLFVGRQDLFDQLEAKIYSLSEMKPR
SIIVSGFEGIGRRTFLKEAFLREK)}*

$3832=158(171):
>gi|1113414911|gb|OJW77318.1| {<Bacteroidetes(B)>}hypothetical protein BGO69_18210 [Bacteroidetes bacterium 46-16]
{(METKH)---VFISHHSAD-EENIQKMKDLLLSKGY--
QIknsSIDSSKPNDAmeeNYIkSLLRpRIEWASTMVVLIGPDTHTR------
DWVNWEIEYANsLSKRIIGVYINGAGdsDVPDSFNDCGDAL-----VGWTGD-------
RIIGAIEGE(IDNIDNNWQKPDGTPRPTISLGGSEC)}*

$3833=283(283):
>gi|1113324256|gb|OJV88390.1| {|80(292)|<Bacteroidetes(B)>}hypothetical protein BGO34_18770 [Bacteroidia bacterium 44-10]
{(MYNCTKWKDYVQHIININNANIKIFPIALFKYSYDFNAKLGRTQFITLKTYSIKDNWTEFQTRIFDGLIRF
ITGKGANKL)--KLFISHSKKdpDNIGEQKATELKAF-
LRSDTKLDSFFDANDILDGYdfeeQIIENVKNSLLII-LKTNTYSEREWCRIE--ALAGKRNK-
VPGIAVSLISGSVKRnfPYLGNTPLIRFNNNwND--
IINLLLRTALDQYYQEQLLDE(IKNYMGMDNYYILPFAPELLSFCLIKKEKKILYPEPPLGREEIVFLKEFND
EISFITPMQAYS)}*

$3834=149(162):
>gi|1113342508|gb|OJW06377.1| {<Planctomycetes(B)>}hypothetical protein BGO49_03035 [Planctomycetales bacterium 71-10]
{(MGKRRSSTSADARDSGPGKAHQ)---VFVSHATAdKWLATMLCEKIEQAGAT------
TFRDDRDINGGDdipeRLRAEIKRSKELVVLLTPESYDRPWVLIEVGAAWGGRKN-
FRIVaILCHVTVEKIPDMLKSKKAFSLND---------VGDYLEELAQRVRGRHG()}*

$3835=221(226):
>gi|1113353558|gb|OJW17290.1| {|71(0)|<Bacteroidetes(B)>}hypothetical protein BGO48_06965 [Mucilaginibacter sp. 44-25]
{(TLFKAYFHDGLVSMLRGEDTPEPSEGIIYLDFSLPTQLTPDLFEGKLDMIPEVTHRDVFKETKTSINFIFT
YEQLMENKP)-KRIFLSHKSAdKPLVRSYRDVLKTLGFEPWM------
DEDDMKAGDklhrSIAQGFKDSCAAIFFITGNYIDEKYLATEVDYAMTEHYA-
KGDRFKIIVLIIAEPGENTKVPELLQQFVYK--QPKTQFDGLTEIIRALPIKIE(HITFTD)}*

$3836=181(191):
>gi|1113342484|gb|OJW06353.1| {|0(22)|<Planctomycetes(B)>}hypothetical protein BGO49_03030 [Planctomycetales bacterium 71-10]
{(MGKSKKVF)--DVFISHALEDAPLADRIASHCRAGGLE-VFTPA-
ELTAGSDVGDAIWDALYESRALILVLSAEGLT--PMMTvELGGAWGTN-----
KPVYGIVTDPLAdhGGAVMKKVALYPKERI--
SDVVLAIQKKVREFSDRDREVL(AEVYAQAPATVDRLIADPAEVESLTDAFAERSGRRVDGDRLTS)}*

$3837=301(292):
>gi|1113404473|gb|OJW67253.1| {|10(673)|<Bacteroidetes(B)>}hypothetical protein BGO68_01530 [Candidatus Amoebophilus sp. 36-38]
{(YVTFLIILCILSTLKAGTPSNQPVASHLRASNMVKVGLTDIPEKENFSLEYFIKEKAYLKLMERDILPYLK
RKKLKPPTC)----FISYAWGDRYheyWVKRFCEMLHKAGIE-VLLDRWSVRKGNNLNEFVKK-
IEKADWVVVVGTKLYLEkynNRAANSkEKEDAARlegqlieylvrysteRG-----
NRIVPILLEGTPeeSLPFMLRQKISSEFTN--NDYFEELL--
RLIRDLYNIDHR(DQIFDEFIEKLQKYAIAAATNVTEGERQAYEQKRMEEIVALDSRISKEINVYKEEAFKLK
GEVEEDKYIPVS)}*

$3838=223(212):
>gi|1113410413|gb|OJW72965.1| {|0(904)|<Bacteroidetes(B)>}hypothetical protein BGO68_02610 [Candidatus Amoebophilus sp. 36-38]

{(MERDILPYLKRKSRKVPTC)----FISYAWGDRYheyWVKRFSEMLNKAGIQ-VLLDRWVIKKG-NILNEFVRKIEETDWVIVVGTKLYLekynkratgskEKEHVARlEGQLIehlvrysTERS-----NKVIPILLEGTLeeSLPFMLRHKISSEFTK--NDYFEELLKLIHDLYNLDNRDR(HFEKIIEKFRRYAIAAGEHITEAERKAYEEKKKDGILALDKGIKEEIDSYKEE)}*

$3839=177(181):
>gi|1113487838|gb|OJX48780.1| {|0(203)|<Proteobacteria(B)>}hypothetical protein BGO81_19110 [Devosia sp. 66-22]
{(MA)--DVFISYKSRMRPRVREIAAALEELGLT-VWFDA-ELEAGRSFAAVINSELAKARCVIVCWTPDAFAPEDGSEiSWVEAEAsiAR-----DRkvIVPVMLERVPlkAPWNMFHTERLMDWSP--ALVATHGPWLATLEVIGRHVGR(PGLADYARAGDDPAALAAWARAYPEDPRAPAVPADGKPD)}*

$3840=194(202):
>gi|1113487837|gb|OJX48779.1| {|0(240)|<Proteobacteria(B)>}hypothetical protein BGO81_19105 [Devosia sp. 66-22]
{(MS)--DVFISYKRRLRPQVERVASALRELRLD-VWFDA-ALEPGVSFSAEISHEVRNAGCVLVCWSNDAFPHGgdenGWVLgE-ASIGRKR-----GRLVTVTLED-TefDPP--WNMIHSERLVG--FDPTDFTADRSAWRGMLAAIGR(LIERPGLADYDRAISAGTADALKGWVQSYPKDTLAETVWARIEDIELATVRKRLAEERVQ)}*

$3841=172(179):
>gi|1113349329|gb|OJW13104.1| {|0(6)|<Planctomycetes(B)>}hypothetical protein BGO49_20085 [Planctomycetales bacterium 71-10]
{()MPTphVFISYARSdRRSVLRLNAALLESGRDGWVD-WED--IPLADNYVrEI----EGADALLFLLSDDSIAST-IARqKLDHAV-RHRK----RLVPVIVRDF-DRGAVPDALGLPQWTFLreDDDFAANFAKLLMNSTPTSTGSA(RTPACSSGRSNGKATAGKRVCSSAGVSGNPDRLRVHFES)}*

$3842=237(245):
>gi|1113392610|gb|OJW55581.1| {|172(0)|<Bacteroidetes(B)>}hypothetical protein BGO55_03305 [Sphingobacteriales bacterium 50-39]
{(FKDCELWDATLIDCAISKSTFKTSKRFEAEKSAYLPAKFDLATLKKALTLDRDYLKKYFKVSDDRLFDIAGDYKILPSFH)--SVFISYSFKdKDAAKFVYDYL---KRNKISCFLWEEDAPGGQYLEhLMASEIKKRNKIIFLASQNSLRSSAC-QfELSEGRSKQAA-SwDEVFVPVYLDRY-MF---EVKDYQIRPK-AkaQEYWSNIQ--ELKSINCIDAQAI(SSSRKRGAILANILQAITIKTDKTQ)}*

$3843=181(187):
>gi|1113494349|gb|OJX55225.1| {|0(636)|<Proteobacteria(B)>}hypothetical protein BGO81_07990 [Devosia sp. 66-22]
{(MADETGAGERR)-TNVFISYSRKdAAAADRLHAALSA---EGLNPYLDKHDIAAGEDWKaRLGGLIENADTMVFLISPDSISSEVC-DwEINHA----EL-KGKRILPVVTREA-TDVPERLQRLNYVFMRTpdEETASLPRLAGALAVDIGWIRAH(TRYGEDAGEWDTAGRPSRLLLRGASIGEAEHWRDTR)}*

$3844=192(183):
>gi|1113552318|gb|OJY12415.1| {|0(1173)|<Proteobacteria(B)>}hypothetical protein BGO99_13630 [Nitrosospira sp. 56-18]
{(MLPSSS)-KKIILFYAKEdEAWEQRLQGRLVSRTRNPQLfpagltvESWNNEKL--GELIRspeQLHEQVRDAVYMLFLVSDDLLKSSYFRDlAGMLRLSLGEA-STFRMLRIHLRPL-

VFSLDWLESAMLLGPESpaLAEIPREQCDSELDRITDAVITR(AAAAMNTNLSENPGGPHVGDDDHATEKPHH
LSGVSSG)}*

$3845=233(223):
>gi|1113552381|gb|OJY12478.1| {|224(0)|<Proteobacteria(B)>}hypothetical protein BGO99_13640 [Nitrosospira sp. 56-18]
{(AANRTRLSGSDSLRKAWLIEQPQPEEGFTCFLNSYGGGTKIISDISPVEFVAELHRRWTQKYGGSKTTADF
VANPVAPRK)-PTFFISYCRRtdQFSAEKLYKALlDMTASEG--
DVWFDHDsIEPGDDFPeRIRDGILNCRYFLPLIShkADALDEAYFYREWGEASERKKAIrGRPFIIPIIVDqd
fqPT-AYHRVPFDWTDIQDFAHapEGQPDDKLRKRLTDLLRDVRREG(RLS)}*

$3846=169(179):
>gi|1113628283|gb|OJY87177.1| {|0(176)|<Bacteroidetes(B)>}hypothetical protein BGP13_14475 [Sphingobacteriales bacterium 40-81]
{(MDGL)---VFVSYSRDdKSRVERLVNYLA----
ENNIPLWWDADLQGGDNFSnVISDKIEKAACVIVVWSKTSVVKDWVRSEADEARKRH------
IFMPVLLDAD-
IKIPKPFDIYHHYNFTDenFISGKEIEKMLRALVKIIKYGKP(VNVYVEELSSGWSINRAISATDEVRELVNT
LRSIT)}*

$3847=175(185):
>gi|1113552342|gb|OJY12439.1| {<Proteobacteria(B)>}hypothetical protein BGO99_13775 [Nitrosospira sp. 56-18]
{(MTN)---IFVSY-S--
RTDIDLIRPIVALLRINASFVFHDIDSIPPGKKWksEIDNALAKTDLVVLFWCEHA-NKSENVKkEWSQAV--
---LqDKD-ILPILLDYT-
VLPGELSQYQWIDFREGLGTKHVKKILEPGIGVGAVIGSV(ILAPLGSVLGARVGSQLLIDEDKSLEEMAGEI
EKEIIRRQDR)}*

$3848=204(198):
>gi|1113454964|gb|OJX16415.1| {<Proteobacteria(B)>}hypothetical protein BGO82_01340 [Devosia sp. 67-54]
{(MGLVVEDRVRARARQVTAATNLSMREVLTRHARPEKP)RYDVFLSQA-LkdeelvLGIHTIL-------
TEDLGLTVFCDwieaghSDHAGTTPADaaFIRDKMLASTGLVFVDSEHAAGSNWMSWEIGWFHGAKGRIcvlP
IVKDGSEHYR---
GRQYLGLYPVAEEDAQHVLRVSVPLAKAAAGYPVGEPLGL(ATSLPMKVWTSLEKLPRYFLP)}*

$3849=247(254):
>gi|1114428942|emb|SHH94122.1| {|490(19)|<Proteobacteria(B)>}Ras family protein [Vibrio aerogenes CECT 7868]
{(QIAGASGQALAEHLIESIMEKTHLGQKPTVHWLKGSDNQKEDAMTQQDSQEKSFSAIGTAGVAPDALPNVY
FSYAWGDET)------------
DERQAVCDEIYQQLSEHdDLNVLRDRNVMKAGDSIeAFERQIGRGDFIVLLVSEKSLRSIDCMHELAMIYHCS
LR-
QRddfvQRVIPVVLSDAKIGGIEDCLEVAALWAEKKKKLKKMVSQVGSENAGTQSVKV(QQKLTEITGCCVDA
LSWISDLITERQPDLQAQAT)}*

$3850=235(222):
>gi|1121336105|ref|WP_073644004.1| {|0(157)|<Cyanobacteria(B)>}DNA transporter [Nostoc calcicola] >gi|1115264960|gb|OKH28621.1| DNA transporter [Nostoc calcicola FACHB-389]
{(MN)--QIYISYAW-
KDnkselgsQREELVDKICEALISERYNLIRDrNHLTLGKSIQSFMQEIGRGNYIVIVVSDKYLRSEYCMFEA
VEIMKH-KD-

YQQKVFPVVLQDANVYTKEGKSEYIKYWEEQREKiekfisdeLSYNEHSamHNVAKKIRDISVK(FDEFIFFI
ADKLSIDPNYKFNDFINQLTDTIRNDAVQLRSKKSILVAGTGNFNIPNEVNWCAQKLGEKIAEDNYNLITGG)
}*

$3851=241(246):
>gi|1121332844|ref|WP_073640743.1| {|809(20)|<Cyanobacteria(B)>}GTPase
[Nostoc calcicola] >gi|1115273825|gb|OKH37189.1| GTPase [Nostoc calcicola
FACHB-389]
{(ARQYEIQCQKSFQMVDVRKLIDDVMFQSIKADGEFNPQVAQLQSELEQEKNESLTHQLRNSEQREKDIMNS
QSPMEFEKE)---IFISYAW-
GGESEQFVNQLDETFQVKGIKIIRDKRDLGyKGLIKAFMERIGRGKCAIAVISDKYLKSPNCMFELVQIAKNG
KF-YDRIFPIVLADAQIYKPIARLKYIKHWEDEI-
KELDEGMRGVSAANLQGFREEI(DQYTEIRNAIAELTNLLKDMNTLNAD)}*

$3852=259(256):
>gi|1121296646|ref|WP_073608470.1|
{|129(330)|<Cyanobacteria(B)>}hypothetical protein [Phormidium tenue]
>gi|1115284882|gb|OKH48017.1| hypothetical protein NIES30_10940
[Phormidium tenue NIES-30]
{(GMNHKIGDRAAIEFAKGFYRGVGAQMAYKDAFALGKNQIDLQSIPESSTPVLKARIQTRRQAAKPSRPSPQ
PPPTSPPSA)AAQIFISYRSQapDIDLAQEFHDALKAAGHN-
VFMASKSIRLGENWPQRIDAELKQADYFLLLLSHQSVTSEMVTEeVRQAKHLQD-----
TRqdgrpvILPIRVNLPMdvPLNYDLRGYLQRIQQG--
QWESPADTPQLLGDILELIAEG(KPQLPVQALEPIAPPLEIPDHPPLPVAEPELPGGQV)}*

$3853=300(300):
>gi|1121296413|ref|WP_073608237.1| {|142(294)|<Cyanobacteria(B)>}ATPase
[Phormidium tenue] >gi|1115285177|gb|OKH48308.1| ATPase [Phormidium tenue
NIES-30]
{(RAAIEFAKGFYDGLGYNRTYPDAFEFGLTAIDLEGIPETATPVLKQGKPQLIKPDLAPESTKSRNFSPVFT
PQPQAETQV)--RIFLSYkrgVEPDEPVALDIYAALSSQ-HQ-VFIDQ-
AMVVGTPWVEAIKREIAQADVLIVLLSATSLQSEMVQQeIELAYEAAN-----
DRhglpkIFPVRLAHTApfPYPlnQYLDPLQWAFLeTP--TDTPQLIT--
QLQSALAGNSLP(IDTEIAKQKLLQRTVPISLAPPTPLAQPPAYHTPLAIPLEPPEGSMDTESEFYIERAEDT
KALSALQRKGGTVTILGPRQ)}*

$3854=281(285):
>gi|1121270509|ref|WP_073584587.1|
{|563(0)|<Proteobacteria(B)>}hypothetical protein [Vibrio quintilis]
>gi|1114431799|emb|SHO57541.1| hypothetical protein VQ7734_03311 [Vibrio
quintilis]
{(QEQDPFTQPGHIDIRIAGASGLDLAQHLVTSVTQNSYLGQQPEVRWVKGGEKQEESSMCEQNNQPPFSVIG
RAQAAPDAL)-PNVYFSYAWGdEtdqrqvvcdQIYQKLQAH-----RLNLCR---
DRNVMNTGDSIeAFERQIGRGDFIVLLVSEKSLRSVDCMHELAMIYGFSQR-QRDdfvqRVIPVILAD-----
-----AGIGALPKRLkvTLYWKNQK--
EELQALVDEVGTE(LAGTETVKALAKLTDITGCCVDALTWLNDLITERNPELQADATVEFVLQKINQWQETRK
DRENDQ)}*

$3855=184(189):
>gi|1121337487|ref|WP_073645386.1|
{|0(730)|<Cyanobacteria(B)>}hypothetical protein [Nostoc calcicola]
>gi|1115252386|gb|OKH16887.1| hypothetical protein FACHB389_35255 [Nostoc
calcicola FACHB-389]

{()MTEAFISYSRKdKEFVKRLYEALNQQKR----
EVWVDwEGIPSTADWRkEIYEGIEAADNFIFIISPNSVASEVC-GeEIAHALKHNK-----RLVPIVWQDA-
TGVNSELAKFNYIFFQEsnDFNTALKLLIEALDTDLAHVKEH(TRFLMLALEWDRKKRNSSYLLQGSQLEEAE
QWFTKGAGKNPFPTLTQKE)}*

$3856=171(176):
>gi|1121284211|ref|WP_073597318.1|
{|0(766)|<Cyanobacteria(B)>}hypothetical protein [Phormidium ambiguum]
>gi|1115266920|gb|OKH30477.1| hypothetical protein NIES2119_30845
[Phormidium ambiguum IAM M-7
{()MTDIFISYSRKdKDFVKELHLRLIKLRR----
DVWVDwEDIPLTADWKeEIYRGIEGTDNFLFILSPDSIKSLYCTEELEYAIKNHK-----RLIPVVYREV-
NPQDVHPTLAALNWIFIrnEDDFHSNFEKLLEAIDTDLDYVR(IHTRLLKRAIEWHSEKYDSSFLLRGKDLKT
ALEWLT)}*

$3857=256(253):
>gi|1121282033|ref|WP_073595140.1|
{|111(336)|<Cyanobacteria(B)>}molecular chaperone Tir [Phormidium
ambiguum] >gi|1115272509|gb|OKH35892.1| molecular chaperone Tir
[Phormidium ambiguum IAM M-71]
{(LGDVAHLEGGEVKGHGVNMAARLQTAAEAGGICISQTVYDVVKNRLPLLGIYREVREIKGIEELVTIYQIP
ALNKEELTN)-KKVFISYrSHDpdLSLAEEFYKALKAAGHEAFMA---
GQSIRLGEGWPqRIETELKQSDYLLLLLSEKSVTSEMVIEEVRRAKELRDR-NpkaKPVILPIRVNFP-
LSSPLNYNLREYLNFIQqrEWRNAEDTQTIVQEILEIVTEGR(EQEETQEGQKHPVAISDSQAKPLPVAIPEF
PQI)}*

$3858=231(228):
>gi|1121308086|ref|WP_073619594.1|
{|0(208)|<Cyanobacteria(B)>}hypothetical protein [Calothrix sp. HK-06]
>gi|1115288913|gb|OKH52003.1| hypothetical protein NIES2101_16175
[Calothrix sp. HK-06]
{(MFLLPESAKPV)--KIFFAYSPsdQdEQLKIVLEKHLMMLEELDVVMVWYQHR---
SENTYeDYENsyHLQTADVIVLLISSDFLSliqNRIDWNnEIAKLQEKHFL-GEVVAIPILLYQV-
QGWQKLLGNIAHLPKNGmpVKSWQNPD--
AALADISQGIEAT(VVEIKKYQQRLQEYRYSYGAAIVQEYPLRAKALNNLNDIKNYLGIKNQDADLVQQEVTA
QAQREYKQKLIQYKQELD)}*

$3859=186(184):
>gi|1115295886|gb|OKH58884.1| {|0(129)|<Cyanobacteria(B)>}hypothetical
protein NIES2130_12395 [Scytonema sp. HK-05]
{(MTMSQY)--KVFLCHNSKdKPQVREIRDQL---EQQG-
IETWMDeQDIKGFDDWKkKIKENLSQMNaVAVLLGSSGF---GEWQKyEIIYTeQEINRR-
EQNKLLPlraglVILKSC-
QQTFQKIRDRHYDTPYGwlFINPHMVDLRASKAPMKQLIEAI(TEQRWHVDYTRLENLLKAGQWKEADQETLA
VMLKTAGR)}*

$3860=236(229):
>gi|1121318562|ref|WP_073628701.1|
{|172(0)|<Cyanobacteria(B)>}hypothetical protein [Scytonema sp. HK-05]
>gi|1115296658|gb|OKH59649.1| hypothetical protein NIES2130_07190
[Scytonema sp. HK-05]
{(VIRAAGIPESLTPKLLSKNQLWTENANTTPPFFADSVAEPQFTSSTSNIKQGTQTQVNYTHPQDLLPSENW
KQANIENME)---
VFLSYSPNdESLRKQLEKSLSILESQSIIRIWYQSKIAPGKNKKvEIEQHLNSARIILLLISPNFLSSVYHLN

yEVKRAMERREC-EGTLVIPVFLSSVASWQTFKLGKfnigdLQPLPKNGkfVTDrryWKSQN--
EAFVTIAEEFAEE(IGKISGELP)}*

$3861=263(253):
>gi|1121282005|ref|WP_073595112.1|
{|14(332)|<Cyanobacteria(B)>}molecular chaperone Tir [Phormidium ambiguum] >gi|1115272481|gb|OKH35864.1| molecular chaperone Tir [Phormidium ambiguum IAM M-71]
{(YEDVGKYLSDLERKVFVGSWQGLTYEQIHPEHREYVEKDVAFRLWKKLSKVLGEDVRKKNLQGAITRAHKAKTYQAKKTF)----
FISYHHQqpdtglaRDLERSIDKLGGQAYRFPLSfnnAVEGNSTPYQAEGDWfaEINHKLQKSDYFLLLLSPESAVSEMAIEILRRVRELRDRqlNnqlTLLTIRVNCPT---
NTILNHDLRGYLAEIKQREWNSFHDTPDLVEEISNLLSGK(VVKWENSKPSSTDHITPAIPLPVAEPEIPSGQV)}*

$3862=195(181):
>gi|1121676096|ref|WP_073888887.1|
{|0(1)|<Actinobacteria(B)>}hypothetical protein [Saccharothrix sp. CB00851] >gi|1115673502|gb|OKI33045.1| hypothetical protein A6A25_04320 [Saccharothrix sp. CB00851
{(MSGYE)-FDLFISYaRR-
GTVQQWLLNHFLRKLKDCLADQFaptpkvyVDREMPRAVHWPsNLQHALRHSKIMIQLLSPQYFASRWCLAEWHSMLEREKMLGlaspekpQGLIYPILYSDSDNFPLEGKTRAWWNFKEFAYPDPVYQQTHEFVRFHHEVNEL(AADLVALLQQVPPWRPDWPDVDPPDPVLPPTPPMPR)}*

$3863=209(199):
>gi|1121605728|ref|WP_073831729.1|
{|0(187)|<Actinobacteria(B)>}hypothetical protein [Micromonospora sp. TSRI0369] >gi|1115775051|gb|OKJ33986.1| hypothetical protein AMK25_29865 [Micromonospora sp. TS
{(MTSERPALPGPPYFFLSYVPPPT)-HNDRTLGD---HWVRRFYHDLNLAIDGRpgdRLRRqgFADFM-
VRPPEARteRKRIALAEAEVFVPLYSPAYLNRNDSRREREWFRQRLIRAgRppdGDNILPVLWIPSPasvhAPDQARAVAMAADVDAYAANGMSALCRLQKFQHAYKEVLG(RLAQHIVDTAEQTPLQPAEPPTGPTDLPSSPTSEVP)}*

$3864=207(215):
>gi|1121676100|ref|WP_073888891.1|
{|0(91)|<Actinobacteria(B)>}hypothetical protein [Saccharothrix sp. CB00851] >gi|1115673506|gb|OKI33049.1| hypothetical protein A6A25_04340 [Saccharothrix sp. CB0085
{(MS)EYDVAVSFAE---EQRSAVEGVVEAFKQRGLTVLHGPEQTHEWWahkeGGdlpD-------
ARVRffVPFVSE--------VDDFTTAALRAVKDGDEHVLPVLFGEVTvppgL-VHPHVAYLRGTAHRP-
DQLTEALV-
TRIEAAEAVGRER(APVAEVVTRAKGVVAVAEEPEPVVPVTFSRYTEQDATLRYLGAQFAAALPSLERRGFVGTAHVGETRIAVRVE)}*

$3865=281(278):
>gi|1121563558|ref|WP_073793464.1|
{|91(104)|<Actinobacteria(B)>}peptidase [Streptomyces uncialis] >gi|1115617213|gb|OKH90803.1| peptidase [Streptomyces uncialis]
{(GRGLRLIPVLCGDADLPAFAGTRVWVDFRELSGPAFDAKAAELANVLRGGEQPRKPGADEEVLDLARPRTRPPTDSAQPA)---FVVCYARADAAYGERLVDQLSGSGLP-VW-
SLGHLKWGDDYLWTIRQRLRHALAVVVLMSPEA-EESEDVTrEILEGQRHS-----
REFFPVLLrgernyllasswyLDARngDLPGPEAMRLLRRLHD--

SHRSERRSPAPGTPPVLPLPAA(RPVPAVRSPARGSLATLEALLAEHETEHADLLTTTLLLEAVGRLEAGWLR
RSDGRRLP)}*

$3866=217(229):
>gi|488795825|ref|WP_002708231.1| {|0(86)|<Proteobacteria(B)>}TIR
protein [Thiothrix nivea] >gi|386420463|gb|EIJ34298.1| TIR protein
[Thiothrix nivea DSM 5205]
{(MIAVIPKSHAANRMT)--DIFISYSRNDQQWVAKLAKALEDVGYS-VW---
WDTQllAGDDFHRTIPAVLEEARCAIVVWSKVSI-DRQWVRaE---AYRAN-----
ERevLVPVRIEEVQipLPFNLLHAEDMKRWNG--KADDPAFR--
RLLGAIARHCPL(TQSIVADEPLPKPESKPDSHPPKRSMTGWLAMAGIATVVAIAIFLFLDRISGKQTVTPPA
VVEEAAKPAPPPEP)}*

$3867=195(179):
>gi|494725022|ref|WP_007460888.1|
{|0(218)|<Actinobacteria(B)>}hypothetical protein [Micromonospora lupini]
>gi|385885044|emb|CCH19057.1| Protein of unknown function [Micromonospora
lupini str. Lupac
{()MPLFFLSYAQEdNRnsQIQEFFVDLSEAVAGLAALHWDeagfiDGHILLGSTWPdTLSDALSTSACLIAL
TSPRYVVSDYC-GrEFQVFADRIDE-HERQfnvrppaIVPVQWIPC-
DRLPKAVAGFQYRNFQTgtAYAQDGLLTLKQRKRHEDDYKDF(VDALARHIVQTARDHTLHRPAQRPVLSKVT
SAFDRPARP)}*

$3868=179(165):
>gi|488794540|ref|WP_002706946.1|
{|0(272)|<Proteobacteria(B)>}hypothetical protein [Thiothrix nivea]
>gi|386419148|gb|EIJ32983.1| hypothetical protein Thini_0325 [Thiothrix
nivea DSM 5205]
{(MV)-
PDIFVSYAHVdnqpfsggeKGWITHFINNLRIevsrrIGRAEHYQLWMDFRLKGSDELTpEIEQQLAKTDMLV
ILMSQGWLASEWC-QrELDIFLQTHPD-TVGRIFVVEVDSV-
ETASKPERLHDLLSYRFwqQNDRDEIHQLGYPVPQKTDAEYF(NRLVNLSHHLAQSVRAAKQAAPV)}*

$3869=150(146):
>gi|488796654|ref|WP_002709060.1| {|0(811)|<Proteobacteria(B)>}SEFIR
domain protein [Thiothrix nivea] >gi|386421313|gb|EIJ35148.1| SEFIR
domain protein [Thiothrix nivea DSM 5205]
{()MPKVFISYSHDsDEHRDFVRGIADRLRNEGV-
DCLIDQYINgfPPEGWQrWMENQIEASDFVLLVCTETYLRRYRGQEtHGGKGVNFEGV-VISQHLYDTYYQN-
AKFIPIIPEQGNYDHVPvpLKSYSTYRLPDEYDDVYRVLTGQ(AKYEQP)}*

$3870=168(175):
>gi|488796732|ref|WP_002709138.1| {|0(55)|<Proteobacteria(B)>}TIR
domain-containing protein [Thiothrix nivea] >gi|386421394|gb|EIJ35229.1|
hypothetical protein Thini_2692 [Thiothrix nivea DSM 5205]
{()MSDIFLSYANNdQSRLLRLVT---ALEAKGWAVFW-
DRAISTGKTWHdTLGNELDACACVVVVWSQHSVRSRWVYE---EAEEGRKK-G--VLFPVILDQG-
VVPPVGFRALQTADLSQwqGEPGHPGFMQLTTEIAACFGGNK(ACHDPVIQTPDAPATLVRQLDPAFASQPGL
DEAAL)}*

$3871=195(213):
>gi|488797486|ref|WP_002709892.1|
{|0(114)|<Proteobacteria(B)>}hypothetical protein [Thiothrix nivea]

>gi|386422163|gb|EIJ35998.1| hypothetical protein Thini_3486 [Thiothrix nivea DSM 5205]
{()MRSAFISYSLKdEAKVIPYENRLKTL----
DIAVWRDKSgLRQGQLWPmRLGEAIAEQDVLLLFWSANAAASHFV-TlEWNTAIALKK-----TLIPVLLDE-
-----------QPLPNVLkpYHCFTDVS--
QLSTHVLSLIPAQ(QPPQNQTLLTELKAAQEDAGQALDIVKHHIQQHIQQDNWRVGGNVYQVQGDLHIGSSGQGDSSWPNRWSLWLG)}*

$3872=225(228):
>gi|495620207|ref|WP_008344786.1|
{|35(0)|<Proteobacteria(B)>}MULTISPECIES: TIR domain-containing protein [Burkholderiaceae] >gi|413939770|gb|EKS71738.1| pentapeptide repeat-containing protein [Burkho
{(LNEIEWLMMIHPKIIDMAKNELLIETLPLKGQRRKTKDFAVAPVLTLYTDQLVITSEEIVRKFDEVGMSVDILSEDEQHQ)--TVFISYGG--P-DQPVVDRINQFLVSRGVKTWYFPVNKLPGQKLhrMMSEGVKTHD-
RVLVVCSKNSLSRNGVLnEIERVLEREAREgGSEILIPLAIDDH-
LFSDEFRPARLDLDQLKSRVAAVVPDPLMDAVGFEREMSL(LLTALRRG)}*

$3873=174(183):
>gi|488796499|ref|WP_002708905.1| {|0(1294)|<Proteobacteria(B)>}TIR protein [Thiothrix nivea] >gi|386421156|gb|EIJ34991.1| TIR protein [Thiothrix nivea DSM 5205]
{(MSADSR)--HVFISHSA--A-DDAFVQALCEKLELHHLNIWVDSRNLRGGDKLwpEVAEAIR-
TARYVVMVISANTVNSSWVRkEVRLAEQEGIT-----VIPLLLPGI-
LPAALGMWFDEEPAGEKIELDIGSLQEAMPRILAALGERL(PDDLPPKQEIANKPIAELLLELSEPTFTRDANGAEQL)}*

$3874=192(177):
>gi|488798055|ref|WP_002710461.1|
{|0(257)|<Proteobacteria(B)>}hypothetical protein [Thiothrix nivea] >gi|386422757|gb|EIJ36592.1| hypothetical protein Thini_4100 [Thiothrix nivea DSM 5205]
{(MS)--
DIFVSYARIndqpfsGQKQGWVSHFINNLRNETSQRIGRAENyslwmdfKLSANDAVtpEIEKQLRDAHILIVMMSRGWLESEWCVRELEYFCATHDdlAGrifIINQDGLPREE---
QPEILHDLVSCPLYEKTPHDQIQRLGYPVPDVADREYFGR(IVELSFQLEKSLRRFAQALVLKSAEPRSVETKATA)}*

$3875=230(229):
>gi|1117467995|ref|XP_019417491.1| {|270(0)|<Streptophyta(V)>}PREDICTED: uncharacterized protein LOC109328467 [Lupinus angustifolius]
{(TFLLVWYKKMGQELGQMISVRQPSPNSNNDPYSKQRYLCLKDMVVEFIKSHHHVLNNVLILILMANNSNSNANATHQLNY)EYDIYLSYSG-
LFTLFNFPSFFSQILRDKGFQVFHNTCCNqeDNKNPLFSLRIMGQSRILIIILISKWYAYSTSCLDELVHILNFFKLKNQL-VCPIFYEVEASDIRYLRNSFGEAMAQHEDTFGvhSDKV-KQWKSALYDVSNF(SGWHLQTNR)}*

$3876=168(162):
>gi|1117372587|ref|XP_019459858.1| {<Streptophyta(V)>}PREDICTED: TMV resistance protein N-like [Lupinus angustifolius]
{(MENSNAIQHLNYE)-YDVYHSYgRC-LSLR-SFPYSLHSVMNQKGLTYNHNNEyymsIRRNDniP-
IspPRLKAMQESRIFIVSIGANYASSVFCLNELVEILNCIKfR-
NNQLLCPVFVNLDPSDLRYLRNRVGEamAQHEHRFGRNSQEV-KKWKTALFELSNL(SGWHIQTQG)}*

$3877=224(230):

>gi|1160749780|ref|WP_079608205.1|
{|59(0)|<Actinobacteria(B)>}hypothetical protein [Mycobacterium abscessus] >gi|1116074055|emb|SIE97298.1| TIR protein [Mycobacterium abscessus subsp. abscessus] >gi|
{(DPDYKNAKERISKIYHIYTEAGAKRRSDIISDDLLLNFNSASILPPDMEYLQPLGFALPTREFLHEHAVEPFASFAPRPK)---VFLSHNS-D--
RKDEVGQLQEQFSAKRIATWFDKTDIEYGQTLvsEIERGIESSGAVVFWWSEGFLKSNWCEYEVDGFLDKIAR-LGRSRVRIFSIVDDNISDDRLPRKIRETKYL-RVGANTSVSEIVNSFARPIYAF(LENIPQDPDA)}*

$3878=272(299):
>gi|1160631826|ref|WP_079493693.1|
{|60(227)|<Actinobacteria(B)>}hypothetical protein [Mycobacterium abscessus] >gi|1116103242|emb|SIM34498.1| ftsk/SpoIIIE family protein [Mycobacterium abscessus subs
{(FDPSHQEAGSILEIYAIYTEAGAKRRTDIIVDDLIINFDFVSVMATELQPFQNFNFPLPSREQFKEYGMNPLVQFAARPR)---VFLSHNEDkkPQVRQMQEQFLAKQVPT---
WFDETDIGYGELLISRIEDGIEESGAVVFWWSQGFLKSNWCTHEykAFIGKIARLGESRVRIFSVVDEDVPmSELP-------RIVKDTkYL-------------------
S(VTPSTSIDYIVNELAHPIRAFLNGITSGDEEDIPGESVRNMPLNNYDEFSRLYGFPLDAYTPKSYWESKTVNDPLYIRV)}*

$3879=266(285):
>gi|1123617557|ref|WP_074308146.1|
{|0(147)|<Actinobacteria(B)>}hypothetical protein [Micromonospora cremea] >gi|1118768465|emb|SIM50874.1| FxsC C-terminal domain-containing protein [Micromonospora cr
{(MSGIGAAAAPVATDQSTYFFLSYAHSAPMLEDPRLDPDKWVKVFFDDLSAAVRGQISAPDDRRVG)----------------------------------FFDAQ-
LSSGADWeeTLTVALGNTQVFVPLYSPAYLSNSWPLGELEAFRRRLRASpIVEKyrhIVPVLWIPLPswdqTAETREALElagdVAGYAENGlRALCMLASYRPAYEQVLGRIATE(VTRIAQRYPLPPSVVPKVAEARHTSRADAQFVVVVAAPTGAEVPVDHEAGVYAERSEQWRPFGQDQALPAAKYVASTAER)}*

$3880=181(180):
>gi|1123643717|ref|WP_074325499.1|
{|0(219)|<Actinobacteria(B)>}hypothetical protein [Mycobacterium abscessus] >gi|1116923957|emb|SIB57247.1| Uncharacterised protein [Mycobacterium abscessus subsp. ab
{(MYMAL)--NIFVSYKREdLEIAKKLERTL----
HANGLRAWRDLVFLPfGHDTEaEVRNAItEEVGGFALIVTENSLFSDFIWRvELPTALERARARgGTFPIIPIFVGDS-
LVSKFARKSVSELGHDLsiQNGIVLHDEDDEEGLNRLAGRLL(ESLITDQPYTRISLFTHSEIPRLDPHDIVVDIREG)}*

$3881=182(186):
>gi|1119587983|ref|WP_072521076.1| {|0(75)|<Firmicutes(B)>}hypothetical protein [Blautia sp. Marseille-P3087]
{(MNI)--DFFISHS--
KETKTTLAIPFAQSLSALGFNVWIDRNGISTGQYIytNIRESIANSTYCIAIIDHTFLAHSWTLEEL-
RLFHLRED-
SQTCIIPIYVNIGKSEVYTTLQWLEGRAFEKINQKDFNAITHTEILCRIVGRYY(KDCILDLQLNSIEYDLLRTKSFPCKETLLTLLDSKAYYSQDFR)}*

$3882=155(168):

>gi|1123830122|ref|WP_074372516.1| {<Proteobacteria(B)>}hypothetical protein [Vibrio sp. CECT 9026] >gi|1119188528|emb|SIO93964.1| hypothetical protein VSP9026_01645 [Vibrio sp. CECT 9026]
{(MADKN)---VFISHYGKDDESV-QSLKSLLSKNGY--
TLknsSIDSTKPNdasNEDYIkSLLRDkMRWAGSAIVLIGPETHTR------
EWVNWEIDLAYrLGKPIIGVFIQGAK--DSDVPDSF------------
QKYGDALVgwnsDKIIDAINGV(YSGWSKVDGSPWESKLSSKREVC)}*

$3883=131(142):
>gi|1123830904|ref|WP_074373298.1| {<Proteobacteria(B)>}hypothetical protein [Vibrio sp. CECT 9026] >gi|1119187702|emb|SIO94773.1| hypothetical protein VSP9026_02503 [Vibrio sp. CECT 9026]
{(MA)-KKIFVSYNFNdKEIVHSVKNMLQSNGgkINGQFSFVTS--
DFSAQGNDainkEIKKVMSDCDAALFLVGDNSHNSPWINREVQLAKSLNLH-IVLTRLPDSRGAApKGLTDS-
--------------YYPEAEWKANDLAYCLNKCQ()}*

$3884=213(220):
>gi|1119394783|ref|WP_072370601.1|
{|24(0)|<Proteobacteria(B)>}hypothetical protein [Hyphomicrobium sp. NDB2Meth4]
{(GGQHGHNLVIHLTPEQLGQIPLATQRDTAERLRADVNAACSSIQNEYIANVHLEMDDGTALGESATLGLTL
PAFWKAGHL)--RLFLSHRDSHKHVAHALAQELEEYGIS-
SFVAHDTIEPDAEWLVEINRALNTMEIMLALISDD-FHESVWTNqEvgfalgWAATL----------
NVAVALSAARtiSAVLRSCSGGRLRTPV--SAENSSASRAASLRDLEPQFSA()}*

$3885=218(220):
>gi|1119797222|ref|WP_072692194.1| {|16(0)|<Actinobacteria(B)>}TIR domain protein [Rhodococcus fascians]
{(AANCPACRGQSRRSYSSYVPYSPTPRYSGGASSTGGGSSSSGHSRRLRGGGSVSYSSTEWRTMEPLAQKAE
LVAQSHPER)-RDLFLCHAWADRESAAKdLCDLLISHGAT-
VWFSEYDVGLGKSLLQEIDRGLANSRIGIVLVTPALLEglksTSGIAGkELSALLA-T-----
DRVIPVAHDTTfdAlrDISPLLAAR--SGLTT--GDTSMSDVAKKVAGAAAAEGSV()}*

$3886=247(255):
>gi|1119188526|emb|SIO93962.1|
{|81(331)|<Proteobacteria(B)>}hypothetical protein VSP9026_01643 [Vibrio sp. CECT 9026]
{(DYNWKEYYLKLFDNDSIIIPIALDKDYSFKLGGDFSYLNFIRECDFSPEIKMTEFLVSTVQSILKKLNIVP
DTNGDSATI)--KLFLSHAKNDSWAEDIAKFLKNIIDNTSLDNFFDaQNIHAGFQFSeEIKRGIEKSTLI--
SIQSDSYSSRYWCQkEIHYAKEKN--------IPIIVVST-
LKFGEDRIFPYSTNVPSinIDANTPLTKEDAYNILEASLLET(LKIIYNRSMLSKYSDKNTHVLSRSPEPFDL
KCIHR)}*

$3887=214(220):
>gi|1119246828|ref|WP_072237169.1|
{|0(612)|<Proteobacteria(B)>}hypothetical protein [Campylobacter coli]
{(MI)-
KKIFYSYSHKdKEILSKLRVHLALLIQQYQIEEFVDTQINCGNNLDeNIMTNLEESDLVILLLSPDYLASEYC
-QkEMNIAIGANKA------VPIVARTC-DWKTTPLINIKALPEDGkpIVEYESVD--
TACQYITDKLRNK(INASLYTIKCRKEFSQKLQEIDFLHPNKSTITLEDTFVQPNINSNEGKRYDTIQKIINN
DKYREILFHGSSYSGKSAI)}*

$3888=197(220):

>gi|1119604824|ref|WP_072532340.1|
{|691(0)|<Bacteroidetes(B)>}hypothetical protein [Bacteroides sp. Marseille-P3208T]
{(NMCGEGVEITDEPVRELTWQGSLADCQFGVSVKDGFRASVLIGTVVVSVNGIPAGKMLFKTNVVRNPQNLY
TNVECRTFH)--KIFISYSHKdEAQ----
VKYIAEAYRAQGVDYFFDRHyLKAGDIYPvMIRQYIDSSDLFILCWSKNAAESEYV-
TlERRQALALAypqvdpEK-ATLTIHPISIKP------------------------
HAEYPEDMATVYNFEEV()}*

$3889=176(184):
>gi|1119611848|ref|WP_072536569.1| {|0(950)|<Firmicutes(B)>}hypothetical protein [Anaerococcus mediterraneensis]
{(MDKSNYR)---IFCSYSHNdEDKFMNIFQKINSLGY----
DYWIDRNgIRAGEYFNeEIVEAINGSDIFLAFLSKSYIVKDYCRREFNQAF----S-ENLRIITVCIDDV-
NESINNNSSFIFSSLAGtnILRYGKGVGDSNEDIDSLSEEID(KAIQGYQFDLVNDLLRKKLSEEYTNNGQYF
LSEISPE)}*

$3890=233(219):
>gi|1119614548|ref|WP_072538783.1| {|0(96)|<Firmicutes(B)>}hypothetical protein [Enterococcus faecium]
{(MLNKS)---IFISYAW--
EKDEPldkkvknFSQWLAIYLQSWGFQVFLDVLENHPGTKLdkFMQDGIDNSHF-
VICICTKTYLKKTtdsatGVYnEIELI----KQQaDSPFIIPiIEQESF-
IDLPDFFKGKYVsqlkfdnPYSQANKKGIFELVStLRDEMVPSKKSET(KTDAKARIESYYNSVEKIKFSNDS
ANLMNFNPQSEDKISFQYLLNGGDFKLGTSPMDFITHWSTSGVNTIHSYN)}*

$3891=202(220):
>gi|1120629921|gb|OKO76833.1| {|0(151)|<Proteobacteria(B)>}hypothetical protein AC628_17130 [Bradyrhizobium sp. NAS96.2]
{(MESQ)---------------ADNIAQSLRNCDHD-
VFFSHDDLPPGDSFDARVEKAIESSDFMVFLISPESVTKGRYTLsELAFARSkwpnPS-----
NRVLPVMMAPTPldQVPTYLKAVTILEPQG--SAAAETRA--
AVDRMLEAKSEQ(SRGQPNLPFLVLALASAIACSLVTTYALNVLQYSFVVSDAGGITILPGVIFGAAIAACNV
MFGAKDRFQLALVVAI)}*

$3892=229(213):
>gi|1122621792|ref|WP_074109586.1| {|0(149)|<Firmicutes(B)>}hypothetical protein, partial [Paenibacillus sp. P26E] >gi|1121035133|gb|OKP75896.1| hypothetical protein A3844_30920 [Paenibacillus sp. P26
{(MSTVP)-DKVFISYSwsS--
PEHKAWVIELATNLERDGIEVILDDWDTTEGQDLnkFMEKMVNDSDIKRVLVISDKIYAEkadgryGGVGtET
TIASIEVYKDsDQSKFIALIAEvddngNA-
YLPTYLKGKKYIDLSspQNFEEGYRKLIRnLYHKPENPRPERG(TAPKWLMHDEKKNLSNSSTHLNILKKIAD
SRPNKLDNAFNQFLSAFLNDYKSYATRFESNTELDRIAF)}*

$3893=178(177):
>gi|1122358406|ref|WP_074042450.1|
{|0(68)|<Bacteroidetes(B)>}hypothetical protein [Culturomica massiliensis]
{(M)---IFLCFSS-KD-RYTIVESIMYHLK-
NyGLSVWYDYHKLilgDDRDYRNLIEGIEQNNYAIVLLTENVFDCVCANDELNVIHRQYIN-
GRIHVFPIFYKITALELPLNYQWLCKLIYNEvddkTGTLMTCNQIILKYLLDKNALYE(TKVLTDYIKIPVQD
TYIKEMISIYFEVDYDNINVRM)}*

$3894=155(164):
>gi|1121610263|ref|WP_073835714.1| {<Actinobacteria(B)>}hypothetical protein [Micromonospora sp. CB01531]
{(MSN)QHNVFISHRHEDDALVAG-LKDLLKRQGA--
NVRDSSvtsetpNRAKDPDYIkSLLaERIQWAGKIVVIVSP--------
DTkkHHWVDWEIEYANrHpDKRIIGVWAPN--AHGCDLPEPLERHADA-VVAWR-------
ADAIIRALNGE(NNWQAPNGSDASARTISRIGC)}*

$3895=194(199):
>gi|1121468168|ref|WP_073706570.1|
{|0(6)|<Actinobacteria(B)>}hypothetical protein [Glutamicibacter sp. 0426]
{(MT)--KVFLSHSSIdKPIVRQVMAELLMMG---ADPFY---
DEVSITNGAviphDIDMALGITDTFVLFWSKDAAVADWVTSERVTAWGMfHNQ-
PSRFVVVILDDTDLPPTLKFKKWIDARNDHS--
HIASEILGLNNDTKLIMAVQK(TFESWGIEVAYVPGYGAYVGCPDCGAVISDVEQWSQVDHNREELYVGVRCNKCKWED)}*

$3896=217(229):
>gi|1121679472|ref|WP_073892208.1|
{|0(162)|<Actinobacteria(B)>}hypothetical protein [Saccharothrix sp. CB00851]
{(MPGPEGVTMT)---IFVSYTRADESVVRALRDDLGRMGHE-VWLD-
HEIHGGELWWREIIRTIQAARVFVFALSQASWKSVP-CHaEFDYAQQLG-----
VPVLPVHVGPLDnlRIPIMERQLVDYRERD--VDAIVALA--
GALTELAARPRA(LPNPMPPPPKVPFEYLYRIAELLGPQPIAPDRQEIIIGQLRRHLRTERDEVARADIIKLLREFRERGEITNLNSREIDE)}*

$3897=258(259):
>gi|1121434565|ref|WP_073685840.1|
{|84(317)|<Firmicutes(B)>}hypothetical protein [Streptococcus salivarius]
{(SFLSGSSLSLINHIQHEKSILPIVVEEISEDIFNEQVGFYHLLYFNDQTFSEKLEHFINEDFDPYRPLVENPALNKLMSH)-
PNIFLSYRKKdHTKALELLNIIHSQPKFEALSVWYDDFLVAGENYNdNIDTQLKSCDFMVMCITKNILEPDnYVLKvEYPKAIAL-----GKKIIPIAMEEI-
DVVLLEKLCPRIGTVISvsDTSKICSAFSKVIDDLNIKLKSL(TPEEMGQLGKFYLEGKGGFERNYSLGIEFLRKAATLGDS)}*

$3898=236(224):
>gi|1121202104|ref|WP_073541872.1| {|0(247)|<Firmicutes(B)>}hypothetical protein [Bacillus cereus]
{(MEQNKEFGSRPPK)---VFISYSwtS--
PDYKEKVISLANRLTENGIEVVIDVWDLKPGHNMfaFMESMVTDSTIdKVLVLCNKSYAERassynGGVGtETQiIGPEVYTKVkQEKF-IPVILErdenekDY--
IPVHMKGLIYIDLHMNFEEGYESLLRlLYEQPLLRKPKLG(SPPAYLFDEKKTKFKVSGVLSSIRDAIVRHPNRVKGLCRDYKQAFIQDLEAFKLTYEELKEPFDEKITNLI)}*

$3899=202(220):
>gi|1123974392|ref|XP_019598710.1| {|327(0)|<Chordata(M)>}PREDICTED: interleukin-1 receptor accessory protein isoform X5 [Rhinolophus sinicus]
{(EDLKRNYVCHARNAKGEVEKPAKVKQKVVAPRYTVELACGFGVTVLLVVILIVIYHVYWLEMVLFYRAHFGTDETILDGK)EYDIYVSYARNaeeEEFVLLTLRGVLENEFGYKLCIFDRDSLPGGIVTDETLSFIQKSRRLLVVLSPNYVLQGTQALlELKAGLENMASRGNINVILVQYKAVKQTKIQ---------------------
WKQCLTSFRGAEG()}*

$3900=205(215):
>gi|1123487906|ref|WP_074218012.1|
{|0(233)|<Proteobacteria(B)>}molecular chaperone Tir [Halodesulfovibrio marinisediminis]
{(MNIFM)------SYAS---EDRHYAALLENALIKAGHKIIKATGKVNS--SKsfaQKLNR-YIQSADalIVLISWATLKSKWIRHEVESMAIQDFSTNEKKIIPIKLDSSPmpS-YLTNYDYLD--STSS-DGVIEKLI-QSLSTGEKKDVFE(SASGAKEQNTLHYSKELSKKLKTGRLTLVCGAGVSIGAGIPTWDDLLFRLLSSMIRNASEARSIPFDEAS)}*

$3901=133(146):
>gi|1124041807|ref|WP_074434014.1| {<Proteobacteria(B)>}hypothetical protein [Escherichia coli]
{(MSGAVH)--------AT---EDKDsFVRPLAELLKAKGINVWYDEFSLG--WGKslrKTID--YGlANSRfgVVVLSKSFIKKDWTEYELNGLTARELSGEDQVILPIWheVSKSDilK-FSPTLVdkmALNTSINTI-DEIAEQL----------ESLLK()}*

$3902=227(222):
>gi|1123876292|ref|WP_074406157.1|
{|0(645)|<Bacteroidetes(B)>}hypothetical protein [Aquimarina megaterium]
{(MT)-YKAFISYKHVtssqfaYYLELHLKRYGKTLFQKPD-RIFRDEQFLKVGSDLsaSIKKALKESEYLILLASKEASESPWVLEELKIWCeELQRSDkiiIIQTSGNIAID---DDTKKINWDETNALPIYLKEFLPELP--LFVDLSWLESKD(YKRLSNIRYKTAVNSIVSRLRNISPNELNGIEWKIRRRNLRLAFSVSLVLIALLITTIIGTVILKNQRDTLDRELKISNS)}*

$3903=263(263):
>gi|1121486971|gb|APP89574.1| {|296(335)|<Rhodophyta(E)>}mitogen-activated protein kinase kinase kinase 1 [Pyropia haitanensis]
{(QISSVLLPLSGALLSPSLRKHFVKAAVDVSVGWMQMGLPLKTVKILLPETDITKDIETFAKVKSKTAAAAAAAAAASMEI)RHDVFFSYaEADRPSAQQMMAYLRSKNENIRVYDAEpREVSVrarGVVNEAAMGAL--NtSRIFLSLLSDDYFHSVECTEAFSLAYCRDFESQGNFIIPLFWRSCDLTPLARRLVATEGVDCR----EAAFESATMLLDEILSALS(GLSAGPADAAEPDAFAEGTDSSSAGPGAAGTAGGGKLLLADKL)}*

$3904=272(263):
>gi|1125059624|gb|OLA24182.1| {|75(102)|<Firmicutes(B)>}hypothetical protein BHW29_02855 [Faecalibacterium sp. CAG:74_58_120]
{(AHWGAVISRFGIEYVEDPATHQRIPTCHRVQVASILTDEDYLAAVENAPDEESRRIYQEEAARIAEIQKGILAISANEKP)-YDVFICYEE-TDengqrTRDSqWAQDVYYGLTEQGLKVFFSRItlEDKLGQQYepYIFAALNSAKVMVVIGSRpEYFNAVWVKNEWSRYLSLMKHDHKRLLIPCYRDMDPYDLPEELSMLQSQDMSKIGFMQDLLRGVQKVMQQPTSAPQ(GVRVETATLETNAPGVTSLLKRAALFLEDGDTASAREYYDRVL)}*

$3905=249(255):
>gi|1124912384|gb|OKY85349.1| {|76(53)|<Bacteroidetes(B)>}hypothetical protein BHV69_05940 [Bacteroidales bacterium 52_46]
{(LAKHQQEEDALVNIFAMLGKKRRILLHFEANDYFPMHQGGMRALYNALIPLRGSIAAICIHDSYNNSMVKRINLTFMNKI)---VYVSYKH-NEATDSRIQAIINGLVTNHINYSIDKEHVGyRDSIRKYEEEIGQGARIIVVITDDYLESPHCMYELTQIWKN-KN-VKERVFPIIDIEKYPRDSEGLLKSKAYWTNE-RSKKCALLAKETGSIELLQREL(ADINDIIDNINNVWTYIHDINSLDMMTLTKDNAQY)}*

$3906=273(263):

>gi|1124998244|gb|OKZ66630.1| {|74(441)|<Firmicutes(B)>}hypothetical protein BHV88_15990 [Clostridiales bacterium 41_12_two_minus]
{(AEAYWGIVLCKYGIEYVEDPNTYKRVPTCHRTQLESVLTDVDYLSAIENADSNQRLIYEQEAKEIDKLQKD
ILSIVHNEK)PFDVFICYKE-
TDEngKRTVESVLandiYYQLTQEGLKVFYAAITLenKLGQEYepYIFSALNSAKVMLVVGTkPENFNAVWVK
NEwsryLKLMSNDRS----
KTLIPCFRDMDAYDLPDEFSHLQAQDMSKIGFINDIVRGIKKLVAPAKKEPT(NTVVVESNNTDIAPLLKRAF
IFLEDGNWKDADEYCERVLDIDP)}*

$3907=260(250):
>gi|1125043750|gb|OLA09246.1| {|74(0)|<Firmicutes(B)>}hypothetical protein BHW21_04540 [Eubacterium sp. 45_250]
{(SEAYWGIILCKYGIEYVEDPTTYKRVPTCHRTSYDAITADEDYKLAIQYADISQKIIYEAEAKAIDEIQKG
ILTISQNEK)PYDVFICYKE-
TDEsgKRTqdsvLANDIYHQLTQEGFKVFYAAITLedKLGQEYepYIFAALNSAKVMLVIGSkPEYFTAVWVK
NEwsryLKLMKADR-----
SKlLIPCYKDMDAYELPEEFAHLQAQDMGKIGFINDIVRGIKKVINKDEPKSA(VKETAAAGQTVNSGVAPLL
KRISLFLEDGN)}*

$3908=213(220):
>gi|1125000334|gb|OKZ68525.1| {|54(0)|<Firmicutes(B)>}TIR domain protein [Clostridiales bacterium 36_14]
{(YDIPRENHVEKMMELFENAGLISRNDLTFSMLMKSMGDRRFNSCTFLSEKEVDLQFESMFIFSEIEVLEEC
GYYMRPFGK)EKQIFISHSSMdKKDVEMIIPYLNGQDLPVW------
FDKYSIPVGTsiteQVQKGIEESDMVIFWVTDNFLKSNWCKMEM---KVYIGR-
MIEENIRMFIVMDDEIEIKKLPLFLRDIKHL--RREHRSVIEIAEEIAGIIKQM()}*

$3909=191(190):
>gi|1125000762|gb|OKZ68917.1| {|0(643)|<Firmicutes(B)>}hypothetical protein BHV87_15045 [Clostridiales bacterium 36_14]
{(MN)--KIFLSHSSHdKQYVEYIATKL------GKDI--
AIYDTFSFESGLktfdEILKELDVTDLFVIFLSNSALESEWVKKELNISNELKqkeklKQ-
IYPIIIDSslsySDPRIPKWLNNNGFNSYNLRHIS--
SPKLAYRKIKNQLIFLNNQSN(NNLHKIYIGHEDLIKKFDEEYYIATNAPKCIIACGIEGIGRESFIREC)}*

$3910=181(184):
>gi|1125009870|gb|OKZ77181.1| {|0(640)|<Firmicutes(B)>}hypothetical protein BHV87_01725 [Clostridiales bacterium 36_14]
{(MDGILQK)KNKAFISWSgNKaRTCADFLSDLLNKIFEENEIIFCSR----
QITDGRmwiqEIDKALKESKIGIILVTREDISRPWLNFEAGALYA--SE-
YDSLVIPVFIDVDNKNRIAEHPLRLFQSRIE--
FNLEGILHLCGILYKQMEWKH(ILGADEIISEEFERFSKEKESVFQKDDLEKFLFSKGV)}*

$3911=247(255):
>gi|1125023456|gb|OKZ90089.1| {|118(38)|<Firmicutes(B)>}hypothetical protein BHW18_10005 [Eubacterium sp. 36_13]
{(MLVNMEQLGIAPSKVILPVLGSGNQNIELCYIIPPLINQCMRALAEIECLEKITFCDYDIEKVKKLVSMLE
STDNINKNS)--DVFISYCSAQREYADCLRKMLTERGVK-
CWMAPYSIPTGSSYQTEIPSALSNTPNVLLVLSKEA-ETSRWVQkEIGCAIGAR-----
HKLIPIRSYMYEisPQFNFLLDGEQIYDVD--
NKLDMEENCAKAADYLLKIIKS(DDIEKLCENNKKLNNEEINNKKACGLKTYKNVSKN)}*

$3912=167(173):

>gi|1124572501|ref|WP_074855040.1|
{|0(397)|<Proteobacteria(B)>}hypothetical protein [Thioclava dalianensis]
{()MKNVFLSYSRSdTELAFSLAAEIELLGYS----
IWIDREIGGGQNWWaEIISAIRNCDVFVFCASGDSLTSEICRTEFDYAVALRK-----VILPISLAEM-
EVVHPMLADRHVISFLSadYGHRKEVAKALLGIPPPHPAPIP(EPDSPQLPAMPLVEISRRLRMAVLTLEEQE
DIL)}*

$3913=251(255):
>gi|1124999598|gb|OKZ67877.1| {|474(25)|<Firmicutes(B)>}hypothetical
protein BHV93_15225 [Clostridiales bacterium 52_15]
{(SKKRIKQYLRRCALEAYKWHDLTLINEIVKTESMMREKNLIIHFDFDELTENVETRDVYEYFLSILKEKNL
KIGDVIVPK)-KTIFLSYAHAdEEDANRLDKCLQNfgydVKRDvRDVKHWGDLR--------
AFMRSIRKEDYVVLLVSDTYLRRDNCMYEVMQLL-KDES-YQQRSFPVVIDFS-
KEEKEKRSKAGRAQSMFdlSYSPEITLFWQERKDAFGKEIQK(IQMEYRCEATKKYGELCNITQSVGEFMNSF
FQNEL)}*

$3914=180(188):
>gi|1125000662|gb|OKZ68829.1| {|0(974)|<Firmicutes(B)>}hypothetical
protein BHV93_11760 [Clostridiales bacterium 52_15]
{(MMENIPSGHAD)---VFLSYSHDdR----
ELVEKIAGLISAADFSCWIDKeRLRAAEKFNsSIDIAIDDSNVFVAFLSKTYVNKPYCIHEFDLAIQR-----
EKSIVAVCLDDV-
SESANRQSAYMFSFCAGhdVLGFGSGINESSDGIASFAREIT(ESVPLTQLKRFLCSGDEKDLPPIDTPEYLM
ARLRLYH)}*

$3915=228(234):
>gi|1125041262|gb|OLA07003.1| {|100(0)|<Firmicutes(B)>}hypothetical
protein BHW12_07020 [Coprobacillus sp. 28_7]
{(FAYRDTKPLDYENYLLTLTNNATKTEINEIIKLIISNVKLREKEIVYKVSNFFYGNDYDEAIEKSLLQRTK
EIELFSDIK)RD-IFICWQS---KDKDKAIEVLNALENDGYTCWISLRNIPWDtDNYwsNITKAIKSCDI-
FLCISSINTIQSRDCVkEVEIASSLNKKKR----IEYKIDDS-
KDITLFKAFFTGQWITNIDDLLLKVYELKNKENKLKNNAI(QFLENKKLYLTMKK)}*

$3916=278(288):
>gi|1125042645|gb|OLA08213.1| {|99(438)|<Firmicutes(B)>}hypothetical
protein BHW12_05295 [Coprobacillus sp. 28_7]
{(LYVNRKSNPFAYESFLEHLNNEATITEMDEIVDIIVSSTQKREEEKIRKLTNHFYGAKYNKDIDKALIKRQ
EEIELFSDI)RRDIFICHSS--TDINKVNEIL-KKLEEDGNTCWISSRNIPwDSDNYwlNIEKAIKSCDI-
FLCINSINTMQSNDCRrEVEIARNLNKKR-----IEYKLDDA-KDITLFKKF----
FTGQWITNTNDLLEKVYDLKNKEDKLL(NKIKDLIDNNKLDEAKASLEELNTITSNENTLTICKSLIKAINLI
NEKMYNEAKEVLLDSADINYTKS)}*

$3917=189(186):
>gi|1125086670|gb|OLA49328.1| {|0(292)|<Firmicutes(B)>}hypothetical
protein BHW40_13610 [Firmicutes bacterium CAG:65_45_313]
{(MQEDRIPK)---VFISYSW--SSD-
AQVLSLAQRLIAHGVDVVLDKWDLKEGQDKyaFMERCVNDPEItKVLIVCDKQYADKannrtGGVGdEtVIIS
SEIYGNIkQEKF-IPIITERD-
ENGVAYVPTYIKTRIYIDLSDDSKYEEEYEKLLRNIYEKP(LFRKPALGKKPEWLDEEKTDFFPLKDLIKQIR
GSVNSN)}*

$3918=223(219):
>gi|1124912181|gb|OKY85152.1| {|0(538)|<Bacteroidetes(B)>}hypothetical
protein BHV69_06535 [Bacteroidales bacterium 52_46]

{(MEKKE)-YTAFISYSHDdtywaDTLQKKLEHFyipvalreANPDIPATVRPIFKDDTDLTPGT-LsdRISNALDNSDFLIVICSIHSAQSEWVSKEVEHFIRAGRSSriiPVVV-GDM------ADGSSIDRCMPEAL-RALKG--DDQL--LWIDLRAEGIDR(TVVKIIAVILNVSFDSLWQRHAKERRRRMRRRVCLCSAFAVIAAGITLFGVTQCSRLKLNEFTTLQNDTAAHAQ)}*

$3919=151(160):
>gi|1125388957|gb|OLD25652.1| {<Acidobacteria(B)>}hypothetical protein AUJ04_07205 [Acidobacteria bacterium 13_1_40CM_3_55_6]
{(MPVRRVKRKAETKAVKAAQS)EYIVFISYSTPdSWIAHVMKEKVEALGA--KAYVYEKDMIGGGVIVKEIIDAIDACNEAIVRISPNTVQ----------KPDWVVFEIGAVRGQHKRVTPIlNNVDPQQLGPTKDIKGVeLNNFEHFLHQLRRRITQAARRKN()}*

$3920=228(224):
>gi|1125180267|gb|OLB34417.1| {|65(0)|<Acidobacteria(B)>}hypothetical protein AUH01_01285 [Acidobacteria bacterium 13_2_20CM_56_17]
{(CEGVAMNPQWLFDSVKGVAVRAGYPGERTHDAADLKFSGRVFLYTEDLFSAEQVGKLEQRTKEQGLDVVFRTPTSALERS)RYEKPLAFIS---HDwrdkKDIAQPIALGLSRMRCPVWYDEYsvKVGDSLRAsveKGL-----KESKkcVLILSSNFLSNTgWTKTEFDSIFTRQILEGSDVVLPVWCGVTKqqI-YEYSPSLLDRLAVNW-DLGIDEVLRKLHRAIEPPISNY(TPIP)}*

$3921=126(144):
>gi|1125388956|gb|OLD25651.1| {<Acidobacteria(B)>}hypothetical protein AUJ04_07200 [Acidobacteria bacterium 13_1_40CM_3_55_6]
{(MAYS)---VFISSSYKdRDLARDVARRVKEAGVSV-----TSEVT--LSAGSeyekIIMDQLKNADEIIVILSGNSVDNLWMMFEIGAASSLRKK-ITPVVVGLEQEELPPVI-KQLKYIRYNEVSD--YISKLER---------GAQAA()}*

$3922=270(290):
>gi|1125393536|gb|OLD29740.1| {|60(45)|<Thaumarchaeota(A)>}hypothetical protein AUI62_02325 [Thaumarchaeota archaeon 13_1_40CM_2_39_7]
{(LEKQDFDLEHRTLRIRDPKVMRYGEPTILPKDVVWLKEFLTNYHDEEKIFPITSTIVEQYAKDAATLVGIQILEEQEDEG)--KVFISYSFDdKDIMEKI-----TLSLNTRNIgYYT--AEHDTQYGNpltaKIQNAIKDSDALIAVITKDHPSSSVDQ-EVGFALNAGIQ-VIPFVEEG-----------AKVGFMLNDIEQ--MRFKKDK--IEDSSDKIAMYI(VEKLKHRKSSEEIFINDHILIKEHGDVVYSSNFQKGDIIRGNIESDLPITIYVVDNQNLEKYNENEEFDY)}*

$3923=167(175):
>gi|1125396816|gb|OLD32609.1| {|0(67)|<Thaumarchaeota(A)>}hypothetical protein AUI61_02545 [Thaumarchaeota archaeon 13_1_40CM_2_39_13_2]
>gi|1125523524|gb|OLE40938.1| hypothetical protein AUG16_02005
{(M)--KVFISYSSAdEDIMEMMKK---SLKKQGIESFTAKHDIKYGSSQSeSIKDAIKNSNAFVVIITRD--NPSLSAAqEVGFAL----N-EDISVIPIVEEGA-KVGFMLGDSAQIRFTRSqiEKACEKASKFISEETGEEQEYNE(KLVDERKRIEDYEAYGFNFEKGDYISGSIHSDVP)}*

$3924=129(141):
>gi|1125183178|gb|OLB36993.1| {<Acidobacteria(B)>}hypothetical protein AUH11_09775 [Acidobacteria bacterium 13_2_20CM_57_17]
{(M)--QVFLSYA---PSDEAFAKALSSQLTRRGLSVWSPGEEVLPGDNVwlRIGEALKNSRA-MIVLVSPDSMRSENVRrEIEYALG---QPnYEGRLFPVQVRPT-NDIP------WVLRRFKTFDARQSAAKVSESIVNALSQVA()}*

$3925=176(168):
>gi|1125837724|ref|WP_075132514.1|
{|0(22)|<Actinobacteria(B)>}hypothetical protein [Actinophytocola xinjiangensis] >gi|1125635928|gb|OLF12323.1| hypothetical protein BLA60_10150 [Actinophytocola xinj
{(MSGY)KFDVFISYcRR-
GNVRKWLENHFYQTLVDclsdqlAAAPTVYVDWAMprGVHWPSELKKAVRHTKIMVPVFNPPYFESRWCMAEM
RSMRERERMLGLRSVEKPQTLLYPILFADSVNFPPESRELSWQDFKEHAHPHPSFQDTVEYLPF(HKRVVAFA
EDLAELLAQVPEWRPE)}*

$3926=196(195):
>gi|1126176133|ref|XP_019620323.1| {|0(126)|<Chordata(M)>}PREDICTED: uncharacterized protein LOC109466898 [Branchiostoma belcheri]
{(MGQAPYKTG)-PSVFLCHAGEDKDRFVEPLYKALVANGlkpD--
DIFYDKFSIQAGHDIkKSIESALASPNLQLVVTV--LSR-
NLLCehKYWPKREFELAQrYGLPIFPVWLGHDFGDFDTLVRKYSPTLKGIRALSVTCDSQTGIVEDKIIQDVA
(DNIIKELPKTTKRSNAGFGVLALVAAIVVVIAVISQFITGCPIPNR)}*

$3927=252(256):
>gi|1126164361|ref|XP_019646234.1| {|142(0)|<Chordata(M)>}PREDICTED: uncharacterized protein LOC109486782 [Branchiostoma belcheri]
{(PQSEKTASSPLIPGPPDVSSRVDNRVGHSKTCAVNIISQIDPSFDAGPEFIGSIDGHVKELEAKIKASNSS
SEGPLKPRT)-YDCFIIYSRDdeDIVYNKLIPFF--
ISNNIKYCEHQEHFELGKDIFDNVNICIKHSRRVVAVLSRSFFASGHCMYDLKSARGYAADNGysvQDFLISI
---KITnFEFPNKY----RDIGNStYADLSKDVNDerK-WLQIKKAL--
T(EFSLASVVIEMGAKYSTPKLRLPVQDVSVVSMVTEV)}*

$3928=265(269):
>gi|1126169556|ref|XP_019616732.1| {|23(0)|<Chordata(M)>}PREDICTED: uncharacterized protein LOC109464235 [Branchiostoma belcheri]
{(SPLIAPAVSSKVQQTQNASTGGACSSRVEHPCAVNIISQIDPSFDAGPELIGTVDGHVKELEAKIRASAYS
SEAPLKPKT)-YDCFIIYSRDdeDFVYDKLIPFFNSNN--
IKYCEHQEHFELGKNIFDNVNTCITQSRRVVAVLSKSFFASGYCMDDLNAARGYAASNGysvQDFLISI---
KITnFVFPPKYCD----ISNFtYADLSEDINDerK-WLQIKKAL--
T(ECRLASVVMRIDMDAKYCTPKSRLPVQDVSVTASKYGTFEAIVRVHYQK)}*

$3929=281(291):
>gi|1126171077|ref|XP_019617566.1| {|156(0)|<Chordata(M)>}PREDICTED: interleukin-18 receptor 1-like [Branchiostoma belcheri]
{(CVGELKGRSDSKTIQLLRGEPREGLPLAVSLGIAAAGVAALVTMVTGFAVHRRRHDPDLGWDKPDPEAEYN
IPYENELPK)-YDIFISYSSKdlAFAKDVLLKEL--
TCRGYEVCVDFKDFTPGVFIVDNIKEGVFRSRKTIIVLSSNFRRSGFGRFELQMAGARKYLERKDVLVVVKID
Q---------CKVPTMLIGRtFLDWT-NENVrpHFWQRLENAI--
G(PDEKVRNRKDNKETPEEEEEKEEEETITAAGDHNHRDNAVNGHIANGGPHVLGLPETDLLENDERPLLPVV
)}*

$3930=276(299):
>gi|1126202427|ref|XP_019634610.1| {|122(461)|<Chordata(M)>}PREDICTED: uncharacterized protein LOC109477705 [Branchiostoma belcheri]
{(VQISSPSSQQAARPTWESRNLPAGHQVSAVKEEGKPSYFIPTEEEDSERAKSASLPPYPSEERGSDSSGPD
LEDDDDVEY)-YDYFLVFSEKdkKHAHKIM-DLLKSHK--
LKGCLLEKDFPAGVSPFKNIADAIERSTYTVLVLTENFTKDRWCEKKLQTSLMEAIENpgKYNCVIPIIPHPd
lMPrKKIPSELRTVSQL-------------------------

D(MMSDYFPDKIKKTFKTKEREKRERKMSKSRREVTRKPLRSVCSVPSSLSSKTDFESLSAGKSSQSLSQLDR
RYSSPVDG)}*

$3931=184(193):
>gi|1126209534|ref|XP_019638486.1| {<Chordata(M)>}PREDICTED: myeloid differentiation primary response protein MyD88-like [Branchiostoma belcheri]
{(MLYTSRPGFGMAEQEIATDAPESKTEDSSQYE)-
YDLFISCHVKqlPWIIEVLLPALENPPLSLKVYVFERNSLPGEVIADALTTAAHTSRKSILVFTEDYLEGEWN
QFEANMAQATSPGSNTRC-IPLKLHPCE--IPDRFQSYHPLDCT-------SPIMrrFFWFKLLKAL--
D(CGVDHHAHLLQMKYPFRTPSH)}*

$3932=223(244):
>gi|1126212212|ref|XP_019639922.1| {|0(1067)|<Chordata(M)>}PREDICTED: uncharacterized protein LOC109481772 [Branchiostoma belcheri]
{(MSKGPLHFKSISCLCFADGIAPTTL)-----VSYCDDakQWVVAELTRRLEEQEKTL-
VKIKDRDYDAGISQLRSFCEWLDVCTNVVLVLSRGYLQSGLCQRELELA---LASERR--LITVLLEE-----
---GCTLPAALQGRlCLHLRQD--GQFWPRLIAA---
T(GPQPCKAAGPGHHTVPRMRPPKLPSRCFIRDAAIAQVTKLISTSARELSRASSIRHIGDNVVGIWGMGGAG
KTVLALMV)}*

$3933=218(225):
>gi|1126218291|ref|XP_019643233.1| {|527(0)|<Chordata(M)>}PREDICTED: tetratricopeptide repeat protein 22-like [Branchiostoma belcheri]
{(QVCRYLVDHDSFQLSRICLKCFRDHNSRTFRTEAESMLSEIHGKTACGEMVTPSAQYRPPEARAKLPVAVD
KPRHSQDFQ)-YDFFVSHSSKdeDWVNYALLPALE-
VDLRFKGCIADRDFIAGKDVFANIIDSIENSFKTLLILTPDFVNSEWCLYETKHALMESLKGKTGRVIPIMLK
E---------CDVPASVRTItYLDVSHDAIGsYDWRRLKEALEAT(PPNSA)}*

$3934=234(238):
>gi|1126164467|ref|XP_019646734.1| {|165(0)|<Chordata(M)>}PREDICTED: uncharacterized protein LOC109487192 [Branchiostoma belcheri]
{(SNATVQSRPPNATLRRPQHSMVLSPRINLQGNGTMVKVKMDTKIDSHNGSPRFIQAILHKMEHVKDQVKAV
ALSEHITPK)RYDCFIIYCGKdsAVVNGIIIPRL--
TELKITYCEHQEHFIAGRDIFTNIQDCISQSRKILAVFSEDFFASDWCRKDLDMAMVHATEKQIEHFIApIKI
DDC-VIPDKYGD-----LKNItYSDLTSYPKDWGEQIRCSLASIR(VASMEMEICMCTTSADSI)}*

$3935=229(243):
>gi|1126171750|ref|XP_019617935.1| {|255(0)|<Chordata(M)>}PREDICTED: uncharacterized protein LOC109465233 [Branchiostoma belcheri]
{(VHITGGVVETLTVPRDQCVDGTWTLQVSEGITCNTEYNVRVTGGTETDLGPSSDTICLVTVGPGRREDSTE
YVKDFWIIG)---------HQddRSRIEKLCKALEKH-
HELTGGTDYRDSEGGDSVFDSLDNAISTSKIIIVVLSNN-
TKGGWVKYKIQTVRHCLMDTNeeeqqtSRRLIPVRLTRC-GKP--------HCLQTLeTLDNGDKY---
FWRKLLATFNKG(SCNEDDRTTETAPLVQNNGPHVV)}*

$3936=209(214):
>gi|1126193509|ref|XP_019629799.1| {|0(922)|<Chordata(M)>}PREDICTED: uncharacterized protein LOC109474030 isoform X4 [Branchiostoma belcheri]
{(MAEGRGSRNRSSSRLSLLTGSSLSLTVPFTT)--DVFVACADKdqAVVKSRVIQPLESRDTTCRY--
PQRDFIPGHSEHNSVVEAMKTSRKTIVVLSENFKDYRLSRVILALSNQLITQ---GRVIPVKIDED-
TEMDGRFRDVVTLNMTAiDEATFIAKLEEAVTLPDQDLSSA(SRSPGSLLDVAAVPELYTPEAESTTSSETSS
DSSTGHTDLPQQ)}*

$3937=222(235):
>gi|1126199935|ref|XP_019633258.1| {|0(1067)|<Chordata(M)>}PREDICTED: uncharacterized protein LOC109476693 [Branchiostoma belcheri]
{(MEKARHDSVCQRPADDTADYL)-YDILLSYCDRnrPWVLYTLASELERD-NNLVLCLRDRDYESAKPRLQNFVDSARISARIVVVLSAAYLADGPCQKELDHAIASGRTMVAVR--------------FDGCEIPAQFRKgagGvVLDRSDVD---FWDKLHRFLHRE(QLNFSTSHSGACCEKKGRNTSFPVMPGVVVERPHDVDAVVVLVQITMETLKERTNGRREGHVVGVVGEPGAGKT)}*

$3938=240(239):
>gi|1126209139|ref|XP_019638268.1| {|26(0)|<Chordata(M)>}PREDICTED: myeloid differentiation primary response protein MyD88-like [Branchiostoma belcheri]
{(KNMKMDDVAELLLEKLTEERTLANVTDDVGDKVEASAEHMEEQEEDKEVGASGVTVGMSFFKRFQKLFISSPVFRWLRKF)KFDAFVSANQRdfPWV-DEWVQQLESPPHNLKICVHHRDFKSGVPIPHNIDWAVANSRKILIVLSPNFLSSNWCKFELKAAIEKELKSDKYCVLPVLKSKC-EIPDELALLTYLDCTDGnVDVAKLASDIKSKVRKRASFASS(PKRKRFKYDHKERDYGPSF)}*

$3939=192(194):
>gi|1126168251|ref|XP_019616027.1| {|0(344)|<Chordata(M)>}PREDICTED: uncharacterized protein LOC109463615 [Branchiostoma belcheri]
{(MPPPTHKKKHLITTEPR)---VFVIHAG-ED-KDSFVRPLVSSLQEKGLQekdIFFDEVAIKPGDIIrdRIMSTLSseSLELAVIVVSASFlNKSYWPRLEYETCLKNN-----KHIFPIWVDANEDNFKAFSESVGKYSPTLKQMSGRCVQRQEVANELPNIAAE(IVQRLSALRQGHHATQPPAIQMLLYPGPPSSSSDSSD)}*

$3940=220(226):
>gi|1126188584|ref|XP_019627119.1| {|77(0)|<Chordata(M)>}PREDICTED: uncharacterized protein LOC109471996 isoform X3 [Branchiostoma belcheri]
>gi|1126188586|ref|XP_019627120.1| PREDICTED: uncharacteriz
{(FGAFHEPMEFHTTTFNTEVKKTGKQKKEKKEKDTDDDQKEKKSGIKDKVKGFVSSVLTSKPSPEEDLTQRMERTTVSDLG)PVQVFLSYQW-DHQKQV--TQLHDMLEEKGHSCWMDIKEMGGGDQLyaAIDKGMRQAKVVISCVTPKYTESANCRNEVALAHSLK-----KTIVPVLLEKTAWPPEGPMAMPFAQLLYIDFTKEPKRNPWQGPHLDELMTSV(EQSLKE)}*

$3941=253(261):
>gi|1126189603|ref|XP_019627671.1| {|798(70)|<Chordata(M)>}PREDICTED: LOW QUALITY PROTEIN: uncharacterized protein LOC109472379 [Branchiostoma belcheri]
{(GASSGSGKFWPDSSFYELLGQLAYHAASDQSLVTDEYRNWTPSVEKKEEKEASPESESVKKEKTSSPPVEEKEVEPVVNP)--EVFLSYQW-GHQPQVKA--LYSQLTSQGFTCWMDIQQMGGGDALygKIDKGIRSAKVVVSCVTPKYTLSANCRREVSLADALR-----KPIVPLLLQDTSWPPEGPMSMAFTELLYIDCRDERTQTDWEGAKFEELMQQV(SRHATPAPKPQRSKSPPVEQNATKPAAPEQGNGEATTEKTK)}*

$3942=253(261):
>gi|1126189605|ref|XP_019627672.1| {|798(31)|<Chordata(M)>}PREDICTED: uncharacterized protein LOC109472380 [Branchiostoma belcheri]
{(GASSGSGKFWPDSSFYELLGQLAYHAAPDQSLVTDEYKNWTPSVEKKEEKESNPDNESAKKETKSSSEIKEKEVEPVVNP)--EVFLSYQW-GHQPQVKA--LYSQLTSQGFTCWMDIQQMGGGDALygKIDKGIRSAKVVVSCVTPKYTLSANCRREVSLADALR-----

KPIVPLLLQDTSWPPEGPMSMAFTELLYIDCRDERTQNHWQGAKYEELVQQV(SGYSTPAPRPAEEGTKEPAD
GAIKAGDNKTQTVQHTDATPR)}*

$3943=255(261):
>gi|1126189605|ref|XP_019627672.1| {|604(223)|<Chordata(M)>}PREDICTED:
uncharacterized protein LOC109472380 [Branchiostoma belcheri]
{(FLAVRLGYGPEDIRAWATNADPTMAMLNEWYNTHRSSEATYAVLKSLQEMGRTDAAGIIETAMEEAGAAVP
KPAADEPTN)PPPIFISYQW--DIQDQV-
KLIKEHLHMAGYDSWMDIGQMGGGDQLyaKIDKGMRGAKVVLAMVTPKYAESTNCNHEINLANLLN-----
KPIIPLLVENTPWPPPGSMSMIFSQLLYIMFHGASSGSGKFWPDSSFYELLG(QLAYHAAPDQSLVTDEYKNW
TPSVEKKEEKESNPDNESAKK)}*

$3944=277(278):
>gi|1126193867|ref|XP_019629994.1| {|62(38)|<Chordata(M)>}PREDICTED:
uncharacterized protein LOC109474183 [Branchiostoma belcheri]
{(AATTEVLMKALYNADLAGIVDALVGRYPELAVYQMPPVQPPREQPTMVQPHPAPRVQPSQVPRRQPSHWDE
PASQGEKKL)----FIVHGG-ED-
KDTLVEPLAQEIVQQGMpreNVFFDKWSIGHTQGIkqSIASAIHdsSCKLAVIVISEDMMKKYWPKKEFEEFL
KRG-----
IRFFPIFYGVNPADVRTSYSPTLADtrgKEIPRTGHRVDGALIAGTANEILTVLQ(ELGGSYTCSQDVMMKYF
KVIAKEMPADWKELALNLGVPWAQIKAVNLHNSADPWTSCH)}*

$3945=204(212):
>gi|1126198246|ref|XP_019632330.1| {|0(1163)|<Chordata(M)>}PREDICTED:
uncharacterized protein LOC109475966 [Branchiostoma belcheri]
{(MAWTEPR)---VFVIHAG-ED-
KESFVRPLVTKLLQQGLAeedVFFDEHSIQPGQGIrdMIMSALSCerLELAVIVVSKSFLEKpYWPKLEFETC
IRNG-----KAIFPIWIDDN------
DDNFRTFSQSVQRYSPTLKQMRAMCVQRRDATDRL(TNIAAEVVKRLSPPGIRALLYLGLSKLSRFFTGQSRH
TEANNGEQIEDKLKERRLELIRTWGEDQ)}*

$3946=225(222):
>gi|1126199137|ref|XP_019632820.1| {|142(0)|<Chordata(M)>}PREDICTED:
uncharacterized protein LOC109476337 [Branchiostoma belcheri]
>gi|1126199139|ref|XP_019632821.1| PREDICTED: uncharacterized protein
{(QPKQETTGQPSSEQPTSWRQPQQETSEKPPQDTTEPKQQSTGEQTSSDTSRQPQQETTGPPSTGGTSSVLE
SPPCSDYEG)PKKLFIIHGG-ED-
KDTLVEPLVHEIVDQGVPkedVFYDKWSIEDAQSFkeSIALAIqdRSCKLAVIVISEHMMKKHWPRKELEEFL
KRG-----IRFFPIFYGVKPDDVRKEFSPMLADTrgvEIPRTGEAVDEILIAGTASKIRKIFQ(KP)}*

$3947=222(231):
>gi|1126199647|ref|XP_019633101.1| {<Chordata(M)>}PREDICTED:
uncharacterized protein LOC109476555 [Branchiostoma belcheri]
{(MYQILMTWKEKEGPSVTMDSLGGTLQELGLYELASALNPEKAEADEKDGSSGSKPPTPTARVQPIVRSVET
PAKQENT)---VFLSYQW-DHQDRVL--
LLHDRLQERGYGCWMDIKQMGGGDSLyqMMDKGIREAKVVVSCVTPPYVESPNCQDEVALARTLK-----
MPIIPVMLEKTTWPPPGPMSIPFAQLLYINMANSQDDDPWKGPLFEELIRMI(EYFVLDATDGSPD)}*

$3948=240(244):
>gi|1126204157|ref|XP_019635553.1| {|0(1026)|<Chordata(M)>}PREDICTED:
LOW QUALITY PROTEIN: uncharacterized protein LOC109478460 [Branchiostoma
belcheri]
{(MDSGTDTVQQDIGQRGAERKPEQAESEPAEPR)---VFIIHAG-ED-
KESIARPLVSALHEQGLDkhdIFFDEHSIKPGEGIrdRIVSTLKSdaLELAVIVVSTSFLkKDYWPKLEFETC

LENN-----KRILPIWVDDNQDDFKAFSELVGKYSPTLKQRKAIRVQ--
RNSVADELTNLA(SQIRKCLPPPAIQALAYLQPTGTPSSHSGSSDEEIGIGRKRRYDIWDGDEEVIEKKLKEE
KTRELTDLQRLH)}*

$3949=224(233):
>gi|1126205513|ref|XP_019636297.1| {|97(0)|<Chordata(M)>}PREDICTED:
uncharacterized protein LOC109478932 [Branchiostoma belcheri]
{(NVPPSSSSNFPPWRMTTMPAETASSRQTAAQPLPTNVPPSSSNNFPPWRMTASSRETAAQLYPSRHTTYPD
MTTYPKENA)---VFLSYQW-DHQDRVL--
LLHDRLQERGYGCWMDIKQMGGGDSLyqMMDKGIREAKVIVSCVTPPYVESPNCQDEVALARTLK-----
KPIIPVILEKTTWPVSGPMAIPFAQLVYIDMARSQDDDLWKGPLFEELIRRI(ECFMLDSKDKSAG)}*

$3950=225(228):
>gi|1126205677|ref|XP_019636386.1| {|26(0)|<Chordata(M)>}PREDICTED:
uncharacterized protein LOC109478990 [Branchiostoma belcheri]
{(VEKLIPYFIQRRKLDLSDKQVIMSKVTTRGKAEALLDILIENGKCSPDEFVEILQKGDHKHVADQLRRTST
QNETTEGPH)---VFICHAG-PD-KGRFVRPLVDKLLE-
GLpaeTIFYDEISLQPGDAIddKIIATLSspSLKLVVIVISRHvLNDRYWPKLELELSLLAN-----
KKFFPIWLDQNDDHFAAFGDKLRKYSPTLKGIVGTKVLADRARGEIQKIAED(IVTKLETA)}*

$3951=245(254):
>gi|1126211924|ref|XP_019639774.1| {|184(21)|<Chordata(M)>}PREDICTED:
ankyrin repeat and death domain-containing protein 1A-like [Branchiostoma
belcheri]
{(NTRDRELVLQGRKEVLKALKGEPIDDTVVESKTPAQVKAAQKKRKRKKKKPAGQIVQEPPDQGTQKQEESD
SGESYIEPV)---VFISYQW-DIQSDVVQ--
LRDRLEEAGYPCWMDIGQMGGGDALyeEIDTGMREAKVVVSCLTPKYIASKNCSMEVNLANDLG-----
KPLIPIIFQPVDWPPRGSMGPILAGHLYIDLTCEGGGHGGGVLHASIANKQQ(EILSRIQQKIRPTGRPRKRA
GVATATQEPGVEEV)}*

$3952=247(256):
>gi|1126214061|ref|XP_019640924.1| {|962(33)|<Chordata(M)>}PREDICTED:
uncharacterized protein LOC109482588 [Branchiostoma belcheri]
{(NSDRNPANWVELANGLGYTSQKTETFMKSADPASALLRDWLDGSGEAAMDHVIRTLENMNRDDVLQVIRGR
RGAADAQPA)---IFISYQW-DIQDRV--
RDLRDRLETAGYPCWMDIGQMGGGDALyeKIDQGMRASKVVIACLTTKYSVSQNCSKEINLANLLG-----
KTIIPIMFQKIDWPPPGGMSLIFSPLLYIDMTSPGGGHGGAGIHSKLETKFQ(EVVERIQTTIRPVGTGKGGS
MEPESPGKESDKQASS)}*

$3953=225(252):
>gi|1126224542|ref|XP_019646633.1| {|2354(254)|<Chordata(M)>}PREDICTED:
LOW QUALITY PROTEIN: uncharacterized protein LOC109487105 [Branchiostoma
belcheri]
{(TGLLLAHMLYINMQDPKLYKDKIAEVLSQLEMKTTKKQTQQFPPCFISYCWANSEEACNMGQVTRYAGAVG
RVDPRQLKS)------------------------HLEQKDVKCWMDIERVGKGG-
LfeDIAEGLRHAKMVVACVSDEYVRSKNCQMEFRFaAVTLR-----
LPIVLAVVGTGYQWESTECGMLSLGFNKINFQHGGEVEMDRLVELVQEKCKT(EDEATNDSADDDKSSNSASF
QELYELAQRKFL)}*

$3954=242(254):
>gi|1126224742|ref|XP_019646741.1| {|6(231)|<Chordata(M)>}PREDICTED:
uncharacterized protein LOC109487201 [Branchiostoma belcheri]
{(FYVQPPKPVQINVIEPTSVFADTAPQSDSQDTQFSLNVKYSQQRHTASSSTSKERLLDSSPSTSFLPNKPE
DNMASSQDT)--DIMISGDG-ND--RDFIKFLSSQLSREGLRVW--NEETK--

DSNpsQRAQAIVEAKVFVIVLSSNAVQSQHCTDEVSLAYISN-----
KAIFPVAKEPFDKIYKDLAFGMKLTLAKLNWMFFTSDEESQKSFPTLVTTIR(SSLNKLSKTEGVVSTYEVSQ
LYVKTEETQEKQPG)}*

$3955=226(226):
>gi|1126226570|ref|XP_019647720.1| {|231(0)|<Chordata(M)>}PREDICTED: platelet endothelial aggregation receptor 1-like [Branchiostoma belcheri]
{(VCDKPAPANGGQDCTGEDKENQTCDTGQPCKEIFGKLGEALSGDIVPTILIGLLVFVVLFVPWCLNRTIKA
IAAVLPFSS)PKKLFIVHGG-ED-
KYSLVEPLVQEIVDQGFpkaHVFYDKWSIGYAQGIkeAISSAIQdsSCKLAVIVISEDMVKKYWPRREFEEFL
RQD-----KAFFPIFYGVSPAEVRQRYSPTLADKRGVEVPRTGERVDDMWIAATAGMIRK(TLEGTL)}*

$3956=210(198):
>gi|1132134353|ref|WP_075825072.1|
{|0(108)|<Actinobacteria(B)>}hypothetical protein [Streptomyces acidiscabies] >gi|1125621591|dbj|GAV43030.1| hypothetical protein Saa2_05977 [Streptomyces acidiscabi
{(MTGRPPFFFT)SYAERRADS---
DLVEQFHARLQREVEIKrgrSApnEGFLNTGAVRLGQGWrrKIGTALGRTRFLVVLLTDDYLTSPWCAREWAV
MQERVRLAePAEpvALLPLFWTGVsRelpaEVADLQLRMATLGAAYTDRCLVDLMREDPRGYEKFVIGLT(DH
MVKEASTELPEMEGELAASYPPAFGLAAPEGEEEAAQIMRAEPELK)}*

$3957=210(219):
>gi|1125837721|ref|WP_075132511.1|
{|0(91)|<Actinobacteria(B)>}hypothetical protein [Actinophytocola xinjiangensis] >gi|1125635925|gb|OLF12320.1| hypothetical protein BLA60_10130 [Actinophytocola xinj
{(MDETAT)-YDIAVSFTE---EQRAAAGEVVEACRQRGLTVLYGPDHTHEWWARktdGDLPDA---
RVLffLPFVS--------TTDEFTSAMLRAVRAGDEHVLPVLVDGVAvpAGlLHPHITYLRSVEYRA-
DQLAEALG-
ERVEAAEWERAAV(GDVVARVLASASPAEEKPAEVAVPATFSRYTEQDRTLRYLGEQFAAAMPKLTRDGLVGT
VNSGHSRIAVRVER)}*

$3958=188(196):
>gi|1126169327|ref|XP_019616607.1| {|0(1643)|<Chordata(M)>}PREDICTED: uncharacterized protein LOC109464114 [Branchiostoma belcheri]
{(MAGRLQDRQTGERVFIIH)--------AG---EDKAqFVEPLVHALQDEGLSqqdVFYDGWSIE--
IGEsihDRIMSTLASESlElvVTVLSQNTLNHSWPKLELETAL-----RHNKQLFPVWLDDNQddF-
QEFNGMVGKYCPTLK-
SIRGYKVPWSRAREEVGKVARE(IVSKLKKRDEPDTDDVPIEILLRGPKMVKSYKDACHQG)}*

$3959=200(220):
>gi|1125535895|gb|OLE51286.1| {|29(0)|<Acidobacteria(B)>}hypothetical protein AUG51_23985 [Acidobacteria bacterium 13_1_20CM_3_53_8]
{(DKEKASVINEYLANDFQTLRGMWLEGKNVPSHLGRHILFGEAQDYKDMLMNDISEIEAAAEQYLLNEPTIS
KPEVEYVYD)---VFVSFSSLdQELANTLYDAITKA---DGKAFLSSKHLH---
PGDdfaeEIRNQLTASRELWLLVSPNSLKSEWVLTEWGAAWMAGKK-IVPII-------------
HRCKPEDLPDRLK--RFQCIDFYNYMRLVERTFKTV()}*

$3960=204(210):
>gi|1125537488|gb|OLE52642.1| {|0(61)|<Acidobacteria(B)>}hypothetical protein AUG51_16860 [Acidobacteria bacterium 13_1_20CM_3_53_8]
{(MKIGKEDMKSFVKMKMLEQYHEQQAEKAKRRRQVGY)--KVFISYSSVdQELAIHIGNILEKNNVTY----
--FLDSKDIGWGQniteQVASGLKNCTHLIVIISPASLKSSWVAYEIGQAAALRKV-

ILPYLIHPSLDVPTFIQQYHYKLKLKELEAY--
FKHSSISSEELKEVFDSLKTH(LPSDVHWYLFCPEESNQKKIVWRSKTPQKVPWMS)}*

$3961=210(222):
>gi|1125546547|gb|OLE59994.1| {|0(143)|<Crenarchaeota(A)>}hypothetical protein AUG17_00470 [Crenarchaeota archaeon 13_1_20CM_2_53_14]
{(MEF)----FISYRYEdRHLAGRVARLLEASGQKC----
FLAHDSMKISLDFedEIRRQIDQCTALVAVVTPNFVESIYANQEVGIAMGKAKP-
IIQLWFPAVDKSKLGFLRSLNAIQ--TTEEG--LNTVISK--
VLDAAGQQINDS(FVRPLEFVEAEKIVDMRLEARKDPYYRALVRPQGLYHMFPISRENVDWLSSNLPELFRGLRTRPSPNGLSFSYDELYGE)}*

$3962=247(237):
>gi|1126176070|ref|XP_019620289.1| {|17(171)|<Chordata(M)>}PREDICTED: uncharacterized protein LOC109466871 [Branchiostoma belcheri]
{(SDLRFRDIRGYLLDKGVLSGDNLNEIESKKLRVDQAKALLNILPTRGLNAFSVFRDALKLHYPHLARLLDDESQHEPLKG)EPRIFIIHAGEdKEhFVRPLVSALRQKGLKEDDIFF---
DEVSIKPGEhireRIMSTLSSDALQLAvivvsnsLLSKNYwpkLELEKCLNKKCVFPIWLDE-
NEDNFKAFSELVGNYSPTLKQMSARRVQRTS--
VESELANIATEVVQQLSALEK(SQPTAIQMLLHAAPTSA)}*

$3963=215(220):
>gi|1126561909|ref|WP_075197400.1|
{|53(0)|<Proteobacteria(B)>}hypothetical protein [Cronobacter turicensis]
{(AVNLQKDPVFFEEWLRTHYPGKLKRHNIFTKDIYIYNVISLIDEDMVDFHLTEEGSFPFIYPERKYFEENNPQYRDIMKK)DSKVFISHSSKdKiKIVNPLNAYLQGNDIA------
TWLDSYEIDYGDniflKVNEGIQKSDVGLFILTDNFFDS--QSGWPLAEFSTFFM-
SLMKSNKKVLMVDAGVSPENMLEMMRAYKYI--NWNNGAGLPEIANAIKKIMTK()}*

$3964=139(166):
>gi|1125594266|gb|OLE97347.1| {<Cyanobacteria(B)>}hypothetical protein AUG75_09415 [Cyanobacteria bacterium 13_1_20CM_4_61_6]
{(MRKHCDTLTKGYENFKEEVSLMQLSR)-KDFFVSYNKADRYWGEWIAWQLEEAGFS-
VVIQAWDFRPGSNFILEMQKAATEAERTIAVLSNGYL-DALYTQpEWAAAFASS-----LR------------
------CFTWAKMA--SVSGSFFGGHSWPPFSDSSPAC()}*

$3965=207(220):
>gi|1125630359|gb|OLF06822.1| {|0(238)|<Actinobacteria(B)>}hypothetical protein BLA60_30200 [Actinophytocola xinjiangensis]
{(MTGA)--DVLIVNSRVDESWSGWVADVARNTGHP-VSRLQDGLANFLDVATIIDRETRAAEQVVVLLSR--
-TGPDFLAeAVAAAHRTG-----TPLLPVHVDD-RpaSYPRPLGHLPAVDLTG--VPLATAAD--
RLRAALDGTHTG(LVRPNHVRRPPPTTDPFVGRESELARLHTALFADRAAVVTGEDGIGKSALAARFVRTHRGRWEHVLWSTPARFADD)}*

$3966=125(142):
>gi|1125543480|gb|OLE57461.1| {<Cyanobacteria(B)>}hypothetical protein AUI36_14625 [Cyanobacteria bacterium 13_1_40CM_2_61_4]
{(MR)---IFLSYSAAdKKTAAELAKSLSKLDY----
DVWFaDQDLLPGDNWSlGIGKALEDADSMVVLVSPASAKSES-QSrEVQYALASPNY-QG-RVVSVLVPP---
--------INRIPSDSVPWILNKLTVIESGKTISETSKR()}*

$3967=254(253):
>gi|1126207161|ref|XP_019637190.1| {|41(257)|<Chordata(M)>}PREDICTED: uncharacterized protein LOC109479645 [Branchiostoma belcheri]

{(DLRFRDIRRRLLNSGILDGENLDDIENEQSRGDQVNALLDILPTKGPNAFSVFRDALNDSGYPHLARILNA
ESQQAPTRE)-PRVFIIHAGEdKESF--
VRPLVTTLQQQGLAEkdiFFDDVSIKPGEVIRdRIISTLssESLELAVVVVSTSFLNKPYWPKlEFETCL---
-R-NNKRIFPIWVDAN-
DDGFKAFSELVGKYSPTlkQMSARRVQKDHVTDEMANIAAEV(VQRLSTLGRSTPQPAAIQTLFYLTPSPSPS
DSG)}*

$3968=269(268):
>gi|1126207183|ref|XP_019637202.1| {|18(464)|<Chordata(M)>}PREDICTED:
uncharacterized protein LOC109479656 [Branchiostoma belcheri]
{(DLRFRDIRRRLLDSGILNGENLDEIEKRQSRGDQVKALLDILPTKGPHAFSVFREALNDSGYPHLARILND
ESQQAPPRE)-PRVFIIHAGEdKESF--
VRPLVTTLQQQGLAEkdiFFDDVSIKPGEVIRdRIISTLSSKslELAVVVVSTSFLNKPYWPKlEFETCL---
-K-NNKRIFPIWVDAN-
DDSFKAFSELVGKYSPTlkQMSARRVQRDHVTDELTNIAAEV(VQRLSTVGRSTPQPAAIQTLWYLTPLSSRG
DSGSSDDETSQGTHAAGV)}*

$3969=287(294):
>gi|1126224590|ref|XP_019646660.1| {|0(203)|<Chordata(M)>}PREDICTED:
uncharacterized protein LOC109487131 isoform X2 [Branchiostoma belcheri]
{(MGSGPSSQKSQPPTLLPIQVVHNLENERFKNGSLEPLQPQAVQNTANEQLEDDTHTRGQKDLKRLESSSEF
GQD)---VMISYSHQdMDFMRKMR---GSLESAG-
ISVWVDEQgLGAGVDFLnKIGHAIVEAKVFLSLLSPNSVTSKYCKDE----LALAYI-
SNKPIFPVAMatRDG-LRKGMDFGmQLTLAPMEWvdFSDRDKFD--
VSFAALTQKIKAA(TNDGDESKVGKPKLERQPTRQNLNKMTRLNSNPLDDESDFWERAFKDAKDVPWSKFKEA
FDKQYGSLLDGMQISSDAENK)}*

$3970=169(175):
>gi|1126551510|ref|WP_075187741.1|
{|0(259)|<Proteobacteria(B)>}hypothetical protein [Alteromonadales
bacterium BS08]
{()MSKLFISYSRYnVEAVQILAADLEAVAH----
DAWYDSALTGGQRWWnSILANIEKADIFVFALTKDSLESTACRSELKYAFELNK-----RILPVLLADD-
VNLGLLPKALSEFQVVKylRDDKHSLAALIKAIHNIPPAGAL(PETPPVRPEVPISYLVTVKERIDSSETLGY
KEQLS)}*

$3971=227(224):
>gi|1126190840|ref|XP_019628351.1| {|20(0)|<Chordata(M)>}PREDICTED:
uncharacterized protein LOC109472926 [Branchiostoma belcheri]
{(KWDDLARELGLERGEIETIRRDSQYPGPDDKCREILERWLEKTTSTDPLRDLKTALIDIRERRTAQSLDIG
ATATPTGAK)---VFVSHAS--EDKEEFVEPLVqellqpNRLQKS--
DVFYDKHSLKPGDDLwtEIEAALRNPALKlFVFVISRHVLSQKTWPkYEYELAHARGVRiFPIWLVREEDEDF
-SQKVSEYDTMRGLERLLAERVHVNEVEEKLPSIAGKIIAQ(PQLQ)}*

$3972=184(187):
>gi|1126218102|ref|XP_019643131.1| {|0(837)|<Chordata(M)>}PREDICTED:
probable ATP-dependent RNA helicase DHX58 [Branchiostoma belcheri]
{(MASSHQGK)--TVFVCHAG---
ESKPFVRQLVDRLENDGLtkeEIFFDEMSLDPGDELdSIIKVIESPSLKlFVFVVSKYCFDneRTWIRaEWEA
ALTNNKQ-----VFPIWLDDN-
NDDFKAFGNLVRKFSYKLRKLIAERVNPQETATALPSLSG(KICSLICGDDEPAKKSPPPSPDDAVQTSRQNI
MPDVLPA)}*

$3973=156(159):

>gi|1125787222|ref|WP_075102661.1|
{|0(18)|<Proteobacteria(B)>}hypothetical protein [Psychrobacter sp. Rd 27.2] >gi|1125666440|gb|OLF39739.1| hypothetical protein BTV99_12340 [Psychrobacter sp. Rd 27.
{(MPV)----FISYRHTdrntaFAIARKLSANNISYYLDII------
DEESKSTDDIteVITKNIKKSTHLIAVISSNTVGSWWVPFEIGEATIANRRIcsyAIRVDSPNLSL---
MSYRQITRLLPEYLQKWPVLVSSIDLEEFINQYRADNITF(GIEESRNKQFDSLSNN)}*

$3974=156(159):
>gi|1125804895|ref|WP_075117584.1| {|0(7)|<Proteobacteria(B)>}molecular chaperone Tir [Pseudomonas chlororaphis] >gi|1125686999|gb|OLF56239.1| molecular chaperone Tir [Pseudomonas chlororaphis]
{(MPV)----FISYRHTdrplaININKRLEEAGIQTYLDV-----QDIESLS-
TEDItaVITRRITECTHLLALVSDKTALSWWVPFEIGEATISNRRIcsfRTGASELPEYL---
DKWPKLSKASDIELFIDTYRNESALKRSLSLESVDGSESL(HTHNKTNADHFHAELK)}*

$3975=236(234):
>gi|1126191085|ref|XP_019628485.1| {|0(183)|<Chordata(M)>}PREDICTED: uncharacterized protein LOC109473034 [Branchiostoma belcheri]
{(MASSETTEEDTEQDFT)-YDSAVYYeEDDPKTAQILdIRaRLESDRWELTLCDPYRDGDGtqmGRFEHS--
----
AKnSRHALVFLSPSLLerikKKQANNAQFNVETFLCNILKrrdntmKRKVMPVMFDASEGDVtPLILSGYTP-
-FHP----GEVG---
FWRKIYRTLSRE(PVPDAFMLQVSKKVQQVKHLQLEAVLDVAAKLGLPEEVVDSIKVEYAGSQARLRQVFYCW
KAHLGAEATLEVVDDVIR)}*

$3976=239(251):
>gi|1126193507|ref|XP_019629798.1| {|0(892)|<Chordata(M)>}PREDICTED: uncharacterized protein LOC109474030 isoform X3 [Branchiostoma belcheri]
{(MAEGRGSRNRSSSRLSLLTGSSLSLTVPFTT)--DVFVACaDKDQAVVKSrVIQPLESRDTTCR--
YPQRDFIPGHSEHNSVVEAMKTSRKTIVVLSENFKDYRLSRVIL--ALSNQLIT-QGRVIPVKIDE-
DTEMDGRFRDVVTLNMTA----IDEAT--
FIAKLEEAVTLP(DQDLSSASRSPGSLLDVAAVPELYTPEAESTTSSETSSDSSTGHTDLPQQEFYEAEPLHS
AAIRNAEDAVALFNDLHRSD)}*

$3977=221(229):
>gi|1126199055|ref|XP_019632773.1| {|590(0)|<Chordata(M)>}PREDICTED: uncharacterized protein LOC109476300 [Branchiostoma belcheri]
{(QVQEYLSQLVETCDENNDFCEAKVYRTLVTAFGGTVDRNRSTECNLAVARIQAGVSEVIASDVYRETFHGV
IAPPGDHSC)RYSVFVMYdPKDQTQADELV-KLIEVDCGLHCCYESRDFEPGLFPMDNIERCLSRsCS-
VLHVLTLTYARN--CLQYTEKTRRLVRERGHGRVAAVVYKHYPG--PRELEGCTVIDNEE----
FTDSSQGQKTRRWAEFREA(VFRALLPID)}*

$3978=235(243):
>gi|1126221476|ref|XP_019644975.1| {|228(0)|<Chordata(M)>}PREDICTED: hemicentin-2-like [Branchiostoma belcheri]
{(CVAWNMLGEKLSQELQLTAKPPDGGLPMLIIIAVASGVAVFVVVTVTATIFVYRRRRQFKHCRLESESDEE
EGDFSDSVA)KYDSGVHCcEKDRSWTEKMVNKLEKE--
KLRVYFSPRDDIGGGSVFQYLSEGIAKSRNVIIVFSNAADSDEWFKkgYQTAVVEQLDRNLR-
GRVIPVLMKGfERTNLPLELKDCVALSVED----PK-----
FFERLLRSLSKD(SAYESDRQSSVSTEEVLDTIIVL)}*

$3979=228(234):

>gi|1127360481|gb|APR81884.1| {|395(0)|<Proteobacteria(B)>}Hypothetical protein A7982_07233 [Minicystis rosea]
{(LLASFRAVDARSKASDGGPLMTLAITLDDAAAKKHRAQRTWGEWLRLARRLHRPSTDDVTWRIAESLLDARRLGLRTPVR)---LYIAYADDdRARREQFESHLgTAIARSTWLSVWHRERVPAGaplDVWQtE----LARADVVVLLVSVDLLTD---QIdEVERAMTLHEE-NRALVLPVLLRSC-EWQET-LGALTPLPADQtyIAAHADPD--GAFRDVVQSLLIA(TFDFMLGPRPEAPA)}*

$3980=213(203):
>gi|1127366334|gb|APR87737.1| {<Proteobacteria(B)>}hypothetical protein A7982_13086 [Minicystis rosea]
{()MPTqplhVVFCYAPQdEDLCQALEAHLSLLVRQEYITGYSSRKVGSGVDVRaEIDRQMDRADLILLLVSADFLASDHIYElELRRALIRRAS-HPEEVIGVLLRPC-DWKHGELALLDMHPREArggeavpLTQWPSLD--EGLKRIAETIRER(AKHRVGSLSLNPPSAHPRRASFGWAQTRRRRSPVFATRPSRRRRTSRVVSTSWTSCASSSMRV)}*

$3981=176(181):
>gi|1129432041|ref|WP_075393354.1| {|0(46)|<Actinobacteria(B)>}hypothetical protein [Actinomyces oris] >gi|1128427473|gb|OLO59569.1| hypothetical protein BKH22_12770 [Actinomyces oris]
{(MTSL)--KVFLSYneTKMgEQTAAGLKTALSAAHIDAIMAR---HSITPGAEWEpTIRNYLEESSALICVATSGYSARPWC-QqEVGWALGRH-----APILWIQYEPN-EKPCGFLASQQALVPDNaeNQPEIAASVATWLANNSNTRDDM(AETLLNALENAETYDESRHVAKLLAKLNWLTTDEWAR)}*

$3982=166(167):
>gi|1129449358|ref|WP_075410115.1| {|0(56)|<Actinobacteria(B)>}hypothetical protein [Actinomyces naeslundii] >gi|1128450968|gb|OLO82450.1| hypothetical protein BKH13_08800 [Actinomyces naeslundii]
{(MSSL)--KVFLSYneSEAgEQTATELKEALSAVHIDAVMAR---HSIAPGAEWEpTIRHYLEESSALLCVATPGFSTRPWC-QqEVGWALGRHAP-ILWIQYEAKEVPC-GFLASQQALIPDDPNNQsqVALSILEWLAENSNTRDDLADTL(LDALEKAKRYDDARSTAELLAIL)}*

$3983=175(185):
>gi|1129423919|ref|WP_075385405.1| {|0(581)|<Firmicutes(B)>}hypothetical protein [Bacillus pseudofirmus] >gi|1128409487|gb|OLO42407.1| hypothetical protein BTR23_04090 [Bacillus pseudofirmus]
{(METTIK)---VFLSHAS--KNYDVMEL-IANQLEAKNYTCWFAPRNIPYGDNYltKILDAIDTVDV-VILLHSQEVHESKYVFrEIQYADNKNK-----LIIPLLIDDA-PVARHLELIINTMQMLKLSQFASVELVIEELHQRMVEYQI(AIQDGVRSSSWIGRLDKNVVRSNIIQLGQSTAFKLSPSK)}*

$3984=201(187):
>gi|1126829128|ref|WP_075219925.1| {|0(318)|<Proteobacteria(B)>}hypothetical protein [Acuticoccus yangtzensis]
{(MTKAVTAMAYTPGYE)-YDVFVSYASVddfnliddgdgwiTELVELLDRMIRsRLGGGDDLTIYFDRHRLASNHGLsTLLDAVRQSATFFAVASSAYNQREWTQREIAEFCRQPDYSnrlFAIEYLPLLPG---EQYHSILESRKRLAFWERDSAFNVEQTLVRGSATYRARCT(QVAQQIATQLFAMRFGGGAAAAPSAGTQERDP)}*

$3985=250(246):

>gi|1128080988|ref|WP_075250184.1|
{|0(701)|<Actinobacteria(B)>}molecular chaperone Tir [Actinomyces oris]
>gi|1127310273|gb|OLL13874.1| molecular chaperone Tir [Actinomyces oris]
{(MKRDLVDKLTSIVKVQWMRWKHRRVGN)-
FDVFISYSHDpsrtlaFALHDSLEKLSRNWYSFRALNVFLDRSSLGPTMMLekELESIIKRSEWLVYICSENA
AKSVPVIKELNWYIEHNGVDkliLIVDDGVIRWD---REAERFSRT-TTNIPNEVLSKIKDEP--
LWIDLRGRNDAQ(ESLESYPDYLDAVLSISSRVQNTSRKALLDRENKRQKRLVRFRWTFFVVLSAFLVISVAV
SWVAVSARNEALSNLNVAT)}*

$3986=250(246):
>gi|1129409538|ref|WP_075371462.1|
{|0(697)|<Actinobacteria(B)>}MULTISPECIES: molecular chaperone Tir
[Actinomyces] >gi|1128417481|gb|OLO50039.1| molecular chaperone Tir
[Actinomyces oris]
{(MKRDLADKLTSMVKVQWMRWKHRRVGN)-
FDVFISYSHDpsrtlaFTLHDNLEKLSRKWYSFRALNVFLDRSSLGPTMRLekELESIVKRSEWLVYICSENA
AKSDPVIKELNWYIERNGVEkliLIVDDGVICWD---RETESFSKT-TTNIPREILNKINDEP--
LWIDLRGRSESQ(ESLENHPEFLDAVLSISSRVQNTSKEALLDRENKRQNRLVRFRRLSFSILAILLTISVAI
SWFAVSARNEALSNLNVAT)}*

$3987=249(245):
>gi|1128417688|gb|OLO50235.1| {|0(702)|<Actinobacteria(B)>}molecular
chaperone Tir [Actinomyces oris]
{(MKRDLVDKMTSIFKVQWMRWKHRRVGN)-
FDVFISYSHDpsrtlaFALHDSLEKLSRNWYSFRALNVFLDRNSLGPTMRLekELENIIKRSEWLVYICSENA
AKSAPVIKELNWYIEQNGVDkliLVVDDGVIRWD---REAERFSRT-TTNIPNEVLNKIKDEP--
LWIDLRGRNDTQ(ESLESYPDYLDAVLSISSRVQNTSRKALLDRENKRQNRLVRFRWTFFFVISVLLVISVAV
SWSAVSARNEALSNLNVA)}*

$3988=250(246):
>gi|1129432043|ref|WP_075393356.1|
{|0(697)|<Actinobacteria(B)>}molecular chaperone Tir [Actinomyces oris]
>gi|1128427475|gb|OLO59571.1| molecular chaperone Tir [Actinomyces oris]
{(MKRDLADKLTSIVKAQWMRWKHRRVGN)-
FDVFISYSHDpsstlaFTLHDNLEKLSRKWYSFRALNVFLDRSSLGPTMRLerELESIVKRSEWLVYICSENA
AKSDPVIKELNWYIERNGVEkliLIVDDGVICWD---REAKSFSKT-TTNIPREILSKINNEP--
LWIDLRGRSESQ(ESLENHPEFLDAVLSISSRVQNTSKEALLDRENKRQNRLVRFRRMSFSILTILLTISVAI
SWFAVSARNEALSNLNVAT)}*

$3989=244(243):
>gi|1129200019|gb|OLQ03347.1| {<Alveolata(E)>}hypothetical protein
AK812_SmicGene13670 [Symbiodinium microadriaticum]
{(MEDSDQAFGRAASTKSCTSSSSYIDVDDLAGFITPDYRYSGTVTPDYLTRGKSKRSTPSSRSGFVGEASSV
PYSKCTW)--DFFLSHKQSNAQDAVQNLRMAISERMP--ACSFWLD-
IEQDPTVpGMCHGVSNSKNFLLFLTQGVTE------
SKFCQMEMRWAMqSDKNIVLVaeTDERHGkpVLGELIercPEDLRNLfEEHVIIPWyrDPDLRSVCVTKILKK
CVFE(VDAEKASISSKLQRPLMFSNNVAGM)}*

$3990=250(256):
>gi|1129178879|gb|OLP86753.1| {|1731(214)|<Alveolata(E)>}Tumor necrosis
factor soluble receptor [Symbiodinium microadriaticum]
{(SGLAIQVRVMPWRTSLANFIDALASASVAILLVGSSLLTVLTAPDIGLLQTWVSLHLLAIFGIFAAVVFNY
IRQWFRSKK)-YQVFISHH------KGSAAALARWFKtcmlaqqRLQLKIFLDSDDLLSVDVLfdTV--
AHQTQSV-ILILTKEYFGRPFCMGEFVSAIQSG-----

VPIVAVKCKDCETLNTELIVEHVRSIWKESHKAMLSSLGITEDLIAKAIAHL(QHNIIPVVDLDRSQSETEQV
KVVSATMEACHLGTFT)}*

$3991=134(145):
>gi|1129389099|ref|WP_075351996.1| {<Bacteroidetes(B)>}hypothetical
protein [Algoriphagus sp. am2]
{(MGLQQ)---VFISYAwgG---ESEQVANELEAILKSNQIDLIRDKN---DLGFKgliREFMKQIG-
KGDfvVLIISDKYLKSKNCMFE---LMEVAKRESFlDRIFPIVLPDAAiyD-GLGILGYLKYWDKKL-KDL--
----NSSAKELDSLEKN()}*

$3992=266(264):
>gi|1129071746|gb|OLP17758.1| {|111(324)|<Cyanobacteria(B)>}molecular
chaperone Tir [Oscillatoriales cyanobacterium 'hensonii']
{(HLGDVEFRDGDVMGDVVNIAARLQPLAAPGGVCLSQAVYDAVMSGLQVSVEDGGLQNLKGIPHPVRVYDIP
PPPPVTITR)-RKVFISYRAKepDLGLAREFHHALTAAGHE-
VFMAGESIRPGEDWVQRIDAELQQCDYLLLLLSEKAVASEMVTEeVRRAKglrdLSPD-----
RKpvILPIRVSFPRtaPLNYDLRGFLNRIQQR--
EWESSEDTPALLEEYLDLIERG(ALLESVEGSELTLTPVVERSDLPPLPVAEPELPGGQMDVASAFY)}*

$3993=259(256):
>gi|1130427927|ref|WP_075596674.1|
{|110(337)|<Cyanobacteria(B)>}molecular chaperone Tir [Oscillatoriales
cyanobacterium 'hensonii'] >gi|1129074617|gb|OLP20350.1| molecular
chaperone Tir [Oscillatoria
{(IHLGDVEFTGSDVMGSGVNLAARLQREAPARGICLSQTVYEVVKGRLPRPAMFMGRRQLKGIPEPVPVYEI
APPYPIETV)SRKIFISYRSQnpDFGLAQQFYAALTEAGHE-
AFLAGESIRLGENWPQRIEAELRQSDYLLLLLSAR-SATSEMVMeEVRRAkdlqeqhPEGK-----
PVILPIRVNFPLgmPLTYDLHHYLDQIQQR--
EWQSDADTPALLAEVLQLITNG(PTQPVSGQPSLPSPSPASDRPTPAAEPELPTGQVNL)}*

$3994=304(285):
>gi|1129202609|gb|OLQ05397.1| {|764(87)|<Alveolata(E)>}hypothetical
protein AK812_SmicGene11389 [Symbiodinium microadriaticum]
{(RNPDFRPWVQSYLGSRAEAAGSAAAVAALVGDSSVDKVLEMARMHFHTVRADQICFEDLQSNEPDAEIGMK
SRKTRLGEA)--DAFISHSwHDdpveKW--RALQA----WRRK-----FRQENH-
GVEPKLwidkycidqnDVENSLAclpiylaSCSRLVVICGETYLERLWCITELFVFLEMGGSPsnlEVIVLPD
PCdkiaqkiknfAP---
ENARSFDMETTDILLGVLeAGRFGLEGIRKLVQDRFVPGSW(QLVFETDPKAVTARSSSSLAAPDASGNDAKA
VALRSTSSLKVPEAMINMVMQTKAYAAGSSSPKA)}*

$3995=191(191):
>gi|1129379030|ref|WP_075343133.1|
{|0(682)|<Bacteroidetes(B)>}hypothetical protein [Tenacibaculum sp. HZ1]
{(MRFINKIKKTLFG)-YDIFISYSRKdsldygYAIAA--------
YFMKKGYECYIDQlSSISPGKKLplNIKNAIKRSSSFVLIGSKESQLSKPILEEIELFLLNNKNKpliPISIE
NNMNEK---
SIWYKNIKGLALINDSSKNLLSGAPDKDVFDRIHNALKFT(KKAVRLRQISLLTLFVLLITTTIAFLYSKQKS
NEAYSA)}*

$3996=173(190):
>gi|388499312|gb|AFK37722.1| {<Streptophyta(V)>}unknown [Lotus
japonicus]

{(MSSSSTNIANLESIIDR)---VFFSYTL-ADIEvEPFISLLHKKLSIP-TCCF-DNINKVKGNPPrrE----IKGSGVSVVVFSERYGGSSRC----------HRV-QGQVVLPVFFGVDVCDVREQKGIFGQEFELM-VKRRSVEEDtvLTWRKALSEIASF(TGYSAPPDRFRYDPHLKYYIDSFDQLPIRLFSY)}*

$3997=234(229):
>gi|388521307|gb|AFK48715.1| {|0(119)|<Streptophyta(V)>}unknown [Lotus japonicus]
{(MSSDTLDKI)-YDVFLSFN--DDAKRSFTGNLYNALRhKK-IKTYFldkNDDSDEDYDQLspSDLKAIQGSKISIVVLSQYYASSSRCTDELGIILECMRM-KNQLVWPILYKADISNVRPEedgADQVRTYFNMK-PKFSSDSDErvQRRKQALWEVSNL(NRRLYRGTDDECEYDFIQNIVESVVKSLPHYDIFISFTGEDTRHSFTGVLYHALHREGFKIFMDDEELEGGNQISKTLLE)}*

$3998=215(219):
>gi|497063012|ref|WP_009453252.1|
{|0(374)|<Proteobacteria(B)>}hypothetical protein [Leptothrix ochracea]
>gi|388591402|gb|EIM31653.1| hypothetical protein LepocDRAFT_00003860 [Leptothrix ochracea L12
{(M)-PAIFVSYRPQDQPDwLLSQVRARVQKD-------IAVDWVEAG------GSLESCQMVLVMIGPQWLsaadvDGRLLIQdpQDPVHVTLKRALeRGLRLVPVLlDGTTMPRSHELPTEFQALVRRPAMSLHPQRLDADMGMLVSMIRRT(LESSFGSVVASSVSAHAHAHAKSEAQALPPPPAPAVAPPVVTPAPLAPPLRAPNTLRGLAEPEELPELAHLFAPPPPP)}*

$3999=191(195):
>gi|494918135|ref|WP_007644172.1| {|0(123)|<Proteobacteria(B)>}molecular chaperone Tir [Cellvibrio sp. BR] >gi|387937598|gb|EIK44154.1| TIR domain-containing protein [Cellvibrio sp. BR]
{(MHPK)---VFVSHAS--EDKERFVIDFATRLRKNGVDAWLDKWEMLPGDSLvdKIfEEGIKDAAA-FIVILSTSSVNKPWVHeELNAA--VVKRInNGSKLIPVVLDNC-EVPEVLASTLWERVPDLNSYNASFDRILASIFNISTKPPL(GQLPLYTESPITLISGMHKNDSLLLKVSCENALLTGSMSIDPKKLFFVDAN)}*

$4000=288(243):
>gi|1129965965|ref|XP_019742319.1| {|299(0)|<Chordata(M)>}PREDICTED: interleukin-18 receptor accessory protein-like [Hippocampus comes]
{(TALHLNHMYTCMASNSVGNTSATVNLKRKNKKKWPSLVGYPIASLLLVAGVGIVLHVKWLELQLIYKSCFQSGKLDEDAK)EFDVFLSYV-Wttpdeqasltlssrsgpesddypstl EPFNFQhviddlrppemLIPMVLEDLWGYRLCLLERDMLPGGAYTNDVVHAIRRSQILICVVSADYLsNSNAVF-VLESGIQVLLQSSTLKLLLIRTsrtskCFV-EPDSPLPSVVRRALTVLpSLDWNLGQSErvngKFWTLLRKALPKN(KVRPVTGVISHAHFGHNDHRNQE)}*

$4001=145(157):
>gi|1129854776|gb|OLS22530.1| {<Asgard group(A)>}hypothetical protein HeimC3_30710 [Candidatus Heimdallarchaeota archaeon LC_3]
{(M)------SHTHQdKEIAERIKGLIETSGAKAN------LLTYEVDPSQtiieKVKNGIKKCDLGIILWTKNSEKKEWIIQEAGALAITEKP-IIVLMESSINP--PGAMLEGIHYVRFGDIEG--MKSLVEWLKQRVQNEELWKII(LILGGGLFLIWYLFSK)}*

$4002=214(221):
>gi|1132188606|ref|WP_075867671.1|
{|21(0)|<Actinobacteria(B)>}hypothetical protein [Kytococcus sp. CUA-901]
>gi|1130548329|gb|OLT31441.1| hypothetical protein BJF82_11990 [Kytococcus sp. CUA-901]

{(EMQREIDRVNRANQKAVDDYNRKAAQHNRKADRHNEKVMAEINRRLGAAPAGRTVTYQPVERQLVERVHDA
IDFDDGRDY)--DVFLSYARIDgEQTAQSLAAALEDCGVS-
VWFDAVAIQPGKSQSLQMDAGLRKARAGIAVLTAAYLAGRFWTQrE-LGALLNK-----
ATLIPVLHGVTFddVKDYSGILPDLAGFTT--EQDDIETIASKIAPAVSVTAIG(G)}*

$4003=211(220):
>gi|1132126267|ref|WP_075818441.1|
{|36(0)|<Actinobacteria(B)>}hypothetical protein [Serinicoccus sp. CNJ-927] >gi|1130558652|gb|OLT41295.1| hypothetical protein BJF86_15915 [Serinicoccus sp. CNJ-927]
{(KVVDDYNRRAQAHNRRVIADYDRQVDAHNRKADAHNRRVLDDLNKQLSQGRSTTVRYTEPERRLADRVHEA
VAQVDPREY)--DTFLSYARIDgSEVAAELRSVLEDLGVA-
VWFDHVSIQAGKSQSLQMDQGLRKARSGIALLTPAYLAGRFWTErELGALLG-K-----STLIPVL--
HNVtfEDVQEYSGILPDLAGF--TTSTDSVSDIARKIAAAVTVTR()}*

$4004=120(141):
>gi|1129846428|gb|OLS14594.1| {<Candidatus Lokiarchaeota(A)>}hypothetical protein RBG13Loki_1786 [Candidatus Lokiarchaeota archaeon CR_4]
{(M)------SYAHAdKS---RVYPELKRLHEQGY-
NFWYDEGIPPSTDWPeTIGTAIARSSYFVVFFSKAAI-SNEHAKkEIYFACDKRKN-----IIPIYLEET-
TLTDG--LELQLGPKQA-----ILKHRISEQDFLKLNFDS()}*

$4005=172(174):
>gi|1130421198|ref|WP_075591553.1| {|0(274)|<Bacteroidetes(B)>}molecular chaperone Tir [Labilibacter marinus]
{(M)--KIFISSSKLdSEEVDFLLKNLSKSEHEFFI---
GDLNIKAGDNWFrKINEKIEGLDVIIIIISRNTLKSKFALKeIESIILNDISR-KDTRIIPITFDGT-
IPPGYLSNYQIIDLSYDkeLGFQRLLAKLSKENIFDEEFSEK(EERIKSDNLKRNNQFIKTLTNSLKDGRLTL
VCG)}*

$4006=241(245):
>gi|1132235535|ref|WP_075903699.1| {|331(814)|<Cyanobacteria(B)>}type IV secretion protein Rhs [Moorea bouillonii] >gi|1130579812|gb|OLT62048.1| type IV secretion protein Rhs [Moorea bouillonii PNG]
{(LLKELNQDALYYEEHKLLLVKAIKWLSQNRNSSILLRGHNLNYYENWLKVNRQRLEHPPLGLHEEFLAASR
NQQHESYLD)---VFISYSRTdSDLARKLNEALQL---QGK-
TTWFDQEsIPPGSDFQeEIYRGIEHSDNFLFIISPASVNSRYCAGEVDYAQTLNKR-FVTVLSSQVPAKM-
LHPGLASIQWIDFNRHGgdFYPNFSELVRTLDTDREHVRSHT(KWLQRAREWEQSNQDTDLLLRGSEF)}*

$4007=268(271):
>gi|1132235535|ref|WP_075903699.1| {|126(992)|<Cyanobacteria(B)>}type IV secretion protein Rhs [Moorea bouillonii] >gi|1130579812|gb|OLT62048.1| type IV secretion protein Rhs [Moorea bouillonii PNG]
{(QLMNSIRKHADYVEQHTRFLVKALEWDRNQQQTNHLLIQSERIQAESWLKVRFKKEQAPCVPTDLHCEFIC
ESTKNANNL)MTQVFISYSDKdQDIKDKLSK---TLMREG-
ITIWKpETDIKTGIDFQaAINQGIEEADNLIYLISPNSSQSKYC-QqELEHAFAHNK-----RIISLLIEDT-
DIDQIPpkISSLQFLDFTKsnHEGNYRLMAAQLLKELNQDALYY(EEHKLLLVKAIKWLSQNRNSSILLRGHN
LNYYENWLKVNRQRLEHPPLGLH)}*

$4008=222(223):
>gi|1129859243|gb|OLS26851.1| {|209(0)|<Asgard group(A)>}hypothetical protein HeimC2_13840 [Candidatus Heimdallarchaeota archaeon LC_2]

{(DNEVNYHKSIFSTVDFVSFNLTKALNINTIFHRRKGAVDYDTLMISKNLPPSFYFGYGISQMFIDNLPSLQ
STSISFYDC)----FISYA---EIDEQFSKRLYDGLTSAGIRCWRWKEDLRYGQTIryGIESSIKKH-
EKLILILSKDSLESQAVIdEIDAgiyqENELRKTNkTYDYIFPIMLDKT-
IMSWDHPNQRFIMQKHIGDFCDWKQEDAFQLSLQRLIKDL(KKI)}*

$4009=278(273):
>gi|1129994929|ref|XP_019712450.1| {|304(26)|<Chordata(M)>}PREDICTED: TIR domain-containing adapter molecule 1-like isoform X1 [Hippocampus comes]
{(PTSSEAPGLEQSKTDAASGTERGKHQDRRFNMKQSCHQSITPTTGSKPSLPFSTNQVLPRRTVSVGAHLSQ
FGEEEDEVT)-FYSFvILHAPEDsEVAESMKEKLET--
IiGSKGAIFSDDFaVPGKSTLRCIEDAINNSAFTILLLTQNFNTRMLEVKTNSALINSINKQHKYNTVIPLLp
keNRMPRHrMPIVLQTIVSLEeNKSFETKIKKAL-
SPAKINRQRSIWL(EEQRTHQRRLPQGCEAAHMLENLNFLGSVDFSSAPSVLQQHPNIHIENASYVM)}*

$4010=190(185):
>gi|1130971997|ref|WP_075735766.1| {|0(437)|<Actinobacteria(B)>}TIR protein [Streptomyces acidiscabies]
{(M)-WDVFLSYSR-ADVES--
VRPLARALRGEGLRVFTDETGVAPFEGIseTIRRELARSTALLAYYSAGYPEREACQWELtcAYLAGLAEGDP
RRRVMvvnpePTASHIHPVELRDARHADPGDPAGLVADVVARLARLDGPMPLPRAAVR(GPHAEFLGRLPELW
RIHSALHAHAAPLTAGRVPARTVQIRGMAG)}*

$4011=171(192):
>gi|1134342274|ref|WP_076581794.1| {|0(32)|<Euryarchaeota(A)>}nucleoside 2-deoxyribosyltransferase [Haloterrigena daqingensis]
>gi|1131822318|emb|SIR77318.1| TIR domain-containing protein [Haloterrige
{(MAGEQIYVSHAPADLE)---------------LVQELFSTVKNFPF--
GTHIALEEVESGRSRqRLEGRLANSDVVVAVLTAES------
STSRWVNQEVGYALaKGIPVIPLADSDRHLGGFLENADSVAITRENPSRTIYDLISRLRSELAPLGALS(VPN
WYIRFPCTIPDCGHPVTLEIEQDQSKLWKRYEH)}*

$4012=168(158):
>gi|1131033001|ref|WP_075792558.1| {|0(34)|<Proteobacteria(B)>}TIR domain containing protein [Massilia putida]
{(MAI)RYGCFFSYAHGrhelmqrfkATLADALRCYLEPYFDNEDE-
LFVDTEQLGGGDDLdrKIARALCESACMVLIYTPKYEAHAYTRREYAAMRLIEAERstwYTLPSQLIIPV---
VMTRHPVSLPPQISESTFYVDFSRFTMATADLKSNPDFLP(DIDKMVRRIVTHYQY)}*

$4013=250(261):
>gi|1134251528|ref|WP_076492411.1| {|14(44)|<Proteobacteria(B)>}TIR domain-containing protein [Aeromonas veronii]
>gi|1131977963|emb|SIQ24807.1| TIR domain-containing protein [Aeromonas veronii]
{(VELQSRMTFSDIDVYFSGHKVPTENNYPTNSKRSYSKDMLSKVSDEVIFKIAEELSLEIPMANAMSTKEVT
FWKAGHFKL)----FLSHLA------SFkvqTSHLQSALRKyA-
ISSFVAHEDIEPTKQWqeEIEAALHTMDALVAILMPGFQESRWCDQEVGFAVGR-----
DVLVIPLRKGLDPYGFIGKYQGIQAQNKTI-
GDVAEAIFRTLIKSPKTKNKIL(MAMSGAVSNASSPEEALDKVSILKSLDNISVELLDSLKQEV)}*

$4014=247(254):
>gi|1134265577|ref|WP_076506221.1|
{|152(9)|<Bacteroidetes(B)>}hypothetical protein [Chryseobacterium

shigense] >gi|1131981881|emb|SIS33094.1| Putative DNA-binding domain-containing protein [Chryseoba
{(KFLLDNPKRINVNKYTIREVFANEISLSEAEQLIEKLKETKYVKSYNDRYIGMSIDTAVFLEDGGFSGKSPNSLQSSIKS)---IFLSYSW-K--QKEAASKLYNFLKANGYNPSMDDHNLAYKDKLstFMES-IRTSDYAILIISDDYLKSANCMTEVLHILnerESY-----SRILPIRHENVKIFSSSDRMKYIHFWKTQFEESTAVIENLDRISTIEESKKL(KITSRIYHDIGDFLITIADMITFTIEKEEEVLFS)}*

$4015=199(187):
>gi|1134327150|ref|WP_076566937.1| {|0(2)|<Proteobacteria(B)>}TIR domain containing protein [Janthinobacterium sp. TND4EL3] >gi|1132053704|emb|SIQ45161.1| TIR domain-containing protein [Janthinobacter
{(MEIRYGCFLSY)AHGQYAFMN---KFKNDLIEALACYLEPHldrEEVLFIDSEQLGGGDDIdlRVARAMCQSVCMIVLYTPKYEAHGYTRREFAAMQlIEQERRgWYDlpshLIIPIIMTRHPdglpPQITESGLYVDFSGYTLASGDLKSNPQYLPDIERIVQRIA(THYHLLKRSTPPGHDCSRFVLPDIPPEWRAVPPPHF)}*

$4016=246(254):
>gi|1134152724|ref|WP_076395556.1| {|99(40)|<Bacteroidetes(B)>}hypothetical protein [Chryseobacterium gambrini] >gi|1131988668|emb|SIT24131.1| TIR domain-containing protein [Chryseobacterium gambrini]
{(RIFPISLYEKAYTISSELGKIQFTNARVFLNQKLDLELEEDLTKAIEKIKSEILHDCSRLIMDFQKIADDTETDRINSPV)--KLFLSHAKKdGEIlAIKFKQFIEN-NLKFDVFF----DTVDIANGYdfaeQFSQNISESALLVF-HTDEYSNREWCRREV----LLAKK-HkSPIVVVHSIKNGESRAFPYLGNMPTTVLTE--ENDYNFYKIVYLTLLQVLNNL(YQHRLLSQYKEFTKKQVYTLTSPPELINLVHLEM)}*

$4017=203(201):
>gi|1134134944|ref|WP_076377939.1| {|0(148)|<Bacteroidetes(B)>}hypothetical protein [Mucilaginibacter lappiensis] >gi|1132061772|emb|SIS02685.1| TIR domain-containing protein [Mucilaginibacter lappien
{(MAKRPTTSSTTNKSESKELIINSKAPL)---VFISHDTRdGDLAEEFSNLLRNASAGALKSFRSSDRkgTQGIEYGEewypAIMDKIDEASDVVCLLTKHSVDRPWILYEAGVAKGkLDKK-VLGIALGIPLRVAVSGPFAQFQNNDGDVESV--TNLVMTLVKKVPGLDPDQTQV(KTLVQGFVVRCIEIIDNQNSGVAAESVEENAVAK)}*

$4018=167(161):
>gi|1133787837|ref|WP_076168322.1| {|0(31)|<Actinobacteria(B)>}hypothetical protein [Amycolatopsis coloradensis] >gi|1132635725|gb|OLZ43517.1| hypothetical protein BS329_38970 [Amycolatopsis coloraden
{(MSDYK)-YDLFISYsrHG-S-ASKWLLNHFYPKLEEYLIdeiypgpKIFIDKGMSRGVV-WspELKSALRHSKIMIAVLSAPYFGSNWCMAEWDSMAAREAF-LGLASEDRPQGLIYPVRYSDSTHFENEGRLRSWWDFKGLDGADRAFHGSKDWHA(FQQKVRDCARDLAELL)}*

$4019=210(220):
>gi|1133787839|ref|WP_076168324.1| {|0(84)|<Actinobacteria(B)>}hypothetical protein [Amycolatopsis coloradensis] >gi|1132635726|gb|OLZ43518.1| hypothetical protein BS329_38990 [Amycolatopsis coloraden

{(MEEIPS)-YDLAISFAE---DRRSGTRELEDASRQRGLTVLHDPERTHEWWARksaGDLPDV---HVRffVPVISA--------LDDFTTAMLRAVATGDRHVLPVLADDVVvppeL-LHPHVSYLRDGDHLI-EELAGR-V-ERAEAAGWERGRL(ADVVTRAREIEPPAAVPVAFSRYVEQEAALRYLGEQFAAAIPRLRNRGFAGTTHHGDERIALRIERAGDIVYAL)}*

$4020=237(239):
>gi|1133120964|gb|OME12110.1| {|74(46)|<Firmicutes(B)>}hypothetical protein BSK60_18920 [Paenibacillus odorifer]
{(PSGDEMVVAFVYQLLSFFRLNPNKLRGLVMFYTSEKSYKDKYQAFTRKTVAPFINYILSYLEVCLIDAEDEDSITHNIKN)-KRVFLSYCSKdGVIADivdkKLQEVLTPHGVHV------SRDERDVMYKEsfrdFMNS-IDEHDFVVMIISDSYLKSRACMYEVLETMKNRNY-ENKILFMILSENERIRYSGINDDFLVGANVY--DISGRVSYAKYWAQEEKKLQE(LIESIESPLNQIEPIKELK)}*

$4021=234(240):
>gi|1133860335|ref|WP_076235623.1| {|61(42)|<Firmicutes(B)>}hypothetical protein [Paenibacillus sp. FSL H7-0331] >gi|1133217724|gb|OMF07394.1| hypothetical protein BK127_29175 [Paenibacillus sp. FSL H
{(STTFQLTNILNVAISKLSSPIILDFCFLNFKGENIFVIKVEKATVKILCDEEYYIYKSNGVLKIANKTEQYDEQIPDKPT)---LFLSYREIdTPIVNIIEDNLKRLTSDGINI----SRYTRIPYKAsfkeFM-NGIQDHDVVLCVVSDGYLRSQACMYEVGEIIKDHHF-NEKLIFVVLSENERKYYPEGFTEKIAANIYG--SEVKRLQYVTYWKEKYDELNE(TIRGIDDYEAISDATRSLKE)}*

$4022=197(205):
>gi|1133813981|ref|WP_076192820.1| {|0(324)|<Actinobacteria(B)>}hypothetical protein [Mycobacterium sp. GA-1841] >gi|1132943253|gb|OMC39018.1| hypothetical protein A5740_02975 [Mycobacterium sp. GA-18
{(MRGADYFDAKDVSDNVVPPMNRRFGPG)--RVFLSHSSNDADRAALLCLQLENEGIS-CWLAPRDIPAGHRYSEAIVDGIDECSVFVLLYSEAA-SNSTHVVnELEHSASKG-----KRILVIRTDDADpgSSRRVSLFVRSHQWFD--ASHGSLSGHLGHLVHDLRLLLD(EDTEERQPSHDYVPAARVPETSPAVSARPAAVDVPRSI)}*

$4023=219(220):
>gi|1133814001|ref|WP_076192840.1| {|0(666)|<Actinobacteria(B)>}hypothetical protein [Mycobacterium sp. GA-1841] >gi|1132943263|gb|OMC39028.1| hypothetical protein A5740_03030 [Mycobacterium sp. GA-18
{(MA)--DIGISYAHVDASLAIGVRRAFVECGYS-VWMDeageaaESAESIGLPWGQAHWDVITsefAAANVIVVIDTAAWRQSRYCQdEylfvrdwgkWVEFIDSR-----SGVVP---DATRigEIAALVAARQPI---------TSAHA--RLVEAARTAAPR(QTSRLERLLHHGEERDAQVVLASSSAVDGVTVTERMAPYAQTAIERSRRARKRLRRIAVSATAVLAALALVGVGALMF)}*

$4024=222(222):
>gi|1133451140|ref|XP_003383414.2| {|395(0)|<Porifera(M)>}PREDICTED: toll-like receptor 6 [Amphimedon queenslandica]
{(ILVSDWTIGQYQCSHLDVVRQVEVTATKRKSPSSSLAIGAVSLIAAIIIIVIIMTAVTALLRCYKATRYARTKEPPYEGF)QWDAFLSYHTEaeEFVIRHIKQVLE--ERGYKIFWHHSDFIGGKTITDNINDAVNESRKVIFVFTRNFVNSEYCMMELHSTLDRLQRTRTRCMIPIALEDE-TTVPIELKSTVTYWPVLtEEEMNTEKLICLIGSSIRNKLQE(KK)}*

$4025=209(195):

>gi|1134229602|ref|WP_076471329.1| {<Actinobacteria(B)>}hypothetical protein [Micromonospora avicenniae]
{(MRAPLFFLSHSRSPARRAAVGPSEY)--------------
LLRFYEDLSGHVSELvgpETGVdpgFMDTAMTG-
GERWtpELLRAAGTCQVFVPLLSSALLRSDWCGMEWHAFSRRRIERrRDDvsahetAIIPVSWSPTEgarlPk
vvgeiqrfSPTPMPAEIVAQYQREgVYGLLTLQMENAYRAVVWRLAQR(IVAIHRAYLVEPQVPAGVSELRNL
FAEESG)}*

$4026=224(230):
>gi|1134104044|ref|WP_076347252.1|
{|775(0)|<Planctomycetes(B)>}hypothetical protein [Paludisphaera borealis] >gi|1133584406|gb|APW61697.1| Ubiquinone/menaquinone biosynthesis C-methyltransferase UbiE
{(QEHFDAARARHDAMLDLIHRDTIDETLQMLDRAGFQVEGAPVPSYLGAVVVIKAVKVREPAVQVAVAVEKK
TAPETQTEL)-RDVFVSYSSEDKPIADQIRACLEDQAIS-
CWVAPLDIHAGLDFAAEIVHAIKSSRVMVLVLSPSA-NKSQHTPrEVALAAKRG-----
IPIIPFRAENVDpsEaLAYYLSNVHWLDAFP--PPISQHFPRLGETVKSLLSTIA(GEAVEAEDAK)}*

$4027=218(229):
>gi|1133775386|ref|WP_076156044.1| {|64(0)|<Firmicutes(B)>}hypothetical protein [Paenibacillus sp. FSL R7-0337] >gi|1133308453|gb|OMF96817.1| hypothetical protein BK147_11640 [Paenibacillus sp. FSL R7
{(QEGHIKTMIDRLSALGLRYRPDITYDDLFDEIANRRFDLKTISKGDEVESNFNSGIFYFAEKDYLQSQGYV
IKPLGNKVP)---VFLSHSSRdKPEIEDLIPYLNGAG----
LPVWFDKvSIDYGESITeAIERGIDKSGAVLFWITKEFLNSNWC-KtERRNFLSRHSGYNDVLVISIISDDI-
DI-RTEVPTFL---RDLkaLVRPKNQD--ISL--IAREIIPV(LKKYLEVMQ)}*

$4028=228(234):
>gi|1134104387|ref|WP_076347595.1|
{|125(0)|<Planctomycetes(B)>}hypothetical protein [Paludisphaera borealis] >gi|1133584602|gb|APW61893.1| Secreted effector protein PipB2 [Paludisphaera borealis]
{(AFFQGSICRSTNFANLDLSEAQGLDETVHHGPSSVGVDTLFASKGRIPEAFLKGCGVPEALIVQLPSLIGS
MNPIQFYSC)----FISHSS--A-DKEFARRLHSRMVQENLRVWFDEVDMRSGKKIheQIDEAIRLYD-
RLLLVLSPNSMNSEWVKtELRKAFKKIEK-ReGKRKLFPVGLAPY-
SAMRDWECFDADHGKDLAVEVREYFIPDFSNWKDHDAFEA(QFGRLLRDLKSEAT)}*

$4029=176(179):
>gi|1133698395|ref|WP_076084006.1| {|0(665)|<Firmicutes(B)>}hypothetical protein [Paenibacillus sp. FSL R7-0333] >gi|1133297458|gb|OMF85924.1| hypothetical protein BK146_28310 [Paenibacillus sp. FSL R
{(MK)---AFLSHS-SsdkelvRAIANELGRQFC---------IF-
DEQAFSTGVDFitSIEEGLSDSSVFVLLASQKALESVWVElelHEVAYRKIQRQIEktlVFIIDSTVDIS---
AMPAWLLRGKIERQITPKLISRKIRQHLDNLMRDQQFPYY(VGRLSSIQEAENKMMPIDGSSPPKIVSIFGLR
GIGRR)}*

$4030=237(238):
>gi|1135561349|emb|SCX26116.1| {|63(52)|<Proteobacteria(B)>}hypothetical protein DSM25558_3934 [Agrobacterium sp. DSM 25558]
{(HETMSQRGDADVYMAFGDPAGAHPLRADARGHSALQYTRRDKWKENLAPAQLEIHLLLHVVFRIRDHLKQI
FGQTRDEPL)----FVSHAkldG-DDTATAIVNYVNSTDNDVPLQTFYDAKELSPGENFaeEFRHAIHRGT-
LLAIVSDAYDSRPWCVFELTEAKRAKRPIVLADVGRARISRTYPYGANLPKVRVTANDKETAWIEPLLVECLS
EGLRCDLFKL(EVAQFKTADESTMVLPRP)}*

$4031=244(253):
>gi|1134971674|gb|APX86878.1| {|102(51)|<Proteobacteria(B)>}hypothetical protein BV511_20555 [Methylobacterium extorquens]
{(AGLRARVFPVAIDGAFGSTGLMQQAVRWDSWRRVKQADRRRRLVSDLTYQFGRMLRAYLERLRRPHEPEEE
LDRYLRKVQ)---IFLSHskHD-PDGER-IARAIRDALyKGDGLTTFFDVHDIPTGVRFddVILRQVRVSAV-
VAIHTDGYSSREWCRRE---IIEAK-----
RWNVPLVIANCISEGDERGFPYMGNVPIVRLDPRAVDRIDHVVGRLLDEVLK(DFLWRCRIELARADAPDDAV
FLPRPPELISLAG)}*

$4032=196(217):
>gi|1143116077|ref|WP_077106349.1| {<Proteobacteria(B)>}hypothetical protein [Agrobacterium sp. DSM 25558] >gi|1135561334|emb|SCX26052.1| hypothetical protein DSM25558_3919 [Agrobacterium sp. DSM 2555
{(MSYQWDVFLSYPRSQQVLPWIEKHFLPLLQGQLENLLDYEPK)----------------------------
----IFVDFAQAT-
GVRWpnNIRDALLRSRVMVAIWTPPYFRSDWCMAEWESMLEREKYLydsgviqAKGLVYPVVFSDGKnfhpRA
KDTMHKDLSHLNYR-
HDCFRDSPKYLEFDDAIRAIAEE(LEGHISAAPNWNSAFPIINPDDVGIMNTSIKLPRL)}*

$4033=215(220):
>gi|1134350189|ref|WP_076589662.1|
{|66(0)|<Proteobacteria(B)>}hypothetical protein [Vibrio ostreicida]
{(EKDDRDFESWLSKHHPHKVRAYNIFVNELIEAAFRKGYSLATFLDSANVDDIMTVNKNELFLFPDRDVMYD
IWGRPVIEK)KKKIFLSHSSRdKNIVDTYFNEIQKSELKA------
WYDKEEIIAGDsitaKVSEGLDDCELGVIFLSNNFL-SKHSGWTEAECNYFITR-
RMKKNKTLIVVNLGVEHDNMPPLLQDYLYID--ASRDNAASDLIHAIRKQLERF()}*

$4034=261(264):
>gi|1143116078|ref|WP_077106350.1|
{|242(122)|<Proteobacteria(B)>}hypothetical protein [Agrobacterium sp. DSM 25558] >gi|1135561335|emb|SCX26055.1| Septum formation inhibitor-activating ATPase [Agroba
{(AVPAEKLVQASTIPYVPFWSFGERIAVVEDKSVDNSGINYSMENIAALIAHRLGQTRLLAESRDEYVSAAQ
RLKPNQRTS)---VLISHAHEDSGVAVQIARQLERQGLS-VSMDVHD-------
SDLRHDELRvpngdtnSYVHLVALMSPssvhsNYFRDILRTFfREAASDESD-----
RLFIPVLLPGVQetDLSLQIRQYRHVRSTN--
PKNTADTIASWIRPRSLPKPNN(TDLMVRVTSDGNVPLAGAHVCALSDNGTTIEIEVANDGTASVQL)}*

$4035=180(186):
>gi|1134715167|ref|WP_076606958.1|
{|0(107)|<Proteobacteria(B)>}hypothetical protein [Comamonadaceae bacterium H1]
{(MPHTHDF)----FISHSS--AT-KELARHLFYNSIANGLFPWYDEALLNLGDQLeqELQLGI-
EASELFLLLHSKAAIEKKWVPfEMKAAEAIYK-NDPTFrLIAVKLDDE-
PLPDFWQQFLYHSWSNADQPRSILRLLSELTGRNPIVQIA(AAAVLSTAPSDAFVNSSSTVAEHARNYVLYYL
GHVKQLL)}*

$4036=276(285):
>gi|1137170832|gb|OMO50715.1| {|309(882)|<Streptophyta(V)>}hypothetical protein CCACVL1_30303 [Corchorus capsularis]
{(TNDHVAEIGMPDWLRKNVKEGEFVVMKAEAEVVEDLVRNKVIGLVDELFLECKPQAQGLVGRKNMSKRAYW
ECLALYGKL)--------RD-EDTRKNFTDHLYSALtRKG-
IKTFRDDESLERGGDFapELLRAIGESWGSIIVLSEGYAFSNWCLDELVEIMKQREE-
CGHRVYPIFFYVEPSDPRHQRKTVEQAFKQH-

EMRYNKDTNLATCFKTIFIEEI(VNVISETLDTEILDEQEVVKMKSTFQTIQVVLEDAEDKQYMEKGEAVKVW
FDKLKHIAYDIDDVM)}*

$4037=176(181):
>gi|1137201298|gb|OMO71915.1| {<Streptophyta(V)>}hypothetical protein
COLO4_27943 [Corchorus olitorius]
{(MPLIGPLPIMSVPSSSSSSSSASHPRKSR)-YDIVLSF-A-AGTHKNFTDDLYSALERERIHTFRDDE---
GGEDSasELLGTSIKGSWGsIIIISEEYVFSGRCLDELVEIMNHREKWGR--
VYPIFYyNVDPSDLTHQRNNIEEAFEQH-EMRYNRAKTQTWRLALKQVAEI(RSRWAFRDYADE)}*

$4038=234(220):
>gi|922900817|gb|ALA99937.1| {|617(0)|<Mollusca(M)>}Toll-like receptor 4
[Cyclina sinensis]
{(RNKTTLSLSRINIIRSQLKYDCSLWIVMTSCVTGFVGLLLILSLITLLYHRRWQLRYLWYIGRKKIDPFHH
PDEQQSRLP)QIDVYISYEQHydvtdgvtlhQTVTDYVYPFFERRGY--IVKIRE-
EFEATDKLYRVIPDTVNKTRKVVVFLTPSYCKDYWNTFEFNIAAYEGIYTKRNIIIPVLIGDFSdQNFtPEIR
SFVNSKIKSKeVLRFPSQancEHRinTFNEQL-EHWLQY()}*

$4039=246(254):
>gi|501716869|ref|WP_012623499.1| {|19(43)|<Actinobacteria(B)>}TIR
domain-containing protein [Pseudarthrobacter chlorophenolicus]
>gi|219862116|gb|ACL42456.1| hypothetical protein Achl_4505 (plasmid)
{(LEELGTFEWDSNRINVLLREFKLEPFTGQFDDPSLAEIVADVTDEMLTELYALVMGIDVDAVADIVESATD
DGPWKPNYA)--RVFLSHSAKhRKFVGEVADELALIGIHG----FVAHDTM--
AFSKpwqsQIEQALRSMQAFVAIIHPEFNTSPWCQQEVG--WARGRR-
VPFYAIRYGVDPAGFIGRDQWPSAAGQSPAE--
VAAVISSWTASLPELGAAVIE(GLFESLRTAPSFIDAGTAAKRITSLGALTNEQWA)}*

$4040=287(300):
>gi|1140826595|ref|WP_076683890.1|
{|91(100)|<Actinobacteria(B)>}hypothetical protein [Streptomyces
silaceus] >gi|1137364734|gb|APY85649.1| hypothetical protein A7J05_07925
[Streptomyces silaceus]
{(VLIGDAPLPPLAASRVWRDFRHMDGSGYEEKVDELVAAIRGETVRGGSDGSAAPGHEENIAAAQPLPPRPL
TPPDQPAVV)-----LCYAAADAAYGQALADQLRRAGLP-VWSVA-
DLRPGDSHVWSAQQQLDFAAAVIVLMSPSSQESEDVTRMILEAMHHR-----RPFFPVLIEGQRN--
YLLAHTWYVDardgrLLN--EDEVGLLK--
DLDAASRNGASP(DGTPTVIPAPPSQPAVETVRVPASVSLGRLDSYLAERNFAYADLQTTAIVLEAADRLDEG
WLSTRHVRRVPAEVLARVDG)}*

$4041=171(174):
>gi|1140829246|ref|WP_076686421.1|
{|0(97)|<Bacteroidetes(B)>}hypothetical protein [Polaribacter
reichenbachii] >gi|1137427629|gb|APZ47708.1| hypothetical protein
BW723_16025 [Polaribacter reichenbach
{(MGQSILT)----FISHSSDdKISL--IEPIVADLENCY-
INVWLDkRKIMPGDNLRkSIfRDGLDKADVALIFFTEKSLKSSWV-DkEIKHVLREESK-
KGNDFDLNKIISI-
FDSKETYDEISLRYPELtdDLMHLMPTKYTKIQLGQLISAIW(SKYLSLQGGDVEVQRQLLSKEKEIFKK)}*

$4042=167(169):
>gi|501598951|ref|WP_012598970.1| {|0(108)|<Cyanobacteria(B)>}molecular
chaperone Tir [Cyanothece sp. PCC 7424] >gi|218171293|gb|ACK70026.1| TIR
protein [Cyanothece sp. PCC 7424]

{(METDSS)-PQVFISYawRG-D--
SEKIANQLEQAFQNQGITIIRDKTNLGYKGLIKEFMEqIGQGKCILVIISDRYLKSENCMFELVEIAKHGDF-
YDRIFPIVLEDANIYQATGRLKYIQHWEQQISELESAMKQGNLANLQGITDDLN(LYTEIRNNIAQLTNLLKN
MNTLT)}*

$4043=296(300):
>gi|186701253|gb|ACC91279.1| {|234(37)|<Streptophyta(V)>}disease resistance protein [Capsella rubella]
{(IDRLVFLDLISLKNPVVIRRLLELVQSRRILLVLLGSLEYYNHVSFLNPSKSFSLKFFYSNVNLYYCSVGF
HFEPYLSTM)------SLGE-NHLGKFLVTSLKGELEKN-------QIEVDSEDETK--
SRTEESGVAVVFFSNKYPKSENCLDELVEIKKLMDA-
GSIDAFPVFYGLKDEQVGKLKGWFHNRLLKIenevRNNIKTRDDKsvldteakiWGWRDAISSIASR(QGLSY
LSCSTDDVFVSDIVTKVKKLFVSKETRETRAATTVVNNIDAALFYSQTSFLHAINRDITDFEGLTDTHNGLVV
LR)}*

$4044=238(231):
>gi|501316556|ref|WP_012348191.1| {|0(406)|<Proteobacteria(B)>}TIR domain-containing protein [Leptothrix cholodnii]
>gi|170777305|gb|ACB35444.1| conserved hypothetical protein [Leptothrix cholodnii SP
{(MADPRPAPDRPAPSTRKA)---IFISYRSADGKKDAARLAEDLRdiFGAP--
QVFFDKHDLQGGSSWrEQIGQALGQQPVVLLIISeqffGARNEQgLRIDqpDDPVRMEVIDSMrVGARLLPLL
cEGVRMPSSASLPVELRSVPEAHALKLRTEDWRADLLKLADRLVAL(GIAPLDDDWREQFTAPPGTARRGRLR
QSLGVAAVLLALGGGGAYWFHTEQLQREAAAVEARRLRALQHVSDGL)}*

$4045=210(224):
>gi|497650785|ref|WP_009964969.1|
{|0(144)|<Verrucomicrobia(B)>}hypothetical protein [Verrucomicrobium spinosum]
{(MTSSP)-
FDLFISYQKAqaAWVEvfardleSRLVVEERGGRRPFKCFYYTDSILPGDHWHRVMIQHAETAQRFIFIFTEE
SYLSNYCILELDARLHRDIYE---TTLFVLRDGTRlSQTPMRFRKHLDLTQPDqY------------------
---
H(CGVEKIISWLCHRKPSDKFTIRPLHGTAPAQPAHLPHPTLDLHTATRKIVQKIGTLVDRNSQEPLGPAWRS
AHNEFLCD)}*

$4046=185(191):
>gi|501598858|ref|WP_012598894.1|
{|0(1188)|<Cyanobacteria(B)>}hypothetical protein [Cyanothece sp. PCC 7424] >gi|218171216|gb|ACK69949.1| WD-40 repeat protein [Cyanothece sp. PCC 7424]
{(MASF)-YDVFISYGR-ADSKF-
FATHLSQRLIEKGLKVWFDKNNIPLAVDFqtQINDGIEKAHNFIFIIAPHSVKSSYCSQEIELAVKYN-----
KRIIPILHVNSDSYLDVMHPTIRKLNWLFMSEDPEVFEKGFTDLLQLINHHS(DYVKQHTKFLNKALKWLRTY
KQTQYLLFNHERNEAESWLKKKFEDEQ)}*

$4047=174(181):
>gi|506437015|ref|WP_015956732.1| {|0(1189)|<Cyanobacteria(B)>}type IV secretion protein Rhs [Cyanothece sp. PCC 7424]
>gi|218174417|gb|ACK73150.1| WD-40 repeat protein [Cyanothece sp. PCC 7424]
{(MKDYF)--DTFISYGR-ADSK-
AFATRLHEKLTQKNLKVWFDQNDIPLAVDFqkQIDEGIEKSHTFIFIIAPHSVNSCYCLKEIELAIKYK----
-

KRIIPLLHVEQISQEIWLERNPNGSDEEWKDYQAKGLHSSQVNMHPVISKLN(WVSFREEIDDCQANLEKLIN
LLQQDTDYIKQHTHFL)}*

$4048=275(277):
>gi|501598226|ref|WP_012598530.1| {|676(0)|<Cyanobacteria(B)>}signal transduction protein with Nacht domain-containing protein [Cyanothece sp. PCC 7424] >gi|218170851|gb|ACK69584.1| putative signal tr
{(KTLKIFLESLLDENNEVRMYAQLALRQVGNLEAIPYLFSLCVQKNYIWELDELIETISTIQNRFKFYKYPQ
YKIKMIEIF)-----ISYAW---EsangDGKESEDMANQLEQ----
VFKDKGYTIIRDKKdlgykgnieEFMQRIGRGKCIliV--ISDKYLKSENCMFELVEIAK--
NGDFYDRIFPVVLPDAKiyK-AIDRDEYIDYWDHRI-EELNEKLK-
SRSGSFANRQSIF(DELNLYDEIRRNIDQLTETLKNMNTLTPTLHTSTEFTAIIEAVKNKLENDLSADERG)}
*

$4049=223(221):
>gi|501315201|ref|WP_012346836.1| {|0(277)|<Proteobacteria(B)>}TIR protein [Leptothrix cholodnii] >gi|170775936|gb|ACB34075.1| TIR protein [Leptothrix cholodnii SP-6]
{(M)SKRVFISYkrnTALDQPLAHRLYDSLGKAGHE-VFLDA-
EILLGEDWVMRIQREVEAADFLILLLSEHSIESQMVAEeVRLAEHARkssgK-----PSILPVRV--
VYegSLPYDLGAILNRLNYA--LwrspaDDDELLV--
QLSAAIGGDALP(APVLAGGELPCDRPMPAANPRAVLELPEQVLEAASPFYIERDGDRVLAAEQTQPGYTLTI
QAARQTGKSSMLGRVLERGV)}*

$4050=171(177):
>gi|501317174|ref|WP_012348809.1|
{|0(314)|<Proteobacteria(B)>}serine/threonine protein kinase [Leptothrix cholodnii] >gi|170777923|gb|ACB36062.1| PASTA domain containing protein [Leptothrix cholodnii
{(M)AHDVLISYSSHDRLEADAICNRLESQGIR-CWIAPRDVPPGSEYADSIVQAIESARVMVLVYS-
AHADGSKQVRrEVERAVSIG-----RTIMPVRIQNAEmsKAFKYYVGSIHWLDAI--
TPPLEQHIDRLGRDIQAVLSAQ(PAAPGPAAATTTLGLPRTARPDPAPAAEASGTSRKG)}*

$4051=186(190):
>gi|501592494|ref|WP_012595971.1| {|0(694)|<Cyanobacteria(B)>}signal transduction protein [Cyanothece sp. PCC 8801]
>gi|218167967|gb|ACK66704.1| putative signal transduction protein with Nacht domain
{(MTFDAIVKSNLPKSRPDYD)---VFISYSRRdKEFVRLLWEKL--LEANY--
TVWVDwQDIPPAEDWRqEIYQGIEASHNFLFIISSHSLTSVFCQEELTYAINHNKR-LIAIIIQEPQNED-
INPDLAKINWIFCSGCNgqECDNFKQSFQQLLQALETDFNYV(KYHTRLLMRAKEWENKQYNSDFLLLGTNLE
E)}*

$4052=161(168):
>gi|501345614|ref|WP_012377249.1| {<Verrucomicrobia(B)>}TIR domain-containing protein [Opitutus terrae] >gi|177843483|gb|ACB77735.1| hypothetical protein Oter_4464 [Opitutus terrae PB90-1]
{(MNDAVQPARL)--TVFVSYSVHdTSLVDTINGHLLPHAR----
ALFWGQDKVPGQDAWgTIYSWIDSADLVVVVLTGKTLSRALSVGnEVGYA----RK-AGKRIIPLVGPEV-
PKGELGCLEGITYIRLDaeDPQRSISQLHDALARFAKEQETG(RALALLGLVALGIIAFSK)}*

$4053=179(183):
>gi|501600559|ref|WP_012599374.1|
{|0(86)|<Cyanobacteria(B)>}hypothetical protein [Cyanothece sp. PCC 7424]

>gi|218175412|gb|ACK74141.1| hypothetical protein PCC7424_5577 [Cyanothece sp. PCC 7424]
{(MIDSYEFD)---VFLAYNSVdKPLVKAIAKYLKS---KGL-RLWFDEeQIKPGDPIAdRVIEGIFQSPVALLFIGTSGFG-KFQRLwELNTLIMLCCQ-ENIRIIPILLPGV-DLNSRQLGLFYGIECLQfhQSVDETEKLEKLVQAIRGDQRLT(SNVCIIDEGLGSFVVNIMGRLIEGGDIRSQNTPIG)}*

$4054=137(142):
>gi|506436525|ref|WP_015956242.1| {<Cyanobacteria(B)>}hypothetical protein [Cyanothece sp. PCC 7424] >gi|218173924|gb|ACK72657.1| conserved hypothetical protein [Cyanothece sp. PCC 7424]
{(M)--KVFISHSHTdTKLAKKVADTL----RQAGFEVWDESQLLPGENWAkSIGNALEESDAMVVLLTPSSVHSSYV-NnEVGYALGTRKY-KG-RLIPVIAAPSEqlDREDIPwifnLEQFQMIH---VPNLEQDE--EGLKKIAQALKAV(A)}*

$4055=246(254):
>gi|501598858|ref|WP_012598894.1| {|325(802)|<Cyanobacteria(B)>}hypothetical protein [Cyanothece sp. PCC 7424] >gi|218171216|gb|ACK69949.1| WD-40 repeat protein [Cyanothece sp. PCC 7424]
{(LIKKIQEDEYYYKQHKMLLTKALKWQLQNYNPSLLLRGYNLQHYQSWMKIAKNHPDHPPIDLQVEFIGVSSNQPPDSSIE)---VFISYCPKdIDFARKINENLQELGK----TTWFDQEnIVVEKEGRqEIYQGIANSDNFVLIISPDSITSSDCAEEFNYAKQLNK-----RIIPILDQEI-DPLILHPDLKKIAKINFnqNERDFLANFNELVRTIHLDRDHV(HSHTKWLQKAREWELSQKNPDLLLRITEFSLAKN)}*

$4056=249(257):
>gi|506437015|ref|WP_015956732.1| {|359(755)|<Cyanobacteria(B)>}type IV secretion protein Rhs [Cyanothece sp. PCC 7424] >gi|218174417|gb|ACK73150.1| WD-40 repeat protein [Cyanothece sp. PCC 7424]
{(LIKVIKEDSRYYETHKLFLIQAIKWERQNHNPSLLLRGSNLSHAQAWLKVGKTKSQHQPTPLQEEFITASLQHPPLGCLD)---VFISYSRSdSDFARQLNEALQEIGK----TTWFDQEsIACGADFQeEIYRGIELCDNFVFIISPKSVNSPYCADEVEYAQKLNK-----RFVTILHRPL-SVEDKQKQPPTLVNMQYidFNQQGRDFAANFYELVRTLDTDR(DHVRNHTEWSQKALKWKNKHQEEALLLRGSEFAVAEA)}*

$4057=266(269):
>gi|506437015|ref|WP_015956732.1| {|154(943)|<Cyanobacteria(B)>}type IV secretion protein Rhs [Cyanothece sp. PCC 7424] >gi|218174417|gb|ACK73150.1| WD-40 repeat protein [Cyanothece sp. PCC 7424]
{(KLINLLQQDTDYIKQHTHFLVQARQWEKSQKQTRYLLIGSERIEAEKWLKTEFKNQQPPCEPTDLHCEFICESIKNAKNL)MSEVFISYGTEdKLIMLKLRKTL----MRHCITVWTNHtDIQSGLDFEqQIKQGIEQSDNVIYLISLNALFSQYC-QmEFNYALAYNK-----RIIPLLISSI-DLHKIPpqLSALQFIDLTDseNEEKYNYNIDQLIKVIKEDSRYY(ETHKLFLIQAIKWERQNHNPSLLLRGSNLSHAQAWLKVGKTKSQHQPTP)}*

$4058=259(264):
>gi|501597201|ref|WP_012598120.1| {|154(935)|<Cyanobacteria(B)>}type IV secretion protein Rhs [Cyanothece sp. PCC 7424] >gi|218170440|gb|ACK69173.1| WD-40 repeat protein [Cyanothece sp. PCC 7424]

{(KLLHVFQQHQDYIQQHTQLLLKALKWEKHQKQHQYLLSGQERIEAETWLKTEFINEQRPCTPTDLHCEFIC ESIKNAKNL)MSEVFICYSDLdKSIMIQLRQSL----
MRHSITVWTNQtDIQSGTQFEqEIKQGVEQSDNIIYLISSNALASEYC-QmELDYALSYHK-----
RIIPLLIESI-
NLDKIPPQILGLQFIDLseSRTEDYYNQTIDKLLKALKQDAQ(YYEDHKLILTKAIKWQRQHHNPSLLLRGYN LSHAQAWLKVGNTK)}*

$4059=248(256):
>gi|501597201|ref|WP_012598120.1| {|359(741)|<Cyanobacteria(B)>}type IV secretion protein Rhs [Cyanothece sp. PCC 7424]
>gi|218170440|gb|ACK69173.1| WD-40 repeat protein [Cyanothece sp. PCC 7424]
{(LLKALKQDAQYYEDHKLILTKAIKWQRQHHNPSLLLRGYNLSHAQAWLKVGNTKTQHQPILLQEDFINASL LQPPLESLD)---VFISYSRVdADFARKLNDALQEIGK----
TTWFDQEsIASGEDFKqEIYRGIESSDNFVFIISPKSINSPYCADEVQYAQKLNK-----RIIPILYREV-
LSNQLHPALAKVQWINFnqYGGNFAANFNELVRTLDSDRDHV(RNHTKWSQRAKEWQTQQESEDLLLRASEFI LAKNWL)}*

$4060=171(176):
>gi|506435524|ref|WP_015955241.1|
{|0(776)|<Cyanobacteria(B)>}hypothetical protein [Cyanothece sp. PCC 7424] >gi|218172912|gb|ACK71645.1| WD-40 repeat protein [Cyanothece sp. PCC 7424]
{()MTDVFISYSRKdQEFVKILHQALSQSHR----
DSWIDwQNIPKGTQWWqEIESGIEGANTFIFVISPDSIKSEVC-GlEINHAI----N-NGKRLLPIIRREV-
SEFDDNNTAHQALKDHNwlFFREEDDFQTSFEELIKTLDTDL(NYVHQHTRLLVRAKEWDKKGRRDSFLLRGE ELKEAE)}*

$4061=184(176):
>gi|493578716|ref|WP_006531813.1| {|0(138)|<Firmicutes(B)>}MULTISPECIES: hypothetical protein [Streptococcus] >gi|171282411|gb|EDT47836.1| SEFIR domain protein [Streptococcus infantarius subsp. infant
{(M)KKKIFISYAW--EENSEkdkkvkmFTQWLAVYLKKWDFEVLLDVYENHPGTKLdsFMLEGV-
NTSRFVLCICTETYTKKmtkigTGVNtEFTL----LQENaDSKFIIPIIEKGK-
FVNLPSFFRGKFVSELNFSEPYSQDNRNNIFELISTLRDE(ALSVKGVEPKKRIENYYNNVEKFKLLADTIDL MNF)}*

$4062=201(204):
>gi|501594054|ref|WP_012596863.1|
{|0(205)|<Cyanobacteria(B)>}MULTISPECIES: hypothetical protein [Cyanothece] >gi|218168868|gb|ACK67605.1| Tetratricopeptide TPR_2 repeat protein [Cyanothece sp. PCC 88
{(MSEGFEFD)---VFLAHNS---
VDKPHVREISNKLRERGLKPWLDEEQIPPGMSFqdEIQKAiplIKSAAI----IIGTQGLGKWQIMeLRSL--
-ITKFVnLKIPVIPVLLPGV-
NNIPGdllfLQELNWVKFEQIDDATAFYRLEWGITQVKPELHPK(TVQLTAEEWFNLGYNKGESGDNQGAIAD FNQAIKIKSDLAEAYYNRGLAKSNLGDY)}*

$4063=218(223):
>gi|506437675|ref|WP_015957392.1|
{|178(0)|<Cyanobacteria(B)>}hypothetical protein [Cyanothece sp. PCC 8801] >gi|218169527|gb|ACK68264.1| hypothetical protein PCC8801_4342 [Cyanothece sp. PCC 8801]

{(QFYTEVPSGPMLFFQDVLLSGFYCAGISSRELPWLVIIDDPNINNDMYDVFNAEFERIWELSSTNRERPSS
ELYNYAHSY)----FISYS-SqdkeiaDHIELLL------WRKNRL--
VIRDERDLTSGQNLseDIESVIGKSQTFLFLCSQSSNQSDYCRGEIDVAFEYKKLKeqqGNNGQEGIQRI---
VVISLDGQKPQDLRLRSYLRLQGENRTERESSIRRIIDEE(ERI)}*

$4064=161(160):
>gi|1137243647|gb|OMP03555.1| {|0(1211)|<Streptophyta(V)>}hypothetical
protein CCACVL1_02371 [Corchorus capsularis]
{(MALKRKHFSEAACSSSSNL)KYQVFLSFRG-
EDTRKNFTDHLYTSLVNAGINTFRDDDQLqrGKIISSELLKAVQQSKISLVIWTSSKGGGATHPIAIQSRTEI
VLGLLSMESPDVGIVGIYGMGSIDDRLKDMFLDIACFFIGMDEEYVMTILKGCGYFPV(I)}*

$4065=154(157):
>gi|1137228795|gb|OMO92690.1| {|0(643)|<Streptophyta(V)>}hypothetical
protein COLO4_17386 [Corchorus olitorius]
{(MLSLPSSSSSSFYVSRK)KYDVFLSFRG-DDTRNNFTDHLLAALkRSG-
IITFKDDENLEAGESIapGLSKAIQESW--HESEFIGDIVRRISHKLFLAYSSVPDD-
LIGIHSRLNELLSKIDTRKDNIRIIGICGMG-GIGKTTLARVVYSQMSPHFEGK()}*

$4066=210(238):
>gi|1137228803|gb|OMO92696.1| {|0(748)|<Streptophyta(V)>}hypothetical
protein COLO4_17384 [Corchorus olitorius]
{(MLTLPSSSSSSHFYVSRK)KYDVFLSFRG-DDTRKNFTDHLLTALKRsG-IITFKDDEKLEAG--------
-------------------WCLDELAEIVKQKNE-GGLKVFPIFYDVDPSDLRKQTGKVAEAFVKH-
DERYKENKHkiENWRSALTEVANL(KGWHRKDRPEAEFIADIVRKVSAKLFQAYSSIPDDLIGIHSRLEELLS
KMDIGEDDIRIIGICGMGGIGKTTLARVVYTQ)}*

$4067=166(167):
>gi|1137228794|gb|OMO92689.1| {|0(217)|<Streptophyta(V)>}hypothetical
protein COLO4_17385 [Corchorus olitorius]
{(MLSIQSSSSCSYVSRK)KYDVFLSFRG-EDTRNNFTDHLYAALvRRG-
IVTFRDNERLEAGESIapELFKAIQESWGSVIVFSETYAFSGIGIDALIKKSLIKVY-
GDKDKYLWMHDLLQEMGRKIVKQKSLEEPGK-RCRLWEGRDVYDVLTKNTASAN(SRQLNIVLPGS)}*

$4068=221(238):
>gi|1139783103|gb|ONI16930.1| {|0(721)|<Streptophyta(V)>}hypothetical
protein PRUPE_3G130700 [Prunus persica]
{(MAATRAQQDASPSSHWCT)-YDAFLSFRA-TDTRKGFADHLYRALeVAG-
IHTFRDDDEIERGANIlaELQKAIQESRVSIIVFSKDYASSRWCLDERVTIMDRRET-
NEHMVMPIFYDVDPFH------------------
RFNKETDkvEKCRKALRDVADL(GGMVLGDRSESQFIQEIVEVIGNKLDHTWNRRLRVDPYVVGIDYRVKGLN
MWLEDGSSDVGVAVVYGMGGIGKTTIAKTA)}*

$4069=196(193):
>gi|1141002217|ref|WP_076844665.1| {<Actinobacteria(B)>}hypothetical
protein [Frankia sp. BMG5.30] >gi|1139644642|gb|ONH33280.1| hypothetical
protein BL254_20205 [Frankia sp. BMG5.30]
{(MSGY)RFDVFLSYRR-AGTvlpwvRNHFhqmlVKCLTDELDRKpEI--
FIDVDRIPVGDYWpdVLAEAHSRSRLLVAVWTPSYFGSHWCVAEWRSMrqrevvfgLDKPLN-GCRLVYPI--
-------RLKDGH---RFPAD-
AQRSEHWDMKEWNVSSSSFEQT(PAYVGFERKVAEIAEELAARLDVVPEWRPNLPILRPEPEPXHRADVPRL)
}*

$4070=147(165):

>gi|1139755923|gb|ONH91380.1| {|0(807)|<Streptophyta(V)>}hypothetical protein PRUPE_8G110800 [Prunus persica]
{(MDSNTTELLGAPSPPSSSSSTNSWT)-HDVFLSFR----------DHLHKNLVQKGIRTFIDD-ELPRGEEIsqNIVNEI--------
SLQVLNDTHINVAKYQVGIEARVQDLHKVLDVDGNDVRMVGIWGNGGIGKTTLAKAVYNSLAHMFEGSCFLEN
VRERSIP()}*

$4071=193(198):
>gi|1142746564|ref|WP_076994104.1|
{|0(657)|<Actinobacteria(B)>}hypothetical protein [Actinosynnema sp. ALI-1.44] >gi|1140153002|gb|ONI70528.1| hypothetical protein ALI144C_48720 [Actinosynnema sp. AL
{(MNDPA)---VFLSCRW-TD--EGAADGIERALRDRGLAVFRDKD-
IHLFDGVtdSTWRALDSSAVLIAFYSRRYHAKFACQWALTRAFLAARRLGDPLDRVLIVNPEPDDRHIAPGDL
ADAHHFSWRRESDTEPLADAVAGKVERAQG(PLCAESADRLGRLRGRLSRPRRFSGRYREMWSIHSELRSRAR
VVVVEAPAGTG)}*

$4072=194(191):
>gi|1142747288|ref|WP_076994828.1| {<Actinobacteria(B)>}hypothetical protein [Actinosynnema sp. ALI-1.44] >gi|1140153729|gb|ONI71255.1| hypothetical protein ALI144C_48680 [Actinosynnema sp. ALI-1.44]
{(MY)EYDVFISYQrnG-TTVPAWVRTHFYPRLsellddqLDHQAKIFFD-
ENVPTGANWplELRNALQRTRILVPVCSPKYFLNEWCLAEWHSMVRREEiaamatpGRPQGLIYPvIFCD---
----------SENFPSY-
AHERRMQDLKTWNLPYPQFQDT(IEYLGFHREVERIAVELKDLIDQAPEWRPDWPVHEPPPDPPRTPRLPRF)
}*

$4073=221(220):
>gi|1139748329|ref|XP_019918925.1| {|142(0)|<Mollusca(M)>}PREDICTED: toll-like receptor 13 [Crassostrea gigas]
{(TLFKFKTFESSLNILQKQCASYLGGYIGCSVTLAIFITFIISLALVKNKWKIRYLIYKSKQIFGIVTPFNR
YLPSGMHYE)-HDAFLSYSGQelMFVLKEVIPRLE-
VNKSLKLIVRDRDYLPGIPKVDSIMNCLQERRRTICIVSKKYLESKWRDYELNMAKVEAIEDRGslDYVILIM
LPEVYnDGIP---HKIMDLIKKDrFIEYPMESCAynDFWERLMGML--D()}*

$4074=238(239):
>gi|1139806120|ref|XP_019923808.1| {|405(0)|<Mollusca(M)>}PREDICTED: uncharacterized protein LOC109619040 [Crassostrea gigas]
{(KCQFPENIKGRRLRDLTDNDIGCGFKMLPIIVVLSILICFLLIFLFLIIRYRLQIRLFCAQRLSGISSDNS
IDMDAEKSF)KFDALICHGLFdeEWARSTFIENRHKLLSHLKLGFYREDATDQKNNFEKLIDQMKSSKYVVVL
LSRQFLEGEFLTPGFQEALQQSNEHLTRKRSILVLMDD-IPTQEETICLRRSLQTFtCIHKNDTR---
FTDKFLYLLSSK(GDLAWCEYNEGNRQQYVAI)}*

$4075=253(244):
>gi|1139845710|ref|XP_019919373.1| {|486(0)|<Mollusca(M)>}PREDICTED: uncharacterized protein LOC109617541 [Crassostrea gigas]
{(NSNHNELYCKASGGNIPIISFNFDALDCAVDQFLIYIAIALALFLIILSVGMFVLYNYRYELVLIHKNPTI
GRENMVSML)-YDIYIVVNENndEallWTKTVLTHKLKR--
LGYNTYFPCRDNILGDLREEQIIQMLSKSRDVIVLLTSEFNSDEenlntlYANLEWRHAWNNFKTNSSERNLI
VINFDQ-
LKAGDFDVSpLKAFLRLKmTLDFADRSHSLMRDLLARIGPPS(RVNKKPIYKQTHRDHTQFIELYFE)}*

$4076=232(222):

>gi|1139808543|ref|XP_019924335.1| {|634(0)|<Mollusca(M)>}PREDICTED: toll-like receptor 13 [Crassostrea gigas]
{(EDWKGKTLLSFDETKIECNLVSVKVTVGIIVTVIILFVVLISISYWYRMHILFITYSTKNRIRRYFTRRNQ
NYTELVNME)-YDAYILYSEDdeDskWIKTNLLKEFDDGskddqnfHGKYKLYFGDRDTL-
GSADNLqTSVNTMENSRKIIIVVSK-YLESYRLRDLF-IGEALDLKGNSLhDIIVITVGNISFAEIPR--
LLHSKVRIGdHVEWRDDDIYrrVFKVKMEEKLMNN(ST)}*

$4077=253(245):
>gi|1139753474|ref|XP_011426659.2| {|3102(404)|<Mollusca(M)>}PREDICTED: uncharacterized protein LOC105327744 [Crassostrea gigas]
{(QDEKCRDMFLYIKEQLFKKFAIAVIRESLQWKHSELGMKIGQPKMCMIRTPQRFATKINDLKDIVEDHIAG
LEMMKSDHP)--DIFISYCW-QNSQEavnngTVARHgalgygdprkIKEFLEKKGLSCWLDIEQMgKYG--
LyqEIAIGLQNCKVIVACVSDEYVESDACMMELRFAVCNM-----
NLPLVVVVVGTGDKWKRSEVSLLMRRSDKGANKVYMQKENPEGFEALHKFVI(DVLPTYTDEILSEEKRKKDA
KKKRK)}*

$4078=255(261):
>gi|1139793216|ref|XP_019923264.1| {|821(53)|<Mollusca(M)>}PREDICTED: uncharacterized protein LOC105329486 isoform X2 [Crassostrea gigas]
{(LSSGEENQDWLALAKLMGHKESQIEEFLEDLNPSLALLTDWIITSGNTSLSVDMLIAFLEKMNRDDVVEII
EKANDTNKD)PPQVFISYQW-DSQDEVKA--
LRDKMEKSGYSCWMDIGQMGGGDFLsaKIDQGIRNCKVVLACITPKYVVSHHCNRELSLADLLN-----
KTIIPIMFEEVPWPPPGGMSLIFSQLVYINMKGVGGHGGTGIHADLKDKYKE(IVQKLMNYIVPDLTKYFDTE
TVISKQFSNDNSSITSEIYSP)}*

$4079=247(253):
>gi|1139818160|ref|XP_019926484.1| {|957(250)|<Mollusca(M)>}PREDICTED: uncharacterized protein LOC105336989 isoform X1 [Crassostrea gigas]
{(RLLASRLGYSPDDIRGWATQSDPCMALLSEWYATHRTFEASRGVLTILQEMNRLDAAIIVENAMKAAEGVV
TDEPVDYPE)PPEIFLSYQW-GH--
QNEVKLICRHLEMAGYKCWMDIGQMGGGDKLfeKIDSGIRAAKIVISCVTEKYAKSPNCNREVNLSVNLG---
--
KPMIPLLMEKMAWPPAGSMGPIFSEYLFIRFFQRPGEETNDDRIWPAAKFTE(LLMQMNCLKIMPDEENIATM
YKNWWMPVVEEIV)}*

$4080=245(253):
>gi|1139818160|ref|XP_019926484.1| {|1154(55)|<Mollusca(M)>}PREDICTED: uncharacterized protein LOC105336989 isoform X1 [Crassostrea gigas]
{(GEETNDDRIWPAAKFTELLMQMNCLKIMPDEENIATMYKNWWMPVVEEIVIPKRNEKKGENKAASSGQENV
NRESQVVSP)--DVFISYQW-GKQKEIIA--
LFQRLTSLGFTCWLDIRQMGGGDSLydKIDRGVRGCKVMLSSVTTKYALSANCRREVSLADALK-----
KPIIPLLMEKIDWPPTGPMSMVLTQLLYVNFSKDESIQLSWEGKYFDELLSK(IKQHVPTAKIEEIQTNSSKQ
EKNTKDENSVTNG)}*

$4081=265(254):
>gi|1139828909|ref|XP_019928725.1| {|1105(423)|<Mollusca(M)>}PREDICTED: uncharacterized protein LOC105342840 [Crassostrea gigas]
{(FEKDETCHDMFLYVANQLRKKVLVVVVRPSLAWQQTDLGMKIADALLCMIRTPDRYKTKIADLQGMIEKDL
MEMEQNKRK)PPEVFISYCW-QNSKQAEAKNtviknrkslgavdprdIKDFLMEKKLSCWLDIERIGT-
YGLylDIALGLQRSKMVVCCVSDEYADSENCMMELKYAVSLK-----
MPVVVVPVGSGNRWDTKVKIFLMKLDGDPIILPMQDKDRRPELLEKVKEVLA(STNQKKDETAEKKRLEAEAK
RNQEKKAQDLKFSY)}*

$4082=266(274):

>gi|1139738936|ref|XP_011429536.2| {|287(91)|<Mollusca(M)>}PREDICTED: LOW QUALITY PROTEIN: uncharacterized protein LOC105329774 [Crassostrea gigas]
{(EKLREIDLQLHIKRFYYTLGGLLDDRSFPQTYDRARVITYDEQQSGFVLADVVLNEMKKDLYSPNEKKVPP QHFQHAECE)-FDAFVFIHEGdENTSDEITHFLEGKGV--KLMRNDR--IGQRIKYQXRIK---- RCRWiIPILSKTSLKDPVFAHCCLMLVGDILEKKELRMIPVLTgGAAYEDVPEVIRMVTLTVADN-- PEYLNRLHQTIKGNTIPFETD(SLLPAGDVVYVLAWNYAVNYLEKVMPGLERVIAETLQKHNKRPTSCRKKLY LIV)}*

$4083=161(188):
>gi|1139828671|ref|XP_019928674.1| {<Mollusca(M)>}PREDICTED: myeloid differentiation primary response protein MyD88 [Crassostrea gigas]
{()--------------------
MEAPPYNLKFCIPWRDDLPGPSRYEVSAHMIATRCRRtLVILSPDFLKSAAADFQLKFAHCLSPGARSKKVVP VFSAPCK--MPDILRALSFVDFTN--PGLRD---
WNWPRLNAVLQCP(LNPDPRDYMSEAELEELKLNAGVITTRMWYSTGVLTMNFPEESEDNTP)}*

$4084=289(298):
>gi|1139648351|gb|ONH36706.1|
{|138(1407)|<Actinobacteria(B)>}hypothetical protein BL254_05460 [Frankia sp. BMG5.30]
{(GIEPLLRLLTAQPVETEPPIGTVPVLPARPAVISGRTIETAGTGGASGTGGAGATAATAATAATSATSATG GIGPAARPV)---VFVSYTEADERWAAWIIHQLTAEGYR-
PRATTWQPPPGEGAAAWLRRELAAAAFVLPVLSPDY-VNSDWAAaXWQAALAtmtrdGR-----
PLLVPVRVAPC-EAPPLLRGVGRVSLVE--QAQAQARR--
TLLDGLSAAAGR(SAQPALPPAYPGLVRGPAREPADADGAEPPAYPGPDPQKITSVFVRWVEQAARVRYPDAT IQRMTADNGTVYFDVNGL)}*

$4085=225(232):
>gi|1140989419|ref|WP_076833570.1|
{|295(0)|<Actinobacteria(B)>}hypothetical protein [Frankia sp. CcI49]
>gi|1139664734|gb|ONH52506.1| hypothetical protein CcI49_33825 [Frankia sp. CcI49]
{(AWLTLPGRPLPAVPGVAAVAATPAGSPSGSPASTQTPAAPVSQKGPGSPDGSSGLVDPPSPNGLDDPDGLD APVRPDGTK)-WDFFVSVLETDAAWGDWIAGLL-QNDYL-VRYDAWNP-
AGTNDYWALDEGLNFSKHMIVVLSQAYLASDK-VQaSWGTAFKRD-----
PAGLGRRLIPVRiekcEPGGLLGGIRYIDLVP--FkkDPENARK--
HLDEQIERSVKG(YYRPPEAPPFPG)}*

$4086=229(229):
>gi|1140985786|ref|WP_076829961.1|
{|50(0)|<Actinobacteria(B)>}MULTISPECIES: hypothetical protein [Frankia]
>gi|1139670034|gb|ONH57727.1| hypothetical protein CcI49_25030 [Frankia sp. CcI49]
{(MINDLLEKGILRGGRKLLLAAAHDDWPANPAFEPSMFPDIDDTPAESVSPAGGFPSPGPTVPVRAAGRPEP REPAAEEYG)---
FFVSYVtgdEKDRAWARWIAGELAQLGQSSrvppsVCLPDADAPLASGMVDRTKQDAPRSAWVVAVLSPAYLG PDDDGAtEWGAVLnatrRHT-----DRVFTVIVADCAPA-GVLAELPWINLVG--LDKDTASR--
TLHREVLQIVNV(ATKCPPERR)}*

$4087=203(214):
>gi|1142796676|ref|WP_077042512.1|
{|0(475)|<Actinobacteria(B)>}hypothetical protein [Rhodococcus sp.

MTM3W5.2] >gi|1140139877|gb|AQA24410.1| AAA ATPase domain protein [Rhodococcus sp. MTM3W5.2]
{(MAN)---VFISHASADHAWAEEIRRWLSEDAHE-VFFDRdkRDgIPAGDEWEPRLYERLRWADAVVCIVTPSYLE-SVWCAaEIGAARALG-----SELLPVRATSESVNHRLLKLKQYVD-VA--RDPVDARE--RLRSRLSVIDGG(GGRGWPDDQSPYPGLRAFETSEHRVFFGRSREITQIAERLRSPAERAARTILTVVGPSGCGKSSLIRAGVL)}*

$4088=236(221):
>gi|1142746558|ref|WP_076994098.1|
{|0(83)|<Actinobacteria(B)>}hypothetical protein [Actinosynnema sp. ALI-1.44] >gi|1140152996|gb|ONI70522.1| hypothetical protein ALI144C_48685 [Actinosynnema sp. ALI
{(MSDRA)APRVFVSYANDSpehtaqvRRFAEFLRTQV---GLD-AHLDIWYRDRRRDWSVWATDHLTKAEFVLVVASPQYklqaegeAAPDESGRaELEAAILRDNLAKNlrdgtERILPVVLPGRSvrEIPAFLNP-HSATSFR--VDEftVGGIS--DLLAAITGQGQY(PLPELGQWPGDAGADERPVTTLLAGMRWLTSSPGLRPGSAKINGVRYDNSVVSRHRPSTGEPRVFVEVDLDRAYRR)}*

$4089=176(182):
>gi|1143093722|ref|WP_077084782.1|
{|0(580)|<Bacteroidetes(B)>}hypothetical protein [Porphyromonas gingivalis] >gi|1139926345|emb|SJL31681.1| TIR domain protein [Porphyromonas gingivalis]
{(MKPTLA)----FISHrsTHKD----FVGKIVDTVKRDNCIVDEFDFY--PATKTMdEIVRNLNHAPIFVLLISKDALASNWV-QlEMSKARQLYDS-GKIKVFlPFIIEED-VKLVELPLWMTRDWALNvkTITSPKIIGQFIIEAQRKIRCNE(NREYGNLVNTFIGRSNELDEFQRAMYDSVCMPHRSL)}*

$4090=207(191):
>gi|1142746555|ref|WP_076994095.1|
{|0(272)|<Actinobacteria(B)>}hypothetical protein [Actinosynnema sp. ALI-1.44] >gi|1140152993|gb|ONI70519.1| hypothetical protein ALI144C_48660 [Actinosynnema sp. AL
{(MLVNGSDVLDSEPARA)-PRVFVTYSHEsEEHKDMVHRFASFLQEEvGVhitLDLWSDRgRI----DWSlWAMKALKTADFVLVIASPEYrrradgdAAPDEGRGaQFEAAMIRNEltrdlaE-WTRRVLPVVLPGM-SIDDIPTFLNAYSTTRYvvSEFTVEGAEALLVPLTGQSRYPV(PPVGVFRPTSRTVQQRVVVTKAIPTVAEGAVLAPG)}*

$4091=284(299):
>gi|1139816448|ref|XP_011438569.2| {|359(90)|<Mollusca(M)>}PREDICTED: uncharacterized protein LOC105336085 [Crassostrea gigas]
{(FFSPYAVGIVCLRSEIMFLLLDISIDHFDAITENLCLDVSVVPCCRVPQETKVEDEQAALAVIKILKGMPLRISEEDVFL)-YDVYVSYDEADypWILGELYPKLERLG--VTAWLTDKDSIPGRPRSEEIVSCVTESRKVMFVVSERFMDKGWFSYAVQMAITHAFHNQRQRSIVVLIkDGVpLERLPD---EMKNIWwCIEHFRWPEND-------------G(SCSDEGSQIPAIFLESYSLLTQQITTVIHEAHSCIAKGWIRIFLDPLLDLCILKKEDCDQQKNIFKKRHISVIYSFLFA)}*

$4092=222(222):
>gi|1139853211|ref|XP_019920955.1| {|617(0)|<Mollusca(M)>}PREDICTED: toll-like receptor 4 [Crassostrea gigas]
{(INQNVFLSNFFQEQTFKKFELICQSDVWLLGSSFALVFVIIILLTTAAIKRYRVHVDYIILRMRSRWKGVMCMANSEKFR)-FDAFVTYTEEDyqLACITFYQTLVRLG--

FKISMPDKDFLPGISEAKQLLQCIDQSRKVVIIVTENFIENGWNSYAVQMVVTHAFHNQRErSIIVIIKDDIs
LERLPR---DLKYIWwSIVSIRWPNGDE-TmdkFCEKICAALR-P(DP)}*

$4093=221(220):
>gi|1139854561|ref|XP_019921223.1| {|614(0)|<Mollusca(M)>}PREDICTED:
toll-like receptor 4 [Crassostrea gigas]
{(SNNLTVELFNENKWLLKFELICQATDWLIFSIVLIVSTLTMLIILAAIKKYHVHLEYVILRVKQRLMPVGD
HVCVEGDFQ)-YDVYISYNDDDtsWVANNLNPKLESLN--
IKAWFKEKDSIPGGWESEEIVNCINDSRKVMFIVSESFLDKGWHSYAVQMAITHAFHNQRQRSIMVIIkDGLp
LERLPKEF---KHIWwCIEHLRWPEDET-NdetLLLNLSNVLVSE()}*

$4094=221(220):
>gi|1139854566|ref|XP_019921224.1| {|184(0)|<Mollusca(M)>}PREDICTED:
toll-like receptor 6 isoform X2 [Crassostrea gigas]
>gi|1139854568|ref|XP_019921225.1| PREDICTED: toll-like receptor 6
isoform X2 [
{(GSKSATDELFNESKWRKFELDCQATEWLIFSIVLIVSTLITLIIIGAIKKYHVHLEYVILRLKQRLIPVGD
HVCVEGDFQ)-YDVYISYNDDDtsWVANNLNPKLEILN--
IKAWFKVRDSIPGVSQSEEIIKCINESRKVMFIISESFLDIGWSSYAVQMAITHAFHNQRQrSIVVIIKDGLp
LERLPKEF---KHIWwWIEHLRWPEDET-NeetLLLNLSNVLVSE()}*

$4095=221(221):
>gi|1139854608|ref|XP_019921236.1| {|622(0)|<Mollusca(M)>}PREDICTED:
toll-like receptor 2 [Crassostrea gigas]
{(VESKIPIVQLLYEKEWRKFELNCQTDDWLVFSVCLLFFTIVSLTIIASVKRYRVHLEYVILRLKNRWKGVH
QKSNEDMFL)-YDVYISYNDADcsWVIETLYPKLEVLN--
IKTWFGDKDSIPGRWKSEEIVGCINESRKVMFIMSESFLERGWHSYAVQMAITHAFHNQRQrSIVLIIKDGLp
LDRLPN---EIKNIWwCIEHFRWPENEQ-HdemIFSTLSKILK-P(K)}*

$4096=221(221):
>gi|1139854606|ref|XP_019921235.1| {|608(0)|<Mollusca(M)>}PREDICTED:
toll-like receptor 4 [Crassostrea gigas]
{(CVENKSSIVKLFNEQTWRKFELNCQTNELLIFSVGLLALTILTLSITAAIKRYRVHLEYVILRLKNRWKGI
NPQCKNEFL)-YDVYVSYNDADysWIIRILYPKLNVLN--
IKTWFGDKDSIPGRWESEEIVSCINESRKVMFIMSESFLERGWHSYAVQMAITHAFHNQRQgSIVVIIKDGLs
LDRLPN---EIKNIWwCIEHFRWPEDDN-SdeyILSKLSSILR-P(D)}*

$4097=225(225):
>gi|1139854519|ref|XP_019921215.1| {|606(0)|<Mollusca(M)>}PREDICTED:
toll-like receptor 4 [Crassostrea gigas]
{(VESKSAVSELFNDHNWRSFELQCQTVAWLILSVGLLVLTVLTLTITAVIRRYRVHLEYVILRIKQRWKGVQ
IYCQEDVFS)-YDVYVSYSDVDypWLIQNLYPKFEKMN--
LKTWLKDKDSTPGSWESEEIVNCINMSRKVMFIVSESFLDIGWSSFAVQMAITHAFHNHRQgSIVVIIKDELp
LDKLPN---EIKNIWwCIEHCRWPEDET-NdeiILSKLCNVLK-I(SNIIE)}*

$4098=279(288):
>gi|1139845351|ref|XP_019919300.1| {|0(101)|<Mollusca(M)>}PREDICTED:
uncharacterized protein LOC105319338 [Crassostrea gigas]
{(MPTPGTARGKSERMSVVNIGGGSLAPPSGVGYLPLKHGSTCFHSPKYLTVTMESLERQRAMAMPRCDTPSD
APPLPVGKD)-YHAFFSFhGEnpDYNWVKAVTRELECRGF--KCCVFPRDLDAGKSL-
SNNIKSVLTkCLRIVTVLSSAYIKNEWSQLEME-IISLG-KLEELVFIPVLIEPC--
DIPPFLKYYRYINATT----AKETW-
WNAFHEQMLGNVP(PLPPRKKHHVFFAHTSSDTKWVNDIVKRLENPVNSIVCCFPARDFKPGASMQESVDSAI
KRSLRVVLV)}*

$4099=243(257):
>gi|1139845351|ref|XP_019919300.1| {|137(0)|<Mollusca(M)>}PREDICTED: uncharacterized protein LOC105319338 [Crassostrea gigas]
{(IVTVLSSAYIKNEWSQLEMEIISLGKLEELVFIPVLIEPCDIPPFLKYYRYINATTAKETWWNAFHEQMLG NVPPLPPRK)KHHVFFAHtSSDTKWVNDIVKRLENPVNSIVCCFPARDFKPGASMQESVDSAI-KrSLRVVLVISKNFLANEWKKYFEK-------RFKQFKTIPIIISDCN--LPIVLDDIVCID-AR----NSGTD-WFKVLLRAIKEED(LNADDDTVTSTSQFIREEVIFTPAAEPMASLSTTSFI)}*

$4100=196(225):
>gi|1142761938|ref|WP_077008902.1| {|74(0)|<Actinobacteria(B)>}hypothetical protein [Saccharothrix sp. ALI-22-I] >gi|1140167380|gb|ONI84744.1| hypothetical protein ALI22I_30005 [Saccharothrix sp. ALI-
{(GHLPFTPGAKRALALAVREALALEHRHVGTEHLLLGVLALDDGVALDLLARCGLEAVDVRDRAFAVLAGID LPAQRPARL)----FLSYRRPQDLHLAGRIADRLDDIGR--SVVGDVG---------------ECDVLLAVVGPEW------APTDLIRAEVDRAAeRSVPVIPVlVDGAVLP-----VDQLRGTGHvADHVTVRHETFRADVARLADVVRYA(QPGIG)}*

$4101=215(218):
>gi|1142734048|gb|ONM48414.1| {|0(1100)|<Actinobacteria(B)>}hypothetical protein B0T46_11910, partial [Nocardia donostiensis]
{(MPLFEYRSEGPIIVS)--RIFISHSSRDNTWAvavrQWLIEQQPDLAQD-IFLDidpEIGIHSGQRWKVALRAAVERCEAVVCLVSRHWLA-SHECEaEYRAAEYLG-----KRIYCARIEPLDGVHDITREWQRCDLYG--DDPVTEVHIDSDREPVTLRTAG(LQQLLTGLRAAGIGADHFPWPPLNDPDRAPYRGWNPFEPVDAAVFFGRDE QVLHAIDELRTMR)}*

$4102=218(222):
>gi|1140942643|ref|WP_076790477.1| {|81(0)|<Chlorobi(B)>}hypothetical protein [Chlorobium sp. KB01]
{(NIADLFQAGVGDTIFTNIDLSVCKGLESLNHVGPSSIGIDTLYKSKGNIPEVFLRGCGLPDEFIAYIPSLI GKGIEYYSC)----FISYSHLdEEFAKRLHNDLQA---KGV-RCWfapHDLKI--GDKIRpTIDDSIRVHDKLLLILSEHSVLSDWV-EhEVEHALDLEKEQKKTVLFPVRIDDA-IMESTTGWAGNVKRQRHigDFTQWKQHDTYQRAFNRLLRDLK(AG)}*

$4103=179(177):
>gi|1142794166|ref|WP_077040002.1| {|0(1267)|<Actinobacteria(B)>}hypothetical protein [Rhodococcus sp. MTM3W5.2]
{()MSRVFITYSSRdRRQAIALKRWLTerepGLAREIFLDVDADTGLPLGIRWKdALRKAKDRCEAVIVLLSS AWESSHEC-KlEYRHAEDLNKP-----VFVVRLEPM-ESGEITREWQRCDLFGDgpQTEASLGDGSEPVVFLTDGLERL(RTGLRATGVGAEYFPWPPPGDPNRAPYRGW EPFEEVD)}*

$4104=224(231):
>gi|1142743308|ref|WP_076990848.1| {|0(637)|<Actinobacteria(B)>}hypothetical protein [Actinosynnema sp. ALI-1.44] >gi|1140160483|gb|ONI77974.1| hypothetical protein ALI144C_31745 [Actinosynnema sp. AL
{(MATTNDDSRDN)-YDVFVSYaSADRDVVAPFIELLKLNG--LKVWFYDQDMHGGPALKKQIADAIKQsTH-TIACLTDEYVRRPWTTFELRNSSHRDPAGDTARTILVRFKPMTLAVPDDVKHLTVSDLTD----QRNYD-RTFQRITKLIKRP(MPVEPDNLAELCEAPFQSAEPDVTLFQIRRANEALSKFLYRREIGELPSNATLDLIIGQ LIVSGKLPGEIVAALATMQSL)}*

$4105=220(225):
>gi|389680317|emb|CCH90982.1| {|0(15)|<Cyanobacteria(B)>}hypothetical protein MICCA_100001 [Microcystis aeruginosa PCC 9432]
{(MTQLINRLKNPDFVTLNNGYTLSKAREALDTIQNKLKYYHPLSQPMNQQ)---VYISYNW-QEDSNEMANQLVQAFAAKGIEIIRDKTHTTYKD-SikNFMRQIGRGKCVVAVISDRYLKSENCMFELVEIARNGDF-Y-QRIFPIILPDARIHKDFERIDYLKYWEDEKAKLQAKYKQIDLAKTNSIMATL(NLYDEIRGNIDNLTDILKDMNTLDINLHRQSEFAAM)}*

$4106=251(256):
>gi|488873701|ref|WP_002785926.1|
{|955(15)|<Cyanobacteria(B)>}hypothetical protein [Microcystis aeruginosa] >gi|389799794|emb|CCI20677.1| Similar to tr|Q8YVS1|Q8YVS1 (fragment) [Microcystis aeruginos
{(IPELLKALEDSNEYVRWNAAFALGKIATETAMTELINRLKNPDFVTLNNGDTLSLAREALDTIQNKLKYYHPLSQPMNQQ)---VYISYNW-KEDSNEMANQLVQAFAAKGIEIIRDKTHTSYKD-SikNFMRQIGQGKCVVVVISDRYLKSENCMFELVEIARNGDF-Y-QRIFPIILPDARIHKDFERIDYLKYWEDEKAKLQAKYKEIDLAKTNSIMATL(NLYDEIRGNIDNLTNILKDMNTLNIDLHRQSEFAAM)}*

$4107=251(256):
>gi|389838597|emb|CCI30625.1| {|170(15)|<Cyanobacteria(B)>}Similar to tr|Q8YVS1|Q8YVS1 (fragment) [Microcystis sp. T1-4]
{(IPELLKALEDSDIYVRRNAAEALGNIGTETAMTELIKCLKNPDFVTLNNGYTLSQAREALDTIQNKLKYYHPLSQPMNQQ)---VYISYNW-QEDSNEMANQLVQAFAAKGIEIIRDKTHTTYKD-SikNFMRQIGQGKCVVAVISDRYLKSENCMFELVEIARNGDF-Y-QRIFPIILPDARIHKDFERIDYLKYWEDEKAKLQAKYKQIDLAKTNSILETL(NLYDEIRGNIDNLTNILKDMNTLNIDLHRQSEFAVM)}*

$4108=250(254):
>gi|488861939|ref|WP_002774178.1|
{|590(65)|<Cyanobacteria(B)>}hypothetical protein [Microcystis aeruginosa] >gi|389764031|emb|CCI09568.1| hypothetical protein MICAD_670009 [Microcystis aeruginosa PCC
{(AIAKLLKALEDSDWGVRGNAAEALGNIGSEAAMTELIKCLKNPDFVTLNNGDTLSQARKALDTIQNKLKYYHPLSQPMNQ)--QVYISYNW-QEDSNEMANQLVQAFAAKGIEIIRDkTHTSYKDSIKNFMRQIGRGKCVVAVISDRYLKSENCMFELV---EIARN-GDfyQRIFPIILPDARIYNDFERIDYLKYWEDEKAKLQAKYKQIDLAKTNSIMATL(NLYNEIRSNIDNLTNILKDMNNLDINLHGQSEFA)}*

$4109=250(254):
>gi|488870438|ref|WP_002782677.1| {|982(65)|<Cyanobacteria(B)>}putative Deoxyhypusine monooxygenase [Microcystis aeruginosa] >gi|389791764|emb|CCI12431.1| putative Deoxyhypusine monooxygenase [Microcy
{(AIPGLLKALEDSEGYVRSYAAEALGNIGTETAMTELIKCLKNPDFVTLNNGDTLSQAREALDTIQNKLKYYHPLSQPMNQ)--QVYISYNW-QEDSNEMANQLVQAFAAKGIEIIRDkTHTSYKDSIKNFMRQIGRGKCVVAVISDRYLKSENCMFELV---EIARN-GDfyQRIFPIILPDARIYNDFERIDYLKYWEDEKAKLQAKYKEIDLVNTNSIMATL(NLYDEIRSNIDNLTNILKDMKTLNIDLHRQSEFA)}*

$4110=199(142):
>gi|488837773|ref|WP_002750179.1| {|0(143)|<Cyanobacteria(B)>}septum site-determining protein [Microcystis aeruginosa]
>gi|389675357|emb|CCH95537.1| putative Serine/threonine kinase [Microcystis aerug
{(MK)--DVFICHTSEdkLLIVEPLVSGLEKADITF--
WYDRAEIKWGDSITAKVNEGLRISRFVIVVLSEAFLNKNFPKRELFAVLNQEIYSDQTKVLPLF----
SdNKQKQKVLESLPLLNDKlYQTWNGNADTIIEALLPHLSEKttlkfkdqksvndflnrdklisdkgvsyskl
rqlleaqswleadqetirllykatdkpeftS()}*

$4111=220(220):
>gi|390358121|ref|XP_003729184.1| {|406(0)|<Echinodermata(M)>}PREDICTED: tetratricopeptide repeat protein 22-like [Strongylocentrotus purpuratus]
{(LVQFAEVKKAQGRLQKQVSILQNIFTHNHGINCCGFECDLSRFDYCSSERPGFVNILSENQCAYVSPISPV
FLRQPSPYK)-FDFFVSFSHIdhKWTVSFVKKLEKDFN--
VRGCIRYRDYAYGATIAENIAENIDESYRTILILSPDSLRDHWCRYELQRAHMESL--KRRCIVPVML-----
----RTCKLPREVENLtHI-----KCNrgQFvlddWYRLQDTLRQdekktkidlsS()}*

$4112=262(220):
>gi|390365362|ref|XP_796009.3| {|74(258)|<Echinodermata(M)>}PREDICTED: uncharacterized protein LOC591349 [Strongylocentrotus purpuratus]
{(ALCCNRQSYRDILAPLYIATVTLPTYMGLVQYSDCRSDPEVQISNTCKQLIKALPLSTQDDSSGNGTTTST
PVEEEEDPF)CYDVFVSYSHCdsE-
AATHIVGVLKELDPSLRIFYDIQELKTGHAWQQTLYHSIDGSRSMLAILSNNYLRSAVCQEEYSLAQMKHLAQ
DKLKLIPLCIENLEkEECSFSEVTMVTATPDVfQDAVATACSSLVSWLHGKADTKadsveglshllagaavsd
rlnienlmedhrkkilkneynT()}*

$4113=250(246):
>gi|391327659|ref|XP_003738314.1| {|972(0)|<Arthropoda(M)>}PREDICTED: toll-like receptor Tollo [Galendromus occidentalis]
{(QKLPSDSKYLGKVFNDMQLDNMLPIVGVATAAVILFFIVVATVVAVAYRKDFRVWVYARYGVRLFERRREE
PEK)LFDAFISYCKKdePFVAQLLAPELECGGStaPYRLCLRYRDLPMSGYVAEAITEAIECSRRSIVVLSEQ
FLRSEWCRFELKAAARdaQIRGTGGSSLVVIVLDKG--
AMRLLDAEARLSLRDAPIIHYEDKRFWEKLRYNLPDAQA(STRKQQVTWPHGLPADATMNRYNVVNSAVKLV)
}*

$4114=261(258):
>gi|390353429|ref|XP_003728107.1|
{|294(245)|<Echinodermata(M)>}PREDICTED: uncharacterized protein LOC100888822 isoform X2 [Strongylocentrotus purpuratus]
{(KACIEQYNIALCCNRRALRDMLAPIYVDSVEMMPTYMGLVQYVDCRPHDPSKIGEACSQLTVSLSVTFHTE
LRVAEFDPL)RYDVFLSYSHRdtEKANR-
FVEMLQKLAPDLKLFFDVQELKTGKSWQRTLYHSIDGSRCMLALISEPYLKSAVCQEEFALAQAKHCAKGKQH
lqLISICLDDL-
DTIQPEFTHIPMVKGTPdVFDDMVKTVCPAVIQWLNGERVD(QTETIKTLFDDQNITTLSADAEMEKFRQSHF
QKEFGTQ)}*

$4115=348(300):
>gi|390354724|ref|XP_001180800.2|
{|253(97)|<Echinodermata(M)>}PREDICTED: uncharacterized protein LOC752978 [Strongylocentrotus purpuratus]
{(QVHCKISGFLTKVNLTLSLATTEEHVVKVTDTTSVVVVVATLTILLSLVVLSWKRYGTDVKLWNHDHRATL
ETEEIDNKL)-YDAYIisCYEGQdkSFVDNILCPSLQD---
QYKLFIRERDATFGSVESEEIVEALTKSRRCIVLLSADFYrhsstsssQSTSGRIETTAAqltssssslssps

spsfsvnnfsgnewhlfeLHHALDTMHPNVIPLLYRDS-
GPTSLSNTDsnaklIDHVLKDVtCLKWKqkgetrlDDW---
AMKRLRLRLPPR(RQSSNLPIEQSSYITMPVMSAATPSNISSSISLTSPSLLTTERPYAVDSSLQHLTALSCP
ESSRSGGLVVGKASGSSRQN)}*

$4116=251(255):
>gi|390359740|ref|XP_003729553.1| {|461(0)|<Echinodermata(M)>}PREDICTED: uncharacterized protein LOC100889426 [Strongylocentrotus purpuratus]
>gi|780020722|ref|XP_011665076.1| PREDICTED: uncharacteriz
{(QVEDAADLRRRAEDHRQKLIATPLDTNGDSFSDDFEHYSRSALNGHVRLLTAADFWVASATGRTRHNPMPY
RGPIPTGQK)KYDFFLSFNERdrIWCL-ALLSKLET-
EYKLRGCTEQRDFEVGADQTENIRSFLRESHRCVIVLSPHFVQDGESRAVLREAFRVGFKRDGPYVLPLVLTD
C--ETDKLDSRLQEVQSIHcELGQIRIH---
HWQNLIRSLRQG(KDLTDDRPPILSTILSTIIVLMTLLSITRSDFPLD)}*

$4117=234(230):
>gi|390367656|ref|XP_790080.3| {|365(0)|<Echinodermata(M)>}PREDICTED: toll-like receptor 13 [Strongylocentrotus purpuratus]
{(SSASLEPLREKPLLEFDPNELCIMNSGLFLLIPLVSISLVVISVLLYHHRWQLKNKLFLLKLAVLGYKEMR
DARDHNDYK)-
FDVTIIFDDDdeEWIREQLRPALEERLPQFQRNVFGDQDLVLGMHYLeSVDYVVSHSYKTIIVLSRAAVHDHW
FILKFRTAMDHVSDTLTEFVVVVFLEDIPDNEMPFLARLY-
LSDGRpYIHWTENVRGqeYFWDELTKDLTIN(LRTNDLIPNE)}*

$4118=252(260):
>gi|390353716|ref|XP_799955.3| {|9(240)|<Echinodermata(M)>}PREDICTED: uncharacterized protein LOC577369 isoform X1 [Strongylocentrotus purpuratus]
{(HKKDKGRISPNTPKSGDPIDGSNHRQECVVEDIPQKRNQDGENQEVTTKSKSTSPQVEGPSAQDSQVGMER
QTERSSSME)PKDLMISYSH-QDRDK--
MRMMIDALEKAGISVWVDETGLKAGVEFlnKIGQAIIDAKLFLSLLSTRSATSKYCKDELALAYVSS-----
KPIYP--
CMIQPHKQVTEVMDFGMRLQLAPIRWFDFSDEDDFQGSFDKLATC(IKQKLAEITDEANRTEGSAKKHKQITR
KETRTRLNVHQIL)}*

$4119=222(228):
>gi|391346209|ref|XP_003747371.1| {|62(0)|<Arthropoda(M)>}PREDICTED: myeloid differentiation primary response protein MyD88 [Galendromus occidentalis]
{(LVSLPEGTLARLQRSNDPVRETLQLWPVEATIGQLVSAMEGIERFDVLDDCMESILEDCRDFIRRKEYWQR
EPSVVQQTI)-FQAFVIHVLDdVVFVREMVTRVEDEGVRLFVPARDFPAAEHNYMYSLI-
EIMQTRCLNvIVVVSRALSEDQEATRLLENAERIHAQDTSRKIVPIVLE----EAPHVGFMIsnlCKINFNF-
-PEAHA---WAWPRLMDSLGVG(RNQDRRLH)}*

$4120=182(184):
>gi|389882452|emb|CCI37083.1| {|0(164)|<Cyanobacteria(B)>}putative Serine/threonine kinase [Microcystis aeruginosa PCC 9701]
{(MKETMK)--DVFICHAS---EDKLLiVEPLVSGLEKADITFWYDRAEIK--WGDsitAKVNEGLRI-
SRfvIVVLSEAFLNKNFPKRELLAVLNQEIYSDQTKVLPLFSDNKQkqK-VLESLPLLNDKLYQT-
WNGNADTIIEALLPHLSEKTTL(KFKDQKSVDDFLNRDKLISDKGVSYSKLRQLLEAQSWL)}*

$4121=173(175):
>gi|504632764|ref|WP_014819866.1|
{|0(650)|<Proteobacteria(B)>}hypothetical protein [Pseudomonas stutzeri]

>gi|390982855|gb|AFM32848.1| hypothetical protein A458_08010 [Pseudomonas stutzeri CCUG 29243
{(M)--KAFLSHSSAdKEsYVG-----IVASRLAEEDIVY---
DEVSFESGEqtinEIIRGLDESTVFCLFISNKSLDSEWVRQEIdGASVRLAsgslKY-
IYPIVIDRSVAHDDPRIPKWLRENYNLRLVT--
RPVIAARRIQQKLRQASWAKH(PKLRERERAFVGRNKQIEAFERRFDDYSQPKPVL)}*

$4122=289(299):
>gi|495472771|ref|WP_008197459.1| {|154(954)|<Cyanobacteria(B)>}type IV secretion protein Rhs [Microcystis sp. T1-4]
>gi|389838382|emb|CCI30831.1| conserved hypothetical protein [Microcystis sp. T1-4]
{(GLISAIYQQQHYVENHSILLVKALNWLRNQQQTNYLLIGEERQAAEKWLKHRFTEEQPPCLPTDLHCEFISESSKNANNL)MTQVFLSSSEKdNVIKEKIGK---
ALMREGFTIWTNQTDIKTGTAFQeQINKGIEGADNFVYLISPEALQSSYC-NlELAHAFATNK-----
RIIPLLIEKT-DIELIPsqLQKLQFI---DLTEHEDRD--
KYRSSIDKLLREL(NSDVAYYESHKVLLVKALKWQQQTHNPSVLFRGYNLQHFEAWLKVAKQRKESPPLPIQEEFIIASLNQHKPSSLEVFIS)}*

$4123=275(277):
>gi|390353911|ref|XP_003728218.1|
{|293(339)|<Echinodermata(M)>}PREDICTED: uncharacterized protein LOC100892549 [Strongylocentrotus purpuratus]
{(SCLEQYNIALCCNRQAHRNVLAPFFVETVTLPTYMGLVQYADCRENMDASIAQACGQLVKSLTATDKVVKVATTDPNHYD)---VFVSYCHAdADQATPIVSQLQ--
KLNPELKIFYDiQELKTGHEWQrTLYHSIDGTSCMIALISPNYLKSAVCMEEYSLAQTKHlSH-
DTLHLIPVCIS---
DGIEKDFAQVSHIPMlDArpGAYAGVVSSICSAVVSWLETNSF(AHLDKLLKKNLDNQVDVPDMMEKLRAARFWAEYAKTFISVKTFPPTFEKLEDAEGKA)}*

$4124=286(297):
>gi|390363797|ref|XP_001197067.2|
{|298(211)|<Echinodermata(M)>}PREDICTED: uncharacterized protein LOC756962 [Strongylocentrotus purpuratus]
{(VDQYNIALCCNRRANRDVLAPVYVHSVDIMPTYMGLVQYVDCRPADQSKIQEACSQVGVSLSVKLHKELAVAHFDPLYYD)---IFLSYSHCdSDKANRFVETFKSLAPELKI-
FFDVQELKTGKSWQrTLYHSIDGSQCMLALISTPYLKSAVCQEEFALAQAKHFS-KGkqhLQLIPICLENL-
DTIQPEFTHIPMV--KGTP--------
DVFEDVVKVICPA(VTQWIKEEELDVGKNLAAIFDKDKIPTISPDDEMEALRAARFQKNFGTSDSLLKESTSFPPPLTDILPHTYAHPPEH)}*

$4125=250(250):
>gi|504595245|ref|WP_014782347.1| {|67(14)|<Bacteroidetes(B)>}TIR domain-containing protein [Aequorivita sublithincola]
>gi|390420335|gb|AFL81092.1| TIR domain-containing protein [Aequorivita sublithi
{(NITIIVALSGLFVFLSLLTILLQGYVATTQEGLPYLAYSKTLLMSALLLIPLEVFIIYLIRKKLYIKKVVDASTDFSNMD)-PKVFISYNHSdSEVA---
LKILDALEKASIEVIIDQEDMMAGADIKnFIEKSIGESTVTVSLVSNRSLKSAWVAlEtIDTFFLERYMK-
NKKFIACYLDDDF-
FQPDYTLKAVTDIDEQLktNQKMIPEYQQKMIDTRDLNIQNT(RLLALRNNLDGIIGRLRDSLCLDVREEPFN)}*

$4126=213(192):

>gi|504596357|ref|WP_014783459.1| {|0(292)|<Bacteroidetes(B)>}TIR domain-containing protein [Aequorivita sublithincola]
>gi|390421453|gb|AFL82210.1| TIR domain-containing protein [Aequorivita sublithi
{(MALIPGYEYD)---
IFISYVHAdNEsetfeedgWIDQFYKYLdtKLNKHSRNIKIWWDSNnLDRSEVFDnSIAEAIDKSAIMICLYS
RLYPQSDYC-KkELDHFYEKVSKEkvgmtvgNRSRIIPVFMSnipHT-
DWLDKLSGTSGFPFHDNddYGDPLKNTSPEFGDQMIQLRNAL(VRIFDDFSKETASLVELAPLPLAEEEDKKF
TIFMSEVTDAFL)}*

$4127=120(140):
>gi|504611058|ref|WP_014798160.1| {<Bacteroidetes(B)>}hypothetical protein [Bernardetia litoralis] >gi|390528989|gb|AFM04719.1| TIR-like domain-containing protein (DUF1863) [Bernardetia litoralis DSM
{()MKTIFIAFAIEdERQRDFLKGQ--
SLNTNSPFEYIDMSVKQAYDsDWKtKVRTRIKRSDGIIVLVSKNSLSST-GQKwEIQCARE------------
-----------EGKKVLGIYAYTDdrTNIVGVNTKVWTWDNIKNFINSL()}*

$4128=143(148):
>gi|389800069|emb|CCI20478.1| {<Cyanobacteria(B)>}Genome sequencing data, contig C321 [Microcystis aeruginosa PCC 9807]
{(MK)---VFISHAF--TEAK-LAKRVADSLKEDGFQVW-DAAEMLPGENWanNIGKALQE-
AEAMVVLLTPESLKSFNVGsEISYAIGEENYQgRVIPVIASSLDNL-
DREHIPWILKKFRMIYVPNLEQDNQQLKEITQALKNVTSS(DGLKVN)}*

$4129=211(213):
>gi|495473040|ref|WP_008197728.1|
{|0(301)|<Cyanobacteria(B)>}hypothetical protein [Microcystis sp. T1-4]
>gi|389838252|emb|CCI30936.1| hypothetical protein MICAI_1510003 [Microcystis sp. T1-4]
{(MPESSEF)--HVFLCHNS---
QDKPEVIKIAQTLKQQGLNPWLDVWELRPGLPWqrELERQIEHINAAAVFVGENGI----
GPWqEMEIEAYLRRFVnKRSPVIPVLLPNAPqkpELPIFLEGMTWVDFRGSQADPIGQLIWGITGSKLSVKPS
S(SSQSQQVKLEEEISNLPRETKPYTVSTPVILAPKSPRIPPLSVAINRRKLLQLIGWTGAGFGATII)}*

$4130=159(167):
>gi|504601799|ref|WP_014788901.1| {<unknown(X)>}hypothetical protein [Thermococcus cleftensis] >gi|390519534|gb|AFL95266.1| hypothetical protein CL1_1063 [Thermococcus cleftensis]
{(MIELLS)---VFISHSS---
SDYEVITKVKETLEVQGFHVYLAEEDVRAGTYLpaKIEEAIRRCDVVLAILTSKAS-
KSAWVQqELGYAKAIGK-----LIIPLVEEGV-
NVTGFLTGVEYIRFDpRNLEPALQKIKEyLTRLKESKEFKEM(LGVLILIALGAFAAYYLTHKK)}*

$4131=274(278):
>gi|390353429|ref|XP_003728107.1|
{|144(382)|<Echinodermata(M)>}PREDICTED: uncharacterized protein LOC100888822 isoform X2 [Strongylocentrotus purpuratus]
{(TGDQGYGEVSMMKGMVEAAVKWMKAGLPLRILKLVLYAKVIDGDLQGHSLRRFSEVLKTFTELKERYEMQL
LLPKAVPLE)-
FDVYLSFsEEDKEVAKVIREKLSGAKDGVRIYDSSHQGINKDTVFQEDMYSIMMkSARVVTVLSPNYLRNKAC
IEQYNIALCCNRRALRDMLAPIYVDSVEM-MPTYMGLVQYVDCRP----
HDPSKIGEACSQLTVSLSV(TFHTELRVAEFDPLRYDVFLSYSHRDTEKANRFVEMLQKLAPDLKLFFDVQEL
KTGKS)}*

$4132=272(278):
>gi|390353911|ref|XP_003728218.1|
{|143(492)|<Echinodermata(M)>}PREDICTED: uncharacterized protein LOC100892549 [Strongylocentrotus purpuratus]
{(TGNQGIDPKVMLEGMVHAAISWVKAGLPIRLLKIVIYANIKDGQATNFFRGVDNNILENFAELKSRYESANLIPQEVTLE)-YDVYLSFsDKDQEIADKIQAQLQEEKSDIRIFTA-RQKVDTEAFWQDQMYDVVSkSARVVTVLSSNYLASESCLEQYNIALCCNRQAHRNVLAPFFVE--TVTLPTYMGLVQYADCRE----NMDASIAQACGQLVKSLTA(TDKVVKVATTDPNHYDVFVSYCHADADQATPIVSQLQKLNPELKIFYDIQELKTGHEW)}*

$4133=274(278):
>gi|390363797|ref|XP_001197067.2|
{|145(376)|<Echinodermata(M)>}PREDICTED: uncharacterized protein LOC756962 [Strongylocentrotus purpuratus]
{(TGDQGYGEASMLKGMVQGAVGWMKAGLPLRVLKLVLYCKVQDGKLQKHSLRCFDTVIATFNELKERFEMQLLLPKEIPLE)-FDIYLSFsEEDGAVAKEIREKLTGAKDGIRIYDGSQQTLDKDSVFQQDMYSVMMkSARVVTVLTPNYLQNKACVDQYNIALCCNRRANRDVLAPV-YVHSVDIMPTYMGLVQYVDCRP----ADQSKIQEACSQVGVSLSV(KLHKELAVAHFDPLYYDIFLSYSHCDSDKANRFVETFKSLAPELKIFFDVQELKTGKS)}*

$4134=188(200):
>gi|390365362|ref|XP_796009.3| {|0(406)|<Echinodermata(M)>}PREDICTED: uncharacterized protein LOC591349 [Strongylocentrotus purpuratus]
{(MS)-------FsPKDQSSVDHIQTALRTIKPDIRIYLSNQKLD-EDAIWQNDIYAVMTkSARVITVLSPNYLSSPDCLEQYNIALCCNRQSYRDILAPLYIA--TVTLPTYMGLVQYSDCRS----DPEVQISNTCKQLIKALPL(STQDDSSGNGTTTSTPVEEEEDPFCYDVFVSYSHCDSEAATHIVGVLKELDPSLRIFY)}*

$4135=268(221):
>gi|1143364100|ref|XP_019935288.1| {|180(0)|<Chordata(M)>}PREDICTED: interleukin-18 receptor accessory protein-like [Paralichthys olivaceus]
{(TPQHLEHTYTCIASNALGKCNVTIRLKQKQVKWASLVGSPIASLLLVVVVGVILHMKWLELQIIYRCHFLHGTHDRDEKR)-FDVFLSYV-WsptsaeieggwtrssrsgpvtdeeacqfsmdplnteEDEATQtplevLLRQVLEEQWGYRLCLLERDILPGGAYTNDVVLAIQGSHMLICLLSADYLcNTDGVF-VLESGVQALLQKSALKLLIIRTCrnsaSI-IQDPPLPPLVQRALKVLpSLNWTSDKPDrgssRFWRSLRKAMPRS(E)}*

$4136=228(218):
>gi|1143089154|ref|WP_077080585.1|
{|0(1181)|<Actinobacteria(B)>}hypothetical protein [Mycobacterium sp. AB215]
{(MS)--RIFLSHSSKDTREAialkTWLTEQRPELRDE-IFIDVdseTGLKLGEHWKTQLFTGKSRCEHFICLLSQS-WANSTECNvEYRTAEGLG-----KRILVARLEDLGVTHDITSAWQRCDLFA--DGEhsdvevpggppvrfnTAALT--SIMEAVDGSGTG(PRQFVWPPRTDPNRAPYRGWEPFEEIDAGVFFGRDAVIARGVDALQGMRFRLLASMSGLKSLFVVLGPSGSGKSSF)}*

$4137=228(236):
>gi|1143687581|ref|WP_077145047.1|
{|0(113)|<Proteobacteria(B)>}hypothetical protein [Sphingopyxis sp. KK2]

{(MNEDPITPDQGKRLSA)----FISHHSSQVATARQIKTLLAARGID-
GWLAPDDIAAGTAFDQAILDQIAKSDA-IVLLFCAQSDQSRHVKrELMLGEDGN-----
KVILPVRLEDVPaqGLAYWLKDYQWIDWFA---GEEQAIE--
RIVAAMTGapaasPARP(IQATSTPVVKPSLRRPLLVGVGALAIAGLFAGGIWWTGSTGKAAGDPDAAVADNP
IEPGIWTSKREVVKILFPEEIAPEY)}*

$4138=173(179):
>gi|1143087887|ref|WP_077079320.1|
{|0(456)|<Actinobacteria(B)>}hypothetical protein [Mycobacterium sp.
AB215]
{()MPKLFVSYARQnQRALNELVDHLNLLG----
FETWVDPSRWGGQNWWdAILRQIAECDTFIAVISRDALSSETC-KrEFDWAQAL-----GKPVLSVAVEPP-
DAAVLQRISIRRIVDYSnpKQRGASTLAGSLSALPQAPSPPP(APLPVPPEAPLGYLTELVDQVSSPEPLTEN
WQREINQQL)}*

$4139=208(221):
>gi|1143691260|ref|WP_077148726.1|
{|0(182)|<Proteobacteria(B)>}hypothetical protein [Sphingopyxis sp. KK2]
{(MTPA)MTDVFISYKREeRARCVAIYNALVDLK----
LSVWFDGRIEPGTDFDrEIEREVRGAGAVLVLWSELAADSDWI-----RAEARTGR-QKDRLVAIRLDDC-L-
PPLEFASVQA-----
IDLFDHRDFQagEGWRQVVGRIGRL(VGRPGLGDYVHCEQEADPGRWRGWIAANAGDPLIDRAKERLAVLDRR
PGGAETVETAPSVAPPPESASPSPRITDRA)}*

$4140=221(225):
>gi|1145329101|ref|WP_077154025.1|
{|0(270)|<Bacteroidetes(B)>}hypothetical protein [Bacteroides sp.
Marseille-P2653]
{(MEYKYD)---
VFISYSRKdYVDANQniipenAITEIQNIFDKNGISYWFDRDgIYSGQEFIeVITSAITESKMLVFISSKHSN
ESIWTAGEIFEAL----D-GEKTIIPVKIDNS-QYN----KKFKLLIRPLdfINYEENPK--
NALNDLLRAINKV(KEDVAQKLREEKELMLKREAEAKKEEIKKEINVLAKDCQRLMLQQLDIVKQISEKQIFI
GNTTKLCPVCDKVVSITSEF)}*

$4141=255(258):
>gi|1143392263|ref|XP_019950621.1| {<Chordata(M)>}PREDICTED:
toll/interleukin-1 receptor domain-containing adapter protein isoform X2
[Paralichthys olivaceus]
{(MLKRHDNVNNMHGWFQNLLKSRTSLASRHEEKSRTSLASRHEENASETSVSTSFSYTSSSSSEPKNPPSAL
SSHLRWRR)KYDLLVCHsqaDSDIEEANGLVSLLEASPRSLRCFLWQRDVSPGSPIFTEFCQAVQESHLQVLL
ITPDFLQEEWCMYMMHQALAEGP--MSRRLIPLIQNLSHDQYPQEIKFCFYINLSR----
NISQGCSLINMTLLKYLED(LIKNERTHDSNVDSSHNRLSGESSSQGDELISNNDPAET)}*

$4142=214(219):
>gi|1143633192|gb|ONN59306.1|
{|0(113)|<Cyanobacteria(B)>}transcriptional regulator [Mastigocladus
laminosus 74]
{(MDDTSP)QFDVFLCHnSEDKEAVIEIAHKLES--HNIRPWLDDWELQPGYP-
WQPELEKQIKnIKAAAVFVG----
KSGIGPWQEDEIHGFLRQFKQRKcpVIPVLLldAPQRPQLPIFLEGITWVDFrQK----TLVYT-
DPWQKLIWGITGR(KPERRPKQNQVIQTDFIQADDLSSEKRIDYTQLRDLLAAGKWKEADEETLAVMLKASGR
EKEGWLNVESINNF)}*

$4143=215(220):

>gi|1146128850|gb|OOE79973.1| {|61(0)|<Proteobacteria(B)>}hypothetical protein BZG72_13140 [Salinivibrio sp. PR6]
{(ERDDRDFDSWLSKHYPHKIRAYNVFVNELMEAVYRKGYSLVTFIDSSNIDEVMTINRNELFLYPDREVMFE
IWGKPVIEK)KKKVFLSHSSKdKDIVDVYYNEIQKAELKA------
WYDKEEITAGDsitsKVSEGLEECELGVIFLSDNFLSKHSGWTEAECNYfinrrMKKNK-
TLVVVNLGVDYDNMPPLLQDYLYIDANNGKA--TDELIHA--VRKQLD----RI()}*

$4144=215(221):
>gi|1148730061|ref|WP_077465071.1|
{|69(0)|<Proteobacteria(B)>}hypothetical protein [Rodentibacter sp. Ppn85] >gi|1147325441|gb|OOF66257.1| hypothetical protein BKL51_01940 [Pasteurellaceae bacterium
{(VNSPMANICFSGGGTLTSDDLELDIVQSNNLNKVREYFYNNLNLNEGDGLFIVNKNLSNLLSEIELNIEKH
KFFLGLTPM)--KIFLSHKTAdKPKVREFKRILDQIG------
FDSWLDEEELTAGAhlekSLFNGMKNSCAAVFFLTENYRDTNYLESEITYAIKRKRE-
DPKFALIALKLSPNATVPDLLTPYVYKEPTS--DLEALSQIIKAIPAKIGKIIY(Q)}*

$4145=208(219):
>gi|1149106712|ref|WP_077683233.1|
{|0(78)|<Proteobacteria(B)>}hypothetical protein [Pseudomonas stutzeri] >gi|1145959644|gb|OOE10231.1| hypothetical protein BSR09_11585 [Pseudomonas stutzeri]
{()MP-VFLSYSHSdAEFVDELA--LQLVKHN--
VHVWIDRwELNVGDSLIdRIQHAVDGASALLVVLSPESVESEWC-KrELSAGLLRElEE-
KRVVVMPVLLRDC-AIPVFARGKFYA---DFR---KNFD--
DGLKAVLAGIAKV(TNASLARIQEDQYHVDWSIQSAYDNAGDVVVKATFIQIPKNEPYTCLTEVVIVCDSAAT
RAFEAVSAAENLDIARLRIW)}*

$4146=153(150):
>gi|1145423944|emb|SJN13039.1| {<Actinobacteria(B)>}hypothetical protein FM113_16610 [Leucobacter sp. 7(1)]
{(MTR)--RVFISYQHAdqmkaRGL--
NLMTYNKNVNVDFTGRHLLDPVKSQDSDYIsrKIKEKIKGSSATIVLIGKETAQSEWVEKEIQWTKEQGKGIv
giRIDPNATIPEG---LTDYGAEILDWNAPSDVNQFDDAIERAIAATSRGRNMPMN(TQTTCGR)}*

$4147=223(227):
>gi|1148306891|ref|WP_077254438.1|
{|0(51)|<Proteobacteria(B)>}hypothetical protein [Klebsiella pneumoniae]
{(MDITTSLCTNYWGAVISSTAVFKQSLANLSGKKENKMH)--DSFISYAI-ED--
NDFVSDLAFGLKRNGLSVWFAPLSLKVGDRLldSIEKGLNESRTGILILSPSYLSKNWTSYEMDILirqnIEG
N-----
KKILPIWLNVTKKDIENKHIGLSGIVGITDTAEVSRVVSQLVEALSDGAPYR(GVIPSWENPEYRFLSGVSEV
NLQSIDGKTTTIFELLVHSNDEQFPFWLA)}*

$4148=199(187):
>gi|1148667103|ref|WP_077407350.1| {|0(2)|<Proteobacteria(B)>}TIR domain containing protein [Janthinobacterium sp. LM6]
>gi|1148317472|gb|AQR70577.1| TIR domain containing protein [Janthinobacterium s
{(MEIRYGCFLSY)AHGQYAFMN---
KFKNDLIEALACYLEPHldrEEVLFIDSEQLGGGDDIdlRVARAMCQSVCMIVLYTPKYEAHGYTRREFAAMQ
liEQERRAwYDlpsHLIIPIIMTRHPdglpPQITESGLYVDFSAYTLASGDLKSNPQYLPDIERIVQRIA(TH
YHLLKRSTPPGHDCSRFVLPAIPPEWRAIPPPHF)}*

$4149=201(206):
>gi|1148604863|ref|WP_077350756.1|
{|0(382)|<Bacteroidetes(B)>}hypothetical protein [Algoriphagus sp. A40]
>gi|1147869203|gb|OOG73037.1| hypothetical protein B0E43_14010
[Algoriphagus sp. A40]
{(MAQQE)---VFISYAwgG---ESENVADELEAVLSAKNIRLIRDK---TDLGFKgliRPFMEQIG-
QGNlvVLVISDKYLKSKNCMFELLEVEKK--GEFFSRIFPVILPDADiyD-SLGIINYLKHWDQKI-
KELNASVKELDNLADTRKVQED(INLYTDIRGAIDDLAGKLSNMNTLTVEIMRAKNYQPLVDSLLKENEIGQP
NPLNPTSPRKK)}*

$4150=161(168):
>gi|1148693650|ref|WP_077432365.1| {<Proteobacteria(B)>}molecular
chaperone Tir [Pseudomonas sp. C9] >gi|1147645101|gb|OOG10362.1|
molecular chaperone Tir [Pseudomonas sp. C9]
{(MP)---VFISYRHTDRAYAMAINTRLTQANIK-TYLDVLDPesQTTEDITTVITRNITECTHLMAVVSEQ-
TAQSWWVPfEIGEATISN-----RRICSYKIGPSAlpQYLDKWPKLSGENDIN--
FFIDAYREELVSKRSMGLESFS(ESVRGTHRRDAELFHDQLKNRIRRGF)}*

$4151=193(198):
>gi|1148784867|ref|WP_077514644.1|
{|0(31)|<Proteobacteria(B)>}hypothetical protein [Rhodanobacter sp. B05]
>gi|1147849961|gb|OOG54065.1| hypothetical protein B0E47_12890
[Rhodanobacter sp. B05]
{(M)ARDIFVSYSQPDRDSAFALVQQLEARGLS-AWIAPRDVSPAADWAAEIIDAIAGAKLMLLVFSGN-
SNDSDQVRrEVERAVHKR-----LRILPFRIEDVLpskSLEYFLSAQHWMDAFP--
PPLQPHIDRLCEHLEAVLGEHA(AQPPPTEHRASLAAAPMHPPGAVDAVRLRRLETELARCIGPVAGHLVRGA
ARGGGDL)}*

$4152=195(202):
>gi|1148789272|ref|WP_077518522.1|
{|0(32)|<Proteobacteria(B)>}hypothetical protein [Rhodanobacter sp. C03]
>gi|1147851414|gb|OOG55509.1| hypothetical protein B0E48_12750
[Rhodanobacter sp. C03]
{(MTH)--DIFVSYSQPDREAAFALVQRLEARGIG-VWIAPRDVSPAADWAAEIIDAISSARLMVLVFSGS-
SNDSGQVRrEVERAVHKQ-----LAILPFRIEDVLpskSLEYFLSAQHWMDAFP--
LPLEPHIDRLCDYLDTALGGYV(ARDLPSEQRPSRVTVPADATHPAASVDAAHLRLLEAELVRRIGPVAGHLV
RRATQGGGD)}*

$4153=180(183):
>gi|1147414733|ref|WP_077216138.1| {|0(285)|<unknown(B)>}hypothetical
protein [bacterium SIT6]
{(MTILIEE)-KQVFLSYSWNdSDEADIVQGNLSGFEG---
ISVLRDRDILGYSSDItRFMKRIKKTDHAILLISNSYLESVNCMFEVSQLI-RDED-YIRKITPILVKNV-
TIYSIEDRLKYAKYWNDkyNYLNEEIRKLHIEDNVSLVEELK(KVKDICSAVDDILSVLNRHQFFKLETHIQN
EFNDVF)}*

$4154=182(192):
>gi|1148784259|ref|WP_077514152.1|
{|0(485)|<Proteobacteria(B)>}hypothetical protein [Rhodanobacter sp. B05]
>gi|1147851088|gb|OOG55185.1| hypothetical protein B0E47_10350
[Rhodanobacter sp. B05]
{(MIREQWDAGIGVAD)---IFLSYKREdRARAGQIVVALRKSGR----
TVWWDEGLTPRQAWDaTIEQEIEAAASVIVLWSTRSVASDWVRSEAHYAQDR------SKLVPVLIEPC-

TVPLAFMLKQSIDLSHWreSPNDPAWRKLIEWLDTQGPATEP(PQLRDRRARWIRWPGRKTLIVAAFAIAALL
AAFFVYRS)}*

$4155=171(178):
>gi|1147855299|gb|OOG59374.1| {|0(486)|<Proteobacteria(B)>}hypothetical protein B0E48_00610 [Rhodanobacter sp. C03]
{()MADIFLSYKRED--RARAAVIVAALRKSGR-
TVWWDEGLTPRQAWDtTIEQEIAAAASVIVLWSPRSVTSEWVRSEAHYAQDR------SKLVPVLIEPC-
TVPLAFMLKQSIDLSHWrgSPNDPAWCKLIEWLDTQGPATEP(LPLHDRRARRLRWPGRKMLIVAAFAIAALL
AALFIYRS)}*

$4156=180(181):
>gi|1148276753|emb|SJM93811.1| {|0(336)|<Proteobacteria(B)>}hypothetical protein CRENPOLYSF2_3630002 [Crenothrix polyspora]
{(MTPEKSKA)-PRVFISYRHVkpdEDLALAIEQKLTG---EGC-
KVFVDRRMLIGTEWAtEIDRQLNAADFFVVLLSSESIRSDMVRQEIKLAHELKQQ-AKLRILPIRVDFT-
GALPYDLGGYLNPLQYVvwQQGQSFHEITETLHLAIRNAREL(PHVGRTGGDSLAEIQELHDVTEKRGAPLPQ
IDP)}*

$4157=226(226):
>gi|1148278147|emb|SJM91264.1| {|0(144)|<Proteobacteria(B)>}hypothetical protein CRENPOLYSF2_2150005 [Crenothrix polyspora]
{(MHKDGSL)-PKLFISYSKDdLAFQKNLLKHLAGLR--
NKIVTWNDQDMLPGEELDdSIKDKLRSADVVLYLVSHNSIATDYIQNvELPLIEDRCRN-
KECVLIPVIVDFC-LWEYLDFAKYNALPNKGlpVTdrSWANQN--
EAWLTVVQGIKRI(IEKVTVESSVTQPSILEQYRQLKISLQPSIVIPEPKPVIIPFALINPYWQSIDQYQVKD
GLAIDTTTGLMWLRFACGQT)}*

$4158=233(236):
>gi|1148368431|ref|WP_077255125.1|
{|0(315)|<Proteobacteria(B)>}hypothetical protein [Klebsiella pneumoniae]
{(MCLFIFSKGEFGMDNKL)--KLFISYCHEdKHHKEEILKYLQP---
NEDIEVWYDKEMLAGDEFDeVISTKLEESDVFVFLITQDFLTSKYCNEiELSKALERkASD-
DELRIIPVILDYC-TFKRSRLSLYNATPSDAkpISSYPNQK--
QAYLEVSEAIEDV(VKYLKEKKTQSLYAVTEETVVERNALRTNFKLHLLDLGFTIQHRRKEILYLDDLFIYPD
LKRTKYEFDDYDVFVNSKNV)}*

$4159=146(155):
>gi|1176437137|ref|WP_082273691.1| {<Actinobacteria(B)>}hypothetical protein [Mycobacterium kansasii] >gi|1148384777|gb|OOK74832.1| TIR domain protein [Mycobacterium kansasii]
{(MA)---IFISYSSQdRSSLEALTAALRRAQQQ----
VWFDQELGGGDAWWnKILEQIRASDVFIVALSNHWLQSK-PSQaELRYAQALQRP-----ILPVQVGPV-
DSVRVNPVSTLQIINYQnpTVDAGIQLVTAIHALREKARRCR(RRCPRSHLYRSAT)}*

$4160=154(160):
>gi|1149900828|ref|WP_077835319.1| {<Firmicutes(B)>}cytosolic protein [Clostridium roseum] >gi|1149407464|gb|OOL87693.1| hypothetical protein CROST_20690 [Clostridium roseum] >gi|1149414754|gb|OOL9477
{(MT)---IFISHKR-EDS--
TIAGYICNELTKLNVKAYLDVLEgnlLLEGKQLtdHIKERINSCTDILVVMSDRTKDSWWVPFEIGMAAQND-
----
FPIVSYLVNKVELPDYLSYWPTLRKSSDLWKYVRAKEKTFNESTIFNKSFDN(AQYYSKTEEFYKKLKSIL)}
*

$4161=175(185):
>gi|1148786146|ref|WP_077515528.1|
{|0(484)|<Proteobacteria(B)>}hypothetical protein [Rhodanobacter sp. C03]
{(MGVA)--DIFLSYKREDRARAAVIVAALRKSGRT-VW---
WDegLTPRQAWDTTIEQEIAAAASVIVLWSPR-SVTSEWVRsE-AHYAQDR-----
SKLVPVLIEPCTvpLAFMLKQSIDLSHWRG--
SPNDPAWCKLIEWLDTQGPATE(PLPLHDRRARRLRWPGRKMLIVAAFAIAALLAALFIYRSKA)}*

$4162=221(231):
>gi|1148787187|ref|WP_077516569.1|
{|0(478)|<Proteobacteria(B)>}hypothetical protein [Rhodanobacter sp. C03]
{(MRLPARKLPGASLDLVGQTAIAFAKLFLTSRSHGPATMIGGPWDAGIGVA)--
DIFLSYKREDRARAGLIVAALRKSGRT-VW---WDegLTPRQAWDATIEQEIAAAASVIVLWSTRSVA-
SDWVRsE-AHYAQDR-----SKLVPVLIEPCTvpLAFMLKQSIDLSRWRE--
SPNDPAWCKLIEWLDTQGPATE(PLLRDRRARLPGWLSRRTLIVAAFAIVALLAAFFVYWPKVD)}*

$4163=199(187):
>gi|1150837811|ref|WP_078030632.1| {|0(2)|<Proteobacteria(B)>}TIR domain containing protein [Massilia sp. KIM] >gi|1150049338|gb|OON62000.1| TIR domain containing protein [Massilia sp. KIM]
{(MAIRYGCFFSY)AHGRHELMN---
KFKSGLADALRCYLEPYfdnEDELFIDTEQLGGGDDLdrKIARAMCESVCMILIYTPKYEAHPYTRREYAAMR
LLEQERstwyelPSHLIIPVIMTRHPeslpRQIAESSFYVDFSHFTMATSDLKSNPDFLPDIDKMVKRIA(TH
YAYQKKHMPPSHDCNQFVLPDVPPEWRDLPDLTF)}*

$4164=234(238):
>gi|1149894061|ref|WP_077829012.1|
{|84(356)|<Firmicutes(B)>}hypothetical protein [Clostridium beijerinckii]
>gi|1149472727|gb|OOM50472.1| hypothetical protein CBEIJ_04420
[Clostridium beijerinckii]
{(WINYIKEIAKEEKVKVIPIAIDQVALNLSSELNNENFIRAYEYDQNFKDEYVFIAIAHEIYRYALNDNFLE
RSLGKNNAL)--KIFLSHAKDgKNGIELAKSLKNFIDNSSMQNFFDS---TDIAAGYrfddEIVGNIKNST-
VIAIHSDVYSSRYWCQREILSAKQHDRP-VIAVDILEEYEDRRFPFATNVPGIHVFIDSE--
PTEKDLLRILSSALLETIRFF(YSKLLLNQYKKIGYIEQD)}*

$4165=254(255):
>gi|1149894059|ref|WP_077829010.1|
{|125(76)|<Firmicutes(B)>}hypothetical protein [Clostridium beijerinckii]
>gi|1149472725|gb|OOM50470.1| TIR domain protein [Clostridium beijerinckii]
{(LTNDMTEQMFIDNYISQEYKENIICIIKETSESLSDLYTDRKKLMRNKFNLIDKTLEFISEYRNQIYSNMF
FIDDNNEAR)-KKVFISYTHKdKAVVYSVKNCFKLSK----
LKIWFDEDnILPAQKISeEIKNGIENSDYFLVFDSENSRDSEYV-KlEIDYATKcFEKF-
GRPKIIPILIGVD-
KPINEEITNTKGITFTNidETAAKVCKAIDSRYIEINTFEFN(ALIKLVNEFRDAVDYTWADPNMDLGEGDFE
MYEML)}*

$4166=177(179):
>gi|1149894065|ref|WP_077829016.1| {|0(121)|<Firmicutes(B)>}hypothetical protein [Clostridium beijerinckii] >gi|1149472731|gb|OOM50476.1| TIR domain protein [Clostridium beijerinckii]
{(M)--KVFISYSRKdKKFAHKLSRLLTKNKIFYFID---
NSEITVGNGLStKIENGLNEATHFIALMSKNYFKSNWC-KkELSYALKKQEdE-KREIIIPILIEEC-

ILPQSISDIFYINAVQGleNSYAEILEVINRLSNDNVGKSYE(SNYIIDWGVEWGMLKGGPSESSFFYLNIDG
VFWEKNKE)}*

$4167=255(257):
>gi|1150013650|ref|WP_077939640.1|
{|230(224)|<Actinobacteria(B)>}hypothetical protein [Micromonospora sp.
Rc5] >gi|1149687480|gb|OON28037.1| hypothetical protein BSA16_28950
[Micromonospora sp. Rc5]
{(RLVCHRADDHAARQLPRLAVSHALQASSAVNRHAIWWSDMDGLLPELSREIGRLQMPRLQFAGLEEARRYV
WSLGRDQGP)---VFLSSAQGdNDLAQLIAQELD-
LRNVEHFHYLFNSDLPRGKAWEhRLLAKVGECRIFVPLVSRHYLSSTWCRKEMDIALELSGQ-
RRMRVLPYFLDHG-
AGASVPIQGTSIMHLDDaaRVRRVVDDVDRALTDLTDRRHRP(RIEVTVSGGRMAPDVDVVVLATGPTEYAAA
ARTLRDP)}*

$4168=206(220):
>gi|1149895175|ref|WP_077830071.1| {|139(0)|<Firmicutes(B)>}hypothetical
protein [Clostridium beijerinckii] >gi|1149470396|gb|OOM48250.1| TIR
domain protein [Clostridium beijerinckii]
{(ANMLLLLAESKRKVVVPLTFLGGAASQAFNRRRYELEDKLGKDYLLLQNEGNILDSDNLQMLLSGFQIPKD
FTKKDELSF)----FISYSRAKPNEA---
DYIENLLRRRNLKVFRDESDFGAGSSIplKITEAIHSSNVFIATWCAEYACSPWCFDEFELALDRyeAGKMql
lIFCTDGT--------------RIVPKRARNLKYIMVSSREQIECAVLNLLERTL()}*

$4169=135(156):
>gi|1150118388|ref|XP_020207008.1| {<Streptophyta(V)>}toll/interleukin-1
receptor-like protein [Cajanus cajan]
{(MACITNNSGSSSRTMN)-FDVFVSFRG-EDTRNNFTDHLFAALRRKGVVAFRDNQnINKGQL-
LepELMQAIKRSRLFIVVFSKNYASSSWCLKELTMIFDWVKE-TGQSVLPIFYD----------
GEFQKAFAEYEES----------FRDDLEMATT()}*

$4170=199(220):
>gi|1150126169|ref|XP_020213324.1|
{|134(0)|<Streptophyta(V)>}uncharacterized protein LOC109797624 isoform
X2 [Cajanus cajan]
{(AGVFNFFLGSLMLAVMKWSLKFALAAAVFCYVLINIRIHSDSDSDGGDMEETDVALIENGGRRRNASEVQV
PPELRPPVM)-YDVFISFRD-KDT-
SDIPEYLACALLKKSLRVYAHPELTGDHVPQTVIEEIGKAQVSVVVFSKSYLETNWCLHEFHFIMACRAR-
YARTVIPVFYNVHRTAIQDYSSNYFA-----------------GLGNVACFKTV()}*

$4171=213(220):
>gi|1150126167|ref|XP_020213317.1|
{|134(0)|<Streptophyta(V)>}uncharacterized protein LOC109797624 isoform
X1 [Cajanus cajan]
{(AGVFNFFLGSLMLAVMKWSLKFALAAAVFCYVLINIRIHSDSDSDGGDMEETDVALIENGGRRRNASEVQV
PPELRPPVM)-YDVFISFRD-KDT-
SDIPEYLACALLKKSLRVYAHPELTGDHVPQTVIEEIGKAQVSVVVFSKSYLETNWCLHEFHFIMACRAR-
YARTVIPVFYNVHRTAIQDYSSNYKRLVCRVGKC---CVLQDRMNFSRVFQIMC()}*

$4172=204(200):
>gi|1151035472|gb|OOP56863.1| {|0(519)|<Planctomycetes(B)>}hypothetical
protein AYP45_06545 [Candidatus Brocadia caroliniensis]
{(MVGNVYM)EYDIFFSYPH-KDAKE--
VQNIFQALQAEGLKVWIDNSEIRDFESItkSIVEGLAHAKVLLAYYSLNYWRSRACQWELTAgfLAAQREGEA

RRRILVI----
NPEEkeghihpVELRDELFQKAPADAEALRKLVQSVKDHISGIKNTIGD(IHALTQPSWYGRKGAGSNRFVGR
LPDMWRIHSALHALKVPVITGAVASAVVQV)}*

$4173=225(205):
>gi|1150198039|gb|OOO02142.1| {|0(297)|<Proteobacteria(B)>}hypothetical
protein USCGTAYLOR_01644 [Chromatiales bacterium USCg_Taylor]
{(MSVEPARTAGKRQESSAGIVRLEGNHCMAYVPGY)KNDIFVSYAHVdniplsGISKGWVETFidnlstklA
QKLGRTDLFKLWDDRL-
LAQNAPLtpEILDALKGSATLVLVVSPGYLKSPWCQREKNAFYdllrsKNRGSNsvfVVHRDRVEHES---
CPEEIRDLLGFRFWEERGKGKEPRIAGEPVPQPTDSLYWD(ELSDLAGKLSQSLEQRRGSAPTEPSSAFMPP)
}*

$4174=236(224):
>gi|1152324865|ref|WP_078205088.1| {|0(242)|<Firmicutes(B)>}hypothetical
protein [Bacillus cereus] >gi|1151478669|gb|OOR10893.1| hypothetical
protein BW897_20545 [Bacillus cereus]
{(MENKLNPGSKPPK)---IFISYAwtS--
QEYQEKVIKFAQRLMENGIDVVADFWNLNPGNSLfaFMESMVTDHTIdKVLVLCNKLYTDKaasyaGGVGtET
QIiGPQIYNKVkQEKFIpIILEREEL-
DKdyiPVHMNGLIYIDLDHNFEKGFEELLRlVYNQPLLRKPKLG(TAPSYLFEEKKTSFKVDGILSSMQDAMI
HRPGRIASLCYDYKRALLEDLEAYRVIELSEPYDEQVYNLIHE)}*

$4175=227(220):
>gi|1152190443|emb|SKA73766.1| {|190(0)|<Proteobacteria(B)>}TIR domain-
containing protein [Thiothrix eikelboomii]
{(QEDKNSLFTKHLLDGLQGKALVTGQYVRVFDLFSYLQPKVTAEQPNQNPLIKAELEENFPVALVANLPAPP
LPKAPNDDY)KYDLFLSYRhiDPdqQWVKKQLVPALTNAGL--
SVCFDGDYkcFRAGEPIIKSMEKAVVNSRYTVAIFTPEYEESGFTELENLMAQHLETEASQVVgknkrLIGVL
YRDCDmQtlSLRLRYKR------FInMLDPAEFTTGIEQLVYECRHPID()}*

$4176=179(179):
>gi|1155016018|ref|WP_078921338.1|
{|0(20)|<Proteobacteria(B)>}hypothetical protein [Thiothrix eikelboomii]
>gi|1152190654|emb|SKA71441.1| TIR domain-containing protein [Thiothrix
eikelboomii]
{(MSTLI)-YDFFISAVEAdqAWVQQHLLPILTAANK--R-
YLTEAEFQPGQPRLLELERAITHSEKTLLILSAAYLQTDSNVIIDLFAMHYGLETRTWPVIPLLLAKLElPPR
LRLVIPLDFSQAAAyPDNYRRLLNSTELPSLPKPLPAQ(GLNPVQKLQLERQQRLTHKLARLQNQYDLETREE
)}*

$4177=220(216):
>gi|1154907019|ref|WP_078812868.1|
{|0(402)|<Verrucomicrobia(B)>}hypothetical protein [Prosthecobacter
debontii] >gi|1152228843|emb|SKA90313.1| 5'-deoxynucleotidase YfbR
[Prosthecobacter debontii]
{(MADNAS)-YDVFLSHNS-RD--KALVKELKTWLNGLGLRSWLDEDELQPGLRWqkLLDKAIK-
CSKSVAVLIGPSGTGPWEIEELenALIyaVKDD-----
RPVIPVFLPGAPENIdlppflsnrgTVDLRPAFAEENIDRLRWGITGE--
RYHIREGAATRN(SSEPVRAISEKELSAAEHVLEALLKQGQQARDEDRSQYVDRVGFTVLIKQLINASPRMQM
EMLAAIRRIY)}*

$4178=178(180):

>gi|1154799830|ref|WP_078715692.1|
{|0(289)|<Proteobacteria(B)>}hypothetical protein [Desulfovibrio gracilis] >gi|1152129256|emb|SKA71223.1| TIR domain-containing protein [Desulfovibrio gracilis DSM 1
{(MLH)--DIFISHAS---EDKDkYVRGLAEALEKENVAVWYDEFslKPGDSLRRsidLGL-----
SKSRfgIVVLSPSFLAKKWPEWELDGLVQRQNSSNDPVIIPVWLDVSAkeI-INYSPSLADKYALQA-
SAGVQSNVQEILKVVKPQGSSL(VVARDRLLSLGYNPPVVTDDWWHKAIEYTGSNELEGT)}*

$4179=189(190):
>gi|1154795683|ref|WP_078711577.1|
{|0(76)|<Bacteroidetes(B)>}hypothetical protein [Anaerorhabdus furcosa] >gi|1152082252|emb|SJZ61449.1| TIR domain-containing protein [Anaerorhabdus furcosa]
{(MEYVSVGGIFITSNIN)--KVFICFAAKdRYLIAQpIVYHLK----
NYGIDTWYDRyEMVMGDNREeKnIKEGVEKCKYAIIILSKFTMFSN-CAVeEITILEKRYKK-
SEIVIFPILYELN-
PDNIPDQFKWLKSLIFKevNKKSGTHEVCNHIACKITTDIKE(KINAKSIDSILFSYKDLIPSLTFELLKTYT
EIDA)}*

$4180=154(151):
>gi|1155017671|ref|WP_078922991.1|
{|0(807)|<Proteobacteria(B)>}hypothetical protein [Thiothrix eikelboomii] >gi|1152189154|emb|SKA84772.1| Formylglycine-generating enzyme, required for sulfatase acti
{(MMHKP)-PRVFISYSHDsEAHR-
ELVLGLANRLRNGGVESWIDQYIPsfPVQGWLrWMKTEVEQADFVLLVCTPRYLQRFNGEDtEGGRGVNFEGV
-IISQTLYNHYYRN-TKFIPVLPPQGCFEHLPleLQAYSAYKLDTDYQDLYRLLTGQ(PKVTPP)}*

$4181=216(200):
>gi|1155016735|ref|WP_078922055.1|
{|0(190)|<Proteobacteria(B)>}hypothetical protein [Thiothrix eikelboomii] >gi|1152190259|emb|SKA75874.1| TIR domain-containing protein [Thiothrix eikelboomii]
{(MRR)-
PKVFISHSSKtPEGITFLEQVYTALDRHGggqSFDVFLDREeIHTGEEWHkKILQNLCTCDAVVILLSKAALN
SHWVRQeAAFSAMRRYGD-LALKLVVVTLDDvtaqeiqaC-
PYLGgvARLHDLQFAPKHKtpEDIIEELAGLTIRPYSPLGDLLL(KMSETLEYIKPATLERAIRLLDCDCVAT
LNWNTHLAHTLALSVASEQYKALKNLSAL)}*

$4182=193(184):
>gi|1154923736|ref|WP_078829267.1|
{|0(122)|<Proteobacteria(B)>}hypothetical protein [Pantoea ananatis] >gi|1152196479|emb|SKA83508.1| TIR domain-containing protein [Pantoea ananatis]
{(MDSKSD)-TTIFISYawggSAEkKEW---
IRDHVVNSIDFKYDVFWDRDSIGFGESIDsMIKNALSSFPLKVFCICdEDYIVSAkkvgSGLHrELQMLANLG
SD-NEVKIIPIILdRKC-
ITELPPPLAGRAYLDLTnlHERGLYLGNAIWHLAENITQAKM(LTWIKLTIEKDDLHRAALDYFYRKPLRFSG
NAITHQIC)}*

$4183=231(235):
>gi|1154908385|ref|WP_078814234.1|
{|164(0)|<Verrucomicrobia(B)>}hypothetical protein [Prosthecobacter

debontii] >gi|1152227444|emb|SKA99840.1| Uncharacterized protein YjbI, contains pentapeptide repe
{(ANLSYAQFGTTVLANVRLSTTIGLESAIHNYPSSIGIDTLYLSKGKIPEVFLRGCGVPDSFITQAKALIGAEEGIQFYSC)----FISYNSKdEAFATRLHARLQ----QDHVRVWYApHDIQGGKKLHeQIDEAIKIHDKLLILLSPNSLKSEWVMTELRKARKAeRKT-GQRKLFPIGLVDY-ETLREWECFDADGGQDLavEVRQYYIPDFTLWKDHDVFEKEY(TRLLKDLRAEAKPIL)}*

$4184=162(175):
>gi|1152149957|emb|SJZ98563.1| {|0(260)|<Proteobacteria(B)>}Tfp pilus assembly protein PilF [Oceanospirillum multiglobuliferum] >gi|1163323616|gb|OPX55513.1| hypothetical protein BTE48_09010 [Oceanosp
{()-------MKIADQIEEDLSRLQ--------LNLRRDVRDLAYKNSIsdFMES-IRESDFAILLISDSYLRSKNCMKEVLHLLKDRNyEDkilPVIVKGIGIYS---TADRIAYTRHWLKEQENLKKVINTLPATSVVSEISELKVV(ESITSQINDFLAYISDKKNLTFEELKAESYSSILD)}*

$4185=175(164):
>gi|1154926164|ref|WP_078831678.1| {|0(327)|<Bacteroidetes(B)>}hypothetical protein [Sediminibacterium ginsengisoli] >gi|1152177585|emb|SJZ92721.1| TIR domain-containing protein [Sediminibacterium gin
{(MQFAK)--DIFISYAHIddeslienqkgwiSEFHRALEiRLAQLLGRRPV--IWRDPA-LQGNHIFdkQIVDQFAQVAVMISILTPRYVKSEWCVREVNEFYDCCNTNigfSINNQARIFKV---IKTPVKVEVHPEKIQNILGYEFYNTDPHTGRVKELAQVFG(QQTEKLYWEKLDDLANDLA)}*

$4186=264(292):
>gi|1152436296|ref|WP_078295928.1| {|13(39)|<Actinobacteria(B)>}hypothetical protein [Mycobacterium sp. D16R12]
{(AAQVLGTQTEADVTLTLSQYGLSTDNPDWYQGDMAAFATDSLSPASDEVLTDIVEFLEGEAAGHAASIPSTGGPWGLNPT)--RVFISHVHADRHLVGGVKQILEARyGID-AFVAHDDIHPSRTWRDQIKIGLNTCHFFVAVVNLDDFHTSQWCDqEVGWALAKR-----LPILPIRP------------------VG--FDRSAARD--GFLEEHQDVCIP(NGVTAPDVWVAHQIFTAVVREPSLREVSVKSLVEAFVNSRSYDTTRTIWANIVHQPNIESEQLRRLEYAVQT)}*

$4187=176(180):
>gi|1154136378|ref|WP_078511933.1| {|0(813)|<Actinobacteria(B)>}hypothetical protein [Streptomyces sp. TAA486]
{(MGGQGSGREPAA)---VFFSYAR-ADRERTLP--LVAALRAAGVSVFVDEHGIDEFEGItqEIRQALAGARLFLAYYSMTYPLRPACQWELLTAFRAAAALGRPSERILVVNPEESHAHIQPVELADPRYLDPADGDGARRLVDLVRGRLVHSRT(PFGDAIESVRPVWRPVEHTGSERFVGRV)}*

$4188=259(251):
>gi|1154245913|ref|WP_078617554.1| {|0(137)|<Actinobacteria(B)>}hypothetical protein [Streptomyces sp. AW19M42]
{(MFSALASVGIMNPNNLAEASVTKIEGFLSYSH)---ESDEFL---QIAEPLHRDLVRAIKLTsnrHIEIFRDRTSIKWGSRWeaAIDNGLANASVLFVIATTDYLNSANCRAEFLDFLNAANSSgnklAKKLILPIVPVNSPvfslDSEDEVARQIAEIQFESiQEAVIEGEKSPAWRRALFKLAER(FIE

VVSNVEEEAFKDQIEGSEEPQNKFSIPASDQTPDGDGGGSEELGFLDAVASVEEDFEEMTSLAEEMGSLLNTV
SEP)}*

$4189=214(220):
>gi|1153981816|emb|SKB64006.1| {|70(0)|<Bacteroidetes(B)>}TIR domain-containing protein [Pedobacter luteus]
{(LNKDRANKIKTLSDKQTKRLTLIQSLNKEEQRERDKIKREQKDILSVQQSITREMERQKALSSASLQLPYS
TSAVQVSKS)-YDVFVSHAS---EDKDdFVRPFVELLQKMQVAVWYDEFElqIGDSLRRsieNGL-----
KNSTygIVVLSEAFFKKEWPQRELDGLFAKEI-EGEKVILPIwhKISKSEviK-FSPIIAdlmAINTTMFTI-
EEVAAQIA-KRV--------KQ()}*

$4190=284(286):
>gi|1160837638|ref|WP_079683292.1|
{|131(26)|<Bacteroidetes(B)>}hypothetical protein [Parabacteroides chartae] >gi|1153985782|emb|SKB55932.1| TIR domain-containing protein [Parabacteroides chartae]
{(DNFNLSADHIIDTDYHRLKKLYCDECKGKISFILVVENKNLSILKKTYRRRVFFDTFDNTDNSLYQSLSHK
KIIYETKMR)RHEVYISYAwgG---ESEIIVTEICQQLQNNKVKYYIDKK---DIAYKdsiREFEERLGAG-
DyvILVVSDKFLKSKDCMYEFLQIKNAGNI--YERIFPIVLEDAKiyD-PNDRIYYIKYWEEKV-
KELDENIKTISPAYLSGLRDDI(DNYTEYRKIIPEITALLSRMNTLSPQVHRNNNYSELIKAIERQHEKDSTS
ISERGNFSADNTITSE)}*

$4191=287(296):
>gi|1154245751|ref|WP_078617392.1|
{|24(87)|<Actinobacteria(B)>}hypothetical protein [Streptomyces lydicus]
{(SPQRTSGVRRLASLPGSPTAPPSASAPSAVPEGDRVTAMRCPTAPVPVRMRGANLRSKERWAHGLMGMPMT
ATTPEAASG)-RRLFVSYARPDSEAVQRLVEVLRALHHV-
PWVDRElESEGGKPWWDRILAEIEACDGIIVVLSPQLFDSVPAERerEYADELGKP-----P--
LPVMVEPVTpdILPRELSARQFVD-FT--RGGPLAGA--
MLASAVAGLPPA(PPLPDPIPKRPDVPSSYLGDLRDRVRAPALSLEEQIHLITELRLRLDLERERQAVVTLLR
ELQQRRDLCHATWQLL)}*

$4192=174(176):
>gi|1160729631|ref|WP_079588872.1| {|0(647)|<Firmicutes(B)>}hypothetical protein [Acetoanaerobium noterae] >gi|1153967175|emb|SKB34309.1| TIR domain-containing protein [Acetoanaerobium noterae]
{(M)--KVFLSHSSSdKDHYVRVVS--KKMERDRII--
YDEYTFEEGVKSIeEIDRGLNSSDLFVVFLSENSLNSHWVkYElFKANTLLTESS-KLERIFPIIIDNR-
IKHDDKRIPDWLRENNLkvVISPNKAVQLIHQRLIEMSFSKH(PKLAEKNRIFVGRNDVIEEFEMRINDFRKK
VPAFI)}*

$4193=182(185):
>gi|1154209969|ref|WP_078582649.1|
{|0(593)|<Actinobacteria(B)>}hypothetical protein [Streptomyces sp. URHA0041]
{(MPTRAGRCRNRRPPWRADMLH)--SVFISYSRRdSSWVDALV---RALTRQGV-
RCWIDREgIPFSVPWRdEVEDAIQACDLFLVCDSGHWRTSSACATEAAYALRYAKV-RLEIPVGQDAGTA-
AEQAARAWRQAARRHGTatELSVRARDWDADGRTRKGLAHWR(LRRKFDALRGQRDLSAVESAYIAA)}*

$4194=278(278):
>gi|1154251504|ref|WP_078623145.1|
{|0(80)|<Actinobacteria(B)>}hypothetical protein [Streptomyces prunicolor]

{(MGEPKACSGLDQYGRASAGAGPGPGTDASETRGRGTVCGMWGADTWGCCHTRAVTVGRTY)---
VFIGSAQKdRQHLAALRRHLTGLIEEGLIEVFDVERIIPGEPRKqVLLSRLESADIVVLLVSADFVSSASCRA
tILEPALVRHNA-RQCVIVPVNLGPV-DLgDDNPLGKLQHIPADRpIVDWrsPSRD--
KAWMAVTSHLRAR(VKEHQQSLPSRSNVSAPSPPDQDQDAIVLDLSARLSARRAVDRQRPTGNGGGDRSTTHG
PTAHGSWDEVRRVLDTTRF)}*

$4195=268(261):
>gi|1155078789|ref|WP_078974714.1|
{|73(241)|<Actinobacteria(B)>}hypothetical protein [Streptomyces
scabrisporus] >gi|1153849381|gb|OPC80455.1| hypothetical protein
B4N89_05385 [Streptomyces scabrispo
{(RHPPGRRADHARHAAGHPGGAPGHAEAANSGHPCGIARDARSGPLRIPVVPPARPRRAVCERLVTIRRVPD
PHGASPGPP)RPRIFVSHST--
RAKPDAVccrcldarEALVARLAAEGFEAVYDKDFLRSGDAWqqEIALELHGSQATVLLL-
SQHAMESANVGyEAGIAD--GRRRvDARYrFIALKLPDV-
RRADLPTSALKAIHLGEVDMCDWADDEITAGRLPAKLCAD(LDQVRLWHLDRSSPTRYAVTTALDGAYEDRLR
AAADVLQVC)}*

$4196=196(220):
>gi|380706420|gb|AFD97495.1| {|677(0)|<Chordata(M)>}toll-like receptor 3
[Labeo rohita]
{(CNTPNAYFNHSVMDFDPLSCKDMTPFKALYILSSTAVLMLLFSAFLVHFQGWRIQFFWNIMVNRMMGSLED
KNNTEGRYA)-YDAYIIHSAEdrPWVERSLLPLE--DEKF-
NFFLEDRDPVPGFSQLDTIVENMGQSRKIIFVITEMLLKDPWGRRfKAHHALHQVMEDNRDSLILIFLQDVTD
YNLNHSLYFCHGML-----------------------NH()}*

$4197=261(255):
>gi|497425264|ref|WP_009739462.1| {|448(0)|<unknown(X)>}TIR domain-
containing protein [Frankia sp. QA3] >gi|392287045|gb|EIV93069.1|
hypothetical protein FraQA3DRAFT_2745 [Frankia sp. QA3]
{(QSLELVAPRDDRAVAAVRRLVTELGARRIGAAEPSAAPATQASAYQFVSWPLGDGAAPSAPPPPTRGPAGA
AAYYFFTSH)AHR---HDR---
DRVVAFHRELEAELRRKvrrRVEPagFLDADRMGGGEHWptALHDAVRTAPVLVALWCDDYFESDWCGREFGV
FQERIRRAtGPGGKPPAGIIPVPwlvrEAEVPEAAQELHIARIDLGRKYDNMPVLDLMRHPVAFAEY(VSLLA
YRVMDVARDQLPPLAADATKLVRSPFHPES)}*

$4198=219(220):
>gi|488442428|ref|WP_002511813.1| {|61(0)|<Firmicutes(B)>}hypothetical
protein [Staphylococcus equorum] >gi|392511125|emb|CCI59501.1| putative
uncharacterized protein [Staphylococcus equorum subsp. eq
{(EKQKELSKLYEDYSKSEKTINETRTKLDKEQAKQQDKLQKSINSSKKDNHNLMAQLLSNSDQINALSKQVK
ATAKQKGLI)EFDVFLSHSS---LDKEeYVSELSNKLTNKGLKVFEDEkvFEIGQS--
QTQMMNMGILNSRfvVVFLSRNFIESGWSKYEFISFLNREINEDNIIILPIW-HEISieE-
VREYNPYlvdkhaLSTSKFSI-DEMTEKI------YTVVEKSKQ()}*

$4199=229(237):
>gi|496889806|ref|WP_009399209.1| {|43(0)|<Actinobacteria(B)>}TIR
domain-containing protein [Frankia sp. QA3] >gi|392284704|gb|EIV90728.1|
hypothetical protein FraQA3DRAFT_0126 [Frankia sp. QA3]
{(VNAVPRLQLLADLQRLNSAGELPDGTVPLRVWLDNASLLTGGGPAEPAIAEAGTWLSAARGGPASRPGDSR
PGASPARES)---IFISHADTtrDRAWAAWTVAVLEGTGRT-
VTAAVWDDVPGGNWTAVAGRALDEGGLALALLSAAYLR-SPRAEqEWTLAARRDGDGG-PRLVPVLIEDVR-
APGFLRVRTGVNLCG--LTESAAVR--ALLTVLGGARRP(AGNAGAPSGVPFPGPAG)}*

$4200=157(157):
>gi|497428334|ref|WP_009742532.1| {<Actinobacteria(B)>}TIR domain-containing protein [Frankia sp. QA3] >gi|392290117|gb|EIV96141.1| hypothetical protein FraQA3DRAFT_6004 [Frankia sp. QA3]
{(MERLTE)-
ASIFLSWCHGdAAAKEALLGQLHLDALAGVrIAWWEDSHLMLGEDWRrQILARLAGCDYGLLLLSPGFFASRF
ILDeELPRLLG---P-GVDKVLPVMLKRV-
PLDGSRDLHGVDTRQIFtgSGGRCFTETRGAGRERFALDLTS(AIRHRVLSDAA)}*

$4201=212(222):
>gi|489691040|ref|WP_003595227.1| {|0(255)|<Proteobacteria(B)>}tRNA-ribosyltransferase [Rhizobium leguminosarum] >gi|392849214|gb|EJB01736.1| queuine/archaeosine tRNA-ribosyltransferase [Rhizobium leg
{(MSD)---VYVIHAS---EDDIITGEIVALLKKH-WDVWWD-DTLVG--
DFvqIVPQQIAKTKCVVAIMSVDSTV-KETVIdEMRLARDAGVA-----ILPLMIDDA-
RLPYTYGSLSRVDF-RLWDRTeghplFQKLLRkISTL-
VPRRVPSK(RPSSVANTSLSLPAVFLSVSSHETQLAPLDAVKALRVFGSPTVLVSAYDLWRDRRPKGIESELE
QIRAADGIVLIDSGN)}*

$4202=168(167):
>gi|495125837|ref|WP_007850648.1|
{|0(35)|<Bacteroidetes(B)>}MULTISPECIES: hypothetical protein [Bacteroides] >gi|392624687|gb|EIY18767.1| hypothetical protein HMPREF1063_04607 [Bacteroides dorei CL02
{(MAHKT)----FISYKYSEAQEL----------
RDRIIKAFGDdasyyQGETSASPDLtdTstenIKKNLKDMMYdtsvTIVIVSPHIKESKWIDWEIEYCLKNIT
RKnrtSHTNGIVGVIM---
KVNGGYDWFKYTSTKSDGCSVSNYYDSKVYDIINNNRYNQ(NPKVYSCNQCKCVSALTGSYIA)}*

$4203=198(199):
>gi|1154951069|ref|WP_078856390.1|
{|0(475)|<Actinobacteria(B)>}hypothetical protein [Streptomyces sp. NBRC 109706]
{(MLNDRLRVSGQSFPCRPEERSRTVATAGLSQA)EWDVFLSYSR-SDEHRIIG--
VVAALRAAGLRVFVDDTAVDDFASItaTITDALARCKVLLALYSADYPRRRACQWELTYAYLTGQHEGDPLRR
TLLINPERSVEHVHPMELRDARHWRWPADQEAAGPLAKRVAEYVDRID(TPMGDIARTPVVPWLPAPARTGST
RFT)}*

$4204=219(213):
>gi|1154965833|ref|WP_078871154.1|
{|0(521)|<Actinobacteria(B)>}hypothetical protein [Streptomyces caatingaensis]
{(MRAFRHEPFREGLIVVPPAAPGGVSALAA)EWDVFLSYSR-TDADK--
ARVLARILREQGLRVFLDDMAVDDFASItaSISEALARSKTLLALYSAEYPRRRACQWELtyAYLAGQREGDP
RRRVLVInperaAHHVHPVELRDAKHWIWPAVPDALQRLAAQVAAHVAAIDSPLGAAG(QLTGTQEPRPIPWL
PAPARTGSARFTNRLAEQWRIHTALVRHRA)}*

$4205=192(194):
>gi|1160843945|ref|WP_079689520.1|
{|0(192)|<Bacteroidetes(B)>}hypothetical protein [Ohtaekwangia koreensis] >gi|1154451432|emb|SKC85961.1| TIR domain-containing protein [Ohtaekwangia koreensis]
{(MDASGYR)-YDVFISHAV-ED-
KIPIANELSARLESAGLKVWYSGYELSVGDHLtkTIVEGIEQCRFGVVIFSPNYISKMWTLHEfhalLARERE

NR-----
KVILPVLFEITPEELAVKHLPIADIFAIRASRGIETVTQLLLEEIDKQRQVE(IRKSEYERSLEIKRKVKRIS
VAVLLGLCILAGFYITNRIFFDGPNAA)}*

$4206=221(220):
>gi|1160733662|ref|WP_079592717.1|
{|0(102)|<Bacteroidetes(B)>}hypothetical protein [Bacteroidales bacterium
WCE2004] >gi|1154413528|emb|SKC39215.1| TIR domain-containing protein
[Bacteroidales bacter
{(MA)--KVFISYAKKDYIAADgsvvpgncvdAILTAFQREGIS-
YWLDREQLAGGDTYAARISRNIKECDVFLF-LSTEAANASEWTLrEISAAVQMG-----
KRILPVRLDASPydDAVSLyLSSVQYVDWVE--LGQKAALE--
RIVALVKHPAFD(TTTGIEFGKFPRLTTFVLYAALVVLTGIYALLTYLFLWSKTLQTSEVIGGLVGFVGETTL
LMSVYYLLRMLRKRRCVF)}*

$4207=174(178):
>gi|1154832201|ref|WP_078745356.1|
{|0(261)|<Proteobacteria(B)>}hypothetical protein [Oceanospirillum
multiglobuliferum]
{(MN)-KNIFLSYSWSdMKIADQIEEDLSRLQlnlRRDVRDLAYKNSIS------
DFMESIRESDFAILLISDSYLRSKNCMKeVLHLLKDRNY---EDKILPVIVKGI-
GIYSTADRIAYTRHWLKeqENLKKVINTLPATSVVSEISELK(VVESITSQINDFLAYISDKKNLTFEELKAE
SYSSIL)}*

$4208=250(257):
>gi|1154956104|ref|WP_078861425.1|
{|0(791)|<Actinobacteria(B)>}hypothetical protein [Streptomyces sp. NRRL
F-5650]
{(MEQAADAVRLLDAQFSTSLFGPTYEHQQYWVGLVAGAGLLAALSSALLRDLASRLGTTSDPFSDASSEPRA
DAPLPEATG)--KVFLSYSRKdGAVARGIRESLEGKLRH----
VWVDwEAIEPSEKWRqAIDEGIRTSDAFILLVSEDSLRSPYCRDECMKAIDLHK-----RILPVIIDPN-
LATGVTAAMNEHGWEPLrsYQSFHMIEGSDEDPDRGIQRIVS(FVTQRHRWDAFHTRLGNLAHEWWESGRKDG
LLLRAEE)}*

$4209=236(240):
>gi|1155007214|ref|WP_078912535.1|
{|51(607)|<Actinobacteria(B)>}hypothetical protein [Streptomyces sp. NRRL
S-646]
{(PSDGPVPRRSCGHGTAGSGRRRGVRAAPPTPGSSPSSEKGSPAAGRTASSPKSTTTHAACSASACAGRCPH
RWRTEVLHH)---VFISYSRRdQAWVDLLLQELAGRE----
VKYWIDRQgIPFSVPWRdEVEDAVQACDLFLVCDSDHWRASAPCATEAAYALRFDKT-RLEVPVGQDLTAA-
AEQVRRMWQHSGRLHGTatELSVRARDWDRDGRKGKGLAPRR(LRRRFEALRGERQLSPIEWA)}*

$4210=239(219):
>gi|1155047461|ref|WP_078952500.1|
{|0(235)|<Actinobacteria(B)>}hypothetical protein [Streptomyces sp. NRRL
F-5755]
{(MCRKIPVTRSRTLRTGGMAMRSNSA)-PRVFVSYSHDsNEHRQRVLGLIQALRRNG-
IDAWADffEESKPPAMWPaWMRTQVGEADYVLTVITETY-
ARRYNQQeEPGIgAGVRWEATlitadiyfstsKQIKYIPVVFSAE-
DTRHIPpplnlTNKYLVVPDDDssLLPLLRHLLQAPEFVPAQLGEAP(DLPSSDGVPTADPAIEPRIASALQL
SKQNPTKAIDTLTALLDSLRGSQRAAAYC)}*

$4211=219(223):

>gi|1155411748|ref|WP_079004258.1| {|41(0)|<Firmicutes(B)>}hypothetical protein [Bacillus cereus] >gi|1154372648|gb|OPD51499.1| hypothetical protein BVG00_06215 [Bacillus cereus]
{(REFVNIISNYAEASEWLECEKNNFFFNLDAIKQKHEDIFGNCTWMKGKILLEPEFDTQKNKAIVNFSATKERIELNQPMK)---IFLSHKG--A-DKKIVREYHKLLLELGFEPWLDEDAMVAGTSLdrALLQGFEESCAVVFFI-TPNYVDDGYLAmEIDYAIEEKRSKgEKFSIITLMLPDK-SGQRGEIPRLLKRFVWKEPENSLEAFKEIIKALPVKLGNQ(MWK)}*

$4212=227(226):
>gi|1160639869|ref|WP_079501522.1|
{|228(0)|<Proteobacteria(B)>}molecular chaperone Tir [Burkholderia sp. YR277] >gi|1154477671|emb|SKC92975.1| SIR2-like domain-containing protein [Burkholderia sp. YR
{(AKNERLSSPRQTVEYLIDPMAARDPALKAFLADFSPATRVVSLEPTVFCAELRSRWGQRHPSGVAQAPSAPGVPRAIEGA)---VFISYSS---QDTDAVQGLARELDAAGIDVWFDRSELRGGDDWerSIRRGVDTSSLFMPVISRatqDPDRRRDFFWrEWMAADERARgMApDEPFLVPVVIDDT-PKYGDWLPDRFRSAQWMTLPGARTDETFVAHVRDLYRQIS(SRCRRS)}*

$4213=250(254):
>gi|1158526528|ref|WP_079058386.1|
{|1005(12)|<Actinobacteria(B)>}hypothetical protein [Streptomyces ossamyceticus]
{(RAGAVDSLQPVELLRGLAPGRFQDETRHVVGLFDLVLTLYAIPFNHPAFQPLRVTLRREWERHRREEHAVVVRSSAPSDT)--EVYLSYSW-SKESNALADELDEAFQARGVTVVRDRRDIGYKA-SikDFMARLGQGKCVVLVISDAYLKSQNCLFELLETARHGEF-TDR-VFPVVLPDARIYRPQDRIGYVRYWEEQINELDEAMRSVSAANLQGFREDI(DLYTEIRAQLPRLADVLRDMNTLSPELHRASEFS)}*

$4214=203(200):
>gi|1158575715|ref|WP_079103826.1|
{|0(619)|<Actinobacteria(B)>}hypothetical protein [Streptomyces aurantiacus]
{(MTVHHGGVAPGPDSL)-YDVFLCYTW-ADGKE-WADDMCTALRAK-GlDVFQDDLSVPEWDEYemlpSVKAALLGSRVLVPLLTPCFDDSDHCWSELHLALSCAKA-LGetemRRVSPLTWKVPPKKVRPKALKKRKLIREG-RSVAELADHVVRVVGDVREGDD(RRFGAAVTDRPSWLVDDLLGDSGFVGRRKELWALHDALREPGGTA)}*

$4215=237(242):
>gi|1159274104|ref|WP_079206895.1|
{|825(30)|<Cyanobacteria(B)>}hypothetical protein [Microcystis aeruginosa] >gi|1158733852|gb|OPF19550.1| hypothetical protein B1L04_09730 [Microcystis aeruginosa KW]
{(AIAGLLKALKDSYSSVRWNAVEALGNIGSETAMTELIKCLKNPDFVTLNNGDTLFQARKALDTIQNKLKYYHPLSQPMNQ)--QIYISYNW-QEDSNEMANQLVQAFAAKGIEIIRDKtHTSYKDSIKNFMQQIGQGKCVVAVISDRYLKSENCMFELVEIARNG---DFYQRIFPIILPDARIHKDFERIDYLKYWEDEKSQLQAKYKQLDLVNTNSIMAT(LNLYNEIRSNIDNLTNIIKDMN)}*

$4216=249(241):
>gi|1158557012|ref|WP_079085137.1|
{|0(209)|<Actinobacteria(B)>}hypothetical protein [Streptomyces sp. RV15]

{(MPLSLLSVPAAGHTAAVTAAPRP)-PRVFISYSHDseDHRKAVLRLAHRLQKAGL--
DVGFDrfVEHEPPASWPsWMRQELNRADFVLLVVTEVYRKRFLGESvvdrGFGVRWEgTLIASeLyyttseKA
RFLPVVLRAE--DRPLIPSPLDQTSWF----
VIGESADADIAELLRVLRRE(PLAGVHRGADPVGWFDGITPALEIGETIGDGDLAAIEDALPHLRGPQWARAV
HFAGVLHRQRGELMQAITCFTRVRET)}*

$4217=269(271):
>gi|1158526239|ref|WP_079058097.1|
{|0(749)|<Actinobacteria(B)>}hypothetical protein [Streptomyces
cellostaticus]
{(MPSTGTFSRAYAGSSEDPWGVRVRLGGLTGARGRPGDVSGEWVFGGAGGGPC)--DAFFSYAR---
TDRERVLPVVEGLRAAGLKVFLDETGIDEFEGITPAIQAALAQARlfVAFYSTTYPTRPACQWELLTAFraAT
ALGEASQRILVVnpeeRADHIEpvE-LR------DARYATVGgEEALGRLV-
ERLRAKAGQSTSP(LGDAVVQERPVWRPAEHTGSPRFVGRVTEFWQLHSYLHALEYAATHDEASAGQAVIAGL
GGMGKTLLAEQYAHRFGSFY)}*

$4218=181(180):
>gi|1159331812|ref|WP_079262123.1|
{|0(219)|<Actinobacteria(B)>}hypothetical protein [Mycobacterium
abscessus] >gi|1155603598|emb|SLF65572.1| Uncharacterised protein
[Mycobacterium abscessus subsp. ma
{(MSMAL)--NIFVSYKREdLEMAKKLERTL----
HANGLRAWRDLVFLPfGHDTEtEVRSAInEEVGGFSLIVTENSLSSDFIWRiELPAALERART-
GGgaFPIIPIFVGDS-
LVPKFARKSVSELGHDLsiQNGIVLHNEDDAEGLNRLAGRLL(ESLIGDQPYTRISLFTHSEIPTLDPHDIVV
DIRES)}*

$4219=279(264):
>gi|1158502072|ref|WP_079033935.1|
{|71(250)|<Actinobacteria(B)>}hypothetical protein [Streptomyces sp.
JHA19]
{(FGASLEDHGPNVVLYRAGPQTARRAGSLRGHDHSRQSSATPKPLRRPQGKATHHSHLFIKISPLGRCHSDE
GRQMQSKPI)-PRVFVSYSHDsDEHRES-
VFHLIQALRRNGVDAWADFfvESDPPSNWPvWMQNQVEEADYVLMVVTETY--
ARRFNQkEVPgvGAGVRWEgtlitadiyystD-NKVKYIPVIFSPE-
DFEHIPAPLKVTNRYVIspEDDSSLIPLLRHLLQAPEFVPAQ(IGEAPPLISRESTPSANLAIDPRIASAIHL
SESDPKAALDDLTQ)}*

$4220=247(251):
>gi|1158521579|ref|WP_079053437.1|
{|0(182)|<Actinobacteria(B)>}hypothetical protein [Streptomyces
phaeochromogenes]
{(MKITLSCIPWLGRFQSPGRTGNTVPEHPVRIA)---VF--YARAdERHREKFRTSIADMVRHG-
IEYFDDRGIAPGQPWKeKLFKNLESADVIVLLLTPEFIASRFCMEeEVPHALRRHAA-GACEILPVkVASFC-
LADESPLCEIQWFPSDKpVTERGNATA-
KAWVEVAKELQRV(IKRVRSEERDRDSRLPPNGAQVARGVKYGPPSAAQTDSGDSPLARLERVTQGISFAVFV
IGALIALIFFLAHSADREKR)}*

$4221=265(271):
>gi|1159277459|ref|WP_079210249.1| {|359(772)|<Cyanobacteria(B)>}type IV
secretion protein Rhs [Microcystis aeruginosa]
>gi|1158728777|gb|OPF14608.1| type IV secretion protein Rhs [Microcystis
aerugin

{(LLKEFNSDVAYHESHKVLLVKALKWQQQTHNPSVLLRGYNLQHFEAWLKVAKQRKESPPLPLQEEFIIASL
NQHKPSSLE)---VFISYSRAdSDLARRLNEALTSL---
GKLTWFDQESIASGEDFRrEIYQGIEISDNFVFIISPKSINSPYCSDEVEYAQKLNK-----RIIPILHQKV-
AAKDLPaaLASIQWLDFSQhgGDFLTNFTQLVRTIHTDRDHVHN(HSKWLQRAKEWENSQENADLLLRGSEFV
LAQTWLKKIEQQNKQPPATELHK)}*

$4222=289(299):
>gi|1159277459|ref|WP_079210249.1| {|154(953)|<Cyanobacteria(B)>}type IV secretion protein Rhs [Microcystis aeruginosa]
>gi|1158728777|gb|OPF14608.1| type IV secretion protein Rhs [Microcystis aerugin
{(GLIGAIDQQQHYVQKHSIFLVKALNWLRNQKQTNYLLIGEERQAAEKWLKHRFTEEQPPCLPTDLHCEFIS
ESSKNANNL)MTQVFLSSSEKdNAIKEKIRK---
TLMREGFTVWTNQTDIKTGTAFQeQINKGIEGADNFVYLISPEALQSSYCYLELAHAFATNK-----
RIIPLLIEKT-DIELIPsqLQKLQFI---DLTEYEDRE--
KYRSSIDQLLKEF(NSDVAYHESHKVLLVKALKWQQQTHNPSVLLRGYNLQHFEAWLKVAKQRKESPPLPLQE
EFIIASLNQHKPSSLEVFIS)}*

$4223=271(264):
>gi|1158494983|ref|WP_079026932.1|
{|119(238)|<Actinobacteria(B)>}hypothetical protein [Streptomyces scabrisporus]
{(APSKLRCTDHAQPAAGQSRGSPGFHDTAPGVVARREIADDAHPRPRRIPLVPPADGRRVLREPPVTIHHLP
EPRGAAPVP)RPRIFISHSTRAkPGQVccrclDARDALVQQLEAEGFEAVYDKDFLRSGDAWqqEIAIELHGS
QATVLLLSRHA-LDSANVGyEASIAD--GRRRaDARYrFLALKLPDV-
QRAELAESALKAIHIGEVDMCDWADDEIVGNRLPDKLRTD(LAQIRLWHLDRSSPTRKAVTYALAEAYEDRLR
AAADLLGVCERL)}*

$4224=233(221):
>gi|1159315791|ref|WP_079246583.1| {|0(245)|<Firmicutes(B)>}hypothetical protein [Bacillus thuringiensis] >gi|1158657314|gb|AQY42600.1| hypothetical protein B4918_32465 [Bacillus thuringiensis]
{(MENKIRSNPRTPK)---IFISYAwT--
SEDyQEKVIKFAQRLRESGIDVVADFWDLNPGNSLfaFMESMVTDPTIdKVLVLCNKIYADKaaaytGGVGtE
TQIiGPHIYNKVrQEKF-IPIILERE-
ELdteyiPVHMNGLIYIDLDRNFEKGFEELLRlLYNKPLLRKPELG(TAPSYLFEEKKTRFRVDGILSSMQDA
IIHRPGRIASLSHDYKRAFLEDLETYRIMELTAPYDEQIYNL)}*

$4225=237(233):
>gi|1158517887|ref|WP_079049749.1|
{|0(792)|<Actinobacteria(B)>}hypothetical protein [Streptomyces torulosus]
{(MPWSSAARAAAATRRRGHGTILPWQRRSCPARCHAPTGSARTDEGAAVGSAGAEGPARSGH)RYDVFLSYA
WEdidlaVRFQELLEA--------
RGLSVFRDVTGMRDYDDIptVIRAALDGSRTLVALYTPSFPESQYCRWEMYTALTrsyhlSGQTRrvmAVVRG
MRFSQV---
LSSRLKCLRLPAVTAALADVSDSVHRLVDGMDHRTFGDAP(EPPVPSWYPEPAVGSRHFRGRLQELWQIRDAF
)}*

$4226=238(234):
>gi|1158553742|ref|WP_079082063.1|
{|0(798)|<Actinobacteria(B)>}hypothetical protein [Streptomyces scabiei]
{(MPSSSAARAAAATRRRGHCTISPWQRQSCPAWCRAPTGRTGRTGEGEAVGSAGAEGPARSGH)RYDVFLSY
AWEdidlaVRFQELLEAHG--------

LSVFRDVTGMRDYDDIptVIRAALDGSRTLVALYTPSFPESQYCRWEMYTALTrsyhlSGQTRrvmAVVRGMR
FSQV---
LSSRLKCLRLPAVTAALADVSDSVHRLVDGMDHQTFGDAP(EPPVPSWYPEPAVGSRHFRGRLQELWQIRDAF
)}*

$4227=194(186):
>gi|1158991741|ref|WP_079161555.1| {|0(455)|<Actinobacteria(B)>}TIR
protein [Streptomyces puniciscabiei]
{(M)-WDVFLSYSR-GDVER--
IRPLVRALSDGGLRVFTDETGVASFAGIsdTIRRELGRSKALLAYYSAAYPEREACQWELttAYLAGLEEGDP
RRRVMVVnpepstghIHPVELRDARHASADDPVALVDDVREHVARLAGPMTIRERGGGQGR(HRAPEPHPEFL
GRLSELWHVHSALHAHAAPLTVGRSATRTVQIRG)}*

$4228=194(186):
>gi|1159083638|ref|WP_079173296.1| {|0(455)|<Actinobacteria(B)>}TIR
protein [Streptomyces sp. MUSC 1]
{(M)-WDVFLSYSR-GDVER--
VRLLVRALRDGGLTVFTDETGVASFAGIsdTIRRELGRSKALLAYYSAAYPEREACQWELttAYLAGLEEGDP
RRRVMVVnpepttghIHPVELRDARHASADDPAGLVADVREHVARLARPMTLRERGSGQGR(FRAPEPHPGFV
GRLSELWRVHSALHAHAAPLTVGRSATRTVQIRG)}*

$4229=248(235):
>gi|1154972952|ref|WP_078878273.1|
{|0(126)|<Actinobacteria(B)>}MULTISPECIES: hypothetical protein
[Streptomyces]
{(MGSSGPCGEGPGAGAGRGAAVTAAAEPVP)--RVFVGHSADDReAFVEPLARELAAQGVR-
AWVEFWDLRLGDSLTGTvFTDGMDEADCFVLVVSP-LWHRKPWLReQLEATAHRR-----
IRdrrtLIPVLLDGVQapsplshlkplSCPRshegaaTAARRIADQLYG--
VDPRPAVRPRRGHRPYPGAGQS(PAPAPTAHVPLAPGRPAFPGLADAEVAVLQRIMTVALREGTLGPLPWSEV
GPALRDAEPDTARTVR)}*

$4230=223(232):
>gi|1158973981|ref|WP_079145621.1|
{|0(86)|<Actinobacteria(B)>}hypothetical protein [Streptomyces lydicus]
{(MGMPMTATTPEAAPSG)-RRLFVSYARPDSEAVQNLVEVLRALHHV-
PWVDRElESEGGKPWWDRILAEIEACDGIVVVLSPQLFDSVPAERerEYADALGKP-----P--
LPVMVEAVTpdILPSELSSRQFVD-FT--RGGPLAGA--
MLASAVAGLPPA(PPLPDPMPKRPDVPSSYLGDLRKRVRAPALSLEEQIHLITELRLRLDLERERQAVVTLLR
ELQQRRDLCHATWQLL)}*

$4231=213(222):
>gi|1158991400|ref|WP_079161214.1|
{|0(822)|<Actinobacteria(B)>}hypothetical protein [Streptomyces
puniciscabiei]
{(MTVFRE)--KIFISYQRDSAALAQRIHDALEAAGFD-
AWVDTERIRHTDRWATSIDQALRSADRLVVLLTEKATE-SREVFnEWFFFYDHR-----
KPIHVVRIDGCElhYQLVPFQRLEWTAQAD--ESEWAARTS-
ALVTALRGSFSW(PHAASADDAVVTTPFAPPRTLLGAFAALEEAIRNPDRSVALTDQQLHDIRDHPSRSVREY
MLSCYARWCTPRYQLD)}*

$4232=250(257):
>gi|1158952047|ref|WP_079123770.1|
{|63(708)|<Actinobacteria(B)>}hypothetical protein [Streptomyces sp. NBRC
110611]

{(THPEHVRWVLEWLDGQAYGALFGPSYGQWYWPPLVAGAGLLAGGASAALREWSARLAASRDPFDERLRTAD
APEPVTRTE)--KVFLSYSRKdTALARRLVARLEG--RVG--
DVWVDwHGIKPSAQWLeELSEAIRSSDALVVLISPDTLRSPYCWDECKQAIDL-----GKRVLPVIVSPE-
LAQGTTAALRAAEWDELtaYQYLGMSTPEAFEAGIDAIAAFV(SREHDWVAFHTRLGLQAHAWKVNGGSDGLL
LRPHEVR)}*

$4233=210(220):
>gi|1159088103|gb|OPG02600.1| {|234(0)|<Actinobacteria(B)>}hypothetical protein B1R27_31915 [Streptomyces sp. GKU 895]
{(GQFVPTMPIVSPRAPGTPEETLYGGSPTGHRKAIVRWDDPDSLVAAVEPHLRVIDEQPRFIGSTAQALDYF
RSAAKRNER)---VFLSYASAnHDHAATFSQLLN--
ERfQNVFDFRRHGAIGVGEDWLdDLMGNLAKSAVGVLLLSKEYMESKYCMLEARELYRHSIE-
GDVRLVPVCLEKL-D--------LPDFLQRTqyRNLARHTPQGIVTELLDQLPPSA()}*

$4234=188(178):
>gi|1159761998|ref|WP_079308407.1|
{|0(228)|<Actinobacteria(B)>}hypothetical protein [Streptomyces sp. GKU 895] >gi|1159094161|gb|OPG08480.1| hypothetical protein B1R27_09805 [Streptomyces sp. GKU 895
{(MAR)-
PRIFVSHSSSeRRCAERgcaCVTHreavTALLDELGCDAVVDRGVLRAGDQWHaKLMRELACCQGMVLLISPH
ALDSYYVFEEAMLSMFLWEvtE-ERFLVLPVMLPGA-
GRTELQRSRMGKLDLGRldMVDWTAAAAHTPPGKLGERLQSL(VERHGSLPYPLVTEYVAARVEDVSAATLSD
IAREL)}*

$4235=175(176):
>gi|1159802482|ref|WP_079343989.1|
{|0(381)|<Actinobacteria(B)>}hypothetical protein, partial [Streptomyces antioxidans]
{(MSAMNDSDA)EWDVFVSYSR-SDARK--
VGALVPALRDRGLRVFVDDTAVDDFASItaTITEALAHSRVLLALYSADYPQRRACQWELTYAYLTGQREGDP
LRRTLVVNPEGTTDHVHPVELRDARHWPWPTTPETIGQFVESVAAHVRDIP(HPMGDAAAAPFVPWLPAPART
GSIRFT)}*

$4236=261(275):
>gi|1159759465|ref|WP_079305963.1|
{|973(0)|<Actinobacteria(B)>}hypothetical protein [Streptomyces sp. GKU 895]
{(RTGAAEAVLPDELRGLLPGEFQDDFWYSRYVVALFDLVLTLYAIPFDHPAFGGLRGDMELEALRFRLAVPE
PLSSMQSAD)---VYLSYSW-SEESNALADELDAAFQKRGV-
VMVRDRRDAGYKasIGRFMERIGEGRCVILVISDAYLKSQSCLFELLQVARHGDF-RD-RVFP----
VVLSDARIHRPQDRVGYVRY-W----
EEQIAELDEALKSVSSA(NLQGFREDMDLYTEIRAQLPGLADILRDMNALTVDLHRESAFTELFEAVLARLAV
)}*

$4237=229(221):
>gi|1159847613|ref|WP_079382309.1|
{|0(596)|<Proteobacteria(B)>}hypothetical protein [Pseudomonas aeruginosa]
{(MK)---VFLSHSSKdkESYVRLVADKIGNGNYV----
LDERTFEDGEITKDEIIKGIDESEMFAFFISNTALDSKWVKSEIDRAEIRLNSEQlkkiypiiidqsikyDDP
RIPIFLkEHYNlKLITRPTVAARRIKSKLrELYWqksPEHKKRqtIF----------
V(GRNEILSDFENRMDDIDFPKPACVIASGISKIGRSKLVARALAKSNIVADNFSPAKIILDRVDSIEDLIIK
IIDTGLTC)}*

$4238=266(255):
>gi|1159633105|gb|OPJ66778.1| {|367(662)|<Chordata(M)>}sodium/hydrogen exchanger 4 isoform B [Patagioenas fasciata monilis]
{(TERDFRSNFTCFAENSVGNATAVIQLKRKQRVFLLYVLCSAISTLFAFLLCTALIYQHWIEIVLMYRSYLV
LNKTTGDGK)EFDAFVSYAKVdscesdstliseEKFALELLPDMLENKYGYKLCILERDIIPGGAYTDEVVTA
IKQSRRVIIILSPAYISGPSIF-ELQAAVNCALEDKNIKLVLIRDTVG-
SSQDMRLHVFTLDYDYVqIPYEVTLWILLASLAKIGFHLYH(RLPRLMPESCILIAIGALVGAIIFASDHKSP
PVMN)}*

$4239=217(220):
>gi|1159648364|gb|OPJ77699.1| {|0(70)|<Chordata(M)>}TIR domain-containing adapter molecule 1 [Patagioenas fasciata monilis]
{(MPTSEPDSGEL)KFYTFVVLHASeDELVAQRVKNLLEKmgvPNGAMLC---
EDFsIAGRSRITCFQDALENSAFIILLLTKNFLCSQ-CRFQTDTALMESILKPSKRDSVIPFVPK-ENPLE-
RSQIPSVLGTLtPLDENSPM---
FSRTVQNTFKPS(RIKERKDMWALMQRRKLQLYEEQTRQQLAALNLGSLPQVPLSATRPLLLEQSSHQWGPPT
GHPTPAPVG)}*

$4240=216(220):
>gi|1159845766|ref|WP_079380462.1|
{|227(0)|<Proteobacteria(B)>}hypothetical protein [Pseudomonas aeruginosa]
{(DNPVEAERKYYIDILGLSPEEAELTEDGSKLKRNIYGRLSFLALVKGADDPTYVKLILKMAEHDSTPPKFI
KSIKAEYKM)-YDVFLCHAS-ED-
KEEIVKPLYDELEKLGVHAFYDAKEISWGDSLvgTINKALLKSKFVIAVMTDNSVGKAWPQQEINSVLNgdis
gGT-----KRLLPLIYGDKDSIL---NNNFLMSDKLYVEWKGNPEEIAQKVKDLLTQKAQ()}*

$4241=315(297):
>gi|1160388157|ref|WP_079432574.1|
{|0(247)|<Proteobacteria(B)>}hypothetical protein [Zoogloea sp. LCSB751]
{(MRRRAPPRRPSPGPPAAPLPLDAQRRRIRPPRKPRPAVVVPDHLRRLAAALRPAGPVHPPVAGLRPMSFER
QVFISY)AHLDNQPLtadqL---GWVSLFHSTLQTLLSQRlgaPADIWRD-
DKLRGNDVFsdEILDQLARTALFVSVLTPRYLKSEWCTREITNFCQRAETEhRlvvgnkarviKVLKLPI--
DGSGALP-
EAVGQLLGYEFYEfDEDRTPRELDPAYgdqpRQAFLRKANK(LAWDLAAQLEQLQQQPAANDATDTSKPAVFL
AECSRDRRDARECIEAELACCGYTVLPDRRLPDDEDDHRAAVAELLERC)}*

$4242=228(230):
>gi|1172485971|ref|WP_080858354.1|
{|282(0)|<Proteobacteria(B)>}hypothetical protein [Agrobacterium genomosp. 7] >gi|1160260543|emb|CUX55620.1| putative RNA-directed DNA polymerase (Reverse transcript
{(ALLRMKLHSARVHGAQKAAKLWLKNTDNPDHFWRHIEGWLAYISQVRGQDDPVTAKLCKMAVLSGLSGAEW
IESYADMVR)EFDVFLSHAT---EDKPIVLKLKTKLESLGLKVFFDAD--SIKWGDsivEKINHGLL-
KSSffVPFLSDTFSAKGWTNKELNSAIAMNISRKG-RILPICESSFSiaD-NYPLLNEILYKRWPA-
DTAEEATFVTTVADEILKIVQD(EQKKPNDLSG)}*

$4243=175(183):
>gi|1160022456|gb|OPL19088.1| {|0(1051)|<FCB group(B)>}hypothetical protein AVO35_12500 [Candidatus Aegiribacteria bacterium MLS_C]
{(MKRT)--QLFISYASQDRKTADHVVSFFESRGIG-CWIAPRDIPPAADWAESIIDGIDSSSCMVLLLSR-
HSNRSPQVRrEVERAVNNG-----IGIFPVVIEEIElsKWMQYYISPHQWRDAT--
DVPLDRRLSELLDPLRKELSLS(TGAPCERELSELLENDLAAISSALDYDAGEGSRIMLNDE)}*

$4244=158(162):
>gi|1172456409|ref|WP_080830746.1|
{|0(10)|<Proteobacteria(B)>}hypothetical protein [Agrobacterium genomosp. 6] >gi|1160329075|emb|CUX71654.1| conserved hypothetical protein [Agrobacterium genomosp. 6
{(MP)-RKVFVSHKKEDSGTASKVADRIKANGIA-
VYLDVIDdalASDGPDLADHIRKRMSECDQLVAVLSTA-TKDSWWVPwEIGVASEKD-----
FRIASYAEQYISlpTYLQKWPVLQTMSEVD--
LYCEYSKRNEQVTVRKMDEAYS(TDRKSTIFKSGIGEFHKDLA)}*

$4245=172(176):
>gi|1159613803|ref|WP_079269759.1| {<Firmicutes(B)>}hypothetical protein [Streptococcus suis]
{(MFFDRLTCELTIRSLFTLPPKSKYLEAGFKEERVFK)-PKLFVSYCHAdKAEVHSIISDL----
REYGLDFWlDEEQIDIGDRILdKVEEGMRGSDIPIIFISHSTKNSLF-AShELK--TYFSDI-
IYSQSTSKPWFII-KLDDVNPDDIVKGLGMFmyYDFSSSVHDIENLANRIKRKLRK()}*

$4246=202(210):
>gi|1159761253|ref|WP_079307751.1|
{|0(796)|<Actinobacteria(B)>}hypothetical protein [Streptomyces sp. GKU 895]
{(MDASSVLARPEPSPSPHPRRMLPRKRPGAAADPGKH)---VFLSYSRRdLAYVEQLEERLA----
ERKVTAWIDlSDIPVSARWReEVADGIRRSDVFFCVVSPHWLASAPCREELSLALEF-----
GKRIVPLLHGTA-
PEDLPVALADRNCLRDGvdAQVVADALALDLERVHHHTRLLV(QAHEWSVSGRRRSALLRGAELRAARAWLAGATAG)}*

$4247=216(200):
>gi|1160400737|ref|WP_079443842.1|
{|0(355)|<Proteobacteria(B)>}hypothetical protein [Pseudomonas veronii] >gi|1159380766|gb|AQY66448.1| hypothetical protein PverR02_15805 [Pseudomonas veronii]
{(MNMIAPSQPPTPALNEQLPADFA)-
FDIFISYRRSdgtqaaRRLRQKLQRYDIhsrlKHLRREKLKVFLDTVyERGADDFYerNIRPALLSSRRLIVLATPDAISRPgaddWIQREVQDFRAHRGAEnilVVRAAGELFET---
LPADLDVTAPNIQIIDLRRSGFFSMMSPLHTSRLADEWIK(LVAPLFDVPTADMPRLRREQELAQQRVLAIVLGGSAG)}*

$4248=239(219):
>gi|1160400733|ref|WP_079443838.1|
{|0(127)|<Proteobacteria(B)>}molecular chaperone Tir [Pseudomonas veronii] >gi|1159380762|gb|AQY66444.1| molecular chaperone Tir [Pseudomonas veronii]
{(MSYVGDPFI)-
HDLFISYSHGydeglgighlqpwsAAFAKELERELRAEPKIRNhLAIFRDGDyrlghGVDPMAPLtqQLCEQIGRTALLVILMSPDYLKSAWCADERDWWYRHQSgldstcDEriaVVRI-----WP---
TDDDPWPKLLTDNRGHSLVGFHFHAP--
VFGKERPLGWVE(MPAFGSDFKKEILNIVARIYPKLDALKQRADEHARLKMEIGKLSDEMGQTIYLHGRAEHFAIWQKMALAL)}*

$4249=178(174):

>gi|1159765092|ref|WP_079311471.1|
{|0(25)|<Actinobacteria(B)>}hypothetical protein [Streptomyces sp. GKU 895]
{(MRHAGGGTVDYD)-YDVFISYSHRghvrdwvknhfsRELQLYLEDLL-----PSDPRVFVDF-EIPAGSPWpqRLEQALLRSRCLVAIWSPPYFRSDWCMAE---WKSMQLRQeslPQVNGQAPTLV---YPINFMDGEHFPEEAQKIQQYKELTKYGYDGPQFRDTPAY(LGFQDRMRTVAEEIAACLACAP)}*

$4250=154(165):
>gi|1159660391|gb|OPJ86617.1| {<Chordata(M)>}hypothetical protein AV530_006750 [Patagioenas fasciata monilis]
{(MSSAPSRRGFT)-YDFSIWYcPNDDPIASRISERLRKEGFRGYA--EHQDQVAGTSVVLTAVEVIEASRVAILLLSAKSLGDPWCRRVSEWNLHHSIHRRGTKMIPVYVDVTKAEGPPFLRHLNELQYQT--------E-FFFERLLSSLRTT(SRRARAATSSRAKR)}*

$4251=185(189):
>gi|1160741468|ref|WP_079600113.1|
{|0(100)|<Proteobacteria(B)>}hypothetical protein [Bradyrhizobium erythrophlei]
{(MGAKQVR)---VFISWSG-ELSRQlseAIRDWLPSALQS--VRPYFTPSDIdkGARWATEISEALSASKVGIIVLTRDNLRSNWIMFESGAISSTLDKS---RICPILFDLEPTDLQGPLAQFQVTRFAEDDFRKLFHTINSQLGEHSLIENV(AQAVFDKWWPDLNEELSKIMQGHTAKADVPKIRSDRDLIEEM)}*

$4252=180(186):
>gi|1160776157|ref|WP_079630427.1| {<Actinobacteria(B)>}hypothetical protein [Mycobacterium abscessus]
{(MRSSRELSTCRGASSCPPREWLEQRGITPIAQFQRRPKT)----FLSHNQ-D--RKDEVRRLQEQLLSRSVATWFDEIDVEYGQTLvsEIEKGIESSGAVIFWWSQGFLSSHWCTYELDAFIDRVAR-LGKSKIQIHSIVDPEVDPERLHRKIREYPWLRFGPHTNVNEIAAKLAPALQRLD(SAGMNTA)}*

$4253=269(269):
>gi|1159033721|gb|AQY56780.1| {|914(10)|<Mollusca(M)>}Toll-like receptor [Sepiella japonica]
{(CLNVTTSGNYSAQPSPVIEMKVLLALAIFSAVFITLMVAIISVICYREELKVWLFTQYGWRVGDDWAKLDDSNR)KYDVFVAYTSKnaMFVEHELAPRLERRDPPYRVCLTYRDYDVDISYAQNTINSINNSKRTLMLVSNDFFCTEWFRYDFQINNHDVLKALSDRLIVVLMEKI--DKKKLNCDLMFYSRSKKYLKYHDARFWDKLYYMLPKVRG(LPLIPQTPESVVSAGELRDQCCNNIAFSKTSLESGYEEINFVRKNVYPNSDQNLY)}*

$4254=149(173):
>gi|1160035586|gb|OPL20379.1| {<Mollusca(M)>}wd40 protein repeat-containing [Mytilus galloprovincialis]
{(MNK)---------------------LKDNLELNGITVWVDVIGLTAGVDFlqKIGQAILDAKLFITLLSSNSVKSKYCQDEVALAYISQ-----KALFPVAIESRETIQEHMDTGMKLQTASIEWSMFVDPDNFTGDFQKLLSKLK(LELKDQGEGMQNITTKKQRTHRNISPVQLI)}*

$4255=220(222):
>gi|1160389628|ref|WP_079433963.1|
{|250(0)|<Proteobacteria(B)>}hypothetical protein [Zoogloea sp. LCSB751]
{(RIPENVYWTFGERLPVVEDQPAANDPLSINQPMMNIAALLARDLSGVQQLMLSRDAYLASAEQNRRKILLNMRSSQTAPQ)---VFLSAPF---DEAEFAESLARELGERGADVQGDwkgVGKRGDNWKHdllRELRN-----ARylIVLIVGKFDDRQDAAVES-FFSLSVRGRDDGRIIPVLLNEesmASvpS-ILRSLTFVDGRDRSV-ETVAFKCS-RIIWADSDVEPLA(LV)}*

$4256=213(217):
>gi|1167880397|ref|WP_080176002.1|
{|0(608)|<Proteobacteria(B)>}hypothetical protein [Photobacterium sp. H01100410B] >gi|1160591709|emb|SKA52397.1| TIR domain protein [Photobacterium sp. H01100410B]
{(MIKAF)-----LSHSS---KDKAsYVRNVANWLDKDQIV--YDEytFEEGE---RsldEILLGL--DKAElfVLFISEHSLNSEWVKTEISEAKQRLDTQALSKVYPiiidssIKFDDPRipD-WLRTYNMKPITRAPV---AARRIH-TKLREICWKKHPK(FKLRDNFFAGRNTETDHFEGRLLDFFKDKPLAIFCSGFPGVGRRTLLHYVLKKTDVTQDPFKPASIAIDRDA)}*

$4257=230(228):
>gi|1160742285|ref|WP_079600899.1|
{|0(94)|<Proteobacteria(B)>}hypothetical protein [Bradyrhizobium erythrophlei]
{(MMPSEDAVVPTDNF)EYDVAFSFHS---LDENIAFEINDRLQDR-YRTFLYSEKQKDLAGRngeDKFNEIFRAKAKfvVILYRKEWGETPFTKIEQRAIRDRAYDEGYGFTLFIPTENKPtmppW-VERTRLYYSLERFGV-DGAAAVVE-SKLQELGTAPVIE(GARDRAQRLERSLIFKTEKENFEHSEAGAQAGSKAYIAVLTLMQGQVADLALSSPRLKPLHMEQRNNYWLLYGL)}*

$4258=196(204):
>gi|1160391713|ref|WP_079436015.1|
{|0(325)|<Proteobacteria(B)>}hypothetical protein [Zoogloea sp. LCSB751]
{(MDETSPARVL)--KVFISYSRRDLEFTEQLCAALEALGYV-ITIDRKGIHGAERWEDRLGQLIRESDSILFVLTPT-SASSEVCRwELEQALSQR-----KRVLPVLAAPLGdvQPHTALRDLNYIHCYP--EASVPGSGWGHGLARLHAALSV(DIEWIREHTRVAEISRRWQISEAPDLLARGAELDGLQRWRDRRPANAPELTDAQ)}*

$4259=219(224):
>gi|1160747592|ref|WP_079606092.1|
{|0(1211)|<Proteobacteria(B)>}hypothetical protein [Bradyrhizobium erythrophlei]
{(MIPLTTGAIV)--RIFVSFASKDRECVRRFVAGLcaRKPDAS-LFFDERDLHGGVYWIPKLGQELRKADVVILILGS---RIGPWQElEYYEALQlsrqteGR-----PRIIPIII---TdsPAPGLafLSTIQHIFAVD--LTSRNAID--AVDRALSDVRHE(TTLEPWKQFQPYKGLPALTSTDAAFFFGREKETAEILDLLARARGRLITLIGQSGVGKSSLVMAGVMSRLKSQL)}*

$4260=212(220):
>gi|1160943997|ref|WP_079782919.1|
{|30(0)|<Proteobacteria(B)>}hypothetical protein [Pseudomonas sp. LPH1]
{(NRENRRIAESNARKVQTAVNAYNRKVEAYNRQAAQHNSKVIADLNRSLRSATSTVRYSDSERQLADRVHRAVAASDPREY)--DSFLSYARIDGAsIAGELRNCLEALGVA-VWFDEVAIVPGKSQSLQMDAGLRKARTGIALLTPAYLTGRFWTErE-LGALLSK-----STLIPVL-HGITfdDVKQYSGILPDLAGFT--TANDSVADIAEKIAAAVLPSEE()}*

$4261=160(163):
>gi|1160391787|ref|WP_079436089.1|
{|0(527)|<Proteobacteria(B)>}hypothetical protein [Zoogloea sp. LCSB751]

{()MADIFLSYAHPdRARVEALGKVLS-----
EHFSVWWDADLDAASTWRsELEEQIDGARCVVVLWTEAARTSDFVRSEASRAAQRRTL-LQVCLDGDGVPLG-
FTESQWLDLRAWTGDPAdpLLRDNFVVPIEKRVRGARSALLV(GLDAYAHAAGFQVSASASANVDA)}*

$4262=262(255):
>gi|1160805845|ref|WP_079655644.1|
{|1001(9)|<Proteobacteria(B)>}hypothetical protein [Vibrio nigripulchritudo]
{(RNQQQIQSFSRPGYIDITLSGKGSDSLAKHLVESIQETNHLGKAPNVCWLLGTQQQDKDSQYQSKMEPFSDMGEATAVQK)-PKIYFSYAWGdEtderqAMCNEIYSKLSEMDWLEVYR--
DKNSMELGDSIDEFERNIARGDFIVLLVSEKSLQSEHCMKELG-LIYSHCQ-GQqkefvSKVIPVVLADA-
KIGSLMDRLEIVEFWAEhlTKLDSMIKKVGAINAGAESVRSL(QITQSFISSCADSLTWLADLVTERQPELQVDATLE)}*

$4263=239(236):
>gi|1160926286|ref|WP_079767766.1|
{|0(281)|<Actinobacteria(B)>}hypothetical protein [Mycobacterium abscessus]
{(MSSGRTSETLALRCRRPGKCWIGTVSEKPKLCHPSTRTASSPGPRLPQKVWNVEAAILHAPYAWTVTAPSGGDVTRPN)-
PTCFISYSHDsEAHKQAVVELAQVLREQHGIDVMIDRfvEHQPPAYWPqWMVQQIEQSDFVIVVVTETY-
AERFKMQqEDPRVGRGVTW-EGAVINSHVYSNF-
GRSSKFIPIVFNENDRAhiPFPLSETSSYVVPGLEPQDLEGL(LRQLNQVPVVVPAPLGQP)}*

$4264=198(193):
>gi|1160684594|ref|WP_079545292.1| {|0(283)|<Firmicutes(B)>}hypothetical protein [Christensenella massiliensis]
{(MGFMEDKIPK)---IFISYSW--VV-
SERVLELAERLVANGVDVVLDKWDLKEGQDKyaFMEQCVVNTGIdKVLLICDKSYCDKansraGGVGdEtVIISPEIYNQVnQEKFLpIIFEVDEY-
NKPYIPAYIKSRIYFDLSTKDDQYEVEYEKLLRNIYNKPL(YKKPALGKKPDWLENDTVDLSSIRDLIRQTRGYTGGNKTKADF)}*

$4265=202(204):
>gi|1162578602|ref|XP_020426506.1| {|0(733)|<Streptophyta(V)>}TMV resistance protein N [Prunus persica]
{(MAAEANSSPSSSSSSSCRDRRW)KYDVYLSCSG-DDMPRSFTDPLDVALTGQGIRTFR---FRNGGA-
SnppvIFLKAIENSRFSILVLSRKYAHSIRCLNELEKIVECRKE-
MGQAVLAVYFDVDPAETFTILFVALDLKRYKAQNIELIVKVLRAELRSSLPISL(HEWNSKLLEKKKSDAVKMIIQGLNDNEKRIFLDIVCFFKGME)}*

$4266=271(300):
>gi|1162533776|ref|XP_020415536.1| {|89(243)|<Streptophyta(V)>}TMV resistance protein N-like [Prunus persica]
{(WKSDLHHYFEAFDDPQVALQEGCPKELEGREDSWAWLCAHFQAPAFVSKIFFLRHKHKLNIQMAATRAQQDASPSSHWCT)-YDAFLSFRA-TDTRKGFADHLYRALeVAG-
IHTFRDDDEIERGANIlaELQKAIQESRVSIIVFSKDYASSRWCLDERVTIMDRRET-NEH------------
----
MRFNKETDkvEKCRKALRDVADL(GGMVLGDRSESQFIQEIVEVIGNKLDHTWNRRLRVDPYVVGIDYRVKGLNMWLEDGSSDVGVAVVYGMGGIGKTTIAKTA)}*

$4267=158(161):
>gi|1167492381|ref|WP_080005790.1| {<Firmicutes(B)>}hypothetical protein [Enterococcus faecalis]

{(MFAEYPKSYYERAGYSFDTPR)KKKLFVSYCHKnKQQVHKIIDDLRSYGLDF------
WLDEEQIDEGDhlldRIDAGMREADIPIIFLSRATKESLFAQQELKTFFLqiinqasIKKK-
WFIVKLDSINPDDILMGLGGFKYFDIENHsvED--LaNRLKEK--FEHL-------E()}*

$4268=197(219):
>gi|1167634180|ref|WP_080063459.1| {|0(672)|<Firmicutes(B)>}hypothetical protein [[Clostridium] hungatei] >gi|1162540076|gb|OPX45117.1| regulatory protein AfsR [[Clostridium] hungatei]
{(M)-----------------WIAETLENNDYS-TIIQAWDFKVGGSFVNDMNEAIKICKKVILVLSEKYLK-
SDYCIaEWQNFFVDD-----PigkntLIIPIRIDDVKPE-GLLKARTYIDLCG--ITEAAeAAK--
ILLEKVVNGSAK(RKSRGFPGAVISFPGGLPFNNLPNRNIYFTGREKLLKSIESAFKQKKGAFLTQAISGLGG
IGKTQIAIEYAYRYAGQY)}*

$4269=137(140):
>gi|1167636760|ref|WP_080066039.1| {<Firmicutes(B)>}hypothetical protein [[Clostridium] hungatei] >gi|1162537341|gb|OPX42505.1| hypothetical protein CLHUN_36300 [[Clostridium] hungatei]
{()MINIFLSCSSKdYDFIKSIISHMRLLPDKYrLIFYMQPYNINTSMDF--
ILDRLRESDLFVLFISRNSLESEFVRLELDEAIRLLMN-SKIKeILPVVI-----
DESINVNEDLRIPRAIvaRKVFYSNNPGKVVKMLEEQILKY()}*

$4270=226(212):
>gi|1167828415|ref|WP_080140510.1|
{|0(127)|<Proteobacteria(B)>}hypothetical protein [Bradyrhizobium sp. BR10280] >gi|1166978777|gb|OPY93576.1| hypothetical protein A5906_17405 [Bradyrhizobium sp. BR1
{(MASLAPEFKHD)---
VFVSYAHGdKrkkgkALLKRWSQALASELEEELALDWDDpkvfldtRDIDQHGELDkEIREAISASATMLVLM
SSPYLQSVACKQeralwfdkisQEAVAEARDRV-LFARVLPI--ADS-EW-PTEFRDASGGP--
PISRWFFEQPGGPRTRPFGWPDPT(GTSGKFRDNVVDLAGDVSVQLREVKESIERKKREAAHAEKMRAASGQA
LYLHARQRDPSQW)}*

$4271=208(219):
>gi|1168759201|ref|WP_080329814.1|
{|0(81)|<Proteobacteria(B)>}hypothetical protein [Enterobacter cancerogenus] >gi|1167730062|gb|OQD48028.1| hypothetical protein BWZ29_18750 [Enterobacter cancerogenu
{()MP-IFISYSHQdKEFVDKLA--LQLINRN--
INIWLDRwELSVGDTILdKVQDALDGASALLVILSKASTSSEWCKKeLSSGLLRELEE-KRVVVLPVLLEDC-
TIPIFARGKMYA---DFR---NNFD--
HGFTQIIESIAKI(ITPSLNRSIQGPECVTDWSIDHGDVNGQLAIVLNYVQHPLKLPFTCLINIEILANEYAT
DIFHEIAKKNGIDFAKMNVI)}*

$4272=215(237):
>gi|1168764065|ref|WP_080334111.1|
{|82(0)|<Proteobacteria(B)>}hypothetical protein [Burkholderia cenocepacia]
{(TPVDGQIEEMKKSMVACGARYRSDLVNDDLFSELANKRFQYAATAGPDSIDFVTPSLFWYKDRSEVASTIS
MRPLGKALP)---VFLSHSSHnkPFVED-LIPYL--
NGKGLPVWYDKVNIQYGETIVTAIQSGIVTSGAVIFFITPEFLESSWCKLEMEGFLSRFGGGHNILILCVIAK
DVShDKLPiflqmKKYVRLSESLS------------------------V(EHVANELIPALKDHLKL)}*

$4273=212(219):

>gi|1168827866|ref|WP_080397630.1|
{|0(667)|<Proteobacteria(B)>}hypothetical protein [Pseudomonas syringae]
{(MQNKMLAKRHHNSLLFANREKIISMHRKRMESKMKF)----FLSHSSVdkHIVGKVY------
EQLGASLC-
HYDvaTFDNTSFLPAQIYEALNESSHFILFASKKALNSDWVKGELRVAFSQWMKAKIRHAMVFILDEA-
ERSEVPEWLQDYVIIEHpSPHHISCRILSAFDEWESKNENS(PPFYRFQEAMQLEKRVSVEPSKLPTTLMLCG
IDGYGRKQLVNE)}*

$4274=270(222):
>gi|1168901612|ref|XP_020493528.1| {|293(0)|<Chordata(M)>}LOW QUALITY
PROTEIN: interleukin-18 receptor accessory protein-like [Labrus bergylta]
{(TPQHLNNTYTCIATNSVGNSSITIKLKKKIKVQWPSLVGYPIASLLLVGGLGIILHVKWLELQLIYRSHLQ
SGKHDGEEK)EFDVFLSFVwsdsstetegvltlsSQsgpdtdeevclssfdllnsegkvthEPLEV-
LFPQVLEEQWGYRLCLLERDVLPGGAYTNDVVFAIQRSRMLICFVSADYLsNSNAVF-
VLESGVQALLQNSSPKLLLIWanggsAALL-
QPDPPLPVLVQRALKVLpSLDWTSGKPArasnNFWKSLRKAMPDH(RE)}*

$4275=292(243):
>gi|1168961111|ref|XP_020463991.1| {|297(0)|<Chordata(M)>}interleukin-18
receptor accessory protein-like [Monopterus albus]
{(TSQHLNHTYTCIASNTVGNSAVTIKLKKKIKVKWPSLVGYPIVSLLVLAGLGIVLHVKWLELQLIYRSHIH
CGKRDREDK)EFDVFLSYV-
WshpsaeadgcvslspqsgpdtdeEaclssmnplntkegqstQTPLEvLLPRVLEERWGYHLCLLERDVLPGG
AYTNDVVLAIQRSRMLICLLSADYLcNSNAVF-VLESGVQALLQNSALKLLLIWTnrasaSLI-
QPNPPLPTLVQKALKVLpSLDWTSGKSAratcNFWRSLRRAMPNC(TEKNWFSSDHANDLPVTSIQYIY)}*

$4276=215(220):
>gi|1168836629|ref|WP_080405217.1|
{|54(0)|<Proteobacteria(B)>}hypothetical protein [Burkholderia ubonensis]
{(VNLQDDPTKFENWVRDKYPEKLKRHNVFLNSLMSFNLMTFIDEPMIDLAFIDEGNGPLFHWIERDLLESKH
PQYKPMKPA)QSRVFISHSSKdKElIVNPINAYLQAQGIA------
TWLDSYEIDYGDniyvKVNEGIEKSEVGIFVLTNNFFDS--SSGWPITEFSTFFM-
ELMKGSKKVIMLNAGVDPEKIHSMMKAYRYL--DWNDGKGLPELGNAIKRKLSP()}*

$4277=198(197):
>gi|1168873620|ref|WP_080425210.1|
{|0(178)|<Proteobacteria(B)>}hypothetical protein [Burkholderia
ubonensis]
{(MATRRTPAKKKLPATPIDSSSDNGELLILSAHKPL)---
VFISHDSRdADLAEAFANLLVDVSGGTLKSFRSSDKkgGTGIEFGTewynAIMSRLSEATDVVALLTQRSLDR
PWILYEAGVAKGRLGT-NVFGVALGVPLEKVSTGPFGQFQNCEADEDS--
LTTLLLQLLERNPDASPREEA(VRLQVSAFLKKTADANALGTGS)}*

$4278=191(183):
>gi|1168764780|ref|WP_080334826.1|
{|0(127)|<Proteobacteria(B)>}hypothetical protein [Stenotrophomonas
maltophilia]
{(MDTPWRED)-ASVFISYAwggqfHAkEWIRSDIVRNLDWKYR----
VFWDRDSVGFGQNIDkLIERAFAERPLIVfCLCDSDFLSAmsrpGSGLNrELAMLEKHVDQ-
SDVRIVPVILDAE-
ARTALPPVLQGRAYLDLteLHSESIPLGEFLLAATDGSTQAQ(ITQGVRQRLEYHALSKRALCWLQHHGVRFT
GSART)}*

$4279=213(224):

>gi|1168785143|ref|WP_080355178.1|
{|0(79)|<Proteobacteria(B)>}hypothetical protein [Stenotrophomonas maltophilia]
{(MGRWI)MP-VFISYSHQdKDFVDQLAVQLV----
QKNVHVWVDRwELSVGDSLIdKVQQAVEGASALLVVLSPESVQSEWC-KrELSAGLLRElEE-
KRVVVMPVLLRDC-DIPIFARGKFYA---DFR---TNFD--
EGLKAVVDGIAKV(TNASLSRVQEDTYHVDWSISSGLDDNGNVAIKATFIQIPKYEPYTCLTEVVIIGDDAAS
QEFKEIAAADNAEIAKLRVW)}*

$4280=157(153):
>gi|1168846324|ref|WP_080414680.1| {<Proteobacteria(B)>}hypothetical protein [Burkholderia ubonensis]
{(MAEG)RKHVFISHVHEddhrlgP-LKDLLGRAGME---VRDGSINSDKPNGASNPDyiksniLapRIEWA-
---SVFVVLISPETKNSEWVTWEIECAQRMG--KrivGVWDHGEAQCD---
VPDALDRYADAIVGWQGERVKEAIEGKINNHETPDSELTQ(ARPLPRYTC)}*

$4281=251(256):
>gi|1171559328|ref|WP_080602288.1|
{|6(16)|<Cyanobacteria(B)>}hypothetical protein [Microcystis aeruginosa]
{(IPGLLKALKDSNKYVRSQAAEALGKISSETAMTELIKCLKNPDFVTLNNGDTLSQAREALDTIQNKLKYYH
PLSQPMNQQ)---VYISYNW-QEDSNEMANQLVQAFEAKGIEIIRDKTHTTYKD-
SikNFMRQIGQGKCVVVVISDRYLKSENCMFELVEIARNGDF-Y-
QRIFPIILPDARRIHKAIERLQYIQHWEGRIQELENAMHSGGLANLQGITDD(LNLYTEIRHRIASLTDILKD
MNTPSIDLHGQSEFAA)}*

$4282=195(201):
>gi|1171039195|ref|WP_080589088.1|
{|0(334)|<Proteobacteria(B)>}hypothetical protein [Rhizobium leguminosarum]
{(METHLLLHSVFKIRHHLKGLLNQKEGEPL)----FVSHAkldG-
DDTATAIVDFVNSRNNDVPLRTFYDAKELSPGDDFaeEFKEAINSGTL-
LAIVSDAYDSRPWCVFELTEAKRAK-----
RPIVLADVGRVRISRTYPYGANVPKVKINPVTGETAWIEPLLVECLSEGLRC(DLFNLEVSGRLGKPDDDVLV
LPRPPELFDLID)}*

$4283=276(281):
>gi|1169088733|ref|WP_080443888.1| {|51(0)|<Firmicutes(B)>}hypothetical protein [Clostridium botulinum]
{(IVFGVKDDGKNLTIKNFPFNIKEGDIRNLLDEHVKFECVIFNYMEHHLAYINVNKNDIVVKFNNVAYVFNN
KMEVEKMVV)-KKVFLSYCHKdSCIADIIENKIMEIAKSKIEI---SRDTRKVKYKDsldeYM-
QSIKEHDFVISIVSDRYLRSIPCMYEIAELMRDRNY-YDKLLFVIISDEDIKF--YENKDIKIKADIY--
SNNRFDY--
IKywEDEKIKIDNK(VAEFKNPALMLELTEESKQLEIISLNVGTFIARLKDGLGESFQNMLSSDFKEIISIIL
DSK)}*

$4284=260(272):
>gi|1169111436|ref|WP_080463512.1|
{|12(34)|<Actinobacteria(B)>}hypothetical protein [Actinomyces sp. pika_113]
{(AAILECTTRLDELDATVVEVILTEYGMKSVAPVGRHIPRESLISVLHAADTQTLLGLAEFLMHSRALLLDE
ELPFHGRGL)--RLFFSHLSRSHGFVESVSTILEDFRIE-VLIAHRDIRPSHDWQAFLASGLDQCDGLVA-
FVEAGFRESEWCDqEVGWALGRH-----RPILSL----FReqTVPYGLAGRIQGQSYE--
GDTPSTVAAKIISWAGEEDSLR(SRLLESLVQTLETSPNFDTTREVVKLLRELPATTLSTDLVDRLLAAATSN
PQ)}*

$4285=169(174):
>gi|1172436754|ref|WP_080812595.1|
{|0(424)|<Cyanobacteria(B)>}hypothetical protein [Halomicronema hongdechloris] >gi|1170997298|gb|OQM35437.1| hypothetical protein XM38_21630 [Halomicronema hongdechl
{()MTDVFISYSRKdKEFVQILNRALV----
ESKYDAWVDwEDIPLTADWWaEIQAGIEAADTFIFIISPHSIASTVCRQEIDHAIEHNK-----
RLVPIVRQEG-
FDMASVHPAISRTNWLFfqEEHDFKLAFQSLVNTLNTDLVHI(KAHTRLLVRALEWEKKHRQDDYLLRGGDLA
EAER)}*

$4286=171(176):
>gi|1172433292|ref|WP_080809242.1|
{|0(769)|<Cyanobacteria(B)>}hypothetical protein [Halomicronema hongdechloris] >gi|1170998803|gb|OQM36903.1| hypothetical protein XM38_11550 [Halomicronema hongdechl
{()MTDVFISYSRRdKAFVQVLNQALV----
ESKYDAWIDwEDIPLTADWWeEIKAGIEAADTFIFVISPDSIASKVCRQEIDHAV----A-
NNKRLMPIVRREG-
FDMALVHPALGKANWLFfkEEDDFNVAFQSLVSALDTDLTHV(KEHTRILVKALEWEKKRRREDLLLRGQELE
EVIQWL)}*

$4287=171(176):
>gi|1172433298|ref|WP_080809248.1|
{|0(823)|<Cyanobacteria(B)>}hypothetical protein [Halomicronema hongdechloris] >gi|1170998805|gb|OQM36905.1| hypothetical protein XM38_11565 [Halomicronema hongdechl
{()MTDVFISYSRRdKAFVQVLNQALV----
ESKYDAWVDwESIPLTADWWeEIKAGIEAADTFIFVMTPDSIASTVCRQEVDHAVE-----
NNKRLVPVVRREG-
FDMSLVHPALGKHNWLFfrEADDFDTAFQSLVATLNTDLAHV(KTHTRLLVKAQEWDKKQRRDDLLLRGDEFV
IAENWL)}*

$4288=183(187):
>gi|1172437731|ref|WP_080813550.1|
{|0(121)|<Cyanobacteria(B)>}hypothetical protein [Halomicronema hongdechloris] >gi|1170996844|gb|OQM34996.1| hypothetical protein XM38_24570 [Halomicronema hongdechl
{(MNTAPTTSYRSPFVST)----FLSHS-SadsdlaAAVTKRLGR--------
RGVLTWLDKNELLEMGSLdgVLKQAVQQQATLTLFLSEASLKSEWCRDELRWAIEaQAGTDhllPVYLGDPLK
LI---
KSHPLLRERFLHADGNRVNQLGYACQQEAGEPNPDAIAQK(IAATAYXRSIPDPWSEVVIVLDQRGSGPRRG)
}*

$4289=218(230):
>gi|1172431909|ref|WP_080807859.1|
{|0(75)|<Cyanobacteria(B)>}hypothetical protein [Halomicronema hongdechloris] >gi|1170999381|gb|OQM37474.1| hypothetical protein XM38_08860 [Halomicronema hongdechlo
{(MTSYQNKSDDY)---VFISYRRTDfdWVKAYLLPKLKV--
WKVNYVIDHQDFLPGQRIAGTIRDYIRSSRKVIFVCTREFLDSEWCREELETARAEDPGSIQGKSIPVVLDKL
gVPDLLSDI-----IWcDLS------NDQ-NdanEWRKLCVSI--

G(GSWDGYRAIQDLVAAAIGAEYSVYKQLPKIKLKKLENHFDVNGGAYKRINNLLVQHSQKKWIISNEHNPST
YLILDIQV)}*

$4290=154(187):
>gi|1172294400|ref|WP_080677679.1|
{|0(76)|<Proteobacteria(B)>}hypothetical protein [Bradyrhizobium japonicum]
{()----------------------------------
MDESEIQLGNSLrvKIDAGLVHSRFGIVILSPSFFSKVWTKSELDGLVA-
KESDGTKVILPIWHQLSREDVQKHSPILAGRLAARTSEGIRAVASKIIKVVSGSGPTS(RKGTPIFAGRLTKK
KLLELPEGSFFLSNTYNADLTPALAKEIPSHHA)}*

$4291=152(152):
>gi|1172423029|ref|WP_080798989.1|
{|0(161)|<Actinobacteria(B)>}hypothetical protein [Gordonibacter sp. Marseille-P3078]
{(M)QFDVFISYAW-
GGPENIahreWVRLLAAHLKQIGFHVGIDSEVDYGEDLTGFMRKVEDAKHVLMIVDEGYVERADNHPDSGVGR
ETK---
TIRDVVDGRPSNWLSVLFVRNEKRLLPKWMEGRDPKYFDFSYRDRDGSFPGSEQ(IDDLWRWLAGL)}*

$4292=215(220):
>gi|1172305397|ref|WP_080688676.1|
{|97(0)|<Proteobacteria(B)>}hypothetical protein [Aeromonas hydrophila]
{(QAIDITANEYMDIDISLLEGGRYSVTNFIAARPVYIYAESDLSPEEESKITSLGSRYSCEVILRGRNYSME
RLLKDTPVA)----FISHDS-ND-
KASIARPIAEGLTKMGLSVWFDEYSLKPGDRLreSIEKGMKDCKKCILILTPNFLKNmGWTAAEFNMiftreI
IEEQ-----SLVIPVWAGVNKKE--VYEYSPGLLNVLGAIWPEDASKQEKTIIDIASSLDV()}*

$4293=197(204):
>gi|1172256903|ref|WP_080646242.1|
{|0(285)|<Actinobacteria(B)>}hypothetical protein [Salinispora arenicola]
{(M)----FISHTQDDAHWARRLAEGLELRGVQ-
TILQERDIEPGQIRLRRIEEAIATARAAILVLSASALRDPR-LYdEYAALLGqsaeRG-----
LRLIPALHGSMSiQMPPLAGTRLWADFRG--
LGPAEHDDKVAALAAVIPNTPC(DEPQVVGGRAIPMEVLEAARSARTEPHDTPPPPAFVITYARPDAEYGDRL
VEWVTNSGLPAWS)}*

$4294=173(174):
>gi|1172283833|ref|WP_080667113.1| {<Proteobacteria(B)>}hypothetical protein [Bradyrhizobium elkanii]
{(MSPCGNGHCNADSAIVRIQFWAVSF)AVRIFLSFPSELESQADSIAQSLRNCDHD-
VFFSHDDLPPGDSFDARVEKAIESSDFMVFLISPESVTRGRYTLsELAFARnkwpNPS-----
NRMLPVMMAPTPldQVPTYLKAVTILEPRP--ASMREPCRSRAMPRTRPRPPRR(SATIPAWDF)}*

$4295=242(236):
>gi|1172295695|ref|WP_080678974.1|
{|0(256)|<Actinobacteria(B)>}hypothetical protein [Salinispora arenicola]
{(MRPDEVGYLVADNNAW)---VFISHTQDDAHWARRLAEGLELKGVE-
TVLQERDIEPGQVRLRRIEEAIGTARAAILVLSASALGDPKLYDeY--AALLHQ-----
SaerglRLIPALHGSLRiRMPPLAGTRLWADFRG--LgpteydDKVAALA--
AVIpdpppvepRAVSGRAIP(VEVLEAARSTRTEPPDAPSPPAFVVTYARPDAEYGNRLVEWVTNAGLPAWSI
ADLAWGSRWVYEIRQRLRHALAVVVVMS)}*

$4296=258(267):
>gi|1172297688|ref|WP_080680967.1|
{|152(42)|<Proteobacteria(B)>}hypothetical protein [Mesorhizobium loti]
{(HPDRMLVLPVRLGDRSAIPPFDDIQSIPTSLFPGSGTSDTRWMRRLVLTVVAHIGNYLRRLEQHEKDPTVP
IDEIQLSRF)--QLFLSHAKRDAmPVVTAIRDHLANNNYG-VdtFIDAKDLPAGLGYEKAFSAAIR-
AGAVVAIRSDAY-SSRPWCRwE---TLEGK-----RLLRPIIVLDLFrdTEPRVNPYGGNVPALR--
LVDPAAANETAQAEMDRAVLAI(MTEAMRIMVWNRRVTSLKEATSKKYPGKDIKILPRPPELVDIANIRI)}*

$4297=244(233):
>gi|1172267350|ref|WP_080656689.1| {|577(0)|<Bacteroidetes(B)>}miro-like protein [Porphyromonas gingivalis]
{(RETNRKKRPIQDLYLSCAQADKDLTESHYIHLGTLDDESKTTARIAAYPLKNGVIDKERVREVSTRPYKHL
SVNKNLATA)-
KQIFISYSKEdQTELETCLQFFKPLEKNGQIEIYYDKLTKFETPIHpEIRKRIVEADCIIALISQSYLTTDYI
LDhELPVFREYNK-----TIVPILIKPC-TFEDDEFlrekyfaqkAQIINLGKEGktIKAYDSitasahRD--
DNWVAVVREFKEK(ILRITKQEVNTDE)}*

$4298=222(219):
>gi|1172298947|ref|WP_080682226.1|
{|0(761)|<Proteobacteria(B)>}hypothetical protein [Sorangium cellulosum]
{()MPEpvrLFISSVPEdAASRAALEAQLKPLLRQRLIEIWHGGKVEAGGEPAaQARAQLEAAEIMVLLVSPD
FISSDRHMHeEAALALARRAA-GEVEVVPVLVRDC-AWDWTEFGKLVPLPKDGpIGGAPDRD--
QAWTEVARGLRRV(VDGVVARRVAAGGWRTLAAGLPSAELCIGRDATVDALAAALCREPPGRALLSGAAGIGK
STVSLAVLHRPEIVTRFGER)}*

$4299=248(255):
>gi|1172299189|ref|WP_080682468.1|
{|0(62)|<Proteobacteria(B)>}hypothetical protein [Sorangium cellulosum]
{(MTRNCPRCGTSRPPSATQCDCGHEFRELQGYRVAVL)-------YAPAdEPYMSQLSKHLAPLAREGV-
NVWHAGDIAPGSNHSaETNLHIDTDDVLIILASADYVASDDCYSrQLYDALLNRNK----IIISVILRPC-
DWLRTPIASRDIYCSSRkgpaiISKLANPD--
DAWIEVANHVRKR(LGEERTYEPWYSSPSGNISRAERLSSEKYAILENTSQNGTRVYRLGEFNTIGRSIKNNI
AIVHPSVSRQHCIISMRTRA)}*

$4300=225(228):
>gi|1172311040|ref|WP_080694319.1|
{|0(655)|<Proteobacteria(B)>}hypothetical protein [Morganella morganii]
{(MRLTLNSKV)--KAFISYSRDdENHRDNIIRYLETNKD---
LEIWYDKKLLGGDKFNdIIDNKIKECDIFIFLISQSFLNSKYCNEiELGGALERKYN-NDAvKIIPVILDYC-
TFSSSVLSEYNALPDDAqpAASYENIN--
KAYLDIAKGINKV(ISEIKGNNVVVTCAQNVNSNKKKVSLNSVFEDYLSDLGFTIQHRRKEYLYLEDLFIYPD
LKKYSYQFDNYDLFVNSKIA)}*

$4301=241(236):
>gi|1172380258|ref|WP_080761497.1|
{|0(96)|<Proteobacteria(B)>}hypothetical protein [Pseudomonas fluorescens]
{(MSLPRYRRLGKMTEVENRGH)---IFISYAwgngFEnKEWVRQ--RVLTQISWNHDV-
FWDRDSIAYGDSIDgVIAKALSKRPVqILCLCDQDYIRAAqqkgSGLHrELKMLAEIAYE-
PGVRIVPVILEAgC-A-DQLPEPLIGRLYLDLqpLHQLNiDLG--
TAVMEVAEGRTPA(QVQSGVNARLAAFKLRERALKYFQTRPVEVWGNGRNHEVTVYREGTPPTLLQPAPWMWE
SNNWNYMLSDDSPTFCP)}*

$4302=187(191):

>gi|1172435143|ref|WP_080810984.1|
{|0(270)|<Cyanobacteria(B)>}hypothetical protein [Halomicronema
hongdechloris]
{(MIEPMPHSSDNRSRSHVSTAFIRSND)---VFISYSRKdKAFVETLEVAFRQASRDPWID-WDD--
IRKGEDWWqSIQRGIEQADTFVFILSPDSVASEVCRDEVEYATQCHKR-FLPLVRREGFEMS-
RLHPSISRHNWLFFRETddFKAAFQELLTALDTDLDYVHAHT(RLLVRSHEWQTKGCDRSYLLRGTDL)}*

$4303=207(218):
>gi|1172469884|ref|WP_080843669.1| {|0(568)|<Firmicutes(B)>}hypothetical
protein [Bacillus gottheilii]
{()MSEIFISYSTKdSKVAEQLYT---
SLKEQGFTCWFAPNDVPPGGDYAsRITQAINKFRYILVLVSKHSCISEHVQSEVALAVQ-----
NKKIIIPFRIDDflS-DWMKYYISRVSWIQADS----ENMD--
SSIETLIQKIKGM(DNLSDSDSPVHSHYGKARQEVKNLKASSTQIYQIQQQEIAKVQHLFIPDPTFESGKSLL
GEQRVLILHSNQHTGKYTA)}*

$4304=179(183):
>gi|1172334937|ref|WP_080718214.1| {|0(667)|<Firmicutes(B)>}hypothetical
protein [Lysinibacillus sphaericus]
{(MEEEFFIFKA)----FLSH-T--NSDKEFVEKVAGKLGRE--RVVFDKYIFPEGEDFrdSIMKYLKESDL-
FVLFASKESLKANWVKyEIDNAemlLIEEKLKkGLVFLIGDDISHS-
QLPKWCQKSLVKRISKHNLVADIIEEKLLEINPKYSDVYI(GRGIEKEKFTDHLFKSKIKHQVLVFYGLNGIG
R)}*

$4305=218(222):
>gi|1036638318|gb|ANK58237.1| {|136(0)|<Mollusca(M)>}myeloid
differentiation factor 88 isoform 2 [Haliotis discus discus]
{(FKELSPGLSGAAVQTQAVEQGDVRNQEMCASGSSESNPISRPIAINTRREEKSSTVTKSGSRPSNRKILST
DDDGTTCAM)-
YDAYINCaENDILLAKEMLNTLESSPHNLRLFLPERQLMPGGCKYEDIADVLEDrCRRkVVIILTRNYVDSEA
CQFVTNFARALDPGAREKQIIPIIYEDI-DETPRMLIGLQFIRKQR----DEKRG-
WFWSKLREAIKNN(QR)}*

$4306=135(145):
>gi|488428192|ref|WP_002497577.1| {<Firmicutes(B)>}MULTISPECIES:
hypothetical protein [Staphylococcus] >gi|394249888|gb|EJD95095.1|
hypothetical protein HMPREF9987_04709 [Staphylococcus epidermidis NI
{(MLGKL)----FVSYRADDeGSKYKNLLVAwsknpnknfyHLKFEDT--
SIGISINSTDAIYIKrRIKDKIKESNKVICIVGKNT------HTSDWVNWEIDTAKeLNKPIVAIkIDRF---
---------YHTPTRLYNKQVKWAYSFTYESIKKALLEI()}*

$4307=223(217):
>gi|488737177|ref|WP_002660569.1|
{|0(348)|<Bacteroidetes(B)>}hypothetical protein [Saprospira grandis]
>gi|395321814|gb|EJF54735.1| hypothetical protein SapgrDRAFT_3088
[Saprospira grandis DSM 2844]
{()MVNVQILAVDSqQEHQATLEQFLAPLKASGQIQIVGEHQENPFEAYEeK-DLAVN---
ILILLLSPAYLEDAQLEAaPLAQLLARKTQ-KRREywVQPILLEAC-
DYGHKPYADYPVFPmpngqKTAlpVLdpSWSSTA--
EAWKRAVEGVELG(VTYIEQQRSNAPTAVGQQQKLLLLTANPKNTQALDLNIEINAIKNALRRSDYRDNFEVE
LSLDIQKKALLETLLQEK)}*

$4308=231(228):

>gi|488737789|ref|WP_002661181.1|
{|0(262)|<Bacteroidetes(B)>}hypothetical protein [Saprospira grandis]
>gi|395322217|gb|EJF55138.1| hypothetical protein SapgrDRAFT_3502
[Saprospira grandis DSM 2844]
{(MYIKATYTL)--
NIFIAYHPDdRSKFEELYQHLRELDEQNetyqVNKIWYSKTKLESSDYEQLAEMMEAADLLLLLLSEASILSP
LFSGrNLQTALALHRK-EKTLVVPIILNTC-WWEDTAYRELAVLPRAGlpIYDSEDvKN--
ELFDQLVQDLNQK(LLKVRQQKFDLEDTFKDLLEEADALFANWEQQPERLRSALPLYKDAVEHWREGFVPHRE
LLEAKIDRCHREIDFRHYAR)}*

$4309=178(170):
>gi|1173294139|ref|WP_081152727.1|
{|0(26)|<Bacteroidetes(B)>}hypothetical protein [Niastella vici]
>gi|1172824143|gb|OQP60590.1| hypothetical protein A3860_32775 [Niastella
vici]
{(MAY)KYDIFISYRR-
DNlTRKwietHFVPLLehHINLELGRIPVIYIDTLLenGTTWPIALGNALGASRTIIPLWTKTFLNSVWCSCE
IGHMLERERKFGFRTIQNPGGLIFPTIIHDGETMPVNLTTIQKIEIQECYNVRMSIDSPKAEVLD(DRLRPLG
KDIADAINNAPVWQQDWQIV)}*

$4310=186(188):
>gi|1172911291|ref|WP_080980800.1| {|0(63)|<Firmicutes(B)>}hypothetical
protein [Streptococcus pseudopneumoniae]
{(MLEDR)---VFFCFSG-KD-
RLVYAQSLNFHLKNLGIEVWYDYEKLylGDDGDYvNIEEGVEQSKIFLLFISTQLFQSTGAVLELDAIKAKID
KYQGIVIIPILCEITSSQIPKKYNWISKYIYGEMKLDISSGTYDLSIDILKRLLYE(QLKSVEIKNLLTLENI
TTDKFINIIIDKYNYIDKNCFSTRITM)}*

$4311=204(197):
>gi|1173286870|ref|WP_081145465.1|
{|0(236)|<Bacteroidetes(B)>}hypothetical protein [Niastella vici]
>gi|1172830229|gb|OQP66524.1| hypothetical protein A3860_13650 [Niastella
vici]
{(MPIPDEWLKYAPKPKALGANE)KWNVFLSYRS-V---NRFwVLNLYDVLKElD-
YKVFLDQYELLDGDELiaKLQDGLAMSQAGVLIWSTAAADSKWVFKEFQTM-ETRAT-
NDEKFrfvpvkldgqqLPVFAQNRVFEDFSSYPDGPNGGELL-
RLVYAITGQTMSAEAIRFANDQ(TTLTKDIITELNAAKIIGDAEGIIKLAKANTLPWRT)}*

$4312=175(184):
>gi|1173114728|ref|XP_020528643.1| {|0(424)|<Streptophyta(V)>}LOW
QUALITY PROTEIN: TMV resistance protein N [Amborella trichopoda]
{(MA)---VFLNLCD-EDTRKCFTALLNKALIDScISTTFFSPAQQQ----
PSIVqEAIKECAVFIAVFSVNYASSFSCLHQLCYLLSLPR---
RRTILPVFYDVDPSHSNDWDPWNGLAKEVFDKIYSKFHGSCFLSDIRESSKTN(GVVNLQRQLLKELFDEVDP
SIFNADRGINVIKNKIGSKKVLV)}*

$4313=212(220):
>gi|1173146532|ref|XP_020518485.1| {|0(292)|<Streptophyta(V)>}TMV
resistance protein N [Amborella trichopoda]
{(MA)---VFLNFCD-QDTGNTFTAHLSKSLIDSgITNFFFSHTRKQPSD---
IQRAIEECEVFIAIFSCNYASSFFCLDQLSYLLKLPR---RVPILPVFYNVEPSDVGRQKGPFEGAFEDH-
KNNncFDEETLQKSRNGLNEVAEF(SGWDMKNYRYDQISWSNFKNYCSHNRKLQNPKNSGNGSKRHEVVYMVS
EYLLADHDIKGWWVDSGANRHIAISKAVFE)}*

$4314=206(210):
>gi|1172964787|ref|WP_081026357.1| {|0(69)|<Firmicutes(B)>}hypothetical protein [Blautia obeum]
{(MIINKKYTELKILNKGDDTINNCSYSL)----FICFAA-ED-
RYSIVEPLVYHLKNYGIKTWYDRDKLLmgdNREEINLEKGVWKCNYALVVISKFTVDSVCAMEELSIIESKYR
Q-
GKIIVFPVVYELSPNDIPDRLCWIKELIFKEVDRHSGTREVCSHIVCKITGNIL(NNCIHQKVRDIISTSQEI
LPSGVYDIIRSYLQIDHANLNSRIS)}*

$4315=152(154):
>gi|1173202477|ref|WP_081093033.1|
{|0(276)|<Actinobacteria(B)>}hypothetical protein [Actinomyces oris]
{(MSSAELQIDPYGP)---VFISYRHSDGKSIADRVSTLLKSVGV--
PVWIDSEAMQVGHIEkRVDRAIEQTISGAIVISTEDTAHSDVISknEVPGFLQLLDNDPTFPICIINVNDKDA
VREGLDYSAPDRRMRTVTVESDLRGPLSLIKQFGAEEED(L)}*

$4316=186(195):
>gi|1172938239|ref|WP_081002209.1|
{|0(665)|<Proteobacteria(B)>}hypothetical protein [Pseudomonas chlororaphis] >gi|1181637879|gb|ORM49425.1| hypothetical protein B6D51_08165 [Pseudomonas chlororaphis
{()MSKAFLSHSSRdkEFVRpvaDELGSALSHYDE--------
RTFEPTGESAGEILEALSNSSVFVLFLSKNALASPWVKQEIRYAQHMYFEGKIQQVAIFPLDET--
TRDSLDVWLR----
PFVIQILKKPKLVALRLRSILAMND(TPASRRFIGRDKQVSDLKDSLRDKSSSRPSTVLVSGIQGIGKRRLLS
RAYQDLYP)}*

$4317=300(294):
>gi|1173294142|ref|WP_081152730.1|
{|247(105)|<Bacteroidetes(B)>}hypothetical protein [Niastella vici] >gi|1172824144|gb|OQP60591.1| hypothetical protein A3860_32780 [Niastella vici]
{(EQIKIPQIDYFSFGEKLAVVEQGTNDPEGMGFIFSKIAKLLSSDFFDIESFVGVDYYKKKKKEFAESNPSD
KKQDETEYL)-
YDVYLSYSREvyQWVRELLIPALTeylNDELGYNpqIYFDINEFNIGESISINIETAMLKSKTFVYIITDSGL
DSSFLNIVLNSIFKREKDSNRNLLFPVLYsqSNI-QKNDSIPPELrdRQYIDFSnF-KYEDTV---
RSTKLRAQFGQE(VEKLSKAIAKSLLQKAQQKKTEKPGKRKTNQEEGIEELLLLAKEYEEVRKKMPGGNPRTR
VMENIVSKMKSVMN)}*

$4318=214(220):
>gi|1172665304|ref|WP_080937933.1|
{|315(0)|<Proteobacteria(B)>}hypothetical protein [Aeromonas salmonicida]
{(NLLEAEIRFIKKRYNLSDSEIKNRKLNGSIYKRAIYGNLSFIKMIKGEEHSPYLTLCKQVLELDNNPPYFV
RKLKGIFDM)-YDIFICHAS-ED-
KDKVAIPLYDELIKRDINTFIDCFAIKWGDSLvsKINTALKKSKFVIAVISDSSVKKSWPMKELHAVLSSE--
---IGSEETKLLPLMVGDGEALLKELPLLADKLFVAFDNNIEEIADKVETLLKKI()}*

$4319=217(220):
>gi|1173324494|ref|WP_081181993.1| {|143(0)|<Firmicutes(B)>}hypothetical protein [Enterococcus villorum] >gi|1172715063|gb|OQO71649.1| hypothetical protein BH747_02125 [Enterococcus villorum] >gi|1172
{(IHTSENYEEILKIVKKANMSVVDDQGLEEKIEEMSYGYKTMFPVPPHASEELVLALFGFGFSFPSLKEEQE
KGLIKNKKI)KKKVFISYCHKdKKKVHYIVDQLKSFGLNV------

WLDEQEIDFGDnileKVSMGLEECDLALVFLSNYTHKAKGARHELNALFSKiiyqesLKS-
WIIVRLDEIDPNKIYFGLGNYKYFdyQLGAEEM--LYTIVDK--KLKK-----VEA()}*

$4320=256(242):
>gi|1173191221|ref|WP_081082902.1|
{|0(61)|<Proteobacteria(B)>}hypothetical protein [Burkholderia cepacia]
{(MRFQNLEDEEARARRDAGFFASVRIKHSYMLGASMPSDRKPL)---
IFISHIHEeGELAKLLKDAIETeFG-
GFVEVFVSSDHqgiaqGVGIAAGAhfveAVEDGLVNCVAALYLISAKSISRPWVNFELGAVWCrgavqkANGK
-DRIMVLPichsGTSRSSLPAPLNAMNAINGNNSAD--
LEFAMKSLQRVLGVAGAPLRT(DFEALAKHIGEKEKRYTVGDDYRKFVWALSGERPNVELFVETCKEQAALGI
HELTLTVGD)}*

$4321=261(257):
>gi|1173289036|ref|WP_081147625.1|
{|1083(0)|<Bacteroidetes(B)>}hypothetical protein [Niastella vici]
>gi|1172826980|gb|OQP63364.1| hypothetical protein A3860_23740 [Niastella vici]
{(QKIANLEALIDNEACRPLDLYISKDNKNFVSYSQLCGSDGASSINAYEKSSETENLTEKINIVEAHPFQVF
TKAKLKKAF)--
KAVISYSKQdLDHVLKFRQYLEPLREQGLLDrHWFCTLLLAGEEWNeKIKKEFDEADIIFFMVSENLMSTQYV
KEnEIKNAIDKYDKGGEIFIIPILLVPY-NFKgkgKYDLTRFSSLPYSLkpITEFRNQH--
IAWHVVSELIRIT(LERNLKPETTNDQFYPQLDRYLAAIINGDKIADSDKL)}*

$4322=224(220):
>gi|1172903611|ref|WP_080973120.1|
{|68(0)|<Proteobacteria(B)>}hypothetical protein [Aeromonas hydrophila]
{(GETASIAQNLNAIFDVFAGLYIGMGNDLNPTGTSVDSFTMPYAPKILLYTDGLKISMQSLISEFQDMGHQI
EVVDESSMY)-NSIFISYGG--P-DQEAATILNRDLKAVGVSTWFFPEDSIPGQKLhrMMHEGVNKHD-
KVLLICSRESLTRNGVLnEIERVLEREAKEgGADILIPVTLDDYIfnewnperaDIAEQLKTRVIInvdPTSD
NYGQAIEKLVKsLKSSG--------()}*

$4323=175(183):
>gi|1173664352|ref|WP_081346711.1|
{|0(1049)|<Proteobacteria(B)>}hypothetical protein [Nitrosospira multiformis]
{(MGEHVSIDQG)-YDVFISYAD-ADNEvpdsaqagwvTSFAHYLNIGPNVLKKNLFIDHHLKSGDV-
FsdDLIKKVENSKLLVLILSQNYIDSEWCGKELEYFIQAHSN-----------------
DPEKPADVFVVEIFPYDELTGVPPNIDRIRKHQIDAKF(WYKPPNAIAPLLSGYPTPRESGAEDFYWNALNQ)
}*

$4324=199(201):
>gi|1173242150|ref|WP_081103310.1| {|0(54)|<Firmicutes(B)>}hypothetical protein [Streptococcus oralis]
{(MYGILVIINSYRQERDVR)EFQYFISY-S-
RDIYYEFVNNLNNTLNEIGLKTWIDKFEINIGDEIidNLYTVLDKSADYygiIFIFDETFFNKAWCIKELDYA
MK-----
KRIPFFPILYKIKKEDIPNEYTFLKNYNLLTVSSLKETDYIVDRFILLHLNNII(CRNISNFSIDSYVGNFLL
EQIRHTTVNSIDAIFILDNFSQYLR)}*

$4325=136(146):
>gi|1173672933|ref|WP_081355011.1| {<Proteobacteria(B)>}hypothetical protein [Pluralibacter gergoviae]

{(MNI)----FVSYTTRdeN-
VDRKLLERVSEVVSLYGHCYIDLLHNTEKDKQRHVEFMLSQSNLLIVIASSSIFTSKWVQWELDEA-------
KRCRIPIIMVEAK-SNMSNILKNLKSILTTNaYLLSNRRSEKIENSSRNTRNLFD(GNV)}*

$4326=213(220):
>gi|1173232809|ref|WP_081100762.1|
{|308(0)|<Proteobacteria(B)>}hypothetical protein [Aeromonas salmonicida]
{(GLLEAERKYLSLKKGWTPEKLNSTKLDGSYFKKAIYGRLGFIKSIQGEDSSPYLSLCKKAITLDLNPPKFI
KVAKEKLDM)-YDVFICHAS-ED-
KEAIVFPLEKALIAKGVKVFVDSSAIKWGDSLvdKINHGLRVSKFVIAVLSPASVNKAWPKKEINAIMsmeis
TEK-----KKLLP------LLAGSDDLINEFPLMADKLYKKFDDNVDEIVEGLCEMLQEN()}*

$4327=174(181):
>gi|1173230403|ref|WP_081098358.1|
{|0(129)|<Actinobacteria(B)>}hypothetical protein [Rhodococcus
erythropolis]
{(MGSVIV)--KVFISHAAIDAPIAAYLTESLgRNLGLT-
FFLMPRDSPPGLPWIDQIKHGLDQCDVLWSIVTPN-SADRPWISaEWACFWYQS-----
KPTVPLLAGVGFeiLWEPMKAYPSVTLDDV--
TSVTQFLRSVADETKSQPQSGV(VPLAAEIVREVPAIQQRQLVANLEEVFGRLGRDIR)}*

$4328=195(221):
>gi|1173402088|ref|WP_081252977.1| {|0(167)|<Firmicutes(B)>}hypothetical
protein [Bacillus thuringiensis]
{(MS)--------------------WILEDAGYT-TIVQAWDFKSGQNFAVLMHQAASKSRHTLALVSNNYL-
GSQFTLaEWISAFADD-----PtgerqKLIPIRIEDIK-LEGLLKAILYIDLVG--KGHEEAAA--
AILEGVQTKRKK(TTTPPPFPGIIPKNTEVQSFSVEGEWYKPWVESRLKEIEIKGPYHKIKDGAKLVVHLIPI
EEITSSKTYDLKELRLPLD)}*

$4329=214(220):
>gi|1173664593|ref|WP_081346952.1|
{|83(0)|<Actinobacteria(B)>}hypothetical protein [Mycobacterium chelonae]
{(RHNDEVVRQAERARQQNIRVIAEHNRQIDGVNQHNRRVDAQNKAAIADLSRRLNTTRYSPQEEELAQRVAT
AVAAQDARE)-WDVFLSYAQIDGAEvGRQLCAHLQDRGVR-
VWFDEVALKPGVSQALQMDKGLARAHCGVALLTPAYIAGRFWTErELGVLLGKK------
TLIPVLhgVTFKQvaEYSGILPDLVGFETTG--HSVAEIAD--KIAEAVLRPKAA()}*

$4330=218(224):
>gi|1173659430|ref|WP_081341791.1|
{|0(650)|<Proteobacteria(B)>}hypothetical protein [Agrobacterium vitis]
{(MTIVNHTLPILGNPLYRHGLMNSLVEVCRGQMIHLTVVFLHGGAFIMHC)----FLSHSSAdK------
RGYVSILaEKFGDRAIYDAYTFEAGMKTAdEIMKNLNRTDLFVLLISDTALNSDWVKTeIVKSKRLLDSG-
KLKQILPLIIDRK-
ITYRDERIPDWLRENYNlrVVPRPNAAFRIINAAFSKLSIDS(NPKSLRARRLFVGRNDQLKDFEARMDDYEK
PLPCA)}*

$4331=233(236):
>gi|1173683590|ref|WP_081365551.1|
{|0(648)|<Proteobacteria(B)>}hypothetical protein [Citrobacter freundii]
{(MCLFIFSKGEFGMDNKL)--KLFISYCHEdKHHKEQILKYLQP---
NEDIEVWYDKEMLAGDEFDeVISAKIEESDVFVFLITQDFLTSKYCNEiELSKALERkASD-
DELRIIPVVLDYC-TFKRSRLSLYNATPSDAkpISSYQNQK--
QAYLEVSEAIEDV(VKYLKEKKTQSSYEITEETVVEKNALRTNFKLHLLDLGFTIQHRRKEILYLDDLFIYPD
LKRTKYEFDDYDVFVNSKNV)}*

$4332=173(181):
>gi|1173685417|ref|WP_081367338.1|
{|0(207)|<Cyanobacteria(B)>}hypothetical protein [Microcystis aeruginosa]
{(M)--SIFISYSRQdQAYVNKLVEAL----
REQDLPWWLDNKIDYGDQWPrVIEENLRKSQVFMLVMSPRSKNSHWV-NcELALALELKKP-----
IFPLLLEGS-
RWLEVGIIQTVDIQGGKlpPASFFDRVRSELNNKVRSKLNHK(TVQLTAEEWFNSAYNKGESGDYQGAIADYN
QAINIKPDYA)}*

$4333=211(212):
>gi|1173338757|ref|WP_081195524.1|
{|0(106)|<Proteobacteria(B)>}molecular chaperone Tir [Halomonas sp. BC1]
{(MHPK)---VFVSHAS--EDKERFVTNFATKLRENGVDAWLDKWEMLPGDSLvdKIfEEGLKEAQA-
VIIVLSNYSIKKPWVReELN--ASIVAKLsKGTKIIPVILDDC-
EVPQSLTSTLWesIPDLKNYDQNFKRVLSsIFGATDKPALGKA(PAHTISVYHEIGGLTKADNLVLKESCEYA
IENDDIMINPESLFGDGEKLGFSRAEIKDCIEVLEQNGY)}*

$4334=204(208):
>gi|1173603355|ref|WP_081315189.1|
{|0(121)|<Proteobacteria(B)>}molecular chaperone Tir [Pseudomonas putida]
{(MLIIRNHQNTRMDMSPK)---VFVSHAS--
EDKDRFVLDFAKQLRAHGVDAWLDKWEMLPGDSLvtKIfDEGIKEAQA-VIVVLSKNSVSKPWVKyELDAA--
CVKRIsTGSKLIPVVIDDC-
EVPEALKSTLWESIEDTASYQSSLERIIASIFGFSDKPAL(GKPPAYVGSFGVSVAGHNNVDSLVCKLACDFA
MNTGSRHIDISAFCEDGEL)}*

$4335=155(152):
>gi|1173543704|ref|WP_081288320.1| {<Actinobacteria(B)>}hypothetical protein [Mycobacterium asiaticum]
{(MSAL)-HNVFISHRHEddrflSQLRDLLARHGCEIHDSSV------
NSSNPNGANSesYIKSILAdriqRSGKIIVLVSPDTKNHEWVDWEIEY-ANRFPDKriiGVWAPGAAQCD---
LPSGLEEFADAVVGWNGQAIIDALNGADRWETPDGSVPAA(RSIPRAKC)}*

$4336=250(256):
>gi|1173835223|ref|XP_012079710.2| {|0(909)|<Streptophyta(V)>}disease resistance protein TAO1 isoform X1 [Jatropha curcas]
{(MSPDLKIPTFTVASNSMSSLMASRSSLLPQRSNFGC)KNHVFLSF---ISANNNFVTSLHNELSlKG-
INIIKDE-----GKSLetD-FTAMEESLFLVVVLSENYISSAECLDKLVKIIDfSETT-
TGHVVVPIFFNVDPSDVKNQRRMISEAFTRH-
EENFKEEMEkvQRWRQVLTKVATI(CGWESQQWEEMVLIEQIVTDISDKLDDASSNNTNDFVGMGSHIIEMEK
ILCLESNVVHMVGIWGMGGIGKTTVARVIYDM)}*

$4337=241(243):
>gi|1173835219|ref|XP_020537381.1| {|0(960)|<Streptophyta(V)>}TMV resistance protein N isoform X1 [Jatropha curcas]
{(MASMSARVVSPSSLLPYSSTSDW)KYPVFLSFTNTEQNQTNFVTSLCRELSLK-GISSIKDEGRSLEKE-
QTCEAFEESRFLIVVLSEKYVSSVRCLDLLAKVMDFCGR-MGKFVIPIFYNVDPCDLRNQTGRVAEAFIKH-
EENFREDVEkvKGWKDALTKVASI(CGWDISQWEEAILIDKIVRDISDKIVYKSSSDTSDLVGMGSHIKEMEK
KLCLDSNAVLMVGIWGMGGIGKTTIAKIIYGM)}*

$4338=200(195):
>gi|1173936886|gb|OQR87763.1| {|0(696)|<Stramenopiles(E)>}NHL repeat containing protein [Achlya hypogyna]

{(MAPF)KYHVFLSHDWGtDEAGrdnHTRVSIVNDALKAKGIVTWFDGDRMAGNIKKKMADGIEGSALAVIFI
TQRYmekvngsNENDNCQLEFTMISDTQ---TRARMIPI---
VMEARMRAQKEWHGAFKLSLATLLYMDFTGDALDEATRDLVDK(VQNMLADTNLFPNQADVQIVETAVIAKSA
VSAPNTGTGWVAPADTPPRPTA)}*

$4339=260(254):
>gi|1173941142|gb|OQR90379.1| {|28(406)|<Stramenopiles(E)>}hypothetical
protein ACHHYP_05582 [Achlya hypogyna]
{(YAPYETGIVANGLDGDVLAHLPPEQLPALLDTLGITNPIHMTKLRSLYDTHRPSLLKRTLSMAQRLDEKLN
QSPRKPKAF)--
DVFLSHNWGhDELHrdnHARVSKINAFLRARKLRTWFDEDQMAGNILKAMANGIEESQIVIVFVTQSYQNkvn
gdnaNDNCQLEFGMANATQ---
TPQWMVPVVMEPCMLSPAHWSGQLKLVLGNQLYIDLAGDDATSFTHGCQNLLQR(IEALLGKLGMPPMHSHRS
SQDTLPPSSPRTNEDS)}*

$4340=243(247):
>gi|1173947460|gb|OQR95751.1| {|0(534)|<Stramenopiles(E)>}hypothetical
protein THRCLA_07605 [Thraustotheca clavata]
{(MGAGASIPDQLYDKTQCQTVCGDLFNEELWRQSSTNGMISHEKLVGLFNSLT)--
DVFLTHDWGvDGINHQHVRSVNKLLRDRGITTWFD---------
DERMEGNENARCIVVFVTQRYIDkvggsnaEDNCQLEFNYAARRK---
TATKMIPVLTDPSPTLKNPQnwTGEVGFVLGGNLYIDISSAFDDPVMLENKINELL(VKMQSIIGLPLQKRLE
HAMASLPTIAPIALVSAATPIPPKVSTTFATVPLRELTK)}*

$4341=278(275):
>gi|1173956866|gb|OQS03538.1| {|417(777)|<Stramenopiles(E)>}hypothetical
protein THRCLA_04144 [Thraustotheca clavata]
{(GFAIIDKIKDGFLRPSLSSSCDLWLKELSENVNDGQTFDKAQCRAICGELFDDALWAQHSCKDTGTISSEK
LQMILSSLT)--
DVFLTHDWGtDGTNHRNVSVINRLLRDKGITTWFDEEKMEGNIKKKMISGIENARCIVVFVTQRYmdkvggdN
GEDNCQLEFNYASMKK---
TNAKMISVLMDPTIKNTRDWTGPVGMTLGGTLYIDFSSAIGNPELLEARMEELL(EKLNSIIGKPLQQRYTQV
ATQNTVPLELLSKEQVSTLLTSLEYSNYCEVFLKNEI)}*

$4342=260(254):
>gi|1173961077|gb|OQS07145.1| {|164(397)|<Stramenopiles(E)>}ankyrin
repeat [Thraustotheca clavata]
{(YSPYEVNILANDVDGDVLAHLQPEQLPMLFNTIGVTNTIHQTKLRLLYNDHRPLILKHSMTISRRMEDAIG
NAPCKNKVF)--
DVFLSHNWGmDELNrdnHARVAKINAFLQFKMIKCWFDEDQMSGNILKSMAEGIEESLIAVVFVTQLYQSkvn
gdnaNDNCQLEFGMAKATQ---
TAQWMIPVVMEPGMRATATWSGQFKLILGNQLYIDISSDDPKAFEHGCHNLLSR(IEALKTKALPPLSTRRSS
KDTMPSSTPRVEIPVT)}*

$4343=165(165):
>gi|1174095887|ref|WP_081434184.1|
{|0(161)|<Actinobacteria(B)>}hypothetical protein [Eggerthella lenta]
{(MLSYFSGATGGAGM)QFDVFISYAW-
GGPENTahreWVRLLAAHLKQIGFHVGIDSEVDYGEDLTGFMREIEDAKRVLMIVDEGYVERADNYPDSGVGR
ETK---
TIQDVVDDRPNNWLSVLFVRNEKRLLPKWMEGRDPKYFDFSYRDRDGSFPGSEQ(IDDLWRWLAGL)}*

$4344=206(212):

>gi|1174495643|ref|WP_081485874.1|
{|0(300)|<Proteobacteria(B)>}hypothetical protein [Thiothrix nivea]
{(MRHGRSIWGCCCCWWWLTNWRVGKFEVSAAVFGATA)----YISYAR-
ADERRvQVVSQLHQAFANEWVHLKVDANEVqPRESFEDFINEIGKADCVVVVFSESYLRSFYCMLELTRILRH
GNA-V-QRVYPVFAEDGFRLDGVRQHWKNYWQDKF-
DHWHNQSNDEPDEVVDAFGSVD(ECNEILANLDSVFEHFARCLAGQVPACNAEVIGWVK)}*

$4345=254(262):
>gi|1173942436|gb|OQR91442.1| {|0(1093)|<Stramenopiles(E)>}hypothetical
protein ACHHYP_04691 [Achlya hypogyna]
{(MSASCSHCSSCNTVVLQQRALVVCGDCEHINGAEDPAAHPTFAIYDRPKLHIFLSCLRKSTIEAITHSVDG
HDRY)-------------QQLALRLKDQLQERGH--
EVWVDVEKLTPGVDWeEGIANgltwvkeAQENGRILLLMTPHALRRPDG----
YCLNEIARATqLKLHIFPTLVCESEPPPaiNMLPYFDMQDCVPSIEVAQTLDYKSKEWQAAMATHLN(SSLFL
TKSLKLFALLEMCDSMTNNMVTVVAGVENLGLFADGPASPKS)}*

$4346=244(247):
>gi|1173951396|gb|OQR99285.1| {|63(212)|<Stramenopiles(E)>}hypothetical
protein ACHHYP_07131 [Achlya hypogyna]
{(LHRKILLLHLSKLKSQPSDGAALDGIPFRRSAPTLAPAQVLCQLFSYQGIHLIDPDDMDSVVAKILPLMRP
QDTMLPDNY)--DCFISYRVSSEKEVAEKLYLHLKAKGC--
SPFLDRMSLKNAEpWKdGFLRGLAHSRVFLALISEAGLEKARDRT--
FNHREDNLLLeYEVALDIAYAGRLVIFPIYVASSNNGIFTKFQGFSTELYASSLNEDDAHRRGSV(PAASSSG
HDVGKRRQSEQVPRSLPSIA)}*

$4347=262(255):
>gi|1173956268|gb|OQS03091.1| {|63(187)|<Stramenopiles(E)>}hypothetical
protein THRCLA_21240 [Thraustotheca clavata]
{(LHRKILLLHLSKLKVIKAPDKDPSSSSHQSVPTLSPAEILAKLFEFQGIHLIDPDDMESTVAKIIPIVPPA
DALHAPESY)--DCFISYRVATEKDIAEKLYLHLKSKGF--
NVFLDRMCLKNGEpWKdGFLRGLETSRVFLALVSEAALTRARDRNisheDDNLLLEYEVALriatsKQLKIFP
ILVASNSNGNFVKFSGFSPDLYSPTIAPQGADDNRRRFSVPIPELED(RRKYAAHRPSVQTLPTISSQKNLGI
EPDINRKLSL)}*

$4348=206(218):
>gi|1174066413|ref|WP_081424296.1|
{|0(108)|<Cyanobacteria(B)>}hypothetical protein [Anabaena sp. wa102]
{(MAEF)--DVFLAHNSIDKPVL-KVIYEALKKRGI--
TSWLDGEEIRPGSlFQdAIQKGIPLSKSAAIFIGKSGLGMWQGLE---LKVILSMCLkQNIPVIPVL----
LPGVSDLPPDMLFLKEFNYINI---
YDGEIVNKIVNKRVLD(LLQWGIKGQKPEDYSAEQIKIKPGKIPNKEKLVDLVIQDIYDRKDNPYADATIWFD
AIEKIIDDLVDDLVDAIA)}*

$4349=202(206):
>gi|1174222257|ref|WP_081443437.1| {|0(94)|<Firmicutes(B)>}hypothetical
protein [Lactobacillus coleohominis]
{(MRWIMTDKSRA)--DIFISYSwgEGNGENFATWLNQFLiKNYDV--
NVFFDKTSTHPGSNLkNFMSKATNVKYIICVCTNEYLKRmEVESSgvKYEVDRIKV--NHDSHIIPLVEASK-
----
ELPEPFEDLKYIKLDMSNPTNPENIKAYYELLNYIF(PDEVNHVSTEYKRQVRKVKNIDKIKHSLAFNPNDKG
VVTLSLDVNDGKVDIGRGK)}*

$4350=221(222):

>gi|1174543054|ref|WP_081490190.1|
{|19(348)|<Actinobacteria(B)>}hypothetical protein
[Pseudopropionibacterium propionicum]
{(TKTWHVVWVAASVASNSDGVRSGCGNRAWGGARPRERDEMVVACPSVLHDGLSESWAGFGGIMGDRCGTAH
GNRYDPTGP)---VFISYRQSDGTEHAKRLDRFLRAGGL--
VPWRDLVDLPPGETArRVHEAfEEGIAAAVLIVTPEIQQSQFVPAeeLPELLKLEQEPHDFSLLVLNTIPKQD
ETASNDGRSQGDGVDAGQPSTDDAPADHLLGGCGRSKKPI(DV)}*

$4351=226(217):
>gi|1174732253|ref|WP_081498921.1|
{|0(315)|<Proteobacteria(B)>}hypothetical protein [Paraburkholderia
terrae]
{(MEISANDTPCRECAAAVHTHGVLDVQARGIRRPRAFAAESPRRCGTGDAQENKRGTDV)-
PAIFISYRRIDSKAYAGRLFDRLSQHFgaD--
HVFMDIEgGIQRGeDFAdALQRAVESVDAMIILIGRQWLtctghNGRRRLDqpDDWVRPGNGHRAAARHSGPA
CTGRRREHAGRGRPARGHPRACAASGERDQRHSLELRRIADRRDPR(TQAAPLTVAPRAGTPRGHS)}*

$4352=230(229):
>gi|1173900804|gb|OQR71808.1| {|973(0)|<Arthropoda(M)>}slit3 protein-
like [Tropilaelaps mercedesae]
{(TECHSSTDDSFSAGQHQFQHIGDFVPMFFVLAVFLLLVGMAILMVTFYRRQVSVWFFSRYGVRMFRRTTIE
EEK)LFDAFVSYSKKdeAFVAQILAPELECGNPPYRLCLHYRDLPmVTGYLSEAIEEAVESSRRTIVILSEHF
LKSEWCRYEFKSAHHEVLSNSNFKLVAIFLGRV--
SYRDLDPDIRMWLKRSTFLHWGEKNFWDKLRYSLPDARN(RKIMRSDLNSVAVHI)}*

$4353=230(229):
>gi|1173911530|gb|OQR75252.1| {|928(0)|<Arthropoda(M)>}slit3 protein-
like [Tropilaelaps mercedesae]
{(ECFGLSEATSLVRPFHIRSMGDLLPVVAVLGVLLVVVSLMAVAVGIVYRRKLSVWFFSRYGVRVCHRAPAE
EEK)LFDAFVSYSKKdeAFVAQILAPELECGSPPYRLCLHYRDLSMaGGYLSEAIQEAVESSRRTIVILSEHF
LKSEWCRYEFKSAHYELLNNTSHKLIVIFLGRV--
SYRELDADIRMWLKQSTFLHWGEKNFWDKLRYSLPDARH(RKNLRSDLNTVAVHI)}*

$4354=281(232):
>gi|1174681794|ref|XP_020568474.1| {|305(0)|<Chordata(M)>}interleukin-18
receptor accessory protein [Oryzias latipes]
{(VMPQHLKHTFTCFATNSVGNSSVTIKLKQKIKVNWPPLVGYPFAAFLLVVWLGILLRVKWLEIQLMYRSHF
QHRRHDGEK)EYDVFLSFV-
WsslsaegtqgltlpsrsgcdcneeEHLSNKklmsckeskstlkplevLLPHVLEVHWGYRLCLPERDLLPGG
AYTNDVVLGIKRSRMLVCVLSADYLaDSSAVF-VLESGIQALLQKSSLKLLLIRtseTSAF-
LTPShlPLPRLVQTALKVLpRLNYSSKTSLravdNFWRSLRKAMPQS(RKRQVATMECGL)}*

$4355=252(261):
>gi|1173894275|gb|OQR70236.1| {|1011(105)|<Arthropoda(M)>}hypothetical
protein BIW11_11759 [Tropilaelaps mercedesae]
{(EAAKLIGSDWVALAKRLGYTDRELSRFSEEPAAGLALLRDWRESNGATMYSTDVLISCLQQMEREDIARLI
QSDEAPQPL)---VFISYQW-HSQEAVLS--
IRQRLEAAGLPCWMDVGQLGAGNSLygKIYEGISRCKLVLCCLTARYAASASCARELSLADVLR-----
RPIVPVMIEPTPWPPPGPMAVILSSLVYVDLCGVGGHGGYGRDADWNSRCQD(IIERANVLIHSTPLAPGPHL
PGAPNRRHQITGATSGGPAPV)}*

$4356=273(279):
>gi|1173944242|gb|OQR93003.1| {|699(139)|<Stramenopiles(E)>}transient
receptor potential Ca2 channel (TRP-CC) family protein [Achlya hypogyna]

{(GVGHAVIAALIAAGAEVDFADDAGNTALHVALMNENRVTAVSLLKCGANIMATNVEGKRAVDCTTSTELQF AVKKEAGSR)--DVMISY---
THAHAPIAKRVRDHLVDHaRLTCWMDTMDpsgIGGGAVWreEIARGIFNAKVVVAVVCDGYARSEWCLKELSF AKISR-----TP--
AVVLVVDPNGMSAEIERLVPSEFILPFPVSDAMLADADAWTAAFETAL(APIREALKGAPPTRLLPLYNFDVH APARRVFVYYTASLTPELLRRVEDGIQARGFVPLL)}*

$4357=247(252):
>gi|1173947925|gb|OQR96094.1| {|623(188)|<Stramenopiles(E)>}hypothetical protein ACHHYP_17005 [Achlya hypogyna]
{(LSQELLSAIVQSGGNINDALAGSGDAALHIAMKRGKKTTALALLGAGADIMQVNGDGCRPLDCTTSTELQF AVKRAAGSR)--DVMISY---
THSHMEFAKKLRTSLENNHITTWLDLMDpsgIGGGSVWreEIARGISNASCVVSILTEDYPKSEWCLKELALA KQVG-----
TPVLALSTENVKIGEELQVYLYARQIVPFESAITAIDKANPRNITYEYNDVA(FARQFRSLLDGVRDEIEKKR QALAKANAGGMG)}*

$4358=249(254):
>gi|1173961401|gb|OQS07434.1| {|155(185)|<Stramenopiles(E)>}transient receptor potential Ca2 channel (TRP-CC) family protein [Thraustotheca clavata]
{(LSKEILAALVAKGGNINYSHPTSRDAALHIAMKRGSKETALALLAAGADIMQVNSDGCRPLDCTTSTDLQF AVKRAAGAR)--DVMISYTH-THTE--
FAKKLRTTLENNHITTWLDLMDpsgIGGGSVWreEIGRGITNASCVVCILTEDYPKSEWCLKELALAKQNG-- ---
TPVLAISTENVRIGEELQVYLYARQIVPFESAITAIDKTNPRNIMYEYNDVA(FARQFRSLLDGVRDEIEKKR QALAKSSDNRSGWA)}*

$4359=217(220):
>gi|1173902297|gb|OQR72151.1| {|27(0)|<Arthropoda(M)>}myeloid differentiation primary response protein MyD88-like [Tropilaelaps mercedesae]
{(AELVLPHERLQQLQLTQDPLAETLKAWPSEATIGHVLTALEAVERFDVIDDCIESIQKDCKDFQNRRRVWG MPSSVHATC)-FQAFVVHAQTdNEFVKQLIDRLEKKE-
GLSLFIPVRDFPAAMANYlYKLMQVMDNQCRKiIIVISKALAADDNATQLLEkaeqlRAQQMQTGFTSSRIIP VILEDC----PVVGsSLISISPVNF--RSHPE---WAWPKLISALKM-()}*

$4360=225(233):
>gi|1174228323|ref|WP_081449500.1| {<Proteobacteria(B)>}hypothetical protein [Rhodomicrobium vannielii]
{(MAARYKRSLLSRRPLLLISFTPEFLHGQPGGRCHSTIDFSQA)--
DFFISRSGADAAIAERIAHILEDAGYQ-VILQQWDFG-
NRNFMQAMHEALASDARVIALLSRDYLESDHCLGeALNkiahDPLNKK-----SRLIVMRVANCTPT-
GLFAALAYWDLVK--LtDPALLRD--
VVLAAVKPGRAK(TAHDAASPFLKPPGTVLHDHRRGLPLPPGRLFHRSSSSAHARRAQPRARYP)}*

$4361=188(192):
>gi|1174252606|ref|WP_081465947.1| {<Proteobacteria(B)>}hypothetical protein [Stigmatella aurantiaca]
{(MAKNRRSGASALEGVTKQPGSTQQQGWVAVSDE)SKNICISHAPQDGAVVQELKGLLAMHGYS-
LQHSSSESPllSGAAGAHRMTPRIDRAGALLVLISPA-IRTRPWTDkEIEYAERQG-----
KRIVGVYVLGGQdaDLPVSFQMYGDALVNC--
QVERVVDAVNGTINTWCNPDGS(NLVPERDIERYNCGEPTPE)}*

$4362=249(260):
>gi|1174309813|ref|WP_081476010.1|
{|148(0)|<Actinobacteria(B)>}hypothetical protein [Verrucosispora maris]
{(ATLSLHGYSGAPVLVGDGRQAAAGVIRWNPQRPDAPDLAAGGTVYATPISAILMRSNHLQSLSESQLTIAA
GGEPRDGYC)-----ISHSNVDLEIARWVGGVLRGERIG-
ARVRGEYILPGMNYVPLLRAAHEKYPDTLLVLSSSTLkcdSPSHMMEmDWM-----K-----
GGEIPGIVLPVRvgSakMPNFLYDIEPIDLRGY-KTEDEVRE--
ALIAGLVATERP(RLPAPRFPAERLISQKPTTLSRSRLAQVTLADRQRIEGGV)}*

$4363=248(253):
>gi|1173930478|gb|OQR83304.1| {|650(170)|<Stramenopiles(E)>}transient receptor potential Ca2 channel (TRP-CC) family protein [Achlya hypogyna]
{(DATLLKLLVSNGANINYQLRSNGDTALHLAIKHEKQETAISLVSCGANIMTTNHAGLRALDIATSTALQFA
IKSAAGHRD)---VMISYTHShAEFALKLRKSLEA----
NNITAWLDTMdpsgIGGGSVWReEIAKGIANATLLVAILTDDYTRSEWCLKELACA----KQ-
VGTPVLPISTEHV-
RLNEELQVYLYTRQIVPfePAITAVDNHNPRQVTYAYDDDKY(QQQFRLLLDGIRDEVEKKRKAVALRSMDTL
SPT)}*

$4364=250(258):
>gi|1173932156|gb|OQR84258.1| {|651(204)|<Stramenopiles(E)>}transient receptor potential Ca2 channel (TRP-CC) family protein [Achlya hypogyna]
{(RERVLYLLRDYGANIHAKTATTGETALHLALKQGNIQTARTLVQMGLDVAATDASGTRALDCAASTDIQYA
IKKAAGQRD)---VMISYCHShASFAHKLRASLEA----
HYITTWIDQMnptgISGGSEWReEIARGILGASVVLAVLSEDYPQSQWCMKELAFAKQHN--------
IPVVGVTC-
GRVVIGDELEVYLWTRQlvDFRPAITAKVRQGTTVRFEFDEA(KYDGQIALLLDGLRDEIEERRVLTKAGNPR
TSAASHAS)}*

$4365=249(254):
>gi|1173955770|gb|OQS02690.1| {|577(173)|<Stramenopiles(E)>}hypothetical protein THRCLA_04955 [Thraustotheca clavata]
{(IDAAIFRLLRENGLPEFGTMSNGDTPLHIAVKLHNERTALALVRSGSSTTITNEEGVRAIDCATSTELQFN
IKKEAGQRD)---VMISYSHAfQPFALKLRQSLE----
DNFLTTWMDLMdptgINGGAVWRdEIANGIQNAAVVLCILSETYPESQWCMKE----LAFAKA-
NNVPVVAVIAQTT-
EMTDELQVYLWSRQLVDfrSAILPRSPSSKEDIVVDNIIYNQ(QLASLLDGLRNEVELHRVEAGRRPTILTRQ
LSMQ)}*

$4366=250(258):
>gi|1173956700|gb|OQS03423.1| {|668(760)|<Stramenopiles(E)>}transient receptor potential Ca2 channel (TRP-CC) family protein [Thraustotheca clavata]
{(REKVLILLRDYGCNIHAKMVTTGETALHLALKRGNIMTAKILVRMGLSVTAKDNDGIRALDCAPTSDIQYS
IKKVAGQRD)---VMISYCHShANFANKVRQ---ALEKQY-
ITTWIDQMdptgISGGAEWReEIARGIIGASVVLAILSEDYPRSQWCMKELAFAKQHN--------
IPVIGITC-
GRVVIGDELEVYLWTRQivDFRSSIVSKIRSGNAVRFEYDEE(KFESQFALLLDGLQDEIEERRIMFKNQESA
DIHLDRVS)}*

$4367=248(253):
>gi|1173957963|gb|OQS04396.1| {|598(171)|<Stramenopiles(E)>}transient receptor potential Ca2 channel (TRP-CC) family protein [Thraustotheca clavata]

{(IDVKLLKLLVSNGANINHQVISGDTALHIAIKHEKKDTAVALVSCGANIMATNYEGYRAIDIATSTSLQFAIKSAAGYRD)---VMISYTHShSEFALKLRKALE----DSNITAWLDTMdpsgIGGGSVWRaEIAKGITNATLLVAIVTEDYTRSDWCLKELACA----KN-AGTPILPISTEHA-RLNEEMQVYLYTRQIVPfePAIKAINNTNPRNVTYEYDDNAF(QLQFRLLLDGIRDEVEKKRKKALRTLKTMKSTV)}*

$4368=200(207):
>gi|1174091015|ref|WP_081429314.1| {|0(192)|<Chlorobi(B)>}hypothetical protein [Prosthecochloris aestuarii]
{(MAPIAQEWFFSIQSLYCINGVTSFFLSVYKLS)MADIFISYARED--LKRIKLLVEALTKQGWSVFW-DRRIRAGQKFSsVIENELQNAQCAIVAWSEHSVNSDWVHDEATEAKERN------ILVPVLLDVI-KPPMGFRRIHAADLTVFgtGKRDSVLEQLFEDIREIVGEPSR(LHRDVEEPIKRKPEERMEPFDLVDVDGATYKTIQI)}*

$4369=164(169):
>gi|1174099319|ref|WP_081437194.1| {|0(77)|<Proteobacteria(B)>}hypothetical protein [Xanthobacter autotrophicus]
{()MSFMFLSYAREdEP---RVATLLEALRAEGL-SIWWDRDIPAEAPWEqVIEEKLHSAALVLTCWTASSVNSQFVK---AEARFAQQA-GKLVQVFLEKTNA-PIFLSYAERQGFELICWngRRADDAFRRLVDFCKGTFHPPHL(ISTRQTKIHALVERVRLLHAALVQTLVAF)}*

$4370=264(271):
>gi|1174227731|ref|WP_081448908.1| {|29(611)|<Actinobacteria(B)>}hypothetical protein [Geodermatophilus obscurus]
{(LTGRRVSAMPVGYPRQAGPERRAPTSRGPRREGRPDPGGRRHERQGPKNAGKKVEKQYQDAPSWLKMRSDAIPHGGGTTM)--WLFASYSRKdETAVRGL---ISDLQRAHL-SVWHDQELRGGDPWWqDILRRIRQCDVFLFLLSDNSLASKPCLTELSYA----RA-LGLPILPVQVGPVGNIRLTPVADIQVVDYRErtLAKGMELLDAIQQAVTERHPLPE(PLPEPPSVPFEYLLRLGSAIGAAQLTPDQQGDFIRQLREVLETEDDEGVKD)}*

$4371=174(161):
>gi|1174231596|ref|WP_081452770.1| {<Verrucomicrobia(B)>}hypothetical protein [Verrucomicrobium spinosum]
{()MPSpeplrLFISYAQRdIRHLDVFKQNLKVMQMNGLIKT--------GSEWDqDIRRELDDADIIVFMVSTNFLASDYIRGvEMARAIQRKEA-SEAELVTLLLEPdC-GWKDphqlklrgtdqtitCNLKKYQAIHPRNrkAARWPTTA--NAFNHVEPALTKL(RETILARRPVGEFLPRHRPTG)}*

$4372=167(168):
>gi|1174505800|ref|WP_081487518.1| {<Proteobacteria(B)>}hypothetical protein [Rubrivivax gelatinosus]
{(MALAKHELQLASLRQPHRKLQAYNAWQR)--TAFLCHSHLdKELAEGLQVWLA---EQGVdlyID-WKDNTMPetPNRETAaRIKRRIQSSHLFLFLATANSKASRWC-PwELGYA--DGEI-NWSQIVIVPTADS-SGTHGNEYLQLYRRLDSnsLGQLFIFDPGSVQGTAVRTARFA()}*

$4373=179(185):
>gi|1174735650|ref|WP_081499998.1| {|0(581)|<Proteobacteria(B)>}hypothetical protein [Komagataeibacter europaeus]

{(MRA)----FLSHSSKdKGYVERVAKLLKP----GTFEL-
DSTTFDAGRLNSfVISEALKRSDLFCLFLSEDSVESPYV-KfETLFGMEQFSK-GNIdRFIVICIDQN-
SFDHVDDNIKLFSIVRRtiSIEASSWLIQGTLLSAASSDTKL(SHPFIGRETALRELEIQVTAPDRPPQKALY
VSGNFGAGRRAL)}*

$4374=194(188):
>gi|1181031795|ref|WP_084443364.1| {|0(301)|<unknown(X)>}hypothetical
protein [Hymenobacter roseosalivarius] >gi|1174201409|emb|SMB81390.1|
SEFIR domain protein [Hymenobacter roseosalivarius DSM 11622
{(MPTTTDAPK)---VFISYSwnP--
PQHKERVIELAKRLSNDGVHVILDVWDLAPGQDKhsFMEQTVTNPDIkKVLLICNKSYKDKadgrqGGVGtES
LIMSKEVYAKaDQTKFIPLVFEAD-
EQGEPCVPTFIHSRIFFDLRETEEYEDNYEQLIRNIFDKP(IHARPPLGMRPSYLEVETPSTLPTAHRVAAIK
RAFLDER)}*

$4375=198(192):
>gi|1174232644|ref|WP_081453818.1| {|0(282)|<Firmicutes(B)>}hypothetical
protein [Aerococcus viridans]
{(MNKLVQEVDYVPK)---VFISYAwtS--
EEYTENVVSLAERLVHDGIDVVLDKWDLKEGQDKykFMEQSVQNSEIkKVLILCNSVYRERadnrtGGVGdEt
LIITPEIYSSSnQTKF-IPVIFDNT-
DNKSVIPTYLNSRIYIDLSNMNNQRDYEKNYEKLVRNIWN(KPEFEKPKLGAMPKWLDSNEKNQSLLQTYVRN
YQNESSL)}*

$4376=205(210):
>gi|1174704702|ref|WP_081494409.1|
{|0(137)|<Proteobacteria(B)>}MULTISPECIES: hypothetical protein
[Bradyrhizobium]
{(MVLRFGLCDVQEAVCLQLRLVVKVVAR)---IFLSYSK---
SDANFALQLAERLKSLGNEITVDVESLTAGQDWrtRLSEGLR-
RAELFVVLISQSSIRSPSVLqEIGAARAYAVESDRMLVIPVLIDDI-
SIPSVVQDILVLMAADRDLDKIVSDISVSISQFVGRKAAK(DERAAEVAKRIEINAASYIDDAVLSQSNAEAR
NRVMGIIWYVA)}*

$4377=250(250):
>gi|1174983082|ref|WP_081656227.1|
{|1092(12)|<Cyanobacteria(B)>}hypothetical protein [Fischerella sp. PCC
9431]
{(KPYSYSLDNLYRRFNAGRYQIECDKSYEMVDVRRLIDDVNLQSFDITQERKLPVIPLQRELEESRDKSLAH
QPQNSDQKE)---VFISYTW-
RDQEsKPLVEKIEQEFKTKGINIIRDTNAIGyKQKFQEFMQRLSHGKCVILIISDGYLKSENCMYELVEIAKY
GEF-HK-
RIFPIVLEDAKIYQAIERIKYVEYWEDKkkelDAAMKKVSGDNLQGFREELDLYN(EIRNTFAKLTNLLKDMN
ALTSDIHIQSNFE)}*

$4378=193(194):
>gi|1174925457|ref|WP_081598677.1|
{|0(282)|<Actinobacteria(B)>}hypothetical protein [Corynebacterium durum]
{(MVTQKRFPIEWCGCNSEPSTKFSYPCVWVEGTCNMLNVRTAESAKRDPNGA)---
VFISYRRDDGTICANKIGALLRAAGL--
RVWRDIYNMPARKMQeTLERVLgKETSAGVIIVTPNTGQSDMMINleVPMLLSLANDPDFPLVIANTVQKLEG
GIDYDAPYRYLKIDAKTSSDINQFNGSNKDGITKLVSEML(NSR)}*

$4379=206(208):

>gi|1174923693|ref|WP_081596913.1|
{|0(28)|<Cyanobacteria(B)>}hypothetical protein [Fischerella muscicola]
{(MVDVRGLIDDVNLQSFDITQELNSQVTPLQRELEQSRNKSLMHQPQNSD)QKEVFISYTWRdKQsepLVKK
IEQAFQAEGI---DII---RDTNAIGYKQkfqeFM-QRLSRGKCVILIISDQYLKSENCMYELVEIAKYGEF-
YNRIFPIVLEDAQIYKSVARIKYIKYWEDRT--
KELNDAMKEVSGANLQGFREE(LDLYTEIRKTFAELTNLLK)}*

$4380=198(204):
>gi|1174951926|ref|WP_081625145.1|
{|0(48)|<Proteobacteria(B)>}hypothetical protein [Hyphomicrobium denitrificans]
{(MSPSSLALIPSRGRCPDTPSIGESKYWLTDHL)--RVFISHVHTaKTSAHNLREVLKRYGI-------
SSFVAHDdIKVSAkwrdEILRSLHSMDAFVAIITKDFSQSNWTDQEVGIAVCRDVL-
IIPLDKGAAPYGFIGELQAEKTGGDAVEVAE--
KVFTAIKDSPKTRDRIIECLS(KTIAASADSTFASFRVDKLASIENVPRDAWET)}*

$4381=113(142):
>gi|1174979171|ref|WP_081652316.1| {<Bacteroidetes(B)>}hypothetical protein [Cyclobacterium qasimii]
{(MA)--KIYILHSKEnNSFVRSLMRNLVNNG---HEIILSDFE-----
VGHdinmSIKKAMQSADIHIALISNKSLNSNYFINEL---------------------
SQIRNYSVHSNLRKLFIPV--FFQIFHSKHYPTQSKISEGYL()}*

$4382=248(254):
>gi|1175124687|ref|WP_081673314.1|
{|0(390)|<Proteobacteria(B)>}hypothetical protein [Phaeospirillum fulvum]
{(MVAAMQAISMRGSVPFGSRPVPVCASFRSIKPPAHDAGSDRWGDRAMQLDEPAINGNLFPIVGLPRKFKMT
HELAAPGL)---IFISHSSAdKPFVESVIRK-----IPKSHLFYDIDT---
INPGShtvdALDDGLFKCSVFAMFVSKNTKESCWVDYETGVAEVekiKRRQ-
IKIVAIPIGEASYKDAPEWMRKYMAVPPGYS--
ASDIARLLKYLYEDALRAQGV(LNESPFIGRENLCNNIILQSRTKPAETGIPLNFVI)}*

$4383=138(150):
>gi|1174934359|ref|WP_081607579.1| {<Actinobacteria(B)>}hypothetical protein [Rhodococcus triatomae]
{(MKA)----FVSYSRRDNTLGRLlsIESQL---GES-
VELyiDDLHFPRGADRRTSVLKALMEAESLIAVLSSNYLHT-PWTVfE---ARQAC-----
VRGIPVFAFSESgeLFETRYEDLSTYCERR--QTGHGFTERARNCRASELPRAE(ALYGVIA)}*

$4384=202(225):
>gi|1174954217|ref|WP_081627436.1|
{|0(189)|<Acidobacteria(B)>}hypothetical protein [Acidobacteriaceae bacterium KBS 96]
{(MIRSAV)-----LCHAPEDRAFAENLARFLAmNCPIE-VSVAEGEIRHGKDLIDRIQWA-
ACADMAIVLLSPE-SACKEWRReRWEPVFAEQ-----ASSLALLL--LRdcRFPKLLRAGHFFD---------
-ASR--
NLLAAQRALKRW(ILARCGRFESNVQLPKHDEPYSAAPDFLEQLRQSVADRPGIELDVPRNCALAFAIAFRDD
FDAVLWTDCAYRDEAGILG)}*

$4385=217(221):
>gi|1180912178|ref|WP_084352408.1| {|309(0)|<unknown(X)>}hypothetical protein [Pseudooceanicola flagellatus] >gi|1175026565|emb|SMC72929.1| TIR domain-containing protein [Pseudooceanicola flagellatus]

{(TLLFALMTIAPFFLVAVIDLQYRSVWPPSWTAIVSAAIGVGLVAGLYVWWIRDSLSRLGLVPSSNRQFDKL
DAPELVGYD)-KKIFLSYSSTDAKAARRVldGFHMASPGVK-
VFEFRRDIPIGVSYQSRIDEALAESDRVLCLLSPEYIQSPECQEeLMVARLRNK-----
RAGFEFLWPVYWreVHGGLTDWLMILNLAD--CRESSVAQLDRTVTRLASELLK(N)}*

$4386=205(209):
>gi|1180910440|ref|WP_084350889.1| {|0(1056)|<unknown(X)>}hypothetical
protein [Pseudooceanicola flagellatus] >gi|1175027685|emb|SMC55787.1|
WD40 repeat [Pseudooceanicola flagellatus]
{(MST)---IFISHSSADSALAKQLEARLRQQNHV-
CVFLDLDPtkgiVAGRSWERTLYRKLRACRVVIAICTESYLQ-SHWCFaEIALARMEG-----
KPIMALLADPLApdaKLPSIITEKQFIDLRA--
NPEDGYRRLWHGLSELDLLGAV(GDWDPRSSPYLGLNAYHEEHAPVFFGRENEVRAGVELLDRGAPGLIMVLG
ASGSGKSSLARAGILP)}*

$4387=291(300):
>gi|1180940635|ref|WP_084364023.1| {|194(193)|<unknown(X)>}hypothetical
protein [Rhizobium sp. RU36D] >gi|1175092162|emb|SMC60583.1| TIR domain-
containing protein [Rhizobium sp. RU36D]
{(AFGSDWRVVREFYEALRDGRPLFANLGDKLGSVTLALADEDEAFWERDPDAVTRDLAERLGGGETSQEPEA
ALNAPSEKV)--DLFISYSTRNEAEAREINGYFEDAGYS-TIVQFKDFGIGSNFVTEMQKGL-
QAERMIAVLSPAYVA-SMQCQaEWNAAYNRD-----PSGEQRYLVPLLiektELPPLAKQIVYKSLVG--
LTGDARRS--
AVLEAIGKLRRP(EVSGVRSPYEFELTPNQTITAVAGAMNTMSVLPSRDPGDARKRLEAAKQLANDLIAGLMD
NRFQVGRHYIKELENYRDRL)}*

$4388=213(220):
>gi|1175164133|ref|WP_081712055.1|
{|35(0)|<Proteobacteria(B)>}hypothetical protein [Halomonas sp. PBN3]
{(KKIIDDHNRKVDAHNRKVIADHNRQVRRHNQRADAHNRKVIAELNRQLRSSSSPVRYTEPERQLADRVHQA
VASLDPREY)--DSFLSYARIDgSEVAGQLRGELESLGVA-
VWFDEVAIVPGRSQSLQMDAGLRKARSGIAVLTPAYLTGRFWTErE-LGALLNK-----
STLIPVLHNVTFddVKQYSGILPDLAGFTT--EHDSIEDIAKKIAGAVLMEDDE()}*

$4389=181(177):
>gi|1174908418|ref|WP_081581638.1| {<Proteobacteria(B)>}hypothetical
protein [Pararhodospirillum photometricum]
{(MRFIHLNRVNVIGPDLPWQKIGGKDGI)-PNIFISYSSEdLKYVEEI---
LNTLRDRVNIKLpstfWMDKwKIRHGSVIVqEVSKGVNEADLVVLFASQNSLKSGWVKNeWMKKYKEEIET-
DSVKVITAIIDDL-NQSNLPSELTGKLAITMseYEKSASIDQLAESICYHLSNSLE(MDAKKNRENK)}*

$4390=123(150):
>gi|1174911094|ref|WP_081584314.1| {<Bacteroidetes(B)>}hypothetical
protein [Alistipes obesi]
{(MTKFVD)---------------------------
DNILDIWWDKNITAGYDFWeQIDFHIENRDIICLFISANYLASKACKEEMRRACELRTQ-
NGICVVPIILSSC-SWlDYDNLKPLLAIPTDGkaISSFPNPD--DGWHDVYNHMKKV(IKDF)}*

$4391=258(264):
>gi|1174926255|ref|WP_081599475.1|
{|155(1090)|<Cyanobacteria(B)>}hypothetical protein [Prochlorothrix
hollandica]
{(ISQTLERQRDYVQQHTQLLVQANHWAQHQKQPSDLLQGEALHQAQQWLGRRFTAEQAPCQPTDRHSEYICT
SLSEAQGGA)-TRVFLCHSEQdIDITETLRW---SLLREGM-

PVWTNRAnIRTGADFQqEISRGIERADTLVYLLSAHSLASPYC-QqELAYGRQLNK-----RIIPMLMADV-PLEQIPQDQRSLQFINNqnFSDPASYAIALDTLLQTLKRHQD(YDYWHKTLLVKALHWDQRERPKGLLLRADELAVAEHWIQLQQQQ)}*

$4392=244(230):
>gi|1174944318|ref|WP_081617537.1|
{|0(193)|<Actinobacteria(B)>}hypothetical protein [Amycolatopsis methanolica]
{(MPHVGHPFPDPDRPYVLTGNAARAGEWASCPLFVQRDFRTAPGTVLYRFPVGSTV)--TVFISYAHDdEQHRATVREFAASLRAQGIdadIDSWADTDRRDWQAWAtE---HILSADFVLVVASKvyremgDGLGpADRHRGvQAEAALLRdllsedREK-WTRKVLPVILPGH-RIDEIPLFLQPYNVDRYdvSSPGGVEYLLRTLTRQPEHVRPP(LGPPVVLPPKSGPGAVPAGRSPQWRRLARPATVVW)}*

$4393=198(205):
>gi|1174963344|ref|WP_081636489.1| {<Proteobacteria(B)>}hypothetical protein [Methylobacterium sp. 285MFTsu5.1]
{(M)------SYCHAnEIQQAKLQIHLAQLKRDEV-ETWFDGDMEAGDRLNtEISRKLREADIFVALMSPEYIASHWC-QlEYRRAMGRRAR-GSMRIVVVVVRPC-AWKDTGASDLKVLPRDGrtVSDWRSMD--HAFANVAEGIKGA(VKAVRVALPPAVPARPAKAMRSAAAPVAKKRPVSAKGGRSASTKVSPGAGAAKGKRPARSRATG)}*

$4394=205(218):
>gi|1174973477|ref|WP_081646622.1| {|0(143)|<Firmicutes(B)>}hypothetical protein [Butyrivibrio sp. AD3002]
{()MMSIFLSYCHKnKAIADLIDKALQT--ECGIIAERDERDVGPWNSFSEFMEQAGTVDIVMPIISGDYLKSRNCMYeALETIRDRKYN-DK--IFPIVLEDD-IYNRMNHIQY-------VKFWQEKS--ESFENELDGIRAE(NLGPLGDELKFSKNIAESMAEFLSVVTDRNNPSILKGMERVTDFVKERLGLEKDTEIIKEEDKDYFEEYGIEKKRLPK)}*

$4395=184(169):
>gi|1180912650|ref|WP_084352795.1| {|0(312)|<unknown(X)>}hypothetical protein [Pseudooceanicola flagellatus] >gi|1175026368|emb|SMC77413.1| TIR domain-containing protein [Pseudooceanicola flagellatus]
{(MVENTISS)-TSLFVSYCHRdARHRPRFNSSrmgeLLADVRYELGADGSDSRyqlvkdtegaLRTGDWIDdKIAEEIAKSDIGLVFLSENYCRSSSC-EkELRTFIKLGKS-LILVELDDHWTTS-DDHPMVALRDELRGLLSirFWAEQAGKSVRFGHPLPQTATGL(AQEGYRQQLQRLVDDIKTMSN)}*

$4396=152(153):
>gi|1180948893|ref|WP_084371545.1| {|0(439)|<unknown(X)>}hypothetical protein [Reichenbachiella faecimaris] >gi|1175032101|emb|SMD32911.1| WD domain-containing protein, G-beta repeat-containing protei
{()MSEIFISYIPEdKRRARQLAEVLDEFGWS----VWWESAIADDEHFDaQIESALKNAHCIIVLWSPGAINNSSILEqAQLGLKKKNLV-SLVYDEVQNEQTL-PTEAYTNLSHWKGDAADksIRILLGDIYDLIGHPPKRTTDKL(FRNLVTFVSLMLV)}*

$4397=234(221):
>gi|1174959299|ref|WP_081632518.1|
{|0(249)|<Proteobacteria(B)>}hypothetical protein [Oxalobacteraceae bacterium AB_14]

{(MTATTQHPKA)----FISYSwsS--
VEHEQFVLELATALRNHGVDAILDKWDLKPGQDKyaFMESMVTDSAVsRVLVICdriyqQKANARAGGVGtES
QiISQELYGKVkQTKF-IPVICeydedDQA-
CLPVFMKHLIYVDVSSDerYGAGLDELLRlIYEQPLHEKPRLG(APPAFTSNAGQPYAKELAAAARAIQDGKP
NRQGLEHLLLKSVLGEIERLYSEPEGNDYHEKIYAAIQATKG)}*

$4398=216(214):
>gi|1174983041|ref|WP_081656186.1|
{|0(317)|<Cyanobacteria(B)>}hypothetical protein [Fischerella sp. PCC
9431]
{(MQQFEFD)---VFLAHNS---
KDKPQVRAIAEQLTRYNLRPWFDEEQIFPGDNIqqVVSQGISQSKVGAFFISQNGL---
GTFQeNLELGAIIqffLRKHnQQGFrVIPILLginDIP-
DSLFYLSQWAWIQFpSSNDEAALKRLIRGIRGRGAEVQAQP(VIQIPDTPLIIEPKPPQSLSTEQQRRLQQKR
DGLQQEWDLRSEKISRIRKALAIENSATVKFQLEQQ)}*

$4399=247(252):
>gi|1180807493|ref|WP_084281258.1| {|22(144)|<unknown(X)>}hypothetical
protein [Novosphingobium sp. B1] >gi|1175032835|emb|SMD06130.1| TIR
domain-containing protein [Novosphingobium sp. B1]
{(DDALDEVMNSFAEEVHGKAASVVDPETGKHTPVFVRRIGKQGWTVFTSGSPAFARALEQRLGLDRGEVHGM
NEPADRERL)---VYLAHAS---
EDKPIVKPLAEGLMQRGIDVWYDNWEIGYGDSLrrKMEEGLGNCTHFVVLL-
TGTSIAKPWVNeEIDAGLMSAVEGTAKFIGLRHDLPL-
SSVSRFLKTRLTPEYRPGEDGIEALAAEIFGVSRKPPLGQ(TPRYVQQHESGSTWSSSARVVAEFFVRNSEQG
)}*

$4400=166(157):
>gi|1181011734|ref|WP_084424779.1| {|0(23)|<unknown(X)>}hypothetical
protein [Kibdelosporangium aridum] >gi|1175112446|emb|SMC61516.1| WD
domain-containing protein, G-beta repeat-containing protein [K
{(MWPLLPCRMPGDER)RYDAFISYSYQtdgprlaPAVQRGLQRLTVPWYRRSPRRIFRDHTSVPAGSNLgqS
IEEALADSRYFVLMASVAFSPDGRLLASGSGDNSVRLWDvatREQVSDPILGH---
SRPVRSVKFSPDGRSLASGGEDTMVRMWDVGYTVDVVKSL(CDK)}*

$4401=176(179):
>gi|1175120603|ref|WP_081669230.1|
{|0(259)|<Proteobacteria(B)>}hypothetical protein [Photobacterium
halotolerans]
{(MNK)--NIFLSYSWSdmkiaDQIEEDLSRLQ--------LNLRRDVRDLAYKNSIsdFMES-
IRDSDFAILLISDSYLRSKNCMKEVLHLLKDRDyEDkilPVIVKGTEIYS---
TAGRLSYTRHWLEEQENLKEVINTLPATSVVSEISELKTV(ETITSQINDFLTYISDNKNLTFEELKSESYSS
ILDC)}*

$4402=136(141):
>gi|1175178687|ref|WP_081726600.1| {<Proteobacteria(B)>}hypothetical
protein [Legionella oakridgensis]
{(M)------SHDS-RD-
KNDFVRPLCENLRSRLCTVWYDEFSLRVGDNLreSIEDGIKKCNKCIVIISPAFISNTgWSKKEFESIFQREI
SDGKTVVLPIWHNVTRNQVYEYCSSFTNVVALNSAEGIDLVANKLFDILMNPKPRS()}*

$4403=303(296):
>gi|1175287575|ref|WP_081835370.1| {|75(0)|<Firmicutes(B)>}hypothetical
protein [Lachnospiraceae bacterium AC2029]

{(EAYWSIVLCRYGIEYVEDPATHKRIPTVNRAQYKSIYSDEDYKSAIEHADMIQRQVYEAEAKTIDDIQKGI
LAISNKEKP)-FDVFICYKE-
TDengkrTRDSvIANDMYHQLEKEGFKTFFAAITLEgkLGTAFepYIFAALNSAKVMIVLGTKpEYFVAPWVK
NEWSRFLAIIKNDSSKILIPAYRDMDPYELPEEF-SFLQAQDMG-
KIGFMNDIIHGIKKVLVKEKEK(AAPQEKITRSVSIGSKTDADTLLERAFLFLNAGNWENATVYCDKVLDVDP
GNAKAYLGKLLASKKTEESIRSFGPE)}*

$4404=176(180):
>gi|1175354575|ref|WP_081902367.1|
{|0(736)|<Actinobacteria(B)>}hypothetical protein [Lechevalieria
aerocolonigenes]
{(MEDHIA)----FLSFSG---
QRLSRVKKIHEELVSRGHDPFFDREKVRPGQGLtpEIEKGLRRSKLFVVHYSSCYAISHSCQLELIHAFLSDE
aEGGESRILVINPEKDRAHIQPVALQDKLYIEDRGQDVSEIVDEICARARDVRGTFP(GINVAVTPPRIHGMS
PTVDVRVRRHGDMWRVRTA)}*

$4405=239(242):
>gi|1175303244|ref|WP_081851039.1|
{|0(1058)|<Proteobacteria(B)>}hypothetical protein [Caballeronia
grimmiae]
{(MRAQARQAADIAISGPDRVVAAFCCCYKSVIEAHIRTSAACHRRDRLIERPSSMERPMRSL)----
FISYHRGNRPW-AIAMFQHLERHGY--DVifdFLDVADI---AYErIARTQIQTCAHFVVLLTPSALRHLSG-
PDDRVRREIETALdARCHVIPLVVegcGFESPAIGGQLKGKLAKLAQHDPLTIDAHRFDEGIGQIHRRCQH(Y
QPRDAWRAPLPADGIDLAHDAALNERAAARSARAVAIEEL)}*

$4406=219(249):
>gi|1175168910|ref|WP_081716831.1|
{|335(0)|<Cyanobacteria(B)>}hypothetical protein [Leptolyngbya sp. Heron
Island J]
{(LQVLPKDAKPVTSWSDQDEALLNVTQGIRRVVASMQDKKVKQSLIAAPAASLKQSQEVGRFPSPTEGSLPS
PALAQDAYE)-FHVFISYqaTGEdkQWVRKLLLPHLESAG--LKVCLDTR-
FPLGVPIITSMERAVQQSQYTVVVLSPNYLESDFSDFESLLSQHLGLEQSKSRLILLKREHCEpR------
LGLRMLM-------------------------M(LDMTDEDEFETNINRLVHQLRLPPLGSSR)}*

$4407=202(211):
>gi|1175246753|ref|WP_081794548.1|
{|0(662)|<Proteobacteria(B)>}hypothetical protein [Stenotrophomonas sp.
RIT309]
{(MLIRSGFMEASLQGGL)VSKAFLSHSSMdkAFVSKV-ADSLGSA-----LC-
HYdeRTFEPAGESADEIIQALQNSDLFVLFLSENSLKSKWVQTEIRYARHLYFSGEIKSVAIFMMGDS--
LRERLDVWLR----
PFVVQTLMVPKLVGFRIRSLLSTAD(ASHVSRFIGRDDEVARIKDSLRDLSASRPSSVLVSGPAGIGRKRLLT
RVYQDLYP)}*

$4408=273(263):
>gi|1175273121|ref|WP_081820916.1|
{|79(572)|<Firmicutes(B)>}hypothetical protein [Lachnospiraceae bacterium
AC2031]
{(AEAYWGLVLCKYGIEYVDDPKTGKKIPTCHRSSFDSVMDDSNFDLVMENSDTVARGIYREEAKQIEELRKG
IVEVSSKEE)PYDIFICYKE-
TadDGQRTIdsviAQDVYQELTDKGYRVFFSRITLedKLGQEYepYIFAALNSAKVMLA-
FGTcyDYYNAVWVKNEwsrFLQLMESgqK-----
KTLIPCFKDIDAYDMPKEFARLQAQDMGKVGATQDLLRGIDKIFGRDKKQVE(TTAVLKSSGDSNATVKRGYF
ALEDGEWEKAIQYFDQAMDSDPE)}*

$4409=221(228):
>gi|1175294090|ref|WP_081841885.1| {|53(0)|<Firmicutes(B)>}hypothetical protein [Lachnoclostridium phytofermentans]
{(YKLSKGRSLESFIDEASTSLIIQLSYDLVEHYEIVKQDLSSDMIKDEQYIKVKTILDKFSYLTARVEENSL
AFRTPMLKK)-YDVFISHAT-QDKLESV-
NDLRNGIMSMGVDVWYDSDSIEWGDSLsdRIDDGLKNCEFGIVVLSKHFFDRPWCEKELHKLaerqeLENR--
---KTILPLLLNI---DVNTVISKYPFLENIKMIEYKSGEE--KD-VALLFARVL(IKRLKQGN)}*

$4410=273(263):
>gi|1175302289|ref|WP_081850084.1|
{|98(459)|<Firmicutes(B)>}hypothetical protein [Ruminococcus sp. HUN007]
{(AELFWSIVLCRYGIEYVDDPVTKNKIPTCHRTQYKSILEDPDYLTALDCADTMQRNVYEREAKIINNIQKG
ILEISNKED)PFDVFICYKE-
TDAngRRTadsvLAQDLYYGLVQEGFKVFFSRITLesKLGTEYepYIFAALNSAKVMVVVGTkPEYFNAVWVR
NEwsryLQIMQKDK-----
NRIlIPAYRDMDPYDLPNELSLFQSQDMSKIGFLQDLIRGIKKITGHDNAAPA(IQEKQVIREDVSVQGEISS
ESGKKNKTVVYVLAGIIALLCISF)}*

$4411=171(182):
>gi|1175343872|ref|WP_081891664.1|
{|0(1194)|<Verrucomicrobia(B)>}hypothetical protein [Verrucomicrobium sp. BvORR106]
{(MIRFEHPPMTKSIKYKT)----FLSHNS-LD--
KGYVRALEGWLKQHGISCFLDDQVLLPGDVLtdRLSEAMDQSESAVICIGPQ-GEGPWQREEVNSLLNRA---
--
IKLSQEKDEFRLIPVLFPGADRSKLRWFMDTRLWVDLKNGITDDEAPLHQIK(RAILGEHSAEGRTTETKNPY
QGLDA)}*

$4412=221(220):
>gi|1175166449|ref|WP_081714370.1|
{|107(0)|<Proteobacteria(B)>}hypothetical protein [Mesorhizobium ciceri]
{(SRLRMKIHSVKRFGATEAAKIWLEKGADEQKMWSHITGNLAFIRQVRGVDDTVLAKLCKDAVVAGLTEPTW
VLKMAELVR)EFDVFLSHAS---EDKEKIRRLKDKLEHVGIKVFFD--ETSISWGEsivEKINHGLL-
KSRyfVPFLSQKFSEKGWTNKELNSAISMNIDRKG-RILPVIDSNFSvdD-
NYPLLGETLYKKWPtdnasegqfI-DEVADAIL------HLVETKPV()}*

$4413=224(220):
>gi|1175332701|ref|WP_081880493.1|
{|269(0)|<Proteobacteria(B)>}hypothetical protein [Paenirhodobacter enshiensis]
{(INRLRMVIHSAKKVGFEEASKVWLPDGKNNLREHILGWLAHIRQVRGASDPVLAKLCLQAHGAGLTKVGWI
EELADMSKD)-FDVFLSHAS---EDKDRVRKLSDALTAGGVKVFFD--ESSIKWGDsivEKVNHGLL-
KSRffIPFLTTTFSKKGWTNKELNSSISMNISRKG-RILPIKSEDFEigDRypILNETLYKTWPKSSV-
DETA-FIQ-EvvdsiiKLIEEDKAAPSV()}*

$4414=179(182):
>gi|1175294091|ref|WP_081841886.1| {|0(221)|<Firmicutes(B)>}hypothetical protein [Lachnoclostridium phytofermentans]
{(MQVVLRQVDTLIALMHVQNNKEEGFTMSEHP)--
KVFISYSHQdAKYEKKVLDFsnrLRSEGIDANVDLYEESPAEG--
WPRWMENQINNADFVLIVSSKSYYQKCYSDIKKGKGISWEVN-
IVYQHIYDATSQNTKFIPIYFDTDDEQYILT--PLKPFTFYNIGSQEGFDKLYW(RLRGLAKTQRP)}*

$4415=241(243):
>gi|1175343634|ref|WP_081891426.1|
{|0(113)|<Verrucomicrobia(B)>}hypothetical protein [Verrucomicrobium sp. BvORR106]
{(MSSVTLTRHSASMLITAPVIGTACRCDKQMKP)ETKIFISHATEdKvEFVRPLAEKLKRL----SHVWY--
-DEYSLPFGAsifgSISAGLKDCDFGVVVLSDPFFQKKWTQAEIAGLFALENV-
GTRKIVPIWKGVSKSEVEKFSPIladrRAIDSAQE--LDKVVAC--
IVEAIEAASVES(IYVHEPIVSDPRKCFQTLGSRLSEYVATRAMNQSPGAPNLVHAAQSQFVSQLNAEVEQIA
LANPKFGISWS)}*

$4416=251(243):
>gi|1175197470|ref|WP_081745380.1|
{|0(91)|<Proteobacteria(B)>}hypothetical protein [Pseudomonas sp. URIL14HWK12:I7]
{(MFTRHLSKAVSGPNNNGVNTMNSERDRGA)---IFISYAWggelQNKEWVRRCIVVHLDWNYR-
VFWDRDSINFGQSITEAIDTALaERPLLVLCLCDQDYLDAAERTNsglyrELQAltevANSPG-----
VRIVPLLLSEECegQLPAPLDGRLYLDlrplhLRG--LDLGAALL--
AMVEGVSQAQVQ(RGIQSQVAMAQLRQRAYDYLRKQPLSLFGDGLTHDVMVRPEGQAHFPLLAPQWMWDSKEW
GFMLGDENSTYCPV)}*

$4417=216(222):
>gi|1175224678|ref|WP_081772481.1|
{|114(0)|<Actinobacteria(B)>}hypothetical protein [Cellulosimicrobium cellulans]
{(RQQAQRRVDAYNREVSRVNAHNQQVNQQNQRANARNRQAVADLNRRLRSASATSGPTYTPAEQGLADKIQQ
QAARFPDRD)-WDAFLSYARIDGAEvGAALREELNALGVS-
VWFDEIAIAPGRSQSLQMDKGLRSARCGIALLTPAYLTGRFWTErE-LGALLHK-----
SMLIPVLHNVTFaeVAEYSGILPDLAGFET--RRDDVVVIAEKIAAAVMPHDDE(ED)}*

$4418=217(228):
>gi|1175295071|ref|WP_081842866.1|
{|0(76)|<Actinobacteria(B)>}hypothetical protein [Actinomadura madurae]
{(MTSAVPATSG)--RVFLSYAYDDHALAQLVSQALAQSGIR-IT--
SWDASPGENLVERLREAIRASDVVILLVSPA-VVGNLGASaELEFALSRDLG---
QRGVEVVPVLAAptDLPPTLRNWSMVDLT---KDFNAGLR--
QLVEQVQATSQA(DFSTLSPHEFEDLVASLLRSAGFRLDEVQNRPDTGVDLRATYQRDDPFGASETEEWLVQV
KLYSHQRVSVDEIRRLAG)}*

$4419=215(219):
>gi|1175303657|ref|WP_081851452.1|
{|0(708)|<Proteobacteria(B)>}hypothetical protein [Bradyrhizobium sp. URHD0069]
{(M)-RRWFLSYHSPDRALGERLKAALErmDTDSS-
VFFAPSSLRAGGFWSKALAEEIAQADAFILLVGEKGIGD--WQVlEYDEALDKR-----
VKSpdFPVILVLLHgqTAPGLpfLRRLHWVITPD--PSSEKDVA--
RLFDAASGSSTR(SGDLWRYTSPYRGLAAMEEKDSDYFFGRERETMDVLSAPAVAMDRLPVLLGNSGVGKSSL
AQAGILAALKRQAWPEGV)}*

$4420=239(225):
>gi|1175354848|ref|WP_081902640.1|
{|167(0)|<Actinobacteria(B)>}hypothetical protein [Lechevalieria aerocolonigenes]
{(VESSIRPGSYSAWMVLLTSADRTSEKFRLTSRFDPELADGDDFGTVTASREFSIELAGPDLAPQVDTSRTS
GATFGLPAR)STRIFICYAHEDDAHKKMVrdfADLLRACGLD-

PRIDSDYEGPRQDWSQWAVRQIKEADFVAVIASPtcQAVGDGTYQGkDRAGIRHElgvisnllqmepdwA---
--RRVLPVILPGLTvnDIPMFLRPRTMDHYPV--EALIKSELGTLLKAVDTAPPWQ(GWERP)}*

$4421=252(257):
>gi|1175168961|ref|WP_081716882.1|
{|162(667)|<Cyanobacteria(B)>}hypothetical protein [Leptolyngbya sp. Heron Island J]
{(IQGLIGLLKTEAAYVHQHTKLLDQALTWMDYQNQTRYLLTGKACQSATDWLLSKNELVSCYPSGLHSEYICQSIKNAQNL)MCQVFLSSAAEdEEVAQQIRRSL----
LQHAITVWTNRtDVVTGEDFLsATYRGIEEADNLIYLISPDAIESHFCRDELNYALSLNK-----
RIIPVLVRPT-
NAAKIPREIRGIQYIELmeLTENTEKRDYFIDESQLLQILKK(DEPYHREHKELLVKALKWERQEKNPSILLKRHSLNYY)}*

$4422=252(257):
>gi|1175169071|ref|WP_081716992.1|
{|166(943)|<Cyanobacteria(B)>}hypothetical protein [Leptolyngbya sp. Heron Island J]
{(DLKQIFHRHQDYVENHTRFLNDALAWERQQKQTEFLLLGETRQLAETWLKQLFKSEQPPCIPTDLHGEFITESIKNANNM)MTQVFLAYAQAdAEVMDSMRR---
ALWRQGFTVWSSQTDIPTGEAFKqAVNRGIEEADSMVYLLSSDALKSKYC-QyELKYALALNK-----
RIIPLLVNPL-
GAAESPSELASLQYIDLtsPLSGEEYQLDDRDLIKALNEDAV(YYNQHKILLVKALKWKRQHQNSSVLLRGYDLRHAEAW)}*

$4423=209(221):
>gi|1175201204|ref|WP_081749111.1| {|202(0)|<Firmicutes(B)>}hypothetical protein [Butyrivibrio sp. FCS014]
{(NTAISKEKSSRPDYVLTIQNYQYYRAKIMIQKSQYEMEKKLFLLQQKIDELQMLSSEKDVEERDDDRNEPHAYDGESRYS)----FVSYAHTdKTDVYNIIGKLQK-KRLN---
IWFDEGIQAGEEWPeVVAKRIVNSDTVLVMLSSNSIQSANVRREVNLAISENKT-------VIVVRLD-
DVTLSPGMKLQFSLYQMvnRDLDDERSLVEKLASAIMMEESN(E)}*

$4424=262(272):
>gi|1175225459|ref|WP_081773262.1|
{|42(32)|<Bacteroidetes(B)>}hypothetical protein [Prevotella sp. P6B4]
{(GGIDTYNIVLTVPTDLFKSLRRKNVIEQAEAELDKCYADAMRGEGESLQIASVVIRPTTADVSQLGNNYNDRMWKPGYFR)---LFISHLSEcKESASNLKECLQNFGIDGFVAH---
EDITPSKEWEqEIEAALFSLDALCAIVVPGFEKSNWC-DqEVGIALGRKK-----MVFSIKkgKDPY-GF---
-
FGKYQALPSQRnaNDMAQELTKAICVNEFTRGEYFT(KLVNLVLNASSDTIALKFIQVLFEAEHLDKQYVSLLHDNFANNQTIMKENVL)}*

$4425=213(220):
>gi|1175278798|ref|WP_081826593.1| {|60(0)|<Aquificae(B)>}hypothetical protein [Persephonella sp. KM09-Lau-8]
{(ELFEVGRKLLQKVAIKELSLLTTKQLIKYSSTRRFDSISIPADINLIQMMFPSLFRFPERKYLIEKGYGFLDDISIFPPN)--TIFFSYA-
NKKLRNDIIKIKSLLTAETYPVFFDIQSLFPGDNLEdKIKSYIVDAQAIVFFIDDSFMRSKFC-
KqELDITLE-NNI-KHMFII----DKN-
LKEEYKRSEPKLQFTNSlfIETDFNNINHLELYKKIKEFINS()}*

$4426=232(240):

>gi|1175291040|ref|WP_081838835.1|
{|0(517)|<Chloroflexi(B)>}hypothetical protein [Thermogemmatispora carboxidivorans]
{(MGDQGSLSSGLSTAALSLRPVL)------CYAQEdREFASRLIKHLSA----
NQISVSDlPKELPAGHDWRaVLRRRIGEANLVIVLIGPDFLQD---HRfELQLIEERLPA-
NDWRFIPILAREV-PRQGIPlLERCQLLPRSEkaLASLkaSQRD--
KVLKEISEEIRML(IERRPARQSLQGGVSAFAREEKSAQVPVLPREQTAAVEGGSGATAEIQPALPVESRKRWRQFAAWKVVAPLALFILLI)}*

$4427=284(290):
>gi|1175313295|ref|WP_081861090.1|
{|111(846)|<Firmicutes(B)>}hypothetical protein [Butyrivibrio sp. AE2032]
{(MDKDYPFALEKHIPVLPLLMENSIKEVYGRADKFGELQYLSPVNTDSTKISYEEKLRKYLESVLVSNDLVSRIKQAFDAY)---
IFLSYRKTdRKLANDLMKMIHNNPECEDIAIWFDEFLTPGESFReGIDRILKDSKLFTLLVTPSLLekndngTPNFIMAqEYPAAVNL-----GKTIFPAEMVYT-DYEEL----IKAYP--GipVCADTNDE--
QGFNKQLLDALVG(IAVSENNNDPEHNYLIGLAYLEGIDVEVDLDKGLKLITKAAEAGLIDAIRKLASMYKNGNKVNADYRKSL)}*

$4428=231(209):
>gi|1175336268|ref|WP_081884060.1| {<Actinobacteria(B)>}hypothetical protein [Micromonospora parva]
{(MFRPRGEGGGAAVSTVDAPSGARAPLF)----
FISYSRApvgratgkpaeyvSRFFEDLSVHVSELVGpvTGVDAGFLDTSIAGGERWSpELLQAAGTCQVFVPLVSSALLGSEWC-GrEwdafsRRRITPRHSSAsvHETAIVPVTWSPT-
NGTALPrvLRDIQRFSPTAmpDPDIAVHYQRDGVYGLLTMQWEN(AYRAVVWRLAQRIVAIHRSYLVEPWVPASVDELCNRFAEEPS)}*

$4429=256(220):
>gi|1175343870|ref|WP_081891662.1|
{|0(136)|<Verrucomicrobia(B)>}hypothetical protein [Verrucomicrobium sp. BvORR106]
{()MPhRVFTSYAVLdRS--
TQMEKFMKLFhevlrtkvgayeaEEIEKLVFFDFRGIRPGNVWTqALADEAGEAELLVCLVSPTYLNSPWC-
GqELEVFHQRHtqwessnpgRA-GQAtFVFPV------
WWERDPFrplpdklSKFQAVtfdsPRGYkgglrqladLSKYRDAF--
KSFVEaLVTNIRTA(LSSRSKLPTIKVDFATVANAFMDQPRPYDIHTWFAVPGGEAWLPSGVGRPICQEVAASAALMRVSLHGFNPSALLGSQIS)}*

$4430=189(199):
>gi|1175389788|ref|WP_081937578.1|
{|0(312)|<Actinobacteria(B)>}hypothetical protein [Streptosporangium roseum]
{(MISSGGRPARPTRHSYCCAVAGYA)----FISYSRRdRRYVERLASHL---KRAGV-
RTWFDDEIPIGDRFEkVIRSKIDACDAFVVVMTPDAEESRW----VHREITRAET-KGKEILPLLLAGD-
VFFRINDANCEAVTDERmpTAKFVDQLKALVSADDSTPGLVE(SPAPAKTWRRSIWFSPFLAIPLGIIAVAALAIPLA)}*

$4431=254(240):
>gi|1175421018|ref|WP_081968805.1|
{|0(264)|<Bacteroidetes(B)>}hypothetical protein [Phaeodactylibacter xiamenensis]
{(MKRNTLRIMPIQPIPETQDRS)-
TNIHIAvlYARAdQEAWEELEKQFTSINaRYPNVHIWTATDIDIGGSVDrQLKDELKRADITLLLFSPEFVND

D-IVDeEVRDLLttyarfsdsDAYRK-EERFIMPILLNHlY-GWddiydQHFDIENLRVF--DKlpEAR-ESRG--
KVYESIAQSLEEY(IARINAKSVQIALPTWIGFIPSIMYNGGFARNENTPLFQKYRREVHFELNDHLDQLCDDFKAGQIDMMWCTIDRLPYVA)}*

$4432=181(189):
>gi|1180636840|ref|WP_084122050.1| {|0(844)|<unknown(X)>}hypothetical protein [Aquiflexum balticum] >gi|1175435061|emb|SMD45311.1| Putative peptidoglycan-binding domain-containing protein [Aquiflexum
{(MAAPVSSEPIQLLLF)-------YAREdQSLAFYLKELLSKYGYR-IIEL--
DEGLEYGKSWEtQLSRNLEIADGFIFLITKSNIKSDF-LNiELEQAYKHLSG-KRKILLPIIDSKL-
PIPSIIENINVIRFSPLddFSGIAENIHQALVYWQRRKTEGS(SSGTASNKTTSTKSDQRDSRNENTLEIQKILKEL)}*

$4433=180(184):
>gi|1175169761|ref|WP_081717680.1|
{|0(142)|<Proteobacteria(B)>}hypothetical protein [Hyphomicrobium nitrativorans]
{(MSFA)---VFISYGG--P-DEKFAAALDEFLRGNNIDTFFFPKSAQPGDRLyrVMRNGIEEAD-
KVVCVCSKTSLGRPGVLnEIDLSLAREAREgASTILMPITLDDY-
VFSDWEHANAAIVGAVRDRVIADFSGVRWEQEGFEEMGRR(LIDAILNDKEYIVLEDMASIQFDDDYGHVATVTERKKIII)}*

$4434=197(202):
>gi|1175172096|ref|WP_081720014.1|
{|0(19)|<Euryarchaeota(A)>}hypothetical protein [Methanobrevibacter arboriphilus]
{(MYWVIKCIIKNKEELFISEQKTPI)---VFISHAT--
EDKERFVEEFAKKLRKDGIDAWYDNWEMNAGDSLteKVFEAIGNCDF-FIVILSPISIKKPWVKeELNAAL--
MQKIkGKLKLIPIPIDSD-
VGIPISIDHLYRFNMSNTKDYEEQYQNLVMDIFGITKKPP(LGERPSYITDRIHLDLEDVDFLIFETLGNMTKNEGLNF)}*

$4435=197(206):
>gi|1175215623|ref|WP_081763495.1| {<Proteobacteria(B)>}hypothetical protein [Thiorhodococcus sp. AK35]
{(MTVKMILLAVLCALLIFLSEHNYMDGLDTLLMVVGAASGLILVSSVIGSRSSKVVRK)---VFILYA---
PEDTEFVSKLYEALKISPYRILWDKKEVKVGDHIekK-
TNQLLASSSDVIFVISSHSTNPEWFEpAFAKVAEQNKRL-----LPVLIDDS-
EPPEALKEFMFADFRESFDDGYLSLRDsLREVKRGRPVSKK(PSTDEVAAN)}*

$4436=191(192):
>gi|1175299770|ref|WP_081847565.1|
{|0(627)|<Proteobacteria(B)>}hypothetical protein [Bilophila wadsworthia]
{(MR)---VFLSHSN--NDRKGYVEYVANNLSSD--EVIIDTRSFIPGRSSsdEMTRLVEECDIFVLFISS-
SSLENKNVKyEIS---QYKKSfFnDNKKFIPLIIedgikyDDC-
RIPKWMRDYNLKRVFRPKKAVACIDEVIRLMTWKKYEFIK(RKDSIFIGRNEYVEKFERRFNDRTLGRAICYCVSGLEAIGRTSFLAYCLK)}*

$4437=199(182):
>gi|1175224011|ref|WP_081771814.1|
{|0(279)|<Proteobacteria(B)>}hypothetical protein [Paraburkholderia nodosa]

{(MAYVTGYN)-HDVFISYAHIdnepigAGdgwisiFASHLKKFLDKGLGCKDASIWMDH-
ELTGNEVFstKIEEALRESATLLVIASPSYVSSKWCARERNAFLdsvreKSRAGSrifRVDIDKLKYAE---
LPTEFGELLGYTFWALDAGGNPRTLGHPIVDPKGEPEYFT(ALNKLRTELGSELKRLRQTQSESRTSARSQPA
IF)}*

$4438=175(171):
>gi|1175282753|ref|WP_081830548.1|
{|0(1284)|<Actinobacteria(B)>}hypothetical protein [Rhodococcus sp.
UNC363MFTsu5.1]
{(MCTPPTCDGSAMS)--RVFITYS-SrdrrqaIALKRWLAEREPGLARE----
IFLDvdvDTGLPLGIRWkdALRKAKDRCEAVIVLLSPAWESSHECKLEYRHAEDLNKPVfvvRLEPMESGEIT
---REWQRCDLFGDGPQTEISLGDGSEPVVFLTDGLERLRTGL(RATGVGAEYFPWPPPGDS)}*

$4439=199(184):
>gi|1175287408|ref|WP_081835203.1| {|0(865)|<Firmicutes(B)>}hypothetical
protein [Lachnospiraceae bacterium AC2029]
{(MQMADREYT)-
YSAFISYKHNpdgdfaATIQNQIERYVVPKeYRNsplvegnRMKKVFRDSTEFSGSHNLaeAIQTALSKAAFL
IVIVSEETKNSPWVAQEIEFFLKDHERDhilTVISSGSTPSK---
NYPEALINVEMPDGRIEKVDPLAVDYRKYNPKRGRRAHGN(RSVYSSEFPRIIAPLLGISYDDLYRRRERYKR
RRI)}*

$4440=157(162):
>gi|1175305308|ref|WP_081853103.1|
{|0(685)|<Proteobacteria(B)>}hypothetical protein [Bosea sp. 117]
{(MMKFP)--RAFLSHGHVdknivREVARDLGRAA----------
VIYDEFEFQTGDEFrdAITKGIDRSDVFILFASRAALGRDWVKFEIELASEALIKKalsKVTTYIVDQEL---
THADLPEWMRVTLIRKETQPGLIATDIRRIISQRLRERIP(TYFVGRRAEIEQSLEII)}*

$4441=170(178):
>gi|1175718811|ref|WP_082138787.1| {<Proteobacteria(B)>}hypothetical
protein [Burkholderia contaminans]
{(MASYSVRASSDANELFLFPDRKILASIWGRATSASSFT)---VFLSHSS-RD--
KPIVDGVFNELHKAGIRAWYDRYEIdpGDSITDKINEGLASSQIGLLFFSKNFIDSKsgWPTKEANYFFQKLM
RSGKKNFVVLNIDLSIDD------LPPLLQDFRFIDMKSESSTREIIDLLRKSEAS()}*

$4442=256(246):
>gi|1175511439|ref|WP_082054086.1|
{|20(315)|<Proteobacteria(B)>}hypothetical protein [Methylococcaceae
bacterium 73a]
{(LFLRSDRAVRIVAQEYGIQPAFSTHGPQSASGSEPVGSSRETFLTLSEGVRIIKRTSRACMARRWRIQGAR
LRRRRKAEM)-HDVFISYahddngipRG-ASLPLGWVTLLAENLNVGpGVRrkdIFIDHR-
LQPGDAFagDLVAKVEGSAVLVVLLSQNYVDSVWCGKELEHFVRSHAN-
DPQRPADVFVVELHPFEELERVPEIIQQLRGQLIHAKFWFQPTDSPSPFLAGYP(SPLDGGEAGKDHYWRELN
KLRVAVDS)}*

$4443=296(298):
>gi|1175871043|ref|XP_020589250.1|
{|18(590)|<Streptophyta(V)>}uncharacterized protein LOC110030716
[Phalaenopsis equestris] >gi|1175871045|ref|XP_020589251.1|
uncharacterized protein LOC110030716 [Ph
{(SASTCSLPPNLSPPKIANSHHLSPSFIDPPLFSSTTIPVVGSVTQVSSTRAMIYNTNISSSSERRLEGNDS
ETERLRSCD)---VYIGYcdRK-
TPSLKRFVNWLRAEMVMHGFSFFMSYLTRCSDARSHAIARtaMEEAALGVVIVTRKSFSSPWSIEEL-----

KLLL-GRKKLIPIFFGLKqkdclSRDIIEKKGEiWGKYGGTL-WKKYEAIE-
EEWSVAVDGLSQI(DVKFEVTTSNMRDCVLDAISFIAKKLGRGDALEKLMKWKNRVATEEFPFTRNLNFIGRK
KEFLKLKLTLFGDDQMECD)}*

$4444=196(220):
>gi|1175511315|ref|WP_082053962.1|
{|411(0)|<Proteobacteria(B)>}hypothetical protein [Methylococcaceae bacterium 73a]
{(SLNFIYPMVTLVAAGIVAASVMTALYLKRRLQWALFHAERIESTGDYDVFISYSRTEPEHIAWVAANVVEP
LRTQARKPD)--------------------------
GSPLKVFFDKESIRLGDDWptRVAHDVNSSRVFVPIYSRDYFSKPVCKYEMEQALKMYVML-KNLIVPVM--
RSQepiaIPNAYDGINYIDAIREPEFAKRLVERVSQAIAEVELRAVV()}*

$4445=187(178):
>gi|1175885556|ref|WP_082152415.1|
{|0(302)|<Proteobacteria(B)>}hypothetical protein [Candidatus Rhodobacter lobularis]
{(MTKRIFLSY)SRRDDQPFGDQQeGWVTRFRRELETDLAQScelACEVWMDVRDLPENRPFddEIDSELTRS
SLFLPVLTPNWFRSEHCTAELAGFEQ--
KRGgHDGRVFPARKRHFPselfKQHFDKRNSYVFHESHDDDPELAMELKIDGDPLSPFRRAL(RNLTVKITRR
LREDAEAEALARAAEAAEP)}*

$4446=124(141):
>gi|1175527904|ref|WP_082070545.1| {<Proteobacteria(B)>}hypothetical protein [Gynuella sunshinyii]
{(M)-----------------FVRPLAETLENIGVKVWYDEFtlKVGDSLRKsidQGLV-----
RSRfgTVILSSAFCSKNWTQYELDSMVAREMNGHKM-ILPIwhKITKTEviN-FSPAIadkVALNTSLQSI-
EEIAGELA-EVILAS-----KA()}*

$4447=215(222):
>gi|1175498145|ref|WP_082040793.1|
{|22(0)|<Proteobacteria(B)>}hypothetical protein [Aeromonas allosaccharophila]
{(NPRDGQVEKMLKLMTDVGARQRPDIPINELMMEMGGKRFNSMTVSNVSSIEQVKHDLFDFKERKDIAQIIS
IKPLGKRLP)---VFLSHSSSdKPFVEDVIPYLSSQGLP---VWY---
DKVSIDYGEsilsAVQSGVKDSGAVIFFITKSFINSSWCKLEMEGFMSRYAG-
GQGVLLLSVVSNDVSHEELPFFLQNIKYLRL--SGEQNPKLVSSELLPALKKHF(KL)}*

$4448=193(195):
>gi|1175569318|ref|WP_082111017.1| {|0(99)|<Firmicutes(B)>}hypothetical protein [Paenibacillus algorifonticola]
{(MLMFYFWFSLEITSQDYLIKTGGYSMSRL)---IFISHIHEeQELAILFKDALEEEFSGFVDIFVSS-
DGTSIPAGAhflkKIEKNLIECSAALFLISPFSVNRNWINFELGSVWIRNQL-
NELNQGPAIPAVPICHSGMDFSKLPHPINSL--
NAIQANMPSQIEFAFRSIQAA(VGGRGKLKTDFFDLASKISRFEGRYT)}*

$4449=251(245):
>gi|1175904150|ref|WP_082163148.1|
{|418(36)|<Actinobacteria(B)>}hypothetical protein [Kytococcus sedentarius]
{(LVRTWAQTLINRRSTIAKEDKIKLAETTAAANSEYAKRISQSIDKLNIPGKDAEGLKQEVHLGLTRGDPLT
DEGQAEAKE)EYRVFISHASQdKVLADIIYNTLLEHGAESNEIFYSSRDKLtvsdkkrYVQLGPmiheAITSS
ATK---ILYLTSTSFCNSIYCCFEGGAGWATRAV-ADYELITTRYRLKPDWLDHDLPTTELIDHQG--
KLKLTRDSYLLFTQAINHLID(HLNVGRTIQGRELITPISETYLPSD)}*

$4450=223(232):
>gi|1175886615|ref|WP_082152766.1|
{|127(0)|<Proteobacteria(B)>}hypothetical protein [Candidatus Rhodobacter lobularis]
{(LPSREQETSNHGKVKLDSFRRKMSEATLVKFWENPSQLGAEVAFSLMDIQRRHPSSGWVRGANQTDKEESP
NTRVPIGSE)---VFISVSSKDLDWGDWIAWKLRDLGYH-
VRYHRWELGIGTDVAAWMADAVASSQVAVLLFSKHTEAASEISLeQRFIAnvglDQKA-----
IKVLPILVDPVK-IPRIFANLYRVELFS--KNQDEAAK--SLLKVFSGFSGG(YSKEPLFPGGNS)}*

$4451=227(227):
>gi|1175921695|gb|OQW42185.1| {|237(0)|<Proteobacteria(B)>}hypothetical protein A4S08_01950 [Proteobacteria bacterium SG_bin4]
{(KPRLSAVKGKTDWFIERTASTPEFTTFLDHYSRSTKHFPLEPVEFVARLAKELPQDLPDTATTRTQAIPRN
PDRDILEGA)---VFLSYASEDRVIVERIHKALDDSGID-
CWFDRQQLKTGDDWESKIIRNVNACSLFVLILSRHTLtQQDRFFRhEWNEAFkrsigfaANA-----
KFLIPVAIDDIQpnDPALPLELHKTNWERS--PGGEMTPAFLEMITIAFQQAQL(RAHGSTP)}*

$4452=230(220):
>gi|1175906786|ref|WP_082165784.1| {|51(0)|<Nitrospirae(B)>}hypothetical protein [Leptospirillum sp. Group II 'CF-1']
{(VINQNKIDWLRFSKPNWIFSSDPFKLSFPQYSQEDWLHGEFVVDFERSTHSGPVPMGGVSSDTTFCRFYIT
KDLIMDQLP)-KKIFLSHKFVnKDLVREFKKTLEAVG----
LDPWLDEDeMPAGTALHrGIRDGFRNSCAAVFFITPDFVDEKFIANEINYAMEEKTE-
KEERfsiitlvfedpirkkgIVPKILKQY-VWKEpknylEAFREiIRALPLKAetNWKWKE----------
ISPPDPP()}*

$4453=180(175):
>gi|1175919503|gb|OQW40085.1| {|0(744)|<Proteobacteria(B)>}hypothetical protein A4S08_05825 [Proteobacteria bacterium SG_bin4]
{()MPTdqVFISYSRAdRAACVQLRTEL---EKAGLSAFRDEDNIQPGDEWIaELEKALEQCTAFILLIGKD-
-GVQRWVSaEYHVALRRHyaktdAA-NSLPIFPVLLQDA-
QSVSLPAFLSSFQSVRWnaSESLPPQLIAAIKNRAARSGNAI(TFEGCPFLGLSAFGRQHASLFFGRRQETLK
ALEGL)}*

$4454=231(218):
>gi|1175524694|ref|WP_082067335.1| {|0(246)|<Firmicutes(B)>}hypothetical protein [Paenibacillus terrae]
{(MTTESKKPP)--TVFISYSwtT--
TDHEQWVYELATRLMSDGVMVKLDKWDLKDGNDTlaFMESMVTSTEIdKVLIICergyqAKADKRTGGVGtET
ELITPKIYGNvGQEKFIPVvseRDEEG-
KpfLPTFCSSRMYVDLSSEYEKGYERLLRtLHQAPMHRKPALG(SVPSFLFEQEVSHYESSNLIKLLNSAIEN
NPKRVRGITSSFIDTFLGELKQLEITSIDNLLDEKVKEKI)}*

$4455=207(220):
>gi|1175545881|ref|WP_082088510.1|
{|137(0)|<Proteobacteria(B)>}hypothetical protein [Burkholderia sp. USM B20]
{(SANMLLQVADGKRKHVLPFPVLGGAAKLSYERRRYELEDKLGLNFIKLQDHDVEDSLESMINAFAVGRDSV
LDAPSNEKI)----FISYPRArqaeaDHVETLL--------
RRRNITVFRDESDFGAGHSVveRIREAVFEATVFIALWSAEYACSPWCFDEFELALDRHSQEK-----
MDLWI---IRTDETRIVPTRARDLIYHTALTRQDLESRVIALIDGRIR()}*

$4456=235(236):

>gi|1175823013|ref|WP_082145764.1|
{|0(311)|<Proteobacteria(B)>}hypothetical protein [Microvirga massiliensis]
{(MSGTRGGYNGAGMPMASQTS)-
FDAFISYRRAdgyavaRWLRRQLQDFRVPAILRRPygrkLSVYLDTAFERGTADFyedSIRPALLESRFLIVI
ATPAAVRRPgvdWMQREINDFTAGPNGRnvlLVRGSG----------------AFADPLPGDLSNRFPNME--
I-
IDLRGAGTLA(GFNPVRAVHLSKEKLKLLGPLIGISPSEMPLLHQEEEHRQQVRFGTLTGTAAGVLVAISGLG
VLTLQSRNAATRAL)}*

$4457=193(170):
>gi|1175886574|ref|WP_082152751.1| {<Proteobacteria(B)>}hypothetical protein [Candidatus Rhodobacter lobularis]
{(MTYQ)-YDAFFSYkRHSesddwhKELKNKIE-
FWLSHELGRQANIFLDTEDIQTGSRWrsRIAGALSESRCLVCIWSPMYFQSKWCVAEWRTFEEREARLncdLV
IParfhDGrhypQAAKD---RQASDF-RDFVSTVPtfweTRLAAEFEkrklrpfTEE--
LAASVRAAPEFS(GDFPIIDDPGAEHVTRRTAIWRISNV)}*

$4458=165(193):
>gi|1175994706|gb|OQX02703.1| {|0(280)|<Proteobacteria(B)>}hypothetical protein BWK73_41895 [Thiothrix lacustris]
{(MCARPDDQPFANQEKGWITH)-----------------FTNHLRNELSRKLgrkdnYNFWKDFRLQGNDA-
VtpEIEEQVTQAQTLVILFSTGWMASPWCQNELELFLQSHPD-----------------
VKGRIFVVELERVAMADKPVIMQDLLNYPFWRVTDKDR(VHQLGYPVPQATDTDYFERLVDLSHDLSATLKT)
}*

$4459=173(175):
>gi|1175963188|gb|OQW82593.1| {|0(303)|<Proteobacteria(B)>}hypothetical protein BVN30_08265 [Proteobacteria bacterium ST_bin16]
{(MAKNPVK)---VFVSYSW-EqEKQTGVVDELERLCRQRNIQLIRDKNALQHGELIrdFMDRLSGGEHI-
ITLFSDAYFRSTWCMYELLTMWQKGDFHSRTHPMVVDAIDLQNTDYRIAVTDFWKEKYKATESSLKGRDPAVF
IDEHKRVKY(YRDYYQNINELLNFAAGRLTTPLGQLKQ)}*

$4460=170(172):
>gi|1175996191|gb|OQX03988.1| {|0(310)|<Proteobacteria(B)>}hypothetical protein BWK73_37685 [Thiothrix lacustris]
{(MAEAIN)---VFVSY-S-
WDVerKTGIVDELEKLCQYRNIRLLRDNNVIKHGELIqQFMDKLTGGEHVITVFSKPYFQSPWCMYELLKTWQ
KGDFHLRTHPIIADDCDLQNAAYRIDVVDYWVAEHQKIEALLQGRNAALFVKEYEKANM(LRDISQNASELMN
FAAGRLTTALVEL)}*

$4461=136(149):
>gi|1176161043|gb|OQX77447.1| {<Bacteroidetes(B)>}hypothetical protein B6D64_08260 [Bacteroidetes bacterium 4484_276]
{(MTDG)---------------
HEFAESLFSDLQNKGIRAWYAPHDLQAGKKVveQIDVGIKYTDKLLLILSDGSMNNEWVKTEITKAFKKSREI
GKQVLFPASLTKFENLRNWECFGADYGHVLAKEIREYHIQKKLLINEIIKFHSY(YFYFL)}*

$4462=206(226):
>gi|1176002121|gb|OQX09231.1| {|0(276)|<Proteobacteria(B)>}hypothetical protein BWK73_23305 [Thiothrix lacustris]
{(MTAAFGATAYVSYA)--------RA-
DEQRVQVVSLLHQDFVGEWVHLKVDANEVQPRESfEDFIGKIGGADCVVVMFSDSYLRSFYCMLELTRIMQQG
DA-VK-RVYPVFVE---------EGFRLDGARQQ-

WKNYWQEKSDRWHEQGHDEPDE(ITDAYGSVVECSEILANLDAIFDAFARRLAGQVPACNAAIIAWVKQTYFQRIQGNPVFRKSIQYLRGCHDKT)}*

$4463=169(179):
>gi|1176122132|ref|XP_020632390.1|
{|0(128)|<Cnidaria(M)>}uncharacterized protein LOC110069235 [Orbicella faveolata]
{(MGKH)---VMLSY-QWNTQELAEQVYQGLRSHGI--
NAWMDVHGGVKGNVNdSMAEGVDGAAAICCFMTSD----YFGSRncK----
KELNYADaQAKAIVPLMCEKGFKATGWLGIITAGLLWIDFREGTKFKNSLESLAKEIINSLE(NEDDVKMKPSERPAAADATTPQIDIKKKPGRAFLH)}*

$4464=143(146):
>gi|1175935837|gb|OQW55823.1| {<Proteobacteria(B)>}hypothetical protein A4S17_05820 [Proteobacteria bacterium HN_bin10]
{(MTDRL)----FISHASEdeA-VANKIVAYLEARGV--
ACWISSRDIPPRAIYADAIVEGMQSCTACAVLVSAASNASKAVKREVELASHEDKAFIPIGTVARLTTSGQlLPTSMSRAPGRRARSSHtYRIVRNCSKLHLSWVEPSMTERV(S)}*

$4465=226(239):
>gi|1176056702|ref|XP_020602500.1|
{|356(0)|<Cnidaria(M)>}uncharacterized protein LOC110041540 [Orbicella faveolata]
{(SSPHWIRPTRSQTTHFVGEPSPSSNAMADVDLLRTMLFIALGLLAGLVAMAVIITLWRRRKRQPPGSLRLPPDMDENDFK)-YDVFVTYSRKdfPWVDGELLRLLRENQVNY--
CVDHVHFELGKAFVESMVESVYQSRKVLAVWSGSYASSKYCKQELDYAIQRSFQNSDCSVIVIRLDRTDrKQLPKP-------LRARtFLDYSDSVERKGWEK-------R(LIKHLKGPDQKNHAIATIV)}*

$4466=217(235):
>gi|1176059428|ref|XP_020617034.1| {|149(0)|<Cnidaria(M)>}toll-like receptor Tollo [Orbicella faveolata]
{(FVQSQTATQVTESVTTSTRLSSNLGSGRRREWLLPIIIISAASVTVLFMVFVPFCVKKRWKSSYNIPNYQYSKEDLINRL)----FISYSSKdfGWVTDNLMSILEKHSIDYS--
IHSRDFEIGRPIVQNMADSVYGSRQVLIVLSENYLASNFCREELHMAVQRGLDEGDSSLILVKINNMKkKQLPAalRKKKLVDFDKYKtTLDW------------------E(ERLISEILKSKNASV)}*

$4467=171(200):
>gi|1176059434|ref|XP_020617065.1| {<Cnidaria(M)>}toll-like receptor 6 [Orbicella faveolata] >gi|1176059436|ref|XP_020617072.1| toll-like receptor 6 [Orbicella faveolata]
{(MMLFVRFVVKKKRKSSYNMRKPGLREEDLINRL)----FISYSSKdfGWVTDNLISILEKHSIDYS--
IHSRDFEIGIPIVQNMADSVYGSRQVLIVLSENYLASNFCREELHMAVQRGADAGDSSLIFVMIKNLKkKKLPAALRKKRLL--------------------------D(FDKHKRKQDWEEKILREISQGKLTSSV)}*

$4468=218(247):
>gi|1176059559|ref|XP_020617650.1| {|341(0)|<Cnidaria(M)>}toll-like receptor 2 [Orbicella faveolata]
{(PTASTTTKQRTTSNQLSTVLGSPTYLGSGRDWLLPIIILSAGSVAVLIMLFIRFIVKKKWTSSYNIKKHGLVETDFINRL)----FISYSSKdfGWVTENLISILEKHSIDYS--
IHSRDFELGRPIVQNMADSVYGSRQVLIVLSENYLASNFCREELHMAIQRGLDVGDSSLILVKINDLKkKKLPTALRKKRLL--------------------------D(FDKHRKKQDWEEKILREILEGKLTANV)}*

$4469=259(273):

>gi|1176078015|ref|XP_020609053.1| {|309(0)|<Cnidaria(M)>}fibroblast growth factor receptor 3-like [Orbicella faveolata]
{(IIVTLSGRTTNRDYITLEVKAEDKEKRLKTWMIAVISVVASILFLVLIAMIIVVCLRKRREEKLLAIAQKYDQEEHVFEK)--DLFISYSTKdyDWVQQSLLPVFDENGINY--
IIHSRDFVPGKAWLDNMADSVYNSRKVILVMSINYLSSAFCKDEMRMAIHRCSERDDSSLIVVRIDNIKsSDIPKS-------LRHRtFIDVTSKEETSIWQR-------
R(ILEYMRSIVSSGSVNSDESFNDTETKSLLSMFKLKRKTKRNNAGRELENQAKA)}*

$4470=226(239):
>gi|1176092359|ref|XP_020616663.1| {|420(0)|<Cnidaria(M)>}hemicentin-1-like [Orbicella faveolata]
{(ALNTSMGLSSATLVLAQMKSKTDYSSGDTEENDSTVLIIILFPVLAGGLLLALTAVVFIRKLLKKRHEWKPEEAASDGEIK)-YDVFVTFSSKdsQWVTSNLTPLLERNRLKY--
CIHSRDFELGRALVDNMAESVYSSRKVLAVMSKNYMDSKFCRGELDMALHRSRGAHKGSLLIVRIDGIKkK-------KLPKALREQtFVDYHSDKERTSWEK-------K(LLRFLVETEVDQSRYVTKL)}*

$4471=218(227):
>gi|1176105050|ref|XP_020623352.1|
{|305(0)|<Cnidaria(M)>}uncharacterized protein LOC110060883 isoform X2 [Orbicella faveolata]
{(STEYRTTSSESNNFLDHSDSKSTESTESHRKWFQAVLISSVGIVIIILALLFVWFVAKERRTSSSSKTKYCPVEKEFINQL)----FISHSSKdfGWVTENLIPIIEKHSIAYS--
IHNRDFELGKPIVENMADSVYGSRQVLIVLSENYLASNFCREELHMAVQREADADDSSLLLVMVNKLKkK-------KLPAALRKKrVLDFDKHKKKqdWEEKMLREIL--D(GELISGV)}*

$4472=273(254):
>gi|1176078691|ref|XP_020609404.1|
{|405(136)|<Cnidaria(M)>}uncharacterized protein LOC110047989 [Orbicella faveolata]
{(KPVIPVVVGSSFDWMMTVVGLLIAGEIYIHFSNKDVQESKMTELLKAIKKSVPQVRVPDSMGSVQELQSTEATTENRTLG)PADVFVSYCW-
VNsekaykskqVKDFIGSQFADPRKVHaglaknnRFSLWIDTERLKTTNQndeslgMfaQIAEGLGKAKVVLAFVSKQYANSENCRMEIQFALKSlK-----
KPVVPVIVGADNDWQSTVVGLLVAGQENQPINLQAVDSEQELQQKIQEIQDS(ILPLLGLKEEIRSYRAPVIGDHVISHHQKWAYFP)}*

$4473=217(224):
>gi|1176085747|ref|XP_020613138.1|
{|1230(0)|<Cnidaria(M)>}uncharacterized protein LOC110051451 [Orbicella faveolata] >gi|1176085749|ref|XP_020613139.1| uncharacterized protein LOC110051451 [Orbicella
{(LENIEADNPRLSERCYCVLKRWTEVFGSAATYECLARALQHPMVKRWTLAVKYCGVCRDGYQVADTAPISSQAVDVSTSG)-CPVFLSYQR--DLQDTVKR-
LKSKIEESGIQCWMDIRQMGGGDALfaKIDAGIRACKVFVCCVTAKYCQSDACQGEATLAHSLK-----
KPVIPLLFEDISWPPEGQLSLVFTKLLYIKITEERGTIPDGEFQQLMRKVEQ(FVNS)}*

$4474=217(225):
>gi|1176090577|ref|XP_020615708.1|
{|871(0)|<Cnidaria(M)>}uncharacterized protein LOC110053769 isoform X3 [Orbicella faveolata]
{(KHKTQSCAQSDCIHFLPISNDGKLICTKTFGARSRLDVPGLDEWRGSTSKTDELSGLPDVQKTSEDNTDSAGPSVLDVRP)--EVFISYQW--DLQDAV-
KLLKNKLEESGFQCWMDIGQMGGGDSLfaEIDAGIRASKVVICCVTKRYCKSEMCHREVTLADALR-----
KPIIPLLFEFLDWPPAGQLALIFAKLLYIDMSQGPTSYFPEDKLNELCLKVK(GHIER)}*

$4475=253(252):
>gi|1176105497|ref|XP_020623595.1|
{|498(0)|<Cnidaria(M)>}uncharacterized protein LOC110061107 [Orbicella faveolata]
{(LRPNMQTILGSCPAVNFEGNSYEESMDILLYHLKPFRNMPAVILEQEFLDRMVEGLKPLRVLATLSDKSSVDLEEISPRP)--NLFLSYHW--DVQNKVQ-VLVQYLESHGYQCWTDVSNANKRhatshspnhENApnQIQRNIKAATAVVCCVTPKYICSENCTKDLSLAASMN-----KPVIPVMLQWLAWPPEGGRVRRILAPLQCIDMSNESLFRRNLSTVENHLKKF(AGKQSPRDGHLVEHVRIAKNPRLVQQKSQQKV)}*

$4476=188(199):
>gi|1176118408|ref|XP_020630408.1| {<Cnidaria(M)>}uncharacterized protein LOC110067425 [Orbicella faveolata]
{(MDSFDMEDELVNRQELRRLNECLGSQEKLRKYLESNILPLVVKKIKYTRFLPQFGF)----FLSHKS-KD--KPLMRTFRNGLKFLGYQTWLDEEDMPMGG-LvgALKVAVEKCDCFVAWLNQEYFESDYCKAELLHARRNG-----KIIIPFGVYGKIKEHLTGEFEFLTDLYIYNTTISSFFEVLRRIDESLFNFES(LTK)}*

$4477=227(232):
>gi|1176108175|ref|XP_020625016.1| {|135(0)|<Cnidaria(M)>}myeloid differentiation primary response protein MyD88-A-like [Orbicella faveolata]
{(KLGYRNDYIKYLGSTDEPVKTLIKEKGDMKILELIPLLEDMGRNDVVEELQKSLKATSTPKQIEERVRRRENKLMTTCEH)-YDAFICFGQNdRQFVDSLTKKMEGRPYKLHLCLSERDFLPGGSIYDITAEAIE-RCSKfVVILSKHFDNSDGARYESQIAMSLAPGAKEKRLIPVLIdEDYRGKIPRSISHITYLDRLR--QE-EN---DFWKKLATSLGWK(EHKNGCKKKHTS)}*

$4478=214(227):
>gi|1176121419|ref|XP_020632012.1| {|44(0)|<Cnidaria(M)>}myeloid differentiation primary response protein MyD88-like [Orbicella faveolata]
{(LYFECRREPVLELISSYESKGKTISELLSLLEEMERFDVIEDLKKFVEATPTLEEKQREWKQCQTEAAEDTKAEAISECH)--DVYIAYAKDdQVFVNEIVTTLERPPYCMKVCTDYRELS------DTAAKAIETQCSKvLVVLSESFNRCPDADHMVNFALHLSRDLRKTILIPILYKSC--EIPRKLQIFTRLDYTS--DRERQ---YFWKKLAASLGYD(DKCNGKT)}*

$4479=218(220):
>gi|1176185219|gb|OQX98294.1| {|212(0)|<Bacteroidetes(B)>}hypothetical protein B6I24_05665 [Bacteroidetes bacterium 4572_128]
{(RFFIRTISYEPYSKGEKVRFIAEDHNENNSEFNTFLLHYKAEIFKIPETDISNSINFINNLYDKWMEYNRKHVRKRYKGL)---VFLSFNS---KDRDIVFNIKNKLEESGVNVWYD---EAKLDAGavyEKQINKQIKNCKlfIAFVSENsisdkskYVyKKEWRLAESRRLFLEGDSDEKSFIRPYILDNT-----SYTDNRIPETFRN-VTMQYLNEVNIIREIINELEPN()}*

$4480=151(161):
>gi|1176013082|gb|OQX19470.1| {<Euryarchaeota(A)>}hypothetical protein BWK75_05640 [Candidatus Altiarchaeales archaeon A3]
{(MN)---IFITYNTKdKKIVEALKsNLNLFLGLKT-DI---SVKNMR--SGKslseKIRKTIDDSDVVMVLLTKQGSKSISVNQEMSYAKSAGKK-IIPIVENGVNTDMLE--DIEYILFDRTNPLK--SIEKSTQ--ILENLKIEKEIL(SLIISTILFIMIIESAGKK)}*

$4481=208(224):
>gi|1175942239|gb|OQW62025.1| {|0(215)|<Proteobacteria(B)>}hypothetical protein A4S17_08260 [Proteobacteria bacterium HN_bin10]

{(MATTA)----YLAFEPDDAEAARSVAAELESHGWA-V------
IRSGpEHRVQDklgsLVQAVGGAAIVVVLASTAA-QESAWIRrEVAAALNNG-----
RSIIVVRTADLpAdsWINQTLDAASFIDL------RRGARS--
EVLAQIVDRART(AAGRGRVIAMLNIKGGVGKTVLAANLFAAAHLADKRAISFIDLDPQHNLTQYFLSPGERN
RIRDRNHTLYGILNARGDH)}*

$4482=211(220):
>gi|1176155107|gb|OQX72226.1| {|122(0)|<Bacteroidetes(B)>}hypothetical
protein B6D61_14565 [Bacteroidetes bacterium 4484_249]
{(RIYFTNKRFFIRGKDSTITLSTDDFEFDGKTTSINNENELLNEIISRTLSKNVRIVFPWIKDKLLNHYPTK
EPSTSSDRY)--DVFLSHKSQDYSIAKGIYDYLEANNYK-
TFLSEVCLPklGSADYMKKIDEALDASQHMILVGSSVEHVLASWVEaEWRVFINEK-----
RsgrkkgNFLTIVSNDINpsDLPISLRyyEVIPFDIDN--FD------------RILPYLKE()}*

$4483=171(178):
>gi|1175936305|gb|OQW56268.1| {|0(135)|<Proteobacteria(B)>}hypothetical
protein A4S17_05225 [Proteobacteria bacterium HN_bin10]
{()MADIFISYKREnQDAVQRIVQGL----
RNAGLSVWWDQDIAPDAPWEqTIESELEKAKVVIVAWSEAAVAS-----ENVKAEARRAR-
NQGKLIQIYVEPC-
EPPLFFGERQGVDLSNWngNAGDNRFQAILTAVRAILAGKRP(PEGVGYRPRQRAPWASLTAAFVLVSAVLGF
ISNLGGAR)}*

$4484=171(178):
>gi|1175936444|gb|OQW56404.1| {|0(94)|<Proteobacteria(B)>}hypothetical
protein A4S17_13885 [Proteobacteria bacterium HN_bin10]
{()MSEVFISYKREdKARVETLYALLLDL----
DVPVWFDAGIEVGVEWEeRILEQIEQARAIVVCWSFAAVSSHWVMREARIGLDR------GILVPVTIHPC-
ALVPPFDALQSADLTQWegAPDDPEIQKVLLRLGLLTGKKNL(ARNARLRGGGQKEALVDLMRALLIERALSG
GPPFTYKE)}*

$4485=165(171):
>gi|1175938457|gb|OQW58343.1| {|0(90)|<Proteobacteria(B)>}hypothetical
protein A4S17_11840 [Proteobacteria bacterium HN_bin10]
{()MPDVFISYKKSdADRVRPLVEHL----
RAAGLDVWWDEGIQPSTSWRaEIAKQLGAAKCVVAVWTQDSVD-----HeQGSWVMEEATH-
GQARKILAPVRLD-
LVSPPLGFGETQYADLAawSGAADDPRFQHFLGVVQAIVRSE(PIPLAPRPAHFEAPGIKLQGVTMENVNIQV
G)}*

$4486=207(218):
>gi|1175941327|gb|OQW61145.1| {|0(4)|<Proteobacteria(B)>}hypothetical
protein A4S17_01965 [Proteobacteria bacterium HN_bin10]
{()MADVFISYSRLdHERVQPIADRLTSLGY----
TIWWDKHLRAGQVFVdEIERQLDGAKAVLTAWSNNARNSTWVYAESSRGLDANKF-LQLRLDAVRL-------
PLPFEALQVADmSGGKSEWGTLE--
NSLTQIVRDRRPP(PPRERLPTAGPLATPPQAGMPHVLTAATTATLLAYSGAVSATYNGVMTPDQLQLAMVGV
LGVGAACTALTAQRLLAIR)}*

$4487=142(143):
>gi|1175943680|gb|OQW63435.1| {|0(364)|<Proteobacteria(B)>}hypothetical
protein A4S17_07200 [Proteobacteria bacterium HN_bin10]

{()MADVFISYKREeRQAVERLALELRKLN----
LDVWFDASLNAGEAFSdEIDREAHAAKAILVCWSPTARESQWVKSeALIGFEQRKLA-ACYVAGPDGFYPP-
TPFNSIHAEDLRAWVAApsNTHAGFKSVLRRIGALCGRADIE(SYG)}*

$4488=212(219):
>gi|1175952573|gb|OQW72175.1| {|0(119)|<Proteobacteria(B)>}hypothetical protein BVN33_13645 [Proteobacteria bacterium ST_bin13]
{(M)--KVFISWSGDrSKALAIALKEWIPLILQYA-
KPWvSEKDISAGERWAqAISGELESSNFGILCITPENLSSEWILFEAGAL--SKSM-LDAKVIPLLfgLELS-
DLSG-PLSQFQALKIDQqgVM---DVI--KA-
INVVSEIKAS(DTTIDQLVPALWPQLQQKVDNIPGKAPAGKNMRPHGEILEELVSQVRGLGARVRDLDPDFMD
RELKIASRSGRDLDPR)}*

$4489=176(171):
>gi|1175993940|gb|OQX02055.1| {<Proteobacteria(B)>}hypothetical protein BWK73_43995 [Thiothrix lacustris]
{(MPKFKESEEKFSSRLDPIEPTKTF)--
KLFISYSRVdRVHKLTLEKHLRLikeaLKHQAKLDIWSDHLMDAGEGVNdQILPELRSADLILLLVSPDFLDP
ErYSRGvELPIALERFKK-EGTPVAPIIIRHT-ENWNELLGHLTVpTTENAdpLENWPSTD--
KFWGSVEKGIRRQ(VEKLLQT)}*

$4490=191(184):
>gi|1175995494|gb|OQX03385.1| {|0(39)|<Proteobacteria(B)>}hypothetical protein BWK73_39700 [Thiothrix lacustris]
{()MPDLFLSYAHVdKHWVDTFAALLEQRVNQYVgrakpDRLWKDNRFSGNTAISpEIDAQLAQAHCLVSFIS
PGYLASEWCIYELATFSTRVGA-NSGRIFCVELDHI-
PPEHKPAAVAPILGYRFwwQDTQSKRTYPLTASQPDFENRLI(DLAKDITVMLQKESPAPTIANAPPSSTSLD
KGRPGGVSSPTRRN)}*

$4491=174(180):
>gi|1175997653|gb|OQX05258.1| {|0(409)|<Proteobacteria(B)>}hypothetical protein BWK73_34100 [Thiothrix lacustris]
{()MHDIFLSYSTKD--
RERLQPLFQALERQGWSVFWDHRTIDIGEHWSkKINHAIRTSKCVVVVWSKASVESDWVLEEANIGKQRK---
---VFLPIQIDEV-
DFPVGFTMLQAGNFIHWngDVNDPQFIRLAEKIRELVEQHDT(AQAKREAAETAEQERLAQEKSVADQQARDH
AEQQRRLEEE)}*

$4492=161(162):
>gi|1175999832|gb|OQX07174.1| {|0(37)|<Proteobacteria(B)>}hypothetical protein BWK73_28770 [Thiothrix lacustris]
{()MHDIFLSYSTKD--
RDRLQPLFQALAQQGWSVFWDHYSISTGDNWHqKIDQAICESGCVVVVWSENSIYSEWVLEEASKAKNRDVL-
LPIKIDSVELPLG-
FGMRQTGNFTCWNGSSDhpVFIELAEKIYERLNDEQQRPQEQ(KFGCGFWVVILIALFWYVSNKP)}*

$4493=171(177):
>gi|1176006400|gb|OQX13171.1| {|0(273)|<Proteobacteria(B)>}hypothetical protein BWK73_12960 [Thiothrix lacustris]
{()MYDIFLSYSTQD--
RERLMPLVSALEQQGWSVFWDHRSIPVGGDWHdMIGDAIRECRCVIVAWSTHSVDSKWVRE---EALEGRAR-
--SVLFPILLDDI-
PQPFGFKIIQSANFTRWnqRADHPEFLKLDTEIRRVLVHQLP(PQQSTVAEPSPQRLPISTPPQISYKRLWGS
GLAAIAV)}*

$4494=156(158):
>gi|1176006739|gb|OQX13491.1| {|0(773)|<Proteobacteria(B)>}hypothetical protein BWK73_11960 [Thiothrix lacustris]
{()MTNVFISYSHDsDAHRERVLA-LSNRLRADGIDCDIDQyNPAPEQGWPrWMMQGIQQAD-YVLLVCTE----TYCKRlELEEEKGKGLG-VKWEGSLIFNILY-RNETFNTKFIPLVFDAEnkAHIPDILFGYDYHDLSQPRGYDS(LYRHIRGELGAEKPLLGK)}*

$4495=287(293):
>gi|1176114002|ref|XP_020628081.1|
{|292(357)|<Cnidaria(M)>}uncharacterized protein LOC110065298 [Orbicella faveolata]
{(YNMALCINRRSHGTVLAPFYITSIDYMPTYMSLIQYEDCRPKDKEKLQAASNDVADSLKKTEEEVSSSVPTMTSKASGYD)---VFISYSHQnKTHANKVLETLKSL--APDLRIFIDTAgLNTGTSWQqILYNALDASRSTMALLSPDYIKSKVCYEeFcLSHALFVDKD-RCMDLVSLLVEPV---QELSLWCQQPVPADCVSSHLDTD--QVLSSICADLVNR(LKGTNNKTDDILTQRVHDLKKEDLTLELSMDNYRKSCFVLRSGVLKTPVEFTSSREKESLRTSHASDVVMSVD)}*

$4496=255(254):
>gi|1176187733|gb|OQY00633.1|
{|352(1071)|<Proteobacteria(B)>}hypothetical protein B6I26_07385 [Desulfobacteraceae bacterium 4572_130]
{(YIEQDKNSNFKLTDPIFAAWLNKKNAFIPYNAKIFIEHENPIKTIKEDRMDNISQNSVSEEKGYESKLDLKLQGKNPEIL)--NIFICYSKNkAlkqKFKEKLQKRFDTAKTDFKIKLKSDKDIPTGKDIDkTIlNEMIQDCNGAILLLSTNFLNSEYIKNkELPIL----KE-KQVPLFPVLFGSL-DIERQNLLGVEKLLIYYqeNKGKKLSFLKIADKEKESQIDEF(INEFAGNIEDRINEIKKGNTQIEKKTFETLYPIL)}*

$4497=177(171):
>gi|1176188584|gb|OQY01448.1| {|0(966)|<Proteobacteria(B)>}hypothetical protein B6I26_03730 [Desulfobacteraceae bacterium 4572_130]
{(MPKDNAI)--KLFISYASNdKfivqAFKNRFNKYCEKNKNKKIsIIHWSMDDLIVGDNFDyKIKESLKSSNYGLALISEAFFDSPYITDvELKYLLKQKKL-LPIGLESKIKGKN-VDIKKFRKSIIKKYPNKskIKKLFETQIFYLKDRKGDFYSDC(DENAQDLFIRNLYIDIQKAVLQIK)}*

$4498=180(188):
>gi|1176215883|gb|OQY26408.1| {|0(752)|<Candidatus Cloacimonetes(B)>}hypothetical protein B6244_13620 [Candidatus Cloacimonetes bacterium 4572_55]
{(MSPPKSPAIARSKD)---IFLSYSRRnTPLVLKLYDALSANDRS----VWYDKySIPPSADWQaEIQDGIEKAHSFIFVISPDSIMSKECAKELNHALKFKK-----RIIPIIFQDA-EDVPPDISSLNWIFMREeeDFDPGIQSVLDSIDTDLKYNQTH(TRLALLALEWERNHYDKGSFLSGGALYEWEQWLV)}*

$4499=190(197):
>gi|1176226163|gb|OQY35913.1| {|0(825)|<Chloroflexi(B)>}hypothetical protein B6I38_00950 [Anaerolineaceae bacterium 4572_5.1]
{(MSRPGLFPDRGNMTDQEQSAASS)--NVFISYSRKnKKFAQKLNDSLDS----SGIEAWVDwEGIPLSSDWMdEITRAIEGADAFLFIISPDSLASEVCAEELELSLKYNKK-----LIPILHIEP-

EKGSTMHEALAATNWVYmrEQDDYDATLPGLIESIHTDLGWV(RQHTRLLKRAKEWESKNKNSSFLLQGSDLN
EAET)}*

$4500=196(184):
>gi|1176240652|gb|OQY49073.1| {|0(303)|<Proteobacteria(B)>}hypothetical protein B6247_24225 [Beggiatoa sp. 4572_84]
{(MSVYVPHYQHD)---
IFVSYAHVdnqippgvnEGWVTTLIKSLKIelgrkLGSTDIFSLWMDYELRGNSAATpETLEHLTNSATLVLI
LSPGYLASSWCHLELNAFLDQVDT-HSGCVFVIEHDDV-
EERLPQLSDLRGYRFWRrdDTNKTRTLAKPQPDPKEEREYYN(LVEDLGRELVDKLKQLKKSARAPSAPSQHR
TPT)}*

$4501=176(176):
>gi|1176247362|gb|OQY55329.1| {|0(75)|<Proteobacteria(B)>}hypothetical protein B6247_08120 [Beggiatoa sp. 4572_84]
{(MCLII)-KKIFLS--
AMpTTLINGLKIELgKQLGRTDAYSLWMDYQLRGNEAVTpDIDEQLNNCATLVLIFSRGYLASSWCLLEMNTF
LSKVGN-GSGRIFIVEQDFI-
PREEKPPEFQDLLGYSFweRNSDTGRTRSLGIPKPNPDREPE(YYQRLNELARDLTDKLNQLKNEALAAEAKD
E)}*

$4502=127(158):
>gi|1175988653|gb|OQW97469.1| {<Proteobacteria(B)>}hypothetical protein BWK73_54380 [Thiothrix lacustris]
{(ML)----------------------------AAGFAVFRDVIGLKPGDVWyrTLEFELENSDV-
MVLVVSEKVRTSKWVHnEVSMAEEIG-----IPVIPVLAEKV-
RYPLWLRHLQSLDFCGVVDWSLLLGAIGHHVGSGGVTPVG(QTMQGRINTGVMRIWW)}*

$4503=176(185):
>gi|1176001116|gb|OQX08322.1| {|0(395)|<Proteobacteria(B)>}hypothetical protein BWK73_25710 [Thiothrix lacustris]
{(MSSTTSGK)--HIFISHAS--A-DDTFVKQLREKLELHHLNVWVDSRNLRGGDKLwpEVAEAIR-
TARQILMVISTNTVNSSWVRkEVRLAEQAGVT-----VIPLLLPGI-
LPAALGMWFDEEPAGEKIELEVGKLQEAMPRILAALGERL(PDDLPSEQTVENKPLAELLLELSEPSLTLNAD
GSEQL)}*

$4504=220(227):
>gi|1176008723|gb|OQX15359.1| {|0(96)|<Proteobacteria(B)>}hypothetical protein BWK80_41035 [Desulfobacteraceae bacterium IS3]
{(MSEFSDKKSPLIVLCYAR)-------------
EDIEKVNDIYDRLKQAGFNLWFDVADILGGQNPdiELRKVISKSGCVLIFLTKSSVHKHGVIHkCIRYASECQ
KEIreDAVFTIPVKLEEI-EEsemPDSLLKLQSVDLYI--
PDGFAKLVNtLKSKFGSDSYEQQ(KILRDRIENYFKIRSNVIVLDGFPTVEKNFTDDLKEKYAVFSITFQGLR
DLKQLDLFVYDLSVQLTKSD)}*

$4505=175(179):
>gi|1176017868|gb|OQX23807.1| {|0(160)|<Proteobacteria(B)>}hypothetical protein BWK80_24045 [Desulfobacteraceae bacterium IS3]
{(MDKA)---ILISHSS---
RDEEFIIKLRQAMEAHGLYNWVDSRDLRGGVDLtmKIRAEIEKSRAFIVVLSADA-
FNSAWVAeETRYASKIKENRqENYPMIALLREGI-
ELGALKWIFSREPIAIRVKNSPTGISEAMPQILAALGEKP(SGDPKSAKNSPSEPSEELLLELKFPYIMEKDG
KHR)}*

$4506=175(179):
>gi|1176246566|gb|OQY54585.1| {|0(151)|<Proteobacteria(B)>}hypothetical protein B6245_21010 [Desulfobacteraceae bacterium 4572_88]
{(MSRS)---IVISHSS---RDKLFIVDLCEAMEAQGLFSWIDSHDLRGGSELtlKIHSEI-EKARAFIVIISPDAFNSAWVAeETRYAAKVKESRqDNYPIIALLREGV-ELGALKWIFSREPVAIRVKDGPKGIYDAIPHILSAMGERP(VADPRQQSQIPPEPVEELVLELKSPHMREKEGKRR)}*

$4507=177(181):
>gi|1176250819|gb|OQY58543.1| {|0(156)|<Proteobacteria(B)>}hypothetical protein B6245_11245 [Desulfobacteraceae bacterium 4572_88]
{(MVMDKA)---ILISHSS---RDDEFVAKLCQVMEAHGLYNWIDSHDLRGGTDLtlKIQEQI-EKAKAFIVVLSSDAFNSAWVAeETRYALQVRAERpESYPIVALLREGV-ELGALKWIFSKTPIAIRLKNNLDDIYRVMPHILVALGESL(PNPGEDTPQTGPVEELVLELNFPYIMKKEGRRCAV)}*

$4508=228(221):
>gi|1176185768|gb|OQX98793.1| {|0(706)|<Bacteroidetes(B)>}hypothetical protein B6I24_04320 [Bacteroidetes bacterium 4572_128]
{(M)KYKAFISYEKNekfysrvIALDNNLRKYAKKSFEEP-LKIYSKKTNETSEYLLpeATQDALDASEYFIYIAEKETCMSPMVEAEIQYWCEeLKKKDkilITHLDDKINLQ---EETFKINWDKTNALPEILENYIYKLP--FYVDLTWAKKVD(YDLNNIKYKNIINIISSHIRGITPEEMNRIEISAIKSNKKTKNFTIIGTIISLVMVFGLIFWQFKQTKNEKIIVENTIKK)}*

$4509=196(179):
>gi|1176245810|gb|OQY53885.1| {|0(273)|<Proteobacteria(B)>}hypothetical protein B6247_13570 [Beggiatoa sp. 4572_84]
{(MPVYVPNC)KHDIFVSYAHVddeplaGAdkgwvttLINGLKIIlGQKLGRSDAFSLWMDY-ELRGHTALtpNVIEQLEGSATLVLILSPGYLASQWCHLELNAFLAkvGSNSGrvfVVEHEQVERPA---ELSDLLGYKFWVSDDAGHPRTLAIPKPNPEEVEYYQRLYD(LARQLTDQLKELKEQAKATPVAVSSPTNITP)}*

$4510=265(284):
>gi|1176114002|ref|XP_020628081.1| {|138(533)|<Cnidaria(M)>}uncharacterized protein LOC110065298 [Orbicella faveolata]
{(IMPLLASGDQGYSKRLMLRLIIDSAIHWIKAGLELKTLKIVLFSRHPERHDKSHEPLFECFQKMKDQYGDKELTYETEIV)-YDVYMSHsPKDHDSALQIAKFLR-TEKKVRVFQEFQELNDNES-WQEEIFDIMGkCARVIALLSPGYLETESCIEQYNMALCINRRSHGTVLAPF--------------YITSIDYMP----TYMSLIQYEDCRPKDKEKL(QAASNDVADSLKKTEEEVSSSVPTMTSKASGYDVFISYSHQNKTHANKVLETLKSLAPDLRIFI)}*

$4511=251(254):
>gi|1176543665|ref|WP_082370657.1| {|140(12)|<Cyanobacteria(B)>}hypothetical protein [Hapalosiphon sp. MRB220]
{(QTPYTYPLNELHDFLKNGAYHIQCRKSYKMVDVRRLIDDVNLQSIDVKRERDLQATSLQQELEQSRDESLTHQPQKSAQK)--EVFISYTlR-DDNSENFVKQIEQVFQEKGIKIIRDKNAVGYKQ-KfqEFMQRLSRGECVILIISDGYLKSENCMYELVEIAK-HGE-FHERIFPIVLEDAKIYQAIERIKYVRYWEDKKKELDAAMKKVSGDNLHGFREEL(DLYNEIRNTFAELTNLLKDMNALTSDIHIQSNFD)}*

$4512=201(203):
>gi|1176377489|ref|WP_082220633.1|
{|0(69)|<Euryarchaeota(A)>}hypothetical protein [Haloferax gibbonsii]
{(MSNSSTEDDSN)-HDVMISYSH-SDSKQ-
VAELLYEELTAYGLDVWYDGVELTIGSNIrdSIDKALTSSDHAVILISPTYFEG-
MSQWELDGIVQRHNSSEHDVILPLLHELEFSEVREKSPSLANIIGEKVTDDNIDDIAAKLYTAIQSSDAN(DE
PDIGDIDSGSRVDIQVSLEDLADISKGSKVTINEWRTEGTPHDRGVRAVK)}*

$4513=307(298):
>gi|1176643573|ref|WP_082470554.1|
{|426(117)|<Actinobacteria(B)>}hypothetical protein [Curtobacterium sp.
S6]
{(FKNHYGERFHRGFVVYTGDRSVAFDDNLWAIPLSSLHDERLWVPQESPSTEDLALPLPSASPESEPMSSMT
DATVFISYT)-HKDQESaTG---
GDIRQFALDIQDTMEGRfgrSIQLFLDVQSIHWGQQLqrSINDGLRTSTFFLPFITPRYVKSEACQDEFTQFS
DAAQRSgSENLLLPLIWiTPRElgsaPAQTPLFTRLRSIKYLDaHKGRVSDRSSAEYRQAVEEIARR(LDEVM
TERESQPVASDESPEGASGDHDEPPRGFMEYMEAAESLMPQAQKDILEFLEDFDRLGREFRTIPPRIAAAGLD
)}*

$4514=270(273):
>gi|1176343625|ref|WP_082190802.1|
{|0(71)|<Proteobacteria(B)>}hypothetical protein [Bradyrhizobium
pachyrhizi]
{(MNWVNAASAANAGSGAHSSTANATMVAGHFMIDLTLRKRALQRGFGNCQNSILGGKF)AMRIFLSFPSELE
SQADSIAQSLRNCDHD-
VFFSHDDLPPGDSFDARVEKAIESSDFMVFLISPGSVTRGRYSLsELAFARnkwpNPS-----
NRVLSVMMASTPldQVPTYLKAVTILEPQG--SAAAETRA--
TVDRMLEAKFEQ(SRDRSNLPFAVLSLASAVVCTLATTYALNVLQYSFVASDPGGITILPGVIFGAAIATCNV
MFGARDRFQLALVVAI)}*

$4515=211(219):
>gi|1176623986|ref|WP_082450967.1|
{|0(453)|<Proteobacteria(B)>}hypothetical protein [Sphingomonas sp.
Leaf22]
{(MVELFPARAKYARATRPLCLTKYIGKSVAGCLGDQAVT)--DLFVSYKSKDRPRVAPLVAALEADGIC-
VWWDA-HIGGGTEWRETIGRELAAARCVLVVWSNRSTGpEGSFVRdEATRAL-RR-----
GAYMPVCIDPVEppLGFGETQALPLIGWKG--
NSNDPRYLAVLAAVQAVLAGRE(RPAPPVQMPKRGIDRRLLIGGGAAAVVAAGAGGWWRMRGTD)}*

$4516=182(182):
>gi|1176543777|ref|WP_082370769.1|
{|0(368)|<Cyanobacteria(B)>}hypothetical protein [Hapalosiphon sp.
MRB220]
{(MQEFEFD)---VFLAHNSKdKPQVRAIAEQLT----
RYNLRPWFDEeQIFPGDNIQqVVSQGISQSKVGVFFISQNGLGT-FQENlELgaiiQFFLRKHKN-
QGFRVIPILLGIN-
DIPDSLFYLSQWAWIQFtsSNDEAALQRLIRGIRGRGAEVQP(PPVIQISDTPLIIEPKPPQNLSTEQQRRLQ
QKRDG)}*

$4517=242(244):
>gi|1176565928|ref|WP_082392918.1|
{|58(131)|<Actinobacteria(B)>}hypothetical protein [Nocardia arizonensis]
{(VDQDRRLMSIMLWRACYSPLIGIRPAETVSALRCRRSGRVATPSRSSTCRRHPGYRRHPSFSEQWRMIGGG
GNSFVREAR)MRQLFISYAREnRPDVEALVRDLDVLGYQ----

AWVDSSLRGGQTWWgEILQRIAHSDVFVAVVSEETLSSVAC-KrELEWAMALDKP-VLPLSVGRLPDAL-PRTLALWQIIDYSTSEKtaALALAGALGSLRAAPPLPDPLPE(PPPAPLSYLSELVEQVDQPDELSH)}*

$4518=196(204):
>gi|1176576397|ref|WP_082403387.1|
{|0(299)|<Actinobacteria(B)>}hypothetical protein [Saccharothrix sp. NRRL B-16348]
{(MSAGATCPGPEGVTM)--TIFVSYTRAdESLVRALRDDLG---RMGQ-EVWLDHEIHGGELWWrEIIRTIQAARVFVFALSQASWKSVPCHAEFDYAQQL-----GVPVLPVQVGPL-DNLRIPIMERQLVDYRErsADAIVSLAGALTELTGRPRVLPN(PMPPPPKVPFEYLYRIAELLGPQPISPERQEIIIGQLRRHLRTERDEIA)}*

$4519=218(219):
>gi|1176612050|ref|WP_082439031.1| {<Proteobacteria(B)>}MULTISPECIES: hypothetical protein [Sphingomonas]
{(MTASVADMASARE)-ASVFVSYSHLdDDFRRRFETHMAQLHREGV-RTWFDGDITPGAELDpNIRRALKQADIFVALASPNYLASRYCFEtEYGFALRKAAR-KKLFVVVVLLRQC-QWRHTRMARYKLLPRDAkpIDQWARRG--DAWENVVEGIRAV(VKQFRLVPQAPDAPKKRKAPAPTRTTKTKPTSVRPSGKAAPAAKPKPKRSVKPTGKRTRPFKPRGS)}*

$4520=229(232):
>gi|1176626175|ref|WP_082453156.1| {<Proteobacteria(B)>}hypothetical protein [Sphingomonas sp. Leaf208]
{(MAVSTRKTSAPSPKAGDKGKRLISAHRPAKPAKPPEIR)---IFVSYSHRdAAAREKLETHLAPWKRDDV-TVWYDKNIEPGAQLDpAIARELRNAHIFIALFSPAYLDSNYCWNiEYRRAMDRRAR-KLLRVVGVVVKPC-GWKHTRAAGFKLLPDDGrePERWSSSD--AAYVNIAEGIGAV(IKTVRRELAAAVPKRPPTPAASATKPKPPVSKPPTPGKARAKQPPKARARSRKA)}*

$4521=220(226):
>gi|1176358285|ref|WP_082205434.1|
{|103(0)|<Proteobacteria(B)>}hypothetical protein [Desulfocarbo indianensis]
{(FNVNFSHVTLYKTEFYDVDLRTVAGLESASHVAHSYIDLDTIYQSKGQIPESFLRGCGVPEDFIKLIPSLTAQPFEFYKC)----FISHSS---LDLDFCKRLHADLQNEGVRVWFFPEDARTGRGVwdEIDRSIKIYD-KLMVVCSENSLNSGPVLrEIERALQ-REDReHKHILFPIKIDDY-LFEEWDHPRKADVLDKVVGDFTRWKDHDSYQKALDRLLRD(LKAGEE)}*

$4522=146(149):
>gi|1176543678|ref|WP_082370670.1| {<Cyanobacteria(B)>}hypothetical protein [Hapalosiphon sp. MRB220]
{(MSNQPQFD)---VFLVHNS---QDKPQVRAIAQELKRCGLKPWSDEEQILPGRSFqdVIQKAIQsvKSAAIFIGL---QGLGRWQIMeLRSL---IVKFLeADIPVIPVLLPEV-EEVPhnliFLQQWMWVSFRNGLDDSEALKNLVWGITGERPILLR(Y)}*

$4523=222(223):
>gi|1178558845|ref|WP_083075394.1|
{|95(271)|<Actinobacteria(B)>}hypothetical protein [Mycobacterium heidelbergense] >gi|1177090804|gb|ORA71396.1| hypothetical protein BST25_16850 [Mycobacterium heide
{(APCGPSPTDVHAIRATAKSSDAAIALLVHIPSGSPAFSGIVAARGRLGRVSTRAATVLLPQNRFPPLGPLPNGAVRGDGP)---VFVSYRQSDGTAAAESLEGLLRAAGL--VVWRDRSDLRAGTTTdRLEQALTQGlSAAVLVMTPDIVYSEIVREreLPRLLQLDADPGFSLCIANHVARQPDNSRCDFEAPDRLLQTAPARTLADKKQTNMLDAAGEVEIAR(DIL)}*

$4524=164(169):
>gi|1176760987|gb|OQY79195.1| {|0(49)|<Chloroflexi(B)>}hypothetical protein B6D42_15590 [Anaerolineae bacterium UTCFX5]
{(MTGQTG)---IFISHAHAdQEIADVIRKKLRRFfanGVP---IFLSPNT---
IAAGKswlpEIEKALKCRVLLV-LLTPNSLSRPWLWFEVGACWDAHRG-
DRTTILPVTVGLNAEDVPAPLDQIQTPDLTA--
KDGCKAFFSSLHDIFAPLGIV(EDMRNNQFIKALQAKFKPSVEML)}*

$4525=237(247):
>gi|1176670640|ref|WP_082497620.1|
{|0(720)|<Proteobacteria(B)>}hypothetical protein [Pseudorhodoferax sp. Leaf267]
{(MSTGCGFKSRTSAESRGATRRVAKLPACSYASPMA)--DIFVSYSRKDQAFARWLTQALLARGRE-
AWVDWEGIPPSAAWLQEIRHAIDAAPTLVFVVSPD-SAASEVCAlEVAHAAAQN-----
KRLVPVVCVDTPprALPDAIARLNWIFLRE--HDDFEAGL--
ALLQSALDTDLD(AARTHARLLVRAREWEARERDASLLLRGSDLRSAEQWLLLAAAKGATPTSVHAEFLVASR
KAAARRERLTLL)}*

$4526=223(239):
>gi|1176673389|ref|WP_082500369.1|
{|0(54)|<Actinobacteria(B)>}MULTISPECIES: short-chain dehydrogenase [Nocardioides]
{(MTSTGPTTAPTSPTTDPATDPATDPARFLAGRTALVTGSSSGIGAA)------------------
IATDLAAAGAH-VVVSGRDVARGTAVVERLRAAGGRADWVAADLTASYADLRRFAGEVTEVLGGG-----
VDVLvhnaavcpPVDTLGLTdeDLDTALAVNVRAPHVL--
TAALAPGMLGRDGACIVVIGSW(MAGVGHPFVGLYSATKAAEAQLARSWAAEFGPRGVRVNTVSPGPTRTPIN
DSD)}*

$4527=179(185):
>gi|1176712281|ref|WP_082537891.1|
{|0(24)|<Actinobacteria(B)>}hypothetical protein [Angustibacter sp. Root456]
{(MT)AQKMFVSYSSADREAADIVREQVEASGRS-VWMAPRDVPAGLSYPEAIISAIRDCDCGLLVLSEQ-
SNASPHVLrEVERLAADG-----KQLFVVRLEPVElsDGLAYFASMLSGSTAR--
ATSSCATRPLCWLPSSAGCRRP(SSECHRRREQPRARYRPLPSTRRRWTSRRSPTPSGGCSRRSVW)}*

$4528=174(180):
>gi|1176782731|gb|OQZ00604.1| {|0(323)|<Planctomycetes(B)>}hypothetical protein B6D35_06050 [Candidatus Brocadia sp. UTAMX2]
{(M)AHDVFICYSYEDKLTADLVCNFLERNGVR-CWIAPRDVVPGKEWAVAIIEAIEKCKIFVLIFSQNA-
DASPQVLrEVERGVSKS-----KIIVPFRIANVIpsKALEYYLSVPHWLDAL--
TEPIEQHVDKLVNTILLFLRRT(ISHVPESEPTTQAKTALSTNIQTVLATETKEPSSKKPVF)}*

$4529=201(205):
>gi|1176841151|ref|WP_082597936.1|
{|0(1240)|<Actinobacteria(B)>}hypothetical protein [Mycobacterium sp. Soil538]
{(MS)--RIFLSHSSRDNRQALALKRWLERAEpgllDE-
IYLDIdpkTGIQTGAKWTEALWRVNARCEAVICLLSRN-WASSKECHaEYRQAEGMH-----
KPIFCARIEDFAdeDV--TLAWQ-RCDLFG--
AGPATEVDLDDGGPPVRLSDDG(LQRLLAGLRAVGIGADSFAWPPPGDPDRSPYRGWRPLESVDAAVYFGRDA
QIVRALDELRWMR)}*

$4530=241(245):
>gi|1176656674|ref|WP_082483655.1| {<Proteobacteria(B)>}hypothetical protein [Methylobacterium sp. Leaf87]
{(MAARTRNRAAKAPSVAKPTTARGSSAKARPSDKAPRVKSIKPEVR)---IFVSYSRVhEVAREKLEIHLAPLTRDNVV-PWYDGEMEAGDKLTtKISNAIRDSHIFVALLSPEYLHSDWC-QkEYRRAMSRRAR-GLMRVVATIIRPC-LWKETPAADLVVLPDGGrtVIEWRSAD--TAYLNVAQGIGRV(VKSVRKDMSVAGATSAIPPKRVKQPTVRKRTPAYDAPGKPVKTKVAAKKTVARKPRPVVR)}*

$4531=205(206):
>gi|1176670611|ref|WP_082497591.1| {|0(410)|<Proteobacteria(B)>}hypothetical protein [Pseudorhodoferax sp. Leaf267]
{(MRSLTTRKADAMGGA)---LFISYSHVdRRWMQAIRMHLEGAM-QAQCRVWTDRNIRPGATWEsVLLTALKGAGTALVLVSPEYLVSPWCRReLASLHQARMKG-DLAAVYWLLLRPC-AWSWTELHDLQAVQEPPtlALESLPPGLERDQMLVELCARVT(TDALQLSTEEDPDVRSVRELLLRADEATPYRPTRILGKDSSNFSIVCRGLD)}*

$4532=175(179):
>gi|1176721884|ref|WP_082545195.1| {|0(679)|<Proteobacteria(B)>}hypothetical protein [Sphingopyxis sp. Root1497]
{(MV)-PQVFISLSGQdMEFASAVERALpRGLAR------FYPKTFATGADLIsEMENGVEESAIFVLLVSKKSLASHWVRFELDVARVRQIQ-KKIKILPFLIEPG-VDIADFPDWMRGFWVSPyvWGPKDIGRIIQEEIFSAHFSNSK(LYQRVIGRGALFDKANREILGVAAENGRTPNVIILSG)}*

$4533=190(195):
>gi|1176822295|ref|WP_082585619.1| {|0(132)|<Proteobacteria(B)>}hypothetical protein [Hydrogenophaga sp. Root209]
{(MLRWCADSGATTGWSTTMR)---VFISHAWEdKPLA------LSLAELPDFIDAWVDvRELLGGEKLDpTIIAAIEDSHVFIVLISRISLAKAWVAQEVNWAMAReAQK-DRVFVLPVVIEAG-IDLAASAPPFQQFAQRLfiDASDRSDSGTDKARAAIAQTLFH(WASDWLDRMEPKGDSNRRFVDTLERDLVEFQTRLYA)}*

$4534=170(176):
>gi|1177080402|gb|ORA61318.1| {|0(327)|<Actinobacteria(B)>}hypothetical protein BST24_11040 [Mycobacterium franklinii]
{()MRKVFISYARAnKPDVDQLVEHLTELN----CQTWVDSSLHGGQDWWqEILRRIADCDVFIPVVSHESLNSGACAREFDWA----EN-LGKSVLPVALERP-SPALPRRILSRQIVDYSnpEERDRAARKLGGGLLSLPPAPPL(PHPMPQPPAAPLSYLTDLVEKLSSPHALSHEEQRQI)}*

$4535=179(177):
>gi|1178513642|ref|WP_083033545.1| {|0(1226)|<Actinobacteria(B)>}hypothetical protein [Mycobacterium insubricum] >gi|1177083014|gb|ORA63886.1| hypothetical protein BST26_20055 [Mycobacterium insubricu
{()MARIFLSYSREnKQAAHALKNWLQAqdpdLADDIFMDVDQQSGIRPGEQWRnSITRAIDRCEVLIGCVSPDWETSTMC-QaEYMLAL----S-KGKRVIPVHVVPM-

AEVGSTADLNGIPIYPDsaDDTVRVDATDFSLTGLHRLLSDI(SSENIKFEFPAGRTPYRGLEPLGEEDAAVF
FGRDGDL)}*

$4536=184(183):
>gi|1176663229|ref|WP_082490210.1|
{|0(224)|<Proteobacteria(B)>}hypothetical protein [Methylobacterium sp.
Leaf122]
{(MEPL)RPLVFISYTT--KDRDEVAPY-
YDYLIRQGHDVWMDYRRIKPGQNWdfEIKRALSKAAIIVLFVSSNSVNRRGYAQrEIKIALDkLDEKLiDDIY
LIPVLVDDN-
AAIPEELKNVQFTRASSENCMADIDDAIKHQIDQLKQSTL(QAQEQSSVRWSKSFYKDTWNGIPGFEIELQMI
NLRSDEY)}*

$4537=211(215):
>gi|1176856927|ref|WP_082613706.1|
{|0(186)|<Proteobacteria(B)>}hypothetical protein [Bosea sp. Root483D1]
{(MAESEPL)---IFISYAS--P-
DRARVDPFVDDLERANFNIWMDHRRLVAGQNWdaEIRRALGRSAVVILFMSENSVDRRGYAQrEIKLALLKAe
EKLtSDIYIIPVLLDEL-PAYPD--
SVAHLHMVRAWESNAAAKIDAAISEQLNQIGQH(VRSVQVDNRIRWVERNYKEKWDGLPGYEAEFNIMELSSS
EIDNVSDINGIVRGVILKSVSKQREVKFS)}*

$4538=216(210):
>gi|1176782615|gb|OQZ00492.1| {|0(1478)|<Planctomycetes(B)>}hypothetical
protein B6D35_06510 [Candidatus Brocadia sp. UTAMX2]
{(MEPAVQNQTYIN)--DVFISYSRKdkefaSKLEKALKAYKPPKGLKgapfRNLVVFRDEEDIT-
GVEYyqSIEKHLKDSKKLIIICSPNSRKSDYVNGEIQAFAKIHGANniiPILLSGIPN-----
NEAKKPEQEDEMAFPEALCKSMQMPLATSYLGYAVQKDKI(NKGAFSGSWYTLLANVYDASRSEIEQREKKKQ
ARRRNILVGMAIVSVIILSGLTSWIF)}*

$4539=256(253):
>gi|1177483006|ref|WP_082673989.1|
{|781(18)|<Proteobacteria(B)>}hypothetical protein [Thiohalocapsa sp.
ML1]
{(GEQAGALLENLIERIGELNEHHGWAGRLERAAPGAPDTRPDPARSALEPPAHARRPQRHGPDGPPPLHLTA
PPPSPPAQP)--EVYVSYNwR-
REAASPLVQRLCDALLARdGIRVIRERDEMNWGDSIrRFMQQLAAGRCVLVILSAEYLRSPFCMTELHGIWra
agERDEA-
FRRRIVPLVQDDLSLRSLPDRVAHLEHWRHECQRLEALLSNYSMTDALRLLGQD(GVAEWQAITGFHQDIVKM
LTVANDMLIPRQPEL)}*

$4540=224(228):
>gi|1178575225|ref|WP_083091122.1|
{|57(0)|<Actinobacteria(B)>}hypothetical protein [Rothia nasimurium]
>gi|1177266482|gb|ORC22067.1| hypothetical protein A7979_00690 [Rothia
nasimurium]
{(GSEDSAKKFFLEVGEKFNSIGSVYRGDLTHKNLIDAMGKRQLNYHTIAKGGPILAEFAFNTKEFLKKNGFN
FERWDSSSK)---VFLSHQS-E--
RKNRAQMLQQSLSANGVSTWFDAVDIDYGENIatAISKGVKESKVVIFWISPKFLESDWCRYEFERFHSRYAG
SNDVLILSIVEQNCIQYLPTFLQELKYLKIDEDLSVDRIAHLLVPSIYNYFKKVS(VKRWSEGV)}*

$4541=163(172):
>gi|1177295465|ref|WP_082650660.1| {<Cyanobacteria(B)>}hypothetical
protein [Mastigocoleus testarum]

{(MRSRPNTNPTPVTANPKHISSSPESSDF)--DVFLAHNSSDKPQV-EAVATELRQRGI--
KYWLDKEQIPPGRWFqYVIQeaipNVKSAAIFIGVNAVGKWQKL--ELRSFINRCV---EADIPVIPVL----
LPGVNKIPENLVFLQEFSWVKFSQSINEKEALDNLEWGITG(KRPQ)}*

$4542=167(176):
>gi|1177544846|ref|WP_082691096.1|
{|0(1176)|<Actinobacteria(B)>}hypothetical protein [Mycobacterium sp. GA-1199]
{(MI)--EVFISYSDADESAV-DALFEDVELAKM--
QVWRDVDSERRHAWWsKTLERIRQATVFVFALSDQAL------
RSEGCRTELAYAEaLGLPILPVQIGEVAGGRSDPVFTAQILDYRETSRRSGIEFVAALLELARERERS(ADPP
PAPRIPGEYLLELGDWVYDDAELSPSTQMQ)}*

$4543=221(222):
>gi|1177704222|ref|WP_082793800.1|
{|8(359)|<Actinobacteria(B)>}hypothetical protein
[Pseudopropionibacterium propionicum]
{(TKTWHVVWVAASVASNSDGVRSGCGNRAWGGARPRERDEMVVACPSVLHDGLSESWAGFGGIMGDRCGTAH
GNRYDPTGP)---VFISYRQSDGTEHAKRLDRFLRAGGL--
VPWRDLVDLPPGETArRVHEAfEEGIAAAVLIVTPEIQQSQFVPAeeLPELLKLEQEPHDFSLLVLNTIPKQD
ETASGDGRSQGDGVDAGQPSTDDAPADHLLGGCGRSDEAI(DV)}*

$4544=183(189):
>gi|1178526940|ref|WP_083045679.1|
{|0(650)|<Proteobacteria(B)>}hypothetical protein [Enterobacter cloacae]
>gi|1177260544|gb|ORC16182.1| hypothetical protein B1H42_23235
[Enterobacter cloacae subsp.
{(MSK)---AFLSHSWAdK----
EFVRSVAHDLGRQYCVFDEQMFDTSESFKDSIEKHLSDSSIFVLFASKSSLESIWVDFEITEARYQTLLKNME
KAIVFIIDSE-
VSFGDLPPWLNRAKVLSvNIPKLVSREIRYQIDRNLMATHK(QYFEGRTADLERFQIMMTPIGLPAPKVASIF
GLPHIGRRTFLTHAA)}*

$4545=221(214):
>gi|1177704318|ref|WP_082793896.1|
{|0(790)|<Actinobacteria(B)>}hypothetical protein
[Pseudopropionibacterium propionicum]
{(MSTPPTNPESPTPPENPTPSRRYSVFVSY)SRRDDA--G---
KWVTRLVEALRDAakddFEVRcdpGKEVFFDRDDLKLGEEWktQLARAIREAEVLLVCVSKPYFESKFCLWEF
QAHETKPKGPdDPSGIVPLLLEDTSrdDQPDEKHGRWHDRIYRMHGHDLRNVFTRLTVTAVPDELAA(QALLL
SDELYQQKQEFRRRDTVTGNLERGTSRFVGRSQELTKLGD)}*

$4546=152(160):
>gi|1177561025|ref|WP_082707269.1|
{|0(700)|<Proteobacteria(B)>}hypothetical protein [Achromobacter denitrificans]
{(M)--KIFLSHSSNdKAIVKKVYDELGAALCHYDAATFS-------
PTGNvseEIYKALSESTHFVFFASEASLNSQWVKGELERAFDGWMR-
GTPKRAMVFLLGTVEVHHLPPWMQRYIMREP--
PSYRHIICRIQSEIDKDNRGS(HKVPPFYRQNELKQLETKL)}*

$4547=228(231):

>gi|1177675813|ref|WP_082765471.1|
{|93(0)|<Proteobacteria(B)>}MULTISPECIES: hypothetical protein
[Phenylobacterium]
{(ERISRAESAERSAARAADEKRHRDDERARRALADANSKLRDDYEARVANLEAQIAAQIESQASRTVPFEVS
AAYGETEPY)--DFFISHAWADKE--EFVDGFVrkaESAGLR-
VWYDNFALVWGDPIRQKIDEGLRSAYFGVVVLSPNFF-SRPWPNyELdgiiQRDLSGR-----
GRLLPIWHRLTQddVEKHAPSLAGRLALST--ASSSSDAIVKELIVVRDRFRDA(VKRNENPTRDG)}*

$4548=194(202):
>gi|1178578006|ref|WP_083093903.1|
{|0(325)|<Actinobacteria(B)>}hypothetical protein [Mycobacterium
mantenii] >gi|1177128947|gb|ORB08072.1| hypothetical protein BST30_05630
[Mycobacterium mantenii]
{(MSSLHLTKETSMA)---LFISYSSQdRATVDALTSAL----
RRGQQQVWFDQELGGGESWWnKILEQIRSCDVFIVALSNNWLQSkPSQAElRYARALNR-------
PILPVRIGDV-
DsMRVNPLAALQIIDYREptVDAGIQLVTAVHALQGKPVPLPD(PLPEDPPVPFGYITRLGNILAEKELSPQQ
QTQLLIELRSGLDEDGEDPS)}*

$4549=169(178):
>gi|1177173745|gb|ORB50809.1| {|0(52)|<Actinobacteria(B)>}hypothetical
protein BST42_18765 [Mycobacterium rhodesiae]
{(M)----FISHVHAdRHLVGGAKR---ILEARYGIDAFvaHD-
DIHPSRSWRdQIKIALNTCHFFAALINlTDFHASQWC-DqEVGWALAKR--------IPILpIRPA-
GFDRMAARDGFLEEHQDvcIPDSATNPEYWVAHQIFSAVVRE(PSLREVSVKSLVEALVNSRSYDSTRLIWAH
IASQPNI)}*

$4550=164(175):
>gi|1178609952|ref|WP_083123598.1|
{|0(396)|<Actinobacteria(B)>}hypothetical protein [Mycobacterium tusciae]
>gi|1177192322|gb|ORB68784.1| hypothetical protein BST47_02535
[Mycobacterium tusciae]
{(MA)---VFLSYHNS---
RLETVAHVANYLARHQIDTWYApRDIPSGAVWGqAIANAIRNCDALVLLFDASADTSKHIHREVDLADRAH--
------IPIHWLRL-
DDVEPDELNYYLGTIQWidWLDKRDEALDRLVRTLTRDVRKT(PVILPAPSPPPEEDPLRKPAVLAPYVEWQP
RHL)}*

$4551=172(181):
>gi|1177223809|gb|ORB84170.1| {|0(331)|<Actinobacteria(B)>}hypothetical
protein B1987_10615 [Mycobacterium kansasii]
{(MA)---IFISYSSQdRSSLEGLTTALRRAQQQ----
VWFDQELGGGDAWWsKILEQIRGCDVFIVALSNHWLQSK-PSQaELRYAQALQRP-----ILPVQVGPV-
DSVRVNPVSTLQIINYQnpTVDAGIQLVTAIHALRDKVPPLP(SPLPEEPPVPFGYLMRLGDTLAEKELSPQQ
QLQMLVELR)}*

$4552=242(243):
>gi|1177317046|ref|WP_082652241.1|
{|0(9)|<Proteobacteria(B)>}hypothetical protein [Aureimonas sp. AU12]
{(MRSAGDPKVGTESRTVDEEVQMTI)--
TIFVSYSHPdEPFRKQLDKHFAPLKHEGDVEFWHDDRIVPGTGLDpDIRKALRRSKIFLALVSAHYLASDYCF
RrEYAYALNRARR-KTMRVVVALVGTC-TWKQTRMAYYKCLPKDGkpVDQWGNRN--
KAYVDIAEGVRRV(VVEVRKELKAASASEAPSPAFPKRKWVVGVSASSRAARSAGSKTATAGNAGNAGKTTIK
KPARRKPSSPHARKSVAPSR)}*

$4553=235(251):
>gi|1177549423|ref|WP_082695673.1|
{|0(166)|<Actinobacteria(B)>}hypothetical protein [Mycobacterium sp. IS-1742]
{(MCSRSPQRCDECAHTTLNRLSRRPAWIGDRVAKT)----FISYARRdKADVDQLVAHLHLLGNS----PWVDSSLRGGQQWWdEILRQIAACDVFIATVSRSALESSAC-QrEFDWAEALHKP-----VLPVALEPL-P---------KALPRrfSMrhIVDYSDsgaRD--RAALSLAGGIGAL(PAAPSLPRPLPKPPAAPLSYITDLVDLLYRNTSLNHEQQRHIVQRIGPALQSSDAEERQAGKDILRMLSSRSDLYAD)}*

$4554=211(214):
>gi|1177654650|ref|WP_082744310.1|
{|0(490)|<Proteobacteria(B)>}hypothetical protein [Erythrobacteraceae bacterium CCH12-C2]
{(MYINWQGRQADLRDTAWRVGVFGCNTAFQGLGQGVSAVPSSDDSQPGQSSSR)-PSVFLSYSRGdQA---RARQVIDLLEAHG-FDVWWDGRLEGGENYLqTTETALETADCVVVLWSAAAVASHWVRDEAQRGRERGCL-VPLTIDGTMAPLG-FRQFQLLDISSWDGASTspEAGRVMVAVRAKAGAGSQGPAQV(VPLSVPAQTAAALPHTSGPALS)}*

$4555=237(233):
>gi|1177295492|ref|WP_082650687.1|
{|0(395)|<Cyanobacteria(B)>}hypothetical protein [Mastigocoleus testarum]
{(MHLAFTSNHRILSSHECQLNIYIVVNLQIMGNYRQNH)RPQLFISHSN---EDYAFERIYRKKLcswfKKKGFKPWLSKEEGRINTEWvvKLVKGLQKSDYFFLAISGK-SNNSKWVRaEID-AWHLMCGLsKIYNIIPFDIgdEEY-KPNEFHPAISSIDWVKYIYKRDDSITKLDKYISEIYDSSN(NSIHFTKAKTTNCGEFAEKLIKILAKLDAPLFWQCADYAYKYFLKDVKEDIETRKI)}*

$4556=267(273):
>gi|1177556858|ref|WP_082703102.1|
{|11(225)|<Actinobacteria(B)>}hypothetical protein [Mycobacterium sp. IS-1496]
{(RHGTGARHAFAEVRQRKFCPRPAQRVTVSQSAAIVPSGLRSGENGHVFLSHSSRLKPKAATNQEVDELDEHDTWQQHRHR)---------------KAFLASLIPKLETklfdRAFDVWVDKTFLSGGSSTdtVIAEAIAKSTVVVLLIDRDALRAD-YLTrEASIAM-YRRTIeDDVRVIPVLLggvsDDD-LKASPLAKRSGVDVLLPFRPSSKKWNAKTADDVAEQIARE(VADTTFNRTARGDSLTRRWIEDVTYLLSEVPELFLGRAADELGIDRRVWLKHP)}*

$4557=240(242):
>gi|1177574190|dbj|GAW49414.1| {|53(89)|<Actinobacteria(B)>}hypothetical protein PD653B2_1736 [Nocardioides sp. PD653-B2]
>gi|1177578656|dbj|GAW55072.1| hypothetical protein PD653_2491 [Nocardioides s
{(VAVVLVGPTAKDSGVLASGVRRAREDGLVILPVVDDLATFHAQVPESLSQFNGFEWSGQEPERRLARVVLEQLDIEERDR)--RVFISHKRSdglaaaEQLHDELTHVR--------FSPFIDRFGVPPGGDVqgRIADALERFAFLLLLETPEAHLSDWVFDEVDYALAHAMGLlilQWPGEPTPIPG---SVGIPRLKLDPTDIQSNAHNYDVLTPDAVDRIIREVEAAH(AHGIVRRRRMLLRSVQEAAEGR)}*

$4558=172(192):
>gi|504676042|ref|WP_014863144.1|
{|0(29)|<Euryarchaeota(A)>}MULTISPECIES: TIR domain-containing protein

[Natrinema] >gi|397681647|gb|AFO56024.1| hypothetical protein NJ7G_0776 [Natrinema sp. J7-2]
{(MTGEQVYVSHAPRNL)--------------DLVQDLFSTVKNFPF--
GVHIALEEIESGRSRkRLEGRLANSDLVVAVLT--------
EDAagNTWINQEVGYAVaKGIPVLPVRDDGVSCRGFISDIEGVTIDRDNLSVTIFNLLCRLRSELAPLGALS(
VPNWYIRFPCTVTGCDQPVTLELERGQTKLWKLHSHG)}*

$4559=199(187):
>gi|498085739|ref|WP_010399895.1|
{|0(2)|<Proteobacteria(B)>}hypothetical protein [Janthinobacterium lividum] >gi|1080110621|gb|OFJ49950.1| TIR domain containing protein [Janthinobacterium lividum]
{(MEIRYGCFLSY)AHGQYAFMN---
KFKHDLIEALACYLEPHldrEEVLFIDSEQLGGGDDIdlRVARAMCQSVCMIVLYTPKYEAHGYTRREFAAMQ
LIEQERrawyelPSHLIIPIIMTRHPdglpLQITDSGLYVDFSGYTLASGDLKSNPQYLPDIDRIVQRIA(TH
YHLLKRSTPPGHDCSRFVLPAIPPEWRAIPPPHF)}*

$4560=164(174):
>gi|1178439257|ref|WP_082961633.1| {|0(1175)|<Actinobacteria(B)>}cyclase [Mycobacterium sp. 852002-51152_SCH6134967]
{()---MFISYSDADESAV-DMLFEDVELAKM--QVWRDVDSERRHAWWtRTLERIRQATVFVFALSDHAL--
----
RSEGCRTELAYAEaLGLPILPVQIGEVAGSRSDPVFTEQILDYRGTSRRSGIEFVAALLELAQEREPA(DPPP
APRIPREYLFQLGDLVYDDAELPPSTQMQI)}*

$4561=198(206):
>gi|1177736014|ref|WP_082825485.1|
{|0(29)|<Proteobacteria(B)>}hypothetical protein [Marinomonas sp. TW1]
{(MGCINLKGFIV)--KAFISYQTTdKLVAAQIKQLLSEVNID------
SFMAHEDIHVSDewrlKILEEIENSKVFLSLWSRNYCSSFWCIQESGiASYHKDMI-IIPLSLDGTTPQ---
AFSSSIQSIKVDPNNV--
LLEDLLPGLRRVDPNIPIKII(FKSIAEARSYRGAEARFKPILSYKDALTNEQGKELLEISLRNDQVCNAGLC
AQTY)}*

$4562=260(272):
>gi|1177738840|ref|WP_082828309.1|
{|86(17)|<Proteobacteria(B)>}hypothetical protein [Tistrella mobilis]
{(YIKICGDKSRHADLIKESIRAVMPARPSNKVDVNILTHDLSPPVDDDPWRTIPQYQFGKDIETLAPDVVKG
LWGDPEILH)---VFISHKSDQSRRARNLKHQLLDYGIS-
SFVAHENIAPNTEWQMSVQQTIFSCDALVALVCDN-FSPSPWTDqEVGIALG--------
RQIPVHCVRLSttAKPAGFLAKVQAFPHQ--
GNIVEHLLPHFITDPRTVRPAI(SSLLSILPYRADGRPVDEVALLLLKAPATAWQTTQKDRFSRLYHRRPVIR
KS)}*

$4563=216(219):
>gi|1178421292|ref|WP_082943876.1|
{|0(252)|<Actinobacteria(B)>}hypothetical protein [Mycobacterium sp. 1274761.0]
{(MM)AGHLFLSYSTQDRDVAELVRATLERHGRR-VWMAPRDVPPGMSYPEAIIGAIRDSDSAVLILSAS-
SNQSPHVLrEVERLSNDT-----KRIFVIRIEPVQlsDGLAYFASMIqwveaSREQLQ--RDPVGALR--
SILEPAHGGGGG(PAAPPRPGTVSPFGVAASLTVDPPPTSFSALAEAQRRMLTTFGVAVLDFICQRPDPAAPI
RRRDLLAELVRAAPTVF)}*

$4564=171(178):

>gi|1178024770|ref|WP_082896871.1|
{|0(399)|<Proteobacteria(B)>}hypothetical protein [Sphingomonadales bacterium EhC05]
{()MTDIFLSHSSKdASVTQF---YADAFEREG-FDVWWDTSIKSGETYDeAIDNTLKAAKVVVVLWSPNSVISRWV-----RAEATVAD-RNKTMMPVMIEPC-ELPIMFELIHTTDLVHWdgTSDDQAWAVFVTDVRQMVAKEQQ(LSVSELAPTSSTALHPILTSGKTDGRGFMPSLAILPFT)}*

$4565=243(251):
>gi|1178460535|ref|WP_082982872.1|
{|0(323)|<Actinobacteria(B)>}hypothetical protein [Mycobacterium sp. 1423905.2]
{(MSRRAHRPRPKSARRINSSDARSHRCARLPKLIAEGRAMRYRRIGFAALAPLCPPIEEISMA)---LFISYSSQdRSSLDALTTALKRAQQQ----VWFDQELGGGDSWWnAILEQIRACEVFIVALSNNWLQSK-PSQaELRYAQALNRP-----ILPVQVGPV-DsVRVNPVSDLQIIDYQNptVDAGIALVTAIHSLRAKTAPLPE(PLPPEPPVPFGYIMRLGNTLAEKELSPQQQLQLLVELRSGLDEDGDDPS)}*

$4566=185(191):
>gi|1178470544|ref|WP_082992879.1|
{|0(172)|<Actinobacteria(B)>}hypothetical protein [Mycobacterium sp. 1245111.1]
{(MLVDPPGPPPRSMP)MRKVFISYARSnKHDVDQLVEHLQTTGY----EAWMDTSLRGGQRWWdEILDNIAGCDTFIAVVSEESLKSTAC-ErELDWA----ES-LSKPILPVLIQPL-AANTLPSRIALRHFIDYgePTARDRVALQLAGALNTMDPAPS(LPKPLPAAPEAPTHELVAIIDLISKPENLDHEQQREI)}*

$4567=172(181):
>gi|1178529467|ref|WP_083048095.1|
{|0(329)|<Actinobacteria(B)>}hypothetical protein [Mycobacterium shinjukuense]
{(MA)---VFISYSSRdRSSLDALTTALKRAQQQ----VWFDQELGGGDAWWnAILEQIRSCEVFVVALSNNWLQSK-PSQaELRYAQALNRP-----ILPVRVGPV-DSVRVNPVATLQIIDYQnpTVDAGIQLVTAIHALRAKTQPLP(SPLPDEPPVPFGYITRLGNTLAEKELSPQQQLQLLVELR)}*

$4568=172(181):
>gi|1178597015|ref|WP_083111742.1|
{|0(327)|<Actinobacteria(B)>}hypothetical protein [Mycobacterium angelicum]
{(MA)---IFISYSSQdRSSLEALTTALKRAQQQ----VWFDQELGGGDAWWnAILEQIRSCDVFIVALSNNWLLSkPSLAElRYAQALNR-------PILPVRVGPV-DSMRVNPVATLQIIDYTnpTVDAGIQLVTAIHGLAAKAQPLP(SPLPEEPPVPFGYITRLGNTLAEKELSPQQQLQLLVELR)}*

$4569=251(257):
>gi|1178606996|ref|WP_083120818.1|
{|11(52)|<Actinobacteria(B)>}hypothetical protein [Mycobacterium rhodesiae]
{(RAAQILGAQSESDVVLTLEQHGLQIGNPDWYEGDMAAFATNVLGSASDQVLTDLVEFLEGEAAGHPPAIASSGNPWGSNP)-TRVFISHVHAdRHLVGGAKR---ILEARYGIDAFvaHD-DIHPSRSWRdQIKIALNTCHFFAALINlTDFHASQWC-DqEVGWALAKR--------IPILpIRPA-GFDRMAARDGFLEEHQDvcIPDSATNPEYWVAHQIFSAVVRE(PSLREVSVKSLVEALVNSRSYDSTRLIWAHIASQPNI)}*

$4570=237(249):
>gi|1178447183|ref|WP_082969559.1|
{|0(327)|<Actinobacteria(B)>}hypothetical protein [Mycobacterium kyorinense]
{(MSDRPHRDIPTTRAQRRPTMLAGPVSARGQCRLNVRWQESADLDETFRRPNAENPRTHSAARKVML)----FVSYAS---QDRAAINHLLTTLRHARHQVWLD-DELAGGEAWwrTILERIRGSAVFIIALSNNSLASKP-CKaELRYAEALRRP-----ILPVQIGPV-DSVRVTPLAATQIFDYRAPTRQANAKLLAVVQKRQAEFAP(LPSPLPAEPPVPFAYLMRLASTLARPELNGQQQAELVWELKTR)}*

$4571=198(193):
>gi|1178467310|ref|WP_082989645.1| {|0(40)|<Firmicutes(B)>}hypothetical protein [Christensenella sp. AF73-05CM02]
{(MGFMGDKIPK)---IFISY-S--WIVSERVLELAERFVANGVDVILDKWDLKEGQDKyaFMEKCVVDPEIdKVLLICDKSYCDKanlraGGVGdEtVIISPEIYNQVnQEKFLpIIFEVDEY-NKPYIPAYIKSRIYFDLSTEDDQYEVEYEKLLRNIYNKPL(YKKPALGKKPDWLETETVDLSPIRDLIRQTRGYTGGNKTKADF)}*

$4572=200(218):
>gi|1178469835|ref|WP_082992170.1|
{|0(638)|<Proteobacteria(B)>}hypothetical protein [Paraburkholderia tropica]
{(MSSLSSSVIKPRTRPMNKA)----FLSHNS---FDKDFVGAVFDRLGAL--QSIYDKETFKRNCDLaeQIREGLYDSKVYVLFL-SRAALDSGWVRnELDLANELKTKWqIKEFLI-----------FQLDDTNWSDL-PAWASRFVVSCPPSPGQVALRIRDV(LRKEKIEEQEPYGRDLDIRKINEMVLHRDTPPKFLFLSGPTGIGRRTIANAVFKSFYKT)}*

$4573=200(204):
>gi|1178691797|ref|WP_083200327.1|
{|0(12)|<Proteobacteria(B)>}hypothetical protein [Thauera sp. ZV-1-C]
{(MPPDRSGSVEATPSSPADIALGSADASP)--EVFVSYAW-GGESEALVDRLERVFAARGIAVVRDKHALGYKA-DihAFMQRLGRGRCVIAVIGDKYLRSPHCMFELMQVAARGDF-QDR-VFPVLLDDAKIQRAAERVRYVQHWEAQIRELDEALKTVSAADLKGFREEI(DLYTEIRRTIAELTAILRNMNTLSTQMHSETDFVAL)}*

$4574=296(298):
>gi|1179532523|ref|XP_020687409.1|
{|18(609)|<Streptophyta(V)>}uncharacterized protein LOC110103155 [Dendrobium catenatum] >gi|1179532525|ref|XP_020687410.1| uncharacterized protein LOC110103155 [Dend
{(TACGRSLSPNLSPPKITNSYIFSSSFPDPPLFSSTSVPAVDSASQVSSTSTILYNNSISSSSARKLEGNDSEIERLKSCD)---VYVGYcgRK-KTLLIRFVNWLRAEMVMHGFLCFVSYRATCSDAKSHVMARIamEEAPLGVVIVTRKSFSSPWSVEEL---KILLGK-NKL--IPIFFGLKqgdcfSRDIVEKKGEiWGKYGGTL-WKKYEGLE-EEWSEVVDGLSRL(DVKFEVSTSNMRDCVFDAIYLIGKKLGRGDALEKLKWKSRVAIEEFPFTRNLNFIGRKKELLELKLMLFGDVQMEGDE)}*

$4575=227(231):
>gi|1178869792|ref|WP_083249312.1| {|0(559)|<Firmicutes(B)>}hypothetical protein [Bacillus solimangrovi]

{(MIPNCYRRSELLG)--NIFISYSSADTKQMLELV-NRLESYEL--
KCWYAKRNIGPGEnYAvEIIEQIASCKVFIILISRKAFESRH------
VATEISLAYrRDMLIIPLLiedlahDNSEWPK------
DFRYFLEtaqmFNGVHLTEEDWQQIVDKICEQLELE(DNVTITSSKIPEFKLESHKVNGADFLFSPMEKTKLD
FIESVYEPNPNYLELQETLQNHRVLVLYNQEQTGKSTAGIHI)}*

$4576=267(269):
>gi|1179492360|ref|WP_083332425.1|
{|0(823)|<Proteobacteria(B)>}hypothetical protein [Hyphomonas sp. Mor2]
{(MSNAWWQKPRSTVGQSKCLQAREVLELGQGDAGAIKALTASCCTNTVLKVLSILRFPLWSLSSAWGACLMV
VEQLK)---VFISYSRTD-SDFRERLAASLEENRF--
HVVFDVQDndtsdpeltLTPQdKWWdQLKSMIAACDVVVFIVSPAS----SQSRvcDD----
EIAHAQnLGKRIIPILWQA--
LDIHTAPERLRSLNIDLDFRADRDKPFDEAFPALIHELNR(DVHWEREGSFFARLADRWHQNNRSEGLLLRSE
TIQAYDSWVFRRPAWAPEPGT)}*

$4577=214(212):
>gi|1179488906|ref|WP_083328972.1| {<Proteobacteria(B)>}hypothetical protein [Psychrobacter sp. AntiMn-1]
{(MRTTSGIGELSPGLIGFNSIDSSNINQIREKIGKHFQATPSDAVFIMSDDGSNLESEIKNITQKHEYALGL
T)PMKIFLSHKG-ID-KPTVRQ--
YKELfTTLGFEPWLDEDAMDAGVELerALLQGFKESCAAVFFITPNYVDENYLAAEINyAIAEKR-----
KRgnkfsIISIVmkkdgLKGKVPELLEPYVWKEPETDLEAFKDIVKAL--PISVG-NVYWKY()}*

$4578=224(226):
>gi|1178862921|ref|WP_083242442.1|
{|269(0)|<Proteobacteria(B)>}hypothetical protein [Ruegeria sp. PBVC088]
{(INRLRMIIHSAKKVGFEEASKVWLPDGRNKLREHILGWLAHIRQVRGASDPVLAKLCLQAHDAGLTKVGWI
EELADMSKD)-FDVFLSHAS---EDKDRVRKLHDALTAGGVKVFFD--ESSIKWGDsivEKVNHGLL-
KSRffIPFLTTTFSKKGWTNKELNSSISMNISRKG-RILPIKSEEFEigDKypILNETLYKTWPKSSV-DE-
ADFIQ-EVVDAIIKMIEED(KAAPSV)}*

$4579=190(191):
>gi|1178698415|ref|WP_083206945.1|
{|0(42)|<Bacteroidetes(B)>}hypothetical protein [Tannerella sp. oral taxon HOT-286]
{(MFTASRLN)--KVYISYKHDSSYSKALEAI--KNGLKKNNIEY-SIDLQDIKYRDdiveY-
EKEIGTAVRVIMFITASYLKSIDCMFEMTEIFkksdVMERV-
FPIVDITPISRNRKGLEEIKDYWQKQKVEAS--
AQMQTEVGSSDYIIDELRKIN(AIIKALDEFWYYIVHINTGVFKTLLENDAALLMEEIQKTTPYR)}*

$4580=132(148):
>gi|1178786362|ref|WP_083233404.1| {<Planctomycetes(B)>}hypothetical protein [Planctopirus sp. JC280]
{(MRPKPRYQ)---VFVSHATAdKWIATTICEKIDAIGA-------
TSFrDDRDINGGDsipqSIRTEIQISRELVVLLTPDSIERPWVLLEVGAAWGRRKD-
YRIVpVLCHVTFDAIPDIIEGKKAFHINDFDKY-LAELKRR--VKK---------()}*

$4581=205(203):
>gi|1178984863|ref|WP_083291376.1|
{|0(86)|<Actinobacteria(B)>}hypothetical protein [Rhodococcus sp. 1139]
{(MSVAARIMSTMPNALIVQNLGRFAVP)DSKVFISHAYAdLALAKLVRDTLIRGGVPNNRVFFSSSRSTGIP
TGTdvvgHLREQLDAAGLVIELISQTFITRPMCLMELGGAWSLKKS-

TFPVVIPPLTRAQAIEAIGSFQMADLGDASS--
ARDMWFELQDRIKAALHIDIG(ASAWSEAIRDFADAFAALPASASSDADAQVAPEIPSQ)}*

$4582=215(220):
>gi|1179614994|ref|WP_083383187.1| {|126(0)|<Firmicutes(B)>}hypothetical protein [Traorella massiliensis]
{(AEDQNKIEAIRNYLNSCGIVLYSDKTLKDLFEEMKSRLWNCCSLHHKADEEYIRLSFQELFYFLEDSELEQ
RGFHLTPEY)QPKLFISYSWKdNEIVQRIVDHLEDIGL---NIW---
IDKKNIEGGEhlheAIRKGIWQCDMVLLIISKNYEMSLNAKAELKTAFanvvANKKD-
WRILKLDDIDPDSIFPSLSQYKYYSINEENE--LIE-----SILRGLDNIKNHK()}*

$4583=287(295):
>gi|1178876486|ref|WP_083255620.1|
{|173(202)|<Proteobacteria(B)>}hypothetical protein [Methylobrevis pamukkalensis]
{(PSLDRLWQSFLRSLLVRNMNWQVWIDWYEARIRGDSVNESLEIARVNLPAEIWDEGPAVVNAEIQRLIELH
SPSPAKYQT)--DFFLSYSTKNESTARKIDAVLREAGYS-
VFAQFRDFRPGSNFVTEMNLGLERCDRVIALLSPDYVA-SDHCQaEWNAAYASD-----
PSGARRRLVPilIRpaNLSPLARQIVYASIVE--RSEEEARQ--
IILDAVRRDPET(EAGVMRRRIADAASPQPEISEDGVLGQHRNPIYDDVAVDAELPSLPRRQCDLIEQILGDE
SEARQPGANAELPWI)}*

$4584=191(194):
>gi|1178889468|ref|WP_083267887.1|
{|0(158)|<Actinobacteria(B)>}hypothetical protein [Lentzea guizhouensis]
{(MARSRTCEPGRVHSAGTPDE)-WDFFISHASEDKRLvAGPLAHYLQSVGFS-
VWYDDFTLRVGDSVLREIDRGLGSSRYGVVVLSEAFFA-KQWPQrElagLMATAGGD-----
RRVLPVWHGMNVadVARYSPLLADVKGVST--
ARGLHVVAGKLVRAAFPERVGE(LPRADSPEERLTVVEAAREPLRAVLAAGGGCDDV)}*

$4585=193(202):
>gi|1179492441|ref|WP_083332506.1|
{|0(1377)|<Proteobacteria(B)>}hypothetical protein [Hyphomonas sp. Mor2]
{(MLNTNTVGIFVLDKSVG)---IFLSHSTRNKSATKKLTQALKK-NFT-
VYYSEHDNQLGESWVKRLTDEMRQCAVVVIYLTKDWLKNKKWCRaEFYAALMAG-----
KKIVVVNAMRGKdhDDPILPLLQDFRTTRR--
GKVDFDDIVTEISKLVPASSVS(VWPGDRSPYPGMNALDIEDAPVFFGRDEVTQKLVDKVNRSIDLFE)}*

$4586=301(291):
>gi|1179622595|ref|WP_083390787.1| {|29(237)|<Actinobacteria(B)>}TIR domain-containing protein [Frankia sp. NRRL B-16219]
{(SEDGAGQLHPTCAGVRSAATAGRGRGATRCRGRASAGKLPRTGPRRTANPRGFAASRKRRHLHRLTPAGMP
EAWDAGPLY)---FFFSYARVDSRddplleqfFRDLRAEVRRHAGHpdadS-
VgFLDLSSIQPGEAWSAELGAALCRCRTFVAMCSPSFFA-SEYCGrEWgmfdgrlrAAAAAGRECP--
PALLPVIWTPLRDPPELLARLQY-DHSG--LGETYARF--
GLRYLLQLKRNH(DEYQEFLLTLAVRIVQLAEDSPLAPQPEIPALHQVPNAFRVLTGPASMPDEGDPPTVEEP
GAVAPTPSMTD)}*

$4587=237(242):
>gi|1178860216|ref|WP_083239737.1|
{|0(132)|<Proteobacteria(B)>}hypothetical protein [Hydrogenophaga sp. RAC07]
{(MHWRRSDPEALQEFVSRDATHPGTLSRRSWSTQSTAHRDKSRRSRVDTARGCADSGAKSRGGTTMR)---
VFISHAWEdKPLA------

LALAQLPDFIDAWVDvRELLGGEKLDpTIITAIEDSHVFIVLISRISLAKAWVAQEVSWAMAReAQK-
DRVFVLPVIIEAG-
IDLAASAPPFQQFAQRLfiDASDRSDSGIEKARALIAQTLFH(WASDWLDRMEPRGDSNRRFADTLERDLVAF
QTRLFA)}*

$4588=161(157):
>gi|1179443448|ref|WP_083326589.1| {<Actinobacteria(B)>}hypothetical
protein [Actinomyces sp. HMSC035G02]
{(MADI)--
KLFISWAHAdSELASSFLQLLKPrlnIVRNHNFTLWEFSLIPIGEEWEqEILKQWNKANYIIQLVSPHFLNSE
FIRKhEIPEIGDATRK-KSLPVMLDDVPLD-
KSMNLHQIDERQIYRGSfnHPHSYISCTTEYERRQFVNGFVE(KLLERVDRKTGYR)}*

$4589=220(202):
>gi|1179618007|ref|WP_083386199.1|
{|0(144)|<Proteobacteria(B)>}hypothetical protein [Methylomonas sp. LWB]
{(MFIHGKRIFRSGPVCDNNNHK)-
AQVFISYAHAdNQtfdkealgWVSNFVDHLTkaiPMKPGGKaVSCWMDHRLEPQRSVDeTLRARIQESACIVA
FMSPAYLSSPWCRLEMDLFINLVGG-
GKAsnRVFLVELLETEreDWHYGIRSISVVQFWDKaiNQPEPITLGWPEPDTKNDRKYWL(ELSNLASVLARQ
IQNLSPTEPTPQPPKTSPVSRPQALPPWP)}*

$4590=189(191):
>gi|1179680061|ref|WP_083418495.1|
{|0(1215)|<Actinobacteria(B)>}hypothetical protein [Frankia sp. BMG5.36]
{(MVSPAGYRDPVWRSP)MARVFISYARGdAVVGQLLRKKIVAAGHDVFLDTAHDRGIRIGEEWEkRLYRELR
AADALVAVITSSSVASNWCLAEVAVARSR-----GAVVLPLQVEKG-
VELPLLGSLHYLDYVADpdGAVEAVVDELVRVDLGSGRGWAA(GRNPYPGLEAFADDRRAVFFGRDDDTQHVL
SLLTAS)}*

$4591=251(243):
>gi|1179681701|ref|WP_083420135.1|
{|0(174)|<Actinobacteria(B)>}hypothetical protein [Frankia sp. BMG5.36]
{(MSSSNVVTAPVPANGVANGVPALSAAAPGVATNSGNAPGTRSPKV)----
FVSFSsHDnvnqsaTTFVSDLtARLAEKFPGTKKPHVFYSNKSLKNGQTWesELLKELDRCDYFLPLCSANFF
GSPWCSKEWSYFRRRllsstpagqRALDrliPV-------WW---
RKIGDGRKKDKENLHPVLTPYHVGYPDDTPERPGLQRLLR(SNPAKYHAYVETIAELIHTDFARRPDPLDPLG
LTNFKSLEPAFSPEAAQRKTLETNLI)}*

$4592=218(233):
>gi|1179682536|ref|WP_083420970.1|
{|0(109)|<Actinobacteria(B)>}hypothetical protein [Curtobacterium sp.
MCBA15_009]
{(MSRAFRWRGLRCRNVWNVKI)----FISWSGEperavaEALKAAL-----
PTLCAVEVDVFVSSKSIAKGADGieVINAELRSAAYGVVLISKSNQEAPWLNYEGGWLASLGCPVsTVLLDLR
IS-------------DITTPLAPRQATNFEDEN--
DMADLLKQVATA(ANKNLPDQAFEALLAVVWPSIRDSWKPLEGDDGNEPIRSDSDMLSELVERVRRIEQTGEA
AFSLNRKRSEFSQ)}*

$4593=234(240):
>gi|1178890049|ref|WP_083268468.1|
{|0(405)|<Actinobacteria(B)>}hypothetical protein [Lentzea guizhouensis]
{(MTARAEFAFRVGDGGRRVMD)QFDVFVSYaSVDRAVVRPFVERLRADGFRV--
WFDEQRMAGGRPAMQQLADGIENsAH-

LVACLTDAYLQRAWTGFELQVSRYLDPAGRSGRTIPVLFERLTGPVPAEIRFLALCDLTD----DATYD-
LEYRKIVGSITRP(VPSASPEPLQDACVAPFQHLDEPAVALFKTHQAVSALLRHQDGTGGPTLEKLVHSGEVP
PHLRAPLATVQAYARGTEDDA)}*

$4594=257(256):
>gi|1179969063|ref|WP_083491417.1|
{|46(12)|<Proteobacteria(B)>}hypothetical protein [Thauera sp. 27]
{(AGVNWKSFGEDIELTVRDGAVHARSSCKVSTTVFDFGRNRQNVEAVFRALESSLARLPAGRPDTIVGVVPA
AATGRAPVP)---------G-ELTREVFVSYawggesealverLERAFAEHGITVVRDKQALGYKA-
DirAFMERLGGGRCVIAVIGDKYLRSPHCMFELMRVAAQGNF-QDR-
VFPVLLDDAKIHRSVDRVRYVQHWEAEIRELDEALKTVSAADLKGFREDI(DRYAEIRRTIAELTSILGNMNT
LSPQMHSESDFAAL)}*

$4595=141(141):
>gi|1179993777|ref|WP_083516131.1| {<Firmicutes(B)>}hypothetical protein
[Alicyclobacillus sendaiensis]
{()--DLFFSYYFKNKDEY-DGIQRLLSERGY-----
FDYrngslDEDNPAKTTkEIEEDIQSQINWagtvVVLIGPGTYTRR------
WVNYEIDYAAeKGKRIVGVYLRG--
QSSSRIPASLRRLYNEGYkelalVRWNADSIVAAIRGKNSWDEGD(K)}*

$4596=247(248):
>gi|1180111226|ref|WP_083630611.1| {|0(306)|<Cyanobacteria(B)>}molecular
chaperone Tir [Scytonema sp. HK-05]
{(MAVAAEMERSAAKAAFIIPTQVLVTAWSELGQAFSLETVKPASQSAK)---
VFISYRSQDpDLTLAQQFYEALKAAGH--EAFMAGESIRLGEnWPeRIDRELQQSDYFLLLLSPKSATSEM--
----VTEEVRRAKqLqdlrpdGKPVIlPV--
RINFPLSSPLNYDLRgYLQRIQQREWKSSADTPKILQEILTILAE(GREAVFSEEELTTSPAVETLESPPLPV
AEPELPEGQVDLASAFYVERPPIESHCYEAILKP)}*

$4597=229(234):
>gi|1180309349|ref|WP_083828152.1| {|0(47)|<unknown(X)>}hypothetical
protein [Frankia symbiont of Datisca glomerata]
{(MTSTSSRHWLLPVLLTRKVDRTSSISDVNCLRLYETTRELRDQVFVNKILHRLFRHVTDDDS)PLTVLLSH
AS-TD-GRGVALAVRKYLHEcTDVGDFLDVHSIAAGERLveAIDRAI-
SASAFLAIVTDSYSSRGWCRHEVIRAKREL-----
GPIVLLDGVEDCDERRSPYLGNIPVLRWKPDSPLVMERLLGLLLAESFRHRY(FPRQVAWLSRLQRVDRPYYC
LSRPPEPLTLLE)}*

$4598=222(221):
>gi|1179993765|ref|WP_083516119.1| {|0(214)|<Firmicutes(B)>}hypothetical
protein [Alicyclobacillus sendaiensis]
{(MAILL)--
KVFISHSHEeKDLALAWQTLLQHVSQGAIEVWLSSDtkPSGGMRIGAewrdNIYQKLAAAHFVLAILSPRSMD
RPWIMWECGVASGTNKERgVIPVVYSMPLSEFDGPL-ATYQAYQGDDREK--VIEICER--
LMVEAGLTPRRE(YWGPIIDTYMASVRVHKPPRLATASAVAVWMRRIDSYVNEGRTSELPGLMDAMYASLGTT
SPIDIQIHDLFSKVFL)}*

$4599=254(253):
>gi|1180009500|ref|WP_083531854.1| {|45(22)|<Firmicutes(B)>}hypothetical
protein [[Clostridium] neopropionicum]
{(IVFGVQDDGKKLRVKGYKFNVDIDSIKAKLSDNVNLTFFDLPHETAALKCINVEKCENVVVFSDAPYILDH
NRNVAELHI)-KKVFLSYSHNdTCIADLVDERLNFFGKGRLAI--TRDKRT-LEYKSdiekFM-

QTVSSHDFMVSIISDSYLKSQGCMYEvselMRNRAFNDKL-LTIILSEADENFYPKDKKPKQVKADVYSLNR--FEYLKYWETEKGKVNKLNTEI(SDLALKQGLVDEIKQINTISTNISELIEIFKKS)}*

$4600=183(188):
>gi|1180166268|ref|WP_083685651.1| {<Firmicutes(B)>}hypothetical protein [Paenibacillus sp. FSL R7-0333]
{(MAIRGRNFALRNSQGYFESFSAQNFTGKNVC)---IFLSHISVdKTAATKIGDYIQDAGFN----IYLDINDEDLQravannDAArvttAIEKGVSNSSHMMCIVSEATKNSWWVPYEVGYGKKSNKE-LSTLAL----RNVTSLPDYLKITYKIEGVIG--LNKYLNS--IRKGSTSYEFLT(ESANYHHSLNPYLKTDR)}*

$4601=229(238):
>gi|1179927107|ref|WP_083449462.1| {|0(1227)|<Actinobacteria(B)>}hypothetical protein [Actinoplanes rectilineatus]
{(MPRRAAPHHSGPVTLPAP)---VFVSHASADDTFVAELRILLEDCRIP-VWVDSRELRGGSRLAPEIEAAIRDAAHVVAVLSPATI-NSPWVRrEITSALAVG-----RPVIPLLLPGITpaALtmwfpEEPVAIEVDRGPGG--L--SAAAP--RILAAL-GRRLP(DDAPPFREAGTKPVEELVLKLTDPTFEEAGGRRRVRATATLIYEPAARGARHVESRRFVLTAPWDSIAADDLAWYLQSYY)}*

$4602=222(230):
>gi|1179951568|ref|WP_083473922.1| {|0(648)|<Actinobacteria(B)>}ATP-binding protein [Frankia sp. R43]
{(MTDGGQGSTEAPTS)--DFFVSYAQEDVSWAEWIAWQVELEGFS-VILQSWDFLVGQNRVARLDEALAASTRVIAVVSTSYL-NSEWDLlQSYATLGRD-----PGgrlrmLFPVAVEDCRIE-GILSSVVGIEIWG--IGENAARE--RLQQAVRSLATD(TRGKPDRLPPYPLLQSRSARFPAKSPIIWGVDWPRNPNFVGRSQEISLLRERLLEDRGTVSVLPQALHGMGGVGKT)}*

$4603=272(275):
>gi|1180113588|ref|WP_083632973.1| {|0(151)|<Proteobacteria(B)>}hypothetical protein [Bradyrhizobium sp. NAS96.2]
{(MNWVNAASAAYAGRCSGTQSNSANATMVAGHFMIDLTPRKQALQRGFGNVRIQFWVVSF)AMRIFLSFPSELESQADNIAQSLRNCDHD-VFFSHDDLPPGDSFDARVEKAIESSDFMVFLISPESVTKGRYTLsELAFARSkwpnPS-----NRVLPVMMAPTPldQVPTYLKAVTILEPQG--SAAAETRA--AVDRMLEAKSEQ(SRGQPNLPFLVLALASAIACSLVTTYALNVLQYSFVVSDAGGITILPGVIFGAAIAACNVMFGAKDRFQLALVVAI)}*

$4604=185(185):
>gi|1180117238|ref|WP_083636623.1| {|0(334)|<Cyanobacteria(B)>}toll-Interleukin receptor [Oscillatoriales cyanobacterium 'hensonii']
{(MPVYLIPYP)-RRVFISYRtqEPDATLAQQFHDALEQQGHQ-AFLAQKDLHLGESWPQRIELELKQADYFLLLLSEQA-ATSEMVMaEVRSATRlqeaqKR-----PVILPIRINFPIdsPLNYTLRSYLDPIQQR--EWTTEKQSPRLIQEVLTLLLEG(RIFEARCSKLPALSPSVPAYSLIPQPFADMPVLEIP)}*

$4605=190(196):
>gi|1180117728|ref|WP_083637113.1| {|0(203)|<Proteobacteria(B)>}hypothetical protein [Marinobacter sp. C18]
{(MSYQRKDIVSIQQ)-KDIFICHASEDKPDVVRpLYEKLESEGFN-IWYDEAEIKWGDSITERVNWGLIHSKYVVVVLSESFLE-KRWPNrEFNSVVNTE-----

SSSGVVKVLPLVvgDGVIERKIIEAYPLIN--
DKRYLKWSNNQTEIVEELYKIF(PEKSKRNFETDAKKINQKNFSDQDVLCRSNVSVNVQKVSCLSG)}*

$4606=186(195):
>gi|1179934040|ref|WP_083456395.1|
{|0(507)|<Proteobacteria(B)>}hypothetical protein [Desulfosarcina cetonica]
{(M)--KIFISYARGdSAQVKELARFLSDSGH----
DAWMDSRLLPGQDWKqNLSSQIVAADLFTSIISNRSVASEWCRWELGQAFLL-----GKPVLPILLDSV-
TIPD-
EIKHLHAVPYNLrdRIMGGIAAASAQGAGHEIERKFD(FPSSPRGRPSRVLGEDPSALRHLHKRNINAGRTWD
LGPDDIYKFSGRIVGIDFG)}*

$4607=249(257):
>gi|1179950641|ref|WP_083472995.1|
{|56(157)|<Actinobacteria(B)>}hypothetical protein [Frankia sp. R43]
{(SENLRQQVEVVRQADVVVVGATLSQSSALPAEITSLPVEGPWVEICEGGRNITPLLARLDTLMAARTQTEE
QVTEKLPIR)---VFISHSSDdKlasDVRVELERH-------
GMDVFWSEQSIGVGEDWLaTITTSINESDVFVLLLSPNVIQKPDWVYqELLLAKEAKR-----
RIIPVILKPI-
PGIPQNFAFLIRNLHFInlDPGFEVGIGRIVEAISGQAGAPS(KIDGFRAKVHRTSEKARRVAHEHELGKKAK
KYGSAAL)}*

$4608=120(141):
>gi|1180051534|ref|WP_083571706.1| {<Bacteroidetes(B)>}hypothetical protein [Chitinophaga sancti]
{(M)-------------LIEGLQDHLSV-
KTGAKIEFLRDKDIGMGADWKaTIGENIRKADAAIILVSAAHLSSPFIRKeESGEFFKRATA-
DGSLILPVLVRNC-DFRAfEELSGLQFFP--------LIIMNMGISLSAIASAAG()}*

$4609=242(245):
>gi|1180152792|ref|WP_083672175.1| {<Proteobacteria(B)>}hypothetical protein [Sphingomonas sp. W16RD]
{(MAVKSPNTTSATKVAKPVKRRSVADKRASQTKGTSKPDVR)---
IFMSYSHLdAAAQVKLNRHLAPWRRDGV-
TVWYDESIEPGNALStEIGQELRRAHIFVALFSPAYLDSHYCWDiEYKRAMSRRAR-GLMRVVAVVVKPC-
GWKQTRASGFKLLPKDGipPESWSSSD--
AAYVNVAQGIGEV(IQAVRRELIAKPTTPARATAKPTQKLKPRPTAPKAMIARSKKASVGPATKVGKSPPRGR
KALTKP)}*

$4610=177(178):
>gi|1180160012|ref|WP_083679395.1| {|0(103)|<Firmicutes(B)>}hypothetical protein [Paenibacillus amylolyticus]
{()MMNIFLSYSFKdKEIAD----
HVSLQIKESNVGSINEYELNVSEiHILdELRYNIRKSDYVIFFISNNFLNSNYSMLELSEALNELRI-
RKINIIPILIEKC-
SIPSELLEIGIIDFSKSfeKGMEKLLTKLTIKKKIDLEEISP(WVYEKIVAIFLKEYGFKIIQGENNRDIGVD
YICEYNAK)}*

$4611=243(240):
>gi|1180183236|ref|WP_083702588.1| {|0(736)|<unknown(X)>}hypothetical protein [Pseudonocardia sp. Ae717_Ps2]
{(MDRKEWGPFQPISRTRPRGRAVKNDQSSSPAARQ)-
YAAFISYSHRsdkrtgPLIESALEQFPATWHRRRRRRIYLDSGDLSASDDLwrSLEEALTDSDRLILIASPES

AESRWVQREVTWWLDNRSIStiiILHSKGDLEFD---AEGK----
ISTDALPAVLLQSMNAEPNVIFLPPIPKRQID(PADPQWNAVLAAVAANLDGVSKDALIGEHVQNRRRWQILR
SVTIALLSVLLVISLAATAIAVVQRD)}*

$4612=196(197):
>gi|1180348793|ref|WP_083867593.1| {|0(195)|<unknown(X)>}hypothetical
protein [Fulvivirga imtechensis]
{(MAFKQYHMNSNTY)KYDVFISHAV-ED-
KMSIANDLCELLKQAGLKIWYSGNELGIGDRIekSIQKGLDQSRFGIVIFSPYYLSKTWTIREL-
YSLMAKERAGQKVILPVLHNVTIDDLKAKDLTIADTFGISSEKGIEIVAASLISEIRKSKKSA(SEENENNRL
TLGLSFSFPRLIGIILLLLLGFSSFFYLNRDIPSD)}*

$4613=157(160):
>gi|1180406574|ref|WP_083925357.1| {|0(203)|<unknown(X)>}hypothetical
protein [Flexithrix dorotheae]
{(MTM)--KVFISYSW-DsEAHKSWVLHLANSLIQNGIDVLLDQyELRLGRNLNHFMESsiIEADKI-
ITVFTENYKLKSETRDGGIGYEYSM---
ISNELYKSQINNTKVIPILRAGSPELSIPIFMSSFLWLDMRDDSQFESALENLI(REIYGELQLKKPALGNK)
}*

$4614=222(217):
>gi|1180410823|ref|WP_083929606.1| {|0(28)|<unknown(X)>}hypothetical
protein [Catelliglobosispora koreensis]
{(MPGNRSQERSTFRLAEPRPQIAPLAGVGVLPTPASAPIDPQWLIRCFPRWEGADPLADYE)-
YDVFLSYRR-
AGSVNGWMhNHFYPKLVESltdELNYrpeiYLDTEQDVGDRWPERLEyALLRSRIMLCVWTPSYFGSDWCLAE
WESMKLREQA-LGMANRQAPGGLVYPIRLKDGESFPKEARET-
QYLDMKPWAYSSPVFQQSVKFL(EFEEKVAGIAEDLARRL)}*

$4615=255(251):
>gi|1180435957|ref|WP_083953409.1| {|9(114)|<unknown(X)>}hypothetical
protein [Thauera butanivorans]
{(YLYPIHLAIRFSISIVKLNVFPSLARCFRMASPCGVASSRFVSASTGMQPRGGLCNDDFLEKTSNHQGFSR
GDSVQPFTF)----FISYRRQDTAPIALLLKSEIEKRlqFV--
RVSVDVDEMKPGDrFPdRLRALIDGAHATIVLIGKNWMPRQEASQeipatPDWIVEELLYSRtAEIKLPEPDR
FGLAGRAIIPLFADCARDFRQFCLRPELAFLAEMHSEHIDYAGW(PSAIGPLLDRLAVRLSLKKRPDADEYPK
PDL)}*

$4616=231(229):
>gi|1180399306|ref|WP_083918103.1| {|246(0)|<unknown(X)>}hypothetical
protein [Methylosarcina fibrata]
{(RLTNQTRLADKKTRAWLVEDDRHQPGLTSFFKGFSRETEFLSDVSPETFVAELHRRWLARRTPGEEKIDEA
VKAPGPTLF)----FISYSRATDLPAAQKLFESLRQQGV--
GeneIWFDRNAIEPGqDFQnSILDGIRGCRYFLPLISAAA------
DRfdEKFFRREWKQAIdRSGAIagrdfvVPLIVDADYDPLAYKRVPRDWTEHIDFGHAPAGVPDPRTRNRLNK
LIRD(ERRGAADSV)}*

$4617=222(227):
>gi|1180306553|ref|WP_083825356.1| {|0(135)|<unknown(X)>}hypothetical
protein [Clostridium sp. DL-VIII]
{(MEGLHMKPT)---IFFSHSSKdRDSLMKLRELIEEKTGGTINIFMSS-
DGQSIPFGSnwmyKIEEGLKDAKIMFVFVTPKSIDTGWIYFEAGYAYSKGIE-VIPIGINIDIGSIKPPI-
SLLQGFNLISEES--INNIIHI--

INKKFDYSIKAN(FSRADFNQINYRSDETESLIGIKDSVDYITTNVFSYKTIDKEVSIDCKKLFEQIKDYLAE
KNVEFVSENNNVLANGLK)}*

$4618=243(234):
>gi|1180224631|ref|WP_083743471.1| {|0(1108)|<unknown(X)>}hypothetical
protein [Mycobacterium sp. AB57]
{(MPARRFGGLRERGVHVVS)--RIFLSHSSKDTREAValklWLTGQRPELGDE-IFIDV-
DTQTGlklgEQWKTQLFTGKSRCEHFICLLSQN-WADSTECNvEYRTAEGLG-----
KRILVARLEDLGhnDI---TSAWQHCDLFA--GGEcsdievpggppvrfnTEALT--
SIMDAVDGNGTG(PRQFVWPPRTDPGRAPYRGWEPFEEIDAGVFFGRDAVIERGLDALLGMRFRLLATMSGLK
SLFVVLGPSGSGKSSF)}*

$4619=255(254):
>gi|1180323729|ref|WP_083842532.1| {|97(367)|<unknown(X)>}hypothetical
protein [Bradyrhizobium sp. ORS 375]
{(GQHPPTGASPHRVLPVALDADALQLAPTLNHVSFVRLDVRHGEDRDRHLVLHIAVRGLRALQDLPQGGGKP
ADVLPDIPV)--RLFISHAKKDLPadpaaIAEgpvkAILATLTQLPVQ-
GWYDSADIPAGGRFPEEIRNGVLNSSALVAVLTDS-WSSREWCRrELLEAKLAA-----
RPLIVVDAIEARviRLFPYLGNAVTLRWRA--
AIASSDIMDTAWVELRKNWEAE(DAALVIEAALLEALRYQYEHRRLLRSIAGNEVAL)}*

$4620=214(220):
>gi|1180363010|ref|WP_083881810.1| {|105(0)|<unknown(X)>}hypothetical
protein [Paraglaciecola chathamensis]
{(VNDYNRKVAEQNRSNFQRVQSFNSEVNRQNTQNDNSNKRLLSGIKQQLRNPASSIRYTPTEERLADRVYEA
ISKSDSKEY)--DVFISYAKIDGyEIASQLRDELKKLEVS-
VWFDEVAIIPGRSQSLQMDSGLRKARAGIAVLTPAYLAGRFWTErE-LGVLLNK-----EILIPV-
LKGCTfdDvkeysgiLPDLAGFVVDSD-------ETAETVALKIAPAVKVEELV()}*

$4621=213(208):
>gi|1180394922|ref|WP_083913719.1| {|0(371)|<unknown(X)>}hypothetical
protein [Nocardia concava]
{(MIGPIGDIEHSREQ)AWDVFISYAREDYEAAKDLHAALRSCVTRQgkqpeVFLDREpgATRLGSDWSMSLE
NSIINSRYFVALFSPSYFLKDICQReLHVALDQSR-----PNspVLPLVITRIEdsDVPYLARNRHRISTTK-
-
PNWFEELRNALDLRPKLDQQPR(KLLWDGSIEDIPANHTLPEIRVIISGADGQPAPPGPDEEVSIIAVPSTTG
LLGR)}*

$4622=160(171):
>gi|1180410616|ref|WP_083929399.1| {<unknown(X)>}hypothetical protein
[Catelliglobosispora koreensis]
{(MM)---IFISYARKDQTVESlrRVDAQLAQLG-T-
TYIDDLHGYSAPDRLAAVRDALAAATVFVGIHSPSYLAT-PWTRvEMSAALL--------
RGIPVFALLDRwrLATDQSPEWPWQAQIS--
DLSQSPARWPSDDSPAYPHHSV(PAANPVTPNASKISATLLGYSSGTNLARG)}*

$4623=289(296):
>gi|1180467979|ref|WP_083977223.1| {|190(1022)|<unknown(X)>}hypothetical
protein [Herbidospora sakaeratensis]
{(SGSAVFARGVFIGMVSRHHRSDGLGVLTATRVDGWLTRHDRGLLAGLCALTGLPSAPEELVDVLGELGDAE
AAVLAPVAP)SRTVFLSYEVHDRASADQVARLLMRHSIR-SLTD-----
PASSADDPLGGSLEQTEAVAVLVGPEGITGERsasiRAAvEYASRERSN-----
LRIVPILLPGASarLLPAFLSRQLAVDLHtA--LGQPRTRA--

ELVATVEGLAPE(RSQTTLPDDPAPFPSLRAFTSTEASLFFGRSAETARLVDRVRGASFTAVVGASGSGKSSL
VMAGLVPAVGERTEVV)}*

$4624=286(284):
>gi|1180487983|ref|WP_083992410.1| {|0(142)|<unknown(X)>}hypothetical protein [Bradyrhizobium embrapense]
{(MNWVNAASAANAGRCSGAHSSSTAHATIVAGHFVIDFMIDLTSRKQALQRGFGNARIQFWVVSF)AMRIFL
SFPSELESQADNIAQSLRNCDHD-
VFFSHDDLPPGDSFDARVEKAIESSDFMVFLISPESVTKGRYTLsELAFAKGkwpnPS-----
NRVLPVMMAPTPldQVPNYLKAVTILEPQG--SAAAEARA--
AVDRMLaakdeQSGDRP(NLPFVVLALASAIVCSLVTTYALNVLQYSFVVSDAGGITILPGVIFGAAIATCNV
VFGARDRFQLALVVAITTAAWIAAY)}*

$4625=183(191):
>gi|1180224525|ref|WP_083743365.1| {|0(324)|<unknown(X)>}hypothetical protein [Mycobacterium sp. AB57]
{(MA)---LFISYSSQdRTTVDALTTAL----
RRGQQQVWFDQELGGGDSWWnKILEQIRECDVFIVALSNNWLQSKPSQSELRYAKALNRP-----
ILPVRIGDV-
DsMRVNPLAALQIIDYREptIDAGIQLVTAVHQLSSKPVPLPD(PLPEEPPVPFGYITRLGNQMAEKELSPQL
QLQLLVELRSGLDEDGDDPS)}*

$4626=183(191):
>gi|1180227270|ref|WP_083746110.1| {|0(329)|<unknown(X)>}hypothetical protein [Mycobacterium sp. AB215]
{(MA)---LFISYSSQdRATVDALTTAL----
RRGQNQVWFDQELGGGDSWWaKILEQIRDCEVFIVALSSNWLQSK-PSQaELRYAQALNRP-----
ILPVRIGEV-
DsMRVNPLAALQIIDYRDptVDAGIQLVTAVHQLRSKPVPLPD(PLPPEPAVPFGYITRLGNQMAEKELSPQQ
QIQLLIELRSGFDEDGDDPS)}*

$4627=240(235):
>gi|1180260134|ref|WP_083778939.1| {|0(122)|<unknown(X)>}hypothetical protein [Desulfarculus baarsii]
{(MDGQIFLHVFQGITVLSGGSDPRRHCLAIACSLVAKPGGETRMPPANRR)--KVFISYCHEdKDLaRDRL-
--LACLEAGGVEAIIDIKHFKGGTTLAgQMDQHQDDADQQVLLISKKYLGQPNC-
QrEMARAIAMDpdfthglAG-NGPKVLPVLLGDV-
ERAELPAGLTRPNPLMPdlRDKRPCAEGETEWDRLVKLCGAD(LGTSPTAWLKARDKIRRLMRNDNPVCLYVN
DNRSVNWRGLIDNVCS)}*

$4628=248(257):
>gi|1180264836|ref|WP_083783641.1| {|0(233)|<unknown(X)>}hypothetical protein [Candidatus Solibacter usitatus]
{(MSCRPDVSPIEPGAAGAARKDVPEFGTTGRGSTGVCERPGAYAGSAGAEGAGEGAEGHHEGGPPVT)-
TDIFVSYARQD--REEVAKLVAFLQAQGW-
TVWWDPKLQGGDAFDkAIGTALAAARCVIVMWSRHSIESDFVFDEARRAYKRGAA------LPVLLDKV-EI-
PLGFGRIQAVSCESsaDYPKIAEGVAKILGQAPKGVTQK(EENPKARKWRWWVAVAVVAAGIAAILMFSSRTD
YRDLFLASRPGGSSGASA)}*

$4629=223(219):
>gi|1180273331|ref|WP_083792136.1| {|0(622)|<unknown(X)>}hypothetical protein [Alteromonadales bacterium TW-7]
{()MPplNFFYSYCHQdHDYRTTLDSHLSMLVRNKVINQWYDGKITAGSPWEdKIIENLENADIVVFLVTKNW
LNSEACIEeWDKAKLFAKEQ-PNKRLIPIIAAEC-AWKDFDDMKSKlVLPQDGkpVSQWKPMD--

SAWSSVYDGIKDV(ASEVKKNFKLKTKFETELKSIEFCSTNSKDIFLEDVFVFPSLTAYSDDLNGVELAVKSF
DELIEEPLQFIVGENQSGKT)}*

$4630=183(190):
>gi|1180274723|ref|WP_083793528.1| {|0(540)|<unknown(X)>}hypothetical protein [marine gamma proteobacterium HTCC2148]
{(MEKPFSAYRGHD)-PYVFICYAHRdSTV---VYTDLVSLKERGV-
NVWYDEGIPGGTSWRaEIAESLRGSRRLLFFISPRSLTSEHCMKEVRFAL----D-ESIDIVPIFLEDC-
KLPAEFALALGGVQAIYrySDILYAQHLEAAAKGKLAGESSA(GRRRPEKKSFITPFALALTLLVLVGIAALY
FKPGSGEI)}*

$4631=206(215):
>gi|1180280183|ref|WP_083798988.1| {|0(60)|<unknown(X)>}hypothetical protein [Coleofasciculus chthonoplastes]
{(MTSIHPPSNHSLPPRFTKKLLSLFQSRFRKLDIQERGSM)---LFFSYSHKdKVFVQKLYVALSP-----
NYEIWVDwEDIPPAVEWWeEIKRGIEDSDYFLFMLTPNSAISKVC-GqELEYAL----S-
QGKRIFPIVIEQP-
DPVPLSLRSINWMFFTPeeDFGVCLTKLLNALNINPEDARLH(TKFYGLWRDWVFHDRDRSYLLRGKQLWEAT
AWLVAS)}*

$4632=241(244):
>gi|1180298167|ref|WP_083816970.1| {|18(697)|<unknown(X)>}hypothetical protein [Micromonospora sp. L5]
{(RSDGRPTDGLWWDRRPAHGGVWSPAAVAARTRLCSRPCGLGSGVVTAPTTCKASPTPPGSGSIYASVHPLL
ERRRTEMLH)--SVFISYSRSdLKQVDDIVAAVESLR----
IPVWVDRRdIPVTVPWFeEVQAAIEAAALLVVCDSPRWRASEACRLELEVARKKHKV-VCTVDLTAMPVPD-
IVRQIAETYGAISPQQRvhTEVTARAAMWDRQNRTSSRLIVG(DLLARARRLMQEGVPPLNMTARDF)}*

$4633=156(162):
>gi|1180305187|ref|WP_083823990.1| {<unknown(X)>}hypothetical protein [Desulfosporosinus sp. OT]
{(MSKYIACILVYTHINIQRRLIK)MINIFLAYCSKdKTLFATILDNLQPLSK--
CCEFWYynqDSDPITMDEI--IKTKLPRTDLFVVFITNNSLNSDHI----QREIFEAKN-LSKEICPIII--
D-NSITANSDARIPEFINSiyYASPPINAIQIIEDELEKINVLR()}*

$4634=199(181):
>gi|1180319735|ref|WP_083838538.1| {|0(364)|<unknown(X)>}hypothetical protein [Desulfitobacterium dehalogenans]
{(MGCCG)MPKkkIYFSYSweeKEnKKWIIRLKNDLS---
NEGLEIIFDQDELDKGTiNIDrFISDNMNDADFIIIVITPSYINKSNIKndgngnrkrswvEiENDYIIKRKH
N-NSKSLIPIIMKKK-
ESNLPDNISGLTYYDMSneNLYKEELQKIRNIIHSEKVKTDI(PLITREHPISNLIENIDILFNYQDNPPQVS
AVVQYG)}*

$4635=208(220):
>gi|1180362521|ref|WP_083881321.1| {|233(0)|<unknown(X)>}hypothetical protein [Thauera phenylacetica]
{(TLFRRCYPGFDAGPEVIFSMGRCTDNLTLACSFDDTCAADASYVASHAVDQETCFELLALDDDLPSLQPAR
PMVTGRRYA)----FISYAHVdTA---RMLAVRGILDSSG-
IETWHDTSIDGGEDWNaVLEGQIRNCSLFVLVLSPAAAASR-IVRrELRFA-------DALEKPLLCIRLG-
ETSLTDGLAMLLLPLQWidADRPDSAERISTAARKLLQIRDV()}*

$4636=205(220):

>gi|1180381784|ref|WP_083900584.1| {|287(0)|<unknown(X)>}hypothetical protein [Thiothrix disciformis]
{(NIHDDDIKNIIWRDKPINLEFLVSSKIENTVDPFAGTLTVTSYGILIADVKFLINFSNLDNKSFRYRKNSISIKGNFSPR)--SAFASYSSED--RAEVFKRIQGIKSIvPDIDIYIDvLKLRAGEQWEeQLLLHIAEKDIFYLFWSELAMKSKY--VEKEWRLALEKR-GISYISPIPLE-------------NPSKAPPpkELASLHFSDTFLAYIDYYQEHMN()}*

$4637=233(227):
>gi|1180381867|ref|WP_083900667.1| {|534(0)|<unknown(X)>}hypothetical protein [Thiothrix disciformis]
{(HLQDKTLSNQGCLQFSLRGASLEHLLHALCKLVKEVSPHNRYSESLQKGLHDQPTPLPEFQEEETMSSRLDAAPAKQAIQ)---VFISYSKEdKEHRVELEKRLKLISRQFKVKAWTDAQLMAGGLVHeDIKKELVNADVVLLLISRNFLLTDYCYEvELPIALERYHA-KKNVVIPIIVgSTP-DWteqtiDGFKLGNITALPTKGvpLSKWgkGKQE--EFWDEVQIGIKKL(IMHLLES)}*

$4638=210(219):
>gi|1180410978|ref|WP_083929761.1| {|0(160)|<unknown(X)>}hypothetical protein [Catelliglobosispora koreensis]
{(MPPEVTEAPESATTVQSRIKCLGDQCNGSAPASRVPCVGGH)---VFISYSRRdRHYVDKLATHLS----EANVTVWYDYEMAAGQRFGaVIQQQIDECAVFIVVLTPEAAGSKWV-GrELSYA-EAHNK-P---IVPLLLEPC-RVPIEVADLHHEDVTNGevPSSRFLARLRQLLPAVTPPPPAP(QQPARSPQAATARQAPAPLPVPAQPKEPLRQASPGPMP)}*

$4639=240(224):
>gi|1180461135|ref|WP_083972400.1| {|0(2)|<unknown(X)>}hypothetical protein [Actinoplanes awajinensis]
{(MDDPPSWTAAIVGSHRGGRRGWGGRDVTSSDRTPVF)----FISYAHRpqrarptrdahlvRDFYDTLYGHVDELLglQAGQEAGFMDAEMDGGQRWSdDLAYAIGHCQVLVPLISPRFAGSEWCAReWhaftrrphrtFPTATSSH---GATPIIPV------SWTPFPIEQLPGEIRAVqlFTPALPQPEMARLYQSEGLYGLL(QLGERGLEVYEAVVWKLAQWIADAFWTHDVETDKDVDFRGLATEFGED)}*

$4640=256(247):
>gi|1180281981|ref|WP_083800786.1| {|0(875)|<unknown(X)>}hypothetical protein [Geobacter sp. M18]
{(MTNATFRKLNMQNDDNHDNCDKKSNMLPSTARTFR)---IFISYGH--DEYAALCEQLKDDLTARGHHVWFDAERLKAGVDWerYIEEGIdwvcsDKETAKCILVMTPHSVRRPN----GYCLnELARLIaRSVTIIPIMAVQS-EPPLSIFRLQYLDMQDSckparskelYEQRLNRLIKaLEEDKRDFEGVQS(RLLTTLSPISFDADINKLLSNFTGRHWVIEAVDIWLKDLKGSKVFWIKGPPGVGKSSIAAWLRDNRREVVGY)}*

$4641=227(232):
>gi|1180375432|ref|WP_083894232.1| {|136(0)|<unknown(X)>}hypothetical protein [Nocardia tenerifensis]
{(IRLSSTTLADMDIGPFCTAEHVGHSNPSTIDARAVMKTYTSPRLKGFMIECGVPPIFAEYMIDCARALGEPLIGRLMQST)----FISYGG--P-DAKFAAKLYEALKAHGAIVFYFPESATLGERIdnEIYRRLSEHD-RVILICSRASLDRPGVLhEIQETFDREARDgGATYLLPIMLDNY-VLTDWKKAHPALAERVGRRVVGDFRGADKSAAKFAAALNR(LIDALKTSRPAV)}*

$4642=189(190):
>gi|1180380120|ref|WP_083898920.1| {|0(514)|<unknown(X)>}hypothetical protein [Robiginitomaculum antarcticum]

{(MLHLFHWDGVLFLID)---VFLSYQN---EDRRLAETLSKALLDMGYSVWWDV-
DLLPGDDFqsEITSVIENAKTVIVLWTSKA-VDSHFVRdEATLALKLKKLIsTSYDIeideIPLGFKQT-
QTLDIKPAFDAPKFSLPDDIIQAIEKKVGSESPGEHEPTE(TENLELEAGPPNQIQAWKKIESKKYDKSSYEN
FLE)}*

$4643=181(180):
>gi|1180261862|ref|WP_083780667.1| {|0(343)|<unknown(X)>}hypothetical
protein [Bradyrhizobium sp. BTAi1]
{(MESVMALAPAAMAAAARSVNDLGPYRPAGV)----FISYA-SedhdvaQAVYQSLQALSETIFDR--
VKIFLDSKSIEASDEIrqDLRAGLRKSDFLVVLYTGQLKKSHGYTGWELGFFEALMEEekkQGDGETARRII-
--YLYCGETPPTGEGILGINMEVDTDDLSGPRADYVQKSTQS(IDDPDPLARV)}*

$4644=191(193):
>gi|1180267399|ref|WP_083786204.1| {|0(779)|<unknown(X)>}hypothetical
protein [Actinomyces sp. oral taxon 848]
{(MTCLCRIGMCVRMRMW)--------RVARAVARLVEGYVvagsvGGVVKRRGFRVFRDVEEL-
ASGELgvLIESALAESEFLVVVCSPRTAGSVWCMREVEFFSRVRGVDhvlAVVVEGDPGVV---
VGDVVGGDVLAVDVRAGAVRSEGFGGFEGCGSGVVDRLVR(QSVGVLRRDGIDRLMAGVVGVSLGDLRQRQRE
RRMRR)}*

$4645=214(228):
>gi|1180275097|ref|WP_083793902.1| {|0(727)|<unknown(X)>}hypothetical
protein [Pelagibaca bermudensis]
{(MIKSIGSISRKILEALFG)-YDVFISYAHRdgvayaEH----LDSILGKTH-----
TIYLDKRDLLAGADLgaLTKLRVRQSRLLVVVGRKHALtASKWVCREVDCFRDTGRLPiiiDV----------
--DHAIEYAAGLPD--KRYLSGWLSAKP--
VAGFGEDTLADN(LLRVVDPGPDNGSDRLPASNVAPEILKRFSGVRSETRRLSVITGALAVLLALTFASVVFA
LQSNIATRHA)}*

$4646=170(163):
>gi|1180393022|ref|WP_083911820.1| {|0(682)|<unknown(X)>}hypothetical
protein [Nocardia takedensis]
{(MA)--DIAISYSHAdretaEAVREEL--------
RTAGYSVWMDETGeysrtaeavnLPVGQQHweVIQSEFAAADLVIVLDTPRWRASPYCRKEFEFLLQWGKWTa
fhVPGSGPVVAHA---
TELVARRRVTGAHTRIVALARSNDIQSRSLVERLLRRSEA(EDAEIVLSADAPAAGISVDET)}*

$4647=224(217):
>gi|1180477533|ref|WP_083984690.1| {|0(436)|<unknown(X)>}hypothetical
protein [Altererythrobacter ishigakiensis]
{(MCVSLAVTVGNLGRLVT)--DVFISYARStqdqaRAAARNLESLG--------YSVWID-
DALPAHRAFssVIEEKLRESKATLVLWSADARNSRWVPAEAEL---
AHNEDkliQISLDGALpplpfnrlqcelmqGWD---
GNPSHPSWAKIEASVSQLAGEAATPQPSKTGSVRQPTRSG(EMLLAVLAFDNISADPDLNYFCDGVAEEIQRT
VARGTQLKVVARTSSFQFRGPEKETGKV)}*

$4648=193(193):
>gi|1180487971|ref|WP_083992398.1| {|0(322)|<unknown(X)>}hypothetical
protein [Bradyrhizobium embrapense]
{(MPETRRRTASAGPGIFRCQKHCAERTTMAQTGV)----FISYNHKdkkiaEAVVESLA------
GLSTNLEVFIDRAGIEGGDDYeaKISESIQKSQWFVFICSggnKSEKDMSWCFYEAGQFRAKLERAdqkEAVR
DRICFLY---
DVDRPDQLSRYQGSLVSIRNRNDVLLNVDADNDDSLAYEN(TELFDFFENLLTNSTGISFR)}*

$4649=222(222):
>gi|1180686360|ref|WP_084171540.1| {|119(0)|<unknown(X)>}molecular chaperone Tir [Blautia schinkii]
{(HKYNQPSEDWINDFEIFYNKYLKEHALGSRIKTILFHRNLDAYRQLVSCLKSISKDQEFIDKMNGIEKTTV PVYQANMLP)EYDVFLSHAN-KD-
KADLVDELNDSLEKLGVKIFYDKKSLEWGDKWkdRILDGTKKAEFAIIVISENFFDREWTEKELNEFLNRQNR NGQKLILPIVHNTTNEDLRNKYPSVADIQAIDSKAYSCDEIALLFARQLIKRLKA(QN)}*

$4650=191(186):
>gi|1180697027|ref|WP_084182197.1| {|0(285)|<unknown(X)>}hypothetical protein [Mycobacterium austroafricanum]
{(MAHRTRQHPHAAAYRCALRRRATPLPLRPRTNLAHSEQTVKAEPA)TPDptgpIFISYRHSDGIDLAAELA GLLRAAGI--
PVWRDKDDLPPGDTEhRLTQAISAGISGAVIVTTADIANSTVVKflEAPLLLQIHDDYPEFALAVANAIENEH GSLDYTAPDKLLGVKPGTLAGVDQHRVDRAELLHIVDRL(L)}*

$4651=160(171):
>gi|1180584507|ref|WP_084070757.1| {<unknown(X)>}hypothetical protein [Desulfobacterium vacuolatum]
{(MKLIYTKNIFQVFKQIRYNKFLWRALMSTAY)KYDVFISYSHKdrLWVTKELLPKLEQ--
HRFKIFIDFRDFQSGRFSVTEMERGVNSSKRVLLVLTDDYIKSDWGTFENVMGQTTDPAAINRKRIPVLRGSC -------DIPLRLQIIHYrDLRNNDSQ---QWDLL--IRDLI()}*

$4652=176(183):
>gi|1180739754|ref|WP_084222136.1| {|0(670)|<unknown(X)>}hypothetical protein [Pseudomonas sp. MOIL14HWK12:I2]
{(MNVKF)----FLSHSSAdkHIVEKVY------EQLGASLC-
HYDvaTFDNTSFLPEQIYEALNESTHFVLFASRKALESSWVKGELKRAFSLWMKAQIRHAMVFLIEDG-
ERSHVPEWLQDYVIIEHpSAQHISCRILSAFDDWESKNGNE(PPFYRYQEIIQLEKHVSVEPRKLPTTLMLCG TDGYGRK)}*

$4653=273(273):
>gi|1180783911|ref|WP_084261087.1| {|296(206)|<unknown(X)>}hypothetical protein [Micromonospora rifamycinica]
{(VRLVCHRPDDAVRQLPRLAVSHALQASSAVNRHAIWWSEMDVLLPELGREIGRLQMPRLPFVGLPEARRYV WSLGRDQGP)---VFLSSAQ---GDNDFAQLIAQELDLRNVEHFHYLFNSDLPRGKawehRLLAKV--
GECRifVPLVSRHYLDSAWCRKEMEIALELS-GRGRMRILPYFLDHhagASvpV-QGASVVHLDAAARVR-
RVVDDVDRALTDLTDRRHRPRI(GITVAGGRTALDVDVVVLATGPTEYAAAARTLRHPAPVVGTAEHPNHFDW LGG)}*

$4654=250(245):
>gi|1180547948|ref|WP_084039576.1| {|402(45)|<unknown(X)>}hypothetical protein [Demequina sp. NBRC 110053]
{(LRGLVRPIVLGLINKRDRIATEMSQQAKAAKQRADGLAKQQFSDQLEVDLARHREIPEETRAELQTVIVNK IEGDVAVKR)DYRVFISHSTDdLPFARFIDEALRTRGATPDEIFFTSRPGETAQVLDdralgiVIKECITSaN TLVFYLTSKHFRASEFCLFEGGAGWATRAV-GDYLKLNMEFDSIPAFLTNGRPELSMIVGDS--
IELTATLHNYIVAGVLNPMVE(HLNRGRAIAGSDLIAPFSTALLPTE)}*

$4655=159(163):
>gi|1180665532|ref|WP_084150719.1| {<unknown(X)>}hypothetical protein [Azohydromonas australica]
{(M)--KVFVSWSgEAgHALGYRINEWLPSI-VPDAKPFFSAE----
VPGGYkwfeFLVAAITDARFGIICLTPESQRSPWLFFEAGALWQQGSQ-

IIPVTVGLETSNIAGPFAQVQAKRFERRDIR--
SVFQQIATATHLDQKRFQLNF(EGIWPNIERDVRNDLAPYGITV)}*

$4656=159(162):
>gi|1180666153|ref|WP_084151340.1| {<unknown(X)>}hypothetical protein [Acidovorax oryzae]
{(M)--KIFLSHQQTdSALALR----
IQGYLKNNHNIDCyLDVIDPGFKNGEdiaaHVRKELGKCTQLLAVVSDATKTSWWVPWEIGVATEKDFP-
LATFGGNTELPEYLKKWPYLKSTSDLDKYAE--
ASKDASKSVVTAGVESASSRS(AVFRGDATKHFYKTLRTKLGQ)}*

$4657=267(273):
>gi|1180704325|ref|WP_084189490.1| {|0(200)|<unknown(X)>}hypothetical protein [Tessaracoccus bendigoensis]
{(MDGTLERRAGKDAEVRLPHHGSVAVHPRGGWGGGVPGGIIGPAGPVRQEGAKGRKPMINQSRTN)---
LFVSSSLAeAHLAESVITRLSNAF--DGDLQFTHARDLG--
AGDmwksWIRKSLNECDAGLFLVTPRYRGSQWLSAEFTAFWLSEKP-
IFILLFPDIEaDDLFTPMRDDYQATEIGDTVG--VKKFLTN--
LAGLCGRQRVPF(EHADLICYYAQEAFEECGAAAPEPGIVDPSANAPADSTYPRRHILLDQTWDLALGADRDS
LRATSTRKE)}*

$4658=251(254):
>gi|1180822333|ref|WP_084295956.1| {|101(41)|<unknown(X)>}hypothetical protein [Rhodobacter johrii]
{(YVKRIREAISDRGNTDFIIPFGSPSGDAPLPTDAAASIQYTYRKAWAQAGMSASARDSRLLLHTLYQIRRQ
LALIGGGDG)QEKIFVSHAKAdGDtTASAIVTFVND---KSQDVtLHTFYDAKELNPGEdfseRFAEEI-
ESGTLLAVVSDAYDSRPWCVFELTTA---KRK-RRPIVLADIGQRRTSRTYPYGANLPKVRIFP--
KPDDTSWIEPLLVETISEGLR(CDLFERQAARVVRNSKSVLLLPRPPELFDVIDAD)}*

$4659=243(251):
>gi|1180607964|ref|WP_084093600.1| {|0(405)|<unknown(X)>}hypothetical protein [Amycolatopsis rifamycinica]
{(MLGSAGEASADGGKVARPGEDDRGSAGGADAESHPVT)--RIFLSHTGADSDIAGRLSEAMRERGAE-VY-
-DYGDRPGQEFVRGTQSALENADYFVALISPAAME-SDWCDlERQTAYHRQ-----
IELKRQFVFVVQvqRTPRTgtgfLRVASWIDLLE--PQTPDKLT--
KTLAALGFGDGA(PAPGTGPTPWTVFHNREDELNRLINALTTRGTPDLWLVVSPPKLGKTWFLQEVLRRRAAL
DPPCRIQLVDLRSL)}*

$4660=231(234):
>gi|1180608232|ref|WP_084093868.1| {|0(190)|<unknown(X)>}hypothetical protein [Amycolatopsis rifamycinica]
{(MDDNDRRIPPPVGPLRGLGP)--KVFLSYSFRDRAFVDTIAEDLRAHGMQ-
VRMEDERSLLGSRLDVALPARIADCEVFIQVVTET-SANSAWVArefEWAtsdhAGHTRP-----
PVVLPVAVDGARvpDRVAswvYLSVNTPIDLAS--L--EVIRA--
VAMRAVVPLPLD(PDAPYQLAERELVEYCGSNLEEQRRVVLDSGGLVPNLVRETARFVAGLTESHGDKVLAAL
TQQVRSHQRLKRYL)}*

$4661=225(237):
>gi|1180647771|ref|WP_084132958.1| {|0(661)|<unknown(X)>}hypothetical protein [Frankia sp. Cc1.17]
{(MAFGRSGILSRVVSEAAASEDAHR)AVDYLVSYDEVDEAWATWVAAVLEASGRS-
VRLRAWDAAPGTNLVVWISEQMRLARQTVAICSEAYF-SSHWTSpEWASA-MRS-----GKLLPIRVEDCQP-
PAVLTAIVFCDLHG--VGEEVATQ--

RLTEATGLAPVS(RVSSGFPGRDKGRNESDEVSSTLTPNPPIWNVPLRLRTFTGRADIFDRLISALPSGLPVA
VIALHGLGGVGKT)}*

$4662=182(190):
>gi|1180648162|ref|WP_084133349.1| {|0(490)|<unknown(X)>}hypothetical
protein [Clostridium grantii]
{(MEERSMDY)--DVFISYSTKDKKIAETVCSTLELDNVK-
CWIAPRDILPGTNYGEAIVEAIEKCRIMVLIFSDNA-NKSRHVTnELERALNYN-----
KIIVPFKIEDVVpcKKIEYFLSASQWIEAL--
ANPIKSKIVELSTIIRRILNEG(GEVEGNSKNPSKVTSKINENRKNIKNTNNNFLAIALKHLKKF)}*

$4663=280(289):
>gi|1180660545|ref|WP_084145732.1| {|0(369)|<unknown(X)>}hypothetical
protein [Amycolatopsis jejuensis]
{(MLRQRAAGLGEHAVQAVEGFGQQAFVRAEPDGGDPAGQLDVQFPGKGSLGHTHLLHSGGEDGCDQICNEDG
RRIE)SVDVFVSYDRSDAAPVQRIVSALEAAGLR-
VFVDVEQIRQFESISDRLRRALGESRVLLAYYSRSYGSRRACQEeFTTAYLASP-----
ERVLVVNPEPNTehLAP---HEILDVLLPG--HPAlKAALA--
TLVEAVCERVET(LSGTIGTKPTPERPGDLPLPAKFTGRWTELWQLHSVLSHGRTAVLHGPVDSGKSTLARAY
VHEFGRTYNQVLLG)}*

$4664=214(222):
>gi|1180772774|ref|WP_084251857.1| {|0(949)|<unknown(X)>}hypothetical
protein [Bradyrhizobium sp. AT1]
{(MA)--DFFVSYTSSDRNWAFWIGQQLDNIGHV-ARLHDWEISAGGDIVRWMEERHKLADHVLCVISEAYL-
NAPYSSwERRAAQMAA-----NKdrpnfAIPVYIEPCEpsP---LLKVFRHCELYG--LDEEQARQ--
RFEDFLKPAIKP(SSPVLFPGQTKLKPAAPPTAPVMFPGTSPASSGEQSQAPAGIVPLEEEPTRPAASAPARR
IEEDAADTSNVQPRDLRMES)}*

$4665=221(225):
>gi|1180785748|ref|WP_084262688.1| {|59(0)|<unknown(X)>}hypothetical
protein [Actinomadura formosensis]
{(TNRVHNWTGVGGCGDRPLPPGMAAYAFVMGIVGSVAPVIAALAGVVGAFFTWVGVRHQLRRPKRRVSPLPS
PGDGRDGGY)--DAFIAYAEEDTAWVAGFIQRLQAKGVK-
LAYDKVVLQPSRKITHTLEQAVLESTHGIIVFGKA-SKSPHWVEaMYETLMRRsieqD-----
RKFVPVLIADVQ-LREFANNHVPADFRG--LAEDEPRydhlvD--LVAQAVRRRGTP(QASAS)}*

$4666=228(234):
>gi|1180882533|ref|WP_084327106.1| {|10(0)|<unknown(X)>}hypothetical
protein [Salinarimonas rosea]
{(DKVARELQARYRYDEIDQFLAEYGVNPPTEVHYNSKWVYSKVALRGVENAKLLRIAQDLGIFSEAVGFGDD
SPELWRDAQ)SMRIFISHVSEARPRAVRLAECLAAHSCS-
GFVAHAAISPTREWTDELHRALRHMEALIAVCTP-GFAQSSWCQqEVGFGVARN-----
VLIIPLHMGENPtgFVARKQAILRRGRQAE--EIVADIAAVLKQDDRTKDRFAQ(AQQKPSEYDDDIPF)}*

$4667=241(258):
>gi|1180501611|ref|WP_084001629.1| {|0(444)|<unknown(X)>}hypothetical
protein [Altererythrobacter dongtanensis]
{(MVRRLHRRRVRKGCAQSACVPLSCPGTLRWGEVRMQSVENDTAEADRAV)--SIFFSYSRQD--
RDRALPIIRMLEGRG-FNVWWDGLLEGGTAFArTTEAALESADAIVVLWSETSVQSHWVRDEATRGRDR----
--GRMVPVSI----DGNQPPigFRQIQVL---DLTKWKGKE--
NAPEMDELVRAVL(AVSGNSDLQLPVTKAAPTAGPGRASRRAVLVGGGAALVLVGGGLAWWQFRPPADDGRTG
IAVLPFENLS)}*

$4668=291(292):
>gi|1180526200|ref|WP_084022243.1| {|0(1001)|<unknown(X)>}hypothetical protein [Chlorobaculum limnaeum]
{(MPCHFSIFVLRHFPNRSDQTIMPEHQRQEHASAELSQDASNVEQAMKPALAEMADYMSSEAVEIKAKIKPRIE)---IFLSYAHYdnsvkKRFAEELKARLGSSKR-
YQFVLHDDKGLLCGDRWHdELQDWLSRCDYGILLVSSHFLASTYIQEhEIPVLQDKS--------
FPVALKPI-DLKNQELHGLEHLQIfflDEkpFSgvSGPNLQ--
NFVNKLADEIEQR(IQKHREESIKTDEADADVEYAACFADGARKLTEGHLKYKGPLYECERFIEGRANPGVISHQSKSESAADTVLARPYILN)}*

$4669=219(212):
>gi|1180526339|ref|WP_084022328.1| {|0(234)|<unknown(X)>}hypothetical protein [Chlorobaculum limnaeum]
{(MVVV)-
PDIFISYAHRdnqpvsgeqKGWVTHFVNNLfnEVSRKIGRVEhyqSWMDYCLKGSDEVTpEIEKQLSDAYALVIFLSSGWLASEWCRRELAIFTKNLKQ-PKGRVFVI--------------
ELDRINRDEkpIILqelrayrfWRETD--
EKKVRQLGYPVPQ(TTDSSYYDRLADLSNELASVIKYKNSTAEKAPLKATVYVAPVNDALYEHRERLISELRQFSIDVLPRN)}*

$4670=223(229):
>gi|1180636845|ref|WP_084122055.1| {|0(85)|<unknown(X)>}hypothetical protein [Marinomonas polaris]
{(MKSSGKSMSQEKSIRIYIDNFVGDDG)-----------
EAFATTLKEKLAGLKIDSMYETFSFTDLGEGNEADpnneanTIS-
AINNSDIVIPIVTTSFISYvtpriEYAYNsIIG-------S-KNRYFFPVLLKAT-
DWsSHEWLVRSQLIPRDAtpLYEHSDNEQENSINELIKSVKTS(IVRLSKEFHEDVEKPAAQGNGKGLVFISHDHDDADFAELLKLRLEKEGIESWIDTERLKIGQD)}*

$4671=176(186):
>gi|1180657158|ref|WP_084142345.1| {|0(182)|<unknown(X)>}hypothetical protein [Psychroserpens burtonensis]
{(MLTILNNKRL)---IFLSYSSKdIEKAKVLS---SALEKSGL-
SVWWDRQIPSGSTFDdVIEDALGKAQCVVVLWSSHSVKSEWVRNEVQDGVDRK------IMIPVLIENV-
KIPLAFRRIQAANLIGWkgDTETKPFQDLINDIKTHTSQNGK(RLDKTLETQTTKEPIEYISKKRQPSKATEKNINNSF)}*

$4672=190(192):
>gi|1180772647|ref|WP_084251730.1| {|0(94)|<unknown(X)>}hypothetical protein [Bradyrhizobium sp. AT1]
{(MR)---
VFISWSGDqSRALAETFRNWIPSALQYVKPYFTPADIEKGSKWAtEIFKELSASSICILVLTRDNLASNWIMFEagaISSALDR------AKVCPVIfdLEET-DLQG-
PLAQFQATKFNKedIRKLFFTINAAAGDQKLSDAVLT(TVFEKWWPDLEAQLHLVLTHHKAARPEKNVRSDRDIIEELLLLTRKLSEA)}*

$4673=212(222):
>gi|1180795032|ref|WP_084269567.1| {|0(339)|<unknown(X)>}hypothetical protein [Patulibacter minatonensis]
{(MERPTPPGYPRALMECTAAIPEKERQPKAPSLVKPQFQPHEGPEPYC)----
FVSYSHAnSDHVYSILNDLA----EHNFRMWFDDTMEIGDDFRkELHDRIAACDAFLLFVSKESMNSKYC---GMEIITAHAL-GK-RIYPVQLDAS-
AEIPAALKLVLENLQHVkaHTGEDKYVQRLIKSLPPDTMRRL(IIEDGIVTSCADNGTDVLVPEGITGIGEAAFKECV)}*

$4674=255(255):
>gi|1180812465|ref|WP_084286161.1| {|230(263)|<unknown(X)>}hypothetical protein [Solirubrobacter soli]
{(SLRLLHLSAEIDREALGPLVSGQLLRGVSVVDDPVVIWQDLDEAREKVRRQLLPLWSSRKQFSTLSDGLKYFRGQGRKGR)--KVFVSNFNDaNSLAERLSARLN----EEDIEHFHYRfnnaAIPLGANWKqMLDQLVHESDLFVVLVTRGYHDSPY-AQkEFLEATKAAKA-GSLRVVPYLLDKS-VGAEVEEQGKDLSDLDDeaRVETIVRDVDDLLVRERVAQPVT(MDQSRSVDVAFVTILPEEYAAVLAHVDRHEEARPT)}*

$4675=215(225):
>gi|1180820558|ref|WP_084294181.1| {|0(268)|<unknown(X)>}hypothetical protein [Mycobacterium flavescens]
{(MKSVPVAPPRRVSTVA)---VFISYSSRdAD----VVGHLAGALRRGHHDLWMDDELSGGDAWWrAILGKIRDCDVFLAALSQNMLASKAC-QaEMRYALAL-----GKPILPVLVGPV-DsMRTNPLAGMQVIDFQQrsIEAWMELD--EAVRAKQASAPAL(PDPLPAEPPMPFAYLMRLGGQISGSELTPAQQTVIVAELKAALEDDGDDAGARRDICALLRQLRDRPDV)}*

$4676=225(226):
>gi|1183808935|ref|WP_085011300.1| {|0(640)|<unknown(X)>}hypothetical protein [Geothermobacter sp. EPR-M] >gi|1180850930|gb|ORJ57584.1| hypothetical protein B5V00_13295 [Geothermobacter sp. EPR-M]
{(MESGLKL)--KLFISYSHLdEGLVNEFIKHITPLKNNGVIDEWYDRKILGGDDFQdTIDNNLDNANIICLFISKNFISSNACIKEKEKAFYLKCS-KGVIVLPIILSPC-AWlDDMELRKVLAYPTDGkpVISHSNND--EAWVDVYNGLKKI(IDEMSKYKQLNLKTDFIEFLNSAELLSKAHYKKERITLDDIFIYPYFEKYDYVKGNEKKINSKELIEKILLIEKILLAG)}*

$4677=192(190):
>gi|1180716752|ref|WP_084201888.1| {|0(307)|<unknown(X)>}hypothetical protein [Microbacterium aurum]
{(MGDVPG)-DHVFISYIT---EDSDQIDELQGALEAADFIVWRDKDKLWPGDDWqrEIRDAIRSGSFVFLACFSSNLAKreKSYQYeELTLAAEEYRlRPpGASWLMTARLDEC-EIPEIDLGAGRTLGHSIHRADLFGTQKTAQISRLVVAIQR(AMGSTPGIPPASVSAVANNAALAESDVVERLRFLLRNPQLIMDY)}*

$4678=184(176):
>gi|1180607797|ref|WP_084093433.1| {|0(30)|<unknown(X)>}hypothetical protein [Amycolatopsis rifamycinica]
{(MSGDSHRVSEPSSQPGLVPLLDGYH)-YDVFLSYRRSgpgnaaRWVQQHFHRMLVDCLADQIgqAAIFIDKSAETGVHWPTmLQQALARSKMLVAVWSPPYFASQWGLVELNTMVAREQAVggvTQLVYPLIYSD---GETFPETARKRQGRPVHKVSNPFPSLEGSHRQADLYDETC(AIAIELAGLLA)}*

$4679=223(219):
>gi|1180608274|ref|WP_084093910.1| {|0(198)|<unknown(X)>}hypothetical protein [Amycolatopsis rifamycinica]
{(MR)RYWAFMSYNWSdkrraRSLHRRLERFevprpvrpvirAPGLSDRRLRPVFRDDDEMTGSGVLdeRLRQAIENSSAMVLLASPASARSPYVDLEVAHFVATHGTErlaIVVTGG-------------DPNRPPELPPTLREQ-RQEF--

LWVDCRVKRRLK(RHSLVRVVAAILAVHFDVLWRRHRRRQKMIAAWCAVVTVVIAVVGTALWQQRAAEQRSP
EQQQVAFEQWITTELRK)}*

$4680=227(222):
>gi|1180683624|ref|WP_084168804.1| {|0(664)|<unknown(X)>}hypothetical
protein [Hellea balneolensis]
{(MAHNTVNNFLKTRTQRLSKSHGLKQNRTELSESSRSRTIRRAASNIGG)-
YDVFISYRHTgnllyaLELERRL--------KAKGLLVFRDESDEDVGTSLdtFIKRACS-
ARTFVILVTDNVYESSSVFEEFSAYLivrlgrwNLRPFSrviPVNVDRAIERA---
PEEPKIWQHLSDFVFVADTKLAVKQGIPSDLVVQKIVRSS(SFLRSWLRFFAAILISGIVLAATILGTSGYLY
NT)}*

$4681=211(220):
>gi|1180822335|ref|WP_084295958.1| {|134(0)|<unknown(X)>}hypothetical
protein [Rhodobacter johrii]
{(RIGASAELLLKLAHSRGRLIIPLAGLKGASARFLESHRSEYDDILGDDIQALHEIEADKDFSSILSKAIHK
QSPRKMANR)--RAFISYAkaHAddadfvEV-----------
TLRRRGFDVVRDEASFSPGHNIptEIREKIFSSEVFIALWCAAYACSPWCFDEME------
LALdrlEAGKIALWILQ---LDDTRIVPPRARDKLSYPAQNRDKLKGALEVMIASGLAKP()}*

$4682=232(236):
>gi|1181045085|ref|WP_084454380.1| {|0(402)|<unknown(X)>}hypothetical
protein [Algoriphagus terrigena]
{(METSDSTSTEITFSKLRSSTDAILSPRKRTNPKPNKHLQGSPEGLPFLLPLRQKKLTFSKKDFSDKMGQQ)
--QVFISYAW-
GGESELVANELEAALSSASILLVRDKTDLGFKgLIREFMQRIGEGNLVILIISDKYLKSKNCMFELLEVAKNG
KF-HDRIFPIVLGDADIYDSLALVRYLKYWDEKI-
KALNASVKELDNLADTRKVQED(INLYTDIRGAIDDLAGKIGNMNTLTL)}*

$4683=256(263):
>gi|1181039118|ref|WP_084448553.1| {|0(952)|<unknown(X)>}hypothetical
protein [Desulfovibrio inopinatus]
{(MIPPQIRRQAVMSDAERVGKGRGGAEFCKILILRHTFFLPSVRAFRYHFRNIDNCVVFRAHQKKSSHIMTS
HEFSP)-PRVFVSYSRSDGRECAEDFEEKIQKDEL--
TSWRDLKSVVGGEDiRpQVLRAIEESEHLVLILTECAL------
HSDWVKREWTHARkHGVMVSPILADHSITRSD-
LPHWARRAEIYDLSEPERWNKLVQVLQGPGKTRRV(SYMTGPSTANYVCRCKEYETLKQAVMENGDNVVGLTT
ALQGAGGYGK)}*

$4684=185(178):
>gi|1181251144|ref|WP_084566259.1| {|0(74)|<unknown(X)>}hypothetical
protein [Mesorhizobium sp. LNJC380A00]
{(MWERQALLG)--DVFINYRRQTDSGVAGRVYDNLnrALPDA--SIFM-
VDKLNPGdDFEvALARSLASCKIMLAIVGPQWAaltdeQGQVRLEspDDFVRKEIRTASrLGCASSPCSSTAP
ACQRKAPCHPICRSQASSHGGPPRALQRRYGGDCVGDSEAS(GAAAEALAAARAGRNDRARHRRDNGGVVA)}
*

$4685=230(251):
>gi|1181239807|ref|WP_084556806.1| {|327(0)|<unknown(X)>}hypothetical
protein [Hamadaea tsunoensis]
{(VAVEAATMAPDVELRMSRAVPSLGVAPRRPVPADRRPEGPVFHLSYAHTGQGNKDVLRFFDHVVADLDQLI
TAVPGQSLG)---------------------------------FTDRM-
LSPGLDWqrELLEVLNTCQVFVPLLSERYLhRSPWCVKEWDVFSRRAVRNgdgsatVRSGILPVLWAPVGsgi

pHRVQAVQGFAPERAALRNRYAAEGVLGLARMSEKDYREVV(WLIALEISRIAAEVVVEPGTTSTTDGLMSQF
)}*

$4686=250(254):
>gi|1181022057|ref|WP_084435102.1| {|0(78)|<unknown(X)>}hypothetical
protein [Kibdelosporangium aridum]
{(MWHSSGPSSTYAVTHTLPCGRAGRCTETRAGRPAAVRSAGLLPNGRKNVLHRIKAARPR)---VFLSFAG-
--DNRAQARLLAKEFKARGFEAFVDDDSIP--LGRdvvIAINNALS-
QYDyfVLLWSGHAVDRRWVELEWTAALAREVNERRSFVFVVLLDDTDppL-VLSPRRYLVARDGWA-
KVANELVGTWQRDQRLDMPVLP(APYPQASTGLPMIVIHVRNRALKVAHVLPVLAGTTGAELLGLVRSALALP
QEEKE)}*

$4687=183(184):
>gi|1181148670|ref|WP_084496183.1| {<unknown(X)>}hypothetical protein
[[Clostridium] methoxybenzovorans]
{(MMANNMIINSGVINGNVIQKSAAGTSLTAEKESQKTGGGISSKY)KYDVGISYAS---
EQERYVTRVAKILEKENLRVFFAPNREEEFLGRdmiTEFYDIYRYESMfvACFVSRDYLDKDITMHEAKTAML
REKEEKRNCLIPVYFGGSRlkE-LDPDIHFLDADRLRE-VEVAEKIK---IIIHSFEKGNR()}*

$4688=221(229):
>gi|1181200913|ref|WP_084543587.1| {|0(184)|<unknown(X)>}hypothetical
protein [Actinomyces viscosus]
{(MRTTRIHLSRSGAL)-KVIFISHSSSdIITTRKLVFELNYLGAK------
TWFDETEITAVSpvfgSIEQAIRNCDVVVLVASVDSLESDYVKREIKLALQLDKP-
LAIVRVPGSNVDISGDL-ADYKHFNLSDFPS--FSRAARE--
IIKKDNLKKRGE(SPEEFWGVCRGVDPLLNDSVRSNIHALKSVVNQSATQEPLVRLLREDYTDLVQSFDVVHD
IDVSWLPPELRRDFI)}*

$4689=157(163):
>gi|1181348155|ref|WP_084649040.1| {<unknown(X)>}hypothetical protein
[Paenibacillus sp. FSL R7-269]
{(MQTISEDEIREKAPR)--KVFLSHKGAnKPLVRKYYQLLKELGFDP------
WIDEEDMPAGAqlmrSIRQGFKQSCAVVFFITPEFKDEKYLEKEINYAINEQLN-
RKQFTIITLQFEDEQGNKGLIPEMLEDYVWK--TPRTELEAFKEIIKSSPIKVG(QTIWKIER)}*

$4690=158(161):
>gi|1181003450|ref|WP_084416582.1| {|0(6)|<unknown(X)>}hypothetical
protein [Massilia alkalitolerans]
{(M)--KVFISHQQADALKAGEIARHLNyQHGIS-
TYLDVVDPMIGTHqgpeLAEHVRREMDNCDSLLAVVSP-YTANSQWVPwEIGIATEKD-----
FPLATFASGNVAlpEFLQKWPYIKSILELD--
KYANAVKQMAGRSRATIVRKNY(TTESFNQSMRTTTSEFYRKL)}*

$4691=202(202):
>gi|1181052407|ref|WP_084461030.1| {|0(886)|<unknown(X)>}hypothetical
protein [Nocardia kruczakiae]
{(MVDTVT)APQVFLSYAGSERSRAMAVRAELTSRGID-VIMDD-
EFEPGRSVMVNIGRALHRATVVLALVSSQYL-DRHFTEiEVSTTLANR-----
DsNLIPIVIgadpEPETdagkDLLRVIKGRTYFRLEM--
TPSSFDKLVSAIRENYRYPERQ(QAEVQVDSISSADFTVVYDWEDSHLVDTAHERLVKLGIDDVKYYSAGVAR
SAGGEH)}*

$4692=215(228):

>gi|1181058183|ref|WP_084466804.1| {|0(96)|<unknown(X)>}hypothetical protein [Nocardia arthritidis]
{(MPRSNRAGGGM)--RVFLSHTSYDAGVVRYLRSVLEhDVGVG-
FFVLPDDAPHGLPWIEQIKLGVENSDELFTVITPESVHR-PWVSaEWACFWMRG-----KPTTPLLLE-
VGvdQLWEPMRAFQTVDLLD--V---SSLT--
RFLRTIADRTGC(EPTSGVTPVAHQMVREIPRIRERQAVADLERTLERLQRTVTANHTNVRAGDVRALIARDR
LDDLIAIAISAQAAQVK)}*

$4693=277(280):
>gi|1181068770|ref|WP_084477314.1| {|7(652)|<unknown(X)>}hypothetical protein [Actinokineospora enzanensis]
{(RARAASGRTGGKPKYNPVRAASRPHAQRPRGSRIPRTRPAGQRLGGDALSPFCRSGPAGVSGMVEWTRWPE
GRMDAAENP)--QVFLSHTWVDEAAVDVVEQALTARGIP-VIRDKR-IHPGVP------
GALDSAALVVAFYSRRYPVQSVCQWeLTRALLAGA-----
DRVLIVNPEPDNrhIVPALpafswrddgprLADAVAERLDG--
IDGSLGDPDTDSRGRPCRFVGR(YRELLDLRHALRVHGKVLVTGAAGMGKTALAEQYAYLFADAYPGGRAWTS
FAGVAPEAAR)}*

$4694=135(141):
>gi|1181343509|ref|WP_084644394.1| {<unknown(X)>}hypothetical protein [Microbacterium gubbeenense]
{(M)----FLSYARIDGSRvATELKSEVEELGVR-
VWFDEVAIVPGRSQSLQMDKGLRAARSGVVLLTPAYLTGRFWTErELGVLLG-K-----STLIPV-
LNEVTfkDvaeysgfLPDLAGFETARDSVQ--VIAEKIAT--AVMPA-ESEASM()}*

$4695=248(245):
>gi|1180967395|ref|WP_084386018.1| {|0(624)|<unknown(X)>}toll-Interleukin receptor [Methanoculleus horonobensis]
{(MGDNTKSFGSKKLILGENCLEQKSGL)--
KIFISYSHQnntaeEQYIEDFIKHIAPLKENGLVDVWYDRLIVPGEDLHeMINHHLEDADIVCLFVSSDFLNS
GECRGEKKKALELRKM-KGVQVISVILSPC-GWEDDPdISPLLVLPTDGkpVSEFKDKR--
TAWQDVYGGLKRV(IEKELKIRQLIISDDFRSFLHDAEMLTKAHPKKENVSLDDVYIDVKLDKFNNLKEGKET
INSNELLNILFTDRRIIVAG)}*

$4696=174(180):
>gi|1181007054|ref|WP_084420143.1| {|0(640)|<unknown(X)>}hypothetical protein [Henriciella litoralis]
{(MKC)----FLSHSSKdKlSYASIVANALKP------
NVEYDEYTFEEGMENFeEILRALERSSVFVLLISDHSLNSEWVRREISEAQHRLST-GDlKRFFPIIIDPN-
IAHTDPRIPDWIQTAYNlrPITRPTIAAKRIKERLVEVSWQT(HPMLKKRDQIFVGRNAQLQEFEQRFDDVTR
RQPIAVF)}*

$4697=202(194):
>gi|1181026812|ref|WP_084439168.1| {|0(109)|<unknown(X)>}hypothetical protein [Sphingobium sp. TCM1]
{()MVFVFLSHRSTdKPRILGFVKRLEAAR----
LAFWIDSPsafgsdapmapgIEAGRDWPeQIDQALMKARAIVVFWSISWENGGAYLSmEHAVALSRHRA-
GTATYIPVLL----
DGQRALASQVQALRADRhdLVQAINIARDGERGWDQLFELLR(QTLPKPLEHPDGKATARALDPSMKWAEILR
APPDDEKERARLLLTLPKGPPIDT)}*

$4698=227(231):
>gi|1181163983|ref|WP_084511494.1| {|150(0)|<unknown(X)>}hypothetical protein [Desulfatibacillum aliphaticivorans]

{(SGADFKKAEFGYTIITCVDLKGAKGLENCIHNGPSSIGLDTIYKSGGNIPEVFLRGCGVPDNFIEYMRSLV
GQKLDIYSC)----FISYSHAdEEFAERLH---SALQSSGV-
RCWYaPHDMQGGKKLYdQIDSAIRVHDKTLLCLSENSMGSKWVETeIRRTRKAMDKT-GKQKLFPISIVDF-
NRIKEWECFDVNTGSDMadEVREFYIPDFSNWKDHDSFQKEF(EKLLRDLKRDA)}*

$4699=231(209):
>gi|1181296989|ref|WP_084597941.1| {<unknown(X)>}hypothetical protein
[Micromonospora chokoriensis]
{(MFRPRGEGGGTAVSTVDAPSRTRAPLF)----
FISYSRApvgratgkpaeyvSRFFEDLSVHVSELVGpvTGVDAGFLDSSIAGGERWSpELLQAAGTCQVFVPL
VSSALLGSDWC-GrEwdafsRRRIIPRHSSAsvHETAIVPVTWSPT-
NGTALPrvLRDIQRFSPTAmpDPDIAVHYQRDGVYGLLTMQWEN(AYRAVVWRLAQRIVAIHRSYLVEPWVPA
GVDELCNRFAEESE)}*

$4700=183(193):
>gi|1181306818|ref|WP_084607703.1| {|0(503)|<unknown(X)>}hypothetical
protein [Sphingomonas jaspsi]
{(MSCRHRRWGARAVSD)---IFVSYKKDdRAIAERLV---AALRATGK-
DVWWDDALTPHEAWDaMIERQIAAARVVIVLWSPRSVHSDWVRSEAHYAQDHHK------MVPVMVEHC-
SIPLAFMLRQAVDLSDGrfDDSNPNWTKLLAWIGAVQAGDAD(AIPEGNPVPAPVAAAAAVPAKTSSGERWLG
PAKGPKMW)}*

$4701=227(220):
>gi|1181058017|ref|WP_084466638.1| {|136(0)|<unknown(X)>}hypothetical
protein [Nocardia arthritidis]
{(ALLSATNLAQLDLGPFCNAESLRHSSPSNIDSRAVVKSYRHMNLKRFMLECGVPPIFAEYMIDCARALSEP
FLRSLMQST)----FISYGG--P-DEAFATKLYESLKAHGSIVFFFPTSATLGERIdnEIYRRLKEHD-
RVILICSQASLDRPGVLhEIQETFDRESRDgGATYLLPIMLDDYVlndwkkvhpSLAERIGRRVVGDFRGadk
srtKFDAALGRLIDaLKTAR-----PAI()}*

$4702=300(293):
>gi|1181139709|ref|WP_084488533.1| {|4(811)|<unknown(X)>}hypothetical
protein [Nocardia niwae]
{(LRRPNPNVHRRTRRVRPRGAGDRTDVRRYDRLHGRRRQTVGCRGLRWHPSRVPQVRGSGRHIREPVVLNEA
STPTSESRP)RFDAFISYRAQtsskaaHQLQRALVALSKRHRPDGDLiSLFLDTQSLVAG-
RLsgEIKDALEDSRCLIVLLGPDTYKSPWVDLEIRHWLDNGGdVSrlfLVRHDPsvDLKWD---DDRNDF--
AEPRLLPSALSKRFTEEQ--
KWLELQSSLTGT(DETALVGLYAPIAGIRPEDLLLAEANYQQRRQRQTRAIVSALSILLVMALVAATMAFSNW
RSSESNRVRADRE)}*

$4703=244(245):
>gi|1181171122|ref|WP_084518633.1| {|0(295)|<unknown(X)>}hypothetical
protein [Bradyrhizobium sp. th.b2]
{(MRLLGASSAISGWPTSRRHNPRAGFLARGSKTEIFQYAPARRDLMGSVSASVFRRLWRHGRSWRIGAVRFR
SLLWERLFG)-HDVFISYSRTdssayaRALHERL--IAKK------
FRCFLDEHEFAIGEALsgATRHHVTASSAMVVIASENAMLSPHVRAEVTGFLATGRPLvpiSVGKALQRSGN-
--EQPWIAVRKLIWFDEDALALTSGPTAGAIDALANSFNVTK(RQTQRARVFLFAAVVVSVLAAMALA)}*

$4704=226(228):
>gi|1181306004|ref|WP_084606889.1| {|176(0)|<unknown(X)>}hypothetical
protein [Lyngbya confervoides]
{(ITKLSGTKFNSANLSGTIFAGVDLSSSQGLEDCRHSSYSHVDHQTLLNNSQLPLSFLRGVGLPDTLIDYMP
SLLNKPIQF)-YSCFISYSHKdeefaKRLHADLQDKGVRcWYAP---------

EDLPIGAKIraGIDQAIRRHDKLLLILSEQSVNSPWVESEVESALEKEQESgclVLFPVRIDDAV---
MDSSDGWAGLIKRSRNIGNFCNWKNYDVYQSVFDRLVKDL(KRSLSETS)}*

$4705=226(228):
>gi|1181306333|ref|WP_084607218.1| {|243(0)|<unknown(X)>}hypothetical protein [Lyngbya confervoides]
{(ANLRHADICNAVLIETVFADVDLGKVKGLAECLHHGPSTIDYRTLARSGQLPESFLRGIGLSNTFIDYIPS
LLTNQPIQF)-YSCFISYSHQdeefaKRLHADLQDKGVRcWYAP---------
EDLPIGAKMrvDIDQAIRKHDKLLLILSEHSVNSQWVESEVESALEKERQSnklVLFPVRLDDTV---
MTSSDGWAGLIKRSRNIGNFCNWKDYDAYQQVFERLLRDL(KPALTEQA)}*

$4706=246(241):
>gi|1181346274|ref|WP_084647159.1| {|570(20)|<unknown(X)>}hypothetical protein [Frankia discariae]
{(MALVRHALAQPGPGTLDTMLQALEAQDPGDPSVRQFRWNVENARPRWGRSAGGGRIDSRFLVEQNAEVGVG
RPDERPPYF)----
FLSYARRpdrdlvEFFHRRLEvEIGRRISREVNSSGFLDRLSLEGGAHWraELREAARSSSVMIALCSDDYFT
SDWCGREWAVFQERINRAtpsGGVPPAAIIPL---
PWLPLTRERPKPVRDVQMMPLHAITTEDLPVMDLIQEDDA(LVRRLMRLLVERMMKITASAL)}*

$4707=252(259):
>gi|1181738589|ref|WP_084824024.1| {|90(24)|<unknown(X)>}hypothetical protein [Bacilli bacterium VT-13-104]
{(ESKEEVSYVYQLLKYIDKTGNFVGIIMGYGSGRKIQDHLDAFNHQVVIHLINHIREYIEDTIYGIEPLKPE
FVESSDPKI)----FISYCW-ED--KLFADLIDEDFKKLGFTLTRDERDLRFKE-
SikSFMESIGKHDFVISIISDNYLKSVNCMYEISEVMRLREY-
KDKMLLIILNESDINLLPDSVLKPKNFSANIYEVVSHIEYIKFWEGEERNLAEK(ISMISEDVNKIQPLNELK
RIQSIKSNISDFLADISDWNN)}*

$4708=208(209):
>gi|1181482932|ref|WP_084778339.1| {|0(63)|<unknown(X)>}hypothetical protein [Saccharibacillus sacchari]
{(MNYLEVLRCLKSILLQMYKEG)-YMIFFCFSS-KDRHAIVEAILYHIRNYR-
IPVWYDRDQMLLGDERnykNFVEGVENSSYGIIILSPNSIQSHCANEEIDLMYDKYLN-
GSMHIFPIFFNLSANEVPEKYGWMKQLVYKEILPSIDVYSTCNHIICKFLSDEL(IQHESQSLISLEKRMKSI
KCDLFILSLIQSYLELDEKNFNSRIALLYA)}*

$4709=237(243):
>gi|1181435383|ref|WP_084730938.1| {|0(284)|<unknown(X)>}hypothetical protein [Microvirga vignae]
{(MPYQAASRCSNFDIELRGRTAEGAAVVSSIFLSYARADNAVPNDDPKSVGWVRR)----------------
-
FYEFLDTGLRARGFLnaaVWRDVADINPSEQFarEIEEAVQRAELFIAIASRAYTQRPWCLEELEKFLRAAKv
rsiADPRERVIAVFkqYLRDVERQRLPTGLQGQEGFHFYEQDPETNEYVPFAEFGRLMNEQ(RYLRTMTRVIN
AIEERLLDSPSEQEPETTVRSDGPIVFIAKPAKELEND)}*

$4710=250(254):
>gi|1181760386|gb|ARI83740.1| {|1155(51)|<unknown(X)>}hypothetical protein BH695_4461 [Microcystis aeruginosa PCC 7806SL]
{(AIAGLLKALEHSDWDVSGNAAEALGNIATETAMTELIKCLKNPDFVTLNNNYTLSQAREALDTIQNKLKYY
HPLSQPMNQ)--QVYISYNW-
QEDSNEMANQLVQAFAAKGIEIIRDkTHTSYKDSIKNFMRQIGRGKCVVAVISDRYLKSENCMFELV---
EIARN-

GDfyQRIFPIILPDARIHKDFERIDYLKYWEDEKAKLQAKYKEIDLAKTNSIMATL(NLYDEIRDNIDNLTNI
LKDMNLLNIDLHRQSEFA)}*

$4711=152(166):
>gi|1181356437|ref|WP_084657322.1| {<unknown(X)>}hypothetical protein
[Thiomicrospira kuenenii]
{(MAVLLRIALGTAAAIAALAILNKKKK)---VFVSYYYDEDKHYKRLLnawsankRFELEFDDI--
STDVSIKSENKTYIRkRISEKIKACDIFVVFVGKESHER------EWISWEINQAKtFNKKIVAI--------
----KEKRNYASPKPLLSSGATWVFGLNEKKIREAIEG()}*

$4712=157(170):
>gi|1181507007|ref|WP_084799564.1| {<unknown(X)>}molecular chaperone Tir
[Bradyrhizobium sp. Ai1a-2]
{(MGGGSGGGGSNRLGDTRALEEKAKRVLQGG)RKNVFISFAHEDLDEVNLLRGQSknensdIEFNDY--
SVKEPYDSVRAEYIKsRLTERISQSSTTVVYLS--------KDTpqSRWVRWEVEKSIeLGKRVIAV-
HAGQSapTPPTWISQN-----KIKVVPWSSLAQE---------LNKS()}*

$4713=147(153):
>gi|1181405533|ref|WP_084701088.1| {<unknown(X)>}hypothetical protein
[Cryptosporangium arvum]
{(M)------SHAS---EDKSgLVRSLAERLRRWGIKVWYDEYSLSpgDSLSRsldKGLAAT-----
RygLVVISQHFITKPWTEYELRGLTAKEMLGRDTVIIPDKIAITTtdK-SLDDVTNLGTHRGYF-
RNSRAAVPPIQCVRNEDGTILY(AGPEPTDEAIRT)}*

$4714=191(192):
>gi|1181370277|ref|WP_084670053.1| {|0(154)|<unknown(X)>}TIR domain-
containing protein [Duganella zoogloeoides]
{(MIQESNVLDSSAGKAVNPL)---
VFISHDTRdAELAEAFSNLLKSVSAGVLKSFRTSDRrgNQGIEYGIewypEIIRSIQAASDVVCLLTERSINR
PWILFEAGMA--KGKL-DTPILGVALGVELKSASTGPFAQFQNCGDDE--
ESLTKLVFQLVNRIPGSEPDR(DTIKFQVGKFKKTVEAILISTNKNLSGTSDSEK)}*

$4715=237(239):
>gi|1181448214|ref|WP_084743726.1| {|67(28)|<unknown(X)>}hypothetical
protein [Tolypothrix campylonemoides]
{(VAQEKVPIPGHPNIVVDYQNLLIYEQKGLPIIPEGLTETFDARQLLNGIEPEEQRRERQRDREDKFMGASK
STRSSKPEL)DKEIFISYTWRdehsKPYVEQLEQTFQAKGIK---II---RDTNAVGYKArfqeFM-
QRIARGKWVIAVISEQYFKSENCMFELVEVANNGDF-YKRIFPIILLDANIYDDLQIIEYIKYWEDKK--
KKLNEAIKEVGSEFLEGIREK(IDLYSKIRNTIAKLIKILG)}*

$4716=193(195):
>gi|1181505984|ref|WP_084798541.1| {|0(14)|<unknown(X)>}hypothetical
protein [Pleomorphomonas koreensis]
{(MNQLERMLRLYLQHPQEGVVANQL)---IFLSHIHEeRELAVLIKNAVQEeFG-GFIDVFVSS-
DGTTIPAGAnflsRIEDGLATCAAAIYLISPKSVKRNWINFELGAVWIRNVL-
NQPSAGTKIPTIPFCHSGSTPSELPAPLNNL--
NGIIATESSQLEFAFRSLQSA(LGGKGALRTDFDKLAAAALAFQTSYTIGASI)}*

$4717=237(245):
>gi|1181462686|ref|WP_084758159.1| {|0(149)|<unknown(X)>}hypothetical
protein [Sphingopyxis macrogoltabida]
{(MEGRRAQRALPLAVTRLPPPHRYATGRTACPLSAETGPSLLQSPIALPFRARHDAHRGIAMPA)--
QVFISYSSKDRTVANMVCALLEERGHR-CWIAPRDILPGASWGEAIIDGLKESAVFVLVFSQNA-

NASPQILrEVERAVHLG-----LPIIPFRIEDVLpeRSLEYFMSVPHWLDAL--
SPPVEDHIVRLCDVVGQLVNGA(AGRGDYIPRKARGTKSVSWNNALVRAGASGAVLALLLLAAWL)}*

$4718=221(226):
>gi|1181500890|ref|WP_084793707.1| {<unknown(X)>}hypothetical protein [Actinokineospora bangkokensis]
{(MPASHPCPRPARATYARAGPVAAQGEWLGVYQY)--DAFISYQRGSRA-
GEWVKTHFEprlralldeNSLHE-VRVFTVDSgRSGGRWPDDLRHALLHAKVLIPVCTPKYFTNE-
WCKaEWASMEGRE-----------QLLGLGvnDPRGLIHPVVFGDGDL--
YPDWASRRHMTSFKDYAHPEPQ(FANSQGYFDFVKLMLTFVEDLVIALDDVPPWEDGWPIHTPDVEPLGPSPL
PRL)}*

$4719=210(223):
>gi|1181400455|ref|WP_084696010.1| {|0(76)|<unknown(X)>}hypothetical protein [Arenimonas donghaensis]
{(MDVP)---VFISYSHQdKDFVDKLAVQL---VRQNV-
HVWVDRwELSVGDSLIdRIQDAVDGASALLVVLSPESVQSEWC-KrELSAGLLRElEE-KRVVVMPVLLRDC-
EIPIFARGKFYA---DFR---TNFD--
DGLRAVIDGIAKV(TNASLSRVQEDTYHVDWSINSGFNDEGNVAVKATFVQVPKSEPYTCLTEVVIIGDGEAS
EEFRRVTDQANVETARLRVW)}*

$4720=298(297):
>gi|1181402728|ref|WP_084698283.1| {|78(448)|<unknown(X)>}hypothetical protein [Roseomonas aerilata]
{(IHDAMLKHERGSALVIPVIARPSSWQSTAFAKILALPRDASPITSWRSEDEALTNVTEGVRRAILGRLERR
HNTEEHRVR)---IFISYSHKdLSDLEKLRPQLAAVF-
DGEPDlaqpiIWDYGSILPGELFQeQIRTRLQSADIVLTLVSPEFFRSDWVRAEMSWIRQIPEK-
KRPRIIPIILRPV-NWVDSPIGHYQALPRDGkaISTWHYPD--
EAWLDVAKGILNA(TRNVRRGSQSPQKATKQNSEGITRFEIGEVFKTAGTPTINYEPPNEFGQCRAELAIFGK
GLVVEGPSGIGKTTMIRR)}*

$4721=221(222):
>gi|1181402728|ref|WP_084698283.1| {|0(603)|<unknown(X)>}hypothetical protein [Roseomonas aerilata]
{(MA)-
TRVFCLYVSEdQRLFERMSRSLATLEQAGLTEALGGASVNLTGGLIgEAKEEYERAQIILMLVSADFLAAGHV
SQLIHDAMLKHER-GSALVIPVIARPS-SWQSTAFAKILALPRDAspITSWRSED--
EALTNVTEGVRRA(ILGRLERRHNTEEHRVRIFISYSHKDLSDLEKLRPQLAAVFDGEPDLAQPIIWDYGSIL
PGELFQEQIRTRLQSADIVLT)}*

$4722=169(167):
>gi|1181421653|ref|WP_084717208.1| {<unknown(X)>}hypothetical protein [Saccharothrix syringae]
{(MRLEVGSSGKPLVR)---
VFLSFVGRdEALVLRLWDLLTeamVIDQEYEFQLWrSDDAILVGEDWDvRIRGALSTAEVGIVALSNAFLGSK
YITGvELPALV---DV-PGKHVVPLLLRQVSkqaDWRGLKSKQIYGY---GrpFSEArgTSAQ--
DAWVNRLVVEVHR(VLARYAMRDDRQR)}*

$4723=198(205):
>gi|1181507005|ref|WP_084799562.1| {|0(793)|<unknown(X)>}hypothetical protein [Bradyrhizobium sp. Ai1a-2]
{(MVTPTAPVDLHRSSGAQAAVPELLAAEGLI)--KVFISYSRHdAPFVEQLGAAL----
RARGIDAWIDRReITPTADWLqRIVQGIVQSTFFIFVVSPRSVASEVCAVELRHAVELNKP-----
LVPVILAEA-

AVPDLLQAVQWCDFRDPaaFDAALTSLEAALRRDPEWLAIHG(RLAAAATEWQARKRPWAALLHGGSITEAMQ
WLAAA)}*

$4724=211(214):
>gi|1181448302|ref|WP_084743814.1| {|0(37)|<unknown(X)>}hypothetical
protein [Tolypothrix campylonemoides]
{(MTEISDTPEQPKSEEFD)---VFLAHNT--A-
DKPEVRTIAHQLKTRNIKIWLDEEQIPPGRSFqyEIQKAISlvKSAAIFI------
GLKGLGKWqEMEVRSLTTRCVeKSIPLIPILLPGV-
TKLPeklvFLKEYTWVDFSKSIDDVQALNKLVWEITATQSSPQQ(QPSELWFNGDRLQQFQKALLSAFPTPGK
LKQMVRYQLNENLEAIAAGANLSEVVSNL)}*

$4725=247(252):
>gi|1181462707|ref|WP_084758180.1| {|56(90)|<unknown(X)>}hypothetical
protein [Sphingopyxis macrogoltabida]
{(DDAFDEVMNSYAEEVHGKAASVVDPATGKHAPVFVRRIGKQGWTVFTSGSPAFARALEQRLGLDRGEVHGM
NEPADRERL)---VYLAHAS---
EDKPIVKPLAEGLLQRGINVWYDNWEIGYGDSLrrKMEEGLGNCTHFVVLL-
TRTSIAKPWVNeEIDAGLMSAVEGTAKFIGLRHDLPL-
SSVSRFLKTRLTPEYSPGEDGIEALAAEIFGVSKKPPLGQ(TPRYVQQHEAGSTWSSSARVVAEYFVRNSELG
)}*

$4726=242(228):
>gi|1181465365|ref|WP_084760838.1| {|0(235)|<unknown(X)>}hypothetical
protein [Cohnella panacarvi]
{(MLPSTKSSYNSEVKVLKRYTVP)--TVFISYCwtS--
EEHKKWVKDLASRLRQDGIDAFIDEWRLKPGQDRfaFMEAAItSEHTTKVLVICdenykLKADRREGGVGiES
QIISSKTFTKvDQRKFIPIisqrsNTGDV-
YIPTYMEHMIYIDLSSTvlFESGYKQLIYAIYETSEHEEPPL(GDAPDYVIAKLQNVERVSGSSSKEQIIPLG
IKESQYFNQMYVSDDKSHKHNGKLISLNSFLPNQHD)}*

$4727=204(208):
>gi|1181489705|ref|WP_084785112.1| {|0(121)|<unknown(X)>}molecular
chaperone Tir [Pseudomonas sp. DRA525]
{(MLIIRNHQNTRMDMSPK)---VFVSHAS--
EDKDRFVLDFAKQLRAHGIDAWLDKWEMLPGDSLvtKIfDEGIKDAQA-VIVVLSKNSVSKPWVKyELDAA--
CVKRInTGSKLIPVVIDDC-
EVPEALKSTLWESIVDTASYQSSLERIIASIFGASDKPAL(GKPPAYVNSFGVSVAGHNNVDSLVCKLACDFA
IKTGSRHISISAFCKDGEL)}*

$4728=225(225):
>gi|1183700153|ref|WP_084930044.1| {|0(612)|<unknown(X)>}hypothetical
protein [Streptococcus mitis] >gi|1181961070|gb|ORP07478.1| hypothetical
protein B7692_04595 [Streptococcus mitis]
{(MPKIF)-----LSHSS---ANKEQVKKIYSKLVTSlgEESVVMDcFNFQE---GRnteSEI--
IYNLDiSDlfVIFFSNDALDSQWVHQELRIAESRIAKDRYYQICPiivadkIKYDDERipK-WLRNQYNIQM-
IKSN-KKIVDIIL-
ERYIEISRQKHNA(IKDRQDLFVGRNSFLDDIERRIDDFNLPKPVALFASGLEGVGRRTFLKKSLIKTNLVKP
TYPFSELAMERNESIEDFILK)}*

$4729=196(201):
>gi|1183132190|gb|ORW50478.1| {|0(735)|<unknown(X)>}hypothetical protein
AWC20_24350 [Mycobacterium parmense]

{(MSL)---IFLSHASRDNREAlalkKWLADQRPELAGE-IFLDI-
DPQTGlrlgERWAGQLVTSNSRCEYLICLLSRSWLNSAE-CAvEFRTAEGFG-----
KRILVARLEDTGagDI---TSHWQRCDLFG--
GGAQTSIEVAGGPPVVFNTAAL(DELKKAIEGSGIGPEAFVWPPRDQPDRAPYRGWEPFEDVDAGVFFGRDAA
IARGVDEL)}*

$4730=203(186):
>gi|1184649472|ref|WP_085209921.1| {|0(134)|<unknown(X)>}hypothetical protein [Methylocaldum sp. 175] >gi|1182709460|emb|SMF93387.1| TIR domain-containing protein [Methylocaldum sp. 175]
{(MSAPT)-
AQIFVSYAHAdNqlfdagtkgwitEFVDKLEKAIGMKPGGNNAKCWIDYRLEPQRKVDeALLGHIRESRFILA
FMSPRYLESEWCAKEMHAFVELAGG-HaDDRVFLVELlptkREC--
WHPGVQAIAELKFWTSslDQPDPMILGWPVPDPKGDRPYWN(ELNRLATIIARQIQDLPPIPPDPPPPPDCPE
PPTPPSGPLR)}*

$4731=208(219):
>gi|1182713480|emb|SMA50966.1| {|0(79)|<unknown(X)>}TIR domain protein [Endozoicomonas sp. S-B4-1U]
{()MP-IFISYSHEnKEFVDKLAMQLV----
QRNINIWLDRwELNIGDSLIdKVQDAVDGASALLVIMSKASIASAWCKKeLTSGFLRELEE-
KRVVVMPVLLEDC-DIPIFARDKFYA---DFR---TNFD--
EGLKTVIEGIQKI(TNPFLARTKEPEYHIDWSIDWGDINGNFAIILNFVEQAKDQPFTCLTNIEALASDKATE
IYNLNSKNHGEEYARSYIIS)}*

$4732=183(191):
>gi|1184637792|ref|WP_085198900.1| {|0(332)|<unknown(X)>}hypothetical protein [Mycobacterium fragae] >gi|1183037982|gb|ORV58643.1| hypothetical protein AWC06_19850 [Mycobacterium fragae]
{(MG)---LFVSYSSQdRSAIEALTTALRRARQQ----
VWFDEDLGGGETWWnAILEQIRSCEVFIFALSNHSLQSKPC-QaELRYAQALQRP-----ILPVQIGAV-
DsMRANPLAALQIIDYRNptVDSGIQLVTAVHTLAAQNPPLPS(PLPDEPPVPFAYLMRLSSTLAERELDPRQ
QTHLLAELKSGLDEDGGDPT)}*

$4733=172(181):
>gi|1184594430|ref|WP_085159249.1| {|0(331)|<unknown(X)>}hypothetical protein [Mycobacterium lacus] >gi|1183089431|gb|ORW08925.1| hypothetical protein AWC15_18630 [Mycobacterium lacus]
{(MA)---LFISYSSQdRSSLEALTTALKRAQQQ----
VWFDQELGGGDAWWsAILEQIRTCEVFIVALSNHWLQSK-PSQaELRYAQALNRP-----ILPVKVGPV-
DSVRVNPVANLQIIDYQnpTVDAGIQLVTAIHALRGKVQPLP(SPLPEEPPVPFGYITRLGNTLAEKELSPQQ
QLQLMVELR)}*

$4734=183(191):
>gi|1184525564|ref|WP_085095573.1| {|0(329)|<unknown(X)>}hypothetical protein [Mycobacterium paraense] >gi|1183111793|gb|ORW30778.1| hypothetical protein AWB91_17490 [Mycobacterium paraense] >gi|11831
{(MA)---LFISYSSQdRPTVDALTTAL----
RRGQQQVWFDQELGGGESWWaKILEQVRSCDVFIVALSTNWLQSkPSQAElRYARALNR-------
PILPVQIGDI-
DsMRVNPLASLQIIDYREptVDAGIQLVTAVHALQSKPAPLPD(PLPDEPPVPFGYITRLGNTLAEKELSPQQ
QLQLLVELRQGFDEDGDDPS)}*

$4735=185(192):

>gi|1184708170|ref|WP_085267279.1| {|0(292)|<unknown(X)>}hypothetical protein [Mycobacterium parmense] >gi|1183145008|gb|ORW63019.1| hypothetical protein AWC20_04605 [Mycobacterium parmense]
{(M)--TVFVSYSSRdRDAVKSLVQNLQDADEQ----
VWMDQRLAGGEAWWrAILEQIRGCDVFVFALSQNSIESKPCQAE----LQYGQA-LGLPILPIQVGPV-DsMQLNPLGAVQAIDYRLptPSTAMRISAALHRERARRHPLPE(PLPGEPPVPFEYLIRLYTIISNPEYLSPRDQAALVAQLQVGLREDGQHEAA)}*

$4736=201(220):
>gi|1184654816|ref|WP_085215263.1| {|244(0)|<unknown(X)>}hypothetical protein [Methylocaldum sp. 175] >gi|1182712442|emb|SMF96369.1| TIR domain-containing protein [Methylocaldum sp. 175]
{(ERLSNRRRDQISEYIADDSATLSQSTMLYFDQAVQTTRLIGGNPAEFTYELFSRWQQSYGVETDDRRFLDSLPAEMPSGA)---VFISYAN---
EDLPTALDIARALHSEGVPIWIDRRHLQAGQRYdeKLEAAVKLRSSLFIALVSPASESSPtrYVQnERRWAAMRQGDLsD-EFYLPV------------------LHGLPSPEAVRVEPENVRKKLSGKKEFM()}*

$4737=265(270):
>gi|1183404649|gb|ORY29213.1| {|197(832)|<unknown(X)>}hypothetical protein BCR33DRAFT_857744 [Rhizoclosmatium globosum]
{(KIQEGFHDIKSYDIFAKHPRGVMQQITEGHNEVLRDAFVSSLEFVLKVSNPSVSPYTMAAALPNSVSNPVLSIDPNEAMF)--DVMISYN--
QKSSNDQVNDICEALENKipGIKIWRDKDQMKTDINEGMCEGVIDSKAVIVCLSKGYLTSQSCKKEIRWADELK---
KPLIPAYMFAKDEDPVVLKQNKNFAIFFFYISSALYADFKESRKNSPVFETALD(VVVGQIKEKVLASKRMDPVNVINVDAVKQWLDPVDFSNDLLAYKSEYVAG)}*

$4738=262(266):
>gi|1183411694|gb|ORY36168.1| {|14(112)|<unknown(X)>}hypothetical protein BCR33DRAFT_485584 [Rhizoclosmatium globosum]
{(LQKEIQKNIQGIEIEDKIYFVDWETSKKTIVDENCIEEICKNGNRLLIERAHIQPETVTKPVFLDPRPVVTNKEWTTEKK)KFDVMVSYSW-ETIQQ--
VESLCAALMQEipGLTIWLDQNKLYSNSYDHMAKAVLNADAIIVCLSQPYLMSDNCRREIHFAGDIK---
KPLIPAYMFGSLDSKEIQNLQKNPNLAGAFLLTAGQQHGNFGDSVPGSEQFNVA(VKIVVNQIGCNVSRFRNSSISEQKSMLENWLNPVDFSADLAEYEKD)}*

$4739=165(186):
>gi|1183416997|gb|ORY41380.1| {|0(466)|<unknown(X)>}hypothetical protein BCR33DRAFT_826312 [Rhizoclosmatium globosum]
{(MATATMQCWAQKET)------------------
VVEIANFLTAHGLRLWRDDHQMSGDIVKRMVEGITQSKLILVHMSKDYEKSKNCQRELNFSWDEE---
KQLIPIRLDKGPFPITRFIVGTSLYYDFSDCLDDEVKKGDLKISLLKEIYQALG(YAPTIALPRSVAAPVSLMVVKQIAANKELKGI)}*

$4740=162(172):
>gi|1183416998|gb|ORY41381.1| {|0(70)|<unknown(X)>}hypothetical protein BCR33DRAFT_345373 [Rhizoclosmatium globosum]
{()----MMSYSW-AQ--
KDIVVEIANFLSDHGLRLWRDDHEMTGDIVLRMVEGITKSKLILVNMSKDYEKSKNCQRELTFSWDEE---
KPLIPIRLDKGPYPVTRFIVGTSLYYDFSDHLNNEAKKADLKMSLLQEIYRVLE(FTPSVPLPKHVAVPKSLMVVNSFAQKVPKDFD)}*

$4741=249(255):

>gi|1183419974|gb|ORY44325.1| {|133(718)|<unknown(X)>}hypothetical protein BCR33DRAFT_765973 [Rhizoclosmatium globosum]
{(KIKKTLDLSGNYKIYAVEIQNSTSASVELIDEDNVKDICRNFSNYRLEISRDLLVASKLKTSVDSKPDKQQVIIGSQNDD)EYDVMISYSW-HT--
KDQVDDLYNALLEAfpGIKIWIDRERMKTDIFDGMTEGIVCSNAIIVCLSAPYLKSVNCQREIKFAGDLN---RPL--
IPAYMFGPNDKVTEFREDKKLGVPFLLTAGALYADFKSQRAGSPEWNRA(FQVITGQIQAKVPRLRRIATELSQELPNIANETVE)}*

$4742=257(263):
>gi|1183424403|gb|ORY48719.1| {|119(448)|<unknown(X)>}hypothetical protein BCR33DRAFT_848072 [Rhizoclosmatium globosum]
{(LSAVFRNYQTLCYNMMTMESVLNSDSTLERVYTEATSKKVLPSVFVITAQNPNTSIIPTTVTSKPKIISTTQDISSEKSF)--DVMLSYSW-ST--
KHLVKSLHTSLLQHTptLRIWIDDREMDTDIYKGMVEGVLKSSLLLICLSRAYLSSANCNREIKFAADLQ---KPMVPVRMFDKMAGDDVGALMASRELAVPFLITSGLMYVDFEGLVPGTEGWEGA(VRAVVEQVESRLAKVSGSVGGGVLKRAGIEEEDGVGRVLLKNW)}*

$4743=279(262):
>gi|1183414859|gb|ORY39270.1| {|87(0)|<unknown(X)>}hypothetical protein BCR33DRAFT_740969 [Rhizoclosmatium globosum]
{(QILQNLDQFPNEPHFQNPMSQRNLPVREIVKILCQKWGHFVNITDPDIEGLAAKISTDVTNLEQKDLRWKWLNNTYTQPF)--DAFISYRVATEKKLARNLFYVLStqrqkvqhlERGL--
TIYLDQIRLVNGEnWEtGFINGLRNSSMVLVLVSRNAVEGFKTAHvrPDNFFLEMEVAfdildhrLdRGIVIQPVFVDGYIPNPQDFPDECHSHQSSPRMSTISNLIKRLVHLQGLRAYVT(QEEGELGACESIAPQVLDRLKDKWEVQRKQAERILLDGFIEE)}*

$4744=265(252):
>gi|1183416356|gb|ORY40746.1| {|236(590)|<unknown(X)>}RNI-like protein [Rhizoclosmatium globosum]
{(PVPLDSNHITSPPPAVPAIPPSSVLNRFKNALTSPNASLSSTSFSAAATTTLQPNQDAQALEDDPTASIAACGGWFLSPE)TYDFFISYRVASDARVAMELYFRLStqrvldefgnSRQV--
RVYWDKECLKKGqDWRdGFVQGLKNSRCVLMLVSEGAVAAMEQSYkrGDNVLLEWETAVmAGRKSICIPQPVFINNPSKPQQAIDAVFAFKASPLCPKRRPASSQETTHKWSAY(TTLSAITQLQGIQLDTTQVAWSIPDLIKALQT)}*

$4745=265(252):
>gi|1183425533|gb|ORY49840.1| {|296(915)|<unknown(X)>}RNI-like protein [Rhizoclosmatium globosum]
{(SSAMFGTQEPVATVPTLSPVAEKQDKNAIPASVTSASETKTGPQTATTASDSKPDTEISGLPDDPSASLQACGEWFKSAE)TFDFFISYRVATDARVAMELYFRLKdqrivdeyghSRQV--
KVYWDKECLKKGqDWRdGFVQGLKNSRCVLMIMSVGAAERMKESDkkGDNVLLEWETAIlAGKKNICIPQPVFINNPKEGVSALDFYSKFRKTCPTTRPDQPDAHRVSCKTTIG(AATNLQGIQLDVNEISWSIPELIRALQTYNAN)}*

$4746=247(254):
>gi|1183412532|gb|ORY36987.1| {|134(879)|<unknown(X)>}WD40 repeat-like protein [Rhizoclosmatium globosum]
{(MLHFYVATEIKFYRCDLMSNQVLPITTSSSLKKTLTSYSCDLVFETPGQRSLQLQTSSQLPQEDTLTMSASSDFKSKEVF)--DVFISYCW-ADM--NIVELFYAELKTSfpQLKVWKDNK---
GGmtSDVytAMPQGISTSKIVMAFFSEAYFKSENCNREIKYAADLK-----
KNIMPVHLSNDNVPNLVALRDEFPAPFFITAGKLYHELKGTDATTDQWKNLI(HNTYKEVCGKGSLSSVDPTLAPLYLWLRPVDFSK)}*

$4747=246(256):
>gi|1183412647|gb|ORY37102.1| {|135(1127)|<unknown(X)>}WD40 repeat-like protein [Rhizoclosmatium globosum]
{(IVEHFGVGSNVRIYYQNSETDAKTIIKNEKSLKLGLINSGRVYFELLETKTIPDEIKLSTSTAISTLTQRDAKSYRDLGL)----FISYNW-S-TKQNVNAVV-NKLSVLKpdLPIWRDTEKMES--DIydSMGSGIAGCQVVVACFSLGYLESPNCMLELGFAQDLR-----KPIIPVFFFEEGEDITTLRQKYSKAFLIIASKKYSDFKRWDALDSNWEAAFD(IFVKEVECALNKVALSAVVVPNSPLEVWLVPESFDA)}*

$4748=262(254):
>gi|1183413818|gb|ORY38246.1| {|199(127)|<unknown(X)>}kinase-like protein [Rhizoclosmatium globosum]
{(LCTNQMPFEGKTREQIAAIIQTETERPEMPEEVNPILWDLMNKCWSLSPSARPNAFEIREILATNFRDEIGEAVNKIDWT)---IFISYAW-ANSKEAFLkgstksmaacgpcdpRELARKLTASGHRTWLDVDRIKAGKPLfeNLVDAMKPAKLAVICVSNEYAESENCMREFKFLRELK-----IPYVLVAVGSNVGYANGQEWRQTVVGFLSGDKLYIDTKGSSKDKFSSEVYAT(ILDAVEDELHTSASMAKHTTLKRSTTTVSDVERV)}*

$4749=254(263):
>gi|1183417688|gb|ORY42063.1| {|252(293)|<unknown(X)>}hypothetical protein BCR33DRAFT_825036 [Rhizoclosmatium globosum]
{(ELLDNITLHFGCERSVKVYHQNKKSYELESVTNQDSLKRAFLDSCDVLFEPIKRTEESSQVVDPEHKNGTPTAFKYNKVR)--DLFISYSW-STTAEVDA--IVNKLeTETKLQIWRDKKDMN--ENIyeSMGGGLAGCRAVVAFLSEKYLTSENCKNELYFARELK-----KPIIPVYLFEESTDIESLKRSDSGPFFILTGKLYFDFKRIDANEPKWLSKFN(ELLAGICSTLDQTKPVALSSDLLETWLDPVLFEDDLEAYKAEY)}*

$4750=247(254):
>gi|1183418151|gb|ORY42520.1| {|740(60)|<unknown(X)>}hypothetical protein BCR33DRAFT_851581 [Rhizoclosmatium globosum]
{(DGPFTWTALITSGLLYTYIGAKELSDETRWEQAIDGLAKELAAVVGPRQPEHHAGGLPHSDTDLFTAAVEEAKKKDLELF)--DCMLSYNW-S-QQQTVLR-IFKSLEDRGLKVWMDLDEMSG--NVytKMSEAVLGSAVVVSCLSDTYEASGNCRRELNFAysqVPNG-----KKIVAVELTDGPFTWTNDVTQGLNRFKISDTELQASEKWESLMTELATEIRA(KAESGRVEAKRAAARAQEEPSIPVIPLPPVPQPL)}*

$4751=286(284):
>gi|1183418151|gb|ORY42520.1| {|569(192)|<unknown(X)>}hypothetical protein BCR33DRAFT_851581 [Rhizoclosmatium globosum]
{(SKVIQPHMKVLTRVSTDPVVKFAGVQESLDDVMNAFSGLSLRGLSDKFKSSMQSLGINPDDPFFAAVEAAMEKESEAIKV)-YDCMLSYNW-TQQPTVIR--IRDSLVKRGFTVWLDMEQMSG--NVysRMCEAVLGSKVVIPCMSKTYEASGNCKRELGFAadqTNAG-----KKIVPVRLDDGPftwtalitSGLLYTYIGAKELSDETRWEQAIDGLAKELAAVVGPRQPE(HHAGGLPHSDTDLFTAAVEEAKKKDLELFDCMLSYNWSQQQTVLRIFKSLEDRGLKVWMDLDEM)}*

$4752=247(254):
>gi|1183418148|gb|ORY42517.1| {|718(63)|<unknown(X)>}hypothetical protein BCR33DRAFT_718212 [Rhizoclosmatium globosum]
{(LDDGPFTWTALITSGLLYTYIGAKQVSNDQNWEEAMDGLAKEVSAVVGARHPPSALPPTDDQLFEAAVQESTQKQNLNLF)--DCMLSYNW-AQQKAVIR--MRDSLEQRGLKVWFDLDNMSG--NVysKMSEAVLGSKVVVSCLSTTYEASGNCRRELNFAysqVPNG-----KKIIPIEFSEGPFTWTNNVTQGLHRYSMTETVIESSDLWDILMDELVVKIRE(LVSESSAVIPAVTAAPANTVPAASKETVVVQPVT)}*

$4753=235(260):
>gi|1183418148|gb|ORY42517.1| {|568(225)|<unknown(X)>}hypothetical protein BCR33DRAFT_718212 [Rhizoclosmatium globosum]
{(PVVQQGGVKESLDNVMNSLRGVSLAALSDKFTSSMKSLGINPDDPFFAAVNETLAKGDIKEYDCMLSYNWSQQPTVIRIR)-----------------------DSLVKRGFTVWLDLEQMSG--NVyvRMCEAVLGSKVVIPCMSKTYEASGNCKRELGFAadqTNAG-----KKIVPVRLDDGPFTWTALITSGLLYTYIGAKQVSNDQNWEEAMDGLAKEVSA(VVGARHPPSALPPTDDQLFEAAVQESTQKQNLNLFDCMLS)}*

$4754=239(256):
>gi|1183420020|gb|ORY44371.1| {|0(30)|<unknown(X)>}hypothetical protein BCR33DRAFT_219974 [Rhizoclosmatium globosum]
{(MIAPSTENSTAHAVIPPPINQSPKISQQAKNAKKVFISYCWSNSRSKTLKDIEAGVASTEDLDKTGFWDPRKET)----------------------YEIIQQCGYEPWLDEFELKVGEDLfsQLARALKnEAALVIIHVSDEYAHSDNCRDEFLYANKLK-----LPIIPLIVGVpgasksmEPWDVTSLGFQLSKYLYIDARDYHFIESKKEALIAAIKGKME(ALAAASNDDVIPIATLPPVIASTHKPKKVFVSYSWANSRNQK)}*

$4755=232(220):
>gi|1183421553|gb|ORY45892.1| {|21(0)|<unknown(X)>}hypothetical protein BCR33DRAFT_715922 [Rhizoclosmatium globosum]
{(EQLHQRLPLLRRHCDSLVDVMNQSTDVALLVKLNNELGAAEAKFNECATEIQRIESMTRCFPSNWRQVRYLRTPNATSDW)--DVFISYCW-LNSAeaQTLAqkdnnqdatqdhvgkcdpRELNRILKKRGFSTWLDVDRMTGGDVLphQLMHGISRSKFAIVCVSDEYVRSPNCQKEFKYL--SK-----RRKIPHVIVVVGHNKHSQWDKSGLVLWMEIISAVRRHVSPRLNIASSELGST()}*

$4756=241(248):
>gi|1183421723|gb|ORY46062.1| {|0(225)|<unknown(X)>}hypothetical protein BCR33DRAFT_159571 [Rhizoclosmatium globosum]
{(MKQFQLSNQDFTIHYVDSKTNNKVIVDKAEIFERACANSYNLVFELPIQTTNILSVTSSFRNTPPQDPKSRQQ)-YDIFISYSW-S-TKAQVAAIVHELKTRMpHLTIWMDHNKMQG--NIysAMSGGISSSKVVIVCLSKGYLESANCNYEINFAADLK-----KRVIPVYLFEDGEDINELKQSYGVPFLITAGALYSDFKRYAVYSEEWSRAFD(SLRKEVENAFGGQVSESDMGTSLMNVKSWLNPVDS)}*

$4757=248(255):
>gi|1183421718|gb|ORY46057.1| {|153(708)|<unknown(X)>}hypothetical protein BCR33DRAFT_716067 [Rhizoclosmatium globosum]
{(NLLKAEIMKQFQLSSKDFTLHYVEKQRKVAVDKAETFERACQNANVLFFDLPPVAHPTSLVRSQTMSQDVLQQDPRSRQQ)-YDIFISYNW--GTKAEV-TAIVDQLKVVmpHLRIWMDHNKMQG--NIysAMSGGISSSKIVVAFLSKGYLESVNCNHEINFAADLK-----KSIVPVYLFENGENVTALKQAYGVPFLITAGSMYSDFKRFSVGTEAWSEAFN(VLSKEVENALSGRTSRATWLKPVDATDDIKAYESE)}*

$4758=249(256):
>gi|1183423581|gb|ORY47903.1| {|71(1104)|<unknown(X)>}WD40 repeat-like protein [Rhizoclosmatium globosum]
{(LKTEIMKQFQLPNQGFTLHYIDSKTQRKVAVDKSEVLERAILSSSVLVYELATGFTPLSVSSGAISLQDSFQQDAKSRQQ)-YDIFISYNW--GTKSEVTALVEQlKLSMPSLKIWMDHKQMQG--NIysAMSGGISSSKLVLACLSKGYLESVNCNMEINFSADLK-----KPIIPAYFFDEGEDVTGYKQSYGVPFMITAGSLYSDFKRYDINSPKWQSAFS(VLCNEIRHVLHPEPIVEEVTSDSLKLWLNPVDFVDD)}*

$4759=234(260):
>gi|1183423575|gb|ORY47897.1| {|387(342)|<unknown(X)>}HCP-like protein [Rhizoclosmatium globosum]
{(GLGTTKDFKQALEFYTKSAQQGNPSAQYAEGTLYRDGLGVKQDRERARRLFTLAAEGGDEDAQMALEIFDL
WAQKDLVVE)---------------------IHDFLTSQGLLVWRDDEKMTG--
NTvdRMVEGIQDSKVILINLSSTYETSVNCQKELNFSLSEY-----
KHIIPLRLDHGPFPRSLIKTGADQLTSIDFSDDSTKSDSLFDLLSEILNAPD(AENIDEGAAQGNAECQNRMG
IMWKNGHVGRKPDQLTAFRF)}*

$4760=227(222):
>gi|1183429014|gb|ORY53301.1| {|0(11)|<unknown(X)>}hypothetical protein BCR33DRAFT_192610 [Rhizoclosmatium globosum]
{(MRKSKVSERRHYRSSTDASNSKSTTLTPPIKDSVAKTGDLGATPVPSWS)---VFVSYCW-
RNSKDAFVRgqidsdfgcgscdprVLATRLSAAGFPCWIDMEQLEVGEPLfeDLVDTIKRSKFAVICVSTEYA
DSENCKDEFKFI---R-----
KIKIPKIIVVVGPTHSVRDWSTTVIGFMASDAVYIDARGPSSFGLSEPTFQR(ILTAVQHGLLKPNNSEQVHA
EFSLTDMRTMFLS)}*

$4761=246(255):
>gi|1183429644|gb|ORY53928.1| {|86(227)|<unknown(X)>}hypothetical protein BCR33DRAFT_11774 [Rhizoclosmatium globosum]
{(LDNITLHFGCERTVKVYHQNKKSSELESVTNQESLKRAFLDSCEVLFEPIKHIQNLPQAVTQITEHNHATP
NTFKYNKLR)--DLFISYSW-S-TSAEVDAIVNKLETDTKLQIWRDKRDMN--
DNIyeSMGGGLAGCKAVVVFLSEKYLASDNCKSELFFARDLK-----
KPIIPVYMFKEGTDIESLMRSGSGSFFILSGKLYFDFKRMDANEPKWFSKFN(ELLAGIYSILDQNIPAALSS
DLLETWLDPVVFEND)}*

$4762=208(220):
>gi|1183408207|gb|ORY32716.1| {|160(0)|<unknown(X)>}hypothetical protein BCR33DRAFT_791614 [Rhizoclosmatium globosum]
{(KFFAKESDDQPEGNKSVGEENPCEITHQAPATIIERKLNLPENTSKAMNQCTHETALQLGSVGFRESTRSY
CHAYKFDAM)-----ISLASTdRRLGEELYTLLISSGY--------
SVWVYWIHMKGnmdkSMVDGIKQSSLFIPILSDDYYGRTDCMFELQTASADSKR-AVILKKPG-
PLPPTILAHLGRLQYIEVGDHL--DDVTACRILELMETSILRHTP()}*

$4763=206(220):
>gi|1183408204|gb|ORY32713.1| {|564(0)|<unknown(X)>}hypothetical protein BCR33DRAFT_771351 [Rhizoclosmatium globosum]
{(TFNHPEHLHETKPLRRPIPPRGSSRSRNNNILRPGSNFDADNDRSSINTLESVEIAMRIGAVGLREATRQY
SHSYPFDAM)-----ISLASSdRTLGQELYDLLSSNGFK--------
VWVYWVNMSGnmdrSMMEGITRSALFIPILSDDYYGRMDCMFELQAASSQCKR-
SLVLKKPGVLPANVLAHLGRLQYIEVNGELD-----GHTRDRILGVMRDSVPEL()}*

$4764=146(148):
>gi|1184696393|ref|WP_085255745.1| {|0(363)|<unknown(X)>}hypothetical protein [Mycobacterium saskatchewanense] >gi|1183154687|gb|ORW71669.1| hypothetical protein AWC23_12910 [Mycobacterium saskatchewa
{()MP-LFISYSSQdRSTVDALTTALRRAQQQ----
VWFDQELGGGESWWnKILEQIRSCDVFLVALSNNWLQSKPSQAeLRYARALNRPI-LPVQIGDIDSMRV-
NPLATLQIIDYRKPTVDagIQLVTAVHALQSSPVPLPDPLPE(EPAVPFGY)}*

$4765=172(181):

>gi|1184690029|ref|WP_085249645.1| {|0(334)|<unknown(X)>}hypothetical protein [Mycobacterium riyadhense] >gi|1183167517|gb|ORW84133.1| hypothetical protein AWC22_14205 [Mycobacterium riyadhense]
{(MA)---IFISYSSQdRSSLEALTTALKRAQQQ----
VWFDQELGGGDAWWnAILEQIRSCEVFIVALSNNWLQSK-PSQaELRYAQALNRP-----ILPVKVGPV-
DSVRVNPVSTLQIIDYQkpTVDAGIQLVTAIHALRDKAGPLP(SPLPDEPPVPFGYIMRLGNTLSEKELSPQQ
QLQLLVELR)}*

$4766=183(191):
>gi|1184603176|ref|WP_085166464.1| {|0(29)|<unknown(X)>}hypothetical protein [Mycobacterium sherrisii] >gi|1183167841|gb|ORW84453.1| hypothetical protein AWC25_25035 [Mycobacterium sherrisii]
{(MA)---LFISYSSQdRGKVDSLTNAL----
RRGQNQVWFDQELGGGESWWaKILEQIRDCEVFIVALSSNWLQSK-PSQaELRYAQALNRP-----
ILPVRVGDV-
DsMRVNPLAALQIIDYREptVDAGIQLVTAVHGLRSKPVPLPD(PLPEEPPVPFGYITRLGNTLAERNSARSS
SSRCWSSCGRVSTRTVTIPA)}*

$4767=222(205):
>gi|1183397612|gb|ORY22403.1| {|0(747)|<unknown(X)>}hypothetical protein BCR33DRAFT_162027 [Rhizoclosmatium globosum]
{(MNTNALWRCNNHLSGE)-HDVFISYRVSseaqvaKWLASLIERVGRR-
RCNKDIRVFLDSNCLNKGEDWeqGFLNGLRNSKLIVYLISaasletliekTNANKVDNVLLEIETGLKMKAAD
qarllPIFV-GSINAD---
GSYSPFDTSYFSGSKYPDTIHAGSKDNVRDSMALLFKIQG(DFIKHPPSNENALYLAADSILKLVAPHQITQA
TDQQLPPIAKSFMSHNT)}*

$4768=222(205):
>gi|1183399185|gb|ORY23910.1| {|0(876)|<unknown(X)>}TPR-like protein [Rhizoclosmatium globosum]
{(MNTNTLWRCNNHFSGE)-HDVFISYRVSseaqvaKWLASLIERVGRR-
RCNKDIRVFLDSNCLNKGEDWeqGFLNGLRNSKLIIYLISaasletliekTNANKVDNVLLEIETGLKMKAAD
qarllPIFV-GSIMAD---
GSYSPFDTSYFSGSKYPDTIHAGSKNNVRDSMAHLFKIQG(DFIKHPPSNKNALYLAADSILKLVAPHQITQA
NDQQLPPIAKSFMSHKT)}*

$4769=224(207):
>gi|1183413911|gb|ORY38336.1| {|0(172)|<unknown(X)>}hypothetical protein BCR33DRAFT_741550 [Rhizoclosmatium globosum]
{(MGNRERIGENITGHLSSE)-HDVFISYRVSseaqvaQWLASLIEGVGRR-
RCNKTIRVFLDSNCLNKGQDWeiDFLNGLRHSRlivYLISVASletliekTEANKIDNVLLEIENGLKMKAVD
qagllPIFV-GSVMAD---
GSYSPFDTSYFSTNKYPDSIHAGSKENVRDSIAQLFRIQG(EFIRHSPCNKNALHLATDSILKLVLPYQTTQA
NNQQLPPIAKSFMSHGT)}*

$4770=225(208):
>gi|1183419874|gb|ORY44228.1| {|0(1135)|<unknown(X)>}TPR-like protein [Rhizoclosmatium globosum]
{(MDTNNTTRLWRCNNHLSGE)-HDVFISYRVSseaqvaKWLASLIERVGRR-
RCNKDIRVFLDSNCLNKGEDWeqGFLNGLRNSKLIVYLISaasletliekTNASKVDNVLLEIETGLKMKAAD
qarllPIFV-GSIMAD---
GSYSPFDTSYFSGSKYPDIIHAGSKDNVRDSMAHLFKIQG(DFIKHPPSNENALYLAADSILKLVAPHQITQA
TDQQLPPIAKSFMSHNT)}*

$4771=210(220):
>gi|1184746169|ref|WP_085295399.1| {|139(0)|<unknown(X)>}hypothetical protein [Clostridium botulinum] >gi|1184018772|gb|OSA94314.1| hypothetical protein B2H93_09185 [Clostridium botulinum]
{(MLLELAQSSRKIIIPLSIWDGAAKECYYNMEYAFKARLQNDNYITTWTNPNELFKDSNFYNILNKICNEGKDFEEQHNKN)-YKIFISY---
PREKNNDADFIEVLLRRRKLNVFRDESEFEAGRSIqqEIIDKLNESNIFIALWCTEYACSPWCFDEFELALDKYENNENSIKLWIFCLDETRIVPKRERELRTLNVKS-REDIEREIVK-------LLNDL()}*

$4772=216(220):
>gi|1184770266|ref|WP_085317724.1| {|239(0)|<unknown(X)>}hypothetical protein [Derxia lacustris]
{(SGQAATIAMGVARAINSIVIRPADEGMASIFLQSVRLSSADLASRIRWVVGGIHTARAVNNSKGKSGLQSSSQAASRDMV)-YDLFVSHAS-KD-
CKSFVDDLVAELRKRGRIIWYAKEQLKNGDDVaqGIDKGLKASKFVLAVLSKNYFVSGWAMAEVYAELDFSKSTGRKRVLLV---RLNMSQEKLYDDSRLFYALLSANSEDGMANVVDTLESAMSPAQ()}*

$4773=163(184):
>gi|1183551386|emb|SMG51683.1| {|0(135)|<unknown(X)>}hypothetical protein SAMN05660862_0019 [Sphingobacterium psychroaquaticum]
{(MKTFLSYSY)---------G-DDT--TVIRNL---
LTENGIEIFDSIKEMQYGNSLqqTIKNAVNNCDFVFFIYSKMNPHIAF---EVGLAVALN-----
KPIFTVFAGGEEDTFLLDSTYVHAYPTESAKIGFSLELFLKNLSVKKRKRKT(DSSSLPKFYGGGQPIPAKNNFDIIERYNTATGKSG)}*

$4774=240(241):
>gi|1183554735|emb|SMG12895.1| {|0(108)|<unknown(X)>}TIR domain-containing protein [Sphingobacterium psychroaquaticum]
{(MEEKKQEKQIDSNKYSKSESPF)---
VFISHDTRdAEIAEAFSKLLKSVSAGMLKSFRSSDNkgTQGIEFGIewypKIIESLQDSSDVVCLLTDFSIDRPWILFEAGMA--KGKL-NTPIlgiaIGVPLTSVSTGPFAQFQNC--DDDNSS--LTKLVKQ--
LVRKLPNSEPDE(DVIKQQVEIFKIKTQNIIAKRLEKTQNNEETKPLDNNESARLFEEVKLMFKELPTRIENVSTKRTINSRRHLHPAMVEE)}*

$4775=264(257):
>gi|1184127570|ref|WP_085049863.1| {|689(7)|<unknown(X)>}hypothetical protein [Vibrio gazogenes]
{(LTQEHIDSFSRPGHIRIHIAGAKGGDLAEHLVDSINDIQALGAKPQVQWLKGAPKQEESMASRSSKEPFSEMGEAIQPPV)-PKVFFSYAwgaESdERQQrcDEIYQYI---
EQQAWLDVYRDKEsMSIGDSIDEFERQIGRGDFIVLLISEKSLHSAHCMKEMGHIYQRCQR-
QKEefvgRVIPVVLSDA-
KIDSLKDRLSIASYWKKecTELNAQVEDLGAAIAGAESIKEL(EIMRSFLDSCVNTLKWVSDLVTERHPDLQVDATVELI)}*

$4776=171(180):
>gi|1184754380|ref|WP_085302414.1| {|0(326)|<unknown(X)>}hypothetical protein [Mycobacterium koreense] >gi|1184188573|gb|OSC35026.1| hypothetical protein B8W67_04320 [Mycobacterium koreense]
{(M)---LFISYASQdKAVIETLVT---TLRRSGQ-
RVWLDEELGGGETWWrAILEQIRRADLFVVAVSEHSLASKPC-QaELRYAQALKRP-----VLPVQIGAV-
DSLRLTPLATTQIFDFRhpTARSREQLLAAASTLIGRDAPLP(TPLPAEPPVPFAYLMRLATTMSSPELSRYQQAELVTELK)}*

$4777=250(257):

>gi|1184232295|gb|OSC69659.1| {|161(779)|<unknown(X)>}hypothetical protein B5181_11920 [Streptomyces sp. 4F]
{(EPVTQQLGKLARWIDARIASSVFGTTYDRQRYWTLLVGATGLLAAVCCLLLRNALLRATAETDPFADPPGAHGIPTEPTG)--KVFLSYSRKdSDRARRLCACLEG--RLG--ELWVDwQAIRPSEQWReSIAEAIRTADAFVVLLSRDALASTYCRDECRQAIALRK-----RILPVVIDPE-LEPGTTTGLMREHGWEDltEYQNLSLTEPGDEELAQAVEDIV(SFVHQHHRWVAFHVRLGVLAHQWWESGRNDGLLLRAD)}*

$4778=279(289):
>gi|1184529017|ref|WP_085099026.1| {|10(274)|<unknown(X)>}hypothetical protein [Mycobacterium fallax]
{(SGARAQRAAGYAPIGASCTRLSPRHQPLRRALVLGQFSGVLCTQVSARRGDGEPDRFYRHTSGQRCRIELTRSDRRATVA)---VFISYSSRdAQIVGELSAAL----RRAHHDLWIDDQLRGGDAWWrAILAQIRDCDVVLAALSQNMLASKAC-QaELRYAQAL-----GKPILPVQVGPlT-SVRTNPLAGMQMIDFQDrsIDAWMALD--EAVRAKRSAPPPL(PDPLPSEPQMPFGYLMRLGGQIGEPDLTAQQQLLIVAELKAALEEDGDDDGARQDIRELLAQLRDRGDV)}*

$4779=248(257):
>gi|1184712021|ref|WP_085271130.1| {|0(300)|<unknown(X)>}hypothetical protein [Mycobacterium parmense]
{(MGLRLIGRATDNSDADKMPTWCATFAYRPRGPLVDRAQRKSFGGSRIGHPVRKQSGPRVCCVSSAAGPSSFEGVHDMG)---VFVSYSSRdKEAVTRLTQDL----RDADEQVWLDQRLAGGDAWWhAILEQIRGCDVFIFALSQNSIQSKPC-QaELHYAQNL-----GLPILPVQVGPV-DSMQLNPLATVQTVDYRtsTSTAGMRLLSALHRARAQRQPPP(SPLPAEPPVPFEYLIRLYTAIAGTDQLSPRDQAALVTQL)}*

$4780=196(192):
>gi|1184768916|ref|WP_085316375.1| {|0(367)|<unknown(X)>}hypothetical protein [Derxia lacustris]
{(MVSR)-KSVFVSYSgriPSdKDFAKRLLVRLC----EHFLDPWvfeqRGGEVPLGENVDeYCRKKIRQADLFVAIISESSLESESTRSEVAFALKFLDP-REIIQISTAVRPH-DSWDDPYRQLTPYKRIEviANDSADLERCIEDICCHAGINYL(GPNNGSPRLPLISRLTEEVKAAKPRYSRLEVGIFSELRLLALHASQAY)}*

$4781=163(170):
>gi|495254048|ref|WP_007978803.1| {<Proteobacteria(B)>}molecular chaperone Tir [Pseudomonas sp. GM33] >gi|398147986|gb|EJM36677.1| TIR-like domain-containing protein (DUF1863) [Pseudomonas sp. GM33]
{(MP)---VFISYRHMDRAHAVKINARLIQANIK-TYLDVLDPesQTTDDITGVITRNISECTHLLAVVSEK-TALSWWVPfEIGEATITN-----RRICSFKTGPTElpLYLDKWPKLTSDRDID--FFIDAYRNEATLKRSMSLESIT(GSESVRSVNRSNADRFHAELKSRIIRGF)}*

$4782=234(219):
>gi|495084548|ref|WP_007809372.1| {|0(245)|<Bacteroidetes(B)>}SEFIR domain-containing protein [Flavobacterium sp. CF136]
>gi|398070856|gb|EJL62140.1| SEFIR domain-containing protein [Flavobacterium sp
{(MNKK)---IFISYSwgN--SEDQEWIVNLATRLMNDTVDVVLDRWSLKDGHDIysFMEEMVKSEEIFRVLIISnkkyteKANIREGGVGtETQIITpNIYSKEkQEKF-IPVVLerdengEPF--

LPIYLKSRKYIDFsrVESFEDSYEELLRniLEVPSIPKPKLGI(KPPVYITERTVNLSETNNKLRTIENQLKK
NNSVSTKELNNFIDLFLENLWNFEQKDTPNNFNLYGEQLFNSLISY)}*

$4783=192(171):
>gi|495271371|ref|WP_007996126.1| {|0(175)|<Proteobacteria(B)>}molecular chaperone Tir [Pseudomonas sp. GM49] >gi|398181781|gb|EJM69329.1| hypothetical protein PMI29_01952 [Pseudomonas sp. GM49]
{(MSYVGEPFT)-
HDLFISYSHGydrghgvghlqpwsAAFAQELEQeLRTQPGVRELLQIFRDGDHRPgcgvdPMAPLtqQLREKL
NHTALLVILMSPDYLKSSWCSDERQWWHEHQTDLaltCEERIAVVRIW---
PTKNDVWPKLLTDERDHPLVGFHFHAPISGEERPLGWVDI(PAFGSDFKKAIIEIVSNINPKL)}*

$4784=173(179):
>gi|498260205|ref|WP_010574361.1| {|0(43)|<Spirochaetes(B)>}hypothetical protein [Leptospira kmetyi] >gi|529321821|gb|EQA53964.1| TIR domain protein [Leptospira kmetyi serovar Malaysia str. Bejo-Iso9]
{(MK)---AFISYSTLdkyvaAKIQNILDRFGIES--------
FLAHEDIQVSSEWqsVILNELKRSDLFVALLSKDYETSPWCEQEagIAAFREM-TLVFLSLDGTIPKG---
FVGKTQSAKVREETLSICDLIPGIIRCNFSFGTNLIVDLI(GKSHSFRQAEKNFQLILPYVSQMKKSQIKELL
KRSAN)}*

$4785=174(175):
>gi|1185394817|gb|OSI16669.1| {|0(57)|<unknown(X)>}hypothetical protein BV912_11395 [Neisseria sp. 124861]
{()MIKCFLSHSSKdKEHYVRIVS--SNLRRD--
AQKFDEITFEKGMGNIeEIKSSLDDTSLFVIFLSNHSLNSDWVKNELAYAKEKFDS-NQInRIYPIIIDNN-
ISYSDDRIPEWMKKDLNiqSLQSPKIAARKINVRLTEISWKF(HPKLKERQEIFVGRNELIQQIEERLDDFDQ
PYPKA)}*

$4786=206(203):
>gi|1185447851|gb|OSI68570.1| {|0(553)|<unknown(X)>}hypothetical protein BSZ22_20595 [Bradyrhizobium canariense] >gi|1185457425|gb|OSI78018.1| hypothetical protein BSZ23_19595 [Bradyrhizobium canarien
{(MMTGTSPNN)---IFISYGR--
QDAAEFVDKLVKDLQRAGFAVWRDTAELQSPRAWdaQIESAIKQADVVVAVLTPHSVRSGRG-
SESDGADSVcLDELaFARFsppptpIVPILLRHC-
EPPFVIYRLQYIDFLGATEDEARYGVALDELIRSINGLKT(GIRPSYRGPKLEPLDFDLYLNLKTRGFVGREW
LIDELFERLQRADGSTAVILAC)}*

$4787=233(221):
>gi|488095539|ref|WP_002166936.1| {|0(245)|<Firmicutes(B)>}hypothetical protein [Bacillus cereus] >gi|401220039|gb|EJR26686.1| hypothetical protein IIG_05243 [Bacillus cereus VD048]
{(MENTIHSNLRPTK)---IFISYAwtS--
KVYQEKVIKFAQRLRENGIDVVADFWDLNPGNSLfaFMESMVTDPTIdKVLVLCNKIYADKaaaytGGVGtET
QIiGPHIYNQVrQEKF-IPIILERE-
ELdteyiPVHMNGLIYIDLDRDFEKGFEELLRlLYNKPLLRKPELG(TAPSYLFEEKKTRFRVDGILSSMQDA
IIHRPGRIASLSYDYKRAFLEDLEEYRIMELSEPYDEQIYNL)}*

$4788=236(224):
>gi|446359386|ref|WP_000437241.1| {|0(247)|<Firmicutes(B)>}hypothetical protein [Bacillus cereus] >gi|401242746|gb|EJR49118.1| hypothetical protein IIO_06602 [Bacillus cereus VD115]

{(MEQNKEFGSRPSK)---VFISYSwtS--
PDYKEKVISLANRLMENGIEVVIDAWDLKPGNNMyaFMESMVTDPTIdRVLVLCNKSYAERassynGGVGtET
QiIGPEVYTKAkQEKF-IPVILERD-
ESEEDyipvhMKGLIYIDLHRNFEEGYESLLRlLYEQPLLRKPKLG(SPPAYLFEEKKTKFKVNGVLSSIRDA
AVRHPSRVKGLCRDYKQAFIQDLEEFKLTYEELSEPFDEKITNLI)}*

$4789=221(225):
>gi|447146539|ref|WP_001223795.1| {|43(0)|<Firmicutes(B)>}TIR domain-containing protein [Bacillus cereus] >gi|401276350|gb|EJR82306.1| hypothetical protein IKA_05438 [Bacillus cereus VD169]
{(HIFRIKRETENLKWLSFEDASFYFNLDLMKVEYPTLFGECNWMKGKVKIAPSNATCITTDRTVISFSMTKE
EIESTFPQK)---IFLSHKG--AN-
KPMVRKYFNLLKELGFDPWMDEKDLVNGDPLdrGLLKGMKESCACVFFI-
TPEFKDEDYLAdEINYAKREKKEKrERFNIITLQFANE-
KGEKGVVPDILTYEVYTSPNSELEAFNDIIRSLPIRLGNV(HWIKR)}*

$4790=250(246):
>gi|489877000|ref|WP_003780496.1| {|0(690)|<Actinobacteria(B)>}molecular chaperone Tir [Actinomyces naeslundii] >gi|399903017|gb|EJN85786.1| TIR-like PF08937 domain protein [Actinomyces naeslundii str
{(MKRDFVDKLTSMVKAQWMRWKHRRVGN)-
FDVFISYSHDpsrtlaFALHDSLEKLSRNWYSFRALNVFLDRSSLGPTMRLekELESIVSRSEWLVCICSENA
AKSDPVTKELNWYIERNGVEkliLIVDDGVICWD---REAKSFSQT-TTNIPNEVLSKIKDEP--
LWIDLRGRNESQ(DSLENYPEFLDAVLSISSRVQNTSKETLLDRENKRQNRLVRFRWMSFSVLIIILLTISVAI
SWFAVSARNEALSNLNVAT)}*

$4791=135(141):
>gi|495657939|ref|WP_008382518.1| {<Proteobacteria(B)>}hypothetical protein [Rhodovulum sp. PH10] >gi|402501875|gb|EJW13518.1| hypothetical protein A33M_3502 [Rhodovulum sp. PH10]
{(M)AYHVFVSHGWHDR----WIARQFArairhDAGAE-
PIVDIFDVKKGDRIDDRIRELLPKCEELVALITPW-SVTRNWVWnEIGAAWILD-----KRIVAV-
LYGVTlaEIDvekGGRACLTSRNVVL--LDEfDDYVA--ELAERVKG--VS()}*

$4792=126(144):
>gi|495657940|ref|WP_008382519.1| {<Proteobacteria(B)>}hypothetical protein [Rhodovulum sp. PH10] >gi|402501876|gb|EJW13519.1| hypothetical protein A33M_3503 [Rhodovulum sp. PH10]
{(MGAH)---VFLSYASADRALADEITRSLGEAGLT-
VTRpDTIAAPTDGPSLDALKTALTNADVMIAIVPPTGTPGSNNVSfEVGAADALD-----
KPVVAVtndvsdaidfgRFQPVSakDLPDLVR-----------------------HVRALLKGQ()}*

$4793=242(231):
>gi|495662866|ref|WP_008387445.1| {|0(45)|<Proteobacteria(B)>}receptor activity [Rhodovulum sp. PH10] >gi|402499503|gb|EJW11206.1| putative transmembrane receptor activity [Rhodovulum sp. PH10]
{(MANDSAKSLSAKAV)---
VFISYSRKdEpeppgdgevAWLTYVQSHLAPAVKRGIFDIFTDQDMEIGTDWRkVIGQKLDTCDICVLLASRH
SLASDFILDvEIARLLARQKA-EGTPILPILVSAV-SKVTTPewVWALNVRPKGLkpLALFPpaERD--
AVMAEIVDEIAGI(TTAIAAKKREREDLPDLAAKSFEIDRLRKGLRGEVEAAIARQADVFRQEKFDWGKPVGS
RKLSDHEGAEFLRDAGVW)}*

$4794=146(140):

>gi|495661920|ref|WP_008386499.1|
{|0(389)|<Proteobacteria(B)>}hypothetical protein [Rhodovulum sp. PH10]
>gi|402499954|gb|EJW11641.1| hypothetical protein A33M_2944 [Rhodovulum sp. PH10]
{()MPEtlhIFISYAEEdSELASSFRDALGEAFGPGLLRVSFAPEFALGTPWRkAIEDELNDADILLALSTGQ
QKDSHSYTGfEVGFFRKSVLA-RPRMEQFESDRLI-
VSVSLLGPAPLTLADVQgvVILPSAARGVTLASSDGFVAAME()}*

$4795=219(223):
>gi|447149621|ref|WP_001226877.1| {|41(0)|<Firmicutes(B)>}hypothetical protein [Bacillus cereus] >gi|401657047|gb|EJS74559.1| hypothetical protein ICY_03462 [Bacillus cereus BAG2X1-3]
{(REYLDIISNYTKTSEWLECERNTFFFNLDKIKQEYKGEFDDCTWMKGKIALNPEFDNQKNRVIVNFSATKE
EIELNQPMK)---VFLSHKG--A-
DKDIVREYHKLLFELGFEPWLDEDAMVAGTSLdrALLQGFEESCAVVFFI-
TPNYVDDGYLStEIDYAIAEHRSRnEKFSIITLMLPDK-
DGKRGTIPGLLRRFVWKEPKNSLEAFKEIVKALPIKVGEA(IWR)}*

$4796=167(163):
>gi|488115166|ref|WP_002186563.1| {|0(35)|<Firmicutes(B)>}hypothetical protein [Bacillus cereus] >gi|401294643|gb|EJS00270.1| hypothetical protein IKM_04449 [Bacillus cereus VDM022]
{(MA)-YKTFISYKYSeakelrERILDALGADAKYY-----------
QGETSESPDMsdRtteyIKEKLKDMIYStsvtIVIISPNMKKSKWIDWEIEYSLKQIKRGdkkSNTNGIVGVV
M---KYNGDYSWLRTMITNSDGHTAIRINNNYLYDIIINNRFNQ(EPKEYTCEICKDIDSFTGSFI)}*

$4797=128(147):
>gi|446291924|ref|WP_000369779.1| {<Firmicutes(B)>}hypothetical protein [Bacillus cereus] >gi|401657057|gb|EJS74569.1| hypothetical protein ICY_03472 [Bacillus cereus BAG2X1-3]
{(MDNNKSV)----FISYSWKnakeaRNLANLLKSYNyKPWI---------
DVENLDLTGDLysQISKAITQSNYFVLVFSSLTLDSQWVEFEI-----------NTAEKKLDNN---
NIYYFDLEKKIIQHKINNKSNQENIKFLNTLQVIKNLILP()}*

$4798=198(211):
>gi|499965018|ref|WP_011645736.1|
{|0(237)|<Proteobacteria(B)>}MULTISPECIES: nucleotide-binding protein [Hyphomonas] >gi|114740430|gb|ABI78555.1| nucleotide binding protein [Hyphomonas neptunium ATCC
{(MSH)---VYISHSG---KDPDALLALHEALRAASVTVFYP--------
GEdtsREEADAAIDKAFavIVLVSAAAVRSKWVRQDVEAAKALGLKR-----IPYQVDKARlnG-
FFKQEVQPDLRLSSsmP-DGLAQLVS-
ETQEAYRKKCPVI(AVMNLKGGVGKTTVSGQVFGAWQAAFGGRVLLIDLDPQYNLTQTFYPMELADASSAQDR
SVISLFERS)}*

$4799=209(217):
>gi|501818303|ref|WP_012647786.1| {|0(117)|<Proteobacteria(B)>}TIR domain-containing protein [Geobacter daltonii]
>gi|221565086|gb|ACM21058.1| hypothetical protein Geob_2708 [Geobacter daltonii FRC-32
{(M)--KVFLSHASSEKKMAKTLRRKLEVAGHE-VSTSD-
SMEQGDDITSAIGKLIERAEAFVIIVSPKGLGNERlmweWRTvLENSWKEQG-----RPIFGVLVGNVVP-
PPFLMGRQLVTISS--LDDiDTAAE--

QLLGRLQGNPID(EMVVEDLKARGREIISHLSSEIQKFEPSPEQLEKQRKRLQEKIHELSAAPASGDLARLNL
KLVNVIKTLAEKGLQV)}*

$4800=179(179):
>gi|493409808|ref|WP_006365795.1| {|0(274)|<Chlorobi(B)>}TIR [Chlorobium ferrooxidans] >gi|110341190|gb|EAT59658.1| TIR [Chlorobium ferrooxidans DSM 13031]
{()MIKVFISHSSQdKKFLDRLTT---ALKNDGV-
QTWRaDKEVTAGENIQeKINDAISNTDYFIVLLSKHSTSSSWV-NfELSAILNREVS-KQQRIIlPVLIEDC-
DIPFSLRDRLFADFRFSfeEGYEKLIRALKQKETKSYQGVKR(EVEKFQPDSYEFQIRNLREEYNRGNLSLFC
GAGISYDAG)}*

$4801=157(157):
>gi|499925912|ref|WP_011606646.1| {<Actinobacteria(B)>}MULTISPECIES: TIR domain-containing protein [Frankia] >gi|111152459|emb|CAJ64195.1| hypothetical protein FRAAL5562 [Frankia alni ACN14a]
{(MEQPAE)-
ASIFLSWCHGdLAAKEALLSHLHLGTLAGVrIAWWEDSHLLLGADWRrQILARLAACDYGLLLLSPGFFASRF
ILDeELPRLLG---P-DADKVLPVMLKRV-
PLDGSRDLHGLDTRQIFtgSGGRCFTETRGAGRERFALDLTT(AIRHRVLSDAA)}*

$4802=208(221):
>gi|497537476|ref|WP_009851674.1|
{|0(69)|<Proteobacteria(B)>}hypothetical protein [Mariprofundus ferrooxydans] >gi|114552593|gb|EAU55053.1| hypothetical protein SPV1_06909 [Mariprofundus ferrooxydans
{(MSNR)-KRVFLSYCHAdIEVALDIERRL----
RDDSIEVLSSFDLDPGSNIQtNLKYFVESSDIVLLLLSKDYFNSVY-SGkELYEFLNESNK-
RKVSVIPVAIERCNiPWQL----------KNLntINLYNNID--
SGIESLKRQINVL(THVSFDNFSAENFEEFTYLLLKEYGFKNIRREFHSSDYGIDFVAEAFTKNPFGFTQKET
WIVEVKFYRNERFSINTI)}*

$4803=209(218):
>gi|501818327|ref|WP_012647793.1| {|0(258)|<Proteobacteria(B)>}TIR domain-containing protein [Geobacter daltonii]
>gi|221565093|gb|ACM21065.1| hypothetical protein Geob_2715 [Geobacter daltonii FRC-32
{(MAD)---IYIVYAK---ENKKIAIMLFELLSQQ-WDVWIDKKIVG---SFrqAIEKELPGSGC-
VVAVNSMAARVKATYAaELQIAENYKVPM-----IPVRLDDS-DLPYPFGENSHVD-
MQDWtgeadHSGIMELKCkLASVVPPRVPPQR(PNAIAGGKIPLPNLFMSVSSFETQISPAGAVSVLRTFESK
TILVSAYDLVPRYKKDPQGIIDELKEYQDNGGFIL)}*

$4804=155(151):
>gi|499919105|ref|WP_011599839.1| {<Actinobacteria(B)>}hypothetical protein [Rhodococcus jostii] >gi|110824880|gb|ABH00163.1| conserved hypothetical protein (plasmid) [Rhodococcus jostii RHA1]
{(MSD)RHNIFISHRHEDD---ALVDRLKDLLDARGCDV-
RDSSIYSATPNDahAegyIRTILADCiRWagkIIVIVSPETRNHEWVDWEIEY-
ANRFPDKriiGVWAPGADQCD---
LPEPLADYADAMVSWDAEAIIDAIGGADNWQAPDGSPGPE(RVISRVGC)}*

$4805=275(278):
>gi|404251504|gb|AFR54116.1| {|63(0)|<Mollusca(M)>}myeloid differentiation factor 88a [Mytilus galloprovincialis]

{(MTKEMRKFEQHESPTTALLNDWSTRLKMSPTVDTLLKYLVEIQRPDVVLDSEKSIRRDVEHSKQPARAPLP
PKPKVPESD)RYDAFVVYgntERDIQFMKDMVNILEGEDHNIRLYVPGRDDLAGDEKYVVTAEMIANrCRRVI
VVLSRGFEDSEDCDFALKLAQSLSPGAKTKRIIPILLDNVK--IPLILNFVGTVNFTN----PMQRE-
WVWPRVAATIKCP(LVPSIQDWQCTIDDLKNMKYDSKHVQHLLYGIGVACQEFPPEEEEEKKGKKGEKKKKT)
}*

$4806=208(227):
>gi|405974572|gb|EKC39207.1| {|42(0)|<Mollusca(M)>}hypothetical protein
CGI_10024612 [Crassostrea gigas]
{(KICDYIWQEPVQLKDVNDLVNKPEELFEVTQKATQKHRNKPLKNEQKTQLPHSIMSGRGSSNTPDVMISYN
LGSKEAALK)---------------------
IRDNLQQNGITCWIDvdveaDSYMAGSLQEAIAAAIENCTVFLTCYSEKYSQSKLCEKEAEYANSRK---
KTIISCNLEADFDPSGTLNTIIGSKLLFNFSGKYPFEKQMEKLIREIRLHLKDN(DNQARML)}*

$4807=199(198):
>gi|405957682|gb|EKC23876.1| {<Mollusca(M)>}Toll-like receptor 6
[Crassostrea gigas]
{(MCQSDAWLIGSTFALFFVLTILLTTAVVKRYRVHVDYVILRLRSRWKGVIHVSNSDEF)RFDAFVTYTEEd
yQLACITFYQALTRLGF--
KISIPDKDFLPGISEAEQLLQCIDQSHKVVIIVTENFLENGWNSYAVQMVVTHAFHNKRErSIIVIIKDDIPi
ERLPRD---LKYIWWTIvSLRWPNGIENmdLFWEEIAAAI-LT()}*

$4808=220(220):
>gi|405957683|gb|EKC23877.1| {|5(0)|<Mollusca(M)>}Toll-like receptor 13
[Crassostrea gigas]
{(IYETTVYSILDWVTPEPCTFSPHTDGCGNHGWMWQCGHRNLSVIPNAAIKQYRVHVDYVILRMRSRWKGVI
HVANSDDFP)-FDAFVTYTEEdyP-ACIPFYQALTSLGF--
KISIPDKDFLPGISEAEQLLKCMDQSRKVVIIVTENFLENGWNSYAVQMVVTHAFHNQRErSIIVIIKDDIPi
ERLPRD---LKYIWWTIvSIRWPDGNENmdLFWEEIAAALRTN()}*

$4809=229(220):
>gi|405949977|gb|EKC17987.1| {|29(0)|<Mollusca(M)>}hypothetical protein
CGI_10017728 [Crassostrea gigas]
{(DSKPVVRNQVSLDPSASAWNVSSVLTGSLLFVTISSLILFVVFRKRIEYRVYRVKQNLRRRTRHPGNRGTG
RPFLQEDKK)-
YDVYLSYSEEdyPWVREHLLSLIDEWEteennvtafGTYKMYCSDRDSHHGKSELENMCENIELSRVAVVVLS
SSYTQKYLHTVELEHLLYSKDISVIQDIVIIMIEELKfKQIPTALHH--QIKQDKvY-
RWEAGKTKqkKFYGDLLRALK--()}*

$4810=176(149):
>gi|405953749|gb|EKC21350.1| {|0(1031)|<Mollusca(M)>}hypothetical
protein CGI_10004069 [Crassostrea gigas]
{(MDNGRGSSK)---VYLIYSEEspeerRFAEGALRTRLEGRGLTVR---
SEADFLAGTNLVRGRSGAVQDCSKALILLTENSNNSSWCTLELFLALEKSYRLNKVSLMVLKISASRkWKFQL
ALEMLNVVPRVDlSSFTELSEDEGKFNELARKLQDtsrmediqpvgniahaqvwshytgllS()}*

$4811=248(194):
>gi|405960051|gb|EKC26003.1| {|0(11)|<Mollusca(M)>}Toll-like receptor 4
[Crassostrea gigas]
{(MARICQNDKIEEIQLIPSSLNLDSDETEVKTVLNSENKGELSTIVTKPLPPGKE)-
YHLFMSYSSEdsDDA-NTLRQHLEER-
FHLKCLYYERDFQIGKNIDESITEGLRKSVKTLLLLSPFYIQSHWCVTEAREACKLSFADmENHNVIPVLLRQ
LHkE--------

LPPFLNSYvYIDAQREMDVAGKINKAFNQPAlscngtllirkraikadfynhglayrflpleafeekkicqng
vdlvgdyllsllkrlrqnD()}*

$4812=183(157):
>gi|405960727|gb|EKC26620.1| {|0(284)|<Mollusca(M)>}hypothetical protein
CGI_10011483 [Crassostrea gigas]
{(MDNGRGRSEVYLIYPEE)--------SPEerRFAEGALRARLEGRGLTVR---
SEADFLAGTNLVRGRSGAVQDCSKALILLTENSNNSSWCTLELLLALEKSYRLNN--
VSLVVLKISAsREWKFQLALEMLNVVPRvDLSSFTELSEDERKLDELVRKLqeierliketeyykldpsrfpl
klyelipercncpR()}*

$4813=142(141):
>gi|405963272|gb|EKC28861.1| {<Mollusca(M)>}Toll-like receptor 13
[Crassostrea gigas]
{(M)------SYDEEdrD-VALRLTEMLETKKQK-
KCCLPERDFMPGTNMFDNVINAVHVSRKVVCIMSENYMISYWPMFEFQIgqmdAMHSSYRKNKKVFIAVLLRR
SLrVETMQPAILGVLESDCCiACNSDENILFVCSEKLDNLLQLdM()}*

$4814=160(141):
>gi|405970910|gb|EKC35773.1| {|0(154)|<Mollusca(M)>}hypothetical protein
CGI_10018640 [Crassostrea gigas]
{(M)--------------------------------
ISGRDFLPGTWICKNIHHEMRKSVNVLLILTPSFLKSLWCDVEARLAVQMSDDPNFNlQIIPLKLRDLGaDSD
LPPFLKPFEYIDAQrESDIPARINKALYHIGSELTSSknshsmlndlvhwsrmrkrrknclfmepdaqeevcd
knrknellyklpP()}*

$4815=231(220):
>gi|405970910|gb|EKC35773.1| {|83(0)|<Mollusca(M)>}hypothetical protein
CGI_10018640 [Crassostrea gigas]
{(YIDAQRESDIPARINKALYHIGSELTSSKNSHSMLNDLVHWSRMRKRRKNCLFMEPDAQEEVCDKNRKNEL
LYKLPPGKK)-YHIYISHAAAqeTQA-KLIGKALESR-
FLLRCMISTCDYSSDQTICDNMLHELLKSVSVLLLLSPEFFKSKLCDVEARLAVQLSYGLKFDlKIIPVLLQD
LNaNTELPLILQPFACIDAQrEADCSAQIKEAFYKSGIIVISKkfsgkeidiY()}*

$4816=189(141):
>gi|405971791|gb|EKC36603.1| {|0(3)|<Mollusca(M)>}Toll-like receptor 6
[Crassostrea gigas]
{(M)------SYSSEdrDDA-NTIRQHLEER-
FHLKCLYYERDFQIGKNIDDNITDGLQKSVKALILLSPFYIQSHYCVTEAREACKLSFTDmENHNVIPVLLRP
LPkD--------
LPPFLNSYvYIYAQREMDVAGKIYEAFNQPGfldplqmnksglpcngtllfrkigtrakyldhglaqcvrrle
nlpvafldttvicrsrsP()}*

$4817=143(141):
>gi|405978563|gb|EKC42943.1| {<Mollusca(M)>}Toll-like receptor 13
[Crassostrea gigas]
{(M)-----ISYSPKelSFVLTDVIPRLEN-
EARFQLYVKDRDEPLGIPLGGAIMEAIQKSKRVVCLVTRSYLKSTWQDYELNMSRMEAIEDREnlNFVHLILF
PDVYnGSIS---IQTLDLVRQEcFSEYPEEECAfdDFWETLIAHIKEldrC()}*

$4818=197(146):
>gi|406943639|gb|EKD75595.1| {|0(662)|<unknown(B)>}hypothetical protein
ACD_44C00090G0001 [uncultured bacterium]

{(MTSYAK)---AFVSHSSSdkP-LLNEVVKQVSAARWE----
IDSLSFEEGRTSANEIFQSMSRSSLFVLFASQEAMNTEWVKSELEIAQHLYYAKKIGGLLVFIIDEMPaQSLP
D-
WIRLHVFARTKnVTRIANMIRSRLMQLDSLRGAEqrpfvsrsnlrseiekklsdlsnpvraiyvsgtdgigrr
alvsnslqtllpgsdamG()}*

$4819=254(257):
>gi|762107823|ref|XP_011436527.1| {|962(0)|<Mollusca(M)>}PREDICTED:
toll-like receptor 7 [Crassostrea gigas]
>gi|762107825|ref|XP_011436528.1| PREDICTED: toll-like receptor 7
[Crassostrea gigas] >gi|4
{(LCENNGKEMIRVAPRYFNCLGKTAARSSDHKGVIVAAVILLCAALLVFASCIYFRRDIIAVFGTKLNIKCF
RHQYEAHSI)-YDVFLMYDFNdvkgsDWANKELIPRLNQFGHKLTTSDST-DLANSLLETENSK--
IKESKCALFVVTKYVGNNQYYMNCFRVAAKHAREHSQFKVIVVVVGDI--
DFTSLEPGLKKMLVSGnYITARSRC---
AWERLEYELPKR(RSRLEPFSQDSEIGICDTETIIYNAARKDKGDECDVE)}*

$4820=179(181):
>gi|405952907|gb|EKC20661.1| {<Mollusca(M)>}hypothetical protein
CGI_10005641 [Crassostrea gigas]
{(MQSLGYEWTIGLRVRSSTTFWYVAPRYVMAYDEGTDVDITH)--
DVFLIHANEpkedkVFALLKLKPLLER--
QNLKVLVDEEDFLGGETVFTNIARAVRRCQKSLIVLTEHSRESPWCSLELLLALEKSHRRNIMSVVVLRKGNS
-VEQQLKGNELKNC---ClLGTLRMHKFGLITLDCCSIFFQV()}*

$4821=287(287):
>gi|762128024|ref|XP_011447106.1| {|314(0)|<Mollusca(M)>}PREDICTED:
uncharacterized protein LOC105342021 [Crassostrea gigas]
>gi|405969326|gb|EKC34302.1| Sushi, nidogen and EGF-like domain-
containing
{(QYGFVGKICNETILESDVEEQVSGGSSNWYAWMLGVCALILVVLLVTVVIIPYLLWRRGFILIMKIVHYLQ
PTEDNDETM)-YDALIAYSAEsdrdaTVATDVIYPYLRHQCG-FKVKLRGNQKASEDD-----
NGGIQKCHRLVLLVSSDFIYREWPLLSSEIS-EESFERMRKRIVPIFFGEA-
SKlCYSADKRFQDLVSKSvFITWsglekDKTK---
NLTRVKYSLPKK(TRFPKETADKEPDRKENPDDSGLQKDPENGTIHSNGGNHLLWKDKIDFLQSVDFNHNPYN
MPYFKGN)}*

$4822=263(262):
>gi|405978517|gb|EKC42897.1| {|0(207)|<Mollusca(M)>}hypothetical protein
CGI_10028870 [Crassostrea gigas]
{(MAGNHDSGALLHHESLVKSDFTGVCEENSCISHYDSDDASFVDCIHFDPPEDSLTPTESVEVDIFYTAPPL
KDLY)RYHIFFSHSLEdsKWISG-LVHRLESEPYSYKC--
HYNDSVTQtkQNVHQTLMSAVILSERVVIVLSPSYVKSNWN--EFQELLQNltTCSLYQQRMLVVLLKDC-
NIPDHLQPLGFLDARECdFFDCLVRHLRSAVGNGQVLSTAH(ISFCGAWNAHLRYTEDDDVPTSLCCHGIGMN
YARYMEILKSMTSVGG)}*

$4823=120(149):
>gi|405970911|gb|EKC35774.1| {<Mollusca(M)>}hypothetical protein
CGI_10018641 [Crassostrea gigas]
{(M)-------------------------------
LFDRDFLPAKPIDVNVQHELEKSVKVLLLLSPDFLKSQWCDMEARLAVQMCYDPNFKLKIIPVLVrglNVDSN
LPP-FLKPFRYIDAQkESDCPAKINEAFYQIGNTANLIN(IFSNFFKP)}*

$4824=162(193):

>gi|405951545|gb|EKC19449.1| {|0(242)|<Mollusca(M)>}hypothetical protein CGI_10008481 [Crassostrea gigas]
{(MIGK)----------------------------
KGIHAWTADSEATAGTDIfnAIGRIIVDCKIFVFIMSDSSVASKLCQDQLALAYVSK-----
KNIFPVQLNEREDLLAHMDNGMKLQLAGFEWFSLNNDLESESQEFVDRVWEE(VQSEIEQEKADGSKKETKNP
GENKATAARKTFKRQKTLTNLNKSLSKTK)}*

$4825=152(159):
>gi|762135078|ref|XP_011450781.1| {<Mollusca(M)>}PREDICTED: myeloid differentiation primary response protein MyD88 [Crassostrea gigas]
>gi|405958985|gb|EKC25063.1| Myeloid differentiation primary resp
{(MKVDFD)-YDAFVICNPEgkdLEFANKLVDTMQASPYNLKLCAPWRNA--GACSPN-
TRECTRDRCRKfLVVLSNNFYKSEEAVSHLHFAFSMSPGYKERRILPICLE--RCEIPTYLQLVTKLDVSN--
SRTRK---WMWEQLYLSLKPR(ETGTHSEVCEQQH)}*

$4826=210(220):
>gi|405960727|gb|EKC26620.1| {|257(0)|<Mollusca(M)>}hypothetical protein CGI_10011483 [Crassostrea gigas]
{(EKLREIDIQLHIKRFYYTLGGLLDDRSFTRTYDRARVITYDEQQSGFVLADVVLNEVKKDLCSPNEKKIPP
QHFQHAEYE)-FDAFVFIHEGdDNTSDEITRFLEGKGVKLM-----RNDRIGQSILNISD--
VIKRCRWiIPILSKTSLKDPVFAHCCLMLVGDILETKELRMIPVLTgGAAYEDVPEVIRMVTL---TV--
ADNPEYLNRLHQTIKGTTDVI()}*

$4827=202(203):
>gi|405969477|gb|EKC34446.1| {<Mollusca(M)>}Myeloid differentiation primary response protein MyD88 [Crassostrea gigas]
{(MVPLDNQIN)RYDAYVIAGDteEdKLFLETMKSNLESSAFNLRLYIKSRDG-
PGDFDHQKESEIIMNSCEKiIVVLSRDFTQNEQCMYLLRVAISKSPGFHLRHIIPIHREECL--
APSCIRFLTANDYTK--
EDIRDLCESDSEDEDEDSSFE(SVVFDFSTEDIPKHEASRDCLLIFFKRRAAKPRRQHGGSTVINKNMQKMTK
NTF)}*

$4828=267(271):
>gi|405975511|gb|EKC40070.1| {|91(0)|<Mollusca(M)>}Myeloid differentiation primary response protein MyD88 [Crassostrea gigas]
{(KHLQTIGRSDDVITECAHLIKKDVDRYKQCHKDITGSKDMIQDPSVSQGPNRPSCDYVPESDKLGLVTIDD
VKEKGDTLY)-
YDAFVIYNPvgKdLEFVKELAGKMEAPPYNLKFCIPWRDDLPGGSRYEVSAHMIATRCRRtLVILSPDFLKSA
AADFQLKFAHCLSPGARSKKVVPVFSAPCK--MPDILRAVSFVDFTN--PGLRD---
WNWPRLNAVLRCP(LNPDPRDYMSEAELEELKLNAGVITKRMWYSTGVLTMNFPEESEDNTPYNG)}*

$4829=205(209):
>gi|405975513|gb|EKC40072.1| {<Mollusca(M)>}Myeloid differentiation primary response protein MyD88 [Crassostrea gigas]
{(MQEFHNDNSDDRVITTGDTLY)-
YDAFVIYNPvgKdLEFVKELSGKMEAPPYNLKFCIPWRDDLPGPSRYEVSAHMIATRCRRtLVILSPDFLKSA
AADFQLKFAHCLSPGARSKKVVPVFSAPCK--MPDILRALSFVDFTN--PGLRD---
WNWPRLNAVLQCP(LNPDPRDYMSEAELEELKLNAGVITTRMWYSTGVLTMNFPEESEDNTP)}*

$4830=244(246):
>gi|405958346|gb|EKC24482.1| {|1347(286)|<Mollusca(M)>}Collagen alpha-6(VI) chain [Crassostrea gigas]
{(NMAICCTRRKGKDFLVPLYFETIQIIPVSISLVQFLDCRVRKEGDTPALKMESVCTRLMLTETEGGESNVS
QADDNVNHD)---VFISYAHKdPKQAQLL---

LDEFEKQYpDMEVFFDRQdLKVGGMWQkGLYYSLDTVKCVVALVSPSYLSSVVCQEEFNIAIYRSQN-KANVASWVKANKR-PNYCSNTASKVALFDFKrmSEIRKKEVYDSYNIKTNSVERKS(SFSVPPLDQSENHVVVSVSQNDLSLA)}*

$4831=189(194):
>gi|406916718|gb|EKD55672.1| {|0(895)|<unknown(B)>}hypothetical protein ACD_59C00077G0006 [uncultured bacterium]
{(MAEINEMDQITPPFAAYAGNE)-PYIFISYSHKdTKIVFE---QIDRYQKMG-FRIWYDEGISPGTNFNrVIANKLCSCSLFMLFVSHNSMASEYV-EnEINLAI--NEK-KELMVIHIKETSI-PPEIKILISKLDHIYMNkmSDEAYMLRIKKLPAILKAGESIN(AELLYKTVMTDYPSIISSCYTNNYSLCNEGVEN)}*

$4832=191(196):
>gi|504772505|ref|WP_014959607.1| {|0(122)|<Proteobacteria(B)>}molecular chaperone Tir [Desulfobacula toluolica] >gi|405108930|emb|CCK82427.1| predicted Toll-interleukin receptor domain protein [Desul
{(MNPKT)----FISHAS--EDKDRFVIDFSSKLRSKGIDAWLDKWEMFPGDSLvdKIfEEGIKNADAFVIIL-SQHSVNKPWVReELN--AGFVKRIsIKTKIIPIVIDDC-EVPECLKSTLWERIQNLESYDENLNRIVQSIFGISEKPQL(GKPPKHIKTVIDVLPGLTKVDTIIFNQSCKITIEKGDKNINLSEIYDSLKE)}*

$4833=151(150):
>gi|405957684|gb|EKC23878.1| {<Mollusca(M)>}Toll-like receptor 6 [Crassostrea gigas]
{(MHVSNSDEFR)-FDAFVSYTEEDyqLACITFYQALTSLG--FKISLPDKDFLPGISKSEQLLQCIDQSRKVVIIVTESFLENGWNSYAVQMVVTHAFHNQRErSIIVVIKDDIsIARLPR---DLKYIWwSIVSIRWPDGNE-DlnkFWEEIVAALRTD()}*

$4834=151(150):
>gi|405961797|gb|EKC27542.1| {<Mollusca(M)>}Toll-like receptor 1 [Crassostrea gigas]
{(MQYEPKENYQ)-FDVFLSFSDEDyaWVVNILYNELTKQG--IHVSFPDKDFVPGISKADELLRCIDDSRKVVFVITEEFLACGWESYVVQMTVTHAFNTHREgSLIILIKNNIaLERMPK---DLKYVWwAVQTVNLSDFQN-NpddLWHHVCTMIRSS()}*

$4835=196(177):
>gi|504911397|ref|WP_015098499.1| {<Actinobacteria(B)>}hypothetical protein [Saccharothrix espanaensis] >gi|407880743|emb|CCH28386.1| hypothetical protein BN6_10580 [Saccharothrix espanaensis DSM 4422
{(MSGY)KFDLFISYarRG-SVQKwllNHFHDKLLECLADQ-LaptpRVYVDRD-MSRAVHWpsSLQHALRHSKIMIQLLTPHYFQSKWCLAEWYSMLERERM-LGLaspdrpqgLVYPILYSDSdnfPLEGKVRSWQNFKDFAHP-DPPYQETREfvRFHREVNRVAADL(VALLQQVPPWRPDWPYVDPPDPVLPPTPPIPRF)}*

$4836=180(174):
>gi|504913602|ref|WP_015100704.1|
{|0(22)|<Actinobacteria(B)>}hypothetical protein [Saccharothrix espanaensis] >gi|407882949|emb|CCH30592.1| hypothetical protein BN6_32880 [Saccharothrix espanaensis D
{(MAYE)-FDVFLSYRRMkewpEFVRRHFLpkfDHwLAAELGQE-PRIFYDADVLETGAAWpqRLAAGVSSSKVMVCLWSREYFASDWCKAELGHMLARRNA-VSRLNDPLPLVLAVVIHDGKDFPVLLGEIQQ-FPLQDCASPWIAAGSPKAELLS(DKVMKLAGDVRQAIERAPEHDPAWRDLAID)}*

$4837=198(220):
>gi|504916267|ref|WP_015103369.1|
{|75(0)|<Actinobacteria(B)>}hypothetical protein [Saccharothrix espanaensis] >gi|407885615|emb|CCH33258.1| hypothetical protein BN6_60020 [Saccharothrix espanaensis D
{(GHLPFTPRAKRAMDQAVRQATALGHGRISTEHLLLGVLDEDEGVAAEVLVRSAVSLDDARERLLAVMSGYE
LPAPRIPAA)---VYVSYLRPDDRHVAGRIADWLhhSLGGD--QVVLDAE-----------
EAVEGSDAVVVVVISPQWAPHWPMS------QELEVAAaWGIRIVPVLvDGARMP--
AVLPEGLAALVEPPAIEIGHDTFRQDMTALTERLQ--()}*

$4838=204(212):
>gi|504911401|ref|WP_015098503.1|
{|0(94)|<Actinobacteria(B)>}hypothetical protein [Saccharothrix espanaensis] >gi|407880747|emb|CCH28390.1| hypothetical protein BN6_10620 [Saccharothrix espanaensis D
{(MS)EYDVAVSFAE---EQRSAVEEVVEAFKQRGLTVLHGPEQTHEWWahkeGGdlpD-------
ARVRffVPFVSE--------VDDFTTAALRAVKAGDEHVLPVLLGDVTvppdL-LHPHVTYVRGTVHRP-
DQLTEALA-
ARIETAEAVGRQR(APVGEVVLKAKGSTPAAEPEPVVPATFSRYAEQDATLRYLGEQFAAALPRLEQRGLVGT
AHVGDARIVVR)}*

$4839=216(211):
>gi|504912384|ref|WP_015099486.1| {|0(92)|<Actinobacteria(B)>}TIR domain-containing protein [Saccharothrix espanaensis]
>gi|407881730|emb|CCH29373.1| hypothetical protein BN6_20520 [Saccharothrix espa
{(MS)QYDLAVSFAG---
AQRASAKKFAWECESLGLAVFFDENVTEDMWGRdfvTEFRRVYGGElARyvVPFLSKEYFDSDYPMDELSAAV
TYGLQrRHDPYLLPIVVGDVKipEEfVRSSIGYLQLEKYSV-EQLARIAF-
KRMKGVDSPGLTP(GHWSGPAPAPAGALRLPKVAPIDFSPFGTLESGLARLGERFRQDASSLVRFGYECHART
GDSSVDVRVE)}*

$4840=179(182):
>gi|407881726|emb|CCH29369.1| {|0(223)|<Actinobacteria(B)>}hypothetical protein BN6_20470 [Saccharothrix espanaensis DSM 44229]
{(MPEVNDLDP)---IFLSYRHSdG---
DALAAKLVWVLRSYGIPVWHDQTHLAPGQFHrRLDEALNSGlSGAVLLITPDIVHSEIVRDrELPALLAM-
EA-NPAFTFAIGNVIT-
DAADVLDYGAPDRLLPQpgWTLQGFDQFPVTDHSEMAKLAGE(MAVRRVRHAAPDGQLRLDIQTRLPALGTLN
APL)}*

$4841=222(220):
>gi|407907629|gb|AFU48617.1| {|451(0)|<Mollusca(M)>}toll-like receptor i [Mytilus galloprovincialis]
{(VTCQTKEGDVHKIIDVTESLLDCNYDTEIGLAVGFGILLILVVIGIVWAKRCPYETKVLVFKFFRIHPSDK
YKIDEEVKS)-YDAYISFDDEniHirqWVTRKLKKRLEEKKQELFIPVIHATL--
GEDRADQIVRSMENSKRVIIILSDKYDENEWSVFECQQA--EMLNPNEGRIIFIKYhpeaEDM-VKKEPW---
-KSRVKDRkVLAIGEkssehRW---FWDKLKYELPVK()}*

$4842=296(300):
>gi|498156234|ref|WP_010470390.1|
{|89(94)|<Actinobacteria(B)>}hypothetical protein [Streptomyces somaliensis]

{(IPLLIDEATLPPFAANRVWRDFRNVGEQEYEEKIEELCTVILGDTAGGTAKSSDTQLREENLASALPSPAR
VITEPDQCA)---FVICYAPADAAYGLELAGQLRTAGLS-VW-
SAADLRPGDSQFWTIRQQLAFATAVIVLMSPHSQDSDDITRmILEATL-HQ-----
RPFFPILLEGKRnyhlahtwyvdARDGRLLNPAEIDLL---RELDATRR--
AGRPAAPSPVLP(APLTRPTVGTVRVPPDVSLERLDSYLAEGELAHADIHTTAVVLEAANRLDEGWAGARHVH
GIPASVLAGIDAIWADHTHG)}*

$4843=262(261):
>gi|408506242|gb|EKK07957.1| {|873(5)|<Proteobacteria(B)>}leucine rich repeat protein [Acinetobacter baumannii Naval-72]
{(FYRPKNDSSSINFIPEDLYISIDNLNYVNYNDLIHHNISMPLIKSSTFHKTDNFIKQIFPNKEIAVSPFSI
FTEKEFTQV)-KNVFISYSRRdVEFKDDLSLYLKTTFEGSGHEVWDCGDLDIG-
KWDdQIQLKLEESDLVIFMLSINFFASKYIIEnELIPVLKAAEE-GKKKIMCLIVKSF-
PWDTFAQLGKKAQLKDPnlTNTDQFENQIVNAKSEITNYQFL(PYTRTITSSINSDNCEQLTPLNHLSQPERE
KIYGDIVKRVA)}*

$4844=287(297):
>gi|497242264|ref|WP_009556489.1|
{|358(713)|<Cyanobacteria(B)>}hypothetical protein [Oscillatoriales cyanobacterium JSC-12] >gi|410712073|gb|EKQ69579.1| WD40 repeat-containing protein [Oscillatoriale
{(KLIKILNQDAGYYEEHKILLTKALKWDRQHRNPSILLRGYNLRHAEAWLKVAHHREQHHPIPLIEEFIQES
LRQPEGVPL)--DVFVSYSRKD-SEFARRLNDALQIHGK--
TTWFDQENIAAGspDFQqEIYRGIENSDHFLFIISPSSIGSpyCFG--------EVDYATrLNKRIITVL--
AESVKLESLPPALATVQWIDFSR--
HDSaFDASFSTLLRTLDTD(PEHLRTHTRLLVRALEWENRDRDESLLLRGSSLKEIQQWLLASPHKNPQPTRL
QRDYVTASNATEIQRQRATLRLQR)}*

$4845=222(223):
>gi|504883486|ref|WP_015070588.1|
{|95(274)|<Actinobacteria(B)>}hypothetical protein [Acidipropionibacterium acidipropionici] >gi|410823069|gb|AFV89684.1| hypothetical protein PACID_18840 [Acidipropio
{(SPSGPSPIDHVAMRATAKDSSNPIALLVFAPGETDAFFGLIARRQRFGRATTKEAIISLPPPRFDPLGPLP
DGAVRSDGP)---VFISYRQPEGNAQADALEDLLRAAGF--
VVWRDHTDLPPGTTIdRLEQALTGGlSAGILVVTPDIANSVIVREreLPRLLQLDADPNFNLCIANTVARPGV
DSECDYGAPDRLLRLAPARTLAGKKQTNMRQPSGEIEIAC(DLL)}*

$4846=206(218):
>gi|504893693|ref|WP_015080795.1| {|0(63)|<Cyanobacteria(B)>}TIR domain-containing protein [Anabaena sp. 90] >gi|413971558|gb|AFW95647.1| hypothetical protein ANA_C12948 [Anabaena sp. 90]
{(MAEF)--DVFLAHNSIDKP-VVKVIYEALEERGI--
TSWLDGEEIRPGSlFQdAIQKGIPLSKSAAIFIGKSGLGMWQGLE---LKVILSMCLkQNIPVIPVL----
LPGVSDLPPDMLFLKEFNYINI---
YDGEIVNKIVNKRVLD(LLQWGIKGQKPEDYSAEQIKIKPGKIPNKEKLVDLVIQDIYDRKDNPYADATIWFD
AIEKIIDDLVDDLVDAIA)}*

$4847=168(170):
>gi|410527833|gb|EKQ02695.1| {<Firmicutes(B)>}hypothetical protein LCA211_1668 [Lactobacillus casei 21/1]
{(MKREQSELLKSQFLSDDASGTRDSK)EYDVFISYAH---EDQEYAEKLTSAIQAMDLSVWID--
EQSMGWGAsqiQSMDDGIRN-SKfsIVLLSPDYFDKYWTTHEYHSMLIKAKNQH-DLILPIYHNVTAdqV-
KDYNLELADRHALNT-SISTVEEIAHKLHELIASTSST(DSENS)}*

$4848=215(220):
>gi|491050318|ref|WP_004911969.1|
{|79(0)|<Proteobacteria(B)>}hypothetical protein [Providencia rettgeri]
>gi|414092918|gb|EKT54590.1| hypothetical protein OOC_13579 [Providencia rettgeri Dmel1]
{(KDIARKTSELHRYQTQLAKDEENERKKLESERKKQEREQLDFQRKLTREMEAKTRAIQAQISLTKQRISED
NHQEHFDSP)EYDVFISHAS---EDKEsFARPFAEHLKQLGINVWYD--ELSLQWGDslrTKIDQGLAN-
SRfgIVVISKAFIKKNWTEYELNGLVAKEIN-GSGRILPIWheISKSEvlK-FSPSLAdkkALTTSDYTI-
EEMAEQLL---------PMLKE()}*

$4849=254(259):
>gi|497454682|ref|WP_009768880.1|
{|20(337)|<Cyanobacteria(B)>}hypothetical protein [Oscillatoriales cyanobacterium JSC-12] >gi|410709099|gb|EKQ66614.1| hypothetical protein OsccyDRAFT_4402 [Oscillato
{(YLNDLERDVFVGSWQGQTYEDIYPTNPEYVEKYVGYKLWQKLSTVCGEKVTKKQVRGAVERAWQRHSHTNP
VAASSSSQV)-QHVFIScrYQEPDASLAKQLCEAIAAAGHR-ACMTA--
PSHETDWLLQLESNLRQCDYFLVLLSPQA-AVSEMVLeELQHAkkLSRD-----
HRDRPLTILPIWvnCPLNLRLTHDLQTFLQ--
DLPCYEWHSPTDTPPLIQVIHH(RLNGTPSPPLPVSPSSSSIPYLLNPLPSSPPLPTPTADP)}*

$4850=211(206):
>gi|447011683|ref|WP_001088939.1|
{|0(1293)|<Spirochaetes(B)>}hypothetical protein [Leptospira interrogans]
>gi|410019844|gb|EKO86654.1| TIR domain protein [Leptospira interrogans serovar Grippotyphos
{(MNSSKV)-TSLFICYSYDqIDWVRKIGTQIHSIF-
GGEIEAWWDEKKSAGDYWDsTINHRLSEAEAFLVILSPSFFISDYIQNvELPIIIRRHES-
EKIRIIPIVAEQI-
DqeqFNSSWLIDIDVFPHRGlktISQLLPEELDNWIKNLSIEPVWK(RMEKEFKRITMRTLDRPFQYDLRLPN
GTGYIIERNEIEEIENWKAKPNDQKIAFLTGEAG)}*

$4851=259(264):
>gi|497242264|ref|WP_009556489.1|
{|154(945)|<Cyanobacteria(B)>}hypothetical protein [Oscillatoriales cyanobacterium JSC-12] >gi|410712073|gb|EKQ69579.1| WD40 repeat-containing protein [Oscillatoriale
{(DLLAIFERDRVYVRQHTAFLAKALEWQHHHHEPQYLLIREERQQAETWLKTRFQDRQPPCIPTDLHCEFIT
ESTKNANNL)MTQVFISWARDdRALMEQFRR---
SLLRENLTVWVSKTDIQTGADFQqEINHGIEEADNVVYLISPAALKSPDC-QqEIQYALSLNK-----
RIIPLLIRFT-
DLNLIPSELRSLHFINFadNTTQAQHERDVAKLIKILNQDAG(YYEEHKILLTKALKWDRQHRNPSILLRGYN
LRHAEAWLKVAHHR)}*

$4852=205(201):
>gi|488852078|ref|WP_002764484.1| {|0(1302)|<Spirochaetes(B)>}TIR domain protein [Leptospira mayottensis] >gi|410796375|gb|EKR98511.1| TIR domain protein [Leptospira mayottensis 200901122]
{(MYPSKI)-ASLFICYSFDqIDWTRKIGTEINTIF-
DGKINAWWDERKSTGDYWDnVIKEKLSEADAFLLILSSGFFKSDYIQNtELPIIIRRHES-
EKIRIIPIIATKI-
NFErfnELWLNEIDVFPHRGlrTISEILSDEIDNWIKNLSIEPVW(RRMEIEFRRISIRALGRPFQYDLRLPN
GSGYIIERNEIEELENWNVKENDQKIAF)}*

$4853=265(271):
>gi|504892450|ref|WP_015079552.1| {|359(426)|<Cyanobacteria(B)>}sensor-like protein [Anabaena sp. 90] >gi|413970301|gb|AFW94390.1| transmembrane sensor-like protein [Anabaena sp. 90]
{(LINILHQDAAYHHQHKLILTKALKWERQKRNPSILVRNNDLRNAEAWLKVAKPKTQQPSLPIQEEFIAESLKQPPNAVLD)---VFVSYSNAdADFARKLN---DTLQIQNK-TTWFDQEsIISGTDFEeEMYKGIELANNFLFIISPNSISSNYCATEVEYAIKLNK-----RIVTVLYLPV-DVDslHPGLAKVQWIDFRKhgGDFLTNFGELTRTLDADPDYVKN(HTRLLLKSKEWEQQKRDDSFLLRGKDLAASEEWLKESENKQPSPTNLQLEY)}*

$4854=259(264):
>gi|504892450|ref|WP_015079552.1| {|154(637)|<Cyanobacteria(B)>}sensor-like protein [Anabaena sp. 90] >gi|413970301|gb|AFW94390.1| transmembrane sensor-like protein [Anabaena sp. 90]
{(GLLELLTRHQEYVHQHTYFLAKALEWEREQQQSSCLLIGDERVEAEKWLKIRFKEEQPPCEPTDLHCAFICQSVKNANNL)MTQVFLSYSDKeREFMEKITK---TLMRESFTVWTNKTDIQTGTDFQaAINRGIEEADNLVFLMSPSSLASEYC-QqELTYALSLKK-----RIIPLLIQPL-DLDQIPQELRNIQFIDFtdKEEEGKESADTDNLINILHQDAA(YHHQHKLILTKALKWERQKRNPSILVRNNDLRNAEAWLKVAKPK)}*

$4855=195(202):
>gi|10177890|dbj|BAB11222.1| {|0(1056)|<Streptophyta(V)>}disease resistance protein-like [Arabidopsis thaliana]
{(MEPPAARVTPSIKADCSHSVNII)--------CE-ETVLHSLVSHLSAALRREGISVFVDAcGLQETKF-FsiKQNQPLtDGARVLVVVISDEVEFYDPWFPKFLKVIQGWQN-NGHVVVPVFYGVDSLTRVYGWANSWLEAEKLTSHQSKFVYIILFSFVKNKILSN(NVLTDSELVEEIVRDVYGKLYPAERVGIYARLLEIEKLL)}*

$4856=211(231):
>gi|75170592|sp|Q9FHE5.1|P2A07_ARATH {|0(121)|<Streptophyta(v)>}RecName: Full=Uncharacterized protein PHLOEM PROTEIN 2-LIKE A7; Short=AtPP2-A7 >gi|9758984|
{(MSEVAVVLEKQEGGGGGGPSSIQCPMLKSTNYTVWDMRMRSTLKVHKSQE)-----------EDVSKGLINFLEPVLQNKNINVFIDEEEVRGKGLKNLFKRIQDSKISLAIFSESKCD----FNDLLKNNESADE-----AIPIFYKVDATGDLADLQNSVKCKKDLINSAVEEMSKLLANISVGVYREK(EANSKCFMVPARKLQMSHSEKLINWTWSSIYETPNDAAIEV)}*

$4857=219(220):
>gi|498289372|ref|WP_010603528.1|
{|0(90)|<Bacteroidetes(B)>}hypothetical protein [Pedobacter agri]
{(M)--KAFISYSHKdVGYLDKLTVHLAQIKREGLITEWTDQEITAGSSLDdSISSALSSSQLFISIVSPDYIASHYCYEkEFTTALKLQEE-GRITVIPIIVEPC-DWKSTPMGAIKALPDDGkpVSEWTNPN--NAFAKVAQEIRKL(LNIGMVNQGITVDHIKQPTSPGRNYRAEKIFTEVDRLNFKEKSFEVIKNYFKLAMSEFNGIENLQAKLLDEDKKFITCL)}*

$4858=265(262):
>gi|504944024|ref|WP_015131126.1|
{|16(0)|<Cyanobacteria(B)>}hypothetical protein [Calothrix sp. PCC 7507] >gi|427352608|gb|AFY35332.1| hypothetical protein Cal7507_4981 [Calothrix sp. PCC 7507]

{(FLQILEVQKAGYGIRVPVDLVIELEEKQKEVASLEARLIQLQGLQPQEANREFNPQVTQLQWELERNSNES PKHRLPESQ)KKEVFISYTW-RDEQSpKVVAQIEQAFQETDIKIIRDSNTVGYKG-RfqEFMQRLSRGKCVIVVISDEYLKSPNCMYEMVEIEKNGEM-YDR-IFPIFLAdaqiHDPVQALKYIGYWENKTQELDEEMKKVSGANLQGFQEKLNLYT(KIREKSADIIKLLSDMNA LTLDIHSNSGFQTLRTAIENRLNE)}*

$4859=281(272):
>gi|551673955|ref|XP_005839733.1| {|74(326)|<Cryptophyta(E)>}hypothetical protein GUITHDRAFT_161280 [Guillardia theta CCMP2712] >gi|428183896|gb|EKX52753.1| hypothetical protein GUITHDRAFT_161280 [Gui
{(RKPVFATKLDSYFNPTGSVAAICKAYGTWFNSWDELAKELKVADSFTFEDADTVEKENSFEETKAGESPES EKNEELEQR)-HDLFFSAC--EDEGRLIAKHLEQDVLVQGLFSHIQRSTGHMDR---DMDLIKRSKLVVFVITEGTAWSSLQLIHLEAARSWKRQ-----LLPIkregvplgdgwlslalagklYYEIDPSDLSKLRVPFKENPNSPIRIADSTPAIEMLRAAQALMLLA(NR KVIDYDHLFEERNEQIVSIYKQEALAAGVKEEEAETICNHLDVSPASSVL)}*

$4860=284(282):
>gi|493566253|ref|WP_006519590.1| {|133(317)|<Cyanobacteria(B)>}hypothetical protein [Leptolyngbya sp. PCC 7375] >gi|425757588|gb|EKU98442.1| hypothetical protein Lepto7375DRAFT_7727 [Leptolyngbya sp.
{(CVIGMNQPIGDVAAISFARGFYTALGHSSSYEAAYEMGRTAIDLDDIAEEVATPVLRTRAGKVIDDRFGST ASPSDSRTA)TRRIFISYRdEAPDKELAQDFYDAFQAAGH--EAFMAAESIKLGEnWRkRISTELHQCDYFLLLLSPLSATSEMVTEevKR--ARSLRDQReNNRPVIlPIRVQFPMDDP--LNYELRgYLQQIQQREWHSAADTPALLQELRSLMQE(QKAQRDIGPADDETESSVPIRPVADSIDQPPLPVAE PELQREPGGSVPLKSGLYVDRPPIET)}*

$4861=176(167):
>gi|490321160|ref|WP_004210645.1| {|0(34)|<Proteobacteria(B)>}MULTISPECIES: hypothetical protein [Sphingobium] >gi|425711051|gb|EKU74067.1| hypothetical protein HMPREF9718_03184 [Sphingobium yanoikuya
{(MSGDMAMDY)QYDVFVSYrrnsfSLKwieeEFL-PELVPRLElELGREPKVFWDEASVDDGQVIQTRILDALRDSRCLLSLLSGPYFTSIWCSAEWHTFRERAERAG LLANGRTLTIPI-QWHDGDNYLDLLQGGRGpHTLNFSRFRLNGAWRGTPVHVEF(QNELNGLADSIRRLIEGT)}*

$4862=248(254):
>gi|493561655|ref|WP_006515065.1| {|359(897)|<unknown(X)>}putative transmembrane sensor domain protein [Leptolyngbya sp. PCC 7375] >gi|425759806|gb|EKV00659.1| putative transmembrane sensor domain pro
{(RAVDELIRTLRTAADYVEQHKRLLVKALAWERQKRDRKFLLRSGDFAMAQAWLSEPGPPGSSPTDLHTAYI QASQGINQF)-YDAFISYGR-VDSKS-FATDLWQRLKDQGFQVWFDQNDIPLGVDFqeQINDGIEKSHNFIFIISPHSVHSPYCAKEISLALKYN-----KRIIPILQVEEISYATWKDRNPTGSEEQWQAYKAAGKHSSFPNMHPQIGKIN(WIYARDGIDDLTAAIDGLVG LCHRHEDYVHQHTE)}*

$4863=258(258):
>gi|551633291|ref|XP_005819503.1| {|959(77)|<Cryptophyta(E)>}hypothetical protein GUITHDRAFT_121310 [Guillardia theta CCMP2712] >gi|428163453|gb|EKX32523.1| hypothetical protein GUITHDRAFT_121310 [Gui

{(DVIKHKIMIKAITCTRGHTTTVEEVLAFNGSSSPCPSCLKDKRVPHFFSRPKCLSMLARDLTAKIYCDGCQ
REMDVLDVT)PPQVFLSYQW-
GHDNSTqkMVREVKRRIEEQTsLQCWFDVEGgINPGEDHmqKMELGVSRCEVFVVFMSDKYVKSGNCRREFAR
ACEAG-----
KYIIPVLVPVLYEASRDSKDLESESGWKGDKGGEDWWKRIAELEKETSEAAN(KVKVDWTYLASFSKPLEVQA
KREQQEQQQQEKDRGGLH)}*

$4864=226(258):
>gi|551643672|ref|XP_005824629.1|
{|971(121)|<Cryptophyta(E)>}hypothetical protein GUITHDRAFT_116126
[Guillardia theta CCMP2712] >gi|428168708|gb|EKX37649.1| hypothetical
protein GUITHDRAFT_116126 [Gu
{(SPCPSCLKQKTVPHFFSRPTCLSKLARDLAAKIECPTCGMHVDVFDVTPPQVFLSYQWGHKCSTQEMVKQV
KRRIEEQTS)------------------------------
LQCWLDVEGgINPGEDHmqKMELGVSRCEVFVVFMSDKYVKSANCRREFARACETG-----
RYIIPVLVPVQFQPDQDSASGLESESGWKGDKRHQDWWKRISGIEQGEGAAL(EVEVNWRHFKSFPQTFELEA
EMRGEKVLVKRGVQELVA)}*

$4865=258(258):
>gi|551660012|ref|XP_005832774.1|
{|940(72)|<Cryptophyta(E)>}hypothetical protein GUITHDRAFT_108245
[Guillardia theta CCMP2712] >gi|428176912|gb|EKX45794.1| hypothetical
protein GUITHDRAFT_108245 [Gui
{(DILKYKVMMNTISCTNGHETSVKEVLAFNDSSSPCPLCLKEKRLPHFFSRPKCLSMLARDLTAKIRCDGCQ
REVEVLDVT)PPQVFLSYQW-
GHENSTqkMVREVKRWIEEQTsLQCWFDVEGgINPGEDHmqKMELGVSRCEVFVVFMSDKYVKSANCRREFAR
ACEAG-----
KYIIPVLVPVLFEPGQDSDRLESESGWKGDHRHADDWWKRVAVIEGSGGAVQ(EVKVDWRRLADFPKTFEIGA
EWLEQQQQQDKAEGGLNI)}*

$4866=254(254):
>gi|551666850|ref|XP_005836186.1|
{|970(135)|<Cryptophyta(E)>}hypothetical protein GUITHDRAFT_105281
[Guillardia theta CCMP2712] >gi|428180338|gb|EKX49206.1| hypothetical
protein GUITHDRAFT_105281 [Gu
{(LEVEGAMETVLSCSKRGHLSTLADVIDFNNSCAPCPLCVKHNRVPFFFNRMECISKLYGDLTACVTCGECA
GSINVVDIT)PPQVFISYQW-
GHggVTQTLARQAKRFIeTRTELLCWLDVEGgiLCGEDHImKMKLGVERCDVFIAFMSDKYVKSENCRREFAQ
ACEAN-----
KFIIPVMVPILFDERSDSDELRESESGWKGETATDDWWKRISAMEGTREVEV(RWSHLASFQRRVELRAEKQE
EGGEVRADESCLDL)}*

$4867=199(187):
>gi|491910622|ref|WP_005666225.1|
{|0(3)|<Proteobacteria(B)>}hypothetical protein [Massilia timonae]
>gi|425719544|gb|EKU82476.1| hypothetical protein HMPREF9710_02103
[Massilia timonae CCUG 45783] >g
{(MAIRYGCFFSY)AHGRHALMQ---
RFKSALADALRCYLEPYfdnEDELFVDTEQLGGGDDLdrKIARAMCESVCMILIYTPKYEAHAYTRREYEAMR
LIEAERgkwytlPSRLIIPVVMTRHPeslpPQISDARFYVDFSHYTMATPDLMSNPDFLPDIDKMVKRIA(AH
YACQKKHTPLDHDCNQFVLPAVPPEWRDVPELTF)}*

$4868=186(198):

>gi|493561281|ref|WP_006514697.1| {|0(206)|<Cyanobacteria(B)>}Rossmann fold nucleotide-binding protein [Leptolyngbya sp. PCC 7375]
>gi|425759423|gb|EKV00276.1| putative Rossmann fold nucleotide-bindin
{(MNQIYISYAWKDNKTEQ)---------G---RQREaLVDRICAVLLARRYHLIRDRNHLT--
LGQsieKFMREI--GRGNyvILVISDKYLRSEYCMFEALEIMKR--KDDQQRVFPVVLSDANiyS-
REGQVDYIQHWQTKK-
EQLSTLMGPNPSDEYSAMVDVA(NKIAEIATNVDDFIQFVRDKLSIDPAKNFDQFIDHLIGNIK)}*

$4869=138(143):
>gi|504976215|ref|WP_015163317.1| {<Cyanobacteria(B)>}GUN4 domain-containing protein [Pseudanabaena sp. PCC 7367]
>gi|427988088|gb|AFY68343.1| GUN4 domain-containing protein [Pseudanabaena sp. PCC 736
{(MSP)EYDAIVFYSA---DDRREVIEICEKLKEKGIKLWLDIWELRP--GTdwqKELDNVFRF-
AKsaIVFVGASSV-SPWQNLETRAFL-RESTKTMMPIIPVILESAPkapqlpA-FLSYYSWVDFRSKSP-
DPIEQL------MWGITGQKVT()}*

$4870=239(253):
>gi|551633949|ref|XP_005819824.1|
{|1168(0)|<Cryptophyta(E)>}hypothetical protein GUITHDRAFT_175538 [Guillardia theta CCMP2712] >gi|428163790|gb|EKX32844.1| hypothetical protein GUITHDRAFT_175538 [Gui
{(QELVREAIPCPQCVFKGRAPPFCFERERCLLFFSEENRNRVGALFCEYCNNNGDNPNIRVLDIMPPSVFCS
YNWGYENRT)-----------QQLVRSFVKQVEL----
STRVFCWLDIAGGIGGGQdhlqAMQEGVRRAKIFLLFLTDQYCKSINCKFEFCQAVKTGKY-
IIPVLLPEFSWTSHNNQGVEYYQHAAQASED--
ANTAQQGFEVPWRALKRFVPV(PVSPEHISDGYLLQDSEPHFEILRRILSRMYRE)}*

$4871=258(265):
>gi|504949038|ref|WP_015136140.1| {|166(1384)|<unknown(X)>}YD repeat protein [Leptolyngbya sp. PCC 7376] >gi|427357703|gb|AFY40426.1| YD repeat protein [Leptolyngbya sp. PCC 7376]
{(LVDIFERQSNYVRQHTEFLDGALKWERGQKQSQLLLDEEQTLRAQHWLQTKFHGEQPPCVPTDLHCEFITE
SLKRLPNGM)SQLFF-AHANSDTETMQSLRQQMRRAGYS-
VWTPEADIQSGQDVQAEILRGIEGADSFIYLMSSQSLESSD-CQsQLQYAQTLK-----
KRIIPIRLESIGsdTLPANQQKFSWIDCTS--
DEAASKQLAIAQVLKAIKEESS(FYEQHRQILVKALSWDRQKRPKSLLLQGQDFTLAEQWLALSKTEQ)}*

$4872=273(283):
>gi|504949203|ref|WP_015136305.1| {|169(1313)|<unknown(X)>}TIR protein [Leptolyngbya sp. PCC 7376] >gi|427357871|gb|AFY40594.1| TIR protein [Leptolyngbya sp. PCC 7376]
{(ILERQSNYVRQHTEFLHAALVWKKQQKQSQFLLSEEQTLRGQHWLQTKFHHEQSPCQPTDLHGEFITESLK
QLPNGMPQI)---FFI-YIPENSDRMALLRQQLWREGYS-
VWTEEADIQLGKDFDTEVLRGIEGSDSLIYLASAQCIESAE-QQaQIQYAQKLN-----
KRIIPVKLEAIApnNIPNNQQQLAWLDFTG--
TEAATKQLVISQILKAIKEDAG(YYEQHRQILVKALSWDRQKRPKSLLLQGQDFTLAEQWLALSATEQDSRAT
DLQQIYINTSKAM)}*

$4873=243(221):
>gi|551664245|ref|XP_005834886.1| {|0(424)|<Cryptophyta(E)>}hypothetical protein GUITHDRAFT_137268 [Guillardia theta CCMP2712]
>gi|428179033|gb|EKX47906.1| hypothetical protein GUITHDRAFT_137268 [Guil

{(MDAPSGTYE)-WDIFLSFRRcwtgynKEEEIAERIYEELTRKGLR-
VFhvprcLGEREDRHGRDWDRKYLKALANSLVVVLLITRETFTGREFDMkkyvwsskqdellmEWELVLELY-
----DRGRTREILPCLigDLTTLKKQkamsriVDRLGLKN--LDERAMIA--
RMRELFNYVDRD(GSGFIDEEELLEAFTKFGVEISREELSERLQEVDDGNCKLDVFEFEDLLFAVLEDKAGIF
FDGRREKGGRKK)}*

$4874=258(256):
>gi|505012286|ref|WP_015199388.1| {|123(329)|<Cyanobacteria(B)>}TIR protein [Calothrix parietina] >gi|428236973|gb|AFZ02763.1| TIR protein [Calothrix sp. PCC 6303]
{(SNLPMLRLRVKANSQQVQGGYSGSPVFNAETQQVIGAIAAYDNSDGALAVPLSTVSQQWSNFSRFINILPV
VPPLPASEI)-KRVFISYRSEDpdLSLAQNFYEEIRNAGHQ-
AFMAGESIRLGENWAKRIDRELESCDYFLLLLSSKSATSEMVTEeVRRAKELQD-----
LRpekkpgILPIRVNFPIssPLNYDLRGYLNQIQQR--
EWKSSADTAKILQEVLTLLAEE(NPSPNLSPTGEALKSCMIESLENPPLPVAEPELPEG)}*

$4875=275(274):
>gi|504988930|ref|WP_015176032.1| {|32(315)|<Cyanobacteria(B)>}toll-interleukin receptor [Oscillatoria nigro-viridis] >gi|428240946|gb|AFZ06732.1| toll-interleukin receptor [Oscillatoria nigro-viridis
{(IRGTLTEIEAESGVGADTVSKIKQGREGADLSKIRQLFAAFEMTLAASDHRSAKLEEAENLNELDNLPPAG
ETASSSRIV)--KVFVSYRSAepDRELGKQLETALSAIGHT-
VFTAENNIRLGDNWFDRINFFLQECDYLLILLPPTGNQSELLTYqVQQAKELQD-----
SRpdrkpiILPIRVGFPLnaPLNHDLRGYLYRIKQR--
EWKTSADTPIIIEEVLNLLAET(PAPVIEETEDLAQKQLLEISSDEIPLPAAEPELPGGQVDVASQFYVERPP
IEER)}*

$4876=259(264):
>gi|493559497|ref|WP_006512935.1| {|154(982)|<Cyanobacteria(B)>}hypothetical protein [Leptolyngbya sp. PCC 7375] >gi|425755544|gb|EKU96409.1| WD40 repeat-containing protein [Leptolyngbya sp. PCC 7375]
{(GLLAILQKQKKYVRRHTILLKQALYWEYHQKQSGYLLIGKERQTAEAWLTQPFKDEQPPCLPTNLHCEFIT
ESTKNAHNL)MTQVFLAHAEEdISFREQLRQRL---MREN-
ITVWTNKtDIPTGTDFKrALKVGIEETDNIIYILSPAALESTYC-QrEIAYAQLLNK-----RIIPLLVKPL-
DMQRLPPRQRSLQFINFsdNTSLEQHEGDIAKLLRILRHGAD(YHSEHKQLLAKALKWKRQHRNPSILLRGYN
LRHAETWLKVAQDR)}*

$4877=173(181):
>gi|493559497|ref|WP_006512935.1| {|0(1222)|<Cyanobacteria(B)>}hypothetical protein [Leptolyngbya sp. PCC 7375] >gi|425755544|gb|EKU96409.1| WD40 repeat-containing protein [Leptolyngbya sp. PCC 7375]
{(MTHYQDA)----FISYGRAdsKAFATELNDQL---VDQGL-
DVWFDfEDLPLGVDYQkQIDTGIDRADNFLFIISPHSVNSSYC-GlEIERAISR-----GKRIIPILHVEE-
ISREIWQQRNPQGADAAweAYKAAGKHSSFLNMHAAIAKINW(IYCREGIDDLEQSFQGLLAILQKQKKYVRR
HTIL)}*

$4878=252(257):
>gi|493560826|ref|WP_006514248.1| {|154(947)|<Cyanobacteria(B)>}WD40 repeat-containing protein [Leptolyngbya sp. PCC 7375] >gi|425758969|gb|EKU99821.1| WD40 repeat-containing protein [Leptolyngbya sp.

{(DLHQIFHRHQDYVENHTHFLNQALGWERQQKQTEFLLLGEKRQLAETWLKQRFKDEQAPCEPTDLHCEFIT
ESIKNANNM)MTQVFLAYAQVdADVMDNIRR---
SLRRQGFTVWSNQTDIATGEAFKrAVDRGIEEADSMVYLLSPDSLRSKYC-QyELKYALTLHK-----
RVIPILVRPL-
GSAESPPELASLQYIDLtdNLDQDDYQLDENGLIKTLNQDAV(YYNQHKILLTKALKWKRQQGNSSVLLRGYD
LSHAEAW)}*

$4879=254(259):
>gi|493561652|ref|WP_006515062.1|
{|159(997)|<Cyanobacteria(B)>}hypothetical protein [Leptolyngbya sp. PCC
7375] >gi|425759803|gb|EKV00656.1| WD40 repeat-containing protein
[Leptolyngbya sp. PCC 7375]
{(LLDIFERQQDYVHQHTTLLAQALAWQRHQKQSQYLLIGQNRQQAERWLRQKFNAEQQAPCEPTALHCEFIT
ESIKNANGL)MTDVFLSYSEKdRELMDRLRL---ALMREG-
FTTWINRtDIRVGGDFQtEINKGIEEASYMVYLMSPKSLESKYC-QqELDYARKLNKK-----IMPLLMRPV-
DLDLMADDLRALQFINFvnKNDADGSAYGKDIAKLVKLLQED(GDYYQQHKQLLVKALKWQRNEQNKSLLLRN
HNLRHAEAW)}*

$4880=295(298):
>gi|493561655|ref|WP_006515065.1| {|589(620)|<unknown(X)>}putative
transmembrane sensor domain protein [Leptolyngbya sp. PCC 7375]
>gi|425759806|gb|EKV00659.1| putative transmembrane sensor domain pro
{(GLVGLCHRHEDYVHQHTELLMSALAWERHQNQVSYLLMGEDRLRAQNWLLTEFSAEQPPCRPSLSQATFIT
ESIKAADGG)MTQVFLSYAQEdLTSK-ELIRY--NLIQAGV-
TIWTDTvDIHMGEDFEaAIQRGIEEADNIVYLISQASLASEYC-QmEIDQALDLNK-----RVIPILVEEI-
NVSLLPdkIRKIQFvnLTDNQtaVDLEQDVA--
ELLRTIRQDARYF(NQHKRLLVRALKWERQKQNPSLLLQHRMQDTFLAWVQTAKDRVVGGVLPLQLAFLEASQ
MQSPDQTLGVFFIHHIDDV)}*

$4881=259(264):
>gi|493561655|ref|WP_006515065.1| {|159(1086)|<unknown(X)>}putative
transmembrane sensor domain protein [Leptolyngbya sp. PCC 7375]
>gi|425759806|gb|EKV00659.1| putative transmembrane sensor domain pr
{(LQAILTRQQPYVHQHTQLLVRALDWQRQQRQSRYLLTDETALAEARTWLDTRFKDEQPPCTPTDLHCEFIT
ESLKLANDQ)MTQIFISYSEEdMEIQEKVRKRL---MREG-
FAVWINTvDIPTAEDFQvAINRGIEKADNIVLLLSPNSLTSKYC-QqELAYGRRYHK-----RIIPLLVQSM-
DVATLPTDLRMLQFIDFssLASDIHFDRAVDELIRTLRTAAD(YVEQHKRLLVKALAWERQKRDRKFLLRSGD
FAMAQAWLSEPGPP)}*

$4882=248(254):
>gi|493563626|ref|WP_006517009.1| {|165(54)|<unknown(X)>}TIR domain-
containing protein [Leptolyngbya sp. PCC 7375]
>gi|425761780|gb|EKV02633.1| TIR domain-containing protein [Leptolyngbya
sp. PCC 7375
{(NIIHLQTYTRLLARANEWDKQGRPQSYLLTLEDLKEVKKQKRWIETHKLGAQFKFTKIQKVFLETVDRAHE
TSEYFSKSP)-PDIFISYSRSnKYFVEKLNQSLK----
DERWKVWIDRdRIPVAANWRdEAEEGIRYAHTVIFIVCPDSLTSKNC-QwEF----EKAKK-
YKKRIIPVISCND-
YNREVFQAIGLSSVQYVsfVRHGQSFDQSLQQLLDALKTNLD(DLKVYRQVLVKAYEWSDRERSDRFLMGRHE
FKEV)}*

$4883=258(264):
>gi|504945184|ref|WP_015132286.1| {|156(988)|<Cyanobacteria(B)>}WD40
repeat-containing protein [Leptolyngbya sp. PCC 7376]

>gi|427353775|gb|AFY36498.1| WD40 repeat-containing protein [Leptolyngbya sp.
{(LIGLMHKQEDYVHQHTFLLTKALEWDRNHRQTRLLLTGDERKAANKWLSHRFKDEQPPCEPTALHCEYICESEKNSNNQL)-TDVFLSYSEHdQNVMQVVARRL----
RREAQTIWTNTtDIKTGADFQqEINHGIERADTIVFLISEDSLNSIYC-QqELEYAAELNK-----
RIISLLVCPT-
DIEKIPEKWRSLQFIDIanTENPEQYEQGLTRLIKSLQEDSV(YHYQHKAILVQALKWKEQDGNASILYRGFNLKNAQSWVTLAAKH)}*

$4884=265(271):
>gi|504945184|ref|WP_015132286.1| {|360(777)|<Cyanobacteria(B)>}WD40 repeat-containing protein [Leptolyngbya sp. PCC 7376]
>gi|427353775|gb|AFY36498.1| WD40 repeat-containing protein [Leptolyngbya sp.
{(LIKSLQEDSVYHYQHKAILVQALKWKEQDGNASILYRGFNLKNAQSWVTLAAKHPRYPALPIHHEFLEASAAQPPDQSLD)---VFISYSRAdTDFARKVNEALQI---
QGKTAWFDQESIASGADFQqEIYKGIEQCDNFLFIISPDAITSEYCVDevEFAASLNK-------
RFVTILHRPV-
AEEkvHPAFANIQWIDFRRhnGDFNANFSDLIRTLDLDRDHVKS(HTKWLQRSLEWKEKGKTKDMLLRGSEFTLAEAWLKSAEIEQKNPPATELQQ)}*

$4885=292(299):
>gi|504947194|ref|WP_015134296.1|
{|154(631)|<Cyanobacteria(B)>}hypothetical protein [Leptolyngbya sp. PCC 7376] >gi|427355811|gb|AFY38534.1| putative Chase2 sensor protein [Leptolyngbya sp. PCC 7376]
{(DLLSLFQRNRDYVHQHTALLNRALEWDSNSRQPQHLLVDETLQASTAWLKTQFGDRQPPCLPTKLHGEFIAESLKYSQDG)MTEIFLCHAASdQDILGKVDEAIS---
RAGLTAWSSERDIKSGVDFQdAIKQGIETTDNVVYLLSAESVKSSYC-QfEIDYALSLNK-----
RIIPVLIESI-DLKtiHPGIQKLQFI--DLtpLAEGKDGT--
VAMGELLKTIQQE(ADYFKTYKRLLVKALRWERQLQNPSILLRGHELQRYAAWQQVAQKRSQHQPLPLQTEYIETSLKQPPDVELNVFIISHA)}*

$4886=260(271):
>gi|504948923|ref|WP_015136025.1|
{|366(837)|<Cyanobacteria(B)>}hypothetical protein [Leptolyngbya sp. PCC 7376] >gi|427357584|gb|AFY40307.1| WD40 repeat-containing protein [Leptolyngbya sp. PCC 7376]
{(SLLKFLDQDFYYHNRHKYLLAKSLTWQDQREIPSLLLRGCILQQYEDLQKIAEQRKYHQFTELQNSFLDASLAQATTQTQ)-TDVFLSYAPQdIDFATKLNK---
TLEEQGKVTFFAPFHADHDSESDaDIQQGLIDAANFIYVISPDSVAKDDCLAQ----LQRAKG-
VGKRILGILCR------
TTELEAFQGICAEDqlIDFSRHGLDFQAGLSQLVRLLDT(DKAHVQSHTKWFRRAIEWEQKKKTRDLLLRGNELAIAQYWLKESDEGQKSP)}*

$4887=259(264):
>gi|504949038|ref|WP_015136140.1| {|595(954)|<unknown(X)>}YD repeat protein [Leptolyngbya sp. PCC 7376] >gi|427357703|gb|AFY40426.1| YD repeat protein [Leptolyngbya sp. PCC 7376]
{(GLMTLCRQHEDYVKQHTEILAKALSWESHQKQSQYLLVGENRIEAEKWLLTRFTETQAPCFPTDLHCEYITESIKNANNL)MTQVFLSHSDEdQEIEDKVRR---
SLMREGYTVWSSQRDIEAGVDFNaAINRGIETTDNIVYLLSPNSLASEYC-QkEIDYALSLNK-----
RIIPLNIAAV-

DETNLPAAIQSLQYIDLtnGIEDETYQARLNELLKILNQDKA(YFEEHKAILVRGLKWQAQQKNNSILLRGHN
LREAEAWLKLAAKR)}*

$4888=254(259):
>gi|504949203|ref|WP_015136305.1| {|595(906)|<unknown(X)>}TIR protein
[Leptolyngbya sp. PCC 7376] >gi|427357871|gb|AFY40594.1| TIR protein
[Leptolyngbya sp. PCC 7376]
{(RLMKLCRQHEDYVKQHTEILAKALSWQSHQQQSQYLLVGEDRIAAEGWLLTRFTDTQAPCFPTDLHCEYVT
KSIRNANNL)MTQVFLSYADEdNEMEAKIRR---
SLMREGYTIWSSQRDIEAGVDFAeAIKHGLEATDTIVYLLSPHSLGSEFC-QrEIAYAQSLNK-----
RIIPLSIAPI-
DETDLSPTIRAIQYIDLscLDENEEAYRKGINVLLKLLTQDK(IYYEQHKTILVRALKWQAQQKNTSILLRGH
NLREADTWL)}*

$4889=283(298):
>gi|504949203|ref|WP_015136305.1| {|801(671)|<unknown(X)>}TIR protein
[Leptolyngbya sp. PCC 7376] >gi|427357871|gb|AFY40594.1| TIR protein
[Leptolyngbya sp. PCC 7376]
{(LLKLLTQDKIYYEQHKTILVRALKWQAQQKNTSILLRGHNLREADTWLKIAAKRSEYPATALHQEFIQTSL
DQPEDVALD)---VFVSYSRAdSDFVRQLNDALQVQEK----
TTWFDQEsIASGSDFQqEIFHGIESCDNFLFVISPNSVNSDYC-GgEVEHAAKLNK-----
RIVTVLHRSVNpASLHPELAKIQ-----WIDFHNNDG--
DFYANFSELIRTL(DTDREHVRNHTQWSQRALAWRQKDNEDLLLRGSELAIAQNWLDKASRTKIIPLTTALQR
EFIEASHALETRIEVEKTK)}*

$4890=233(232):
>gi|493563626|ref|WP_006517009.1| {|0(234)|<unknown(X)>}TIR domain-
containing protein [Leptolyngbya sp. PCC 7375]
>gi|425761780|gb|EKV02633.1| TIR domain-containing protein [Leptolyngbya
sp. PCC 7375]
{(MSLISRMTQYKLRLAYQRPKEDRLRRIAVVKPRNND)---IFISY-T--
PQDGDFVRRLDQAIRDQGLDPWIDFDDipdfnhfLDGDSTYekQIKDGILGADV-
FVLVLSNAALGNGKIMqGLRLAQRLNKLIvLlyqNSFA-----DIH-
ELTLSLDDLQYLDLNSPLVGKVFEQVACNIIHLQTYTRLL(ARANEWDKQGRPQSYLLTLEDLKEVKKQKRWI
ETHKLGAQFKFTKIQKVFLETVDR)}*

$4891=203(204):
>gi|504942258|ref|WP_015129360.1| {|0(113)|<Cyanobacteria(B)>}GUN4
domain-containing protein [Calothrix sp. PCC 7507]
>gi|427350827|gb|AFY33551.1| GUN4 domain protein [Calothrix sp. PCC 7507]
{(MNQPQFD)---VFLAHNS---LDKPQVRVVAAALKLRGLEVWIDEEQISPGQIFqvEIQKAIQK--
VKTAVIF----IGKQGLGrwqTIELPTLYSQFMqAENVttVIPVLLPDV-
KEIPkdllFLTQHHWVSFANGIDDVEALYQLEWGITGRKPERKT(QPISQPIQPVIPSDDLSSEKGVDYRRLR
DLLAAGKWKEADEETLVVMLKASGREKEG)}*

$4892=273(271):
>gi|504934904|ref|WP_015122006.1| {|129(309)|<Cyanobacteria(B)>}ATPase
TIR domain-containing protein [Rivularia sp. PCC 7116]
>gi|427374498|gb|AFY58454.1| ATPase,TIR domain-containing protein
[Rivular
{(GMNKAIGDKAAIEFAVGFYDALGAGRTIEDGFEFGCTSIDLESIPESSTPVLKCRNTVNHQQTSSSQDNSQ
QDNSQAGKR)---IFISYKR--DVqpDEEIALQIVQALSAE-HQVFIDQKILV-
GTSWaeLIETEIRHSDFLIVLL-SEHSVHSEMMEkEITMAHEFAQERsGKPAILPVRLAyrqqfHY-

PLSAYLNHINWAYWSHEQDTPRLIEELKQAIYDRELGVST(QKKIELLPSKEPSDLPRPSPSAQPGALEIPEG
TMKLKSAFYVERQGDRIAL)}*

$4893=256(257):
>gi|504936070|ref|WP_015123172.1|
{|272(337)|<unknown(X)>}serine/threonine protein kinase [Synechococcus sp. PCC 6312] >gi|427375676|gb|AFY59628.1| serine/threonine protein kinase [Synechococcus sp. P
{(VYERYNSADEVLQALHNLESAPPVWKRLGNFLRTPVQDLLRPNSPPPVSPILANPPPTRQPTSASESDLPP
LKAYGQGRN)---VFISHSNG-
SEEQELAEEFFAALVAAGHQPFMASQSIQMGEGWaqRIDQELQLCDFFLLLLSSQ-
SAQSEMVLeEVRTVRNLQTQRpERRpFILPIRVNFP-
LDLPLNYELRGYLHRLQQRFWHSSTDTPALLQEVLSLVNA(TAEPTQAPEIPSGSGASLLPPPPTIAEDAPPL
PVAEP)}*

$4894=234(220):
>gi|115772393|ref|XP_001199453.1| {|648(0)|<Echinodermata(M)>}PREDICTED: toll-like receptor 3 [Strongylocentrotus purpuratus]
{(SSASLEPLRGKPLLEFDPSKLCGLNIALVCLTPLAGIFLVVIVIVLYHYRWQLKKKIFLLKLMVVGYKEMR
DARHHNDYE)-
FDLNIIFYDDdeEWVREQLQPALEEWLPQFqRNVVGDQDLIIGMHYLDAVDYVVSHSYKTLILLSRAAVHDRW
FMLKLRIAMDHVSDTQTEFVVVVFLEDIPdEDIP--
FLARLYLSDGRpYLHWTEDVRGlaFFWNNLVKNLTInlrtndlipkE()}*

$4895=248(256):
>gi|501444917|ref|WP_012468366.1|
{|91(334)|<Proteobacteria(B)>}hypothetical protein [Geobacter lovleyi] >gi|189419611|gb|ACD94009.1| hypothetical protein Glov_0280 [Geobacter lovleyi SZ]
{(YINNLKLTRTMRAVPVALSHEGLGHGGGGSLKNINFIRVYEWQKEYMVQNTVLAMAHELYRHGFIEIEEQD
HGKSSSIKL)----FLSHAK-SgDTGRLHAESIKRFIDTTNMSHFFDATTISPGFKFdeEIIKSIKESTV-
LAICSDDYSSRYWCQREILSAKEHH-----
RPMIAINCLEEFEDRIFPAGSNIPCVHISPESPLKESDVLKILTAAILETIR(HCHALSSLEHYQARGWIDSR
CALFSRPPEIRQLLTL)}*

$4896=148(145):
>gi|500040805|ref|WP_011721523.1| {|0(31)|<Firmicutes(B)>}MULTISPECIES: hypothetical protein [Clostridium] >gi|118134213|gb|ABK61257.1| conserved hypothetical protein [Clostridium novyi NT] >gi|658073
{()MPKVFISYSHDSQELEEKVKNLADILIEYGIQTEIDQyTTNPVQGWPqYMLENILKSDYILCVCTENYKK
RFENEEkIGVGLGAKFEG-KYITQILYSDEYN-
EKVLPILFTDNDSIPLAlqPYTYYKLYQNGEFEKLYRYITGQ(PLTKK)}*

$4897=283(286):
>gi|82468375|gb|ABB76627.1| {|84(0)|<Mollusca(M)>}MyD88 [Azumapecten farreri]
{(DPEPKVGNLISFLCKLERFDVISDCRTMIERDVEKWRQNQLSLQKINDDPTFREPQSRPRSPQSEETQTRA
DVGSKDEIY)-
YDCFVIYnPDasdQLEFVKQMSRILEGPEYNFRLFIPWRDDLVGTAMHSVSAAIIEKkCRRCIVVLSKSFNNS
PAADFQLKFAHALSPGARQKRVIPVLIENI--EPPSILNFVAPAPFYN----VGIRD-
WQWPRVAATIKSE(LRPDPETWLPKVDAWNIALDTSDVTKRELWGITVATQIYPEEDPVGIPDVDAQRKKHKN
KNKKRGE)}*

$4898=283(298):

>gi|470112958|ref|XP_004292696.1| {|117(36)|<Streptophyta(V)>}PREDICTED: uncharacterized protein LOC101300219 [Fragaria vesca subsp. vesca]
>gi|764536462|ref|XP_011458784.1| PREDICTED: uncharacterized
{(KYKDEPEKVIRWKAALTEVANLSGWNSKGRCESDLIKDIVKDVWQKLHPGLPFHSPSQLAVNKIGSTRYSSSSSSSHQKK)KCDVFISFRG-
KDTRSNFFSHLYNALVDYGISTYRDDIHLerGKSIPSELVKAINEARICLVIFSPNFTSSTWCLDELSQILQKRNR--------------VSVVRNGT---LESFGEHEDvcLSNREKV-
EKLMPTLRHAAMF(NRGASCQLTKLRLLVNYNGMWTNAGYIGGKTKGILVSEDITYQELEDRVYRIVGVDPREYKMIMKAKYESKDPTQPVE)}*

$4899=206(191):
>gi|469474097|gb|AGH33839.1| {<Streptophyta(V)>}Prv splice variant B [Cucumis melo]
{(MAAGSPSHPSQMA)-FDVFLSFNK-
NEENDGyrrFIKCLYETLSEGGIKMFMDDDKkmfmddikmlkddgkkMFMDDEVnlSddIVKAIEGSITSIVVLTKGYASSKWGLKELVKIIDQKNK-TKHQVLPLFYRHLVHHVYHQS----QDVVRH-
QSQDVVQHQSQDVVRRQSQDVR(KFFRSMAEKDYSEVNYRTSLAMSEFCRLPGIYISRKNS)}*

$4900=227(212):
>gi|469474108|gb|AGH33849.1| {|0(939)|<Streptophyta(V)>}Prv splice variant B [Cucumis melo]
{(MAAGSSSHPSQMA)-FDVFLSFnRD-EedDGYRRFIKCLYETLSEwG-
IKMFMDDDKkmfmddikmlkddgkkMFMDDEVnlSddIVKAIEGSITSIVVLTKGYASSKWCLRELVKIIDQKNK-TKHQVLPLFYRHLVHHVYHQS----QDVVRH-
QSQDVVQHQSQDVVRRQSQDVR(KFFRSMAEKDYSEVNYRTSLAMSEFCRLPGIYISRKNSNTMFQIKSTAKQIIDRLLSLK)}*

$4901=228(212):
>gi|469474117|gb|AGH33856.1| {|0(939)|<Streptophyta(V)>}Prv splice variant B [Cucumis melo]
{(MAAGSSSHPSQMA)-FDVFLSFNK-NDENDGyrrFIKCLYETLSEwG-
IKMFMDDDKkmfmddikmlkddgkkMFMDDEVnlSddIVKAIEGSITSIVVLTKGYASSKWCLKELVKIIDQKNK-TKHQVLPLFYRdlVHP-YVYHQS----QDVVRH-
QSQDVVQHQSQDVVRRQSQDVR(KFFKSMAEKDYSEVNYRTSLAMSEICRLPRIYISRKNSNTKFQIKSTAKQIIDRLLSLK)}*

$4902=227(212):
>gi|469474119|gb|AGH33857.1| {|0(939)|<Streptophyta(V)>}Prv splice variant B [Cucumis melo]
{(MAAGSSSHPSQMA)-FDVFLSFNR-DkenDGYRFFFKCLYETLIEwG-
IKMFMDDDKkmfmddikmlkddgkkMFMDDEVnlSddIVKAIEGSITSIVVLTKGYASSKWCLRELVKIIDQKNK-TKHQVLPLFYRHLVHHVYHQS----QDVVRH-
QSQDVVQHQSQDVVRRQSQDVR(KFFRSMAEKDYSEVNYRTSLAMSEFCRLPGIYISRKNSNTKFQIKSTAKQIIDRLLSLK)}*

$4903=227(230):
>gi|470113294|ref|XP_004292859.1| {|0(147)|<Streptophyta(V)>}PREDICTED: protein VARIATION IN COMPOUND TRIGGERED ROOT growth response-like [Fragaria vesca subsp. vesca]
{(MASLIASAPW)KYDVFLSSSS-QDAG-NFSYLLHKALKDeG-ILTLRDSQEVE-
GAEStaSLEVGIRQSKVAIVIISPDYASSPRLLEQLVQFLEANRD-
SSSLIFPGFHLVDPSDVRKQKGTIGASFAKH-EQ--
EQDSDkvKRWRAALTSVASL(TGWHTESHKDQTALINDIVQSLQRCIFRKETENIIEQSVDEVSTNTSVEEKIIEQPVDEVSRNTSVKDKIITVKHEEQKA)}*

$4904=251(251):
>gi|591355953|ref|XP_007072279.1| {|322(133)|<Chordata(M)>}PREDICTED: TIR domain-containing adapter molecule 1 [Chelonia mydas]
>gi|465951201|gb|EMP24220.1| TIR domain-containing adapter molecule 1 [C
{(TTVTAELQELGERMVQKPEQESPTELPRLCTPGPAAGSIRVPVEDSCVLTEKADTASFSSSEGCPPPQAAAAPTPELDCG)-EKRFFSfvvlHASEdVAIACRVKEMLESMGVPDGATFC---EEFLIP-GQsqltCFQDAIDNSAFTLLLLTQNF-QSRFCVHQMNTALMDSLQ-RRPKYNSVIPFVPKENSSKKCPIPSMLVGVV--PLDESSPVFSRTVKNTFRLRR(ISEQKAMWSQLQHIQEHHRKQQQHQEHLQML)}*

$4905=209(205):
>gi|490606005|ref|WP_004471014.1| {|0(1303)|<Spirochaetes(B)>}TIR domain protein [Leptospira santarosai] >gi|464206838|gb|EMN22892.1| TIR domain protein [Leptospira santarosai serovar Arenal str. MAVJ
{(MELFAHPPEI)-ASLFICYSSDqIDWARKIGNKINV-NYDGKISAWWDERKSTGDYWDsIIKQKLSEADSFLLILSPGFFKSEYIQNiELPIIIRRHES-EKIRISPIIAEQI-SFEqfnESWLNEIDVFPHRGfrTINQLLPHEINNWIKNLSIEPVW(RRIEKEFKQISIRTLGRPFQYDLRVPNGSDYIIERNEIKEIEDWNLNANDQKIAF)}*

$4906=181(180):
>gi|490618915|ref|WP_004483919.1| {|0(1325)|<Spirochaetes(B)>}TIR domain protein [Leptospira santarosai] >gi|464354475|gb|EMO31434.1| TIR domain protein [Leptospira santarosai str. HAI821]
{(MHPSKI)-ASLFICYSSDqIDWARKIGSKVNTA-YDGKISAWWDERKSTGDYWDsIIKQKLSEADSFLLILSPGFFKSEYIQNiELPIIIRRHES-ERIRIIPIISAEI-NFEQFNEYWLNEIDVFPhrGLRTINQLLTHEIDNWIKDLSIE(PVWRRMEKEFKQISVRTLGRPFQYDLRLLNGSGY)}*

$4907=205(201):
>gi|464392287|gb|EMO61010.1| {|0(553)|<Spirochaetes(B)>}TIR domain protein [Leptospira borgpetersenii serovar Pomona str. 200901868]
{(MHPSKI)-ASLFICYSFDqIDWTREIGSKINTI-YDGKINAWWDERKSTGDYWDnIINQKLSEADVFLLILSPGFFKSDYIQNtELPIIIRRHES-EKIRVIPIIASKI-NfeqFKESWLNEIDVFPHRGlrTISEILPDEINNWIKNLSIEPVW(RRMEMEFRRISIRTLGRPFQYDLRLPNGSGYIIERNEIEELENWNVKENNQKIAF)}*

$4908=220(204):
>gi|464452885|gb|EMO95433.1| {<Spirochaetes(B)>}SEFIR domain protein [Leptospira interrogans str. UI 13372]
{(MTEEP)RPKVFISYSwtT--PDHQRFVMELATELrEKLGIDIILDRWNLKSGHDInyFMEKSIIKEEIKVLIICDKAYCEKadkreGGVGtEtYIITSEIYENVeQEKFVAAVVEKDE-NGEPYLPDYLksrmYIDLSDplNRDEGLKQILHWSFGRDLYPKPEL(GPVPSFNKQEISLVKTELRSKDYIENIKLGKISSQVYIQEYLDHFLSHFDSIRISKGKK)}*

$4909=221(220):
>gi|471180491|gb|AGI05196.1| {|614(0)|<Mollusca(M)>}toll-like receptor T precursor [Mytilus galloprovincialis]
{(YKCTLLNGTVITVLEAYETMHDLFSNCRNSIWLTVSSTLLGTSIIGVVLLIIYSKRWNIAVFFYRKFRKMVEKKYGKSYT)-YDVFVSYGGDciPWITQCLIPKLET-

EWKLKICLKDRDFLPGESYFDIEAESIESSRHVIFLLTPKFKNSHECLFEIERVKHEKRMKNIENII-
VISKDMTlKDVP---VELANIWNYViFIQWPAGDCDdldVTWQKLKKWISSD()}*

$4910=258(280):
>gi|475605769|gb|EMT26193.1| {|0(24)|<Streptophyta(V)>}TMV resistance protein N [Aegilops tauschii]
{(MASRLSASAEAMRASEGGEQRHAVLARRVEYDEESLAGEPPIRRVHQPPAAWTRSGRWRAW)---------------------
LYDPRAQGGIRGFLDNMSMRPGDRLreTISAGISECSVAVAIFSPSYFDSEYCLWELATLVESR-----
KTIIPIFYNIKPSDLVLPEALAASddylPQDVERYKYALREA--
KNTVGLTYDSAT(GDIAELVSAAADAVLYHMEKMITNLGSVVWHAVLQPNDPNSIPGIDMEGGVVWRRGGIDSKPGKVDTLVYALKMDRWKK)}*

$4911=166(174):
>gi|490279381|ref|WP_004175318.1|
{|0(677)|<Proteobacteria(B)>}hypothetical protein [Nitrosospira lacus]
>gi|475054147|emb|CCU61607.1| hypothetical protein EBAPG3_6300 [Nitrosospira lacus]
{(MHFPHRGQNMT)--KAFLSHSSSdKEFVRAVAN-----GLGRQ--FCL-
FDEQVFDTGEtfklSIEKHLDDSSIFVLFASSESVKRMWIEFEINEAWYRILE-
GRISRALVFLIDSSIGHEALPPWLSRAKVSR--
TNAPKIVAREIRQHLDELIRT(EQHPYFEGRSDDIGHLQKLLRPI)}*

$4912=146(141):
>gi|489615453|ref|WP_003519894.1| {<Proteobacteria(B)>}TIR domain-containing protein [Agrobacterium tumefaciens]
>gi|474939757|gb|EMS99433.1| hypothetical protein H009_02433 [Agrobacterium tumefaciens
{(M)---IFISYAK---
EDAAYAQKIFLALKAQELAPWMDKppaphnyEGLQIGQKWqtVLQTQLSNADYIILVLSPRSVAKKGYVQvEFRTALDRMNYLpDDGvLVLPILIEAC-HVPslsvgnINLRDLQWdiisDEHIEEFALGLAQRIKeL-----------A()}*

$4913=221(220):
>gi|471180483|gb|AGI05192.1| {|648(0)|<Mollusca(M)>}toll-like receptor P precursor [Mytilus galloprovincialis]
{(LCTLKDGTVMTISEVMQKYHDLFIDCNSGHWLRIGIALLVSFVVLTIPVALVVNFRWQLAYWIYRRFRLVIEERMKLKSK)-YDIYISYGDQSlhWTINTLIHKLENS-
WKMITCLDDRDILGGEICSDAIAESISNSRNVIFVIDDSFQDRIWGKFEIQRAKYEKYAGNLQHIIIIKKHNItVQDFPS---ELRQIWdDVIFIEWWDNDD-SvGWDRLRIALFQkvL()}*

$4914=226(220):
>gi|471180493|gb|AGI05197.1| {|623(0)|<Mollusca(M)>}toll-like receptor U precursor [Mytilus galloprovincialis]
{(YNCTIHDKQAVLREVIQNTKEYFSNCKSTIWLHVGIVLVASTFCILIPIIVIYNYRWMIILTIYRKVRRAVEKDLHENYM)-YDAYVSYEDRSvaWIKKFLLPKIE-
GEWGLKVCLHDRDILPGNLTADAKAESIQKSRHFVFIITEQFTEGKWGRFEIERAVYEKY-
TTNLRKIIVILQNIqVQDIPE---EIVKISnDVCFIEMPLheneiyDKR-DsqsKWLKLKDLCYLN()}*

$4915=221(220):
>gi|471180495|gb|AGI05198.1| {|623(0)|<Mollusca(M)>}toll-like receptor V precursor [Mytilus galloprovincialis]
{(CYLDDQSRTSTTWVYSRYHDLYSHCDAIVWMRVGVICIMSSFLLIGSSAIVYQFRWRMAYFCYRKLKAKYLNNYQKEDFK)-YDVFIAYAFDSekWLITTLVPKLEK-

EWGLCVCVKDRDFPAGADRTDTVISGIRNSKSVIFIITPAFSKRQWGRFEIEMAKYEMFAKQRrqKKIIVIMK
NGTkIEEVPFEFSLIWK--DVTLINWPDENI-StenVWYDLK-LQIS-()}*

$4916=212(220):
>gi|489540492|ref|WP_003445200.1|
{|87(0)|<Bacteroidetes(B)>}hypothetical protein [Psychroflexus
gondwanensis] >gi|476539742|gb|EMY79918.1| hypothetical protein
pgond44_14538 [Psychroflexus gondwanens
{(KKIAEYQKKIADKSIELGKKKQDLRNSEDTEIKKQNKQQEDFQRSLQRKIDEQKTQLDILIHQNYSSQTTF
ENVEVKENK)RYDFFISHAS---EDKDdIVRSLAGSLKENGFEVWYDEFElkIGDSLRKkidVGLTN-----
SRfgIVIISPSFVKKNWTEYELNGMVAREMNGHKV-ILPI-----CH-KISKDEVLKFSPTLA-
DKMALNTPIHSTKDIIKALKEL()}*

$4917=202(212):
>gi|489098502|ref|WP_003008373.1| {|0(18)|<Spirochaetes(B)>}TIR domain
protein [Leptospira weilii] >gi|476504197|gb|EMY76396.1| TIR domain
protein [Leptospira weilii serovar Ranarum str. ICFT]
{(M)--KAFTSYSTAdKYTAGKIQNLLDHFGIS------
SFLAHEDIKVSSewqtVILNELAKSDLFVALLSADYERSPWCVQEAGIAAFQEMT-LVFLSLDGTIP--
KGFVGKTQSAKIKEETVS--ISDLIPG--
IIRCNFTVGTNM(IVDLIGRSRSFRQAEKNFQLILPYVDQMKKSQIKDLLKRSANNRQVYDAGLCKKRYIPEL
LKDYRYLLPKK)}*

$4918=285(295):
>gi|495587563|ref|WP_008312142.1| {|47(714)|<Cyanobacteria(B)>}WD40
repeat-containing protein [Leptolyngbya sp. PCC 6406]
{(SLANWLMRRYEATLEEIADRIDQEPAAVQATLDALIHRGLLKAVEGEQGLVYRVNLAPKTGRQMPKDIFKV
LDNASEQAN)---VFISYSRRNKPFVERLYTALSATDRE-
VWVDWENIPLAVDWWQEIELGIELADTFIFVLSPDSVQ-SQVCRqEIDHAVQHN-----
KRLVPVVYQDVQpdQVHPELARLNWIFLRP--EDDfDGGLR--
GLLAALDQNIDY(VRTHTRLLLRALEWDNNGRDASYLLRGKDLHRANEYLVQGREEEPKPTPLHHQYVLASAE
VEAAARQAEMVHQAS)}*

$4919=254(259):
>gi|495589628|ref|WP_008314207.1| {|154(988)|<Cyanobacteria(B)>}WD40
repeat-containing protein [Leptolyngbya sp. PCC 6406]
{(GLLGLMERHHAYVHQHTYLLDRALTWKRGQKQSRYLLIGEERQQGEAWLTTRFQEEQPPCFPTDLHCEYIT
ESIKQANSL)MTQVFLSYAQEnQAVMQKIRN---
SLRREGFTVWTNTTDIQTGEAFEkAILRGIEQADNLVYLLSPDSLQSSFC-QqELNYALSLNK-----
RVIPVLVTAT-
DPEVVPTVLRELHYIDLtdNVEEQDYWLDESQLVKILRQDAD(YYSQHKGLLIRALKWQQQNDNASLLLRGYD
LRRAEAWLK)}*

$4920=183(189):
>gi|490158470|ref|WP_004057137.1| {<Firmicutes(B)>}TIR domain-containing
protein [Eubacterium plexicaudatum] >gi|476610142|gb|EMZ17505.1|
hypothetical protein C823_05925 [Eubacterium plexicaudatum ASF
{(MCFEPYEGKEAY)---IFVSYGHKdY---QKILPILKLLHNKGY-
RIWYDKGIEWGSEWPdTIAKHLSDSTICMAFHSVTSVQSLNCRQEIYYAIKQKKS-----ILSVYLDDvnlC-
EGLDMQLSPIQSVALFKykDYTDFYKLIENTKIPQKWCDLSK(GGEISDEHVVSVLDFNIDDAIDEKFKKVFA
EEKKKHF)}*

$4921=219(224):

>gi|479884154|gb|ENU34783.1| {|72(0)|<Proteobacteria(B)>}hypothetical protein F989_00205 [Acinetobacter parvus NIPH 1103]
{(HTGSSGMMHINGIGDISFLFPNQTINESNFVAFRQKIIGHFDLKKKDLIFVVKNDLSNLQAEILFNLKNQE
LCMNLQPIK)---IFLSHKS---VNKPKVREFKKTLELLGFQPWLDEDAMHAG--
VELERALltgMQDSCAAIFFITPDYVDENYLSsEITYA--IQRKRaDPNFaIIALNIEEN-
GREGIIPMLLTPYVWKKPSSDLEALQEILKALPVKVGEVF(FKQS)}*

$4922=248(221):
>gi|490455413|ref|WP_004326217.1|
{|0(118)|<Proteobacteria(B)>}hypothetical protein [Thauera aminoaromatica] >gi|479289866|gb|ENO77966.1| hypothetical protein C665_18642 [Thauera aminoaromatica S2]
{(MSYVGEPF)RHDLFVSYSHGddgsggaclapwsAAFAHELERELRADRRFRtSLSVFRDQDHRPAQSldpm
aPLtaQLQEQIAGAALLVVLMSPDYLASKWCADERDWWLQrqaalgLPHGEriaVVRI-----WP---TDTP-
WPAALCDARGEPLVGfafypadGVPPRP--
LgWTDLPGAFGSE(FRKALLDMVGRLYPKLEAMRARTEELRRQREEAERLVRDGGQLLYLHGRSDHAAAWEEA
ALKLVDKGFAVVPG)}*

$4923=255(213):
>gi|490513925|ref|WP_004379508.1|
{|0(111)|<Proteobacteria(B)>}hypothetical protein [Thauera phenylacetica] >gi|479307052|gb|ENO95152.1| hypothetical protein C667_20479 [Thauera phenylacetica B4P]
{(MSYVGEPF)RHDLFVSYSHGddgsggaylkpwsAAFAHELERELRADRRFRtSLSVFRDQDHRPAQSldpm
aPLtaQLQEQIAGAALLVVLMSPDYLASQWCADERDWWLErqaalgLPHEEriaVVRIwPTDTPWP---
AALCDargeqlvgFAFYPADAVPPrplgwtDLPGAFGSEFRKALLDMVGRLYPK(LEAMRARAEDLRRQREEA
ERLVRDSGQTLYLHGRSDHAAAWEAAALKLVDKGFAVVPGEPDRVDS)}*

$4924=157(159):
>gi|490806467|ref|WP_004668590.1|
{|0(16)|<Proteobacteria(B)>}hypothetical protein [Acinetobacter sp. CIP-A165] >gi|479880846|gb|ENU31745.1| hypothetical protein F991_00514 [Acinetobacter sp. CIP-A165
{(MPV)----FISYR-AkdreiaLKIARKFREYSIEFYLDVI-----
DEESINSTSNIteIITKNIKRCTHLIAIISPNTKGSWWVPFEIGEANIINRRIcsfAYNTNGYSLTK---
SNMYLYKSFLPEYLHKWPILLNEKDVDNFILRYNQDNRNN(VLLDSINGDSNRFGTL)}*

$4925=216(220):
>gi|491292511|ref|WP_005150527.1|
{|75(0)|<Proteobacteria(B)>}hypothetical protein [Acinetobacter sp. ANC 3929] >gi|480145119|gb|ENW82249.1| hypothetical protein F909_01326 [Acinetobacter sp. ANC 3929
{(SELQKKKTDIDNKLLKEKQNLIKAEVLERKKLDALSKKQQKDEIEHQKKLKREIDALKASTQYISNVSVSS
IDKASEVEP)EYDLFISHAS---EDKEdFVRPLAETLQDLGVKVWYDEFtlKVGDSLRRkidSGLRN-----
SKygTVVLSTDFIKKDWTNYELDALVAREMNGHKM-ILPIWHKITKndV-LeyspnLADKVAISTTMYSI-
EEIAHQL--------ADVILTK()}*

$4926=162(159):
>gi|491224115|ref|WP_005082404.1| {<Proteobacteria(B)>}hypothetical protein [Acinetobacter haemolyticus] >gi|480078791|gb|ENW17408.1| hypothetical protein F927_02118 [Acinetobacter haemolyticus CIP 64
{(MTTDI)--
NIFISHCHtndEdQKFFNDILMFINPaIKSMDNVKLWSDKQLLTGQHIDsEVENALNDMNLMICLVSPEYLNS

NYCIEkELKDALAKRQI-GKANIFPIILRKC-VWKHTFFGQVLCQPKDGkpLKEWTDKD--
DALSDVTDALMDV(IQYLKKQQISEKKN)}*

$4927=221(221):
>gi|491394503|ref|WP_005252365.1|
{|0(557)|<Proteobacteria(B)>}hypothetical protein [Acinetobacter sp. CIP 102136] >gi|480182199|gb|ENX18562.1| hypothetical protein F893_03333 [Acinetobacter sp. CIP 1
{(MEHS)-
TKIFISYCHAnDIHFNALMSHLGAIAKFNNIEIFTDQQVDIGQDLDgTIQEHLQSADIVVCLVSTEYLNSDYC
IRkELEVAIEKQVI-DNSKIFPIIAEDC-LWKRTYFGKIKCAPEDGgpVSKYENIS--
SVYLTVVDQLMSQ(IERNRTVDIEKKKDHLASALIFDQSEKFHNYIHDLGISIKHPHKDHVILDDVFIYPDLE
HINSNDVINYKQISSESI)}*

$4928=152(151):
>gi|491810416|ref|WP_005612448.1|
{|0(193)|<Proteobacteria(B)>}hypothetical protein [Ruegeria mobilis] >gi|798777130|gb|KJZ23253.1| hypothetical protein TW79_13190 [Ruegeria mobilis] >gi|1041942372|gb
{(M)--KVFISWSgPRsKDMANALRDWL-
PMVLQYVQPFVSDKDISAGDRWAqTIASELEAANFGIICITPENLASEWVLFeSGALAKSMLDG-
KVIPLLFGLEMSD-LSGPLKQFQAQKVEMLGimEVAKAINNVAESKADDKIIDRTV(PALWPQLEAA)}*

$4929=221(227):
>gi|565477117|ref|XP_006296199.1|
{|0(160)|<Streptophyta(V)>}hypothetical protein CARUB_v10025364mg [Capsella rubella] >gi|482564907|gb|EOA29097.1| hypothetical protein CARUB_v10025364mg [Capsella rub
{(MTSPTAS)KYDVFLSFSG-QDTRRTFSSFLYHELVGRNIRTFKDDKDLeiGQMISPELERAIGDSRIAV--
-----------LWSLRTTLRLVAQDDHGFRGPGFDHCEAHLLRCGSVQFEDAdgvvaeqFKKHEAREDHEKV-
LSWRRALTNLSYM(AGPCSWICEDDMKLVDEIADNISNMLITVTSKTIRNGRYIVGSDKHMKYLNRLMDLNSK
RGLTMVGIWARGCNARSALAK)}*

$4930=153(175):
>gi|565447769|ref|XP_006284939.1| {<Streptophyta(V)>}hypothetical protein CARUB_v10006238mg, partial [Capsella rubella] >gi|482553644|gb|EOA17837.1| hypothetical protein CARUB_v10006238mg [Capsella ru
{(MASSSLTQ)---VYLSFHG-PDLPRVFLNHLHDHFARKGMTTFK--EVKTGQT-
IgpESLKAIRESRVSIVVLSKNYISSRWCLDELVEIFKCNEA-SGQMI----------------
GEFGRCFiERTCQGETEEVK-KRWSSALAHVGSL(RGDYSLFWDDDAAMVGNIAARVSRILK)}*

$4931=194(220):
>gi|565431030|ref|XP_006279848.1|
{|0(543)|<Streptophyta(V)>}hypothetical protein CARUB_v10028394mg, partial [Capsella rubella] >gi|482548552|gb|EOA12746.1| hypothetical protein CARUB_v10028394mg [Cap
{()-----------ETLRCSFVPHLSAAFGRKGVSVVTADKHDQSY----------
MSIASILIFSENYVSSKESLDEFVKTIQRRHE-EGHIVTAIFYRFSRSYVQEQMGNFSQAFLEHC---
TSDQV-
SQWRNALAEITSL(PGHETSISQSDYESTENIARDLYKKIFPNERIGIYSRMLPDVEKLIKKQQRGVKSIGFW
GMPGIGKTKLAQALFDQKSGD)}*

$4932=268(294):

>gi|565440868|ref|XP_006282986.1|
{|443(1235)|<Streptophyta(V)>}hypothetical protein CARUB_v10003971mg [Capsella rubella] >gi|482551691|gb|EOA15884.1| hypothetical protein CARUB_v10003971mg [Capsella
{(TCLDGEIGSIIKLTYDSLCGEDKELSHHHISCLHDMELNERMEQYLAMGVRKKCIITNQDLKEDIYTSETAFKGMSNRLL)-HTVYI-YCA-DTLQYSFASHLSMEFRRKGIYAFVNCN--------ETLDVIERVSASVVVFSKSCFSSTSDLD-IVRIFHCRRK-TNQLVVPVFYGISLSDMVVQEHE------------SADQI-REWSSTLHELREL(LGHQSRKECSERELVEEIVKDVYEKLFPTEQIGINSRLLEIEQLLRKQTWGIRRIGIWGMPGIGKTTLAKAFFD)}*

$4933=261(282):
>gi|565451241|ref|XP_006285972.1|
{|209(0)|<Streptophyta(V)>}hypothetical protein CARUB_v10007493mg, partial [Capsella rubella] >gi|482554677|gb|EOA18870.1| hypothetical protein CARUB_v10007493mg [Cap
{(SGCSPDDVIDFLRTRQFSGSIEALVGRLITASEAHGLIPENNPFRSLAVTSYLKAASGVTHETECILIFSCNDNLDVDET)----------------YFIESISKELLKREVIPLTYNLLGRENL---DMEMIYRSSVGIMILSDSYACSRQSLDHLVAIMEHWKA-TDLIIIPIYFKVTLSDICGLKGRFEASFQLLEKSLKEDRI-QKWRAAVTKIVSI(GGLEWTKGTQFMLAEEVVRNASLRLYLKNSKSLVGILALLNHSQSSDVEMVEILSTTYVVLK)}*

$4934=249(245):
>gi|551610554|ref|XP_005786395.1|
{|1017(181)|<Haptophyceae(E)>}hypothetical protein EMIHUDRAFT_229070 [Emiliania huxleyi CCMP1516] >gi|485639672|gb|EOD33966.1| hypothetical protein EMIHUDRAFT_229070
{(CVLFLGYSYIYVFERFEALPVERGVVVWVMTFASPDAVATAMVVVVAVLATLALGVTIRDALAATSASVFLLCETRRPPE)-----LSMHG-GQRFHLFLSHTwntgqdqcaaikrQLQLHLPGIKVFLDVDDLR--DIGALEEHVEESMAVVVFLSRGYFASRNCLREVEAITSQRKP-VILVLEADEAKGGLTLEQAMLECPEKYRDYLFKHSDGQPRLPYRWHRVREFQMR(SLCLIASEMLGVSPSYARYCKRRPD)}*

$4935=230(237):
>gi|551562075|ref|XP_005767845.1|
{|364(220)|<Haptophyceae(E)>}hypothetical protein EMIHUDRAFT_211623 [Emiliania huxleyi CCMP1516] >gi|485618391|gb|EOD15416.1| hypothetical protein EMIHUDRAFT_211623 [
{(LGAVIQLCGDDNDLCYQLVGIQSGRRASEFVVVLTVAMLVASLLIVFFKTATAVRVPTLRLVSTGRAPILELPAACHFHA)----FISHAwgtG-QDQTHTVVRRLQLLL--PGVRIWLDVDNLDDVGK--LEESVADSATFIIFLSSGYFRSWNCRRELYAALASVRP-FIVVHEADTAKGGASLAALRDECRDSCVEVAPPAYPSYSGPGEVLTRVFEEAEP(IVWVRVHDFQLESLKEV)}*

$4936=244(255):
>gi|551614889|ref|XP_005788342.1|
{|896(446)|<Haptophyceae(E)>}hypothetical protein EMIHUDRAFT_110524 [Emiliania huxleyi CCMP1516] >gi|485641788|gb|EOD35913.1| hypothetical protein EMIHUDRAFT_110524 [
{(TVIQLCESAAYEDMCKTLVGFESARGASEFVIVLTAAMLAASLLVVLFKTVSAVRAPTIRLCSSGRPPVLELSPECHFHS)----FISHAwgtG-QDQTHTVVRRLQLLL--PGVRIWLDVDNLEDVGK--LEESVADANTFLVFLSAGYFKSFNCRRELYAAL----G-

KDRPFIPIFEADVAKGGASIEALKAECRENCVEVAPPAYPSYGGPDEMIARVFE(EAKPIVWVRVNIFQLVSL
KAVALRMLLHSPYYASR)}*

$4937=190(183):
>gi|490694639|ref|WP_004558431.1| {<Actinobacteria(B)>}hypothetical protein [Amycolatopsis vancoresmycina] >gi|486081674|gb|EOD64804.1| hypothetical protein H480_30101 [Amycolatopsis vancoresmycina DS
{(MYE)-YDVFISYLR-TAgdisawIRHHFHPRLLEVLgnNRYeDVRIFFDDQ-
VRVGSRWpaELSSALQRSRVLVPVCSPKYFRDEWCLAEWASMEEREAL-
TGARRRPLIYPVIFCDSRHFPDWAHARRPRDLQQWNHPFEHFQTAPAYLDFNQQ(IKQIAAELEDLIDGAPEW
RGDWPVRTPAPAPPSRARMPRF)}*

$4938=297(299):
>gi|565431160|ref|XP_006279889.1|
{|2338(97)|<Streptophyta(V)>}hypothetical protein CARUB_v10025731mg [Capsella rubella] >gi|482548593|gb|EOA12787.1| hypothetical protein CARUB_v10025731mg [Capsella r
{(GAAGRSPDGGDKATSPQPPIAKSRFSWAEYRSGSKNSERERHIKELEVDAQLSGEPHHHSGNIIPMVASSS
EAGIQQSHS)--QIFISYHG-
AELQRTFFRHLVSTLTDAGVTVFTDNDKGNGiKQLIQLYQRIEESKIALVIFSKSYVESNVCLNELVMMDELV
KE-
GKLLVIPVFYNVRSSDVRNIKGEFGQHFKEMRERYKDGLERvPKWETSVKSIAKT(IGIHSEVHCEDASLVEA
TVEAVQRALTKISEGNPKYLGSVNTPTDHVFALFVTFLFCLFMSPLLFYQSYSFPSGIWLMN)}*

$4939=237(238):
>gi|565450680|ref|XP_006285808.1| {|0(13)|<Streptophyta(V)>}hypothetical protein CARUB_v10007284mg [Capsella rubella] >gi|482554513|gb|EOA18706.1| hypothetical protein CARUB_v10007284mg [Capsella rube
{(MERQTGPLAFLDQFRRKPL)-PRLFVSFRG-
EELRNSFVSHVVRALKEAGVNVFIDSHEVRGRDIRILLRRIECSKVALVIFSERFAESEWCLREATKIAEQVR
A-
GCLVAIPVFYRVRTDDVKMLEGKFGECFVETLEKGGGVDHPlgVRWMNSVRFIANM(TGFTSEVHSIDNDLVE
EIVKAIKRQLPYQSPASKALEREFIRELLSAAIVSCICYFLIPTLFTDLKFFASPEWYLVVVM)}*

$4940=189(204):
>gi|565431176|ref|XP_006279894.1|
{|0(1222)|<Streptophyta(V)>}hypothetical protein CARUB_v10025742mg [Capsella rubella] >gi|482548598|gb|EOA12792.1| hypothetical protein CARUB_v10025742mg [Capsella ru
{(MTNCENDAGFVCISCV)-----------DEVRCSFVSHLSEALRRKGINYAVVDVS-----
SDELFskesrEKVEKARVSVIVLPGNCEPSRVCLDNFAMVLECQ---RRMV-
VPVLYGDSPLREEWLSELDLKGLSPVHKSRKECSDSTLVEEIVGDVYEK(LFYSGRIGIYSKLLEIEKLVGNQ
PFGIRCVGIWGMPGIGKTTLAKAVF)}*

$4941=283(275):
>gi|551534955|ref|XP_005756303.1|
{|716(53)|<Haptophyceae(E)>}hypothetical protein EMIHUDRAFT_439327 [Emiliania huxleyi CCMP1516] >gi|485603142|gb|EOD03874.1| hypothetical protein EMIHUDRAFT_439327 [E
{(HVPEQIAGTWAIWNSSEKVWLDAADLHIQADASARAAAERVANEPLPLPKAQAEEAIMVVEEARPQASVVS
ISSQQVEQR)-HDVFLTHDW-
GTDGEGRraqerVAAITRALRGRGLRVCFDDERMQGNVVAKMCSGIDDSDVIVVFVSQNYIErvagragpQDH
CKKEFEYAERTK---

GADRLLSVVVEPAVRDTRSWRGGVGMVLGSRPFFDLSVEEASREWEAAVSALYD(EVLKLQGFEVYASEAHPA
AGSGLAGLPGAGGGATAVAAGGAGGGLAATPADGQLT)}*

$4942=250(243):
>gi|551626929|ref|XP_005793475.1|
{|141(0)|<Haptophyceae(E)>}hypothetical protein EMIHUDRAFT_222110
[Emiliania huxleyi CCMP1516] >gi|485648078|gb|EOD41046.1| hypothetical
protein EMIHUDRAFT_222110 [Em
{(GRVRGDGICDVCHAGVRGGAFVDACAQCDFWICQQCQLKLGKYGAPAKSAASVHGNKMAPLSSPPPRVTVP
QLPLPDGCL)-YHAFLTHSW-
VKDGKGRdtharVSRINDHLKARGMVTWFDGDRMEGEIADKMCEGIDDSAAIVVFVSKAYldkvaskNPNDNC
KKEFKYATQRK---
SATKMVPVPMEPDCLMPGNWKGQVGMELGGTLFKANFAEDADFEEQAEALFDEI(LRVAGVNTPPSAPTHPHA
RKTRM)}*

$4943=301(299):
>gi|565431160|ref|XP_006279889.1|
{|69(2362)|<Streptophyta(V)>}hypothetical protein CARUB_v10025731mg
[Capsella rubella] >gi|482548593|gb|EOA12787.1| hypothetical protein
CARUB_v10025731mg [Capsella r
{(VSVNKFEPPKDDGNQNQKLKDDGNKEPKDDGSNKEEPDKNTYFFKSYMESLGFERGILGGIVQAKLKSRRD
DDLPGEAKP)-DETFINFvenkrvldvvpvteeKN-LDLSHDFVRHLVKALENNGVNV-------
RRGDLHFEIK--ESTTTALIIFSSRYAESSWCLNELVKIKELREE-
GKLSVIPIFYELEQSEVTQLKGDFGVRFWNS-
WRINRDHHIVEWKEALESVANI(KGFYWKEEDCSEFITLTIKEVMEKGEIASLFLSTGEGKSSDFQTGRGEKH
KTYLNKCHLFGIEQRLEQLERKLEFDCNE)}*

$4944=153(151):
>gi|565486651|ref|XP_006300965.1| {<Streptophyta(V)>}hypothetical
protein CARUB_v10021364mg, partial [Capsella rubella]
>gi|482569675|gb|EOA33863.1| hypothetical protein CARUB_v10021364mg
[Capsella ru
{(KV)-DQVFFSFRG-
QQLRYNFVSHLSDAFQRNEIKFFIDKDVKKGQDLKNLFVRIEESSIALAIFSTRYPDSRWCMDELVKMKKLVD
Q-GNLLVIPIFYKVKAKDARNPKcsSEFGRNFWKL-
AEsvssTSRKYQIAHWLEALHYKKRV(ILQHRKLLQ)}*

$4945=190(220):
>gi|565430944|ref|XP_006279820.1|
{|0(173)|<Streptophyta(V)>}hypothetical protein CARUB_v10028098mg,
partial [Capsella rubella] >gi|482548524|gb|EOA12718.1| hypothetical
protein CARUB_v10028098mg [Cap
{(R)-----------------
FISFLVPALKDQNINVFIDEQEERGKYLTSLFDRIGESKIALVIFSEDYTESKWCLDELVQIKECMDQ-
NKLRVIPIFYNLIPP-------------
WDLEYRYQHKPEIpQKWKEAVVSVCQT(FALSLSENIDNNLCGWIQSIRKANEYHLMTYPIRLIVEEVSKVLP
EERNGQIGDIEVDNYSVPTSNLTLTLCEAPKTQL)}*

$4946=217(246):
>gi|551560103|ref|XP_005766950.1|
{|169(131)|<Haptophyceae(E)>}hypothetical protein EMIHUDRAFT_196897
[Emiliania huxleyi CCMP1516] >gi|485617346|gb|EOD14521.1| hypothetical
protein EMIHUDRAFT_196897 [

{(DNVDDIVNTLIALVFVMLAFFLVSAIFAARRIARKPKILLVSSNQPPELTLARHMKWHLFNSHIWSTGQDA
AAVIKNQLK)------------------------LLLPGIKVFLDVDDL--
ADIGALEEYIRRTQVILIFLSKGYFKSRNCLREVRASIEQRKP-
LVLELREECPPELRAAVFDRGWRRVLWLRAL-
LLHTPDFRREASLPLFVPGELR(REELSFPKHVLLWVSPVNAGAQELAD)}*

$4947=208(213):
>gi|551590068|ref|XP_005779478.1|
{|0(205)|<Haptophyceae(E)>}hypothetical protein EMIHUDRAFT_366755
[Emiliania huxleyi CCMP1516] >gi|485631997|gb|EOD27049.1| hypothetical
protein EMIHUDRAFT_366755 [Em
{(MISLVVFALVVFLIGAVVAARRVAKVPTVRLVSTKQPPELSIGTGLT)-WHLFLSHiwsTG-
QDAVGNIKSELQ--LLVPGLKIFLDVDDLK--
DIGSLEEYIQRSQVILFFLSCGYFRSKNCLREIRSSLEQSKP-
IVLVQEADPAKGGATLQALRGECPEDLQQDI-
FEKGWAHTTYMRVEEFQRVSLK(TIVEAVLLCSPNYLNQTSLPLCVPGE)}*

$4948=267(271):
>gi|551541289|ref|XP_005758941.1|
{|1187(290)|<Haptophyceae(E)>}hypothetical protein EMIHUDRAFT_106757
[Emiliania huxleyi CCMP1516] >gi|485606844|gb|EOD06512.1| hypothetical
protein EMIHUDRAFT_106757
{(ALVINVCNLSADTCETFGMGRTSYLPALVFVVFGTAMLVSAFLLLAWAAGRYATTLPTLRLVENGLEPPLT
IAQGDKWHL)----FVSHVWSTGQDQAANIKRALQviLPGS--RIFLDVDDLAD---
IsALESEIGQSALVLMFLSKGYFS----SRNC--
LREIRCAVqLRKPIVLVHEaneakGGLTleESMSECPDELRDRVFDSRRRLGMAGGRAIQWHRIADFQKM(SL
KLIAEQLLLASPQYASTCDALPLYFPGEISADALSFELPVCLRFSRHNS)}*

$4949=243(254):
>gi|551580103|ref|XP_005775610.1|
{|275(275)|<Haptophyceae(E)>}hypothetical protein EMIHUDRAFT_195816
[Emiliania huxleyi CCMP1516] >gi|485627554|gb|EOD23181.1| hypothetical
protein EMIHUDRAFT_195816 [
{(ALVINVCNLSADTCETFGMGSTSYLPALVFVIFGTVMLVAGVLLLVWAAGRYASALPTLRLVENGLEPPLT
IAQANKWHL)----FVSHVWATGQDQAANIKRALQvtLPGS--RIFLDVDDLED---
IgALESEIGQSALVLMFLSKGYF----TSRNC--
LREIRCAVqRRKPIVLVREANEAKGGLTLEDSWHECPEELREGVFDGRHAIDWHRIADFQKMS(LKLIAEQLL
LASPQYASTHNALPLYFPGEMSVDM)}*

$4950=241(252):
>gi|551611141|ref|XP_005786663.1|
{|1001(168)|<Haptophyceae(E)>}hypothetical protein EMIHUDRAFT_98641
[Emiliania huxleyi CCMP1516] >gi|485639965|gb|EOD34234.1| hypothetical
protein EMIHUDRAFT_98641 [E
{(LATLTLNSCSMGTCGEFGLDQFELATIFLSFTTALLGIGLILVLWLYTAASRAPTFRLVSSGREPLRDGGT
LTEGMRWHL)----FLSHIWSTGQDAAAVIKNQLRLllPGV--EVFLDVDDLES---
IaALEEYISSSRVVLLFLSRGYLRSLN------
CQREVRAALaNNKPIVLVQEVDPEKGGGALAELRAECPAELQNGVFDNRQPIPWHRIREFQLVS(LKLIAEAT
LLQTPRYFGKSNLPLYLPGELRSE)}*

$4951=241(252):
>gi|551620397|ref|XP_005790776.1|
{|937(120)|<Haptophyceae(E)>}hypothetical protein EMIHUDRAFT_224744

[Emiliania huxleyi CCMP1516] >gi|485644400|gb|EOD38347.1| hypothetical protein EMIHUDRAFT_224744 [
{(NSCSLLETDTQTCADEFGLSQLGLSYMFLSFTTALLGIGLILVLWLYTAASRAPTFRLVSSGREPLRDGGT
LPEGIRWHL)----FLSHIWSTGQDAAAVIKNQLRLllPGV--EVFLDVDDLES---
IaALEEYISSSRVVLLFLSRGYL----RSVncQ----
REVRAALaNNKPIVLVQEVDPEKGGGALAELRAECPAELQNGVFDNRQPIPWHRIREFQLVS(LKLIAEATLL
QTPRYCGKSNLPLYLPGLLDKQ)}*

$4952=286(289):
>gi|483502481|gb|EOA95601.1| {|332(77)|<Chordata(M)>}TIR domain-containing adapter molecule 1 [Anas platyrhynchos]
{(VPTKDSSVPVSTSSSAPASTSACSFPPHTYSSSTPSPPLYATPYSLPHPSPFRSPPSPACPPPFQTAKAAS
AAEPDSGEG)KFFTFVILHAGeDERVACRVKNLLEGMGVPNGATFCEDFLVaGHGRLTCFQDALENSAFIILL
LTKNFL-CHLCMFETNSALMESIQRPSKHNSVIPFVPK-ENPLE-RSEIPSILSVLvPLDENSPG---
FAKVVKNTFTHR(KINERKAMWCQIQQVREHKRRQQLFKDHYQTLQNLGALNLGSFPQVPLSETPWQQLGQFV
PHQLSQQSC)}*

$4953=244(255):
>gi|551534531|ref|XP_005756119.1|
{|489(210)|<Haptophyceae(E)>}hypothetical protein EMIHUDRAFT_108081
[Emiliania huxleyi CCMP1516] >gi|485602900|gb|EOD03690.1| hypothetical protein EMIHUDRAFT_108081 [
{(TVIQLCESAAYENMCYTLVGFDSAHGASEFVIALTAAMLATSLLVVFFKTVSAVRAPTIRLASTDRPPALE
LCPECHFHG)----FISHAwgtG-QDQTHTVVRRL--QLLMPGVRIWLDVDNLDDVGRLeeAVADAMSF----
LVFLSAGYFKSFNCRRELYAALASN-----
RPFIPIFEADATKGGASLAALQAECRESCVEVAPPAYPSYRGPDEMLARVFE(EAEPIVWVRVNAFQLESLKA
VALRMLLHSPFYASH)}*

$4954=243(254):
>gi|551535809|ref|XP_005756668.1|
{|1098(213)|<Haptophyceae(E)>}hypothetical protein EMIHUDRAFT_107847
[Emiliania huxleyi CCMP1516] >gi|485603633|gb|EOD04239.1| hypothetical protein EMIHUDRAFT_107847
{(MVIQLCESAAYEDMCKALVGFESARGASEFVIALTAAMLAASLLVVLFKTISAVRMPTIRLSSSGRHPVLE
LSPECHFHG)----FISHCwgtG-QDQTHTVVRQL--QLLLPGVRIWLDVDNLEDVGRL--
EESVRDATTFLVFLSAGYFKSFNCRRELYAALGSN-----
RPFIPIQEADVDKGGASIEALKAECREHCVETAPPAYPSYSGPSEMLARVFE(ATPPIVWVRVNAFQLESLKA
VALRMLLHSPYYAS)}*

$4955=240(251):
>gi|551535877|ref|XP_005756702.1|
{|711(230)|<Haptophyceae(E)>}hypothetical protein EMIHUDRAFT_221430
[Emiliania huxleyi CCMP1516] >gi|485603667|gb|EOD04273.1| hypothetical protein EMIHUDRAFT_221430 [
{(TVLVFKMCDSYSDACDVFGFSAEGIFSFFVLFGICLLVLHVLLSLTHLVFHAAATTPALRAVSTNSPPLLS
MPKEVHFHL)----FVSHvwkTG-QDQTHTLVRQL--QLMLPGIRIWLDVVNL---
DDIgKLEEAVADAAVVLIFLSSGYFASRNCRREVYAALANN-----
KPIEAVIEADKEKGGATVEEMQAEARRCFAPQVPGIPSAEVVVARVFAQDPV(VSLKEIALRVLRQLPYYISI
PSDLDRGLTLP)}*

$4956=243(254):
>gi|551536516|ref|XP_005756952.1|
{|1926(224)|<Haptophyceae(E)>}hypothetical protein EMIHUDRAFT_453848

[Emiliania huxleyi CCMP1516] >gi|485604058|gb|EOD04523.1| hypothetical protein EMIHUDRAFT_453848
{(MMIQLCESAAYEDMCKTLVGFESARGASEFVVALTAAMLATSLLVVLFKTVSAVRAPTIRLASSGRPPVLTLPRTCHFHG)----FISHCwgtG-QDQTHTVVRRL--QLLIPGVRIWLDVDNLEDVGRLeeSVRDAMTF----LVFLSARYFMSFNCRRELYAALGSN-----
RPFIPIQEADVDKGGASIKDLKAECREHCVETAPAGYPNYSGPGEMLARVFE(EAAPIVWVRGNAFQLESLKAIALRMLLHSPYYAS)}*

$4957=244(255):
>gi|551543115|ref|XP_005759744.1|
{|710(194)|<Haptophyceae(E)>}hypothetical protein EMIHUDRAFT_198748 [Emiliania huxleyi CCMP1516] >gi|485607871|gb|EOD07315.1| hypothetical protein EMIHUDRAFT_198748 [
{(LCPDPGASAAYEGMCYTLVGFDSARGASEFVIVLTAAMLAASLLVVLFKTVSAVRMPTIRLASTGRPPALELCPECHFHG)----FISHAwgtG-QDQTHTVVRQL--QLLLPGVRIWLDVDNLEDVGRLeeSVADAMTF----LVFLSAGYFKSFNCRRELYAALASN-----
RPFIPIFEADAAKGGASIEALQAECREHCVEVVPPAYPSYGGPGEMIARVFE(EAKPIVWVRVNAFQLESLKAIALRMLLHSPFYASR)}*

$4958=244(254):
>gi|551546779|ref|XP_005761285.1|
{|1201(162)|<Haptophyceae(E)>}hypothetical protein EMIHUDRAFT_453001 [Emiliania huxleyi CCMP1516] >gi|485610004|gb|EOD08856.1| hypothetical protein EMIHUDRAFT_453001
{(GRWALPASTTEECSSNDDSTTLIVVVCSVVGAVLLLGCCCAAYVVRRLQRQRHRPNQQFLCRRTHLPPDIAPVGADEFHL)----FLSHTW-LSGQDQIAmikqQLL--GFLPAHVRVFRDVDDLENIDRL--
EEYVAKSQVVLLFLSKGYFLSRNCLREVRAAVTSS-----
KPVILVHEHAEAKGGDRLEVLRQECPEELRELVFDSKRPLVRWHRLSDLNRV(SLLQIASLLHTFGRSLNALYLPNSLTEETISVSH)}*

$4959=244(255):
>gi|551548523|ref|XP_005761904.1|
{|472(215)|<Haptophyceae(E)>}hypothetical protein EMIHUDRAFT_197925 [Emiliania huxleyi CCMP1516] >gi|485610782|gb|EOD09475.1| hypothetical protein EMIHUDRAFT_197925 [
{(TVVKLCESAAYEDMCKTLVGFDSARGASAFVIALTAAMLIISLLVVVLKTASAVRMPTIRLASSGRPPVLELPPECHFHG)----FISHSwgtG-QDQTHTVVRRL--QLLLPGVRIWLDVDNLEDVSRLeeSVADAMSF----LVFLSVGYFRSFNCRRELYAALGSN-----
RPFIPIFEADAAKGGASLAVLQAECRENCVEAAPDAYPSYGGPDEMIRRIFW(ETEPIVWVRVNAFQLESLKAVALRMLLHSPYYVSD)}*

$4960=243(254):
>gi|551571775|ref|XP_005771748.1|
{|1174(180)|<Haptophyceae(E)>}hypothetical protein EMIHUDRAFT_118025 [Emiliania huxleyi CCMP1516] >gi|485623078|gb|EOD19319.1| hypothetical protein EMIHUDRAFT_118025
{(ICTDAGGASVASEECAAYFGIDSAEDITTVAIVLGGAMLAVLSLVVTWRIVASARDPTIRLVFSGREPILELPKGCSFHG)----FLSHvwgTG-QDQAHTIVRQL--QLLMPGVRIWLDVDNLSNLASLesCVAD-----
SAAVLVFlSAGYFKSGNCRRELYAAMEQG-----
KPLVVVREGNEEKGGGSAADFRAECAAYCSDAAAGVAEAVFARELIVWLRAH(EFQRESLKQIAHQLLAASPSFARPAGVHQLAPGL)}*

$4961=244(255):

>gi|551576767|ref|XP_005774027.1|
{|1843(230)|<Haptophyceae(E)>}hypothetical protein EMIHUDRAFT_117061
[Emiliania huxleyi CCMP1516] >gi|485625797|gb|EOD21598.1| hypothetical protein EMIHUDRAFT_117061
{(TVVQLCESATYEDMCKTLVGFENSRGASEFVIALTVAMLIASLLVVVFRTVSAVRAPTIRLVSSGRPPVLE
LSPECHFHG)----FISHAwgtG-QDQTHTVVRRL--QLLLPGVRIWLDVDNLEDVGRLeeSVRDAMNF----
LVFLSAGYFKSFNCRRELYAALASN-----
RPFIPIFEADVAKGGASIEALKAECREQCDNSISRDPESDAREREMLERVYP(NYSGPDEMLARVFEEAEPIV
WVRVNIFQLVSLKAV)}*

$4962=274(277):
>gi|551580785|ref|XP_005775913.1|
{|413(196)|<Haptophyceae(E)>}hypothetical protein EMIHUDRAFT_239574
[Emiliania huxleyi CCMP1516] >gi|485627933|gb|EOD23484.1| hypothetical protein EMIHUDRAFT_239574 [
{(HGHGTCTDNGACSCDLLYYGGACAYGGPVVVAAVASTVTLFVACCVLVIATRMCTRRRSLGLRVEKTGLLP
VLSLAEGHR)-YHLFLSHiwaTG-QDQvaviKRRLATLL------
PGASIFLDVDDLLSADNLeaSAydlpassNAAhIAESTVVLVFLSAGYFKSRNCLRELRAALKEE-----
KPLLLV-HESNPS------
KGGAPLEQLQQECPLELRDAVFGPPPPDQRLSG(DHGAMRIVPWHRLNRFQLESLRLIAEATLSHTPHYRKKG
RSPRLYFPERSDPADYEF)}*

$4963=243(254):
>gi|551581218|ref|XP_005776109.1|
{|651(213)|<Haptophyceae(E)>}hypothetical protein EMIHUDRAFT_469461
[Emiliania huxleyi CCMP1516] >gi|485628171|gb|EOD23680.1| hypothetical protein EMIHUDRAFT_469461 [
{(MVIQLCESAAYEDMCKALVGFESARGASEFVIALTAAMLAASLLVVLFKTVSAVRMPTIRLSSSGRPPVLE
LSPECHFHG)----FISHCwgtG-QDQTHTVVRQL--QLLLPGVRIWLDVDNLEDVGRL--
EESVRDATTFLVFLSAGYFKSFNCRRELYAALGSN-----
RPFIPIQEADVDKGGASIEALKAECREHCVEVAPPGHPSYSGPDEMLARVFE(ATTPIVWVRVNAFQLESLKA
VALRMLLHSPYYAS)}*

$4964=243(254):
>gi|551581979|ref|XP_005776469.1|
{|459(221)|<Haptophyceae(E)>}hypothetical protein EMIHUDRAFT_207102
[Emiliania huxleyi CCMP1516] >gi|485628573|gb|EOD24040.1| hypothetical protein EMIHUDRAFT_207102 [
{(LGTVIQLCGEDNDQCYQLVGLNDARRASEFVIGLTAAMLVASLTIIIILKAVTAVTAPTLRLVATGRAPILE
LPSGCHFHG)----FISHAwgtG-QDQTHTVVRQL--QLLMPGVRIWLDVDNLDDVGKLeeSVAD-----
SATFIIFlSAGYFRSWNCRRELYAALASK-----
RPFIVVHEADTAKGGASIQALRDECRESCVEVAPPAYPSYSGPDEVLACVFD(EADPIVWVRVHDFQVESLKE
IALRMLRHTPYYAR)}*

$4965=207(218):
>gi|551584755|ref|XP_005777728.1|
{|0(213)|<Haptophyceae(E)>}hypothetical protein EMIHUDRAFT_254651
[Emiliania huxleyi CCMP1516] >gi|485630008|gb|EOD25299.1| hypothetical protein EMIHUDRAFT_254651 [Em
{(MLIASLLVVLFKTVSAVRMPTVRLTSSGRPPVLELSPECHFHG)----FISHCwgtG-QDQTHTVVRRL--
QLLLPGVRIWLDVDNLDDVGRLeeAVADAMNF----LVFLSAGYFRSFNCRRELYAALASN-----
RPFIPIFEADVDKGGASIEALKAECRENCVEAAPAAYPNYSGPGEMLARVFE(EAAPIVWVRVNAFQLESLKA
VALRMLLHSPFYASR)}*

$4966=244(255):
>gi|551586358|ref|XP_005778270.1|
{|1251(189)|<Haptophyceae(E)>}hypothetical protein EMIHUDRAFT_100757 [Emiliania huxleyi CCMP1516] >gi|485630625|gb|EOD25841.1| hypothetical protein EMIHUDRAFT_100757
{(TVIKLCESAAYENMCYTLVGFDSARGASEFVIALTAAMLATSLLVVLFKTVSAVQAPTIRLASTGRPPALE
LCPECHFHG)----FISHAwgtG-QDQTHTVVRQL--QLLLPGVRIWLDVDNLDDVGRLeeAVADAMSF----
LVFLSAGYFKSFNCRRELYAALASN-----
RPFIPIFEADAAKGGASIEALKAECRESCVEVAPPAYPSYRGPDEMLARVFE(EAEPIVWVRVNAFQLESLKA
VALRMLLHSPHYASH)}*

$4967=243(254):
>gi|551590801|ref|XP_005779735.1|
{|154(215)|<Haptophyceae(E)>}hypothetical protein EMIHUDRAFT_114743 [Emiliania huxleyi CCMP1516] >gi|485632265|gb|EOD27306.1| hypothetical protein EMIHUDRAFT_114743 [
{(MVIQLCESAAYEDMCKALVGFESARGASEFVIALTAAMLAASLLVVLFKTVSAVRAPTIRLASSGRPPVLE
LSPECHFHG)----FISHAwgtG-QDQTHTVVRQL--QLLLPGVRIWLDVDNLEDVGRLeeSVADAMTF----
LVFLSAGYFKSFNCRRELYAALGSN-----
RPFIPIQEADVDKGGASIEALKAECREHCIETAPAAYPSYSGPDEMLARVFE(EAEPIVWVRVNAFQLESLKA
IALRMLLHSSYYAS)}*

$4968=243(254):
>gi|551601292|ref|XP_005782284.1|
{|337(215)|<Haptophyceae(E)>}hypothetical protein EMIHUDRAFT_233421 [Emiliania huxleyi CCMP1516] >gi|485635162|gb|EOD29855.1| hypothetical protein EMIHUDRAFT_233421 [
{(LGVVVQLCESDDDMCKALVGFESARGASAFVVALTAAMLAASLLIIVLRIASAVRAPTIRLASSGRRPALE
LSPECHFHG)----FISHAwgtG-QDQTHTIVRRL--QLLLPGVRIWLDVDSLDDVGMLeeSVADAMTF----
LLFMSAGFFKSYNCRRELYAALASN-----
RPFIPIHEADPAKGGASIAALKAECRENCVEAAPPAYPSYGGPEEMLARVFE(EAEPIVWVRVNAFQLESLKA
VALRMLLHSPYYAS)}*

$4969=244(255):
>gi|551611342|ref|XP_005786763.1|
{|1162(240)|<Haptophyceae(E)>}hypothetical protein EMIHUDRAFT_462567 [Emiliania huxleyi CCMP1516] >gi|485640065|gb|EOD34334.1| hypothetical protein EMIHUDRAFT_462567
{(LCPDPGASAAYENMCYTLVGFNSARGASEFVIVLTAAMLATSLLVVLFKTVNAVRAPTIRLASTGRPPALE
LCPECHFHG)----FISHAwgtG-QDQTHTVVRQL--QLLIPGVRIWLDVDNLDDVGRLeeAVADAM-----
AFLVFlSAGYFKSFNCRRELYAALASN-----
RPFIPIFEADAAKGGASIEALKAECRKSCVEVAPPAYPSYRGPDEMLARVFE(EAKPIVWVRVNAFQLESLKS
IALRMLLHSPFYASR)}*

$4970=279(286):
>gi|551616922|ref|XP_005789304.1|
{|345(319)|<Haptophyceae(E)>}hypothetical protein EMIHUDRAFT_201153 [Emiliania huxleyi CCMP1516] >gi|485642808|gb|EOD36875.1| hypothetical protein EMIHUDRAFT_201153 [
{(FSVAIASAAVETGDTDPVVSVLVFESLENIAVLCFAAMAVLALGLSALTVRSMVLRGKDELLRLKATKARP
QVSIARGLK)-YHLFLSHIW-GTGQDQVA-VIKRQLLRMvpDLVIFLDVDDLEDIGNLegYIDETM-----
AVLMFlSRGYFTSRNCLREVRSTAERK-----
LPVILVHeRDVNKGGLPYEASMDECPEDLREFVFGKEGAMRDVLEWHRVTVFQ(LCTLKEIARVVLDYTPLYL
DDQILDVYLKTDCEVEDLTFASPVAAYASPNNVGCRRAIRELAKTFR)}*

$4971=166(169):
>gi|486084086|gb|EOD66685.1| {<Actinobacteria(B)>}hypothetical protein H480_20334 [Amycolatopsis vancoresmycina DSM 44592]
{(MRPIREENPAEPVLAEVLPFERPGAAVRY)EYDIAVSFSG---
RQRSYVHETVLAARQLGLRVFYDEDVNGDWWGRnilVEMRRVYRTGTRfvVPFISEDYLASRFGMDELWFATS
TALHRPGPYILPVVFGDIElpADvLHPYTVYLDAGKHAP-HEIA-------AHMSRMAGAGE()}*

$4972=244(249):
>gi|551600793|ref|XP_005782066.1|
{|677(273)|<Haptophyceae(E)>}hypothetical protein EMIHUDRAFT_233722 [Emiliania huxleyi CCMP1516] >gi|485634894|gb|EOD29637.1| hypothetical protein EMIHUDRAFT_233722 [
{(PAYTTLGCSTRLAGAFWVFLLVLILAEAVLDDLPLSLYILTLPCYLTTMLGCLLLGAFAFIRPPTRFGRLL
ALAALRQPE)AYHVFVSYGGreaADRASA--IAYTLNSGGVN-
VWLDVQRLENGCSVLHAAHAAARDAAFLVLLLTPQYLSCPRRCVELLAALRRP-----
PQHTLVWLDASAdwSGCAQAPESGADFAAR--
LQEWLAAQGFRVVTTSLATLLR(SLDAALMSREDYHAEWWRRQRVHEQLQLH)}*

$4973=225(212):
>gi|486085389|gb|EOD67739.1| {|0(940)|<Actinobacteria(B)>}ATPase [Amycolatopsis vancoresmycina DSM 44592]
{(M)----FISYAPESGSAADdltvrelWV--FLRSCGVD-ARLDLSAEHR-
QDWSLWLAEQIREADHVLVIASPRYREGAEGRAagdvgrgvQWEARLIRDAFYRDphalDRFLPVVLPGQSld
GVPDFLAaastTVYRVDRFS--LAGAESLL--
RLLTDQPAYPEP(PVGTAPVLPPVDLTPDPASRPPRFPLAVHRTVLVVDVEGFGDHHRANHHQVAVRDTMYET
LRRAFRQAGIS)}*

$4974=244(233):
>gi|482527887|gb|EOA10675.1| {|570(0)|<Bacteroidetes(B)>}miro-like protein [Porphyromonas gingivalis JCVI SC001]
{(RETNRKKRPIQDLYLSCAQADKDLTESHYIHLGTLDDESKTTARIAAYPLKNGAIDKERVREVSTRPYKHL
SVNKNLATA)-
KQIFISYSKEdQTELETCLQFFKPLEKNGQIEIYYDKLTKFETPIHpEIRKRIVEADCIIALISQSYLTTDYI
LDhELPVFREYNK-----TIVPILIKPC-TFEDNEFLRKKYFAYKAramnkgkedktikaydsITASAHRD--
DNWVAVVREFKEK(ILRITKQEVNTDE)}*

$4975=197(181):
>gi|490694640|ref|WP_004558432.1| {|0(126)|<Actinobacteria(B)>}SEFIR domain-containing protein [Amycolatopsis vancoresmycina]
>gi|486081675|gb|EOD64805.1| SEFIR domain-containing protein
[Amycolatopsi
{(MSEPDP)-PKVFFSYSHDtEEHKElvrRFATFLhSRIGLEVVLDQWHDnHRI----
DWSlWATRNLKAADFVIVVASPLYRAradgeapPDEGRGaQFETAIIRNkltrdlED-GTRRVLPVVLPGR-
SIDEIPEFLNPYSMTRYeiAEFSDEGVADLLAAITGQGRHPM(PRRGVWRGGARAPRRVPAGDLPWTCTSPGV
RRGEA)}*

$4976=230(216):
>gi|498412630|ref|WP_010722075.1| {|0(95)|<Firmicutes(B)>}hypothetical protein [Enterococcus faecium] >gi|486634361|gb|EOF74966.1| hypothetical protein SGC_02542 [Enterococcus faecium EnGen0136]
{(MLNKS)---IFISYAW--EKDEPldkkvknFSQWLAIYLQSWGFRVHLDVLENHPGTKLdeFMKNGI-
DSSHFVICICTQTYLKKisdsaTGVYnEIEL----IKKQaKSPFIIAIIdIESF-

TDLPDFFEGKYIsqlkfdtPFSQEKKKGLFELIStIRDELFLNIKVET(KPDAQDRIERYYNSVEKIKFLNDS
ANLMNFKPQSEGEISFQYLLNGGDFKLGISPMEFITHWTSAGSDTIY)}*

$4977=298(284):
>gi|551625185|ref|XP_005792731.1|
{|474(26)|<Haptophyceae(E)>}hypothetical protein EMIHUDRAFT_471377
[Emiliania huxleyi CCMP1516] >gi|485647075|gb|EOD40302.1| hypothetical
protein EMIHUDRAFT_471377 [E
{(CSFRRRMTQKELLHVAEAEFRCIRFDRLCFEDLAECSRGGANSAPSSRREGSATCPSPVGPKAGAADFAER
TERCGLGEV)--DCFISHSwHDppaakwEALKAWASTFEDRHGRTPRLWF--DKACLD---
QDDITRALpclpifiAGCRSLLILAGPTYASRLWCVMELFAYLKMGGRReaiTVVPiaacateEGLQTVS---
ESLAAFDAQQARCVLPADRHHFLAVIESSYGSLSQFNLWA(RTVLEERSTRRSPALQRSAARSCGTEASSGGT
PRRSVVSTAATVVSVAEPAVATDVGGEDADAA)}*

$4978=184(190):
>gi|493214208|ref|WP_006197955.1|
{|0(154)|<Cyanobacteria(B)>}serine/threonine protein kinase [Nodularia
spumigena] >gi|119462941|gb|EAW43895.1| serine/threonine kinase
[Nodularia spumigena CCY9414] >
{(MDNWNIFKKRF)--DIFLSHAS-ED-
KETFVEKLYNELTDAQYKVWYDKNELYWGDNLqqKITEGLKKSSYGIVVISPSYFDkhKEWTFLEFNQILTTN
-----N-
ILPILHNINMQTIKDLHPQEHEQIKNWLAISSEKGIDYIIQQAKKKIGDR(ESEKGIDYTRLRDLLAAKNWKE
ADKETYRVMIKAVDKEE)}*

$4979=203(230):
>gi|501134086|ref|WP_012182790.1|
{|0(23)|<Actinobacteria(B)>}hypothetical protein [Salinispora arenicola]
>gi|157917062|gb|ABV98489.1| conserved hypothetical protein [Salinispora
arenicola CNS-205]
{(MTEDEFRVDPRAPVFFLSYARARHRPGEPPRDTNQKVFQLYVDLSDHVSELLGLPAGSTAG)---------
-------------------------FLDRV-
LDGGQVWadDLAFAAGNCQVFIPLVSPQYLRSVWCAREWNAFVRRRQVRrPDARATPGEQPVIPvnwsVLGRR
RDLPAAIRRRQVFSPTGLPPDIAPQYQQEGIYGLL(SLGRNGKDAYDAVVWRLAQRVVRAVDTHW)}*

$4980=197(219):
>gi|119445000|gb|EAW26296.1| {|0(622)|<Proteobacteria(B)>}hypothetical
protein ATW7_14011 [Alteromonadales bacterium TW-7]
{()-----------------------
MLVRNKVINQWYDGKITAGSPWEdKIIENLENADIVVFLVTKNWLNSEACIEeWDKAKLFAKEQ-
PNKRLIPIIAAEC-AWKDFDDMKSKlVLPQDGkpVSQWKPMD--
SAWSSVYDGIKDV(ASEVKKNFKLKTKFETELKSIEFCSTNSKDIFLEDVFVFPSLTAYSDDLNGVELAVKSF
DELIEEPLQFIVGENQSGKT)}*

$4981=184(192):
>gi|494441352|ref|WP_007234631.1| {|0(762)|<Proteobacteria(B)>}adenylate
cyclase [marine gamma proteobacterium HTCC2080]
>gi|119460028|gb|EAW41122.1| putative adenylate cyclase [marine gamma
proteobac
{()MPDIFISYSSKdRAF---VVPMVEYFERTGL-
EVWWDQRIDAGSSYArDIEDALDAAQTVVVLWSRHSVQSDWVR---AEANEGHTR-G--ILVPVLIDDT-R-
PPLIFRALQGIPCRFgnNADFAELIAAIGQIAPALKKKQV(ALTEKITASENQKHWSTPPFVGRNNLLNSLTA
ALSQSAQGSGQVVLLSGEPG)}*

$4982=291(293):
>gi|194348554|gb|ACF51677.1| {|82(242)|<Proteobacteria(B)>}hypothetical protein Smal_1973 [Stenotrophomonas maltophilia R551-3]
{(AGHTPDVFVFACTEVIAARDKDAAVKIVKDEFGWRLDLFDLERLRVQLSGSQRHLLAQHPSIFVPPFFPQR
GGESIVSSH)-DTILIDHVT---
SDHAIAAWLSRRLSLSGFRTWCRGTAPLAGEDAdeTVRTLIAQRAQRYLPVFSSASLADPVFLeRCAIATARS
----PDFVIPckgIISEDW-
VMPSRLNKLVAADFSSSWREGLSAVLDsLSSSGIGPSLAAE(RGAQIALRDFLPSQVTIAKPEPVFANVFAMT
VPSSMLDIRLPRTLTMVERYELSRKWAFYIANELTVIAFDLP)}*

$4983=180(200):
>gi|501187213|ref|WP_012230242.1|
{|0(269)|<Actinobacteria(B)>}transcriptional regulator involved in sugar metabolism [Candidatus Microthrix parvicella]
>gi|499275764|emb|CCM65492.1| putative transcri
{(MKVFFSHSSHYKPLLREL)----------------------
RALLPNHIDSWIDEDKLliGENITENLEVAIQsDSDFVILFIDSVAARSAWVRKEVEWAMAAEKRNGRTFLLP
VVLDMDAWEGFEPAELCKRKHLSCNDFTEKGIGLLADEIAGSLFAWL(SRDAESAKSDTNSGDRADLLGDADR
FLRSVASEIRTIVYPHR)}*

$4984=226(238):
>gi|498957537|ref|XP_004524167.1| {|801(0)|<Arthropoda(M)>}protein toll-like [Ceratitis capitata]
{(LLAMRCVMKIDSIGVQFGSLTVVNVISRMICLSAKYLKLGLLFFIAFLLMYVILRKHCENEDHDYCPCMIE
RNVYPNLLE)---AFIIYSKLdeHFFTE---------PTEFK---
NTRDLNPPKKRPDNIVvkdikQYAKKSKRTVILLSENFVKDIWAVKKYRSALQNFIPKIIDRVIIILYG-I-
KCTKSLDSVMMKCFKGAtFYVWGEPN---FWDQMRAELPQI(ADKKAIDKYMVKLEVREL)}*

$4985=219(224):
>gi|498480747|ref|WP_010783926.1| {|0(155)|<Firmicutes(B)>}TIR domain-containing protein [Enterococcus faecalis] >gi|486987318|gb|EOJ16485.1| hypothetical protein UMM_00181 [Enterococcus faecalis EnGe
{(MEKPT)---IFFSHSSKdRDLILPIKNKLSEITSNVINIFMSS-
DGQSIPFGNnwihKIEEGLRDSKIMFVFITQNSINNAWVYFESGFAYSKGIE-VIPVGI-
GINVGDLKPPLNLLQGFNVTSGDS--LNNFVDI--
VNQKFDLNFKET(FSDIDYDYIKALNLRTEYDISQIFESANYSTYSQYSSKDDGLIRYDLNTAFETYKKYLNE
QKIKFSIGTDSFSSEILLV)}*

$4986=217(221):
>gi|498531690|ref|WP_010830437.1| {|0(77)|<Firmicutes(B)>}hypothetical protein [Enterococcus faecalis] >gi|487092447|gb|EOK18241.1| hypothetical protein WU5_02925 [Enterococcus faecalis EnGen0329]
{(M)--KLFLSWS-KdesKQYANVFKEELPTL-FPNTEIFMS---
DTDISMGSfsipTIMQELENSTYGILFITPDNKDEPWINFEAGALskGLSDMH-
VTPIGFRGIEMEYLSSPIKNYQGF-LFEELK--FKKTMNE--
INRLTDTPLPKK(TFDVLLNSVWESLSDKIEKITDDYKIDKKSKEDPVEGKLNLILSELTKMNRNKNKKYTIY
TTSEIRKFLPEFDSFINENE)}*

$4987=187(186):
>gi|501180259|ref|WP_012223386.1| {<Actinobacteria(B)>}hypothetical protein [Candidatus Microthrix parvicella] >gi|499279723|emb|CCM62196.1| hypothetical protein BN381_100083 [Candidatus Microthrix pa
{(MHGPSHTPTTSRQPAFGDATDADYQG)----
FVSYAHAnQAAAWQLLTEIDVASKvaappgscFPRVSGWWDDQIAIGQgQWRqQIAAAVARCDFGIFLLSNDL

LLSEFVRRhEWEPI---RDD-PTKIVIVAGLAPV-DFGRARMHGF-----DDeqVYRWrerPNQH--
HHWFTELGPRNRR(RFAMGLIEGLRDALTQRRTS)}*

$4988=197(188):
>gi|501180261|ref|WP_012223388.1| {|0(1139)|<Actinobacteria(B)>}putative Histone acetyltransferase [Candidatus Microthrix parvicella]
>gi|499279724|emb|CCM62197.1| putative Histone acetyltransferase [
{(MPTEAQAER)--TIFACYRVRD-
FRDQVgpneeeRKFLQVLgERLGDLPGWSlrwDDHLQSGQKFHpQIQQWIKDAGAFIVLVGPQHGGSPYIRDh
EFPAILREHEA-RGVPVFWVHLRTT-
DLQALARAYPELGGDLDrtFDALGDLSQSVFQNDLAPGSRPA(TADTRRMDAVLSQLCTELAVRNPPTGERSP
GADPTDEEP)}*

$4989=276(276):
>gi|507031109|ref|WP_016102820.1| {|46(0)|<Firmicutes(B)>}hypothetical protein [Bacillus cereus] >gi|500352482|gb|EOO70344.1| hypothetical protein IIC_04786 [Bacillus cereus VD021]
{(IILGIKDDGITLSIKKFVFNLNIDNILDLLDGAVKIEYDRFTYEGNTLFHISIDKSDELVKVNNIPYKINK
DGDVEEMTI)-KKVFISYAHKdSDLVNILEEELKQYEN---
IKITRDIKVTAYRDSLdEFMKTIRDHDFVISVVSSAYINSLNCMYEVMHLMQDKDY-QEKLFF-IIVSRD-
DVEyykeknryDGFEAKIYDVIDRLkyITHWRDKK-----
AELERSINEA(ALSPELMVNLAVDMRKLNSVIPPMDDFIKLLSDKVGRSFKEMYEDDFKEIVDTINR)}*

$4990=279(272):
>gi|546298010|ref|XP_005710386.1| {|698(0)|<Rhodophyta(E)>}TIR domain-containing protein [Chondrus crispus] >gi|507106463|emb|CDF40092.1| TIR domain-containing protein [Chondrus crispus]
{(KCRLDLNGCGGEWTASFQLPGDWVENDTKARLPNLDGECASPCEEAWRLWELMPEIVEDCRPEASGMMQQS
SKVVRMEAM)--DVFISHAG-TDKSFIAGPVFHEVQKEG-
LNCFLDKARIEPGDTGsdEMTAAMESAKIGVFILSPEFAARRWTMRELRCFLCRRRE-
ARRtgvmppTILPVFYRLSICECRdfkpeQYVDEKGKSLFER-
EKFYSAERQNEATMAMVKAELR(EITEITGIENMEWASNNVHDLLAHDYRERLVKRIVMWVRKKWGEYWQQER
AV)}*

$4991=253(241):
>gi|546319716|ref|XP_005717096.1| {|0(1320)|<Rhodophyta(E)>}WD40-repeat containing protein [Chondrus crispus] >gi|507113661|emb|CDF37277.1| WD40-repeat containing protein [Chondrus crispus]
{(MQESFRQETGPSRKRRKIRSP)--DVFISHAG-PDKLH-IARLLSQKLEDqG-
FNCFLDKTSLKTGGHGpdTMKKAMESAMVGVFILSPEFAARKWTMRELRCFLRRRReamargM-
LPPTLIPVFYRLSIRECRelepdRYRDKEGKNIFEV-
EKFFSEERQreastDMVKRELKELADI(TGIENDVRATNLPGDKFGHLAREHLVEQVFLEVKKLGEEDSSDVV
TEGVHGPLEEEAKRSDAIGDARHQMRTTSAEKDPK)}*

$4992=277(263):
>gi|546321195|ref|XP_005717498.1| {|311(0)|<Rhodophyta(E)>}Protein containing a DNA replication factor 1domain, rfa like [Chondrus crispus]
>gi|507114041|emb|CDF37627.1| Protein containing a DNA repli
{(CPHIEILHAKVHFWLGDWGDRSQVVLKVDSDATALIRFENPAVSEKDASLSPTSVHELAKSLANLDASGEA
TKIDSETAH)RYTVFISHAS-AN-
KEKAAKPLHDYLKQRRIKSFLDSGRSRSGDQIsdSRMAGMKHAMVGVFVLSPEFVASKWTMHELRCFMDRRRE
-DMRKgrnppeIVAFFHRWSVADCRNLaffsiRGEDGEDVFRN-EGFFSEERQknfpvtdvSRY---
LTELADL(MEVKITDTEHRRAWGARDRRMVGGLLDAVTEGLQNLIAEADKQ)}*

$4993=295(278):
>gi|546321418|ref|XP_005717561.1| {|1063(0)|<Rhodophyta(E)>}TIR domain-containing protein GTPase [Chondrus crispus]
>gi|507114109|emb|CDF37690.1| TIR domain-containing protein GTPase [Chondrus crispus
{(TGCGGEWTAKFEMPGDWVENATKARLPNLEGECPAPCGESWRLLDLMPEIEKYYGPADPKALKALAVIPRSSKEVGMKAV)--DVFISHAG-VQKGKGgkggdklhFARPLHEDLcKRG-MCCFLDKVSLRPGDDGvdKMTVAMETADIGVFILSPEFACQRWTMKELECFLMRKRK-RRKReasetkkpgqspgqsvpiLIPVFYRLSLQECRR------FDPEKY-SKLFRDEDFYDEARQREMSTAT(VTALLKELSWTTGIENDGGASNDNGDPFASLARESLVERVAMAISEKYANIQANRNEL)}*

$4994=261(256):
>gi|546307815|ref|XP_005713456.1| {|897(23)|<Rhodophyta(E)>}Sel1-repeat containing protein [Chondrus crispus] >gi|507109847|emb|CDF33637.1| Sel1-repeat containing protein [Chondrus crispus]
{(LSREWLLERNGKRGVFDSELLKRLAQIMGRYQESLPVEGLCCRSCFRPWTISDVEHSLGLTQTCHPGGVSPLQRVMPPNH)-YDVFISHAG-PD-KMAMANPLYQSLTRHNVNVFLDFEGLQILDSTasEEMnRAMNEARIGVFILSPEFAAREWTMRELRCFLDRDetcrlEKRSRPVLLPIFHRLSLTECSRCDELVYNENSEHYELLQAERFYEQSRQDICTTEGA(IAALKGLRRHAGVVKQRLGGGDYLSSAELVETAAER)}*

$4995=263(258):
>gi|546316461|ref|XP_005716157.1| {|196(27)|<Rhodophyta(E)>}TIR domain-containing protein [Chondrus crispus] >gi|507112669|emb|CDF36338.1| TIR domain-containing protein [Chondrus crispus]
{(PRKETTLERKDNCSGDNCVPQKSTKDKRSNPSANHKKSCLPEPKRDPKATPEACDDKKTHCHTKPGEKNVAKGTRVKAKP)EFTVFISFCD-LD-KNTIAIPLHARLTKKGISSFCGKQSLIPGEDVreARNRALRSAAIFVFILSPEFAFRETNQEDLSYALKRSKYAKKSktagpILLPVYHRMTTEEANKELFDLQAPRNQYSTNSHRSASAASIVTVCRKEGFQ(ERVERGEAKFADVRKLFEELDGIADVGPGMNSSNAKHP)}*

$4996=259(256):
>gi|546327552|ref|XP_005719309.1| {|532(49)|<Rhodophyta(E)>}TIR domain-containing protein GTPase [Chondrus crispus]
>gi|507115986|emb|CDF39398.1| TIR domain-containing protein GTPase [Chondrus crispus
{(MKFRLMFDIPSLAKHRQTEEGDVEVAWWDVMDLDVESVSRLCKPALCRNGNCANVFRYTDAMTISVPSNKPGAVRTGVPD)QYAAFISHAG-TD--KEWAVYLAEALERKCLRTFVDRLELICSSETgdELMeKAIQTARVGVFITSPEFFARRYTMHEMRTFLrrnETSDANEKVVIYPIFHRLSYNEASSGDLASHEQYAPLFREEGFFTEKRQDACSTQEALAA(MQRMSRYPGLEKKLPELHNTVFRKHPWTYRGRMVWR)}*

$4997=213(215):
>gi|546297040|ref|XP_005710104.1| {|0(1154)|<Rhodophyta(E)>}WD40-repeat containing protein [Chondrus crispus] >gi|507106134|emb|CDF39810.1| WD40-repeat containing protein [Chondrus crispus]
{(MQESFQQEAGPSRKRRKIQSP)--DVFISHAG-PD-KLHIARPLSEKLQAQGLNCFLDKTSMEPGDCGgdEMTKAMESAKVGVFILSPEFAARKWTMRELRCFLRRR-----SgmrppILIPVFYRLSIRECRELEPELEPDRYRDDEGNNIFEVEKFFSVERQREAST(DTVKRELQELAGITGIENHDRATNLPGDEFGPLARAHLVERVFLWVKKKSEEQE)}*

$4998=218(215):

>gi|546297046|ref|XP_005710106.1| {|0(1203)|<Rhodophyta(E)>}WD40-repeat containing protein [Chondrus crispus] >gi|507106136|emb|CDF39812.1| WD40-repeat containing protein [Chondrus crispus]
{(MQESLRQETGPSRKRRKIRSP)--DVFISHAG-PD-
KLHIARPLSKKLHAQGLNCFLDKTSMEPGDCGgdEMTKAMESAKVGVFILSPEFAARKWTMRELRCFLRRR-----
ReaigsemrppTLIPVFYRLSIRECRELEPELEPDRYRDDEGNNIFEVEKFFSVERQREAST(DTVKRELQEL
AGITGIENHDRATNLPGDEFGPLARAHLVERVFLWVKKKSEEQE)}*

$4999=200(197):
>gi|546297702|ref|XP_005710302.1| {|0(1358)|<Rhodophyta(E)>}WD40-repeat containing protein [Chondrus crispus] >gi|507106357|emb|CDF40008.1| WD40-repeat containing protein [Chondrus crispus]
{(MQESFRQETGPSRKRRNIRSP)--DVFISHAG-PD-
KLHIARPLSQKLEDQGFNCFLDKISLKTGGHGpdAMKKAMESAMVGVFILSPEFAARKWTMRELRCFLRRR-----
EeairsgrqppTLIPVFYRLSIRECRELEPNRYRDKEGKNIFEVEKFFSEERQREASTDMVK(RELKELADIT
GIENDVRATNLPGDKFGHLAREHLVE)}*

$5000=189(186):
>gi|546297742|ref|XP_005710313.1| {|0(1169)|<Rhodophyta(E)>}WD40-repeat containing protein, partial [Chondrus crispus]
>gi|507106370|emb|CDF40019.1| WD40-repeat containing protein [Chondrus crispus]
{(MQGSYRQESI)--DVFISHAG-PD-
KLYIARPLSIKLEAHGLNCFLDKTAMQPGDCGpdAMTKAMESAKVGVFILSPEFAARKWTMRELRCFLRRR-----
ReaisdgmlppTLIPVFYRLSIRECRELEPSRYRDNQGKNIFEAEKFYSEKRQREASTEMVK(RELQELADIT
GIENDEEATNLPEDSFGHLARRELVE)}*

$5001=271(256):
>gi|546298021|ref|XP_005710390.1| {|1059(25)|<Rhodophyta(E)>}NB-ARC protein [Chondrus crispus] >gi|507106467|emb|CDF40096.1| NB-ARC protein [Chondrus crispus]
{(NKCGGEWTAKFEMPGDWVENATKARLPYLEGECPAPCGESWRLLDLMPDIEKYYGRADPKALKALAVIPRS
SKEVGMKAV)--DVFISHAG-
VQKGKGgdklyFARPLYNDLCSRGMCCFLDKFSLRPGDDArpKMTSAMETADIGVFILSPEFACRRWTMKELE
CFQARK-----
RKasetekpkesvpvlpiLIPVFYRLSLQECRRFDPEKYSKLFRDEGFYDEARQRKMSTATVTALLKE(LSWT
TGIENDVGASNDIGDPFAPLARKSLVEIVAMA)}*

$5002=288(279):
>gi|546298510|ref|XP_005710537.1| {|12(50)|<Rhodophyta(E)>}TIR domain-containing protein [Chondrus crispus] >gi|507106635|emb|CDF40243.1| TIR domain-containing protein [Chondrus crispus]
{(LNGCGGEWTASFRLPADWAKNGTEKGLWNLDGECTDCEEAWRLSQLMPEMEKYYGPARPEALKPLTVIQRT
SKEVGMKAV)--DVFISHAG-PD-
KWTIASPLFDKLRGEGFSCFLDKKSMKAGDCAseEMIAAMESAKIGVFILSPEFAARRWPMKELRCFLrryrd
aqSSG-----
ATlpiLIPVFYRLRISECRsfkpeqYVDENGRDLFEAEKFYTEERQNEATMAMVKDELREV(TTLLGIENEAA
SNDVLDPLASGFRKMLVERIVYSVKEAREARQQQEAKGVGIPQTGREG)}*

$5003=218(215):

>gi|546304949|ref|XP_005712496.1| {|0(609)|<Rhodophyta(E)>}NB-ARC and TPR repeat-containing protein [Chondrus crispus]
>gi|507108819|emb|CDF32725.1| NB-ARC and TPR repeat-containing protein [Chondrus
{(MQESFRQETGPSRKRREIQSP)--DVFISHAG-PD-KLLIARPLSEKLQAHGLNCFLDKTSMEPGDCGedEMTKAIESAKVGVFILSPEFAARKWTMRELRCFLRRR-----
ReaigsemrppTLIPVFYRLSIRECRELEPELEPDRYRDDEGNNIFEVEKFFSLERQREAST(DTVKRELQELAGITGIENHDRATNLPGDEFGPLARAHLVERVFLWVKKKSEEQE)}*

$5004=266(262):
>gi|546306135|ref|XP_005712860.1| {|997(9)|<Rhodophyta(E)>}Sel1-repeat containing protein [Chondrus crispus] >gi|507109230|emb|CDF33057.1| Sel1-repeat containing protein [Chondrus crispus]
{(VALDCVKSLGGCDTPGAVEIELTKQWLLRKGEISLPLPQLPCGSCLTSMPLEVVSMSLWPKDSASPDAAGMQRVMPEGRF)--DVFLSHAS-PD-KLALAQPLYQSLTWQRVRAFLDMEVLQILDNTapeEMDRAMREAPVGVFILSPEFVARPWPMKELQCFLKrdrvcrleggAR-----
PVILPIFYRLTLKDCSECDTLIFGEGSRHHDTLVSAGFFEQDHQNVLSTEDA(IWMLKEVKNHSGVEVSRVSGGEDLSSAGVMTEIVRRVVDYMR)}*

$5005=200(197):
>gi|546306158|ref|XP_005712869.1| {|0(811)|<Rhodophyta(E)>}Sel1-repeat containing protein [Chondrus crispus] >gi|507109239|emb|CDF33066.1| Sel1-repeat containing protein [Chondrus crispus]
{(MQESLRQETGPSRRRRKIQSP)--DVFISHAG-PD-KLLIARPLSKKLQAQGLNCFLDKISMEPGDCGgdEMTKAMESAKVGVFILSPEFAARKWTMRELRCFLRRR-----
SeaigsgmrppTLIPVFYRLSIRECKELEPDRYRDDEGNNIFEVEKFYSEERQREASTDTVK(RELQGLAGITGIENDEEATNHLEDEFGHRARERLVK)}*

$5006=200(197):
>gi|546308786|ref|XP_005713805.1| {|0(1049)|<Rhodophyta(E)>}WD40-repeat containing protein [Chondrus crispus] >gi|507110208|emb|CDF33986.1| WD40-repeat containing protein [Chondrus crispus]
{(MQESFQQETGPHRKRRKMHST)--DVFISHAG-PD-KLHIARPLFEKLQAHGLNCFLDKISMEPGDCGadEMTKAMESAKVGVFILSPDFAARKWTMRELRCFLRRR-----
SeaigsgmrppTLIPVFYRLSIRECRELEPKRYWDDEGNNIFEVEKFFSEERQREASTDMVK(RELQELAGITGIENDERATNLPGDVFGHDARANLVE)}*

$5007=200(197):
>gi|546309366|ref|XP_005714016.1| {|0(1181)|<Rhodophyta(E)>}WD40-repeat containing protein [Chondrus crispus] >gi|507110431|emb|CDF34197.1| WD40-repeat containing protein [Chondrus crispus]
{(MPESFPQETGPSRKRRKIQSP)--DVFISHAG-PD-KLHIARPLSEKLQAHGLNCFLDRMSMEPGDYEqdEMTKAMESAKVGVFILSPEFAARKWTMRELRCFLRRRIEAIGsgmrpPTLIPVFYRLSIPECGELDPDRYWDDAGNNIFEVEKFFSEERQREASTDTVK(GELHELAEFNGIENEVGATNLPGDRFGHGKRAHLVE)}*

$5008=259(256):
>gi|546316416|ref|XP_005716146.1| {|48(50)|<Rhodophyta(E)>}TIR domain-containing protein [Chondrus crispus] >gi|507112656|emb|CDF36327.1| TIR domain-containing protein [Chondrus crispus]

{(ETGGVMVSFVSQKRHIEWKVFARDPVSVAKRCLIDVEAFSSRLWQLMPEMEKYHGPARPEALRSLTVIQRTSKEVGMKAV)--DVFISHAG-PD-KLTIANPLFDKLRGEGFSCFLDKKSMKAGDCAseEMIPAMESVKIGVFILSPEFAARRWPMKELRCFLRRR-----RdarssgaappILIPVFYRLGVSECRSFKPEQYMDENGSNLLEAEKFYTKERQKEATAWVFP(LQVQPQTAKEGGCSTVPKGSVELRFPKWDLRKDSPA)}*

$5009=274(266):
>gi|546316458|ref|XP_005716156.1| {|269(0)|<Rhodophyta(E)>}TIR domain-containing protein [Chondrus crispus] >gi|507112668|emb|CDF36337.1| TIR domain-containing protein [Chondrus crispus]
{(VSFEAEKPKPKPKPKPKPTIHEEGGRPSKTLESEHHHKARARNDVKQKACRSNRHQEQPDRHAEKSVEKRESLRKSPEFT)---VFISHCG-ED-KQKMAIPLHKKLTELSISSFVDHLSMKAGRNGheAMNHAMNVAQIGVFIVSPECVPREWPIKELScflkRLEEAK-----KnntkkpILIPLYYrmNVEESKHRLFGMPSCHgggtACRLEGFRKRVErgEA--SYADVRKCFQDL(AKTTGIEYTAIENKLASPNANTPEAQQNRDKLLKDVVKAIVSHMSV)}*

$5010=212(203):
>gi|546317324|ref|XP_005716400.1| {|0(1062)|<Rhodophyta(E)>}WD40-repeat containing protein [Chondrus crispus] >gi|507112930|emb|CDF36581.1| WD40-repeat containing protein [Chondrus crispus]
{(MHST)--DVFISHAG-PD-KLLIADLLSEKLQAHGLNCFLDKISMEPGDYArdEMTKAMESAKVGVFILSPEFAARKWTMRELRCFLRRRIEAIRsgmrpPTLIPVFYRLSIRECRELDpNRYWDDErknifEVEKFFSDERQREASTDTVKRELQEL(ADFTGIENEVGATNLPGDRFGHDKRAHLVEQVFLWVKKKCEEDSSDEVREVLNASGQVG)}*

$5011=161(168):
>gi|546317419|ref|XP_005716428.1| {|0(1139)|<Rhodophyta(E)>}WD40-repeat containing protein [Chondrus crispus] >gi|507112959|emb|CDF36609.1| WD40-repeat containing protein [Chondrus crispus]
{(MQESFRQESGPSRKRRKIQSP)--DVFISHAG-PD-KLHIARPLSEKLQAHGLSCFLDKISMEPGDCGedEMTKAMESAKVGRNIFEVEKFFSEERQHEASTDTVKG-----ELKELAGITGIENDGGATNLPEDEFAHLAREHLVKRVFLWVKKKSEEDSSDE(QEADHLH)}*

$5012=208(205):
>gi|546320773|ref|XP_005717376.1| {|0(1460)|<Rhodophyta(E)>}WD40-repeat containing protein [Chondrus crispus] >gi|507113908|emb|CDF37505.1| WD40-repeat containing protein [Chondrus crispus]
[truncated: 1,756,655 more chars]
